# Enhancement of the production of _L_-glutaminase, an anticancer enzyme, from *Aeromonas veronii* by adaptive and induced mutation techniques

**DOI:** 10.1371/journal.pone.0181745

**Published:** 2017-08-16

**Authors:** S. Aravinth Vijay Jesuraj, Md. Moklesur Rahman Sarker, Long Chiau Ming, S. Marylin Jeya Praya, M. Ravikumar, Wong Tin Wui

**Affiliations:** 1 Centre for Pharmaceutical Sciences, JNT University, Kukatpally, Hyderabad, Telengana State, India; 2 Faculty of Pharmacy, Lincoln University College, Petaling Jaya, Selangor Darul Ehsan, Malaysia; 3 Department of Pharmacy, State University of Bangladesh, Dhanmondi, Dhaka, Bangladesh; 4 Pharmacy, School of Medicine, University of Tasmania, Hobart, Tasmania, Australia; 5 School of Pharmacy, KPJ Healthcare University College, Negeri Sembilan, Malaysia; 6 Faculty of Pharmacy, Geethanjali College of Pharmacy, Cheerial, Keesara, Telengana, India; 7 Non-Destructive Biomedical and Pharmaceutical Research Centre, iPROMISE, Universiti Teknologi MARA, Puncak alam, Selangor, Malaysia; University of Nebraska Medical Center, UNITED STATES

## Abstract

Microbial anti-cancer enzymes have been proven to be effective and economical agents for cancer treatment. *Aeromonas veronii* has been identified as a microorganism with the potential to produce _L_-glutaminase, an anticancer agent effective against acute lymphocytic leukaemia. In this study, a selective medium of *Aeromonas veronii* was used to culture the microorganism. Strain improvement was done by adaptive and induced mutational techniques. A selective minimal agar media was incorporated for the growth of the strain which further supports adaptive mutation. Strains were also UV-irradiated and successively treated with N-methyl-N'-nitro-N-nitrosoguanidine to find a resilient strain capable of producing _L_-glutaminase efficiently. The Plackett-Burman design and central composite designs were used to screen and optimize additional carbon and nitrogen sources. Adaptive mutation resulted in promising yield improvements compared to native strain (*P*<0.001). The mean yield of 30 treated colonies from the induced mutation was significantly increased compared to the non-induced strain (*P*< 0.001). The economically feasible statistical designs were found to reinforce each other in order to maximize the yield of the enzyme. The interactions of nutrient factors were understood from the 3D response surface plots. The model was found to be a perfect fit in terms of maximizing enzyme yield, with the productivity improving at every stage to a fourfold output of enzyme (591.11 ±7.97 IU/mL) compared to the native strain (135±3.51 IU/mL).

## Introduction

Enzyme therapy in cancer is effective and has been pursued for long time. The significance of microbial enzymes is overwhelming and microbial cultivation and production of enzymes has been found to be both economical and eco-friendly. _L_-glutaminase (EC 3.5.1.2) is an amido hydrolase enzyme which catalyses _L_-glutamine to _L_-glutamate and ammonia [1]. It is useful in the treatment of acute lymphocytic leukaemia [2], and exhibits its anticancer effect by depleting the _L_-glutamine from the cancerous cells, since these cells more avidly consume _L_-glutamine for their energy needs and proliferation than normal cells [3,4]. It is also understood that the cancerous cells cannot synthesis their own _L_-glutamine and this is the Achilles heel that is exploited by these amino acid depleting anticancer agents [5].

Other than in pharmaceutical applications _L_-glutaminase has been employed as a flavour enhancer in soy sauce and other fermented food preparations, providing a characteristic taste [6]. It has also got applications as a biosensor and analytical agent. It is used to analyze the content of _L_-glutamine in culture medium and also to measure the reaction rate in the synthesis of threonin [3]. Thus, it is important to know effective, feasible and eco-friendly techniques for the large scale production of this enzyme. In this regard, *Aeromonas veronii* (*Av*) is a gram**-**negative rod shaped bacterium that has been identified as a potential agent in yielding _L_**-**glutaminase from a total isolate of fourteen microorganisms isolated from different areas of virgin rainforests [7].

Mutational studies by physical and chemical means have a key role in strain improvement since they are able to improve productivity and economically feasible technology allow us to draw the results effectively [8]. Strain improvement based on adaptive mutation [9] allows the organism to thrive in stressful conditions, such as starvation in respect to a particular nutrient, slowly making the organism more resilient and allowing it to be modulated into a more desired form [10]. William et al. first reported the induction of _L_-glutaminase in *Bacillus licheniformis* [11]. Stanley, meanwhile, concluded that a nitrogenous substance was the agent to induce this enzyme in an experiment with *E coli* [12].

Statistical designs lead to improved yields, less laborious and more precise in their results than the experiments optimized with one factor at a time, where the interactions among the factors are also ignored [13]. The Plackett-Burman design (PBD) is a statistical design which uses less resource compared to one factor at a time to identify critical factors that are needed for the experiment. Central composite design (CCD), meanwhile, involves optimizing the screened parameters. Effective analysis of this design over the various factors allows a full understanding of the significance of individual effects and their interactions. It also furnishes unbiased information regarding the linear relationships with the nutrient factors as the nutrient factors in the composition of the medium is an indispensable factor in deciding the outcome of the experiment [14]. The paramount objective of our investigation was to produce a robust strain by mutational studies and to optimize the nutrient factors that enable the mutated microorganism to produce copious amount of _L_-glutaminase.

## Materials and methods

### Microorganism and culture conditions

The strain was identified from among fourteen isolates collected from rainforest soil of Kalikesam, Tamilnadu, India (8.418534, 77.391876). Several soil specimens were collected in aseptic screw capped bottles. About 1gm of soil specimens were diluted with distilled water and identified for _L_-glutaminase yielding strains. They were found to be positive for _L_-glutaminase by the rapid plate assay method [15]. Morphological and biochemical characterization were done to screen the microorganism, which was identified as *Aeromonas veronii* (*Av*) by 16S rDNA gene sequencing and phylogenetic analysis. The sequence data was submitted to the DDBJ database under the accession number LC020025. Selective minimal agar (SMA) medium, which comprises of 5 g/L _L_-glutamine, 1 g/L KH2PO4, 0.5 g/L MgSO4. 7H2O, 0.1 g/L FeSO4.7H2O, 0.1 g/L ZnSO4.7H2O and 0.5 g/L KCl, was the primary medium employed to culture the microorganism. Additional carbon and nitrogen sources were identified after screening and these nutrient factors were optimized. The culture conditions were 37°C, pH 7.4, an inoculum volume of 1 mL and agitation of the submerged fermentation at 150 RPM. The fermentation medium included all the nutrients as given in the SMA medium with variations as specifically stated below.

### Estimation of _L_-glutaminase

The estimation of the enzyme L-glutaminase was done by the principle of nesslerization, primarily adopted from IMADA et al[15]. The activity of the enzyme was determined calorimetrically from the proportional release of ammonium ions after adding Nessler’s reagent to the sample. Half millilitre of the sample enzyme preparation was mixed with 0.5mL of 0.2M glutamine. After 30 minutes of incubation the mixture reaction was stopped by one mL of 10% TCA. Enzyme mixture preparation, Nessler’s reagent and distilled water were added in a proportion of 0.1, 0.2 and 3.7mL respectively. The enzyme activity was calculated from the absorbance spectra using spectrophotometer at 450 nm.

### Adaptive mutational study

The isolated strain *Av* was subjected to adaptive mutation with an SMA medium. The strain was sub-cultured every three days for two months in order to attain 20 successive subcultures. For each of these subcultures, 48 hours’-worth of separate culture was grown from inoculums loopful of cells for the estimation of enzyme yield. The medium was maintained at pH 7.4 with a phosphate buffer and incubated in an orbital shaking incubator (Remi CIS 24BL) at 37°C and at 150 RPM. The cell density of the culture was measured from diluted samples of the 14^th^ sub-culture of the fermentation medium by taking the optical density at 600 nm and at 12, 24, 48, 60, 72 and 90 hours. The enzyme yield of the mutated strains of the SMA medium was compared to native strains grown over nutrient broth at the end of every subculture.

### Amplification and alignment of glutaminase genomic DNA sequence

DNA was extracted from a loopful of the wild and mutated cultures of *Aeromonas veronii* by utilizing Qiagen DNeasy tissue kit. The saline suspended isolates were centrifuged in a refrigerated centrifuge at 10000 RPM. The pellets obtained were resuspended in saline solution and were lysed by protienase K to render DNA as per the manufacturer’s instructions. Glutaminase gene was amplified from the identified the left primer 5’CTGAACCCCATGATCAACGC 3’ and the right primer 5’TCCTCGTCGATGATCCTGTG 3’ utilizing primer3 program for the enzyme glutaminase. Polymerase chain reaction was carried out with the primers using thermocycler of Applied biosystems (USA). Montage cleaning kit (Millipore) was employed to purify the sequence and analyzed by Big-Dye terminator cycle kit over DNA analyzer. The glutaminase gene sequences were aligned by employing T-Coffee program.

### Induced mutational study

Physical and chemical mutagens were successively employed to determine which were associated with better yields of the enzyme. _L_-glutamine rich agar medium was prepared using SMA medium and agar, and was used to grow *Av* for about 12 hours in a Petri-dish by uniform streaking. The grown colonies were then irradiated by ultraviolet rays at 254 nm from a light source located 30 inches above the colonies. Samples were collected every 30 seconds for about 5 minutes. During the treatment a gradual increasing kill rate was observed. Those resilient strains that survived a 99.95 and 99.99% kill rate were isolated and streaked in nutrient agar plates and preserved. The prospective samples were identified based on their morphological feature and by the rapid plate assay method [15]. They were streaked into _L_-glutamine rich agar petri**-**dishes and incubated for about 24 hours at 37°C. The colony which produced the maximum yield was fermented in nutrient broth to give out considerable biomass and was then used for chemical mutagenesis. The UV irradiated strain was centrifuged out from the liquid medium and washed with a pH 8 phosphate buffer. About 1 g of biomass from the strain was introduced into 50 mL of pH 8 phosphate buffer containing 5 mg (100 μg/mL) [16] of N methyl-N'-nitro-N-nitrosoguanidine. After half an hour, regular samples of 5 mL were carefully pipetted out at 10 minutes interval for about 60 minutes. The samples collected were centrifuged and the pellets were further washed twice in 10 mL of pH 8 phosphate buffer. The samples were then serially diluted and plated on a nutrient agar medium. The colony forming units that had produced higher yields were selected and sub-cultured. Thirty colonies of the mutated strain were randomly isolated from and subjected to undergo submerged fermentation for the estimation of enzyme production [15] against a set of 30 colonies of the native strain.

### Screening for ideal carbon and nitrogen sources

The Plackett-Burman design (PBD) was used to select important carbon and nitrogen nutrient materials that might enhance the enzyme yield [17]. PBD uses fewer experiments compared to one factor at a time and reduces experimental variations, time and cost. We have selected a total of eleven carbon and nitrogen sources such as glucose, fructose, sucrose, lactose, maltose, _L_-glutamine, casein, sodium nitrate, yeast extract, beef extract and peptone in to form the incubation medium by the submerged fermentation process. Twelve experiments were conducted to screen the nutrient factor as per the configuration of the design. The nutrients were taken in two levels of high and low values. The nutrient factors were mentioned in coded values in the table and the real values are given below. Each of the experiments was done three times, with the mean value for the enzyme activity being used as the result. This statistical design followed a first order polynomial design (Eq 1).

$$Y=\beta_{0}+\sum \beta_{1}P_{1}$$ (1)

Where, Y was the enzyme yield, β_0_ was the intercept of the design, β_1_ was the linear coefficient and P_1_ was the level of the independent factor.

### Optimization of screened components using central composite design

A total of 54 experiments were conducted to optimize the five screened nutrient factors based on CCD [18]. The experiments were undertaken in five levels which were coded as -2, -1, 0, +1 and +2. These included both the cubical and the axial points. Twelve experiments were replicated to determine the pure error of the design. Ten experiments, from 41 to 50, were undertaken to determine the axial points that may fall outside the cube. The nutrient factors were mentioned in the table as coded values and the actual values of the nutrient factors ranged according to the coded values which are presented under subtitle “o**ptimization of screened nutrient factors by Central Composite Design” in result section.** The quadratic model for the optimal value of the nutrient factor in this design was given by the following Eq 2:

$$\begin{array}{l} Y=\beta_{0}+\beta_{p}P+\beta_{q}Q+\beta_{r}R+\beta_{s}S+\beta_{t}T+\beta_{pp}P^{2}+\beta_{qq}Q^{2}+\beta_{rr}R^{2}+\beta_{ss}S^{2}+\beta_{tt}T^{2}+\beta_{pq}PQ+\\ \beta_{pr}PR+\beta_{ps}PS+\beta_{pt}PT+\beta_{qr}QR+\beta_{qs}QS+\beta_{qt}QT+\beta_{rs}RS+\beta_{rt}RT+\beta_{st}ST \end{array}$$ (2)

Where, Y was the predicted response (enzyme yield IU/mL); P, Q, R, S, and T were the independent nutrient factors; β_0_ was the intercept; β_p,_ β_q,_ β_r,_ β_s_ and β_t_ were the linear coefficients; β_pp,_ β_qq,_ β_rr,_ β_ss_ and β_tt_ were the quadratic coefficients; and β_pq,_ β_pr,_ β_ps,_ β_pt_β_qr_β_qs,_ β_qt,_ β_rs,_ β_rt_ & β_st_ were the interactive coefficients of the design. Three-dimensional plots were constructed to identify the main and the interactive effects by response surface methodology. Minitab^™^ 16 statistical software was employed to construct the CCD analysis and for the response surface graphs. The optimum values of the nutrient factors identified were used to validate the design.

### Statistical analysis of the data

The experimental results presented in this manuscript are means of ± S.E.M (standard error mean) of three independent trials. The data were analyzed by paired *t*-test for 95% confidence levels. *p* values which less than 0.05 were considered as significant. The native and adaptive mutational strains were also analysed by Two-way ANOVA for strain, time as two independent factors against the dependent factor the enzyme yield with the pair-wise post comparison.

PBD and CCD statistical experimental designs were employed for screening and optimization of nutrient factors. The screening and optimization of the nutrient factors were achieved by solving the regression equation. The models were constructed based on the degree of variability of ANOVA calculations which determines the tests for significance.

## Results

### Augmentation of _L_-glutaminase production by adaptive mutation

**Fig 1:**
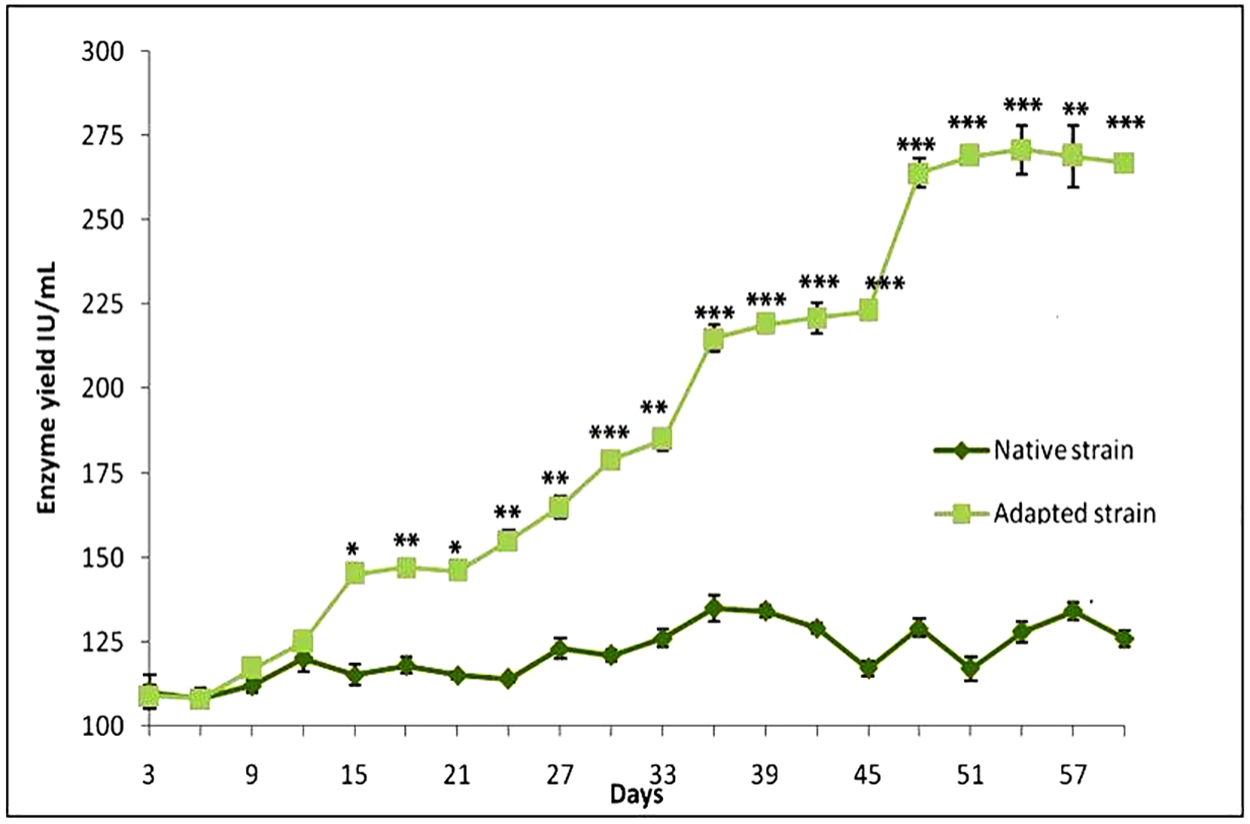
Enhancement of _L_-glutaminase production by adaptive mutation of *Av*. The isolated strains of *Av* were repeatedly sub-cultured every three days in SMA medium for 60 days to ensure adaptive mutation. The yield of the enzyme in the mutated strains was significantly higher when compared with the native strain. The data are shown as means ± SEM of three independent observations (* P<0.05, **P<0.01 and ***P<0.001).

**Fig 2:**
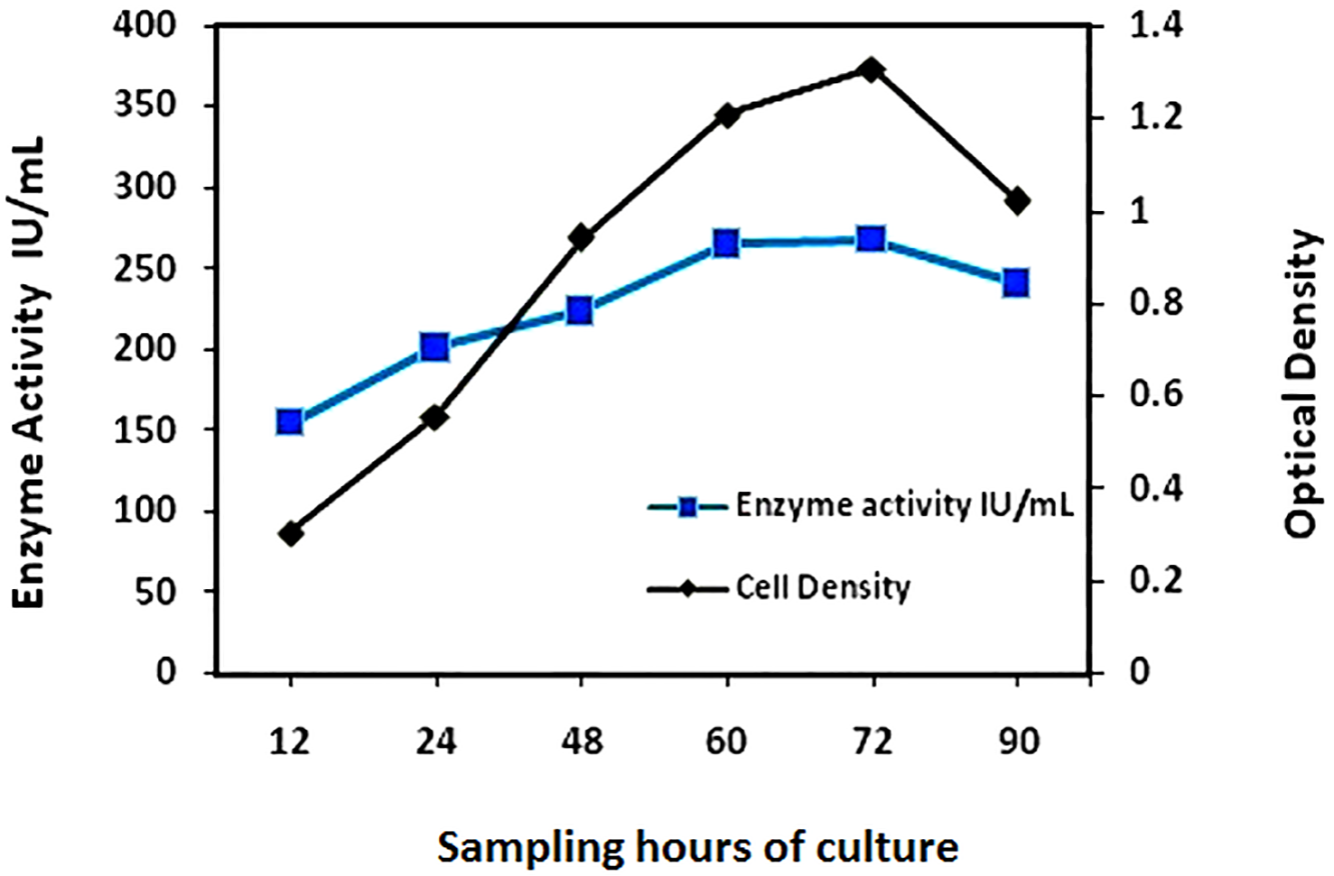
Cell density and corresponding enzyme activity of the adapted strains of *Av*. Growth pattern of the adapted culture showing cell density in terms of optical density and enzyme activity in IU/mL. Cell density and the enzyme activity proportionately correspond to each other.

The culture started to produce larger amounts of enzyme from the 15th day, and this enzyme differed significantly from the native strain (*p<0.05). The production of enzyme quantity was continuously increased in the subsequent cultures for up to 60 days (*p<0.05, **p<0.01, and ***p<0.001) (Fig 1). The adapted strain and the time duration were found to have significant effect in the production of the enzyme (p<0.001). Their also existed an interactive effect with the adapted strain over the time duration of the culture with the SMA medium (p<0.001). The highest production of enzyme (264–271 IU/mL) was observed between the 48^th^ to the 60^th^ days. The native strain grown in nutrient broth fluctuated in terms of enzyme yield between 108 IU/mL and 135 IU/mL while the adapted strains productivity was consistent across the subcultures, showing a gradual but steady improvement. There was a significant leap in the enzyme activity of the adapted strain from the 13^th^ to 14^th^ subculture, as well as from 15^th^ to 16^th^ subculture. The cell density of the microbial culture increased considerably to sub maximum and maximum at 60 and 72 hours respectively (Fig 2). The enzyme output attained its maximum value (268 IU/mL) with the sole carbon and nitrogen source of _L_-glutamine at its stationary phase (Table 1).

**Table 1:** Two-way ANOVA results of strain, time and strain and time interaction.

Source	Mean Square	F	Sig.
Corrected Model	9806.185	871.016	.000
Intercept	2940322.133	261168.509	.000
Strain	139810.133	12418.369	**.000**
Time	7353.519	653.162	**.000**
Strain * Time	5416.537	481.114	**.000**
Error	11.258		

Table 1 shows two-way ANOVA study for strain and time as two independent factors and enzyme yield as the dependent factor. R Squared = 0.998 (Adjusted R Squared = 0.997). ‘*’ denotes the interactive effect.

### Mutational analysis

**Fig 3:**
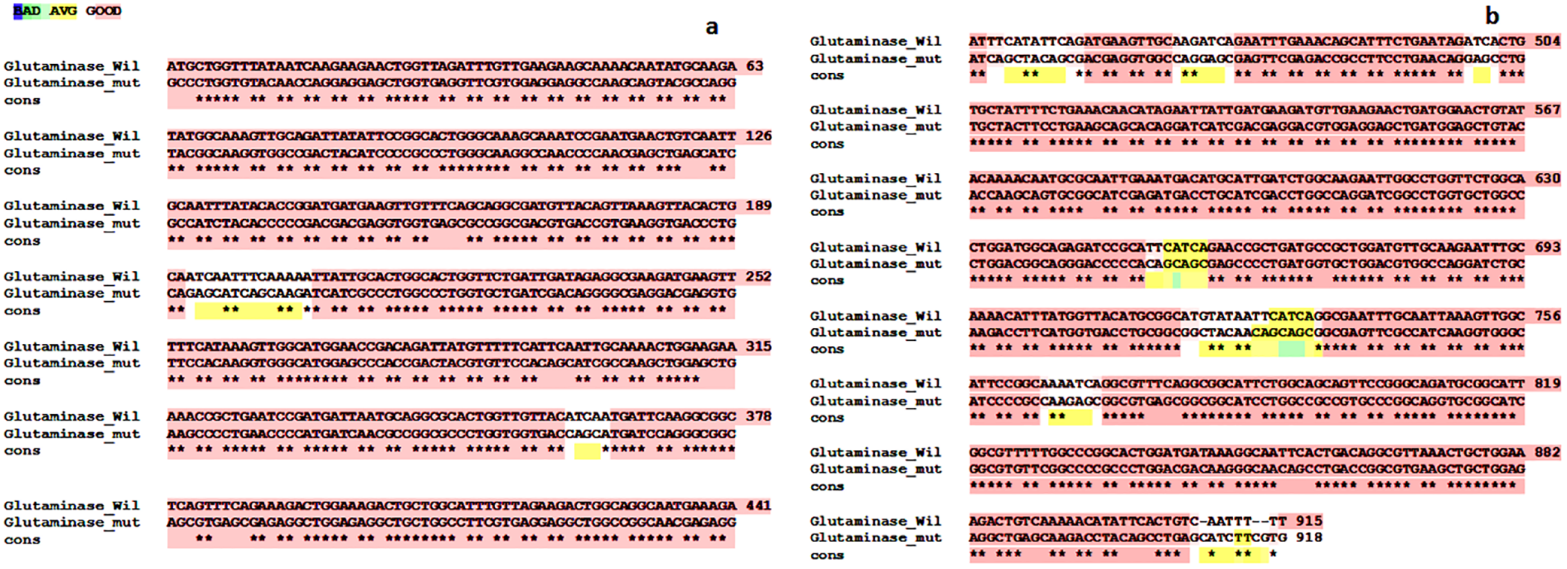
Glutaminase gene sequences of wild and mutated organisms of *Aeromonas veronii*. a)Alignment of the gene sequences from 1 to 441 nucleotide bases of wild and mutated organisms of *Aeromonas veronii*.b)Alignment of the gene sequences from 442 to 915 nucleotide bases of wild with 442 to 918 nucleotide bases of mutated organisms of *Aeromonas veronii*. The sequences were aligned for similarities by T-Coffee program. Glutaminase_Wil—Glutaminase gene sequence of wild organism, Glutaminase_mut- Glutaminase gene sequence of mutated organism and cons—highly conserved regions of the sequence.

The wild and mutant genes of glutaminase were aligned to reveal the conserved and mutant regions (Fig 3a & 3b). The mutant strain when compared with the wild strain, retained significant amount of highly conserved regions. The star marks of the base pair revealed the conserved regions, but dissimilarities were found at base pairs ranged from 193 to 204, 361 to363, 446 to 452, 446 to 470, 499 to 500, 651 to 657, 720 to 732 and 766 to 770. Strong dissimilarities were found at two regions of the base pairs of 654 and 728 to 730.

There were gaps at the wild gene sequence at the base pairs of 916, 915 and 909. The wild sequence consist about 915 base pairs whereas the mutant form had 918 base pairs. The ratio distribution of nucleotide A: T: G: C of the wild strain was 33:27.5:23.5:17 whereas the mutant was 21:14:35:30.

### Enhancement of _L_-glutaminase production by induced mutation on adapted strains

**Fig 4:**
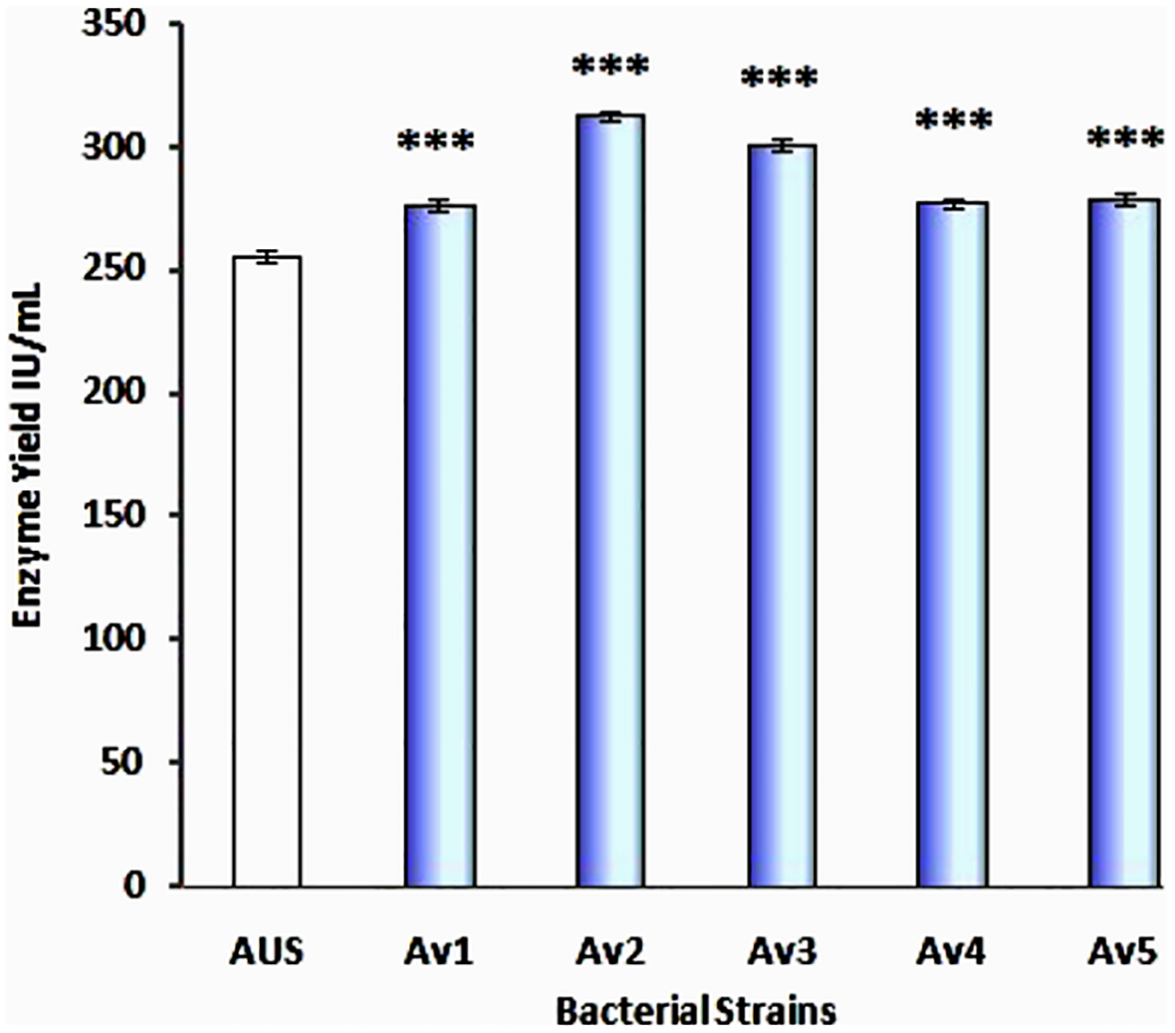
Survived UV-induced strains of *Av* and the corresponding yield of the enzyme. Comparison of the yield of enzyme in IU/mL between the UV induced strains (Av 1–5) and the adapted but un-induced strains (AUS). Av 2 was found to produce the maximum yield relative to the others (312.67 IU/mL).

**Fig 5:**
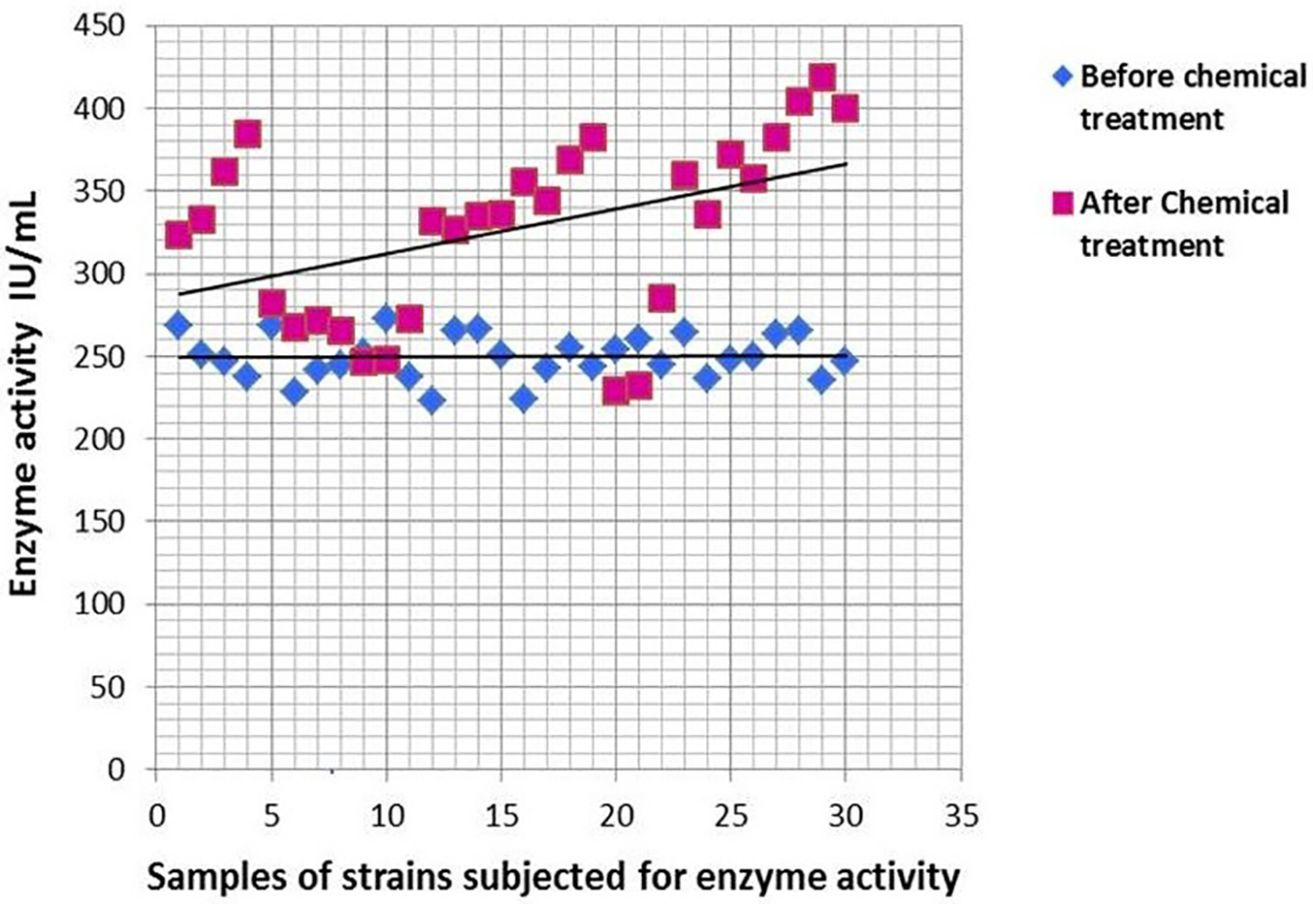
Comparison of enzyme yield before and after chemical treatment. Scatter plot showing the yield of enzyme in IU/mL compared between 30 samples of chemically treated strains to that of 30 sets of non-chemically-treated adapted and UV-induced strains. The yield can be seen to increase slightly towards the upper end of the chemically treated strains compared to the control.

The kill rate of the adapted microorganism *Av*, reached 99.95 and 99.99% after 180 and 210 seconds of UV irradiation, respectively. Five colonies (Av1, Av2, Av3, Av4 and Av5) were isolated, which produced between 276 and 313 IU/mL of the enzyme, with an average production of 289 IU/mL (Figs 4 and 5). An increment in enzyme activity of 13% was identified after adaptive mutation. The 30 samples of the treated strains which had undergone genetic modification had an inclination to produce the enzyme higher than earlier. The *P*-value was found to be <0.001 and the 95% confidence index ranged from 56.026 to 99.040. The yield of the mutated strains were found to average at 131.07% compared to that of the native strains.

### Screening of nutrient factors using the Plackett-Burman design

**Fig 6:**
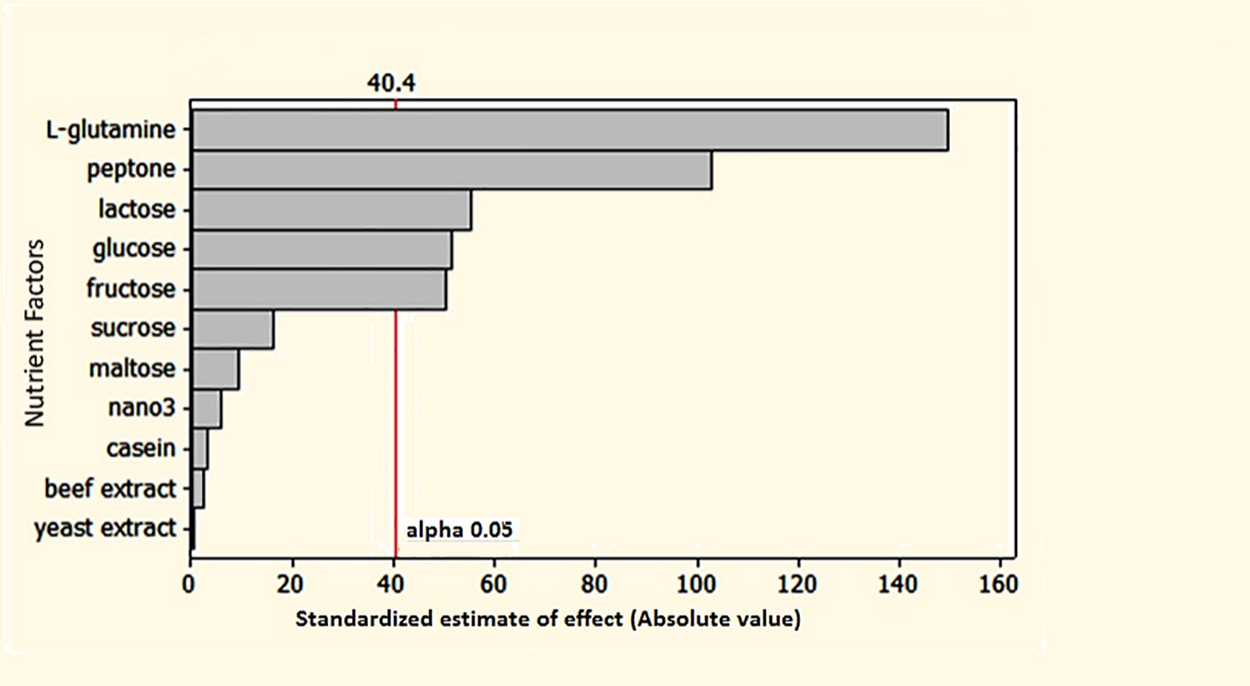
Pareto plot of the PBD showing the significant nutrient factors affecting the yield of the enzyme _L_-glutaminase. The nutrient factors were ranked in order from highly significant (top) value to the least significant. The nutrient factors greater than the alpha value 0.05 were considered as significant.

In order to improve the yield further, additional carbon and nitrogen sources were screened using PBD [19] (Table 2). With the help of twelve experiments five out of eleven nutrient factors were screened. This was identified from the Pareto plot, where the alpha value was set to 0.05. In the Pareto plot the alpha value cut across at 40.4 (Fig 6). The significant nutrient factors identified were peptone, _L_**-**glutamine, lactose, fructose and glucose. The enzyme activity varied from 41.29 to 329.14 IU/mL. The following regression equation was derived from ANOVA and the nutrient factors were selected based on its significance. Table 3 details the effect, coefficient, t and *p* values.

**Table 2:** Design matrix of outcomes from a Plackett-Burman experimental design for the screening of significant nutrient factors. N1 to N11 represent the nutrient factors in the following order with the low and high uncoded values in brackets: casein (1&7 g/L), peptone (1&7 g/L), lactose (1&5 g/L), sucrose (1&5 g/L), fructose (1&5 g/L), maltose (1&5 g/L), nano3 (0.5&5 g/L), glucose (1&5 g/L), _L_-glutamine (2&6 g/L), yeast extract (0.5&2g/L) and beef extract (0.5&2 g/L). E.A = Enzyme activity IU/mL, P.E.A = Predicted Enzyme Activity IU/mL.

Sl. No	N1	N2	N3	N4	N5	N6	N7	N8	N9	N10	N11	E.A	P.E.A	Error
1	1	-1	1	-1	-1	-1	1	1	1	-1	1	303.84(±4.46)	302.11	1.7317
2	1	1	-1	1	-1	-1	-1	1	1	1	-1	331.59(±2.46	330.2	1.3917
3	-1	1	1	-1	1	-1	-1	-1	1	1	1	296.23(±8.85)	297.96	1.7317
4	1	-1	1	1	-1	1	-1	-1	-1	1	1	71.73(±3.34)	70.34	1.3917
5	1	1	-1	1	1	-1	1	-1	-1	-1	1	84.71(±1.46)	82.98	1.7317
6	1	1	1	-1	1	1	-1	1	-1	-1	-1	194.33(±2.77)	192.6	1.7317
7	-1	1	1	1	-1	1	1	-1	1	-1	-1	329.14(±5.04)	330.53	1.3917
8	-1	-1	1	1	1	-1	1	1	-1	1	-1	86.89(±2.75)	88.62	1.7317
9	-1	-1	-1	1	1	1	-1	1	1	-1	1	164.18(±4.97)	165.57	1.3917
10	1	-1	-1	-1	1	1	1	-1	1	1	-1	139.64(±6.95	138.25	1.3917
11	-1	1	-1	-1	-1	1	1	1	-1	1	1	189.37(±1.89)	191.1	1.7317
12	-1	-1	-1	-1	-1	-1	-1	-1	-1	-1	-1	41.29(±3.54)	42.68	1.3917

**Table 3:** Estimated effects and coefficients from the Plackett Burman design. The table shows the regression analysis of the nutrient factors with their corresponding *P*-value. R Square 99.99%, R Square predicted 98.42%, R Square adjusted 99.88%, Significant values *, and non-significant values ^.

Factors	Effect	Coefficient	T value	P value
Constant		186.08	174.18	0.004*
Casein	3.12	1.56	1.46	0.382^
Peptone	102.97	51.48	48.19	0.013*
Lactose	55.23	27.62	25.85	0.025*
Sucrose	-16.08	-8.04	-7.52	0.084^
Fructose	-50.16	-25.08	-23.48	0.027*
Maltose	-9.36	-4.68	-4.38	0.143^
nano_3_	5.71	2.85	2.67	0.228^
Glucose	51.24	25.62	23.98	0.027*
L glutamine	149.38	74.69	69.91	0.009*
Yeast extract	-0.34	-0.17	-0.16	0.9^

Y=186.08+74.69 N9+27.62 N3+ 25.62 N8+51.48 N2-25.62 N5 (3)

### Optimization of screened nutrient factors by central composite design

A CCD comprising 54 runs was used to analyze the significance of the proportion of nutrient factors. The experimental and predicted yields of the enzyme were rendered in Table 4. The enzyme yield ranged from 432.11 to 551.23 IU/mL. Table 5 depicts the coefficients, T values and *p* values of the linear, squared and interactive factors. The quadratic equation for the production of the enzyme _L_-glutaminase from *Av* was given by multiple linear regression analysis (Eq 4):

$$\begin{array}{l} Y=481.88+9.63P+6.61Q+6.23R+12.45S-6.34T-3.59P^{2}+1.66Q^{2}+3.59R^{2}\\ +2.34S^{2}+2.03T^{2}+1.44PQ+2.62PR-2.09PS-2.21PT+3.50QR+2.19QS-4.10QT\\ +2.90RS-1.69RT-3.17ST \end{array}$$ (4)

**Table 4:** Full factorial central composite design matrix showing predicted and real values in the optimization of _L_-glutaminase yield with the screened nutrient factors. E.A = Enzyme activity IU/mL, P.E.A = Predicted Enzyme Activity IU/mL. Corresponding five levels of the actual values were _L_-glutamine (1, 3.5, 6, 8.5 & 11 g/L), Lactose (0.5, 2, 3.5, 5& 6.5 g/L), Glucose (0.5, 2, 3.5, 5 & 6.5 g/L), Peptone (1, 3, 5, 7 & 9 g/L) and Fructose (1, 2.5, 4, 5.5 & 7 g/L).

_L_-Glutamine	Lactose	Glucose	Peptone	Fructose	P. E. A	E. A	Error
-1	-1	-1	-1	-1	454.55	453.11	-1.4447
1	-1	-1	-1	-1	474.31	473.14	-1.1683
-1	1	-1	-1	-1	461.73	468.55	6.8211
1	1	-1	-1	-1	487.23	491.31	4.0800
-1	-1	1	-1	-1	452.38	457.1	4.7211
1	-1	1	-1	-1	482.59	482.92	0.3251
-1	1	1	-1	-1	473.55	473.17	-0.3755
1	1	1	-1	-1	509.51	504.61	-4.8991
-1	-1	-1	1	-1	479.79	483.54	3.7469
1	-1	-1	1	-1	491.21	494.5	3.2934
-1	1	-1	1	-1	495.71	498.81	3.0977
1	1	-1	1	-1	512.87	505.11	-7.7633
-1	-1	1	1	-1	489.2	491.31	2.1078
1	-1	1	1	-1	511.08	511.74	0.6617
-1	1	1	1	-1	519.11	515.67	-3.4439
1	1	1	1	-1	546.74	551.23	4.4925
-1	-1	-1	-1	1	464.21	470.1	5.8943
1	-1	-1	-1	1	475.1	475.1	-0.0018
-1	1	-1	-1	1	454.99	449.51	-5.4773
1	1	-1	-1	1	471.63	468.29	-3.3409
-1	-1	1	-1	1	455.29	450.13	-5.1574
1	-1	1	-1	1	476.65	480.42	3.7741
-1	1	1	-1	1	460.06	465.01	4.9485
1	1	1	-1	1	487.17	482.12	-5.0476
-1	-1	-1	1	1	476.76	481.39	4.6309
1	-1	-1	1	1	479.32	473.91	-5.4051
-1	1	-1	1	1	476.29	471.55	-4.7358
1	1	-1	1	1	484.59	491.22	6.6307
-1	-1	1	1	1	479.43	479.04	-0.3857
1	-1	1	1	1	492.44	488.87	-3.5743
-1	1	1	1	1	492.94	495.99	3.0451
1	1	1	1	1	511.71	505.23	-6.4809
0	0	0	0	0	481.78	480.54	-1.2399
0	0	0	0	0	481.78	489.33	7.5501
0	0	0	0	0	481.78	477.27	-4.5099
0	0	0	0	0	481.78	479.39	-2.3899
0	0	0	0	0	481.78	489.38	7.6001
0	0	0	0	0	481.78	475.16	-6.6199
0	0	0	0	0	481.78	472.28	-9.4999
0	0	0	0	0	481.78	487.32	5.5401
-2.366	0	0	0	0	439.28	432.11	-7.1694
2.366	0	0	0	0	484.85	491.38	6.5318
0	-2.366	0	0	0	475.81	471.17	-4.6440
0	2.366	0	0	0	507.09	511.1	4.0064
0	0	-2.366	0	0	487.53	484.22	-3.3083
0	0	2.366	0	0	517.04	519.71	2.6708
0	0	0	-2.366	0	465.81	464.7	-1.1079
0	0	0	2.366	0	524.7	525.17	0.4703
0	0	0	0	-2.366	485.81	480.22	-5.5884
0	0	0	0	2.366	455.79	460.74	4.9508
0	0	0	0	0	482.16	481.22	-0.9405
0	0	0	0	0	482.16	483.35	1.1895
0	0	0	0	0	482.16	491.39	9.2295
0	0	0	0	0	482.16	475.87	-6.2905

The corresponding five levels of the actual values were _L_-glutamine (1, 3.5, 6, 8.5 & 11 g/L), Lactose (0.5, 2, 3.5, 5& 6.5 g/L), Glucose (0.5, 2, 3.5, 5 & 6.5 g/L), Peptone (1, 3, 5, 7 & 9 g/L) and Fructose (1, 2.5, 4, 5.5 & 7 g/L) (E.A = Enzyme activity IU/mL, P.E.A = Predicted Enzyme Activity IU/mL).

**Table 5:** Estimated regression coefficients in the optimization of nutrient factors by central composite design. The table shows the regression analysis of the nutrient factors with their corresponding *P*-value. R Square 94.38%, R Square predicted 83.89%, R Square adjusted 90.97%, # denotes significant values, and ^ denotes non-significant values.

Factors	Coefficients	T value	P value
Constant	481.88	273.986	0.000^#^
L Glutamine	9.63	10.344	0.000^#^
Lactose	6.61	7.1	0.000^#^
Glucose	6.236	6.699	0.000^#^
Peptone	12.445	13.368	0.000^#^
Fructose	-6.344	-6.814	0.000^#^
L Glutamine*L glutamine	-3.59	-4.531	0.000^#^
Lactose* Lactose	1.66	2.095	0.044^#^
Glucose* Glucose	3.595	4.537	0.000^#^
Peptone* Peptone	2.339	2.952	0.006^#^
Fructose* Fructose	-2.03	-2.561	0.015^#^
L Glutamine* Lactose	1.437	1.328	0.193^
L Glutamine* Glucose	2.616	2.418	0.021^#^
L Glutamine* Peptone	-2.085	-1.928	0.063^
L Glutamine* Fructose	-2.214	-2.047	0.049^#^
Lactose*Glucose	3.498	3.234	0.003^#^
Lactose* Peptone	2.186	2.021	0.051^
Lactose* Fructose	-4.098	-3.789	0.001^#^
Glucose* Peptone	2.896	2.678	0.011^#^
Glucose* Fructose	-1.686	-1.558	0.129^
Peptone* Fructose	-3.171	-2.932	0.006^#^

Table 5 shows the regression analysis of the nutrient factors with their corresponding *P*-value. R Square 94.38%, R Square predicted 83.89%, R Square adjusted 90.97%,

^#^ denotes significant values, and

^ denotes non-significant values.

‘*’ indicates the interactive effect between different factors or the squared effects in the case of same factors.

Analysis of variance (ANOVA) was done to evaluate the adequacy of the design. Table 6 rendered the ANOVA of the design. The significance of the design was given by both the F and the *p* value. The predicted values were obtained from the model whereas the error is the difference between the actual run and the predicted value. It was observed that the experimental values were close to the predicted values. The effects were significant since the T values and *p* values were higher and lower respectively. It was found the coefficient of determination, the R Square value, was near to one (0.94) and thus, the error was less (0.06). R Square predicted and R Square adjusted closeness represented the fitness of the model. All three Linear, squared and interactive effects were found to be highly significant. This was understood by the high F values and the lower *p* values. The linear contribution was the highest followed by the squared effects and the interactive effects. The “lack of fit” of the design was found to be insignificant (0.591) as it is desired to confirm the fitness of the model.

**Table 6:** Analysis of variance of the quadratic model. The table shows the outcome of ANOVA for the quadratic model. DF -Degrees of Freedom, Seq SS- sequential sum of squares and Adj Ms-Adjusted Mean square.

Source	DF	Seq SS	Adj MS	F value	*P*-value
Linear	5	16002.5	3200.45	85.49	0.000
Square	5	2381.5	476.29	12.72	0.000
Interaction	10	2344.1	234.41	6.26	0.000
Residual error	33	1235.4	37.44	-	-
Lack of fit	22	798.4	36.29	0.91	0.591
Pure error	11	437.1	39.73	-	-
Total	53	21963.3			

Table 6 shows the outcome of ANOVA for the quadriatic model. DF -Degrees of Freedom, Seq SS- sequential sum of squares and Adj Ms-Adjusted Mean square.

**Fig 7:**
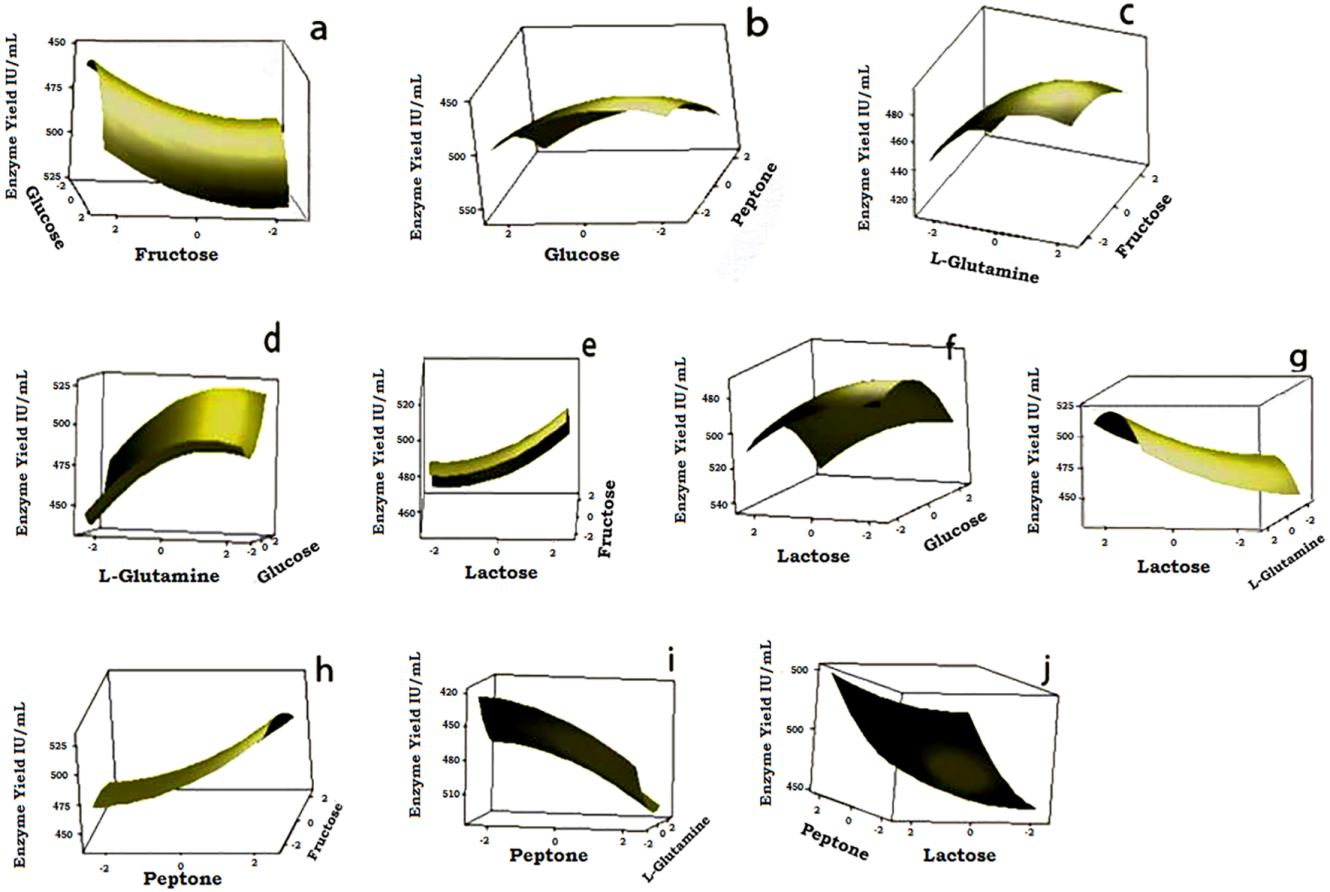
3D response surface graphs for the possible interactions of nutrient factors. X and Y axis of the Fig. 7(a): Glucose vs. Fructose, (b): Glucose vs. Peptone, (c): _L_-glutamine vs. Fructose, (d): _L_-glutamine vs. Glucose, (e): Lactose vs. Fructose, (f): Lactose vs. Glucose, (g): Lactose vs. _L_-glutamine, (h): Peptone vs. Fructose, (i): Peptone vs. _L_-glutamine, (j): Peptone vs. Lactose.

Three dimensional response surface graphs were drawn by taking two nutrient factors at a time in the X and Y axis, with the Z axis then being the enzyme yield IU/mL (Fig 7). These graphs depicted the linear and the quadratic effects by varying the levels of the nutrient factors, while the other factors were kept at a zero level. There were prominent interactions among six of the plots (Fig 7b–7f and 7h) as understood from the *p* values in Table 5. The optimum un-coded values of the nutrient factors were deduced from the regression equation. The nutrient factors L-Glutamine, Lactose, Glucose, Peptone and Fructose were 8.2 g/L, 6.5 g/L, 6.5 g/L and 2.33 g/L, respectively. The model proved to yield 591.11(±7.97) IU/mL which was close to the predicted value of 582.36 IU/mL.

The optimum levels of the components were used in the verification of the model. It was found that the optimization model yielded out 80.73% of the excess _L_-glutaminase compared to that of the experiment with un-optimized parameters. Thus the total productivity had been increased to 4.11 fold against the un-optimized native strain (135 ± 3.5 IU/mL).

## Discussion

_L_-glutamine plays a vital role in cellular nitrogen metabolism since it serves as an important nitrogen carrier. Microorganisms derive most of their nitrogen from the conversion of glutamate to α ketoglutarate by the action of glutamate dehydrogenase. Glutamine would be generated by glutamine synthase with glutamate and nitrogen, but at the expense of energy [20,21].

When abundant glutamine is available in the culture medium, however, _L_-glutaminase, the putative enzyme, is produced in copious amounts to convert _L_-glutamine to _L_-glutamate. Thus glutamine, the medium nutrient factor, is an excellent way of inducing _L_-glutaminase.

The optimization study revealed the successive utilization of PBD and CCD designs to be crucial in optimizing the yield of the enzyme, although previously there have only been a few reported experiments using PBD and CCD techniques on the enzyme _L_-glutaminase [22,23]. This paper is also the first to record the significance of adaptive mutation in the production of _L_-glutaminase. Furthermore, there was no report on the use of these experimental designs with the microorganism *Av* in the production of the anticancer enzyme _L_-glutaminase.

The cultured *Av* grown over SMA medium showed a remarkable improvement in the yield of _L_-glutaminase. The strain was found to be well adapted to the restricted nutrients of the SMA medium. Since _L_-glutamine was involved in nitrogen assimilation of the microorganism, and was administered as a sole carbon and nitrogen nutritional source, it kindled the productivity of the enzyme _L_-glutaminase. The steady increase in the production of the enzyme over time signified a positive correlation with the nutrient supplement _L_-glutamine. It should be noted that the enzyme output increased profoundly at latter stages of the culture. This explains the existence of interactive effects between the strain and the time duration of the cell culture. The induction of _L_-glutaminase was, however, specific for the nutrient glutamine, as can be seen from the results for the control strain. It has also been shown that there should be a glutamine transport system in the organism as noted by Takenori et al., in *Bacillus subtilis* where it assimilates glutamine by special operon that switches on when abundant glutamine is available [24].

The experiments showed that there was a proportional increment of the enzyme activity as the cell density increased. The enzyme activity was found to increase with the growth of the strain but to then decline after the stationary phase. Thus the release of the enzyme was found to be an anabolic function.

Adapted strains were subjected to UV irradiation and then rendered for chemical mutation by N methyl-N'-nitro-N-nitrosoguanidine, resulting in a remarkable yield. The impact of the physical and chemical induction elicited a yield of about 100% but this was still less than with adaptive mutation. It was found that the mean enzyme activity between the native strain and the mutated strain was statistically significant.

The mutant form of the organism yielded more of the enzyme was clearly observed over the modification on the Glutaminase gene. The amplified complete genome of *Aeromonas veronii* of strain TH0426 consist of about 4923009 base pairs and the Glutaminase gene was found to be 1197 base pairs (NZ_CP012504.1) (S1 File) [25]. Our wild strain possessed 915 base pairs whereas the mutant strain possessed 918 base pairs. The primary mutations here it occurred in the organism were substitutions, missense mutation and Insertions of base pairs occurred. Overall in the genomic sequence substitutions had occurred. Missense mutation might change the aminoacid that the sequence codes, such changes could both affect the conservative and non- conservative states, in our case that probably affected to restoration and more release of the enzyme. Frame shift mutation that usually occurs at gaps in the gene might produce stop codon. Insertions or deletions should occur not at the multiple of three base pairs. In our mutational study insertions were occurred at the terminal end of the sequence but at the multiple of three. So this type of mutation was not occurred.

A large variation in enzyme activity was observed between the PBD experiments combining the eleven components, allowing the importance of the individual nutrient factors to be inferred. Peptone, _L_-glutamine, lactose, and glucose were found to possess a positive effect and fructose was found to have a negative effect. Although the outcome of PBD experiments through the pareto chart clarified the significance of the nutrient factors that influenced the yield of the enzyme, the positive and negative effects of them were only determined after the CCD experiments. This denotes that the positive effect of the nutrient factors will be effective on higher amounts and the negative effect hold good for low values.

The prediction of PBD was found to be good since the adjusted R Square was high and the error was small. The possibility of error that could not be explained by the model was only about 0.22%. The predictability was also explained well by the adjusted R Square value (99.88%) and predicted R Square value (98.42%). The software provides the enzyme activity theoretically after we key in the original experimental values through computational analysis based on the design algorithms. The predicted enzyme values would match with the real experimental values may be with marginal error. The prediction was good enough since the two values were quite close.

The addition of carbon and nitrogen sources led to better yields than when the microorganism was grown with limited nutrients. It was evident from the research that a balanced mixture of nutrient factors was needed for its growth, maturation and the eventual yield of the enzyme. The design could not explain only 5.62% of the outcome due to error or factors unrelated to the medium components.

There were three linear-quadratic interactions (Fig 7d, 7e & 7h) and three quadratic-quadratic interactions (Fig 7b, 7c & 7f). Fructose in all the plots showed a high enzyme activity with low levels than the other interacting nutrient factor. Fig 7b, 7d, & 7f showed the interactions of all the nutrient factors that had a high influence. Fig 7c, 7e & 7h showed the nutrient factors interacting with fructose, which had a low influence. Fig 7b, 7d & 7f showed those factors with a positive association to the yield of the enzyme, whereas, Fig 7c, 7e, & 7h showed factors with a negative associations to the yield. Peptone and _L-_glutamine had a good impact in the delivery of the enzyme, _L_-glutaminase. Peptones contain a cocktail of amino acids, peptides and protein essential for the growth and maturation of the microorganisms. They consist of sulfur-containing amino acids like cysteine and methionine, which are needed for cell development and metabolism of the microorganism [26].

## Conclusion

In our investigation, adaptive and induced mutational methods created a platform for screening and optimization processes using PBD and CCD for a colossal yield of the enzyme _L_-glutaminase. The CCD was precise in optimizing the screened nutrient factors, as shown by its higher R square value. Both the PBD and CCD designs reinforced each other in screening and optimizing nutrient factors. The Pareto ranking of PBD was quite closely matched with the CCD optimization ranking. Our study demonstrated promising enhancement of _L_-glutaminase of *Aeromanas veronii* by adaptive mutation. Further, the production of this enzyme was significantly augmented by the induced mutation technique. The phase-wise improvement of enzyme yield culminated with the model guided experimental designs, CCD and PBD. The mutational techniques and the optimized nutrients that we pilot here can be applied to up-scale the production of _L_-glutaminase to economically viable levels. The produced enzyme could be employed as a decisive tool and can be guided as nanoparticles to target cancer cells in the domain of cancer chemotherapy.

## Supporting information

S1 File Complete genome of *Aeromonas veronii* strain TH0426. The complete genome of *Aeromonas veronii* TH0426 consists of 4923009 nucleotide base pairs.
(PDF)
