# Supplementary material for: Enhancement of the production of L-glutaminase, an anticancer enzyme, from Aeromonas veronii by adaptive and induced mutation techniques
Source: PLoS One. 2017 Aug 16;12(8):e0181745. doi: 10.1371/journal.pone.0181745 (PMC5558970; doi:10.1371/journal.pone.0181745)
Supplement: S1 File — The complete genome of Aeromonas veronii TH0426 consists of 4923009 nucleotide base pairs. (PDF) [file pone.0181745.s001.pdf]

Aeromonas veronii **strain TH0426, complete genome**

>NZ_CP012504.1 Aeromonas veronii strain TH0426, complete genome **4923009**
ATGATGGCCTGCTTAACCTGGCGTGTCACACTCTGGGTCAACTGAGTACTCCTGTACAAGAGCCGAGCAG
TCTACCATTACCCCCTGACCCGCAACAATAAAGCGCCGCTTATTAAGAAGTTAATCAATCAATAACGATT
TCTTCTGCGCTGGCAGGTGAGTCCTTGCTCACCCCTTCTCCAATTCGTCGAGGATCGGGCACTCGGGATT
TTCATCTCCGTGGCAGCAGCAGACCAACCCTTCCAGGGAGGTAAGCATGGCCTGCAGCCCGGCAATGCGG
GCGCGCAGATCGACGATCTTCTCCTGCGCCAGCTTCTTCACCTGGGCGCTGGTTCTGTGTTCGTCCCGAT
AGAGCGCCAGCAGCTCCTGACACTCTTCCAGATTGAAGCCGGTCTCCCGCGCCCGCTTGACGAAACGCAG
CTCGCGCAGCGCCCCCTCGGTGTAACTGCGATAGCCGTTGTCACTGCGTACCGGGGCTGTGATGAGGCCA
ATGCTCTCGTAATAACGGATGGTCTTGGCGGTCAGCCCAGTGGCCTTGGCAACCTTGCTGATGTTCATTT
ATGCCTCCTTACTGATCGGGTTGGATGCTGGCCCGTGCGCCGCCCTGGGCTGCCAGCGTTTCAATAACAG
GGAATTGCTCAAAACCGTGATACTGCTCAGGGCCATGGCCGCCCCCGCCAGCTCGGGGCTCAGGTAGCCC
AGCGCCGCCAGCGGAATGCCGATGATATTGAACACGAAGGCCCAGAACAGATTCTGCTGTATGGTGCGCC
AGGTCGCCGCCGAGATGTCGATGGCATCGGCCACCAGCCGCGGGTCAGATCGCATCAGGGTGATGGAAGC
TGTCTCCATCGCCACATCGGAGCCGGAGCCCATGGCGATGCCGACGTCCGCCGCCGCCAGCGCCGGAGCA
TCGTTCACTCCATCCCCCACCATGGCCACCAGCCCGCGGGTTTGCGCCCGCAGCGCCTCCACTTTCTCCA
CCTTGCCCGCTGGCAGCACGGAGTCAAAGGCCCCGGCCAGCCCCAGCTTGGCGGCGATATGGGCCACCGG
CGCCGGTGCATCGCCACTCACCAGCCAGCTGGCAATGCCACGCTGGCGCAGGGTGGCGATGGCCTCCGGA
CTTTCGGGGCGCAGGGTATCGGCCAATGCGGCGATACCAGCCACCGTGCCGTCGATGGCCACCCAGACCC
GGGTCGCCCCATCGGCGGCTTGTTCATCCTGTTGCGGCGGTTCAATGCCGAGCTGGGCCAGCAGGGAACC
ATTGCCGATGGCGACACTGTGGCCCGCCACCTGACCGACTATGCCGGCACCGACCCGCACCTCGACCGCC
TCCGGCTGAGGCAGTGCCACCCCGCTGCCGACCGCCTCGCGCATGGCCAGCGCCAGCGGGTGTTCGCTCG
CCTGCTGCAGGGCGGCGGCCAGATGCAACTGCTCGTCGTTGCCGCTCCAGCTAGCCAGCACAGGCTTGCC
CTGAGTGAGCGTGCCGGTCTTGTCGAAGATCAGCGCCTTGATGGCGTGGGCCTTCTGCAGGGTATCCACA
TCCTTGATCAGAATGCCGTGGCGCGCCGCGACGCCGGTACCGGTGACGATGGCCGCCGGGGTCGCCAGCC
CCAGCGCACAGGGGCAGGCGATCACCAGCACCGCCACCGCTGCCAGCAGGGCCTGACCGAAGTCGTTGCT
GATGGCGTACCACACCAGCAGGGTGAACAGGGCGATGGCCATCACCACCGGCACGAACACGGCGGAAACC
TTGTCCACCAGCTGTTGCAGCGGGGCCTTGCCCATCTGGGCACTCTCCACCAGTGCAATGATCTTGCTGA
GGCTGGAGTCCTCCCCCACCGTAGTGGCTTCAATATCCAGCACGCCGGAGCGGTTGATGGCGCCACCCAG
CACCTTGTCGCCCACATTGCGCGGTACCGGCAGGCTCTCGCCGGTGAGGATCGATTCGTCCAGTTCGCTG
GCGCCGCTCACGATTTTGCCATCCACCGGCACCCGCTCGCCCACCAGCACCCGCAGCCGATCGCCGCGCA
GCACCTCGTCGATGGCGACGCTTTGCCAGTTCTCGCCACGCCAGACGGTGGCGGTTTCCGGCCGCAGCGC
CATCAGTTCGCGAATGGCGGACTGGGTGCTGCGTCTGGCCCGTGCCTCCAGCAACTTGCCGAGGGAGATC
AGGGTAATGATGATGGCGCTCGCCTCGAAATAGAGCTTGCCGCTGGCAGCCATCCCCAGCGTCAACAACA
GATAGAGGCTGTAGAAGTAGGCAGCGCTGGTGCCGGTTGCTACCAGCACATCCATATTGGCTGCGCGGTT
CTTGATGGCGAGCCAGGCACCGCGATAGAAGCGTCTGCCTACCCAGAACTGCACCGGGGTGGCGAGCAGC
AGCTCCAGCCAGGCGGGTAGATGCCAGGGGAACAGTCCGGCCATGGCCAGCATCCCCACCAGCAGCGGCA
ACGTCAGCAAAGCCGAGACGATGACCCGGGTTTGAGCCTGACGGGCTGTGGCGGCCTCCTGCGCTTCCCG
CTCCAGCAACTGCTGGCGGCGGCCGCTGGCCGAGCCTTGTGCCAGCTGGGCGCCAAAGCCGAGCGCTTCG
ATCTGCTCGCGCAAAGCGGCGGGCGTCTGCATCCCGGGGACCAGCACGATGCGGGCCTGTTCGATAGAGA
AATTGACCTCGGCCGAGATAACGCCGGGCAGCGCGGCCAGCATCTTGTTCAACCGAGCCGAACAACCGGC
ACAAGTCATGCCGGTAATTTGGAACAAAAAGGTCTCGCTGCCGTAGTCGTAACCCTGACTCTTGATGCTG
TCGAGCACCGCAGGCAGGCGATCGGGGCTGGCCAGCTCGATGGCCGCCTGCTCCAGCGCGAAGTTGACTG
TGGCCTCGACCCCTTCAAGCTTGTTGAGGGCGGTCGCGATGCGGCCTGCGCAGTTGGCGCAGCTCATGCC
GCTGAGCGGCAACTGCAGATGAATGGGTGATTGAAGATGAGTGGTCATGTTGTTCCTCCCCGGAACCCGT
CGGCACAACAAAACGCCGACTGGCAAACCCTAACCTTTACTGTAAGGGCAAGCTCAAGCCACATGATCCA
CTTTTTGCAGATGAAAAAAACGGCGCCCGTTTGCTGCCAGAGAACAAACGGGCGCCGCATTCAGCTATTG
CGCGGTGCGAGCGGAGCTCAGATACCGTACTGGGCGCGATAAGCCTTCATGGCCGCCAGTTGCGCCGCCA
GCTCCGGCTGTTCGGCCTGCTTCTCTTCCAGATACTGGATCAAGTCGCTCATCTGGATGATGGCGATGAT
GTGAGCGCCGTAGTCACGCTCCACTTCCTGAATGGCGGAGAGTTCGCCCTTGCCCTTCTCCTGACGGTCC
AGCGCGATCAGTACGCCAGCCAGCGAGGCGCCGTTGGCCTGGATCAGATCCATGGATTCGCGGATGGCGG
TGCCAGCGGTGATGACGTCATCCACCAGCATGATGCGCCCCTTGAGCGGGCTGCCCACCAGATTGCCACC
CTCGCCGTGATCCTTTGCCTCCTTGCGGTTGAAGCACCAGGGCACGTCCACATCGTGCTGCTCCACCAGC
TGCACCGCGGTAGCGGAGGCGATGGGGATCCCCTTGTAGGCCGGGCCAAACAGCACATCAAACTCGATAC
CGGCATCCATCAGGGCGGCGGCATAGAAGCGGCCGAGACGGGCCAGATCGCGGCCGCTGTTGAACAGACC
GGCGTTGAAGAAGTAGGGGCTTTTACGACCGGATTTCAGGGTAAATTCACCGAATTTGAGGACCTGTTTC
TCCAGGGCGAACTCGATAAATTGACGCTGGTAGGCTTTCATTCTGGCTCCTTGCTTGGCTTGTCGTTGTC
TTGTGTGGGGGTATAAAAAAACGCGGTCAATGCCGCGTTTTTAAAATCAGGAGAGCGACTGCTTCTGCAG
CGCAATGATGTCGGCGATACCGCCCTTGGCGAGCGCCAGCATCTCCAGCAGCTCTTCATGGGTGAAGGGC
TCGGCCTCCGCGGTGCCCTGCACCTCGATGATGCGACCATCTTCGGTCATCACCACGTTCATGTCGGTCT

CGGCGGCGGAGTCTTCGATGTACTCCAGATCGCAGATGGCTTCACCCTGATACATGCCGACGGATACGGC
GGCGATCATCTGCTTGAGCGGGCTCTGCTTGAGCATGCCCTTCTCGACCATCCAGTTCAGCGCATCCACC
AGCGCCACACAGGCACCGGTAATGGAGGCGGTACGGGTGCCGCCATCGGCTTGCAGCACGTCACAGTCAA
CGGTGATGGAGTGTTCGCCCAGCGCAGTCAGGTCTACCGCCGCGCGCAGGGAGCGGGCGATCAGACGGGA
GATCTCCAGAGTGCGGCCACTCTGCTTGCCGGAGGCCGCTTCGCGGTTCATCCGGGAGTGGGTGGAGCGC
GGCAGCATGCCGTATTCGGCAGTTACCCAGCCCTGATTCTTCCCTTTCAGGAAGCGCGGCACGCTGGTGT
CAACGCTGGCGGTGCAGAGCACGCGAGTGTTACCGAACTCCACCAGCACTGAGCCTTCTGCATGTTTGGT
GAAATTGCGGGTAATGGTAACCGGGCGAAGTTGCGCGGCACTGCGATCGCTTGGACGTGACATGGAACGG
ACCTCTGGGCTGGCTCAAATTGGCGAGGGATTATAAGTGATCCCGCCCCAAATATCAGCCGCTAAAACCA
CGGCATCGGCTGACGAGCAGGGCCGGGCTGAGTATACTGGCCGCACACTTCATCAAGCCCGGAACAATCA
ATGATTCACAGCATGACCGCCTACGCCCGCAAGGAATTCAAGGGCGACTGGGGCACAGCCGTTTGGGAAA
TTCGTTCGGTCAACCAGCGCTATCTGGAAACCTACATTCGCCTGCCGGAGCAACTGCGCAGCCTGGAGCC
AGTACTGCGTGAGCGTTTCCGCGCCAAGCTGCAGCGCGGCAAGGTGGAGTGCAACCTGCGTTTCGAAGCC
GCTCAGGCCGCTGCCAGCCAGCTGCACATCAATGAAGAGCTGGCCAAGCTCATCATCGACAGCGCCAACT
GGGTAATGAAAGAGGCGGGTCAGGGCCAACTCAATCCGGTCGACGTACTGCGTTGGCCGGGCGTCATGGC
TGCCGCCGAGCAGGACATGGACGCGGTCGCCACCGAGCTGCTCGGCGGTTTTGACCAGACCATGGAAGAT
TTTCTCGCCTCCCGCGCCAGCGAAGGGGCCAACCTCAAGGCGCTCATCGAACAGCGTCTGGCCGGTATTC
AGGTGGAAGTGGAGAAGGTGCGCGTCCACCTGCCGCAGATCATCGAGTGGCAGCGCCAGAAGCTCACCGA
CCGTCTGGCCGAAGCCAAGGTCGAGCTGGACCCGGCCCGCATCGAGCAGGAGATCGTGCTGCTGGCCCAG
AAGATCGACGTGGCCGAAGAGCTGGATCGTCTGGAGATGCACATCAGCGAAACCAACAAGATCATGAAAA
AGGGTGGCGCCTGCGGTCGTCGTCTCGACTTCATGATGCAGGAGTTCAACCGCGAGTCGAACACCTTGGG
CTCCAAGTCCATCAACGCCGAAGTGACCCAGTCCGCAGTCGAGCTGAAAGTCCTCATCGAGCAGATGCGC
GAGCAGATCCAGAACATCGAGTGATGGGTCACTGACCATCCGGTTGTTACCTGCAAAAGAGCCCTTGATT
CCTCAAGGGCTCTTTTATTTTCAGGCCATGGGGTTTGCCGCCTGTGAGCCAACGAGCAAGAGAAGCCCTA
GCCTATCGGCATCACCAGCGCCGGTTGGTGCGGGTCGCTCAGCCGCTCGGCCAGATAATCCACCAGCAGT
CGCACCTTGGGGGAGAGGTGGCGGTTGTGGGGATAGAGGGCCCAGATCCCCTCGGCCGGAGCCCGCAGTT
CGGGCAGCAGCTCGATCAATTCGCCTCGGGCCAGATGGTCGCTCACGTAGTAGTCCGGCAGCTGGATAAT
GCCGATCCCCTTGAGCGCCGCATCCAGCAGAGCGTGGCCACTGTTGCACTGCAACAGGCCGCGCACCCGC
ACGCTGTGGGGGCGACCGTCCAGAGTGAAGTGCCACTCGTCGCTGTTGCCCACCAGACAGGTGTGGCGGG
TCAGCTCGGAGAGGCTGTTGGGCATGCCGTGGCGGGCGGTGTAAGCCGGTGAGGCGCAGACATAGGGCAC
CCGCTGCGCCAGCTTGCGCGCCACCAGCGTCGAATCCTTGAGGTGGCCAAGCCGCAGCGCCAGATCGTAG
CCTTCGTGCACCAGATCCAGCAGCTGGTTGGTGAGGTTGAGGGTCACCTCCAGCTGGGGGAAACGCACCA
TGAAGTCGTTCACCAGCGGGGCGATGCGGCTCTCGCCGTAGGAGACGGGGGCCGTAATGCGCAGCGACCC
TTGCGGCACCGCCTGATGGCGCCCCACCGCCAACTCCGCCTCGTAGAGGTTGTCCAGCAGCTGACGGCAG
TGGCGATAGTAGATCTCGCCAAGCTCGGTGAGCCGCACCTGACGGGTGGTGCGCAGCAGCAACTTGGCCT
GCAGCCGATCCTCCAGCTGTGCCACCTGACGGCTGACTTGCGCCACCGACACTCCCAGCTTGCGCGCAGC
CGGGGTAAAGCCCCCCTGCTCCGCCACGCTGACAAATTCACAGATCCCTTCCCAGCCGAACATTATTACC
TGTGTGTAAAAGTGATTTGAGATTTGCCCTCATTATCACCAAATCGGCGGGAAACTACACTGAATGCCCG
AACTCATCTCTGTTCACCCTGCTCAACAAGAAGGATGTCATTCATGGCACAGGTTCAAAGCATCAAGTGT
AAAGCCGCCATCGCCTGGGGCCCGGGTCAGCCCCTCTCCATCGAAGAAGTAGAAGTCATGCCGCCGCAAG
CGGGCGAGGTGCGGGTGCGCATCGTCGCGACCGGCGTCTGTCACACCGATGCCTTCACCCTCTCCGGTGA
AGATCCGGAAGGGGTCTTCCCCTGCATCCTCGGCCACGAGGGGGGCGGCATCGTCGAATCCGTCGGCGAA
GGCGTCACCAGCGTCAAGGTGGGCGATCACGTGATCCCGCTCTACACCCCCGAGTGCGGCGAGTGCAAGT
TCTGCAAATCCGGCAAGACCAACCTATGCCAGAAGATCCGCGCCACTCAGGGCAAGGGGCTGATGCCGGA
CGGCACCACCCGCTTCTCGAAAGATGGCCAGCCCATCTATCACTACATGGGCACCTCCACCTTCAGCGAA
TACACGGTACTGCCGGAGATCTCCATCGCCAGGGTGGACCCGGCCGCCCCGCTGGAAGAGGTCTGCCTGC
TCGGCTGCGGCGTCACCACCGGCATCGGCGCAGTGATGAACACCGCCAAGGTGAAAGAGGGTGAGAGCGT
CGCCATCTTCGGGCTGGGTGGCATCGGCCTCTCGGCAGTGATCGGCGCCCGTCTGGCCAAGGCCGGTCGC
ATCATCGCCATCGACATCAACGAGAGCAAGTTCGAACTGGCCCGCAAGCTGGGCGCCACCGACTGCATCA
ACCCGAACGATTACGACAAACCGATTCAGGAGGTGATCGTCGAGCTGACCGATGGCGGCGTCGACTTCTC
CTTCGAGTGCATCGGCAACGTCAAGGTGATGCGTGCGGCGCTGGAGTGCTGCCACAAGGGCTGGGGCGAG
TCGGTCATTATCGGCGTCGCCGGAGCAGGTCAGGAGATCAGCACCCGTCCGTTCCAACTGGTGACCGGTC
GGGTCTGGCGCGGCAGCGCCTTCGGCGGGGTACGCGGTCGCAGCGAACTGCCGAGCTATGTGCAGCGCTA
CATGCAGGGCGAGTTCCGCCTCGATGACTTCATCACCCACACCATGCCGCTGGAGCAGATCAACGAGGCA
TTCGATCTGATGCATGAAGGCAAGAGCATCCGCACCGTCATCCACTACTGAGCATGATCGCCCCGCCCGC
CATCGTGGGCGGGGTACAGGAGTCCTTCGATGAATCTGGAGATTATCAGCAGCAACAAGAGCTTCGGCGG
CTGGCACAAGCGCTATCGCCACAGCGCCAGCGTGCTGGGCTGCGAGATGAATTTTGCCATCTATCTGCCG
CCTCAGGCCCTCACCGGCCAGAAGGTACCGGTGCTCTACTGGCTCAGCGGCCTCACCTGCACCGATGAGA
ACTTTATGCAGAAGGCGGGTGCCCAGCGCATCGCCGCCGAGCTCGGCATCGCCCTGGTGGCGCCGGACAC
CAGCCCCCGCGGCGACGGCGTAGCGGACGATCCCGGTTATGACCTCGGCATGGGCGCAGGCTTTTACGTC
AATGCCAGCCAGAGCCCCTGGCAGGCGCACTACCAGATGTATGACTACGTCACCCGCGAACTGCCTGACT

TGATAGAGACCCACTTCCCGGTCTCGACCCGCCGCGCCATCAGCGGCCACTCCATGGGCGGTCATGGTGC
GCTGATCGCCGCGCTGCGGGAGCCGGGTCGCTACCGCTCGGTCTCGGCGTTCAGCCCGATCTGTCACCCG
AGCCGCAGCCCTTGGGGCCAGAAGGCGCTCGGCGCCTATCTCGGCAGCAATCCTCTGCTGTGGCAGGAGT
GGGATGCCTGCCAACTGCTGCGCCACCAGCCGGTCACGCCGCTACCAACCTTGGTGGATGTGGGGCTGGA
TGATCCCTTCCTCGCCGAGCAGCTGCATATCGATGCGCTGGCAGAGGTGGCCGCCAGCAAGGGGTGGCCG
CTGGAGCTCAACCGCCACGAGGGCTATGACCACAGCTACTACTTCGTGGCGAGCTTTATCGAGGCGCACC
TGCGCTTTCACGCCCGCCACCTCTGATCAAAACAGGCCCGTTGCGGGCCTGTTTCAGTTGTGGTGATCGG
TTGATATCAGCTACCGGCGCGGGTCACTCCGCCGGAGCCCACGATCTGCTGCTCCACCTCGGGGTCACCC
CAGTAGGTGATGTTGCCAGTGCCTGCGATGCCGATCTTGAGCGTTTTCTGGGCCCGCACCTCCACATCAC
CGCTGCCGGCGATATTGACCTCGACCTCGTTGCCACGCATTTCGCTGCCCAGCACATCGCCAGCACCGGC
AATGTTCAGCTCGATGCTAGTGGCAGAGCCGCTCCCCAGATCCAGGCTACCTGCACCAGCGATATTGCCT
TTCAATCGCTGTACCTCCAGCTGGCTGGCGACGATACCACCGGTTCCCGCCACTGACAGCTCCAGCGCCT
CACCCTTGAAGCCACTCAGATCGCCGTTGCCGGTACCTGCCAGCGCCATTTCACTGAGCTCTGGCAGGGT
GATGGTGATCTCCAGCGGTTCGGTAGGCCGCAGTCGGTAACCATCCTTCACCTCGATCTCCAGCTTGCTG
CCGCGCTCGGTCAGCTCCAGATACGGCAAGAGATTTGCCTGCCCCTTGATCAGGATCCCGCGCTGCTCCC
CCGCCACCAGATGGAGTGTAGCTGGAGTTCCCGCCAGCACCCGGGTCACCCGCTCGCCCAGCGCCTGCTG
GCGCTCGACCGGGACACCTTCCCCCTTGATCTCGGTGTCGGCAAAGACGTTGCAACCTCCCATCACCAAC
GCCACCAAGACGCCAATCATCCACCGCATAACTGTTCTCCTTTTTATTTATTGATAAGCCACCTGACAGG
CCCTGTTCGAAACCACAAAAATAGAGGCCGTATGAAACCACAGATAGCAATGGCCATGCCAGAATCTAAC
TATATGATTTTCAATAAAAATAAAATCAACAGTAAGAAGTGGTACGCATTAAAAAGAGCCATTTAGACCA
CCATTTGGCGAATCTGACCATCCCCTAACAGGCAGGTGGAGAGCTGGCTGGCGAGCCGTCCTGCCGGTCG
CTACGCTGACAAGCCGTGGAAGTTTTGGATTGCCCTGCCGGTCTGCTGAGCCAATAGTGGTACTTCGTGC
GTACCAAGCTCTCCATTGCGGTAATGTCATTACCACTGTCTTGTCAGTCTTCACTGCCAACAAGGAGTCA
GAGCGTGACCCAAGCAAGCCCTCTCTATGCCACCGCCACCGGTCTGCCGGAACCGACTCGCACCCCGCAG
CTGACCGGCAGCGATCCCGAGCAGAAACGGCGCGAGCTGCTCGCCTACTTCTGCCAGACCTTCGATCTCT
ACGATTCGCTGTTCGACTGTCTGGCCGATGAGCGGGCCTGGTTCAACAAGGCGATCCCGCTGCGCCACCC
GCTCATCTTCTACTACGGCCACACCGCCGCCTTCTTCGTCAACAAGCTGCTGGCGGCCCGCCTCATCGAG
GGTCGGCTCGACGCCCGCATCGAGGCCATGGTCGCCATCGGGGTGGACGAGATGAGCTGGGACGATCTGG
ACGAGACCCACTACGACTGGCCCAAGGTCAGCGAGCTGCGCAGCTATCGGGCCAGAGTCCGTACGCTGGT
GTGCGACTTCATCCGCCAGATGCCCCTCACCCTGCCCATCGACTGGCAAAGCCCGGCCTGGGTGATCCTG
ATGGGGATAGAGCACGTGCGCATCCACCTCGAGACCTCCAGTGTGTTGATCCGCCAGTTGCCGCTCGCCT
GGGTGCGGCCGCAGCCTTATTGGCCCGCCTGCACTGAAGCCCGCCACCGCATTGATCAGGTGCCCGCCAA
CAGCCTGCTGCCGGTGGCTGGCGGCAAGGTGCGCCTTGGCAAGCGGGACGCCACCTACGGCTGGGACAAC
GAATATGGCGAGCGCCATATCGAGCTGGCCCCGTTTCAGGCCAGCCGCATGCTGGTGAGCAATACCGAGT
ACCTCGCCTTCGTCCAGGCGGGCGGCTACGGCCAGCAACAGTGGTGGGATGAGGAGGGGTGGGGTTGGTG
TCAGTACGCCAAGGCGCAGATGCCTACCTTCTGGGTGGGTGACCCCGCCGAGCCCGAACAATTGCAGCTG
CGGCTGATGACCGAGCAGGTCGCCATGCCATGGGACTGGCCGGTGGAGGTCAACCAGCTGGAAGCGGTTG
CCTTCTGTCGCTGGAAGGCGGCCCGGACCGGACTCCCCATCCAGCTGCCGAGCGAGGCGGAATGGATGCT
GCTGCGCGAGCAGCTACCCGGCGATCAGCCGGTCTGGACACAGGCCCCCGGCAATATAAATCTGGCCCGT
TTCGCCTCCTCCTGCCCGGTGGATGCCTGCCCGCAAGGGGAGTTCTTCGATCTGGTGGGCAATGTCTGGC
AGTGGACCAGCACCGCCATCGACGGCTTCGACGGCTTTAAAATCCACCCGCTCTACGACGACTTCTCCAC
CCCCACCTTTGATGGCAAGCACACCCTCATCAAGGGCGGCAGCTGGATCAGCACAGGTAACGAAGCGCTC
AAATCATCCCGTTACGCCTTCCGGCGCCACTTCTTCCAGCATGCGGGTTTCCGCTATCTGGTCTCCCGTT
ATCAGGAACCCGTTATCGTGAATCCCTATGAAACCGACACGCTGGTCGCTCAGTACCTCGACTTCCAGTA
CGGCCCGAGCCACTTCGGGGTAGCCAACTACGCCAAGACGCTTGCTGATCTTGCTGGCGAGCTGTGCAGC
AGCCACCAGCGGGCGCTGGATATCGGCTGCGCCACCGGCCGCGCCAGCTTCGAACTGGCTCGCCACTTCC
AGCATGTGGACGGGGTCGACTACTCGGCCCGCTTCATCGACGTGGCGCTGGCCCTCGCCAGCCAGGACAG
CTTCCGCTACGCCATTCCGCTGGAGGGTGAACTGGTGGAGTATTGCGAGGCGCGCCTGTCGAGCCACGGG
CTGGGGGCGAGTCAGGCTGAGCGCATCCACTTCAGTCAGGGGGATGCCTGCAACCTCAAGCCCAAATATG
ACCACTATGATCTGGTGCTTGCCTCCAACCTGATCGACCGGCTGCGGGAGCCCGCCCGCTTCCTGCGGGA
TATCGCCCCCCGGCTGCGCAGCGGTGGCCTGCTGGTGCTCACCAGCCCCTATACCTGGCTGGCGGAATAT
ACCCCGCCCGCCAACTGGCTCGGCGGCATTCGCGAAAACGGCGAGGCGCTCACCACCTATCAGGCGCTGC
AACGGCTGCTGGCGGCCGAATTCGAGGAGCACTGCCCGCCGCAGGATGTGCCCTTCGTCATCCGCGAGAC
CGCCCGCAAATACCAGCACACGGTGGCGCAGCTGACCGTCTGGCGCAAACGCTAATCCGCCGGTCGGCAT
ACAGATGAGAAGAGGGAGGCCAACGGGCCTCCCTCTTGTTATGACAGCCAAAACGACACGAGTATCACTC
TCCCTGCTCGTGATAGCGGCGCGCCAGCACGGCGCAGACCATCAGCTGGATCTGGTGGAAGATCATCAGC
GGCAGCACCATGACGCCGACGCTGGCGGCCGGGAACATGACGTTGGCCATCGGCACCCCGTTGGCGAGGC
TCTTCTTGGAGCCGCAGAAGACGATGGTGATCTCGTCCGCCTTGTCGAAGCCGAGGCGACGGCTGATCCA
GACGTTCCAGCAGAGCACCAGCGCCAGCAAGACGCAGCTCAGCAGCACGATACTGGCCAGATCGTACCAG
CCGACCTGGTGCCAAATCCCCTGCACCACCGCCTCGCTGAAGGCGACATAGACCACCAGCAGGATCGAGC
TCTGATCCAGCTTGCCGATGAGCGGACGGTGGCTGTCGACCCAGCGACCGAGCAGCGGGCGCGACAGGTG

GCCTACCACGAACGGCAGCATCAGCTGCATCATGATGGACTGGATGGAGTGCCAAGTGTCGATCCCCTCC
CCCTGCACGTGGATCATCATCCCCACCAGCACTGGCGAGAGGAAGATCCCGAGGATGCTGGAGGCCGAGG
CGCTGCACACCGCCGCCGACACATTGCCCCCTGCCATCGAGGTAAAGGCGATGGCCGACTGCACGGTGGC
CGGCAGGGCGCAGAGATAGAGGAAGCCGAGGTAGATAGCCGGGCTCAGCCAGCTGGGCACCAGCGGGGTG
AGCAGCAGCCCGAGCAGCGGAAACAGCACGAAAGTGCTGAGCATCACCACCAGATGAAGCCGCCAGTGGC
CGAGACCTGCCAGCAGGTTCTGGCGCGACAGCTTGGCGCCATGCATGAAGAACAAGAGCGCCACCGCCAG
CTTGGTCAGCAGACCGAACCAGACGGCCACCTGCCCCTCGCAGGGAAAGAGGGAGGCCAGAGTGACCACC
GCAATCAGCACAACAGTAAAGGGATCCAGACGCACGAAAGGTCTCCATCAGAAGGGCATCCCGCAGCCAT
GGCAGCAGTCAGGATTGGGGAGGACAAGAAACAGAGCGGGAGATGTCTGGCACCTCCCGCTAAAACGAAC
CTCCAGCTTACCCCGATAACGGAGAGGCGCAAAGTTGAGCGGCGAATAATTGTTCATCAATGCTTAACGC
CTTCGCCATATCGAGTAGCGAAAGTTGACGATAAAGCGGCCCTGACAGCTCCCAAACGGCTAAAAATGGA
TAAAAAAACGGGCGACCAAGGCCGCCCGTTCCGCTTTTTCACTCAGCCGATCAGCGCTGGCTGGCCAGCT
TGCCGTTGCCGCCTTTCTTCTCATTGCCGGCAAGCTTGCGGGCCTGCCGCTTCTCGAAGCGGGAGATCCA
CCAGTAGGCCAGCGCCGAGAAGACCGCGGTCATAAAGACGTTGATGAAGAAGGCACGCACCAGATCCACC
CCGGCCGGGAAGGCGGAGGCACTCATGCTCACCACGAAGATGGCGATCAGCACCGACACCACGCCCATCC
CGAACTTGCGCGACCCCATGCGGAAGTCGCGTGGGGTGTCGTCATGTTTCCAGCGGAACACGAAGTAGGC
CACCATGATGAAGATCGGCGGCAACATGGCGGTGCCGGCGGTGAGGTTGATGGCGGTGTTCATCATCTGC
TGCACCGACTCGGAGCCGAAGCCGTTGACCACCAGCATCGCGAACACGAAGACAAACTGCCACCAGGCGG
CGCGCACCGGCACGCCGTGCTCGTTGAGCTGGGTGGTCTTCTCGCCGTAAACCCCCTGCGGGATCTCGGA
GAAGTGGATCTTCACCGGCGCCGAGGTCCACATCATCATGGAGCCGAACATGGCGATGAACAGCACCACG
CCGACGAAGCGGCCCACCAGCGCCTCGGAAAGCCCGAAATACTGCGCCATGCCGGTGAAGATCTCCACCA
TGCCGCCGGCATAGGTGAGGCTCTCGCGCGGTACGAACACGTTCACCAGCAGGGAACCGACCGCATACAT
GGCACCGATGACGATACCCGCACTGATGATCACCTTGATGAAGGACTTCTGGCCGCCCTTCACATCATTG
AGATAGGCCGCTGCCGTTTCGGCACCGCCCGCCGCTTGGAAGATCCAGCACATGATTCCGAGGGTCGCCC
AGTTGACGGTGGGAGTCATCGCCTCCAGCGTGATGGGCTGGGCCGGCACATGATCGCCACTCAGAGCCAT
CAGGGCACCCAGCGCATAGATGGCGAACAGGGCAAACACCCCGTAGGCCACGATCTCGGCAATCTTGCCA
AGCCAACTCGCCCCCTTGGTGGAGATGTGGGTCGCCGCCGCAAACAGCACGATGGAGATGATCGAGGTGA
CCATGGGCGAGAAGCTGTACTCATAGCCCAGCATGGCGTAGGAGGCGTAGGCGATGACGTTGGGCAGCAG
GGAGACGAACCAGAACAGGTTCACGAACCAGTAGAGAAACGAGCCCAGATAGGCCATCCGGGTGCCGAGC
GGTTTTTTCAGCCAGTCGTACATCCCCGACTCGGAGTTTTTATTGGCCGAGACGAACTCGGCAATGATGA
AAACGAACGGAATGAAGTAGACGATGGTCGCCAGCAGGAAGATGGGGGCGGAGCTGAGCCCCAGCTCAAT
GTTGTTGTTGACGATGTTGCGCACGTTGAACACGGCGGCAAAGGTCATCGACAACAGGGCAAACTTGCCT
ATGGTGCCGCGTACGGATTCAGACATGGTGTCCTCCAGTGGACTCGCGCTCCCTGCGGGTAACGCTGTGA
CTCTGTGATGTGTTCGGCAAGGGTCTGTCGGCCCCGCGCTTTACTCTCTTGTTATGATCGGGCCGCGTGG
ACGGCCTCGTTGCTGAACCGGTACCGTCTCCCGCACCGGCTCGCAATACCATTCAGACGATCGGAGTACC
CGGCGCCATCGGCACCCGCGGTTGCAACAGCACGCTCTGGCTCTCGTCCGGGGTCTCGGCGCAGAGCAAC
ATGCACTCGGATCGCTCACCGCGCATCCTGGTGGGCTTGAGGTTGGTCAGTACCACCACCTCGCTGCCCA
TCAGCTCCTCTTCGCTGTAGTAGGGCACCAGACTGGTGACGGTCTGCAGCGGCCGCTCGCCACCCACGTC
GATCTGGACGATGTAGAGTTTGTCGGCACCGGGGTGGCGCACCACCTCCAACACCTTGCCCACCTTCATC
TCGACCCGTTTGAATTCGGCAAACTCGATCGTCTCGCTCATGCTCTTTTCCTTTTCTGGTTCAACGGTTT
GCGGGTGGCCAACGGCCACCCGGTTTGATTGGGATTCAATTCAGTGGCTATGGCGTCTGCATGGCAACCT
GCGCTGTCGGCTGGGCCTCTTTGGCACGCGCTTCGGCACTGAGGGAGGCCTCGTATCTGTTGTATTTCCA
CCAGGCGAAGCCAAGGAAAATCACGATCCCGCCGACGTTGTAGAAGATGATGGTCATGATGTCGGCACCG
GTCGGGAAGGTGGAGGCGAGGAAGCCCACCGAGAAGATGGCGATCAGCACCGAGACCACGGCGATGCCGG
TGGTGCGCGAACCCATCTTGAACTCCCGCGGCAGGTGATCGAGCTTCAGACGCAGATTGAGGTAGGCCAG
CATGATGAACAGCGGCGGCAGCATGGAAGCGGCGGCGGTCATGTTGATAACAGTGTTCATCAGATCCTGC
GCTGTGTTGGAGCCCAGAGTCGGGATCACCATCAGCGGCAGCACGATGAGGTACTGGATCCAGGCTGCAC
GGGCGGGCACGCCGTTCTCGTTCAGCTCGACCGTCTTCTTGCCGAAGATGCCGGCCGGGATCTCGGAGAA
GAAGATTTTCACCGGGGTCGCCGTCCACATCAGCAGGGAGCCGAACATGGCGGTGAAGGAGACCAGCCCG
ACGAAGCGGTTCATCAGGATCTCCGGCAGGCCGAAGTAGGCCGCCAGCCCCTCAAATACCTGCACCGAGC
CGCCGGTATATTTCAGGGTACCGCTCGGCACGAACACGTTGATCAAGAGCGAGGCGATGGAGTAGAGCAC
GCCGATAAAGATCCCGGCGATGATGATCACCTTCACGAAAGACTTGGAGCCCCCTTTCACGTCATTGACG
TAGACCGCCACCGACTCGGCGCCACCGGCTGCCATAAAGATCCAGGTGGTGATGCCCAGGAAGGCCCAGC
TGAAATCGGGGATCATGGCCTCTACCGTGATGGGGTCGGCCGGTTGCACGCCGCCCACCAGTGCCGCACC
TGCCAGCAGGATGTAGGAGAGGGTCAGCAGCAGCATCAGGGACGAGGTCACCGAAGTGATGGGGCCGAGC
ATCTTGGCACCGTTAGTGGAGACGTAGGTGGCAAAGGCAAACAGCACCATGCTCAACGCCGTGGTCGCCA
CCGGAGTGAGAATGTACTCGTAGCCGAGGAAGGCATAGGAGGCGTAGGCGATGACCCGCGGCAGCAGCGA
GGTGAAGAAGAACAGGTTCACGAACCAGTAGGTGTAGGCGGAGATAAAGGCCCAGCGCCCGCCGAGGGAG
CTCTTCACCCAGGCATAAACCCCCGCTTCGGAGTTCTTATTCAGTGACACGAACTCCGCAATGATCAGAC
AGAACGGAATGAAGTAGAAAATGGTGGCCAGAAAGAACATCGGGGCCGAGGCCAGACCGATCTCGATGTT
GTTGTTGATGACGTTGTTGAAGCTGAATACCGCCGCGAAAGTGAGCGACAGCAGACCAAACTTGCCTATC

GTATTGCGTTTGGAATCAGACATGGTGCATCTCCAGACCTGTAGTTATTGCCGCGAAAGGGCGGGGCATG
ATTTGTAGGGTGAACCCCGCCCTTCCGGCTTTTATCTGTGCAGAACCGGCGGCTTGTTATTTGTTCTTGA
AGTAGCCGCCTTCGACGGTGACCTTGATGATCACCTTGCGCACCGGCCCGTCGCCGACGAATCGGTAAGC
CTCGTCGTTCTCGAAGATCACCACCTGCCCGTTGCCCACCTGCTGGCGCTCACGCACCTCGCCAGCCAGC
CACTCGCGGTCGGTGGTGTCGTCATAAGCCTGCTCGACCGCCAGCTCCGATTTGGGGGCCCACTCCACCG
TCTCTGCTCCTTCCAGGTAGTAGTGAACGTCGAAGTAGCGGCGCTGCCCCTCGAACAGTGAGGTGGCCGG
GCTGGCCCCCTCCTGCAAGCGGTAGGTGAGGGAGTCACCAATGGAGTGGCAGATGCCGGGCTTGAGGTTT
GGCAGGTTGTTGATGGCCTCCACGCAGCGGCCCCACTTCTTGCCTTCCCGGTAGATGTTTTTGAACAGGG
TCAGGTTGTCCAGGGTGATCATGCGAGGTTCCTCCCGCCGTTGATGGTGAGATCGAGCCCGGCCAGCGCA
GCCGGGTTGAGGTTGCCACGGGCGATGGGCAGCAGGGTCAGGCCGAAGCTGAACGGCTCGAAGCGCACCC
GCCAGGAGTCCAGCACCTCGGAGCCCCAGGAGTTGGAACCCAGCCCCAGTACCTTGTGATCCAGATTGAG
GGTGAGATAACCACTCGGCTCCAGCTCGTTGATGTGCTGCGCCGCGTGGATATGCTCCCAGCTGTAGGGC
CAGACGCTCAGGTTGATGGGGGCATCGGGCCGCACGTAGAGACCGGCGCCGTGGCGGTTGGTCAGGGTCG
CCCAGCGCACCTGCTGGCGGTTGCCGTTGTCCTGCGGGAAGGGGTAGTGCTCCCACATCTCGCTAACTGT
GCTCTGGAAGCTGTCGATCCAGTTGCTCTGGCGGCTGTCCTGATAGTTTTCGCCCGGCCCCATGCCGTAG
TAGTGCACCCGGTCGAGATCCTGACGAATGCCCAGCTCGAAGCCGATCTTGGGGATGATGTCGTCATAGC
CGCCGTAGGGCTGACCGGACAGCGCCACGTGCAGCTGACCGTTCGGGCTCAGGGTGTAGACGTAGCGGCA
GCACATGCCGAAGTCGAACACCGGCGGCGCGATCAGACTCTCCACCGTCACCTCGACCGCCTCGCCCAGC
TGACGCCAGCTCAGCGTGCGGAAGTGCTCCTGCATGATCTGCAGGTGGTTCGGGTGCCACAGGCTCTCGT
ACTCCTGCTTATGGTTGTCGATCATCGGCTTGAAGAAGGTGAGGCGCGGCGAGCGCTCAATCAGCTCGAT
ACCGTCCTGCTGCCAGCTCACCAGCTTGCCGTCGAGGCGCGAGAAGCGCAGGGCAAAGCCGCTACCGCTC
AAGGTCAACGCGAGGCGCTCGTCGGCAATCTGCAGCGCAGTAGCGTTGGGGTTGGCAAAGGGCTGCAGCT
GGCGAGTCGATGCCTTCAGTTGGAACTGGTACTGGCCCAGCTCGTGCTCCGCGGCACTGTAACGGGTGGC
GGTGGTCTTGATGACCCGCAGCTGGACGAAAGTTTCCCGCGTATCAAGCGTGGGCAGTTCGAGCTGCAGC
TCAGTGGTGGCACCGGGAGCAACCCCTTCCAGCCGGATTTGCTGGCTGGCCAGCAGTTCGCCTTCGGCCT
TCACTTCGACCAGCAGGCGGATATCGTCGAGAGTGGAGAACCAGTAGCGGTTTTCCACCGCCAGCTTGCC
GCTGGCCAGATCCAGCGCCCGCACCTTGACCGGACAGATCACCTGCTTGTATTCGCGCAGGCCAGGCCCC
GGGGTCTGATCCGGATAGATGAGGCCATCCATGCAGAAGTTGTAGTTGTTGGGGTAGTCGCCATAGTCGC
CGCCGTATTTGTAAACAGGGCGCCCCTGCTCGTCCTGATCCAGAATGCCGTGATCGCACCACTCCCAGAT
GTAGTGACCCTGAATGTGGGGATGACGGTCGAACACCTGCTGGTATTCGCTGAGGCCACCCGGGCCATTA
CCCATGGCGTGGGCGTATTCGCAGAGGATGCGCGGCTTCGCCATCGGGAACTCGCCGAAGCAGTTCATCT
GGGAGACGCGCGAGTACATGGTGCTCACCACATCCACCACCTCGGCATCGCGATCCTCTTCGTAGTGCAC
CAGCCGGGTCGGATCGAGCGCCTTGCAGCGCTGGTACATAGCTCTGATGTTGCAGCCGTAGCCGGACTCG
TTGCCGAGCGACCAGATGATGATGGAGGGGTGGTTCTTCTGGGCGTGCACGTGGCGCTCTATGCGATCCA
CGAACACCGGCTCCCAGAAGGGGTCATCGGTGATGCGGCTGAGATCGCCGACGTTGGCAAAGCCGTGGGT
CTCCACGTCGGTCTCCGCCATCACGAACAGACCGTACTGGTCGCACAGCTCGTAGAAGCGCGGGTCGTTC
GGGTAGTGGGCAGTGCGCACCGAGTTGAGGTTGTGCTGCTTCATCAGCACGATATCGCGTTCAACACGGT
CCATACCGACCGCGCGACTTTTGAGGTGGTCGTTGTCGTGACGGTTCACCCCGTGCAGCTTGAGGTAGTG
GCCGTTGACGTAGAAAAGACCATCCTTGACCTTGATTTCACGCACCCCGACCCGCTGCGGGATCACTTCC
AGCAGCTCACCCCGACCGTTGTAGAGGGAGAGTTGCAGCTGGTAGAGGTAAGGATCTTCGGCGCTCCACA
GATGGGGATTGGCCTGATCCAGCGTGAAGCGGCAATCACACTTGCCGTCGATGGTGAGATGATCGAGGGA
GCCGCTGGCGATCTCGCTGCCCTGATCCAGCAGCGACCAGACCAGACGGTGATCACGGGCAGCCGCCCCC
AGACTTTCCAGCTGGATATCGCAGCTCAGGGTAGCGCTCAGATTGTCATCGGCCAGCGCGGTGCGGATGA
AGAAGTCCTGCACATGGGCGGCCGGTTTGCCCACCAGATAGACGTCGCGAAAGATCCCCGCCATCCACCA
CATGTCCTGATCTTCGATATAGGTGGAGTCGGCCCACTGCATCACCCGCACCGACAGCAGGTTCTCGCCG
ATGTTGGCGTAGGCACTGATATCGAACTCGGCGGTGAGACGGCTCCCCTTGCTGAAACCGACATAGTGGC
CGTTCACATAGACCTCGAAGTAGGTCTCCACCCCGTCAAACTTGATGATCACCTGCTCGCCGTCCCAGGC
CGGGCTCAGCGTGAAGCTGCGCTGATAGGCGCCGGTGGGGTTGTTGGTCGGCACGAAGGGCACGTCGATG
GGGAAGGGAAAACCTTCGTCGGTGTACTGCAGCTGGCCATGTCCTTCCATCTGCCACATGTTGGGCACAG
TGATCTGGCCCCACTCGCTCATGGGGCTGTGATAGAAGGCCTCCGGTACCTGCAGGGGGTGGTCGAAGTA
GTGGAACTGCCACTGGCCGCCAAGGCTCAGAAAACGGCGGCTCAGCTCGCGCTGCATGGTGGCCGCCAGC
GCGTGGTCGGCATAAGAGAAGAAGTAGGCACGCGGGGCCAGCCTGTTTTCACCAACGAATTGGACGTTTT
CCCAGTTATTCACATGTCCTCCCGGAACAAGGCAACGTTAACTGGGTAAACTCTAGATAAACATTTACTA
AATATCAGCTTTGGATTGGTAAAGTTTTAACCTGATCACAATGAACAGATGGTTTTGTGAGCGAGATCAG
AAGCGGGAGATAAAGGAGGGCAAGATATGGATGAGTGGCATAGATGTTGATAGCCGTTGGCTGAACTGCA
ATAAAATCAACTATAAAACAATACGATATGACGTCACGGGATGATATGGGAAATAAGTTTACTTGGGAGA
GGAGTCGCACCACCGCAAGATGGCACGACTAAAAAGCAGAGTATTTTACTAAAATCAGCGAGTGGTGCCA
CGCAGGGTCAACTTGCTCGGTACCAGTACCCGAAGGGGAATATCACGCTCGTCACGCAGCCGCTCAACCA
GCAAATTGACCCCCTGGGAGCCCATCAACTCGGAGTGGATCCGCACCGTGGAGAGCGGCGGGAAGGTGAA
CTTGGCGGTCGGGATATCGTTGACGCTGATAAGCTCGATCTGCTGGGGAATCGCCAGTCCCTTCTCATGA
ATGGCACGCAGCACACCGATGGCGATGGAGTCGGAGGCGACAAACAGCGCCTTGGGCCAATCCCCTGCCA

GCATCTCCTTCGCCAACTGGTAGCCGGAAGCGCTGGAGAAGTCCCCACGATAGAGATCGCTCTCCTGCAC
CACCCCCAACCGCTGGCCATAATCCCGGAAGGCCAGTTCGCGCAGATCCGGATTCTCATCCTGGCCACCG
ATATAACCGATACGCTGGTGCCCCTGCGCGATGAAGTAATCGACGACCTGCTGGCTGATCAGTGCGAGAT
CCACATCAACCGAGTCGAACTCTCCCTGTGAACGGCAGTCGACAAACACCAGCTGGCTGGAACAAGCCCT
TAGCTGGACGAGACGTTCGGCTGTGAGTGTGCCGACAACCAGAATGCCATCCACGGCGCTCAGCTCGCGC
CCTTCACCACAGTTGTAGAGATGAGTCAGCTCGATATTGAGGCGGGCACTCTGGGTCTCGATGCCATAAC
GGATGGCGAGATAGTAAGGGTCGTTGACCTCGGTACTCTGGGGATAGGCATAGACCACCAGAAAGTGCAG
CTTGCTCTTGTGTACCCCCTTGCGGGCGCTGCTGGTCTTGTACTCCAGCCGTTCGGCAATCTCGAAGATC
CGCTGCTTGGTCTCTTCCTTGACGCTCAGGCTGGGATCCTCGTTCAGTACCCGGGATACCGTGGCGAGAG
ACACCCCCGCTTCCTGTGCAATTTCCTTCAACGTAGCCATCAATACCTCGCTGGCAGCATCACGTGTTAA
TCGGCAACGGCTAAAGAGTAACGGGCAATCCCTTGATAGCCCAGCACTTTTACTAAAATGCCGGCAGCAT
GACAGATTTTTGGAACGCAGAAATGCAAAAAGGCCTCCCGAATGGGAGGCCTCTTCTAAATAGGTGCCTG
ACAGTGTCCTACTCTCGCATGGCGAATGCCACACTACCATCGGCGCTACCGCGTTTCACTTCTGAGTTCG
GCATGGGATCAGGTGGTTCCACGGCGCTATAGCCGTCAGGCAAAAGCTTTTAAGACTAACTTGAAAATGG
TGCTGATACCCAGAATCGAACTGGGGACCTCATCCTTACCACGGATGCGCTCTACCGACTGAGCCATATC
AGCACACTAACCGGTAAAGACCACGCTGGGCGTAATCTTGAAATTGGGTGCCTGGCAGTGTCCTACTCTC
GCATGGCGAATGCCACACTACCATCGGCGCTACCGCGTTTCACTTCTGAGTTCGGCATGGGATCAGGTGG
TTCCACGGCGCTATTGCCGTCAGGCAAAAGCTTTTAAGACTAACTTGAAAATGGTGCTGATACCCAGAAT
CGAACTGGGGACCTCATCCTTACCAAGGATGCGCTCTACCGACTGAGCCATATCAGCACACTAACCGGTA
AAGACCACGCTGGGCGTAATCTTGAAATTGGGTGCCTGGCAGTGTCCTACTCTCGCATGGCGAATGCCAC
ACTACCATCGGCGCTACCGCGTTTCACTTCTGAGTTCGGCATGGGATCAGGTGGTTCCACGGCGCTATGG
CCGCCAGGCAAATTCTTCAATCTGAGAAAGCTGACGTAAGTAATTCGTTCGTATTTGCTACAAGCCTTAG
AACACTTCTTGGGTGTTGTATGGTTAAGCCTCACGGGTAATTAGTATGGGTTAGCTCAACACGTCGCCGC
GCTTACACACCCCACCTATCAACGTTGTGGTCTCCAACGGCCCTTTAGGACCCTCAAGGGGTCAGGGATG
ACTCATCTCAGGGCTCGCTTCCCGCTTAGATGCTTTCAGCGGTTATCGATTCCGAACTTAGCTACCGGGC
AGTGCCACTGGCGTGACAACCCGAACACCAGAGGTTCGTTCACTCCGGTCCTCTCGTACTAGGAGCAACT
CCCTTCAATCATCCAACGCCCACGGCAGATAGGGACCGAACTGTCTCACGACGTTCTGAACCCAGCTCGC
GTACCACTTTAAATGGCGAACAGCCATACCCTTGGGACCGACTTCAGCCCCAGGATGTGATGAGCCGACA
TCGAGGTGCCAAACACCGCCGTCGATATGAACTCTTGGGCGGTATCAGCCTGTTATCCCCGGAGTACCTT
TTATCCGTTGAGCGATGGCCCTTCCATTCAGAACCACCGGATCACTATGACCTACTTTCGTACCTGCTCG
ACCTGTCCGTCTCGCAGTTAAGCTGGCTTATGCCATTGCACTAACCTCCTGATGTCCGACCAGGATTAGC
CAACCTTCGTGCTCCTCCGTTACTCTTTGGGAGGAGACCGCCCCAGTCAAACTACCCACCAGGCACTGTC
CGCGAGCCCGATTCAGGGCCCTGCGTTAGAACATCAAACATACAAGGGTGGTATTTCAAGGACGGCTCCA
GCGCAACTGGCGTCACGCCTTCAAAGCCTCCCACCTATCCTACACATGTAGGTTCAATGTTCAGTGCCAA
GCTGTAGTAAAGGTTCACGGGGTCTTTCCGTCTAGCCGCGGGTACACCGCATCTTCACGGCGAATTCGAT
TTCACTGAGTCTCGGGTGGAGACAGCATGGCCATGGTTACACCATTCGTGCAGGTCGGAACTTACCCGAC
AAGGAATTTCGCTACCTTAGGACCGTTATAGTTACGGCCGCCGTTTACCGGGGCTTCGATCAAGAGCTTC
GCTTGCGCTAACCCCATCAATTAACCTTCCGGCACCGGGCAGGTGTCACACCCTATACGTCCACTTTCGT
GTTTGCAGAGTGCTGTGTTTTTGATAAACAGTCCCAGCCATCTGGTCACTGCGACTCCCAACTGCTCCAT
CCGCAAGGGACTTCACTGTCAAGAGCGAACCTTCTCCCGAAGTTACGGTTCTATTTTGCCTAGTTCCTTC
ACCCGAGTTCTCTCAAGCGCCTTGGTATTCTCTACCCGACCACCTGTGTCGGTTTGGGGTACGATGACTT
GTAATCTGAAGCTTAGAGGCTTTTCCTGGAAGCAGGGCATCAATGGCTTCCACACCGTAGTGTGTTCGTC
TCGTGTCTCAGTGTTGTGTCTCCGGATTTGCCTAGAAACACCACCTACGCACTTTCACCAGGACAACCGT
CGCCTGGCCCACCTAGCCTTCTCCGTCCCCCCATCGCAATTACAAGTCGTGCAGGAATATTAACCTGCTT
CCCATCGACTACGCCTTTCGGCCTCGCCTTAGGGGTCGACTCACCCTGCCCCGATTAACGTTGGACAGGA
ACCCTTGGTCTTCCGGCGAGGAGGCTTTTCACCCCCTTTATCGTTACTTACGTCAGCATTCGCACTTCTG
ATATCTCCAGCATACCTCTCGATACACCTTCGCAGACTTACAGAACGCTCCCCTACCACTCACACATAAG
TGTGAATCCGCGGCTTCGGTGCCTGGTTTGAGCCCCGTTACATCTTCCGCGCAGGCCGACTCGACTAGTG
AGCTATTACGCTTTCTTTAAATGATGGCTGCTTCTAAGCCAACATCCTAGCTGTCTGAGCCTTCCCACAT
CGTTTCCCACTTAACCAGAACTTTGGGACCTTAGCCGGCGGTCTGGGTTGTTTCCCTCTTCACGACGGAC
GTTAGCACCCGCCGTGTGTCTCCCGGATATTACTTACTGGTATTCGGAGTTTGCATGGGGTTGGTAAGTC
GGGATGACCCCCTAGCCCAAACAGTGCTCTACCCCCAGTAGTATTCGTCCGAGGCGCTACCTAAATAGCT
TTCGGGGAGAACCAGCTATCTCCGAGTTTGATTGGCCTTTCACCCCCAGCCACAGGTCATCCCCTAACTT
TGCAACGTTAGTGGGTTCGGTCCTCCAGTTGATGTTACTCAACCTTCAACCTGCCCATGGCTAGATCACC
CGGTTTCGGGTCTACACCTTGCAACTAGACGCCCAGTTAAGACTCGGTTTCCCTACGGCTCCCCTATACG
GTTAACCTCGCTACAAAATGTAAGTCGCTGACCCATTATACAAAAGGTACGCAGTCACCCCGAAGGGCTC
CCACTGCTTGTACGTACACGGTTTCAGGTTCTATTTCACTCCCCTCACAGGGGTTCTTTTCGCCTTTCCC
TCACGGTACTGGTTCACTATCGGTCAGTCAGGAGTATTTAGCCTTGGAGGATGGTCCCCCCATATTCAGA
CAGGATGTCACGTGTCCCGCCCTACTCGATTTCACATCAAGGTCGTTTTCGTGTACGGGGCTATCACCCT
GTATCGCCGGCCTTTCCAGGACCGTTCCACTAACTTCCAAGATGCTTAAGGGCTAATCCCCGTTCGCTCG
CCGCTACTGAGGGAATCTCGGTTGATTTCTTTTCCTCGGGGTACTTAGATGTTTCAGTTCTCCCGGTTCG

CCTCTGTTACCTATGTATTCAGTAACAGATACCCGAGTTATCTCGGGTGGGTTTCCCCATTCGGAAATCT
GTGAGTAATAGCGTCTCTTACCGACTTCTCACAGCTTATCGCAGGTTAGTACGTCCTTCATCGCCTCTGA
CTGCCAAGGCATCCACCATGTACGCTTAGTCACTTAACCATACAACCCCAAGAAGTGTCGTCGAAACAAC
GCTTGCTTGTTGTCGTACAACAAGGACCAAATAAAATTTGGTTTTCGCCAAGAAGTTTCCAAAGCACTTG
TAACAAATGTTTGAGAACTACTTTTTAAATCAGCTTTCCAGATTGTTAAAGAGCATGTTTGCAACGGCGC
AAGGCCGAAGAAAACAGAGTTAAGAATCAGTTCTTAACTCTGCATTCTTGTTAGCAAGAAGAGAAGTGGC
GTCCCCTAGGGGATTCGAACCCCTGTTACCGCCGTGAAAGGGCGGTGTCCTAGGCCTCTAGACGAAGGGG
ACCCAAAATCATCTTTGCGCTGCGTCAGCAGTGCAGATTTTGGGTAATGGCGAGTGCCGAGCCTGCGAGG
CCGAGCGCCATTATTCACATCCCAAAACCCAATGATAGGGCTTCTCTCATCTGACCGAAGCCAGATAAGC
ACTGACACTTTCGCATCAGGTCTTTGCTCTAACTACTTTGAATCAAGGCAATCTGTGTGAACACTCAACA
ACTTCGACATCTTAAGGTAAGGAGGTGATCCAACCCCAGGTTCCCCTAGGGTTACCTTGTTACGACTTCA
CCCCAGTCATGAATCACACCGTGGTAAACGCCCTCCCGAAGGTTAAGCTATCTACTTCTGGTGCAACCCA
CTCCCATGGTGTGACGGGCGGTGTGTACAAGGCCCGGGAACGTATTCACCGCAACATTCTGATTTGCGAT
TACTAGCGATTCCGACTTCACGGAGTCGAGTTGCAGACTCCGATCCGGACTACGACGCGCTTTTTGGGAT
TCGCTCACTATCGCTAGCTTGCAGCCCTCTGTACGCGCCATTGTAGCACGTGTGTAGCCCTGGCCGTAAG
GGCCATGATGACTTGACGTCATCCCCACCTTCCTCCGGTTTATCACCGGCAGTCTCCCTTGAGTTCCCAC
CATTACGTGCTGGCAACAAAGGACAGGGGTTGCGCTCGTTGCGGGACTTAACCCAACATCTCACGACACG
AGCTGACGACAGCCATGCAGCACCTGTGTTCTGATTCCCGAAGGCACTCCCGTATCTCTACAGGATTCCA
GACATGTCAAGGCCAGGTAAGGTTCTTCGCGTTGCATCGAATTAAACCACATGCTCCACCGCTTGTGCGG
GCCCCCGTCAATTCATTTGAGTTTTAACCTTGCGGCCGTACTCCCCAGGCGGTCGATTTAACGCGTTAGC
TCCGGAAGCCACGTCTCAAGGACACAGCCTCCAAATCGACATCGTTTACGGCGTGGACTACCAGGGTATC
TAATCCTGTTTGCTCCCCACGCTTTCGCACCTGAGCGTCAGTCTTTGTCCAGGGGGCCGCCTTCGCCACC
GGTATTCCTCCAGATCTCTACGCATTTCACCGCTACACCTGGAATTCTACCCCCCTCTACAAGACTCTAG
CCGGACAGTTTTAAATGCAATTCCCAGGTTGAGCCCGGGGCTTTCACATCTAACTTATCCAACCGCCTGC
GTGCGCTTTACGCCCAGTAATTCCGATTAACGCTTGCACCCTCCGTATTACCGCGGCTGCTGGCACGGAG
TTAGCCGGTGCTTCTTCTGCGAGTAACGTCACAGCTGGCAGTTATTAGCTACCAACCTTTCCTCCTCGCT
GAAAGTGCTTTACAACCCGAAGGCCTTCTTCACACACGCGGCATGGCTGCATCAGGGTTTCCCCCATTGT
GCAATATTCCCCACTGCTGCCTCCCGTAGGAGTCTGGACCGTGTCTCAGTTCCAGTGTGGCTGATCATCC
TCTCAGACCAGCTAGGGATCGTCGCCTTGGTGAGCCATTACCTCACCAACTAGCTAATCCCACCTGGGTT
CATCCAATCGCGCAAGGCCCGAAGGTCCCCTGCTTTCCCCCGTAGGGCGTATGCGGTATTAGCTACCGTT
TCCAGTAGTTATCCCCCTCGACTGGGCAGATCCCCAGGCATTACTCACCCGTCCGCCGCTCGCCGGCAAA
AGTAGCAAGCTACTTTCCCGCTGCCGCTCGACTTGCATGTGTTAGGCCTGCCGCCAGCGTTCAATCTGAG
CCATGATCAAACTCTTCAATTTAAGTTTGGTTGCTTCGAAAAGCGGCTCAATGAATTGCTGAAATAAACT
GTTCGACTTCTTATTGCTAAGAAATCGTCTTGGTCACTTCACCAGACATTGAAAATCAAAAATTGTTTTG
ATGTTCGATGCTGTGAGTGCCCACACAGATTGCTTGATTCAAATTGTTAAAGAGCGTCACGCTTGTTGCG
TTGAGGAGGCGCATATTACGCCTATCACTTCGAAAGTCAAGCGATTCTTTTCGAAAAACTTTCGGCGCCC
ACTTCGCTAGGAAGCTGGCTCATCAGTTCGGCGTGTTCCGCCGTGCTGGTAGGGGCGCATTATAGGGAGC
CGCGCCGGGATGACAATAGTTTTTTTGAAGAAAATCACGAAAATTTCAAATTATTCAATTAGAAACAGCT
TATTCAGTGTATATATACAATGTTATCCACAATCCGGTTATTTCACTCCCATTTTAGCGTCATTTATGTC
GCCTTTAGTGACCTTGGGAATGCCATACTGGAGCCGATAACCTCTCCCGAGTGGTCCATGGTAGCGCCAA
TCAGGAAAGCTGGTTACCATGGAGGTGAATATAGTGTTGCACCTTTTTTGCTTTCATTTACAAAAGGTCT
TTTAAGATGAATTTATCCAAGTTTCCCGTATATATGCAGGCCGCAATCCTTGCTCTGGTATTGGCAGTGG
TTGGCTACCTGGTTGCCCAGGCCCTGCAGTTCTCTTTGAAAGCGGAAGTGATTTTTGCAGCAGGTCTGGC
CATCGGCGGTTTCGTGGTACCGCTGTTCCTGAAGCGAACCTCGGCTAATGTCGCCGAGTCGATCGCCAAT
CAGGAGACTTGCACCTTATATGTAGGCAACCTGCCCTATCGCGCCAATGAGATTGCAGTGCGCGAATTGT
TTGCCGAACAGGGCCAGGTCATTTCCGTGCGGTTGATGAAAGACAAGGCAACCGGCAAGCGCAGAGGTTT
CGGTTTCGTGGAGATGCCCGCCGCCGATGCCGCCAAAGCGATTGCCGCTTTGAATGATAAAGAGTACCAG
CAGCGCACCCTCAAGGTGCGTGAAGCGAATGACAAACGTGATAAAGAGGAAAGAGAAGGTAGCGAAACTG
ACGCCTAACCGCTCGTCAGTACTTCACCTCTTCCAGTGATGACAGCCCCCATGCTTGCAAGGGGGCTGTC
AGTTTTTGAGCCTCCCCATCCAGCCGGGTACAGTAAGCCCTGGCAATTACCTCTTCTATATGTACAGGCT
GCACGATGGCATGCCCGCCATTGTTCAGCAACCAGGCCACCCGTTTGGCAACGGCACTGCCCGAGTCAAT
AAGCTGACATTGCGACATCAGTTGCCCAATCTCTTCATTGAGTAGCGGGAAGTGAGTGCAACCAAGCACC
AGCGTATCCGGCTTTTCTTCCCCCTCCAGCCAATCGGCCAATACCTCGCGCAGTAGCGCCATATTGACCG
GTAGGCCCGCCAGCTTGTGTTCGGCTTCTATGACCAGCTCGGTGGTGCCCTTGAGCAGCACCTGCTTGCC
GGGAGCAAACTGGGCGATCAGTTCATGGGTATAGTCGCGGCTGACGGTGCCCGGCGTAGCAAGCAGACCG
ATACAACCGTTGCGAGTCTGTGCAGCGGCGGGCTTGATGGCTGGCACCACCCCCACCACGGGAATATCCA
GTGCCTCACGCAAGGCCGGCAGCGCTATGGTGCTGGCCGTGTTGCAGGCAATCACCACCAGATCGATATG
ATGACGGGCCACCATGGTGGTCACCAAGCGGGTGCAGGCAACAATCAGGGCGGGCTCGCTCAATTCACCA
TAGGGGAAGTTGGCATTATCGAAGCAGTAGAAATAGTTGTGTGCCTGCAGAACGCGGCGGATCTCGCGGT
AGATGGTCAGGCCACCCATACCGGAATCAAACACGAGAATATTGGCCACTTGAGCTCCCCTTTGGTGCAA
GGCGGCGATCATACTCCCGCCTGCCGCCAATAGAAAAGGCCCAGCCAATGGCAGGGCCCCTGATTTTTCC

ACTTGGCCGGGTCCGGCTTAGAGTGAGTAATCCATCGTCACGTAGTAAGCGCGCTCATCGGCCGGATAAC
CCACCGCAGTCTGATACTCCTTATCAAGCAGGTTGTTGATCTTGCCAGATACCTTGAGCGCCGGAGTGAC
CTTGTAGCCGACCGCCAGATTCCACACGCTGTAGCCACCAAGTGTTGTGGTGTTGGCCACATCGCCATAG
CGCTCACCCTGATAGATCCAGCTCAATGAACCATCAAACTGCTGCCACTGCGCCTGCGTCACCCACTTGG
CCCCTTTGCGGGAGATCAGCTGCAACTGCTTGTCGGTTACCTTGTCTTTGGGATCCTTGTATTCGGCAGA
GACCCGATGACTCACCCAGCCGGTATCAAACTCCCCTTCCAGCTCGATCCCTTCAATTCGCGCCTTGCCA
ACGTTCTGTGGTGTCCACATACCCTGACTGTTGGGTGCCCACTGGATCATCTGCTCGAAATCATTGCGAT
AACCGGTGGCCCGCCACTCGACACCCGCAGTTTGACCATCCAACATCAGCTCGCTGCTCTTCGACTCTTC
CGGCTTCAGGGTAGGATTGCCTGACCCCGGATAGTAGAGGTCGTTAAAGGAGGGGGCGCGGAAACCGGTG
CCGTAGCGGGCACTCAGGCGGTAATCCTCGACAAAGCGCCAGCCTGCGCCGGTTTGCCAGGTGTTGTGAC
GACCAAACTGCTCGTTGTCATCACTGCGGCCGCTCAGCTCGGCCTGCCAGGTCTGGTTGTCGAACTGGGC
AAGGGCATAGAGGCCGGTATTGTCCCGCTCTTTGGCGTCGGCCGGATATGTGGCGCCGGAGGAGCGGGAG
CGATCATCCAGTCGATCACTCTGCCAATCGGCCCCGCCTCCCAGCGTCCAGCTCTCGTTCAATTTCAAGC
TGTTGACCCAGTTCAGGCTGTACTGGCGGGTGTAGGTCCGGTTATTGGCCTGATCGCGGCCACTGTTGTC
CTGATAGTCGTACGCATCCTGTTTGGAGAAGGCGCCACGCAACTCACTCAGATAGCGATCATCGTGATAG
CGCGACCCCAGCTGATAGCTCTGGTTTTCAGTCCAGGTCTCGTTGCGCTGATGGGTGGCAGGCCCCCAGG
CGGAAGCCGCAGAAGAGCGGTCGTACTGGGCGACATTGCGGAACCAGCGGGTTGTACCGAACAGATCCCA
ATTGCTGCCGAGCGCCTGCTGGTAGTCCAGCATGGCGTTGTAGCCCTGATGCCCGTGACGGTCACCATCG
TTGATGCCGGGCAAGGGGTGTACGTTGTAACCCTCTTCATCATCAAAACCGCCAGCCACCTTGAGCTGAC
CCGCCTCACCTACCTGGCTGGCACTACTGAAAGCGGCCTGACGCTGTTGATGGGAACCAGCACCGATATT
GAACTTGTGCTGATTTGTCCCCTTGTCGGGACGGGTAATGATGTTGATCACCCCACCGATGGCATCGGAG
CCGTAGATGGTGGCGCTCGGGCCGCGGATATACTCGATCCGCTCGACCTGATTGAGTGGCAGATTGTTCA
GTTCGGTCATCCCGGCGGTCAAAGAGGCACTGCGCACCCCGTCCACCAGCACCAGCGTCTGAGAAGAGGT
GCCACCACGCACCCGCAGCGAGCTGAGCTGGCCTCGGCCACCATTGCTGACCACTTCCACACCGGGCAGG
GTCTTGACCACATCGGTCACGGTCTGGGCTTGCAGGCGATCGATCTCGTCCCGGGTCACCACATTGACCG
GGGCAAGCACGCTGGAAGCAGGTTGGGCGACGCGGGTAGCAGTCACCACCATGGTGGGATTGACGGGAAT
GGTCAGCTGCTGGGCAAACGCCGCCGTCGGCAACAGGGCAGCCGCCAGAAATTTCTTCGACATGAGAGAT
CCTTAAGTTCGCGTAAGTATCTACTTCGTGGCCGGTTTCTCTCGGCCCGAAATACGAACTCTGGCAGGTC
TTCGGGCTCGGGGGCTGATAGCCTACCGCGACGGCTTCCCGCCTGAAGGCAGTGCCCTGATGTCGCTTCG
TTCCCCCTTACCGCTGCGCGCCAGCTCCGGATTCTCACCGGATTCCCTATTAAGCATGATGCGCCAAAGC
GGGAGGATTATAGGCAGAGCAAAGCTGGATGTATAGCCATCCAGATTTCTATTTCTTGCCAGCCAACGCC
GCCAGCCGCTGCTGGGCCCCCTCCATCACCGCCTGTACCCCGGGATGGGAGAGATGGCGCCCTGCCGAGA
CCAGATAATAAGAGAGGGTAACGTCGGTGAGGGTCGCAATCCGCTCCACTCCGTAGAGGCGACCAATTTC
GTCAGCCATCGCCAGCGGCGCCGGGAAGATCCCCATCCCCGCCTTGCCGAACGACTTCATCAGGGTGGAG
TCATCAAACTGCCCCACCAGATGGGGATGAATGCCCTGCTGGCGATACCAGGCCAACAGCCGATCGTGGG
TAGCCGACTTCTCTCCGGGGATCAGCAACGGCAACCCCTCCAGCGAGGCGGGAAAGCCATCCCGCGCCCG
CGCCGCCAGCTCCGGGCTGGCAAACAGCGCCAGCGGGCTGCGCCCCAGCTCGTGGTTATAGCCACGCACG
CCGCTGTCGGGAGGAAGCGGGCGATCGATCAGCACCATGTCGAGCTTGTGGCGGGCCAGCTCGCTGAACA
GCTGCTCGGCCCGCTCCTCGTGACACACCAGCCGCAACGGAGTCGCCAGCGCCATGGCGGGGGCCAGCAG
CTCGAACGCCAAGGTGCGGGGTACCGAATCGGCGATCCCCACCTGCATAGTCAGCTCCTGACTGGCGTGG
CGCAGGGTATGCTCCAGCTCGGCACCGAGCGAGAAAATGGCCCCCGCCTGCTCCTGCGCCTGCCGACCCG
CAGCAGTCAGCACCAGACGCCGCCCCACCCGGTTGAACAGCGCCACCCCGAGGGACTGCTCCAGCTCTGC
CACCTGACCGCTGATGGTCTGCGGCGTGAGATGCAGCTGTTCGGCGGCCTGCGTCACGCTGCCGCTGCGT
GCCACATGCCAGAAGTAGTAGAGCTGTTTGTAGTTGAGCATGCCGTTCCCTATCAATTTTTACCGAACAA
CCATCAGCGAGAATCTGATTTTATCGAAACATTGCGGGCTCTAGCATGCAACCATTATCTGAACTGCCCT
GCGAACCATCATGCTGGACGTTGTTGTACTCTTCTTTCTGTTTGGTCTGCTGGCCGGTCTGGTGCGCTCC
GAGCTCAAACTGCCTCCTGCCCTCTACGACACCCTCTCTCTCTTTCTGCTGCTGGCCATCGGCCTCAAGG
GCGGCGTCGGTCTGGCCCAACAATCCCTGCAACCTCTGCTGCCACAACTGGCGTTGGTGATCCTGCTCGG
CGTGGTGCAGACCCTGCTCGGCTTTGCCGTGCTGCGCCTCAAGATGAACCGGGTGGATGCCGCCGCCACC
GCCGCCCACTATGGTTCGGTCAGCGTCGCCACCTTTGCGGTCGGGGTGAACTGGCTCACCGAGCGGGGCA
TCAGCTTTGAATCCCAACTCTCGATCTTCCTCGCGGTGATGGAGATCCCGGCCATTCTGGTAGGCATAGT
GCTGGCCCGCGGCATCAGTCGCGAGACTCGCTGGCGCATGCTGGCCCACGAGACCTTCCTCGGCAAAGGG
GTCACCCTGCTGCTGGGCGGCATGGCCATCGGCTATCTGGCCGGGCCGGACGGAATTGCCCCACTCAAAC
CGCTGTTTGTCGACCTGTTCAAGGGGGCGCTGGCGCTGTTCCTGCTGGAGATGGGCCTCATCGTCGCCCG
CCAGAGTCAGGACTTACGCAAGCATGGCTTCTTCCTGCTCGGCTTCGCCCTGTTGATGCCGCTCGCTTCA
GCGGGACTGGGATTGGCTGCTGGCCAGCTGATGGGGCTCTCCCTCGGCGGCCTGACTCTGCTGGCCACCC
TGGCGGCCAGCGCCTCCTATATTGCCGTGCCGGCCACCCTGCGCATCGCCCTACCCCAGGCCAACCCGGG
GCTCTCCCTCAGCGCCGTGCTGGGGGTCACCTTCCCGTTCAACATCATGCTGGGGATCCCGCTCTACCAC
AGCTGGGCCCGCCACTTCACGGAGTAGCCACATGCAGACCGAGACCCGCACCCTGCTGACAGTCATCACG
GAAGCCAATCTGGAGCCGACCCTGCTGCGCACCCTCACCCGCGAGGGGCTGCGCGGCTACACCATCACAG
ATGCCCGCGGCAGGGGGGATCATGGCGAACGCAATGCCAGCTGGAGCGAGAGCGGCAACATCCGGCTGGA

GGCGATCTGCAGCCGGGAGCAGGCGGAGCGGCTGCTGGCCCACCTGCAGAGTCGCTACTACCCCGATTAC
GCCATGATCGCCTTTCTGCAACCGGTCGAAATCGTGCGACCCGAGAAATTCTGAGCCGGCTTGCCGGCTC
TTTTTGTTTACCAAAAGCAAACGTTTGACAGCAAGGAACCAATACACATGAACACCCCTCCCCTGATCGG
CCTGGTAATGGGCTCCGACTCCGACTGGCCCGTGCTGCAGGCCGCCGCCCGCATCCTCAAGGAGCACGGC
GTGCCCTACGAGGCGCAGGTAGTCTCGGCCCATCGCACCCCGGATCTGCTGTTCGAGTATGCCGCCACTG
CCCGCGAGCGCGGACTGCGCGCCATCATCGCCGGGGCCGGTGGCGCTGCCCATCTGCCGGGCATGGTCGC
CGCCAAGACCACTCTTCCGGTGCTGGGGGTGCCCATCCCATCACGCCATCTCAAGGGAATGGACTCGCTA
CTCTCCATCGTGCAGATGCCCAAAGGGGTGCCAGTCGCCACCTTCGCCATCGGCGAAGCGGGGGCTGCCA
ACGCCGCCCTGTTTGCGGTCTCTCTGCTCTCCGTTGCACAAGACGACGACGCCCCACGCTACCGGCAGCA
GCTCGACGACTTCCGCACCCACGAGCAAGATCGAGTGCTGGCACTGCAACTGGAGGAGCCGACATGATCC
TGCCCCCCGCCACCCTCGGCATGCTCGGCGGCGGTCAGCTTGGCCGCTACTTTGTGATGGCCGCCCACCG
CCTCGGCTACAAGGTGGTGGTGCTGGATCCCGATCCCGCCAGCATCGCCGGTGCCGCCGCCGATCACCAT
ATTGTCGCTTCCTATGATGACCAGACCGCCCTGCATGAGCTGGCTAACCGCTGCGAGGCCATCAGCTGCG
AGTTCGAGAACGTGCCCGCCGCCACCCTCGCGCTGCTCGAAAAACAAAAACCGGTACGCCCGGCCGCCAG
CGCGGTGCGCATCTGTCAGGATCGCAGCGAGGAGAAAGCCTTTCTGCGCAGGGCCAACATCCCGGTTGCC
CCCTCTCTGGCGCTACTGCCGGAAGAGCCCATCCCCGCACAGGCCAGCGAACTCTTCCCCGCCATCCTCA
AGACAGCCCGCGAAGGGTACGACGGCAAGGGGCAGTGGCAAGTGAACAATCTGGATGAGCTGGCGACGGC
GCTGGCAGCCAGTGGTGTGCCCTGCGTGCTGGAAAAACGGCTGGCGCTGGAGGGGGAGTTTGCCCTGACT
CTGGCCCGCAGTCCGAGCGACACCATCAGCGCCCTGCCGCTGGTGCAGAACTGGCACAGCAGCGGCATTC
TCGATCAGACCCGCTCCCCTGCCAATGTGCCGGCGCTCGAGAGCGAAGCCAAAGGGATCGCCGAGCGGCT
TATCGCAGCCCTCGATTACGTGGGGGTGCTGACGGTGGAGTTCTTTCTGGTGGAAGGCAGATTGCTGGTC
AATGAGCTGGCACCCCGCCCCCACAACTCGGGTCACCCCAGCCTCGACAACGCCGCATGCAGCCAGTTCG
AGTTGCAGGTGCGGGCCCTGTGCGATCTGCCGCTGCCGGAGCAGATCGCCGTGCGCCCCGCCCTGTTAAT
CAACCTGCTGGGGGATCTCTGGCAGAACGGCACGCCAGACTGGGCCGCCCTGCTGGCGCTGCCCGGCCTG
CATCTGCACCTCTATGGCAAGGGCGAACCCCGCCCCGGCCGCAAGATGGGCCATATCACGGTGACCGGTG
ACGAGTGGTCAACGGTGGAAGAGAGCTGCCGTCAGGCCCGCCAGCTGCTGGGACTGGCGCCACGCTGATC
GAGCATCGGAAATGCAAAGACCCGGGCATGGCCCGGGTCTCTGTTTTTGCTTGCTGTGGCAGGGAGACTT
ATCAGGCCCGCTCGACCGCTACCGGTGCCGGCAGGCGGATGGTGAGGGAGAGCACCAGCGCCACCACCAG
CAACACCAGGATCAGGTGGAAGGTCGCCATAAAGCCGCCAAACAGGGAGGCCACGATGGAGCCGATGATG
CTGCCCAGACCAAAGCCCAGATAGATGACGCCGTAGTTCTTGGTCAGGTTGTTGAGACCGAAGAAGTCGC
TCACCAGCGAGGGGTAGACGGTGATGGTGCCACCGAAGCTGAATGCCACACAGGCGACCGCCACAAAGAA
CAGGTTGGCGTTGAGCGGCACGAACAGCAGCAGCGCCATGCCCGCCAGGGTAATCAGCTGGGCGATGGTG
ATAACCCGGATGCGGGACATCTTGTCGGAGAGGATACCCAGCACCAGACGGCCACCCAGGTTGGCCATGG
CGATGATGGCGACGGCGTTGGCGGCGACCACGGCAGGCAGACCCACCATCTTCTCGCCGATATCCTTGGC
CACGCCGATGACGTAGAGGCCGCTCATGCAGGCGGTGAGGAACATCAGCGCCAGCATCCAGTACTGCGGC
TTGCGCATCGCCTCGGCCAGGGTGAAGTCACGGCTCTCGCTCTGCTGCACGGAGGCGGCCTGCTTGGGCG
CATCCTTCATCAGCATACCGCCCACCAGCACCATGGACATGGCGATCAGCCCCCACAGCTGGAAGGTGGT
CTCAAGGCCGGAGCTGGAGAGCAGCAGCAGGTTGATGTATTTGAAACCGAGACTGCCGAGGCCATAGGCA
CCGATGGAGCAGGCGGAGATGAGCCCCTTGCGATCCGGGAACCACTTCACGCAGTTGGAGAGGGTCATCA
GGTAACCGGTACCGTCGGCAAAACCGACCAGAATGCCCGCGCAGAGGTAGAGCATCGCCAGATTGCTGGC
ATGGGCGGTGAGGAAGAAGCCGATGCCGAGCAGCACGCCGGCGCCCAGGGTCACGTTGCGCACCCCGAAC
CGCTCCTGCAGCTTGCCCGCCATGGAGGAAGCGACCTCCAGCGAGAGGCTGAGCAGGCCGAAGGCGAAAG
CGACCTGACTCACCGGCTCGTCCAGCTTGTCGGAGAGCTGGGCGTTGAACAGGCTCCAGGTATAGACAGA
ACCCAGTGCAAACTGGGTGATGATGGTACCGATCAGTGTCAGGTACCGGGTGCGGTTCATCTCTTTGGTC
ATGATGCGTCTCGGTCGATGGCCTTAAGGAAATTGAGGCCACTATAGGCAAGACGCAGCTCGCGCCGATC
GATTAAGGCATGAAATGCAGATCGGCGGGAATGAATGACAATCAGAGCCGCATCAGCTGACGGAACGCCT
TGATGTTGCTGCGACTGACCGGCACCTCGAACGGCAGATCATGGAGCCGGATGAGATAGGTGCTGTTAAA
CCAGGGGACGATTTCGCGGATCTTGTTGATGTTGACGCAGTAGGAACGGTGGCAACGGAAGAACCCCTCG
GCAGGCAGCCGGCTGACAAACTCGCTGATGGTCATGGTCATCACGTAGCGGTCATCACGGGTGTAGACAT
AGGTGAGCTTCTCGTCGGCTTCCGCGTAGTAGATCTGCTCGCAGGGGGTGACGATAATGCGCTCCCCCTT
CACCAGATTGACGGTGCGATTGTGCGGGCTATGCCCCTCACCGCTCGCCTCCCCCGTGGTGGCTGGCTGC
GACTTGCCCGCATGCTCGAGCTTTTGCAGCAGATTGATGATGCGGGGCTCGTTGTAGGGCTTGAGGATGT
AGTCGAACGCCTCCAGCTCGAACGCTTCCACCGCAAACTCCTTGTAGGCGGTAACGAAGACGATGTGGGG
CGGATGGCTCGACTTGTGCAGGTTCTTGGCCAGCAGCAGGCCATCGATGGAGGGGATCTGGATATCCAGA
AACACCACATCCACCTCGTGATCCTGCAGGTATTTGAACGCCTCCAGCCCATCCTCAAAGCTCGCCACTA
TCTCGATCTGGCTGTGCTGATTCACCAGATAGATGAGCTCTTCACGAGCCAGATATTCATCTTCGACAAT
AATGGCTTTCAGCATGACAACACCTCTCGCGGCACACACTTATCCTCAACCATGATGGCACTCAGCATGG
CATCGACTCCTGATCCGGCAGATAGAAACTCACCTCGGTACCCGGCTCCAGCCGCTTGAGGTGCAGGCCG
TCGCCATAGAGCAGCTTGACCCGCTGATGCACGTTCATCAGGCCGATGCTGCGGCTCTCCACCCGACCGG
CGGCTACCCCGTCGATCACTGCCTGACTGATGCCGTAACCGGTATCCCGCACCGATACCCGGATGCCGCC
CGCCAGCTGCTTCACCTCGATGGTGACCCGACCCGGCGCGCTGCGGGGCTGGATGCCGTGCAGGATGGCG

TTTTCCACCAGCGGCTGCAACAGCAGGCTCGGTACCTGAATGTGCACATCGTCCACCTCGAATACCACCT
CCAGCTTGTCGCCGAAGCGGGCCTGCTCGATGGCCACGTAGTCGCGCACCTGCTGCAGCTCCTCCTGAAT
GTCGATGAGCTCATCCCCCTTGTTGAGGTTGTAGCGCAGGTAGTCCGCCAGATTGGCAATCAGCTGACGC
GCCTGCTGGGGCCGGATGCGGATCAGCGACGAGATGGCGTTGAGGGCATTGAACAGGAAGTGCGGGTTGA
TCTTGCTCTGCAGCGCGGTGAACTCCGCCTTGCGAGTCATCTCCTTGAGCTGCTCGATGCGCGACACCTC
CATCTGGGTGGAGATGAGCTGGGAGAGGCCCACCGCCATCTCCCGCAGGGAGCTGGTAATGCTGTGGGTG
CGGCGATAGTAGATCTTGAGGGTGCCGCTCACCGAGCCGTTTTCTCGCAGCGGAATAATGATGACCGAGT
GGAAATCGGAGAGGTGATACTGGCGCAAATCGTTGTTGATGATGATCTGGTCGAGCAGCACCGCCTGCTG
GGTCATGCCACTGATGGCGTGGTGCTCATCCAGCTCGTAATAGTCCTTGCCCACCCCGACATAAGCCAGC
ACGTCATGGGTATCTGTGATGGCCACCGCATCGGCGCTGATGTCGCGCCGGATCACATCACACACCTGAC
TGAGGGAGTGGCGATCGATGTTCTGGAAGAAGGGCAGCGTCTTGTTGGCGATATCGAGCGCCAGCTTGGC
CTGCTTGGCAGCAATCAGCTCCTTCTCGTCATCCAGATCCTGCACCAGCTTGATGATGAGGCCGATGCAG
AGCGCCCCGGCGATCATCGGATAGGCGATGTGGTTAACGATCTCCACCCCGACTTCGTGGGGCTCGGTCA
GCAGCCAGATCAGCAGCATGGTCAGCCCTTCGCAGAGCATCCCCGCCAGAATGCCGTAGAGCCAGAGCCG
CGACTTGCGACAGCGCAGGTGCAGCCAGGTGGCGAGCAGACCGGCGATAAGGCAAGGGATGGAGGTGTGG
CCATCCATGTCGATGAGATAGCGGTGCAGACCGGAGATGACCCCGGCCGGAATGCCGACCCAGGGGCCAA
ACAAAATGCCGCCGGAGATAATGGCAATGATCCACACATTGATCAGCGCCCCCTCTACCGGAATGCCGGT
GTAGGTACTGAACACCGCAAACAGGCCGAAGATGGAGGCCACCAGCACCAGCTCCGCCGGGGTGTGATCC
CGCTTCTGGAACAGCCGCTGGAACGGCCGGGTGCGGGTCAGGAAGAAGAGGGTCATCAGCATCAGCGCCG
CCCGTTCGAACACCGCCAACAACATCATCTGATTCTCAAGCATCTGCGCCCCCTTGCGGCAACCAGACCG
ATCACCCTAACCCTGACCAATAAATCGGGGCCGGATAAACCAGCCCCTGTGACATCAACAGTACTGACCG
GCCACCCAGCCGGAGGACCAGGCCCACTGGAAGTTGTAGCCACCCAGCCAGCCGGTCACATCCACCACTT
CGCCGATGAAGTAGAGGCCCGGCACCTTGCGCGCTTCCATGGTCTTGGAGGAGAGCTCGTTGGTATCGAC
GCCGCCCAGGGTCACTTCGGCGGTGCGATACCCCTCGGTGCCGTTGGGCAGGATCTGCCAGTCACCCAGC
AAGTTGGCCACCGCTTCCAGTTCCCGCTCGTTGTACTGACGCATCGGCTTGCTGACCAGCTGACCCAGCT
CCACCAACTTGTCGACGAAGCGCTTGGGCAGCTCGCGCCCCAGCACGGTTTTCAGCTCCTGCGCCGGGTG
TGCCTGCGCCCACTCACGCAGGGCGGCCGGAATATCCAGCTCCGGCAGCAGGTTGATGTGGATCTTCTCG
CCAGCGTGCCAGTAGGAGGAGATCTGCAGGATAACGGGGCCGGAGAGGCCGCGATGGGTAAACAGCATCG
CCTCCTTGAAGCGGGTGCCATCCTCGGCGGTCACCGCCACCGGCAGGCTGATGCCCGCCAGATCGGCGAA
CGCCTCTTTCTCTGCCTGATGGAGGGTGAATGGCACCAGACCTGCGCGGGTCGGCAGCACCTTGAGGCCG
AACTGCTCCGCCAGCTTGAAGCCATAAGGGGTCGCCCCCAGCTTGGGCATGGAGAGACCGCCGGTCGCCA
CCACCAGCGAGTGGCAGCCGATCTCCCCCTTGCTGGTCGTCAACACAAACCCTTCGCCCTGTTTGCTGAC
GGTGAGGATCTCGGTCTGGAACTGCAGGGTCACCCCGGCCCAGTCACACTCGGTGAGCAGCACATCGACG
ATATCCTTGGCACTCTCGAGACAGAACAGCTGCCCCAAGGTGCGCTCGTGATAGTTGACCCCGTGGCGGT
CCACCAGATCGATAAAGTCCTGCTGGGTGTAGCGGGCCAGCGCCGACTTGCTGAAGTGGGGATTCTCCGA
CAGGTAGGCGTGAGCGCCAGCCTGATGGTTGGTGAAGTTGCAGCGACCGCCGCCACTGATGAGGATCTTG
CGGCCCGGCTTCTTGGCGTTGTCGAGCACCAGCACGGAGCGGCCACGGTAGCCCGCCTGCGCCGCACACA
TCAATCCGGCCGCACCGGCCCCGATCACCACCACATCGACCTGCTTCATCATCACTCACTTATCAAAATA
GAAACGGCACCTGGCCAGAACCGAGATTGCCTCTGGCTGACATCACAACGGTGGCTGCCCACCAGAATAA
ACAACGGCCCTTAAAAGGGCCGTCATTGTACTGGCTGAACACTAAGTGTCCACGACTATCAGGCGTTGCT
GTCCATGATGCTGGCAGGGGCGCCCTTGGCCAGTTCGGCCAGCTCCTTGTCGATGAAGTAGAGGCCCTTG
CCATCTTCACCCACCAGACCCAGACGGTCGACGATGCTCTTGAACAGCTTCTCCTCTTCATGCTGCTCGG
CCACATACCACTGCAGGAAATTGAAGGTGGAGTAGTCCTGGGTGGTGAAGGCCACGTGGGCCAGACCATT
GATGCACTTGGTGATGTGATACTCGTGCTCCAGGGTGGTACGGAACACGTCGCCCAGCGACTTGAACTCG
TGGGGAGGGGCATCGATGGCACCCAGCTTGGGCATGGCGCCAGTCTCGCTCACATAGGTGAACAGACGCT
CCATGTGCTGGCGCTCCTCGACCGCGTGCTGGCGCAGGAAGGTCGCCGCCCCTTCAAAGCCCTTGTCTTC
GCACCAGGCGCTCATCTGCAGATAGAGATTGGAGGAGTAGAACTCCAGGTTAATCTGCTCGTTCAACTTC
TCGATCATGGCTTGGGCCAGCATCTTGTTTCTCCTCAGTGATTTGGTATCGATTGTCGCCACACTCCCGT
CACGAATCAACCCGCCAACGAGAGGGAGATCCCGTTTCTTTCAGCTCAGGGGGTCAGCTCCCCAAGGGAG
GCGACATCCACCGGCGCAGCATAATCGACGCCCGGCAGCTCGAAGCCGTTGAGCTGCATGAAGTCCTGCC
GCAGCCCGGCAAAATCCCCCAGCACGTGGAAATTCTCCGGCGTCACCTTGGGCCAGAGCGCCGAAACGGC
GGCCTGAATGGCCGGGTCGAGCTCGTGATCATCCATCCGGATCAGCCGGTTGCCATCGGCCACCACCCCA
TTGGGCCCATACATCTTGGCTGCAAACAGCCGCTGCATCTGCTCGATACAGCCTTCGTGTACACCGCGCT
CCTTCATCACCCCCATCAGCAAGCCGAGATAAACCGGCAGCACCGGAATATAGGCGCTCGCCTTGGTTAC
CAGCGCCTTGCAGGCCGACACCCAGGCATGGCCGCCAATCTCGGCCAGTTGCAGGTTGATGGTCTCGGCG
GTGGCGTGCAGATGCTCCTTGGCATAACCGATGGTGCCATCCCGATAGAGGGGATAGGTGGATTCGGGGC
CAATGTAGGAGTAGGCCACGGTTTGCGCGCCGGGAGCGAGACAACCGGCCTGCTGCAACGCCTGCATCCA
GAGCTGCCAATCCTCGCCCCCCATCACGGTGACGGTATCGCGGATCTCCTCTGGCGTGGCGGGCGCCAGC
GACTGCTGCACCAGGGTATCGCGCTCCAGATCCAACCCCCAACCGCTGAACGGCTGGCCGGTGGTCTTGA
GCACCGAGCGCACTTGCGTGCCGTCGGGCAGCACCCGGATGCCGCTCGCGAGCGAATAGATCACCAGATC
CACCTGCCCCAGCTGCTGGTGGATGGTATCGATGGCCTGCTGGCGCATCCCGTCGGAGAAGGCATCGCCG

ATCAGGTTGATGGCGATACGCCCCTCCTGCTCCGCCTCCTTGCGAAACCAGATGTTGTTGTACCAGCCGG
CGCTACCCAATCCTTTGTCGGAAGGGCCGCGCTCAAACGAGATGCCAATGGTGTCGGCACCGGCACCAAA
GGCGAGTGCAATGCGCGACGCCAGTCCAAACCCGGAGGAGGCACCCAGCACCAGCACCCGCTTGGGGCCG
TTGAACGGGCCAGCGGCCTTGATCCGCTCGATCTGCTGCTGCACGGCGGCGCGACAGCCGATAGGGTGAC
AGTTGCGGGCAACACAACCTTGAATCTGGGGATGAATGATCATGGGAAGGCCTCCAAAGAAACTGGCTAC
AGCATAACCAAATCGAAAGAGATTAACTTGATGCCGGATCGCTATCAGTCAGAAAAGCCGCGGGAATTGA
GGCAGAAAAACAAAAGGGAGCACGTGGCTCCCCAAAGCGGTATTCAGATGCTTTTTGTTGTTTTGTGTTC
ATTGCGAAGCACGGGCCCATCCTATGCCTCTCCCCCCTGCTCGGCAAGCCGAGGGGTGTGACCGGCGTCA
AACTCTTGTTTAATTTATAGGCAAATAGTGGTTAATAACTGAGTGGCTGACCCTGTGTCACACGTCATTA
AACTCATTTAAATCCCAAAACTGTGCCTGAGGCACCATTTTAGTTTCAGCAGAGATTGGTATAGTGGGCT
GAAGGATCGGTAAGGAGAACCCCCATGAGTGGAACAACCTATTACAAGCATATTCTGGCTGCGATCGACC
TCTCTGAAGACAACCGCAAGGTGATAGACAAGGCGGTGGACAGAGCCCGCTCCAACGGCGCCAAGCTGTC
GGTTATCCATGTCGATGTGGACCTCAAGGATCTCTACACCGAGATGATCGATATCGACATCGACAACGTG
CAGGATCAGGTGATCGCCGAAGCGAAAGAGAAGCTGGAGGCCTTCCTCGCCTCGGTGGACTACCCCATCG
AGAAGAAGCTGGTGATCTGCGGCGATCTGAGCGAGCGGGTCAATCAGGCGGTCAAGGAGTACGAGATCGA
CCTGCTGGTGTGCGGCCATCGCCAGAGCTTCTGGAGCCTGCTCACCTCCAGCGCCCGTCAGCTGATGAAT
ACGGTGCCGTGCGATCTGCTGGTCGTCCCCTTGCAGAAGTAATGAAACCGGCGTAGTTTGATTTGCTATG
AATATGACCGCATTCAATCACAACTACTGGTGGTGCTGCTCCTAAACAGGCGGCACCGCGTTTCTATTGT
AAATTTCCCAAAGAAAACACCGTGACCGCCGAGAGGCGGTCAACTGTTTTTATCCCTTTGTCGCCTCCCG
GCTCACCCAACTGGAGAGAGTGACATGCACAACCCCGTAATCCTGACTGGCGATCGTCCCACCGGCAAAC
TGCACATCGGCCACTATGTCGGCTCCCTGCGCCAGCGCGTCGAAGCCCAGCACCACTATCGTCAATTCGT
GATGATCGCCGACTTGCAGGCGCTGACCGACAACGGCCACAACCCGGCCAAGGTGACCGACAACGTACTG
GAAGTGATGGCCGACTATCTGGCGGTCGGCCTCGACCCGGCCAAGACCACCTTCTGCCTGCAGAGCGCCC
TGCCCGCCCTCGCCGAACTCACCTGCTACTACCTCAATCTGGTCAGCGTCGCCCGGCTGGAGCGTAATCC
CACCGTCAAGGCGGAGATCCAGCAAAAAGGGTTCGAACGCACGCTGCCGGCCGGTTTTCTGGTCTATCCG
GTGAGTCAGGCCGCCGACATCACCGCCTTTCGCGCCAGCCACGTGCCGGTCGGGGAAGATCAGCTGCCGA
TGCTGGAGCAGACCAACGAGATCGTCCGCCGCTTCAATTCTCTGGTAGGCAAAGAGGTGCTGACCGAGTG
CCAGCCGATCCTGAGCGACACCGGCCGCCTGCCCGGCATCGACGGCAAGGCCAAGATGTCAAAATCTCTC
GGCAATACCATAGAGCTCGGCATGTCGGCGGAAGAGGTCAAACAGGCGGTGTTCGCCATGTATACCGACC
CCAACCACCTCAAAGTGAGCGATCCGGGTCAGGTGGAGGGAAATACGGTGTTTGCCTATCTCGATGCCTT
CCACCCTGACAAAGCGCTGGTCGCCGAGATGAAGGCCCACTACCGGCGCGGTGGACTGGGGGACATGCGC
TGCAAACAGGTGCTCAATGACTGCCTGCAGACCCTGCTCGCCCCGATGCGGGAGCGCCGGGCCGCGGCCA
TTGCCGACAAAAAGATGCTGCTGGAGCTGCTCTATCAGGGCACCGAGCAGGCTCGCATCATCACCGATGA
GGTCTTGGTAGAGGTGAAAAGCGCCATGGGGCTCGACTACTTCGCCAGCATCCGCTGAGCGGGAGTCCCG
AGCGCATCGTCGCCGTAGAGGTCGAACACCAGCTGGTGCAGCAGGCTCTTGAACCAGCCGATCGCCTCGT
CATGGGCGGTCAGCGGGTGCCCCGCCATCAGGTAGACGATAGGGATGGCGGGGTCGAGCTGGTGCATCCG
CAGCGGGTAGTAACGGCAAAAGTGGCGCCCGATCATCTCGGGCACCACCACCAGATAGCGGCCGGTCGCC
ATCAGTGGCGGCACCGCCATAAAGCTCGGCAACCGCACGCCCAGCTCCCGCTCCACCCCGAAGCGGGCCA
GCCAGAGCTCCACCATCGCCTGACTGTGACCCCAACTGGAGGTATGGATGTGGGGACGGCTCACCAGCTC
GACCGGAGTCAGCACATCGGGCAATTCGCTGCCAAGCGGCGACAGGCAGACCAGCGGGTTGTTGAACCAC
TCCTCCCGCCACAAGCGGGGTGAGAGGTGATTCGCCCCGGCAAACCCGATCACCAGATCCAGCTCCCCCT
TCTCCAGCTCCCGCTCGTAGTTGCTATCGGCCAGCTCGCACACCTCAAGCCGACAGTGAGGGGCCTGCCG
ATGGAGCCTCTCCACCAGCACCGGCACCAGCCCGTGCTCCACCGAGCCCGGGGTGGCGATGCGGAAAATC
CGCCGCGCAGTGGCGGGATCAAACCCCTTGGCCTGCCGCAATCCCTGCTCCAGCATCGCCAGCGCCTGGC
GCACCGGCCCGGCCAGCGCCTTGGCCCGCTCGGTCGGCATCATTTCGCGCCGACTCATCACGAACAGCGG
ATCATCCAGCGCCACTCGCAGCCGCCCCAAGGCGTGACTGACGGTGGATTGGGAGAGATGCAGCCGTTCG
GCGGCGCGGGTGACATTGCGCTCCTGCATCAACATGTCGAATACGGCGAGCAGGTTGAGGTCAAGACGGG
CGAGCGAGCTATCCATCGATATTAGCCATAGTTAAATGCCAACAATTCATTTTTACCATAGTAACAGATT
GCCTAGAATGCGCTGGCTTAATACATCGGAGATTCAAATGCGTAGCTATCTGTTATTGCTGGCCATTGGC
CTGCTGTGGGGTTCCCAATTCATTTTCATGCACCAGGCCGTTGCCGAGTTGCCCCCCATTCTGGTGGCTG
CCGGTCGTGCCCTGTGCGGCAGCCTGACCCTGGGTCTGCTCTGCCTGTTCATGCGGCTCAAGAGTGAGCA
CACCCCGTGGCGCACCTATATGCTGATCGCCCTGCTGGATGCCACCATCCCCTTCATCATGGTCGCCTGG
GGCCAACAGTATGTGGATAGCGCCATCGCCGCCGTGGTCATGGGTTGTATCCCGTTTGTGACTATTCTGG
CTGCACCGCTGTTTATTTCTGGTGAAAGAATCACCAAAACCGGTCTGCTCTCCGTGATCATCGGTTTTGC
CGGTGTGCTCACCCTGTTCTGGCCGAAACTCTCCCAGGGCATGAACGCCGGTCTGCTTGGTGCCATGGCC
ATCCTGCTGGGTGCCAGCTGCTTCGCCATCGGCCTGCTGATGATCAAGCGCTTCGCCAAGGACCATCCGG
TCGTGGTCGCCCGCAATATCCTGATCTCCTCGGCAATCCAGTTGCTGCTGGTCGCCCCCTTCATGGTTGA
CCTCGGCAGCCTGACCCTGCCGAGCACCCAGGCCCTGAGTGCGATCACCGTGCTGGGTACCCTCTGTACC
GGTCTGGTCTACTTCCTCTACATGGCGCTGATCCAGAAGGCCGGCCCGACGTTCGCCTCCTTCAGCAACT
ATCTGGTGCCGCTGTTCGGCGTGATGCTGGGTGCCCTGTTCCTCGGCGAGCAGATCCACCCCACCACCGG
CGTGGCCCTGGCCCTGATCCTCGGCTCGGTCGCCATGAACCAGTGGGCGCAACAGCGTCGCATGCAGCGC

GAGGTCTCCCTATGTCAGGCATCCTGACCACACTGTGCGGTGGTCTGGTACTGATTGCCGGCTGGCAGTA
TCGCCAGCGGCTGGCCGGGATCTTGGGCCTGCTGATGGTACTGCTGCCCTGGTTCTTCGATAGTAAGCTG
CAAGCCATGCTGAGCTATGCCCTGAATGCCTGAAGTCCATCTCTGAATGTGTGACGAGAGTCTATCTCTC
CTGTTTTTGGGACTGTTTTAAAAAACCTCCGATTTGTCCCCCGCTACCGGCGCTCTGCGCCGGTTTTTTT
ATGCCTGCCAACCGGCGTCAGATCAGATAGCGATAGCTGAGTCGCTCGTTCTTGCGCGGCCCGGTGATCT
GCCAGCTCAGCAGAAAACCACCGTCGATCTCGGTCAACTCGGCGCTGTAGTGATCCGCCCCGCACAGATG
GGGCTCGGCGGTCAGCCAGCGGCCATCCCCCTGCGGCAGCAGCTCGAATAGTTCAACCGGCGCTTCATAA
CGCAGGTGAGAAAGGCGGATCCCCCGCTCGCTGCGTTGCCACCAGAAGCGGTTATGCATGGCGAGCTCCC
TGCCATGAGGCGTGATGTAACGGCCCTGTTCGTTAAACAGCCAGCCTCCCTGATGATCGGTCACCTGCAC
ACGCCCCTCGCCCCGACCATTCCAGTCGGTCGCCGACCCCTCGCCATTGCTGGCGGTAAAGGAAAATGCT
CCGACCTGTGGCAATCGCGCCCATAAACGCTGGATTGCCGCTTCCATCTCGGCCATCATAGCCCCCTTAA
AATTGACAACTGACGATAAATCAAACACATGAACTACTTGATAGGGGTCACCCTGCTCTGGTCATTCTCC
TTCAGCCTGATCGGGGTCTACCTCGCTGGTCAGGTCGATGCCTACTTCTCGGTGCTGACCCGCATCGCCC
TCGCCAGTCTGGTGTTTCTCCCCTTCCTGCGCCGCCGCTGGCTGCGCCCCGATCTGGTGGCCAAGCTGAT
GGCGCTGGGCGCCATCCAGCTCGGCATCATGTATCTCTTCTACTACCACTCCTTTCTGCTGCTGACGGTG
CCGGAAGTGCTGGTCTTTACCATCTTCACCCCCATCTACGTCACCCTGCTCCACGACCTGCTGGAAGGGC
GCTTCAAGCCCACCTATCTGTGGGGCGCCCTGCTGGCGGTGATCGGGGCTGCCGTCATCCGCTTCGACGG
CCTGACCGAAAGCTATGTGCTGGGATTTATGGTGGTGCAGGGGGCCAACCTCTGTTTCGCCCTGGGTCAA
GTGGGCTACAAGGTACTGCTGGCAAGGGAGGCGGAGCAACCGCCGCAACTGGCGGTGTTCGGCTGCTTCT
ATCTGGGAGCGCTGGTGATCGCACTGCCCGCCTGGTGGCTACTGGGCAAGCCGCAATACCCAACATCAGG
GTTGCAGTGGGGCATTCTGCTCTGGCTGGGGGTGGGTGCCTCCGGCCTCGGCTACTTCCTCTGGAACAAG
GGGGCGACGCTGGTCACCAGTGGGGTGCTGGCCATCATGAACAATGCGCTCATTCCGGCCGGCTTGCTGG
TCAATCTGCTGCTCTGGGGCAAGGATACCGACCTGACCCGGCTGACCATCGGCGGCCTGCTGATGCTGGC
CTCGCTCTGGATCTGCCAGCGTGACAAGCGCGGCCAACCCTGACCACACTCAGCCGACGACGGTGTTGTG
CTCCCGTCTGGCCTGACTGTGACAGCTGGCCAGCCGGGCCAGCAGATGATCGAGCTGCTCGCCAGCCTGA
CTCTGCACCAGCCCGATGCTGACGGTGACCGGCCGCTGTGACAGCAGGCTGGCATCGGCAATCTCGAGGC
GCAGCCGCTCGGCGATCTCCAGCGCACTCATCCGCTCGGTCTGGGAGAGCATCAGGATAAAACCGTCCTC
ATCCCAGCGGGCGAAGGGATCTTCCCGCCGCAGCTCGTGGCGCACGATACGCGCCAGCTTGGCCAGCATG
GCGTCGCAAGCCGCTTCGCCAAACAATTGCTGGATTTTGGCCATGTGATCGACCGAAAACTGCAGCAGAC
TGAAAGTGCCACTCTCGGTCGCCAGCCGCTCCTCCAGCAGTTGCTCGAAGCGGCCACGGCTCTCCAGACC
GGTCAGCGGATCGGTGCCATGTCGCTCCTCCACCAGCACGGCAGCGGGATCGAGGTGACGGACGACCCCC
AGCAGATAACCCCGCTCATCACGACGCATCCGCTCTTCCAGCCGGATGTAGTGACCGTGATGATGCAGAA
TACGATATTCGAGGCGCAGACCGCTGCTGCGGCCGCTCTGGCAGGCGAGCAGGGCATCTTCCAGCCGGGC
AACATCGTCGGGATGCACCCGATCCAGCCAGCCAAGATCATCCGGCCAACTCTCTCCCAATCCCAACAGG
CGCAGGCAGGAGGGATCACGCTGCAGTCCATGTTGGGCTGACCAGCTCCAGATCCCCGCCTCCAGCAGCG
CCATGGCATCAGACAGCAGACCCGGATCAAGCCGGGCAGTCACATCGACCAAAGGTGTCTTGAGCATTTC
CATGGGGCGCCTCGAAACAGGATCTGAATACCTGCTCAATAAGTAACATTGGCGTTCACGCCGAGCAACA
GGGTCAGATCGCTTTTCCCTGATACCCGTTCATCAAGGTCTGCCAGTTCTCCGGTACGAAGTGGAAACTG
GTATGCAGTTGAGACTCTTTATTGAATGAACGGAGCAAGCGTTCCAGATTGGCCTTCTGCCATTCGCCGG
GGGAGCGGATCGCACCCTTGTCAAAATCGATCACCCACACCTTGCCCTCTTTATCGAGCAGCAGGTTGTG
GCTGTTGAGATCGGCGTGATAGACCCCGGCATCGTGCAGCTGGCGCACCGTCTGACCTATCTTGTGCCAC
ACCTCGGTCGCCACCGGCCCCTGCTTGAGCAGCGCCACCATATCCTTGGCACCGCGAATTCGCTCAATCA
GGATATCGGCACGATAGAATGGGCCCTGTTTGACCATGCGGGCACCAAACGGACGCGGCACCGGCAGCCC
CAGCTCGCACAGCTTGGCCAGCAGGGTGTATTCGGCCATGGCACGGCTCGACTCGACCCCTTCGAACCAG
AAGCGATCCCGCACCACCTTGCCCACCATGCCGCCGCGATAGTAGTGGCGCAGCACCAGATGGCGGGACT
CATCCTTGACGAACCAGGTCACCCCCCGCCCGATGGAGGTTCCCACCACCTGACGGTTGTGCTGCCACCA
GGCAGGATCGAATAACTGGGGAGAGGGGTCGCGAAAGGCCCCTTCGGCATACCAGCAGATCTGGTTGTGT
TCGGTCTGTACTCGCATCGTGGAGATCCCGCTCGGTGGGTCATGGATCGCAATTTTTCAATTGGCGATGA
GGCTGCCGCTCTGGCATGCCCGCAATAGTTGCCTTTAATCAACGACGTGCAGAAACAGGTCATGGCGCTG
GGCCCGGCCTATCCCGGCTGACGATTGTGCGCAATTTTACAATCCAGAGGGGAGTTTGCATAATGCCATC
CACCCTTTTTGCCAGCCGTGCGCCACAACATGCCGCTATTTCAAACGCCCCCCTCCTCCATCTGCATCCT
GCGGCTCTCCGCCATCGGGGATTGCTGCCACGCACTGGCGCTGGTGCGCGTCATCCAGCGGGAGTGGCCA
CAAACCCGCATCACCTGGATCACCGGCAAAATCGAGGCAACCCTGTTTGCCGATCTGCCCGGCGTCGAGG
TAATCCTCTTCGACAAGAGCAAGGGGTGGCGCGGCTATCGCGATCTCTGGCAGACCCTCAAGGGGCGCAA
ATTCGATGCCCTGCTCCACCTGCAGGCGGCGATGCGAGCCAGCATCGCCACCCTCGGCATCAAGGCCAAG
GTCAAACTCGGGTTTGATCGGGAACGGGCCAACGACGGCCAGTGGCTGTTCACCAACCACAAGGTGCCTT
CGCCTGCCTCCCCCCATGTGCTGGACGGTTTCCTCGCTTTCGCCAAAGAGCTCGGCATCAAGGATCTGAC
CCCGGACTGGCAACTGCCCATCAGCGCCGAGCATCAGGCGTGGGCAAGGGAGAAGATTGGCGGCAAACCG
ACCCTGCTGATCTGCGCCGCCGCCAGCAAGGCCTTCAAGAACTGGACGGCCGCAGGCTATGCCGCGCTGG
CGGATCACGCCGCCAACAAGGGCTTTCAAGTTTATCTCTGTGGCGGCCTGCCAAACTGGAGCGAGATCT
GGCTGCCGAGATCCGGCAACTCAGCCAGAGTAAACCGGCCGATCTGGTGGGGCAGACCAACCTCAAGCAG

TTATTGGCACTGATTGGCGAGGCGAGTCTGGTACTGGCCCCCGATACCGGCCCCACCCATATGGCCACCA
TTGTCGGCACGCCGGTGATCGGCCTCTATGCCCACCACAATCCGGCGCGCACCGGCCCATATCTCTGCCG
CGACTACGTGGTCAGCGTCTATCAGGAGCTGGTCGAGCAGGAGACCGGTAAGCCGCTGGCCGAATTGAGC
TGGCGCACCCGTCTCAAAGACCCGGAGGCCATGCGAAAAATCACCAGCGAGCAGGTGACCGCCGCGTTCG
ATCGGCTCTGTGCAGATCGTCAACTGCCGTAGTAACAGGGCACCTCGGGAATTCCGAGCCATCAGGCAGG
GATAACCTGATAGCCTCTGCCCTCAGCCAAAGGCACAATGCAGGCTGATGTGGGTTACGCTCCCGCAGCA
AGCTACGAATACTTTGATCGGCTGGAATATGATTCCGGCCGTTTTTTTATTCGCCGCATTCTGGTAAAAT
TCGCCGCCTTTACATCCCCCTGACTCAAGAATTTTCCGCGTTTTGCGTTTGGCTGTCTCCCTTTCGGGCA
CAAGACGGCGGTCTCTGCTACAACCAAAACAGCGGTTTCGATTAAAGGTAATAATATGGCGCAACAAGGC
ACTCTCTATATCATCTCTTCTCCGAGCGGCGCGGGTAAATCCAGTCTGCTCAATGCCTTGCTGACCAACC
ACAATCAAGCTGGCAAGATGCAGCTATCGGTTTCCCACACCACTCGCGCCACCCGCCCGGGCGAAGAGCA
CGGCGTACACTACCACTTCGTTCAGGTGGAAGAGTTCAAGGCGCTGATCGAGCGTGGTGATTTTCTGGAG
TGGGCCGAAGTATTCGGCAACTACTACGGCACCTCACGCGCCGCCATCGAAGCCTGCCTGGCCAAGGGGA
TCGACGTATTCCTCGACATCGACTGGCAAGGTGCCCGCCAGATCCGCGAGCAGATGCCGACCAAATCCAT
TTTCATCCTGCCCCCCAGCCGCGAAGAGCTGGAGCGCCGCCTGATTGGGCGCGGTCAGGACAGCGCCGAA
GTGATTGCCGGCCGGATGGCGAAAGCCATTGCCGAAATGGTGCACTACGACGAGTATGACTATGTCATTA
TCAATGAAGACTTCGAGCAGGCGCTGTTCCAGCTCAGGTCCATTATTGAGTGCCAACGGCTGGAGATGCG
TCACCAGCAACAGGCTCAAAAAAACTTGCTGCAACAGCTACTGGCAGAGTAAGCCAAAAACAAGTAAACT
TTTGCGTCATTTTTAAACACTGCTTTTCTAAATACAGAAAGCCCAAACACTGGAGTCCTGCATGGCACGC
GTTACTGTAGAAGATGCTGTAAAACAGGTAGGTAACCGTTTTGACCTGGTGCTGGTCGCCGCGCGCCGCG
CTCGTCAAATCGCGGTACAAGGCAAAGATCCCCTGGTCGACGAAGAGAACGACAAGCCGACTGTGATCGC
CCTGCGTGAGATCGAGCTGGGTCTGGTGAACAATCAAGTGATGGACACCCAGGACCGCTACGAACAGCAA
GAGCAGGAAGCCGCCGAGCTGGCTGCCGTTGCTGCCATCGCCGAAGGCCGCGGTTAAGTTATTCCTCCGG
TTGGCACCCGCCCGGGTGCCGACTGTTCATCCCCATTTACAACCGCTCGAAAACCTCGCAAACTCAGGGC
TATACCCTCAATTCCACGAATTGCCGTTTCACCGCTGCGGGGACTGTCTTGTATTTATTTGAAAACCTTA
AAGAAATAGCCAGTTCCTACCTGCCGCCAGAGCAGGTTGAGAAGCTCAAGCAAGCCTATGTGGTGGCCCG
TGATGCCCACCAGGGCCAGATGCGTTCCAGTGGTGAACCCTATATCACCCACCCCGTTGCCGTTGCCCGA
ATCCTGGGTGACATGCGCCTCGATCATGAAACCCTGATGGCCGCCGTGCTGCACGACGTCATCGAAGACA
CCTCTGTCACCAAAGCCGATCTGGCCGAACAATTCGGCAATGCGGTTGCAGAACTGGTGGCCGGGGTCTC
CAAGCTCGACAAGCTGAAATTTCGCGATCGCAAAGAGGCGCAGGCCGAGAACTTCCGCAAGATGGTGATG
GCAATGACCCAGGATATCCGGGTCATTCTGATCAAGCTGGCCGACCGCACCCACAACATGCGCACCCTGG
GCTCCCTGCGCCCTGACAAACGCCGCCGCATCGCCCGGGAAACCCTCGAAATCTTCGCTCCCATCGCCAA
CCGCCTCGGTATTCACACCATGAAGAACGAGCTGGAAGAGCTGGGTTTCGAGGCGCTCTATCCGATGCGA
TCCCGCGTGTTGCGCGAATCGGTGCGCCGCGCCCGTGGCAATCGTCGCGAGATCATCGACTCCATCCAGA
GCGAAATCAGCGGTCGCCTGAAAGAGGCGGGGATCGAGCATCAGGTCAGTGGCCGCGAGAAGAATCTCTT
CTCCATCTACAACAAGATGGAGAAAAAAGAGCTGCAATTTCATGAGGTAATGGATATCTACGCCTTCCGG
GTAAAAGTGAAGGACATAGATACCTGTTACCGAGTGCTGGGGCAGATGCACAGCCTCTACAAGCCGCGCC
CCGGCCGCTTCAAGGATTACATCGCCATCCCCAAGACCAACGGCTACCAGTCGCTGCACACCTCTCTGGT
GGGGCCGCACGGGGTACCGGTGGAGGTGCAAATTCGTACCGAGTTCATGGATCAGATGGCCGACAAAGGG
GTTGCCGCCCACTGGGCCTACAAGCAGGATGGCGACAGTTCGGGCACCACTGCCCAGATCCGCGCCCAGC
GCTGGATGCAGAGCCTGCTGGAATTGCAGCAAAGCGCCGGCAGCTCGTTTGAATTTATCGAAGGGGTCAA
GACCGACCTCTTCCCCGACGAGATCTACGTCTTTACCCCGGAAGGGCGCATCATGGAGTTGCCCGCTGGC
GCCACGCCGGTGGACTTCGCCTATACGGTGCACACCGACATCGGCCACGCCTGTGTCGGTTCGCGGGTCG
ACCGCCACCCCTACCCGCTCAGCAGCCCACTGCACTCGGGCCAGACGGTGGAGATCATCACGGCGCCGGG
CGCCCGCCCCAACGCGGCCTGGCTCAACTTTGTGGTGACCACCAAGGCACGATCCAAGATCCGCCAGTTC
CTCAAGAACCTGCGCACCGAAGAGTCGGTGGTGCTGGGTCGCCGCCTGCTCAACCATGCGCTGGGCGCCA
AGAATATCGAAGACATTCCCGAGCCACGCATCAAGCAGGTGCTGGCCGATACCAAGCATGAGAACCTGCA
GGGGCTGCTGGCCGATGTGGGCCTCGGCAACGCCATGAGCGTCATGGTGGCACGCCGCATGCTGGGGGAT
GAACTACCGCCGGAAGAGCAACCCAAGCCCAGCAGCAAGAAGATGCCGATCAAGGGTGCCGACGGCATGC
TGGTCACCTTCGCCAACTGCTGCCGTCCGATCCCGGGAGATGCCATCATCGCCCATATCAGTCCGGGCAA
GGGTCTGGTGATCCATCAGGAAGCCTGCCGCAACATCAAGGGCTACAGCAAGGAGCCGGACAAGTATCTG
CCGGTGCAGTGGGAAGTGGACAAGGAGCAGGAACAGGAGTTCCGCACCGGTATCAGCATCGAGATCGTCA
ACCATCAGGGTGCGCTGGCCGAGCTGGCCAACGTGATTGCCGCCACCGGCGCCAACATTCACGCCATCAG
CACCGAAGAGAAGGATGGCCGGGTCTATCAGGTCAGCCTGCTCATCACCACCAAGAGCCGCATCCACCTG
GCCAACATCATGCGCAAGATCCGCGTGATGCCCAACGTGCTCAAGGTGAGCCGACAGAAGAACTGAACAC
AGGCGGCGCAAGCCGCCTGCTGCTTTTATAGAGACAAGCCATGACCCCCGAACGCTTCAAGCGCATCACC
GATATGCTGGCCCAGCGTCAGCTCGATTTGACCGTCTGTATGGAAGAGGTCCACAAACCTCACAATCTGG
CCGCCATCGTGCGTACCGCCGATGCCATCGGCATCCACCGGGTACACGCCGTCTGGCCCAAAACCTGGAT
CCACAAGCGCAAGGGCACCGCCCGCGGCAGCCAGAACTGGGTGGATGTAAAACTGCACCCGGACATCGGC
AGCGCCGTTGCCGAGCTGAAAGCCTCCGGCATGCAGATCCTGGCGACTCACCTCTCCGACAGCTCGGTCG
ATTTTCGCGCCATCGACTACACCAAACCGACCGCCATTCTGGTGGGTCAGGAGAAACACGGCATCGGTGA

AGAGGCGCTGGCGCTGGCCGATCACCATATCGTCATCCCCATGGTGGGCATGGTGCAGTCCCTCAACGTC
TCGGTGGCTGCCGCCGCCATCCTCTACGAGGCGCAGCGCCAGCGCGAGCTGGCCGGTTGCTATCAACGCG
GCTGCCCGCTCACGCTGGAGGAGCAGAACAGCATCCTGTTCGAGGGGGGTTATCCTATCTATGCCCAGCT
CTGCAAAGAGAAAGAGATGCCCTACCCCCAGCTCGGCCCGGCCGGTGAAATTCTGGCGGACGAAGAGTGG
TGGCAACGGATGCAGCTGACCCGCAAAGGGTGGGCGCAGCAGAGCGAGCAGGACGATCCGCAGGAGGAGT
GATGGAGAACCCTTTTGGCAGCTGGCTGGAATCGCAAATCATCCGGGATCACCTGACCAACCCGAACATA
GAGGCCGGCGACTACAGCTACTACTCCGGCTACTACCACGGTAAGCCGTTCGAGGATCACTGCGTCCGTT
ACCTGCTCGGCGATGGCTCGACCCGCGACACCTGGGAGAGCGGCCTCTGGGGCGAGGTGGACCGGCTGAT
CATCGGCAAATTCTGCTCCATCGGCTCGGGCGCCACCTTCATGCTGGCGGGCAATCAGGGCCACCGGCTG
GATTGGGTCTCCACCTTCCCGTTCAATCCCGACACCTTTGGCGAAGGGGCCCGCAGCGGCTTTCTGCGCA
AGGGGGACACCCGGATTGGCAACGATGTCTGGATCGGCTCGGAAGCGATGATCATGCCCGGCATCACCAT
AGGTGATGGCGCCGTCATCGCCACCCGCGCCGTGGTGACCAAGGATGTGGCCCCTTACACCATAGTCGGC
GGCAACCCGGCCCAGCCGATCCGCCGCCGTTTCAGCGATGAGCAGATCCTCATGCTGCAAGAGATGCAGT
GGTGGGACTGGCCCCTGCCTCGCTTGCAAGCCTGCATGGCGCTGCTCTGCTCGGGGGATATCGCCGCCCT
TTATCACCATTGGCAACAGGAGTGTGCCTGATGAAACCCCTCTCTCTTTCCCTGTTGGCCCTGACCCTGT
TTTCTGCCGGAGCCCTTGCCGTGACCAATACCTATCAGCTCACCAGCGAAGCCGATGTACCTAGCCTCTA
CCAGCAAACCCTGCCCGACTTCTGGCGCCAGCATGCGGTGGAAGGAGAGTTCAAGGGCAAGGATGGCGTA
ACCATCCGCTATGCCGCGCTGCGGCAAGCCAAGGTCGATCGCGCCATCCTGATCGTCAATGGCCGGGTCG
AGAGCTACCTCAAGTATCAGGAGCTGGCGTGGGATCTCTGGCGTCAGGGCTACAGCCTCTATCTCATCGA
CCATCGCGGTCAGGGGATGTCTGACCGGATGCTGAATGACCCGCAGAAGGGATATGTCGACCAGTTCGAC
GATTATGTGGTCGATCTCAAGCAGTTTCACGACCAGATAATCATGGCGGACCAACCTGCCAAGCTGTTCC
TGCTGGCCCACTCCATGGGCGGCGCCATCTCGGCCCGCTATCTGGAACGCTGGCCCGATGATATCAAGGC
GGCCGTGCTCTCCTCCCCCATGCTGGGCATCAATCTCGGCGGTCTGCCCAAGTGGCTGGCCAAGGGATTG
GCGAGCACCATAGGCACAGTCGGCGGCTGGCTTGGCGAGCCTCCCTACGGCCCCGGTCAGGGCACCTATC
AGGATCACGGCTTTGCCGATAACGAGCTGACACACAGCCAGTCCCGCTATCAGGCGTTCCGTCAGCTCTA
CGAACAGCACCCACAGATCAAGCTGGGTGGTGCCACCGCCCACTGGATTTATCAAGGGATCACCGGTGCC
GATGCCGCCATCGCCGATGCGGGCGCTATCAAAACCCCGCTGCTGGTGCTGCAGGCTGGCAGCGACAGCG
TGGTAGACAATGCCGCGCAGGATCGCTTCTGCACCATTGCCAAGTGTGAAGGGGGCAAACCGCTGCGCAT
CGAGGGTGCTTGGCACGAGCTGTTTATCGAATCCGACGACAAGCGCCAGCCCGCCCTGACCGCGATGGTC
GACTTTTTCGCCCGCTTCTGAGCTGGCTATTTTTTGTAACCCTAAATAAATGGGGTGACAGTGCGGGCTA
AGGTAAACTGCCCCTGTTTTCAATCGATGCCGAGGTAATGATGTTCAAGGTAGTTGTATCCGATCTCGAT
GGCACTCTGCTCAACAAGCAGCACCAGATCTCTCCCCGTACCCGGGATACCCTGCACCGCCTGGTAGAAC
AAGGTGTGAAATTCGTGGTCGCCACCGGCCGTCATCACGTTGACGTACGCAGCTTCCGCGATGCGCTGGG
GATGGACATCTACCTCATTACTTCCAATGGCGCCGTGGTCCATGACAAACAGGATCAGCTGATTTTCAAC
CAGCCGCTGCCGACCGAGATTGCCGCCGAGCTGATTGCGCTGGAGCGCGATCCCTCTATCCACCTCAATG
TCTATCAGGGTGATGACTGGGTGGTGGAAGAGGAGCTGCCCTGGCTGCTGCAGTTCCACGACGAGTCAGG
GTTCACCTACCGTCTGGTCGATGACCTGAAGCAGGAACCGAAAGAGCAGATCAACAAGGTGTTCTACATC
GGTGACCACGAGAAACTGCTGAAGATCGAGGCGCACCTCAACCAGCGCTACGGCGATCAGCTCAACGTCA
CCTTCTCATTGCCGGACTGCCTCGAAGTGATGCACGCCGGAGTGCATAAGGGCAATGCTGTGCGAGCCGT
GCTGGAGCAGAATGGTTTCGATATGTCGGAGGCGATCGCCTTCGGCGATGGCATGAATGACTTCGAGATG
CTGACCATGGTGGGACGCGGCATCGTGATGGGCAACGCCCATGACCGCCTGAAAATGGCGCTGCCGGAGT
ATGAGCAGACCCTCACCTCCGACGAAGATGGCGTCGCCGTCTATCTGGAAAAACTGTTCGAGCTGGAGAG
CGTCAGCGCAGCCTGATCCTCTTCATCCCGACATTCGATTCAACAAAGCCTCACCATGTGAGGCTTTGTT
TTTTCAGGGTCTCCAGCCAGGGATTGGGCCGCTGCCAGCTGAAGCTCTGGTTCGCCCCGCCGTAGCATTC
AGCCAACCGCAGCGGCGTGCCGGGGGTATGGCGTCGCCCGGCCACATCGAGGCAACGGGCAACCTTACTG
TTGAACAGCCTGTCTTTCTGCCACTCCCACTGCGCCTGACCGTCACAGGGTGCCAACATCACTTCGCTGT
CGTTTTTGGCTTGCAAACAGAGCTCCTGCAGGCGCAACTCACCCAGATCCCACTCGAACAGTTGATCGTC
TTTTCCGCTGCAAGGTTGTGCCAACACCTTCTGTTGGGACACCGCAACGCAAAAATCACCGCTGGTCAGC
AAGGGGGAGAGAGAACGCTCCTCCTGCGGTTGTGATGCAATAGAGCCCATCATCACATCGGTGGATGGCG
GCTTGATGGTACAGCCACCCAGCAGCAAGGCCGCCAGCAGGGCAGGAGAGCAAGCAAAGGAAATTTTGTT
ATTCATGGAAAAATGCCTGAAATGGGCCGATGACATCCGGTTGTCAGTGACTCGAGGCTGAAGCATAACC
GAAAGGCGGGGCGCGCTGCCCAAATAGAGTGTAAACAGACGTAACAGGGAGAGAGTACTGGCACGGCGGA
GGCCAGAAAGCAAAAAACCCCGCCGAAGCGAGGTTTTTCTAAATGTGGTCGGTGATAAAGGATTCGAACC
TTTGACCCTCTGGTCCCAAACCAGATGCGCTACCGGACTGCGCTAATCACCGAGGCTATTTCTTTCAAAA
TAGTTTACTTCAAAAAAACAAAGACGACCTGATGGTCATCCTCGTCATACAGCAAGTATGGTGCGAAGAG
AGGGACTCGAACCCTCACACCCGGGGGGCACTAACACCTGAAGCTAGCGCGTCTACCAATTCCGCCACCT
TCGCGTACCTGACAAGCTGTAAAATGGGGTGGCTGATGGGGCTCGAACCCACGACAACCGGAATCACAAT
CCGGGACTCTACCAACTGAGCTACAGCCACCACAGCTGTTTTCGTTCCACAACAACGCCCGTTTAACCGA
CGCACTTTTTATGGTGCGCCCGACAGGATTCGAACCTGTGACCTCTGCCTCCGGAGGGCAGCGCTCTATC
CAGCTGAGCTACGGGCGCTACGCTGTCGAACGGGGCGCATTATTAATGATGGTTGCTGGTTTGTCTACAC
TTTTCTGTCACCGAAGCACTGTTCGTTGTTTTTTCAAGCACATTGCGCACTTTTCTTCCACTATTCCCCT

GCTGCCAGCCGAAAAGGGGCAGATCGCAGTCGCAAATCGCCTTGCTGCCTTGACTTGATTTGATGCCCAT
CTAGAATGAATGAACGTTCATTCAATCGAAAGTCACACTGATGATTCTGGATAAAAAGAGAGCATCTTC
AACGCGGCCCATGAGGTGCTGGGTGAACGGGGTTTTCATGGTCTGTCGATTGCGGAAGTCGCCAAGAAGG
CCAATGTTGCCACCGGCACCATCTATCGCTACTTCGGCGACAAGGATGATCTGATCCGGCAACTGCACCA
GCACACTATCTTGCAGTGTGTGCCCATGGTCATGGCCGATGTAGCAATCGATAAAGTTTCATTTCAACAA
TTTCGCCAATTATGGCTCAATATTCATGCCATTTTTGTTAACGAACCCAACGCTATCCGGTGCAAATTGC
AGTACGAGAGCTCTCCGCTAGGGGCAGAATTGGAGACCAATCCTGTCATTCTGGCTGCCTGGGAGCCTCT
GGACCGATTTTTTGAACAGGGCGTTGAACAAGGACTGTTTATTGATTTACCGATACGGGCACTCCAGGTG
TTGAGTCTGGACAGTGTCATGCATCTGGCCCTGCAGTGCAGGGTTCACAACATCACGCTGACCGAATCGC
AGTTGGAAACCGCGATCCGGGCCAGCTGGAATGCCATTTTATCCCCCAATCTTTCCACCTCAGGAGCCTG
TTCATGAAAAAGTGGATGGCCATTATGTTGCTGATAGCGATCGCCTTGTTTGGCAGCGTCATCGGCTTCA
ATTTGTTCAAACAGAAGATGATTGCCCAATATATGGCCAACCGGCCGGAACCCGAGTTCCCCGTCACCGC
CATGGTGACCAAGGCGCAGGATTGGGTACCGACCATCGAAGCGATCGGCTTCATCGAACCGAATCAGGGG
GTGACCCTCTCCACCGAACTGGCAGGTACCATTGACGCCATCACCTTCGAATCAGGCAAACCGGTCAAGG
CTGACCAGCTGCTGCTGAGCCTGGACTCCACCGTGGAGCGTGCCAATCTGCGCGCCTCCCAGGCCAAGCT
GCCGGCGGCAAAAGCCAAGTTCGATCGTTTCCAGAACCTCTACAAGACCAGCTCCATCTCCAAAGAGCAG
CTGGATGAAGCCGAAGCGGCCTATCGCTCGCTGGAAGCGGATATCGAGAGTCTGAAGGCGACCATTGCCC
GTCGTGAAGTGCGTGCGCCCTTTAGCGGCGTGGTTGGCCTGCGCAATGTGTTCCTCGGTCAGTATCTGCA
GCCGGGCACAGATATCGTGCGCCTGGAGGATACCAGCGTGATGCGCCTGCGCTTCACCGTGCCCCAGACC
GATATATCCAAAATCAAGCTGGGCCAGACCATCAAGATCAATGTGGATGCCTATCCGCAAACCCAGTTTG
ACGGTCATATCACCGCCATCGAGCCTGCGGTCAACTTCCAGAGCGGCCTGATCCAGGTACAGGCAGACAT
TCCGAACAACGACGGTCAGCTCCGTTCCGGTATGTTCGCCCGCGCCAGCATCATTCTGCCGACGGTGAAA
GATCAGATCGTGATCCCGCAGACCGCTATCTCCTTCACCCTTTACGGTCAGAACGTCTACGTGCTCAAAG
AGGGCGAAGAGACCGACAAAGAGGGTAACAAGGTGAAAGTGCTGCGAGCCAAGCAGGTTGTGGTCAAGGC
CGGTGAGCGCCGTGGCAACGATGTGCATGTGCTCTCTGGCATCCAGGCCGGTGACCAGATCGTACTGTCG
GGTCAGGTTCGCCTGAGCAACGACACCAAGGTGCATGTGGTCGAGAACGATGCTCTGGCCGTTCCGGCAC
AAACCCCGATGCTGTAAGCGCGGAGATCTGCGATGCGATTTACTGACATATTTATAAAACGGCCTGTGCT
GGCGATCTCGCTCAGCTTCCTGATCGCCTTGCTGGGCTTTCAGGCCATCTTCAAGATGCAGGTACGGGAA
TACCCCGAGGTGACCAACACGGTGATCACCGTCAGCACTGGCTACTATGGCGCCAGTTCTGATCTGATCC
AGGGGTTCATCACCCAGCCGCTGGAACAGGCGGTCGCGCAAGCCGACAACATCGACTTCATGACCTCCTC
CAGCCAGCTGGGCAGCTCCACCATCACTGCGTACATGAAGCTCAACACCGACCCCAACGCCGCGCTGTCC
GACATCCTGGCGAAGGTGAACTCGGTGCGATCCCAGTTGCCGAAAGAGGCGGAAGACCCGTCGGTCACCT
CCTCCACCGGTTCAACCACGGCGGTGCTCTACCTCGGCTTTACCAGCCCGGAGCTCAACTCCAGCCAGAT
CACCGACTATCTGGAGCGGGTTATCAAGCCGCAGCTCTTTACCGTGGGCGGGGTCTCCAAGGTTGACCTC
TACGGCGGTGTCGAGTTTGCCCTGCGGGTATGGCTGGATCCGGCCAAGATGGCGGCCTTCAACCTGACTG
CCAGTGACGTGATGACGGTGCTCAACAGCAACAACTATCAGTCCGCGACAGGTCAGGCGACCGGTTACTT
CACCCTGTTCAACGGCAACGCCGAGACCCAGGTCAAGGATGTCGACGAGCTGAAGCGTCTGGTCGTTGCT
ACCCGTGACGGCAAGGTGATCCGCCTTTCCGACATTGCCAAGGTGACCCTCGAGAAGAGCCACGACATCT
ATCGCGCCAGCGCCAACGGCCGCGAAGCTGTGGTCATGGCGGTCAACGCCGCGCCGACAGCCAACCCCAT
CAACATCGCCCACGATGTGCTGGCGTTGCTGCCGAGCCTTGAGCGCAATATGCCGAGCACCATGCGTCTC
AACGTGATGTATGACTCCACCGTGGCGATCAACGAGTCCATCCACGAGGTTATCAAGACCATCCTGGAGG
CAGCCGCCATCGTTCTGGTGGTCATCACCCTGTTCCTCGGTTCGTTCCGCGCGGTCATCATCCCGATCAT
CACCATTCCGCTCAGTCTTATCGGCGTAGTGATGATGATGGATATGTTCGGCTTCTCGATAAACCTGATG
ACCCTGCTGGCGATGGTGCTGGCGATCGGTCTGGTGGTGGATGACGCCATCGTGGTGCTGGAGAACGTCG
ACCGCCATATCAAGGAGGGGGAAGAACCGTTCCGGGCCGCCATTATCGGTACCCGTGAAATTGCGGTGCC
GGTTATCGCCATGACGGTTACTCTGGCGGCGGTATACGCGCCTATCGCCCTGATGGGGGGCATCACCGGC
TCCCTGTTCAAGGAGTTTGCCCTGACGCTGGCTGGCGCCGTGTTCGTCTCGGGCATCATCGCCCTGACTC
TGTCCCCAATGATGTGTTCCAAGATGCTCAAGGCCAACGAGCAGCCAGGCAAGTTCGAGAGCACAGTCCA
CCACCTGCTGGAGCGGATGACCGATCGCTACGACAGCATGCTGCACGCCGTGATGAAAAAGCGCCCCGTC
ATCGTGGTGTTCGCCGTCATAGTCTTCGCCAGCCTGCCGCTGCTGTTCAAGTTCATCCCGAGCGAACTGG
CACCGTCAGAGGACAAGGGGGTAATGGCCGTGCTGGGTACCGCACCGTCCAACGCCAACCTGGACTACAT
CGAAAACACCATGGCGGATGTGAACAAGATCCTGGACGACCAGCCCGAGATCGCCTTCTCCCAGGTCTTC
TCAGGGGTGTTCAACGCCAACCAGGCGTTCGGTATCGCCTCCATGGTGCCCTGGAGCCAGCGTGAAGCGA
GCCAGAAAGAGGTGCTGGACCGGGTCGCCAATCTGGTGAAAGACATCCCGGGCATGGCCATCACCACCTT
CCAGTTCCCGGAACTGCCGGGTGCATCGAGTGGTCTGCCGATCCAGTTCGTCATCACCACCCCGAACAGC
TTCGAAAGCCTGTTCCTGGTGGCCGGTGAAATTCTGGCAGCGGCGCAGAGTAACGGTCAGTTCGTCTATT
CGGATCTCGATCTGAACTACGACTCCGCCACCATGAAGATCCGCATCGACAAGGACAAGGCGGGTGCCTA
CGGCATCACCATGCAGGATATCGGAATCACCATGGGCACCATGATGGCGGACGGTTACGTCAACCGTATC
GACCTGGATGGCCGCTCCTACGAGGTGATCCCGCAGGTCGAGCGCAAGTACCGTCTCAATCCGGAGTCCA
TCAAGGGCTACTACGTGCGGGCCGCAGACGGCAAGTCGATCCCGCTCGGCAGCCTGATCAGCATTGAAGT
GGTTGGCGAGCCCCGCGCCCTGCCCCACTTCAACCAGCTGAACTCCGCCACCATTGGCGCCGTTCCGGCT

CCGGGCGTGGCCATGGGTGATGCGATCAACTGGTTCCAGACCACCGCCGACGAGAAGCTGCCGCAAGGCT
ATCGTTACGACTTTATGGGTGAAGCCCGTCAGTTCGTCACCGAAGGCAATGCACTCTATGCCACCTTCGC
GCTGGCACTGGCCATCATCTTCCTGGTGCTGGCAATCCAGTTCGAGTCGGTGCGTGACCCACTGGTCATC
ATGGTCTCTGTACCGCTCGCCATCAGCGGGGCCTTGATTGCCCTAGCATGGGGTATGGCGACCATGAACA
TCTACTCCCAGGTGGGCCTCATCACCCTGGTGGGTCTGATTACCAAGCACGGTATCCTGATCTGTGAAGT
AGCAAAGGAAGAGCAGTTGCTGCGCGGCATGAACCGGATGGAAGCCGTGATGCAGGCCGCCAAGGTGCGT
CTGCGTCCGATCCTGATGACCACCGCCGCCATGATTGCCGGTCTGATCCCGCTGCTCTACGCCGCTGGTG
CCGGTGCGGCACAGCGTTTCAGCATTGGTATCGTCATCGTGGCCGGTCTTGCCATCGGTACCCTGTTCAC
CCTGTTCGTGCTACCAGTGATCTATACCTATCTGGCTTCCGAGCACAAACCCTTGCCGGTATTCGATGAA
ACCATTCCGCCCAAAGCAAACGGCGGACATTAATCATCAATGACATGCAAAGGCCCCGTATCGGGGCCTT
TTTTATGCTTTGCAGGAAAGACGACATAAGTGCATGAAGCAGTTAAACTAAAAAACACCACATCCAGACC
GCAAGGACGCCATGTCAGGACTCTTCTCCAAGACCGCCAGGGCCGTAGGCCGCAACGGACTCTCATATCG
CCTGCTCTCCTACATTCTGGTCTGCAGTACCGTATTGGCGATGATCATTACGGCGCTGCAACTCGCGTGG
GATTACCGCAAGGATGTCGCCGTCATCGAAGACAGCATCGGCCAGATCGAGGCCTCGTTCCTGCAACCCA
TCGCCGCCAGCTTGTGGAACCTGGATGAAGAGCAGGTCAAAGTGCAAATTGAAGGGATCATGAACCTGCC
CAACATGCAGTTCGTGATGGTCAAGGAGATGCTCGGCAACTCCGAAGTCCCGCTGCTGACCCAGGGGGTC
GAGCGGGAAAGCTATGATATCTCCCGAGAATTCAACCTCACCTATCAGGGTGAAATCGTCGGCAAGCTGT
TCGTTGCCGCCTCTCTCGAGCAGATCTACCAGCGCCTCATCGAAAAATCGGTGCTGATCATGGTCAGCCA
GACCATCAAGACCCTGGTGGTCTCATTTTGCATCCTGATCATCATTTACTATCTGGTGGTCAGGCACATC
AACCGCATCGCCAACTACGCCCAGAAGTTCAACCTCGATCGGCTCGACATGGCGCTGGAGCTGGAAGGCC
GCCCGCAGCCGCGCAAGAAACCCGATGAGCTGGATACCCTGGTTTCCACCCTCAACCAGATGCGTACCCG
ACTGCGCGACGAGCTGGTTGCCCGCCATCAGGCGGTCGAACAACTGCAAAAGGAGCGGGACTTCTCCGCC
ACCCTGATCAACTCCGCCAACATGGTCATCTGCTGCATGGAACCGGATTTGACCATCGCCAGCATCAACC
CGGCTGCCATACTGCTGACCGGCTACCACCAGCAAGAATTGTTGCAGCACAACTGGCTGGATCTCTTTGT
CAGCCCGGCGCAACGGGAAGAGCTGAACGATATTCTGGCAGCGGAAGGTTCACTGGCCGACAAAGAGGTC
ATCATGCATGACCAGCAGGCCCATGAGCTGGTACTGCAATGGACCTTCGTACCCTTCTACGATGGCCCCA
ACCTCAAGTACCAGATCGGGTTTGGCTACGACATCACCCAGCTGAAAAAGGTCGAGCGGGAGATAATCCA
ACTCAACGAACAGCTGGAAGGCAAGGTGGTAGAGCGCACCCACAGCCTAAGCGAAGCCAACAACCAGCTC
GGCAAGGCGTACGACGACCTGAAACAGGCCCAGCAGACCCTGGTGGAGTCGGAGAAGATGGCCTCCCTCG
GCTCGTTGGTAGCCGGTGTGGCACACGAGATCAACACTCCGATCGGTATCAGCGTGACCGCCTCCTCCTA
CCTGCAGGAGCGAGTCGCCGACTTCAAGTCCCATATCGACTCCAAGCAGCTGTCGCGCTCCTATCTCAAC
GAGTTTACCGTCAACCTCGACGAGTCGATGCAGCTGCTGCAGAGCAACCTGCGGCGCGCCTCCGAGCTGA
TCGCCAGCTTCAAACAGGTGGCTGTCGACCAGTCATCGGAAGCACGCTACAACTTCAGCCTCGCCGACAA
CCTCCATCAGGTGGTGGTCTCGCTTGGCCACAAACTGAAGAAGAGCCAGTGCGAAGTGGATATCCAGTGC
GATTCCAAGCTGTCAATCTTCTCCTTCCCGGGCAGCTTTACCCAGATCTACTCGAACCTGATCCTCAACT
CCATCAACCACGGGTTCGACAGCTGGGACAAACCGAAGAAGATCACCATCAAGGTGGAACAGCAGGGAGA
GGAGCTGTTTATCGACTATTCGGACAACGGCCGCGGCATCCCGCCCGAAATCTTGCCGCGCATCTTCGAT
CCCTTCGTCACCTCCAAGCGGGGTCAGGGGGGCAGCGGTCTGGGCACCCACATCATCTACAACCTGGTGG
TGCAACTGCTGAAAGGCCGTATCAGCTGTGCCAGCGAACCTGGCCAGGGCGCCCAGTTCCATATTCGCCT
GCCGATCCAACATAACTGAGCGCTGGCTCTTGTGAGCCAGCCGCTTTATCCCCATCATGCTGTGATTGCA
TTCTTAATGAGCGGAGAACCTCGCAATGGCACAGCATTTTGACTATATCGCCATCGGCGGCGGCAGCGGC
GGCATCGCCTCTGCCAACCGGGCTGCCATGTACGGTAAGAAAGTTGCCCTGATTGAAGCCAAAGAGTTGG
GTGGAACCTGCGTCAACGTAGGCTGCGTACCGAAGAAGGCCATGTGGTATGCAGGCCAGATCGCCGATGC
CCTGAAATATGGCGCCGACTACGGCTTTGACACCACCCTCAACCACTTCAACTGGGCCAAGCTGGTCGAG
TCCCGCCAGGCCTACATCGGCCGCATTCACCAGTCCTATCAGAACGTGCTGGGCAAGAACCAGATCACCG
TCATCAAGGGCTTCGCCCGCTTCGTCAGCAGCAACACCATCGAGGTTAATGGCGAGCACTACACGGCTGA
TCACATCCTGATCGCCACCGGTGGCCGCCCGGAAATTCCGGCGATCCCGGGAGCCGAGCTGGGTATCGAC
TCCGACGGTTTCTTTGACCTGCAAAGCCAACCCAAGCGGGTTGCCGTGGTTGGAGCTGGCTACATCGCAG
TCGAGATCGCCGGCGTGATGCAGGCGCTCGGCTCAGAGACCCATCTGGTGGTGCGCAAGCACGCACCGCT
GCGCAACTTCGACCCCATGCTCCACGAGACACTGGTCGAAATCATGGCGCAGGAAGGGCCCAAGCTGCAC
ACCCACGCCATTCCCAAAGCCGTGCTCAAGAACGCTGACAACAGCCTGACCCTGCAACTGGAAGATGGTC
GCCACCTCACCGTCGACTGCCTGATCTGGGCCATCGGCCGGGTACCGGCTACCGACAACCTCAATCTGGC
TGCCGCAGGCATCGCGCTGGACGAGAAGGGCTTTATCCCGACCGACAAGTTCCAGAACACTTCCGTCGCC
AACATCTATGCGGTGGGTGACAACACGGGTCGCATCCAGCTCACGCCGGTAGCCGTCGCGGCGGGCCGTC
GCCTCTCCGAGCGGCTGTTCAACAACAAGCTGAACGAACACCTCAACTACGATCTGGTGCCGACCGTGGT
GTTCAGCCACCCGCCCATCGGCACCATCGGTCTGACCGAACCTGAAGCCATCGCCGAATATGGCGAGGAC
CAGGTGAAGGTCTACCGCAGCCAGTTCACCGCCATGTACTCGGCACTGACCCAGCATCGCCAACCCACCC
GGATGAAGCTGGTCTGTGTCGGCCCGGAAGAGAAGGTGGTCGGTCTGCACGGCATCGGCTTTGCGATGGA
CGAGATCCTACAAGGTTTCGGCGTCGCCATGAAGATGGGTGCCACCAAGGCGGACTTCGACAACTGCGTG
GCTATCCATCCGACCAGTGCAGAAGAGTTCGTGACAATGCGATAAGCACGTTTGAAGTTGGCCTGTCATT
ATCAGGCCAACTTCAACAACTGCGTTCCTACGCCTCATCCCACCAGCGCGGAAGAGTTCGTCACTATGCG

TTAAGGGCAGATGAAGTTGGCCTGCCACGAGCAGCCGGCTTCTACAGCCATGTAATCCCTTCATCCAAAC
CCGGCTCAGGCCGGGTTTTTTGATCACTCCTCGCTATTTCTCTCGCAAATCCGTCGTTCTTTACGCCGAT
TTTACGCCTGCCACATGGCGCTTTATGGCGCCTTTACGCCTGTTTTACATACCCTTTTCAGCGTCAATGA
CACCAACAGAGTCGATATGGTCTTACATCGGCTCTCGCAGCTAATTATCTCAATGGAGTCGATATGAATT
GGCTATATCTGCTGCTCGTCCTCGCCTTGATGGCCTATCTGATGCGCGCACTCACGCGCAGTCATGAGTG
AGGGAGTACCTATATGCAGCAAGAAATTATCTACCCGGTGGCCAGCAAACTGCCGAGCCTCGTGAGCAAT
CCAATGCGTGCCCTGACGCACCGTGACCACGAGTGAGGAACTAGAACATGTGGCAAGAAATAATCTATCC
GGTGGCCTTCGTGCTGCTGGTTCTGCTGCCGGCACCGCTGCTGGGCAACTACATCTACCGGGTATTTGAA
GGCAAAATCCGCTGGTTGGCACCGGTCGAGCGCGCCACGCTGGCTTGCTGTGGCACCGATGGACGGGAGC
AGGACTGGAAGTCCTACGCCCTCAGCCTGCTGGCCTTCAATGGCGCCGGTTTCGGTCTGCTGTTCCTGAT
CCTGATGGCGCAGGGGTTGCTGCCCCTCAACCCGCAACAACTGCCGGGTCTGAGCTGGCAACTCGCCTTC
AACACCGCAGTCAGCTTTATGACCAACACCAACTGGCAAGCCTATTCCGGTGAGGCCAGCCTCTCCTACT
TCAGCCAGATGGTGGGCCTGACCACCCAGAACTTCGTCTCCGCAGGGACGGGCGCCGCCGTCGCCATTGC
CCTGTTCCGCGGCATCGCCCGCCAGCAGACCATCAACCTTGGCAACTTCTGGCAGGATCTGGTGCGTTTC
TGCCTCTATGTGCTGCTGCCCATCGCGCTGATCATGGCGCTGGTGCTGGTATGGCAAGGTGTTCCGCAAA
GCCTCTCCGCCTATCTGTCGTTCCACGGTGTGGAAGGTCAGGAGCAGCTGCTGCCCCTCGGCCCGGCTGC
CTCGCAGATCGCCATCAAGCAACTGGGCTCCAACGGCGGCGGCTTCTTCGGCATCAACTCGGCACATCCG
TTCGAGAACCCGACCGCCCTCTCCAACTGGCTGGAGATGGTCGCCCTGCTCACCATCGCCGCCGCCATGG
TCTTTACTCTGGGCCGCTATGTGAAGGACATGGCCCACAGCCGCGCCATTCTCGGTGCCATGACCCTCAT
GCTGGTGCTGGGGCTCTTCATCTCCCTCTCCCAGGAGCTGAAACCGGATCCCGCCCTCGCCCAGCTCACC
ACTGACGCCAGCAACATGGCTGGCAACTGGGAAGGCAAGGAGAGCCGCTTCGGCCCTGTGCTCTCCAGCA
TCTGGGAAGTGGCGACCACAGCCGCCTCCAACGGAGCCGTCAACGCCATGCACGACAGCTTTACTCCGCT
CGGTGGCATGGTGGGCATGATCAACATGCTGCTGGGTGAGGTCATCTTCGGCGGGGTCGGCTCCGGTATC
TACGGCATGATGCTGTTCGTGCTGCTGACCGTGTTTCTGTGCGGCCTGATGGTGGGTCGCACCCCGACCT
ATCTGGGCAAACGCCTCGGTATCACCGAGATGAAGTGGGTGGTCGCCAGCATGCTGGTGATGCCGGTGGG
GGTTTTGGTGATCGGAGGTGTCACCCTGTTGATGCCAGATGCCAGCACCATCATCGGCCATGATGGCCCG
CACGGTCTGTCACGCCTCATCTATGCCTATGCCTCGGCTGCCGGCAACAACGGCTCCGCCTTCGCCGGTT
TTGCCGCCGGTGACAACTGGCAGTGCATCGCCATCGGTCTGGCCATGCTGCTCGGCCGCTTCGGCTACAT
CATTCCGGTACTGGCCATCGCCGGTCAGCTGGCCCGTGCGCCCCGTCAGGAGACGAGCGAAGGGGACTTC
CCCATCAGTGGCCCGCTGTTCGTTACCCTGCTGATCATCACCGTCCTGCTAATCGGTGGCCTCTCCTTCC
TGCCGGTGCTGGCGCTGGGTCCGGTGGCCGAACATCTTAGCCTGATCGGAGGGGCATTCTGATGAACCTG
ACCCTGTCGATCACCATCCGGATGCCAATGGCCAACTATCTGATGCAGGGAGCTTTCCAATGAGCAAGTC
CACTGCAATGAGCACCGACACCATGTTGGTGAAGGGCAAAAAACAGAAATCACAGATCATCCAGGCAATG
GTTGAGGCCCTTTATCGCTTCAATCCCAAGGCGCTGTTTGGCAGCCCCATCCTGTTCACCCTCTGGCTGG
CCGCAGTCATGGCGACGGTGGAGTCCCTGCTGGGGCAGCCGCTCTCCGGCGTCGCCCCTTCGCTGGCCTG
GCAGTTGACCGCCTGGCTCTGGCTGACCCTCTGGTTTGCCAACTTCGCGGAAACCCTGGCCGAGGGGCGC
GGCAAGGCTCGCGCCGACAGCCTGAAAGCGGGCATGAGCCAGCTCAAGGCCCGCCGGGTGGCTAACGCAA
AAGATGGTCAGGGCGAATGGGTACCCGCCACCAGCCTGATGAAGGGGGACCTGGTGCTGGTACGCAGCGG
CGAGATGATCCCGGCCGACGGCGAAGTGATCGCCGGCATCGCCTCGGTCAACGAGGCGGCCATCACCGGT
GAATCCGCCCCCGTCATCCGCGAGTCGGGTACCGACCGCAGCGGCGTGACCGGCAACACAACCGTGGTCT
CCGACGAGATCTGGGTGCGCGTCAGCAACAATCCGGGCGAGAGCACGCTGGATCGGATGATCGCACTGGT
GGAAGGGGCCAAGCGCCAGAAGACCCCCAACGAGATGGCGCTCGACGCCCTGCTGGTGGGCCTGACCCTG
ATCTTCCTGTTGGTGGTCGCGACCCTGCCCTGGTTCCTCGACTACAACGGCACTCAGGTACCGCGTCTCT
ACCTGATCGCTCTCTTTATCACTCTGATCCCGACCACCATCGGCGGCCTGCTCTCCGCCATCGGCATTGC
CGGTATGGATCGATTGGTGAAGCTGAACGTCATCGCCAAGTCTGGCCGTGCCGTCGAAGCGGCCGGTGAT
GTGCGCACCCTGCTGCTGGACAAGACCGGCACCATCACCTTCGGTAACCGGATGGCGGATGAGCTGATCC
CGGCACCGGGTGTCGACCCCGCCCTGCTGGCACAAGCGGCCATGCTGGCCTCGCTGGGTGACAACACCCC
GGAGGGCAAATCCATCCTGACTCTGGCCAGCAAGAGCATGGCCAAGCCGAGTCAGCTGGAGAGCGACAAG
GTGATCCCCTTCAGCGCCGAAACCCGGCTGAGCGGACTGGATCGCAACGGTCATCAATATCGCAAGGGTG
CGGTGGATGCCGTGTTGAACTACCTCAGCCTCGACCGCAAGGCGGTGCCTGAGCTGGTGCTCAAGTCAGT
CGACAGCATCGCCCGTCAGGGTGGACTCCGCTGCTGGTCTGCACTCAGGAGAAACTGCTGGGGGTGGTC
TACCTCAAGGACATCATCAAGCCCGGTATCAAGGCTCGCTTCCAGATCCTGCGCCACATGGGGATCCGTA
CCGTGATGATCACCGGTGACAACCCGAAAACCGCCGCCGCCATCGCCGCCGAAGCCGGGGTGGATGACTT
CATCGCCGAAGCGACGCCGGAGAAGAAGCTGGCCTATATCCGTCAGGAGCAGGCCGATGGTCGTCTGGTC
GCCATGTGCGGTGACGGCGCCAACGACGCACCGGCGCTGGCGCAGGCTGACGTGGGGCTGGCCATGAACG
AGGGCACCCAGGCCGCCAAGGAGGCGGGCAACCTGGTGGATCTCGACTCAAACCCCACCAAACTGCTGGA
TGTGGTGCTGGTCGGCAAACAGCTGCTGGTGACCCGTGGCGCCCTGACCACCTTCTCCATCGCCAACGAC
GTGGCCAAGTACTTCGCCATCCTGCCAGCCCTGTTCATCGCCGCCTACCCGCAACTGGGGGCACTGAACC
TGATGCAGCTGGGCAGCCCGCAGAGCGCCATCCTGTCGGCCATCATCTTCAACGCCCTGATCATTGTGGC
GCTGGTGCCGCTGGCACTGCGCGGTGTCGACCTGAAAGGCTCGGCCGCCAGCCTGCTGCGCCGCAACCTG
CTGATCTACGGTGTCGGTGGCCTGCTGGTGCCGTTTATCGGCATCAAGCTGATCGATCTGGTCATTACCG

GCCTGGGTCTGGTGTAACGGCATAACCGGGGTGCGGGTCAGCCCGCATCCACACAACGAATTCGGAGCAT
ATCCATCATGATCGCAATCAGAACCCTGCTGACCCTGACCCTGTTGCTGGGAGTGGGTTATCCGCTGGCC
GTCACCGGACTGGCCCAGCTGCTCTTCCCCTGGCAGGCCAACGGCAGCCAGCTGACCGACAGCAAGCAGC
AACTCATCGGCAGCGCCCTGCTGGGACAGAAATTCGAGCGGGCTGACTACTTCCATCCCCGCCCCTCAGC
CAGCGATTTCAACACCATTGGCGGCAGCGCCAGCAATCTGGGGATGAGCTCACCGCTGCGGGCAGAGTTT
GCCGAACAGGCAGCCAAGGCACAGCCGGGAACCAGCTCCCCCGCCATGCTGACCCGCTCCGCCTCCGGCC
TCGATCCCCACCTGCCGCTGGATGCGGTGCTGGCTCAGGTCGACCGCGTCGCCAAAGGACGTGGACTGGA
TCCCGCCCAACTGACGGAGCAGGTCAAGTCGGCCACACAAGCTGGTATCCTTGGCCCGCAGGTGGTCAAC
ATTCTCCAGCTGAATCTGCAACTGGATCATAAAAAGGGCGCCTGATGGCGCCCATTCCGGTTTGGTGATG
ACCTCGATTTTCGCAACGGGCGGTTGGCTCGATAGGGATAACAATGGCTGATGTAGCAAGCGCTGACGCC
CTGCTGGCGAGTCTGATGCAGGAGCATGGGGATGCGTGATGAGGTAAGGGCCGACGCCCTGCTGGCGAGC
CTGGAACAGGAGCACAAGGGCAAGCTGAAGGTGTTTCTCGGCGCCGCTCCCGGCGTCGGCAAGACCTACG
CCATGTTGCAGGCGGTGCGGGAACAGGCCAAGGAGGGAGTCGACATCCTGATCGGCGTGGTGGAGACCCA
CGGCCGTCAGGAGACGCTGGCCCTGCTCGACAAGCTCGCCATCATGCCGCGCAAGATCCACATGCACCGC
CAGCAGCGACTGGAGGAGATGGATCTCGACGGCCTGCTCGCCCGCAAACCGGCCATCGCCGTGGTGGACG
AGCTGGCCCACAGCAACGCCCCCGGCAGCCGCCACGAGAAGCGCTGGCAGGATGTGGAGGAGCTGCTCAA
CGCCGGGATCGACGTCTACACCACCCTCAACGTGCAGCATCTGGAGAGTCTCAACGATCTGGTGTGGCAG
CTGACCGGGGTCAGGGTGAAAGAGACCCTGCCCGATCAGGTGCTGCTGGAAGCCGACAGTCTGGTGCTGA
TCGACCTGCCCCCCAAGGAGCTGCTCGGCCGCCTCAAGGAGGGCAAGGTCTATGTGCCCCATCAGGCCAA
GGCTGCCCTGCAATCCTTCTTCTCCCTCAACAACCTCACCGCATTGCGGGAACTGGCGATGCAGACCGCC
GCCAGCCATGTGGAGGGCGATCTGCAACTGCAGTGGCAGGCAGCAGGCAAGAGCACCTTGCCGCTGCGGG
GCAAGCTGATGGTTTGCCTCGGTGACCTGCACGGCTCGGCGCTGGTGCGCTACGGCCACCGCTTTGCCCA
GCGCCGTCAGCTCCCGTGGCTGGTGGTGCATGTCGACAGCAATGGCCGCCGCTCTGCCGATGTGGAGCAG
GCGCTGGCGCTCGCCTCCCACCTCGGTGCCAAGGTGCTGACGCTATCCAGCCCGGCGGTCGCCAACACCC
TGCTGGAGACCGCCGAACAGCAGCAGGTCTCCCAGCTGCTGCTCGGCAAACCCCACAAGGCACCGTGGCG
CCGCTCGCTGGTGCAACACCTGCTCAAGCAGGCGCAGGGGCTCGAAATCACGCTGGTCGATACCGAGAAG
CAGAAGCCCGAAGTGGGAATGCGCGTCCCGACCCCGAAGGATATTCACCACAGCCTGCTGGCGGTGACCA
TCATGGGGATCACCTCGGCCGCCGCCTGGGGGCTCTCCTACTGGCTGCCACCCGCAGCGTTGCCGCTCAT
CTTTGTACTGGGGGTGCTGGCGACTGCCCTCACCACCAGTCTGGTGCCCGCCACACTGGCGGCGGTGCTG
GGCTTTATCGCCCACAATCTGCTGTTCACCGCCCCCTACTTCTCCCTGAGCGTCGCCAACCACAGCGATC
TGCTCACCCTGTTTGTGCTGCTTATCGTCGGCATGACGGCGGGACGGCTCGCCTCCCGCCAGCGGCAACA
GCTGATCGGTCTGCGCGAGACCCAGCAGCTGGGCAATGCCCTGCTCTCCCTCAGTCAGGGGCTGGCCACC
GCCAGCAGCCCGGAGGAGGTGCTGAGACAAGGTGCCCAGGCCATTTCGCTGGCGCTCGGCCAGCCGACCG
TGGTGATCGACAAGGAGTTCAACGATCGGCTCGGCAACGAGAAGCACAGGCCCGGCCAGACCGACAAGGC
GGCTATCGACTGGTGTCTGTCCCAGAAGCAGAGCGCCGGGGCCCACACCGCCACCCTCAATGCCGCCGAG
GCCCAGTATCATCATCTCAGCGACGATCTGGTGATCGGCATAACACTGGCCAATCCGCTCTCCTCCTCTG
CCGAGCATCAGCTGCAGGCGCTGCTCACCGATATTCGCGCCGCGCTGGCGCGGATCGACCTCAACGAGCG
GCTGGCCGACAGCCAGCTACAGGCGGAGACCGACCGGATGCGCGCGGCCCTGCTCTCCTCGGTCTCCCAC
GATTTGAAGACGCCGCTGGCCACCATCATGGGGGCTGGCAGCACCCTGCTGGAATATGGTGACCGGATCC
CCGCCGAAGACCGCGACGAACTGCTCCACTCGGTGCAGGAGGAGGCGCGCCGCCTGCACAGCTATGTGCA
GAATCTGCTGGATATGACCCGTATCGGATCCCCCGACTTCCAGCTGAAACGGGACTGGGTCGATCTGGCG
GATCTGGTGGAGAGCGCCCGCAAGCGGCTCGACTCCTCCTGGCGCCAGCACAAGCTGCTGGTCCATTTCG
ATCAGCATGTGCCCCAGCTCTATGTCCATGGTGCCCTGATCGAGCAGGTGCTGGTCAATATCCTCGACAA
CGCCTCCCGCTACTCACCGGCAGGCACACCCATCGAGTTCAAGGTGATGGTGGCCGAACCCGACCTCATC
ATCGACATCATCGATATCGGGGTCGGTATCGCCGAGTCGGATCGGGAGAAGATCTTCAACCTCTTCTACA
CCCAGCCGGTTGGCGATTGCGGTAGCCGTGGCACCGGGCTTGGCCTCGCCATTTCCCGCGCTATGATAGA
GGCCCACGGCGGCAAGATTTGGGCCTTCTCCGGCCCCAATGGCAACGGCACCTGCATGCGCATCTCCCTG
CCGCTCGCACTCAATTCACCGGAGGTTTGATGGCGCACATTCTGGTCATAGATGATGAAGCGGCGATCCG
TCGCTTTCTGCGGATCAGCCTGATTGCCGAGGGCCATCAGGTGAGTGAGGCGGTGGGGGTCAATGAAGGG
CTGGAGCAGTATCGCCACCTCAACCCCGATCTGGTGATCCTCGATCTCGGCCTGCCTGACGGCGATGGCA
TTCAGGTGTTGCAGGCCATTCGCGAACACCACCCCAACCCGGTGCTGGTGCTGTCGGCCCGTGATTCGGA
GCAGGAGAAGGTGCGGCTGCTCGATGCCGGCGCCAACGACTACATGAGCAAGCCGTTCGGCATCGGCGAG
ATGATGGCCCGCATCCGTGTGCTCTTGCGCCAGCGGGCTGGCGTCAGCAGCGGCGAGCGGCGCCACTTCC
CCCAATGCGGCCTGACGCTGGATATCAGCAGCCACAAGGTGACCATGAGAGAGGAGGAGATCCCCCTCTC
CCGCAAAGAGTTCGCCCTGCTGCAGGCGCTGGTGTGCCACCCCGGCAAGCTGGTGATGCAGACCCAGCTG
CTCAACGACATCTGGGGGCCGAGCCACAAGGAGGACACCCACTACCTGCGCATCGTCATCGCCAGTCTGC
GCAAAAAGCTGGGGGATAACCCCCAGCAACCGACGCTTATCGAGACCGAATCAGGTATCGGCTACCGCTT
TATCGGCAACTGATGTTTCACGTGGAACGACGCCCACTGCAAATCAGCGCCAATAGACCGCAGCCACTCA
GCAACAGGCCGAGATCCTGCAATCCGGCCGCTACCGCCAACCCCAGACTGAGCAGCAGGTAGTAGCCAAG
CCCGAACAGGGCGCCAGCGCTGCCTGCCACCTCGCGATAGGCCAGCAGCGCCTGACTCAGCACATTGGGG
ATCGCCAGCCCAAAAGCGATGACTACACCCATCATGGGCAACAGGAACCAGAGGCTGGCCTGAGTCGCCC

ATACCAGCAGCCCGGCAACCAGCGCCAGAGCGCAAGCCAATCTGACCAGAGATGACGGCAGCCACCCCCT
CCCCAGCAGATGCTTGTTGAGCAGGCTGCCGAGCAGAGTCGCCAGCGCCAGCAAGATCCCCGAATAGCCA
AACTCGCCGGTGCTCAATCCCAATCCGGCAAACAGGAAGGGGGCCAGACTGTAGTAGCCAAACAGCATAG
TGTTGAACAGGGCGACCAGCATGGCACTGCGCCAGAGTCCGCCATCCTTCATCATCCGACACGCCAGCGG
CCAGAGCGCGACTCGCTGGCTGGTGGCCGGACGGGTTTCCGGCAACTGCCAGCAGGTCACCAGCAGCAAA
CCTACCGCCAGGACCAGCAGACCGCTGAACACTCCGAGATAGCCGAACCCGCTCGCCAGCTGGCCACCAC
TGAGCAGCCCCAGCACTGGGCTCAGTGAGAGCGCAATGCCCATCACCGAGAAGACCCGCGCCAGATCGCT
ACCCTGATAGCTGTCGCGCAGCATGGTCTGGGTCACCACAGAACCCACGGCGGCGCCAAAAGCCGAGATA
ACCCGGGCCACAAGCAGGGTTTCAAACTGATTAGCCAGCCGCGCCAGCAGGGTGCCGACACCATAGGTGA
GCAGACCCGCCAGCATGGCGGGACGCCGCCCAATCAGATCGCAGAGCCGCCCCCAGCAGACCACGCCTAC
CGCAAACGCCAGAAAATAGACCGACAGGGTCTGGGATGCCTGCCCTTCGCTCACCCGAAACTGGCTGGCG
ATATGGGTCAGGACCGGGCTGTAGATGGTCTCCACAATTTGGGGAAACATCATCAATCCCACCACCAGCC
AGAGCGGTGGCAACTTGTTGTTCATGACAAACCTCCTTCACAAGAGGTCGCCATTCTAGAGAGATGCCGA
TTGTCTGATTACAATAAGCAGGACTATAAACATCAAAAATCGGACATATGGCCTTTATTCTTCCCGATCA
CCCTTTTGAGCCCGACCAGCTGACGGGCAGCGTGATCGGCATTGCCTCCGCGCTCGGTTGGCACGACTCG
GGCCTGCATCAACATCAACGCCATCAGCTGCTGTTCGCCCAATCCGGCTGCATGACCATGGAGCTGGAAG
GTCGGGTCTGCCTGCTGCCGCCCACCCGTGCCGCCTGGCTGCCTGCGGGCACTGCCCACCGGGTATTGAT
GCGTGGTGTGGTGGCCTACCGCTCACTCTACTTCCAGCCGCACCCAGAGTTGCCCGGCACGGTGGAGGTG
CTGGCGGTCAATCCGCTGCTGCACGAACTGATCGAGCGGATGGCGCTCTGGCCGTGGGACAAACCGCAAG
AGCAGCAGCAACGGACGGTGGCACTGTTTATGGAGGAGTTGGGGCAGGCGCCACGGGAGTCGTGGCAGTT
GCCGCTACCGACCGATCCCAGACTGGGTGACTGGCTACAGCAGTTGAAACGGGGAGATGCCCTGCCCGAT
CGGCTCAACCGGCTGGCCGAACGGGTGGGTGCCAGTGACAAGACCATTGGCCGCATCTTCATGCGGGAGA
CCGGCATGAGCTATCAGGCGTGGCGTCAGCAGTGGCGGCTGTTGCGCGCCATGGAGCTGCTGGCCGAAAG
CGAACCCATCAGCCGGATTGCCGCTGCGCTGGAGTTCTCCAGCGACAGCGCCTTCATCAGCTTCTTCAAA
CAACATACGGGACAGACCCCCCTGCGCTACCTCAACTCTCGGGGTGAGTCGCCACAGCCTGACTCGCCTC
CACCACCAGGGTATCCAGCGCCAGCGGCTTGAGATTGTGCAACGCATCCTGCACCACGGCATGAGATGCC
TGTTGCAACTCGCTCTGCACATCTTGCTCCAGTTGGGCACTCTCCAGCTCGGGCCGCGGGTTTTCGTTGG
CCATCACGTTCAGGCTCAGGGCACTCACAATCAGGGTAGCGATCAGGCTTTTCATCTGGTTTGCTCCGGT
ATGGTTGAGATGACCAGATAGCTATTCCAATAGTTGTGCCAATCCTCACGCAATTCATAACATTATGATT
TTGTTTAAATTAACTATATATCGATACCAAATTGAGTCGCAAAATGCATCCCGCTCGGTCATTTTTAAAC
CAACAGTTGGTGCTTTTAACCACGCTTGATTTTTGCACCATGTTCCACGTGGAACGTCCCATCTTTTTGA
TCGACACCCGCTTTTAAACTGAGCTATGGTGCTACATCCCTGTAACTCAAATCTGGATCTGTCACTGAAT
GGAATTTTCCTTGTTCGGCGAGAAGTTCACTCGCCACGCCGGTATTACCCAACTCATGGATGACCTCAAT
CAGGGGCTGACCAATCCGGACGCCATCATGCTGGGTGGCGGCAATCCGGCCCCCATCCCCGCTATGCTGG
AACGCTTTCAGGCCGAGGCCAAGAGCCTGCTCGACAACGGCGAGCTGGTCAAAGCGATGGCCAACTACGA
CGGCCCACAGGGCAAAGACAGGTTCACCAAAGCGCTGGCTGCCCTGCTCAGCAAGGAGTTGGGATGGGAT
ATCTCGGCCCGCAATATCGCCCTGACCAACGGCAGTCAGAATGCCTTCTTCTACCTGTTCAACCTGCTGG
CTGGCGAATTTGCCGATGGCCGCAAGAAGAAGGTGCTGTTCCCGCTCGCCCCTGAATACATCGGCTATGC
CGACTCAGCGCTGGCCGACGACTACTTCGTGGCCTACAAGCCCACCATCGAGAAGCTGCCCGATGGCCAG
TTCAAATACCACGTGGATTTCGAGAGCCTGCAGGTAGGTGACGATATCGGGGTGATCTGCGTCTCCCGCC
CGACCAACCCGACCGGCAACGTGCTGACCGACGAAGAGATCGAACACCTCGACCAGATCGCCCGCGACAA
GGGAACCCCGCTGCTGATCGACAACGCCTACGGCGTTCCCTTCCCGGGCATCATCTTCAGCGAAGCCAAG
CCGTTCTGGAACGCCAACACCATCCTCTGCATGAGCCTCTCCAAGCTCGGCTTGCCGGGCACCCGCTGCG
GCATCGTCATCGCCGACGAGAAGATCATTCAGGCCATCACCAACCTGAGCGGCATCATCAATCTGGCGCC
GGGCAGTCTGGGCCCCGCCATCACCATTCCGATGATCGAGAGCGGCGAGATCATCGCCCTCAGCGAACAG
GTGGTGAAGCCCTTCTATCAGCAGAAGGCGGAGCTGGCGGTGCAGCTGCTGCGCGAAGCGATCCCTGACC
CGCGCTTCCATATTCACAAACCGGAAGGTGCCCTCTTCCTCTGGCTCTGGTTTGAAGGCTTACCGATTCA
CTGTCAGGAGCTGTACGAGCGACTGAAGGCGAAGAACCTGCTGATCGTGCCGGGTCACTACTTCTTCCCC
GGCATCGATGACCCCGAGTGGCGCCACAGTCAGGAGTGCATCCGCCTCAACTACTCTCAGAGCGAGGAGC
TGGTGCGCCGCGGTATCGCCATTCTGGCCGACGAGATCAACGCCCTCTACGCTGGTTAATCCAATCCGGC
GATACAAAAACAGAAAGGCCTGCAGATGCAGGCCTTTATTGTTTATCGCGATCCGGTTTGCTTAGCCGGG
CAGCGCCAGCTGCTCGGGTTTGCCGATGGGGATGGCTCTGGGCTTGAGCGCCTCCGGCAGCTCGCGAGCC
AGATCGATGGTCAACAGACCATGCTCCAGCTTGGCGCCCTGCACTTCCACGTGCTGGCTCAGCTTGAAGG
CGAGCTCGAAGTCACGCTCGGCAATTCCCTGATGGAGGAACTGACGCTCACCGGCCGCAGCCCCTTTGCG
CCCCTTCACCTTGAGGGTGCCGTTTTCGGTCTCCAGCTCCAGCTCTTCGCGCTCGAAGCCAGCTACGGCG
ACCGTGATGCGATAGCCATCCTGATCGCGCTTTTCAATGTTGTACGGGGGGTAAGCAGCGGTCTTCTGAT
CGAACAGACCTTCCAGCTCACGGGCCATCCGCTCGAAGCCGACGGCGTTGGAATAGAGATGAGAAAAATC
GAGGTTACGCATAATCCTATCCTTCTAATAAGCAACAGGTTCAACTCTCCACAGGCCCGTCATCGGCACC
CGGGAAATTTTAAAATTTCAGTAAATTCAGGCTATCAGCCGTTGATCTCGATCTTGCGCGGCTTCATCGC
CTCGGGCACTTCACGCACAAGCTCGATGTGCAGCAAGCCGTTTTTCAGGTCGGCCCCTTTCACCCGCACA
TAGTCGGCCAGCTGGAAGCGGCGCTCGAAACCACGTTCGGCGATCCCCTGATAGAGATAGTTGCGCCCGG

TGTCGGCCTGCTTGTTGCCCTTCACCGTCAACAGATTTTCCTGGAAGCTCAGCTCCAGCTCTTCCTGGGT
AAAGCCCGCCACCGCCATGCTGATGCGGTAGTCGTTGTCACCCAGCTGCTCGATGTTGTAGGGGGGATAA
CCGGCATTGCCATTGCTGGCCGCAGATTCGATGAGATTGGCCAGACGATCGAAACCGATGGCAGAACGAT
AGAGCGGAGAAAAGTCGATAGAACGCATAGTATTACCCTCTTATGAGCGATAACGGATTGTTTGCCCTCC
TTATGGACGGGCGCCTGGTCGAAACCTCTGTCGAGCGTTCCGGCCTTGTTACCTATATAGGGGCTCACTT
TTTCCTTTCAAGGGGATTTTTTCAAATTTTTTAAATGGCCGCGCCGACCTGTAGAACATCAACCTGTTGG
CCCGATAGTGATGATAATCGACCCGTGGCCACTGATGGCAATGCTCTCCGCACCCCATAATTGGGTGCAT
ATACACCAGCCAACATGGAGCCATCACATGAAGATTACCCAGGTTCGCAACGCCACCTTGCTGCTCGACT
ATGCCGGCACCCGCTTTCTGATCGACCCCATGCTCTCGGCCAAGGGTGCCTTCCCCGGCTTTGAGGGCAC
CGCCAACAGCCAGCTGCGCAATCCGCTGGTCGAGCTGCCGCTACCAATCGATGAGCTGGTCGATGTGGAC
GCCGTTATCGTCACCCACGACCATCCCGACCACTGGGATGACGCCGCCAGAGCCCTGATCCCCAAGCATC
TGCCGCTCTTGGTGCAACATCAGAAAGATGCTGATGCCATTCGTGCCAGTGGATTTGAGGATGTGCGCCT
GCTGAGCGACCAGCCCGAGTTTGCCGGCATTAGCCTGCAGCAGACCGGTGGCCAGCACGGCAGCGACCAG
TTGATGGCGGTACTGGGCGAGCGCATGGGAGAGGTGTGCGGCGTGGTCTTCTCCCACCCCGATGAACAGA
GCCTCTATCTGGCCGGTGATACCATCTGGCATCCCACGGTCGAGCAAGCCCTGCAACAGCATCAACCCGA
GGTCATCATCCTCAACTGCGGCGATGCACAGGTGCCCGGACTCGGTTCCATCATCATGGGCAAGGAGGAT
GTCGCCGCCGTTTATCAGGCCGCACCGCAAGCCACGCTGATCGCCAGTCATATGGAGGCGGTCAACCACG
CCGTGCTGTCACGCCAGGAGCTGCGCGACTATCTGGTGGACCGGGGCATGATGGATCGGGTACGGGTGCC
GGAAGATGGCGAGGCGATATCGCTGTGATCCGCAGAAGGGCCATGCCAGACATGGCCCTTCGGCTTTCAC
TGCCGAACGCTTATGATGAGAGGTCTGCGCGCAGAGAGGAGTCAACATAATGTCCACCCCGATCGTTGCC
GTGGTGACCTTCGATTACTTCAGCCCTTTTCACTGCGCCGTCCCCTGCCTCATCTTCGGCGACATCCTGC
CGGGCCAGCCACTGTTCGAGCTCAAGATCTGCTGCGGTGATGGCAACCCTCATTCACGCTCGGCGCAAGG
CTTCACCATTCAGGCGCCCCATGGGCTGGAGGTGCTGGCCGAGGCGGAGATCATCGTCATTCCCTTCTGG
CACGACCCGGCAGCACGACCACCAGCACAGCTGCTCGATGCCCTGATTGCCGCCAGAGCCAGAGGTGCCC
GACTGGTCGGGCTCTGTCTCGGCACCTATGTGCTCGCCTACGCCGGGCTGCTCAATGGTCGCAAGGCATC
GACCCACTGGGAGTATGAGCAGGATTTCTGCCACCGCTTTCCCTGCGTGCAGCTCGATACCAACGCCCTC
TATGTGGATGACGAAGGGCTGGTCACCTCGGCAGGCACCGCTGCCGGGCTGGACTGCTGCCTCTATCTGG
TGCGCCAGTACCACGGCTCAACCATCGCCAACAAGGTGGCGCGGCGGCTGGTGATCCCGCCCCACCGGGA
AGGGGGTCAGGCCCAGTTTATCGAGCAGCCGGTGCCCGCCTCCACCCGCGATGTCCGGATGAACCAGTTG
CTCGACCATCTGCGTGCCCATCTGGCCGCCCCCCATACCCTGGATTCTCTGGCAGATCTCACCCTGATGA
GTCGACGCACCTTTACCCGCCGCTTTCATCAGGCTACCGGCCTCTCGGTGGGTGAATGGCTGCTGGCGGA
GCGGCTCAGGCGCAGTCAGGAGCTGCTGGAGGTAAGCGGCCATTCCATCGATCGCATCGCCGAGCTGGTC
GGCTTCAAAAGTGCCACCTCTCTGCGCAATCACTTCCGCTCCCGCTTTACCATCTCCCCCAGCGAGTGGC
GACGATCTTTTCGTGGCAACAATGTCTGATGACATCCTCTATACCCGCTCACTCCGCTATGGCAGGCTAA
CCAGAAATCCCGAGGAGTCATCCCATGTCCCGTTCCGAACGCCTGCTGGCCCTGCTCCAGCTGCTGCGCG
GCCACCGTTACGCCATCACTGGCGCGGAACTGGCCAGCCAGCTAGGCATCAGCCTGCGCACCCTCTATCG
GGATATCGCCACCCTGCAGGCACAAGGAGCCCATATCGAGGGCGAATCGGGGATCGGCTATGTGTTGCGT
CCTGGCTTCATGCTGCCACCACTGATGTTCAGTGAAGAGGAGCTGGATGCGCTGGTGCTTGGTTCGCGTT
GGGTCGTCGCCCACGGTGACGGAGCCTTGAGTGCATCGGCCAGTAGTGCGCTGGCCAAGATAGAAGCCGT
GCTGCCCGCAAACTATCGCCAGCAGCTCCAGAGCAATGCCCTGCTGATCGGCACCTCCCGCAGCGACCGC
CTCACCGATGAAGCCCCGCTGCGCTGCGCCATCCGCCAGCAACGCAAGGTGACCCTGAGTTATAGTGACC
TCAAAGATGAGGCCTCTGAGCGTACCGTCTGGCCCTTTGCCCTCGGCTACTTCGAACAGGTGCGGGTACT
GGTAGCCTGGTGCGAACTGCGTCAGGGCTTTCGCCACTTTCGGGTAGATCGCATCACCCGGATGCAACCG
CAAGATTGCCACTATCCCAAAAGCCGGGAGAGCCTGCTGAAGGAGTGGCGCACCACCCAGCAGATCCCGC
AGCCGAAATTCATTGCTGACAGAAACTGACAGCAGGTAGCGACATCCTGAGTGGGTCGGCGCAGATGTGC
TATTTCATCTGTTAGGTCATATACATCGAAACGAGGTTGCTCAAAAACATGAACAATAAAAGTGGTTATA
TCGGCGTCAACGGTGGTGAGTTGTATTACGAACTTTCGGGTAATCCTGATGGTGAGCCTTTATTGATGCT
TCATGGTGGCCTTGGTTCCCTTAATGAACTAATACCCATTCAAAGATATGTTGCCATGGATTATCAGTTG
ATCAGTGTAGATTTCCGTGGCCATGGAAAATCCCCTCTAGGTAGTAAGCCACTGAGTTACTTGCAGTATC
AGCAAGATATCCAAACACTCCTCGACTATCTGGGCGTTGATAAATATTCAATATTTGGCTTTAGTGATGG
TGGTATCGTAGGTTATCGACTAGCCGTACAAGAACCCGATAGCGTTTCTTGTTTGGTTACACTTGGTGCA
ACGTGGCGATTAGAGCCTGATGATCCATCACTCGGGTTGCTAAGCGGCTTAACAGCAGACTTTTGGATTT
CAAAGTTTGCCGATGATGTCGCGCTATACGAGGCGTCAAATCCCGAGCCTGATTTCCCAAAACTGGTCGA
TGCTGTAAAGGCTGTTTGGCTTGATACAACACCATCTGGCTACCCGAGTAACCTAGTTGAAAAAATTCGT
TGTCCGACATTAATCATGAGGGGTGATAACGATGTCATATTTTCGCTTGAGGAGTCTGTGGTTTTAAAAG
CAAAAATCGCAGATTCCAGTTTTGCCAATATTCCACTCGCGGCTCACTCAGCTCATCAAGAATCGCCGGA
ACTGGTAGGTAAAATGCTTCAACAGTTCATGTCACAGCATAAAAACTATTAACTCTTCCATAGCACACTG
CATATAAAGAATCAGCTCTGTGCAAGTGAACTCATCATCAAAATGCCGAACATCAAATTCGGCATTTTCT
TTATCTCAGCCCGATCGCCCATCCTTTCAACTGCCAGTGTTTCACGTGGAACGTCGGGCGGCATCCGGCT
GGGCCGTCTGTGCTATCATCCCCGCTCCTTACTCTCTGGAGAAACAATGACCACTCGACATAATCCGCTC
GAACTGTACCAGCCGGACAACAGCTCCCCCGATAAACTGAGCGAACGACAGCTGATGCTATTTGCCCTAC

TACAACAGCGTGAGCAGGAGTTTGGCTTCAACCAGAACGTAATCGTCCACTACCCGGAAGCGAGCTTTCT
CACCGCTCCGCTCGATTATCTGGATCAGCTGATCCTGGAACAGCAGCAACAGGGTCACCTCATCAATGAG
GTGGTGATACTCACCACTTACCCTCAGGTGCACACCCCACTGGTCGAGCGTGACTATCTGCATGAAGCGG
TTCATATGCCCGCAGGTCCGGATTACACGGCAATCTTCAGATTGGATTATGGTGCTAATCCGGCAGGCAA
AACCCTCTATATCGAGGCCGTCAATGAGCAGGATCCCAAAATCCGGCCGACCTTTGTCTTGATGCTGCAT
GATGAGAATGGTGAACTGCAGGGAGGTATGTCCGGGTCTATCTACTCCACAGACAATCGGCGATACGCCT
ATATCGGCACTGTGGTGGTGCAACCTGGGCAACCCGCAAGAACCGGCACCCTGCTCGCCAATACCGTGCT
GGAGTACCTGACGCAGCAAGGGGTTTACGAGGTCAATCTCGGTACCCAGACCGCCGAGCCCTTCTATGCC
AAACTGGGCTTCCAGACCATTCACCACATAGTGCCAGCGTTGCGTCACCGCACAGCCAGCGATGGCGCTG
TGCTACCCCACAACCTGGTCATCATGAGCAAAATGCTCTGATAAAGCAGACTACAAATGTAAAAAGCCG
CTCTTGTGAGCGGCTTTTTATTGCAATCAGCGAGTCTGATTAAACGTCGAGGTTGGCCACGCGCAGGGCG
TTGGTCTCGATAAAGTCACGACGGGGCTCAACGGCATCACCCATCAGGGTGGAGAAGAGCTGGTCAGCAC
CGACGGCATCGTCGATGGTGACGCGCAGCATGCGACGGGCTTCCGGATCCATGGTGGTTTCCCACAGCTG
GTCCGGGTTCATCTCACCCAGCCCTTTATAGCGCTGGATGTAGATGCCGCGTTTGCCTTCGCCCATCAAC
CACTCGACGGCTTCCACAAAGTTGGCAACCGGCTTCACCTTCTCGCCACGCTTGATGAAACCACCTTCCT
CGATCAGGTTGGCGATCTCCTTGCCCAGCGAAACCAGCTGACGGTACTCACCGGACTGCAGGAAGTCGTA
GCTGAGCGGATATTCGCGGTCAACACCGTGCTGACGAACCACGGCGTGCGGAACGTACAGGCTGCGCTCT
TCATCACGACGAACCTGGATCTGGTACTGGATACCGTGGTTGCTCTCGTTGAGCACGGCTTCCATACCCT
GACACCAGGCCAGCAGATCACTCTCGCTGTTCAGCAGCGCTTCGTTCAACTCCGGCTGATAGATCAGCTG
GGTCAGTACCGCTTCCGGCGCACGACGAGACATGCGGGTGATCAGGTGAGTCACCGAGCGGTGCTGGTTG
ACCAGACGCTCCAACGCCTCGCCACCGATAGCCGGGGCAGACTCGTTGACGTGCAGAGTGGCACCATCCA
GCGCCATGGCGGTCTGGTACTGCAGCAGCGCGTCTTCGTCTTTCAGATACTGCTCCTGCTTGCCCTTCTT
CACTTTGTAGAGCGGCGGCTGGGCGATGTAGACGTAGCCGCGTTCGATGATTTCCGGCATCTGACGATAG
AAGAAGGTCAGCAGCAGGGTTCGGATGTGTGCACCATCGACGTCCGCATCGGTCATGATGATGATGTTGT
GATAACGCAGCTTGTCCGGGTTGTACTCGTCGCGACCGATACCGCAACCCAGTGCGGTGATCAGGGTGCC
CACCTCTTGCGAGGAGATCATCTTGTCGAAACGGGCCTTCTCCACGTTCAGGATCTTGCCCTTGAGCGGC
AGGATGGCCTGGTTCTTACGGTTGCGACCCTGCTTGGCGGAACCGCCAGCAGAGTCCCCTTCCACTATGT
AGAGTTCGGAAAGAGCCGGGTCTTTTTCCTGACAGTCAGCCAGCTTGCCGGGCAGACCGGCGATATCCAG
CGCGCCTTTGCGGCGAGTCAGTTCGCGAGCTTTGCGGGCCGCTTCACGGGCACGGGCCGCATCGATGATC
TTGTTGACCACGATCTTGGCATCGCCCGGGTTTTCCAGCAGGAAGTCGGCCAGCTTCTCACCCATCGCCT
GTTCAACGGCGGTCTTCACTTCGGAAGAGACCAGCTTGTCCTTGGTCTGGGAGGAGAACTTGGGATCCGG
CACCTTGACAGAGATAACGGCAATCAGACCTTCACGCACGTCGTCGCCACTGGCGGCAGACTTGGCCTTC
TTGCTGTAGTCCTCTTTGTCCATATAGGAGTTCAGAGTACGGGTCAGCGCGGTACGGAAGCCAACGAGGT
GAGTACCACCATCCCGCTGCGGGATGTTGTTGGTGAAGCAGTAGACCCCTTCCTGATAGGCGTCGTTCCA
CTGCATCGCCACTTCAACGCCGATACCATCCTGCTCGGTGGTGAAATGGAACACCTTCGGGTGGATCGGG
GTCTTGTTCTGGTTCAGGTATTCAACGAACGCCTTGATGCCACCCTCGTAGCAGAAATGCGCCTCGCGGC
CATCACGCTCGTCTTGCAGACGGATGGAGACACCGGAGTTGAGGAAGGAGAGCTCGCGCAGGCGCTTGGC
CAGGATCTCGTAGTGGAACAGGGTATCGCTGAAAATGGCAGGGCTCGGCCAGAAGCGAACTTCGGTACCG
GTGCCGGTGCTGTCGCCAATCTGCTTGAGCGGCGCCTGCGGCTCACCCAGATGATAGGTCTGCTCGTAGA
CGTGGCCGTTACGACGAATGGTCAGCAACAGCTTGTCAGAGAGGGCGTTAACTACCGAGACACCCACGCC
GTGCAGGCCGCCGGATACCTTGTAGGAGTTGTCATCAAACTTACCGCCAGCGTGCAGCACGGTCATGATG
ACCTCTGCGGCGGAGCGGCCCTCCTCCTCGTGCATGTCTACCGGGATACCCCGGCCATTGTCACGCACAG
AGACAGAACCGTCGGAATGGATCTTGACCAGAATATCGGAGCAGTAGCCAGCGAGCGCCTCGTCAATGGA
GTTATCGACGACCTCGAACACCATGTGGTGCAGACCCGAGCCATCATCCGTATCGCCGATATACATCCCC
GGGCGCTTGCGGACCGCATCCAGGCCCTTCAGCACCTTGATATTCGAGGAGTCGTAAGTATTTTCACTCA
TAGCTTACTCTCTGCCTTCTCTAACGTTTCGGAGATTTTACCCTGTTCCACGTGGAACAACTTGCAGTCA
TTCGCATCCATCATGTCTGCGAGCTGACCGGTGTCGATGGCGGTGATAAACACCTGGGACGCACACTGCT
TCAGCCTTGCGGCCAGCAAGCGGCGCTTGTCGACATCCAGTTCAGAGGCAAAATCGTCAATTAAAAAAAT
ACAGCCACGGCTGCTATGTTGATTCAAATAGAGCCCTTGGGCCAATCGCATGGCGCAGACCAGCAACTTC
AGCTGACCCCGGGACAAAATATCCTGAGCGGGCACCCCGTTCGCCTTGAGGCGCACATCGGCTTTTTGTG
GACCGACACCGGTATAGCCCAGCGCCCGATCCCGCTCAAACCCCGCCTCCAGCAGATCGCCGAGGGGAGT
ATCTTTCTCCCAGCCCCGGTAAAAGCCGAGACTGATATCGAATTCCGGCAGAAAATCCGCCGTCATCTCC
TTGATCAGGGGCGTAATGGCCTGACAATAACTGGCGCGAAACTCGGCCAGCTCACCACCCAGCCGTACCA
ACTCCTGATCCCAGAACGCCAGCTGGCGGTACTGGGTGCTCTGGCGCAGCAGCGCATTGCGCTGTTTCAG
CAGCCGCCGCACTCGCCCCCACAGGGCAAAGAAGGTCGGCTCCTGATGGAAGACCCCCCAATCGAGCCAG
GCGCGGCGAGCCTGAGGCCCGCCAGTCAGCAGGTTGAAGCCATCGGGATGGATCAACTGCACCGGCAACA
GCTCCGCCAAATCAGCCAGCCGCTGGGCCTGCGCCCCGGCAATCTTGAGCTGGGTCTCGCCACTCTTGTC
TTTGGCAAGACCGATAGGTACCTGACGCCCCTCCAGCTCGCACTGGGCAAACAGGGTAAATGCCCTCTCC
CCCTGGCGGATAACCCGCCCGGTCAAATGAGTGCGAAAGGAGCGCCCCAGACCAAGGTAGTGAATGGCTT
CCAGCACGCTGGTCTTGCCGCTGCCATTGCACCCGACCAGAATGTTGAGGCCGGGCGACAGCTTGAGACT
GGCTTGCTGGATATTGCGAAAGTCGCTGAGCTGGAGTTTGACCAGGGACACGACTAGATCCGCATCGGCA

TCACCACGTACTGAGCATCCTGATGTTCAAAATCCTCAATAATGGCACTGCTGATGGAATCGCTCAGGCT
AATCCGAATTTGGTCACATTTTAACGTATTCAGCACGTCCAGCACATAAGAGACGTTAAAGCCGATCTCC
ATCTCGCCAGCGGCATATTGTACGTCAAGCAGCTCTTCGGCTTCTTCCTGTTCCGGGTTGTTGGCGGTGA
TGCGCAGCAGGTTTTCCTGCAGATTGAGACGCACCCCACGGAATTTCTCGTTGGAGAGGATGGCGGCGCG
GGCAAACGCCTGACGCAGATCCTCACGGCCGGCGATGAGCGCCTTGTCGCTGTTGCGCGGAATGACGCGG
CGCCAGTCCGGGAAGCGGCCATCCACCAGCTTGGAGGTGAAGATGAAGCCGTCCAGCGCAGCACGCAGGT
TGCTGCGACCGATCTGCAGACGTACCGGCTTGTCTTCCGCTTCCAGCAGACGCACCAGCTCCAGTACCCC
TTTACGCGGCAGGATAACCTGATGATCGGGCAGCGATTCCCCAACTACCTCGCGGCGGCAGGTAGCCAGA
CGGTGACCGTCGGTCGCCACGGTACGCAGACCGTGACCTTCGCTCTCGAACAGCATGCCGTTCAGGTAGT
AACGCACGTCCTGGCTGGCCATGGAGAACTGGGTCGCATCGATCAGCTTCTTCAGCTCCAGCTGGCTGAT
ATCGAACTCGAGCACGGATTCCCAATCCTCGATGTTCGGATACTCGGTCGCCGGCAGGGTCGAGAGGTTG
AAGCGGGAACGACCGGACCGGATGATCAGGCGCTCGCCTTCGGTATTGAGACGGATCTCGGCGCCTTCCG
GCAGACCACGGCAGATATCCAGCAGCTTGCGCGCCGGTACTGTGGTGCGGCCATCTTCGCTGGCCCCGTT
CAGATAGACCTGACCGATCATCTCTACTTCGAGATCGGTACCGGTCAGTGACAGCAGACCGTTGCTGACA
TCAAGCAGCACGTTGCCGAGGATCGGCAGGGTCGGTCGGCCGCCGAGGGCGCCGGACACCAGTTGCAGAG
GACGTAACAGGGCCTCACGGCTAATTTCGAGCTGCATCTGTGCTCTCCGTTGTGATTAGGAGGAAAAGAT
TCGGATCAGGTTGGAATATTCGCGCTGCATCTGTCCACTCGATTCCGTTATCAGGAAGAAAGGGTACGGA
TCAGGTTGGAATAGTCCTCCTTGATGTCGTGACTCTCCTCCTTCAGCTGCTCGATCTTGCGGCAGGCATG
AAGAACGGTAGTGTGGTCACGGCCACCAAAGGCATCACCTATCTCCGGCAAACTGTGGTTGGTCAGCTCC
TTGGCCAGTGCCATCGCCAACTGGCGCGGACGGGCAACGGAGCGGGAACGACGCTTGGAGAGCAGATCCG
CCAGCTTGATCTTGTAGTACTCCGCCACCGTCTTCTGGATATTGTCGATGGTGACCAGCTTCTCCTGCAG
TGCCAACAGGTCGCGCAGCGCTTCGCGCACGAAATCGATGTTGATGGCACGGCCGGTGAAGTTGGCGTTG
GCGATCACCCGGTTGAGGGCGCCTTCCAGTTCACGGACGTTGGATCGCAGACGTTTGGCGATGAAGAAGG
CCACTTCGTCCGGCAGATGGATCTGGTTCTCATCAGCTTTGCGCATCAGGATCGCGACGCGGGTCTCCAG
CTCCGGCGGTTCGATCGCCACGGTCAGGCCCCAGCCGAAGCGGGACTTGAGGCGATCCTCGACACCGTTG
ATCTCCTTCGGATAGCGATCCGAGGTGAGGATGATCTGCTGGTTACCTTCCAGCAAGGCATTGAAGGTAT
GGAAGAACTCCTCCTGGGAGCGCTCCTTGTTGGCGAAGAACTGGATGTCATCGATGAGCAGGGCATCGAC
GCTGCGGTAGTAACGCTTGAACTCTTCAATGGCGTTATTTTGCAGCGCTTTCACCATGTCCTGAACAAAC
CGCTCGGAGTGCATATAGATGACTTTGGCATCTTTTTTACTCTCCTTGATGGCATTGCCAACGGCGTGCA
GCAGGTGGGTTTTACCCAGGCCGGTGCCGCCATAGAGGAACAGCGGGTTGTAAGCACCACCCGGATTGTC
CGCTACCTGGCGGGCAGCCGCACGCGCCAACTGGTTGGACTTACCCTCGACGAAATTCTCGAAGGTATAG
TTCACGTTGGTATTGCTCTTGTGGTTCGGCTCGGGCTTGGCTTCAGCCTTGCTTTCCCAGCTCTTCTGCA
AGTTATTCACAGGCGCGGCACCCTGTACCGGCGCTCTGGCGACAGCAACAGGATGGGGACGATTGCCTAT
ATCAAAACGCAGGGCGGGGCCGTCGACCCCGCAGAGTTCGTTGATGATGCCGTTAACCCGGATGAGATAC
TTGTCTCGTACCCAGTCCAGAACAAAGCGATTCGGTGCATACAGGGTCAGAGTATTGTCACTCAACTCCG
CTTGAAGCGGCCGGATCCACATGCTGAATTCTGCCGAGGGCAACTCATCTTGCAGCCGGTTAAGGCACTG
CTGCCATAGCGAAGCAGTCACTCTAAACTCCTGTCGATTGATTAACTAAAGACCGGCTATTCTACGTTTT
TAGCCGCCGGATCTCCAGCCATCGCCCGGTGGATCCAGCAAATAAGATCCTTGACTTTCAAGGATCCAGC
GCCTTTGGATCGGATTTCATCCCCAAAGTTACCCACAGCTTGATCCACCGTCCGGATTGGTCGATCCCGT
GCTTCTCTCCTGTTATTTCATATAACCAAATGTCATTTATTTTTTAAATTTTATTCCAATAACATCAGGG
CCAAACCAGTTATTACAACTACAAATACAAGACATTGAGAGAGAGAAACACCATGAAAACATCCATCAAA
CTGATTTCCGGCGCAGCCATCCTGCTCGCCACCCTGTCCGGTCAGGCCATCGCCCAGGAGACCATCAAGG
TCGGCATGTCTGGCAAATATTTCCCGTTCACCTTCGTCAAGCAGGACAAGCTGCAAGGCTTTGAAGTGGA
TATGTGGAACCAGATCGGCGAGCGTACCGGCTACAAGGTCGAATTCGTGACCGCCAGCTTCTCCGGCCTG
TTCGGCATGCTGGAAACCGGCCGCATCGATACCATCTCCAACCAGATAACCATCACCGACGAGCGCAAGG
CGAAGTACGACTTCTCCCAGCCTTACGTCTATGACGGCGCCCAGATTGTGGTACGCAAGGGCAACAGCAT
CATTCATGGCATCAAGGATCTGGAAGGCAAGAAGGTGGCCGTGAACCTGGGTTCCAACTTTGAAGAGCTG
CTGCGCAAGAATGACCCGAACAAGAAGATCGACATCCGCACCTATGACTCCGCCTTCGAGCAGGATGTCG
CCCTGGGTCGCATCGATGCCTTCGTGATGGACCGCGTCTCCACCGCCCAGCTGATCAAGGAGTCCAAGCT
GCCGCTGCAACAGGCTGGCGCACCGTTCGAGACTATCGAGAACGCCCTGCCCTTCCTGAAGACCCCGGAG
AAACAGGCGCTGCTGAAGAAGGTCGACGAGGCCCTGACTGCCATGCGCAAGGATGGCTCCCTGCGTCAGA
TCTCCGAGAAATGGTTCGCTTCCGACATCACCGACAAATAAATGATGGAATTTGACCTCGAATACACGCT
GGGGCTGTTTCCCATCCTGCTCAAATACCTGGGCACCACGATGGAAATGGCCCTGTGGGGATTCGTGCTG
GCACTCGTACTCTCGCTGGCCCTGGCGATAGTACGGGTGTTTCGCGTCCCTGCGATTCACTGGCTGGCGA
TGCTCTATATCTCGTTCTTTCGGGGTACACCGCTACTCGTTCAGCTGTTCCTGCTCTACTACGGACTGCC
GCAGCTGTTCCCAATCTTCGTGGGGATGGATGCCTTTACTGCCGCCATCGTCGGTCTGACCCTGCACTTC
GCCGCCTATATGGCCGAGTCGATCCGCGCAGCCATCCTCGGCATTCACAAAAGCCAGATGGAAGCGGCGC
TCAGTATCGGCATGAGCCGTTATCAGGCGATGCAGCGGATCATCCTGCCGCAGGCGGCCCGTATCGCCAC
TCCGTCGCTGATGAACTACTTCATCGACATGATCAAAAGCACCTCGCTGGCCTTCACCCTGGGAGTGGCC
GAGATCATGGGCAAAGCGCAGATGGAAGCCTCCTCCTCCTTCAAGTTCTTCGAGAGCTTTATGGCGGTGG
CGCTGATCTACTGGGTGGTGGTGATCTTCTTTACCCGCTTGCAGCACCTGCTGGAAATTCGTCTCAACCG

TGCCTATTAAAGGAGGGCTCTCATGATCAAGCTAACCGGCCTCTCCAAGGCATATCACGGCAATCAGGTA
CTCAACGGCATCGATCTGGAGGTCAAAAAGGGGGAAATTGTCGTCATCATCGGCCCCTCCGGCACCGGCA
AGTCGACCCTGTTGCGCTGCCTGAACCTGCTGGAGACCCCGGATGCGGGCACCCTTGCCATCGACGATCT
CGAGCTGAATCTGGCCAAGGCCAGCGAACAGCAGGCGATCGAGCTGCGCAAACGGGCCTCCTTCGTGTTC
CAGAATTACGCCCTGTTTGCCAACAAGACGGCGCTCGACAACATCGCCGAAGGGCTGATCACCGTCTGGA
AGCAGCCAAAAGCGGAGGCCCGAGCCACAGCGCTCGGCATCCTCAAGGATATAGGCCTCGCCGACAAGCA
GGATGCCTATCCGGCCTCCCTCTCCGGCGGCCAGCAGCAGCGGGTCGGCATTGGCCGCGCCATGGCGGCA
CACTCCAAGGTGATCCTGTTTGACGAGCCCACCTCGGCCCTCGATCCCGAGTGGGTCGACGAGGTTTTGG
GACTGATGAAAGAGTTGGCCCGCCAGCACCAGACCATGGTGGTGGTGACTCATGAGATTGAATTTGCTCG
TGATGTTGCCGATCGGGTGATCTTCATGGAAGGTGGGCGGATCGTCGAGCAGGGCCCGCCGGATCAGATA
TTGGTCGATCCCAAGGATCCCCGTACCCGGGAGTTCTTGCGGAAAGTCCTTAAATAGCTCTCTCTTTGGC
GGCCTGGCCGCCTTTTTTATTGAGGGATCCCTTGTTTAACCCCGGGTTGCCAGTATAATCCGGAGCCCTT
GATCCTCTGGATCCTCACGCTCGGCAGCGGCCTTTTCAAGACCACGGCAGGATGAGAGGGAACAGCGGAT
TGACTAAAAGCCGAACTCTGTTTAGAATTCGGCCTCTTTTGCTAGTCGTCACACGCTATAGCTGGCGCGT
CGGCTAAGTGTGACAATCAAAACTGTACAGAAATACGGAATCGAATCATGAAACGTACTTTTCAACCGTC
CAACCTGAAGCGCAAGCGCTCTCACGGTTTCCGCGCCCGCATGGCAACTGCCAACGGTCGTAAAGTTCTG
GCCGCTCGTCGTGCCAAGGGTCGTGCCCGTCTGGTAGTTTAATGCCTACCCAGCACACCTTCTCTCGGGA
GTTACGTCTGTTAACTCCCGAACACTTCAAACGCGTTTTCGCCGAGCCTGTCCGGGCTGCTTCCCCGCAA
ATTACGCTTCTCGCCTGCCCCAATTCACTCGAACACTCTCGTCTTGGTCTCGCTGTGCCCAAAAAAGCAC
TCAAGCGTGCCGTCTGGCGTAACCGTGTCAAACGGGTGGTCCGGGAAAGTTTCCGCCTCAAACAACATCA
GCTGCCCGCTATCGACATCGTGGTGATCGCCAAGGCAGGTGTGAAAGAGATGGATAACGAGGAACTGTTC
AAGTTACTGGAAAAGCTATGGCGCACTCTGTCACGCCGTTGCAATGGGTAGCTATCAAACTGATACGCCT
GTATCAGCTAATCATTAGCCCTCTGCTCGGGCCGCGCTGCCGTTTTACTCCAACTTGTTCCCAATTTGCG
ATAGAAGCCATCCGACTTCACGGTTTCATAAAAGGCGTTTGGTTAGCTAGCAAACGTCTATTAAAATGCC
ATCCCTTATCCGAGGGTGGTTATGACCCGGTTCCGCAACCAAAGCGAAGAAACTGAAGACTATGGAATCA
CAACGCAATCTACTCCTGATCGGTTTGCTGTTCGTGAGCTTTCTGCTCTGGCAACAGTGGGAGTCCGACA
AGGCTCCGAAGCCGGCCCCGACTACCGTGGCCCAAACCGAACATTTCGTACCTGAAGCCGGTCAGACCGG
TGATATTCCTCAGGTTTCCGAGCAAGCCAACACGACTGCCGCACGCAAGCTGATCACCGTCTCCTCTGAC
GTGCTCAAGCTGACCCTGGATACCCAGGGTGGTGACGTCGTGTCTGCCGAACTGCTGTCTCACAAACTGG
AAGAGGGCAAGGACCAACCGTTCGTGCTGCTGACCAGCAAGCCTGAGCACCTCTATATCGCCCAGAGCGG
TCTGGTGGGTCGCAACGGTCCGGACAGTCAGGCCGAAGGTCGTCCTACCTATGAAGCCGCCCAGACCAGC
TATCAGCTGGCCGATGGTCAGGATCAAGTCGTCGTTCCGATGACCTGGACCGACAGCAAGGGCGTGGTCT
TCACCAAGGAGTTCATCCTCAAGCGTGGCGACTATGCCGTTGGCGTTGATTACAAGATCGACAACAAATC
TGCCGAGCCGGTTCAGGTGCAGTTCTATGGTCAGCTGAAGCAGACCGTAGCAACGCCGAAGGATCAGGAA
GGTCATGCCATGGTAGCCAGCGCTTACCGTGGTGGTGCCTTCTCCACTGAAGATACCCGCTACAAGAAGT
ACACCTTCGACGAGATGAAGGATGCCGACCTGAACAAGACCACCAAGGGTGGTTGGGTCGCCATGCTGCA
ACACTACTTCGTCTCTGCCTGGGCACCGAATGCCGACGATACCAACAGCTTCTACAGCCGTGTGATCCCT
GGTAAAGGCCAGGCCATCATCGGTTACAAGGCGCCGCTGGTTGACGTGGCCGCCGGCCAGCAGGCCGAAG
TGACCAGCAAGCTGTGGGTTGGTCCCAAGCTGCAAGACCAGATGGCCAAGGTGGCTAACCACCTCGACCT
GACCGTCGACTACGGCTGGCTGTGGTTCATCGCCCAACCGCTGCACTGGCTGCTGACCGTGTTCCACGGT
TTCGTCCAGAACTGGGGTCTGGCCATCATCATGCTGACCCTGCTGGTGCGCGGCATCATGTTCCCGCTGA
CCAAGGCCCAGTACACCTCCATGGCCAAGATGCGCATGCTGCAACCCAAGATTGCGGCCCTGCGCGAGCG
CTTCGGCGATGACCGCCAGAAGATGTCTCAGGGCATGATGGAGCTGTACAAGAAGGAGAAGGTCAACCCG
CTGGGTGGCTGCCTGCCGATCCTGGTTCAGATGCCGATCTTCATCGCCCTTTACTGGGCACTGATGGAGT
CCGTTGAACTGCGTCACGCGCCGTTTGCCCTGTGGATCACTGACCTGTCAGTGAAGGACCCCTTCTTCGT
GCTGCCAATCCTCATGGGTGCCTCCATGTGGTATCTGCAGAAGATGAGCCCGACCACCGTAACCGACCCG
ATGCAGCAGAAAGTGATGCAGTTCATGCCGATCATCTTCACTTTCATGTTCCTCTGGTTCCCGGCTGGCC
TGACTCTCTACTGGCTGGTGAGCAACGTCATCTCCATCATCCAGCAGACCATCATCTATCGTCAGCTGGA
GAAGAAAGGACTGCATTCACGCAGCTAATCTTCCCGTGATGACAAAAGAGGCGGCCTTCGGGCCGCCTTT
TTTATTACAATGTCTCCCACTACGTTTTCCTATCAGCGAACAAACGAGCCAAGCAAGATGACAACAGATA
CGATCGTGGCCCAGGCCACCGCGCCAGGCCGTGGTGGCGTCGGCATAGTCCGGGTCTCCGGCCCCGCCGC
CGAACAGGTCGCCGAGATAGTGCTCGGCAAGTGCCCGCGGGTGCGCTACGCCGAATATCTGCCGTTCAAG
GATGAGCAAGGCCAGGTACTGGATCAGGGCATCGCCCTGCTGTTCAAAGCCCCCAACAGCTTCACCGGCG
AGGATGTGCTGGAGCTGCAGGGCCACGGCGGCCCCGTCATCATGGACATGCTGATCCGCCGCATTCTGAA
AATCGATGGCATCCGCCCTGCCCGCCCCGGCGAGTTCAGCGAGCGCGCCTTCATCAACGACAAGCTGGAT
CTGGCCCAGGCCGAAGCTATCGCTGACCTTATTGAAGCCTCCAGCGAACAGGCCGCCCGCAGCGCCATGC
ACTCCCTGCAGGGGCAGTTCTCCAGCAAAATCCAGCAACTGGTGGAAAGCCTGATCCGACTGCGCATCTA
TGTGGAAGCGGCCATCGACTTCCCGGACGAGGAGATCGACTTCCTCTCCGATGGCAAGGTGGCGGGCGAC
CTCTACGCCATCATGTCCGAGCTGGACGATGTGCGCGGGGAAGCCAAACAGGGCGCCCTGCTGCGGGAAG
GGATGAAGGTGGTGATCGCCGGTCGTCCCAACGCCGGTAAATCGAGCCTGCTCAACGCTTTGGCAGGGCG
CGAGTCAGCCATCGTCACCGAGATCGCCGGCACCACCCGCGACGTGCTGCGCGAACACATCCATCTCGAT

GGCATGCCGCTGCACATCATCGACACCGCTGGTCTGCGCGACACTCAGGACAAGGTGGAGCAGATCGGTA
TCGAACGCGCCTGGGCCGAGATTGAACAGGCCGATCGGGTGCTGTTTATGGTGGATGGCACAACCACAGA
CGCGGTTGACCCGCGGGAGATCTGGCCGGAATTTGTTGATCGGTTGCCAAAAAACATCGGCCTCACCGTC
ATTCGCAACAAGGCCGATCTGACAGGCGAGGATCTGGCTCCGAGTCAGGAGCTGGGCCACGCCGTCTACC
GCATCTCCGCCAAGACCGAGCTGGGACTGCCTGCCCTGCGCGAGCACCTGAAAGCCTGCATGGGCTTCCA
GGGCAACACCGAAGGGGGCTTTATGGCCCGTCGCCGCCATCTGGATGCGCTGGAGCGTGCTGCCGAGCGG
CTGCTGGTGGCCAAAGAGCAGCTGGAAGTGTTCGTGGCCGGCGAGCTGGTGGCCGAAGAGTTGCGCCTGG
CGCAAGAGTCCCTCTCCGAGATCACCGGCGAATTCAGCTCAGACGATCTGCTGGGCCGTATCTTCTCCAG
CTTCTGCATCGGCAAGTAACTCTCCGCACAGCAGGCAACAAAAAGCCCGGGTTCATCCCGGGCTTTTTGT
TGTGCGTTTTTTGTCCGCAAACCTAGAGGCGACGCAGGCACCACATGAAGTAAGCGCCCCCTAACAAGGT
GGATACCAGCCCGACCGGCACCTCCTGCGGGAAGAGGATCTGCTGGCCGACCCAGTCCGCCGATACCATC
AACAGGGCGCCGGAAGCGGCGGCCCCAAGCAGGTGCCAGCGTGCCCGCACCAGCCCCATCAGCTTGGCCA
TATGCGGCGCCAACAGACCGACGAAGGAGAGCGGCCCGACCACCAGGGTGGCACTGGCGGTGAGCACGGC
CACCAGCAGCAGGATGGCAAGCTGGGCACGGTTGAGGCGAATGCCCAGCGCGGTCGCTACAGCACTCCCC
ATCGGCAGCAGATCGAGCCAGCGGCTGACAAGCAGGCAGGCGGCCAGCATCAACAGCGCCAGCCCCACCA
ACCCATAGGCCACCGGCAGGGTGACGTAGTAGGTGGAGCCGGACATCCAGGAGAGCAGTTGCTGCACCCG
CATGTCACCATTGGCCAGCGCGATGGCCTGCAGCGGCTCAAACAGTGCCGTGATGGCAATACCTGAAAGC
AGGATCCGCTCCGGCTGGAAGCCGTGCTTGCGATTGACCACCACTAGCAGCAAGAGACAAGCGAAGGCAC
CGAGCAGGCCACCGGACAGCATCAGCGGCAGGGGCAATGCAGGCAGCAACAGCGCCGTCGCTATCACCCC
CATAAAAGTGCCGCCGCTCACCCCCAGCAGCTCAGGGCTCGCCATAGGATTGTTGCTGACCCGCTGCAAC
AGAGTGCCCGCCAGCGCCAGTAGCACGCCAGCCGCCCCCGCCGCCAGAGTGCGCGGCAACCGCCACTCCA
GCTGGGCCTGCCAGCGGAGCCAGCTTGGCCAACTCCAGCCCATCATTCCCTGCCCAACCAGCAGGGAGGC
GACTACGGCCAGCATCAGCCCGACCAGCAACAGGCCAATAAGGCGAGCCGGCGCCGGGTGGCGGGCCACC
AGCAGGCCGGCGTTTGCCTTCGGCGCACCCGACTTGATGCCAAGGCGCGGGATCAGCCAAAGCAGCAGCG
GCGCCCCCAGCAGAGCGGTCATTGCGCCGGTGGGGATCAATACCGGCCAGAAGCGGCTCAGGCTCTGCAG
CAGCAGATCGGTCGCCGCCAGCAGCAGGGCGCCGAGGATCGGAGCCCACAACAACCGCTGGCCGAGCTGG
CGGATCCCGAGCAGCCGCACCATAGCGGGCGCTGCCAACCCGATAAAGCCGATCAGCCCAACCACGCTCA
CCACGCAAGCGGTGACAAATACCGCCAAGCCAAGCCCCGCCAGTCGCAAGTGTTGGAGGGAGACCCCCAG
ACTGCGGGCGCTGGCATCATCCAGTTCCAGCACCGCCAGCGGGCGCACCAGTACCGCGGCCAGCAAAGCG
GCTGCCAGCAGGCGGGGTAACAGGTAACTCACCCCGCTCCAGCTGTTCTGCACTAGCGAGCCACTACCCC
ACACCAGCAAGCCTTTCAGCTCCTCCTGAAAGAACAGCAAGAGTCCCATGCTGATGGCCGCCAGATAGAG
GTTGATCACCAGCCCGGCAAAGACGATGACCACCGGATTGAGCTGGCGACGCCAGGCCAGGGCAAACACC
AGTGCCATCGCGAGGCTGCCACCGGCCATGGCGATCCACTCCCGCCCCAACAGCAACCAGGAGGGGGCAA
ACAGGGTCACCATCATCAATGCCAGTTGGGCACCGGAGGCCACCCCCAAGGTGGTTGGTGACGCGAGCGG
GTTGCGCAGCACCTGCTGCATCAGGGTGCCCGCCAGCCCGAGCGCCGCCCCGGCCAGCAGGGTGACAACC
AAGCGGGGCAGCCAGCTGAAATGCACCACTGCTTGGCTGACGTCATCCAGATTGGGGGTGAAGAGGCTCT
GCCACCAGAGCGCACCAGGCATCTGGCGAGCCAGCTCCCAATACGCGAGCAAGAGGGTCAACCCCAGCAG
GATCGAGACCGGCCGCAGCAGCGGCGATGTGAGCAGTCGCTTCATCGACCAGCCTCCATACGCTCGGCAT
CTTGCAACAATGCATCGCTGAGCAGACCGGCGAAACGGCGGGCGGCAGGTACGCCGCCAAAACTCCAGAC
CGCAGGCAGATGCAGCACGGGGGCTTGGCGTACCAGCGGCAGATGCTGCCACAGCCCAGGCTCCTGCAGC
CGTTCGCTCACCCCTACCGGGATGGGATCGACCACCACCAGCCGCGCGGTTGGCAAGGTCATAAACTGCT
CGATGCTGACCACCGAGAAACCCCAGTCATTGGTCTGCTCCTGCCAGCCATTACGCAGCCCAAGCCGCGT
CATCACCGCATCGAACAGGCTGTGGCGGCCAAACACCCGCACATGGCGCTCATCGATCAACTGTACCACC
AGCAGGGGCGGCAGATCCGCAGGCAATCCGGCCTGCATACTGGCAAGATCCCGATCCAGCCCGGCCAGCA
GCCGCTCGCCCTCGGTGGTTTTGTCCACCATACGGGCGAGGGTCAGGGTCACCTCGTGCAGCCGCTGCCA
CAGATCGGTTTGCCCATCATAGAGGGCGATGGTGCTCACCGGTGCAATGCGGGAAAGAGTTGGCGCCAGC
GGGGCTGCCAGCGGCGAGATGAGGATGCGATCGGGTTTGAGCTCCGCCAGCAGCTCACGGTTGGGCTGGG
TGCGCAGGCCGATATCCACCACCCCGGCAGGTAGCCGTGGCTCGCCGACCCATGCCTGATAGGGGCCTGC
ATCCCCAACGGCCAGCGGCGTCACCCCGAGTGCCAGCAGGGTCTCGGCTATGGTCCAGTCGACGGTGGCG
ATACGGGGTAGCGCGCTGGTGGCCTCGTTTGTTGCGGATGATTGTACAGCCTGCAACGGGAGCGCCAGCA
GCAGGCTGACGAGACAAGGGGCCATCTTCAATAAAAGGTTCAAGGATGCCCCCGGTCAGACGATGGCGAT
GGGATGAGCATGAGCCGGGTGGCGAATCACCTCCATCGGAATGCCATAGATAGCGTTCAGGGTCTCGCTG
GTCATGAGCCCCTCAGGTTCACCGTGGGTAAGCAGGCGGCCGGAGTGGAGCGCCACCAGCCGATCACAGA
AGCGGGAGGCCATATTGATGTCGTGAATGACGATGATGACGCCGAGGTTTAGCTGACGGCTGAGCTGCCT
GACCAGGGTCAGCACCTCCACCTGATGGGCCACGTCCAGCGCGGCCAGCGGCTCGTCCAGCAGGATGAAG
CGGCTCTCCTGGGCCAGCAGCATCGCCAGCCAGACCCGCCCCCGCTCGCCGCCGGAGAGGGTATCCACCA
ACCGATCGGCAAAGCGTTCGGTATGGGTAAGGGCGATGGCCCGATCGACCTGACGGTGATCCTCCGCCCC
CATCCGGCCCAGCAGGCCGCGCCAGGGGTAGCGGCCCATCTCCACCAGCTCCCGCCCGAGCAGATTCTCG
GCGCTCGGCAAATGCTGGGGCAGATAGGCCACCTGACGGGCAAACTCCCGATTGCCCCAGTCGGGCAGCG
GCTTGCCATCGAACAGGATCTCGCCGTCGCTGAGGGGTTGCTGGCGTGCCAGTAGCTTGAGCAGGGTCGA
TTTGCCCGACCCATTGTGGCCGATGAGGCCATAGACCTGTCCCTGCTCGAAACGCAGGTCGGTGGGGTAC

AGCAGCGTGCGATCATTGACCGTGAAGCTGGCTGATTTCATCTCGAACATAAGCAATTCCTGTGCTGGCT
TGCCGCCAGTCTCATCGGTGCCGGCATCAAGCCGGCACCTCGCAATCCAGATGGGCGATCGCATCGCGGC
TCACCTCAGTACGGCGCGCGCCGGTCAGTTCGGTGAGCTGGCCCTGACGCATCTCCAGCAGGCGATCGGC
CAGCGGGAAGTAGTGATCGTCGTGACTGATGGCAATCACCGTCTTGCCCATCTCCTTGAGGCGCGGCAGC
AGCACCTGATAGAAGATGCGGCGGAACTGGGGATCCTGATCCGCGGCCCACTCATCAAACATCATCACCT
CGCGCTGCTCGGCCAGCGCCAGCAGCAGGGCGACCCGCTTGCGCTGCCCTTGAGAGAGATCGATATCGGC
GATAAAGCCATCCTCGATGGTGAGCTTGCTGCCCATCTGCAACCGCTCCAGCCACTCGGCCACCAGCTCA
TCCTTGGGTTCCGGCCCCAGCAGGTGCTGGAACAGGTGATAGTCGGTAAAGATGGCGGAGAAGAGAGCCA
GATAGCCGTTGCGATCGGTGATCGGCTCACCATCCAGCAGGATCCGGCCCGATACCGGCTGGTAAAGGCC
GGTCAGCAGCATGGCCAGGGTCGACTTGCCGGAGCCATTGCCGCCGATGATGAAGATCTGCTCCCCCTTT
TCGATGACCAGATCGATGGGGCCAACGGCAAAACCGCCCTCCCCCTGATAGTGAAAGCTCACCTGCTCCA
ACTCGATACGCTGCCAAGGGCGGATGGCGGGCGGCAGCGGGAAGTCCGCATCCGGCTCGGCCAGATTGAG
GGCGGCGATCTTGTCGAACGCCACCTGAGCGGCAAGCAGGGTCGGCAGGGCGCCAATCCCCTGCAACAGC
GGGGTGCGCAGGAACAGCAGGGTCAGCGCAAAGGTGGCGGCCACATTGGTGTTGGCCCAGCCAAGGCCGT
TGGCCAGAAAGAAGACCAGACCGATGGCCCCCAGCATCATGATATTCGACCAGTTCACCGCGCTCAGGTG
ATAGGTGTCGGCGCGAATGATCTGCTGGCGGTAGTCACGGGCATTTTGCTCATAGAGCTGGTGGTAAACG
AAGTGAGCCCGCTCGCGGTTTAGCGCCAGCTCCTTGCTGCCGGCGATGATGGCCTGATAGTTCTGATAGA
GCCGATCTTCGGCCTGACGCATGTGGGAGAGATGGCGATAAACACGGTTCACCAGCAACCAGCCCACCAC
CATGGTGAAGGTGACCCAGAGGGCGGTCACCCCGAGCAGGGCCGGAGAGAGCCAGCCGAGGTAAAGGGCA
GAGCCCAGCGTGAGCACCAGCCCCTGCACCAGCTCAGGCAGGCGCACGAAGGCAATGGTGATCTGGCCGA
TATCGCTGGAGAGGGAGGCCAGCAGTGGCCCCTGACCAATCTGGCGGATGCGGGCGACATCGGTATCCAA
CAGTTGCTTGAGCAGACGGTCGCGCAACCGGTAGACAAAATGGTGACCGAGAGTGGTCAGGGCCAGCTGA
GATCCGAGAGTGATCACCAGCAGCAACACAATCAGGCCGACCAGCTGACCGAGGATCGGCAGCGGATCAC
CTATAGATTCGATAAGAGACTGATTGATAAAGGCAATGACGCCAATCCCGCTGACTGCACTGAGCAGACT
GAGCAGGGTAATGGCGATGAAGGGCCAACGATATTGGCGATAAACCAGAGACAAGAGTTCCATAACGGAC
CGGTATTCCAGAGAGATCAGAACAACTGCGGGTGAAAAGAAGGGCGGGAATCCGGTTCCCGCCCGACTTC
GATTACCAGCGGTAGTTGATGCTGCCCACTACGCTGCGCGGCTCGCCGTAGTAGCAGTAGTAGTCACAAC
TGGCCACGTAGGTTTCACCCAGCAGGTTGTTGACGTTGAGCTGGGCACGCCAGTTCTTGGCAAAGTCATA
CCCGATCATCGCATCCCAGACGGTATGGGCCGGCACCTCGGCAGAGCCGTAGATGGGGGTGAAATCCGCA
TTCACGGCGCCAATACCCACGCTGGAACCGATATAACGTACACCGGTACCCAGCTCCAGACCCGAAGCCA
CCCCTTGCTGCACCTTGTAGTTACCCCAGAAGGAGGTCTGGTGGCGCGGGATAAGGCTGGCACGGCGATC
GCCACTGCCGGTGTCGTTGGTTTTGGCATCTGTGTAGGTATAAGCAGCGGTCAGACGCAGCGCATCGGTC
ACCTGCACGCTCCCTTCCAGCTCAATCCCCTGAGAGGTCACTTCACCCGCCTGGGTCTGCTGGCCGGAAC
CGGTAGAGACCAGCGAGTTGGTCTGGGCCAGATCGAACAGCGCCAGATTGATGTAACCGTCGAGGAAGGC
AGGCGCATACTTCACGCCGGTCTCGTAGAGCTTGCCCTTGAGCGGCTTGTAGGAGTTTCGGGTGTTGGGA
TCTATCCCCGGCAGCACTTCGAACGACTCGGCATAGGCAAAGTAGGGAGAGAGGCCGTTGTCAGCCAGAT
ACATCAGGCTGCCCGAGAGGGAGAGCTCGTGATCGTACTGGCGCTCGTCCTTGCTGGTATTGGTGTTGTG
GGTCTCCACATAGTCAAAACGGCCACCGCCAGTCAACAACCAGCGATCGTCCCAGCGGATCTGGTGCTGG
GCATAGAGGCCGCTCTGGCCCTTGGAGCTCTTCTGGTGAGTGGCGGTCGACTCGTCAAAACCGGGGAAGC
CGCTGTAATCCGGGTTCATGGTATTGATGGAGCCCATACTGTAGTTGTCGGCCTCAACCCCTTCATTGAC
GTGACGCTGCAGATCCACCCCCAGCAGCAAGGTCTGCTCCGTGCTGTCGGTGTTCCAGTAACCCACCATC
TGGTTATCCATGGTGGCACTCTTGGTATTGCCATCGCGGTAAACCAGACCACGACTGGCGGTTGTGCCTT
CATAGGTCGGGAAGATATAGGTCTGGCGCAGCAGCAGATCGTTGTAGTTGAAGCGGACGTTCTGCTTGAA
CTCCCAGGCCTGGTTGAGCTGGTGGGCCAGCTCATAACCGGTGGAGATCTGGGTATTGCGGTTGCGATCG
TAATCCGGCTGGCTCAGGTTGGTGGAGGGATCAATCTGGCCACCTGACGTATTCAGGGTGCCATAGACCG
GGAAAAAGCCGTTGGTCGGGACCCCTTCGTCGTGCAGGAAGCTGGCCAGCAGGGTCAGGCTGGTACGATC
CGAAAGATCGAAAGTGACGCTGGGCGCCAGATAAACCCGATCGTTGTAAGTACCGGTAAGCACCCCATCA
CGCTCTTTATAGAGAGCAACCATGCGGTAACGGCTGTTGTCGGAGAGCGCGTCAGAGACGTCCACCCCGA
CCTGACGATGATCCCGGCTGCCAAGCTGCAGATCCACCTGCCCTTGCGGCGTGGCGGTCGGCCGCTTGCT
GATGGCATTGATCACCCCGCCCGGAGGCGCCTCACCGTAAAGGATCGAGGCTGGCCCCTTCAGCACTTCC
ACACTCTCCAGACCAAACGGTTCTGGCGTCCAGACGTAATAGCCGGTGCCAAACAACCGGTTGCCATCCA
GATAAGAGGCGGCATCGAAGCCACGTACCTTGAACCAGTCGGTGTTGTTGTCGGAACCATAAAGACCGGT
CAATACGCCGGAACGGTAGCGAATCGCTTCATCCAGCTGCAACACGGCGTGCTGATCCAGTTCCTCGCGA
TTAACCACGGAGACGGCGCGAGGGGTCTCGGCCATCGGCACTTCCACCTTCATGGCCGTACCGGTCACCA
CCATGGTTTCGCTGGCTTTGGGCAGCTCAGCGGTGGTTTGTGCGGCCATCAGCGGCAGGGAGACGAGCAA
ACCGGAGAGCGAAAGCACGCCGAAGGCGCCCTTCTTGCCGGGTCCTGTAGTGTATTTTTGCGCAATCATA
TTAAAGTCTCGTTTATCAATGAGTTATGTTGTTTTAATTGTTATTTGTGGTCGTTTGTCACGACATCACC
CTGTTCCATGGGCGAATAAAGCAGCGCAATCCTACTACAGGATCCGTGATCGAATCGATTCAATAATTGC
TAATCATTTGCATCTACATTGTTATCTAGCTGCATCATTGGCAGGATCTCGCAGAGGATGGGAGTCATCA
GGAGAACCATGAGCGGAGAAGAAAATGAAGAGGAATACAAAGAAGCTGAAAAATGATCGGCGGGATGGAT
CCGGTGGCATTATCTGACTGGATCCATTATCCCCAAGCTTTATCCATATTTATCCACACCCTTTGGCGGT

AGAATCCGGCTTTCCGCTTCTTTGTCTGAAATCAGGATCCATTCCATGGCCAAACTCAATCTCGTCGTCG
GCTCCATGCTGGGCGCCGCCGAATATGTCGCAGACCACGTCGCCAACCTGCTGGAACAGGCTGGCCACCA
GACCCGGATCCACAACCCGGCCAAGCTGGCAGAGGTACTGGATGATGCGGGATCCATCCTGCTGGTGGTC
ACCTCGACCCACGGTGCCGGTGATGTACCCGACAACCTGCAACCCTTCGCCAAGGATCTTGCCGAGCAGC
ATCCGGATCTTAATTCATTGAAATATGGCGTGATCGCCCTTGGTGACAGCAGTTATGACACCTTTTGCCA
GGGCGGCAAGACGCTGGATCGGCTGCTGGCCAAGTGCGGTGCCAGCCGGATCGGCGAGCGGCTCGACATT
GATGTGACCCAACACGAGATCCCCGAAGATGCAGCAGAAGTGTGGATCCGCGACTGGATGACGATGATCG
CCTGATCAGAGTGCGGGAGATCCTGAAAAAAAGCTTGATCTCCCCACTTATCCCCAGATCGACTATCCAC
CATGTTATTAACAGATTGATCCGGGTGAGTTGTTCTACAAGGTTACCCACAACGCCCAGGGGTAATGTGT
ATAACTACGGTGTAAAGCTCTGCAAAACCTATAGTTATCCATGTATTAGATCCAGACTGACAGACCTCTG
TGTATAAAAGTCCTGGTTATCCCCTCGTTCACCGGGCCAGGATCCAAGCTTTTCCACAAGCTAGGATCGT
CCTTAAATTAATGATCTTTATGATCAAAAAGTGCTTATCAACAGAAAGGGTGGATCCTAATAAGAGATCC
AGTAAAAGATCGATCTAAAGATCCTTAAGATCTAATTAGTGGATCCTCCCTTCATACTGAACGTTAAGTA
GACGTTCCCCTCAGGACGCGAAACAGATAGAATGTTCCGCCCTTTAGCCCAGGCAAATAAAGATCTTCAG
GCGTGCAGATCAAGGTGATCCCAATGCAATACCATGAACAATTTGATGTGATCGTCGTCGGTGGCGGTCA
TGCCGGAACCGAAGCAGCAACAGCGGCAGCCCGTATGGGCATGAACACCCTGTTGCTGACCCACAATATC
GATACGCTGGGACACATGTCCTGTAATCCGGCGATCGGCGGGATCGGCAAGGGACACCTGGTCAGGGAAG
TTGACGCACTCGGCGGGATCATGGCGCGTGCCATCGATCTTGGCGGGATCCAGTTCCGCACCCTCAACTC
CTCCAAGGGCCCGGCAGTACGAGCCACTCGTGCCCAGGCGGATCGCCTGCTCTACAAGGCTGTCGTTCGC
CAGATGCTGGAGAACTACCCGAACCTGAAGATCTTCCAGCAGGCCTGTGACGATCTGATCATGGACGGGG
ATCGTGTTGCCGGTGTGGTCACCCAGAGCGGGATCCGCATCAGCGGCAAGACTGTGGTGCTGACGGTCGG
TACCTTCCTGAATGGTTTGATCCACATCGGGATGGAAAACTACAAGGGCGGTCGTGCCGGGGATCCTCCC
TCGATCGCCCTCGCCCAGCGCCTGCGTGAACTGCCGCTGCGCATCGATCGTCTCAAGACGGGCACACCGC
CGCGCATCGATGCCCGCTGCGTCGATTTCTCCGTCATGCAGGAACAGCACGGCGACGATCCGCGCCCCGT
CTTCTCGTTTATCGGCGATGCCAGCCAGCATCCCCGTCAGGTGCCCTGCTACGTCACCCACACCAACGAG
CGTACCCACGAGGTGATCCGCAATAACCTGGATCGCAGCCCCATGTACGCCGGGGTGATCGAAGGGATCG
GCCCGCGCTACTGCCCGTCGATCGAAGACAAGATCACCCGTTTCGCCGACAAGACGGCGCACCAGATCTT
CGTCGAACCGGAAGGCCTGACCACCCACGAACTCTATCCGAACGGGATCTCCACCAGCCTGCCGTTTGAC
GTGCAGGTGCAGATCGTCCGTTCCGTGCGCGGCTTCGAGAACGCCCACATCACTCGCCCCGGCTATGCCA
TCGAATATGATTTCTTCGATCCGCGGGATCTCAAGGCCAACATGGAGAGCAAGTGCATTCCAAATCTGTT
CTTCGCCGGCCAGATCAACGGCACGACCGGCTACGAAGAGGCTGCGGCACAAGGCCTGATGGCCGGCCTG
AATGCGGCCCTGCGTGCCCAGGACAAGGATCCATGGAATCCTCGTCGGGATCAGGCCTACATGGGCGTGA
TGATGGACGATCTCTCCACCCTGGGGACCCGCGAGCCTTACCGCATGTTCACCAGCCGCGCCGAATATCG
CCTGCTGCTGCGGGAAGACAACGCAGATATCCGCCTGACCGCCATCGGTCGCGAGTTGGGTCTGGTGGAT
GACGAGCGCTGGAGCAAATTCAACGCCAAGATGGAACAGGTCGAGCTGGAGCGTCAGCGCATGCGTTCAA
CCTGGATCCATCCGCAACATCCGTCACTGGAGGCAGTCAACGCCCTGGTCAACACCCCGCTGACCCGCGA
ACAGAGTCTGGAAGAGCTGCTGCGTCGCCCCGAGGTGACCTATGATGCGCTGATGGCCATCGAAGGGGTT
GGCCCTTCCGTGACCGATCCTGCCGCGGCCGAACAGGTTGAGATCCAGATCAAATATGCCGGTTATATCG
AGCGTCAGCACGATGAAGTGGAAAAGCAGCTGCGCAACGAAAACACTCTGCTGCCGCTGGATCTGGACTA
TCGCGAGGTGAAAGGCCTCTCCAATGAGGTGAAAGCCAAGTTGAACGATGCCAAGCCGCAGACCATTGGT
CAGGCTTCCCGCATCTCCGGCATCACCCCGGCGGCCATTTCTATCCTGCTGGTTCACCTGAAAAAACACG
GTCTGCTGCGTAAAACCGCCTGAGGAATCCCATGTTGGAAAGATTGAACGGCCTGCTCATGCAGGCCGGA
ATTGTTATCACCGATACCCAAAAGAGCCAACTGGTGCAGTTGGTCGAGTTGCTGCACAAGTGGAACAAGG
CTTACAATCTCACCTCGGTGCGAGATCCGGACGCCATGCTGGTCAAGCATATCCTCGATAGCCTGGTGGT
AAGCCCCCACTTGCATGGTGAGCGCTTCATCGACGTGGGGACAGGTCCCGGCCTGCCGGGATTGCCTCTG
GCGATCATCAATCCGGACAAGCAGTTCGTCCTGCTCGACAGTCTGGGCAAGCGGATCAACTTCATCCGCC
AGGTGATCCAGGAGCTGGGTCTGACCAATGTGACGCCGGTCAAATCCCGGGTTGAAGAGTATCAACCCGA
GGTGGGATTTGATGGTGTACTGAGTCGGGCATTTGCCTCGCTGGAGGACATGCTGAGCTGGTGTCACCAC
CTGCCGTCAGAACAGGGTTGCTTCCTGGCACTCAAGGGACAATATCCCGAGCAGGAGCTGGCCCAGTTGC
CTGCCAATATCCGTCTGGTAGCCTGTCATGAGTTGCGGGTGCCGGAGCTGGAAGGCGAACGTCATCTGCT
GGAATTCAAACATATCCAGCCCGAATAGGGTCGCAGTTTCATTTAGGGGATCGTGTGGGAAAAGTCATCG
CCATAGCCAACCAGAAGGGTGGAGTGGGTAAAACCACCACCTGTGTGAATCTGGCCGCCTCCATGGCTGC
CACCCGGCGCAAGGTGCTGGTGATCGATCTGGATCCGCAGGGCAATGCCACCATGGGCAGCGGTGTCGAC
AAGTATGACGTCGAGCGCACCGCCTATGAGTTGCTGATCGAGGATGCCCCCATCAGCGAAGTGATCATCC
CCGAAACTACCGGGGGTTATCACCTGATCGCCGCCAATGCCGACGTGACCGCGGCCGAAATCCGTCTGAT
GGAATTTTTCGCCCGCGAAATCCGCCTACGCAATGCGCTGGCCTCGGTGCGGGACAAATACGACTACGTC
TTCATCGACTGCCCGCCCTCTCTCAACATGCTGACCGTGAACGCCATGGCCGCTGCCGACTCCGTGCTGG
TGCCCATGCAGTGCGAATATTACGCGCTGGAGGGGCTCACCGCCCTGGTCGACACCATCAGCAAGCTGGC
CGCTGTGGTCAATCCGGAGCTCAAGATCGAAGGGGTGCTGCGCACCATGTTCGATCACCGCAACCGGCTG
GCCAATGATGTGTCCGATCAGCTAAAACAGCACTTTGGCGACAAAGTCTACCGCACCATCATTCCCCGCA
ACGTTAGACTGGCTGAGGCCCCGAGTTTCGGTGCCCCGGCCATGCACTATGACAAATCATCGGTGGGCGC

CAAGGCCTACCTGGCACTGGCTGGCGAGATCCTGCGCCGCCAGGAGCAGGAACGTCAGGCGACCATCGCA
GACCGAGAGAGCATATGACTCCGAAAAAACGTGGACTGGGCAAGGGGCTGGATGCCCTGCTCAGTACCAG
CACGGCTGCCCGCCAGAAACAGGTGATGAGCGATCAACGTACTGAAGAGGCCATGGCCCCCACCAATCAG
GGCGAGCTGCGCAAGCTGCCGGTGGAGTGGTTGCAGTCCGGCAAGTACCAGCCCCGCAAGGACATGTCCC
AGGATGCACTGGAAGAGCTTGCCAACTCCATTCGCGCCCAGGGGGTGATCCAGCCCATCGTGGTACGCCC
TCTCGGCGAACAATCCTTCGAAATCATCGCCGGTGAACGGCGCTGGCGCGCGTCCCAGCTGGCGCGGCTC
GAGGTGGTGCCCTGCATCGTCAAGGATGTACCCGACGAGGCTGCCGTCGCCATCGCGCTGATTGAGAATA
TTCAGCGGGAAGACCTCAATGCCATCGAAGAGGCGGTCGCTCTACAAAGACTGCTGACCGAATTTGAACT
CACCCACCAGCAGGTGGCCGAGGCGGTGGGCAAGTCCCGCACCACGGTGACCAACCTGTTGCGTCTCAAC
CAGCTCAACGACGACGTGAAGCGTTTCGTCGAGCATGGCGACCTCGACATGGGCCATGCCCGTGCCCTGC
TGGCCCTGAGCGGCCAGGCCCAGTCTGAACTGGCCAAGCTGGTTGCCCAGAAAGGGCTGACTGTACGCGA
TACCGAGAAGCTGGTGCAAAAAGCGCTCGAACCGGCAAAAGCCAGGGTCGAACCGGCCCGTGATCCACAG
ATCGGCTACCTGGAGCGCCAACTGGCAGAAAAAATTGGCAATCAGGTGCAACTTCAGCCTGGCAAGCGGG
ATAGCGGGAAGTTAGTAATAAGTTACGAGAATTTGTCCGATTTGGACCGTATATTGGGCTATTTTGGCGT
GTCGGAATCGTGATTTTTACGCTTTTTGTAGTAATAAAACATCCATTCCTTTTGATATGACCATAACCCT
GATTTGCCGCGGAAAGCCGCAAAAATTAAGGATTTTTGTCATATTGCAATAGCTGCCCTCAAGGGTATAA
TTTGACCGGCTTTAAGAGCTGTTAACATGGGGTCTTCGGATCCCGATTTTTTACAGTTCTGTAATGTTCA
GGGGTAGGTTGGTGAAGCAGAAAATAGCCTTGGAAGGTTTGACACTGGCCTTGGTTATGCTCGTTTGTCA
GCTCATCCTGATCCTGGCGATGAGTGCCGTCTGGTTTTACCTGACTGGTGCCAGCGCCGGTTATTCCGCT
CTCTACGGAGGGGGGATGTACTGGGTTCCTCAAGCCTTGTTTACTGTCTGGGTGCTGCGCCGCAAGGTGA
CAACCGAGAGTGTGGGCCTGGTTTTGCGGGACTTTTATGGTGGGGCAGGGATTAAGCTCATTTCTACAAC
AGGTCTATTCGCCCTGGTGTTTGGTGCGGGAGTCGAGGTCGTTACCGTCTCGTTTTTCGCCACTTATATC
CTCGCCCTCGTTGTACAGTGGATGGTTTCATTCACGCTTAACAACCACTATTAGGAAAACTCATGTCTGC
AACCGGAGAAGTCCTGACTCCTCAGGGATATATCTCCCACCACCTGACCCACCTTCAAGTCGGGTCCGGG
TTCTGGACGGTCAATATCGACTCAATGGTATTTTCCGTCGTGCTAGGCGCCCTGTTTATCCTGCTGTTCC
GCAAAGTAGCCGTTAACGCTACCAGCGGGGTGCCGGGTAAGCTGCAGTGCTTTGTGGAGATGCTGGTCGA
GTTTGTGAATGGTAACGTCAAAGACATCTTTCACGGTCGCAACAAGGTCATCGCACCTTTGGGGCTGACG
GTGTTTGTGTGGATCTTCCTGATGAACTTCATGGATCTGATCCCCGTTGACTTTATCCCTCACGCTGCAC
AATTGATGGGTATTCCCTATCTGCGTGTGGTTCCCTCTGCTGACGTTAACATCACCATGTCCATGGCTTT
GGGCGTGTTCTTCTTGATCCTGTATTACAGCATCAAGGTCAAGGGTATCGGCGGGTTTGTGAAGGAGCTG
ACGCTTCAGCCTTTCAACCACCCGGCGGCCATCCCGGTTAACCTCATCCTGGAAACTGTTACGCTGATTT
CCAAACCTGTTTCTCTGGGTCTTCGACTGTTCGGCAACATGTATGCTGGCGAACTGATCTTTATCCTGAT
TGCTGGTTTGTTACCCTGGTGGTCACAGTGGATCCTGTCCGTGCCTTGGGCAATTTTCCACATCCTGATC
ATCACTTTGCAGGCATTCATTTTCATGGTTCTGACCATCGTCTATCTGTCGATGGCGCAGGAAGACCATG
GTTGATGCAACTCAAATAATCCTTTTTATACAATCAATTACATCTAAATAACTGGAGATCCTCATGGAAA
ACTTGAACATGGACCTGCTGTACATCGCTGCTGCAATGATGATGGGTCTGGCTGCTATCGGCGCTTCCAT
CGGTATCGGTATCCTGGGTGGCAAGTTCCTGGAAGGTGCTGCTCGTCAACCGGATCTGATCCCTGTTCTG
CGTACCCAGTTCTTCATCGTAATGGGTCTGGTGGATGCGATCCCGATGATCGCCGTTGGTCTGGGTCTGT
ACGTGATGTTTGCGGTTGCTGGCTAAGTCGTTACGACTCTCCAGGCATAACCCATTAACTAACTTAAGAG
GACATCGGCTGTGAATATCAATGCCACCCTCCTCGGCCAAGCAATTGCTTTTTTCTTCTTCGTAGTGTTT
TGCATGAAGTATGTATGGCCGCCGCTGATTGCTGCCATCGAAGCCCGTCAGAAAGCTATTGCTGACGGTC
TCTCTTCTGCCGAGCGTGCCAAGAAAGATCTGGATTTGGCCAAGGCCAACGCCACCGATCAGCTGAAAGA
AGCCAAGCTGCAGGCTGCTGAAATCATTGAGCAGGCTAACAAACGTAAAGCCCAGATCATTGATGAAGCT
GCCGCTGGTGCCCAGTCTGAGCGGGAAAAAATCCTGGCTCAGGGCCGTGCCGAGATCGAAGCTGAACGTC
ATCGTGCCAAAGAAGAACTGCGTAAGCAGGTTGCTGCTCTTGCCATTGCTGGTGCAGAGAAGATCCTGGC
GCGTCAAATCGACCAGGCTGCCAACAGCGACATCGTCGACAAACTGGTAGCAGAACTGTAACGGGAACGG
GGCTATGTCTGAACTGACTACAATCGCTCGCCCCTACGCCAAGGCTGCTTTCGAGTTTGCCGTTGAGCAC
AAGGCGGTTGACCAGTGGCTGGGTATGCTCGGTTTTGCTGCGCAAGTAGTGGAAAACGAGACCATGCACA
ACCTGGTAAACGGCTCTGTGGCCGCCGAAGAGCTGGCAACGCTGTTTGTCGGTGTCTGCGGTGAGCAACT
CGACGTGCATGGCCAGAACCTGATCCGGGTGATGGCCGAGAATGGTCGCTTGGGTGTGCTGCCGGCGGTC
GTTGCTGAATTTGTAGCGTTCAAAGCTGAACTGGATAAAGAGGTTCAGGCTGACGTGATTTCGGCGATCG
AACTGACCGACCAGCAGAAAGCCAATATTCAGGCTTCTCTGGAACAACGTCTCGCGCGCAAAGTGAAGCT
GAATTGCAGCATCGATGCGTCCTTGATGGCCGGGGTGCTCATCAAGGCCGGTGATCTGGTAATCGACGGC
ACAGTCCGCGGTAAGCTGGATCGCATGGCTGACGCGCTGCAATCTTGATTGGGGTATTAGAGCATGCAAC
TGAATTCCACTGAAATCGCCGAGCTGATCAAGCAGCGCATTGCGCAGTTTGATGTAAAGAGCGAAGCGCG
TAACGAAGGTACTATCGTCTCTGTGAGCGACGGTATCATTCGTATTCACGGCCTGGCTGATGCCATGCAG
GGTGAGATGATCGAGCTGCCGGGCAACCGCTACGCTCTGGCATTGAACCTGGAGCGTGACTCCGTCGGTG
CGGTGATTATGGGTTCCTATGACGGTCTGTCAGAAGGAATGAAAGTAAAAGGTACTGGCCGTATTCTGGA
AGTGCCGGTAGGTCGTGGTCTGTTGGGCCGGGTGTTGAACACCCTGGGTCAGCCGATCGACGGTAAAGGT
CCGATCGACAACGACGGCTTCTCGCCGATCGAAGTGATCGCACCGGGCGTTATCGAGCGTAAGTCTGTTG
ACCAGCCGGTCCAGACCGGTCTGAAAGCCATCGATGCCATGATCCCAATCGGTCGTGGCCAGCGTGAGCT

GATCATCGGTGACCGTCAGGTTGGTAAGACCGCCATCGCGATCGACACCATCATCAACCAGAAAGATTCC
GGCATTAAGTGTGTCTACGTTGCGATTGGCCAGAAGGCCTCCACCATCGCCAACGTGGTACGCAAGCTGG
AAGAGCACGGCGCCCTGGCCAACACCATCGTGGTTGTTGCCTCTGCCTCCGAAGCGGCTGCTCTGCAGTA
CCTGGCTCCGTATGCCGGTTGCTCCATGGGTGAGTACTTCCGTGACCGCGGTGAAGACGCGCTGATCATC
TATGATGACCTGTCCAAGCAGGCCGTAGCTTATCGCCAGATCTCCCTGCTGCTGCGCCGTCCGCCAGGAC
GTGAAGCTTACCCGGGTGACGTTTTCTATCTGCACTCCCGTCTGCTGGAGCGTGCTGCTCGCGTTAACGC
CGAGTACGTAGAGAAGTTCACCAAGGGTGAAGTGAAAGGCAAGACCGGCTCTCTGACCGCGCTGCCGATC
ATCGAAACCCAGGCGGGTGACGTATCTGCGTTCGTTCCGACCAACGTGATCTCCATCACCGATGGTCAGA
TCTTCCTGACTTCACAGCTGTTCAACTCCGGTATTCGTCCGGCGGTTGACCCGGGTATCTCCGTATCCCG
TGTAGGTGGTGCTGCTCAGACCAAGATCGTCAAGAAGCTGTCCGGTGGTATCCGTACCGCGCTGGCCCAG
TATCGCGAACTGGCGGCATTCGCCCAGTTCTCTTCCGATCTGGACGAGGCGACTCGCAAGCAGCTGGATC
ACGGTGTGAAAGTGACCGAGCTGATGAAGCAGAAGCAGTACTCTCCGCTGAGCGTGGCTCACCAGTCTCT
GGTGCTGTTCGCCGCCGAGAAAGGCTATCTCTCCGATGTCGAGCTGAACAAAATCGTCGACTTCGAAGCC
GCTCTGCTCTCTTACGCCAATACCCAGCATGCCGAGCTGATGGCTGAAATCAATGCCAAAGCCGACTACA
ACGACGCGATCGTTGGCAAGCTGACTGCGTTGCTGGATAGCTTCAAAGCAACCCAGACCTGGTAAGCGTG
TGTGGCAGCGTGAGCTGCCACTTGATGGAGAGTAAGAGATGGCCGGCGCAAAAGAGATACGTAACAAGAT
CGGGAGTGTGAAAAACACTCAGAAGATCACCGGCGCTATGGAAATGGTGGCAGCAAGCAAAATGCGCCGT
GCGCAGGAACGCATGTCTGCCAGCCGTCCGTACGCTGAAACCATGCGCAAGGTGATCGGCCATATCGCTC
AGGGGAACCTGGAATACAAGCACCCCTACCTCATTGAACGTGAGGTCAAGCGAGTGGGTTACATCGTCGT
CTCCACCGACCGTGGCCTGTGTGGTGGTCTGAACATCAACCTGTTCAAGGCTGCCCTCAATGATATGAAG
CAATGGAGCGCGAAAGGTGCCAAGGTTGACCTGGCATTGATCGGTAACAAGGCCTCCAACTTCTTTGAAC
GGCACGGCGCCAAGGTTAAAGCTCATGTCGCAGGACTGGGCGATAGTCCGAGCGTCAATGACCTGATTGG
TTCAGTCAAGGTCATGCTGAAGGCTTACGACAACGGTGAGATTGACAAGCTGTATCTGGTGTACAACAAG
TTCGTCAACACCATGGTGCAGCAACCACGGGTCGATCAACTGCTGCCTTTGCCCGTAACTGAAGACAGCA
AGCTCGCCAAGAAACACCACTGGGATTACCTCTATGAGCCGGATCCCAAGAAGCTGTTGGATACCCTGCT
GGTTCGCTACGTCGAATCTCAGGTTTACCAGGGTGTAGTGGAAAACTTGGCAAGTGAACAGGCAGCCCGA
ATGGTTGCCATGCAAGCCGCGACTGACAACGCCGGTAACCTGATTAACGATCTGCAACTGGTATACAACA
AGGCCCGTCAGGCCAGTATTACCCAGGAGCTGACAGAGATCGTATCCGGTGCTGCTGCGGTGTAAGGCAA
GGGTTATCAAAGGTTTAGAGGATTTGACATGAGTAACGGTTTCATCGTCCAAATCATCGGCGCTGTGGTG
GACATCGAGTTCCCGCAGAATGCCGTGCCCCAGGTGTACGATGCACTGAAGGTCGTTAGCGAAGGTCAAG
GCCTGGGTCTGGTGCTGGAAGTTCAGCAACAGATCGGTGGCTGCGTCGTTCGTTGCATCACCATGGGCTC
CTCCGACGGTCTGCGTCGTGGGTTGGAAGTAGTGAATAGCGGTAAATCCATCCAGGTTCCGGTCGGTACT
GCGACTCTGGGTCGGATCATGAACGTACTGGGCGAGCCAATCGACGAAAAAGGTCCGATCGGTGAAGAAG
AGTGCTGGTCTATCCACCGCGCTGCCCCCAGCTACGAAGATCAGTCCAACAGTAACGATCTGCTGGAGAC
CGGCATCAAGGTTATCGACCTGGTATGCCCGTTCGCCAAGGGTGGTAAGGTTGGTCTGTTCGGTGGTGCC
GGTGTAGGCAAAACCGTAAACATGATGGAGCTGATCCGTAACATCGCGATCGAGCACAGTGGTTACTCTG
TGTTTGCCGGTGTAGGTGAGCGTACCCGTGAGGGTAACGACTTCTACCACGAGATGATGGAGTCCAACGT
ACTGGACAAAGTATCTCTGGTTTACGGTCAGATGAACGAGCCGCCCGGAAACCGTCTGCGCGTTGCGCTG
ACCGGCCTGACCATGGCCGAGAAGTTCCGTGACGAAGGTCGTGACGTGCTGTTCTTTGTGGACAACATCT
ACCGTTACACCCTGGCCGGTACTGAAGTATCCGCACTGCTGGGCCGTATGCCTTCCGCAGTAGGTTACCA
GCCGACCCTGGCCGAGGAGATGGGTGTTCTGCAAGAACGTATCACCTCCACCAAGACCGGTTCCATCACC
TCCGTACAGGCCGTTTACGTGCCTGCGGATGACTTGACTGACCCGTCTCCGGCGACCACCTTCGCCCACC
TGGATGCGACCGTCGTACTGTCCCGTCAAATCGCGGCACTGGGTATCTACCCGGCCGTTGACCCGCTGGA
CTCTACCTCTCGTCAGCTGGATCCGCTGGTTGTTGGCAAAGAGCACTACGAGACTGCTCGTGGCGTTCAA
ACCGTACTGCAGCGCTACAAAGAGCTGAAGGACATCATCGCCATCCTGGGTATGGATGAACTGTCAGAAG
AAGACAAACTGACCGTAGCCCGTGCCCGTAAGATCGAGCGTTTCCTGTCCCAGCCGTTCTTCGTAGCAGA
AGTGTTTACCGGTTCTCCGGGTAAATACGTGCCGCTGAAAGAGACCATCCGTGGTTTCCAGGGCATCCTG
AAAGGCGAGTATGACGATCTGCCCGAGCAGGCGTTCTACATGGTTGGCGGTATCGACGAAGTCGTCGAGA
AATCCAAGAAACTGTAAGCCTCGACAGGAGGGCCCATGGCAGAGATGATCTCCTTTCACCTGGACGTGGT
GAGCGCCGAAGGAAAACTGTTTTCCGGTCGCGTGCAATCCGCTCAGGTATCAGGCTCCGAAGGTGAGTTG
GGTCTCCGCCACGGTCATGCTCCGTTGCTGACTGCCATCAAGCCGGGCCTGGTCCGCTTGGTGAAGCAGA
ACGGAACTGAAGAGGTGCTGTACATCTCCGGCGGTATGCTGGAAGTACAACCTGAATCCGTGACCGTGCT
GGCGGACACTGCGATTCGTGCTGACGACTTGGATGAGGCGAAAGCCCAGGAAGCCAAGCGCGCAGCCGAA
GAGCGGATCCACAGCTCCCATGGTGATATCGACTACGCCCAGGCCGCCACCGAGCTGGCCAAGGCGATGG
CTCAACTGCGAGTCATCGATATCGTCAAAAAGAATTACCGCTAATAACGGTGATGTAAAAAGCGACCTTC
GGGTCGCTTTTTTTTTACCCGTTGGATAGACTCCGCCGGGTATTTCCACACAAGTTCAAGGTCGAAGTCA
TGTCTCTCAACGTCGTGATCCTGGCTGCGGGTAAAGGTACCCGTATGCGTTCCGTTTTGCCCAAGGTACT
GCACCCGGTAGCCAACAAACCCATGGTTTCCCATGTGATCGATACTGCCCGTCAGGTAGGCGCTGAGCAG
CTGCATCTGGTCTATGGCCACGGTGCCGAGCTGCTGAAAGAGCGGATTGTGGCGGCCGACGTGAACTGGG
TGCTGCAGGCGCAGCAGCTGGGGACAGGTCACGCCGTCGCCCAAGCTATCCCGTTCTGGAAGGATGAAGA
CAACGTGCTGGTGCTCTACGGCGATACCCCGCTGATCCAGCCCGAGACCCTGCAGCGACTGCTGGCTGCC

AAGGCTGAAAACGGGATGGCATTGCTGACCGTGGTGCTCGACAACCCTACTGGCTATGGCCGTATCGTCC
GTGAAAACGGTCAGGTAGTCGGTATCGTCGAGCAGAAGGATGCCAATGCCGAACAGCTGGCGATCCGCGA
GGTGAACACCGGCGTGCTGGTGGCCAACGGCGGTCAGCTACGCAGCTGGCTATCCCGTCTGGACAACAAG
AATGCCCAGGGCGAATTCTACCTGACCGACGTGATCGCCATGGCGCACGGCGATGGCTGTCCGATCGCGG
CGGTCCATCCGGAGCGTGCCGCCGAGGTGGAAGGAGCCAACAATCGGGTGCAATTGGCCCAACTTGAGCG
CAGCTATCAGCAGATGCAGGCCGAGAAACTGATGATCGCCGGTGCTACCCTGATCGACCCGGCCCGCTTC
GATCTGCGCGGAACCCTGGAGATTGGTGAAGAGGTGGTGATCGACGTCAACGTCATCATCGAAGGCAAGG
TAACCCTCGGCAACCATGTTCGTATCGGTGCCGGTGCCGTGCTGAAGGATTGCGTGATCGGCGATCACAC
TGAAGTGAAGCCCTACTCCATCGTTGAAGGGGCTCAGGTGGCAGATCAGTGTTCGGTCGGCCCGTTTGCC
CGTCTACGCCCGGGGGCTGTGCTCGAGCAGGATGCGCACGTTGGCAACTTCGTCGAGATGAAGAAGGCCC
GTCTGGGGGTTGGCTCCAAGTGTGGTCATCTGACCTATCTGGGGGATGCCGAGGTGGGCGCAAAGGTGAA
TATTGGTGCCGGTACCATCACCTGCAACTACGACGGGGTCAACAAATTCCAGACCATCATCGAGGATGAT
GTGTTTGTCGGTTCCGATACCCAACTGGTAGCGCCAGTGCGCATCGGCAAGGGGGCGACTCTGGGGGCGG
GTTCTACCATCACCAAGGATGTGGCCGAGAACGAGTTGGTGATCACCCGGGTGCCCCAGCGTCACATCAA
AAACTGGGCTCGTCCGGTCAAGAAGAAGTGATGCCCGATCAATAAAATAGAACCGGCGCCTGATGGCGCC
GGTTTTTTTTTGGCTTATGACTGATGGTTGGTGTCACTGGTCGATGACTTGGGGGCCGCCCATGTGGTCG
CGGGTGCGCTGTAGTCCATCACTTGATACCAGTCGAGTTTGCGGGTCAGCAGCATGATCAGCGCAAGGGT
GGCGAACAACAGCACGGCCCCGAGCAGCAGGGCGATCTCTTCGGCCTGCAGCAGGCTGTAGAGGATGGCA
TAGACCAGCCCGAGCAGCGCCGCGAAGCCGATCCCCTGCTCCGGGCCACCCAGCACATGACTGAGGTAGA
AGCCGTTGAGCAGCACGCTGGCCAGCGCGGCTACCAGATAGGCCCAGCCAAAGCCGAGGTGCTCGGTCAG
GGCCAGCAGCACCAGATAGAAGATAGCCAGCCCCATGCCTACCAGCGCGTACTGGATCGGGTGAACCCTC
AGCCCCTTGAGCAGCTCGAACATGAAGAAGCTGATAAAGGTGAGGCCGATGAAGAGCAGGGCATATTTGG
CCGCCCGTTCGTTGAGCTGATAGTGATCCACCGGCTGCACCAGACTGACGCTGAAGGTGGAGAGATTGCT
ATCGCCCCCGTTCATCATCCGGTTGAAGCGGGTCTCCATGTCGCTGGCGAACCAGCTGGTTTGCCAGTTG
GCCTCAAACCCTTGTGGGCTCAGGGTGCGACTGCTCGGCAGAAAGTCACCGATAAAGTTGGGGTAGGGCC
AGCTGCCCGCCAGCTGCAGGGTGCTCTCCTTGCCCAGCGGGACTATGTCGAGGGCGTTCATCCCCTGTAG
ATTGAGCTCCAGATGGAAGGTGCCATCCTGCTGCGGCAGGCTGTCGATCGGGGCATGGATACCCGCGAGC
GCATCACCGAGGCGGGCACCGGGGGCAAACGGGATCCGCTGTGTGCCGAGTTGCAGTTCGGGGACGCTGT
TGATACCGCGGGCATCGGAGAGCACCAGGCTCAGGTAGGGTTTGCCCGCCACCAGCTCGGCCGTTTCACC
GAGCGGTGAGGTCGCGTCATTCAGCAAACTCTGCCGGGTGGGCAGGCGGCCGGATAGGGTGAGGCGGGTC
TGGTAGACCTGCGCCTGATAGATGCCCAGCTTGCGCGGGGTGACCTCCATGTTGGCCTTGATATCCAGTT
GCTCCGGCAGCTGGTAGAGGTAGATCTGCTCCTGACGGACGAAGCGCTTGCCCTCCTGCTCGACCGTCAC
CGAACGGGTATAGGGCTGCACGATAATGGGCCCCATCAGTCGTTGCGGGCCGCTGTTGCTGGCCATGATG
CTGTGAATGGCCTGACTGCGGTAAGCCTGACGCTCCCGGTTCAGCTCCATGATGGATTGCACCGGCACCA
TCAACAGCAGGCTGAGCAACAGCAGCAGAAAGATCTTGTGGGTGAATATCTGTTTGACCATGACACGTCT
CCTTGTTGGATGGAGTGAGTCTGTCAGTCCGGCGTGAAGTGGTGATGGGCTTGTGTGAAGTGGCGGTGAA
CTGTTGAAAAACGGCGCCATCAGGCGCCGCGGGATTGTTCAGGGGCGGGAGCGCACCAGTCGGGCGGCTT
GTGCGAAACGATCGGTGTGGCCGTCGATGAGTCCGTGCATAAAGTCGATGTAGTAGACCAGTTCCGGCAT
CTCCTCAATGGAGCTAGAGGTCCAGTAGGGGGCTGAATTTTCCCCGTTGTCATGGATATCGGAGAAGAAA
CGCGGGTCTGTCATCGGCGCCCGGCAATCTTCGTCGAGCAGGGTGAAGAGCTCCTCGATGGTGGGGAGTC
GCCACCCCTCGCCAAGGCGGGCTGCTTCATCTTTGGCTTCAAGCAGCGTCAGCAGGGCCGGTGTGCCCAC
GCACCCTTTCCCCTCCTGCCACTGGCTGCCCAGACTGCACGCCTGCCATTCGAGGTGGTTTAGCCGATCA
TGGATGACTTCTCCCTGCCGTGTCAGGCGGTCGGAGAGATCACAGGCAGCCAGCGCCATGCTGGCCGGAA
GCCAGAGCAGCAATAGAAATGATTTCATGTTTGTCTCGTTTACGAATGGCGGTCAGCCCGTCGCTGGTGG
GCTGACCGTGGTTATCGGGGTTTATCTGGCGGGAAGCGAGCTCACTGCTTTTTAAGTTGTGCAGGCAGGG
CAGCCAGCACCTCTTCTTTCGCCTTGAGGGAGGCGAACTGCTTGCCGGGCTTTTGCACCACGGTATGGAC
GAAGATCAGCTGTTTGCCCGGTTTGCTGGCCCAGCCGACGAACCAGCCAATCTGCTGATCGCGGTTGAGG
CTGCCATCCAGCTTCTTCGGGTAGCCCATGCCGGTTTTGCCGTGGATCTGCCAGCCGTCGATCTCGCTCA
CCTTGAGGATATTGGCGGTGTACTGCAGGGTCTGCGCCGAGACCGGCAGCTTGCCGCTCAGCATCTTGCC
GAGGAAGCGAGCCTGCTCCTCCGGACTGATGGCGAGGCTGGAGCTGAGCCAGGCCTGGGTCAGGCCGTCA
TGTTTGCCCGGATTGCCGGAGAGATCCCGGTTGCCGTAGTCGAAGCGGTCGACGTATTGCTGGAAGCGCT
CCATCCCCAGCCATTCGGTGATCTGCTGGGAGAACCAGACCACCGAGTAGGTTTCCCAGCGGCGCGGGGT
GGTGGTTTCACGCCAGGCGGGCAGCCAGCCGTCATAGCTCGGTTTGTAAGGCAGTGCCGGATGCTCTTCA
TCCACCAGATAGCCGCTGTCGTAACCCATCAGCGCGAGCGGGATCTTGAAGGTGGACGCGGGTGGCAGCT
GGCTGGAGCAGTCCCCTTCGCTGGAGAGGGTCTGGCCGTTGCCGTCAGCATAGAGAAAGCAGCCGCTGGC
GGCGGAGGCAGGCAGGGCTGCGAGCAGACCGGCAGCCAGCAGGCTGGAGAGAAGCAGGCGGGACATAGCA
TTTCCTTGAGCAAATGGATGAGAGAAAGCGGCAGTCTAATGCCGCTTTGTGTGGGGAATGTGAAGTTGCG
GGGGACTAGGCGAACGGCAGCCAGAGTTCCGCCAGCGCGCCCCCTTCCGGCCGGTTGCTCAGGGTCAGAC
GGCCGCCATGCTGGTGGGCTACTTCATGGGCGAAGCTGAGCCCCAGACCGCTGCTCTTCCCCTTGTCCGG
TCGTGGCAGGGAGTAGAAGCGCTCGAAGATGCGCGGCAGGGCGTAGTCGGGAATGCCGGGGCCGCTGTCG
GTGACCCGAAAACAGTAGCCCTCCTCCTGCTGGCTGCCGCTCAGGGTGACCTGCCCGTTGGCGGGAGAGA

AGTCGATGGCGTTGTCCAGCAGGTTGCCGATGGCCTGCTCCACCAGCAGCGGATCCCACTTCTGTTTCGG
CGCGCTGGCCAGCTCGGCCACCAACTCGACCTGACGGCGCTGGGCAATGATCTGGCGGGCGTCGAGGGTG
CGCTTGCCCAGTTTTGCCGGTTCCACCGACTGGCGATCCAGCCCCTGCCCCGACTCCAGCCGCGCCAGTT
GCAGCATTCGCTCGATCAGCTGCTGCATGCGCGCGGTTTCCTGATTGATGTTGCCGATAAACCGTTTGGC
AACTTCGGGGGGCGGCGTTTCGGCCAAAATCTCCCCTGCCCCGCGAATGGCGGCCAGCGGGCTCTTCAGC
TCGTGGGTCAGGCTGTGCACATAGGCTTCGATGTAGCTTTTGCCATCGAGCTGGTGGCGCATGGTCTCCA
GCGAGCGGCCGAGCCGATCCAGCTCGACGGTGTGCAGCCGTGGCAGCGGTGCCGCCTCCCCCTTGCTGAC
CGAGTCGGCGTAGTGCACCAGCTTGCCGATGGCGCGGTTTAGCCACCACACCAGCAGGCTGCCGATCAGC
AGCGAGATGAGCAGCATCTGGCCGCCGGCCCGCAGCAGCTCCTGTTCCCCCCGCTCGATGGCAGGCATCA
GAGTGCGATTGGGTTTGGCGACGGTGAGGGAGCCGATGATCTCGACCCCTTCCCGCAGCGGCGCCGCCAC
ATACATGGTCGAGCTGGCAGCATCATCGGGATCGGTGCGGGTGCTGCGGGCGCCATACTGGCCGCGCAGG
GTGCGGTAGACATCGTTCCAGCGGGAATAGTCCTTGCCCAGATCCGTGCCGTCGGAGTCGTAAATCACCA
CACCTTGCCGGTCAGTGACATAGATGCGGTACTCCGCCTGCTGCTTGAGGTGGCCATCGATCATTGCATT
GATGGGATTCTGGTTGAGGCGGGCGAAGGCGCTGGCCAACCGCCCGTTCATCATATTCCCCTCTTGCAGA
TCATCGAGGGTGAGCGGCGCCAGCAGCTGGGTCATGTCTACCAGAGTATCCTCGGTGGCGCTGCGCACTC
CCGGTTTTATCTCCTCGACGAAGATTTCCAGCCCGAACCAGGCGGCCAGCGCGAAGATCAGCAGCAGGCC
GCACAGCAGTTGCAACCCGAGTCTCATGCCAGCTCCAGGCTGTAGCCCAGCCCGCGGTGGGTGAGGATCA
GGTTGGCTTCTGGATCATGTTCGCGCAGCTTGGCGCGGATGGTCTTGATGTGGGTATCGACGGTGCGATC
CAGACTCTCCTCTGCATCCTGCCACACCAGATCCATCAGCTGCTGGCGGGAGTAGACCCGCCCCGGCGCC
AGCATCAGGGTCTTGAGCAGCAGATATTCGTAACGGGTGAGGGCCAGCGGCTGACCGCGAAAGTGAATGC
GGGCACGCTCCTCATCCAGCACCAGCAGGGTGCTGGGCTGGGGGGCGACGGGCTGGCTGCGGCGCAGGAT
GACCCGCACCCGGGCACACACTTCCCGTGGCGAGAAGGGTTTGGCCACATAGTCGTCGGCACCGATCTCC
AGCCCGATCAGTCGGTCTATCTCCTCGCTGCGGGCGGTGAGGAACATCAGGGGAATATCGGTCAGCGCCC
TCACCTGCTTGCAGAGTTCGAAGCCGCTGATGTCGGGCAGCCCCACATCGAGGATCAGGAAGTCGGGTCG
CGTCTGTTCCAGTCGTGTCAGCAACTGCTGGCCCAGCATGAACCACTCCACCTCGAACCCGTCGGTCTGC
AGGGAGTAAATCAGGGTGTCGGCGATACTGGCCTCATCCTCCACCAGCCAGATGACTCTCTTTTGCATGG
CATCCTCGTCGCAAATTGGCACCCCATTCTGGCATCGAACCAAATCGGATCCTATTTGCTGTTTTATTTT
TCCGAAAAAGGGGCTAATATTTCGATGCTCTTTCGAATCGAAACTTAAAAATGACCAAACGTAATACTCA
ACAACGCCGCCACACCATAGTGACCCTGGTACAAGAGCAGGGTGAAGTGAGCGTCGACGCCCTTGCCAAA
CACTTTTCTACCTCCGAAGTGACCATCCGTAAAGATCTGGCGGTACTGGAGACCGGCGGTCTGCTGCTGC
GCCGTTACGGTGGTGCCGTTCCCTTGCCAAACGAGATGGTGGTCGAGGTCAACCCTGAACAAGTTTCGAA
CCGAAAGTTAGCTATTGCCCGTGCGGCGGCCGAGCGGCTGCGTGATCACAACCGCGTCATCATCGACAGC
GGCAGCACCACCAGCGCCATGATCCCCATGCTGGGCAACAAGCGCGGCCTCATCGTGATGACCAACTCCC
TCAATGTGGCCGGTGCCCTGCGCGAGCTGGAAAATGAGCCTACCCTGTTAATGACCGGTGGCACTTGGGA
CCCTCACTCCGAATCCTTCCAGGGGCAGGTGGCGGAACAGGTGTTGCGCTCCTACGACTTCGACCAGCTC
TTTATCGGTGCTGACGGCATCGACCCGGAGCGTGGCACCACCACCTTCAACGAGCTGGTGGGCCTGTCGC
GGGTCATGGCCGAGGTGTCGCGGGAGGTCATTGTGATGGTCGAATCGGAAAAGATCGGCCGCAAGATCCC
CAATCTCGAACTTCCCTGGTCGAGCATCCATACCCTGGTGACCGACGAGGGGCTGGCCAGCGAGGCCAGA
GAACAGATTCAAGCCCAGGGGGTGACGCTTATCTGCGCCCCGGTCACTTGCTGACATCGAGCTCTGAACC
ATCCAACTGACTTTACGATTTGCATCTCAAGGAGAAGTGTATGTGTGGCATCGTCGGGGCTGTTGCCCAA
CGTGATGTGGCTGAAATTCTGGTAGAGGGCCTGCGCCGTCTGGAGTACCGCGGTTATGACTCAGCCGGTG
TGGCCGTGTTCAGCGCCAACCAGCCGTTGCAGCGGGTTCGCCGTCTGGGCAAGGTGGCCGAGCTGGCCAA
GGCGCTCGACGAGCAGTCCGTCCACGGTGGTACCGGCATCGCCCATACCCGCTGGGCCACTCACGGCGAG
CCTTCCGAGCGCAATGCCCACCCCCACGTCTCCGAGCATATCGTGGTGGTACACAACGGCATCATCGAGA
ACCACGAAGAGCTGCGGGAAGAGTTGAAGGCGCTGGGCTATGTGTTCAGCTCCGACACTGATACCGAGGT
GATTGCCCATCTGGTCCATCACGAGCTGAAGAGCGCCGGCAGCCTGCTGGCCGCCATGCAGACTGCGGTG
AAGCAGCTGCGCGGTGCCTACGGTACCGTGGTGATGGACAGCCGCGACGACAGCCGCGTGGTGGTGGCCC
GCTCCGGTTCACCGCTGGTGATCGGCCGCGGTATCGGCGAGAACTTTATCGCCTCCGACCAGATGGCGCT
GCTGCCGGTGACCCGTCGTTTCATCTTCCTGGAAGAGGGTGATGTGGCCGAAGTGACCCGTCGCGATGTG
CACATCTTCGATACCAACGGCAACGCCGTGGTGCGTGAAGAGCAGGAGTCAGAACTCTCCCACGATGCCG
GTGACAAGGGTGAATACCGTCACTACATGCTCAAAGAGATCCACGAGCAACCCAAAGCGATCACCAACAC
CCTGGAAGGGCGTCTGGGCAGCGATCACGTGGTGGTCGAATCGTTCGGCAACGGCGCGCGCGCCATCTTT
GACAAGGTCGAGCACGTGCAGATCGTCGCCTGCGGCACCTCCTATCACTCCGGCATGGTGGCTCGCTACT
GGTTCGAAGAGATCGCTGGTGTCTCCTGCGATGTGGAGATCGCCTCCGAGTTCCGCTATCGCAAATCGGT
AGTGCGTCCCAACAGCCTGCTGGTGACCCTCTCCCAGAGCGGCGAGACCGCCGATACCCTGGCGGCGCTG
CGTCTGGCCAAGGAATCCGGCTACATGAGCTCGCTGGCTATCTGCAACGTGCCGGGCTCCTCGCTGGTGC
GTGAATCGGATCTGGCCTTTATGACCCGTGCCGGTGCCGAGATCGGCGTAGCCTCCACCAAGGCGTTCAC
CACCCAGCTGGCTGGCCTGCTGATGCTGGTGGCCTCGGTCGGTCACTGCCGTGGTCACCTGAGCGCCGCC
GCCGAGGCCGAACTGGTCAAGGCGCTGCAAGCCCTGCCGCTGCGTATTAAAGAGAGTCTGGCGCTGGCCA
AGGATATCGAAACCCTGGCCGAAGAGTTTGCCGACAAGCAGCACAGCCTGTTCCTCGGCCGTGGCAGCCA
GTACCCCATCGCCATGGAGGGGGCGCTCAAGCTCAAAGAGATCTCCTACATCCACGCCGAAGCTTACGCC

GCTGGCGAACTGAAGCACGGCCCGCTGGCGCTGATCGATGCCGAAATACCCATCATTGTGGTGGCGCCGA
ACAACGATCTGCTGGAGAAGCTCAAGTCCAACGTGGAAGAGGTGCGCGCCCGTGGCGGTATCCTCTACGT
GTTCGCCGATGCCGATACCGGCTTCAAGAGCGACGAGACCATGCGGGTCATGAACCTCAACCATGTGGAA
GAGGTGATCGCCCCCATCGTCTATACCGTGCCGCTGCAGCTGCTCTCCTACCACGTTGCCCTGATCAAGG
GCACCGACGTGGATCAGCCGCGCAACCTGGCAAAATCGGTGACCGTTGAGTAAGTCACTCTCTCAAATGA
AAAAGAGCCGCAATTGCGGCTCTTTTTGTTTCGGCTGAGGGTTACGACAGCCGCAGGTTGGTGATGGTCA
CCTCGTCGAGGGAGGGCACTATGATGCCGCTGGTGATCCCTTTGGCGATGGCCTGACTGCGATTCTTCGA
GTCGGTTTTGTTGGTCACCTGGGTCAGGTGAAAGTTGACCGTCCGCTCACTGATGCCGAGGATGGTGGCG
ATCTCCCAGGAGGTCTTGCCCTCGCTGGCCCAGAACAGGCACTCCTTCTCCCGATCCGACAGTTCGGTCA
GGTATTTCATCATATCCGGTCTGCTTTTGATCAGTTGCAGTGCGGAGTTGAAGATGTAGCTGGCGCAGAA
CGACAGCAGCGGAACATTTTCAAACATCAGATCGGAGTTGGATTTCTCCTTGGTGATGAATGAGAGGATG
CCGTGCTCCCCCTGCGGGGTGTGCAGCGGAAAGGAGACGCCATTGCGTACCCCGAAATCAGCGGCCAGGT
TCATCACGTCGCGGCTGGCGCTAGGCAGCCAAGGCGAATCGCAGTCGAGCTTGTTCCAGAAGATCGGCTG
GGTCTGTTTCAGCCCCAGATAGACCACCGGATCCTGGGTCAGATACTGGTTTTCGCTATAGGCGTCAAAC
CAGGTGGTCGGACAGTTGTTGAACAGTGCCACGTGGGATTTTTGCATGCTGATGGGGAAAATAATCAGCA
GCCGAAAGTAATCGAAGCCCATTTGATGACTGAAGCGCTCCAGCAGTTGCTGGATATCGCTGGTTTTTGT
TGCCTTGGTGAACTTGTCGATATGTTCCCAGATCGCATCGTTGAGCATTACTTTCTCCTTTTTCGATCTC
TATCACACTGATCGAAAAGATTTTTTCTCGTTATTTATATTTGTGCGGTGGCTATTGGCGCCACTTATTC
CGTGCGTGCTTAAAAATTATCCTGTTTTTGTGCATAAAGGCCAAGAAAATCCTCTCGTTGCCAGTCCGCC
CAGCATTTGAGATAATGATAATAGTTATCATTAGTAAGATGTATTTCAATGAACAAATTATGGGGTGTGG
CCCTGCTGCCACTGTCAGTCCACTTTGCTGCCATCGGGGATGAGCTGGTTGCTCCCGCCCTCAAGAACAG
CATAGCTGCTGGGAAAGCTTCCCAGCAGCGGGTGGAAAAAGCCGCCGATGCCGCCGTTGCCGCACGACTG
GAGCTGCAAAATGCCCAGCTGCAACTGCGCGACCTCGAAGCCTACAACCGCTACATGCAATCTCTGGTGG
CCGATCAGCAAAATGAGCTGGGTAAATTGAGCCAGCAGCTGGAAGCGGTGCAAGAGACCCGTCAGGGCTT
GATCCCGCTGATGCTGCAGATGCAGGCAGATCTGGAACAGCTGGTGCAAAATGACATCCCGCTGCGCAAG
GAAGATCGTTTGGCGCGGGTTGAACAGTTGAAAGCGACCCTGGCTCGCGCAGACGTTAGCGAGGCAGAGA
AATTCCGTCAGCTGCTGCAGGCCTATCAGATTGAAGCCGAGTACGGCAGCAGGATGGACAGCTATTCCGC
CGAGCTCGAGCTGGATGGTGCCCCGCGTCGGGTCGATGTGCTGGCGCTGGGTCGGGTCTCCCTGCTGGCG
ATGACGGCCGATCGCAGTCAGGCATGGCGCTGGTCTGCCGAGGCGAAGCAGTGGCAAGCCCTCGACAGCC
AGTGGCTCTCCCCTATCGCCGAGGCGATGGATGTGGCCGCCGACAAGAAAGTTCCCCAGTTGCTCAATGT
CCCCCTCTCTGTCAGCCGTAGTCAGGAGGCCCAATCATGAGAGTCACCATGAAGTGGTTCCCCCTCCTTA
TTGCCCTCTCCTGCGGTACCAACGCTGCTGTCGCCGATGAGTGGCAGCAGCAGGAACAGGCTCGTGAACA
GCAGGCTCAGCAAGATCTGGCGACCGTTGCCAAGGAGCTCGGCACTGCCCGCACCAGACTGGCCGAGGCC
CAGCGCCTGAGCAAGGAGCTGGCTGGCAAGTTCGCCAGCAACGAGAAGCAGCTGGTCGAGCTTAACGCCC
AGTGGGAGCAAGCCTCCGGCGACATGAACGAGATCTTCGCGGTCACCCGTCAGGGCGCCAGCGATGCGGT
CAAGCTGCTGAGCGAATCCGCCGTCGAGGGCCAGTACCCGGAGCGTATCGCCCCGCTGAAAGCCATGGCA
CAGGATAAACAGGTGCCGGATCGTGCTGCTCTCGCCCTGCTGCCCGCCACGCTGCTGCAGGAGATCCGCG
AATCTGGCCGGATTGCCCAGTTCAACGGCAAGGTGCTCGATGCCCAGGGGGCTGCCAGCGAGCAGTCGCT
GACCCGGGTTGGCAGTTTTGCCCTGCTGGGGAGCGAAGGCTTCCTGCAACCGACAGCCGAAGGCTTGAGC
CCGGTACTGGGTCTGCCCGGTAGCGTGCTCTCTGCCGTTGCGGCTTATCAGGGTCAGGAAGGGGAAGCGC
TGCCGCTGGATCCTTCTCATGGCACTCTGCTGGCGATGCTGGCACAGGCACCCACCTTCTGGCAGCAGGT
ACAGCAAGGTGGTCAGGTCGGCGCCATTATCGTGCTGCTGGCCGCTATCGGTCTGGGAATTGCTGCGGTA
CGGCTGTGGAGTCTCTCGCGCGAACTGACTCTGGTGCGCCGCCAGCTCAGGAGCGGTGAGTATCACGCCG
ACAACGCCCTCGGCCGGGTGCTGACGGTGGCAGACAAACACCCCGAGCTGAGCATGGAGACCCTCGAGCT
GCGTCTTGATGAGGCGATCCTGCAGGAGACTCCTCGTATGGAGCGCGGCATCGGCATGGTCAAGGTAATC
GCGGCCATCGCCCCCATGCTGGGTCTGCTCGGTACCGTGACCGGGATGATCGGCACCTTCCAGGCCATCA
CCCAGTTTGGTACCGGCGATCCCAAGATCATGGCTGGCGGCATCTCCATGGCGCTGGTCACCACGGTACA
GGGTCTGGTTGCGGCCATTCCGCTGATCCTGGCCCACAGCCTGCTGCAATCACGCTTCACCGAGCTTTCC
AATGTACTGGAGCAGCAGGTAGCCGGCATTCTGGCCGAGCGCGCCGAGAGCAATAACGGCGGGATGGAGC
GAGCAGCATGATGCTCGACATCTGGCAACAGTTGCAGAGCTTCATGGGCCGTGGCGGCCCTGTGCTCTGG
GTCATTCTGGCCCTGCTGGTGCTGATGTGGATTTTGATGATCGAGCGGCTGCTCTACCTCAATCTGGGTT
TCGGCCCGCTGCAGCAACAGCTGCTCGGCCGCTGGCAAGCCCGCAGCGAGCGCCACAGCTGGCACGCCCG
GGCGATCCGCAGCCGCTGGCTGGCGCAGGCGGAGCTGGAACTGAACCGTCATCTGATCTTCATCCGCACC
CTGGTGGTGCTCTGCCCCATGCTGGGGTTGCTGGGCACTGTGACCGGCATGATCGGCGTATTCGATGCGT
TGGCTGCGGCCAACCGCTTTAACCCGGAGAGTATGGCGGGCGGGATCTCCCGCGCCACCATCCCCACCAT
GGCAGGCATGGTGGTGGCGCTCTCCGGTGTGTTGTTACTCAGTCGGCTCGAGAGTCAGGCCAAGCGTGCG
CTGGCCAAGACCCGGGACGGCCTGCGAGAAGAGAAGGTAATGCAATGAGACGGCAATCCAAACGCCAGCG
TGACGAAGTGCAGATCGACATGACCCCCATGCTGGATATCGTGTTCATCATGCTGATCTTCTTTATTGTC
ACCACCTCCTTCGTGCGCGAAGCCGGGCTCGAGGTACACAGACCCCAGGCCAGTCAGGCCAAGGTGCAGA
AATCTTCCAGCATCATGCTGGCGATCGGGGCTCAGGGTCAGATCTTCCTCGACCGCAAGCAGATCGATGT
GGAGCGGGTACAGGCCACCATCGTCCGTCTGCTGGCGGAGCAGCCCGATGCCAGCCTGGTGATCCAGGCC

GACGAGCGAGTGCCCCATGGCAAGGTGGTGCGGGTGATGGATGAGGCCAAGGCGGCGGGTATCGCCAATA
TCGCCGTGGCGGTGGCGCCGAAATGAAATACAAGCTGATGAGCCTGGGGCTGGGGATCGCCCTCAGTCTG
GGCATCCTGCTGTTCATGGCCACGCTGGTTGAACCGCCCCACGGCGAGAAGGTGGCGAGTGAACAGAAGC
CCATCGTCATCAATATGCAGAACGAGGTGACCGAGGTGCAGGTACGCGAGCGTCCGGTACCGCAGGAACC
CGAGCCTCTGCCAGAGCCTCCCGCGGCATTGGCCTCGACACCTCTGCCGATGCCTGCCGCGGCCCCCTTG
GCGGCCGTTGAGCCGAGTCTGGATCTGGTTTCCAGCCTCAGCGCGGTGCAGGTCTATGCCCCCGGAGTGG
CGACCAGTACCGCACCGGTATTGAGTGGCAACTACCACGGCCAGCAGCAGGGAGCGGGTATCGGCGCAGG
TGACATGCTGATGCCGCTGCAACGGATCGAGCCGGTCTACCCCTATCGTGCCCAGCAGTCAGGGATCGAA
GGATTCGTCACCCTGCGCTTTAGCGTGAATGCTGAAGGTGGCGTGCAGGATGTGGAAGTGGTCGAGGCCA
AGCCCAAGCGTCAGTTCGAGCGGGCGGCCATTCAGGCGATCAACAAGTGGCGCTATCAGCCGAGACCGGG
CGCTACCGACAAGCTGGTGCAAGTCATCACGCTAAAATTCAAACTGGAGTCATAACTATGTTGCGGATCT
GGATCTGTCTGCTGGCCCTGGTCGGCCAAGCCTGTCTGGCGATGGAGGTCAGCCCTCACTTCTCCCGTCA
GCTTGAACCCGTGATGAAGCTCTATCAGTCCGGTCAGTGGCAACAAGCCCAGAGCAAGGCGTCCGGCCTC
AAGCCAAATAGCGATGCCGAGCGTGCCTGGCTGGCGCAGTTGCAAGCCTCGCTGGCGGCTAATCTGCAAC
AGACCGCCCAAGCGAGCCGTTATGTCGAACAGGCTCTTGCCTATAAAGAGTGGCCCGAGCAACAACAGCT
CCAGCTGCTGCGACTGCGCGGCGATATCCAGGCCCAGCAATCCAACTGGTCTGGTGCCATCGCCAGCTAC
AAGGCGGCACTGGCACTCAAACAAGACGATGGGCTGCGCCTGCGCCTAGCCGGCCTCTATTACCACAACA
AACAGTACGGTGACGCTGCCAGCCAGAGCGAACAGTTGTTGAAGAAGGGTTGGCAGAAGCAGGCCGCCAT
CATTCGTCTCTCCGCCCTCACCGCTCAGCAACGTTATGGCGTGGCTGCCGATCAGGCGGCCGAGTTGATC
AGCCACGAACCCAAAGAGAGCAAATGGTGGCAGCAGGCGGTGTCGCTCAACCTCTCCGCCAAACGCGGCG
ATCAGGCGTTGGCTCTGTTGCAAACAGCTATCGACAGAAAACTGATGGGTGATGCGTCTGCCCGCAATCA
GCTGATCCGCCTCTATGCCTGGCAAGGGCTGCCTTATCGTGGTGCCCGTCTGCTGGAAGCGGCCATGGCC
AAGGGACAGATGAAGCAAAGTGCTGAAAACCGCCAACTGCTTGCCCAATTGTGGGAAGGTGCCCGGGAGT
GGTCACAAGCGGTCGATAGCTGGCAGCTGCTCGCCAACCAGCACGGTCAGCCCAAAGCCGCCATGCGCGC
CGCCGAACTGCTGCTGCAACAGGGCAAGACTGATGCTGCCATGACCCAGCTCGCTGCCATCAAGTCGGTG
AAGGGCGAGCAAGGCAATCGCGCCAAAGCGTTGCTGGTGCAGGCACACCTTAATAAGGAGCAGTATGCCC
AGGCGCTGGAACTGGCCCGTGAGCTGCAGCAACAGGATAACTGGCAGCAGAAGGCAACCAGCTGGGTCAA
CTATATCCGGGCACAAAGCGAAGAATTGAGCAAAAAAGCGGCGTAAAAGAGATGCCAAGGGGTTGTTTTG
GGGCGTTCAGTTCACTTTTTCAGCAAACGAACGCATTTTTGCAAAAAGCCCTTGTCATCCCGGCGTGGCG
CCCTATAATGCGCCCCTACCAGCACGGCGGAACACGCCGAACTGATGAGCCAACTTCCTAGCGAAGTGGG
CGCCGAAAGAAAAGCGAAAACAATTGCTTGACTTTCGAAGTGAGAAGCGTAATATGCGCCTCCTCAACGC
GATAAGCGTGACGCTCTTTAACAATTTGAATCAAGCAATCTGTGTGGGCACTCACAGCATCGAACATCAA
AACAATTTTTGATTTTCAATGTCTGGTGAAGTGACCAAGACGATTTCTTAGCAATAAGAAGTTGAACAGT
TTATTTCAGCAATTCATTGAGCCGCTTTTCGAAGCAACCAAACTTAAATTGAAGAGTTTGATCATGGCTC
AGATTGAACGCTGGCGGCAGGCCTAACACATGCAAGTCGAGCGGCAGCGGGAAAGTAGCTTGCTACTTTT
GCCGGCGAGCGGCGGACGGGTGAGTAATGCCTGGGGATCTGCCCAGTCGAGGGGGATAACTACTGGAAAC
GGTAGCTAATACCGCATACGCCCTACGGGGGAAAGCAGGGGACCTTCGGGCCTTGCGCGATTGGATGAAC
CCAGGTGGGATTAGCTAGTTGGTGAGGTAATGGCTCACCAAGGCGACGATCCCTAGCTGGTCTGAGAGGA
TGATCAGCCACACTGGAACTGAGACACGGTCCAGACTCCTACGGGAGGCAGCAGTGGGGAATATTGCACA
ATGGGGGAAACCCTGATGCAGCCATGCCGCGTGTGTGAAGAAGGCCTTCGGGTTGTAAAGCACTTTCAGC
GAGGAGGAAAGGTTGGTAGCTAATAACTGCCAGCTGTGACGTTACTCGCAGAAGAAGCACCGGCTAACTC
CGTGCCAGCAGCCGCGGTAATACGGAGGGTGCAAGCGTTAATCGGAATTACTGGGCGTAAAGCGCACGCA
GGCGGTTGGATAAGTTAGATGTGAAAGCCCCGGGCTCAACCTGGGAATTGCATTTAAAACTGTCCGGCTA
GAGTCTTGTAGAGGGGGGTAGAATTCCAGGTGTAGCGGTGAAATGCGTAGAGATCTGGAGGAATACCGGT
GGCGAAGGCGGCCCCCTGGACAAAGACTGACGCTCAGGTGCGAAAGCGTGGGGAGCAAACAGGATTAGAT
ACCCTGGTAGTCCACGCCGTAAACGATGTCGATTTGGAGGCTGTGTCCTTGAGACGTGGCTTCCGGAGCT
AACGCGTTAAATCGACCGCCTGGGGAGTACGGCCGCAAGGTTAAAACTCAAATGAATTGACGGGGGCCCG
CACAAGCGGTGGAGCATGTGGTTTAATTCGATGCAACGCGAAGAACCTTACCTGGCCTTGACATGTCTGG
AATCCTGTAGAGATACGGGAGTGCCTTCGGGAATCAGAACACAGGTGCTGCATGGCTGTCGTCAGCTCGT
GTCGTGAGATGTTGGGTTAAGTCCCGCAACGAGCGCAACCCCTGTCCTTTGTTGCCAGCACGTAATGGTG
GGAACTCAAGGGAGACTGCCGGTGATAAACCGGAGGAAGGTGGGGATGACGTCAAGTCATCATGGCCCTT
ACGGCCAGGGCTACACACGTGCTACAATGGCGCGTACAGAGGGCTGCAAGCTAGCGATAGTGAGCGAATC
CCAAAAAGCGCGTCGTAGTCCGGATCGGAGTCTGCAACTCGACTCCGTGAAGTCGGAATCGCTAGTAATC
GCAAATCAGAATGTTGCGGTGAATACGTTCCCGGGCCTTGTACACACCGCCCGTCACACCATGGGAGTGG
GTTGCACCAGAAGTAGATAGCTTAACCTTCGGGAGGGCGTTTACCACGGTGTGATTCATGACTGGGGTGA
AGTCGTAACAAGGTAACCCTAGGGGAACCTGGGGTTGGATCACCTCCTTACCTTAAGATGTCGAAGTTGT
TGAGTGTTCACACAGATTGCCTTGATTCAAAGTAGTTAGAGCAAAGACCTGATGCGAAAGTGTCAGTGCT
TATCTGGCTTCGGTCAGATGAGAGAAGCCCTATCATTGGGTTTTGGGATGTGAATAATGGCGCTCGGCCT
CGCAGGCTCGGCACTCGCCATTACCCAAAATCTGCACTGCTGACGCAGCGCAAAGATGATTTTGGGTCCC
CTTCGTCTAGAGGCCTAGGACACCGCCCTTTCACGGCGGTAACAGGGGTTCGAATCCCCTAGGGGACGCC
ACTTCTCTTCTTGCTAACAAGAATGCAGAGTTAAGAACTGATTCTTAACTCTGTTTTCTTCGGCCTTGCG

CCGTTGCAAACATGCTCTTTAACAATCTGGAAAGCTGATTTAAAAAGTAGTTCTCAAACATTTGTTACAA
GTGCTTTGGAAACTTCTTGGCGAAAACCAAATTTTATTTGGTCCTTGTTGTACGACAACAAGCAAGCGTT
GTTTCGACGACACTTCTTGGGGTTGTATGGTTAAGTGACTAAGCGTACATGGTGGATGCCTTGGCAGTCA
GAGGCGATGAAGGACGTACTAACCTGCGATAAGCTGTGAGAAGTCGGTAAGAGACGCTATTACTCACAGA
TTTCCGAATGGGGAAACCCACCCGAGATAACTCGGGTATCTGTTACTGAATACATAGGTAACAGAGGCGA
ACCGGGAGAACTGAAACATCTAAGTACCCCGAGGAAAAGAAATCAACCGAGATTCCCTCAGTAGCGGCGA
GCGAACGGGGATTAGCCCTTAAGCATCTTGGAAGTTAGTGGAACGGTCCTGGAAAGGCCGGCGATACAGG
GTGATAGCCCCGTACACGAAAACGACCTTGATGTGAAATCGAGTAGGGCGGGACACGTGACATCCTGTCT
GAATATGGGGGGACCATCCTCCAAGGCTAAATACTCCTGACTGACCGATAGTGAACCAGTACCGTGAGGG
AAAGGCGAAAAGAACCCCTGTGAGGGGAGTGAAATAGAACCTGAAACCGTGTACGTACAAGCAGTGGGAG
CCCTTCGGGGTGACTGCGTACCTTTTGTATAATGGGTCAGCGACTTACATTTTGTAGCGAGGTTAACCGT
ATAGGGGAGCCGTAGGGAAACCGAGTCTTAACTGGGCGTCTAGTTGCAAGGTGTAGACCCGAAACCGGGT
GATCTAGCCATGGGCAGGTTGAAGGTTGAGTAACATCAACTGGAGGACCGAACCCACTAACGTTGCAAAG
TTAGGGGATGACCTGTGGCTGGGGGTGAAAGGCCAATCAAACTCGGAGATAGCTGGTTCTCCCCGAAAGC
TATTTAGGTAGCGCCTCGGACGAATACTACTGGGGGTAGAGCACTGTTTGGGCTAGGGGGTCATCCCGAC
TTACCAACCCCATGCAAACTCCGAATACCAGTAAGTAATATCCGGGAGACACACGGCGGGTGCTAACGTC
CGTCGTGAAGAGGGAAACAACCCAGACCGCCGGCTAAGGTCCCAAAGTTCTGGTTAAGTGGGAAACGATG
TGGGAAGGCTCAGACAGCTAGGATGTTGGCTTAGAAGCAGCCATCATTTAAAGAAAGCGTAATAGCTCAC
TAGTCGAGTCGGCCTGCGCGGAAGATGTAACGGGGCTCAAACCAGGCACCGAAGCCGCGGATTCACACTT
ATGTGTGAGTGGTAGGGGAGCGTTCTGTAAGTCTGCGAAGGTGTATCGAGAGGTATGCTGGAGATATCAG
AAGTGCGAATGCTGACGTAAGTAACGATAAAGGGGGTGAAAAGCCTCCTCGCCGGAAGACCAAGGGTTCC
TGTCCAACGTTAATCGGGGCAGGGTGAGTCGACCCCTAAGGCGAGGCCGAAAGGCGTAGTCGATGGGAAG
CAGGTTAATATTCCTGCACGACTTGTAATTGCGATGGGGGGACGGAGAAGGCTAGGTGGGCCAGGCGACG
GTTGTCCTGGTGAAAGTGCGTAGGTGGTGTTTCTAGGCAAATCCGGAGACACAACACTGAGACACGAGAC
GAACACACTACGGTGTGGAAGCCATTGATGCCCTGCTTCCAGGAAAAGCCTCTAAGCTTCAGATTACAAG
TCATCGTACCCCAAACCGACACAGGTGGTCGGGTAGAGAATACCAAGGCGCTTGAGAGAACTCGGGTGAA
GGAACTAGGCAAAATAGAACCGTAACTTCGGGAGAAGGTTCGCTCTTGACAGTGAAGTCCCTTGCGGATG
GAGCAGTTGGGAGTCGCAGTGACCAGATGGCTGGGACTGTTTATCAAAAACACAGCACTCTGCAAACACG
AAAGTGGACGTATAGGGTGTGACACCTGCCCGGTGCCGGAAGGTTAATTGATGGGGTTAGCGCAAGCGAA
GCTCTTGATCGAAGCCCCGGTAAACGGCGGCCGTAACTATAACGGTCCTAAGGTAGCGAAATTCCTTGTC
GGGTAAGTTCCGACCTGCACGAATGGTGTAACCATGGCCATGCTGTCTCCACCCGAGACTCAGTGAAATC
GAATTCGCCGTGAAGATGCGGTGTACCCGCGGCTAGACGGAAAGACCCCGTGAACCTTTACTACAGCTTG
GCACTGAACATTGAACCTACATGTGTAGGATAGGTGGGAGGCTTTGAAGGCGTGACGCCAGTTGCGCTGG
AGCCGTCCTTGAAATACCACCCTTGTATGTTTGATGTTCTAACGCAGGGCCCTGAATCGGGCTCGCGGAC
AGTGCCTGGTGGGTAGTTTGACTGGGGCGGTCTCCTCCCAAAGAGTAACGGAGGAGCACGAAGGTTGGCT
AATCCTGGTCGGACATCAGGAGGTTAGTGCAATGGCATAAGCCAGCTTAACTGCGAGACGGACAGGTCGA
GCAGGTACGAAAGTAGGTCATAGTGATCCGGTGGTTCTGAATGGAAGGGCCATCGCTCAACGGATAAAAG
GTACTCCGGGGATAACAGGCTGATACCGCCCAAGAGTTCATATCGACGGCGGTGTTTGGCACCTCGATGT
CGGCTCATCACATCCTGGGGCTGAAGTCGGTCCCAAGGGTATGGCTGTTCGCCATTTAAAGTGGTACGCG
AGCTGGGTTCAGAACGTCGTGAGACAGTTCGGTCCCTATCTGCCGTGGGCGTTGGATGATTGAAGGGAGT
TGCTCCTAGTACGAGAGGACCGGAGTGAACGAACCTCTGGTGTTCGGGTTGTCACGCCAGTGGCACTGCC
CGGTAGCTAAGTTCGGAATCGATAACCGCTGAAAGCATCTAAGCGGGAAGCGAGCCCTGAGATGAGTCAT
CCCTGACCCCTTGAGGGTCCTAAAGGGCCGTTGGAGACCACAACGTTGATAGGTGGGGTGTGTAAGCGCG
GCGACGTGTTGAGCTAACCCATACTAATTACCCGTGAGGCTTAACCATACAACACCCAAGAAGTGTTCTA
AGGCTTGTAGCAAATACGAACGAATTACTTACGTCAGCTTTCTCAGATTGAAGAATTTGCCTGGCGGCCA
TAGCGCCGTGGAACCACCTGATCCCATGCCGAACTCAGAAGTGAAACGCGGTAGCGCCGATGGTAGTGTG
GCATTCGCCATGCGAGAGTAGGACACTGCCAGGCACCCAATTTCAAGATTACGCCCAGCGTGGTCTTTAC
CGGTTAGTGTGCTGATATGGCTCAGTCGGTAGAGCGCATCCTTGGTAAGGATGAGGTCCCCAGTTCGATT
CTGGGTATCAGCACCATTAAATAGAAAGCCTCCCTGAAAAGGGAGGTTTTTTTGCATCTGTCAGTTTTCT
TATATCCCCCTACATTCCCTATTCCCGCAACCCCCCTCTGCTTGCAGTCGACACAGTTTATCTGCTGATT
TGAGTCCCGGTGTGTTACTCATTTGACTGCTATTGATGGTTTTATCCAGAGATTTAACCATGATTGATTG
ATGTAAAACGTTTTACATGTAAAATCCGGCGCGTTTTGCCTATTACGTGGAGAGAGAGCATGGCGCAGGA
TAACGAATACTGTTTGGGGGCGGTACCCGCCAAAGGGCGCAAAGGTGTGCTGTCGCTGATGCTGGTGATG
CTCGGCCTGACCTTCTTCTCTGCCAGCATGTGGACCGGTGGTGCGCTTGGTACCGGTCTGGTGTTCGATG
ACTTCATCAAGGCGGTACTGATTGGCAACCTGTTGCTGGGGATCTATACCGCCTGTCTCGGTTATATCGG
TGCTTCAACCGGTCTCTCTACCCATCTGCTTGCGCGATACTCCTTCGGCGTCAAAGGTTCCTGGCTGCCA
TCCCTGCTGCTGGGCGGTACTCAGGTCGGCTGGTTTGGCGTGGGCGTGGCGATGTTTGCCCTGCCGGTGC
AAAAGGCGACCGGCATAGATGTGAACTGGCTGATCGCCATTTCGGGCATTTTGATGACGGTGACCGTCTA
CTTCGGGATCTCTGCTCTGATGGTGCTGAGCTTTGTGGCCGTACCGGCTATCGCCCTGCTCGGTGGCTAC
TCGGTCTGGCTGGCGGTGACCTCGACCGGTGGCATGGCTGCACTGCAGCAGGTACAACCAAGCAATCCCA
TCGAGTTCACTACGGCGTTGACCATGGTAGTCGGTTCCTTTATCAGTGCAGGAACCCTGACCGCCGACTT

TGTCCGCTTTGGTCGCAAACCCTGGGGCGCCGTCATGATTACCATGGTGGCGTTTTTTTTGGGCAACACG
CTAATGTTCGTCTTCGGTGCAGCTGGTGCGGCGGTAACGGGGCAGGCCGATATCTCGGAAGTGATGCTGA
TGCAGGGGCTGCTAATCCCCGCCATTCTGGTGCTGGGCCTCAATATCTGGACCACCAACGACAACGCGCT
CTATGCCTCCGGCCTCGGTTTTGCCAACATTACCGGCCTCAAGAGCAAGCCCCTCAGCGTGATCAACGGC
CTCATCGGTACCGCTTGCGCCCTCTGGCTTTACAACAACTTTGTCGGCTGGCTGGCCTTTTTGAGTACGG
CAATTCCGCCGATCGGTGGCATTATCATCATGGATTTCCTGCTCAATCGTGAGCGTTACCGTCAATTTGC
CGATCAGCAGTTCGAGACGGTGCGCTGGCAGGCGCTGGTTGCCACGGCAGTCGGGGTGGCTGTCGGCAAG
TGGCTGCCTGGCATAGTGCCACTCAACGCGGTATTGGGCGCCGCCTTCACCTATTTCATTCTGGTTCGCC
TCACCCAGCGGCCGGTTCTGGTACAGGAAGCTAACAAATGCTGATTCAACATATTCGTTTGGCCGATCGG
GAAGGGTTTTGGCAGATCCGCTGTCAGGATGGGTTCATCACAGCCATCGAACCCCATGACGAACATGCCG
TCGCCGGTCGGGTGCTGGACGGGGAGGGCGGTCTGGCGATCGCTCCCTTTATCGAACCCCACATTCACCT
CGATACCCCCCAGACGGCGGGCGAGCCGAGCTGGAACCTCTCCGGCACCCTGTTCGAGGGGATCGAGCGC
TGGGCCGAGCGCAAGGCGCTGCTGACCCATGAGGATGTGAAACAGCGCGCCATCCAGACGCTGAAGTGGC
AGATCGCCAACGGCATCCAGTTTGTCCGTACTCACGTCGATGTCTCCGATCCCAATCTGGTGGCGCTCAA
GGCGATGCTGGAGGTGCGGGAAGAGATGAAGGAGTGGGTGGAGCTGCAGATCGTTGCCTTCCCGCAGGAG
GGGATTCTCTCCTACCCCAACGGCAAGGCACTGCTGGAGGAGGCGCTCAAGCTGGGGGCCGACGTGATTG
GCGCGATCCCCCACTTCGAGTTTACCCGGGAATATGGGGTCGAAAGTCTGCACTACATCTTCGATCTTGC
CGAGAAGTATCAGGTTCTGGTCGATGTGCACTGTGACGAAATCGATGACGAGCAGTCCCGTTTTATCGAG
ACGCTGGCAACTCTCGCCTACGAGCGGGGTATCGGCCATCGGGTGACGGCCAGTCACACCACCGCCATGC
ACTCCTACAACGGCGCTTATGCTTCCCGCCTGTTCCGTCTGCTCAAGATGGCGGATATCAACTTCGTGGC
CAATCCGCTGGTGAACATTCACCTGCAGGGGCGGTTCGATACCTATCCCAAACGCCGCGGCATTACCCGG
GTGAAGGAGATGCTGGAGGCGAACATCAACGTCTGTTTCGGTCACGACGACGTGTTCGATCCCTGGTACC
CCATGGGCACCGCCAACATGTTGCAGGTGCTGCACATGGGGCTGCATGTCTGCCAAATCATGGGTTACGA
GCAGATCAACGACGGTCTGAAGCTGATCAGCTGCCACAGCGCCCGCACCCTGAATGTGCAGGATCGCTAT
GGTATCGAAGTGGGCAAACCGGCCAACCTGTTGGTCCTGCCCGCCGACAACGGCTTCGATGCGGTGCGCC
GTCAGGTGCCGGTGCGTTATTCAATTCGTCACGGCAAGGTGATTGCCCAGACCCGCCCGGCCCAGATCGA
AATCGTGCTGAGTCAGCCAGAGCCCATCGATTTCGCCGCTGAGATTTCAATCAGGCTGGATGTCGATTA
GTTCCGCCTCTGGGCACAAGGGCCGCCATTGCGACATAATGGCGGCCTGACCCTGAGGGAGTGACACCAT
GCAAACCTGGTTCTATGTTGCTGCAGGGGGCGCCATCGGCGCCTGCCTTCGTTTTGGGATTGCCGAACTG
ATGGCCTTGCTGCTTGGGCGCCACTTTCCTTACGGGACTCTGGTGGTCAACGTGGTGGGCTCTTTCATCA
TGGGGGTGGCGTTCGCCCTCATCAGTCATGGTCATGTGGTAGAACACCCGATGAAGCCGCTGCTGATGGT
GGGGATCCTCGGTGCCCTGACCACGTTTTCTTCCTTTGCCCTCGATACGGTAGTGCTGGCTCAACATGGG
GCCTATCTGAAAGCGCTGCTCAATATCGGTCTCAATCTCTTCCTCTGTCTGGCCATGGTGGTGCTGGGCA
TGCAGTTGGTTGCGAGCCGGGTTTAAGGTGTCCGGACGCCAGTGGCTCTGGTAAACTGGCGGCCATTCTT
TGCTCTATGGGTGTTTAAGGGATGTCAGAACAAGCAACGACAACGCATTTTGGCTTTAAAACCGTGGCCG
CGACCGAGAAGGAAACTCTGGTAGCAGGTGTCTTCCATTCGGTGGCAGCCAAGTATGATCTGATGAACGA
TCTGATGTCGTTCGGCATCCACCGCCTGTGGAAGCGCTTCACCATCGACTGTTCCGGTGTGCGCAAGGGC
CAGAAGGTGCTGGATCTGGCCGGTGGTACCGGTGACTTGACCGCCAAGTTTTCCCGCATCGTCGGCGAGA
CCGGTCAGGTGGTGCTGGCGGATATCAACGACTCCATGCTGAAAGTGGGCCGCGACAAGCTACACAATCT
GGGGGTCGCCAACAACGTCTCCTACGTGCAGGCCAATGCCGAGGCGCTCCCCTTCCCGGATAACCATTTC
GACGTGATTACCATCGGCTTCGGCCTGCGCAACGTCACCGACAAGGACAAGGCGCTCGCCTCCATGTTCC
GGGTGCTCAAGCCGGGTGGCCGTCTGCTGGTGCTGGAGTTCTCCAAGCCGGTCAGCGAAGTGATCGCCAA
GCTCTATGACCTCTACTCCTTCAAACTGCTGCCGAAAATGGGCGAAATCGTCGCGAACGACAGTGAAAGC
TACAAATATCTGGCAGAGTCCATCCGTATGCACCCTGATCAGCAGACCCTGGCTGGCATGATGGAAAATG
TCGGGTTCGAGCAGGTGGAGTACTTCAATCTGACTCAGGGTGTGGTCGCCCTGCACCGCGGTTACAAGTT
TTAAGGTTGCCTATGCCGATGGATGCCATGGTCACCGCGGTGATCGAAACCAGCCTCAACCAGTTGCTGG
CGCTGGACAAACAGAGCCCGGAGCGGCTGCGCAAACTGGTGGGCAAGGTGCTCAAGCTGGAGCTGCGTGA
ACTCAAGCCGCTCTGGTTTGTCTTCTCCGAGCGTCGGCTGGATGTGCTGGCCCAGCATGAAGGGGAGGCC
GATGCGGTGCTCAGCCTGTCGCTGACCGCCCTTGGTCTGCTGAAAGACCCGTCCGCCCTGACCCGTTATA
TCCGCGAGGAGAAACTGGATCTGAGCGGTGATCCCCAGCTGGTACAGGCATTCAGCGTGCTGCTGGGTGA
GCTGGATATCGACTGGGAAGAGGAGCTGTCGCGCTATACCGGCGACGTGCTGGCCCACACCCTGTTCAGT
GGTGCCCGTCAGGCGCGTCGCCTGGTCGGTCGCGAGCTGTGCCGCACCCGCAGCCAGCTGGCGGAATATC
TGACCGAAGAGGCCCGCCTAGCCCCCGGTCCGCTGGAAGTGGCGAGTTTCAACGATGACGTCGAGGTGCT
GGCCCAGCAGCTCAAGGCCGTCGAACTGCGGCTGGCCCGCTTTGAACAGCAGGTGACCTGATGACCCCGA
AGGAGTTCAAACGCCTCTATCGCATCATCACCATCCTGCTGGAGCAGGGCATCGACGAACTGGTGCCCGC
CCGCTATCAACCCTGGCCGGGTCGTCTGGCCCGTCGCTCCCTGTTCTGGCTCAAGAACAAACAACCGGAT
CTCAGCCGCGGTGCCCGCATCCGCCTCGCCTTCGAGGCTCTTGGCCCCATCTTCATCAAGTTTGGCCAGA
TGCTCTCGACCCGCCGCGACCTGCTGCCGCCGGACATTGCCGAGGAGCTGGCCCTGCTGCAGGACAGGGT
GCCCCCCTTCTGTGGTCAGGCGGCGCGCCAGCAGATTGAGCTGAGCCTGGGTTGTCCCATCGAAACCCTG
TTTGACGACTTCGACGAGACGCCGCTGGCGTCGGCTTCCATCGCCCAGGTGCATACCGCCCGCTTGAAGG
AGAGTGGCCGCGAGATCGTCATCAAGGTGATCCGTCCCGACATCGAGCCGGTTATCGAGGCTGACCTGCG

GCTGATGCAGGCGCTGGCACGTCTGGTGGCCCGCTTCGTGCCCCAGAGTGCGCGGCTGCGCCCCATCGAG
GTGGTGGAGGAGTATCGCAAGACCATCCTCGACGAGCTGAATTTGATGCGCGAAGCGGCCAACGCCATCC
AGCTACGGCGCAATTTCACCGGCTCCGAGGCACTCTATGTGCCTGAAATCATTACCGACCTCTGCCGCGA
GCAGGTGCTGGTGATGGAGCGCATCTACGGTATTCCGGTCTCCGATATACCGGCGCTGGAAGCCAACGGC
ACCAATATGAAGCTGCTGGCCGAGCGCGGGGTCGAGGTTTTCTTCACCCAGGTGTTCCGCGACAGCTTCT
TCCATGCCGACATGCACCCGGGCAACATCTTCGTCTCTTACGAACATCCGGAAAACCCGCTCTGGATCGG
CATCGACTGCGGCATCGTCGGTACCCTCAATCGGGAAGACAAACGCTATCTGGCGGAGAACTTCCTCGCC
TTCTTCAACCGCGACTATCGCCGGGTGGCGGAGCTGCATGTGGAGTCGGGCTGGGTACCGGCAGACACCA
AGGTGGACGAGTTCGAGTTCGCCATCCGCACCGTGCTGGAGCCCATCTTCGAGAAGCCGCTCTCCGAAAT
TTCGTTCGGTCACGTGCTGCTCAATCTGTTCAACACTGCCCGCCGCTTCCATATGACGGTGCAGCCACAG
CTGGTGTTGCTGCAAAAGACCCTGCTCTATGTGGAAGGGTTGGGGCGTCAGCTCTACCCGCAGCTCGATT
TGTGGCAGACCGCCAAGCCGTTCCTCGAAAACTGGATGCACGAACAGGTAGGGCCCAAGTCGGTATTGAA
CGCCATCAAGGAGAAGGCGCCATTCTGGGCCGAGAAGCTGCCGGAGCTGCCGGAGCTGGTCTATGAGACC
CTGCGCCAGACCCGTCATCAGCAGCGTCATTTCGACCAGATGTTTGCCGAGTTCCGCCGTCACAGCCGTC
GGCAGGGGCAGGCCCGCTACCTCTTAGGGGTTGGAGCCAGCCTGCTATTGGCGGGTGTATTCTTGCTGAC
CCAAAAACAACACATTGAGTGGGGACAAGCCAGTCTCGCCGGTGCAGCCCTCTGCTGGCTGGTCGGCTGG
TTGCGAACCCGTTCCCATTAATCAACAACCGCGCCGTCAGGCGCCTGTTCTGGAGAGAAAATCATGGGTG
GTATCAGTATTTGGCAATTGTTGATCATTGCAGTCATCGTCGTGCTGCTGTTCGGTACCAAGAAACTGCG
CGGTATTGGCGGTGATCTGGGCGCGGCGGTCAAAGGGTTCAAGAAAGCCCTCTCCGACGAGCCGGCTGAT
GCCAAGGCCGAGCAGAAAGATGCCGAGTTTCCCCCGAAACAGCTGAACGAAGCGGCCACTCAGAGCAGTG
AGACCGCCAAGCAGAAAGACAAAGACCAGGCCTAAGCCATGTTCGATATCGGTTTTTGGGAGCTGGTCGT
TATCGGTGTGGTAGCACTGGTGGTACTGGGGCCCGAGCGGCTGCCGGTTGCCATCCGCACGGCTACCCAC
TGGATCCGCCTCATTCGTTCGACCGCGAACTCGGTCAAGTCCGAGCTGGAGCAGGAGCTCAAGTTGCAAG
AGCTGCATAACGACCTCAAGAAGGCCGAACAGTTGCAGATGAGCAACCTGAGCCCCGAGCTGCAGGAGTC
CATCGAACAGCTCAAAGCGGCGGCCCAGTCGGTCAATCGCCCCTATCAGGTGGAGAACGAGATCCGTCCG
CCGCGCCCCGAGCCGGTCGCCCAGGCGGAGCCTGCCACGCCGGTGGAGCCTGCGGTGCAACTGAGCCACG
ACGTGCCGCCGGTCAACAGCCAGCGGGAGAGCGAGCCGGTGCCGGTCAGCGACTCCGTAGTAAAAGGGGA
GGTGAAGCCATGAGTCAGGCCGAACAACCCCTGATCAGCCATCTGGTTGAGCTGAGAACCCGTTTGCTGC
GCTCCATTACCGCCATCCTCGTGGTGTTTCTGGCGCTGATCTACTTCTCCAACAACATCTACGACTTCGT
GGCCCAGCCGCTGCTCAGCCAGTTGCCGGAAGGGACCAGCATGATCGCGACGGATGTGGCGACCCCCTTC
CTGACGCCGATCAAGCTGACCCTGGTGGTCTCCTTCTTCGTGGCGATCCCCTATCTGCTCTATCAGGCCT
GGGCCTTTATCGCCCCCGGTCTGTACCAGCATGAACGGCGCCTGATCATGCCGTTGGTGGTCTCCAGTGC
CCTGTTGTTCTATGCGGGGATGGCGTTCGCCTACTACGTGGTGTTCCCGCTGGTGTTCGGTTTCTTCACC
AGTACCGCCCCTGCCGGGGTGACGGTGGCGACCGACATCGCCAGCTATCTGGACTTCGTACTGACCCTGT
TCTTCGCGTTCGGGGTGGCGTTCGAAATTCCGGTGGCCACCATACTGCTCTGCTGGACCGGCGTGACCAC
ACCGCAAAACCTGAAGGAGAAACGTCCCTACGTCGTTGTCGGGGTGTTTGTGGTCGGCATGCTGCTGACG
CCGCCTGATGTCTTCTCCCAGACCCTGCTCGCCATCCCGATGTGGGCGCTGTGGGAGATAGGTCTGTTCT
TCGCCCGCTTCTACGTGAAGAAAGAGGATGAAGAGCAACAGGAACAACCGGATGTGGAGAGCTGAGGCTC
TGTTCGTCACCTGTTTGCCGGACGGCGAGAGGATGACGAAGTCCGCAGGAATAGCAGAACGAGGGGAGCT
GAGGCTCCCTTCCTCTTTTCATGCTCCCGTTCGAGGCGTATGGTAAGACCCCATTCGGTAGATGGTTTTC
AAATACCTGATATGTATTCCTTTTGTTCGGAACGTCTCATCCCATGATCGATATCGGTTTAAACCTGACC
AGCAGCCAGTTCGCCGGCGAGCAGGCTGAGCTGGTGGCCCGTGCTCGCGCCGCCGGAGTGGAAGCGCTGA
TCCTGACCGGTACCGATCTGGCTGGCAGCTGCGAGAGCGCCGAGTTGGCCGCCCGCTGGCCGGGCTACTG
CTTCTCCACCGCCGGGGTCCATCCCCACGATGCCAAGAGTGTCGATGAGGCGACCCTGCCCGCCTTGCGC
GAGCTGGCGGCGTTGCCGCAGGTGGTGGCTATCGGCGAGTGCGGCCTCGATTACAACCGCGACTTCTCCC
CCCGTCCGGTGCAGGATGCGGTATTCGACGCCCAGCTGGCACTGGCTGCCGAGCTCGGGATGCCGGTATT
TCTCCACTGTCGCGATGCCCACGCCCGTTTTATCGAGATCCTCCGTCCCTGGTTGCCGAAACTACCGGGA
GCCGTGCTGCACTGCTTCACCGGTTCCGACGAGGAGCTGGACGAGTGTCTGGCGCTGGGGCTGCATATCG
GGGTGACCGGCTGGCTCTGCGACGAGCGCCGCAGTCAGCTGCTGCGCGAACAGGTGGCTCGTATTCCGGC
CGGTCGGCTGATGATCGAGACCGATGCCCCCTATCTGGTGCCGCGAGATCTGAAACCGCGCCCGAAACGC
AACGAACCCGCTTTTTTGCCGCACATCGCCCAGGTGGTGGCGGCCTGTCGTGGTGAAGCGCCCGAGGCCT
TGCTGGCCCATACCCGGGCCACCTCCGCCGCGTTCTTCCAACTTCCTCTCCAGGAGTGATTCATGGCTAC
AACTCTTCCGGGCGCCTTTCCGGGCCGCCGCCTGCGCCGTCTGCGCAAACATGATTTCAGCCGTCGTCTG
GTGCGCGAGAACGTACTGACCGTCAACGATCTCATCTATCCGGTGTTCGTGCTGGAGGGGGAGAACCGTC
GGGAAGCCGTTCCCTCCATGCCGGGGGTGGAGCGTCTCTCCATCGATCTGCTGCTGATCGAGGCTGCCGA
GCTGGTGGAGCTGGGCGTGCCTGCCATCGCCCTGTTCCCGGTGACCCCGCTGGAGAACAAGAGCCTGATG
GCGGAAGAGGCCTACAACCCGAACGGGCTGGCCCAGCGCACCGTGCGTGCCCTCAAGGCCCGCTTCCCGG
AGCTGGGGGTCATCACCGACGTGGCGCTCGATCCGTTCACCACCCACGGTCAGGATGGCATCATCGATGA
CGAAGGCTATGTACTGAACGACATCACCACCGAGATCCTGATCAAGCAGGCACTCTCCCACGCCGAGGCC
GGGGTCGATATCGTCGCGCCGTCCGACATGATGGATGGCCGCATCGGTGCCATCCGCGCGGCGCTGGAAG
AGAACGGCTTCATCAACACCCAGATCATGGCCTACTCCGCCAAGTACGCTTCCTGCTACTACGGCCCGTT

CCGTGATGCGGTTGGTTCTGCCGGCAACCTCGGCAAGAGCAACAAGGCCACCTATCAGATGGACCCGGCC
AACAGCAACGAAGCGTTGCATGAAGTGGCGCTGGATATTCAGGAAGGGGCCGATAGTGTGATGGTCAAGC
CGGGCATGCCCTATCTGGACGTGGTTCGTCAGGTGAAGGATCAGTTCGGCGTGCCGACTTTCGCCTATCA
GGTCAGCGGCGAGTACGCCATGCACATGGCGGCGATCCAGAACGGTTGGCTGAAAGAGAAGGAGACCATC
ATGGAGTCTCTGCTCTGCTTCAAGCGGGCCGGGGCCGACGGCATCCTTACCTACTTCGCCAAGCGCGTAG
CGCAGTGGCTGAAAGAAGATGCCCGTTAAGCCGGGTTGAAGAGATAAACGAGAGGGGGAGCCGATGGCTC
CCCCTCTGTCGTTATGGCTCTCTTTTGGTCACGAGCGGATGATTACAGCTTGATCCAGGCATCGCTCCAG
TTGCTGCCGGTACCCGGTGCATAGTGGCTGGTCGAGCCGCCACACCACCCACTGTAGGGCCAGGGTTTGC
ACTCGTAGTCGCCACCGGCATTGCTGACCCGTTCGCCACTCTGATAAGCGGTGCCCGCAACATAGGCCGG
GTAGCTGCCGTTACCACCGTCACTGTCACTCCCCGGAGCCTGTTCCACCAATGCCAGGGTGAAGCGGCGC
TCGAAGCCCTGCTTGCTCCCCTCTGCCATGCCCGCCAGCAGCAGCTCGTAACTGCCGGCCGCTAGGCCCG
ACTGTGCCAGCTGCAGGGAACCCTGACCATCGATCAGGGCGCTGCCGGTGGCGACCGGATTGCCATCCTG
CAACAGGGTCGCGGTGACGGTCACCGGGTTGGCGGTGGTCAGCGTCAGGCTGAAGTCGATCGCGCCGTTG
ACGATGAGATATTCGGCCGCCAGATCGGTGACGGTGAAGTCACTGCTGCTACCGGTCGTGGGCAGAATGA
AGTCTCCGCTCAGGTAGGTCAGACCGGTCCAGCCGGAGCTGGTCAGCCAGTGCTTGCCATTGGTCTGATA
GACGTTGGGCAACTGGGTGCACTGTTTGTTGTCATTGACGAAGCCGATACCGTAAAAGCCCGCCACATTC
TTGTAGCAGAGCTGGCGACCATTCTGGTGCAGCACTGGCTGATCCAGCTTGCCCTTGTGGTGGAGTGACA
GCCAGTCAAACTGGTTGATGGCCGAGACCAGCGGCACAAACTGGCCATCGGGCTGACGATAACGGATCAG
GGATCCGGCATCACAGAGCAGACCATCAGCCTGCTGTTCGACGTAGCCGTAGTACTGCTCGCCATCCTGG
AAGGTGAATCGGCAGAGATTGCCTTTCTCCGACTCCTTGGGGATCCAGCCCATCAGCAGTCGCTCCTGAT
TGGTCAGGGCCGCCAGCTGCCCGGCACTGAAGTCGCTGCCGTTGGCTACCCAGCCTTGCGGCATGGTGGA
GAATTGCATGAAGTAGTGGTCGGTATAGTACATGTGGCTGTCAGCGCGGTGCTTGCGAGCCCCCTGGCTG
ACCGAGTTGTAGCGATAGATGTCGTTTTCCGGCTTGTGGAAGCCGTTGAGATCATCGTCCAGATAGGGCA
GGTCGCCCTTGCGGATATGGTAGTTGGCCATGCCCGTCATCAGGGGATGCCAGCCATGGCTGCCACGGGC
AAACGCGCTCTCATGGCTGTAGGACTTCCCATCGAAGCCATGGCCAAATTCGTGGGCAATGATGCCCCAG
CTACCGTAGTAGTGGCTGACAAAGTTAAAGGTATCGACCCCGGTGATGCCACCACTCCCCAGCCCGCCCC
CCATGGCGGTGATCCCCAGGTTGAGGTAGGGGCGCGTCATCAGGCTGTGGTAGTAGTAGTCATAATCGGC
TGCGGTAAAGATCCCGTTCGGCACATAGCTGTTGCCGCCATTGCCGAACATCTCGTAGCCGTTCACCGCC
TTGAAGTTGTACCAGACGTGTTTGAACTCCTCGGTCGAGACCATGTAGGCTAGGTTGGTGGCGATGATCA
GCCACTCGCGGGCGTAGAGGGCATTGATGGGGCGCCAGGAGCCGCTGGTGTTGGTGGCGAAGCGCACGTT
CCAGCTTGGCTTGAGGCGTGCCAGCTTCTGGGCAAACGGATCCGAGTTGGAGCTGAACCTGTGCTCGAAC
TGGGCGGGCAGATTGATGCTGTTGTCGAGCAGGTTGTTGAGATCAAACACGCCATCGCGGCTGGCGCTGT
TGAAGCGTCCCTGCTCGCCAAAGGCGCTCAGTGGCAGGGTGATTTGGGCATAGGCGGGCAGGCGATCGAG
CTGGCCCAGGATCAGCCACTTGTTGATACCGTTCAGGCGTGACTCGATATTGATGTCATGCAGCTCTCTG
GCGGCATAGTTGGTGATGCGCACCGTGTCGTTATCAAGCAGCTCGGTCTTGACCAGCGAATTGATGTTGA
CCGAACGCTGGGCGCGATCGATCAGCACCTCGTAGGGTTCGTCATTGCGCAGTATGTAGCTGACTGCGGC
CGGCATCAGCTGATCGAAGATCAGCGGGCGAGCCGGCAACTGCTCGTCGGCCGTGAAGCTGATCTCCTTG
CTCAGCAGCTGGTTGAAACCCAGGCCGCGCTCGTTGGCGGTGACAAGCGCCGAGCCCGCCAGCTGGAGAT
CTTTCAGTGCCGCCACGGGCTGCTGGTTGATCTCCCGAACACTAAAGGTCGGGTTCTTGAACTGAAAGTC
GTTGGCGATCAGGGTATCGGCATCATGCCACTCGATGCGGTCCTTGAAGTTCACCACCAGCCGATCGCCA
TCCATGTCGATTTTTTGCAGATTGCTGTCGGCGATCACCTGCTCAGGCGCCAGCGGTGTGTTGCTGGCGA
TGGCATCAGCCAGTTTGCGCAAGTCATAGATGTAGTAGTTTTTGCCGGCGGTATGGGCCAGGAGATAGTC
ACCCACCTGATGGAGTTGCACCAGACGGTGGCTGTATTTTCCTTCAAAGGCGAGCCGACCGGCGATGGGG
GTCTGGAGGCTGTTCTCCGGCGTCACATCCTGCTGGCGGTAGACCTTGATCTCGCTCAGCGCATCGGCCA
CCAGTACGTAATCCTGATTGGCAAAGACGGCCTGGGCGCGGTGCAGGCTGTTGCTGCCGGAATCTCGACC
GGTTCCCAGGGTGGTGACATGGCGATGTTCATCGGCCAGATCGAAGATATCGACCCGGTGCGAGTAGCGC
GAGACCACGTAGAGCAGGTTGTTGCGGACAAACAGTTCGCTGACCTGGTTGTAGGCCGTGGCACTGCCGG
TTGCGGTGCCGCGGATCTCGCCAATCTGCTGGCGGCTCGGGATATCGATAATGGCCACATGGCCCGGATT
GCCGTTCTGGCTGACATAGAGCCGATCCTGATAGCGCTCGACCGCCGTGATTAGGTTGGCGTCCAGCCCC
AGCTGATCGGCCGTCAGCTCCTCGCCTTTGTCCAGATATTGCAACGAGGTGGCAAACGAGTTGACGCTGC
AGGAGCTGGTCTCCATCACCACCCGGGTGCGTTCGCCATTCTGGATCTGGCCGTGGGGGAAGTACTCCAG
CAGACAGCCGTTGCCGTAGTCATAGTTGCCAAAGCGTTTGTTGACCGCAACGACCCCACCGTCGTACCAG
GTCAGCTCCCACTCCTCATCGAAGAAATCAAAGACGTTACGAATGGCTGTTTCGTCGAAAAAGTGGGGTT
TGGGCAGATCGAACAGGATGTGGTTATCCGCTGTCAGCGGGTAGAGCAGGCCGTTGATGGCAAGGTGTCG
CTTGCCATCCTTGATGATCACGTTCTGCTCGTCAGCCGCACTCTGGTGCAGCGCGGCGAGGCGCGCCGTC
ATCTGCTCGTAGTGCTCCTGATAGGTTTGTTTGAAATAGGCTTGTTCGCGCTCATCGGCCACGACATGGG
CCGACAGCAGCAGACCGAGCAGGGTGGCAAGCTTGGTGGTTCTTGGCATGGTGATTTCCTTATCTTGGTG
GTTGTTGGACGTTTTTTGGTGGTTGGCAAGCATGTAAAAGAGAGGGGCTAACCCGTCCGGGTCAGCGCAT
CCTGCCCATCGGCAGCGTGGCCAACGGGCAGGATATGGGCGGGGGCCTGTCGGCCTCCGTTAGTGGTTGG
GGCAAGGTCAGAGCTTGATCCAGGCATCGCTCCAGGCAGAGCCGGTACCCGGCGCATAGTGGCTGGTCGA
GCCTCCACACCAGCCGCTGTATGGCCAGGGTTTGCACTCGTAGTTGCCACCGGCATTGCTGACCCGATCA

CCGGACTGATAGCCGGTGTTGGGGGCGTAGGCCGGGTAGTCACCTTCGCCACCCCCGCTGTTCTCTTCGG
TCAGCGCAACCGGGAAGCGCTGTACCACCGGCTGCTGGCCGTCAGCCAGACCGGTGATGACCACTTCGTA
GCTGCCATCGGTGACGTTGCTGCCCTGCAGTACCAGGGTGCTGGTGCCGTTCACCTGGCTGCTGGTGGTA
CCGTGGCTGTGGCCGCTCTGCTCCAGAGTCGCGGTGATCTGTACCGGGTTGGCGGTGGTCAGGTTGAAGC
GGATGCTGACCTGACCGTTGACTATGGTGTACTCGCTCGCCATGGCGCTGACGGTGAAGTCGCTGCTGCC
TTCGGCGGGTGGCGTTGTGCCGCCGCAGTCGCTGTCGCCCTGTACGCGCCAGACGCCCCAGTCACCCGTG
GTGCCCGGCTCTTCGTTCTGGGTCCACCAGCCTGCCAGCCAGTTCTTGCCGTTGTGGCTGACGATGTCGC
CGCCCACGTAGACCTTCTTGGCATCCCAGCTGTTTTCACAGACGGCGCCCTGCTCCCTGACGGTCACGTT
GCGCTGCTGGGTTACCTGCTGACCGGCGCTGTCGCTCACCTGATAGGTGAGGGTGTAGCGGCCGACCTTG
CTGCTGTCGACGTTGCCGCTGACCTTGATTTGGGTGGTCAGGTCGCCATCTTCTTCATCATGGACGCTGA
CGCCGGTCATCGGATCGAAAGCGGTACCCAGTTCCAGCGTGGTGTCGGTGACCCCACTGAATACCGGCTT
CATGCTGTAGACCTCGATGGTGCGTACCTGCTCGGTGACGTTGTTGTCGCGATCGCTCACCTTGTAGGTG
AGGGGGTAGAGGCCGATGCGCTGGCTGTCTACCGAGCCTTCTACCTGGATCTGGCCAGTCAGGTCGCCAT
CTTCCTTGTCGTTGGCGCTGACACCGGCCAGCGGATCAAAGGCGGTGCCCTGCTTGATGCGGGTATCGCT
CAGGCCGCTGAACACCGGCTTGCCGTTGTCGACCGGGGGCGTGATCTCCTGATCGTGGATGAAGGGGCCG
TAGCTCTTGATGAAGGATTCGTTGTAGGACTGACCGGCGGCGTTGCGGCCCATGTCCCAGTTGATGGACC
AGGTCATCACGCCACGCAGCGGTTGCCCCTCGCTCTTCAGGCGGTTGAAGGCGTTGTAGAGCTTCTGCGG
ATCCTGCACATAGCCGGTGGCGGCGGCGTCGATGTTGGTCGGAATGCCGAACACCAGCTTGTCATGGGGG
ATCTTGTAGAAGCCGCGGGTACCGTTGCTCAGCGAATCCGCCATGTAGTAGATGAACTCTTCTTTCAGGG
CATCGTTGTTCTGGGCAATCCAGCCGATGCCATCGATGTAGATACCGTCGCCACCCTGGTTGTAGAACTG
GGGATTGATCCAGTCGTAGTAGCCTTCCAGATTGCGGATATAGGGGATGTAGGCGCCAGTCTGGGTCAGA
TAGGGGAACTCCGGCGCCATGGTGATGAGGAAGTTTTTCCCCTGGGCGCGGTAGTGATCCTTGACCAGCC
GCAGGGCCGCCGGGATCACGGTCTGGTTGTCGGCGGCAGTGATTGCGGCCTGTTCCAGGTCGATATCCAG
CCCGTCGAAGCCATAGGTCTCCACCAGACGGATGATCTCGTCGGCAAAGGCCTGCTCCTGACCCTTGCGC
AGCTCGATGTGCGCATCGGCACCGCCCAGCGCCAGCAGCACGGCACGGCCCTGACGGTTCAGTTCGGCGA
TCTGCTCGATAAACTGGCTTTCGCTCATGCTGATGGCCGGGTCGAGTTTGAAGGTGGGGATGGGATCGCT
GCCATACACCTTCATGAAGGAGACATCGACCACGTTGTATTGCGGGTTGACCTGATCGAGCTTGACGCAG
GGGGCGATACCGCCCTTGTAGCCCGAGCCGTCACACCAGTTGTGCCAATAGCCGACCACTACGCCGGACT
GGGGGTTGACCATCGCCTGACCGGCACTGGCGGCATATGCCATGGGGGCGCCCATGCCGAGCAGGCAGCC
AAGCAGGGATAATTTGAGTTTGTTCATCGTCTCTCCGTACGGTGCAGATGGGGCGTCGGTTGTGACCGGA
CGTCCCCGGGGGCAGCCCCGCTGTCATAGGTGTTTTCCCTTAGACCGGTGGCTTTGCGTCGCGCCCTTTC
GGGTCACTTTGCCTTTTTCACCCGATTTCTGTGCAGCACAGAGAATCAAGTGACCTGCATGGCAGGTTGC
CTATGATAGATGCTTGAAAATAAATTAATCAATAGTTGGTTTTGATTCTAATTAACTGAAAAATATTGAT
AATTTGTTACAGACACCACATTTTGTCCGTTTCCATGATTGAGCATGACGGTACTTGGCTGGCTGCTGTG
GCGCCGGTAAATGGCGACTTTTTGCCCCTTGAAAGTAGCGGTAAGAGCCAAAAAAGGCCCACAGATTCAT
TCTGTTACTGGCTGTTGATGGCAAAACTGCAGCAACGGCAGAGATCCGGGTCGCAATGGCTGGAAAAGTA
CAGGGTTGCGGCGGATTCCCGCTTGATTATTGCGTGGCTTGGCCACATGTTAGAAAGCCATACAGAGCTG
CTAAAGCGGTCTGTTCAAGACCAAACAAGAGGTAACCGGGATGAATAACCCTTTGCTGACAATGGATTCG
CTGCCCCCTTTCAGCCAGATCAAGCCGGAGCAGGTTCAGCCTGCCGTGATTCAGGCGATTGCTGACTGCA
AGCAGAAGATCAGCGATGTGCTGGCCCAGCGTGATCCCCATACCTGGGATAGCCTGATTGCCCCGCTGGA
AGAGGTAAATGACCGTCTGTCACGGATCTGGTCACCGGTCAGCCACCTCAACTCCGTACTCAACAGCGAA
GCGCTGCGCGAGGCCCATGATGCCTGCCTGCCGCTGCTCTCCGAATTCCAGACCTATGTCGGTCAGCATG
AAGGGCTCTATCAGGCCTATCTGGCCCTCTCCCAGAGCGATGATTTCCCGCTGCTGAGCGGCGCCCAGCG
CAAGGAGATCCAGAATACCCTGCGTGACTTCCGCCTCTCCGGCATCGGTCTGCCTGCCGAGGCGCAGCAG
CGTTACGGCGAGATTCAGGCCCGCCTCTCCGAGCTGGCATCCCGTTTCAGCAACAACGTGCTGGATGCGA
CCCAGGGCTGGCACAAGCTGGTGGCCGATGAAGCCGAATTGGCCGGTCTGCCCGACAGTGTGCGTGCCGC
TGCCCGTCAGATGGCTGAATTTAAAGCCAAAGAGGGGTGGCTGTTCACTCTGGATATCCCTTCCTACCTG
CCGGTGATGATGTATGCCGACAACCGCGAGCTGCGCGCCGAGATGTACGAAGCCTTCACCACCCGCGCCT
CCGATCAGGGCCCCAATGCGGGCAAGTGGGACAACTCCGCCATCATGAGCGAGCTGCTCACCCTGCGCCG
CGAACTGGCCCAGCTGCTGGGCTTTGCCAACTATGCCGAGCTCTCCCTGGCCACCAAGATGGCAGACAAG
ACCGAACAGGTGGTGAGCTTCCTCACCGATCTGGCGGCCAAATCCCTGCCGCAAGGCAAGGCCGAGCTGG
AGGAGATCCGCTCCTTCGCCGCCGAACAGCACGGTCAGAGCGAGCTGGCCGCCTGGGATCTCGCCTACTA
CGCCGAGAAGCTCAAGCAGCACAAGTTCTCCATCTCCGATGAGCAGCTGCGCCCCTATTTCCCCGCCAGC
AAGGTGGTGAAGGGGCTGTTTGAAGTGGTGAAGCGGGTGTTCGGCATGAAGGTGCGCGAGCGCCTCGGCA
TCGACACCTGGCACCCGGACGTGCGCTTCTACGACATCTTCGATGCCGACGACGAGCTGCGCGGCAGCTT
CTATCTCGACCTCTACGCCCGCGAGCACAAGCAGGGCGGGGCCTGGATGGATGTCTGTCTGGGTCGTCGC
TACCGTCAGGATGGTTCACTGCAAAAGCCGGTGGCCTACCTGACCTGCAACTTCAACGGCCCGGTGGATG
GTAAGCCTGCACTGTTTACCCACAACGAAGTGGTCACCCTGTTCCATGAGTTTGGCCACGGTATTCACCA
CATGCTGACCCGCATCGATGTGGCCGGTGTCGCCGGTATCAACGGCGTGGCCTGGGATGCGGTGGAGCTG
CCGAGCCAGTTCCTCGAGAACTGGTGCTGGGAGTCCGAGGCGCTGGCCTTTATCTCCGGCCACTACGAGA
CCGGCGAGCCGCTGCCTGCCGATCTGCTGGAGAAGATGCTGACCGCCCGCAACTTCCAGGCGGCGATGCA

GATGCTGCGTCAGCTGGAGTTCGCCCTGTTCGATTTCCGTCTCCATCAGGAGTTTGATCCGGCCAATCCG
GCACAGCTCCCGGCGCTGCTGGATGAGGTGCGCAGTCAGGTAGCAGTGATGACGCCGCCAGCGTTCAACC
GCTTCCAGCACAGCTTCTCCCACATCTTCGCCGGCGGCTATGCGGCGGGCTACTACAGCTACAAGTGGGC
TGAGGTGCTCTCTGCCGATGCCTTCTCCCGCTTCGAGGAGGAGGGGATCTTCAACCCGGCGACCGGCCAA
TCCTTCCTGAAGAATATTCTGGAGAAGGGGGGCTCGAAGGAGCCGATGGAGCTGTTCCGAGCCTTCCGTG
GTCGTGAGCCCAAGGTGGATGCACTGCTGCGACACAGCGGGATCGCTGCCTGAGATTCCTGACCCGCCGC
CTGTGTGAGCTTCAAGCAGGTACACTGTTTTGCGGCATAGTGGGATCGAGTCGTGATTTCTGCGACAAAA
AGGGACCTTTTGGTCCCTTTTTTCAAGGCTTTTTAATATCTTGCTGATTGCATATTTGCACTGCCCACGG
CTCTTCAGTAGAGTCGTGTTCTAACCTTCAAGGAGAGAAAGCCACTTGCCATGAGGCGAGCACTCGATTC
CGTCTATCAAATCATCTCAAGTCTGGCTCATTCGAGCGGCCAGGGATATTTCGATACCTTTGTGAGTGAA
ATCGCCGCTGCTGTGATGGCTGATGTCGTCCTGATCGCCAGCTGGGAATCAAATGCTTCCCATCTTACGG
TTCAGGCTGCCTACCCCTCCTCGCTTGACCCCGATACCCTGACGCTGCCACTGACTGACTCTCCCGATGC
CATTACCTGGCAGGAGGGGGAAGCCTGGTTCAGCGATCGTCTGTTGCAGCTGTTCCCCGCGATGCCGCTT
GCCGATGCGTGGCAAGCCCAGGCCTACGCCGGGATCGCCCTCAAGGATGCGGATGGCGAAACCATAGGCA
TCTGCTGCCTGCTCTATCGTCAGCCGCAGGCCAATATGACCAAGGTGATGGGGCTGCTCCGGTTGCTGGG
GCCGCTGGCCGGTCGCGAACTGGCGCTGATGCTGCAACGCCAGCTGCCGCAACTCTTGCCTGATACCCCC
CATGTGCTGCAGAGCATGCTGTTTCAGGCCCCGGTGGGGATCGGCAAACTCGATCTCACCGGCCGTATTC
TCTGGGCCAACCCGGCCATCGAGAAGATGCTGGGTTATCCCCTCGACGAGCTGCTTCATCGCTCCATCTC
CGACATCAATCACCCGGAGGATTGGCAGCGCAGCCTTGAGTGCTACGAAGAGATCCGGCGCGGCGAGCGC
AGCAGTTTCACCCAGGAGAAGCGCTACTTCTGCAAGGGGGGCGAGATCCTGTGGGGGCGAGTGACTGTCA
CCCTGATCCGCGACGAGCAGCAGCAGCCGGACTACCTGATGGTGCTGCTGGAGAATATCGATCCCCTCAA
GCGCCATGCGGAGCAGCTCAAGCTCTCCCACCGGGTCTACGACAACCTCTCCGAAGCGATTCTGGTGTGC
GATGCCGACAGCCGCATCATCTCGGTCAACCCGGCCTTCGAGCAGATCACCGGCTACAGCAGCGACGAGG
TGCAGGGCCAGCGTCCCAGCATGCTCAAGTCCGGGCTGCACGATCAGACCTTCTATGCCGACATGTACCA
CGCGCTGGAGCGGGTGGGGGTGTGGCGTGGCGAGGTGTGGAACAAGCGCAAGAACGGCAAGCTCTATCCG
CAGCAGCTGATGATCAGCACTCTGCGCGAAGCCGGCAAAATTCAGCAATATATCGCCATTTTCAGCGATC
TTTCCCAGACCAAGGTGGCCGAGCAGAAGATCGCGACCCAGGCCAACTACGACAACCTTACCAGCCTGCC
CAACCGCTGGCTGTTTGGCCGCTGCCTCACCCGCTTCTGCGAGCGGGGCGAGCGCTTCGCGCTCATGGTG
CTCGACCTCAACAACTTCAAGGCGGTCAACAACAGCATGGATCACCATGCTGGCGATGCCCTGTTGCGGG
AGGTGTCGGATCGACTGGTGAGCCGGGTGCGCACCGAAGATTTGGTGGCCCGTATCGGTGGCGATGAGTT
CGCGTTCCTGGTGCCCGGCATCGTCACCCGCCGTCAGGCCGAGGTGTTTGCCAAGCAGGTGATCGGCAGT
TTCGCCCGCCCCTTTATGCTGGCCAACCAGCACCTCTATGTGACCGCGACCATCGGCATGACTCTCTGTC
CGGTCGATGGCAGCGAGAGCGACGAGCTGCTGCGAAATGCCGAACAGGCGCTGTTTGCGGCCAAGCGGCA
GGGAAGGTTGCTCGGAGCCTACTGCTCATCCATGCGGGAAGAGGTGCGCCAGCGCCACCAGATGCAGCAG
GATCTGGCCGAAGCGATCAAGCTGGAGCAGCTGACCATGGCCTACCAGCCGATCTGGGACAATCGCAGCG
GACGGGTCGCCAAGCTGGAGGCGCTGGTGCGCTGGTATCACCCCCACTGGGGGCAGGTATCTCCTGCCGA
GTTTATTCCGCTGGCGGAAGAGGCGGGATTGATTCAGGGGCTGGGTGCGCTGGTATTGTGGCAATCGTGC
AGGGATCTGGCGCGGTTGCAGCAATCCGGCTTCCCTGATCTGCAGATGTCGATCAACCGCTCCACCATGG
AGTTCCAGACCATCGACCCCGAGGCGAGTGAGTGGCTCAAGGTGATCCAGCACTTTGGGCTCGATCCTGC
CGACATCGTCATCGAAATCACCGAATCTTTGCTGATGGAGAGCAGCGATCAGCACAGGGTGCGGATCGAT
GCCCTGCGCGAGGCGGGCTGCAAGCTGGCCATCGACGATTTCGGTACCGGCTACTCGGCCCTCAACTATC
TGCGCACCTTCCCGGTCGATCTGGTCAAGATCGACCGCTCCTTCGTGCGCCATATCCCCTTCAATGAGCA
GGATCGGATGCTGCTCGACGGCATCATCAACATAGTGCACAACCTCGGCATGCAGGTGGTGATCGAGGGG
GTAGAGACCCGCGAGCAGCTCAACTTCCTCTGCCAGAAGGGGTGTGCCTTCACCCAAGGTTATCTGCTCA
GCCGCCCGCTGGCCTATGACGATCTCACCGAATATCTGCAGCTCAATGACAAAGGGATGTTTCTGCAGAT
CAGCTAGTGGCGCTTACTCCTTCGATGTCATCTGTTCCTGTTTGGTTTTCAATGTCCGGTAATCCTCGCC
GAGATAGCGCAGATAGATGCGATCCAGTACTCCCTCATCCTGCATCTGTTGCAGCGCCTTGTTGTAGGCA
GGCAGCAGGTCAGCCTTCTGTTTGCCAAAACAGAGGTAGAGCGGCGTCGAATCGATAAAGGGGGTGAGGG
CGCGGATCCTGACGTCCTGCTCCCTCGACAGAATGGGGGCAGAGAAGTAGTCCAGGATGATCATATCGAC
CCGGCTCTTTTCCAGCATCAGTAGCAGCTGTTTGTCGGAGTGAACCTTGATGGGCTCCCAGCCGGTGAAG
TTCCTGATCTTGGCCGTGTAGTCATACCCCCTGACCATTCCTACTTTCAGCCCCTTGGCCATCTCCCACG
AGAAGTTCTGTAACGGACTGTCGGTACGCACATAGGCGGCCAGAGGATAGAAGGCGGTGGCAAGGTCAGA
GTGATAGCAACCGGGGAGAGGGGCATTGTCGAGAATGCCATCCGCCAGCTGGGTGCTGACATATTGCTCG
CAGCGTCCACAAGGCAGTTCGAGCAGGGTGAGGTTCATGCCAAGGCGGCGCGCCACCTCTTGATGGATCT
CCACCGAAATGCCCTCTTTCAGGTTGCCCTGTTCGGAATAAGAAAAGGGAGGCCAGTCTACGCCGCAGAC
CTTCAGGTTTGTGCCGCTCTGGGCGGCGAGGAGCAGGGCAATGAACAGGTTCATGGGATTTCCTCCCTAC
AGCGACTTGAAGGGGCTGTGGTACACTCGGTTCACTGAACTTAGAACGCATCCAAAGACCATGCAAATCC
TCCTTGAAGACCCCGCCAAGGCCGCGCAGGCAGACGCGCTGGCCAGCCAGCTTGCCCAGCTCGACCTTCC
CCAGTTTGCCCTGGTGTTTACCCCGGAAAGGTTGGAACTGCGCAAGCTGGATGAGCCCAAGCTGGGTGCT
GTCTATGTGGACTTCGTGGAAGGGGCGGTGGCACATCGTCGCAAGTTTGGTGGTGGTCGCGGCCAGTCCA
TCGCCAAGGCAGTGGGGCTCAAGGCCGGTGCCACACCGACCGTGGTGGATGCCACTGCCGGTCTGGGGCG

CGACGCCTTCGTGCTCGCCTCCCTCGGTTGCAAGGTGACTCTGATCGAACGCAGCCCTGTGGTGGCTGCT
CTGTTGCAGGACGGGCTGGCGCGGGCGGCACAGGATCCCGAGATTGGCCCCTGGGTCAGCGAGCGGATGC
ACCTGCTGCAAGGCCCGGCGGTGGACAACTTGCTGGCGCTGCCGGAGCGGCCCGAGGTGATCTACCTCGA
CCCCATGTTCCCGCACAAGCAGAAGTCGGCGCTGGTGAAAAAGGAGATGCGGGTGTTCCAGTCGCTGGTG
GGGCCGGATCTTGATGCTGATGCTCTGCTGCCAGCTGCGCTTCAAATGGCAGAGAAGCGGGTGGTGGTGA
AGCGGCCGGACTACGCCGGCTGGCTCAACAAACAGAAGCCCAGCATGGCTATCGAGACCAAGAGCAACCG
CTTCGATGTCTATGTGATGGCGGCGCTGGCGGCGAGTTAACGGTCGGTGCGAGTTGCGGATGCAAAAAAG
GAGGGCCAACGTCATGTTGGCCCTCCTTTTTCGTATTGGATTCAGTTGGTTGGCGCGAAGCGGATCAGCT
TCTCTTCCCCTTGCCAGTTCAGGCGCAGGGTCGCCAGTTCTGCCAGACTGAGCCGCTCGCCACTGTGCAG
CAGCAGATACTCGACCCCTTCGCTGGCTTCGATAGTGCGGATCTTGTCATCCCATTGGCGATCATCGCGG
CTGGTGAGGGTCACCGGCAGTTGCCAGCTGGCAGCAATTTCGAGCCAGTCGTAGAGATCGCAGCTGATGG
GTTGATAATCCGGCATGATCGGCTCCAAAACAGGCAGGTTGCTGAGTCAGGCTTCCTGTACGCGGGTCGG
CCAGCCCATCTTCTGCTGGCGTTCCACTTCAAAGCGCAGCTCACTGGCGCGCAGGTAGTGTTCATCCATC
CAGCCCTTGGGCAGGGTCAGGGTCAGTGACTCCTCTTCTGCCTGCAGGGTGAAGCTGGGCACTGTGCCCT
GGGTACGGCGCATGCAGAGGATCAGCGCGATGCGCAGGATACGGGCCAGCCGGATCGCCTGACGACCGGT
GACTGCACCCTGTTTCTCCAGTGGTTCCAGCTTGAATTCGTCCCGCTGGTTGAACAGCAGGGCCGACAAC
AGCTTCTTTTGCGCAGGGGTAAAGCCCGGCATGTCAATGTTGTCGATGATGTAAGCGGCGTGTTGCGGCG
CCTTCTTGTATTCGATACAGAGGCCGATCTCGTGCAGCAGGGCGGCATAACGCAAGATTGGGCGACCGTA
GCGTTTGGAGAGTCGCCAGGCCGGTTGCAGCTGGTTGAACGCCTCGACAGCGATATCGCGCACCCGTTCG
GCGTGCTCTTTATCCAGCTGATAGCGGCTGATCAGGCTGTCGGCGGTGCGATCCCGGGCATCACAGTCGT
GATTGTTGCCAAGCAGACCGTAGATCAGCCCCTCACGTAGGGCACCACCCGCCAGGGTCATGCTCTCGAT
ATCGAGGGTTTCGAAGATGGCGATCAGGATGGCGAGGCCGGAGGGGAACACGGTCAGCCGCTCGGCCGCC
AGCCCTTCCAGCTGCAGTCGGTCGAGCTTGCCGCAGGCGATGGCCTGACCCATCAGCTCCTGCAGTTTTG
GCAGGGTGACCCGTTCGCTCTTCCCTTGCGCCAGCATGATCTCTTGCAGCGCCTGCACCGTGCCGGAGGC
GCCGACACAGGTACGCCAGCCGAGGGCGCGGTAATCTTCCGCCACCTTCTCCAGCACCGCCTTGGCGGCA
GCAATCGCCTGCTCGAAACGGGCTTCGGAGAGCTCGCCATCACCGAAGTGATTGTTGAGCCAGGTAACGC
AGCCCATGTGCAGGCTGTTGAGCAGTTTTGCTTCGCTATGTTCGCCGATCACCAGTTCGGTACTGGCACC
GCCGATGTCGATCACCAGCCGGTTGCCTTCCCCGGCAGAGGTCCAGGAGACCCCTTCATAGATGGTCTTG
GCCTCTTCCTCGCCCGAGATGATCTCGATGCTGTGGTTGAGAACCCGTTCCGCTTCGCTGAGGAACTCAT
CGACGTTGGTGGCAAGCCGCAAGGTGGCGGTGCCCACCACCCGGATATTGTCGGCCGGAATGTCCTGCAA
CTGTTCGGAGAAGAGACGCAAACAGTCCCAGCCCCGCTCCATGGCGGCACGGCTGAGACGAAATTCCGGA
TCGAGCCCGGCTGCGAGGCGAACCTTGCGCTTGACCTTGGCGACGGTGCGCAGAGCACCGGCGACCTCGC
GCACCACCAGCATATGAAAGCTGTTGGAGCCGAGGTCGATAGCGGCATAGAGAGGCGAGTTGTGCAACGG
GCCTGTCCTTAGCTCTTGCGCGGCGGACGACGACGGTTGTTGCTGTTGCCACCGCCCTGACGATCCCGCA
TGTTGCGGTTCACCGGCTGACGGTTGCGGAACACCCGTTTCGGCGGGGTCACGTCATCCAGCAGCGCTTC
GCGATCATATTTGCTGACCGGAATGGCGTGGTGGATGTACTCCTCGATGGCCGGCAGGTTGAAGGCATAG
TCTTCACAGGCCAGGCTGATGGAGTGACCGCTCTTGCCGGCACGACCGGTACGACCGATGCGGTGTACAT
AGTCTTCGGCGTCGTCGGGCAGGTCATAGTTGAAGACGTGGGTCACGTCAGGGATGTGCAGACCACGGGC
GGCCACGTCGGTGGCGACCAGAATATCGAGCGCACCCTTGGTGAAATCCTCGAGGATCTTCATCCGCTTC
TTCTGTGGCACATCACCGGTCAGTAGACCGACGCGGTGGCCGTCGTTCTCCAGCCAGGCATGGACGTCTT
CACAGACATGCTTGGTGTTGGCGAAGACGATCGCTTTTTCCGGCCACTCCTCTTCCATCAGGGAGAGCAG
CAGCAACATCTTGTCTTCATTGGAGGGGTAGAACAGCTCCTCCTTGATCCGCACGCCGGTCATCTGCTCC
GGCTCTACCTGCACATGTTCCGGGTGGTTCATGTGTTCGTAGGCCAGCTCCTGCACACGCAGGGAGAGGG
TGGCGGAGAACAGCATGCTCAGACGCTCGGTCGCGGGCGGCATGCGGCGGAACAGGAAGCGGATGTCCTT
GATGAAGCCCAGATCGAACATGCGATCCGCTTCGTCCAGAACCACGACCTGAATGTTGCTCAGATCGATG
ACCTTGGACTTGAAGTAGTCGATGATGCGGCCTGTGGTGCCGATCAGGATGTCGACGCCCTGCTCCAGCA
CGGCCAGCTGCTTGTCGTAACCTTCGCCGCCGTAGGCGAGGCCGAGTTTCAGGCCGGTAGAAGCGGCGAT
GCTGTCGGCATCGTTATAGATCTGTACTGCCAGTTCCCGGGTGGGTGCCATGATGATGGCTCTGGGTTGG
TTAACCAGACGGGGCTTGGTCAGCGGGTGAGTCAGCAAATAGTTGAATGTCGCGGCCAGGAAGGCAATGG
TCTTGCCGGTTCCTGTCTGAGCCTGACCGGCGAGGTCATGACCTTCTACCAGCAGTGGCAGAGACAGTGC
CTGGATGGGCGTGCAATAGTGAAATCCCTTTGATTCCAGACCAGCCAGAACTTCGGGTTCGAGGCCCATT
TGGGCGAATTTGTCGGTCGTTAAGTGTGTTTTGCTCATGGCGGTAGGTTATCAGGTTAGGCTTGCAATAA
TAAACAGGATCATTTCAAATAGGGCAACTATTGCCTTTGTTGATATGTCAATAAAGCCTTAATTACTGAC
TGGAGTTGGAGATGAGTGACAAGATTGTTCAGCTGAGCGATGCCAGCTTCGAGGCAGATGTAATCAAAGC
CGATGGCGCCGTTCTGGTCGACTTCTGGGCCGAGTGGTGTGGTCCGTGCAAGATGATTGCCCCGATCCTA
GAAGAAGTTGCCCAAGAGTACGAAGGTCGTGTGACCGTAGCCAAACTGAACATCGACCATAACTCTGATA
CTCCGCCCAAGTACGGCATTCGTGGTATCCCGACCCTCTTGCTGTTCAAGAATGGCGAAGTGGCAGCGAC
CAAGGTCGGTGCACTGTCCAAGACTCAGCTGAAAGAGTTCCTGGACGGCAACCTGTAATAATTAAAGAGG
AGCGCATTGCAATAATGCGCTCCTTGTGTTTTATCACCTAGACGCTCATCCCGATTGGTGCTAGTTTATC
GCTCGTTTGCTAACCTTTTTGCCTGACTCTTTGCTAACCACAGCCTGGCAATCAAATCCGATCAGCATCA
ATCCAGCAGACTGTCTCCTCATTTCAATCCTTGCTTACAGACTTTCACCACTATGAATCTGACTGAATTA

AAGAACACTCCTGTGTCCGAGCTGGTCAAGCTTGGCGAATCCATGGGGTTGGAAAATCTGGCTCGGGCGC
ACAAGAAAGATATTATTTTTGCAATCCTCAAGGCGCACGCCAAGGGTGGTGAAGATATCTTTGGCGACGG
TGTGCTGGAAATCCTGACCGATGGTTTCGGCTTCCTGCGCAGTGCAGACGGCTCCTATCTGGCAGGCCCT
GACGATATCTACGTCTCCCCGAGCCAGATCCGCCGCTTCAACCTGCGTACCGGCGATACCATCTCCGGCA
AGATCCGGCCACCGAAAGAGGGCGAACGCTACTTCGCACTGCTCAAGGTCAATGAGGTCAACTACGACCG
TCCGGAAAACTCCCGCAACAAGATCCTGTTTGAAAACCTTACCCCCCTGCATGCCAACGAACGTCTGCGC
ATGGAACGGGGTAACGGCTCTACCGAAGATATCACCGCCCGTGTACTGGATCTGGCTTCCCCCATCGGTA
AAGGCCAGCGTGGCCTGATCGTGGCGCCGCCCAAGGCCGGTAAGACCATGCTGCTGCAGAACATCGCCCA
GAGCATCGCCTACAACCACCCGGACTGTGAACTGATCGTTCTGCTGATCGACGAGCGTCCGGAAGAGGTG
ACCGAGATGCAGCGGATGGTGAAAGGTGAAGTGATCGCTTCCACCTTTGACGAACCTGCTTCCCGTCACG
TTCAGGTTGCCGAAATGGTGATCGAGAAGGCCAAACGTCTGGTCGAGCACAAGAAAGATGTGGTGATCCT
GCTGGACTCCATCACCCGTCTGGCCCGTGCCTACAACACCGTTATCCCGTCATCCGGCAAGGTACTGACC
GGTGGTGTGGATGCCAACGCCCTGCACCGTCCCAAGCGCTTCTTCGGTGCCGCGCGTAATGTTGAAGAGG
GCGGCAGCCTGACCATCATCGCCACCGCGCTGATCGATACCGGCTCCAAGATGGACGAGGTTATCTACGA
AGAGTTCAAGGGTACCGGCAACATGGAACTGCACCTCTCGCGCAAGATTGCCGAGAAGCGCGTCTACCCG
GCTATCGACATCACCCGCTCAGGCACCCGTAAAGAAGAGCTGCTCACCTCAGGTGACGAGCTGCAGAAGA
TGTGGATCCTGCGCAAGATTGTCCACCCGATGGGCGAGATCGACGGTATCGAATTCCTCATCGACAAGCT
GGCGATGACCAAGACCAACGACGAGTTCTTCGACGCCATGCGGCGCCAGCAGAAGTAAGTCCGGTCAGAA
AATGACGAAACCGCGGCCATGGTCGCGGTTTTTGTTTATCTGTTTTCCGGTCGCCCTAGTAGTATGAGAT
CCCGATGTCACATGGATGGAGACGTCATGAAGCCTGTTTTCTGGTTATTGCTGGGGTTGCTGAGTGCAAT
GCCAGCCTGCGCGCAGAGTGCACCGGTTGCGGTGGCAGCCGAGACGTTGCAGACTCACCAGCGCTATCTG
GCTGGCCGTCTGCAGGAGGGGCAATTTGCAGCCATCGACTCTCTGCTGGCGACCCTGAGTCGCGAGCGGC
GGGAGTTCCTGCTGTTGCAGCTGCTGCGGGATATTCCCGCGACCACCCCTGCATCCCCTGCGTTGCAACG
CTGGGTCGAGGCTCAGGCCGACAAGGCCCCCGAGTGGCTCATGGAGCAGCAGGTCGATGGTTTTCTGGTG
CAGCAACCTGTCTATGATTTTGCCGCGCAGGCCCGTCAGTTGCTGGGGGAGTGGCAGCTGCAATCACTGG
TGCAGCGCTATCGGCAGCAGCTTGCCCAGGGCAGCTTCCAGCTAAAGTCCATCTACTACAGCAGTAACCC
CGATCTCGCCATGCAGCAGCAAGCCCTGTTGCTGGCCCTCGATACCTTGCCGCAACCGATCTGGATGCGG
GAGGCGCAAGCGCTGGCCAGCCAGAATATCTATCTGCCCGACAATCATCTGTTGTTCCACCTGTTGCAGC
GCACCGGGGAACCGGCACTCTATAGCATGCTGTGGCGGCGGCCGGCAGATCAGGATGCACTGCGTGCACT
CTCCACCATCAACCGCTTCCATCACGGCAGTGCTGCCAGCGATTTGCTGATTGCCGCCAGTGGCAACGAA
GGGTTGAAGGGAGCCGCCCTGCGCCAACTGAGCGCACTCTCCCCCTTGCCGCCGCAAGCCCAGAAATACC
TGCTGGCGGAACTCTCCAACCGTCAATACGGGGCGCTGGTGGCCGGTCTGTTGATGGAGGTGGATGAGCC
CCAGCTGCTCTCTGCGCTGGCTAGACGCCTCGGCCAGCGTGACATGCCACCCATTGCACCGAGTCTGCTG
CCCGATTCGGGTACACCCGATGGGCAGCCAGGGTTATAATGCGTGACCTCCTGAACCCGGTTGGGAACTT
GCATGAAATACAAGGATTTGCGTGATTTCATCGCGCAGCTGGAACAAAACGGACAACTCAAGCGCATCAC
CCGCGAGATAGATCCCTATCTGGAGATGACCGAGATCAGCGATCGCACCCTGCGTGCGGGTGGCCCGGCT
CTGCTGTTCGAAAATCCTAAAGGCTACACCATGCCGGTGCTGACCAACCTGTTCGGTACCCCGGATCGGG
TTGCCATGGGCATGGGCCAGCCCGATGTGGGCGCCCTGCGTCAGGTCGGGGTCTGGCTCTCCTATCTCAA
GGAGCCCGAGCCGCCCAAGGGATTCAAGGAGCTGATGGAGAAGCTGCCGATCTTCAAGCAGGTGCTCAAC
ATGCCGACCAAGCGACTCTCTTCCGCCCCCTGCCAGCAGGTGGTGCAGGAGGGAGATGCGGTCGATCTCG
ACCTGATCCCGATCCAGCACTGCTGGCCCGGCGATGTGGCGCCGCTGGTCACCTGGGGCCTCACCATCAC
CCGCGGCCCCTACAAGAAGCGCCAGAACCTCGGCATCTATCGCCAGCAGAAGATCGCCAAAAACAAGCTG
ATCATGCGCTGGCTGGATCATCGTGGCGGTGCCATCGATTTTCGCGAGTGGCAGGAGGCCCATCCCGGTG
AGCGATTCCCGGTGGTGGTGGCGCTCGGCGCCGATCCGGCGACCATTCTCGGCGCGGTGACGCCGGTGCC
GGATACTCTCTCCGAATATGCCTTTGCCGGTCTGCTGCGCGGCAGCCGTACCGAGGTGATCAAGGCCATC
AGCTGCGATCTGGAGGTGCCCGCCAGCGCCGAGATCGTGCTGGAAGGCTATCTGGAGCCGGGTGAGATGG
CGCCAGAAGGCCCTTATGGCGACCACACCGGCTACTACAACGAGGTGGACGAGTTCCCGGTCTTCACCAT
CACCCACATCACCAAGCGGCGCGATGCTATCTATCACAGCACCTACACCGGCCGTCCGCCCGATGAACCG
GCGGTGCTGGGTCTGGCGCTGAACGAGGTGTTTGTGCCACTGCTGCAGAAGCAGTTCCCCGAGATTGTGG
ACTTCTATCTGCCGCCGGAGGGGTGCTCCTACCGGATGGCTGTGGTGACCATCAAGAAACGCTACCCGGG
CCACGCCAAGCGGGTGATGCTGGGGGTCTGGTCCTTCCTGCGCCAGTTTATGTACACCAAGTTCGTGGTG
GTGTGTGACGATGACGTCAACGCCCGCGACTGGAAGGATGTGATCTGGGCCATCACCACCCGGATGGACC
CGGCACGGGACACCACATTGATCGAGCACACCCCGATCGACTATCTGGACTTCGCCTCGCCGGTCTCCGG
CCTCGGCTCCAAAATGGGGCTGGATGCCACCAACAAGTGGCCGGGCGAGACCAATCGGGAGTGGGGTCAG
CCCATAGTGCAGGATGAGGCGGTGAAGCAGAAGGTCGATGCCATCTGGGATGAGCTCAATATCCTCGGAT
GAGGCAAACATGACAGCTTTGCTGCAAAAAGTGATGCGATCCGCACTGATTTGAGTATTCTCGACTGACG
GGGCCAGGGCTGCTGGTCGTCACTCCATAGTGAAACAATGAGCGAAGCGGGCCGAGCGAGCCCGCCACAA
GGACAAGAATGCAACGTATCAAATGCCGCGTAGAGGAACTGCGCGAATATGTCGACACCATCTGGCATGT
GGCCCTGACGCCGCAGCAAGCGATCAGCTTCAAGCCGGGCCAGTATCTGCTGGTGGTGATGAGCGATTCG
GACAAGCGCCCCTTCTCCATCGCCAACTCGCCGACCCGCCCGGGCGTGCTGGAGCTGCAGATCGGCGCCA
CTCCCGAGAACGCCTACGCCGGTCAGGTGCTGGCCCGCATGCGTGAGCAGGGGGAGATCGAGGTCGAATT

GCCGGCTGGCAAGGCGTTCCTGCGCGATGAATCCCCCCGTCCACTGATCCTGATGGCGGGCGGCACCGGT
TTCTCTTACGCCCGCTCCATTCTGGAGTACCTGATCGACACGGGCAGCAAGCGCCCGGTGTTCTTCTACT
GGGGGGTGCGTCAGGCTCACTGGCTCTATGAGCTGGAGCAGATGCAGCAGTGGGAGCGTGACTATGCGCC
GCTCACCTTTATTCCGGTGGTGCAGGAGCCGGATGCTGACTGGACCGGCAAGAGCGGGCTGGTACACAAG
GCGATCATGGATGACTTCGTCAGCCTCCACGACTACGACATCTATGTCGCCGGCCGTTTCGAGATGGCGG
GTGCCGCGCGTGAGGAGTTCAAGATCCTCGGCGCCGAGCCGGAGCGCATCTTTGGTGACGCCTACGAATT
TATCTGACGGGATCCCCGTCTGGATGACGCAGCCCCCGGTTTTGCCGGGGGCTTTTGTTTTTAGGGCTCC
CGCAGTACCTGATCGTACCGAATGGTTGGATTATCGCGGCGTTATCCCATTATTTTTCAATATTTTGGCT
ATGAATGGCTACTAATCGAACGTAGCTGATGGCATCTTTGTGGGGGATTGACAAGAGCATCCATAGAGCA
AGCACAAGGAGGCGCAGTGTTGAGTGAAGAGGGCAGGGCATCTGCGGAGGATGACAGGGGAGAGACCGGC
TCTGTCGAGCGGGTCGCAACGCTGATCCCGTCCCCTCATGGCGGTGAAATGGCCCGTTTGCAGGCAGAAA
TCGCCGCTTTGAAGCATGAAAACGGCCTGTTGCAGGAGAAGCTCAATGCCGCCCTCGATGGCACCGGCAT
CTGTCTCTGGCAGGGCACCATTCCCACCGGCGAGCTGCGGGTCTTCAATCTGCAGAACTTTCAGGCCGGT
GACATGGCCCCTCATTTCGATCAGTGGAGCGCCAAGCTCCATCCCGACGATCGCGCCCATGCCATGGGCA
GCTACTTTGCCCACCTTGAGGGCAAAACCCCCTTCTATGAAGCCGAATACCGCACCATCAGCCCGGCCGG
AGAGATAACCTGGCTGTGGGATCGGGGCCGGGTCGTCGATCGGGATGGCGATGGCAAGCCGCTGCGGATC
ATGGGCTCCCATATCGATATCACCCAGCGCAAGGCTTATGAGCAGCGACTGGCGGAGCGCGCCAACAGCG
ACGCGCTGACCGGCTTGCTCAACCGGCAGGGGTTTGGCAAGGCGTTCGCCCAGCTGCAGCCGCAAGGGGC
GGGGGCGCTGCTCTTTATCGATCTGGATGATTTCAAGGGGATCAATGACCAGCTGGGGCACGCCTGCGGC
GATCTGGTGTTGCAGCAGATGGCGGACTGGTTGCGCCTCATCATGCTTGGCTCGGCCCTGTGTGGCCGTT
ACGGCGGCGACGAGTTTGTGGTGCATCTGCACCGGGATGTGTCGAGCCATGGCCTGGCGCAACTGGCTGA
CGCCCTGATCAGCCGGATGCATGGCTATGTGCCCAAACCCGGTTGCTCGCAACGGGTGGGGCTGAGTATC
GGCATCGCCCTGTGGGAGGAGGAGCCATTGAGCTTCCGGCAGGCGCTGGAGGTGGCCGACAGGGCCATGT
ATGAGGCCAAGGCACGCGGCAAGCGGGCCTGGTATCTGCTTCAGGTGTGATGTTTTTTACAGGTTCAATA
CAAAGCAAAAACCCCAGCCGAGCTGGGGTTTTTGTTATTCGCCTTTGGCGCCGAACTGCTGCTCGAGCTG
AGCCAGCTTGCTTTCGAGGGCGGCCAGTTTTTCACGGGTACGCAGCAGCACCTTGGTCTGCACGTCGAAC
TCTTCGCGGCTGACCAGATCCAGCTTGCCCAGCTGGGCTTGCAGTACCTGACGCATCTTCTTCTCGGCCT
CTTCACCCATGCTGCGAATGCCGGGCGGCAGGGAGTTGTGCACCTGCTTGGCGATCTCTTCCAGTTTCTT
CGGGTCGATCATGATGTCCTCCTTAAGCGTTGGCGGACATTCTACCGACAATTCCCTCACTTTGCCGAGA
TCGCGGCGGCTTTTTAGTCGTGATGGTGCCCGCTGGCTGGTCGGGTCTGGTGATGGGGCCACAACTGCTA
CCCTTGCGCCCCTTTGTTAACCTGAAACCATGACATGCACCACAAAGCTGAGCCAGAACTGTCCGGCATG
AGTCTTATTTGGTGTTGGTTTTATGTTAACGTTTTGCAGTCGAGGTCGTGATGACCCCGTTGGCTTGTGA
TGGATAACAACCTTATAAAAAGAGAATAAAAAATGAAGAAGAAGCTAATTTATGCAGCTGTTGTCAGCGC
CCTGCTGGCCGGATGTGGTGGAGATGACAACAAGGGAGACACCACCAGCTCCCTCGACTACGTCCTGAGT
GGTGCCAACGGGGTGCGCCCGAGCGTGCTGGCCCCCCGTGCCAGCGACGGTACCCTGAAGTTTTCGACAG
AAACTGCTGATCTGTCGAACCCGGTATCTGCCCTCTCCACCCTGGATGGCTGGTCAACCACCCAACCCGT
CCAGCTGGTACCGGCGACCGTGACCGGTGTCTCCGTGCCGGCACCGGCGGCCAGCGAGTATGCTTCTGCC
GTTGCGCCCCTCTACCTGCTCGAGCTCACGCTGGATCCGGTCACTCTGCAACCCAACGGAGTCAAGGGCG
GCAAGCCACTGGTTTATGGGACCGACTTTGTGGTCACTGGCGGGGACGGCAAGCTCAATCTGGTTCCGCT
CAAGCCGCTCAATCCCTCTTCTTACTACATGATCGTAGCGACCGATGCCCTCAAGGACAGTCGCGGCACA
CCGCTCAAGGCGGGAGGCGATTACAGCAGCTTCAAATCGACAGCTGGCGCAACTACTCAGGAGCAGACCA
TCAACGGCCTGATCTCGCTGCAGGAAGGTCTGTTCAAAGCGGCCACCGGGATCACCAGCGATCGGGTGAT
CTTCTCCGACTGGTTTGCCACCCAATCTGGTGCCGATGTGCTGCTGGCAGTGAAAAGTGCCGCCGCTTCC
GTGCTGAAGAGCCCGAGCCTGGACGCCGCGGCGCTGTGGAAACAGGATGCCAGAGGCAACACCAGCCTGC
CAGCAACCTATACCATCAGCGGTCTTAATAGCGGGGTGGATTTTCTGACCCAGCTGGCGAATGAACCATT
CTTGCCGCAGGATCAGAAAGATGCACTGACAGCCGCCTTTGGTGTTGGTACGCCGCTTAACGGGGTTGCC
CAGCTAACCAAGGTGTATACCGGCACCGTCAAGCTCCCTTACTTCCTCTCTACCCCGGCGATAGCTGGTT
CATGGGACAAGGCCAAGACCCAATCCTGGCACGGCGCCATTCCCAGCCTCTATGCCATCGCCAACGCGCT
GAAAGCGGGCGATAGCGAGGTGATCGGTGGACTGGTCGGGTCGGGCGTGGACCCGGCATTGCTGGGTGAG
CTGATCGCCGACCCGAGCCGTCAGAGTGAACTGCTGACCGAGGCAAGCAAGCTGATCGGGGTGACTCTCA
CCTCCGGTGGCAAGCCGCTGGATGCCGAGCGCAATATCGGCCGTTTCAACCCGTTGCCGACCCTGACCGA
AGTACAATCGGTACCGCTGCGGATCTTTGCCGCTGCACCGCTCAATACCATTACCGATGTGATCATCTAT
CAGCACGGGGTTACCTCCATCAAGGAGAACGCCTATGCGCTGGCGCTGGGCCAGATTGGTGCTGGTCTGC
AAGTCACACCAACAGCCAAAAATGTCGCTGTGGTAGTGATCGATCATCCGCTGCACGGCGAACGTGGCTA
CGCCTTGAGCGGCAGCATGGATACCGTCACCACCTCCGAGAACCCGACTCCCTACCTGAATCTGGGCTAT
CTGACGGTGGCGCGGGACAACCTGAAGCAGAGCGTGGCCGATCTGCTTGGCCTTCGCCTGGCTGTCGGTC
TGGCCAATGCCAAGGGGCTGGGTGGCCAATTGGGTGGTGCCGGTCTGAAGGTGCATTTCCTCGGTCACTC
ACTCGGTGCCATTACCGGGGCCAATCTGCTGGCTGTCGCCAACCAGACCACCGGCAGTGCTCAGGCAGAT
GCCCTGTTCAAGTTCGATACCGGTGGTCTGGCGATGCCGGGTGGCGGCATCGCGCCGTTGTTGCTCAACT
CGCCGAGCTTCGGGCCGACCATCAAGATGAGCGTGCTTACTTCGGGCAGTCCGGCGCTGAAAGCGGGCTT
TACCGCCTATGCACCGAATTGCAAAACGGCTGCCGCCACTTGCTTCGTCAACGAGTTCCTGCCCAGTCTG

GATGCGGCCACGCAGGCGGGTGCGGCGAGCACCTTGCAGAGCTATACCTTTGCCGCCCAGTCAGTGCTGG
ACTCTGCTGACCCGATCAATCTGGGCAGTGGCGTCGCCAAGAGCTTCCCGCTGTTCGCCACCGAAATTGT
TGGCGATGGTGCGCTCAATCTGCCGGATCAGGTGATCCCCAACAGCATCGCCTCTGCGCCGCTGGGTGGC
ACCGAGCCGCTGTTCCGGGTGCTGGGTTTGCAAGAGCTGAAGGGTTCCGGCGTGCTCCCGGCGGGTCACC
ATGCTGTGCGCTTCCTGAAAGGTGGCCATAGCTCGCTGCTGGCTCCTGACGAGAACTTCGATCAGGCTGG
CGCGGTCACCACCGAGATCCAGACCCAGTTCGCCAGCTTCTTTATGAGTGGCGGTGCGGCGGTCAAGGTA
ACCGATGACACCCTGATCAAGCAGTAACTGCCTATCGCGTAAAAAAACGCCGCCCTTCGGGGCGGCGTTC
TCGTTTCTGCAACTGCTGAAAAATCAGGTCGGCTGGTGGTTGCGATCCAGCCAGCCTTGCAGCAGGCCGA
CCAGCAGACCGGTCAGGTGGGCGATGTTGGCGGTGCTGGTGCCGAGCATGTCGAAGAAGCCGAGCACCAT
CCAGAACAGCATAAAGCCCATCAGTGCCGGTGGCAGAGTGAGGCCGCAGGCCGGTTGCAGCTTGCTGCGC
AGCCAGCAGTAGCCGAGCAGGGCATAGACCACGCCGGAGAGGCCGCCAAACAGCGGGCCGCTGGCAAAGA
ACTCGGCGATATTGGGCAGGGCGGCGCCCACCAGCAGCAGGATCAGCAGCTTGCCGCTGCCAAGGCGCTG
CTCGACCTGGCCTCCCAGATACCACCACCAGAGCAAGTTGAACACCAGATGGAGCACCGAGAAGTGGATC
AGGGCGGGGGTGAAATAGCGCCACGCCTGCCAGTCGGTGAACTGGGCCAGTGTCGGGTGGAATGCCAGTA
CGTCGTAGATGGGCAGGCCGATGGCCTCGATCCCGTAGATGGCGAGACAGGCGATGATGACCACCAGCGT
CAGTGGCCCTGCCTGATGGAGGAAGTCGGTGATGGGATCCGTCATCCCCTTGCTGTAGTCGATGTGGGCA
TCTGCGCTACCAGATTGCCAGGAGGCCTCCATGTAGCGCGGGTGGCCCGGCTCGGTCAGAAAGCGCTTCA
CCTCCAGCTGGGCCTGAGCGAGGCGTTGCTCGTCCGCCAGCCAGATGGTGACCCCTAGCTCGGACTGGGT
CAGCTCGCAGGAGATCCCCAGGGTCGCCAGATAGTCCACCAGCGCCTGCGCCATGCGGGCGTTGTCGAGC
ACCAGCAGCTGGATCATGCCTCGCCTGCCTCGATGGGAAACTCCCGGCGCCACGCCTCGAAACCGCCATC
CAGACTGTAGACCTCGTCATAACCCTGTTGCAGCAGGTATTGGGCCGCCCCCTGACTGCTGTTGCCGTGA
TAGCACATCACGATGACCGGGGTATCGAAATCCACTTCATTCATAAAGCGCACCAGCGTGCCATTGGTGA
GGTGGAAGGCGCCGACCGCGTGGGCCGCTTCAAAGGATTGCGGGTCGCGAATATCCACCAGACGGGCTTC
ACCCGCGCTCAGTTTCTGGTGGGCATCATGGGCATTGATATGGGCAAACTGGTCCATCGGGATGTCCTGC
GAATAGGTTGATTACGGGCCAGTGTACCGCAAGCTTGGGCGCAAACTAACCCCACAAAAGATAAGGGAGC
CAGCGGCTCCCTTGTTGATATGGCATGCGGGGCTGTGGGTTAACCGCCCAGATAGGCCTGCCGCACTGCC
GGGTTGGCCAGCAGGGCGGCGCCCGTGTCTTGCAGCACCACGTGGCCGTTCTCCAGCACATAACCGCGGT
CGGCCAGCTTGAGCGCCTGGTTGGCGTTCTGCTCCACCAGAAAGATGGTCATCCCCTTCTGGCGCAACTG
CTCTATGGTGTCGAAGATCTGCTGGATGACGATGGGGGCCAGCCCCAGCGAGGGTTCATCCAGCAGCAGC
AAGCGCGGCTTGCTCATCAGGGCGCGGCCGATGGCCAGCATCTGTTGCTCGCCGCCGGACATGGTGCCTG
CCCGCTGTTCCAGTCGCTCATGCAGGCGCGGGAACAGGGTAAACACCTGATCCAGCAGGCCATGCTGCTC
GGCCTTGTCAACGAAGAAGGCGCCCATCAGCAGGTTCTCCTCCACCGTCAGGCGGGCGAAGATACGGTGC
CCCTCCGGCACCACGGCGATATCCTTGCGCATGATGCGGGCGGTGGTGAGGGAGCTGATCTGTTCCCCCT
CAAACCAGATGGAGCCGCTGCTGGGCTTGGGTTCGCCGCAGAGGGTCATCAGCAGGGTGGTTTTGCCAGC
GCCGTTGGCACCGATCAGGGTGACGATTTCCCCCTCTTTCACCTCGACGCTGACATCGTGCAGTGCCTGA
ATTTTGCCGTAGTGGGTCGATACGTTGCGAAGTTCTAACATCTTGTTTTCCTTACGACTCGCCCAGATAG
GCCTTGATCACTGCCGGGTTGTTACGGATCTCATCCGGCTTGCCATGGGCCAGCGGGGTGCCCTGGCTGA
CCACGAAGATACGGTTGGAGATGCCCATCACCAGCTTCATATCGTGTTCGATCAGCAGCACCGAGACCCC
GAATTCGTCCCGCAGGCTGATGATCAGCTCGTCCAGCTCGTCGGTCTCCTTCGGGTTGAGGCCGGCAGCC
GGTTCGTCCAGCATCAGCAGCTCGGGCCGGGTCGCCATGCAGCGGGCGATCTCGAGGCGGCGCTGTTGAC
CGTAAGCCAGGTTACCGGCGGCGCGGTTGGCAAGATCCTTGAGGCCCACTTTATCCAGCCAGAAGGCGGC
CCGCTCCAGCCCCTCCTGCTCCGCCCGGCGGAAGGCCGGGGTCTTGAACAGACCGGCGATAAAGCTGGTG
TTGAGGTGCTGGTGCTGGGCAACCAGCAGGTTCTCCACCGCTGTCATCTCCTTGAATAGCCGCACGTGCT
GGAAGGTACGCACAATCCCCTTGCGGGCGATGAGGTGGCCGGGCAGCCCCTGGATCTCCTCACCCTTGTA
GCGGATGGTGCCGCCGCTCGGGCAGTAGAAGCCGGTCAGGCAGTTGAAGATGGTGGTCTTGCCCGCGCCG
TTGGGGCCGATGATGGAGATCACCTCGCCCTTGTCGATATCCAGTTTTACCCCGTTGACGGCCAGCAGGC
CCCCGAAGCGCATCGACAAATCAGAGACTTCCATTAACTTCTGATGTGTACTCATTTCTTCAACTCCAGA
TGCGGACGGGTCATCGGCAGCAATCCTTGTGGCCGCCAGATCATCATGAACACCATCAAGAGGCCGAACA
TCAGCATGCGGTACTCGTTGAATTCGCGGGCCAGCTCCGGCAGCACCGTCATCACGATGGCAGCCAGCAC
CACCCCGATCTGGGAGCCCATACCACCCAGCACCACAATCGCCAGCACAATGGCCGACTCGATAAAGACG
AAGGATTCCGGGCTGATAAAGCCCTGCCGGGAGGCAAAGAAACTGCCCGCAAAACCGGCAAAGGTAGCGC
CTATGGTGAAGGCGGTCAGCTTGATGATGGTGGGGTTGAGCCCCAGCGATTTGCAGGCGATTTCGTCCTC
CCGCAGCGCTTCCCAGGCGCGGCCAAGGGGCATGCGCAGCAGGCGGTTGATGACGAACAGGGTCACCACC
ACCAGCACCAGCGCCATCAGGTAGAGGAAGATCACCTTGTGGTTGGCGTTGTAGGTAATGCCGAAGAAGT
TGTGGAAGGTATCAAAGCCCCCATCCTTGACGGTGCGGTTGAACTCCAGCCCCCCCAGCGTCGGTTTGGG
AATGCCGGAGATCCCGTTCGGGCCGCCGGTCAGGGTGGTCATGTTGTTGAGCAGGATGCGGATGATCTCG
CCAAAACCCAGGGTGACGATGGCCAGATAGTCCCCCCGCAGCCGCAGCACCGGGAAACCGAGCAGGAAGC
CGAAGGTAGCGGCCATCAGCCCGGCGATGGGCAGGCACTCCCAGAAGCTCAGCCCGAAGTAGGTGTTGAG
CAGGGCATAGCTGTAGGCGCCGACCGCATAGAAGCCGACATAGCCGAGATCGAGCAGACCGGCGAGGCCG
ACCACCACGTTCAGCCCCAGCCCCAGCATGATGTAAATCAGGGTGAGGGTGGCAAGATCGATGGCGCCGC
GAGAGGCCATAAAGGGCCAGCTCACCGCAAACAGCAGCACCAGCACGGCGGTGAGGTTATAGAGCTTGGG

CGCCTCTTCCGGGCTCGGCAGTACCAGCTTGGTGAAGCCGACCGAGAAGCCGCTGATGGAGGCCTGAAAC
AGCTTGGCAATCTGGCCGCGCAGCATCTGGAACCAGAAAACGAGGGTGGCGCCCCCCAGTAGCCAGGCCA
GCGACACATCGAGGCGGCTCACTACCTGCAGGCTGGAGCCATCGGTCTCCAGCTTGAAGCCGAGCAGCCA
GAAAGAGAGCACCAGCAGCACCAGGCTGGCGATACAGGCGTGGATAAATTGATGTTTCATCACACTTTCT
CCACTTCCGGACGACCCAGAATGCCGGTCGGCATAAAGAGCAGCACAAAAATGAGCAGGGCAAACGCCAT
CACATCCTTGTATTCGGTGCTGAGATAACCGGCGGTGAGCGCTTCAACCACGCCGAGCAGCAGGCCGCCC
AGTACGGCGCCCGGAATGCTGCCGATACCGCCCAATACAGCGGCAGTAAAGGCCTTCAGACCGGCCATAA
AGCCGAGGTAGGGGTTGATGACGCCGTAATACATGCCGAGCAGCACCCCGGCGACGGCGGCCAGCGCAGC
CCCCAGCACGAAGGTGATGGAGATGACCACGTGGGTGTCGATGCCCAGCAGGTTGGCCATCTTGATATCT
TCCGAACAGGCGCGGCAGGCGCGGCCCATGCGGGAGCGGCCGATGAACTGGGTCAGCGCCAGCATGGTGA
TGAAAGTCACCACGAAGATGATCAGCTGCATATAAGAGAGGCTGGCCTGAAAACCATCCTCACCCCCCAG
GGTCCAGCCACCGGAGATCAGGCTGGGCATGGCGATATCCCGCGAGCCCTGCGCCATCCGCATCATGTTC
TGAAGGAAGATCGACATGCCGATGGCGGAGATAAGGGGGATCAGCCGGTTGCCACCGCGCAGTGGCCGGT
AGGCGACCCGCTCGATGCTCCAGCCATAGGTGCCGGTGATGATGATACTGACCAGGAAGGCGCCTCCCAG
CAGTAGAAAGGTGCTGTCGATGCCCATCATCATGAGCCCCGCCATCACCATAAAAGCGACATAGGAGCCG
ATCATATACACCTCGCCGTGGGCGAAGTTGATCATGCCGATGATGCCATAGACCATGGTGTAGCCAATGG
CGATCAACGCATAGGTGCTGCCTATGGTTAATCCGTTCAGCAGTTGCTGAACCAGATAGAGGAGGTGTTC
GGACATACTGCGTTCCCTGTACTTGCCCGCAAAGAGAGGAGGGGGTGAGCGTACTCACCCCCGATCGGCA
GATTATTTGACTTGGCTGGAAGTACCGTCAGCGTGCCACTGGAACACACCGAATTCGAAGCCTTTCAGGT
CACCGTTCGGTGCCCAGGTCAGGGGACCCATCACGGTATCGATCGGTGCGGCCTTGATGGCGGCGGCAAC
GGCAGCCGGGTCATCTTCACCCGCCTTGGCGATACCGGCGGCCAGGGTTTGCACCGCGGCATAGGTAGTC
CAGACGAACGGGCCGGAGGAGTCCTGACCCTTGGCCTTGAACGCCGCCACCAGCGGGGCGTTGGCAGGCA
CCAGATCATACTTGTTCGGCAGGGTCACCAGCAGGCCTTCGGAAGCCGGGCCACCGATGGCGGAGATATC
CTTGTTACCCACCCCTTCCGGGCCCATGAACTTGGCTTTCAGCCCCTTCTCGCCAGCCTGACGCAGGATC
AGACCCAGCTCAGGGTGATAGCCGCCGTAGTAGACGAAATCGACGTTCTCTTTCTGCAGCTTGGCGATCA
GGGCGGAGAAGTCCTTGTCACCGGCGGTGATCCCTTCAAAGGCCACCACTTTCACGCCAGCCTTGTCGAG
CGCGGCTTTGACGCTGGAAGCGATCCCTTCACCGTACTGCTTCTTGTCGTGGATAACAGCGACGCGCTGG
GGTTTGACCTGCTCGATGATGTACTTGGCAGCAGTCGGGCCCTGATCGCTATCCAGGCCGATGGTGCGCA
GGGTCAGCTGGTAACCGCGCTCGGTGATCTTGGGGTTGGTGGAGGCGGGGGTGACCATCAGGATCCCCTC
GTCTTCATAGATGTCGGAGGCGGGCAGGGTGTTGTCGGAGCAGAGGTGACCGACCACGAACTTGACGCCA
TCGTTCACCACCTTGTTCGCAACGGCCACCGCCTGCTTGGGATCGCAGGCATCGTCATAGACGACCGCTT
CGATCTGCTTGCCATTAATGCCGCCAGCCTTGTTGATCTGCTCGACCGCCATCTTGCCGCCGGTGAACTG
CATGTCCCCGTACTGGGCTACCGGACCGGTGACCGGGCCTGCGATACCGATCTTGATGGTGTCTGCCGCG
TGAGCGATGGAGATCGATCCAAACAGAGCCAAAGAAACCGCTGCGCCAACTGTGATTTTGCTCAACTTAT
GCATTGATTCGTCCCTTAATCAGTGGATTGTTTCATTTCCGTGTGCCATTCCGGATCTACATCCCGTGCA
TCCGGCTATAGGTATGGTTCTATCATTCCTCACGGGGCAATAACAAGCTGTTAAAGATACTAACCAGAGG
GGGGTGGATAGGCGGATTTTGAAATAAATGACCTGTTATTTTCGGCTAATCAGAGCTTAATTTTGATTTG
TTGACTTAACATCTTGGTAACGAGAGGCGATTCCGACAAACGCCAGAGGATTAATCTGGCGATATTTTAC
TCTGACAACGCTCCCATTTTGACCAGAATCCATTTGCGCAAACAGAAATGAGCGCCTAAATTAGCCGGCA
TTGACAATTCCGTGGCAGAGCTCGTTAAAACCAAGCCTTTATTCGGGAAATCTGCTAATGTTCCGCCTTT
GACAGTCAGGAGACAGAGATGGCCACTATCAAGGATGTCGCCTCGCGCGCAGGCGTATCCATCTCCACCG
TCTCACATGTGCTCAACCACACTCGGCGGGTCAGCGAAGACGCGACCCAGAAAGTGATTGAGGCGGTGGC
AGAACTCAACTACGCGCCCAACTCGGTAGCGCGCAGTCTTAAAATAAATTCGACCAAGACCATTGGTATG
CTGGTGACCACCAGCGTAAACCCCTTCTTCGCAGAAGTGGTGCAAGGGGTTGAAGCGTACTGCTTCGAAC
AGGGCTACAGCCTGATCCTCTGCAACACCGAAAACCAGCCGCCTCGCCAACAGCATTACCTGAGAATGCT
GATGGAAAAACGGGTGGATGGTCTGCTGGTATTGGGTACCGATATCGACACCCCGTTGCGCGACATGCTG
CGCATCCACAAGAACGTGCCGCAGGTGGTACTGGATTGGGGCACGGAGTGCGATTTCGCCAACGTCATCA
ACGACAACGCCCGCATCGGCGCCCGCATGGCCGTACGCCATCTGCTGGAGCTGGGGCACCGCGACATCGT
CTGCATCACTGGCCAGCTCTCCAAGCCGACCACCCAGCAGCGACTGGACGGGGTACGTGATGCGCTGGGC
GAACACGGTCTGACCCTCAAAGATGAACAGGTCTTTGAAGGGGACTACGAGAGCCAGAGCGGCTTCGATG
CCATGCAGCGCATCCTGGCCCTGCAGCCTCGTCCGACTGCGGTTTTTGCCTTCGACGACCCCATGGCGAT
CGGCGCGATTTGCGCTGCCTGGGAAGCCGGAGTCAAGGTGCCGGACGATATCTCCATCATTGGCTACGAC
GATGTGGAGATGGCGCGCTTCTCCAGCCCGCCGCTGACCACCATCCGCCACCCGAAGGCCGAACTGGGCC
AGCTGGCGGTGAAGCATCTGGTCAGCCGGATCCGCAACAAGGAGCTGGAAGTGGAGTCGATGACGGTACA
GCCCGAGCTGATTGTCCGTCGCTCGGTCAAACCGCTGCGTTAATCCGCACCCGTTGCCAACACGAAAAAC
CCCCTCTGTCACGAGGGGGTTTTTGTTTATGGGCGGGTTACTTTCAACCTGTGGCTCGCAGTGCCGATCT
CGGTCTTGTGAGTGAATATTCCCCCCGTTTGACCCACCTCGCATTTTGCCTGTTAATCGTTTGATCAATA
TCAACAAAGGGTTAAGTTCTGATGGACTCCCGACTTAACCGTTCCATCCTGTAGGTTGGGTATGACTGTC
AAAGATGGTGTCTTATGTTCCCCCCTCGCAAGCTGGGTCACCAGATCACGCTGCTGGCAATCGGCGACTC
TGATCGCCCAGCGGCCGGATGCAGCCAAGGCGGTGGCCGACAAGGATTGGCAGACCCTCGCCAATCTGTT
TGATCCGCTCTGGCCCGCTATCAAGGCGCAGGGGATCGAGCAGTTCCAGTTCCACTTGCCGCCAGCCACT

TCCCTCTATCGGGTACACAAGCCTGAAAAACACGGTGATGACCTCTCTGCCATTCGCCCCACTGTGGTTG
AGGTGAATCGCGAGCATCATGCCGTTGGCGGGCTGGAGATTGGCGTGGCTGGTACCGGTTTGCGCGGGGT
CGTGCCGGTCACCTTCAACGGGCAGCCGGTCGGTTCGGTGGAGTTCGGTCGTGCCCTGGATGAAAAGCTG
TTTGGCCAACTGCTGGACAAGCAGGTGCAGCTCGCCATCTATCTGTTTGAGCAGGGCAAGCTGGTGACGC
TGGTTGGCACATCCCGGCTTGGTGAGGCGATGGAGTGGTATCAGCCGGTACTGGCCGGTGAGGAACGCTT
CGTCAATATGACGCAACAGGGGAGTGATTTGCTGGTGATGGCGGCCCCCCTGCGCGATTACTCCGGCAAG
GCGGTCGGTATCATTGAATTGACCCGGGATCACAGCCTGGTCGCGGCCGATCTGGTTCGTCAGGGCTGGC
AGATGGCGATCATCTCTCTGGTCGGGGCTCTGGTGATTGCCATGGTGATGGCGTTCGCTCTCAATCGCAT
CAATCGACCGCTGCTCGACAGCGTCAAGGCGCTGGAGGCGCTGGCCAATGGCTCCGGCGATCTCGGCAGC
AAGCTGCCGGTGGCGGGGCCGGAGGAGATCCGCCGGCTCGGGTCGGCGTTCAATGGCTTTGTTGCCAAGA
TCCGCGACACCATCAGCCAGCTCACCGCCACTCTGGGCGAACTGGCTGGCGAGAGCGAGCGGCTGTCGCG
CAGTGCGGCGGGCAACCTTGGCAGCATGACGCGCCAGCAGGAGCAGACCACCCAGCTCGCCACCGCCATG
ACCGAGATGACCAGCACGGTACACGAGGTGGCCAACAACACGGCACAGGCGGCCGAAGCGGCCAACGAAG
CGGACAATCAGGCGGTCACCGGCGATGAGGTGGTGCAGCAGAGCGTCAACATGATCGAACAGCTGGCGGA
GGAGATCGCCAGCGCGGGCAACACGGTGAAAGAGGTCGCCAAGGCCAGCGAGCAGATAGGCTCTGTGCTG
GCGGTGATCCAGTCCATCGCCGAGCAGACCAACCTGCTCGCTCTCAACGCTGCCATCGAGGCGGCCCGGG
CCGGGGAACAGGGGCGTGGTTTTGCGGTGGTGGCCGACGAGGTGCGCTCGCTGGCCGGGCGCACCCAGCA
CTCCACCGCCGAGATCCGCGGCACCATAGAGCAGTTGCAACGGGCGGTGGGCACCACGGTGACCATGATC
GAACACAGCGTCTCGCGAGCGGGGGAGTCGGTGGAGCGGGCCAATCAGGCGGGAGAAGCCCTCGGCCGGA
TCAAGGGGGCGGTCAACAGCATTCGCGACATGAACACCCAGATCGCCGCCGCTTCGGAGGAGCAATCCTC
GGTGAGCGACGAGATCACCCGCAATATCGTCAGCGTCCACGAGATCTCCCAAGCCACCACGCTGGTGGCG
CGGGATACCGCGCATATCGCCAGCCATGTGGCGACCCTGGTGGACAAGCTGGGGGATCTGGCCGGGCAGT
TTCAGGATGGTTCCAACGTGCGGATGGTGCTGAGTCGCGCCCGCGCCGCTCACATGGGCTGGAAGACCCG
GGTGCGCGCCTATCTGGATGGGCAGGAGGGGCTGTCGCGCAGCGAGGCGGTCAGTGACAAGGAGTGCCGC
CTTGGTCACTGGTACTTCAGTGAAGGGCAGGCATGCTGCGGCGATCAACCCGCCTTCAGGGCGATGGCCG
CCCCCCACGCCAGACTGCATCAGGTGATCCAGCAGATTATCGAGCTGAAAAATCAGCACAAGCATGAAGA
GGCGGAGGCGCTCTACCGCGAGATCGAGCAGAACTCAGAGCAGGTAGTGGGCTGCATCGATCAGCTGCTG
CGGGAGATGACCAAGCCGGAGCGTTAATGACGAGCGGATCGGATAGCCAAAGGGCTGACAGCGATGTCAG
CCCTTTTTCATGGTGCGGGTTTGGGGTGAGGTTACTGCTCCGGGCGCAGCTCCTGATAACGGGCGGCGGT
GATGAACTGGCCGAAGGTTTCACACCAGACGATCACGCTGCTGTAGCGGGCCGGATCGATCGTGGCGGGC
AGCGGCACGATAAAGTTGTCGAAGGTCTTCACCGGCCCGACTCGCACCATTTGATCCTTGAGACGGGCAA
AATCGGCTTCGGTCTCGACAAACTCGGGGGAGAGGTAGAGCTGGTAGTCCGGCCCGGGGGCCAGCTTGCC
CATCAAACTGACGGCATCCGGCCCTATGCTCACCTTGCCCTCCCCCCAGTGCAGACGATCGCTGTCTTTC
AGATCCCGGCGAAAGGTGCCGTGGAATCTCGCTTGCTGCTGGCTGGCCGCTACCTGCTCGGTATCAGGGG
CGGGTGGCGCGATCAGGATTGGTAGGGTGTAGACACCGGCGGCAAACCCTGCCGCCGCGACCAGCAGATG
GGTGCCCAGCAGCAGGGCCATGGTTTTGCGTTTCATGATATCTCGGTCGTTGATGAATTGATGGCAAAAC
CATACCAGTCCGGGGGCCCGCTGCCCAGTTTGCTCAGGCCAGAGGCCTGGTTATCCATCTATGGTGACGT
GGGGTGTGGTCACGCCAATGGCTCAAGGCCGGATGGTGCCGTCCTGCCCTTTGTTGATCTCGCTGGCGGC
GTGTACCAGCTGTTCCAGCTGGTGGTGGATCTGGGTGCGATCCAGCTGCTGCAAGGGCTGACGACTCCAC
AGCACCGGCGTCAGGCTCTCGGTATCGAAACCCAGTACCGGTTTGCACGGGTCCTGGCTGAAGAGGTTCT
GGGCCGCGGCTTGGGCGCTGGTGGCCGCGTCGAGGCGGGTAAACATCAGCAGATAATCGGCCGGGTGTTC
GGTCAGGTGACAGGTCAGTTCGCCGATCTGCAGGCTGGTGACGCTCTCCTCCTGTTGCGGCGCAGGCAGG
GAGAGGGAGAGAAACAGCTGGTGGATGGCGGCGTGATAAAGAGAGTTCATTGATGGCTCCAGATTGATGA
TGGCGCCATTAGAGCAGGTCACCGCCAGCGGCACTGTTAATGGTGGCTCTCTTTTTTTAGCTTTTCGGTC
AGCGAAATAGCGCACCGCGCTCAGTACAAAAATCGCGACCAATCCCGATAGTCCCTGTTGATGGCCTCTC
CTAGACTGGCGGCGAAACATCACAAGAAGACAATCATCATGCAGATTCAAGCAAACACCGTCGGCACACA
GGCCGTCGCTCACCACAGTGATGCAACCACAGGGGTTGGCCGGATGGGCCAGCTGGAGGCTCGTCAGGTT
GCCACCGGACAAGATGCGATCTTGCTGGGCAACCGCAGCGAGCCGCAGAAAGGGCAGGGGCTGCTCTCGC
GATTGGGGGCCCAACTGGCCCGTCCGTTCGTGGCCATCAAAGAGTGGATTGGCAACCTGCTGGGGAGCGG
CAAGAGTGCCGCTGCGCCGAAGGCGCAAACCGCCACGTCCCCCGAGGATCTTCAGCGTCTGATGAAGCAG
GCTGCATTTGGTAGCTCGCTGGGTGGCTTTGCCAAGGCGGACGTGTTGAACAACATCGCCGCCGAACAAC
TGGGCAAGGAGCACGCCACTCTGGCGACCGGCAACGGCCCACTGCGCTCGCTCTGCACCGCGTTGCAGGC
CGTTGTCATCGGCTCCGAGCAGCCGCAACTTCGTGAGTTGGCTGCCGGGCTGCTGGCTCGCCCCATCGCC
GGTATCCCGCTCCAGCAGTGGGGCACTGTGGGGGGCAAGGTGACCGAGCTGCTCGCCAGCGCCACCCCCA
AACTGCTGCAGGAGGCGATGAGCCAGCTGCACACCGCGATGGGTGAAGTGGCCGACCTGCAGCGGGCCGT
CAAGGCTGAAATCGCTGGCGAACCGGCGCAAAGCGCGACCACCACTGCGGATGCGACGTCCGCCCAAAGC
GGTGATAGCAAAGGGGCTGCTCGCGAACAGGTCGCCATGGCGCGGCAGACCCCGGCCACCGGCTACAAGC
TGGCGCTGGATCTTATCAGCTATCAGGCCAGCTACCTGCTGCGGGATCAAACCTCGACCGAGGTGACCCT
GAGCAGCAGCGATCTCAACGCCCTGCATCAACACATTGCCGATGGCTCCATCAACAGTTCCCACATGGCC
AAGTTGCAGACCCGCGGCGATCTGCAAACCCTGCGTACTCTGGCGCTCTCTCTGGCCAACGGCAGCGACG
CCAAGGGCTCTTCCCTTGGTCATGCCCTCGACAGTCTCGCCTCGGCGCGTCCCAACCAGCGGTTGGTGCT

GGGCGGCCTGATGCAGTTTGCCGGTCAGACCGATCAGGCGTGGGTGAACCATACGGCTGGCAAGCCGGAG
GATCGGCTCGATGCCGGGGCGCGACTGCGCTTTGACACCGGCCATATGCAGGCGGAGCTGGGGCGTCTCG
GCGACAGCGAGGCACGGCAGGTGCTGCAACAGCTGGAAGGGGCTTTTGGTGATCGGGCCAGGGCTATCTG
CGACTTCGCGGTGGCTCAGGTCTCCGGCTTTGCCGATAGCGAAAGCAGTCCTGAGGCCGTGCTGGTCTCC
CGCCTGACCCGCATGGGCAATCTGGTGGGCAGCCTGACCGATGCGCTCAAGGATCGGCTGCAACTGCCCG
GGTCCGCCCGTGGCGAGCCGACCATGATCGACTCCGCCAGCCAGCTGACACCGCTCGAACTGGCTGCGCT
GGCTCATATCGGCGTCGATGAGAGCTATCTGTAAACCGTCAGCACACAAACCCCTCTGGGCAAGGTGATA
TGACATAAAAACGGACACCCTTGGGTGTCCGTTTTCTTATTCGGGCGGGAGTGGGCTGAGGGGAGCAGGG
CAGGCCGACATGGCCTAGAGGCTGGCGAAATCCACTGCGGGCAGGTTTTCAAAGCCGGGGCGGGCAAACA
GATAACCCTGCATCAGGCTGATACCCATCTCCCGCAGGCAGGCCATCTCTTCGCGGGTCTCGATTCCCTC
CGCCAGCGGGATGATCTTCAGCTCCGCAAACAGCGCCAGACAGTGGCGCACTATGGCTTGCCGGGTCGGG
TCCTGGTCGATGCCGCGCAGCAGGTGCATATCGAGCTTGACGATGTTGGGGTGAAAATCGGCCAGCAGGT
TGAGGCCCGAGTAACCGGCGCCAAAGTCATCGATTGCCGTCTTGAAGCCGAGGGCCTGATAGTGATCAAC
GATACTGCGGAGATGCTGGCTATCCTGCACCTGCTCCACCTCGGTCAGCTCGAACATGATCTGCTCGATG
GGGAAGAGGTGCTCGCGGGCCGCCTGCAGGGTGGTGCGGATGCAGCGTTCCGGGCGGTAGACCGCATTGG
GCAGAAAGTTGATGCTCAGCATGCCCCGCATATCGAGCCGGGCGGCCAGCGCGATAGCCTTGATCCGGCA
CAGCTGATCGAAGCGGTAGCTGTTGCCGTCGTTGACCCGGGCAATCACGCTCTGCGCTGGCTCATTGTTG
AGCCCCCGCACCAGCGCTTCGTAGCCGAAGATGGTGCGCTTGCTGCAATCGACGATGGGCTGAAAAGCCA
TGGTGAAGTCGAAGTCGAGATCTGCTTTCTCCTGACACAGCTGGCAGATGGCGGGGGACGGGGCGGCAGG
TATATCCATGACTCTCCTCTCGGGGCGTGGCGGCGCTGGGGACTGTCTCAAATGATAACAGCTGATCACC
CCCCTGTAGGTTCGATTAGCGAAGCGTAATCGGACGAACGAGACGCTTTGAGCGTACAGCAGAGGTGGCC
AGCCGTAGGGGGAAATCGACTGGCCGTTATGGCGAGAGAACCGATTTACGCGAACGTCCGATTACGCCTA
CCGGCTAATCGAACCTACGAGTTGCCCCCTTCTCCCCTTGTGGGAGAAGGGTTGGGGATGAGGGGGCGTT
ATGCCAACCCAAGGTCAATGTTGGGCTTCTCGATGTTCGGCAGCGACCGGCATGCACCATATCTCACCGC
CTTATGTAGGTTCGATTCGCGCAGCGTAATCGGACGAACGAGATGCTGTTGTTCCGGCCGTTCACGTGAG
TCAGGGTTGGCCAGCCGGAGGAGAAATGCGGGTGGCCGTGATAGCGTGAGAATCCATTTACGCGAACGTC
CGATTACGCCTATCGGCTAATCGAACCTATTGGCTAACAGTGGCGATTCCGTACAATTCTGATGGCATCA
CCAGTGATTAAATCTTCTGGTGATAAGTTAAGAACTACGTTTATAATATCAGCCATGGCATTATTACTGA
ACCGAAAGTTATTTGGCAAGTCATTATCATCATCGAAATAATTGTCAAATGAAATAACAAAGTCTTTTAT
ATCTGACGCTCTGCCATCTAATACTTTTTTCGGGAGTTCAATATGTTCTGGAGCATGTTGATTGCGTTCG
AAAACACAGATGCTATCAATTATTTCAGCTCTTGATGGCCAGTATTTTAGATCATATCTACAGCTGAGAG
ATTCTAACTGGTCAGCAATGTGACTTGTAAATAAATAGTTGTCACTTCCCGCATCAACCATCATATCTAA
GGCAGACTGATGTTGACCCCTTATAAACCCAGAATGTTGATAAATATCATCCTGCTCCCTGATTAAACTA
GCAAGTAATGAAGAGCAATCAATGATTTTTTCTTGAATGGCGATTAACTTTTTCCTGTAATCTCTAGCTT
GAACTATTTCCTCTTTACAAAAATCACCACTCATCCAAATATGCTCTAGCGTTAGCCATAGATAAACCCC
CTTTGGAGTCCGATATTCATAAGTATCAGAGTAGCCAAAAGCGTCTACTAACTCTTGGAAAGCGATGCTC
ATTTCTTCTGCTCTAGAGACCATCCTTTCCATTAATTCCCATCTAGTCATCCAGATTTTCTTTTCTTTGA
AGTCAGATAGTTCTCTTTCAATAAAGGATTTGCAAAAAAGAGTCGGATTTGTGGTGTTCAGAGTCATAGA
GCCTCACAGTGTTATCTAATCATTGTCTATATAGTACTGCCTCAAGATAGAAGTGCCGTATCTGAACTAA
AGACTTTTTGTTGCTTCAGATCGCTGAAGTGGAACAAAAAATTGGGTATCGTATGCACTCGTTTTGATTA
AAAAATGGCCTTCTGTGTGTTTTAACTCGCAATTAAAAACGGCGCCGTAAGGCGCCGTTTTTAATATCAC
TTACTCAACCAAGATCACTGCAGCAGGGAGATATCCGCGACTTGCAGGAACAGGTTGCGCAGGTTGTTCA
GCAGCGCCAGACGGTTGGCCTTGAGGGCTTCGTCGTCGGCCATCACCATCACTTCGTTGAAGAAGGTGTC
GACCGGCTCGCGCAGGGCGGCCAGACGGGTGAGGGCAGCCTGATAGTCACCGGCAGCGAACAGCGGGGCC
AGTTCGGCTTGCAGTTCGGCAACCTGGGTGGCCAGCGCTTTCTCGGCGGCATCGACCAGCAGTTCCGGTT
TGACGGCAGTCGGCAACTCGCCTTCAACCTTGGCCAGAATGTTGCTCACGCGCTTGTTGGCGGCAGCCAG
TGCCAGTGCGGCATCCAGAGTGCGGAAGTGGCTGACGGCCTTGACGCGGCGATCGAAGTCGAGCGGACGG
GTCGGGCGGCGGGCCAGTACGGCCAGCACCACGTCGGCACCGATGCCTTCGTCCTGATAGGCGGCGCGGA
AGCGGCCCAGCATAAAGTCGACCACGTCGGTGACGACGGTTTTGTTGGTCAGTTTGTCGCCGTAGACGCG
TACCGCTTCTTCAACCAGTTCAACCAGATCCAGATCCAGCTGCTTCTCGGTCATGATGCGCAGGGCACCG
ATGGCGGCGCGACGCAGGGCGAACGGGTCCTTGTCACCCTTCGGCAGCATGCCGATGCCGAAGATACCGG
CCAGGGTGTCGAACTTGTCGGCCAGCGCGACGGCGCAGGCAACCAGACCGGACGGCAGGGCATCACCGGC
AAAGCGCGGCATGTACTGCTCGTTCAGGGCCACGGCCACCGCTTCGTCTTCGCCATCGTGACGGGCGTAG
TGCATCCCCATGACACCCTGGGTGTCGGTGAACTCGCCGACCATGTTGGTCATCAGGTCACACTTGGAGA
GCAGACCGGCACGCTTGGCCTGAGCCACGTCGGCGCCGATACGCTCGGCGATAAAGCCGGCGACGGTCTC
GATGCGCTCGACCTTGGCCTTGACGGTGCCCAGCTGCTGTTGGAACAGCACGGTTTCAAGGCGCGGCAGG
CGGGAGGCCAGGGTCTGCTTGAGGTCGGTCTTGAAGAAGAACTCGGCGTCAGAGAGACGGGGACGAACCA
CCTTCTCGTTGCCGCTGATGATCTGGCTCGGGTCTTTGGACTCGATGTTGGTGACGAAGATGAACTTCGG
CAGCAGCTTGCCGCTCTTGTCGTAGACCGGGAAGTACTTCTGGTCACCCTTCATGGTGTGCACCAGCGCT
TCAGCCGGAACGGCCAGGAACTTCTCTTCGAAGTTGGCGGTCAGCACCACCGGCCACTCGACCAGTGCGG
TCACTTCTTCCAGCAGGGCGTCGTCCAGGTCGGCAACGCCACCGAAGGCGGCAGCGGCCGCTTCGACGTC

AGCCTTGATCTTGGCCTTGCGCGCCATAAAGTCGGCAATCACCATGCCGCGCTCCTGCAGGATCTGCGGG
TACTGGCTGGCGTTGTCGATGGTGAATTCGCTCTCGCCCATAAAGCGGTGACCGCGGATGGTGCGGGCAG
AGTCGATACCCAGAATGTGGGCGGGCACCAGATCCCCATCCAGCAGCAGGGTCACGGTGAACACCGGACG
CACGAACTGGATGGTCTTGTCGCCCCAGCGCATCATTTTCGGGATCGGCAGCTTGGCCAGTGCTTGGCTA
ACCAGCTCGCCCAGCAGATCTTTGGCCGGACGGCCTTCGACCTTGGCGGTGTGAACCAGCCACTCACCCT
TGTCGGTGACCAGACGCTCGGCCTGCTCAATGGTGATGCCGTTGCCACGGGCCCAGCCTTCGGCAGCCTT
GGTCGGCTTGCCTTCGGCATCGAACGCCTGCGCCACGGCCGGGCCGCGCTTCTCGACGCTCTTGCTCGGC
TGTTCGCCGGCCAGCTCGCTTACCTTCAGCGCCAGACGGCGCGGGGAGGCAAACCACTCGACGTCGCCGA
AGGCCAGATCGGCCTTGGTCAGTTCGGCTTTGAAGTTGTCGGCAAAGGCTTCGGCCAGGGAGCGCAGGGC
TTTGGGCGGCAGCTCAGCAGTACCGATCTCTACCAGAAATGTATGTTGTGCCATGTCCGTTCCTTAACCT
TGCTTGTTCTCACTGCGCTCACGCATGTCGTCGCTACGCTGGCACATAGGGAAGCCCAGCGCTTCGCGGG
AGGCGTAGTATGCCTCTGCCACGGCCTTGGTCAGGGTGCGAATGCGCAGGATATAGCGCTGACGCTCGGT
GACCGAGATGGCCTTGCGGGCATCCAGCAGGTTGAAGGAGTGAGCGGCTTTGAGAATGCGCTCGTAGGCA
GGCAGCGGCAGCGGTTTTTCCAGTGCCAGCAGATTCTGGGCTTCCTTCTCGTACTGCTCGAAGCAGTGGA
ACAGGAACTCCACATCGGCGTGTTCGAAGTTGTAGGTGGATTGCTCCACTTCGTTCTGGTGGAACACGTC
GCCGTAGGTGGTCTTGCCCAGCGGGCCGTCAGACCAGACCAGATCGTAGACGCTGTCTACGCCCTGAATG
TACATGGCCAGACGCTCGAGACCGTAGGTGATCTCGCCGGTGACCGGCTTGCACTCGAGGCCGCCGACCT
GCTGGAAGTAGGTGAACTGGGTCACTTCCATGCCGTTCAGCCACACTTCCCAACCCAGACCCCAGGCACC
CAGCGTCGGGTTTTCCCAGTTGTCTTCCACGAAGCGGATGTCGTGGATTTCAGGATCCATGCCCAGCTCT
TTCAGGGAGCCCAGATAGAGTTCCTGAATGTTGTCGGGGGAAGGCTTGATGATCACCTGGAACTGGTAGT
AGTGCTGCAGGCGGTTCGGGTTTTCACCGTAGCGGCCGTCAGTCGGACGGCGGGACGGTTGCACGTAGGC
GCAGGCGATGGGCTCCGGGCCCAGGGCACGCAGACAGGTCATGGGGTGGGAAGTACCGGCACCCACTTCC
ATATCCAGCGGCTGAATGATGGTACAGCCCTGGCGGGACCAATAGTCCTGCAGCTGCAGGATCAGGCCCT
GAAAGGTTTTGACATCGAATTTTTGCATAATTTTTTTCGCGCGAAGTGAAAATCAGAGCAAGATTTGCTG
AATGTGAACCGAGGGAGTATACCGTCTGGCATGGTCGGATAATAGGCGAAATTGCAGCGGATTCGCGGGA
TGGTGCTGCGCCCTGGCTGGTCGGGCGGGATGTCCGGTATATGATGTGAGCAGTGCCGCCTGCAGAGTGG
GCAGGGTCGGGCAAACCGCTGAAATAACGAGGGAGAGGAAATGGAACTGCGCTGCGCCTGGGTGACCAAA
GACCCGGAATACATTGAATACCATGACAAACAGTGGGGCAGGCCGGTCTATGACGCCCGCGAGCTGTTTG
CCAAGCTCTGCCTCGACGGCCAGCAGGCTGGGCTGTCGTGGATCACCATCCTCAAGCGTACCGAGAGCTA
CTACCGCGCCTATGCCGACTTTGATCCGGTGCAGATTGCCCGCTTCGACGAGCAGGATGTGGAGCGGCTG
ATGCAGGACAGTGGCATCATCCGCAACCGGCTCAAGGTACAGTCGATCATCAGAAATGCCCGCGCTTATC
TGGCGTTGCAGGAGCAGGGGATCGACTTCGCCGACTACCTGTGGGGCTTTGTTGGCGGCGCCCCCATCGT
CAATCAGCGGCAGGGCAACGGCGATATTCTGGCGACCTCGCCGGAGTCGGATGCCATGTCGAAAGCGCTC
AAGAAGCTCGGCTTCAACTTCGTCGGCAGCACCATCTGTTACGCCTTTATGCAGGCGGTGGGGATGGTGA
ACGATCATCTGGTCAGCTGTCCTTGCCACGCCGAGTGTCAGTTACCGCGCTGACAACCCGTCGATTGCCC
AAAAAAACATCACTCTCGCCATCTGTTCAGGCTTGCGTCAGACAATGGCGTGCCAGCGCGTTACAATCCG
GCCCCCGGCATGGTGCCGGATTGTTTTCACAGAGGTTTTTTCATGCGAGCCAAGATCCTGGTGCCTGCTC
TGGCACTGACCGTTGCCCTGAGCGGCTGTACCACCAACCCCTACACAGGTGAATCCCAGACCTCCAAAGG
TGCATGGGGCGCGCTGGCTGGCGCCGCTGCCGGTGCGGCCGTAGGTGCGCTCTCCTCCAGCAAGGGTGAC
CGCAAGAAGGGCATCCTGACCGGCGTTGCTGCTGGCGCGGCGCTGGGTGGCGGTATCGGCTACTACATGG
ACGTGCAGGAAGCCAAGCTGCGCGAGAAGCTGCAGGGCACCGGCGTCAGCGTGACCCGCAATGGCAATGA
GCTGATCCTCAACATGCCGAACAACGTGACCTTCGACAGCTCCAGCTCCCAGCTGAAAGCGGCCGGTGCC
AACACCCTCTCCGGCGTGGCCATGGTGGTTGCCGAGTTCGACAAGACCCGCCTGAACGTGGTCGGTCACA
CCGACAGCACAGGTTCCCGCGATCTGAACATGAAGCTTTCCCGCGATCGTGCCGATGCCGTTGCCGCTCA
GCTGATTGGTCAGGGCGTAGCAGGTAGCCGCATCTCCACCACCGGCGTGGGCCCGGATCAGCCGGTTGCT
ACCAACAGCACCGCCGCTGGCAAAGCCCAGAACCGCCGCGTGACCATTACCCTGACCCCGACCGCCTAAG
TCGCTCAGGTTTCGGTAACGAAAAGGCCCCGCCAGTGATGGCGGGGCCTTTTGCTATCTCAAGCTCAAGC
CGGGAAAGGCTTAGACAGCTGCTGTCAGCTGTACTTCGACCTTGTAGCCTTCGTGGGCCAGACGGGCTTG
TACACAGGCTCTAACCGGTGCTGTGCCAGCAGGGATCCAGGCATCCCACTGCTCGTTGAAGGCAGCGATG
TCACCAATGTCTTTGAGATAGATATTGGCCATCAGGATCTTGTCTACGCCGGAGCCGTTGGCTTCCAGCA
GGCGTTGCAGGCTCGCCAGCACTTCGCGGGTCTGCTGGTGGATGTCGGCCTCTTCGCTGACGGGCACTTC
CACCACGTAGAGGGTGCCATTGTGGATGACCACGTCTGACCAACGCTGGGTGGTGCCGATTCGGGTAATG
GTTGTCATGGAGTGCTCCGGTACAGGGGCTTATTGACCCTTGCGGATCAGATAGCGGTAGGGGGCCTGCT
CTGTCTCGCTGGCGACCAGGGTGTGATCCATAAAGCGGCAGAAACTGGGGATGTCGCGGGTGGTTGAAGG
ATCGTCGGCGCTGACCAGCAGGGTCTCGCCATCCGCCATCAGCCGCACTTTCTTGCGAACCATCATAACG
GGTTCCGGGCAACGAAGCCCGATTGCGTCCAACTCGTGGGTTGCGCCGCAGAATAAATCACTCATCTATT
ATCAACCTTCAACCTATTCGGCTGCCAATGATACTCAGGGCTAAAAAATAATCAAACAAACGCCCCATCA
ACCAGCATATGTAACCGAAATGTTATTCTGCCCTATCCAGCTTGCGCCGGACGAGTAATATAAAAATTGA
ACCATATTCAACAAGGAGGTGAATATGCAGGTCTCCTACGGCAGATTACAAGCCTATGCAGGCATGGGGT
TCTTGTCCTTCGCCATGATAGTGATGGGCAGCCCGATCGGGAACACCGGTACCTGGTTCGGCTCCATGGT
GATGGTCGGGTTGGGGCTCTGGATCGAGCTCAGCGATTACTATGACGAGGATGAGGACGAGAACGATCAA

TCCTGAATCGGATGACGATTCAACAACAAAGGCGGAGCAATTCCGCCTTTGTCATTTCTGCCGTTTGGCA
ATGTGCCCGGATCAGCGGGTCGAGCGCATCGCCCGCTCCAGATGCAGCGCCGTCTGCTCCGGGTTGGCTT
TGGGGTCGTTGAAGAAGTTGCTCAGCACATCGAGGATTGCCTGCCGCATATTGGTCGGCGTCGCCATCCC
TTCCGCCATGCTGGGCAGCAGGTTGTCCTGCTTTTCCGCCAGCAGGAAATCCTGATAGGAGCGGACGGCG
CAGCGGTCGAATTTGCTCATGTCGGGATTGGTCAGCGCCGGAATAGACCCCTTCACCCGATTGAACTCCT
CCTGGAATTGCGGCGTCATCAGCAGCTGGGCCAGATCCCCTTGCGCCTGCAGCTGGGCCGGATCCCGCTG
TTTGAACATGGCGATGGAGTCGAGGTTGTAGCTGAACAGTCCGGCGCTGCCCGGGCTCGGCAGGCAGGCG
ATATCCTCACCCGGCCGGTAGTTACCGGCACTCAGTTCACCCTTCACCCAGTCCCCCATCACCTGCATGG
CGGCGCCACCGCTCTCCAGCAGATTGGTGGCCTGATGCCACTTCAGACCGGCGTATTTTTGCGGCACATA
GGCGCGCAGCTGCTGGAAACGGGTCAACACCCGCACCATGTCAGGGCCGGTCAGGGTGGCGATGTCCTGC
TCGAGGAACGCCTTGCGATAGAATTCTTTACCGCCCTCACCAAGCGCCACCGTTTCAAACAGTACTGCCA
GCTGCCAGGGCTCGTTGCCAATGGCGAGCGGGGTGATGCCCCGTTTTTTAAGCTGGTCGGCGACGGTGAC
AAATTGGGCCCAGTCGGTGGGCGGAGTCAGCTTGTTGCGCTCGAAGATCTTGCGGTTGAGCCACAGCCAG
TTGACCCGGTGGATGCCGGTGGGCACCGCCATCAGGGCGCCATTCTGGCTGAGGGTGGCACGCACCATGG
GCGGCATCTGACTGTACCAGCCGAGGTGTTCGGCCATCGGGCTGAGATCGCGCAAAAAGCCGAGTCCGGC
CCACTCCTTGAGCTCGTAGCCCTTGAGGTGGGCCGCTTCCGGCGGGTTGGCCGCCAGTGCCCGGCTCTTC
AATACCGTCATGGCACTCTTGCCGCCGCCACCCTGCACTGCAAAGTCGTTCCACTGGTTGCCCTGTTTGG
TCCACTCGGATTTGAGCACTTCGACCGCTTTCGCTTCGCCGCCTGACGTCCACCAGTGCAGTACCTCCAC
CTGGGAGGCGGCAACGGGAGTAGAGAGCAGGGCGATGACAGAAAAAGACAACCACTTCATAAGGTTACTG
CCTTGGTAGGGAGAGCGTGGCCTGCAGGCCACCTTGCGGACGATTTTCCAGCACCAGATCGCCGCCGTGG
GCGTGGGCAATGTTGCGGGCGATGCCAAGGCCGAGGCCGGTGCCGGCCTGATCCGTGCTGAGCCGGTAAT
AGGGTTCGAAGATGCGCTCGAACTGATCGTCCGGCAGGCCGGGGCCCTCATCCATGATAAAGAGGATCAG
CATCTCTTCGTCATCCATGATGATGATGCGCACCTTCTGGCCGTACTTGATGCCGTTGTCTACCAGATTG
CCGATGCAGCGCTTGAGTGCCAGCAGCTTGCCGCGATAGGGCCACTTGCAGTGCCCTTCGATGGTCAGCC
GCTCGTCGTGCAGGTTCATCGACTCCGCCATCTGCTCCAGCAGGGCGTTGATGTCGATCTCCTCGATATT
TTCGTGAATGTCGGTCTCTTTGACCGTCTGCAGCGCCCCTTTCACCATCAGCTCCAGCTCGTCGAGATCC
TTGTTGAACTTGGCGATCTGTACCTCGTCATCGAGCAGTTCGACCCGCAGCCGCAGCCGGGTGATGGGGG
TCTTGAGGTCGTGGGAGATGGAGCTGAACAGCCGCTCTCTATCGTCGATATAGCGACGAATGCGGTGTTG
CATGATGTTGAAGGCGCGGGTCGCCGCGACGATCTCGGAGGCACCCTCCTCCTTGAGCGAGGGTTGGTCG
ATATCCTTGCCCAGATTCACCGCCGCCTTGGCGAGACGGCGCAGCGGGCGGGTCTGCCAGCGCACCAACA
TAAAGGTGAAGAAGCAGAGGAACACCGTCATCAGAGCGATGAAACGCACCTGGTTGCTCGGCATCACGGT
ATCGTCGAGGGTCATGTAGGGGGCGGGCAGCAGGGCCGCCAGATAGAGCCACTCCCCCTTTTCAATCTCG
ATCTGGGTCACCAGAACTGGCGGATTGAGCGGCTCCAGCGACAGGGTGTAGCGGGCCCAGGAGGAGGGGA
GATCCGCCAGCAGGGTGTCGTTGTTGAAGACGTGGAGGTCATCGGGCCGGGAGAAGTCCACCTTGATCGC
CATGGCGTCGGAGAGCTTGCGGGTCAGCACTCCCTGCACCTCCTCCAGCACCTGCGTCTTGCGTTCGCTG
TCGGGGATGGCGTTGATGTGGATCTCTTCCTCGTTGAGCGAGACGAAGAAGCGGCTGCCCCCCATGTTGC
GCAGTTGGTCCAGAGCGATATGGCGATACTGCAGCGGCAGGGATTTGAAGAAGTTGACGGTGGAAGCGGC
AGACATGGCGAGGTTGCGGGTGGTGCTGAGCATCCCCTCCAGCTCCCGCTTCTGGATCTGCTGCATCCAG
ATGCCGGTCAGAATGGTCTGGGAGAGCAGGATGGCCAGCAACAGCAGCAGGATCATCCGTGACAACAGGG
AGCGGGGCACCAGCGCCCCCCAGGACCACTTGTTGCTCATGGCAGCTCGTGCACCTCGGCGATCAGCATG
TAGCCACTGCCGCGAATGGTCTTGATGATGCGCGGGCTCTTGCCGTTGTCGCCCAGCTTCTGGCGCAGGC
GGCTGATCTGCACGTCGATACCGCGCTCCATCGGCAGGCTTTCGCGGCCGCGGGTGGCATCCGAGATGGT
GTCCCGATCCAGCACCACGCCGGGCTGTTGCAGGAACATGCCGAGCAGCATGGCGTCGCTGCCGGAGAGG
TCCAGCAGGCTGCCATCGTCATGGGTCAGCTGGTGGCTCAGGGTGTCGAGGGTCCACTCGGCAAAACGCA
GGCGGCGGCTCGGCTCTTTCTCTTTTTCCGGCTGGCGGAATTCGGCGCGGCGCAGCAGTGCCTTGATGCG
GGCCTGCAGTTCACGGGGGCTGAACGGCTTGGCGATATAGTCGTCAGCGCCGAGTTCCAGGCCGATCACC
CGGTCCGCTTCGTCGGAGGCGGCGGTCAGCATGATGATGGGCACCTGGGAGTCACGACGGATCTGCTGGC
AGAGGGTAAAACCGTCCTCGCCGGGCATCATGATGTCCAGAATAATCAGGTCCGGGCTGTGCTGGGCGAG
TTGCTGGCGCATCTCGTTGCCCTCCGCCGCCGTCAATACCTGAAAGCCCGCCCGGGTCAGGTACTCATTG
AGCAGCTCGCGGATCTCTTGGTCGTCATCGACGATCAGCAGTTGTTTGCTCATTGACATATGTATGTGCA
TCCACCCATTTTTTTGTGCAGATTGAACCTGTCGGGGGAGTAAAAAGCAAGCAAGCCCCCTATCTAATGT
GAGGTTTAAGGCAATAACAGCCTATTGATAAGATTAAAATGAAAAAAGGCCGACCCAAACGGGACGGCCA
ACAGACAGCTTCTGACCACTGACGAGCACCCCGTGTGGCGATCGTCACAGACTTGAAAAGGGGCCCGTCA
GACTGGCCCCGAAAAGGGACAACTCTCAGTAGTGCATCCCGTCGATCCGGCTGGTTATCACACCCGTTCA
AACACGGTGGCGATGCCCTGGCCCAGACCGATACACATGGTGGCAACCCCCAGGGTGGCATCCTTCTCCT
CCATCAGATTGATGAGGGTGGTGGAGATGCGGGCGCCGGAGCAGCCAAGGGGGGTGACCCAGCGCAATGGC
GCCGCCGTTCAGGTTCACCTTCTCGTCCACCTGATCCTGCAGACCCAGATCCTTCACGCAGGGCAGGGAC
TGGGCGGCAAACGCCTCGTTCAGCTCGAACAGGTCGATATCACCGACAGTCAGACCGGCACGCTTGAGCG
CCTTCTGGGTCGCCGGCACCGGGCCGTAACCCATGATGGCGGCGTCACAGCCGGCGATGGCCATGGCACG
CACCTTGGCGCGCGGTGTGAGGCCGAGGGCCTTGGCGCGATCCGCGCTCATCACCAGCATGGCGGCGGCG
CCATCTGACAGCGCCGAGGAGCTGCCGGCGGTGACGGTGCCGTTGACCGGATCAAACACCGGGCGCAGCT

GGCTCAGGGTTTCGACCGTGGTCTCTGGGCGGATCACCTCGTCATAGTCGTAGAAGAAGCGGGCACCGCT
GGCGTCATGGCCTTCCAGCCCGACGATCTCCTTGGCAAAGCGCCCTTCCACGGTGGCGGCGTGAGCGCGG
CGATGGGAGCGGGCGGCAAACTCGTCCTGCTGCTGGCGGTTGATGCCGTGCAGCTTGCCGAGCATCTCGG
CGGTCAGCCCCATCATGCCGGAGGCTTTCGCCACCGACTTGGCCATGCCCGGGTGGAAGTCGACGCCGTG
GCTCATCGGCACATGGCCCATGTGCTCGACGCCGCCGATGATGAAGATATCCCCATCACCCACCTGAATG
GCGCGGGAGGCGTCATGCAGCGCCTGCATACTCGAGCCGCACAGGCGGTTGACGGTAACCGCCCCCACCT
GCTTGGGAATGCCGGCCAGCAGTGCGGCGTTGCGGGCGATGTTGAAGCCCTGCTCCAGGGTCTGCTGCAC
GCAGCCCCAGTAGATATCCTCGATCTCGTTCGGGTCGAGGTTGGGGTTGCGCAGCAGGATGGATTTCATC
AGGTGCGCGGACAGATCTTCTGCACGCACGTTGCGGAAGGCGCCGCCCTTGGACCGGCCCATCGGGGTCC
GGATACAGTCGACAATGACTACGTCTTTCATGAATGGATCTCCTTTCCGCAATCAGTAGAAGGTTTTGCC
CTGGGCGGCCATCTCGCGTAAGCGGTCGCTGACGCGGTAGAGCGGGCCCAAGTCGGCATACTGGTCGGCC
ATGGCCACGTAGCGATCCAGACCGATAGTGTCCAGATAGCGGAACACGCCGCCGCGGAAGGGAGGGAAGC
CCAGACCGTAGACCAGTGCGATGTCGGCTTCGGCTGGCGTGGCGACAATGCCCTCTTCCAGACAGAGCAC
CACCTCGTTGATCATCGGGATCATCATGCGAGCGATGATGGCGTCCTTGTCGAAATCCTGTTTCGGCTTG
GCGATGGGGGCCAGCAGCTCGTAAGCAGCTGCGTCGGCTACCTTCTTCGGCTTGCCCTTCTTGTCCTGCT
CGTAGGCGTAGAAGCCCTTGCCGTTCTTCTGACCGAAGCGGTTCACTTCGTACATCACGTCGATGGCGGT
ACGGCCTTCCTTGCTCATACGTGCCGGGAAGCCCTGCGCCATCACGTCACCGGCATGGTGGCCGGTGTCG
ATGCCGACCACGTCCAGCAGGTAGGCGGGGCCCATGGGCCAGCCAAACTCCTTCTCCATCACCTTGTCGA
CGGCAGCGAAGTCGGCGCCATCGGCGACCAGCTTGTTGAAGCCGAAGAAGTAGGGGAACAGCACGCGGTT
GACGAAGAAGCCCGGGCAGTCGTTGACTACGACCGGGGACTTGCCCATGGCGGCGGCGTAGGCCACCACG
CGGTTGATGGTTTCATCGGAGGTCTGCTCGCCGCGGATGATCTCCACCAGCGGCATGCGGTGCACCGGGT
TGAAGAAGTGCATGCCGCAGAAGTTCTGCGGGCGCTTCAGTCCCTTGGCCAGCAGAGAGATGGGGATGGT
GGAGGTGTTGCTGGCGAGCACGGCGTCTTCGCCGATCACGCCTTCCACTTCACCCAGTACGGCGGCCTTC
ACTTTCGGGTTCTCGACCACGGCTTCGACCACGACGTCCACGCCTTTGACGTTGTCATAGCTGAGGGTCG
GAGTGATGGCAGAGAGCACCTGGCCCATCTTGATGCCATCGATGCGGCCCTTTTCGAGCTGGCCGTTGAG
CAGCTTGGTGGCTTCACCCATGCCAAGTGCCAGCGCCTTCTCGTTGATATCCTTCATCACCGCCGGAATG
CCCTTGCTGGCAGACTGGTAGGCGATGCCGCCACCCATGATGCCGGCACCCAGTACGGCGGCGTGGCCAG
TGGCCTTGCTTGCCTGCTTGGCAGCCTTCTTGGCCAGCGCCTTGATGTGTTGATCGTTGAGGAAGATGCC
GACCAGCGCCTTGGCGACATCGGTCTTGGCCAGCTTGATAAAGCCCTGTGCCTCAACGACCAGCGCTTCG
TCACGGCCCATGCCAGCAGCGGCTTCGACGGTTTTCACCGCGGTCATCGGCGCCGGGTAATGTTTGCCCG
CCACGGCGGCAACCATGCCGGCGGCGGTGGTGAAGCTCATCATTGCTTCCAGTTTGGAGAGGCGCAGCGG
GGCTTTCTTGGCGGCACGACGGCTCTGCCAGTCCAGTTTGCCCGCGATGGCGTCCTTGATCATCTGCAGT
GCAGCACTCTGCAGCGCGTCAGGGGCGACCACGGCATCGATGGCTCCCACCTTGAGGGCGTCATCGGCAC
GGTAATCCTTGCCGGTGGTGATCCACTCGAGCGCGTTGTCGGCACCGATCACTCGTGGCAGACGGACGGT
GCCGCCAAAGCCCGGCATGATGCCGAGTTTGGTCTCGGGCAGGCCGATTTTGGCGGTGGTGTCGGCCAGA
CGGAAGTCAGTGGAAAGGAGGGTCTCGCAGCCGCCGCCCAGAGCGTGGCCCTTGATGGCAGAGAGGGTCG
GAACCGGCAGATCTTCGATGGCATTGAAGATGTCGTTGGCCTTTTTCAGCCAGCCGAGCAGATCTTCTTG
CGGCAGATCGAACAGTTCCAGGAATTCGGTAATGTCGGCACCGACGATAAAGGCGTCTTTGCCGGAGGTG
AGGATCAGACCTTTTAGTTCACGTTCTTGTTGTAATGCGGCGATCGCTTCGCTCAGGGAGAGCAGGGTAG
CGCGATCCAGCTTGTTGACTGAACCCGGGGCATCAAACCTCAGCTCGGCAATGCCGTTTTCGAGGTAGCT
GACCGACAGGGTTTCGCCTTGGTAGATCATGAGAGTGTCTCCTTGTTCCCACTCAGTGGGGAGTGGTGCT
CCTTAATCCCCAGCAGGGGGCGAGGCTCACTATTGATCCAAAAGGAGACATTTTCAACACCTATTTAAAA
CATTTGTTTAACAGTTGTCGAGTAAAAGCTCCTCATCACAAAACGGGCAAAAGAGAGGCGCCTTGCGGCG
CCCCGGATGGATGTGAGTTTGTTATCAACTGGCCAGTTCCGGCCGGTTGCGATACTGGTTCAGCACTTCG
GGATCGGCGAGGGCGTGGGTATTGTTGACCGGCCGGTTGTGCACCACTTCCCGTACCGCCAGCTCGACGA
TCTTGCCGGACTTGGTGCGTGGGATGGCATCCACCTGCAGGATGCGGGCGGGCACGTGGCGGGCGGTGCA
ATGTTGCTTTATTTGCTGGCGAATGCGCTCGCGCAGCGGCTCGTCCAGCTTGCAGCCCGCCTTGAGCTTG
ACGAACAGCACCACCCGCTCGTCCTGCTGCCACTGCTGGCCGATGACGATGCTCTCCTCCACCTCGTCCA
GCTGCTCGACATAGCGGTAGATCTCGCTGGTGCCGATGCGCACGCCGCCCGGGTTGAGGGTGGCGTCGGA
GCGGCCGTAGAACAGAATGCCGCCGGTCGGGGTCAGCTCGATCCAGTCGCCGTGGCACCAGATGTTGTCG
AACCGCTCGAAGTAGGCGGCATGGTACTTGTCGCCATTCTCGTCGCCCCAGAAGTAGATGGGCTGGGCCG
GGAACGGCTTGGTGCAGACCAGCTCGCCTTTCTCCCCCTGCACTGGCTGGCCCGCCTCGTTGAATACCTG
CACCGCAAGACCGAGGCCGCGCCCCTGACTCTCGCCGCGATAGACAGGGCTGAGCGGGTTGCCGATGACA
AAGCAGGAGCAGATATCGGTGCCACCTGAGATGGAGCTGAGCTGCACATCCTGCTTTATCTGCTGATAGA
CGTAGTCGAACCCTTCCGGCGACAGCACAGAGCCGGTGCTGCAGATCAGCTGCAAGTGCGGCAAGTCGTG
GCTCTTGATGGGGGCGTAACCCTGCTTGTGCAGTGCGTCGAGATACTTGGCCGAGGTACCGAACAAGCTG
ACCTGCTCGTCACGGGCGAGATCCCACAGCACGTTGCCATCCGGGTAAAACGGCGAGCCGTCATAGAGCA
CCAGGGTGGCGCCGGAGGCGAGGGCGCTCACCAACCAGTTCCACATCATCCAGCCGCAGGTGGTGAAGTA
GAAGATCCGCTCCCTCGGCTTGATGTCGCAGTGCAGCTGGTGCTCTTTCAAATGTTGCAGCAGGGTGCCG
CCAATGCCGTGCACGATGCACTTGGGTTTGCCGGTGGTGCCGGAGGAGTAGAGGATATAGAGCGGGTCAT
TGAAAGCCATCGGCTCGAAGGTGAGCTGGGCATCGTGTTGGCTCGCCAGCACTTGCTGCCAGTCGTGACC

AAGCTGCAGCGGGTTGCCGAGCAGGGGGATCATCACCGTTTGCTCTATGCTGCCGATCTGGCTCACTACG
CTGGCGACCTTCTGTTGAATGTCGATAGCTTTGCCGTTGTAGCGATAGCCATCTACCGCAAACAGCACTC
GTGGTCGGGTCTGGCCGAAGCGCTCCACCACGCTGGCCTCGCCAAAGTCGGGGCTGGTGGAGGTCCAGAT
CGCCCCGAGGCTGGTGGTTGCCAGCATGGCAACCACGGTTTCCGGAATATTGGGCAGATAGGCGGCGACC
ACATCGCCACGACCAATCCCCTGATGACGCAGCCACTGAGCCAGCTGGGCAACCTGATCACTCAGTTCAC
CCCAGCTAAGAGTACGGCTTGGGCTACCTTCGATACGGGAGATGATGGCCGGGCTCTCGTCCTGACGGCG
CAGCAGGTTTTCGGCGAAGTTGAGGCGGCTATCGGGGAACCAGCGAGTGCGCTGCATATCCTGACGGTTC
TCCGCCACTATGTTGCCGAGCTCGCCCTTGACGCCGCAGTGCTGCCATACCAGCGGCCAGAAACGGGTGG
TCTTCTCCACCGACCATTGATAAAGCTGCGGGTAGTTCTGCAACTGCAAGCCGTGGAAACGGTTCACCTC
CGCCATAAAACGGTAGAGGTTGGATTGCTGCATCCCGAGCGGATCCGGTTGCCACAGGCAGAGGTTGTCC
ATGAGTAATCCCTGACTTCATGTATCTACCGGCAGTGTATTGAATTGTTTTTAATTTGTTAATGGTTGGT
TTTCTGTTGATAACAAACACGTTTGTAACGTATGCGTTACCTCACGAAGCGGCAGCTTGACGGGTCGAGC
CGCCAAGCGCTTGATGTTATGCTGCAATCACATTCACCTCGGAGCAGGACGCCATGAGCAGCTACAGCCA
ACTTTTTGCCCAGCACCTCGATACCTTGCAGCAGCGCACCCGCGACATTCTGCAACAACAGGGGCTCAGC
GGACTGGCCATCCACTCCGGCCAGACTCACCGCATCTTCCTCGATGATCAGGATTACCCGTTCAAGGTAA
ACCCGCATTTCAAGGCCTGGTTGCCGGTGCTGGACAACCCCCACTGCTGGCTGCTGGTGGACGGGGTGAA
CAAACCAATCCTGCTGTTCTACCGCCCGGTGGATTTCTGGCACAAGGTGGCGGATCTGCCCAACGCCTTC
TGGGTCGAGTTCTTCGATATCCGTTTCCTGACCCGGCCGGAACAGGTGGCCGACCATCTACCTGCCAACA
AGCAGGAGTGGGCCTATCTGGGGGGCCATCTGGAAGTGGCTGAACTGCTGGGGCTGACCCAGCCCAATCC
GGAGGCGGTGCTCAACTACCTGCTCTATCACCGCGCCTACAAGACCCCCTACGAGCTGGAGTGTCTTCGT
GAGGCGAACCGGATCGGGGTACGCGGCCATATCGCCGCCAAGGACTCCTTTATGGCGGGGGCGAGCGAGT
TCGAGATCAATCTGGCCTACATGAAAGCGGTCGGACAGGGAGCCAATGAGGCGCCCTACGGCAATATCGT
CGCCATCAACCGCAATGCAGCCATCCTTCACTACACCCATTTGTCCGCCGTGCGGGTGCCCGATGCGGAA
CGTCACTCCTTCCTGATCGATGCCGGCGTCGATTTTCACGGTTATGCCTCCGACATCACCCGCACCTGGG
CGTGGCGGCGCGGCGAGTTCGCCGATCTGATTGCAGCCCTCGACGAGCAGCAGCAGGAGATCATCAAGGA
GATCAAACCGGGACGCCGCTACAGCGAGCTGCACCTGCAGATGCACCACAAGCTGGCGCGCCTGCTGCAA
GCGACCGAGCTGGTGGACATGTCGGTGGACGAGATGATCAACACCGGAGTGACCAACGTCTTCTTCCCTC
ACGGCCTCGGCCATTTCCTCGGTTTACAGGTACACGATGCGGGTGGTTTCATGCAGGATGAGCGCGGAAC
CCACTTGGCGGCGCCCGAGCTGTTCCCCTATCTGCGCTGCACCCGGGTGATGGAGGTGGGTCAGGTGTTC
ACCATCGAGCCGGGCCTCTACTTCATCGACAGTTTGCTGGAGCCGCTGCGTCAGGGCGAACAGGGCAAGC
GGGTCAACTGGAACAAGGTGGAGGCGCTGCGTCCGTTCGGCGGCATCCGCATCGAAGACAACGTGGTGCT
GCACGCCAACGGGGTGGAGAACATGACCAGACAAGCAGGACTCTGATGGCGGCAGCGTACTCCATTCCGG
CTGCCTCGCTGGAGTTGACCGAAGAGATAAAGAAGAGCCGCTTCATTACCCTGATCGCCCACACCCCGAC
CGTCGAGGCGGCCAAGAGGTGGGTCAACGAGATCAAACGCCAACATCCGGCCGCGCGCCATCACTGTTGG
GCGTTTGTGGCTGGTGCGCCGCAAGATAGTCAGGTTTATGGCTTCAGCGATGATGGCGAACCTTCCGGTA
CTGCGGGCAAGCCGATCCTGGCCCAGTTGATGGGCTCGGGGCTCGGTGAAATCTGCGCCGTGGTGGTGCG
TTACTACGGCGGCATCCAGCTTGGTACCGGTGGACTGGTCAAAGCCTATGGGGGCGGCGTCGGTGCCGCC
CTCAAGCAACTGCCCACCCTGCTCAAGGTGCCACGCACTCCCTCTCTCATTCACTGCGATTACGGTGACA
TGGGGACGGTCGAGTCGCTGATCAGCAACCATAATGGCGAATTGCTGGCGGCCGATTTCGGTCAGCAGGT
CACCTTGCAGGTGGCGTGGGAGTCCTCGGTTTGGGCTCAGGTGGACCGTGAATTGACCGACCGGACTCAT
GGGCGGGTATCCCTCAGTCCCTTAGATGGCTAGAATCCATCGCTCTTTAATTGGGGAAGGGGAGCTGTAA
TGCACATTCTGAGCATTATTCGCATTGTTGGCCTGCTGGTTGCGCTGTTCAGCTCGACCATGCTGTTGCC
TGCACTGGTGGCCTTTGGCTATCGGGATGGTGGTGGTGCCGAGTTCGTCAGCTCTTTTCTGATCGCCCTG
CTGCTCGGCGTGGTGCTCTGGTTTCCCAATCGCTATCACAAGAAGGAGATGCGCTCGAAAGAGGGTTTCC
TCATCGTGGTGCTGTTTTGGACCGTGCTGGGCAGTGTGGGGGCGGTGCCCTTCATCATGAGCGACGTGCC
GGATATGTCGGTCTCGGAGGCCTTCTTCGAATCTTTCTCCGGCCTGACCACCACTGGCGCAACGGTACTG
ACCGGGCTTGATAATCTGCCCAAGGCGTTCCTGTTCTATCGCCAGTTGCTGCAGTGGCTGGGGGGGATGG
GGATCATTGTACTGGCGGTGGCCATTCTGCCACTGCTGGGTATCGGGGGCATGCAGCTCTATCGCGCCGA
GATCCCGGGGCCGGTGAAGGATACCAAGGTGACGCCGCGCATCGCCGAAACGGCCAAGGCGCTCTGGTTC
ATCTATCTGCTGCTCACCGCACTCTGTGCGCTGGCCTACTGGCTCGCCGGAATGACGCTGTTCGATGCTG
TGTGCCATTCGTTCTCGACTCTGTCGGTCGGTGGCTTCTCCACTCACGATGCCAGCATCGGTTTCTTCAA
CAGTCCGGTCATCAACCTGATTACCGTGGTGTTCCTGCTGCTGGCCAGCGTGAACTTTGCGCTCCACTTT
ACCGTCTTCTCTCACCGCCCGGTGCGGCTCGGTCGCTACCTTTACGATTCTGAACTCAAGGTGTTTCTGG
CCATCCAGGCGACTCTGGTGATGATCTGTTTTGTCGCCTTGCTGACCCACGGACACTACGCCACCTGGCA
AGAGGCGCTCAACCACGGTCTTTTCCAGGCGGTCTCTCTGGGGACCACCACCGGCTACAGCACCACGACC
TATGCCGACTGGCCCTCGTTCCTGCCCATGCTGCTGATGTTCTCCGCCTTTATCGGCTGTTGCGCGGGCA
GTACTGGCGGCGGCATCAAGGTGATGCGTTTCATGATCCTGTTCATGCAGGGGATGCGCGAGTTGAAGCG
GTTGGTTCACCCCAGAGCCATCTATACCTTGAAGCTGGGGAATAGGGCGGTGCCCGAGCGCATCGTCGAG
GCGGTGTGGGGCTACTTCGCCACCTATATTATTCTCTTCTTTATGTTCATGCTGCTGTTGCTGATGACGG
GGCTGAACGAAGTGACCGCATTCACCGCTGTGGCTGCGGCTTTCACCAACGTAGGTCCGGGTTTGGGAGA
GGTATCAGCCAACTTCGGCAATATCTCGCCAGCGGCCCAGTGGGTACTGGTGTTGGCTATGTTGTTTGGC

CGATTGGAAATTTTCACGCTTCTGGTACTGTTTACTCCTGCATTCTGGCGTAGTTGAGGTTGTTATGGAC
AAGATTCTGGTGCTTTATTCCTCTCGGGATGGCCAAACCCGCAAAATAGTCGATGTCATGCTGGAGCAGA
TGCCGGGATGCGAAGTGGTGATGCACGACCTGCACACCTTGCCGATGTGCAACCTCAGCAAATATGCCAA
GGTCTTGATCGGCGCCTCCATTCGTTATGGCAACTTCCACCCCAGCCTGTTCAGCTTTATTCACGCTCAT
CAGGAGCAGCTGGAGGTGGCCAATGCGGCCTTCTTCTGTGTCAACCTTACGGCCCGCAAGCCAGAGAAGC
AGACGCCGCAAACCAATGCCTATATGAAGAAGTTCCTGCGCCTCTCTCCCTGGAAGCCGAAAACCTTGGG
TGTGTTTGCCGGTGCGCTGCAGTACTCCCGCTATAACTGGTGGCAGACGCGCATCATCCAGCTGATCATG
AAGATTACCGGTGGCAGTACTGACACCAGTAAGGACATTGAATTTACCGATTGGGAAAAGGTGCGCAGCT
TTGCACGCGAATTCTGGTCAAAAAGGTAGCAGCGTGATGTGAATTCAGGGTTTCTGCCCGAAACAGCCCC
GCCGATGCGGGGCTTTTGTTTAATAAAACAACGGTTGAACCGGAAAACTGTAAAAAGTGCCATTTTTTTG
CAGTTTTCCTCTTGTCATCCCGGCGCGGCTCCCTATAATGCGCCCCTACCAGCACGGCGGAACACGCCGA
ACTGATGAGCCAGCTTCCTAGCGAAGTGGGCGCCGAAAGAAAAGCGAAAAGAATCGCTTGACTTTCGAAG
TGATAGGCGTAATATGCGCCTCCTCAACGCAACAAGCGTGACGCTCTTTAACAATTTGAATCAAGCAATC
TGTGTGGGCACTCACAGCATCGAACATCAAAACAATTTTTGATTTTCAATGTCTGGTGAAGTGACCAAGA
CGATTTCTTAGCAATAAGAAGTCGAACAGTTTATTTCAGCAATTCATTGAGCCGCTTTTCGAAGCAACCA
AACTTAAATTGAAGAGTTTGATCATGGCTCAGATTGAACGCTGGCGGCAGGCCTAACACATGCAAGTCGA
GCGGCAGCGGGAAAGTAGCTTGCTACTTTTGCCGGCGAGCGGCGGACGGGTGAGTAATGCCTGGGGATCT
GCCCAGTCGAGGGGGATAACTACTGGAAACGGTAGCTAATACCGCATACGCCCTACGGGGGAAAGCAGGG
GACCTTCGGGCCTTGCGCGATTGGATGAACCCAGGTGGGATTAGCTAGTTGGTGAGGTAATGGCTCACCA
AGGCGACGATCCCTAGCTGGTCTGAGAGGATGATCAGCCACACTGGAACTGAGACACGGTCCAGACTCCT
ACGGGAGGCAGCAGTGGGGAATATTGCACAATGGGGGAAACCCTGATGCAGCCATGCCGCGTGTGTGAAG
AAGGCCTTCGGGTTGTAAAGCACTTTCAGCGAGGAGGAAAGGTTGGTAGCTAATAACTGCCAGCTGTGAC
GTTACTCGCAGAAGAAGCACCGGCTAACTCCGTGCCAGCAGCCGCGGTAATACGGAGGGTGCAAGCGTTA
ATCGGAATTACTGGGCGTAAAGCGCACGCAGGCGGTTGGATAAGTTAGATGTGAAAGCCCCGGGCTCAAC
CTGGGAATTGCATTTAAAACTGTCCGGCTAGAGTCTTGTAGAGGGGGGTAGAATTCCAGGTGTAGCGGTG
AAATGCGTAGAGATCTGGAGGAATACCGGTGGCGAAGGCGGCCCCCTGGACAAAGACTGACGCTCAGGTG
CGAAAGCGTGGGGAGCAAACAGGATTAGATACCCTGGTAGTCCACGCCGTAAACGATGTCGATTTGGAGG
CTGTGTCCTTGAGACGTGGCTTCCGGAGCTAACGCGTTAAATCGACCGCCTGGGGAGTACGGCCGCAAGG
TTAAAACTCAAATGAATTGACGGGGGCCCGCACAAGCGGTGGAGCATGTGGTTTAATTCGATGCAACGCG
AAGAACCTTACCTGGCCTTGACATGTCTGGAATCCTGTAGAGATACGGGAGTGCCTTCGGGAATCAGAAC
ACAGGTGCTGCATGGCTGTCGTCAGCTCGTGTCGTGAGATGTTGGGTTAAGTCCCGCAACGAGCGCAACC
CCTGTCCTTTGTTGCCAGCACGTAATGGTGGGAACTCAAGGGAGACTGCCGGTGATAAACCGGAGGAAGG
TGGGGATGACGTCAAGTCATCATGGCCCTTACGGCCAGGGCTACACACGTGCTACAATGGCGCGTACAGA
GGGCTGCAAGCTAGCGATAGTGAGCGAATCCCAAAAAGCGCGTCGTAGTCCGGATCGGAGTCTGCAACTC
GACTCCGTGAAGTCGGAATCGCTAGTAATCGCAAATCAGAATGTTGCGGTGAATACGTTCCCGGGCCTTG
TACACACCGCCCGTCACACCATGGGAGTGGGTTGCACCAGAAGTAGATAGCTTAACCTTCGGGAGGGCGT
TTACCACGGTGTGATTCATGACTGGGGTGAAGTCGTAACAAGGTAACCCTAGGGGAACCTGGGGTTGGAT
CACCTCCTTACCTTAAGATGTCGAAGTTGTTGAGTGTTCACACAGATTGCCTTGATTCAAAGTAGTTAGA
GCAAAGACCTGATGCGAAAGTGTCAGTGCTTATCTGGCTTCGGTCAGATGAGAGAAGCCCTATCATTGGG
TTTTGGGATGTGAATAATGGCGCTCGGCCTCGCAGGCTCGGCACTCGCCATTACCCAAAATCTGCACTGC
TGACGCAGCGCAAAGATGATTTTGGGTCCCCTTCGTCTAGAGGCCTAGGACACCGCCCTTTCACGGCGGT
AACAGGGGTTCGAATCCCCTAGGGGACGCCACTTCTCTTCTTGCTAACAAGAATGCAGAGTTAAGAACTG
ATTCTTAACTCTGTTTTCTTCGGCCTTGCGCCGTTGCAAACATGCTCTTTAACAATCTGGAAAGCTGATT
TAAAAAGTAGTTCTCAAACATTTGTTACAAGTGCTTTGGAAACTTCTTGGCGAAAACCAAATTTTATTTG
GTCCTTGTTGTACGACAACAAGCAAGCGTTGTTTCGACGACACTTCTTGGGGTTGTATGGTTAAGTGACT
AAGCGTACATGGTGGATGCCTTGGCAGTCAGAGGCGATGAAGGACGTACTAACCTGCGATAAGCTGTGAG
AAGTCGGTAAGAGACGCTATTACTCACAGATTTCCGAATGGGGAAACCCACCCGAGATAACTCGGGTATC
TGTTACTGAATACATAGGTAACAGAGGCGAACCGGGAGAACTGAAACATCTAAGTACCCCGAGGAAAAGA
AATCAACCGAGATTCCCTCAGTAGCGGCGAGCGAACGGGGATTAGCCCTTAAGCATCTTGGAAGTTAGTG
GAACGGTCCTGGAAAGGCCGGCGATACAGGGTGATAGCCCCGTACACGAAAACGACCTTGATGTGAAATC
GAGTAGGGCGGGACACGTGACATCCTGTCTGAATATGGGGGGACCATCCTCCAAGGCTAAATACTCCTGA
CTGACCGATAGTGAACCAGTACCGTGAGGGAAAGGCGAAAAGAACCCCTGTGAGGGGAGTGAAATAGAAC
CTGAAACCGTGTACGTACAAGCAGTGGGAGCCCTTCGGGGTGACTGCGTACCTTTTGTATAATGGGTCAG
CGACTTACATTTTGTAGCGAGGTTAACCGTATAGGGGAGCCGTAGGGAAACCGAGTCTTAACTGGGCGTC
TAGTTGCAAGGTGTAGACCCGAAACCGGGTGATCTAGCCATGGGCAGGTTGAAGGTTGAGTAACATCAAC
TGGAGGACCGAACCCACTAACGTTGCAAAGTTAGGGGATGACCTGTGGCTGGGGGTGAAAGGCCAATCAA
ACTCGGAGATAGCTGGTTCTCCCCGAAAGCTATTTAGGTAGCGCCTCGGACGAATACTACTGGGGGTAGA
GCACTGTTTGGGCTAGGGGGTCATCCCGACTTACCAACCCCATGCAAACTCCGAATACCAGTAAGTAATA
TCCGGGAGACACACGGCGGGTGCTAACGTCCGTCGTGAAGAGGGAAACAACCCAGACCGCCGGCTAAGGT
CCCAAAGTTCTGGTTAAGTGGGAAACGATGTGGGAAGGCTCAGACAGCTAGGATGTTGGCTTAGAAGCAG
CCATCATTTAAAGAAAGCGTAATAGCTCACTAGTCGAGTCGGCCTGCGCGGAAGATGTAACGGGGCTCAA

ACCAGGCACCGAAGCCGCGGATTCACACTTATGTGTGAGTGGTAGGGGAGCGTTCTGTAAGTCTGCGAAG
GTGTATCGAGAGGTATGCTGGAGATATCAGAAGTGCGAATGCTGACGTAAGTAACGATAAAGGGGGTGAA
AAGCCTCCTCGCCGGAAGACCAAGGGTTCCTGTCCAACGTTAATCGGGGCAGGGTGAGTCGACCCCTAAG
GCGAGGCCGAAAGGCGTAGTCGATGGGAAGCAGGTTAATATTCCTGCACGACTTGTAATTGCGATGGGGG
GACGGAGAAGGCTAGGTGGGCCAGGCGACGGTTGTCCTGGTGAAAGTGCGTAGGTGGTGTTTCTAGGCAA
ATCCGGAGACACAACACTGAGACACGAGACGAACACACTACGGTGTGGAAGCCATTGATGCCCTGCTTCC
AGGAAAAGCCTCTAAGCTTCAGATTACAAGTCATCGTACCCCAAACCGACACAGGTGGTCGGGTAGAGAA
TACCAAGGCGCTTGAGAGAACTCGGGTGAAGGAACTAGGCAAAATAGAACCGTAACTTCGGGAGAAGGTT
CGCTCTTGACAGTGAAGTCCCTTGCGGATGGAGCAGTTGGGAGTCGCAGTGACCAGATGGCTGGGACTGT
TTATCAAAAACACAGCACTCTGCAAACACGAAAGTGGACGTATAGGGTGTGACACCTGCCCGGTGCCGGA
AGGTTAATTGATGGGGTTAGCGCAAGCGAAGCTCTTGATCGAAGCCCCGGTAAACGGCGGCCGTAACTAT
AACGGTCCTAAGGTAGCGAAATTCCTTGTCGGGTAAGTTCCGACCTGCACGAATGGTGTAACCATGGCCA
TGCTGTCTCCACCCGAGACTCAGTGAAATCGAATTCGCCGTGAAGATGCGGTGTACCCGCGGCTAGACGG
AAAGACCCCGTGAACCTTTACTACAGCTTGGCACTGAACATTGAACCTACATGTGTAGGATAGGTGGGAG
GCTTTGAAGGCGTGACGCCAGTTGCGCTGGAGCCGTCCTTGAAATACCACCCTTGTATGTTTGATGTTCT
AACGCAGGGCCCTGAATCGGGCTCGCGGACAGTGCCTGGTGGGTAGTTTGACTGGGGCGGTCTCCTCCCA
AAGAGTAACGGAGGAGCACGAAGGTTGGCTAATCCTGGTCGGACATCAGGAGGTTAGTGCAATGGCATAA
GCCAGCTTAACTGCGAGACGGACAGGTCGAGCAGGTACGAAAGTAGGTCATAGTGATCCGGTGGTTCTGA
ATGGAAGGGCCATCGCTCAACGGATAAAAGGTACTCCGGGGATAACAGGCTGATACCGCCCAAGAGTTCA
TATCGACGGCGGTGTTTGGCACCTCGATGTCGGCTCATCACATCCTGGGGCTGAAGTCGGTCCCAAGGGT
ATGGCTGTTCGCCATTTAAAGTGGTACGCGAGCTGGGTTCAGAACGTCGTGAGACAGTTCGGTCCCTATC
TGCCGTGGGCGTTGGATGATTGAAGGGAGTTGCTCCTAGTACGAGAGGACCGGAGTGAACGAACCTCTGG
TGTTCGGGTTGTCACGCCAGTGGCACTGCCCGGTAGCTAAGTTCGGAATCGATAACCGCTGAAAGCATCT
AAGCGGGAAGCGAGCCCTGAGATGAGTCATCCCTGACCCCTTGAGGGTCCTAAAGGGCCGTTGGAGACCA
CAACGTTGATAGGTGGGGTGTGTAAGCGCGGCGACGTGTTGAGCTAACCCATACTAATTACCCGTGAGGC
TTAACCATACAACACCCAAGAAGTGTTCTAAGGCTTGTAGCAAATACGAACGAATTACTTACGTCAGCTT
TCTCAGATTGAAGAATTTGCCTGGCGGCCATAGCGCCGTGGAACCACCTGATCCCATGCCGAACTCAGAA
GTGAAACGCGGTAGCGCCGATGGTAGTGTGGCATTCGCCATGCGAGAGTAGGACACTGCCAGGCACCCAA
TTTAACAGCGATGTGAAAAATCACATCACTGGCTGCGAGGATGACACCTCACTGACGCGGCAAACAATTT
AGCGCAAGCTAACCGAATGCGGAGCGGTAGTTCAGTCGGTTAGAATACCGGCCTGTCACGCCGGGGGTCG
CGGGTTCGAGTCCCGTCCGCTCCGCCACTAATTTTAAGGCCTTCCCGATTGGGAAGGCCTTTTTGTTATG
GGCGGTTTGTCCATAAGACCCACAGGTTTGCATACCAGCACAAATACCAAAGCCCGGCTTAATGACCGGG
CTTTGGTATTTTTAGCGTGTTAAACAGCCAACGTTTGGTTTTTACCTGCCAAGAGAGGGCCAGATAGCCG
GTTATTTCCACCATTTTCTGGCTATATTAGAGCCTTGTTAGCTTCATTCGCGGCCAATTGAGCGAGTCTA
CAGGCGGTCAAATCAGCCTTCTGCTGTCTGCTGACTACAGGGCAGTTCCATATTGACCACCCCCTCTTCC
ATTTGCACGTCGATCTTGAAGCCAAGCCGTTTGGCGAGGTTGACCATGCCGCGATTGCTGGGCATGGTCA
TGCCGGATAGCTGGCCAATCCCTTGCTCTTTGGCGTAACGGATGATCTTCTCCATCATCAATTTGCCGAG
CCCCACCCCTTTCAAGTCCGATCGCACCAGAATGGCAAACTCGGCATCGCTCAGGTCCGGGGTCGAGATA
GCGCGTACCACGCCGAGGATTTGATCCTGCAGCTCGCCATCTTGCCCCACAGCCACGAAGGCCATCTCCC
GGTCGTAATCGATCTGGGTCATGCGAGCCAGCTCTTCGTGGCCCAGCTCGCCCACATCGGCGAAGAAGCG
TTTGTACCTGTCTTCATCGGAGACTTGCAGTACGAACTGCTTGTGAGCAGGCTCATCTTCCGGCCGGATC
GGGCGCAGCAGGATATGGCGCTGATCTTTAAGCCAGGCACCCTCCTCCAGTTCGGTAGGATAGGGGCGGA
TCGCCAGTCGGTTGCTGGTCGGGATGGTGGGGTCACTCAGTGTGAGGCTGGCATCCAGCACCACCATTTC
GGCGCCGTACGCTTGCAGCGGGTGCAGGTCCAGCTCCTGAATCTCAGGAAAGGCGAGCAGCAGTTCGGAG
AGCTGGCAGAGCACCTGACCGAGGGCATCGATATCCAGTCGTTCCGGTGTGGCCTGCTCACGGATCTTGC
GGCTCTTGATCGCACCGATGATCTGGTAACGAGCCAGTGCCTGATTGAGCGGTGGCAGGGCAGCCACCAT
CTCCTGCGGTTCCGCACCCGACTCACCCAGCAGAATGACGGGGCCAAAGATGGCGTCTTGCTGGATCCGC
ACCCGCAGCTCCAGGCCGCCGCTGCGGCGAGCCATGCGTTGCACCAGCAAGCCTTCGATGCGGGCGCCCG
GATCATGCTGTTTGACCCTGTCCAGAATGGCATCCGCAGCTTGCGCCACTTCGGCCGAGGTGCGCAGATT
GAGCACCACCCCGTGCACGGCAGACTTGTGCAGGATGTCGGGTGAGCGCAGCTTCACCGCTACCGGATAG
CCGATCTGCTCGGCGGTCAGGGTCGCTTCGATGGCGTCGGTGACGATCCAGGTTGGCAGGGTCGAGAGAC
CGGCGGCTTGTAAAATCGGGTGCACCAGATGGGTATCCAATCTTAGCTGTTTGCGCTCCTGCGCTTGGCG
GATCAGCTGCTGGCACAGCTCGACATTGAGCTGGTTGCCATGTAGTGACGCCGGTGTCTCCATCAACTGC
TTCTGGTTGCGGCGGTATTCCACCATGTGCATGAAGGCGGCCATGGCGCTTTCCGGTGTGCGGTAGGTCG
GGAAGCCCGCTTCGGTAAAGAGTTTGCGTCCCTCCTGCGCCGAGTATTCACCCGCCCAGTTGGTGAGGAT
GTTGAAACGGCGCGCTCTGGGGTGCTGTTTCAGATGGGCAATAAGATGGCTGGCCAGCGGCGCGCTCGGT
GCCGTGAGCGAGGGGGCGTGGATGATCAGGATGGCGTCGATGCCATCTTCCTGCAACACGTTGTCCAGCG
CCTCCTGATAGGTGGTGATGGTAGCATCGCTGCCAATATCGAGCGGCGGGCGAGCCAGCTTGCCGCCCCG
CTCCAGCAGCACATCCACCGCCATATTGGCCAGCCCGCGGCCGTTGGAGATGATGGCGAGCCGCTCTCCC
TTGAGGGTCATGGAGTGGCTCAAGGTTTCTACCGCGGCAAACAGTTCGTGGGTATCCCGTACCCGCAACA
TGCCGGCGCGGCGGATGGCGGCATCATAGACGTTGTCGAGACCGTGGGCCGGTTCGTGGCGCCCGCTTTT

CAGCACCAGGATCGGCTTGTTGCGCGAGGCTGCCCGCGCAGCCGAGAGAAAGTGGCGGGCGTCGTGCAAC
TTGTCCATGTAGATCAAGATGGCGCGGGTGACGCTGTCGCGGGAGAGCTGATCCAGCAGCTGGCCGAAGT
CGATGTCGCAGTGATCGCCAAGGGAGATAAAGGCGGAAAAGCCCAGCTCATGCTGCTTGGCCCAATCCAG
AATGGTGGTGCTGACGGCGGCGGATTGCGACAGAAAGGCGACCTGCCCCGGCTTGGCGGCAGTGGGTGAA
AAGCTGGCGTTGATCCCCTGGCCGGGCAGCAGCATTCCCATGCTGTTGGGGCCGAGCAGGCGAATGCCGT
ATTGTTGGCAGACCCGTTTCAGCTCCAGCCGCTCCTCGGGGGAAAAGTCGGCGGCCAGTATGATGGCCGC
CCCCGCACCCTTCTGGCCGAGCGCTTCCAACAACGGCAAGACCCGCTCCCGCCTGGTGCAGATGATGGCG
AGATCCGGGGTCAGCGGCAGGCTGGCGATATCGGGATAGGCCAGCACGCCGGCGATGGCCTTGTTGCGCG
GGGTTACCGGGAGGATAGGCCCTTTGAAGTGGCCCGCCAGCAGGTGGCGCACGATTACGTGACCCGCCTT
GAGCGGGTCGGTCGAGGCGCCGATCACGGCGATTGAGTGGGGTTTGAACAGCCGATCCAGACCGCTTGCT
TTCATGGGCACTCCCGACAAAAGGGGGGAGATACCAGTCTAATACGCCTTTGCTGCGGGTTCTGTGACCG
GCTCCTGATCACCGTTTGTTCAGGGCATTGGCACGCAGATAAAAGCGCAGTTCGCCTTGCACCCGACGAA
TGGCACCCAGGTGGCGTTCCTGCAGATAGATATCCTGCTCCCGATCGAGCGCTGTACTGGGCACGCCCAT
GCTCTGCAGATACTCGTCAAACAGGGTTGAACCCTTGATCTCTACCAGATGCTGTTTCATGGCTCTCCTC
CCTGCGCCTTGGCGTGTGTTGATGAACGCCCAGTCTGCGATGACCTGCCGGGTTTGCCTATTGTCACTTT
TGACACGAGATTTGACAGGAGAGCGCTCACACATCTTTCAGAGAGGGCGCCGTTGAGGTGCTGGCTGACG
TTATAGTATGGCGAGGAGCATGAGGCACGGATGGTGCGCAGGATGATCAGAGTCTGGTGGTGGTGCATGG
CACTGTGGACGGTTGGCGCAACGGCAACCGAGCAGCCTATCCGCGTCTGGAATCTGCTGCTGGATCACCC
CAACCCCGCCGTCGTCCAGTTATTGCGCCTCTCCCTCGACCTCACCGTGCCCGAATATGGCCCCTACCAG
CTGGTCTCCAGCCCGCAGATGGAGCAGGGGCGGGCGGTGAAGGAGCTGCGCAGCGGCGAGCTGGTTCAGG
TCGGTGTCTTTGCCCCGGATGGTCAGCGAGAACGGGAGTTGCTGGCGGTCCACATTCCGCTGGCCAAGGG
GCTGCTGGGGTGGCGGGTGTGTCTGGTTCGGCAGGGGGATGAGGGACGTTTTGCCGCGATCGGGTCGTTG
GCAGACTGGCATCGCGCCGGGCTCTCCATCGGTCAGCACAGATCCTGGCCAGACACCCAGTTGCTGCGGG
CCAACGGCCTCAAGGTGACGGTGGGCAATCTTTATGAGGCGCTCTTCAACATGCTGCGCAAGAAGCGCTT
TGACTGCTTCCTGCGCTCGGTCATCGAGGTCGAGGATGAGCTCAGGCAGCATCCCGATCTCGCCATCGAG
CCCCGTCTGGTGTTTCGCTATCCGCTGGCGCTGCTCTTCTTTGTCTCGCCAAAATATCCGGAGCTGGCGC
AGCGCATCGAGCTCGGCCTGCTGCGGGCGCGGCAGTCTGGTGCTTTTGACCGGGTCTTTGAAGAGGGATT
TGGCAGCACCATTCGGCGGCTGAAACTGGATGAGCGGGTGCGTCTCGAGCTGCACAACCCCGACTTGCCG
CAGCCCAGTCGCGTCATGATGGCCGATCCGGCCCTTATCTATAAACCGGTGTCAGCGCAGTGAGCCCTGA
TTCTGCGCGGTATCCCAGAAGGCGCGGATCAGCGGTTCTTCCAGCCGCTTCTTGAGCACCCCGATCCCCA
CATCAAATGGCTTGAAGGCGGGTTTGAGGTCGATGATCCGCACCTTGTCGCCGAGCGGGCTGTGGTCGAT
CACCGCCGCCGGCACCAATCCTACGCCACAACCGAGCGCTACCATGGCGACAATCCCCTCATTACCCGCC
ACTTCGGCATAGATGTTGGGGGCGATCCCCTTGTTGCGAAACCACTGATCACTGCGTTTGCGGGCCAAGC
CCTGCTCAGAGAGGATGACCGGCAGCTGCTCCCAATCCGGCTCGCCCTGACGGAACCACTGGTTCAGTTG
CGGGGCGTTGCGCGGCGCGATAAAGACCAGCGGCACCTGCTGCAGGCTGGCGAACTCCAGCTTGGCGGGC
AGGGCGTCGGGGCGGGCGGCGATGGCGAGATCGGCCTCGTCGGCCAGAATTTTGTCCACCGCCAGCGCCG
GGTCGCCGGTCTCCAGCTTGATCTCCAGCTGGGGATAGCGGCGGCGAAAGCGCTCCAGTACCTCGGGCAG
CAGAAAGTAACTGGCGGTGACCGAACAGAAGACCCGCAGCCGCCCCTGCAGCGGTTCATCGCTGCGTTTG
AGTTCCTGCTTGAGCTGACGCCACTCCTGCAACAGGCCGCCGCCAAATTCGCGCAGCTTCTCGCCGGCGG
GGGTGAGCCGTACCGAGCGGTTGTCCCGCTCGAACAGGATGCAGCCGGTCTCCTGCTCCAGTCGCTGGAT
GGCGCGACTCAGGGTGGAAGGGCTGACCGCCATGGCATCGGCACTCTTGCCGAAGTGGAGGGAGTCGGCG
AGGTGGAGAAACAGCTCGAGATTGCGAAGGTCCATCTTTCAGCTCCTGGCGCGGCTTGTGTGCCCGATAT
CCAGCGGTTGGCGGCTTGCTTCTGCCATCTCGCGGTACATTCAGCAGGCGATTATGAAAGGCTAGCGTTT
CAGTTATTGCAACGTACTGTTTCGAATATATCAGTTCAGGCAATAAGTGTCCCGCGCTATAGTGGTTCAG
ACAACGCCGCTATCCGGCATGGAATCAATCGAACGGAGCAATCACCATGGCTAACTATTTCAACACCTTG
AACCTGCGTCAGCAGTTGGCCCAGCTGGGCAAGTGTCGTTTCATGCAGCGCGAAGAGTTCGCGGACGGCT
GCAACGTGCTGAAGGGCAAAAAGGTGGTGATTGTGGGCTGTGGTGCCCAGGGTCTGAACCAGGGTCTGAA
CATGCGCGACTCCGGGCTGGATATCTCCTACGCCCTGCGCAAGGCTGCCATCACCGAGAAGCGCGCCTCC
TGGCAGAAGGCTACCGACAACGGCTTCGCGGTCGGTACCTATGAAGAGCTGATCCCGACCGCCGATCTGG
TGCTGAACCTGACTCCGGACAAACAGCACTCCGACGTGGTGAAAACCGTGATGCCGCTGATGAAGCAGGG
CGCTGCGCTGGGCTACTCCCACGGCTTCAACGTGGTGGAAGAGGGTCAGCAGATCCGCTCCGACATCACA
GTTGTTATGGTGGCGCCGAAGTGCCCGGGTACCGAAGTGCGTGAAGAGTACAAGCGCGGTTTCGGCGTAC
CGACTCTGATCGCTGTTCACCCGGAAAATGACCCGAAAGGTGAAGGCATGGCCATCGCCAAGGCGTGGGC
CTCTGCCACCGGCGGCGACCGTGCCGGGGTGCTGGAGTCCTCCTTCGTGGCGGAAGTGAAATCCGACCTG
ATGGGCGAGCAGACCATCCTCTGCGGCATGCTGCAGGCCGGTTCCCTGCTCTGCTACGACAAGCTGGTTG
CCGAAGGCACAGACCCGGCCTACGCCGGCAAGCTGATCCAGTTCGGCTGGGAGACCATCACCGAAGCCCT
GAAACAGGGTGGTATCAGCCTGATGATGGACCGCCTCTCCAACCCTGCCAAGCTGCGCGCCTTCGAGCTC
TCCGAGCAGCTCAAGACCCTGATGCGCCCGCTGTTCGAGAAGCATATGGACGACATCATTGCCGGTGAGT
TCTCTCGCGGCATGATGGCCGACTGGGCCGAAGACGATGCCAAGCTGTTCGGCTGGCGCGAAGAGACCGG
TAAATCCGCCTTCGAAAACGCCCCGGCGTTCGCTGGCAAGATTGCCGAGCAGGAGTACTTCGACAATGGC
GTGGTGATGGTGGCCATGGTCAAGGCGGGTGTCGAGCTGGCATTCGAGACCATGGTGGCTTCCGGTATCT

ACGAGGAGTCCGCCTACTACGAATCCCTGCACGAGTTGCCGCTGATCGCCAACACCGTTGCCCGCAAGCG
TCTGTACGAGATGAACGTGGTCATCTCCGATACCGCCGAGTACGGCAACTACCTGTTCGCCAACGCCGCC
GTGCCCTTGCTGCGCGAGCACTTCATGCCGACCCTGAAAGCGGGCGATCTGGGTGCCAGCAAGGCGGAAG
GGCAGAGCGTGGATAACCTGGCGCTGCTGGCGGCCAACGAAGCGACCCGCAACCACCCGATCGAAAAAAT
CGGCCAGGTACTGCGCGGTTACATGAAGGACATGAAACGCATCGCCGTTGGCGGTTAAGTCAGCATCGGA
CTGGAGGATGAAGGGGCGGGGATGACGATCCCCGTCGCCGGTCGGACTGGACACCCGACCGGCCCGCCAG
AAAACCCAGCCTGACAGCCATAAATAAAAAAGCCCCGCTCATCGAGCGGGGCTTTTGCATTCAGAAAGGC
CGGATAAATCAGGCTTTCCACGCTTTCCAGGTGTTGATCAGACCGTTGGTGGAGCAGTCGTGGCTGGTGA
CAGCCTCGTTGTTGCCCAGCTCCGGCAGGATCTTGTTGGCCAGCTGTTTGCCCAGCTCCACGCCCCACTG
GTCGAAGCTGAAGATGTTCCAGATTGCGCCCTGTACGAAGATCTTGTGCTCGTACATGGCGATCAGGGCA
CCCAGGGTTTTCGGGGTCAGGCTCTTGAACAGGATGGAGTTGGTCGGGCGGTTGCCTTCGAACACCTTGA
ACGGCACCAGATCCTTGACCTGCTCCAGGGTTTTGCCAGCCGCCAGGAACTCGGCTTCAACCTCTTCCTT
GCTCTTGCCGAAAGCCAGCGCCTCGGTCTGGGCGAAGAAGTTGGCCAGCAGTTTCGGGTGATGGTCACCG
ATCGGGTTGTGGCTGATGGCCGGCGCCAGGAAGTCACAGGGGATCAGCTTGGTGCCTTGATGGATCAGCT
GGTAGAAGGCGTGCTGGCCGTTGGTGCCCGGCTCACCCCAGATGATGGGGCCGGTCTGGTAGTCAACCGG
CTTGCCGTTGCGATCCACGTACTTGCCGTTGGACTCCATGTTGCCCTGCTGGAAGTAGGCAGGGAAGCGG
TGCATGTACTGGTCGTACGGCAGAATCGCTTCGGACTCGGCACCGTAGAAGTTGTTGTACCACAGGCCAA
TCAGCGCCAGCAGCACCGGCACGTTCTGCTCATAGGAGGCAGTGGCGAAGTGCATGTCCATCTCGAAGGC
GCCTTGCAGCAGGGCTTCGAAGTTCTCGAAACCGACGGAGAGGGCGATGGGCATGCCGATGGCAGACCAG
GAGGAGTAGCGGCCGCCAACCCAGTCCCAGAACTCGAACATGTTGTTGGTGTCGATGCCGAACTCGGCCA
CGGCCTTGCCGTTGGTGGAGAGCGCGGCGAAGTGCTTGGCAACGTGGGCCTTGTTACCGGCGATGTTGAT
GAACCAGTCGCGGGCGGTCAGGGCGTTAGTCATGGTCTCCTGGGTGGTGAAGGTCTTGGAGGCCACCAGG
AACAGGGTGGTTTCCGGATCGATCTTCTTCAGGGTCTCGGCGATGTGGGTGCCATCGACGTTGGAGACGA
AGTGCATCTGCAGGTGGTTTTTGTAGGGACGCAGAGCCTCGGTGATCATCACCGGACCCAGATCAGAGCC
ACCGATACCGATGTTGACCACGTGCTGGATCGCCTTGCCGGTGTAGCCTTTCCACTCGCCGGAGATGATC
TTCTCGCAGAAGCCCTTCATCTTGGCCAGTACGGCGTTCACTTCCGGCATCACATCCTTGCCGTCGCACT
CGATGGGGCGGTCGGAGCGGTTGCGCAGGGCCACGTGCAGTACGGAGCGGCCTTCGGTCATGTTGATCTT
GTCGCCATTGAACATGGCATCGATCGCGCTGCGAAGATCGGTCTCTTTGGCCAGATCAACCAGCAACTTG
AGGGTTTCTTCGGTGATCAGGTTCTTCGAGTAATCAACCAGGATGTCGCCGCCGAAGGTAAGGGAAAACT
TGTCAAAGCGCTTCGGGTCCTGCGCGAACAGCTCCTTGAGTCGGGTCTCTTTGTTTGCTGCAAAGTGGGC
TTCCAGTGCCTGCCAGGCCTGGGTTTGGGTTGGATTGATATTTTTCATAGGATTCCTGAAATGCCAAGTT
GTGTTTGAACCTTGCAGGCAAGGTCCCGCCAAACCTTATTATCCGAGCATTATAGCGAGCCGATCGGGCG
GCCCGTGTAACAGATTTTTACCGATTGTCTTTTACCGCAATGGGTCACTGTCGGGAGCATATAGCCAGAA
CATTGGGATTATGTGTCACGAAAGCAAGGCGGATCTTGCGCTTGCTCATGATTGGGGCCACCCCGATAGC
CTGAAATTGTTTTCATCCGGTTGGCGTGGTTATGCACCGACATGGCCATGGGCTGGGGCCAGAAACGACA
ATGCCAGCCCGAAGGCTGGCATTGTCTGTTGCAGCAGAACCGCGATCACGCCTGCGGTTTTTGCATGTGC
ATCACCATCTGCGGGAAGGGGATCTCGATGCCTTCGGCATCAAAGGTCAGTTTCACCTTCTCGTGAAAGT
CGAACCAGACACCCCAGTAGTCAGCGGTCTTGACCCACGGGCGCACCACGATATTGACCGAGGAGTCGGC
CAGCGCGGCAACGGCGATGGTAGCGGCCGGATCTTTCAGGATGCGCGGCTCTTCGCTTACCAGACGCTCG
AGGATCTGTTTGGCCTTGCGCAGGTCAGAGCCGTAACCGATACCGAAGGTCATATCGACGCGGCGGGTAT
CCATGCGCGAGTAGTTGACGATGGTGCCGTTGAGGATGGCGGAGTTCGGTACTACCACCATCTTGTTGTC
ACCGGAGGTGAGGGTAGTGGTAAAGAGCTGAACGGACTGCACCACACCCGCAGTGCCTGCCACTTCGATA
AACTCGCCCGCCTTGATGGGGCGGAAGATGATCAGCAGGAAGCCGGCAGCGAAGTTGGAGAGCGAGCCTT
GCAGCGCCAGACCGATGGCCAGACCGGCGGCACCGATAATGGCGACGAAGGAGGCGGTCTGCACGCCGAC
ACGGCCCAGCGCGGCGATCACTACGAACACCAGAATGGCGTATTTGAGGATGCTGCCGACGAAGTGGGTG
ACTGTGGTGTCCAGCTTGCGGGCCTGCATCACCTTGACCACGCCACCGCTGATGAGGTTGGCGGCGATAT
AGCCGACCAGCAGGGTCAGCAGGGCGGCGGCGATATTGACGCCGTATTCGATCAGCAGTCCCTGATTATT
GACCAGCCAGGTCTGCGCTTCGGTAATGATTTCGGGTTCCATAGGATGTCCTGTCAGGTTCTAAAAGGTT
TATGGTGGATAAATCTAACACCGCAGCGCGACAGTACGGGTCGCATCTGGTACTTCTTTTCTGCCATAAA
AACCTGACACAAAAAAGAAGGGAGGCCAAAGCCTCCCTTCTTTTTGCTGAATGATGCGAAAAAACCATCA
GCTCTGCAGCAGCTGGCTGCGGATAAATTGCCACTGATCTTCAAAACCGGCGGTCGGCTTGAGCTTGAAC
TCGGAGCGCACGAACTGGCTGATGCGGCCTTCGGTGTAGGCCAGCAGCAGATTGGCCAGCATGGTTTCGT
CGAGCTGGAAGCCTTGCCCCTCGCGCAGCCGCTTCTCGCGCAGCACCTGTTTGATCTGGGTCTCCAGCTT
GTCGAACAGGATGCTGATGCGATCCCGCAGCCGATCGTGCTCACCCAGCAGGACGTCGCCATTGAGGATG
CGGGTGATGCCGGGATTGCGCTCGGCGAACACCAGCAGCAGCTGCAGGATATGGTGACAGCGCGCCATGG
TGTCCTTCTCTTCCGCCATGATCAGATTGACCCGTGACAGCAGGGAATCCTCGATAAAGTCGATCAGCCC
CTCGAACATCCGCGCCTTGCTGGGAAAATGTCGATAGAGGGCTGCTTCAGAGACGCCGACCTCGGCCGCC
AGACGGGCCGTGGTGATGCGCTGACCCGGACTGGTTTCCAGCATATGGGCGAGCGCCTGCAGGATCTGAT
CGCGCCGACTCTGTTTCTGATTACTGCTTGCCATGGATTCCCCTAAAAAAACAATGGCTTGATTCTATTA
TTAGGCACCGCCCATCGGGCTGGTGTGGTCTGTTATTGGCGACCAGAAGAACCAAAACCGCCTTCACCCC
GTTCGCTCTGATTGAACTCATCGACCAGTTGGAAGCTGGCCTGAACCACCGGCATGATCACCAGCTGGGC

GATGCGCTCGCCGGGTTGCATGGTGAACTCATCTTTGCCGCGGTTCCAGACCGATACCATCAGCTGACCC
TGGTAATCGGAATCGATCAGACCAACCACTATACGATAAAAAACTGTCTCAATTTCTTAACCACATGAAA
TTAAAGTCTTTTTATCTTGGTGTCGGAAAAAGTGTCTCAGGCCCCAGGTTTTGGTATGTTGGCGGTCTAT
GCTTTACCGGTACTACCGAAACCACCACTACCACGCTCGGTTTCGGTGGAGAATTCCTCAACAACACTCA
ACGACACCTGTACAACCGGTTGGAACATCAATTGTGCGATCCGGTCACCAGGCTGTACGGTGTAAGGGTC
TTCGCCATCGTTTGCCAAGATGACGCCTATTTCGCCATGGTAGTCCGCATCAATGACACCAATGCCTTGA
GCTACGCGAATACCATGTTTCAATGAGAGCCCAGAACGGGAAGCTACTATGGCTACTAAGCTCGGGTCCA
TCATATTGATAGCGAGTCCAGTTTTCAGAAGATGGCGAGCGCCTGGTGCAATCGTCACGGGTTCAGCGAT
GCATGCGCGTAGGTCCATGGCTGCGGAGCCATCGGTCGCGTAGGTCGGCATTGGGAGCTGTTCTGTGCCC
AGAAGGTTATTGACCATGCGTGCTTCTATGCGTCGATACATCTTGTGTTCCTCGATAAACTTGAACTAGC
AAATTCAAAAATGGGTACAGCGTTACAGGCGCGAAGAGATGGGGCTTGGCTTTTGTCAGCAAAGCAAAAC
AAAAAGCCATGAGGTCTCAGCCGCTGACCGGGGGCTAAGATACCAGATTGCCTCCCTGCATGGCGAGGGT
AGGGTTTACCAAAACCCTCGTGGTCAGCATCATCAGAGAGACAGGAGCAAACAGAGAGGGATTCATTTTG
GTCATTTATCTGTTGGCAGGTAGTGAGCCGCTATAGGTATTTCATTTTCGCTAATTTGTTCGATAGTTAA
GCATTGTTGCTGTTGGATAAAACCATGTCTTGCAGTTCTTGCATATGACAATTGAGCTTTGGAATATCTA
AAAAGTGCCCTCTTGCAAAACTCGATGCAAGAAATGAAATAAGGTCTGGATAGTTTTTTATGTGACCATG
CCTAAGAAGCAAATTAAATATGAAAAGGGCTAGTCTTTTTTCAGCGGTGTCACCATCTAATTCAGACAAT
GCAGTATGACAAGCATGATTGAAAACATTTTCGGCATAACCATCATATTCTGAATTATAGCATCGACCAC
CTTTTAGAATACTGCCTTCCAAGCAAGCCATAACAGTGGCATTTCCTGTGACCTCAAGCATAAATGATTG
TAGAGCCTGTGGGGACTGAAAGGAGGCACCATATTTTTCTATAAACTTTGCAACCAAATACACGTGTTCA
TGAATAGAGGATAAGCTTAATTCTATAACATCACTGTACAATATAGGAACTGGTGTCCCAGCAAATAAGT
TCAGCTTAATGCTTTGTAGCTTAGCATTTAAGGAAATATTCTCAACTACATCAATAACAAACGAGTTTAT
GTTTACTGTAGGATAATTAGACTGTTTTGCGAGCATGCACATAACATGATCAATACATAACCACGGTATA
TTGTTAGCAACAGAGAGTTGAAAAGTAGAATAAACTGTCCCGTCAATCATATCTCTAATACTAACTAATT
CATTCGGGGTGTCAGATGGGATGAGTGATTCAACCTTTACAACTGACATTAGCGCTTTCAATTCATTTAT
AAACTGAAATGAGTTCTGTTTAATATCTTTGTGTGTATATTTACGCATCCCATCATCAGTATATTCAATG
GCTAGATAGTCTTCTCTGAGGATGTCAGAAAGCCAATGCTCATAAAATTTCTTTGATTGCTCTGATATGA
CTATTTCAACTGATGAGTTGGATAAGTAGGTGGAGAACCCCATTAGAGAAAAATAGACGGCAGTATATAA
ATCTATAACTACTTTACTCGGTTTTTTAATTCCGTCATCATATAGCGCAATGTATTGGGTCGAATCTGCT
GATGTCAAATGAGTTACTGCGCCTTTTATTGGATTATCTGGCGCCGTATATCTACCTCTCATAGTAAGAG
GGATTTTATCACTCCGTAATACATCATGTTTTTTTCTATCCTTTTCTGATAGAGCTTTTAATATCTTTTC
GAAGTATGGTATAAATTCATCTTCACTATCTGGTACAGTATATAGCTTAAATACATCTGTACCATCATTG
ATTCTATCCCTCAGCTCAATAGCATGTCTAAATGCCGCAATATAAGGTGACAATCGTTCAATTACGGTAA
ACTCTCCATATATAGGGTCATTTATCGTCTCGCCCTCCTTCATGCTATCTAGAATTTTCCCTGTTGGGGA
TTCTGCATCGAGTAGAGATGGATGTGTAACATCTAGGTTTTTGACAATAATGCGATTATAAGTCTTGAAG
CCATCACTATACGTTATTCCATCACCGCAATATTCAGGTGTGTATGGATTTTCTGTTATTTCTGGTCTGT
TTGATAAGGAGGAGACATGAATTTGAGTTAGTGGTTTTGCGACTATTTCTGGTGCCTTCACGAACCAGTC
AACTAACACTTTTTCGGCAAAGTTAATATCAACAAAAGATGCTATTTCGCTAATTAAGGACAGTTTGGTT
TCATGGAACTTTAAAAAAATAGAATCTGGTATTTTTCGTAATATTTTCTTACGTTCATCAACATTAACCC
CACTATTTCGCAGTGTAAACAACAATAAGTTCCAAGCGAAATGATCATCTGGATCAAGAGCTATTGCTGA
TCTGGATGCATTTATAGCGTATTGATACTGATTGAGTCGCTCATGAACTTGAGCCTCTAACAACCATACA
AATGAGGTTTTTTCTTCTGGAGATATTAGCGCAACTTGAGACAAGAAAAGCTGATGCTTTTCACTTGCAA
CAAGGGCTCGTAAATAACATTCGAACAATGGTGATGCCCATGGTTCTTTAGGTATCATCGGGCTTAAGTA
GTCTACAGCGAGAAGAGGGCAGTCCATGTCTATAAAGAGATCACTCAACTCAATAAGTGCTAATGGGGGG
AGTTGAATAAATAGATCATCATTCAATTGTAATAACTTATTTGCCTCATCTACAAGTGAAACTATTTTTT
TGTTATCCCCATCCTTATGTATTAAGGCCTTTAAATACAGCATGTAAAATGAATTTACATAACTCTCACC
CGTATCGATGCTCCCACCTTGATTTATCCACTGCTCTACGATGGAGCCTTTGATAGTTTCCTTTTTAAG
GCATCAATAAGAACTAGATAGCGTTCAAAATCAATAGATATTTGAGAAAAATCTAATTCAGATTTTTTAA
ATGACTTAGATAGCAAAGCTCTATTGATTTTATCGGATATGTCACTATCAAACGTGCTGATCTTATCAGC
ATGTTTTTGCCCCATTATGATTAGGTCAGTGTCTGAAAATAAAGTTATTTTAAGTAGATTTATTAATACA
ATTGCTAATCTTGCATCCTGCGTATCTATCATATTAGACACTTGAACTATAAGCCTATCCACGCGTATTT
TTACGCTTCTATCAAGTGTCCAGATGTGCTTAGAGTGGCCTTGACTAAGTAAGAGATAGCTTTCGCAAAT
AAGTAAAAGTATTTGAGAGTTGCTCGATGGAAATACATCGTGAAGATGTGTGCATATTTCCAAAGCTAAT
TTAAAACTATGAACTCTTAATGCTCCTCGGACTAACCCACATAGTTCATCTTCTAACAAATCTATTCTGT
TGCTGGAATTGAAGTGCTCTTTAATATCCGATTCTGTAGCGAGGCGCTCTAGGTATACTTCAATAACATG
AGATGATTTTTCAGTAATTGAATTGTATCGTTCATGAGCTAAAGATGGCTGTGTTCGAGACTCTAAATCA
ATCAAGATTGAAATGACCACTGATGTTATATAATCAGAGTTACTCCTGTCGCGCAAAAAATATAATATCT
CTTGTCTCGATGACTCGCTTGGAAGTGAGTTATCTATTAGATTGGTTTTTATTAACAAACATTCCAGCAC
AGTTTTTACTTCACTTTCAAGCGAGCCTATATTATTTATTACTCCAATTTTCTCTCTTGCTCTTGATGTC
TCTCTATGACGGATGTCATGCATTATGTTTTCCGCAACACTCTTCAAGTCCTGAGCCTTAATGCTACGTA
TGATATACTCATACTTATTTTCTACGTTGTCGCCAGAACCATAATGCATTTGTGTAGTCATAGGTGCTTA

CTTATTGTATACGGTTTTATTTACAACGTTATCACCAGAACCATAATGATTTTGAGTGACGGAACTTTGA
TTTCTCTCTGGAATTATCTTTTGCAGATATGATTGAATCTCTGATGCTTCATTTATTTTTTGCTCAATCG
CACGCTCAGATAAATCATCTAAATGCATAGATGCAAGCTTATGAGTTAATTCATGTAATGTGTTCTCATC
GAAAATCCATCCTGTAAGTTTTTCCTTAAGAAGATCAATCGTTAATAGGGCCCCGCTTTTAAATCCATCA
TAAAGTAATCCAGACAAAATCGTGCTCGTTAAAAAATCCATATATTCCCACTATAATGACATTTAAGTAT
CCATGTATAATAATGGCTAACTCATTAATAAATATCAACATCTTTTGTAGCGCATTGAGATGGAAAGCTA
TGTTCGCAAGAGGAGATGGTTGCGACAATATTTGAATGCTTAACATTTGCATCAACGCTCAGATGTAGGG
GAGGCTGACTGATTGATGAGCCGCTAGGGTGGTAACGTTATGGAAAATTCAGGCATCCAGATCTGTTCAC
AGATGTTGCTCTACCTCTCTATAGATTGCTTTCTCTTTTCTTAGGTGTGGTGATCATCCAGATCACAGTC
AAGATTCGCTCAACAGCAGCTAGCTTGCCCTGTATTTCACGGCTCTCCTCAACAGCATCGCTGACAGCTT
TGTCGATTATTGGTGCTAACAAGGGAAAGTGAGAGCGCAGTTCGTTGTATTCTGCAAGCTTGAGGTCGTT
CATCTGCAACGTTTGACGGTAGTGTTGTTCTCTCGTACCCAACTTACTCTTAATGGATTCAAGTTCATTC
TTTACCCTCTCGATCTCCCTGTCTTTCTCCTTATCCTGCTCTGACACCAGAGCCTCCGGCCCAATAAATT
CCTCCTTGAAATGCACTTCATCGAACTTGGGAGTCCTTATGAAATGAGAAAAGCGCTTCCCATCATCAAC
ATGCTTCTTTTTTAGAAGTCCCTGATTTACGAGCTTGATCAGATGCCGATAAATGAAGGTATAACGCCGC
TGACGTATCTGCGATTCTGCTTTGTCACCAAAAATGGCATTTACAAATCCTGTCGCAGTGAAGTCATCCA
TCTGCCATCGAATGAGTGCGGTGTAGAGCGCGGTTGGTAACCACACAGTCTGGGAGTCCATAAGCAGCCC
TCAGTCTCGAAAGACAAATTTAGTAGGAATTAAAATTAGGCCGCGGATTATAGTCCCTAGTTGTAGCTCA
GACAACCTAGAAAATAATATTATGAACCTACTGTGTTACCCATAGCCCCTATTGGGGCCTAGGCCAAAGG
CTTAAATATTTTTATGGAAAATAATTGTTCATATATAAATAAATAGATTTATAAAACTAATTTGCTTATG
TAATTCTCACTATCATACGGGTACAGATATCAATTATTTTGATACTGGCCCTGACCCCACGACCGGAAGA
GTCAAGTTCTACGGCCTGGCGTCCGGTTTCAGAAGCTGGTTTGTTCTTCAACACTACAAGCGCTTTGCCT
ACAAGCCATTTTTGACCGTCATTGAACTCAGCTATCCATTCCAAGGGGAACTAAAGTGAGCGCAGCCGTG
AAGACAGAGAGAACATGGGTATTCGTGTTGTTCATGCTCTATCACCTCTTGAATGCATCGATCCTGTTCA
TCCAACCTGTCTATGATTGGTTTAACGATGAATACTCACTGACTGGCCTATATGTACTGCTCATGCCCTA
TCTCTGGGGTTTGATAACGATACTGGGGGTGATTTTGTTCTTGTCAGACCGCTCTTGGAAATACCTGGCA
GGCAATCTACTCGGTGGTTCGATCCTTCTCTTCTGGTGGCTTAGACTGGCATCCCAATATTAGTGAGTAT
CGTCAGGCATATGAATGAGTAGACCGAATGGCATTTTCTTGGGGGTGGATTCATGCAGCGTAAGCTGCAA
GCTCACAAGATCTATTCGAAACGAAAATGAGTTCCCGCCTTGTCTATCTACTGGTTGCTGTAGCGCCACA
CAGCAGCAATAAATCGAGTTTTTCATTAGCTCCGGTCTGAGATTTTCAGCCTAGATCGGCCCCTCAATAC
CGGCTCTGTTGGGTAACAGTTCGCATCATTTCACGGGGGGGTGAAAGTATGAATCTGAGCATTTTAACCT
TTGCCACCGCCTCCACAGCCAACACCTCAATGTTGCCATTTGTTGTGAGCGTGTAGGCATCGTCACCGGA
AAAAAATCCCCAATCAGGTGCGGTATGTCGGATGAAGAACAACTGCGCCATTTTACATTGATTCCATTTG
ACTATGCCTTTAGCATAATGAAACTCCCGAGGTGTAGAGGAGATCGTATAGATAGCCTCTGCTGTCCAGC
AATGGATGACAGTAACAGAGAAACGTGCTTCTGCTCGTTTGGTTAACCTGCTTGCTCCAGCAAGCGATAG
ATGCTCATAGATGAGACCTCGGGAGCCCATTCTGGAGATGAACTAGTGTTACAAGAAACTCTTAAAGCAA
GAATATGGTGGACCAGTAGAGCAAGAATGCAGACTGAGAAAAGGTTGTTATCCAATCATTTTCAAGCTCA
AGTTGTTTTACTTTATTACTCATTTTTCTCTACATGTATAGCTATTTATTATTTAAAATTTGATAATGCC
AATGATTTAGCTAACGTCAGCTGGCTTTCCTTTTCTATTCTAACAATGATTGCATCTATATTTATTAGCA
CCCTAAGATATAAAGAACGAGCTGATAGTGTAAAGCAATGCTATGAGTCCTTGACGTCAATATACAGTCG
AATCCATCAAGATACTTCCCTCTCAGAGTTATACGCAATCGAAGATAAATATAATTACACCTGTTCACTT
TGCGAGAATCACTCATCTTCCGATTATGGTGCTGCGATCATCAGTGAATATTCAAAAAACGATAAAGATG
GCAGACAGCGCTTAAGTAAAAAGCCAACGTATCATCTTCGGATAGAAAACAACTTAAATAAAATCAAACG
TTTTGTGCTATTGATTGCAATATATTCCCTGCCAATTATAGTGCTTCTATTACCGTCATACATAGCACCT
TCCACACAGACTTATAAAATCGAAATTCCTTCGCTTCATTATGTGCGGTGACAATAAAAATGAAATCAAA
GTCATTTGAGAGTTTTTTTGAAATTGAGAGGCTTGAAAAATGCTATTCCGAGAAAATAGCTAAATCCATG
TCTTCTGGGATTGATAATGTCACTCACGAAATCTTTAGCCAAACCTTAGAGCGAGAACTGACAACAATAA
ATAGGAAGGTGTTAAACGATAAATATCGTTTCACAAACTATAAGCTAAAACTAATCAGTAAAGGAAGAGG
GAAGATACCAAGAGAAATTTCCATTCCAACGATCAGAGATAGAATTACCTTGCGGTGTCTTTGTGATTAT
CTTCTCGATACATATAAAGAAAGTATAACACAAGCACTACCGCAACAAATGATTAAGGATGTCATTTCAA
CAATAGATGATTTCGATACGTTTATTAAACTCGATGTGAAGGATTTTTACCCTAGCATCAGGCATGATAT
TTTAAGTTCGATATTGGAAACAAATATACCAAAGAGCGCTTGCAGGCTTATCATGGCCGCAGTCTCTACT
CCCACTGTCAGTTGTGCAGGAGGAGCAAGTACCCCAAGCTTTATTGGCGTTCCACAGGGAACATCAATCT
CTAATATCCTTGCATATATATATCTTTTTGATACAGACAAAAAATACCATTATCAAGAAACGTGTAAATA
CTTTAGGTATGTTGATGATATTCTAGTTTTGTGCCAACACTCCGATAGTCATAACATTGCAGATAGTTTA
AAGTCTGACTTTGTAGATTTAGGCCTAAATACACATGAAATAACAACTTCCTCTGAGAAGTCAAAGATAG
GCTCTACTACCGAAAAACTTTCATATCTTGGTTATGAACTCAGTTACAAGAACCACAAACGGATTATTTC
ATCCAGAAAAAGCTCTGTAGATAACCTTAAGAACTCCCTGGCATCTATATTTACCTCATATAAATATGCC
AAAGATAAAAGAATCGAGTATCTAAACTGGAGATTACATCTTAGAATAACTGGGTGCATATATGAAAAAC
AATGCAAAGGATGGTTGTTCTTTTTTTCTGAGATGAATGACTTCTCTCAACTTTACATCCTCGATAATTA
TGTAAAGAAACTAATTACTAGATATAAAGTTCCAGCTTCCCCACCTAGCTTCAGCAAGGCAATTCATGAA

ATTAAGCACAATAGATACGAATCAAAAACAATCATGAATTACGATCTCTTTGATGTTAAAGATATGCGTA
AATTTCTTATCGAGGTTTTTGGTATAAAAAATATTGCAGCAGCAAGGGATAGCGACATTGAAGCTTTGTT
TCATAAAAAAATGAAGCGAGAAACAACAGACCTTTTAGTCGACATTCAAAGCTTTAGCTAGATAACTATG
AGTAATGTAATACTAAGATGTACTTGCTCTGTTTGTTGTCGTGGGGGGATTTACGCCCCCACATTTGCTT
CCTAGGCGCTCATCTGTTCAATTATAAAAAGACTACCTCAGGCAGTCCTTAAAATGCATATAGGTTTATT
ATTCTTCTGCGCTATCCGCCAATACTTGATGCCCTAATACATAAACCCGTCATTCCGTACCTAAATTTGT
ATTTTAAATGGTGATGACGTTCACACAGGCAGAATTGAATTGAGAAAACCTGTGACTTCACTCACGTTGC
CACGTAATAATGCTCTGTTGGGTAACCGTTCACATCATTTTATGGGGGGGAAAGTATGAATCTGAGCATT
TTAACTTTTGCCACCGCCTCTACTGCCAACATCTCAATGTTGCCATTTGTTGTGAACGTGTAGGCATCGT
CGCCAGAAAATAATACCCAATCAGGTACGGAATGTCGGATTGAAACCCTGGCACGTTGAATGAGGAAATA
ATGGAACCATTGTGGATTTGGCAACAAGCTGACTGGCCCCATTTTCGTTGGCGGGATAGCGAGATATTGC
CCAGATTACGGCATGTTCAACGCAGGCTGGGAATTCTAATAGGTAGTCATTCCAGGTTGGGGAACCCCGA
CCAAACGTTAGATACGTTACTGACTAACATCATCTCCTCATCCGCTATTGAGGGTGAGCGTCTCAATGCC
CAATCCGTCCGTTCCTCCCTTGTTTGGCGGTTAGGGGGGCCACAAACTCAATCCTATCCAGTGTCAGATC
GCTCAGAGGGGCTCGCGGCCATGATGCTGGACGCCATCGATAACCATACACAGCAGTTATCGATTGAGCG
GTTTTACCAATGGCATCGTTGGTTGTTCCCCGTCAACGAATGGTCGGTGCAGCAGCTGAATGTAGGACAG
TTGCGAGGCGATGAACCGATGCAGGTTGTCTCTGGACGTGTTGATCGACCCACCGTGCATTTTGAGGCAC
CGCCCAGAGCAACTCTGGACGATCAACTGGCCGAGTTCATCCTCTGGTTTAACCAGACTCAGCATGACCC
CACGATGGATCCACTGCTTCGTGCGGCGATCTGCCATTTCTGGTTCGTGACCCTGCATCCCGTTGATGAT
GGCAATGGGCGGCTAGCACGGGCACTGACCGATCTCGCACTTTCACAGGCTGACAGCAACAGCATCCGGC
TGTATGCCATGTCAGTTGCTATTCTAGAGCGGCGAGGTGACTACTATCTGGCGCTTGAGTCGGCGCAGAA
GGACACGCTGGATATTACCTCCTGGCTCTGTTGGTTTCTCGACACACTGGATTACTCCATAGAGCTCGCA
TTACAGAGTATTGCTCGTGGCCTTGCCAAGTTATGCTTCTGGCAGCGGCACTGTCATGACGGTCTGAGTC
CAGAGCAAACCAAAGTGCTGAATCGTTTACTGGATGGCGGCGAACAGGGTTTTGAAAATGGCATCAGTGC
CAGTCAATATCAAAAAGTCGCCGGAGTTAGTAAGCCCACGGCTACACGCCATCTTGCGCAACTGTTAGAT
AGGGGGTGCATTGAGAAATTACCTGGTGGCGGACGCAATACACGCTATCAGATCTGCTTACACACCGAGG
GAGTTTGAAAAAACATCGCCATGGGAGCGTTAATTTCGTTTGGTAGATGAGATCTGCGATTAACGTACCG
ATGACGATCTTTAGTTAAATATTGACACCATGATGAGTGCCGTCAGGCATATGAACAAATAAACTCAACC
GACAGTCATGGCTCTCAACTGATTTTTTGAGCGACAACAAACCATCGAGAACATCGGTATCTATCGTAGG
AACATCACCCGTCCCGAGATTTGCAAGCAATTTAGTCACACTCAGACGAACCAATTGCTCGTCTGCGATA
TCTTGGGCTCTCTGGACAACCTCTGGTGATTCAGACTTGATGATATCCTCTAATGTCCTCCCCATACGGT
GCTCCTACTACAGATAGAATTAAAGGCCCACGCCGAGCAGGCATGGGCTCTCAGCACTAGATCAAAAACT
CTTCCAATGCACGACCATTAGCCACTGCTTCTGAGATGGTCTTGGGCATGCGGCCCTGACCAGTCCAGGT
CTTTTCCTGGCCATCTTCGATGTATTTGTATTTTGCAGGACGGGGAGCACGTTTCACTTTAGCTGAACCG
TCAGCCTTCGCCGGAGCCGCTGTGCCAACAAGCTCACTCGGATCGATGCCAGCGTCTCTCAGCATCTCGG
TAAACTCAGCCAGCTTTTGCAGGCGCTCGGCAGATTCGGCTTTGATTTTGTTTTCTTGCTGCTCACGCTC
AGCAAAAACCTCATCCAATTTCTCTTTAACTTCGACCAACTGCTCAAATGACAACTCACGCAACGCAGCT
CGCAGACTACGAATATTCATCAGTGTTTTCAAAAAATCGTTCATTTTTATCCTCATGTATCACATTTTTA
TATCAGGAAAATTATCTCTCAATCCCAGCATCAGCTCCACTGTTTTTGGCACCAAGATGATTCGAAAAAG
CATCATTTAAAATTCGTGAGCTAAATTGCGTTTGGATCTACTTATTGGCCTAGCTCCAATCTGCTATGAA
ACTATGTAGCTAAAAATTAAATCCAAGTGGTAGTGAGCGTTGAGACCACCTCACCAGATGAGCCGATGAA
GGATCTTGTTCGGCTCACGAAATGAGCCGATTGAAAAATATCATTGGCTCACAGAATGAGCCGATGATAA
GAGATTTTTATCTCCACACAAACTAGAGTATGGGAGCATATTACTCATATTATTTCCCTATTAGAATAAT
CACGCCCAAATGTGTCTGGGTTTGCAGCCTCATAGTTATCGTCAAACATATGTTCCCCTCGATGCATCTC
CCTCATTGTTGCGAGTGTACCGAAAAAGCACATTTCACACAGATGCACCAGATAACGCTCACCATCGTGA
CGGGAACCATAGCCAAAATCCGCCGATAACGAGCCGTATTGATTGGTTCCAAAATTCGTTTTTGTACTAC
GACCACAGACATCACAGAGAATGTCAACGACCTGTTCTGTTGGCACAATTTCTTTTACGAGCATGCTACG
CTCCTTATTTAAGGCTTTATGAAAACTATAATATCTTCTGCTTCTGATGTGGGAGTAACCTGGGCTGGCT
ATTTATCACTGGCATAACCAGTATTGAACTCTCATTTGTTATTTCTGGATGTCTTTGCACTCAAATTTTG
GAGGTGTTTTCCAACCTGTAACGCGCCCGCTGCGTTTGTTGATCCTGCGGTATTCATTCCCCAACAGGAT
TTCCCATTCAGAGCCGTTTTCCGATAGAACCTGAAAAGTAGTCCCAGGCTCAATGCCTAAACCAGGTTCA
GTACAGACCAACTCGGCCATCAGCTCTCCTCAATGCAGGGGCATTCTGCTGTGAATCTTCCACTCAAAGC
GACAGTTTGGAGTAGGATAGCTCATGAGAGGTCGTGTTTGTACAATGAGGAATGCTACATGATGCCCTAT
GCAACCTATCATCCTAGTAGTGACGCAGCAGCTACTGCGAACTATCTCTTGGAAGCCCTTGGTATTCCTT
GTGACCCACTAGGAGCACATCAATGGATCGTGGCTGGCGTAGCACCGGACATAGTCTATCGTCTTGCGGA
ATTGATGAAGACGGATGTGGGAACGGTTTGCCAATGGGCTGGTATTAGCCGGAGTACACTGGCCCGCAAG
CTCCGGATTGCTGCCCCCTTATCAATATCGCAGGGTGCCAGGGTATACGGAGTGGCGCAGGCCCTCGATG
CGGTACTCGTCTTCCACAGGAACGATACCGTTAAGACACTGTCGTGGTTAAACCGCACTGCCTGGGGTCT
AGGTGGTGTAGCGCCAGCCCAACTGCTGAATACCCCGATGGGGGTTCAAGCTGTTATCGATCTTGTGGGA
CGAATTCAGCATGGGGTGTGCTTATAAATTTAGGCAGCCTGCGGGCCTCATCTTTCAGTTCAAAAAAACA
TCTCCCTTCTCTTTGGTAAATCCAGGTGACATCCACACATGCATAGAAACGTGGATCTGATCATCTGGTG

TAAACTTGTACTGTGTACCGTGCAGATAGATTGATATCTATATGCCAATGAAAAATCAAGTATCACTCTT
TTTGTTTAACAAACAGAAATCTACAGCCATATCATAAAGATTCATATTGTTATACCATGGAAAAATCATC
TGCATATTAGTCACCATCTTGCAATAATACCTTATTTTTTTGTAATTATCTTTTTTTATATTTTTATGAC
GTAAATAAAGACTTCTTGCTGATATCGTGAAGCCAAGTTGTTTAAATTTTTTCACAACGCAATCTGTTAT
TGTTCCTTGGTTATCCGCTTCGCAGTATATTGCTCTTTTTTCATTACATGAACGTTCAATAGCTACATAA
TATGCACCTAGTGTGCCCAGCCAACTTGCTTGTGTCCCCGTAAACATTCGCTTGTGAATGGTAAACCTAC
AAATGTTATTCAGAAGAATTTCCACTTCTTTCTTTCCAATAGCATCTGAGCATCTGAATAAAATGCCAGT
TATATCTTCATTTCCGAAGAAGAAATCATCTATAAGTTTTTTGTAATAATCAGCTGCGTGTTTTTTTTCT
ATGCGGGTCATTGAGGGCGCTAGGTCAACTAAAGAAAGCTCTCTTTTATTTTTTTTATTCAAGAAATGTA
ATGCTCGCTCCCTCATTATCTTCAATTCAAAAATTAGACCTGCTCTTATAAGCATGTCTGACACATCACT
GTCATCATCATGAATTGAACAATCATCTTTGGCAGAGTATTTTGCACACGATATTATCCCAAGTTGTAGT
AACTCTCTATAGATCAACAACGGTCCATTGTTAAATTCAATCGCAAAGCAGCAATCAACAAGGCGAAATC
TTACTATATCAGGAATACCTAACAATGAATCATCGTGGATCAAATTTTCGTAGGTTTCGGCCAAAACTTT
GGTGTCTAAGCAATTATAACTTGCTATTGCAAATAGTTGAGCGATGCAAAAGCTTCTTGTGTATTCCATA
ATTTTATTATGCTCATCCTCGGAAAATGGAGGTGGATTTTCTGTAAATTTTTCCATTTGTGAATACAGGG
AATCTCTCACAGATAAATGAATTTCCAAAGAGCTTAAGAGATAGTGCTTCGCTATATGATACTTTTTATC
GCAAAAGCCTAAGTCCAGTAGCCTCTTATCTACGGCTTTCAAAATCCGCTCATACCAGATGAAATCAGGA
TGAGTGTGCATAAATCGCAAGTACACCTGCATAAAGTGTAAGTGATCAATCATGTTGAAAGCGGCTCTTT
GCAAGTTCGTCAATGCTGGCATAGTTAGTGTCAAAAAAAGTAGGAAGAATTTTTACCATTATAAGCACTA
AGTTATTTGTCTACAATTTTCGACTCCTGATTGATTTTCGGGAGCAACAATTCTTTCACACAGATTCACA
TTAGAGGGGTTCAATATGAGCAATGATGTAGTAGCACTGACAGTCCCACCAGTTCTTGATGTAGCGCGTG
CGGCTCAAACCGGAAACGTTCTGAAACTGCATGATGCTGAAGTTAAGTTAATGAACAGTGGTTCGAGAGG
CATGCAGCGCCTTGCTGCAATGCGTACACAGGATCTGTTAGAACAGTTTCCTGACATGAATGAATCCGAC
GCAGATCTGATGGGTAAAGGTTATGTAGTGCTCAAAGTCATGGGAATGAATGGGCAACTTATTGACACGA
CTCAAGCTGAAGTTCAGCGTCTTGAGAACAAATAATCTGAAAATATAACAGGTCTCTATATTTTTAGAGA
CCTGTTTTCATATCGAAAATTTAAGCCACTAAAAGTACATTGTACTAAAAAAATATTTTCATGGTATCGCT
TTATATAGTTTAGCAAATTGATGTGTTCCGTACATAAACTCTTATAAATTGCCTAGTAAAAAGAGGCGAT
TAAAATATAACACTCTCTTAGCTAAAAACATTTTTCAATCCTAAAGTGAATACTTTTTTAATCTCATTAT
GAAAATCTTTACCAAAGTCAACGTTGGTCACATCTGTACGCCATATAACGTGAGCAGCTCCCTCTCGTTG
TTCTTTAAAGGTACTAACTATGAACTCTTCACCAATAATAGTGAAGTCAAATGTAGGTCGAAAACGAGGG
TATGAAGTCCCTGGAGTATCAAACCCTATATTAAGCCAAGAGTGACGAGGTTCATTTTTATAAAATCCAG
TTCTTACGGGCAATAAATATCCACCCATAATTTCTATCATGTCATCTCGAGTAGAACATATGGCATTGAC
AGCATTGTTAATTATAGAGAGGTAATTCTCATTTTGCCCTGCCCCCTGAGCCTCCAACCTATTTTTTATC
TCTAAAAGAACATCTTCTTCTGTCTGTGTCGAATCATTATCTTTCATTCTATTTAATTCCTGTTTAAGTT
CTTGTGCAGATTGAAACCTTCTACTCAATAGAGGTGAAAAACCACGATCAAACAATGTAAACATTGGAAG
AGCCCTACTGTTAGTGGCATCTCGAAGACGTTCAGGTATCTCTTCACGTTGATGGGGGCGGCGACCTGTA
TCAGAATCTTCTAGTACACTTGGATTTTTCCCCGTCAATACATAGAATAAAATCCCAACTACAAAAGTAA
TATCACTTCGAGGGTCTCGCTTATTGGGGCTGTCCGGCGCTAGTTCATTAAGCCGCAAGAAGCGATTACC
CAGTTCTTGGCCATCATCGGTTGCGTAGCTGTCCTGATCTTGAGGCTGCCATGCGAGACCGAAATCTACC
AATACAGCATCTGCGGGCTCCCCCCCCCGCAGTATGATATTATCTGGCTTTATATCCCTATGGATGCCGT
CACTAGAATGGTAGTATTCTACTACATCTAATAGGCGATCTGTTATAGCAACAGCATGTTCAAAATTCAT
TGGCCCGTAATCTTCAATGTACTCACGCAATGTCGGGCCTTGAACTAATTCTGTAACCAAATAAAGCTTA
TAATCTTTGTTTTGATAATGATGGCAGTTACTCTCTATAAACGCTGGAATTCCTGGGTGTTTATAGGTGG
AAAGAGCAACAGCCTCTCGATACATACGAACTCGTCGCTCCGAGTCATTTTGACTTTTCAATAATTTTAA
AAATGCACTTTCCTCAGAACGAATCACTGGCCACGCCTCGCCTTGTCCACCACTTTTCTTCTCACCAGTA
GTTACCCAGCCAGCACTCTTCCAATTCAAACAATCTCTCCATACGCCGTTAGTCATCTTCCCCCCTGTCC
GTCATAGTATTATGTATCGAGTACCGTTCGTTACACCTCTTTACTTTACGAATTATCTTATTTTTAGGAT
GTGAATGTAAGACCGATTGAACAAAATTTACCTCTATATGCCATTTCATCGGTCATCGTCACATAAACAT
TTACTGCCTGAACCTATTATTTTAGCAAGGCTAATCTAGCATCCAGAATCATTTTTTGGAGTACATTGTA
CATCAGCCATTGAGCAGCTTCGATTTGGGGAAAAATGACACTACGATTCATCTTAATAAGATGAGTTACT
TGGTTTTTCTATAAAGTAGCTGACTATAGTAAAAATAAACACGTCTTTCTAGTCGCCTGATCTCCTGACT
AAATATACATCCAGCTCGATTATCACTTGGTGATCCTGGTACTACGACAAATAAACCACAGTTTGGAGGA
TATAGTAGGCCATGCTTCTTGCCACGTTTAAACATCCAATTACGGTTTACATATTTTCTCACGACCTCAT
TAATTTCTTTTGATGTTGAATATTTCATGATAAACCTATTTTTAATCTGAAATTTATTTTTTGGTTATTT
CCTCAAAGAGAAGCTCAATCTTCTCATCATAAGAGTGAATATCCTCCTCCCATGCCAGCTGTAGTTGTCT
GGTTATATTATTAGCTGCATCTTCTATGCCTTTTTTAGACCATGGTGCAGCTCGATAGCTACCATCAGGC
ATTAGTTCAACTAAAGAAATAGTGTAACTTAGATTGTAAAAGCTAAGTTTTGCACGTTTTTCCGCAGATA
ACTTCCACGAGTCACAGGTTAAAGTAATTCTTCCATTATAAGCATCGACTTTCCATGAGACCTCTGGGAG
ATGATGGAGATGAGCACGCAACCCCTCTGCTAGACCACCAGTAAGCTTATTAACAATCCAGTAGTGAGCT
CTACCACTAGCTAAAAAGCCATCAAACACCGAATCTTCTTGGTCAGTTAACCAACCTTCGGAATATGCAA
TCCTGCTCTTAGCATGATCAGTTATCTCAATGATTTTCAGTCTTAAATTTGTTATGTCTGATGGATAATT

AAAACCATCATTTAAGGTTACAAAATTAGCTTCATCAAATGCTATTTGGTCTAGCTCAGACAACATCCAT
TTATCAGCCTCACTGGGTGATACCCATTTTAGTCCATCAGGCTTCGGTAATGTCCTTGAGAAAATGAAGG
TGGTTTTATGGAAATCACTAATTCTTGGATGGATAAGATCTATGTGTATCCAGGGATATGCATGCTTAAC
GTTGACATGCATATCATCTGTATAGAAATGGCCCACCTTCTTGATGACATCTCGTACCTCCCACATAATC
TCAGTAAGGCCATACTCCAAACTTGTACTTATTGAATCCATTACAGTCTTACGGATAAACCAGTGACCAT
GTACATTGGCAGGATTTAGAATTTGCTCGCTACTCCAACCTGTAATATCTCGAAGTTTTATTAACTCGTC
AGGTTGTCCAATTAATTGAGCCAGTTCTATTTGGATGCCAAGATCTCTTGCAAAATCATAGATATCTTTG
TTCATATCAGGACATTGACTATTATATTTGCTTTGCCACTCACAAAACTCATTATTAAGTGCTTGGTTAT
TGCCAAGTTCCAGCTGTGCTTTTAGTTGTTTGACTAATTTATCTATATAGTGATAAGACTCAGGAGTATC
TTTTCCTTGTAATACCTCTACGGTTGTTTCAAGCACTTGAGCTAGAGCATTTGCCAAAGCCTTGCTGGTA
TGTCCGTTCTTTTCTACTCGTTGGTAATGACCAATGAGGGTTTTTGAAGCGGCCTCTGGAGAGCGTCCCA
AAATCTTGTAAGCCTTACTCATCAACACTTCTTGGGTCAGGCCTGATTCAACGCGCAGACCCTTCAGCCT
TTCAGGATTGATGACAAACGATGTTCTTCCCATATGAATGAGCTCCTAAGTGACGATGCACATATCATGG
TGGAAATAATAAATGTTGTATATACATCATAAATACCACATCTATTTTTTTGCGTAAGCCGAGCAACAGG
GTTCGCTGAGCTATACGCTTGGTCTTGGCCTTCGTAGGTGCGTGAGAGGTAACTGCGATAGTCAGCATCA
GTTGCAATCTTCATCACTTGCTGTGAGCAGATAGAGCTTTACTTCGAGTGGTGAGAAGTAAGAGGTTTAG
CCTGGTGTGCAGCCAGTTGGCTTCACAGGCCCAAACCACAACTTAGATTCAGCTCGGTGTGATCGCCCTC
TCATCTAGTTTCTCTACTCATAGGTTTGTGACCAAACAACGCCTGTAATCTGTTACGATATCACTCATT
TGGCACCACAACTTGTGGGCAAGTAAATAAGTCGCATGACGCATTAATTAGAGAGTTGTTAATGAATGAT
TCCATAGCCAAGCTCTCATTTAACTTCATTGGGAAAATCCTTGGCTCAGATACTATTGTTGTGCAGGGAG
ATAATTTAGTATCCTCAAAAAAAGATACTATTTTAGAAAATGACTCTGCATCCGATTTCAGGAGTTTTGC
AACTTTTGAACGAAAACTCCTAGGCGGAATACTTACCTACAAAATTGGAAGTAAGACTAAGAAACACAAA
TTTATTAGATGCGCTGATTCAGATAGCTTCGTTGAGTCGCTAAATACTTTAATAGCAAAACACATTACAA
CAACAATAGAGCAGAAAGTTACTGAGTTTAATAGTCTTGCTTTTGACGAGTACCCAAGAGATTCTTGGGT
GATCAATCTTGCTCAGATATGTATGTCATTGAGCCGCGACTATGAGGCCCAGAGCGAACAATGGGAAAGA
TATTTAAATCCTGAACTAATAGAGAAGGTAAAAAACCTAATATCATACCATCCTCTTAATATTGACTACA
TACGCGAGCAGCACGAAGAGTATCAACTTATAAAAAGAAAAGAGTTCTTTGATGTAGTAGAATCAAATCC
ATTAACTAATGAACAGCGTTTAGGCGTATTACGTTCAAATGATAGGAATATGGTACTTGCAGCAGCTGGT
ACTGGAAAAACATCCGTCATGGTTGCTAAGACCCTCGATTTGATAGACCGAGGATTAGCGAAGCCATCGG
AGATCTTGGTTCTTGCATATAATAAGACTGCTGCGGGCGAACTAAGTGAACGCCTCGAAGATAAAGCTAA
GAAGTCTAATATTGAGCTAGAATCGACTCCAGAGATTGCTACTTTCCATGCGTTAGGCAGGATGATTCTA
CGTAATTCAAACGTTGATACCAACATATCTATTTTTACTGAGGATGACGTAAAGCTAAAACTATGGGTCA
CCTCATGGCTTGAAGAATATTTATCATCAGATATCGATAGAATTTATGACTTTATAAACATGTTCCCAGA
ACCCGTTAACCCTTTTTATTTTAAGTCAAAATCAGAATATGAAGCGTATATCAGGGACAACGAATTTAGA
ACCCTCAACAGTGATCTCGTTAAGGGTTACCAGGAACTCCTTATAGCAAATTTCCTTTATGAGAATGGTG
TTGAGTATAAGTATGAGTCTCCATATGTGACCAAACGGCGTATTGATATTGGTTTTGACTATCGCCCTGA
CTTTAAAATCATAGAACCTGAACTTTATATTGAGCATTTTGGTATAGACCGTCATGGAAGAACCAGGCCA
GATATAGACCGTGTTTCTTATAATCAGTCCATTAATAATAAAAGAATGCTTCATAATGAATGTGAAACTG
TTTTAATTGAGACATTCCATTATGAATGGATAGAGGGTGTCTTACTGGAAAACCTCAAGAAAAAGCTGTT
AGATAATGGTGTCGTTCTTAATCCATTATCTTCTGATGAACTGTTCAACAAGCTGCGTGATATGGGTCAT
ATCACAGGCTGGGGTGAACTTTTGACAAAAGCCCTCCAATCCATTCGAGTTGAACGCCTCACGAAAGAGT
CCATACAGAATAGGTTCGATACTACTGGAGTGCATCATTCCAAGAAGCTCTCAGAAATCTTGGATTTGCT
TCATCATGCATATATCAGTGAGCTCAGAGAACAGAATGCAATTGACTTTGATGACATGATAATCAGAGCG
ATTCAGGAGGTGAATGCTGGCAAATATATTCCTGAATGGCGATACATACTGGTTGATGAGTTCCAGGATA
TATCAACAGCTCGAATGGAGTTCATCCAGACCATAATTAAAAATGGGCCACAGCCATCACTAACTGTCGT
TGGAGATGATTGGCAGTCAATTTATCGATTTAATGGTGGAAAGTTAGAGCTAACAACTCGCTTTGGTGAA
ATCGTTGGTAGCTACACGCTTACTAAACTACAAAAGACCTTCCGCTATAACAATAGCATTGCCGATACTG
CTGGTCAGTTCATTATGGAAAATCCTGAACAGTATAAAAAGTATATAGAAACGCATACTGTGGTTGAGGA
TTCACAGATTTATCTGCTTGATGACAAATCCCCAGAAAAAAGAGGTTTGAATAAAAGAGTCGCTGAGGTT
GTTCAAAAGATTAGAGAGAATGATGCGACTGGAAGCATTGCAGTTATTGCTCGTTATAACTATCTTTTAA
ATGAGGCTAAGAGTGCATTAAAACAGACGAATAGTTTACGCAATGTTAACTTCTGGAGCTTCCATAAATC
TAAGGGGTTAGAGGCAGACTATGGAATTTTGATCGGCTTTTTTCAAGGACAGATGGGATTCCCTAATGAA
AATAAAGACAACCTTATAGTTGAATCCCTGCTGCCATCTCTAGACCCATATCCTCATTCTGAGGAAAGGC
GTCTCCTGTATGTAGGCATTACCCGAGCAAGGAAAAAAGTCTACATTATTGCCAATCCTACAGCACCATC
CGATTTTGTGACGGAGTTACTCTCGCCAAAATACAAGATAAATGTTGTTTCAAAAGCATTCAGTGAGGAA
TCAAAAAAAAGGTTCAAGTGCCCATACTGTACAGATGGCTATCTAAGGTTGGTCAAGGGGCCGTATAGTG
ATTTCTATGCTTGTAGCACAGGTTTTGGCTGTCCTGTAGGAAAGGCTAGAGTGTGTGAAAAATGCCACTC
ACCCTCAATTGATACAAGGAATGAGAGTGTTTGCAATAATTCTCTTTGTGGTTGCAAAATTAAAATATGC
CCTGAGTGCGGACGACCAATGAAAATCCGTAATGGTCAGTATGGTGACTTTTGGGGATGTACTGGTTATG
GGATTAAGGATGACCAGTGCAAGGTTACCATCAAGGTCAAGGCAGGCTTATGATAGCTAATTACGTGAGT
TAAGGTAGGGGAACATATGGATACGTATTCCGAAATCATAAATGAATTCAATAGTACGTTTTCTACCAAT

GCTAGCCTGTGTGAAGATTTAAAGGTTGGGTGGGATCTGGGAGATTGTAGGTCTTTTGCTTTATACCAGT
TAGTAGAAGACCAACGTAGCGCCCCCTTTGGTACGGTTCTATACCACCATATCGGCAGCTACAACACTGG
TGAGGTATACGAGGTAGAGGGGACAGCAGGCTTCAGGCTCAGCTCACGGCTCGATAGTATTGAAAAATTT
TTCCCTTTGTCTTCAAACAAGGCGACTCGAAATTTAGAGATTGGGTATCGGTCACCCTGGCTTGGCGGTA
GCTGTGCATTTTCCTCTATTCCGTTTAAGCGGTGGTGGGTTGATTCATTTAAAACATTGTGTGCAAATGT
TCCTGCCCAGGCTGAGCTGGTTAACTCATTCTTGACGCGAGAGATAGAGGTTCTTGCGGACAAAGCTAGA
AAAAATAACGATAAAAGTGGCTCAGTTTACAAGTGCTTCGTAGAACGGTTGGAGTACCTATCACTTCGTG
TAAATCAAGAATTTCTTGATTCAACAAACTACATTCTTCATCCTGAACTGTTTTTTAATGTAGTTTCAAA
TAATTTGGTTAGTTTGAATGAACAAGAGAAAAGGGAGTTGATGAACAAGGCTAGAAGAGATTGCCATTTT
GATGACCCACTTAAGAAGTGGTGGTAATCAGCGATGGCAGTCAGCAGTTCTACGCAATCATCAGACTTAT
GTAAGCAATGGTTGTCTCGACAGGTTGCTTGGTTGCAAATAGTTATCGATTGCGCCCCTCACTAGGTTAA
CTCATTGGCCTTTAGAGGATGGGCAGGGGCAGGCAGTCCAACAGGGAGGGTCATGCAAGCAGAACCCCCT
ACAGCATGTTTAAAAGGGCTGGCCTTGATACCAATGTCAGTGCGCACGAAAGTAGCGTAAAAAAGACACC
AGTTTGAGGTCGGTGTCACGTGACATTACGCTAGTATGAATGGATAGTTAAGGCATTAGCTTGGTCACGT
TATCACTTATAATTTTCTGTAGTCGATTATTTTCTGCCTGCAATCGTTCATTATCCATCCATAATAGCTG
GATTTGGTTAGCTCTAAGATTATTAATTTTTTTTTATTTCTTGCTTGCATTCATTAAGCTCCGCACGAAGC
TTTTTCACCTCTGATTCTCGTTGCTGATATGCCTCAAGTAACGTTATAGGAGCATTGTCAGTTCGTTGGA
GCCCATTTTTTCTCAATGCCTCAAACGCATCATTTAGGTCTTGAAATTCATCAAGATATCGATAAATGGT
TGCTCTGCTAACCCCAGCTTCTCGAGCTAAATTAACACTCGTTATTTTGCCATTTGTTTTGGTGGATACC
CCTCTGGCTAATACATCCAGTGCATCAATGATAGCCTGATGATTTTTATCTGCCATCATTCCGCCTCTTT
TAATTCTTGAACGACACTCTCTAAACTCAGAATTTCAGCTTGGAGTTGGTGTTTCTGAAATACAGAGGTC
TTCGGGTGCCTGTAAAACTCATGGGCTTGAGTTAACTGAGCTTCCCATAGGGATTTATGGCGTTTTGAAA
TGCAGGCATTACTACAATTACCTGGTTGACAGAAATTTATTATGGGTTTCTCTATAGATGGGTTTCTTGA
TGTGCAAATCGCCTTGGTCGGGTCGAAGAAACAAAAATTGAATTTACCTACAAATAAAGCTTTATTTGTG
TTTGCCAACCATTTTGCTATCTGTGATGGTGGATAATCCTCGGCCCGTCCACGAAATTCTGCATTGAACT
GCGATTTCAGATGGGTGCCAAATTCACCAGCCACCATTCCCTGTGATAGATCCATTGCTAGTTCACCCAA
AATATCGACATTCGCAAGAACTTTTTCCTGTTTCAGCAGCTTATTCCACTCAGGTTCTTCACCAGCATAT
CCCTGGAATATAGCCACCTCAACGTGCTTGTATTGCAGCATGCCAGCAATGACCCCGAATGGTTGCCTAG
CTATGAATCTAGCTAATGTTCTACGAAATTGCCTAGCGTTGAACGCCCATGGCTCCGATACGCCAAATTC
ATTGCACCAATCGGGTATCGGTGGGCGGTCTAGGTTTTTATTACAGTGGTCTCTGAAATTGTTAATTTGG
TAAACAATAGCTTCCATCGTATACTCGGAGAAGGTCTTTCCTGTAAATTTTTTATTTACACTAAAGCTGC
TGTTTGAACCATCTGTCAGCATTAGGCGTTGATCATTTGCGGCTTCCCTAATTTTTATTGTTAGAGTTTC
GATAGTTTTAATGGCAGTGACGACCTCCTCGATGACAACCCATGTATCTTTTCTGCCATTACCTGTATGT
TTATGTAAAATAGTATTGATATAGTAGATAGATTGACCATCTACACTAAAATCTTTTATGTAGCATCCTG
CGGTCAAGGATAGCAACTCTGTGGTGCGAATACCCGAGTAGGCTAGGATAGTGAGAGTGCATGCTGTGCG
CAATTGCTGAAGTTCAACATATAGATCCCCGTATGTCTGTATTGGCTTTTTCAGCCAGCCAGAATTAACG
TCCTTCATTAATTTTTTGGCGACTATAGTGAACCGCTTGGTTTTCATGCTCTCGCTTAAATCTAGGTCTG
CAATTTCACTATGCCATAAAATGTGCAACTGCTCCCAAGTGTCCAGGATAACATGGCTGTATTCCAACAC
GTAATCTAATGCTGCACTCATAATGGCGGTGTAGATGGGTTCAGGAATCGGCGGTGTCCGATTCTCAGTA
TCAACCCGATCTTTTTTATTGGTGAACATTTTATTGAATGGTCTATCGAACGGCATGTTGGTGAGCGGCA
TAGTAATCCGGCTGCTGTACTCCCAAAGGCGATATATGATGCGAAATATATTATATATTCTATCGTCCGT
CTTACACTCAACATACATAGTATAATCCAGGTATCGTTCGAGCAGGTTGTTATCAACCTCGTAAAAACCT
TGTCGGCCCTGACTTGCACAGTATTTAGAGAAGCTTTTTAGAATGTATAGCTCATTATGTAGAGTTAACC
ATTCACTTTCTCGGTCATGAGTTGCGACTGCGTTGTATAGTCTGGCATATGCATACTCAGCCATTGCTAA
TGAGAATCCATATGGATAGCCAGGAACGTCTCGAAAATCCCAGGTCTTATCGGACTCTGATATACCAGGG
GTACTCACGAGCGAGGACATCTTCCATAGTGCATTGCTAAATACGGATTGGGTAGAAACAGCCGTGTCAG
GAGAGACAAACCTACTGGTTGTTAGTCCTGCTTCAAGCAGAGAGGCGGTTGGGTTCAGTTTCATTGCAAT
ATTACGTCCTGATATCCAGTGGGATGTAAAAAATCTGCTCCTGAGCTAGACGCTCAGCTTCCTCAATAGC
ATCGGATGAAAATCGTGGCAGAACATCATGGGTTATTACATGCCAGAGGAACGAAAATTTCCCATTCCAG
TCATCTGCGGATAATTCGGTACGGGCCTGTATAATCAGGTCGCGAAGTGCTATTACGCGAGGCAGCACAT
GGCGTGTAATTATGGCATTGGAGCATATGAAGCACCCCCATGGTTTATCACATGGCGTATTCGGCTGACC
ACCAGGCCGATTGTAAAAATCCAAGCAGGATGCAAATAATACATCCTGTTCACCAGTGGAGATTTTTCCT
GCGGTAGTTATATCAGTATCTAGAACTACAGCGATCTTTTCAGGATTGTCATCTGGCACGACCTGAGGGC
GAGCTAGATGCTTCACCTCATAAAGTGCATTTCTGATAGCCTGCTCATGGATATGCTTGGTGGAATCATT
CGCAACATAATCGACAGTCGTAGTACCTAGCGTATGTGCTGCATCCCGAGTGACTCTGCTCAGTTGTCCA
TACCTTTTATAGCGGTTGGTGAGATATGTTACACGCCAGCGTCTCATCGATACACGTAGCGGCTGATCAT
GTTCATCTGCGAGTTCATGTTGGTCACACCAACCACCAACGCAGTTCATCTGAGCCTTGATATAGCTAGG
ATCCAGTTTTTTAACTGGTTCTCGTCCCTGTCCCTCTACAGTTAGTCCTAGGAATAGATATTTTTGATCC
GCCAAGGGGGCATATTTGACTAGTGGTTCTGTGACTTGTAAGAGCACCTGTATCAGCGCAACAGGAGAGA
ATTGCCCTTCATTATCAATTATTTTCTTATATCTACGTGCAGCACCACGATTTTTCTCATACCAGAGGAC
AAAGCGCCCATCGTATTCCTGTAAACAGTCTCGAGTCAGATTCAGAATTGATGATTCATTGCGGCCGGTT

TTTTTTGCTAGCAGATTTATAAATGGTATTAGATCGGTTGTGAGGGGGTATAGCAGAGAGTATGTGTCTT
TTTTTCTATATGGAACATCATAACATCCCATCATAACATTATTAAATGTGCTGTGGCCGCCAGCAAGTAG
CGCTGATCTTTTTAGGTACTCCCCATTCATGACATTGACGATGTACCACAAAATATTGGGCAGTTGCAGC
CATGGTCGCTCACTGGGGTCGAGACGCAACATTCGACCAGTCTGCGGGTCGCGTCTATTATTATGCCCTC
GTGGATCAGTACCAGTAGTGAGTAGTTGTCTCCCAGTATCGAGCCGAGCCAGCAGTGCTCGTATATCGCT
GCGGCAGGCGTGTTCCAGATCGAGGGCCTCACCAGCTGTATATGGCTGTATCCGGTCACTATCGGATTTA
TAAGCGAAATTTCGAGGGGGGGAGAAATCTGAACGTGCGAGGTCAATAGCCTGTGCCGCCGCGAGTAGCC
TGATTAGGTTACCGAAATGCCGACGTGAATGGCTGGATTTAAAACCACTCGAGGTTAATTTGATGTGTGC
TCGGTATTCCAATAAAAATTCATACGTGATTTCCATCATTCGGAATTGCTCAGGTAGTTTTTTTTGATCT
ACGTATTCCAAAAACTCCTTAATGGTTTGGGCATAGGTCCAGATTGTGAGGTCACTTGCTATCCGGCTGG
GAGAGGTGTGTTCATAGAGTGCAGCAATAAAATCCCGTGCTAATACCTGGTGTGAGGAAATGTTAGTGAG
ATCTAGATGTGCCCGGTCGCCCAATTCGTTACGTTTATCGAATACTAGGCTGCCTAGGCTGCGGATATTG
TTGTTTGTTTGGGGGATTTTTTCGAACTCTACCCTGGACATAACGTCTGCTTGCACCAAGTCAGCCTAAA
CTGGAATGTAGACTAGCCAAGAAAGTTTTTGCTTTCAATTGGTGTAAGTTATTGATTATTTTACACTTTA
ATAATTATCGGAGTCTAAGGAGATTTGATTTTTTCTATCTAAATCAATCATTTGTTGAAGTCTATATTCA
TAAAGCGAGACATATATAACGCTTTGCTTTGGTGATTTTCTGGAATTATATGCTTGATCTGGCTTAATTA
AGTTTCATTTGTTCTGCACTATTAATGGGTGTCATTCACTCGCAAATTCTATGTTAGCACCTCCAGACCG
CCACGATTTTGGATACAACACTGTCAAACTTGTTCTAATTACTGCGGTCGAGTGAGCAGCTGTAGTAGAC
CTCTTAGACCTGCCTGCAAGTACAAGGGGGAGCACTTAGTGGGGGATAACCTTCGGGTTAGGGATTTTCG
TTTTCCCATTTAGTCCAATCCCCCAAAGATTGATCAACCAGAGCCTCAGAGTCCTCTAGGAAGTCGAGGT
AAATGTAGGTAGTCGATATGTGGGAGTGCCCTAACAGTTTCTGTAACTCTCTGAGTGGGTCGTTCAAGAT
CTGTCGATATGCATCGGACCCATTCTCTCGGAGCTCCCTGAGATTTTGGGCCATGCGTTTAATCATGGCA
TCCAGCTGATAGATAGCGAAGGTATGACGGCAGCCATGGGGGGTTAGTCGTATACCAGCTCTGCTGGATA
TAGCAGCAAAGAAACTCTCCACGGTGCGGCTAGAGAGAGGGCGCCCTGTTCGTGCTAAAAAGATTTTGCT
GGATTGTTTCGGGGACTTTTTGCGCGTGTGGTCAGATTTTGCCCGAGTGAGGATCTCTGCTCTTTCCTCA
TCACAATAGAATCTGATTGCTCGAACGATCCTTTTTGGAATCCGAACATCGCGGCTTTTTTTGCCCTTCC
CAACTACTTTAAGCGTCACACTTCTCAGTCCGGCATAGCATTGGTCATCTGGATCTGGGAGCTGATGAAC
GTCGAGGTTTAGCGCCTCTGAAATACGTAACCCTGAGACTATCAGGGTATCAATTAAAGCCAGATTCCGT
TGAGAGTTCTCGCCGTTGGCTATAAGAGGGCGCCAGATGCGTTGGTAGTTAGTGATGCTGATAAAATTTA
CTGGTTCTGGAGTGTATTTAGCAGATAAATCAGCAGTTTCTACATGTTTCCCGCCGAACCTAGCCTTCGA
ATGTCGGTACTTGAATGGAGTGCTATCGATGAACCCGTTATCACGGGCGTATTCATACAAGTGCACAATG
GCGCTTTTAGCCACATTCCATGAGCGATCTTTTAGGGCAGGTTGATTCTGGTATTCACCACTGGTTCGGA
TGGTGTAGTAGAGGTCTAAGTCACATTTTGTTGCCTGTTGCCAAGATTTTCCTTGGGCAGTCATAAACCG
AATGAAGATGGAGACCTGATTGGCGTATGTCTTCCAAGTCTGAATACTAGCACGTTGAGGACAGAGTAGG
TATTTACAAAAGCCGTCGAGTATCTGGTCTGGCAGGCCATTGTCATCCAAAAAAAAAGGGCCGACCATTTG
GAATTATATTTGTCTTAAGAAGAGCCCTGAACTTGGTTCTTTCTACTGGGGGCAGCTGATCAAAGACACC
CTTATCAAAGTAAAACAGTTCAGCCATCGCTCAGCTCCAGATGATGAGACTATTGAGACACTCTATCCAT
GTGGCTTATAAATTCATGTGAACCATTGAAAGGACAATATTCTCTACTATGCAACGAATGAGACAGCTTC
ATTAATGACGTATAACGACCAGGTTGCCCAGCACGATGCCGTGCTTATGGCCGAGGCCGGAGCGGGGCAG
GATGGTGGCACACAGACCCGGATCCTGGATGTGAATGGCCAGACCGGTCGGCACCAAGGTGGTATCACCC
GGAGCCAGAGTCAGCGGCGCATCCAGCAGTGCGCGCAGATCCATGCCGGCAGAGCCCGGTGTGGCGTAGG
CGGGCAGCGGGTACTCGGTACCGATACGGGCATCCAGAATCTTCAGTTCAATTTGTGTAGTCATGAATCG
CTTTTTCAGTTGGATCTTGTATTGGCGCCCCTCTTGGCAGGCGGGGTGCCGTGAGTCTTGTTCTTTCGGG
ACGCTCAGTGCCGGTAGTGGCGTGCAATCAGGGCGATCAGGTGACGAGCCAGCGTCAGCTTGTCGGCCAG
CGGCAGCGGGGCCTGGCCCCCTTGCCAGAACACGGTCAGGGCGTTGTCATCGGCGTTGAAGCCTTGCCCC
TCGCGGGACACATCGTTGGCGGCGATCATGTCCAGCCGCTTACGCTGCAACTTGTCGAGGGCGTATTGTT
CCACATCCACGGTTTCGGCGGCAAATCCGACGGTGAAGGGTTTGTCTGAAAGGGCACCAACGGCCGCGAT
GATGTCGGGGTTTTTCACCAGCTTCACCACCATCTGGTCGTTGTCGCCGGTTTTCTTGATCTTCTGGCTG
GCCACCTGCTCAGGACGATAGTCGGCTACCGCCGCGCAGCCGATGAAGAGGTCGCACTCGCCGACCCGGC
TCATCACCGCCTGATGCATCTGCTCGGCGCTCTCGACATCGACGCGGGTAACACCTGCTGGCGTAGCCAG
CGTCACCGGGCCGCTCACCAGCGTCACCTCAGCGCCTGCTTCACGGGCGGCGCGAGCGATGGCGTAGCCC
ATCTTGCCGGAGCTGTGGTTGCTGATGTAGCGCACCGGATCCAGCGCCTCGCGGGTTGGCCCTGCGGTCA
GCAGCAGTTTGACGCCTGCCAGCAAGGGCTCGGCGGCCAGTTGCTGACAGCAGCGATCCACCAGTTCGAG
GGGGGCCAGCATCCGCCCCGGGCCCACATCGCCACAGGCCTGGCTGCCGGCATCCGGCCCCCACAGCAGA
ATGCCGCGGCTGGCCAGGGTCTTGAGGTTGGCCTGGGTCGCGGCGTTGAGATACATCTGCTGGTTCATGG
CGGGCGCCAGCGCGATGGGCGCCGGCGTGGCCAGACAGAGGGTGGTCAGCAGTTCGTCGGCCATGCCTGC
AGCCATCCGCGCCATCAGGTTGGCGCTGGCGGGGGCGATCAGTACCAGATCGGCCCACTTGGCCAGCTCG
ATATGGCCCATGCCCGCTTCGGCAGAGGGGTCCAGCAGGCTGTAGGCCACCGGATGACCGGAGACCGCCT
GCAGGGTCAGCGGGGTGATGAACTCCTTGGCGGAGCGGGTCATCACCACCCGCACTTCGGCGCCCTGATC
TTTCAGGCGGCGTACCAGCTCGGCACTCTTGTAGGCGGCGATCCCGCCACTGACACCCAACAGGATGCGT
TTATCGGCCAATCTCATCTGCTGTTTCTCTATCCGGCTCATTCTGACTGGTCGGCTAAGATACCACACGC

AATGGATGACGACAGGGGTTGGCATCAAGGGGTGTGGTCGGGCGCGCGCAATTTTTTGCTATGTTTTTTC
TCAACAGGGATGAGGGAGAGACAAATGAGCATCAAAGAGTGGCCGGAAGATGAACGCCCCAGAGAGAAGC
TGCTGCGTCAGGGGCCCGCGGCGCTCTCCGATGCCGAGCTGCTCGCCATCTTTCTGCGCACTGGCGTCAA
TGGCCTGAGTGCAGTGGATCTTTCACGTAATCTATTGAATCAATTCGGCTCTTTGCGCAGCTTGTTGGGG
GCGGATCAACGGGCATTCTGCGAGGCGCACGGCCTTGGCCCGGCCAAATATGCCCAGTTGCAGGCGGTGC
TGGAGATGGCCAGACGCCATCTGGCCGAGCAGCTGCAACGGGGTGATGCGCTCACCTCGCCCCAGCTGAC
CCGCGACTATCTGCAGGCCCAACTGCGGGATCGGCCGAGGGAAGTGTTTGCCCTGCTGTTGCTCGACAAT
CAGCACAGAGTCATTCAATTTGTTGAGCTTTTTTTCGGCACAATCGATTCGGCGAGCGTCTGGCCAAGAG
AAATCGTGCAGATCGCGCTCAAACATAATGCAGCCGCGGTGATTCTGGCGCATAATCACCCGTCCGGCGT
CGCCGAGCCCAGCAGGGCCGACCGCCAGATCACGGATCGGATCACGGCCGCTTTGGCCCTGATCGATATC
AGAGTGCTGGATCACTTGGTGATCGGTGATGGCATCACGGTTTCTTTTGCCGAGCGCGGTTGGCTATAAC
TGTGCTCTGGCACCGTATTAGTTGATCTTTGATCACGGATGCTGTATAAAATGCCGCCTTCTGTTTGCCC
CGCAATCGAAGGCCAGCGGGGCAGATATTTGCTCGAGCAATGAAGTAAGATTTGGAGAGACTGACATGTC
TAGAGTATGCCAAGTAACCGGCAAGCGCCCGGCAGTTGGTAACAACCGTTCGCACGCTAACAACGCCACC
AAGCGTCGTTTCCTGCCGAACCTGCACACTCACCGTTTCTGGGTTGAGAGTGAAAAACGCTTCGTAAGCC
TGCGCGTATCCGCAAAAGGCATGCGTATCATCGACAAGCGTGGCGTTGAAGTGGTTCTGGCTGAACTGCG
CGCCAGCGGTGTTAAGGTATAAGGAATTAGATCATGGCTAAAGGTATTCGCGAGAAAATTCGCCTGAACT
CCAGCGCCGGTACTGGTCACTTCTATACCACTACCAAAAACAAGCGCATCATGCCCGAAAAAATGGAGAT
CAAAAAGTTTGATCCCGTTGTTCGTCAGCATGTGATCTACAAGGAAGGCAAAATCAAGTAATTACTTGAT
GCTGCACTCCCAAAAACCCGGCTTCGTGCCGGGTTTTTTTATGCGCTGAATTCAGTACACTGCCGGTTCA
AAGGGAGGCTTGCCATGTTCAAATTGAGCCGCAAAGGCTGGAATAACGTGATCATTGTGGTGGTGCTGAT
CGTGATCACCCTGTTGCATCGTATGGAGACTGCCCAGCAGGAGAACAGCGCCAAGCGGGCTCGCCCCTTG
CTGCCGGATGCGGCCGTGGTGCTCACCTGGCAGGGGCCGAGCTGGCAGATTGAGCGGATCGGTCAGGGCT
GGCGCAGCGTGCCGGATTTGGGGCTCGACACTGCTCAGCTGGATGCCCGTCTGGCGCGCTGGCAGAGTTG
GTTGCTGCACCCCGGCGAGGTGGTGCGCGGTACCCCGGTGACCATCAAGGTGTGGATTGCCGGCCAAAGC
GATCCGGTCGAGATCGGCCTCTATCAGAACAGCGGCCAATATGGCGCCCAGCTGCCGTCCGGCCTCTGGC
TCACCCTCACTGCCGAGCAGTATCGCGATTTGCTGCGTCCTGCTCCCTGAGTTTCTTTTCCGTGCGAGGT
GCCCATGCCCGAATTGCCAGAAGTTGAAGTCAGTCGTCAGGGGATCTCCCCCTGGCTCACCGGTATCAAG
GTGACGCGTGTGGTGGTGCGGGATGGCCGCCTGCGCTGGCCGATCCCCGGTGAGATTCAGGAGCTGGTGG
ACCTCACCATCTATCGGGTGCGTCGCCGTGCCAAATACCTGCTGCTGGAGACCGATTTCGGCACAGCAAT
CCTCCATCTGGGGATGTCCGGCAGTCTGCGGGTACTGGATATCGGCACTCTAGCCGAGAAGCACGACCAC
GTGGATATCGAGCTGGAGAATGGCAAGCTGCTGCGGCTCAACGATCCGCGCCGTTTCGGCGCCCTGCTCT
GGACCCGCGAACCGGCCGAAGCCCACGCGCTGCTCGCCAAGCTGGGGCCAGAGCCGCTCACCGATGCCTT
CAGTGCTGATTACCTGCAGGGGCGCGCCAAGGGGCGCAGCACCGCCATCAAACAGTTCCTGATGGACAAT
CAGGTGGTGGTCGGGGTGGGCAACATCTACGCGAACGAGGCGCTCTATGCTGCCGGCATTCACCCCAAAC
GGGCGGCGGGCAATATCAGTGCCGAGCGGCTGGGAACCCTGGTGGCCGAGATTAAGCGCGTGTTGGCCGA
GGCGATTCGCCAAGGCGGCACTACCCTCAAGGATTTCACCAGTGCCGATGGCAAGCCGGGCTATTTCGTG
CAGCAGTTGCAGGTCTACGGCCGTGGCGGTCAACCCTGTTTCCACTGTCATACCCTGCTGACCGAGACAA
GAATAGGCCAGCGCACCACGGTTTTTTGCAGTCATTGCCAGCGCTGAGCGCACTTTTTGCCCCGATCCGG
GGGAAGTTGTAACAGCGTATTTCCACTCTGTTGAAGATTTGCACAGCTGTCACTAGTATCCCGACCGTTC
AGGCGGTTTGAGAATATAAAAATGACAGTTAAATCAATAGACTGGGAATATTTATCCCGGCAGGAGCGCT
ATTGCGCAAAATCCATGGCCCGCTTCCTCTACCCCGAGGGATGTGACCGAAAAACCTTCAATCGCGGCAA
GTTTGTGCTGCGCAGCCTGCTCTATCGCAAGTCGCTGAGCCGGGTCTTCAGCCTGTTTCAGTGCGACTCG
CTCAAGGCGCTGCCAGCGGTCTATCCCGAGCTGCTCGACAAGCCGATGCGCCCCTATCGCTTCGCCTGTG
CCAGCGCCCGTGAGCGGGCCGAGATGCTGGAAAATCACTACCGTTTGCTGCTGGAGTGCTACCCGCAGCT
GATCGACCCCCTCTATCTGGGCGAGGGGATCGAACTTGGGCACTATCCCCAGAGCGGCTGCCGCATTCTG
TTGCGCCACGATGGCACCTTCCGCCGCGAGGCCGAGCTGGCGCTCTCCATCGTCAACGGCGAGGGGCAGC
GCCTCTACAGTTGCGCCTTTTCGCTGGCAGGTAGCGTGGATCGTTTGATTCTGATGATCGGCTCCATGCA
GGGGCCGGAGCCTGCGGTCGACAATGCCCAGGATCGGGTGCGCGAGCTAACCAAAGAGGGACATGGCCTG
CGGCCCAAATCCCTGCTGGTGCAACTGGTGCTGCAACTGGCGCAGAGCATGGGGGCGGCAGAGGTGCTGG
CGGTGCGCAAGCGGGCCCACGTGTTTCAGGCCAAGCGCTACAGCAGCAAGCAGAAAGCCAACCTGCAGGC
CGATTACGACGAGTTGTGGCAGGAGTTCGACGCCCGGGATGTGGATGCCAACTTCGTCGCTCTGCAAGCC
CAGCCGCGCAAGCCGCTGGAGGAGATCGCCTCCAAGAAGCGGGCCATGTATCGGCGCCGCTACGAGTGGC
TCGATCTGCTGGTGCAGGAGATGAATGAGCGGTTTGCCCGTCAGTAACCGGCAAGCAGGCTGACCTCTCC
ATGCGATAGAGAAACGACAAGGGCTACCCGTGGGTAGCCCTTGTCGTTTCTGCCATCAGGGGAGGAATTA
GCGCTGTTGCTGTTTGGCTGCGATGGCTTGGGCCACCACGGGGTCGACAAACTGACGAATGTCGCCACCG
TGCAGCGCCACCTCTTTCACCAGAGTGGAGGAGATAAAGGAGTTCTCCTCCGCCGGGGTGAGGAAGACGC
TTTCCAGCTCCGGCATCAGGCGGCGGTTCATATTGGCCAGCTGGAACTCGTACTCGAAGTCGGAGACGGC
GCGCAGGCCGCGGATAAGCACGTTGGCCTGATGCTCTTTGGCAAAATCGACCAGCAGACCGGAAAAGCCC
ACCACCTTGACGTTGGGAAGCGTGCTGGTCACTTGCTGGACAAGCTGGACACGTTCTGCCAGATCGAACA
GCGGGCGCTTGCTCGGGCTGTTGGCGACGCCGACGATCACCTCGTCAAACAGCTTGGCGGCGCGGCCGAT

GAGATCGGCGTGGCCATTGGTAACGGGATCAAAAGTCCCGGGATAAATTACCCTGGTAGTCATCTTTGCG
GTCCTGCAACCTGGAAATTTGGTCGCATTATAAGGTCGTCGCTTGAGAATAGCAGTGCATCAGGCAAGAA
TACGCCATCGCGCATCAAGCCTCCTTGCATTGGTGACAGGGAGCAAAGGGATTTTTTATGAAACGTATTC
TGGTCGTTCGCAATGACAAGATTGGTGACTTCATGCTGGCGTGGCCTAGCTTCGCCATGCTCAAAGGCTC
CATGGAGTGTCATGTGACTGCCCTGGTTCCGGCCTACACGGCCCCGCTGGCCCGGCTCTGCCCCTGGATT
GACGAGGTGATCATCGATCCGGGCGTGCGAGCCGACAAGGAGCAACAGCGGGCACTGCTGGCCCGGATCA
AGGCGGCGGCGTTCGACGCGTCCATCTGCCTCTTCTCCAACTCCCGCAACGCCATGCTGGTGTGGAAGGC
ACACATCCCTTACCGGCTGGCCCCGGCGACCAAGCTGGCGCAGGTGCTCTACAACCAGCGACTGGTGCAG
CGTCGCTCCCGCTCCCAGAAACCGGAGTCCGAGTACAACCTGGAGCTGATCCGCCGCTTCCTGGCCGATC
AACAGGTGGCCGTGGTGGAGCCCAAGACCCCCTTCCTGCGGTTCGATGACGAGACTCTGCGGCAGGTGCG
TGAGCAGCTGGCACTCAAGCTCAAGCTCGACGCCAGCCGCCCCTGGCTGATGGTACACGCCGGCAGCGGT
GGCTCGGCCAACAACCTCTCCATCGAGCAGTACGCCCGCATCATCATCAAACTGAACCAGGCCTGCCCCG
AGCTGCAGTGCGTGCTGACCGCCGGCCCGGGCGAGGAGAAGATAGCCGAACAGCTGGCGGCCGAGTTGCT
GGCCCACGGTGGCAACGGCTGGATCTACCGCTCCAGCGATGGTCTGCCGATGTTCTGTCAGGTGCTGGCC
AACGCCGCCCTGTTCGTTGCAGGCAGTACTGGCCCGCTCCATATCGCTGGCGCGCTCGACGTGCCGACCA
TCGGCTTCTTCCCGATGCGCCGCTCCGCCACTCCGCTGCGCTGGAAGCCCCTCAACAGCGAAGGACGCCA
CCTCGCCTTCAGCCCGCCGGAGGATAGCGACCATCCTGAGGATATGAGTCAACTCGATCCCAAGGCCATG
GTGCCGGTGATGGCCCGCTGGGCGGCGCCTTTCTGGCGCAAATAACGTATGCAGCAATGCAAACAGGGCC
CCGCGGGGCCCTGTTTCGTATGACGGTTATTCCGGCTGCTGCGGTTGCTGGCGCACCCAGAGGTCGGCGT
ATTTGACGAAGGTGGAGTGGGCGGAGAGGATCGCCAGCAGCAACCCCTGCTTGCCATCGAGGAAGCCTGC
CTTGAGCAGATACATCTTGATGAAGCAGCCGACCGCGTGCATCAGCCCCTGACTGAGGGAGCCCTTCTTG
CCGCGAGCGGTGCGCTGATCGGCCCAGGCGCGGGCATAACCGGCGGATTTCACCAGATAGTGCTCCAGAT
CCCGATAGGTGAAGTGGAGCAGGTCACCATGAATGTTGACGATCTTCATGTTGGCCCGTACTTCCACCTT
TTCGTGCACCAGCGCCTCGTTGTAGCTGGTGAGTGCCTTGGGATAGAGGCGCAGCACCCGATCCGGATAC
CAGCCACTGTGGCGGATAAAGCGGCCAAAGACCCAGGAGAGGCGCGGGATCGAGTAGATGGTATCGCTGG
CCGGATTGGTCATCACGTTGGCTATGGCGGTTTTCAGCTCGGGGGTGAGCCGCTCGTCGGCATCCAGCCA
CAGCACCCAGTCGGATTGCACGTGGGACTGGGCCAGTCGGCGCTGTTTGCCAAAGCCGGGCCACTTGGCG
TTGACGAAGAAGCGGGCCCCAAATTCGGCGGCAATGGCCGGGGTCTCGTCCTGGCTGCCGGAGTCCAGAA
TGACGATCTCGTCCACCAGTCCGTCGAGGGAGGCGAGGCACTTGCGCAGGTTGTCGGCTTCATTCTTGAC
GATCAGAACGGCAGCCAGAGTCGGTTTTTTCACGAATGTGGGCTCACGGTTATTGCCCGGCTGGCGGGTC
GAATGCGTATTGATGGGCCATTCTAACGGAAAAACGGGTCAAAGAGAGAGCCGCCATTGCCCGTTTCTAT
CAGCTGTTTGCCTGTCTCGTCTCGTCCGGTTGGAAGCGGGGCACCGCTGGAAAAATGGGGTGACGGCAGC
GTCTGCCAAGGATCTTCTGTTATTTATCCAGTCATTCTTGGGGCATAATCTCGCCCAGTGATGATTAACA
GCCGGGCTAGTCCCGAGAGAAGACGCGTGATGAAAATATTGGTGATCGGCCCCTCCTGGGTCGGCGATAT
GGTGATGTCCCAGAGTCTCTACAAGGCGATCAAGGCCAACCATCCGGAGTGCGAGCTGCACGTGATGGCC
CCCGCCTGGTGCTGCGCCCTGCTGGAGCGGATGCCGGAGGTGGACAAGGCGATCACCATGCCCCTTGGCC
ACGGTGACTTCAAACTGGTGGCGCGCCGCCGCCTCGGCAAGCAGCTGGCGGCGGAGAAGTACGATCAGGC
CATCGTCCAACCCAACTCCATGAAGTCGGCGCTGATCCCCTGGTTTGCCCGCATTCCGGTCCGTACCGGC
TGGAAGGGCGAGAGCCGCTACGGTCTGCTCAACGACCTGCGCAGCAACAAGTCCGCCTTCCCGCTGATGG
TGGAGGGCTATCTGGCGCTCGCCTATCCCAAGGAGCAGATGAAGAGCCGGGCCGATATTCCGACCATTCC
GCATCCGGCGCTCCATGTCGATCTGATAAACCAGAGCAAGGCGCTGGAGCGGCTGGGACTGGATCGCGCC
CGTCCGGTGCTGGTGCTCTGCCCCGGTGCCGAGTTCGGCCCGGCCAAGCGCTGGCCGGAAGGGCACTACG
CCGTGGTGGCCCAACAGCATCTGGACGAGGGGTGGCAGGTGTGGATCTTCGGTTCCAACAAGGATGTGCC
GGTGGCCAATACCATCCGGGATCGGGTCAATCTGCTGACTCGCCCCAACTGCCACGTGCTGGCGGGCAAG
ACCAGCCTGCACGAGGCGATCGATCTGATGGCGCTGGCCAGCCGGGTCATCTCCAACGACTCCGGCCTGA
TGCACATCGCCGCCGCCCTCAACCGGCCGCTGGTGGGTGTCTATGGCTCCACTTCGCCGCTCTACACCCC
GCCGCTGGCGGATCGGGTCGAGATCGTCCACACCGACATCGAGTGTCGCCCCTGCTTCAAGCGCACCTGC
AAGTTCGGCCATCTCAAGTGCCTGATCGACCTGGCGCCGGAGCAGGTGGTCGATGCCTGCAAGAAGCTGG
AGCAGAGTCAGTAAGATGCCCTACCGTCTGCTCTACAACCTGCTGATCCATCTGGGATTGCCGCTGGCGC
TGCTGGCCCTCTACAAGCCTAAGAAAGGCAAGCCCGGCTTTGGCAAGCGCTGGGCCGAGCATCTCGGGCG
GCTGCCTGCCACCGGACAGGAGGCGCCGCTCTGGATCCATGCGGTGAGCGTGGGGGAGACGCTGGCTATC
ACGCCGCTTATTCGCGCCCTCAAGGCGGAGCGGCCCGATTTGCCGATCCTGCTCACCACCACCACCCGCA
CCGGCGCCGATCAGGCTGCCAAGCTGGGGGATCTGGTGATCCATCGCTACGCGCCGCTCGACTACCCCTG
GGCCATTGCCGCCTTTCTCGACACCTTCAAGCCGCGCGCGCTCTGGGTGATGGAGACCGAGTTGTGGCCG
AACTGGCTGGCCGCCTGTGAGGCGCGCCACCTGCCGGTGACCATCATCAACGCCCGCCTCTCCGAGCGAT
CCTGCCAGCGTTATGCCCGTTTCCACGGCGCTTTCGACGCCCTGAGCCGGCCACTGACCCATCTGCTCTG
CCAGCATCAGGATGATGCCGACCGCTTCGCCCGCCTTGGCATCGATCGTTCGCGGCTGGCGGTGACCGGC
TCCATCAAGTTCGATATCCAGTTTGGTGACGAGGTGCAGGCCAAAGGTCGCGCCCTGCGTCAGCAGCTGG
GGGCGGATCGGCCGGTCTGGATCGCCGCCAGCACCCATCTGGGTGAGGACGAGCAGGTGCTGGCCGCCTT
CGATCTGCTGCTCAAACGCCATCCCGCTGCGCTGCTCATGCTGGTGCCGCGCCACCCCGAGCGGTTCGAT
CGGGTGGCCGAGCTGTGCGCCCCTTATGGCTGCGTGCGGCGCACTGATGGTGCTGCGGTGAGTGCGGAGG

ATAAGGTCTATCTCGGCGATACCATGGGCGAGCTGCCGCTGATGCTGGCGGCGGCCGATGTGGCCTTCGT
TGGCGGCAGTCTGGTGAAGATTGGCGGTCACAACCTGCTGGAGCCGGCGGCCCTCGGCAAGCCCTGCCTG
ACCGGTCCCGCCTACTTCAACTTCAGCGACATCACCCGCCAGCTGGTGGCTCAGGGGGGCGCCGCCATCG
TGGCCGATGCCGCCGAGCTGGGCGAGCAGGTGAGCCAGCTGTTTGCCGGCGGTAACGTCCGCCGCGAGAT
GGGGGAGCAGGCCCGCGCCGTGGTGCTACGCAATCAGGGTGCGCTGGCGCGCACCCTCTCGCACTGCCTC
ACTGCGCTGGAGAACGACTGAACCCATGATGAGCACCGCTCTGGCTCTCGATTTTCAACTGCAAAAGCGT
CAAAGCAGCTGATCCCCGTTTCACACGTCATCAAAGAGGCAACCATGTTCAAGGCTCTGTTACTGGAAAA
CCAAGACGGCAAGACTGTTCCGACTCTGACCCGGCTGGCCGAGAGCCAGTTGCCGGAAGGTGAAGTGCGG
GTAGCGGTCGCTTACAGCTCCATCAACTACAAGGACGGGTTGGCCATCACCGGCAAGGGCAAGATAGTAC
GCCAGTGGCCGCTGGTGCCCGGCATCGACTTTGCCGGTACCGTGCTTGAGTCTGCCGACAGCCGCTATCA
GACCGGCGACAAGGTGGTGCTGACCGGTTGGGGCGTCGGCGAAGGGCACTGGGGCGGCATGGCCGAACAG
GCGCGGGTCAAGGGGGACTGGCTGGTGCCGTTGCCCGCCGGGCTGGATGAGCGCCAGGCCATGTGCATCG
GTACCGCCGGTCTCACCGCCATGCTCTGCGTGCTGGCACTGGAAGAGGCGGGTATTACTCCAGATAGCGG
CGAGGTGCTGGTCACCGGTGCGGCAGGCGGCGTGGGGGCCACCTCGGTTGCCCTGCTGGCAGCCCTTGGC
TATCAGGTGGCTGCGCTGACCGGTCGTCCGGAAGAGCAGGGCGCCATGCTCACTGCGTTGGGTGCCAGCC
GCATCGTGCCGCGCAGCGAGCTGCTGGTGCCTGCCAAACCGCTGGAGAAGCAGCTGTGGGCCGCTGCCAT
CGATACCGTCGGCAGTCAGGTGCTGGCCAAGCTGCTGGCCCAGATGAACTATGGCGGCGCTGTGGCGGCC
TGCGGGTTGGCGGGCGGTTTCGATCTGCCGACCAGTGTGATGCCATTTATCCTGCGCAATGTGCGGTTGC
AGGGGGTCGATTCGGTGATGTGCCCGTTTGAACGTCGTCAACTGGCGTGGCAGCGACTGGCACGGATCT
GCCCGCCAGCTTCTTTGAACAGGCGGTTACCGAGATTGGGCTGGAGCAGGTAGTGGAGGCCGCCGAGGCC
ATCACGCAAGGCCAGATCGCCGGTCGCACTCTGGTCAAACTTTGAACTCCCTCTGACTGGCGAGCCCGAC
AATTCCGGATAAAATTGGAGTGCGGGTGAACTATCCAAGGAGAGTGAAGATGGGTGGACACAAGCCGGAT
TACGACTCCTCTGATGATGACTGGGAGCCGGATTCCTGGGAAGAAGAGCTGGATCAGTGGGAAGATGAAA
GCTGGCAGGATGATGACACTTTCGATGACCAGTGAACCACGGCATTGAACGAACAGGCGGGCTTTGGGCC
CGCCTGATTTTTTTGCCGCGCTCACATGGCTGAGCGTTCGTCCTTGGTCAGGGGCGGGCCGCTGGGTAGA
ATGAACTGCCCAAATGACGCTCAAGGATGACTATGCGACTCTTCATCATATTGTTGGCTGCCCTGCTGGG
TGGCTGTGTCTCCAACGATGATCCCTGCGAGAAGGTATGGAGTGAGGTTGGGGAGGCGGATGGCAAGCTG
GGCTTCACCACCGAGCGGATCGAGTTCCATCAGGGCCAGTGTGGCAAGAAGGTGGATGCCGAACTGTGGA
AGCAGGGACATCAGAAGGGATTGGCATGGTATTGCCGCCCCGAGCATCTCTATCTGGCTGGCCGCTCAGG
CGAGGAGTATCGCGGCGTTTGCCCGAATGATACCCAGGCACGCAGATTGTTCGAACAGGGGCGTCAGGGG
TGGACCGATCAATAACGCCCTGACAGGGAGGGGCGTTACTCCTCTTTCAAACGCAGCGGCTGGCAGAGGT
AACCCTTGTCATTGGCACTGCGGGCGCACTTGCTGCAGACAAACTTGGGTTTGCGGACTATCTTGCGCAA
CGCCTTGAGATCGGTGGGGATCTCGTGGCGGCGCCATTCACACAGGGTTTTGCTGCCCATAACGACTACC
TCTACGACTTGACGGCCGTATTGTAATCGATTTTCATTTCGAATAAACCGGCAAAGCTCGCTTATTGGCC
GATATGCCAATAAGCGATTGTTTCGGGAGGGATTACTGGTAGAGGAACTTGGTGAAGATAAGCTCGCGCA
CCGCTTTCTTGCCGGTCTCTTTCACCAGCAGTTCGTTGACGGTTGTCTCGCACTCCTTGCGCAGCTCTTC
TCGGTTGGTCAGGGACTTCACTTGATCGGCATTTTTGCTGCCAATCAGGCGGATGATGGCATCGCGGATC
AGCGGGTCATGCTGCTCCAGCAGCTTGAGATCGGCTTCGTTTTCCGCCATCAGCTCTACCGTGACCCGGA
TATAACCGAGGGTTTTGCTATCACTCAGGTAGTTGGTGATGATGTCGGGATCGAGCGCGTGGTAGGAGAA
ACCAGGCTTGGCAGCCCCCTCTGCGCCCTCGGCGGGCGGCTCATCTTCAGCCAGAGCTTGCTGCTGCAGG
GCGCCACAGCTCAGTAACAACACCAGCAGAATTTTGAAGATCTTCATATAGATGGCTCATCAGCAAGAGA
TAACCCATTTTTGATGTTGCCATCGGGGCCGTCAAGCACGGATGTTCTTACATTTCGCTATTGATTTACG
GATAATAAACCGGATTTTAACCTGAACACGGAAATCTCTGGATGGTGAAGCTGTGAAGTCCGAGTTGACT
GTTCCCCTCACGCCGTTAAGCGGGTGGCTGGCCCCCGAATGTTGCGAATTGCCCGCCAGCTTGCGCCCCT
GGCTGCTGGAGCCCGGCTCCATGACCCGGCGGCTGCGTCAGCACAATCGCCACTTTTCGGTGGAGTGGCT
GGGCAATCACCCGGTCGCCCTTACTGCCGATGAGCAGTGGCTGGTGGATTCGGCCTCGCCGGCCGGCACC
TGCCGCGAGGTGATCCTGCACGGGGATCGTGGGCCCGCCGTGCTGGGCTGGACCCTGTTTGCCGATGACG
CACTGCAAGGGAGTGGCATTGCCGAGCTTGGCGAGCAGCCGCTGGGTGAACGGGTATTTGGCCATGAGCC
TGCCCGCCGTGATCACCTGCAGCTGGCCCGTTTCGATATTGCCGCCAATCCCCGTTGCCCGGCCGCCACT
GTCTGGGGGCGTCGTTCCCGTTTGTTTCTGGGGCAGTGGCCACTGCTGGTGCACGAGCTGTTTCTGCCCT
CTCTCGTCTGTGACAAGGAGCTGGAGTGAATCTGTTAACCAAGGAGCGGGGACTTGCCTATATGCAGTTG
GCCCGCGTCGACAAACCCATAGGTACTCTGCTGCTGCTATGGCCAACCCTGTGGGCGCTCTGGCTGGCCG
CTGGTGGCCTGCCCAATCTCTGGGTGCTGACCGTGTTTGTGGTCGGCGTCTTTCTGATGCGCTCGGCAGG
GTGCGTGATCAACGATTATGCGGATCGCAACTTCGATGGCCACGTCAAACGCACCGCCGGTCGCCCCTTG
CCCACGGGCAGAGTCAAGCCGTGGGAGGTGCTGGCGCTCTTCTTCGTGTTGGCACTTATCTCGTTTGGTC
TGGTGCTGACCATGAATCCGCTCACCATCGGTCTGTCGTTTGTCGCCCTGCTGCTGGCGGTGAGCTACCC
CTTCATGAAGCGCTTTATCTCCAATCCCCAGCTGGTGCTCGGGATGGCCTTCTCCTGGTCGATCCCGATG
GCCTATGCGGCGCAGGCGGATGCCCTGCCGCTGGTGGCCTGGCTGCTCTTCTTCGCCAACCTGCTCTGGA
CCGTGGCTTACGACACCCAGTACGCCATGGTGGATCGGGATGACGACCTCAAGATCGGCCTCAAATCCAG
CGCCATCCTGTTTGGTCGTTACGACAAGCGGATCATCGGCGCACTGCAACTCTCGACACTGCTGCTGTTG
CTGGCGGTCGGGCAGCTGATGACGCTGGGGGGCTGGTATTACTGGGGATTGCTGGGGGCGGCGGTGCTGT

TTGTCTATCAGCAGCGGCTTATTCGTGAGCGGCAGCGGGAAGCCTGCTTCCAGGCGTTTCTCAACAACAA
CTATGTGGGGGCGCTGGTGTTTGCCGGTCTGGTGCTCGATTACCTGCTGGCGTGACATGTTCCCACGCAG
ACTCCGATAAAAATGAAGGCCGCATCATGCGGCCTTCTTTGTTTGCGTTGCAGATTAGAGCTGATTGCCT
GCCTGAATGGTGCGGCGGATCTTGTTGCTCAGCAGCGGCACTATGTTGTCGGCCAGCGCCTGGAAGCGGC
CGAGATCCGGCTTGCACTCTGGGGAGAGGTAACCCTCCTTGCGCAAGGTGTGGATCAGGGTGCTGAACAG
CTTCTGGTCGAAGAACTCGGGGGCGTTGATGCCGTGCAGGGTGCCGAGGCGCTCTGCCATCATCAGGCCG
TCCGCCTCCAGCTGCTCCGCCTCGATATGGGGCTGGGCCAACACCCGGGTCAGCACGATGGCGTAGCGTT
GCAGGGTCTCCTGAATGCTCTCCGCCAGCAGCAACAGGCGCATCTGGTTGGCAGGATTGACCCAGTAGCC
GTCATCGCGGGTCTCGATCAGTTGCTGGGCCTGCAGCGCGTCGAGCAGGCTGTCGACCAGCGCTGGCAGC
TCCTCCTCTTCATAACGCAGGAACAGCTCGGTCTTGAGCAGCGGATAGATGTCGATGCAACGGGCCATGA
TCTCGCTGCGGGAGATCCCTTCGCACCGTTCGATCAGTGCCGCCACCAGAGAGGGCATGGCGAACAGGTG
GAGGATGTTGTTGCGGTAGTAGGTCAGCAGGATCGCCTGATAGCGGTCGAGACTGATGATCTGACCCAGC
TTGTCCTCGCTCACCTCGAACTTGTTGAGCTCCATCGCCTGATCCAGCAGGGTCTTGGCATCCTCATCCG
GAATGGTGCTCTGCGGGCTGTAAGGCACCGCCTTGAGCAGATCCAGATAGGTGTTGAGCTGAGCCTGCAA
CTCGTCGCGGGTCAGGGCGTGGCGCTCGGCGGCCAGCAGCGCCAGCGCGCTCAGGGTCAGGCCGTTGACC
GCGGCAGCGCCGTTGATGCGGGTCATCAGCAGTTCGGCCAGATTGTTGACGGTGCCCGCCATCCACTCCG
GACGCTCCTCTTCCCCGATGTGCTTTTTCCAGTCGGGGATATGTTCGCTCAGGTAGCTGTTGAGGGTCAG
CGGCTCGCCGAAGTTGACGAAACCACGGCCGTAGTTGCGCAGCTTGCGCAGGATGCCGAGCACCTGCAGG
AAGCTCTCTTTCTCCTTGCGGCTTCCCTTGAGCTCGTTGTGGTAGGTGTTCACCTCCATCACGTGCTCGT
AACCCAGATAGACCGGCACCAGAGTGACCGGCCTGTCCAGTCCGCGCATCATCGCCTGCAGGGTCATCGC
CAGCATGCCGGTCTTGGGCGGCAGCAGGCGACCGGTACGGGAGCGACCGCCTTCGGTGAAGAACTCCACC
GAGTAACCCTTGGCAAACAGCAGGTTGAGGTATTCCCGGAATACCGTGCTGTAGAGCGGGTTGCCCTTGA
AGGTGCGGCGAATGAAGAAGGCACCGCCGTGGCGGAAGATCGGGCCCGCCGGCCAGAAGTTGAGGTTGAT
ACCCGCCGCGATATGGGGCGGCACCATCCCCTGGTGGTAGATGACGTAGGAGAGCAGCAGGTAGTCCATG
TGGCTGCGGTGACAGGGAACGTAGACGATCTCGTGCCCTTCCTGCGCCAACTGGCGTACCTTCTCGGCGC
CATTGACCGACAAACCGCGGTAGAGCTTGTTCCACAGCCAGCCGAGGAAGCTCTCGCCCAGGCGGATCAG
CCGGTAGGAGAAGTTGGAGGCGATCTCGTCCATGTAGCCATGGGCACGCTTTTGCGCCTTCTCGAGGCTG
ATCCCTTCGCGCTGGGCCTCCTCTTCAATCGCCTGCTGGATCACCGCGGAGTCGAGCAGCTGCTTGAACA
GCAGGTTGCGGTTCGGCAGTTTCGGGCCGGTGGCGGCCAGCTGCTGGCGGCTGAAGTGGGTACGGGCCAC
TCGCGCCAGCTTGTGGGCGATCGCCTCGTCAGTGCCGTGCTTGTCCGCCATGTAGCGCAGGGAGAGCGGC
GGCGAGAAGCGCACCAGATTCTCGCGACCCTTCAGTATCACGATGAGCGCCTTCTTCAGCCGGTTCGGCG
CCAGACTGCTGATGATGTTGAGGCCGGAGGCTTCTTCTCCTTCCCGACCCGGAGCGCGGCCCCAGAACAG
GGTGACGGGCACCACCTGGATATCCAGCTCGGGATCCTGCTTGTGCAGATCCAGCAGCTGGTGGAACTCC
TGCAGGAAGGGGAGCGGTTTGCTGCGTTTGCCAAACAGGGGAGGCGGACGATCCAGACAGACATAGCGCG
GCAGCAGCTGGCCGTTCAGTTCCAGCGGCGTGAAGGGACCGGGCAGACCGAGATCCTCGCAGCACTGCTG
CAGGGTCATCAGGTCGGTGATCGAACTGGTTTTCAGGGCATAGACGATGGGTCTGGCCGGATCCAGATTG
AGTTCTGTGATGGGATTTTCCGGCAGGCTCTTGTGATTGACCAAGCCGCTGACCGGCCATTGCAAGATGG
CTCTGGAAATCTTCTGTCCTAGGGACATGTGCGTCTGCTCTCCAAAAAAATGCGGCGTGAGCATAGCAAA
TCGACAGCCCCTTGTCGCCGAGAGGTACGATGAGTCGGCTGCGCAGGATCAAAACAGCAGTAAATGAGGG
GTTGGTTGCGACAAATGTCAGCTGCGCCTTGCCTTGGCCACTGCACTGTATATACTGCCAGTGTGCTGTA
TAAAAAAACAGGTGCCAATTCTATGAAACCCCTTACCCCCCGCCAGGCCGAAGTGCTGGAGCTGATCAAG
GTGAACATGAGCGAGACCGGCATGCCGCCGACTCGTGCCGAGATTGCCCAGAAGCTTGGTTTCAAGTCGG
CCAATGCGGCCGAAGAACATCTGAAGGCCCTGGCCAAAAAAGGGGTCATCGAGATCATGCCGGGCACGTC
CCGCGGCATTCGCCTGTTGCTCGAAGAGGAAGAGGTGCTGGAAGAGAGCGGCCTGCCGCTCATCGGCAAG
GTTGCCGCCGGTGAACCCATTCTGGCTCAGGAGCATATCGAGAGCCACTATCAGGTTGATCCCGCTCTGT
TCCATCCGCGCGCAGATTTTCTGCTGCGGGTACAGGGGATGAGCATGAAGAACATCGGCATTCTGGATGG
CGATCTGCTGGCGGTGCACAAGACCCAGGAAGTGCGCAACGGTCAGGTGGTGGTCGCCCGTCTCGATGAG
GATGTCACCGTCAAGCGCTTCCAGCGCAAGGGCAGTCAGGTATGGCTGCTGCCGGAGAATGAAGAGCTCT
CCCCCATCGAGGTGGACCTCTCCTGCCAGCAGCTCACCATCGAGGGGCTGGCTGTCGGGGTGATCCGCAA
CGCCGACTGGATGTAATCATCCCCGCCAACTGCGTTTGCCACACGGCCTCCTTCGGGAGGCCGTGCTGTT
TCTGGCGCACTTTCCTTGTCATATCAAACAGATCATCGGTGGCGGCATTTTTGTCCTGCGCGACCGGCTA
CCAATCATCCATTTCAGATATATGCTCAGATCAGTGCCCTGTCGCCGAGACAGGGCTTGGCACCTCCCTC
TGACCATCGCAATGCGCCGTGACGGGAGGGCCGGTAACAACGAGAATCAGGCAGTACACCTTCACGGGAT
ATGAAGGGGGAGTGGGGAGATCCCGAGCCTTGTCGGGCAAAGGGGACCACTCGTAATGGTTGCAGATAGT
CGTGACGGGCCTGTTCCCAGTTCAGGCCGACGAAGCAACATGACCTGTCGGTCAAGGAGGACTGGTATGG
CATCAAGATCGCCATGGGATTCAGAGGCTGGGGCTGCGCCAGCGGAATCGTCAATCACCCGTTTCGTCAA
AGGTAGCCGGATGACCTGGTTTTACGGTCTCTGGCTGCACCATCGTGGTTCGCACTGGACGCTCTGGCTG
GGATGCCCGAAAGAGAAGGTGGTGCGTCTGGTGCTGCTGTTCAAACGTGGTCATGGCGATCGCCAGGGGG
AAGAGGGCTGGCTGGCCCCCGTGATTCCCATCGGCGTGCGGGCCATCGGCAAGATGTTGCAGCAGGGGCG
TAACGCTCGCCCCTGAGCAGAAGAGACAAACAAATCTACCCATAACCGGCACCCGAGGGGGCCGGTTTTT
TTATGGCCGCAACTGGCGATTAATTATGCCAAATGGTCGACTCCTGACAGGGCGTTGCACAAGGTTGAAC

CACCTTGGCGGCTCGCTGGGGTACAATGCCCCCCTATTGTCCGGTCACTGCTGGCCATCTGCCTCGAACT
GAAAGGGTCTCTTGTTATGATGAAGCTCCGCTATCCCCTGCTCTTTCTGCTGCTGACAGCCTGCCTGATC
CTGGTGTTCTACGCCACGCAGCCAGAGCAGCCGGAACAGGCCGATTATTATCCGGCGGGGCGGGACATCA
ATGATTTCGCCTTTACCGATGCGGACAACCAGCCGTTCACCCCTGCCAACCTGAAGGGGCACTGGACCTT
CCTGTTTGTCGGCTACACCTTCTGCCCTGATATCTGCCCCACCACCCTGGCAGATATGCGCTCCGTCTAT
GACCAGCTGAAGAAGATGGCGCCCGACAGTCAGGTAGTGTTTATCTCGGCGGATCCCCAGCGTGACGATA
TTCCACGCCTCAAGACCTATACCGCCTTCTTCAATCCCGAATTCAAGGCGGCCACCGCCCCCCACGACAA
GCTGTTCCCGTTTGTGCGCAATCTGGGGCTGATCTACTCCATCGCCAATCAGGGGGAGAAGGATTATCTG
ATCGATCACTCCGCCTCCATCGTGCTCATCAATCCCAAGGGCAAGCTGCAGGCGGTATTCCGCCCGCAAG
TGGCGCCGGGTCAGGTACCACGGGTGCAGATGGCCACCATGCTCTCTGACTTCTCCCGTATCCTGAAGCT
GACAGGCAACTGAGCGTGACCCCAGCTGTTGCCCCCTGGTGGCGAGATCCCTCTCGCCACCGCGCCGTCT
TTGCGCTCGCTCTGCCCATGGTTTTTTCCAATATCACCACCCCCTTGCTGGGGCTGGTGGACACCTGGGT
GATCGGTCATCTCGGTCAGGCCTGGTTTCTGGGGGGCGTCTCGGTTGGTGCGACCCTGATCAACCTCATC
TTCTGGTTGCTCGGCTTTTTGCGGATGTCGACCACCGGCTTGACGGCGCAGGCGCACGGCGCCGCCGACG
GTCGTGCCCAGCTCGATACCCTGCTGCGGGCGCTGGGGCTGGCCATTGCTCTTGGCCTGGCGCTGCTGTT
GTTGCTCTTCCCCCTGTTGCCACGACTGATCGCCCTGAGTGGCGGCTCGCCCGAGGTGCAGCTCTATGCG
GGCCAATACGTGGCGGTACGGATCTGGAGCGCCCCGGCGGCGCTCTGCAATCTGGTGGTCATGGGCTGGC
TGTTGGGGATGCAGGATGCCCGCAGCCCCATGGTGATGTTGATCCTCACCAATCTTGTCAATATGGCGCT
CGATGCTCTGTTTGTGCTGGGGCTCGGCTGGCAGGTGCGGGGTGTGGCGGCGGCCTCGGTCATGGCGGAC
TACTGCGCGCTGGCAGTCGGTATCTGGCTGGTACGCCGTCAGCTGCGGCAACTGGCGCCGACGGTGTGGC
AGGATGGTTGGCAGCGCTGGCGGCAGCTCGCGCCCATGGTGCGGTTGCTCGGCCTCAACCGCGATATCTT
CCTGCGCTCCCTCTGTTTGCAGCTCTGCTTCGCCTTCATGACTCTTCAAGGGGCACGGCTGGGGGATGTG
GCGGTGGCCGCCAATGCGGTGCTGCTCAACTTCCTGATGCTCATCTCCTATGGACTCGATGGTTTTGCCT
ATGCGGTGGAGGCCATGGTAGGACGAGCCATCGGTCAGCAGGAGCGGCAGAAACTGCGGGAGGCCATCGT
GCTCAATCTGGGCTGGGCCGCCCTCATTGCCAGTGGTTTTACCCTGGTGTTTGCCCTCTTTGGTGCCCAC
CTTATCGGCTATATCACCGACATTCCGGCTGTGGTGGCGGAGGCAAACCGTCAGCTTCCCTGGTTGATCG
CCATGCCCTTGCTGGCGGTCTGGTGTTTCCTGCTGGATGGCGTATTCATCGGGGCGACTCGGGCGCGGGA
GATGCGCAACAGCATGTTGCTGGCCGCGTTTGGCGGCTTCTTCCCGGTCTGGTGGCTCTGTCAGTCGTGG
GGCGTGGCCGCGTTATGGGCGGCGATGGCAGCGCTGATGGTGGGGCGCGGTCTGTCGCTGGGGGTCACTT
GCTGGCGGCTGGAGCGCAGCGGGCATCTCCTCGACGCCCGCCACTGATCATCGGTGGCGCTTACATGGCA
CCGTCGTAGCCGAGCTGGCGCCAGCTCTCGTAGACAAATACAGATACCGCGTTGGAGAGGTTCATGCTGC
GGCTCTGGGGCACCATAGGGATGCGTAGCCGCTGCTCGAATGGCAGACTCTCGATGATCTCGATGGGCAG
GCCGCGACTCTCCGGGCCAAACAGCAGGGCGTCGCCAAGGGCATATTGTGCTGCCGAGTGCGCGGTGCGC
CCCTTGGTGGTGCAGGCAAACACCCGTTTGGGCGCCACGGCGGCCAGAAAAGCCTCATAGTTCGGCCAGC
GCTTCACCTCGGCAAATTCGTGATAGTCGAGTCCCGCCCGCTTTACCCGCTTGTCATCCCACTCAAATCC
CAGCGGCTCGATCAGATGGAGCCGGTAACCGGTATTGGCGCAAAGCCGGATGATATTGCCGGTGTTGGGC
GGAATTTCCGGCTGGTAGAGGACGATATCGAGCATGACGAGCTCCTGCAAAGAGATGTAAAGCCAAGAAG
GGGCGCCAGCATACCCAGCGCCCGTCCGGATGAAAAGGATCAAGCGATGGGCAAGCGGATGGTGATGGTC
AGCCCGGTGGGATGATTGTTGCTGGCCGCAATGGTGCCGCCGTGGCGCTGGATCGCCTCCGAGGCGATGG
CCAGCCCCAGCCCGGTGCCGCCGGTCTTGGCGTTGCGGGCATCATCGACCCGGAAGAAGGGCTGGAACAG
CAGCGCCTGCTGCTCGTCAGGCACCCCGGGGCCGTCATCCCGAATGGTCATCACCCACTCCTGCCCCTCC
CGATACCAGTCCACCTTGATGAACTCGCGGGCATATTTGAGGGCGTTGCGCAGGGGGGTTCTCCACCGCCC
GGGCCAGCAGCTCGGGCCACATCTGGATCGACTCGGCAGGCAGCGGCGGCAGTCTGAGCTGTTTGCAGCT
CTGGTTCGCTTCGAACATCGCATCTTCGAGGATGGGCTCCAGCAGGTCGGCCAGCGGGTGCATCACCGGC
GCCTCCACATGCTGCTGCACCCGCGACAGATCCAGCAGATCGCCGATCAGCTTGTCCAGCCGTCCGATCT
CCGATTCGATCCGCGCCAGCTCGGCGCTGTCACCCTGCTTGCGGCGGATCAGCGCCTGCGCCAGTTGCAT
CCTTGTGAGGGGGCTGCGCAGCTCGTGGGAGATATCGGAAAGCAGCTGTTTCTGTTTCTGGATCATGCTC
TTGAGCGTCTCCACCATGCGGCTGACGTTGTCCGCCAGCTGGCCGATCTCGTCGCCCCGTCCCAGGTTGG
GGATCTGTACCTCCAGATTGCCGGCCGCCAGTTGGGAGACCGATTTCTGCAACTGCAGGATGGGGCGGGT
CAGCCGCCAGGCGAGCAGCAGGCAGAGCGGGGTGCTGACCAGCATCGAGATGCCCAGAATGCGGATCGGG
TGATCCAGCACCTGAATAAACAGGAACAGGTAGGGATTCTCGGCATTGAGGCCGAAATAGACGTGATAGC
GATGACCGTGGTGCTCCATCAGGAAGGGGCCGGCGATCGCCTTGCGATCCTCGTTGCCCAGCATGGGATT
GGCCGGGTCGTCAGCATCCACCATAAAGCGCACCACGAACTTGCGCGGATGGGTGGTGGACTGGATCTGC
CCCTTCTCGTCGCGCAGGTAGACGTTATCCACCGGCAGCATGGCGATCTTGTCCATCGCCTTGGCCAGAT
CGAAATCCTGATCCGGCTCGGCGGCGTTGAGCAGGGCCTGCACGTTGTTCTGCATCCGCGCGACTTCGTG
GGTCGGCAGCGGGATCTGGTTGCGGGGATCCAGGGTCGGCAGGCTGATCACGGCGGCCAGCATCACCAGC
AGCATGAACCAGAACCAGAGGAAGATGTGGGTCGAGATCCCGTTGAGGGAGTTGATGCGCTCTATTCTTG
CTCTAGCCACAGGTACCCGCGCCCCCGTACGGTTTTGAAGCGGGGCTGGCCATCAGTGCGGTCCGGCAGC
TTCTTGCGCAGGTTGCTCAGGTGCATGTCGATGGCCCGATCGAAGGGCTCCAGCTTCTTGCCCAGCACCT
GCTCGGAGAGGTGGCTCTTGCTGACGATCTGGCCGGGATTGCGCAGCAGACACTCCAGCAGCCCGAATTC
GGTGGCGGTCAGCTCCAGCAGCTGTTCGCCGCAGCGGGCCTGCTGCAGGCCGGGTTGCAGCACGATATCC

ATGAAGTGCACTTCGTCGGAGGAGTTGCCGCGCTGGGGCGGTGCGCTCTGGGTACGGCGCAGGATGGCGC
GTACCCGGGCCAGCAGCTCCCTGTCATCGAATGGCTTGGCCAGATAGTCGTCGGCACCCGCTTCCAGACC
GTTAACCCGATCCTGGCTGTCACCGCGGGCGGTCAGCATCAGCACCGGGGTATCGTGGGTCTTGCGCAGC
TTGGTTAGCATGGCAAAGCCGTTGAGTTTGGGCATCATTACATCCAGCAACACCAGATCGAAGCTCTGCT
CCGCCAGCCGTTGCAGCCCCTCTTCACCATCCTCGGCGACCGTGACCTGAAATCCCTCCAGGGTCAGAAT
TTCGGTGAGCAGTTGGGTCAATTCAAGATCGTCGTCGACCAGGAGTATTCTGTTCATCTGGTGTTATTCC
GCAGATTTTCGCGCCAAACTGACGCCGGGGTCAGGGTATAATAAGTTTTTCATCGCCAGAGGAAAGCCGG
TTTTGAGCCATCAACAATATTCCCGTTGGGTGACCTTTGCCAGCATAGCTGCGGTCACCACCGCTACCTT
GCTGATCATCGGCAAACTGATCGCCTGGCTGATGACCGACTCGTCGACCCTGCTGGCGTCGCTGACCGAC
TCCTTCATGGATGTGAGTGCCTCCATCATCAACCTGCTGGCCATTCGCTATGCGCTTGCCCCCGCCGATG
ACGAACACCGTTTCGGTCACGGCAAGGCGGAGTCGCTGGCGGGCCTCATCCAGTCGGCCTTTATCTCCGG
CTCCGCCTTGCTGCTGGTGATGCACGGAATTTCATCCTTGCTCAATCAGGCGCCTATTCTGCGGCTGGAG
GCGGGTCTCTGGGTCAGTGGCGGCTCCATCGTGCTGACCCTGCTGCTGGTCAGCTTCCAGAGCGTTGTCA
TTCGCAAGACCAACAGTGTGGCCATCAAGGCCGACATGCTGCACTACCGTTCCGATCTGCTGCTCAATGC
CGGTGTCTTGCTGGCGCTGGTGCTGGCGGGGCAGGGGTGGTACTGGGCCGACGGCCTGTTTGCCATCCTG
ATCGGACTGTTCCTGTTGTGGGGGGCGGGACACATCGGCTACGAGTCGGTGCAGGCGCTGCTCGATCGTC
AGTTGCCAGCGGAAGAACAAGCGAGAATAATGGCGCTCTGCTGTGCCGTAGAAGGTGTACACGGCGTACA
TGACCTGCGCACCCGTCAATCCGGGCCGACCCGATTCGTACAGTTGCATCTTGAACTGGATGATCAGATC
CCCCTGGTCAAGGCCCATCAGATCGCCGATGAAGCCGAACTGGCGGTACGCCAGTCATTCGAGCGGATGG
ATGTCATCATCCACATGGACCCCATCTCGGTGCTGACAAAAGAACAACAGAGCCCTCACCCAGAACAACA
GTAGGCTGCAACATGGATGAACTGGTTGCCAAAACCTGGCACAAAATTCAGCAAGAAGCACAGTGCATGG
CGGCCCAGGAGCCGATGCTGGCCAGCTTCTTCCACTCCACCATTCTCAATCACAAGAACCTGCGTTCGGC
CCTCAGCTTCCAGCTGGCCAACAAACTCGACAGCGCAACCATGCCCGCCATCGCCCTGCGCGAGGTGATC
GAGCTGGCCCTGCGCAGCGAGCCCGAGCTGCTGACGGTAGTGGCGGCCGACATCTGCGCTGTGCAGGAGC
GGGATCCTGCGGTCGATCTCTTCTCCACCCCGCTGCTTTATCTCAAGGGGTTCCATGCCCTGCAGGGGTA
TCGGGTCGCCAACTGGCTGTGGCGTCAGGGGCGCCGCTCGCTGGCCCTTTATCTGCAAAACCAGATCTCG
GTGGTGTTCGGGGTGGATGTGCATCCGGCCGCCCGCATCGGCAAGGGGATCATGTTTGACCATGCCACCG
GCATAGTGGTCGGCGAGACAGCGGTGATTGAAGATGATGTCTCCATCCTGCAGAGCGTGACCCTCGGTGG
TACCGGCAAGGAGAGCGGCGATCGCCATCCCAAGATCCGCGAAGGGGTGATGATAGGGGCGGGCGCCAAG
GTGCTCGGCAATATCGAAGTGGGCGTGGGCGCCAAAATTGGCGCGGGCAGCGTGGTGATCAACCCGGTGC
CGCCTCATACCACGGTCGCTGGCGTACCCGCCAAGATCGTCGGCCGTCCGGAGTGCGACAAGCCTTCACT
GGATATGGATCAGTGTCTCTGATCTCCCGGCCGGAGTAGTCGATGGCCAAACGTTGGTCGGGTTATCTGA
TTTTGGGGATGTGGCTGCTCAACCTGCTCCATCTCTATCTGGGACTCTCCCCCTGGCCTGCGGCCATTGC
GGCCTGGGGGGCATCACTGCTCACCTGGCCCTGGCTGGTGGGGGCCGCCAAGCGGCAGGCGCTGGCCCTG
TATGGTGCCGCCCTGCTGCTGTTGCTCTTCTCCTGGTGGCAGGGCGCGACCCTCACCGCCGGTGACTGGC
TGCTGCCCAACATCAATATGCTCACCATGTTTGCCGCGGTGAGCACCCTCAACCTCGCCACCTCGGGTCT
CTTGACCGGCACCACCTCCTGGACCGGGCGAAAGGGGCTGTGGAGCACCATGGCCAGCCTCAATCTGCTG
GGGGCGGTCATCAACCTCTCGGTGCTCTTTATCATCGGCGATCGGCTGGAGCGCAATGGCACCCTGGAGC
GGCGTCAGGTGATGGTGCTGAGCCGGATCTTCTGTGCCGCTGCCTTCTGGTCACCCTTCTTTGTCGCCAT
GGCGGTGGCGCTTACCTATGCGCCGGGCTTGCAGCTCTCTCATATTCTGCCGTTCGGGATCCTCGCCGCC
TTGCTGGCGATGGGGCTGACTGCCTGGCAGGTGGGGCGGATCGGGGTGGCTGACTTTGCCGGTTATCCGC
TGCGGTTGCAGACCCTGGGGTTGCCGGTCACGCTGGCGCTGCTGGTACTGCTCATCAAGCAGATCTGGCC
CCAGCTCGGCATTCTGGCGGTGATCGCGCTGGCGGCCCCGCTGCTGGCGCTGCTCTTTATGCCGAGGGTT
GGGCGCAAGGCGGCCCTCAAGCGGCAGGTGGTGGAGCGCTTTCCCGCCATCGGCGGTCAGCTGGTGCTGT
TTCTGGGGGCCGGTTTGCTGGCGGCAGGCATCAACAGTGCGTTGTCGGTCATCGATTTCGGCTCCGGTCA
CGGCCTGCCGCTGTTCAATCACTTTGGTTGGCTCGAAGCATCCCTCACTCTGCTGCTGATCCTGCTGGTG
GCCATCGTTGGGGTGCATCCGCTCATCAGTATCTCCGGGCTGGCGCCACTGCTCTGGCCGCTGGCGCCGG
ATCCCAGCCTGCTCGGCATGTGTTTCCTGCTGGGATGGGGGCTTGCCACCGGCACCAGTCCGCTCTCCGG
CTCCAATCTGGCGCTGGCGTCCCGCTACAACCTCCGTTCCGGGCTGATCCTGCGCTGGAACCTGCTCTAC
GGCCTGCTGATGTATGTGGTCGCCTGTCTGTTGCTGGGGGCTTATATCGCCTTGCATGGATAACGGCGGC
GCCGTCAGGAGGGTGCAAATGATGCGCGGGCGGCGGGGTCTTTTACGCACGGTTCAGTGTGTTGTCGCGG
CTCGGGCGCAGGATGCGAGAAAAATAGACGCATATTTAGACAATACATCTATGGAAGGCGCATTTTACAG
CGCACTGGCTTGTTTGTGCCGATGCGCTCGGGGTATGCTGGCTTCACGCGCTTGAATCGCCCTCTGTGGG
GCATCGCAATTGAACAAACAGGTATGCATATGTCACTAGAAGTCCTTGAGCAACTCGAATCCAAAGTACA
ATCCGCCGTCGACAACATCTCCTTGCTGAAGATGGAGCTGGACGAGCTGAAAGAGCAGAACAGCAAGCTG
CAAGACGAGAACCATCAGCTGCGCAACGAGCACACTGCATGGCAGGAGCGTCTGCGTGCCCTGCTGGGTA
AAATGGATCAGATGGGCGAAACCATCTAATTCATCGTCTTCAGGCAGAATTGAAAACGGGCCGCTCCATG
AGCGGCCCGTCTGTTTTTGGCGGGATGGGAATTACAGCGCCAGCAGTTGCTTGACTGTCTTCTCGATGCC
GTCCGCCGCTTCGACTATGTTGTTGGCCAGCATGTAGGCGGGGGTGGAGATCACCTTGCGCTCCTGATCC
ACCACGAATTCGTTGACCGGGCAGTTGAGATGGCTGCCACCCATCGCCTCGATGGCGTTGGCGGTGCCTT
CGTCATGGCCGATGGTCGCCAGCGTCCCGGCACCATAGATCAGCGGGATCATGGCGGGCGCGATGCAGAT

ATAGGCGGCCGGTTTGTTGGCTTTGGCAAAGCTCAGGCACGCTTTCAGCACATCGGGCTGGATCTGGCAC
TCGGCCCCCTTGATAGCAAAGTCGCACAGGTTCTTGGCCGCGCCAAAGCCGCCGGGCACCAGCAGCGCGT
CGTAATCGGCCGCATCCAGCTCGGCCACATCCTTTATCTGGCCACGGCAGATGCGGGCCGCTTCGACCAG
CACATTGCGGCTCTCTCCCTCGCTCACTTCACCGGTCAGGTGGTTGATGACATGGAGTTGCGACACGTTC
GGGGCGAAGCACTGGGCGGCGGCTCCCTGACGGGCCAACGCCAGCAGGCTGATGACGGCCTCGTGGATTT
CGGCCCCGTCAAAGACACCGCAGCCACTCAAAATCACGGCCACTTTTTTCATGGCAAACTCCTCGTTGTT
GAAAATGTGAGCCAGGATGGATTGTTGTACTTTTGTATGGTTATCGCTCGCCCTCCGCTGGTCAAGTCAT
CTTGCTGTGCTAGGATTGTTTCGTTTTGGATCCTGCCGTACCTACTCCCTTAAATCTTTTTCCACATTGA
TCAAGATCATTCTAACTGTCTCACGATCGGTTTTTATGATCTCAATGCATTGTTTTTAATGGCTTTTTAT
ATTATTATGCACAGTTTTATCACTGTGCGCTCTGATGCGCCCCATTTTTGAACACAGAGTTATCCACAGC
TTGAGTCCCTTTATCAGGCGCTGTGTGCTGTTGCTGCGCCCCTTGGTGTGAGGGGGGCCCTGTGGCATCC
TAGCGGCTGTTTATCTCTGGCAGGACGATTTCATGGCCCCTAGCAACCCCCTTATTCTGGTCGACGGCTC
CTCTTATCTTTACCGCGCCTTCTTCGCCTCCCAGCAGGCTGATCTGCGCACATCGACCGGTTTGCCGAGC
GGCGCCGTCCGTGTCATGGCCAACATGATGCGCAGCCTGCGCAAGCAATATCCTGACTGTCATGTGGCCG
TGGTGTTTGATGCCAAGGGCAAGACCTTCCGCGACGACATCTATCCCGAGTACAAGGCGACCCGCGCCAG
CATGCCGGACGATCTGCGCAGCCAGGTCGCGCCTATCCACCAGATGATCAAGGCGATGGGTTTCCCCTTC
CTGATGGTGGAGGGGGTGGAGGCCGACGACGTGATCGGCACCCTGGCCCGCCAGGCGACCGAGAAGCAGC
TGCCGGTCCTGATCAGCACCGGTGACAAGGATATGGCACAGCTGGTCTCCGACCATGTCACCCTGATCGA
CACCATGAAAGATGTGAAGACCGATCGGGAAGGGGTGATCGAGAAGTTCGGCGTACCGCCCGAGCTCATC
ATCGACTATCTGGCCCTGATGGGTGACAAGGTCGACAACATTCCCGGCATGACCGGTGTCGGCGAGAAGA
CCGCACTGGCCCTGCTGCAGGGGATTGGCAGCATCGACGAGATTGCCGCCAACCTCGACAAGGTGGCCGC
GCTCGGCTTTCGTGGCTCCAAGGCCTTTGCCGACAAGTTCCGCGAGCAGGAGGAGCAGGTTCGTCTCTCC
TATCGACTGGCGACCATCAAGACCGACGTCGAACTGGAACAGAGTCTGGAAGAGCTGTTGCTCAAGCCGG
TCGACAAGGAGTCCCTGCTGGCGGTCTATCGCGAATACGAGCTGCGCAACCTGATCAAGGAGCTGGAATC
CGGTGGTGAAGAGGGGAGCGAGAGCGCCGGACAGGACGAGGCTGGCGATGAGAGCGCCGCTCCGGTTGCC
GCCATCGAAACCGACTACCGCTGCATTCTGGACGAAGCCGAGTTTGACGGCTGGCTGGAACAGCTGAAAG
CGGCACCGTTGTTTGCCTTCGATACCGAAACCACCAGTCTCGACTATATGGAGGCGCGGATCGTCGGCGT
CTCCTTCGCGGTTGAAGCGGGCAAGGCTGCTTATGTACCGTTTGGTCACGACTATCTGGGGGCACCGTTA
CAGCTGAGCGAGGCCGTGGTGTTGGGCAAGCTCAAACCGCTGCTGGAAGATCCGACCCGTCTCAAGGTGG
GGCAGAACCTCAAGTACGACCGCAACGTGCTGCTCAACCATGACATCGACCTGCAGGGTATCGCCTACGA
CACCATGCTCGAGTCCTACGTGCTCAACTCCACCGCCAGTCGTCACGATATGGATTCGCTGGCCAAGCGT
TACCTCGGGGTCGAGACCACCTCTTTTGAAGATATCGCGGGCAAAGGAGTGAAACAGCTCACCTTCAACC
AGATCGAGCTGGAGCAGGCCGCTCCCTATGCGGCGGAAGATGCCGATATCACCCTGCGTCTGCACCAGAC
CTTGTGGGGTCAGCTGGAGGCCGTGCCTGGCCTCGCCAAGGTATTCAGCGAGATCGAGCTGCCGCTGCTG
CCGGTATTGGCACGGATGGAGTTGCTCGGCACCACCATCGATCCCAAGCTGCTGCACCAGCAGAGTCAGG
AGATCGAGCTGCGGTTGGCGGAGCTGGAGAAGCAGGCTCACGAGCTGGCCGGGCAGGAGTTCAACCTCTC
CTCGCCCAAGCAGCTTGGGGAGATCCTCTTCACCAAGCTGGGCCTGCCGATCATCAAAAAGACTCCGAAG
GGTGCGCCATCCACCGCAGAAGAGGTGCTGGCCGAGCTGGCGGAGACCTACGAGCTGCCGCAGCTGCTGA
TGGAGCACCGTGGGCTGGCCAAGCTCAAGTCCACTTACACCGACAAGTTGCCGCTGATGATCAAGCCCCG
GACCGGGCGGGTGCACACCTCCTATCATCAGGCGGTAGCGGCTACGGGCCGTTTGTCATCATCTGATCCC
AACCTGCAGAACATCCCGGTGCGCAACGAGCAAGGGCGCCGGATCCGGCAGGCCTTCATCCCGAGCGCCG
GTTACAAGCTGGTGGCGGCGGACTACTCCCAGATCGAGCTGCGCATCATGGCGCACCTCTCCGGTGACAA
GGGGTTGCTGACCGCTTTTGCCGAGGGCAAGGACATCCACAAGGCGACTGCCGCCGAGGTGTTCGGCGTG
GCGCTCGATGCGGTCACCACCGACATGCGCCGCAGCGCGAAGGCGATCAACTTCGGTCTTATCTACGGCA
TGAGTGCCTTTGGTCTGGCCAAGCAGCTGGGGATCGGTCGTGCCGAAGCGCAGAAGTACATGGATCTCTA
CTTCGAGCGCTACCCAGGCGTGCTGGCGTATATGGAGCGCACCCGCCAGCAGGCGGAAGCCCAGGGGTAT
GTCGAGACCCTGTTCGGCCGTCGCCTCTATCTGCCGGATATCAAGTCCCGCAACGCCGGTCTGCGCAAGG
CAGCCGAGCGGGCCGCCATCAACGCCCCCATGCAGGGCACGGCTGCCGATATCATCAAGCGCGCCATGAT
CAATGTGGACAGCTGGATTCGGGGCATTAAAGATGAGTCGATCCGCATGCTGATGCAGGTACACGATGAA
CTGGTGTTCGAGATCCGCGAAGAGAAGCTGGAGGAGTATATCGCCATCATCAAGGAGAAGATGTCTGCCG
CAGCCGAACTGCATGTGCCGCTGCTGGTTGAAGCGGGTACCGGTGACAACTGGGATCAGGCCCACTGATT
TCCGGTCAGCCGTTCTGCACGGCTATATAGCTATATAAGAAGCGAAGCCCGGCATTATGCCGGGCTTCCT
GTTTCTGCCAGCCCTATCAGGTCTCTTGCAGATACTCATCCTTGAGCAGGACATAGTTGTCGGCCGAGGT
CTTGAGGAAGGCGATCTCGGCGGGCTTGAGCGGGCGAGCCTGTTTGACGGGATTACCTATATATAGAAAG
CCCGACTCGAGTCGCTTGCCCGGTGGCACCAGCGAACCTGCGCCGATCATCACATCATCTTCAACCACGA
CGCCATCAAGCAGAATGGCACCCATGCCGACCAGCACCCGGTTGCCGATGGTGCAGCCGTGAAGCATCGC
CTTGTGACCCACAGTGACATCTTCACCGATCAGCAGCGGATAGCCGGTCGGGTTGCTGGCGCTCTTTCGA
GTCAGGTGCAGCACAGTGCCGTCCTGAATATTGCTGCGGGTGCCGATGCGGATATGGTTCACATCCCCCC
GCGCCGCCACCATGGGCCAGATGCTGGCATCATCGGCCAGCTCGATATCGCCCACCAGTGTTGCGCAGGG
GTCTACATACACCCGTTTGCCCAGTTGCGGGCGTTTTCCCTTATAGGGTCTCAGTCGCAAGTCCATCTTG
GCCTCCTTGTTTTCGGCCATTCTAGCAATCCGAAGGGTGATTCTGTGGATAAGTTTGTATAAAAGCTGTG

TTTGGATATGAATAACTAAAAAAGTGCTATTTTTTGCAATTTTATTGCAGATAAGGGGTTGCCAGAAAGA
ATCTGTTCCCTATAATGCGCCTCCATCGACAGGGCAAGCGGCAACGCAAACAACCTCGTCGATAACCTCA
AAAAGCCTTTGAAAATAAGGCTTGACTCGAGAAGGCGGTTGAGTAGAATTCCACTCCCGCAGCAACACAC
TGTTGCGTCGCTCTTTAACAATTTGAATCAAGCAATCTGTGTGGGCACTCACAGCATCGAACATCAAAAA
CAATTTTTGATTTTCAATGTCTGGTGAAGTGACCAAGACGATTTCTTAGCAATAAGAAGTCGAACAGTTT
ATTTCAGCAATTCATTGAGCCGCTTTTCGAAGCAACCAAACTTAAATTGAAGAGTTTGATCATGGCTCAG
ATTGAACGCTGGCGGCAGGCCTAACACATGCAAGTCGAGCGGCAGCGGGAAAGTAGCTTGCTACTTTTGC
CGGCGAGCGGCGGACGGGTGAGTAATGCCTGGGGATCTGCCCAGTCGAGGGGGATAACTACTGGAAACGG
TAGCTAATACCGCATACGCCCTACGGGGGAAAGCAGGGGACCTTCGGGCCTTGCGCGATTGGATGAACCC
AGGTGGGATTAGCTAGTTGGTGAGGTAATGGCTCACCAAGGCGACGATCCCTAGCTGGTCTGAGAGGATG
ATCAGCCACACTGGAACTGAGACACGGTCCAGACTCCTACGGGAGGCAGCAGTGGGGAATATTGCACAAT
GGGGGAAACCCTGATGCAGCCATGCCGCGTGTGTGAAGAAGGCCTTCGGGTTGTAAAGCACTTTCAGCGA
GGAGGAAAGGTTGGTAGCTAATAACTGCCAGCTGTGACGTTACTCGCAGAAGAAGCACCGGCTAACTCCG
TGCCAGCAGCCGCGGTAATACGGAGGGTGCAAGCGTTAATCGGAATTACTGGGCGTAAAGCGCACGCAGG
CGGTTGGATAAGTTAGATGTGAAAGCCCCGGGCTCAACCTGGGAATTGCATTTAAAACTGTCCGGCTAGA
GTCTTGTAGAGGGGGGTAGAATTCCAGGTGTAGCGGTGAAATGCGTAGAGATCTGGAGGAATACCGGTGG
CGAAGGCGGCCCCCTGGACAAAGACTGACGCTCAGGTGCGAAAGCGTGGGGAGCAAACAGGATTAGATAC
CCTGGTAGTCCACGCCGTAAACGATGTCGATTTGGAGGCTGTGTCCTTGAGACGTGGCTTCCGGAGCTAA
CGCGTTAAATCGACCGCCTGGGGAGTACGGCCGCAAGGTTAAAACTCAAATGAATTGACGGGGGCCCGCA
CAAGCGGTGGAGCATGTGGTTTAATTCGATGCAACGCGAAGAACCTTACCTGGCCTTGACATGTCTGGAA
TCCTGTAGAGATACGGGAGTGCCTTCGGGAATCAGAACACAGGTGCTGCATGGCTGTCGTCAGCTCGTGT
CGTGAGATGTTGGGTTAAGTCCCGCAACGAGCGCAACCCCTGTCCTTTGTTGCCAGCACGTAATGGTGGG
AACTCAAGGGAGACTGCCGGTGATAAACCGGAGGAAGGTGGGGATGACGTCAAGTCATCATGGCCCTTAC
GGCCAGGGCTACACACGTGCTACAATGGCGCGTACAGAGGGCTGCAAGCTAGCGATAGTGAGCGAATCCC
AAAAAGCGCGTCGTAGTCCGGATCGGAGTCTGCAACTCGACTCCGTGAAGTCGGAATCGCTAGTAATCGC
AAATCAGAATGTTGCGGTGAATACGTTCCCGGGCCTTGTACACACCGCCCGTCACACCATGGGAGTGGGT
TGCACCAGAAGTAGATAGCTTAACCTTCGGGAGGGCGTTTACCACGGTGTGATTCATGACTGGGGTGAAG
TCGTAACAAGGTAACCCTAGGGGAACCTGGGGTTGGATCACCTCCTTACCTTAAGATGTCGAAGTTGTTG
AGTGTTCACACAGATTGCCTTGATTCAAAGTAGTTAGAGCAAAGACCTGATGCGAAAGTGTCAGTGCTTA
TCTGGCTTCGGTCAGATGAGAGAAGCCCTATCATTGGGTTTTGGGATGTGAATAATGGCGCTCGGCCTCG
CAGGCTCGGCACTCGCCATTACCCAAAATCTGCACTGCTGACGCAGCGCAAAGATGATTTTGGGTCCCCT
TCGTCTAGAGGCCTAGGACACCGCCCTTTCACGGCGGTAACAGGGGTTCGAATCCCCTAGGGGACGCCAC
TTCTCTTCTTGCTAACAAGAATGCAGAGTTAAGAACTGATTCTTAACTCTGTTTTCTTCGGCCTTGCGCC
GTTGCAAACATGCTCTTTAACAATCTGGAAAGCTGATTTAAAAAGTAGTTCTCAAACATTTGTTACAAGT
GCTTTGGAAACTTCTTGGCGAAAACCAAATTTTATTTGGTCCTTGTTGTACGACAACAAGCAAGCGTTGT
TTCGACGACACTTCTTGGGGTTGTATGGTTAAGTGACTAAGCGTACATGGTGGATGCCTTGGCAGTCAGA
GGCGATGAAGGACGTACTAACCTGCGATAAGCTGTGAGAAGTCGGTAAGAGACGCTATTACTCACAGATT
TCCGAATGGGGAAACCCACCCGAGATAACTCGGGTATCTGTTACTGAATACATAGGTAACAGAGGCGAAC
CGGGAGAACTGAAACATCTAAGTACCCCGAGGAAAAGAAATCAACCGAGATTCCCTCAGTAGCGGCGAGC
GAACGGGGATTAGCCCTTAAGCATCTTGGAAGTTAGTGGAACGGTCCTGGAAAGGCCGGCGATACAGGGT
GATAGCCCCGTACACGAAAACGACCTTGATGTGAAATCGAGTAGGGCGGGACACGTGACATCCTGTCTGA
ATATGGGGGGACCATCCTCCAAGGCTAAATACTCCTGACTGACCGATAGTGAACCAGTACCGTGAGGGAA
AGGCGAAAAGAACCCCTGTGAGGGGAGTGAAATAGAACCTGAAACCGTGTACGTACAAGCAGTGGGAGCC
CTTCGGGGTGACTGCGTACCTTTTGTATAATGGGTCAGCGACTTACATTTTGTAGCGAGGTTAACCGTAT
AGGGGAGCCGTAGGGAAACCGAGTCTTAACTGGGCGTCTAGTTGCAAGGTGTAGACCCGAAACCGGGTGA
TCTAGCCATGGGCAGGTTGAAGGTTGAGTAACATCAACTGGAGGACCGAACCCACTAACGTTGCAAAGTT
AGGGGATGACCTGTGGCTGGGGGTGAAAGGCCAATCAAACTCGGAGATAGCTGGTTCTCCCCGAAAGCTA
TTTAGGTAGCGCCTCGGACGAATACTACTGGGGGTAGAGCACTGTTTGGGCTAGGGGGTCATCCCGACTT
ACCAACCCCATGCAAACTCCGAATACCAGTAAGTAATATCCGGGAGACACACGGCGGGTGCTAACGTCCG
TCGTGAAGAGGGAAACAACCCAGACCGCCGGCTAAGGTCCCAAAGTTCTGGTTAAGTGGGAAACGATGTG
GGAAGGCTCAGACAGCTAGGATGTTGGCTTAGAAGCAGCCATCATTTAAAGAAAGCGTAATAGCTCACTA
GTCGAGTCGGCCTGCGCGGAAGATGTAACGGGGCTCAAACCAGGCACCGAAGCCGCGGATTCACACTTAT
GTGTGAGTGGTAGGGGAGCGTTCTGTAAGTCTGCGAAGGTGTATCGAGAGGTATGCTGGAGATATCAGAA
GTGCGAATGCTGACGTAAGTAACGATAAAGGGGGTGAAAAGCCTCCTCGCCGGAAGACCAAGGGTTCCTG
TCCAACGTTAATCGGGGCAGGGTGAGTCGACCCCTAAGGCGAGGCCGAAAGGCGTAGTCGATGGGAAGCA
GGTTAATATTCCTGCACGACTTGTAATTGCGATGGGGGGACGGAGAAGGCTAGGTGGGCCAGGCGACGGT
TGTCCTGGTGAAAGTGCGTAGGTGGTGTTTCTAGGCAAATCCGGAGACACAACACTGAGACACGAGACGA
ACACACTACGGTGTGGAAGCCATTGATGCCCTGCTTCCAGGAAAAGCCTCTAAGCTTCAGATTACAAGTC
ATCGTACCCCAAACCGACACAGGTGGTCGGGTAGAGAATACCAAGGCGCTTGAGAGAACTCGGGTGAAGG
AACTAGGCAAAATAGAACCGTAACTTCGGGAGAAGGTTCGCTCTTGACAGTGAAGTCCCTTGCGGATGGA
GCAGTTGGGAGTCGCAGTGACCAGATGGCTGGGACTGTTTATCAAAAACACAGCACTCTGCAAACACGAA

AGTGGACGTATAGGGTGTGACACCTGCCCGGTGCCGGAAGGTTAATTGATGGGGTTAGCGCAAGCGAAGC
TCTTGATCGAAGCCCCGGTAAACGGCGGCCGTAACTATAACGGTCCTAAGGTAGCGAAATTCCTTGTCGG
GTAAGTTCCGACCTGCACGAATGGTGTAACCATGGCCATGCTGTCTCCACCCGAGACTCAGTGAAATCGA
ATTCGCCGTGAAGATGCGGTGTACCCGCGGCTAGACGGAAAGACCCCGTGAACCTTTACTACAGCTTGGC
ACTGAACATTGAACCTACATGTGTAGGATAGGTGGGAGGCTTTGAAGGCGTGACGCCAGTTGCGCTGGAG
CCGTCCTTGAAATACCACCCTTGTATGTTTGATGTTCTAACGCAGGGCCCTGAATCGGGCTCGCGGACAG
TGCCTGGTGGGTAGTTTGACTGGGGCGGTCTCCTCCCAAAGAGTAACGGAGGAGCACGAAGGTTGGCTAA
TCCTGGTCGGACATCAGGAGGTTAGTGCAATGGCATAAGCCAGCTTAACTGCGAGACGGACAGGTCGAGC
AGGTACGAAAGTAGGTCATAGTGATCCGGTGGTTCTGAATGGAAGGGCCATCGCTCAACGGATAAAAGGT
ACTCCGGGGATAACAGGCTGATACCGCCCAAGAGTTCATATCGACGGCGGTGTTTGGCACCTCGATGTCG
GCTCATCACATCCTGGGGCTGAAGTCGGTCCCAAGGGTATGGCTGTTCGCCATTTAAAGTGGTACGCGAG
CTGGGTTCAGAACGTCGTGAGACAGTTCGGTCCCTATCTGCCGTGGGCGTTGGATGATTGAAGGGAGTTG
CTCCTAGTACGAGAGGACCGGAGTGAACGAACCTCTGGTGTTCGGGTTGTCACGCCAGTGGCACTGCCCG
GTAGCTAAGTTCGGAATCGATAACCGCTGAAAGCATCTAAGCGGGAAGCGAGCCCTGAGATGAGTCATCC
CTGACCCCTTGAGGGTCCTAAAGGGCCGTTGGAGACCACAACGTTGATAGGTGGGGTGTGTAAGCGCGGC
GACGTGTTGAGCTAACCCATACTAATTACCCGTGAGGCTTAACCATACAACACCCAAGAAGTGTTCTAAG
GCTTGTAGCAAATACGAACGAATTACTTACGTCAGCTTTCTCAGATTGAAGAATTTGCCTGGCGGCCATA
GCGCCGTGGAACCACCTGATCCCATGCCGAACTCAGAAGTGAAACGCGGTAGCGCCGATGGTAGTGTGGC
ATTCGCCATGCGAGAGTAGGACACTGCCAGGCACCCAATTTAAGAACCCCGCTCATCGAGCGGGGTTTTT
TATTGCCTACAATTCATAAAGTCGTCTAATAACTGACCTTTTATTCCCTCTTCCCTGCCGTTTTCCCTGT
TTTTCCTCCCCTATTTTCTCCGGTTCTGCCATCGCGGCTGGCGGTGGTTGCCGTTATAATGGCCGCCCTC
AAGCAAGGTGGTGGTATGAAAGCAGAATATCTACAGCGATTCGGGGGCATTGCCCGTCTTTATGGTCAGA
ACGCCCTGCGTTCCTTTAGCCAGGCCAAGGTCTGTGTGGTGGGGGTTGGCGGCGTGGGCTCCTGGGCGGC
AGAGGCGCTGGCACGCGCTGGTATCAACCAGATCACCCTGATCGACATGGACGATATCTGCATCACCAAC
ACCAATCGCCAGATCCACGCCATGAAGGGGACGGTTGGTCGCCTCAAGACCGAGGTGATGGCAGAGCGCA
TTCGCGCCATCAATCCGGATTGCGAAGTGATCGAGGTAGACGATTTCGTCACCGCCGACAATCTGGCCGA
GCATATCAAGCGGGAGTTTGACTATGTGGTGGACGCCATCGACTCGGTCAAGGCCAAGGTGGCGCTGATT
GCCTTCTGCAAGCGCAACAAAATCCCGGTGATTGTGGCGGGTGGTGCTGGCGGACAGATGGATCCGACCC
AAATCACCGTGGCCGATCTGGCCAAGACTATTCAAGACCCGTTGGCGGCCAAGGTGCGCTCCGACCTGCG
CCGGTTACACAACTTCAGCAAGAATCCGGCGCGCAAATTCGGGGTGGAGTGTGTTTTCTCAACCGAGCAG
CTGAGCTATCCAGATGGCAATGGTGGCACCTGTCAGGCCAAAGCGGCCAGCGATGGCACCATGAAGATGG
ATTGCGCCTCCGGCTTTGGCGCCGTGACGCCGGTGACGGCAACCTTTGGGCTGGTTGCCGCGTCCCGGGT
ATTGAAGAAGATTGCCGAGCGGGGTGCCCGCGCCGAGAAAGAGGCAGGGAATGAAGATGTTGGGTCGATT
GGCGAAGCCTAATCGACCACTGAACAAGGCGGGTTAGACCGCTTGTTCCCGGATCGCCAGCACGATCGCT
CTGAGCCCGTTACCTCGTGACGGGCTCAAGTGTTTCTCAAGTCCCAGTTCGCTGAAATACCCTTCCATAT
CAAAAGCCTGGATCGCCGCAGAAGATTGGTGGTTGAGTGCCGCCAGCACGATGACGATAAGGCCCCGCAC
GATGCGAGCATCGGAATCACAGGCAAAGTGCCAGCAGCCATCCTCGCTTTGTTCTCCCTTCAGCCAAGCC
TGGCTTTCACACCCCTTGAGGCGAAACTCCTCCTGCTGCCATTCGCCGGGTAGCGCTGGCAGCTGTTTGC
CGAGCTGGATGATGAGCCGGTACTGGTTCTCCCAGCCGTGGGCGGCGGCAAACTGCTGGCGGATGGTGTC
GGCGTTGGGCTCAAGGCCAATCTGGGTATAGCTTGCGAGGTCACTCATTCGGTTGTCTGCCTTAGAAGAA
GTCACTGAGTTTGTGCAGGGCTGCCAGCAGGGTGTCGACATCGTCCCGATTGTTGTAGCAGGCCAGCGAG
GCGCGCATGGTGCCGCCGATCCCGAGTGATTCCATCAACGGCATGGCGCAGTGATGGCCGACTCGCAGGG
CGATACCCTGCATGTCGAGCAGGGTAGCAGCATCTTGCGGGTGGATGTCGTCGAGCAGGAAAGAGACCGC
ACCGGCGCGTTGGCCGGGTTCGCCGACCAGACGCAGCCCCGGCACCTGTTGCAGGCCAGCGACCAGATAG
TCGGTCAGTGTCTGCTCATGGCTGGCGAGCCATTGCCGATCTTGCCACATCACAAAGTCGATGGCGGCGG
CGAGGCCGATAGCGCCAGCGATATGGGGGGTGCCTGCCTCGAATTTGAAAGGCAGGTTATTGAAGGTGGT
GCCGCTGAAACTGACCCGATCGATCATCTCGCCACCTGCTTGCCAAGGCGGCATCCGCTCCAATAGCTCG
GTGCGGCCCCACAACACACCGATGCCGGTCGGGCCATAGAGTTTGTGGCCGGAGAAGGCGTAGAAGTCGC
AGCCGATGGCTTGCACATCCACCTCGAGATGAGCCACCGCTTGGGCGCCGTCGATCAGCGTCACGGCGCC
AACCGCTTTGGCGGCAGCCACCATCCGGGTGACCGGATTGACAGTACCGAGGGCATTGGATACGTGGGCC
ACGCTCACCAGCCGGGTGCGTGGTCCCAGCATGGCGTGATAGGCGGCCAGATCGAGATCGCCCCGCTTAT
CGAGTGGGATAACCTGGATCACCGCGCCGGTACGCTGGGCGACCAGCTGCCACGGGACGATATTGGCGTG
GTGTTCCAGCGTGCTGAGGATGATCTCGTCCCCCGCCTTGAGCTCGCTCATCCCCCAGCTCTGGGCCACC
AGATTGATCGCCTCTGTGGTGCCGCGGGTCCAGATAGCTTCGTGGCTGCGGGGCGCATTGATAAAGCGGG
TGACCGTCTCGCGGGCTGTTTCGAAGGCGCGGGTCGCGCGGCCACTCAGGGCGTGGGCGGCACGGTGCAC
GTTGGCGTTGTCGACCCGATAGTAGTGAGCGATGGCATCCAGTACCGCTTGCGGCTTCTGGGTGGTGGCG
GCGTTGTCCAGATAGATCAGAGGGTGGCCGTTTACCTCCTGCGCCAAGGCCGGAAACTGGCTGCGCAGTT
GGGCGTGCTGTTGCTGATTCATGCTCCCCTCATCATCTGGAAAAAGTCATCATCTCGAAAGGTCGGTCAT
GATCCTGAAAATAGCGCCCGAACACAAGGCGCGATAAGCCCGATTGGCGGTTGCCTGCCGTGCAGCAGGT
ACGGCTCGTCGGGTGATTTCTGGTACAATGCCGCCCCTCATCATAAGCCAAGAGATCCGGCCCATGAAGC
TCAACCCCAATCAGAACGAAGCGGTCAAGTATGTCTCCGGCCCTTGCCTGGTGCTGGCGGGTGCCGGATC

GGGCAAGACCCGCGTCATCACCAACAAGATTGCCTATCTGGTGCAGCAGTGCGGCTACAACGCGCGCAAC
ATAGCTGCGGTCACCTTTACCAACAAGGCGGCGCGGGAGATGAAGGAGCGGGTCGGCCAGACTTTGGGCC
GCAAGGAGGCGCGGGGGCTGATGGTCTCCACCTTCCATACCTTGGGGCTTGATATTATTCGTCGTGAGCA
CAAGAGCCTGAATCTCAAGGCGAACTTTTCCCTGTTCGATGACACCGACCAGCTGGCGCTGCTGAAAGAA
TTGACTGAAGACGAGCTCGACAACGACAAGGACAAGCTCTCTGCCCTTATCAGCCAAATCTCCAACTGGA
AGAACGATCTTATCCTGCCCGCCCGCGCCATGCGTATTGCTCGCGGGCCGGAAGAGGTGCTGATGGCCCA
GCTCTACGAGCGCTATCACCGCCAGATGGTGGCCTACAACGCGCTGGATTTTGACGACCTCATCGTGATG
CCGACCCTGCTGCTCAAGAGCAATCAGGAGGTGCGCGAGCGTTGGCAGAACAAGATCCGCTATCTGCTGG
TCGATGAGTACCAAGATACCAACACCAGCCAGTACGAGCTGGTGAAGCAGATCGTCGGCGAGCGCGCCCG
CTTTACCGTGGTGGGGGATGACGACCAGTCCATCTACTCCTGGCGTGGGGCCAAGCCGCAGAATCTGGTG
CTGCTGAGCGAAGATTTCCCCAGCCTCAAGCTGATCAAGCTGGAGCAGAACTACCGCTCCAAGCGGCGGA
TCCTCAAGTGCGCCAACATCCTGATCGCCAACAACCCCCACGTCTATGACAAGGCGCTCTTCTCCGAGCT
TGATGAGGGGGTGAAGCTCAAGGTGCTGTTCGCCAAGAACGAGGAGCACGAAGCGGAGCGGATCGCTGCC
GAGATGATGGGCCACAAATTTATGAACCGCACCCGGTTCAAGGATTACGCCATCCTCTATCGGGGCAACC
ATCAGTCGCGCATCTTCGAGAAGGCGCTGATGACCAACCGGATTCCCTATCGCATCTCCGGCGGCACCTC
GTTTTTCTCCCGCACCGAGATCAAGGACATCATGGCCTACCTCAAGTTGCTGGTGAATCCGGACGAGGAC
ACCGCCTTCCTGCGGGTGGTCAACCTGCCGCGCCGCGAGATTGGCCCCACTACCCTCGAAAAGCTCGGCC
AATACGCCAATCAGCGCGGCAAGAGCCTGTTTGCCGCCAGCTTCGAGCTGGGGCTGGAGCAGCACCTCTC
AGGCCGTGGCCTCGTGGCCCTGCAGGCCTTCACCAACTGGCTGGTACGCCTTGGCGATCAGGCGCTGCGC
GGCAATCCGGTGGAGGCGGTGCGGGACATGATTAAGGAGATCCACTACGAAGAGTGGCTCTACGAGACCT
CCCCCAGCCCGAAAGCGGCCGAAATGCGGATGCAGAACGTCTCCACCCTCTATCGCTGGATCACCGAGAT
GCTGGAAGGGGATGAGCTGGAGGAGTCGATGACGCTGGCTCAGGTGGTCACTCGCCTCACCCTGCGCGAC
ATGATGGAGCGCAACGAAGGGGAGGATGATGCCGATCAGGTGCAGTTGATGACGCTGCACGCCTCCAAGG
GGCTGGAGTTTCCCTATGTCTACATGGTGGGGATGGAAGAGGGCTTGCTGCCCCACCAGACCAGCATCGA
CGAAGATAATGTGGAGGAGGAGCGGCGTCTCGCCTACGTGGGGATCACCCGGGCTCAGACCGAACTGACC
TTCACCCTCTGCAAGGAGCGGCGCCAGTTTGGCGAGTCGGTACGTCCGGAGCCAAGCCGCTTCCTGCTGG
AGCTGCCGCAGGATGACCTTGAATGGGAAAACCAGAAGAAGGTGACTGCCGAGGAGCGCCAGCAGAAGGG
ACAGGTCGGTGTTGCCAACCTGCGGGCGTTGTTCAAGAAGGATTAACCCTAAGGATTGGCCGGGGTCAGT
AGCGGTACTATGTCTGCTCCCGGCATGGGCGGTGAGATATAGCGCCCTTGCCCCATCCGGCAGCCGATAT
CAATCAGCTTCTGCAACTGCTGCTGGTTCTCGATCCCCTCGACCACCAGGGTGTAATCGAGATCCTGACA
GATATCCCGCATGGCGCGGATCAGCGCCAAATCTTTGGGTGATTGCAGCATCCGCTGGGTAAAGCTGTCA
TCCACCTTGATGTAATCCACCGGGAAGTGGAACAGGGCGTTGAGGGAGGAGAAGCCGGTGCCGAAATCAT
CGAGGCTGATCTGGATACCCTTGGCCCTCAGGTCATGCAGGGATGCCAGCGCATCGGAATCTTGCCGCGA
CAGCTCCCGCTCGTTGAATTCAAAAATCAGATAGCCGGGGGCGACCCCTTCCTCTTCGATGATCCCCATC
AGTCGCATGATGTGGTCATGGTTGACCAGATGCTTGCCCGACAGATTGATGGCGACCTTGAACTTGCCCT
GCCCGGAGATTGGCAGCCAGTTCTTCAGCTGTTGGCAGGTGTGGCGCAGGATCTGGTAATCCAGCTCCAG
AATCAGGCCGCACTGCTCCGCCAGCGGAATAAAGTCGAACGCATCCTTCAGAATCCCCTCCTCGGTTAGC
CAGCGCGCCATCACCTCCAGTCCGGTCAGCCGGCTATCCTGCAGCCGGATCACAGGCTGGAAGTAGGGGA
CGATACGCCCCTCCCGCAGGGCTCTGGCCAGCATCTCTTCCGGCGAATTCCAGTTCTGGTTGATGCAATC
ATCCGAGTAGCTGACGATGCGACTGCGACCATTGCGTTTGGCCTGATACATGGCCAAATCGGCCTGATGG
AGTAGCTGGCTGATATCTGAGGTTTGCTCGGATACGCTCGATACCCCAAGGCTGACGGTAATGCTCAGCT
CGTTGCCGAGTAGCCGGAAAGGGTAGGAGAGACGGCTGATAATGCGGTTGAGCACCGGGCTGATGTCATC
GTCGCTCTTGCTGCTGTCCAGATAGATGACGAACTCGTCGCCCCCCAGTCTGGCCACCAGATCGTTTTGC
CGGATGCAGGTTTGCAGGCGGCGGCTCACCTCCTTGAGCAGCTGATCGCCAGCACTGTGGCCCAAGGTAT
CATTGATCTGCTTGAAGTGATCCACATCGATAAAAATTACCGCGAATCCCGGCGATTTGTAGCGGTTGTA
GTGGGTCATGGCGTGGTTGAGGTGGTTCATCAACAGCGCGCGGTTGGGAAGTTGGGTCAGCGGGTCGTGA
AGCGCATCGAATTGCAGCCGCCGTTCCAGCTCCTGATGCTGCTTGAGCTGCTTTTGCAGCCTGAGGTTGG
TCTGACGCAGCTGTTGTAGCCGCTGGTTGATGGAGTCTTCGGTATTGATGTCGTTGAGCTGCTGGCGGAT
CCGTCTCAGCTCCAGCAGCAGGGTGAGCTGGCCACGCAAGAAGTCGAGAAACAGCTTGTCGTCGGTCTCC
AGCGGTTGAGCCGTGCTGATGATGAAGTGGGCGAAGTACTGTTGTTGCAGCCGCAGCGGCATGCTGTGCC
AGTAGCGTGACTCCCTGCTCACCTCGATATTGCTGGTGCTGTGGCACAAGCCGCTGATGAACTCGTCTCC
CTGATGGGGATGGGCAGGGTAGATCTTTTCCAGCTTCTGACTGGTTAACCTGACCAGCAGCATATCCTCA
ATGGTGAGCATCTGGCGCAGTACCGCTTCCAGTTGGGAGAAGAGCGCCTGAGCCGGCAGGGTATTGGCGA
GCCGCATGATGCGGCTGATGCTCTCGATTTTCTTTTTCCAGTTTGGGTCTTTGCGGATCAGCGGCGAAGG
CAGCTCGGTGAGATGGGATCCCGCCTGGGTTTCGGTCAGCCGTTTAGCCAGACACTCCAGCAGCAGACGC
AGGGTCGGCAGGTCGGCTGGTGTGCCACGCGCCAGCAACCACTGTATCTGGCCATGCTCGATGGGGAAGA
CGCACCACCCTTCGGCTTCATCCAGCTGGGTGAGTTGATCGGTGATGCGGGACTGCTCCCGGCCCAGCTG
TTGCTTGAAGATGTCGCTGACCTTGTAGGCGTAAAGCCAGTTCAGACCGGTATCGGAGAGGCCCAGCATG
CCGAAATGTTGCAGCGGGCAGAGTTGGTCAATCTGCTGGATCAGGGCATCGTAATCGTCAGATGTGGCGG
TTTGTGGCGCCGGGAAGTCCATAATGGCTGAGACCAACGCCAACAGAGGAGTTGAGTAGTGGGGTTCGAT
GGATTGGGCCGACATGTGCAACTCCCGATATGTCTACCTTACAAGAGTAGTCGGAAGCCGATAGATTGAA

AGGGCCGCAATTGCGGCCCTTTCAAATACTTACAGCTTGTCGATCATGTAGTGGATGGCGTTTTCAATCT
CTTCATCACTACAGTTCGCACAGGTACCGCGCGGAGGCATCATGCCGGACTTGCCGGTAAAGCCTTCAAT
GGCATGTTTCTTGAGAGTATCAATGCCCTGGGCGATGCGAGGATCCCAGGCTGCCTTGTCACCCCGTTTG
GGGGCACCGGCGGCGCCGGTGTCGTGACAGGCAAAGCAGGCGCCCTTGTATACCGTTTCGCCATCGCGGG
GACCGGAAGAGGCGGCCGCAGTCGGTGCAGCACTCGCGCCGGCAATACCTTCCAGATCCTTGGCGGTATA
GACCTGACCGATTGGTTTGATCCGCTCGGCAATGGCCTCCGGTGACATCTCATCGGCAGCAAGCACTGCC
CCAGACAAGCTGGCGGCCGTCATCCCGACAGCCAGCAAATAGCTCAGTTTTTCAACACGCTCATATACT
CCCAGCAGTCTTTTATTATCCATGTAAACGACGGGATTATAACCGAATAGTTACAGTCATTTAAACGCTT
GTGTGTGAAGTGTTAAACACTCTGCGTACAAAGTTTTGGCCAATATCACGGCAATTTGGGTGGTAGAGAT
AAAAAGAAACCCGGCATTCGCCGAGTTTCGAGTAACCAAGGGGTGAAATGGGTTATTTATCCCAGCTCAG
GATCACCTTGCCGGAGTGGCCGGAGCGCATCGCATCGAACCCTTGCTGGAATTCGTCGATGTGGAATCTG
TGGGTGATGATGGGGGAGAGATCCAGCCCGGACTGGATCAGGCTGGCCATTTTGTACCAGGTCTCGAACA
TCTCGCGGCCATAGATCCCTTTGATAAACAGCCCCTTGAAGATCACCTTGTTCCAGTCGATGGCCATGGT
GGAGGGGGGGATGCCAAGCATGGCGATCTTGCCGCCGTGATTCATCTTGTCGAGCATCTCCTGGAAGGCG
CTCGGGACGCCGGACATCTCCAGCCCCACGTCGAACCCTTCGGTCATGCCCAGCTCGCTCATCACGTCGG
CCAGCTTCTCCTTGGCCACGTTGACCGCGCGGGTGGCGCCCATCTTGCGGGCTAGCTCCAACCGGTATTC
GTTGACGTCGGTGATCACCACGTGGCGCGCACCCACATGGCGGCAGACGGCGGCGGCCATGATGCCGATG
GGGCCCGCACCAGTGATGAGCACATCTTCACCCACCAGATCGAAGGAGAGGGCGGTGTGCACCGCATTGC
CGAACGGGTCGAAGATGGCAGCCAGCTCGTCCGGGATATTATCCGGCAGCTTGAAGGCGTTGAATGCGGG
GATCACCAGATACTCGGCAAAAGCACCCGGGCGGTTGACGCCAACACCAATGGTGTTGCGGCACAGATGA
GTGCGGCCAGCGCGGCAGTTACGGCAGTGACCGCAGGTGATATGCCCTTCACCGGAGACCCGATCGCCGA
TGGCGAAGCCGCGCACCTCCTGACCGATGGCGACCACTTCCCCAACATATTCATGGCCGACCACCATGGG
TACCGGAATGGTCTTCTGGGACCACTCGTCCCAGTTGTAGATATGGATATCGGTGCCGCAGATGGCGGTT
TTGCGGATCTTGATCAGCAGGTCGTTGTGGCCGAGCTCGGGGGCCGGCACATCCGTCATCCAGATGCCGA
CCTCTTTGTGCAGTTTGGAGAGTGCTTTCATGGCTGTTCCTTCAATATGAATGCAGAAAAAAGGGGCTGC
AGCCCCTTGATTCAAATCAGATAACGCCGAGTTCACGGCCGATGCGGATAAAGGCGTCGATCGCCTTGTC
CAGCTGCTCGCGGGTATGGGCGGCAGACATCTGGGTGCGAATGCGGGCCTGACCCTTGGGCACGACCGGG
AAGGAGAAGCCCACCACATAGATGCCGGCTGCCAGCATGCGGCTCGCCATTTCGGAGGCGAGTTTGGCAT
CCCCCAGCATCACCGGAATGATGGCGTGGTCGGCGCCTGCCAGAGTGAAGCCGGCGGCGCTCATCCGCTC
GCGGAAGTAACGGCTGTTCTCTTTCAGACGTGCGCGCAGGTCGTGACCTTCCGCCAGCATGTCGATCACC
TTGATAGTGGCGGCAACGATGGAGGGGGCCAGCGAGTTGGAGAAGAGGTACGGGCGGGAGCGCTGGCGCA
GCCAGTCGATCACCTCTTTCTTGCCGGAGGTATAGCCGCCGGATGCGCCGCCGAGCGCCTTGCCCAGAGT
GCCGGTGATGATGTCGACCCGATCCATCACGCCGCAATATTCGTGGGTGCCACGACCGTTTTCGCCGATA
AAGCCGACGGCGTGGGAGTCATCCACCATCACCAGCGCATCGTATTTGTCTGCCAGATCGCAGATGGATT
TGAGGTCGGCGATGACGCCATCCATGGAGAAGACACCGTCGGTGGCGATCAGCTTGAAGCGGGCGCCGTC
GGCATCAGCCTGCTTGAGCTGGGCTTCCAGCTCGGCCATGTCGTTGTTGGCGTAGCGATAACGCTTGGCC
TTGCACAGCCGCACGCCGTCGATGATGGAGGCGTGGTTGAGGGCATCGGAGATGATGGCATCTTCGGCGC
CGAACAGGGTCTCAAAAAGACCGCCGTTGGCATCGAAACAGGAGGAGTAGAGGATGGTGTCTTCGGTGCC
GAGGAAGGCAGACATCTTCTGCTCCAGCGCTTTGTGCTGATCCTGGGTGCCGCAGATGAAGCGCACCGAG
GCCATGCCGAAGCCGTGGCTGTCGAGTCCCTGATGGGCGGCGGCGATCAGATCCGGGTGGTTGGCCAGCC
CAAGATAGTTGTTGGCACAGAAGTTCAGCACCTGCTCACCGCCGACGGCGATGCTGGCCTGCTGGGCGGA
GGTGATGATGCGCTCCTGCTTGTACAACCCTTCAGCTTGCACCTGGTTCAGTTGCTCGGTCAGGTGACGA
TAGAATCCGTTAGACATCCTCGATCCTTAGTGGCTATTTCAGCTGCGTATCAACAGAGGGCGACATTTTA
CTGCAATCTGTTGCGGTAAATTCAAATGATTGTGCTGATATTCAACAAATGTGAAACAGATCTCACCCAG
CTGGTTGGGCCAGCTGACGGTAGTGCTGTTCCAGTTGCTGGATCTGTCCGGCCACCTGGGGTTGCACATA
GGGGCGCAGCAGGAACAGCACCTTTAATACGCCCTGATAGAGCAGCGGTTTGGTGATGCGGGTGTTGGGG
GCCTGGGCAATCTGGAACGGGATCCAGCAGGTCACCACCATCTTGATAGTCTGGCCCAGATCCGGCAGGT
CTTCATCGCTGACATCTATGATGCCCCCTTCGCGCAGGCTTCTGAGCAGGGCGACCACGGAGGAGCGCAT
CTGCTCCTGGGCCTTGAGGTACTTCTGTTGCAGTGGTTCGTCACGGCTCAGGATGTCCGGCAGATTGGCG
TAGAAGAAGCGGAACTGCCACATCAGATAGAAGATGGCATCCAGATAGCCCATCAGATCCTGCAGGGTGA
TTTCACGGTTGCGGGTCGGGACGAAGCTCTCGCTCAGGTGACGGGCATACTGGTCGAAGATGGAGTGGAT
GATGTCTTCTTTATTGCGGAAGTGGTAGTAGAGGTTGCCCGGGCTGATACCCATGTGCGCCGCAATATGG
TTGGTGGTGATGTTGCGTTCACCCTGATCGTTGAACAGCTCGGTCGCGCTGTGAATGATCCTGTCTCTGG
TTTTCATCACTCATTCCTCCAACAGGTTCCGCTCGGGGTAGTTTTGAACAAGGTCGAATCCCCGGACGGC
CCGTGATAAACTTTTCGGCAATTTCAAATTGGCTCACTATACACAGCTTTAACAAGCAGTCACATGTAGC
CATCAACGTACGAGAGGCAAACTCCATGATCGTAGTAACTGGCGGTGCTGGCTTTATCGGCAGCAATCTG
GTCAAGCAGCTGAACGCTCAAGGACGGACCGATATCGTGGTCATTGATGACCTGACCGACGGCACCAAGT
TCGTCAACCTGGTCGATCTGACCATCGCTGACTACATGGACAAAGACGAGTTTCAGGCGCGCATCGTCTC
CGGTGACGAGTTTGAAGAGTGGGATAACGGCATTGAGGTGATCTTCCACGAAGGCGCCTGCTCTGCCACC
ACCGAATGGAACGGCAAGTTCATCATGGAGGTCAACTACGAGTACTCCAAGGATCTGTTCCACTACTGCA
TCGAGCGTGAAATCCCGTTCATTTACGCCTCTTCTGCCGCGACCTACGGTGGCCGCAACGACAACTTCAT

CGAAGATCCCAAGTTCGAACAGCCGCTCAACGTCTACGGCTACTCCAAGCAGCTGTTTGACCAGTACGTG
CGCCGCTGGATGCCGGAGATCAACTCTCAGGTTGTCGGTCTGAAGTACTTCAACGTCTACGGCCCGCGCG
AGCAGCACAAGGGCTCCATGGCTTCCGTTGCCTTCCACCTCAATACCCAGGTGAAGAAGGGCGAGAATCC
CAAGCTGTTCGAAGGTTGCGATGGCTTCCCGAACGGTGGCCAGATGCGTGACTTCATCTATATCGACGAC
GTCTGCAAGGTAAACCTCTGGTTCTGGCAGAACCCGCAGCACTCCGGCATCTTCAACTGTGGTACCGGCC
GTGCCGAGCCGTTCCAGAACGTGGCCGAAGCGGTGATCAAGCATCATCAGAAGGGAGCTATCGAGTACAT
CCCCTTCCCGGATCACCTCAAGGGTCGCTACCAGAGCTTCACCCAGGCTGACATGACCAAGCTGCGCAGC
GTGGGTTATGATGCTGAGTTCAAAACTGTTGCCGAAGGGGTAGCGGATTACATGGCCTGGCTCAACCGGG
CTTAATTATTCGCTCTTGCGGAATGGCCGCCAATCAGGCGGCTTTTTCATTATTTTCTATGTGGTAAGCA
TGAAGAAAAAAGTTTTATTTATAATGGAAACATTGGGCGGAGGAGGCGCCGAAAAGGTCCTTATTGAAAC
GCTTAAGCGTTTGGATTTTAGTAAGTACGCTATAACTTTGCTTTTATTGAGGCGTGAGGGTGTATATTTA
TCTCAGGTTCCCCAAAATGTAACAGTAAAATTTCTGTTGCCGACCGAACCATCGGAGTGGTTTCAGCGGA
AAATTTTATTTTCTATTAAAAAACGTTGGTGGAATTTTGTTGTCAATACCAAATGGTTGGCCAGTGTCAT
ATTTAGCGAACGATATGATGTCGGTGTGGCATTTTTGGAAGGGAATAGCTCTCTGTTGTTATCGCACCTG
AATTCTGTTGTTCGAAAGATAGCATGGGTCCATATAGACTTGTTGTGCCATCAAATTCTTTCCAGAAAAG
TTGAGCGTACCGTTTATAAAAAAATGGATAACATTGTCTGTGTCTCTAAAGGTGCACAGAATGCATTGTT
GAAATTGTATCCTGAAGTAAAACCATATACTCAAGTAGTGTATAATCCTGTTGATATAGAAGGCATAATG
CGGAAAAGTCATGAGCCTATTACGAGTGAGGGGAAAATAAAAGTTATTGCTATTGGACGGCTATGCAATG
CCCAAAAAGGATTCGACATCTTACTGGCTGCTCATAAAATAAATATTGATGCAGGGGTTAAATATCACCT
TACTATTTTGGGAGAAGGTGATGATAGAGCTGGGTTAGAGTCATTTATCAATATTAATAATATTGGTGAT
AGTGCTAAATTACTTGGATTCAAGGAAAATCCATATCCATATCTTGCTGATGGCGATCTATTCGTTATGT
CCTCACATTATGAGGGTTACCCAGTTGTACTCGTAGAAGCAATGGCGTTGGGGAAGGCCATTGTATCGAC
AGATTGTACTGGGCCTAATGAAGCTCTCGATGGCGGGAAATATGGTGTTCTCGTTACTGTCGGGGATGCA
AATGCGCTTGCATTGGCAATACGAAAAATGATTGATAATCAAAAAATGCTATCAACATATAGTTCCTTAT
CGAAGGAGCGAGCCAAGATTTTCAATCTCCGAGAGAGTATGAGGCAGATCGAGCAATTATTAGATGGGGA
GAGCAAACATCCTTCCAGTTGCTCTCTTCATTCGAGTCTTGTTAACGGAGGTCTAGGGTGAATATATTGA
TGGCACTCTCCCAGCTCGAGGTAACGGGAGCTGAGGTCTATGCCACTACGGTCGGTAACGAACTGACCCG
GCGTGGTCACAACGTTTTTTACGTTTCAGATACCCTGACCAAGCCGACTCTTGGTCTTGTCTTCAAGCTT
CGATTCAACAAGCGCAGCATTCTGCGCCGCTTCTGGCACGTCTTCTACCTTATCTATCTCATTAAGAAGC
ACCATATCCAGTTGGTACATGCTCATTCCCGCGCCTCCGGCTGGAGCAGCTATGTCGCCTGCAAGCTGAC
TGGCACCCCGATGATCACCACGGTACATGGTCGTCAGCCGGTACACGCATCCCGCAAAGCCTTTCATGCC
CTCGGCTATCGGGCGGTTGCCGTTTGTGAGGATATTGCCCATCAGATTGTCGACAATCTGGGGGTCGATC
CTGCGAGCGTACAGGTGCTGCGAAACGGTATCGAGACCGACAAGTTTCAGCCTGTGCCGGCGCCGGACAA
CCCAAAGCCGGTGATCGCCATCATAGGTCGCCTGAGCGGCCCCAAGGGGGAGCTTTGCTACCGCCTGCTC
GACGAAGTACTCGATCTGGATGCCTGCAAGGTACGGATTGTCAGCGGTACCCGCGTGCCCGAGCGTTTTG
CCCGTTTTCAGGATCGTGCCGAGTTTGTCGGTTATGTCGATGATGTGCCTGCTCTGCTGGCGGGGTGTGA
TCTGGTGATCGGTGCCGGTCGGGTGGCGATGGAAGCGCTGCTCTGCGGCCGGCCCGCCTTTGCCATTGGC
GAAGCCAAGGCCATCGGTCTGGTGACTGAACAGAATCTGGATGAAGCGCTGGCCAGCAACTTCGGCGATA
TCGGTCCCAAGGATCTGGCCATCGATTTTGCTGCCCTGAAGGGCGAAATCGAATCCGCCCTCGCAAGCAA
GGTGGTGCCGGAAGCCATTCGCCAACGCATTCAGGATGAGTATGGCTTGCAGGGGGTAGTATCTGGCCTC
GAAACCATCTATCAGGATGCCGTAGTCGAAACACTGCGCCGCGAGATGCCGGTCATCATGTACCACCGTT
TTATCGAGCAGGATAGCGAGAAAGGAGTGCATGGCACCTGGATGCCGATCGCCATGTTCGAGAAGCATCT
GCGATTGATGAAGTGGCTAGGCTACGAAACCCTGACGTTCCGCGATTTGGCGGACAAAGGCTTTATCCAC
CGTCTGCAATACGGCAAGAAGTACCTGATGATCACTGCCGATGACGGCTATCAGGACAACCTGACCCGCA
TGCTGCCGCTGCTGGAAAAATATGGTTATAAAGCCATGGTGTACGTGGTTACCGGTGAAGGCTATAACCG
TTGGGATGTGGAGCATCCGACCAATCCGGATACCAAGGTTTCATTGATGAGTGGTGAGCAGGTCAAGGTG
CTGGCTGCCTGCGGTCATGTGGAAATTGGTGGTCATACTCTGACTCACCCCCGTTTGAGCAAGCTCACAC
CCGAACAACAAGCCCATGAAATTCAGGAGAATAAACGCCAACTGGAGGCCCTGCTTGGCCATCCGTTGCT
CTCGTTTGCCTACCCTTACGGAGATATGAACGAGAGTGCCAAGGAGCAGGCTATCGCCGCCGGGTATCGT
TTTGCCGTCGCTACCAATAGCGGCCCCAAAGCAATGCACCAGGACCCTTTCCAGATCCGCCGGATCGCCA
TCTTTCCCCGCACAGATGTGTTTGGGTTATGGCGCAAAATTCGCGGAAATTATGTCTTCAGGAAATCAAA
GTGATATCAAAAAAATGCCATAAGCATGGAGCGATTTGCTCTTCTGATCTTTTCATGCACCCTGATTTTG
CATTCAGCGTAATGTTTTTCTTTACTGATGGAAAAACATATCTATCTAACCTCATTGTTGTGGCATCCAT
AATTGGCATGGTTTCTTTTTACATCCATCGTTATCCGGTGGGGTTTAAAAATCGTCATATAATATGGGTT
TTATTATTCTATGCCATTTATCTATTTGTAAATAGACAAATTCATGGTGATCAATATGGCATTATGCGTG
CCATAATTTATGTCATATTTTTCACATTTTTAATTCCACGGAATGTTTTTTTATTAAAAGCTGGTTGTTA
TGGGGTTATTCTTGGTGGATTTGGCTTGGGGGTTATTAGTATATGGCAGTATAATCATGGGGTCGCTAGG
GTAGAGGGTTTTACAAATGCGATAATTTTTTCTCAAACAGCCCTAACATTGGCTATTCTCAATTTTTGGT
TTTCGGTTTCTTTCTCAGAGAATCGCCTTGAAAAATGGTTTCATATCATTTCGGCATTATTCTCTCTGGG
GGCACTATATCTTTCACAAAGTCGTGGTGTTTGGCTAGCATTCATGGTTGTGATTACATGTTTTTTATTG
TTTAAAATAAAAACAAAACCACTGAAATATATTTCAATCACGCTAGGTTTGTTGTTAGTACTCGGAATGC

TATGTTTAAACAGCAGTATCGTGAAAAATAGAATAGACTCTGGACTTTCTGATGTTGTAAACATGGAGAA
TGGAAATTACAGCACATCTCTTGGGCTAAGAGCGGTAGCATGGGAAAGCGCTTGGTTGGGATTTCTCGAC
TCGCCATTAATAGGTGTGGGCACTGAAGGTTTTGATGCGTTAAAGCAAGAGCAAGTGAAGCTGGGTCTTG
TGCCACAAGCGTTGCTAAACCCCGAACTTGCGCATGCACACAACCAGTATTTACAGACTTTAGTTATTCG
GGGTGGTATAGGATTCATTTTCTTACTAGGTATACTTATATTGCCGGCAATATTTTTTGCGAAAGAAATG
GGGTTTGTGTCTGCAGGCGTCTTTATCCCTATATCATTCGCTGTCGGAGCAGTTTTTGATGTGCCTTTTG
AACACCAAAGCATGATGTATTTATACTCATTCAGTCTGATTTTTATTTTATTTTGTCATGAACTCAAGCA
GGATTTATTATAGCTATGATACCCGTATTTATTATTAGTTTGACACGTAGTGCTGACAGACGGGCCATGG
TAGAGCGCCAGATGTCTCATTTGGGGATTAACTTTGAGTTTTTCGATGCTGTAGATGGTAAATCATTACC
ATCAGATCGCTTGGCGAAAGTAGATTTCAATCTGGCAAGAGAGACTTGCGGTCATGATCTTAGCTTGGGT
GAGGTCGGTTGTGCCATGAGTCATATCAATATATATGAAGTGATGGTGGAGCGAGATATCCCACGCTGTG
TAATTTTAGAAGATGATATATACGTCCACATGCATTTCAAAGCTATCGTCAACGAGGTCACTAATAAAAA
TAGCTCTGACATTGTATTCTTACATCATGGCAAGGCGAAACACTTCCCAATATACTCATCTTTGCCAGAG
GGGTATCGTTTGGCAAAATATTTAACACCTAGTAAAAATTCAAAAAGAGGAATTATATCGACAGGTGGAT
ATATTCTTACATTGGCTGGCGCTCACAAGCTGCTGAAAATAGCTTATCCAATCAGAATGCCTGCTGATTA
CCTCACTGGACGGCTGCAGTGGAATAAATTGAGTGCCGCTGGTGTGGAGCCGTGCTGTCTGGATGTTGGA
CTATTCCAATCGACGATTGATGACAGAAATTATGGTCAGCATATAGAGTGATGGAGGTGATTGCTATGAT
TAAAATATTTCAATTATGGAGGGACGTCCTTCGCCGCAAGCTGGGTATTTTACTTTTTGATCGTCATTCA
TCAGTGAGAGCGTCATCACATGGTGCCACTGTTTTTGTTAGGTGGGATGCTAAGCTTGGTGATGCAATCG
TCTCTTCCTGGGTGGCAAGGGAGATTAAAAATAAATTTCCGGAGCGGAAAGTCTATGTCATTACCAGCTC
TGAAATGGCGCCATTTTTTAGAGATTTTTTTAACTTTGATTGCGTCTTTGAAATGCCCAAGAGGGCTGGC
TATATAGAGCTCAAAAAATTAGCCAATGAAATTGGGGGGGTAGATTATCTTGTGCATTTTAGCCAAAAGA
TGAAGATGAAAGACCTTTATTTCATCTCCAAAGTTGGGAGCAAAAATGTAGCAGGACTCGATGATGAGCT
TGACTGTATTAATATCAAGTTGGGCCAAAAAACAGCAGGGATGCACTTCGCTAAGAAATTTAAGGTGCTG
CTGGAGCATATGGGGATTGCTAACCCTGATACCAGTTATATAGTACCTTCGTGTCAGGGGGCTGAGGATA
GGGTTGGCTCTTGGTGGCCAAGTAATAAAACGTTGTGTTTCAATCCATATGGCAGTGGGACATCCCGTTG
TTTCAGTGTTGAAAAAATTATAGCGTTAATTAACACAATGCTTGATTCATCAAATTACAACATTTGTCTC
TTGTATCCACCTGGGCGTGAGAGGGACGTTGAACGAGTTTTATCTCATGTTTGTGACCCAACCAGGGTTG
TTAGTGATCATGAGAAACCATCTCTGGCTGCACTCTTTGCACAGACAAGGCAGTGTGCCGGTATGGTGTC
TGTGGATACAGCAACGGTGCATATTGCAACAGGTTTGAATAAACCTGTTCTTGGTATTTACAATGATAAT
TTCGGGATGCCAGAAAATGTGGAGTGGCATCCCAATAATCCTCGCTCAAGTGTTATCTACGCAGAAACTA
GGTCTGCACAACAAGACGTAAATTTTATAAACGTTGATGAATTTAAAACAGTATTTAACAAATGGGTGGC
TGATTATCTATAGAGAGTTGGGGTTTTCCCCAACTCTTTCTCATAAAGATATAAACTGTTCGGTCACACT
TTCACTAGAAAATTGTTGATAACAGTTTGAATCAATGGAAATTCTCCTTCGATCAACACCTGTAATTGCC
ATGGCCAGTCCATGGATATCTTCTATCGGGATTAGAAATTTATCGAGTGGTTTCGTCAATATCTCTGCCG
GTCCAGACGGGCAATCCACGCTGATCACCGGGGTGTGGCAGATAAGTGCTTCAATAAGCACTGTTGGCAG
CCCTTCAGCATCGGATGTCAGGATGAGCGCCTGAGCTGCTTTGATATAGGGGTAGGGGTTCTTCTGAAAG
TCGATGATCTTCACCTTGCTTGCAAGATTCATCTCCTCGATCATGGCTTCAATGTAAGGGCGTCGCTCCC
CTTTGCCCACCAGGACGAGTGGCGTATCAACACCGCTTAGCTGATATGCCTGCAAGAGACGATCATGGCG
TTTCCGCTCTTCGAAGGTGCCAACATGGATAAAATACTTGTCGAATGGCTTGCTGAAAGGCTCATTGGCC
AACGCCTGAATTTTCTCGATATCAAACGGATTGTAGATAACCTGACACGATTTGGGTGTAGCTTTTACAT
TTTGCAAAATGTCGTCCGCGATGCCATGAGATACGCAGATCAGTTTCTTGCCATTATAGAAGCGCTTGAA
TTTTCCATATCCTTTGGCATGGTCTTTGGCAAAGCCGGTGACATTTCCGTGGATCCAGTAATAAATATTC
AGTGTATCAGGCAGGTGTCTGGTAATTTTGTCACATGAGCATGAGATAATTACGTCAGGCGAGATCTCTT
TGACTTTTTTAAAGATCAGAGATGCTTGTATTTTTTCAACCGCAACATTGGATATGGCTTTAGTCAGCTT
TGTGACAATGCCAAGATTAATCGGGTGAATTTTGTCAGGTAGCGTATGGTCTATTTTTTCCCGCAAGGTA
AACAAGGTTACATCTGCACCGGCATCTTTTAGCAATGATGCGGTATTGAGCAAAACCTTTTCCGCACCAC
CACCATACAAGTCTTCAACAATGAATAAAAACTTTTTAACTGGTGGGGTCATAACTGCTCCGCGGGTCTC
ATAACTGACTGGTAAATCTACTAAAAATGTGAATCGTAAACGAGCAAAATCAGGTGGCAACTGTTAAAGT
CGTTAGCAATTTTTTCAATAGGTGTGGATCAATGATTGGTCTTAGTGGGCGCTACCATAACGCATTATTC
GCGGCCTGTATATTTGCCGGGCTGCTGCTTTCCTGGTTGTTTTCCGATCACCAACTCCTCACCGGCGACC
AGACCCAGATGCTTTACAAAGGCTACCTTGGTGCTTATGAGGGGGTCTGGCTCAGCTATGGCAATGCGGC
GAGTGCGGTGGGCAATGTGCCGGGGAGTTTGTCGGCCTGGCTGATTGGTGGGCCGCTGCTGCTGTGGGAT
TCTCCCTATGCCCCCATGGTGTTGCTGCTGGCGATGCGGTTGGTCGGATTCCTGCTGTTTGATGCGGTGA
TCCGTCAGGTGTTTGATGACAGGGTGCGTCTGCTGTTTCTGGTGCTCTGCTGGCTCAACCCCTGGTTCCA
GTACGAGAGTCTGCTCTACAACCCCTCCTATCTGTTCCTCTTCTCGGCCATGCACTGCTGGTCTGCCTGG
CATATGCGCGAGCGGGCCTCCTTTTGGCACATGATCGTTCACCTGCTGGCCATCGGTATGGCGATGCAAC
TGCACTACTCCTGGCCGCTGCTGGCGGTAATGTCCACCTATCTGTTCTGGCGCCGCATTCTCAAGGTGAG
CTGGAGCGGGGTGGTGGTGGCTACCCTGCTGATCGGTGCTTCCCTGATCCCCTACGCCATGGAAGTGATG
AGCAACAGCCATATTACCCAGAACGTCGATCCCGAGGCGCGCCAGCGCTATATCGGCTGGGGGCTGGTGC
ATGTCTATCCGGTGCTGAAATCAGTGCTCTACTGGCTGCGTTACGGCTCCTGGCTGTTTGCCAGCAAGCT

GGTCAATGACACCCAGTTCATCTGGCTGGCGGGGCACGAATATCTGCAGATGGCGGCAGTCTGGCTTTGG
CGGGTGGTGATTTACGGGGTAGGCTCGGCGACTGTGCTGCTGGCGGCCAAGGCCAACTGGCAGCTGTGGC
GCGAACTCAAGCCACGTCTGCTGCGTAGCGACCGCGCGCCAGTGGATGGTGAAAGCTGGCTCGGCCTCTA
TGCGCTGGCGGCGGTGCTGGCGGTGCTGGTGAGTGCGGCGCTCTCGCCCATCATCTTCAACTACTGGCAC
CTGATGCTGATCTTCCCCTACGCTCTGTTCCCGATCCTGCTTTTGTTGGTGCGCTGGAGCCGTCGTCATC
CACAGTGGGTTGGCAAGGGGCTGCTGGCGGCAACCCTGTTCTGCACCGCTGTGAACCTGATCGCGGCGTG
CGACAGCACCAAGTTCTCCTATCAGGCGGACTACAAAACCCTGGTGCTGGAGTATCTGCAAGAGATGAAG
CTGCAACCCAAGCAGTGAGTCATCCAAGGCCAGCTCCCCTATCTCGGGGGGCTGGCTGTCACTATTCGAA
ACGGTCCATATGGGCGAAGTTTTTGGCTTCGGCAACCCAACCCTCACGCCCCTGCTTGTCCCGAATTTTT
ACCCACCACTGGTTCTGCGGTGACCGGCCGTATCTGATCTCTTTGCGCGCATAGTCATAATCGAGAATAT
AGGTTTTGCCCTTGTACCAGAACAGGCTATTGCCTTCGCCCAGTCCGCCGAGGATATAAAGCGTTTCCCC
TGCCTTCATATTCAACAGCGGCCCTTTTCCCTCCTTGCTGTAGCCCAGCTTGACCGATCTGAGTATCTCG
ACAATCCCCGGTTGTTCGGTAATGACGACCCCGGTCAGCCCTTCCACCTGCTGGCCGCGGGGTAGCCGGA
ACAGCGCTTTGGCCGTCAGGGATGGCTCGGTTCTGACGATCACCTCTTGCTTGGTGGTCCACGCCTGATA
GGTACAACCCTCAAACGGGCAGCGACCCCAGTCGTAATAGGGCAGGCGCGGCTTTTCATCAGAACTTACA
GTCGTAGCAAACAGCGACAACAACAGCAAAAACAGTCTCATGAATCTACCTGTCCGGCGGGGCGCTCACC
CGCCGGTTTGATGAATGGGGCCGCACTATATCTCAAGAGACCAAAATCCATCAAAGCCGCGGGATTATTG
ATTTTCACCGCAATAAAACGGGGACCCATTGGTCATTGGAGTAGGGGGCGCAAGAGGTCGAAGCGCGGCG
GGAGCGGATAGCCCGCTCATTTGCCTTGTGCCATGCTGATGGCTATCAGGGCATCGGGTCGGCACCTATG
GGCATCTCTCGTTCGGCTATGCGTTTGTTATTACTGCTATCGGCACTTCTTCTCCCTCTTATGACCATCA
GCCAGGCCGTGGCGATCAACGCGCCACTGCGGGGATGCGTCTTTCAGCTGGCGCCGCTGGTCACCATTGA
TCAGCAGGGCGAGGTCTCCGGGCCGGTGATCGACTACTTTCGCCGTCATATCGAACCCGCGATCGGGCGT
ACCATCGAGTGGCAGGGGCTCAATGTGGCGCGGCTGCTCAGCAACCTCGAGAGCGGCGAGTGCGATCTGA
CCCCGCTGCTGAGTTACAGTCCCGAGCGTGCTCGTCACTTTCGCTATATCGACCCCCACCTGTTGCTGCT
GCGGCCAGTGCTGGTGGTACTGGTGCCATTGCGACTTCACTCGGTCGAACGGCAAGAAGATCTCTTCGGC
ACCACCATCGGCTGGTCGAGTCAGGCGATCGTGCCGCCGCTGTTAAAGCATCCGCAGATCCACATCGACA
CCCTCTCCCAGCTCAGCTGGGAGCATGGCAACATGCAGAAGCTGCTGCGCGGGCGCATCGATGCCGCGCT
CTTCTCCAACGATATTTCCGCCAACCATTTTCGCGCCAAGTTCCCTGCGGCGACCCGCCTCATCTACCTG
CCGGTCACCCCACTGCAGGTGCATATCGTGCTGAACAAGTCGTTGGGGGATGGCAATCCCGAGCTGGTGC
AACAGATGCGTCAGATGGTGGAGCGGGTGCCGCTCGCCTCGCTGGTGGAACCAACCCCGCGGCTGTGGGG
GCGTTGAGAGGGGGTTGCGTGCAATAATCTACAAGTCCAAGGCCCGCTTCTGGTGTATCTATAACCCATG
AATACCACCGATTCCCTTATCCGCTACTGGCAATCCCCGCACCTGCCGGGGGTCGAGCTGTCACACTCCC
ACCACAGGGCGTTCAGCTACGGCCGCCATGTCCATCTCGACTACCATATCGGGCTGGTGCAGCGGGGTGG
CCAGAAGTTTATCCACAAGGGAAATAGCCACCATCTGGTGCGCGGAGTGCTCTCTACCGTCAACCCGGAT
GAGGTGCACGACGGCCTCAGCCTGCTGCCGGAAGGGTATGAGGTGCGGGTCTTTGCCATCGATCCGCAGC
AGCTGGCGCTCTGGCTGCCGGAGCAGCCGGAACCCTTCTTCGACAAGGGATTGCAGAGCCGCCCCGACCT
CTATCAGGGCTTTGCCCAGCTGCACAGCTATCTCGACAACCCCAAGGCCGATAGCCTGCTGATCGAGAGC
CAGCTGCTTGCCCTGCTGGGGGAGCTGCTGCATCTGGCCCCCGAGGTACACCAGCTGGCGGATCCTCAGT
TGCGATTCCTGCGCGACTACCTGATGGCCGCCCTCGACGAGCCCCACTCGCTGGAAGAGCTGGCGGGGCT
GCTCGGGCTCGACCGCTTCGCCTTCCTGCGCCAGTTCAAGAAGCGCAGCGGCATGACCCCCTACGCCTGG
CTGAAACGGCTGCGATTGGAGCAGGGCAAGCGGTTGCTGGCGAGCGGGATGGCGGTGAGCGAGGTGGCGC
TGGCGGTCGGCTTCTTCGATCAGAGTCACTTCCACCACGGTTTTCGCCAGGCATTCGGTCTGACACCCGC
CGAATTTCGTGCCCAGATGCAATCTTTTACAAGCCGCGCCCCCCGCTGATCCGCCATGCTGGCTCTCCGT
CGTTAAAGCGAGAGTTGTTATGCTGACCAGTCTGTTCGTTACCCTCGGCCTGATGCACCTGATTGCACTG
GCCAGCCCCGGCCCCGATTTCGCCCTGATCCTGCGCACCAGCCTGCACCGGCCGACCGCCCTTGGCGCGG
CGCTGGGCATCTCGCTGGCGATCATGGTGCACGCCACTCTGAGCCTGACCGGGATCAGCATGCTGATTGC
GAGCCATCCGCTGCTGTTTATGGTGATCAAGTGCTGCGGCGCCCTCTATCTGGGCTGGTTGGGGTGGGGC
GCCCTGATGGCAGCTCGCCAGCAGAGCAGTACAGTGATGGCCAGCGCCGCCAACGAACTGCGCGGTTGGG
GCAGGGGTGTGCGCACTGGCCTTGCCACCAATCTGCTCAACCCCAAGGCGCTGGTGTTCTTTATCGGTCT
GCTGGCCGCCATGGTGACACCAGAGGTGGATGGGATGACCCGCAGCCTGCTGGTGGGCGAGCTCTTCATC
ATCTCCCTCTGCTGGTTCGGGCTGCTGGCGTGGAGCCTCTCCACCGATCGCGCCCAGCGCCTGCTGCTGC
GGGTGCAGCGGCCGCTCAATCTGGTGACCGGCGTGCTGTTTGCGGTGGTCAGCCTCTCCATTCTGGCGAG
CATGGGCGCCGAGCTGGTGGCCTGACGAGTCGGCGCATGGGCCTATACTCCAGCAAGGTTTACCTGTCAG
GAGTGCCCGGCCATGCGTCTACTGCCTCTTTGCTGGTTGCTGGGGATGACCCCGGCGCTGGCGGCCCCGG
TTCAGCTCAAATTGCTGGAGTGGGGCTATCTGCCAGCGGGTTATGAACAACAGTTTGCCGACTACGCCAA
AGCTCATGGGGTGGAGGCAACGGTCAGCCGGATCGAGCCGCTGCTGACCGATTTCGACAGCGTCTACCGC
GCCCTGCGCACCCGCAGCGCCGATGTGGTGCTGCCGACCAGCTACTTCTATAAGGCCCATCACCAGCCGC
TGTTCAAGCTGCTGCTCCCCATCGATTTCAGCCGCCTGCAGCACTACCCCGAGGTGAAGCCCGCCCTCAA
GAACACCGAGTATGACAAGCAGGGAGAGCGCAACTACTCGGTGCCCTTCTCCTACGCCATGTTCAGCCTC
GCTTATGACAGCAAGCAGGTGCCGCAAGCGCCCACCAGTTGGCAGGTGCTCGCCAACAGCTCGCCGCCGG
GCCAGTTTGCCGAGTTCTGCGACCAGTTCGAACCGCTGCTGTTCTCGGTGCTGCTCTCCCTTGGCAAGTC

GCCCGCCCTGTTTGCCAGCGGCGAGATGCTGGCCGACAAGACCTTGCAGCAAGAGGTGGAGCAGCAGCTC
TCGCAGCGGCTGCGAGCCAGTCGCGGCTTCTGGTTCAATCAGGCCAACCGCTGCGAGGATCTGCGCCAGC
AGACCTTTGCCACCACCTGGGGGTTGGAGCTGGTGGAGTGCAACAAGCAGAACGATCAGCAGTGGCGGAT
CGCCATGGTGCAGGAGGGGGCCATCTATGCCCTCGATGCGATCTCCATCGCCCGCCATGTCGGGGATGAT
CCCGCACGGCTCAAGGCGGCCTACCTGCTGGTGGATTTTCTGCTGAGCGCGCCGCTGCAACGCCAGATGC
TGGTGGGCACGCCCACCATCTCGGCCAGCAATATTGCCTCCCGGCCGCCCCTCAGCCCGCAGGAGCAGGC
GGTGCTGCCGGATCGCCCGCTGCCGGAGTTTGACGAGCGACTGCTGGTGCCCGCGCTGCCCGCCCATATC
AAGAATCGCTACAAACGGATGGTGCAGCAGGCGATCGTCGCCAGCGGCAAAGAGGGACTGGTCAAGCAGT
GCCCCTGGGATCTCGATAACTGATGAATGAGCCTGCCGCTTCTCCGGCGGCAGGCAAGTTTGCGCAAGAT
GGCTGATGATCGGTCATCGCCCGTTCCAGGGCATTCCCCCGTTCAACTCACGCCGCTCGCGGGCCCCCAT
CGTTTGAAAAAACTGCGCGCTTTCCGCAGCCCCATTTAGCCGGATTAAACAACCGAACCCTCGTCAGATT
GCCGTTCATACCCTGGCTGACCCTGTACGAAAGTGCCGCAATTGTGACAACTTGGATGTGAGCTGAACAA
TCCTGATCTGTCCCTTGTGGGAAGGTGACGAATAGGTTATTTCTCTGTGACATTGGCGGCATCCGGCACG
GGTTCTCCAGCCATGTGGTGCGAAATCCACAGCAAAAACAAACAGTAAAATGCAGGAGCAAATATGAAAA
AGGTCGTGATGGTTGCCGTGGCTGCGGCCCTTCTGGGTGGCTGCGCCGTCAAGAATCAGGTATCCCACAG
CTTGCAGGCGATCCCGCTGACAGCCTTCCAGCAGAGCGAGAGCAACCCCAATCAGCGCGTCTACAAGGAG
CCGGGCGTTGATCTGCGCCAATACAATGAGGTGCTGCTCGATCCGCTGCAGTTTATCCGGCAGGAGAACG
GTCAGTGGTATCTGCTGACCGCCAACGAACAGAACCAGATTGGCCGCTACTACCACGACAAGTTCCAGAG
CGAGCTGATCAAGCAGGGCGTCAAGATCGCCTCCGCCCCCGGCCCGAAAGTCATGCGGATCCAGGCCGCT
GTCACCAACTTCGACCTGACCCGCCCCGACCCCAAGCTGCGTGACCTGCTGCCGGCCAAGATCGCCATCA
ATGTCACCCGCGAAGTGATCGGCAAGGAGCCCTATCTGCTCAAGGTGGGCAGCATGGCCCAGCTGCTCGA
CTCCCGGAGCGGCAAGCTGCTGGTGCGTGTGATGGATGCCCGCGAGAGTACGGATACCACCCACAAGGAT
GAGCCCATCACTGCCGAGGAGATGGAGAAGATGATCGACCAGTGGGCTGCCAGCCGCGCCAAACAGCTGG
CGGACAATCTGGGGCGCTGATCGATTGGTGCGTCCAAGCGGGGCGCTGCGGGAGCCTTTTGGGCTGCAAT
AATGGCAAGATGTTGATAAGGTGGTCATTTTGCAGGCAGGAGAAAATGATATGAAACGTGTGTTGTTACC
CATCCTTGTTGCTGGCCTGTTGAGTGCCTGCAGCACCTCGTTACCCACGCCGGAGTGTGCACCGATGGCG
GGTGGCTGGCAGAGTCAGCCGTTGCCCAATGCGGAAGGGGAGGCTGCGCTGGCCAGCGTGCTGGCTCGCA
TGAACACGGCCGCCAAACTGCAGAGCATCCGCGAGGTGCGCAGCCAGGTGGTCTCCGGGGTCAACTACGA
CATCGAGTTCCAGCTCGACAACGGAGAGGTGTGGAACACCCGTGTCTATCGGGATCTTGCCGGCAACTAC
CAGATGACCCGCCCGGCTGCACAGGGCGAGCTTCCTCCCTTGCCTTGCGTCAAGGCCAAGTAATTCCTGC
TTGTTGAGCCCCTCATCAGAGGGGCTCATCTGACCAGAAAATTTCTGCTTTTCTCCCCGCATTCTGACAG
TCAGGATGACCCTTCCCGTGACTGCATCCTCATGGAAAAGGAACCGCTATGAATCGCCACCCCTGCATCA
AACACTGGAGCGAGCTGCAAGAGCAGACGCCGCGCTACTACTCCGGCAGTCAGGAGCCCCTTTCTCGAGG
CTGCGCCCTCGGCAAGCACTTCGGCTTTAGCCGCATCGGCATTCACCACGAGATTGTCGAACCCGGCCAT
CGCACCAGCTGGCCCCACGCCGAAAAGACCGAGGATGAGTTCGTTCACGTGCTGGAAGGGACGCCGGATG
TCTGGCTCGATGGCGTGCTCTATCGGCTGCAACCGGGGGACTCGGTCGGCTTCAAGGCCGGTGACGGGCT
GGCCCACGCCTTTATCAACAACACCGAAGAGACTGTCCGGCTGCTCTGCATTGGCGATACCGATCGGGAG
GACAACCGCATCCACTACGCCGTGCATCCCGAGCGCAATATCACCCTCGGCAAGCTGCACTGGGATGACG
TACCCGAGCGTGAGTTGGGTGATCACGACGGCCTGCCCGACAAGCTGCGCGAGAGCAACGGCCCCTTCTA
GGGCGCAGCTCTTCTGCGCACCAGATCTGCACCACCCGATATAAAAAAGCCTGCCGCGTGGCAGGCTTTT
GCTATCTGTTTGCCGCCTGTGGCGAGCGGCGCTTATTGCAGGTTGAGCCCGCCCAGATAGTCACGCTTGC
CGAGATCGACCCCGGCGTGGCGCAGCAGGGCGTAGGCCATGGTGAGGTGGAAGTAGAAGTTGGGAATGCC
GTACTCATGGACGCAGGTATGGCCATCCATCCCCTTGCCGCCCGCCCAGTGGGGCACGTAACGGCCGCTG
GCGTACTGGCTGTAGTCCGCCTCCAGCTTGCCTTGCAGATAGCTGATGGTCTTGCCGATATGGGCGCGCA
GATCCGCCATGGTTTTGGGGTCATCGCCCATGGCCGGCTGCTCGCTGTGGCTGGCGGTGGCGACCACGGC
GCGGGCCGAATCGCAGCAGATCAGCACCTGCTTGCTGAAGGGCAACATATCGACAATCAGCCGATCGCCG
AAGAAGTTATCCACGTTGAACTGCTTCGCCGCGGCGTGGGCCTCGGCCTTGGCCAGACAGTGATCCAGAT
TGGTCAGCATCTTGATGAACTGGGCGGTGATATCGCGATTCATGAGCACTCTCCATCTTGTTGAAACAGC
GGCCCGCTTGCGCGGCCACGGTGAATGAGACTGGCTAATCGGCCACCAATATACCCCAACCGCTGCCGGG
TTCGAGCCCGCCACGATGAAAAGGGCAGGGCAGCGCTACTTCGCGCGACAACCGTTGGCCAAGGGGCCAT
GCTCGCTTTCTGAACCTGTGAGTCCCTTATCGCCCCTTTTTTGATCTGTGACATACATCACCTTGTTTCA
GGGGCGTAGGCTGAATGCCGTTGTCCTGGACAGGCGCCCGCGCATTCGCCAGGACACAAGAAAAGGAGAA
TGAGATGAAGATCTATGACCGTAACCGCAATGTGCTGAATCAGGGTCAGCGCGTGATGATTGCCGCCACC
GGCGCAGTCGACTTTCTGAAAGAGGCCCACACCGACAATATGACCCCCTATCAGGCGGAGCATGAGAAGT
GCGTGCTGCTGGCAAACTCCGGAGAGCGTTACGCCCCCATCGAGTTGATTAGACTAGGCTGATCCCTTTC
CCTGTCACCCTACTCTTTACAATTCACCTGGCCGCCGCGTCCCATCTGGCGGCCGGGTTTCTGAGTTCCC
CCCTTCATCCCACCAAATGCCGAAAAATTGAGCCGGGTCACACCCCGACCGATTTGAGCGATTGTCAGCC
AGCGCAGGCTCATGCGAACCTTTAAACCCCGTTAACTCACTAAAAAATAACGACTATGTTCAGGAAAATA
ATGATGAGTGCCGCCTTGCTATCGGCGGCGCTGCTTTCCGGTTGTGCTTCCGTACCCATGGCCGATGCCA
CCGCAGATGCGCAGGCCAAGCAGTTTGTGGCGCCCAAGGATGCGGCCAACCTCTATATCTATCGCAACGA
GACCTTTGGTGCGGCGGTGAAAATGCCGCTGCTGGTGGACGGTATGGCGGTCGGCGATACCGTGGCCCAC

ACCTACATCCTCAAGCAGGTCACGCCGGGCAGCCACACTATCATCTCCAAGTCGGAGAACGATGCCACTC
TGACCCTCAGCACCGAGGCAGGTAAAAACTACTACGTCTGGCAAGAGGTCAAGATGGGCCTGCTGATGGC
TCGCTCCAAGCTGAGTCAGGTTAGTGAAGAGGAGGGCAAGCAAGGGGTGATGGAGAGTAAATTAGTTAAA
CAATAAGGAAATTTTCCAACTGAAAAATTTAAGGAGTAAGTGACATGGAAGTCTCATTCAATATAAATGA
TGTCAATGATCATATTGTAAAGTGTATAGAGAAAGGTGCATCTGCTGTTGAAGTAATAAACAAGATCTAT
ATAAATAATACTTTCCTTGGGAAGGAGTCTGTTTTATGGGATTATAAATCTGAATTAGTTGCTGATGACG
TATCTCTTTCTAAAACTATTTGTCAAATAGCCTGCTTCCATAATAGCTATGGTGGCTATTTGATTTATGG
GGTTCAAGAAACAGTTAAAGATAAAAAGTTTTCGCCTGTTAAGTGCGATTTAAGTAAAATTAACACGGCT
CAAATACGAAGCTTAATAGAATACTATCTGGACACTCCGATTGACTTTACATTTTCTACTACAACTATCT
CATATGATGGAGATGAGTTTGATATTGGTGTTATTTATATACCCAAGAGAGGTATAAACAATAGAATTCC
AGTTAGATTTATTAGGGATGCGAAAAAGGATAGAAAAAACAGAGATTCAAAACCAGTTTTTCTTGATAAT
GAGGTGCATTTTAGGCGTTTGGATACATGTATCAAAGCGGAAACCCCACAAGATTGGCAGTTTTTATTTT
CAGATAGAGTATTGAATCAGTTCAAGAGCAACAGAATAAGTATTAGTGTTGAGCATAACTTGCCGGATAA
AAAAATGATTTGTGCTGATTTTTTCGGTCGCAAGGACATACTTAATATGCTGTGGGAGTGGTTTTCTGAT
CCTTTTGAATATACAAAAATACTGGCTGGTGATGGAGGTAAGGGGAAAACATCTATTGCCTATAAGTTCT
GCCAAGAATTTATAGAGTGCCCTCCAGTTGGATTTGAGAGGGTCGTTTGGCTCAGCGCAAAAGAAAAGCA
GTTTTCAGGAATTGAGAATAATTACTTTGATTTGCGGCAGATAGATTTTTATGACTACAGTAGCTTTTTA
AAAGCTCTTTCAGACTTTTGTGCAATAGACACAGATGAAATAAGTTCAACTTCTGCTCATAATATAAAGA
AAAAACTTAGAATGGCGCTCCCTATATTCCCGGGGTTGATCATTGTTGATAATATAGATTCATTAGCACA
CAATGATCAATTACAAGTTGTAGATGCATGCAGACAACTAGGTAAAGATGAGAATCGATTCTTAATTACC
ACTCGCAATAGATTCTCATATTCTGAAGATGCTTGTATTGAAATTAGTGGTTTAGAACGCAGTGATTATG
AAGACTTCTTGAATGCAAACGCCACAAAATATGGATTGCCTGTACCAAAGAAGAAACAGATAGATAGTAT
ATATATAGCTACTGATGGTTCACCGTTGCTTACGCAGTCTATTCTTAGGTTATGCAAGCTTGGTGATAAT
TTCGATGTTGCTGTATCTGATTGGAAAGGGCAGGCAGGGGAGGATGCAAGAAATGCCGCATTGCAGAGGG
AAATTCAAGGGCTTACATTTGATGCCAAAAGAGTAATTCTTTGTATCTTTTATTTTGATAGTTGCTCTAA
GTCTGAATTACAGCAAGCATGCGGGTTAGGAAAAACAAAGTTAAACGATGCTTTACAAGAACTTCAGTCT
CTTTTTTTAGTGAATGCACCAAGGTTCATTGATAATGAAGACCGGTTCTCAATTTCCACAACAACTAAGT
TAATTGTTGATAGTATTCAGGATAACTTAGCGACAGATTATTCAAAGTTAAAAAAAGAGATTCAAAGGTC
TAGGTCTGGTTTAAATGCCTCTGGCAAAAAAGGGAATGTAAAAAGAATTGGCTTTGCAATTAGTCAAGCA
TTGGCGTTGATGAAAGAAAAGAGAGAAGTAGAAGCAATAACTACAATAACAAATGAATTGAAGAGGCAGC
CAAATAATCCAGATCTATTACTGGCTCATGCAAGATGTATTATTCAGTCAGCAAAACCAGATTATGAGAC
AGCAAGGAAAATATTAAAGAGCTCTTATGATAATGGTCAAGACAAGGAGATGTTATTTGACTTTTGGTAT
AATTGTGAAGGTTATTTAGGCTCAATTGCAGGAGAAATTGAGGTATCTAAGCTAGCTATAAGTAAAAATA
AATTCAAAGAGTGTAAATGGCTTTATTATTTGGCTAAAAGTCTGGTTATTCGTTCTTCATTACGAAGTGG
GCTTGATAAGATAAATGATCTTATGGAAGCCAGCTTGTATCTGTCTCAAGCTATTAAAGATCCACAAAAC
AGGAATAGGGATGAGCAGAAATTGGAATCGAATGAGATTCATAACATTATATGGCGACTATTGACGAGTG
ATGTTTCTATTAGCTGGGCTCAATCTTTTGATCATGTTATGAACATTATTGAGCATGGTGACCGAAGGAC
AAATATGTATATGAATGCTCATGAGTGTATTGTAAAGGCTGAGGTCGATGCTAAGACAATTAGAGCAAAG
ATGCACATTGTGAAGTTAAAAGAGAAATTTAATAAAGCAGTTGAGTTAAGAGGGAATATGAATGGTGTGT
TGCGTGAGCTTATTTTCGATATTCCAGTGGTTTATAGTTAGACTGTAGAACAAAAAATTGAGATAGACAT
ATCAGCCAGCCAGACTAAATCGTCTGGCTGGTTTCCTCATAAAAGAATATGAAACTAAAGCGCTTCCAAT
CTGGCGTAGGCCGTCACCAGCCACTTGGCGCCGACGTCGTCGAACTGGATCTGCACCCGGCTCTGCTGGC
CCACACCTTCGAAGTTGAGTACCACCCCTTCACCGAATTTTGGATGGAGCACCCGCTGGCCAAGTTTGAT
GCCGCTGGCGTCAAAGCTCTGCTGCACCTCGTTCTGGCTAAAGCGACCGTACTGGGTGGGCGGCTGACC
TGAGTGCGCAGCCGGATCTCTTCCAGACACTCGGCGGGCATCTCGCGGATAAAGCGGCTCGGCTTGTGGA
ACATCTCGCGGCCGTAGATGCGGCGGCTCTCGGCATAACAGATGTAGAGTTTCTCCATGGCGCGGGTCAT
GCCGACGTAACAGAGGCGGCGCTCCTCTTCCAGCCGGCCCGACTCCTCGGTGGATTGCTGGCTGGGGAAC
ATGCCTTCTTCGACGCCGACCAGCAGCACCAGCGGGAACTCCAAGCCCTTGGCGCTGTGCAATGTCATCA
GCTGCACCGCATCGGCATATTCGTCCGCCTGATTCTCGCCGGATTCCAGCGACGCGTGGGCGAGGAAGGC
GGAGAGATCGCTCATATCCTCCAGCTCGTCCGGGCGCTGGTACTGGCGGCAGGCGGTGACCAGTTCGTCC
AGGTTCTCTACCCGCGCCTGGGATTTCTCGCCTTTTTCCGCTAGATACATCGCCTTGAGGCCGGAGTTCT
GGATGGCGATATCCGCCTGCTGGTGCAGCGGCAGCATGGCGACCTGCTCCTCCAGCGCGTCGATCAGCTC
GACAAAGCCGCGCACCGCGTTACCGGCGCGACCGGTCAGCACCTTGTCGTTGAGCAGGGCCTTGGCCGAT
TGCCACAGGTTGAGCCCCTGATCGCGGGCGTTGCCGCGCAGGATCTCGAGGGTGCGATCACCGATGCCGC
GGGTCGGGGTGTTGACCACCCGCTCGAAGCTGGCGTCGTCGCCACGGTTGTTGATAAGCCGCAGGTAGGC
CATGGCGTCTTTAATTTCCTGCCGCTCGAAGAAGCGCAGGCCGCCGTAGATGCGGTAAGGCATGGCGTCC
TGCATCAGCGCCTCTTCCAGCACCCGCGACTGGGCGTTGGAGCGATAGAGGATGGCGCAGTCGGCCAAGA
GGCCGCCCTTTTCCTTCCAATCCTTGAGACGGCCGACCACGAAGCGGGCTTCGTCCACCTCGTTGAAGGC
GGCGTAGAGGGAGATCGGCTCGCCCTCGGCCCCTTCGGTCCACAGCTCCTTGCCAAGGCGCTCGGCGTTG
TTGGCGATGACGCTGTTGGCGGCTTTGAGGATATTGGCGGTGGAGCGGTAGTTCTGCTCGAGGCGGATGG
TCTCGGCGCCCTGATAGTCGGTCAGGAAGCGCTGGATGTTCTCGATTTTGGCGCCGCGCCAGCCGTAGAT

GGACTGGTCATCGTCGCCGACGATCATCACCTTGCCGCTGTTGCCCGCCAGCATCCGCAACCAGGCGTAC
TGGATGCCGTTGGTATCCTGAAACTCGTCCACCAGGATGTTCTGGAAGCGGTCGCGGTAGTGCTCGAGGA
TGTGCGGCTTGTTCAGCCACAACTCGTGGGCGCGCAGCAAGAGTTCTGCGAAGTCCACCAGACCGGAGCG
ATCGCAGGTCTCCTGATAGGTCTTGTAGATCTGCTGATAGGTGCGGGTGACGGGGTCGCCGTAGAGGTCG
ATGTCGCCGGGGCGCAGCCCCTCATCCTTCTTGCCGTTGATGTAGCCCATCACGGCGCGCGGGGCCCAGT
GCTTCTCGTCCAGATTGAGCGCCTTGAGCACCCGGCGGATCAGCCGGTACTGGTCGTCGGAGTCGAGGAT
CTGGAAATCCTGCGGCAAACCCGCATCCAGATGGTGGGCCCGCAGCAATCGGTGGGCGATACCGTGAAAG
GTGCCGATCCACATGCCGCGCACGCCGTCGCCGATCACCTTCTCGACCCGGCCGCGCATCTCGGCCGCCG
CCTTGTTGGTAAAGGTGACCGCGATGATGGAGAAAGGTGAACAGCGTTCCACCTGCATCAGCCAGGCGAT
ACGGTGCACCAGCACCCGGGTCTTGCCCGAACCGGCACCGGCCAGCACCAGCAGGTTGCTGCGGGGCGCC
GCAACGGCATCTCTTTGCTTGTCGTTGAGCCCGTCGAGCAGGGTTGAAACGTCCATCACCACTCCTGTTT
ATTTATACAGGCGGGGATTATACGGCCATCCCCCCTGCCAGACCAAATCCAAACTCGGGGGGAGGGGCGA
GATGGGGTCTTGCAGGGGCGCCGGTGCGGCCATTTTTGGCTGGCGCAGAGAGAACGGGACGGGGAGGTTT
CCAGCTGTAGCCAACTCTTGCTCACGCGGTTTGTGGGGATTTTGCGAGCCGGATCACGGGCTTGCCGGGG
GGCTGGATGAATAAGAGGGGAAAACGGTTTACAGTAACTTGCTGAACAATAAGAGAAATGTCGTTTGGGT
CTTTTGGTTGTTAGCATAAAAAACTGAAAAGTAGCTAAAAAACAACTGCTTATAAGGTTATTTGCTTAGA
ATTCAAACCGTTAGCGCAAACGTTTTTCCCTTATCATTTGCTTGACCCGCATCGGGGCTGACTTAGAGTA
AGCCTGCGTGCTAGATAGCCTGCTCTGACAGGCAAGCAAGTTCCCTCACTTAATAACAATCAAGGATACG
ATCGTGACTAAAGTGCTCAAGCTGGCCACTGTGGCCTCCCTCACTCTCGCTGCTCTCTCTGCTCAGGCTG
CTTCCGAGCCAGCCGTCATTTATGACACCGCGGGCAAGTATGACAAATCATTCAACGAAGCCGTGTTCCG
TAACGGCGTCGAGCTCTACAACAAGGACAAGGGCGTCAAGGTCAAAGAGTTTGAACCCCAGAACGAAGCC
CAGCGCGAGCAGGGTCTGCGTCGTCTGGCCAGCCGTGGCAACGGCCCGATCGTGGCAGTCGGTTTCAACA
TGGGTTCCGCGGTAGAGAAGGTTGCGACCGAATTCCCGAAAACCCAGTTCACCATCATCGACATGGTGGT
CGACAAGCCGAACGTCCAGTCCGTCATCTTCAAGGAGCATGAAGGTTCCTTCTTGGTGGGCGCACTGGCT
GCCATCGCTTCCAAGAGCGGCAAGGTCGGCTTCGTGGGCGGCATGGATATCCCGCTGATCCGCAAATTCC
AGTGCGGCTACGAGCAGGGTGCCAAGTTCGTCAATCCGAAGATCGAAGTGTTCCAGAACATGGCGGGCTC
CACCCCGGCCGCGTTTGCTGACCCGGCCAAAGGTGCCGAGCTGGCCAAGTCCCAGTTCGCCAAAGGTGCT
GACGTGGTTTACGCCGCAGCTGGCGGTACCGGTATCGGTGTCTATCAGGCAGCCAAGGACAGCGGCAAGT
TCGCCATCGGGGTGGACTCCAACCAGAACCACCTGCAACCGGGCACCATGCTGACCTCCATGGTCAAGTC
TGTGGGTCTGGCCGCTTACCAGACCTGGGATGACGCTGCCAAAGGCGAGTGGAAGCCGGGTATCAAGAAC
CTGGGTCTGGCCGAAGGCGGTGTGGATTGGGCGCTCGACAAGAACAACGAGAAGCTGATCACCCCGGAAA
TGAAAGCCAAGGTGGATGCCATCAAGGCTGAAATCATCGCTGGCAAGGTCAAGGTTCATGACTACATGAG
CGATAACACCTGCAAATACTAATTAGATAGTTGCTACGACTCTCAACGCACCACCGGCCCGCACGGGCCG
GTTTTTAGTCTTATTTCATGAAACGCAGGGCTGCATCATGGACCAGACAGATCGCTATGCCATCGAATTA
AGAGGTATCGATAAGCGGTTCGGCGAAGTGTACGCCAACAAGCTGATCGACCTTAAGGTTCGCAAAGGCA
GTATCCACGGCATTGTCGGCGAAAATGGCGCCGGTAAGTCCACCCTGATGAGCATCATTTATGGTTTCTA
CCATGCCGACAAAGGCGAGATGCTGATCGACGGGAATCCTTTCAAACCCCATGGCTCACAAGATGCGATT
GCCGCAGGCGTGGGCATGGTTCACCAGCACTTCATGCTGGTCAATAACTTCACCGTATTGGAAAACGTCA
TCCTCGGCGCTGAGAACGGCTGGCACCTTCAGCAGAGTGTGGCGTCAGCCGAAAAGTTGCTGGGCGAACT
GGCGCGAGACTATGGCCTCGACGTGCCGCTGCACGAGAAGGTCGAAGATCTGCCGGTTGGCTTGCAACAG
CGGGTCGAAATTCTCAAGGCGCTCTATCGCGGCGCCCGCATCCTGATCCTCGACGAGCCGACCGGGGTAC
TGACCCCGCAAGAGGCGGATCACCTGTTCGAGGTGCTGAAAAAGCTGCGGGATCAGGGAGCGACCATCAT
CCTCATCACCCACAAGCTGCGGGAGATCCTGGCCATTACCGATCAGGTCTCCATCATGCGCCGTGGCGAG
ATGGTGGCGCACGTCGCCACCAAGGATACCGACAAGGAGCAGCTGGCCGAGCTGATGGTGGGCCGCAAGG
TGCGCCTCAAGGTCGACAAGGGGCCAACCCAGCCCGGTGAAGCCAAGCTTAAGATCGAAGGGCTCAGCTA
CTTCGACGCCAGCAAGGTGGAGCGGGTCAAATCGGTCAGCTTCGAGGTGCGGGCCGGTGAAGTGGTCGGC
ATCGCCGGGGTCTCCGGCAACGGCCAGTCCGAACTGCTCAGCCTGCTTGGCGGCATTCTCAAGCCGAGCA
AGGGTGGCTTCACCATCAGCAGCCGCAGCGGCGTGCACAAGGTGAGCGCGGAGCACCCGGCCGATCCCGA
GCTGGTGCGCAACTACGGCCTCGGTCATGTGCCGGAAGACAGGCACAAGATGGGGCTGATCAACCGCTTC
GAGGCGAAAGAGGCCTTTATTCTCGGTTATCACCGTCGCCCGCAATACAACAAGGGGTGGCTGCAGAACA
AGGAGGCGATCCAGCAGGATTGTCAGGCCAAGATGGAGAAGTGGGACGTGCGCCCGCCCCATCCCGATCA
CAAGACCGCCAACTTCTCCGGTGGCAACCAGCAGAAGCTGGTGATCGCCCGTGAAGTGGAGCAGGACCCG
GATGTGCTGTTGATCGGTCAGCCGACCCGCGGGGTCGACATCGGCGCCATTGAGTATATCCACCAGCAGA
TCATCGCCATGCGTGACAAGGGCAAGGCCGTCTTGCTGGTCTCGGTGGAGCTGGACGAGATCATGAGCCT
CGCAGACCGGATCCTGGTCATTGCCGATGGTCGCATCGTCGGCGAGCTGGATGGTGCCAAGGCGGATGAG
CGCACCATTGGTCTGATGATGGCAAACATAGTGCCGGACGAAGTTGCCAAGGAGGCCCACTGATGAGCCA
AGTACGTATTCCTGCCTGGGTCTCCGTAGGCGTCTTGTCTGCGGTCAACATCCTGCTGGCCTTCCTGGTG
TCGGCCATTCTGTTCTACTACCTCGACATCAACCCGGTCGATGCCGCCAAGATCATGTGGTACGGCGCGT
TCGGCACCGGTGAAGGGGTCGGCTTCACCCTCTACTACGCAACCGGCTTTATCTTTACCGGTCTGTCGGT
GGCGGTCGCCTTCCACGCCGGTCTGTTCAATATCGGTGGTGAAGGTCAGGCCTATATCGGCGGGCTCGGT
GTCGGTCTGGTCTGTCTGACGCTCGGCGATGTGCTGCCGTTTGCCCTGCTGTTGCCGCTCTCCATCATTG

CCGGTGGTCTGTTCGGTGCGGCCTGGGCTTTTATTCCGGCCTGGCTGCAAGCCAAGCGCGGCAGCCATAT
CGTGATCACCACCATCATGTTCAACTTCATCGCCGCCTCGCTGATGGCCTATCTGCTGGTGGAGGTGTTC
AAGCCGCCCGGTTCGATGGCGACCGAGAGCAAGGTGTTTGCCGAGGCAAGCTGGCTGCCCAAGATGAGCG
GGATGGCGAGCCTGTTCGGGATGGAGATCCCCAACAGCCCCCTCAATATCAGCTTCTTCTGGGCGCTGAT
CTGTGCGGCGCTGGTGTGGGTCTTTATCTGGCACACCCGCTGGGGTTACGAGATCCGCTCGGTCGGCGCC
AGCCAGAGTGCCTCCGCCTACGCCGGTATCTCCTATCCCAAGGTAGTGATCCTGGCCATGGTGATCTCCG
GCATGCTGTCGGGCTTCTTCGCCCTCAACGTGCTGCAGGGTGAGCTGCACCAGATAAAGCTCAACTTCGT
GGAAGGGTTCGGCTTCACCGGTATCGCGGTGGCGCTGATGGGGCGCAACCACCCGGTCGGCGTCATCATC
GCCAGCCTGCTGTTCGGTTTCCTCTATCAGGGGGGCGCCGAGCTCAGCTTCGAATTCGGTGTCGACCGCA
ACATCGTCGTGGTGTTGCAGGGTCTGGTGATCCTGTTCTGTGGCGCCCTTGAGCACATGATGCGTCCGCG
CATCGAGCAGCTCTATCTGGCGTTTGCCAACCGCTCTGCGCAAGTCAAGGGGGCCTGATCATGTTTGAAA
TTCTGATCCTGATGCTGGATGCCACCATCCGTACCGCGCCGCCCCTCATTCTGGCTGCCATGGCCGGCAT
GTTCTGCGAGCGCTCCGGGGTGGTCAACATTGCGCTGGAAGGCAAACTGCTGGCGGCGGCGTTCGCCAGT
GGTGCGGCGGCTGCGGTCTCCGGTTCCGCCTGGGTGGGACTGGGTGCCGGGATTGCCATCTCGGTGCTGT
TTGCCCTGATGCACGGCTTTGCCACCATCACCCACCGCGGTGATCAGGTGGTGAGCGGCATGGCCATCAA
CATTCTGGCGGCCGGTCTGACCGTCACCCTGGGCCGTTACTGGTTCGATCAGGGTGGCCAGACCCCGGCG
CTCTCCGGTGATGCCCGTTTCGCCCCCATCGATCTGCCCTATGCCAAGGAGCTGTGGGATGTGCCGGTGA
TCGGCCAGCTCTACAGCGAGCTGCTGAGCGGCCACTCCCTGCTGGAGTACGTGGCCTTCGCCTGCGTGCC
GCTGGCCTGGTGGGTGATGTTCCGTACCCGCTTCGGTCTGCGTCTGCGGGCGGTGGGCGAGGCGCCCGCA
GCGGTCGATACCGCCGGTATCTCGGTGGTGCGGATGCGCTACACCGCGCTCCTCATCGGCGGTCTGCTGG
CCGGTATCGGTGGCACCTATCTGGCGGTGGCCCAGACGGCCCAGTTCATCCCCAACATGAGTGCGGGCAA
GGGCTTTATGGCACTGGCGGCGCTGGTGTTCGGCAAGTGGCGTCCCTGGAATGCCATGGCCGCCTGTCTG
CTGTTCGGCTTCCTCGATGCGGTCGCCATCCGCCTGCAAGGGGTCAGCATCGGTGGCTTCGCCATTCCGG
TGCAGGCCATCGAGGCACTGCCCTACATCCTGACCGTGTTCCTGCTGGCCGGTTTTATCGGCCGCGCGGT
GGCACCCAAGGCACTTGGTACACCTTATGTGAAGGAGCGGGAGTAACGCTCTTAAGTGCGATATTCCCGA
TCACACAGATAACAGCAGGCCCACTTCGGTGGGCCTGTTTTTTTATGGGCGGTATGGGGCAGGCCAGCCG
GGAGCTGGATGGTGTGATAGAGGGAGGCGATGGCGTGAAGGGATAAACGGGCTGCGCGATCTGCTGCTGG
TCGTGGTTGGCGTACAGGCGCTGACCCCCTTGTCAGCTCCATCAAAAACCCCCTCGTATCAGGAGGGGGT
TGCAGAGGTTATTGCCAGATTAGCCCTGAGGATCAGGGGGCCAGCACCAGCTTGTAGACGCCAAAGCCGG
GGTGTTTGGCATCGGTGGGCGGCAGGGTCTCCACATAGTTCAGCTTGCCACCGGTCAGGGCTTGCGCCTC
GCTGCGTGGCGAGCTGTAGATGCGCAGATCCAGATTGGCGGGCAGGGGGGCCAGCTGCCAGTTGTTGTCT
GCCGGGGGCTCGAAACCGGCTGGATTCTTCTCTGCCAGATCCTTGAGGTATTCGGAGAGCACTTCGCGGG
TTTCGAACTGATCTTCATGGACGATCACCTTGGCGTTGATGCCCGGGAAGTTGCCACCACCGCTGGCGCG
GTAGTTGTTGGTCACCACGTAGAACATCTGGGTCGGGTCGATCGCCTTGCCCTGATAGGTGAGGTTGGTG
ATGCGATAGCTGTCGCTCACCTTGTTGCCATCCACATCGTAACGGGCGGGTTTGGTCACATCGACCTGAT
AGTTCACCCCGTCGATCACGTCGAAGTTGTAGGTGCGGTACTTCTCGTTCACTAGCCACTGGGTCGCGGT
GCTGGCGGCATCGATCTTGTTGAACTGGACGGCACTCTTCTCCAGCCACTCCTGTACGGTCGCGCCATTG
ATGGCGACCACCTGCAGGGTGTTGGGGTAGACATAGAGGTCGGCCACGTTGCGCAGGGAGATATCGCCAG
CCGGTACATAGGTGAAGTCGGTCGGGCCGTTGCGACCGCCACGGAATGGCGCAGCGGCGGAGAGGATGGG
CCATTTCTCCTTGAGCTGACCATCCTGTTGCAGTTTCTTGGCGTGCCACAGCTGGGCGTCGCTGACGATC
TGGATGGAGGGGTCATCCTGCACCAGCGCGAAGAAGCTGTGGATCGGCTTGGTGATCTTGCTGAGGGGCT
CGTCCAGCCACTGGTTTGCCTGCTCGTGCTCGGCAATGACCTGATCGACCACCGCCTGATCTTCGGTGCT
GCTGGCGCTACTGTCGATCTTGCGCAGCTGGGACTGGCTGGAGGCCACTTCCCAGCGACCCTCTTTCCAG
GTCATGTGCAGGTCGATGATGCCGAGGTGGTTACCCCAGACGCCCGGCATCACGGTCGGTACGCCCGCCA
GGGTGCCTTTCTCGTTGTCCACGTCGGTCATGTTGGGGTAGTCACCCGGGAAGGTGCGGTGGGCGTGACC
CAGCAGCAGGGCATCGACCCCTTTTACCTTGGCCAGCGGCAGGGCTGCGTTCTCCATCATGGGTTCGCGC
GGGTCACCAATGATGCCAGTGTGGGCCACGACCACGACGATGTCTGCGCCCTTGGCGCGGATTTCGGGTA
CATAGTGCTCGGCGGTCTCGACCATATCGGCCACCACCACCTTGCCTTCCAGATTGCGCTTGTCCCACTG
GGTGATCTGGGGAGGAGTCAGCCCCAGCACACCGACCTTGAGGGTCTGGGTAGTGCCGTTGGCATCCTTC
ACCTGACGATCCAGCACCACATAGGGTTTGAACTTCTGCTTGCTCCAGTCGATGGTGCCCTTGCCATCAC
GGCTGAAGGCATCGGCCTGGAAGACGTTGGCGTTGATGTAGGGGTAGTTGGCCCCTTTCAGGGTCTCTGA
GAGGAAGTCGAGACCGAAGTTGAATTCGTGGTTGCCCAGGTTGCCGACATCATAGCTCATGGTGTTGAGC
GCCTTGATGGCGGGGTGGGTCTGCTTGCTCAGCCACTGCTGTTTGACCTGCTGGTCGTGGATATAGTCGG
CCAGCGGGGTGCCCTGGATCAGGTCGCCGTTGTCGATCAGCAAGTTGTTGGCGTTCTCTTCGCGGGCCTT
GCGGATCAGCACAGCGGTGCGGGCGAAGCCCAGCGAGGCATCCGGCTTGTTCTGGTAGTAGTCGTAACTC
AGCAGGTTGGAGTGGATATCAGAGGTTTCGATCAGCCGCAGGGTGAGATCACCGGGGGCCTGAGAGTTGT
TGTCATCACAGCCGGATAGCAAAAAGATAACTGCCACCGCGCTCAGTTTGAGGGTGGTTTTCATTGTTGG
ATTCTCTTGTTATGGGTATGCCAGCGTGGTGCATTCTATCGACAGCAAAAGAATCCGATTGTGAGCAGGC
TGGCAATTCGGGCTAATTACCCTCCTATTAATAGAGTTATGACTCTGATTTTCATCTGTTTTTTCGGGTC
GCTACCCTCGCTGCTTTTCCGCCGTTAGTCACCCTGTCGTCACCGCCGTGCGGTCGGGGCAAGCGGCACG
GCCAAGCGCTTCTGCGAGGCTGTAATGGTTGGTTACAGATCTCCATTCGACTGATAGATGACCTTTCCCT

TAAATTCAGCGGCGATTGATTGCATCTAAAAAATTACAAAGCGACAGCAAGGAATAAAAACTCATGAAAA
TGCACAACAAAGCGCTGCTGGCCATCGCCTGCGCTGCGGCCCTGTCTGCCTGTGACAGCGAAAAAGCTGC
CAACAGTATCGCCAAGCGGGTGCCGCTGGCTCTGCCGGAGGCGGGTCTTTACCAAGCGAATCTGATGAGC
CGCGATGGCGACAAGGCGACGCCGCGCATGATCAAGGATCTGGATGGTCTGGCGCTGGTCTACCCGAAGG
GTGAAACCGTGCAGTACTGGGGTGTCTGGGTGGATCATCAGGTGGGCAAGGTCGAGACCAACAGCCAGTG
GCTGGGTCAGGCTGACCAGAAAGCCGACAAAGACGGGATCTACCCGGTGCAGTTGATCCGCAACAGCGAA
CGGCTCGGCACCAGCACGGCGCTCAGCTCGGCGACCAACGACCACAACCTCATCACCTTCCAGGATCAGC
CGGTCATCGATCTGCAGGGCAAGGAGATCAAGCGCTGGGTTTTTGATTTCACCCGGACCGGCACCACCTT
CTCCGACAACTCCCCCATCTATTCCGGTTTCAGCGGTCATGTGGCGGTGACAGCCCTGACCACCAAAGCG
GTAACCACGGCCAGCTGGAGTGCCACGGACAGCGATGGCTTCAGCAGCGACATGGTGGGCAAGGTGGATA
CCACCAACAACGGCGGCAAGCTGACGGTAGCGATCGAGTTTCCGGCGGCAGGTTGTACGCTGGTGGGCGA
GGGGAGTGCCACTGCCGGGTTGAGCAAGCTGACCATGACCGGGTTTGGCAAGTGCAACTTCAAGCAATCC
GCTGCCGCCACGCCGATCGAAAACCTCTGGAATGCCGCTCTGGCCCGGGCGATGGATAACCGCGTCGCCT
ATGTCACCACCTTTACCACGGATGCCAAAAAAGAGGCGCTGGTCATCGGCTTCCCCGACACCAATGGCTT
GCTGATCACCGCTGACAAGCGATAAGCAGCCCAAAGGCAGGGGTGGGCAAACGCGCCTGAAACAACAAGG
GAGCCAGTGGCTCCCTTGTTGTTTTCAACGCACGGTTTACAGCAGGAACAGGGTCGCCAGCCCGAGGAAG
ATAAAGAAGCCGCCGGTATCGGTGATGGCGGTGATCATCACGCTGGCGCCCACGGCCGGATCGCGGCCGA
GCCGGTTCATGGTCATGGGGATAATCACCCCCATCAGCGCCGCCACCAGCAGGTTGAGCACCATGGCAAG
CGTCATCACGGCGCCCAGCGCCGCATCCTGATAGAGCAGGAAGGTCGCCACCCCCATGGTGCCGCCCCAC
ACCAGCCCGTTGATCAGCGCCACCCCGAGTTCGCGCAGCAGCAGGAAGGAGAGGTTGCCCGCCTGAATGT
GCTGCAGCGCCAGCGCCCGCACTATCATGGTAATGGTCTGGTTGCCGGTGTTGCCGCCGATCCCGGCGAC
GATTGGCATCAGGGCCGCCAGCGCCACCAGTTGCGAAATGGTGTGCTCGAACAGGCCGATCACCCGCGAG
GCGACAAAGGCGGTGCAGAGGTTGATGGCAAGCCAGGCCCAGCGGGTCTTGACCGCCTTGGAGACCGGGG
CGAACACATCCTCCTCTTCACTGATACCCCCCATCCGGCGCAGGTCGGTGTCGCTCTCCTCATAGACCAG
ATCCACCACCTCTTCGACGGTGAGGCGGCCCATCAGCTTGCCCTTGCCATCCACCACGGCGGCGGAGAGC
AGGTCATCCCGCTCGAAGGTGCGGGCCGCCGCTTCGTCGTTATCCTCCGGATCGAAGGTGACCGGATCCC
GCTCCATCACGTCGCTCACCAGGGTGGCGGGCTTGTGCAGCAGTACCGTGGTGAGGGCCAGCTCCCCCAG
CAGGTGGTTGCGGCGATCGATGACGAACAGCTTGTCGGTGTTGGCGGGGATCTTGCCGCGCAGGCGCAGA
TAGCGCTGCACCGTGGCGAGCGTCACCTCGGGGCGGATGGTGATGATCTCGAAGTCCATCATGGCGCCGA
CGGTGTGCTTGCCGTAGCGGATCACCTCCCGCACCCGATCCCGCTCGGCGGGGGCCATATAGGTGAGCAG
CCGCCCCATCAGGTTGCGCGGCAGGTATTGGCCCAGATAGATCTGGTCGTCGATATCCAGGGTGCGCAGG
GCGTGCAGCAGCTCCTTGTCGCTCATCCCGCTGATGAGGCTGTCCCACACCGTCTCCGACGCTTCGACCA
AGATCTGGCCGCGTTTCTCCTCCTCCACCAGCGCCCACAGAGCATGACGCTCATCGGGCGGCAGCGACTC
CAGCGCATCGGCGACGTCGGCGGCGGGCAACCCGTTGACCAGCGTTGTGAGTTCGCTGGTCTGATCGCGC
TGCTCGGCATCCTGCACCACCTGCGGATCGAGCAGGCTATCGGTGAGGGCATCATCGCGCAGCAGCAGGG
TGAGAATGCGGGATCTGTCATCCGCGCGCTGCCGGGCGCTGTTCTTGATGGGAAGAGACATGGGATGAAT
TCCTGAGATGGTAGCCAAGGGCTCAATGCCCATAGGATACCAGACTTAAAACAACAGGTTATGACCGTGA
TGGGAGAAGAGGGAGGTAAAAGTGAGGCCACCTGCTGGTGGCCTCATGGTGAGTTGCCGAGGATACCTCA
GGCGAGGGCTCGCTGAGCCTGTGGTGCCTCGCCAAACCAGGCGAGCCGGTCGGCCAGGGTCACCACCGAG
CCCACCATGATGAGGGCAGGACTCTCGACCCCCACCGCCAGTGCGGGCAGCTCATCGAGAGTGCCGCGAA
TGACCCGCTGGCAGGGCTGGGTGCCGCGCTCGATCAGCGCCACCGGGGTGTCGCCTGCCTTGCCGTGGGC
CAGCAGCTCTTCACGGATGGTGGCGCAGGATGAGAGCCCCATGTAGAAGACCAGCGTCTGCCGGTCTTTG
GCCAGCAGTGGCCAGTCGAGATCCCGGGCGCCCCCCTTGCCGTGGGCGGTGACGAAGCGCACGCTCTGGG
CGTGATCGCGGTGGGTCAGCGGAATGCCGGCGTAGGCGGCGCAGCCGCTGGCCGCAGTGATCCCCGGCAC
CACCTGAAAGCCAATCCCAGTACCAACGAGCGTCTCCAGTTCCTCGCCGCCACGGCCGAAGATAAAGGGG
TCGCCCCCCTTCAGCCGCACCACCCGTTGCCCCTTCTTCGCCTCCTCCAGCAAGAGCTGGTTGATCCCCT
CCTGCGGCACGCAGTGGTTGCCCGCCTGCTTGCCGACGAAGATGCGCTTGGCGTCGCGGCGCACCAGCGC
CATCACTTCGTCAGAGACCAGCCGGTCATAGACCACCACGTCAGCCTGCTGCATCTGGCGCAGGGCGTGC
AGGGTGAGCAGGCCCGGATCGCCGGGACCGGCACCCACCAGCACCACCTCGCCAGCGCTCTGGCTCTCGT
CGGCAAACAGGCTGTCGGCCAGCTGGTGGGCAGAGGCGCTATCGCCCCGCGCCAGCGCCTGACCGAGCCG
ATCCGCCCCGAGCAGCCGCTCCCAGAAGCGGCGCCGCTCTCCCATGCTGGCGAATCGCGCCTTGACCCGC
TCCCGCAGGCTGCCGGCGAAGGCCGCCAAGGCCCCCTGATGCTGCGGCAGCAGGGCTTCCAGTTTTTCCC
GCAGCAGCCGCGCCAGCACCGGCGCCTTGCCGCCGGAGGAGATGGCCACCATCAGCGGCGAGCGATCGAT
GATCGACGGCATGATGAAGCTGGAGCGTTTCGGGTTGTCCACCACGTTGGCAAAGATCCGCGCCCGGTTG
GCGCTCTGGTAAACCAGCGCATTCACCTCGCGCCGATCGGTGGCGGCCACCACCAGCCACTTGCCCGCCA
GTTGTTCCGGCACAAATTCACCGGCCAGCCACTCGATGCTCCCGTTGGCAGCCAGTTCCGCCAGCTCGGG
GTCGAGCTCGGGGGCGACTACTGTGAGCTGGGCACCGGCATCCAGCAGCAGGCGCGCCTTGCGCTCCGCT
ACCTCGCCGCCGCCGACCAGCAGCACGGGTTTGTTGTCGAGTCGGCAAAAGATGGGCAAGTAGTCCATGG
GGCCTCCGGATCAGGCGGTTCGGGTCTGGTTGGCGGTGGGGGTGGTCTCGCTCACTGCGACCGGGCGTTC
AGCTTTCGGGGTGGCATACCAGTAGCCGAGGCCCATAAAGAGGACGCCGGAGACGGTGTTGCCCAGCGTC
ACCCACAGTAGGTTGTGGCCGATGCCGCTCAGGGTGTAGGCCTCCGAGTGGGCGCCAAACCAGGAGAGGG

CGAACAGGGTCATGTTGGCGATGGAGTGCTCGTAGCCGGAAGCGATAAAGGCCAGCAGGCACCACCAGAT
GGCGATGAAGCGGGCCGCCCCTTCGGTGCGCAGCGCCATCCAGATGGCGAGGCAGACCAGCCAGTTGCAG
AGGATCCCCTTCATAAACAGGGTCAGCACCGGAGCCTGGGTTTTGGCCAGCGCCACCTTGTGGGCCAGAC
TGCCTGCGTCCGGCAGCAGGCTGCCGCCGTAGGAGTAGATGAGCGCCACCACGATGGAGCCAATCAGGTT
ACCGGCCCAGGTCTGCGGCAGGATGCGCAGCAGGTCGGCCATGGTGATCTTGCCCGCCTTCACCCCGAAG
GTGAGGAACATGGTGTGGCCGGTGAACAGCTCGGAGCCGGCGATGATCACCAGCGTCAGGGCGATGCCGA
AGGTGGCCCCCATCACCAGCGGCCGGATGGCGGGATCCACCAGATTGCCGAGGGTGAAGATGAGGATGAT
GCCGAGGCCCACATAGGCCCCCGCCATGGCGGAGCTGAGCCAGAAGCCGAGCTTGTCGCTGCGCTCGAAG
CGGTTGATGCGGGCTGCGTTGGCGGCGCACTTGTTAATGGTTTCCGTGTACATCTTGTGAGTCCTTGAAA
GGGCTGCCCACAGGCAGCCCGAAAGAGAGTGAATTTGGTTTTAGACGGCGACCCAGATCTGCTGGTCGCG
TACTTCAACGGGGTAGCTTTGAACCGACATGGCGGGATTGTCGAAACAGTGGCCATCGCTCAGGCGAAAG
CGCTGCTTCTTGAGCGGGCTGGCGACCCAGAGCTCGCCGTCGTGTTCGCAAATCAGGCCCCGCGACAGCA
CGTTGGCGGCGAAGAAGGGATCCCGGTTGTCGATGGCGAACACCTCGGCCGCCGCGCTCGGGCGGAACAG
AGCGATCTGGCGACCTTCAATGAGTGCGCAGACGCCGGTTCCCGGCAGGATGTCGTTGAGTTGGCAGGCA
AGTTTCATCATTTGGTTTCCACCTCGATCTGGTAGACGGGGATGCGCTCGGCCGGAGTGGCGGGTCTGTG
CTGGTCGCGTTCGCTGACGAACTGCACGTCCGGGTCGCGCTTGTCGCTATTGATGAAGTGGCTGAATCGC
TTGAGTGCCTCCTCTTCGTTGAGGGTGCGCGACCATTCGCACTCGTAGCTGTCGATGAGAGTCTGGATAT
CGCGCTCCAGCGTCTCGGCCAGCCCCAGCTTGTCGTCGACGATCACCTCGCGCAGGTAGTCGACGCCCCC
TTCCAGATTGCCGAGCCAGACCGAGGTGCGCTGCAGTTTGTCGGCGCTGGTGACGTAGAACATCATAAAG
CGGTCGATCAGCTTGATGAGGGTCTCGCGGTCGAGGTCAGAAGCCAGCAGGTCGGCGTGGCGCGGTTTCA
TGCCGCCGTTGCCCCCCACATAGAGGTTCCAGCCCGCATCGGTGGCGATGATGCCGACATCCTTGCCCTG
CGCTTCCGCACATTCGCGGGTACAGCCGGAGACACCGAACTTCATCTTGTGCGGGGTGCGAATGCCCTTG
TAGCGGTTCTCGAGGAAGACCCCGAGGCCGACGCTGTCCTGTACGCCGAAGCGGCACCAGGTGCTGCCGA
CGCAGGTCTTGGCCATGCGCAGTGCCTTGGCGTAGGCCTGACCGGTCTCGAAACCGGCCGCCAGCAGCTT
GCGCCAGATGGCGGGCAGATCATCCTTCTGGGCCCCGAACAGGCCGATGCGCTGGGCGCCGGTGATCTTG
GTGTAGAGCTTGTAGTCGCGGGCCACTTCGGCCACCGCCAGCAGCCCTTCCGGGGTCACTTCACCACCGG
CCATGCGCGGGATGACGGAGTAGCTGCCGTCCTTCTGCATGTTGCCGAGGAAGATGTCGTTGGTGTCCTG
CAGCGGGATGTTGCGCGGGCTCAGCACATGCTCGTTCCAGCAGGAGGCGAGAATGGAGCCGACGGTCGGT
TTGCATACCTCGCAGCCATAGCCCTGACCGTGTTTGGCCAGCAGCTCGTCGAAGCTCTTGATCCCCTCCA
CCTTGACCAGATGGAACAGCTCCTGACGGGAGTGGGGGAAGTGGGCGCAGAGGTGGTTGTTGATCTCGAT
GCCCTGCTTCACCAGCTCGGCGTTGAGCACCTGACTGATGAGCGGCACGCAGCCGCCGCAGCCGGTACCG
GCCTTGGTGTGCTGCTTGATGGCGGCCAGGGTGGTGTGACCCTCGGCGACGGCCTGGGCGATGTCGCCCT
TGGAGACGTCGAAGCAGGAGCAGATCTGGGCGCTCTCCGGCAGGGCATCCACCCCCAGCGTCGGCTTGGC
ACCGGCGTAGGCGGGCAGGATCAGGGTATCCGGGTGGCTCGGCAGCGGCAGGGCGTTGAGCATCATCTGC
AGCAGGTTGCCGTAATCTTCCACGTCACCCACCAGCACGGCGCCGAGCAGGCGGCTGTTGTCTTCGCTCA
CCACGATCTTCTTGTAGACGCCAGCTTGGTCATCCTGAAATACATAGCTGTGGCTGCCTGGGGTGCGGCC
GTGGGCATCACCGATGGAGCCGACGGAGACGCCGAGCAGCTTGAGCTTGGCACTCATGTCCGCCCCCTCG
AAGCGGCTGTCGCCGCCGAGCAGGTGGTCGACGGTGATCTGGGCCATCTTGTAGCCCGGCGCCACCAGCC
CGAAGAAGCGGCCCTGCCAGGCGGCGCACTCGCCGATGGCATAGATGTCGGGGTCGGAGGTGAGGCAGTG
ATCGTCGATGACGATGCCGCCGCGCTCGGCGATGGCGAGATCACCGTAGCGGCCCAACGTATCTTGCGGA
CGAATGCCGGTGGAGAAGACCACCACGTCTACTTCCAGCTGGCTGCCGTCAGCGAAAGCGAGGCGGTGCT
TCGCCTGCTCGCCGCCATGCATCAGGATCTCGCTGGTGCTCTTGCTGGTGTGTACCTGCACCCCCATGGA
TTCAATCTTGCGGCGCAGCAGCTGGCCGCCCTGACCGTCGAGCTGCTCGGCCATCAGCACCGGAGCAAAC
TCCACCACATGGGTCTCGAGACCCAGTGCCTTGAGGGCACCGGTCGCTTCGAGGCCAAGCAGGCCGCCTC
CGATCACCACCCCGCTCTTGCCGCTCCTGGCGGCGCTGCGGATGGCTTTCAGATCCTCGATGGTGCGGTA
GACGAAGCACTCGTGGTGCTGACTGCCCGCAATGGGCGGCACCCAGGGGTAGGAGCCGGTGGCCAGCACC
AGCTTGTCGTACCCGACCACAGTGCCCTTGTTGGAGTGCACTTCGCGGTTGGCCCTGTCGATTTTTTTCA
CCGCTTCGCCGAGCAGCAGGCGGATGCCGTGCTTGTCGTAGAAGCCGGGTTTGACGAGGGAGAGATCTTC
ACTGGTGTGATGGGAGAAGTAGGAGGAGAGATGGACGCGATCGTAGGCGGGACGGGGTTCTGCACCGAAG
ACGGTGATCTCGTAGCGGCCCGGGTCGGCCCGCTCGATCAACTCTTCGATGAAGCGGTGCCCCACCATGC
CGTTGCCCACAACGACCAGTCTGACTTTGCTCATGATTGCCTCTAGTTTGGAGTAATAAAACATCATTCT
GGGGCTAGGCTACCCAGTCAGTCGGGGCGGGCAATTGATGCAAATCAAGTGTCTATTTATATATCTCGTC
AGGGGTAATTCGATAAAATTCAAAGATATACAAAATAGGTATACCAGTTGAATGTTTTGCTGCTGGGTGC
GAGTGGGACATAGAGAGAAGGGGGCTGGGGCTTAAAATGCAAAACGCCCCCATTTCTGGAGGCGTTTTTT
TACAAACAGAACTTCGCTTACAGAATAAAGCGGCTCAGATCTTCATCCTCGACAAGCGCACCGAGGTGGG
CGTTGACGTAGTCGGTGTCGATGACAAAGGTCTCGCCGGACTTCTCGGAGGCGTCGTAGGAGATGTCTTC
CATCAGGCGTTCCATCACGGTGTGCAGACGACGGGCACCGATGTTCTCGGTACGCTCGTTGACCTGCCAG
GCCGCTTCGGCCAGACGGCGGATGCCATCCTTGGTGAACTCGATATTGACCCCTTCGGTGGCCATCAGCG
CCTTGTACTGATCGGTCAGGGAGGCGTTCGGTTCGGTCAGGATGCGCTCGAAGTCATCTGTGGTCAGAGC
GGTCAGCTCGACCCGGATCGGCAGACGGCCCTGCAGCTCGGGGATGAGGTCGGACGGCTTGGCGATCTGG
AAGGCGCCGGAGGCGACAAACAGGATATGGTCGGTCTTGACCATGCCGTGCTTGGTGTTGACGGTGCAAC

CTTCAACCAGCGGCAGCAGGTCGCGCTGTACCCCTTCACGGGAGACATCGGGGCCCGAGCTCTCGCCGCG
CTTGCAGATCTTGTCGATCTCGTCGAGGAAGACGATGCCGTTGTTCTCGACGGCGGCGATCGCTTTCTGC
TTCAGCTCTTCCGGATTGACCAGACGGGCAGCTTCCTCTTCGATCAGCGCTTTCATGGCCTCTTTGACCT
TGATCTTGCGCTTCTTCTTCTGGTTCTGGCCCAGATTCTGGAACAGTCCCTGCAGCTGGTTGGCCATCTC
TTCCATGCCCGGTGGGGTCATGATTTCGACGCCCATCGGGGAGGCGGAGAGCTCCAGCTCGATCTCCTTG
TCATCCAGCTGACCTTCCCGCAGTTTCTTGCGGAAGATCTGGCGGGTGTTGGAGTTGTCGGCTTTCTCTT
CCTCACCCCAGGTGTTGCGCGGGTTGGGCAGCAGGGCGTCGAGGATGCGCTCTTCGGCGGCTTCCTCGGC
GCGATATTTCATCTTCTCCATCTCGGTTTCGCGCACCAGCTTGATGGCGGCATCGGTCAGATCGCGGATG
ATGCTGTCTACTTCCTTGCCGACATAGCCCACTTCGGTGAACTTGGTCGCTTCCACCTTGATGAAGGGGG
CGTTGGCCAGCTTGGCCAGACGACGGGCGATCTCGGTCTTGCCGACACCGGTCGGGCCGATCATCAGGAT
GTTTTTCGGGGTCACTTCATGGCGCATCTCTTCGTTGAGTTGCATCCGGCGCCAGCGGTTGCGCAGGGCA
ACGGCAACGGCGCGCTTGGCATCGGCCTGACCAATGATGTGTCTGTCCAGCTCGTGGACGATTTCTCTCG
GGGTCATCTCGGACATGATGGTGTTCCTGTATCCGGGGCGGGCAGTGCCCGCCCCTTGGGAAATTGGGTT
GGCTGGCTGCCTTATTTGGCCGACTTATTTAGCTAAATAATCCAGCACTTCGATGGTGTGGTTGCCGTTG
GTGAAGACGCAGATGTCGCCCGCAATTTTGAGGGACTTCTCGACAATGGTCCTGGCATCCAGCTCGGTGT
TTTCCAGCAGCGCGATGGCGGCAGACTGGGCGAAGTTGCCGCCGCTGCCGATGGCGATCAGGTCATGCTC
CGGCTGCACCACGTCACCGTTGCCGGTGATGATGAAGGATTTGTGTTCGTCGGCCACGGCCAGCAGCGCT
TCGAGGCGGCGCAGGGCGCGATCGGTGCGCCAATCTTTGGCCAGCGCCACGGCGGCGCGCTCCAGATTAC
CCTGATGGGCCTGCAGTTTTGCTTCGAAACGCTCGAGCAGGGTGAAGGCGTCGGCGGTACCGCCAGCGAA
ACCGGCCAGCACCTTGCCGTTATAGAGGCGATGAACCTTGCGGGCGTTGCCCTTCATGACGGTGTTGCCA
AGAGAGACCTGGCCATCGCCACCGATGACGACCTGACCGTTGCGGCGAACTGAAACTATGGTAGTCACTT
TACTTACCTCGCTTGAGCCGAATGACGGCCTGACTGTGCATGGCAAGAAAGATGGGGATGGCAAACGGGC
TTTTCAACCCTGCCTTTGGTCGGGATAAACGGGGTAGCAGACAGGGTGAGTGCGAGTGACACCAAAAGAA
CAGGGCCGGGTGTTGCCACCCGGCCCTGCGTCATCGGTAAGCGGATTACCCTTTCTTGGGAATGCAGCCG
CTGATGCCGGAGCTCTGCAGCTTGTTGTTGGCCGCGTCGGCGGCCGCCTTGCCGTTGAAGGGGCCCACCA
TCACCTTGTAGACGGTGCCGTTGGCGCCATTGATCACCTGCAGGCTGGAAGAGAGGCCGGCGGTGAAGGC
GATACGTGCCTTGAGCGACTCTGCGGAATCCTGCGAGCGCAGGGCGGCGCACTGCATCATGTAGCGACTG
CTGTCGGCGGCTGCGGTCTGGGCAGGTGGGGTCGCCGCTTTGGCCTTGGCCGCTTCGGCGCGCTCCCGTT
CCAGCTCGGCACGGATCTCCCGCTCCATCCGTTCCCGTTCTGCCTTGCGGTCGATCACCTGCTCTTGCGC
GCTGGCAACCGGCGCTCCGGAGGTGGAAGGGGTGGTCGCCGGATTGGGCTGATAGGGCTTGGGCTGGCCG
ATGGGCGTTCCCGGTTGGGGAATGTCGGTCGGCACCGGCTGCGGCAGTTTTTCCGGTTGCAGGGTCACGG
TATCTGCCGGTGGTGGTGGCAGCACACCCGGCTGGGTCGGCGGCTCTATGATATCGACCTGCTTGTTCTC
AAGCCGTTCGATGTAGCTCCACTTCTCCTGAGGCAGCCCGTTATTCTGCGCTTTTGGCTTGTTGGCCTTT
ACCTGCTCCTCAATGGTGGGGGCGTCGTTGCCCTTGCCATTGATCATGTAAAGGAAGCCACCAAAGCCTG
CCAGCAGTGCCACGACCAGCAGCACTGGAATAACAGGAAAGCGGCGCGGAGCCGCTTTCTTGGTATTGCG
GCCACCCGCCCGACGTCGCGGGCGGCTGCCGACATAATCCCGGGTTGCCATCTATTACATGCGCTCCAGG
GTATGGATGCCCAGTACGCCGAGGCCCAGTTTCAGCACCTTGGCGGTGGCGGCGCACAGCAGCAGACGGC
TCTGGCGGGTTGCTTCGTCCACGCCATCCTTGTTGATCGGGCAGGCTTCATAGAAGGTCATGAAGTTGCC
GGACAACTCGTACAGGTAGGTACAGAGCAGGTGCGGCATGCCTTTGTCGGCCACGCCGTTGACCGCGTCG
GAGAACTGGATCAGCTTCTGGGCCAGCACCTCTTCGGCCTCTTCGTTCAGGGTCACGTTGCCGGTCAGCG
TTTCGGCATCGATGTTGGCCTTGCGGAAGATGGACTGGATACGGGTGTAGGCGTACTGCAGGTAAGGGGC
AGTGTTGCCTTCAAAGGAGAGCATCAGATCCCAGTCGAAGATGTAGTCGGTAGTGCGGTTCTTGGAGAGA
TCCGCATACTTGACCGCGCCCATGGCGATGGCGTTGACCACGTTGGCCTTCTCTTCGGCAGAGAGGTCGC
TGTTGCGGCTCTCCAGCAGGGCAGCGGCGCGCTCTTCCGCTTCGTTCAGCAGGTCAACCAGCTTGACGGT
GCCACCGGAACGGGTCTTGTAGGGACGGCCATCCTTGCCCAGCATCATGCCGAAGGCGTGGTGCTCGAGC
GGCACGGATTCCGGCACATAACCCGCCTTGCGGGTGATGGTCCAGGCCTGCATCAGGTGCTGGTGCTGAC
GGGAGTCGATGAAGTACATGACACGGTCTGCGCCCAGGGTTTCATAACGGTATTTGGCACAGGCGATGTC
GGTGGTGGTGTAGAGGAAACCGCCATCGCTCTTCTGGATGATGACGCCCATTGGCTCGCCATCCTTGTTC
TTGAACTCGTCTAGGAAGACAACGGTGGCGCCTTCGCTCTCGACCGCCAGCCCCTTGGCTTTCAGGTCGG
CAACGATCTCCGGCAGCATGTCGTTGTACATGGACTCGCCCATGATGTCCTTGTTGGTCAGGGAGACATT
CAGGCGGTCGTAGTTGATCTGGTTCTGTTCCATGGTCATGTCGACCAGCTTCTTCCACATGGTACGGCAG
TACTCGTCGCCGCCCTGCAGCTTGACCACGTAGTTACGGGCACGCTCGGCGAAGGCTTCGTCTTCGTCGT
AGCAACGCTTGGCCTGGGTGTAGAAGGCTTCCAGATCCTTGAGTGCCATGTCGCTGGCGTGCTCGTTGGA
CATCTTCTCCAGATAGGCGATCAGCATGCCGAACTGGGTGCCCCAGTCGCCGATGTGGTTGGCACGGATC
ACTTTGTGGCCGAGGAATTCCAGCGCGCGCGCAGCTACATCACCGAGTACGGTGGAGCGGATGTGGTGCA
CCGCCATCTCTTTGGCCACGTTGGGGGCGGAGTAGTCCACCACCACGGTTTGTGGCGCCGGCAACCGTAC
GTTGGCGTTGGCAGAGGTCACCATGGCCTCGACCTGACCGGCCAGCCAGCGCTGGTCAAAGAAGAAGTTG
ATGAAGCCGGGACCGGCGATCTCGACCTTGGCGATCAGGTCGGAGGCAGGCAGGTGCTCGATGATGGCAG
CCGCCAGCTCACGGGGATTCTTGCGGGCTGGTTTGGCCAGCAGCATGGCCAGGTTGGTAGCCAGGTCGCC
GTGAGCTTTTTCCTTGCATCGGTCTACTTGCACGCGGGCTTCCAGGTCCGCCGGCAGGACGCCTGCTGAC
TTGAGATTGGCTACCGTTTGTTCAAGTAGATGATGAATATGCTCTTTCATAACTGACCACCAAGGTTGCG

CGTCGAAAAATGACTGATATATAAGGGCGTGAATTTTACCCGCATCGGCCCGAGAAAACTATAGGGATGA
CCCGCCGCTCGCTGTCGGGCGGATATTTCACTGCGTTTAGTGCCATATTCCAGAATATTGGGCGGAAATT
ACGGTGCTTATCCATTTCTACTGCATATCGAAGCGGCTTGTCCAGGCTATTTTCCCCTGATTGTTAAGGG
TTTTTCCTTTCCGCTGACGGGAATGATCTCCTCCCTCTGGCCCGGCGTATCAACAGAGGTTCCCAACCGA
GGAGTTTGCGATGCGACTGGCGCTGTTTCCCCTATCTGCCCACCTGCTGCCCGGCGGCGTCATGCCGCTG
CGGATCTTCGAACCGAGATACCAGCGGATGATCGCCGAGGCGGGGGAGTCTGGCTTTGCCCTCTGCATGG
TGGACCCTCGCCAGCCCGACGCCCTGCGCAACATGCTGCCCATCGCCACCCGGGTCACCATCATCGATTT
CGACCGGTTGCCGGACGGCATGCTGGGGATCACGGTGCAGGGGATGGAGCGGGTGCAGATAGAGGATCTG
TGGCAGGAGCCCGATGGCCTGCGTCTCGGCGAGGTGACGCCGCTGACGGCCTGGCCGCCCCGCCGACTCC
ATCCCGATCAGCAGCCGCTGGTCGAGGCGTTGCGGGAGGTGTTTGCCGATTATCCCGACTACGCCGCCCT
CTATCCCGCGCCCCGCTGGGACGATGGCAACTGGGTGGCCCAGCGCTGGCTGGAGGTGCTGCCGATCCCC
CCCGAGCAGAAGCAGTTGCTGCTAGCCGCCGCCGACAATCAGCCCGCCATCGCCCTGCTCAAGGGGGTGC
TGGTGGCCAGCCACTGATTTTTTCTGACCCGCTGATCCGTCAGCGGCGTAGCGCCGTATAAGTCATCACC
ACAGCATGTTCGACAGCACACCAAAGGGATGGCGCAATGGCATATACTGGCCAACCACACTCGACCATAC
GCAGGACCTCGCCCGTGATGGATAACCCGGATGGCGACCTCAAATCTCTGCTGATCAGGGTGGCAGAGCA
GGCCGACAAGGCTGCTTTTGCCCGCCTGTTCCAACACTTTGCGCCGCGTATTCGTAGCTACGGCCTGCGC
CACCTCGGCAGCGAGGCCAACGCCATGGAGCTGGTGCAGGAGACCATGCTGCTGGTGTGGCAGAAGGCAC
GCCTCTACCACCCCGAGAAGGGGGCGCCGACTACCTGGATCTACACCGTGATGCGCAACCAGTGCTTCGA
CATGCTGCGCCGTCGTCGTGCCAGCAAGGAGGACCTCTGTGCCGAAGAGTTGTGGCCGGTACTGGAATAT
CAGGCGGAAGAGGAGCACCTGAGCGGTGGTGAAGACGCCGTACTGACGCGGCAGATGGCCCACTATCTGG
GCACCCTGCCGGAGCCGCAACAGCAGGTGGTGCGCGGCATCTATCTGCAGGAGTTCTCCCAGCAGGAGCT
GGCGGAGCGACTCGGCGTGCCGCTCGGCACCATCAAGTCCCGCCTCCGGCTGGCATTACAGAAACTGAAA
GAGCAAGTGGAGCAAGGCCATGATTAAAGCCCATCCGACAGACCCTATGCTACGCGCCTTCGCCGCCGAC
GAGCTCCCTCTGCCGCTGGCGGTAGGGCTCTCCGCCCACTGTGAACTCTGCCCCGATTGTGCTGCCCGAC
TAAAGACATTCGAAGAGGAGCTGGCGCAGCAATATCTTGCTTCGCCAGCAGAGCCCGAGCTGGCCTCCCG
CGATGAAGTAGTGCTGGATGCCGGATTCGACGCCATGCTGGCGGATATTCTGGCCCAGCCGCTTGCAGAG
CCCCCCTCCGAGCCAACACATGACCAGCCCCGAACGCAGCACGGCGATCTGGAGGTGGCCGGGCACGCCT
ACCGTTTGCCACGGGTGCTGGCCCGCTACCGTTCGCCCAAGTGGCGCCATATCGGGGCCATTCGTCAGCA
GAGTCTGCCGCTGGACGAGATGGGCGCGCGCGCCAGCCTGCTCCATATCGAGGCGGGTGGCCGCATCCCC
GAGCACACCCATCAGGGCTATGAGCTGACTCTGCTGCTGGCGGGCAATATTCAGGATGGCGACACCCTTT
ACAAGGCGGGAGATTTCATCTGGCGCGATGCCAGCCACTCCCACAGCCCGCACACGCCCGATGGCTGCCT
CTGCTACACGGTGCAGGATGCGCCGGTGCAGTTCACCAAGGGGCTGTCGCGGCTGCTGAACGGCATCAGC
CAGCACCTCTACTGAGCACTATGACACCATAGAAAAAGGGACCAAGTTGGTCCCTTTTGTCATTTCATGG
CTGTCGGTCAGGCGACAAATACCAGATAGACGGCGAATGCCACGATAAAACGGTAGATGGCGAACGGGAT
AAAGTCGATGCGGCGGATCAATGTCAGGAAGGTCTTGATCGCAAACATCGCCACCACGAAGGCAGTGATA
AAGCCCACGGCAAACATCGGGAAGTCGGCCATCGAGAGGAAATCGCGGCTCTTGTAGAGATCAAGTCCGC
TGGCCGCCACCATCATCGGCACCGCCATGATGAAAGAGAACTCGGCGGCGGCGTGACGGCTGATCCCCAT
CAGCATGCCACCACTGATGGTGGCGCCGGAGCGGGAGAAGCCGGGCCACAGGGCCAGACATTGGAACAGA
CCGATGCCGAACGCCTGCTTGTAGCTGATGTCATCCAGGGTTTCGGCTGTGACTGCAGGGCGGAACTTCT
CGGCGATGATCAGCAGGATGCCCCCCGCGACCAGCGCGTACATGACGGTCTGCGGACCAAACAGGTTGGC
CTTGATCCAGCTGTGGATGGCCAGACCGACCACGATGGCGGGCATCATGCCGAGGATGACGTGGATCAGC
GACAGGGTGGCGTGATCCCGGGACGGCTTCTGGCCAAAGTGGATGCCAATCAGGCCAAACAGGCGACGCC
AGAAGACGGCGACCACGGCGAGGATGGAGCCAAGCTGGATCACCACTTCGAAAGTGGCAGCCTTGGGGCC
TTCAAAGCCCAGCAGATGGCCGACGATAATCATGTGGCCGGTGGAGGAGACGGGGAGGAACTCGGTCAGT
CCCTCGACGACGCCCAGAATAAAGGCGACCAGCAGGCTATAAGTGTCAGTCATCGAACAAACAATCCTTA
ACTTCGTTGATGTAAAAACGCCCCGGGAGGGTCTCGGGGCGGCTTATCTTGCTCGATAGTGACTGCTGAG
GCTATGTAAAAATCATGAAACCTTTAAGTCAGTCGCGGATAAAACGGTAGCGGGTTTGGTGGTGATAACG
CCCGCCTGGCAGCAGCCAGGGGTTGCCCAGCTCGGGGCGATTGGGGCTGTCCGGCAGCTGTTGCGCCTCA
AGACAGAAGCCGTCGTGATCCCGGTGCGACAGGCCGCTGCGGTTCGGGGTATTGGCCAGCCAGTTTCCGG
TGTAGAACTGTAACGAGGGCTGGTTGGTATAGACTTCCATCGCCAGCTGCCTGTCCCCTGATACTACGCG
AGCAGCCCACTCTTGCGTTGGGCCGTTGAGCACAAAGGCGTGATCGTACCCCTTGGCCTGTTGCTGCTGG
GGATGGCTCAGCCAATCCTGTCCGACAAGGCGCTCGCGGCGCAGATCGAACGGCCCCTCCACCGGGGTGA
TGGCGATCGGCAAACCGCTCGGGTCGGTCGGCAGGAAGTGGTCGGCATTGATGCTGATGAGGTGGTCACG
GACATTGCCACCATCGTTTTGCCCATCGAGATTGAAGTAGGCGTGGCTGGTAAGGCTGACCGGGGTCACT
GCATCTGTGCTGGCCACAAAATTGACCACCAGATCTCTCTGCTCAAGCCGGTACTCCAGGGTGACCCGCA
GATTGCCGGGAAAGCCCTGATCCCCCGCTCGGGAGAGCAACGCCAGCTTGACCCGATCTGCTTCCAGTTC
CCTGATCTGCCAACGCTGGCGATGAAAGCCTGCCTGCCCGCCGTGCAGACAGTTGGGTGCCTGATTGGCA
TTCAGCGGCCAGCGCTGTCCATCACGCACCAGCTCGGCACCACCGATGCGGTTGGCAAAACGCCCGGCCA
CCGCACCCAGCCACACCTGCTGGGTGGGATAGGCGTCGTCGGCACAGCCCAGCAGCACTTCACGCCGTTT
GCCCACCACCGGCAGGGTGAGGGAGGTGAGGGTGGCGCCGAAGTCGAGGCCGCGCAGCCGCAGCCCCTCT
TCATTTTCCAGCTCGAACGGGGTCATGGCGCTGCTCATAGCTTGCCTGCACCGGCGCTGGCAGAGCAGAC

GTAGCAGTCGGCCTTGAGGCCCGAGATGGCCGGATACTCCGCCTCGACGGCGGCCATCACCTCGGCCACT
TTCTCCGGGCGCAGCAGGGCGACCACACAGCCGCCGAAGCCGCCGCCGGTCATCCGCACGCCGCCATCTG
TGCCGATTTTAGTCTTGATGATCTCCACCAGACCGTCGATGGCGGGCACTGTGATCTCGAAGTCATCGCG
CATGGCGGCGTGCGACTCGGCCATCAGCTTGCCGAGGCGGGTCAGGTCGTGGCTCGCCAGCGCATCGGCG
GCCGCCAGAGTGCGGGCATTTTCCCCAACGATGTGGCGGGCACGACGATAGCAAGCATCATCCAGCCCGG
CCTTGCCCGCTTCCAGTCCGGCCAGATCGAGGTCGCGCAGCGCCTTGACTCCGTAGTGACGGGCGGCCGC
TTCGCACTGCTGGCGACGGGTGTTGTATTCGCTGTCCACCAGACCGCGGCGCACATTGGAGTTGACGATC
AGCACCGCCAGATCCGCCGGCATGGGGATGAGGCGAGTCTCCAGCGAGCGGCAGTCGAGCAGCAGGGCAT
GATCCGTTTTGCCGCTGGCGGAGATCATCTGATCCATGATGCCGCAGTTGCAGCCGACGAACTTGTTCTC
CGCCTGCTGGCCGTTGAGGGCGATCTCGGCCTGGCTGATGTCGAGCCCCAGCGCCTCTTTGAAGGCCTGA
CCGATGGCAACTTCCAGTGAGGCGGAGGAGGAGAGTCCTGCCCCCTGCGGTACGTTGCCGGAGACCACCA
GATCAAGGCCGAGCAGGGCATAACCGCGCTCCTGCAGATACTTCACCACGCCGCGGATGTAGTCGCTCCA
GCGTTGGCTGGGGTGGTGCTCGATGGGCTGGTCGAGGTCGAACTGGTCGCGCTGGTTGTCGTAGTCGGCG
GCGATCACCTGCACTTGCCGGTCATCGCGCAGGCCGATGGCGACGCAGGTCTCGTAGTCGATGGCGCAGG
GCAGCACGAAGCCGTCGTTGTAGTCGGTATGTTCACCGATGAGGTTGACGCGGCCGGGGGCGCGCACCAG
CAGATCGGGCGCTTTGGCAAATTGCTCGGCAAAAACGGTGCTGACGCGTTGGCTGGGGGTCATTGGGCCT
CCTCATTGGTGTGGGTGGTTTGGGTGTAGTGAACAGGGCTCAGGGCACGCAGCCGCTCGGCAGCCTGCTC
GGGGGTAAGGTCACGCTGGGTTTCGGCCAGCATCTCGAAGCCCACCATAAATTTGCGCACGGTGGCGGAA
CGCAGCAGGGGCGGATAGAAGTGGGCATGCAGTTGCCACGCCTCGGGGCAATCCGATTTGGGCGGTGCAA
AGTGCCAGCCCATGGAGTAGGGGAAGCTGCACTCGAACAGGTTGTCGTAGCGGCTGGTGAGCTCCTTGAG
CGCCACCGCCAGATCCTGTTGCTGGGCCTTGGTCAGATTGAGCATGGAGGGGACGTGCGCCTTGGGCAGC
AGCAGGGTTTCGAACGGCCAGGCGGCCCAGAAGGGGACAACGGCAAGCCAGTGCTCGGTCTCTACCACGA
TGCGAGAGCGATCCGCCAGCTCCCGCTCCACATACTCCAGCAGCATGGGGCGGCCATGCTCCGCCAGCCA
GTCGCGCTGGTGCTGCTCTTCGCGGGCGATCTCGTTGGGCAGGAAGTTGCTGCCCCAGATCTGGCCGTGG
GGGTGCGGGTTGGAGCAGCCCATGACTGCCCCTTTGTTTTCAAAGACCTGCACCCACTCCCACTGGGCCG
ACAGCTCGGCGACCTGATCCATCCAGGTGGTGATCACCCCTTCGATGGCGGGCAGCGGCAGCTCGGGCAG
GGTCTTGCCGTGATCCGGCGAGAAGCAGATGACCCGGCTGGTGCCGCGGGCGGCCTCCAGTTTCAGCAGG
GGATCGCTGGCGCTATCGGCGGCCGGGGTATCCTGCATCAGAGCAGCGAAGTCGTTGGTAAAGACGAAGG
TGCCCTTGTAGTCGGGGTTGATGTCGCCAGTGACCCGGGTGTTGCCCGCGCAGAGGAAGCAGTCGGGATC
GTGGGCCTGTTTGGGGGCCTGACTCACCTTCTCCACCTGTCCCTGCCAGGGGCGTTTGGCCCGATGGGGG
GAGACCAGCACATACTGGCCGGTCAGCGGGTTGAAGCGGCGATGGGGATGATCGACGGGGTTGAACATCA
TCTCTCCTGAATAAGGTATTGAAAAATAGGGTCATCTATTAACAGGGAGCAGGCAGGCCTACTCCTCGTA
ACCGTTCGGATTGGCGGATTGCCAGCGCCAGCTGTCGGCGCACATGGCGTCGATGTTGCGGGTCGCCCGC
CAGACCAGCTCGCGCTCGGCCTTGGCCGGATCAGCCCAGCAGGCGGCGATATCGCCCGGGCGACGCGGCT
CGATGCGAAATGGCAGCGGCTTGCCACAGGCGGCAGAGAAAGCGGCGACCAGCTGCAACACGCTCTGGCC
CTGACCTGTGCCCAGGTTGTAGGTGTGCACGCCACCCTTGCCTACGCAGTGTTGCAGCGCCTTGACGTGT
CCTTCGGCAAGATCCATTACATGGATGTAGTCGCGCACGCCGGTGCCGTCCGGGGTGGAGTAATCGTTTC
CAAAGACAGACAAGCAGTCGCGACGGCCGATGGCGACCTGGGTGATATAGGGCATCAGGTTGTTGGGGAT
CCCCTGCGGGTCTTCACCCATGGTGCCCGATTCGTGGGCGCCGACCGGATTGAAGTAGCGCAGCAGGGTC
ATGCTCCAGTGGGGCTCGGCCAGCTGCAGATCTTCGAGGATCTCCTCGATGATCAGCTTGGATCGTCCGT
AGGGGTTGGTGGCGCTGCGAGGGAAATCCTCGCGGATTGGCAGGCTGGCGGGATCGCCATAGACGGTGGC
CGAGGAGCTGAACACCAGATTGTGCACGCCAGCCCGCTTCATCGCCTGCAGCAGGGTGAGGGTGCCGCCG
AGGTTGTTTTCGTAGTAATCCAGTGGAATGCGGGTCGACTCGCCGACCGCCTTGAGGGCGGCAAAGTGGA
TCACTGCATCGATCTGCTGTTCAGCGAAGAGCCGGTCCAGCAGGGCTGCATCGCGAATATCCCCTTGATA
GAACAGGGGGTTTCTGGCCGCTGATGGCGGCGATTCGCGCCAGCACGCTGCGCTTGCTGTTGCCGAGGTTG
TCGACCACCACGGGATTCATGCCTGCCGCTTGCAGGGCCAGACAGGTGTGGCTGCCGATATATCCGCAAC
CGCCGGTGACCAGAACGTTCATCTTGCTCTCCACTGAAAAAGTGTCTGGAAGCAGTCTAGCCGAGGGTGT
GGCGACCAAACTGTGAGCTTGCTTGCAAAACAGTGTAAACGATTACACTTTTTTTGTGGTCATCCTGACG
GCAGATATGCGTTTCACCCCTGTTTTCAAAGGGTTTGCTGGGTATAATGGCGCTCTATTGCACAGTGAAA
GAAGTGAAAACAGCATGAGCACCATCAAGGATGTCGCCCGTTTGGCCAATGTGTCAGTGGCAACGGTTTC
ACGGGTGATGAACAACTCCCCCAAGGCCAGCGCCGCGTCTCGCGAAGTGGTGCAGAAGGCCATGGCCGAA
CTGGGCTACACCCCCAACGCCAACGCGCGGGCACTGGTGAGCCAGACTTGCGACACCATGGGGGTGGTGG
TCGGTGACGTGGCCGATCCCTTCTTCGGTGCACTGGTGAAGGGGGTCTCCGGCATCGCCGTCGAGCAGAA
CCTCCACCTGCTGATGGGTAACGGTTTTCACAAGGCGCGTCAGGAGCGTGAAGCGATCGAGCTGCTGATC
GGCAAACGCTGTGAGGCGCTGGTGGTGCACAGCAAGGCGCTGAGCGACGAGGAACTTTACGATTACGCCA
TGCGGGTGCCCGGTATGGTGCTGATCAACCGCGATATTCCTGCCCTCAAGGGGCGCTGCATCGCTCTCAA
CAACCGGCTGGGGGCGGCTACGGCGACTCGCCACCTGATCGAACTGGGCCACCGCCATATCGCCTTTATC
AGCTCGGATCACGCCATCGAGGATGCCACCTTGCGTCTGCAGGGGTATCAGGATGGGCTGATGGAAGCCG
GTCTGGCAGCCGATCCCGAGCTTATCGAGAGTGGCACCCCGAACGAGGAGGGGGGGGAGCGCGCCATGCT
CAACCTGTTGGCCAAGGGGCTCAAGGTGACCGCCGTGGTTGCCTATAACGATGCCATGGCGGCGGGCGCC
ATCTCAGTGCTGGCCGACAACGGCCTGCGAGTGCCGGAGGAGGTCTCGGTAGTGGGCTTCGATGACATCA

TCTATGCCAGATATTTGCGCCCCAAGCTCTCCACCATGCGCTATCCCATCGAGCTGATGGCAGCCCAGGC
GGCCAAGCTGGCCATTCAGCTGGCGGCGGGGGAGAGTGACGCGACCCAGAGCCGGATCTATACCCCCACC
CTGATCAATCGCCACTCGGTGGCACCGGTTCGGGCCGGATAGCCCCCTTTTGCGGTCCTGTTCGGGGCCG
TTTCTTGCACGGTTATTGTTTTACCTATTTTTAATCATTTTTACTCCGCTTTACTTAACAAAATCAGTGT
AATCGTTTACATCTGTGTGAGCGAGATCGCCGTTCCCTGCGTCATACTTCTCTAGACTGAATACGTTTTC
CAATCGGTAGGGAGAGAGTGAATGCTCAGGGAGATAGTGGCGCGTCAGGATTGGCAGACTCAGGCTATTA
CGTCGGTAAACCGGCTGGCAGCCCATACCCCCATGTTCAGCTGGCGCAGCGAAACGGATGCCAAGGGTGA
TCTGGCCTCTTCCTCCCGTCTCTTGCTCGATGGCGAGTGGCGCTTCTCCTTCTTTGCCGCTCCCGAGCGG
GTGCCCGAGAGTTGGCTGGCGCAGGATCTGGTGGATGCTTGCCCCATTACAGTGCCGGGCAACTGGCAGC
TGGATGCCGCCTATCCGGCTGCGCGGCCGCTCACCGATGGCCCCATCTACACCAATATCAAGTACCCCTT
TCCTTGCGATCCACCGCGAGTGCCAGCAGAGAACCCCACCGGTTGCTACTCACGCGAGTTCGATCTGCCT
GCCGGCTGGCTGGCCAGCGGCCAGAGCCGGATCATCTTTGACGGGGTGAGCAGTGCCTTCTATCTCTTCT
GCAACGGCCGCTGGGTCGGCTACTCCCAGGACAGCCGGCTGCCCGCCGAATTCGATCTGACTCCTTATCT
GCAGAGCGGTCGCAACCGGTTGGCGGTGCTGGTGCTGCGCTGGTCCGACGGCTCCTATCTGGAAGATCAG
GATATGTGGTGGCTGTCGGGGATCTTCCGCTCCGTCAGCCTGCTGCACAAACCGTCCCGCCATCTGATGG
ATATGCGGGTGACCCCCGAGCTGGATGCCTGCTATCGGGATGGTCGTCTCAAGATTGCCCTGCAGGTGGC
TAATGGGGCCGGTCTGTCGGTGGAGGTCAATCTCTATGACGGCGGCGAGCGCGTCGCGACCCTGCGCCAG
CCGATCGGCACCCAAGCCATTGATGAGAAAGGGGCATATGACGATCGCGCCGAGTTCTGGCTCGACGTGG
TTGCGCCGCGCAAGTGGAGCGCCGAGACGCCCCATCTCTATCGCCTGACCCTGACCCTACTGGACGAACA
GGGCCAGGTTATCGAGAGCGAGGCATGTGATGTGGGCTTTCGGGTGGTGGAGATCCGTGGCGGCCTGCTG
CGGGTCAACGGTCCGCCGCTGCTTGTTCGCGGTGCCAACCGTCATGAACACCATCCCGCCAAGGGTTATG
CCATCGATCGCGCCACCATGGAGCGGGATCTGCTGCTGATGAAGCGGCACAACTTCAATGCGGTGCGCTG
CTCTCACTATCCCAACCACCCCGACTTCTATCGCCTGTGCGACCGGCTCGGCCTCTATGTGGTGGACGAG
GCGAATCTGGAGACCCACGGCATGACCCCCATGGGGCGGTTGGCGCGGGATCCCGCCTGGAGCAATGCCT
TTCTGGAGCGAGCGACCCGCATGGTGGCACGGGATTTCAACCACCCCTCGATCATCATCTGGTCGCTGGG
CAACGAGTCGGGTTACGGCCCGGCCCACGATGCCATGTATGGCTGGATCAAGCGCAGCGACCCGAGCCGT
CCGGTGCAGTACGAGGGGGGCGGGGCCGATACCCCGGCCACCGACATCATCTGCCCCATGTATGCCAGAA
CCCATCAGGATCAACCTTTCCCGGCGGTGCCCAAGTGGGCGCTGGCCAAGTGGATCGGCCTGCCCGGCGA
GCACCGGCCGCTGATCCTTTGCGAATATGCCCACGCCATGGGCAACAGTCTCGGGGGCTACGCCCACTAT
TGGCAGGCGTTTCGCGATCATCCACGTCTGCAAGGCGGATTTGTCTGGGATTGGGTCGATCAGGGGCTCG
ACAAATACACCGCCGATGGCCGCCACTACTGGGGCTATGGCGGCGATTTTGGTGATGCGCAAAACGATCG
TCAGTTCTGCTGCAACGGCCTGCTGTTCCCTGATCGTACCCCCCATCCCGCCCTGTGCGAGGCGAAGCGT
GCCCAGCAGCCGTTCCGCTTCACCCTGCTCGAGCACACCCCGCTGCGGGTGCGGATCGAGAGCGAATACC
TGTTTCGGGAAACCGACAACGAGCGGCTTTGCTGGCAGCTGTGCGAGAACGGCCATGTGCAGCTGCAGGG
GGAGAGCTCCCTTGAGCTGGCGCCACAGGGAGTGGTCGAGTTGACCCTGAGCGAGACACTGCCCGCCTTT
GCGGCAGGCTTACTGGCCTGGCTCGATCTGGCCATCGTTCAGCCGCAGGCCACCCCCTGGTCGCAGGCGG
ATCACAAGGTCGCCCGTCAGCAGTTCACCCTGCCGACCCCCCTTGCTCTGCCTGCTCCCGCCGCTGCCGC
CACCCTGAGCGAGCAGCCGGATGGCTGGCGGATCACCGCCGCAAACAGCCAGTGGTGGCTCGACAAGCAG
AGCGGCCGTATCACCAGTTGGCAAAAATCGGGGCAGGAGCAGTTGCTGGCGCCGATCATGGATCACTTCT
ACCGGGCGCCGCTCGACAACGACATTGGCACCAGCGAAGCGGATCACGCCGACCCTCACTCCTGGATTGC
CCGCTGGCAGGCGTGTGGTCTCGGTCAGTGGCTGCACCGCTGTCTGGGGATCGCGGTCGCTGGAGGGGAT
ATCCGGGTCAGCCATGGCTACTTCCACCACGATGAGCTGCTGCTGGTGAGTCACTGGCATCACCGCTTTA
CCGACGATGGCACCATGAGCCTGGAGATAGAGACCGAACTGGCGCAGGTTCTGCCGTCGCTGCCGCGGAT
TGGCCTGCTGCTTCATCTGGCCGAGGTACCGGCGCGAGCCGAATGGTTGGGACGCGGCCCTCACGAGAAC
TATCCGGATCGTCTGCTGGGCGCTGATCTGGGGCGCTGGAGCCTGCCGCTGGAGGCCATGTACACCCCTT
ACATTTTCCCGAGTGACAACGGCTTGCGCTGCGATACCCGTCAGCTGCAGCTGGGCAGCACGACGGTGAT
CGGCACCTTCCATTTCAGTGCCAGCCGCTTCAGTCAGCAACAGCTGGCCGTTGCTCGTCACCAGAGCGAT
CTGGTGGCCGAAGAGGGGCTGTGGGTCTGCCTCGATGGCGCCCATATGGGGGTGGGCGGGGATGACTCCT
GGAGCCAAAGCGTGCGTCCCGAGTATCAACTGCTCGAGCGTCGTTACCGTTGGGGCTGCACTCTCCGCTA
AGCCAGCTATTGAGGCGACTGTCGAGCCAGTGCGCCATTTTGACAACAGACCAGTAACGACTAGCCTCAA
TGCTGTGGGAAAGATTGAGTCTCAAATCTGATTTTTATTTATATGTATTTGAAAAATAAATATAAAGTTT
CAATGTAACAGAGTGTAAACGTTTTCATTTTGTCGTCATGATCACAGAAGAAATGACAACAAACTGATTA
CATTGATTGGCGCTTGCTCAATTGCTCGCCACCCTACAACACAGATAGACACATCGGAGCACACATAATG
AAAAAACTGACCACAGTTGCCGCCCTGCTGATGGGCATGATGGCTGGAGCCAATGCCCAGGCTGACACCC
GTATCGGGGTAACCGTCTACAAATACGACGACAACTTCATGGCGGTCGTGCGCAAGGCCATCGAGAAGGA
GGCGGCAGCCACTCCGGGGGTGGAACTGCTGATGAACGACTCCCAGAACGACCAGTCCAAGCAGAACGAC
CAGATCGACGTACTGCTGGCCAAAGGGGTGAAGGCGCTGGCCATCAACCTGGTTGACCCGGCTGCGGCAC
CGGTGGTGATTGAGAAAGCCCGTGGTGAAGATATCCCGGTGGTCTTCTACAACAAGGAGCCGAGCGCTAC
CGATCTGGCCAGCTACGACAAGGCCTACTACGTGGGCACCGACTCCAAGGAGTCAGGGATCATCCAGGGT
CAACTGATTGCCAAACACTGGAAAGCCCATCCGGAGTGGGATCTGAACAAGGATGGCGTGATCCAGTTCG
TGCTGTTGAAAGGGGAGCCGGGCCACCCGGATGCCGAAGCGCGTACCTCCTACGTGGTCAAGACCCTGAA

CGATGGCGGCGTCAAGACCGAGCAACTGCACATGGATACCGGTATGTGGGATACCGCCATGGCGAAAGAC
AAGATGGACGCCTGGATCTCCGGCCCCAACGCCAACAAGATCGAAGTGGTCATCGCCAACAACGACGGCA
TGGCAATGGGCGCAGTCGAATCACTGAAAGCCCAGGGCAAGACCGCCATTCCGGTGTTCGGCGTCGATGC
GCTGCCGGAAGCGCTGGCCATGGTCAAGAGTGGTGCCATGGCGGGTACCGTGCTGAACGATGCGGCCAAC
CAGGCCAAAGCCACCTTCGAGCTGACCAAGAACCTGGCTGAAGGCAAACCGGCCGGTGAAGGCACCAAGT
GGAATATCGTCGACAAGGTAGTACGTGTTGCCTACGTCGGTGTGGATAAAGACAACCTGTCCCAATTCGA
GTAATTCCCTCCTCCAGGGCAAGGGAGATCCCCTTGCCCTTTTTTCAACCAGATGTGATGGCTGATATGA
CAACACAGCAACAATCAGAATGGCTGTTGGAGATGATTGACGTCAGTAAGAGCTTTCCCGGCGTCAAGGC
ACTCGATAATGTCAATTTACGGGTCCGTCCTCATTCTGTGCATGCGTTGATGGGCGAGAACGGAGCGGGA
AAATCCACACTGCTCAAGTGCCTGTTCGGCATTTACGAGAAGGATCAGGGCAAGATCTTTTTCAAGGGGC
AGGAGATCAACTTCACCTCCTCGAAAGAGGCGCTGGAAAATGGCGTCTCCATGGTGCATCAGGAGCTCAA
CCTGGTGTTGCAGCGTACCGTGATGGACAACATGTGGCTCGGCCGCTACCCCACCAAGGGGTGGTTTATC
GACCACGGCAAGATGTATGACGACACCAAGCGCATCTTCGATGAGCTGGATATCGACATCGACCCCAAGG
TGAAGGTCGCGACCCTGTCGGTCTCCCAGATGCAGATGATCGAGATCGCCAAGGCGTTCTCCTACGATGC
CAAGATCGTCATCATGGACGAACCCACCTCCTCCCTGACCGAGAAAGAGGTGAATCACCTCTTCAAGATC
ATCAACAAGTTGAAGGAGAAGGGGTGCGGCATCGTCTACATCTCTCACAAGATGGAGGAGATCTTCCTGC
TCTGTGACGAGATCACCATATTGCGGGATGGCCAGTGGGTGGCAACCCAGCCGCTCAAGGGCATGACCAT
GGATCAGATCATCGGCATGATGGTCGGCCGTGAACTGACCCAGCGTTTCCCGGAAAAGACCAACCAGCCG
AAAGAGGTGATCCTCGAGATCGAACACCTGACGGCGAAGAACCAGCCCTCCATTCAGGACATCACTTTCA
ACCTGCACAAGGGGGAGATCCTCGGCATCGCCGGACTGGTGGGGGCCAAGCGCACCGACATCGTCGAGAC
CCTGTTCGGTATTCGCGATCGCAGTCAGGGCAGCATCAAGCTGCACGGCAAAGAGGTAGCCAACCACAAC
GCCCACGAAGCGATCCGCAACGGCTTTGCGCTGGTGACGGAAGAGCGCCGCTCCACCGGTATCTACTCCC
GACTCGATATCGCCTTCAACTCCCTGATCGCCAACATGGACAGCTACAAGGGCTCCATGGGGCTGCTCAG
CGACAACAAGATGAAGAGCGATACCCAGTGGGTCATCGACTCCATGCGGGTCAAGACGCCGACCCAGCAG
ACCCAGATCGGTTCCCTCTCCGGGGGCAACCAGCAGAAGGTGATCATCGGTCGCTGGCTGCTGACCCAGC
CGGAGATCCTGATGCTCGACGAGCCCACCCGAGGCATCGACGTCGGGGCCAAGTTCGAGATCTACCAGCT
CATCCTGGAGCTGGCGAAGAAGGACAAAGGGATCATCATCATCTCGTCAGAGATGCCGGAATTGCTGGGG
ATCACCGACCGCATCATGGTGATGAGCAATGGCCGGGTCGCCGGGATCGTCAATACCAAAGAAACTGACC
AGAGCGAGATACTGCGCCTGGCATCTCTGTACCTCTAGAGACAGGAAGACATTTATGGAGTCGGCTAAAA
ATTTCAATCTGATGCAGGCGCTCAAGGAAAACGCCATCTATCTGGTGCTGCTGGTGCTGCTCATGGTCAT
CGTGATCCAGGAGCCCTCCTTCCTGAGCCTCACCAACTTCAGCAACATCCTGACCCAGTCCTCGGTGCGG
GTCATCATCGCGCTGGGGGTGGCGGGCATCATCATCACCCAGGGGACGGATCTCTCGGCCGGTCGTCAGG
TCGGTCTGGCGGCACTCATAGCAGCAACCATGTTGCAGGCGTTGACCAACGTCAACAAGGTGTTCCCGAC
GTTGGGGGAGGTGCCCATTCCTGCGGTGATCCTGCTGGTGTGTGTGGTGGGGGCGCTGATTGGTCTGGTC
AACGGTCTCATTGTGGCCTACCTGAACGTTACCCCCTTCATTACCACTCTGGGCACCATGATCATCGTCT
ACGGTATCAACTCCCTCTACTTTGACTCGGTAGGGGCCTCGCCGGTGGCCGGGTTTGACCCTCGCTTCTC
CAGCTTCGCCCAAGGGTTTATCCGGATAGGAGGATTCAAGCTCTCCTATCTCACCTTCTATGCGGCCATC
GCCAGCGTCTTTGTCTGGGTGCTGTGGAACAAGACCCGCTTTGGCAAAAACATCTTCGCCATCGGCGGCA
ACCCTGAGGCTGCCAAGGTCTCCGGCGTCAACGTGGCGGTCAATCTGGTGATGATCTATGCGCTGGCTGG
GGTCTTCTACGCCTTCGGCGGCATGCTGGAAGCGGGCCGGATCGGTAGCGCCACCAACAACCTTGGCTTC
ATGTACGAGCTGGATGCCATCGCGGCGTGCGTGGTGGGCGGCGTGTCGTTTGCCGGCGGGGTGGGAACGG
TCGCTGGTGTGATTACCGGCGTACTTATCTTCACCCTGATCAACTATGGTTTGACCTATATCGGCGTCAG
CCCTTACTGGCAGTTCATCATCAAGGGCGGCATCATCATTCTGGCGGTGGCCCTGGACTCCATGAAGTAC
GCCAAGAAGAAATAACGATTGGCAGGGAGGGTTGGCGTTGCCAGCCCTCTCTTCTGGAGGGAACCCATGC
CATTCAAGGTCAAGATTTACCTCTTGCTGCTATTGGCCATTCTGGTCACTATCGGCACCGCCGGGCTCAG
CGTGAATCACTTCATCAGTGGTTACATTCGCACTCAGGCCACCCACAACATCAACGAACAGATCACCCTG
GTCAAAGAGAAGCTGGCCAGCGACATCAATCAGAAGATCCTGTTGGCCGCCAACCTCAATTTCGGGGTGA
CCAACGTCAAGAAAACTCTGCAAGAGACCGGCTTTCACAACATCGTCAAGGTGATCGGCAACATGGCCTT
CGACGTGAGCGGTGTCATCAACGACAGCGCCAAAGTGGATGCCCTGCGCAAGCTGATCCCCGCCGCCAAC
AACCAGATCACCGTCAGCCCGCTGCAACAGGTGGATGGCAAGCCGGTCATCACCATTCTGGTGCCGCGCG
GTGCCGATTCTGCCTATCTCTACTACCTCGACATGAGCGAGTTCAAAAGCCTGCTGGAGAAGATGAGTGG
CGCTGGCCGCTACTTCTCGCTGGCTGACAGTCAGGGCAACAACCTCTATGACAGCAAGCCGCAGGGGGAG
AGCGAAGCGCGCCCCAACCAGCTGGATATTCGTGGCAGCAGCTGGACCCTGACCGGCTACATCGACCTTG
ACTACATCCGTGCCATGACCCGGTCACTCAATGGCCAGATCATCATGGCGCTGTGCGCTGTGCGCTGTGC
GCTGTGCGGTGTGGCGATCGTGGTGATGGTGCTGGCGATGCTGGCGCTCAATGTGGCCTATCGACCCATT
GTCTCGTTGCGGGATCTGGTGCTGGAGCTCTCCCGTGGCAGCGGCGATCTCACTCGCCGGCTGGCGGTGA
CCAGCAAGGATGACCTCGGTCAGATCTCGGCCGGGATCAACCGCTTTATCGAGCGGCTGCAGGAGATGAT
GGGGGAGGTGCGGCAGGCGAGCGGCCAGCTCAACAGCGGTATCGAGGGGCTGGCCAGCCAGACCGGCTCC
GCCCAGTCTCTGCTGGCGGACCATGTACGCGAGACCGAGCAGGTGGTGACCGCCATCAACGAGATGAGCC
GCACCGCGGTCTCCGTCTCCGAGAGTGCGGCCAGCACCGCCCAACTCACCGGCCAGAGCCAGCAGCTGGC
GAGCCAGTCTCGTCAGGTGGTGGATCAGGCGATGGCCAGCGTGATGGCGCTGGTGGATGAAGTGGAAGCC

ACTGCCCGCTCCATCGAGGCGATGCAGCAGGATGTGCAGCAGATTGGCTCTGTGCTGGGGGTGATCGGCA
GCATTGCCGAACAGACCAACCTGCTGGCCCTCAACGCCGCCATCGAGGCGGCCCGCGCCGGTGAACAGGG
GCGCGGTTTTGCGGTGGTGGCAGACGAGGTGCGTTCCCTCGCAGCCCGTACCCAGCAGAGTACAGCCGAG
ATCCAGACCATGCTGGCCAGTCTGCAGCGGGGCACCAGAACTGTGGTGGATGCCATGGGCAACACCAAGC
AGAGCTGTCAGGGGGCGGCCGAGAACACTACCAGGGTCAACGAGTCGCTGGATCTGATGGCAGGGGAAGT
GGTGGAGATCAACGATCTCATCAGCCATATCGCCACGGCGGCGGAGCAGCAGAGTGCGGTGGCCGAAGAG
ATCAATCGCAACATGAGCGCCATCGCCGAAGTGATCCGCCAGCTGACCCACAATGGCGAAGCGACGGTTG
CCAGCTCCGCTGCGATGCAGCAGACCTACGACCGGCTGCGGGAGATCGTGGGCCACTTCCGGCTGGAGTG
AGGCCGGTGTTCAGTCAATAACAAAACAGGGCCATCCGGCCCTGTTGTTGTTTTCCTGTCTGCCTGAATC
AACCGGCCTTGCGGCGCAGATAGACCCCGGCTTCCATGTGATGGGTCCTGGGGAACTGGTCGAACAGGGC
GAAACGGGCGATCTCGTGGGTCTCGCCCAGTACCGCCATGTTGGCCTGCAGAGTCTCGGGGTTGCAGGAG
ATGTAGAGGATGTTGTCGTACTCCTGCACCAGCTTGACGGTGGCATCATCGAGACCGGCGCGGGGCGGGT
CGACGAAGATGGTGTTGCACTGGTAGCTCTTGAGATCGACCCCCTTGAGGCGGTTGAACTCCCGCTCGCC
ACGCATCGCCATGGTGAACTCCTCGGCGGACATGCGCAGGATGATCAGGTTGTCGACGCCGTTGGCGGCG
ATGTTGAACTGGGCGGAGTCGACGCTCGGCTTGGCTATTTCGGTAGCCAGCACCTTGCGGAAGTTCTGCG
CCAGCGCGATGGAGAAGTTGCCATTGCCGCAATAGAGCTCCAGCAGGTCCCCTTCGCTCCCCTTGGTCAC
ATCCAGCGCCCAGGCCAGCATCTGCTCGTTGATGGCGGCGTTGGGCTGGGTGAAGCTGTTCTCCACCTGC
TTGTAGATGAGCTGGCGACCCGCCACGTTGAGCTGTTCGATGACGAAGTCATCCCCCAGACAGATCTTCT
GCTTGTGGGCGCGGCCAATCAGCTGTAGCTCGAACCCCTTGGCGCGCAGATCGGCCTGCAGCACTTCGGC
GGCTTGTTGCCATTCGGCCTCCAGCTTGCGGTGATAGAGCAGGCTGACAATGATCTGGCCGGACTGGGTG
GAGAGGTAGTCGATCTGGAACAGCTTGCGGCGCAGCACCGGGTGTGGGCGCAGCCCCTCCAGCAGCAGCG
GCATCAACTGGTTGATGAGGCGGCTGGCGGTGGGGAAGTGGTCTATCTTGATGATCTCTTTGGTCGCCGG
GGCATACATGCAGTGGAAGAGGTCATCCCCCTCGTGCCAGATGCGAAATTCGGCGCGCATCCGGTAGTAC
TCCGCCGGCGAGCTGTGCACTTCCAGCGCCGGGAGGGTGAAGCCGGCAAACAGCTCGGTGAGGCGAGCGC
GCTTTTCGTCGAGTTGGGCCTGATAGTCGGCCGCGGTTCTAGGCGCCGGAGCGTTCGCGGAAGGCTGTTG
GGATTGGGTCATAAATACCTCACATGCACACCGGCCGGGCCGGGTCGAAATCGGGGGAAGGCAACAGGCC
ACCCAAAGGGGCCCGATTTTAAAAGCCAGCCGCAGATTGTCCAGCATTTTGTGGTCAAGAAGTGTTCAGT
CAGGCCGATAGCTGATCTACAGGCATGGTGGGAGGACACATTATGCAGATCGAAGGCGTGGGCGCAGCCC
AACCCAAGGGAGTGGGTGTTTCTCCCAAATCGCCCCAACAGGCGGAGCAGAAACAGGTCAAGAGCGAGGA
GGAGGTCTCCTTGCACAAGCCGCCCAAGTGGGCGCAGGTGCTGATCCAGCAGGCGTTGCAGTTCTCGCTG
GAGATCAACGGCCAGGGCTATCAGGCGCCGAAGGTGAAAGAGCCCGAGGAGATCACCCCGGAGACCCTGT
TCGACTTCCAGTCGGTGGCGGACAACGTGCTGCAGTTTGTGACCGGGCGGCTTGGTGCCGCGCGGGCCGA
TGGCAAATCTGACGACGACCTGACCGGGATGATGGAGCAGGCCCGCAAGGGGGTCGACAAGGGCTTTGAG
GAGGCGAAGAAGCAGCTCGGCAAGTGGGCGACCGACAACGACGATATCAAAACCGGTATCGATCAGAGCT
ACAAGCTGATCCAGAAAGGGCTCGAGGAGTTCGAGAAGGAGTTCTTCGGCAAGGTCTCCCCGACCGACAT
GGGACAGGCCGAGATGGCCAGTCGCCAGCAGGGGTATCTGGAGATCCAGACCAAGGATGGCGACAAGGTA
GTGCTGCGCTTCAACGACAGCTGGCAGACCAAGTCCCAGAGCAGCGAGAGCGGCAGCCAGTTCAGCCTCA
AATCGAGCCAGAGCTTCAGCTTCTCGCTGGAGGGGGATCTCAACAGTGACGAGATGGAGTCCATCGGCAA
GCTGGTCAAGGGGATCGACGAGCTGGCGGGGAACTTCTTCTCCGGCAACTTCGAGGATCTGCTGGACAAG
GCGGGCGAGCTGAAACTGGATGACAGCCAGTTGGCCTCCTACTCCCTCAAGCTCAAGCAGAGCGTCAAGC
TGAGCCAGACCTATCAGGGGGCGCACTCCCTGCAGGAGCTGATCAAGCCGTTTGCCGACTATCTGCCGAA
GCTGGATCAGGTGCAGAAACAGGCGGATAGCCTGCTGCCACCATCCCAGCAACAGGCGCTGACCCCGGCC
GTGTTGGCCGCGAGAGGAGAAGAGGAACCGGCGCAGGTCGATCGCTTCACCAGCTTCAACCAGCGGATGC
TGGAGGCGCTGCGGATGATTAACCAGTTCCAGCCCTCCACCGAGCCCGCCAGTAAGAACGTCTGAGCCAC
AGCGCGTTACGCAAGTAATGACCTGCCGTTTGCCAGCCCTTCCCGGGGCTGGCAAACTGGCCTTTTCTCC
AATCCGGTTGCCCCATGAACCCCAAGAAGCCAAGAGACAAGCGCGAGCTCTGCTACCCCTTTATCTTCGA
AACCTCGCTCAAATGTGATTTTGAAATCGCCACCGACGAGCTCTGTTGTCAGGTTGGGGCCGTGCTGTCA
CACCTTGCGCCGGATGCCGATACCGAGCCCATGCTGGCGGATCTCGCGGCGTTGCAGCGACTTATCTACA
ACCTAAACGGCTCGGTACGCGGCAAGCTGGGGATCTTCGATGCAGATCTTGAGTGGCTCAAGGCCCGTTA
CGACCACTACAACGAGGCGAGTCGCGGCAGCGTCACCGGTTTCGTGCTGCCGCAGGGGCCGCAGCCCATT
CCCACCCTCCATCTGTGCCGCTGCGGCAGCAAGAAGGTGGTGCGGCTGCTGGTCAGGCTGGAAGAGAGCG
AGGTGGCCTTCGATCCCATTCTCTATCGCTTCACCAACCTGCTGGCCAACTTCTTCTTCGTGCTCACCGT
CTACCTCAAGCAGCGCTGGCAGGTGCCGGAAGTGCCCTACGTCAGCATCAACTACTGATGGCGGCTGGTT
AGGGGCTAGACGTCATGGTGGATTGAACGGAATAAAAAACCGGAGCCTTGGGCTCCGGTTTTGTTTTTCA
GCGCGTCAGTTGCCCGCTCAGCTGGAGATCAGCACCCGGGCCGAGTTGGCGGGGATGGTGAGCTGTACCT
CGCCCTGCACCACTTCAAACTTCTCGCCGTTGAAGGCGTCGGTGAAGCGGCTGGCAGGGCTGGCGGTCTG
CCACAGGGGCAGGCTGATGGTGCGCGGCTCGCTCGGGTGGCGGTTGCAGGCCACTACCACCTGATCGCTT
TGCAGGGTACGGGCAAACACATAGCTGTGGGGGCCGGCGTAGAGGGTCTGGATATCGCCACGGCGCAGGG
AGGGGCGCTGGCGGCGGATCTGGATCAGCCGACGATAGTGGTCGTGCAGCACGTGATCCCAGTCAGTGGT
ATCCCACGGGAAGCAGCGGCGGCAGTCAGGGTCGTTGCCGCCAGAGAGCCCCACTTCATCGCCGTAGTAG
ATGGAGGGCACGCCGATATAGGTGAGCAGCAGGGTGGCGGCGAGACGCATCCGCTGTTTGTCCTCTCCCA

GCAGATGGAGGAAGCGGGCGGTGTCGTGGCTGTCCAGCAGGTTGAACTGGGCCAGCTGGTTCTTGAACGG
GATGTGGGCCCGCGCCAGCTTGAGCCAGCGATCCAGCTCTTCGGCGCTGATCTTGATGGGGTGGTAGGCG
ATGTCCTTGCCAGCCAGGAAGGCGCGGATCGGATGGGCGAAGCCGTAGTAGTTCATGGCACCGTCCTCCT
GATCTCCTTGCAGCCACTGGCTCGCCTCGAAGAAGTGTTCGCCGAGCACGTAGGCGTCCGGGTTCTCCTG
CTTCACTGCCCCGCGAATGGCGCGTACATGGCTGCGGTTACCCTCGGCGGTGCCGCGCTCACCCAGCATG
TGGATGACGTCAAAGCGCCAGCCATCGATCTGGTAGGGGGCGCGCATCCAGTAACGGAGGATGGCGTCGT
CAGCCCGATAGACGGCGTCTTGCACCTTCTCGCAGGAGAAGTCGAGCTTTGGCAGGCTGCGAATGCCCTT
CCAGCTGACGTAATCCCCTTCTTTGTCAAAGGTGTAGAAGCTGCGCCACGGGGAGTCCGGGTTGCTCTGG
GCCCCCAGTGGCGGGCAGAACCAGGGGTGCTCGGTAGAGGTGTGGTTGACCACCGCATCGAGGATGATCT
TCATGCCACGCTGGTGCAGGTTGCGGGTCAGCTCGGCGAACTGTTCGTTGCTGCCGAGATGGGCATCCAC
CTTGAAGTAGTCCTGGGTGTCGTACTTGTGGTTGCTCGGCGAATCGAAGATGGGGTTGAGGTAGAGCGCC
GTCACCCCGAGAGATTGCAGATAGTGCAGCTTGGCATCGATCCCTGCCAGATCGCCGCCATAGAATTCGC
AGGCGCCGTGGCCCTCTTCGTGGCGGCTGACCGGCTCGCCCCACGCCTTGCTGATCACCGCCTTGCCACG
GTACTCGTACTCGTGATGCTTGACGCTCAGCGACGGATCGCCGTTGCAGAAGCGGTCGGGGAAGATCTGA
TAGAACACCTGATCCTGCACCCAGGCAGGTGGCTGGTGCAGGCTGTTGAAGCGAAAATGTTGCTCCCGTG
GCGGCATGGACGCGCTGACGCCCGCCCCGTGCAGCCACCACTGCTCCTCGTCGGTGAGGCACTTGAAGCT
GTAGAGGTGGATATCTTGTCCCTTGCTGAGCGGCAGGGTCGCCTGCCACACCTGCAGCCGATCGCGGGTG
GAGACTGCCGTCATCGGGATCAGGTACTCCTCGTTATCCGGCTCGTGGCGCAGCCACACCTCGCGGATCG
GCGCATCCAGCTCGCTAAAGAGGGTCAATTGCAGTGCGTCGGCGCTCTCTTTGAACCAGGGCGCCACCTG
GGGATGAAACAGAAACGGATGGGTCATGATGCTTATTGAGTCAGAAGATCGGCGAGGGCATTACCCTGTT
CATGCAATGTTTCGAGCTGGCCTGCCAGCGACTTGGGATCCAGTCCCAACTGGTCGGCGAGCGGTTTGGG
GAAGCGGGCGATGATGGCCTGGGCAGGCACCCCGGCGTTCCAGGCGAATGAGATAAACACGGCGAGGCGG
ATCAGCACCGCCTCCTTGCTGACCGGATCGTGGAGAGCGGGTCAAGCTGTTGGGAGATGGCATCGACGA
AGGCCTCGGAGAGGTTCCAGCGTCGCGCCAGCTCGGCACCGAGCTGGGCATAGTCGTAGCCCAGCATCTC
CCGCTGGGCCTCGACCCGGCTGCTGCCGTTCTGGAGCGCCATGTTGATGAGCTCGGCCTCTTCCGGCAAG
GTGCTCTGGATAAGCAGTTCGCCGATGTTGTGGATCAGGGCGCAGGTGAAGGCGGTCTCGGGATCCATCA
CCTTGGCCTCCTGCGCCAGGGTTTTGGCGATGGTGGCGACCTGGAAGGTCTCGGTCCAGAACTTGTTCTT
GTCGAAGCTGGGCGGGGCCTTGAAGGTGCCGATGATGCCGGAGGCGACCACCACGGAGCGCAGCCGGTTG
AAGCCGAGCCGGATCACCGCCTGTTCGATGGAGGTCACTTCGTTGCCCCGGCGCAGGGCGGCCGAGTTGG
CCATCCGCAGCACCTTGACGCTGATCACCTGATCCATGGCGATCTTGGCCGCAATCTCGTCGATGCGGGC
ATTCTCGTCGTTGAAGCTCTGCATCAACTCGTGCAGCAGCTTGGGAATAGTGGGGAGTTGTTTGATCTTG
TCAAACAGGCGATCCATCGACATGGCTGAGGCAGCCTCCGTTGCGAACTCTGCCTCATATTGTGCCACAG
CCGGACAACATGTTCCTGTTTGTGATGCGGCTTTGTGAGCGCTAGAACACCCAGTCGCGCAAGGTGTATT
CCAAACGGTGCTGTTTGCCCTGCAGGATCAGGTTCTGGCTGGTGCCGCTCAGGGTCGCACCCTCGGTAAG
GGTGGTCAGTACGGCCTGTTCGATAGTGTCGAGCGGCGGCATGCAGGCCATTTCGGTGCTGCCCAGACTG
GTGACCCACAGTTTGTCATCTTCCAGATGGCCCTGACCGAAGTAGCGGTTGCAACCGGCGATGCCGTTGA
CGGTGAAGTGCTCGCCGATCTCGAAATCGGGGGGATTGTCGCGGGGGGCCTTGATCGGTTCGCCATCCAG
TTTGCTCAGTACCCAGTGGTGGTGTTGCAGATCTTGCTGAACAAAGGCGGTCGAGCTGCAGCCAGCGAGC
AGTAACAGGGGGATCAGCAACAAAAAGGGCAATCTGGGCATGAGGGGGCTCCGGTTGGGGGAGCGTCAAG
AATACCCTTTTTGGGCAGTCGGGAGCAGGGCGGGAGAGGGGACGACCGACCGCTTTTGTCGTCCCTTGTC
CCGCACGACTGTCGCTTAATGGCAACACCTTGGTGATATTGCCATCAATTTACGCTATCTCAGGGATCAG
TTGGTGAATAGACGCTCGGCATAACGGGCGAGACCCGCAGCCACCGAGCCAAAGTCATCGCCACCCTCAA
GCGGGATGGAGGGGAGCTTCTGGCGGATAAAGCTGGCGATCAGCGGCGAGCGGGCGCTACCGCCAGTGAC
GAAGATGCGATCCGGCTGCACACCGGCAGCAGCAATCGCTTCGTCCATCAGCTCGCCAATCTTCTCCAGC
AGCAGGGCGGAGGCGTTGGCCAGCTGTTCGCGGGTCAGTTCGGCTTCCAGCCCTTTTTCCAGCTCGGCCA
GCGACTGGCGATTGAGCTCCTGCTCCGAGAGGGCGATCTTGCCCTGCTCGGCACGCCATACCAGCTGGTG
GCTCAGCTTGTGGGCCCGCAGGCTGGCCAGACGTCCCAGCTTGCTGCCGGGCTGGCTGTCGAGCTGCAGA
TCCTGCAGCTGGCGGGCGGTGTCGAGGCTGTAGAAGCGGCTCTGGGCGCTGACGTCATTGATGGCGGCCG
CATCCCAGAACAGCGGGTGGGGCAGCGGCTTGCCGGTCTTGAGGGTCTCGTGCATGCCGAGCAGCGGCAT
CATCCCCTCCACCGTCAGGCGGATATCGAAGTCGTTGCCGCCGATCCGCTGGCCGCTGTGGCCGAGCAGA
TCGCCGCTGCGATCCAGCTGATCGCGGTAGCTGGGGCCCATCCGCAGCATGGAGCAGTCGGTGGTACCGC
CGCCGATATCCACCACCAGTACCACGGCGTCTTCGGTGAGGCGCGCCTCGAATTCAAACCCGGCCGCCAC
CGGTTCGTAGAGGAACTCCACCTGCTCGAAACCGGCCAGACGGGCTGCCTCGGTCAGGATGGCGATAGCT
TGCTGGTTGCTCTCTTCCCCCGCCAGACCCTGGAAGTTGACCGGACGGCCGATCACCGCCTGGCGGATCG
GGGCGCCGAGCTGCTGCTCCGCCTGCTGGCGAACGTGGAGCATCATGGCGCAGACGATGTCTTCAAACAG
GGCGATCTGGGGAGCCTTGAGGCCATAAGCACCGAGGAAGGATTTCGGCGATTTGATGTAGTAGCCGTCG
TCCGGCTCTTCCAGATAGCGCTCCAGCGCGGCACCACCAAAGCTCAGCTCGTCGATCAGTCCCTCTTCGC
GCATCTGGCGGCGAACCATCTGGGCGCGGGTGACCACGGGGCCACGCAGCTTGAGGTACTCCGCCTGCTG
GGCGGGTGCCAGCCCTTCGGCCAACAGACCGGCGATAGCATCCCGGTGGGGGGCATGCAGGGTGGAGGGG
AGATAGCGGGATGCACCAAGATTCAGCAGCCTGGGGCTACCTTGTTCCATCACGGCCACGGCACAGTTGG
AGGTGCCGTAATCAAATCCGATAAACATGGGCGCCAGACTCCTGAATAAAAAAGGGGGGCTAGCCTACCC

GAAGGCGCCGGGTCTCACAACCCGGCGCGTGACGAAGAGAGGGCGAGCCCTTATTCCACTTCGTTGTCGC
AGCGGCCCTGGCTCTCGATGGCGCGGATATTGCCGAGGGTGGTATCGGCGATGGCGTGCAGCGCCTCGCG
GGTCAGGAATGCCTGATGGCCGGTGAACAGCACGTTGTGGCAGGCGGACAGACGGCGGAACACGTCATCG
GTGATCACTTCGTTGGACTTGTCGGAGAAGAAGAGATCCTCTTCCTCTTCGTAGACATCAAGCCCCAGCG
CGCCGATACGGGAGGTCTTGAGTGCCTCGATGGCCGCGTTGGAGTCGAGCAGGCCGCCACGGCTGGTGTT
GATGATCATCACCCCGTCCTTCATCTTGGCGAATGCCTCCTTGTTCAACAGGTGGTAGTTCTCTTTGAAC
AGCGGGCAGTGCAGGCTGATGATGTCGGATTGGCGGAACAGAGTATCGAGATCCACGTACTCGGCCCCCA
GCTCGATGGCTGCCGGATTCTGGTAAGGATCGTTCACCAGCAGGCGAACCCCGAACCCCTTGAGGATACG
CAGGGCGGCCAGACCGATCTTGCCGGTACCGATGACGCCAACGGTCTTGCCATGCATGTTGAAGCCGACC
AGCCCTTCCAGCGAGAAGTTGGCGTCGCGGGTGCGCTGATAAGCCTTATGGATGCGGCGGTTGAGGCACA
TCATCATGCCGACAGCATGCTCGGCGGTCGCCTCGGGGGAGTAGGCGGGCACTCGCACCACTTTCAGCCC
CAGCTCTTTGGCAGCGGCCAGATCCACGTTGTTGTAACCGGCGCAGCGCAGGGCAATCACCTGGGTGCCG
TTGGCAGCCAGTTCGGTCAATACCTCGCGGTCGGCATCGTCATTGACGAACAGACACACTACCGGGAAGC
CGTGAGCCAGTCGGGCTGTTTTGGCCTCCAGCCGCACATCGAAAAATTCCAGGTCGAAGCCGTACTGGCT
GTTGGCCTGGTTGAAATGCTCTTTGTCATAGCTCTTGGTACTGAATACGGCGACTTTCATGGTATGTGCT
CCGCAGCGTGTGTGTCGTCAAATTACAACAAAATGGGTCAGCTATCACCGGACAAACCGGCTGGTTATTG
TCCCCACGGCATGATGGGAACGGCGCTGACTGCACTTTTGTACGAACCATCCACCAGTTTGGTGCTGTAG
GTCAGGTAAACCAGTGCATTGCGTTTCTTGTCGTAAAAACGCACCACTTTCTGCTCCTTGAACACCAGCG
AGGTGCTGACATCGAACACCTCTTCCCCCGCTTTCAGCTTTTCGGGCAGGGTGATGGGGCCGATCTGGTG
ACACGACAGGGAGGCGTGGGAGGGATCTTCCGCCAGTCCCAGCCCGCCTTTTACACCACCGGTCTTGGGG
CGTGCCAGATAGCAAGCGACGCCGTCGATGCGCGGGTCGTCGAACGCCTCCACCAGGATCTTGTGGTTGG
GGCCGAAGAGCTTGAAGGCGGTGCTCACTTCACCGACCGGGTCGGCCTTGTCGGCCAGTGCCGGGCCGGC
AAACAGGGCCCCCAGTAGCAGGGGCAGGGCGCGGCGCAGGGCGTTATGTCGCGTGTGGTTATGTGTCATG
GTCTCTCCTGTTGGCCCGGCACTCCTCAATAGGGAATGCCCAGGCGACGATAGAGGCTGGCAAGCACCCA
GGCGGGGCCTATCAGCAGAAATTGCAGGTCGGCGAGGAAGGAGGGGCGCTTGCCCTCGATCTTGTGGCCG
ACAAACTGGGCTATCCAGAGCAGCACGAACGCTGCCACGGCGGGCCACAACAGCGGCCCCTGCCATTGGT
GGCAGCAGGCCAGCCCGAGCGCTGTCAGCCCGGCCATCCCCGCGAACAGCGAAAACGACAGCTGCAGATA
GAACAGCAGCACCGGAATGGCCGCCACTGCCGCCCAGTTGAGGAGCTCTGGCAAGGGCCCGTGTGGCAGA
CACCAGAGCAGCGCCAGCGAGCAGAGATAGATGCCCGGCACCGCCAGCTTGTGGATGGCGACGTTGACCG
GATGGCGATGGCTCTGGCCGTACTCCTCAAACCACTCCTGGATTCCCTTCATGGCACCTCCCTGGCGCAC
TGTGTTCTCGCAGTCTGACCTTCGTGGTCAGGCGTTCGTGATGCTTATCCTTGCGGTGGATACTCCCAAT
ATTGGGCCGGAATGGATTTTAGCAACGGGGTCATATCCGGATTGGGCAGGGCGATGGTGACCCGCTGATC
GAGGTTGGCGCTGGCGAGCGCTTCGCGGATCTTGGGGTTGTCATGGAAGAACTTCATAAAGCTGCCATCG
GCATAGGCACGCTCCAACCCGGTCTGAATCGCCTTGGCCAGCTTGGGGTATTCGGGGCTGACGAAGAAGA
ACTGGGCGAAGGGGTAGCGGATCAGCAGATGCTGCTCGATGGCGAGGTTGGGATACTGCGCCACCAGCTC
GCTGCGCTCGGCGAAGATCTCCACCAGGGCGCGCGGGAAGAAGTCGATCTTGTCGCCGTCGTTGATAAGG
CGAAACAGGCTGCTGTATCGGCCGGAGAAGGTCGGGATACCCGCATTTTCCAGAATTTTGTTATCGGCCC
AACCGACCCCTTGCGCCGCCTTCAGCTGTTTGAGGTCGGCCAGGGTATTGATCTGGTTGAACTTCTCTTG
GGCATCTGCGTGGATGAAGGACAGGCGCAATCCTTCCAGCCCGCGAAACAGCGGAATGGGGACGGCAATC
AGGGAGGATTCGAGCTCGCTGGAGGTGCCCATCCAGAAGACGTTGATGGTCTTGCCCTTGCTCAGCTGCT
GCTGGAGGGTGAACTGGTTGAGGTCGAGTTCGACAGGCTGGATGCGAAAGGGTTGCCCGGAGCGGGTCAG
GGCCAGTTCCAGCAGCTTGTACTGGTACTCCTGGTCAAGATTGTTGACGGGACGGCTGGGGACCTTGACC
GTCAGAACCTGTGCTTGTGCCAGCAGTGGCAGGCACAAACACAGCTTGATGAGCCATTTCACTCTCTGTT
CTCCTGTAGATTCCGGAAGGTGTCCGGAGTCTAACAGAACTTGTCACCATCTTGCTGCGAGGCGTTGTGC
AAGGTTTGAGCGCGGAGCCCTGCCTTGCTGGCGTCTGTTGCACCAGAATGGTGACGGTTCTTCGAAGAAT
CAGGCGACGCGACGGAACACGTGGGTATCGTTAAATTCCTTGGTGCTCTGGCATTCGACGCAGAAACGGG
ATTCCGGACGGGCATGCAGCCGCTTGAGGGAGATCTCGTCACCGCAAGATTCGCAGTAACCAAAATCCCC
TTCGCTGATGCGCTTCAATGCGCCCCGCAGCTTGCGCAGGTGCAGCTTGTCGTGCTCGATGCGGTTCAGC
TCCAGACGGCGGGCTTCCTCGATACTGGCACGGTCAATTTCATCAGCCATCTCGTTGGTGTCGGTGGCGA
TGGCCTGGCTGGCCTGGGAGTTCAGGCGCTCTTCGAGCTCGACGATCTGTCGCTCGATCTGCCCCTTGTA
AAAGGCCAGTTGGTCTTCAGTCATGAAATCACACATACGATTAACTACCAGTCAGTTTACTAACCCAATT
ATCCCTGTTTATGGGGGCTGTCTGCCTACAGACCAAGCCCTTGTATCCCGATTTGCACCTGTTTCATGCA
AGAGAGTGCAAATCAGGGCGGGCCTTGTACCAAAAAAATGCAGCCAAGCAAAGCAAATAGGATAAGGATT
GTGCGCCCTTGCAAAGAAATGGTCCGTTCCTGCGGGGGATTGGTCGGGATCTGGCGTCCGCTTCACCCTC
TGTTGGCATCGCCTTTGCAGCGATGAGAAGGGTGGGCTTCGTACGGATTTGAGTGTTTAGTATTTGGTGC
CGCCTTGGTCATTATATGCGCCGTTATTTATAGTTTTTTAAACACGCCCGCTTGACATTGCCCTTGATGG
CGGGCTTTTATGGTGATGTTGATTACAATTTTATAACAAGGGTGTTCCATATATTAGACACCCTTGCCAA
TGGCAACCGTAAAGTCGGGGTTTAACATGGACAGTATCAAAGGCTGGATGGAGAGCAATCGCATCACGGA
AGTGGAGTGCCTGTTCCCGGATTTCACCGGCAACGCCCGGGGCAAGATCATCCCAGCCAACAAGTTTTTG
CGCGAAGGGGGCATGCGTCTGCCCGAGGTGATTTTCACCCAGACGGTGACCGGCGAATATCCGGATGACG
ACAGCATGATCGACCCGCTGGAGCGGGACATGCAGCTCTATCCTGACCCCAACACCATCCGTTTTGTCCC

CTGGGCGCTGGAGCCCACTGCACAGGTGATCCACGACTGTTTCGACGTCAACGGCAAGCTGATCGACATC
GCGCCGCGCTCCGTGCTGCGTCGGGTTCTGTCATTTTACGAGAAGCAGGGCTGGAAGCCGGTGGTGGCAC
CCGAGCTGGAATTCTATCTGGTCACCCGCAACGAGGACCCCGACTATCCGCTGGTGCCGCCGGTCGGTCG
CAACGGCCGCCCTGAGACCGCCCGCCAGTCGTTCAGTATCGATGCGGTCAACGAGTTCGACCCCATCTTC
GAGGATATGTATGACTACTGCGAGGCGCAGGAGCTGGAGGTGGACACCCTGATCCACGAGTCGGGCGCCG
CCCAGATGGAGATCAACTTCCTGCATGGTGATGCCATCGATCTGGCGGATCAGGTGTTCCTGTTCAAGCG
CACCATGCGTGAGGCCGCTCACCGCCACGGCATCTATGCCACCTTCATGGCCAAGCCGATGAGTGACGAA
CCGGGGTCGGCCATGCACATCCACCAGAGTCTGGAAGATGTGAACGGCCACAACCTGTTCGGCACTCCGA
ACGGCGGCAACAGCGAACAGTTCATGCACTTCATCGCCGGTCTGCAGAAATACACCCCGGCGGTGACCGC
CCTGCTGGCCCCCAACGTCAACTCCTACCGCCGCCTCACCTTTGGCGAGAGCGCGCCGCGCAACGTCCAG
TGGGGGGTCGAGAACCGCACCTGTGGTCTGCGGGTGCCCTTCTCCGACCCGAGCGCACGGCGGGTGGAGA
ACCGTTTCGCCGGTGCCGATGCCAACCCCTATCTGGCGCTGGCCGCCACTCTTGCCTGTGGCTATCTCGG
CATGATGGAGAAGCTGGAGCCGCTGCCGGAGATGAAGTCGAGCGCCTATGACCTGCCCTACAGCCTGCCC
CGCTCGCTGGAGGAGGCACTCAGCCATCTCGAACAGTGCGAGCCCATCAAGGAGATGCTGGGGGAGCGCT
TCACCCGCGCCTTCGTCGCGGTCAAACGCAAAGAGTACGAGACCTACTTCGGGGTGATCAGTCGCTGGGA
GCGGGAGTTCCTGCTGCTGAACGTTTGATCCCCCTGTAACGGGGAGGCCGGAGTCGGCCTCCCCGGCCCC
TGTATCGAAGAGACCTGAACGCGAGTCAGGTGGCGGCAGCCACCTGTGAGTGGTTGTGGCTGCGAGCATG
CAAGCCCGCCTGATTACCCACCTGACTGTTTGACGACTGAATCTTGCTAAATCGAAGGAACCGAGGAGCA
ACAAATGCAGCAGACAACCCGTGAGTGGCAACAGCTGGATGCCAGCCACCACCTGCACCCCTTTACCGAT
TTTCAGGCCCTGAACAAGAAGGGCTCGCGCATCATTACCAAAGCCAGCGGCGTCTATCTGGAAGACTCCG
ACGGCAAACGCATTCTCGACGGCATGGCGGGCCTGTGGTGCGTCAATATGGGCTATGGCCGCCAGGAGCT
GGTGGATGCCGCTACCCGTCAGATGCAGCAGTTGCCCTACTACAACCTCTTCTTCCAGACCGCTCACCCG
CCGGTGATCGAGCTGGCGACCCTGCTGGCCGAGGTGACGCCAGCCCATCTCAACCACGTCTTCTTCACCG
GCTCCGGCTCCGAGTGCAACGATACCGTGCTGCGGATGGTGCGCCACTACTGGGCCAGCAAGGGTCAGCC
TGACAAACAGGTGATCATCAGCCGCCACAACGCCTATCACGGCTCCACCGTGGCGGGGGCGAGTCTGGGT
GGCATGAAGGGGATGCATGCCCAGGGCGGCCTGCCCATTCCCGGCATAGTGCACATCGATCAGCCCTACC
ACTTCGGTGAGGGGCTGGGGATGAGTGCCCACGAGTTCGGGCTGGAGCGGGCCCGTCAGCTGGAGCAGAA
GATTCTGGAGCTGGGGGTGGACAAGGTGGCTGCCTTTATCGGCGAGCCGATCCAGGGGGCGGGCGGCGTC
ATCATTCCGCCTGACAGCTACTGGCCGGAGATCCAGCGCATCTGCGATCACTACGGCATCCTGCTGATCG
CCGACGAGGTGATCTGCGGCTTTGGCCGTACCGGCCACTGGTTTGCCAGCGAAGGGTTTGGCATCAAGCC
GGACCTGATGTGTCTGGCCAAGGGGATCACCTCCGGTTATATGCCGCTCGGCGCTGTGATGGTGAGCGAC
CGGGTTGCCAAGGGGTTGGTGGAGGAGGGGGGCGAGTTCAACCACGGCTTCACCTACTCGGGCCATCCGG
TCTCTTGCGCCGTGGCGGTGGCCAACATCCAGCTTATGCAGAAAGAGAGCATAGTTAAGCGAGTGCATGA
TACTATCGGCCCCTATTTGCAGCGCCGTTGGGCGGAGCTGGCCGATCATCCGCTGGTGGGAGAAACCCGT
GGCAAAGGATTGGTCGCCGCGCTGGAAATCGTTGAGGACAAGCAGACCAACCGGCGTTTCCCGGCTCAGG
CAAATGCGGGGATGACTTGCCGGGAGTTCTGCTTTAACAATGGCTTGGTGATGAGGGCGGTAGGCGATAC
CATGATCATATCGCCCCCCTTGGTCATCACCGAGGAACAGGTTGATGAATTGGTCGATCTGGCCAGGCGC
AGTCTGGATCTGACAGCCGCAAAGCTGAAGGGTTGAGGCAAGTCGGGCAGGTTGGCATGCGCATACAATT
TGCTTAGGAGACAAGGATGATGTCTTTCAAAACAGGAGTGATCGCTGCCTCTGTGGCTCTGGTCATGGCA
AGTGCCGGAGCAATGGCTGAAGAGAAGGTGCTGCACGTCTACAACTGGAGCGACTACATAGCGCCGGATA
CCCTGGATAACTTCCAGAAGGAGACGGGGATCAAGGTGGTCTATGACGTGTTCGACAGTAACGAGGTACT
GGAAGCCAAGTTGCTGGCGGGTAGCTCGGGCTACGACATCGTGGTGCCATCCAACCCCTTCCTGGCCAAG
CAGATCAAGGCGGGCGTGTTCCAGAAACTGGACAAGTCCAAGCTCTCCAACTGGCAAAATATGGATGTCG
ACCTGCTCAAGGCGCTCGAGCCGAGCGATCCGGGCAACCAGTACTCCATCCCCTATCTGTGGGGCACCAT
CGGCTATGCCTACAACGCCGAAAAAGTGAAAGCCGTGCTGGGGGCCGATGCCCCGGTCAACTCCTGGGAT
CTGGTGTTCAAGCCGGAGAACATGGCCAAGTTGAAAGAGTGTGGCGTCTCCTTCCTCGATTCACCGACCG
AAATGCTGCCGGCGGCCCTGCAATATCTGGGCTTCAAACCGGATAGCCAGAAACCGGCCGAGCTGAAGAA
GGCTGAGGAGCTGTTCCTCTCCATCCGTCCCTATACAGCCTATTTCCACTCCTCCAAGTACATCTCGGAT
CTGGCCAACGGCAACCTCTGCGTAGCGGTCGGTTACTCCGGTGACTTGCAGCAGTCCAAGGCGCGGGCAG
CCGAGGCCAAGAATGGCGTCGCGCTCACCTACACCATTCCGAAAGAGGGGGCAGGCAGCTTCTTTGACAT
GATCGCCATTCCGGCAGATGCCAAGAACGTTGAGGCGGCCCACACCTTCATCAACTACCTGATGAAGCCC
GAGGTGATCGCCAATATCACCAACGTGGTGCAGTTCCCGAACGGCAACAAGGCGGCGACCCCGCTGGTGG
ATGAGGCGATCCGCACCGATCCGGGTATCTACCCTGATGCGGCGACCCTGCAGAAGATCTACACCTTCCC
GGATCTGCCGGCCAAGGTGCAGCGTCTGATGACCCGCAGCTGGACCAAGATCAAGTCCGGCAAGTAAGTA
ACGAATGCCCCTCCCGGTCCGCAGTTCGGCCGGGAGGGTAGTGATGCCATCCATCCGGATGGGCCGCGCG
AATGGATGCGCAGACGGTCAACCACTGAAACAGGAGAACTGACACCGTGAATACAACCAGATTGCTAACC
ATAGCGCTGACCATGGGATTGGCGAATGTGGCCCAGGCTGCCCCTGTGCTGCACTTCTACAACTGGTCTG
ACTATATCGGCGAGACCACCCTCAGCGATTTCCAGCAGGCGACCGGGATCAAGACCGTCTATGACGTGTT
TGACTCCAACGAGACCCTGGAAGGCAAGTTGCTGGCGGGGCGGACCGGTTACGATCTGGTGGTGCCCTCC
AATAATTTCCTGGCCAAGCAGATCAAGGCTGGAGCCTTCCAGAAACTGGATAAATCCCTGCTGCCCAATC
TGGCCAACCTGGATCCCGTACTGCTCAAGCAGCTCGACAAGGTGGATCCGGGTCATCAGTACGCGGTGCC

CTATCTGTGGGGGACCAACGGCATCGGCTACAACGTCGACAAAATCAAGGCGGTGCTGGGGGTCGACAAG
ATTGACTCCTGGGCCACCATCTTCGAACCGGAAAACATGGCGAAACTCTCTCGCTGCGGCGTCGCCTTTC
TCGACTCTGCCGACGAGATGATCCCTGCCGCCCTCAACTACCTCGGACTCGATCCCAACAGTCATGCGAC
GGGGGATATCAAGCAGGCCGAGGCGCTGCTGCTCAAGGTGCGGCCCTATGTGGCCTACTTCCACTCCTCC
AAATACATCGGCGATCTCGCCAATGGCGACATCTGCGTGGCGGCCGGTTTTTCCGGCGACATCCTGCAAG
CCGCTGCCCGCGCCGATGAAGCTGGCAAGGGGGTGAAAATTGCCTACAGCATTCCGAAAGAGGGGGGCAA
TCTCTGGTTCGACATGATGGCGATCCCGTCCGATGCCACCAATGTCAAAGAGGCGCACGCCCTCATCAAC
TACGTCCTGCAGCCCGAAGTGATCGCCAAGGTCAGCGACTATGTGGGCTACGCGAATCCCAACTCAAGGG
CGGGCGAGTTTATGGATGAGGCGGTGCGCAACAATGAGGAGGTTTATCCCCCTCAGGCCGTGCTGGAGCG
ACTCTATGTGCAGGAAGCACATCCCCGCTCGGTACAGCGTCTAATCACCCGGAGTTGGACCAAGATCAAG
TCCGGAACCTAACCTTGGTGCCCGTTCGGGGGCGCCAAATCGCTTTTATGGATCGGCCGCAAGGCCGGTA
TCACGTTGGGGAGTTTGGTAATGGCGATAGCCTCCAGTGCCTACAAGAAAGCCCTCGAGGGCAGTCAGCA
GCGCAAAGAAGTGCTGGTGAAAATTGATCGGGTAAGCAAGCAGTTCGACGAAACGCTGGCGGTCGATAAC
GTATCTCTCAATATTCACAAGGGAGAGATTTTCGCCCTGCTCGGCGGGTCCGGTTCCGGCAAATCGACCC
TGCTGCGGATGCTGGCCGGTTTCGAGCGCCCGACCGAGGGGCGGCTGCTCCTCGACGGGGTCGACATCAC
CGATATGCCCCCCTATGAACGCCCCATCAACATGATGTTCCAGTCCTACGCCCTGTTCCCCCATATGACG
GTGGCCCAAAACATCGCCTTTGGCCTCAAGCAGGATGGCCTGCCCAAGGCCGAGATCGATGCCCGCGTGG
AGGAGATGCTCAAGCTGGTGCAGATGACCCAGTATGCCCGCCGCAAACCGCATCAGCTCTCTGGTGGCCA
GCGCCAGCGGGTGGCGCTGGCCCGTTCCCTGGCCAAGCGTCCCAAGTTGCTGCTGCTGGATGAACCCATG
GGGGCGCTCGACAAGAAGCTGCGTTCCAAGATGCAGCTGGAGCTGGTTGAGATCATCGAGCGGGTCGGGG
TGACCTGCGTCATGGTGACCCACGATCAGGAAGAGGCCATGACCATGGCGGAGCGGATCGCCATCATGCA
CCTCGGCTGCATCGAGCAGATCGGCAGCCCGATGGATATCTACGAGACCCCGGCCAGTCGGCTGGTGTGC
GAGTTCATCGGCAACGTCAACCTGTTCGATGGCCTGCTGGTGGAGGATGAGCAGGACCACGCCACCATCG
CCTGCGCCGATCTGGAGCAGCCCATCTATGTGGGCCACGGCATCAGCTCCCGCGCCGAAAACAAGAAGGT
GACCTACGCCCTGCGGCCGGAAAAACTGCTGATGAGTCTGCAAAAGCCGGAGGAGATCGAGCACCCGGAC
TTCAACTGGACCAAGGGTGAGGTCTATGACATCGCCTATCTGGGCGGTCACTCGGTTTATCACATCCAGC
TGCCCTCGGGCAAAATCGTTCAGGCTTTCATCGCCAATGCCGAACGCCACGGCAAGCGGCCAACCTGGGA
TGACCAGGTGTTCCTCTACTGGGAAGATGACAGCGGCGTGGTGCTGCAATCATGAAGTTCTTGCAACGAC
TGAAGCGGGTCGGGGGGCGCCGACTGGTGATTGGCATCCCCTTCTTCTGGCTGTTCCTCTTCTTCCTGCT
GCCCTTCGTCATCGTGGTGAAGATCAGTTTTGCGGAAGCGGATGTGGCGATCCCGCCCTACACCGATGTG
GTGAGCTGGGCCGACAATCAGCTCACCATCCTGCTCAACATGGGCAACTACCTGCTGCTGCAGGAGGATG
AGCTCTATATCGCGGCCTATCTGGGCTCCCTCAAGATGGCCTTCGTCAGTACTTTGCTCTGCCTGCTGAT
TGGCTATCCGATGGCCTACTCCATCGCTCGGGCCAACAAGGAGACCCAGACCGTTCTGCTGCTGCTGATC
ATGATGCCGACCTGGACCGCCATTCTGATCCGGGTCTATGCGTGGATGGGGATCCTCAGCAACAACGGCC
TGCTCAACAGCGTCTTGCTGGGGATCGGGGTGATCGACGAGCCGCTGCAGATCCTCAACACCAACCTGGC
GGTCTATATCGGCATCATTTACTCCTACCTGCCCTTCATGGTGCTGCCGCTCTACGCCAATCTGGTCAAG
CATGACCAGAGCCTGCTGGAGGCCGCCTCCGATCTGGGGGCCCGCAACCTGACCCGTTTCTGGCAGATCA
CGGTGCCGCTCTCCAAGAACGGCATCATCGCTGGCTGCATGCTGGTGTTCATTCCGGTGGTGGGGGAGTT
CGTTATTCCGGAAATGCTGGGCGGCCCGGAGACCCTGATGATCGGCAAGGTGCTGTGGCAGGAGTTCTTC
AACAACCGCGACTGGCCGGTAGCCTCTGCACTGGCGGTGGTGATGCTGGCGGTGCTGATCATTCCGATCA
TCCTCTTCAACCGTAACCAGGCCAAAGAGCTGGAGGGCAAGGCATGATGAGACGTTTCGGTTTTGCCAAA
CTGATGCTCTGGCTGGGGCTGCTGTTTATCTACCTGCCCATGATCATCATGGTGATCTACTCCTTCAACG
CCTCCAAGCTGGTGACGGTGTGGGGTGGCTGGTCGCTCAAGTGGTACTTCGGCCTGCTCGATAACAAGCA
GCTGATCGGGTCTGTGTTCCGCTCGCTGGAGATCGCCTTCTACACCGCGATCGCCGCGGTGGCGCTGGGG
ACGGTGGCAGCCTTCGTGCTGACCCGAATTCCCCACTTCCGTGGTCGCACCCTGTTCGGTGGCATGGTGA
CGGCGCCGCTGGTGATGCCCGAGGTGATCACCGGTCTGTCGCTGCTGCTGCTGTTCGTGGCGATGGCCCA
GCTGGTGGGCTGGCCCGCCGAGCGGGGCATGCTGACCATCTGGATCGCCCATACCACCTTCTGTACCGCC
TACGTGGCGATTGTGGTGTCGGCGCGGTTGCGGGAGTTGGATCTCTCCATCGAGGAGGCGGCGATGGATC
TTGGCGCCAAGCCGTGGAAGGTGTTCTTCCTGATCACCATCCCGATGATCGCCCCCTCGCTGGCGGCAGG
CGGCATGATGTCGTTCGCCCTGTCGCTGGATGATCTGGTGCTGGCGAGCTTCGTCTCCGGCCCGGGCTCC
ACTACCTTGCCGATGGAGGTCTTCTCGGCGGTGCGGCTAGGGGTGAAACCCGAGATCAACGCGGTGGCCA
GCCTGATCCTGTTGTCGGTATCGCTCTTTACCTTCGGCAGCTGGTATCTGATGGCCAGGGCGGAGAAGCG
CCGTCGTGCCGAGATCGCCATGACCACCCAGATGCAGGCCGTCGTCTGACGCCATGATAAGATCCGCCTA
CTCTCGTCAACCTGGAGGATGAGTATGCGGATCTCCCTTTTTAGCGGTCGTGGCACCGAGCTGGTGCCCG
GCCTCACCAGCGCCATTCACAAGCAACCGATCTCTGCACCAGTTCGCTGTACCTTTGCCGGTCTGGAAGG
GGATGAGCAGTGTGACCTGCGCCACCACGGTGGCCCGGATCGTGCGCTCCACTACTATCCTGCCGATCAC
TATCTCTGGTGGCAGACCTGGCAAACCGCGCTGGGCTTGCCTGCGCCACGTACACCCTGGCAACCTGCCG
CCTTTGGCGAGAACCTCTCCGGTCTCGGGCTGACCGAGGCGCAAGCCTGCATTGGCGATGTCTATCGACT
CGGCGAGGCGCTGATCCAGATCAGCCAGCCGAGATCCCCCTGCTTCAAGCTCAACCAGCGCTTTGGCTAT
GGCCAGATGTCGCAGGTGATGCAGCTAAATGGCCGTTGTGGCTGGCTGCTGCGGGTGCTGGAGGAGGGCA
TGAGCGCGCCTGACACGGCCATGGAGCTGGTAGACAGACCCTATCCCGAACTGACGGTGAAGCGCACGGC

GGACATTCTGTTCAATCAGGTGCGCAATGAAGCGGATCTGCTGATGTTGCTGGAGAATCCGGCCCTCTCC
CCCAACTGGCGCCAGCATGCAGCAGACTGGCTGGAGCATGGCACTGTGGCTGACTGGCAGCGCCGTCTGC
TGGGGCCAGCATCCCTGCGCCTGGCCTGACGGCCGGTTACCGGAATGTCACCTGCTGGCTGCAGTCCGGT
AAACGGGTGAAAAGAGTGCTGAATAGTTGAGCAAATGGCACTTTTTTTCCTGCCAAACGCAAAAAAGGCT
TGTGTCGGGTCTGGGCTTTCGGTAAATTCCCGTCCCGCAGACATCGGTGTGTAGCGCAGCTTGGTAGCGC
ACTTCGTTCGGGACGAAGGGGTCGGAGGTTCGAATCCTCTCACACCGACCAAATTTGAAAAGGCCGCTCA
ATAGAGCGGCCTTTTTGCATTTATCCTTCCTGTTTTCCCGATTTTCTTTAGCTGCTGACCTGTTAATTGG
CCAAGTGATCTGACTTGATGTATGCAGGGTCGAAGCAAGCCCACAGACATTAAAAGGCCACCGCTCGGT
GGCCCATCGGTGGAGTCTGATCCTGACCCCGGCACTCTAGATTGTGGCAATCTCGCTATGGGTCTTGTCT
ATGGTGCCCTCCTTGCGCGCCAGGCGCTTACTCAGATATGCCAGTGCTGATGCCGCAAAGCCTTGAAACT
GTGTTGAGGCAGCAAGGCCGCGTGCTTGGGGTACTCACTAATGAAATGTTCTATTCCCTCCATCAGTTGT
GCACGTAATGAAATCACGGGCTCATGCCAGGCTTGCAGGCATAAATGGCCGGGGATCAACTGCTCCTGAT
AATCTTCCAGCAGCTGTTCTGGCCAGGGTTCAATACCGGAATTGCGTTGCAGCAAATAGTGGGCGGCGGG
TTCACGGGCATAAATGAACTGAATTTGGGCCAGATCGCGTTGGCATAGCGTTTGACTGTTGATGTGACAG
GTCAAATATTTTCCCGTTTTCAGATCCAGAAAGTGACCGCGTTGCCGACAGGGAACGATGCGATACCAAG
GCAAGGGTGAGGAGAGAGAATACCCCTCAAGCTCCTGTTCCAACCAGTGGCGACACTCGGGTGTCTGCAC
CTCGTCGAGCATGGACTCGAGCTGTGTCAACAAGGCTCTGGATGGTTTGATGGCCAGTTTCGCACTCATC
GCCTGCAATCCTCCCTCTGCACACAATCCCGACTATCAGTATGGACCTTGTTCGGCCAGAGGGGGTAAGG
AATCTAATGATTAAAAGTGGATTAGCCGCGTGGTTTTTATGCACTAACTTGGTTCAACCCACCAGATAGG
TACCAGTGCCGAAGGCGATGTGGGTGCCCTCTTCGTTGTGCAGCTCCATCCGGGCGACGGCGACCTTGCT
GCCAGCCCGGATGATGTGGGCGGTGGCGATAAACTCGTTGCCGCGCCCAGGGCGCAGGTAGTCCACCCGC
AGATCTATGGTGCCTAGGCGGGCGAAGCGCTCCTTGAGATAACTGGGGGAAAACTCCTCCAGCTCGTCGA
CGCAGGAGGCCGCGACGATCATGCCGCCCGCCACATCCAGCAGCGTGGCGGTGACGCCGCCGTGCAGGAT
GTTGTGGAAAGGGTTGCCGATCAGCTTCTCGTCCATCTTGATGCGCAGTTCCACCTTGTCGAAGTCGTAG
TGGGTCACCTGCATGCCGATCAGCTTGTTGAACGGCATCTTCTCGGCAAACAGGTTGGCAATGCCACTGA
GCGCCATCTTCTTGAGCATCTGTTTCATTCTCTGATCCCAATCCTTGTCATCAAAGCCGAGTAAAAACTC
CAGCCAGTCTGCAACAGTTCCTTAACAGTCGCAATGGCAGGTTTATTGTGGGCGGCGGCTGCTTTAGAAT
GGCGACCCCAAAGGAGAGCCCATGACCGCGATTGCCGAAACCCTGCCAGATCCTCAGCCAGAGAGTCCGC
TTGCCCTGCTGCAGGCGGTCTTCGGCTATCAGCAGTTTCGGCCGGGTCAGCTCGAAATCATCGAGCAGAT
CGTCTCGGGGCGCGATGCGTTGGTGCTCAAGCCCACCGGTGGCGGCAAGTCGCTCTGCTACCAGATCCCG
GCACTGCTGCGGCCCGGGCTCGGAGTGGTGGTCTCTCCCCTTATTTCGCTGATGAAGGATCAGGTCGACA
GCCTGCGCGCCAACGGCGTGGCGGCGGTCTACATCAACTCGGCCCTCAGTCGGGAGGAGATGATCCAGAA
CTTTGCCGCCATGCGCCGTGGCGAGATCAAGCTGGTCTATGTCTCCCCCGAGCGACTGTTGCAGCACGAG
TTTATGGATCGGCTGGCCGAGCTGCCGCTCGGGCTGTTCGCCATCGACGAGGCGCACTGTGTCTCCCAGT
GGGGCCACGATTTCCGGCCTGAGTATGCGGCGCTGGGGCGCCTCAAGCAGTGGTTCCCGCAGGTGCCGGT
GGTAGCGCTTACTGCCACCGCCGACGAGGCGACCCGCAGCGACATGCTGCACCGCCTAGAGCTGAACGAT
CCCTTCATCCACACTGCCAGCTTCGATCGTCCCAACATCCGCTACAGCCTGGTGGAGAAGTTCAAGGCCG
CTGAGCAGTTGCTGCGTTACGTGCAGAGCCAGAAGGGCAACTGCGGCATCGTCTACTGCTCCAGCCGCAA
CCGGGTGGAGGAGGTGGCCGAGCGGTTATCCCGCCACGGCTGCAAGGCCGCGCCCTATCATGCGGGGCTA
CCGCTCGAGCAGCGCCAGCAGACCCAGGATGCCTTCCTCAAGGATGACATCGAGATAGTGGTGGCGACGG
TGGCGTTCGGTATGGGTATCGACAAGCCCAACGTACGCTTCGTGGTGCACTACGACATTCCGAAGAACAT
CGAATCCTACTATCAGGAGACCGGTCGCGCCGGGCGCGATGGCACTCCTGCCGAGGCGCTGCTGCTGTAC
GATCCCGCCGACATCGGTCGGGTGCGCCGCCTGCTCGACAACATCGAGAACCCGCAGCAGTTGCAGGTGG
AGCAGTACAAGCTCAACGTGATGGCGGCATTTGCCGAGGCGCAGACCTGCCGCCGTCAGGTGCTGCTCAA
CTACTTCGGCGAGTACAACGACAAGCCGTGCGGCAACTGCGATATCTGTCTCGATCCGCCCAAAAGTTAT
GACGGCACCGAAGATGCCCAGAAGGCGCTCTCCTGCGTCTGGCGGGTGGGGCAGAATTTCGGGGTGGGCT
ATGTGGTGGAGGTGCTGCGCGGTTCGCTGAACCAGCGCATCAAGGATCACGGCCACGACAGGCTCTCGAC
CTACGGCATCGGCAAAGATCAGAGCCATGAATACTGGATGAGCGTGATCCGCCAGCTGATCCACAAGGGG
CTGCTGACCCAGAACATCACTCGCAATCTGGTGCTGCAGCTGACCGAAGCGGCCCGCCCCGTGCTGCGTG
GCGAGGTGAAGCTGGAACTGGCGGTACCCCGCCTGCAGCCTATCTCCAGCCGCAAGGAGAAGCGCAACGG
TCTGCTCGACAATGCCAACTATGACAAGCGGTTGTTCAAGGAGCTGCGCGGTCTGCGCAAGCAGATTGCC
GAGGATGAAGAGGTGCCGCCCTACGTGGTGTTCAACGATGCGACCCTGGTGGAGATGGCACAGCTGATGC
CGATGACCGAGGACGAGATGCTGGCCATCAACGGTGTCGGTCACCGCAAGCTGGAACGTTTTGGCGAAGC
CTTTATGGACCTGATCATCGACTACTGCCGTCGTTAGGGTCGATTAGGCAGCCTAATCGACCACGGTAAG
CGCGGCTCGGAAGAACCTGGGGTCAGACGCGAGGTTATTTCAGGCTGGCGTAGAAGGCCGCCAGATTGTC
GATATCCGCATCGGTCAGCGGCGCCATGAAGGGGTCCATCAAGGGCTCTTTGCGAGTGCCATCCTTGAAC
GCTTTCAACTGTTTCACCAGATAGGCGGCATTCTGACCCGCCAGATGGGGATAGTTGGGGATCATCGCCT
TGCCTTCTACGCCGTGACAGGCGGCACAGACCGCCGCCTTGGCCTTGCCTGCCTCGATATCTGCAGCCTT
GGCCTGATGACCCCACGCCAGTGCCAGCAAGGGCAGGGCATATACCCATTTTTGCATCTTCTTCTCCTTG
AATTGAAAAATGATGGTTGCCAGTGAGCACAAGCATGGCTGACGGCAGTATAAAGAGTGACACGAGCATA
CGCAGGGACGAATGGCACAACCATGAAGTTGATCAACACTTTTGCGACCGAGCTGCCTTGGGCCTGTGAA

CCCGTGGTCCCCCAGCCGCTGCAGCAACCCCGGTTGCTTCATCTCAACCGGACTTTGCTGGATGAGCTGG
GATTGGGAGGCGTCAGCGAGGCTGACTGGATCGCCTGTTGTGGCGAGGGAAAAGTGCTGCCCGGTATGCA
GCCGGTAGCGCAGGTCTATGCGGGCCATCAGTTCGGGGGTTATAGCCCCCGGCTTGGGGATGGTCGCGCG
CTGCTGTTGGGTGAGCAGCAGGCGCCGGATAGTCAGCGCTGGGATCTGCACCTGAAGGGGGCAGGAAAGA
CGCCGTTTTCCCGCTTTGGTGACGGCCGCGCCGTGCTGCGCTCGAGCATTCGCGAATATCTAGCCTCCGA
GGCGCTCCATGCCCTCGGCATACCGACCACCCGGGCGCTGGTGCTGGTGGGGAGTCAGGAGCCCGTTTAC
CGCGAGCAGGTGGAGACGGGAGCGACCGTGCTGCGCACCGCCCCCAGCCATCTGCGCTTCGGCCATATCG
AGTATTTCGCCTGGAGCGGGCAGGGAGAGAAGATCCCGCCACTCATCGACTATCTGCTGCGCCACCACTT
CCCCGAACAGGAGAGTGGTGCCGAACTGTTTGCCGAAGTGGTGCGCCGCACCGCGCGGCTGATTGCCAAA
TGGCAGGCGGCGGGCTTCTGTCACGGGGTAATGAATACCGACAACATGTCGCTGCTCGGCTTGACGCTGG
ATTACGGGCCATACGGCTTTATCGATGCTTACGTGCCCGATTTTGTCTGCAACCACTCGGATCCTGCGGG
CCGCTACGCGCTGGATCAGCAGCCCGCGGTCGGTTACTGGAATCTGCAAAAACTGGCGCAGGCGCTGGCT
GGCCATGTAGACGGTGATGCGCTGGCTGCAGCGTTGGCCCAGTACGAGCAGCAGCTGATGTTGCACTACT
CCGAGTTGATGCGGGCCAAGCTCGGACTGGCGGTGTGGGAGGAGGATGACCCGGCGCTGTTCCGCGAGCT
GTTCCGGTTGCTGGCGGCCCACAAGGTGGATTACCACCTGTTCCTTCGCCGTCTCGGTGAGGTGACGCAA
GAGGGGGCGTGGCCCGCATCTCTGCTGGTCCTGTTGCCTGAACCACTGGGTTGGCAGGCGTGGCTGGAGC
GCTATCGGGCGCGGCTAATGCGCGAAGGCAGCGAGGATGCCGTGCGCAAGGCGCAGATGGATGCCATCAA
TCCCAAGTATGTGCTGCGCAATGCGCTGGCTCAGCAGGTGATAGACGCCGCCGACGCAGGGGATATGCGG
CCGTTCGAGCGGCTGTTCGCCGCCTTGCAGCGCCCCTTTGATGAACAACCCGAATATGAGGATCTCGCCA
CCCCGACTCCGGCATGGTATTGCGGTGGTGAGCTCTCCTGCAGTAGCTGAGAGACAGGGAATGAGTTGGC
AAATCAAACAGGTAGATAGCGAAGCTGGCGTAGTGACGTTGGATTGTCCGGCGCTCGAGTGGGACTCGTT
TCCGCTGTTGGCGCAGGCCTTGCTGCGAGATTGGGAGCTGCAGCCGCTCGAGCAGGATGCGGGGGCCGAC
CGCCATAGCTGGTTGCTGGAGTTTGAAGGGAGCCAGTTTCGGCTGGAGTACGAACACTACAGCGGCTGCT
GGCTGGAGGCGGTGCGCCCGGTAGATCGGGAGGCGCTGCTCTGGCTCGCCAGACAGCACAAGCTGTGACT
ATCGCCCACAAGGCCTGCTCTGCCGCTGGCCAACCTGGACAAGCGTAATCGCATAAAGATTGAACTATCG
GTTTGGGGGCGTATAATCTCGCCCCTCGATTATCGCCAATGGTGGCGATATGGGTTCCCTAACCCCATTA
ACCAAAAAGGTCACAAGATGAATCTGACCCCTGCGCAGTACCAAACGGCGCTCGTTCGCCTGTCGTTGTT
TCACATCCTGATCATTGCCAGCAGCAACTATCTGGTGCAGCTGCCGATCACCGTGTTTGGCTTCCACACC
ACCTGGGGTGCGTTCAGCTTCCCGTTTATCTTCCTCGCCACTGACCTGACGGTGCGGATCTTCGGTGCCG
GTCTGGCGCGCAAGATTATCCAGACCGTGATGCTGCCAGCACTGGCGGTCTCCTATGCGCTCTCCGTACT
CTTCTTTGAAGGGAGCTTCCAGGGCGTTGGTCATCTGGCTGAATTCAACCTGTTCGTGGCGCGTATCGCA
CTGGCCAGCTTTATGGCCTATCTGGTAGGGCAGTTCATGGATGTGGTGGTGTTCAACCGCCTGCGTCTGC
TCAAGCAGTGGTGGATTGCGCCAGCGGCCTCCACCCTGTTCGGTAATCTGGTTGATACCATCGCTTTCTT
CAGTGTGGCCTTCTGGCGCACCACAGATCCTTTCATGGCAGAGCACTGGGTCGAGATCGCAACCGTTGAT
TACGTATTCAAATTGCTGATAAGCCTTGGTTTGTTCGTGCCGCTCTACGGTGTCTTGTTGCGCTACCTCA
GCCGCAAGCTGGTGGGCGAGGCCGGATTATCCAGCCTGCAAACGGCCACCAGGCAAGCCTGACCAGATTG
ACCACCCTTCGCGGTGGTCAATTTTTTTGCACTTTCTGCAAATAGCGCTTGCCTAGTTAAATGAGAATGA
TTATTGTTAACTTGTTTTCCGGGAAAGACTTCATTCGTTCCAACGTGATGGACTTCTTGTGAAGGCACGA
CATTGCTCACATTGCTTCCAGTTAAGGGCCAGTTTATCTGGCCCTCTTTTTTTGTCATTTTTCAGCAAGT
TGCAGGCATAAAAAACGGGAGCCAAGGCTCCCGTCGTCACTCTGTCTGTGGCTTACTCGATATCGAGTGC
CTCGGGGGAGAGGATCACCCCTGTGGTATCGGCGTAGAGGTGGTCGTCAGGCAGGAAGGTCACGCCACCG
AAGTTGACCGGCAGATCGGTCTCCCCTACATCCTTGCTGTCGGCACCGACCGGAATGGCTGCCAGTGCCT
GAATGCCGATATCCAGATCTTCCAGAATATCGACTTCGCGGACGCAGCCGTAGCAGACAATCCCTTCCCA
ATCGTTCTCGGCCGCCGTGGTGGCGATGTCGCTGTCGATCAGGGCTCGGCGCATGGAGCCACCGCCATCG
ATCAGCAAGACGCGTCCCAGTCCGTTCTCTTTGACGAGTTCACGGATCAGGCCATTCGATTCGAAGCATT
TGACGGTGGTGATGACGCCGCCGAAAGAGGCGCGGCCGCCGAAGGAGCAGAGCATGGGTTCAAGCACATC
CACCATGTCCTGATAGATGTCGCACAGTTCAGAAGTATTGTATTCCATAGTGTCAAATCCACGGCTGGAA
ACAGGTGCAACCAGTATAAGGAGTGGGTGTGGCATTTCAATGAATATTCCTTGCACAGATCACCTGTCGC
CGCTTTTCTGCATAAGCTCAATGCAGTTCACTGCCAGTTTGCGAGAGGTCCATCGTGCTTATCCGTCATA
AGTTGATCCTGAATACCGTGCTGGTTGCCGTTGCCATGCTGGTACTGAGCGGCCTGTTTATCTACTCCAG
CCAGGTGCACCGTCATCTGACCGAAGCCCAGCGTAATATCGACCAGCTGGAGATCGCCATGCTCAACCTG
CGGCGGGCCGAGAAGGATTTTCTGATCCGCAAGGATATGACCTTCGTCGAGCGCTTCAATCAGGGGCTGT
CTGACTTTCACCAGCACAATCGGGAGCTGGTGACCGATGACAATCTGGCGGCCGTCGAGGGGGAGCTGAC
CAGACTGGATCAGCAGATCAGCGGTTATGGCAAAGCCTTCAACGATCTGGTGGCCCAGCAGCAGAAGATT
GGTCTGGGGCCTGAGGAGGGGCTCTATGGCAAGCTGAGGGCGGCGGTGCATGAGGTAGAGAATGGGCTCA
AGCAGCTGGATCAGCAGGGGCTGCTCATCACCATGTTGCAGCTGAGACGGGCGGAGAAAGACTTCATGCT
GCGCTCCGATATCCAGTATCTGGAGCGTTTCGAAACCTTGCACAAGAGTTTTCAGGATAGCCTTGCCACT
TTGCCGGAGGCGGGTCGCGGCAAGCTGGAGCAGGCCAGCAAGCAGTACCGGCAGGACTTTGTGGCGCTGG
TGCAGGGGATGCAGATACTGGGGCTGAAAGAGGATGAGGGGCTGCGCAACCAGATGCGGGATCTGGTGCA
TCAGACCGAACAGGGGTTTGCCGCCGTCGATCAGCACATTGTCGCTGAGCTGGAGCGGCAGAGCGCTCGC
CTCAACAGCCTGATGCTGGTCTGTGGCGGGGTGATTGTGGTGCTGATCGGCATCATGAGCGTGTTGCTGG

GGCGCAGCATCGACCGTCCCATTACTCAGGTGAATGACACCGTCAACCGCATTCGTCAGGATAATGACCT
GCGCCTGAAGATAGAGCTGCGCGGGGCCGATGAAATGGCCCAGCTGGCAGGCAATCTCGACGTGATGCTG
GGCGGGTTTCGCAACCTGATCGGCGATGTGAAGCAGAGCGTGCACACCCTGACAGAAGCGGCTGATCACC
TCTCGGCCAACGTCAAGCGCACCAGTGAAGGGGCGGCGCGTCAGCTGCAGGAGACCGACATGGTGGCAAC
CGCTTCCACCGAGATGGGCTCCACCATCGAAGAGATCGCCCGCAACACCGAGCAGGCGGCCAACAATGCG
CAGGCGACCAACAACAAGGCGATGGAGGGGCGGACTGCGGTCGAGAATACTGTCGAGCAGATCCGCTCGC
TGGCGGGCAATCTGGAGAGCTCATCTCGCGAGGTGAGCCAGCTGCAAAAAGAGAGCGAGACCATCGGCTC
CGTGCTCGACGTGATCCGCGGCATTGCCGAGCAGACCAACCTGCTGGCCCTCAACGCAGCCATCGAAGCA
GCTCGGGCAGGGGATCAGGGACGTGGTTTTGCGGTGGTGGCCGATGAGGTGCGCAATCTGGCCATCCGTA
CCCAGAAATCGACCCAGGAGATCGCTGGCATCATCAGGTCGCTGCAGAAACAGACCGGCAGCATCGTCGA
GGTAATGGCAACCTGCCGCGAGCAGGGCAACCGCAGCTCGGATCAGGCCAGTACCGCAGGGGAGTTGCTG
GGGCAGATCACTCAGGATGTTACCAACATCATGGACATGAGCACCCAGATTGCTGCGGCTATCGAACAGC
AGAGTCTGGTGGCCAACGAGGTGAACCGCAACGTCAACAACATCCGCGACATCGCCCAGGAGTCGAGCAT
CATGGCGGAGGAAAATGCGAGATCCAGCGAGGGCCTCAACGAGCAGGCCCAGCGGCTCAACAAGGCGGTG
GCAAAATATCGGGTTTAGTGAGCGGACTGGCATCCTTGTGAGGTTTTTACACCAGAGTGGCAATGATTTG
ATCGTAATGGCTGTTATTGGGGAAATTCGCTCATATAATCGCCGCCACTATGATGATTCGGAAGGATTCG
ATGGCAAAGTTGCTGGTGGCCGCATTCGGGCTGCTCTGTTCCTGTCTGGTGTTGGCAGGATCGGCGCCTC
AGGCTACCCCACTCAAGCCCGGTATGGAGCCGCTACTCAGCTGGTTCAACGGTGCCTTTACGAGCCGGGA
GCAGGCCAGTTACGACAGCCGCTTTACCAGTGCCGAGCTGCGCCTGATGGAGATTTGGCCCGGCTATCAG
GGCTATCGCTGGGTCTATGCCGAGCAGTATCTGAGCATGCGCTCCGTCCGCCCCTTTCGCCAGCGCATCT
ATCGCTTCTCACCTGCCCCTGACGGGCGGATCCTGATGGCCGAACTGACCATGCCCCGTGCCACCGATTT
TGCCGGTGCCTGGCATGATCCCGCCCTGCTCGACAGTCTGACCCCGCAGCAGCTCTCCTTGCGCCACGGA
TGCGAGATCTGGTTGACCCGTACCCCCTCCGGCGAATATGCCGGTCACACCCGGGTGGGCCACTGCTCCA
CCGATTTTGGCGGTGCCACGACGCTGGTGCAGCATATCTGGATTGGCCCCGACAGCGTCAGATTGCTGGA
TCGCGCCTATGACAACGCAGGGAGTCCGCGCTGGGGCAGCCCGGGTGAGGGGTATGTCTATTTGCGTCGT
TCCAAGGGGTTTGGCCAGTAGGGGAGAGTGCGTCTGGCTGCGGGAACATGAAGGATGAAAAACAGGGATC
GTAAAAAAGGCGCCCGATGGGCGCCTTTTTCAGAATTCTTCAGAAGTAAAATTACTTCTTGGTGGTCATC
GCACCGAAACGCTTGTTGAACTTGTCTACGCGGCCGCCGCTAGAAACTTCTTTCTGCTTGCCGGTGTAGA
ACGGGTGGCACTCGGAGCACACGTCCAGGTTCAGATCTTTACCGATGGTAGAGAAAGTGTTGATGACGTT
ACCGCAAGAGCACTTGGCGGTGATGGCTACGTAGTCAGGATGGATACCAGCTTTCATGGACAACCTCAAT
TAATAGGCCGTGTCGCCATCCGATCTCTTGTCGGACACCACACGATTGTTGAACACAGTTTCAAAGGCTG
CGAATAATATAGGAAATGCCTGAGATCAGGCAAGTGGCTTCGCAGAAAAAATAGGCAATCCCTGGTGAAT
TCTTCCCAAACGCTGGATCTGGTTCGTGTCGCTGTCCCGGTACCGTTACGCCGTCACTTCGACTATCTCT
CTCCCCTGCCGTTACCTGCCCCCGGTTGCCGGGTCGAGATCCCTTTCGGTCCCCAGACCCTGGTCGGGCT
GGTGGTGGATCATCCGGCCGACAGCCAGGTGCCCCGCAACAAGCTCAAGCCCATCAAGCGCGTACTCGAT
GCGACGCCGGTGCTGGGGCCGGATATTCTGGCGCTGATGAACTGGAGCGCCAGCTACTACCAGCATCCGC
TGGGGGAAGTGCTGCCCCACGCCCTGCCGGTGTTGGTGCGCAAGGGGGAACCTGCCGCTTATCGTGAACT
GGAGTTCTGGTTTGCCACTGAGGCTGGCATGGCCATCGACCTTAACGAGCTCAAGCGGGCGGCCAAGCAG
CAGCAAGCGCTCGCCTTGCTGCGCAAGGGGCCGCAGACCCCCTCTGCCCTCAAACAGGAGGAGATCCAGA
GCGCAGCTCTCACCGCGCTGGAGAAGAAGGGGCTGCTGGAGAAGCGTTCGCTGGAGCCGAGCCACGATGG
CGATTGGGCTGCCCGCTTCGAGCCGGGGGAGGGGCTGCGCCTCAACGGCGAACAGGCATTGGCCGTCTCT
GCCGTTACCAGCCAGAGCGACAAGTTCGGCGCCTTCCTGCTCGATGGCATCACGGGCTCCGGCAAAACCG
AGGTGTATTTAAGCATCCTCGAGCCGTTGCTCAAGGCGGGCAAGCAGGCGCTGGTGATGGTGCCGGAGAT
CGGCCTCACCCCGCAGACCATCAACCGCTTTCGCCGCCGCTTCAACGTGCCGGTGGTGGCGATGAACTCC
GCCATGAACGACAGGGAGCGGCTCGACGCCTGGCTCGCCTGTCGCGACGGCGGTGCCGCCATCCTGATCG
GCACCCGCTCTGCGGTGTTCACCCCGTTTCACAACCTCGGCATCATCATCATCGACGAGGAGCACGACGG
CTCCTTCAAGCAGCAGGACGGTTTTCGCTACCACGCGCGGATCTGGCGGTGATGCGGGCCCATCGTGCC
GGTATCCCCATCCTGCTTGGCTCGGCTACCCCATCGCTGGAGACATTGCACAACGCCCGCACCGGCAAGT
ATCACCATCTCACCCTGACTCGGCGTGCCGGTAACGCCCAGACCGCCCGTCAGGTGATCCTGGATATCAA
GAGCGTGCGGCTGCAGGCCGGGCTCTCTCCCCAGCTGGAGCAGCTGATGGCAGAGCATCTGGCGGCGGGC
AATCAGGTGATGCTGTTTCTCAACCGCCGTGGCTATGCCCCGGCTCTGATCTGTCACCAATGTGGCTGGA
GCGCCGCTTGTGAGCGTTGTGATGCCTGGTACACCTGGCATCAGGCCGGACGACGGCTGCACTGCCATCA
TTGCGACAGCGTGCGCCCCTTGCCCCACCACTGCCCCGAGTGTGGCAGCAACGAGCTGATCGGATCGGGA
GTGGGCACCGAGCAGCTGGAACAGCTGCTCACCACGGTGTTCCCGCAATATCCGGTGGTGCGCATCGATC
GGGACAACACCCGCCGCAAGGGGGAGCTGGAGACCCATCTCGGCGACATCAAGGCGGGCAAGTACAAGAT
CCTGATCGGCACCCAGATGCTCGCCAAGGGCCACCACTTTCCGGATGTGACGCTGGTGGGTCTGCTGGAT
GTGGATGGTGCCCTGTTCAGCGCCGATTTTCGCGCCGCCGAGAAGCTGGCCCAGCTTTACACCCAGGTGG
CGGGCCGCGCCGGTCGCGCCAGCAAACCCGGGTTGGTAGTGCTGCAGAGCCACCATCCGGAACATGCCCT
GCTGCAGGATTTGACCCAGAACGGTTACGGCCACTTCGCCGACACGGCGCTGAAAGAGCGTCGCCTGCTG
GGGCTGCCGCCCTTCAGCTATCAGGGGCTGTTTCGGGCCGAGGCCAACGGCCGCGACGAGGTGCAGCTGT
TCCTCTCCCGCCTCGCCGATACCCTGCGCAGCGCCCGCTATCCCCACGTTCAGGTGCTGGGGCCCATCAG

CGGCTTTATGGAGCGCAAGGCGGGCAAGTTCCGCATGCAGTTGCTGGTGCAGGGGCCGAACCGGGCACCG
CTGGCCCAACTGCTGGATTGGGCGGTGAAGGAGCTGGAAGGCTGGCCTGAGACCAAAAGGGTGCGCTGGA
GTCTGGATATCGACCCCACCGAGCTGAGTTGATGGTGGCACCGGAGCCGGTCGCTGGCAGTTATCGTCAG
CGACCGATGGGTGATTTCATTTCAAGGCTCAGACCCGCAGATCGATCTGCTGCACCCAGCCTCCTTTACC
CTGTTCGGTGAGATAGAGGCCGGATTGGCGCAGCACCCCCTGATTGCCGCTCTCCCCCAGATGGTTGAAG
GGGGTGTCTAGGGGGCGTAGCAGAATGGCGCCGATCCCCAGTTCGACCAGCGCGATAGGTGCGTGCTCCG
GATGCCAGAACTGCAGCGCTGAAAACTGGCTATCCCCTTCGTCCAGCACTCCGTTGCCATCCTCATCCAG
GGTGGCCAGCTCGCCGAAGCCATTGCCGGAGCGGGCGCCAAAGAGCTCGCCGCCGTCATCTATGGTGCCG
TTGCCATTGCGATCCAGCGCCACAAAGCCGCTGCCTTCCCGCAGACCCGGCAGGCTATCCTCTTTGCCAT
CCTGATCAATATCGAAAGTGACCCGTGCCTCGGTCAGATCGGCAATCTTGCCATTCAGACTGAGCACCAG
CGGATCCTTGAGGGCATTGAGGGTGGTGCGTCGCTGGCTCACCTCCAGCTGACGCCAGTCGAGCACAAAG
CCGAGATCGAGCTGAATAGTCTGACCATCTTGAGTCGCTACGCTGGCCGATGCCACCACGTTGAGCTGCT
CCTGTTGCAGCAGGGTGTCAGTCACCTCCACCAGTTGATCCGGCTCGGCGGCTTGAGCAGCGGCTTCTCC
TTGCGTCCCCCCTTCGCTTCCCTCTTCGGAGGGTTTGATCGGCTTGGGCAGCTCGATTTCGTGGCCCAGC
ATCGCTTCAATCATCAGCTTGATCAGCCGCTGGCGGTTATCCAGCCCATCATCGGGAATACCATCCTGCT
CTTTGCCGACGTCCGCTGCGGGCGGGGCGGGAGGTGTTGCTGCTTGCTGACTGGCGGCGGATTGCCTCGC
GGTTTGTAGCGTCAGGGCGCTGGTGAGGGTCAGGGGGCTGCTGCGTTCGCCGGCCACTGCCGCGTTGTTG
CCTTCCGGTGGATTGACGACGGGTGGCACTGGCCCGAGGTTGCGCACCTTGCGGCTCTGGGCCTGCTGTT
GCCGGTATTGCACCTCCCCCTGGAGGTCGTAGTGATCGATGCGCATACATTCGCCCTCGGCGCTATACAT
CTGGTCAGTTATCGACCGGTTAGTGAGCAGGGCTTAACACCAAATCGGACTAATCAGGGAGGTGGTTAGT
ACTAGGTTGCAAGGTGGTGGCGTGGCAGGATGATGGGCATGCGGGTGCTGGCCCGTTTGCCCAAGCGCAG
GGCGCAACAAGTGAAGAGGAAGAGAGGATGAGAAATTGTCTGGTGATGGGAGCGGCAGGCTATATCGGTT
CCTATCTGGTGCCCCACCTGCAAACGCTGGGCTACAGGGTGATTGCCGGGGCGCGCCGTCCCTGCCGCTT
GCCTGAGGGAGTCGAGTTTCGCCTCGCCGACAGCCTCAAACCCATCACACTGCTGCCTGCGCTGGCGGGC
ATAGATACCGTCTTCTATCTGGTGCATGCCATGGGGGCGGGGGCCGATTTTCACCGGCTGGAGCAGCAGG
GGGTGAAGAACTTCGCTGCCGCTGCCCGCGCCGCCGGGGTGCGCCGCATCATCTATCTCGGGGCAATCCA
GCCCGAGCAGTGCAACAGCCGCCATCTCAACTCCCGCCGCCACTGTGGCGAGCTGTTTCGCGAATCCGGC
GTACCGACGGTGGAGCTGCGCGCCGGCATTATCATCGGGCCAGGGTCAGCGGCGTTCGAGGTGATGCGGG
ATCTGGTGTTCAACCTGCCGATGATGGTGACGCCCAAATGGGTGCGCTCGCGCACGCCGCCGATTGCGCT
GTCCAACCTGCTGCACTATCTGGGTGGGCTGGTGGAAGCCGACGAGGTGGATGGCCGCATTTTCAATGCG
GTCGGCCCCGAGCTGCTCAGCTATCAGCAGCAGTTGCAGAAGTTCGCCGCCCATATCGGCAAGCGCTGCC
CCATCATTCCCATCCCGTTTCTGAGTCCGCGCCTCTCTGCCTGGTGGCTGCAGTTTGTCACCTCGGTGCC
GCAGCCGGTGGCCAAGGCGCTGGTGGGTGGCCTCAAGCACGATATTCCGGCCGACGATGGCCCGCTGCGC
GCCTTGCTGCCCCAGACCCTGCTCAGCTTCGACGAGGCGCTGGCGGAGAGTCTGGCGCTGGAGCAGAAGC
TGGGTGCCGAGCAGCAGGGGCAGGAGACGCCGCTCGGCCTGCGCTGGCGCCATCCCGAATATGGTTTCTA
CGATCGCATCGCCAGCGGCGAGGCCATCTGCCTCGCCTCGCCCGAGGTGGTGTGGCAGGTGTTGCAGCAA
CTGGGGGGTGAGCAGCGCTACTTCTACATGAACGAGCTGTGGGTGGTGCGCGAATGGATGGATCATCTGA
TCGGCGGCCCGGCCCGCACCCGTGGCCGCACCAATCCGGATCGTTTCGTGAAGGGGGATATGCTCGACTC
CTGGCAGATCCTCGGGGTGGATGAGGGGCGCAGGCTCGATCTCTTGTTCAACATGAAGGCCCCGGGCGTT
GGGCGGCTAGAGTTCAACATCCTGCCGGAGGAGTCCGGGTTGACCCGCATCCGAGTCACCGCTCACTGGC
ATCCACAGGGCGCCTGGGGGCTGGCTTACTGGCTCGCCATGCTGCCGTTCCACCTCTTCATTTTTCAGGG
GATGACAGAGGCCATCGCCCGTCAGGCCGAGGCGAGGGCAGGTATCCCGGTGATGGATTAACCCTCTTCA
CATCATTGCCGCTGATAGAGCGCCCCGTTCGGGGCACTTTTTATGCGGCGGCCATGCTCGGTTATGGCAG
ATAGTGGTAGGCGTCTCATGGCAAAGGCTGGCATCATGCCGCGGTCTTGTTTACCGCGGATAGATAATCA
GTCAGAAAAAGAAGGGATTATGAAGAAAAAACCGTCAATTTTGGGGGGCGCCTGCATCATTGCCAGCGTC
TGCATGGGGGCCGGCATGCTCGGTCTGCCGAGTTCGGGGGCGGGGGCCTGGACCCTCTGGTCGGTGTTGG
CACTGACCGTCACTATGGTGATGATGACCCTCTCCGGCTGGATGTTGCTGGAATCCTTCCAGCACCACGA
TATGCGGGTCTCCTTCAATACCGTGACTCGCGAGCTGCTGGGGGAGAAGCTCAATCTCCTCAACAACCTC
TCGGTCTACTTTGTTGGCGGCATTCTGCTTTACGCCTATATCACCTCCTCCGGCGCCATCCTGCAGGGCA
TTCTGGGGATCGACAGCAAGCTCGCGTCCGTGCTGTTCGTGGCGCTCTTCTCTGCGGTGGTGTGGCACTC
GACTCGCGCCGTTGACCGTATTTCGGTGGTGCTGATCGCCTTTATGATCCTCAGTTTCGCCCTCGGTGTG
GCCGGTCTGCTCGGCAGTATCAAGCTGCCGACCCTGCTCGATCTGGCGGGCAGCGCGAGTCGCTACGCCC
CCTATGCCATGGCGATGTTGCCAGTGGCGCTCACCTCGTTCGGCTATCACCACTCGGTTGCCTCCCTGCG
TGCCTATTACCAGAATGAGCAGCAGGCCAGATGGGCCATTCTGGGGGGAACCCTGATCGCGCTGGCGCTC
TACGTGCTCTGGCTGCTGGGGATCTTCGGCAACCTGCCTCGTCAGGATTTTGCTCCGGTGATTGCCAAGG
GCGGCGATGTGAGCGTGCTGCTCTCGGCCCTCTCCGGGGTCATTGAATCGGAGAAGGTGGCCAGCGCCAT
CAACGGCTTCTCCATGGCGGCGATCCTCTCCTCCTTTATCGGGGTGGGGCTCGGGGTGTTCGACTATCTG
GCTGATCTGTTCAAGTTCGACAACAGCCGCGCCGGTCGCTGCAAGACCTGGGCGGTCACCTTCCTGCCGC
CGCTGGTGCTCTCCCTGCTCTTCCCGTTCGGCTTCCTGCTGGCTATCGGTTATGCCGGTGCCGCCGCCAC
CATCTGGACCTGCATCATCCCGGCACTGCTGGTGTTGCGCACCCGGGCGCGGGCCGAGCGGGGCGAAGTG
ACACCCCACTTCGTCACCCCCGGCGGCAAGCTGATGGCTTGGCTGCTGATCCTGTTCGGATTGGCGACCG

CGCTGTTCCACTTCCTGTCGATGGCAAATCTGTTGCCGGTCTATCTGGGCAACTGAGTCAGGTCTACAGC
TCTGACAGAAAGGGCGGCAAGGCCAACTTGCCGCCCTTTTTGCGCCATTTTTACCCCACATGTCAGGATC
TATAACTAAAGTGGTAGGTGTGGGTGGGGTAAGGTGCTGAAATAACTGGTCTGATCTGGCTGGCGGCAGC
ATGATCTGCTGGGCATGCCCGCCGCTTTCTTTTATGATCCCCGCCCTTCTGCGTGCCCCCTGTCGGCACC
TCATTTGCACCAACCACATCGGGGTCCCCCATGTCCGATCTGCGTCAAGAAGCACTCGATTACCACGCCC
AGCCCGTACCGGGCAAAATTGCCATCGCCCTGACCAAGCCTGCCGAAACCGCCCGTGATCTGGCGCTGGC
CTACAGCCCGGGTGTTGCCGAGCCGGTGCGCGAGATCGCCGCCAACCCGGCCGATGCCTATCGCTATACC
GGCAAGGGCAATCTGGTGGCCGTCATCTCCAACGGCACCGCCATTCTGGGTCTGGGCAATCTGGGGCCGC
TGGCCTCCAAGCCGGTGATGGAGGGCAAGGCCCTGCTGTTCAAGCGCTTTGCCGGTATCGACGCCATCGA
CATCGAGGTGAAGCACGAGACCAGCCAGCAGTTCATCGACACGGTAGCGGCTATCGCCGATACCTTCGGT
GGCATCAATCTGGAAGATATCAAGGCGCCGGAGTGTTTCGAGATTGAAGAGGCGCTGATCGCGCGCTGCG
ACATACCGGTGTTCCACGATGATCAGCATGGTACCGCCATCGTCACCGCCGCCGGTCTGCTCAACGCGCT
GGAAGTGCAGGGCAAGCAGATCAGTGACAGCCGCATCGTCTGCATGGGGGCGGGGGCTGCCGCCACCGCC
TGCATGGATCTGCTGATCAAGTGTGGCGCCAGAGCGCACAACATCTTCATGCTGGATCGTCAGGGTGTTA
TCCACACCGGCCGCAGCGATCTCAACAAGTACAAGCAGCGTTTCGCCAACGAGACCCCGCTGCGCACCCT
GATGGATGTCATCGAAGGGGCGGATGTGTTTGTCGGCGTCTCCGGCCCCGATGTGTTGCCCGCCGAGGCG
GTCGCCCTGATGGCGCCCAACCCGGTGATCTTTGCCTGCTCCAACCCGGACCCGGAGATCAAACCGGCGC
TGGCCCACGGGGTGCGTAGCGACCTCATCATGGGTACCGGCCGCTCCGACTACCCGAACCAGATCAACAA
CGTGCTCTGTTTCCCCTTCATCTTCCGCGGTGCCCTCGACGCCCGTGCCAGCCGCATCAACGATGCCATG
AAGATTGCTGCGGTCGAGGCGATCCGCGCGCTGGCTAAGGAGCCGGTGCCAGCCGAGGTGCTGACCGCCG
CCGGGGTCAGCGAGCTGGCCTTCGGCAAGGATTACGTCATTCCCAAGCCGATGGATGCCCGTCTGCTGCC
GCGGGTTGCCCGCGCCGTGGCGCTGGCGGCGGTGGCCTCCGGCGTCGCCCGCATCGAGCTGCCCGCGGAC
TACATGCTGTAATCCGGCGTTGCAAACGGCCCCATAGCGGGGCTGTTTTTATTTCTGCTTCTGGCGACAG
CCAGAGACGCCAGCCGGGTGACAGGTTGGGGCAGATGGAGGCAAATCGGGTGCAAAGGTGGTCAAGCAGC
GCTCTCCTCTGACACAATATTGCCACTATGACGCTAACGAGAATCAAGATGCAGCAGCCTGTCAGCGGCC
GTTTGGCCCACGCCCTCTCCTTCTTGCGCCATGATGGTCGCCACTTTGCCCAGTTTGTCTGGGGCCGTTT
CCAGCAGGACAGATTGACGGTTACCGCCGGTTATCTGGCCTATGTCACTCTGCTTTCACTGGTGCCGATG
ATCGCCGTGGTCTTTGGCATGATGTCAGCCTTCCCGGTGTTCCAGAGCCTCAAGCAGGCGATGGAGCAGT
TCGTCTACCACAACTTTGTGCCGACCGCGGGCGACATGCTCAAGGAGTACATCGACGGCTTCGTCGCCAA
TGCCACCAACACCACAGCAGTGGGGATTGGGGCGCTGGTGGTGGTGGCGCTGATGCTGATCTCCGCCATC
GACAAGAACCTCAACTACATCTGGCGTTCGACCCAGGGCCGCCCGCTGGCGCAGGCGTTTGCCATGTACT
GGATGATCCTGACCCTGGGGCCCGTGCTTATCGGGGCCAGTATCGCTATCTCCTCCTATATCTTCTCGCT
GCGGCTGTTTGGCGCCGAGAGCCTGTTCGGTATCGGTTATCTGCTGTTGCGCGGTTTGCCGTTCCTCTTC
TCGGTGCTTACCTTCCTGCTGGTCTATACCGTGGTACCCAACTGCAAGGTGCGGCTGATCCACGCCTTTA
TCGGTGCGCTGGTAGCGGCGACCCTGTTCGAGCTGGCCAAGCGCGGCTTTGCCATCTACATCACCAACTT
CCCCTCCTATCAGGCCATCTACGGCGCGCTGGCCACCATCCCGATCCTGTTTGTCTGGGTCTATTTGAGT
TGGCTGGTGGTATTGCTTGGCGCAGAGACTACCGCCTGCCTCGGTGAATATGAAAAACCGGCGAGCGAAG
AGCTGGGCTAAGCCATCGCCTTGCCCACCGGTTTATTGGTAGCATCGACGACCGCCCCTGCGAGGGGGCG
GTCGTTCTGTTTTCAAAGGAGAGGGATGTGATTGCATTGATTCAGCGGGTCAGCGAGGCCAGCGTGACTG
TCGAGGGGGAGGTGACCGGCGCCATCGGTCAGGGATTGCTGGTGCTGCTCGGGGTGGAGCAGGGGGATGA
TGAGGCCAAGGCCGACAAGTTGCTGCATCGGGTCAGTGGTTATCGCATCTTTCCGGATCAGGAGGGCAAG
ATGAACCTGAATGTCAGCCAGATCGGCGGCAGTCTGCTGGTTGTCTCCCAATTCACGCTGGCGGCGGATA
CCAAGCGAGGGATGCGTCCCAGCTTCTCCTGCGGTGCTCACCCGAGCGAGGCCGAGCGGCTCTATGACTA
CTTCGTGGCCAAGGCTTCCGCCAGCGGCATCCCGACCGCAACAGGGTGTTTTGCCGCCGACATGAAGGTG
GCGCTGGTCAACGACGGCCCGGTTACCTTCTGGCTACAGGTCTGAGGATGCCTGATCCCGCGGTATGACG
TGGACGATCACGTTGATGAGCCCTGCGACCGGGTAGGGGGTAGCCGGGCCAATCCACTGCTCGATCAGTG
TCAGGGCGTGGGTTAGCTCTAGTGCGGTACGCGGCAGCAGGTTGATCACCAAGGTGCCGCAGAGGCGGGG
AGCCAGCGCCCGGTAGAGCCGCTCCTGAAACAGCAGCAGCGGGTTGCCATCCTGACTGAAGAGATCGAGC
AGGATGAGGTCGTACTGCTGCTCGCTCTGCTCGAGAAAGGCGAGGGCATCGGCCTGATGGAGTGCCGGCG
CGGCGTGTTCATCGCTCTCCTGATGCTGGAAAAACTGCTGATAAACGGTCAGCACCTCTTCGTCGAGGTC
AACGCACTCGTGGCGAGCATCGGGCCAGCGCTGACTCAGATGGCGGGTGAGATCGCCGCCACCCAGCCCG
AGTTCGAGGATCTGCCCGGCCTGTTCTGGCAGCAGGCAGGCGATGACCCGCTGATGGGGTAGACAGAGGG
CGGCGGGGTTGGCGAGCGACATGGCGGATTGCACCACGCCATCTATCTCCAGCCAGCGGTGCTGGTCATT
TTCCTGCACGGTGAGGCGGCGCTGGGGTTTGACGCTGAGGTGCAACAAGTGATCGGCTTGGTACGCTTGC
AGATTGAAGTCCATGAATTTGCACGGCAAGATGGTTTCCGACCAGTGTAACTCAAGGATGAAACTATGTA
CCGGGTAGTCACCCCTCAGACAGTCGCCGAGCTGGATGCCTACTACCAGTTGCGCTGGGAGCTACTGCGT
AAGCCCTTCAATCTTCCCGTCGGTTCCGAGCGGGACGAATACGACACCGTCGCCATTCACCGGCTGATGC
TGGCACCCGATGGTACCCCCATCGCGGTGGGCCGCCTCTTTATCGGCGGTGACGAGGCGCAGATCCGCTT
TATGGCGCTGCGGCCCGAATATCGCGGTCAAGGGCTGGGCGCCAAAATGGTGGAGGATCTCGAGGAGCTG
GCCCGCAGCGAGAAGGTGAAACGGCTGGTGATGAACGCCCGTCAGGAAGCGGTGGAGTTCTATCGCAAGT
GCGGTTTTCTCGAAGTGGGGGGCGGCCCCGTCTCCTTCGGCCGCATTCCCCATCGCCAGATGATCAAATC

CCTGAGCCCGTTGCAGACCATCCAGTATCGCCCGGAGTGGTGTCAGGATCTCACCACCCGCTGGTCGCGC
GGGGTGCCCATCAGCGAGAAGATGGGGATGCACATCAGCCACTACGACGGCCAGACCTTCCATCTCAAGG
CCAATCTGGCGGCCAATTTCAACGTGCATGGCAGCATGTTCGCCGGCAGTGTCTACAGCCAGTGCGTGCT
GGCGGGCTGGGGGCTGATCTGGCTGCAGCTTAAAGAGGAGGGGCTGGTGGGCGAGCCGGTGTTGGCCGAA
AGTACCATCAAATATCATGGCCCGGTCGATGAGGAGCCCGAGGCGCGAGTGGCCCGCGAGGGGATGCCCG
CCGTGCTGCAGCCGCTCAAACGGGGCGAGCCTGCCACCTTCAGCCTCAAGATCCAGCTGTTCAGCGGCGG
CAAGCTGGCGGCGGAGTTTTGCGGTCACTATGTGGTGCAGCCGCCCCGCCGCCCGGGCCAGTAACCGCGT
CGTTTCCCCATCAAACAACAAGGCCTCATCATGAGGCCTTGTCTGTTTTTACCCTTGCTGGCTGTCGCTT
GTCTGCCCGGCTTGGGTCTGCACTTGGGCCTGGTTTTGGGCTTTCGTTGCGGGAGCCGGTCGCCAGAGCG
GGGCGCTGTTGCCTTGCGGCGGATAGCGTACTCCCACTTCCCAGCCGCAGGCGTCCTGCTTCTGCGGGAA
GCTCCAGGCCAGTGTGGGTTCCGTCAGCGGCCCGCGCCACTGGCCGAGCAGCTCGGCGCAGCGGTTGCCA
TTGAGGATCCCCAGATCCAGCTGCCACTGCTCGTTGACCAGATCGATCCCCCCTTGCAGCGCCAGCAGAT
GGGTGATGGTGGAGGCGCCAGCCTGCTCGACTTGTAGTTGCCCCTTGTCGATCTTGCCCTGCAGCTTGAT
GCGGTAGAAGGGGGTGTCACCCTCCTGCATCGCCTGCCACAGCTGATCCGGCGTCAGGGTGGGCGGGGTC
GTCCCTTTGAACCACTCATCCAGCAGCGGATCTATCTTCACCTTGTCCCAGAACGGCTCGTCGGCATTGA
GGGCAAAGCTGCCTTGCAGGGTCTGGCGCCACTCGCCCGCCGTTTTGCCGTGTTCGGCTTGCAGGTCGGC
GCTCACGTCCGCTTTGCCGGCAAAGGAGACCGGGGAGTGGAGTATCTTGCCCCACTGCTGCAGATCCAGC
TTGGTTGCCTGCAACTGCAGGCGGCTGCTCTGGCCCGGTTCGCGGCCCCACTGGCCACTCAGGCTGAGGC
TGCCCTCATCCGGCAGCGAGCTGGTGAGTTTGCCCAGTTGCCAGGAGCTGGGGGTCAGTTCCCCATCCAG
CTCGCCGTTACGGGCGCTGAGGCCATCCCACACCAGCTCGAACCACTTGCTCTCGATTCGGGCACGGCCG
AGCAGCGGGCCGGGTTGGTTATCGCGCAGGGCGAGATCGCTCAGATAGAGCTGCCAGCCGGTCAGCGACA
GGGGCAGGGTATCGTCGAAGGAGAGCACCTTGAGCTTGTCGAACGCCAGCTTGCGGATATCGACCTGCTG
CGGGCGCCAGCTCTGCCAATCCTGCCACCACCCTTGCGGCAGGCTGATATCCATGCCGCTCAGCGCCATG
TCTTTCAGGGTCAGCTTCTGCTGCTGGCTATCGAGGGATAACTCGACATTGAACTCCCCTTCATAGGCCT
TGCCCTGCAGTTCGCCGTCGATCTGCTGGGGCGAGAAGGCGAGCTTGCCCTTGATCTCCTCCAGCTGGAA
CAGGCCACGGCCCATGTCCCCCAGGGTGCCGCTCAGATAGCCGGTGGGCTGGTTGCCCGCCTGCCAGTTG
AAGGCGGTGAGCTGGCCGCTGAAGTTGTTGAAGGAGAGCTCCTGATTGATGTCGTTGAGGCTGACGCCGT
CCAACTGACCGCGATCCAGCGACACGCTTTGCAACGGGAAGCGCTGCGGGAAGCCTTGTTGTGTCAGATC
CCCCAGCTCCACCCGCATGCCGCGCGCCTTGAGATCGTGCAGTTGCAGAGTACGGGTGGGCCAGTCCAGC
ACCATGCCGGTCTCCAGCAGACCGTCGAACAGGTTGGTCACCAGCTTGTCGGTGCGCAGCACTTCTCCTT
CCAGCCTTCCTTGCAAGTTGAGACGGGCGAAGGTGAATTGCGGGGTCTCCAGCTGGCCGATATTGAGGCT
CAGGTTGGCCTGTGGCAGCTGTTTCGGATCGATCAGCTCCCAGTCGCTCAGGGTGGCGTTGAGACCGCGC
AGGGTCAACTCGGGGCTTTCCAGACTGAGCCGGTCTATGGTGCTGTCGCTGATGGTGAGGGTGTGCACCG
GCAGGTTCGGCAGCTGGTTGGGCAGCGGCAGCTTGATGGCGGGACGAATGAGATCGAGATGGGCGATCCG
CACATCGCGGCTCAGCCAGTCGATGCTCTCGATCTCCAGATAGATCTTGTCGAGGGAGACCGCATCGCCG
TAGCGTACCTGCTCTGCCAGCAGGGTGTAGGGGTGGAGCGGGTTGAACACCAGTCGCCCTATGGTGACCG
GTACGCCGGTCTGCTGGCTGATCCAGCTGGCGATGGGGCCTTTGGCGCGCTCGGCGTCGAACAGGCCGAG
GGCAACCCAGACAAACAGCAGCAGGAAGGCGAAGGTGTAGAGCAGGCCGCGTAGCCAGGAATTCATGGTT
GCTTTCCTCCTGTTTTGGGATTTGTTGTCAGGCGTTTGAGTAGTGATCTCGCAGACCAAGCCAGCGCCGT
ATCAAGGGGTCGACATGACTCGGATGCCTATCCATAAGGAGATGTGCCCATCTTCTGCATCTATCTCGAT
CAGGCATTGGAATAGTGATCCCGTAAGCCGAGCCAGCGCCGTATCAAGGGTTCGACATGGCTTGGGTGCC
TATCCATCAGGAAGCGCGCCATTTTTTGCACCTGTGGAATAAGCGGCTGATCCCGCACCAGATCGGCGAT
CTTGAGGTCGGCCAGACCGGTCTGGCGGGTGCCGAGCAGTTCCCCCGGGCCGCGCAGTTCCAGATCGCGC
TGGGCGATGAGGAAGCCGTCGTTGGTCTCACGCAGCACCCCGAGCCGGCTCTGGGCCGTCTTGGACAAGG
GGGCATGATAGAGCAGCACGCAGTGAGAGGCCACCGCACCCCGGCCAACCCGGCCGCGCAGCTGGTGCAG
CTGCGCTAGCCCGAGACGTTCCGGGTTCTCGATGATCATCAGGCTGGCGTTGGGCACGTCCACCCCCACC
TCGATGACGGTGGTGGCGACCAGCAGTTGCAGTATGCCCGCCTTGAACTCCTGCATCACCCGCTGTTTCT
CGACCGGACGCATCCGACCGTGCACCAGCCCGATGTGCAGGCCGGGCAGCAGGTTCTGCAGCTCGGCGGC
GGTATCTTCGGCGGCTTGGCACTCCAGCACCTCCGACTCCTCGATCAGGGTGCAGACCCAGTAGGCCTGC
TTGCCCTCGGTGCAGGCGAGCTTGACCCGCTCGATCACGTCGCCGCGACGGCTGTCAGGCAGCGCGACTG
TGGTGATGGGGGTACGGCCCGGCGGCAGTTCGTCGATGACCGAAGTATCGAGGTCGGCATAGGCGGTCAT
CGCCAGGGTGCGGGGGATGGGGGTGGCGGTCATGATGAGCTGATGGGGATGTACCCCTTCCCGCTCCCCT
TTCTCGCGCAGTGCGAGGCGCTGATGCACCCCGAATCTGTGCTGTTCATCGATGATGACCAGCGCCAGAC
GCTGGAACACCACCTGCTCCTGAAAGATGGCGTGGGTGCCCACCACCATCTTGACGCTGCCATCGGCGAT
GGCGGCCAGCGCCTCCTCCCGAGCCTTGCCTTTCTGTTTGCCAGCCAGCCAGCCGACTCCGATGCCGAGC
GGCTCCAGCCACTTGGCGAAGTTGATGGCGTGCTGTTCCGCCAGCAGTTCGGTCGGCGCCATCAGCCCCA
CCTGACAGCCGTTGCCGATAGCCTGCAGGGCGGCCAGCGCCGCGACCAGGGTCTTGCCAGAGCCTACGTC
ACCCTGCACCAGCCGCATCATGGGGTGGCTCTGTTGCAGATCCTTGCTTATCTCCGCCACTACCCGGCTC
TGGGCGCCGGTGGGCTTGAACGGCAGGGCGCTGAGCAGCTGTTCGACCAGCTCGGGCGCCGGTTTGAGAG
CGCGAGCCAGCTGGGTTTGTGCCTGAGCACGCACCTTGAGTACCGAGAGGTTGTGCGCCAGCAGCTCCTC
CAGCACTAGCCGTTGCTGGGCCGGATGCTGGCCGCTTTCCAGCAGCGGCAGCGCCACCGAGGGAGGCGGG

CGATGCAGCAGCTTCAGGGCTGCGGCCAGTTCAATCTGATGGGGATAGAGTCCGGCGGGCAACAGCTCCT
CCACGCCGTAGAGGTCGAGCTGGGCCAGCGCCTGATCGGTGAGGTTGCGCAGGGTGAGCTGGCGCAGTCC
TTCGGTAGTCGGGTAGACGGGGGTGAGCGCCTCTTCGGTCTGACCGGCCTGCTCTTCGCCGAGCAGCCTG
TACTCGGGGTGGGCCATCTCCAGCCCGTATTTGCCGGGGCGCACCTCGCCAAAGCAGCGGATCAGACGGC
CCGCCGCCAGACTGTTTTTCTGGGCGGCGGTGAAGTTGAAGAAGCGCAAGGTGAGGGTGCCGGTGCCATC
GCTGATGCGACAGACCATCATGCGGCGGCGCCCCATCACCAGCTGAGTGTCCTGGATTTCGCCCTCGACG
GCGCCGTGCAGGCCGGGCGGCAGATCGCCGATGGGCCACACCTGGGTGCGGTCCTCGTAGCGCAGGGGAA
GGTGGAACAGCAGATCCTGTACCGTGGCCAGCCCCAGCCGCTCGAGCTTCTCCAGCATCTTGCTGCCGAC
ACCCTTGAGGCTATCGAGAGGAATTTTATCCAATTTCATCAATTAGATGCCCGAAAACACTGTAAATATG
TCCAGATGATAGGGGTTGTTGAGGGGCCGATGCAAGCCGAGTTGCTGCTGGCAGGGCCGCCAAGGCGGGG
GAAACGGCGGATATGACAAAGGCCTGCCGAAAACGGGAGGCCTTGGTGCGGCTGCCAACTGGCAAGGTGC
GGGCGCATGGCGCCCAAGGTCAGATGATGCGTTTAAGCAGCAAATGGGCCTTGACCCGATGGATCCGTCC
CAGCAGCTTGCGCACCTGTTCCGGGTAGTCCTGGATCTGCTCCACTTGATCGAAGTGGCTGATCCGCTTG
GTGTTGGTCAGGATGTTCTCCCGCTCCGCGTTGAACTGGGGACGCAGCAGGTGGCGACGATCGTGCACCA
GCAGGCCATTCTCCAGATCCAGCGCCCAAGCGCGGGGGTTGAGGTTGTTGCCGGTGAGCAGGGCCCACTC
GTCATCCACGAAGATCCCCTTGAGATGGTAGGAGTTGCGCTCGCAACACCAGAGCATCAGGTTGAGACGC
CCGTCGTCGATGTGACGCTGGTTGCGCTCGGCAAACTTGCGCAGGTTGGTCTCGTAGAGATAGGGCAGGC
CACCGATGGTGGAGAACTTCTCTTCCGGCGGAATGAAGAAGTCGTTGGCGGTCTTGTCGCCCACGACGAT
GGTCACCTTGCAACCGCGCTGCAGCAGATCCTCGATATCCTTGCTCAACTCCTTGGGCGGGTTGAAGTAA
GGGGTGCAGATGAAGATCTCACGCTCGGTGGCGCGCACCAGATTGCGGATCATCAGGTTGAGCTGATTGT
TGCGCTTGCCCAGACCCACCATGGGGGTGACGGCCACCTCCTGGGTGTTGACCGGCATGGTGCTGAAGCG
GTACTGGCTCTGGCGCAGGTTCTGCTTGAAGCGGCGGATCGGCCCCTTCAGCTGTTTGGCGCTGGGGATA
TCGCTGTGATCCAGTCGCTGCACCGCGTCGCTCTTCACGAACAGGTCATCCACATAGTTGACCATGCTGC
GGGCCAGCTCCGGGCTGGTGATCTGGTGATAGCGATCGAAGCGGTAGCGATCCTGCTGATGCAGGTAGAT
GTCGTTGAGGCTGGCACCGGAGTAGAGCACCACATCATCAAAGATGAAGCCCTTGAGGTGGAGCACGCCG
AGCAGCTCGCGCCCTTTGACCGGCACGCCGTAGATTTCGATCTGGTGCTCGTGTTCGGCAGCCATGGCCT
GATACATCAGGTGGTTGCCGCCCTGCTTGCCTTTGCCGATCAGGCCGCGCTGGGCACGGTGGAAGTCGAC
GAACAGCTTGATGTCGAGGGCCGGATTGCGCTGCTTGGCGGCGTAGAGGGCGGAGAGGATCTCCCGGCCT
GCTTCGTCATCCTGCAGATAGAGCGCGACCAGATAGATGCGCCGGGTGGCACTGGCGATAAGCGCAAGAA
TGCGGCGTTTGAAATCCTGGGCGCTGTAGAGCACTTCAAACTGATCCGCTGCGACGGCGATCTGGGGCAG
GCGCGCCAGCAGTTGTCGATAATGGGCCGATGAATGCATAGATAAACCTGAAATCACGGGGTTAAGCGTT
CCACTCGGAGCGATGAATAGGCGATTCTAGCAATCTCGCCCTCTCAGCCAAAGAAAGAATCATCAGAAAG
ATGAAAAAGGCCTCACGAGTGAGGCCTTTTTGTCAGCATTTCGCCCTTGTCAGAGGAAGCTATAGATGAT
CATCGCCATCACGCCGCCGGTGGTGCCGATGATGGTTTCCATCACGGTCCAGGTCTGCAGGGTTTGCTTC
TCGGTCAGGCCGAGGTAGCGGTTGGCCAGCCAGAAACCGGAGTCGTTGACGTGGGAGAGCACGATGGCAC
CGCCGCCGATGGCGATGGTGACGGCCGCCAGCTGGGCTCCGTTCAGGCCGAGGGGTTCCAGCATCGGCAG
CACCAGACCACAGGCGGTCAGTATGGCGACGGTGGCAGAACCCTGAATCACCCGTACCGCGGCGGCCAGA
ATGAAGGCCAGCGCGACGATGGGCAGACCGGAGTCGGCCAGCATGTTGCCCAGCGCGGCGCCTACGCCGG
AATCAACCAGCACCTGCTTGAACACGCCACCGGCACCGGTCACCAGGATGATGACACCGGCGGGCTGGAT
GGCGCTGTTGCAGACGCTCATCACCTCGTCACGGGACATGCCGCGGCGAATACCCAGCAGGTAGAAGGCG
GCCAGACAGGCGACCATGATGGCGGTGAAGGGGTGACCGATGAACTCCAGCCAGTTGTGCAGGGTAGAGC
CCGCCTCGACGAAGCGGCCAACGATAGTCTTGAGGCCGATCAGCAGCAGTGGCAGACCGATGAGACCCAT
GGCCAGCCCGAAAGAGGGCAGGGATTTGCTGTCATCGTGGATACTGTTCTGGGCATCGGCAGGCAGCGGC
ACATCGACGATCTTGCTGATGAAGTCACCAAACAGCGGGCCAGCAAGGATCAGCGCCGGTGCGGCAGCAA
TCAGACCGAACAGGATCATGTAGCCGAAGTCGGCACCCAGCTGGGATGCCACCAGGATCGGGCCCGGCGC
CGGGATAAGGAAGGCTTGGCAAGTGGCGATACCGGCCAGCAGAGCGATACCGATCTTCACCACGCTGCCG
CCACCACGACGGACGATGGCGAAGGCGACGCCGATCAGCAGCACCACGGCCACGTCAAAGAAGAGCGGCA
GGGCGCAGACAAAGCCGGTCAGGGCCATGGCCCAGTTGGCGCGCTTGACGCCGAACTTGCCGAGCAGGGT
GTGAGCCACCCGGTCGAGGGCACCGGTCACTTCCATCACCTTGCCGAACATGGCACCCAGCGCCACCACT
ACCGCGACGAAGCCGAGGGTGCCGCCCATCCCTTTCTGGATGGTGGCGGCGATGTCGGCCGGGTTCATGC
CAGCGGCGAGACCCGCCACCATGGAGACCAGGATCAGGGCTACCACGGCGTGGAGCCGGGCCTTCATCAC
CAGGAAGAGCAGCAGCACAATCGAGCCGGCGGCTGTCATGATCAGGGAGAGGTCAGACATTGTTATGCGT
TCCTTTTTCCAAGTTCAGTCGTGACACCTTGCCGATCCGTCGGCAAACCATATCTTTATGATTTATTTAG
ATTTATGGCAAGGGCTCAACATCATATGCCCAGGTGACTATGGGTTTGCTCAGTCTGTGGCAGACTTCAC
ATTTTCAATCGTTACCGGTAACATGTTACCGGTAACGTGTTAGATTAGAACCCATCAACAGCTGGTTTTT
TGAAAAGTGGGACAGAGGTCAAATTATGGCGGGTAAGTGCATTATCGTGATGGGTGTGTGTGGTAGCGGC
AAATCCAGCGTGGGTCTGAAAGTGGCCGAAGCGCTGGGCGCCAAGTTCATCGATGGTGATGACCTGCACC
CCAAGGCCAACATCCAGAAGATGGCTGGCGGCAACCCCCTCAACGATGAGGATCGTGCCCCCTGGCTGGA
GCGGATCCGCGACGCCGCCTACAGTCTCGAGCAGAAGAACGAGACCGGCATCATTGTCTGTTCCGCCCTC
AAAAAGAAGTACCGCGACCAGATCCGCGAAGGCAACGAGTCGGTCAGCTTCATCTTCCTCGATGGCAGCC
AGCCGCTGATCCTCGAGCGGATGCGTGCCCGCAAGGGCCACTTCATGCGTGAAAGCATGGTACAAAGTCA

GTTCGATACCCTGGAGCGCCCGGATGGCGAAGCCGGTGTATTCCACATTGATATCGACGGCACCTTCGAA
CAGGTGGTTGACCGCGCCGTCACAGCATTGGAGCAAGCCCAATGATTCATCAGGTAATTTCCGACGTCAC
CGCCCGCATCCGCGAGCGCAGTGTCGCCCGTCGTCAGGCATTTCTGGCCCGCATCCAGCGTCAGGCAGAG
CAGGGCAAGACCCGCGCCGCGCTGGCCTGTGGCAACCTGGCCCACGCCGTGGCCGCCTGCAGCAGTGACG
AGAAGGGTCGCATTCTCGATATGACCCGCGCCAACGTCGGCATCGTCACCGCCTACAACGACATGCTGAG
CGCCCATCAGCCCTATCAGGGCTATCCGGATCAGATCAAGGCAGTGCTGGCCGAGCTGGGCCACAGCGCC
CAGGTTGCTGGCGGCGTGCCCGCCATGTGCGATGGCGTGACCCAGGGTCAGCCGGGGATGGACATGTCCC
TCTTCTCCCGCGACCTGATCGCCCAGGCGACCGCGCTCTCTCTTTCTCACAACACCTTCGATGCCACCCT
GCTGCTCGGCATCTGCGACAAGATCGCTCCGGGCCAGATCATGGGGGCCCTCTCTCACGCTCACCTGCCG
ACTGCATTTGTTCCTGCCGGCCCGATGGCGAGCGGCATCAGCAACGACGAGAAGGTGAAGGTGCGTCAGA
AGTACGCCGCCGGTGAAGTGGGTCGCGACGCCCTGCTGGAGATGGAGTGCGGCGCCTACCATGCGGCAGG
CACCTGCACCTTCTACGGCACCGCCAACACCAACCAGCTGGTGTTCGAGGCCATGGGCCTGATGCTGCCG
GGCTCCGCCTTTGTGCATCCGCACAGCGAGCTGCGCCGCGCCCTGACCGCCGAAGCGGCCCGCCGCATCA
GCGCCATGATCCCAGGCTCTCCGGCCTATCGCCCGCTGAGCGAGGTGCTGGATGAGCGTTCGCTGGTCAA
CGGTCTGGTGGCCCTGCTCGCCTCCGGCGGCAGTACCAACCACAGCATCCACATGGTGGCGCTGGCCCGC
GCGGCCGGTTTGGTACTGACCTGGGATGACATCAGCGATCTCTCTGACGTGGTGCCGCTGCTGGTTCGCA
TGTACCCGAACGGCCCGGCCGACGTGAACGCCTTCGAACAGGCAGGCGGCGTGCCCGGCCTGATGCGCCG
TCTGGCGCAAGAGGGGCTGATCCACATGGATGCCACCCCGGTCTTTGGCGAGATGCAGGATTACCTCAGC
CGTCCGGCATTGGTCGATGGCCAGCTGGTGTGGCAGCCGGTCGGCGAGAGCGGCGATGCGAGCGTGCTTT
CACCTTCCGGCAGCGTATTCCAGGCCACCGGCGGCACCAAGCTGCTGGCGGGCAATCTGGGCCGCGCCGT
GGTCAAGGTCTCCGCCGTGGCTCCCGAGTATCGGGTGATTGAAGCGCCGGCGCGGGTCTTCTCCTCCCAG
CATGCGGTGGAAGCGGCCTACAAGGCGGGCAATCTCAATCAGGATGCCGTTATCGTGGTGCGCCACAATG
GTCCGGCCGCCAACGGCATGCCGGAGCTGCACAAGCTGATGCCGGTGCTCGGCAACCTGCAGAAGGCGGG
TTACAAGGTGGCGCTGGTCACCGATGGCCGCCTCTCCGGTGCCTCCGGCAAGATCCCGGCCGCCATCCAC
GTCACCCCGGAAGCGCTGCACGGCGGGGCCATCGGCCTGCTTGCTGACGGCGACCTGCTGCGGGTTGACG
CGGTAAACGGCAGCCTCGACTGCCTGACCGATTTGAGTGGCCGTACCCAGGCCGAGATTGACCTCACTCT
GGAACAAGAGGGGTGGGGCCGTGAACTGTTCAGCGTGATGCGTCGCGCGGTATCGAGCGCCGAGTGTGGC
GCTACCATCTTTGACTAAGGAAGAGCTATGCAGAACTGGAAAGTGACCCCGGCCGAGGTGTTTGCCGCCT
CCCCGCTGGTACCCGTGATGGTTATCAACGAGCTGGATCAGGCCCTGCCGATGGCCAAGGCGCTGCTCGA
CGGCGGCATCTCTGTCTTCGAAATCACCCTGCGCACCCCGGTGGCGCTCGATGCCATCGCCCTCATCGCC
AAGGCGATGCCGGATGCCATGGTCGGTGCTGGTACCGTGCTCAACTGCGAGCAGTTTGACGCAGCCGTTG
CCGCCGGTGCCCGCTTCGTCATCTCCCCGGGGATGACCCCTGCCCTGCTGGCCCACGCGGCCAAGAGCAC
AGCGCCGCTGATCCCGGGTGTGGCAACTCCGTCTGAAGTGATGCAGGCGCTGGAAGCGGGCTATGACCAC
CTGAAGTTCTTCCCGGCTGAAGCGAACGGCGGTACCAAGGCGCTCTCCGCCATTGCCGCGCCGCTGCCCC
AGGTGAAGTTCTGCCCCACCGGCGGTATCGGCCCGAAAAACGTGGCCGACTATCTGGCACTCAAATGCGT
GGCCACCGTCGGCGGCTCCTGGATGCTGCCGGCCGATGCGGTCAAGAGCGGCAACTGGAAGAGGTGACC
CGCCTCTCCCGCGAAGCGGTTGAGCTGGTAAAACGCTAAGCATCAGTTGTGTGCAGAAAACCAAACAGGC
CACCCTCGGGTGGCCTGTTTGTTATCGGCGTGACTTAAGTGGGTGAGGGAAGAGATTACCCCAGACTCTC
CCCGATATAGAGGGAGAAGCCGATATCCTTGCGGGTGTCGGGTTGGGATTGGCCGCTGAGACGCGCCAGC
AGCATGGTGGCCGCCTCCCGACCGATCGCTTCCCGCGGGGTGGCGATACTGGCCAGCTTGGGGGTCATGG
CGTGGCCGATGTCGAGAGCGTTGCAACCGGCGATGGCGATCTGCTGGGGCACCGGGATCCACTTGGCCTG
ACACTGCAGCAGGGCGCCCACCGCCAGGTCATCGTTGGTGCAGAACAGGCCGTCGAGGTCGGGATAGCGC
ACCAGCGCCTGATCCAGCAGCTCGCCCCCCAGAGTGAAGCTGGAGGATTGTTCGGTCAGCAGATGCTGGC
CGGTGAGGCCGAAGTCGATCATTGCCTGATAGTAACCCTCCATCCGCAGCTGGGTACGTACATCGAGCCG
CGCCCCCAGATAGACCACCTTGCGACGGCCCCGGCGCAGCATCTCTGTTACCATGGCCCGGGCGGCGGCT
CTATGGTCCATGCCGACCACCATGTCGATGGGGGTTTGCGGCAGATCCATGGTCTCCACCACCGGAATGC
CGGCGGTGGCGATCATCTTGCGAGTGCGCTCTGTGTGCTGGCTGTCGGAGAGGATCAGCCCATCCACATG
GTAGGAGAGCAGGGACGCGACCTGCTCCTCCTCCCGCTCCGGGCTGTAGCCGTAGTGGGCCAGCAGGGTC
TGGTAGCCAGCCGGTCGGGTTACCGACTCGATGCCGTGGATCACCGCCGAGAAGACCTGGTTGGAGAGGG
AGGGGATCAGGATGCCGATCGCCTTGCTGGTCGCGTTGGAGAGAATATCCGGCGCTCGGTTGGGGATATA
ACCCAGCTCTTCCACTACAGCTGCGATCCGCTCGCGGGTCCCCTCCGCTACGGTCTGCGGGTCGCGCAGA
TAGCGGCTGACAGTCATCTTGGTCACGCCGGTTTGGTCGGCGATATCTTGCAGGGTGGGGCGGCGCTTGC
GGGTGGGGCTCATAGGGGTGTCCAGCGTGGATGTTACATGTAACATTACCGTTTTTGGCGGATAAAGTCA
GCATATGAAGCCGTTTTTGCAGGAGTTTTCCGATGATCAGAGGCAAACAGTTCGTGGATGTCCGGTTCGA
GCAACAGGATGTGGAGGGGGAGCAGTTCTCGGAGTGCCGCTTTATCGGCTGCAACTTCTCTTGGCTGGAT
TTGAGCGATAGCCGCTTCATCGACTGCAGCTTCTACGATCGGGAGAGTGAACGGGGCTGCCTGTTGCAGG
GTTGCGATCTGCGCGAGGCCAGCTTCCTGCGCTGCGATCTCACCATGGCCGATTGCAGCCGCAGCCAGTG
TCTGGGGTTGGAGATGCGCGACTGTCAGGCGGTCGGTATTAACTTCGGCCACGCCAGCTTCGCCAACCAG
ATCACCGCCAAAAGCTACTTCTGCGAGGCCCATCTGACCGGCAACAACTTCAGCTACGCCAGCTTCGAGG
GCTGCCTGCTGGAGAAGTGCGAGCTTACCGGCAACCGTTGGCAGGGGGCCAACCTGTTTGGTGCCTCGCT
GGCGGGTTCCGATCTCAGCGGCTCCGAGTTTGGCCAGATTGACTGGACCAGCATCAAGCTGCAGGGCTGC

GATCTGCGCCAGTGCGATCTGCCGGGGCTGGATCTGCGCCGGGTCGATCTGCAGGGAGTACAGATCAACG
AGGATCAGCAGCAGACCTTGCTGGAGCAGATCGGTCTGGTTGTCTTCCCCTGAGCCGAGCGAAAAAATAG
GTAATTTTTCATCAAATGGGAGTGACAGCGGGTTGCTGTACTCCTGAAATGACAAAAGCCTGCGTGAGGC
AGGCTTTTGCTAGTGAGCGTGTGACGCGTTTGTCGGCCGGGTTAACTCAACCCTTGGCCAGCCATTTGGT
TGGGTTGATCGCCTCGCCCTGATAGCGAATTTCGAAGTAGAGGCCCGGTCTGTCCTGACCGCCACTGTCA
CCGACCAGCGCCACCGGCTCACCCTGTTCCACATTCTGGCCAACTTGGCGCAGCAGCGACTGATTGTGAC
CGTAGAGGCTCATGTAGCCCTTGCCGTGGTCGATCACCAGCAGCATACCGAAGCCGTCGAGCCAGTCGGC
GTAAACCACTTGTCCCGGTGCGACAGCCTTGACCTGGGTTCCTTCGCTGGCGCCGATCAGGGTGCCTTTC
CACTTGAGCTGGGCGGTACGGGGCGAGCCATAGGAGATGAGGATCGGTCCCTGCACCGGCCAGCGCAGGC
TGCCATTGGTCTTGAGGCCGCTATAGTGACCGGCACTGCTGATGCCCGAGCCGCGCTCCGGTTTGCTGCT
GGCCACCTCTTCGGCAGGTTCTGCCGGTTTGACGGTTTTATTCTGCTGGGCGGCGAGTCGTTGCTGCTCG
GCGCGGCGCTGTTCGGCCAGCTTGCGCTGACGCTCCTGCTCCGCTTTCAGCTTGGCCAGCCGTTCCCGCT
CGGCCCGTTCACGGCGCTCCTGCTCTTCCCGCTTGCGACGCAGCTCTTCCAGCCGGGCTTTCAGCGCTTT
CTCCGCCTTGACCAGCTTGGTGAGCTTCTGCTCGTCATCCTGTACCGACTGCTGCAACTGGTTGCGCACC
TTGGCCCGGCTCTGCTGGCTGGCCAGCAGGGTCTGGTGATGCTCCTGCTGTTCGGCAAGCAGGGTCTGCA
GTCTGGTTTCGGTCTCTTTGGTTGCCTGCTGGTTTTTCGCCAGCTTGTCGCGGGTGGCGCGCAGGGCGTC
GATGGCCTCGATGCGGGCCTTGTTGAGGTAGTCGTAGTAGTCGAGTGAGCGGCTCAGTTTGGCCGGATCC
TGCTGATTGAGCAGCAGTTTGAGGTAGTCGTGGTTGCCCGCTTGAAAGGCGCTCTCCGCCTGTTTGGCCA
GCAACTGTTTTTGATGTTTGGCTTGAACTTCCAGCCCCTGCTTGTCTTTTTGCAGGGTCGCCAGCGTCTG
CTGGTTCTGCTTCAACTGCTGCCTGGTCTGGTTGAGTTTGGCGGCGGCGGCCGAGATGGCCTGCTCGTCA
GCCTTGAGCTGGGTATTGAGCTTGGCAAGCTCCTGCTTGCTGAGCTTGATGGTCTGCTGTTGTTCCTTGA
TCTGGGACTGCATGGTCCCCAGCTGCTGATTGGCGGCCAAAGCCGGTTTGCTGGCGCCAAACGACAGGCA
AAAAAACAGCGCGCCGGCCAATAGGCCGACGCGACTGGTTTTCTGTGAAATAACTTCGCATCCCCGCATG
GGGAAGGATTATTTCAGAATCATCAGGGGCTTGCCAGTCATTTCCGGCGGCACCGGCAGACCCATCAGGG
TCAGCATGGTGGGGGCCAGATCGGACAGTTTGCCACCCTCAACTACCTCGGCTTTGCGACCGAAATAGAT
GAGCGGAACCGGCAGATTGGTGTGGGCGGTGTGGGCCTGACCAGTCTCTTCGTCCAGCATCTTCTCGGCG
TTGCCGTGGTCGGCAGTGATCAGGCACTCGCCACCCACTTCGGCCAGGGCGTCGGTGACGCGACCGATGC
AGTGGTCAACGGCTTCACAGGCTTTGACGGCGGCATCGAATACACCGGTGTGACCAACCATGTCGCCATT
CGGGTAGTTACAGATGATGACGTCGTACTTGCCGCTCTTGATGGCCGCAACCAGCTTGTCGGTCAGCTCT
TCGGAGCTCATTTCCGGCTGCAGGTCATAGGTAGCCACTTTCGGGCTGGCCACGATCTCGCGATCTTCGC
CAGTGAAGCAGGACTCTTCGCCACCGTTGAAGAAGAAGGTGACGTGAGCATACTTCTCGGTTTCGGAGAT
GCGCAGCTGGGTCTTGCCCTGCTTGGCCAGCCACTCACCCAGGGTGTTGACCAGAGCGGTCGGCGGGTAG
GCACAGGCAGTTTTGATGTCGGCCGCGTATTCGGTCAGCATCACGAAGTTCAGCTTCGGCTCAACGGCAC
GGGCGAAGCCGGTGAAGTCGGTGTCAACGAAGGCGCGGGTGATTTCACGGGCACGGTCGGCACGGAAGTT
CATGAAAATCAGTGCATCACCATCTTCCATCGGTGCAGCTTCGCCGATGCGGGTGGCTTTGACGAACTCG
TCGTTCTCGTCGCGGGCATAGGCGGCAGCCAGTGCTTCGGTGGCGCTCTCGGCGGTGAACTCGCCCTTGC
CCTGGGTCATCAGGTCATAGGCTTGCTGCACGCGATCCCAGCGGTTGTCACGGTCCATGGCGAAGTAGCG
GCCGATCATAGAGGCGAAACGACCCTTGCCCAGCTTGGCGAACAGGGCGTCGAACAGCTCGATGGAGGAC
TGGGCGGAGCGCGGCGGAACGTCGCGGCCATCCAGGAAGGCGTGCAGGTAGATCTTCTCGGCGCCACGCT
TGGCAGCCATTTCGATCATGCCCATGATGTGCTCTTCGTGGCTGTGTACGCCACCCGGGGACATCAGGCC
CATAATGTGAACGGCTTTGCCGTTGGTGACGGCAGCGTCAACAGCCTTGGCCAGCTCGACGTTCTGGAAG
AATTCGCCGTCCTGGATGTCTTTGGAGATGCGGGTCAGCTCCTGATAGACGATGCGACCGGCACCGATGT
TGACGTGGCCCACTTCGGAGTTGCCCATCTGGCCATCCGGCAGACCGACGTCCAGACCGGAGCCGGAGAT
CAGGGTGCTGGTGTAGTCACGGGCCAGACGATCGAGGTTGGGAGTCTTGGCGGCAGCGACGGCGTTGTGC
TCTTGCTTGGTGCTGTAGCCCCAACCGTCCATGATGACCAGAACCAAAGGTTTCTTAGCTGTTGACATAA
GACTATGTCCTCTCGGATTGGAAAAAATGCGGGAAATGAAACTAGCGTAATATTACTACATCTGCCGAAA
TAGTCACCTGATAGCCAAGGGGCGTTCCCGCGGCCCCGCAAGCCACGCCCTTCCTGCCATGCTCGCTGTC
CAATGATGATGGGAGCCTGCTCGCGCTTTGCCGCCAAGATGGCTGATCTTATCAATACCCCCATGTATAC
TCGGGCCCTTGTCTTGGTCCCTGGATAGTAGAAGATGCAAGAGTATTTGGATTTTGCGGCCCGTAACCCG
CTGCTGACCGCAGCCTGGTTGGGTCTGGCTGGCACCCTGGTTTACACCACTGTGCGTGCCCGTTTGTCCC
CGGTAAAAACCGTCAACAACCACACCGCGACCCTGCTGATCAACCGCGAGAATGCCACCGTGGTGGATAT
TCGCAGTCAGGAAGAGTATGCCAAGGGCCATCTGGCCGGTGCCCAGCACCTGCCGCTGACCCAGATCCAG
AGCAATAATCTGGGCCCGGTTGAAAAGCATAAAGATGCCCCCATCATAGTGGTGTGCGAGTCGGGAATGA
CTGCGGGCGGCGCGGGCCGTCAGCTGAGCAAAGCTGGCTTCAAGCAGGTTTATGTGCTCGGCGGTGGCAT
GGCCCAATGGCGTGCAGAAAATCTGCCGGTAACCAAGAAACGCTGATTTAAGCAGGGCCTGAGCCCACTT
ATCCATATAACAAGGAATCAAACGAAATGGCTGACGAGATCAACAACCAGGAAGTACAACCGGAATTCCA
CATCCAGCGTGTTTACACCAAGGATGTCTCTTTCGAAGCGCCGAACACCCCGCACATCTTCCAGAAAGAG
TGGCAGCCGGACGTCAAGCTGGATATGGATACCAAGACCAACATCCTGGCTGAGAACGTCTACGAGGTTG
TCCTGACCCTGACCGTGACTTGCAAACTGGAAACCGAAACCGCCTTCCTGTGCGAAGTACAGCAAGCCGG
TATCTTCACCATCGGTAACCTGCCGGAGCCGCAACTGGCTCACTGCCTGGCTGCCTTCTGCCCGAACATC
CTGTTCCCGTATGCCCGCGAAGCCGTGGCCAACATGGTGAGCAAGGGTTCCTTCCCGCAGCTGAACCTGG

CACCGGTCAACTTCGATGCCCTGTTTGCCCAGCACATGGCGCAAGCACAGGCCCAGCAGGCGACTGCGGA
AGCCTGATAATGGCTGACCAGATCGCTATCTCGGTCTTGGGGGCGGGGTCTTATGGCTCCGCCCTTGCCA
TTTCTCTGGCGCGCAACGGTCATCCGACCCTGCTGTGGGGTCACGACCCCGTTCATGTCGCCGAGCTGGA
ACAAGACCGCTGCAACAAGGCGTTTTTGCCGGATGTCCCTTTTCCTGCCGATCTGCAGCTGACTGCGGAT
CTGCAACGTGCGGTACAGGCTGCCCCCGTGCTGCTGCTGGTCGTGCCGAGCCATGTATTTGGCGATGTGC
TGACCCGGATCAAACCCTTCCTGCGCCCCGATACCCGTATCGCTTGGGCCACCAAGGGTCTGGAGCCGGA
CAGTGGCCGTCTGCTGCAGGACGTGGCCCGTGAAGTGCTGGGTGACGAGATACCGCTGGCGGTGATCTCC
GGTCCCACCTTCGCCAAGGAGCTGGCTGCCGGTCTGCCGACCGCCATCTCGGTCGCTTCGACTCACGATG
AGTTCGCCGACGATCTCTCCCATCTGCTGCACTGTGGCCGCTCGTTCCGGGTCTATACCAATCCGGACTT
CGTCGGTCTGCAGCTGGGTGGGGCGGTGAAGAACGTCATCGCCATCGGTGCCGGTCTCTCCGACGGGCTC
GGGTTTGGCGCCAATGCCAGAACCGCGCTCATCACCCGGGGTCTGGTGGAGATGCAGCGTTTGGGTGCGG
CGCTGGGGGCCGATGCCAAAACCTTTATGGGGATGGCGGGGCTCGGGGATCTGGTGCTCACCTGTACCGA
CAACCAGTCGCGCAACCGTCGCTTCGGGCTGGCCCTCGGGGCTGGCAAGGATGTGAACACCGCCATGACC
GAGATCGGTCAGGTGGTGGAGGGATATCGCAACACCAAAGAGGTGCATCTGCTGGCGGCCCGCTGCGGCG
TCGAGATGCCCATCTGCGAGCAGATCTTCAAGGTGCTTTACGAGGGTAAGAACCCGAAAGAGGCGGCCAT
CGCCCTGCTGTCGCGAGACAAGAAGGACGAGTAATCTGCCGATTGCCCATGCAAAAGGCCCCTCCAGATG
GCGGGGCCTTTTCTTTGCTGCAAGGGCTGCAAATCAGTAGTAGGAGTGGTCGCCGTGCTGGTGCTCGGTG
GCGTCTTTCACACCGTTGAGCTCACCCGGGAACTTCTCCAGCAGCTGCTTCTCGATTCCCTCTTTCAGGG
TGTAATCGACCATGGAGCAACCGTTGCAACCACCGCCAAACTGCAGGATGGCCAGTTTGTCTTCAGTCAG
CTCGACCAGGGTGACTTTGCCACCGTGACCGGCCAGCATGGGGTTCACTTCGGACATCAACACGTACTCG
ATGCGCTCGATCAGCGGCGCGTCGTCGGCCACTTTACGCATCTTGGCGTTCGGTGCTTTCAGGGTGAGCT
GGGAGCCCATCTGATCGGTCACGAAGTCGATGGTGGCATCCACCAGGAAGGGGGCGCTCAGGGGATCAAC
CATGCAGTCGAAGCCGCTGAAGGGCAGGTGTTGATCTTCCGGGTCCACGGCATCGGGCGGGCAATACGAT
ACGCCACACTCGGCATTCTGGGTTCCCGGATTGACCACAAACACCCGGATATTGGTGCCATCAGGCTGTT
TTTCCAGCAGTTTACGGAAATGGGCCTGGGCGGCGTCGGAAATGCTGATCATCACTATCCTCATAAACCT
GAGTGTACGACTAGGGTATTCATGATAACGCGGTTATTAATGCTTGGGTAGCATGCCGATGGCTACTTGT
TGTGCCGCAGGGTTCGACAGATTGCCCATACTTCTACCTTTGTGATGCCGCTCTCATGGAGCAAACGGCT
GAGCTGCCCAGCGGTGGCGCCTGTGGTCACGACGTCATCGAGCAGGGCGACATGGCGATAGTGGTGGGGG
CGGATCCGGAATGCCCCGCGCAGATTGCGCCGCCGTTGCCCGGCGCTCAGGGTTGTTTGTTGCGGGGTCG
CCTTGATGCGCTGCAACAAAGTGTTGTCGCAGGGAATGTCGGTGAACTCGCCAAGGGCTTGCGCGATCTC
CTCCGCCTGATTGAACCCTCGCCGCCACTGCCGCCACCAGTGCAGGGGAACCGGAATGATCGCCTCCGGC
GGCTCGCGGCCATCCAGATGATCGGCCAAGAGGCGTGCCAGTAGCGGCGCCAGCAGGCTCTGGCCGGAAT
ATTTGAGCCGGGGGATCAGCATGGGGTAGGGAGCCTGGTAGTCGCCGATCACCTGCAACCGCTCCCAGGG
GGGAGGTCTGCGCTGGCAGCGGCCGCAGACCGGTATGCTCTCTTCTATATAGTCAGCCAGCGGTGCGGCG
CAGAGGTGGCATCTCGGCTGTGACTGGTGCAGCGCCTGCCGACACCAGCTGCAGAGCAGGGGCTCGTTGT
CGCAAGGCTGATGACAGAGGAGGCAACTCCCTTGCCAGTCGCCGTGGTTGCCGAGCAGCGGGCTTGCTTT
AGCGAGCAGTCGCTTTAACATGCTGACCCCAAGAGCATGAACAGGGATCTTGAGTCTAGATGAGCATATC
GGTAGAGCAGTTTGGCCAGGGGCCCGATCTGGTGCTGTTGCACGGTTGGGGGATGAACGGTGCGGTCTGG
CATGGCATTGCCCAACTGTTGGCGCCCCACTATCGGGTTCATCTGGTAGATTTGCCCGGTTTTGGCAACA
GCCTGCTGGCAGACGAGGTGGATTACAGCCTGCCCTGGCTTGCCGAGCAGGTGGCCGCCATCTTGCCGCA
GAAGTGTCATCTGCTTGGCTGGTCGCTCGGCGGTCTGGTGGCAAGCCAGTTGGCGCTGACTCGTCCCGAT
CGGTTGCATTCCCTGATCACGGTAGCCAGCTCTCCCTGCTTTATGGCGCGGGACGAGTGGCCCGGAATTG
CCCCCAAGGTACTGACCGGCTTCAACCAGATGTTGGCGGGGGATTTTCGCCAGACCATAGAGCGCTTTCT
GGCGATCCAGGCAATGGGCAGCGAACATGCCCGAGATGATATCCGTCAGTTGCGCCACTGGCTGGCCGAG
CGCCCGGCGCCCCAGTTTGCCGCACTTGAGGCCGGGCTGACGCTGTTGGCCGACATTGACCTGCGCGAAC
CGCTGCGGGACCTGGATCTGCCCTGGTTGCGGATCTATGGCCGACTGGATTCATTGGTGCCCAAGGCGGC
CATTCCCCTGCTGGATGATCTCTACCCTGCCAGCCGCAGCGTGACTCTCAATAAGGCCTCACACGCACCT
TTTATCTCCCACCCTGACGAATTCATTGAAATCGTTCGAGATTTTGTTGGCTAAAAAACAGCCGGCAAGC
TTCATTAATCCTCTGGTCTGGCGGATAATTAAGCTGTGACCAGAGGAGTAACACCATGCGTATTGATTCT
GCTTTCAACAGCGGGGTACAGGGATTTCAGCGAGCCGAGCAGATTGCCGACAAGGCATCCAATCAGATCG
CCCGCCTCAACACCCCCAGCGGGGATCAGGTGCAAGTGACCGATGAGCTTGTCAATCTCAAGGTGGCCGA
GCAACAGGCTGGCGCTTCCGCCAAGGTAGTGCAGACTGCCAGCGATATGATGGGAACCCTGATCGACATC
CGGGTGTGAGATGAACATCAATGTCAGTCTGCCTCCCCTTATCCCGACACAGCTGCAACCCCAGGTGGAA
GCGGCGAGGACGGATAATCGCCGGGCAGAACTGATCCCGCAGGCCCGAGCGGGTCAAGCCTCCGTTGCCG
AATCCGAAGTCGGCTCCCAGAAAGAGAAATCCAAAGCCGGTCAGACCTCCAGCAATCAGACCTCCCAGAA
CAGCCGTGAAACCGGGCAACCGGCGCCGACGACACTGCCAGATAATCGCTTTGTCGAGGCAACCGGTGAG
GATGGTCAGCGCAAGCAGCAGGATCCCAAACAGCAGGAGCAGCAACAGCGGCAAGAGCAGCAGGTTCAGG
ATCTTGTCGAGCGTGACAAGGAGGTGCGCACTCACGAGCAGGCGCACCAGTCTGCGGGTGGAGAGTACGC
CAGCTCGCCCACCTATCAGTTTACGCAAGGGCCGGATGGCAAGCGTTATGCGACGGGTGGCGAAGTCCAG
ATCGATACCAGTGTCGTACCCGGTGACCCTGCCGCTACCATCGCCAAGATGCAGCAGATCCGCTCTGCAG
CGCTGGCGCCTGCCGAGCCTTCCGCGCAAGATCTGGCCGTCGCGCGCAGCGCCGCCGCCAGTGAAGCCAA

GGCGCGCAAAGAGCTGATGGCCGAGCAGAGCGCAAAATCGGGCGGCGTGCTGAGTGCCTATATGGACAAG
CGCAACAGCGTCATCGCGGGCCACTATCGACAGGCTGTCAGCCCGGCCTCGACCCAGTCCCTCAGTCTGC
ATATCTGAATCCCCCTCACCAAACTTTCAGCTGCTTTGCCTGTTTTGCAACTAACCTGAAGATCAGGCCC
ATTCCAATTTCACAGGAGCTGTCATGCATTTCGATGAAGTCAAAGCGGAAGATTTCACCACCTTCTCCCG
TGTACCGCCCCCCCACCTGCAGATGGAGCAATTTCTGATGCAGCTCGGAGGAGGGGAACTGAAGGAACC
AGTTTCAAGAAGAAAGTGATGCTGGCGGCTGGCTGGAGCCACACCGGCGTCGTCTCTTACGGCAAGTATC
CGCAAGAGGCCTGCAAGGCATTCAACCGACTGCGGGAGGTGCTGGCCCAGCATGGAGAGCCCGAGAGCAT
TCTTGCTGCCCTGGCGCAGTGATCCTGAGCTTCGCGTGTAAAAAAGCCCCTTGCGGGGCTTTTTGCATTC
AGAGGTGTTCTGGAAGGTGTCGTTACTTTTTCGCTTTGGAGAGCGCCGCCGCGAAGGCGTTGCCCATGGC
ACCACCCATATGGGGGCTTTGGCGCGGCTCATTTCTTGGCTTGGTGTTGCCAGCACTGCTGTTGCGCGCT
TCAGCCGGCTTGCGGGCGGGCTGCCCTGCCTTTTCGTCGAGGCGCATAGTGAGGCTGATCCGCTTGCGCG
GGGCGTCCACTTCCAGCACCTTGACCCGCACAATGTCGCCCGCCTTGACCACCTCACGGGGATCCTTGAC
GAAACGGTCGGTCAGGGAGGAGATGTGCACCAGACCGTCCTGATGAACACCGATATCGACGAAGGCGCCG
AAGTTGGTGACGTTGGTGACCACCCCTTCCAGTACCATCTCTGGCACCAGGTCGCTGATCTGCTCCACAC
CTTCCTTGAAGGTCGCGGTTTTGAACTCGGGGCGCGGGTCGCGGCCCGGCTTGTCCAGCTCGCCGATGAT
GTCGGTGACGGTAGGTACGCCGAATTGCTCATCGGTATAGTCGGCCGGTTTCAGGCTGCGCAGCAGGCTG
CTGTTGCCGAGCAGGCTCTCCACGGTCTGCTCCAGCTTGGCGAGGATCCGCTCCACCACCGGATAGGACT
CCGGGTGTACCGCTGAGCTGTCGAGGGGATTGCTGCCGCCGCGAATGCGCAGGAAGCCGGCGCACTGTTC
GAAGGCTTTGGGGCCGAGTCGGCTCACTTTCAGCAATTGCTGACGGCTCTTGAATGCGCCGTGCTCATCC
CGATAGGCGACGATGTTCTGGGCCAACGTCTGGGAGAGGCCGGAAACCCGGTTGAGCAGGGCGACCGAGG
CGGTGTTCACATCGACCCCGACCGCGTTCACGCAATCCTCAACCACGGCATCCAGCCGGCGGGCCAGCTG
GCTCTGGCCGACGTCGTGCTGATACTGGCCGACCCCGATACTCTTGGGATCGATTTTGACCAGTTCGGCC
AGCGGGTCTTGCAGGCGGCGGGCGATGGAGACGGCGCCACGGATCGAGACGTCGAGATCCGGGAACTCCT
GGGAGGCGAGTTCGGAGGCGGAGTAGACCGAGGCTCCTGCTTCGCTCACCACTATCTTCTGCGCCTTGGC
GGCGGGGTAGCGCTCGAACAGGTCGCTCACCAGCCGGTCGGTCTCTCGCGAAGCGGTGCCGTTGCCGATG
CTGATGAGCTCGACCTTGTGCTTCTGGCACAACTCGCTCAGGGTCTTGAGGCTTTGATCAATCTGGCGTT
TTGGTTCGAACGGATAGATGGTGGCGTGATCGACCAGCTTGCCGGTGGCATCCACCACGGCAACCTTGAC
CCCGGTGCGGATGCCCGGATCCAGCCCCATGGTGCAGCGCATGCCCGCCGGGGCCGCCATCAGCAGATCC
TTGAGGTTCATGGCGAACACCTTGATCGCCTCCTCCTCTGCCGATTCGCGGATACGGCCGATCAGCTCGG
TCTCCATCTGAAGCGACAACTTGATCTTCCAGGTCCAGCTCACCACCCCTTGCAGCCATTTGTCGGCCGG
GCGGTTCTGCAGATTGAGCTTGAGGTGGTGGGCAATGATCCCTTCGCAGGGGCTGGCACTCTCATCGTCA
CCGGTTACCAGCGCCAGATTGAGGATCCCTTCATTACGGCCGCGCAGCATGGCCAGCACCCGGTGGGAGG
GGGCCTTGTGCAGTGGTTCGTCGTGTTCGAAATAGTCCTTGAACTTGGCCCCTTCCTGCTCCTTGCCTGC
CACCACGCGGGCGCGCAGGGTGGCATTCTGCCACAGGTAGTCGCGCAACTTCTCCAGCAGGTCGGCCTGC
TCGGCGAAGCGCTCCATCAGGATATAGCGGGCGCCGTCGAGGGCAGCCTTGCTGTCGGCAATGCCCTGTT
CGGGATTGAGGAAGGTGGCTGCAGTTTGCTCGGGATCCTGCATCGGGTCGCTGAGCAGCAGGTTGGCAAG
CGGCTCCAACCCGGCTTCGATCGCCATCTGCCCCTTGGTGCGGCGCTTGGGCTTGTAGGGGAGATAGAGG
TCTTCCAGTCGGGTCTTGCTGTCGGCGCCATTCAATTCGCGCATCAGCTCGGGGGTCAGTTTGCCCTGCT
CTTCGATGGAGCGAATGATCACCTGACGGCGATCTTCCAGTTCACGCAGATAGCCGAGGCGGCTCTCCAG
AGTACGTAGCTGGCTGTCGTCCAGACCGCCGGTCACCTCTTTACGGTAACGGGCGATAAAGGGCACGGTC
GAGCCTTCATCCAGCAGGCGCACTGCGGCCTGCACCTGCTCCGGGCGAGCATTGAGCTCACCGGCAATCT
GTCTCTCTATGGTTTGCATCATGATGTTGTTGAGGGCCTCTGTGGTCGAGTCCGCGCCATCAGGCGCCAA
GTGTGAGCACTATATGGGGTCGTGTTGCGGGATGCCACCCTGCGCCGGGGTCTGCACCCGTCAGACTGAT
AAAGTCGCAGGGGAGGGCGTTTATCTTGCCATTGCACCTGTGACTCAGGATGTGCCTGTGGCAAGCAGTC
CGGCTATTTGGGGTAGCTGATCTCGTTGATATACCAGCAGACGTCACCAACTGGCGTACGGACGATGGCC
TCATCACCGACCTCTTTCTTGAGCAGGGCGCGGGCCATGGGGGAGTCGATGGAGATGTAATCCTTGCGCT
CGAAGATCTCGTCATAGCCGACGATGCGAAAGCGCTTGGTCTCGCCCTCATCGTTTTCCACCTCGACCCA
GGCGCCGAAGAAGACCTTGCCCTCCTGCGCGGGGGCATAGTCGACGATCCCCAGCTCTTCGAGGCATTTG
CGCAGGTAGCGCACTCGCCGGTCGATCTCCCGCAGCCGCTTCTTGTTGTACTGGTAGTCGGCATTTTCGC
TGCGATCCCCCAGGCTGGCGGCCCATGCCACCTTCCTGGTGACCTCGGGGCGCTCCTGACGCCAGAGGAA
ATCGAGCTCTTCTTTCAGCTTGTTGTAGCCTTCGCGGGTGATCAGCTTTGTCTTCATGGTGATGCGGACC
TGGTCAAAGAGGAGTATTCATCGCTAACTGCTCGGGAATATTAGCTTTTGTCCTCTTGCTGTCGAGGGAA
ATCGTGATGGCTCGGCTGGCGCCATTCAGGGAGTAGCTGGCCAGCGCAGTGACTTGATGGCCGTCACGCT
CTCGGGATGGGTAATAACCGGTAACATATTTGTGCTTTGCTCCCGCTATATCCCCCCCTAGTATTGGGGG
CAGCTAAAGAATGAAGAGGCAAAGCAATGAGTCAGCAGCAATACAAGGTCCTGGTCGTGGACGATGACCT
GAAATTACGTTCCCTGTTGGAGCGCTATCTCACCGAGCAGGGATTCGTAGTGCGCGGCGTGGCCAATGGC
GAGCAGGCTGATCGCCTGCTGACTCGTGAAAACTTCAGCCTGATGGTGCTCGACCTGATGTTGCCGGGGG
AGGATGGGCTCTCCATCTGCCGCCGTCTGCGGGAAGCGGGCAACCAGATCCCGGTGTTGATGCTCACCGC
CAAGGGGGATGAAGTGGATCGCATCGTCGGGCTCGAGATGGGGGCCGATGACTATCTGCCCAAGCCGTTC
AACCCCCGCGAACTGCTGGCGCGGATCCGTGCCGTGCTGCGCCGTCATACCGTCGAGTTGCCCGGAGCCC
CCTCGGCTGCAGAGAAGGTGGTCAACTTCGGCGCCTTTCAACTCAATCTGGCGACCCGCGAGCTGAGCCG

GGATGGCGAGCCGATGGCACTCACCAGCGGTGAATTTGCGGTGCTGAAAGTATTGGTCAGCCATCCGCGC
GAGCCGCTCTCCCGCGACAAGCTGATGAATCTGGCCCGTGGCCGTGAATACACCGCTACGGAGCGCAGCA
TCGACGTGCAGGTCTCTCGCCTGCGCCGCCTGCTGGAACAGGATCCGGCCCAGCCCCGCTATCTGCAGAC
CGTCTGGGGTCTGGGGTATGTGTTCGTGCCAGATGGTGACGGTGCATGAAAAAGGTCGGCAGCATGTTCT
CACGCATGCTCTGGTTTCTGGCCGCCGTTCTGGTGGTCTATCAAGCAGTCTCCTACGTCACCTTCTACAA
CTATCTGCTGGCGCCAACCGTCAAGCAGATCAGTCACTTGCTGGCCAATCAGGTCAAGCTGATCTTTCCG
GTCAACACGGAACAACTGGCGCTGAGCGAAGATGCCGAGGCGCTGGTCTATGTGACGACCGGTACCGATA
TCTATACCCAGAGCGAGGCGGTGCAACATGGCTTCAACGAGGCCAAGCCATACCCCTATCTGGAGGAGAA
CATCAGTCGCGAGCTGGGTGGCCCGGCCAAGGTGCGGGTCGAGATCCAGGATCACTACATCATCTGGATC
CAGCCGCCGCAGGCCAGCAAAATCTGGGTGCGGATCCCGCTGACCGAGATTGAAGATGACGATATCGAAC
CCCTCATCATCTATCTGAGCGTGCTGTTGGTGATCACCCTGTTTGCGGCCTGGCGCTTTGCCCGGCAGCT
GGTGCGCCCGCTCGAGGATCTCGAGGCTGGTGCCGTTGCTGTTGCCAGGGGCCATTTCCCCGAGGCGCTG
GGGGAGCTGGGATCACGGGAAGTGCGGCGATTGACCCGCACCTTCAACCATATGTCGCACTCCATCAAGA
GCCTGATCGAAGAGCGTAACTTGATGATGGCTGGGGTTTCCCACGATCTGCGTACTCCGCTGACCCGCAT
CCGGCTGGCGACCGAGATGATGTCCTCTGGTGACGAGTACCTCAAGGAGAGCATCAACGCCGATATCGAT
GAGTCCAATGCCATCATCGATCAGTTCATCGATTACATCCGCCCGGATGAGGGCCAGGATCTGGAGCCGA
TCGACCTGACCCAGCTGGTGCACGATACCTTGCTGATCTACTCGGAGCAGGGACTGGAAGTTGATCTGGC
GCTGCCGCAAGAGGCGGTGCGGGTGGCCTGCAATCCGGTCAGTATCCGGCGGGTGTTGAACAACCTCTCA
AGCAACTCGCAGCGTTATGGGGCGAGCCGCTTGCATGCCGAGCTCAAGGACGACGGGCACTGGGTGCGCT
TGCGCCTTTCCGATGATGGCCCCGGCATTCCGGAGCAGGACTACTCCCGGGTGCTCAAGCCGTTTACTCA
GGGTAATGTGGCGCGGACCGTCGGCGGTAGTGGGCTGGGGTTGGCCATCGTGGCCAAGATAGTCGCCCAG
CACCACGGCACTATCCGGCTGGGAAGTTCGGATCTCGGCGGCCTGCTGGTCGAGATGCGGCTGCCGAAAC
ATCAGCCACAGGAGTGGATGGATTGAGAGAAGGGCGTATCCGTGGATACGCCCTTCTGCATGGTCAGCTC
TTGGTTTCGACTGATGCTCGATAGCGTTTGCCGTACTTGAGCTGCTGGATCAGCAGGGCGGCAACGATGA
GCGCCAACCCGATGGGGGTGGCCGGATGGATGGCTTCGCCGATCAGCAGATTGAGCAGCACCAGTGAGGC
AAACGGCGAGATAAAGATGAGGTTGCTGATGGGGGCCGTGTTGGTGGCATGGCGCAGCGCCATCAGCCAG
AGCACGAAACCGAAGCCCATTTCGAACAGTCCCACATAGATGGCTCCAGCCCATCCCTGCCAGGCGGTCA
TGGCAAAATCGGAGAGCAACCAAGTCGCCACGACGATAAAAGGCAGGCTGATCATAAAGCCGAGCAACAG
GCTGACGATGGGATCGCCCTGATTGCGGGTGTTGAGGATCCAGTAGCTGGCCCAGATGAGGGTCGAGACC
AGCGCCAGCGTCACGCCGAGGGGGCTGTCGAACTGGAGGCCGAATACATCCCCTCTGGTGGCGATCACCA
GTGCGCCAAAGTAGCCGAGCACGATGGCGACGCCATCGCGCCGACGCAGCTTCTGGCCGAGGAAGGGGAC
GGCCAGCAGACTGAGGGTAATGGCCCAGGTGTAGTTGAGGGTCTGTGCCTGCTGGGCAGGCAACAGGTCA
TAGGCCTTGAATAGCAACAGGTAATAGAGAAAGGGGTTCAGCACGCCCAGAGTCAGGTAGTAGAAAGGGC
GGGCCTTGATGTAGCTTCCCAGCAGCGGCAGTTTGCCCTGTTTGGCGACGACGGCTGTCAGCGTGAGAGT
CGAGGTGAAGGCGGCCACAAGCAACAGTTGCACAGGTTCGAAATAGGCGAGGGAGATCTTGAAGGCTGTG
GCGACCGTCGACCAGAGGGCAACGGCGCAAAGGCCATACTGATAGGCTTTCTTTTGATCTTTGAGCATGG
GATGTCGTTCCACTGACAGAGGCAAGCGCACAGTCTACCAGTCTGCTTGTAACATTGTCGTTACCTGGGT
GTTGGCAGCAGAGTGCGTTGCCGCAGGCGAGATCCATCTGTCACATAAAACTGCGCATAATCTGTTCGTT
CTCCCTTGCGCAAATTTGAAAAATCCGTATAATCCCCTCCGCTTACCCATGAGGTAAGCCAGACGATATG
TCTGCTGCTGAGTTTCCTGTTTACAGGAGCCAGCCATACAGTCCTCCCGATATGGGGGAAACGTGAAAA
ATCACACCTCTGGCGGGAGAAATCTCGGTCGGGCGGTGTTGATTTATGTTCTTTTATCTATTGGAGCTCT
GTCAATGCAGAACCAAAGAATCCGTATCCGCCTGAAGGCTTTTGATCATCGCCTGATTGATCAATCCACT
GCGGAAATCGTTGAAACTGCTAAGCGCACTGGCGCGCAGGTTCGCGGTCCTATTCCGCTGCCGACTCGCA
AAGAGCGCTTCACCGTCCTGATCTCCCCGCACGTCAACAAAGATGCGCGTGATCAGTACGAGATCCGCAC
TCACAAGCGTCTGGTAGACATCGTTGAGCCGACTGACAAGACCGTAGACGCCCTGATGCGTCTGGATCTG
GCAGCTGGTGTAGACGTCCAGATCAGCCTGGGTTAATTACGGAAAGAGGTTGATAACAATGACAATCGGT
CTTGTAGGTCGCAAAGTGGGTATGACTCGCATCTTCACTGAAGATGGCGTTTCTATCCCGGTGACTGTTA
TCGAAGTTGAAGCTAACCGTGTAACTCAGGTCAAATCTGTAGAAACCGACGGCTACAACGCTATTCAGGT
TACCACTGGCGCCAAGAAAGCCAGCCGTGTGACCAAGCCGGAAGCTGGCCACTTCGCCAAAGCTGGTGTA
GAAGCCGGTCGCGGTCTGTGGGAATTCCGCCTGAACAACGGTGAAACTTTCACTGTTGGTAGCGAGCTGA
AAGTAGATCTTCTCGCTGACGTTAAGCTGGTAGACGTTACCGGCACATCCAAAGGTAAGGGCTTTGCTGG
TACTGTTAAGCGCCACAACTTCCGCACCCAGGATATGACCCACGGTAACTCCTTGTCTCACCGCGTTCCG
GGTTCTATCGGTCAGAACCAGACTCCGGGTCGTGTATTCAAGGGCAAAAAGATGGCTGGTCACATGGGTG
CTGAGCGTGTAACGACTCAGAACCTGGAACTGGTTCGTGTTGACGCTGAACGCAACCTGCTGCTCATCAA
AGGTGCAGTACCGGGTGCAACCAACGGCAACGTGATCGTCAAACCTGCCGTCAAAGCGTAACGTCTGAGG
AGATAGTAATGGAATTGGTAATGAAAGACGCCAAGAGCGCTCTTGAAGTTTCCGAGACTACCTTCGGGCG
CGAATTCAACGAAGCTCTGGTTCACCAGGTCGTCGTTGCATATGCCGCTGGTGCCCGTCAGGGTACTCGT
GCGCAGCTGACTCGCTCTGAAGTGTCTGGTGGCGGCAAAAAGCCGTGGCGTCAGAAGGGTACTGGTCGTG
CCCGTGCTGGCTCCATCCGTTCGCCCATCTGGCGTTCCGGTGGTGTGACTTTTGCTGCTAAGCCGCAAGA
TCACAGCCAGAAAGTAAACAAGAAGATGTACCGCGGCGCTATCCGTAGCATTCTGTCCGAGCTGGTACGT
CAAGAGCGCCTGATCGTTGTTGAGAAGTTCGGCATCGAAGCTCCGAAGACCAAAGAACTGATCGCCAAGC

TGAAAGAAATGGAACTGACTGACGTTCTGATCGTGACTGCTGAAGTCGATGAGAACCTGTTCCTGGCTGC
TCGCAACCTGTACAAAGTTGACGTGCGTGACGTTGCCGGTATCGACCCGGTCAGCCTGATCGCTTTCGAC
AAGGTTCTGATGACTGCTGACGCAGTTAAGCAAATCGAGGAGATGCTGGCATGATCCGTGAAGAGCGTCT
GCTGAAAGTTCTGAAGGCTCCGCACATCTCTGAAAAGAGCACCATGGTTGCAGAAAAGCAGAACACTATC
GTGTTCAAAGTTGCTGTTGATGCTACCAAGGCGGAAGTCAAAGCTGCAGTTGCGAAACTGTTCGAGGTCG
AAGTTGAGACCGTCCGTACCCTGAACATGAAGGGTAAGACCAAGCGCGCAGGTGCACGCGTAGGCCGTCG
TTCCGATTGGAAAAAGGCTTATGTGACCCTGAAAGCTGGTCAGGACATCGACTTCATGGGCGCAGCCGAG
TAAGAGGAGTTCTCAAAAATGGCAATCGTTAAGTGCAAGCCTACTTCTCCGGGCCGCCGCCACGTCGTCA
AGATCGTCAATCAAGAGCTGTATAAGGGCAAGCCCTTCGCCGCTCTGCTGGACAGCAAAAGCAAGTCCGG
TGGTCGTAACAACAACGGCCGTATCACTACCCGTCATATCGGTGGTGGTCACAAGCAGCACTACCGTATC
GTCGACTTCAAGCGCGACAAAGACGGTATCCCGGCTAAAGTAGAGCGCCTGGAATACGATCCGAACCGTA
CCGCTAACATCGCTCTGGTGTTGTATGCAGATGGTGAGCGTCGTTACATCCTGGCTCCGAAAGGCCTGAA
AGCTGGTGACGCGATCGCTTCTGGTGCTGATGCAGCCATCAAGGTAGGTAACGCCCTGCCGATGCGCAAC
ATCCCGGTTGGTTCTACCGTTCACGCTGTAGAGATGAAGCCTGGTAAAGGTGCCCAGCTGGCTCGTTCCG
CTGGTACTTTCATCCAGATCCTGGCTCGTGAAGGTAACTATGTAACTCTGCGTCTGCGTTCCGGCGAAGT
ACGTAAAGTTCTGGCCGAGTGCCGCGCTACCATCGGTGAAGTCGGTAACTCCGAGCACATGTTGCGTCAA
CTGGGTAAAGCCGGTGCAAACCGCTGGCGTGGTATTCGTCCGACCGTTCGCGGTATGGCGATGAACCCGG
TTGACCACCCGCACGGTGGTGGTGAGGGTCGTAACAAGGGCATGCAGCCTGTGTCCCCATGGGGCCAGAA
GGCTAAAGGCTTCAAGACCCGTAAGAACAAGCGTACCGACAAGTACATCGTACGTCGTCGTAACAAGTAA
TTGTTAACATAGAGGAATCACCATGCCACGTTCTCTCAAGAAGGGTCCATTTATTGACCTGCACTTGCTG
AAGAAGGTAGAGAAAGCGGTGGAAAGCGGGGATAAAAAGCCGGTTAAAACCTGGTCCCGTCGTTCAATGA
TCATCCCGAACATGATCGGTTTGACCATCGCTGTCCATAATGGTCGTCAGCACGTTCCGGTTTTCGTTAC
CGAAGAAATGATCGGTCACAAACTGGGTGAATTCGCACCGACTCGTACTTATCGCGGCCATGCTGCTGAT
AAGAAGGCTAAGAAGCGCTAAGGGATAAATGATGGAAGCTATCGCTAAACACCGTTATGCCCGTACTTCT
GCCCAGAAAGCTCGTCTGGTAGCCGATCAAGTACGTGGTCTGTCCGTAGACAAGGCTCTGAATATCCTGA
CCTTCAGCCCGAAAAAGGCTGCAGTGCTGGTTAAGAAAGTGCTGGAATCTGCCATTGCAAACGCTGAGCA
CAACGAAGGCGCCGATATCGACGCGCTGCGTGTTGCTACTATCATGGTCGACGAAGGTCCCTCCATGAAG
CGCATCCGTCCGCGTGCCAAAGGTCGTGCGGATCGTATCCTCAAGCGTACCGCTCACATCACTGTAGTGG
TATCCGACGCTAAGGCTGGGAGATAAGCAATGGGTCAGAAAGTACATCCTAATGGCATTCGCCTGGGTAT
TACCAAGCCTTGGAATTCTACCTGGTTCGCGAACACCAAAGACTTCGCTGACAACCTGTACAGCGATTTT
CAAGTACGCCAGTTCCTGACCAAGGAACTGAAAAACGCGTCCTTGTCCAAGATCACCATTGAGCGTCCGG
CCAAGAGCATCCGTGTGACCATTCACACCGCTCGTCCGGGTGTTGTCATCGGTAAGAAAGGCGAAGACGT
TGAGAAGCTGCGCAAAGCTGTAGCCACTATTGCTGGCGTGCCTGCTCAGATCAACATTTCCGAAGTCCGC
AAGCCGGAGCTGGATGGCAAACTCGTTGCCGACAGCATCACTTCCCAGCTGGAGCGTCGTGTAATGTTCC
GTCGTGCTATGAAGCGCGCCGTACAGAACGCAATGCGCCTGGGTGCCAAGGGCATCAAAGTGGAAGTTTC
CGGTCGTCTGGGCGGCGCTGAAATCGCGCGTACCGAATGGTACCGTGAAGGTCGTGTGCCGTTGCACACC
CTGCGTGCCGACATCGACTACGCAACTTCTGAAGCCCACACCACTTACGGTGTGATCGGCGTTAAAGTTT
GGATCTTCAAGGGCGAAGTTCTGGGCGGTCTGGCTGCTGTTAACGCTGCCGCTGCTCAAGAGCAAGCTCC
TGCAAAGCCCAAGCGTGACAACAAGCGCCGCGCTGCTAAGTAAGGAGATTCGGTAAATGTTGCAACCTAA
GCGTACTAAATTCCGCAAGACCCACAAGGGTCGTAACCGCGGTCTGGCCACCTCTGGTAACGAAGTATCC
TTCGGTACCTTTGGCCTGAAGGCGACATCCCGTGGTCAACTGACTGCTCGTCAAATCGAAGCAGCCCGTC
GTGCCATGACCCGTCACGTTAAGCGTCAAGGTAAGATCTGGATCCGTGTGTTCCCGGACAAGCCCATCAC
CGAAAAGCCCCTCGAAGTTCGTATGGGTAAAGGTAAGGGTAACGTGGAATACTGGGTTTGCCCGATCCAG
CCTGGCAAGGTTCTGTACGAGATGGACGGTGTACCTGAGGCTCTGGCGCGTGAAGCGTTTGCCCTGGCCG
CAGCTAAGCTGTCTGTTCAGACCACTTTCGTAATTAAGACGGTGATGTGATGAAAGCCCAAGATCTGCGT
CAAAAGAGCGTCGAAGAACTGAACCAGGAGCTGCTGGGCCTGCTGCGTGAGCAATTCAACATGCGCATGC
AAGCGTCCACCGGCCAGCTGGCTCAGACTCACACTCTGAAACAAGTGCGTCGTGACGTCGCACGTATCAA
GACTGTACTGACTGAGAAGGCAGGTGCGTAATGACTGACAAGATCCGTACTCTGCAAGGTCGTGTAATCA
GCGACAAGATGGACAAGTCCATCACTGTTGCTATCGAGCGCAAGGTAAAGCACCCGATCTACGGTAAGAT
CATCAAGCGCACTACCAAGCTGCACGTACATGACGAAAACAACGAGTGCAAAGCTGGCGACCTCGTGGAA
ATCCGCGAATGCCGCCCGCTGTCCAAGACCAAGTCTTGGACCCTGGTTGCCGTTGTAGAAAAGGCCTAAT
CGGTACTTTGTAGTCCAAGCGGCCCCTTTCAGGGGCCGTTTGTTTTTTAGGCTACCTTTTCTGAAAATCC
TGTGTTATAGTTTCGCGCCTTTTGAAGGCAGCCAGATCCCATTAGGGATAAGAAGTAAACAAAGCGGAGC
ACTAAGATGATCCAGATGCAAAGTATGCTGGGTGTTGCCGACAACTCCGGCGCTCGCAGTGTAATGTGTA
TTAAGGTTCTGGGTGGCTCGCACCGCCGTTACGCCGGCATCGGCGACATCATCAAGGTTTCCATCAAGGA
AGCCATTCCTCGCGGTAAAGTGAAGAAAGGTGATGTGTATAACGCGGTGGTCGTACGCACCCGTAAAGGC
GTTCGTCGTCCGGACGGCTCTGTCATCCGTTTCGACAACAATGCGGCTGTGTTGCTTAACAACAACCAAC
AGCCAATCGGTACTCGTATTTTTGGCCCGGTGACCCGTGAACTGCGTAATGAGAAGTTCATGAAGATTGT
GTCCCTGGCACCCGAAGTACTGTAAGGAGTCGAACGATGGCAGCTAAAATCCGTCGCGAAGACGAAGTGA
TTGTTCTTACCGGCAAAGACAAGGGCAAGCGTGGCAAAGTCACTCAAGTCCTGATTGCAAAAGGTAAGGT
AATCGTGGAAGGCATCAACCAGGTGAAGAAACACCAGAAGCCGGTACCCGCACTGGGTCAGGCTGGCGGT

ATCGTCACCAAAGAAGCCCCCATCGATGTATCAAACGTAGCGCTGTTCAACTCTGCCACCGGCAAAGCTG
ACCGCGTTGGTTTCCGGATTGAAGACGGCAAAAAAGTCCGTTTCTTCAAATCCACCGGCGAACTTGTGAA
GTAATTGGAGTTTAACGATGGCGAAACTGCATGATTACTACAAATCCGATGTAGTAAATGAACTGTCCAA
GCAGTTCGGTTACAAGACTATCATGCAAGTCCCTCGGATCGAGAAAATCACCCTGAACATGGGTGTTGGT
GAAGCGATCTCTGACAAGAAATTGCTGGAAAATGCTGCTGCCGATATGGCTGCCATTTCCGGTCAGAAGC
CGCTGATCACCAAAGCTCGCAAATCTGTTGCGGGCTTCAAGATTCGTGAAGGCTACCCGATAGGTTGTAA
AGTAACCCTGCGTGGCGAGCGTATGTGGGAGTTCCTGGAACGGCTGATCTGCATCTCCGTACCGCGTATC
CGTGACTTCCGTGGCCTGAACGCTAAAGCGTTCGACGGTCGTGGTAACTACTCCATGGGCGTGCGTGAGC
AGATCATCTTCCCGGAAATCGACTATGACAAAGTCGATCGCGTCCGTGGTCTGGATATCACCATCACCAC
TTCCGCGAATACCGATGAAGAAGGCCGTGCTCTGCTGGCTGCCTTTAACTTCCCATTCCGCAAGTAAGGT
TAGGGTTATGGCTAAAACATCCATGAAAGCGCGCGAAGCAAAGCGTGCCAAGCTGGTAGCCAAGTACGCG
ACCAAGCGTGCCGAGCTGAAAGCTATCATCATTGACATGAACGCTTCTGAAGAAGCGCGTTGGGATGCTG
TACTGCAACTGCAACAGCTGCCCCGTGATTCCAGTCCGTCCCGCCAGCGTAACCGTTGCAATATTACTGG
TCGTCCGCACGGTTTCCTGCGCAAGTTTGGCCTGTCTCGCATCAAGGTGCGTGAGCATGCCATGAAGGGC
GAAATTCCGGGCCTGAAGAAGGCTTCTTGGTAACGAATCTCGGGAGTTAGACTTATGAGCATGCAAGATC
CGATCGCGGATATGCTGACCCGCATCCGTAACGGTCAGGCGGCGAGCAAAGTTGCGGTTTCCATGCCTTC
TTCCAAGCTGAAAGTGGCTATCGCCAAAGTGCTGAAAGAAGAAGGTTACATCGCTGGCTACTCCGTAGCT
GGTGACGTGAAGCCGGAACTGGAAATTGAACTGAAATATTTCCAGGGCAAGCCAGTTGTAGAACTGATCC
AACGCGTAAGCCGTCCAGGCCTGCGTATTTACAAGCGCACTACTGATCTGCCGAAAGTTATGGGCGGTCT
CGGTGTTGCTATCGTGTCCACGTCTAAAGGTGTGATGACTGACCGTGCTGCTCGCAAAGCAAGCATGGGC
GGTGAGATCATCTGCTACGTCGCTTAATAGGAGGTAGGAAATGTCTCGTGTTGCTAAGGCACCCGTCACT
ATTCCTGCTGGCGTAGAGGTGACTCTGAACGGCCAGGAACTGACCATCAAAGGTGGTAAAGGTTCTCTGG
TTCGCTCTATTCACGCCGGTGTAGAAGTGACCAAAGAAGACAATGTACTGAAGTTCGCCCCGCGCGGCAG
CATCGCTGGTGCTGACGCTCAGGCCGGTACTGCCCGTGCATTGGTCAACAACATGGTAATCGGTGTTACC
CAAGGCTTCGAGCGCAAGCTGCAGCTGGTTGGTGTAGGTTATAAAGCCTCCATCAAGGGCAATGCTGTTG
CGCTGGCTCTGGGCTTCTCTCACCCGGTAGAGCATGCGCTGCCGGCTGGTGTGACCGCTGAGTGCCCGAC
TGCTACAGAAATCGTTCTGCGTGGCGTCGACAAGCAGCTTGTTGGCCAAGTCGCCGCCGATATCCGTGCT
TACCGCGCTCCGGAGCCCTACAAGGGCAAGGGTGTACGCTATGCCAACGAGCAAGTGCGTACTAAAGAAG
CTAAGAAGAAGTAAGGTAACACTATGGACAAGAAAGCAGCTCGTCTCCGTCGTGCTACTCGTGCTCGGAA
AAAGATGCAGGAACTGGGAGCTACCCGTCTGGTTGTTCACCGTACACCGCGTCATATCTATGCGCAGGTT
ATTGCAGCCAACGGTTCCGAAGTTCTGGCCTCTGCTTCCACAGTAGAGAAAGCCATCAGTGAAGCTCTGA
AATACACCGGTAACGCAGATGCTGCTACCGCTGTAGGTAAAGCTATCGCTGAGCGTGCTATTGCCAAAGG
CGTTCAGAACGTATCTTTCGATCGTTCCGGTTTCAAGTATCACGGTCGCATCGCTGCCCTGGCAGCTGCC
GCACGTGACGCTGGTCTTCAGTTCTAAGCTAGGAGTCGAAGATGGCTAAAGTCGAATCTCAAGCCGGCGA
ACTGCAAGAAAAGCTCATTGCAGTAAACCGTGTTTCGAAAACTGTTAAAGGTGGTCGTATCATGTCCTTC
ACCGCGTTGACCGTGGTGGGTGATGGTAACGGCCGTGTAGGTTACGGTTACGGTAAAGCCCGTGAAGTTC
CGGCTGCTATTCAAAAAGCAATGGAACAGGCTAAGCGTAACCTGAACAAGGTTGAGCTGAACAATGGCAC
TCTGCACCACCCGGTGCGTGGTGTTCACTCTGGCTCCACCGTGTTCATGAAGCCGGCCTCCCAAGGTACT
GGTATCATTGCAGGCGGCGCTATGCGTGCTGTTCTGGAAGTTGCCGGTATCCACAACGTGCTGGCCAAGA
CCTACGGTTCCACCAACCCAATCAACGTTGTTCGCGCTACTGTAGACGCTCTGGTTCACGGTCAATCACC
GGAACAGATCGCTGCCAAGCGTGGTCTGCGCGTTGAAGAAATTCTGGGGTAATGCGACATGGCTAACACT
GTAAAAGTAACTCAAACTCGCAGCTCTATCGGCCGTCTGCCAAAGCACAAGGCGACTCTGCGTGGTCTGG
GTCTGCGTCGCATTGGTCACACCGTTGAGCTGGAAGATACTCCTTGCGTACGCGGCATGATCAACCAGGT
TTATTACATGGTTAAGGTGGAGGGCTAAGCATGCGTCTGAACACTCTGTCTCCGGCCGCTGGTTCCAAGC
CTGAAAAACAACGTCGTGGTCGTGGTATCGGCTCCGGCCTGGGCAAGACTGGTGGTCGTGGTGTTAAAGG
CCAGACCTCTCGTTCCGGTGGCGGTAAAGTCCGCAACGGTTTCGAAGGCGGCCAGATGCCTTTGAAAATC
CGTCTGCCGAAGTTCGGTTTCTTCTCTCGTAAATCTTTGGTTTCTACCGAAGTGCGTCTGAACGAAATCG
CCCTGGTAGAAGGTGATGTGGTCGACCTGAGCACTCTGAAGCAAGCCGGTGTTGTTACTAAGAACATCGT
GTTCGCTAAAGTTGTTCTGTCAGGCAACATTGACCGTGCTGTGACCGTTCGTGGTCTGTCCGTCACCAAA
GGTGCACGCGCAGCCATCGAGGCCGCTGGCGGTAAAATCGAGGAATAATCAGTACAATGGCTAAGAAACC
AGGATTGGATGTTAAAGCACAGGGTGGTCTGAGTGAACTGAAGAGCCGTCTGCTCTTCGTTCTCGGGGCG
ATTATCGTTTTCCGTGCAGGCTCTTATGTGCCGATTCCTGGTATTGACGCCGCCGTACTGGCCGAGTTGT
TCCAGCAACAGAAGGGCACCATCATTGAGATGTTCAACATGTTCAGTGGTGGTGCACTCTCGCGTGCTTC
TATTCTTGCGTTGGGGATCATGCCGTACATTTCGGCGTCGATCATTATCCAGCTGCTGACAGTGGTTCAC
CCCGCTCTTGCTGAACTCAAGAAAGAAGGTGAATCCGGCCGTCGCAAGATTAGCCAATATACCCGTTGGG
GCACGCTGGTTTTGGGAACTTTCCAGGCCATTGGTATTGCAACCGGTCTGCCGAATATGATGCATGGACT
CGTGACTAATCCGGGTCTAGGTTTCTATTTCACAGCGGTTGTGAGTCTGGTCACCGGTACCATGTTTTTG
ATGTGGTTGGGTGAACAGATTACCGAGCGTGGGATTGGTAATGGTATCTCGTTGATTATTTTCACAGGTA
TCGTTGCTGGTCTGCCGCATGCCATTGGCGCTACGGCCGAACAGGCACGTCAAGGGGAATTGCACATCCT
GCTGTTGTTGTTGCTGGGCTTGATTGTTTTCGCAGTGACTTACTTTGTGGTGTTCGTGGAGCGTGGTCAG
CGTCGTATCGTCGTTAACTATGCCAAGCGTCAACAAGGCCGCCAGGTATTTGCCGCGCAAAGCACACACC

TGCCGTTGAAAGTGAATATGGCTGGTGTGATTCCTGCGATTTTCGCATCCAGTATCATCCTCTTCCCGGG
GACCATTACCTCCTGGTTTGGTCAGGGTGAGGGTAAAGTAGCTGATATCCTGCAACAGGTCTCTATGGTC
CTGCAGCCGGGTCAACCGCTTTACGAGATGCTGTATGCAGCAGCCATTATCTTCTTCTGCTTCTTCTATA
CCGCGCTGGTGTTCAACCCCCGCGAGACAGCAGATAATCTGAAGAAGAGTGGAGCCTTTATCCCGGGGAT
TCGCCCGGGCGAGCAGACAGCACGTTACATCGATAAGGTTATGACTCGCCTGACATTGGCAGGTGCGCTC
TATATAACCTTTATCTGTCTGGTACCCCAGTTCTTGATGACTGCCTGGAACGTACAGTTCTACTTCGGTG
GCACTTCGCTGCTGATTATCGTGGTAGTTATCATGGACTTCATGGCTCAGGTACAAACCCATATGATGTC
TCATCAATATGGCGACGTCCTGAGGAAGGCCAACCTGAAGGGCTACGGCCGCTAAAGGTCGGCGTAGTTA
CGGAGTTAAGCAATGAAAGTTCGTGCTTCCGTTAAGGCAATCTGCCGTAACTGCAAAATCATCAAGCGTC
ACGGTGTGGTGCGTGTGATTTGCAGCGAGCCAAAGCATAAACAGCGCCAAGGCTAATTTTTTACGGTTAG
TACTTGCAACAAATAGTTGTGCTGGCTAATATAGCCAGCCAACTTTTGTTGTGTATTGGTTGACCGCCAC
GTATCCGCTCACGGGCTTTGTGGTGGTCGTAATCTACTATATGTAGGAGTGAATAGTGGCCCGTATCGCT
GGCATTAACATTCCTGACCATAAGCATGCAGTCATCGCTTTGACTGCGATTTATGGCGTCGGTCGTACCC
GCTCCAAGGCCATCTGTGCCGCAGCCGGTATCGCTGAGAATGTGAAAATTAAAGACCTGGACGAAGCTCA
GATCGAGAGTCTGCGTGAACAAGTAGGTAAATTCACCGTTGAAGGTGACCTGCGTCGTCAGATTTCCATG
AACATCAAGCGATTGATGGATCTGGGTTGCTACCGTGGTTTGCGTCACCGTCGTAGCCTGCCTGTTCGTG
GTCAACGTACCAAGACCAACGCTCGTACCCGCAAGGGTCCGCGCAAGGCTATCAAGAAGTAACGGGAAGG
TAGAAAATGGCTAAAACTCCGACTCGTGCTCGCAAGCGCGTTAAAAAGCAAGTGAGCGACGGTATTGCGC
ACGTTCATGCATCTTTCAACAACACAATCGTGACCATTACTGACCGTCAGGGCAATGCTCTGTCTTGGGC
TACTTCTGGTGGTTCCGGTTTCCGTGGTTCTCGTAAGTCCACCCCGTTCGCTGCTCAGGTAGCTGCCGAG
CGCGCTGGTGAGGTGGCCAAAGAATATGGCGTCAAGAACCTGGAAGTCATGGTCAAAGGTCCGGGTCCCG
GTCGTGAGTCTTCCATCCGCGCCCTGAACGCGGCTGGTTTCCGCATCACTAACATTACTGATGTGACTCC
GATCCCGCACAACGGTTGTCGTCCTCCCAAGAAGCGCCGCGTATAACGCTGTAGCTACTTGGTCTAGGAT
TGTTGGAGAAAGAAGATGGCAAGATATATCGGTCCTAAGCTTAAGCTTAGCCGTCGTGAGGGCACCGACC
TCTTCCTGAAGTCAGGTGTTCGTGCGATTGATTCTAAGTGCAAGATTGACACCGCCCCTGGTCAGCACGG
TGCGCGTAAAGCGCGTCTGTCCGACTACGGTGTTCAGCTGCGCGAGAAACAAAAAGTTCGTCGTATCTAC
GGCGTATTGGAAAAACAGTTCCGCAACTACTACCGCGACGCTGCCCGTCAAAAGGGTAACACCGGTGAAA
ACCTGTTGCAGCTGCTGGAAGGTCGTTTGGACAACGTTGTTTACCGTATGGGCTTCGGCGCTACCCGTGC
TGAATCTCGTCAGCTGGTGAGCCACAAGGCCATCATGGTAAACGGTCGCGTTGTGAACATTCCTTCTTTC
CAGGTTTCCCCTGAGGACGTGATCTGCGTTCGTGAGAAGGCTAAGAAGCAAGCGCGTATCAAAGCTTCCC
TGGAAGTTGCTGGTCAGCGTGAGAAGCCGACTTGGGTAGAGGTTGACGCCGCTAAAATGGAAGGTGCTTT
CAAGCGTCTGCCAGAGCGTTCCGACCTGTCTGCCGACATTAACGAACAGCTGATCGTCGAGCTTTACTCC
AAGTAAAGCTAGCTTCTAAAGAGAGGACACAATGCTGGGTTCTGTAACAGATTTTCTTAAACCGCGGCTA
GTTGACATCGAGCAAGTTAGCCCGACTCATGCGAAAGTGACTCTTGAGCCACTCGAGCGTGGCTTTGGTC
ACACGCTGGGCAACGCCCTGCGTCGTATTTTGCTGTCATCTATGCCCGGTTGTGCAGTTACTGAAGTCGA
AATTGACGGGGTACTGCATGAGTACAGCAGCAAAGAAGGTGTACAGGAAGACATTCTTGAAATCCTGCTC
AACCTGAAAGGTATCGCTGTGAAGCTGGAAGGCAAGGACGAGGTAACTCTGTCCCTGACCAAGTCTGGAA
CAGGCCCCGTTACCGCTGGTGATATCACTCACGGTGATGAAGTCGAGATCGTAAACCCGGAGCACGTAAT
CTGCCATCTGACTGGTGCCAATGCTGAAATCAGCATGCGCCTGAAAGTTCAGCGCGGTCGTGGTTATGTG
CCTGCTTCTGCTCGTGTTCACAACGATGACGAAGAGCGTCCTATTGGTCGCCTGCTGCTGGACTCCGCGT
TCAGCCCCATCGTTCGTATTGCCTACAATGTTGAGGCAGCACGTGTGGAACAGCGTACCGACTTGGACAA
GCTGGTCATCGACATGGAGACCAATGGTACCCTGGATCCTGAAGAAGCCATCCGTCGTTCTGCCACTATC
TTGGCCGAGCAACTGGAAGCCTTCGTGGATCTGCGCGATGTCAGCGTGCCCGAGAAGAAAGAAGAGAAAC
CCGAGTTTGATCCGATTCTGCTACGTCCTGTCGATGATTTGGAGCTGACAGTTCGTTCTGCGAACTGTCT
GAAGGCAGAAGCTATCCACTATATTGGTGATCTGGTACAGCGTACCGAAGTTGAGTTGCTTAAGACTCCT
AACCTCGGTAAGAAGTCCCTGACTGAGATCAAGGACGTGTTGGCCTCCCGTGGTCTGTCTCTTGGCATGC
GCCTTGAGAACTGGCCGCCCGCCAGCATCGCTGACGAGTAATCCAGATTACGGGTTTCACAGATTTAGTT
AGAAGGATAAGGTCATGCGCCATCGTTTGAGTGGTCGTCAACTGAACCGGAACAGCAGCCATCGTCAGGC
AATGTTCCGCAACATGGCCAGCTCCCTGGTTCGTCATGAGATCATCAAGACGACTCTGCCTAAGGCAAAA
GAGCTGCGTCGTGTAGTTGAACCGCTGATCACCCTTGCCAAGACTGATAGCGTTGCTAATCGTCGCCTGG
CATTTGCCCGTACTCGCGATAACGCGATCGTGGCTAAGCTGTTCAATGAACTGGGCCCGCGCTACCTGGA
GCGCGCCGGCGGTTATACTCGCATTCTGAAATGCGGTTTCCGTGCAGGCGACAACGCCCCTATGGCTTAT
ATTGAGCTGGTAGACCGCCCGGCCGCTGCTGAAGCAGCTGCTGAGTGATAAAGAAAAAGCCGGGCTTGTC
CCGGCTTTTCTTTTAATCCATTCTGATACTGCCTCAACTGCCTCAACTGCCTCAACTGCCTCAACTGCCT
CAACTGCCTCAACTGCCTCAACTGCCTCAACTCATTATTCAACTTCTTCTATATAGGAAGTAAGAGTTGT
TCCTGAACCTGGCGAAATGGTGAATTGTCTGTTCATTTCATCCGAACATCGCTGGCCAGCTGGATATCTC
CCACTAGGTTGCGCAGGGATCTACATATACCCGTTTGCCCTATTGCGGGCATTTTCCCTTGTAGGGTCTC
AGTCGCAAGTCCATCTTGGCCTCCTTGTTTTCGGCCATTCTAGCAATCTGGAACCAGTTTCTGTGGATAA
GTTTGTATAAAAGCTGTGTCTGGATATGAATAACTAAAAAAGTGCGGTTTTTTGCAATTTTATTGCCGAT
AAGGGGTTGCCAGAAAGAATCTGTTCCCTATAATGCGCCTCCATCGACAGGGCAAGCGGCAACGCAGAAA
GCCAAGTCGATAACCTCGAAAAGCCTTTGAAAATAAGGCTTGACTGGAGAAGGCGGTTGAGTAGAATTCC

ACTCCCGCAGCAACACACTGTTGCGTCGCTCTTTAACAATTTGAATCAAGCAATCTGTGTGGGCACTCAC
AGCATCGAACATCAAAACAATTTTTGATTTTCAATGTCTGGTGAAGTGACCAAGACGATTTCTTAGCAAT
AAGAAGTCGAACAGTTTATTTCAGCAATTCATTGAGCCGCTTTTCGAAGCAACCAAACTTAAATTGAAGA
GTTTGATCATGGCTCAGATTGAACGCTGGCGGCAGGCCTAACACATGCAAGTCGAGCGGCAGCGGGAAAG
TAGCTTGCTACTTTTGCCGGCGAGCGGCGGACGGGTGAGTAATGCCTGGGGATCTGCCCAGTCGAGGGGG
ATAACTACTGGAAACGGTAGCTAATACCGCATACGCCCTACGGGGGAAAGCAGGGGACCTTCGGGCCTTG
CGCGATTGGATGAACCCAGGTGGGATTAGCTAGTTGGTGAGGTAATGGCTCACCAAGGCGACGATCCCTA
GCTGGTCTGAGAGGATGATCAGCCACACTGGAACTGAGACACGGTCCAGACTCCTACGGGAGGCAGCAGT
GGGGAATATTGCACAATGGGGGAAACCCTGATGCAGCCATGCCGCGTGTGTGAAGAAGGCCTTCGGGTTG
TAAAGCACTTTCAGCGAGGAGGAAAGGTTGGTAGCAATTGAAGAGTTTGATCATGGCTCAGATTGAACGC
TGGCGGCAGGCCTAACACATGCAAGTCGAGCGGCAGCGGGAAAGTAGCTTGCTACTTTTGCCGGCGAGCG
GCGGACGGGTGAGTAATGCCTGGGGATCTGCCCAGTCGAGGGGGATAACTACTGGAAACGGTAGCTAATA
CCGCATACGCCCTACGGGGGAAAGCAGGGGACCTTCGGGCCTTGCGCGATTGGATGAACCCAGGTGGGAT
TAGCTAGTTGGTGAGGTAATGGCTCACCAAGGCGACGATCCCTAGCTGGTCTGAGAGGATGATCAGCCAC
ACTGGAACTGAGACACGGTCCAGACTCCTACGGGAGGCAGCAGTGGGGAATATTGCACAATGGGGGAAAC
CCTGATGCAGCCATGCCGCGTGTGTGAAGAAGGCCTTCGGGTTGTAAAGCACTTTCAGCGAGGAGGAAAG
GTTGGTAGCTAATAACTGCCAGCTGTGACGTTACTCGCAGAAGAAGCACCGGCTAACTCCGTGCCAGCAG
CCGCGGTAATACGGAGGGTGCAAGCGTTAATCGGAATTACTGGGCGTAAAGCGCACGCAGGCGGTTGGAT
AAGTTAGATGTGAAAGCCCCGGGCTCAACCTGGGAATTGCATTTAAAACTGTCCGGCTAGAGTCTTGTAG
AGGGGGGTAGAATTCCAGGTGTAGCGGTGAAATGCGTAGAGATCTGGAGGAATACCGGTGGCGAAGGCGG
CCCCCTGGACAAAGACTGACGCTCAGGTGCGAAAGCGTGGGGAGCAAACAGGATTAGATACCCTGGTAGT
CCACGCCGTAAACGATGTCGATTTGGAGGCTGTGTCCTTGAGACGTGGCTTCCGGAGCTAACGCGTTAAA
TCGACCGCCTGGGGAGTACGGCCGCAAGGTTAAAACTCAAATGAATTGACGGGGGCCCGCACAAGCGGTG
GAGCATGTGGTTTAATTCGATGCAACGCGAAGAACCTTACCTGGCCTTGACATGTCTGGAATCCTGTAGA
GATACGGGAGTGCCTTCGGGAATCAGAACACAGGTGCTGCATGGCTGTCGTCAGCTCGTGTCGTGAGATG
TTGGGTTAAGTCCCGCAACGAGCGCAACCCCTGTCCTTTGTTGCCAGCACGTAATGGTGGGAACTCAAGG
GAGACTGCCGGTGATAAACCGGAGGAAGGTGGGGATGACGTCAAGTCATCATGGCCCTTACGGCCAGGGC
TACACACGTGCTACAATGGCGCGTACAGAGGGCTGCAAGCTAGCGATAGTGAGCGAATCCCAAAAAGCGC
GTCGTAGTCCGGATCGGAGTCTGCAACTCGACTCCGTGAAGTCGGAATCGCTAGTAATCGCAAATCAGAA
TGTTGCGGTGAATACGTTCCCGGGCCTTGTACACACCGCCCGTCACACCATGGGAGTGGGTTGCACCAGA
AGTAGATAGCTTAACCTTCGGGAGGGCGTTTACCACGGTGTGATTCATGACTGGGGTGAAGTCGTAACAA
GGTAACCCTAGGGGAACCTGGGGTTGGATCACCTCCTTACCTTAAGATGTCGAAGTTGTTGAGTGTTCAC
ACAGATTGCCTTGATTCAAAGTAGTTAGAGCAAAGACCTGATGCGAAAGTGTCAGTGCTTATCTGGCTTC
GGTCAGATGAGAGAAGCCCTATCATTGGGTTTTGGGATGTGAATAATGGCGCTCGGCCTCGCAGGCTCGG
CACTCGCCATTACCCAAAATCTGCACTGCTGACGCAGCGCAAAGATGATTTTGGGTCCCCTTCGTCTAGA
GGCCTAGGACACCGCCCTTTCACGGCGGTAACAGGGGTTCGAATCCCCTAGGGGACGCCACTTCTCTTCT
TGCTAACAAGAATGCAGAGTTAAGAACTGATTCTTAACTCTGTTTTCTTCGGCCTTGCGCCGTTGCAAAC
ATGCTCTTTAACAATCTGGAAAGCTGATTTAAAAAGTAGTTCTCAAACATTTGTTACAAGTGCTTTGGAA
ACTTCTTGGCGAAAACCAAATTTTATTTGGTCCTTGTTGTACGACAACAAGCAAGCGTTGTTTCGACGAC
ACTTCTTGGGGTTGTATGGTTAAGTGACTAAGCGTACATGGTGGATGCCTTGGCAGTCAGAGGCGATGAA
GGACGTACTAACCTGCGATAAGCTGTGAGAAGTCGGTAAGAGACGCTATTACTCACAGATTTCCGAATGG
GGAAACCCACCCGAGATAACTCGGGTATCTGTTACTGAATACATAGGTAACAGAGGCGAACCGGGAGAAC
TGAAACATCTAAGTACCCCGAGGAAAAGAAATCAACCGAGATTCCCTCAGTAGCGGCGAGCGAACGGGGA
TTAGCCCTTAAGCATCTTGGAAGTTAGTGGAACGGTCCTGGAAAGGCCGGCGATACAGGGTGATAGCCCC
GTACACGAAAACGACCTTGATGTGAAATCGAGTAGGGCGGGACACGTGACATCCTGTCTGAATATGGGGG
GACCATCCTCCAAGGCTAAATACTCCTGACTGACCGATAGTGAACCAGTACCGTGAGGGAAAGGCGAAAA
GAACCCCTGTGAGGGGAGTGAAATAGAACCTGAAACCGTGTACGTACAAGCAGTGGGAGCCCTTCGGGGT
GACTGCGTACCTTTTGTATAATGGGTCAGCGACTTACATTTTGTAGCGAGGTTAACCGTATAGGGGAGCC
GTAGGGAAACCGAGTCTTAACTGGGCGTCTAGTTGCAAGGTGTAGACCCGAAACCGGGTGATCTAGCCAT
GGGCAGGTTGAAGGTTGAGTAACATCAACTGGAGGACCGAACCCACTAACGTTGCAAAGTTAGGGGATGA
CCTGTGGCTGGGGGTGAAAGGCCAATCAAACTCGGAGATAGCTGGTTCTCCCCGAAAGCTATTTAGGTAG
CGCCTCGGACGAATACTACTGGGGGTAGAGCACTGTTTGGGCTAGGGGGTCATCCCGACTTACCAACCCC
ATGCAAACTCCGAATACCAGTAAGTAATATCCGGGAGACACACGGCGGGTGCTAACGTCCGTCGTGAAGA
GGGAAACAACCCAGACCGCCGGCTAAGGTCCCAAAGTTCTGGTTAAGTGGGAAACGATGTGGGAAGGCTC
AGACAGCTAGGATGTTGGCTTAGAAGCAGCCATCATTTAAAGAAAGCGTAATAGCTCACTAGTCGAGTCG
GCCTGCGCGGAAGATGTAACGGGGCTCAAACCAGGCACCGAAGCCGCGGATTCACACTTATGTGTGAGTG
GTAGGGGAGCGTTCTGTAAGTCTGCGAAGGTGTATCGAGAGGTATGCTGGAGATATCAGAAGTGCGAATG
CTGACGTAAGTAACGATAAAGGGGGTGAAAAGCCTCCTCGCCGGAAGACCAAGGGTTCCTGTCCAACGTT
AATCGGGGCAGGGTGAGTCGACCCCTAAGGCGAGGCCGAAAGGCGTAGTCGATGGGAAGCAGGTTAATAT
TCCTGCACGACTTGTAATTGCGATGGGGGACGGAGAAGGCTAGGTGGGCCAGGCGACGGTTGTCCTGGT
GAAAGTGCGTAGGTGGTGTTTCTAGGCAAATCCGGAGACACAACACTGAGACACGAGACGAACACACTAC

GGTGTGGAAGCCATTGATGCCCTGCTTCCAGGAAAAGCCTCTAAGCTTCAGATTACAAGTCATCGTACCC
CAAACCGACACAGGTGGTCGGGTAGAGAATACCAAGGCGCTTGAGAGAACTCGGGTGAAGGAACTAGGCA
AAATAGAACCGTAACTTCGGGAGAAGGTTCGCTCTTGACAGTGAAGTCCCTTGCGGATGGAGCAGTTGGG
AGTCGCAGTGACCAGATGGCTGGGACTGTTTATCAAAAACACAGCACTCTGCAAACACGAAAGTGGACGT
ATAGGGTGTGACACCTGCCCGGTGCCGGAAGGTTAATTGATGGGGTTAGCGCAAGCGAAGCTCTTGATCG
AAGCCCCGGTAAACGGCGGCCGTAACTATAACGGTCCTAAGGTAGCGAAATTCCTTGTCGGGTAAGTTCC
GACCTGCACGAATGGTGTAACCATGGCCATGCTGTCTCCACCCGAGACTCAGTGAAATCGAATTCGCCGT
GAAGATGCGGTGTACCCGCGGCTAGACGGAAAGACCCCGTGAACCTTTACTACAGCTTGGCACTGAACAT
TGAACCTACATGTGTAGGATAGGTGGGAGGCTTTGAAGGCGTGACGCCAGTTGCGCTGGAGCCGTCCTTG
AAATACCACCCTTGTATGTTTGATGTTCTAACGCAGGGCCCTGAATCGGGCTCGCGGACAGTGCCTGGTG
GGTAGTTTGACTGGGGCGGTCTCCTCCCAAAGAGTAACGGAGGAGCACGAAGGTTGGCTAATCCTGGTCG
GACATCAGGAGGTTAGTGCAATGGCATAAGCCAGCTTAACTGCGAGACGGACAGGTCGAGCAGGTACGAA
AGTAGGTCATAGTGATCCGGTGGTTCTGAATGGAAGGGCCATCGCTCAACGGATAAAAGGTACTCCGGGG
ATAACAGGCTGATACCGCCCAAGAGTTCATATCGACGGCGGTGTTTGGCACCTCGATGTCGGCTCATCAC
ATCCTGGGGCTGAAGTCGGTCCCAAGGGTATGGCTGTTCGCCATTTAAAGTGGTACGCGAGCTGGGTTCA
GAACGTCGTGAGACAGTTCGGTCCCTATCTGCCGTGGGCGTTGGATGATTGAAGGGAGTTGCTCCTAGTA
CGAGAGGACCGGAGTGAACGAACCTCTGGTGTTCGGGTTGTCACGCCAGTGGCACTGCCCGGTAGCTAAG
TTCGGAATCGATAACCGCTGAAAGCATCTAAGCGGGAAGCGAGCCCTGAGATGAGTCATCCCTGACCCCT
TGAGGGTCCTAAAGGGCCGTTGGAGACCACAACGTTGATAGGTGGGGTGTGTAAGCGCGGCGACGTGTTG
AGCTAACCCATACTAATTACCCGTGAGGCTTAACCATACAACACCCAAGAAGTGTTCTAAGGCTTGTAGC
AAATACGAACGAATTACTTACGTCAGCTTTCTCAGATTGAAGAATTTGCCTGGCGGCCATAGCGCCGTGG
AACCACCTGATCCCATGCCGAACTCAGAAGTGAAACGCGGTAGCGCCGATGGTAGTGTGGCATTCGCCAT
GCGAGAGTAGGACACTGCCAGGCACCCAATTATAGCGCCGTGGAACCACCTGATCCCATGCCGAACTCAG
AAGTGAAACGCGGTAGCGCCGATGGTAGTGTGGCATTCGCCATGCGAGAGTAGGACACTGCCAGGCACCC
AATTAAACAGCGATGTGAAAAATCACATCACTGGCTGCGAGGATGACACCTCACTGACGCGGCAAACAAT
TTAGCGCAAGCTAACCGAATGCGGAGCGGTAGTTCAGTCGGTTAGAATACCGGCCTGTCACGCCGGGGGT
CGCGGGTTCGAGTCCCGTCCGCTCCGCCACTAATTTTAAGGCCTTCCCATGTGGGAAGGCCTTTTTTCTA
TGGGCGGTTTTTGCCCATAAGTCCCACAGGTTTGCACACCAGAACCAACACAAAGCCCAGTCTCACGACC
GGGTTTTGATGTTTTAGCCTGCTGGTGAGCTGCCGACATCCACCACGATGCCAATGCCTTCCCCTATTGG
CGTTCTTGCTACTATTTCCACCTCTGTATCGCCGCACATCCCATCTTGGCCATATTGTTCTGCTGCGAGC
TGATAGAGCTTGAGGATTAAATCAGGCCATCTTGCAACGGTACGTCATCGCTTCAACTTGTTTGTCTAAT
CCCTTTTTTGCTTATGGATTTATTTCATTAAAACAGTCTCTTATCAAGAATGAGCTGTTTTGCAGCAGGA
ATGCAACACGATATAAATGATAATGATTATTGTTTGAATGGTCGAGGTGGGGTCGTTACAATGCAGGCTG
ATTAATAAAGTGCTTTTCAAATGAGCCTTTTCTAATGCTGCGTCTGGCCGGATGCCAGGGCGTTGTAATG
AGTGAAGTAAGGGAATACGTCACCTTCCTACTAATAATGTTCAGGCTAAAACATGACAACAGTGAAACCG
AACAAGCTTGCCTTGCTGATCTGCTCCCTTCTTGCGACCCAGGCCTATGCTGCCAAACAAGAGGTGATGG
TCGTCACCGCTTCCGGCTTCCAGCAGAAGATTGAAGACTCTGCTGCCTCCATCTCCGTGATTACCCGTGA
GCAGCTGGAAAAGCGGGCCTATCGCGATGTGACCGATGCCCTGAAGGATGTGCCCGGTGTTGTGGTGACC
GGCGGTGGCAGTAGCAGTGATATCAGCATTCGCGGTATGGGGTCGAAATACACCCTGATGCTGGTTGATG
GCAAGAAGGTGGATAGCCGTGGCACTCGCCCGAACAGCGATGGTCCCGGTATCGAGCAGGGCTGGCTGCC
GCCACTGCAGGCGATTGAGCGCATTGAGGTCGTCCGTGGCCCCATGTCATCCCGTTATGGTTCCGATGCC
ATGGGGGGCGTGATTAACGTGATTACCCGCAAGACCACCGGTCTGGCCTGGCAGGGCTCGGTTCATGCTG
ATTCGACCTTCCAGGAGAACAAGGATGCTGGCAACAGTTTCCAGACCGATGCCTATACCGCAGGTTCACT
GATCGATGGCGTGTTGGGGCTGCGTTTCAATGGTCAGCTCGCACATCGTGGTGAAGACAAATTTATCCAG
GGTTTTGCCGAGCAGGAGACTCGCAGTGGCACGGCCGTGCTCAGCTTCAAGCCTGATGATCATAACCGTT
TCGATCTGGAAGCCAGTCGTAGTCTGCAGGATCGGGATCGCACGGCCGGCAATTCGCTGGCTGCCAAGGC
GAAAGATAGCCTTAACCGCTACGAGCGCACCAACTATGCGCTGACCCATGATGGTCAGTATGATTTCGGC
TCTTCCAGCAGCTATCTGCAGCAGGAGACCAACACCAATCCCGGCCGCCAGATGGAGCTCACCAACACCA
TCGCCGATACCCATACCCAGTTTATTCTGGGTGATCACTACCTGAGTGTCGGTGGTCAGTATCGCTATGA
GGATCTGCACGATCAGGGCAATACCCTGAAAGTGGCCAACCCTGCCGAGCAGCTGACCCGCTGGAGTTGG
GCGATGTTTGTCGAGGATGAGTGGTCACTCACTGACAGCTTTGCCCTGACAGGGGGCGCACGCATGGATC
GGGATCAGAATTACGGCTCTCACTGGAGCCCGCGCCTCTACGGCGTCTGGCATCTGGATGATAGCTGGAC
CCTGAAAGGGGGCGTATCAACCGGTTACCGCTCCCCTGACTTGCGGATGTCGGCGGCCAACTGGGGCCAG
GTCACCGGTGGTGGCAGCCTGGATGGCATGCTTGTCGGTAATCCTGATCTCAAACCCGAGACTAGCGTGA
GTGAAGAGATCGGCCTGCTGTGGAACGGTCATCAGGGCTTCAACGCCGGTGTTACCCTGTTCAACACCGA
CTTCAAGAACAAAATCTCCGAAGTGCGTCGCTGCACCAGCAAGAGCGATCCTGCCTGTACCCTCAATGGT
TACAGTTATGATTTTATCAGTGATCGGGTCAACGTCGACAAGGCGAATATGCGCGGTGTCGAGGGGATGA
TGGATTGGGCCATCAATGAGGATTGGTCGGTCAGTTCCAGCTACACCTTTACCCAGTCCGAGCAGAAGAG
TGGCCCACTGGCGGGCCATGCCCTCAACCAGATGCCCCGTCACATGTTCAACGGCAGTGTTGACTGGCAA
ACCACCGAGGATCTCAGCCTCTGGTCACGGGTCAACTTCCGTGGCGAGACCTCCGAGTACCTGAGCCGGG
TCAAGATGGCGGAGGGGACCCCTTCCTACACCTTCTTTGACATGGGCCTGGTCTACAAGGCCAACAAGCA

TCTGGATCTGACCGCCGGGGTCTACAACCTGTTCGACAAAACAGTGGATTACAGCAGCTATGGCACTGTG
CTGGATGGTCGCCGCTACAACCTGGGCGTGACCTACAACTTCTGATAGGGAACCTGTTATGCGGCCGCCA
TCTTCCGATGGCGGCCGTTTTTATTGCACGTCCGTCAGGGGGCTATGCCTGATAGGGGCCGGCTTGCCAG
GTATTGCGCCCCAGTCCCTTGGCCTGATACATGAGGGCGTCAGCCTGCCGCAGTAGTTGGTCGGCATCAG
CCTCTCTCTCTGCGGCGACCCAGACTGCCCCGGTGCTGATGGAGAGGGTCACCTCGCCGCTGCTGGTGTG
CAGGGTTTGCCGGTTGCTCTCCAGGCAAATACGCTCGGCCCATTGCTCTGTCTCAGCCAATGTGGCCAGC
GGCAGCAGCAGGCAAAACTCCTCGCCGCCGATGCGGGCCAGCAGAGCCTCTTCGGGCAACAGGTTCGTCA
CAGTCGTGGCCAGCTTTTCCAGCAACAGATCTCCGATGCCATGACCAAAGCGGTCATTGGTCTCCTTGAA
GTGATCCACATCGAACAGCAGCAGAGCACAGGGCTCCCCGCTTGCCAGTCGTTGCTCCATGGCGGGCATG
AATGCCCGTCGGTTGAGCAGGCCGGTCAGGGGATCGGTACTGGCTTGTTGCTCCAGTCTGGCGTTGGCGC
GGCTCAACTCGGTGGTGCGGCTGGCAACAGCGCGGCGCAGTTGCACTATGTAGGCCACCATGATTAACAC
TCCGGCAAACAGTAGCCAGGGACCGAGATGGATGGGGTAGACGGTCTCAACATGCATCCACTTGCGGATG
ATCCGCTCCCGATCGCTCTCTTTGATCTTGGCGAATCCCTCTTCCAGCCGGTTTAACAGGGCAAGGTCGC
CCTTTTTCACTGCAAAGCGGATCTGGTCGGTATAGAGGTGGCGAACCGGAATAAAGCGGGTGGGATCGTC
CGAGGTATAGATGTAGAAGTTGGCGACCTGCAGATCGGCGATAAAGGCTTGTATGTCGCCCCTAAAGGCT
GCCGCCAGCAGGGTGTCATTGTCGTCGAAGGTCTGCAGTCTGGTGCCGGGGAAGTAGCGTCTGGCGTAAT
ACTCCTCGTAGCCACCGGCTACCACTCCGACCGTTTCATGGAGCAGGTAGTAGTGGACATCGGTGCCGCG
CAGTTCGCTCGAGAAGAAGAGTTGGGAGTCGAGCTGGGCGAGAGGCCGGCCATAGTCGAACAGTTTGTCC
CGCTCCGGTGACCAGAGCAGTCCGGCGTGAACGTCCGCTTTCCCCTCATGGAGCAGGTCGAGGGAGCCTT
GCCAGTCGACCAGCCTGAACTCGATCTTCTGGTCGGTCACCCGGGCATATTCACGCCACAAATCGATCAG
CAGGCCATCCGGCTGGCCCGACTCATTGATATAGGAGAAGGGCTGCCAGGCTTTGGAGTTGGTAATGATG
ATCGGCTCTTCCGGGGCTTTTGCCCAGGCAGGCAGGGCAAGGGCGAGCAGCAGGAGCCCCCATCTGATAC
TGACTATGACTCCTGTCCTGGAAGAGGCCATCCTTGCTCCAATCCGTTGTCGTATATCGTCCCGTGGTCA
CCGATTATCACCGTCTTTCTGATCGAAGAACAAGTGCTCAAATTATGAAGTTTGCATGATGCAAACGGCG
CCATGACAAGGCTGGCGGATAGAGTGACTACTTACAGCGGCAGGCTTATCCGCGCCTCCAGCCCCCCTTG
TGGCCGATTGAGCAGGGTCAGTTCGCCACCATGCTGATGCACCAGGGTACGGGCAATGGAGAGCCCCAAC
CCCACGCTGCCGGACTCGGTGTTGCGCGCTTCATCGAGTCGCACGAACGGCTTGAACACATCCTCGAGCC
GCGCCGGATCGATCCCCGGCCCGTTATCGCAGACCCGGATCAACAGCTCCTCCTTGCGCCGCTCCAGCGA
CACTTCGGCGCTGCCGGCATATTTGATGGCGTTGCCGATCAGGTTCTGCAGCACCCGGCGCAGGGTGTTG
GCCCGACAGGCGTAGACCTCTTTGCCCTCCGCCAGACAGGTGACCGGCTGGCCGGTATCGGCATAGTCAT
CGCACAGGCTGACCAGCAGGCTGACCAGATCCAGTTCGCGGGTAGCCTCCTGCTGTCCCTCCTCGCGGGC
AAAGCTGAGGGTCGCCGCCAGCATCTGCTCCATCTGTTGCAGGGTCTGCTGGATCCGCTCCTTGTCCTCG
CCATCGGCCAGAAATTCGGTACGCAGCCGCAGGCTGGTGATGGGGGTGCGCAGATCGTGGGAGATGGCCG
CCAGCATTCGGGTTCTGTCCTGCACGAAGCGGTCGAGCCGGGTGTGCATCCGGTTGAAGGCCTGAATCGA
TTCGCGGATCTCGGTCGGCCCCTCCTCTTTCAAGGGCTCGATATCCTCGCCCCGTCCGAGCCGCTCTGCG
TTGCCTGCCAGCTGCTTGAGCGGGCGAGTGGCACGGCGAAACAGCAGCACCATCAGCCCGATCACCAGAC
TGGCGACCAGCATAAGATTGAGGGTGGTTTGCCACGACCAGCTGCCCGCCTCCTTGTCGACCAGTGAGCT
GAACTGCAGCCAACCTCCCTGCGGCAGTTCGATGGAGCCATAGAGCCGCATATCCCGCGGCGGGGGCGGT
CCCTTGCGATCCCGCATCATGCGGGCATGATGTTCCCACGCCTTGCTGCGCTGGGTATCCTCTTTCAGCT
CGGCGCTGATCTCGACGGTGAGGCCGGGGGGATAGTTGAGGCGCGAGCGCACCAGCTGCTCGAAGCGGGG
GCTGCGGTTATCCGGTTGCAGCGGTTCGTCGGAGAGGCGCAGCAGCAAGGTCTCGCTGCGGCTCGCCTTG
AGGATCTCGTCATAGAGGGACGGGGGGGAGAGGGAGAGCAGCCTGACCACCGAGATGATCCGCTGCATGG
CATTGCGGCTGTTGACGAAGCCGAGCGCCTCCTCCCGATCCGTGCGGTAGATCTGGATACTGAGCAGCTG
GATCAGGATCAACCCCGCCAGCGCCACCAGAATGATCTGGCCGGTCAGCCCCTTGGGCCACATCCGTGCC
CACAGTCGTCGCAAGGTGCCCACTGCTTAAACCTGCGTCACGTCGGCGGAGAACATATAGCCGCCACCCC
AGATGGTTTTGATCAGCTCCGGATTCTTGGGATCCCGCTCCAGCTTGCGGCGCAGGCGGCTCACCAGAGT
ATCGATGGCGCGATCGAAGGCCACCGCCTCGCGGCCACGGGCCAGGTCCAGCAGTTGATCCCGGCTCAGC
ACTCGCTGCGGGCGTTCCAGAAATACCTTTAGTAGATCGAATTCGGCGGTGGAGAGGCTCAGGCCCACCC
CATCCTCATCGACCAGCTCCCGGCGATTGACGTCGAGCAGCCAGCGATCGAAGCGCAGATCCCGGGTCAG
GGCCTCGGCGGCGGGTTGGTTATCCGCCTGGGTGCGGCGCATCACCGCCTTGATGCGCGCCAGCAGCTCG
CGCGGATTGAAAGGCTTTGCCAGATAGTCGTCGGCCCCCATCTCCAATCCGATGATGCGGTCGGTCTCTT
CCCCCATGGCAGTCAGCATGATGATGGGCAGGTTCGATTTGACCCGCAGCTCGCGGCAGAGGGTCAGACC
ATCTTCACCCGGCATCATCAGATCCAGCACCAGCAGATCGAACTCCCGCTCCTCCAGCAGTCGCTTCATC
TCCTTGCCATCCCGGGCGCAGCTGACCCGCAGGCCGTGCTGTTCGAGAAAGCGCTTCAACAGATCGCGGA
TCTCGCTGTGGTCGTCGACCACCAGAATGTGGGTGTTGCCCTGGTTCATGAATACCTCCCCTGCTTGGCG
TTGGCACGAGTATACGAAAATACGCTGCACGGTTTGTGTCAAAGTGTGCCAGATCCGCCTGATGCCACAC
TCTGCTACAAAAAAGGGCGATGCTGGCAAAGTTCAGACACCGACAGGGTGTTTACTTGAGGGCAAGGCAA
GAGCGCCGAACCAGAATCATCCCTACAGGAGATAAACCATGAAAAAGCTGACCAAAACCCTGCTGACCGC
CACCCTGATCCTTGGCCTGCCGCTGGGCGCCTATGCTGCCAGTGAAGCGGCCCCCACCGGCCAGCCCGGT
GCCATGGTGGATTGCCCGATGGACGGTGGTGGCATGATGGGTGGTAAACACCACGGTGGTCGCCACGACA
AGATGGCCCGCATGCAGAACATGACCCCGGAACAGATCCAGGCCAACCTGCAGCAACGTTATGACCACAT

TGAAGATCCTGCCAAGAAGGCGGAGTTCATCAAGAAGCTCAACGAGCGTGCAGCCGGCATGACCAAGCAC
GCTGAAGCGATGAAGCAGTTCGCCGAAGCGAACCAGTAACAACGCCAGCTGTTATCAACCCAAGGGTGGC
CGAGGCCACCCTTTTTGCGCTTACTGCGTTGAGCCGCAGCTTGGCTGAGCGCTGGTGATTGCCAATAAAA
GAGGGGGAGCATGATGCTCCCCCTCTTGCTATCTGTGTCGGTGCGAGTGTGCAACCCACTTCACCGGACG
AGCGAACTTAGCCCTGAGCCTTGTCGGTCAGTTCTACTTTCTTGACGGTTTCGCCTTCGAACTTGATGAC
CACGTTTTTCTTGGCGGCGTCGGTATCTTTCAGCAGGGTTGTGGTTGTCTCTGCCAGCAGTGGAGAGGCC
TTCTTCAGGGCATCCAGATCATAGGTCCACACTTCCAGACCGGCCTCGGTCTTGGTAGAGGCAGGTGCGC
CCAGCTTCTCTTTGACTGCCTGCTTGTTGGAGCCGCCTTCCTTGATATCCAGAGCCTTTTTGATGGCTTC
GTTGGATGCACTGTCCATGGCACCGGATACGGCAGTGCTGGCCGCATCCTTGACGCTGCTGGTGACGCTG
TCGGTCAGCGAATCCATCAGGCCGGCATTGGCAAACAGGGGCAGGGTGGTGAGGGCAACCAGCAAACTCT
TTTTCATTTTCTACAGCTTCCATGGGTTCAATAAGGCGGCTATGAAATCAGCAAAGATATTGGGCGTCAA
GTGAGGCATGCAATTGACATTTCTGTTAACTGGTTCTCATTTCTCACCAGTCGCAAACAGTTTTGGTATC
TCCCGCACTAGCCAGCTTTTGGCCTTGCCCATGGTGTCTCGCCGCCAGGCCAGCACCACCTGCAATTGAT
GGCGATATTCGGGGCTGATCACCTTGAGCCATCCGGCCCGTATGTCCTCCTCAATCCAGCTCTGTGGCAT
GGTGCCGACCCCGATCCCTGCCAGCAGGGCATCCCGTTTCTCCGTCATGGTGCTGACAGTGAGGCGCGGC
TGCTTGTCGAGCAGGCGGTAGGTGAGAACCGGTTTGCGCAGGGCTGTGTCGGCCACCGCGATGGCGCGGT
AGCGGCGCAGGGTTTCGTCAGCCAGAGGCTCCGGCTCCTGATGCAGCGGGTGATCTGGATGGGCGACGTA
GAGCATCACCTCGTCCTTGATCACCTGATGCTTGATGCCTGTCATCATGATGTCCGGGTTGATGGAGGAG
ATCAGCAGGTCGGCGCGACCATCTTCCAGACACTCCCAGCTACCCGCCAGCACCTCGGCTCGCAGCCGCA
GTCGGGTATCGGTCTGCTCGGCCAGCTTCTCCACCAAGGGGTAGAGGGCCTGCACCGGGACCAGCGCATC
GACGGCGACTGTGATGTCGGTCTCCCAGCCACGGGCCAGCGCCCGGGTCTCGCCTACCAGATGCTCGGCT
GCCTCCAGCAGCACCCGGCCGCGCTCCAGCAGCATGCGTCCGGCTGGGGTGAACTTGGTGCGGTGGCCGC
TGCGGTCAAACAGCATGGCATCCAGTTCGTCTTCCAGCTTCTGCACCGTATAGCTCAATGCCGAGGGCAC
CTTGCCCAGCTCGTCGGCCGCCGCCGCAAAGCTGCCGCGGCGGTCGATGGCGTCCAGCACCCGGATCGCT
TCCAGGGTGAGTGCGCGTTCCTTGTTCGACTCCTTGGCCATTTTTCACCTTCAATTTTTTTGAATATGTT
CGCCAGATTATCTCGCTAACAAGTTAAGCCAAGCACCTTTAATATTCAATCATCGCTTAGGCAGGAACGT
CTGGCGGCCAAATATGTGGGAAGTTCCGCACTGTGACAGGCCAAGTCGGCATGTCTGATGTCGGTGAAAG
AACAACACACTATTTGAATGACCTCTTGCATGGATGCATTGATTCTGGCCGAGCTGGCCTTGGCAATGAC
ATATAAAACAAGAGAGTGAATGAACATGAAAAACGTAATCAAGATGCTGGCCGTTGCAGGCCTGATGACC
ACAGGTTTCGCCCACGCCGCCGTACCGGCTGACTTCAACGCCACCCCGGGTGTGCTGTTTGTGAAGTGGC
ACTCCACCGCAGCGCTGGATGGCAAAGCCAACCCGATGCTGGAAGTGCGCGACGAGTCTGGCGATCTGCT
GGCATCGGTCCACGCCAACCTGATGGGCACCCAGCAGGTGAAGATCCCGGGTCGTACCCAGGGCAACCTG
ACTGTCAGCCTGGCTGGCGAGAGCAGCGAGTATCGCATTCCGTTCGGTCTCGGCGGTGGTAAAGATCGCT
AAACCGAACCCCGGATAGATGAGGAGCACACCATGATGAAATTGAGAGCCGCAGAGGCGCGAGGCAAGGC
CAACTTCGGCTGGCTGGACGCACGCCACAGTTTCTCCTTCGGTCACTACTACGACCCGGAGTGGATGGGA
CACTCGGTGCTGCGGGTGATCAATCAGGATCTGGTTGCGCCGGGTGGCGGATTCGCCACCCATCCTCACG
CCAATATGGAGATCCTCACTTGCGTGCTGCAAGGCACCATAGAGCACAAGGACAGCCTCGGCCATGTCGA
GCAGATCTCGGCAGGGGATTTCCAGCTGATGAGCGCAGGCAGCGGGATCCGTCACAGCGAGTACAACCCC
TCGCAGACGGAACCCCTCTCCCTGTTGCAGATCTGGATCGAGCCGAATGAAGTCGGCACGCCACCCGGAT
ATCAGCAGAAGAAAATTGAGTCCAGACCGGGTATGACGCTGGTGGCGTCACCGGATGGTGAGGCGGGATC
CTTCGTGATCCGCCAGCAGGCCCGCGTATGGCGGCTGGAGCTGGCCCCGGGCGAAACGCTCAACTGGGCA
CTGGCTGGCCGACACGGTTACCTGCAGATGATTTCAGGGGAGCTGACCGCCAACGGTCTGGCTGCCCGAC
CCGGCGACGGGGTACTCAGTCGTGATGAATACCGGTGGGAGTTGGTTGCGACAAGTGCCACTCAGGCCCT
GTTATTTGACCTGCCCTGATTGGTCAGTGGCGATACGAGTTAAACGAGGAAAGTGAAATGGACAAGATGA
AAGATGTAGCGCTGCTGGCGGGTCGTGTACTGCTGGCACTGATGTTCGTGATTGCGGGCTGGGGCAAGAT
CGGTGGTTACGCCGGCACCCAGGGCTATATGGAAGCGATGGGGGTGCCGGGCTTTATCCTGCCGCTGGTG
ATCCTGCTTGAGCTGGGTGGTGGCCTCGCCATCATGCTGGGTCTCTTTACCCGCAGCCTGTCGGTGCTGA
TGGCCGGTTTCACCCTGATGGCCGCCTTTATTTTCCACTACCAGCCTGCCGAACAGATGCAGATGCTGAT
GTTTATGAAGAACGTCTCCGTCGCCGGTGGTTTCCTGGCGCTGGCGGCAGCCGGAGCAGGGGCTTTCAGT
GTGGATGCCCGTCTCGGCAAAAACTGGTAATGCCCTGAAGAGTCAAGTTTTCCAATGCCTTTTGGCCGGT
GCAGTGCACCGGCTTTTTTCTGTCTGCGTTTTCCGGGTCGATGAGGCTGCTGCACAAATCTGCGGCCCCC
CTCATGGTTCGGCGGTTTGCCCGCCTGCTGGCCACAAGTGCCTTTTGCATCTTATCCCCCGATCCCCGAC
AATTCCCTCTCTTGTGAAGTGACGGAAATGGCTCGCCTTTTCCCTCGCCCCCTCTCATCCACAGGCGGGA
ATTGCTCATGTTTACCCTCTACCACTCCAATCAGCTGGATCTGCTCAAAGAGCTGCTGGTGAGCCGCATC
CGGCAGGCCCCTCTCTCTCACCCCTTCGATCGGGAGCAGATACTGGTGCAGAGTCCGGGCATGGCCCAGT
GGCTCAAGCTGGAGCTGGCCAAGGCCTTCGGCATCGCCGCCAATACCGACTTTCCGCTGCCCGCCAGCTT
TATCTGGGAGATGTTCACCCGGGTGCTGGCGGATGTGCCGCGCCAGAGTCCCTACAACAAAGGCTCCATG
AGCTGGCAGTTGATGACCATTCTGCCTGCCTTGCTGGATCGCCCCGCCTTTGCCCCGCTCGCCGCCTATC
TGGGTGGGGGAGATGAGAAGGATCCCGCCAGGGATCCCGAGCAGGTGCGGCTGTGGCAGCTCTGCCAGAA
GGTTGCGGATCTCTTTGACCAGTATCTGGTCTACCGGCCGGACTGGATCGCCCGTTGGGAAGAGGGGGAA
GGATTGAGTCAGGAGCTGGCCGGGGTGAGCGGTCAGGATTGGCAGCCGGAGCTTTGGCGTGAGCTGGTGG

CCCGCACGTTGGCGCTTTCTCCGAGTGGCTACCACCGCGCCAACCTCTACGAGGAGTTTATCCACGAGCT
GGAGCGCACCGCCGATCTGCCGGGCAAGCTGCCGCAGCGGGTGTTCGTGTTCGGCATCTCGGCGCTGCCG
CCGCGCTATGTGGAGGCGCTGCTGGCTCTCGGCAGTCGGCCGGAGGTGGAGGTGCACCTGTTCGTCACCA
ACCCCTGCCGCTACTACTGGGGAGATCTGCTGGATCGCAAGACCCTGGCGCGCCTCGAAAACAAGCTCAA
ACCCGGCACCGATATCGAGACGTTGCAGGGCCCCGCCAACCCGCTGCTCGCCTCCATGGGCAAGCTGGGG
CGCGACTACCTGCATCAGCTGATGGAGCTGGAGGTGCCGCAAATCGAGGCCTTCGTCGATATCGACGAAG
TGGATGGCAAGGGCAATGTCAGCCTGCTGCGCGCCATCCAGAAGGATGTGCTGGAGCTGGCCGAACGGGG
CGCCGGTCAGTTCGATCTCGACAGCAGCCACCACAAGAGCCCGATCGATCCGGCGGATGGATCCTTGCAG
ATCCACGCCTGCCACGGCCCGATGCGCGAACTCGAGGTGCTGCACGACCGGCTGCTGGCATTGTTCGAGC
AGGACCCGACCCTCACCCCGAAAGATGTGGTGGTGATGATGCCGGATGTGAACAGCTACGGCCCCTACAT
TCAGGCGGTGTTTGGTGCGCGCGGCCAGATCCCGTTTGCGGTCTCGGATCGGGCGGCGAGTCAGGAGAGC
CCGCTGCTGCAAAGCTTCCTCGCCCTGCTCAGGCTGCCCAACGCCCGTTTCGGGGCAGGGGAGCTGCTCG
CCATTCTCGAAGTGCCCGCCGTGCTGCGCCGCTTCGAGCTGGATGACGACGAGTTCAACCAGCTGCGGGT
CTGGGTACAGGAGACAGGCATTCGCTGGGGGCTGGATGACGGCTATCCCGTCCGTTTCGATCTGCCGCCC
ATGTCCGGCAACAGCTGGCTGTTCGGTCTGCGCCGCATGTTGCTCGGCTTTGCCATGGGGGATGGCGAGC
CGGTGGCGGGCATTCTGCCCTACGCCGATATCGAGGGGCAGCAGGGGCTGGCACTCGGCAAGCTCGCCTG
GTTTGTCGATTCGCTGGCCGAATTCCTGCCCCGCCTGCAACAGGCGCAGCCGCTGGCCGATTGGAACGCC
TGCCTGCTGGCGCTGCTGGAGCGCTTCTATCTGGCCGACGAGGATGAAGAGCGTCAGCTCCAGCTCATTC
GCAGCCAGCTGGCCGAGTGGCAGACCCGGCTTGGCGAAGCCCGCTTCGAGCAGTCCATCACCGCCGATCT
GTTGCAGGATTATCTCGGCAGCGTGCTGGGGGAGTCCCGCTCCAGCCAGCGCTTCCTTGCCGGTCAGGTC
AACTTCTGCACCCTGATGCCGATGCGCTCCATCCCCTTCAAGCAGGTCTGCCTGCTCGGCATGAACGACG
GCGTCTACCCGCGTACCCTGGCCCCCATGGGGTTCGACTTGATGGCGGTGGCGTCGCGGCGCGGGGATCG
CTCCCGTCGTGATGATGACCGCTACCTTTTCCTCGAGGCGCTGCTCAGCGCCCAGCAGGGGCTCTATATC
AGTTATCAGGGTTTCAGCGCGCAGGACAACAGCGACAAGGTGCCCTCGGTGCTGCTGGCGGAGCTGCTCG
ACTATGTGCGGCAGGGCTTTGTGCTGGCGGGGGATGAGGATCTCGACGACGAAACCTCCGGCAAGCGGCT
GCTCAACCACCTGATGGTGGCGCACCCGCTCACCCCCTACAGCCGCAACTACTTCTATCCGGAGCCGGGT
TCACGCCTCTTTACCTATGCCGCCGACTGGTTGCCCGCCCTCAATCCGGTGCGCGGTGCGGCCAACTTCC
AAAGCGGCGAGCTGCCTTTACCTCCCGAATGGGGCAAGGAGCAGGGGCTGGAGCTGGCGGAGCTGCTGCG
CTTCTATCGCCAGCCCACCAAATATTTCCTCAACCGCCGCCTCAAGGTGTGGTTTGAGTTGAACGAAGCC
AACATCGAGGACAGCGAGCCGTTCGAGCTGGATGGCCTGCAACATTATCAGCTGAAAGATCTGCTGCTCG
ATGCCCATCTGGCGGAAGGTGAAAGTACCGAGCGTCGCGAGCGGTTGCAGCTTACCGGCCTGCTGCCGCA
AGGGTGGTTCGGCAGCCTGCTGCTGGACGATCTGGATGCCGAGATGGGCAAGCTGGCGGATCGGCTGCGG
CCATGGGTTGCTGCCAATGAGTCAGAAAAGCAGGCGGTGGAGATCGATATCTCCCTCACTCAGGGCCAGC
TGCAAGGGTGGATCGACACTCAGAAGGGGCGCTTGCTGGTGGTCAAGCCCGGCAGCTTCAACGGCAAGGA
TCTGCTGCTGGGCTGGATCCAGCACCTCTGCCTCTCATTGAGCGACGCCCCCGGCGACACCCTGCTGTTC
GACGCCAAACAGAGTCTGCGCCTGCCTGTACTCGCTGCCGACGAGGCGCGGCCCAAGCTGGCGGCGCTGG
TGGCGTGCTGGGCGCAGGGGATGAAGCGGCCGCTGCCCTTCTTCCCCAACACCGCCTGGGATTGGCTCAA
GACGATCGAGAAGGATCCCGACAAGCCGGAGGCGGCCGACAAGGCGGCTCTCACCCGTTTCAACGGCGGT
TGGATGATCAATGGGGAGGGGCAAGATCCTTACGTTGCCCGCTGCTTCCCCGAACTTGATGACGAGGTGC
TTGGTCAGTTGCAGGCGCTGGCCCGCGAACACCTGACCCCGCTGCTCAAGACCATGGAGGAGCTGAAATA
ATGGCTAACCCGCTCAATACATTGCGTTTTCCCCTCCATGGTGAACGACTGATCGAAGCCTCCGCCGGTA
CCGGCAAGACCTACACCATCGCCGGTCTCTACCTGCGGTTGCTGCTGGGTCACGGCCCGTTCATTGAGGA
GGGTGCCGATGCCGGTCAGCCCAGCGCCCACGAGCGGCCACTCTCGGTGACCGAGATTCTGGTGGTGACC
TTTACCGAAGCGGCCACCGCCGAGCTGCGCGGGCGGATCCGCGCCCGCATCCACGAGGCGCGCCTCGCCT
TTATGCGCGGCCATAGCAATGATGCCCTGCTGGCCCAACTGCTGGCAGAGGTGGAGGATCACGAGCTGGC
GGCCCGCCGCCTGCTGGCTGCCGAGCGGCAGATGGACGAGGCGGCGGTCTTTACCATCCACGGCTTCTGC
CAGCGGATGCTGAAACAGAACGCCTTCGAGTCCGGCGCCCTCTTTGAAACCGAATTTCTCACCGACGACA
GCCAGCTCAGGTTGCAGGCGGTGAGCGACTACTGGCGCGCCGAGTTCTATCCGGTGGATAAAGCCCTCGC
CAGCGCCGTGCGCGCGCTCTGGCCGAGCCCCGCCGCCCTGCTGCGGGAGATGGGCAGCTGGCTCGACAAC
AGCGAGCTGGAGATCCACCCCGCGCTGGGGGACGAGACCCTCGCCGCCCGCCATCAGGCGGCCATGAGCC
GCATCGCTGCGGTGAAGAGCGAGTGGCTGGCGCAGACGGACGAGATCCGCCGCCAGACGGATGGGCAAGT
AAGCCGCTATACCGGCAAGAACTACGAAGGGTGGCTTGCCAAGATAGCCGACTGGGCGCAGGACGAGAGC
AGTGGCTACGCCATTCCCAAGGAGCTGGAGCGCTTCGGCCAGACGGTGCTGGAGGAGAACCTCAAGAAGG
GGGGCGCGGTGCCGACCCTGCCGCTGTTCAGCCAGATAGACGAGTTGCTGGCGAGCCGCCCCGGTATTCG
GGATCTCATCCTGCAACGGGCCGCCAAAGTAGTGCGCAGCCGGATGCAGGCCAGCAAGCGGCAGGCTCAT
CAGCTCTCGTTTGATGACCTGCTTAAAGACCTCGACGGGGCGCTCGGCTCCAGCTTGGGCGAGCGGCTCT
GTGAGCGCATTCGCGCCACCTACCGGGTGGCGATGATCGACGAATTTCAGGATACCGATCCCCAGCAGTA
CCGCATCTTCCATCGCCTCTACGGCGGCCACACAGACACGGCGCTCTTGATGATCGGTGACCCCAAGCAG
GCCATCTACGGCTTTCGCGGCGCCGACATCTTTACCTACATTCAGGCGCGGCGAAATGTGAGCGCCCACT
ACACCCTCGGGCGCAACTGGCGCTCCAGCAGTGCGCTGGTGGGGGCGGTCAATGGCCTGTTCGAACGGGC
CAAGGATCCCTTTATCTACGAAGCGGATATCCCCTTCCTGCCGGTGGAGGCGCAGGGCAAGAGCAAGGCG

CTGCTGCTCGATGGCGCCACCGCCCCTGTGCTCCACTGCTGGCAGCTGAGCGGCCAGCCCACCTTCAATC
GGGGGGATTACCAGAGCAAGATGGCCCGCGCCACTGCCGCCGAGATCCACCGCCTGCTGACCCTGGCCCG
CGAGGGCAAGGCGCTCATCGGTGACAAGGCGGTGAAGGCCGGAGATATCGCCGTGCTGGTGCGCACCGGC
GCCGAGGGCAAGCTGGTGCAGCAGGAGCTGGCGCGCCTCGCCATCGCCTCCGTCTATCTGTCGAACCGCG
AGAGCGTGCTGGAGCAGACGGAGGCTCGGGAGATCCTGCTGATTTTGCACGCCTGCCAGAACCCGAGCGA
GGAGCGCAGCCTGCGGGCGGCGCTCGCCACCGGCCTGTTCGATCTCGACGCCAAGGCGCTCGATGGACTG
GCCAGCGACGAGCGGGCGTGGGAGAGCGCGGTGCAGGAGTTTATGGAGTACCGCAAGATCTGGCACCGCC
GCGGCGTGCTGGCCATGCTGCGGGCCCTGCTGCACCGGCGCAATCTCGCCTCTTCGCTTTTGGCGAGCCC
CTATGGCGAGCGGCGGCTGACCAACTTCCTCCATCTCGGCGAGCTGCTGCAACAGGTGAGCAGCGAGCTG
GATGGCGAATACGCCCTGCTGCGCTGGCTGGGGGAGGCGGTGAGCCGCCCCAACGGGCAGGATGCTGAGC
AGGTACTGCGCCTCGAATCCGAGCGGAAATTGGTGCAGATCGTCACCATCCACAAATCCAAGGGGCTGGA
GTACCCGCTGGTGTTCCTGCCCTTTATCTGCAGCCACAGAACCGCCGAGACCCCGCTCTATCACGAGGCG
GACGAGGCGGGCAATCGCACCATTCTCGATCTGACCGGCGCCGACTCGTCGCTCGCAGAAGCGGACAGGG
AGCGCCTTGCCGAAGATTTGCGGCTGCTATATGTGGCGCTGACCCGGGGCGTCTACGCCACCTGGCTGGG
GCTGGCGCCGGTGCGGGCGGGGGCGGGCAAGTCGGAGAAGACCGATCTGCACCACACCGCCATCGGCTAC
CTGCTGCAAAAGGGCGAGGAGGGGGATGCGCAGACGCTGGCCACCGCTCTGACCGAGCTGTCGCAGGCGC
TGCCCGGGGTGGCCGTCGGTGAGCCCTCGCTCACCCGTCCGGCCCCGCTGCCGCCAGAGGAGGAGCAGCT
GGGCGAGCCACAGGTGCGCCACTTTAGCGGTTCCCTTGAGCGGGACTGGTGGATCAGCTCCTACTCCGGA
CTGGCGGCGCAGGGTCACGGTCACAGTAAGGGCGTACTCGCCAATCCCGGCTTCGATGACGAGGTAGTCA
CCGAAGCAGCCGCAGCGCTGGCGGCGGAACAACCGGACGAGGCGCCCCAGCCCTCCATCTTCACCTTCCC
GAAAGGGGCGCGCCCCGGCACCTTGCTGCACAGCCTGTTCGAGACCATCGACTTTCAAAGCGCTGGTGGC
GAGCCGCTGGCAGAGCATATCGCCAGCTTGTTGCTGCAAGACGGTTTCGACGAGAGCTGGGCCCAAGTGC
TGCAACAGCAGGTGGAGGCGGTGCTGGATACCCCGCTCGATACCGGCTTTGGTGATCCGGTGCGGCTGCG
GGATCTGGCCCCCGAGCGCAAACAGGTGGAGCTGGAGTTCTTTCTGCCCATGGGTCGGGTGACAGCACCG
GTGCTGACTGCGCTCTGCCAGCAGCACGACCCGCTGTCGCGGGGCAACAAGCCGCTCAGCTTTGCCACCG
TGCAGGGGATGCTCAAGGGCTTTATCGATCTGGTGTTCGAGTGGCAGGGGCGCTGGTATCTGCTCGACTA
CAAATCGAATCACCTCGGCATGAGCCCCGCCGATTACAGCCGTCCGGCGCTGGAGCGGGCGATGGCCGAG
CATCGCTACGACCTGCAATACCAGCTCTACTCGCTGGCGCTGCACCGGCTGCTCACCTTGCGCCTGCCGG
GCTACGACTTCGAGCAGCACTTTGGCGGCGTTTTCTATCTGTTCCTGCGCGGCATGCCGCAGGGGGGCAT
TTTCCATACTCGCCCGAGCCGTGAACTGGTTCTGGGGCTGGATAAGTTGTTTCGTGAGGGCAGTGAGCTG
CCAGCTGCGGGATCCCAAGTATTGAACGAGGTAGAAGCATGAGCGGCACCATGATCAGCACCGCAACCAG
CGCAATGATGGTTGAACGGCTCAAAACACTGGCGCTGGCTGGCCGTATCCGCCAGCTGGATCTGCAATTT
GCCAAGCTGGTGGCCGATCTGGGCGGCAGCCCCGAGCTGGTGCTGGGGGCTGCGCTCACCTGCTTCGAGC
TGGGGCGCGGCCATGTCTGCCTGCCCCTAGAGATGTTGGCGAGTGGAGCTTTACGGCCGTTCGGGCTGGA
TCCACAGCAGAGCCGCGATCTGCTGGGGGATCTGGCGGATCCTGCCCGCTGGCCCGCCCTGTTTGCAGAG
AGTCCCCTGGTTGGTACCGAACAGGATGAGGGGCTCGCCCCGCGCTGGCCACTGCGGCTGTGGCACGGGC
GGCTCTATCTCACCCGTTACCACGATTTTGAACTGGGGGTGGCCCGCTATCTGCGCGCTTTGAGCGAGCG
CGCCGAATGGCCGGAGATTGCTCCCGCCCTGTCGCGGTTGTTTGCCCGCGACTACGGCCTCGTGTTTGGG
GCGCTGCTCAAGGCCCGCCTCGCCCCCGACTTTTCGGCCCGCCATTTTATCGAGAAGTATCTCGATCTAG
TGTTCCCGAACGAAGTGGACTGGCCCGCCATCGAAGGGCTGCTGGCCAGCGCGCAGGCGGCGAGCGACTT
GAGCAAGCTGGATGCGCTGGTGCCCGAGGTGCTCTGCTGCAACGGCCAGAAGCTGGCGGCGGCCACCGCG
GCGGCGCGCCCCTTTGCGGTCATCTCCGGCGGCCCCGGTACCGGTAAGACCACGACTGTGGCCAAACTGC
TCGCCATTCTGGTGGAGACCGGCCTGCAAGCAGGCAAGGCCCCGGCGATCCGGCTGGTGGCCCCCACCGG
CAAGGCGGCGGCGCGCCTTACCGAGAGTATCGGCTCTGCCCTGCAGGCCCTGGATCTGCCCCCCGAGTGG
GTCGAAGCGATCCCCACCGAAGCGGGCACCCTGCACCGGCTGCTCGGGGTCATTCCGGGGCGCAGCCAGT
TCCGCCACCACGCGGGCAACCCGCTGCACCTCGACTTGCTGGTGGTGGATGAGGCCTCCATGGTCGACCT
GCCGATGATGGCGCGCCTGCTCGATGCGCTGCCGAGCCACGCCCGCCTGATCCTGCTCGGCGACAAGGAT
CAGCTCGCCTCGGTGGAGGCGGGGGCCGTGCTCGGCGATATCTGCAGCTTTATCGAACAGGGCATCAGCC
CGGCGCAGGCGGACTGGCTCTCCCGCCAGACTGGCTACCGCCTGCAGGGCACGCCCGCGGGGGCCCCGGT
GCGCGACAGCCTCTGCTTGCTGGCCAAGAGCTGGCGCTTTGATCAGCACTCCGGCATCGGCCAGCTGGCG
CGCGCCTGCAACAGCGGCGATGCCGCGGCGGTGGAATCGGTCTGGGGCGCAGGATTCGCCGATATCAGCC
TGCACCCCTGGGCCGGGGAGGCCTACGATGCCCTGATTGCCCAGGGGGTGGCCGGTTATGGCAGCTATCT
GAAGGCGGCCCGCGCGGGGGAGGCGCCCGCCGCCGTGTTCAAGGCATTCAACAGCTTCCAGATCCTCTGC
GCCCTGCGTGACGGCCCGTTCGGGGTGTTGGGGCTAAACGAGCGGCTCGAACTGGCCCTGTCACGGGCTG
GCCTGATTGACCGCGACGGCGACTGGTATGCCGGTCGCCCGGTGATGGTGGTGCGCAACGACCACGGGGT
CGGCCTCTATAACGGCGATATGGGGCTCTGCCTGCCCGATGAAACTGGGCGGCTCAAGGTGTGGTTTGAG
CAGCCGGACGGCACCTTGCGGGCCCTGTTGCCCAGCCGCCTGCCGCCCCACGAGACGGTCTACGCCATGA
CCATCCACAAATCCCAGGGCTCGGAGTTTGCCCATACCGTACTGGTGCTGCCCGACAGCCCCAGCCCGCT
GCTGACCCGCGAGCTGGTCTATACCGGCATTACCCGCGCCAAGGCGCGGCTCGATCTCTACTGCGATCGT
GCCCTGCTGGCACAAGCGGTACGGAAAAAGACCGAGCGTTATTCCGGATTGGCGGAGAGGTTGGGGGTGT
GATTGATGGCGGCGGTAAAGGGATTTACCGCAGTTTCATTACGTTTTAGTAACTGAAGCCGAAATTAAAT

AAATGAATAGGGGGAATATAATATATGAAGTATGAGAAAACCCTAAAAGCTCTTTGTCGAAGTCCTAAAC
TGACAGAAGAACAAATTCAATTAACATTCAAGAAGCGGGATTTTACTGATGTTGAGGTGTCGAGGGATAG
TTTAACTCGAGCTGGCTTTAATATTAAAACCGATGAAGGGTTATTTTATGTAACAGAACGCCCTATATCA
TATATGAACAAGCGATGGGGAAGGGTGACGTCACTCCAACAACGTATGTTGAGTTTATCTATACCTGTAC
CATTATTTCTTGGAGAAGGGGATATTGTAAGTGATATAAGTCGTATAAATAAATCAAATAACCCTCATAA
GTCGGCCAGTAAATGGTTGCATGAAAACTTTACCCCAGAAGTATTTGCAACATATTTCAATAAATATTTT
TCTGTAAGTGACTCATTAATCGAGTATAAGACAATAATACTTGAGGCTATTGAGGCCTATCATATGGGAT
TAGACCATATAGCGATAATGTCTCTTTTTCCTGTTTTTGAGGCTGGATTAAGGAATGTTCAAGTTAGTAT
TCTAAATCAAGGTGTAAACAATGTAAGTGCTGTGGGGTTTGAAAAAGGTCTTAAACAACTAATATTAAAT
CATGGTCGAAAGCAAATGTCTAAATATGACTGGCATCCAGGGAAGGGCTATAACAGTGAGGTTGAGATAG
ATTTTTTAACGCATGTTAACCCACAATGTGATGTAATAAATGCATTCAGAATTTTCTTTTCCTTGGTCTT
ATACAAGCCAAGTCGGGCAGATGGGAAAATGAATGGTTTTAATCGACACCTAATTATGCATCTTTTAAAA
AATGATTTTAATGATCCATCCAACTTCCCTAGAATAATCTTGGCACTAACTCACATTATGTTTATTGAAA
GTTTGAAAAATGAAAAGGTTCCTTTTTCTTGGCCGGGAATTGATAAAGTCGATATGGACTTTGGTGGATA
TTTACGTAAGTTAACAGATGTGGTTTTCATATCTCGCAGAAAGTTACTAGACTCACTAACCACATGTGCA
TATTAAATCGACAAATAACAAATTTATTTCATCTTGTATGTTGGAATTAGCGTATTTCGGATGTTGGGCT
TCGCAGGCTCAGCCACAACCTACCGATTGTGTAGGCTGTGGCTGAGCGTAGCGAAGCCCAACGTTTACCG
CGTAATTTCTTTTTTCCTCCCCCTCATCCCCGACCCTTCTCCCGCAAGGGGAGAAGGGAGGTTTGGCCCC
GTGGCTATATCTCGAATTTACACGCGATTGCCAACACCATATCCCCGATGCTGCTTTATCCCCTCTCCCT
CTGGGAGAGGGCTAGGGTGAGGGGCGCAAGTTGGCACAATGCCAATTTTGCCCGCTTCGTGAAATCCGGT
AGGGTGCAAAGAACGAAGTGAATTGCACCGCTGCTATTTATCTGCCACAAGGTGTCATGCGCTGCGCTTA
TTACACCCTGCATCCAACCCACACACGACAGGATGTCATTGCTTGAGACTTGAATTTAACACTATCGATC
TGGATCGCGATTACGAACTCTGTGTTCAGGCGCGCAGGGATGCCTATTTTTGCAGCTTTCATACCCATGA
CTGCTTTGCCGACTTTATCGACGGTTATCGGGAGCGGATCCGCGAAAGGCTGGGGCAAGCGCAATGGCAC
TACCTCCATGTCTGGGCAGGTGAACAACGGGTGGGCCAGCTGGAGTTTCGCAGTTTTTCCGATGAGCCCG
AGACCGGTTATGTGCAGCTGATTTATCTGTTCCCCAAATATCGTGGCAGCGGTCTGGCTGCCGAGTTGCA
GGCATATATCCGCCAGCAGTTGCTGGGGGCGGGTTGTAAATGGGCCATTCTCTCCGTGAGTCGCACCAAT
GTGCGGGCCTTGAATCATTACCGGCGCTGTGGCTGGGTATTTCTGCGCCCCAATCCGAAACATCGGGAGA
CGGATTTTTATCGACTGACTCTGGCCGAGTGACCTCTGTGGTGGCCTCTGGGTATCCCCCTCATTTATTC
CCATCCCCCAAATGGCTGAATCGTGAGTTAAATCCGGTAAATGGGGCGCGCTTGAGCCACACTTATGGCC
ACCCTGATTCAGCCTGTGCCGTGAGTGGCCGATAACCGGGGAAACGTGGTGCGGTGGGGTTCTGGCTGAC
AGATCTGTTGCAATATGCCAAGGTAGCGCCTCACGACAACAGCGCGATTCCCCGTGGTGGCGCGGGGCGG
AAGAACAAGGATAACTGCTATGGTGCAGGACAGTGACACGATCAGGATCTCGGATGTCAGCGAGGCCGTG
GTGCATGGCATTCTGGAGGTGACCAGCGATGGCATCTGGGATTGGCATGCCAATACCGGTTTTGTCTATC
GCAACCCCGGCTGGTACCGGATGCTGGGTTACGAGCCTCACTCCCTCGGCAATACCGTCTTCACCTGGGA
GAACGTGATCCACTCCGACGATCTGGCGGCGGTGATGGCGAAGTTCGATCTCTGCCTGAGCGGCAGCGCC
CATGAGTATCGCGCCGAGTACCGTTGCCGCTGTCGGGATGGCAGTTACCTCTGGATTGAGGATTGCGGCC
GCATCATTGAGCGCAATCCCGACGGGTCTGTGGCGCGGATGATCGGCGCCGCCAGCCGCTATCGGCTGGA
GCGGGCGTTGCAGCTGGAGTGCGAGCGGGCGCGCCGCTTCCATCAGCCGTTTACGGTGATCGCCATGGAT
GTGGACAACTTCAAGCAGATCAACGACAGCTATGGCCACAATGCAGGGGATCAGACCCTGATCAACATAG
TGGCGCTGGTGCGCCACCATATCCGCGAGATCGATCTGCTGGCCCGCTGGGGTGGTGACGAGTTTATGGT
GTTGCTGCCCAACACCGGCCGCGAGGATGCGCTGGTGGTGGCGAGCAAGTTTCAGGCATTGATGGCGAGC
GAGCCCATTTGCCCCCATGCGCCCGCTACCATGAGCTTCGGGTTGGCGGAGTATCAGCAGGATGAGCAGA
AGGAGAGCCTGATGGTGCGCGCCGATCGGGCGCTCTATCAGGCCAAGCAGCTGGGTAAAGACCAGATCTG
CTAACGCGCAGGGCACGGAAAGAGAGCAGTAAAAAGGGCCGCAGGTTTGAAACCTGCGGCCCTTTTGCAT
TGTTACTGACGCGGCGCGACTTAGAAGTGGTAGCGCAGCCCCAGCTTGAAGGTATCCAGAGAGTAGTGCT
CTTCACCCAGGCTCAGGGCGTCGAATTGGCGCTCGTAACCGATACCGGCAGAGAAGTGGTCGTTGAAGGC
GTAGTCCAGACCCACTTCCAGATTGGGGTGTACCTTGCTCTTGGTTGCGCTCTTGCTTACCTGATCGCTG
AAACCGAGGAAGGTGGCGTTGCCGACAACTTCCAATTCGGTGCGGGTGTAGGCGATACCCGGTTTGGCAT
AGGCGCTGAAGCCCCAGGCCAGCGGCTGGCTCAGCTTGGCTGCCAGGCTGATGCTGCGGGTTTCGATGGT
GGTGTCGGCAAAGCCGCTTGCAATCGGGGAGGTCACCCAGGTCAGCTGATCCATCTGTTGGGCGTGCAGG
CCACCTTCAACCGCCAGCCAGGGTTTGAAGCTGTAACCCACGATCAGGCTGCCGCCGTCGAGCTGGTCGT
CGATCACTTTGGCGTCATAGGCGTCTTTGGCTGCACCATCGAGATCCATCTTGGAGGTAGTCAGATCCAG
ACCTGCATACCAGCCTTCAGCATGGGCGCCGCCATTCATGGCCAGAGCAAGCAGGGAGAATGCGGCAATT
TTATTAAACTTGTTCATGGATAAATAACCTTGTGTTGCGTGAACTGCGTGTGATTTATATTGCTCAATCC
ATGAAGATTTTTATATTTCCGTCATTGCTCTGCTTTCACAGAGGGGCCGCAGTATAGTCAGCACATTTCT
GAATAACAAGAGAGTGATTTCCCCATTATTCTAAATATGACTGGCTGTAAGCAGGGTCATTTTGTTTAAT
AAATGGTGTTATTAGAGTTTTAGCTTTATTGAGGTTTAAATAATTAACTGGTGAGGCAAATGACAGGCTG
CGAGTTCGATTTTGATTCTATGTGATTGATTTGTATCAGCATTAAATTTTTATTCGAGCGTGGCCGTGAT
GAGTTGTCTAAAAATTGACATCTCAGATGTCACTGATTGGCATCAGGTTCTTTTCGATGATTTTTTGAAC
CCATCACAAGGTGAAACCGTGAGAAAAAAATCTATCTTTTTGCCTGTTTTTTGCTCTTTTGGAGGTTTGG

GATTGTTCCTATTGTGGCTTTTGGTGGTCTTTTGAGGCTGTTTATGCGTAGGCCATGTTGCCCACCAGAT
ACGCGGATGGGTTCTTGTGGGTGACTTTTCATTTCTCCCGGCAGCACTCGTTGGGGTGGCATGATGTGTG
ATAAGCAGATATGGGGCGTGCGGTCTGATTAATTTTGTTTGCTTATATATACAAGCCATTTAAAAGATAG
GTTTTTGGAGTTTTAAATAACTTTACTATATTCGTTTCTATATTTGGCGAAGCTTTCCAAGCAAGCCATT
TAAATATATTGAATGGCAATGAAACGCTATTGATGATCGTTTAGGATACCTTGCCAGAGAGGTCGTAGTG
GCTGGCGCATCGGTTGGCGGCGGGTGGCCTGTGACACTTCCCCTTGCCGGGGCCCTTGACCTGACCTGCG
AATTCAGGGAAAAGAAAAGGGCGGTGCTATCAGCGCCGCCCAATGCCAGGAAAGAGAAAAAACCTTACGG
TTTAAAAGATAAAAATTTGGAAGCAAACAATCGATTTCTCAACCAGGAAGGGTCTTGAATTCGATACGTT
TCGATTATGCGCGATGTTCTATGCAGCACTAGCAAAATGTTTGCGGGTTGAAAGATATGCACTGTCTGGT
GTCAGATTTTGCGACCTCATCGTTCAACTGGTCTGTCCTGATAAGCGTTGTAGGCGATTTTTTCTCTAGG
CTGTTTTTTCGGTGTCAATATCTGAGCCTTGAAGGTAAAAACAGGTCAGAAGCTGACTCGGCATAACCAT
GCAATGAAAGTGCGATAACAAGGAAGAGTGGCATGACCTACAGCCAGACCCTGGCCAACAATATTCTCGA
CACCCTGAAGAAGACCCTGACCGACAAGGGGATCCGCTATCAGACCCTGGCAGAAGCCATGGGTGTCTCC
ATCGCGACCGTGAAACGGATGATGAACAAGCCCTCCCTGCCGTTCGATACTCTGCTGGAGATCTGCCATC
TGGTGGGGCTCTCGTTCGAGGAGTTGCTGGATCGCACTCAGGACGCCCAGAGCCGCCGCGCCGTCTTCAC
TCAGGAGCAGGATGAGGCGTTTCACAACGAGCCGGGGCTCTACGCCTTTCTCGCCAATATCTTCTGGCGC
GACAAGACGCTGATCGATCTGAAGCGGGAGTACGGCCTGACCGATGCCTCCTGCTACCTCTATCTGCGCA
AGCTGGAGAAGCTTGGCATTTTGCAGCTTGGTATCGACAACAGTTACCACTTCCTGATCAGCGAGCGGAT
CAGCTTCGAGCAGCACAGCCGTTTCGCCCGCCAGCAGGCGCGGCAGGCAATGTCGGTGCTGGGGGAGTAT
CTGGTGGAGAACATGCACAAGTCGAACAACTATATGGCGCTCTGCCAGCTGCACCTCAACGAGGGGGAGG
CGATGGCGCTGATCGACCGGATGAAGGAGTACTGGCATCAGGAGATCAAGCTCAACCGCCCAGCCATCGG
CCAGCAGGAGCACGCCAAGACCTACACCATGTCGCTGCAACTGGCCGACTGCGGCTACCAAAGCTTCGAC
GACATGATCCCCAATATCGACAACTGATCCCGGCGGATCCTGCGGGATCCGTCTCTGCTCGTGAGTATGA
GAGTGGTTACTCTGCCAGTTGCATGGGTTGCGCTTTGGCGATGATGGGTTGCAGCGCCCGGGTATCGAGA
CAGATACGAATGGCATCCGGCCGCCAGTGTTCGATATCGCAATCCACCAGAGCACCCCGCTCGTCGTAGT
TGACCCGCACCAGCCGTAGCACCGCGCTCCCCTCTGCCAGATTGAGGGCGCGGGCGATGGCGCCGCGCGC
TGCCGAGGGGGTGATCTCGAGACTCGCTCCACCCTGGCCTATGCCATAGCGCTGCTGATAGAGCTCGGTG
AGGGAGCTGGCCAGCGGCGCGCTGGCGATATCGGGAAAGCGGCTCGCCAGCAGGTGGTGGCTGACATGGA
GCACAGGACGGCCGTCGATCAGCCGCTGGCGGCGGATCTCATAAAGTGGGGTGAAGGGTTCCAGCCCCAT
CCAGCGGCACAGTTCGCTGCTGGCAAGTTCGCTGCCGTGGGCCAGCACCCGGGTTTCGGCGGTGCGCTGC
TGCTCGCCAATCCACTGGTGAAAGTGGGTGTGGATGGCGGGATCGTAGGTGAGGCGGGGCGGCGCCACAA
ACCAGCCGCGCCGCTCTGCTCGGTAGATAAGGCCATCCGCTTCGAGGCTGGAGAGCGCCTCCTTGATGGT
GATGCGGGTGGTGGCGAAACGCTCGCTCAGGGCCCGTTCGGCGGGCAGCTGCTGCCCGGCCCCCAATTGG
CCGCTGCCGATATAGCGGCGCAGGGTCTGGCAAATCTGTTCGCACTTGGAGGAGACGCGGCCATCGGCCA
TGCTCTGTTCGTTCACTCTCTGCTCGCTCAATCTCGGCTCGGTCACACTCTTGTCCCTGTTGCTTATTCA
GAAGGCGGATGACTGGAGTAGTCCAGCCTCACTCGGGTTCAGAGTTGTAACTGCTGTGGATGACAGCGCC
ATGACAGGGTGATTGTCATCAAACTGCCATTAAAGCGCCCCGCCACCGTCACAATTCCCCTCTAGCATCC
CTCTCGAACCTTAACTGACCTAGTCCAGAGGGATGGATGATGAAAAGCGTGATCGCAAGTGCACTGGCC
ATGGTGGCCATCAGCCAACCGTTGTTTGCCACAAATAGCTATGCAGCCGAAAATATCGCGGATCTCGAAA
AGGCCGCCCGTGGCGAAGGCCAGCTCCACAGCATCGGCATGCCCGACAGCTGGGCCAACTGGAAGGATAC
CTGGCAGCAGCTGGGCAGCATCTACGGCATCAAGCATCAGGATACCGACATGAGCTCGGCGCAGGAGATC
GCCAAGTTCGCCGCCGAGAAGGCCAATGCCACCGCCGATATCGGTGACGTGGGCGGCGCCTTCGGTCCGG
TTGCGGTCAAGCAGGGTGTTACCCAGCCCTACAAACCCTCCACCTGGGCTGATATTCCCAACTGGGCCAA
GGACGCCGACGGTCACTGGATGCTGGCCTATACCGGCACCATCGCCTTCCTGATCAACAAGGATCTGGTC
AAGGCCGCCCCCACCAGCTGGCAGTCCCTGCTGGGTGGCGACTACAAGGTGACCCTGGGTGATGTGGGCG
TAGCCTCCCAGGCCAACAACGGTGTGCTGGCGGCGGCCTATGCCACCGGCGGCAGCGAGAAGAATCTGAA
ACCTGCACTGGAGCTGTTCGGCCAGCTGGCCAAGCAGGGTCGTCTCTCCCTGAACGATCCCACCATCGCC
AATATCGAGAAGGGCGAGGTGGAAGTGGGTGTGCTGTGGGACTTCAACGCCCTCAACTACCGCGACCAGA
TCGACCCCAAGAAGTTTGATGTGGTGATCCCGAGTGACGGTTCAGTCATCGCCGGTTACACCACCATCAT
CAACAAGTGGGCCAAGAACCCCAACGCCGCCAAACTGGCCCGTGAATACATCCTCTCCGATGCCGGTCAA
ATCAACCTGGCCCGCGGCTATGCCCGCCCGATCCGCAGCAACGTTGCGCTGCCGGATGACGTGAAGGCCA
AGCTGTTGCCAGCCGAGCAGTATGCCAAGGCCAAGCCGGTGCAGGATCACGCCGCCTGGGAGCAGAGCTC
CAAGGCCCTGCCGCGCCAGTGGCAAGAAACCGTCATGATCAACATGCAGTAAGGGAATCCAGTGCAACAC
AAGGTGATAGTGGTACTTGTGGATGGCCTCTCTGCCGGGGTAGCCCATGGGATGGGCTACCTCGCAGGCA
TGGTGGAGGCGGAGCGCGGCCTCTATACGACCCTCAGCTGTGCCTTGCCATCCCTCTCCCGGCCTCTCTA
CGAGTGCATCCTGACCGGTGTACCGCCGGTGGCGAGCGGCATCACCCACAACGGGGTGAGTCGTCTCAGC
CTGCATGACTCCATCTTCCATCTGGCCCGCGCCGCGGGTAAACGCACCGCAGCGGCGGCCTACTACTGGG
TGAGCGAGCTTTACAACAAGAGCCCCTGGCTGGCGGCCCGCGATCGCTTTACCCACGATGAGCAGCTGCC
TATCCAGCACGGCTGCTTCTACTGGGATGACAACTACCCCGACAGCCATCTGCTGATGGATGGCGAGTGG
CTGCGCAGCCAGTACGATCCCGATTTTCTGCTGATCCACCCGATGGGAGTGGACGATGCGGGCCACAAGT
TTGGCCTCGATTCCCGCCAGTACCGCAATCAGGCACGCCGGATGGACAGCCTGCTGGCCGATCTGCTGCC

CCATTGGCTGGCGGAGGGGTATCAGGTGGTAGTCACCTCGGATCACGGCATGAACAACGACCTGAGCCAC
GGCGGCACCCTGCCCGAAGAGCGCACCGTGCCGCTCTGGCTGTTTGGCGATGCCTTTATCGAACGCTGGC
CGGATGGAGTGGTGATCGCCCAGACCCAGCTCTGCGCCCTGATGGCCGATCTGCTGGGCATCCCCCACGA
CAAGCCGGCAGCGCCACCGCTGCTCAAGGCGGCCTTTATGCGCGAGGTGCACTGATGATGCCTGCCACCA
CCGAGATTCTCGTGGATCAACCCGTGGCAACCAAGCGCAAGGTGCGCCGCCACTGGCTGCCCGCGCTGGT
ATTGCTGCCCTTTATCCTGTTGATGATCCTGTTCCAGCTCGCCCCCATGGTGTGGGTGCTGGTGGGCAGC
TTTCAGACCCCGGATGGCTGGGGCATCGACTACTACCGGGAGATTTTGAGCTCCCCCTTCTACCTGCAAT
CGTTCGGCAACTCGTTGCGTATCGCCGGTATCGCCAGTCTGGCGGGGCTGGCTATCGGTACCCTCGGGGC
AGCCGCGCTGCGCCACAGCGGCGAGCGGGTCAAACGGCTGCTGCTGGCCTTTGTCACCATGACTGGCAAC
TTCAGCGGGGTGCCGCTGGCGTTCGCCTTCATCATCATTCTCGGCATCAACGGTGCAGTGACCCTGCTGC
TGAAATCCTGGGGATTGATCGACGGCTTCAACCTCTACTCGGGCAGCGGCCTTATCCTCATCTACACCTA
CTTCCAGATCCCGCTCGCCCTGCTGCTTCTCTATCCGGCCTTCGATGCGCTGCAGGATGAGTGGCCGCAG
GCTGCCGCGTTGCTGGGGGCGAGCAAGGGGCAGTATGTGTGGCATGTGGCGCTGCCGGTGCTGACGCCTG
CCCTGCTCGGCACCCTGATCATCCTGTTTGCCAACGCGGTGGGGGCTTATGCCTCCGCCTACGCCCTCAC
CACCGGCAACTTCAATCTGGTGACCATCCGGGTGGCCAGTCTGGTCTCCGGCGACATCTTCCTAGAGCCC
AATATGGCGGCGGCGCTGGCGGTGATCCTGATGGTACTGCTGGGGGTAGTGACGGCCGCCAACCAGTGGC
TGCTGAAAAAGAGCTATGTGAACAAGGGGCAATCCCATGCATGATTTGACGCCGCGCTGGCCCAAGTGGG
TGCTGGGTGGGCTGGTTGCCACCCTGCTGCTACCGCTGCTGGCCACCTTTCTCTACTCCATCTCCACCCG
CTGGGGGGCGACCCTGCTGCCGGACGGCTTTACCCTCGACTGGTATCTCAAGCTGTGGGGCGATCCGCGT
TTTCTGGCGGCGTTTGGCCGCAGCCTCCTGGTCTGTTTCGCCACCCTGCTGCTGGGGACCCTGGTGCTGG
TACCGACCGTGCTGGTGGTTGCCTACTACTTCCCAAAGCTGGATCCCTGGATGAACCTGTTGATCCTGCT
CCCCTTCGCGGTGCCACCTGTGGTTTCGTCAGTGGGGCTGCTGCAGATCTACGCCGATGGTGTCCTGCCG
ATTATCGGCACGCCGTGGATCCTGATCGGCACCTGGTTCACCGTGGTGCTCCCCTTTATGTACCGGGCAC
TGGCCAACAGTTTGCAGGGTGTGCCGCTGAAAGATCTGATGGATGCCGCCCACCTGCTGGGGGCAAGTAG
TCGGCGCGCCTTCCTGCTGGTGGTGATCCCCTGTCTGCGCAAGGGGCTGCTGGCGGCGCTGTTTCTCTCC
CTCTCCTTCCTGCTCGGCGAGTTCGTGTTTGCCAACATGCTGGTGGGCACCCGCTACGAGACCCTGCAGG
TCTACCTCTACAACATGCGCGCCACCTCGGGCCACTTCACCAGTGCGCTGGTGATGAGCTACTTCCTGCT
GACCCTGCTGCTCACTTGGGCGGCCAACAGATTTCATCGGTGATCCAAGATGTTTCATTTAGAAGTCAAT
CAGTTGCATAAATCCTACGGCGATACGCCGGTGTTCGAGGGGATCGAATTCGGCATTCGCAAGGGGGAGC
TGGTGACCCTGCTCGGCCCCTCTGGCTGCGGCAAGTCCACCTTGCTGCGGGCGCTGGCCGGGCTCACCCC
GGTGGATGGCGGTCAGGTGCGGGTGGCCGGTGAGGAGATCACCTGGCAGGCGCCCCAGAAGCGGGGCATC
GGCATGGTGTTCCAGAGTTATGCGCTGTTCCCCAACATGACGGTGTGGGACAACGTCGCCTTCGGCCTCA
AGATGAAACCCCAGCCGGGGCGGGAGCTGGCGGCCAGCGTGCAGGAGGCATTGGCGCTGGTGGAGCTGAC
CGGTTTCGAGCGCCGCTATCCGGCCGAGCTCTCCGGCGGCCAGCGCCAGCGGGTGGCACTGGCCCGGGCG
CTGGTGGTGCAGCCGCGGATCCTGCTGCTGGACGAGCCGCTCTCGGCGCTGGATGCCCGCATCCGCCGCA
GCCTGCGCCAGCAGATCCGCGAGATCCAGCAGCGGCTCGAACTCACCACCATCTTTGTGACCCATGATCA
GGAGGAGGCGCTCACCATGTCGGATCGGATCTTCCTGATGAACAAGGGATCCATCGTCCAGAGCGGCACG
CCGGAGGAGATCTATACCCGCCCCGCCAGCGAGTTTGTGGCGGGTTTTATCGGCAACTACAACCTGCTCG
GCGCAGAGCAGGGCAAGTCGCTGTTCGGCCCCCGTTTTCAGGGCAAGCTGGCGCTGCGGCCGGAGTCCAT
TACCCTGCTGCCCGCAGGGGTGACCAGCTGCGAACCCAGCCTGCCCGGCATCATTCGTCAGCAACAGCTG
CTTGGCAACGTGATCCGTTATCAGGTGGAGTGCGAGGCAGGGCTGTTGACGGTGGACTGCCTCAACCGCT
CGGCTGGCGATCTGCTGGCCACCGGCAGTGCCGTGACCCTGGCGGTGGCCCGGGATCAACTCTGTGAGGT
AGCATGACGCCTCGTTTCATTTTCGTTGTTACCGCAAGGAGCCCTCATGGCACTGGCACTGTTTGATCTG
GACGAGACCCTGATTGCGGGGGATTCCGCCAGCCTCTGGCTGGAGTACATGGTGGCCGAGGAGCTGGCTC
CTGCCAGCATGATCGCGGAGGAGCAGGCCATGATGTCCCTCTACCATCAGGGCAAGATGGATATGCACCA
GTACATGGCCTTCACCCTGCAACCGCTGGCGGGCAAGACGCGCCAGTGGCTCGACTCGCTCTCTACCCGC
TTTGCCGAGCAGGTGCTGCGCGAGCGCCTCTACCCCGAGGGGTTGGCGCGTATCGAATGGCATCGGGAGC
GGGGCGACGATCTGGTGCTTATCTCCGCCTCCGGCGAGCATCTGGTGGCCCCCATGGCGAAGATGCTGGG
GATGGATCACTGCGTCGCCATTTTGCTCGATGAGGAGGAGGGGATGCTGACCGGCCAGACCCGCGGCACC
CTCAGCTTTCGCGAAGGCAAGGTGGCCCGCATCAACCAGCTGTTTGCAGGCAGGGACCATCTCTGGCAGG
GGAGCTTTGGCTACAGCGATTCCCACAACGATCTGCCGATGCTGCGGGCGGTGAGCCATCCCCATGCGGT
CAATCCGGCCCCCGCGCTGCGCCAGCAAGCCGAGGAGCTGGGCTGGCCGGTGTGGGAGTGGCAGCGCTGA
GTCGCGCGCCACACAGGCTTAACGTGTGACAAGAAGCACGAAAAACAAAGAGAGAGGCTGGAGCCTCTCT
CTTTGTTTTTGCGGATAGCGTTGTGGTTGATGCTGGCCGCTGTGGCTACCCCTCGTTGGTTTGTTGTCGG
GGCTCGCCAAAGGCGGGCACTTCCTCCTCTGCACTCTGGTGAGGGGCGTGGCGCAGCGGGAAGCAGATAC
GGTTTTTCCCCTCCCGCTTGGCTTGATAGACCCCCTTGTCGGCCAGCTCCACCAGCTCTTTGGCCGAGAG
CCCGGAGCGGCTGAAGGCCACCCCGCCGCTGATGGTGATGGTGAGATCGGGTTCGCGCCACTCCAGCTGT
TCCACCCCGAGCCGGAACGCTTCCAGCAGGGAGGGAAGCATCGGCTGCTCTTCGCCGTAACGGAAGATCA
GCACCAGCTCCTCGCCGCCATAGCGGCAGAACCAGCCCCACTCGTGGAACAGATTCATGCCCAGCTTACT
CACCCGCTTGAGCACCACATCCCCCGCCTGATGGCCGTGGGTATCGTTGACCCGCTTGAAATCATCAAGA
TCGAACATCACCACCCCGAAGGGGGCGCCACTGTTGAGCTGCTTCTGCAGGCACTCGTCGAAGAAACGAC

GGTTGTAGAGTCCGGTGAGCTTGTCCTGATGGGCTTGCAGGCGGATTTGGGCAAAGTCCTGACTGGCGGC
ATCCATCTCTTCATAGACCTTGGCCAGCTCCAGCATGCGGCGGCCGTTGTAGGCGATACCGCCGGAGCGG
CTGGCGCGGATCAGCAGGGTGATCTCCCCCAGCATCCGGCGGAAGTAGCGGGACCAGAGCAGGGCCAGCA
GCAGATAGAGAATGCAGGCGACGGCAATCATCAACTGCACGTTGATGGAGACGCTGTCGATCACCTGCTG
CATGCTGGCAGTCGGCTCGACGCTGAGCAGGATCCAGCGCTGCTCGGGGATGCGGTAGTAGGAGACCATC
AGGTCGCTGGCATCATCGAGGAAAAAGCCCTGCGGCTGTTGCATCCGGGCCAGCCAGTCGGGGTGGGCGA
GGGGTTTGAATACCCGTTCGCGATCCGGATGGGCCAGCACCAGCCCGTCATGGTTGAGCAGGATCTGATA
GCCATTGCCCAGATGGTGACTCTGATCATGCCGACTGTTTAGCCAGCTGGCGAGGGGGGTGAGCTGGGTA
TCCAGCGAGAGTACGCCAACCCGCTGGCCGTCAGGATCTTCAATCACCCTAGCCAGCGCCATGGTGAGGG
TGCCCAAGGTGTAGTCGTAATAAGGATCGGTCCAGACAATCTGTCCGGGATGGCTGGATGCCAGCAGATA
CCAGGGGCGGGTTCTGGGATCGAACTTCTCGTCGGCCTGCCAGGATGGGTGGATCAGCATCTTGCGATGC
TCGGTGCCTACATAGACGTAATCGAGCTCCATCACCTTGTGATAGGCCTGCCACTGTTGCAGGTAGCGCT
CGCCGTTAAGGATCTCCGGGCCGCTGAAATCCCGATAATTTACCTGCTGATAAATGAGCGTAAACTGGCG
TTTGAGGGTCGCCAGATAGGGTTCGATCAGGGTATGGAAATACTGGGTGTTGCGAGCAATCAGATATTGT
TGCTGCTCGTGGCGCAGGGTGCGCTCCATATGCAGGTAGTAGATGCTGCCGAGCAGCAACACAGGGATAA
AGAGCAAGAGGAGGCCCAGCCGCATGCGGGTGATCAAAGACCAAGTGTTCTTGGGCTTGGCGGGGGACAT
GAACAGATCCTTTTCGTCTCGGGTGGCACCAGCATATATGATGCTGTGCGGCTCGTCTAAAGGCAGAAAA
TGAGATGTTTCCGATGCTGAACCACACTCTGCTCTCAATGATTCATTATGGCAGCATAGCGGTGCTAATC
CTTGCCCTCTGTGTCGAGTATCTGATCTTTCGGCGCGGCCTGAACATGCTGCGAGTGCGCCGCCTGCTGC
TGGCAGATGGGGTGGCGGCGCTGGCGCTCTTCTCCATCTTTGCCACCGGGTGTCTGCTGGCCCTCGAGCA
CGCCAGCTCGGTGGCAGAGATCTTTGCCCATCCTGCCCACGGCCTCAAGATGGGGTTGTTTCTGCTGATT
GTTGGCTCCCTCGTTTATCCCAGCCGCCTCTTCTATCGCTGGCGTCGCGCCCTGCGTCTGGGCAAGGCGC
CGATGATCTCCATCCAGCAGCACTTCCGGGTGGTGTGGATCCTGCGGGGCAATCTGCTACTGATTGCCCT
GTTGGCCGTGTTCACCAAGCCCTCGCTGCTGTAACACTCGCGGTGCTCAAAAGCAGTGCGATGCAGCGCG
TTGAGAGCGAGAGCGAAGACAAAGAGTGGAGACAAGAGGATAGGGAGGCGGGTATCTTGGATCGGATAAC
CCGTCACGCGCCGATTTAAGGAAGAATCGCCATGCGCTCCAATCTGATGTTGCTGATGGCTGCCACCATC
TGGGGTATGGGCTTTGTCGCCCAGCGCCTCGGGATGGATCACATGGGGCCCTATACTTTCAATGGCCTGC
GCTTTCTGCTCGGCGCCCTCTCCCTGCTGCCGCTGCTCTGGTGGCTCAAATCCCGACAACCTGTTGCCAT
TGCGGGCGACCATCGCCAACTGCTGATCGGTGGTGTGCTGGCCGGGCTGGTGCTGTTCTGCGCCGCATCC
CTGCAGCAGGTGGGGCTGCTCTACACCACGGCCGCCAAGGCCGGTTTCATCACCGGGCTCTACATCATTC
TGGTGCCGGTACTGGGGCTACTGCTGCGCCACAAGAGCGGACTCAATACCTGGATCGGCGCCGTCATTGC
GCTGGCCGGTCTCTATGTGTTGAGCGTCACCGACGAGTTCACCATCGGCTTTGGCGATCTGTTGCAGGTA
GTCGGCGCCCTGTTCTGGGCTGTGCATCTGCTGCTGGTGGATCACTACTCCAACCGGGTTGCTCCCATCA
AGCTCGCCGGAGTGCAGTTCGTAGTCTGCGGCCTGCTCAGCCTCGCCACCGCTTTTGTTATTGAAACTCC
GACTGTTAGCGGCGCTGTGGCCGGTTGGCAGGCGCTGCTCTATGCCGGGCTGGTGTCGGTCGGGGTCGGT
TATACCCTGCAGGTGGTGGGGCAGCGGGGCGCTCATCCGGCCCACGCCGCCATCATCCTCAGTCTGGAGA
CCGTGTTTGCCGCCGTGGGTGGCGTGTTGCTGCTGGGGGAGACGCTGGACGAGCGCGCTATCGCCGGTTG
CGCCCTGATGTTGGCGGGAATGCTTATCTCTCAAATCCGGCTGCGCTGGTGGTGGAAGTCGCGCCGAGAC
AAGGTGCCTGTGACCAAGTAAACCGCGCTCGCAGCATAACAAAACGCCCCGACTGTCGGGGCGTTTTGCA
TGGTGGCTGTTTGGATCAGGGGCGCTGATCCAGCTCGCGCTCCAGCTCTACCGCCTTCTGACGGGCCGGA
TGGGTATTGAGGCGCTGCCAATAGGCTTGCAGGTGCGGGAAGTGGGCCAGCTGACCACTTTGCGCCAGCA
GATCCACCAGGAAGGAGTTCATGATGTCGGCGCCGGAGAGGCGATCCCCCACCAGATAGTGGCGACTCGC
CAGTACCTCATTGAGGTAGCCCAGCACCTTGGCACACTCCGCCTTGGCGTAGTCCGGCAGGAAGCGCATC
GGGCTGCCATCCTTGCGCACGAACATCTCGAGCAGCAGCGGCAGGATGCCGGAGCTCTCGGCAAAGTGGA
GCCACTGCAGATATTCGGGATACTCCGGGCTATCTGGTGCCGGTGCCAGCCGCTCGGCGGCGTAGCGGGC
GATCAGGTACTCGGTGATGGCGCCTGATTCCGCCAGCACCCGGCCGTCGAACTCGATGACCGGCGACTTG
CCGAGGGGGTGGATGGCTTTTAGCTCGGGCGGTGCCAGAAAGGTGGTGGCATCGCGCTGATACGCCTTGA
TCTCATAGGGCTGGCCCAGCTCTTCGAGCAGCCAGATGATGCGCTGGGAGCGGGATTTGTTCAGGTGGTG
CAGGACTATCATAACGACCTCGATTCGGTGGCAGGATGGGGTATACGAGCGGACTAGGGGGGCGGTTCAC
CGATGGCTAAAAAAGAGCGGGCTGATGCCCGCTCCCTTGCTGACTAACTCAGGCCTCCAGCGCCTGATCC
AGATCCGCCAGTATGTCCTCGATCGCCTCGATCCCTACCGACAGCCGGATCATCTCGGGTTTCACGCCAG
CCTTGGCCTGCTCCTCCTCGCTCAACTGGCGATGGGTGGTGGAGGCGGGGTGGCAGGCCAGCGACTTGGC
ATCGCCGATGTTGACCAGCCGCTTGAAGATCTTGAGCGCATCGTAGAAGCGCACGCCCGCCTCGTAGCCA
TCCTTCAGACCGAACGAGAGGATGGCCGAGGGGCGACCGGCCATATACTTCTCCGCCAGTAGATAGTGCG
GGTGGCCCGGCAGACCGGCGTAGCTGACCCAGGCCACCTTGGGGTGATGCTTGAGGTGATGGGCAACCCG
CAGGGCGTTATCCACGTGGCGCTCCATGCGCAGGCTCAGGGTCTCCAGCCCTTGCAGCAGCAGGAAGGCG
TTCATCGGCGCCAGCGCCGCGCCGGTATTGCGCAGTGGCACGGTGCGTACCCGACCGATAAAGGCAGCGG
GGCCAAAGGCCTCGGTGTAGACTACCCCGTGGTATGAGGGCTCGGGTTTGGTGAGTTGTGGGAAGCGCTC
GGCGTGATCCGCCCAGGGGAACTTGCCCGAGTCGACGATGACGCCACCCAGCGAGTTGCCGTGGCCGCCG
ACATATTTGGTCAGGCTGTGTACGACTATGTCAGCGCCGTGTTCGATGGGCTTGCAGAGTACCGGGGTGG
CGACCGTGTTGTCGACGATAAGCGGCACGCCGCGGGCGTGGGCTGCCTTGGCCAGCGCGGCCAGATCGAC

GATGTTGCCCGCCGGGTTGCCGATGCTCTCGCAGTAGACCGCCTTGGTCTTGTCATCGATCAGGGCGGCG
ATGGCTTCGGCGCTGTCATCCTTGGCAAAGCGCACCTCGACCCCGAAACTCGGCAGCATGTGGGCGAACA
GGGTGTAGGTGCCACCATAGAGCTGGGGCGTGGAGACAATGTTGTCACCGGCGGCGGTCAGCGCCTGAAT
GGCGTAGGTGATGGCGGCGGAACCTGCCGAGACCAACAGCGCGGCGATCCCCCCTTCCAGCGCGGCCATC
CGCTGCTCCAGCACATCGTTGGTGGGGTTCATGATGCGGGTGTAGATGTTGCCCGGCACCGCCAGATTGA
ACAGATCCGCCCCGTGCTGGGCGCTCTCGAACTCGTAGGCCACCGTCTGGTAGATGGGCACGGCGACCGC
CTTGGTGGTGGGGTCGCTGGAAAAGCCGTGGTGCAGGGCCAGGGTCGCATCTTTCATCACACACTCCTTG
TTGGATGTTGCTTGTCGCAGTTATAGATGGATAGACGTCCAATCTATCAGGCCCGTGGCCGATGTAAACA
GCAAGATCCGGCAAAGCAATATCGCCCGCAGGGGATGCGCGTCCTGAGTGGCTGGCATGGCCCGTACTCA
GAGACGACGGCGATACTCCTTGTCATCGATCTGGTTCATATCCCACTGCATCAGGCGGTTGAGCTGGCTC
ATGGGATCTTCGATGAGCGACAGCGGGCGTGGCAGGATCCGCTTGAAGTAACGGCGCAAGGTCTGCTGGA
TCCGGTTCGGTTTGAGGGGGTTGGCGTGGCTCATGATGGTGCTCCTCTGCTGTGGTTCTCACGTTCATCA
GCAGTATTGAGCTAAAATGCGGAAGTAAAAACTGATGACTTACAGCAACAGGATTTCCTCTTTTATGCCG
ACCGGTCGCCTCTACCAGCAATTTCAACGACTCGCCCAGCTGCTTGGCAGCGACGAGCGGGAGATCAGCC
TCGCCGAGGTGGCGGATCTGCTCTGCTGCACCCCGCGCAACGCCCGCCTGCTGCTGCGGCGGATGCAGGA
TCAGGGCTGGCTCAGCTGGTCGGCGGAAGCGGGGCGGGGTCGGCGCTCCCGGCTCACCCTGCTCGACAGT
CAGGAGAGCCTGACCCGGCGCCGCTTGCGGGATCTGCTCAGTCAGGGCCAGCTGGCGCAGGCGGTAAGGC
TGGCCGAAGATCGCCTCGATCTGCTCACTCCGCTGCTGATCGAACAGCTCGGTCAGGCCACCCGCGAGGG
GCGCCAGATCCTGCGGGTGCCCTACTACCGGCCGTTGCCGCAGTTGCTGCCCACCGGCCCGATGCGCCGC
TCCGAAATTCACCTGAGCCGCCAGATCTTCAACGGCCTCACCCGGCGAAATGAGGAAAATGGGGAAATCG
AAGGGGATCTGGCCCACCACTGGGAGTGTTTGGGTCCTTGCCACTGGCGTTTCTATCTGCGCCCGGCGGT
GCGTTTTCACCACGGTCGCGAGCTGGTGGTGGAGGATGTGATGGAGAGCCTGCTGGCCCTGCGGGATCGC
CCGCTGTTTTCCCATCTGGTTCGGCTGGAGAGCCCCTGGCCGCGCACGCTGGATCTCTATCTCGACAGTC
CGGATCCCTGGCTGCCCCACCTGCTGGCGGAGCCGGTGGCGACCATTTTGCCACGGGAGCTCAAGGGGGA
GGCGGGCTTTGCCCTGCAACCTGTGGGCACTGGCCCCTACCGGGTGGCCGCCAACGATCCCCTGCATCTT
TGCCTTGCGGCGTTTGACGACTACTTCGGCCTGCGCGCCCTGCTCGACGAGATCGATATCTGGATGCTGC
CTGAACTGGCGGAGCGGCTGGAGACCCACCTGCAGCTGGGGCGCGACAGCCCTGAGCTCGGCACCATGCG
TGGCGAATCGGAGCTGGAGTCGGGATGCTACTTTCTGCTGCAGGACGATCGCTCCGGATCCTTGCAGGAT
CCCGCCCTGCGCCAGTGGCTCGGCCAGCTGCTCAATCCGGTAGCTCTGATGGGGCGGGTCGCCCCCGAGT
TGCAACGGGGCTGGACCAGCGCCCTGGGCCTGCTGCCCCACTGGCGCGAAGCACTGCCCGCGCCCTTGCC
CACACCGGCGCAGTTGCCGACGACCTTGGTGCTTGCCTGCTTCAACCAGCATCTGGAGTTCAACGAGTGC
GCCAACGCCATGAGTAGCCTGCTGGCCGAACAGGGGATCAGGCTCGAGGTGCGCACCCTCGATTACGGTC
GCTGGGTGAGCGGCGAGGATGAGGATGTGGATCTCTGGCTCGGCACCCTCAACCTCGAACATGCCGGCCC
CTTTGCCCCCTACGCCTGGTTGCAGGGGACCCCGTTGCTGCGCAAGGTGTGGAGCAGTCAGCATGAGTTG
TGGCAGGGGTTGATGCAGTGGCGCGCCAGCGGTGAGGAGCCTGGCCCCCGCGCCCTGCTGGCAACTGTGC
AGCAGCAGAGCTGGTTTGTGCCGCTGTTTCACCACTGGCTGGAGCTGGAGAGCCGGGTCGGCGTGCACGG
GGTGCGGATGACGGCGCTCGGCTGGTTCGATTTTCGCAGCGCCTGGCTGCGGCCCGAGCCGCCCCATCAG
CCCGCAGAGGCGCCATAGTTCATCCAATTTCTGTCGCGGCCGATGGGCGCTCCGCGGCCGCTTGCGTGAA
AGATCCGTAAATCGGCCGCTGCTGCACAGTTTGTGTCACGCCTCTGTGCCATGCTGTTTCAGGTTATGGA
CAGCAGAGCAAATGAGGAAAACGATGAGCAATAATTCAGACAGTTCCGCAGGAAAATGCCCGGTCATGCA
CGGGGGAACCACCTCCGTCGAGACGTCCAGAATGGAGTGGTGGCCGAAGAGCCTCAACCTCGACATTCTT
CACCAACACGATACCAAGACCAATCCGCTGGGGGCCGGTTTCAACTATCGGGAAGCCTTGAAGGGGCTCG
ATATGGCGGCCCTCAAGCAGGATGTGACCGCCCTGATGACCGACAGTCAGGAGTGGTGGCCCGCCGACTG
GGGCCACTACGGCGGCCTGATGATCCGTATGGCCTGGCACTCGGCCGGGACTTACCGCATCGCCGATGGC
CGCGGCGGCGGTGGCAGCGGCAACCAGCGTTTCGCCCCCCTTAACTCCTGGCCGGATAACGCCAACCTCG
ACAAGGCCCGTCGCCTGCTTTGGCCCATCAAGAAGAAGTACGGCAACAAGATCAGCTGGGCCGACCTGAT
CATTCTGGCGGGCACCGTAGCTTATGAGTCCATGGGGCTCAAGACCTTCGGCTTCGCCTTTGGCCGCGAG
GATATCTGGCACCCGGAGAAGGATATCTACTGGGGATCGGAGAAGGAATGGCTGGCGCCCAGCGGCAAGC
CGGGTGGTCGTTATTCCGGCGAGCGGGATCTGGAAAACCCGCTGGCGGCGGTGATGATGGGGCTTATCTA
CGTCAACCCGGAAGGGGTGGATGGCCAGCCCGATCCCCTGAAAACCGCGCAGGATATGCGGGTCACCTTC
GCCCGCATGGCGATGGATGATGAAGAGACAGTGGCACTGACCGCCGGTGGCCACACCGTCGGCAAGGCTC
ACGGCAACGGCGATGCCAAACAGCTTGGGCCCGAGCCTGAAGGGGCCAGCCTCGAGGAGCAGGGGCTCGG
CTGGATCAATCACCACAGCCGCGGCATCGGCCGCAACACAGTGACCAGCGGCATCGAGGGGGCCTGGACC
ACTCACCCGACCCAATGGGACAACGGCTACTTCGATCTGCTGTTCAAGTACGACTGGTGGTTGCAAAAAT
CTCCGGCGGGGGCCCATCAGTGGGAACCGATCAATATCGCCGAAGAGGATATGCCAGTGGATGTGGAGGA
CCCGTCGATCCGCTGCAAACCCATGATGACGGATGCCGACATGGCGCTCAAATTCGACCCCGAGTATCGC
AAGATTGCCGAGCGCTTCCGCGATGATCCGGCGGCCTTCTCCGACGCCTTTGCCCGCGCCTGGTTCAAGC
TGACCCACAGGGATATGGGGCCGAAGAGCCGCTATGTCGGCCCCTATGTGCCGACAGAAGATCTGATCTG
GCAGGATCCGGTGCCAGCCGGACGTGCCGATTATGACGTGGCGGCGGTGAAGGCCAGGATTGCCGCCTGC
GGCCTCTCCATCAGCGAGCGTGTGAGTACCGCCTGGGATAGCGCCCGTACCTTCCGTGGCTCCGACAAGC
GGGGCGGTGCCAACGGCGCCCGCATCCGGCTGGCGCCGCAGAAGGATTGGCCGGGCAATGAACCGGCGCA

GCTGGCGAAGGTGCTGGCGGTCTATGACAAGCTGGCGACCGAGTGTGGCGTCAGCGTCGCGGATCTCATC
GTGCTGGGGGGCAACCTCGCCATCGAGCAGGCTGCCCAGGCCGCCGGGGTGACGGTGAGCGTGCCGTTTG
CGCCGGGCCGGGGGGATGCCACTCAGGCTCAGACCGATGTGGCCTCGTTCGAGGTGTTGGAACCGCTGGC
CGACGGCTTCCGCAACTGGTTGAAGCAGGACTATGTGGTGACGGCGGAGGAGCTGCTGCTGGATCGCGCC
CAGCTGATGGGGCTCACCGCCTGCGAGATGACGGCGCTGGTCGGCGGGATGCGGGTGCTCGGCATCAACC
ACGGCGGTACCGGGCACGGGGTCTTTACCGATCGGGTCGGGGTGCTCAGCAACGACTTCTTCGTCAACCT
GACCGACATGGGCTACCAGTGGCTGCCGAAAGCGAGCAATCTCTACGAGATCCGCGATCGCCGCAGCGGC
GCGCTGAAGTGGACGGCCACCCGGGTGGATCTGGTGTTCGGCTCCAACTCGGTGCTGCGCGCCTATGCCG
AGGTCTATGCCCAGGATGACAACAAGGAGAAGTTTGTCTGTGACTTCATCACCGCCTGGAACAAGGTGAT
GAATGCGGATCGCTTCGATCTGCTCGGCTAGTCCCCGAGCCGTTTGGTTATCTGCACCAGCCCCGCACAG
CCAAGATGTGCGGGGCTTTTTACTGCCCGCTTTTCTGTGCAGCCCTACAATCCGCCGCGCTGCGACACCC
GCTGGCCGGTATCGCAGGGCCGCTGGCATCCCTGTGCTTGGGCCAGCAGTATCATGTAACATGCTGATCC
GCTGGTGATGCCCGGCGGCCACCCATTGGCCACTGATGTCCAATCAACAGGACAAGGACAGAACAAAGAG
ACAAGGAGTCAACCCATGCTGGAAATGCAGATCCAACCCCGCTTTCAGGAGACGGATGCCCTCGGCCATA
TCAACAATACGGTGCCGGCGGTCTGGTTTGAATCGGCGCGGGATCCCCTATTCCGGATCTTCTCCCCCGA
TCTGGATGTGCATAACTGGCATCTGATCATCGCGGGCTACACAGTGCAGTTCAAACGGGAGCTGTTCTAC
GGCCAGCCGGTCACCATCAAGACCGGCGTACGCCGCATCGGCACCAGCTCATTTACGGTCTGGCAGGAGG
CCTGGCAGGGCGGCGAACTGGCCGTCACCGCCGAAACGACCCTGATCCATTTTGACTACCAGGCCCGCCA
ATCCCTGCCCCTGAGCGATGCTCACCGGGAAGGGCTGCGGGCCTGGCTGACAGAGAGTTAACCACGGGCC
GGATGGCCCATCACAGGAGTGCTATGTACAACTCAATGCGCAACACAGCGATCGCGCTGACCCTGGGATT
CTGCTCCCTCCCTTCTATGGCCGCTTCTCTCGCCGAGCAGTTTACCCTGATGGAGAAGGGGGCCGAGAGC
GCCCTCGATACCCGTCAATTCAGCCATGACGGCGTCGATATCAAGGCCTGGATCGATGGCGCCCCCGTCA
TCATCGCCGTGCCCATCATGAACGAGCTGGGCAAGCTGGAAGGGGAGAGCCGCTACTACTTCAAGGGCGG
CAAGCTGTTCGGGGTCAAAGAGCCCGCAGCCCAGTTCGCCTTTGACGCCAGCGGCAAGCTGACCCAATGG
CTGGACGAGAAGGGTCAGCCCGCCGAGTTCGTCAGCAAGATGAGCATGCAGCAGCGTCAGGAGTGGCTCA
CCAAGCGGGCCGCCGAGCTGAGCAAGCTGTTTGTGGTGAGCGCGGCCGAGCAGAAGGCGGCCAAGGGGGG
CGTCAAGCTGAAGGGGGCAGATCTCGCTCACTGGCTCTGCAACGGCAAACTGATGGATCTCGCCCATGGC
GACAAGGTGATCTTCGAGCAGGATAAGCTGAAGATTGGCGAGCAGGGGATCGAAGGGGAGGTCTCCCTGC
GTCAGGAGAAGGGGTGGCAGGAGCTGAGCCTCAAGTGCGAGGTGCAGGGCACTCAGGTAACCCGCCTCAC
CTGGCAGCCGCTGCCGGGGGCCAACAAGCCGCAATAAGCGCAATAGCAAGGGGGCCCATCACGGGCCCCC
TTCTCATTCGGTGTGTTGCTCCCCCAGCTTGAGCCGGGTAAGTGCCTCGACCCGGCGCCACTGTCCGGGC
TCGAACCAGCGGGCCAGCAGCTCCTGTTGCTGTTCGGCCCGGGCCTCGTTCGGCAGTTGTCTGAGCTCGG
CCAGAAAGCTGCTGAGCTGCTCCTCGAACCCTTGCCGGTTCTGCTCCAGCTGGCGCATCCGATCCACCGC
TTCCGGTCCCCCCGTCTCCAGCAGCAGAGCCTCCTGTTTGGCCGGGTCCATCTGTGCCAGCTCTTGCAAC
TCCTCAAGCAGACGGCTGTTGGCCTCCGCCTCGCGAAACCAGTCCGGTTGCTCCGCCAGCCAGAGATCCA
GCTCGCTGGCCAGCTGCGACTTGTCGCTGTCTCTTGCTTCCGCCAGCGCCCTGCGGCGCAGGGTCAGCTG
CTCCATCAGTCGATCCTCGGCAAACAGCTGGGCCTGCTCCGCCGGGGTAAAGCTCTGCTGGCGCAGCTGC
TCCCGCTCGTCAAACAGGATCTGCAGGCTGGCGAGGGAGATCTCGGTGGGCACCGTCAGGCTGCTCTGGG
CGGAGACAAAGGTGCGATAGCGCGCCTGCAGGGCATCGGCGGCCTCCTGCTCGGGATCGTGCGGGTGGCT
CTGTGCATGCATGCTGGCTTGAGCGGGGGATGAGTCTGCCGTCGGTGCCGTGGGCCAGAGCAACACCCCG
CCAAGCAGCAGGGTGATGGCGCAGGGCACGACGAGCAGGGTGGCTATCTTCATAGTCCCAGCTCTTTGAG
TCGGTTGGCATGCTGGCGATAGAGGGTGACCGGATCGGTCTCGAACAGATGGTGGATGCCGAAGGACTGG
TTCACTTCGTCCAGATGGTTCATCCGGTAGCCGGTGCCGATCACCTTGCCGAGGTGGCTGCTGCAGACCC
CGACCAGTCCGTCATTGGGCTCCTTGAAGAAGGCGCCGGTCAGCGCCAGCGCCGGATCCACCGGATCCAG
CAGATTGGTCAGGTTGCCGAGCCCGCTCCAGGAGAAGTAGTAGACGCCGTTGGTCGCCTGCATCGGCCCC
TCGCCGCAATAGCGGCTGGGCAGCCCCTCCGGGTAGCGCTGGTTGAACTGGCTGGCCCCCTTGCTGGTGA
GGGCATCGAGGGCGGCGACTGGATCTTGCGGCAGCTGGCTCCCGCCCGAGAGCAGTGAAATCATGCCGGA
GAGGGCGTGGGCCGCGGCGGCAGCCAGCTTCTCGGCGGCGCTGCCGGGGGCGGCATTGGCCCGCACCAGA
TCGGCAATCTCTGAGCCGTAGTTGACCCCGCCGACGCTGGTGGCGGAGGCGACCAGCTCGGGGGCGACCG
AAGCGACATAGCGGATGGTCGGGCCGCCATGGGAGTGGCCGATCAGGTTGACCTTCTCGGCGCCGGTGGC
GGCCAGCAGCAGTTCAATCTGGGCGAGCAACTGCTCCCCCCGCTGCTCGGAGCTGGCGGTAGCAGATACC
TGAGCCACGTAGACCTTGGCGCCATCGCGGCTCAGGGCTTGCGGAATGCCGTAGAAGTAATCGACCCCGA
GCAGCTTGTCGAAGCCGAACAGGCCGTGCACCAGCACTATGGGGTAGCGGGTCTGGGTGTATCCGCTGGC
GTGTGCCAGCATGGGCAGGGTGCAGAGCAGCACCAGAAGCCATCTCTTCATCATCAGCATCCTTGTTGAT
TCGTGAGGAACAGATTGTGTGGCGTACTCCATGTTCGCCGGTGCCAGTATGGTGGCTGCTGACGGGAGAG
GCTGTGAGGGGGATCGCAACTCGGGATTCAAAAGGGATTCAGGGTGCGGGCGGGGAAGCTGAGCTCCCCG
CCCTGCGGCTTATTGCCACTCCACCAGGTGATACTGCTTCTTGCCCCGGCGCAGCAGCAGGTAGCGACCA
AACAGCCGATCATTGGCTCTCAGCTGATCATCTTGCTTGAGCACACCATTGAGGCTGATGGCGCCTGCCG
CCAGCAGTTCGCGGGCGATGCGCCTGGAGTTAGCCAGACCGCAGCTGACCAGCAGGCTGACCAGATCGGT
CTCGCCGCTGACGGTGCTGCTCGGCATGCCATCCTGCGCCAGCTGGGCCAGATCGCTCTCGCCGAGGCGG
GCCACATCGCCGCTAAACAGCAGCTCGCTGATGCGCTGCACCGCCGCCAGCGCCGCCTTGCCGTGTACCA

GCTCGGTCAGCTCGTTTGCCAACACCTGCTGGGCCTGCTTGCGCCCCTGCCGCTTCGCGTCTTCCGCCTC
CAGTGCATCAATCTCGCTCAGCGACATAAAGCTGTAGTAGCGCAGGAAGCGGTAGACATCCTCATCCGCG
GTGCCGAGCCAGAACTGGTAGAAGGCGTAAGGGGAGGTGAGCGCCGGATCGAGCCAGACCGCGCCCCCCT
CGGTCTTGCCGAACTTGGTGCCGTCTGCCTTGGTGATAAGGGGCAGGGTCATGCCGTGCACCTGAGCCTG
ATGGAGGCGACGGGTGAGATCCATGCCGCTGGTGATATTGCCCCACTGGTCATTGCCGCCAATCTGCAGG
GTGCAGCCGAGCCGCTGGTGTAGCACTGCAAAATCGTAGGATTGCAGCAGAGCGTAGGAGAACTCGGTGA
ACGAGATGCCCTGATCCGGACGGGCCAGCCGCTGGCGCACCGATTCCCGCGCCAGCATGGCATTGACCGA
GAAGTGCTTGCCGATATCCCGCAGAAAGTCGAGGGCGGACATCTGCCCCATCCAGTCGCCATTGTTGACC
AGTTGCGGGGCCGACAGGCCCTCATCGGCGGGCAGCAGCGCCCTGATTTGGGCAGACAGGCTGGCGACCC
AACCCTGCACGGTATCGGCACTGTTGAGGCTGCGCTCGGTCGCCTTGAAGCTGGGATCGCCGATAAGGCC
GGTTGCCCCACCCACCAGCGCCACCGGGGTGTGGCCCGCCAGCTGGAATCGGCGCAGCATCAAGAGCGGC
ACCAGATGACCGATATGCAGGCTGCCGGCGGTGGGGTCGAACCCGCAATAGACGGTGCGCGGGGTGGCAA
GATGATCGGCCAGGGCCGCCGGGTCGGAATTCTGGGCCACAAGGCCGCGCTCGGTCAGCTCATCGAGCAG
GTGGGGGTTTATCATGGGTGCTCCAACAGCAGGGGGAAAAGTCGTCATCCTCGCTGTTCTGGCTGGTGGC
GGATATGTCCCCTGAGGGACAGTTTTGCCACTCTCGTGCTAGCATCCAGCCATAGTGGGGAGCTGCTCCC
CGCCACCGGGAACCCACTCGCATGACTCAACAACCATGACCCAGCCATTCACCACAGATGCCTTGACCCG
CAGCATGACCGAGGTGGTCGATGCCCTGCACAGCAGCGCCTTTGCGGCGGCGCTGGTGCGGCTGATCCAG
CAGCAGGTGGCGTTTGATTGCGCCCTGCTGCTCGGGGTGGGGCAGCGCCCCGTCTACCTCTACGACAACC
TCAATCATCAGCGGGCTCTGCTCTTTGATCGCTACCTCACCAGCCACTTCAGTCAGGATCCCTTTATGCA
GGCGCTGGAGCAGGGCTTGCCTGCCGGCGTCTGGACGCTGGCGCAGGTCTCCCAAGGGCGGCTCGATCCC
GAATATCGCTACCACTTCTATCAGGCCACCGGCTGGCAGGAGGAGGTGGGGCTGATCCTGCCAGTGCGGG
ACGGACTGACGCTGATGCTGTTTCTGGGGCGCCTCGACAAGCGCAGTACTCTCAGCCGTGACGAGCTGGC
GAGGCTGGAGCAGCTGTTCCCGCTGGTGCATTCGCTCTGTCGTCAGCAGTGGCGGCAGAGCCAGCCGCTG
CTGGCCCAGAGCACGGCGCTGCCAGATAGCGCCAGCCTCAAGAGCGCGGTGGAGCTGGCGATGGCGAGTG
TCGGGGGGGATCTGCTCACCCGGCGCGAGCGGCAGGTGGCGGAGTTGCTGCTGCAGGGACTGGATACGGA
GGCGATTGCCGCAGCGCTCGGGATTGGCAGCGGCACGGTGAAGAATCACCGCAAGCATCTCTATGGCAAG
CTGCGGCTCGGCTCGCGGGCCGAGCTGTTCAATCTCTTTCTGAACCACCTCATTACGGCGCCGGTGGAGG
CATCACAGGCGTCATCGGCGGGAGATAAAGAGAACCCGCCATAGGGCGGGCCGGAGTTACTCGATGCGCG
GGATGGTGGGAGTGGTTTCGGGCAGGATACTGAGCAACTGGTAGCGCTTCATCAGCTGCTTGTATTCATC
CCCCTGTCTGACCTTGTCCAGCGCCTGTTGCAGGCGGGCAACCTGTTGCTTGTTTGCCCTCAGGTTGAAG
GCGAAGCAGTTATCCTGCTCTTCAATAATCGCGACGGTGACAAAATCCTCGCTGGGGAAACCCAGCTCCT
TGGCCTGATGGCGCAACATCACCTCGTTGCCCGCCACCAGATCGGTGCGTGCCATGATCAGCTGGCGCAC
CACCTGCTCCATGCGCTCCACCGACATCATATTGCCCGGGCTGATCCCGCGGTTGAGTAGCAACTGCTCC
CCGACATCCGCCTGCATCACGCCGATCTGCAGCTTGGCGAGATCCGCTTTGCTGGCCACTTTGGGGGCAT
CCTGCTTACGGCCGACCAGCGCGACCCTGGAGTGGCTGATGGGGCAGACCCAGAGGAAGTGGGGATCCCG
TTCCTTGGTGTGTGCCGTGGTAAAGAGCACGGCCTCTTGCTGTTGGGTCAGCAGATACCAGCCGCGGGCC
CAGGGCAGAAAGTGGATAGGTTGTTCCGGCTCCCCCATTTCGTGCCACATCAGGCGCAGCAACTCGACCG
CCAACCCCACAGGCTGACCATTTTTATCCTGGCCATTGAGTGGCATGTTCTGCTCCGAATAGTAGATAAG
TGCCGGAGCAGGGAGCGCGATAAACAGGGCGAGAGAAGCGAGAGCAGGGAGCAGACGACGCATGATGCAC
TCCTTGTGCGGGTAGTTACCTAACAGTCTATGTGGCCTTTTTCATAACGTCATGATCCGGTAGCGGGATA
AAAAACAGGCCACCTTGCGGTGGTCTGTCGGTTTATTTGAGCAGGGCTTCCATCTCCGCCTCTGCCTTGG
CGCGCGCCTTGGCTTCCGCCGCTCTGGCGGCATCGGCGACGCTCTCTCCGGCCGTATGAGAGGCTGCACT
GACAGCCTGTTTGGCTGGATCGGCAACGGCCTTGGTTGCCTCTTGCGTCACGGTAGCGGCGGCACTGCTG
ACTGCCTGTTTGGCCGGAGCGGCAACGGCTTTGGTCGCTTCTTGTGTCACGGTTGCTGTAGCGCTGCTGA
CGGTCTGTTTGGCCGGAGCGGCAACAGCCTTGGTGGCCTCTTGCGTCACGGTCGCAGCGGCGCTGCTGAC
GGCCTGTTTGGCAGGAGCGGTAACGGCTTTGGTCGCTTCTTGCGTTGCGGTCGCAGTGGCGCTGCTGACG
GCTTGCTTAGCAGGAATGGCAACGGCTTTGGCCGCTTCTTGCGTCGCGGTCGCAGCGGCGCTGCTGACGG
CTTGCTTGGCAGGAGCGGCAACGGCCTTGGTGGCTTCTTGGGTCGCGGTCGCAGCGGCGCTGCTCACCGC
TTGTTTGGCGGGTGCAGTGACTGCGGTGGTTGCAGTGCGGGTAAGATCGGTTGCGCTTTGGTTCACCGCT
TGGGCGGCCTGTCCGGTGGCTTGTCTGGCGGCCTGCCCTGTGGCGTTGGCGGCTGCGCCGCTGATAGCCT
GGCTGGCGCTGTTGGTCAGCGAGCCGGTGACGCCGCGAGTCACTGTGCCCAGCGCGCTGCCAAGGGAGAA
GGGGGAGGCGCCACTCTGACTGGTCGCCGCCCCAGTCAGGGCATTGGTAGTCGGGGTGAGCTGGCTGGTG
GGGGTCTGCCAGCCCTGCATGGCTGTTGGCCAGGTCGGTGCCGTGCCCGCCAGATTTTCGCCAAAGGTCA
GGGTGGTGCCATCCCCTTTTTGCAGCAATACCGACATCTGGTTGGTTTGCGAGTTCCACACCATCCCCGC
CTGCTTGGCGATATCCGGCACCGGCGCATACTCGGAGACCACATAGAAAAGCGGCCCCTCCTGCTGTAAC
TTGTCGGCAGTCTTGAGCAACTGCATGGTGCTGTCGTAGTTGGGACCAAGGATGGCGGTCATCCGATCCT
TAATCAGGGGTTGTTCGAACAGATTGACCTCGTTCGGGTCAAAGCCGACCAGCGGCTTGAGCAGGGCCCA
CATCAGCTGGCTGGTGGGGCTCATCATCAGCAATGGGGCAACGCAGGCGGACAACATCAGGCTCAGGCAG
GCCGCCTGAAGCAACGGCATGATACGCATTGGGATAATATCCAAATAACGATTTGATAAATAAGGTTATA
TTTTGTCAGGACGTGACAAATTCACCGGAAGTTTGCGCGTGGCAGCGAGAGACTGCCCGCAGGCGCTGGC
AGGCTACCATATTGGTTCCACAATGCCAGCAGGCAGTATGATTGTCAGTCAAGTAAACCCCGGGGCTGGT

CGACTCTTCGAGAATGCCTTCCATCGAAACAGGATCCCTCTCATGAAAAATGTACTGCTCTGCCTACTGG
CCTCCCTCTGGCTCGCCCCCGCTCTGGCCGCGTCCAAAACCACTGTGGTGGGCGAGTCTGCCAGTCCCGA
GCTGGTGTTGCCGCTGGGCTCATTGCCCGAGCGGGTCTGCTGGTTTGCGGATCAGAAATATTCCGAAGGG
GCCCGCATTCTGCAGGGAGATATCTGGCTGGAGTGCGGCCCGCAGAACGACCTTGAGAGCAATGGCGCGC
TGGCCTGGCGCTCTCCTGCGGTGGTGTTGCCTGCCACCCGTGGTGCGAAAGCTCAGAAGACGATTTCCGT
GGGTCAGTGAGGCTTTTCTTGATCCAGCTAAAGACATGGACGTGATCCCTGTCGCATAATGCAGCTCCTT
TCTTCTCTTGGCTGGAACCTAAGAAATGGCTCGTCGCTCTTCCAATGCACAACGTCGTCGCAGTAGCTGG
TGGTACAAACGCTTGTTGGCAAGGGAGCGGGAAGAGCGTGAAGCGCGTTCCGTCCGGGATTGATGCAGCA
TTGAAAAACGGAGCCTCGGGCTCCGTTTTGTATTTATCACCAGCTCGGCGGTGCTCTTCCAATGCACAGC
CTAGTTGCCGTAACGGGTGGTACAAACGCTTGTTGTCAAGGGAGCAGGAAGAGCGCGAAGCGCGTTCCGT
CCGGGATTGATACAGCAGTGAAAACAGAGCCTTGGGCTCTGTTTTTTTATTTATCAAATACCGTCATGAC
GAGAACGGGGGCCATCAGGCCCCCGTTTTATTTTTGTAGAGTTGTCCTGCTTGCGGCTTAGCGCTGCAAG
GCGGCCAGTGCCAGTTCGACCCGCTTGGCATACTCGGGGTCGGCCTGCTCGAAGTGTTTAGCTGGGTCG
CGACGATGGTGTCATCCTCCACATTGATAAGGGAGCGGGCCAGGTTGGCGGCGAGCCGTGAACGCTGCCC
CTCGTCCATGATGCGGAACAGGTTGCCCGGCTGGCTGTAGTAGTCGCTGTCGTAGTCACGGAAGTCGTAG
TGCAGGGCAGCCCCTTCCAGACTCAGCGCCGGTTCGTGCTGCTCGCTCGGTTGCTGGGTACCGAAACGGT
TCGGCTGATAGTTGGGGCTGGCGCCGCCATTGCTGTCCGCCCGCATGGCACCGTCACGATGGGCGCCGTG
GTGGAATGGGCAGCGCGGGGCGTTGACCGGCAGCAGGCCGTGGTTGACGCCGAGGCGATAGCGCTGGGTG
TCGCCGTAGGAGAAGAGTCGCCCTTGCAACATGCGATCCGGCGAGAAGCCGATACCGGGCACCACGTTGG
CCGGGGTAAAGGCGGCCTGCTCCACATGGGCGAAGTAGTTGTCCGGGTTCTGGTTGAGCTCCAGCATGCC
CACCGGGATCAGCGGGTAGTCGCTGTGCGGCCACACCTTGGTCAGGTCGAACGGGTGGATGTTGTAAGTC
TGGGCGTCGGCCTCCGGCATCACCTGCACATAGACCTTCCAGCGCGGGAAGTCGCCCCGCTCGATGGCAT
TGAACAGATCCGCCTGACTGGTCTCCCGATCCTGCCCGACGGCGGTGGCGGCCTCTTCATCGGTGTAGAA
GCTGTGGCCCTGCTCGGTCTTGAAGTGGAACTTGACCCAGAAGCGCTCGTTGGCATCGTTGATAAAGCTG
AAGGTGTGGCTGCCGTAGCCGTTCATCTCCCGCAGGCTCTTGGGGATGCCGCGATCGCTGAACAGTATGG
TGACCTGATGCAGGGATTCCGGATGGCGTGACCAGAAGTCCCAGGCGGCGGTCGCGCTGCGCAGGTTGGT
ACGCGGATCCCGCTTCTGGGTGTGGATGAAGTCAGGGAATTTCAGCGGATCGCGGATGAAAAAGACCGGG
GTGTTGTTGCCCACCAGATCCCAGTTGCCCTGCTCGGTATAGAACTTGAGGGCGAAGCCGCGCACGTCGC
GCTCGGCATCGGCGGCGCCCTTCTCACCGGCCACGGTGGAGAAGCGCAGGAAAACAGGAGTCTGCTTGCC
GATGGCGCTGAACAGATCGGCCTTGCTGAACGCTCTGATGTCGTGGGTCACGGTAAAGGTGCCGTAGGCG
CCCGAGCCCTTAGCGTGAACCACCCGCTCGGGAATGCGCTCGCGGTCGAAGTGGGCGAGTTTCTCCATCA
ACCAGATATCTTGCATCAATACGGGGCCGCGCGGGCCGGCGGTGAGGCTGTTGTTGTTGTCGACGACGGG
GGCGCCGTTGGCGCTGTGCAGGGTCTTGTCAGTCATGGCTTCTCTCCTTGTTGATATGGGGTCAGCCTAC
GGATCTGCGCCGTGCTCGGCCAATGGGCCTTTCCTCGCCCTTTGATAGGCAAGACAAATGGGTGCGGGTA
GGGGATGTGGAACCGCGCCGGATATTGCATGATGGTGGCTTCGAGAAGGAGCCTCATATGCAGATTACTA
TCCGTCATGCCGAATCCGCCGATGCCCCCGCCTTGCGGGATCTCTATGCCATGCCCAATGCCCAGGCGGG
CACCTTGCAACTACCCTATCCGGCACTGGGTGTGTGGCAACAACGGTTGGAGCAGAGTGGTGTGGTGGCG
CTGGTCGCCGAGGTAGAGGGGCTGCTGGTTGGCCAGATCGCATTGCATGTGGAGTCTAATCCGAGACGCA
AGCATGTGGCCGGGATTGGCATGGGGGTACGGGATGACTGGGCCGGCAAGGGGGTTGGATCTGCCCTGAT
GGCTGCGGCGCTTGATCTGGCCGATAACTGGCTCAACCTGCACCGGATTGAGCTGACCGTCTATACCGAC
AATGAGGCCGCACAGGCGCTTTATCGCAAGTTCGGTTTTACCGAGGAGGGGCGAGCCCGTGACTATGCCT
TCCGCCAGGGGCGTTATGTGGATGCGCTCTATATGGCCAGAGTGGCGGGGAGCGTCAGTCGCTAACGGCA
TGGTCGATTATCAGGCCAGATTGCGCCCGGTGATCCGGGCGCTGGAGCAGAATCCCGAGCTCTGCCTCAG
TGAGGCGGCCAGTCTTGCGGCGCTCTCCAGCTATCACTTTCATCGGGTCTTTACCGCCGTCACCGGTGAA
ACCCCGGCCGAGATGCAGCGCCGCCTGCGGTTGGAGCGGGCTGCTCGCTCTCTCTGCTACAGTCGCGACC
CGATCACCGAGATTGCCTTCTCGGCCGGTTTTGCCAGCTCACAGGCCTTTGCCAAGGCGTTTCGCAAGCA
GTTTGCCATGGCACCGGGGATGTTGCGCCGCCAGCCGTCACTCTTGCAACTGAGCAAGAATGGACACGCG
GCTGCCCGGGTCTCTGATTATGCTGAAGGCCGTTCCAGTGAAAGGAGGCTACAGATGAAAACCGAACAGA
TGGCTGCGCGCACTCTGGCCTATATCCGGGTCACCGGCCCTTACGGCATAGGCTATGAGCCGGTGTGTGA
CCAGCTCTATCAATGGGCTGTCGGGTTGGGCTTGGCCGAAGGGGAGTGGATCTTCATCTACCACGACAAC
CCCGAAGTGACGGCTCCCCAGCAGTGCCGCACCGATATTTGCGTCACCGTGCCGGCGGGGACCAAAGGCC
GCGGCAATGTGGAGATCCAGCAGCTGGCGGGCGGCCGCTATGGCCAGTCACGTTTTACCGTGACCGACCC
GGCCCAGTACCCCGCACTCTGGCAGCAACATATCGGTGAGGTGGTAGCGGCCGGTTTCGAATTTGATGAT
CGACCCTGTTTCGAGCTCTATCACAGCTTCGATCCGCTGACCAAGGTTGCGGATGTGAGCTTTTGCTCCA
GCCTAAAGGGTTAGGGCACAACAAGACGTAAGGGATGGTACAGAGCATCGCGCAGAAATTAGAGAGCCGG
ACAGTGTCCGGCTCTTTTGCATCTGGCGGTTGGGTGGCGTTTAGCGCTGGAACCCCTTCTGGGCGAACTT
GGGACGTGCGTTCATGATCTGCTCCACCTGTTCGTCGCTCATCTTGCCACGCGACAGCCCCAGCACCTTG
TAGGTGAGCAGCGGTCGGGCGGGCAGGGCGATCATCAGGGCGCGGATCTGGTTCTCGTCATCCTTATCCC
GGGTCGGCTTGCCGTAGGCATAGGTGAGGACGCCGGCATCCATCAGGTTTTTGACGGCGGGCTCGGTGAT
CGGCAAGTTTATGACGCTTTTACGCTGTAGCACGAACTCGCGCAGCACCGCCTTTTCGTTGAAGTCGAGG
AAGCCGATCATCTGGGCCAGCTGGTCCTGCTGACGGCGCGCCTGCCACTCTTCGCAAGCCCACTCCCAGG

CGATCAGGAAGCCCTGACTGCAGAAGTAGGAGATGGCGATCAGCATGCCGACCCCCAGCCAGGCCGCATG
ATCCGCAGCCCACTGGCTGACGGCCCCTTTCATCAGCCCGGCCGGAAACAGCAAAATGATGCTGCAGCAG
AGCAACAACCAGACCATCAGCCAATTGAGGCGGTTCAGTTGATAGAAATGTTGGATCCACCGTTTCATTG
TTCATCCTTCCCAAACTGTTGTGGATAGTGAACTACCTACAGCAATCGGTGTGCCAACAGCGTAATGGCG
CCTGGGAGGGGAGGCTGGCGATACATTTCCTTACAACTTATTGAATGAGTTGTCAGTAATTTGCCTTGTG
AGGTGACGGTCGAGTTGGTAAAACGTCCCCTCAGTCATTGGGGAGTAGCAACCCGCCCGTATTCCGGGCC
GGGCCCATGTCAACACTCTTGGATCCTCGCATCCATGGCATGGGCGGCGATAGGCACTATCGCTCTGCAA
GACCTTTGGCCTCGATATGCACGCCAGCCGGGCGAGCCATGTCGGGTCCATCGGTTATTCTGCTCGCCTG
GCGGTATCTATTATGTTGATTACCCTGCTGTTATTTCTCGTCTCTGCGGTCACTATCTATCTTGCTTGCG
AATACTTCGTCAACGGCATCGAGTGGACCGGACACCACTTCAAACTCTCGCAAAGTGCCACTGGTTCGCT
GCTGGCTGCACTGGGTACGGCCCTGCCCGAATGCATCGTCACCCTGACCGCCCTGCTGATGGGCAACACG
ACCGCCCAGCAGGAGATCGGCATTGGCGCCGCCCTCGGCGGGCCGCTGGTGCTGGCCACCATCGGCTATG
GTGCGGTCGGTCTCACTCTGCTGCTTTGCATGGGGCGTACCGAGCGCGCCATCAATGGGGAGGTACAGCG
CGAGATTGGCCGGGATCAGCGCTGGTTTATGGGGATCTTTTTGGTGGCGACCATGCTGGGTCTGGTCGCC
TTTACCGGCAAGAGCTGGTTGGCGCCTCTGTTCGTGCTGGTCTACCTCGTTTATGTGAGGCGCGAGCTGA
AACAAAGCGACAGCCATGAGGTCGAGCTGCCGGAGCCCCTCAAGCTGGCTCCAAATGCCGGGATCCCACC
CCTTGGCAAGGTGCTGCTGCAGACGCTGGCGGCGCTGGTAGTGGTGGCGCTGGCGGCGCAGCTGTTTGTT
CATCAGCTGGAGAGCATGGGCGAGCTGTTTGGCCTCTCGCCGGTGCTGGCGGCCCTGCTGCTGAGCCCGG
TGGCGACCGAGATGCCGGAGTTGATGAACGTGCTGATCTGGGTGCGACAGGGTAAGAACCAGCTGGCACT
GGCCAATATCTCCGGGGCCATGATGATCCAGGGCACTATCCCCAAGGCGTTCTGTCTGCTCTTTACCCCC
TGGCTGCTGGATGGCGCCATGATGGCCTCCTCGTTGGTCACGCTACTGGCGGTAGGGTTGCTCTGGTGGG
GCTTCAGAAAGGGAGTGATGACCGCAGAGCGGTTGGTACTGGCCGCGCTGCTCTATTTCGGCTTCTGCGG
CTGGCTGCTGGTGAGCTGAAACGGGAAGCGCGGATATGAAAAAGCCCCCTTGGCAGGGGGCTTTGTTGTA
TCGGTTAGGGCGGTGCTTACAGCGCGGCGATCTCGGCCTGCTGGGCTTCCAGCTTGGTCAGCTGGAGACG
GTAATCTTCCAGCTTCTCGCGCTCTTTGGCGATGACCGCTTCCGGCGCCTTGGCAACGAAGGCTTCGTTG
CCAAGCTTGCCTTCGATGCGGCCGCACTCGCCCACCAGACGGGCAACCTCTTTGGCCAGACGGGCCAGTT
CGGCGTCCTTGTCGATGAGACCGGCCATCGGGATCATCAGTTCGGTAGCACCGATCAGCTTCTTCACTGA
CAGCGGGGCGGTTTCACCGTCCGCCAGTACGCGGATGGATTCCAGACGGGCCAGCGACTTGAGGAAGGCC
TCGTTGTCAGCGGCGCGCTGGGCATCCTTGGCATCGCACTGGAGCAGCACGTTGAGGGGCACGCTCGGGG
CCACGTTCATCTCGGCGCGGATGTTGCGGATGCTGACGATGAACTCCTTCACCCACTCCTGATCCGCCAT
CGCGGCTTCGTCCACCTTGGCGGCGTCGAACTCCGGGAAGGCCTGAATCATGATGGTGTCGGCGTGTACG
CCAGCCAGCGGGGCGACCGATTTCCAGATGGTCTCGGTGATGAACGGGATGATCGGGTGCGCCAGACGCA
GCAGGGTTTCCAGCACGGTCACCAAGGTATGGCGGGCAGCGCGCTGCTCGGCCTCGGAACCCTTGGTCAG
CACCGGCTTGGTCAGCTCCAGATACCAGTCACAGAACTGGTTCCAGATAAATTCGTACAGCACCCCTGCC
GCGATGTCGAAGCGGTAGGTATCGAGGGCGGTACGGAAGTCACGGATGGCCACTTGCAGCTGGGACTGGA
TCCAGCGATCCGCCAGGCTGAACTGCATCTCGCCGCCGTTGAAGCCGCAATCCTGATCTTCGGTGTTCAT
CAGCACATAGCGGGAGGCGTTCCACAGCTTGTTGCAGAAGTTGTTGTAGCCGTCCAGGCGCTTCATGTCC
CAGTTGATGTCACGACCGGTAGAGGCCAGTGCAGCCAGCGTGAAGCGCAGGGCGTCGGTACCGTGGGCAT
CGATCCCTTCCGGGAACTGCTTGGCGGTACGCTTGGCGATCTTCTCGGCCATCTGCGGCTGCATCATGTT
GCCGGTGCGCTTCTCCAGCAGGTCTTCCAGCGAGATGCCGTCGATCATGTCGAGGGGGTCCAGCACGTTG
CCCTTGGACTTGGACATCTTCTGGCCCTCTTCGTCACGGATAAGGCCAGTGATGTAGACGGTCTTGAACG
GGACTTGCGGCTGGCCGTTTTCGTCCTTGATGAAGTGCATGGTCATCATGATCATCCGGGCAATCCAGAA
GAAGATGATGTCGAAGCCGCTCATCAGCACGTCGGTCGGGTGGAAGGTCTTGAGTGCCTCGGTATTGTTC
GGCCAGCCCAGAGTGGAGAAGGTCCACAGAGCGGAGCTGAACCAGGTGTCGAGCACGTCTTCGTCCTGAC
GCAGTACGGTTACGGCCGGGATGCTGTGCTTGGCGCGCACTTCATCTTCGCTGCGGCCAACGTAGACGTT
GCCGGCGTCGTCATACCATGCCGGGATGCGGTGACCCCACCACAGTTGACGGGAGACGCACCAGTCCTGA
ATGTCGCGCATCCAGGAGAAGTACATGTTTTCGTATTGCTTCGGTACGAACTGGATACGGCCATCTTCCA
CCGCTTCGATGGCGGTCTTGGCCATCGGTGCCACGCGCACGTACCACTGGTCGGTCAGCATCGGCTCGAT
GGGCACGCCACCGCGATCGCCGTACGGCTGTTGCAGTATGTGATCCTTGATCTCGTCCAGCAGACCCAGC
TCTTCGAGCTTGGCCAGAATCGCTTTACGGGCGGCGAAACGCTCCAGACCGGCGAATTCGCTCGGCAGAG
CTGCATCGTAGGCGGTGCTCGGGTTGCCGTTGGTATCGACCACTTCGGCCTCGGCACGCACGTGGGCGTC
CAGAGTGAAGATATTGATCATCGGCAGGCTGTGGCGCTTGCCCACTTCGTTATCGTTGAAGTCGTGGGCC
GGGGTGATCTTCACGCAACCGGTGCCCTTCTCCATGTCGGCGTGTTCGTCGGCGACGATGGGGATCAGAC
GGTTCACCAGCGGCAGCAGGATGTGCTGGCCGATCAGCGCCTTGTAGCGCGGGTCTTCCGGGTTCACTGC
CACGGCGGTATCGCCCAGCATGGTCTCCGGACGGGTGGTGGCGACGATCAGGTAATCCTTGCCTTCAGCG
GTCTTGGCGCCGTTGGCCAGTGGATAACGCAGGTGCCACATGTGGCCCTTGATCTCGCGGTTCTCCACTT
CCAGATCGGAGATGGCGGTGTTGAGTTTCGGATCCCAGTTCACCAGACGCTTGCCGCGGTACATCAGGCC
ATCTTCGTAAAGACGAACGAACACTTCCTGCACGGCGGCGGAAAGACCCTCATCCATGGTGAAGCGCTCG
CGCTCCCAATCCACGGAGTCGCCCAGACGACGCATCTGGCGGGTGATGGTGCCACCGGACTCTTCCTTCC
ACTGCCAAATCTTGTCGATGAAGGCATCGCGGCCATAATCGTGGCGAGTCTTGCCCTCTTCTGCAGCGAT
CTTGCGCTCAACCACCATCTGGGTGGCGATACCGGCGTGGTCGGTACCTGCCTGCCACAGGGTGTTCTTG

CCCTGCATGCGGTTGTAGCGGATCAGGGTATCCATCAGGGTCTGCTGGAAGGCGTGACCCATGTGCAGGC
TGCCGGTGACGTTCGGCGGCGGATCATGATGCAGAAGGAGTCTTTGCTGGTGTCGCCGTGGGGCTTGAA
GTAGCCCTGAGCTTCCCAGTGCTGGTAGAGCGCCTGTTCGATGGCGTTGTGATTAAAAGTCTTTTCCATG
GTCTCGCCGTAATCTGGATGGATGGAGTCTGGTGTTGATTCGTCGTGGATCACAGGCGGCGCAGTCAGGG
CTGCGCCGGATCCGGTGCGGCAAGGACGGGCTGATCCCTCATCTCGAGGGCATGGCCTGCCTGGCGATAG
TGCTTGTAGCGCTCGCGGGCTTGCTGCTTTTGTGTTTCGTCAGTGGGGACAAAATCTACCACTTGAGCGA
AGTGTCCTGCAAACAAAGGTGTCTGTTTGGCCAGATTGATGAGGCGGGCGTAGCGCCGCCGTGGCGGCTG
GTGGCCGATCTCCACCGGCGCCTGACCGCTTTCGCCCTGCAGCTGGTGCGGCACGAAGCGCTCCGGCGGC
AGTTGCCACAGCAGCTCGTCGAGGCGCTGAGCCTGAGCCTCATCGTCGCAGAACAGGTAGATATGGCCCG
CTCCCCAGCTATCGGCTACGAGCTGGCAGGCGAGCCGTTCGATGGCCGACGCCTGCGCGTCATCCTGTTC
GCTCATCAGGTAAAAGGTCACTTGGCTCATCAGGTTGATCCCGTGTTGCAGTGACGGACTATTGACCGCC
TCATGAGGCTCGCGGGGTGTTTCTCCGCTCGCAACTCACTGATTCAACAATGAAACAAGGAAGAGGCGGG
CCTCTTCCTTGTGATTCAGGGTAACGCCAACCCCGTTGATGCACGCATCAGACAGGGTGGGAGAGTTACT
CTTTCTCTTCGATATCCACGCCAGCCCGGTTCAGCAGGAACTGGGTCAGCAGGGGCACCGGACGACCGGT
GGAGCCTTTGTCCTTGCCACTCTTCCAGGCGGTCCCGGCGATATCCAGGTGGGCCCAGTTGTACTTCTTG
GTGAAGCGTGACAGGAAGGCGGCCGCAGTAATGGTGCCGGCCGGACGACCGCCGATGTTGGCCATGTCGG
CAAACGGGCTGTCGATCTGCTCCTGATACTCGTCGAACAGCGGCAGACGCCATGCGCGATCACCCGCCTG
CTCGGAGGCGTTGAGCAGCTCGTGGGCCAGCGGGTTGTGGTTGGCCAGCAGACCCGAGGTGTGGTGACCC
AGCGCGATGACGCAGGCACCGGTCAGGGTCGCCACGTCGATCACGGTTTCCGGATCGAAGCGGTCCACAT
AGGTCAGCGCATCACACAGCACCAGACGACCTTCCGCATCGGTGTTGAGCACTTCGATGGTCTGGCCGGA
CATGGAGGTGAGGATGTCACCCGGGCGATAGGCGTTGCCACCCGGCATGTTCTCGCAGCCTGCCAGCACG
CCGATGACGTTGATCGGCAGTTGCAGCTGGGCCAGCGCATGCATGGTACCGAGCACGGAGGCGGCGCCAC
CCATGTCGTACTTCATCTCGTCCATGCCGTCGGCCGGTTTGATGGAGATGCCGCCGGAGTCAAAGGTCAG
ACCCTTGCCGACCAGCACGATGGGCTTGGCATCCGGATGACCCTTGTATTCGATGATGGCCATCATGGCT
TCGTTGTCGGAGCCGCGGGCCACCGCCAGATAGGAGTTCATGCCGAGTTCGGCCATCTCCTGCTCGCCCA
CCACCTTGGTGGTGATGTTGTCGTAGGCATCGGCCAGACGACGGGCCTGGGAGGCCAGATAGGCCGGGTT
GCAGACGTTGGGCGGCATGTTCGCCACGTCGCGACAGACCCGCACACCCTTGGCCACGGCCAGACCGTGA
GCGATGGCTTTTTCACCAATGGTCAGTTCCCGACGCGTCGGCACGTTGAACACCAGCTTGCGCAGCGGGC
GACGTGGTTCGGCTTTGTTGGTCTTGAACTGATCAAAGCTGTAGAGGCCCGCCTTGGTGGTTTCTACCGC
CTGGCGCACCTTCCAATAGGTATCCCGACCCTTGACGTGCAGCTCGGTCAGGAAGCAGACCGCTTCCATG
GAGCCGGTTTCGTTCAGGGTGGTGATGGTCTTGTTGATGATCTGCTTGTACTGGCGTTCGTCGAGTTCCC
GCTCCTTGCCGCATCCGACCAGCAGTACGCGCTCGCTGAGCACGCCCGGTACTTGATGCAGCAGCAGCAT
TTGGCCTGGTTTCCCTTCCAGGTCGCCACGGCGCAGCAGCGAACTGATATAGCCATCGCTGATCTTGTCC
AGTTGTTCGGCAACCGGAGAGAGGCGACGAGGCTCAAATACGCCGACTACGACGCAGGCGCTCCGTTGCT
TCTCTGGACTGCCACTCTTTACACTGAACTCCATGGAATCTCCTACATCCTAAAGACAAAAGGCACCTTT
TATTGAATAATGCGGTGCTTGTTGGGGTATCAGAGGCGGAGCGAACAGCCACAGGCTCCGCAGTTCAAAC
TGGTAAAAATCCAATATACGGCCCTATTTTTCAGGCATTCGACTAAAAACACAAGTTTACTTGGGAATGA
CTGTGATCGTTTTCCGATATTTGTTCAGGGAAACCCTGAAAACCCAGCTGGCAGTGTTATTCGTATTGCT
GCTTATCTTTGTCAGCCAACAATTTATCAAGATCATCGGTGACGCCGCCGATGGCGAGGTGCCGACACGT
CTGGTCTCCACCCTGCTGCTACTCAACTTGCCCAATATGGCGTTGTTGATGCTGCCTATCAGTCTCTTTT
TAGCCATTTTGTTTGCCCATGGTCGCCTTTACGCCGAAAGCGAGATGACGGTGATGCATGCGGTCGGCTT
CAGCCACCGTTTCGTGATGCGCAGCGCCATGTTGCTGGCCTTGCTGACGGCGGTGGTTGCGGCGTTCAAC
ACCGGCTGGATCGCCCCGCAGGCCAAGGAGCGCGAGTCGCAGGTGATCGACCAGTTCAAAGCCGATCCGG
GTATCTCCTTCCTGCAGCCTGGCCGCTTTATGGAGCTCGACAAGGGGCGTCTGGTTGCCTATATCCAGGA
TTTGAACGACAACGGCTCAAAACTGCAAAAGATCTTCGTGCTGCAGCGGGCGGAGGGGAACAGTCCTCCC
TCGGTGGTGGTGGCGACCGAAGGGGTGGTGACGGTCGATGAGAACGGTCTGCAGTGGCTGACCCTGATGG
ATGGCTCCCGCTACGAGGGGAACTTCTCCGGCAAGCAGTTCCAGATCTCCGATTTTCGCGAATATGGTCT
GGTGATCCGCCAGCAAGAGATGGAGCGTTCCAACCGCAAGGCGGCGGCCAAGCCGACCATGGAACTGTTC
GGTACCCAGGACAACGCCATGATGGCGGAATTGCAGTGGCGTATCTCGCTGCCCCTCTCCATTCCGGTGC
TGACCCTGCTGGTGGTACCGCTGGCGCGGGTCAACCCCCGTCAGGGGCGCTACGCCAAGCTGTTGCCGGC
CATCATGCTCTATCTCTCCTACTTCCTGCTGCTCAGCGCCGCCCGTTCGGCCATCGACAGCGGTCGGCTG
CCCTACTGGCCGGGGATGTTCCTGGTGCCGCTCGGTTACCTGCTGTTTATCGGCCTGCCCCTCAATCTGC
AGGGCACCAGCTGGTGGAACGGGGTGAAAGGGCGCTATTTCAACAGAAAATCAGCATGATGCTGCAACAT
AGGAACTTCTGCTGATGTTTGGCATTCTCGATAGATATATCGGTAGGGTCATCTTTATGTCGATCCTGCT
GTGTGAAATCACGCTCGTTGGTCTGGCGGCGCTCATCAAGTATGTGGAGCAGCTCAAGTCGGTCGGTGAC
GGCAACTACGACATGATCAACGCCCTCTACTATGTGCTGCTCTCCATGCCCAAGGAGGCGGTACTGTTCT
TCCCGCTGGCCGCGTTGCTGGGTGGCCTGATTGGTCTGGGTCAGCTGGCGACCAGCTCCGAGCTGGTGGT
GATGCAGGCGGCGGGTCGCTCCAAGATCAGTATCGTGATGGCGGCGCTCAAGACGGCGATCCCGCTGATG
ATCATGGTGGGGCTGATGGGGGAATATGTGGCCCCCGCCGCCAAGCGGATAGCTGATGACATCAAGGCGG
GCGCGCTGTCCGAAGGTCGTCTGACGGTTTCCGCCTACGGGGTGTGGGCTCGCGATGGCAACAACTTCGT
CAATATCAACGGGGTGCGCAACGATGGCGCCCTCACCGGCATCACCCTCTATCGCTTCACCCCCGAGCGC

AAGCTGATCGACGTGGTGCAGGCGCAGGAGGGGGTGTTCGAGCGCCACCACTGGCTGCTCAAGCAGACCC
AGGTTACCCGCTTCGACGATCCCGCGCAGATCCGCAGCGAGCAGCATGACAAGATGGAGTGGAGCTCGGA
GCTGACGCCCAAACAGCTGGGGGTGGTGAGTATCGACCCCGAGAACCTCTCGGTCTCCGGTCTGCTCGAC
TACATCGGCTATCTCGATGCCAACAAGCAGGATGCCGGTCGCTACAAGCTGGAGATGTGGCGCAAATTGC
TGTCACCACTCTCGGTGGTGGCGATGATCCTGCTGGCCTCCTCCTTCATCTTTGGCCCGCTACGCTCGGT
GAGCATGGGGGCGCGGATGCTGATGGGGATCATGACCGGCTTCGCCGTCTACGTGAGTGATCGGGTGTTT
GGCCCCATCAGTCTGGTCTATGCGGTACCGCCCATTCTGGCGGCTATCGCGCCCAGCCTGCTGTTTGGCG
GTATCTCCTTCTACATCCTCAGCCGCAAGCAGTAATCCGCACGAGGTTATGAGGAAATGCAAAGAGGGCA
CCTGTTGGTGCCCTCTTTCGTTATTGCATTGATCCGGGATTACTTGTTCAACAATTCCAGATTTGCCTGC
TTGCTCAGCACGATCACTTCGCACTCGGCCCAGATATCCTGAAACGCCCGCGGGCGCTGGCGGTTGAACA
GGCACATCAGGTTGCCCAGACCAAAAGCGGCAGTGGCGAGACGGATCAGCGCCTGGGTAACGCGGATGTG
GCTGCCATCCTCGTTCTGAATGCGCAGCCGCCAGGCGCGCATACCGACGGTCTGGCCAGCGCGGGTCCAG
AACCAGGTGTAGAAACCACAGATGACGAAGGCGAGCCACAGGGTGTAGATCAGGCTCAGGTGGTGGCGGG
TCAGCAACCAGACGGTATCTTCACCCGCCGGTACGCTGGCCATGCCGGTGACCAGCAACAGCTTGGCGAC
ACCCATGCCGATAAAGCCGGCGATGATCAGCAGCGAAGTCACCACCAGCGCATCATAGATCAGGGCGCCG
AGGCGACGCAGCAGACCCGCTGACGGGTATGGCTGAGTGGCGGGAGAGGGGGGATTTTTGTTGGACTTTTT
TAGGCTTGGCCATCGTTTTTATTCCATTAAGATGATGGCGGCATTCTATGTAACTGGCCCTGCATCGGCA
AGGCGTTGAATAAATGAACAAACGGGAGTGTTCAATCGAAAAACACCTTGCATGGCATCGCGCCATTCGT
ATAATCCCTCGCAGTTGCCCGGGTGGCGAAATCGGTAGACGCAGCGGATTCAAAATCCGCCGATGAATAA
TCGTGTCGGTTCGAGTCCGACCCCGGGCACCATTAGAATTCAATGACTTAAGGCCATCCTAGCGGATGGC
CTTTTGTTTTTCTGTGTCATGGTGGCGGCAAAATGGCGGCAGGTATTTAGATGATCGGCGTAAACCGATG
CTCGATTCTTCTTTCCAGTGACTCAGTTCATCTCAATTCCAAGTGTGTCCTACCCATTTTTTTTTAGCCT
TCTGCTAGAATGCTCCCCCGTCTTGAGCATCCAACTGAGATTTTTTCGTGACAGTAAAAGAGTGCCCCGA
TCAGCCTGATTGGAAAAGCATGGAGTTGGAAGACCTGAAGGGCCTTGGGGTAGAGAAGAGGCTTCCTGCT
ATTGAGCAAGAGCCGCCGGCTGCTACTCAAGAAGATGCAGTTGCGGTGTTGGTAAGTAAGTTTGGTTTTG
TCGGCAACGAAGCATCAGTGATGATGGCTACACCAGTCGGTGATATTCAGGTGTGTCGGGACTCTTTACC
GCACATTGTTGAGAAAAGGCCGCATGCCAGAGAACGTTACGTTAATCATGCTATCGCAACTCTGCAGAAT
CCCTTTGAGGTTTGGGAGGTTGCTTATGACGACGGAAGCTATCGGTATGCCTTTATAGGTACCTTTGAAG
AACGAAACATGATGCTGGTTGTTGCCACAATTACACCAGATAAGATCTTGTGGAACTTCATGCAAAGTGA
AGCAAAATCGATGAACAAGCATAGACATGGCAAGCTGGTGTATGCTCGTCATAAATGAAAAGAGGCAACC
TATGGTTGCCTCAATTTTGAGCTTTCATTCTGCTTGTACTTTGTACTCACCCAAGCTGGTGAGGAAAGGT
CTCACCCTCACAATGCAGTTGGACATTCCGGGGTTCCTCTATCGGTCATTGCCCCAACTGGCCACCGACT
TTGCGCTTTCGAATACAACATTATTAAAAAATGAGGAATGCGTCAAGAATTTGTAGGCGGTTTCCGCCTA
GTTGGATGGGGCTTTTGGTTACAAATTTTCTACATTTGCATTTCAGCGATAGATAGAAATGCTTCACTCG
TGAAATTATGGATCCTTGCCAAAACCGCCCGCCAAGCCTTGCCGCATAAGGCTTAACAGTTTTTCCCTTG
TGGTCGCACCAGATCCACCGTGCCGACTCAGATCCTCACGGAAAGTAACCGCAATCCGTTTTCCATCAAT
TGCTTACGCTTTTCTCGCCGCCTTGTGTGTGTGATCAAACCATCCCAAAACGATCATCAAATGACGTTTT
GTGAAATTCCGAATTCCCCCCAAGAACCCAGCCACGGCGCGGGCTAGACGATAGATTTGCACTATTTTAT
TTGTGCAAAAAACGCCACATATTTAGCCCGAAGGTGGGGGGGGAGGAGTGCGGATTCCGTGAGCGGCGCG
TTTCCTCCGTCGCCACTGGGCTGCAGGGCAGGGGGCTGGCCACAAAAAAGCGACGCTGGTGCGTCGCTGT
TGTGTGCGGGGTGATAGTCGGCGGGTTACTGGCCGGCGCTCCATCCCATCTTCTGGAATGCCTTGGCGGC
AATAGCCAGCAGTTCGTCGTTGTTGGCCTGGGGGAGCATGGGGCGGGCGGGGGTCTCTACCGTCCAGCGG
TGCTTCCTGGTCTCTCCAGTGGTGGTCCTGATCAGGATGGCAGCCCGGGTAACACTGACATGTTCCCGTA
TCCACTTGAGGGAGGGCTTGCGGTAGCGTCGACGAGCGCCCCGGTCTGACAGCACCTTGTACCCCAGCGC
ACGCAGACGCTGCGCCTGGCTTCGGGTACAAGGGCTATCTGTTGGTTTGAATGGTTCTTTCTTCTTGCCC
TGTTCTGACTTCCTCGGTTGTGCTGGCTTGGGCGGCGCTGCCGTGAATGGCTGTGACATCCCCTGGTGGT
TCTGGTTGGCGATCATCCCCGCAAAGCGGGAGTCAAAGTCGATGATGGCCGCATCCTCGGTACCACGAGA
GCGCAGGCGCTTGGAGAGGCCCGTGAGCCGCTTGTGTTTGCGTTTGCTCTTGGTGGGGGCCCACTTTGTA
CCATCAGGTGCTTTGCCTGCACGGATGCGCTGGCGGTTTACCTTGGCCACTTCGCGGCCTATCTGCTGGG
TGATGCGCCGGCGTTTCGCCGGCGGCAGAGAGGCTGCGTCCAGGCGCGCCAGAAGGTGGTGCATGGCGCC
CTGATTAAGGTTCAGTGTGATCATGTGCGCATCCCTATTCGATGATGGGGGAGAGCTGGTCGGCCAGTTG
TTTGGCTTGTTGGCCATGTCCGGTCATCGCCCCGCTGTTACCAGGCACCGGGCCGCTGCCGTGGGTGTGG
CTGGCCGTGGCAGCGGCCAGCTTCTCCACCACGTTCATCAGCTGCAGCAGCAGTCGGAAGATGTTCACGC
CCTCGGTCCCCATCCACGAACGGGGCGCCTCCAGGTGTTGCAGCTCGCCGGCAACTGCCCGGCGCAGTTG
TCCCACTACCTCAACCAGATCCCCCGCCGTGGTCTGGCTCATGTTGCCCAGACTTCCCAGGGTGAGATCA
TCGCCGGCCAGCAGCTCGATGGCACCGAGCGCCTCGATGAGCTTGAGCCCGCCCACCTCCTCGATGCTGT
GCTGGGCTATCTGCAGCCGGTGCTGGCCATGCTCTCCCAGGTAATCATCACTCTGGTGGTGCATCTGCAG
GGCGCGGTCATGCAGGCGTCGGTCGGTGTGGCGGGAGAGATTGCCCACGGTGTCGGTGCGGCTGGCCACC
TCGGCCCGTTGCTGCTGCAGCTGCTCGCCCGGGGCGATGTCCGGCAGGGGCCAGCCACTGCCGAGCACGG
TGCGGATAAAGGGCCGGTCGGCCCGCCCGAAGGCGAACCCCAGTTCAACCAGTGTCCCCTCGATGGGGAA
TTGCATCAGCCCCTGCTCTGGCCCGCCAAACTGCACCGGCAGCGGTACCGCCCGATAAAGCGGGGTGGCC

TGGTCCGGCTGGCCATCTTCGCCCAGCAGCTGTACGTCCACCGCATAGCGGGGGCGAAAGGGGTCATTGA
GCTGGCCAGCGGCCGCGCTGTCGCTGATGGCCTCAACCCGCCCGAACTTGGGCAGGTGCATGTTGTCGGC
CAGCTCCGGGAACTCCCCCTCTATCTTGCGCCGTTCCGGCGTTTTCACCGGTTTGCCAGGTGTTGCTGTG
GTCAGGGTCATCTGGTCGCCCTTAAGGCGCACCCGCATCACCCGCTTGCCGTTGACGATAGCGCCTGGGC
GCATGGTCGGGATCGGCGCCGTGGTCAGCGTGTTGCCCGCTTGATGGCTCGAAAACGCCGGATCCAGCGC
TACGGCACGCTCAGGCCAGCGGCTATGGGCATGGCTGCCGACGAAGATGGTGCCATCGGGTTGTTGATGC
CAAATGAAGTCGGGGACCGCGAAGGCGCGACCGGCATGATCGAGCAGCTGATAGCCGGTACCGGCGCTGG
TGAAGTTGGGGATAGGCCGGTCGGTATAGTCAGCCCCATCAGGTAACAGGAAAGTAAGGCTAGTCTGGTC
AGTCAGCCAGGCCAGCAGGCGGCGCAGGGTGGCATGTTGGAGGCTGACCGGAAAGCGGCTGCCCAGTATC
CCCGCCAGCTCACGGCAAAGCAGTTTGTTGGTGCCATTGGCGGCGGGTTGCACGTCATACACGTAACCGG
TGAACCAGCGGTGCAACTGGTTGTTATAGCCGGTGTCCACGGTCAGGGTCTGCCCCTTCTCGGCCTGGCC
TTCGATGGTGAGAGCGGCACGGCCGCCGGCGTTGAGGTCCAGCACCATATCATGGTCGACCAGGTGGACA
GGTTGACCCGCCAGGGTCAGGCGAGTGGACAGTTTCACGCTAAAATATCTCCTACCTTGCCATCAACCCA
TTTAAGGCCTTGTTCAAAGTGGTTCAGTTTGTCTTGTTCTGGTGCGTCGTTGCCCTTTACTGCGGTGGTA
TTACTGGTCCCTTTTCCGACAGAGGCAGTCGTTTTTGGTAGGCGTTGTTCCCGCTTCTCGGGCACCGAGT
TGTGTTCGCGCAGGGTGAACTGCACTTGCCACGCCAGCAGCCCCTCCTGCTCGCTGGCGGTGATGCGGCC
GGCAAACTTGGCCTGGCGCACCTTCACTGACTTGGCCAACAGCGAACCGACCCGGTAAATATGGCGCTGA
CCGCCATCCCCCTTGGCGCTGGCCAGTTCAAACAGCCGGCTCAGCATCTGCTCATCCTTGAACGGGATCA
GGCCGGAGACATCGAGCTCCTTGGCCTTCTCGCCCTGCTCCGAGCTGCTGGTTGCGCTGGTCTGGCCGCT
GTTGTCCTTGTCCTGAAACTGCATCGATGCGGAGACCCGCATCGACTTCATCACAATGGGCTCACCATCC
AGGGTAAGCATGGCGGTTCGGCTCATCGGGTTAACTCCTGCCAGAAGGTGAGTGGGGAAGGGGAGAGCAG
CAGGGCGCCGACCGTCATGCTCTGACTATGGTCTGGCGGGCTGCTCTGGCCCAGCTGGGCGGCCAGGCTG
GCTGCATCCCCCTGGCCCTGCCAGTGCCAGAGGGTACCGGAGAGTGAGCCCAGCCTGGCCAGAGCCTCGG
CGAGGGTGGCCAGGCGGTCGGCACGGCGTTTCGCCAGCCCCTGCAGCTTGGTGATCGGGGTCTGGCTGTC
CCTGCAAAGGCTCTCCAGTTGGGCCAGCTCTGCCCCCAGCGCCAGGCGGGCCGGGCGTAGCGGATCCCAA
CTCAGAGGCTCGTCTGCTTTCCAGCGTGGCACCTTGGCGGCGGTGGGCTTGCTCATGGTGTCGTTGTTGG
CGGTGAGTCGGCGCAGGGTGGCGCACCACTCTGGCAGCGGCAGGATGGCGCAAAGCGGCGTCAAGGCATC
GGCCAGCGCGGTCTCGCTGTTGCCGGTGACCAGCCAGGCGAGGGCGTGCAGCTGGCCAGTCGGTAGCAGG
GGATCGGCCCCGTCTTGCAGCTTGGCGGCAAGGGTGGCCACCGCATTGGGGGCAGCGAGGCTGTACTGGT
TGCCCTGGTGCTGGCCCACACCATGCTGATACGCGGTCACGGTCAGGCAGCGTCCGGTGACCAGTAGGCG
GTCGAGCTCGCTGCGCAGCCCTGCCAGCGCGGCGGCGGTCTCGCTGAGCGGGTGGGGCCGGTACTGAGCC
CGCCCCGCCAGCCCTTGCAGGCGGTTTACTGCGGCTGATTGCGCGCCCGGGATCTGGCTTGTCACCCCGT
TGGCCGCCTCATGGATGGCGTCAGCCGATGCGGGCCATACCAGATGGCCCTGGTGCCATGTCATGCCTCG
GGCTCCTCGGTGGGCTCGGTCGGCAGGATCGGCTCAGGGTTGACCCACTCTTTGCCGTCAAAGGTCGCGC
CAGCGATCACCCAGTCAGGGCAGGGCACAAACTTGGCGGCCAGCTCAGCCACATGCACATCAGCCGGGGT
GATGTTCAGGCCACCATCGTTCCAGACTTCCGTCACGCGATCTTTAATCAGTCTTGCGTAGTAATTCATC
CCAATACCTCCACAATCACGGCCCCCGGGCCCCCTCCGCCGATATTGGCCTGATGTGCAGCGTTGCTTGT
ACCCTGGCCAACTCGGCTATAGACAGAGAGATAGTTGCTGCCGCCGATCGCCTGGAGCGTGAGATCCGCA
TCCAGTGGCAGGTCGTGTGGGGTGATCCCCAGTCCCCAGCCATCGTCGTACAGCAGGGGGCGAAGCCCAT
CGACCCAAGAGCCCCCCACACCACCCGCATTCGGGGCGTTCGCGTTGTCTAGCCCATTGCCACCGGCGCC
ACCGAGGCCGTAATGATGGCCTGAAGCGCCGCCACCACCGCCACCACCAGAGGCCGCATCAAAGGCGTTA
CCGCCAGCACCGCCAGCGTGATTGATGGTGCCACCGATGCCGGCTCCTCCAGCTCCGCCTTTACTGGCGA
CCGCGGTGTTATCGGTGGCGGTCTTGCCGCCGGTGGCGGAGAGCAGCGCGCCGAACGAGGTGGTGCCGCC
GTCCTGATTCAGGTCGCCGCCCGCCCCCACGGTGTAGGTGAACGTTTGGCCAGGCACCACATCCAGCTCG
ATTTCGGCATAACCGCCGCCTCCACCACCGCCGCCAAAACCGTGCTGGTCTTTGTTGGTGACGTTGCGGC
CACTGCCACCGCCACCCACCACGGCGACCCGGATCTTGCTCCAACCGGTCGGGATAATGAGCTGGCGCGT
GCCAGGGATGATGTCCAACACTTCAATGCGGTTTCTAAACCGCCCAAATCCAGTGCTTGTTCCGGCACTG
CCTCCCAATGTGATCATGCTGCCCTCCACTGTCCGGCAATCTTCTCGAAAATAAACTCGCGACTGGCTTG
GCGTAGTCTGATTTGACGGTCAGCCCCGGCGCTGGTGCTGATCGTCCCGCTGGTTAGCTGCACGATGCAG
TCCCCTGCCGCCATATCCACGCCGTGATCGACGATCACGCCGAGGCGGCTACCATCCGGCCATGTCTCAT
CCAGGGTCACGACGGCAGGTTTCATAAATCTCAGGTGTGACGGGTATTTCCCGTTGGCGGGCAGCGAACT
GTCGGCAGTGATCGCCGATGCACTGAACCCGGCGCGCAACCCATCGCTGATCGCGGTGTTGATGTAGTCG
GTGAAATCAAACTGCCATGTCTCCGGCGTGATGGTCACATCCAGCGCGGCGCTGGCGCGGGCGAATTTCA
AGATAAAATTACGGATCAGGGTGTCGCCCTGCTGGTAGGCATCCTTGGCCACCTTGACCGTATCAGGCAG
ATAAGAGACTGCGACCTGGGTGCCATCGCTGGCTTCCAGCCCCATCCAGTTGAATGTAAACGGCCCCACG
GTCGGTTCCAGCAGCAGGGAATAGACCACCTTGTCCGGCGATATCGCGGCGGCTCTGGTGACCGGCCCCC
GATATTTGATTTCCCCCGGTGCGGGATCCGGTTCATCCGGATTCACCGGGTCGGTGTAGTTCAACCCCGG
CTTGTAGGCCAGCACAACCTTGTCGATGTGGGTGGCCACGTGCAGGGCGAGATTCTGGGAGATGAGTGCC
ATCCCCCGATTCAGGATTTCGGCCATTTAGCCTCCGTAGTTACATAGCTGGCAGAGAGCGGCTGGGCCGC
TATCACGCAATGCCCCCAGACCGAACCGGCCAGTTCGACATCCGTTGATGCGGTGATGGTTTGATAGCTG
GCCGAAAACTCGGCGCAGCCCAAGAGCTGCTGGTCGGTGAACGCGGTTACGCCCACGTCATAGCGACGAC

AGGTGCGGCGGTACTGCTTCACCAGCTCGTGGGCCAGCCGCTCCGTCAGTTGGTCGCTGTGCTCATCGAG
CAACAGCAGGATGATGTCCCAATCCATGCCGTCGATGCGCTCCATCTGGGCGCGCAGGGTGATATCGAAC
CGGCCAAAGATATCGACAAAGCCTGCGGTACCGCCCGCGTCTCTGGCGTTGATGAAGGCGAACTTCACCC
GTTTGCGAAACAGGTCGAGCGGTTCGCCCTTGAAGCGCGTGATATCCCGCTCCCAGGCGAGCAGTGCCAG
CAGGGACTCGGAGCAGGTCAGCGGGTCTTGTTGCGCCAGCGGCTGTACCAGCCAGCCTCGCAGTCGGCGC
CACAGGGCCATGATGCCCCTGGCCAGGAAGGCAGGCTCCTGCACCCCGTCCGCGATGCTCTTGCCATCTT
CCCACCACGGGGCGGTGCTATCCGGCAGCTGGGGCGCGGCGCTCAGATGGTCGATGCCGGTGGTTTTATT
CATGGAGCGTCACCTCCAGCCCCGAGAGGCGTGGGATAGCGAGCCCCGAGAGGATATCCAGCTCGGTGAA
GTGCAGGCTCTTGATCTCGGGGAATGCCTGATGCAGCTCACGGCCCAGCTGGCTTAAGGAGAAACGGGAC
TGCGGCCATGTGCGAGTCACGGTCGGATAGTCAGCCGACAGCCGGAACGCCGCATTGACCAGCTGCCTGA
CCCCCGCGACCAGGGCGGCCCGCTGGGGCTCGCCAAGGTTATCGACTGGCCACAGCTCCAGATGCAAGCT
GTGCTCGGTTTCCGGTATCGCCATCACCTGCAGATCATCGCCGTGGCCGTGGTTGCCCTGGTTGGTCACG
TAGTCGTTAAGCTTGCTGATGAGGCTGGCCGGTGTGGTCCCCACTTCCAGCAGGATGTAGGCATTGGCGG
TACCTGGCCCGCGCGGGGCGTCGTGCTCGAAGAAGATATGATCGGCGCGAATACCCGCGACGCTGGCCAG
CATCGAGCGATAGACGGCGTCGATGTGATAGCGCCCCACTGCCGAGAACTGGTTCTTGATGCGCAGACCC
AGCGCATCGTTGCTCTCGGCATCGGCGCCCGGGGTGGTGATCCACTCCTTGTCATCGTTGCGAGCCGAGA
GGATGCCGGTCACCGGTTCACTCAGCAGGTGGTAATAGCCCGGGGCCAGATTCCAGGCCGCGCCCGCGAA
CTCGGCCTCGCACACCACGCGGGCCACCGCTTCACCGGCGGGGCTCACCACCGCTTGCAGTGGCTTGACC
CGGTAGATGGTGCCGTTGATGCGCTCGGTGCTGACCCAGATATCGGCCGGGATGGTGACCGCTTCGCTCG
GATTGGCTTTGACGAAGTTGATTACCCCGCGGGTCTTCTGGTCGCCTTTGCGGGTCAAGTCCACATCCCA
CGCCTTGAGGTCGAGATAGGTATCGGTGGCAGTGGCGGCAAAGGTGCTGGGCAACACATGGCCAGCCAGC
AGGGTACGGACAAGCCAAAGCGCCGGGGTGATGACCACGCCGCGCACCAGTCGCCAAAATGGGCTCACAT
CGCTGTCGTTGGTGATAAGGGAGCCGGCGGCCACCACCTCCTTTTTAAGCTCGGCCTCCATGGCCTGCTC
GGTGGTCGGGATGCCGGTCTCGGCCAGCAGGGCCATAAAGTCCACGTTCGGGCGCAGGTTCACAGGGTTA
CCTCCAGATCGCCAAAGTCATACGTGCGGGCCGTCACCAGAATGCGCTGCGGGTCTTCCTCACGAATGAG
GATGGTGCCTGGCACCAGTCGCTCGTCGTTCTCGACCAGCAATTCAATCTCGGTCATCACATCGCTGCGC
AGGGTTGGGCTGCGCTCGCCAATCAGCTTGCGGGCCAGCCCCGATTCCATGATGCGGTGCTTGATGTCCT
GGCCGATGCTGTGGCGGTCCTGGGTGGTGCGCGGCTGGCCGCCGGCATCGAGCAGCCAGGCGCCGTTCAC
GACCAGCAGATCGATGTACTTGGGTTCGCTCATTTGGTCGTTATCCACATTTCTTGCTCCATCTCCCCAG
GGGTGATCGGGTTGGCAAAGTGGTTGTGCACCTCGCCGATGTGCATGGCCTTTGCCGGCTTCTGGTTGGT
GGTGGTCGCCGCCGCGTTAGCCTGGATCAACTGTTGGCCAAGGCCCCCGGATGGCACCTTGCTCTGGTCC
TGCTGGCGGTAGCTGGCCAAGGGGCTGTTCACCACGGTGGCGGGCGGGGTGATATCCGTGGGGGAGGCCG
CCCATTTGGGCAACACATCCACGTTCAAGGCGCCGGTGTCGAACTGGGTGCCCAGGAACTGATTCGAGGC
ATCGAGCGCCGTCTTGATGAGCGCGATCAGTGCCCGCCACGGGGTGAGTAGGGTGTCGAATACCGCGCCA
AGGATGGCCCCCATCCGGCGGCCGGCGTCGGCCACGTTGTCGATCCCGACGCTGGCCTGGTTGGTTTGTT
CAAAGAAGCTGCCAAGCCAGGCGATCCCCTGACCGAGCAGCCCCACGATGAGGGTGAAAAAATCCGCCAC
GGGGGCCAGTGCTTGCGACGCGAGCGGGCCGAAGGTCTCGGCCAGGCCGCCAAAGAAGGCGCCCAGCAGT
TCAAAGTCAGTCAGCACCGCGAAGGTGGCGCGCAGTTCGTCCCAGTAGATGATGGCCAGCGCCACGGCGG
CGACCAGTGCCAGGATCGCCCCGATGATGATCACCGCCGGGTTCAGACTCATCGCCAGGTTGACGGCGAA
GATAACCGGGCGCAGGGCGGCGAGGGCCTTCTTGAACAGAAGGATGGGGCCGGTGAGACCGCCGAAGGTC
AGCGCCCAGGCCAGGGTGGCCAGCTTGGCGACGCCGGCCAGCATCAGCCTGGCACCGGTCACGATGCTAA
GGCCCGCAATGGCCAAGATGACGGCCCCCACCACCTTGGTGAGGTTGGGAAAGAGCCGGGTCCAGCGCAG
TACCAGGTCACCGCCATCGGCAAAGGCGCCCACCACCTTGTTGATGGCGGGGAGCAGTGCCCCAAAGGCG
GCAGCTCGGATCGCGAACCAGGCCGATTTGACCCGTTGCCACTGGTCCACCATGGCCCCGGCCATCTGCT
CGGCCTTGCCCATGCCGTGGGTGTTGGCCAGGGCATTGATACTGGTGGCCAGGCCCTGGGTGTTGCTCAT
CAAGAGCTTGATCATCGAGACCGCCTCGTCTGAGCCAAACGCCTTTTTCAACTCGTCGCTGCCGGCCACG
GTGAGGGTGTCACCATAGCGGGCCTTGAGCTTGTCCAGGATATCGAGCACCGGCAGCATGTTGCCGGCCG
AGTCGGTGAACTTGAGCCCGAGTGCTTTCTGGGCACTGCCGACGCCGGCCAGGAACGACTTGAACTTGGT
ACCGGCCTCGCCACCGCCCATGGTGGCCTGCAGGTGGCCGAGCACGGCGAACTGCTCATCCATCGAGATC
CCGGCGGCGGTGGCGTTGGCGCCAATGCCCTTGAAGGCGTCGGTCATGCCCTGGCCGGTGGTTTTAAACA
TCTGCACCGCTTGCGCGGTCTTGCCGGCGACATCCTCCACCCAGTTGGCCTTGCCCATCTTCTTGGCCTG
TTGCTCGAAAATGCCATACATGGTGCCCATGTAGTTGGTGATGGTGGCGGTGTCGGCCTTGGTAGCCTTG
GCCAGCACGCCAGAGGCACGGGCAAAGGAGGGCAGTTCGTTGCCCTCCAGCCCCGCGATGGCGGATTGGA
TATCGTAGGAGGCGCTGACAAACGCGCTGGCCGATTCGCCGTACTTGACGGAGAACATCAAGGCGGTGTC
GGAGAGTTGCTTGAGGGTCTTTTCATGGACATCGAGCGAGGCCACTTCGGCCAGGGCCGCGTCCATTTCC
AGAGCTGGCCTCAGTGCATTCTGGATCGCCATGGTGCCGGCCAGCATGGCGGCGCCACCGCCGGCGATAT
TGGCAAAGCCGCTTTGCGCGGTCTCTTTCACCCGGTTCAGCTGGCTGTTGATGCCCTCAAGCGGCCGGGT
AACGGCATCTATCAGGGCAATCCTCATGATCAGTTGTTCGGTGACAGAAGCCATGATTTATCCATTGAAG
GCGGTGGCGATGCCCTCGGCGACGGCATGGGCGAGGCGTTGGGCGTGGTACTTGTCTAACCAGAGGGCGC
GAGCCAGGGTGTCGAGCTCGTCACCTTCATGGGGCAGGTAGTGGCGGCGCAGCGCCAGTACCTGTTCCAG
TTGGTTGTGCTCGATGGCCTCGGCGCGCGCGGTCAGTTTTTTACTTCGATATCCAGATCGGGGGCGTACT

GCTCGTTGACCTTGGCGGCGATCTGCAGGGCCGCGCCCGGGCGGGCCAGCAGGCCATCCAGCGCCTCTTT
GCTGTCGGCGTCGACGATGCGCTTGAGGTACTGATGGGACGGCGCCACCTTGTCGGTCATCGACAGGCCG
TTGATGTAGCTGTTGTAGGCCACCATGGTGGGCACAAAACGGATGTCGGTGCCGGCGATGGTCAGGGTGA
TAGGTGTGATCTTGCTCATGGGGTGGTTTCCTCTTGTTCAATCCAATGGTTCAGGGTGGTGATCTGGGTT
TGGCAGCGGCGCAGCGCCGTCTGCAGAGTGGGGATGAACCGCACGGCCTCGCCGTAGGTGCTCCCCGTAA
AGTCAGGTTCCGGGCAGTGGGGCACCAGCCCCGGCGGTGGCAGGCGCTTGACCACCTTGGTTTGCACCAC
CGTGGTGGGCTGGCTGGAGCAGGCGCAAAGCACCGCTAGGCAGAGGCTCGCGAGCACAGTCCGGACGGCC
CGCCGGCGGCGTGGCCAAGGCTTGTTGCAGTTCATCGGCGGTCTTCCTGTTCTGTTTGTCGAGCTCAGTC
AGTGCGTCGTTCTGGTTGGCGAGCAGCAGACGCAACCCCTTTGCCTGCATCAGCAGTTCTTGGAAGGCAG
TCGCCTGTTGGGTGTTGACGGCCTGCAGAATGTTGATGGTGTCGTTGGCACTGGCCAGTTCCTTGGTGCG
CTGCGTGAGTCGTTCGCCTTGGGTAAACAACAGGGTGCCCAGTACCAACCCGATGATGGTCGGCAGGAAT
CGGATGAGCGTGCTCATGCCAGCACCCCACCGAACTCGGTGAACTTGGCCAGCAGGTCCGCCAGTTTGTG
CTCGCGCTGACCGTAGCCGGCCCCGGGCAGGCTGGCCCAGATGTTGGCGCACTTGGCCACCGCCTGGGGG
ATGCGGCCCTTGAGCACATCGTCCAGCGCCTTGCGCTCGCGGATCAGCTGGATGGCCCAGGTGTCTTGCG
ACGCGGGGCCGAAGTCCGGCAGGCCGAGCTGGTCGCGGTAATGCGGCCAGTGCTTGGACAGATGCTGATA
GCGGCCGGCGGCGGTGCTGCTCAGTGTCTTGTTGACCTGCACCAGCACGTTCGGGTGGGTGCGGTAGTCG
GTGAAGAACCCCGCCGGATTGACCAGCTTGTTGTAACCGTCATCCCCCAAGCCCTTGGTGCCTTCGGCAT
AGGCGAGCAGGTCGAGAAAGGCGGCCACTTGTGGGTGGCAGTTACTGCGCGGCATTGTTGTCTCCCTTCT
TGTTGAACACGGTCTTGGCCCGCTCGCGGATGATGTCGATCCCAAGCAGTCCCACCATGCCACCCAGCAA
CGGGGTGGCTTCCTGGGGGATCCCAAGCAGATGGGTACCGGTGGCGGCCGCCAGGGTGATGAGGCCGCAC
AGCAGGGATTCGATCAGGCGGCGGCGGCCCCGCCCGCCGGCGTAGGTGATGCGCAGGAAGGCGATGGCCA
GCGCCAGCAGTGCCCCATAGACGGTGGGCCAGTTGTCCATCAACCAGGCCAGCAGGGCAGCACCGATGAT
GGGGTCTTTGTTTGGCATAGGGTTCATGTCCGTTGCTCTGATTAGCGACCCAGCCGCTCAAGGCGGGTCT
TGCAGGGGACGCACAGGCGCACCCCGGGGATGTGGCGGCGGCGTTCTTCCGGGATGGGGTCGTCGCACTC
TTCGCACTGGTGCAGGCTCTCCCCCTGGTAACGCCCCTTGCCCACCTGGTTGGCCAGCTGAGCCGCCAGC
ATGCGGGCGGCATGGTGATTGGCGCGGTCGATCTCGTCCAAGGCGCGCCCCTTAATCCATCAGGTGGCGG
GTGTCGTCCTTGGAGAGGTACGGCACGCCATCGAGGTGAACAAAGTCGGGGGAGGTGACGAAGCCTTTCA
CCTTGTGCACGGTCTTGCTGCCGCCTTTGCCATCGACGTCGAGCAGGTCGGACGCGTTCAGCTTGACGCC
GTAGGCTTCCACCTTGATCTCCTCGCGGCCGGTATTGGCGTAGAACAGCACATCGTCCGGCTCCATCCCG
CGCCAGCTGCCGGCCCGTTTGGCGGCGGCGATCAGCAACTTGAAGTTCTTGGTGTCGAGCTCGAATTCAC
ACTCGGCGGCGACATCTCCATCCACATAGCCATCAGGAATGCCCCGGGTTTGCGCCACCGCGCTGTTGTC
GGTGATGGAGAGGCTGGCCTTTTCGACGTGGACCATGGCCCCCATCAGGGTGGTGTCGAAGTTGATACCG
GAAAAGCGTTTGGTCACGGGTTAACCCTCCCCGTTGTTGAGGCTCAGATCGAGCAGGATGTTGACGGTGA
TCCCCTTGGGGCAGTCCACGGTGCGCACCACCACAAACACCGAGACCAGGTTCTTGGCCGTCCACTGGAT
ACGGATGTCGCCATCCTGGGGGGACGCGATGTCACCGGGGAACAGCTGGCCGTTGATGGTGGTCGCCTTG
GCCATGGCGCGCAGGTCTTTGCCAAAGTAGGTGATGGCGGTGGCGGTACTGCCCGGGGTGGAGTTGAACG
AGCGATCCCCGATACGGGCGATGGCGCGCAGGCGCATCCGGCGCGCCACCTTGTAGGCAATGCGCAGGTT
TTCGATCACCTGGTAGTCGCCGCCCTCGGCGTCCAGGGTGCGGCCGTCGGCCCAGTAGGTACCGTCGAAA
TCTGGGTACCACATCGGTACCGAGAACCGGTTTTGCTCCAGGGTCTGCAGGGTGGCCAGCGGCAGCGGGA
TCCCGTCCTTGTCCACCGGCTTGTTGCCCAGGCCGACCAGGGCGCCGGTCTTCACCCGGCAGGGGCTGTC
GGCGATGCTCACTGCCCGGTTGCACAGGCGCCCCGCATAGGCGCCGGCGAGGTTCGGCCACAGCTGCGGT
ACCAGAGACACCGAATCGGCTCGGATGCCATCTTGCAGGGCAGCCAGCGCCCCCTCGTACTCGCTCCAGT
CTTGACCCCCCTCGGCGGTCGAGACGATGCCTGGCACGGCCAGCAGCATGAACTGCCAGCGCCCCCATTT
GGCGATGAGCTCCTGGTTGAGGGCGTGGGCGGCGTTGATTTTCGCCTCGTCCCACGCTTGCCCCAGTACC
ACGACCCCCTCAAACGACTGGGTTTTCTGGGCATCACGTACCGCGTCTTTCCAGTCCATTTCAGCGGGCA
GCACGTAGGCGCCGGCGCTCCAGTTCTGACCGGCGTTGGCCATGGCGGCCTGCAGGTTGGCTTTCAGTTC
GCTGGCGGCATCACCCAGCAGCTTGTTGAAGTCGCTCTGGGTGTTGAGGGAGAGCAGCTTGCCGGCGTTG
TTGGCGGCGTGGCCGATGAACAGCAGGTGGCGTTCGACCTCCGTCACCGGCCCCTGCATCTGGTTCAAGT
TGTTGATCTGTACATAAGGCCACATGGCGCGGGCTCCTTAAGTCTCGGGGTTAGCGGTGAACGGGGTGGG
CGCTGCCCGCCACCGACAGGGTGATTTCATCGGCCACCCACACTTCAAAGGGGGCCACGGTCCAGGTCTT
ATCCAGCCAGCGGACTGCACCGTCCGGGTCTTCCACGATGCGCAGGGGCTCGGCGAACTTCACTTCCAGA
TCCAGGTCGGCGCTGTGCTCGTCATTGGGCACCACGTCATAGGTGGGATCGGGCAGGGCGAAGCGTTCGC
GGAAAGGGTCGTTTTCCTGTATCCAGCTGGCGATCACCGCCAGCAGGGTCTCGGTGCGGCATTCCCGAAA
CGGCAGGTTTTCCAGGGAAAACACGGCGGTGTATTTGAGCCAGGCCAAATCCATCCCGGTGACCCCCATG
TCCTTGGGTTCCAGGCTGATGGTGCCGCCTTCCATCCAGCTGTCGAGGGAACGGGCGCAGCGGGCTGGTA
GCACGCGCTCAAGCTCACTGTGCAGGGCTGACAGGAAATACCCCTGGGGATGCAGGGCCTCGTCGGTTTC
GCTCATATCATCGAGACCCCGACCCGGTGCTTGTTCTTGATGCTGCGCACCAGCTGCTGGCTTTCGGCCA
GCAGTTGCTCACGCAGATCGGGGGCGCGCTCGGCCATGTCTTTGCGGGCGTCCCGTTCGGTGACCGTGGC
GAACTCGGGCAGCAGCATGGCTTTGGCCCGGGCAAAGACGGCGGCCAAGTAAGTCTCGGTCAGCGCGTTG
TTGCCGCCGGCGAGGCGGGGGCCGGGCACCTGACCCGCCTCGGTGTAGCCCTGGGCTCGCAAGGTGGCCT
GGTGACGGGTCAGCTGCAGGTTGATTTCGCTGATGGCCGCCAGCAGGGCCGCACTGATGGCGTCGCTGTC

CATGTCAGCGGGCAGGGCGCGCCGGCGCTCGAAGTCGCCACACACCACGTCAGGCCAAAAGCCGTCATTG
GTGATGGTCTGCTCGCTGTAGCGGATGCTTTTGCCTGAAATCATGTGCTGCCCTATCAATTGGGGCGCCC
CTGAGGCCACGGGATTGAGCGCGGTGCGAGCGGTCAGCTGCAGGCTCATCCCCGCCGGGGCGCGGTGGCG
TGGGAGTCGTTATTCCGGGTTGAGTGCTCGCAGGCGCATGGCGATCTTGTGGCGCAAGGTGCCGACCCCG
ATCTTGCTGTGGGTCTTCTCGGCCAAAGCCAGCCAGTGGTCGGCCTGCTCCAGGGCGGCGTTGTCTGCCA
GCGCGCTGGGGCGAGGTACCCCGTCCTTGTCACGCAGCAGCAGGCAACCGGCCAGCTTGTACCATTTGGC
GGTGGCCTGTTCGTTGAGGCGCCAGTCACCCCGCACCTTGTCGAACACCCGGGAGAACCAGGGCTCGACG
CCATGGCCCAGCGCCGCTTGTTCCTCGGCCCAGCCCAACACGGTGTCGGCGGTGAAATGGGCCCAGTCGC
GCTTGATGTTGCCCGGGGTGCGCTGGTTCTGCTCGATAGCCAGCAGCGCCCAGCTGATGGCGGTTTCCAG
ATCGCCGATATCAAACGCCCAGACGATCAGGCGTTGAAACAGCGCGTTCTGGTACACGGTGCCGAGCGCC
GCCAGTTCCAGGTACTTGTCCACGTAGGGGCGATATTTGGGGAACAACTCGTTCCGTTTCATCGCCACCT
TGTCGCTCATCCTGGCCAGCGCCTTGAGCCGTGCCATGTCCTGTTCCAGGGCAATCAGCTGCAGGTGCAA
GCTGTTGGCCACAAGTCCTGTGGCTTGCCCGGTGGTGGCGGCTTGCGCCGCCCCCAACTGGGCCTGGACC
TGCTGCTTGTGACGCATGCCAGGTGAGAGGGCCATGGTTAGGCCTCCGGTTCGCTGTGCGCGTCAGTACC
TGCCGGCGGGGCGGCAGGGCGCGCCCCGACCGCCACGTCGGCCTCTTCGTAGCTGCCAAAGGCTTCATAG
TCGCCGATGGCATAGCCTTCCTGACGCCAGTACTGGTTTTCAAAGCCCTTGGTGTCGTCGTTGTCGGCGG
CCTTGCGCTTGCGGGTGTTGCGCTGGGTGTAGATGTGCAAGTTGGCCAGGGTGGTGACCCACATCCCGTT
CGCTGGGAAATAGGGCGGGATATAGGCCGGGCGGCCGGCAATGGACTTGGCCAGCTGCTGGGCAGCGATC
TGCTCGGTGGGCTTGGTGGCCTCGCTGTAGAGCTTGGCCTGGGCTGCGGCCACCAGGTCGGTACCGACCA
GCACCACCAGACGGGGATCGGTGCGATAGAGCGGGTTGATGGTGGCGTTGATGAGGTCAGAGGCCATTTC
ATCCAGGGTCTTGAAGTCGCCATGGCCGTCCGGGTCGAAATAGATCTTGTCGCCGGCGGCGGCTTTCACC
ACCTGGCTGCCCTTGTTCCACTCACGCGCCAGCTGCTGCCAGCCCTTGTTAACGTCTTCACCCAGCGGGT
ATTTGACCGGATCGGAGTCATCGGCGATGTGGGTACCGTTAAAGCCCACCCGCAGCATGTCGAGGCCAAA
GGTCTGGTTGGTGAAGTCGGACACCAGCTGCACGAACTGGCCTTCGTGGCCGGCGTTGGCCCAGGTGCAC
AGAGTGCTCCAGTCCAGGCGCACGCAGGAGTCGGTTTCGGTCAGGCTGTACTTGTTGCCGTCGACCCCAA
TCTTGCGACGAAAACGCCCTTCAACCTTGCGGCCGGTGTGCAGGGCACTGGCGCCGACCTGCACCACCTG
GCCGGTCAGTTGATCCACGTCCAGACAGGTGATGAGTTTCAGAAACTCGGTGGACTCGAGGATGGCGCTG
CGCAGCACGGTTTCCATAGGGGCGCTGACCGAGAACTGCTTGGCCAACGCGTTCTCGGGTACGTTGAAGG
CCTTGGCCAGCAACGCGTTGTAGTGATTCAGGCACTTCTCAGCCTGCGGGGTTTTGGTTTGACTCACAGA
GGGTTCCTCAGCAAACGGTCGGGTGGGTGTCGCTGCCGCCCAGGGCATCGGGGCGCTGGTCCGGAGCTTC
TACCGAGAATTTTTCGATCTTGGCGTTGAGGCCGGTCAGCTGTTCGCCCAGGCCTTTGAGGGTCTGTTCG
AAAGCTGAAAACTGCTCGGTGGTGACGGCAACCGGTGTGGGGTCGGTCACGGTGGCCGGCTCGGTGACGG
CCGGTTGCTTGTCTGCCGTCAGCGCGTCGACTTTGGCGCCAAAGGTTTCAATCTGGGTGCCCAGCGCGGT
GAACATCCCCGCCAGCTTGTTCATCTGTTCTTCGTTCATGTCGGTACTGTCCTCGGGTTGGGGGCGGGTC
GGTGACTCAGTGGGGAGCGCTGCGTTCGGGGCGCCGTGACCGCCCAGGAAGTTGAACACTTTGTGCAGGA
GTGAGACGCGGTCGGGGTGGTTGTGCTCGTCGGCAGGCAGCGAGAAGCCGGACAAGTCCAGCGGCTGGCT
TGTCCCCACAGCCGGCAATGGGTGCTTGGCGGAAAACTTGAGCATGGTGGTGCCGGTGCTGGCGGGGATA
TCCGTCACGCCCACCGCAAACAGGTAGGTTTTGCCCTGACCGGTGAAGTTATCCAGCGGCTCAATGGAGC
AGAACTTGTACTGGCCGCGCTGGTTCTGATAGATGAGGTCGCGGGTCGGGCAGAGGATGGCGAACAGGGT
GTGCTTGCCGTCCACCTTGTCAGCCTTAAGGGCCTGCACGTAGCCCAGGTTCTCGCCCCATTTCTCATGG
TTTGGCCAGAGCTGTGCGCAGTAGAAGGTGGGATCGTAGGTGTCGGCCATGTCGGTGATCCACTTGGCCG
AGATCTCGCGGCCATCCACGGACTGGCCCTCAGTGGCAATCACCACCCAGCCGGTGCGCAGTGATGAGTC
TATCGGTGTTGGCACGGCGGCCCCCTTGTGTCGAATTGGCGTCAGGTTATCGGGGCAATGCCGGGGTAGC
ACTCAGATGCGTTCTGGCGATTTCGGATAGAGGGGGTTATCCGAAATCATCCGAATGAAGGCGGCGGGAT
GGGGGGAATTGGCTCAGTATCATGAGCCCCAGCTTTATGACTGGGGAGCCTCATGGCCTATACAGAAGAG
GTACGACAAACCGCCAAGCGGCTTTATCTGCGCCACTGGAGCGCACGGGAGATCAAGGAAGAGCTCGGCC
TTGGCAGCGTGCGCGTGGTGTACCTGTGGGCCGAGAAATACGGCTGGACGGAACTCCTTAGCGATGAGGC
GTTGGAAGACGCGATCACCCGGCGCTATCAGGCGCTGGCCGCCAAGCCGAAGAAGAACCACGCCGACCTG
GCCGAGATGGACAGGCTTATCAGTCACCACGTCGCCCTCAAGGCGGAGGCTGTCAAGCTGGCCGAGCGCG
AGCAGGCCCTCAAGGCTCGTCGGCAGGCGACTCCGTCAGATGAGGCTGACGAGGTGGGCGAGCGTGAAGG
GCGTCGCCAGCGCGGGGAGAGCAAGCCGAAGAAAGGCGGCAAGAAGACCAAAAACTGGGTCAATGACCTG
GGCCCCGAAGACTTCGAGGGGTGGCTGGCCTCCCTGTTCCCGCACCAGCTGTACGTGCGGGAGGTCAAGA
ACGACCCGGCTATCCCGCGCACCCGCAATATCCTCAAGTCCCGGCAGATTGGCATGACCTACTATTTCGC
CGGCGAAGCGTTGGAGGATGCCATCTTGACCGGTGGCAATCAGGTGTTCCTGTCCGCCACCCGGGCCCAG
GCCGAGATCTTCCGCTCCTACATCATCAACATTGCCCGCAAGTTCCTGGGGGTGGAGCTCAGCGGCAACC
CCATTGTCCTTTCCAACGGCGCGCAGCTGGTGTTCTGCTCGACCAGCGCCAACAGTGCCCAGGGTTACAC
CGGCAACTTCTACGCCGATGAATACTTCTGGATCAAGAACTTCAAGGCGGTCACCGATGTGGCCACCGGC
ATGGGCTCACAATCCCACTGGCGCAAGACCTTTTTCTCCACCCCATCCAGCAAGGCCCACGGGGGTTATA
AGCTGTGGACCGGGGATGACTGGAAGGGCAAGGATCCGGCCCGCCAGGCCATCGAGTTCCCGACCGATGC
CGAACTGCGCGACGGTGGCCGGGTGTGCCCTGATCGGGTGTGGCGCTACATCCTTACCCTGGAAGATGCC
GTATCCAAGGGGTTCACGCTCATCGACATCGAGGCGCTGCGCGAAGAGACCGCCATCGAGGTGTTTGATC

ACCTCTACATGTGCTCCTTCGTTGATGATGAAGCCTCGGTGTTCAAGTTCCAGCACATGGAGCGGGCCCA
GACCAGCATTAGCAACTGGAGCGACTACACCCGGGGACACCCCGAGCCATTTGGTAAGCGCGAGGTGTGG
CTGGGCTATGACCCGAGTCGTACCCGCGATAATGCCACCCTGGTGGTGGTCGCCCCGCCGTTGTTCCCGG
GCGAAAAGTTCCGGGTGCTGGAGAAGCATTTCTGGCGCGGGATGAACTTTCGCTACCAGGCGGACGAGAT
AGAGAAGATCGCCAAGAAGTTCCGGGTCACCTATCTGGGGATCGACGTCTCCGGGGTGGGCAGCGGGGTC
TATGACCTGCTGCAGCCCGTGTTCAAGTCAACCATCACCCCCATTAACTACAACGTGGAGAGCAAGGCCC
GGCTGGTACTCAAGATGGTGGATGTGGTCGAGTCAGACCGCATCGAGTGGGATCAGGAAGACATCGAGAT
CCCGCTGGCCTTCATGAGTATCAAGCGCAGCACTACCGGCGGCGGCCAGCTGACGTTCCGGGCCTCGCGC
AGCAGTGAGACCGGACACGCTGATGTGTTCTTTGCCATTGCTCATGCGGTAGACAACGAACCGCTCGACA
CATCACGCCGCCGTAAATCCACCTGGGCCATCAGTAAACGAGGAAAACATGAGCCGCAAACAGCGCTTTC
ACCCGCGGGCCGAGCGGCCCACCCCCAGTACAACCAGCAACCGGCCCACGGTCAGCTTTTCCATTGCCGA
ACCCATCGACCCTACAGCCTGGATGACCGATTACACGGACGTGTTCTACAGCCCCTGGGGCGAGTATTAC
ATGCCGCCAATCGACAGGCAGGGGCTGGCCAAAGTCGCCCGCGCCAATGCGCACCATGGGGCCATCCTGA
TGGCGCGGCGCAACATGGTATCGGGCCGGTTTATCAGCAGCGAGGGCGTGCCCAGGGAGGTGATCACCGC
CTTTGTGCATAACCTGCTCCAGTTCGGGGATGCAGCCCTGCTGAAATTGCGCAACGGGTTTGGCCAGGTG
GTGGGGCTCTATCCCCTGTCGAGCCTGTATCTGCGCCGCTGTCAGGATGGCAATTTCCTGATGTTGCAGC
GGGACGGCAGTTACAAACACTACCGAGCCGAGGACATCATCTGGCTGGCCCAATATGACCCGGTGCAACA
GGTTTATGGTCAGCCTGACTACTTGGGTGGCTTGCAGTCTGCCCTGCTCAACCAGGATGCAACCATGTTT
CGGCGTAAATACTTCCTCAACGGGGCGCACATGGGGTTCATCTTCTACGCCACTGACCCGAACATGGACG
ATGACCAGGAAGCGGAAATGAAGGAGATGATCGCGAGCTCCAAGGGGGTTGGGAACTTCCGTTCGATGTT
CGTCAATATCCCGGGCGGCAAGCCCGATGGCATCAAGCTGATCCCGGTGGGGGACATCGCGACCAAGGAC
GAGTTTGCGGCCATCAAAGCGATCACCGCCCAGGATGTGCTTACCGCACACCGGTTCCCCGCCGCACTCG
CCGGCATCATCCCGGCCAACGGCAGCGGGGGCCTCGGAAACCCAGAGCAGTACGACCGGACTTATGCCCG
CAATGAGACGATCCCGATGTGTGAGCTTATCCAGGATGCAATCAATGGGGCCAACCTTCCGCGCCGGCTG
CAGGTGGATTTCAATCGGTCGCTGGAGGCCGGTGTTACTGTATAGAGATCCAGCTTAGGGTATAATCGAG
CGGTTTTTTATCGATGGTATCAGGGGGGGATTTATGCGGGTATTTTGTCGGGAATGTGGGGAACTAGGGC
GCATCACCAAGACGCACCGCCTGAGTCGTGATACCTCGGATCTGTACTGCCAGTGCACGGATGCAGAGTG
CGGGCACAGCTGGGTGTCACAGGTGTCATACAGCCACCCACTGAGCCCATCGGGGCGAACGACCAGCCAA
CTGGCCTTGAGCCTTATCAACTCGCTTAGCCAAGAGGGGCGACAGGCCCTGCAGCGGGAACTCAACCTGG
GACAATGAAAAAGGGGCGTATTAATGCGCCCCTTTTGCAACTGTTATGTAGAGCTTAAGGGTACAAATGC
CAGTTCCACGAGGTTTCTAGGTCTGTCTCTTGGCAAATTTGGTTACAGTTAGGATCTTTACTTAAGGCTA
TATTGAATGTGGCAAGAGTTGGTTGTGGAACATCTGGCGTGTATAGCGTGAAATAGTTATACCAGAGCAA
GAAATCCTTAGTGGTGAAATTGTAATATCCAAGGTCAATCATGCCTAGCCTGTCTTGCGAAGTATTTACC
CACTGTTCCTGCCCATATTCTTCCACGACGAGCAGTATATTAATATGGTCAAGTTTCATTGTATACCCTC
ATTATGTAGTATCACTAATGTCTTAACGAGACATAGGAGGGTGTGAGTGCGAACAAAATACTTTCTAAAA
GATGATGTCATTAGTTTGCTACGCTATTAATTCTGGATTATTCTAATGGCAGGGTTGGTTTATATAGGTA
TAAGCTTGTTATTGCATATATCCCCATAGTTTATGTTGGTGTTTATAAATCTATGGGGATTTTTATAATG
GATTATGTTGATTTATATTTCAACAGTCATTTGTCTTAATTTTCTTCTGAGATAATCCACTTTGATGAAG
CATTGTCAGGGTTGCCTTTTTCTGTCAAATAAGCCCCCTTGCTATTTTTATCATACCATACATATTGGTT
CCAATATGTATTTTTAAGATTGAAAATATTATTGCTTCCTAGTATTTCAAATTCAGCTTGATTCGGAGAA
TGCCAAAGTTTTATGCGTCCATTTTTTTTATCGCTATAGCTGAAGAATAAATATGGATTATCTTTACTAC
GCAAATAAAATGATTTACCTCCATCATTTGGGACTCTTACGAGGCTTAAAGGTGTTTTTGCTCCTGTAAC
AATAGAATAGTTGTTTTCAATGCTGTCAACAGTTAGCTCTTGGTTGTTTGTCAATTCTTTAATTTTTATG
ATCTTACCTGTATCTCTATCAAGCTGCTTTTGTTGCCATGATGCGTTAGGTACTAGCCAACTGTTAGACT
TCCAGCGCCAGTTAAATGGGATGTTAAGGTAAGGGTCATTAGCATCAGCATAACCGGGTTCAGCTTCTAT
ATTGAAAAGACAGTATCGCCGACTATCATCCAGCACATCTACTAGTTCTTTCTGTGTTTTGGGTTTAATA
GTCGCGCCGTATCTATTTTTCGCTGACAAACCAGACATACATTCTTTTTGCAGTTTGCCTTTACCTGTGT
CACCATCAACTTTTACATATGTGTTGTCACGGTTATTCTCATTGTCATACCAGTGTTCCATTTCATCTTT
ATCAAAAGACATCCATTTGTCTATCAACACCTGAGCTTCATCGCGAGCATATTTTTCACGTTCGTTAATA
TCAACGGACATTGTTCTCATAAATGCAGCCCAAAGGTCTTTACTGGCTTCCATCGCAACCAAAGCAACGG
GTTTCATATTGCTTGCTTTGTATCCTCCCCAGCCAGTATCTGTCTTAGTCCCGACCTTCCAGATCACATC
GCCAGATGTATAATCTAGAACAGCACCGTTTGAGTGGGCATGTTGTTCAGCACCTTCCGTGCAAATATAA
GCCTTAACTTGGCGAACCTTTTCATAATTATCACTTGGACTCCTTACTACATGCTCAGGACTAAAAGAAT
CCTGGAAAAGGTGTACTGCTCGTCCAAATAGAAAATAGTTATAGTCGACATCGGTTTTGGCAGAGCTACC
ACCGCCATCCCATACGACAATACGTTTATTAGTTGCCATCGCGGCATTGATAAAGTGAGTAATAAATCGT
TCTTGTCCTCTTTGGGCTGATTTAACTCCCCCTTGTTGGCCATTATCATCATATCGACGCATGAAATGGT
CTTGTTGTATATCTGCGGGTTCTTGAGAAACCGCATCGAAACAGTCTGGCCCTGTTTTACTTATCAAACT
ATTAGCAACATTCATCCCTCCGAGATCAACCCAGCGTTGACCCACAATAGCGGAGTAAACTGCATCATAC
CTAGGAGCATAAAGACTATTGTCATTAGTGTGGCTCTTTATCTTGGCGACTTCATCATCAGCTGTGCTAA
TATCGACATTTTTAGCCAAACCACTGGTCCAGGTATTGCGTGGATCATTTTGGTCAACACTGATTTTATG
CTCAGCATTCAACACTTCGAGCGCAGATGTTCTGGTTAACCATTCATGGCCCATTGGGGTTATACCTCCA

GCGCCCCATTGGGTAAATGCGTGTACTTGTGTGGTGGCAAAAAAGATGCACAAAGACAACTTCTTTATAT
TCATAAAGTCTTACCCCTAAAAAGTCTAGTTGTTTTTTAATTAAGGTAGTATGTCAATAATCACCATGTT
GTCATGGTGAAAAAAAACATATACTGATATTTAATTAGTCACAATGATTATTTTTAATGTTGAGTTTGTT
ACTATTCGGTGCTCTCGATTTCTAATCGAGATGTATCTACAATTACTGATTTTTATTAACATTTTTCGTT
TGAGTCTGTCTTTGTTCTGTTAGCTTTAAAACAGCTAAAAACCAATAGTTTAGTCAACTACATTCTAGTT
TTTATGGTCTCAATTTAAATCGTGCTTCAAGGTGTCTAAATATAATCAATTGTGACTCATTTGTGGCTGT
TTTGGTTGTGTTATTTGTTGTTTTTTTTGAAAAAAACTAGTCTAATCAATCACTTTTTGTTTTTTTTTAG
ATTTTTCTATTATTGTGAGTGGTTGTAACTTGGATTTGTTTATTTTCAATAAGTAGAGCTGAGTAAGCTA
CTGTTAATGACTTTGGGTAATGTATTTTTTTGTTATCAGGCTGGTTCGTTTTGGATGGGAGGATTTTTAA
GAAAGCCGTTAGGTTTCTCAGTCATGAGGTGCTTGCTATTAACTGATTGGTAAGTATGAGCGACTATGAG
TTACCAATCTGCTGGCGTATGAAATTTACATTCACAGTGCCTAATCAGTTAGAGAGAAAGCCGTTACGAG
TGGACATTTTAACTATATAGCTAAATCGGTATAGCTCTAAGTTTATAGGTTGTTTTTCTGATAAACTTAA
CGTATGTATTGAAGTAAAGGTATTTTAAGTGAGATGTTGGTTACGAGGTGCCCATAGAACATGGTGATTT
GCTCTATATAAATGTCTGGTGTTATCTTCTTGTCTTAAGACAAAGTTTGTTGCGCAACAATATGAAGCAG
ACTATATAGCAACATTGGGTAATGTACCTATGGAGCTGTTATCTAAATAGCCCGTTGTTCTGGGAACTAT
TAATATAGTTAGGTGGAAAGTAGATATCAGCAATTGTTTGACTTGTTATTGAGGCTGATTTTACATCGAT
TATCGTTAGATCAATAGGCATGTTTCCGTAGGTCATCAGCTGGCAGCCCGAAGAACTCGGCATTGCTGAT
CGTCTCTTGCCGGTGCTCGACTGGTGGCAGTGGCCACGTCCGCAGATCGACGGTATCGCGCAGCCGGTCA
AGCTGGACGTGGGCTGTCTCCGCGTCCCCATCTCCCAAGGCCTCCAGCAATACCAGTGCATCTTCGGCGC
CGGCGGCTGTCATGCTGGCCAGCCAAGCCGCTGTATCCCCGGACTCGGCCAGCAAGCACCGCACATCCTT
GAGCTCCGTCTGGCGTTTTGCCTCCCGGCGTCTGGCTTCGGCTTCAATTTGATACGGTGATGGCTCGTTT
GGTGATTCGAACGGATGCCGTTCGGTCACTTTCAACTCACCATCCCGAATGCAAACAGACCGGTCCCCCG
CTCTGACAATCAAGCCGCATCGGACCATTGCCACTTCTTCATCACATAGCCCCAAATGGAATAGATTCAT
ACCGCCCAACGGATCCTCTTTAGATCCTTCGAAATCGTCGCGTACAGTTGTTGCCAGAGCTCCAAGGGGG
AAACCCCCTTCGCTTCGCTCGGTACCCACTAACTCGCTTCGCTCGCCCACAGACCAAAACCCGCTGCCTT
GGGCCTCGGTGGTACTCTGTGAACCACATTTACGCAGCACCCATTCCCCCACACGGGTCTGCTGGATGAG
GCCGTCAGCGGCCCGCACGCCCACAAGCTTGGTTTGCGGTTCGCCGTACTGATTGGGTTAGGCAAAAGCG
GTGCGGTGGATACTCAAGGGGCGTTCATCGCGCCGGCAGCAGGGGCCGCCCATGGCTTGGGTGAAGTTCT
TCCAGTCCACCGCGTCGGCATAGCGCCGGCACTCTTCCATGATGGGGCTGGCCAGTGGGGCCACGACGAT
CCCCTTGGCCTCTTGCAGGCGGCCAGGTAGCCGGCGCAGCTCCCGCCAGATCCCGACCGGCGGCCCTTTC
AATGGTTGGAACTGGCGCAGGCCCCAGAGCGAGGCCCAGGCCCGCACTCGGCGGGCGCCTTCGGTCGCCG
CGGTCTCGGCTTCCAGATCGCCTTCGTCGCCGACCCGGTACCCGTCGATATTCTTGGCGATGTATTTGAC
GATGTAGCCGACGGCGCCGCCCTTCTCCTTGTCCATTACCTTCCAGTCAAAGCGGGGCGTGATGTCGCTA
TAGGGCTTTTTGCTGTCCGGGTGGCGTTTGCGTTCCAGGTCGCCTTTGTCATGGCTCAGGGCATAGCGCT
GCAAGATGCCGATCAGCCGGTGCTGATGCTCAGGTTTAACCCAGATCAGCAGGTGCCAGTGGGGTGTGCC
GTCGTGGTGGGGTTCGACCACCCGAAAGCCGAAATAGTCGATAGGGTCGGCCACCAGGAATTCCCCCCGC
TTGGCCAGGTTGCGATCGAGGGCAGCACGGCAGCGCTTCCACAGCTCGCTGATGTAGTGTTGTGAGTCGC
GAGGGGATGAGCCGTCGTGCTTGGGGTTCTCTTCTGTGCTGTCTGGTCCGCCGGTTTGGGAGGCTCGTAC
CGTCTTCCAGGGGTGGAAGCGGCTCGGGGCCGTCCAGGTGAAGAACAGGCCCACATAACCCATTTCGTCA
GCCACATCAGAGAAGCCGCGCGCTCGAACAATCAACTCATGGCGGCGGTTCTCCGGGTTGGCGATGGAGG
CTTTTACCGCATCCTCCAGACTGATGATGATGTCGTCCTGGGCGTTATAGGCTTCCATGTCTTTGAGCCA
AGCGGCGGCCATATGCTGACGCTCGATAAAGGCCTGCAGGCCCTGGCTGGACACGTAGGCGGATACCCCC
TTACGCACCTTGCCGAGCAGGATGGCGCAATGTTCGTTGTATTGATCCCAGATTTTGCGCATGCGCCTGG
CCCACCACTTGGGGTCGAGCCAGCGGATAAGGTGGTGGGCGATGAAGTCGTCGCGGCTTTCGGCCGTTCT
GAACTTGGGCAGGCGGGGCAGCATGCCCCAAGCGGCCAGCGGTTGGCGGCACGCCTTCCACAAGCGGATC
GCCGGTACCTGCTCGGCGCCGAAATCGGTGATGGCGTTAAACAGCCGGGTGACCCGTTCGGCGTAGTTGA
CGGCCAGGCGCTCGCGACTATCTTTGGTGCGCAGCGCTTCGAGCGGTTCGGGGATCACGTTCTGCACCTG
ACGCAGGGTGGTGATGCGGCTTGATAGCCAGCGGTTGGCGTCATAGCAGATCGCCACGCCTTTGAGCACA
TCCGGGGCGCGACGGGCGCAGTAACCCACGAACAACTCGGCGATCAGATCGTCGGGCAACTGCACCCCTG
GCCCGCCGTTCTCACGCGGGATCGGCCGCTCCAGCAGGTCAAGCGCCCAGTCCAAGGCATAGGCGCCAGG
GATGCCGACAAAATAGCTCTTGATGGCCTCGACCCGGGTATTGATATCGCCGCCGGCGAGCTCCCTGCGC
CGAGCGGCGGGGAGGCGGGATGCGTCGAGTGGACAGAGCGAGACACGACGACAAGCCATGATGCGACGCG
CCATGGCTTTGGTGGATGCTGCCAATTTCCAGGTAGCAGGTTGCCTGGGGGATATGGGTTTGGCGTCGAA
CAGCTCGGAGTGGTAGTCCCGCATTAGAAGGGCAGGGGCTCGTCACCGCATAGGTGGGCAAGATACCGCT
CTTGGTGGGCGACGGTGTCTAACACCTCGATACGGGCACGGCGCTCCTCTCTGCATTCGAACAGCAGGCG
GCACAGGGCGTATTTGGGGTCTGGCAGGCGGGCCTCTCGTAAAGTGAGGAGCTGGCGTTGGTACTGACGA
AGGGTTGCCAGTTTTACCTTGGCGATGGCGGACAAGGTGGCTATGTCGGCCTCTACTTCCGCTTGCAGTG
CTGAAAAGTCCGATGTGTTCTCGGTCATCGGATCACCTCGCCCAGTCCATGCAGAGGAGCGCACTCTTTC
CACCAGTCGCCGATTTCGGTGGCAAGCGGAGTTTCCCCCTGGCCGAGTGCCAGCCAGTAGATGGAGCGGA
TCGCCCCAAGGGCGAGCACCTCAGCCGCATCAGTCCCATAGGTGGCGTTCGAGGCGCCGATGTAATCGGC
TCGGTAGCTCTGCCAGTGGAGCCATAGCCCGCTTTTCGGCTGGTCCGGCTCACCGGTTTCGGCGTATTCA

TTATCGCTGCTGTCATCATCCAGCAGCGCGTTTGGGCTGCTTGTGGCCACAAGCTGGATCTGAATGTGCT
GCGGGCCAGACCATACGCTGCCCAGGCACACCCGATTGTCATCAGCCCGGGCGGCGAACATGTCGGCCAG
CAACCCCTCGATGAGCTTGGGGGCCTGGCTGGCTATCTTGATTGCGTCACTCATAGCTACTTCCTCAACG
CTTTCTTACGGTCAGTTTGTTGTACCGTTCGTGGGTCATCAGCTGATAACCGCTGGCCCCACAGGGCCGA
AACAGGCGGTATCGGTAGCCCACTGCAATGACATCCCCACACCCGGATTGCAGCCGGCGAGGGCGTTGAC
AGGCCAGCACTACTGCCGCTTTTGCCTGTATCTGGGCCGGTATCCGGCCAATGCTCTTGAATCCTTCCAT
GTGCATTGCTCCAGGGGCTGCCGAAGCAGCCCCTTGTTGGATTAGTTGGGATGGGCCGGGCGGACTTGAT
CGGGTATGATCAGAGAGCCTTTCATCAGCCATACGGCGTAATCAGACAGGCCAGGGGTATTGATGATGCG
CAGCAGCAGGCCGCCGCCAATCTCGCGATAGCCCAGCTCGTAGTTCTTGAGGGTGGCGGGCGGGATATCT
AACTGCGCTGCAAATTTGGGACGACTCATTCCCAGAAATTCGCGCAGTTGGCGCAGGCGTTGGCGGGTAC
TGGCATTGAGGGCAGTCATGGACGGCATTTCAGAGTTGTGCAGGGTCATGGTTAGGCTCCTTGATGGGCA
TTAAGGCGATTGATGTGACCAAACAGAGAAGCCCAGACCAGGGCGTTGGCGCGCTGGCTCAGGATCGTCA
GGTCGTTTTCCGAGTAGCGGGCGGCATATGGGCCAGCAATACGGCTGTTCTGGATCTTGCGGTTGCGCAG
CGCGCAGGGGATTGCTAGAGTTGCCATGTCGACTTCCCTTCCTAGAGTGGTTGATAGAGAGCCCCGTTAG
GTGTTTGCGGCACCAGTGATCGGGGTTTTTTCTTGTCCGCAATTTGCGGTTTGTGGGTTGTTGACCAGTG
TCATCAACAAGCCCTTGCTCTTCGCCAGCCGGTCGGCTGGTCCTGTTACCTCTGGTTTTTCTTCTGGTGG
TGCTATTAAACCGGTTCGCCATTCTTCGAGGCTGACCGCGCCGTGAAACAGGTGCTGGTACCCAAGGCGG
CGCATGGCCAGACAAAGGGCCTCGCGGTCGCGCTCCCCAAGTGGGAATTCAGGCCTTGCCATCGAGATCG
GTAATTCGGCTTCATTGCAGATGGCTTTGCGCTGTGGCTGATTGAGCTCGCCCCAATACTTCGCAACCCT
GCAATTGGCGTGGTAAAGCGCTTCACGCATTTCCGCCAGCGCCTTCTCGGCTGCACTCGCTCGTAAATTC
ATCTTTTCCTCCTTCTCACATCGCCAGTGCCGCAAGGGGCGACACCACCTGAAATCGCTGTTCGACGTCA
TTGATCAGCAGCGCCACTGAGCCCATGGCCGCTGCGGCGATGCTCATGAAAGAGCGGTGCTCGTTGCTGG
TGATACGGCCGCGTTCTGTGAGCTCCAGGGTGCGCTGGCCGATGCTCGCTATTTGCGAGTTGAGCTGGAT
CACCTGGTGGGTCAGGGATGGCGCCCGCTCGGCATCGGGGATGGCGACAGCAGTCAGGCCACACTCAAAC
AGCAAGGCATCGAGTAGCGTTTCGTCTTTGGTCACCTGGTAAAGCGCGATCAAGTCGTCACCCGACAGCT
TGTGTTTCTGCTGCTGATTGAACTTGTTGCGCAGCACATGGGCCGACATACCCATGCTGGCGGCCAACTT
TTCTACGTTGCGATCCTTCACGAAACCGGCGCAGGCACTGCGGTAGTGGGGGTGTAGGTTTCTGGTGGTA
TTCGACACTCTGATTTCCCTCCAAAGGTCGTTATGGTTAAGCCGCCAGCCTGAACTGGACTGTGGGGAAA
GCACTGGCCATCTGCTTGGCGATAAATACATGCACTTTCACCATGACCAGCTCATTGCTGCGGGTCTTTT
CCAATACATCTAGCCGGCCGCTTTTGACCCAGTGGCGAGCGGTATCGACAGAGACGCCGACCCACTCGGC
AAATTTGGCGATGGGCATCAGCGGTGGAGTGTCAGGGGAGATCACGAGATTGATCGCGCTGACGGTCGTC
GGCGGAGCATGCGAAACGGCAGGGTTGATATGACGTGGTGGCATAGGGCAACCCTTATTGCACGGTACGA
GGTTGTGCGGGCGAGGCTTTCAGCTTGCCGCCAGTCAGTGACTCGAGCTTGTAAGCGCTGCTTTCTGGGA
TAGTTTCATCCCATTGGGAAACTGCAGATCTCGTAATCCCAAGCGCATTTGCTGCTTTGCTCACACCACC
GAAATACTCAAGGACATCACTTTTTTTCACAGAACCCTCCAGCGTCAGAATGCTTAACTTTGTCAAAATC
GCTTACCTTTCTGGATGGTAAGACCGCTTAACATCGATCGCAAGCACTTTTGACATTGTTTTGTTTAGAG
TGCTAAACATGAAAAGTGAACGCATCAAAGAACTAAGGAAGAAACAAGGACTTACCCAGCAAAAGCTGGG
TGAACTTGTTGGGGCTAGCCGAGCATCGGTATCGCAATGGGAAAGTGGTGAACATTCGCCTAGTGGGGAT
TACCTGATTGCACTAGCGAAAGCGTTTGGGGTCTCCCCTCATTGGCTTTCTACGGGCAAGGGGTCGCCTG
AACTGTCGAATGTTGAACCAGCCGTGATCCCGCAAGGGAACCGAGTGCCGATTCTGAGCTATGTTCAGGC
TGGTCACTGGCGTGGGATGTGTGAACAGGCCACGAGCTTTGATGGCAATGTGGAGTATGTGTCTGCCAGC
GTCGATATCGGCCCTTGTGGGTTTGGTCTTTGGTTGCGTGGTAATTCTATGACCCCGTTTTTCAACGAAG
GGGATCTGGTGATCGTTGACCCTGACGAACAGCCGCGCCCTGGTGATTATGTAGTGGCCAAAAACGGCGG
CGAAGAAGCCACGTTCAAGAAGTACCGGCCGCGTGGCATTGATGAGAACGGGCAAGAAGTTTTTGAGTTG
GTCCCCCTTAATGATGATTACCCCACCATGCACTCCGATCGGCAGCATATCGAGATCATCGGAGTGATGG
TCGAGCATCGAAGTTTCAGAAAACGATAAGAGTGATTTTTTAACGGTCCCATAGCGACTTCATCTGGCAA
AGAGCGTTAGGCAGCCCCATTGATTGAGACTATTAACTTCCCCTCTCGGAGAAGCTTCTATGATGCCAAT
GATTTTGAACTGGCAATTAAGAAGCTGAAATTTAGTGAATTGCCTTTTATAAGTCATGTGGAGATAAAGA
TAGGGCGTGACTGCAAGTTCTTAGCTGATGCCACGTTGAAGTTGCTCTGTCTTTGCCATCATGTGGTGTC
TCTTGGTAGAAATTTGACGCTGACTTTTGAAGGGGGCGCGTGCAACTGCTTTACATTTCTAGAGCGCTGC
GGTTTTTTCCACTCACTACCGCAAGAAACTGTTGTTACTCCCTACAGGCCACAGGCACAGGATGCATCAA
TCGCATACGCAGGTCAGAGCGCTAACCTGCTCGAAATTCGGGCGGTTAGCCCAACGAATCCCAGCGCCAA
CAGGCCGATACCTAGCCAGTTGACTGATGCGTTAGGTACTTTAATCAAAACTGATTGCGATACATTCCTG
TCAAACATTCAGACTGTCGTCAGTGAGTTTTGTAGCAATGTGTCTGAACATAGCCAGGTATCAATCCCTG
GGTTTGTATACATGCATCACTATCCGGCGTCAGGGAACATATACATTTCTATCGCTGATCTGGGAGTCGG
GCTGCTCAACAGCCTGCGTGAAGGTTTGCAACTCCGGAATCACTGGGCTAAAGACCTGCCTGATCAGAAC
CTGATTTTAGAAATGTTTAATGAGGGGCTGAGCCGCAAGCATGATGGGAGAGGAACTGGTCTGAACCGAG
CCGCAAATATCGCGATCCAGTTTGATGGAAGACTCCATGTAAGGCTTGATCGACACGTCATCGACTTGTA
TCCCAATGGCTCTCGATTTGAGGCTGCAGCCACAGCCACGGCGTTAATGAGTACTGATTGCTTTCTGGCG
AAAGGACACACATAACGCTCTGTTTTTCTCGGACAAAACTGTAGTGTTTCCTTGCTATCTCAACCTCAC
TCTGATTCAATAAGTTTTATGAAACAGATAATCGCACGTATCAAACTCATCGATTTCATGGGCTTTGCTG

AGCCGAATGCTGATGGAACCAAGGATGGATGGGGAGTTACTGATGGTGAGGCCGTCGCGGCAGCACTCTC
AAACTACATCTCTAATTCTCCGGAAGTGGATATCTATGATATCTCACTTGAAGGTGTCAGACGCATCGAT
GCATCCTTCCCTCGTGAGGCGTTCATCATGCTCGCAGCAAAGTCCGTGGGTAAACGTGGCTTTTTTGTCA
GTGACGTTGCTAATGCTGTCTTACTAGACAACATTCGTGCTGGAGCTGATAAGTTGAGCTTTCCCTTGCA
TGCCTGCTGTCAGGGACGTGTGGAGGTTTTGGGGCCGGAACCGAAAAGAACCCAAAAAGAGCTGTACAGC
TATGTTGTGAGCAAAGGGTGGGTGACCGCGTCGGAGGTTGCAGAGGTATGCGACCTGAAAATCAACAATG
CGAGCAACAAGCTAAAAGAGCTGGTTGATGAAGGTTTTTTGCTTAGAAAAGAAGGCGTTGCTGAGACTGG
GGGGATTGAATTTTACTACTTCCCTATCTATGGGCAGGGTCAGCAGAAAACTGCTTCATAAAATTTCTTG
ATCGAGAAGATCCGCTGTGTTACTTTAGCCAATAAGGAGGTCTAGTTCGGCCTCCTTGAAAAACAAAGGC
CATTCATTTTTTTTAGTGAAAGGCCTTTTCAAAGGTAGGGTGTTCATTGTTTTTTGTGAATGCCATACGG
CGAGCAATCGTCTTTATCAAGAGAATTGAAATTATGTTTAGCAAAATCAAAGATGTACATGTATGTGCCT
ATCTGAGGCGGCGTAAAGGGCGTTTGGAGTATGTGACTTCACATTGGCGTTCTTCGCCGGGGAGTTAGTA
ATGTTTTGTGTTCTAGAAAACAAAAAAGCCCAATGTGGGGACATTGAGCTCCTTTCTTAGACACACACCG
TTGAAATTCGGACTGCGCCTGTTTGTTTTTGCAAACTAACTATAGTCGCAGTCCGATTTTTTGCCAACAA
GTTTTAATGAGTCTCGGCTTGTCTTGGCCAGGTCTCAATTTACAAAATGGCTTAGTCAGTAGCTTAGTCA
GTAGCTTAGTCAGTAGCTTAGTCAGTAGCTTAGTCAGTAGCTTAGTCAGTAGCTTAGTCAGTAGCTTAGT
CAGTAGCTTAGTCAGTAGCTTAGTCAGTAGCTTAGTCAGTAGCTTAGTCAGTAGCTTAGTCAGTAGCTTA
GTCAGTAGCTTAGTCAGTAGCTTAGTCAGTAGCGATGACATGGAATAGTTTGAAGTCTTTGAGCATTTAT
TGGCAACTTAGTAACAGGTTCACCACGCGATGACAGTTCGCAAACTTGATGACGGCAAGCCAAAACCCTG
GCTTGCCGAAGTTTATCCTCAGGGGCGTGATGGCCCTCGTAAACGCAAGCGCCATGCCACTAAGGGCGAA
GCCTTGGCGTGGGAAAAGCACATGTTAGCCCAACCTTGGCAACAGCCAGAGGCGGGGAATGTGGATGAGC
GGCGGCTGGGCGATTTGGTGGCTCTTTGGTATGGCCGACATGGCCAGACCCTGACCGATGGCGACCGGCG
CCGCGACAAGCTGAATTGGCTGGCGGATGCGCTAGGCAATCCCATAGCCACTGAATTTACAGCCCAACAG
TTTGCGGCCTATCGTGAGCGGCGACTTGCCGGCGAACTTTATGTGCCTGGTCAGCGCAAGCAGGTATCAC
CGACCACTATCAACCGCGAACTGCTCTACCTGCAGGCGGTGTTCAATGAACTTGGCCGCTTGGGTGAGTG
GACTCGTGGCAATCCGCTAGAGGTGCTGCGTCAATACAAGGTACAAGAGTCCGAGTTGGCATTCCTGAAC
CAGGATGAGATTGAACGGTTGCTGGCTGCCTGTGCGGGACTGAAAGACTTGTGGTTGATCGTGATGCTAT
GTCTCTCGACTGGTGCCCGCTGGTCCGAGATCGAGAAACTGACCCGCTCACAGGTTGGCATCGGCCGGCT
TACCTTCACTCGCACCAAGGGTAAGAAGAATCGCACCGTACCGGTGGCGGGTTGGCTGTTGGCACTACTG
CCCAAGCGAACCGGCCGACTGTTCGGCGACTGCTACTCTGAGTTCGAGAAAGCGATACGGCGTGCCGAGA
TTGAATTGCCCGCTGGGCAGAGTACTCACGTTCTGCGCCATACGTTTGCGAGTCACTTCATGATGGGTGG
CGGCAACATCTTGGTGCTACAGAGGATCCTCGGCCATACCGATATCAAGATGACTATGCGTTACGCCCAC
TTTGCCCCGGACCATCTTGAAGACGCTGTCAAATTAAACCCGATCACTGCGCTGAAAAATGGCGGCAAAA
TGGCGGCAGCGCTCACCAGCGCTCACCAGCGCTCACCAGCGCTCACCAGCGCTCACCAGCGCTCACCAGC
GCTCACCAGCGACACCCAGTGAGCCTCGTTCATTCCTATGTAACCCCCTGAAAACACGGCAAGTCATTGA
TTAAGAATGGCTTGCGATGGTTTCAAAATCCGCCGGTGAATAACCGTGTCGGTTCGAGTCCGACCCCGGG
CACCATATCACAGTTTCATGCCGTATCACGGCAGACCTGAAACCCGCATAGCTTAAGGCTCTGCGGGTTT
TTTGTTATGTCTTGTCGTATCATTTGGTCTCATAGCATACCCCAAATACTGGGGGCCTTTCTGGGGGCTC
AATGTTAAGCCCCCAGAATTTAGGCCCCCAACTGTGGGCCAACTGTTAACCTGCAAGGCTGCTAGCTCGA
TGCTTCCGCCACAATCTGCAGTAACTTAGGCCCCCATCATTGGATATGGAGGCCCCCAGATGCCTTTAGA
TAACATCACAGTCAAGCAGGCTATATGGAGTGGCAAGCCGAGCGGCGACAAGCTAACGGATGAAAAGGGC
ATGTTCCTGCTGGTTAACAAGTCCGGCAAGTATTGGAAACTCAAGTATCGTCATGCGGGAAAAGAGAAGA
AGCTCGCCCTAGGTGTCTATCCCGATGTGTCCCTGAAAGAAGCGCGAGCTAAGCGGGATGATGCTCGCCG
CCTGATCGCCGAAGGGTATGACCCGAGTCTTGTCCGTAAGCAAGAGAAGATCGCCAGTAGGCTTGCCAGT
GAGAACAGCTTTGAGGTACTGGCTCGGGAATGGCACCATAGCCAACTTGCCATTTGGAGCTCTGGTCACG
CCACCCGAGTAATAGAGTCGTTGGAGGCGGATGCCTTTCCTGAGCTGGGATTAGTCCCTGTCACAGAACT
GACGGCCCCTATGATGCTGGATACCTTGCGTAAGGTGGAAAAGAGGGGGGCAACGGAGACAGCAGGACGG
ATATTACAGCGGTTTAGTGCCATCATGCGTTATGCCATCCAGACTGGGCGAGCCAAGTACAATCCGGCCC
AAGATCTTAAAGGGGCCCTAAAAGCCACCAAGCAGGAGCACAGACCGGCCTTACCCAGAGTGGAGCTGCC
TGAGTTTTACCGCCGCCTAGCAGTTGAACCACTGAACCCTGCGACCCGCCTTGCCTTCCATTTGCTGATG
CTGACCATGACCCGCCCCGGTGAGGTGCGCTTTGCCCGCTGGGCAGAGTTTGACCTGGATCGGGCGGAGT
GGCGGATCCCTGCTGAGCGGATGAAGATGCGAGCCCCCCATATCGTTCCTCTCTCTCGTCAAGCGCTAGC
CGTGCTTGATGAGCTGCGCCAACTTACAGGACATTGTGAACTGCTATTCCCGAGCGAGCGCAGCCTGACC
AAGCCTATTAGCGAGAACACCCTGAGCTATGCCATGGGGCGTATGGGCTATGCAGGCATTGCTACGCCGC
ACGGGTTCAGGGCGCTGGCCTCCACCACCTTGAACGAGGAAGGGTTTGATCCTGATGTGATAGAGCGTCA
GCTCGCACATGCAGAACGCAATAAGGTGCGGGCGGCCTACCATCGTGCCGAGTACCTGGACGACCGCCGC
AAGTTGCTGCAGTGGCTGGCAGATTTTTATGAATCCCAGCAGGGTGGCAACGTGCGCCCAGTGAATCTTA
CATCAGTGGACTAACAACATTGAAAGGTGGCTCGATGACAACAACTCAGAATAAGCGGAACATTCTGAAA
CAACTTGAATACTGTAGATTAGATAGGGCTGCAGAGTTGCTAGGTTGCGATGTTTCAGATCTCATCCACT
TTGGGGCTACTGAGAGAGTAGCAATATATTTATATTTAAAAGACAACCCTACAGCTTTGGTTTCTTTTCC
AAAAATAACAGATTCTTTTCCTCAGCATCAAGACTTATGTGAGAAAGGAATATTTACATTTGACCTTAAT

ACATCCATGCGCCCTTTTGGCTTTGGAACAATAGAAAGTGATGATGATGGGAATATTATTGGTTTTGAAT
CTGAATCTGAGTTCAAAGCCGAGCTTTATGGACTTTGGCAGTTACCACCTTATATAGTGACTGACATTGA
AAATGACATGCTTCCGGATATGGAATACCTTCTTATACGAGTGTCGGCTGTAACCAGTGATGGTGATTTT
GCATATGCTGACTTGAAAGAGTGGGGGTTCCGCCCCACAGTTCATGACTGTTATCTTAAGAATGTCGATC
TAATTCGGATAGATGAATGCTTTACCAATAACGGTAACACTAAACCTTTGCTAAGGATTAGTAAGTCAGC
TAGCGAGCAAGAGGCACAATCTAGACCTCCGAAAGAAAGTAAACCTAATGGTGTTGCAGAACGCCATGCA
GTAAATCGTGAGATTATATTAGCTGCTGCGATCTATGCAAAGATAACTTGGCCTGACGAATGTGGTATCA
CAGCGAAAGAGTGGGCGGCCACAATATTAAATCATGAGCATGTATTGTTCAGTGAAACAAATAGTGGTAA
AGCCATATTGGCAGACCAGCAGATAGAGCGGATATTAGGCGCTGCTATGAATTCTGGTCGCCCTCACAAG
AGCAAATGATTATACGAATTTCATGTTGGCTCGATTATACGTTGCTTAATGTAAGGGGGGGGTGATGTTT
ACCTGATGTTAGGTAAGTTGAATGATGTTTACCTAATGTTAGGTCGATTTATTGATACATACCTTACATC
AGGTAGGCCGATTAATGCTTACCTGATGTAAGGTATGGCCAACCTATCATCGATACGACCTCAACTTGTG
TTTGAATACCCTTAACACGTTAAAACAATAAGGGAGTATTCAAGATATGTCGATGAACGAAGCCCATGCA
ACTAACCAGCATATATTACCAATGGGAGGTTATATCAGAGCTAAAGCATTAGCTCCTCTGCTGGGGATCT
CCGTTGTTACCCTGTGGCGCTGGTCTGCCTCTGGCAAGATCCCTAAGCCTGTGAAGCTAAGTGAGCGAGT
GACCGCATGGTGGATGGAAGATGTGCGCAACTGGATGAATACTCGTAATAATGGACAAGCAACATAGGGG
CAGAGACTTTGAGTGTTCAATCTGCCGGGCTGCATGGTCTCTTTTTAATGAGGTGCTGAAAGCAAGGCTA
TAAGTTGAAACTGTAAAGAAGCCATGGCATTAGAAAAGGAGAAATATCATGGCAATGAAGGTCTATCGAA
TAAACGATGTTGTAGAAGGATGTGTGTTATCCAAAATAACCATATATCGACTAATGAACAATGGGCTGTT
CCCGAAGTCAATCAAGTTGACTGGAAGGAGCTCTGTTGGATGGCGAAGATCAGATGTCGACCATTTGGTA
TCAACAGTTGGTTCTGAAGCTGAGGCTAGATATGAATAGACTATCTATCCGCAGAGATCTTTGTAATCTC
ATGGAATCGCCTGATGATACAATAATTGAGACTGAGAAACGGATTACACATCTTCTTAAAACTGGTAATG
AAAAGCATCAACAAATAGCTTGGAAGTTATGGTCTTGCTTAGAAGAGGACGGTCATGATTGTAACTCTCT
AGCATGCAAGCAGTGCACTAGAACTAAGAGAATGAAAGTCATCGAGTCAGTATTACCTAAGGTTATTGGT
AATAACTATCGGTTTGTCACTGTGATCCTTTATCATGATGTATTGGCGAAGAGTGAGTTGCATACGCTAA
AACCGCAGAAGTTGAAAGAACAACTCTATCGGGATCTCAAGAAAGTAGGAATCACAAGTCCAGTATATGG
AGCATTGGAAGTCGACTTTAATGAAGGAGAGCAAGTATGGCTACCACACTTTCATCTTCTTGTTGACGCA
GATGAGGACAAGATGAAGTCGCTGAAGGCAAAGCTTAAGCGTCGGCATAACATTGAATCATGGAATGATA
AGATACCTAGACCTGTGAAAGTAGACCCTGTCACAGATGCTATCAGGCAAGTGAGTTATATATACAAGTT
TATGTGGCAAACGGTACTAGTTAATGGGTGTAAGGTAAGAGGAGAGCTTAACGTATATTGTGCAGCATTA
GCATACCTTGATTCATTGCCCATAGAGACCTTGCAAGTTCAGTATGGTGTCAGAAGGGGAAAGTAAGGAA
TGACCATTTGGAGTGTCTACGCATATGTATAGTGTGTATATACTACAATATAATGACTTAAGGTAATAAC
ACTACAGTGAGATGAAAGGTAAGGGGACGGTGAAAGTAGTACTTCACCTTACCTCTCGTATTTGGTTAAA
TATATTGAGCTAGGAAGATAGTGAAAATGGCTTGCCAAGGACATTTCGCCAATTACTGATGCGGCTCCAC
GAGCACCTCAAAGACCTCGACAAACAGGTCCACGAGTTGGAGGACACGATCGTACGCTGGCATCAAGACA
GTGCCGCCAGTCAAAAATTGGCAAGTTGCCAGGCATCGGGCCGCTCACCGCCAGTGCACTGATCGCTTCC
ATCGGCGATGCGAAAAACTTCAAAAATGGCAGGCAGTTGGCCGCATGGCTGGGGTTAGTCCCTAGACAGC
ATTCCAGCGGTGGCAAGCAAACGTTGCAGGGGATCAGCAAGCGCAATGACAGTTATCTGCGGACATTGTT
AATTCTTGGCGCTCGGGCCATGTTGCGGGTGGCAGAGCGTCGTTTGCAGGTAGCTGGCTGGCCGGGGTGA
TAGGGCGGCGCAACGACAATATAGCGATAGCTGCGGTTGCACAAGCCAACAAGACGGCCCGAATCACCTG
GGCGTTGCTGGCACACGACAGAGAGTTCGATGCCTATTATGGCGTGGCAGCATCATGATGAAAAGTATCC
TTATCAAGGAAACGGCACATGATGATCGCTCTTGTTCAGTAGGTGACAACCCGTGAATCATCAGCCAGAT
TGTCCAATGGAGTACGAGATAGTTCCGTACTAGCCAACATCAGATGGCATGATCGATGGCATAGCGAAAA
TCGCACATAAAGAAAAATCCCAAGCAATTGAATTGCTTGGGATTATCATTTGGCGGTGAGGGAGGGATTC
GAACCCTCATTTTGCACATCACCACTCAAATATAACCATATTTTTCAACTACTTATGATATATTTTTACC
TGTAAAACTTATGCGATTGTCTCATGACATTGCCGTGAGATTTGTAGCTTATTTATACTACAAAAGCTAG
GGAAGTCAACATGATGGGACTACTGTCGCACGCTTAAAATTTCTAATTTTAATTCAACAGCAACCGAAGG
CAGTTCGTTCAAATAAAGAGTCAGGAAGTGCCATTCCCCCAGCTGTATCCCGTGAAACTCGCTGAATCGA
CAATCTCAAGCGTTTGCGATTCTTGGTAGCACTTTCCGAACCAGCCTTCGTATACTATCGTTATAGTAAT
CTTTCCTCGTACTTTCCAAAAGCCTTGATCATTTAAACGCGTTGTGCCGAACGAACAGGATGTAATCGCC
CCATTATGTAACACTGTTATTTGCTGTGTATTTGAGAAGCAGGCAAGCCAACGTTTCTTGTTTTCTTTTG
TTGCATCTTTACCAAGAGCGTCTTCAAGAGTAGACTCATCAACATTGAGACGGATATTCCTTGCAGGAAT
TGAGCCTGAGTTTAAAAGTACAAGATTATAGGCAATGCATTCTAAACCAGCCATTTTCGTTCTAACCGCA
GCGGTCACGATCGGACGAAACTGCCTTCGCCAATAATAAATTGACAGCGTCAGTGCTATTACTGATACAG
CTAGTGCAACAAGTGCGATGACCGTATCAACGTTGTTCTTAAGTTCGACTTCACAAAAAATGAATTCCAT
ACAGTTCTGCCTTGTAACACCCTAGCGTTTAATTAAGATGTTAATTTTAACTTATACTATACCTAAAATA
TAGCAAACCATACGACAGCCGATGTTGCGTCAATTTAGTTTAACTAATTTTGTATAGCCTTAAGAATGCC
ATCATCGAAGTAATAAAAACTAAGACCACCCGTAAAAGACCTATATACCCATTGAGAGTGGGTGCCATAC
TGAGTTTCTGTTTTGTTAATGTCAATAGGGCTCCCTATAGCAAGACGAACCATAGTAGATGTCATTCCGA
GAGTAACCTCGCCTTGCTCAATTTGTTTCCATACCTTGGCGGGCCAGTCTTTATAAACGTCTCTTGGGTT
ATCCATATGCCAATCTAAATTAAATCTGGACAATCCATTTTTCATGTCATAGTTAATGTATACAATTTCA

TTCGTTGCTCTTTTTAAGAACACACGCGCCTGATTTTTTTTACCTATTCCATCAGGAATGGTAACATTAT
AGACGGTTAACTCTTCAAGATTTTTAAGCAATGAAGCATCAGATACACTATCGACTTGATCAATGTGCAC
AATTTTTTCTGGTTGTGACCAGACTTTTTTCCCAATAAGAGACTTGAAAGCAATGAATGCTTCCTTCATT
TCATCTGCAAACCTCTCATTACCTAGCGCCCCGGCTACATCGGCACTATTTGTGTAAATTATATCCGGTA
TGCCTTCAACTCTGAGTGCAATAACATGTTGATTAATGCTGCTTGGACCAGGAACATTTTCATAACATAC
AACTGTTGCTTTTTTTACCTTCGATCATTTCTTTTGGAACAAATTTATTATAGTTTGCCCACATTGCTTTG
TTATATCGATAGTAAAATGAGTAGCTATTTTCTGATTTAACTACGAACGATTGCCCATCATAAGAGGACG
CCTCAGTGCGGGTACACGGAGGTTCTATGGCGGTTTCTGGAGGGCTTACACAGCTACTGAGCACAGCCAT
TGTCAGCAATATTAAAAACTTACCTCTCATTTCATCTACCTTATATTAATGTCGCGTGATGATATTAAAG
ACCTATTCCGAGTGATTCGAACCCTCTTGTTCGTACTTCACAGCACAAATATAACCTTCAATTTCAATAG
CTTATGCAATGCTTTGTCGCAATAAAAGTAGCGTGATTGTCTCAGGTAATTGCTATGAGACTTATCGTAC
TCTTATATAGAAAATTAATTAAATGTCAACCCCATAAATTGTTGCAATCTTAACTATGTTGTAGTGCCAG
GCGGCCCCATTCGGGGTTGTCTGGGGTTTTGCCAACAAGACCAATGTACATTCATCAATCACTATGTTTT
GATGTAAGCTTAGTCCAATCCAACAAGGGCTCACTTATATTCAGTCGTCTGGCAGACGGTATTCATATCG
AACAGCTCATTATCTTTGACGACGTTAGACACAAGGTCTTAAAATCTGACGTTAGAAACAGGGTCTTAAA
ATATAATGGATCTTTGCCTAGGCCCTCTAGAGCCAGATGTACAGTTTTACGCTCCCTTGGCTGCTTTTAA
GCCGATGACTATCAACAACTGGTAAAATACTACATGTCTTTGCTCAATCACAGGTCTGTAAATTTAGGGG
GTGACCCGATACAATAAGAAGCTACAGCATTCACCACGCTTATCAAGACAGACTGTAGTGCTGAATGTTA
GTGAAAAGTCAGAAAACCAACGCAGAGAAATTATCAGTCTTACGTAATGGGCATCTGGCAGAAGGCTCAA
CAACTTTTAAACTCATTGTAGTAAAAGTGCTGTTGGATAATACCCGATTCTTCGTCACATAACGTTTGCT
CTATAAATGAGTTAAAAGAAGGCCCGCAAATGAATAAAGAACTCACTATCAAACTACTAATCACGGCGGG
TCTATTTGGGCTTGTCGCCGTTGCATCTACATTAAATAAATATAGCACAACAGAGACAACTACTGTGCCT
CCCAAATTAGATTACAGCAGAACAATGTTCACCGGCCGTAAGTCAACAATTTGGCACAACATGAGCGACT
TAGAGAAAGGCTGGATTGGAGGAATGGTCATTTCTTCCGCGGGGCCGTTCGGATATTCAGGGGGATTTAA
AATACTGGAGATGAAGGCAGCACGTAGCCTAGATGAAAATACAGTAGTGGCATGCGGGCGCCTGCAACAT
GGTGATAAGTTTGGCATATTCTCTGTATTATATGATCCAGCAACACTTTCACTTAACGGGCATGTCGTAT
ATCGCATCCAAGACGATATTATGTCCGTCAGGCACTCAGTGGAAACGGAAAAAATGGCGGTTTCACCGAT
GGACTCCGCTTTAATAATGGCTGACTGCAAGTATGTCGGACTCTTGTAAATAGATACTCATGATAGTAAA
TACCCCCGGCAAAGCCGGGGGGTATTTACTTCTATTTTCCGACTGACTAGAGCCCAAAACCCGGTTCTAG
GTTGATTTATAAATCATTAGATTTAAATATTGACATTATATTTTTCGTTTTATCTAAATCTGCACCATAC
ACAGGGCAAGAACATGAACGTTTTTGTTCAAATTGCTCCAGTTGTTGACCCCGAAGATCGTGGTGTGATC
TTATGCTCTCTTTTTGAGGGGCGTTGTTGCCCAAATCGATGCAGCTTGAATGGAACTAATTGAAAACCCA
GTGATCTGATCACTACCATCCTCTTTAAGTCTGCATCATGGGCACGTCGCTTCACCCCATCACCAACTGG
CCTCAATACAACAAGTCTTTGATTAACCGTGGTTCACTCACCTTCTGGGTTGATGCCGAGGCCATGAGCA
ACTGGTTTCACCACGACCATCATGGGCGGCGGGGCCGCAGCCAGCTTTACACTGACCAGACCATCTGTAC
CTTCCTGATGCTCAAGGGGATTTTCAGCCTCACGCTGCGAGCCACTCAGGGGCTACTCGACTCCCTGTTC
GAGCTGATGAACGTGCCGTTGTGCGCACCGGACTATAGCTGTGTCAGCAAGCGAGCACGCACGGCGACGG
TGGCTTATCGACAGTCCCCAAAGGGACACATTACCGACTTGGTTATCGACTCAACCGGGTTGAAGGTGTT
TGGTGAAGGCGAGTGGAAAGTCACGATAAAGGGGAATTCTATGCCAGCGGGTGATCTATAACGTGTCTCA
TACCAAGGAGCTCATCATGGGGGCCTTTTTGGGGGCCCAAGGTTGGGTTGAAAGAAAAATTCCGCTTAAA
AATCAATTATTTTATCCATTAATTCGATAGAGACCCCGGCACCATCAAATTCATGACAAAAGGCCATCAG
CAATGATGGCCTTTTGTTTTTTGGCGTTCCTGCGCATTGGTGCGTTGCTGCTGAGCAGGCAGTACTTCCC
GTTACCTCTTGGTATCCCTTCTCTTTTTCCGCTCTCCATTATTTCCTTTTCTTGATGTGGCCGCTGACGA
CATCACCGCCAGCGGCCTGTTTCTATTGCGGTTTTCTTTCCAAACCTTGCTGCATCAGATCATGATTGCT
GCGTTACCCCAGCAGGGCAAACAGCAGGGTGCCGAGGGTGCCAGCGAGTGTCAGCGCTATGATGGCTCTG
GGTGTGCCGTGCAGCTTGGTCCACATAAACAGGCTGGTGAGGGCGAGCAGCAGCAGGGCCAGGGCGGCGA
TATCGCCAAACAGCTGCCAGCCGAGGCCGGTGCCCATACCGCGGTGCAGCCGGTTGAGGCTGGCGGCAAA
GTTGGCCAGTTGCAGGTCGGTACGTACCAGCAAGGTGCCCTCAACATAGCTGGCGGCCACGCTCTGGCTG
AGGGTAACGCCGCGCATCTCCCACTTGGCGGGCAGGGTGAGCGGGCCGTTCGGGGTGGGCAGGGTCTGGG
CCGGGGTGATCATGGTTCGCCCGTCGGCAAAGGTATCGGGATAGCGCTGTTGCAGCAGGCTTTTCAGCTG
CTCCGGGCTGGCGAGCGGCGCCTCCAGCACTATCTCTTCACTCGATTTGTCGGTGTGGGGGCCGGGGATG
GGCAACACGGCACGGTGTTGCAGCCAGAGGCCGGTGATGCCGTACATCAGCATCAGGGCGAGGGTAAAAA
AACCGGCCCAGGCATGGAGGCGGCGTACCCATTTGTTGTTGTGAAGGGGAACTCTTCTGCGTTTCATCGT
GGTTGCTTCATAAAAAACTGGCCAGCAAGGGCTGGCCAGTCGGGGTGGTTGTCACAGAGATCAGAAACGG
TAGGTACCGGTCAGGCTCAGCTGGCGGGGTGTGCCCGGCATGATCTGGGTGGCACTGGTGGCGCTGACGA
AGTACTCGTGGTCAGTCAGGTTCTCGGCGCTCAGCTCGGCTTCCCACTGCTGCCAGCGATAACCGGCGCG
CGCATCGAAGCGGGCGTAACCGGGCAGTGTGGTGGTGTTGGCGTTGTCGGCGAAGCGATCGCCCACCGCC
GTTACGCCGGTTTCACCGAACCAGCCCTGCTCGCCACTCTTGTAGCCGAGGTAGAACTGGCCATTCTGGC
GGGAGACGTTCATCGGGCGCTTGCCCTGCAGATCCGCATCCGCCTTGACCTGCTCCGCATCCTGAATGGC
AAAGCCGCCACGCAGATAGATCTCGTCGGTCAGCTCGGCGCGGGCGCTCAGTTCGACCCCTTCGGTACGC
TGCAGGCCGGTGAGGATCACCTTGGTGGGATCCAGCGGATCCTTGGTGCGCTTGTTGTACATCTCCAGTC

GATAGAGGGAGAGAGTGGTCGCCAGCTTGCCGTTCAGCCAGTCACTCTTCACACCACCTTCGTAGAGGCG
GGTGTGCTGGGGTTCCAGCCCGTTGTTCTTGTCACCCGGGGTGATGCCGATCAGCTCGCCGCCCACTGGA
GTGAAGGTCTTGCTGTAGGAGGTGTAGAAGGCGTGATCCTCGAGGGGATTCCACACCAGACCGAGGCGCG
GGCTCAGGCTGTTGACCGAGACGCTCTCCTCCTTGTTGAGGTCGTAGCGGCGACTGGTGACGGTGAAGTC
GTCACGGCGCAGCCCGCCAACCAGATGCCAGTCACCCAGGCTGAGCTGATCCTGCAGATAGAGGCCATTG
CCGCGCACCTTGTGTTTGGCATCGCTGGAGAGCTTCATGGCGCCACTGTAAGTAGGCAGCGAGCCCGGAT
CGTAGAGATTGCCAGACGGGATGGCGGTGGCATTCTGGTAGAGCTTGGGGGTGCGCTCCTGCCAGTTCTG
CTCGAAGCCAACCAGCAGGCGATGTTCCACCGGGCCGGTCTGGAACAGACCCTCGGCCTCGGTGTTGCTG
ATCAGGTTCTTCGCTTTCAGATCCTGCTGCCAGCGGGAGCGGGTCACCTTATCCCCTTTGACGCTGGTGA
CATAGGTGTTGTCAAACTGGCTGTCGAGGGTGGAGTAACCGAGCTGCTGGCGCAACTGCCACTGATCGTT
GATATCCCAGGAGAGGCGGGAGCGGGTGGATTGCGCCACGTCATCTATGAAGTCGCGGCTGGTATCGCTG
TAAACATACTCTCTCGGCACATCGGCCGGACGGCCATTGACCCCGGGAATGCCGCGATCTGGGGTGCGGT
CATGTTCGTTGCGCTCGTACTGCACCAGCCAGTTGAGGTTGTCGCTGATCTCCCAGTTGGCGGAGGGGGC
CAGCAGGGTGCGTTTGCTCTTCACACCGTTGCGGAAGCTATCTTTGTCCTCTTGCGCCATATTGAGGCGC
AGTTGCACCTGCTCACTCGCCTCGCCATTGAGGTCGGCGGCCAGTCGCTGACTGTCAAAGCTGCCGACCC
GGGCGGTGACGCTGGACTCCTGCCCTTTTTGCGGCTTCTTGCTGACCCGGTTGACGATGCCCCCCGTGCT
GCCGCGGCCGTAGAGCACGGCAGCCGGGCCCTTGAGCACTTCCACCCGCTCGATGTTGCCGAGATCCCGG
GTGTACTGCATGTCGTCGCGGAATCCGTCGAGATAGAAGTCGTTGCTGGCGCTGAAGCCGCGGATATTGA
CCCCGTCGAAGCGGGTATCACCGCTGGCGTTGACCCCGGGAATGCCGCTCAGAGCTTCACTCAGCGTAGG
TTGGCCGAAGGCGGTCAGCTCGCTGACCTTGACGCTGTCGATGGTCTGGGGCACCAGCTTGGCTGGCGTA
TTGGTACGGGTTGCGGTGGCAACTTCCTGATGCTGGGATCGTTTGCCAACCACTGTCATGGTTTCATCGG
CGGCCTGGGCAGTGAAGCCGGTGCCCAGCAGTGCCAGCGAGACCAGCGTTCTGCGAAAGTGAGGGTGCTG
CATATTCAATATTGACTCTTTGGATGTCGACCTGACGGCTCGAACATATTGATTTAATTATAATTAGCCT
GACCCGCTTAAGCGGGATGCCAATGATATTGATTTTCATTTGTCTGGCAAGGTGAAAATGGAGGGGGCTT
TGGGTGCGTGTTGTGTGGTCGGCAGAGCTCGGCTGAAAAGCAGAGCCATTACAACAAACCGCGAGAACGA
AGCGAGGGAAAAGGGCGTAGCGGAAGAAAAAGGTGCGAAATGTGAAGCGATATCCGGCCCGATAGGACGG
GCCGGATATCGGACTCAGCGAGTGCTCAGCAGTCGGCGGGTGAGGGGGATAGTCGCCAGCAGGCGATAGA
CCAGCAGAGACGATCCCAGCAGCAGCGGCACCTTGGCGATCTCCCACAGGATGGATGCGGCCTGCGGGCC
AAACACGAAGAGCCAGACCACCAGCATCATGTGCAGGCAGTAGATCCCGAGCACTTTGGGGGCCTGGCGC
TCAAGCCAGCTGCCCTTGCCCAACTCGGGGTTGGCCAGCAGCAGGCCAAACAGGCCGATAGCCCAGGGAA
GGGAGCCGAGCAGGAAGTCGTGGCGGGTGAGCGGCACTGCGGCGAAGTGCTGCAGCGCCCAGGCTTCACA
GCCATAGAGCGCTACGCCCGCCAGCAGCAGTTGCCAGCCGGTGCGTGCATCGGGCTGCCACTGACGCTGG
CGCAGCAGGGCACCGAGCGCCACAAACAGTAGGGAGAAGAAGGGGCCGTTACGGGTGAGAAGTGGCCACT
CGGAGCCCAGCAGCGGCTTGGCATAGGAGCCCCCCAGCAGAGCCAGCAGGTAGAGGCCAAGACCCAGCCA
GAGGGCGAGTTCTGGTCTGCCAAGCCGATAGCAGAGTGCCAGCAGGGCCACCGCCATCACCAGGGCTGGT
AGAAACCAGAGGTGGATCATGCCGCCCACCAGCCAGCCATTGAGGGGGTCGCCGAGCTGGAATTGCCACT
GCCCGCGCAGGGAGGGCAGATAGCCTTGCTGCACGGCAGCAAGCGGATTGAACGGCACTAGCAGGTAGAC
CAGACTCCACACCAGCCAGAGCATCAGCAAGGGGCGGCAGTAGCGCCAGGCCACGGTCAGCGGCGCTTCG
CGACTCAGCCTTGGCTGCAGAAAGTAACCAGCACAGAGGAAAAACAGCGGCACGGCAAAGCGGCATAACT
GGTTGATGGTGCCGCTCAGCCAGAGCCAAGATCCACTTCCCCACAGGGCGGGATTGAAGGGATTGGCGAA
GGGGGATACGTGAATGCTCATCACGGCCAGAATGGCCAGTACCCGCCCGCACTCGATACTGGAAATACGC
ATGGTGGCTCGTCGCTACAGGGGTTGTGACAGCACGCTAAAATTCTTGCCGAAAACTTACAACCAATTGA
TTCTATTAGTGTGAATCACGCTGTTGAGTGGTTACATTTTGTAACGGGATGGTGGAAGGTGTTACCTGTA
ACGGGAGCGGGCTCCCGTTTTTTGGGCGCGCTCATGCAGATAAAAAAAGCGAGGCGGGCGCCTCGCTTGG
TTGGGGATCTCGAGCTTACTGGTGAGTCAGCATGCTGCTCTGGTTGGCTGCCAGGGTGGTTCTGCCCGAG
CTCTCCAGCCAGGTTTTGATCCGCTTGGCGTCGGCGACCCGATCGTTGGTGGTCTCGGCATTGAGCAGCA
CCATGATCACCGGCTCACGATTGACCTTGGTGCCCAGCACCAGGCAGCGGCCCGCTTCGCGGATATAGCC
GGTCTTGGAGAGGGTGAACTCCCATTGCCCTTCGCGGATCAGCCGGTTGGAGTTGAGGTAGTGCAGCTGA
CGCTTGTTGGTGCGCACATAACTCTGCTCGTCGGTGGAGTACTGGCGGATCAGCGGATAGGAGGCCGCTG
CGGCGGCGAGTTTGGCGAGATCCTGCGCGGTGGAGACATTGCGCGGGTTGAGCCCTGTGCTGTCGGCGTA
GCGGGTATTCCACATCCCCAGCATGCGGGCTTTGGCGTTCATCGCCTCGACAAAGGCACGCTGGCCGCCG
GGATAGTGGCGGGCCAGCGCCGAGGCGGCGCGGTTTTCAGAGGACATCAGGGCGATATGGAGCATTTCGG
CGCGACTGAGCCTGGAACCTATGGCCAGGCGCGAGCCGGTTCCCTTGATGCGGTCGATGTCGGCATTGGT
CACGGTGAGCATTTCGTTGAGGCGCAGGTTGGCATCCAGCACCACCAGCGCCGTCATCAGCTTGGTGAGT
GAGGCGATGGGCATTACCCGGTTGCCGTTGCGCTCGGAGATCACCTCGCCGGTTCTGTGGTTGGCCACCA
CGAAAGCGGCCGAGCTGAGGTTGGGCACATCCCGTCGCTCGAGAAAGCCGCTGCTGCTCTTGCTGCTGGC
GGAAAAGGGGTTGGCGTGGGCACTGTCCATAGTGGCCGGCAGCATTACTGCGCCAAGCAACAGGGGAAGG
AGGGAACGCATCATCATGATCAGCTCTACCGGACGAAATACAGGGATATCTCGCTAAGTTATATGTCGTG
AACTGGCGCTTCACAATCATTCTCACCTTGATATTGATAATTTACGGCGTTGAGCGCGCTCTGGCGGCGA
GGAGTGCGTAAAAGAGGTCTCCATGGAGTCTTTTTTTACATTGAAACTATGGGCTGGTCGTGATGGCGAG
CGGAAGCCGGCGCTAAAAAAATCGGGACCAGAACGGTCCCGATTGGCATGGAGCATTGCTTACCGTTTAG

TGGGCATCTTCCTGCTTGATCTTGAAGCGCATGGCGATCACCACGAACAGCAGCGGGCCAATGATGGTCC
AGACGCCGGTGGAGATATCGCCGGAGATAAAGGGGGCTACCACGGTAAAGACGTTGCCGAAGGCGACCAG
GATCAGGCTGATGATGGTGGCCAGCAGTACCGAGCCCGCGCTCTTGAAGAAGATCAGGTCCTTGCCCAGC
GCCGGGTTGTTGCGATAGCGATACCAGGCGTAGATGAGGAACATGTAGGGCAGGGTCATGCCGACGTTGG
TCATGGTGATGATCAGCTCATAGAGGGCGGCGGCCCCTTCCGGGTTGATCAGGGTAAAGATGGACTTGGC
GGCAATGAACAGACAGACCACCCAGGCTTGCACCTTGAGGGCTTCAATGCGCACACCATTCTCGTTCTCG
CGTTGGAAACGCTCCGGCCAGAAGTAACTCGGGGTGCCGGTGATGAACTGGCGCAGCGGCGCGTAGGAGA
GGGCGATAAAGGCACCCAGATAGCTCAGGAACATGCCAAGGCCAGCAAAGCGCATGAATACCTGTCCCAG
CCACTCGCCACCAAGATGGCGCCCCAGCTCCAGCATGATGATGAAGAGGGCGGAGAGAGAGGTGATCCCC
TCGGCCGGGAAGTCGCTCCAGCGCATGATGGAGCCGACCGCCAGAAAGCCGATGATGTAGCAGAGCACGA
TAAAGGCGCCGGAGAGGAAGATGCCGCGCTTGAGGTCACGCTCGGGATCTTCCAGCTCGTCAGCCACCCC
GGCCATCGCCTCGACCCCGCCGTAGGCGAAGATGGCGAACACCATAAAGCCGAGGAAGGGGATGATGTCG
TTGAACGCGGCATTGGGAGAGGCTACCAGTGATGCCTGGGTGAGGGGCTCCTGCAGGGTCATGCCGCTCA
CCGCGAAGGTGAAGACGCCACCGACCAGCAGCAACACGTTGAGCGCCACCACGGAGAGGCCGCCGATGGC
CGCCACTTTGGCGAAGCGGGCGCTACCCAGGGTGATGAGCTTGGTGACGGTGGCAACCAGCGCGATGGCG
ACCACTCCCAGAATAAAGGGGCCGAAGTCGACCCCAGTGCCCAAGGTGACCGGGATGTCGCGGCCAAACA
GCATGAAGGAGAGGGGCACCCACATGGAGAGCGCCTTGCCAAACATCCAGATGACGTAGGAGGCGAACCA
CATGGTCAGACCGATAAAGGCGAATCGGGTCGAGACCGAGTGGCGCATCCAGGTAAAGATGCCGCCCGCC
TCTTTGCGAAAGCCGGTGCCGAATTCGATCATCATCAAAATGAACGGGATGAAGAAAAACAGACCGCCAA
TGACGAACATCGGAATGGCGGCATATCCCATTCGGTAAAAACCGATCCCGATATTAGTGACGCCAAATAC
CGATGTTAATATCATCAATATAAAGGCGCCCAACTTCAGCTTTTTTGCTGACATATTAAAAACTCCCTGA
TGGTGCTGGATTTAAAATAATCAAAAATTAAACGGACGAAACCGGACCCATTAATAACGCGGTAGCGCCA
TGCCATCCTGCTGTGGCGGGCGCGATCGGGCCATCTCTGACCGGGCTGGCTTACCCGTCAGGCGGTCAAT
TAATGTATGGGCGGAGCGGCATGGTCTGTGCCATGCGCGGTATTGGTCAGCGTTGCCGAAAATTAACTGT
GAACGATTTTATCATTAATGCTGCTGGGCAGGTAAGTTTCCCGAATGAAGCTGTGGTTGAAATAAACAGA
CCTGCAAGTTAATTGATACGTTGCGGATGTAAACATTTACGCCCGGGCCGACACTTATTTCCATTATTGG
TATTCGGCATATTTGGCGGAGTGAATTACGCCATCCTGTCAGATAAATCTGCACCAATGGCGACAGCGCT
TATCCTCCCAGCCGGGTCAGCAGCACGCCGCCGGTGATAATGGCCGCCGCCGTCAGCCGCATGCGCCCGA
GCGGCTCTTTCAGCAGCCAGACCGAGAGCAGCATGGCAAACAGCACACTGCTTTCGCGCAGAGCCGCGAC
CACGCCAATGGGGGCTTGGGTCATGGCCCAGATAACAATCCCATAGGCGAGCAGTGACATAGCCCCTCCG
GTGGCGCCAACCAGTGCATATTGACGGATCTGGCGCCATGCCTTGCGTTTCTGGTGGACGAGCAGCACCA
GCAGCATCGCCAGCCCGGTGCAGGCAAAGAGCCAGAGGGTGTAGCGTACCGGTTCACCCCCTGCACGGGC
GCCGGCACCATCCGAGAGGGTATAGGTCGCCGTGAACATGGCCGTGATCAGTGCCGCCCGAACCGCTTTG
CTGTCAAGGCGCTGCTGCCCACCTCGCCAGGCCATCAGCAAGACCCCCATGATCAGCACGCAGACGCCGA
CGATGCCGCCGTAACCGGGGTAGTCCCCCAGCAATAAGGCACTTAACACCATGGCGAGCAGCGGGGCACT
GCCACGGGCCAGCGGATAGATCTGGCCAAAATCGCCCTGATTGTAGGCATGGCTGAGAAACAGGCAGTAA
CCGATATGGAGCAGGACTGAGAGGCCGAGCCAGGGCAGCTCGGCAGCAGAGGGAATCCCCATGGGCAGCA
GCGCCACGGCGGCGATAAGCCCTGAGAAGATCGCCACCAGTGATACCCCCACCAGCCGCTCTTCGCCAAT
TTTGACCAGCGCATTCCAGCTGGCATGAAGCAGGGCGGCCAGCAGAACGGCAAAAAAGACGGTGGTGCTC
ATGGGAAGGGGCTCTCGGGCGATGGGGGTCAACAGCATACCCGCCCGGAGCCTGATTGGCCGAGATCTTT
ATCAAGGCTTCGGCTTTTCGGGGCTCGCCTCCTGCGTGGTGGCGTTACCCAGTTGCCCCAACACCAGTTT
GGGATGGGGATCGGTCTGCAACGGAATATCTTGCAGCCCGGTTCCCTGCTGGTGGCGATGGATGATCCCG
GCGGCCATCTTGCCCAGTGCCTCGTAGCTGCTGATGGTGCCAACGCTACAGCCGTGCTCGACGAACAGCT
CGGCACTGCAGATGGTAGGGATGGCCGCTGCGTTGAGCGCATTCAGCAAACGGGGGGCGATGGAGAGCAG
ATAGCTGTCTGCGGCCAGAAAGACGATGTCGTTTTTGGCATCCTGTTGCAGCCGCTTCAGTTCGTTGTCC
AGCCGCCGTGGGTCAGGGGCCATTCGCCAGGTGACCATCTGCCACTGATAGCGTTTCGCTTCATTGAGCA
GCATATCGGCCACCAGCAGGGTGTTCTGCTCACGGGGGTTGAAGGGTAGCAGCAGCCGTTTGCCGGTGGC
CAGATGCTCGCTGGCGTTGGCCAGTTGCAGCGCCATCGGCACCATGTTGCTTACCCCGGCTACCATGCTG
TTCTGGGCTTTGTCTTCCGAGAGAATGTCAGCCCCGACCGGATCGGAAACCACATTGAAAATCAGAGGCT
TGCGGTTGGCCACCAGACTCTTGGTCATGGTGGTGGCGGTGGTGCCAAAAGTGTAGATGTAGTCGTATTC
GGCCAGTTTCGGCAGCATCTGCTGGCGCAGCATGGTTGCCAGCGCCGTACGATCCTGATCGGCGTTATAG
GTGGTGATGGTGGCCTGATAGCCAAGGCGGGTCAGCTCTGCCCGAAAGCCCGCTTCGGCTGCGGTTTCAC
CGCGCCACAGCACCATGGCGATAGAGAGGGCGTGAGCGGGAAGAGCCAGCAGCAACAGCAGGACGAAAGG
CAGCATGACCATGATCATCTCTCCGGCGATACATCTACTGTTGAGCCTAGGCCACTTTCTCCGGTCGTGA
GCGACGCCCAGCAAAAAGCGCGATGGCAAGCCATCGCGCTTTGGGGATCCGCAAAACCTCGATTAGAAGT
CGTAGCGGGCAGCCAGCTGGAAGTCATCCTTGACGTTGAGCTTGCCGCTCTCCTTGCTGTCCAGATTGTT
GATACGGTACTCGCCAATCACCCGCAGGTTCTTGTTGAATTTGTACTGGGCACCCAGGGTGTAGTAATCC
ACGTTGTCTTTCTTGATGCCAGCCTTGTCCTGCTCCTGTTTGTTCCACAGCGCCATCAGACCGAAGCCGT
TCTCGAAGTTGTAGCCCAGCGCGGCTTCCCAACCGGTGTGATCGGTGCCGGTCGCCAGGAAGTCGCTGCC
ATCGGCATAGCTCAGGGCGGCGTAGGCGGCCTTGTTGTCGAACTTGGCGGAGATCAGCCACAGCTTGGCA
TCTTCTTCACCGCTTTTGTCGCGGATACCGACGTTGTAAGCGGTACCCAGAGACAGGTTGAACGGGAAGG

TGTAGGCCACGGCAGCACCGTAGGCGGCGTCGCTATCCTTGCTGGTGGTCTCTTCGGTGTCACCACCGTC
GAACTTGTAGCTGGTGTCGAGCTGGAAGCCATTGAACAGGCCGGAGTATTTCAGTACGGAACCGGCGCGG
TTGGTGGCGAAGGTGTCGGTCTTGGCGCCCAGCGCGTCGTTGCCGTAGCCGTCGGAGAGGGAGACATCAG
TCCAGTCGGCCAGGAAACCCGGGGCCCCTTTCTGGCGACCGAAGGTCACGGTGCCCCAGTCAGCGCCGAC
ACCGGCGAACGCCAGACGGGTGCGCAGGTTGTCGGAGTTCTCGCTGATGGTCTTCTCGCTGTCATTGATA
TACATCTGTACTTCGTATTGAGCCAGAGCCTTCAGGCCGCTCTGGATCTGGGTCTCGGCCTTGGCGCCGA
AACGGACAAACTGGTTGGCACCCTGCTTGTCGCTGTATTCGCTTTTCGGTTTGCCGCTGGCATCCAGTTC
CTTCTTGTCACCAAAGAAGTGGCCAGCGTAGATACGGCCATAGAGATCCAGCATGGAGGTGTCATTTTTG
TACACTTCAACGGCTTGAGCCTGGAAGGCTGCGGTGAGAGCCAGTGCCACTACAGTCAGCTTTTTCATCT
TAATCCCTTGTGGTGTTGCAAGCTTTTGTTTTGAAAACGGGGTCATGCTAGGCAGCGGGGATGACAGCGG
GGTGACAGTTGGATGAAGAGTCGATGGCAATTGGGTTGCAATTGGATGAATTGGCTACATTCTTGCTCGC
AGCGGCTACTTTTGTGCGCAAAGGTTTGCCCTTGTGGTAGAATGCCGCCCGTTTTCGAGCTCCATAGGTT
GGATATGCGATGAGACGTGAACTGGCAATCGAGTTTTCCCGAGTGACGGAAGCGGCTGCACTGGCTGGCT
ACAAGTGGCTCGGACGTGGTGACAAGAATATCGCTGATGGGGCGGCCGTAGCGGCCATGCGCCACATTCT
CAACCAGATCGAGATCGATGGTGAGATTGTGATCGGCGAAGGTGAAATTGATGAAGCGCCGATGCTCTAT
ATCGGCGAGAAGGTTGGTACTGGACACGGCGACGCGGTGGATATCGCGGTCGACCCCATCGAGGGTACCC
GCATGACCGCCATGGGTCAGGCCAACGCGCTGGCCGTGCTGGCGGTGGGCGACAAGGGTTCGTTCCTGAA
AGCGCCTGATATGTACATGGAAAAGCTGATCGTCGGGCCCAAGGCCAAAGGCGCCATCGACCTGGATCAG
GCTCTCGAGCTGAACCTGAAGTCCGTGGCCAAGGCGCTGGGCAAGCCGCTCTCCCGTCTGACCGTCATTA
CCCTGGCCAAGCCGCGTCATGACAAGGCAATCAAGGAGATGCAGGGTCTGGGCGTGCGGGTGTTCGCCAT
TCCTGATGGTGACGTTGCCGCCTCCATCCTCACCTGTCTGCCGGACAGCGAAGTCGACATGCTGTATGGC
ATCGGCGGCGCACCGGAAGGGGTGATCTCCGCGGCGGTGATCCGCGCGCTGGATGGTGACATGCAGGCCC
GTCTGGTGCCGCGCCATCGCGTCAAGGGCGACAGTCCAGAAGTGCGTGCGCTGGGCGAGCAGGAAATTGC
CCGCTGCGAAGCGCTGGGCATCGATGTCAACAAGGTGCTCAAGCTGGAAGACATGGCGAAGAATGACAAC
GTCATCTTCTCCGCCACCGGCATCACCAAGGGTGATCTGCTGGATGGCATCACCAGCGATGGCGTGATGG
CGACCACCGAGACTCTGTTGATCCGTGGTAAATCCCGTACCATTCGTCGTATCAAGTCGATCCATTACCT
GGAACGCAAAGACAGCGTGGTACGTGACATCATCCTGTGATGATGGCTCGCTGAACCGGCTGTGTCTGAA
GCCCGATCGCCCGTGCGGTCGGGCTTTTTGTTGTGCGCCAGCTACGTTTGTGCCGCCGCTTCGGCTATGG
TGGCTCAGATCGTCCCAAAGGAGTGGAGTCTATGGAGTGTCGTAGCGGCTGCGCTGCCTGTTGCATCGCC
CCCAGTATCAGCAGTGCCATTCCCGGTATGCCAGATGGCAAACCGGCTGGCGTCCCCTGTATCCAGCTCG
ATAGCGCCCTTGGCTGCAAGATCTTCGGTCAACCGGAGCGGCCAGCGGTGTGCGGCGGTTTTCGCCCCAT
GATGGATGTGTGCGGCAGCCACCGTGCCGAGGCCATCTGGCTGATCGGCGAGCTGGAACGGCTCACCACC
TGACGAGGGCAGGGCGAAGAGATAAAAAAAGAGGCCGCATCATGCGGCCTCTCGCGTTTCTGTTTGCCGG
GGATCAGACTCCCGCTAGTTCCAGTGCCTGACGATAGTACTCGTTGGCCTTGTTGGTGAGGCGGCGCTTG
TCCATCAGCTGGCCGAGGGCGTGGTAGGCGGCGGCGCTCGGCGCCCGCTCCACCTCCTGCTCCAGCAGCT
TCTGGGCATCGTCCAGCTGACCTGCCAGCAGATAGAGCTGGGCCAGTGCGGTGTCCACCTCCGGTTGCCC
CTGCTGCTTTTGCAGCAGCGTCAGCAGCGGCTGGTAGCTGTCGAGTTTCAGCTTGGGCAGGCGAGCCAGC
AGCTGTGGCATCGGCTGTTTGCGCAGCGCCTCCTGCCACAGGGTGGCGGCCTCGCCATCGGCGCCCAGCT
GGCGCAGCAGATCGCCGTAGCAGGCCAGCAGTTCTGGCTCAAGGCGCAGTTTGCGCGGCAGATCCTGCCA
TACCGCCTTGAGGGTCTCGAGGCTGCCGGCAGCCTGTTGCAGACGACCACACCAAGCCTGTTGCAGCAGA
TCTTCTTCCTGTTTCGGGGTCAGTTTGCCCACCTTGTGCAGTGCCGGGATCAGGTCGCACAGGGCGCTCC
AGTCCTGACGGGCCAGATGTACCTGACGCAGCTGATCCAGCACCATGGGGTGGCGCGGGTTGAGGGCGTA
GACTGACTCCAGCATCGCCAGCGCGGTATCGTACTGGCCCTGCTCGATCTGCAGCTGGGTCTGGGTCAGG
GTGAGCGCCAGCTCGGCCTTGGGATTCTCCTCCTGCGCTTTTTGCAGGTATTGATCCCGGCGGGCGTCGT
CACCACGGGCCTGTGCCGCCTTGGCGGCGCTCAGGTAGTTGAGCAGCGGGGTGTCGCTGTTGCTGGCCCC
CTTGATCATCAGCTTTTCAGCCTGACTGTAGTGCCCTTCGGCCATTGCCAGGGTGGCGGCGACGGTCTGT
TGATTGGCCTTGCGGCGGCGGCGCGAGCCGTACCAGCCGAGGGTCTTGCGGCGCAGGCCGAATACCCGAC
CCAGCAGCCACTCGACGATCAGCAGGGCGCCGTAGAACAGCACGGCCAGGATCACCGCGCTGGTTACGGA
AGATTCGATGGTGTAATTGCCAAGGGCAATCAGCACATAGCCCTTGTTGCCGGAGGCTTGCGGGCCAAAG
ATCAGCCCTGCGACCATCACGGCGACCAGAATGATTATACGGATCATGGTGCCCTCCTCAGCTGCTGGCC
AGAATGCGTTGCAGGCGTTCGGTCAGCACCTGCTCCAGCATGGCCTGGGTCTTGAACTGGGCCGGATAGT
CGATCTGGATCTGCTCACCCTTGAGCTTGTCGATCTCGCCCTGCATGTAACGGGTGGCGCTGTTGTCGGT
GTCGAAATACTGGGTCAGCCAGCGCTGGGCCTTGTCCAGGCTGTCTTCGTAGAGCGCCTGCTGCTCGCGA
TAGACGGAGAGCTGGGCCTGCAACAGCTTGGTTTTGAGGTTTTCACGCAGGAAGATCTCCTGTTGCGGCG
AGAGCAGCGCCTCGACCGCGCCATCGCGGCGACGGATGGTGACGAAGTTCTCGGTGAACTTGACCCAGTT
CTTCTTCAGGTTGCTCTCCCACTCGTCGGGATTGGTGCTGACCGCCTGATCCGGCTCGGCTTTGGCTTCC
GGCATGCTGACGGTGGAGAGGGGCAGCAGCTCGATCTGGTCTGAGAGCGCGGCCAGCTTGAGGGTCAGCC
CCTCGCGGTCGATGCGCGGCATCCCCTTGAGCTTGGCGATATCCTCGGCCAGCGCCTTGCGGATCGGCAT
CAGGCTGGGGTCGTTGAGGGCTGCGATACGCTCGTCGGCATTGCCGAGCAGGGTGATGGCGGAGACGAGG
TCATGTTCCAGCCAAAGTTTGCGGCCCGCCATTCGAACCAGATACTCGGATTCTGCCAGCATCCAGTCAT
TGGGGCGCTTGTCGTTGAGAGCCAGTACCCGGTTCTGCAGACCCTGCATCTCCCCTTGCAGCCGCTGCTG

GGTTTGATCCAGCGCGGCCAGCTGCTCGGCATCCTTGCTGCTGGTCTGGTCGATCTTGCTCAGGGCGCTC
GCCAGTTGCTGCTTGAGCTGGGCCAGTTCAGCCTGCTGGGCAACGGCGTTCTTGTGGCCGTGCAGATAGA
GACCGCCGGTCAGGCCAAGGGCCAGCAGGATGGCAACCCCTCCCAGTACGGCACCGGAGCGGCTCTTGCT
GCCATTGACCGCAGCGGTCGGCTGGGGTTTGACCTGGGATTCTTGTTGTTCTGTCATTCCATCTTCCTCA
GTTCCAGTGCGGCCGTCAGGGCCTGGTTGGATGCACCTTGGGCAATCGTGATATGGATAAAGCCCGCGGA
GTGGGCCATCTCGGCCACGCGCGGGCTGGGGACCACCAGCAGGCGGTCAAAAAGCCAGGGCCGCTGGTGG
TCGGGAACCAGTTCCAGCAGCCGTTGCAGCAGTTCACCACTGGTAATGAGCAGGCTGTCCAGCCCTGCTG
CCTGCCAGTGGTGGGTTAATGTATCCCCGTCTAGCTCGGGGTAGTGGCGCTCATAGGCCGCACAATAGTG
CACCAGAGCACCGCGTGAGGCCAGTGTGCTCGCGATGAGATCCCGCCCGCCATTGCCCCGCAGGATCAGT
ACCCGTTTGCCTGCTACCTGCTGCAGGGCGGGCAGGGCGAGCAGCCCTTCGCTGCGCGGGTCATCCGGGC
AGCTTGCCGTGATGCCGATGGCGGCAAACGCATCTGCGCTGGCCTGACCCACCGCAAAGTACTCAATATG
TGGCCAGGTCTGACCAGTTTGCAACAGAAAATCGTGTGCAAAATGAACGGCATGAATGCTGACGGCGATA
ACCGCGTCAGCACTGCGCAGCAGGTCGGGCAGGAGAGGGAGTTCACTGCCCGCCACGGTTTCCAGCAGCG
GGCAGCAGAGTGGTGCATGGCCGTGCTGGCGCAGCAACTGCACCAGCTCGGCGGCCTGAATGGCCGGACG
CACCACCAGCGGGATCATGCGCGGTAGACCTCGGCCAGGATCTCGTCGGCACCGGCGGCCAGCAGGCGCT
GGGCCAGCTGCTCGCCGAGGGCCTCACCATCGGCCAGCGGACCGCGGATCTCGTCGCGGATGACGTAGGT
GCCATCCGGGCTACCCACCAGACCGCGCAGCCATATCTGCTCGCCTTCGACCAGTGCGTAGGCTCCGATA
GGCACCTGACATCCCCCTTGCAGAGCGCGGTTCATGGCGCGTTCGGTCAGCACCCGGGCGCGGGTCTCGG
TGTGTTCCAGCGGCGCCAGCAGGGCGTGCAGTTCAACATCGTCGAGGCGGCATTCGATGCCGACGGCGCC
CTGACCGTTGGCCGGCAGGCTCTGCTCCGGCTCGATAAAGGCGGTGATGCGGTGCGCCATCTCCAATCGT
TTCAGACCGGCGGCGGCGAGGATGATGGCGTCGTACTCACCGGCATCCAGCTTGGCGAGGCGAGTGTTGA
CGTTGCCACGCAGGTCGCGGATCACCAGATCCGGGCGGGCGGCGCGCAGCTGGCACTGGCGGCGCAGGCT
GGAGGTGCCGACCACGGCCCCTTGTGGCAGTTCGGCAATGGCCTTGAAACGATTGGAGACGAAGGCATCG
CGGGGATCTTCCCGCTCGCAGATGGTGTGCAGGCCGAGCCCCTCGGGGAACTCCACCGGCACATCTTTCA
TGGAGTGAACGGCGATATCGGCCCGTCCTTCCAGCATGGCGGTCTCCAGCTCCTTGACGAACAATCCCTT
GCCGCCCACTTTGGCCAGCGGGGTATCGAGGATCTTGTCACCCTGAGTGCTCATGGGCACCAGTTCGACC
TGCAACTGCGGGTGCAGGGCTTCGAGCCGGTCTTTGACAAAGTTGGCCTGCCACAGGGCCAGAGGGCTCT
TGCGAGTGGCAATTTTCAGGGTTCGGGCTGCCATATCAATCAGTTGTTTCATTTTTAGAAGGAAATCGAT
ATTACCACCCTTTGCCCGGGCTTTCATGGCGGAGCCGGCAAAAGCGGGGCCAAGCTCAAAAATGCATGCT
GAAAATGATTTTCATTAAGCAAGTGGTGTTGATTAATTCATTGAATGTATTGGAATTTAATGATTTCCCC
TGATACTTGATGTGGTCATACCAGAAATAATCTGACGTTTAGCTGAAGGCTTGTTTACCACCGCTGTTTG
CATTGTTAACATGATCACACTTTTTTGACGTATCCCCCTTTGGTGCGTCATTTTCACTTAGGTGATAGGC
AGGGCATTGCAGGAACACATCGATACGCTGGTTGCGCGTTATGACGACATTACCCGTCTCAAGACGCAAC
GGGCGTTAGGCCTCATGAGTCGCTATGGGCAGCAGGTATTCCAACTGCTGCCTGTCATGCTGCACTTCAA
TCATCCCCTGCTGCCCGGTTATGTCTCCGGTGATGTCCCTCATGGCATCTGGAGCTTCAGCGCCACCGCC
GAGCAGCAAGCCTTTATCGATGACCTCTGCCGCAACGCCAACTGTCAGAGCGGCCTTGCCACCCATGATC
GGGCCATTCAGGGGCTTTACTCCATGGGCAGCACCTCCTCCATCGGTCAATGCTGTCACTCCGATCTCGA
TATCTGGGTCTGCCACGTGGCCGGTTTGTCGCAGGAGCGGCTCGATCTGCTGGAGCTGAAGTGCCAGCAA
CTCTCCAAGTGGGCCGAACAGCGCGGGGTCGATCTCAACTTCTTCCTGATCCCGGAAGACAAGTTCCGCC
AGACCAACGATGCCCAGATGCAGGGGGAGAGCTGCGGCAGTGCCCAGCACCTGCTGCTGCTCGACGAGTT
CTACCGCAGTGCCATGCGTATCGCGGGCAAACGCTTGATCTGGTATCTGGTGCCCACCGAGTGTGATGAT
CACTACGATGCGTATGTGAACGAGCTGTTCGAGAGCGGCAAGCTCAAGCAGGAAGACTGGCTCGATCTCG
GCAGTTTTGACCGTATTCCGGCCGAAGAGTATTTCGGTTCCGCCTTGTGGCAGCTCTACAAGGGGATCGA
CTCCCCCTACAAGGCGGTGCTCAAGTCGGTGCTGATGGAAGCCTACTCCCACGAGTATCCCAACACCCGC
CTGCTGTCGATGATTGGTCGCGACTGGTTCCAGCACAACGAGGGGATGCACTACCGGCTCGACAACTACT
GTCTGATGCTGGACAAGGTGACCAACTACCTCAAGTCCATCGGCGACATGGCGCGGCTCGATCTGGTGCG
CCGCTGCTTCTACCTCAAGGTGTGCGACGGTCTCAGCCATCCCAAGGATGATCACTCCCCCGAGTGGCGC
CGCGAGCAGATGAGCCAGCTGGTCTCCTACTGGGGCTGGAGTCAGGACAAGCTCTATCAGCTCGATCATC
GCCACGAGTGGAAGATCGAAGAGGTGAAGATCGCTCACGCCGAGCTGCTGGAAGCCCTGATGCAGAGCTA
CCGCAACCTGATCCAGTTCGCTCGCCGCAACAACATCAGCGAGTCGATCAACCCGGAAGATATTGGCATT
CTGTCGCGCAAACTCTACGCCGCGTTCGAGAGCCTGCCGGGCAAGGTGCAGCGGGTGAACCTCAAGATCG
CGCCGGATCTCAGCGAGCCGGATCTCAGCTTCGTGCAGGTGCCCCACGGTCGTCTTAACCGCGCCGGCTG
GTATCTCTACAAACATTCGCTGGAGCCGGTGGACATCATTGGTCGCGCGCCGCTGGAGTTCAACGGTTAC
ATCAGCAAGCTGGTCTCCTGGGCCTACTTCAACGATCTGCTGACCCCCCAGTCGCGGGTGCACCTGTTCA
ATCAGGGCTCGGATCTGCACATCGACAATCTGCACCAGTTCTGCCGCGATCTCTCCGGCTGCTTCCCCGA
GAAGTACCCGCTCGCTACCAACCTGGCCCTCAGTCGGCCGTGCGAGATACGCCAGCTCTCCATCTTCCTC
AATCTGGAGATGGACCCGACCAGCCATTGGGTCGGTCAGGTGATCGAATTTGACGCCAACACCGTGGATG
TCTTCTCGTTCGGCCGCAATCAGGAGTGTCTGGTCGGTTCGGTGGATCTGGTCTATCGCAACTCCTGGAG
CGAGATCCGCACCCTGCACTTCCAGGGGGATGAAGCCGTGGTCGATGCCCTCACCACCATCCTCGGCAAG
ATGCATCAGGATGCCGCCGCCCCCGAGATGATCGAGGTGTTCTGCTACAGCCAGCACTTCCGTTCTCTGG
TGCGCTCCCGCTTCCAGACTTTGGTGGCGGAGTGTATCGACCTGCGCCTTGCACGCGACAAGCAACAGCT

GGTCAAGACCATGGCCCTTGGCCGCGAGAAGTACGGCATCTTCTTCGAGCGGCGCGGGGTGTCGGTGAAG
AAGCTGGAAAACGCCATCGATTTTTACCGTCACATCTCCCACAACAAGCTGGATCACCTGCCGCTGCGGC
TCGACAAGACCCACAGCCAGCATCTGCCGAGCATCGTCGATGCCTACGCCAGCGAAGGATTGGTGCAGTT
CTTCTTCGATACCCGGGATGCGGGCACCAACATCTATATCCTCGATGAGGCGAACCGGGTGGAGATCTAC
CAACACTTTGCCGGCAACAAGGATGAGCTGGTGGCGGGGGTCAACCGCTTCTACACCTCCAATCACGAGC
GGTTCAGCGATGATGGGCAGTTCATCAACTTCAACCTGCCCCAGTACTACGAGATAGTACAAGTGAATGG
CGAGCTGGATGTGATCCCCTATCGCAGTCAGGGGACTCTGCGCGAACAGGTGCCGCCCGCTCGAGAAGTG
GGCTGACCCGTCAGTTGGAATCGCAATAAAAAACGGCCTGCAGATGCAGGCCGTTTTTTATTGCGGTTGC
TCAGGCGCCGAGGCGAGCCAGCAGCCAGCTGCGAATGTCGCCCAGCTGCGGGCGGCAGATCTCGTGACGC
ATCGGGTACTCGTGCCACTCGGGGGCGAGTCCGGCGCTTTCCAGCCGGTTCTTGGCATCCCAACCGAGCG
ACAGGCTCACCACATCGTCATAGATGCCGTGCATGTAGCAGATGGGCTGGGTGCGAGCAGCCTCGCTCAT
CTCGCCGAGCAATTTGTCGGGGGCCGCCAGATAGGTGGACATGCAGAGCAGGCCCGCCAGCGTTGCCTGA
TAGCGCAGCGCGGTGAAGGAGGCAATCACCCCGCCCTGAGAGAAGCCTGCCAGCACGATATGCTCGGGGG
CAAACCCGTCGGCCACCAGCTGATCGAGCAATGCGGCAATCTGGTCGGCTGATTCACGGACATGGGACTC
CACGGCCCGCTCGGCGGGATCCTCGAAGCTCCTGATGTCGTACCAGCCGCGCATCTTGTAGCCCATGTTG
ATGGTGATGGGGCGCTCGGGGGCATCCGGCAGCAGGTGACGCACCGGTAGCTCGGTGGGCAGATCCAGCG
CATCCACCAGCGGGGCGAGACCGGCGCCGGAATCACCCAGCCCGTGCAGCCAGATCACCGCATGACGGGC
GCCCTGCGGGTGCAGGTCGATCATGGGAACACCACATCTTCGCCCGCCTGGGCGGAGCAGGATTGGCTCA
GCAGCGCCTTGAGCTCGTGGCCGAATCGGTTGTCGATCCAGGTGTCACCCCGCAGTTCGAAGTGGAAGCC
GTTCTCGCGGGTGGCGACCCAAATCTGGTGCAGTGGCTCCTGCTTGTTGATGATGACCTTGGTCTTGTTC
TCGAAAGAGAGGGTCAATACGCCGCCGTTGCGTTCGCAGTCGATATCCGGATACCCCTCGTCGAGCGTGT
CTTCCACATATTGGAAAAAGGCGTCGGTCAGGGCGTGATACTCGTGATCCTTCATCGGTTTATCCTGTTG
CTTTTAACAATGTGAGTGCGATTATAGAGGGCCAAAAATTTTTTCGCAGCGAACCATTATGATCACCCAG
TTCACCAAGTGTCTGCTTGCTACTCTTCTGTCCCTCGGGTTGGCCGCGTGCGGTCTGAAAGGGCCGCTCT
ATATGCCGCCCCCCGAGCAGCCACAGAGCCAGAGTACACCGCAAGCAGCACCGCCTGCGACCTCCGAGGC
TGCCACTGAGGCCGCGCCGCACTAATGCCGCACCCATTCTAGGAAGGAACCGCCCCCTTGGATCACTTTA
ACTACGACGCAGACGGCCAGCTTCAGGCCGAGCAAACCTCGCTGCAACAGCTTGCCGAGCAGTACGGTAC
CCCGCTTTACGTCTACTCCCGTGCGACCCTCGAGCGCCACTGGCACGCCTTCGACAAGGCCGCTGGCGAT
ATTCCCCATCTGATCTGCTATGCGGTGAAGGCCAACTCCAATCTGGCGCTGCTCAACCTGCTGGCCCGTC
TAGGCTCGGGGTTTGACATCGTCTCCGGTGGCGAGCTGTCGCGGGTACTGGCGGCGGGTGGTGATCCGGC
CAAGGTGGTATTCTCAGGCGTGGCCAAGAGTGAAGCGGAGATGCGCCTCGCGCTGGATAAAGAGATCCTC
TGCTTCAATCTGGAGTCGGAAGCCGAGCTGGAGCGCCTCAATCGGGTGGCGGGCAGCATGGGCAAGAAGG
CTCGCGTCTCGGTGCGGGTCAATCCGGATATCGATGCCGGTACTCACCCCTATATCTCCACCGGCCTCAA
GCAGAACAAGTTCGGCATTCCCATCGAGCTGGCGCCCGCCATCTATCGCAAGGCGGCCGCGATGGCGAAC
ATCGAGATCGTCGGGGTCGATTGCCATATCGGCTCGCAACTCACTGAATTGAATCCCTTTATGGAAGCGG
CTGACAAGCTGCTGCGGCTGATCGACGGTCTGGCGGCGGAAGGGATTCATATCCACCATCTGGACGTGGG
CGGTGGCCTTGGGGTCAACTACGGTTCCGAGCAGCCACCCCATCCCACCGAATATGCCGAAGCGCTCAAG
CAGAAGCTGGCGGGCCGGGATCTGACCCTGCTGTTCGAGCCGGGCCGCGCCATCGTCGCCAATGCCGGTG
TGCTGCTGACCCGGGTCGAGTACCTGAAGCCGGGCGAGACCCGCAACTTCGCGCTGATCGATGCTGGCAT
GAACGACATGCTGCGCCCCTCGCTCTACGGCGCCTGGATGAACATCATCGAGGTGGATAGTCGTGCCGGC
CATGCTCCCGCGCTCTATGACGTGGTGGGCCCGGTGTGCGAGACCGGTGACTTCCTCGGCAAGGAGCGCA
CCCTGGCGATTGCCGAGGGGTCGCTGCTGGCGGTGCGTTCGGCCGGTGCCTACGGCTTTACCATGTCATC
CAACTACAACACCCGTCCGCGCGCAGCCGAAGTGCTGGTGGATGGTGCCGAGGCGCACCTGATCCGCGAG
CGTGAGCAGTTGGCCGATCTCTGGCGCGGTGAGCACCTGCTGCCCTGATGCAGTAGATAGAGATGACAGA
ATTTTACAGCCCTGACAGCACAGGAGTGCGCGATGTGTACAGATTGTTCCAGGATGCATGGGGCTTTGGA
CAACGACTTCATAGTGGTTGACGGTGAGTTTTTACAGCCCTAACAGCACAGGAATGCACGATGTGGATAG
ATTGTTCCAGGATGCATGGGGCTTTGGACAACGACTTCATGGCGCTTGACGACCCTTGGTTCGGTGAATT
TTTACAGCCCTAACAGCACAGGAATGCACGATGTTGATAGATTTTTCCAAGATGCATGGCTTGGGCAACG
ACTTCATGGTGGTGGATGGCGTCACCCAGAAGGTATTTTTCAGCAATGACGTCATCAAGAAGCTGGCAGA
TCGCCACTTCGGGATCGGCTTTGACCAGCTGCTGCTGGTGGAGCCGCCCTATGACCCTGAACTCGACTTT
CACTATCGCATTTTCAATGCCGATGGCAGCGAAGTGGAGCAGTGTGGCAACGGTGCCCGCTGTTTTGCCC
GTTTCGTGCGTCTCAAGGGGCTGATCAACCGGGATCGCATCGCGGTCAGTACCGCCCGGGGTCGCATCGT
GTTGCAGCTGGAGGGGGAGAATCAGGTCACGGTCAACATGGGTGTTCCCCAGTTTGAGCCGGGCAAGATC
CCGTTCCGGGCCCAGAAGGCAGAGAAAACCTATCTGCTGCGGGCGCAGGAGCATACCGTGATGTGTGGCG
TGGTCTCCATGGGTAATCCCCACTGCGTCATCGAAGTGCCTTCGGTCGCCGATGCGCCGGTCGAAACGCT
GGGTGCCATCATGGAGCGCCACGAGCGTTTTCCCGAGCGGGTCAACGTCGGTTTCATGGAGATGGTCAAT
GCCACCGAGATCAAGCTGCGTGTATTCGAGCGGGGCTGTGGCGAGACCCTGGCCTGCGGCACCGGTGCCT
GTGCGGCGGCGGTGATTGGCATCAGTCAGGGCAAGCTCAAGGAGCGGGTCACCGTCAGTCTGCCGGGCGG
CAAGCTGACCATCGCCTGGAAGGGGCCGGGCCAGCCGGTCTATATGACAGGTCCCGCCGAGCATGTGTTT
GACGGCCAGATAGAGGTTTAGCCGATAAAGGTTCAGGTACGGGGGGCGCTGGCCCCCTTTGCCCATGATG
TCGGGGCAGCGAGCCCCATTTCTCTGATGGATCAACCAATTTATGAGCGAACTCAAACGGCAGGAGCAAT

CGGTGGATGAAGCCACTGTGCTGGAGTACCTGGCCCGTTATCCCGAGTTTTTCCAACGCCATGCCGCCGT
GCTGGACCGGATCCGCATTCCCCATGAACGTAAAGGGTCGGTCTCGCTGGTCGAGCGTCAGCTGGAGCGT
CAGCGCGAGCGGATCGAACAGCTGGAGCAGGAGATCACCGAATTGATGGGGATCGCCAGCGAGAACGAGC
GGATCTTCCGGGTCTATGCCGATCTCTACGGCGAGCTGTACGGTTGCCAGACCCTGTTCGAGGTGAGTGC
CGTGATGGGCAAGGCCTTTGTCCAGCGGCTGCGCCTGACCGGGCTGCGGCTCTGGCTCAATCCCAAGAAG
TTTGCCCTGAGCGGGCCCGAGCAGCGTTTTTTGGCACAGGGCAAGCAGCTCGAATCCCTGCTGGGTCAGC
GTTTGCCGGGGCAGGATTACTACTTTGGCCGCCTGAGCCAGAGCGAGAAGCAGGCGCTGTTTGGCCACGA
TGTGCTGGTCAACTCGGTGGCCCTGATGCGGCTCGGCGATCTCGGTGTGCTGGCCTTCGCCAGCTCGGAT
CCCGGCCACTTTACTCCCCACAACGACACCCTGCTGCTGGGCCAACTGGGGCGCCTGCTGCAACTGCGTC
TGCCCGAGTTGACTCATGGCTAGAACACCGGCCAGGGCGCTCGCCAAAGCTCCCGTCGTCGCACCGCCAC
TGCCTGCGGCACTGGCGGACGAGCTGGCGGCCTTTATCGAATACCTGAGGGTCGAGCGTCAGCTCAGCCC
TCATACTCGCAATAACTATCAAGCCCATCTGGAGGCGATGACCGCCGAGCTGGTGGCCCTGGGGCTCACT
GACTGGGGTCGGCTCGAGGCGAGTCAGGTGCGCACTCTGGTGACCCGGATGCACAAGGGGGGACTTGCTC
CTCGTTCCCTTTCCACCAAGGTCTCGGCCCTGCGCAGCTTTTGCGACTGGCAGGTGCGGCAGGGGCGGCT
GACCGCCAATCCGGCCCGCGGCATCGTCACCCCCAAACAGGGGCGGCCGCTGCCGAAAAACCTCGATGTG
GACGAGATGCACCAACTGCTCAACATCACCGACGAGCAGGATCCGCTGGCGGTGCGGGATCGCGCCATCA
TGGAGTTGATGTACTCCTCAGGGCTGCGTCTGGCCGAGCTGGTGGGGATCAATCTGGTGGATCTGAAGCT
CGACGACCGTCAGCTCAAGGTGACCGGCAAGGGCTCGCGGGAGCGGGTGCTGCCCATAGGCCGGATGGCG
GTCGAGTGGCTGCAGAAATGGCTCAAGGTGCGCCCGCTACTGGCCGGGGATGAGAGCGAGGCGCTGTTTG
TCTCCAGCCGCAAGCAGCGGCTGTCGGCCCGTTCGGTGCAAGCCCGCCTCGATGGCTGGGGCAACAAGCA
AGCGCTCAACGCCCACGTCCATCCCCACAAGCTGCGCCACAGCTTCGCCACCCATATGCTGGAGTCTTCC
GGCGATCTCCGGGCGGTTCAGGAGCTGCTGGGACATGCCGACCTCTCTACCACCCAGATCTATACCCACC
TCGACTTCCAGCATCTGGCCAAGGTCTACGACAGCGCTCACCCAAGGGCCAAGCGTGACCCGGATGCACA
GGACGATGAATAACGGGCCCTGAGCCCCGTTGCAGAGGAGAGTCCATGCACTTCTATCGCCGCTGGCAGC
CGGTTGCTGCCATCTCGTTTGACCTCGATGACACCCTCTATGACAACGGCCCCGCCATCATGCGCGCCGA
GCAGTGGATGCTGAGCCATCTGCGCAGCGAATATCTGGCCACCGCCATGCTCGACAAGCCGCGCTGGCTG
GAGTTAAAACGCGCCGTGTTGCTGGCCAACCCCGAGTTGCGCCACGATGTCAGCCTGGCGCGTCAGCAGA
CCATCCGTACCGCTATGGTCGAGGGGGGGATGGCCGACCAGCAGGCCAGCCGCGAGGCGGAGCAGGTGTT
TGCTGCGTTTCTGGCAGAGCGCTCGAAGATCGAGGTGAGCGAGTCCACTCATCAGCTGCTGGCCAGACTA
GCCGAACGCTACCCGTTAGTGGTCATCACCAACGGCAATCTGGATCTGGCGCAGGCAGGACTGGAACGCT
ACTTCACACTGGTCTGCAAGGCGGGGGGGGGCGCCAGGATGAAACCGGCGCCGGATATGTTTGAGCAGAC
CCGCAGTGCCCTCAAGCTGCCAGCGGGGCGGATCCTCCATGTGGGGGATCATCCCGAGACGGATGTGCTG
GGTGCCCGCTTGCACGGCTTTCGCGCCGCCTGGCTCAACGAGCGGCAGCAACCGTGGCAACAGTTGCAAC
TGTTGCCGGACGTGGAGCTGACGGCATTGGAGGCGCTGGCCGAGTTGCTGCTCTGATGGCGCTGGCGGGT
GGCCCGGCTCAGGCGGTAATGGCTGTCATCGAATAGCGATATATAGCAAGGGGTTACGCCCGCTATTTGC
GGCAAACCAACCCGAGCAGGGTGTAGCGGTCGCGCTGGTTGACGCGGCAGTAACTGATGCCGACGGGTAG
CGGCTGCTGCAGGGTGATGGCGCTGTTCTGATAGCGGCAGACCAGATAGAGCGGGCGCAGGCTGCTATCG
AGCTGACTCCAGTAGTGGATCTCGTCTCTGTCGCCATTATCCGGTTTGAGTTGTGCCAGCTCGGCAGGAT
CTCCGTCGAACAGGGCCAGATTATCGAGCCGGTGGCTGAGGGTTTCGTCCGGTTTGCCATCGTCATTGCG
GGTGAACGCCTGCCAGCCCTGCTGCGGGGTCAGCAGCTGTTGGCGAGTCTCTATGGTGGCGGGACAATCA
AATTGCAGGCTGGCGGCAAGGGCCAGGGTTGTCAGTAACATGGCTGCGGTTCTCTCTTCACGTCCTCAAT
ACGATGTCATGCTACGTCATCTTCCGCCGAACAGCTTCATCAGCTTATCAAGCAGATCAAGCTGTTGTGG
TGGCTGTTTCATGGCTTCCGCTTCCCGCTCCAGCAGCGCCTGCTGCTCCTGCTGGCGGTCGCTCAGCACC
CGATAGAGGGGCTCGACCAGCGCCTCGCGCTGCATCACCAACTCCTGAAACATTGCCTGATTGATGCGAT
AGGTTTCGACGGTGGCCACCGCCCGCACCGTGAAGGAGCTGGGTTCGCCGGTGAGCAGGCTCAGTTCGCC
AAAGAAGTCTCCCGCGTTGAGCGTACCAAGCTTCAGCTCTCGCCCTTCGACATTGACCACCATCTCGGCT
TCCCCTTTGACCAGCACGAATAGCCAGCTGGAGACATCTCCCTGCTCCAGCATGATGTCGCCGCTGACGA
AGGGGGCGAACTTCACCTGATCGGCCAGCTGGAGCAGCTCGCTCTCCTGCAACATGGCAAACAGCGGCAG
CTTGCGCAGGGTATCGGTTCGTTCGGCAGTGTGGCGCTTGTGGCGGGCATCCAGATGCTGCTTCTCCTTG
GCCATAAAGACGTTGAAGATCGGCGGGGTGAGGCGGCGATCGTTGCGGCGCAGGGCGGCATCGATCAGGC
TGCGCACCATGGAGTCGGTCGGATCGTCGCGAGCCAGGTCGGTCAGCCAGTAGCGCACCGCATAGCGGGC
GATGCCCCCTTCGACGTTCATCAGCAGGCAGTCCGGTTGCGGATGCTTGGCGACGCAGGGGAGCTTGGTC
TCTCGCAGGGATTTCTCGATCAGCGCGATGATCTGGGTCGGCAGGGTATCCAGGGTCAGATCGAACCAGA
CCCAGCGCCGCCACTGCAAGGGTTCGCCACGGCGGCGCCCGAGCACGGTGAAGCGCTGTTTGAGCAGGTG
GCTGTTGGGGATGACCACAGTCTCCCAGTTGCGGGTCTCGATGGTGGTAGCACGCCAGTTGATCTCCACC
ACCCGGCCCTGGGTGGTGTCGACTTGCAGCCAGTCACCGATGGCGATGGAGTTGTCGAGCTGAATGGAGA
CCCCGGCCAGCAGGTTGCCAAGGGTGTCCTGCATGGCGAAGGCGATGACAGCGGTGATGACCGCCGAGGT
GGTGACTATCTCCCCGAGCGCCATGCCGGAGGCGTAGAGGCGAAACAGACCCCAGCCGATATAGCCGATG
GTGATGACGATATCGGCGATGATCCGCGGGATCCGCACATGGCAGAGGGGGAGCAGAAACTCGAACAGCA
GGATCCCCCATATCTTGACCAGTATCAGGCCGGCCAGCAGGGTGGAGATCTCGATCAGCAATTGATGGGG
CAGCGGCAGTTTGAACCAGCCCCCTTCCAGCTGGATCAGCAGGATCACCACCGCGCTAATCATCACGAAA

CTGAGCTCGGAGAGCAGCTCCCGTCGCTGTCCCGGACGGCGCCAGAAGTGGATCAGCATCAGGGAGAGGG
TGAGCAGGGTCCAGAACGGGTGTTCGCCAATCCAGATCAGCGTCGGTAGCGCGGTGAGGTTTTCGCCAGC
CATAGCAGCACCTTATTGTGGTTTTATTTCCAGCATAGCTGCTGCGGCGCGGGGAAATGGTGCGTTGGGG
CGGCAATTTATTTATCCTGTGCAGAACCGGGCAAAGGAGTGCGATTGAGTGAAATTTTCAAAGAAAATCA
TCTGTTTGATGGCCCTGTTTCCATTGGCTGGCTGCGACCTGCTGACCGAGAAGTATCGGGTCGAATTCCG
GGATGGCTCCTGGCGTGAAATAGCCATTACCCCCTTCTCCAGCGAGGCGCGCTGGCGTGAGGGGTGTGCC
ACCGGCGAGGTGCTGAGTGACATGAAGGTGCCCATCAAGCGGGGTGACACGGTCAATGGCCGCGAGCAGT
TGCTGATCGGTGGCGAGAAGTTCGAGCTGGATGACAACAACCTCTACCGTAGCAACGGCATGGTGTTTGG
CCATTCGCCTGACCATATCCAGCGCGGGCTCAAGGGGATGGATATCTGCCGTACCCTGCGCCCCGAGCGG
GGCGGACTGCCCAAGCTCAAGGCGCGCGGGTTTGGCGACTGAATCGATGAGGGATTAACTCGCTGCCATC
AATGCACTGCGTTGGCGGCAGGCATCGCCAAAGGCTTGAAAGATCTTGATGGAGTGGGGGTTTTCATTGG
CCTTCCACTCCGTATGCCACTGCACTGCCAGCAAAAAGGGTGACAGTGCGGGGGCATGAAGTGCCTCGAT
CAGCCCATCTTCTGCGTGGGCCAACGGCTCCAGACCCTTCCCCAACCGGTTGATGCCCTGCCCATGCAGG
GAGTTGACCTGAATCTGTTCTTCCCCCATCAGTTCGGCAAACCAGCTGCCGGGCAGCAGTTTGATCTGAT
GGCTCGGCCCATACTGCTCGTCGACCGGATCGTCGGCATCCTCCCGGTGATCGTCATAACCCGGCTCGTT
ATAGACCTTCTGATGGATGTCTCCCCCCAGCGCCACGTTGATCTCCTGCATGCCGCGGCAGATCCCGAGG
ATCGGCAAACCACGGGCAAGCGCCCCCTTGATGAGGGGCAGATCGAACAGATCGCGATCCCTGTCTTGCT
GTTTTTCTGGCGTGAGGTTCTCCTGGCCGTAGAGGGCCGGATCGATGTTGGAACCGGCGCCGCTCAGATA
GACCCCATCGGCGAGATCCAGATACTGCTCCAGATCGGTGGTGCCGCAGCAGGTGGGCACCAGCAGCGGG
ACGCAGCCGCTTATCTCCACCAGCGGCATGATGTATTTGTGGGTCATCACCTGATAGGCGTGGCCGTTGC
GGGGCTGGGCGCCCATGGTCATCAGCACGACAGGTTTACGGGTGGCGGGAGGCTGGGGATTTGGCATAGG
GCACCTTGATTATCCTTGGCGTTGAAGTCTCATCCTGCGCCTTGGGCAGGCCCTTGCAAACCGGCAGGGC
AAGGGCATGCCTCAAGGCATGGTCATGGGATCCCGCGTCGGGCCGGGTGACAGAATCGTTGGTCAGCTTG
GGCAGCGACGGCATGGATCCTCTCTTCAGTCTGCCAAGGCCGTTCAATATGTTAAACAAAATGATGCCAA
ATTGTTAACGTGTCTGTTTTGGTCTGCTCTGAAGCGGCGATTTAACGGGGCTGTTGAGATGGGCCGTTAT
GGCTGGGGTAGCAAAATGGTGGATATATTAAACGCGCAGGGATGGCGGGGAGAGGGGAGAAGGCATCAAT
AGAAGCACGCCTCCGCAAGGGGAGGCGTGTTTCGGGGATCACAGCAGGTCTTCGATCTCGCCGCGCAGAT
ACTGGTACATCTCGCGGTAGGCGACCGGCGGCTTGTTCAGCTCCCGCTCCTTGTTGGCGGAGCGGATCAG
CTGGCGCAGCTTTTGGCGATCCAGCTCGTGGAACTGGGACATCAGCTCGTTGAGGGCGCTGTCACCTTCG
GTGATCAGGCGCTCGCGCCACTGCTCCAGCCGATGCAGGCGTGCATTCACGGTGGAGTGACGGTTCTTGA
TGATGGAGAGCGCTTCGGCAATCGGCTCGATATCGCGGCTGCGCATCAGGCGGCCGATGAACTGCAGATG
GCGACGGAAGGACTCATCCTTCTTCGGCTTGAGTTTGCGACCCAGCTCGACAGCTTCTGCAAGCTCTTCA
TCCAACGGGATTTTTTCCAGCTCGCTGTGGCTGAGGGAGACGATCTCTTCCCCCATTTTCTGCAATGCTT
CGGCATCGCGCTTGAGCTGGCTCTTGCTGGGGCCCCAGTCTTCGAACTCGTTGTCGTCTTGATATTGACT
CATCGGTCTCTTGATTCCTGTCATTCACGTTTGGCGGATATGTTACCAGCTTCTCCCGCTGGGCGCAGGG
GTCTGATATGCTTACCCCCCTTTCCTTCTTTCATACAGATGTGAGTTATGGATCAGCAAGACCAAATCCG
CCAGGATCAAGCCCATCTCGAGGCCGTGGTTGCCGAAGCCCTGGAGATAGCCAAAGGACTGGGCGCCTCG
CTGGCCGAAGTCTCCATCAGCAAGCAGACCGGGTTGTCGGTGAACACCCGGGGTTGTGAACTAGAAAGCA
TTGAATTCAATAAGGATGGCGCCCTCGGTATCGCCGTCTATCGCGATGATTGCAAGGGATCTTCCTCGAC
CACCGATCTGAGCAAACCCGCCATTCGTGCCGCCGTTGAAGCGGCCATCGAGATTGCCCGCCACACCTCG
GCCGATGATTGCGCCGGTCTGGCGGACGCCGAGCTGCTGGCTTGGGATGCCCCCGACCTGCAACTGTGCC
ACCAAATCGAACTGGATCCCCAGCGCGGGATCGAGCTGGCTATCGAGTGCGAACGGCTGGCGCTTGGTCG
CGATGCCCGCATCAAGCACTCCGACGGCGCCGGTTTCTCCAGCCATCTAGGCATCAAGGTCTATGGCAAC
AGCCACGGCTTCGTCAAAGGTTATGCGGCGTCGCGCAACTCCCTGAGCTGTGTGCTGATCGGCGAGCAGG
ATGGCGACATGCAACGGGATTACGGCTACTCCTCCAGCCGTACCCTCGACGGGCTCTGGAGCCCGCAGCG
CATTGCGGACGAAGCCGTTGAGCGTACCGTCTCCCGCCTCGGCGCCCGCAAGATCTCTACCCGTCATGCG
CCTGTGCTGTTCCACCCGGACACAGCCTCCGGTCTGTGGGGCCATCTGGTGATGGCCATCAGCGGTGGCA
ACCTCTATCGCAAGTCGAGCTTCCTGCTCGACAAATTGGGCAAGCAGATCCTGCCCGAGTGGCTCACCAT
TCAGGAGTTCCCCCACCTGCTGGGCGGGCTTGCCAGTACCCCGTTCGATGGCGAAGGGGTGCGTACCCGC
GACATGGATATCATCCGTAACGGCGAGCTGATGAGCTGGCTGCTGACTTCCTACTCCGCTCGCAAGCTGG
GGCTGACTACTACGGGTCATGCCGGTGGCATCCATAACTGGCAGGTCTCCAATACTGGCCAGGATTTTCA
GGGCATGCTTAAAGAGATGGGCACCGGTCTGCTGGTGACCGAGCTGATGGGGCAGGGGGTCAATATCGTC
AATGGCGACTACTCCCGCGGCGCTGCCGGTTTCTGGGTCGAGAATGGCGAAATAGCCTATCCGGTCGAAG
AGATCACCATCGCCGGCAATCTGGCCGAGATGTTCAGCCATATCGTGGCGGTGGGTCGTGACGAGGATGA
GCGCTCCAGTCTCAAGACCGGCTCGGTGCTGGTAGAAAATATGAAGCTGGCGGGTCACTGATCCCGGTTT
CAACTGAACCGAAACCATGAAAAAGGCCCCCTCGCCAACGCGAGGGGGCCTTTTTATCGGCTAGCGATCA
GGGGGCTATCAGCCCTTGATGCGTTCGATGCGGCCGCCCAGACCCTGCAGCTTCTGCTCGATGTCTTCGT
AGCCACGGTCGATGTGATAGATACGGTCGACGATGGTGGAGCCTTCGGCTACGAAGCCGGCCAGTACCAG
ACTGGCGGAGGCGCGCAGATCGGTCGCCATTACCTGGGCACCCTTGAGCTGCTCGGTGTCACGGCAGATG
GCTACGTTGCCCTGCAGTTCGATGTCTGCACCCATCCGTACCAGCTCGGGAACGTGCATGAAGCGGTTCT
CGAAGATGGTCTCGGTGACCATGCCGGTGCCTTTGGCGACGGCGTTCAGCACGGTGAACTGTGCCTGCAT

GTCGGTCGGGAAGGCCGGGTAGGGGGCAGTCTTGATGGTGACCGGCTTGAGGGTGCGGCCAGTCATGTCG
AGGGTGATCCAGTCTTCACCCTTCTCAATCAGGGCGCCAGCCTCTTCCAGCTTGACCAGTACCGCTTCCA
GCAGGGTCGGGTCGGTCTTGCGGCAGGTGATCTTGCCGCCGGTGACCGCTGCGCCCACCAGGAAGGTACC
GGTTTCGATGCGGTCAGGCTGCACGCTGTAGTTGCCGCCGTGCAGACGCTCGACACCGTCGATGGTCAGG
GTGTCGGTGCCCGCGCCCTGGATCTTGGCACCCAGGGTATTGAGGAAGTTGGCCAGATCGACCACTTCCG
GCTCGCGCGCGGCGTTTTCGATCACAGTGCGGCCATCAGCCAGGGTGGCGGCCATCATCAGGTTTTCGGT
ACCGGTCACGGAGACCATATCCATCAGGATGTGGGCGCCTTTCAGGCGGCCATCGACACGAGCCTTGATG
TAGCCATCTTCGATGGTGATCTTGGCGCCCATCAGCTCCAGGCCGTGGACGTGCAGGTTGACCGGACGGG
CACCGATGGCGCAGCCGCCGGGCAGGGAGACGTCAGCCGCGCCGAAACGGGCAGCCAGCGGGCCCAGCGC
CAGAATGGAGGCGCGCATGGTTTTCACCAGCTCGTAGGGAGCGACATGGTTGTTCACCGCACCTGTCAGC
ACGGTGACGTTGCCGTTGACCTTGGTGGTGGCGCCCAGCATCTCCAGCAGCTTGATGGTGGTGCCCACAT
CTTTCAGGCGCGGCACGTTGGAGAGGTGTACCTCTTCATCACAGAGCAGGGTCGCGAACAGGATCGGCAG
CGCGGCGTTTTTGGCGCCGGAGATGGTCACTTCGCCGTTGAGAGTACAACGACCATCGATTTTGAATTTG
TCCATTTTTACTTCGTTACCAAATCAGGAGGGCAATAGGAATTTACGTTCGCGTTTCCACTCGGCGTCGG
TGAACGCCTTGATGGAGAGGGCGTGAATAGCGTTGCTGGCGATCTTGTCCATCAGCGGGGCATAGATGGC
CTGCTGCTTCTTGACCCGGCTCATGCCGTCAAACATGTCACCGACGGCGATCACTTGGTAGTGACTACCG
TCCCCTTTGACATGGACTTCGGACAATGGCAGAGCCTCAAGGAGTATCGCTTCAATTTCAGAGACTTGCA
TTATGCGTCCTCAGTTGTGAGCGGGGTTGACTGAAACAGCCCGGCCACCCCGTAGAGGTTGGCCAGGGTA
TAGAAATCACTGGAGGCGCCCACCAGTTGCGGTGTGGCGCCTCGCGCTAATACGGCTTTGGCCCATTTGA
CCAGCAGGGCCAGTCCGGCAGAGTCGAGGGTGGTGACGCCGCCGAGCTCAAGCTGGTCGTCCTGCCACCA
TTCGGCGCGGCGTTGCCACAGGTCATTGACCTGTGGCGCCTGCAGCTCACCGCTGAGAGTCATGGTTTCA
ACACCAGCGGCTTGGCGTTGTGTTCGCGCAACTGGGCGATCACCGCATCGATGCCGTTCTGGCGGATCAA
ACCGCCGAGCTCGCTCTGTTTGGCGGAGAGCAGGCTGATCCCCTCTGCCACCATGTCGAAGGCTTTCCAC
TCGCCGGTCTTGTTGTTCTTGCGCAGCTTGAACTCGAGGAAGATGTCCGGCTTGCCGGCCTCTTTCACGC
TGACGTTGACGGCGGTGATGTTGCCGCTGGGAGCTTTGCCCGGCTCGACCTTGACGGTCTGCTTGTCAAA
ATGGGCCAGCGCATCGGCGTAGGAGCTCACCATGTACTCGGTGAACACCTCGACAAAGGCATTGCGCTGC
GCTTCGGTAGTCTCTTTAATCTGGTTGCCAAGCACCTTGTAGGCGGCGAAGCGGTTGTCCACATAGGGCA
GCAGCTCTTCGCGGATGATCACCCGCAGGTGATCCGGATTGCTTTTCACCTGGGCCTGATCGGCCTTCAG
GCGGGCAAAGGTCTGCTTGGCAGCCTGATCCACCAGCGCGTTGGGGTCGGTGGCATCGACTGCGGCCTGG
GCAGACAGGGAAAAGAGCATGCTGGTCATCAGGCCCAGCAGCAGGGCGATTTTCTTGAACATGATTACTC
CTTCTTGCTGTCTGATTTGCTGTCGGAGGATTGGCTGTAGAGGAACTTGCCAATCAGATCTTCCAGTACG
ATGGCGGATTTGGTGTCTTCAATCAGGTCGCCATCCTTGAGCATCTCGGTGCCCATCTCCTCATCGCTGA
AGCCGGGGGCCAGACCGATGTACTGCTCGCCGAGCAGACCGGAGGTGAGGATGGAGAGGGAGCTGGTATC
GGCGAACTCGCCAGCGCTCTTTTGCATCATCAGGGTGACTTCGGGCACCTGGGTTTTCGGGTCCAGTACG
ATGTCGCTGACCCGGCCTACCACCACGCCGCCGACCTTGACCGGCGAGCGCACTTTCAGGCCGCCGATAT
TATCGAAATGCGCGCGTAGGGTATAGGTTTCTCCTTCCGGTTTGAAGCTCAAACCGGCCACTTGCAGGGC
CAGTGCCAGAATGGCACCGATGCCAGCCAGCATGAAGGTGCCGACCAGGAATTCTACTTTGCTGAACTTC
ATCTTTACCTTATCCAAACATGACTGCAGTGAGTATAAAATCCAGGCCGAGCACGGCGAGGGATGAGTGG
ACCACGGTTCGGGTCGTGGCCTGACTGATCCCGGCCGAGGTCGGCTTGGCATCATAGCCGTTAAAGAGCG
CAATCCAGGTGACCACCAGGGCGAAAATCAGGCTCTTGATGGCGCCCTGCATGATGTCTTCATGCCAGTC
GACGGAGGCCTGCATGGCAGACCAGAAGCCCCCTTCGTCCACGCCGAGCCAGTCCACGCCGACCAGCTTG
CCGCCGAAGATGCCGATCAGGCTGAACATAAAGGCCAGCAGCGGCATGCTGATGACGCCAGCCCAGAAGC
GGGGGGCAACCACCCGGCGCAGCGGGTCGACCGCCATCATCTCTAGGCTTGAAAGTTGCTCGGTGGCCTT
CATCAGGCCGATTTCAGCGGTCAGGGCCGAACCGGCACGGCCGGCAAACAGCAGCGCCGTGACCACAGGC
CCCAGCTCCCGCAGCAGGGAGAGGGAGACCAGCGGTCCCAGGCTCTGCTCGGCGCCGAAATCCTTCAATA
CGTTGTAACCCTGCAGCGAGAGCACCATGCCGATGAACAGGCCGGAGACCAGAATGATGGCCACCGACTG
TACGCCCACCACATACAACTGCTGGATCAGCAGCGGGAAGTGCTTGCGCGGCTCGGGTTTGCCCGCCAGC
GCATGGAACAACATGATGGTGGCGCGACCGACGGAGCTGATAAACCGCACTCCGACCCGCCCCAGCTTGC
TTATTTGACTTATCAACATCAGAGATCCTGTAGCAGATCGCCTGCCGGAAAATGAAACGGAACAGGACCA
TCGGGCAATCCTTTGAGGAACTGCTCGACTTCGGGATTGTGCTCCTCGCGCAGCTGGTCCGGCGTGCCGT
GCGCCACCACCTTGCGATTGGCGATGATGTAGGCGTAATCGGCGATGCTCAGCACCTCTTTCACGTCGTG
GGTGACGATGACGGAGGTGATACCGAGGGCATCGTTCAGCTCCTTGATCAGCTTGACCAGCACCGCCATG
GTGATGGGGTCCTGCCCGACGAACGGTTCGTCATACATGATGAGGTCGGGGTCAAGGGCGATGGCCCGAG
CCAGCGCCGCCCGGCGCGCCATGCCACCGGAGAGTTCGGAGGGCATCAGTTTCGCTGCGCCGCGCAAGCC
CACCGCCTGCAGCTTCATCATGACGATGGTGTGGATCAGCTCCTCCGGCAGGCCGGAGTGCTCCCGCAGC
GGGAAGGCCACGTTGTCGTAGACCGTCATACCGGTAAACAGGGCGCCGCTCTGGAACAGCATGCTCATCC
GTTTGCGCACTTCATAGAGGCGCGAACGGGAGAGGGTGGGGATATCTTCCCCGTCAAACAGAATGTGACC
GGACTCCGGCTTGAGCTGGCCACCGATCAGTCGCAGCAGGGTGGTTTTACCGATGCCGCTTGGGCCCATG
ACGGCGGTCACCTTGCCACGAGGAATGGTCAGGTTGATCCCGTCATACAACAGCCGATCTCCGTGACTGA
AGGTCAGATCGGAGATGGTGATCAGGTCATTGTTCAAACAATTGTCCATCCGGTGTAAATGAGGTGCGAA
TTTTATGGGGTAGTCTAGGTCTAAGCAACAGCAAGGAACAACTATTGAACAGGCTGCAAGCCCATTTGCA

CGACAGTTTTCTGTAACAAAGTGCGTCATTGAGAGCGGGACTTGCCATCCGATTCGCTTATCCGGGCAGA
CTTTTTTATAATCCCGCAGCTAATTTCGGCCCGGAGCTGTGTCTTTATGTCTGTAAAGCCTGAGAAAGTG
TCTGAACTGTTCGATTTTCGTCGCAGTGCCCGCGCGGTGCTGGACACAGAAAAGCAAGCCATTGATGGAC
TCTATCAGTATCTGAATGACGCCTTCGACAAGGCGTGCGAAATGGTGCTGCGCTGCGGTGGCAAGATAGT
GGTCACCGGCATGGGCAAATCGGGCCATATCGGCAGCAAGATCGCCGCCACCCTCGCCAGCACCGGCACA
CCGGCCTTCTTCCTTCATCCGGGAGAAGCGAGTCACGGCGATCTGGGGATGATCTCCAGCGGCGACCTTA
TCATCGCCATCTCCAACTCCGGCGAGAGTGACGAAATCCTGGCCCTGCTGCCGGTACTCAAGCGCCGCGG
CATTCCGCTCATCTGCATGACTGGCAATCCCGCCTCCACCATGGCCAAAGAGGCCAATGTGCACCTGTGC
ATCAAGGTGGAGAAAGAAGCCTGCCCGTTGGGTCTGGCACCCACTTCCAGTACCACTGCAACCCTGGTGA
TGGGTGATGCCCTGGCAGTGGCTCTGCTGGAAGCGCGCGGTTTTACCGCCGATGACTTCGCCCTCTCTCA
CCCGGGCGGGGCGCTCGGCAAGCGTCTGTTGCTGCGGGTTGGCGACCTGATGCACAGTGACGATCTGTTG
CCCCGGGTGGGGATAGATGCCACCATCAGCCAAGCGCTGCTGGAAGTGAGCCGCAAGGGGCTCGGTATGA
CGGCGGTGGTCGATGGCAATGGCCTGCTGGCCGGGCTCTTTACCGATGGCGACCTGCGCCGCATTCTTGA
CCAGCAGATCGATATCCATCACACCCCCATCAGCCGTGTGATGACGGCCAACTGTGTTACCGTAGGTCCA
GAGATGATGGCTGCAGAAGCCGTCAAGCTGATGGAAACGAGAAAAATCAACGGATTACTGGTCGTGGATG
AAGAGAAACGCCCGCTCGGCGCCTTCAACATGCACGACCTGCTGAAAGCCGGAGTAATTTGATGCAAAAC
GTACCGACCCTCTATGGTCCGGTGACGCGCAGCCAGTTCGACAAGGCGGCGCAAATCCGCCTGCTGGTGT
GCGATGTGGACGGGGTGCTCTCCAACGGCCTCATCTACATGGGAAACAACGGCGAAGAGCTGAAAACCTT
CTGCACCCGCGATGGTGCGGGCATGCGAGCATTGCTCAATGCCGGGATCGAGATCGCCATTATCACCGGG
CGCAACTCCCGTATCGTCAGCGATCGGATGAAAAGCCTGGGGGTGGCTCATGTGGTGCAGGGGGCTGATA
AAAAGCTGCCTAACTTCGAAGCGCTGCTGGCCAAGCTGGGGCTGACTGCTGAACAGGCCGCCTATATGGG
CGATGACACCATCGATCTGCCGGTGATGGCCGCTTGCGGCCTGGGTATCGCGGTGGCCGATGCCCATCCG
CTGGTGCTGAGTCGTGCCGATATGGTGACCCGGCTTGGCGGTGGCATGGGCGCGGTGCGCGAGGTGTGTG
ACCTCATTTTGCAGGCGCAGGGCCACGACGATTATCAGCCCGAGGCCTCGGTATGAGCAGACAGACCCTG
TTGTTTGCCCTGCTGTTTCTGGTCGCGCTGGTTGCCTGGCAGTTGGGCAAGGTGGAGCTGGTGCCTGAGA
GCGCCGTCAAAATCGAAAACTATCAGCCGGATTTTGTCGCCAAAGATCTGGTGACCACCCGTTTTGATAT
CAATGGCAAGCGCACCGAGCGGCTGGAGTCGACCTACGCCGAGTATTATCAAATCCTCGAGCAGGCCACC
TTCGACAAGCCGGTGGTCTACATGTATGACAATCAGGGTGCCGCCGAGTGGAAGGTGACCGCCGAAACCG
GCGTGCTCAACACCGGCGACAACGTCATCCTGCGGGACAAGGTGCATCTGGATGGTCTGCTACCCACCTC
CTTTATCTCCACACTGGATACCCCCTATCTGGAGCTGGATCTGGTGACACAGGATGTGCGCAGCAACCGG
GAAGTGAACATTCTGGGTCGGGATTTCCAGACCCAAGGGGTTGGCCTCAAGGGCCATCTGGACCGCAAAT
ATTTTGAGTTACTGGATCAAGGCCATGCCATCTATTTCAATGAAAAACGCTAATCTGGCCCTCGCAGCCC
TGTTGTTGTGTGGTGCGGTGACTGCCAAAGAGTCCGACTTTGCCGAGAAGGTCTACGTCGATGCCGTCAA
ACAGGTGGCCGAGATGCAGGACAACCGCATCACCTTCGAAGAGGATGTGACCATCAATCAGGGCACCATC
CGGATCAAGGCTGACAAGGTGGTAGTGACCCGCTCCGGCGAGAAAGGGGCGGAGGTGATGACTGCCTACG
GCAAGCCCGCCACCTTCTTCCAGATCCTGGATAACGGCAAGCCGGTCAATGCCCACGGCAACACTATCCG
CTACGAGCTGAAGAACCGGCTGGTGACCATCACCGGCAACGGTCAGCTCAAGCAGGAAGATAGTCAGGTC
AATGGCGATCTGATCCGCTACGACATCGTCAAACAGAAGATGATCGCCGAGAGCAAAAGCCCGAGCCAGC
GGGTCAAAACCGTGTTCCTGCCGGATCAGGTAGAGAATTTCGACAAGCCCGCCGACCAGAAGCAGCAGGG
AAAATAAGCCATGGCAGTGTTAAAAGCAGTGGGCCTGCAGAAGAGCTACAAGCGCCGCATGGTGGTGAGT
GACGTCAGCCTTGAAGTGGGCACTGGCCAGATTGTCGGCCTGCTCGGCCCCAACGGGGCGGGCAAGACCA
CTTCCTTCTACATGATCGTGGGGCTGGTGCAGCGCGATGCCGGTCTCATCACCATCGACGATCAGGACAT
CAGCCTGCAGCCCATGCATGTGCGGGCGCGCAACGGCATCGGTTACCTGCCGCAGGAGGCCTCCATCTTC
CGTCGCCTGTCGGTATTCGACAACCTGATGGGAGTGATGCAGACCCGCAAAGGGTTGAGTCGCGAGGAGC
GTGAGGACAAAGTTCAGCAACTGCTGGAAGAGTTCAATATCACCCACATTCGCGACAACCTGGGTCAGAG
CCTCTCCGGCGGTGAGCGCCGTCGGGTCGAGATCGCCCGTGCGCTGGCGGCCGAGCCGCGTTTTATTCTG
CTGGATGAACCCTTTGCAGGGGTTGACCCGATTTCTGTGATAGACATCAAGAAGATCATTCAGCATCTGA
AAGATAGAGGCCTTGGCGTCTTGATTACCGACCACAACGTCAGAGAGACTCTGGACGTATGTGAGAAGGC
CTATATCGTCAGCCACGGCAAGATGATTGCCGAAGGTGCACCGGCTGACATCCTTGCCGATGAGCAGGTC
AAGCGCGTCTATTTGGGCGATCAATTCAGTCTATAGTAAGGGGGCATGCCTTCATTTCCTTTGACGAGTG
CCTCGCTGAACATAAGGATATCCCGACGTAGATGAAGCCGACATTACAGTTGCGTCTCGGTCAGTCCCTG
ACCATGACGCCGCAATTACAACAAGCGATTCGTCTGCTCCAGCTCTCCACTCTGGAACTTCAGCAGGAAA
TTCAGCAAGCGCTCGAAAACAACCCTCTGCTGGAGCAGGAAGAGAGCGAGGCGCAAGAGACTAGTCCGGA
AGCGCCGGTCGATGCCAGCCAGCTGGACTCCAGCGATGCCATGGCTCAGGAGCAGATGCCGGACGAGTTG
CCGGTGGATACCACCTGGGATGAGGTTTACTCTGCCGGCACGGCCCAAAGTGCGGGCCCCATCCACGATG
GGGAAGATAGCGTCTATCAGGGTGAAACTACCGAAAACCTGCAGGATTATCTGCTGTGGCAGATGCGGCT
CACACCTTTCAGCGATGTGGATGCCGCCATCGCGCTCGCCATCATCGATGCCATCGACGAATCCGGTTAT
CTGACCGTTGCGCTGGAGGATATTCAGGCTGCCGTCACCAGCGATGACGTAGAGGTGGAACTCGATGAGG
TCGAGGCGGTACTCAAGCGGGTGCAGCACTTCGATCCGGTCGGCATTGCTGCACGTTCTGTGCAGGAGTG
TTTGCTGATCCAGCTGGATCAGTATGACCCGCAGACCCCTTGGCTCAACGAGGCCAAGGAGGTGATCCTC
GAGCACATGGAGCTGCTCGGCAACCGGGATTACCGCACCCTCGCCCGCAAGACAAAACTCAAAGAGAGCG

ACCTCAAGGAGGTGCTGGATCTGATCCAGCTGCTGGAGCCGCGTCCCGGTAACGGCATCATCCAGAGCGA
CTCCCAGTACGTGATCCCCGACGTCTCCGTGGTCAAGAAGGGCAGCAAGTGGGTGGTCGAGCTCAATCCC
GACTCCGCCCCCCGCATCCGGGTCAACGAGACCTATGCCGCCATGGCACGTAACGCCCGCAGCAGCACAG
ACACCCAGTTTATTCGCTCCCATTTGCAGGAAGCCAAGTGGTTCATCAAGAGCCTGGAAAGTCGCAATGA
CACCCTGCTGAAAGTGGCCAGTTGCATTGTCGAGCAGCAACAGGGCTTTTTCGAGTATGGTGAAGAGGCG
ATGAAGCCGATGGTGCTCAATGACATCGCCGAAGCGGTGGAGATGCATGAGTCGACCATCTCCCGGGTGA
CGACCCAGAAGTACATGCATACCCCTCGTGGTATCTTCGAATTGAAGTTTTTTTTCTCCAGCCATGTGAA
CACCGAAGGAGGAGGAGAGTGCTCATCGACCGCAATTCGCGCGCTCATCAAGAAGCTGGTCACGGCAGAG
AATCCTGCCAAGCCGTTAAGTGATAGCAAGATCGCGGATTTGCTGGCCGAACAGGGTATTATGGTGGCGA
GACGCACGATCGCTAAGTATCGCGAATCCTTGTTTATCCCACCTTCCAATCAGCGCAAACGCCTGGTTTA
GGCAAACAAAAGGATGATGTTATGCAAATTAACCTGACCGGACACCATGTCGAGATCACCGAAGCTCTGC
GTGATTATGTGAATAACAAATTTGCCAAACTCGAGCGTCACTTCGACCACATCAACAATGTTCACGTGGT
GCTTAGCGTGGAAAAACTGAACCAGATAGCGGAAGCCAAATTGCACGTCAGCGGTGGCGAAGTGTTTGCC
AACTCTGAACATGCCGATATGTATGCTGCGATAGACACCTTGCTCGACAAGCTGGACAGGCAGATCATCA
AGCACAAAGATAAGTTAAATAAAAAATAACCATGCAACTTGAACACATACTGAGTCGGGACTGCACCAAG
AGTGCAGTCCCTTGTACCAGCAAGAAACGCGCCCTCGAAATCATCAGCGAGCTGGCCGCCGAACGCCTTG
GCACCTCCCGTCAGGCGCTGTTTGAATGCTTGCTGGCCCGCGAAAAAATGGGTAGTACCGGGATCGGTGC
CGGTATTGCGATCCCCCACGGTCGCATTGACGATGAATTCCCGCCAACCGCGATCCTGATGACCTTTGCC
GACCCTATTCCTTTCGACTCCATCGATAATCAACCGGTTGACCTGCTGTTTGCCTTGCTGGTGCCGGAAA
GCGAGTGCAAGCAGCATCTCAAGACCCTTTCTCTGATGGCTTCCAAGCTGGGTGACAAGGCGGTTTGCCG
CCAGTTGCGTCAGGCAACCAGCGACGAAGAGCTCTATCAGATCATGACCTCGGCCTGATAGCAGGCCAAG
GATGAGACACCCGTTTTGGCGTGAGGGAACAAGATGCAGTTAATCGTGGTAAGTGGACGTTCGGGCTCCG
GTAAAACGGTAGCCCTGCGAGTGCTGGAAGATCTGGGTTACTACTGTGTGGACAACCTGCCGGTCAATCT
GTTGCCCCAGCTGATAGTTTCGGTGGAGAGCCAGTACGACAAGCTGGCGGTCAGTATCGATGTGCGCAAC
CTGCCGAGCGATCCGGAGAAACTGGAAAATCTGCTGGCTCAGGTGCGCAATGAGGGGCGGGTTGAGTTCT
CCAGCTTTTTCTTCGACGCTGACAACGGCACCCTGCTCAAGCGCTATGGCGAGAGCCGCCGCTTGCACCC
CCTCTCACGCAACAAGCTGTCGCTGGATGAGGCGATTCGGGAGGAGACCCACCTGCTGGCGCCCATCTCC
TCGACCGCTGACCTGCGCATCGACACCACTACCCTCAGCATTCACGACCTGAGCGAGCTCATCAAGACCA
GGGTGCTTGGGAAGAAAGAGAACGAGCTGGTGCTGGTGTTCGAGTCGTTCGGCTTCAAGTACGGCATTCC
CAAGGATGCCGACTTCGTGTTCGATGCCCGCTTCCTGCCCAATCCGCACTGGATCCCCGAGCTCAAACCG
TTTACCGGCAAGGATGAGCCGGTCGCCAACTACCTCTCGGCGCAGGCTGACGTGATGCTGTTTACCCAGC
AGATCGAGAACATGCTGGTGACCTGGTTGCCCCATCTGGAGCGCAACAATCGCAGCTACCTTACGGTCGG
CATCGGTTGCACCGGCGGTCAGCACCGCTCGGTCTTTATTGCAGAACGGCTGGCCAGTGCATTCCGTTTG
CTCGGCAAGAACGTGCAGATCCGCCACCGCACTCTCGACAAGAGTGCCCCCCAATCCTGACGGACCAATA
GGACGACCCGATGACTGTTTCCGCCTCGGTTGAAGTGAAAAACAAGCTGGGTATTCATGCCAGACCAGCC
ATGATGCTGTTCGAGCTGGTGCAGGGTTTCGATGCTCACGTGCTGCTGCGCAATGAAGATGGTGTGGAGG
CCGATGCTGACAGTATCATCGGCCTGCTGATGCTGGATTCGGCGCAAGGGCGGCAGGTGGAAGTGCAGGT
TGAGGGGCCCGAAGAGGTCGAGGCTCTGGCCGCCGTGGTGGAGCTGTTCACCTCCGGCTTCAATGAGGAG
TAGTCACTCCTCGTTATATGTTATGAACCGGTGCGCGCCAGCGCTTTGAAGCTGGCAAAATCCCGACAAG
CCAGCAGATTCCTGCTGGCTTTTTCGTTCATCAGTGCCAGAAACAGGTCGTAGAGACCCATCGCCTCCTC
GTAGTCAGCCTTGCTGATGGCCAGCACGAAGATGAGCGTCGCTATTTCCCCGTTCCCCCAGTCAATCCCC
TGCGGGGCCAGCACGGTGTAAACCAGAGTGCGGCGCGCCAGCAGACCGAGGGAGTGGGGCAGGGCGATCC
CTTCTCCCAACATGGTGGAGACGATCCGCTCCCGCTCCTGCAGTGAGGCTCTGAAGCCGCTTTCCACCAG
ATCTTCCGCCTCCAGCTGATCACAGATACGGGCAAACAGTTCGGCCTGGGTAAGGGGTTCTTTGACCACC
ATGAAGTGGTCGGCATCGAAATATTTGTCGAGCATGTAGGGGCGGGTGCGGTCGATCAGCACCAGCTTGC
CAAGCTGCTCCAGCTGGTATTGGGTCGGGAAGGGGGCAACGAGTACCACGGGGGCGTTTTTTTCGTTCAC
CTTGACGGTACTGATGACAAAGTCCTGCGCGATCTGCGCCAGTCCTTCGTAATCCCGCACCGATTCAAGC
GTGGTCAGCTGTAACTGGGGATACTCCCGCTTGATGCGTGCTTCCAGTACCCGGAAGGTGGCGTTGCCCG
CATCGCAAAGCAATAGCGCCTGCGGGCGGCGGGTATAGCCGATATCGTAATGGCGCTCCAGCCCGACCCC
GATATGGATCACCAGATAGCCGATCTCGTGGTGGGTCAGTCGGTAGGGGGTCTGCTTGATCCACTCCGAA
ATGGCCGCCAGCGTGATGTCGTAGGCGAGGGGGTAGTACTGCTTGATGTGATCGGCCAGCGGGTTATGGT
TGCCAATCTGGTATTTGACCCGTAGCAGCATGGCGCTGATATGGGTTTGCAGGTCGGCCCGCAGCTGTTG
GTCGCCTCGCAGGTCGTAAGGGTAGTGTTGATGAATGTAGGTCAGCAGATGCTCCACCAGCTGTTCGCTC
TCGGCGGCCATCGCCGGGCTGAGCTGGGGAGTATCGGCAATCAGGCGCGCCTGGATCTGGATGGCGAGAC
CGGCGATCTCGTCCTCTACGGGGGGCGTCAGGGTTGGCAATACCGCATATATATCGCGAGATACTGCCAG
CAGCGGCAGAGTCAGATCCTCGCTGGCGAAGTGGCGCAACTCGTGACCGGATGCCTGACGCAGCAGGGCA
ACGGCGCAGTAGATGGTGAGCTGCTGCAGGCTCTCGTCAGCGAGACGCAGCTGATGTCGGGCAAGCTGGG
CATGGATGTGATTGCCCACTACCTCCATCGTCTTGCTGGGGCAGAGGTTGGGTAGCAGGGTTTGCAACTG
GTTGTTGTGCAGCAACTCCTGATAGAGCAGCTGGGTCAAGCAGGCACGGATCGCCGTCTCCTCCCCCTGA
ATGCGCATCCCCAGGCGGGGTTTGCTCTCGACGCTGAGCCTGAAGTGGCTGAGCTGCTCACGGACTTCCG
CCATATCCCCCTGCAGTGCCGCCCGGCTCAGATACCAAGTATCAGCCAGCTCATCGAGCTTGATCCCCTG

CTCTGCCGTCAATAGCAGGATCTGCAGGTGCAGAACCCGCTCGCGGCTGGTGCGTGGCAGGCCGCTATCC
TGACCCTGGGCCGCCAGCAGAGCCTCGAACAGTCCCTGATTGTAGATTTTCAGCTGGTAGCCGCTGCCAC
GCTGGTGGATCAGATGAGCGCCGTGCGTGGCAATCAGTTCGTTGAGGGTCGCCACATCGGTGCGCACCGT
GCGGGTGGAGACATTGAGGCGGCGAGCCAGTTCCTCCTGTGGCAGTGGTTCGGCGTGGAGCTGGATCAGC
AGTTGATGGAGCCTGGGGTGTGGCAGCTTGATCATAGGGGGTATGCAAGACTCCTGTTGCGAGATAAAGG
GTCGATGTTAACAAGTGGGTCATCAACAAGCCAGGCACCGGTCAGTGATTGAACCGGTGCCGTGACTGCC
ATCAGGCCAACAGTCGTTTCACTATGGCCAGCAGAGTGCGAACATCCTCGGGGCGAGTGTGCCCGCTCGC
GTTGTCGATGATGGAGCTGTAGACGTGCGGGATCACCTTCTCGACTCCGGCATCCAGCGCAATCCGCACT
ATCTCCTCGAAGTTCTCCAGATCGATGCCGCCGGTGGGCTCTAGCCAGAAGTTGTTGCGGGCACAAGCCT
CGGCTACTGCCTGATACTCGTCGCGGCAGGCCAGCCCGCCCATGGGGAAGAACTTGACCGAGCTGCCGCC
CATGTCCTTGAGCAGGGCAATGGCGGTATCGATGGGCACTATGCCATCGGGCTGCCCGGCGCTGAGTGGA
CCGGTGGAGATCTTCACCATGCCCGGCATGCCGGTAGGGGAGAGCAGCCCGTTGACCAGTGTCTCTGACT
GGCCGAGCAGCGCCCGGCTGGCACCAACACCGGTGAACACCTGGTTGACGTGCTGGGGCTGCAGTTGGCA
GGCCAGTTCGGTCACCATGGCGGATTGGCGCGGATCGCCGGCGCCCAGCCCGATGGAGAGGGCGTTGTCG
ATCAGCTTGGCGTACTCCTTCATATCGGCAACTGCGGAGGGAATATCCGGGTAGTTTTTGGAGAGCACAC
CGACCAGCACGTGGCCTTCGGCTGCGTCGTACACCTCGACGGCATTCTCCTTGGATCCAGCCAGCACGTT
GAGACAGACCCGATCACGGTAGTAGTTGGGGGTAAGGGACATCAGCGATTCTCCTGAACGAGGTTGGCAA
TGGCAGAGTGGATTTGGCCGAGCTGGGGCACGGTGACGCTGCGCACATCGATCTCGATGTGCCCCTCGTT
GGCCTTGTAGCCGCGGCAGTAGATGGCAGGCGTGCCCTGTTGCAGACGGGTGACGACCTGCACTGTGGTC
ATGCCGAGCTGCTGGTGGTCAAAGGCGATATCGGTGCGGGCAATGTCGCGTCCTGCGCTGTCCCACACCA
CCTTGGCGGTGACGCCGGGCAGCTGGTTTAGGCGTTCGATGAAGGGGCTCATCTTGGCGACCATGGCGGC
GCCGCTCTCTTTCTCTTTGGCCAGATAGCGCTCGATGGCGTGGGTCAGGCCGAGGATCCCCTCCTTGCCC
ACTTTCATGGCGCGACCGATGCCATTGGCTTGCTGCTTGACCCACTCCACGTACTGACGCTTGCCAACCA
CCAGCCCGCTGGTGGGCCCTTCAATGGCCTTGGCGCCGCTGTAGATGACCAGGTCCGCACCCTGGTTGTA
GTAGAGGCGCAGATCCTCTTCGGCAGCCGCATCGACGATGAGCGGGAGCCCGTGGGTCTTGGCGACCTCG
GCGGCCTCTGCCACCGACAGAATGCTCTTCTGCACGCAGTGGTGGGATTTGATGTAGAGGATGGCTGCTG
TGTTGGCATTGATGGCAGCGGCCAGCTGGGAAGGGGAGCACTCGTTGGCGTAACCGGCTTCCACTACTTT
GCCGCCACCCAGATTGACCATGGTACCGACCGGGGCGCCGAAGTTGACGTTATGCCCCTTGGGCAGCACG
ATTTCACTCGGCACCTCATGGGGATGGGCATGCAGGTGCTCGAGGCGGTAGGGATCGCCGCGTACTATCA
TGGCGGCGACTGACTGGGCGATGCCGGCGCTGGCGCAGGAGACCACCACCGCATCTTCCACATCCAGCAA
ACCAGCCAGATAGGCGCCGGTCTTGTTGACCAGATCCTTGACCTCGAAGTAGTGGCCCAGGCCAAAATTG
ACCGCATCGACCACCTCCTGCTCGGGGGTGGAGACGCCGAGAATAGTCATGCGCCCGGAGGCGTTGATCA
CCGGTTTGAGCTGATATTTCTCATAAACAGAAGGCATGGTGTGATTTCCCATAGTGAGTCAGAACCAGTT
CGCCACCGACCAGGGCGGCGAGAGGCTGCAGTTGTTGGTCACCGCTGCGGGACTGGGCTTCGGTATCCAT
AAACAGGGTCGGGCCGCTGGTGACCTCGAGCAGGGTCAGATCGGCATCGGCTCCCACCTCGAGCCGCCCC
TTGCCGGAAAGGCGCAGGGCATCAGCGGCCTGATGGGTGACGCAGGCGAGCACCTGTTCCAGCGTCATGC
CGATGGCGAGAAACTTCGACATCACGTGAGCCAGACTGTAGACCGGCCCCTTGATGCGGTTCTTGCAGTA
GATGTCGGAGCTGATGGTGCGGGGTAGGATCCCCTGTCGGATAGCCAGCTCCGCCACCTCGAAGCTGAAG
CTGGCGCCGCCGTGGCCGATGTCGAGCAAAAGGCCCCGTTTCATCGCCTCGCGCACCGCGACCCGCAGCT
CGCCAGCCGGAGTCAGGATGCGGTTCGGTTTGCCGTTGAAGCAGTGGGTCAGCAGATCTCCATCCCCCAG
CAGGGCGACGATCTCGTCCAGATCCGGCGGTGTGTTACCCACGTGCACCATCAGCGGCAGCTGGTCGTGA
GCCTGTTGCAGGGCCTTGGCCATGCGCAGCGGTTGCAGGCCGTTCTCACCGACCACGCTACCGCTCATTC
GCGCCTTGATGCCAACGATAAAGTCGGGGTGGCGGCGAATGGCTGCCGAGGCGAGATCCATATCGAGGTC
CCGGCTATCCGCCAGCTCGTTCTGGCGTAGCAGACCGATGCGGGAGATGTTGAGCAGGGCATGCACCCGG
GTCTTGCAACCCGCAGCGATCTGCTGGAAGTCATCCACATCATCGGCTCCGGTGCTGCCGGCGTCGATCA
CGGTGGTGACGCCGGACTCTACGCCTACCTTGTCCGGCTCATCGTTATAGATCGGGGAGGCGGGGTAGCA
GTGGGTGTGGCCGTCGATCCAGCCTGCGCTCACATGCACCCGGCCGCCGAGATCCAGGATCTGCCGCGCC
GTTGCCATCTTCGGCAGGGAGCCGATGGCGGCGATTTTGCCCTCCCTGATGGCGATATCGACGAGCTGGC
CATCTCCCAGCCGCCCCGCTCTGATGATGATGTCATACATGCTGATAACCTCTGGCGAGCCGCCCTGCAG
GGCGGCTGGTACTTACACAAGGGCGACCGGGAAGAAGGTGCCCAGCAGCATGGCTCCCAGAATGGCGCCA
CCGGTGATGGGTTTGCCCCACACGTAGAACAGCAGGGCACCCAGCAGGGAACTGATACCGATGGGGATGG
AAGCGCCCATGGCGGAGAGGATGATGAGGGGGCTGAGGAAGCGGCCAGAGCTGTTGCCAGCACCCATCAT
CACGTCAGCACCGTAAGTGGAGTTGCTCTGGTTGATGGTGAACTTGCGCGCCAGAATGATGATGTAGCCG
ATGGCCAGACCAATCCCCATACCGGTTGCCAGCGAGGCGCCGAAGTTCTCCACCGGATAGACGATGCCGG
CCCCCAGCAGCAGGGCTGGCACTCCGAGGCCAACCCCGGTCTGGATGGCTCCGCCGATATCCAGGATCCC
CACCAGGGATCCCTCGATGATGCGGGCGAACAGGAAGCTGGCACCGAACGCGGCCACCGCGCCGTAGGCA
CCCGTGTCCATTCCGGCTTTCAGCATGGCGACGAAGGCTACCTCGTTGAAGGCACCAACCCCGTACAGAT
AGAACATGTGGGTACCGGCAAAGACCCCGGCCGAGAGCAGGCCGACGAAGATGGGGAAGAACCAGTCGGC
ATACCAGAAGTTGTTTTTCAATTTCTCATCCATGGTGTGACCCCTTATTTGCCGCTCAGCAGGTTATGGA
CGTTTTCCAGCCACAGGGGGACGCCCATATGGAAGGATTCAATCAGTTTCATGTCGAAGCCGCGGAAGAA
GCCGCTCAGCACGAACAGCAGGACGATGGCTGCCATCATCACCTTGGTGACATGGTTCCAGCCGCTCTCT

TCCACACCCTTGCCGATCAGGATGCCGAGCACCAGACCCGGTACCGCGTTACCCATGATGAGCTGTGCCA
GACCGCCGAAGATGGTGGCCCAGAAACCGGATTTCTTGCCCGCTTCGATGGCGGCCAGCCAGAAGATCAC
CGGCATGACGGTGTTGACCAGCAGGTTGGCTGCCGGTACCAGCACCTTGACCGCAGTGACCTGCAATGCA
GCAGGGACGGCTGCGGCCGTGCTGTTGAGGAAGGCAACCACGATGGCACCTATGATGCCGCCGGCGATGG
CCATTTTTTTCGGGTCGTGCAGAGTGGTGGCGAGATCCCGATTTTTGAACAGCAGTGCCGCCGTGGCCCA
GTGCGGGATGATGCGGTGATCCACATCCTGGGTGAAGGCGCCTGCCGCCACGGTCGAGGCCCAGGCGTTG
AAGAAGAAGCCCAGACCAAACGAGAAGTGGGAAGCGGGATCCCCTTCGCAGGAGTTGAGTTCGCCCAGGG
TGCGGAAGGCACCCATGCCCTGTACGGTCGGGGCGTGGAACATCCGCGCCGCGCCGGCCCCCACCCCGAC
GCCAACCAGGCCGCCAATGATGAGTGACTTGATAAAAATGATTAGAAACATCCAACGTATCCTTCTTGTG
GGGTTGTGCCTGCGCAGGCATCACACCTGGGTGAATGTCACCTTGGCGACATCCAGACTGGTGACCTGAA
CCTTGATCTCCAGCTGGACGCGGTATTCGCGGCGCTGGCGCGGCAGGAAGAAGAACAGGAACTTCTCCAC
CCGGACCAACTCCTCGGCTTTGAGCACCTGTACATCGACCGGCTCAATGCGCAGCAGTACCTGCTGGTTG
TCCTTGAGCAGTGTGGGTTGCACCTGGCTGAGGGCGGAGGCAAACGCCTGCTGGCGGGTCGTCCCCTTGC
CGTTGACGATGACCTGGGTGGTGAAGTTCTCTTTCATCAGGCGCGTCCGTGCTTCTTGGCGAAGGCTTCG
ACCAGCTTTTGGCCCAGCTCTTCCTTGTCCATGAAACCGAAGCCCAGCACCACACACCCTTCGTTAATTG
CGGTGACGCCCTCTTCAACAGAGCGCATGCCGTAGCGGCACTTGTAGCCGTATTTGGTCTGGGCGGTGAT
CGCCCCGGCGCCGCCGCTGCCGCAGAAGGAGATGCCGAGATCCGCCTGCTCCTTGTTCATCACATCGCCG
AGCTTCATGTCAGCGGCCATGCCAGGGATGATGATCACGTTGGCCCCGGCAGCTTCCGCGCCTGCACCCA
CTTTCTGACCCTTGCCGAGACGATCTCCGATTACGAGTTTGATGGCTGTCATATCGATTTCTCCTTGAGT
TACGGGTTCTGCACCGAGCTGAGTTCGTTTTCTTTGGCGACTTCAAAATGGACGGACAAGAGGTGTGCCT
CCTCATAAGCAAGCTGATCCAGCCAGCTGACGACCTGTTCGGCGAGACGCAGTGAGAGGGGGGAGATCTC
TTCAAACAGGCTGAGGTCAACCTCCGGCAGGGGCTCGCCGCTGTGGGAGCGCCACACCATGGCTTTGACA
TGGGAGGCCAGCATCTGCCGTTGCACCTCGTTGGGGATGATGCCCTGTTCGGCCAGCAGGGGATAAATGC
GCGCGAGGATATGATCGGTCTGGCGCAGCAACTCCGGGCTGTTCGGGGTCAGGGTCTGGTCGTGAGTCTC
TGTTTGCAATGTCTGCTCTCCAAGGCTGGCAGAAGGTATGGCGTTACCCTAAGCCTGCGCGTCAATGTGG
AGGAGGCAGCGTTTTTCCGATTCGAAGCGGAAATTCCTGATGAAATGGCCCCGAAATGATCGTTTGGATA
CTAAAAAGGTAAGGAGAGTGCTTATTGAAGCTGGTTTTTTGTGATCGGTAGCGCACAAATCGGGGGGCGG
GGGCAGGGCAAGCGTTGCGGGAAACTGCGAGTCATCTCACGGTAAGAGACGTTGCCGAGAGGGCAGATAT
GGAAAAGGGCGGTTTCCCGCCCTTCTCATAAAAAGGAGTGACGCTAGCTGGCCGATGCGGCGCTCGAGCT
GGCCGTTCTCTGCTGATAGCGCAGGTCTTCCTCTATGGTGACCACCACTTCCAGCGGCTTGCCGGAGCGG
AATACCGAGATGGTCAGCTTGGTGCCGGGGCGGCTCTCGACAATCTTGTCCATGGCGCTGCGTACGCCAC
TGATGGCCTCTCCGTTGATCTTGAGCAGCACATCCCCTCGCTGCAGGCCGCCTTTGGCCGCCGGGCCGTT
GGGATCCAGACTCTCCACCACCAGACCACGCAGGTCGCCGAGGTTCATCATCCGCGCGACTATGGGGTTG
ATCTCGACGCTGGAGATCCCGAGGTAGCCGCGGATCACCCGGCCGTTGGCGATCAGCTCATCCATGATCC
GTTTGGCCAGCCGGTAGGGGATGGCGAAGCTGATGCCGTAGCTCTCCTGATTGCCGTTGAGGTGATAGGT
GGCGGTGTTGATACCCACCAGATCGCCACGGCCGTTGACCAGCGCGCCACCCGAGTTGCCCTCGTTGATG
GCGGCATCGGTCTGCAGCAGATCCTGACGGCCATTGCTGTCAGGTCCCATGCTGGAGAGGCCGGTACGGC
CGGTCGCACTGATGATGCCCTGGGTGATGGTCTGGCCGACGTTGTAGGGGTTACCGATGGCGAGCACCAC
ATCCCCCACTTCTGGCAGTCGCTCCGGATCCTGCGGGATCACCGGCAGGTTGTCTGACTCGATATAGAGC
ACGGCGAGGTCGGTGAGCTGATCGGTACCGACCAGCTCGGCACTGAAGACCCGACCATCCTGCAACGCGA
CGATGATCTGGTCGGCATCGGCGATGACGTGGTAGTTGGTCAGTACATAGCCGCGCTGGTTCATGATGAC
GCCAGAACCCAGCCCTTGCGGGCGCAGTTCCCCCTGATTGCCCTGATTGGGGGCAAAGCTGCGGGTGTAG
ATGTTGACGACGGCAGGGCCTGCCCGATGGGCGGCATAGGAGAAACTCAGTTCACGGGCATTGGTGATGA
TGCCGGGCAGTTGGGCACCACCGCGAAAATGGGGAAACAGCAGCAACAGCAGCGCCGCGACGGTCAATCC
AAAACCGACAGACTTGCCCAAGTAACTGACTAAAGGGGGGATTTTCATGTCATTCTCATCCGTACAGGCT
GCGCGCAGAGAATAACACTATCGATGGATAAAAATCACCCGCGGTGAAGGCCGCGGGTGATGGAGGGGTT
GGCGAACAACTTGAGCGTTAGCGGATCACCAGATAGAGCGAGGAGTCGCCGCGCTGGATGTTGAGCGCCA
GCACGTCAGGCTTGTTCTTGAGCGCCTTGGTCAGCTCGCCCAGGGTGTTGATGCGCAGGCGGTTGACCCC
GATGATGATGTCACCCTTCTGCAGGCCGGAAGCGGCTGCCGGAGAGCGAGGATCAATATCGGCCACCGCG
ACACCGGAGACCGGGTCGGTGGTGGTGCTCAGCTTGGCCCCTTCCAGTGCCGGATGCAGCGCGCTGGCGC
GTACTTCGCTGTCATCGGCCTGCTTCAGGGTCACTTTGGCAGTTTGTGACTTGCCATCGCGGATCAGCCC
CAGTTCGACCTGCTTGCCAGCGCCCATGGTGGCAATCTTGGCGCGCAGTTCGCCGAAGGAGCGAACCGGT
TTGCCATCGATGCTGACGATGATATCGCCGGCCTTGATGCCCGCCTTGTCGGCCGCCGAATCAGGCATGA
CCTGATTGACGAAGGCACCATCCTTCTTGTTGTAACCGAAGGTTTTGGCGATGTCGGAGGTGAGCTCGGT
GCCGGTAATACCCAGCTGGCCGCGACGCACTTCACCGTACTTGACGATCTGCTCGGTCAGGTCCCGCACC
ATGTTGGAAGGGATGGCAAAGCCGATGCCGATGTTGCCGCCGTTCGGGCCCAGAATCGCTGTGTTGATAC
CGATCAGCTCGCCACGCAGGTTGAGCAGGGCACCGCCGGAGTTACCGGAGTTGATGGCCGCATCGGTCTG
GATGAAGTTCTCCAGATTCTCGATGTTGAGGCCGCTGCGACCCAGCGCACTGACAATACCGGAGGTGACG
GTCTGGCCCAGACCAAACGGGTTGCCGATGGCCAGCGCATAGTCACCTACCCGCAGCTCGTCGGAGTCGG
CGAACTTGATCTGTACCAGATCCTCGGCCTTGATTTGCAGCAGGGCGATGTCGGACTGCTTGTCTTCACC
GATCTTCTTGGCCGCATACTCACGACCATCCTTCAGGTTGACCTTGATCTCGTCCGCCTCGTGCACCACG

TGGGCGTTGGTGATCACGTAACCCTTCTTGGCATCGACGATGACACCGGAGCCGAGCGCCTGGAATGGCT
GCTCGCTCACCTGTTCATCGGGCATGTTGGGGCCGAAGAAGAACTTGAACTGTTCCGGCAGACGCTGACG
GGTAATTTTTTTGCCAGAAACCGAGATATTGACCACCGCCGGGGTGACCTGTTCGAGAACAGGCGCCAGG
CTTGGCATCTCCTGATTTGAACCTAACAGGGAAGGCAGTGCAGCCTGAGCAGGGGCCGCAGACATGGCGA
TACCGACACTGAGGGCTAGCACACTGAGCATGGAAAGAGGTTTACGCATATGAAAAACAGCTCCCAGAAA
AAGTTGCATTGCAATCGTCAAGGTCAGACCACGGGACTGGCCAATTAGTTCCGCGAGGAACTAGTTTGGT
TGCTTGAGCAGTCCGGAAGAGGCGCCAGCATAGTCGCGAGGCGGAACGCCCGGCTCTTCTGCCACATTGG
CAGGGGCTTCTTCTACCAATGATTCGCGGAACAGCGGTTCCTGCGCCTTGGCCAGGAATTTGCTCTGCTC
GGCCATGTGGCGATAGAGGGTCTGATACTGCTCGGCCAGTTGCTCCATCAGGGCGGCACTGTCGGCGAAG
TGGGTGTTGATCTGGCCCTGATAACTCTCGAGCTCCTTGTGGGCCTTCTTCAGTTCCTGTTCCAGACGGC
CAGCATCGCGGCTACGCACCGAAAAACGGCCGAGAACGATACCGATAATCAAGGCCGCGACAGCCAGCAG
GATCCCAGTTAACAGACTCATAGATGCTCCTTGTTACAGCTAAAACAGAGTGGGTTTATGCGGTCGTCAT
AACCGCCATGCCTTGATGGCACTATACCGTGCGTCCTGAGGCCGTGATACCATTTTGTCCTCGATTCAGG
GACTTGGAGAGGCGCTCAAGGATGACACCGCAGCAAAAATACCAGCAGGATCTGCTGCGCCCCGGCTTTG
TGGCCGACCCTGCGCAGGGGATGGCCATTAGCAGGCTGGAGCGACTGTATCAGGATTTGCTACGGCGCCC
GACTCCGACCAGATCTCGAGGGCTGCTGGGTTGGCTGCAAAAGCCCAAGCCGCCGGAGCCTGTCCTCGGT
ATCTATATGTGGGGCGGAGTGGGCCGAGGCAAGACCTGGCTGATGGATACTTTTTTCGACAGCCTGCCTG
GGACGCGCAAGATGCGCAGCCATTTCCACCGCTTCATGCACCGCATTCACGATGAGCTGCAGGGGCTGAG
CGGGCAATCCGATCCCCTCAAGCTGGTGGCCAGCAAGCTTGCCAGTGAAACAGACATCATCTGTTTCGAC
GAATTTTTTGTCTCCGACATCACCGACGCCATGCTGCTGGGCACGCTGTTCCAGGAGCTGTTTGGTCACG
GTGTTGTGCTGGTCGCCACTTCAAATATTCCGCCGCAGGATCTTTATCGTAACGGGCTGCAGCGAGCCCG
TTTTTTGCCTGCTATCGAGCTTATCGAGCGTCATTGCGAGGTACTTAACGTGGATGGCGGTATCGACTAT
CGTCTGCGTACGCTGGAACAAGCCGAGATTTATCACTGCCCGCTGGATTTGCAGGCAAAAGCCAATCTTG
ACCACTATTTTCAGCAATTAACCGGTGGGCACACGGCTTGCGAGGGGACGTTCGAGGTCAATCATCGTCA
GCTGGCGTCGCTCGGGATGGGGGAAGGGGTGCTCTATATGGAATTTGAGCAACTTTGCTGTACTCCCCGC
TCCCAGAACGACTATATCGAGCTGGCCAGACTGTTCCATACCGTTCTGCTGGCCAATGTTCAGCCAATGG
GCACAGGCACAGATGACGCAGCTCGCCGCTTCATTGCCATGGTCGATGAGTTCTACGAACGGCACGTCAA
ACTCATCATGTCGGCGGCGGTTCCCATGACAGAACTCTACGGCGAAGGGCTGCTGAATTTCGAATTCCAA
CGCTGTCTTTCGCGGCTTCAGGAAATGCAATCGCACGAATACCTTGCCAGAGTGCACCTTCCTTAGTCAC
AATACCGCGCCTGCCAGTCGGATGGGCATCTGCGGCAGGTAGGGCCGGGCGACATTTGCTGATTTCATGG
CAGTGAAATTAGCAGGTGATATTTAAGGCAACTTCACTTATAATCGCTCGGCCCACGTTACAGCTGTCGA
TAAGACAGCAACACTTTAAGCTTTACTTGTATTCGAAGGGGTACAGGTAGGCCATCGTTTGTTGTTTGTG
GGAGAGCCCCCATAAGCAATTGGGTCTGTAACAGGTAATTGGGTTTAACTTAATGAAAACTTTCGTTGCC
AAGCCAGAAACCGTAAAACGTGACTGGTACATTGTGGACGCAGAAGGTAAAACTCTGGGTCGTATCGCAA
CCGAGATCGCTGCTCGTCTGCGTGGTAAGCACAAAGCTGAGTACACTCCGCACGTTGACACCGGTGATTA
CATCATCGTTGTAAACGCTGAGAAGGTACACGTAACCGGTAAGAAATTTACCGACAAAATGTACCACGCT
CACTCCGGTTTCCCGGGTGGTATCAAGTCAATCAGCTTCGACAAGCTGATTCAGCGTAAACCGGAAATGG
TAATCGAAGCTGCCGTCAAAGGCATGCTGCCGAAAGGTCCTCTGGGCCGTGCCATGTTCCGTAAACTGAA
AGTTTACGCAGGCGCCGAGCATGCTCATGCTGCTCAGCAACCTCAAGTACTGGATATCTAATCGGGAACT
GGCAACATGGCAGAAAATCAATACTACGGTACCGGCCGTCGCAAAAGCTCCACTGCTCGCGTATTTATCA
AAGCGGGTAGCGGCAAGATCGTAATCAACCAGCGCTCCCTGGAGCAGTACTTCGGCCGTCCGACTGCCCG
CATGGTAGTTCGTCAGCCGCTGGAACTGGTTGAGATGACCGAGAAACTGGACCTGTACATCACCGTTAAC
GGTGGTGGCATCTCCGGTCAAGCTGGTGCGATCCGCCACGGTATCACTCGTGCTCTGATGCAGTACGACG
AAACCCTGCGTTCCGAACTGCGTAAAGCTGGCTTTGTTACTCGTGATGCCCGTAAGGTTGAGCGTAAGAA
AGTTGGTCTGCACAAAGCTCGTAAGCGTCCGCAGTACTCCAAGCGTTAATTCGCTTTTGGTACTCACTCT
TTCGAGTGTCAAAAAAGCCTGGCAACCGCCAGGCTTTTTTTATTGGTAATATTACCGACATCAATGCTAA
CCACGCGTCACAAAAGACAAAAAATTGTTTCTTTATTGTTTTCATTGCTCACTGCTCTTTCTTGTCAAGT
TGTTATCTTTTCTTTAAAATTTCCCCTGTTTTCTGTGGCAATTGTAAATCCAGCTTCCGCCGCTGTTGTC
ACGGGAAAAATGGGGAACAGGGACACTATAAGAATGTGCGGAGTCCACATGGGAGAGTTTTTGGATGAGC
AATGCGCCAGTTGATACCGGTCGCCGCAGATTTCTTACCTGGTCAACGGTTGCCGTTGGTGGGGTAGGAG
CTGCGTTTACCGCAGTGCCGTTTATAAAGTCATGGAATCCGAGCGCCAAGGCCAAAGCAGCCGGTGCGCC
GGTTGAAGTCGATATCAGCAAGTTGGAACCGGGTCAGCTCATCCGTGTCGAGTGGCGAGGCAAGCCTGTC
TGGGTGGTGAAGCGCACCAAACAGACGCTGGATGCCTTGGCCGCGCATGATGACAAGCTGCGGGATCCCT
CATCTGAAGAGCCGCAGCAGCCTGACTATGCCCATAACGGGTACAGATCGATCAAGCCCGAAATTTTTGT
CGCGGTCGGTATTTGCACCCACTTGGGGTGTTCACCTTCCTATCTGCCTGACAGCTTTGGCGAGCAGGTG
CAGGGCGTTACGTCCGGCTTCTTCTGCCCGTGCCATGGCTCCAAGTTCGACATGGCCGGCCGTGTGTTCC
AGGGGGTGCCTGCACCGCTGAACCTGGTCATTCCGCCCTATCAATTCATTAATGACACCACCATCCTGGT
CGGTGCCGATGGCAAGGAGGCCTGACCATGTTGAGTAAACTGATGGGCTGGATCGATTATCGATTCCCGC
TCACGGCCATGTACAACGACCACATGGCCAAGTATCCGGCACCGAAGAACCTGAACTTCTGGTACTTCTT
CGGCTCGCTGGCCATGCTGGTGCTGGTTAACCAGATCATCACCGGTATCTGGTTGACCATGAACTACAAC
CCCTCTGCGGAAGGTGCGTTTGCCTCCGTGGAGTACATCATGCGCGATGTGGAGTACGGCTGGCTGCTGC

GTTACATGCACTCAACCGGTGCGTCTGCATTCTTCATCGTGGTCTATATGCACATGTTCCGCGGCATGAT
CTATGGCTCTTATCAGAAGCCTCGTGAGCTGCTGTGGATCTTCGGCATGCTGATCTTCCTGGTGCTGATG
GCGGAAGCCTTTATGGGCTACTTGCTGCCTTGGGGTCAGATGTCATTCTGGGGTGCCCAGGTCATCATCT
CGCTGTTTGGCGCCATTCCGGTCATCGGTGACGATCTGACCCTGTGGATCCGTGGTGACTACGTGATCTC
CGGTGCGACCCTTAATCGCTTCTTCGCGCTGCACGTCATCGCGTTGCCGCTGGTGCTGGTGATGCTAGTT
GCCATGCACATTCTGGCGCTGCACGAAGTGGGCTCCAACAACCCGGACGGTATCGACATCAAGAAACACA
AGGACGAGAACGGCTGGCCGCTGGATGCGGTGGCGTTCCACCCTTATTTCACCGTCAAGGACATGATCGG
TGTGGCGGGCTTTCTGTTCTTCTTCTGCGCCATCATTTTCTTCAAGCCGGATATGTGGGGTTACTTCCTC
GAGAAGCCGAACTTTGAAGTGGCCAACGGCCTGAAGACTCCTGCTCACATCGCGCCTGTGTGGTACTTCA
CCCCCTTCTACGCGATTCTGCGTGCGGTACCTGACAAGCTGCTTGGCGTCATCATGATGGGCCTCTCTAT
TGTGGTGTTGTTCCTGCTGCCTTGGCTGGATCGCTGCAAGGTGCGTTCTGTTCGCTATCGCAGCAAGCTG
CACAAGCTTAACATCGCCCAGTTCGTGGTCTGCTTTATCATCCTGGGGGTGCTGGGTGTACTGCCGTCGA
CGCCGACCCTGACGCTGGTTGCCCAGATTTGTACCTTGGGTTATTTCGGTTTCTTCGTGCTGTTGTTCTT
CTACAGCAAAAACGAATGCACCAAGCCGCTGCCGGAGAGGGTTACATTTAAATGAAAAAAATAATATTTG
CAGTGCTGACCCTGCTGCCATCACTGGTGTTTGCCAATGCTGGGGCCGTGCATCTGGACAAAGCCAATTA
TGATCTGAGCGACAAGGCATCCCTGCAACGTGGAGCTGCCACCTTCATGAACTATTGTGCAGGCTGCCAC
TCTACCCAGTATCAACGCTACAACCGGGTTGCTGCCGATCTGGAGATCCCCGAGGAGCTGATGAAGGAGA
ACCTCATCTTCACCGGCGCCAAAGTGGGTGATCTGATGAAGAGCGCCATGTCCGAGAAGGATGCCGCCAA
GTGGTTTGGTGCTCCCCCTCCGGATCTGACTCTGGTTGCCCGGGTACGTGGTGCGGACTGGATTTACACC
TATCTGCGTTCTTTTTACGTGGATCCGACTCGTCCGTTTGGGGTGAATAATGTGGTGTTCCCGTCTGTGG
GTATGCCTCATGTGTTGGAACCGTTGCAGGGAACCCCCCGTGCCGAGTTCGTCACTCACACGGTCGATGG
AGTCGAGACCCAGCAGGTTGTCAGCATCAAATCTGACGGTAATGGTGAAATGGATGACGAAGAGTATGAT
CAGACGGTACTGGATCTGGTAAACTTCTTGGTCTACTCGGCTGAACCGGTACAACAGGAGCGTGAACGCA
TGGGCTTCTGGGTGCTTGGTTTCATCGTGATCTTCTTCATCTTCACTGTGCTGTTGAAGAAGGAATTCTG
GCGCGACGTTCACTAAGCGACCAGATACGGGTAATATAATGCACGGCAATGAAGACCAAGATGCTTCATT
GCCGTTTCTGCTTTTTAATGACGGGAGGGTTCAATGGCTGTAGCTGCCAATAAGCGTTCGGTAATGACGC
TGTTTTCCGGTGCCAACGATATGTTCAGCCATCAGGTGCGTATTGTCCTGGCGGAGAAGGGTGTTAGCGT
GGATATCTGCCAGGTTGATCCAGCCAATCTGCCGGATGAGCTGGCCGAGCTGAACCCGTACAACTCTGTA
CCGACCCTGGTTGATCGTGAGCTGGCGCTCTACACCTCGCGCATCATCATGGAGTATCTGGATGAGCGCT
TCCCGCATCCGCCCCTGATGCCTGTTTACCCGGTTGCCCGCGGTAACAGCCGTCTGATGATGCACCGCAT
CGAGCTGGACTGGTACTCCCTGGCCGACAAGATCATGGCCGGTACCGATGCTGAAGCGGCACGCAACGAA
CTGCGTGACAACCTGCTGGCCATCGCGCCGATCTTCGGTGAAATGCCCTATTTCATGAGCGAAGAGTTCG
GTCTGGTGGATTGCTACATGGCACCGCTGCTGTGGCGTCTGCCGAGCCTTGGCATCGACCTGACCGGTCG
TGGTGCCAAAGAGCTGAAGGCCTACATGGTTCGTCTGTTCGAGCGTGAGTCCTTCCAGGCTTCCCTGACC
GAAGCCGAACGCGAGATCCGTGCTGGCGTATGAGCATGGAACCGACAATGACACCGAGCCGCCCGTACTT
GTTACGGGCGTTCTTTGACTGGTTGCTGGACAACGATCTGACCCCTCATCTGGTGGTCAACGTCAACATT
CCCCATGTGATGGTGCCGATGCAGTTTGCTCAGGACGGGCAAATTGTACTGAATATCGCACCCCGAGCCG
TGATGCAATTTCACATGGATAACGAGGCTATCAGCTTCAGTGCCCGCTTTGGCGGCGTCTCCCAGCAGGT
TTACATCCCGATGGCGGCAGTGCTGGCTATCCATGCCCGGGAGAACGGCGTGGGCACCATGTTCCCGCCC
GAGCCGGGTTACGATATCTGGCTGGAGCAGGCTGAAGCGCCGGTGGAGCCTGAGCCGGAACCGCCGCGTC
CAAGCGGTCGTCCGACCCTGAAGGTGATCAAATAAAAAACGCAGCCGATTGGCTGCGTTTTTTTATGTGC
GGTCGAGCTTAGTTCTGGGCGAACTCGAAGGCCTTGATCACGCGCTTCACGCCGGTGACATGGCGGGCGA
TGTTGACTGCCTGCTCTCCCTCTTGGCGAGTCACCAGACCAATCAGGAACACCTCGCCGTTCTCGGTGAC
CACCTTGACCTTGGTGCTGTCGAAGTTCTTGGTGCCGAACATATCGGCTTTCACCTTGGAGGTGATCCAG
GAGTCATTGCTGCGAGTGGTGAAACTGGCAGGTTGGCCGATGCGCAATTCGTTGTAGACGTGGCGAACCC
CTTCGATGCGACTGACAATTTTGCCCGCCTCAATCTTGTAAACGTCAGAAGGTGTCTGGCCGACCAGCAG
GACGCGGCCATTGTTGCTGTAGACGCTGATCTTGCTGGCTGCGCTGAGGGGTTTGTTGTCGGCCAGCAGA
TTGGCCGCTTTCAGCTCTATGGTCTGGTCATCCCACTGCGCGCCCAGGGTTCGGCGATCGCCAGAGGCCT
TGGCAGTCCCTGCGGCGCCGCCAACAACCAGGGCTGCGCAGCCTTGCAGCAGCAGGGTGCTAACCAGCAG
ACCGAGCAGGATAGTGTGCTTTTTCATGGTTTCAATCTTCCTGTTGCGGGAAAAGTGTCTGGTCGATGAG
ATCACACAGGCAGTGCAGGGTCAACAGGTTCACTTCCAGAATGCGAGGGCGACGGGCGGAGGGAACACGG
ATCTCCACATCGTTCGGGCCGAGCAGACCAGCCATTTCGCCACCGTCCCCCCCGCTCAGCACCACTATGG
TCATGTCACGGGAGAGGGCAGCCTCGGCGGCCTTGATCAGGCTGCGGCTGTGGCCGGAGGTGGTGATGAC
CACCAGAATGTCGCCAGGCTGGCCCAGCGCGCGGATCTGCTTGGCGTAGACCTCTTCAAAACCGTGATCG
GTGGCGATGGCGCTGATGGTCGCCATGTCTGGGGTCAGGGCCATGCCGGGCAGGGAGGGGCGCTGGGTCT
CGTAGCAGTTGACCAGTTCGGAGATGAACAGTTGCGCCAGTGCGGCAGAAGGACCGTTGCCGCATGCCAG
CACCTTGTGGCCATTGAGCAGGCAGATGGTGATCATCTGGGCTGCAGTATGGATGGCATCCGGCAGCGCC
TCGGCGGCGGCTATCTTGGTCTGGATGCTTTCGGTGTAGTTCTCTTTGATGCGGTCAGTCATAGGGTCCT
CTTAAAATGCGTTCTGGATCCAGGCTGGCTGGTCGCCCTCGAACGCAATCACATCGAATCGGCAGGCCTG
ATTGGCCTCGTTGATGGCATGTTGCTTCAAATAGTGGCGCGCTGCCAGCATGATCTTGTGCTGTTTGCTG
CGGGTGACCGAGCTGGCGGCCCCGCCATGGCTGCCGGTGGCCCGGTAGCGCACCTCAACAAACACCAGGG

TCTGGCCCTGGTGCATGATGAGATCTATTTCGCCGCCACGACAGCGGTAGTTGCGAGCTACCGGTTGCAG
GCCCCTAGCCTGCAACCAGCGTTCAGCGACTTGTTCAAAGTGCTGTCCCTTGCTCGTTGGTGAGGGGAAC
AGGCTCGGCAACTGCTGGCGCATCCGTTTCCATAGTTCCTTCATTGGAGGGCTCCTCCAGCTGCTGAACG
GCGTTCTCGTCGACCAGTTTGCCGTTCTGGTAAGTACCCCAGCTCAGCATCCGCACGATATTACCCTGGG
CATCCACGCTCAGCTGACCGCTCATGCCGGCCTGCTGCATGCCACCCACCTTGCGCATCTGCTGCAGGTT
CTCTGCCAGACTGACGGCATCGTAGCCCATGGCAAACAGGCGCAACAGGTCACCCTGGGTCTGCGGCCAG
AGCAGGGCGATCTGCTCGCGCTGTTTCTCGTAGCCGCCCAGCAGCAGCGGCATATCGGCGATATGCATGC
CGTTGAGCTCGGAGGCGACCTCGGTGGCGGCGCTGTCGTAGCCACGGGAGCTGGTGTAGATGGGCAGGCT
GCCCATCGGATTGACCGAGACATCCACGTAGGGCTTGATCATCCGGGTTTCCAGGCTGTTGGCCACCACA
TAAACGGCATCGATGCTGCGTGGTGCGGCATCAGAGAGTTGCACTACCTCGCCAGCACGGGCGGTAGAGC
GGCCGCTCAGGGCATTCTTGACCATCCCCTGCACTTCGTTGCGGGCACCGAAGGTGGCAACGACCGGCTT
ATCCTGGCTCAGGGTGGCCCAGCTCTGCTCGAACGCCTTAATACTGCTGTAACCGATCCGCCCCTGAGCG
GCGATCAGCAGCGGTTTGCGGTAACCCTGGGTGTAGAGGTACTGGGCTGCCTGCGCGGCATCTGCGGCGG
CAGAGAGGGAGAAGTAGTAGGTGGTGTCGTTGACGACCGGCTTGTCCAGCTCGTTGAGTGCCAGCACCGG
CACGGTCGGATTGGCCTTGAGCAGCTCCTCGACCCGATCTTTCAGCAGCGGGCCGATGATCATGTCAGCC
CCTTCCTGAATCGCCTGCTTGTAAAGGTCGCCGGTCGACTTGCTCTGGGTGTCGTAGAAGTTCAGGGTGA
ATTGCCCCTGATTTTCCTTATAAGACATCAGCATACCGTTACGGATGGCGGCGCCCTGCGGCTCCAGATT
GCCGGAGAGGGGCAGCAGCACAGCGATCTGTTGCACCGAGGTGCTGGCAGTGGCCGCCAGATCGCCCAAT
CCTGCCGGCATATCCTTCTGGGCTGGATGGTTGGGGAAGTCGCGCTTCCAGCCTGCCAGTTGGCGGGCCA
GCAGGTTCGGCTGGGCGCCAAACTCGTTGACCAGTGCCGCCAGTCGCAACCAGCCGGTGGTCACATCAGG
GGCCGGAGCCTCTTCCAGCGCTTGCAGGGTGGAGGGGGTCATGCTTTTGAGCAACGACCAGATCTGCTGA
TGGTTGGTCGCCTGCTCGCTCTGATTGAGGTAGGGCTCCAGTGCAATCAACGACTTGGCGGCACCAAACT
TGTTATTGATGTCGAGCTGTAGCACCACACGCTGGCGATACCAATCCTTCTGGGTATCGGCATCCAGCAC
CACCTCGGGTTTGCCTTCCAGCAGGATCAGCGCCTGATTGGCGTGGCCTTGCGCCAGCAACAGATGGGCT
TGCAGCAGTTGCAACTGCGCCTTCTGAACGGGATCCTTGGCCTGCTTCTGCAATTGCTGGAACAGTGCCG
AGGCCGGTTTGGCCTGGCCCAGCGCCAGATAGCTGCGTGCCGCCAGCATCTGCCAGGTAAAGGCCTCGGC
CGGCTTGGCCGGATCCACCTGCTCAAGATACCACTGGGCATTCTTGGTCAGATTGGAGAACGCAGAGGGC
ATGCCTGACTGCCCTGACGGTTGATTGGGCTCCGACGCACAGGCGGCGAGCAGTACTGCCGCGAGTATGA
TGCCGAAGAGTCGTGATACACTTAGCTGCTTTGTAACCCGGTTCAAGTTCATTCCCCGACCAGTGTTGAA
GTTCCCCATAGTGTAAACGGGCCAATATGGCGACACAATTGTCGGGGCACTCGGAGATCCCATGAGTGAC
ATCCCAACCCTGTATATAGTCCCCACTCCGATCGGTAATCTGGCGGACATCACCCAGCGTGCATTGGACA
TTTTACGCAGTGTCGATCTGGTGGCCGCCGAAGATACCCGTCATACCGGTATCCTGCTCAGCCACTACCA
AATCTCGGTGCCGACCTTCGCCCTGCACGATCACAACGAGCAGCAGAAGGCCGATGTACTGATCGGCCGG
ATCAAAGAGGGCAAGAGTGTTGCGCTGGTCTCCGATGCCGGCACTCCGCTCATCAGCGACCCCGGTTATC
ACTTGGTCACCCGCTGTCGTGAAGCCGGGGTCAAAGTGGTGCCGTTGCCCGGCCCTTGTGCCGCCATTAC
CGCCCTGAGCGCTGCCGGTCTGCCCACTGATCGATTTGCCTTTGAGGGCTTCCTGCCCGCCAAGAGCAAG
GGACGTGACGATCGCCTGCAAGCCGTTATTGAAGACACCCGCTCGCTGGTGTTCTACGAATCCCCGCGCC
GTGTGCAGGATACAGTGGAAGCGATTGCTCGCATTCTCGGCGAGCGTCAGGTGGTGGTGTGCCGTGAGCT
GACCAAGACCTTCGAGTCGATCCACGGTCTGCCAGCCTCTGAAATGCTGACCTGGCTTGGTGAGGATGAC
AACCGCTGCCGCGGCGAGATCGTGCTGGTCGTGGCTGGTGCCTCCAAAGATGAAGAGGATCTGCCAGCCG
AGGCGATCCGTACTCTGGGTCTGCTGGTGACTGAGCTGCCACTGAAAAAAGCGGCTGCCCTGACCGCCGA
GATCCACGGCGTGAAGAAGAATGCCCTCTATAAATACGGTCTCGAGCACTATTGAGCCGATGAAATAGGC
TGGCAGACTGCTTTTTGACCTGTGCAATCCCCCGAACCGGCGAGCATTCAGCCTGTAAACGGGGGCCAAA
GCGCGATAAATGGCTGTAAACGGGAATTGCCGGTTTACCCCGTGCGTATCGCAAGGCTATAATCCGCGCC
TCGGAGTTGGCTGGGCAATCGCTGCTTCGTCGTTGTGTCCCTCGGGACAGACGGGCGGAGGGGAGGAAAG
TCCGGGCTTCACAGGGCAGGGTGCCAGGTAACGCCTGGGGGGCGTGAGCCTACGACCAGTGCAACAGAGA
GCAAACCGCCGATGGCCCGCAAGGGATCAGGTAAGGGTGAAAGGGTGCGGTAAGAGCGCACCGCGCGACT
GGCAACAGTTCGTGGCACGGTAAACTCCACCCGAAGCAAGACCAAATAGGCCCCTATTGGCGCGGCCCGC
GTTGGGGGCGGGTAGGTTGCTTGAGCCAGCGAGCGATTGCTGGCCTAGAGGAATGATTGCTACCCGGGGC
AACCCGGTACAGAACCCGGCTTACAGGCCAACTCCGACCTATTTCAAATCAAGAGCTTAGCGGTAAAACG
CTAGGCTCTTGTCATATGTGGAACAAACTGAGTAACAAAACGGGGAACATCCCTCACTATCTGACCCGCC
GCAATGGCGTTTTCTACGCCTGCTTCCGTCTGCCAACAGCACTGACCTACCGACCAAAAGTGATTCGTCG
CTCTCTCCATACCCGTGATGCTGGATTGGCTCGTTTGCGTCTGGCAAAGTGGTTGCCAATCTTTCAGCAG
ATCAAGGAAATGGCGCAGGTGAAGAGTGAAAAGGAGCAGTTAGAAGATCTTGTCCCTTCTATCCAGCGAT
TGAATGAGCTGACCGCCCAAGATGAACAGAGCGTGGTCAAGGTGGTGGCTGATCTGAATGCCCCTTTCGA
GGATTTCCCAGAAGAAGGACTGTATGCCGAGCAAGATGCCATAGATGACTTTATCGCGCCGTATCTGAAA
GAGAAGCTGACTCTCTTGGAAAGTATTGAGACGGAGGACGATGCCGAGCGCATCTTTGCGCAATCCCCGC
TCTCTGGCCTTGATATCGCCGCTTCTGAGCGGGAAGCCTACAGGGATGAACTCCTTTACCTCAAAGCGCG
GCTGGCGAAGTTACAGGGGGATTCTGGCCTCTATGAGCTGATGCGTAACCAACTGGTGGAGCGGTATCTC
ACCCCTGAACTGGTGGCGTATCGTGAATGCGAAGCCGCCAAGGAAGCCCAAGACAAGATGCTTGCTGTTG
TCGCCAGAGAAAGTATTGCTGGTGGGGTTAGTCGTGCTCCTGCCATGACCGAAACATCCCTGTTACTGTC

TGAATCGTATAAGAAATTTATCAAATTCAAAAAGAATCTAACGGGAAAAAATGATTAAGGATTACGACCGT
TATCTCGATTTTACGCTGGCGATGTTAGGGGATATTGAAACCCACAACGTCACCAAGCAAGCATTACGTG
AATGCCTTGAGGTCTATCAGCAGATGCCGAAGGGGAATCACTCGCCCTATAATCGGCTGAGCATGGCTGA
CAAGGTGGATTTGGCAAAAGCAGGAGAGATTGAAAAGGATCACTTGATAGCTGCCAAGACAGCAGGAGAG
TTACTCAAATTTTATCAAAGTTTCTTTAGTGCTTTCTTAACGGATAAAGAGGATATTTTCACTACATCCC
CCACAACCGGATTGAAAAAGCCACCATCGGAAGATGTGAGCTGGGGGGCCTATACCAAGGCTCAGGGGCG
CCAGATATACGAGTATTGGCTGGCACAACCTGATAGCCCCATGCGCTGGGTGTTTCTTCTGGTACTATTT
ACGGGAATGCGCCGTAGCGAAATTGCCAATGTTTCTCGTGACTCATTAAAGCAGGATCAGGATTCTGGCC
GCTATTACCTGTGGATACAAGAGGGTAAAACTGCTGCTGCCACCCGTCCGGTAACGATAGCGAAAGAGCT
TGAGGCGCTGGGGTTTGTCGAATATCTACAGTCACTGGATGATACGCTCTATCCGGCGGAAAGGCTGAAC
GACATTACCGAGAGAGCAAACAAAGTTCGTCAGTTTCTCATGATCGGTGAGACAGAGGAAAATGGCAAAG
GGCGCTTGGTGTTCCATAGTTTTCGTCACTCCTTTGTTACGTTCAAGCAGGCAAAAAAAGTGGATTTCCA
GCGTTTGCAACGATTCGTTGGTCATGAGGGTACAAGAAGTATCACTAAGCGTTACACACACGATCATATG
ATCACAGATTATAGTGATATCGCAGATGGCGCATGGTGGGGATAATAGGGGGGCCGGAATTACTGTAAAA
CCGCTCTAAATAGAGCTGTATTGCATTTTATGGGGTAGGGTAATACCTACCCACACAGAACCACAACAAA
ATCAAAATTAGCGCGTATATTTTATTTTTTTTAGATGCATGGAAAATCTCTGTCGATATCTTGAATTTTT
TAAGTTTATTAGTTAACCTCTGCAATTGAATCAGCAGAAAACAACTCACCCCATTAAAATATGCTCACCG
GCCTTTTCGTCTGTGGGCTTTGCTGCATTCTAAATGTAGGTTTTAATATTATCTATATCTATATCTATA
TCTATATCTATATCTATATCTATATCTATATCTATATCTATATCTATTTATACAAGAAAGTTAAGTTAAA
TTAAATGTAAGGAATAACAATATAGGCACAGGAGGAATATGAAAAATATAGTTCAATACAAACTAAATGA
TATGGGAGAAGATGACAATCTTGATACTACAACCATAACTAAAATGATCCATCAGATTGAGACATTATCG
AATAAATACAGTAAGTTATTAGTGGTCAGGATAGATCTGCATGTGCCAGATGATGTGTGGTTAAATGATT
CAAAATTGATAGGTAAGTTACTGAAAAACATAAGAAAAACAAAATGTAAAGATCACTTGTTCGGATACTT
TTGGGTATTGGAACGAGAGAAAAAGAAGAAAGGGCATTATCATATGGTGTTCTTCTTTGATGGTCAGCAG
TGGCAAAGTCAGTGGGCCGCACAGACTATGATTGAGGGCTGTTGGCAGCGAGTTGTCAGCGGGGGAACAA
TACACCGCACCAAGGGGGCTGGCACTGGGATGCTATTGCGTAGCGATCAGCAGAAAAGAGCGGATACCAT
TTACGCCCTCTCTTATCTCTGCAAGAAACGAGGCAAGAAGCGTCTTAAAAAGGGGCAAAGGTTATACGGA
TTCTCTCGTTTACCTAAACTGCCAATAAAACCCCGTCAAATAGCGGCCTAAAAAGCCCTGCAATCGCATT
CACAACCCTACCCTGTCGGGGGGTATTGGGTAGGGGTTGGAGTTGAAAATAGCGCGTTTTAGTAGTTTTG
TTTAGCAAGAGGCTCCCTCTACCCACGTCCATTATTTTAAAATGGTGATGATATCATATTTTTTATTGTC
AGCACGCAATGTGATATTTAAATAAATGGCGAGTGCCTAGTAAAAAGTACATTTTTTATTTTTATCAAAT
TTTTCTCTGCATTGGTATTTCCCGTTTTTAATGTTCACCTCTCTTAGGTTGAAAAGAAAAGTTGGTTCAT
GACCAAGTAGAGATAGCTTTTCTGTCAGTTCTTTAATCTCAATTACTGCATTTAAAAAATCTTCAATGGA
AATATTTTCTTTTCTTCCCTGCTCTATGCTTCGGATGTAGAAATCAAGTTCAGAAGATATTTGTGCCTGC
TTGTGAATTAGTTCTTCCCAGTCACTCTTTTTTATTATGGATCTGAATATGTTCATTTTTATCCTTTGGG
ATTGTAAGTCTGCGGTTTTATGTTTTTGCTAAGGTTTTATTTGGTGATGTGATGTGCCTTAATTATAAGG
CTCTATAAACTTCAATTAGCCTTGACATACAGAATGTTGTTAGTGTTATAGTTGTTTATAGTTCGCCGAA
GTTCATTTTTTGTATGGTTTTTTCCACAGACTGGCATGAGTCAGGGCTGGAACCATCTATTTGAGATTGC
ATATTCAATTTCAATGATTCCGCAAATATCATTAAATACTTATTTGTCAATCCTCGCTTATCCATCCAAG
TGCCAACGTGCTCTAGTTCTTTTGAGACGTCAAGCTCACAACCAATAGCACGACCAAGAATGGTTGAGTA
AGTTGTCATCTTATCAATATCTTTTTCTTCGGCGGAATATAAATTTAATGAAAATAAAGAGCAGAAAAAA
ATAAACAGTGCCTTCATAATTTACCCCATTTATTAATTACAGAGTTTTAGCTACTTTAGCAATTGTTGCT
CGGCTGCATTTATACATATCCTCAATCTTGCTATAAGACCATCCAGCAGACAAGGCCAATTGAATCTTTT
CGTGAAGTGCCGCATCTATCGGGCGGCCTTTATAGCGTCCTTCTGCCTTGGCTTTGGCGATGCCTTGAGC
CTGACGGCGGCGACGGTCGGTATAGTCTTTGCGAGCAATGGCGGCGAGCATATCGAGCATCATACTGTTT
AAGGCGTCCAGCATCCGGCTGGTGAACTCATCCCCTGTCGTCATAAGCTGGTGGCTGGTGGGCAAATCCA
GTGCCACGATCCGCACTCCTTTAGCCTTGATTATGGCTTTCAGGCTTTCCCAGTCCTCACCGTTAAGGCG
GCTGATACGGTCTACCTGTTCCACCAGCAGGCAATCCTCTTGCTGGGCTATATCTAGCAGCCTGAACAAC
TCGGGACGTTTGAGACTGGCCCCTGATTCATTTTCAACAAACCATCCGGCAATCTGTTGCCCTTGGCTTT
GGGCAAACTCGATCAACTGTTCTTTTGCGCGGCTGGCGTCTTGTTCGTCGGTACTGGCTCGCAGGTATCC
ATAGATCCGGTTACTCATAATGCCCTCTGGTTTCCTTTATATGGTTTCATAATTGTAGTTTGTTTTGAGT
GGTTTAGTAAATGAGAGGCCAGAGAAAATCGAGATGAAAAACCAGTTTCTTTTAGGTATACCCAATGAAA
CTAATGAAAACCTGTACGATATTTCAGCTTTATATAGAGAACGCTAAGTGGGGGAGGTTATGAGTCCATT
AGGTGGCTTTGACTGCCTTCTGAGTGGTTAAAAGGTTGTCAGATTAAAAGTCAAGCAGTTCGTGAAGTGT
ACGAGCACTGATAACCAGAAGAGATGACCTGAGAGCTAAGTTGTGTAATAATATGTCACCCCGTATTTGA
AGGGGTATAGATATATTTTATATAACCTATTGATTTTAAACTAAAATCGCTGGGTTTTTGAGATTGGGAT
AGGGCGGCTTACAGGCCGACTCCGACCTCATTCCGCGTGAACCGTCACGCTAAAATCCCGGTGCCTAACG
GCACCGGGATTTTTATTATTCACGCCGAGATGCACCGCATGGCGGCATTCTGGAATTTGTAATAAAGTTC
TCACCCTTTTTGGTCGATATACGTCGGCTGGACTGTCTGTATCTATCACACTGCTGGCCTGATGAGCGAC
CCTGTGGCAGTAAGGCGAGATGAAATGGTTAAGGTGGATTATTAATGTTATCAAGCAGCGCAATGCTCAC
ACCTTCCCGGCTGCTTAGCGCAGAACGGGGCAGAACCCTGCCTCATTATGATCTGTTGGGTGTCGAGCGG

TTAAACCACGCCCGTTTGAAGCAGGTCAATGGCCAGTTGAGCACATTTTCCCTGCAAGCTGCGAGTTGTA
TGGCCCAGCTGTTTAATAGTGCGGAGTGCGAACTGAATTATGGCGAGTTCGCCTTTTCCGATCCGGCAGA
GATAGATGACGCTCATATCTGGGTCGAAGCGAGAAGTCCGCTTGAACCACAGCGCGCCGTCTATTTCTCG
ATCGCACCTCTGGTCATCTATCATCTTGCGGTCCTCTTTTTCGGCGGCACCTTGCAGCAAAAAGAGGAGC
ATGTTCAGTCTGGCAAAGGGTTGACCGATACCGAACAGCGGCTGTTATTGCGGCTTTGCCAGTATCAGGT
TGATATCTGGTGTGCTTGTCTGGGGTTGGGGGATCTGAAGTGGCAGTTGCATGTCGTGCCACGTGATTCC
ATCGCCCACCGTCAGCTCTGGACCACGCAGGCAACCCTGCAGGCAGGTAATTGCACTCATCACTGGCAGA
TGTGGTTGCTTCCCCCCGAGGAGTCCATTTCTCAGGTCAGTCATTCCCTGCCGGAAGGTCAGCTGGAACA
GGCTCTGACTCATGTCCCGGTGCGTCTGCGGGTGGTGATGGGCCAATTGTCCATGACGCTGGCGGATCTT
GAGTGTCTCAAGGTGGGTGATGTGCTGTCACTGGACCTGCCTGAGGTGGTGCCAGCACTGATTGGCAGCC
GTCCCTGCTTCAGTGGACGAATCGCAGAACACAAAGGCAGCCTGGTCTATCAGGTTGCGACGGTTGTAGA
GGAGTAAATTGAATCATGAGCGAGCACAATATGAATGATCCGCTGGATCTGGATGGACTGGATCAACTGT
TTGGTGACGACATCAGTATGCCTAGCGCTGCAGACTACTTGGCCCCCACCAGTAGCAGCAAACAGCGGGA
TCTCTCCTTCTTTCGCCAGATCCCGGTCAAGGTGACGCTGGAAGTGGCCAGTACCGAAGTGCCGCTCGGC
GATTTGATGCGCGTCGAGGAGGGGGCTGTCATTGAGCTGGACAAGCTGGCGGGTGAACCGCTGGATGTGC
GGGTGAATGGCCGTCTGCTTGCCAAGGGCGAAGTCGTCGTCGTGAATGGCAAATATGGTCTGCGCCTGGT
CGATGTTATCGATACCAACCTGCTGGGTGGCATGGACGACTGATGCGCTATCTGCTGCTGACGCTGCTGT
TATTTTCGCTGCCTGGCTGGGGCGCGGACATGACCCTGTTCTCGGTATCGCCAGGCGCTTCAGGGGGCGA
GGACTACAACATCAAGTTGCAGATCCTCTTCTTCATGACCCTGCTGGGTCTGCTCCCGACCTTGTTACTG
ATGATGACCAGTTTTACCCGGATCATCATCGTACTGGCCATATTGAGACAGGCACTTGGCTTGCAGCAGA
GCCCTCCCAACCGTGTGCTGATCGGCATCAGCCTCATCCTGACTGTGGTGATTATGCGTCCGGTCTGGAC
GGACATGTATCAAAATGCATTTGTCCCTTATGACCAGAATGAAATAACCCTGTCAGAGGGGATCGCCAAG
GCAGAAAAACCCCTGCGTAAATTCATGCTGGCCCAGACCCGTGAACCGGATCTGGAGCAGATGCTGCGCA
TCTCTGGCGAACCAACTACGGTCGCCAAAGAGGATGTGCCTTTCGGGGTGCTGTTGCCCGCGTTTGTATT
GAGTGAGTTGAATACCGCATTTCAGATTGGCTTTATGCTGTTCATTCCCTTTCTGGTGATCGATTTGGTG
GTCGCCAGCGTCTTGATGGCGATGGGGATGATGATGCTCTCCCCGCTGATCATCTCCTTACCCTTCAAAC
TGATGGTGTTTGTGCTGGTCGATGGCTGGTCGATGACCGTCGGCACCTTGGCCGCCAGCTTTGGTGGGGG
AGGCTGATATGACCCCTGAGCAGAGCGTTGCCCTGGTTGGTGGCGCGGTCAATCTGGTGGTGACCATGGT
CTCGGTGCTGATTGTACCGGGCCTGATCGTAGGTTTGGTGGTCAGTATTTTTCAGGCGGCCACCCAGATC
CAGGAGCAGACGCTCAGTTTTCTTCCCCGTTTTATCGTGACTCTGATGACCCTTATCTTTACGGGTAACT
GGCTGTTGTCGCAGATAACCACCTTGTTCCAGGATCTTTTTCTTAAAATCCCCGGGTTTATCGGATGAAT
TCGCTGCTGTCCCTCAGTGCTGCTGACCTGACCGCATGGTTGGGACAGTGGTGGTGGCCGTTTATCCGGA
TCGGTATGGCATTCTGGGTGATGCCGGTCTTTGGGGATACCCGGGTGCCTGCGCAAGTTCGCCTGATGCT
GGCGTTCATCATCAGCCTGCTGCTCGCCCCCATGATGAAGGATATGCCGGTTGTCGATCCTTTCAGTGCC
GGTGCGCTGGTCCTGGCGGTGGAGCAGCTGCTGTTTGGTTTGCTGTTTGGTCTGTGTGTCCAGTTGCTCT
ATATGGTGATGACCACTGCGGGGCAGATCCTCTCCATGCAGATGGGGCTCGCGATGGCGGTGATGAATGA
TCCGGTCAATGGGGATTCTGCACCCATCATCAGTCAGGTCATGATGGTGTTTTGCGCCTTGCTCTTTCTG
GGGCTGAATGGCCATCTGGTGGTACTGGATATTCTGGTGCAGAGCCTGCGTAGCTGGCCGCCGGGTTCAT
CTCTGGATCTGATCGATATGAAAGGTGTGATCGGCTTGTTTGGTTGGTCTATCGGTGCCGCCCTGTTGCT
GACGTTGCCTGCCGTTGTCGCCATGTTGATGGTTAACCTGACCTTCGGGGTGATGAACCGGACCGCCCCT
TCGCTCAATATCTTCTCTCTCGGTTTTCCCATGACCCTGTTGCTCGGGTTGCTCACCATGGCGCTCTCCA
TCAGTGGCGTGCCCTCCCGTTATCTGGATTTGGTTACCCATGTGCTTGAACAGCTGCGAGGCTTGACCCC
GGCATGAGTCAGAACAGCGCACAGGACAAAACCGAAGAGGCCTCCAGCCAGAAACGCCAGAAAGCGAGAG
ATGAGGGGCAGATCGCTCGCTCAAAAGAGCTCAGTTCTGCCGGTTTGCTGCTGCTTGGCGGGTTGGTGAT
GCAGTGGATGGTGCCGGGGTTTGGCCAGTTCTTTGCCGAGTTGATGAAGCGTCCGCAGCGTTTTGACTGG
CAAGGTGCCCGTGACCCGCAGATGATGATCCATTGGTTGGGTGAAGCGCTGATAAACATGCTGTGGGTGC
TGCTGCCCCTGTTTGGTTTTCTGGCCCTGATGCTGGTGTTGCTCAGCATAGTGCCGGGCGGTATGGCGTT
GACCTGGAAAAAGCTGCTACCCAGTGGCGAGAAGATGAACCCTTTGAAGGGGCTGGGGCGCATGTTCTCC
AAGAACAGCCTGATGGAGTTGCTGAAGTCACTGCTCAAGGTGACGCTGATCGGCGGCACGCTGGCGTTGA
TGTTAAATCATCAATGGCCTTCCTTGTTGATGATGAACCGGATGCCCCCTCAGGCGGCCCTCGAGCAGGC
GCTACGGATCCTCTCCCAGGCATTTATTTTGCTGGGGAGCGTTCAGATGCTGATTGCCGTGCTGGATGTG
CCCTATCAGCGCTGGTCGCTTAACAAGCAGCTCAGGATGACCAAACAAGAGGTCAAGGATGAGCACAAGA
ACAGCGAAGGGCGTCCCGAGATCAAGGCCAAAATCCGCCAGGTACAGGGAATGCTTGCCCGCGCGCGTAT
CGAGCAGCGGGTACCGCAGGCAGATGTGATTATCGTTAACCCGACCCATTATGCGGTGGCCATAAAATAT
GATGCGGCTCGTGCCAATGCTCCTTTCGTGATTGCCAAAGGGGTCGATAGCATGGCCGAGCGGATCCGTC
AGGTCGCCAAACAGCATGACAAGATGGTGATGAGCTTGCCCGAGTTGACGCGAGCCATCTATCACTCGAC
CAGGGTGGATCAGGAAGTCCCCGCAGGGCTGTACACGGCAGTTGCCTATGTATTGAACCATGTATTGCAG
TTAAGGGCGTATGAGGCAGGCAAGGGGAGGAAACCGACCCCTCTTGCCCCGTTATATGTACCGCCCGAGT
TACGTCAACCTGAGAGGTAAGTTGTGAACTGGCGTGTATTCACCCAATCCTATACCGCAATTCCGCTGAT
GTTATTGGCCATTCTGGCCATGATGATCCTGCCTCTGCCCGGTTGGCTGCTTGATGCGCTCTTTACCTTC
AACATCGTGTTGGCGGTTCTGGTGTTGCTGGTATCCGTCTCGGCCCGCAGACCACTCGATTTCTCGGTAT

TCCCAACCGTATTGCTGGTTGCCACCTTGATGCGGCTGTCGCTGAACGTGGCCTCTACCCGGGTGGTGTT
GCTGCATGGTCACGAGGGGCATGAAGCGGCCGGTAAGGTCATCCAGGCATTCGGTGAAGTGGTGATCGGT
GGCAACTATGTCGTCGGTCTGGTGGTCTTCGTGATCCTGATGATCATCAACTTTGTGGTGATCACCAAGG
GTGGCGAGCGGATTTCGGAAGTATCCGCCCGCTTCACGCTGGATGCTATGCCCGGCAAGCAGATGGCCAT
CGATGCCGACTTGAATGCCGGCACCATGGATCAGCATCAGGCCAGGGCTCGCCGTGAGGAGATCGCACGA
GAAGCAGACTTCTATGGTGCAATGGATGGTGCTTCCAAGTTCGTTCGTGGTGACGCGATAGCTGGCATGT
TGGTGCTGATCATCAACCTGGTGGGTGGGTTGGCCATCGGTATCTTCCAGCATGCCTTGCCCGTCGCGGA
GGCCTTCCAGCGCTATGCACTGCTGACCATTGGTGATGGTCTGGTTGCCCAGATCCCGTCCCTGCTGATG
TCGACTGCTGCGGCTATTATCGTGACCCGGGTCAGCGATGAGGGTGAGATGGCTGATCAAGTTGGTCAGC
AGATCCTGGCATCGCCTCGCGTTATCTATACCTCTGCAACCATCATGCTGATCCTGGGTCTGGTGCCCGG
GATGCCGATGCTCGCATTCCTCAGTTTCGCAGCTGTACTGGCCTGGACTGGCTGGAGTGTCAGCAAGCGA
GCCGTCAATGTGCCGGATCTGGATGCAGCGCAAGAGCTGACTCGTCAAATGATTCAGCCTGAACAGCAGG
CACTGGAGTGGCAGGATTTGCCGCCAGTCGATACGGTCGCCATCGAGCTTGGTTATCGTCTGGTGCCATT
GGCTGAAACGGATAAAGGGGCTGAACTGCTTGCACGAGTGCGCGGGATCCGCAAAACCCTCTCCGAGCAG
TACGGTTTTCTGCTCCCCGAGATACGGGTGCGCGACAATCTGCAGTTGCAGCCGGATCAGTACCGGATCA
AGTTGTCCGGTGTCGCGTTGGCGATGGGGAATGTGGATTGCCAGCGCTTGATGGCGATCAGCAGTGGTGA
GACCTATGGCGCGCTAGATGGCGAGTTGACCCAGGACCCGGCCTACGGCATGAGTGCGGTCTGGATCTCC
CCTGAGCAAAAGGCCAAGGCCCTTAATCTTGGTTACTCGGTGGTAGATTCGGCCACCGTGATTGCTACTC
ACGCCAGCAAGCTGTTGCGTGAACAACTTGATGCACTGTTTGGTCACGATGAAGTTGGCAAACTGGGCCA
ATATCTCGCCAGTCAGTCCCCCCGGTTGGCTGAAGAACTGAATCTGGCGCTGACCCAGATGCAGCAATTG
AGAGTGTTTCGTCAATTGCTAAAGGAGCAGGTCAGTCTTACCGATATACGAACCGTTGCCACGACGCTGC
TGGATGCGAGCGAGCTGACCAAAGACCCTGTCTTGTTGGCATCCGATGTGCGCTGTGCCTTGAAGCGCCA
GATTGTGCGGCAGGCCATTGGTAATCGCGATGTGCTGGCTACGTTTACACTGGATGATCGCCTTGAGCAG
ACATTGCTGGCTGCAATGAATCAGTCACAACAGCAGGGCAAAATCCAGCTGGACAGCTTCCCGGTCGATC
CGCAATTGCTGGCCCAATTGCAGCAAAATATGCCGTTGGTGAAGGAGCAGTTGCAGCAGTTGGGACATGC
CCCCGTATTGGTTGTTATGCCGCAACTGAGACCGCTTATGGCCCGTTATGCCCGGTCATTTGCAAAAGGC
CTCAAAGTGCTTTCCTACAATGAGATACCTGAAGAGCTACGGGTGGAGGTAATTGGTACACTTGGTTAAT
TACTGTTGGATAAATATCCTGAGGGGAAGTGTTTTTTCCGCTCCTGCTTGAATTGTCGTATGAATACTAT
ATGATGTGGCGAAAATTGACGCAGCAATGTGTGAAAGTTAAATAACAAAAACAATATTAATTGTGTTGCC
ACACGCTTAGCCCATCAGGCTGGGCGTGTTATCACATTGTAAGGGCAAACTGAACTGAAAAGTCAGGACG
CAAAGCCTCCGGTCTAACAGCAGGAAAGCTAATGACAGCGGGGTTACCAAGGTGAGTTTGGATACAATCC
TGTTACCATCTAGATACAAATCTCACTTTTTTAAGTGCGAATTTGTATTTCTCCTTTGTGTCTGATAATT
CAGCTCTATTGATTGTTCTATCCACAATCACAGGTGTCGGACGTCTATGTCTTCGGGATTACCAATAAAT
ACAACAATCGGGTTGTGTGGCGATAGCTTGGCTGGGCCCCATATATTATTGATTCAGCCGCTAATAGTTG
CCGAAAATGAGGTTGAAAAAATCCTATCCAAGGAAGGAATCAGGGTATCAAGCGTTAATTCTGTTAAACA
ATCAAAGGGCATTATTGCTGATGATGATGTCAATTTGATCTTGCTCGATATGGATGCCATGACCGAGCTT
GGGCCGCGTATTCTGCGTGAGTTCGAAGGACTTGATTGCATCATCATGGTCAATGAGCACCAAGCCACCA
TTGCTGCCGAGGCCATGTGTGCCGGGGCTGTTGACTATATTGTCAAACCCTTCTGCCCCCAGCAATTGCT
CGATGCTGTCCGTGGTGCTCTACGCCTTCGTCAGGCGATCCCCAATCTTATCGCTGTGTCGCCCATGAGC
CTGCAGGTCTTGCAACTGGCCCGACGAGCAGCCAAAACCTCTGCCACCATTCTGATCGGTGGTGAGTCAG
GAACCGGTAAAGAACGGTTGGCCCGTTATATTCACGAAATGTCTGTCAGAGCGGAAAAACCATTTCTAGC
GATTAATTGTGCAGCAATTCCTGAGGCAATGCTTGAGTCAATTCTTTTTGGCCACAATAAAGGTGCATTT
ACCGGTGCTGTTTCTGCTCAGCCCGGCAAATTTGAGTTGGCTCAGGGGGGGACCCTGTTGCTGGATGAAA
TATCAGAGCTTCCTTTGAACCTGCAGGCTAAATTATTACGGGTGCTGCAAGAAAAAGAAGTTGAGCGACT
TGGCAGTAATCAAACGATTGCTCTTGATGTTCGAATCATTGCCGCCAGCAATAAAGATCTGCGCAAACTG
GTTGCATGTGGGCTATTTCGTGAAGACCTTTTCTATCGTCTTGATGTATTGCCAATGAATTGGCCCGCAT
TGCGTGAACGTCCTGAGGATATTATTCCGCTGGCCAACTACTTTATTGAGCGTTATGCATCCCAACAGGG
CTATCGCCTGAGTGAACGTGCCTGTCAGTTGTTGCTGTCATATGAATGGCCAGGAAATGTACGAGAGCTG
GATAATGTCATTCAACGGTCATTAATCATGGCGCGTGGTTTGCATATACAGTCCGTCGATTTGATGTTGC
CAAATTGTATTCTTCCCGAAGAAACAGTCGCAGCAGACACGTGGCATGCTCCCGAGGGGTTAAGTGAAAC
CCGGCGGCATGCCGAGTTCCAATTTGTATTAGAGACCTTGCGCCGCTTTCGTGGCCATCGTACCCGCACA
GCCGATGCACTGGGCATGACAACCCGGGCACTGCGCTACAAGCTTGCGGCGATGCGTGAGCAAGGCATTG
ATATTGAACAGCAGATTCAAGCAGAGGGATAAAGAGTGAAGATTCAGATGATGTCATCACAGGTGACGCT
ACTGCAGCAGATGCAGGAGATGCAGCGTGTCGCAAGCCAGACCCCTTCTATCGAATCCAATGATCTATCT
GGAGCATCATTTACCGATGCCATGCGGGATGTGGTCGGGCGCGTCAATGAACAGCAGAATGTCGCGTCAA
AATTAATGGCATCCGTCGATGCCGGGCAGAGCGATGATCTGGTGGGCGCCATGGTGGCGAGTCAAAAAGC
CGGCTTGAGTTTTTCAGCCTTGATGCAGGTTCGCAACAAGCTGATGACCGGTTTTGATGACATTATGCGT
ATGCCGCTCTGAGGTAACAGATGACTGAACTGGTAACGTCTCCTCAGGGGGCAGCCCCTGATTCGCTCAA
AATCAGGCTACAAGAGGGATTACGCAACTGGCGTACCCTCTCCCGTGACAACAAATCGATCCTGACCATT
GCTCTGCTGGCTGCCATTGTGGCCGGTACCATAGTCGTTATCCTGTGGACCTCCAGCAAAAACTATGTGC
CCCTGTATGGCAAACAGGAGCTATACGATACCGCCAACATCATGGAGATGCTGGAGAAAGAGCAGGTACC

TTTCCGTCTGGAAAAGAGCACCGGTCAGGTACTGGTTCCTGAAAACCAGCTGGCTCATGTCCGGATGGCG
CTTGCTGCACGAGGGGTGCGTGCAGCCATGCCGTCTGGTCTGGAAGGGCTGGACAACGTAACCGGATTGG
GTACCAGCGAATTTATGGAAGGGGCGCGTTATCGTCATGCCCTGGAAGGGGAGCTGGCCCGTACCATCAT
AGCCCTCGATGCCGTGCGCAGTGCCCGCGTTCATCTGGCTATTCCGAAACGCACGTTGTTCGTGGGGCGC
GATGAGGAGAAACCCAGCGCATCGGTAATGCTCGATATGCAACCGGGCCAATCCATCGAAGCGGGTCAGG
TCGAGGCGATTGCCAACCTGGTAGCAGGTAGTATCTCCGGAATGAAGCCCGGTGCCGTCACTGTGGTTGA
TCAATCCGGCCAGTTGCTCAGCGCAGAGCTGGGGGACAAGGTCGGTTTTGGCAAGCAGAGCGTTCAACAG
ATGGAGTATGTCCGCAAGCTGGAACAATACATTCGTCAGCGTGCCAATGACATGCTCTATCCCATGCTGG
GCAGCACCAATTTCAAGGTGCAGATTGCGGCAGACGTTAACTTCAACGCTGTCGAAGAGACCCAGCAGCA
GCTCGATCCGCAAGGTGTGGTGACTCGCGAGTCCAACAAGTCAGACAAGACCATCGATGCGTTGGCGATG
GGGATCCCGGGTGCGTTATCCAACCGCCCGCCTGAAACGGCGGCAAAAGATGAAAAGGCTGGTGCTGACA
AAGGCAATGCCAATACCGCGACACAACAGAATGACACTCGTACCGAGCGTCAGGAAGTCAGCAAGCAGTA
CGAAAACAGTCGCACCATAGTGCACACCCGTTACCAGCAGGGACGTCTGGAACGAATTAACGTATCCATT
CTGCTTAACCAGCAGATTGCTCCGAAAGGTGGCTGGAGTGCCGAGCAATTGGAACAGATCCGTCAGATGG
TAGAGCGTGCAGTCGGTTTCGATGAGTCACGTGGCGATCAAATTAGTCTGCAGGCCTTCGATTTCAATGG
TGCAGTACAAGTAGCCGCTCCCGAATCCAGTTGGTGGGAGACCCCTTACTGGCAAGACTCCCTGCGTTAT
CTGGTCGGCGGTTTGCTTGGCCTGACACTGGTCTTCTTCGGTATTCGTCCGTTGGTGAAACATCTGGTTC
GTACCCAACAGCAGCCGGATATGGAAATGGGTGAGACGGAAGAAGAGCTGGAGCCCACAGTCGGATTGTC
CATGCGCAGGGATGACGATGATCCCCAGCTGACGTCTGCTCTTTCAAATAGTCTGATCCGTACTGACAAC
AAGATGGAGCCCCAGTTCTCGTCACTCGATATGGAGTCGTTGCCGGAACCTGGATCCGAGCTGGAGGTTC
AGCTCAAGCATTTGCAGTTGCTGGTTGAAAAAGATACCGCCCGGGTGGCGGAGGTTGTCAGAGCATGGGT
GAGTGGCAATGAACAGCGTTAACTTGAATCAGAGTGCCGTCGATATCGATATCGACAACCTTGAGAAGGC
CGCCATCTTGTTGCTGAGCATGGGGACCGAATCTGCGGCCAAGGTGATGCAGAAGCTCTCCCGCGACGAG
GTGCAGCGTTTGATTTCCAAGATGGCCTCTCTCTCTGGCGTCAGTTCGATGGAGGCAAAGTGGACGTTGC
AGCAGTTCTTCAATCACTACCGTGAGCAGAGCGGAATTGGCAGCGCTTCACGGGAGTATCTGGAACAGAC
GCTCGATCTTGCTCTGGGGCAACGACTCTCCCGTAGTCTGCTCGACAGTCTGTATGGCGATGTGATGAGC
CAGGATATCCAGCGTCTGCAGTGGGTACCTGCCGATGTGCTTGCCCGTTTCTTCCGCAACGAGCATCCCC
AGATGCAGGCTGTGCTGCTGGCCTTCCTGCCGCCCCAGACTGCGTCCGCCGTGCTTGATGCCTTGCCCGG
CTCGGTGCATGACGATTTGCTGGTGCGCGTCGCCAGTCTCAGCAGTGTCAGTGAACATGTGCTGGGCGAA
CTCAGGCTGACACTGGATCGCTGTCTCACCTACGTGGCGGATCAATCCGGTGCCAAGGTAAACGGGGTGC
GGCAGGTAGCCGATATTCTCAACCGTTATCAGGGTGACAAGGAGCAGATGCTCTCCCTGTTGCGCGAACA
CGACGGTGACATGGCGCTTGAAATTGAGAAGAACATGTTCGATTTCATGGCGTTGCGCCGTCAGAGCGAC
GACACCTTGCAACGGTTGGTGCAGGAGCTGCCCAGCGAATTGCTGGCATTGGCTCTCAAGAACACCGAAA
CCAGCTTCCGCAAGGTGGTGTTGGGTGCCATGCCGAAGCGGATGGCTCAGGCGTTGGACGATCAGATCCA
GAACCAGGGCAGTGTCTCTTTGCGCAAGGTCGAGCAGGCAAGGGAAGAGGTCATGCAACTGGTCCGCGAG
TTGGTGGAGCAGGGCGAGATCGAGTTCCAGCTGTTCGAAGAGCCCACCGTCAGTTAACAGGAGGCATTCA
TGAGTCACAATGTGCGCAAATTGATGCCTGGGGCGACCCGTCTGTATCGTTTCCCTGCGCTCGGTCAGAG
TGGGGAAGAGGCCCGGAGTCAGCAAGAACAGTTTGAATTCGGCTATCAGCAGGGGCTGGAGCAAGGTCAT
GACGAAGGTTATCAGGCCGGCTATCAGCAAGGGCTGACCAGCGGCCTGCAGGAAGGCGAGAGTACCGGTT
TGCAACAAGGGCAAACCCGCGGGTTTGCTCAGGGCCTTTCGGAGGGGCGAGCCGCCTTCGAACAGGCAAT
GGTGCCGTTTGCCAAGCTGCAAGAGCAGTGGGAGTCACTGAGCCGGGCCCGGATGGCCGCGCAGAAAGAG
CTGATTGCCCAGCTGGTGGCGCAGGTGGCCAAGCGGGTCATCATGGCCGAGTTCACCCTCAATCCGCAGC
AGGTGATGAATCAGGTCGAGCAGGCGCTGGCTACCTTGCCGAGTGAGTCGGAAGAGCTGGTGATCTATCT
CAATGAGGGAGATCGACAGCGACTCGCCGAGCAGGGGGTGACTCGCTGCGAAGGGTGGCCATTGCAAGTT
GATGCAGCACTTGGGGTAGGCGATGCCCGAATCGAAACGCGCGCTGCCGTCGTCGAGGTAAGCACCAGCT
CCCGTCTGGCAACCTGCATCGAACAGCTCAACAGCTCGCTGGACGTACCGCAAGATGCATGAAGCCTTGC
AGCAGGCACTGACCAGTGCGCTGGATAATCTGGATGAACTGCCCGCGTTTCGTCGTTTTGGACGGTTGGT
GCGAGTGACCGGTTTGACTCTGGAAGTAGTCGGCTGCCAACTTGTGATGGGGCAGCGTTGTCGTGTCAGC
ACGGCAAGTGGCGCCCCCTTGCTGGCCGAGGTGGTCGGTTTTAACCGGGAGATCTCTTACCTGATGCCGC
TGCAGCCGATGAGTGGCCTCTTCGCGGGAGCTCAGGTCGAACCGATCGATGGAGAGGAAACCGTTCGGCT
TGGTCGTCATCTGCTGGGGCGGGTACTCGATGGCCTGCTGCAGCCCCTCGACGGGCAGCCGTTACCCGAT
GTTGGTGACAGTGTCCCCCTGTTTGCTCTTCCCCCCAATCCGCTATTGCGAACTCCGGTACGTGAACCGC
TGGATGTCGGTATCCGCGCCATCAATGGCCTGCTGACGGTTGGCAAGGGCCAGCGTTTGGGGCTGTTCGC
CGGCTCGGGGGTCGGCAAGAGTATGTTGCTGGGCATGATGACCCGCAATACCGCAGCCCAGATTGTGGTG
GTGGGACTGATTGGTGAGCGGGGCCGTGAAGTGCGCGAATTCATCGAACACAGTCTGGGGGCCGATGGTC
TGGCTCGCGCCGTGGTCATTGCGGCGCCAGCGGATCAATCTCCCCTGATGCGGCTGCGAGCGGCCCAGCT
ATGCCACCGGATTGCCGAGTATTTCCGTGAGCAGGGGCAGGATGTGCTGCTGCTGATGGATTCACTGACT
CGCTATGCCCAGGCACAGCGGGAAATTGCGTTGGCTATCGGTGAACCCCCCGCGACCCGGGGCTATCCGC
CCTCGGTATTCAGCATGCTGACCCAGCTGGTGGAGCGAGCCGGTAACGGCACGCATCCGGATGGCAGTCT
GAGTGCGTTCTACACCGTGCTGGCGGAAGGGGATGACCAACAGGATCCGGTTGTGGATGCCGCTCGTGCC
ATTCTTGATGGTCATATCGTGCTGACCCGCCGTCTGGCGGAAGAGGGACACTATCCGGCCATCGATATCG

GTGCCTCGGTCAGTCGGGTTATGCCGCAAGTCGTACCTGCCGAATGGCAAAAGCTGGCGGTCACCTTGCG
CCAGCTGTGGGGACGCTATCAGCAGGTGCGTGAACTGCTGCCACTGGGTGGTTATCAACCCGGGGCCGAT
CCGCAGATGGATGAAGCTGTTCAGCGTTATCCGGGGATTGCGGCATTTCTGCAGCAAGGCTTGCATGAAG
AGATGGCCTTTGAGCAGGTGATGGTTGGTCTCAAGCAGGTGCTGGGGCAGGGATAAGGATTCGTAATGCT
GAAGTCTTATCTGGGGTTGCAACAGGAAGAGCTGGAAAATCTTGGCGCCGAGCGTCGACGCTTGCGGGAG
CTGGCGTTGCGGGAAGAGCAGCGAGCACACAAATTGCAGGAAGTGATCTCCTCGCTGCGACCCGGTTCCG
ACAAGTTTCACCCTTTGCTATGGCAAAACAAGCAACAGATGGATGGCCAGTTGCGCCGTTTGCTCTCCCA
TCAGGTGCAGCAATCTGCGCTGGCCAGACTGGATCTGGAGCGCCACGAAGTGGAACTTGTCCGGCAGTTC
GGGCGGGTCAAGGGACTGGAGCTATTGCTGGCAAGACGTGATGATGTTGCTCGCCAGCAGCAGGAGAGGC
GGGCTCAACTGCAGCTGGATGAGCTGGCGAGTTTGCGTCACCTGACCCGCCAAGAGTAAAGCGGAAGGTT
AATCCGTAATGCTGTCCGGGTCGCGATAACCTGACTCTTGTCCCAACAGTGAATGAATCGTCTCCATCTG
GCCTGCCAGCAGGCGGCCATTGAGATCATTCTGGCGCAGACACTGGTGCAGCAGCTCTTCAAGCTGTTGC
CATTGATTGGCTACCGTGGTGCGCAGGGGAAGTGGCAGCTTGGCTATCAGGGTGGCCATCCCCTTCTCAT
CGGGTTTGAGGCCCAATGTCTGCAACTGCTGGCAACGCAATTTTGCCTGTTGTGTCACCTTGGTCATCAG
CGCGGTCTGCTGCTCGTTATGGACTTGCAGGCCATCGACATCATGGCTTGCCAGCAGTGCATGCTGACGC
AGCAACAGGGCGGTGAGTTCGGTATAGTGGCCGTAATCCTGCTGGATCCCGAGGATCAATGCCTTGACGG
TGGCCTTGGTATCCACGCTCATCGGCGATGCATATCCAGAATGGCCTGACCCAGCGCATCCAGATCGATC
TCCAGCTTGCCTTCACTGATGGCTTTGCGGATCTCGTTGACCTTGTCCATGTCGACGTCGGATGTGGTGG
CCAGCTGGCTGCGGGCGTTGGCTACTGACTGCGCTGTGCTGCTGATCCGTGCTTCGGATGACTGTGCAGA
CCTGGTTTCCGGTTTGGTCGTTACATCCGGTTTGCGGCCAGGCTGATTTTGAATATAGAGCGAGTCAGTA
CGGGTAATTTTCATAACATAAATTCCTGATGGTCTGTTTATGACAAGCGACCCAAGGCTTGTCGAACTTG
AATCAAATTGCGATCTTGGTTCAAAAACGGGTTTCAACTTCCCCTATATCAGTCACCCATGCCTGTATCT
GCTTGCCAGAGCTAAGATTTTTCACTCTGATCCCCTCGCCTATTCCGCCATTATCGAGGGCCTCCCCTCT
GGTTGTGGCGGAGAACTCACCTTTCCCTGCACGAATTAGTACCCCTTCCCCCTGTTTGACCGCCCAACTT
TTTTGTAAGTCCAGCTCACCGATGAGTTGCCCACTTCTCAGATGCCGGTTGCTTTTATGGCCCAGCAGGG
AGTGATTACTCGGGGTGAACCCGCGCTGTATGCGGCTGACATCCAACTCTTTCTCCACCAGATCGCTGGC
GCTGATGGCCTGACCTTTTGCGATATCCCGTGCAGCAACCCAGACCGGCAACAGTAGCGAGACCTCTACC
CTCCCGCGCACTTGCCAGGCGGGATCTGTGCATTCGATGAGATAGGGGCGTCGTCCCCAGGGTTGCCGAT
ACTGGCCACCTGCTTGTAACGAGAGTGACTGTGAGGGACAGGGAGGAAGGTGCTCGGCGGAAGGGGCAAG
CCAGACCTTAAACTCCCCTGAGGCTGCGGGCCATCCCTGCTGGGTGATGTAGTCATCAAGACTCTGGCGG
GCATCTTGTTCAAGCAACTGTTCAAGCATGGCGCTTTGCACCTGCGGGGCACCGTGCGCGAATGCGCTCA
GCAGTAGCCAGAGGATGCCGCTCCATTTCCACACGGGAAGTCGCACTTCCTCATTTTTCATCTGCTTGCG
GCGGATGGTGCATGGTAACCGCTTGAAACTAGGTATCTTCATGGTTGGCATGAGTCTTGATAAGGGTAAC
CCCGGTCTGAGTGAACGATAAAGGATGAAGCATGAGTATCTCATTTGACAGCGCCCTCGGCGTACACCCT
TATGCCCTCGATGTCAGGGCTGAGCGGGCGCGGATTTTGGCGGGGAATCTGGCCAACGTGGATACACCGG
GCTATCTGGCTCGGGATGTCGATTATAAGACGATTCTGGGCCGTGTCGCGCAGCAAGTTGCTGCGGGTGA
AGAGAGCCAGGCCATCAGCCAGGGCGCCATGCGTGACGATATGCGTCACCCCCTCTATCGCATTCCTTAT
CAGGTCTCCATGGATGGTAATACCGCTGAGTTGGGCGTGGAGCAGGGCAAGTTTGCCAACAATGCCACCG
ATTTCCAGACCAGCCTCACCTTCCTCAATATGAAAATTACCGGTATCGCCAAGGCGATTGAGGGCCGTTA
ACCATGTCGTTCAACAAGATTTATGACATTGCTGGCAGTGCCATGCGTGCCCAGACCGTGCGGCTGGATA
CCGTTGCTTCCAACTTGGCCAACGTGGATAGCGCTGCCGCCAGTGAAGACGCCGCGTTTCGTTCCATCAA
GCCGGTTTTTTCCACTCTCTATCAGAAGATTCAGGATGCCGATGGCCTCGGGGCTGCGTCGGTGCAAGTG
GCGGGTATCGTGCAGTCCGACCGTCAGGTCGAGAAACGGCTGGAGCCGACCAACCCGATGGCCGATCAGG
ATGGTTTTGTCTACTACTCCAACGTCAACGTGGTGGAAGAGATGGCTGACATGATGAGCGCCTCTCGTGG
TTTTGAAACCTCGGTGGAGGTGATCAACCGCATTAACAGCATGCAACAGGGGCTGTTGCGTCTTGGTCAG
GGAGTCTAAGTCATGAGTCTGCCACAGATCAGTAGCCTGGATACGGCGCGCGGTACAGTACAGACCAATA
CCGATCCCAAGATCGCCAGCAATGGCAGCGGTGCTGCTCAGGAAACGCAAGCCAACCAGAATAGCGGGAT
GCAGTTGCGTAACGAATTCCTGCAGATGATGGTGGCGCAGATCCAGAACCAGGATCCCACCAACCCGCTT
GATGGTGCCCAGTACGTGGCTCAGCTTGCCCAGTTCAGCATGGTTGAAGGGGTCGAAGGGCTGAAGAAGC
TCCAGCTCCAGAGCAATACCAAGATGGATACCCAGCAGGTGTTAGAGAGTACCGCGTTGGTCGACAAAGA
GGTCATGGTGCCTAGCAAAACCCTCAAACAGCAGCAAGAGCACAAGATCCGCGGTCAGATCGAGTTGCAA
GGTGCGGCGGAACAGGTGGATCTCAAGGTATATGACCAGCATGGCGTGCTAGTAGCGAGCAAGAGCTGGG
GTGACAGCAAGGCGGGTGGGCTGGATTATGAATTGCCCCCTCTTCCTGTCGGGGAGTACCGCTTTGAGGT
CAAGGCCTCGGCGGGCAAGACCCTGGTGGGGGCCAAAAATTATGTGGCGAGCAAGGTGGAACGTGTTGCC
TTGCCCGGTACGGGAGAAATCATGCTGCAGGTTGCCGGTGTGGGCGATGTCCCGCTCTTCAGTGCAATCC
AGTTCGGCAAATCAGCGTAATCAGAGAGGAGCGACACTGTGTCTTTCAGTATCGGATTGAGCGGCCTGCG
CGCCGTAAACCAGGAATTGAGTATTATCAGTAACAACGTAGCCAATGCCAGTACCGCGGGCTTCAAATCC
AGCCGCGCCGAATTTGCGGCAATTTACGGTGGTGGTCAGGCTGGCGGTGTCGAGATGAACAACGTCTCCC
AGAATTTTGACCGCAATGGTGATATGACTCGTACCGGTCGCGGGCTGGACCTGGCGATTTCTGGCAGTGG
TTTTTTCGTGCTGAAAGATGGCAGCGGCCAGACGTCCTATACCCGTGCCGGTATGTTCCAGCGTGATAAT
GCCAATTACTTGACCACTGCAGGCGGTACGCGGTTGCAAGGTTATACCACTGATGACGCGGGTAAGCTGC

AATCCGGTGTGGTTGGTGATATCCAGGTCAAGGCGGGCAGCCTGCCTGCCAAGCCATCCGACAAACTGGA
GTTTGTGGCTAACCTGAAAGCCGATGCCAGTATCATCCCGGCTGACCCTAAGTTTGATCCAAACAAGTCG
AATACCTTCAGCTATTCACAGTCCAGCAAGGTTTATGATTCGCTGGGAACTGAGCATACCGTTACCCAGT
ATTTTGTGAAAAAAAGGACCAATGCCTGGGATGTTCACTACAGCTTTAATGGAGCAGACGTGACGCCTTC
TGGCGGTGCCAAAATGATTTTTGATCCCAATGGCAAGTTGGATACCGGTGCTTCCAGCATCCCCCCCTCC
TTGGCCCTGAAGCCTGCGGGTGCCTCGCCGATTGAATTGCAATTGGACATGAGCCGAGTCAGCCAGTATG
CCTCTGACTTTAATGCCACCCGCAACCAGTCGAATGGTTATACTGCTGGGGATTTGACGGAGGTGCGTGT
GGATGGGGACGGCGGTGTCTACGCCACCTTTACCAACGGTCAGCGCCTGTTGCAGGGGCAGGTCGTGATG
GCGAACTTCACCAATCCCAATGGCCTGCTGCAGACCAATAATACCTCTTGGCAGCAATCTTTCAGCTCAG
GGCAGCCTGTACTGGGGGCACCGGGAACCGGCACCTTGGGCAAGCTGACCGCCGGGGCTTATGAGAGCTC
CAACGTTGATCTGACCGGTGAACTGGTAAGCCTGATGACTTCTCAGCGTAACTATCAGGCCAACGCCAAG
ACCATCAGTTCGGCTGACAAGATGAGTCAGATCCTGTTCAACTCCTTCTAAGGGTAATCCGTGGAAAAGC
TTATCTACACCGCCATGTCCGGGGCACAACATACCCTGATGGCTCAGCAGATCCGTGCAAATAACCTGGC
CAACGTCAACACCGCCGGCTTTCGGGCTGATTTTGAGCGGGTTTCGGCTTATGCCCTGACTGGCGATGGT
TACCAGAGTCGGGTGATGGCCAAGGAGGAGATGGCGGGCACCAATTTCAAGGCTGGCCCTCTGATGCAGA
CCGGTCGCAAGCTGGATGTGGCGATTCGTGGCGAAGGCTTTCTTGCCGTACAGACTGAGGATGGCAAAGA
GGCCTATACCCGTGCTGGTAACCTGGAAGCGGATGCTGACGGGCTGTTGACCCTCAATGGTCGGCCGGTA
CTGGGGGAAGGTGGCGAGCTTGTGCTGCCCGCTTATCGTGACATCAGTTTCGGCAAGGATGGCACTCTGA
GTATCACTCCTCCGGGGGGCGGCGCCCGGCTCGATGTGGGGCGTCTGAAACTGGTCAATCCAGAGCCAGC
CGAATTGGTCAAAGGTCATGACGGTCTGTTCCGGATGCGTCAGGGAGAGGATGCAGAGGTCAGTGACCAG
GTGGTGATGGCATCCGGTTTTCTGGAAGGGGCCAACGTCAATGCCGTGGATGAATTGGTGAGCAGCATGT
CATTGACCCGCAACTTCGAACTGCAGGTCAAGTTGATGAAAGCAGCGGATGATCAGGCTCGTCAGGGAGC
CAAATTGATTTCCGGTCAGGGTTGATTTTGACGGCATCGGGCAGATACCCGATGCCTTGCCACATCCCAG
TGGATGAGGCTTGATGAATAAGGTGGAATAACAATGCACGCAGCATTATGGGTCAGTAAAACAGGTCTGT
CGGCACAAGATGCCAAGATGTCCAGTATCTCCAACAACCTGGCTAACGTGAACACGGTCGGTTTCAAGCG
CGATCGAGTAGCGTTTGAAGATCTTTTCTATCAGGTGCAGCGCCAGCCGGGTGCTCAGCAGGATCAGCAG
AATCTGGCGCCTATCGGTATTCAGATGGGTAATGGTGTGCGGGTAGTGGGTACCCAGAAGGTCTTCACCT
CCGGCCAATTCCAGAATACCAATCAGGAGCTGGATATGGCGGTGGTCGGTGCCGGTTTCTTCGGCGTCGA
GCGCCCGGATGGCGAGACGGCCTATACCCGCAATGGTCAATGGCATCGCAATGCCGAGGGGCAACTGGTT
AACGCGGATGGTTTGCCCATGGTACCGCAGATCCAGATCCCCGATAACGCTACCAAGATCTCCATTGGTA
CGGATGGTACCGTCAGTGCATCTTTGCCGGGTCAGCAAGAGAGTGTCGAGCTGGGCCGTATCACCCTGAC
CAACTTCGCCAACCCGGCCGGTCTGGAAGCGCTCGGTGGCAACCTCTACCGTGCAACCGGCGCGTCCGGT
GAGCCGATTGAAGGTGTGCCTGGTGAGGAGGCGATGGGGGCCGTCAAGCAATTCGCGCTGGAGAGTTCCA
ACGTGAACGTGGTTGAGGAGATGGTCGACATGATCACGACTCAGCGGGCTTACGAGATGAATGCCAAGGT
GGTCTCTTCTGCCGACCAGATGCTCAAGTTTGTCACTCAGGCACTGTAATGATGAAGTCGATGCTGCGTT
ATATCTGGTTGGTATTGTTGCTGGGCGGCTGTGCCACCTATGAGCTGCAGGCACCCGAGCCGGGTGACGA
GAAATGGGCACCATCCCGTCCCAAGGTGACCGCGACTGCGCAGGGCGAAGATGGTGGCCTCTATCGGGGC
AACTACATGATGACCCTGTTTCAGGATCGGCGTGCTTACCGTGTTGGTGACATTCTCACCGTGGTGCTGG
AAGAGCGTACCCAGTCGAGCAAGAAAGCCAATACCAGCATGAGCAAAAACAGCAGCATGAATGTGCCGGC
CCCCTCTATTGGCGGTAAACTGCGCCCCGACTGGGGGGCCGCCCTCAGTGCGGATCGGGATTTTGATGGC
GGAGCGACCTCCAGTCAGCAAAATACCCTGGCAGGTTCCATTACCGTGACCGTAGCAGAAGTGATGCCCA
ATGGTGTGCTGGGGATCCGCGGTGAAAAGTGGATCCGCCTCAATCAGGGGGATGAGTATATCCGCTTGGG
CGGCATGGTGCGGGTTGAGGATATTGATCAGAGCAACCGTATCTCGTCCCAGCGCATTGCCGATGCCCGC
ATCACCTATGCCGGTCGTGGAGCCCTTGCAGACAGTAACCAGATGGGGTGGCTGGGCCGCTTCTTTAGCT
CGGCGTTTGCGCCATTTTGAGGAATCACATGAACAGATGGTTTATCGCCCTGCTGTGTCTCTGCTTGCCC
TGGTTTAGTCAGGCTGCCGCACCGGCCAAACGAGCCTTGCTGGATATTGCCGATGTGCAGGGGCTGAGAA
CCAACCAGCTGGTGGGTTATGGTCTGGTGGTCGGTCTCTCCGGGACCGGCGACAAAAGCCAGGTCAAATT
TACCGGCCAATCCATGGCCAACATGCTCAAACAGTTTGGTGTGCAGTTACCGGACGATATCGATCCCAAG
CTTAAGAACGTGGCAGCCGTTTCGGTTCAGGCCACGCTACCCGCCTTGGCTGGCAAGGGGCAGTTGCTCG
ATGTAACCGTGACTTCCATTGGTGATGCCAAGAGTCTGCGTGGCGGTTCTCTGCTGCTGACCCCCCTGCG
TGGTATCGACGGTGAAGTCTATGCCGTGGCGCAGGGCAATCTGGTTGTCGCCGGGGTGAAAGCGGAAGGG
CAATCAGGTTCATCGGTCACGATTAACGTGCCGACAGCCGGGATTATCCCCAATGGCGGCACCATCGAGC
GAGAGGTGGTCAGCAATTTTGCCGAAAAGCCGGAAGTGACCCTCAATCTGCATCAACCTAACTTCAAGAC
GGCCCGCAATGTGGAGCGTGCCATCAATCATCAGCTGGGGCCGGTTGCCCGCGCGGTGAGTGCCGGGCGG
GTTCAGGTTGCAGCCCCCAGAGACGAGAGTCAGCGCATCGCGTTTATGGCACTGTTGGAAGATATCAAGG
TGGATCCGGGTCAGGAGCGGCCGAAGGTGGTCTTCAACAGTCGAACCGGTACTGTGGTGATGGGGCAGGG
GGTCAAGGTCCACAAGGCTGCCGTTAGTCATGGCAGTCTGACGGTGACCATTGGCGAGAGTCAGACGGTC
AGTCAGCCCAACGCTTTCTCCAATGGGCAAACCACGACTACCCCCAACTCCAAGATCCAGGTCGATCGGG
AACGTAACAACATGTTTGTCTGGCCGGATGGCACCTCTTTGGAGGTGATCGTCAAGGCTGTCAACTCACT
CGGGGCAACCCCCGATGATGTTATGGCCATCTTGCAAGCGCTCAATGAGGCTGGTGCCCTTGAGGGTGAG
CTGGTGGTTATCTAAGGACTATCATGACAAGAATTAGCTCGGATCCCGGTTTTTATCAGGATCTCAATGA

ATTGCAGCAAATCAAAAGCAACCCGGATCAGCGTGCCGCGCTGGATGCTGCAGCCGGTCAGTTTGAAGTC
AGTTTTCTGCAAACCGTGCTCAAACATATGCGCTCGGCGACGGATGCCCTGCAGGATGAGGACGACAAGA
TTATCAAGGGACAATCCCTCTATCGCGATATGTATGACAGCCAGTTGGCGATGAGCATGGTCAAACGTGG
TGGCATCGGTCTGCGAGAGCAGATGGTCAACCAGCTGGCTCCGTCATTAAAGAATCCGGACGGGGTGGTC
GCTTCAGAGCAGCAGACCCTGGCTGCGTTCAGCCAGCCTCTCAACTCGGTAAAAATGAAGAAGGTTGACT
GATGTCACTGCTCAATATCGGCGCCAGCGGACTAAATGCAGCCCAGATTGCATTGAACCTGACCGCCCAG
AATATCGCGAACGTCAATACCATCGGGTATAGCCGTCAGGAGGCCATGATGGGCAGTCTGTCCGGTTTCG
GGCGGCTGGATAATGGTGCCGGTGTCGAGGTGACAGGGGTACGCAGGGTAACGGATGACTATCTGGTAGC
CCAACACTGGCGTAGCCGTGCGGCCACCGGTTCCTCTTATGCCTTTCATCAGTACATCAATACGACAGAG
CAGTTGCTGGGCAGTGAGTCGATGAATATCGCAAAAGGGCTCGACAGCTTTTTTGCTTCGCTCAGTGCTG
CGCTCGACAGTCCGGAAACACCGGCACAGCGCAGTCAGATTGTCAGTTCAGCAGGGGCGCTCGCCAACCG
TTTTACCCAGCTTGACGAGAGCATGGTCACCCAGGAAAAGCAGATCGATGATCAGCTGAACAGTACGGTT
TCCCAAGTGAACAGTTACTTGAAGCAACTGGCTGAACTGAACATGCAGATTGGCGATCAGACAGCCAAGG
CAGTTAATACCGCTGCACTGGAAGACAGTCGCGAACAGATTGTGCGTGAGTTGTCTGCCTATATGGAGGT
GCGGGTCAATCGTCAAAGCGATGGCTCGTTTGCGCTCAGTCTGCCGCAAGGACAGCCGTTGGTCCTGGGA
AGCAGCAGCAGTACCCTCGCGCTTGCGGGCGATAAACTCAGCCTCGCTTTCGGTGGGCAGACGTTTGAAG
TTCCTAAATTGCACGGTGGTTCGCTGGCGGGTGTGATGGATTATCGAACCACAGTATTGCGGCCGACCAG
AGAGGCGTTGAATCAGATAGCGGGCAAACTCGCAAAGGATTTCAATGATAAACAGGCCGGTGGTGTTGAC
CTTCATGGAGTAACCGGGAAACCGCTATTCGCTTTTGACGAAACAAAAGATATCCATGGTAACTACATCA
ATGCGTTGGGAACATTGCGGGTGGCTGATGGTTTTACCGGAGACGACCTTGCCTTTGCCAAGACCGGCGG
CGGTAAGGGCGATAACCGCAACCTGCAGGAGTTGCTGACCATCAAAGATAGCCAGTACAACGCATACAGT
GCGCTGCTGGGCAAGATGGCGGTGCAGAGTGGTCAGGCGAAAGCGACCATGCAGGCCGATGCCAATATGG
AAAAACAGATGGCGGCCAAGCTGAGCAGTGTCAGCGGTGTAAATACAGATGAAGAAAGGGTGAAGATCAT
GGCGTATACCAAGGCTTATCAGGCCAATGCCAAGGTGATCAGTACCTCCGAACAGCTCTTCAACTCCATT
CTGAACATGTTCTGAGAGTCATAACCATGCGGATCAGTACCCATCAAATTCAGCTCAGCATGCTCGACAA
CTTGCAGCATGGCTTCGGCGAGTATGCCCGCCTCGATCGCCAGATCTCCACGCAAAAACGCATTCTTCAG
CCCTCAGATGATCCTGTTGGCAGTGTGCAGCTGCTGGGCTTGCAGAAAGAGCAGGTGGCGATGGCGCAGT
ACCAGAAGAATATCGCCAACGCCAAGAGCCAGCTCTCCCAGGGGGAGTTGCAGCTCGATACCATGACCAA
CATGCTGATGCGGTTGCGTGAGTTGACCCAGACGGCCGCTAACGGCTCCCTGTCTGAAGGGGATCGCAAA
GCGGTTGCCAGTGAAGTCGCCATTATCAAGGATGGTCTGCTGGATTTGGTCAATGCCCGCAACGAGAGTG
GCTCCAGCCTGTTTAGCGGCAGCAAGGTGGATGAGACCGCTTTGGTGAAGGTGACTGACCCAAAAGAGCC
TGACTTTGGAGGTTACCGCTACAAGGGTGATCATCTGGTTCGGGAGGTCGGCATTGCCAAAGGTGTCACG
GTAGGGTTAAACCAGACGGCCGATCAGCTTTTTTTGCAGCCTGACGATTTTTTCAAACGACTCGATAGCA
TGGTGAAGGCAATTAATGACGCAGCACCTGACGCCATCGATCAGGCACGAGCCATGCTGGATCGCAGCCA
AACGCTGCAAGATAATATCAGCCAGGCGGTCAGCACGATTGGAGCCCGGATCAATCTGCTGGATCAGATT
GATGAGAGCCATGCCGAGAAAGGTGTCTATAGCAAGGAAGTGAGCAACCAGATAGAGTCGCTGGACTATG
CCGAGGCCGCGACCCGACAGGCCCACGTCCTGATGGCATTACAGGTGCAGCAGCAAGCTTTTGCCAAGGT
TAATGGTCTTAGCTTGTTCAATTACATGCCGTAACGATCGAAGGAGTCATGATGCAGGTCCAGCAAACCC
GTCTTGGAAGCGTTGCAGAGGCGCAGGCAAAGCCCAAGCTGCAAGGCCGGCAGACTACGCAGGCTCATCC
CCAGGTTGCCAACAAACAAGGCGAACGCCACCCCAATATTATCCGGATGGATAGATGGGCAATGATCGGT
GTCGCTCAGCAGCGCATTGCCCATGCCCAGAGCGGCGAACAGGCGCTTAATCTGGTTTGGGGGGAATTGA
AGCGGCTGGAACGCCAACTGGGTCAGCGCGACCTGGCTACCAGTGACGTGACCAATCGCTTAAAAATACT
GGAAGAGAAAGTCACCCAACCAAGAGGGCCTTTGACGGACGAGCTCAAACCCAGACTTATCGCATCGGCT
CAGGAACTGCGTCCCCGCTTCAGTGCCGAGCATCTGGATCTGCTCAGTGCCAAAAATAGTACCGAGCGAC
TGGTTTTCTCTTTTCCCCAAAGTAGTGGTGCAGTGGAAGTAACACTTCCGGCCGGTGCGCGGGATGGAGA
GGTGGTCGCCAGGCTGGATCGGGCACTGCGCAAAGAACATATTCAGGCTCGGTTGAATGAACTGGGCAAG
CTGGAGTTGTCCGTCGCCGAGCCCCTGCGTCGCAAGCTGGATGAACCTGTGCTGGTGCGTGGCGAGGGAA
TTCGTATTCCCGCGGGCAATCCTGTACCGGTACAATTCAAGCCGAGAGCGGGACGGTTGACTCAACTGTC
TGACGGGATTGGCAAGGGGGAGCTGCGCCAAGAGCGGCAACATCTCCAAAAATTGCTGGTCGAGATTGAG
CAGTCGGTGCGAGAGCTGAAACAGTTTCGCCAAAAGATGGTCAAGCAGCTCGATAAGGTGAAGGCTCGTA
GCCAGCACCTGGAACAGGAAGATCTGCAACGGTTGCAGTCATCGCTCGCCTCCCAATTGCAGCAGGGGGG
ATTCAAGGGGGCGATGTCCGGTTTGCTGGCACAAGCCAATGTCACCCGGCAAGGGGTTGTTGCGCTATTG
ATGTGAGCAGAATAGTGAGTTCAACATTGAAATTTTTGAAATAAAAAACGCCTTCTATTCCCGGAAGGCG
TTTTTTTATCTATGCGCTTCTATCCCTTATTACGTAACAGTGCATAGAGTGCGATATCGCTGTTCAATCC
CGATGCGTTGAGGCTTTCGTGCAAGATGCGGGTAAAGTCACGGGAATGTAGTCGTCTGGCATGATAGCTG
TAAAAGAGCGAGACGATGATGCTCTCATCAGGTTCTTCTCCTTCCTGGGTCATCAGGCAGAGATAGGCGC
GCAGCAAGAAGAAATCAGCCACCAGCAGCAGATATGCCTCTGTCGGTGAGCAGTCTTGTCGCGGAAAGCC
GTGGTGATAGACCCAGTAGAGAAGGTAGTTATCCAGCACATGTGGATGGTTAACGAGCCAAGGCTCCCAT
TTACTGTGCCATTCATGATAGAGGCTGGTCACTTTGTCTTTATCGCATTCTCCCTCGAAATGGCGATAGA
CCCGGTCCATGCATGCTTCCATGGTTTTGTAGCCGCGCTTTTTGCTCGATTGTTCGCTGCGCAACAAATT
GCTCATCGCTTTTAGCCCCCACCAGTGAAGGTCGGCCAGGTACGGCAGTTGCTCAAAATGGGCGGTCAGC

CCGCCGGCATCCGAGATATCTGCCAGTTGATGGAGAAACTCTTCATGGGAGAGTTGGCTCGCATCCCGAT
GGACCAGCATACCGAGGATCCAGAGCCGCTGTTCGACAGGCAAGTCTCGATTCAGCATTAGATGTAGCGC
CAATTGCTGTAACTGGTTATGTGACGGAGGAAACGGGGCTGTGGTGTTATGGCCCTTTTGCTGTTCCAGC
GCTATCTCCATGGCGTGTGGATCCAGCAACACGCATCGTACAGCTTGTGGACACGAGAGCGTCAGGGTAT
GGCGGATCTCTTGATCCTGGGGATAGGAGATGCGTGGATAGCTCTGGCAGGTATTGCTGAGTGCATCCGG
GCCCGCTTTTGCGTGAATAACACATAACCCGGCGTTATCGAGGAAGGGGCAGGCTCCTTGCTCATCCAGT
CTGATCTCTCCCCACTGCGCATCACTCTTTCTGATTTTACGGATATGCGTGGCGGCCATTTTTTTAAGGA
CGGCATCGGCATGGCCAAGATAGCTGCGCAGGCTCTTTTTATCGATAGTGATGCTCCAGCCATGGCAGCA
ATTGTCTTCACAAGCTCCCCCAATACAGCTGAATTGTTGGTAGTAACGGGGTGTCAGATGGGTGACGGGC
ATTCCTGCTCCTTTCTCGTAATGTAAGTTGGTATCGAGAAGATTTAGCAAAATAAGTGCCAACCAGTGGG
TCTGGAGCAAAAAGAAAAAGATGATGTATTGATTTAATTTTTCTGCGTTCAGGGTCGTCTTCTCATTCTG
TCGAGATAAATGTGGCCAATTGGCCGAGCTAACGAACGAGGAACGAAAAAATGGGTCTTTCTATTCATAC
CAACTTTGCCTCTCTGACTACTCAGAACCAGATCAACTCCACCAACAAGATGCTGGGCACTGCCATGCAG
CGCCTGGGTACCGGTCTGCGCATCAACTCCGCAGCAGATGATGCCGCTGGTCTGCAAATGGCGACTCGCC
TGCAGGCACAATCCCGTGGCCAAGCTGTTGGTATGCGCAACTCACAAGACGCCATCTCCATGATGCAGAC
CGCTGAAGGTGCCATGGACGAGATGACCAACATCACCCTGCGCATGAAAGATCTTGCTACCCAGTCTGCT
AACGGCACCTCTTCTACCGAAGACCGCAAGGCCATGGATGACGAGTTCAAACAGCTGACTAAAGAGCTGG
GTAACATTGTTGATAACACCACTTTCGGTGGTCAGAAGCTGCTGAAGGGTGGTGCTTTTGAATCTGACGT
GATCTTCCAGATCGGCGCTTCCAAGGATGAAAAACTGACCGTTGATGTAAAAACTGCGATGAAGGAACTG
ACTGGTACCACAGCAAGTAAAGGTATCGCTTCCAAGGATATTACTGATGGTATCGGTGATCAAACCAAGG
CAAATGTGGCGATTAAAGGTGCTGCAGACCTGATCGACCAGATCGGTGCTGTCCGCTCTTCCTTCGGTGC
CAACATCAACCGCCTGGAGCACACCGTCAACAACCTCTCCAACATGAAAGAGAACACCGATATGGCCAAC
GGTCGTATCATGGACGCTGACTTCGCAGCGGAAAGCACCAACATGACCAAGAACGAGATGCTGATGAACA
CCGGCGTTAATATTCTTAGCAAGTCCAACGGCATGACCGGTATGGTTATGGGTCTGGTGCGTTAATAGAT
TATCTGGCTGCCCCGGTGTGGGCAGCCTTTCTTTTTGTCCGTTGGAAAGTGTCGGGCCGGTATTTTCCAA
CAGGCATGCCTATTAGCTTAATAAGGATGATGTGATGCAGATAGATCCTGCCAGCATGGCGAAGCAAGTG
GTTGCCATTGAGCGCAAGAACATGGACAAGCTGCTGAAAAAGCAGATGGACAATGTAAAGGGGCAGCAAA
CGGCGTTTAATACGCTCAATAGCAAGTTGTCTACGTTCCAAACCATGCTGAAGGATTTGAATCAAGCATC
CAATTTGCAGGCTCAGAAAGCGACCACCTCGCAAGAGGGGGTAATGACGGTCACCAGTAATGGTAAGGCC
AGTTCCGGACAGTACAGCTTCCATGTCGAGCAGTTGGCGCAAGCCCATCAAATCGGGATGGCGTTTGATA
GTGAATCAGCGTTACTGCCCGCAAGTGGAGCCCTCTCGCTCACAGTCAAAGGCAAGGCGGTGGATATCGA
TATGGCCAGCTTGCCAGCCGGTTCGACCATCAAAGATCTGGTCAGTCATATCAATAACGCTAAGGATAAC
CCTGGCGTGAAGGCCTCTCTGGTACGTACTGACGGCAAGGTCAATATGGTGTTAACCAGCAAAGATACCG
GGACTGAAAATGCGATTACGATGGTTTATTCTGGTGACGGCGCAAGTATGCTGGGCGTTGCCCTGGCAGG
TAAAAAAGACATTACCAATGCCCAGAATGCCAAGCTACAAATGGGCGGGAATAGTCCCCTGACCATTGAG
TCTGCAAGCAATAAAATTGAAAATGTGGTGGAGGGGTTGACCTTTCAGCTCACGAAAGCTCAAAAGTTGG
GTGATGCACCGCTACAAGTTGTTGTAGATCAGGACAAAGAAGCGGTGACCGGCTCGTTGAAAAAGTTCGT
CGACAGCTATAACGAGTTGCTCGATGACATTGGCAAGATGACCTCCAGTGACCCCAAATCTCCGGGGGCT
CTCTCTTCTGATAGTGGTGTGCGCACCCTAAAAAGCTTGTTGGGCAACAAGGTGCGCGATCTGCCAAATG
GATTGTCACTAAGTAATCTCGGGATCAAAACAGACAAGGCTGGTAAGCTTTCATTCAACGAAACCGATTT
TAACAAGGCGCTTGAGAAAGATCCCGAGTTGCTCGGCAAGGCATTGCTGGGGGATGATGGCCTGCTCAAG
CGGATGAGCAACGGGCTTGATCCCTATACCAAGCGTGACGGGGTTTTAAAGACTCGTAAAACAGGTCTGG
AATCCAGTCAAAAGCGTCTCAACGAACGGATGGAAGCGCTTGATCGCAAGATGGATGCTGCATACAAGCG
TTACCTAACCCAATTTAATACCATGAATCAGATGATGCAGAATATGGGGTCTCTCTAACCCTGAGCTCAT
AATCGCTGCATCCAACTTTGGAAGTGATGTCTCATGTTTGAATCCAAAGATGGCTATGATGCCTATCGAT
TTGCCGCCACTGAAGCAAAGGCCGCGAGTGCCGATCCATATCAGCTGGTACTGATGTTGATGGATGGTTT
GCTTGACGAGATAGCCAGAGCAGAAGGGCATATCTTGGCCAAACAGTTTGAGCGCAAAGGGTTGGCCATC
AACAAGTGTCTGCAGATCCTGGGTGGGCTGGACAGTGCGCTTGATATGAATAAAGGGGGCGAGCTGGCGG
TTAACCTGCATCGTCTTTATGACTACTGTGGTCAGAAACTGTTCGAGACCAGCGTATCCAACAGTGTTGA
GGGATTTGCCGAAGTGCGCAAAATTCTGCAAGAGCTGAAAGAGGGTTGGGAAGCCATGGCGCGTCATCCT
CGTTAATACGGAAGTAGTACTTCCTTCCCCTCTGTTCTTCCTCACGCCCAACGTATTGAAATTTCTTGGT
TATCGGTCTGGCCCACTCTTTGCTTGTGGGCTGGTTCATCTTCTTTTTGGGGCTCGTCATGTCAGGTATT
GCGCCCGTGTTACGTGAGACCGAACTGCAGACGCGCCAGCGCCAGTTATTGGGGTTGGGTACGCTATTGC
TGCAACAAGCGCAAGCTGGCCAGTGGGAAGCTGTCAGGCTAACCGATGGACGCTTCGCGCAATTTGTCTC
TCAAGTTAGCCGGAATCCCCAACTCTGGGCTGCGCTGCAACCAGCAAGAGATAAAGCCCGGGTGTTGTAT
CGACAAGCATTGCAGCTTTGCGAGCAGGAGACCTTGATTCGTAAACAGGAGTGGCAGCAGCTTTCTTCGA
TTCGTGAAGGCTTGACTGCTTACGGTGAGATAGAGCAATGGGATTGATTCAATTTAGCGGTTTTGATGCG
GTGAATGCGCCGGATGTCACGGCCTTGAACCGTTTGTCCGGCCAAGCCGGCAGTGAAGGGGAGTTATATC
AACCGCTGGATGAGATTGTTGACCCTCTGGTTGCATTGCCCTTGCCCGAGTTGGTTGCCAGTGATGTTGC
GGTAGACGAAACAGAGAGCGAACAATCAGGAGCGGAAGGCGACCCGACTGAACAGCTGCAAGCGTTTACC
AATGTGCCTCCTCTCTTTGTCAGAGTAAGTGATGCCGATCTTGATGGTTTTCGTGTAAGCCGTACTTTTT

CCCGAGCTGAAGGTAGCACTGGTGGGCAGCAGGTGGTGGTGGATAACAAATTGCCGCCCTCTCTTCGTGC
CTTGATGGGGAGTGCGACCACGCTGCCCGAGCAAGAGGTGATGCCGCGTCGTGAGACGGTTTCTGCCGCT
GCGGAACAGGCGCTCTCTACCTTGCTTACCAAATCAAATGGGCAAACCGGTGTATTGCCCGGCCGCTTCA
CCTTGCCGGTTATGGCTGCCGGTTTCGATGCTGGAAATGTGCCTGATACCGCAGTGGTAGAAAACAGGCT
GCCAGATGCTCGCCAGCTGGCAGTTGGCCAACTCGCTGCGACGCCGTTGCGACCTGCCGACTATCAGTGG
GCCCCTGCCCGGTTGTCAGACAACCAGAGCCAGTGGGGGCAACAGTTGGTTAGCCTGCTTCAGGACAAGG
TTGAGCTGCAAGTCAATCAACAGGTGAAACAGGCCCACATCCGGCTGGATCCGCCAGAGCTCGGGCGATT
GGAGTTGACCGTCAAGCTCGATGGTGACCGGCTCAATGTCCAGTTGAATGTGACTAACCCGGCGGTGCGC
GATGCAATGATCCAGTCGATGGAGCGCTTGCGCATGAGCCTGGCTCCCCATCATGCCGGCGGCGTTGAGG
TCAATGTGGGGCAAGGCGGCGAGCAGGGGCAGCAGGATCGGTGGCAACAGGAGAAGATCCTGGCTGGCCG
CCGTCAGTGGTTGGATGAACCAGAGTCCCAATGGGGGACGATGCACGACTGGCTCAATACTCTGGTCTGA
TTTTTAACACGTAATAACGAGAGTGGTTTTACATTATGCGGCGTGTCATGAAGTGGTTGATGGTTGTCGT
CATTGCCCTGTGTCTGCTGGTTTTGCTGGCATATGGTGCCTGGTTGAAACGCGATGTGATTGAAGTCTGG
CTCGGATTGGCAGAGCCAAAGCCAGAGCTGAGCAGCACTCCGCTGTTCAAGCCGATGGAGCGCTTCGTTA
TCAGTCTGGAAGGGGGCGAACAGTCCCATTATCTGGTGCTGGAGCTGGCACTGGTGACCTACAACCCGGA
GCAGGTTGCCAAGTTGGATGAGCTGACTCCACTGATCCGCAATGCCATGGTGCAGTATTTCAGCCACCGC
TCCCATGATGACGTAAAGGTTGAGCTGCAGAATATTTCCAAGCTGCAGGTCTCTTTGCGCGACAAGCTGG
TCACTACGGTGCAGGGATATGGCTACAAGCCCTATCTGGACGAAGTGCTGATCACCAAGGTACTGGTACA
GTAATTACCATATGACTGATTCTGCTTGGGATTCTCAACAAGAGTTTGCGGATACGCCAACAATTCCCCG
TACTGGTGAACAGGCCATTCTGACTCGCTATCTGCCTCTGGTTAAGAGGGCTGCTTGTCACCTGCGTAGC
CAGGTCAGTGCCTGTTTTGATCAGGAGGATATGGAACAGGTAGGGATGATGGGATTGCTGGAGGCGTGGC
GTCGTTATGGTAGTGAACCTGATGCCCAGTTTGAAAGTTACGCCTTCAAACGGATTCGTGGTGCCATGCT
CGATGAGCTGCGGCGTCTGGACTGGCGGCCTCGCCAGTTGCGACAGCAGGTACACGGCCATAATCAGGTG
CAGCGTGAACTGCACAATCGTTTGGGGCGCCCCCCCTCAGAGCAAGAACTTGCTGCTGCGCTCGACTGTT
CGGTGGATGAGGTGCGTCAGTTGTCCTACGCCAGCCAGGCTGAAGCGCTACAGAGTCTGGAAGAGTGGCT
GGAGAATGGTGGCAAGGCGCCAACAACCGAGTCTGATGATGTCGATATGGCGATGACAATCAGCAAGGTG
TTGGCCACTCTGGATAAACGCGAGCAGCTATTGCTTGCCCTCTATTACCAGCAAGAGCTCAATATGAAAG
AGATCGCGCTGGTGCTTGGATTAACCGAATCCCGGGTTTGTCAGCTGCACAAGCAGTGTGTGCTTCAGCT
AAAGCAGCGATTGGTCGACTCCATATAGTAGTGCTTCACCTTTACTGGTGACCCTTCTTGTATCGTGCGG
GAACATTCATGCAAAAACAGATTGGCATCGGCGTCATTTTAGTATGCGTGCTGGGTGGCTTCATGATGGC
CGGGGGGAACCTCGCCGCCTTGTGGCAGCCAGCCGAGCTCATCATTATCATCGGCGCAGCGGTAGGCTCC
ATGTTTCTGGGCAACTCGAAAGAGGTGATGCACGAAATGTGGCGGCAGATCAAAATGTGCTTCAAGCCTC
CTAATAACGAAGAGAGCGAGGTCTATCGTGAGCTGTTGACTCTGATGCATCAACTGCTGGAGGAGACACG
CAACAAGGGGATGAAGGCGCTCGACGAGCATATTGAAAATCCCCAGCAGAGCTCGGTTTTCTTGATGTAC
CCCCAGATCTCTGAAGATCCCCAGTTGCTCGGTTTTATTATTGATAACCTGCGTTTGTTGGGGATGGGCA
AGATCACACCTCATGAGCTCGAAACCGTGCTGGAGCAGGAGATCATGGCCATTGAAGAGGACATGCTCAA
GCCTGCCCATGCTTTGCACAAGACCGGTGAGGCCTGCCCGGGCTTCGGCATTCTTGCTGCGGTGATGGGG
ATCATCATCACCATGCAGCAATTGGACGGCCCCCTGAGTAACATTGGCGTTCATGTCGCTGCCGCTCTGG
TGGGAACCTTTATCGGGATTTTTCTCTGTTACTGCTTGCTGGAGCCTGTCAGTAACGCAATGGAAGAGAT
GGTCAGGCGTCGTATTAGCCTGTTGATGTGTATCAAGTCGATCCTGATTGCCCAGTTGCGTGGCAAGACA
CCGCTGCTGGCCGTCGATTCAGGTCGCAAGATCCTGGAGCCTGAAGTCAAACCAAGCTTCCTTGAACTGG
AAGATTGGGTTAACAACAGGGCAATGTAATGCGCAATCACGAGCAGATTATCGTCAAGCGCCAGCAGCGA
CGTAATAGTTCCCCTAAAGGAGGGGGCGCCTGGAAGGTGGCATTTGCCGACTTCACGTTAGCTATGATGG
CTTTCTTTATGGTGCTCTGGATCCTGGCGGTATCTAACCAGGAGGAGCGGGAAATTGTTGCCAGCCGTCT
GCGGGACTACAGCATCATGGACAGTGAGTCCAACCCGTTTGACATACGCAATAGCCCCTATCCTGTCGAT
TTGGGGGGGAATCCCTCGGTGATCGATGAACTGAACCCCAAGTTCGATCAGCAGGGTCAGCTGATGCCCA
GGCATATGGATCGCCGTCAGAGCATTTTTTCAGATGGGCCTAACGGCGGGTACGAAAATCTGCTGGTTGG
AAAGCTCGAAGGTGAAGGCCAGATGCAGCAATTAGCTGGATTCATCAATGATCTGGCTGAACAAATGCAA
GCTAACAATAACCTCGAGATTCAGATTGTTCCCCAAGGATTGCGGATCCTGATCCGCGATGACGCTGATC
GGGAAATGTATAGCCGTGGCAGTGCCCGTATCACTCCATTTTTTCGTCACCTGTTGATGGCATTGGCCCC
TATTTTCCAGCGTATCGAGAATCGGGTCATGATTTCTGGCCATACCGACAGTACACCGTTTGCGGGTGAC
AACTACACCAACTGGGAACTCTCGTCAGACCGCGCCATGATGGCAAGGCGGGTATTGCTGGCGGGCGGCA
TGCCGTCTGATCGGGTCGCGCAGGTGGTCTCTATGGCTGATACCATGCTGGAAGATACCCGGAATCCGCA
AAGCAGCCGTAATCGGCGCATTGAATTGCTGGTCATGACAACTCAGGCAGAAACCAATCTGGAGCAACTT
TTCAGCTCACAAACACAAGCGGCAGCTAGTCATGCCAAGGAGATGATTTCTCATGGACAGCCCTGAAATC
GAGTTTCCGGTGCATACCTCGCACAATGATCCGCGCCGAAAGGTACCGCGCTGGTATCTGCATGATGGAT
CAACGCCACTTGAGTCACAAAGTTGTGGTATTGCCGCACGGATGCTGATTTCAGGTGTGTTATTTGACAA
AAGTATTGGCCGAGCCGTGATCAAGGATATCGGCCCGGGAGGCGTTGGCTTTCTTGCCCCTGCTCGCTTT
ACGTTACCTGATACGGTGACCCTTTCTCTGTCCCGCCAGATTGTTCTGGATTGTCGCATCACGCATCGTC
GGCCTATAGGTCAGCATCTTTGTTTCTATGGCGCTTGCTGGTCATATCCGGAAGAAGTCGATTTGGGACC
AATATTGAGCCAGTGGCGCCACTGCTTCATGGTGCCGCAATCAGCGGCCGCTTCTGCTGAATCCCAAGAG

GGTCAGGCAAGCGTTCCAGCCTGATCTCGCCTCGACTCATATCAAGCCCCCTGGCAGCTTTCAGCAACAG
GGGATCATGCAGACTGTCCAGTGAGACCGCTTTGACAATGGCGGTCGCCCCTTTCTTCCGGTTGGCTGTG
GGGGCCTTGCCCTTGCTATAGACGATCGGGCAATTGGTTATCCCTTGCGCGACAGGGAGTTGCCAGGCCG
GATTCCAGCGGCTGGCGTCATTCATTGATACGCCCTTTGCCTGAGCCCACGCGGGCAGTGAGCAGCCGTT
CGGATGACGGAATATTTGCAAGTCATTGCCGCTGTGCCATGGCGGAAGTTGAAACTGATAGCTGTCAGCC
ATATCAAGCAGTCTTGCCAGCGCAACAAAGCGCGCGACATAAAAGCGTGTTTCAGTCGGAAGCTCCAGTT
CCCATATATTACGTGTCCCCGCTTTTAAAACCGCATTGAGTACCCGCCCTTCTCCTGCGTTGTAGGCGGC
CAAAGCAAGCAGCCAGTCATTGCCAAAGAAGCGATACAGCCATGCCAAATAGGAAAGGGCAGCCTCTGTT
GCCAATGGCAGTGAATAGCGTCCATCAAATTCGCTGGTGATGGGAATGGCAAATCGGGTGGCGGTATCTG
ACATCAGTTGCCAAGGGCCTGCGGCGCCTGCAGCAGAGACCACATCCAATCGGTAACTGCTTTCCAGTAA
AGGAACAAAGGCAAGCAAGGGCGGCAGTTTGCGTTCGTGAACCTGTTCCGCGACCCAGCGAATAACGGGC
TGTGTCTCCGCAAGACGTTGTTGGAGATAAGGGCGCTGCTTCAACAGGAATTGTTGCTGCTGGCTGACCA
AGTCGACCCCTTTTCCTTGGATCGGGATTGGGAAACCAGCTTGCCAGTGATAATTGTGATAAGCATGGCA
AGATGTTGATATCATTAACATCCCGCTCATTAGCAAAAAAACGAGCCGGGTCATGGGGCAATTGTTGAGC
AAGGATCGCGCAATAATAAAAACATGATATATCTCAACAGTTTTTTGAATTTAAGGTCGTCTGATCATGT
GTAATCCAGGACGTAGCAAGCATGATAACACGAGTAAAAAAGAGCCCAAATTCGGGTAAAGCGATTCAGA
AGTTTTTGCTGTATCTGATACTCGCCAATTTCATTCTGATGGGCTTGTACATTGGTTACGCTCAAGAGCT
GGCATTTGCCACCAATATTCTGCTGACATCCCTGCTGATTATTTACAATCTGTTGCTTTGTCAGCTGGTG
TCTCTGCGTGCTCGCCGTTCGAAAGATGGTTACATCATCTATCCAGTGATATGTGTCAGTATTTTGCTCT
GTGCAGTGGTATTGTTCCGTTTCATCATATCGCCCAGATAGTGACCGCTCACCGACTTGTTGCTTATTAA
TCACTTTCTGTTTTTTCAGCAATAACTCTTGGAAAATCAACCCACTCAATCTGCGGTATGCAATTGCTGC
GTCAATATGTTCCCTCTGTCCCATCTCCTTGCTTGAAAAGCCCCCTGCACGTCCTTAAACTTCAAGGTGG
GGAAAAGTGGATCAAAGTGGTTTTTAGAGGGGCAATCTGCCTGCTCACTCACCTTCAGGGACTGCAATTT
GTTGCGTGGCGCTCATGCCATCAGCCTGGACAGTAAAGGGCGACTGGCAATTCCGACCAAATTCCGTGAT
TGGCTGCGCGACGAAAGCGACGGCCAGCTGGTCTGCACCATCGATATTGCCCATCCCTGTCTGCTGCTTT
ATCCCCTGAATGAATGGGAAGAGGTCGAGCGCAAGCTCAAGACTCTCTCCAGCATGAATCCGCAAGAGCG
CCGTTTGCAGCGTCTGCTGCTGGGACATGCAACCGAATGCGAGCTGGATGGCAATGGCCGTCTGCTGCTC
AGTCAGCCACTGCGCAACCATGCGGGGCTGGACAAGAAAATCATGCTGGTGGGCCAGCTCAACAAGTTTG
AGTTGTGGGATGAAGCCCGCTGGCAACAACAGGTCAATGACGACATCTTGGGTCTGCCCGGGGACGATTG
GGCAAGCTCACCACGACTACAGGATTTCTCTTTATAAATGACCCAAGCCGCTGAACACATCACAGTGCTG
CTGCACGAAGCCGTTGAAGGTCTGGCCATCAAGCCGGACGGTGTCTACGTCGACGGTACTTTTGGCCGTG
GTGGTCATTCTCGTCTGATCCTGCAGCACCTTGGCCCCAATGGCCGCCTGATCGCCATCGACCGCGATCC
CCAGGCGATCGCCGAAGCAGCCAAGATCCAGGATCCCCGTTTCGAAATCGTGCACGGCCCCTTCTCCGGC
ATCGCCTCCTATCTGGATGAGCGCGGTTTGCTGGGCAAGGTGGATGGCTTCCTGCTGGATCTGGGGGTCT
CTTCACCCCAGCTGGATGATGCCGAGCGTGGCTTCAGCTTTATGAAAGATGGTCCGCTCGATATGCGGAT
GGACCCCACCTCCGGTCAGAGCGCCGCCGAGTGGCTGGCCAAGGCCGATGTGGATGACATCGCCTGGGTG
CTGAAGACCTTCGGTGAGGAGCGTTTTGCCAAGAAGATCGCCCGTGCCATCGTGCACGACCGAGTGACCG
AGCCTTATGTGCGTACCCGCCAACTGGCGGAGATGATTGCCCGGGTCAATCCGAGCAAGGAGAAGGGCAA
GCATGCGGCAACCCGCAGTTTCCAGGCCATTCGCATCTATATCAACAGCGAGCTGGACGAGATCGAAACC
GCGCTCAACGGAGCATTGGAAGTGTTGGCACCACATGGCCGTCTGTCGGTTATCAGCTTCCATTCGCTGG
AAGACCGCTTGGTGAAGCATTTCATCCGCAAGCACGAGAAGGGACCGGAAGTGCCTTACGGCATTCCCTT
GACCGAGGCCCAGCTGGCAGGCGGTCGCAAATTGAAGTCGGTCGGCAAGGCACTCAAGCCTTCTGAGCAT
GAAGTATCTGAAAACAGCCGGTCACGCAGCTCGGTGCTGCGGGTTGCCGAACGGCTGGCGGAGTAAGCGA
TGAGCGAAGTGCGTGTTCATCTGGCCAAGGAGATCGTTGCCGATCTCTGGCGGCACAAGCTGCAGATTTT
TCTGTCGGTCGCCGTGCTGGTGACTGCGTTTGCCGTGATCCTGGTGACCAACATGACCCGGGGTCTGACG
GCCAAGCAGAACGATCTGATGGCGGAAGAGGATCGCCTCAATATCGAATGGCGCCACCTGTTGCTGGAGC
AGGGCACCCTGGCTGAACACTCCCGGGTGGCGAGTCTGGCGATGGACAAGTTGCAGATGGGTCGTCCGCT
GGTGACCACCGAGAAGGTAATTACCCAACCATGAAACGCAAGGCTACAGCCAAAGCTGCCAAAGCGAATG
TCAAAGCGCCATCGATCTATCCGTGGCGCTTTCGCACGCTTGCGGTGTTTATCGGCATCGCGTTTGTGGG
TCTGATCCTGCGTCTGGCCTGGATCCAGGTGATCAATCCGGATCGTCTGCGTCAGGAGGGGGACATGCGC
TCCCTGCGTACCACCTCTACCCAGGCGGTGCGCGGCATGATCACCGATCGCAACGGCGAGCAGCTGGCGG
TTTCGGTACCGGTCGAGGCGGTCTGGGCTGATCCCAAGACAGTACATGAATCCGGTGCCATCAATAATGA
GCGAGCTTGGCTGGCACTTTCTGCCGTGCTCAAGCTCGACATCAATACCCTCAAACACCGCATCGCCAAC
CCCAGCAAGCGCTTCGTCTATCTGCAGCGTCAGGTTACTCCTGCGGTGGCGGAATATATCAAGGGGCTTA
AGCTGGGTGGCGTCTACCTGCGCCCAGAAGCGCGCCGCTTCTATCCGACTGGCGAAATCAGTGCCCATCT
GGTGGGGGTGACCAACATCGATGGCAGCGGTATTGAAGGGATTGAGCGCAGCTACAACGAGTGGCTGACG
GCGACACCCGGCGAAATGCGGGTGCGCAAGGATCGTCAGGGCCGGGTCATCGAGCGGCTTGGCATCGTCA
AAGAGGGCAAGAATGCCAACGATCTGCAGCTCAGTATCGATCAGCGGGTGCAGGCTCTGGCCTACCGTGC
TCTCAAGCGGGCAACCGACGAGAACAAGGCGACCTCCGGCTCCATGGTGATGCTGGATATCAAGACCGGT
GAAGTGCTCGCCATGGTCAACACCCCCTCCTATAACCCGAACAACCGCGGCCAGTATCAGAGCTTCCGGG
TACGCAACCGGGTGGTGACTGATACCTACGAGCCGGGTTCGACCGTCAAGCCATTGATTTTGCTCAGTGC

CTTGCAGGCAGGTGTCACCAGCTGGAAAGATTTCATTCCTGGTGGGCCGCTCTTTATCGGTGCCAAGCAG
ATCAAGGACGTCAGTATCCACAAGGCGACCAACCTCTACGATATCCTGCGTTACTCCTCCAACATCGGTA
TGTCGCGCATCGCGCTGCGGATGCCGGCGCAGGAGATGATCAATACCCTCAGCATGTTCGGTTTCGGTAT
GGATACCGGCAGCGGGCTGATGGGCGAGAGCAGCGGCATGCTGCCCCAGCGTCGTCGCTGGTCCGATATC
GAGCGGGCGACCCTCTCGTTCGGTTATGGTCTGCGGGTCACCCCGCTGCAGCTGGCTTCCGCCTACGCCA
CCCTGGCCAACAAGGGCAAGCGGGTGCCGCTCTCCATCGTCAAGGTGACTACGCCGCCCAAGGGGGAGCA
AGTTATTGATCACAACAACGCCGCTGCCATGTTGCAGGCGCTGGAGTATGTGGTCGACAACGCCATTCCA
AAAGCCAAGATCCCGGGCTATCGGGTTGGCGGCAAGAGTGGTACCGCCAAGGTCGCCGTCGCCGGTGGCT
ATGGCAAGGATTACATGGCCTGGTTTGCCGGTTTCGCACCCGCCAGTGATCCCCGTTTCGTCATGGTGGT
GGTGATCAACGAGCCAAAAGGCAGCGCCTATTATGGCGGCGCCGTGGCGACCCCACCGTTTGCAGAGGCC
ATGGGCGGGGTGCTGCAGCTCTTCAATATTCGTCCCGATGCGGTGCCGCCAGCGGTGCCCACCAAACTTG
CCCGTACGGAGGCTTCTCTTGTCCCGCGCACTTGATGCATTACTGCGCCCCTTCGGTATCCAGGCGCCGG
CGCTCACCCTGACCGATATCCAGCTGGATAGTCGGCGGGTTGGCCCCGGCTCGCTCTTCGTTGCCATTCG
TGGTCATCAGATGGATGGCAGACGGTTTATCGAACAGGCGGTGGCTCAGGGAGCGACCGCTGTTGTGTTT
GAGGAGGGTGGTGAATTTATCCCACCATCGACTCCGGTTCCCTGTATCGGGATGCGGGATCTGCCCGCTC
ACCTCTCTGCTCTTGCGGGCTATTTCTACGATCAGCCAGCCCAAAAATTGGATCTGGTCGGGATCACCGG
CACCAACGGCAAGAGCACTACAGCACTGCTGGTCGCCAACTGGCGCACCCTGCTGGGTGGCAAGGCGGGT
GTGATGGGCACCATCGGCAATGGCCTGTTTGGCAATTTGATCGAAGCGGAAAATACCACCGGCAGCGCCG
TGCAGGTGCAAGCCAATCTGGCTGCGCTGCAAGAACAGGGCGCGGATCTGGTGGCGATGGAGGTCTCCAG
CCATGGTCTGGTGCAGCATCGTGTTGCCGCACTGCCGTTTGCCGCTGCCGTTTTCACCAACCTGAGCCGG
GATCACCTCGACTACCACGGCACCATGGAAGCCTATGGCGCGGCCAAAGAGCAGTTGCTGCAGCTGGTCG
ATGAGTCGAATGCGGTGATCAACCGTGATGACGAATTGGGTGCCAAGTGGATTGAGAAATACCCGGCAGC
GGTGGCGTTCAGCGTTAATGGCCCGATTGAACACCATTCTGGCCGTCAATTAACGGCGCAGCAGCTCGAT
TTTCACCAGCAAGGTTTTCGCGCGCAGATTAACTCCAGCTGGGGGAATGGTGTATTATCCGCCCCCTTGC
TCGGGCGCTTCAACGTCAGCAATGTGCTGGCCGCGATGGGCGTGATGCTGGTGCTCGGTTACGACTTCCA
CGAGCTCCTGGCAACAGCGCCTCAGTTGCAGCCGGTAACCGGTCGCATGGAGTGCTTTGGCGGTGGTGAT
ACGCCGCTGGCGGTGGTCGATTACGCCCACACCCCGGACGCTCTGGAGAAGGCATTGCAGGCCCTGCGGG
TGCACTGCACAGGGCAGCTCTGGTGTCTGGTCGGTTGTGGTGGCGACCGCGATCGCGGCAAGCGCCCCAT
GATGGCCGCCATGGCCGAGCAGTATGCGGATCGCGTGATCCTGACCGACGACAATCCCCGTACCGAGGAG
CCGGCCCGGATCATGGCCGACATGGTGGCGGGTCTTCGCGATCCTGCTGCGGTGCAGGTGGAGCACGACC
GGGTCAAGGCGATTGTGCTGGCCATTGGTCAGGCGAGTAAACAGGACATCATCCTGGTGGCCGGTAAGGG
TCACGAGGATTATCAGATCATTGGAACCGACAAGCGGCACTACAGCGACCGGGAAACCGTCGCGGCCGCC
TTGCACACATTATTGGAGTCATTCAAATGATGACCCTCAGGCTTGCCGAGCTGGCGCAGGTACTCGATGC
CCGTCTGATTGGTGACGATGGCGAGATCACCGTCGTCAGTACCGACACCCGCACTCTGGGCGCGGGTGCC
CTGTTCGTTGCCCTGCGCGGCGAGCGCTTCGATGCCCACGATTTTTGCGAGCAGGCCGTTCAGGCAGGGG
CCTCTGCCCTGCTGGTCGAGCGCGAACTGCCGCTGGCAATCACCCAGCTGGTGGTAGCCGATACCCTCAA
GGGGCTGGGCCGACTCGGCAAACTGGTGCGTGAGCGCATCAACCCGAAAGTGCTGGCCATTACCGGCAGC
TGCGGCAAGACCACGGTGAAGGAGATGGCAACCGCCATCCTGCGCAACGAAGGGGAAGTGCTGGCCACCG
CTGGCAACTTCAACAACGAGGTGGGCGTGCCGCTCACCCTGCTGCGCCTCGAACCGCAACACAAGTTTGC
GGTGATGGAGCTGGGTGCCAACCATCCGGGCGAGATCGCCAGGACTACTTCACTGGCCAAACCCGATGCC
GCCATCATCAACAATGTGGCGGCGGCCCATCTGGAGGGCTTCGGCTCGCTGGAAGGGGTGTTTCACGCCA
AGAGCGAAATCTTTGACGGCTTGAGCGAGGGTGGCACCGCCATCGTCAATGCCGACAACGAATTCTGGCC
GAAGTGGCGGGAACGCGGCATTCAGTGCGCCTTCTCCATCGACGATCAGAGTCAGCCGTTCCACGCGCGT
AACCTTGTGCTCAACGAGCAAGGCTGCGCCGAGTTCGTGATGGTGACCCCGCAGGGCGAGCTGAGCCTGA
CTCTGGCGATCCCGGGTCGCCACAACGTGGCCAATGCGTTGGCTGCCGCCGCGGGTTGTCTGGCCCTCGG
CGCATCCCTCGACTCCGTCAAGGCTGGGCTGGAACAGATGGCGCCGGTCAAGGGGCGCTTCTGCGTCCAG
CGCTGGGGCGGTCTGACTCTGGTGGATGATACCTACAATGCCAGCGTGGAGTCCGTGCTGGCGGGCATCG
ATGCCCTGACCGCCATGGGCGGCTTCAAGGTGCTGGTGTTTGGTGATATGCGGGAACTGGGTGAGGAGTC
TGCCGAGCAACACGCCCGGGTTGGCCGTCATGCCCGCGAGCGCGGTCTGGATGCCGTGCTGACAGTGGGG
GAAGAGAGTCGCCACACCGCAGATGCCGCGGCTGGCCGACATTTCGATAACAAGGCGTCGTTGTTTGAAG
TGCTTGCGCAAATGATAAATACACATCAGGAAATTTCGATTCTGGTCAAGGGGGCCCGTGGCTCCCGCAT
GGAAGATATCGTCGAGATGGTCAAAGGCCATCAGGAGTCAGCCACATGCTAGTCTGGCTGGCTGAACTGC
TCACCCCGCACTTCACCTTCTTCAACGTCTTCTCCTACCTGACGTTTCGCGCCATCCTGTCGATCATCAC
CGGACTGGGGGTTGCCCTCTGGATGGGGCCACGCCTGATCCGTCGCCTGCAGATCCTGAAGTTTGGTCAG
GTGATCCGCAACGACGGTCCCGAGTCCCACCTCAGCAAGCGGGGCACCCCGACCATGGGTGGCCTGATGA
TCATTGCTGCGATTTTCACGTCTGTGCTGCTCTGGTGCCGCCTCGACAACCCCTATGTCTGGCTGGTGCT
GTTTGTGCTGGGTGCCTATGGTGCCATCGGCTTTGCCGACGACTACCTCAAGGTGGTGCGCAAGAATACC
GACGGCCTGATCGCACGCTGGAAGTATTTCTGGCAATCCGCGGTAGCGCTGGCGGTGGCGGTGTTCCTCT
ATGCGACTGCAACCCATGAGGGGCAGACCCAGCTGGTGGTGCCTTTCCTCAAGGATGTGATGCCGCAGCT
TGGCCTGATGTTTATCGTGCTGACCTACTTTGTCATCGTCGGTACCTCCAATGCGGTCAACCTGACCGAC
GGTCTCGACGGCCTGGCTATCATGCCGACCGTGATGGTGGCGGGGGGCTTTGCCCTGATCGCCTGGGCTA

CCGGTAACGTCAACTTTGCCAACTACCTGCACATTCCCTACGTGCCCTATACCTCCGAGCTGGTGATCGT
CTGTACCGCCATCGTCGGGGCGGGTCTGGGTTTCCTCTGGTTCAACACCTATCCGGCGCAGGTCTTTATG
GGGGACGTTGGTTCCCTGGCACTGGGCGGCGCGCTCGGCACCATCGCCGTGCTGGTCCGTCAGGAGTTCC
TGCTGGTGATCATGGGCGGCGTCTTCGTGATGGAGACCGTCTCGGTGATCCTGCAGGTGGGGTCATACAA
ACTGCGCGGCGTGCGGATCTTCCGCATGGCGCCGATCCATCACCACTACGAGAAGAAGGGCTGGCCGGAG
CCGCGCGTCATCGTGCGCTTCTGGATTATCACCCTCGTGCTGGTGCTGCTGGGTCTTGCCACCCTGAAGG
TGAGGTAAGGCCATGGTCATCATCATCGGATTGGGCAAAACCGGACTCTCCTGTGTCGATTACTTCCTCG
GCCGTGGCGTCACGCCACTGGTGATGGATACCCGCGCCAACCCGCCCGGCAAGGATCAACTGCCGCCTGA
GGTTGAGCTCATTCACGGGCTCGACGAGGCACTGCTCAAGCAGGCGCACATGATTGTCGCGAGCCCGGGT
ATCGCGCTGGCGACCCCCGAACTGAAAGCGGCTGCCGAGTGTGGTGTGCAGATCGTCGGTGATATCGAGC
TGTTTGTGCGCGAGACCCAGACCCCGGTCATCGCCATCACCGGCTCGAACGGCAAGAGCACCGTTACCAC
CTTGGTGGGCGAGATGATTGCCGAAGCCGGTCTCAATGTCGGCGTGGGCGGCAATATCGGCACGCCTGCG
CTGGAGCTGCTCAAGCAGCCGATGGATCTCTGCGTGCTGGAGCTCTCCAGCTTCCAGCTGGAAACTACTC
ACAGTTTGCGTGCTGCCGCCTCCGTAGTGCTGAACCTCTCAGAAGATCACATGGATCGCTATCAGGGCAT
GGCCGACTATCGCGCCGCCAAGATGGAGATCCATCAGCATTCCAAACATATTCTGGTCAATCGTGACGAT
GCCCAGACCCTGCCCGATGTGGGTCAGGTGTGGCAGAGCTTTGGTCTCGATTGCAATGGCTATGGCCGTT
GCCTGCTGGATGGCCGCTACTGGTTGACCCTGCACGGCGAGCCGCTGCTGGCGGTGGACGAGATGAAGAT
CGTCGGTGCACACAATCAGGCTAACGCGCTGGCCGCCATGGCGCTGTGCGATGCGGTCGGCATTCCCCGC
GCTGCCCAGCTCACCGTGCTGCGCCGTTTTGAAGGCTTGCCCCACCGTGCCCGTTTCGTACGGGAGGTGA
ACGGGGTGCGCTGGATCAACGACTCCAAAGCCACCAACGTGGGGGCCACGCTGGCTGCCGTTGCCGGTGT
GCGCGAGTCGGTCAAGGGGCGCCTGCTGCTGCTGGCCGGTGGTCAGGGCAAGGGGCAGGATTTTGGCCCG
ATACAGGCGCTGCTGGGTAACCAGATCGATCACATGTACTGCTTCGGTCAGGATGCCGAGGTGCTGCTGG
CACTGGGCGAGCAGACCGAACGGGTGGCCGACCTGGCCGCTGCGGTTGCCAAAGCGGCGGCCGATGCCAA
GCCCGGTGACTGGGTGCTGCTGGCGCCAGCCTGTGCCAGCCTCGATCAGTTCAAGTCGTTCGAGGTGCGC
GGCGATCGTTTCACCGAGATGGTGCTGGCACTATGAACGCCCTTCGCGCAGCAGCAGGTTTGTTCCAGCG
CTGGTTGTTACCGGCGCGTCCTGCCGGTCTCTACGATCGGCAGCTGGTACTGCTGGCGCTCTCCCTGATG
GCGGTCGGGGTGGTCATCGTGGCCTCGGCGTCGATCCCCGAGGGGATTGCGCTGGGGGATGACCCCTTCA
TGTTTGTGAAGCGTCACGCCCTGTTTCTGGTGATGGCGCTCGGTATCTCCTGGTTTGTGTTGCAGGTGCC
GATGGCGCGCTGGCAACAGCACAACGGCCCCATGTTGCTGCTGGCGATCTTGATGCTGGTGCTGGTGCTG
CTGGTGGGCCGCAACGTCAACGGTGCGGTGCGCTGGTTGCCGCTCGGCCCCTTCAATCTGCAGCCTGCCG
AGTTTGGCAAGCTGGCGCTGTTTGTCTATCTGGCCGGCTATCTGGTGAGGCGCCAGAGCGAAGTGCGTGA
AGCCTGGATCGGCTTCCTGAAACCGCTGGCGGTGTTTGGGGTGCTGGCGGTCTTGCTGCTGTTGCAACCT
GATCTGGGTTCTACCGTGGTGATGTTCGTTACCTCGTTCGGCATGCTGTTTCTGGCCGGTGCCCGATTGG
GACAGTTTTTGACCCTGATCGGCGCGGGCCTCGGTTCGGTGGTGATGCTGATCATCGTCGAGCCCTACCG
GATGCGGCGGGTGACCTCCTTTATGGATCCTTGGGCCGATCCGTTCGGCAGCGGCTACCAGTTGACCCAG
TCGCTGATGGCGTTTGGTCGTGGCAGCTGGTTTGGCGAAGGGCTTGGCAACTCCATCCAGAAGATGGAGT
ACCTGCCGGAAGCGCACACCGACTTCGTGTTTGCGATCCTCGGTGAAGAGCTGGGTTACGTCGGGGTGCT
GGGCGCCCTGTTCCTGATTTTTGCGCTGGCGGTCAAGGCGCTCAAGCTGGGGCATCGCGCGCTGGTGGCT
GAGCGGCTCTATGACGGTTATCTGGCCATTGGCATCGGCATCTGGTTCAGCTTCCAGACCTTCGTTAACG
TCGGCGCGGCCAGCGGCATGATGCCGACCAAGGGCTTGACCCTGCCGCTGGTGAGTTACGGTGGTTCCAG
TCTCATCATCATGTCGGTGGCGGTGAGCATGTTGATTCGAATCGATTTTGAGTTGCGTCAGGCGACAGCC
CAGGCGCGCGTGCGGGAGGTTTAGTGAGCAAGACTCTGTTGGTGATGGCAGGTGGTACCGGTGGGCATGT
GTTCCCCGGTCTGGCCGTGGCCGATCGCCTGAAGGCCCAGGGCTGGACTGTCCACTGGCTCGGCACTGCC
GACCGGATGGAGGCGGAGCTGGTGCCCGCCCACGGTTATCCCATCTCCTTCATCGATATTCAGGGAGTAC
GTGGCAACGGCATCAAGCGACTGCTGGTAGCGCCCTATCGCATCGTCAAATCGATCCTGCAGGCGCGTCA
GGTACTCAAGACCGTTCGCCCTGATGTGGTGCTCGGCATGGGCGGTTTTGCTTCCGGCCCGGGTGGCGTG
GCGGCCTGGTTCTCCGGCATCCCGTTGCTGCTCCATGAGCAGAATGCCGCAGCCGGCATGACCAACAAGC
TGCTGGCCCGTATCGCCAGGCGGGTGCTGATGGCATTTCCGGGGGCCTTCGCGCCGAGCCGTCGTACCGC
CGTGGTGGGCAATCCGGTGCGTCCCGAAGTGGTCGCCCTGCCGGATCCGCAACTGCGCAGCGGAGATGAG
CCGCTGCATCTGCTGATCGTGGGTGGCAGCCTGGGGGCGCGGGTGCTCAACGAGCAGGTACCTGCTGCGG
TGGTGGCATCCGGCGTGCCCATTGAAGTGCGTCACCAGTGTGGCAAGGGCAATGCTGAGGCCGTCTCTGC
TGCCTATGCCCAGCACGGCATCAATGCCGAGGTGAGCGAATTTATCAAGGATATGGCCAGCGCCTACGCC
TGGGCCGACCTGGTGGTCTGCCGTGCCGGTGCGCTGACTGTTTCCGAAGTGGCTGCTGCCGGTGTGGCCG
CCATTTTTGTGCCACTGCCCCATGCGGTGGATGATCACCAGACCCGTAACGCCCTGACCCTGGTCGACGG
CGGAGCGGCCGAGTTTCTGCCCCAGTCAGAGCTGAACACCGCCTCACTGGCGGCGCGCCTGACCTGGCTG
GCTGGTCGCCGGGAGACCCTGTTTAACATGGCGAAAGCGGCTCGTCGCGTCGCCATCACAGATGCGGCCG
AGCGGGTTGCTGATGAGTGCAAACAGCTCGCAACCGGGCTGCGTGGTAAAGATTGATGAGCAAGGTTGCA
GCGGTGACACCGTGCAGCCTTGTACCTGTATGTATTGATGCCGCCGTACAGCCGCGCTTTGACGGCACAT
GGCCTGCGGCAGACTAAAGAGACGATTATGACCAAGGTTGAACTGGCAAAACTGCGTACTGTGATCCCCG
AAATGCGCCGCGTGCGTCGCATCCACTTTATCGGCATCGGTGGTGCCGGTATGGGCGGATCGCCGAAGT
GCTTGCCAACGAAGGGTATCAGGTAACGGGTTCTGATCTGGCTCGCAACGCCGTGACCGAGCGGTTGGCC

GGACTGGGCGCGCACATCTTCGTCGGCCACAGTGCCGAACACGTTGAGGGTGCCAGCGTGGTGGTGGTCT
CTACCGCCATCAAGCAGGACAACCCGGAGCTGCTGGCCGCCCGCGAACTGCGGATCCCGGTCGTGCGCCG
CGCCGAGATGCTGGCCGAGCTGATGCGTTTCCGCCACGGTATCGCCATTGCCGGTACCCACGGCAAAACT
ACCACCACCAGTCTGGTGGCGAGCATCTATGCCGAAGCCGACCGCGACCCGACCTTCGTCATCGGTGGCC
TGCTCAACAGTGCCGGAACCAATGCCCGTCTGGGCAGCAGCCGCTACCTGATTGCCGAGGCGGATGAGAG
CGACGCCTCTTTCCTGCATCTGCAGCCGATGGTCTCTATCGTGACCAACATCGAAGCGGATCACATGGAT
ACCTACGGCGGTGATTTCTCCAAGCTGAAGGCCACCTTCATCGAGTTTCTGCACAACCTGCCCTTCTACG
GACTGGCGGTGGTCTGCATCGATGATCCGGTGATCCGTGGCATGTTGCCCGAGATTGCCCGTCCGACCCT
GACCTATGGTCTCTCTGATGATGCCGACGTGCAGGTGCTGGATTTTGTCCAGACCACCAACCACAGCTGC
TTCCGTGTTCGCCGCAAAGAGGCCGGTGAGCTGCAGGTGACCCTCAATTTGCCGGGTATTCACAACGCAT
TGAACGCCGCAGCGGCGATAGCTGTCGCGACAGAGGACGGTGTCAGCGATGAGGCCATCATCTCTGCTCT
GGCCAAATTTGAAGGGGTTGGTCGTCGCTTCCAGCAGTATGGCGAGTTTGAAACCGGTCGCGGCAAGGCG
ATGCTGGTGGACGATTACGGTCACCACCCGAGCGAAGTGAAGGTCACCATCAACGCCGCCCGTGCCGGGT
GGCCCGAGAAGCGCCTGGTGATGGTGTTCCAGCCGCACCGTTATACCCGCACCCGCGACCTCTACGAGGA
CTTCGCCGATGTGCTCTCCAAGGTAGATGTGCTGGTTATATTGGAGGTTTATGCTGCCGGTGAGGAGCCG
ATCCCGGGCGCTGATGGACGGGCACTGTGCCGCTCCATTCGCTCGCGTGGCAACCTGGAACCCATCTTCG
TCGCCACACCGGATGACGTGCCAGCCGTGCTGGCCGACCTGATCGGCGAGGGGGATTTGGTGCTGACTCA
AGGCGCCGGGAACGTCGGGGCACTGGCCCGTCGTCTCGGAGAGATGAAATTGAGCATAGATAGCATGAAA
TCTGGCGCCTGAGGGCCCAATGAGTATTTTGACCGCCAAGCCGGGGCCTATATAATGGCGAACCGGTCTG
TCAAAGGCCGGTTTAGCGCGTTACACGGGGATCGATCGGGTGGAGGCAAGGGCACAGGGGCGCACCAAGG
GTGGCTTCATCGCAGGGGTAGCGTTTTTCCTGCTGGTGATATGGGGCTGCTACCGGACGGCGCTGGATGT
TAAAGGTTGGCTGACAGATGCCAACCGGTTGCCCATGAGCGAGCTGTTGCTGCAGGGGCAACACCAGTAT
TTGCAAACCGAAGAGCTGCGGATGGCCGTGCTGGATGGCGCCGAGCTGCGCAACTTCTTCGAATTGGACG
TCAACGAATTGCAGGCAAGGCTCAATGCCTTGCCATGGGTGGCCTCGGTCTCGGTACGAAAGAAGTGGCC
GAACAAGATCAAGATCTACCTGACAGAACAGGACGTGGCGGCGCGCTGGAATGGCAACCGCTTCGTCAAC
ACCATGGGCAAGGTATTCAGTGCCCCGGATCGGGTCAAGACGCCGCTGTTGCAGCTGTCAGGGCCGGATG
ACCAGGCCGCCCGGGTGTTGAAAGAGAGTCGGCAGTACGAGTCGCAACTGGCTGCCAAGGGATACAAGTT
GCTGGGGGTGAATCTGACCCCCCGCCATGCCTGGGAACTGACCCTGGATGGCAATATCAAGCTGTTCCTG
GGACGCAATGACATTGCGCTCCGGCTGCAGCGGTTTATCGATGCCTTCCCGGTTATCGAACCCCGTGCGC
AAGTCGCCTACGTGGATCTTCGATACGACACCGGAATGGCAGTTGGCTGGAAGAAGAGCGAAGAGAAAGT
GCATGACCAACACCGCAGATAGGAATTTGATTGTCGGACTGGATATCGGCACAACCAAGGTGGCGGTTCT
GGTCGGGGAAGTATTGCCGGACGGTGAACTGAACGTCATCGGTATGGGCAGTCACGCTTCTCGCGGAATG
GAGAAGGGCGGGGTCAATGACCTCGACTCCGTGGTCAAGTCACTGCGCCGCGCGGTGGAAGAGGCCGAGA
TGATGGCAGATTGCCAGATCAGCTCGGTTTACCTCGGTTTGTCCGGCAAGCATATCGACTGCCGTAACGA
GACCGGCATGGTGCCCATCTCCGATGAAGAGGTGACTCAGGAGGATGTGGACAACGTGATCCACACCGCC
AAGTCGGTTCGCCTCTCCGACGAGCTGCGCGTGCTGCATGTGCTGCCTCAGGAGTTTGCCATCGACTATC
AGGAAGGGATCAAGAATCCGGTTGGCCTCTCCGGTGTGCGGATGGCAGCCAAGGTTCACTTGATCACCTG
CCACAACGACATGGCCCGCAACATTGAGAAATGCGTGGAGAAGGTCGGCTTGCGTGTGGATCAACTGATC
TTCTCCGCGCTGGCCTCCAGCTATGCCGTGTTGACCGAAGACGAGAAAGAGCTGGGTGTCTGCGTGGTGG
ACATCGGTGGCGGCACCATGGATATGTCGCTGTTTACCGGCGGTGCCCTGCGCCACACCAAGGTGATCCC
GTACGCTGGCAGCACTGTGACCAGCGACATCGCCTACGCCTTTGGCACGCCGCCGCTCGACGCGGAAGCG
ATCAAGGTGCGCTACGGTTGCGCCCTCGGCCGTCTGGTCAGCAAGGAAGACAATATTGAAGTACCCAGTG
TCGGTGGTCGCCCGGCGCGCAGCCTGCAACGGCAGACACTGGCTGAAGTGATTGAGCCCCGTTACACCGA
GTTGCTCTCCATGGTGAATGATGAGCTGGTGCGGGTACAAAGCGAGTTGAAAGCGCAGGGGGTCAAGCAT
CAGCTGGCAGCCGGTGTGGTCTTGACCGGCGGCGCTGCCCAGATTGAAGGCATTGTGGAGTGTGCAGAGC
AGATCTTCCAGAGCCAGGTCCGGGTGGGAGAGCCCATGAACATACGTGGTTTGAGTGATTACGTGCAGGC
ACCGGTGTACTCGACTGCGGTCGGGTTGCTGCAATATGGCCGCACCCATCAAGCCACAGGTAGCAACAAA
GAATATGCCGCCAAGGCAAGCGTCGGTGGCCTGGTCAAACGGGTTAGCAACTGGCTGCGTGGCGAGTTCT
AAGAATTTTGCTGAGTCAGGCAAATAAAGCGGAGAGACAACATGTTTGAATTTATGGATAGCCACACTGA
CGAAGCCGTCATTAAAGTGATCGGTGTAGGTGGCGGCGGCGGTAACGCTGTCGAGCACATGGTTCGTCAA
AATATTGAGGGTGTTGAGTTCATTACCGTCAACACCGACGCCCAGGCACTGCGTAACTCCAGCGCCAACA
CTACCCTGCAGATCGGCGGTGGCATCACCAAGGGTCTGGGGGCCGGCGCCAATCCGGAAGTGGGTCGTGA
TGCGGCCATGGAAGATCGCGAAGCGCTGCGTGAGCTGCTGACCGGTTCCGACATGGTGTTCATCGCCGCC
GGTATGGGTGGTGGTACCGGTACCGGTGCTGCGCCGATCGTGGCCGAAGTGGCGCGCGAGATGGGCATCC
TGACCGTTGCTGTGGTGACCAAGCCGTTCTCCTTTGAAGGGAAGAAGCGGATGGGCTTCGCCGCCCACGG
TATCGAAGAGCTCTCCAAGAACGTCGACTCCCTGATCACCATCCCCAACGACAAGTTGCTGAAGGTGCTG
GGTCGCGGTATCTCCCTGCTGGATGCCTTCAAGGCGGCCAACGATGTACTGCTTGGCGCGGTACAGGGCA
TTGCCGAGCTGATCACCCGTCCGGGTTTGATCAACGTCGACTTCGCGGACGTGCGTACAGTGATGCGTGA
AATGGGTACCGCCATGATGGGTACCGGTTCCGCCTCTGGTGATGACCGTGCCGAAGAAGCGGCCGAGAAA
GCCATCTCCAGCCCGCTGCTGGAAGACGTGGATCTGGCCGGTGCCCGTGGCATCCTGGTCAACATCACCG
CCGGTATGGACATGACCATCGAAGAGTTCGAAACCGTGGGTAACGCGGTCAAGGCCTTCGCTTCCGAGAA

CGCCACTGTAGTTGTGGGTACCGTAATCGATCCGGAAATGCACGACGAACTGCGTGTGACCGTGGTTGCT
ACCGGTATCGGTGCCGAGCGCAAGCCGGACATCACTTTGGTCAAGAGCAGCCAGGTGCAAGAGCGCCCGG
TGCGTCAGCCGCTGGTTAGCGACATGATGCAAGGCCGCGTGATGGACGAAGCCCCGGCCAAGGTGGTCAA
CGCTGAGCCGCAAGCTCGTCGTGAACCGGATTACCTGGATATTCCGGCGTTCCTGCGCAAGCAAGCTGAC
TAAGCCTCTTATGGCTCTCGTTTGTTTGTTGTTCAAGGAATGTGCTAAGATGGCCTGCCAACGACCATCT
GGTCCGGATTTTAAGCTGTTTAGGGTACACCCATGATTAGACAAAGAACCTTGAAAACGAGTGTACAGGC
GACTGGTGTCGGCCTGCATTCGGGCCGGAAAGTCACTATGACGTTCCGTCCGGCGCCTGTGAACACAGGG
ATCGTTTACTTGCGTACCGACCTGAATCCTGTGGTGGAGCTGCCAGCCAATGCCGATCAGGTGCGCGATA
CCGTGCTCTGTACCGCTCTGGTCAACGAAGCCGGTGTCCGTGTCTCTACCATTGAGCACCTCTCTGCCGC
GCTGGCCGGGATGGGGATCGACAACCTCTATGTTGAGGTCGACGCCCCCGAGATCCCGGTGATGGACGGC
AGTGCGCATCCCTTTATCTACCTGCTGCAGGAAGTCGGGATCCGTGAGCAGAATGCTGCCAAGCAGTTTG
TGCGGATCAAGAAGAGCATCCGGGTTGAAGACGGCGACAAGTGGGCGGAATTCCACCCCTATCGCAACGG
CTTCAAGATGGATCTCGAGATAGATTTCCAGCATCCGGTCTTTGAAGGGCGCAATCAGCGTTGCGTACTG
GATTTCTCCGGTGCCTCCTTCGTGAAAGAGATCAGCCGTGCCCGTACCTTCGGGTTCATGCGTGATATCG
AGTACCTGCGTTCCCAGAACCTGGCGCTGGGCGGAAGCCTGGAAAACGCCGTGGTACTGGACGACTATCG
GGTGCTGAACGAAGACGGTTTGCGTTATGAGGACGAGTTCGTCAAGCACAAGATGCTGGACGCGATCGGT
GACCTCTATATGTGTAACCGCAGCATCATCGGTGAGTTCCGTGCCTACAAGACCGGACATGCCCTGAACA
ACAAGCTGCTGCGTGCCTTGCTGGCCGAGCAGGAGGCTTGGGAACTGGTGACCTTCGAGGGTGAGGGCGA
CAAAGCGCCTATCGCTTACTACGATAACGGTCTGGTTCTGGCCTGATAGCTGTGCAACGGAACGTCGGTC
CTCTCTCCGCACCGACGTTCCGTCATTTGTTTACTCCATCCTTTAATACCGATTATCTCCCGGTTTTATT
TCCCGTGCAGGCTCTTGGCCGCACCTGACCGAGCAGACTGATTTCTATGAGCATTACCCTGATCCTCCAC
GGCAAGCAGCGACGCAAGTTGCACCTGCCCAAACAACGTCTGGCCTGGGTCGGCGGTATTCTCTTCTCCC
TGTTGGCCCTTGGTGGTGGTTGGCTCTGGCAGAGCTGGCACCAGAAAATGGAGGCGCTGGAAGTGGCGCT
TGCCGTTGCCGAGCAACAGTCCGTTGACCAGAGTGGTGACGAAGCCCGTGAACAGTTGGCGATGCTGGCT
GCTCAGGTGGGCACCATGCAGGGTCAGCTTGGCCGTCTCAATGCCCTCGGTGAACGATTGACCGAGCAGG
CCGATCTCGATCCCGACGAGTTCAATTTCAAAGAGCTGCCTCCGATGGGCGGCCCTTCTTATGATGAAGA
GTTGGAAGTGAGCCTCAACGAGCTGCAGGCCTCGATGAAACACCTCAGCCAGCAGCTCAGCAGCCGCGAA
GAGCAGCTGTCGGTACTTGAATCTTTCATCATGAATCACCATATCACCGATGCCGGTTTTATTTCCGGCA
CGCCAGTCAAACAGGAGCGAGTTTGGGTCTCGTCCGGGTTTGGTGGACGGGTCGATCCATTCAACGGGCG
TGCCAAGATGCACAAAGGGGTCGATTTCCGTGGCAAACCCGGAACCCCGATTCTGGCCACCGGGCCCGGC
ATTGTCAGCTGGGCGGGCCGTCATCCGGAATTTGGCAATATGGTGGAGATTAATCACGGCAATGGCCTGG
TCACCCGCTATGCCCACAACTCCAAGTTGCTGGTCGAGGTGGGCACTCTGGTTGATCAGGGTCAGAAGAT
TGCCTTGATGGGACGCACCGGTCGCGCGACCGGGGTCCACCTGCATTACGAAGTGTTGAAAGATGGTCGT
CAGGTCAACCCCGCACGCTTTCTCAGTGCCAGCCGGGGTTAGGTTTTTTTCTTCAGGTTCCAGTGACGGA
ACGCTTTTATAACCAGAAATAGTTGGTACCCATGATTAGCACACTGCTTACCAAGATTATCGGCAGCCGG
AACGACAGAACGCTCAAGGCTTTGCGCAAAATTGTAAAACAGATCAATGCCATGGAGCCGCAGTTCGAGG
CTCTCTCCGATGCCGAGTTGCAGGCCAAAACGGCCGAGTATCGTCAGCGTATCGAGCAGGGTGAGACCCT
GGAGCAACTGCTGCCGGAAGCGTTCGCGACCGTGCGTGAAGCCTCCAAGCGGGTGTTTGGCATGCGCCAC
TTCGACGTGCAGCTGATCGGCGGCATGGTGCTGAACTCCAACCGCATCGCGGAAATGAAAACCGGTGAAG
GTAAGACTCTTACTGCGACTCTGCCAGCTTACCTGAACGCCCTGAGCGGCCGTGGCGTGCACGTGGTAAC
GGTGAACGACTATTTGGCCAAGCGTGATGCGGAGGCGAACCGTCCGCTGTTCGCCTTCCTCGGCATGACC
GTCGACTGCAACGTGCCCGGTATGGATGCGTCCCAGAAGCGTGACGCCTACGCCGCCGACATCACCTACG
GTACCAACAACGAATTCGGTTTTGACTACCTGCGCGACAACATGGCGTTCTCGCCAGAGCAGCGCGTACA
GCGTCCGCTCAACTATGCGCTGGTGGATGAAGTTGACTCGGTGCTTATCGATGAAGCCCGGACTCCGCTC
ATCATCTCCGGTCCGGCCGAAGATAGCTCCGAGCTCTATATTCGCGTCAACAAGCTGATCCCGCTGCTGG
TCAAGCAGGACAAGGAAGACACCGAAGAGTACACCGGCGACGGTCACTACACGGTGGACGAGAAGAACCG
TCAGGCCCTGCTGACCGAAAACGGTCAGATCTTCGTGGAAGAGCTGCTCAAGAAAGAGGGGCTGCTGGAC
GAGAACGACTCCCTGTTCTCCGCCTCCAACATCAGCCTGCTGCACCACGTCAATGCGGGTCTGCGCGCTC
ACACCTTGTTCGAACGCAACGTCGACTACATCGTCCAGAAAGACGAGATCGTCATCGTCGACGAGCACAC
CGGCCGTACCATGCCGGGTCGTCGCTGGTCCGATGGTCTGCATCAGGCCGTTGAAGCGAAGGAAGGGGTG
AAGATCCAGAACGAGAACCAGACGCTGGCATCCATCACCTTCCAGAACTACTTCCGTCTCTACAACAAGC
TGGCGGGGATGACCGGTACTGCAGATACCGAAGCCTTTGAATTTCAGCAGATTTATGGCCTGGATACCGT
TGTTATCCCGACCAACAAGCCAATGGTGCGTAAAGACATGGGCGATCTGGTCTACCTGACCGCCAATGAG
AAGTATGCCGCCATCATCGAAGATATCCGTCAGTGTGTGGCCAAGGGCCAGCCTGTACTGGTTGGTACTG
TCTCTATCGAGAACTCCGAGCTGCTCTCCGGCATCCTGACCAAGGAGAAGATCCCGCACAAGGTGCTGAA
CGCCAAGTTCCACGCCATGGAAGCGGAGATCGTTGCCCAGGCAGGTCAGACTGGTGCGGTCACCATCGCG
ACCAACATGGCCGGTCGGGGTACCGACATCGTGTTGGGCGGCAACTGGCAGGCAGAGATTGCCAAGCTGG
AGAACCCGACCAGCGAGCAGATTGCCGAGCTGAAAGCCGCCTGGCAGGTGCGTCACGATGCGGTGCTGGC
CGCCGGTGGCCTGCACATTATCGGTACCGAGCGTCACGAATCCCGTCGTATTGACAACCAGCTGCGCGGC
CGTTCCGGTCGTCAGGGTGACCCGGGCTCATCCCGTTTCTACCTCTCCATGGAAGATACCCTGATGCGGA
TCTTCGCCTCCGATCGCGTGACCGGCATGATGAAGAAGCTGGGCATGGAAGAGGGCGAAGCCATCGAGCA

TCCGTGGGTCACCAAGGCGATCGAAAACGCCCAACGCAAGGTGGAAGGTCGCAACTTCGACATCCGCAAG
AGTTTGCTGGAGTTTGATGACGTTGCCAACGACCAGCGCAAGGTGGTCTACGAGCAGCGCAACGAGCTGC
TGGATACCAACGACATTTCCGAGACCATCCACGTTATCCGTGACGACGTCTACGGCGCGGTGATCGACGA
GTACATCCCGCCCCAGTCTCTGGAAGAGATGTGGGATGTGCCGGGTCTGGAAGCACGCCTGAAGGCCGAC
TTCGCGCTGGATCTGCCGCTGCAGCAGTGGCTGGCGGAAGATGACAAGCTGTATGAAGAGAAGCTGCGCG
AGCGCATTCTGGACGAAGCCACCAAGCTCTATGCCCACAAGGAAGAGCTGGTCGGCAAAGAGGTGCTGCG
CAACTTCGAGAAAGCGGTGATGCTACAAACTCTGGATGGTCTGTGGAAAGAGCATCTGGCCGCCATGGAT
CACCTGCGTCAGGGCATCCATCTGCGCGGCTACGCCCAGAAGAACCCCAAGCAGGAGTACAAGCGCGAAT
CCTTCGATCTGTTCACCCAGATGCTGGAAACCCTCAAGCGCGACGTGGTCAGCATCCTGAGCCGTGTTCA
GGTGCAGGAGCGCGATGTGGAAGCGATGGAAGAGCAGCAGCGCCAGCAGGCGGAAGCCGCTCCCCGTACC
TATACCCACGCCGCTGCCGAGAGCCAGCTGGCCGATGAACAGGCCGCAGGCGAAGAGGGCCACACCACCT
TCGTACGCGACGAGCAGAAGGTCGGTCGCAACGATCCCTGCCCGTGCGGTTCCGGCAAGAAGTACAAGCA
CTGCCACGGTCAGTTGACCTGATCGACGCTATGCCATAACAGGCGGGCCAAGTGCCCGCCTTGTTATTGG
TGGCAACAAGCGAACAGAGGTAATTGATGGAACAGAAAAAACGGATCTGGGTGGCGGTTGGCGTCATCGA
GAATGAACGGGGCGATATCTTCATCGCCAGACGCTCCTCCGATCGCCATCAGGGGGATCGCTGGGAGTTT
CCCGGTGGCAAGGTTGAGTCCGGTGAGGATCTGCTCACCGCGCTGGATCGGGAGCTGTGGGAAGAGATCG
GCATCCGGGTATTGGATTGTGCTCCCTTCATGGAACTGCAACACGACTACCCGGAGAAGCAGGTATTGCT
GGATATCTGGAAGGTGACTCGTTTCAGCGGTGAGCCGTTCGGCAAAGAGGGGCAGGAGTGCCTGTGGGTG
CCGGTGGCCGAGCTGAACAACTACCACTTCCCCGATGCCAATGGCCCGATTGTAGCGCGGCTACAGGGTC
TGCTGGCGTGAGCCGGTAGCAATCAACAAGCATGAAAAAAGGGCCAATCGGCCCTTTTTCATATTGCAGA
GATGAGTGACCAAGGGCTTACAGCCACTGATCGCCCTCTTCCGGCTCCGGCAGCAGATCCGGGTTGATCT
CCCCCGGGATCCGCTTCTCTTCATCGGCCCATTCGCCGAGATCGATCAGCTGGCAGCGCTTGCTGCAGAA
GGGGCGGAACGGACTCTGCGGGCCCCATTCCACTTCGCTCTGACAGGTCGGACACTTTACCTTGGTGACC
ATAAGCCCTCCTTAGCAGCAGGCGAGCTGGAACGGCACGTCGCCAATCGGCTGTTCTGCGGTGGGCAGAA
AGCGCAGGGCGAAACGGTTCTTGTGGCCGCTCAGGGTCGGGTAGCAGAACAGATTGCCCGGCAGCTTGAT
GCGGATCAGCTCGGCGCTTTCGGCGGTGTCCTGGAAGAAACCGTTGCTGGCAACCTGCTCACTGAAGCTG
CCTGATTCGCGCCACAGGCGCAGCAGCAGAGTGATGGCATTCATCAGCAGATTGATATCGCTCAGCCAGC
CCTGCATCTGCTGGTGACGGGTGATGGCCGGGGTACCGAGCCAGTGGTGCAGCTGCGGCACATCGAAGGC
ACACAGACCACCCGGGATGGAGAAGCGCTGGCGGATGGAGCCGAGCAGACGATCCTCTTTCAGGCGGGCA
CCCGGGCGTGGCGAGTTGAGCAGGTTGCGGGAGAGCTGGCTGCTCTCTTGCTGCATCAGTTTGATCTGCT
CCAGATCGACATCGGGCAGGCTGGCCCAGTGGCTCAGACGCTGGCCGAGACGTTCCAGATCCTTGATGAG
GTCGGCTCGCAGGTCACCACGCTCCAGCAACTCCTGAAGATCGAACAGCCCCTTGAAGAAGGCGATATGT
GCCCAGGATTGTTCACTCTCCAGATTGTCGCGGATCTGGGAAAACAGGGACTCCAGACGAAGATAGGTCC
GGCTTTTTTCATTGAGGGGGAAATCGTAAATCATGCCTAATCCTAGCCAGCCATAAACCGGCCTAGTGTA
CTCACACTTGGCGGGCTTGTTGAGAGGCAATTGCCAGATATGTCTCGTGCAGCGCCAAAATCCGCGTTTT
AATCGCCATTTCGCTGCCATTGTTGTTTTCGATGACATCATCCGCCGCAGCGAGACGCTCCGACCGGCTC
GCCTGAGCGGCGATGATGGCCCGGGCCTGCTCGGCCGATACCCCATCCCGGCGGCAGGTACGCTCAATCT
GGGTGGCTTCGTCCACATCGATCACCAACACCCGGTTGGCGAGGCCGGTGAGTTTGTTCTCCACCAGCAG
TGGCACCACCAGCAGGCAGTAAGGAGAACTGGCCGCGGCACATTGGCGCAGCATTTCGCTGCGAATCAGC
GGGTGGAGCAGGGCATTGAGCCACTCCTTGGCCTGTGGATCGCTGAAGACCCGCTCGCGCAGGGCACGAC
GGTCGAGTTGACCGCTCGGATCAATCACATTCGGGCCAAAATGGTCGGCGATGGCGGCAAGGGCCGGGGT
ACCCGGCTCGACCACTTCGCGGGCGATCACATCGGCATCCACCACCTCGATACCCAGCGCGGCAAACTGA
TTGGCGATAGTGGTCTTGCCGCTGCCGATACCACCTGTGATAGCAACTACATGCATGGGGGCCTCTTGCT
CAAAGCAGGGTAGAGAGATAACAGCGGGTGATGGTATCGCCCCATAGCAGGGCGATCCAGCCCGCGATGG
CGAGGTAGGGGCCGAAGGGGATGGGCTTGCCCTGATGATGTTTTTGCAAGGCGATCAGGCTGATACCGAT
CACTGCGCCGACCAGGGATGAGAGCAGCAGAATAATGGGCAGCGCCTGCCAACCGAGCCAGGCGCCCAGG
GCTGCCAGCAGCTTGAAGTCGCCATAGCCCATCCCCTCCTTGCCAGTCAGCAGCTTGAAGGCCCAGTAGA
GACTCCAGAGCACCAGATAGCCCGCCATTGCGCCAATTACGGCATCAGCCAGCGGCACGAAGCCACCTGC
CAGGTTGAACAGCAGTCCGCCCCACAGCAGTGGCAGGGTGAGCTGGTCGGGCAGCAACATCTTGTCGAGA
TCGATAAAGGTGAGGGCGACCAGCACCCAGGTGAGCAGCAGAGCAGCCAGCGTGCCCCAGCCCGGCGTTA
TGACCATGGCAACGGCCACCGAGAGCAGGGCGGTCAGCAGCTCCACCAGCGGATAGCGGGCCGAGATGGG
GGCCTGACAGTCGCGGCAACGCCCTTTGAGCCAGAGCCAGCTCAGCAGCGGGATGTTCTCCAGCGCAGTG
ATGGCGTGGCCACAGTGAGGGCATGCGGAGCGTGGCACCATCAGGTTGTAGCGATCTGCAACAGTGGCCT
CCTCATCGGGGGAAAAGTAGCCGCGATACTCGGTCTGCCACTCCCGCTCCAGCATGATGGGCAAACGGTG
GATGACCACGTTGAGGAAGCTGCCGATCATCAGGGAGAAGAGAAAAACCAGCGAGAAGTAGAGCCAGGGC
AAGCCGTGAGCCAGCTCGAGAAGAAGTGTCATAACCAACCGTCAGTCATTAAATGAGGGGGAGGGCATGG
CGGTGGCGCCATGCCCTGATGAATCAGCCTACCACGTCGCCAAGCTTGAAGATGGGCAGGTACATGGCGA
TAACCATGCCGCCGACCAGCACCCCGAGCACCACCATGATGAGGGGTTCCAGCAGGCTGGTGAGGCCATC
GACCAGATCATCCACCTCCTGTTCGAAGATGGTGGCGACCTTGGAGAGCATATCATCGATGGCGCCGGAC
TCCTCACCGATCATCACCATCTGGATCACCATGTCGGGGAAGAGATCGACGGTGCGCATTGCAACGTTGA
TCTGCATACCGGCCACCACCTCGTTGCGGATCGCCATGACCGCGGTGCGATAGACATAGTTGCCCGAGGC

CCCTGCTGCCGAAATCAGCGCATCCACCAGCGGGATACCGGCGGAGAAGGTGGTCGACAAAGTACGGGCA
AAGCGGGCCATCGCCGCCTTGTGCAGGATCATCCCGACCACCGGAATGGTGAGGACGAACTTGTCGGTAT
TGTCTTTCACCTTCTGGGAGGCCCGCCAGGCCCGCACATAGAGGAAGATGCCAAGGGCGGTGCCACCAAA
GAAGACATACCACCAGTTCTGCATGAAGCGGGAGATGCCGATAACAAACTGGGTGAACACCGGCAGCTCG
GCACCGAAGCTCTTGAAGATATCCTCGAACTGCGGAATGACGAACAGCAGCAGGATAGAGGTGACCACGA
TGGCGACCAGAATGACCATGGTGGGGTAGAACATCGCCTTCTTGATCTTCGACTTGAGCGCTTCCGACTT
TTCCCGGTAAGTGGCGATACGGTCGTAGATGGTCTCCAGTGCACCGGATTGCTCACCGGCTTCCACCAGA
TCGCAATAGAGATCATCAAAGTAGAGGGGATGGCGGCGCAGTGCCTCCGACATGGGGGTACCGGTCTCCA
CATCGGCGGCAATCTGCCCCATCAGCTCGCGCATCGAGGCTTTCTCATGGCTGCGGGCGATGATCTGCAG
GCTCTGCACCAGCGGTACACCGGCGGAGAGCATGGTAGTTATTTGGCGTGAGACGATGGCGATATCCATC
GGTTTGATCTTGGCGCCGCCCTTGGAGAAGAGCCCCTGGGATTTCTTGGCTACCTTGGTGACGTTGACTC
CCTGCTTGCGCAGTTCGGTCTTGACGGTGTTGATGCTGTCGGCCTGCAGCTCGCCGGAGACCTTCTGCCC
CTTGCGATTAACCCCGTGCCAGCGGAAGGCGAAGACTTTCTTGGGGGCATTGTTCTTTTTCGTTAGGGTT
GCCATCTTTGCATCCTGCAATGGGGTTAGTTGTCAGAGTCGGACTCTGCACGTCACCAACCTGGTCAAGG
TGCCCGACTGGCTTGATGCCTGCGGGGTACTGATAGGGTGGGTGGTACCTTCTTGGCGCCAGCCCGGTCA
CTTTAATGCCTGATTATTCTGTCATTCGGGCTGTCATCTATTCCGTGTTCGTGAACGACGAATTCGCTCT
GCCAACCAAACCGGCGGGTCGTTCCGCACACGGATCAGTTGGTGGTGACCCGGTTGACCTCGGCCAGACT
GGTGACCCCGAGCCGAGCCTTCTCCAGTCCCGAGGTACGTAAGGTACGCATCCCCTCTTTCTGCGCGATA
GCAGCGATTTGCAGGGAGTTGGCCCCCTGCATGATTAATTTGGCGATGTTTTCCGACATCAGCATGATTT
CGTATATGCCGACCCGCCCCTTGTAGCCGCCGGAACACTCCTTGCAGCCGACCGGTTTGAACAGTCGCAG
TCCGGCTGCCAGCTGCTGCGGCGTAAAGCCCAGCTCCAGCAGTTCGGCTTCCGGCACCACTTCCGGCGCT
TTGCAGTGGTCGCACAGTTTACGGGCCAACCGCTGGGCCATGATCAGGGTCACCGAGGAGGCGATGTTGA
AGGCCGGAACCCCCATGTTCATCATCCGGGTCAGGGTCTCGGCGGCGGAGTTGGTATGCAGGGTGGATAG
CACCAGGTGGCCGGTCTGTGCCGCCTTGATGGCGATTTCGGCGGTTTCGAGATCGCGAATTTCACCCACC
ATCACCACATCCGGGTCTTGCCGCAAAAAGGAGCGCAGGGCACTGGCGAAGGTGAGCCCCGCCTTGGGGT
TGACCTGCACCTGGTTGACGCCGGGCAGGTTGATCTCCACCGGATCTTCGGCGGTAGAGATATTTACCTC
GGTGGTGTTGAGAATATTGAGGCCGGTATAGAGCGAGACCGTCTTGCCCGAGCCGGTGGGGCCGGTCACC
AGGATCATCCCCTGTGGCTTGGAGAGGGCGCGCAGGTACTGCGCCTTCTGCCGGTCATCAAAGCCGAGTT
GCTCGATATTAAGGCGTGCCGCCGAGGAGTCGAGCAGACGGATAACAATCTTCTCCCCCCACATGGTGGG
CAGGGTATTAACCCGCATATCCATCGACTTGTTGCGCGACAGCTTCAGCTTGATGCGGCCATCCTGTGGC
AGCCGCCGCTCGGCAATATCGAGCCGCGCCATGACCTTGAGGCGGGCGGAGAAGCGGTTGGAGAGGTTGA
CCGGCGGCGTAGCGATTTCATGCAGAATGCCGTCGATGCGAAAGCGGACCCGATACTTGAACTCATAGGG
CTCGAAGTGCAGGTCGGAGGCACCGCGCTTGATGGCGTCCATCAGGATCTTGTTGATGTACTTGACGATG
GGAGCATCGTCATCGGCGGTATTGACGCTTTCGTCCAGCCGACTGTTTTCGTCGGATACCTCGAGATCCG
AAATCTCGGATTCGTCGATATCCTCCATGCCGAGGGCGTCGTTTTCGCTCTCCAGCAGCTTGCCGATGGC
GGCGGTGAGCTTGTTCTCCTCCACCAGCAGGGCTTCAGTATGAAGACCGAAGCTGAAGCCGAAGTCCTCC
AACGCCGAGACATTGGTCGGATCCGACATGGCGATATAGAGGGTGTGGCCCTGGGTGTAGATGGGCAGGA
CGTGGTGTTTCTCAATCAGCTTCTGATTGAGGTACTTCTGCGGGATCTCGGCCAAATCAAAGGCGGCAAG
ATCCAGCAGAGGTACCCCATACTCCAGCTCGCAAAAATCGGCGAGCGCCTTGCTGTCGAGGATCTCGTTC
TCTATCAGAAATGTCACAAAGGGTTTGCGCTGGGCCTTGGCCTGGCTCAGATAGCGCTGGGAGTCCTCTT
CACTGATCAGTGAAGAGGCGGCCAGGCTTAACGCCAGGCCGCTATTAGGGCTAGAGGTCATGCTATAGCT
TTAGCAGAGGCCACTCGTTTTGCAGGCCCCAGATGCTGCCCATGTTACTTTGCCATCAGTTTCAACACTC
GGTGTTAAGATATATGTCATGCCAGTACCAAAAGTAGCTTCTTTACCGGTTGCGGTTATAGCGTTGGTTG
TATCACTTACCTCAACACTTTTAACCTCTCCTGTCCCAGCTGTAAGATTGGAGGGTACACCATTGGACCC
CGCATCACAGGTGCTTAGTGCTCCCGAGGCTGCACCTGTTGTTTGCGCACAAATTTCAATTGCAGTTTTA
TAAGCTCCAGTAGCCGCCACTACTTCAGTAAACTTAGCTTTTTTGGTGTAGGTCTGGTACGCCGGCAGTG
CTACTGCCGCCAGAATGGCCACGATCGCGACCACGATCATCAGTTCAATAAGGGTAAAACCCGATTGTTT
CTTCATTTCGCTCTTCATGGAAAATATCCTTTTATCCTTGGTTCCTGGGAAGTGTCGGTGGAAAATCATC
CGCACGTCAGTATCTTATCCGAGCTGTCAGGGGTGGCAACAGAGATTGCTGCCTTTGTTAACTACTTTCA
TGTCATTTACCAAAAACTTGATGCGAGTCATAAAATAAGTGGCTTCTTATGACTCTTTGTTACGGGTGAA
GGCGGGTCCGTCATCACACTCCTTTGCTGAGGGATTCTGCATAAAGAAAAGGCGCCTATGGCGCCTTTTG
CAAGTCGTTTATCCGGTTAGGTCGAGTATTTGCTCAGGCCTGCGGGGTGAATCTCACTGCTGGATAAAGC
GCATGGAGAGATCGAGCGCCCGTACATGCTTGGTCAGCGCCCCGACAGAGATAAAATCGACACCGGTGGC
GGCAAACTCGGCCAGAGTATCCAGCGTCACATTGCCGGAAACTTCGAGCTTGGCGCGGCCCTGATTGATG
GCGACCGCTTCCCGCATCATGGCAACATCGAAGTTGTCCAGCATCACGATATCGGCCTTTGCCGCCAGCG
CCTGCTCCAGTTCTGCCAGCGATTCCACTTCCACCTCCACCGGTTTGCCCGGATTGAGATGACGAGCCTC
ATTAATCGCCTCGGCAATGCCGCCGCAGGCGAGGATGTGGTTTTCTTTGATGAGATAGGCGTCGTAGAGA
CCGATGCGGTGATTCTTGCCGCCGCCGCAGGTGACTGCGTACTTCTGGGCGGTACGCAGCCCCGGTATGG
TCTTGCGGGTGTCGAGCAGGCGGCAGTCAGTGCCCTCCAGCTCTGCCACATAGCGGGCGGTGAGGGTGGC
GACGCCTGAGAGGGTCTGGACAAAGTTCAGCGCATTGCGCTCGCCGGTCAGCAGTAGGCGGGCAGGGCCA
TGGAGGCGGAACAGCTCCTGATTGGGGGAGAGCACTTCCCCATCCTGCACCAGCCACTCCACCTTCACTT

CGCCACCGAGCTGGGCCTTCAGTTGCAGGAAGACTTCATCCACCCAGGGCTGGCCGCAGAATACTCCTGC
TTCACGGGTGATGACCCGGGCGCTGGCCATCCGGTCGGCCGGGATCAGCTGTGCGGTGATATCGGCGCTG
GCGTCCGGTTGGTCCAGCGTGGTGAGGGCATTGCCCAGATCTTCGAGTAGTGCGGCGCGCACGGCGCGGC
TGACATCCTGTTGAAACATGCCTTATTCCTGCAAGGGTCTGAGCAGCAGTTGCCGCTCCTTTTGAACCAG
CTCCAGCCGACCGAGGGCGTTCATGCCAACCAGCACTTCGTCACCGTCTGCCGCCGGATTGATAAAGATG
GCCAGATCGTACAAGGTCAATGGCCCCATCGCCAGCGTTTCGATGGTGCCGTTGCCAACCCGGATCTGCC
CTGCGGCGGTATTGACCTGGCTGCTGCCATAAGCGGTCAGGCCGAGCCGCTCGGCAACCTGCTGCGGGAT
GGTGATACGGGTGGCGCCGGTATCGAGCAGCAGTTGCACCGGCTGGCCGTTGATGGCACCCTCCAGACGA
TAGTGGCCGCTCTGGTCGGCCCGCAGCAGCAGCTCGCCGCTGGCGGTGACGCTTGGTTGGTTGCGGGAGT
GAAAATAGAGGGTCAGCAGGCCCATCAAAGAGAGCCAGGCCAGTAACCACATCCAGCGTGCCGTGTGCTG
CATACCCCATCCATATCGAGCGGAAGATGACTCAGCCTAACATACCATTTCCCGTTGATTCAGTGAGTTG
GTGCTGGGTTGCCTATCAGGTATCTTCGTTTTCTCGCCATTTATCAGCCAACTCGAGTAGCCGATGACTC
AACTTGCTTCGCAACCTTTTTTCTCTATCGATGCCGCAGGCTGGTGCCAGCAGGCGCGCCGGGTGCCTTC
TCCCCATCACAACGAACGGGCAGCGCCGGATGATATCTCCCTGCTGGTGGTTCATGGCATCAGCCTGCCG
CCGGGGGAGTTCGGTGGCCCCTTTATCGACGATCTCTTTATGGGGAGACTCGACCCCGAGGCTCACCCCT
ATTTTGCCGGTATCCATCAGTTGCGGGTCTCGGCACACTGCCTGATCCGCCGTGATGGCGAGCTGGTGCA
GTATGTGCCGTTCGGCGCGCGCGCCTGGCATGCAGGTGTCTCGAGCTGGCAGGGGAGGGAGGCATGCAAT
GACTTCTCCATCGGCATCGAGCTGGAAGGGACTGACGAAATCCCCTATACCGAGGCTCAATACCGGGCGC
TGGTGGGGCTCACCCGAGCCATTTCGGAGCGCTATCCGGCGATTACCCACGAAAAAATTGTCGGCCATTG
CGATATCGCGCCAGGGCGCAAGACGGATCCCGGAGCCAGTTTTCAGTGGGATTACTACCGGCAATTACTA
AACAGCTAAAGTAAATATTCAAATTTAGTTTGGATTTTAAAATCAATGGCTTGCCTATCTGGCAGGCCAT
TTTTGTCTCTGGTTGCGCAAATGTTATTTGTGAGCGAGGTCAAGAATATTGGTCAGACCATTTTCTATGA
GCTAAATCATGTTTCCCCTGCTTTACCTCTTAAGTGGTGGGGCTGATTCTTGACTCAGATCATCTTTTGA
GTGGACTTTTCAAAAGGCCTCTGCTAATTTTCGCCCCATCCATAAAATTGGTAATACCAATTTAACAAAG
TAGCCTCATGCCCTATCGCAAGATTACGCAACCCAAGTTGGCCGACTCCATTGTCGCCGAGCTGGAACAG
ATGATTCTGGAAGGCAGCCTGCAGCCGGGGCAGAAATTGCCTCCCGAGCGGGAACTGGCCATCCAGTTTC
AGGTATCGCGCCCCTCCCTGCGCGAAGCCATCCAGCGACTGGAAGCGCGCGGATTGCTTTATCGCCGTCA
GGGCGGCGGCACTTATGTGCAGAATGCCCTGAGCAAGGGGATCGCCGATCCTCTGTTCGAACTGCTCAGT
ACCCACCCGGAAGCCCAGTATGATCTGCTGGAGTTTCGCCACGCGTTGGAAGGCATCTGTGCCTACTACG
CGGCCTTGCGGGGAACCGGGTCTGATTTCGAGCGCATTCGTCAGGTGCAAACCACCATCGAAGAGGCGGG
CCAGCTGGGCTCGCTGGTGGCCGAGTCGGCAGCCGTGACCCAGTTCTATCTGGCGGTGGCCGAGGCGTCC
CACAACGTGGTGCTGCTGCACCTGCTGCGGGCCATGAGCCCGATGCTCGAACAGAACATTCTGCAGAATA
ACGAGATTTTGAATCGCCGCCCGGGAGTGGTAGCCAAGATACGTCGCCATCGCGCGAATCTGATCGAGGC
TATTCTTTCGGGGGCTCCGGAGCGGGCCAGAACAGCTTGTCATGAGCATCTGGCCTTTATCGAGGACACC
TTGTTGGATATGCAGAGGGAAGACAGCCGGATCCAGCGATCCATGCGTCGGATCCGTCAGCAGGAAAACT
AATCCCACAAGGCTCTATTGTCGAATGCTTGCGCCCTTCGCCGTGCAATGCCGTGCAATCACAACAACAT
AGGAACCAGCCATGTCTGATATCCTGAAGAACGATGTGGACCCGATAGAAACGCTGGAGTGGCTTGCCTC
TCTGGAATCCCTGCTGCGTGAAGAGGGTCCGCAACGCGCTCAGTTCATCCTTGAACAGTTGGCCGAGAAA
GCCCGCGTAAGCGGTGTGGACGTGGCTGCCAAGGCAAATCGCGATTACATCAACACCATCCCGTCCAGCG
ATGAGCCGGACTACCCGGGTGACCTGGAGATGGAACGCCGCATTCGCGCCATCATCCGCTGGAACGCCAT
GATGATCGTGCTGCGCGCGTCCAAGAAAGACCTGGACCTGGGTGGTCACATGTCTTCCTTCGCTTCTTCT
GCGACCATCTATGATGTCTGCTTCAACCACTTCTTCCGTGCTCGCAACGAGAAGGATGGTGGCGATCTGG
TTTACTTCCAGGGTCATATCTCCCCGGGCATCTATGCTCGCGCCTTTGCTGAAGGTCGTCTGACCGAAGA
GCAGCTGGACAACTTCCGTCAGGAAGTGGACGGCAAGGGTATCCCTTCCTATCCGCACCCGAAACTGATG
CCGGACTTCTGGCAGTTCCCGACCGTATCCATGGGTCTGGGCCCCATCGCCGCCATCTATCAGGCTCGCT
TCCTGAAGTACCTGACCGACCGTGGTATCAAGGATTGCTCCGAGCAGACCGTTTACGCCTTCCTGGGTGA
CGGCGAGATGGACGAGCCGGAAGCCAAGGGTGCCCTGACTGTTGCCGTGCGTGAAAAGCTCGACAACCTG
GTGTTTGTGGTCAACTGCAACCTGCAGCGTCTGGATGGTCCGGTTGTCGGTAACGGCAAGGTCATCAACG
AGCTGGAAGGCCTGTTTGCTGGCGCCGGTTGGGATGTCACCAAGGTCATCTGGGGTCGCAAGTGGGATGA
GCTGCTGAAGAAAGACACTTCCGGCAAGCTGATCCAGCTGATGAACGAGACCGTGGACGGTGACTACCAG
ACCATGAAGTCCCGCGACGGTGGCTACATCCGCGAGCACTTCTTCAACCGTTATCCGGAAACTGCCGCGC
TGGTCAAAGACATGACCGACGAGGAGATCTTCGCTCTGAACCGCGGTGGTCACGATCCTCGCAAACTGTT
CGCTGCATTCAACAAGGCGACCCAGACCAAGGGTAAGCCGACCGTTATTCTGGCCAAGACCATCAAGGGT
TACGGCATGGGTGAAGCGGCCGAAGGCAAGAACATCGCTCACCAGGTCAAGAAGATGGAGCTGGACTCCG
TGCGTCACCTGCGTGACCGCTTCAACCTGCCGGTGGCCGACGAGCAGCTGGCCGATCTGCCTTACCTGAA
GATCGAAGCCGGTTCTGCCGAGCACAAATACCTGCACGACCGTCGCACTGCCCTGAAAGGCTATGTGCCG
ACCCGTCTGCGTGAAAGCACCACCAAGCTGGAAATCCCGGCGCTGGATGCCTTCGGTCCGCTGCTGGGTG
AGCAGGCGCGCGAAATCTCCACCACCATGGCGTTCGTGCGTTCCCTGAACGTGATGCTGAAAGACAAGTC
CATCGGCAAGCGCATCGTTCCGATCCTCGCTGACGAAGCCCGTACCTTCGGTATGGAAGGTCTGTTCCGC
CAGATCGGTATCTACAGCCCGCACGGTCAGCAGTACACCCCGCAAGACCGCGACATCGTCTCCTACTACA
AGGAAGACAAGCAGGGTCAGGTTCTGCAAGACGGTATCAACGAGCTGGGCGCCATGTCCTCCTGGCTGGC

TGCTGCGACCTCTTACAGCACCAACGACTGCCCGATGATCCCGTTCTACATCTACTACTCCATGTTCGGC
TTCCAGCGGATCGGCGACATGGCGTGGGCAGCCGGTGACCAGCAAGCCCGTGGCTTCCTGCTGGGTGCAA
CCTCCGGTCGTACTACCCTGAACGGCGAAGGTCTGCAGCACGAAGATGGTCACAGCCACATCCTGGCGAA
CACCATCCCGAACTGCATCTCCTACGATCCGTCCTACGCCTATGAAGTGGCGGTGATCGTACAGGACGGC
CTGCGCCGCATGTACGGTGAGAACCCGGAGAACGTGTTCTACTACATCACCACCCTGAACGAGAACTACG
CCATGCCGGCCATGCCGGAAGGCGCCGAGGAAGGCATCCGCAAGGGTATCTACAAGCTGGAATCCGTCGC
CGGTGCCAAAGCGAAAGTTCAGCTGCTGGGTTGTGGTTCCATCTTGGGTCACGTGCGCAAGGCTGCCCAG
ATCCTGGCCAGCGAGTACGGCATCGGTTCCGATGTGTTCAGCGTCACCTCCTTCAACGAGCTGGCTCGTG
ACGGTCAGGACGCGGATCGCTGGAACATGCTGCACCCGACCTCTGAGGCGCGTGTTCCCTACATCGCTCA
GGTACTGGGTTCCGAAGCCACCATCGCTGCGACTGACTACATGAAGTCCTTCGCCGATCAGGTTCGCGCC
TTCGTTCCGACCGAGAACTACCGCGTGCTGGGTACCGACGGTTACGGCCGTTCCGACAGCCGTGCCAACC
TGCGTCGTCACTTCGAAGTGAACGAGTTCTACGTGGTGGTTGCTGCTTTGACCGAACTGGCCAAGCGCGG
CGAGGTAGACAAGCAGGTTGTGGCCGACGCCATCGCCAAGTACGGCATCGATGCCGACAAGGTCAACCCG
CTGTACGCGTAAGTGGATCAAGGGTCGCGCCGTATGACGGCGCGACCCTGCCAGGATTTAAAACGGTTAA
CGCGAGATGTGCGTGGCGGCATCAGCCAGTCACGCGGATCGACGCAAGAGGATAAAGCACAATGTCCAAA
CAGATTATGGTGCCGGATATCGGCGCCGATGAAGTTGAAGTCACCGAGATCATGGTGGCCGTGGGTGATA
AGGTCGAGCTGGACCAGTCCATCATCGCGGTAGAAGGCGACAAGGCCTCCATGGAAGTTCCGGCCCCGTC
CGCCGGTATCGTCAAAGAGGTCCTGATCAAGGTGGGCGACAAGGTTGCCACCGGTTCCCAGATCATGATC
TTCGAAGCCGAAGGCGCTGCCGCCCCTGCTGAAGTGGCTGCACCGGTTGCTGCTCCGGCCGCCGCACCTG
CCGCTGCCGTGCGCAAAGATGTGCACGTGCCGGACATCGGTGATGACGAAGTGGACGTGACTGAAATCAT
GGTTGCCGTGGGCGACATGGTTGAAGCCGACCAATCCATCATCGCCGTGGAAGGTGACAAGGCCTCCATG
GAAGTGCCGGCTCCGTTCGCAGGTCGCGTGGTCGAGATCAAGGTTGCCGCTGGCGCCAAGGTCTCTACCG
GTTCTCTGGTGATGGTATTTGAAGTGGCTGGCGCTGCTCCTGCTGCCGTTGCTGCTCCGGCTCAAGCTGC
CGCGCCCGTGGCTGCTGCCCCGGTTGCCGCCGCTGCCAAAGAGGTCAACGTACCGGATATCGGTGGCGAC
GAAGTGGAAGTCACCGAGATCATGGTTGCGGTGGGTGACAAGGTTGAAGCCGACCAGTCCCTGATCGCCG
TGGAAGGTGACAAGGCCTCCATGGAAGTGCCTGCGCCGTTCGCCGGTGTGGTCAAAGAGATCAAGGTGAA
GGCGGGTGACAAGGTCTCCACCGGTTCCCTGATCATGGTATTCGAAGTGGCTGGCGCTGCTCCTGCCGCC
GCGCCGGTTGCTCAAGCTGCCGCCCCGGTCGCTGCAGCCCCTGTTGCCGCTGCTCCGGCTCCGGTTGCCC
AGGCTGCTGCCGCTGCGACCGATTTCGTTGCCAACGACGCCTACGTGCACGCCTCTCCGGCGGTACGCCG
TCTGGCGCGCGAGTTCGGCGTCAACCTGGCCAAGGTGAAAGCTTCCGGTCGCAAGGGTCGTATCGTCAAA
GAAGACGTGCAAGCCTACGTGAAGGATGCCATCAAGCGTGCCGAGTCCGCACCTGCCTCTGCCGGCGTAG
CCGGTGGCAACGGTCTGGGCGTACTGCCGTGGCCGAAAGTAGACTTCAGCAAGTTCGGTGATGTGGAAGA
GGTCGAGCTGACCCGCATCCAGAAGATCTCCGGTCCGAACCTGCACCGCAACTGGGTCATGATCCCTCAT
GTCACCCAGTTCGACGAAGCCGATACCACTGAGCTGGAAGCGTTCCGCAAAGAGCAGAATGCGATGCTCG
AGAAGCAGAAGGCAGACGTGAAGATCACCCCGCTGGTGTTCATCCTGAAAGCCGCCGCCAAGGCACTGGA
AGCTCACCCACGCTTCTGCAGTTCCCTGTCCGAAGATGGCAGCAAGCTGATCATGAAGAAGTACATCCAC
ATCGGTGTGGCGGTAGATACCCCCAACGGTCTGGTTGTGCCCGTGGTGCGCGATGTTAACAAGAAGGGCA
TCATGGAGCTGTCTCGCGATCTGGCCGAGATCTCCAAGAAGGCCCGTGCCGGCAAGCTGACCGCTGCCGA
CATGCAGGGCGGTTGCTTCACCATCTCCAGCCTGGGTGGCATCGGCGGTACCAGCTTTACCCCGATCGTC
AACGCGCCGGAAGTGGCCATCCTCGGTGTTTCCAAGTCCGAGATGAAGCCGAAGTGGAACGGTAAAGAGT
TCGCGCCGCGCCTGATGCTGCCGCTGGCTCTGAGCTACGACCACCGCGTGATCGATGGTGCCGATGGGGC
TCGCTTCATCACCACCATGAACGGCGTGCTGTCCGACATTCGTCGACTGGTCCTGTAAGTGAGAGGGCGG
GCCCAGAGCCTGCCCTTTTTACTTCTCATTTCATTAACATGATCGTAAACTTGCCCCGTTTTTTTCACGG
CTGCAGCCACCCGTTTCGGTATAAGACTGCGCTGTCCGGGGATATAAGAACGACAAAGAGGTCATGATGA
GTAAAGAAATCAAAACCCAGGTCGTAGTACTGGGTGCTGGCCCTGCCGGTTATTCCGCTGCTTTCCGTGC
AGCCGATCTGGGTCTGGATACTATTATCGTCGAGCGTTACTCCACCCTGGGTGGCGTCTGCCTGAACGTG
GGCTGCATCCCCTCCAAGGCGCTGCTGCACGTTGCCAAAGTGATTGAAGAAGCCAAGGCGCTGGCCGAGC
ACGGCATTGTCTTCGGTGCACCGCAGACCGACATCGACAAGATCCGTCTGTGGAAAGAGAAGGTTATCAA
CCAGCTGACCGGCGGTCTGGCTGGCATGGCCAAGATGCGTAAAGTTCAGGTTGTTAACGGCTTGGGTAAA
TTTACCGGCCCGAACACCCTTGAAGTGGAAGGTGCAGACGGCAAGACTACCGTGACCTTCGACAACGCCA
TCATCGCGGCCGGTTCCCGTCCGGTGAAACTGCCGTTCATCCCCCACGATGACCCGCGCGTATGGGACTC
TACCGATGCGCTGGAGCTGAAAACCGTTCCCGGCAAACTGCTGGTTATCGGTGGCGGCATCATCGGTCTG
GAGATGGGTACCGTTTACTCCTCTCTGGGTTCCGAGATCGACGTGGTGGAATTTGCCGACCAGCTGGTTC
CGGCTGCCGACAAAGACATCGTCAAGATCTACACCAAGCGCGTCGCCAAGAAATTCAACATCATGCTGGA
GACCAAGGTCACCGCAGTTGAAGCGCGCGAAGACGGCCTGTACGTCTCCTACGAAGGCAAACACGCCCCG
GCCGAGCCGGTTCGTTACGACAACGTGCTGGTTGCCGTAGGCCGTGTGCCGAACGGCAAGATGCTGGATG
CCGAGAAGGCAGGCGTTGCCGTGACCGAGCGCGGTTTCATCGAGGTGGACAAGCAGCTGCGTACCAACGT
GGCCCACATCCACGCCATCGGTGACATCGTGGGTCAGCCGATGCTGGCGCACAAAGGTGTTCACGAAGGT
CACGTTGCCGCCGAAGTGATCGCTGGCAAGAAGCACTACTTCGACCCGAAAGTGATCCCGTCCATCGCCT
ACACCGAGCCAGAGATGGCATGGGTTGGTCTGACCGAGAAGGAAGCCAAGCAACAAGGCCTGAACTTCGA
AGTCGCCACCTTCCCGTGGGCTGCTTCCGGTCGCGCCATTGCGTCCGACTGCTCCGACGGTATGACCAAG

CTGATCTTCGACAAGGAGAGCGGCCGCGTCATCGGTGGTGCCATCGTGGGTACCAACGGTGGTGAACTGC
TGGGTGAAATCGGTCTGGCCATCGAGATGGGTGCTGACGCCGAAGATATCGCGCTGACCATCCACGCTCA
CCCGACTCTGCACGAGTCCGTGGGTCTGGCTGCCGAAGTCTTCGAAGGCTCCATCACCGACCTGCCGAAC
GCCAAGGCCAAGAAGAAAAAGTAATTTTCTTCCCCGCTTGTCTAAAAAGCCGCTCGAATGAGCGGCTTTT
TGTTTTCTCCTTGCTGCCCCGACTTCGACAAAGCCCAATATGCTAAAGATAGGTTTTGGCACTGACGAGA
GAGGCACGATGCTAGAAGCACCCATTCCACCCGATGAGGCATTGCGGTTGGAGACCCTGCGGCGGCTCTC
TTTGCTCGATTCCGAGGCTGAGGAGCGGTTTGATCGGCTGACCCGCATGGCCCGACGGGTGTTCAATGTC
CCCATCGCGCTGGTAAGTCTGGTGGATGAAGATCGCCAGTGGTTCAAATCCTGCATCGGTCTGGAGGTCC
GTGAGACTGCCCGGGACATCTCGTTCTGTGGCCACGCCATCATGGGCAATGAGACCTTTATCGTGCCGGA
TGCTTCGCAGGATCCCCGCTTTGCCGACAATCCGTTGGTGGTGGGTGGGCCCGGCATCCGCTTCTATGCC
GGACAGCCACTGCGGATCGCCAACGGCTGCAAACTTGGGACACTCTGCATCATCGATACGGTGCCGCGGG
AATTGAGTGAGGAGGATGTGGGGCTGCTCAAGGATCTGGCTGCGATGGTGGTGCGGGAGCTCGAAGCGGT
GCAGCTGGCCACCATTGATGAGTTGACCCTGATCTCCAATCGGCGCGGGTTCGTGATGCTGGCGGAGAAG
AGCCTCTCGCTGGCCAGTCGCTTGCAGATGCCACTGGCACTGCTCTATCTGGATCTCAATGGCTTCAAGG
GGATCAATGATAGGTGGGGGCATGCAGAAGGGGATCGGGCCCTGATCGTCTTTGCCACTCTGCTCAAGCA
GGCTTTTCGTGGCGCCGACGTGTTTGCCCGCATGGGCGGCGATGAGTTTGCCGTCCTGTTGCCCAATGTG
ATGGAGGCGCAGGCGCAACTGGCCTATGAGCGTTTCAATGCCATGTTGGCCGAGCACAATCAGCACAGTG
GTTGCGGTTATGCGCTGACCTGCAGCACCGGAATTATCTGCTATGACGGGCACGGCTGTGTCTCCCTGGC
CGAGCTGCTCACCCGCGCGGATGAGCTGATGTATCAGCGCAAACGGGATGGCAAGGGCTGACTGCTATGC
CGGATTAGCCGCCACCAAAACAACAGAGAGCGCCACGGCGCTCTCTGTTGTTGATGGCTTAGATCAAGGT
CAAGGCCAAGGTCGGGGTCAGAGCCGGAAGTTGCCAACCAGCTTGTCCAGCTGACCGGAGAGCTGCTGCA
GGCTGCGGCTCGATTCGGTCAGCTGGTGGGCGATATCGGCGGTCTCCATGGTGAGCTGGTTGATATCTTC
CACGTTGCGGTTGATCTCGCCGACCACACTCGACTGCTCCTCGGTGGCGGTGGCGACCTGGATGTTCATG
TCGTTGATCTGGGTGATGTGGGCGGTGATCTGGCCGAGGGCTGCGTTGGCCTCATCGGCCTGGCTCACCA
CCTCCAGACTCTGGTTACGCCCCTGTGCCATGGCGCTGACGGCGCGGGCACTCTGCTCCTGCAAGCGGTT
GATCATCCCCTGGATCTCTGCGGTCGAGGCGGCGGAGCGGTTGGCCAGATTGCGCACTTCATCTGCCACC
ACGGCGAAACCGCGACCCTGTTCACCGGCGCGAGCGGCCTCGATGGCGGCGTTCAATGCCAGCAGGTTGG
TCTGCTCCGAGATACTGCGGATGGTTTCGAGGATGCTGCCGATGGAGTCGGTCTGGCTCGCCAGCGACTC
GATAACCCCTGCCACCTGCTCAATCTCGCCGGAGAGGTCGACGATGCCGTGGCGCGCCTGCGCTACCACT
ACTGCCCCTGCATCGGCCTGGTCGTTGGCTTGTCTGGCTACTTCGGCTGCCAGCGAAGCGTTACCGGCGA
TCTCGCTCACCGTGGCCCCCATTTCGTGGATGGCGGTGGCCACCTGCACAGTGCGGTCACGCTGGGCAGT
ACAGTTGTGCTGGGTCAGCTGCGCCTTGTTGGCCACCTCGTTGGCGGTGGCCGCGAGCTGGCGGGAGTTC
TCCGCTACCTGCTCGATGGAGCCGTGGATCTTGGCGACGAAGTTGTTGAAGCCGGTGGCGACCTGACTCA
GCTCGTCACGCCCTTCCACCTTGAGGCGTTGGGTGAGATCACCATCGCCGCTGCCCAGCTCCTTGAACAG
GCGGGCCAACTCGTTGAGCGGGCGGCTGACCGAGCCTGCCAACAGCATCCCCATCAGCCCCATGGCCAGC
GCAATCACCAGCGAGATCATCAGGATCTCGTTGCGGGTCTGATCCAGCTCGGCATAGATCTCGGCTTCTG
GCACCTGGGCCACCAGATACCAGTCGAGCAGCGGGATATAGCTGCTGGCCAGTACCATGGGCTCGCCATT
GACTTCGGCGTTGATGACGGCAAAGGGGCGTTTGGCCAGCAGGCTGCCGGTATCACCGCTGGCAATGTTG
CCAAGTGTGTCACGCTCGATGCGATTAGCCTCGGGGTGCAGCTTCACCTTGCCGCTGCCATCGACCATAA
AGACGATGCCGCTCTCTTCCAGCCGGAAGTTGGAGACCATGGTCACCATGGTGTCGAGGGATTTGGCAAA
GCCCGCCAGCCCGCGCCCGTTGAGCTGCTGGAAGTTGACGAACAGCTTCACCTCGCCGTTGCTCTCGCGG
AAGATGCTGAGGGTCATCTCCTTCTGGCTCTTGGTGTAGTCATAGAACCAGGCATCTTGCGTGGGGGTCA
GGTTGCGCAGAAAGCCATCCTGGTTGTAGTAGGCGGCACTCTGACGATCGGCGAAGGAGGCGGTCACCAG
CCCGTACTGGTTGGCCACATCCTTGAGCTGGTTGATGAGGATCGGCTCCTGCTCTTTGGGCATGCCCCGA
TCCACCCAGTCGAGGATGAAGCGGTCGGTGGCCAGTTGGCGTGCGGCCTGGGCGATGCCGTGGAGATCCT
GTTCAATCTCCTTGCTGATCCGCTGGGTCAGGTTGGGGAGCTCCTGTTCGAACATCCGGTTTTCGATCAG
TTGCTGGGCCGAGCGTTGGCTGAAGTAGCCTACCAGCAGGCAGGAGACGACCACGGCCAGGGTGACCGTG
GCCAGTATTTTCTGTTTAAGAGTGAGGGTTGCCAGCATAAAGGTGCTCAAAAGGTTGAGATTGACGACGC
TCATATTAGCGGCAGCGCGGCGCTACCCATGAGGCTTGCAGCCCTTTATTTGCGGATTACGTTTGCGCTC
ACAGGGGCGGCGGCGTGCCCCTTCGCCATGCAGCACAGGCGGGATGCGGATTGACGGTTGCGCTAACTGC
CGGGCCCCTTATAGTAGAGGCCATCCTTGGTGACTGACCCTCTGAATAATATGGAAAATATGATGCGCAG
CTTAGGTGCGGGATGGACCCGTCTGCTTTTGGTGATGCTGGCGGCCTGCCCGCTGCTGGCTGTGGCGGCA
CAGGGGGAGCGCCCGAAAATTGCGGTGGTGCTCAGCGGCGGTGGCGCCAAGGGATCGGCCCATATCGGCA
TCCTCAAGATACTGGAGCAGAAACGGATTCCGGTCGACATCATAGTCGGCACCAGCATGGGCTCTTATGT
GGCGGGGATGTATGCCATGGGGCTCAGTGCCGAGGAGGTGGAACGCACCACTTTGGCCATCGACTGGAAC
AAGGGGTATCAGGACAAGGTTGGCTGCGACGAGCTGTCGCTGCGCAAAAAGCAGCAGAACGAGCAGTACC
AACTGCGCACCGACATCGGGGTGAATGGCGACACCACCCAGCTGCCGGACGGTTTCTTCCAGGGCCAATC
CATGGCCAGCCTGCTGCGGGGGGCCACCTCCAACCTGCCGGTGCAGGCGAGTTTCGATGATCTCCCCATT
CCCTACCGCGCCATGGCGACCGACATGGAGACGGTCACCCCCTTTGTGCTGGATCACGGCAGCCTTGCCA
AGGCGATGCAGGCCTCAATGTTCATTCCCGGCGCCCTCAAGCCGGTGGAGTGGAGGGACACATCCTGGC
GGACGGCGGCAGCGTCAACAACATGCCGGTGGATGTCGCCAAGGCGATGGGGGCTGATGTGGTGATCGCG

GTGGATATCGGTGCCAAGCTGCGTACCCGGGAGTCCCTCAAGTCAGGGTTGGCGATGATCGACCAGCTCA
CCACCTACATGACCCAGGTGGGTACCGACAAGCAGAAGGCGCTGATGGGGCCCAGGGATATACTGATCGC
CCCCGAGTTTGGCGATATGGGGATCGCCGACTTTGCCCGGATGCCGGAGGGGATCAAGATCGGTGAGACG
GCGGCTGACAAGGTGTCGCTGCAACTCGATAAACTGTCGCTCACCAAGGCGCAGTACAACGCCTATCGCA
ACCAGAAGCTGAGCCGCCGTGCCGAGCGTAGCGGTCAGCCCGCCTACTTCATCGACAAGGTGGAGATCAT
CAACAAGTCGCGCCTCTCCGACGAGACGGTGCGCGCCATGCTGAAAGTGCGGCCTGACAAGGTGCAGACC
AACGAGAGTCTGGAAGCGGGCATTCGCCGCCTCTATGCCCTTGAGTCGTTTGATCGCATCACCTACGAGG
TGGAGGAGCGTGATGGTGAGAACGTGCTGCTGGTGGATGCCAGCGAGAAGAACTGGGGGCCGGGCTACCT
CAACTTCCAGTTCGGTTTCACCGATGACTTCGAGAACACCTCCAACTACAACGTCGGCATGTCCTATACC
CTCACCAACGTCAACGAGTGGGGTGCCGAGTGGCTGACCGAGGCGTCGCTGGGCACGGCCAAACATATCA
AGACCGACTTCTATACCCCGCTCGAACCCTCCCAGACCTTCTATGGTGAGGCGAGTCTGGCCTATGACCA
GACCCTGCGCCGCCTGTTTTTCCCCAATCCGGATCCCGAGACGGTTTCGATAAGCTATCTCGACTCCGAA
TACTCCTTCTTCAGCGGCGATCTGGCGGTGGGCTGGAACCGCCAGCCCTGGAGCCGGTTGTCGATGGGGC
TGGAGGGACAACTGGGCACGATTGATGTGAAGAACTTCAAGGGGCTGGAGATGGATGCCACGGCGTGGGG
CCCCTATGTCCGCTTTGAACACGACAATCTGGATAGCCGCTATTTCCCCTATCAGGGGGTGAAATGGGAT
ATGAAACTGGGCCACTCCCACGTCAGTGTGGATGGACTCGAGTCGGCAGATGGCCGCAGTGAAGGCTGGA
ATTATCAGCTCTCGATGATCAAGCCCTGGTCATGGGACAGGCACAGCCTCAACCTGATCCTCGAAGGGGG
TGGCTCGGGCAGTGAAGAGGTGGTGCCGCTGCATATTCAGGATCTGGGCGGGCTGTTCCGCCTCTCCGGT
TTCCAACGCTACCAGCTGAGCGGTCGCTACAGCCTGTTTGGTGGCCTGCGTTACATCTATCGGGTGGCGG
ACAACGACTTCGGTGCCCTCAAGATGCCGCTCTATCTGGGTGGATCTCTGGAGCAGGGCGGGGTCTGGAA
CAAGGGTGAGGATATCAGTCTGGAGAGCTCGTTTACCGCCGGTTCGCTTTATGTCGGGGTGGAGAGCTTC
CTCGGCCCTGTGTTCCTCGGCTACGGCATGGCGGAGGGGGGCAATGACGTCTTCTATCTGCAATTGGGCA
GTACGTTCGAATAATGCTGAAATGATGCCAATTTGTTAACTCTGGTTGAGGGCGTTCAATTAATTTTCAA
AAAACACCAATCTGTTTAATTGGTTACGATTTTGTGGTGAATAAGTAAACAGGGTTTGGATTTTAATTAG
TCGTTAAAACTTATATAGTGGGGAGCACCCTACGGGGTGTCCCGCAAGGACGATCGCCGCGAGCGTGGGC
GAACCCCAAAAAAAGACCAAAGAGGAATTAGTCGTGCTTGAAACATACCGTAAACACGTCGCAGAACGTG
CCGCCGAGGGCGTGGTTGCCAAACCTCTGGATGCAGAGCAGGTCGCCGCCCTGGTTGAGCTGCTCAAGAA
CCCGCCCGCTGGCGAAGAAGACTTTCTGAAAGAGCTGTTGTCCTCCCGTATTCCCCCCGGTGTTGATGAA
GCGGCCTATGTCAAAGCCGGTTTCCTCACCGCCGTCGCCAAGGGCGAAGCCCACTCTCCCATCCTGACTG
CCGCCGAAGCTGTTGAGCTGCTGGGCACCATGCAGGGTGGTTACAACATTCAGCCGCTGATCGACCTGCT
GGATCATGCCACTCTGTCGCCGCTTGCCGCCAAGGGGCTCTCCCACACCCTGCTGATGTTTGACTCCTTC
CACGACGTGGAAGAGAAGGCCAAGGCGGGCAATGTCCACGCCAAACAGGTGATGCAATCCTGGGCTGATG
CCGAGTGGTTCCTCTCCCGCCCGGAGCTGGCCGAGAAGATCACTGTCACCGTGTTCAAGGTGACCGGCGA
AACCAACACTGACGACCTCTCTCCGGCACCGGACGCCTGGTCCCGCCCGGACATTCCGCTGCACGCGCTG
GCGATGCTGAAAAACGCCCGTCCCGGCATTACCCCGGACAAGGATGGCGCCGTCGGCCCCATCAAGACCA
TCGATGCCCTGAAAGAGAAGGGCTTCCCGCTGGCCTACGTGGGTGACGTGGTCGGTACCGGCTCTTCCCG
TAAATCCGCCACCAACTCGGTGCTCTGGTTCATGGGCGACGACATTCCGTTCGTGCCGAACAAGCGAGCT
GGCGGTGTCTGTCTGGGTGGCAAGATTGCCCCTATCTTCTTCAACACCATGGAAGATGCCGGCGCTCTGC
CCATCGAAGTGGACGTCTCCAAGCTGGAAATGGGCGATGTGATCGACATCTACCCCTATGCCGGCAAGAT
CTGCAAACACGGCACCGAACAGGTGCTGGCACAGTTTGAATTGAAAACCGACGTACTGATCGACGAAGTA
CGCGCCGGTGGCCGTATTCCGCTGATCATCGGTCGCGGCCTGACCGACAAGGCCCGCGAAGCGCTGGGTC
TGCCGTTCTCCACTGTATTCCGTCGCCCGCAGCCGGTGGCCGATACCGGTAAAGGCTTCACCTTGGCCCA
GAAGATGGTGGGCAAGGCGTGCGGCGTGAAGGGCATTCGCCCGGGCACTTACTGCGAGCCCAAGATGACC
ACGGTCGGCTCTCAGGACACCACGGGCCCGATGACCCGCGACGAGCTGAAAGACCTGGCCTGTCTCGGCT
TCTCCGCCGATCTGACCATGCAGTCCTTCTGCCACACTGCCGCCTATCCGAAGCCCATCGACGTACAGAC
CCACCACACCCTGCCGGACTTCATCATGAACCGCGGCGGTGTGAGCTTGCGTCCGGGTGACGGCGTCATC
CACTCCTGGCTGAACCGCATGCTGCTGCCGGATACCGTGGGTACCGGTGGTGACTCCCACACCCGTTTCC
CCATCGGTATCTCCTTCCCGGCGGGCTCCGGTCTGGTGGCCTTCGCCGCCGCCACCGGCGTGATGCCGCT
GGATATGCCGGAGTCCGTGCTGGTGCGCTTCAAGGGCGAATTGCAACCGGGCATCACCCTGCGTGATCTG
GTTCATGCCATTCCCTACGCCGCCATCCAGAAAGGGCTGCTGACCGTCGAGAAAGCGGGCAAGAAGAACA
TCTTCTCCGGCCGCATCCTCGAGATCGAAGGTCTGCCGACCTTGAAGGTGGAGCAAGCCTTCGAGCTGGC
AGATGCCAGCGCCGAGCGCTCCGCTGCCGGTTGCACCATCAAGCTGGATAAAGAGCCGATCATCGAGTAT
CTGAAGTCCAACATCGTGATGCTGAAATGGATGCTCTCCGAAGGTTACGGTGACCCGCGCACCATCACCC
GTCGCATCAAGGGGATGGAAGAGTGGCTGGCCAACCCGACCCTGATGGAAGCGGACAAGGATGCCGAGTA
CGCCGAAATCATCGAGATCGATCTGGCCACCATCAAGGAACCGATCCTCTGTGCGCCGAACGATCCGGAC
GATGCCCGTCTGCTGAGCGACGTGGCTGGCGACAAGATCGACGAAGTGTTCATCGGTTCCTGCATGACCA
ACATCGGTCACTTCCGTGCCGCCGGCAAACTGCTCGAGAAGTATCAGGGCGAGCTGCCGACCCGCATGTG
GATCGCGCCGCCCACCAAGATGGACAAGGATCAGCTGACCGAAGAGGGCTACTACGGCATCTTTGGTCGC
GTCGGTGCCCGCATCGAGATCCCGGGCTGCTCCCTGTGCATGGGCAACCAGGCGCGGGTAGCGGAAGGGG
CCTCAGTGGTCTCCACCTCCACCCGTAACTTCCCGAACCGCCTGGGTAAAGGTGCCAACGTCTATCTGGC
CTCTGCTGAGCTGGCCGCCGTTGCCGCCATCCTCGGCAAGATCCCGACCGTGGCGGAGTACCTGCAATAC

GCCAAGGCGCTGGATGCCACTGCGGCAGATACTTACCGCTACCTTAACTTCAACGAGATCGCCTCCTACA
CCAAGAAGGCCGATGCGGTGATCCTGCAGCAAGCCGTTTAACGGAGTTCTTGCGCGATTTGCGCGATAAG
AAGCTAATAATGCCGCGTATTTACGCGGCATTATTTATTTGTGTGTTATTCACCGTCTTTTTTGAAGATT
GTTTGCACGATCCTTGCTAGAGAGATCCAACCACCTGTTTGGTGGTTCGTTTTATAAACGCTTTGGGTTA
TGTTGTTCGGTTATGTGCGGTAATGATGAGGCATCGTTATCGAGGGAAAGTGGATATCTGTTCACTGGGT
GCTTGCGTTGGCAGGCCTGACGAATTGCGGCGTGGTTCTTTATGTTTCTGTTCAGGATCGATCTCTCCCG
AACCGGGTTACGCTTCGGCGGTGCCTTGCTGGTCTTTACCCTGCCGCTGCTGGTGGGAGCTTGGCTCATC
CACAGCAATTATGTGCAGGAGCTGAAAGCGGAATCCAGCGCCTCGGCCCACAAGGCCAGAACCCTGATGG
AGTCGATGCTGGATCATGCGGAGCAGGCCAATCGCTCCGTGCTGCCTCTGGTGAATGCTCCCTGTCGCGA
CAACCTGCTGGTGCTGCGCAAGAAGGTCGCCATCGAGCCCTTCCTGCGTTCGGTCAATCTGGTACGAAAC
GGCATCATCAATTGCACCTCGCTGTTTGGTGAGGCCAATGATGCCGATGACGGCTCCATCTACACCGGCC
GCAAGCTGCTGCTGATGAGTGGCAACCGGGTGCGGCAAAATCACCCGCTGCTGGTAGTGCGCACCGAGCA
GGGCAAGGATGCCGCCTTGAGCGCCATCGACAGTACCCAGCTCAGCTTCATGCTCTCCATGAGCGGAAAA
TCCAGTGCGCTCTATCTGCAGGTGGGGTCTGCCTGGCTCAATGAGTCGGGCCGCTATCTGAGCGAGGCGC
CCACCCTTCATCGCGGGATCAGCCAGGCCCTCACCTCATCCCATTATCCTTTCACCATCCATGCCGGTCT
ACTGGAAGTCGCTGTGGGCCGCGCGGCATGTGGCGCTGTCGTGGCTGGCGGTTTCCTCGCTTTTGCTGGC
CTGGCTGGTGTGGTGGGTGCTGGGGCGGCCCGGCTCGCCAACAGATGAGTTGCGACGAGCCCTGCGGGCC
AGGGAGTTTGTTCCCTATCTGCAGCCGCTGATTGCCAGCCACGACGGCAAGATGATGGGCGCCGAGGTGC
TGATGCGCTGGGAACATGTCAATGAAGGGGTGATCCGGCCCGATCTCTTTATTCCGCAAGCGGAGGAGTC
GGGACTGATCGTCCCCATGACCACCCATATCATGAAGCAGGTGGCGACACGGTTGAACCGCTCTCAGGAG
CAGTTGCCCGATGGGTTTCATATCAGCTTCAACATCAGTGCCGCCCATTGCCACGATTTTGCCCTGCTGG
CCGAGTGCCGCGCCTTCCTCGGCCACTTCCTCCCCGGCAAGGTGGTACTGGTGCTGGAGCTGACCGAGCG
GGAACTGTTGATGTCAGATCCCCAGACCCTCTCCCTGTTCCGGCAGCTGGACGAGATGGGGGTCAAACTC
GCCATCGACGACTTTGGTACCGGCCACTCCAGCCTGGCTTACCTGCAGCAGTTCCATGTGGATTACCTCA
AGATCGACAAATCCTTTATTGCCCGCATCGGTACCGAGTCGCTCTCGGAACATATCGTGGATAACGTGAT
CGATCTGGCGACCCGGCTCGGTCTGGCGCTGGTGGCGGAAGGGGTGGAGAGCGAGCGTCAGGCTGACTAC
CTCAGGGCCAAAGGGGTCGACTATCTGCAGGGATACCTGTTTGCCCGCCCCATGCCGCTGCGCCAGTTTT
GCGAGGAGCTGCTGCCGGAAGAGGCACAAACCCGCCAGCCAGATGGGGTGCGAGGCATCCCGAACGCGAC
CTGATCTATCGGTGACAGACGGGCGGCCACGCTCCCCTCTGGCAGGGTAGGGGAGTGCTCTCTCCCTATC
CCCCCTCGAGTGCAGGGGCTTGTTTTTCATGCCTTGCGGCTCGCTTTTCGGTAGAATCAGCGCTCATTTT
GACGTCAGTAAAAGATGAGGCGCGGTATGACGGTTCAGTTCCAGGCATTTCACAGCATCAAGGCGATCGG
TAATCGGGAGGTGCTGTTCAACTGGTCGAGAACCAATCCCCTCGGAGCGCTGGATCAGGTGTGGCGCCCG
AAAAGCCGCGACGAGTTGCAAGCGCTGCTGCGGGAACACCCCGAGCGCAAGCTGCGCCTTATCGGCTCCG
GCCTCTCTTTTGAACCCATCCACAGCGTCTATGCCGAGGGCAGTCAGGCGCTGCTGGTTGACTTGCACCA
CCTGCGCGGTCAGTTGGCGCAAACCGCCGATACCGTGACATATCAGGCGGGGACGCCCCTCGATACCGTT
TATGCCGAGCTGATCGCCATGGAGCGGATGCTGCCCGCATCCCCGGGCGTTATCGGCATTCAGACCCTGG
GTGGCGCCCTCAGCACCGGCACCCACGCCCAGGGGTTGCACCAGTCAACCCTCTGCGATGCGGTGGAGGC
GATGACGGTGATGCTGGCCAGTGGCGACATCATCCGGGTGGATCGCACCGACCCGCGCTTCGGCGCCTTC
GTGATGGGGATGGGGATGCTCGGCATCCTGCTCGACGTCACCCTGCGCACCGTGCCAAATCGCATTATGC
GCTGCACCAAGTTCACCACCGATTACCCCTTCCTGCTGGAGCACAACGAGCGGCTTAACCGCGAGCACGG
CTTTGTGAAGAGCTGGTGGTTTGCCTGGACCGGCGAGAGTCACATCTGGCTGGTGGATCCCGCCTCCGAT
GAGGAGGTGGCCCGCTATCGCGCCGGCGGCAGCGAGCCGCTGCTGCTGGATGGCGATATCGACGCCCGCA
TGAATGCCACCATTGATGCCACTTTGCAGAAGATGGCGAAAGACACCAAGGATGAGGCGCTGGCTGGCGA
GCACTTCGAGACGGTGCGCCGCTTCAAGGATGCCTCCGATCTGGTGGGCAATGTCTACCAGATCCTCTGC
AAGGGGATCCCGGCCCCCCAGATCAACTGCGAGGTGGCGGTGCCGCTTCATCGCATGAACGAGGCACTGG
AGACCCTGCAAGCGTGGCAGCAGGCCAACCCGGGTGTGCTGCACTACCCCTTTATCCTGCGTTGCACCGG
CCCCTCCGAGGCCTGGCTCAGTGCCGCTTATGGCCAGCCGGTCTGCTGGATCGGTTTCCTGGTCTATCTG
GCGGCGGACGGCACCTTCGTCAACGGCTCCATGGAGCAGATGCGCGAGCTGCAGCAACTGCTGGTGCCGC
TCGGCGGCATTCCCCACTTCGGCAAGCATCTGGCGATGGATCTCTACGACTTCCCGGCCCTGCTGCCGCG
CTGGAACGACTTCGTGGCGTTGAAAGCCGAGCTCGATCCGCACGGCCGCTTTGAAAACCGCTGGCTCAGC
GACCTGTTTGCCAACCGGTAAGGATCTGACATGACCGATATGCAAGATACCCGTATCCCCGTGACCCTGC
TGACCGGCTTTCTCGGTAGCGGCAAGACCACCTTGCTCAACCACTGGGTCAAGCAGCCGGAGCTCGGCGA
GTGTGCTGTGTTGATCAACGAGTTCGGCTCGGTGGGGCTGGATCACCATCTGGTGCAGCAGGTGGATGAG
CAGGTGATGCTGCTCGACTCCGGCTGCATCTGCTGCTCGGTGCAGGGCTCTCTGGTGGAGGCGCTGCAGG
GGCTGTTTATGAAGGCCATCCAGCGCAAGATCAAATCGTTCAAGCGGCTGATTATCGAGACCACAGGTCT
GGCCGATCCTGCTCCCGTGCTTTTTACCCTGCGCGAAGAGGGCTTTATCGCTCAGCGTTATCGCTTCGAT
GGCACCGTCACCGTGGTGGATGCCGGCCATATCGAGCAGCAGTTGGCGGCTCAATATGAAGCGGTGAAAC
AGGTGGCGCTGGCGGATCTGCTGGTGGTGAGCAAAGGCGATCTGGTGGACGGCGAGCAGCTGGCGCGGGT
CGAAGCCCAGCTGGCGGCCCTCAATCCGGCGGCCCCGATCCAGCAGGTGACCAATGGTGAACTGTCGCCA
GCTGTGCTGGAGAAGCTCGGCGCCTACAACGAAGCGGCTGGGCGCGATGTGCGCCAGCTGCTGGCTTGGC
TGCGCACCGAATCCCCCAAGCAGGGGCTGGCGTCACCGATGCAGCCGAAGATGCAACCGTTCGCCGCCAA

GGTAGGCACAGCTGCGCCTACCCACTTCGAGCACGGCAACATCGAGAGTTTCTCCCTGCGTATCGGTGAG
CCGCTCAAGCCCTCTCGCCTGCTGGCCGCCATCGATGCGGTGCAGGCCCAATACGGCGATGCCCTGCTGC
GCCTCAAGGGGATCTTGCAGCTGGAAGGGGAGAGCCAGCCGGTGGTGATCCACGGCGTCCACGGCCAGCT
CTATCCGCTGCAGGCATTGGGTGACTGGCCGGAGGGAAAGGCCCAGTCCCGACTGGTGTTTATCGTGAAA
AGTCAGGACAAGGCCGGTATCGAGCAGCTCTTTATGGCGACCTTAAAGGCCCCCGAGCCCACTCTGGAAG
AGCGGCTCAAAGCCATGCTGGGTCAGCCCGACAACTGATCTTGCGCGTTGCAGCTCTCCTGCACCGGGTC
AGGACGGCGACGGTTGAACAGCACGAACGCCACCGGCACAAAGTAGAACGAGAGCAGGGTTGCCAGCGCT
GTGCCACCGGCGATGGCGATGGCAAAGGGTGGCCAGAAGCCGCCGCCCCCAGCAATAGCGGGATGAAGC
CGCCCACCGTGGTGATGGTGGTCGAGACGATGTGGCGGCTGCAACCGGCGACTAGGTCACGGATCTGCGG
CAGATTGCCCTGTTGTGCCAGCGGGTCTGCCTTCAGCTCAGCCAGAATGACGATGGCGGCGTTGATGGCC
AGCCCCATCAGTCCCAGCAGGGCGATGATCACCGTAAACCCGAAGGGGTACCCCAGCAGCCATACCCACA
GCAGCCCGAGCCCCGCCGACTGGATCGCTACCAGGGTGACGATGCCCGCCAGCCGGAACGAGTTGAAGCT
GAGCACCAGCACGGCCACCAGCAGCACGGCGACCAGGCTGACATGGGCCATCAGATTGCCCACCGATTCA
TCCCGCTTGGCGGCTTCCCCGCCAAACTCCAGTCGATAGCCCGGTGGCAATGCCAGTGCCTTGGCGGCCA
GCCTCTCTTTTACCTGCTCCAGCACGGTTTGCGGCAGTACGCCGACCGCCAGATAGGCCTCCAGCACATT
GACCCGCTCACCATCCCGACGGCTGATCTCCCCCCAGCTTGGCAGCAACTCGGGAGTGGCCAGTGCCCGC
AAGGTGAGATTGGCAGCCCCTTGCTGGCCGGTGCCGGTGAGCGATAGCGGCACATTGAGCAGCGCATCCG
GATCCTGACGCAAGGGCTCGGCCAGTCGCACCCGAACCGGGATTGACTCGGTGCCTTCCAGCACAGTGCC
GCCCAACCTGCCATCCAGTGCGCCCGCCAGCCGGGAGGCAACATCGGTCAGGCTCATGCCTGCCAGCCGG
GCGGCCTCCTCATCGACCTGCAACCAGAGTTTGGGGGCCCCTGCCTGCAAGGTGGCGCGGGTCTGCACCA
CGGCCGGGGTATTGGCGAGCAGACTGCGAAGCTCCTCTCCCAGACGGTGCAGCTGTTCAAGGTCGGGGCC
AAACAGCCGCAGCTCGACCGGGGCATTGAAAGGCGGCCCCTGTTCCAGCTTGCGCACCAGGATCTGCGCT
TCGGGAAATGTTGCATCGAGCCGCTGCTGCAGTTGTGGAATGAGCCGCTCGGCGGCATCGGCATCCTGCA
GAGTGATCATCCCCTGGGCGTAATCGGCCCGCCCCTGTTTTCTGGGCACCAGGTTGTAGTAGAAGCTGGG
TGCGTTGTCGCCCACCACCCAATCCTGACGAACCAGCTCGGGGAGGCTAGCCAGCTCGGCGGAGATCCCC
AATGCCAGCTCGCGGGTGCGCGCCAGGCTCGCCTGTGGAGAGAGGCTGGCCTCGATGTGGATCATGTCCC
GGTCGGCAGGGGGAAAGAACTGTTCAGTGAGGCGGCCCGCCACCCAGAAACCGATCAGGGGCAGCAGACT
GACCAGCAGCAGGGTCCGCCAGGGGTGAGCAAGCGCCATGGCGATGCTCTGCTGGAAACGGGCGCTCACC
CGGGGCAGGTGCAAGCCATCCTGATACCAGCGCCCTCCCTGATCACTGCCAATCCAGCGGGCTGCGAGGC
CAGCCACCAGGGTGTGGGCGATGAGGTAGGATCCCAGCAGCGAGCAGATCACCGCCCAGGCGATGCCGCC
GACAAACTCGCCCGCCGGGCCCGGCATCAGAATGATCGGCATAAAGGCGAGCATGGTGGTGAGGGTGGAT
CCCAGCAGTGGCAGCCAGAGGTGGCCGATGGCTTCCAGTGCGGCGGCACTCATGGCCATGCCGTGGCGGC
GCTGATACTGCACCCGATCCGCCATGACGATGGCGTTGTCCACCATGATCCCGAGCGCCACCACCAGACC
GGTGACCGACATCTGGTGAATGGGCAGATTGATCAGGTTCATGCAGGCGAATGAGAACGCCACGGTAAGC
GGCAGCGACAGGGCCACGATAATGGCGGCCTTGACCCCCAAGGTCACCAGCAGCACGGCCACAATGATGA
GGAAGCCCTGCACAATGTTGATGAGCAGGGTGTCGAGCCGCTGATCGGTATAACGCTGCTGATCGAAGCT
GGTGACGAGGCCGATATTATCCGGCAAGTCCTGTTGCAGCTGGGCCAGCATGGTGTCGACCCGTGCCGAC
CAGAGGTCGATGCGGTGATCCTGTGCCATGCGCAGGCCGACCACCACGGCCCGCTCACCATTGGCAAAGG
CCAGCTGGCTCTCCGGCTGGTGCAGGGTACGTCTTACCTCGGCCAGATCGCCAAGGCGCAGGGTCTGGCC
CTGCTCGCCGGTACGCAGCGGGATCTGGCGGATCCGCTCCAGGGTATCGAGGGCGCCGCGGATCTCTACC
TGAAAGCGATTGTGGCTGCCCTGCAACTCCCCGGCGGCAACTTTGGCATCGGCACCGCTCAGCGCCTGGG
CAAGCTGTTCGCCACTCATGCCGCTGGCACTGAGGGCGAGGGGATCGAACCGCACCTCGATCTCCTCCTG
CGGTGCCCCGACCCGCTCGACGTATTCGGTGCCCGGCAGCTGGCGAGCCCGGCGTTCCAGCTCGCCGCTG
TAGCGGGCGAGGCGATCCAGCTGCGGCTGGCCCGGCCCGCGCCAGACCACATTGACCAGCCGGGTGAAGG
GGTAGCCCTTCTCGTCATCCAGCTTGGGGGCACTGGCGCCGGCTGGCAGTCCGGCGGCAAGATCTGCCAG
CCGGTCACGGACTCTGGACCAGATGGGCTCGACATCGCTGACCTCATCCTTGAGCTCCAGATTGATGATG
GAGAGGCCGGGGCGAGAGCTCGACTCGATCAGCTTGATCTCGCTCTGGGCCCGCAGCCGGTTCTCGATGG
GCTCGCTCACCAGCGCCTCCACCCGCTCGGCGCTGGCCCCGGGGAAGTGGGTAACTACGCTGGCAAAACG
GCTGGTGAGCTGGGGATCTTCGGTTCTGGGAAGGGTCTGCAATGCCGCAAGGGCGGCCGTCAGCAGCAGC
GCGACCGTCAACAGCAGCAGGCGGCCATTCTCGATAAACGGGCGGATCATGACTGTGGCTCCGGATGCGC
CGCCTGATGATCCACGCTGGTGGTCAGGGTGACCTGCTGGCCCGGCACCAGTTTATGCAGGCCATCGGTC
ACCAGCTGTTCCTGTCCGTTCAGGGCACCACTGATCCAGGCCCGCTCGCCATCTTGCTGCAGCAGGGTGA
CGTCCCGACTGACCACCCGGGGTGGCGTTTCGTCGTTGAGCAGATAGAGGGTCCAGCTGCCGCGCAATCC
CTGGGTCAGTGCGGTGAGCGGTACCTCGTAGGCGCGGGTAGGCTCGCGGGTCTGATGCAGCAGGGTGATC
AGTTGCCCCTCTGCCAGTTGTTCGCCGCCCCGGGTCAGCTGGAAGCGGGCGGTGCGGGTGTGGGTCTGCC
CGTCCACTTGTACCCCCAGATTAAGCAGGCGACCCTCCAGTGATTGCGCACCGCTTTGCAGGGGGTAGCG
TTGGCCGACGATCAGGGTCTCGGCCTGTTTCACCGGCACGCCGATATCGGCCTCCCACTGGCCGCTGCGG
GTCAGAGAGAAGAGAGGCTGGCCTGCGGCCACCACGGCCCCCAATGCCTGCTCGCGGCTGACCACAATCC
CGTTGAAGGGGGCGCGCAGCTGTGATTTCTCCTGTTTGACCCGGTTGGCTTCGAGGGCGGCGGCCAGCTG
GTTGGCGCTTGCCTCCAGCATGCGGACTTTGCCTTGCTGTTCATCCAGCGCCTGACGGGAGCTGAACTGC
TGCTGGCGCAGGGCGGTGAGTCGATCGAGCGTCTGCCGGGCAAGGGTCAGTTCGGCCTCGTTCTGGGCAG

CCCGGGCGGCCAGCTCTTTGGCTTCCACTGCCAGCAGGCGGGTGTCCAGTGTGGCGAGCAGTTCGCCGGC
CGCGACCGGCTCTCCGCGTCCCTTGTGCAGAGTGGCGATCCGCCCTGCCAGCTCGAAGCCTATCTGGCTG
TGCTCGGGGGCGATCACCCGGCCATTGAAGCGGTGCAGCACCGGGTAGTGATCGCTGGCTTGCAGCGGCT
GGGCGATCACCCGGGTCAGGGCGGATGTTTCTTCTGACTCTGTGACCGGGCTGCAGCCACTCAGTAACAG
CGCCAGCAGGATTAAGCAAAGTGTCATGACATCATCCTCTGGTTGACATAACATCAAATCACACCGGTGA
GTCTAGTTTTTCAAGACTAGACTGTCCAGTTTGATTGTGGAGGAAAAATGAACATTTCACCCCGTAGTCA
GCAGAAGCGGGCTGCGATCCTCGATGCTGCCGGGCGGCTCTTCTGTCAGCACGGTCTGCATCTGGTCAGC
ATGGACATGGTGGCCAGTGAAGCGGGCGTCTCCAAGCAGACGGTCTACAGCCATTTTGCCAACAAGGAGT
CGCTGTTTGCCGAAGCGGTGGGCAGCAAGTGCGTCAGCAGCGAGCTGATGCCGGAGGTATTGTTGCGTGG
CAACAATGTGCGGGAGAGCCTGATTGCCTTTGGTCGCAGCTTTCGCAAGTTGATCCTGAGCGATGATGCC
ATCAATATCTTTCGCACCTGCATCAGCTGCAACGATGCCCAGCTGGCCACCCTTTTTTTCGAGGCGGGCC
CGCTCTACGTGACCGAGATGCTGTCCAACTGGCTGGAGCAGCAGAAGCAGCAAGGCCTGCTGTGCCATCA
AGACAGCGAACAGGCCGCTTTGCAGCTGTTGCTGATGTGGCAGGGCAAGGAGCGAATGCTCCGTGAGCTG
GGTCTGCCGTTGACCCAGAGCGCTGCCGAGCAGGATGCCTGGACCCTGAGTTGTGTCGATCTCTTCCTGA
CCGCCGCCGCTCCCCCGCAAAAATAATGGAGGTGGCAATGAGCCACACAGTGACAGATGCCCAGCGCAAG
TGGCTGCCCCTGCTGGCGGCGGTCGGTTTCTTTATGCAGGCGCTGGATGGCACCATTCTCAACACCGCCC
TGCCCAGCATGGCCGAGACCTTGGGAGAGAGCCCGCTGCAGATGCAGTCGGTGATCATTGCCTACATGTT
GACGGTGGCGTTGCTGATCCCGGCTTCCGGCTGGCTGGCTGACAGATTCGGCACCCGCCGCATCTATCTG
GCTGCCATCTCGCTCTTTACCCTCGGCTCGCTCGCCTGCGCCGCCTCCAATTCGCTCTCCATGCTGGTGG
CCGCCCGGGTGCTTCAGGGGATTGGCGGTGCGCTGCTGATGCCGGTGGGGCGGTTGGCGGTGCTGCGGGT
CTATGACAAGCATGAGCTGCTGCGGGTGATGTCGTTTGTCACCATCCCTGGACTGCTCGGCCCGTTGATG
GGGCCGGCACTCGGTGGCTGGCTGGTGGAAGTGGCGAGCTGGCATTGGGTGTTTCTCATCAACCTGCCGG
TGGGGCTGCTCGGCTTTATCGCCAGCTGGCACTTTATGCCGGAGCTGCGGCAGCAGACCGCTCGGTTCGA
CTGGCAGGGATTCGTGATGTTCAGCGTCGGCATGGTGCTGGTGTCGGTCGGCTTGCAGGGGCTGGGGGAG
CACAGCATCTCCACCGGTTGGGCGCTGTTTGCACTGATCTTCGGCTTGGCGGCGATGGCGAGCTACTGGC
TCTATGCCGCCAATGCACAGCAGCCGCTCTTTAGCCTGAACCTGTTCAAGACCACCACCTTTGCCATCGG
TATCTGGGGCAACCTGTTTGCCCGCCTCGGCAGCGGCGCCATGCCCTTCCTCACTCCGCTGTTTCTGCAA
CTGGGGCTCGGTTTCTCTCCGAGCAAGGCGGGGATGACCATGATCCCCACCGTGATTGGCGCCATGCTGA
CCAAGACGCTGGTGAACAAGCTTATTCCGAAGGTGGGCTATCGCCGCATTCTGATCGGCAACACCCTGTT
GCTGGGGGCGATGATCGCCAGCTTCTACTTTATCGACAACCAGCTCCCCTACGGGGTCTTGCTGGGCTGG
CTGGCGATCTTCGGCGCCATCAACTCGCTGCAGTTCTCCGCCATGAACACTCTCACGCTGCAAGATCTCA
GCCCGACCCTGGCCAGCAGTGGCAACGGTCTGCTCTCGGTGGTGATGCAGCTCTCCATGAGTTTGGGGGT
GGCGATTGCCGCCAGCCTGCTCGGGCTCTTCTCGGCGGGTACCTCAGCGGGCACCTTAGTGGCCGAGGGT
CAGTGGCTGGCCGGGTTCCACCTTACCTACCTCTGTGTCGGGCTGCTCTCTATGCTGGCGGCGCTCATCT
TCGCCCAGCTGGCGCGCGATGGGGCTACGCCGTCGGGGCGGGCAGATAGCTGACCTCGCAGGGGAGCTGC
GCCAACACTTCGGCCAGATGCTCGGTCGCCAGCAGAAAGGTCTGCTCCGGGTGGCCCGGAGTCCAGGTGA
AGGCGGTGTGGTTGAGCAGTTGCTGATCCACCAGTAGCGGAATATCGGCGCGGGGCTGGAAGGGGCACAC
AGCCCCCGGCAGGCAGCCGATCTCCGCCTGCATCTCTTCGGCGGTGCAGATGGAGGGCTTGGCACCGAGT
GCGGCCTTGACCGCCTTGTTGTCGAGCCGTCCGTCCCTGTGGGTCAGCAGCAGGGCGAAGCGGCCATCCT
TGAACTTGAGAAACAGGGTCTTGCTGAACTCCGCCTGCCAGCCGAGGCGGGCCTTGACCTTTTCATCGGT
GGCGTAGTCGAGGATGGGCTCGTGCTCATAGCGCTGGTGGGGCAGTTCGGCCAGTAGCTGGCAGTTGAAA
TGGTGAATGGTATCGAGTGGCATGGGGTTCTCCTGTAGTGGGCGCTATCCTGCCAGCCTCGCAGGGTCAG
GCCCAATGACACTTTCTTATCCGTGATAAGCAACCCTGTCCGTGCAAACAACAAGGGCGCCGTGAGGCGC
CCTTGTGGATCAGTGATGCTGCAATTCGCACGCTTCCCAGTGAAAGGGAATGGGGCACAGCTGCTGGGAG
GCCTTGTCATAGGCTTGCCACAGCTCGCCCATGGTGCGGCCATCGACGGGGTGGCGCAGTTTGGGGGCCA
GCTCGGCCAGCGGCCACAGCACGAAGGCGTTGGTGAGCACTTCACCGCGCGGCAGCACCAGCGGGGTGTC
GCAGACCAGATCGCCATAGAGCAGCAGGTCGAGATCCAGGGTGCGCGGGGCGAACTTCTTGGCATCAGGC
TCGCGGCCGTGGGCGAACTCCATGGCCCGCAGTCGCTGGCAGACGGTGGTCAGCGGCAGATCGGTCTCGG
CAGCCGCCACCAGGTTGTAGAAGGGGCGGCCGTTGAAGCCGACCGCTTCGCTCTCGAATACCCGGGAGAC
CCGCAGCTCGCCAAATTCGGCGTGCAGGGCATCGAGTCCGGCGCGGATATGGCGATCCCGCTCGATGTTG
GAGCCGAGGCTGATATAGATGGTGGTCATCAGTTGCTGCTCGCGGCAGGCGTGCGATGACGCAGGAGCTC
GACGCCGACCCCGGCGGCATTGGGCACGGCGCCCGGCTTGGTGAGGGTCACCTTGACCCACTGTACCCGC
TCGTCGGCGAGCACCAGCATGGCAACCTGCTCGGCCATGGTCTCGACCAGCTCGACCACCTTGCCGCTGA
CATGCTCGCAAATCTTGTTGGAGAGGTCGGCATAATCCAGTGCCAGATTGATGTCGTCGGTTGCTGCCGC
GGGGCGGTTGTCGAACCCCATCTCGAGGTCAAAACGGAGCTTTTGGCGAATGCCCTTCTCCCACTCATAT
ACACCGATGGTGGTCAAAACTTCGAGTCCGCGAATAAACACCTTGTCCATGACTGTACTTCTTCTTTACT
CTTGATGGGCTGTGATGACAGCGCAGGGATGCTGGCCGGGCAACAGGCTTGCCGAGGCGCTCTGGGTGGG
CATGGGCCCATGCCAGCGTATGTGGCCCGCATCATACCCCATTCATATCGTGGTAAAAACGACCAAGATG
ACGGCCCTGACGATTTTGATGATTATTCTGGCCTACCTGGGTGGTTCACTATCCAGTGCTGTGCTGGTCT
CCCGCTGCACAGGTCTGCCCGACCCGCGGGATCATGGCTCCCACAATCCGGGGGCAACCAATGTGCTGCG
CCTCGGTGGCCGGATGGCGGCGCTGGTGGTGCTGTTGCTCGATGTGCTCAAGGGCACTGTGCCCGTCTAT

CTCGCCTGGTATCTGCAGATCAAACCCGTCTACCTCGGCTTTATCGGGGTCGCTGCCTGCCTCGGCCATA
TGTACCCCATCTTCTTTCATTTTCGTGGTGGCAAAGGGGTGGCGACGGCGCTGGGGACCATGATGCCCAT
CGGCTTTACCATGGGGGGCGCCGTGATTGGCACCTGGCTGCTGGTGCTGCTGGTGAGCGGCTACTCCTCG
CTGGCCTCCATCATTACCGTGCTGCTGACGCCGCTCTTTACCTATCTGCTCAAACCCGAATATACCCTGC
CAGTCTCCCTGCTCTCCTGCCTCATTCTGATCCGCCATCACGAGAACATCTCCCGCCTGCTCAAGGGGGA
GGAACCCAGAGTCTGGGGGCGAAAAGGCACGGCCTGTGCCGAAGAAGTGGCAGAAATGGACGATGTTGCG
CAAAAAAGAGACGAACGAGACAAAAAGTGAAATTTCCCGTTGACCATCCGCCTTAAAATCCGTTTAATAG
CGCCCACGCCCAGATAGCTCAGTCGGTAGAGCAGGGGATTGAAAATCCCCGTGTCGGCGGTTCGATTCCG
TCTCTGGGCACCAACTTTGGTGCCATGAAAGAAAAAATTTGTTCATGCACAATGGTTTAAAGTGTGCCGG
CTTAGCTCAGTAGGTAGAGCAACTGACTTGTAATCAGTAGGTCACCAGTTCGATTCCGGTAGCCGGCACC
ATTTGCCCAGATAGCTCAGTCGGTAGAGCAGGGGATTGAAAATCCCCGTGTCGGCGGTTCGATTCCGTCT
CTGGGCACCAATTACAATTCCTCCTTAGTTCAGTCGGTAGAACGGCGGACTGTTAATCCGTATGTCACTG
GTTCAAGTCCAGTAGGAGGAGCCAATTCAAAAAGCCCTTGTCGCAAGACAAGGGCTTTTTTCTATTTGGC
GCAGCCATTTTTTAACCATAAAAACAACCACACACGCACAGATGAACCGCAGGATAGATATGGAAAATCA
TGTAGTTATAGAGTCAAAGGGGCGACTTGCCGCCATTCGAAACTGGATGGAACCCGTGCTGGTGCTGCTT
ATTCTTGGCAGCCTGTTCGGTTACCTCGGCAGCCAGATGGGCCTCTCTGCCATGGTCAACACCCTGTTCA
ATACCGCCCACCAGCTGCTGCTCAATACCGTGCTCTTTATCATGGGGATCACGGTGCTCTCCGGTGCCCT
GAGCCAGCTCTTGAGCGAGTTCGGGGTGATCCGCCTGCTGGAGGTTCTGCTGGCCCCTCTGATGAAGCCG
GTGTTTCGCCTGCCTGGCCGCACCGCGCTGGCGGCGCTGATGACCTTCTTCTCCGACAACCCGGCGGTGA
TCAGCCTCGCCAAGGACAGCCGCTTTCGCAAAGGCTTCACCCCGTGGCAACTGGTGTCGCTCACCAACTT
CGGCACCGCCTTTGGCATGGGGCTGATCGTGGTCACCTTTATGGCCACCCTGCAACTGCCGAGCGGTGAA
TCCACCGCCAGCGCGGCGCTGATCGGGTTGCTGGGTGCTCTGGTGGGCTCGGTGGTCTCCACCCGACTGA
TGCAGCGGATGATCCGCCCACTGGTGGGGGAAGTCCTGAACGATGACGCTGTATCGTCCGACGGCCGCAA
GGTAGGGGTGACCAAAGAGGCGGCTCCCGGCTGGCTGCGGATCCTCAACTCTCTGCTCGATGGCGGCAAA
TCTGGCGTGGAGCTGGGGATGGCGGTGATCCCCGGGGTGCTGATCATCAGTACCTTCGTGATGCTGCTCA
CCTTCGGCCCCGGCGACAAGGGCTATACCGGCGAAGCGTTTCAAGGGGTTGCCCTGCTGCCGGTGCTGGC
CGCCAAGGTGAGCTGGCTGTTTGACCTGCTGTTCGGCTTCCAGCAGCCGGAGCTGGTCGCTTTCCCGGCC
ACCTCGCTTGGTGCGGTCGGTGCCGCCATGTCACTGGTGCCCCCCTTTATCGCCAAGGGGTGGATCACCG
GTAACGAGGTCGCGGTGTTTACCGCCATGGGGATGTGCTGGAGTGGCTTTCTGAGCACCCACACCGCCAT
GCTCGATGCCCTCGGCTATCGCCACCTCACCTCCCGCGCCATCGTTGCCCACACCATCGGCGGCCTGTGC
GCCGGTGTGGCCGCTCACCAGCTCTATCTACTGCTGGGCTGAGTCGGGTTCAGACACGGAAACAAAAAGA
GGCTCTTCGGAGCCTCTTTTTGTTTCCCCCTGCCGCGCCCTGCTCACGGGCGCACCAATCACCCTTGCTG
CGTGATGCCCTGCACCTTCAACCAGAATCGCTCCTGACAGTGGTTGTGAAACAGCTTGCTGGCTGGAGTG
ATGGGGATGGGACTGGCGATGACCCGATAGCTGATCCAGCACTCGGCACAAAGCTGCTGCGATGGCGCTT
CGTCCTGTTCGGTTTGCTGGCGGATGACGACCCCATTGGCAAACCAGCTGGTGGTTATCTGGTGCTGGAT
CACAGTGCCGCTCTCATCATGCTCGCTATAGTGTTCACTCTCTTGGTGTGTGAGGGTGACGGGGCTCGCC
TGTCGATGAATGGCCCTGATATAGCTGATTAGTTGATTCATTGCCGATCTTGCCGTGATGGTTGGCCTGC
ACTGGATGAGCTGTACAGGATGAAGAGGGCTAGCGTAACGTGCTGCCTTGGTTGCGGCAATCGCCTCGGC
TGCGCCGCAAGCAAATCTGAAAAGGGGAGAGCAATGGGAATCCCGCAAAACAAGGCCGAGCTGCTACTCG
CGATCGATACCAATTTTAGCAAGTTGTTCAAGGCGTTGCAGGCCGTCCCCGAGAGCCGGGTGCGGGAGCC
GACCCTGGCAGGGCACAGCAAGGGAACCACCATGAGCGTCGTCAATCTGCTGGCCTACCTTATCGGCTGG
AACGAACTGCTGATCAAGTGGATCGAGCGGGATGCGGCAGGTCTGCCAGTGGATTTTCCCGAAACCGGTT
TTAAGTGGAACGAGTTGGGCGGGCTGGCCCAAAAGTTCTATCGGGATTACGAGGGGCTCAGCTATCCGGC
GTTGTTGCAGCGGTTGGCTGCCGCCAGAGGGCACATCATCGAACTGGTGGAGGCGCGCAGCGACCTGCAG
CTGTATGGCTGCCTCTGGTATGAGAAGTGGACGCTCGGGCGGATGATCCAGTTCAATAGCGCCTCCCCCT
ATCTCAACGCCAACGGGCGGCTGCGCAAGTGGCTAAAAGAGTTGGCGAGCAGGGTGTGAAAGGAAGGGGG
TCGCGGCCCGGGTGGGCGGCAAAACGCTTTGCCACCATATTGCCACTACCGCCCGTGGCGGCAGCCATTG
GCCGCCTGACACCCCTTTGCACTCGGCCATCTCCTGCGTGCTGCTTGAACAAATAGATCAGCCATTCAAG
GGATTAGCCGATTCGGTGCGTTTTATCGCCGTGCTGGCCCCGCTTATGCACATCTCTTGGCAACACCATC
AAGGGTCGTTGTCGCGGAGAGTTATCATGATCAGCAGCCTGCTTCCCCTTGCCTCGATCGCCCTCAAGCT
GGGCAGTCAGCTGCTCGGCAGCAGTGCCAAACCTGAGCCTGTGCAGCAAACAGTGCAGGAAGATCGCCTC
GCCCCGACCGTCCAGAGTTTGTTCCAGCGACTCGGCATCAAGGATGCCGATAGCGAACAGAACCGCTCGG
CCATCAACCACTTTATGGGGGAGCTGATGCAGACTCTGGGGCAGGAAAATAGCGGTCAGTCACTCCCAGC
CGCCATCGATAGCGCCCGCAACCAGCTGGCGAGTGGCACTCAGAGCACCCGCCTCGATGCCCTGCAAAGC
GAGCTCGATACCCTCAAGGATTCCCTCGGTGCCAGCAATCTCAATCTCGATCAGCTGCTCGGTGCCTTTA
AACAGACCCTGCCCAATGGCAAGGGGGAGGGGGCGGGCAAACTGCTCAATGCCAGTGTGTGAGTGAAAGT
TCATAAATGGGTTGTGAGCACAATGCAAATATCGGCCCGAAACTTGAACAGTTAATGACTACTTTATTGA
TCTGTGTCAGGTAATTATTTGTATTGTCTATTTCATATAGAAAATATCCATTTGGCAATGTAGGATAATG
TTATTAATCCGTGGTTAAATTATTTCTAATCTGCATTAGGAGGAGGTGTGAAGGAAAACTTAGGCTTTAC
TAGGGTAATGATGGTGATTGGGAGCTTGTCACCATTTTTCGTATTAGCTGCCATTAAAGGTTCAAAAATC
ATTGATGATAAATATCTACTATTAGGGTGTTTAAGCTTTATTATTATTCCTAACTTGATATTGTATATTA

GAATTTCATGTGCGATAAAAAATAATGATAAAAGATCTTTTGTCGTTAAGTCGGTAAGTGATAATAGAGA
ACATTTACTTGTTTATTTATTCGCTGTTTTAGTCCCATTGTATCAAGCTAATTTAGACTCCCCTCGTGAA
ATATCTATGCTGTTAGTGGTTTTATCATTTGTTGTTTTCCTTTTTTTACATCTTAACCTCCATTATATGA
ATTTCGTTTTTGCATTGTTTGGATATAAAATATATTCCGTTGAAGTCGGAGAAGACTTAAAGAAAATAGC
TGTTTTAAGCAAGCGAGATCAAATTAATAGAAATCAAGCTCAACATTTTTTGAGAATTAGCAATACAGTT
TACATTGATATTTGAGGCCGTATGATTCAAAATTTCGATTTCAACAACATAGTAAATATCGAATTCAGTT
TTCGAGTTGAAGATGGAGAGAATGTTAGTTTTCAATCTGCACCTACAGATCGCGAAATAAAAAATATTCT
CAGGCAAATGTTAGATGACACAGTGCGTGAATTTAACATCAATCAAGATGAGATACTGCCATATGAAGTG
TCAGAAAAGTACGCAAGTAAAGAAAAATTACGTGCAAGTATTGTCGATCCTTTATATCAAGAGACCTTGT
TGATAAGGGATGCTGAAGATGCTCAGATAAATTCTGAGGCAATAAATAATCCGGCATCAATCCCATTTTA
TAGGGTTAAATACTATGATTCTGAGGGACGAGTACTTGTTGCTGTAAGAACAGCTTCGTATTTTAAAGCG
ATGCTAAAACAACGTTCAAGATTAGTTCGTCTAGTCAATGATGCATTGGAATCATTTCAAGATAGCATTT
TCAAATTGGATTTTGATTTCGACTTTTTTATCATAGGCAATGATGTCTATATTCTAAGGCCGGCAAATTT
CGAATCAATTACAAATCTAGGTGATGAAATTTTAGAACATGCGGTTGCAAAAGTAGAAGTTATATCTGAG
GCCTTGGGGTTTATTAATTTTGAACATATACAAGAGTTGGCAGGCCGTTCTAAACGTGTGGCACGATTAG
TTGCCTCTGTTAGCTCAAGAGATGATTTAGCACAGATATCAGAACAAAATGTTCGTACGCTATCTCAAAG
AACTGGGGTTGGGTTGGTTGAGAGTGAAAATGGTCTGATGCCTTTAGAAGGTAAAGAGCTTGATTTTCTT
GAAATTCTTGATAGAAGACGATATATCGTGGAATTAATTGATGGAAATCAGGAGGCTTATCGAGCCCAAA
GTAGACAAGCAATTAGGCGAGTTTAATTTATTCACATGATTATTTTGTACTGATCTTCCTATTTATATGT
TGATATCAATAGTGATGACTATGATTTGAAAGAACGGCAACCTGAGGTTGCCGTTTTGCTTTCTATTTCG
GGTGACGACTCCCGCGAATTAACCCACCCGTTTTACCAGCCAAGCTTCGGCGGCAGTCAGGGCTGCCTCA
ACCTGCTCGCGGGCGACGCCGCCTTTGGCAACCCGCTTGGCCAGGGTCGCTTCCAGCTCCAGATTGGGGT
AGACGTCGAACTCGATCACCGGGCTGAAGCGCTGGAACTCGCCGATGGAGAGCTCCTCCAGCGGTTTACC
CACGCTGATGGCAAAGACCACGGTTTCGCCGACGATATGGTGCGCTTCACGGAACGGAATCCCCTTGGCC
ACCAGATAGTCCGCCAGCTCGGTGGCGTTGGCGTAGCCGCCTTGCGCCGCCGCCATGGTGCGCTCGCCGT
TGACCTTCAGATCGATTAGCACCAGCGCCGCCATATCGAGGCAGTCGTGCCAGGTGTCGAGGGCATCGAA
CAGCCCCTCTTTGTCTTCCTGCATGTCCTTGTTGTAGGCCAGCGGCAGCGCCTTGAGGCTCATCAGCATG
CCCATCTGGGCGCCCACTACCCGGCCGGTCTTGCCGCGGATCAGCTCCAGCGCATCGGGGTTCTTCTTCT
GCGGCATCAGCGACGAGCCGGAAGTGACCGCATCGGAGAGCTCGACAAAGCCCGCCTCGCCGGTGTTGTA
GAAGATAAGGTCTTCAGCAAAGCGCGACAGGTGGGTCATGGAGAGGGACGCCACGTGCATCAGCTCCACT
ACGTGATCCCGATCGGAGACCGAATCCAGACTGTTGCGGGTGGCGCCGGCAAAGCCCATATCGAGCGCCA
AGGCTTCGCGGTCGATGGCATAGGCGGTGCCTGCCAGCGCGCCGCAGCCGAGCGGGCTGGTGTCGAGCCG
CTTCAGCGCATCCTGCAGCCGGGAGTAATCCCGCTCCAGCATCTCCACATAGGCCAGCGCCCAGTGGGCG
TAAGTGACCGGCTGGGCCCGCTGCAGGTGGGTGTAACCGGGCAGCACCGCGGCCTGATTGGCGCGAGCGG
AGGCGACCAGCCCCTGCTGCAGGGCGGTGATGGAGCCGAGCAGCAGCTCGCCCTGCGCCTTGCACCAGAG
TTTCAGGTCGGTGGCGACCTGATCGTTGCGCGAGCGGCCGGTGTGCAACTTCTTGCCCAAATCGCCGACG
GCGGCAATCAGCTGGCTCTCGACCCAGGAATGGATATCTTCGGCGTCCGAATGGAGGATCTGCCGGGGAT
CCTGCTGCACCGAGGCGAGCAGCACCTCCATCGCCTGTTGCAGTTTGCCCTGCTCATCGGCGGTCAATAC
GCCCACCTTGACCAGCGCTTTGGCCCAGGCCATGGAGCCCTGAATATCCTGCTCCGCCAAGCGGTAATCG
AACCGCAACGAATCGTTGAACTGCTTGAACCGGCTATCGGCTCCCTGACTGAAGCGTCCACCCCAAAGTG
CCATACTGCTCTCCTTGAATGCGTTGTCGGTAGGTGTTTACGCCTGCCGTACTCGCGCGGCCTCTTGGCC
TGCGCGCAGAAATCTTGCCCTGTATTTTCCTGAATAACAGTGCGGTGGTGATGAGTGCTGTTGCCGTTGA
ACACGTCAAATCCAGCAACTGCGAATCGGTTCCCTCTCCCTCTGGGAGAGGGCTAGGGTGAGGGTAATTC
CCCCCTATCCCCGCCCCTTCTCCCGCAAGGGGAGAAGGGAGAAAATACGTCAGCATCATATTCACACAGC
CCCTGTTATCTCACAGAAGCGGCCGGGGCGCTGCCGCTCTTGTCACACCGCCGATGATGGGTCAGGGGGC
TATGCTCTCTGCCTGTAGAAGAGGGGGCCATGCCCCCTCAACGGAATCAACGGGTTATCGCGCTTGCTTA
GCCGTGGGTGTGATCGCCACCGATGGCTTGATGCTGCTCCTTCATGGCGCGGATCCGGCTGGCCAGGGTG
TAGAGACGGATAAAGCCTTCGGCATGGCTCTGGTCATACACTTCATCGGCGCCGAAGGTGGCGAAATCTT
CGGAGTAGAGGCTGTTCGGCGACTTCTTCTGCACCGCGGTGACCTGACCCTTGTACATCTTCAGCACCAC
TTCACCGTCCAGATCTTCGGCGATGGCGTTGGCCGAGGCGAGGAGCGCCTTGCACAGCGGAGTGAACCAG
CGACCGTCATAGACCAGATGGGAGAACTCGGCACCCAGCTTCTCCCGCCAGGCGCGGGTCGGGCGATCCA
GTACCAGCTCTTCCACGGCGCGCAGCGCGGCGACCATCACGGTACCGCCCGGGGTTTCGTAGCAGCCGCG
GGACTTCATCCCCACCATCCGGTTTTCGGTGATGTCGATGCGGCCCACGCCGTGCTTGCCGGCACGCTCG
TTCAGGGTCATCAGGATCTGGTGCGGGCTGAGCGGCTGATCGTCAACGGCCACCACTTCACCCTGCGCCA
CGGTCAGCTTGACGTACTCCGGCTGATCCGGTGCCTGTTCGGCCGGTACGGTCCACTGCCAGACCGCTTC
GGACGGCTCGTTCCAGGTGCTCTCCAGCTCGCCACCCTCGGTGGAGATGTGCCAGGCGTTGGCGTCGCGG
CTGTAGATCTTCTTCAGGGTCGCCTTGCAGGGGATGTTGCGGGTCTCCAGATAGGAGAGCAGATCTTCCC
GGGAGCGCATGTCCCAGATCCGCCACGGGGCGATCACTTTCAGCTGGGGCGCCAGCGCGGCGACCGCGCC
TTCGAAACGCACCTGATCGTTACCCTTGCCGGTGCAACCGTGGGAGATGGCATCGGCCCCTTCCGCCAGC
GCCGCTTCCACCATCGCCTTGGCGATCACCGGACGGGCCATGGAGGTGCCCAGCAGATAGGTGCCCTCAT
AGACGGCGCCGGTCTTCAGGGTCGGGTAGACGTACTCTTTGACGAACTCTTCCCGCAGATCCTTGATGAT

GCACTTGGTGGCGCCGGAGGCGATGGCCTTCTGCTCGATCCCTTCCAGATCGTCGCGCTCCTGACCGACG
TCGGCCACGAAGGCGATGATTTCAGCGTCATAGTGCTCTTTCAGCCAGGGAATGATGGCCGAGGTATCCA
GTCCACCCGAGTAAGCCAGTACGATCTTGTTGATTCCGCTCATGTTACTTCTCTCCGTGATGATTCGATG
ATGATTCAATAAAAGGGTCGTGATTCAGGTTGAGTGCCGCGTTACTTGCCACCCAGAAGGGTCAGCAACA
CGGCGTTCTGGATATGCATCCGGTTTTCGGCCTCGTCCATGATGAGGGAGGCGGGGCCATCCATCACCTG
CGAGGTGATCTCCAGCTCCCGATGAGCCGGCTGGCAGTGCAGCACGTGCTGGATGCCGGTGCGGTCGAGC
AGGGCCTGGTTGATCTGGTAAGGCATAAAGAGGTCTTTGACCTGCTCCATCGGGGTGTTGTCGCCCATGG
AGACCCAGGTATCGGTGTAGGCCACGTCGAACCCTTCGATATCCGCCACGTCATCACTGATGTGGATGGT
CGCCCCCGACTTGGCCGCCAGTGCCTGCGCCTGCAGGAAAATCTGGGTATCCGGGCCATGGCCTTTCGGG
CAGATGAGGGTGACGTCGGTGCCGAGGGTGGCCCCCAGCAGCAGCAGAGAGTGGCTGACGTTGTTGCCAT
CACCGAGGTAGGCAAGCTTCACCTTCGACAGGTCGTCGTAGTGCTCGGCAATGGTCATAAAGTCGGCCAG
CCCCTGACAGGGGTGGTAGAGGTTGCACAGGCTGTTCACTACCGGCACTGTGCCGTGCGCTGCCAGCTCG
ACCAGGGTCTGGTGATCGAACACCCGGGCGACGATGGCATCACACCAGCGGGACAGGTTGGCGGCGAAAT
CCTTCACCGACTCCCGCTTGCCCAGCGCGCCGTTCTGCTGATCCAGATAGACGCTGTGGCCGCCCAGCTT
GGCGATGCCGATGTCGAAGGTGACCCGGGTGCGCAGGGAGGGCTTCTCGAACAGGGTGACCACACTCTTG
CCCGCCAGCGCCTGGCTGTACTTCTTCGGGTCGGCCTTGACCGCTTTGCCCAGCGCAATCAGTGCCTCGA
TCTGGGCTTTGTTCAGATCACTGTCTTTCAGAAGATGTTGCATCGGATGTTCCTTGTTCCGGTGGAGATC
AAATAGCTACCTGGGTGCCGACCGCGCCGCCAGCCAGCAGCTTGAGCAGCTGATCCGGGTAACGCCAGCT
GGCGACACAGACCGGCTTGCCGAGGGTCTCTGCGGCGTGCAGGGCCGCTTCTACCTTGACCGCCATACCA
TCGCGGATCACCCCTTTCTCCATCAGATCCAGTGCGGTCAGCTTGTCGAGCTGGGGCACCAGCTTGCCCT
TGCCATCGAGGATGCCACTCACATCGGAGAGCATCACCAGATCGGCGCCGAGCGCCTCGGCAATGGCGGT
GGCCGCCTGATCCGCGTTGACGTTCATCAGTTGCCCCTCGGCGGTGATGCCGATGGAGCTCACCACCGGC
AGGAAGCCCTGACCTAGGATGCCTGCTACCAGCGCCGGGTTGCCCGGCTTGCACTCGCCGACTGCGCCGA
GATCCGGGTCGAGCTGGGTCACCTGACAGAGGCCGCCATCCGCCAGGCAGAGCCCCACAGCCGGAATGCC
GGTGGCGATCGCCTTGGCCATCATCTGCTTGTTGGCTGTGCCCGCCAGCGCTCCGGCAATAAAGGGGATC
TGCTCGAACGGGGTCACCCGCAAGCCGTTCTTCTTGGTGGAGGTGAGACCCAGCCCCTTGAGCAGGTCAT
CCACCAGACAGCCACCGCCGTGTACCAGCACCAGCGGACGGTGCTGATCGTCGAGAAAGGTTTTGAGGGT
GGCGAACAGGGCGGTCAGCGCCTCGTCGTTCTCGATAAGGGCGCCGCCCAGCTTGATTACCAACGTCTGT
TTGTCCATGTTCGAGATCCTTCCTAAGTCCTTAAATCAGGCCGGTGGCCGGTTCAAATCCAAATTTGATA
TTTATGCACTGCATGGCTTGCGAGGCGGCGCCCTTGAGCAGGTTGTCGATGGCGGAGACCACCACCAGCA
TGTTGCCCTGCTGTTGCCAGGCGATATCACAGTAAGGGGTGTTTGCCACACCGCGAATAGAGGGCATCTG
GCCGGTGAGGCGCACCAGCGGCTTGCCTTCGTAAGCCTTGAGGAAGGCCTGATCCACCTGGGTGGGGGTG
ACGCCGTCCGCCAGCTGCACATAGATGGTGGCCAGGATGCCGCGCACATAGTTGCCAAGGTGGGGCTGGA
ACAGCACCTCCTTGCCGAGGTGGTGGCTGATCTCCGGCTGGTGTCTGTGATTGAAGGTGCCGTAGGGATT
GAGGCTCACTTCGCAGAAGCTGGTATTGATGGCGGCCTTGCGCCCGGCGCCAGAGACCCCGGAGACTGCG
TTGATGATGGGCTGCCAACCCTTGGCAATCAGGCCATCCTGCTGCAGCGGCTTGAGGGCGCACAGCGATG
CGGTCGGGTAGCAACCCGGCACCGCAATCAGCTGGGCGCTGGCGATGGCATCGGCATTCCACTCCGCCAG
GCCATAGGCGGCCTGATCCAGCCACTGTTCGCTCTGGTGGGTGAAGCCATAGAAGCTGTCGTAGAAGCCC
TGATCCTTGACCCGGAAGGCGCCGGAGAGATCGAACACCGGCAGCCCCTTGGCGAGAAACTTGGGTGCCA
GCTCGGCGCTCACCTCGTGGGCGGTGGCCAGCAGCACCAGATCGGCCTGGGTCAGGATCCGGGTCATGCC
GTCGTCGTCCAGCGGTAGCAGCGGCTGATCGAGGGCGCCGACCCACTGGGAATGCAGGGAAGAGAAGCGC
TTGTGGGCATCCTGACTGCCGGCGGAGACATAGAGGCCGAACAGTTTGAGTTGTGGGTGGTTCTGTACCA
GTGCGGCCAGTTCGGCTCCCGCGTAGCCACTGGCACCGACGATAACGGTGTTAAGCATGTTGAATATTCC
AAGTCAGAGTTCGAGATTCACAGCATGGCGCTGCAGTGCACAAGGGTGTTCGAGCGCCCCTTTGCACCGT
CTTGATAAAACAAGTGCGGAGGGGCAATTCAGGCGGGTGTGTGTCGTCGGTTATAGATCAGGTGACAGAA
CATTAACTTTCCCTTACAACTAGAAGCCTTTGTTGATGAGCCAGATATTCAGGTAAAATTGTAGAATATT
CATGCACTCACTTTGCATTTGTATTTCTAACAGAAACATTCCGGGGACGCAAGGAGGAGCTGATGGCCA
ATCTGGATTTTTTTTCACTTTATAAGAATATTATTGCGATTCCCTCTATCAGCAGCACGGATCCCAAGTG
GGATCAGAGCAACGAGGCGGTGATCCGGCTGCTGGCCGACTGGTTCGGTCAGCTCGGTTTTCAGTGCGAG
GTGACGGCGCTCCCCGACCTGCCGGGCAAGTTCAATCTGGTGGCTACGATCGGTCAGGGGGAGGGTGGTC
TGCTGCTGGCAGGTCACACAGATACTGTGCCGTTCGATGAGGGGGGCTGGAGCAAGGATCCCTTCAAGGT
GACGCAAGAGGGCAATCGCCTCTATGGTCTCGGCACTATCGACATGAAAGGCTTCTTCGCCTTTATCGTC
GAGGCACTGAAAGAGATTGACCTCACCAAACTGACCAAGCCGCTGCGTATTCTCGCTACCGCCGATGAAG
AGACCACCATGGCGGGGGCGCGTGCCATCGCCGCCGCTGCCGAGCTGAAACCCGACTACGCCGTGATTGG
CGAGCCGACCGGGCTGGTGCCTGTGGTGGCCCACAAGGGGCATATGTCGGAGGCGATCCGCATTACCGGC
AAGAGCGGCCACAGCTCGGATCCCGCCAATGGCGTCAACGCCATGGAGATCATGCACAAGGCGATGGGGC
AGGTGCTGCGCTTGCAGCAGGATCTGAAAGATCGCTACGCCGACCACCGCTTTGCCGTGCCGCAGCCGAC
CCTGAACCTGGGTTACATTCAGGGCGGCGACAGCCCGAACCGCATCTGTGGCTGCTGCGAGCTGCATATC
GACCTGCGACCCACTCCGCAGGTGGGGCCGGATGAGTTGATGGGGATGCTCAAAGAGGCGCTCTCTCCCA
TCGAAATTCACCAACCCGGCTGCCTGCATCTGCAACATCTGCATGAACCCATTCCCGCCTACGCCTGCGC
CGACGACTCTGTGCTGGTGCGCGAAGCAGAGAAGGCGAGCGGCCGCGCCGCCGAGTCGGTCAACTACTGC

ACCGAGGCGCCGTTTATCCAGCAGCTTGGCTGCGAGACCATAGTGATGGGCCCCGGTCATATCACCCAGG
CGCACCAGCCGGATGAGTATCTCGACCTCTCCTTCGTCAAGCCGACCACCTCGGTGTTGCAACACCTGAT
CAGACGTTTTTGCCTGTAGCTGCGGCGGTGAAAGGGGCGTAAGAACTGTAAGTAAGTTACATAACAAGCC
GGTTGTGGAGTGGTGATGGAAATACATGTTCTGAATCGGCCTGTTATTTGACCTTGCGCAGACAATCTGG
GTAGATTGTCACCCTAAGATGACGGAGCATATGTTGTAATCGCACAACAATAAGTGAGGTCGTCTAGCAC
CCCGGATGGGTGTCAGGCACCAACAAGATAAGGATGTGGAATGAACGAAAAGTACGCCGCACTACGCGCC
AACGTAGGTATGCTGGGTCAGCTGCTGGGTAAATCCATCAAGGATCATCAGGGTCAGGCCTTTCTCGACA
AGATCGAAACCATTCGCCAATTGGCCAAGTCCTCCCGCAAGGGCAATGAATCAGACCGGGAACGTCTGCT
CGATACCCTGCGCACCCTGAGCGATGACGAACTGCTGCCGGTGGCCCGCGCCTTCAGCCAGTTCCTCAAT
CTGGCCAACGTGGCGGAACAGTTCCACACCATCTCCCGTCGCTGCGAAGAGCAGATCTGCACCCCGGACC
CGCTGGAGCAGATGTTCGACAAGCTAAAAGCCTCCAACCTGTCGCAAGAGGCCATCATTCAGGCCGTGCG
CGAGCTGGATATCGATCTGGTGCTGACCGCTCACCCCACCGAGGTGACCCGTCGCACCCTGATCCACAAG
CACGTGCAGCTCAACGACTGCCTCGAGGCGCTGGAGCTCTCCGACCTGCTGCCCCGCGAGCGGGACAAGA
TCCTCAATCGCATCGAACAGCTCATCAATCAGGCGTGGCACACCAACGAGATCCGCGAACAGCGCCCGAC
TCCGGTCGACGAGGCCAAGTGGGGCTTCGCTGTGGTGGAGAACAGCCTGTGGCCCGCCATTCCCGAATTC
ATGCGCAATCTGGACGAGCGTCTGCAACACCATCTGGGTGTGCGGCTGCCGCTGGATGCCGCGCCGGTCA
AGTTCACCTCCTGGATGGGCGGTGACCGTGACGGCAACCCCTTCGTTACCGCCAAGGTGACTGCCGAAGT
GCTGGAGCTGGGCCGCTGGATGGCGGTGAGCCTGTTCTACAAAGACATCAAGGAGCTCACCTCCGAGCTC
TCCATGTCCGACTGTACCGACGCCGTGCGTGCTCGCGTTGGCGATCACCCCGAGCCCTACCGTGCGCTGG
TGCGCGAGCTGCGTGAAGCGCTGCGCGAAACCAAGGAGCACCTTACCGCCAAGGTGCAGGGTCAGGTGAG
CGAGAGCCAGGATCTGGTCAAGACCACCGCCCAGCTGCGCGAGCCGCTGGAGCTCTGCTACCACTCTCTG
CACGCCTGCGGCATGGGCAACATCGCCGATGGCATGCTGCTGGATGTGCTGCGCAAGGTCGCCTGTTTCG
GCATCCATTTGGTCAAGCTCGATATCCGTCAGGATGGCGAGCGTCACGGGCTGGTCTTCTCCGAGCTGAC
CCGCTATCTGGGTCTGGGTGACTACGCCGAGTGGAGCGAAGATGACAAGCAGGCCTTCCTGCTCAACGAA
CTGAACTCCCGCCGCCCGCTCATTCCCCACGACTGGGAGCCGAGCGCCGAGGTGCGCGAGACGCTGGATA
CCTGTCAGGTCATCGCCGAGCACGACCCCGATGCGTTCGGTATCTACATCATCTCCATGGCCGGTGCGCC
TTCCGACGTGCTGTCGGTGCAACTGCTGCTCAAAGAGGCGGGCTGCAAGTTCCGCATGCCGGTCGCCCCG
CTGTTTGAAACCCAGGATGACCTGATGGCAGGCACCGCCGTGATGGAGCGTCTGCTCTCGGTGGACTGGT
ATCGCGGCTACATTCAGGGTCGTCAGTACGTGATGATCGGTTACTCCGACTCTGCCAAGGATGCGGGCAT
GATGGCCGCTGGCTGGGCCCAGTACGCCGCCATGGAATCCCTGGTGGGGCTGGCCGAAGCCAACAACATC
CGCCTCACCCTGTTCCATGGCCGTGGCGGTACCGTCGGTCGTGGTGGTGCGCCTGCCCATCAAGCCATCC
TCTCCCAGCCGCCGGGATCCCTGCGCGGTGGTCTGCGTACCACCGAACAGGGGGAGATGATCCGCTTCAA
GTTCGGCCTGCCGAAAGTGGCGATCAAGAGCCTCGAGCTCTACACCAGCGCCGTGCTGGAAGGGAACCTG
CTGCCACCGCCCAAGCCGAAAGAGTGCTGGCGCGAGGTGATGGAACAGCTGGCCGCCGACTCCTGCGATC
ACTACCGCAGTATCGTGCGCGGTCATCCGGACTTCGTCCCTTACTTCCGTGCCGCCACCCCGGAGATGGA
GCTCGGCAAGCTGCCGCTCGGCTCCCGTCCTTCCAAGCGCAAGCCCAACGGCGGCGTCGAGAGCCTGCGC
GCCATTCCGTGGATCTTCGCCTGGACTCAGAACCGCCTGATGCTGCCGGCCTGGCTCGGTGCCCACAAGG
CGCTGCAACAGGCCATCGACGGCGGCAAGCTGGCGGTGATGGAGGAAATGAGCGCCCAGTGGCCCTTCTT
CCGCACTCGCCTCGAAATGCTGGAGATGGTGTTCCTCAAGGCGGACGTCTGGCTCGCTGAGTACTACGAC
ACCCGTCTGGTGCCGCAGGAGCTGTGGGGCCTTGGCAAACAGCTGCGTCAGGAGCTCGCCGAATCCATCG
AGGTGGTGCTGAAACTGAGCCCGCGCGGGGATCTGCTGGAGGATCAGCCCTGGATCAAGGAGTCCATCAA
GCTGCGCAACCCCTACACAGACCCGCTCAACGTGCTGCAGGCCGAGCTGCTCAACCGCTCCCGCAACCAC
CCGGAGACCCTGCACCCCGAGCTGGACAAGGCGCTGATGGTCACCATCGCCGGTATTGCCGCAGGTATGC
GCAACACCGGTTGATAGCCAGCTCAATCCAGCAGGCTTCAGCGATATCCATCAAGCCAAAGGGCGCCGAC
AAGGCGCCCTTTGTGTTTGTTGTTCGGAAGAATTTGGACAGATGGGAACCCCGTCTTGATGTGAGGCTGT
ATTTCGTAGGGTGCAATAAGCGCAGCGCATTGCACCACTCGAAAATGTGTTTTGCATGGAGTTATCCTGC
GTTTTATATGGGCAAGTGGTGCAATTCACTTCGTTCATTGCACCCTACGGATGACGAGCGCAGATTGCTT
CTCCGGCTTGCATCCCTCGCCACGCAGGCGCAGACTGTTTGGCCTTGTTTTTATGCGATTCGATTCAAGG
CAACCATCATGACTCCTGCCATCAACCTGCTGAAGAAGCTCAAAATTCCCCACAAGCTCTACCCCTACGA
ATGCGAAGCCCACGACGATTTCGGCAAGCATGCCGCGACCCAGCTCGGGCTGCCGGAGGCGCAGGTGTTC
AAGACTCTGGTCGCCCATCACGACAAGCAGGCGGTGGTGGCTATCGTTCCCTCATCCGGCATGTGCAGCC
TGAAACAGCTGGCCAAGGCGACCGGCCTCAAGAAGGTGGAGATGATGAAACCGGCGGAGGCGGAGAAGCT
GACCGGTTACAAGGTGGGTGGCATCAGCCCGCTGGCCCAGAAGAAACTGCTGCCCACCGTGCTCGACGAA
TCGGCGCTGCAGTTTGACGAGATCCTGGTGAGCGGCGGCAAGCGCGGCCTCTCGGTCGGCGTGGCGCCGC
AGGATATGCTGACGCTGATGAAGTGGATCGCCGCTCCCATCGGCGAAGAGTAGTGCCGGGCACGGTGTGA
ACCTGCCCTCCGGGCCTGTGGCGGCCCGGATGTATCCTTGTTGGCTCACTCCTGTTGGCTGATATAGTGG
AGATGGAATAATTGCCGCCAAGGATATGGCCTATGCGCGCATCTGCCCGCTCCCTGCTCTTTATCGCGTT
GCTGCTGCCCGCCATCGGCGGTGCGACCCCAGCCTTTACGCTGCGAACCTGCTTTGAAAACAGTGACTCC
TACCCCTGGCTGCTGCAATCGGGGGAGGGGGTTGTGCAATATCACCTCAAGGCGGTTGCTACCGCTCTTG
ATGCCGAGATCGTGCTTACGCCGCTACCGTGGAAGCGCTGCCTCTCTCGGGTGAGCAGTGGCCAGATGGA
TGCGGCCATCAAGATGAGCTACAGCGTCGAGCGGGCGACCACAGTGGGGGTCTATCCCATGCGTGAGGGC

AAACCCGACCCTACCAAGCGGCTGCTGACCGAGAGTTACAGTCTCTACCAGCTCAAGGGGGGTAAGAGCC
AGTGGGATGGCACCACCCTCAGGGTCAATGGCATGGTGGCTGCCCAGTCGGGATTTTCCATCGTCGACTT
GTTGCAGGCTGCGGGAGCCGAGGTGGATGACTCCAGCCGTGACCCGCTGATCATTTTGAAAAAGCTGGTG
ATGGATCGGGCGCAGGCGACCGCCATCCAGACCGAGGTGGCTGACAGTATTCTGGCGGCCAATCCCGCGT
TGCAGACTCGCATCGAGCGGCTGTCGCCGGTGCTGGTGGAGAAGCCCTACTATCTGGTCTTCTCTCACCG
CTTCTATCAGGCCCATCCCCGACAAAGCCGACAGGTGTGGGATGCCATCGAGCAGGTGCGCGACTCTGCT
GCCTATCAGCGTTATGCCACAGCGCTAAGAAGCACGCCTCCGGTGGGGGAGTAGGCTGATGGATGCGACT
GGCCGAAAAGGGGAGGAGAGCGTGGGGGGGCAGGGCCGAGCGGCCACGCTCAGGGCTACACTGAAAGTAC
CCCTTCAGCCAAGGTGCGGTTTATGTCACCCCTGCATCTGCTTGCTGCCGCTCTGCTCTGCATGTTGTCG
CTGACCGGACAGGCGGCCACCCGGTTTACCCATATCAGCCCGGAGAGCGAGCGTGATCCGCGCACCACTT
ATGATCGGGAGCTGCTGCGGCTCGCCCTCGACAAGACCCGCGACCAGTTTGGCGACTATCTGCTGGAGCC
TGCGCCGCCCATGACCAAGGCGCGCGAGCGGCTTAGCATGCAGCTCGACAGCTATCCCAACCTCTTTGTC
ATGGACAGCTACAGCAGCACCCGCGAGGATCTGGGGCTCGCCTATGTCCGTTTTCCCATCCATCTGGGGA
TCGTCAGCTACCGCATCTGTTTTGTCAGCCCGCAGCAAAAGGCGGCTGTGAGCAAAGTGACCGACCTCAA
GGGGCTGCGCCAGTTCACCTTCGGTCAGGGCAAGGGGTGGCTGGATGTGGATATCCTGCGTTACGCCGGG
ATGGAGGTGGTGGAAGTGGAGGGGTACGAAAAGCTGTTCAAGATGGTGGCGCGGGGCCGCTTCGATCTCT
TCTGCCGCGGGGTGAGCGAGCTGCGCAGCGAGCAGCTGACCCATAAGGAGATGACGGATCTGCTGGTCGA
TGACGCGCTGTTGATCTACTACCCGCTGCCCCGGGTTTTCTACACCCATCCCAACAATCGCGAGGCGTTG
CAGCGGGTCGAGCGAGGGTTGCAGCTGGCGTGGCAGGATGGCTCGCTACAGGCGCTGTGGCGCAAGAGCT
TTGGCCCGGCCATCGCCTTTGCCAAGCTCTCCCAGCGCCGACTGCTGCGACTGGATAACCCCTTCCTCGA
CGGGATCAGCTTCGATTACCGCCCCTACTTCTACAATCCGCAAACCGATCGCTTCGGCGATTACTGAGGT
GCCTGCCGCTGGCGGCAGAGGGCCAGCCAGGCCTCGGCGGTGCGCGACAGATAGCGCTCGCTCGGCCAGA
TCACCCCGAGTCGCCAGCTCAATTCGGGCTCCAGTGGCCGCAGCACCAGACCATCCGGAGTCAGCCGGTT
GCAGACCGGCTCCGGCAAGAAGGCGACCCCCATGCCGCTGCGTACCATGGCGGTGAGAAAATCCCACTGG
CTGCTGCGCGCCGCCACCTGTGGCACAAAGCCCGCCTCCTGACACGCCTGCTCCAACCGCTCGCTCAGAG
TAAACGCCTGGGTATAGAGCAGGAAGGGCTCCTCCTGCAGGGACTCCAGCCGCAGCGGCGCATCGCCATG
CCAGCGGGGTTGATCAGGCAGCACCACCCGGATCGGGTATTGATCCAGCTCCAGCGCGCTGAGGGCGCCC
CCCTCTTTGGTCGGCAGCATGGTGATGGCGAGATCCAGCTCCCCCTCCAGCACCGCCTGTTCGATGCGAC
GGCCGCCGTACTCCACCAGGGTCAGCTCCACCTTGGGATAGCGGGTGCGGTAGGCGCGGATCAGATCCGC
ATAGACGTGACCCACCATGGGGGGAATGCCGAGGGTGAGGCGACCATGTTGCAGGCTCTGCAGATCCGCC
AGTTCGGCTTCCAGCTGCTGCATCTCCGCCAATATGGTCTGGGCTCGGTTGAACAGCACCTGGCCGCTGT
CGGTCAGGGTGAAGCGGCGCCCTTCCCGGTTGAGCAGGGGTTGGCCCAGCTCCTCTTCGATATTGCGGAT
CATCTTGCTGATGGTGGGCTGGGTGACGAACAGCTTTTCAGAGGCGCGGGTGAAGCTTTTTTCCCGCACC
AGTTCGACAAAATAGCGCAGGGCGCGAATATCCATGGACTCTCTCGACTCGTGTTGGCGGGTACGGGATG
AAGTATGCCTATGTGGCATGAATTTGATAATTTTAATTCATTTCTGGCAGGTTTTGCTCAACTCTATACT
GTGAGCCGGTTCACAGAAAGACGATGATCATGAAAGCCCTCTGCTTGCGCTGGTTACAGACTCCCTTCCA
GATTGTGTTGCTGGCAGCCATCTGGCTGCTGGCCGATACCGCGGTTCGAACCCTACATCTGCCGCTGCCC
GCCAACCTCACCGGCATGCTGTTGCTGCTGGCCTGCATCCTGCTCGGAGTGGTTAAGGCCGAGTGGTTCA
GCGCGGGGGCTCGCTGGCTGCTGGCGGAGATGCTGCTTTTCTTCGTGCCCGCCGTGGTCGCGGTGGTCAA
CTATCAGGATCTGCTGCTGCAGGAGGGGTGGCGCATCATGGTGGTGCTGCTGGTGAGCACCACGCTGGTG
CTCGGCACCACGGCGCTGGTGGTGGACCGGGTTTACCGCCTCGAACTGAAACTGGCCCGCCGGAGTCGCC
GTCATGTCTGATACCCTGCTCGGGCTGATCTGTTTCGCCCTCACCCTGCTCTTCTACTACGCCAGCAAAT
GGCTCTACGGCAAAAAGCGGATCCTGCCGCTGATGCCGCTGCTGCTGGCGCCGACCCTGCTGGTGGCCGT
GGTGCTGCTGTTCCACATCCCCTATCAGGACTACATGGCGGAATCCCACTGGCTGCTCTGGCTGCTGGGC
CCGGCCACCGTCGCCTTCGCGGTGCCGGTCTACGAGAACCGCCAGCTCATTCGTCGCCACTGGCTGTCGC
TGTCGGTCGGAGTGGTCGCCTCGGTCATCATGGCGGTGGGGAGTACCGTGGTGCTGGCCCGCTGGCTCGA
TCTCTCCGACATGCTGCAGCGCAGTCTGGCGATGCGCTCCATCACCACGCCGTTTGCGGTGGAGGCGACC
CGCGCCATCGGCGGTCAGAGCGATCTCACGGCGCTGTTTGTGGTGCTGACCGGCGTCATCGGCATGGCGG
TGGGGGAGAGCGTGCTCACCGTGCTGGCCATTCGCAGTCGCCTCGGCAAGGGGGCGGGGTTTGGTGCCTC
GGCCCACGGCGCCGGCACTGCCAGGGCTTATCAGATGGGTAATGAGGAGGGAGTGGTCTCCAGCATGGTG
ATGATGATCGCCGGTATGGTGACCGTGCTGCTGGCACCCCTGCTGGGCCGGATGTTCTGGTAGGCCGGGC
TTCCCTTTAGAAGGCTGCTCGTCTCGGCGCTACATTCTAGTGAGCCTGTATTTCCGGGATGAGCAGTATG
ACCTTTCATCGTCTCGCTTCCTGCTTGCTGCTGTGCAGTCTTTCCCTCCTTGTCACTCCCCTACGGGCGA
GCGACGTCATCACTCATATCAGCCCCGAGAGCGATCGGGACTTGCGTACCCTCTATTTTCGCGATCTGTT
GCAGCTGTCGCTGGAGAAGACCCGCGACAGCTTCGGTGACTACGAGCTGCGAACCGCGCCGCCGATGAAC
CGTATCCGGATGCGACAGATTGTGCAGACTCAGCAATACCCCAATCTGTTTGTGGCCGACAGTCTGCGTC
CGGCCGCGGAGAGCGGAGCGCTGGATTACGTCCGTTTTCCGGTGGAGCTGGGCATCCTCGGTTATCGCAT
CTGCTTTGTCAGTCCGCAGCAGGCCGACGCAGTGGCAAAGGTCAATACCCTGACCGAGCTGCAGCAATTT
CTGCACGGGCAGGGGCGTGGCTGGCAGGATGCGGAGATCCTGCGTCATGGGGGATTCAAGGTGCAGGAGA
TCGATGGTTACGAGCGGCTGTTCAAGCTGGTGGCGCGCGGTCGCATCGACCTCTTCTGCCGCGGGGCCAA
CGAGCTCTTTATCGAGCAGGAGCAGCACAGTGATCTGCCCGGCCTCACCGTGGATCAGCGAGTGGCGCTA

CACTATCCCTTCCTCCACCTCTTCTACACCCACAAGGATAATCAGGCCGCCATCCGCCGCCTCTCGGTCG
GCCTCAAGCTGGCGTGGCGGGATGGTTCGCTGCAACAGCTCTGGCTGCGCCACTTCAAACCTTCCCTCGA
TCAGGCGCAGCTCCCCAAACGCCTGTTGTTCGAGTTGGCCAATCCGCGCCTTGATAAGGTGGACTTTGAT
TATCGGGCCTACCACTACAACCCCGTCGACGGGACGTTCGGACCCGGGCTGCCTTGATCCTGATGGTGCT
GTTCCCGGATAAAGGGCCGTCTCTGCCGATGTGCTTAAGCATGCCAAAAAGGCATGTTTTTTATTATTTA
AATTCATTTTTTGAAGGTTGGTGCGCTGCATATACTGGGGGTCGCTTCACGGTTCAGGTTTGCTCCTGCC
CGCTTCTGGCTTGTGCGAGTGAGCCTGAATCGTCCTGCTGCGGCGTCTTGATGTTCTGCATGAAGGCGTC
CATCACTGGGCCGGGAGCGATCCCGGCAGGTATTACAGCTGACGGAGCCTTGTTATGTTTAATCCCCACT
CTTGTGCCCTGATTGTCGAAGGGGGCGCGATGCGCGGCATCTTTGCCAGCGGCGTGCTCGATGCCTTCGT
GGAAAGCCATCACTATCCGTTCGATTTCGTCATGGGCGTCTCGGCCGGTGCCACTAATCTGGTCTCCTAT
CTGAGCGAGCAGCCGCGCCGCAGTCACCACATCATCACCGAGCTGGCTTGCAGCTCCCGTTTTCTCGATC
CCCTGCGTTTCGTCAAAGGGGGCGATCTGGTGGACATCGGCTGGCTATGGCAGGCCAGTCGCCATCGCTT
CCCGCTCGATCTCGAGACCTTCTGCGCCCGCGACATTCCCTTCTACGCGGTGGCGACCGATGTGGCGAGC
GGCAAGGCCATCTATCTGCCGGTGCGGCCCGACAACATGGATGAGGTGCTGACTGCCACCTGCGCTCTGC
CGGTGGTGCAGCACGATACCCCCTGCGTCGATGGTCGCCCCATGGCCGATGGCGGCATCAGCGACTCCAT
TCCGGTGATCGAGGCCTATCGGCGCGGGGCGCGGCGGATGACGGTGGTGCTGTCTGAGCCCTGGGGATAT
CGCAAGAAGCCGGCCCGTTTCTGCTGGATGGTCAAAGCCTTGCTGAAAACCCAGCCTGCGCTGGCGCAAG
CCATGCTGGAACGGGATCGCGCCTACAACCGGGCACTCGATTTCATTGCCAATCCCCCTGCTGATTGCGA
CATCGAGGTGATCGCGCCACCGGCCGATTTCATGGTCTCCCGTTTACCCAGGACCCGGTCAAGCTGGAG
TCCGGCTATCTGCAGGGTTACTGGCAGGGGCGCCAGAGTCTGCTGCGGGCCCGTGGAGAGGCGCGAGCGG
TGGCCTGACTGGTTTGCCGGGTGCTGAATATAGAAAGGGGAACCTTGCGGTTCCCCTTTTATCTTCCTGA
TCGGCAGCCGCTGGTTACAGCATCTGCTTGAGGCGATAGAGCAGGTCGAGGGCCTGACGGGGAGTGAGCT
CGTCCGGGCGCAGCGCTTCCAGCTCTTCCACCACCGGATGAGGCTGCGGGGCGAGGGTCACCGGAGCCGG
GCGGCTTTCACCGGCGGCGACCGGGGTGGCGCTCTCCAGCTCATGCAGTTTGTGACGGGCCTGCTGGATC
ACCGATTTCGGCACCCCGGCCAACGCTGCCACTTGCAGACCGTAGGAGCGGCTGGCCGCCCCTTCCTGCA
CCGCGTGCATAAAGGCAATGGTGTCGCCGTGCTCCACCGCATCGAGGTGGACGTTGGCCAGCCCGCTCAT
CAGCTCCGGCAGACGGGTCAGCTCGAAGTAGTGGGTGGCAAACAGGGTGTAGGCGCCAATCTTGCTCGCC
AGCTGCTCGGCACAGGCCCACGCCAGCGAGAGGCCGTCGTAGGTGCTGGTGCCGCGGCCAATCTCGTCCA
TCAGCACCAGACTGCGGGCGGTGGCGTTGTTGAGGATATTGGCGGTCTCGGTCATCTCCACCATAAAGGT
GGAGCGGCCGGAGGCGAGATCATCCGAGGCTCCGATGCGGGTGAAGATGCGGTCGATGGGGCCGATGCGG
GCGCTGTCGGCGGGCACGAAGGCGCCGATATGGGCCAGCAGCACGATCAGCGCGGTCTGGCGCATGTAGG
TGGATTTACCGCCCATGTTGGGGCCTGTGATGATCAGCATCCGCCGCTCCCGCTCCAGCCGGATGGGGTT
GGCGATAAAGGGATCTGTCATCACCTGCTCAACCACCGGATGGCGGCCCGCCTCGATGATGATCTGATCT
TCGTCGATGAGGGTCGGGCAGCTATAGTCGAGGGTCTCGGCCCGCTCGGCCAGGTTGGCCAGCACGTCCA
GCTCCGCCAGTGCGGCGGCGGAATCCTGCAGATCGCCGAGATGGGGCAGCAGGGCATCGAGCAGCTCTTC
GTAGAGCCGTTTCTCCAGCGCCAGCGCCTGGGACTGGGCGGTGAGTACCTTGTCCTCGTACTTCTTCAGC
TCATCGATGATGTAGCGTTCGTTGTTCTTGAGGGTCTGGCGGCGGATGTAGTGGGCCGGCACCAGATGGC
TGTTGGCGCGGCTCACCTCTATATAGAAGCCGTGCACCTTGTTGTAACCCACCTTGAGGGTGTTGATGCC
GGTCAGCAGTTTTTCCCGCTCTTCGATGCGTGCGAGGCTGGCGGTGGCACCGTTGGCCAGGTCCCGTAGC
TCGTCCAGCTCCTGATTGAAGCCGTCACGGATGACGCCACCATCGCGGATCAGCACCGGCGGCACTTCCA
TCACCGCGCGCTCCAGCAGGTCGAGCAGCTCGGGGAAGGTGCTGGCCCGCTCGGCCAGTTGTTGCACCGC
TTCGTGCTCGTTGTCCACCAGCAGGCGCTGCAACTCAGGTAACTGGGCGAAGGCCTGACGCAGGCGGGTG
AGGTCGCGCGGGCGGGCCGATCGCAGGGCGAGACGGGCCAGCACCCGCTCCACATCACCCACCTGACGCA
GCAGGTTGCCCAACTCGTCGTAGAGGTTTTGCTCGATGAGCTCCTTGATGGTGCTCTGGCGACCTTTCAG
GATCACCCGATCGCGGATCGGCTGGTGGATCCAGCGCTTGAGCAGGCGGCTGCCCATGGGGGTAGCGGTG
CGATCCAGCACCTCGGCCAGGGTATTTTCATAGCCACCGGCCAGATTCTGGGTCAGTTCCAGATTGCGGC
GGGTGGCGGCATCCATGATGACCGCGTGATCCGGCTGCTCGAGGCGCACGTTGCGGATGTGGGGCAGGGC
GGTGCGCTGGGTGTCCTTGACGTACTGCATCAGGCAGCCTGCGGCGCACAGGGCGGTCTCGCTCTTGTCG
ACGCCAAAGCCCACCAGATCCTGGGTGCCGAACTGCTGGCAGAGCAGCTTGCGAGCGGTGCCCAGCTCAA
ACTCCCACTCCGGGCGGCGACGCAGGCCGCGGCGACCCTCTATATGGTGCAGGAATTCGAACGATTCGGG
ATAGAGCAGCTCGGCCGGATTGGTGCGCTGCAGCTCCGCCAGCAGGGTCTCTTCTTTCTCGAACTGGTTG
ATGAAGAAGCGGCCGGAGCCGATGTCCATGGTGCCATAGCCGAAGCGGCGGCCATCGTGATAGACGGCGG
CGATCAGGTTGTCCTGCCGCTCGCTGAGCAAAGCCTCGTCGGAGACGGTACCCGGGGTGATGATGCGAAT
GACCTTGCGCTCCACCGGCCCCTTGCTGGTGGCGGGATCCCCCACCTGTTCGCAGATGGCGGCGGATTCG
CCGAGCTGCACCAACTTGGCCAGATACCCTTCGATAGCGTGATAGGGCACCCCCGCCATCGGGATGGGGC
TGCCCGCCGACTGACCGCGCTTGGTCAGGGAGATATCGAGCAGCTGGGAGGCCTTGCGGGCATCGTCATA
GAACAACTCGTAGAAGTCGCCCATCCGGTAGAACAGCAGGATCTCCGGGTTCTCGGCCTTGAGGCCAAGG
TACTGCTGCATCATAGGCGTGTGGGCGCTCAGATTGGCGCCGGGAGCGGAGCTTGTGGCGCCGCTGGCTT
GGTGTTGTGCGGTCATGTTCTGTTCCAGAAGGGGCCTCTGCGGGCCAGTGTCTGCGGTGATGAGCCTTGA
GCTTGATGGCGCCATTCTACACATAAGCGCCGCTACGGGGGAGCGGGTGTTCCGGGCGCGGTTGTTCTGC
ACTATTGAAGTGCATTGCACAGGTACGGCACCTCACATTGCGGCAGGCAAGGGCGGTCTGCTGTGGTTGC

GGGCGGTGGTAGCGGGATCGCATAATGTGCCTCTTGAGCGGATCATAAGGAGTCGTGATGAAACTGGATG
CAGAGATAGAGCACCTTGCCATCGAGCTGGGACGGGCCCTCGGTCAGCGGGGCTGGCTAGCGGCGACCGC
CGAATCCTGCACCGGCGGCGGGGTGGCTACGGCTATTACCGACATCGCGGGCAGCTCCGGCTGGTTCGAT
CGCGGATTTGTCACCTACACCAACGAGGCCAAGCAGCAGATGTTGGGGGTGAGCAGCGAGAGCCTTGAGC
AGCACGGTGCGGTGAGCGAAGCCGTGGTGCTGGAGATGGCCCGCGGCGCGCTGGCCCACTCGTCGGCCTC
CATCAGCGTTGCTATCAGTGGCATCGCCGGCCCCGGCGGCGCCACCGAGGGCAAGCCGGTCGGCACCGTC
TGGTTCGCCTGGGCCGACCGCAACGGCCGTCACCACTCCCTGCTCGCCCGCTTCGACGGGGATCGCCGAC
AGGTGCGCCAGCAGGCGGTGAGACAGGCCTTGTCGGGTCTGTTGGCCCTGCTCAGATAAATTGGGCTTGA
TACTGTATGAATAGACAGTATACTTAGCCCAACTTCACTTCTTACTCACGATTTCCAGCGGAGATTGGCA
TGGATCAGAACAAACAGAAGGCACTGGCGGCTGCGCTGGGTCAGATTGAAAAGCAGTTCGGCAAAGGTTC
CATCATGCGTCTGGGCGACAGCAAGACCATGGATATCGAAGCCATCTCTACCGGTTCCCTCTCGCTGGAC
GTGGCGCTGGGGATCGGCGGTCTGCCGTGTGGCCGTATCGTCGAAATCTACGGCCCGGAATCTTCCGGTA
AAACCACCCTCACCCTGCAGGTGATCGCGGAAGCCCAGAAGAAAGGCAAAACCTGTGCCTTCGTCGATGC
GGAACACGCTCTCGACCCTATCTACGCTGCCAAGCTGGGCGTCAACGTCGACGACCTGCTGATCTCCCAG
CCGGATACCGGCGAACAGGCGCTGGAAATCTGCGACATGCTGGTTCGCTCCAACGCCGTTGACGTCATCA
TCGTCGACTCCGTGGCGGCTCTGACCCCGAAAGCGGAAATCGAAGGGGAGATGGGTGACTCCCACGTTGG
TCTGCAGGCCCGTCTGATGTCCCAGGCACTGCGCAAGCTGACTGCCAACATCAAGAACGCCAACTGCCTG
TGTATCTTCATCAACCAGATCCGGATGAAGATCGGCGTTATGTTCGGTAGCCCGGAGACCACCACAGGTG
GTAACGCGCTCAAGTTCTACGCCTCCGTGCGTCTGGATATCCGTCGTATCGGCGCCATCAAGGAAGGCGA
CGAAGTGGTCGGTAACGAGACCCGCGTCAAAGTGGTCAAGAACAAGGTGGCCCCGCCCTTCAAGCAGGCT
GAATTCCAGATCTTCTACGGCGCCGGTATCTCCAAAGAGGGCGAGCTGGTGGATCTCGGCGTCAAGCACA
AGCTGATCGACAAGGCCGGCGCCTGGTACAGCTACAACGGCGAGAAGATCGGTCAGGGCAAGGCCAACGT
GATGAAGCTGTTCGCCGAGAACAAGGTGATGGCTGGTGAAGTGGAGGCTCGTCTGCGCGAGCTGCTGCTC
TCCGGTGCCGTGCCAGTCGACGACAAGGCTGCACCGGTTGAGGCAGACGAGTTCGATGCCGAAAGCGAAC
AAGAGTTCGAATAAGGAACAAAGGAAGGCCGGGTTGATACCCGGCCTTGTTATTTATGATGGTTGATGAA
TCTGCTGACAAACCTGTCGAACTCACCTTTGAAGAACAACTGGCTGCGGCCCGCGGCTATGCCATGCGCA
GTCTGGCCCGCCGCGAGAGTGCCGAGTCGGAGCTGGCCAACCGGCTGCGCCAGCAGGGCTTCAATGAAGA
GGTGATCGAGGCGGTGGTCGACTACTGCCGCGGTTACAACTGGGTCAATGACGAACGTTACGGCGGTATG
GCTGTACGGGCCGGTGCCGCCAAGGGGCATGGCCCCATCAAGATCCGCTTCGATCTGCGCCGCAAGGGGC
TCGACGATATGCAGATTGATGCCGCATTCGATCAGCCGGAGCTGGACTGGTTCGAACTGGCGTTTGCCCT
GCTCGAACGGCGCGCCAGGGTGGCCGATCTGGCGGATTTCAAGCTGCGGATGAAGTGGCTCAAATATCTG
CTGGGGCGCGGCTTTACCCAGGATCAGGCCCGTTATGCCATCTCGGCCCTGCAGGAGCGGGAAGTGTAAC
GCCACTGCGTGGCAAACGTCGTTTCACCTCTCCTTATGGCAGGTTTTTCCTTCCCGTTCAGTAGCTTGCG
CGCACAATCCCGAAAAAGCGGCCTTTTGTTCATCTCATCTCTCTTTATGCGCTGCTTTTTTGCCTCTCGA
CAGTGAAAAAAGCCACTGTGGGCAGCGGCGCTCTTATCTGGCCGGTTTATGTGCGCTTTTTCGCCCGTGG
CGGCCCTGTCCGTTTTACTTTACCCCGCAGAAAACTTACATTAATGCGGTTAAAAGTCCGTATTCGAATC
AAGCAGGATTTTCCATGTATATGTCCACGTCAGAGATTCGCGCTGCGTTTCTCGAGTATTTCCGTTCCCA
GGGGCACCAGGTTGTCTCCTCCAGCTCGCTGGTACCGCATAACGACCCGACCCTGTTGTTTACCAACGCG
GGTATGAACCAGTTCAAGGACGTGTTCCTTGGCGCAGACAAGCGTGCCTACAATCGCGCGACGACTTCCC
AGCGTTGCGTGCGTGCCGGTGGTAAGCACAACGATCTGGAAAACGTCGGCTATACCGCTCGTCACCACAC
CTTCTTCGAAATGCTGGGCAACTTCAGCTTCGGTGACTACTTCAAGCAGGATGCCATCAAGTTTGCATGG
GGCTTCCTGACCGAGGTGCTGCAGCTGCCGAAAGATCGCCTGCTGGTGACCGTCTACGCGACTGATGATG
AAGCTTTCGATATCTGGGAAAAAGAGGTCGGTGTCCCGGCCGATCGCATCGTGCGCATCGGTGACAACAA
GGGCGCGCCGTATGCCTCCGACAACTTCTGGGCGATGGGCGATACCGGTCCGTGCGGTCCCTGCACCGAG
ATCTTCTATGACCACGGCGATCACATCTGGGGTGGCCGCCCCGGCTCACCGGAAGAGGATGGCGACCGTT
TCATCGAGATCTGGAACGTGGTGTTCATGCAGTTCAACCGTCAGGCTGACGGCACCATGGAGCCGCTGCC
CCGTCCGTCCGTGGATACCGGTATGGGTCTGGAGCGTATCTCCGCCATCATGCAGGGTGTCCACTCCAAC
TACGAAATCGACATCTTCCAGGCGCTGATCAAGAAAGCGGCCGAGATTGTCGGCACTACCGATCTGAGCA
ACCAGTCCCTGCGCGTCATCGCTGACCACATCCGCTCTTGCGCCTTCCTGGTGGCAGATGGCGTGATGCC
GTCGAACGAAGGCCGTGGCTATGTGCTGCGCCGCATCATCCGCCGCGCCGTGCGTCACGGTCGCAAGCTG
GGCGCTACCGATGTCTTCTTCTACAAACTGGCTGCCGAGCTGGCAGTGCAGATGAAGGATGTTGCTGCCG
AGCTGATCGCCCAGCTGCCGCTGGTCGAGCGTGTGCTGCGCATCGAAGAGGAGCAGTTCGTCCGCACCCT
GGATCGCGGTCTGCTGCTGCTGGAAGACGCTCTGGCCAACCTGGGCGATGCCAAGGTTATTCCGGGCGAA
GTGGTGTTCAAGCTCTATGACACTTACGGCTTCCCGGCCGACCTGACTGCTGACGTGGTGCGCGAGCGCG
AGATCGGCATCGACGAAGAGGGCTTCAAGGCCGAGATGGAGAAGCAGCGTGCTCGTGCCAAAGAGGCCTC
CAGCTTCGGCGTGAACTACAACGAGATGCTCAAGCTCGACTTCGAGAGCCCCTTCACCGGTTACAAGACT
CTGACCGAGCGCACTCGCGTTGTCGGTATCTACAAGGGTGTGGAAGAGGTGAGCAGTCTTATCGCCGGTG
ACGAAGCGGTCATCGTGCTGGAAGAGACCCCCTTCTACGCCGAATCTGGCGGCCAGATTGGCGACAGCGG
TACCCTGAAAGTAGACGACGGTATCTTTGCCGTGACCGACACCCTGAAAGCGGGCAAGGCGATCATCCAC
AAGGGCTACATGGAGTTGGGCACTCTGGAGAAGGGCGCCGAAGTGGAAGCCGTGGTTGACGCTGACCGTC
GTCAGGCCATCGCCCTGAACCACTCCGTGACTCACCTGCTGCACGCAGCCCTGCGTCAGGCACTGGGCGA

GCATGTCACCCAGAAAGGTTCTCTGGTTGGGGCCGAGCGCATGCGTTTCGACTTCTCCCACTTCGAGGGG
CTGACCATGGCGACCATTCGCCGGGTCGAGGAGCTGGTCAACAACCAGATCCGCGCCAACCATGAAATCA
CCACCCAGCTGATGGATCTGGAAGCGGCCAAGTCCGCCGGTGCCATGGCGCTGTTTGGTGAGAAATATGA
AGACGACGTGCGCGTGGTGCGCATGGGCGACTACTCCACCGAGCTGTGTGGCGGTACTCACGCCAAGCGC
ACCGGTGATATCGGCTTCTTCAAGATCATCGCCGAGAGCGGTATCGCTGCTGGCGTGCGCCGTATCGAAG
CGGTGACCGGCAAGGCCGCCATCGACTTCATGCATCAGGTTGGCGAGCAGATCGAAGAGGCTGCTGCACT
GGTGAAAGGTGACCAGTTCTGCATCGCCGACAAGGTTCGCCAGATGCTGGACAAGGCCAAGATGATGGAG
CGCGAGCTGGAGCAGCTTAAGGCCAAGCTGGCTGCTCAGGCTGGTAACGATCTGCTGGGTCAAGTGATCG
AGATCAATGGTCAGAAGGTGCTGGTTGCAGCGCTGGAAGGGGCCGATCCGAAATCCTTGCGCGGCATGCT
GGACGAGCTGAAGAACCAGATGAAGTCCGGTGTCGTGCTGCTGGCCACCTGTGCCGATGGCAAGGTCAAC
CTGATCGCCGGTGTCACCAACGACCTGACCGGCAAGGTCAAGGCCGGTGAGCTGGTCAATCTGGTTGCCC
AGCAGGTGGGTGGCAAGGGCGGTGGCCGTCCGGACATGGCACAGGCGGGTGGTACCCAGCCTGAGGCGGT
GCCGGCAGCGCTGCAATCCGTTCACTCCTGGCTGGAAGAGCGCCTGTAATCGGGCGAGGTGATCTGTGGC
ACTCTATGTACAGAAGTACGGCGGCACCTCGGTCGGCACACTGGAGCGGATCGAGGCGGTAGCTGAGCGG
GTCGAGCAGACCCGCGCCCAGGGACACGACGTGGTGGTGGTTGTCTCCGCCATGTCGGGCGAAACCAACC
GGCTGCTGGGCATGGCCCAGCAGCTGGATCCTTATGCCAACCGGCGGGAGATGGATGTACTGGTCTCCAC
CGGTGAGCAGGTCACCATAGCGCTGCTGGCGATCGCGTTGAACAAACGTGGTTGTCCTGCGGTCTCAATG
ACCGGTGATCAGGTGCGGATTCATACCGATTCCGCCTATGGCAAGGCGCGCATCACCCACATCGACACCG
AGCTGGTTCATGCCGAACTGGCTGCGGGCAAGGTGGTGGTGATCGCCGGTTTCCAGGGCCGTGACGAGCA
TAACGCCATCACGACGCTGGGCCGCGGCGGTTCGGATACCACGGCAGTTGCCGTGGCGGCGGCCATCAAG
GCCGATGAGTGCCAGATCTTTACCGATGTGGATGGTGTATACACCACGGATCCGCGCATCGAGCCCAAGG
CGCGTCGTCTCGACACCATCACCTTCGAGGAGATGCTGGAGATGGCCAGTCTCGGCGCCAAGGTGTTGCA
GATCCGCTCGGTGGAGTTTGCGGGCAAGTACCGCGTCCCGCTGCGGGTGCTGTCGAGCTTTATCGATGGA
GAGGGAACCTTAATCACATATGGAGGTGATAGGATGGAAGCTCCCCTGGTATCCGGGATCGCTTTTAACC
GCAACGAGGCCAGCTTGACCATTCTGGGCGTGCCGGACAGACCAACCGTTGCCGCGCAGATCCTGAATCC
GATCAGTGACGCCAACATTGATGTCGACATGATTGTGCAGAACACCCTGGGAGATGGCACCGCCGACTTC
ACCTTCACGGTGAATCGTGACGACTATCGCCGCGCCCGGGCACTGCTGGAGGAGACCGCCACCGAACTTG
GCGCCTCCTGCGTGCAGGAAAATGGCGAAATTGCCAAGGTTTCCATTGTCGGTGTCGGCATGTGGAACCA
TCCCGGTGTTGCGCGCACCATGTTCAAGGTATTGGGCGAGGAGGGGATCAACATGCAGTTGATCTCGACC
TCTGAAATCAAAATCTCTGTGGTGATTGACGAGAAGTATCTGGAGCTGGCGGTGCGAGCCCTGCACGCGG
CTTTCGGGTTGGATCAGGCACCGCAAGAGCAGCCCTTGGGCTAACAATCTCTGATTGATTTAGTTTCAAC
CATTTACCATATCGGTATCTTTGTTAGATAATGTCGCATATCGATATAAAAACAAAACAGGATACAGGAG
CAAGCGAATGCTTATTTTGACTCGTCGTGTTGGGGAAACCCTGATGATTGGTGATGAAGTGACTGTCACC
GTACTGGGCGTTAAAGGCAACCAGGTCCGTATCGGTGTGAACGCACCGAAAGAGGTCTCCGTGCACCGCG
AAGAGATCTACCAGCGCATCCAGGCCGAGAAAGTTCCGGGCCAACCGGGCTTCTAAGCCACTTTCATGGC
TGTGACAAGGCGACACCTCATGTGTCGCCTTTTGTTTTTTCTCGGCCCCTGAAATCGCCCTGCAGGGCGC
GAAACTGCGGGATAAGTGCCGATATTGTTTGAATATTCCACGCTTTGACCAAATGCTGAGCGAACGCACT
TTTTTCTCCAAAAAAGTGTTTGACTAAAAATCCCTGTGAAGTATTATCAGCCCCGCAAACGGAGAGGTGG
CCGAGAGGCTGAAGGCGCTCCCCTGCTAAGGGAGTATGCGGCTTATACCCGCATCGGGGGTTCGAATCCC
CCCTTCTCCGCCATTTGCGCCCGTAGCTCAGCTGGATAGAGTACCTGGCTACGAACCAGGTGGTCAGAGG
TTCGAATCCTCTCGGGCGCACCATTTAAAACGACACAACGATTTATGAGCGCCCGTAGCTCAGCTGGATA
GAGTACCTGGCTACGAACCAGGTGGTCAGAGGTTCGAATCCTCTCGGGCGCACCATATTTAAAGCAGTAC
CAAAAGTTTATGAGCGCCCGTAGCTCAGCTGGATAGAGTACCTGGCTACGAACCAGGTGGTCAGAGGTTC
GAATCCTCTCGGGCGCACCATACAGACAGCCTCGACTTCGGTCGGGGCTGTTTTTTTATGGGCGACATTT
ACCCCTGTCGTGTGGATCAGTGTTCGCTATGGCGGCCGGGTCACCCTCCTGCCTATCTGTCTGTTCTTGT
CCTCTTTTATACCTCTTCTGTTATTGAGCCGCGTTCTTCTGAACGGTACAAGCCTTTGCCTCATCTGCAA
GCAGGCTGTATGCCTGCTGTTGCTGCGGGGGACTGCGCGCAGCTCATCACTCAGGGTTTGGCTATCAACA
TCCTGCAAGGCCTGCATAGGCGTTCTGCGGGCAATAAAAAAGGATGCCGCAGCATCCTTTTGTGTCGAGA
TATTGACTCAGCTTTTCAACCATTTGCGAATGCGGTTGGCATCGCCGATGGGGGTGCGTTTGCCCACCGA
GTTGAGCAGCACGATGGCGAGCTCGCGGCTGTCCGGTTTGGCGCGCATCACCAGACAGCGCCCCGCTTCG
TCAGTGAAGCCGGTTTTGGAGAGGCGCACGTCCCAATCCGGGTTTTTCACCAGCGGGTTGGTGTTGTTGA
ACATCAGGCTGTAGGCGGGGGCGCTGAAGCGCATCTCCCGGTTCTCATCTTGAGTGAACTGACGGATCAG
CGGCTGGCGATAGGCGGCGGCGGCCAGCTTGCCCAGATCCTGGGCGGTGGAGACGTTGCGGGTGGAGAGG
CCGGTGGAGTCGTAGAAGTGGGTATTACGCATACCCAGCTGTCTGGCCTTGTTGTTCATGGCTCGCACGA
AGGCGCTGCGACCGCCCGGATAGTGACGCGCCAGTGCCGAGGCCATCCGGTTTTCGGAGGACATCAATGC
CAGATGCAGGGCGTCGCGGCGGGTCACCTTGGTGCCCATGCGGATGCGGGAGTGAGTGTGTTTGACCCGA
TCCAGATCGTTCTTGTCGACCGTAATGGTCTGGTTGAGATTCTGTTTGGCATCCAGCACCACCAGCGCTG
TCATCAGCTTGGTGAGAGAGGCGATGGGACGCACCTTGGTGGCATTCTTCTGGTAGAGCGTCTTGCCCGT
TTTTACATCCACGACCAGGGCGCTGCTTGAGCGTACTTCCAGTTTGGCAGGGTTGGGTGCCGCGCTGACC
GCAGGCAATCCCAGCATTCCCATCAACCCGACAACCAGCGCTGCTGCGCGAATCCGACACCACATACAAC
CGACTCCATTCCCATCCAAAAAAAGGCGCTAGATTCACTCATTTTTACCCCGGCTTTACAAGGGCCGAGC

GGGACTATTTCTGCTATTTGCGCAAAATTGGCGATCAAAAAAGCCCTCTGGCGAGGGCTTTCAGGTCGGG
CGTTGGGCGAGGGGTCAATCCCGCGGCTGCGCGGCCCTGCGTTTGCTGATCCAGCGCCAGACCAGCGCCA
CCATGCCACCCAGCAGCAGGGCCACCGGCAGATAGATGATGATGTTGAAGAACTGGGTCTGGTATTCGGC
GGCCAGCTTGGTGTGGCTCAGGTAGTAGCCAAACCCGACCAGCGGGCAGATCCACATGATGGAGCTGATC
AGATTGACGATGAGAAAGCGCCCCTGGGGTAGCTCGGTGGCACCCACGATATAGGGGTAGGCGGTGCGCA
CGAAGGCGATATAGCGCGCGGTGATCAGGGTGATGATCCCGTACTTGTCCAGCAGGTGGTAGACCTTCTG
CTGGTGTCGCTCTTCCACGTGGGACATCAGCCGATGGTAGAGCTGGGTGTGGCCAAGGAAGCGCCCCTGC
ATGTAACCGAGCCAGGTGCCGACAAAGGTGGCCATGATCAACAGCGGGATTACCCCGAGAGACAATACAT
CCTGATAGATGAGCACGCCGGTGAGGATCAGCAGGCTGTCACCGGGCAGAAAGGCGGTAGGGATGAATCC
GTTTTCCAGTACCAGAAACGTCAGAATCACACCACAGATCATGAAGGCCATCTTGGGATCGGAAAGTGTG
TTCAGATCGTGATTCATAAAGGCGTGAGCCAGTTGGTAAAACTGCTCCATACTTGCAAACTCCGCAGGTT
AGCTGGGTTGATCGGTCAAGGGCATGGCGAGCACCTTGTCGATGCGCAGGCCATCCATGTCAACAATTTC
CAGTTGCCACTGTTCCAGTGTCACCAGGTCTCCGGTTTGTGGAACACGACCAAGGAGCAGCATGATAAGC
CCTCCGAGGGTGTGATAGTGATGTTTTTCTTCTTCTGGCAGACGAACCAGCTCCAGACAGTCCTTCAACT
CCGGCAGCGGGATCAGGCCATCGAGCAGCCAGCTGCCGTCGGCGCGCCGGAAGGCCCAGTTGTCCTCGCC
ATCCTCCTGCTGGAAATCCCCCGCCAGCGCCTCCAGCAGATCCTGCTGGGTGACCAGTCCCTGCAACTCG
CCATATTCGTCCACCACCAGCGCCATCTGTTCGCTGTGGGTGCGGAAGTGGTTGAGCAGCTCGACGCCGT
TGAGGGTCTCGGGCAGCAGGCTGCCCTCCTGGGTCAGGGCAGCCAGATCGATGGGCTCCCCCTTGATATA
GGCGTGCAGCACCTTGCTGACATTGATGATACCCACCAATTCTGACAGGTTGCCGTCGCAGACCGGAAAA
TAGCTGTGGCGATACTCCACCAGCCGCTGCAGGTTGGTCTCCAGCGGCAGAGCCAGATCCAGATAGCGGA
TATCGCTGCGCGGCACCATCAGGGAGGAGAGGCTGCGCTCGTCGAGACGGAAGACGTTGCGCAGCATGGT
GTGTTCATGATCCTCGATTACCCCGGCTTCCGATCCTTCCACCAGCATGGCGTGGATCTCTTCCTCGGTG
ACGTTGGCGCTGCCGTTCTGGTTGACCCGCAGCAGCCGCAGGCTGGCATTGGTGGAGGCCGACAGCAGCC
AGACGAAGGGGCGGGTCAGGGTGGCGAGCCAGTGCATGGGACGGGCCACCAGACAGGCGATGCGCTCGGC
ATTGAGCTGGCCGAGCCGCTTGGGCACCAGCTCCCCTACCACGATGGAGAGATAAGTAACCACCACCACC
ACGATGATGGTGGCGATCAGGCCACTGCTCTTGGCCGGGAGTCCCCACTCCTGCAACCAGAGGGAGAAGG
GCTGGGCCAGCAGGGATTCGCCGTAGATACCGTTGAGCAGACCTATGCTGGTGATGCCTATCTGGACGGC
AGAGAGAAAATGGGTAGGGTCTTCGCCCAGTTTGAGGGCGATGGCGGCGGAGCGGTTGCCTTCAGAGGCA
AGCTTGCCGAGGCGAGCCTTGCGTGCGGTGACGATGGCGATTTCTGACATGGCGAAGACGCCATTAAGCA
AAATCAATCCCAGCAGGATCAATATTTCCATACAGCAACAGCACTCCTTGAGTGGATAAGATCGCTGGCA
GTGTAATGAATTTGGCGGCGATGCGCTTGTGAAAGCGGGTGATCCGGCTCGCTAATTTGATGACAGCGCT
TTGTGATGGCTGAAAAAGATGGCCGGAAAAGAGAGGGACGACCCCGCCAAACGGCGTAATGGGCGAGGCA
GCATTGCAGGAGTGAAAAGGGCGGGAAGTCCCGCCCTTGCTTGTCACTTGTCAGGCAACCGTTGTCAGAA
CTGCTGGAACCAGCGCTGGATCTGGGCCGGATCCTTGCTCTGGGTCAGGGAGAGCATCAGCAGCACCCGC
GCTTTCTGCGGGTTGAGAGTGCCGGAGGCCACGAAGCCGTACTGGGCATCATCGATCTCGGCATCCAGAG
TGGTGGCGCCGGTCGGCACGCGGGCGGAGCGCACGACGCGGATGCCATCCTTGCTCGCCTTGGCCAGGGT
GTCGAACAGGGTGTGGTAGAGGTTGCCGTTGCCCACCCCGGCGCTGACGATGCCGTCGAACTTGGCATCC
ACCATCGCCTTGGCCGGCAAGTCGGTGGCGTTGGCGTAGCTGTAGACGATGCCAACTTGCGGCAGCTTGT
CGAGCTTGCTCACGTCAAATGGCGTTTTGCTGGTGTGCAGACGGGCCGGGCTGCGCTGATAGTCGATCTT
GCCGTTGTGGATGTAACCCAGCGGGCCAAAGTTGGGGGAAGCGAAGGTCTGCACCCCGGTGGTGTTGGTC
TTGGTGACGTCGCGGGCATCCAGCACGGTGTCGTTCATCGCCACCATCACGCCACGGCCCTTGGAGGCGG
GGTCGGAGGCGGTCACCACGGCGTTGTAGAGGTTGAGCGGGCCATCGGCACTCATGGCGGTGGAGGGGCG
CATGGCGCCGACCAGCACCACCGGCTTGTCGCTCTTGACGGTCAGATTGAGGAAGTAGGCGGTCTCTTCC
ATGGTGTCGGTGCCGTGGGTAATGACGATGCCATCCACGTCATCCTTGGCCAGCAGCTCGTTGACCCGCT
TGGCCAGCTTGAGCCACACCTCGTCGTTCATGTCCTGGGAGCCGATCTTCACCACCTGCTCACCCTGCAC
ATCGGCCACTTTCTTCATCTCGGGCACGGCGGCGATCAGGGTTTCAATAGCCACCTTGCCTGCCTCGTAG
TTGGACTGGGTAGCCGACTGGCCTGCTCCGGCAATGGTGCCGCCGGTTGCCAGAATATGGACGTTGGGCA
GCGCCATCGCCATGGGGGAGAGGGTCAGGCCAACGACGGCGGCGAGCAGGGTCTTGCGGATCGGTAGGGT
CATGCTGCAAATCCTTATGGTTGGAATAATCGCAGCAAGTTTGCCCGTTTAATAACCTGGGTCTGTGATA
AAACTCACCAAAAGACAGGTGAGTACAGATTTTTAACCTGTTTTATACCTCTATGGAGATAATTTAACTC
GCAGAGAAACCCTTGGCTTCGATCAGGGCGATCAGTTCTGCCTTGCTCAGCTCGCTCTCGATAACGGCCT
TGCTGCTGGCGAAATCGACGGTTGCGGTGGTGACCGTAGGCTGGGCCAGCAGCGCTTTTTGCAGACTGGC
GGCGCAGCCACCACAGGACATGCCGGAGACGGTGAGTTCGATGGTCATAAATGCTCTCCTCGGTTGATAT
GGCACAAGGGTAAAGCTGTCGCCTCCGGCAAGGTCAAGCCGTCAGTACATCAACCCCAGATAGTTCATCA
GGATTGTTCCCAGCCGGTGGATGGGTGACATGTGAGCCAAGGTTCATAGTGGCGATGAGGTTGGCAGTTT
CCTCTGGCAGGGTCAGTACATCAGACCGAGGTAATTCATCAGAGTCGCCCCGAGGCTGGTAGTGACCAGC
GAGCCGAGGGTGGTGGTGGCGATGATGTTCGCTGCCAGCACGCTGTCGCCCCCCATGGCGCGGGTCATCA
CATAGCTGGCGGCGGCAGTCGGGGTAGCGCTGATGAGGAACAGAATACCCAGCTCCAGATGGCTGAAGCC
GAGCAGGGCAGCCCCAAGAGTGAACGCAGCCGGAATGAGGCAGAGCCGCATGATACTGGCCCACCCGGCA
ATGGCTGCGCCACCGCGCAGGGCGTTGAGGCTGAGAGAGGCACCGGTGCAGAGCAGCGCCAGCGGCAACG
TCATGTTGGCGAAGTAGCTGCCGGTGGTGAGCAGCAGTTGCGGCATCTTGATACCGAGCAGAGTAAACGG

CAGCGCCGCCACAATGGCGATGATCAACGGATTCTTGATGATGCCGAGCAGAATGGGGCGAATGCCGGTG
GCACCGCCGAGACTGCGGGTGAGGGTGATCACCGCGAGAATGTTGTAGAGCACGGTCACCGCACCGACAT
AGAAGGCAGCCGCCCCCAACCCTTCGTGGCCGTAGGCGTTCTGCACATAGGCGAGCCCGATAATGGCGCT
GTTGCCGCGAAAGCTCCCCTGCACGAAGATGCCGCGCAGCCGCTGCTCCCCGATAAAACGCCCAGCCAGC
AGCTCCAGCACCAGATAGCTGATAACGGTGGCGAGCAGGCCATAGCCGATAAACCAGAGGCTCGGCATGG
TGGCGAAGTTGGTACCCAGAATACTGAGAAAGAGCAGAGCGGGCAGGGTGACTTGAAACACCAGTCGCGA
GGCGGATTCGGTAAAGGGATCCGGCAGTAGCCCCAACCGCTTGAGCAAGATGCCGAGCAGCAGCACCACG
CAGATGGGGCCTGTGATGGAGAGGGAGAAGAGCAGACTGGCCAGCAGATCCATGATGGGCATCCTGTGCT
GTGCCCACACACCCTGACGGCGGGGCAAAAAGGGCATTCTAACGCCATGGGCTTGTATTCACAGGGGTTA
ATCACAACAATGTGGGCAATTCCACCGCCTAGCCTCCTGTGAGCCAATTCATGGACAAGAAACTGACCAT
CCTCGATATCGCCAGACTGAGTGGCGTCGGCAAGTCGACCGTCTCCCGGGTGCTTAATCAGGATCCCAAG
GTGAAGCAGGAGACCCGCGAGCGGGTGGAGCAGATCATCGCCGAGCACGGCTTCGTGCCGAGCAAATCGG
CGCGGGCCATGCGTAGCCAGAGCCAGCAGGTGGTGGGGATCATCGTCTCGCGGCTCGACTCCAGCTCGGA
GAATCAGGCGGTGCGCGGCATGCTGGAGACCCTCTACCAGCGCGGCTACGATGCCGTGCTGATGGAGAGC
AAGTTCTCCCCCGCCAAGGTACAGGAGCATCTGGCGGTGCTGGAGCGGCGCGGGGTGGATGGCATTATCC
TGTTCGCCTTCAATAACTTGGACTATGCCGCTATGGCGCCGCTGAAAGAGAAGCTGGTGCTGGTCGCTCG
TGAATATCCCGGTTTCTCCTCGGTCTGCTTCGACGACGCCGGTGCGGTGCGCACCATGCTGGCGCAGCTG
GCAAGCCGTGGTGCCCGCGATATCGCCTATCTGGGGGTGGATGCCTCGGATCTCACCACCGGCCAACGCC
GCCTAGACGCCTATCTCGCTTACTGCGCAGAGCAAGGGTTGCACCCGCGCAGAGCCCTCGGCGATCTCAG
CCTGCAGAGCGGCTACCGGCTGGCGGCCGAGCTGCTTACCCCCTCCACCCAGGCGCTGGTCTGCGCCAGC
GACACCCTCGCCATTGGTGCGGCAAAATACCTGCAAGAACAAGGGCGTAGCGAGGTGCTGGTGACCGGCC
TTGGCAACAACCCCATGCTCAGCTTCCTCTTCCCCAACGCCCTCAGTCTCGATCTTGGTTACAAAGGGGC
GGGGGAGCAGGCGGCGCGTCAGCTGCTCGGCCAGATTGAGCAGGAGCAACTCCCGCAGGTGACAATCGCC
CCCTGTCGAACCCTGTGACCCCCATCACTCCGGACTGAACAAAAAGTGCGCTTTTTGTGATGCTGGCCAC
GAATTGGGAACGTTCCCATTTCGATCTGGCCAGATTGTCGGCATGCTAAATGGGAACGATACCAAAAACG
CTCCGGCCGACAGACTGGCTCAAAAGGGCGATACAGACCACTATTCAATGGCGTCACAGAGGGAAGACCC
ATGTCCAAGATCAACGCACAGCAGCTTGCCACCCTGATCGACAAGATCGGCGGCAAGGAGAACATCGCTA
CCGTCAGCCACTGCCTGACCCGGTTGCGCTTCGTCCTGAACGATCCGGCCAAGGCCGACAGCAAGGCCAT
CGAAACCATCCCGGCGGTGAAGGGAAGCTTCACCCAGGGCGGTCAGTTCCAGGTGGTGATCGGTAACGAG
GTAGAGCAGTGGTACAAGGCGCTGACCGAACAGCTCGGCGTCAGCGGTGGCAGCAAGGAGACTGCCAAGC
AGGCGGCTCGCCAGAACATGAACCCGCTGGAGCGGGGCATCTCCCATCTGGCGGAGATCTTCGTACCGCT
GCTGCCCGCCATCATCACCGGCGGTCTGATCCTGGGCTTTCGCAACGTCATCGGCGACATCAAAATGTTC
GATGATGGCACTCACACCCTCACCGAGATCAGCCAGTTCTGGGCTCAGGTACACGCCTTCCTCTGGCTGC
TGGGCGAGGCGATTTTTCACTTCCTGCCGGTCGGTGTTTGCTGGTCTGCAGTGAAGAAGATGGGCGGCTC
CGAGATCCTCGGTATCGTGCTGGGGGTGACGCTGGTGTCACCGCAGTTGATGAACGCCTACGGCATTGGT
CAGCAGACTCCCGAGGTGTGGGACTTCGGCCTGTTCGTGATCCAGAAGGTGGGTTATCAGGCCCAGGTCA
TTCCGGCGCTGCTGGCGGGTGTATTCCTCGCCTGGGTGGAGACCAACCTCAAGCGGATCATTCCGGCTTA
CCTCTATCTGGTGATTGTGCCGTTTGTCTCGCTGCTGCTGGCAGTGATCGTGGCTCACGCCTTTATCGGC
CCGTTCGGTCGCTGGATTGGTGATGGCGTCGGTTTCGCGGCCAAGGCTGCCATGACCGGCTCCTTTGCTC
CCATCGGCGCTGCCCTGTTCGGTTTCCTCTACGCACCGCTGGTCATCACCGGCATTCACCACACCACCAA
CGCAGTGGATCTGCAGCTGATGCAGAGCATGGGTGGCACGCCAATCTGGCCGCTGATTGCCCTCTCCAAC
ATCGCGCAGGCGTCTGCGGTCGTCGGCATCATTCTGATTAGCCGCAAGGAGAACGAGCGGGAGATCTCGG
TACCTGCTGCCATCTCCGCCTACCTCGGGGTGACCGAACCAGCGATGTACGGTATCAACCTCAAGTACAA
GTTCCCCATGCTGTGCGCCATGGTGGGGTCCAGCCTTGCGGCCCTTATCTGCGGTTTCGCCGGAGTGATG
GCCAACGGCATCGGGGTGGGTGGCCTGCCGGGCATCCTCTCCATCCAGCCGCAATACTGGGCGGTATTTG
CCATCGCCATGCTGGTCGCCATCGTGGTGCCGGTGGTGCTGACCCTGCTGGTCTACAAGCGCCAGCAAGC
CGCTGGCAAGTTGACCCAAGCCAGCGCCTGAACATAGTTATCAGGGCGACCGATGGGTCGCCCTCTCCAT
TTTCTATTCGAGACAAGAGAAAGACCCATGAGCCAAACACCCTGGTGGCAGAGTGCCGTGATTTACCAGA
TCTACCCCAAGAGCTTTCAGGATTCCGCTGCCCGTGGCACCGGTGATCTCAAGGGGATCATGGCCCGCCT
CGACTATCTCAAGACTCTGGGCGTTGACGCCCTTTGGCTGACCCCGGTCTACGTTTCCCCGCAGGTGGAC
AACGGCTACGACATTGCCGACTACCTTGCCATCGACCCCGCCTACGGCACCATGGATGATTTCGAGGCGC
TGCTGGCCGCCGCCCACGCGCGCGACATCCGCATCGTGATGGATATAGTAGTGAACCACACCTCCACCGA
GCACGCCTGGTTCAAGTCGGCGCTGGGGGATAAAAACAGCCCTTACCGCGACTACTACATCTGGCGCGAT
CCGGTGGATGGCGGCGTGCCCAACAACTGGCAATCCAAGTTTGGTGGCAGCGCCTGGGAGCTGGATCCCG
CCACCGGCCAGTACTACCTGCACCTGTTTGCCCGCGAGCAGGCGGATCTCAACTGGGAAAATCCTGCAGT
GCGCGCCGAGGTCAAAAAGATCATCCACTTCTGGGCCAAGAAGGGGGTGGACGGTTTCCGGCTCGATGTC
ATCAACCTTATCTCCAAGGATCAGACGTTCCCTAACGATGAGACTGGCGATGGCCGCCGTTTCTACACCG
ACGGGCCGCGCATCCACGAATTCCTGCAGGATGTGAGCCGCGACGTGTTCGCTCCTGTCGGCGCCATGAC
GGTGGGGGAGATGTCCTCTACTTCGCTGGAGCACTGCCAGCGCTATGGTGCCCTCGATGGATCCGAGCTA
TCCATGGTGTTCAACTTCCACCACCTCAAGGTGGATTACCCCAACGGCGACAAGTGGACCAAGGCACCGT
TCGATTTCCTCGAACTCAAGCGCATCTTCAACCACTGGCAGAGCGGTATGCATGGCAAGGGGTGGTCGGC

GCTGTTCTGGTGCAACCACGATCAGCCGCGCATCGTCTCCCGCTTCGGTGATGAGGGTGAGTACAGAGTG
GTCGCTGCCAAGATGCTGGCCAGCACGCTGCACGGCCTGCAAGGCACCCCTTACATCTATCAGGGGGAGG
AGATCGGCATGACCAACCCCGGCTATCAGCGCATCGATGACTATCAGGATGTGGAAAGTCGCAACATCTT
CGCCATCAAGCAGGCGGAAGGGATGAGCGAGAGCGAGATCCTTGCCATTCTCGGTGCCAAGTCCCGCGAC
AACTCGCGTACCCCGATGCAGTGGAGCGCTGCGGCCAATGCAGGTTTCACCCAAGGAACGCCCTGGCTCA
AGCCTGCCGCCAACTATTCCGAGATCAATGCCGAGGCGGCGTTGGCCGACCAGCACTCTGTGTTCTGGCA
CTATCGGGATCTTATCGCTCTTCGCAAGGCGCACCCCATCTTCACCCAAGGGGATTATCAGGAGTTGCTG
ACCGGGCACCCGCAGATCTGGGCCTATGCCCGCCGCGCCAACGGTCAGACCCTGCTGGTGGTGAGCAACT
TCTACGGCGAGCCGGTGGAGTTTGCCTTGCCTGCCGAGCTGCAAAGCGGGCAAGGTCGGCTGCTGCTCGG
CAACTACCCGGATAGCCCGGCCCAACCGCAATCGGGTATGCTGCGCCCTTATGAATCCCTGATCTGGCTG
ATGGAGTAGGTAATGGAGAAGGTGGTGCGCGGTACCGAGTGGCAGAAGATGGTGGCGGGGAAGCCCTATG
ACGCGGCTGACCCTGAGCTGAAAGCGGCACGTGAGCGCTGCCGCAGGCTCTATCACACCTTCAATACCAG
CTGGCAGGAGAAACCGGCGCGGGATGCTTTGCTTCATGAACTGCTGGGCGGCCGTGGCCAAGCGTGTGCC
ATCAATCCGCCGTTCTTCTGCGACTATGGCGCCAATATCTTCGTCGGCGAGAACTTCTACGCCAACGTGG
GCTGCACCATCCTCGACGTCTGCGAGGTGCACATCGGCGACAACGTGCTGCTGGCGCCCGGGGTGCAGAT
CTACACCGCCGCTCATCCGGTGGCGGTGGCGCCGCGCATCAGGGGTGTAGAGTTCGGCAAGCCGGTGCGG
ATCGGTCACAACGTCTGGATTGGCGGCAGCGTCGTCATCTGCCCCGGCGTTACTATCGGTGACAACAGCG
TCATCGGCGCAGGCTCGGTGGTGACCAAAGATGTGCCCGCCAACGTGGTGGCGGCTGGCAACCCTTGCAA
GGTGCTTCGGGCGATGACGGCACAGGAGCTTGAAGGCGTCATCCAGCGGTGAATAGGGACCATGAATGCT
ATTGCTATCTGATTGATAGAAAAGCGACTTTCAGATTGTTTTGCATTCAGTGCTTATGTTCGGGGCCGCT
TGGTCGATACTGTTGAGTGCAGGAGGAACCCTATGGATATATCAAGTTCTGCAAATGCGTCGTTTGGTTC
TGCTCTGATGACGGCCAGTCTGGCCAAGAAACAGCAGACCCAAGAGGGCGAAGCTGCGCTGAAGCTGCTG
CAAAACGCAGCCCAATCAGCGCCGGTTGCACCGACATCGGCCAGTGCTTCTCTGGGAAGCAAGATCGATA
TTCGCGTCTGACCCTAATGAAAAAGCCCCCATCTCGCGATGGGGGCTTTTTTATCTCTGCGATTTGGCGC
GAGCCTCTTTCGCTCAGAGCGGGAAGGGGGCTGGCGCCTCGAAGGTCATCCGCTCGCCGCTGCGGGGGTG
GCTGATGGTCAGACTGGCCGCATGCAGATAAAGGTGCGGTGCCGCCGCCAGCGCATCCGGATGGGCGTAG
AGGTTGTCACCCAGGATCGGGTGCCCCAGCTCCAGCATATGGACGCGCAGCTGATGCGAACGACCTGTGA
TGGGGGTTAGCTTGATCAGGCTGTTGCCGTTCTCCACTTTCAGTTTTTCCCACAGGGTCAGAGCGTGACG
CCCCTCCTCGTGATCCACCTTCTGCTTCGGGCGGTTGGGCCAGTCGACGCAGAGCGGCAGGTCAACCTGC
CCCGACTCTGCTTCCGGCTCGCCCCACACCCAGGCGAGGTAGTGCTTCTCGGTCTCCCGCTCGCGAAATT
GTCGGCTCAGTTCCCGATGAGAGTTGGGATTGAGGGGAATGACAATGACGCCAGTGGTGGACATGTCGAG
GCGATGAGCTGCCGCCGCCTTGGGGTGTTGCTGTTTCACCCGATGCAGGACGCTGTCTTCATTCTCGGGG
CCGCGCCCGGGGACGCTTAGCAGGCCAGTGGGCTTGTTGACCACCATGATGTCCTTGTCCTTGAACAGGA
TATCGAGCCAGGGTTCCAGAGGCGGTGAGTATTCAAACATAAATGTCTCGCATCAGAAGGTGGGGGCAAG
CCCCCATCAGTTATGGGTAACCACGACAAAGTGGCCTTGGGTATCCAGTTTGAGCTGGTTCTGGCCGCTG
TCGGTCGGTACCTTGTCAGTTATGGGTGACCACGATAAAGCGGATGGCATCCAGCTTGAGTTGGGTCTGA
TCGATCAGGTGGTGCAATTCTGCCTGCAGATTGCGCAGATAATCTAGTTCGCTGTCTCGCACGTTCGGGT
TGACCGCCTTGAGCGCTTCCAGACGATCCAGATCCTGCTGCAGGGTCTGCGCCATCTGGGTGCGGGCCTT
GTCGACAATCCCGGCTTTCAGCTCTTCGGCAATGGCTTGCCCCTTGCCAATCAGACCGTGGATCACCGGC
TGGGATGCAGTCACCAGCTTGCTGCCGAGGTGGCGATTGACCGGCGTCAGCTGGCGGTTGAAGGCATCGA
ACGCCACTTTCTCACCCAGATTCTGGCCGTTCTTGTCCATCAGCAGACGGATCGGCGTCGGCGGCATGAA
GCGGTAGAGCTGGGGATGGGCCGCAGACTCGGCAACGAAGATCAGCTCAAGCAGGATGGAGCCGATGGGC
AGCGCCTTGTTCTTGAGCAGTGCGACCGAGGTGGCACCGATCTCGGAGCCCAGGATCAGGTCGATACCGC
CGCGCATCATGGGGTGATCCCAGGAGAGCAGCGCCATGTCATCCCGCGACAGGGCTGTGTTGCGATCGAA
GGTGATGGTCATGCCGTCTTGCGGCAGGCCCGGGAAGCTGGAGACCAGCATGTGATCCCCGGGGGTCAGC
ACCAGCGCGTTCTCGCCACGGTCATCCTGATTGACGCCGATCTCGTCGAACATCTTGAGAGCGAAGGAGA
TCATACCAGTGTCGTCATCTTCGGCGGCCAGCTTGTCGACCAGCTGCTGGGCGGCGGCGCCACCGCTGGA
GTGGATCTCCAGCAGGCGATCGCGACCCTGCTCCAGTCTGGCCTTGAGCGGCTCGTGCAGTTCGCGGGTC
TTGACCAGCAGGGCATCGAGGGCCGCCTGATCGCCGGTGTTGGCGGCCAGCAGCTCGAACAGATCGTCAC
GTACCGCTTCGAATACCGGGCGTGCAGTCGGGCAGGTCTGCTCGAACGCGTCCAGACCATCGTGATACCA
GAGCTGCAGGGCGCGCTGGGCTGTCCCTTCCAGATAGGGAACGTGGATCTCGACGGTATTCTGCTGACCG
ATACGATCCAGTCGGCCGATACGCTGCTCCAGCAGATCCGGGTTGAGGGGCAGATCGAACAGCACCAGAT
GGCTGGCAAACTGGAAGTTGCGACCCTCTGAACCGATCTCGGAGCAGAGCAGCACCTGGGCGCCACCCTC
TTGCTGGGCGAAATAGGCGGAGGCCTTGTCCCGCTCTAGGATCGACATCCCTTCGTGGAACACGGCACCA
CGGATCCCTTCACGGGTACGCAGTGCCTCTTCCTGCGCGATGGCGGTCGCCGCTTCGGAGCAGATCACCA
GCACCTTCTGCTGACGGGCTGAGAGCAGCAGCTCCAGCAACCATTCGACACGGGGGTCGAACTGGGTCCA
GGTGGCGTTGTCGCCTTCGAACTGCTGGAAGATCTTCTCGGGGTAGAGGTAGCGCAGGGCGCGGGTCTGC
AGATCGCCACCGTTGCCACCCATCATGCCCATCACCTTGATAGCAGTCTTGTACTGCTCCGGCAGCGGCA
TGGGGTAGACGTTGAGGTGACGCTCGGGGAAGCCCTGAATATTGGCGCGGCTGTTACGGAACAGCACCCG
GCCGGTGCCATGCTGATCCAGCAGCTTGGCGACCGCCTGCTGGCGTGCAGTCGCATCGCTCAAGTCCAGC
CCTTCCAGCTGGCTGGCGAGGATCTGTCTGGCTTCATCGCTCAGGGTCTCGCCATCCAGCAGCTCTTGTG

CGGCGTTGGCGACCTGACCATAGGCCTGCTCCTCGGCGAGGAAGGCCTCGTAGTCGTAGAAACGCTCGGG
ATCGAGCAGGCGCAGACGGGCAAAGTGGCTCTGGTGGCCCAGCTGATCCGGGGTCGCGGTCAGCAGCAGC
ACGCCCGGCACCTGTTCGGCCAGCGCTTCCACCATCTCATAGGCACGGCTCGGCGCCTCTTCACTCCACT
CCAGATGGTGGGCTTCGTCGACAACCAGCAGATCCCACTCGGCTTCCAGCACCTGCTCGAAGCGACGACG
CTTCTTGCGCAGGAAGTCGAGGCTGCAGATCACCAGCTGTTCGGTCTCGAAGGGGTTCTCCGCATCGGCA
AATGCCTCGATGCAGCGCTCCTCATCAAACAGGGAGAAGTGCAGGTTGAAGCGGCGCAGCATTTCGACCA
GCCACTGATGCTGGAGCGTTTCGGGCAGCAGAATGAGCACGCGATGGGCGCGGCCGGAGAGCAGCTGCTG
ATGAATGATCATGCCCGCTTCGATGGTCTTGCCCAGACCCACCTCATCGGCCAGCAGTACGCGCGGAGCG
TAGCGGTGACCCACCTCGTGGGCGATATAGAGCTGGTGCGGGATCAGGCTGACCCGGCCACCGGCCAGAC
CGCGAGTCGGGTTGCGACGACGCTGGTGCTGGTTGACCAGCGCCTCGTAGCGCAGGGTGAAGCGGGACAT
CCGATCGATCTGACCGGCGAACAGGCGATCCTGCGGCTTGTTGAACTTGATAAAGTTGTTGAGAAACACT
TCCTTGAGGGCAACGGGCTCATCATTGTCGGTGCGCACACCTACATATATAAGCAGGCCGTCTTTCTCCT
GCACTTCTTCGACCGTCATGGTCCAGTCTTCGTGACTGGTGATCTGATCGCCCACATTGAAGCTGACCCG
GGTGACCGGCGCCTCTTCTTTGGCATACATGCGGTTTTCACCGGTAGCCGGAAACAACAGGGTAACCATG
CGTCCTTCAACAGCAACGACAGTCCCTAACCCCAGATCCGTCTCTGTATCACTAATCCAACGCTGGCCAA
GTGCAAAAGGCATTAAACTCTCCAAAGCCCAAGAGTGTGTGAATCGCAAAGGGGCGCTATGTTACCCGAA
GGCGTGACCTCGATCACCATGCATATAGATGTAGAGAGACCAAATCGCCACAACCTTGATCTGCTACCAT
CGCCACGCCATCTAGAGGATAGCGAGCTCGCCAAACGGGTGTGTTATTGCAGGATAAATGCGCTTTTTGG
GTGATTAGTGAACGGTTGAACCAAAAATGCGAATTTAATTCGAAAACACCCTTGCAAGCGTGATCCAGAT
CCCTATAATGCGCCTCCATCGACAGGGCAAGCGGCAACGCAAACAACCTCGTCGATAACCTCAAAAAGCC
TTTGAAAATAAGGCTTGACTCGAGAAGGCGGTTGAGTAGAATTCCACTCCCGCAGCAACACACTGTTGCG
TCGCTCTTTAACAATTTGAATCAAGCAATCTGTGTGGGCACTCACAGCATCGAACATCAAAACAATTTTT
GATTTTCAATGTCTGGTGAAGTGACCAAGACGATTTCTTAGCAATAAGAAGTCGAACAGTTTATTTCAGC
AATTCATTGAGCCGCTTTTCGAAGCAACCAAACTTAAATTGAAGAGTTTGATCATGGCTCAGATTGAACG
CTGGCGGCAGGCCTAACACATGCAAGTCGAGCGGCAGCGGGAAAGTAGCTTGCTACTTTTGCCGGCGAGC
GGCGGACGGGTGAGTAATGCCTGGGGATCTGCCCAGTCGAGGGGGATAACTACTGGAAACGGTAGCTAAT
ACCGCATACGCCCTACGGGGGAAAGCAGGGGACCTTCGGGCCTTGCGCGATTGGATGAACCCAGGTGGGA
TTAGCTAGTTGGTGAGGTAATGGCTCACCAAGGCGACGATCCCTAGCTGGTCTGAGAGGATGATCAGCCA
CACTGGAACTGAGACACGGTCCAGACTCCTACGGGAGGCAGCAGTGGGGAATATTGCACAATGGGGGAAA
CCCTGATGCAGCCATGCCGCGTGTGTGAAGAAGGCCTTCGGGTTGTAAAGCACTTTCAGCGAGGAGGAAA
GGTTGGTAGCTAATAACTGCCAGCTGTGACGTTACTCGCAGAAGAAGCACCGGCTAACTCCGTGCCAGCA
GCCGCGGTAATACGGAGGGTGCAAGCGTTAATCGGAATTACTGGGCGTAAAGCGCACGCAGGCGGTTGGA
TAAGTTAGATGTGAAAGCCCCGGGCTCAACCTGGGAATTGCATTTAAAACTGTCCGGCTAGAGTCTTGTA
GAGGGGGGTAGAATTCCAGGTGTAGCGGTGAAATGCGTAGAGATCTGGAGGAATACCGGTGGCGAAGGCG
GCCCCCTGGACAAAGACTGACGCTCAGGTGCGAAAGCGTGGGGAGCAAACAGGATTAGATACCCTGGTAG
TCCACGCCGTAAACGATGTCGATTTGGAGGCTGTGTCCTTGAGACGTGGCTTCCGGAGCTAACGCGTTAA
ATCGACCGCCTGGGGAGTACGGCCGCAAGGTTAAAACTCAAATGAATTGACGGGGGCCCGCACAAGCGGT
GGAGCATGTGGTTTAATTCGATGCAACGCGAAGAACCTTACCTGGCCTTGACATGTCTGGAATCCTGTAG
AGATACGGGAGTGCCTTCGGGAATCAGAACACAGGTGCTGCATGGCTGTCGTCAGCTCGTGTCGTGAGAT
GTTGGGTTAAGTCCCGCAACGAGCGCAACCCCTGTCCTTTGTTGCCAGCACGTAATGGTGGGAACTCAAG
GGAGACTGCCGGTGATAAACCGGAGGAAGGTGGGGATGACGTCAAGTCATCATGGCCCTTACGGCCAGGG
CTACACACGTGCTACAATGGCGCGTACAGAGGGCTGCAAGCTAGCGATAGTGAGCGAATCCCAAAAAGCG
CGTCGTAGTCCGGATCGGAGTCTGCAACTCGACTCCGTGAAGTCGGAATCGCTAGTAATCGCAAATCAGA
ATGTTGCGGTGAATACGTTCCCGGGCCTTGTACACACCGCCCGTCACACCATGGGAGTGGGTTGCACCAG
AAGTAGATAGCTTAACCTTCGGGAGGGCGTTTACCACGGTGTGATTCATGACTGGGGTGAAGTCGTAACA
AGGTAACCCTAGGGGAACCTGGGGTTGGATCACCTCCTTACCTTAAGATGTCGAAGTTGTTGAGTGTTCA
CACAGATTGCCTTGATTCAAAGTAGTTAGAGCAAAGACCTGATGCGAAAGTGTCAGTGCTTATCTGGCTT
CGGTCAGATGAGAGAAGCCCTATCATTGGGTTTTGGGATGTGAATAATGGCGCTCGGCCTCGCAGGCTCG
GCACTCGCCATTACCCAAAATCTGCACTGCTGACGCAGCGCAAAGATGATTTTGGGTCCCCTTCGTCTAG
AGGCCTAGGACACCGCCCTTTCACGGCGGTAACAGGGGTTCGAATCCCCTAGGGGACGCCACTTCTCTTC
TTGCTAACAAGAATGCAGAGTTAAGAACTGATTCTTAACTCTGTTTTCTTCGGCCTTGCGCCGTTGCAAA
CATGCTCTTTAACAATCTGGAAAGCTGATTTAAAAAGTAGTTCTCAAACATTTGTTACAAGTGCTTTGGA
AACTTCTTGGCGAAAACCAAATTTTATTTGGTCCTTGTTGTACGACAACAAGCAAGCGTTGTTTCGACGA
CACTTCTTGGGGTTGTATGGTTAAGTGACTAAGCGTACATGGTGGATGCCTTGGCAGTCAGAGGCGATGA
AGGACGTACTAACCTGCGATAAGCTGTGAGAAGTCGGTAAGAGACGCTATTACTCACAGATTTCCGAATG
GGGAAACCCACCCGAGATAACTCGGGTATCTGTTACTGAATACATAGGTAACAGAGGCGAACCGGGAGAA
CTGAAACATCTAAGTACCCCGAGGAAAAGAAATCAACCGAGATTCCCTCAGTAGCGGCGAGCGAACGGGG
ATTAGCCCTTAAGCATCTTGGAAGTTAGTGGAACGGTCCTGGAAAGGCCGGCGATACAGGGTGATAGCCC
CGTACACGAAAACGACCTTGATGTGAAATCGAGTAGGGCGGGACACGTGACATCCTGTCTGAATATGGGG
GGACCATCCTCCAAGGCTAAATACTCCTGACTGACCGATAGTGAACCAGTACCGTGAGGGAAAGGCGAAA
AGAACCCCTGTGAGGGGAGTGAAATAGAACCTGAAACCGTGTACGTACAAGCAGTGGGAGCCCTTCGGGG

TGACTGCGTACCTTTTGTATAATGGGTCAGCGACTTACATTTTGTAGCGAGGTTAACCGTATAGGGGAGC
CGTAGGGAAACCGAGTCTTAACTGGGCGTCTAGTTGCAAGGTGTAGACCCGAAACCGGGTGATCTAGCCA
TGGGCAGGTTGAAGGTTGAGTAACATCAACTGGAGGACCGAACCCACTAACGTTGCAAAGTTAGGGGATG
ACCTGTGGCTGGGGGTGAAAGGCCAATCAAACTCGGAGATAGCTGGTTCTCCCCGAAAGCTATTTAGGTA
GCGCCTCGGACGAATACTACTGGGGGTAGAGCACTGTTTGGGCTAGGGGGTCATCCCGACTTACCAACCC
CATGCAAACTCCGAATACCAGTAAGTAATATCCGGGAGACACACGGCGGGTGCTAACGTCCGTCGTGAAG
AGGGAAACAACCCAGACCGCCGGCTAAGGTCCCAAAGTTCTGGTTAAGTGGGAAACGATGTGGGAAGGCT
CAGACAGCTAGGATGTTGGCTTAGAAGCAGCCATCATTTAAAGAAAGCGTAATAGCTCACTAGTCGAGTC
GGCCTGCGCGGAAGATGTAACGGGGCTCAAACCAGGCACCGAAGCCGCGGATTCACACTTATGTGTGAGT
GGTAGGGGAGCGTTCTGTAAGTCTGCGAAGGTGTATCGAGAGGTATGCTGGAGATATCAGAAGTGCGAAT
GCTGACGTAAGTAACGATAAAGGGGGTGAAAAGCCTCCTCGCCGGAAGACCAAGGGTTCCTGTCCAACGT
TAATCGGGGCAGGGTGAGTCGACCCCTAAGGCGAGGCCGAAAGGCGTAGTCGATGGGAAGCAGGTTAATA
TTCCTGCACGACTTGTAATTGCGATGGGGGGACGGAGAAGGCTAGGTGGGCCAGGCGACGGTTGTCCTGG
TGAAAGTGCGTAGGTGGTGTTTCTAGGCAAATCCGGAGACACAACACTGAGACACGAGACGAACACACTA
CGGTGTGGAAGCCATTGATGCCCTGCTTCCAGGAAAAGCCTCTAAGCTTCAGATTACAAGTCATCGTACC
CCAAACCGACACAGGTGGTCGGGTAGAGAATACCAAGGCGCTTGAGAGAACTCGGGTGAAGGAACTAGGC
AAAATAGAACCGTAACTTCGGGAGAAGGTTCGCTCTTGACAGTGAAGTCCCTTGCGGATGGAGCAGTTGG
GAGTCGCAGTGACCAGATGGCTGGGACTGTTTATCAAAAACACAGCACTCTGCAAACACGAAAGTGGACG
TATAGGGTGTGACACCTGCCCGGTGCCGGAAGGTTAATTGATGGGGTTAGCGCAAGCGAAGCTCTTGATC
GAAGCCCCGGTAAACGGCGGCCGTAACTATAACGGTCCTAAGGTAGCGAAATTCCTTGTCGGGTAAGTTC
CGACCTGCACGAATGGTGTAACCATGGCCATGCTGTCTCCACCCGAGACTCAGTGAAATCGAATTCGCCG
TGAAGATGCGGTGTACCCGCGGCTAGACGGAAAGACCCCGTGAACCTTTACTACAGCTTGGCACTGAACA
TTGAACCTACATGTGTAGGATAGGTGGGAGGCTTTGAAGGCGTGACGCCAGTTGCGCTGGAGCCGTCCTT
GAAATACCACCCTTGTATGTTTGATGTTCTAACGCAGGGCCCTGAATCGGGCTCGCGGACAGTGCCTGGT
GGGTAGTTTGACTGGGGCGGTCTCCTCCCAAAGAGTAACGGAGGAGCACGAAGGTTGGCTAATCCTGGTC
GGACATCAGGAGGTTAGTGCAATGGCATAAGCCAGCTTAACTGCGAGACGGACAGGTCGAGCAGGTACGA
AAGTAGGTCATAGTGATCCGGTGGTTCTGAATGGAAGGGCCATCGCTCAACGGATAAAAGGTACTCCGGG
GATAACAGGCTGATACCGCCCAAGAGTTCATATCGACGGCGGTGTTTGGCACCTCGATGTCGGCTCATCA
CATCCTGGGGCTGAAGTCGGTCCCAAGGGTATGGCTGTTCGCCATTTAAAGTGGTACGCGAGCTGGGTTC
AGAACGTCGTGAGACAGTTCGGTCCCTATCTGCCGTGGGCGTTGGATGATTGAAGGGAGTTGCTCCTAGT
ACGAGAGGACCGGAGTGAACGAACCTCTGGTGTTCGGGTTGTCACGCCAGTGGCACTGCCCGGTAGCTAA
GTTCGGAATCGATAACCGCTGAAAGCATCTAAGCGGGAAGCGAGCCCTGAGATGAGTCATCCCTGACCCC
TTGAGGGTCCTAAAGGGCCGTTGGAGACCACAACGTTGATAGGTGGGGTGTGTAAGCGCGGCGACGTGTT
GAGCTAACCCATACTAATTACCCGTGAGGCTTAACCATACAACACCCAAGAAGTGTTCTAAGGCTTGTAG
CAAATACGAACGAATTACTTACGTCAGCTTTCTCAGATTGAAGAATTTGCCTGGCGGCCATAGCGCCGTG
GAACCACCTGATCCCATGCCGAACTCAGAAGTGAAACGCGGTAGCGCCGATGGTAGTGTGGCATTCGCCA
TGCGAGAGTAGGACACTGCCAGGCACCCAATTAAACAGCGATGTGAAAAATCACATCACTGGCTGCGAGG
ATGACACCTCACTGACGCGGCAAACAATTTAGCGCAAGCTAACCGAATGCGGAGCGGTAGTTCAGTCGGT
TAGAATACCGGCCTGTCACGCCGGGGGTCGCGGGTTCGAGTCCCGTCCGCTCCGCCACTAATTTGAAGGC
CTTCCCAGATGGGAAGGCCTTTTTGCTATGGGCGTTTTTACCCACTGACCCCGATGGTTGCACAGGAACT
AACACAAAACTCGCTCTCACGACCGGGCTTTTGTATTTTTAGCGTGTTAAACAGCCAGTGTTAGCATTTA
TCTGCAGGAAGAGAACTAAAACCCTGTTATCTCCTCCATTTTCTATCTATATCAAGCCCTTATCTGATGG
ATTGCTGGCCATTTTAACTGGTTTACTTTTGGCCAAACTCTGTTCATGGCGCGCGATTAGGGTTGTATCT
CGTTTATAACTCGGTAGAGTAAGGGCATCTTGAGTTTGCCTGCCCCGCGCCGAGTTTGCCGGGTTCCAGG
TGTAACTTGGCCAGTTTTTATCAACCTTAAGTCAGTGGTATCGATATGGAACAAGTAAACAAGTCCACCT
TCAGCGATGTACTGGAATATGTGCGTATGTCCCGTCGCCAGAACAAACTCAAGCGTGAAATTGCGGATAA
CGAGAAGAAGATCCGTGACAATCAGAAGCGCGTGCTGCTGCTCGACAACCTGAGCGACTATATCAAGCCG
GGCATGAGCATTGATGATGTGCAGGCGATCATCGCCAACATGCGCGCCGACTATGAAGAGCGCGTGGATG
ACCACATCATCAAGAACGCCGAGCTATCCAAAGAGCGTAAAGAGCTGAGCACCAAACTGAAGGGCATGAG
CTCTCACAAATAAGCGTTCAGCTTTCGCTTCAGAGACACCGGCCACTGGCCGGTGTTTTGTTATCAGCTC
GGATCTCTGCCACTCCTGCAACAATCGGCTCCCCTTTTTAGAATTGCCCTGTTACCATCACCACAAAGCG
GTCAATAGTTTCAAGCCATTGACTTGAAAAGACTAAGCTTGAGTGAAACACAATAAATTGCGCAACCATG
GTGGGGCTGATATGGGCGCATCAGAACAGACGGATTTGACACAAGTGGCATCTCAAGATTCATCTGTCCA
GACCTCGGACGGGAACCCAAAGCGCAAGCGTCGTCCCTGGTGGCGTTGGCTGTTCTGTTTTCTCTTTTTA
TTGTGCCTGGGGGCGGTGATCGCTCTGGTTGGCTATGAGATGAAAACCTCGAAGCTGCAATCTCAGGAGA
TCTCCCGCTATGCTGCCAAGCTCACCTATCAGGTCGAGCCTGGTCAGAGCAAGCAGATCGCCTTCCCTGA
GGATGGCCCGTTCGACAAGCGGCTCGGCTATGTGCAACTGCCGATGATGCAGGAGCGTTTGCTTCAGCGG
GGTTATGAAGTCAGCAAGCAGGTGCATTTCTCCGACGCGCTCTATGACTACGCCAGTCGTGGCTTCTTCG
TGCCCTATACCGAGAAGGTGCAGGCTGGACTGACGCTGCACGATTGCCGCGCCGAGCCCTTCTATCAATT
CAAGTATCCGACCCATCACTATCCAGATTTTGACTCCATTCCGCCTCTTGTCATCCAGTCGCTGCTGTTT
ATCGAGAACCGCGACTTGCTGAGCAACGAAGAGCCGTTGGCCAACCCGGCGGTCGACTGGCCCCGTTTTG

CCAAGGCCGCGCTCTCTCAAGTGGGCAAGGCACTCGATATGCAGGATCAATCTGCCGGTGGCAGTACCCT
GGCGACCCAGGTGGAGAAGTATCGCCACTCGCCGGATGGCCTGACCCTGTCGCCGACCGAGAAGATCCGT
CAGATGGCCTCTGCCAGCGTGCGGGCTTATCGTCTCGGGCCGGAAACCCTCGAAGCGCGCAAACTGGTCG
CTTGGGCCTACCTCAACTCGGTACCGCTCTCGGCTGCGCCGGGATATGGTGAAGTGCATGGTCTGGGAGA
TGGTCTCTGGGTCTGGTTTGGTGCCAATGTCGATGACGTGAACCGTCTGCTGGATCCCGCCCGCAATCAG
AATGCGCCGCTGGCCGAACAGGGGCTCGCCCTGCGTCAGGTTGTCTCTTTGATGATTGCGCACCGCCGTC
CCTCTTACTATCTGGCTCAAGGGCGAGAGGCGCTGGCTACCCTGACCGACAGCTACCTGCGCCTGTTTGT
GCAGGAGGGGATGATCACGCCACCTCTGATGGAGGCGGCGCTCAAGGCCAAGATTACGTTCCGTGACTTC
CTGCAGTCCCCGGCTATCACCCGGGTGAACAACAACAAGGGGTTGCAGGTTGCCCGTACTCGCCTGAGCC
GTCAGCTCGGGGTGCAGCTCTATGACCTCGACCGGATGGATCTGACGGCCGATACCAGCCTCAATATGCC
GTTGCAGAACGAAATCTCCCGTTATCTGCAACAGCTGGCCGACCCGACGTTTGCCGCCCAGGTAGGACTG
TTTGGTGAGCGCCTGCTCTCGCCGGAGAAGACCGGCGATGTGCGCTACAGCTTCACCCTGTTCGAGAAGT
CGCCGGAAGGGTTCAAGGTGCGGGTGCAGACCGACAACACTGATCAGCCGTTCGATATTAACGAAGGCTC
CAAGCTGGAGTTGGGCTCAACCGCCAAGCTTCGGGTGCTGACTACCTATCTGGAGATCGTGGCCGAGCTG
CACGACAACTATGCCGGGATGCCGGTTGAGAATCTGCGTAAATTGGCTGCCGTCTCTCAGGACAACCTCA
GCCGCTGGGCCATCTCCTATCTGATGACGGCTCAGGACAAGAGCCTGCCTGTAATGCTGGAAGCGGCGCT
GGATCGCAAATACTCCGCCAGCCCCAACGAAGGGTTCTTTACCGGTGGCGGCATGCACACCTTCAACAAC
TTCCGCCGCGAGGATAACAATCGTATTCCGACGTTGCGTGACTCTCTGCGTGAGTCGATTAACTTGCCCT
TTGTGCGGCTGATGCGCGATATCGTGCGCTACAGCCTCTACAAGGAGGGCAACCGTGCCCAGTTGCTGAA
GGATGACAAGGATCCCCGTCGTCAGGAGTATCTGGCCAAATTTGCCGACAAAGAGGGGCAGACCTATCTG
CTGCGCTTCTGGCGCAAATATCGCGGCAAGACTCCCGAGGAGCAACTCGATACCTTCCTCGACGGTCTCA
AGCCGAGTGCGGTACGTCTGGCGGCGGTTCACCGCTACCTGATGCCGGAAGCCAGCGAAGCTGCATTTGC
TGCCTTCCTGGCGGAGCGGTTGCCGCGGGAGAAGTTGAGCGCCAAGCGGGTCAATCAGCTCTATGTGAGC
TATGGCCCGGGCAAGTATTCACTGCCGGATCAGGGCTACATCGCCCGGGTGCACCCGCTGGAGCTGTGGC
TGCTGGCCTATCTCAAGGAGTTCGACAAGGCGACCTTTGCCGACGCGGTGGCGGCCAGTCGTGACGAGCG
GCAAGAGGTTTACAGCTGGCTGTTCAAGACCCGTCATCGCAGTGCCCGTGACAGCCGGATCCGCACCATG
CTGGAAGTTGAAGCCTTCCTCGATATCCATCAGCGCTGGGCCAAGCTCGGTTATCCGTTTGATCATCTGG
TGCCTTCGCTGGCGACCGCGTTGGGTAGCTCCGGCGATCGTCCGGCTGCACTGGCCGAGCTGATGGGGAT
TATCCAGAATGGCGGTATCCGCTCGCCAACCTTGCGTATCGAGCACCTCTCGTTCGCCAAGGGGACGCCC
TTCGAGACCGAGTTTGAGCCGCAGCACACCGAGGGAACCCGGGTGATGCCGGCAGAGGTGGCGGCAGCCC
TGCGCAGTGCGCTCTCCAAAGTCGTTGAAGGGGGCACGGCAAGACGTATAGCCGGAGCCTTTACCCTGCC
GGATGGCACTGTGCTGCAGATGGGGGGCAAGACCGGGACCGGGGATAACCGGATTGAAACCGTGGGTCGT
TATGGCAACGTGACCAGCTCACTGGCGCGCAACCGCACCGCGACTTTCGTCTTCTATCTGGGTGAGGATC
ACTTCGGTACCCTGACCGCCTATGTACCGGGTAGCCAGTCTGACAAGTTCCGTTTCACCTCGGCGCTGCC
GGTGCAGGTACTCAAGGGGATGGCACCCCTGCTGACGCCTTACCTGCAACCGGGCTCCCATACCCAGTGT
CTGTCGAACCAGTGATGCGCACGAATTACCGCTCATCACGCAGATAACTAACCAGAGAAAAAACGGGGAG
GCTGACAGCCTCCCCGTTTTTATATCCTCATCGGACGCAGCGGCGCTCGATTGCGATCAGGCGTTCAGTA
CGAACTGCTCGATCACCTGAGCCACGCCATCTTCATCGTTGCGGGCAGTGGTGTGCTGGGCGAGAGCCTT
GATCTCGTCGGTGGCATTGCCCATCGCAACCCCGAGGCCTGCGTATTCGATCATGTGGCGATCGTTGCCC
GCATCCCCGACGCAGATCACCTGATCGGCGGTCAGGCCAAGGTGCTCGGCCAGTGCGGCCACGCCTGTTC
CCTTGTTGCTCTGCTTGTTCATGAACTCGAGGAAGAAGGGGGCGCTCTGTACCACGGTATAGCGCTCATA
GAGCTCTGCAGGCAGTCGCTCGATGGCGCGGGAGAGCTGGGGCGGCTCGTCGATCATCATCACCTTCATG
ATCTGCTCGTCGGCAGGCAGGGTGGCGAAGTCGATCAGATTGATCGGCATGCCGATCAGGCGGGATTCGT
GCTCGGTGTAGGTGCTGACACGGGGACTGATCAGGCCACGGGTCACCGAGAAACCGTGGACGTTCACCTC
CAGCTCGTCAGCCAGTTTGGCGACGGCGCTGGCGTCACTGCCGGTCAGCAGCTGGCTGCGGATGATGCGC
TGATCGGCCACCTGTTGCACCAGGGCGCCGTTGTAGCAGATGACGTAGTCATGCTCTGTGGTGAGGCCGA
GCTCTTCCAGATAGCGGCTCATCCCTTCCAGCGGGCGGCCGGAGGCCAGCACAACGGTGACCCCTTTGGC
GCGCGCGGCGCTGATGGCGGTGTGGGTGCGGGGAGTAATCTGGCCCTGCGGGTTGAGCAGGGTGCCATCC
ATATCCAATGCGATCAGCTTGTACATCTCTCACTACCTGAGTCTGGGTGGCCCGCCCGGTGGGGCCAAGG
AAGTGGCGAGTGTAGCGAAAATCGGCGGCGGGATCACGGCCGGTTCAGCGATGGGGCCGTGCCGGTTGAG
CTATTTGTGTCCCTCGTAAGGGGTCATCTCGAGGGTTTCATCATGGATGGTCAGACGGGTAGGGCCTCGG
GTGATGGTAACCTTGCGTTTGAAGCTGGCCATCTCGACCTCGATGCCGGAGTAGAGGTGTTGGGTGGCAA
TGACCTCCATCTGAGCCATCAAGTTTCGCAGTTCCTCCTGGTTGGCGGTGAGCTGCTCATCGAGCTGGGC
CAGTTCGGCCAGCTGATGGTCCCGGATCGGTTTGAGCTTGCGCAGGGTATCCGGATTGCGCTTGTCGCCG
GGCAGTTGCAGCAACTTCATGACCAGCTCCTTGATTTTCTCCAGCTGGGCCTGACACGCCTGCTGGCGCA
GCTTGAGCTGGTGTTCCTGCTCCTTGTGGGTGAAGTAGCCGCCGAGGATCTGCAGCCGGGTATGATTGGC
GGCCTCTGCCCCCAGGCTGGGAGCCATGATCAGGCGATTGGCAATGCTGGTGCCCCCCAGCAGAGTGCCT
TTGCGCATGTTGCTATCGCCCACCTTGATGTCTTGTCCGCTGCGGGAGTAGCAGTGGCTGAGCTGGCTCT
GGATCTGGATATCCCCCCCCGCTTCCAGCTGGGCATATTGGGCGTAGCTGGCGTGGATCTCTCCTTGTGC
ATTGAGCTGGCAAAGGTACTCCCCCTGCTCGTTGCGGCGGCCAATGATGCCGTGGCGTACCGTGATGCAG
CCCCCCGCCTCGATCCAGCCCCCTTCGATAAAGCCGCCGATGACGATATCACCGCTGACCCTGATCTTCA

TGCCCGGGTTGACGTCGCCGCTGACGATTAAGCCCCCTTCAAAATCAATGTGGCCGTGGCGGGCATCAAC
CTGTTTGACACTGAGCACCTCGTCCACCTTCATGCCTGCTTTCTCCTGACAGGGCAGGCCAGCCCGACCG
GCAATCAGCAGGTCGGGATCGTCGGGGGAGATGAAGGTGCCCTCGCCAGCGCTCATCGGACTGTCTTTGC
CATCTTTGGCGGGCAGGTCCTGACCGGTCACGGTAAAACCGGGTACCCCGACGGTGGCCGGATGACGGCG
CATCAGCGGAGCGCCGGCCTTGACGGTGACGATGGAGCCGAGATCCCGCAGGTCGACCTTGCCATGCCCC
AACTCCTGCGGTTTGAGGATCCGTTCGGCCGGGGTCTCCACCAGCCGCTCATAGCGAGTGTCCTGACCAT
TGACGGCAGGCTGGCCGTTTGCAATCACCAGCTTGAGCTGACTGCCGGGGGCGGCCTCCTTGGCCGACTG
GATGGCAGCGGTGAGCAGTTGCTCACTTACCCCCTGTTTGATTTCGCTGATGGCGATCGCCTCTTTCAGC
TGTTCCAGCGTCACATACTTGCCCCCCCAGTCGGCAGTGATCTGGGCGATGGCGCTCATGCGATCCTTCT
CCACTATTACGGCGAGTCGGGCATCCTGACGCTTGCCGACAGGGATGGGGTTGGCGGGGATGGGGGCGGC
AGGGTTGGTGAGCAGGGTATTAAGCAGGCTGATGGCCTGTTTGATCCCCTCGGGCAGCGGCTGATAGCGG
CGACAGTTGGAAGCGAGCAGCAGAGCCTGCACATCCTGTTCCGTGACAGGTTTGGTGAGACCGGCGGCAA
TCCGCAGGCAGACCAGTTCCATATCGCTTGATAGCAGCAACCACTCTTTGCTTAACACCGCATCTCCTTG
CTGGATGAGAACGGAGCGATCGGCTCGCTCCTTTACTGTCATTTAATCTCTCAATGATTAATAAAGCGAG
TCGCCAACCCTGAGAGTGGGCAGTATTCAGATATTTCCTCTGAATGGTCAATGAATTGAAGTCAAATCGG
TGATGGCGGCCAGATAAATGTGGGCGCTGTTTGGAAACGGAGTGAAAAGACGGATCGCAGCGCCAGCGAC
ATAGGGTCGCTGGCAGGGGAATGGCGGTTCTTATCAGCTGCGCTGGGCTGTTCTGGCCAGGGTGAGGTAA
TCGCCGAGCACCGGCTCACCCAGCATATGGCGGCGCAGCATGTCGAGGGCGGCAAAGGCGAGGATCTTGC
GGCGACTCTCCGGGTCGGGGTGGCTGACCTTGACCGTCTGACCGATGTGCATGCCATCGGCGCTGAGCAG
CAGTGGCACCCCCCTCTGCGCCCTCTCGCCCTATGGTCAGTTGCAGGCCCGCATCGGTTGTCGGCAGTTGT
GCCAGCTCATCGGCCAGTTGCGTACTGAAGTTGGCCTCCAGCCGGGGATGGCCGTGACAAAGCGTCAGCA
GGGCGCCGCCACTCTCCCCCTCCAGCAGAGCCAGCGGTTTGCCTTGCAGCAGCGCGAGGATCTGTTGCGC
CGGTTCCGTATCTCCGCTGCCAACCAGCCAAGGTCGGATCGTATCCAGCAGTCGGCGCTCTGCACTCTCC
ATGTCAGCGGTACTGGCGCCGTGGCCGATCAGTTTCAGCTCGATAAGGGGCATGGAGGAGCGGTAACCCA
GCTCGATGCTGGCTGGCCAGGGTTCGGCATCCAGCATATCGCTGAGCGCAGATTCCGAGATGCCGAAGGT
GTAAAAGCGACGCAGCTCGGTATCGATCTGTGCCCCGGCCAGCTGTTTGAGGCGCGGTAGAATTTGCTCG
CGTACCATCTGCTTGAATTCGAACGGCACGCCTGGGGTGAAGAAGAGCTGACTCTGGCTATTTCGCACCA
CAAAGCCGCAGGCGGTGCCAACCGGGTTGTCGAGGATCTCGCTCCCCAGCGGCAACCAGGCCTGTTTGGC
ATTGCTTTGTGCCATGGGGCGACCACGGCTGGCGTAGCGCGCTTCGATGGTAGCGAGCCAGTCGGGGAAG
AGGGTGAGCGGTTGGTCAAAGGCCTCGGCTGCCGCCTGAGCGGTGAGGTCGTCGGCAGTGGGCCCGAGCC
CGCCACACACCAGCACCACCTCGGCGCGGCTGGCACTCTCTTGCAGCACCGTTTTCAGGTCGGCGAGGTT
GTCCCCCACCGTCAGACGACGGCGCACCTGCCATCCCTGTTCCAGCAGCAGAGCGCTGAGCCAGACCGAG
TTGGTATCCACCACCAGACCCGTCACTATTTCGTCTCCGGTACTGATGATCTCGATACGCATAGCAGGCT
CCTTGGATTAAACGGGTGGCAACGGTTGGCCTCGGGCCAACACAATCAGGCTTTGTTGTGCTCGAAGTAG
TAGCTGATCCGGGTACGCGGATTGAGGTACTCGAACACGGCGGGCGGCAGCCGTTTGGGGTGGATCACCT
TGGCGATCGACAGTTCGGCTTGCTGCAGGTCGATGATGGCGGCTTCCTGACGGGGAATGCGCGGATCGTA
GACCAGCACCGAGGGGTAGAGCAGGCGGGAAGCGTTGACCGACATCACCAGCCCCACCTGACCGTTGGAG
AGTTGCACCACGCTGCCGGGGGGATATATCCCCATCAACCGGATCAGCATGCTGAGGTACTCCGGATTGA
ACTGCGCCTTGCGCTGCTTGTAGAGGCCGCTCAGAGCCGAGTAGGGCACCTTGGCCTGCATGTGGCAGAG
GTTATCGTACTCGTTGATCAGCGACACCATCTGGGTCAGCGGATCGAGCTGGCCACCCTTGAGCCCTTCC
GGCCCGCTGCCATCGAGATATTCATGGTGATGGAGCACGATCCCCTTAGCTTCCGACGGGAAATCCTTGG
CCAGATTGAGCAGATCCAGCCCGTAGCGCGGGTGCTGGGCCATCAGGTTCTGCTCCGGCTTGGTCAGCGG
CTCGGTCTTGCGCAGGATCTGGCTCGGGATCTTCAGCTTGCCAATATCGTGGAACAGCGCCCCCATGCCC
ACCTGACGGATCTCGTCGGCCGTCATGCAGCACTCCTTGGCCAGCAGCATGCTGAGCACGCTGACGTTGA
GGGAGTGGAAGTAGAGGTTGTTGCCATCCTCGCTGTCGGAGACCAGATGGAGCACCATCTCGTCCACCGA
GAGCAGTTGCTCGGTCATGGCGTTGACCAGCTCCTGCGCCTCGCCGATGGCATTGAGGGGGCGGGACTGG
ATTTTGCTCATCACGCTGCGCACCTGCGCCAGCGACTGCTGAAACTGTTTCTCGCACTGCTGCAGGTTGC
GGCGATAGAGTTGCAACTGTTCGATGCGGCGATCCTTCTCCTGCTGCATCTGGGCCTGACGCTGGGCGGC
GTCTGCGCTCTCTTGTTGCTGAGGCTGTACCTGATTGAGAGGCTTGGGCGGAACGTCGCTCTTGTCGGGA
ATAATGGTGACCAGTTTCAGGCCGAGACGGCGGATCAGCTCGATCTCTTCTTCACTTTTGATCTTGAAGC
TGGAAAACAGGAACGGGTGTTCGCGCCACCCCAGTGGCAGGGATACATAGTTGCCGACTTGCAGTCGATC
TACGGAAATCTTGATACCGGTCATTATCTTGGAAGCAGGAGACACACTGAGCCGTATGCTAGTCAAAGGC
GGGGGTGAATACAAAAGGTATCTGATGACAGGGCAGGTGCTTTATTATAGGCTTCTGGATGTCCAGACAT
ATTTACCGATGCAGTACAGGGAGTAAGCATGCCGATCAAGATCCCGGATCAGTTACCCGCCGCCGAAGTG
CTGGGGCAGGAGAACATCTTCGTGATGACGGAATCCCGGGCCGTCACCCAGAACATCCGCCCGCTGCGGG
TGCTCATCCTCAACCTGATGCCAAAGAAGATTGAGACCGAGATCCAGCTGATGCGGATGCTCTCCAACTC
GCCGCTGCAGGTGGATGTGGATCTGCTGCGCATCGATGACAGGGAGTCAAAGAACACGCCGCAGGCGCAT
CTGGAGAACTTCTATCACGACTTCGAGCAGGTGCGTGGCAACAACTACGACGGCATGATCATCACAGGGG
CGCCGCTCGGGCTGGTGGAGTTCGAGGAGGTGGTCTACTGGCCGCGCATCGTCGAGATCATCGAGTGGTC
CCATCAGCATGTCACCTCCACCCTGTTCCTCTGCTGGGCGGTGCAGGCGGCGCTGAAAGCCTTGTATGGT
ATGGAGAAGCAGACCCACGGCGAGAAGCTCTCCGGGGTTTACCGCCACCATCGCCTCGATGAGCACGAGC

CGTTGCTGCGCGGTTTCGATGACGAGTTTGTCGCGCCCCACTCCCGCTATGCGGCGTTTGATGGCGATCT
GATCCGTGCCCAGACCGACTTGCAGATTTTTGCCGAGTCAGAAGAGGCGGGGGTCTATCTGGCGGCGACC
AAGGATTGTCGTCAGGTGTTTGTCACCGGGCACCCGGAGTACGATGCCCTGACGCTGGACGGGGAGTATC
AGCGGGATCTGGCGGCGGGGCTCGACCCGGCCATTCCGGTCAACTACTACCCGAATAACGATCCGGGCAA
GACCCCGCGCGCCAGCTGGCGCAGCCACGGTCATCTGCTGTTTGCCAACTGGCTCAACTACTACGTTTAT
CAGCTCACCAGCTATCGGGTGGAAGATATCGGCAAGGTCTTTACCTACGACCAGGGAGGCAAGTAGTCAG
TCGCCCTGCGCCAGCCGCTCGGCCAGCTGGTCGAGCCATTGGCGGTGGCAGGCCAGGGTAAACGCCTTGG
GTCTGTGGGCCTTGATGGCGGCCATCGCCTCTTGCTGTGGCTGGCCGAGGGTCATCAGCAGCGCGGCGGC
GACCAGACCGGTACGACCGCTGCCCCCTTTGCAGTGGATGACCAGATGTTTGCCATCCCCCAGCAGCTCG
CGCAGCTGCGGCAGCGCCTGTTGCCAGGCCGCCTCGAAGTCGGTGTCGGGGGCCAGGTCATCCTCGATGG
GCAGATGGAACCAGCGCATCCCTTCGTGTTCGACCTGCTGGCCGAGCTGCGCCAGTTCGAGTCGGATTAA
CTCGGCGCTTGTCATCAGGGTCACCACTCCGTGGGCACCGGCCAACCGCAGTTGATCGAGCGCCAGGGTC
AGCGGCACCTGCTGGGTGCCGGGGCAGGGGGTCAGCAGCAGCTGACCGTCGACACGGGGAACCTCAAGGC
TCCAGAAGGGGTGATACGGGTGCATGGCGGATCCTCGCGTGGTGGCCGCAGGGCTGCGACCGGTGAATGG
CGCCAAGGATAGGCGGGGCAGGGGGGATGTCAATGGCCGTTGTGTTTGCCCCGGTTTACGGGCTGAGTCT
AAAAAATTGAGCGGTTCAAGGAGTTAAGATGAGAAACCTGGATCAACTGGATTTGGATATCCTGGCCCAT
CTGTTTCGGGATGCGGGCATGACCAACAAGGATCTGGCCGCGCTGGTGGGCGTTGCCCCCTCCACCTGTC
TGGAGCGGGTGCGCAAGTTGCAGCAGGATGGCGTGCTCAAGGGGGCGCACTGCGAAGTGGATTATCAGGC
TCTCGGTGGCCACATTCAGGCCATGGTCTCCCTGCAGTTGGCCCGCCACAGCCGTCAGATCATCGATGCC
TTCCGCGATGCCATTCTGGCGCTGCCCGAAGTGATCAGTCTGTTCCACATGGGGGGCAAGGATGATTTTC
TGGTGCACCTCTGTGTTTCCGACACCGAGCACCTGCGCAACTTCGTCTTCGATCACTTCACCTCCCGCGA
GGAGGTGGTGCACCTTGAAACCTCGCTGGTGTTCGAGCACAAGTTCAGCCGCTCCCTGCCCTGCTTTCCC
CAGCCATAACGGCCAGTGGGCCGACGGTATGGATAGATGGAAAAAACCGCCAGCTGGCGGTTTTTTCCAT
TCTGCTATCGGCTGAATCAGCACAGTTGGCTCAATTGGCGGCGGTGCGTTCATCCTTGGCCGTCTGATAG
AGACTCTTCTGATAGGCCATGCTGCCCCACAGGGCCAGACCAAGCGCATCCTCCTTCAAGGCGTCCGGCT
GCGCTGCCGGGGTCAGCTTCTCGCTGGCACCATCGTAGACATAGCCTTCTGCCGCCCGCTCCGGTTGCAG
GATCACCACATCCTTGCCGCGCATCAGGGCGAAGTTCTTGTCGTACTGCATGATGGCGCGCCCCGACCAG
TCGGCGGGCATGCGCGTCAGATCCTTGCCGAGCATGGGGTAGTCGGCACTGATCCCCATCAGGGAGAGCA
GGGTCGGCGCCATGTCGATCTGGCTGACGATGCGCGGATCCTTTCTCGGCGCAATACCGTCGCCCACGAT
AACCCCCGGAATATGGAAGCGGGGAATGGGCACCAGACTGGCGCCACCGACCCGGCTATCGTGGTCGGCG
ATGACCAGAAACAGGGTATCCTTCCAGTATTCGGATTGTTTAGCCTTCTTGAAGAACTCGCCGATGGCGT
AATCCGCATACTTGGCCGCGTTGTTGCGGGTCTGCTTGGGCTGCTCGAAGAGTTCGATGCGGTTGTCCGG
GAATTCGAACGGATCGTGGTTGCTGGAGCTGAACACCAGACTGAAGAAGGGTTTACCCTCCTTGTGCAGC
GCTTTGAAGGTCTCGTCGGCCTTGGCCATCAGATCCTCGTCGGAAGCTCCCCAGGAGCCCTTGAACACCG
TATTGTCGAAATCCTTCTGCTCGACGATGGTGGTGAAGCCATTGCCGAGGAAGAAGCTGCGCATGTTGTC
GAAGTGACTCTCGCCGCCATAAATAAAGCTGGTGTCGTAACCCCGTTGTTTGAGCAGATCCGCGATGGTG
AAGAAGTTGGTCTGGCTCTTGCCAAGTTTGACCACCGACTGTGCCGGGGTTGGTGTGAAGCCGGTCAGTA
CCGCCTCGATACCGCGCACCGAGCGGGTGCCGGTGGCGTAGAGCTGCTCGAAGGCCCAACCCTGCTGCGA
CAGGGCATCGATGTTGGGGGTGAGCGGCAGGCCGCCGAGGGAGCCGACAAACTGGGCGCCCAGGCTCTCC
TGCAGCAGGATCACCAGATTCTTCGGTTTACCCTGGAAGCTCGCCTCGTTGAAGGTCTGGCTGGGCAGCA
GATCGGAGTTGAAATCACTGGCCGGACGGCCACTCTCCTTGCGCATGACCTCGAGCACCTTCTGCTCCGT
CATCTCGCCGTAAAACTGGCCGGCATCCTCCTCGGCGCCCATCTGTTTGATGGCAAAGAAGAGGGAGTAG
GCGGAGTTGACGGTGAGCGAGTTGACCAGCGGATCTTCGGCAAAGGCGACCATGGCCGGGTTCAGGGCGC
GGTGGCCGAGACTGGAGCGGGCACCGAGAAAACCTATGGCCAGAATGAGCAGGGCTAAGGCCGGACGCAG
ATACCAGCGGGGGAAGCTGAGACCACGTACCAGACGGCCGCTCAGCCACCAACCGCCGGTCAGCACGGCG
CCACTGAAGATTAACGCCAGCAGCAGCTCGGCTTTGCGCCCCGCCCACAGCATGGCGAACACCTCTTTCG
GATAGATGAGGTACTCCACATAGAGGCGGTTGGGGCGCACGCCATACTCGGTGACAAACGAGGGGGTGGA
GATCTCGAGGAACAGCATCAGCCAGAGTCCGATGGTTAGCCACACTCGCATCAGTTGCAGCCAGCCCCGG
CCGATGGCATGGGGGCCTGCCAGCAGCAGAGTGAGCATGGCGGGGACACCCCAGACCCAGCAGAGGGTCG
CAAAGTCGACCCGGATCCCTTGCAGCAGCATCTCCTGCCAGCCGGCCACGGCATCCACCCGTTCCAGTTG
CCAGAGACCGAGGGCGATGCGGCTGAAAGAGAGAGAGAAAAGCGCGAGCGCGCAGAAGATGGCGAAGGGC
CAAAACGGGCCGAGTCGCTGTTTTAGATCTGTCATTTTTGTTCCTGAATGAGACTGTAAGCGCCTGTCCA
CAGACGTTTTTTACTTGTTGTGAGGTTATGACAGATCGAACTGGTTAAGGTTGCATTCAATTTTGTTAAA
AGCCGGAATTAACACATTTGTCATCTAAAAATGTGATGTCGCGCGCTTAATGAGCACAGCATAACCTGCT
GGATAAAATGTGAGCCACAGCGCGTATGGATGGGGGATGGGTGTTTATAATTGCCGCGAATTATCTCAAT
CGGAGCCGAAGGGATGCCGGTCTTTATCGATGTCGAGTATGTGCGGGGATGGCGCCTGTTTCTGGTGTTA
TCGGATGGCACCACGGGTGTGCTGGATCTGACCCCCTGGCGAACCCAGCCGCTGTTTGCAAAGCTGGAGA
GCGAGCGTCACTGCGCCCGGGTCTTCATCAACCGTCACCACCAGCTGGAGTGGCCCGTCGGATGTACCTT
GTCAGCCCATGAGGTCTATCACCTCATCTCTCCCATTGATGAGCGGGAAGCCTTCAGCTAATCCCCATTT
TTTGTGAGCCAACGCCAGATTCCTGTTACTGGAATGTTTGCCCGTTTGCCTTTCTGAGCCAACTTGGGTA
AAACCTGCGCAACATTTTGCCAACTTGCCTTCGCTTCATCTGACAGAGGCTTGCCGCTTTCGCGCCGCAG

GCCTTTTTTGCGGCCAGATGGCAAACCTTTCCCTTACTGAAAGGTTTGGTTGGTTGAATGAAAATAACAA
AACAAACAAATACATAAGGAAAATGTATGGCTTGTCATATGCGCACACCCCTTGCCCTGCTGGTTGGTGG
GCTCGCCTGTCACGGCGCTGCCGGTCAGGAATCGGTTCAACAGCTGGATCCTATGGTGGTGGTGGCAACC
CGCCCCGCTAGCACACTTGAAGTGACACAGGATCCCAAGAAGCCCGGTTCCCCCATGCCTGCTGCCGACG
GCGCCGGTTATCTGAAGAATGTCACCGGCATGAGCATGGTGCGCAAAGGGGGGCTCGGTGGCGACCCTGT
ACTGCGGGGGATGGGGATGTCCCGCCTCAACGTGCAGATGGATGGCGGCATGCTGGCCGGTGGCTGTGGC
GGCCGGATGGACCCGCCCACGGCCTATGTGTTCCCCCAATCCTTTGACCGGATCCGGGTGCTGAAGGGGC
CGCAATCCCTCGAGCAGGGTGCCACCCTGGCGGGAACCGTGCTGTTCGAACGGGACAGACCCGAGTTCAC
CGCACCGGGCCTCAAGGCCGATGCCTCGGCCCTCTATGGCAGTTTTGGCCGCGATGACCAGATGGTGGAT
ATGACGGCGGGTTCGACCGATGGCTATCTGCGCGGTCAGTTCACCCACTCCGACAGCGACGATTACCGCG
ATGGTCACGGTAACAAGGTGCGCTCTTTCTATCGCCGCGAGAACGGCACCGCCCAGCTGGGCTGGACGCC
GGGGGAGCACACCTGGCTGGAGCTCACCGCCGAGCGCAGCAATGCCCGTGCCGCCTATGCGGATCGCACC
ATGGATGGCCCCATGTTTGATCGCGAATCCTTCGGGCTGAAGCTCAAGCAGGAGCAGATCACCACTCACT
GGCAGCGCAGCGAGCTGACCCTGTGGGACAACTACATCGACCACATCATGGACAACTTCAGCCTGCGGCA
AAACAGCGGCATGAAGATGCTCTCCAACCCGGATCGCCGCAACACCGGCGGTCGCTGGGCCAACGACATC
GCCTTGCCAGCAGACTGGCTGCTGACCGCCGGGCTCGACAGCAACCGGGATCAGCACCGCAGTCGGAGTG
GGGTCGACTATGCCACCAAGGATCGTCAGCGCACCCTGCGTTTCGAGCAGCAGGGCGGCTTCGCCGAACT
GGGTCGTCAGCAGGGGGCTCACAGCTACAAGGGCGGTTATCGTCAGGATAGGGTCGAGACCACCCGCTAT
GGCGCCGGTGAACAGACCCTCTACAGCGATACCCTGACCCTGAACAGCGGGTTTGGCCGCTACGAGTATC
AGTGGTCGCCACGCCGGTCCAGCTATCTGGCCTGGGGGCAGGCAGAGCGTGCGCCGGACTACTGGGAGCG
CAGCAAGGACAGCACCCCGTTCACCCTCAACCCGGAGCGCAGTCGCCAGTGGGATGCTGGGTTGGCCCTG
CGCAGTCGCGAGCTGGACCTGACCCTCTCCCTCTTTAGCGCCGATATCAAGGACTACCTGCTCTACAACA
GCAAGTCGAACAAGGTGCGCAATATCGATGCCCAGACCCGGGGTGGCGAGCTGGAGGGGCGCTGGCAGTT
TGCCGAGCAGTGGCGTCTCGATGGTGGTTTGGCGGTGGTCTATGGCGAAAACCAGAGCGACAACAAACCG
CTGGCCCAGATGCCGCCGCTTGATGGCAAGCTGGCGCTGGGCTGGCAGCCAAATGAGGCGCTGAACTTCA
CTCTGCTGGGGCGCGCCGTGGCGGCGCAGGATCGGGTGGATGTGGGCTCCGGCACGGTCGCCGGTCAGGA
TCAGGGCGAGACCCCGGGCTTCTTCACCATGAACCTGAGCGGCGCCTGGCAGTTTGCCAAGGGGTGGCAA
CTCTCGGCCGGGGTAGACAACCTGTTCGACACCTTCTACTACGAGCACCTCTCCAAGTCGGTACACGCCA
GTCAGGCCGGGCTGGGCTACGAGCAGAGCGGCCGCATCCCCGAGCCGGGTCGTTCCTACTGGCTCTCGGT
CAACTACCGCTTTGCCTCGGCCGGTGTGCTCTAACCAGTTGAGTCAGATAACGTTTTCAGTCACAGACAC
CGCTGTGTGAGCATCGCTATCATGGTGAAAAACAGGCGTATCAGACGCCTGCTCTCACAGCGGGAACATA
ACAATGAAACAGGGAACGTTACGCTATGGCGGCCTGCTCGGGGCCGCCCTGTTACTTGCGGCTTGTGGCG
GCAGCGGTAGTTCAGGTGGGGAGAGCGCTCCCCCCGTCGTCAATCCGCCGGTCACCCACCCTCCTGTCGC
CACGGCCAGTCGTGCCTGCTATGGCGACGATCAACTCGCCTGCAACCTGCGCATCTATCAGGTAATGGTG
GAGTCGTTTGTCGATGGTGACGGCAGTGCCAACTACGGCGTCGGCTATGGCCCCTCCCAGCACAACGGCG
ATCTGCAGGGGATCATCGACAGTCTCGACCACATCAAGAGTCTCAACGTCAACGCCATCTGGCTCACCCC
GGTGTTCGACTCCTGCGCGGGGCAAGGCGGTGATAACAAGCTCGATGCCACCGGCTACTTCGCCTGCGAC
TTTTTTACCGTGGATCCCAACTTTGGCAGCAATGCCAAGCTCAAACAGCTGGTGGATGCAGCGCATCAGC
GCGGCCTCTACGTCTTCCTCGACGGGGTGTTTGGCCACGTCAACAAGGTGGGGGTGAGCAAGCCCTCGCC
AGAGGGGCGTCTGCCAGCCCTCACCAGCGGCGGTGCGGGTTATCCCGGCCAGCTGGTGGATTACAGCAAG
CCGGAGAGCCTCGCCTACTTCAAGGAGGTGGCCCGCTATTGGGTCGAGCAGTACGGCATCGACGGCTGGC
GCCTCGATCAGGCCTATCAGCTTGGGCTGGATGATTGGCGCGCCATTCGCAGCGAGGTGGAGCAGGCCAG
CGCAGCGCGCAAGGCCGCCGGTCAGCAGTGGGGCACCCTCGGCTACATGGTGGGCGAGGTGTGGAAGGGG
GCTGACGATATTCGCGCTCAGGCTTATGGCAGCAGCGACAATCCGGCGCTCTCCTCGGCGTTCGACTTCC
CGTTGCGCTACGGCATCGTGCAGGCGCTGGCGGTGGAGGAGTCCGGCAAGGGCGGCCAGGGGGCCAGCGT
GCTTGACGCCAGCTGGAACAAGGTGGAGAACTACCCGAATCATGCCATGCCGAACCTGATGCTGGGCAAC
CACGATCTGGTGCGTTTCGGTGATCTGCTGGAGCGTGGCAACTTCAAGTCGACCGAGTACTGGCAGCGCC
ACAAGGCAGCCTTCAGCTTTATGGCGGCCCGCTCCGGCCCCATCACCTTCTACTATGGCGAGGAGTTTGG
CGACGAGGTGCCGGGCTTTGCCGCCAAAGTCGGCGGTGACTGTGCGGCGCAGGGCTTGTGCGACGATCAT
GTGGCCCGCAGCGATGGCAAGGTGCCCGCTGTCACCGGTTTTGTGCCAAGTAGCGAACAGGCCGAGCTGA
AAGAGTGGCTGGCCCAGCTACTGGCGATGCGTGCCGCGCACCCCGCTCTCTATCAGGGGGAGCGAGTCAA
ACTGGTGGCCGAGGGCAGTCTCTATGGTGACATCAAGCAGACGGCGGGGGAGCAGATAGTCTACCTGCTC
AACCTCTCCACCACGGCGCTCAGCTACAACCTGCCGACGGGCAAGTTGCGCAGCGGCAGTGCGCTGGTAG
ACCTGCAAAGCGGCGAGAGTCTGGCCATTGGCGGCAGCAGCGTAACGGTGGAGATACCGCCCTTGAGCGG
GCGTTTCCTGCGTCTGCAATAAGACAGCCGCAGCACGCTTTGATATGAGAGAGGGCCCCTTCGCGGGGCC
CTCTCTGTCATTGTGGGCGACAAAGTCATGGCGGGTTATGCGGTGGTCTGGCCCCGGTTTTCCAGCCGCT
GGCGGAAGGCGCGGGCCAGTCGCTCGTAGAGATCCTGCGCCTCCTTGTCTTCGATATCGCCGCGGATCCA
GGGGTTGTCGACTATCTCCAGCAGCACGCAGTCACGCCCTTGCTGGCGCAAATCGAGCCCGTAGAGGCCA
TTGCCGAGCTGATGCACGGCACGGCGAGCGGTCTGCAGTACCCGCTCCGGCACCTCGTTAAGCGGCAGCG
CCTCGATGCGGCTCATGTCGAGGGGGTGCTTGCGGCGGCGAATGCGGTTGCCTGGTTCGGGCCGGTAGCG
GCGACAGGCGAACAGCGGCGAGCCGTCGAGAAAGCCGATGCGCCAGTCAAACTCGGCGGGGATCCGGCTC

TGCACCAGCAGCAGGGCGGAGCGGCCCAACCCCTCGTCCAGCAGCCGTGCCAGCTGCTGCTCGTCGCTGG
CCATGGCGAGATCGCGCCCCTGCGCCCCGTCGGGCACCTTCACCATCAGCGGCAGGCCGAGCCGCTGTAC
CAGCTCGTCCACATGGTGGCGGCCGCGGCGGGTCACCACCATGGTGGGCGGCATGGGCACGCCGCTGCGC
ACCATCAGCTCGTAGAGATAGAGTTTGTTGCTGCAGGCGAGGATGGCGCGGGAGCTGTCGATCACCGGCA
TCTTCAGCTGTTCGGCACGGTGGGCGAATTCGAAGGTGTAGTGGCCGACCGCGGTTTCGGCACGCAGCCA
GAGGGTGTCGAACTCCGGCAGCCGCTCGATGGCGGACGGTGGCAGGATCTCGGCCCGGATCCCCTGTGCG
GCGGCAGCCCGGATAAAGCGCTCCAGCGCCAACCCGTCACTGCTGGCACGGCCGTCATCTGGGTCGACCA
GAATGGCGAGCTGGTAGAGCGGCGCCGCCTGCTCCTCGTCACCGGTCCCCACCAGTGCCTGAGCGTGGTG
CAGCATCAACTCTTTCTCGCTTGGGCTCAGCTCCAGCGGCGACAGGCTCTGCTGCTGGCGCACCGACCAG
CCGTGGCGGCCGCGCTTGAGGGTCAGCTCCTGCAGCGGCAACTGGCTCAGGCCATAGTAGTAGCGCGCCA
GCCCCGCCAGCTCGCACTGTTCACACTGGCCGAAGATGGCACGTACCCTGATCCGATCCTCGCTCTGCTG
GGCCAGCCACCCCTGTGCCTTGCGCCACAGCTTGGCCGGTGGCTGCTGGGGTTGCAGTCGGCTGATGGCT
TGCAGACCCGGCAGACAGTGGTGGCCGCGAGCCTGGGCCAGCAGCGAGCAGTAGTAGCCTGCGCTAAGGT
GTTCCAGATTGCGGCAGAGGTTGATGATGCGGGGTCGGCCGCCGGTGGTCGGCACGCCATGTTGCAGATA
GGTGCGGGCGGTCAGCAGATAATCTGCATGGCAAGGCCAGTCGCCCAGCTCTTCGACGATGACATAGGTA
CTCATATTTTCGGCTCCACAGATCAGACTGCCTGAACAGGCAGGATCCCCAGTGGGCTTCGATGCACCGA
CAAGCACGACAATGGCGCGAGGGGAAACAAGAGGTTACAGTGCCTAATATGACGCCTTGGTAACTTGTGA
TACAAGTCGCATTTTTGGTGCTGAGGATAAATAACCGAACAAGGACTCCCCGTGTTGACCGTACGTGCGG
CCCGAACCGATGATCTGGGCGCCATCGTTAATCTGGAACGCTACTGTTTTCCCCCTGAAGTCGCCTTTGG
CCGCAGCCGCTGGCACTATCTGCTGGCCCACGCCAAGGGGCGCACCCTGTTGCTGCTGGACGAGAAACAT
CAGCTGATGGGCTATCTTTGCCTGCTGGAGCACAGAGGCTGGGATCGGCTGGTCATTCAGGCGCTTGCCA
TCCGCTGGACGGTGCGTCGTCTGGGTTGGGCCCGCCGCCTGCTGGAGCAGGTGATCCGCGAGGGCAAGGC
CGCTGGCTGGGGGGCGATTCGCCTCGAAGTGGCGGACGCCAACGAAGAGGCGCTGGCGCTCTATCAGGGA
TTGGGATTTTGTCCCCGGCAGCGGCTGGCGGACTACTATGGTCGCGACCAGCACGCCCACCGGCTGGTGC
TGCCGCTGGTGAGCGACGAGGAGCGTGAGCGGCGCAGAGTGGCTCGCACTCAGGCGCGCAAGCCCTCTGC
ACAGGCCAACTGATCATGTTGAACGAGCAGAATAGAGGAGCAGGATGAGCTGCGACAACTGTCTTGCCAC
CGCCATCAGGGCCAAGCTGGGGCGCTGTCCGGTCTGCGCCGGCCAGACCTTTGCCATCGGCATGGTCGGT
TGGCTGGTCTGGTGGTGGTGTGGCGCCGACACCTCGGTCAACGCCCTCACCGCCCTGCTGTTTGCGCTGG
GTGGCTCGGCCCTCTTGCTGCTCCATCTGGCGGTATTTGTCTGGCGCAAGGCGACCGGACGTGAATTGAG
CGATGAATAAATCAGATTGGTTTCAACAACATAAATAACAATATCGGTTGAGATAATGGAGGTTCAGATG
ATAGCAATGGTAACGGGCGCCTCGTCCGGTTTTGGTGAAGCCATCAGCCGCCAGCTGGTGCAGGCGGGCT
ACAAGGTGATCGGTACCGGTCGCCGCGCCGAGCGGCTGGCTGCGCTGGCTGACGAGCTGGGGGAGCAGTT
CCTGCCGCTGGCGTTCGACGTGCAGGACAAGGCGGCCACCCGCGCCGCCATCGCCGGCCTGCCAGCCGCC
TGGAGCGAGGTGGATCTGCTGGTCAACAACGCCGGTCTGGCGCTGGGGCTGGAGCCGGCCCACAAGGCGG
ATCTGGAAGATTGGGAGCAGATGATCGCCACCAACATCAGCGGCCTGACCCTGCTCACCCGTCTGCTGCT
GCCGGGCATGGTGGCGCGCAATCGCGGCCACGTCATCAACATCGGCTCCATCGCCGGTACCTATCCCTAT
CCGGGCGGCAACGTCTACGGCGCCACCAAGGCGTTCGTGAAGCAGTTCAGCCTCAACCTGCGTGCCGATC
TGGCGGGCACCGCCATCCGTGTCACCAACGTGGAGCCGGGCCTGTGTGGCGGCACCGAGTTCTCCAACGT
GCGCTTCCACGGTGACGATGCCAAGGCGGCCTCCGTCTATCAGGGGGTGGAGGCGATCCAGCCGGAAGAT
ATCGCCAATACCGTGCTCTGGGTGAGCCAGCAACCGGCCCACGTCAACATCAATAGCATCGAAATCATGC
CGGTGGCGCAGACCTTCGGCCCGCTCAATATCAGCCGTCGCTGATCGCTGCATTGGTTGAAACAAAGAGG
CTCCCGTGGGAGCCTCTTTGCATTGGCGAGCCGATTACAGGCTCATGATCTTCTTGAACTCCTCCGGCGA
ATACTTCTTCTCGCTGCGCTTGGTCAGGTCCGTCATCAGCTTGTAGCGCAGCGTTTTGGCCATCTGATAG
GTCTGCTCCAACTGGCCATAGGAGTAGTCGGCAGCCAGCCCGATGGCGGTAGCCTTGTCGCTGGCATAGG
CCTGCTTCAGGCTCTGGTCGAGCACGGCGACCTCCTTGTAGAGCTTGTTGCTCTCCTTGCTCCAGACGCC
GCGCAGCAGCGGCAGGTACTTGTCCGGATCGGCGCTGGCCAGCGCAGTCAGGCTGCGGAACTGCCAGTAG
GCGGAATCATCGGAGTAGCTGTCGCTGCCGGTGCGATAGGGCAGGGGGTAGGATTCCAGCGCGTTGTAGA
TCGGCACCATCACAGATTCGGCCAGCACGCCGTAACTCTGCCAGATCAATCCCTGCAGCTCTTTGGGCAT
CTCCTTGCGCAGCTGGATGACGTGGGATTCCAGCTGGCGATCGACGCGGATCGGCCGCTCCGCCTTGCCT
GCCAGCAACGTGCCGTCATAGGTGGCGCTCAGCACCCTGGCCACGTCCGGCACGGCGATGGGGGAATCCG
GCTTCATAAAGAGCGGATATTGCGCCTGCTGGGTCGCTTGTTTGTGGGAGGCCGTCAGCATGTTCTGGCC
GAGCCACTCCCGATCCACGTTGTAGGGGTCGGCAATCACCCCGAACGCCTTGGCGAAGTTGAAATCTTTC
GGGTCGGCGTTATCCAGCAGTTTGTGCTTGCGCACGAACTCCAGCAGCTTGGGCGAGTGCAGCACATCGG
TGCTGTCGAGATTGACGCCGTGGATGCGCAGGCCGTTGGCCACCATGGCGTAGCTGTCGTCCGGCACCCG
CACCGCGATCCAGTGGTGGCTGGAGCCGATCTCCATCAGCCAGGCTTCGTTCACATCTGCCAGATAGAGG
CTGTTGCCCTCACCGGCGCCATATTGCTCCACATAGCGGCCGAGCAGCTCGACCCCCTCTTTGGCCGTCT
TGGCCTGCGGCAGGATCAGGGTCGGGATGATGGCTTCAATCACTCCCTTCTCGATCAGCGGATCGGCTTT
TTGGGCCTTGTCGTTGACCTCGGCACTGGTGGTGGCCGAGATGGCGACGTTGAACTCGTTGATGCCGCGC
TCTTCATAATATTTGCCTGCCGGGTCGCTGGTGGTGGCATCCCAGTCGTGGATGGCGGAGTAGGCGAAAA
ATGCCTTGGGCATCGGCACTTCCAGTCCGTTACCCAGTTTCCAGACTTTGCTCTCGTTCTGCTGCGCGGG
CCGGAAGGCGAGGTACTTGTTCCAGTTGTTGACGCCAAAATCCTCGTTTCTGGCGATCATCACGCTGCCG

TCGGCGCTGGCCCCCTTGCCGACGATGAGGCCTGTACAGGCGTCGGCACTGGTGCCAAGCAGGGTGAGCA
GGGTGGCGGAGAGAAGACTCAAGGTGTGTTTTTTCATAGGTGTACCCGAAGGGTTGCGGTGAGTGAGAGT
CAGACGAGGGGATGAGGTGTGGGGAGGCTGCAACTCCGAACTGGCAGCGGAGTGAGCACGACAGATTGGT
CTGCCTGTTATTGGTTTTTCGAACAGATCATCCATGACACGGCTGGCTGGTTGTGATGTGCACCAAAATT
GTATTTCCGAGTGGTTGTTTGTTTTGTGATCGAACTGCGGTTCCTGACTTTTCTCTGGCGTCGTGGTGTG
CGGACGGCAAAATGTGCCAAAACAGCGTGAACCGGCAGCAGATGAGTCATGATTCTGACGCCAGCCTTAC
GTGGGGGGCGTGGTGAGATCCGCCATATCTTTGGATCTCGCACTATCTGACAAGTTGGCCTGTAACTTGC
TGGTTTAATTGATAATGATGGGGCCTGACGCCCCATGTGACCGGATCAGCCTGTGCCCATTGCCGCAGAT
GCGCACTGAATTGGAGCCCCCATGGATATCAAACCTTACAGCCAGCAGCTTGCCCAGAGCGCATCCAACA
ACCGCGCCCTCGCTACCCTCAACAGTGCCAGTAGCGGCGGTCAGGGCGGTGGCTGGGAGGAGATGGTCAG
CCTCTCCACCCAGCGCATGACCGGTCAGGCCCAGCAGGTGCTGACCTACGAACATCTGGCCAAGAACAAC
AAGCTGCCGCCGGCGCTGGATATGAGCTCCGACAAGGGGGGCGTGGTACAGCAGCTGGTGGACAAGGCGC
TTGGTTTCGATCGCAAGAAGTTCAACGAGATCGAAGAGAAGCTCAAGACCATCGCCGATAACAAGAAGCT
GACGCCGGAAGAGCGGGCGGAGCAGAGCAAGCCGCTCAACGAACAGCGAGAGGCGATGCTCAAGGAGGCG
ATGGATCGCCTGACCGGCAAGAAGAGCTCATCCAAAGATGAGGCCAAAACTGCCTGATCCTGCCGCTGTT
CTGAGATCTACCCGGTCGGCACCGATGGGCCAGTATCGACCGCCGATGGGCAAGCTTCTGCCACCTCTTT
TGACCTCACCTCCCCACAGGCGCACAATGTCCGCGCATTGAGCCAGTCGAGGAGTCCGCCGTGTACAACT
TCCAATATGCCAATCCGACCCGCATCTGTTTCGGCGAGGGGCAGATCGCCAGCCTGCCCGAACTGATCCC
GCCCGGCAGCCGGTTGCTGGTGCTCTACGGCGGTGGCAGCATCAAGCAGAACGGGGTGTATGAGCAGTTG
ATCCGGGCGCTGGAAGGGGTGACGTGGCAGGAGTTTTCCGGCATCGGCGCCAATCCCAGATACGAGCTGT
TGATGGAGGCGGTGGCGCAGGTGAAGCGCGAGCGCATCGACTTCCTGCTGGCGGTTGGTGGCGGCTCCGT
GGTGGATGGCACCAAGTTTGTTGCGGCGGCCGCCTGCTACGAGGGGGATGATCCCTGGGATATCTGCGGC
AAGCAGGCGAATGTGCACAAGGCGCTGCCGATGGGCTGTGTGCTGACCCTGCCAGCCACCGGCTCCGAGA
GCAATCCCACCTCGGTGGTGAGCCGGGGGGATGTGAAGATCTCCTTCAGAAGCCCGCTGGTGCAGCCGCA
ATTTGCGGTGCTCGACCCGACCACCACCTACAGCCTGCCGCCGCGCCAGACCGGCAACGGGGTGGTGGAT
GCCTTCGTCCATATCGCCGAGCAGTACCTCACCTTCCCGGTGGGAGGCGAGGTACAGGATCGGCTGGCGG
AAGGTTTGCTGCAGGTGCTGGTGGAGAACGGCCCCAAGGCGCTGGCGGAGCCGACCAACTATCAGGTGCG
GGCCAATCTGATGTGGGCGGCCAGTCTGGCGCTCAACGGCCTTATCGGTTGCGGAGTGCCGCAGGATTGG
GCCTCTCACGCCATCGGCCATCAGCTGACGGCGCTCTACGGGCTGGATCATGGCCAGAGTCTGGCGGTGG
TGCTCCCCTCACTGCTGCGTGAGCGGGCGAGTCAGAAGCAGGACAAACTGGCCCAGTTTGCCGAGCTGGT
GTGGCATTCGACCCGTGAGGACAAGGCTCTGCGCATCGAAGAGGCGATCATCCGTACCGAGCAGTTCTTC
CAGAAGATGGGACTGGGCACCCGGCTGGCTGACTATGGCCTCAGCGAAAGCTGCATTCCGGCGGTCTGCT
CCAACCTCAAGCGGGTGGGACGCACCGCGCTCGGTGAACAGCAGGATATCGACCCGGATCGGGTGGCACG
CATTCTCGCCCGCGCGCTATAAGCCGGCGCCGGTTTGTTTCCCTGGCTGATTCATTTTCCCAATTAAAGA
GGGCCGATTGTGGCGGCCCTCTTGCATGATGGGTTAGCCGATCACTCGGTACGCGCCTTGAGATAACCGG
CGTAATCCGGGATCTGCACGCTGAACGCCTGCCGCATATGGGGGCTGTCCATGATGAAGTCGGCGGTCGC
CTCGTTGGTGGCAACCGGGATGTTCCACACCGCAGCCAGTCGCAGCAGCGCCTTCACATCCGGGTCGTGC
GGCACCGCATTGAGGGGATCCCAGAAGAAGATCAGTACGTCGATCTTCCCTTCGGCAATCAGGGCGCCGA
GCTGCTGGTCGCCCCCCATGGGGCCGGAGAAGAGGCAGGTGATGGGCAGTTGTGCCTGCTTGCCGAGCAT
GGTGCCGGTGGTGCCGGTTGCATAGAGGTGGTGGAGCTTGAGCTCCTCCTTGCGGCGCATCACCCAGTTC
AGCAGAGGGGTCTTCATGTTGTCGTGAGCCACCAGTGCAACCCGTTTGTGGGGCGCCATCTCGCGTGCAA
TCTGTTCCATGGAAATCGTCCGTAGCATTGTTATTGGAAGAGAGCCGTATCCTAATCGAAAGCGGCGCCC
GTTACCTTGCCGGGCGCCGTAACTGTGATCACTTCGACTGTTCAATCCACTGCTTGTTCTTCTGCTCCAG
CACCGAGATGGGCAGCATGCCGTATTCCAGCACATGGTGGTGGAATGCCTTGAGGTCAAAGCCTCCGCCC
AGTGCCTGCTCGGCCGACTCCCGCATCCGGATCAGCGCCAGCTGGCCCGCCTTGTAGGAGGTTGCCTGAC
CGGTGTAGGCCATGTAGCGCGGCACCTCGGAGCTGATGTCCCCCGCCCCCAGCGCCGAGTTCTCCTTCAT
GTAGTTGACAGCCTGATCGTGGCTCCAGCCATACCAGTGGATACCGGTATCCACTACCAGCCGCATCGCC
CTGAGCATCTCCTCGTTGAGGCGGCCAAAGTATTGCAACTGGTTGTTGAAGGCGGATGATCCCTCGACCT
TCACGTCTCCCACCGCCGGATCATAGAGTCCCCGTCCCTGATATAGCCCCATGTCGTGATCCGCCAGGTA
CTCGGTGTAGAGCGCCCAGCCCTCCGCATAGGAGGTGTAGGAGGCGTTAGCCACGTACTGCGGGAAGTCG
GGATCCTTGGCGGCCAGCTCCTGGGCGATGGTGATCTGGAAGTGGTGACCCGGCGCCGCCTCGTGGGCCA
GCAGGGTGCTGATGTTCCAGCTCTGCAGACCATAGGGGTAGTAGGTGTTGAGGAAGAACTGCCCCTTGCG
GCCGGTCTGCGGGTTGCCGTCCTGATACCAGGCCACTCCGCCGTATGGCGCATCTTCATAGGAGACGGGC
ACCACCTGGTAATCCAGCTTGGGAATGGTACGTTCGGCATCGAAGAAGTCCGGCAGCACCAGTGCTGCTT
TGCTCTTGAAGACGTTGTACTCCTGCAGTGCCCGCTCGTGTTCGGCCAGCGCCTGCTGCTGTGGCCCGCT
CAGGGTCGCCCCGGCTGCATTGTCCGGGTTGAGGCCGGCGGACCAGACCTCTAGCGGGTTCTGATAGAAG
AAGGCCGGCTCGTTCAGATAGCGGAAGAAGGCGTAGATCTGGCCGCTCTGCTTGCTCTGGCTCACGCCGT
CGCTGTCACACTTGCCGACGGAATGGCATACCTTGACCATGGCAGCGAAGATCCGCTCTACCTCGCTCAA
TCCCAGCTCATGTACCTGCTCGGGTGTCATATTGGTGGTGGTGTGCTTCTTCAGCAGCCAGCGATACCAG
TCGCGCCCGTTGGTCATGCCACAGAAACCGTCGTCACACTCGCCACTACCCAAGCGCACTGCATAGGCGC
CGCCATGCTGGTAATCCAGATAGTCGCGCAGCGCCAGATAGCGGGTGTTGATGCTGTCCAACACGGCTTT

GTAGTTTGCTCGCAGTGTGGCATCGGCATCCCCCGGCAACTGCTGCCAGCCGAGGTAGGAGCCGGCGATC
GCCTCCCCATCCAGCTTGCTGTTGAGCTGGGCCAGCAGCCGGTCTACCAGCTCATCAGGCAAGGTGTTGC
CCTGAACACTGGCCAGCTCGAACTGGTGTTTACCTCGGCAAGATAGACATCATAGGCCTTGAGCCAGGT
GAGGTGTTCCTGATACCAGGCCTGCGGGGTCATTGTTGTCTTTGCTCGAGTTGCCATTGCAAGTGCCGGA
GCTGTGTCTGTGCGGTCGAGATCCCGTCGCCATGTGCCATCACCCGGTTCCACCCGGTCGGCCGAGCTGT
GCTCGAAATAGTCGAAGTCACCGCTGACGGTACGAGCTGCTTTGCCGCTGGCAGGGGCTGCTCCGACGTC
ATCTGCCAGATCCACCACGCGGTTGCGGAAGTGATGGATCGGGATCTGGGATTGGGGTAAATCGTATCCT
TCAAGATTGATCTGCAAGTCCCAGCGGATCATGTCGTAGAACAGCCGATCCCGCTCACTTAGCACCGTGG
GATCCAGAGCATTCAGTCGTTGGAGCAGTGAACTGTCGAGTGCTTTGCGCTTGGCGATGCTGGTGGCATC
GATGGCAAAACCGTAGGGGTCGAGCGACTGGCTCTCGCGCAGGGCCTCATCCATGGTTTGCATCAGGGAT
GTTTTGGTGGCTGCGGGGTCTGCAGGTGCGTTCGAGCTGTTGTCATTGCAGGCGGCAAGAGACAGGGCAA
TGGCGCAGGCTAGCGCGAGCTTGTTGGGTAGGAAGTGCATGATGTAGTCCTTTCACATGTTGTTATTGTA
TGTCCCTGTGAGGGGCCCCAATAGTGCAACTCCCAAGCGTCTGTTTTTGCGATCTGAATAGCAAAAATAC
GCTTATTTTTCATGCTGATAAGTGGTGTGAAAAGCTATGCAGCCAGACATTAAAAAACCCGCCTGAGCGG
GTTTTTTAATCGTTTTTTCATGCCATAACTATTCAGGATGAACCGTGGTGCAGTTGCGACTGCTGGTAGT
TCTCCAGCCCGATGCGATCGATAAGGTCTAGCTGGGTCTCCAGCCAGTCCACGTGCTCCTCCTCATCCTC
CAGGATCTCCTGGAACAGGTCGCGGCTGATGTAGTCCTGCACCGACTCGGCGTAGGCGATGGCTACCTTG
AGGTCGGGGATGGCATCCATCTCCAGCGAGAGGTCGCAGCGCAACATCTCTTCGACGGTCTCACCGATAC
GCAGCTTGCCCAGATCCTGCAAATTGGGCAGCCCTTCGAGAAACAGCACGCGCTTGACCAGCTCGTCGGC
GTGTTTCATCTCATCGATAGACTCGTGATACTCCTTGTGCCCGAGCGCCTTGAGGCCCCAGTCATCGTAG
ATGCGGGCGTGCAGGAAGTATTGGTTGATGGCAATCAGCTCATTGGCCAGCACCTTGTTGAGGTGGGCAA
TGATCTTGGGATCTCCTTTCATCGACAGTGCTCCTTGGTTCGTGCCTGAGGCTTCTATCGTTAGAGCTTA
GTCCAGAAAAATGACAACAAGAGGTCTGGCGAGCCAGCTCGCCTGAGAAAAGGGGTGACCCTGTTGGCGG
GTCGCATCAGAAAAGAGGTGTCAGGATCAGGCCACTTCGTAGTAGAGCACGGGTTGCTCCTGCTCCATCT
TGTCGAGTTCGGCGGCGATAATCTCCATGGTCATGCGCACGCATTTGCCACATTGGGCGCCCACCTCGAG
CGACTGCTTGAGCTGGCGGAATTCGCTTTTACCTGACTGCACCGCTTTGCGGATCTGGGTATCTGTGATG
CCGCGGCACAAACAGACGTACATGATGGTCACCTCTTGTGGAATGAGATGAAATCTAAATGAGAATGGTT
CGTAGTGCAAATAACCATTGGTAATAAGATCCCCTGCTTGCCACTGTGCAACCCCTTGGGGACAATGCTG
GCAACCAGAGGAGATCACCATGCTCAAGAAACTGTTCAGCGCCCTGTTCGGGGGCGAGCCGACCAAAGCG
CCGGAAGTCACTCTCACCGAATATGCCGGCTATCTCATCTATCCGGAGCCCATGGCAGAAGGTGGCCAGT
ACCGACTGGCAGGGCGCATCACCAAAGAGCTGGATGGGGTGCTGCAGACTCACCGCTTTATCCGCTCCGA
TCTGTTTGCCAATCCGGCCGATGCCGAGCATTTCATGGTGCAGAAGGCCCACACCTTTATCGATCAGATG
GGGGAGCGCATGTTCGAGCCCCGCAAACCGGCTGACAGCCAGGCTTCATGACGCCGCCTGAGCTCAACAG
CCTGCGCAATGCGCTGGCCCGCGCCGGTGAGCGGCGCCTGATCTGGCTGGAGGGGGAGGAGGCCGAGTGC
ATTGTCCGCGCCCAATCCCTGCTCGGCGAGACCGTCTTCTGGCTGGGTGCCGGGCCCGAGTGTTATGCCG
CGCAACCCGCTGCCAAGGCGCTGCAGCGGCTCGGTCAGGAGTGCGACACGCTGGTGTTCAACGCCTTCAG
CGGCTTTCACCCCGACGCCTTCGGGGCGCTGGCGGGCACTCTATGCGCCGGTGGCTTGCTGTTGCTGCTG
ACGCCGCCCAGAGTAGAGTGGCCTTCATGGCCGGATCCTGACCGGATCCGGCTGGTGGCCCAGCCGGAGA
AGGCGGGCCGGGCCGGTCACGGCTTTATCGCCCGACTGGTGCGGTTGCTGGCGGGGGATCCTGCCCTCTC
GCTGGAGCCGCCGGTTTGCTCGCAGCCGTGGCAACCGCTTGGGCCCGAGCAGCCATTGAGCTGCGATCAG
GCGCGGGCACTGCCCGCCATCCACAAGGTGTTGCACGGTCATCGCCGCCGGCCGCTGGTGCTGAGTGCCG
ACCGCGGTCGTGGCAAGAGTGCGCTGCTGGGCCTGGCTGCCGCCGAGCTGCTGCGCCAGCAACCCCGGCT
GAACATAGTGGTGACGGCCCCCTCGCAGGCAACGGTAGCGACCCTGTTTGCCCACGCAGCTCGCGGGCTA
GATACCACGCCTGAACAGCTGACCGGACTGAGCTACTGCTCGCCGGATCGGCTGGTGCACGAGGCGATGA
ATCCCGATCTGCTGCTGGTGGATGAAGCGGCTGCCATTCCGACCCCGCTGCTTGAGGCTATGCTGGCCCG
CCACAGCCGCATCGTGTTTGCCACCACCGAGCACGGTTACGAGGGGACGGGTCGCGGTTTTCACCTGCGC
TTCAAGCGGGTGCTCGATAGCCAGACCCCGGAGTGGCAGGAGATCCACCTAGGTGAACCCATTCGCTGGG
GTGAGCGCGATCCGCTGGAGCCGCTGATTTTCCGCCTGCTCGGCCTCAATGCCGAAGTGGCAACTCCCTT
GCCGCCGACCAGCCCGAGCTGGCGTCTGGTCAGTCAGGAGGCGCTGGCGAGCGACGAGGAGCTGCTCAAC
CAGCTGTTCGGCCTGCTGGTGCTGGCTCACTACCAGACTACGCCGAGCGATCTCAGATCCCTGCTGGAGA
GCCCGGATCTCGACATCCATCTGCTGGAGCAGGATGAGAGCCTGCTGGGTGTGGCGCTGGTGGCGCGGGA
AGGGGAGATAGAGCCCGAACTGGCCGAGGAGATCTGGGCCGGTCGCCGCCGTCCGCGCGGCCATCTGCTG
CCCCAGTCGCTGCTGGCCCACGCTGGTTTTAAAACGGCGGGGGGGCGCAGCTACGCCCGGGTGATGCGTA
TCGCCATCCACCCCGAGCTGCACCGGCACGGACTGGGTTCGCTGCTGCTTGGCAAGCTTGAGGAGCACTA
TCGTCAGATTGGCATCGACTACCTCGGCTCGGCCTTTTCGGCCAGCGCCGACCTGCTCCCTTTCTGGCGC
AAGGCAGGGCTGGAGGTGCTGCGCATCGGCCTGCAACGGGATGCCGCCGGCGGATGCCACGCAGCCCTGC
TGCTCAAAGCGCTGAAACCCGAGCATGAGATCGAGTTGAATCAATGGCAGCAGCGTTTCATTCGCCAGCT
GCCGACCTTGCTGGCGGGTGAACTGAAAGGATTGGATCCCGAGTTGGTATGGCAATGTTTGCAAGGCAGC
ACCGTCACCGCGGCTGAGCTTGGCGAGTTCGAGCGTGATGAGGTTGAGTGCTTTGCCCTGCATCACAAGC
CGTTTGAACTGAGCCAGTCGAGCCTGCAGTTGTGGCTGGCGCGCAATCTGGATGTGCTGGCTTCGCTGAC
TCCTGACGAGCGTTATCTGCTGATCGCTACCATCTGGCAATATGCCGATTGGGGAATGCTGGCCGCGAGG

TTGCAACTGGCAGGCAAGCCCGCCGTGATCAAGAGGTTGCGGGCGCTGCTGGCGCGTCTCTTGAGTTCCT
CTACCACTCGATAGGTTTGCCCTGCCAGTCGAGAAAGCTCAGCTCGCCATCCGGTTGCAGCCGGTTCATC
AGGTTAATCAGATGGTCGGCTACCTGCTCGGGACTCTGCACATTTCCTGGCGGTACATTGGCGTGAAACG
GGCAAGAGAGTTGGGTATCCACTGTGCCGGGATGGAAGGCTAGCAATTTGACGCCTGGGGCCCGCCGGGC
CAGCTCGATGGCGGCACACTTGAGCAGCATGTTGAGGGCGACCTTGGATGCGCGATAGCCATACCAGCCG
CCCGCTCGGTTGTCACCAATGCTGCCGACCCGGGCGCTGAGAACGGCCAGAGTGCAAGGCTCGCGGCCAA
ACAGCGGTAACAACTGGCTGATCCAGCGCAGCGGCAGCAAGGCATTGGTCTGGTAGACGCGGGTCATGGC
ATCCAGATTGATTGCTTCGAGTCGCTTCTCTGGCTGGATTTCCCCCTGATGCAGTATGCCATTGCAGATC
ACCACCCTGTGCGGTCTCGGTGCCAACTCCGCGATGCGGGCCACCACTTCTGTCATTTGCTCATCGCTGT
AATCGCAACATAGCCAGTGCAGACCCGGCGATGAGAGGCCAGCAGGTGCGGGCTGGCGGCTGATGGCAAT
CACTGGCCCGACACCCGCCGCCAACCAGTGTGTCACCAGAGCCGCCCCGATACCGCCAGAAGCGCCAATC
ACGACTATGCCTTCACTCATCTCAGGCTCCTTGTGAGTACATGATGGATTTCACCATCCGCCAATGTTGT
CCTGATTGGCAGATACGATCCCGCCAGCCTACGGGATCAAGCGGTTAACGCTGCAATCTTGCCGAGATCC
GAGCCGTTGGGTGAATTCCATTCCTATACTGAGGGAAAACCGCAGCGGTCTGCTGCCCGCGCAACCAGAA
TGAGAGTCACCATGATGTCCAAAGATGGGGAAGAGCGTAAATCTATCCTGACCGAAAGTGGCACCAACGA
GCTTGAGATCATCGAGTTTCACCTCAAGAAGCAGCTACCGGACGGCCGTGAAAAAACCGCTTATTACGGG
ATCAACGTCGCCAAGGTGCGCGAGGTGATCCGGGTGCCGGAGACCACAGATTACCCCAATGCCCAGCGTC
ATATGGTGGGGGTATTCTCCCTGCGGGATCGCCTCATCCCGCTGGTGGATCTGGCGGGTTGGCTCGGCAT
CAAGACCAACCCTGACCCCAGCCACAAGGTGGTGATCGTCACCGACTTCAACAAGATGGCCAACGGCTTC
CTGATCGATAGCGTCAGCAGGATCCATCGGGTCTCCTGGGAGGATGTGGAGTCCCCCAGCCAGTTTCTGG
AAGCGGGGGAGAACGACTGCGTGGTGGCCGTGGTCAGGCGCGATGGCAACCTCATCATGATCCTCGATTT
CGAGAAGATCATCGCCGATATCAACCCCGAGCTCAGCATGGAGAAGTACGACGTCACCCAGGATCGCAAA
GTGCTTATCGACGATCGCATGATGGCGCTGCGACAGGGCAAAACAATTCTGGAGGTGGACGACTCGGCCT
TTATCCGCAAGATGATCGAGACCACCCTGCGCACCGCTGGCTACAACGTGATTACGGCCAAGGACGGGGG
AGAGGGGCTGGAGATGATGACGGATTTCGATCGTCTGGCCCAGGAGGAGAGAATGCCGGTGACCGGCTTC
GTCAATGCGGTGATCACCGATGTGGAGATGCCGCGCATGGATGGCATGCACCTGCTCAAGCGGCTGCGGG
ACACCCCCTCCTACAAGGAGATGCCCATCGTGATGTTCTCCTCCCTGATGAGCGATGACAACCGCCAGAA
AGCGCTGCAACTGGGGGCCAACGATACCATCACCAAGCCCGAGATAGGGCGCATGGTGGGGATGATGGAT
CACATGCTGCTGGGAATTGCCCCGCCTTCGCCCGCCAACAACATCTGACCTGCCATTAACTTGCCATTGG
CCGGTCGAATTCACTGACCGGTTGCCTCAAGCGGCCACTCCTTGCCAGGGTGTGGCCGCGCTGTTTTAGC
TGTTTGCTGCGATTCCCGACGCCCCTTGTCTCCCGGATATGATTCATGTAATGATAATTATTATCATTTG
TTGGTGTGAAGCATATGACAGCAACCTGGGTGAAGGGATGGGTCGAGTCGACCGAAGGCGGCAGCAGCAT
GAGTCACTGGAAACAGCAGACCAGAGAGGGCAACCGGCTGTTTGACTGCGGCGAGTATCATCAGGCGCTG
GCTCATTATCAGCAGGCGCTCTGGCTGGCTCGTCAGCAATTGGTGCAGTGGCATGACGCCGATGAGGCGG
TGGCTGCGCTGGTTGTGGCTCACCACAATCTGGCGGATCTGCTGGTGGCCATGACTCTGCCCGATCGCGC
CGCCGACCTGCTCTGCTATGTGCACGACTGCCTGCTGGCGACCTGTCACTCCCCCGCTTGCTCCTCTCGC
CTGCGAGAGGCGGCTTTGCGCCATACCCGCACAACCCGTGCCGAGTTGCTGACGTTCGTAAAGCGCCACG
GCAGCCACGCCGGGATCATGCGTTCGCTCGGTCACTGCCTGTGTCAGAGCGAGCCCTCATCCCGGCTCCA
TTAATCCGAACAGAGGTCATCATGAGACATACCCTGCCAGCCCTGAACTATGCCTACGACGCACTGGAAC
CCCATATCGATGCTCTGACCATGGAGATCCACCATAGCCGTCATCACCAGACCTATATCAACAACCTCAA
CGCCGCACTGGAAGGGGCGCCAGAGCTGGCTGCACTGCCGGTTGAGGATCTGCTGCGCCGCTTCGATACT
CTGCCCGCCACCTTGCAAGGGGCGGTACGCAACCATGGCGGCGGCCATGCCAACCACTCCCTGTTCTGGC
TGGTGATGAGCCCGCAGGGGGGCGGTGAGCCGGGCGGTGAGCTGGCTCTTGCCATCGAGCGGGAATTGGG
GGGATTCGATGTCTTCAAGCAGGCCTTCACTCAGGCGGCCCTGAGCCGTTTTGGCAGCGGCTGGGCCTGG
CTGGTGGTGGACAAGGCGGGCAGATTGCAGGTGGAGAGCAGTGCCAATCAGGATAGTCCCCTGATGCATG
GCCATACCCCGATCCTGGGGCTGGATGTGTGGGAGCACGCCTACTATCTCAAGTACCAGAACAAGCGTCC
TGACTATATCTCTGCCTTCTATCAGGTGGTGGATTGGGCCGAAGTGGCCCGCCGTTATCAAGCGGCGCGA
GGCTGACTTGGTCGCTGGGAATGGGTGTACAGGCTCACCGCCACAACCCGCGCTGCAATAACAAGACCCG
CCAGTTGGCGGGTCGTCTGTTTATGGCGTGGCAGTGCTGACGCTGGCGGCTGAAGGGGTCATCGGCAATG
TGGGCAAGGCCGTGCTGGGCGTCTCGGCGGTGGTTTCTGCCGAGGGTGTGACGGGAGTGACCGGCTCAGT
GGCTGCCGCTGCGGATAAAGCTGGAGTGACTTGTTCAGTCGTGGCCGAGGCAGACGAAGTTGCTGACTCG
GTGGCAACCGCGGCAGAGACTGCCGCTGGCTTGGCCTCATCCACCGCCGGTTTGGGCTGTTTGAGGCAGG
GTTGCAGACTGCCGATGGTGCTCAGTGACTGGCAAGGCACAGGCAGGCCGATGGGGACCGGTGCCCCGAG
GAACTTGGGCCCCTTGTCCAGTACATATAGGTCGAGCAGCCCCATCAGGCGGGCAAAGACTTCCCGCACC
CGGGTGCGGATCCCCTGATAGGCGCTGGGATCCTCGGCATTAAAGCCGACCGCATCGATACCGAAATGGC
GGGCGATGAAGATGGCCCGCTCGTTATGAAAGCCCTGGGAGACCACGGTAAAGCGCTCCTGGCCGAACAC
CTCTTTGGCGCGAACCACTGAGTCGAGGGTGCGGAATCCGGCGAAATCCATATAGATGGCGGAGGCGGGG
ATACCCGCCTTGATCAGGGCGCGGCGCATCTGGCGCGGCTCGTTGTACTGCACGGTAGCGTTGTCACCGG
AGACGATGATGAAGTCCACCTTGGCATTGTTGTAGAGCTCGGCCGCCGCCTTGATGCGGTATTTGAAGTA
GTGGTTGGGGCCGCCACCGGAGAGATATTTGGAGGTACCCAGCACCACCGCAACCCGGTTGTAGGGCACC
TTGTCCACCCGATCGTAGGTGTAGTGACGGGCATCCGAGACGATCCACTCGCTGTAGCCCAGCAGCATGG

CAATCAGGGCCAGCAGCATAACGAGGCAGGCGAGGAGGATCTTGGCAAACTTCTTCATGCGCGGTGCGGT
CAGGGTCTGAAGTTGGGAGTGGCTATCGGCATTCTATTACAACTCGGCCGCTGGCGTCAGGCTTGATAGC
GGGCGGGCACTTCATTCGGGCAGAGGTTGTCGCAGCAGAGGCAGGGGGAGCGGGAGGATAAAGCGAGGGG
CTGCGGTGGCAGCCCCTCTGTTAGGTGAAGGTCTATCAGTGGGCGGAGTCATTGCCCTTGCAGGCGTCTG
ACTCCTTGTTGGCTTGCCACCATGGCAGTAGATCTCGGGCGAGCCACGCCTGATAGGCGTTGCTGTCGTT
GGCGATCAGCCGGTCCAGCTCCCCTTCGAAGTCGCTCATCGCCTGCTGCCGATCCGGATTGCGCAGCGCC
TGTGCCAGCGCCAGCCAGCTGGCGCCGCGACCGGTATCGTTAAGGAAGCGGTGCACGGTATAGGACCAGG
AGTAGACCATCTCGCCACCCTGGTCGTAGTCAAGGTGGAGGATAGCCGCCAGTGTCGGACGCTGGTTGGC
GGGGCGGTTTGCCACGTTATCCAGACCCCGGGCAAAGCACTGGCCGTGGGCGATGTACTCTGCAATCCCT
TCGGCCCACCAGGTGGTGCGGTTGAGTGGATAGTGGCCGAAGCTGCCGTACTGGTTGAAGCGGCCATCCA
GATAGTGAACGTACTCGTGGCGCAGGTTCCACACCTGGAAGGCAGGGCGTTTCCACTCCGCCTCATAGGC
GAAGAAGCGGGCCTGATTGCCGGGGCGAGCCGGATCCCCCTCGATATAGATGCCGCCATTGTCGGTGGAT
ACGCCAAACAGCGCGCTGCCGTAGCGGCCCCAGTCGGCGGAGGAGTTGAAGATCACCAGCTCCAGCGCCT
CGTTGTGATCATCGGCCACCGGTTGCCAGCCTGTCTCCATCTGCTGGTGGAAGCGCTGCTCTTCATCACC
CAGCTCGCGGCAGATCCCTTCGGCCTGAGCCTGGGTCAGATCCTGAGCGCGCAGACGCAGGGTTGGCGAG
CAGGTGTGCTCGAACGGCAGAATATCTTCCATGCGGATCGGGGTACAAAGCCCGGCGAACTCTCCACTGA
CACAGGCTTCGCTGGTGCGATCCGTGTAGGTGAGGTAGTAGCTGGAGAGGGTCAGAGAGACCTCTTCCAG
CATGGCAGCCGGATGTTGTCCACCCGGGATCAGCCCCTGCTGGCGCATGATGGCGATGACATCGCGATCC
AGCTGTTGGCGATAGGCAATCGCCTTATCCGGGCTGGTGTGATGGAGGTAGCGCATCATCTGGTAGAGGC
GGCTGTGGGCATCGAGGATGTTGTTGAAGATAAACAGATCCCGTGAGCCATCGCGCTGTTTCCAGAGGGC
CAGCTCGCTCAGGCCCAGTTGCAGCAGGCTCTGGCGCAATGCAGCCTCCTGCTGCAGCACAGCTGCGCTG
AAGTCAGCCTGCTGACCGTAAGAGGCGCTCTGATCGACCAGATTGAGCAGCTCGAACAGGCCATCGGCCC
AGGGTTTGTGGCCATTGATCTGCGCCGCATGTTGCACCGCATAGCCGAGCGCCTGGTTGAGCTGGGCCAG
CATGGGGGCGGCACTCTGGCGGAAAGCGGCTTGGCCAAGCTGTCCCTGCGCCGCTACGCTCCAGGCTTCC
AGCACTGTGCCAGCCTGATCTTGCGGTTCCGCGCCCAGCAGCAGGGGGTGGTTGAGCAGATCAATCATTA
CCCCGGCCAGTGCCCGGGCGGCCTGATCACTCTCTTTGGCTACGCCGCCATGGTAGTTGTGGGCACGCAC
ATAGTAGAGCAGCTTGTCCATTGCCGGATGGGTCGGCTGGTTGGCCGCCAGCAGGGCACGCATGCCGCGC
ACCACCGCCATCAGCTTGGCGTCATCGTGGCCGTAATCTGCCTGCCAGGCAGGGCTGTTCTGGCCGCTCA
CGTCGGCCACCAGTTTGGCGGCATCGCTGTCGAGCAGATCCACATCGGGTTGCTCTGGCTCGGAGCAGAG
ACGACCGTTGGCGCGGCGGCACTCCAGCTGCTCCTCCTTGCTGGCCAGCACGATGGGGCGATTGAGCACC
CGACTCTCCTGCCACTCTTTGCTCTCTGGCAGGGGCTGCCACTCGCCGCCGGCGACCGGGTATTGGTAGC
CGCTATCTTGCAACGTTACCTGAACCTCGCTGCCAGCGGGGATCACCTGACGACAGATGCGATAGCGAAT
GGTGTTGCTCCCCTCCTTGCGCTCCTGCGGCTCATCACACTCCTGCTGAGGCGTGAGCTGATCCAGCTGC
CACTGATAGCGGCGCCAGACATGGATCTCGCTGACCTGAGCGGCAGCCTGACTGCCTGCCACCGGCTGCA
GGCGGAAGAAGATCTGCTCCAACCGGCCATCCCGCTCACTCGGGGTCTCTTCAAGTACCTGCAACTGCCG
CCAATCCTGCGGCTGATAGTGCCACTCCGACTCTGCGCAGCGGCCTTGTGCGTTGAGTTCGCAGCGATCC
AGCTCCCTTTCGCTCTGACCCAGGATGATGGCTTGTGGCAGTGGCAGACGATCCTGCCGCTGGGCGGTCG
GCGTGCCCGCCAGCTGCTCCCAGCGACCCTGCTTGAGGCCATAACCAAAGTCATAACTGCTGGCGGGAAT
GAGCTTGCCCGCCGGAATCCGATCTTCACAGCGCACGAAGCGGATCTGCCAGCCCGCATCTACCTTGAGC
TGCGGCTCGCCACACTGCTGTTCGGCCAGTGCGGCGAGATCGCTGCTCTGCCAGTCGAAGCGACGGTAGA
CCCTGAGCTGTTGGTAAGGAGCATTGCTGCTGCGCGCCTTGAGCTCGTAGGTGATCTCGGTGATGCGAGC
GGCCAGTTGCCGGCTCGATTCGCCAGTCAGCTGATACTGTCGCCAGTCATTGGAGGAGTAGTTGGCTTGC
GCCGAGGATGTCGCCAAAAGTAGGGGGCCTGCCAGCAGCAGCCACCCGGTACCTGAACGGTTCATTTTTA
TCCCTTTGTTCTTGTTTGTGTATTGTCTTGCGAGTAATCGCAAGGCAATTTAAACAGTTCCAAAGGGATA
TCAATTGCCAGTTAAAAATCAGATAAATCAATAAGATGGTGACTTTTTGAGTCTGATCGAATTATCGTGG
GTTGGCGCCGCAGTGAGGACTCACATGTTTCTTTTTTGTTTTCAAAGAAGATCGGTAAAATGTGAGTCCG
ATCTCATTCAGCTTGTCGTGCCCGAGTGGTCTGTTGTCGGGCAACCTTGCCCAGCTGTTGGCGGGAGGGT
TTGAGATTACCGCCAAGATCGGGCCAGGGGAGCCAGTGGTCGGGCACCATAAAGCCGGGCAGGGTGTTGC
GGATCCAGGCAGCCAGTTCGGAGTCGGGAACAGGTTCGCCGTGCCAGCCGATAAAGGCGGCGGGGCGTTG
CCCCCACTCGTCGCTCGGTACCGGCACCACCAGCGCCTGGGCGACAGCGGGATGATCTACCAGTCGCTGC
TCTATGGTTTCTGGCTGGATATTCTCCCCGCCCGAGATAAAGAGGTTATCGAGCCGTCCCTCTACCAGCA
GTTCCCCTGCCGTAGTGAACCTCCCCTTGTCGCGGGTATGAAACCAGCCCTCGTCATCGAGGGGCTGTTC
CAGTTCGCCATCTCGGTAGTAACCGGCAAACAGGGTGGCGCCGCGCACGCAGATCTCCTGCTCCCGGATG
CACACTTCCCGTCCCGGTAGCGGTTTGCCAACTACACCGGCATCGCCTGGGATAGCGGTGCAGACCTGGC
TCCCCATCTCGGAGAGACCATAACTCACCTTGGGTTCGAGCCCCATGGCGCAGAGGCGGCTCACCAGCGG
CTGCGGGATGGCGGCGCCACCGAGCAGCAGCTCGCGCAGGCGGGTACGGGTGGGATCAAAGCCCTCTGCC
AGCAGGCGCCAGAGTTGGGTAGGCACCAGAGACAAGTGAGTGATGGGTTGGCACTCCAGCCGCTCCTTGA
GGGGGCGTTCACGTTGATCCAGTACCAGAGTCGCACCCGCCAGAAAGACCCGAAACAGGATGGCGTAGCC
GCCCACATGGAACAGCGGTAGGGAGAGCAGCCAGCCGCAGGCCTCATCGAGGGGTATGAGGGTGGCAGAG
CCGCGCGCAGAGGCCAGGTGGTTGCAGAGCCGGTGCACCACCGCTTTCGGCGTGCCGCTGGAGCCCGAGG
TGAGGATCATGTTATTGAGCTGGGTCGATTCCAGCGGCCACGCTTCCTCGCTTGCTGGCAGTTCGTGCGT

GAAGTCGAACTGCAAGGGCTGCCAGTTGCCTGCCGGGATCTCCCCTGCCGACCAGAAGGCATCGGCATCG
AGTTGTAACGCCAGCTCGCGCTGCTTTTCGAGAGGAAAGGCTGGATTGAGCGGGCAGAACACCACGCCGC
TGCGCACGCAGGCCCACGCCAGCAGCACATCTTCCAGCGCACCACGCACCACCGCTGTCAGATGATCGCC
ACGATGCACTCCGGCTTGTTCCAGCTGTTGGCACAGGCTGTTGAGGCGGGCATCGAGCTGGCGATAACTG
AGGGGCGAGGCTGTGTGGATGGCAATGCGGTTCGGGGCTGTGATTGCCCGGTGGCGTACCGGGCAAGGCG
TCTGTTCGGATGAGCTGGAGGGGTCAATCACGGTAATACAGTTGCTCCAGTCGACTCAGATCCGGTTTGC
CATCTTCCCCCAGCAGGTTGCCTGCCATCCAGCGGCGGGTATCCAGACCCGGCGCCTGTTCTGGCGCCCA
TTCGCCAGCCAGATGAAAGAGCTGGTTCAGGCCCAGATCCGATTCGAAGCAGGAGGAGATGATCACCTTG
AGCCGCAGTTCGCGGGCGCGGGCGATCAGCGCTTCGCAGTTGGCGAGTGAACCGAGCAGGGTAGGCTTGA
GCACCAGCGCGCGCAGTTGCGGGATCGGCTCCCACGGCTTGCCTTGTGCCAGCAGCTCGTCCAGCGCCAC
CGGCATGCCGGTGCGGTTGGCGACAAAGGCGATGTCGGCAAAGTCGGCGCAGGGGTCTTCCAGATACTCG
ATGCGATTCGGGTCGAGATGGCCGCAGAAGGTCCATGCCTCCTCGCGAGTCCAGCCCTGGTTTGCATCCA
GCACCAGCTTGAGGGAGGGGAGGCGGTCGCACAGCAGGCGGATCAGCGCCAACTCGTCACGCATCGGGTA
GCGCGCCACCTTCAGCTTGGCCTTGCCGGGCGTGCGGTGCAGCCACTCTTTCCAGTTGCGCAGCAGCTCC
TGGGGGGAGCCTTGAATGAGGGGATAGGGCTCCGGCAGCGGGTTGCGCTGTTCGGGCCATGTCCGGCGCG
CGCAGTCGAGGCCAAATTGCACCGACGGCAGCACGGGGTTCGGGGTGTCGCCGGCGGCAAGGGCCGCCAG
CGTGGCAAGGGCTTCTGCCTCTGCCTGTTGCAGACTCTCTTTCGAGAAGCCGGGCAGGGGGGCAATTTCC
CCCCAGCCGTCGTTGATGCGCACCAACAGCCCCTCTCGCGCCACCTCCACCTTGCCGTGAAAGGTCAGTG
GCTGGGTAAAAGGCAGTTGATAGCGATACAGGGCGAGTGTCATCAGGGGGTCCGCTTGTATTTGGAGAAG
TCGGGGCGACGCTTTTCGTTGAAGGCGTTGCGGCCTTCCTGACCCTCTTCGGTCATGTAGAACAGCATGG
TGGCATTGCCCGCCAGCTCCTGCAAGCCCGCCTGACCATCACAGTCAGCGTTGAGGGCGGCTTTCAGGCA
GCGCAGCGCCATCGGGCTGTTTTGCAGCATTTCGCGGCACCAACGCACGGTTTCACGCTCCAGTTCGGCC
AGCGGCACGACGGTGTTTACCAGACCCATGTCGAGGGCTTGCTGGGCATCGTACATGCGGCAGAGGAACC
AGATTTCGCGGGCTTTCTTCTGGCCGACGATGCGGGCCATGTAGGAAGCACCCCAACCGCCGTCGAAGGA
GCCCACTTTCGGGCCGGTCTGGCCAAACTGGGCGTTGTCGGCGGCGATGGTCAGGTCACACATCATGTGC
AGCACGTGGCCGCCACCGACTGCATAGCCGGCCACCATGGCAACCACCGGTTTCGGGCAGGTGCGGATGT
CGCGCTGGAAGTCGAGTACGTTGAGGTGGTGAGTCCCCTCGTCATCCCGGTATCCACCGTAGTCGCCACG
GATCTTCTGGTCGCCGCCGGCGCAGAAGGCTTTCTCGCCAAAACCGGTCAGGATGATAGTGCCGATTTGA
TCGTCATAACGGGCATCGGCCAGCGCCTGCAGCATCTCTTTGACGGTGCGGGGGCGGAAGGCGTTGCGCA
CCTGGGGACGGTTGATGGTGATCTTGGCAATGCCATCGTTGGACTTGTGATAGAGGATGTCCTCGTAACC
GGCGGAGCAATCTTGCCATTCAACAGGTGCATACAGTTCAGCTTCCGTCATTGCTTCAATCATGACTCGT
CTCTTCTTGTTGGTTGACCCACTCCGCGAGGAGTCGGGCGAAGTTATCAGGCTGATGGGCGTGCAGATTA
TGGCCGCCATCCAGCACCAAGTGGTTGATTCTGCGATCCTGGTTCGCCAGCTGGCAAGCCAGCTGATGGA
ATTTGTGATCCCGCTTGCCACTCACCCAGGTGACCGGCAGCTGGTTCGCCATGAGCCAGGGGGCGAGATC
CGGCTGCTTGCCGAGCGAAGTGGCGCGCAGCATGGTGGCGATGCCCTGACCGTCGTTGCCAAGGCGGCGG
GCGATCTGGCTGGCCCGGGCCGCATCGTCAAGATCGGCAAACACCCCTTGCCGGTACCAGTCTGCCAGCA
CGTCAGCCAGGGGCTCCTGCTCGAAGCGCTGCGCCCACAGTTCATCGTGCTGGATGCGGGCGGCCCGCTC
ATTGTCTGGTAGCCCCGGGTGGCAGTTCTCCAGCAGCAGCCCCCGCAGCCCGGCGGGCTCGCGGCTGGCG
TGATAGAGGGCCAGTCGCCCACCCAGCGAGTAACCTGCCAGCAGATAGCGTTCAATGTTGCGGCTGGCCA
GCTCGGTGACCAGCCACTGGTGGCTCTGCTCAAAGTCGCTGACGTTGAGATCGCGATGCTGACCGTGACC
CGGCAGATCGAGGGCGATGCAGTGGATGGCGGGCAGCAGGTCGATCACCGGTTGCCAGTCGCCGCTATCC
CCCAGCAGGCCGTGCAGCAGAACCAGCGGCATATTGGCATCTTTATTCGCCACGGATGGCGGCCCCCAGC
GCCTTGAGATCATCGGCCACTTGCTCGCTCGGTACCCGGATCTCGATCAAGGTAACGCCATGTTGGAGGG
CGGCGCGGTAATCGCGTTCGAACTCGGGCAGGGTGGTCGGCGCCCGATAGGCGAGGCCAAACATGGCGGC
AGCATGTTCAAAGCTGAGGCCGTGGGGCAGCCGGTAGTAGCTCTCCAGCAGTTCCCCTTCGGTGGGCACC
GGCAACATACGAAAGATGCTGCCGCCATCGTTGTTGAGCAGGATCACCACCAGCGGTCGGCTCGCTTTGC
TGAGCAGGGCGAGGCTATTGAGATCGTGCAGGGCGCTGAGATCCCCAAGCAGCAGGGTGGTGGGCTCATC
GCTGCCCGGTTCGACCGATTGGGCAAACCCGTAGGCGGTGGCGATCAGGCCATCGATGCCGGAGGCGCCA
CGGTTGGTCATCACCCGGCTCGGCCCCTTGCCCGTTTCGCCGAGCATATCCATCAGTCGGGCTGGCATGC
TGTTGCCGACAAACAGTTGGCCTTCGATCAGGCGGTTGAGGCGATGGCAGACCCCAAGCTCGGAGAAGTG
CTCGCAGGCGGCGGCGATCTGCTGGCTGGCGTTGTGTTGCAGCTCGGCCAATGTGTGCCAAGGGGCCCGG
GTGGTGACCGGATGGGCCGCAGCAAAGGCGCTGGCACTGCAGAGCAGCCGGTTGCGCAGCCGGTAGTCGG
GATCGAGCCGCGCCGGTTGCGGATCCACCAGCCAGTAATCTTGCCATGAATGGTGTTTGATGAACTGGCC
GAGCCGCTTGGAGACAAGCCGCGCGCCAAATTGCAGCAGCACCTCCGCGCGGCCCAGCTCGGCGACGAAT
TCAGGGTCGTTGAGCGCCATATCCATGTGGATGAGGTTGCGGCTATCGAAGCGGATCTGGCTCTGCAGAT
CTGCCAGCAACGGCCAGCCAAGTTGCTCTGCCAGTGCGGCGACCACCTGTGCCTGTTCGGGGTCAGTGAT
CCTGCCGGCGACAATAATGCCGCGCTGTTGGCGAAAGGTTTCCCACTCCGCCTGAGGTGGGCAGCTCGGC
TCGTTTTGCTGCCACGGGCTCCACGGCTTGCGCGAGCCGAGCCAGTCACCGAGGGGGGCCAGATAGTCGG
AGAAATCCTGATAGTGCTCGCCCGGGTAGAGGGGCTCGGGGTACATGCAGTTGAAGTGCACCACCCCGGG
GGTGAGCGCCTGTTTGGCCAGCGCCTGATCCACCGAGCTCAGTACAAACGCCGCCGGAATGGTCGGGGTG
GGGCTCGGCAGGTTCTGCTGATAGACGGGGTAGCGGCCGAAGATCCCCTGCTGGTCGATCGCCTGATTGG

CGCCGTTGTCGATAAGCTCGTGAGGGCGATCGGCGGAGAGAATAATCAGCGGTACGCCGGTGAGCTGGGC
CTCTGCCACGGCGGGCCAGAGGTTGGCCACCGCGGTGCCCGACGTCATGATCACCGCCACCGGCCGGTTG
CTCCCCTTGGCCAGCCCGAGCGCCATAAAGCCGAGGCCGCGCTCGTCAAAGTGCAAGTGACGACGAAAGC
CCTGATGGGCCGCGGCTGCCATGGTCAGCGGCGCAGAGCGGGAGCCGGGGGCCAGCGCGATGTCGCGCAC
CCCGAGGCGAAAGAGCTCCTCCAGCAAGAGGGAGGACCAGACGTGGTTGAAGGTGGCGTGCAGGTTGGCA
AACTCGGTGCAGGCAGACGCTTCGCTGGCAGGCTCTTTACGGGTGGACATAGGGGCTCTCGTAGCGTGAA
AAGGGTGAATGACTATCCCAGCAGGGAGAGCACATTGGCGATCTTGTTGTCGAGTTCGGCCCACTCGGCG
ACAGGCTCGGAACCGGCGACAATGCCGGCACCGGCAAACAGGGTGACGCTGCCCGGTTGAATACGGGCGC
TGCGAATGGCGACCGAGAACTCGGACGTCTCCCGACTCAGCATGCCGCAGGCTCCGGCGTACCAGCCCCG
ATCGTAGGGCTCGAACTCGCGGATAAAGGCGAGCGCTGCCTCGCGCGGAGCGCCGCCCACCGCCGGAGTC
GGGTGGAGGGCGTCGAGCAACTGCCAGTCGCAGACCCCGGGCTTGAGCTCGGCTTCGATATCGCACTTGA
GGTGTTGCAGCAGCCGCAGTTTGAGAATGCGCGGCTCGGTCAGGGTGGCATCGTGGGTCAGTGGCGCCAG
ACGGCTGAGTATGTCGGCGTGTACCAGCCGGTTCTCCAGCCGGTTCTTGCTGTCGGCCAGCAGCTCGGCG
GCAAGTTGGGCATCGACCTCTTCATCGCCGGAGCGGCGAATGGTGCCCGCCAGCGCCTCGGTAAAGAGGT
GGCGATCTTCCCGGCGGTAGAGCCGCTCGGGAGAACAGGCGATAAAGGCCTGCTCGGGGGAGAATTGAAA
ACCGAAGTGGAAGCAGTCCTGGGCGTGGCGGGCCCACTGGTCGAGCATGGCCCAGGGATTGATATCCGCA
TCATGGTTCGGACTGGTCAGCAGGCTCTCCCGCGATAGCACCACCTTGGGAGTGTGCGCCTGAAAATCGG
AGGCGGTGACGCGCTCCACCAGCGTCTGCCACTGGGCGGGGCCGGGGCAATCCTGTCGCTGCCAGGGCTG
CTTTTGCAGGACGGGCAGCGGGGCTTCCGGGCGCAGGGCATCGAGGGCGGCTTCGGCCGCCTGCAGTTCG
GCCTCGAGATTGTTTTCATCCAGCCATAGGTTGAGGCAGAGACTGACGCACTCGCCACGGCGCACCAGCT
CGATGCGGGGCAGCACGAAACGGCAGGGGCCAAACCCTTGCCAGCCGGATTGGGCCGGATCGAACGCCAG
TCCGCCATAGTAGCGAGGGCTGTCGTCATGATGCTGGTTGGCTGTGTTCGTGCACACCTGCTTGATGGCG
TGCGGTTCGGTCAGTTCATGGATGGCGCCCAGCGCGACATACTCGGGACGAGCTTCTCCACGGGCCTGCC
AGTAGATGCGGGGATAGAGGGTTTGCGCCGCGAGCCAGCCCAGAAAGGAGCGGATTTCGATCGCCACTCG
CAACCGTACGAAGCCGCTGACGTTGCTGTGACGCAGGGCGTCCAGTTGTTGCTTGATATGGGTCTGAGCC
AACATGGATTCTTGTCTGAGCCGGGTAGTAACGAGCGATCCGGTAGCTGGATGCCGTAGGTTTATGCCAA
AGGGCGATGATTTCCTATATATTATCTGCCCATTCGTCCATGTAAATCGCCCGCGGGAGCTTCACTCTCC
GGGGCCAAGTATACCCAAATCCGGTTCGATAACCCTTAACACGATCAATATCCGATTGTTTGGCCATCAC
AAAATGACAAATACCTTCTCTGTTTGGTTGCTGGCGGCGCGTTTGCGAACATTGCCGCTCGCCTGCTCTT
CCATCCTGCTGGGCTCGGGGCTGGCTGCCAGTCGTGGTGCCTTCGATGGCACCATCATGGCGCTCTCTCT
GCTGACCGCCATCTTGCTGCAGATCCTCTCCAATCTGGCCAACGATTACGGCGATGCGGTCTCCGGCGCC
GACAACGGCGAGCGGATCGGCCCGCAGCGGGCGGTGGTCTCCGGTCTTATCACCAAAAAGCAGATGTGCG
TGGCGATGGGGCTGACTGCGCTGGCGGCGGTGGCCAGTGGCCTGCTGCTGCTGGGCTGTGCCTTCGGCGG
CCAGTGGCTGCAGATTTTGGCATTTGTGGTGCTGGGGGGGGCGGCCATTGTCGCGGCGGTGACCTACACC
GTCGGCAAGACCCCTTACGGCTATCGCGGGTTTGGCGATATCTCGGTGTTCCTCTTCTTTGGTCTGCTGG
GGGTGCTCGGCTCCTACTACCTCTTCACCAAGACGCTGGAGTGGGATCTGCTATTGCCTGCCACCGCCTG
TGGCCTGCTGGCGACCGCCGTGCTCAATATCAACAATGTGCGCGACATCGAGACCGACGCCGCCAGCGGC
AAGATCACTCTGGCGGTGCGCCTTGGCCGCAACCGCGCCATCGCTTATCACTGGGTACTGTTGGGGGCGG
CGTTGCTCGCAACCATCGCCTTTATCCTGATCCAGCCCGCCAGCTTCTGGCCGTGGATCTTCCTGCCCGC
CATGAAGCCTCTCAGCGATGCGGCCAAGACCCTTCGCGAAAGCTACGATGGCGAGGTGCTGACCGGTTCC
CTCAAGAAGACCGCTATCAGCGCGTTTCTGTTCAGCCTGTTGCTCTCCATCGGGTTGGCGCTCTCCTGAC
CGCTGGTCGCAGCTCGCCAAGGCGAACAATGAAAAGGGCTCCCGATATGGGAGCCCTTTTGGGTTTTGGC
CGGTTTAGCTGGCGCGCGCCACCCTGAGGGGGCGGCGGGAGAAAATCAGGGTATCCAGCAGCCAGATGGC
GACCATGGCGGCGCCCACCAGCGGGAAGGCTATCCCCAGCAGGATCAGCAGGGCGATGGCGCCACGCATC
GGCGGGAGTTTGGCGGGCAGCGGCGGTGCGGCGAGACGGCCCTGCGGACGGCGGCACCACCAGATCCACA
GACCGCTCACGGCGCTGGCCAGCACCATCAGGCAGATAAGCAGCATGGCCAGCTGATGGGGCCAGCCGTA
GAGCTTGCCCATGTGCAGCATGACGCCGCTCTCCACCGTCTTGGCGACCGGGCCGTAATCCTGCCAGCGC
ACATCGGCCAGCACCTTGCCGCTGTATTGATCGATATGCAGGGTGGCGTCGTTGCGTGGATCGTCGGCAA
ACAGGGCGATGGTGTAGACGCCGGTGTCGCCGACCGGCGGGGTGATGCTGTAACCCGGTTGGACTCCCCG
CTCATGGGCGATCTCGACCACCCGTTGCAGCGCAATGCGCTGGCTTGCCATCACCACTCCCTCGTGCTCG
CCGTGGTTCATGGCACCGTGATCGTGCCCCTCATCAACTGGCTGCGAGCTGGGCATGGGGGTGTTCTCCA
CCGCCTAGGGAACCGTCTGCTCATGGGCCTGATTCAGGCTGCGAGCCAGCGGTGCCGATTTGGGTACCTC
GTTCCACATAGCGGCGGGGAAGCGGTTCCAGACCGCGGCAAACTGATCGCCCCAGAAGCCGGTCCAGGTC
ATGCCGCTCAGCAACAACAGCAGCAGGAAACCGGCGCCCCAGAAGCCGATAACAGCGTGCAGATCGCGCC
ACCAGAGGCGGCCACGCTGGCTGACTCTGGGCCAGAGAATACCGCCCACGCCAGGCCCCTTGCGCGGCCA
CCAGAGATAGAGTCCGCTCAGCAGCAACACTATCCCCCAGCCGGCAGCCAGCTCGATCAGCCTATCTCCG
GTGGTGCCGAGCAGCAGGTCGGCGTGCAGGGAGCGAGCCACCGCTTGCAGATTCCACTTGTTATCCATGG
TGCCGAGCAGGGCACCGCTGGCGGGATCCATAAACAGGGTCAGCTCCTGTTCACCCCGATTGACGATGAA
CTGAGCGCTCTGATCGGGTGCGACAGGCGGCAGGTATTTACGCAGGCTGGCATCGGGCATGGTCATCATC
GCCTTGGCCAGCAGCTGGTCGGCACTCAGTGTTTGGCTGGCGGGGGTGGCCTTCATCAGCTCTGGGTACA
TCAGGTTGTCGAGCTGGGGTTTGAACAGATAGACGATCCCGGTCAGTGACAGCAGGATCAGGAAAGGGGC

GACAAAGAGACCGGCGTAGAAGTGCCAGCGCCAGATCCGTTGGTAATGACTCGAGTCCCTGTTTTTCATT
GCACATCCTTGTTGAGGTGCCAAACCTTTCGGTTGGTGGAATGTTTGTGGCGGCGCGGATAATAGCAGTA
ACGTTTCGTTAACTGCAATCACACATCTTGAGATCGGTTCAGTGCGGGATGTCGGTAACATGAGGTGCCG
ACGGTTTGCTGGATAGGATGACCACGCTTTGGGGAAAGCCTGTTTGCTGCTGGACTTTTCCTTATAAATT
ATTACCTTGTGGTAACAATTTGCAGACGATTGTGGTAACGGAATGGGAGCAGTCATGCAGCCACATGGGC
AATTTGACATGGAGCGCAGGGGCCAGATGCTGATCTGCCGCCCGAGTGGCCCCTTCAATCTGGCAGGTGC
CATGGCCTATGAGGCGCGCTTTCGTCACGAACTGGCCGGTCTGATGGATGGCCCCTGGGGAATTGTCGAG
GTCGCGACCGAGTTTGAAGCGGCCGGGCCCGAGGTGCTGGCCCGTTTTCGTCGCCAGTTTGGCTGGTGTG
CCGAGAGTGGCTGTCAGTTTCTGGCTGTGGTTTTCGTAGGGGGCTTCAAGCGCTATCTGGCGGATAACAT
CTTCAAGGGATTGCCCTTTCGGGCGGTACGTTACTTCGAGCAGGAGGCAGCGGCCATCCAGTGGCTGGGG
GAGCAGCTCAACACAGCTCCCTGTGAACCTGCGAGCGCTTGACGACCCCGCTCCCCGCGTGGACGGGTTT
TACTCCAGCTTGATAAACCACTTGTCCCGCTCGTCAACGCAGTCGGGTTCACCCATTTCGAATTCACGGC
AGATCAGCGGTCGCACCTCGTAGATACGGCACATCATGGTGTTGCGGTCGAGGGCGGCGCACCAGCCATC
GTCCAGCCGGCGCATGACCGGTATCTCGCCATCCCGTTCCAGTACATAACGGTCCGGCACGCCGGTATCG
GTAAAGAGCATCACTTCGAGGCGGCAGCAGCAGGCTTCGCAGTTCTGGCAGCTCACATCGGTTTGGATTG
CTTGGGTCATGGTGCCTGTCTTGCCGCATGGCGGCATCGGAGATATGAAACAGGCGCCTGTCAGGCGCCT
GTGGTCTGGCTAACGGCTTGCAAAGAACCTTAGCCCAGTTTGCGGGCGCGGTAGCTGCGACCCTTGATCT
TGCCTTCCTGCAACCGCTTGAGGGCGGTGCTCGCCACGCTGCGCTTCACGGCGACGTAGGAGTACTGCTC
GGATATCTGGATCTTGCCCACGTCGGCGCCGGCAATGCCGCCTTCACCGGTCAGGGCACCCAGAATGTCA
CCGGCCCGCACCTTGGTCTTGCGACCTGCATCGATGGAGAGGGTCACCATCTGCGGGGCGATCGGCTTGA
TGTCGCGGCCGATGCTGTCGAGATCACCCAGCGGCATCGGGGCCTGCTGGTACTCTTCGATGAAGTTGAC
GCGCTGGGCTTCGTTCGGCTGATAGAGGCTCAGAGCCAGACCCTGCTGGCCAGCACGACCGGTACGGCCG
ATGCGGTGCACGTGCACTTCCGGATCATAGGTCAGCTCGTAGTTGATGACGGCGCCCAGCTCCTTGATGT
CGAGGCCGCGGGCCGCCACATCGGTGGCCACCAGAATGGTGGCGCTGCCGTTGGCAAAGCGCACCAGCAC
CTGATCCCGCTCCCGCTGTTCCAGATCGCCATTCAGCGCCAGCGCCGAGAAGCCTTTGGCGGCCAAATGG
TCGGCCACGTCGTTACAGGCTCGCTTGGTGTTGCAGAACACCACGCAGGAGCTGGGCTGGTAGTGGCTCA
GCAGCCAGGTGAGGGCGTCGAGGCGCTGACCGGCAGGCACTTCGTAGAGCTTCTGTTCGATGGCGCTACC
CTCGTGCAGGGACTCCACGCTCACCTCGACCGGATTGCGCTGCACGCCGCGGCTCATCTGGGCGATCCCT
TCCGGATAGGTGGCGGAGAAGAGCAGGGTCTGACGCTGCTGGGGGGCATAGCTGATGACCCGGTTGATAT
CTTCGCCAAAGCCCATGTCCAGCATGCGATCCGCTTCATCCAGCACCAGCGTCTTGAGGCCGGATAGCTC
CAGCGTGCCTTGCTCCAGATGCTTGAGAATACGACCCGGGGTACCGACGGCGATGTGGGCACCAAAGCTC
AGGGTGGCGGACTGAGGTGCGGTCGGGGTGCCGCCGCACAGGGTGACCAGCTTGACGTTGGGCAGGGCGC
GCGCCAGACGACGGATCTCCTGAGCCACCTGATCCGCCAGTTCGCGGGTCGGGCAGAGCACCAGCGCCTG
CACTTCGAGGCGGTTGACGTTGAGCTTGGCCAGCAGGCCGAGGCCGAAGGCGGCGGTCTTGCCGCTGCCG
GTTTTTGCCTTGGCGATCAGGTCTTTGCCATCGAGCACCAGGGGCAGGCTCTGGGCCTGGATCGGGGTCA
TCTGCAGGTAACCCAGGCTGGTGAGGTTCTCTTGCAGGGCGGGGGAGAGATTGAGGCTGCTAAATTCGCT
GTTGTTCACAAAAGGACTCTATTTCGGAGAAAACGGGGATGCGCGAGCGCTAAGGCCGCGCATCATACCA
GAATGTGGCACCGGATGTGGCGGATTTTTGAACGGCTGGTCACGAGGGGGCCATGAAAGGTTCATTGTTG
CCAAACCGATCGCGGGCGAGCACTGACAATGTAATAAGGACTATTTTTCAAACACACATCTAATTATTTA
TCGCGGACAAACGTTTTCTATTTTCAGTGTTTTAATATAATGACTGGAAAATATTCCACGAGTAGTTATT
GCGCCATAGGGAATGTTGCGAAAATGCTGATGAAATATTTTCAAAGAACATAAAAACGGCGCCAACCTTA
CTCAATATGATGTTTTTAAAGGAATTTATATTTTCAATGTTGCGCGATTAATATCAGTTCATTGCACAAT
TAATTTAAAAACGTTTGTATTATCCTGCCTCCATAATATTGCGCGGGATTGTGATGGCTATCTGATTTTT
AAATGTGCAAATGTCTATGATGGCGCCGGTCAAAAATTACAATTTACTCAGGTGCTACAGGTATGGAATA
CGTTCACTTTGTGCTCGGACTGGTGATGGTCCTCGCTCTCTCTCTGCTGGCTAACCGCCACAACTGGAAA
CAGATCAAGCTGCGTTATATCGGCCAACTGCTGGTGGTCGAACTGGCGCTGGCCTGGTTCATGCTGAACT
CGGAAGTGGGTCTGGCGGTGGTGGGGGGCTTTGCCGCCGGCTTCACCAAGTTGATGGAGTTTGCCAAACA
GGGTACCGACTTCGTGTTTGGTGGTCTGGTCAACGAAGGGGCCTTCTCCTTCTTCCTGATGGTGCTGATG
CCCATCGTCTTCATCTCCGTGCTGATCGGTATTCTGCAGTACATCCGCCTGCTGCCGCTGGTGATCCGCG
GTATCGGTACCGTGCTGGCCAAGATCAACGGCATGGGCAAGCTGGAGTCTTTCAATGCCATCAGCTCGAT
GATCGTCGGCCAGTCCGAGAACTTCATCGCCCTCAAGAACATCCTGCCGCACCTGAACGAGAAGCAGATG
TACACCCTGGCGGCGACTGCCATGTCTACCGTCTCCATGTCCATCGTCGGTGCCTACATGCAGCTGATCG
AGCCGCGCTACGTGGTCGCCGCGCTGGTGCTCAACATGTTCAGTACCTTCGTGGTGCTCTCGCTGATCAA
CCCCTACGAGCCGGATAACTCCGTGACCGACATCAAGGCCATTGCCGAAGGTGACGAGCACCACACCCCG
AAGAAAGAGAACTTCTTCGAGATGCTGGGCGAGTACATCATGGCCGGTTTCACCGTTGCCGTTATCGTGG
GTGCCATGCTGGTGGGCTTTATCGCCCTGATCGCCCTCATCAACTACCTGTTCGAGATGGCATTTGGCGT
GAACTTCCAGTTCGTCATGGGCTACCTCTTCTACCCGATCGCCTGGATCCTGGGTATCCCGAGTGGTGAA
GCGCTGCAAGCGGGCTCCATTATGGCGACCAAGCTGGTGACCAACGAGTTCGTGGCGATGATGGATCTGG
GCAAGGTGGCTGCCGAGCTCTCTCCGCGCACCGTTGGCATCCTCTCCATCTTCATGGTCTCCTTCGCCAA
CTTCAGCTCCATTGGCATTATCGCCGGTGCGGTAAAAGCGCTGAACGAAGAGAAGGGCAACATGGTTTCC
CGCTTCGGTCTGAAGCTGGTCTACGGTTCCACTCTGGTCAGCCTGCTCTCTGCTGTTATCGCTGGCATCA

TGATCTGATCCTCTAGTGGTTCGCACCACTGCTTGGGCCCCTGACTTTGTCAGGGGCTTTTTTGTTTCTG
GCTCACAGCCGCCAGCCGCACCACCAGCCGGCGACCAGCAGGGTGAGGGTGCAAAAGCCGGTTTCGATGG
CGCCGCCGCTGCGCAGCGGCCAGCCCGGCAGGCGGAAGCGGCGTTTGATGGGCCAGAAGAGCGGAATGCC
CGCCGGGGTGAGCCAATCCCCCAAAAGGTGGCTGAGGTAGCCGACGATGAGTGCATCCTTGACCCCCATG
GGCAGGGTGTCCGGCGGCAGGCTCTGGTTCAGCCCCCAGATAGCGGCGGCGACCGCCAGCAGACTGTGGG
TGAAGCCGCGATGGCCGAACAGCCGCGACAGAGGTTTCGAGATCCACGGCAGGCGCTGCCCCAGCACCGA
TTTGGGGTGGTCGAGGTCGGGCAACAGGGCGCTGGCCAGCGCGGCAGGGATGGTCTGCCACAGGCTGGCA
TCGGCCAGTGCCGGAGTGAGCTGCAGTTTGTGGGCGATCAGGGTGCAGGTAACGGAGAAGAGCAGGTGCC
CTTGGGCGGTCATGATGATTACGGCGTGGACAAGATGGCGGCGCATCTTACCAGCCTGACGGTTGTGGTC
AGCTCTTATTTTAAAAATCAGGATGTTACGGCGAAAAAGCGCCCAAGCAGGGCGCCTTTAGGGTCTCTTT
ACAGCTTGCCTTTGACCAGCCACTTGTCGAGGGCGTTGGCAAAACGCTGTTTGTCGGCGGCATTGAAGGG
GGCCGGGCCGCCGGTCTGCACGCCGGCTGAACGCAGCTCTTCCATAAAGTTGCGCATGCCGAGCCGCGCC
TTGATGGTCTCGCGGGTGTAGATCTCGCCGCGGGGATTGAGCGCCTCGCCACCGGCATCAATCACCCAGG
AGGCGAGGGGGATATCGCCGGTGATCACCAGATCGCCGGGCTGAACCTGCTGGGCGATGACATGATCTGC
CACATCGAATCCCTTCTCCACCTGCTGGGTTTTGATGAAGGGCGAGGGGGGCACCCGCAATCCCTGATTG
GCCACCAGGGTAGTGACGACCTGGGCGCGGTGGGCGGCGCGGTAGAGGATCTCCTTGACCGGAATGGGGC
AGGCATCGGCATCAACCCAGATGGGCATGGCATGATTTCCTGTAGTGAAAGCGGATGGAAAACGAGAGCG
GCCATGTTAACACAGGCCTCCCCTTCCCGGCGCGGGGCTGAGTCCGCCCAACCTTTGGTGTAAGCTGGGG
CCAGCACATGAAACGAGAAGGAGACGAGATGGATCGGGTGCCCGGTGAAGTGATTTATGCCCTGACCCTG
GATGGCAAGGGTGGCATGGCGCCGCTGATGGCGGGAAGCGAAGTCCCGGCGGCGCATCCCGGCTGGCTGC
ATCTGGATTACGGCAATCCCGATTCGGCGCGCTGGCTGTTGCAGACACCCTTGCTCAGCGAGGCGGCCAG
GGAGTCGCTGCTCGGCCAGAGCAATCGTCCCAAGCTGGTGCGGATGGGGGAGACTGTGCTGCTGATCCTG
CGCGGCATCAACCACAACAAGGATCACAGGCCCGAAGAGATGGTGGCGCTGCGCATCTTCATCACCCCGG
ATCTCATCATCAGCAGCCGTCGTCGTCCGCTGCTCTCCGAGCAGGATGTGTTCAACCAGCTCAAGCTGGG
GGGCGGCGCCGATACCCCGGCCGACTGGCTGGTGGAGATCTGCGATGCCCTGACCGATCGGGCGGGGGAG
TTTGTCGAGGAGCTGCACGACAAGATCCTCGATCTGGAGGAGATGGTGCTGATGCGCGACATGCCCGCCA
ATGGCCGACTGGCGCTGATCCGCAAGCAGCTCATTATGATCCGCCGCTACCTCTCCCCCCAGCGGGATCT
GGTGGCGCGGCTGGCCAACGAGAAGATCAGCTGGCTGGATGAGGATGACCGCCGTCGTCTGCTCGATATC
GCCGATCGGCTGCGGCGCTGGCTCGATGACCTCGATGCCGGAGTGGCACGCACCGCCGTGCTGGCAGACG
AGATCAACAATCTGATGGCGGAGGCGACCAACCGCCGCGCCTACCAGATGTCGGTGATGGCGCTGCTCTT
TTTACCTGCCAGCTTCCTGACCGGCCTGTTTGGCATCAACCTCGGCGGTATCCCCGGGGCCGAGAATCCG
ACCGCCTTCTGGGTCTTCTGCGGATCCTTGGTGGCGCTGGCAAGCGGGCTGGCTATCTGGCTCAAACATC
GGCGCTGGTGGTAAGGAGGCATAACCCATGTGGCAATCAATTGGCCTGACCCTGCTGCTGTTGCTGGGGC
ATACCCTGATCCGGCGGCTTATTCACAAGAGCCTGCTGGCGCTCGGTCAGAGCAGGCAGGTGAGCGAGAA
CCGGGTGCTCTACGTGGCCCATGTGTTCCACTTCCTGCTCGGCTGCGCCACCCTGCTGGTGCTGGCCGGG
GTGTGGGGGCTCGACTTCTCCCGACTGGTGGTGCTCGCGTCCTCCTTCTTTGCGGTATTGGGGGTCGCCA
TGGTGGCCCAGTGGTCGATCCTCTCCAACATCACCGCCAGCATCACCATCTTCTTCGCCTTCCCCTACAA
GATTGGCGACCGGATCCGCATCCTCGACAAGGATGATTCGGTCACCGGCGTCATCACCGAGATCGGCCTC
TTCTATGTGCGGGTGCGTGATGACAACGGCGATCTGGTCACCTATCCGGCCAACCTGATCCTACAAAAGC
CGGTCAGAAAGCTGGAGGGCAAGGTGGCGGAACCGCTGCAACCGGAGTAGAAAGCAACCGGAATAACAAA
AGGCTCCCTCGGGAGCCTTTTTACTGTGCGGTGGGTGGCGCTAGCGTTCGAAGAAGCGGTAGAGGTGCTC
CACCTGAACCGGGCAGTCGGCCACCTGATGCAGATGTTCGCGGGCCTGCTCGACGGTGCCGAAGCTGACC
GGGCAGCCGAGGGTGTCGGTCAGCTCGTAGAGGGCGCCATCCTTGCCTCTGAATTCGATGAGCCAGCCGG
GAATGGCCACGTCGTGGAGTATTTCGGCCTCTTGCAGGATCTGTTGCTGATAGAGCTTACGAAGTTCACT
GACTGTCATGATGCTCTCCTGTGGGGAATATCCAGCTCCAGTCTATGCCTGCCCTTTATATGCGGCAACG
GGATCATCCCCGCAGGACCTCCCATGCCCAGCGGATGCGGACAAAGGTCTCTGCATTGCCGCCCCGATCC
GGATGGTGTTGCAGTGCCAGCTCCCGCCAGCGGCGGCGGATTATCTCCTGACTCGTCCCCTCATTCAGCT
CGAACAGGGCCAGCGCCTCGGCTTTGTGCCCCTCCCCGTCATAAGCGCGCCAGAAGCGGTTGAGCAGGGC
CTCAACTTCGGCACGGTCGGTTTGCCAGAACACCTGCCAGTCGAGATAGTAGCTACGCAGATTTTCGCCA
ACGGCAAAGCCGTGGTTGACCTCCTGGTTCCCGGCCAGCGGTTCGAGCCGGATATCCAGGGTGCTGATCA
CCAGCCACCAACCCTCTTCATAAAGCTCGGCTTGCAACTGGTAGAGGGCGTTCATGATCAGGAAGTTGAC
CCGAAACAGCTGCAGTTGCTCGTCGTCGGCCAGCCGGGGGATCGCCCCCTGCTGGCGCAGGGCGGCCATC
AGTTCATGCACCTTGTAGCTGCCCGCCGCCTGTTGCAATAGCACCAGCAGAGGGGCGATCAGCGGATTAT
GGTTGGCTTCGTTCATTCGGCGCCCTGCTCCCTGGGTGGCTGCAGGGCGATCAGCAGCTCACCCGCCTGC
GGGGTGACGGGGCCGGTAACGATCTGGGGCTTGCCCTTGCCATCCATGATGAAGAGCGGGATGGCGCCGG
GATTGGCGGTCTGATACTGGGCCCAGTCGAACGCCTCGCTCAGGCGGGTCGCCTTGATCTGGCCACCCCG
GCTCAACAGGCCGCTCAGCCGGGCATAGTTGATGTCGGGGCCGAACAGGTTCTGGCGAGCGCGAAAGGTG
GCGCTCTCCCGGTTGGCCTTGCCGTGCTGCTCGCTGGAGCGCAGGGAGTAGACCTGCTCCTCGCCGTAGA
GGTGGGCAAAGTGCAGCACCCCGAGTGCGTTGTTGTGGCGGTTGGGGGAGAGCGCCAGCACGGTACTGAT
GCTGGTGAGCGGCAGGTGCAGCTCGGCATGTTCCGACTGGGGGTTGCCGAAGTAGCAGGGGAGGTTGCTC
ATCCGTGCCTGTTTGCAGAACTCCCAGGCGGGATCGCAGAGCTGTACCGGCACCCCCTGATCCGCCAGCG

CCTTGCCGATAGCCCGCGCCACCGAGTTGGCCCCTATGATGAGCCAGGTGTTGGGGGCGCTCTGGCGCAT
CTTGAGCAGGCTGGCCAGCGGTGCCGAGGTGAGGCTCTGCAACACCACGGTGGAGATGATCACCGCAAAT
ACCAGCGGCACCAGCTTGTCGGCATTGGGGATCTCGGCCTGATGCAGGCTGATGGCAAACGAGGCGCTGA
CTGCCGCTGCGACTATGCCACGTGGGGCGATCCAGCTTAGCAGGGCGCGAGCTCGCCAGGAGAGATCCGA
GCCCCAGGTCGAGACCAGAATACAGAGCGGGCGCGCCACGAACTGCACCACGCAGAGCAGCGCCAGCACC
ATGGGGCCGAGCTGCCAGAGCTGGGCCAGATCGAGGCGCGCCGCCAGCAGGATAAACAGGCTGGATATAA
GGATGACCGCGAGATCCTCCTTGAACGCCAGCACCTCCTCCAGGTCCAGCCCCTCCTGATTGGCGAGCCA
GATGCCGAACACGGTCACCGCCAGCAGGCCGGACTCGTGGCTGAGCCAGTTGGAGAGACTGAAGGTGACC
AGCACCAGCGCCAGCACGCCGAACTTGTGCAGGCTGACCGGTAGCCAGTCCTTGCGAATGAGCAGGGTGG
TGAGCCAGCCTGCCGCTACCCCGATCAGGGAACCGACCAGCACTGTCTTGCCCAGCGCCAGCAGGGTATG
GCTGATGAGATCCCCCTGACTGCCGAGGCGCACCGCCTCGAACACCAGCACCGCAAACAGGGCGCCGATG
GGGTCGATGACGATCCCCTCCCAGCGCAGGATGCGGTCCACCTCCCGGGTCGGCGGAGCACGTTGAGCA
TGGGGGCGATCACCGTCGGGCCCGTTACCACCAGCACAGCGCCGAGCATGGTGGCGACCCGCCAGTCCAG
CCCCAGCAGGAAGTAACCGGCGCTGCCGATGACGGCGAAGCTGACCAGCATGCCGGTGGAGCAGAGATTG
CGCACCACCTTGCCGATCCCCTTGAGCTCGGCCAGATGGAGGGTCAGCGCCCCCTCGAACAGGATGATGG
CCACCGACAGGGAGACCAGCGGAAACAGCAGATCCCCGAGCAGGGCATCGGGGTCGAGCAGGCCGCTGAC
CGGACCCAGAGTCAGGCCGGTGAGCAGCAGAAACAGGATGGCGGGCACCCGCAGCGACCAGGCAAGCCAT
TGGGCCAGTACGGAGACCAGGGCGATCCCGGCCAGAGAGAGGGCAATCATGCAGACCTCGATTCAGGCAG
TGAGTATGGAGCTAGTGTAGTGGCTAAGTTGGTCAGATTGACACCTGAAGGAGCGTGCATCATGGCCGGG
ATGGGGAGATGTGCGACAGTCGGCGTCATGGTGGCTGGCGGTGGTGTGCAGGGCGCCAGCCAGACGCCAA
CCGTGGTGATGGGGTGGCCTCGGGGCCGGATTAGATGGGGCTCTCCTTGTGGAGCTCGCCGACACAGTCG
GCCAGCGCCAGTACCAGTGCGGAGCGCACCATCTGATCGAGCCGGTTGAGCTGCATCTGGTAGAAGGCCC
AATCCTCTTCGCTGGCGGGGGGCTCCAGCTTCATCATGCCGCCCTGATTGACCCCGTGCAGGCGGTCGAG
CAGATCGCGGATCGGCTTGTCGCTGAAGCGGTAATCCTTGGGGTCGCGCACCAGAAAGTCGCGGATCTTG
ATATAGGCTTCGATATCCTGATAGCGCTCGAGTGAGATGAGGCCGAGGGCGAAGATGAGCTTGAGGCGCA
CCTCCAGCTGGCCGAGGGGGCCGCTCTGGTTGAGCAGGGGTTCGATGGCGTATTTGACGGCATACTCCTC
CTGACGGAACGCGCGTCGCATCAGGGAGTCGACCGCCTCTTCCAGGATGGTCACGGCTTCGAGAAAGAAG
CCGCGGGGGCCATCCTGCTCGTTCAGTCGTTCCAGTACTTCATCTTCCTGGTGTGTGGTCATAGGTGCAT
TGGGTCCAGCTGACAGGGAGAGAGTGGGCCTCTCTCCCGGTTTCTATCATATGGGGCACATTCCCTTGCC
ATTCAAGCGCTTGGCGCGCTATCAGTGGTGGCTGAATGCGCCGCAAGCAGGGTCATTACAGGGTTTCGTA
ACGGGCGACGATGGCGCGAACCACCTCGCTCTCTTCATCCAGTCCGGTGACGCGGGCCAGCGCTTTGGCC
GGGCCAAGCTCCGCCAGCAGGGCTTGCAACTCCACTGCCTGCGGGTCATCGGCATTGCGATAGTGGAGCG
CCGCGGCGATCCCCTCCTGCAACTGATCGCTCGGCAGGCCGTACTCCAGGGTGCCCAGCAGCGGCTTCAC
CAGTCGATCTCCCGCCGCCAGCTTGCGCAGCGGCTGACGACCGACCCGGTCAATCTCGTCCACCAGATAG
GGGTTGGCGAAGCGGGCCAGGATCTTCTCGATGTAGGCGGCGTGCAGGCGCGGGTCGAAGCCGTAGCGTT
TCACCAGTACGGCACCGCTCTCTTCCATGGCGCGGCGTACCTTGCTGCGGATCACCGGATCTTCGATCGC
CTCGCGCACTGTGTGGTAGCCCCGCAATTTGCCGAGATAGGCGGTGACGATGTGGCCGGTGTTGAGGGTG
AACAGCTTGCGCTCGACGAAGGCCATCAGGTTGTCGGTGGGTTCCATGCCCGCCACCTGTGGCAGCTCCC
CCTTGAACTGGGTCTGGTCGACGATCCACTCGCTGAAGCTCTCGACCGTCACTTCCAGCGGGTCATCGTT
GGCCGCCGCGGCGGGCGGCACGATGCGATCCACCGCCGAGTCGACGAAGCCGATGCAGGATTCGAGCGTG
GCATGATACGCGACCGGCAGGTACTTGAGTACCTCCCGCTTGAGGTGGCTGGTGCCGCGCACCATGTTTT
CGCAGGCGATGACATTGAGCGGTGTCAGGTTGCCAGCATCGAAACGGGCTTGCAGCCCCTTGGCCAGGGT
GCTGGCAATCTTGTCCAGAATGTTGGGGCCCACTGCCGTGGTGACCAGATCGGCGGTGATGATACGGGCG
ATCACCTCGTGACCGGCGGAACTGACCGCCGCCACGTTGCGCACCACGTCCAGCTTCTGATCCGCTCCCA
CCACATGGACCTTGTACTCCTGACGGTGGTTGAGCTGGTCGATCAGGGTGTCGTTGACGTCGGCGAAGGT
GACCTGATGGCTGGCATCCGCCAGCACTTTGCCGATAAAGCCGCGACCGATGTTACCAGCACCAAAATGC
AATGTTTTCATGATATTAACCTCTTGAACCAAAGACGATGAAAGATGATTACGCAGCCTGTTCGGCACCC
AGCAGATCCAGGAAGGCCTGGGGATCCGTGGTGCTGGCCAGCCGGTCGATGAGACCGGGTTCATCCAGTG
CATTGGTCAGGCGGGTTATCACCTGCATGTGTTCGTCGTTGCGGGCGGCGATGCCGATCACCAGCCGCGC
TACCTCATCGGCAGCCTCGCCAAAGGCGACCCCGGCCGGGTACTGGCAAATGACGATGCCGGTACGCTTC
ACAAACTCCTTGGCGGCGATGGTGCCGTGGGGCACGGCGATGGATTCGCCGAGGTAGGTGGAGACCAGCT
GTTCACGCTCCAGCATGGCGTTGACATAGTCGGGCGCCACGTAACCGCGCTCTGCCAGCAGTCGGCCTGC
GGTCAGGATGGCGGCCTCCTTGTTGTCGGCTTTCAGTCCCAGATGCACGTCATGACGGCTCAGGGTAAAG
ACTTCGGCCGCTTGCGGCTCGTAGCTGTCGTCGTTGGCCGCCACCAGCAGTTGCGGGTTGGCGACGTCAT
CGTTGGCGGCGCGGCTCAGGCTCTGTACCAGCTCCTGATAGAGGCCGTTGTCGAGGAAGTTGGTGAGCGA
GATATGGTGAGCGTTCGGCGCATGGCGACGGGCCCGCTCGGTCAGATCCTTGTGGGTGATGACCCAGTCC
ACCTGATCGTCGAGCTGATCGATGGCGCGGTTGGTGACGCTGATATCGAGCCCCGCGGCCTGCACCCGCT
TGCGCAGCATACCGGCGCCCATGGCGCTGGAGCCCATGCCCGCATCACAGGCGACCACGATATTGCGCAC
TGCCATCAGTTCGCCGCCCGGCTTCTTGGCCGCTGCGGTTTCGGGCTTGCCGCCCTTCATGGCTTTCATC
TTGCGAGTGGCTTCACCGAGGGCATCGGCCTCGTCCGCTTCCGGCTGCTGGGCGCGAACGAATACGGCGG
CGACCAGGAAGGAGACCAGAGTGGCGCTGATGATGGAGAGGGCGACCCCGATCAGGGAGGCTTTCGGCGT

CATCAACAACACGGCGAAGATGGAACCCGGTGAGGCAGGGGAGACCAGACCGGCGTTGAACAGGGTCAGG
GTAAAGACCCCGGTCATGCCACCGGCGATCACCGCCAGAATGAGGCGCGGGTTCATCAGCACATACGGGA
AGTAGATCTCGTGGATGCCGCCGAAGAAGTGAATGATTGAGGCGCCAGCCGCGGACTGCTTGGCCGCACC
ACGACCGAACACCATGTAGGCCAGCAGCACACCCATGCCCGGGCCCGGGTTCGCTTCAATCAGGAAGAAG
ATGGAGCTGCCGTGCTCGGTCGCCTGCTGGATCCCCAGCGGCGAGAAGATGCCGTGGTTGATGGCGTTGT
TGAGGAACAGGATCTTGGCCGGCTCGACGAAGATGGAGGTGAGCGGCAGCATGTGGTTTTCCACCAGGAA
GCCGACGCCACCGGCCAGCAGGCCGGAGAGCACCTTGACGAAGGGGCCAATCACAAAGAAGGCAAGGATG
GCCAGCAGCATGCCGATAAGGCCGGCGGAGAAGTTGTTGACCAGCATCTCGAAGCCGCTCTTGACGCGAC
CGTCCATCGCCTTGTCGAAGCGCTTGATGGCCCAGCCACCGAGCGGGCCGACCATCATGGCACCCATAAA
CATCGGGATGTCGGTCCCCACGATGACACCCATGGTGGTGATGGCACCGACCACGCCGCCACGATCACCG
CCCATCAGCTTGCCGCCGGTGTAGCCAATCAGCAGCGGCAGCAGGTAGGTGATCATGGGGCCGACCAGCT
TGGCCAAGGTTTCATTGGGCAGCCAGCCGGTGGGGATAAAGAGGGCGGTAATAAAGCCCCAGGCGATAAA
GGCGCCTATGTTGGGCATGACCATATTGGACAGGAAGCGTCCAAAATTCTGTATCTTGACTTTGGTATCC
GGTGATAACATGTCGGGACCCCGTTTTAAGCCTGTTTGATTAAAAGTGGTAGTTCGGGATGGCCGGTTTT
GCCGAACCTCCATCCCGGTCGTCTAAGCAACTTCAATCTACCAGCTTGCGATCAAATTAGGCTGAAAACG
GGGTGTCTTGTGATCCAGATCTCGGATACCCAGCGATACCCCCTCCATAATCAGTGACCCACATCACATT
CCCATGCTGTAATTCGGCTACATTTCGATCTTTTCTGTTTTGCATGGTCATTTTTTTGTGACTCAAATCAC
TAAAGTTGTTTTGTGGCTCTCTTGCTGAATAGTGACTTTCGTCACAAATTCAATTTTTGTATTCGAATGG
GACAAATCACTCAGTCACTCATCAGCGGGTAGTGACGATGGATCTATCGAAACACTCCATTGTTTACCAA
TGAATCAATAAATTTGTAACCCGCTTTCAGTTTTTACGGTCGGCGATGAGGTCTCTTTTGCTGCTGGCTG
GTGGGAATGGGCGCCAACATGCAGGATGTTATCGAGCGGCAGATGTTCGGGGCGCTTGCTTGCCGCGCAG
GGCTGATAGCAGATAAAAACAAACCGCCAGCAGGCGGTTTGTCGGTTTTTACGAGCTATCCCTTGTCGGG
GGCTGCGGTTTAGTAGAAACCGGCATCCCCGGGGGAGGGGCGGGTCTTGAAGCGGCGATGGAGCCACATG
TACTGCTCCGGCGCCTTGCGCACGGCGGCTTCAATCTCGCGGTTGGCGCGGGCGGCATCAACCATTTCAT
CCCCGCTCGGGTAGTCGGCGAGCGGCGCGCCGATATGCAGGGTGTAACCCTCGCTGGTGCGGATGTTGTA
GCAGGGCAGGGTGACGGTGTTCTTCACCCGCGCCAGGGTGGCGGTGCCGGTGATGGTGGCCGCCTGCTCT
ACCGCGAAGTAGGGCACGAACACGCTGGCGTGGCTGCCGTAGTCGTGATCCGGTGCATACCAGAGGGCGT
CGCCGTTGCGCAGCGCCTTGATCATCCCCTTCACATCCATCCGGTCTACCAGATATTTGTTGGAGGCACA
GCGCCCGTGGTACTGGGCATACTCCAGCACCGGATTGGTGTTGGGGCGATAGACCCCGACCCCGGGGCGC
ACCAGCCCGAAACAGCGGGCGTTGAGTTCCAGCGTCAGGAAGTGGCAGGAGAGCAGCAGCATGCCCTGAC
CTTTTTCCATGGCGGCGATGACGTGCTCTTCCCCTTCCACCTTCATCAGGTTGCGCATCCGCCAGTCTGG
CCAGAACCAGGCGATCCCCACCTCGAACACGGCGCAGCCCACGCTCTCGAAGTTTGCCTTGAGCAGTTTC
TCCCGCTCATCCCGGGTCAGCTCGGGCATCGCCAGCTCCAGATTGCGACGGGCAATCTTGACCCGGGACT
TCAATATCCTGGCTGCGAGACGACCGAGCTGACGGCCCAGCCACATCTGGCTACGCCACGGCAGCAGGGT
CAGCAGCCAGAGCAGGCCTAGGCCGAACCAGCTGGCCCAGTAACGGGGATGAAACAGGCGGGGCTCCAGG
CGGGGAGCGTTGTCGTGGGCCATGGCTAATCTCTTGTTCGCAAACACAATCGGGGGATTCTAGCCGAAAA
GGGGGGGCGGGGGCGAATGGCTGTGTGCTGGTCAGAGGGGTAGCCTGCGTCCGGTTAGCGTGACAGCGGC
GGCAACCGCGGTCGGCTTTTTCTACACCGGACAAAAGAGTGTGATAAAATCGCCCGCTGATTTTTCAGGT
TTTGAGAGTCGATACAGATGAAGATCACCCTGCCCGATTTTGAAAAAGCCCGCGTTCTGGTCGTTGGCGA
TGTCATGTTGGACCGTTACTGGCATGGCCCTACCGGTCGGATCTCCCCCGAGGCGCCGGTGCCCGTGGTC
AAGGTTGAACACATTGAGGAGCGTCCCGGTGGCGCGGCCAACGTGGCCCTCAACTCCGCCGCCCTTGGCG
CTCACGCTGTGCTGCTTGGCCTGACCGGTCAGGACGAGGCTGCCGATGCTTTGGCTGGCCAGATGGCTGG
CGTCAAGGTGGCCTGTGATTTCGTTCGTCTGGCCGACTTCCCGACTATCACCAAGCTGCGGGTGCTGTCA
CGCAACCAGCAGCTGCTGCGTCTGGACTTTGAAGAGCCCTTCCACTCCGTCGATGCCACCCTGCTGATGA
GCAAGGTTGAGCAGGCGCTGCCAAACAGCGACGTGATGATCCTTTCCGACTATGGCAAGGGTGCTCTCAA
CGATGTGCCCGGTATGATCCAGCGCGCCCGTGCCGCCGGTATTCCGGTGCTGGTCGATCCCAAGGGGACC
GACTTCGAGAAGTATCGCGGCGCTACCTTGCTGACCCCCAACATGTCCGAGTTCGAGGCTGTGGTAGGCA
AGGTGAAGAGCGAAGAGGAGCTGGTCGCCAAGGGGCTGGATCTGGTCAAGCGTTTCGAGCTGGATGCCCT
GCTGGTCACCCGTTCCGAGAACGGCATGACCCTGATCCGCGAAGGTCAGCCGGAGCTGCATCTGCCGGCT
CAGGCTCACGAAGTTTACGATGTGACCGGTGCCGGTGATACCGTCATCTCGACCCTCGCCACCTCGCTGG
CTGCAGGCAAGAGCCTGGACGAAGCCTGTGCGCTGGCCAATACCGCAGCCGGTATCGTGGTCGGCAAGCT
GGGTACCTCTACCGTGAGTCCGGTCGAGCTGGCCAACGCCCTCTATACCGAGCAGGAGACTGGTTTTGGC
GTGATGTCCGAAGCCCAGCTGAAAGTGGCGGTTCGTGCCGCTCGTCTGCGCGGCGAGAAGGTGGTGATGA
CCAACGGTTGCTTCGACATCCTGCACGCCGGCCATGTCTCCTATCTGGCCAACGCCGGCAAGCTGGGGGA
TCGCCTGATCGTGGCGGTCAATACCGACGAGTCGGTTCGCCGTTTGAAAGGGCCGGGTCGTCCGGTCAAC
CCGACCGAGCGCCGCATGGCGGTGCTGGCTGCGCTGGGCGCGGTGGATTGGGTAGTGCCGTTTGGTGAAG
ATACCCCGCAGCGCCTGATCGCCGAGATCCTGCCGGATCTGTTGGTCAAGGGCGGCGACTACAAGCCGGA
AGATATCGCCGGTTACGCCGAGGTGACCGCCAATGGCGGCGAGGTGCGCGTTCTCAACTTCGAGGATGGC
TGTTCCACCACCGACATCATCAAGACGATCCGCGAGCGCGGCTAACGTTCTGCTGGCCCCGCAATCTGGC
GGGGCTCGCTATCGGCGGCTGCAAGCCGACAGAAATACGGATAACAAAAAGGCGACCTTGCGGTCGCCTT
TTTCATGTCCGATAGCGGCTATCAGCCTTTGGCTTTTGCCGTGGCAGGCATCAGACCCTGGTTTACTTCC

ACCAGGTCCTGCGCTTCCAGAATACCGGCAGCCTGTTTCAGGCTCAGGATATTGAGAATGTAGTTGTAAC
GGGCTTCGGAGAGCTTCTGCTTGGCCTCGTACAGCTTGCGAGTGGAGTCCAGCACGTCGACGATGGTACG
GGTACCCACTTCGTAACCGGCTTCGGTCGCCTTCAGGGCGCTGTCAGCGGAGACCACAGACTGGCTGTAG
GCACGTACCGAGCCGATGCTGGCGTTCACGTTGTTGTAGGAGGAGCGCACTGTGCTCTGCACCGAGCGGA
AGTTCTTCTCCAGCAGTTCGCTGGCCGCCACGTAGTTGTACTGAGCCTGCTTCACCTTGGAGCTGGTGGC
GCCACCGCTGTAGATGGGCAGCTTGAAGGTCAGACCGATATTGGCCTGATTGTCATTGCCGCCCGGAATG
CTATCCAGCTTGTAATCGGTATTGCTGGTTTGCAGTCCGGCACCCAGATCCAGAGTCGGCTCATGGCCGG
TCTTGGCCAGATCGATCTGCTCCTTGGCAATGTCCTTGCTGATGCGGGCACTGTGCAGTTGCAGGTTCTT
GTCCAGCGCCAGCTCCAGCCATTTGTCGGAACCGAAGGCAGTCTTTTGCGGGCTGAAGCGATCGGTGTTG
AGGATGTCCAGGTTGCGGTGATCAACACCGGTCAGCTCGCGCAGGGTCTCGTAGCTGTTATCCAGAGTGT
TCTCGGCATTGATCTCGTCAGCCAGCGCCTGGTCGCGGGAGGCTTCGGCTTCATGGACGTCGGTGATGGC
GGTCAGGCCCACTTCGAAACGCTGCTGGGTTTGCTCCAGCTGACGCTCTACCGCAACCTTGTTGGCACGA
ACGAACTCCAGGGTATCCATCGCCTTGAGCACATCAAAGTAGGCTTTGGCGGTGCGCACCATCAGGTTCT
GGATCTCGAGGTTGTAGGCGACATCTGACTGGGTGGCCTGCTTCTCGCTGATATCCAGATTGACCCAGTT
ACTGCGACGATAGAGGGATTGCGACAGCGTCACTTCACCCTTGGCGTTGGTATTGCTCTGGGTATCGTTC
TTGTTTTGCAGATAATTGAGCCCGGCACCCAGATCGATCTGGGGCAGCAGAGGGGCACGGGATTCGTTGA
TCTTCTCGAAGGCCTGGTCGCGAACGGCTTTGGACTGCAGCAACTGCGGGTCTTTGATCTGGGCTTGCTG
GTAGATGTCGAGCAGGTTTTCGGCATGGGCACCGGCACTGACGCCCAGCAACACCATGACTGACAAAAGT
GTTCTTTTCATAGATCGTTTTAACCTTATGTAGTAACCCAAACTGGTTGCTGGAGAGGCAAATATTGTCA
CCGACCAAGCTTGATTGCAGCTTAATCACTCTACTATAAGCAAAATTTAATGCTAAGAGTCTCAGGTCCG
GTGACGGGAACGTCAAGAACCGGACGCAGTTTTCTCCCCGCGTGCCTAGCCAAATGGTCTGTGGCTGCTA
AAATCCACCCCTCATTACCATGGCACGCCGAGTGAATGAACGTTCATTCATGTTCCTCGGATTTTATGGT
CAAACTGATACAAGTCAAGCTGACGGGAGCCCCGGATGTCGCAGCAACCCTTGCCGCAAGATCGGCATTA
CGCCGCCGATGATGTGAAAATCATTGAAAAATCAGCTGGATATAACGGCTTCTTCAAGGTGAATGTCTAT
CGTCTGCAGCATAGGTTATTTGCTGGCGGCTGGAATCAGCCCATCGTGCGCGAGCTGTTCGAGCGGGGTC
ATGCCGCCGCGTTGCTTCCATACGATCCGGTACGCGACCAGATAGTGTTGGTGGAGCAGTTCCGTATCGG
CGCCATGGAGACCAGCCCGACCCCCTGGCTGCTGGAGCTGGTGGCCGGCATCATCGACGAAGGTGAGACC
GCCGAGGCGGTGGTGCGCCGCGAAGCGGTGGAAGAGGCCGGTATCGAGGTTGCCCGCTGCGAGCATGCCA
TCAGCTATCTGGTGAGCCCGGGCGGCAGCACCGAGCGGATCGAGGTCTTTGTGGGCGAGGTGGATGCCAG
CAAGGCGAGCGGCCTGCACGGTCTGGCGGATGAAGGCGAGGATATCCGGGTGCACGTGGTGAGCCGCGAA
CAGGCCTACGCCTGGCTCAAAGAGGGACGTATCGACAATGCGGCCTCGGTCATTGCCCTACAGTGGCTGG
CCCTCAACCATGGCGAGCTGCGCGCCCGCTGGCTGGCGGATCGCTGAGGTGGCGCTGACCCTGCAACAGA
GCAGGCGCTATGTGCCTGATCTGATGTCCTTGCAGCGTACCTGCGAGGTCAATTACATGGCGTTGATGAA
ACTGCTGCCGCCAGATGGGGAAGTCGGGGCCGAGCGCCGCTATCGGGTCGGCAACGATCTGCAGTTCCAG
CTCACCATCATTGGCGAGAGTCGCTTCACCAATCAGGTGATCCTGAGCCAGCACAACCCCGAATTGCCCG
AATATTTACAAGCTCAGATAGAGATTCGTTTGTATCATGATGTGCGGATGGCGGAAGTGTGTGCAGCGCA
ACAGATTTCCCGTTTGCAACCACGTTATGATTATCCCAATAAAAAGATGCACCAGCGGGACGAAAAGGAG
CAGGTAAATCTGTTTCTGGCCGAATGGCTAAAATTTTGTCTCAGACATGGTACCAGCCCGATCAATATCT
CCAGTTGAACAAATAAAAAGAAGCGTGATTTTGGAAACAGAGCTCCCCCAGGCGCAGGACGGCAGTGTGC
GCCTGTTGCAAATTACCGACACCCACCTCTTCGCGAGTGCCGAAGGGCGGCTGTTGGCTGTGCGTACCGC
AGAGAGTCTGGCCGCCGTGCTGGAACAGGTGCAGGCCAATGAACACCCCTACGAGGTGATTCTGGCGACC
GGCGATCTGTCGCAGGATCACAGCCCGGAATCCTATCAGCGTTTCGCCTCCATGATGGCGCCCCTGGGCC
GCCCCATCTACTGGCTGCCGGGCAATCACGATGACAGCCCGCTGATGACCGAATATCTCCATGCCGCCGG
GATCAGCGAATCCAAACAGCTGGTGGGCGACCACTGGCAGGTGATCCTGCTCGACACCCAGGTGCGCGGC
AAACCCCACGGCATGATTGGCGATCACCAGCTGGCCGCCCTCGACCGCGCCCTGCGCCAATATCCTGATC
GCCACGCTCTTATCACCCTGCACCATCAGGCGGTGCCGGTGGGCTGTGCCTGGCTGGATCAACACAATCT
CAAGAATGCCGATGACCTGTTCGCCGTACTGGCCCGTCATCCGCAGCAGAAGACCATCCTGTTCGGCCAC
GTCCATCAGGAGTTTGACGAGGTGCACCGCGGGGTGCGGCTGATTGCCAGTCCATCGACCTGCATCCAGT
TCAAACCGCTCTCTGATGGCTTTGCGCTGGATGAATCCGGCCCGGGCTGGCGCTATCTCACCCTCTATCC
GGATGGGCGGGTGGCCAGCGAGGTGTGGCGCCTGCCGGTGGGACAGTTTGTGCCCGACCCCAACGCCACC
GGTTATTGAGGAGAGAATTCCATGAAGTCGGTTCTCCTTTATCTGCACGGTTTCAACTCCTCACCGGGCT
CCGCCAAGGCGCAGCAGATGGCTGCCTGGGTGGCCGCCAATCGGCCCGACATCGAGGTGATCATTCCGGC
TCAGCCCAATACGCCGGCGGCGGCTTGGGCGGCTATCGAACAGACCATTGCCGATCTGCAGCGTCGTTAT
GGTAGTGAATTCAAGCTCGGCGCCGTGGGTAGTTCCCTTGGTGGCTTTATGGCGACCCGGGTGAGCGAGC
TGTACGGCTGCCGCGCCGCGCTGATCAATCCGGCGGTGCGCCCGCACGAGCTGCTCTGTGACTACCTTGG
CCCCCAGACCAATCCCTATACGGGTGAGCAGTATGTACTGCTGCCTGAACATATGGATGAGCTGCGGGCA
CTGGCGGTACCGGTGACGGCGCCGGAGCGACTCTGGATCCTGCAACAGCAGGGAGATGAGGTGCTCGACT
ATCGCAAGGCGCTGGACGAATACCGCTTTGCCCGTCTCTCGCTGGAGTGCGGCGGCGACCACGCCTTTGT
GGCGTTCGAGCGCTACCCCGCCCAGATTATCCGTTTCCTCGGGTTATAGTCAGGTTTGAGTCAATAAGGG
CGGTCTCGCCCTGCTTGTTCTTCTCCAGATCAACCTTTCGGTGCGGGGAGTTTGTTCTACCCCCGCCTAG
CAGTTAAGATTCCCTCCAAACGTCAATTCGCAAGGCCCTCCATGTCTACTCAATACAATGCGGATGCCAT

TGAGGTCCTCAATGGCCTTGAGCCGGTGCGTCGCCGCCCCGGCATGTATACCGACACCACCCGGCCCAAT
CACCTCGGCCAGGAGGTCATCGACAACAGCGTCGATGAAGCGCTGGCAGGTCACGCCCGCAGTCTGGAGG
TGATCCTGCATGAAGACCAGTCCCTCGAAGTGATCGACGACGGCCGCGGCATGCCGGTCGATATCCACCC
GGAAGAGGGGGTCAGCGGGGTGGAGCTGATCCTCTGCAAGCTGCACGCGGGTGGCAAGTTCTCCAACAAG
AACTACCAGTTCTCCGGCGGTCTACATGGGGTGGGTATTTCGGTCGTAAACGCCCTCTCCGATCGGGTCG
AGGTGACGGTGCGCCGCGACGGTCAGGTCTACGACATCGCCTTCGAGCGTGGCGAGAAGGTGCAGGAGCT
GACCATCACCGGTACCTGCGGTCGTCGCAACACCGGCACCCGAGTGCGCTTCTGGCCGCAGGCGAGCTAT
TTCGATTCGCCCAAGTTCTCGGTCACCAAGCTGGAGCACCTGCTCAAAGCCAAGGCTGTGCTCTGCCCCG
GCCTCACCATCAAGTTCCTCGACAAGAACACCGGCATCAAGCTGGAGTGGTGCTACGAAGATGGCCTCAA
GGATTATCTGGTCGACGCGGTCAAGCAGTTCACCTGCCTGCCGGAGCAGCCTTTTATCGGCTCGTTCAGC
TCCGAACAATCGGCGGTGGACTGGGCGCTCTGCTGGCTGCCGGAAGGGGGCGAATGCCTCGCCGAATCCT
ATGTGAACCTGATACCCACTGCGCAGGGCGGTACTCACGTCAACGGTCTGCGTCAGGGGCTGCTCGACGC
CATGCGCGAGTTCTGCGAGTTCCGCAACCTGCTGCCGCGGGGGGTCAAGCTGACCCCGGAAGATATCTGG
GATCGCTGTGCCTACATCCTCTCCATCAAGATGCAGGATCCACAGTTCGCCGGTCAGACCAAGGAGCGCC
TCTCCTCCCGCCAGAGCTCGGCGTTTGTCTCCGGCGTAGTGAAGGATGCCTTCAGCCTCTATCTGAACAG
CAACACCGAGCTGGCAGAGCAGATCGCCGAGATCTGTATCTCCAGCGCCCAGCGCCGGATGCGCGCCGCC
AAGACGGTGGTGCGCAAGAAGATAACCCAAGGCCCGGCGCTGCCAGGCAAGTTGACTGACTGTAACTGCG
CCGACCCCATGCAGGGGGAGCTGTTCCTGGTGGAGGGTGACTCCGCGGGCGGCAGCGCCAAGCAGGCGCG
GGATCGCGAGTTTCAGGCCATCATGCCGCTGCGCGGCAAGATACTGAACACCTGGGAGGTGGAGGCTGGT
CAGGTGCTCGCCTCACAGGAAGTGCACGATATCTCGGTCGCCATCGGGCTCGACCCCGACTCCGAGGATC
TCAGCGGCCTGCGCTACGGCAAGGTGTGCATCCTCGCGGATGCGGACTCGGACGGTCTGCACATCGCCAC
CCTGCTCTGCGCCCTGTTCGTGAAGCACTTCCGCACGCTGGTGGAGAAGGGCCACGTCTATGTCGCCATG
CCGCCGCTCTATCGGATCGATATCGGTAAAGAGGTCTACTACGCCCTCGACGAGGACGAGAAGAACGGCG
TGCTGCAGCGGGTCGAAGCCGAGAAGAAGAAGGGCAAGGTGATGGTGACCCGATTCAAGGGTTTGGGCGA
AATGAACCCGAAACAGCTGCGGGAAACCACCATGGATCCCAACACTCGCCGTCTGGTGCAGCTCACCATG
GAGCCGGCAGGAGAGAGTGAAGGGGATGCGGCTGACGAGACCCTGCAGCTGATGGACATGCTGCTGGCCA
AGAAGCGGGCGGGTGATCGCCGTGAATGGCTCGAAGAGAAGGGCAACCTCGCCATCATCTAAGCCGTCGG
TCACCTGCCGACCCGCATTTCAGGTGTGCCCTCCTGGGGCGCGCCATAGCAAGAGAAAAACATATGAGTG
ATGCTATCGAGCTCAGTCTGGACGGGGTAGAGCGCCAGCCAATGCGCACCTTTACCGAACAGGCTTACTT
GAACTACTCCATGTACGTCATCATGGATCGGGCCTTGCCGCATATCGGCGACGGCCTCAAACCGGTGCAG
CGGCGCATCATCTACGCCATGAGCGAGCTGGGGTTGTCGGCGCTCTCCAAGCACAAGAAGTCTGCCCGTA
CTGTGGGTGACGTGCTGGGTAAGTACCACCCCCACGGCGACAGCGCCTGTTACGAAGCCATGGTGCTGAT
GGCCCAGCCCTTCTCCTACCGCTACCCGCTGGTGGATGGTCAGGGCAACTGGGGGGCGCCGGACGATCCC
AAATCCTTCGCCGCCATGCGTTATACCGAGGCGCGGCTGTCGCGCTTCTCCGAGCTGCTGCTCTCCGAGC
TGGGGCAAGGCACGGTGGAGTGGACGCCGAACTTCGACGGCACCATGAAGGAGCCGGTGGTGCTGCCCGC
CCGCCTGCCGCACATCCTGCTCAACGGGGTGACCGGTATCGCGGTGGGCATGGCGACCGACATCCCGCCC
CACAACGCGCGGGAAGTGGCCTACGCCTGTGCCGAGCTGCTCGACAACCCCAATGCGGGGCTCGATGTGT
TGATGCAGCACATTCAGGGGCCGGACTACCCGACCAACGCCGAGATCATCACCCCGCGCGACGATATCCG
CAAAATCTACGAGACCGGCAAGGGCTCCATCAAGCAGCGGGCGGTCTGGAGCGAGGAGGATGGCGATATC
GTCATCACAGCGCTGCCCCATCAGGCCTCCGGCGCCAAGATCATGGAGCAGATCGCTGCCCAGATGGTGG
CGAAAAAGCTGCCGATGGTTACCGACCTGCGCGATGAATCGGACCACGAGAACCCGACCCGTCTGGTGAT
CATCCCCCGTTCCAACCGGGTGGATGTGGAGGGGCTGATGGCCCACCTGTTCGCCACCACGGATCTGGAG
AAGAACTACCGGGTCAACCTCAACATCCTTGGCCTCGACAACCGGCCGCAGGTGAAGTGCCTCAAGACCA
TCCTCAGCGAGTGGATCCAGTTCCGCCGCGAGACAGTGCGTCGCCGTCTGCAGTTCCGCCTCGACAAGGT
GCTGGCTCGCCTGCACATCCTCGAAGGCTTGCTGATCGCCTACCTCAACATCGACGAGGTGATCGAGATC
ATCCGCACCGAGGATGAGCCGGGCAAGGTGATGGTGGAGCGCTTCAACATTACCGAGACCCAGGCCGAAG
CGATCCTCGAGCTGAAACTGCGCCATCTGGCCAAGCTCGAAGAGTTCAAGCTGCGCGCCGAACAGAGCGA
ACTGGCCGAAGAGCGCGACAAGCTGGAGCTGATCTTGGGCTCCGAGCGCCGCCTCAACACCCTGCTCAAG
AAAGAGCTGCTGGCCGATGCCGAGAAGTACGGCGACGATCGACGCACCCCGCTGGTGGAGCGTGCCACCG
CCGTGGCGCTGACCGAGAAAGAGCTGGTGCCCAGCGAACCGGTCACCGTCATCCTCTCCGACAAGGGGTG
GGTGCGCGCCGCCAAGGGCCACGACGTGGATGTCGAGGGGCTTTCCTACAAGGCGGGGGACAGCTATCTG
GCCCACGCCTGTGGCCGCAGCAACCAGCAGGCGGTGTTCCTCTCCACGTTGGGCCGAACTTACGCCCTCG
AATCTCACACCTTGCCATCGGCGCGAAGTCAGGGCGAACCGCTCACCGGCCGCTTTGCGCTGGGGGCTGG
GGAAGAGGTGCGCCATCTGGTGATGGGCAACGAGGGCGAGCCGTATCTGATCGCCAGCGACGCCGGTTAC
GGCTTTGTCTGCACCTATGACGACCTGATCGGCAAGAACAAGAACGGCAAGGCGCTGCTCACCGTGCCGG
AAGGGGGTCAGGTGATGGCACCCCAGAAGATCGGCTCGCCAGAGACCGATCTCTGCATGGCCATCTCCAA
CGAAGGGCGCATGCTGCTGTTCCCGCTCAACACCCTGCCGGTACTCGGCAAGGGCAAGGGTATCAAGCTG
ATCAGCATTCCGTCGGCTCGGGTCAAGGCGCGGGAGGAGTTTGTGGTGATGGCCGCCGTCATCCCGCAGG
GTCAGTCGGTCACCCTGTTTGCCGGCAAGCGCAAGCTGACCTTGAAACCGTCTGATCTCGACTACTACCG
CGGCGAGCGCGGCCGTCGCGGCGCCAAGCTGCCCCGCGGTTTGCAGCGGGTCGATCGCATCGAGATTGAA
CCCGCCACTGGAACCGAACCGGTAGCTGGGGAAAATCAGGAAGGGTAATTAATATAGAAGGGCGCGAAAG

CGCCCTTTCTATAACTGGGTTAATATGTTCACCAGTACAGGAGCAAAGGGTTCCAGCTTACCATCTTGTG
GTTGATTCAGTTCATTAATTAGTTCATTCAAGAGCTCTCTATCGTCCTCAATGGATAGCGAATCAGGGTT
ACCATAGTCTTTCCAGATCAAGGTATTGACTAGCTGTTTGCGTTTCTCGATCAAACCCTTGTTGTTTTCT
TCTGTATCACCAAGATTGAGTACTCGTATTGCCTCGCGAGCCCTATCAGTTACACCTTCAACACGTCCAC
TGAGTTTGAAACGGAATTCATTTTCACACTCAGACATAAATGGGGTAAGTGGTAAGTGTTGTGAGTCATG
AGAGCTATCACACTGTTTTGGCGTTGTGCAGCTTGCCACTATGTTACTAAAGTCTAGTGAACGTTCGGGA
GCCAGTCTTCGAGCTACTACATGCTCATTCATTGTATCTTGATTGGCACCACTTATTGCTTGGCAACAAT
ATGCACAGAGATAAAATTGTTCTCTAGAGCAACTTTGACGAATATCCTGACGCTCTACATCAGACAAATC
TTGATAGCGAGCCAGTGGGTTAGTTTTTTTGAATGCTGTGAGTGATACAGGCTCTGGCCCCTTGTTAATT
TTTTTCACGACGCAATGCCTCTTTGCGTAATAACAGACGAGCCTTGGTTAATTCCAAGTTGTCTGCCGGA
AGCTCCGCAGCCAATTTATCAAAAAGTATATTGGCATCATCCAGTTTTTTGTCGTGAACTAGATCCATAA
AATCATTTAGCTGTTTAGATATAGCTTCATTACGGACATAAGTATCCATTACATTCAGTAGGACAGAGTT
AGCGTCTTGCCCGTACTGTGATGGTACTTGAATTACGTTTCCGTTATCAAGAGAATAAACAAGAACATCT
TTACAATCGCTAATCACCAGGGGAGAATGCGTAGTCAAAATAAACTGACAATTAGGGAATGCTCTAGTTA
GTCTAGAGATAATAGTGCGCGCCCACTGGGGGTGTAAATGCATATCTATTTCGTCGATCAGTACAACACC
ATCACCATGTAAAGGATTTTCTAGGTTAGGGTTCATCATTGCTAGACGACGAGCAATATCTCCCACCAGC
GCCATTAAAGATTTCTCACCTTGGGATAATTGTAAAACATTGAGGGTTTGACCCTCTTTATCTATGGACA
TATGCAAGCGTGGCTTCCGACGAATTCGGATATTGCTGAATCCTGGCATAAATGATTCGATTGCAGACCG
AACTGCATTCAGTTGAGGATCTTTAGTTTGAGTCTCAACGTGCTTCATAACATTCATCATTTTTGTGATT
TGTGAGAACTCTTTAGTCAGGGCCTCAATATCAAAATCTTTAATCTGATCTAGACGAGATGAAGCTGACT
CTGCAATCTCCATCATATCATCTATTAGTTGAGTGTTTTTTGAGTAAAAGTCAAGAAAAACATCTCCTTC
TCTTTCATTTTCAATATCTTCTCGCTCTCGAAACCATTCAAAAAATCTACGGAAATCTACCCCTTGATTT
AAAGAGTTATCATATCCATCAAGTTGTTGGAATGTGTGCCTCTCTTTAATTTTTAGAGGAATATCCAATA
CAATCCGTTCGACAGGATAGAAGGCTATTAGAGGTAGACTAGCTGAGTCATTTTCTGCCAAACGCACCCG
GTAATTATCCGCCAGCCTGCTGGCCTCATTAAGTTGAGATTGCCGATTGCCTTTGCGGCCTTTGCGAGTT
TTGGTTAGTAACCAACTAAATTCAGTCCCTTGATCTTGTAAAATAATCTCACTTGTAGTTGTAGGGGTAC
CATTTAAAATGACTTCTTCTGGTAGCGGACTACCACTGCCCTTATCGCTACGAAGACGGGCGACAAACCA
GCTTAAAGACGTTGCCAGTGACCTCAGGATCGAGGTTTTTCCTGCTCCATTATTTCCGATCAGAACGGTT
ACATTTGATGTAACTGTATCGGTGGGAGCAAAATTGATGTCTAGGGATTGAAAACGCCCCAGATTTTCAA
GTTTGAGTTGTCTGATTTCCATTCAATGTTCCTGTTTTAGTACATCATTGGCCTTACTTAACAACTGACC
TTCTAGTCACTGTGCCAGTTAGGCCTTGTTGGATTGATAGTGCAAGTAACTAATTAGTGACATATGGTCA
GCTATTGTCTACGTCGTAGCTATTGGATTGGATTGGGTTGGTGTCATTCTAGCGTCTACAACGAACCTCA
TAAACAGTTAAAACACTTTGTAGTGATATAGTTACAAGCTTTACGTATCCGTTCACAGCCCTGCCTCTCC
AATAATTTAGCCGACGAGTTTTCTAGGGGGGGCTCGGCTTCTGCAATGCCTTGGAGCTCTGGGACTCCCA
CCTTTTTGACACGGATGCCGCTTCTTCCAAGAAAGTTGGGCCGTAGGTCTGATTAGCGAAGCGTAATCAG
ACGTTCGCGAAAACAGGTTGCCGTAATTCTGCCGGTGTTCCAGCTGATCTCTTCTCTATGTCCATCGCTA
CCGGGATTGCGGTGCGGACGATTACGCTTCGCTAATCGACCCTACGCTTTCCTGCTTGATTTAACGTTCT
GCGTCTGGTCGGTGGCTTCTGTTGTGATGTTTTGTTTCCCCTCATTCCCGACCCTTCTCCCCTTATCTAT
GGCAAAGAGTGGGAGAAGGGAGCAGAGATCGCAGCCATTGATAGTGACCGTGGTATGGCGTGATAGCTAA
GGGTGACAGCTATGGCTACAGGTTGGCAGGCACTGGTTCCCTCTCCCCTTGGGAGAGGGTCAGGGTGAGG
GGAGCCGACGATAGGGGTAAACACTATAGCAAGCGCAAGGTTGCCAACCTGAAAAGAACCATCCAACCCG
CCAAAAGCGAGGGCCCCGCCATCCATGATGGCGGGGCCCTTTGCATGATCAGCCGCGTTGCTGATTTAAC
CGTTCGCGCTGGCAGGGGCCACTGAAATGGTTGGCCCGAACCCGATTAGACAGGAATGGCTGTCGCCGCC
GGTGGCGGGGTGATTTTCGAGGCGATCAGCTGGCGCAGGCGGGGCACCAGCAGCAGCAACACCAAGCCGC
CCGCCAGCGAGCCCCAGCCGAGCATATTGAATACCGAGCTGTAACCCGGGTTGGTGGCGACCGGGGTGCT
GCCGATACTTTGCGGCATGGCGCTGGAGACATAGCCCGCCAGCACGGAGGCAACGCCGGTCACCATCATC
CAGCAACCCATCAGCAGGCCTTGCAGGCTGGCGGGGGCCAGCTTGCCAATCATGGCGTAACCGATGGGGG
AGATAAGCAGCTCGCCAAAGCTCTGCAGCACATAGCTGATGGCGATCCACTTGAAGGCGACCAGCCCGTC
GGCCTCCGCCAGATGGATGCCGAGCGGCAGCACCAGCATGCCGAGCCCCATGCAGAACAGGGAGGCGGCA
AATTGGGAGGGGATGTCGATATTCCACCCCTGCTCGCGCAGCCGCTTGAACAGCAGCGCCATCAGGGGGC
CACCGATGACGATGACGAGGGTGTTGATGTTCTGGATCCACTGGGGCGCGACCCGCAGCCCGAACACATT
GAGGTTGATGTTGTGCTCGGAGAAGAGCATCAGCCCCATGGGCGCCAGCTGGTAGAGCGACCAGAAGATA
AGGGAGCCAAGGGCGAGGATCAGGTAGGCCACCATGCGGCTGCGCTCGGCGGCGTCCGGATGGCGGGCGG
TCATCACGCAGAGCAGCACCAGCACCACGGCGCCCAAGATCAGCACGAAGCTGCCGCTGAAGCTGGCATG
GGTCAGCAGCAGGCGCAGCGCCGGCACCAGCATGGCCAGAATGGCGATCCCGGCCAGCATGCGGCGGTAG
AAGCTGCTCCCTTTGACATGGCGCAGCGGGGTGCTGATATCCGCCAGAATCGGCCAGCAGGCGAAGGTGG
CGATGATGGCAATGATGTTGCCCAGCGTTGCAAACAGGAACAGGGTGCGGTAATCCTCGGTGAGCTGGAA
GTAGCCCGCCACCGTAAAGCCGATAAAGAAGCCCAGATTCATCCCGGCGTAGTTCCACAGGAAGGCGCTC
TCGCGGCGGTGATCATCCGGGGCGAAGCGCTGGGTCAGCATCATGTTGAGGCAGGTGACGTTGAGGCCGC
TGCCGGTAAGGAACATGGCGAGCCCCCAGTAGAGGCCGGTGATCCCCATCTCGGCGATGAAGTAGCAGCC
GATCACCTGCAGCACCATCCCCATCACGAACAGGTTGCGGTTGCTGAGGTAGCGCCCGCCGAGATAACCG

CCAAACAGGTGCAGGCCATAGTTGAAGGCCCCGAAGACCCCCATCATGGCGTTGGCCTGGGATTCACTGA
AGCCGAGCCGCTTGGTGGCGTAGAGCACCAGCGTGGAGTAGAGCACGGCAAACCCCAGAGTCGAGAAGGT
CTGGATAAAGAACAGCGCCCCGGAGCCGGGGGGAATGGTGGGGTGTTGATTCGAAGCGCTCACGGTCTGT
CTCCTGTGAGAGAAGAAAATCATAACTCGTCCTGAGAGCGTACCGGTCTGCTGCCGGCTAGCCTTGTGCG
CTTCCATGCTGGTCTGAAGGCCGGTCTGTCATTTAGCGGCCAAGCGGTACAAATAGCCATACCTGAAATT
TAATCCGGCGCGATTAATTGCTTTTTATTGATCTATTCCGGTTTTTTATGCGGTTTTGGCCGCTGGAATG
CTGGCGCTGGCGGGGTGGTCATCAGAATGGTTGGGTTTGAATGGAGCCCATTTTGGGCTGGGCTGATTTG
ATAGTGAGATCGCCATCTCGCTTTTGGCTGCTCTTTCGCCATTGCAGTCGGCATGTTGAGTATCAGCTTA
TTTATTCAGTTTCACTGTTTGAGTGAATAAATATTGAAAATGAGGGCTTGCTCAGAACCTGATGGTGTGG
GGAATTTTTGAGCGTTTCATCGCAGGGGATAATATATATTCTTGTTGTAATAACGTGGTAAAGATTAGTT
TCGATAGATAGGTGACTTAAGATAGTCTGATAATATATCAATCAGAGCATTTTGGGGCTGGGAGTCGTGA
TGCTATCTTTTAGGAAAATATATTTTCTTTGCATGCTTTGAGTATTTTTCTCAGAGATAAAATCTGATTA
TGGTGAGAACAGCTCATAGTCCGGTGTGATAATAGAACCTTTCCGATAGGTCATATAGACAATGGTTCCT
TCCTCATCGACCATGGCATATGGCCATTTTCCGTTGGCTCCGTACATGGCCACATAGAGTGGGTTGAAAT
AATCTTGAGTCTGCCCAGCTGGGTCATACGGGAGTCGAACATGATAGATTGGATATTTACCATCGTTACA
TAACTCGACTCTCTTTAATATAACGCCTCGTTCCAGTAATACCTGTCTGGTCGGATGTTGCCAATTTTCC
AAATCTACATTTTTACAATTATTGGCGACTTCTCGATAAAGGCGGGTATCTGACAGTAGCGGAGTATTGG
CGCTGATAAGCATTAAAAGTGGCAGCAGCATAAAAATCTGACCTGTATTATGACCGGTTTATTCAGCCCT
GAATCAGGGGCGCAGTATAGCTGCGAACAAGGTTTGAATGAAGCCATCTGAACTGGTTGCATCCCGATTT
GTTACGTCAATCGTGGGAGTTGATGAGGTAGATAGAACAACGCCCGGCGGGCCGGGTGTTGGGTTATCCC
ACATTGGGGGCAACAACAGAGGCGGCGAAGCGCGCCCTAGAAGTTGTGGTAGAGCGGCCAGCCAAGCAGC
GGGTGCTTGAGACCGGCGCGCATCTCGATGTTGTCGAGCTTCTCCGGCGAGGCGTTCTCTTTTTGCAGCA
GCTGGTACTGGCGCACAAAGCCGAGGGTGTGCAGCAGGTTCGGCACAAAGCCCGGGCTCAGCTCCTGACG
CAGGGCGGCGGCCATGTCGGCGGGCAGCGCATTCAGCATCAGGGCGAGCTGGGCGGCGAGATAGTCGGTG
TCATACCAGCCCTCGTCGAGCTCAATCTCCCGCGCCAGTTTGCGCTTGAGATCCTGCTTCACCTTGAGCG
GGGCGGCGCCACTGCCCTTGCCGCAGAGGATGCGGTAGAGCAGCGCTTCGGCCAGCTTGTCCTCCATGGT
GGGGTGGTCGCTCTCGCAGGCGAAGAAGTGCTGGTCGATGAGTTCCATCAGGCGGCTCTTGTTGTGCTCG
GTCAGCGCCTTCTCGTACTCCTGCCAACCTTGCCACTCCTCCACGTCGGGCGGGCTCGAGATCTTGTCCA
AGAGCCGCGCATCCACGTCACCGTAGAGGCTGTTGCCAGCCTGCTTGGGGCTGACGGTAATGAGCATCGA
CCAGCCCTTCTGGAACGGCTTGATGGGGCCTTCCGGCGCCTGCAGCAGCGCCAGCGCCTTGGCCGGATCC
CGCGCCGAGAGCTCCTGCAGGCCGAGGCTCACCACGCCGATGACGTCGTGGGCGGCCTGCTCCAGCAGGT
GCATCTTGTACTTGTTGTAGTACTTGTCGGCGAATTTGAGGCTCATCAGCACCGCCTTGCCCTTGATGGC
GGCCAGCTGTTGCTCGGTCAAACGTTCATTGTCGCGGCCAAAGCGCAGCACCTGGGTCAGAAAGGGGCCC
TGGTTGGCATCGCGAATGACTAACTGCATGAATGGGCTCCTTAATCCAGAAAGCTGAACATGTCGTCGAC
GTCGCCGTACTCGTCTTCCTCGACCTTGTTCTTCTCTTTGGCCTGCATCACCAGCTCGGCGGCAAACTTG
TTGATGATGCCGTCCCAAGTGGAGTGCTCGTTGCGCAGCAGCGCCTTGATGGCTTTCTCCACCTCGGGGG
CCAGCTCGCGAATGAGCATCAACAGGCTGCGCGGGTCGTCCAGCGCGTAGTCGTGGACATCCTCGCTCTG
CTGGCGCTCGCTGTAGCGCAGGGACTCTTCGAGATCTTCCTCTTCGGCGTGCAGCACGTCGCGATAGCCT
TCGTCCTCTTCATAGCGATCGCCGCGATAGAACTCGATAAAGGGGATGGCGAGGTAGAAGAGACCGGCCG
GATCTTCGGCCACGCACTCGAGGATCTCACGCTGCTTGGCTTCGCTATCCTGGGTGCGGATCAGGTAGTT
GAGGAAATCGAAGGTGTGCTGTTGCAGCTCGTCGGCGTTGTTCTTGATCGCCTCTTCCCAATAGAGCGGC
TGCTGGTAGACGATGTCGCGGATCCGCTCCGGGGTCATCAGCTCGGAGATGATGGAGGGGAAGGAGATAT
CGTGCTGGCCGTTAATCTGGGTCAGGGTAACGATATCAATGTTTTCAATCACTTCTGCCAGCTGGTCGTC
AGACATGGTCTTGGCGATCAGCTCGAACTTCTCGCTGGCCTGCTTCGGCTCGATAACGGCCAGCTCTTTG
ATCTCGATGGCGGTAATTTCTATCAGACTGTTGCTCATGCCTTGCCCCAATTAGAAACGGACTTCGTCGT
ACATATCCTGATCGCCGTCGTCATCGCCATAGCGGTCACTGCCGCCGTGGTAGTCGGCGTCGGCATCTTC
CCCGTCTTCATCTTCTTCGTCGCGCAGCTCATAGTCGTCATCGCTGCGGTGGCTACCGCGCGGTAGCGGG
TTGTCGAGCAGGAACAGCACGGCGCAGGCTTCCATCAGCTGATCTTCGCTCTGGGCCCGCACCGCCTTGA
CCAGCGGCAGGGCGGCGTTGTTGAAGCCGACGCTCACCTTGAACTCGTCCCAGGAGGGCTGCTCGCCGCT
GGCGAGCCACTTGTTCCACTGCGACTTGGCCTTGGCCGGGAACTGCACCCGGCCATAGAGGGCCAGCGGC
AGATACTCGGGCATAAACTCCAGCATGCTCATGTCGGCCAGCAGCAGGCTCTTCATCTGGTCGGTGAACT
GTTCCAGCCCCTTGGGGTCGTCCTGATCCCGGTAGGATTCGATGTTTTCGCTGGCATCGCTTTGCAGCGA
CTTGAGCCGCGCCTGGTCCCATCTCTTTTGCATAGTTGCTCTTTCTGCCTGCTGTTTCAGGGGTAGAACT
GATCCCACATCTCCTGCATGGCGGAGGCGGGGTCGGTCACGATGACGTTGTTGAAATCCTTGATAAAGGC
GGCCCGCTGTTTCGGATCGGACAGCACTTCATAGGCCTGCTTTACCTGGTGCAGCTTGATGGCTGCCTGC
TCTTTCTCTTCTTCACTGGCCCCGTGCAGCTTGTCGGGGTGGTACTGGTTCGACAGCCGACGATAGGCTT
TCTTGATGTCGTTTTCGCTGGCGTTGGACTTGACGCCCAGCACCCGAAAGTAGTTGTTCATGGCAGATCC
TGCATCACTGGCATCATAGGATTCACGCTGCGAGTGCCTGATGGTGGGGATTGGCAAACCAGGCACGGCA
AAAACCCGTCATTTTAACGGAAACAGCCCGCCAACGCGATGAGTTGGGCGATCAATTAACGGGCGAGATG
AAGAGGTGTCAGTTGAGCGTCTATTGTTGCCAGTCGGTCTGGCCAGTGCAAGGGGCGAGCACGGGAAGGC
GATGAAAAAATGGTCAGGGGCGAGTGTGGGGTGAGATGGCGGCGGCAGCGCAAATCGGGCTGCCGCCGCA

GGGGTTACTCCTCCCAGAAGGAGGCGGAGGTGTGGTAGATCATCTTCTTGCTCATCCCGCCATCGACCAC
CAGATTGGCGCCGTTGATAAAGTCGTTCTGGGGATCCGCCAGAAAGAGGCAGGCGCGGGTGATGTCGTCG
GGGCGCCCGACCCGGCCGCTCGGGTGCTGGCGGTGATCCGCCTCGGTCAGGGTGTGGTAATCCTCCGCCT
GGATCCAGCCCGGACTGATGGCATTGACCGTGATGGGGTAGTTGGCCAGCGAGTTGACGAGGGACTGGGT
GAGGGCGACCAGCGCCCCCTTGGAGGCACCATAGGCATCCCAACCCGGTTCGTTCTGCTGCCAGCGGGTG
GAGGCGATATTGATGATGCGGCCATAGGGCAACCCGCGGTTGGCCTGCACAAAGGCGCGGGCGCAGAGCA
TGGCGCCGCGCACATTGACCGTCATCACCTGATCAAACTGGGCTGCGCTCAGCTCCTCGAAGCGGGTGTG
GAACTTGCTGATGGCGCCGTTGTTGATCAGCAGATGGACGCCGCCATGCTGCGCGCGCACTTCGGCAAAG
AGCCCCACGATAGCCTGCTCATCGCGCAAGTCGAGCGGGTAAAAGTGGATCTCGGGATGGTCGAAAGCGT
GAGGAGCCAGATCGGTGGCGATCACCTGATAACCCGCCTCGGCCAGCCCGATACAGAGTTGGCGACCAAT
CCCGCCAGCGGCGCCGGTGACCAGTGCGATTTGGGGTTGGTTGGTCAGCTCGTTCATTGCGCCGCTCCAT
TGCGTGCCCGCAGCACCTTGATGAGGGCCGACACCTCCTGCTGGCCAAACCCTTGCGCCACCCCTTCCTC
AAACAGATCGGCGGCAAAACGCGGGAAGCGGCTGTCGATCCCGGCATCGGTTGCGTGACCGGCGAGGCGG
CTGATGGCGGCGCTCCAGGTTTTCAGGCTGGCGTAGGGGTGATCGAAATCACCGCTCGCCAGCGTCTTGC
CAAGGTGGGCCACATCGCCACCGAGAATGGGCGAGAGCTCGCTGAACTGGGCGCACACCTTGGCCACCGG
CAACCCTTCCGCCTCGCAGATCAGCGCGCCGTCCAGAGCACCGAGCAGACCGCCAGCAAAGTAGGCGGCG
ACGGCACAATCTTGCGCCGCGGCGGCGCCCACCGCCTCGGCCACATGGTCGAGGGTGGGGGCGAGCAGGC
GCAGCACCGGCTCGGCCCCGCTAAAGGCGGCGCTGGCCCCCGAGCAGATGATGCTGGCATCAGTGGTGCC
GATCTGATCCGGGAAGCAGAGGATGGCGCCATCGAGATAACGGGCGCCGCGAGCGTGACTCCAGCTCTCC
AGCGCTCTGGCCCCCTGTGGGCTGCCGGTGGAGAGTTGCACCAGCAGCTTGCCCGCCACCGCATCGCTCG
CCTCGTCCAGCAACAGCTGGCTGACGGCGTAGTTATCGACGCAGACAAGGGTGATATCGCTTGCGGCAAT
AGCCGCGCCAACCGAGGGGGCGAGATTCGCGCCCAGCGCTTGCAGCGGGGCCGCCTTGGCGGCGCTGCGA
TTCCAGACGGTGACCGGATGGCTCGCCTTGAGCAGGGTGGTGGCGAGGGCGGAGCCCATGGCTCCTAGGC
CGATGACAGAGAGAGGGCGCATGATGGCTCCTGGCTGTGTTGATAGACGCTGATATAGGGAATTGAGATG
CCTTGTGGCAGGTAAGCCACTATATTTGCCCAGTGTCGCCGTGAAAAATGGCTTTTATGACTGTCTGGTA
TAGGAGAATTCTATGGATGTGCGAACCCTGCGTGCGTTTGTGGCTGTCTTCGAGGAGCGCAACATCACGG
CGGCGGCCACCCATCTTTTCATTACCCAGCCCACCCTCTCCACCACCATCAAGACGCTGGAGGATGAGCT
GGGGGTGACCCTGTTTGTGCGGCAGGCGCGAGGGGTGGAGGGGACTGAAGCTGCCCACCGGCTCTATCCC
CGCGCCAAGCGGCTGATCGCCGACACCGACTCCCTCGCCCGCGAGTTTCGTGAAACCTCCCCCTGCCTTG
AGCTGACGGTGGGGATTGAGGCAGACCTTGGCGATGGCGTGGTGGGGACGCTGGTGCAAGGGGCGCTCGC
CCACGAGGAGATCCTGCTGACTCTGGAGGAGGGCTGCTGTGGCGATGTGCGGCTGGGGTGTGAATCCCTG
CGCTGTGGTGACGAGCTCTTTATCCCCCTGCTGGAGGAGGAGTTTGTACTGGTGCTGCCGCCGGATCACC
CGCTGGCGGGCAAGGGGGCGCTCACCCCGACAGCGTTGGCGGATGAACCCTGGGTCACCTGCCCGCGCCA
CGACTCCCACCTGCGGCTGATCGCTATTCTCGGCAATCAGGCGCCGCTCTACCCACGGCGGGCGGGGTCG
CTGCGACTGGCGGCGACCATGGTGGCCGCCGGGGCGGGGATTGCCTGGTTGCCCCGTTCGCTTTGTCCGC
CCGGTTGTACGGTCGCCGAGCTGGCGGCCTCTTCCTTCAAGCGGCGGGTGGGGCTCTGCTATGCCCCCGA
TGCGTTGCGATCGGTGGCGGTGACCGCCCTGCTGGAGCAGGTGCGCGGGATGCAGCTCCCCGCCTGATGT
CGGCATAAAAATGTCGGGCATAAAAAAGGGCCATCAAGTTGGGATGGCCCTGTTGCAGGCAACGAAGGTG
CGTGGCTAGAGCGACGCCCACCAGAGCCGCAGCTGCAAGCCCCACTTCGAGCTCCAGCCGGTGGTGGTGC
TCGCAAGCGTCCCATCCTTGATAACGAGGAAAGTCGGGGTGACCCCCACCTGCCACTGGGCTGCCAGCGC
TCCCGACTCGTCGTTGTGGGTCGGGAAGGTGAGCCCCTTCTTGCTCATCCCATTGGCTAACTGCTCCGAA
TTGCCGGAGCGCAGCGCCACCGTCAGCACGTTTTCCCCCTCTTGCCAGAGCTGCTGCACCGCCGGGGTGG
TGAAACGGCAGACACCGCACCAGCTGGCCCAGTAGTAGACCAGCAGCGGATTGCCCTGACTTAAGGTAGC
GAGATCCGTGCTGCTGCCATCCTGCAGGGTGACGGGGGCGCTCATCGCCCCCTCTGGCAGGGTCGGGCTG
CGCCACAGATCGAGCAGGGTCGAGATGACCAGCGCCATCAGCAGCAGCCAGAGCGCCTCCTTGCCCCAGC
GGCGCCAGCGGGGTTGAGGATAGGTGGTCATCCCTTACCCCTGCTTGGCCAGTTCGGCCTGCACCAGTTG
ATCCAGCTCCTCATAGGGGATGGCGCCCGGTACCAGCTGGTTGCCAATCAGGGTCGCTGGTGTGCCCTGT
ACCCCCAGAATGGTGCCGATGCGCAGGCTGTTGCGCAGCTCTTCGGTCGCCTTGGCGGCCGGTTTGAGCG
CGGTGTTGCCGGTGGCGGCCAGCGCCTTGTTGATGTCCGCCTCGGTCAACATCCCCTGCTTGCTCATCAG
CTTGTCGTGCAGGGTGAGGAAGCGGGCGGGATCCTGCTGCCACAGGGTCAGGCTGTATTGGGCCGCCTTG
GCGGAGGTTTGCCCCTTGAATGGCAGCAGCTTGATCACCAGCCCCAGATCGTCCGGATAGGCCTTGAGCA
GTTTGGTCAGCTGGGGATCGAACTGCTTGCAGTAGGGGCAGTTGTAGTCGGTGAACAGCACCAGCGTCAG
CTTGGGGTTCTTGGCGCCGAGGCGCGGGCTGGTGGCGCTGTTGAACAGCACATCCTTGTTGCCCTTGATG
AAGTTGCCAAGCTGCGCCTCCTGGGCTGCCGCGTTCTGTTTCTCCCAGGATTCGGCTGCCTCGTCGAGGA
TCTTGGGGTTGGCGATCAGGGTCTCGCGGATCAGCTCCTTGATACGCGCCTCCTGCTCCGGGGTGAAGGG
GGCAGCGTGCAGGACCGGAGCCAGCAGGGTTGCGGAAAGGGAGGCCAGCAGGGTCAGGGCGCTAAAAGTC
GGTTTCATGGTTGCTCCTTAAAATCGGGTGGTGCCAGCCTGAAGCAGGCCAGCCTTGTGCAATGTGGTCA
GCAGATCGTCCTTGTCGAGCAGGGGGGACAGCGTGACCCCCTGCGGCAACTCCGGGCCGTAAATCTGGTT
GAAAGGGACGGCCGCCGCGCCCCGCTTACGGAGGAAGGCGGCGATGGTGTCGCTTGGTTTGCTCCAGTCG
CCGCGCAGGGCCACCACATCGGGGGCCGAGAGGGCCTGTTGTACCTCATCGCGCAGCAACACGTTGTATT
TGTTGGCCTTGCAGGTAACGCACCAGTCGGCGGTGACATCGATAAAGACCCGCTTGTTCTGGGCCAGCGC

ATCGGCGATGGCGCGTTCGGAGAGCGGCTGCCAGACCACCTTGTCAACGCTGGCCGAGCCCTGCGCGCTA
AAGGCGCCGCTCAGCAGCAGGGGACTGCCAATCAGGGCGGCGAGGGCGATCGCCAGCGTGAAGCCGCGCA
TGCCGTAACGCCATATGATGCCAGCCAGCAGTGCCAGCAGCATGGCAACTATCAGCCAGCCGGTGGCATA
GCTGCCGAGGTGGTTGTCGAGCAGGCTGGTGAGCCAGAGGCTGGAGCCCAGCATCATCAGGCCGAGCAGG
ATGCGCAGATGGTTCATCCAGCGCCCTGGTTTCGGCAGCCAGAGGGCGAGTCTTGGCAGGGCCGCCACCA
GCAGCCAGGGCAGGCTCATGCCAATCCCCAATGCCGTGAAGATCAGCCAGAGCTGGCCGAGCGGTGCCGC
CAGCGCAAAGGCCACCGCAGTGCCGAGAAAGGGTGCCGAGCAGGGGGTCGCCAGCAGAGTGGCGAAGCTC
CCCTGCAGGAAGTGACCGCCGAGGCCGTTGCCGCCGCTGGTGGCGAGGCGGGTGCTGAGGGCGCTGGGCA
GGCGGATCTCGAACAGCCCCAACAGGTTGGCGCAAAACAGCAGGGTGACCAGCACCATCAAGCCGATAAA
ACCGGCACTCTGGAACTGGATGCCCCAGCCGACGGCACCTTGCGTGGCGCGTAGCAGGCTGCTCATGGCC
GCCAGCACCCAGAAGGAGGCGATGATCCCGGCGCTGGCGGCGAGGAACTGTTTGCGCACCGCTCCCTGTT
CACGCTTCTGATGTTGCAACACAGTTCCCAGCTTGAGCGCCAGCACCGGCAGCACGCAGGGCATCAGGTT
GAGGATCAGGCCGCCGAGCAGCGCTGCTCCCAGCAACCAGAGCTGCGAATGGCTCTGGTTGGGCAGTGCG
AGTGGCGCGCCGATCGTCCCCTTGCCCTGCCACGCCTGTTCGCCGCTGGTGAGCAGCAGGCCGAGGGATT
GGCCGCGCAGATCCGGCGCATCCCCCTGCCAACCATCGCTCACCGGCACCCGGGCGATCAGGGTGTTGCC
CTCGACCTCAAGCGCCGGTTTGCCAAACTCGGCCCCCTCCAGAGCGTCGATAAAGAGCGCTGGTGATTGC
CAGCCCTCTGCCCGCTCGGCGCGCAGTTGCAGCTGGTTGTGCTGATAACCCAGCTCGACCCGGGTTTCGG
CAGGCAATTGCTGGGGCAGGGTACTCATCGCTTTCGCCCAGGCAAAATCGAAGTCGGCCGGGGCCTGCCC
ATCCACCGCGAGGGTGAAGGGAAAGTCGGTCAGGATGCAGACATTGCTACAGGTGGAGAGGCGCAGAGTC
CCCTTGAGCGGTTGCCCGGCGGGGTAGTTGAGGCTAAGGGGGAAGGTCACGTCCCCCTGATAACCCTGGG
TGCTGAGCCCGGCTACCTCGAAGTGGCGTGGCACCGGCCAGCGCCACTGAAAGTCACCGACCGGCTCGTC
CCACAGGATCTGGGGGGCAATGCCCCCTTCGCCCGGGCTGTGCCAGTAGGTTTTCCAGCCTGACTCCAGC
TCGACATCGAGCAGGATGCGGGTCTGATCTGCCGTGCTGCGGTCGGCCTGCAGCCGTACCCGGGCGTGAT
CATTCTGTGGGCTGGTGAGCCAGCCCGTGTCGCTGGCCAATCCCATGGATTGCCACAGCAGACCGCCCAG
TAGCAGGGCCGCCTTGAAGAGATTCGACATCTTTTTGGTCTCGCAAAAATTGAGTGACAGCTAACGAAAG
AACGCTGCCGAACTCATTCCCGCCAGACGCAGAGGGTGAGGTGCAGACGCCGCTCGGGCGGCGAGATGAC
TCTGGGAGGGGGCAGTGGCGGTGTGTAGCGCCAAAGGGGGGCCAGCAGGGCGATCAGCACGGCCACGGCA
AAGAATAGCTGTTCGACGCAGAGCAGGGTGGCGGCGCTTAGCCACTTGCCGTTGAGCTGGCAGCTGGTGG
GTTGCGGGCTGGCCGGTTTGGCATCCTCTTGATCCGTTGCCGAGCCTGGAACGGGCAGTGCCTGACAGCT
GAGGGCGTGATCGAGCAGGGCAAGCCGGGCCAGCGGCTGGGCCGCACAATTGAGCACCAGCAGGCAGACC
AGCAGCAGGAGATGTTTCGCTACCCGTTGTTGCAACATCATGTTCGCCCCGTCCATCAAGATGGGGCCAT
CCTAACGACCAATGGCGGGTGGGGCAAGTCGGCGGCGTATTGATGAGCAGTACTGTGGGTGGCGCAACGG
GGAGGGTGGTCGGTTCTGTTTGGCGAACGCTTGTTCGCTAAAAGGGGGATCAGTATACTGCGCCGAGTTA
AAACTCGAGGTGTTCGATGCTCAAAATTGCCCGTGTGCTGCTGCTTGGTCTGCTGCTCATTTTCTGGTTC
CTGTTCGGCCTGCTGCTCTGTCTGGTTCGTCCGCGCCATCCCAACAACGTCTATGTCTTCGCCCGCATGT
ATCACGCGGTCTGCCCGCTGATCGGACTGCGAGTCAAGGTGGCGGTGGCCGACGAGGTCAAATCGGTCGG
CCCGGCGGTCTACGTCTGCAACCACCAGAGCAACTTCGACATCTTCACCGTGACCGGTGCGGTGCAGCCC
GGTGTGGTGGCGGTGGGCAAGAAGAGCCTGCTCTGGCTGCCGCTGTTCGGCCTTATCTTCTGGCTCTCCG
GCAACGTGCTGATCGACCGCTCCAACCGCTCCCGTGCCATCGGCACCATAGGTCAGGTGGTGGATCGCAT
CAAGAACCGCGGCACCTCCATCTGGATGTTCCCGGAAGGGACCCGCTCCCAGGGGCGTGGCCTGCTGCCG
TTCAAGGCGGGCGCTTTCCACACTGCGGTACAGGCTGAAGTCCCCGTGGTACCCATCGTCTGCTCCAGCT
ACTTCGGTCAGGTCGACCTCAATCGCTGGGACAACGGCGAAGTGCTGGTGGAAGTGATGGCGCCGGTCTG
CAGCAAGGAGCATGGTGCCAGCGGCATTCGCGATCTCTCCGACACCTGCCGCGCCCGGATGGAGGCCAAG
CTGGCCGAACTGGACAGCAAGCTGGGCCACAGCCACGCCGCCTGACGGATAGCGCAGACGAATCGACAAG
GGAGCCAGAGGCTCCCTTTTTTGTTGGTAAAAATCACCACCTGGTTGGCTATTCTCACCAACAATCCCTT
CTTGAATTTCCTCAGTAAATTCCCATCTATATGTAAAACAACAATAATTTTTCTTGGCACGGCTCTTGTA
ATGCAATGGGAAGAGATAGCCATCCCATCAACAAGGAGCCAGACCATGAGCGTATTCAGCCGCCTGACCG
ACGTGATAAATGCCAACCTGCACAGCCTGCTCGACAAGAGTGAAGAGCCGGAGAAGATGCTGCGCCTGAT
GATCGAAGAGATGGAGCTGGTGCAGGTCGAGATGCGTACCCAGGCCGCCCGCGTGATTGCCGAACAGAAA
CAGCTGGAGCGTCACCAGCAGGGGTTGCAGGCGCAGATCCTGGAGTGGTCGGCCAAGGCCGAGCTCGCCA
TCCAGAAGGGACGTGAGGATCTGGCCCGCGCCGCCCTCACCGAGAAGCTGGCGGAGGTCAAAAAATGTGA
ACAGCTGGAGCAGGACAAGGCCCGGGTCGCCGAGGTGCTGGCCCAGCTTACCGCCGATAGCGAACGGCTG
GCTGCCAAGCTGACCGAGGCGCGCGAGAAGCAGAAACTGCTGACTCTGCGCGAGCAGACTGCCCACAGCC
GCATTCGTGCCCGCGCCCAGGTGGACAACCAGCGCATGCATGAGCTGATGGCGCGTTTTGACGGTTTTCA
GGGCCGGGTCGATCAGCTGGAAGCCCAGTTGGAGGCGGCCACCTTGGGGCAGAATCCCTCGCTGGAGGCC
CAGTTCCGCGAGCTGGAGCTCAATGACGAGATCGAGCGCGAGCTGGCTCGCCTCAAAGGCCAGAAGGCGG
AGGTGTAATCATGAGCCCCTGGCCCAAGGATCTGGCGCATCGCAAGCTGACCGGGGTCTGCGCCGGTATT
GCCAGGCGGTTGGGGGTCTCCAGAGTCACGGTGCGGGTGGTGGCACTGCTCGCCCTGATCCTGATGCCGC
CGCTGGCACTGGCCGCCTATCTGCTGGCGGTGCTGGTGATGCCGCCCCGTTATGAATTGAAGTGCTGGCT
GGATTGACCGAAGGGTTGATCAGCCGGCAGAGAAGGTACTGATAAGGAGCAAGTTATGCTGGAATTTTTC
ATATTGCTGGTGGTGCTGCTGGTCATGGCTCTGACCGGTTTTACCCTGGTAGCCATGTTGCTGGTGAGCG

GCCTGTTCGTGCTGCTTGGGGCGCTGGGTGGCATGCTTGCTTTGATCATCAAGCTGGCTCCCTGGTTGCT
GCTGGTGCTGGCGGTTTGCTGGCTGATCAGCAACCGCAGCAAGCGTTCCAATACTTACCTCTAACCCGCC
TGAGGTTGTGCAAGACGTTGTGTGATGCTCCCTGATTTCCCCTTTCCGGCCACTCATAGAGTGGCCTTTT
TTTGCCTGTCGTCCTCAAGGCTGGGTGGCTGCGGGTAACCACTGGTGGCTCCAGTTCTGCCAGAGTACCG
GGCTGTGGATCAGCTGCTCCTTGAAGGATTTCGGCTGCTGCTTGAGGCGATATTCGATGCGCAGGGCCGC
CCCCTTCTCGCCGACTTCCTGTTGCCACGCGAGCGTCAGTGGCCCCTTGCCGCGTAGGGCGCGAGCGCCC
TTGCCACTCTGGTGCTGGCGCAGGCGGCGCAGCGGATCCGTGCTGATCCCGGTATAGAGCAGGCCGGTGG
CGGTACGCACCATGTAGACAGACCAGCGGGAAGGGGCGGATGGTTGCGGCGCTGGAGTGGTGGTCGCGGG
TTCTCTCATTCAAATTCTCTGTGCCAAACTCAGAGGCAGCTCAAGGAGGTGCGATGAGAGTCTGGTTTTG
GTTGTGGTTACTGTTGTCGGTCCCAGCCCTGGGGGAGACGATCCGTCTCTACGATTACCATCAGCATCCG
CCCTTCAACACCGGAGTGGATGAAGGGCTGAGCCCGCGAGCTGGTCAAGTATCTCAATGAAAAGCTGGGG
GGTGAACCCAGAATCGAGCTTTGGCTGGTCAATCGCGCCCGGCTGGCGCTGGAGATGGCTGATCCCGACT
TTGACGGTCTGGTGGTCTGGGTCAATCCCGCCTGGGTGGGGGATCCCCATCGCGAGCGCTATCTCTGGAC
TTTCCCGGTGATGCGCGACACCAACGAGCTGGTCTCGAACATTGACAACCCGGTCACCTATCGCGGGCCA
GACTCCCTCAAGGGGTTGGTGTTTGGCGGGGTGGAGGGCTACCGCTATCAGGGGATCGATGATGCCGCTG
CAAGGGGAGAGCTGGTGCGTCAGGATGCCAAGAAGGTGAAGACCAATCTTGATCTGCTGGTGCGCCGCCG
CATCGATGTGACGCTGGTCAGTGGTTCATCGCTGGGCTTTTTCATCGACGCACAGCACTATCAGGGCAAG
CTGTTTATCTCGCCCTTCCCCCAATCCAGCTATACCCGCCATATCCTGCTACCCAAGTCTCGCCCCGAGC
TACATGCCACCCTCAACGACATCGCCGAGCAGATGGACCGGGATCCCCGTTGGCACAAGGTGCTGGAGTC
CTATCGCGGTACGCCCTGAGGGCGGCGCTTGAGCCGATGCAGTGGCAACAGGCTCACCAGCAGACCTGCC
CAGACAAAGGCGAAACCGACTGCCTTGATGGGGTCCGGCGCCTCACCAAACCAGTAGATGGCGAGCAGGA
AGGCGAGGCTGGGCTCGATATATTGCAGCAGCCCCACCGTGCTCATCCGGGTTCTCGCCACCGCATAGCA
GAACAGCCCCACCGGCAGCAGAGTAGCGGGCGCACTGCCCATCAGCAGCAGCTTGTCAGCCAGATCTCCG
TTCACGAATGCCCCTTCCCCGTGCCATGCCAGCGTGCCGATGATGATCAGCGCGACGGGCAGTTGCACCA
TGGCTTCCAGATAGAGGCTGGTGCCGGTGTCGTAGTGGATTTTCTTCTTGATAAGGCCATAGCAGGCGAA
GTTGGCCCCCATGATGAGGGAGAACCAGGGGAGCTGGCCGTAGCAGAAGAGCATGTAGGCGAGACCCGCC
AGCGCCAGGGCGACCGCCAGATGCTTTTGCGGGGTGAGCCGCTCTTTTAGGAAGATGACGGCGAATACGA
TATTGAACAGTGGGGTCATAAAGAAGGCGAGGCTGGTGGTCAGCACTTGCCCGGTAGTGAGGCACCAGGT
GAACATGCACCAGCTGATGGACATGACGGGGCCCGCCAGCAGAATGAGGCCGAGCTGGCGCGGCTCGCTG
CGCAGTTGTGCCCAGGGTACCTTGTGGCCAAGCAGCAAAAACAGGGCCCCCAGCAGCACCACAGACCAGA
GCACCCGGTGGGAGATCAGCTCCCACATGTTGACCTCGGGCATGAATTGATAATAGAGCGGCAGCATCCC
CCACAGGATGAAGGAGAGGGTCGCGGTGCCGGTACCTAGCCAGTTTTTCATTATTATCCGTTTATTAAAT
TAAACATCCATGGCGATAGTAGGGGGCGCGCCCTGTTTCCACAAGAGGGAAAACAAGGAAATGCTGGTAC
TATCAACGTCATGCAGCGAATCGATAAGTTGTTTGGGCAAATCAGGGCCTGCCGTCTGTGCGAGGCTCAT
CTGCCGCTGGGGCCTCGTCCGGTGATCCGCGGGGCCGAGAGTGCCCGCTTGCTGATCATCGGTCAGGCGC
CCGGCACCCGGGTGCATGCCAGCGGTATCCCGTGGGATGACCCCTCCGGTGACCGGCTGCGTCAGTGGCT
CGGGGTCGATAAGGCCACCTTTTACGACGAATCCCGCATCGCCATCATGCCGATGGGCCTCTGCTATCCC
GGCAGGGGAAAATCCGGCGATCTGCCGCCGCGCCCAGAATGTGCGCCCACCTGGCACGACAAGGTGCTGA
TGCTGCTGCCCAATATCGAGCTGACCCTGCTGATCGGCCAATATTCACAAGCCTACTATCTGCGGGGGGC
AGCCAAGGGATCGCTCACCGAGACGGTGCGCAGCTGGGCCGACTATGGCCCCGGCTGGTTGCCGTTGCCG
CATCCCTCCCCGCGCAATACTCTCTGGCTCAGGAAGAATCCCTGGTTCGAAGATGAGATCGTCCCCCATC
TACAGCAAAGGGTCGCCCCGCTTCTGGGGAGACCCTGAGTATTAGAGCGCTGGCTCGGCCAGCGGTTTGG
GTTTGCTGCGGACCATGGCGATGAGCGGCAGGACGATGCAGATACAGCCCAGCAGCAGCGGCAGACCAGG
CTGCTCGTCAAACAGCCACACCCCGATGGCCACCGCCATCAGCAGACCGGTGTACTCCGCCGAGGCGATC
TTGCTCGCCTCCGCCTTGCGATAAGCCACCACGCACAATCCCGCATAGACCATGATCAGCGCCGAGGAGC
CGAAAGCGGGCCACCACATCTGCCACTGCCAGTCGCCCCCTTCCCATAATGCCAGCGCCAGAATGAATGG
CATGCTCAGCACGTTGGTGAAAAACAGGGTCTGGGCGATGGGCTGATTGAGCGGGATCCGCTTGATCAGC
AGGTTGTTGAGCGCCATCGACAGCGCCACCCCGAGCGCCGCCAGTGCGGCCCAGTTCACTTCGGTTGGGC
GCAGCACGATCAAAACCCCGATAAATCCGATGACTGCGGTCAGCACCTTCTGGCGCGAGATCGCCTCTCC
TGCCATCCACACTGCCAGCGGCACCATCATCAGGGGAGCTGCATAGAAAAGGGCATTGGCAGTGGCGAGC
GGCAAGCGGGTGAGGGCCACCACCATCAGGCAGACGCCGACCGCCCAAATATGGGCCCGCAGCAGGTGCC
ACTTGATGGCCACCGGTGGGTTGCGGCGCCATGACCAGAGCAGCCAGGGCATCAGCAGAATGGCGGCCGA
GAGCTGGCGGAAAAAGACAAACTGGAAGATGGGCTGATCCGGCTCCAGCACCTTGACCAGCGCATCGGAA
AAAACCGCCACCATATTGCCCAGCACCAGATAAAGGATGCCGATCCCTACTGCATTCATACCACCTCCCA
CTTCAGATGGGCGGAGGGTAGCGTGCCGGTTGATATCCAGTAAAATGATAAAAAATGACCACTCATGAGA
TATTTAGATAATGAAGCTTCCTCCTCTGCGTGCGGTGCAATATTTCGAGGTGGTGGCCCGGCTGCTCAGC
TTCTCCCGCGCCGCTCTGGAACTGAACGTCAGCCAGAGTGCGGTCAGCCACCAGATCCGTCTGCTGGAGG
ATTTTCTGGGTGAGCGCTTGCTGCTGCGTCAGGGGCGGTTGCTCTCGTTGACACCCCAGGGGGAGAGTTA
TTTCGAGGGAATTGCTGCTGGGCTCAGCCAGATTGCCCAGAGCAGCGAACAGCTGCGTGGTGGTGGCGAG
ATGCGAGTCCGGCTCGCGGTCTACAGCTCGTTTGCGGTGAAATGGCTGATCCCGCGGTTGGCCGGATTGC
GCCAACTCTACCCCGAGCTGGATTTGAGTCTGGAGATGGTGGCGGAAGACCCCGAGCTCTCCGATCGGGT

GGCGGACTGCTTTATCACAGTCTCACCCCACGGGCGGGGCTACTGCCACGACCTTATCTATCAGGAGCGG
CTCTTTCCGGTCTGCAGTCGCCGCCTGTTGCAGCAGATCAGCGGGGAGTGGCCTGAGGCCATCTGGCAGC
TGCCGCTGCTTTCGGTGCAGTCCATCTACAAGGTGCGGGGGGAGGATTGGCAGCGCTGGGCCAAGGCGGG
TGGGCTGGTGATTCCGGACGAGGCGCGGATGCACCACTTCAGCCATGTGCTGTTGGCGATGGAGGCGGCA
GCCTGGGATCAGGGGGTGGCATTCGTGAATGATTACATGCTGCGGCCCGATGATCCCCAGCTGGTGGCCT
TGCCGGTACACCAGCTGGAGACGGGGGATGCCTTCTACTTCACCTGCAAGCGCTCGCGGGCTCATGAGCC
TGCCATCAGCCGGTTGCGCCAGTGGCTGATGATGGAGACACGCCAGAGCATGCCGCTCTAGCCTGCACCG
TTACGCTCTCTTGAACTGGCTCAGCACAAACAGGCTGGTGGCACAGACGACGACCGAAGGCCCAGCCGGG
GTGTCCTGATACCAGGAGAGGGTCAATCCACCCAGCACGGCGAGACAGCCGATCAGCGCGGCAAAGCCCG
CCATCTGCTCCGGCGTCTGGCTGAAACGGCGGGCGGTGGCGGCGGGGATGATCAGCAGGGAGGTGATGAT
CAGTGCCCCGACAAACTTCATCGCCACGGCAATCACCAGCCCGATGAGCAGCATCAATACGCAGCGCAGC
AAATCGACCCGGATCCCCTCTACCCGTGCCAGCTCTTCCGAGATGGTCATGGAGAGCAGGGGATCCCACA
GTCGCCACAGGGTGAGCAGTACTAGCGCGCCACCGCCGTAGATCCAGAGCAGATCCATCGGTTGTACCGA
GAGCAGGTCGCCGAACAGGTAGGCCATCAGATCCACCCGCACGTTATCCATAAAACTCAAGGTAACCAGA
CCGAGGGACAGGGCACTGTGGGCCAGAATGCCGAGCAGGGTATCGGTGGCCACCTGCTGGTTGCGACTCA
TGATCACCAGCAGCATCGCCATGGCGAGGCAGCAGACCACCACCGCCAGGGTGAGATCGACCTGCAGCAG
GATGCCCAGCGCGATGCCGAACAGGCAGGCATGGGAGAGGGTGTCGCCAAAATAGGCCATCTTGCGCCAT
ACCACGAAGCTACCGAGGGGACCGGCCAGCAGGGCGATGCCGAGACCGGCCGCGAGGGCGTATAACAGAA
AGCTGTCCACTTGAATGTCCTGTTGCTTGCGCTATTTGCTGTGAGCGTTGTTGCGAGAGGGCAGGCGGAT
GACGGGAGCCTGCGGTTCATGATGATGGTGTTCGCCGTCGCAGTGGTGATGGTGGGTATAGACCGCCAGC
ACCTCCTGCTCCGGTCGCCCGAACAGGCGGGCAAATTCGGGGTGGCGCGCCACGCTTTCCGGCTCGCCAT
GACAGCAGACGTGGCGATTGAGGCAGATCACCTCGTGGGTGCTGGCCATCACCAGATGCAGGTCGTGGGA
TACCATCAGCACGGCGCAATTGAACTCGGCGGCGAGCTGACCGATGAGGGCGTAGAGCTCGATCTGGCCA
GTGATGTCCACGCCTTGAACCGGCTCGTCCAGTACCAGCAGCTCGGGTTTGACCAGCAGGGCGCGAGCCA
GCAGCACCCGCTGCATCTCACCGCCGGAGAGTTTTTGCATCCGGTTTTCCAGCAATCCCTCTGCGCCCAC
CCGGGCCAGAGCTTCCTCGATGGAGAGGCGGCTATTTTTGCCCAGTTGCAGGAAGCGACGCACCGTCAGC
GGCAAGGTGGGATCCAGATAGAGGCGCTGCGGTACATAGCCGACTCTCAGATCGCGGCTGCGGGTGATCT
TGCCTGAATCCGGCGTGAGCAGGCCCAGCACCAGCTTGCTCAGGGTGCTCTTGCCAGCCCCGTTGGGGCC
GACGAGCGTGGTGATTTTGCCTTTATTGAGGGTAAAGGAGACCTTGTCGAGCACAGAGCGTCCATCAAAC
GAGAGAGAGACCTCTTTCAGCTCTACCAGTTGGCTCATGACAACTCCGGGGTTTACAGCCAGTGAGAGTT
ATAATATAACAAAAAGCGGCTGTTATATTATCACATACTCACCCCCTGACGAGATTCACCAATGAGAATT
ATTGCGCTTTTCACTGCCCTGCTGGCGGTGAGTTTGCCTGTTCGTGCCATTGAGGTACTGACCACCATCA
AGCCCCTTGGCTTCATCGCTGCGGCGATCACTGACGGCGTGAGTGAGCCCAAGGTATTGTTGCCGACCGG
CGCCTCTCCCCACGATTTCTCCCTGCGCCCGTCCGATATTCGCAGCATCAACAGTGCTGGGCTGGTGGTG
TGGGTTGGCCCCGAGCTGGAAGGGTTTATGGCCAAGCCGCTGGCCAATCATCCCCATGCTTTGGCCCTGA
CTCAGGTTGAGGGAATGCCGCTGTTCAACTACGCGACTCAGGATAGTCATGACTCCCATGACCATGATGA
TCACGATCATGCCGCCCACGAGCACGGTAACCATGATCATGATGAAGGGCATGAGGGACATCATCACGAA
GGGGTCGATCCCCATATCTGGCTTGGTCCGACGCAGGCCAAAGTGATTGCCAAGGCGATCGCAAGTGAAT
TGGGCAAGCTGGATCCGGCCAATCAGGCTCGCTATGACGCCAACCTCGCGGCATTTGATACCAAGGTGGA
TGCCAAAGACAAGGTGATTGCTGGTCAGATGAAGGCGGTGAATGAGAAGGGGTACTTTGTCTTCCACGAG
GCGTATGGTTACTGGGAGCGCCACTACGGCATGAGTTCAAAAGGTCACTTTACCGTGAGCCCTGAGCGTC
GCCCGGGCGCCAAGACCCTGGTGGATATTCGCAAGGCACTGGAAGAGAAGCAGGCGAGTTGCATTTATGC
CGAGCCGCAATTCTCACCGGCTGTGATCGAATCGGTGGCACGCAATACCGGCGCCAAGGTGCTGTTGCTT
GATGAGGTGGGTGAACAGGTACCGCTCGGCCCGGATGGTTATCCCCAATTCATGCAGCAACTGGCAGACG
CATTTGCCCAATGTCGCTGATCAGCGGCTGAGTCTCTCATCCGGAGGGGCTATACTGACCTCCAGCATCT
GACATGGAGTGTCCGGTATGAAATGGTTGTTGATCCCGGCCTGTACTCTGGCGCTATGTTGCGCCAGCGT
GCAGGCTTCGCGTCTTTCCGAATATATTGCCAGTCCGGATCTGCGGGCCAAGCTGCAGGAAGTGTATGGT
GGCCGCGCCATTCAGCGGGCCGAGGCATTTTCTCGCCTGCTCACCCCCAGAGCGGGTACAACCGACCAGC
AGAAGATCGAGCTGGTGAACAACTACTTCAACAGTCTGACCTATAGCGAAGATCCCAAGCTGTGGGGAGA
TGAGGATTACTGGGCCAATCCGCTGGAGTTCATCGGCGCCCGGGGTGGGGATTGCGAGGATTACAGCATC
TCCAAGTACTACACCCTGATGGAGCTGGGGGTGGATGAGAAGAAGCTGCGACTGGCCTACGTCAAGGCGG
TGCGTTACAACCAGTTCCACATGGTGACGCTCTACTTCCCGACACCCAAGGCTGACCCACTGGTGCTCGA
CAACATCAACAGCCGGATCCTGCCGGGCAGCAAGCGTACCGATCTCATTCCCGTTTACAGTTTCGATGCC
AAAAGCATCTGGATCATGAAGTCACGGCGGGAAGGGACGCTGGCGGGGAGTGCCGATCGGTTGGCGCAGT
GGACATCGATGAAGACCCGTTTCTCCAGCGAGCAACTGCGCTCTCCCCGTCGTCAGTTATAGCTTGCCCG
CCGCTTTCAGCTCCTCGAAATATTTAGCTGCGCCCGGATGCTGCTCAATACTCAGCACCGGGCCGGTTTT
ATTGCGTGCCCGGTGAAACATATTGGCGTAGCCATAAGTCTGCTCATTGAACTTGGTCTGCTGGGTCAGC
ATGGCCTTGGTCAGACGATAGACAGACTCTTCCGGCATGGTGGCATTGGTGACCAGGGTGGCGGCAATGG
CAAAGGTGGGGGTCGCCTTCAGATTGCCGGGGTAGATGCGGGCGGGCACCTGACTACGTTGGTAATATTT
GACCCCCTTGAGTAGCTGATCACGGGTGGCCCCATCGATGGGGAGGAAGCTGACTGCGCAACGTTTGGCG
GTGTCGAGCACAATCCGGTTAGGGTGTCCCATCACCAGTGCAAATGCGTCGATCTGCTTGCTGCAGAGCG

CATCCGGCAGGTTGGCCATGCTTTTCAGATCGAGATCCTGCTCTTTCGCTCCCAAGATGTTGAACAGGGT
CAGCGCCGTCTGGCGGGTTCCCGAGTTCTTCAACCCCATGTCGATGGGGGCGAGCTTGAGCTGTTCCAGT
GAAGTGATATCGCCATCGGCACGGATCACTATGGTGAAGGGCTCGGCATGGGTGGCAAACAGGGCGCGCA
GCTCAGGTTGCGGCTGGGTGGTAAAAGGGCCCAAGCCCTGGCTTGCCTGATACAGCCAGTCTGACTGCAC
CATGGCGTAGGAGGTCTCGCCACTCTTCAGCCGATTCAGGTTTTCTATTGAACCCGATGAGCTCTGTAAT
GTGCACGCAAGTTGCTCATCGACCTCATGGGCCAGACGGCAAAACTCTTTGGTTATCTGGTGATAAATCC
CCTGCTCACCCCCGGTGGTGATAGTGACGGTTTCAGCCATCAGGCCGGGTTGCCACAGCAGGGGCAACAG
CATGGCGAGCTTCTTCATGATTGCGCCTCCTTGGCATAACCATCGATACCCCAGGTCCGCAGTTTGTCGA
TGTCGCGACACTCTCCCGATTCTGCCAGCAGGGTATAACCGGCGGCATGCATGGTTTGGGAGAGCAGATT
CCAGTAGAGCGGCGCATCGGTCAGCTGACAGCTGATAAAAATGGGATTGAGCGGGTCAAGTTGATTGAAG
GTATCCGCATTGAGGGGAATGGCGCTGAAGGCGAGCTCACACCCCTGTTCCTGCAACGCAGCCAATGTGG
CTTGGCCGTGGGCGGGAAAGGTGACCACATCCCAGCGCCAATCCAGTGTTTTGCCGCGGCCCTGGCTTAG
CAGGGGGGCGATCTCGGACCATTGCGGGTGATTGAGCAGGGAGAGGGGCAGCAAGACCCCTTCTATATGA
GAGTGCTGCCACTGTTGCAGGGCGCTCTGGATGGTCGCGAGATCGCTGTCGGCTCCGAGGGGCTGGTTGG
GCAAGAAACGCTGATAGAGCGCGATCTCCCCTTGTGTGGAGACAAATTTTTGCAGTTGCCCGCCTTCCGG
CAGCGGGCTCACTCCTTGCAGCGGCTGTGGGGGCGTCAGTGGCAGCTGTTTTCCCAGCTGGGTGATCTTG
AGTCGTTGTTGCAGCAGATCCCGTTTGCGTTCGGAAACCAATTGGTTGATGGAAAGTGCCACTTCTTTGA
GGTCTTGCAACCAGGGAGTGGGCAGGGTGCTGTTGAACTGGTGTTTGCGGAGACAGATCAGCTGCAGGTT
GATCTGCTCCAGGGGCTGCAGCAGGCGTTGGCACACCAGGGAGATGATCAGGGTAAATACGACGAGGCCC
GCCAGCAGCGCCAGACCGGTGCGACTGATGGCATTCCACAGATGCTGATAGGCGTAGCCGCTATGCCCCT
CAAGGGTCAGAGTGCCCAGCATGCGCCAGCCATCGGTCAACTCTTGCTCAATTTTGACGGGGGGCATGCC
GATAACCGAGGGAAGCCAGCCGGGGACATTTTGCTGGGTAGAGTGGAAGACCTTCTGAAACAGCAACTGG
CCATCAGGATCGATCAGGGTGACCGAGCTGACGAAGCCGCCATCGAACATGGCATTGACGATGGTCTCGG
CCAGCACATTGTCGTCATTAAGCAGGGTTCCTTGCAGCACCAGCCCGAGAGCGGTGATGGCGGTTTCCAG
GTTGGCTGACATCTGGTCGAGGATGGATTGGCGGACCGTCGTGACTTCCAGTGCAAACAGCAGCGCTGCA
CCGATCAGGAACAGGACGACAATTATTCTGATTAACAGTTTTGCTAACGACATATCCACTCCATGGAAAA
GTTATCGCAACATTTCAGGTTTCTATTCAGTAGCGAATCCGACCCGGTTCTTAAAAATAGGAAAAAATGG
TTAAAAAAGACGATCATTTCTGCCCATCCGGGCGGGTTATGCATAAAATGTGAGGTTCATGCAGATTGTG
ATGAACTATTTGTAACTATTTGTGTCCAACAAACAGTTTTATACGGTTTTGTCACAACTGACATCGCAAC
TCACACTCTTTAGATCTAAATACAGGATAGCGGATGAAACGTCTCCGTCCCTTGGTAGTACAGGCTGCCA
ATTGGGAACCACCGGTTCCCCGCAAACACTTTTACGCCATGCTGATGCTGACGGCCATGACCCTGTCAGC
AGTGGCATTATTGCCAGCTCCGGGTGACATCCTCGAGCGGCCGCTGCGTCTTGAGTTGCCCATTGATACC
GCAGGTACGGCAACCAACGAAAACAACACCGACTTTAACGAAATTCCCGATCACGAACTGGTTGATCAGA
GTGCGCCGGATGACGACATCCCGGTCGACACCACCCCCCAGTGGCAGGATTACCGGGTCCGCAACGGCGA
AAACCTGACCGCCATTTTTAACAATCTCGGTCTCTCCACCACCACCCTCTACAAGGTGCTGGACGCGGAC
AACAAGAACTATCTGGCGCGCCTCAAGCCGGGCCAGAATATCGAGCTGCTGATCGATCAGGACAACATCC
TGCAGCAACTCAAAATTCGCCTGAACATCAAGCAGACGCTGGTGATGGCACGCAGCGACGATGCCTATAC
CGCCTCCATGATCAATGAGAAGGTCGAGTGGCAACCGCAGCGTTACAACGGCGTGATCAACGGCAGCTTC
TATGTCAGTGCCCGCAATGCGGGGATTCCGGCCAACCATATCCAGAAAATTGCCAACCTGTTCCAGTGGC
GAATGAATTTCGCCAAGGATCTGCAGAAGGGCGACAAGTTCAAAGTGCTGGTACGCCAGGAGACGGTGGA
AGGCAAGAGCACTGGCAACAGCCAGTTGCTGGGTGTGGAAGTGACCAGCCAGGGCAAGACGGTGTCGGCC
TGGCTCTCCGATGACGGCAACTATTACGACGGTCAGGGCAACAGTCTGGAGCGCGGATTCCGCCGCTACC
CGACCCACAGCCGCTATCGGATCAGCTCCAATTTCAACCCGGCCCGCAAACATCCGGTAACCGGTATGGT
GCGTCCTCACGAGGGCACCGACTTCGCGGTACCGGTGGGCACGCCAGTGCTGGCGACCGGCGATGGTGTC
GTCCAGAAGGCAACCAGCCACCCGCTGGCGGGTACCTATATCGTCATCAAGCATGGCCGTACTCTCTCTA
CCCGTTATCTGCACCTGAGCAAGCTGCTGGTCAAAACCGGTCAGCGAGTGAAGATGGGCGACAAGATCGG
CCTCTCCGGCAACACAGGTCGCTCGACCGGTGCTCACCTGCACTACGAGCTGCGGATCAACAACCGCCCG
GTCAATGCCATGACCGCCAAGCTGCCGATGGCTGAACCCCTGAGCGGCAAGGAGCGTCGTCAGTTCCTTG
CCAAGGTGAAGAGCTACCGCAAGGAGCTGGCTGCGGGTTGATACCTGTTGTCACAGCCTGAAATGAAAGG
CCCCGCCAGATTGGCGGGGCCTTTTTACATGCGCCGTGAGGGTCAATAACCCTTGTCGAAGTCGATCTTG
CCATCCAGTGGCTGGCCATCGATAAAGCGGATGTAGTTGCGCACAAAGATCTGGGCCACATCATCCGGGA
AGCTGTAGGCGCTGTTGTGGGGGGTGACGATGAGGTTGGGTTGGCCCCACAATGGACTGTCGGCGGGCAG
CGGCTCCTGCTCGAACACATCCAGCACTGCCATCGCCAGCTTGCCGGTACGCAGTGCGGTGAGCAGATCG
CCAGAGTGCACGGCGCTGCCACGACCGACGTTGAACAGGATGGCGCCCGGTTTGCAGTGTTCGAAGCGGG
CGGCATTGAACAGATGACGGGTATCGCGGGTGGCTGGCAGCACGCTGACGATGACATCGGCCTGCGCCAG
CATCTTGTTGAGCGCAGGCAGTTGATAAACCTGATCAAACCCGGCCCGCTCCCGACCGCTGCGGCTGATA
CCCAGCACCTTCATGCCAAAATGTTTGCCGGTGTGGGCGATATGCTGGCCAATGCTGCCGGTCCCCAGGA
TTAGCATGGTGCGCCCCTTGAGGCCCTGATAGGGGTGGCTTTGCCACAGTCGCTGTTTCTGCTGCTCCCG
ATAAAGGGGGATTTGGCGCATCAGGCTGAGCAGATGACCAAAGACATACTCGCTCATCAGCGGGCCGAAG
ATGCCGCGCACATTGGTGAGCTGATAATCACGGCGACTGTCAGGGTCGAGCAGCACATCGACTCCCGCAT
AGGTGGACTGGAACCAGCTCAGCTTGTTGGCTTTGGGCAGCAGCGGTTTGGCACGGGCCGGTTCGGCCAT

CAGAATATGGGCTTCGCCGATCAGCCTCTCGGCATCGCTCTGGTTATCGGCTCGCAGAATGCGCAGATGG
GGGAGATTGGCGGCCTTGAGCAGCCGTTCGTAGTGAGCATTATCCTGGCTGAGCAAAAGAAGGGTACGTT
GGCTCATACTCTTGTTCCTTATCAGAATTTGCGGCGCAACAAGATGCCGCTCTCGACCCATCTTACCTGA
CTTCATATCGCAACATAGCCGACCAGGGCGCTTTTTCGTTTTCGCCGCGCGCCTCGTGGGCTACAATCTG
CCCCACATTTTGCAAAGGATCCGACTCCAGTGTTTGGTGACAAGTTTTATAAGCGACTGGCCGAGCTACA
GCCTTGGCAACAGACCGTCTACACCCTGGCGCTGGCAGAGCGCATGTACCCCAACTATGCCCTGTTCTGT
CAGGCCTCCGGCTTTGGCAATGCTGGCGCTTTCCGGCATGCGCTGGATCAGATGTGGGTCTACCTCACCG
TCAAGGGATCCCGGGTCAATCTGGGTGCCATTCTCGAAGAGTTCGAGTCTCACATTCCGGATCCCCGCAA
GTTTGAATCCTACGGTGCCTATCCCGCGCTGGATGCCTGTGTTGCGCTGGGCTGCGCCTATAACTCGGTG
ATCTGCCGGGTTGGTGAAGAGGCCAACGATGCCAGCCATGCGTCAGTCGGTACTGTGGCCGGTTTTATCG
AGCTGCTGGAAGAGCGGGATCTGACCGAAGAGGAGCTCTACGAGCACGAGTACATGGAAGCCGAGCTGGA
GTTCCAGGTCGAGCTGCTCAAGCGGGTTGCCCATGAGCGCGACAGCGATCGTATTCTGGCCATCAGAGTA
TTCGCCTCTCAGGATGGCGTCTCCAATATCGGGATCAGCCTGGAAGAGTAATTCTCTGCGGCCCCTGATC
GAAAAAAGCTCCCGTTCGGGAGCTTTTTTATGCCTGAAATTGTTCAGCAGCTTATGCCTGCTCTTCGGT
GAGGGGTCTGACGCTCAGCACCGGGGCTGCGTCGATTTCGTCTGCATTCATCATGGCGGAGATGCGTTGC
AGGGAGGAGAGCAGCAGGGTCTGCTCCCAGGTTTCAAGGGATTGAAAGCGTTGGATAAAGCGCTCTTGTA
GCGGGGTCGGGGCCTGAGCCAGCAGGGCACGGCCTGCATCAGTCAGTGTCGCGTGAACCTTGCGCTTGTC
GGTATTGCTGCGAACGCGCTGCACCAGTTGGCGGCTCTCCAATCGGTCCATGATAGTGGTGGCGGTGGCC
TGGCTCATGTTGGTGTGATCGGCCAGCTGGCGCATGGAGATCTGTCCCAACTCGTCGATCCCTTTGAGCA
GCAAGAGTTGCGGGGAGGTGAGCCCGGCTTCTTTGATCAAGCGCCTGGAGTGCATGTCGATGGCACGAAT
AATCTGGCGCAGGGCGACCAGCACTTCATCATGTTTTTCCATGGGGTGTGCGTCATTGGAAGGCTATGGA
GAGGCGCAAAATTTTCCGCCTAATTCGTCACAAGGTCAATATGGTTTTTGTTGTAATAGTTTCTGCTCTA
ACTAATTTTGTGTTAGGTTGACGGGCATTTCACGGAATTCACCCTGACATTTGAGGAGCGAAATGATGAA
TGACGAGCTGGATATCAAGTCTCTGGTGGCCGAGTTACGTGTGGTCAGAGTGCGCTATGAACTGCCGGAC
ACCTCGGCTTTTGTGCGCTATCAGCCTGCCTATGATGAAGATCCCAACAACCTCAAGGTACACCACTATG
ATGGTGAGTTACCCCTCTCTTGCAGTGAGACAGGTACTTTCAACCTAAAGGAGTTGGTGAATGAATTGCG
CTACGCCTATGTGTTGGTGCACCTCTTTCCTGACGAAAGTGCCATGAACTTCTATCTGACCGGCCTCGAT
ACCGGCCTGGCACAGCTGGAGTTCATCGGTGGCTCTTACGACTTCAACCGCCTCTGGATCACCAGCGGTA
TCTCCAATCTGGGCGTCCCCATGGCCATCTTCCTTGGCAAACGTCACGCCGTGAATGGCCAGCCCTCGGT
TTCGCTGGTGGATCACCGCGAACAGGCAGCTGGCAAACTGGTCTATCGCGCCATCGGCGGTGCCGGTGAT
CATGTGGGCGAAGACTTTTTCAACAACGACTAAGGAGGCTGGATGCGAGCAAGCAAAGGGCTATTGAAAG
GCCTCAACCCCACCATGGCCATGACGGCCCTCACCGTGATCACCCTGTTTTTGCTGTTTGGCGTATTTGT
TCCCGATACCGCGGCCGCCTGGTTTGTCGGTGCCAAGGATCAGATTATTGCCGGGTTCAAGTGGTACTAC
ATTCTGGTGGTCGCCCTGTTCCTGTTCTTCGCTGTCTACCTGATGTTCAGCCGTTTTGGCAGCATCCGGC
TGGGTGACGACGACCAGGTACCGGAGTTCGGCTACTTCGCTTGGTTCTCCATGCTCTTTAGCGCAGGCAT
GGGTATCGGTCTGGTGTTCTGGAGCATCGCCGAGCCGATGAACCATCTGCAGGGCAATCCGTTCATTACC
GAAGGGATGACCCCGGAAGCGGCCCAGATCGCCATGCGCCTCACCTTTTTCCATTGGGGCCTGCACCCCT
GGGCCATCTATGTGATCGTTGGCCTGTCGCTGGCTTTTTTCAGTTATCGCCGCAAGTTGCCGCTGGCGAT
CCGCTCTGTGCTTTACCCCATCCTTGGCGAGCGGATTTACGGTTTTTGGGGCCATGCGGCCGATGTGCTG
GCGGTGTTCGGCACCGTGTTCGGGGTGGCGACCTCCCTTGGTCTGGGGGTCTCCCAGATGAATACCGGCC
TCAACCAGATGTTCGGTATGGACGTATCGCAGTCCAACCAGCTCTGGCTGATTGGCGGCATCAGCCTGAT
CGCCACCCTCTCGGCGATCTCCGGGGTCGGGCGCGGGGTGAAGATCCTCTCCGAGCTCAATATCTGGCTG
AGCGTGTTCGTGCTCGCACTCTTTCTGGTGCTGGGGCCGACCACCTATATCCTCAACGCCTATGTGCAGA
ACATCGGTGATTACCTGCAGAACATCGTCAAACTGAGCTTCTGGACCAACACCGAGGCCAACGGCACTTC
GGCCTGGCAGTCGAGCTGGACGGCGTTTTACTGGGGCTGGTGGATTGCCTGGGCGCCGTTTGTCGGCATG
TTTATCGCCCGCATCTCCAAGGGGCGTACTATCCGCGAGTTTGTGCTCGGTGTGCTGCTGGTGCCCTCGC
TGCTCGGCATGTTCTGGCTCACCGTGTTTGGCGGTACGGCGATGAATCTGGAGCTGTTCGGTGGTGGTGG
CGTGGTCGCTGCGGTCAATCAGGATGTGACGCTGGCGCTCTACAAGACGGTCGAGCTGTTGCACTGGGAT
ACCTTCGGCTGGATTGCCAAAGCTGTGCTGACCCTGCTCATCTGCACCTACTTCATCACCTCCAGTGACT
CGGGCACGCTGGTCATCACCACCCTGCTCTCCATCGGTGATCAGGAGCCGCCGGTACTGCACCGCGCCGT
ATGGGGTCTGGGGCAGGGGCTGGTTGCCGCTATCCTGCTGGTTTCCGGCGGCCTGGCAGCCTTGCAGGCG
GCCAGCATCATAGCGGCGCTACCCTTCTCGGTGATCATGCTGGCGATGTGCTATTCGCTGATGCTGGGGC
TCAAGCAGGAGAGCCAGCGCCAGTTCGAGTATCGTCAGAACCTCAAGCGCCACGATGGCTCATCCCATAT
GAGCCTGATGGAGCGAATTGGTCCGGCATAACGGCACCAGAAGAGGGCTGACTCCCCTTCCTATGTAAAT
CTGGATGCAAAACGGCCGCCCTGGCGATGCTCTCCTTGCGGAGCTCATCATCAGGGCGGCCGTCTGTTAT
GGGGCCGAGGTTACTCCTCGATAAAGGTCCACTCATCCTTGATGCGGCTGATGGCATCGGTGCGCTTTTT
GGCGAGCTGTTCCTTGATCTCTTCCCCTTTGAAACCGGCTGCCACTATCTCTTTCACCGGCACCGCCTGG
GCGGCAGCCAGCGCTTCGGCCACATAGTCCGGCTCGGCGTAGACCCGCTCCTCGAAACCGGTACGGCCAT
GGAAGTCGGCGCGACAGGCATCCAGCAACTGGGCAAAGCGCTCGGGGCGGCGCCAGGCATCCGCCTTGTC
GAACACCTTGAGCAGGGTGGCGGGCTTGAGCTCAAAGGCGATATGGATGTGGGTGTGCAGATCGCTCACC
AGCAGCGCCAGATCCCGGCACTCATTGGGTACCCGGAACCGCTCGCACAGAGCGCGAATAAGCGGCAGCC

CTTTCTGGCCGTGACCGTGATGGCTTGGCCAGAACTCCTTGGGGGTTACTCCCTTGCCAAGGTCGTGGCA
GAGGGCGGCAAAGCGGACGGTCAATTCCGGGGAGATCCGGCACGCCTGCTCCAGCACCATCAGGGTGTGG
ATGCCGGTGTCGATCTCCGGATGCCACTTGGCCGGGGCGGGGACGCCAAACAGCGCATCAATCTCTGGGA
ACAGCGCCTTCAGGGCGCCGCACTGGCGCAGCACCTCGATAAAGATCTGCGGGGTCGGCCCCAGCAGCAC
TTTTTCCAGCTCCTTCCAGACTCGCTCCGGCGTGAGGTGGGCCAGCTCGCCAGCGGCCGTCATCTCGCTC
ATCAGGGCGAGGGTCTCCGGCGCTATGGTGAAGCCCTGAGTGTGGAAGCGGGCGGCGAAGCGGGCGACCC
GCAGGATGCGCAGCGGATCTTCGGCAAAGGCAGGGGAAACGTGGCGCAGCAGCCGTTTTTCCAAATCCTC
GATGCCGCCGTAGGGATCGTGGAGCTGGCCCTGATCATCTTCGGCGATGGCGTTGATGGTGAGATCCCGG
CGCAGCAGATCCTGCTCGAGCGTCACATCGGGGGCGGCGTGGCAGACAAAACCGGTGTAGCCCTGACCCT
GTTTGCGTTCGGTACGGGCGAGGGCATATTCCTGCTGGGTTTTGGGGTGAAGAAACACGGGAAAGTCGCG
ACCGACCTGGGTAAAACCCAGCGCCAGCATCTGCTCCACGGTGGCGCCGACCACCAGATGATCCCTGTCG
CCCTGCGGCAATCCCAGCAGGCGATCCCGCACTGCGCCCCCAACCAGATAAGTCTGCAAACCGTCTCTCC
CGTCATCAATTGTGGGGCCGAGTATATCGTCTTTGTTACCGCTTTGCTGCCTGAGCACCACCTGCCCGCA
GCGCCAGTCAGCGCGGCTTTGACTTGGGCGTGACGAGGCCTTACGCTGCCGCACCAGACAGCTGGCGCTG
TGCAGCGGGATGACTTTCATGCCCGCGCAGGCCAGATGAGCCGAAGGAGCCGCAGAGTTCCCCTTATATC
AACGACATGAGAACAACGTGAATCAATCAACCTTCGCAAGCCGGGTTCTGGAGTGGTATGAGCTTCATGG
CCGCAAAACCCTGCCCTGGCAGCAGGAGAAGACTCCTTACCGGGTCTGGGTCTCGGAGATCATGCTGCAG
CAGACCCAGGTCGCCACAGTCATCCCCTATTACCAGCGTTTTATGGCGCGCTTCCCCGATGTGATGGCAC
TGGCCGATGCCCCTGTCGACGAGGTGCTGCACCACTGGACCGGGCTTGGCTACTACGCCCGGGCCCGCAA
CCTGCACAAGGCGGCCCAGCAGATCCGCGATCATCACCATGGTCTCTTTCCCGAGCGCTTTGACGAGGTG
ATGGCGCTGCCGGGGATTGGTCGTTCTACCGCCGGGGCCGTGTTGTCGCTCTCTCTGGGTCAGCCCCACG
CCATCCTTGATGGCAATGTGAAGCGGGTACTCACCCGCTGGCTGGCGCTGCCGGGCTGGCCCGGCCAAAA
ACAGGTGGAGAACGAGCTGTGGGAGATCGCCATCCGGCTGACTCCCAAACTGGGCGTGGCGCAATACAAT
CAGGCGATGATGGATATCGGGGCGACGATCTGCACCCGCAGCAAGCCTGCCTGTGATCGCTGCCCGGTGC
GGGCCGATTGCCAGGGGCTGTCGCAAGGCAAGCCCACTGTCTATCCCAACAGCAAGCCCAAGAAGAGTAT
TCCGGCCCGCGATGGCGTCATGTTGTTGATCCGCCATGGTGACGAGTTATTGCTGGAGAAGCGGCCGCCA
CAGGGGATCTGGGGCGGGCTCTACTGCTTCCCGCAGGTGGCTTCGCTGGCGGAGGTGGATGGTCTGCTGG
CATCCCTCGGCCTGAGCCATCACGGTTATCGTGAACTGGCCGGTTTTCGCCATACCTTCAGCCACTTCCA
TCTCGATATTCAGCCGCTGCTGATTGACATTGATGAGGCGCAGCCGCTGCGGCTCATGGAAGCAGATAAC
CGACTCTGGTATAACTTACGCCATCCTGCCGAGGTGGGGCTGGCGGCCGCCACGGCCCGTCTGCTCGCCT
ATCCAGAGTTACAACCATAGAGGTCAACATGAGCCGTACTGTATTTTGCCAACGCCTGAAAAAAGAGGGC
CCGGGTCTGGATTTCCAACTCTATCCCGGTGAGCTCGGCAAGCGGATTTTTGACAACATCAGCAAGGAAG
CCTGGGCCGAGTGGCAGAAGAAGCAGGTGATGCTGATCAACGAGAAAAAGCTCAACATGATGAATCTTGA
GCATCGCCAACTGCTGGAAAAAGAGATGGTCAGCTACCTGTTTGATGCAGGTGAAGTGCAGATCGAAGGT
TACACCCCGCCTGCCCAGTAAGCTCTCCGGCTGTTGTACGTGTGAACAGGTGCGGGAATGGCGCCACACT
GGCGTGACCGCTCCTGAAGTCGCAGCAAATGAATGATGGCAGCTCTGCTGCCCGGTTTGAGGAGCCTATG
GGCCGAATTTTTTGGCTGCTGTGCACCCTGCTGTTTCTCTCTGCCTGCTCATCTTCCCCCAAGCCCAGCG
GGAGTGAGGATGACGATCTGGTCAACGGCAAGGACATCCGCGGTTTTGAACACATGATCAGCGCGCTCTC
CCATAACGTGAACGAGATATGGGGGCGGGAGGCGCTGTTTGCGGGCAAGTTTGACTACGTCAAATACACG
GATCAGTACAAGAGCCGGGCCCACATCGACTTCTCCAGCGGCCACATCATGGTGGAGACGGTCTCGGGCA
TCGATCCCCGCGCTCACCTGCGTCGCGCCATCATCGAGACTCTGCTGACCCCGGACGATCCGGCCGAAGT
GGATCTTTACTCCGACCGGGAAATTCTGACCGGTGGCGAGCCCTTCCTCTACGGTCAGGTGCTGGACAAC
CAGCGCCAGCCAATCCGCGCCTCCTGGCGCGCCGAGCGTTATGCCGATTACCTGCTCAACACGGCGATGA
AGAAGCGCACTCTGGGTATTCGCACCATCTTCTTCGTCGATATTCCCATGGTGGCCAACCATGAAGATCG
ACGGGGCTACAAGTATGCGGGGATCATCCGGGAAGCGTCGCGCAAATACGGCGTGCCGGAGAGCCTGATC
TATGCGGTGATCAAGACCGAGAGCAGCTTCAACCCCTATGCAGTGAGCCATGCCAACGCTTATGGCCTGA
TGCAGGTGATCCCGGCTACCGCCGGTCGTGACGTCTATGTGCGGGTCAAGGGCAAGAACGGTCAGCCGAC
CCGGGAGGAGCTGTTCAACCCGGCCTACAACATCGATGTTGGTACCGCCTATCTGCACCTGTTGCAGACC
GTCTATCTGGCGGATATCGAGGACCCGCGCTCCCGTCGCTATGCGGTGATCTCCGCCTATAACGGTGGCG
CTGGCGGGGTGCTCAATACCTTCTCCAGCGATCGCAAGGCGGCGCCGGGCCGGATCAACAGGCTCTCCCC
CGAGGCGGTCTATCAGGTGTTGACGGAGCAACATCCCAAAGAGGAGGCGCGCCGTTACCTCTACAAGGTC
AATCAGGCCGAGAAGGGCTTCAAGAGAGCCTTTTCCAGTCGGGCCGGTTAAGCGGTGATGCTGGGTGAAC
CATGCAAAAGGGCGCTTGATATCAAGCGCCCTTTTAACGTCTGGCCAGTTCGTCGTTGAAGTGGCCTTAT
TTGGCGATAAAGCCGCCCTTGGTCTGCGGAGCCGCGCCCACTTTCTCCAGGGTTTTCACTTCCACTTCGG
TCTTGTTCCAGCTGTGGTCCACTTCACCACGGATGCGGATCTGATCATCTGGGCCCACTTCGGTGCCACG
CCACACATCCTGATCCACTTCGACCTCCACTTCACCGCTGGCATCGCGGAACAGATAGTTCTCGCCGCCC
AGGTGCTTGACCAGTTTCCCTTCCAGGGTGGCCCAGCTGTCATCGGCCAGATCTTTCAGTTGAGCAACGG
AAACCTTGTTCTGCTCTTGCGGGCCGGTATAGGCCGCCATGGCAAAAGAGGAGACAGACATCAGGGTCAA
CAGTCCGATTACGCTTGCGCTTGCCTTGTTCATTTCATACTCCTGTCGTTGGTATGAGGGCATTGTGCCG
GGGGGGGCGTGAAACAAGCGTGAAAGAAATAGGCTGGTGTGATTGTCTGTTTTGTGAGCGCAAGCGATTT
CATCGGTGGCGGAACATGTGTGATTTCATCTGGAACTGTAATCTGTCGCCTTCACCAGAAGTCAGTGGCA

GCAGACTCTTCCCATGCCTGTCGCCACTCGAGGAGAAGTCCGCAGGTCTCACCTGCTTAATCGAAGAGCA
TAACAATATGAAAAAATGGCTTGTTTGTTTATTGGGGTTAATGGCATTGACCGCGCAAGCGGCAGAGGGA
CGCCAACCTTTTTCACGGGTCGTGATGTTTGGTGACAGCCTCTCCGACACCGGCAAGATGTACAAAAAGA
TGCGGGGGTATCTCCCCTCCAGCCCACCCTACTTCAACGGACGCTTCAGCAACGGCCCGGTGTGGCTGGA
GCCGTTGGGTGACGAGCGTTTTCCCGGTCTGGTCGTGGCCAACGAGGCGGAAGGCGGTGCAACCGCGGTG
GCCTATAACCATCTGGGGGCCCTGAATGGCTGGCTCGGTTTCTGGTCCTGGGATCCCAAATATCAGGTGA
TCAACAACCTCGATTACGAGATCGATCAGTTCCTGAAAAAGGACAAGTTCCGTCCCGATGATCTGGTGGT
GATCTGGGTCGGTGCCAACGACTATCTGGCCTATGGCTGGAACACCGAGCGGGATGCGGATCGGGTGATC
GATACCATCCGGCTGGCATCCAACCGGTTGGTGCTCAACGGTGCGCAGCAGATCCTGCTGCTCAACATCC
CGGATCTGGGGCAGACCCCGTCAGCCCGCAGCATGAAGGTGGTGGAGAAGGTGCGCCATGTGGCCAGCTA
CCACAACCAGAAGTTGCAGAACCTCACTCGCGAGCTGGCTCCCCTTGGCATCGTGAAGCTGTTCGAAGTG
GACAAGCAGTTTGACGAGATGATGCGCGATCCCCAGCAGTTTGGTCTGAGTGACACAGAACACGCTTGCT
ACGGTGGCGGCTATACGTGGAAGCCATTCTCCGGCAGCGCCGCCGAGGTGGCTGCGACCCCGGCTTTGAG
TGTCTCCGAAAGAGTGGCTATTGCCGGTAACCCGATCCTGGCGCAGGCGGTGGTGAGCGGGCAGGCAAAA
GGGCGAGCAGCGACTCTCAACTGCGATGAGCATATGTTCTGGGATCAGGTGCATCCGACCAAGACGGTGC
ACAAGGTATTGAGCCAGCGTGTTGCTGACTTTATCGATCAGCACTACGAGTTTGTTCGTCACTGATATCG
CTTCGGGCCAGCATGTTGCTGGCCTTTTCCCTCTCTCGGTCCCCATCTGTGCGTCTTTTTGCGTCACATC
CAGTTCAAATAACAAACGTTTGTTGCTGTTTTTGTAACGTGGTTGCGTTAGAGTGTTTTTTGTTAATTGA
TATCAGTGACTTGAAAATAGGCTAACGTTGATATGCTGCGATCGCTTTTGGGTGGTTGCACCTATAAGAC
GAGGTGATATCAATCAACCTCCTCCTACATAAAGTTGCAGAGGCTCAAACATGAAAAAATCATTGATTAC
CTTGCTGGTTACCGGCTTGCTGGCGGCCAATGCCCAGGCTGCCGGTCAAGACAACACCTGGTATGGCGGT
GCCAAACTGGGTTGGTCCAACTTCTACGGCATTGATGAAAGCAATGCCCTGACCCTTGGCAATGAGACCA
ACGATGCCTTGGGTGCAGGCGTGTTTGGCGGTTATCAGATCAACAAGAATCTGGGGTTGGAGCTGGGTTA
CGACTATCTGGGCAATTACAAATACGATGCCACTACGGCGGCAGGCGCTTTCAGTGATGAAGTAAAAGCA
CAGCTGGCTCAACTGACCCTGAAACTGGGCTTCCCGGTTACCGATAATCTGGATCTGTATGGCCGTGTCG
GTGGCGGTTATGGCTGGGCTGATACCAAGAGCGATGTCTACAAAGATGACAGCCGCTTTGTCATGGTCGG
TGCACTGGGTGCCGAGTATGCATTCAACCTCGACTGGGCTGCTCGCCTGGAGTATCAGTACACCACCCCG
TATGGCAGTCGTGATGATACCGGTTTGAGAATGGATAATGGCCTGCTCTCGCTGGCAGCGGTTTATCGCT
TTGGTCAGGTTGCCCCTGTGGCGCCCGCTCCGGCTCCGGAACCTGCTCCTGCACCTGTGATGGTTGAGAA
GCAGTTCACCCTGAGCTCCGATGTGCTGTTTGACTTCAACAAGGCGACCCTCAAGCCAGAGGCCAGTCAG
GCACTGGATAGCCTCTATAGCCAGATTGAAGAGGCGCGACCCAAGGATGGCGTGGCAACCGTAATTGGTC
ACACCGACCGTATCGGTTCCGATGCCTACAACCAGAAGCTCTCCGAGCAGCGTGCCCAGACCGTTGCCAA
CTATCTGGTTGGTAAAGGGATTCCTGCCGCCAAGATCAATGTGGAAGGGCGTGGTAAATCCTCACCGGTT
ACCGGCGACAGCTGCGTCAGCAAGTCCAAGCGTGAGCTGATCGTCTGCCTGGCTCCGGATCGCCGTGTTG
ATGTGAAAGTCGAAGGTGTCAGCGAGGTGCCGGAGTAATCCTTCACACTGAGCTGATCTTCACTGGGGCT
GGAATGAACAGCCCCTGTCGGAGCAAGAGAGACCAAAAAGCCAGTCAGTTGATCTGACTGGCTTTTTACA
TTGGCATTGCGGTCGGGCAGGGGACTAAATCAGGAAGGGAGCGGCATCACCCGGTTGCGCCCCAGCTCCT
TGGCCTTGTAGAGCAGCTTGTCGGTACGTTCGACCAGACAGTCTGCGCTCTCCTGACCGTTGAACTCGCC
CACGCCAAACGAGGCTGTGATGTGGTCGATGCGGCGGCCTGATTTGCGATCCAGAATCGACATCCGCTCG
ATGGCGCGGCGCATGCTCTCCGCCATCTGTCTGGCCAACGCCAGCGAGCGATGGGGGACCAGCATAGCGA
ACTCCTCCCCACCCAGCCGATAGGCGGTGACCCCCTCCTTGCAGGCATCGCGCAGCCGTTTGCTGAAGGC
GCGCAGTACCTGATCGCCGAGCAGGTGACCGTACTCGTCGTTGAAGTTCTTGAAGCGATCGATATCGGCG
AGGATCAGCGAGAAGGGGTGCTGGCTGCGGATCAACCCCTCCAGCTCCAGATCGAAGGCGCGGCGGTTGA
GCAGGTTGGTGAGGGCATCCTCGTTGGCCAGCTTGCGCGTCTCGTTGAGCGCCGCCCGCAGCTCTTTGAT
CTCTTTCTCCGCATTGCTGAGCTGGTTGCGAAAGTGCATGGTGGACATGCGCACATCCCGCGACTCGCGC
ACCAGCTCCCGCAGGATCCCCATGGTGTCTTCCAGGCTGAAGCCCTCGTCATCAATGCGGCTGAGCTTGT
CAAAACTCTTGTCGAGCATCTCCTGAAACATGCCGGTATCGGAGATGGCGTCCTGCATGGAGTGGCCGAG
CTCGTTGGCCATGGCGGCAAGGCTCAGTTTCATCGCCTCCATCTGCTTCTCGTCCTGAGCGGCCAGATAC
TGGTGATAGAGTCGCTCGCAGGTGGTGAGCGAGCAGGTTCCCTGCATTTTGACTGCCTGATCAATCGCCT
GGTTGAGCTCGGGCATGCTGGCCGAGACATAGTTGTACCACAGGTGGTAATTGTCCGGCGTCGTGGGGAT
CTGGTACTTGATCATCAAGGGAACCGCCTGTTTGAGATAGGCGGCGGATTGGGCAAATGTGTCCTTACTC
ATGAGTTCTGTGTCTCATCATGCCAGCGAACTGAAGATAGTATGACAGAGAGATTGGCCAAAAGGCAGCC
GATGGATGCCATTCGCCCCCCCCTCCGATATAATGGCACCGTTACCACCTACCCTAAGGCCTGCTTATGT
CTCAAAAAGATCCTTTCCTCGAACGCGAGGCCGAAAAATACGATAACCCCATCGCCAGTCGCGAGTTGAT
CCTGGAACTCATCAAGGGCCATGAAAAGCCCATGTCCCGTGAAGAGTTGGCCAAGGTCCTCAAGCTGAAA
GATGAAGAGCCGTTGGAAGCCTTGCGTCGTCGTCTGCGCGCCATGGAGCGTGATGGACAACTGGTATTCA
CTCGCAACCAATGTTATGCCCTGCCGGATCGCCTGAATCTGGTCAAAGGTTATGTAATCGGCCACAAGGA
TGGCTTCGGTTTTCTGCGTCCGGAAGGTGGTGGTCCGGATCTGTATCTGAACAACCGAGAGATGCAACGC
CTGATGCATGGCGATTACGTGCTGGTGCAACCGACCGAGATTGACCGCAAGGGTCGTCAGGAAGCGCGTC
TGGTGCGCCTGCTCAAGTCCCGTGAGGCAGACATTGTCGGCCGTTACTTCGTCGAGAATGGCGTTGGTTT
CGTGGTGCCGGATGACAGCCGTATCGGCCAGGACATCATCATCCCGGAAGGGGAGAACAAGGGGGCTCGC

CAGAGCCAGGTCGTGGTCGTGCGCATCAACCAGCGCGCCACTGCCCGCTTCAACGCGGTCGGTACCGTGC
TGGAGGTGCTGGGTGAAAACATGGCGCCCGGCATGGAGATCGAAATCGCCCTGCGTACCCACGGCATTCC
GCACACCTGGCCGGAAGAGGTGACCAAAGAGGTTGCCGGTCTCGGTGAGCAGGTGCCGGAGAAAGCCAAA
GAGGGCCGCATCGACCTGCGCGCCCTGCCGCTGGTGACCATCGACGGGGAAGATGCCCGCGACTTCGATG
ACGCCGTCTTCTGTGAAGCCAAACGCGGTGGTGGCTGGCGTCTGTGGGTTGCCATTGCTGACGTCTCCGC
TTACGTGAAGCCCGGCACGGCGCTGGATGCCGAAGCGTATCAGCGCGGCAACTCCGTCTACTTCCCCGAG
TTCGTGGTGCCCATGCTGCCGGAGGTGCTCTCCAACGGCCTCTGCTCGCTCAACCCGCAGGTTGACCGTC
TCTGCATGGTGTGCGAGATGACCATCTCCGCCGCTGGCCGCATGTCCGGCTACAAGTTCTACGAAGCGGT
AATGAACTCTCACGCCCGTCTGACCTACACCAAGGTTGCCGCGATCCTCGATGGCGATCCCAAGCTGCGC
CAGCAGTATGATCCGCTGGTGGGCCACATCGAAGAGCTGAACAACCTCTATAAGGCGCTCAAGCATGCCC
GTCATCAGCGTGGTGCGGTGGAGTTCGAGAGCGAGGAGACCCGCTTCATCTTCAACGCCCAGCGCAAGAT
CGACCGCATCGTGCCACTGGTGCGCAACGATGCCCACAAGATCATCGAAGAGTGCATGATCCAGGCGAAC
GTGGCTGCTGCCCGTTATATCGAGAAGAACGAAGCCGCCGCCCTGTTCCGGGTGCACGACCGTCCGGGCG
AAGAGCGTCTGACCGGTTTCCGCGATTTTCTGGCCGAACTGGGTCTGGAGCTCAAGGGCGGTCTCGAGCC
CGAGCCGAAAGACTTTGCCGAGCTGGCCGCCAAGTTTGAGGGGCGCCCCGATGCGGAGCTGCTCTCCACC
ATGCTGCTGCGCTCCATGCGTCAGGCCATCTATCAGGCTGACAACATCGGTCACTTCGGTCTGGCGTTGA
AGTCCTACGCGCACTTCACCTCGCCAATCCGTCGCTATCCCGACCTGATCCTGCACCGGGCGATCAAGTA
TCAGACCGCCAAGGAGCAACAGGGCAACCTGCGCCACAAGTGGACCCCGAGCGGTGGTTATCACTACCAG
CTGGAAGAAGTGGACCCGATGGGCGAGCACTGCTCCATGACCGAGCGCCGTGCCGACGATGCCACTCGCG
ATGTGGCCGACTGGCTCAAGTGCGAGTACATGCTGGATCACGTGGGTGACGAGTTTGACGGCGTTATCGC
CAGCGTCACCGGCTTTGGCTTCTTTGTCCGTTTGGCTGAGATCCATATCGATGGTCTGGTGCACGTCAGC
ACCCTCACCAACGACTACTACCAGTTTGATCCCCTGCACATGCAGCTGATTGGCGAGAACTTCCGCCGTC
GCTACCGTCTGGGCGACAAGGTGCGGGTCAAGGTGATGGGCGTGAACCTGGACGATCGCAAGATTGATTT
CGTGATGGTGGAAGAGCCGCTCAAGGCCACCGGCAAGAACGCCAAAGAGATGGCGCGCAAGCAGGCCGAG
ATCAAGAAAGAGAAGGCCAAGAGCGCCCAGCGCCACGAAAAACGTAAAGAGGGCAAGGATCACGGCAAAT
CCGACAAGGGTGGCCGTGCCAAACCGAGCAACAGACGACCGAGCAAAAAGGCTCGCGATAAGGTGAAGGC
CAAGGGGTGAGCCCTCGGCCTATGGCTTGGGTCCGACGACCCGGGCTGAGGTCGTAAGGGGCTGATGGCC
CCGGGTTACCTTCGCCAGCGCCCGGGTTATCACTGCAAAAGCCTGCGACTCTTGAGATGAAAAATCCGAA
CCCGGTTACCGAGGTTCGGATTTTTTATGGTCAGCGAAAAGGGGAGGGGCTTAGATTCTGGGCGGGGCTC
AGCCAAGATTCGTGCATATGGCTAGGCTGTGTTCTTAAAGTGATGAATATATAATCTCTTATCTTCTCAA
TAGCTTAAGATACTATGCAGTGTCCCGTCGTTACCAGTTAACCGTTGCGCAATGGGGCTTATCGAGCCCC
TTTTTCGATACCAGCGCATAAGCGGACGCCCTACTCGTGAGCCACGCCAAGTGCTTGATGGCATTCTCTG
GATACTTCACACCGGTGCACCTTGGCGAGATCTGCTGGAACGTTTCGGCCCATGGCGCTCGGTCTACAAC
ACCTTTCGACGTTGGCTGAGTACAGGTCATATCGATGCCATCCTTGCTGCGCTGCAACTGCATCTCAATG
AAGAAGGGCTTATCGACTTTGACCTGTGGTGCATTGATGTAGTGGTCAACTAAATCCGGACACTTGGTTA
GGCCGATCCTCGGCTTTTGCTGGCGGTATCCCGTCATTGTGGTGATGTGGACGCCGCCAGTTGTAGTAAT
CCATCAGGTAATAACTGATATCCCGCTTTGCCTCCCGCACGCTTATGTATCCCATAGCCGGAACCCACTC
CGTTTTAAGACTTCTGAACAATCGCTCCATCGGGGCATTATCCCAGCAGTTGCCACGCCGACTCATGCTC
TGGGTCATGCGATAGCGCCACAGTCGTTGCCGAAATGCCCTACTGCCATATTGGCTGCCCTGGTCAGAGT
GAAACAGCACACCAGATGGGCAACCACGCTGCTGATAGGCCATCTCCAGTGCTTTGATTGCCAATTCTGC
ATCAGGTTTATCAGACATCGCCCAGCCCACGACCCGCCGAGTGTGCAGGTCAAGCACCGCTGCCAGGTAA
TGCCAACGGCCACCAGCCCAGACGTAGGTGATATCGCCACACCACACCTGATTGGGTTGCTGGACATCAA
ATTCGCGGGCCAGCAGATTGGGGATATCCGGTCGCTCAGACTGGGCGACCTGATAACGATGAGCGCCCGG
CTGTTTGGATACCAGCCCGGCTTCCTTCATCAGGTTACGTACTTTGAATCGACCAATCTGATACCCCAAC
TCCCGCATCATCGACATCAAGGCGCGGCTGCCTGCAGAGCTCCTGCTCTCTTTGAACAACCGGCGCAACT
CACTGCGTTGCTGTATCCGCTCACGGTTAATCGCCCGTCTGCGGGCCAGATAGTCGTAAAAGCAGGATGT
TGGAACATCGAAAGCGCAGCAGACCAAGGTGATGGGGGCTTGCTCCCTTAATCGGTCTATCAGCGCGTAC
GTGTGAACTCGTCCGCCATCAAGAGAGCAGTAGCCTAATGGGATGGTCTGCTCCACCCACCCCGCTATGC
TGTAGCCAGCATCAAGGAGGCTCATCATGACTCAAGCGCAGATTTACATTTACGGGATCGATTTAGGCAA
AAATTGGTTTCACCTGGTGGCGATGGATCGCATGGGGCATGTATTGACCAAGCAAAAGCTCACGCGTTCA
CAACTGAAACAGTTCATTAGCACGACCCCACCCTGTCGGGTCGCCTTCGAGGCATGTCCTGGTTCGCAAT
ACTGGGGGCGCCTGTTTCAACAGGCGGGGTTCGAACCCAAGATTATCCCTGCCCAATTTGTGAAGCCCTA
CCTCAAATCAAACAAGAATGACTTCAACGATGCTACCGCCATTGCCGAGGCTGGTAGCAGAGGTACTATG
CGCTGTGTCCCGTTGAAATCTCATGAGCAACTGGCATTGCAAGCGACTCATCGCGTCCGTCAGCGTTTCA
TCGTCGAACGTACCGCCACTGTCAATCAGATGCGAGCGTTGCTGCTGGAATATGGGCTCACCGTGCCAGT
CGGCAGAAAAGTCTTCGAGCGCTCTCTGCCGACGATCCTTGAAGATGCCGAGAATGGCTTGCCTGACTTT
ATCCGCGCCCTGGTATTACGCCTGAGAAAACGGTGGCAACAGCTGGATATTCAGATTGATGACATGAGCC
TCATGCTCGGACAAGCCTCAAGCGCATCAGGTTTGTGCCAAAAAATCAGTACCGTCCCCGGCATTGGACC
GATTGTCTCAACCGCGTTGATTGCCGCGATTGGCAATGGCTCACAATTCAAGAAAGCCCGCGACCTGTCA
GCTTGGTTGGGGCTGGTCCCCAGGCAATACTCAACGGGCGGCAAATCTTCGCTCGGCGGGATCAGTAAGC
GTGGCAATAGCTATTTAAGGCAAATGGTTATTCAAGGCGCCAAAGCGCTAAAAATTCACATGAAGCGTGA

CAAATCATCGTTAGGCGAATGGGTCGCTAGGCTGGAGGCTGGCCATCACCATCATGTGGTTCTTATCGCC
TTGGCCAACAAGATAATGCGAATTTGCTGGAAAGTACTGACATCCGGCAGGGATTACCAACCCTATCCCA
ACGCAAGCGCAGCCGTTTAACAGATATAACTCATACCCGGTTTTGCGAGCCAAAAACAGATGACAGAAGC
GTTCACCCCACGACGTTGCAGCCTGGCTAAAAAAGCAGTCTCTCATGACTGGTAGCTTTATTAGGACGAT
GACGTGCGGATCCCATCAAGGCCAGAGCCGGAAAACGGCTCACAGACAGGCCGGATACATTGACGCAAAC
GACTTCTGGAAATCAGTTCAAGCTTGCAGATAAGGAGCAGACCATACATTTTTTAGAATGTCCTTTTCAC
GTTCGAGCCGGTTGATACGGGCTTCCAGCTCCTGAATTTTTTGCTGCTCCGGAGTCAGTGCTTTGCCGAT
TGGGGTTGTGCCAGTTCGTTCGGATTGCAGTTGCTGAACCCAGCGGCGAAGTACGGTTTCACCGACATCC
AGTGAACGGCTGGCTTCAGGGACAGAATATCCCTGGTCGAGCACCAGGGATGCGGCTTCGAGTTTGAATT
CGGCAGTAAATGAGCGACGTAATCTTGTCATTGAACACCTCAGGTCATGGTGGCGACTGTACCACCTAAA
ATGGTGTCCGAGATGATTAGACCACTACATGACGGCTCCAATGTCAGGGCATCAAAAGATGCTGTCGGAG
CGCCTAAAAAAGCACTCCGGTAAATCAAGCTCTTGGTCGCTCTCGTGGTGGTTTTGGCAGCAAAATTCAC
TTGGTCACTGATGGTAATGGTTTACCGCTGGGGTTCTGCCTTTCGATGGGGCAATCAGCAGAAATCAGAT
ATGCGATAAGTGCGTTGGTCATGGCAAGGATCCCGACTTCATCAGGCCTTTATCGCACTCGGCCAACTTA
TCTTGCCGCAGATAAGGCCTATAGCAGCAGGGCTCTGCGGGCTGAACTCCTGCGTAGAAAGATAAAAGTG
GTTATCCCGCAACGCAGCGACCAGCAACGGCACCATAAGGGACGTCCACTGGAGTTAGACAAAGCTCGTT
ATCGTAGGAGAAATGTGGTCGAACGGTGCTTCGGATGGCTGAAGAAGTTTCGCCGATCCTCAACGCGGTA
TGAAAAGCTGACGGGAAGTTTTGCTGCTTTTATCAAGCTGGCTTTCTGCCTTCGTTACTTACGGGAACTA
TTGGTGGACAGAAAACCAGCATTTTGAGAACACAGCCGAGGGACATAATTCTTGCGCTATTGCCAATGTA
TTGGCCGCTATGAGGCGCTCAGAGAGGTGGGTGTCCTATTTCTGTAATAATTTTTTAAATAACATCCTCT
AGGCTTACTCTTTTAAAAACATCCAACTTTCCTCCACGAGTAGCATTAAATATTTCAATTCCTCTCACCT
CTAAGTGAGACTTAAGCAACATGAATTGTTTTGTTATATTTTCAATTCTTGGTTCTGCCCATATGTCACC
AGCTTTTCGATAATTTTCATGAAAATGATTTTGCTCTCCCTCACTAATCTTATATAGATTATCTTTGGTT
TTTGGGGGGAACTTATAGTTATGATCAAAACCGAGAAGAAAAACTCTTTTTGCGCCAATAAATACTGCTA
GCTGAACCATTGCATACACAACACTTTCGCCTTTAAAAAAGCCAACAATAGGATTTTCTTCAAAGCCCAC
TTCATCAAAAGGCACATTGCTTTTATTTGTGTTGTAGTATGTGATATCACGATTAAAACGTGTATATCTT
AAACAGTCGCCAGGGGCATAAACATTTTTCAATGAGTATTTTTTAAAATCATGCAGGTACTCAAATATAT
CTAGAGGATCCTCTACCAGATAATGTTCTGGTCGCCAAGTGGTCTCATCAAAACATAGAAATATTTTATT
ACATGCAATTGTAGTTTTCCCACGTATTAGTTCAAGATCAGAGACTTTCAGGCTAGGGCCATTACCAATT
ATAAAAACATCTTTATTTGCTAAGTGCTGTTCTTCCATTTTTTTCAACGAACTATTCATTGATAGCTTTC
TATATATCTTACTTTTATAAAAAATATTTAAACTCGCCCTCGATAACAAGCTTGTCAATGCAGCCTTGAA
ATTATTTATTTTAAAGAGATTGAAACCTTCTTCTTTGCTATAAAAAACAAACTCTCCACCTAAAAAAAAC
ACCATAGTCACTTGGCAATGACTCAACTTAGGATAAATTTCATCTTTGTAGTTTGGGCTATACAGATATA
CATGTGAACCTACTGGTAGGTTATCATATTTGATTATGTTCCCCCCCTCTTTAAAGTCATCAGCATAACC
ATCTATCTTACATCCTTGCCTTTGCAACTCTATATACAAAAATTGTGCAATGTCATTATATGGTGCGATG
TATACTCTGCCTGAACTCATTGCCCCTCCTCAATAATTTATAATGCGCTTAGTTTTTTGCGCCACCATTG
GTACTGTGTGAAAAAACTGTACACGGTCAGTCTTGGAAAGCGTCGCCTAATCACGCCTACGTGAACATAT
CAGGAATGAAAATGATGCTCTACTTGTACAGATCTAGTTATCACCTGACACCGGCCTCAAACCGGTGTCT
TGTTTACGCTACTTTAGTGCGCACTTCCTTCGCTATCAATTTCAGACCCTTTTTGAACATGCTGTAAGGG
GTCTGCCCCTCCATCATCCTGCCCTGATGTGGCCGCTGGGTGTTGTAGTGCTCCAGGTAGCTATCCAGGT
CTGTCTGCATCTGCTCTACCGACTCATACTAGATCGTCCTCCCCTTGATACGGAAGTGTTCATCCAGCAA
GGTTCGATGCAGCCGCTCGATAAAGCCGTTGCTCTGCGGCCTGCGCACCTTGGGGGTTCGGTGCTCAATC
CCCTCCAGTTACAGGAACAGCTCGTAGGGATGGTGGTCAGGGCGCCCACAGAACTCGCGCCCGTTATCCG
ACAGGATGGTATAGACCCGTGCCTCGTGGGTTTCAAAGAATGGCAGCACCGTTTCGTTAAGCACATGGAC
TGAGGTGATGGGCAGTTTGCTGGTGTAGAGCCGCCCCCAGGCGTGGCGGCTATAGCAGTCCAGCACGGTT
TGCAGATACACCTTACCGACCCCTTGAGCGCGCCGACGAAGAAGGTGTCGACCGCCACCAGTTCCCCGGT
GTAGTGGACCTCAATCTGACGTTCGCGAAACTCGGGGCTGAAGCGCTCTAACAGGCGAATTTGCTCGTCA
TTGAGCTCAATGGTCTGCTCCCGTTGGATTTTCTCAAGGCGCAACAAGCGGTCGTGTTTGGAGAGCAAGT
CGTGACGTTGCCACACGCCACACACGCCGCCGGCACTGACGTTGATACCTTGCAGAGGCAGTTCTTGCGC
AACCCGCAGCGGGCCGTGAGTAGGCCTGGTGAGGGAATAATCGAGGATGGCCTGCTCAATCTCGGGCGCA
ACCCGTTTGAGGAGCCCCTTTACAGCCGGGCAATTTGTCGAGCAATCCCTCGGCGCCATAGGTCTGGTAA
TTACGGCGAATTTCGTAGAACTGCTGACGGCTGTAGCCGATGAGCTTGCAGGCTTTGCTGACATTATTGA
GTTCTTTGGCAAGCTCTAGCAGGCTGAGTTTGCGACGTGCTACTTTCTCGTTGGTGGTCATACGGTGACT
CCGGATAACACGGTAGGGGTACCGTTTTGTTATCCGGTTTGAAGCCGTATGACCACTCCCTTTTGTGGGC
AACTGTCAGGTGAATACCATCTCCATACAAATAAATAAGGTATTGGATTCCTCTGTCATTCGCCGATGGG
GGAGAGCGGGTGTTGGGCTGAGCAGTTTCCTCCTCCCTAGATGCCGGTCTCTCTGGTATGATGCGCCATC
ATTTAGCGACCGGTGACGACGCCACCATCATGGCGGTCAGGCTGCCGGCACGGCCATTTTTCTTCGGGGC
GCATTTATGCGACCCGACCTACGGATCTTGAACATGAGTACTGAACTGATCTACGGCATTCACGCTGTAT
CCGCGCTGCTGGAGCGCACTCCCGAGCGCTTCATCGAAGTGTGGGCCCTCAAGGGGCGGGATGACGACCG
GCTGCAACCCCTGCTGGCCGAGCTGGAGTCCCTCGGTCTCAAGGTGCAGAGCGTGAACCGCAAGACCCTT
GATGACAAAGCGGAAGGCAACAATCACCAGGGCATCATGGCCCGGGTGAAAGAGGCGCCGAAGCTGGCCG

AGCACGATCTGGCCAGCCTGCTGGACGAGCTGGCGGCACAACAGAACCAGCCCTTCCTGCTGGTGCTGGA
TGGCGTGACCGATCCGCACAATCTCGGCGCCTGCCTGCGCAGCGCCGATGCTGCCGGGGCTCATGCGGTC
ATCGTGCCGCGGGACAAGGCCACGGGTCTCACCCCGATCGTGCGCAAGGTTGCCTGTGGCGCGGCCGAAG
TGGTGCCGCTCATTCAGGTCACCAACCTGGCCCGCACCCTGCGCGAATTGCAGGAGCGCGGCGTCTGGAT
TGTCGGCACCGCTGGCGAGGCCGATCACGACCTCTATCAGGCCAAACTGACCGGCCCGATGGCGCTGGTG
ATGGGTGCCGAAGGTAAAGGTATGCGTCGCCTGACCCGCGAACATTGCGACGAGCTGGTGAGCATCCCGA
TGGAAGGGGCGGTATCCAGCCTCAACGTCTCGGTGGCAACCGGCGTTTGCCTGTTTGAAGCCGTGCGTCA
GCGTCGCGGTTAATCATGCTGACTGACAGACCCCCCTTCGATTGGGCGCAGGTCGCAGATAGAGAGGAGG
CCGCGCGCCTCCTCTCTTTTGCCCGCCCGCTGACCCTCAGCCCGAAAAGCTGCCTCTGGCATGCCGGCGA
CAGCCCGGATCTGCTGGTCAGGGTGGAGCAGGGGCTGCTGCGGGCCAAGCTGGTGCTGGAGGATGGCCGC
GAGTACATCAAGGAGTTCTACTGGGAAGGGGACGAGTTTCTCGACTTCCATCACCTGCTCAGTGGCGAGC
CGGCCCGCTACAGCGTGGAGGCGCTGGAGTCTTGCCGTCTGCAGCTGTTCAGTCTGGCCGACCTGCGCCA
GCTGCCCGGTTGGCCGCGCTGGTATCAGCACCTGCTGGAGGTGCAGCTGCGCATCAAGGAGGAGAAGGAG
CAGTTGCTGCTGACCGGCAGCCCGCAGGCGCGCTATCAGCACTTTCTCGACAGCTTTCCCGCACTCGATG
CCAGAGTGCCGGATCACCAGATCGCCGCCTATCTCGGCATTACTCCCATCAGCCTGAGCCGCATTCGCAA
GCGGTTGAGAGCGCTGAACAAGGAATAGTGTCGCCGAGGCGCCATGAGGGGCTGTGTTGCCACCAGATTT
CAGACCTTTCATAGAGCCCGTCTCGGCATTACGCCAGCCAGTCAGTCTGAGCCGCATTCACAAACGGCTG
AAAGAACTTAACAAAGATTAATGCAGGGCGCTGGCGCCACGATTATGCTGGCAGGCCGAACTGATAAGGA
GTTGCCGATGAACTGGATCCTGAAACCCTGGTCTGAACTGACCACTGACGAGTTGTATGAGTTGCTGGCG
CTGCGGGCCGAGGTGTTCGTGGTGGAGCAGACCTGCCCCTTTCAGGATCTGGACGGGCTCGATCGCCGCG
AAGGGGTCTGGCACCTGCTCGGTTATCAGGGCGAGCTGCTGGCCGCCTATGCGCGGATCATGGCGCCCGG
CATCGGCGACGAGAGCGGCGCCGCCATCGGCCGGGTGGTGACCTCACCCAAGGCGCGTGGCGGTGGTCTT
GGTCATAAGCTGATCGGCGAGGCGGTCAAGGCGTGCGAGGCGCGCTGGCCGACCCACAGCATCTGGCTGG
GGGCGCAGGCTCATCTGCAAGGCTTCTATGGCCAACACGGTTTTGTGGCGGAAGGGGAAGGCTATCTCGA
AGATGATATCCCCCATATGGGGATGCGCAGAGTGGTGGCATAACCACTCGTCAGAGTGATTCAGAATGCA
GAAGGGCCATCCCGCGGGATGGCCCTTCTCTCTATCGCCAGGCGATCACTTCTTCTTTTTCTTCTTGGCG
GACTTCTTCTTGTCGACCGGCTTTTTCTTCTTCACCGGTACCCGCGCTTCCTTGTGTTTCGGGCGCAGCT
CGTCGATCACCCGGCGCTTGAGGCGCTCCTCGGTGTAGCGTTCGATCTTGGCGACCATCGCCATGTCGTG
GGCTTCCACCAGGCTGATGGCACAGCCACGGTTGCCAGCGCGGCCGGTACGGCCGATCCGGTGGACGTAG
ACATCGGCGCCGTAGGGCATGTCGTAGTTGATGACATGGCTGACGTTCGGCAGGTCGATGCCGCGGGCGG
CAACGTCGGTGGCGATCAGGAAGGGCACTTCCCCTTCGTGGAACTTGCGGATCGACTCGATACGCTTGCT
CTGCTCCATCTCGCCACGGATCCAGGCGCAGGAGATACCTGCCGCCTGCAGCTGGCCGGAGAGCTCCGCC
AGACGCTCGCGAGTCTTGACGAAGATAATGGCTTTCTGGGTTTCCGGATCTTTCAGGATATGGGTCAGCA
GCGCCAGCTTGTGGGCGGCGTCATCAGCCAGATGGATCCACTGGGTGATGGGGCGACGTTCGCTGCGGGG
CGGCTCGGCGTGCAGCTCCACCGGATCCTTGAGGATCTCGTTGGCGAACTTCTCAAGGCCGGCCCCTTCC
AGGGTGGCGGAGAAGAGCATGGTGTGCTTGCGGTAGCGCGCCTCTTCCACGATGCGGTTCACATCCTTGA
TAAAGCCCATGTCCAGCATGCGATCCGCTTCATCCAGCACCAGCACTTCGATGTCGTGGCTCTCGAACTC
CTCTTTCTCGATGTACTCGAGCAGACGGCCCGGGGTGGCCACGACGATGTCGGTGGTCTTGGTCAGGGCA
GGCAGTTGCTCTTCATGGCTGACGCCGCCGATGATGGTCTCGATGCTGAGGTTGGTGTGAGCCGCCAGCG
CCTTGGCGTGGGCGGTCACCTGCAGCGCCAGCTCGCGGGTCGGGGTCAGGATCAGCATGCGGCAGGGGCC
CGGCTTGCGGCGCGGGAAGTCCAGCAGGTGCTGGAGTGCCGGCAGCAGGAAGGAGGCAGTCTTGCCGGTA
CCGGTCGGAGCGGATGCCAGAATATCGCGGCCGTCCAGGGCTGGCTCCAGCACCATCTGCTGGATGGTGG
TGGGGCGGGTGAATCCCATCTCGGCCAAGGCCCGGTTGAGGGCTGGGTGCAGGTCGAAATCATCAAAGGA
CTGGCTCATGGCAATACTCGGTCATTAAGGAAACGGCAGATTATAGGCGATTATCTCGTCGGCTCCTATT
GGTGAGTGTGTCTTAGCCGCTATAATCGCCCACCATTTTTGCAGATGACGGGGGCCAGATGGGACGTAGT
CGCGGATTTACCTTCAAACAGTTCCATATCAATCACGATCGCTGCGCCATGAAGGTTGGCACCGATGGCA
TTTTGCTCGGTGCCTGGGCGCCGGTCGAACAGGCGCGACGAGTGCTCGATATCGGCACCGGCAGCGGCCT
GATCGCCCTGATGCTGGCCCAACGCAGCAGATCTGACTGCCGTCTTGATGCGGTCGAGCTGGATATTAAT
GCGGCCAGTCAGGCGCGGGAGAATGCGGCTGCCTCTCCCTGGGCCGATCGGGTGACCATCATAGAGAGTG
CCATTCAGGATTATCAGGCTGCCCCTTACGATCTTGTCGTCTCCAACCCTCCTTATTTCGTGGCAGGCCA
GTCATTTAGCGATCCGGCGCGGGCGTTGGCCCGCCATACCGGTGCCCTCGACAGCCCCGCATTACTGGCG
GCCTGCGATCGTTTGCTGAGCCCGAACGGGCAGGTGGCGCTGGTGCTGCCGACGGCGATGGCCGATGAAA
TTTTATGCATTTCGGCGAATTATGATCTGCATGGCATTTGTTATACAGCTGTTATCACCAGAGAGGGTAA
AGAGGCAAATCGTGTTTTATTGCTACTTGGCAGAGGATTAAACAGGTGTGAGAGGGGTGAAATTGTGATC
CATTCTGCTGACGGGGCCTATTCCGACAGATATATCCAACTGACCAACCCCTTCTATTTGAAGATGTAAA
TTTCGTCTTGAACTATTTTTTCACGCCATTATGCTGGTTCCAGATCGAGCGCCCGCTCCTGACCAGCTAA
AACCACTGGTTGCGATGAAAAAACATGCGGGGCTCTTTTTTTATACCCCCTGAACACACCCGGTCAGCGA
CCGATAAAAACTATAACAAGCAATAATTTACAAGACAGACGAGGTTGAACTTGACGAAATCACTCACATT
GTCCGATGTACTCGGATTAGGGTTTATGACCTTCGCCTTCTACTTGGGCGCCGGTAACATCATTTTCCCA
CCGTTGGCCGGTTACATGGCTGGTGAGCACCTCTCCCTGGCGATGTTTGGCTTCCTGGTCACTGCCGTGG
GCCTGCCTCTGGTGACCATTCTGGCCGTGGCGAAAGCCGGTAACGGTTGGGCTGGCATGACCAAGCTGCT

GCCTGCCGGTGTCGCGACTGCGCTGGCCGTTGCCATCTATATCATCATCGGCCCGGCTTTTGCCGCGCCC
CGTACCGGTCTGGTTGCCTATGAAATGGGCCTCAAGCCATTCATCGGTGACATGGGGCAGGCGGGTCTGG
CGGCCTATACCGTGATCTTCTTTGGTGTGGCCATTCTGGTCTCCATGAACCAGGGCAAGCTGATGGACGC
CATCGGCAAATACCTGACTCCGGTACTGATGCTGCTGCTGCTGACTCTGGCGATCGGTGTATTCGTAGCA
CCGCAAGGCTCCATGCCTGATGCGTCCGGTGACTACCAGAACAGCCCCTTCGTGAAAGGGATCCTGGAAG
GCTACAACACCATGGACACCCTGGCGTCCCTGATGTTTGGCGCCCTGATCGTCGACCTGCTGCGCCAGAA
AGGCATCAACGATTACAAGAGCCAGTTCAAGTATCTGGCCATTGCCGGCCTCATCTCTGCCGTTGGTCTG
AGCGTGGTGTACGTATCCCTGTTCCAGCTGGGCAACACTGCGGCCGGTGTGGCGACCGATGTCAGCAACG
GTGGCGCTATCGTCAACGCCTACGTACTGAGCCTGTTTGGTCAGCCGGGTCAGTTCATTCTGGCGGCCAT
CATCACTCTGGCTTGCTTTACCACTGCCGTGGGCCTGATCTCTGCCTGTTCCGATTTCTTCCACAATCTG
ACCGGCATGGCTTACAAGAAGCTGGTGGTGTTGCTGGGTGTTATCTGTGCCGTGGTAGCCAACGTGGGCC
TGAGCCAGTTGATCAGCCTCTCCATTCCGGTGCTGGTTGCCATCTATCCGGTTGCCGTGGCGCTGGTGCT
GGTCACCTTCCTGAAGGGTTACTTCGGCCGTCCCCGTCTGGTGTTCCGTGGCGTGTTGATGGTGGCCTTC
CTGTTCGGTTGCCTGGATGGTCTGGGTGCTGCCGGTATGAAGATGGATGCGTTTGCCTTCTTGCCGCTGT
TCGACAAGGGCCTCGCCTGGCTGATGCCGACCCTGCTGGCCTGCGTAGTTGGCGTGATGGTGCGTCCGAC
CGAGATGGCTGCCGAAGCTGCCTGATCACACCCGATCTGATATTGCCAAGAGCCTGCCCCGTGCAGGCTC
TTTTGTTTTCTGTGAGCCTAGCTTCTCGCCTCGCTTTGCTGCCGTAATTGTCACTGTACGCAGATAGCAT
CTGAGCAGCCGTATCTTGCCGCTATAACCAGTTCCCCCTTCTACAACCCCTTCTCACTTCCCATAGGGCA
CGTGCGACACCTGTTTATGGAGCGCCGAGTTGTTCGGTGATTAATTCCGCAGCGGTAAATTGTTTCAAGA
TATTAATTGGTGATTGAGATCGAACTCCTCTTCTTTATGCTAAAGATGGTGCGCCTCATCCGGTCTGCGG
GCCGTGTGGGGTGAGAGGTATTGTTACCGGTTATCTGCCAGGCAGTGGGTGCAATCAATAACAAGCTGAT
AAGGCAGATGAGGTTGAGTTTGACGAAATCATTGAAGTTGTCCGACGTATTCGGATTGGGGTTTATGACG
TTTGCCTTCTATCTGGGGGCAGGCAATATCATTTTTCCGCCGCTGGCAGGTTTTATGGCTGGTGAGCATC
TGCTGGCCGCCATGTGCGGCTTTTTGCTGACGGCAGTGGGCTTGCCGCTGATCACCCTGATTGCGGTGGC
CAAGGGGAGTTCGGGGGGCGGCGCGAGTGGCTGGACCGGAATGACCCGCTATCTGCCCGCCGGGGCGGCG
ACGGCGCTGGCCATCGCCATCTATATCATCATGGGGCCCGCCTTTGCCATTCCCCGCACCGGTCTGGTCG
CCTACGAGATGGGGCTCAAGCCCTGGCTTGGCGATACCGGTCATGGGGGACTGCTGATCTACACCCTGAG
TTTCTTTGCGCTGGTGATCCTGGTGGCGCTCGATCGGGGCAAGCTCCTTGATGCGGTCGGCAAGTATCTG
ACCCCAGCCCTGATGGCGATGCTGCTTATTCTGGCGGTCGGGGTCTTTATGACGCCGCAGGGCACCATGC
CTGATGCCAGCGGTGAGTACATAGATGCAGCCCTGACCAAAGGAATGCTGGAGGGGTACAACACCATGGA
TACCCTGGCCTCCCTGATGTTTGGCGCCCTGATCATAGGTCTGCTGCGTCAGAAGGGGATCGAGGATTAT
CGCAGTCAGTTCAAATATCTGACCATTGCAGGCTTGATCTCGGCGGTTGGGTTGTCGGCGGTGTATGTCT
CGCTCTTTCAGCTGGGCAACAGTGCCGGTGGGGTGGCGAGCGATATCAGCAATGGTGGGGCCATCGTCAA
TGCCTACGTCCTGAGTCTGTTTGGTCAACCGGGCCAGTTTATTCTGGCTGCCATCATCACCCTGGCCTGT
TTTACCAGTGCGGTCGGCCTGCTCTGTGCCGGTGCCGACTACTTCCATGGCCTGACAGGTTGGGCCTATC
GCAACTGGGTGATGCTGCTGGGGGCGCTCTGTGCACTGGTGGCCAATGTGGGGTTGAGCGAGCTGATCGG
CCTCTCCATCCCGGTGCTGGTGGCCATCTATCCGGTGGCCATCGCGCTGGTGCTGGTGACCTTCCTCAAG
GGATGGTTTGCCCAGCCACGGCTGGTGTTTCGCGGTGTGCTGACGGTCGCTTTTCTGTTTGGTTGCCTCG
ATGGGCTGGGGGCTGCCGGGATGGCGATGGACGGCTTTGCCTTCCTGCCGCTGTTCGACAAGGGACTTGC
CTGGCTGCTACCGACTCTGCTGGCCTGCATCGTCGGCATCCTGGTGCGACCAGGCGGTAAGCTGGCTGCC
GAAGCAGCTTGATTCAGAAACAACGAGATAGCAAAGAGGCCAGCAGCTGCTGGCCTCTTCTGTTTCTGCC
GGATCCGTTACCGGATCAGATTTGCGGGCCTTTCGGCTTGGGTTTGCCATCCAGACCATACCAGTGGGTT
TCTACCCGTCCGGCATGCCACTCGAAGGTGCCCCAAGGCTGCTTGGCTTCCCAGCGCGGGAAGTTGATGA
GCACCCGGTTATCTTCGTACTTGTAGAGGGAGGTGCTGATCTGAATGTCCGACAGGATCAACAGCAGGGC
CCAGCAGATGAAAATCCATTTCAATGCTTTCCACATAGGGTTACTCGAAGCAAATTTCGATAGTGGCGCG
ACCGAAATCCGATTCGAACGGCATCATGATCTTGGTGCCATCGGCACGGTGGGTAATGGTGTGGTTGGGG
CCGGAGACGATGATGGGGGTCGCCATGTCAAACTCGTAGCCCTTCTCGCCCAGCATCCGCTTTGCACCGC
CGGTCACCATGTTGGTGATTTCACCGACCATGTCGGTCACCTCTTCGTTGATGTTGGCGGGAGCTTCCCC
CAGCATGCGGCGCATGATCTCCAGCGCCAGCCCCTTCTCGAAGCTGATGGAGAGGGAGCCACGGGTCTGC
GGGCCGACCATGCCAATCAGGCCTGAGACGTCGCCGCGCGCCAGCTCATCCATCTTGCGTTTTGGTTTGC
CCGGTTTGAGTTCCAGCTGTGCCATGGTCGAGAGCACATTGAGCAGGGATAGCAGGAACGGATTTACAAA
ATCAGCCTTCATGATGACTTCCTACAGTGGGCGTACAACGGGCACATTGGCCATGTGCCTCTATCGTCTT
ATTGGTAATAGTAAAACCATGCAAGTGGGCCTGCTCGGAAAACGCGCTATCGATCGCCGTATCGTGAAGC
TCGACCACATCCCCGCACTCGTCGCAGATCAACAACTGCATCGGGTGAGCGTGATCGAAGTGGCAGCAGA
AGATAAACGCGTTGAGAGATTCCACCTTGTGAGCAAAGCCCTGTTCGAGCAAAAAATCGAGTGCCCGATA
GACGGTTGGCGGCTTGGCATTGGCTTCGGTTTGTTGCAGCTGGGCCAACAAATCATAGGCGCTGATGGCG
TTGCCGTGTGCCGCCAGCAGGCGAAATACCTGACGTCGGGTCGGGGTGAAGCGGATCCCTCGCTGTATAC
AGAGTCGCTCGGCTCGTTGTAAAAGTTGGTCTTGATTCATGATCGCGCAGATACCCTTTCTATTAGCCGG
ATACTATCACAGGGCCTCGCATGGCGCGGCGACAAAATTCATTTTCTGGGGCTGCTTCACGTTGTGATAA
AATCCGCCCCTTTCTTTAGCCAACCCGGATGATTTTTATGCAGGCGCAGCGTTTTTCCATCGCCCCCATG
CTGGACTGGACTGATCGGCATTGCCGTTACTTTCATCGGCTGATGAGCCGCCAGACCTTGCTTTATACCG

AAATGGTGACCACCGGCGCCATCATCCACGGCAAGGGGGATTATCTGGGTTACAGCGAGCAGGAGCACCC
CATCTCCCTGCAACTGGGTGGCAGCAATCCGGCCGATCTGGCGCGCTGTGCCAAGCTGGCCGAGGAGCGC
GGCTATGACGAGGTGAACATCAACGTCGGTTGCCCCTCCGATCGGGTGCAGAATGGCCGCTTTGGCGCCT
GCCTGATGGGGGAACCGGCGCTGGTGGCAGACTGCGTCAAGGCGATGCGCGATGTGGTCGATATTCCGGT
GACGGTGAAGACCCGTATCGGTATCGACGATCAGGACTCTTACGAGTTTTTGCAGGCCTTTATCGAACAG
GTGCGCGATGCCGGTTGCGACACCTTTATCGTCCATGCCCGCAAGGCGTGGCTGAGTGGCCTGAGCCCCA
AGGAGAACCGCGAGATCCCGCCGCTCGATTATCCCCGCGTCTATCGGGTCAAGCAGGATTACCCGGATCT
CACCATCGCCCTCAACGGTGGTGTCACCTCCATGGAGCAGACTCTCGAGCACCTGCAACACGTCGATGGC
GTCATGATGGGGCGTGAAGCCTATCAGAACCCCTACATTCTGGCGCAGGTGGACAATCTGGTGTTCGGCC
AGAACAACGCCGTGCCGAGCCGCCACGAAGTGGTGCGGATGATGCTGCCCTATATCGAGCAGGAGCTGGC
CAAGGGCAACTACCTCTCCCACATGACCCGCCACATGCTGGGATTGTTCCAGAATATGCAGGGGGCCCGC
GCCTGGCGCCGTCACCTGAGTGAGAACGCCTGCAAGCCGGGGGCGGATATTCAGGTGGTGCTGGATGCCA
TGGCCAAGGTGCCGGAGTTTGCCGCCAGCGAGACGGCCGAATAAGCCAGTTGTGCCAAGGCAAGAGCAGC
CAAAAAGCGCGTCAGTTGACGCGCTTTTTTATTACTGTCGCTTCAATATCTGAACTGGTTGATCAGCCGC
GAGAGATCCTGCAGCCGCTCTACCAGCAGGGTGATGCCATCGAGGGCCTGCCTGCTGTTGTCGGCGGTGT
GGCTGGCCAGCTGGTGGATCTGGTGTACCCCTTCATCGAGGGTCGCCGTGACGTCGGCCTGCTGACTTAC
CGCCTGGGCGATCTCGTCTCTGGTGCGGGTTACCTGCCCCAGCATCCGGTTGATCTGGGTGAGCACGTCG
GCGGTGGTGCCCGCGTGCTGCTGGCAACGCACCGAAGTCTGTGCCCCTTGTGTCATCCCGTCCGACAGCT
GTCGTGTAGTCTGTTGCAGCGCCTGGATCATCTCGTGGATCTCGCTGGTCGAGGCACGGGTCTTCTGAGT
CAGCGCCCTCACTTCATCCGCCACCACGGCAAAACCGCGCCCCTGTTCGCCAGCACGGGCGGCCTCGATG
GCTGCATTGAGGGCGAGCAAGTTGGTCTGGTCGGCGATTTGGCTGATCACCTCCAGAATGCCGCTGATCT
TCTGGCTGTCGCTGGTGAGCTGGGCCAGCACGGCATGGTTGGCATCCAGCTCCTGATGGAGTTGGTTGAC
GGCGGAGCGGGTGGTCGCCAATGCTTGCAGCCCCTCGTCGGAGACCTGCTGTACCTCTATGACCAGATTG
CTCGCCTCATGGGCACTGTGGGCCACATCCCGAATGGAGTGGCTCAGTTCGGTGATGGCGGTCGCCAGCT
GCTCGCTGTGGGCGCGCTGCTGCTGCAGGTGGGTATCGATGGTCTGGATATTGCCCCGGTCATCTTCGGC
TGCCTGCAGGATCTGCTGGTTGGTGTCGGCGCTGCGCCCGACCACAGCCTTGAGCTCGGCCTGCTTCATC
CGCAGGGCCAGTTCGACGGCCGCCAAGGTGTCGCGCCGTCGGGTGTAGAGCACCTGACAGAGGCGGGTTC
CCTTGTCTTGATCGGCAAGGGTGAGCAGATGAGCCAGCCGCTGTTGCAATCGCCAGCCGTGGATTGCCTG
TGCCAACAGCAACAAGGCGGTGGGCAGCAGCCATAACAGGTTGCCGGTGGCAAAGTTAGCAAGGGTGGTG
AGCAAGGCAGAAGCGGAGAGCCAGAGGGTCAGCTGACCCGGTTCAAAGTGAAAGCGGCTCAGCCAGGGAA
GCGCCTTGCCGCTGCGCAACAGGCCATAGAGCTGCTCGGCCCAGGCCACATCGCTGGCGGCGGGTTTGGT
GCGCACCGACTGGAACTCGACGATCTCCTTCTGCGCATTGAGGATCGGGGTGACGTAAGCGTTTACCCAA
TAGAACTGACCGCTTTTGGCGCGGTTCTTGACCAGCCCCATCCAGCTTTTGCCCTGTCGCAGCCGCTGCC
ACATGTCGGCGAAGGCCGCTTTGGGCATGTCGGGGTGTCGCACCAGATTGTGGGGTACTCCTTCCATTTC
GGTCGCACCATATCCGGCCACATCACAGAAGTGCTGGTTGGCATAGGTAATATGACTGGCGGGATCCGTG
GTCGAGATGAGTGCGATGTTGTCGGGGAAGTGTATCTCCCCCTCCTGATGGAGTTTGGTCATGTGTCGTT
CCTGAAAAAGCCAAATAACTGATAAATATATTTTTTATTGTCGTTCTCAGTTAATCATTCTAAGGCTCTT
CAGTGACCCGAACTTGGCTTGGGTCAATTAATCAACACAATGAATTGATGAAGTAAGGGTCAGCTGGCCG
GTGTGAAATCGTGAACTTCGCCGCTTCTCGGTTTAAAGCGCGGATCGCTATCTTTGTAGCTACGCACCAG
CTCCATCGTTTTGCAAAAGCTGGTCTGCTTGTCGTACTCCTGACGAGCCAACTCGATGGCCCGTTCGGCC
CGGCGCCGCTCCATCTTGTCAGACTTGCGATCGCTGAACTGTTGCTGCTGGTGCAGCATCCAGCCGACAA
ACTCTTGCTGGCATTGGGATTCGCTATTGGCCTGCGCAGGCAGGGTCCACAACAACGAGAGCAGTATCAA
ATAACGCATGTCTGACTGGGTATTGAAGATTAATTGAACAGAAGGGGCGGCATTTTAGGCTGGAAATCGT
GGTGTGAACAGTATCAATAGCGAGGCCCAGCGGGGCCTCGCATCGTTGTGGCCGGTGTCAGTGTTTGACG
AAGTACAGCTGGCGGCTGTAGGCCACATCTTCCGGGTTGTTGATGGGGTAGCCCTTGAGCCATGGCTTGA
TGAGGCGGGCATTGGTGTACTGGTAGATGGGGGCGATGGGGGCTTGATCCAGCAGGATCGCCTCCGCCTC
GCCAAACAGCTTGCTGCGGCTGGCGGCATCCTGAGTGCTGGCCGCCTCTGCCAGCAGCTGGTCATAACGG
GCATTGGCAAAGCCAGCCATATTGCCGCTGTGACGAGAGCCCCACAGGCCAAGGAACGCTGAAGGGTCGT
TGTAATCAGCGACCCAGGAGGAGCGGATCACCTCGAACTGACCGCTCTGGCGGCTGTCGAGATAGGTTTT
CCACTCCTGATTGGTCAGCTCGACCTCGGCGCCCAGCTTTTGCTTCCACATGCTGGCAATCGCCAGTGCC
ATCTTCTTGTGAATTTCCGCGGTGTTGTAGAGCAGGGTCAGCTTGAGCGGCTTGTCCGGGCCGTAACCGG
CCTCCTTGAGCAGGGCCTTGGCCTTGCTGTCCAGCGCTGGCTGGCTGCTGGTGGAGAGCAGGTTGGCGGC
CGGGGTAAAGCTGGCGGTCACATCCGGAGTGAAGTGATAAGCCGGTTTCTCGCCAGTACCGAGCACTTTG
TCGGCGATCAGGGCGCGGTCGATGGTGTAGGAGAGCGCCTTGCGCACCCGCACATCGTTCAGTGGTGCCT
GACGGGTATTGAAAGCGTAGTAGTAGGTACCGAGCTGATCCGGGGTGTAGACCTCATTCGGGATCTGCTG
CTTCAGCTTCTCGTACTGCTCCTTGGGGAAGGATTCTGTGATATGCAGGTCGCCCGCCAGATAGCGGTTG
GTGGCCGCGCTCTCCTGATTGATAGGCATGAAGATCACCTTGGTCAATACGGTATGGGCCCGATCCCAGT
AATATGGGTTGGGGGTCAGCTCCAGCTTCTCGTTGACCACCCGGTTGTTCAGCACAAAGGCACCGTTGCC
CACCAGTTTGCCGGGGTGGGTCCACAGGTTGCCGTACTGCTCGATGCTGGCTTGATGGAGCGGCGACAGG
CTGGGGTGGCTCAGCAGGTTCAGGAAGTAGGGCACCGGCTGGGAGAGGGTCACCAGCAGGGTGTGGTCAT
CGAGGGCCTTGACCCCCAGCTCGCTGGCAGGCAATTTGCCAGCGACTATCTCGTCCACCTTGTCGAAACG

GGCCAGCTGGGCGAACCAGGCGAAGGTGGCCGCCTCTTTCGGGTCGACCAGCTTGCGCCAGCCGTAGACG
AAGTCGCCGGCCTTGACCGGATCGCCGTTGGACCAGCGCGCATCCGGGCGCAGATGGAAGGTAAAGAACT
GGTTGCCCTTGTTCTCCCAGCTGGCCGCCACCCCGGCAACCGGTTTGCCATCCGGCCCCTGGGTCAGCAG
CCCCTCGAACAGATCCCGCAACACCTGCAACTCCGGCAGGCCGACCAGCTTGAGCGGGCTCAGGCTCGCA
GGTTCATCCTTGAGGTGGCGAACGACAATCTGCTCGGCGGCGAGTTGTGTTCCGGCGGGAATATCGGCAG
CCTGGACAGCAGCACAGCCCAGCAGGGCCAGCAAGGGGCTCCATCCTTTCATGGGTCGGATCCTTCAAAT
AGTGTGAAGCGGGGAGAAAACTCGGCGAGAAAATATCGCCGACAGATGGGAAATCATGATATCCCGTTTG
CTGGTCTGACCGCCAGCATTCGACTCTCACTCCCAATCGGGGTAGAAGAGCGTTCTCGGCGTCTGTGCAG
TTCAATGTGGTGAGGAGTGTCAAACGATGAAAGCATGGTTATTGCTGGCCTGTGTGCTGAGCCAGGGTGC
CTGGGCGTTGGCCCCGTGCCAGAGCGACACGGCGGTGGGCAACTGGTGCGAGATCGGCATCGATGCCTTG
CACCCGACCCAGGGGGGCGTCGGCCAGATCCAGGTCGATGAGACCCAGGCCACATTGGCGGGCATGAGCG
CAAAGCAGCTCGACAAGTTGATGAAAAAGAAAGAGATCCCCGTGGTGATTGCGCCGGATGGCAGCTACTG
GCTGGTGGACAGGCACCATCTCACCAAGGCGTTGTGGCAGCAGGGCGTCAAGGATGCCCGGGTCAAGGTG
ATTGGCCGCCTGCAGGATAAGGCCAACTTCTGGAGCCAGATGCAGAACAACCACTGGGCCTGGCTCAAGG
ATGAGAAGGGGCAACCGCTGACCCCGGAGCAGTTGCCGACCAGCATCGACAAGCTGCCTGATTACCCCTA
TCGCACCCTCGCCGGACTGCTGCAGAATGCGGGCTACTTTCGCAAGGATAAACAGGTCTATTTCGTCGAG
TTTGCCTGGGCCAGCTGGCTCGGCAAGCAGATGCAGTGGGTGCCGGTGGACAGCGCCAATCTGGCGGCCC
GGCTGCAACAGGCCAAACGGCTCGCCTGCGGCAGCGACGCCAATGATCTACCCGGTTATCCCGGCAAGCA
GTGCAGCCTCAACCAGTCCAAAAGCGCGCAATGAGTGGATGTGCGATGACGGCATCCAGAGGATGCCGGA
GATAACAGAGGGCGACCCTGGGGTCGCCCTTATGCTGTCAGCTACTGGTTAGCGGTGGTCACTCATGGCG
CTCCGCCCAGCGGGCAGCCGCCTGTTCAAGCAGGGCGACGTCACCTTGCTGGATCAGCGGTAGCAGATCG
TTGACCTGTTGATCCGGCCAATCCCACCAGGCGAGGGCCTGTAGCCGGGCAATCTCCTCCTCGCCAAAGC
GCATCCGCACCACCCTGGCCGGGGTGCCCGCCACCATGGCATAGGGCGGCACATCCTGATTGACCAGCGA
ACGGGCGGCGATGATGGCGCCGTCGCCGATGGTGACGCCGGGCATGATCATCGCCTCCATGCCGATCCAG
ACATCGTTACCGATGCGGGTATCGCCAGCGGGGCGGTAGCTGCGCTGGAGCGCAGCCAGATCGGCGAACG
GATAGGTGCTGATGAAGGCGGTGTTGTGGGTGTGGTTGCCGCCCATGATGATCTTTACCCCAGCGGCAAT
TTGCACATAGTCGCCGATATGGAGCCGGTCGATATGCCAGAGCGGCTCCCAGCCGGTGTCCGGGCTGCGG
CTGAAGGGATCGCCATGGAGGTAGCGCACCGCCACCTCTTCGAAGGGGCCATCCCAGGCGCCCGAATAGT
AGCTGTGCTGCCCCTTTAGGCAGATATTGGGGTTGGTCACCGTCAGGTGCAGGTATTCGACCTGCGACCA
GTGTTGGCAGGTGTTCATGCTGGTACTCCTTTGCTGAATGGATCGGATGGGTTGCGTTCGCTTGAGCGCA
GCTAATAGATCCAGACCAGCATGCGGGGCACGCAGAGCAGGGAGCAGCCCAGCATCTGCGGGAGGCGAGG
TCGCATTGTGGTGTGCTCCAAAGAAGAGGGAAGGGCGCGATCATAAGGTCGGGCGCCGGGTTTGCCAAGA
TTGCGCAGAGATCCTGCACTAACCCTCTGGTTGATATTTATTCTCAATTAGCCATGATAACACTCCCACC
ATGACGGACTCGGGAGAGCGAAATGACACCGCAAAGTTGGCAACGATACATGCTGGAGGCGGAGCGTGCC
TGGCAGGCCGGTTCGCTGGGGGCGGCGATCTGCTTTTATCAGCAGGCGCTGGGGGATGTTTACGAGATGA
CCCAGGTCGAGTTGACCGAGCTGGCCACCATGCGAGTCGCCACCTGTCACCGGCTGGCCGACTTCTGGCG
GGCGATGGATGAGCCGACTTACGAGCTGCGCTACCTGAAACTTGCCTCCGAGCTGGTCACTGCGCTGGTG
CCGCAGTGCCCCAATCGGGAGTGTGAGGCGCTGATCAGCGAGCTGGGTTGCTGTCGCGGTGCCCTGCTCG
CCTTTCTCAAGCGCCACCCCAATCCGGAAATTGCCCGGCTGATCCAGCTGCAGGACAAGGTGCAGGGCTG
CGAGCTGATCGGTCGGTTTCGTCTCAACTGAGCGGATGTGGGCGCGGCTCAGCCCCGCCGTTTGAGTTTC
TGGGCGATGCGCTGCTGCGCCTCTTCCCCTTGCAGTGCCTGGGCGAAGGCGCGCGCCTCCAGATCGATGA
CAAAGTGCACCGCCTGCTTAAGCTCCTGTTTCAGCAGCGCCTTGCTCTTTTGCAGCGCTCTGGGCGGTTT
GGCCGCCAGCTTCTGCCCCTGCTCGCGGGCAAAGGAGAGCAATCCATCGGCCGCCACCACCTTGTTGGCG
AGCCCGAGTCGCAGCGCCTCCTCGGCATCAATCGCCTCGGCCAGCAGCAACAGCTCGGCTGCCTTGAGGT
GCCCCACCAGACGCGGCAGCAGCAGGCTGGAGGCAAACTCCGGCACCAGTCCCAGCTCGACAAAGGGGAG
CTGCAAGCGGGCGTTATCCCCCAGATAGACCAGATCGCAGTGCAGCAGAATGGTGGTACCGATCCCCACC
GCCGGGCCTGCAACGGCGGCGACCACAGGTTTGGGGAAGTCGGCCAGGGTGTGGAGGAGCTGCAGTATGG
GATCGTCGGGCTCCAGCGAGCTCTTGCCCATAAAGTCGGCCAGATCATTGCCCGCGGTAAAGCAATCTTG
CTGCCCCTGCAACAGCAGGACATGCACCTCCTCATCGGCTGCCGCCTCTCGCAGCGCTGCCACCAGTTGC
TTATAGAGCTCGGTATTGAGAGCGTTGCGCTTGTCGGGGCGGTTGAGGGTCAGGGTGAGCAAGCCATCCT
GCTGCTCGGCAATGAGAATGGGGGCCATGTCTAAATCCTTGTTATTGAAAGGTGCAAAAAAGGTTGCTGC
TGGGGTAACCTTGGCGCCGTTTAACAACATCATAAAAGGATTTCAACCATGTTTAAACGTGCGTTTTATT
CTCTGCTGGTTCTGGGTATGACGGCGCCTGCACTGGCCAAGGTCGAGGCTGTCGACGGCTATGTGCGCCT
GTTGCCGCCGGGCTCCCCCAACACTGCCGCCTTTATGGTGCTGAAGAATGACGCCGACCAGCCGGTCAAG
CTGGTGGCGGCGGCTTCTGCTGTGGCGGGGCGTGCCGAGCTGCACACTCACCTGCACGAAAATGGCGTGA
TGAAGATGCGTCAGGTCGAGAATATCGAGATCCCGGCCAAGGGCGAAGCGGTGCTCAAGCCGGGCAGCCT
GCACATCATGTTGTTCGAGGTGGGCACGCTCTCCGAGCAGACCCCTTTCCCGCTCACCCTCACCATGGAT
GATGGTCAGAAGCTGGATCTGTCACTGCCGGTGAAGCCGATTGAGCCGATGGCAGAGATGAAGCATATGA
AGCACTGACATTTGTCGTCGTTTGTTCAGGATTGACGCTGGGTATTTTGGCTGCACCGAGTAAAATAGTG
CTCTTTTCCATGTATGCAAGAGGAAGTGACGTAATGAAAAAGACGCTGATTGCCGGTTTTGTGCTGGCTC
TGTGCAGCCAGAGTGCCATGGCCGCTGACGACTGGCGTTTTGCCGCCGGTGCCGACGTATGGGCCGCCAA

GGGCAGTGGTCAGGCTTATGGCACCAGTGCTGATGGCAGCTATGACGACAGCTACAACTGGAACGGCTAT
CTGCAACTGGAACACGGCATCATCTTCCTGCCGAACGCCAAGTTTGAAGTCTCCGATTTCAGCACCTCCG
GCGGCAACTTCAAGAACGATCTGACTGCCTACGATCTGAGCCTCTACTACCGTCTGTTCAACAACGATCT
GTTCAAGATCGATCTGGGTATGACCGGCCGTCACTACGATGGCGAGCAGGTCTACTCCGGTCGCAAGGGT
TATGACGAAGATACCCTGATGGCCTATGCCGGCAGCGAAGTGAAGATCCCGGGCACCGGCATCGCCTTCT
TCGGCGATCTGCGTACTCACGATGCGGACAACTATGACTACCGTCTGGGTGGTTCCTACAAGTTCTCCTC
CATGCCGATCAAGCTGCGTGCCGGCTGGCGTGAAGCCAAGGTCGACTTCGCCAATATCGACCAGTCTGTC
GATGGCTGGTTCATGGGCGGCGAATTCACTTTCTAATCTGACCGTCGACTGACGGCGAGGAGCAATCATG
GGCCACATCATAGTGACAGGTGCCGGTTCTGGTCTCGGCCGGGCGCTGACCATAGGTCTGGTTGAGCGGG
GCTATCAGGTCTCCATGATGGGGCGCCGTTATCAGCGACTGCAGGAGCAGGAGCAACTGCTCGGCAGCGC
GGTGATCGGCATCGCTGCCGATCTGGCGCACCATGAAGAGGTCGACGTCGCCTTCGCTGCAGCGGTGGAG
TGGGGCGGGCAGCCCGACATGGTGATCCACTGCGCCGGGGTGGGCGAGTTTGGCCCGGTCGGTGTCTACA
CCGCCGAGCAGATCCGCCGGGTGGTGGAGAGCAATCTCATCTCCACCATTCTGGTGGCTCAGCAGACGGT
GCGGCTTATTGGTGAGAAAGGCGGAGTGTTGGCCAATGTGCTCTCCTCCGCCGCTCAGGTGGGCAAGGCT
AACGAGAGCCTCTACTGCGCCTCCAAATGGGGGATGCGCGGCTTTCTCGAATCCTTGCGCGCCGAGCTGA
AAGGCTCGCCACTGCGGCTGGTCAACCTCTATCCGAGCGGGATCCGCAGCGAATTCTGGGACAACTCGGA
TCACGTGGACCCGAGTGGCTTCATGACCCCGGAAGATGCGGCGGCCTATATGCTCGACGCGCTGGAAGCC
AGAAGCAGCTGCCACGTGACCGATCTCTTTATCGGTCGCAATCCCTGAGGCAACCCTTCATCAGGTTGCC
GTCGTCAGCACACGGAAACAAAAAGCGCGCCAAATGGCGCGCTTTTTTATATCGGGAAGAGGGCGGGATT
AACGGCCCAGCTTGGCCTTGAGCATGGCGATGATGTCGCTGATGGCCACTTCCTGCTTCTCGCCGGTGCG
GCGGCACTTGTACTCCACCACGCCGTTGTCCAGACCGCGATCGCCGATGACGATGGCGTGCGGGATGCCC
AGCAGCTCCATGTCGGCAAACATCACGCCCGGGCGCTCTTTACGGTCGTCGAACAGCACGTCCACACCAG
CGGCTTTCAGCTCGGCGTAGAACTGTTGTGCCTGCTCGGCCACGCGTTCGGACTTGTGCATGTTCATCGG
CACGATGGCCACTTCGAACGGAGCGATGGCGTCCGGCCAGATGATGCCGTACTGGTCGTTGTTCTGCTCG
ATGGCAGCGGCAACCAGACGGGAAACCCCGATACCGTAGCAACCCATCTCCATGGTGACGGATTTGCCAG
CTTCGTTCAGCACGCTCGCCTTCATCGCTTCGGAGTACTTGGTACCCAGCTGGAAGATGTGGCCCACTTC
AATACCGCGCTTGAGCAGCAGCTTGCCCTGGCCGCACGGGCTCGGGTCGCCTTCTACCACGTTGCGCAGG
TCGGCAACTTCGTAAGAAGCGATGTCGCGATCCCAGTTGGCACCGGTCAGGTGGAAGCCGGTCTCGTTGG
CGCCGCAGACGAAGTCCGCCAGATGGGCGGCGCTGCGATCGACGATGATGCGACCGGCAAAGCCGACCGG
GCCGATGGAACCTGCATCGCAACCGGCGGCAGCTTTAATCTGTTCGTCGTTGGCGAAGGTCAGCGGGTTG
GCCACGCCAGCCAGCTTCTCGGCCTTGATTTCGTTCAGCTCGTGATCGCCACGCAGTACCAGCGCGATGA
CGGCCTGTTTGCCGTGTTCATCTTCCTCTGCCAGTACCAGCAGGGTCTTGGCGATGGCGGTCGGTGCCAC
GTTCAGGAAGGCGGCCACTTCGTCGATGGTGTGGACGTTCGGGGTGGCAACCTTGGTCAGGGCTGCGGTG
GCGGCAGCGCGCTCACCAGCCGGCGCCAGCGCTTCGGCCATCTCGATGTTGGCTGCGTAATCTGATGTGT
CGGAGAAGGCGATCAAGTCTTCACCGCTCTCGGCCAGCACTTGGAATTCGTGGGAACCGGTACCCCCGAT
GGAGCCGGTATCTGCCTGCACCGGACGGAAGTTCAGACCCATGCGGGTGAACGTCTTGCAGTAGGCGGCA
TGCATCTTCTCGTAGGTATCGACCAGAGACTCTTTGTCGATGTGGAAGGAGTAGGCATCCTTCATCAGGA
ATTCACGGCCACGCATCACGCCGAAACGGGGGCGCACTTCGTCGCGGAACTTGGTCTGGATCTGGTAGAG
GTTGAGCGGCAGCTGCTTGTAGCTGTTCACTTCGTAACGTACCAGCGCAGTGATCACCTCTTCGTGGGTC
GGGCCCAGCACGAAGGGACGGTTGTGACGGTCGGTCAGACGGCACAGCTCGGGACCGTAGTCATCCCAGC
GGCCGGACTCCTGCCACAGCTCGGCGGGTTGTACCACCGGCATGGAGACTTCGACGGCACCAGCATTGTT
CATCTCTTCGCGAACAATGTTCTCGATTTTCTTCAGTACCCGCAGACCGGTTGGCAACCAGGCATACATG
CCGGAGGCCAGTTTACGGATCATCCCGGCGCGCAGCATCAGCTGGTGGCTGACAACTTCAGCGTCGGAGG
GGGTTTCCTTGAGAGTGGAAAGCAGATATTGGCTGGTACGCATCGCGGTAGACCTTAGCTAGAACGGGCG
CGGGGCCCAGGATAATTTCAAGGGCGGAATTTTAACAGGCTGGGCGTTGCGGCCCAAGGGCTAAATTTGC
CGCAACCCGAAAGGTGGCCACGCCCGGCAGATGGTCAGGGCAATGACCATTGCAACAAGCAGCCTTTTTA
TGCCTGCAATTTGGCCGGATCTCCGCTAAACTCTGCCACCTTTTCTCGATGGGCATGGCTCTGCCGCGTT
CATCACCCCCCATTTTTAGTACTTTTGGAGCCGCTCATGCCGTTAGTGGGACATCAACACCAGATCGAGA
TCCTGGAACTGACCGACAAAGGAGATGGCAAAGGACGCCTGTTCGATCGCCCCCTGTTTGTCGAAGGTCT
GCTGCCCGGTGAGCAGGCGCTGGTGGAGCTGACCGAGGTCAAGCCGAACTATCTGCACGGCAAGGTTGCC
GAGCTGCTCCAACCTTCTGCAGATCGGGTTGCCGATTTCTGCCCCAACACCGAGTGCGGTGGCTGTCAGG
TGCGCCCGCTGGCCTATCCGGCCCAGCTCAAGCTCAAGCAGGCACTGGTGCAGAGCGCACTGGAGCAGGC
TGGGCTGGGTGATACTCCGGTCAAGGATATCCTTGGCATGGAGAGCCCGTTCGCCTATCGCAACAAGGCG
CAATATGCGGTGCGTGGCTGTGACGCCGGTGCCGAGATCGGTTTTTATCGCAAGCACTCCCACGATCTCA
TCACCGCCGATGACTGTGCGGTGCAGGATCCGCTGCACGTGGAGCTCAACGCCCGGGTGCGCAACTGGAT
GCGCGCGCACGACATCGCCGCCTATGACGAAGTAAACCACAGCGGCTGTGTGCGTAACCTGATGACCCGC
AAGGGCTTCAAGAGCGGCGAACTGATGGTGGTGCTGGTCACTTTGGGTGAGGTGCTGCCGTTCGAAGCCG
AACTGTTGGCAGCTCTGAGCGATCTGCCGGTCACAACCTTGGTGCAGAACATCAACGATGAGCGCACCAA
CCGCATTCTGGGCGCGACCAACCGGGGGCTGCTCGGCGAAGGGGTGATCCGCGACAAGATCCACGAACTC
GAGTTCGAGATCTCGCCGCTCTCCTTCTTCCAGAACAACCCGACCCAGACCGATGTGCTCTACTCCAGCG
CCCTCGAGTATGCCGAGCTGACCGGTAACGAGACAGTGTTCGACATCTATTGCGGTATCGGCACCATCTC

CCTGTTCCTGGCTGGCAAGGCCGCCAAGGTCGTCGGGATCGAGTCGGTGGAGAGCGCCATCAGCGATGCC
CGCCGCAACGCCGCCCTCAACGGCATCGAGCACACCGAATTCCACGTTGGCAAGGCCGAGCAGCTGATGC
CCGAGCTGCACGCCCAGGGCGTGACAGCTGACGTGGTGGTAATGGATCCGCCCCGCAAAGGCTGTGACAA
GGCGGTGCTGCAGACCCTGGTCGCTATGGCGCCCCGCCGTCTGGTCTATGTCTCCTGCAACCCGCAGACC
CTGGCCCGCGACCTTGCCTGGCTGGTCAAACAGGGCTTCGTGCTGGACGAAGTGCAGCCGGTCGATATGT
TCCCGCACTCCATGCATGTGGAAGCGGTTGCCCGCCTGAGCCTGCCAGCCAAGGCATAAGTCGCCCCCGA
CTCTTGCCCATAAAAAAACGACGCCAGCTGGCGTCGTTTTTTTATCTCTGCAATCGGCGGATGGGCGATT
AGCTGGCGCTCTGGCGCACCTCGGCGGCGATCTGGTCGAGCAGCTGTTGCCAGCCCTGACCCAGCTCGTT
CACCAGCGCGTTATAGCCATCGGCCGGCGGCACCCGCTCCTGACGGAAGGGGGCATCGCGCAGCACGGTG
CCATCTTCCGCCAGCAGGCGCCAGCGGCCGCTCAGCACGGCGCGGCCGTCATGACGGCCCTGAAAGCGCT
CCACCAGAATGGTCAGCACCGGCCCCTTGTGGGCGCCATCCTGCCGTACCACCCAGCCCGGCAGGCGGGT
GGAGAGGCCATTGAGGGTGAGCTGGTTGAGCTGATCGTCCAGCGCACCGGCCCAGCGATGGTACTCGGTG
AAGTGCAGCTCTTGCCCTTCCAGCTGATAGACCAGGCTGATCCGGTCGAGCTGGCTGGCCAGCGCCACCG
GGCGCAGCACCAGCTGGTGTTGCGGTTGGGCCGGGGCCGTCACGGGCTGCTGGCTGTCGGCATCGAGCGA
GTAGTAGTGCAGGCTTGGCTGGCTGCTGCAACCCGCCAGCAGGCCCAGCAGGGCCAGCGTCAGGATCTCT
TTTTTCATTGTTTGCCTCGCTTGGGCTCAGGGTCCGATGGATGCGCGCGATCGAAGATCAGCGAGCTGGG
CTGCTCGTTGAGGGAGTGCACCAGCGGCTGCAGTTCGCGCATCAACTGATTGAGGGTCTGCACCGACTGA
CGCAGGTCCTGATTGGTCTGCGACTGGGCATCATAGGCCTGCAGGGTCTCCTGCAACTGCTTGAGGCTCT
CGTTGAGCGAGGCGGGCAGCTGCTGGGTGGAATCCTTGGCTGTGAGCTTGTTCAGGTTGGCGCTGACTGC
ACTGATGTTCTTCAGGGTGCCATCCAGGGTGGTGAGTGACCCGTTGACCTTGGCCAGGGTGGTTGCCATA
TCCATGTCGACAAACTTGTCGAGGATGAGGTTCACCTTGCGCTCGATCTGATCCAGCCCGCCCGGCACGG
TGGGGAAGACCTGATAACCGGCCAGCTTGGCCTGCTCGAAACGTGGCGCGTCCGGATAGAAGTTGAGGTC
GATGATCTTGCCGCCGGTCAGCAGGCTCGAGGTCTTGAGGCTGGCACGCAACCCCTCCTTGAACTGCTTG
CCGAACAGGGCACGCCATTGATCCTTCTCCGCCTTGGCATAGCGGTGGGAGAGGCGCTGCACCTCGATGC
GGGCCAGCACCGGGATCTGGCGGCTCTTGAAGATCTGCACGCCCTTGTCCTCGATGAGGTAGGGGGCGGA
GATGACGGTACCGACCCGAATGCCGCGATACTGCACCGCCGCGCCCGGGTGCAGGCCACCCACGTTGTCA
TCGAACAGGAAGACGTAGTCGATGAACTGATCCAGACTACCGGCCAGCACGCTCGCCTCATCCTGGAACA
GGGTGAACCCTTCCATCTGTTTGACCGGCTCGCCAGGCGCCCAACCGGGTGGCAGACCCATGGTGATGCC
GCCATCGATCAGGCTCTCCAGCGAATCCATCTTCACCTTCATCCCCTCGCTGCTCATGGAGACCTCGAAG
CCCGGGGTCATCCAGAAGCGGGAGTTGCTGCTCACCAGAATGTCATAGGGGGCGTGGATGAAGATTTGAT
ACTGCATCTCGCTCTTTTCCGGCAGGAACTTCGCCTCTTCCACCTGACCTATGGTGAAGCCCTGATAGTT
GATGGGGGAGCCTTCTCCAAGCGCGCGGGTGTCGCGACTGCTGAGGGTGAGACGCAGCCCCTTGGCATCG
AGGGAGGCGAGCGGCGGTTTGTCGAGCATGGCGTATTCGTCCCGCGCCCGACCTTTCTTGCCGGGGGCGA
GCTCGATATAGGCGCCGGAGAGCAGGGTGCTCAGACCCGATACCCCTTCACGGCCCACTCTGGGCTTGAC
CACCCAGAACTGGCTGTCACCGCGCAGCATGCCGGAGGCGGCATTGGTCAGCCGTCCTTTCAGCACCGCA
TGGCTGTAGTCATCGCTCAGCTCGACGGCCTCGATGCGGCCGACGTCCACTTCGCGGGAGCGGATCACCG
TCTTGCCAGCGACTATGCCCTCGGCAGTGGCGACTGTCAGGGTGAAACTGGGGCCCTGGGAGTACCAGTG
CTGGAACAGCATCCAGAGACCGATGAACAGCGCCAGCAGGGGCACCATCCAGATTGCGGCGGCAGAGCTC
AGAAAGTCAGAGCCCGGTTTCCACCTTTTTTTACGCTCACTCACGTTCGCGTTCTCCTTGTTGTAAATCC
CAGATCAGGCGGGAGTCGAAACTCATGGCGGCGACCATGGTGATCAGCACCACACCTGCAAATGCGACGG
CCGCAGGGCCGGGATAGATGGTCATCAGATTGTCGAGCCGGATCAGGCCCGCCAGAATGGCCACCACGAA
GACGTCGATCATCGACCAGCGTCCGACAAATTCGGTCATGCGATAGAGTTTGAGCTTGCCGAGGGGGGAG
GTGACATTGCCGCGCTGCACCATATAGCAAAGCCAGAATAGCGCCAGTATTTTCACCAGCGGCACCACCA
CGCTGGCGAAAAAGATCACCGCAGCTATGGGGTATGAGCCCATGGCCCAGAGCAGCACCACACCGCCGAG
GATGGTGGAGGGGCTGTCATCACCGAGAAACACCGTATCCATGATGGGATAGACGTTGGCCGGAATGTAG
AGAATGGCGGAGGTAAACAGCAGCGCCCAGGTACGGTTCAGGCCGCCCGGCTTGCGGGCATGCAGCTTGC
CGCCGCAGCGGGAGCAGCTGTGGCTACCCACCTCGCTCAGGGCGCCACAGATATGGCAGCCCACCAGACC
GGACTCCAGCGCGCCCGCTTCCGGGTCGATCTCCCGCGCCTCGGTCGGGCCGAACAGCCGCTGCCACAGC
CACATCTGATCCAGCGAGGAGATCATCTTGATGAGCAGCACCGTATAGCCGACGAAAGCCCAGAATGACA
GCCCCATCTTGATGTCGGCCATCCCCATCAGCTTGACCAGCGAGATGAGCACGCCCACCAGAAAGACATC
CACCATCAGCCAGGGTTTGATCCGGCAGAGCAGACGGCCGAGGGAGCGCAGGGCATTGCTGTGCAGATCT
TTATCGACCCGCCACAGCACCAGCATGATGGATCCCATATAAACCAGTGGCAGGCCGATCAGGGTCAGGC
TCAGTACCGCGCCGAGAAACAGATAGCCCTCATCCACCAGGGTGGTGATGCTCTGCAGAAAAGTCATCTC
CTGGCCCACCCCTTTGGCCGAGAAGGACATAAAGGTAAAGGCATTGGCGAGCACGAACATGATGAGTGCC
GCGAACCCGTAGGAGATCGGCCGCAACTCCTGCTGTGGCAGGTGGCGACTCAGTGTGTGTCCGCAACGGG
GGCAGCAGCTGGCCTGTCCCACCTCGAGCGGTGTGGCCGGTATCAGCAGGTCACACTCTTCGCAGGCAGT
GGCATCATGGACATAGGTGGTGTGGTGTTGGTGTGAAACCGGATGCAAGATAAAACCCCAGATAAGGCCC
GTGGTAGAGCCAGAGCTGGCGATTATCGCAGTTGTGCTGGTCAGCGACCAGCCCCCGGAGGCCGCTATTG
CTGGCGGGAGTGAGAATGACTTTGACAAGCCAGTGCCCATTTAGCATAATCAAACGCCACTTTTTTTCCG
CGGGACAATAATTTATGACAACTGAATCCCCCAGCTACTCCACGATTGAGCAAGCCTTCTCCCTCGGGAA
TGGGCAAGATTCTGCCCTCAACTGGTTGCGCAAGAACCGTAGTCAGGTCGCTATTTTTCTGGTGAGCGGC

ATCAAGCTGGAAGGCACCATCAGCGGGTTCGACCAGTACAGCGTGCTGCTGACCGATGCTCATGGTAACC
AGCAGCTGGTCTACAAGGCCAAGATCTCCACCATCGCCATGCACACCGGCCGTCCGCAGGTGAATATCCA
GCGTGCTCGCAAGCCACGTGTCTCCATGGCGCCGCGCCCCCACGGTGCGCCGGGTCGATATGACGACAAT
CAGGGCGGCGGCAACAACGAGTAATCGTCTGCCCCGCACTCCCCGTTGACGCGCCCTGCGTCAGCGGCTG
ACGGAGCCTGCTCTCATTTTTCATTCATGTCCCGTATGAGAGCCGGTTCCGGTTATCGCCCCTTGCAGGG
CGATATTTTTTTCCGCACTACGCCGTTATGCCGACCGGTTGACCCTCTTTGACCATGCAGGTCTGCATAC
CCGCCGCTTCTCCGGCCTGTACCCCGATACCGGTATCTTCAAACACTAGACAGCCTGATGGCTCGACCCC
CAGCTTGCTGACGACCAGCAGGAAGGTGTCCGGATTGGGCTTGTGCAGTTCCACATCGTCTGCGGTGACC
ACCACGGAGAAGTAGCGATCCAGCCCGGTGTTGCGCAGCACCGCTTCGGCATTGACGCGGGGCGAGCCGG
TGCCGATCCCCATCGGGATGACGCCATGATAGCGCTCGACCAGCGCCTGCATGGCCGGAAAGACGGTCGC
CTTGTGCAGGTTGGCCACATAGTGGGCGGTCTTGCAGCGGGTGACTTCGAGCGGATCGAGGGGGATCTGC
TGCTCCTGTGCGACCTGCAGGGCTATCTTGCGGCTCGGCATGCCACCCAGCTCATAGAACCAGTCGGCGT
CGAAGTGAAAGCCAAACTCGCGGGCGGTATGTTCCCAAGCGGCGAGGTGCAGCGGCATGGAGTCGACCAG
GGTGCCATCAAGGTCAAAGACCAGGCCTTGGAATCCGGAAAGCGTCATGGTGCGGGTCAGTCTCTGAAAA
AAGGGAACCCATTCTAGCCAGTGGCTCCCCGCCTCGCCATCACCTAGCTGGTGAGAAACGCTTGCCCCCA
CATTTGTGACTGAAAATAGACGGGGGTGAGCTGGGCCGGATCGAGTTGATATTTGGGGGCAAGCTGTTCC
AGCAGGGCCCAGTTGCCCTGTTCATAGGTGCTGACCAGCTGAAACAGATGAAAGAGTGGCCCTTCGTGCA
GTTCGATGGCGCGCTGCACGCTCTTGGGCAGGTGGGCCATCAGCGGCTCCGACAGCAGGGAGCCATCGAG
CACCAGTCCTACCAGAAAGGCCCAGCTGGCCTCCGCTTTACCGAGGGCGGTGAGGGCAATCAACTCGCAG
AAGCGGCCGCGGGCGATGGCCATGCGGGTGCAGGCCTCCGGCATGTGCTGGCTCACCCGGGCCAGACCGT
TGATCAACACGAACTTGCGGATCTCCTGCTCCCCCAGATAGGAGAGGGCGCGGGGTACCGAGGTGATGGG
GGCGGCATGATCGAAGCCGGGGGCGTTGACGTAGGTGAGCAGCAGGCCGGGCAGCCAGGCATCCTGCTCC
AGCAGGCGGGCTACCTGGGCGAAGGAGAACTCCTCGTGGCAGATGAGCTGAAGGATCTGCTGCACCAGCG
CCATATCGGGCTGGTTGGCCCGCTGATTAACCAGCTCCGGACGGGCGAGAAAGCCGCCGGCAAAGAAGTC
GACCCCGGCCTCCCGCAGCGGGAGAAAATCCGCCCAGGTTTCGATGTTCAGCGCCATCCGCTTGTGTTCG
GTAGGCTGGCAGATCCCCTCTTCCCCCATCCAGTACACCAGCAGGCGGCTGGCGCCGAGCCAGCGCGGAT
CCCGGTTGGCGCCATGCACCGCCAGTCTGCGCCGGCTGTCGCGCCACTGCTCCAGCACCGGGAACGAGTT
GGGTTGGCGTTCACTGGAGGCTTCAACCAGCGGGATCGCGTCCTGATCCAGCAGTAGTGGACGCTGTTCA
TCTAGCTGTTCGTGGGTGAAGGGCACCAGATATTGACGCCCCTGCGCCAGCAGGCGGCAATGTTGCGCTA
GTGAAGGCCATTCGGCCGGTGAGCTGAGCTGATAGTCGATGCCGTAGGCCACCAGAGCCAGGCGACGATT
GAAGATGGGGTAGCGACGCAACAGCATGGGATCACCGATAGGCCAGAGTGCGGGCCCTCCCTTGTGACTT
GGCGTGGTAGAGCGCCTGGTCAGCCTGTTCGGTCAGGGTCTGCCATGCTCCGTCCTGGGAGGGAACCAGA
TAGCTAAAGCCTATGCTAACCGTAATGAAATCAGCAACCCGCGAGGCGGGATGGGGCATTTTCAAGCGGG
CAAGACCATCGTGGATCCGTCCGGCCAGCTGTTCGGCCTCACCGGGTTGCGGGGCGCAGAGCAAAATGAC
GAACTCCTCCCCGCCGTAACGGGCGACCAGATTGGTGCAGCGTCGCTCTGCATCCCGCAGCACATGGGCC
ACCTGTTGCAGGCAGAGATCCCCCTTTGCATGGCCGAAGTGGTCGTTGTAGGCCTTGAACTCGTCGACGT
CGATCATCAATACCGCCAGCAGGTTCTGGGTGCGTCGTGCCCACTCCCACTCCCGCTGCATGGTGATGTC
CCACTGGCGCCGGTTGGCGATGCCGGTGAGGGGATCTTCCAGCGACAGCAGGGAGAGGCGGTGATTGGCC
TCCTCCAGCGCACGGTTGGTCTCGGCCAGTTGCATGGTGCGCTCGGCGACCCGGGCCTCCAGCTGGCGGT
TGAGGGTTTCCAGCTGCTCCTGAGTACGTTGACGGATCAGCAGTTGTGACAGGATAGCTGCGTACTGTTT
GAAGATCTCGCTCTGATAGGCTTTGAGCGGCCGGTACCAGAGCAGGTTGTCGGCGGCGATCCAGCCGATG
GGGGTGGTGCCATCCCATAGCGATACCATGGCGTTCCAGCCACGGCCCACCTGCTGCTTCTCGTAGTAGA
GGGGGGTATCTTCCATCACCAGTACGTAGTCCTTGCGCCTGAGCGCCTCTTGCACCGTGGGGTGATCCGG
AATGGGGCTGATGAAGTTGTGTTCATCCATCAGCTCGCCGTCCTCGCTGGTGCCCCAGGTGCCGCGCATG
GTCTTGCCATCGGGTTCGATCAGGAAGATGGCGACCCGATCGAATTGCAGCTCCTTGCGGGCCAGCAGCA
CGGCATCTCGCAGCAGATCGTTCTCGCTGCGACTGCGGGAGAGGGTGACGCTGACCATGTGCAGCGCCTG
CAACATGGTGAGGAAGGAGCCTTGCAGTGCCTGACTCTGTACCAGATCGGTCTGGGTATGGAGCCGCTGG
TCTCGCTCCTGATTGACCTGGCTGGAGAGCAGGTGATTGCTGAGCTCGGCTGTCAGATAGTGGATAAGGA
GCTGGATGCCCTGACGCTGGTGGGAGGAGAGGCGATCGATGCGGCGGCGGGTCTCCAGATAGATGACCCC
TTCCAGCTTCATGTGACGCTTTAGAGGGAAACAGGCGCGTAACTGAGGCTGGGCGGGCTCCACATTTTCG
CCGGGATCCGGCGGTGCTTGGAGCTGCTCGATCACCAGTTGTCCCTTCTCCATCGCCTTGTCGATCAGGT
GGTGGGGCAGGGTCAGTTGACCGAGATGGGCGAGATCAAGCGTGGTCAGCCCGTGATCGAGCTGAAAACT
GTAGAGCTGCGGTTCGGGGGCTTTCAGCCAGAGGTAGAGGCGTTCGGCATCGGTCTTTTCACGGATGGTG
GCGAGGCTCACTTCGCATAACTGAGCAAAGTTGGTGAAGTGTTCCGGGTCCCATTCCTGCATACCAATAT
CCCTTGGATTGTACCCAAGCGAGTATAGACATATTTCTCCTTGATTAATATGTCAGAAAGGAGAGACAGC
GCACATAACGGTTGCTTTCAGCCCTGCTGGTGGAGACATTCACTGCGTTTTCTGCCAATCGTGGATAGAA
CTGTGTGCCATAACGGTATATATGGCAGCTTGTAGGGCGGGATGCCGGGCAAAAAAGAGCCCGCACGATG
GCGGGCTCTCGGCAGATCAGATGATTCTGCGTGGCTTATTCCGCCTTGGGGGCAGCCTCAGCGGGCGTTT
CACTGCTATCGGCTGGCGCACTCTCGGCCGCCGGGGCTTCCGGTGCCGGGCCCGGCACCAACTCCACCGC
CGGCTTGGCGATAAGGGAGCGGGTCTCTTCCACCGCTTCGGTCAGCTTCACCTCGATGATCTGGCCGAGC
TCATAAACCACTTCGGTCTTGTCCAGATAGACGCGGCCATTGTCCCAGTTGCACTCCAGTCGATCCTTGT

TGTCGAGGATGTGACGGGCAGGCAGGAAGACCACTGCTCCGTTTTCCTGCAGACGCAGCTTGAGACCGGC
ACGCATCACGTCGATGATCTCGGCGTTGAACACCTGATCGGTGCCAGCGGCTGCTTTCAGGTAACGGACA
TAGAGCCAGTCGCCGATGTCGCGCTCCACCATGCGGTGCAGGCGGCGGCAGGCGGTCAGGTGCTCGGTGA
GCTCGGTGCTCGGGCGCTCACCCGGGGTCTTGCCGGCGATGACCGCTTTGAGCAGACGATGGTTGACCAT
ATCGCCGTATTTACGGATCGGGGAGGTCCAGGTGGCGTAGGCATCCAGACCCAGGCCGTAGTGGGCGCCC
GGCTCGGAGGACATCAGCGCATAGCTCTGGAAACGGCGGATCTTGCCATCCAGCCAGCGGGTGTCGAGGT
TGTCGATCCAGCGACGCAGGGCGCAGAAGCCGGAGAGGCTGGCGATCTCGTCGCGCTCGAACGGGGCTTC
GGCACTCTTCAGCAGGTCGATGGCACCGTCGAGAGACTCTTCGTCAAAGCCGGTGTGAACGTTGAAGATG
CCGTAACCGACGCTCTTGCCCAGCACGCGACCGGCGCAGATGTTGGCGGCGATCATCGACTCTTCCACCA
TGCGGTTGGCGATGCGGCGCGGCTCGACGTGGATGGCCAGCACTTCGCCATTCTCGCCCAGCTCGAAGTC
GTAGTCGGGACGATCCGGGAATACCAGCGCATGCTCGGTGCGCCACGCGATGCGTGCTTCGGTCATCGCC
TTCATCAGCGGCAGCTGGGCCAGTACGCCTGCGTCGATAGACAGCTCTTTGCCATGCTCGGCCCAGTCGG
AGACGTCGTCATAGTTGAGACGGGCGTGGGAGCAGATGCGGGCGGCGAAGAACTCGGTCTCCTCCAGTAG
CAGACCATCTTCGGCGATGAGCAGACGGGCGCACAGAGTGTTGCGCTCCTCGCCCTCTTTCAGGGAGCAG
AGCTCGTCGGAGAGGGTGCGCGGGATCATCGGCACGTTGCGGCCCGGCATGTAGACGGTAAAGGCGCGCT
GGGCGGCCTCTTTGTCCAGCTCGCTCCCTTCGGCCACATAGGCGGTCGGGTCAGCGATGGCGACCAGCAG
CTCCCAGCCGTTATCCAGCTTGGTGATGGCCAGCGCATCGTCCATATCCTTGGTTTTGGCACCGTCGATG
GTGAAGAAGGGCACCTTGGTCAGATCGCGACGGGGCAGCTCTTCCTCTTCGATCACCTTCCACTCCGGCA
AATCAGCCGGCTCGACCTGGGCCAGATTGTGGCGCGCCAGCACCACCCACCAGGGCACATTGTGATCTTC
CTGGGCGGCGATCTTCTGGATGATCTCGGCAGAGAAGTTGCCGTCGACCAGCGCATGGCGCTTGAGGGTA
GCCACCACCCAGTCACCTTCCTTGAACTCTTTCTCATCCAGACCCTTCTTGACGCGGGCCTTGATGGCAT
CCTTGATCAGCGGATGGTCGGCCACCACGTTGAGGCGGTCGCGGAACATCTTGACCCGGGCCACGAAGCG
GGTGACGGACTGCTCCAGCAGGGTATCCGGCTCGGCCACTTCCTTCTCTTTTTCGGTGCGGATCAGGGCG
GTCACCCGGTCACCGTGCATCACCTTCTTCATGTAGGGAGGCGGCACGAAATAGCTGGTCTTCTCATCGA
CTTCCAGAAAGCCGAAGCCCCGATCGGAGGCGCGGATAACACCCTCTTTCTTCGGGATGTTTTCGCGGAT
TTGTTGCTTGAGCTGGGCCAGCAGCGGATTGTCTTGGAACATAAGTGAGGAACCTGAAGTGAAAACCTGG
GTGGAGACCTGATGAAGGATCGCCCAGCACTATACGTTTTTAGCCCTGCAAAGCCAAGGTGAATGGCCTG
CTCAGCGTGGTTTCAACTGCGATCTGGCATGGTCGATAAGGCGTTGGGAGAGCTGCTTGTGAACCCCTTT
TTCCAGTACCCAGCGGTGCCAGTAGAGGCGTTCCGTCAGAACGTGGTCGGGACTGAGGTTGAGCAGTGAC
CCGCCAGCCAGCTGGCCTCTTATCTGCAATTCGGGAATGAGACAGTAGGCCAACCCCTGCTCGGCCATGG
CGACGAAGGCCTCTGACGAGTGGACCGTATGGCAGGGATAACCGCCCGGCTCCAGCCCGAAGCGTTCTCT
CATAAAGCTCACATGCATGTCATCGCGTTGATCGAACGCCACCGCCGGTGCCTTGGCCAGCGCCTCACGG
GTCAGGCCGCGAGGGAAGTGGCGGGCAACAAAGGCGGGGCTGGCGGTAAGCTGATAGTTCATCTCACCGA
GCTCATCGACGCAGCAACCCGCCAGCGGTTGGGGATGCAGGCTGACTGCGCCAAAGGCCTGACCCTCGCG
GACCTTGTCGAGGGTGCGGCTCTCGTCATCGACCAGCAGGTTGAGTTCGATGGGGTGCTGTTTCAACAAG
GGGGCAATGGCGGGCAAAAACCAGGTGGCCAGGCTGTCGGCGTTGACGGCGATGCTGATCCGGATTGGCC
CCTGCGGCTCATCGGGAGTCAGCTCCCCCGCCAGTTCGAGCTCAAGCTGATGTACTTGCCGATAGTGGGC
CAGCAGTTTCTGGCCGAGTGGCGTGGCCTGCAGCGGCTGGCTGCGAATGAGCAGCGGCTCGGCAAACTGT
TGCTCCAACTGCTTGATCCGCTGGGAGATGGCCGACTGGGTGATATGGAGCGACTTGGCCGCCCGATCGA
AGTTCTGCTCCTGCAGTACCGCATCCAGTGCCCGCAGCAGTTTGTAATCCAGCTCTTTCATCTTCTCCCC
CTTCCGTTGCCCAAGGGCTGACTCTACGTCACTCGGCAGGGGACAACAATGGGCGGTGGAAGGGGCGTTG
CGCTGTGATATCTGACAGTGGTGGTCTGTCTTGTAAGGAAGGCATACTGCTAGAGACCGCAGGGGGCAGC
CCTGCGGGGTCATAGCGATACAGCGATACGTTTATCCCGGGCCTGTTTTCCCGGGATTTTTTATATCTGG
GCCGCTAACTCATGCGAGCGATAGGTGGCAGTTTGATAGTGTCCGATGAGCGGCTGCGAGACCGCCTGCG
CGAAGCGCTCATCCAGATGGAAGCGGATGCCAACGCCCCTTACCGCCCCCAGATCGACCGCCTGACGATG
ACCGAGCCGCTCGATGGGCAGGCCAAGTCGGCGGAAGATGCGCTCCACTGGCAGGCTGACGACGGCGACC
AACTCCCGGATCCCGTGGGCATGGGCGAAGGCATAGACCTCGCGGAAGATGACACAAGTGAGTTCGCTGA
CGCCATTGCTCATGCGCGGTGCGCGCTGGGCATCGATAGCCAGACGGGTGAGTTCCCAGACATCGGGGGT
GCGTGGCGCGCTTTCACCGGCAAGGGCGGTGGGGAAGATGGTGGGCAGCATATAGGCATCGGCACAGTTG
AGTAGACGAATACAGCCACACAGCCCTTGTTCGTCCTCGATCAGAACCCAATGGGTGTCTGGCGTGTCGA
AGCTGTCACGCTCCAGGCCCCGATGAGACTCGACATCCCAGCCTAGCCGGTCGGAAAAGACGCGATTTCG
AAAGCGATAGAGTTCATTCTCCACCTCGTTTCGTGGATGTTCTCTCAATTTTCCTTTAAAAACCAACATT
ATGGACCCTCTATCTTGCTATTCGCCGTTATAATCACATGTCTGGCCAAGGGGGCTTTCATATGAACAGA
GACCAACTGCTTGAGTACCTCGAACATTTCACTTCGGTGACAGACGGCAATCGTCTGGCCGAACTGATCG
GTCGCTTTACGCTCGGGATGGGCTATGACTACTATCGCTTCGCCCTTATCATCCCGATGTCGATGCAAAG
GCCCAAGGTGGTGCTGTTCAACCAGTGCCCCGATTCCTGGGTGCAGGCCTATACCGAAAACAACATGCTT
GCGTGCGATCCGGTGATCCGGCTGGCACGCAAGCAGACGCTGCCCATTTACTGGAACCGCCTCGATGAGC
GGGCCAGTTTTTTGCAAGAGGGAACCATGGACGTTATGGGGTTGGCAGCCGAGTTCGGGTTGCGTAACGG
CATCTCATTCCCTCTGCACGGTGCAGCCGGGGAGAACGGTATCCTCTCCTTCATCACTTCCGAACGGGCC
TCGAGCGATCTGTTGCTGGAGTCCTCGCCGATCCTCTCCTGGATGGCCAATTACATCTTCGAGGCGGCCA
TTCGGGTGGTGCGTTTCAGCATGATGGACTGTGCGGGTAAAGACCCCTTGACCGAGCGCGAGACCGAGTG

CCTGTTCTGGGCCAGCGAAGGAAAGACCTCTAGCGAGATCGCCTGCATCCTCGGGATCACGGAGCGCACG
GTCAATTACCATCTCAATCAGGTCACCCGCAAAACGGGCTCCATGAACCGCTATCAGGCCATTGCCAAAG
GGGTTAGTACGGGGCTGTTGCTACCCAATCTGGAGCAAGTCGTGGTTACCAACTTCCCCAGATTGCTGCA
GTAGGCTGAGCCTGCAGGCGCAACAGATTCTCCTGCCGCACTCTGAGGGTCACAATTGTCAGATCCGGTA
ATTGACAAGGGCTGGAACAGCGGCGGGTATGCCGTTTTTTATTAGCAAAATGAATCAATCAGTAAATCAA
TGAATTTCTAAGAAGGAACCACCATCGATATAGTGCCGCTCATTCATCATTTCGAACGGTACTCTCCATA
ATGCTCGCAACGACACTGCAAGGCTTCACCATCGGTCTGGCCATGATTATCCCCATCGGCGCCCAGAATG
CCTTTGTGCTGAGCCGGGGCATCCATCGCAATCACCATCTGCTGACGGCCACGCTCTGCAGTTTCTGCGA
TCTCACCCTGATTGCTATCGGCGTATTTGGCGGGGCCAATGTGCTGGCCGCCAGCCCCTTCGGCATGGCG
CTGCTCACCTGGGGTGGTGTGCTGTTTCTCGGCTGGTTTGGTGGCCGTTCCCTGCTCAGTGCCTGGCGCG
GTAAAGGGGAGGGACTTGCCGATTCTGCTCAGCAGATGGGGGCCAAAAGCGTGCTGGCGATGACGCTGGG
AGTTACGCTGCTCAATCCTCACGTCTATCTCGATACCCTGATGCTGCTTGGCTCCCTTGGCAGTCAGGTG
AGCGAGTCCTTGCGTCCGGCCTTTGCTGCGGGGGCCATGCTGGCCTCGTTGGTGTGGTTCTACTCGCTGG
CGCTGGGTGCTGCGGCACTGGCGCCCTGGCTGGCCCGCAGCCGGGTGCAGCAGGGGATCGACCTGCTGGT
TGGCATCATCATGCTGAGCCTGGCTATTCAACTGGCCATAGGACGTTGAGGAAGTTAAGGCGGCAGGAAT
GAGCGACCGGTAACAAAAAGATGCCACTCACATGAGTGGCATCTTTGCTATTGGAGGGGCTCAGACTCTG
AACTGGGAGAGCAGGCGCTGCTGCAGCAGGCTGGCCTGATGGAGCGAGGAGGCGAGCTGCAGACACTCCT
GCGCCTCCTGTTCGCCGGTTCTGGCTTGCAGACTGATACCGGAGACCTTGTCAGAGGAGTGGCTCAGGGT
GTGTGACAGTTGCCCGGTAGCCACGGCCACCTGCTGCACCATGTCGGCCAGTTGGTCGATGGCGCCTTCT
ACCTTGAACAGGATCTGTTTGGTGTTCTCGGAGTCCTGCATGCCGGACTCCACCGTCTTGAGGTTGCGCT
GCATCATCAGATAGGCGGTCTCGGTCTGCTGCTGGATGCTGCTGATCACCTGGCTTATCTCGGCGCTTGA
TGTCGCCGTGCGATTGGCCAGGGTGCGCACCTCGTCCGCAACCACCGCAAAGCCACGCCCCATCTCCCCC
GCGCGGGCCGCTTCGATGGCCGCATTGAGGGCAAGCAGGTTGGTCTGCTCCGAGATACCGCTGATGGTGG
AGATGATCTGGCCAATCTGACCCGCCTGCTCCTTGAGGGAGTTGATGGCGATGGCACATTCGCTCACCTC
CTGTACCACCCGCTGCATGCCCGCGCTGGCGGAGGCGACATACTGGCTGCTCTCGGTGGTCAGCTGGCGC
ACCTCATTGGCATTGTGGGCCGCCATGCTGCAGTTGTCCGAGATACCGGTGGCGACTTGCACCATCTGTT
CACTGTCGTGGTGCAGGGCCAGCGCATGTTCGGCGGTCTGGCGGCTGCTCTGGCTGATTGCCTCCACCCG
GTTGACCGAGGTGTGAACGTGTTCGCTGATGTGCTGGTTGTTGTCCATGGTGGAGCTGATCATCTTCTGC
AATTTTTCGATGAAACGATCCACGCTGTTGGCCAGCGAGCCTATCTCGTCATCCCGTTGCAGGTTGATGC
GGGCGTTGAGATCTCCCTCGCCGGCGGCGATATCCTGCATTCGTTTGTCCACTTGCAGCAGCGGGGTCAC
CAGCTTGCGGGTCAGCCAGAAGCCGCAGAGGGTGACCATGATCAGGCTCAGCAACAGGCCACCCAGCAGC
AGGGTTCGGATCTGGCTGATGATGCCGTTGAGCTGGGCCACCTGATCCTCGACGGTGGCATCGCCGCTCT
CCTCGATGGCAACCAGCTGTTGTCGCAGCTCATCGGCACTGACCTCATAGACGGACTGCTGGGCCTGATA
GCTGCCGAGCGCATCGATCCACTGTCCCATCAGCTGCTGATGCCGGGCCAACAGGGCGCGATACTCTGCC
GACAGTGTCTTGCCGGCAAATTCGCCGGTTCCCTCGATATCGAACAGCCCGGTGCCGAGCATGCTGTCGA
TTGCTTCGTGGTAGAACTCGGTCGCTTCTTTCAGCTCGGCCTTGATCGCCTCTGTGACTCCTTGTCTGCG
CATCTTCTCCAGAGCATAGAGCTGGCGCAAAAAGCCTATGTTGGATTCCATCCCCCCGTCGGCCGCTTCC
CAGCGAGTGGAGAGGCCGCTGTTCCAGCTGATGGTCTGGTCGGGGGATTGGGTCAGCGCCTCGACCGCGC
CGTCGCCGATCACCTCCAGCTGGGTCGAGAGGCGAGCCATCAGATCGGCGTTGTGATGCAGGGCCTGATC
CACCTCGGTATAGGCGTGATAGAGCGCCAGCAGCTTCTCTTGTTGCTGTTGATAGTCCTGCTTGTGCTGT
TCCATGGTGGCAAGGGCTGCACCATCAATGAGCAGCCCCTGGCTGACCCGGGCCAGCGCGTCATCGGTCG
CCGCCTTGGCAGCATCGAGTTCGGCGCGATTGAGCGGGGTTCCGTTTTGCATCTTCTGAATCTGTAGCTG
TTGGTTGCCCAGCTCTATGGAGCCCTCCATGGCACCGTCGGCGGTCTGCCAGGCGGCGCCCAGGACATAG
GAGAGCAGATTGGCTAGCCGCTGGCTGCCATACCAGCCAACGACCCCCATGCCCAGCGTACAGCCGAGCA
ATATGGCAAGACCCAGATTGGAGGTGGTAACAATTTTCATGTGATGGCCTTCCTTGGTATCGACATTGGC
ACCAGTTTCGGTTCCGAACGGGTTTAGTGTAGTGGTTTATCCCCCGCTCGGGGGCCGGTGCGGCGCTGAA
ACATCCCGTGCACTGGCTGCACCCGATCAAGCATGGTGGAAAATTGGATAAAAAGCCTCTCTGACCATGC
GGGTGTTGCTGCCCTATGCTGGATACTTATCCAGTTATTCTTTATGCTATGAGCCAATTTACGAATAGTT
GTATTGGTGAGGAGAGAGCCGTGATTGGTCGCTTGAGAGGCATTGTCATTGAAAAGCAGCCCCCCGAGGT
CCTGCTGGAAGTGGGCGGGGTTGGCTATGAGGTACAGATGCCGATGAGCTGCTTCTATGATTTGCCGGAG
ATCGGCAAGGAAGCGACCATCCATACTCACTTCGTGGTGCGGGAAGATGCCCAGCTGCTCTATGGTTTTA
ACCACAAACAGGAGCGGGCGCTGTTCCGCGAGCTGATCAAGACCAACGGAGTCGGCCCCAAGCTGGCGCT
GGCGATTCTTTCCGGCATGACAGCTACCCAGTTTGTGCTGAGTGTCGAACGTGAAGAGATAAGCTCTTTG
GTCAAACTGCCGGGAGTCGGCAAGAAGACCGCCGAGCGGCTGGTGGTGGAGATGAAGGATCGCCTCAAGG
GCTGGGTCAGTCACGACCTCTTCTCCCCGGCCATGGTCACCTTGCCAGCCAGTGAGCCCGAATTGCGGGC
ACCTGATGTGACCGAAGAGGCGGCCAGTGCGCTGGTGGCCCTCGGTTACAAGCCGCAGCAGGCGAGTCAG
ATTGTCAGCAAGATCGCCAAAGAGGGGATGTCGGTGGAAGATATCATCCGTGAATCCCTGCGTAGCCTGG
TATGAGGTAGTTGATGATTGAAGCAGACCGCCTCATCTCGGCCAGTGCCGCCCGTGAGGATGACATTATT
GACCGGGCTATCCGCCCCAAGAAGCTGGCTGACTACACCGGTCAGGATACCGTCTGCGAACAGATGGAGA
TCTTTATCGAAGCGGCCCGTCAGCGGGGTGAAGCGCTCGATCACCTGTTGATCTTCGGCCCGCCGGGTCT
GGGCAAGACTACCCTCGCCAACATCGTCGCCAACGAGATGGGGGTCAATATCAAGACCACCTCCGGCCCG

GTGCTGGAGAAAGCGGGGGATCTGGCGGCGCTGCTCACCAACCTCGAACCGAACGATGTGCTGTTCATCG
ACGAGATCCATCGCCTCAGCCCTGTGGTGGAGGAGGTGCTCTATCCGGCGATGGAGGATTACCAGCTCGA
CATCATGATCGGGGAGGGACCCGCAGCTCGCTCCATCAAGCTGGATCTGCCTCCCTTCACCCTGATTGGC
GCCACCACTCGGGCAGGTTCACTGACCAGCCCGCTGCGGGATCGCTTCGGTATCGTCCAGCGGCTGGAGT
TCTACAACGTCAAGGATCTGACCGATATCGTCTCCCGCAGCGCCCGTTGTCTGGGGCTCGACATGACGGA
AGATGGTGCCATTGAGGTCGCCCGTCGCTCTCGCGGCACGCCGCGGATTGCTAACCGTTTGTTAAGGCGG
GTGCGCGACTTTGCCCAGGTGAAATCCGATGGCCGGATCGACGGCCCCATTGCTGCTCGCGCCATGGACA
TGCTGGATGTCGATAATGAAGGCTTCGACTTCATGGACCGCAAGTTATTGTTGGCTGTGATAGACAAGTT
TATGGGGGGCCCGGTGGGTCTCGATAATCTGGCCGCCGCGATCGGGGAAGAGAAAGATACTATCGAAGAT
GTGCTGGAGCCTTATCTGATCCAGCAAGGGTATCTGCAACGTACACCCAGAGGACGGATTGCGACCACAC
GAGCCTACGCTCATTTCGGTCTTCAGCGACCAGATGAGAAGTAAAAACAGTCGTTTGAACATGAAACCGC
CGTCAAAGGCGGTTTTTTATTATCCGGGCAAAAAAAAGCCCACGGCGGATGCGGTGGGCAAAGACAATTC
AGGATGGATGTTCACAGACAGTGAGTGCGTCTGGACACAGACCGGAAAATAAACAGGTGAGCCGTCGCGA
GCTCTCAGAGTCGGTTCAGTTGATAGAGCTGAACAAAGGAAAGCTTGTAGTAACGGATGGTTTTAGCAAC
AAACTTTTTCATCTTCGATTCCAATAGACAGGTTCAAATTGATCGTCGAATACGTACCGTGAACCCGGAT
TAACCGGCCTGTATCCAAGGGTATGCCCTCACTGCGAGGCGCATTGTAGGAGTTCGAATCGACCCCGGCA
AACAAGAAAAATTGCACTCTTCAAATTAGTTTATCTAATGTCTTTATTGATGAAATTGGTTCGCATGAGT
ATGCATTCTTTGCAATTTTAAATTGAGTTTTCGAATCAGATTGCCTGACTCGGATAACATGGAAAAAGGC
GCGTCATTCACGCTTTTCAAATTAAACCGAGTGGCGGGATGTTAAATTCTTAAATTGATTTAAATCAATG
TTTTACGCTTTATCGTGTTTTGTGATCTGGCTCGCGTCAATAATCGTGAAATAGATTAAAAATGCCTTTC
AAAGCCATGTATGGCGGGTCCTTTTACATTGATTTCAATCAAATAAAGGTGATATAAAAACCCGGTCATC
ATGGGCGAGAATAAATTTAGGCTGATTGTTTTGGAATTGTTAAACATTAGCTTTATTTGTGTTCTCGTAT
TGCCTTGTTGTTAATGCCAGTGGTTACTGAACGCGTTTTGTTGTGACACGTCCCAATAAGGCTCATTCGA
TAACCTCCAACGGAAGCTTTGTGCTTAAAACTTAAAATAGCCGTGCCAATAATCGGTGAGGAAACATCAT
GATTGCTGAGCATGTGGTAGACCTATCGCGGTTCCAGTTCGCTGCGACGGCCCCTTTATCACTTTTTGTTT
GTACCCCTGACGCTGGGAATGGCCTTTCTCCTGGCGATCATGGAATCCGTCTATGTGATGACCAACAACA
CCATCTACCGGGACATGACCAAATTCTGGGGTAAGTTGTTTGGTATCAACTTTGCCTTGGGGGTCACCAC
AGGTCTGGCCATGGAGTTCCAGTTCGGGACGAACTGGGCTTACTACTCCCATTATGTCGGCGATATCTTC
GGGGCCCCGCTGGCTATCGAAGGGCTGATGGCCTTCTTCCTGGAGTCCACCTTCGTCGGTATGTTCTTCT
TTGGTTGGGATCGTCTGAGCAAGCATCAGCATCTGGCTGCCACCTGGTTGATGGCGCTGGGTACCAACCT
CTCCGCCCTCTGGATCCTGGTCGCCAACGGCTGGATGCAGAACCCGGTCGGTGCCGAGTTCAACTTCGAA
TCCATGCGGATGGAGATGATGAGCTTCGCTGACGTGGTGCTCAACCCGGTTGCTCAGGTCAAGTTCGTCC
ACACGGTGGCGGCTGGTTATACCTGTGGCGCCATGTTTGTGCTGGGTATCTCCTCCTACTACCTGCTGAA
GAATCGTGATGTGGCGTTCGCCCGTCGCTCCTTCGCCATTGCCGCTGCGTTCGGTATGGCTTCCATCGTG
TCCGTGCTGATCCTCGGTGACGAATCCGGTTACGAGACCGGTGAAGTGCAGAAGGTGAAACTGGCCGCTA
TCGAAGCTGAGTGGGAAACTGAACCTGCTCCGGCCGCCTTTACCCTGTTCGGTATTCCGAATCAGGAAGA
GATGCGCACCGACTACGCCATCAAGATCCCCTATGCGCTGGGCATTATCGCGACCCGTTCTCTGGACGGT
CAGGTAACCGGTCTGAAGGATCTGAAGAGCGAGCACGAACTGCGCGTCCGTAACGGTATGACCGCTTACG
ACCTGCTGACCAAGCTGCAATCCGGTGACAAGTCTGACGAGACCCGCGCTCGCTTCGACGAAGTGAAGCA
GGATCTGGGCTACGGTCTGCTGCTCAAGCGCTACACCAGCAACGTGACCGATGCTACCGACGAGCAGATC
AAGGCCGCCGTGGATGACTCCATCCCGCAGGTTCTGCCGCTGTTCTTCGCCTTCCGCATCATGGTTGCCG
TTGGCATGCTAATGCTGGTGCTGATTGGTATGGCCTTCTATGACAGTGCCCGCCACCAGATCGGCAAGCG
CAAGTGGCTGCTCAAGGGTCTGCTGTGGGGTATCCCGCTGCCGTGGATCGCGATCGAAGCTGGCTGGTTT
GTGGCTGAATATGGTCGCCAACCCTGGACCATTGCAGAGGTGTTGCCGACTTCCCTCTCTGCATCCAATT
TGCCTGCAGCCGAGATTTGGTTCTCTCTGATCGGTATCTGCCTGTTCTATACCGTACTTCTGGTTATCGA
GATGTACCTGATGTTCAAGTATGCCCGTCTTGGCCCCAGCAGCCTGAAGACTGGCAAGTATCACTTTGAA
CAGATCAAGGCATAAGGAGAGCGACCATGTTTGATTACGAAACTCTGCGTGTCATCTGGTGGGCTCTGGT
CGGCGTACTGCTGATCGGTTTTGCCATCACTGACGGCTTCGACATGGGGGTAGGCGCACTGCTCCCGTTC
GTCGGCAAGAAAGATGTCGAGCGCCGCGTGATGCTCAACACCATGGGGCCGCACTGGGAAGGTAACCAGG
TGTGGCTGGTCACTGCCGGTGGCGCCTTGTTCGCCGCCTGGCCGATGGTGTATGCCGCGACCTTCTCCGG
CTTCTACATCGCCATGATCCTGACCTTGATGGCGCTGTTCCTGCGTCCGGTCGGTTTCAAGTACCGCTCC
CTGCGTGCTGATGCAAAATGGCGCAGCAACTGGGACTGGGCCTTGTTCGCCGGTAGCGCAGTACCGCCCA
TCGTCTTTGGTGTGGCCTTTGGCAACCTGCTGCAAGGCGTTCCGTTCGAGTTTAACCAGTTCATGATGTT
GACCTATCACGGCAACTTCTTCGGCCTGCTTAACCCGTTCGGCATTCTGGCTGGTCTGGTGAGCCTGTTC
ATGCTGCTCACCCAGGGCGCAACCTGGTTGATGATGAAGACCGACGGTGAAGTGCTGGCGCGTTCCCGCA
CTGCCGCCATGATCTGCTCACTGCTGACCCTGGTGCTGTTTGCTGCCGCAGGTTGGTGGGTAAGCGGTAT
GGAAGGGTATGTCATCGTCAAGGCTGCTGCGACTGACGCGGTATCCAACCCGATGAACAAGGAAGTTGTC
CTGCAGGCTGGTGCCTGGATGAACAACTACACCCTCTATCCGTGGATGGTGGCCGCCCCTGTACTGGGTC
TGGCCATGAGCCTGCTGACTGCGCTGTTTGCCCGCATGAACATCGGCTGGCTGGCGTTTACCAGCTCCTC
GTTGGCGATTGCCGGGGTCATCCTCACTGCCGGTTTCTCCATGTTCCCGTTCGTGATGCCCTCAAGCCTT
GAGCCGTCACTGAGCCTGACCATGTGGGATGCTACCGCCTCTTTCAACACCTTGCAGGTGATGACAGTGG

CTGCAGCGATCTTCGTACCGATTGTGCTGGGTTACACCATCTGGACTTACATCAAGATGTTTGGCCGGGT
AACCAACAAGCACATCGAAGATAACCAACAATCTCTCTATTAAGAGGTGCGCTGACAATGTGGTATTTCA
CCTGGATCCTGGGACTGTTGCTGGCCTGTGCATTTGGCATCATCAATGCGCTGTGGCTGGAACACACCGA
GAACATGGATCGTCAGATCGATGAAAACAAGTGATCGACTCGCCAGGCTGGCCGCTCCACTGGAGCGGCA
GCCGTGGCAGGGGTTGATGTTACTCGCGGCGGCCTTGTTGGCCGTCGCTATTTTACAGGCCCCCAATCTG
ATCGCGGCCAACACCAGCGAGCATGGTCTCTGGCTTGCTCCCTGGCTGATGTGGGCCGTCTGTTGTGGCG
TACTTAACGGGCTTGGCTTTCATCTGAAAAGCTTGCCCGGACGTGTGCTGTTCAACCCCTGGCTCGCCTG
GCCCAGTTTGTTGTATGGCCTCTGGCTGACACATTCATATTTCCTTTCATAAAAAACGGGGGGAGGCAGG
ATTTCCCTGTCTCCCCCTGCTTCTTTCCTATTCAGCCCTGCGCTATGATAGCCCCCCATCTGACTGTCGA
ATGGATCACAGATTGTTATGGGGGAAGTCTTTGAGTTTCCGGTGCGTGTCTACTACGAAGACACCGACGC
CGGGGGCATCGTCTACAACGCCAATTATCTCAAGTTTATGGAACGTGCTCGCACCGAGTTTCTTCGTGCC
AAAGGGGTCGAACAGGACGTCTGGCTGCAGGAGGGATATGCCTTCGTGGTGAGCCGAACTGAGATCGATT
TCCGCTCGGCGGCCCGTTTTAATGAACTATTGAATGTATTAACGCAAGTGATTGAAGTGAAACGCGCATC
CCTGCGCTTTTCGCAACAGATCCTGGCGGCTGATGGACGACTGATTACCCAGGCCATGGTACAGATAGCC
TGTGTCAGTCATTCGCAAATGAAACCTGTAGCTATACCTGAAAAAATCAAGGGAGTGCTGTTAAGTGCAC
GCTGAAATTTCGTTTATTGGCCTGTTTTGGCAAGCCAGCCTGTTGGTAAAACTGGTCATGATGACCCTGA
TGAGCATGTCTATTGTCTCGTGGGCGCTGATTATCCAGCGTTCAAAGGCGATCAAGGCGGCCAATCTCGC
ATCGGAACAGTTCGAAGATCGCTTCTGGTCAGGGGTCGATCTCAATCGCCTCTATCAGGAGTCCTCTTCC
CGCCGCGATGATATCGAAGGCATGGAAGATATCTTCTACTCCGGCTTCAAGGAGTATGCCCGCTTGCTCA
AGGCCGGTGCCCGTGGTCAGGATGCGGTGATGGATGGTACCTACCGCGCCATGCGCGTCTCCCTCTCCCG
CGCGGTGGATGATCTGGAATCCAATCTGCCGGTGCTGGCGACCATCGGCTCCATCAGCCCCTATATCGGT
CTGTTCGGTACCGTATGGGGGATCATGAACGCCTTTATCGCCCTGGGTCAGGTGCAGCAGGCTACCTTGC
AGATGGTGGCACCGGGTATCGCGGAAGCGCTGATCGCCACCGCCATGGGTCTGTTTGCCGCTATCCCGGC
GGTTATCTTCTACAACCGCTTCTCCAACAAGGTCGAGCGGCTGGAGAACGCCTACGCCAACTTCATGGAC
GAGTTCACCACCATTCTGAACCGTCAAATTGCACAGCAGCAGGGTGATCAGTAATGCAAACCTATCAGCG
GATCCGGCGCAAGAAGGTGGCCGAGATCAACGTGGTTCCCTATATCGACGTCATGCTGGTGCTGCTTATC
ATCTTTATGGCGGTCACGCCGGTGATCACGCAAGGGGTCAAGGTGGATCTGCCACAGGCTGAGTCCGAGC
AGTTGCCGGAAGATGCCAAGCCGCCCTTCATCGTCAGCGTCAATACCGAAGGCGAGTATGTCATCAAGGC
TGGTGAAGCCGATGATGAGCCGGTGCCAAGCGGCACGCCAGAGGCGATGCAGCAATACATCACCGAAAAA
GCCATGGGCTATCTGGCGATCAACCAGAATGCCCCCGTGGTGGTCGCGGGTGACAAGGCCGTGCGCTACG
AAGAGGTGATCCTGTTGATGGTGGCCCTCAAGAAAGCGGGTGTGCCTCAGGTTGGCCTGATGACAGATCC
GGTGAACTAACGGAGATGGATGTGAAGCGTGGTATTTCCGGTTATCTCATCGCGTCGTTTCTGCTGCACC
TGATCATCGGCATCATCCTGTTTGTCGGGCTCGATTTCAGCAAACCGAAACGACCCGAATCGAAGGGGCA
GATCGTCAATGCCGTCATGCTCGATGAGAACTTCCTCAACGAACAGGCCAAGCAGATCCAGCAGCAGAAA
TCTCAGCCCAAGCCTCAAAAGGTAGAGAAAGAGAAGGATAAGGAAGACACGGATCTCGCCAAGCGCGAGC
TGGCTCAGAAACAGGAGCAGCTTCGCATCGCCGAGACCAAGCGCAAGCAGGCCGAAGAGGCGACTCGCAA
GGCCGAGGCCGAGAAGCAGAAGAAGGTTGCCGAGCAGAAGCAGGCGGAAGAGAAGGCCCAAAAAGCCGAG
GAAGCCCGCAAGCTGGAAGAACAGAAGGCCAAGAAGGCCGAAGCCGAGCGTAAAGCGGCGGAAGCCGAGA
GCAAGGCGTTGGCGTTGAAGAAGAAAAAGGAACAAGAGAAGAAAGAGGCCGAAGAGAAAGCCAAGGCGGC
AGAAGCCAAGGCCAAGAAAGCGGAAGCTGACAAGAAGGCCAAAGCCGAAGCCGAGAAGAAAGCCAAGGCA
GAAGCAGAGAAAAAAGCCAAAGCCGAGGCCGAGAAGAAGGCCAAGGAAGAGGCTGAGAAAAAGGCAAAAG
CTGACGCAGAGAAGAAAGCCAAGGCCGAGGCCGAAAAGCAGGCCAAGATCGCGGCTGAGCGCAAGCGTAA
AGCCGCCGAGCAGGCCAAGCTGCAAAAAGAGATGGAAGAGATGATGCAGCAGCAGCTGTCGGCTGAGGCC
AATGCCCGCAGTCAGGCGGCATCGGCAGCAGCCCAGGGCGAAGTCGACAAATATGCGGCGCTCATCAAGG
CCACCGTTGAGCGGTATATGATTTTGGATCCCACCATGCGGGGCAAAACATGTACTATCGGCGTACGTCT
TGCGAGCAGTGGCTTTGTCATATCGGTAGATAACGGCCAGGGTGACCCGGCGGTCTGTCGTTCCGGTAAG
GCAGCTGTTCTGAAAGCCAATCAACTGCCTGTGCCCAAGGATCCTGCGGCATTCGAGATGTTGAAGGAAT
TTAACCTGAAGTTAGAACCGAGTATCTAAGCGTGATAAAACGAGGTTGCTTGCCTCCGGTTGGCAACCTC
TCGCCGAGAAAGGAATTTAACCTCTCATCTATATTGGAACCGAGTATTTAAGCCATGATCAGAAAAGTAT
TTTTTGCTTTGGTTGGCTGTTTGTTGCTGGGCCAGAGTGCCCAGGCGGCCCTGGACATCGTCATCACCGG
CGGTATTGATAGCGCACGCCCTATCGCCGTCGCCCCCTTCAAGTGGGAAGGCAACGGGCAGTTGCCGCAG
GACATCGCGGAAGTGGTCTCCAACGACCTGATGCGTAGCGGCAAGTTCAAGCCGCTGGCCCGTGGCCAGA
TGCCGCAAACCCCGAGCAGCAGCAGCGAGATCAACTTCGCTCCCTGGGCCTCCCAAGGGGTTGAAGCCGT
GGTGGTGGGTTCCATTTCGGCAGTCGGTGACGGCACCTACAAGGTCAACTTCGAGCTGGTCGATGTGCTG
AAAGGCCAGCTGGCCAAGGCGCAAGGTGGCGAGAGCAACGGTTACATCCTGGATAGCCGCATGGCGACCA
TCCCGGGTGCCCAGATGCGCCAGTTTGCCCACCGTATCTCCGACATCGTCTATGAGCGTCTGACTGGCGA
GCGCGGTGCCTTCCTGACCCGTCTGGCTTATGTCTCCGTCCAGCAGGGTACCCAGTTCCCGTACCAGCTG
CGCATTTCTGACTACGACGGTTACAACGAGAAGACCCTGCTGCGCTCCCGCGAGCCCTTGATGTCTCCGG
CCTGGTCGCCGGATGGCAGCAAGCTGGCTTACGTGAGCTTCGAGAACCAGAAGTCCGAAATCTACGTGCA
GGATATCTACACCCAGCAGCGCAGCCTGATCACCAGTTTCCGCGGTATCAACGGGGCGCCGGAGTGGTCT
CCGGATGGTCGTCGTCTGGCTATCGTTCTCTCCAAAGATGGTTCACCGAACCTCTATGTGGTTGATGTCG

CTAGCAAGCAGCTGACCCGCGTCACAACCAGCCGTGTTATCGACACCGAACCCTCCTGGATGCCGGATGG
GCAAAATTTGTTGTTCACCTCTGAACGTGGTGGCAAACCCCAGATTTATAGCGTAAATTTGGCGACTGGT
ATGACTCGCCGGATGACTTGGGAAGGTGAATCCAACCAGGGTGCATCCATCACTCCTGATGGCAAAATCA
TGGTGATGGTGACCCGTGTTCAGGGACAGTATCGTATTGCCCGTCAGGACATGGAAAACAATGCCCTGTT
GGTCCTTACCCAGAGCGCTCTGGATGAGTCGCCAAGTGTGGCTCCCAATGGCAGCATGATCATTTATGCC
ACCATCTATCAGGGTCGTAAGAGTCTCGCGTTGGTGTCGACTGATGGTCGCTTCAAGGCTGTGCTTCCGA
CCAGTAGCGGTGAAATTCGTGCACCAGCTTGGTCGCCCTTCTTGAATTGATAAAAATCCAATTGTTTTTT
GTTTTGTTATAAAGGTTTAAGGAAATAGCATGCAACTCAATAAACTGCTCAAGGGTTTGGCCATCGCACT
GCCACTGTTCACCCTGGCCGCTTGTAGTTCTTCTTCTGATTCCGACGCTGCTGGCGCTGAATCAGGTATG
ACCGACGGCATGGGCGGTGTACAAACCGGCGGCGCCAACGGCATGTTGTCCCCGGAAGAGCAGATGCGTC
AGAAGTTTGAAGCACTGCAGCGTGACAACGTGATCTACTTCCCGTTCGACGGTTACGATATCCAGGGTCA
GTACGCTGATCTGCTGGATGCCCACGCCAGCTACCTGCGCGAGCGCCCGAGCGTTAAAGTCCTGATCGAA
GGTCACGCCGATGAGCGCGGTACTCCGGAGTACAACATCGCCCTGGGCGAGCGTCGTGCCAAGGCTGTTG
CCAAGTATCTGCAAACCCTGGGTGTGCAGGCCGAGCAGCTCTCCATCGTCAGCTATGGCGAAGAGAAGCC
GCTGGACATGTCCCACACCGAGGATGCCTTTGCCAAAAACCGCCGCGCGGTTCTGGTTTACTAAGGTTTA
AACCGAACGAAGTTCGTTTATATGCAAGCGGCCATTTCGATGGCCGTTTTATTTGCACTAAACGCCAAGG
TTTGATCCGAATGAAATCTGCATATGCGAGCGACGATGGCGCTGGCCGTTTTATCTGTACTAAACGCTAA
GGTTTGATCCGAATGAAATCTGTGTACAAGCAAGCTGTCGTTGCGCTGGCCGTATTGGTTGCTCTGAATG
CGCAAGCTGCGGCTCCTGTCAGCGATCTGGGCGGTGACTCGGGTGGTGGCGTTGTCCTTAGTGGCAACAA
CGGCGCCATCGTGCCCACCGGTAGCCTGGAAGATCGGGTTGCCCTGCTGGAGCGCACCCTCAATGCCCGT
CTGCGACTGCAAGCTGAATTGCAGCAGCAGGTCGACTCCCTGCAAGGGGAGGTTTCCGAGCTGCGTGGTC
AGCTGGAGCAGCAGACCTACCAGATGGAACAGTCGCAAGAGCGCCAGCGTCAGCTCTACCAGGAGCTCGA
CAAGGTTGCCAGCAGCCAGCAAGCTGCTCCAGCCGCCCCGGCCCCTATTGCTGCCACACCGGCTGCTGCC
GCCAATTACTCAACCAATCAGGATGAGAACCAGGCCTACGACGCAGCGGTCAACATGGTGCTCAAAGAGA
AGAACTACGACAAAGCCATTCCGGCTTTCCAGGGCTTTATCAAGCAGTACCCCAATTCAGGCTATGTTCC
CAATGCACACTACTGGCTCGGCCAGTTGCTGTTCAACAAGGGTGACAGAGCCGGTGCCTCGGCCCAGTTT
TCGACCGTGGCCAACAAATACAGCAAATCCCCCAAGCGAGCCGATGCGCTGCTGAAGTTGGGTATGCTGG
CGCAACTGGATGGCAAGAAGGCGGAAGCCAAATCCTTCTATGAGCAGGTGATCAAGGGCTATCCCAACAC
CTCGCCGGCCCAGTTGGCCAAACAGAGTCTGACCAAGCTGTAACATTGCTGTATGTCACTGTTTTCGTAA
CGCTACTGTAAAAACAGCCTGCATTTGTACGCACAGTGAGCAAACGGATTTAAATTACGAATTTTCTGTT
GCACCGGAAATTTAAATCCGTAATATAGGCCGCCGTCAGAGCCGAAGCAGTAATGCAGTCGGAACTGGTC
GTGGTGAGGGTCGTTAGCTCAGTCGGTAGAGCAGTTGACTTTTAATCAATTGGTCGCTGGTTCGAATCCA
GCACGACCCACCATTTTCTCCACTCGGTGGAGACAGAATTCCCAGGTCGTTTAGCTCAGCTGGGAGAGCA
CCTCCCTTACAAGGAGGGGGTCACTGGTTCGATCCCAGTAACGACCACCAGTTTGTAAAAAAATGATTTA
GATGGGTCGTTAGCTCAGTCGGTAGAGCAGTTGACTTTTAATCAATTGGTCGCTGGTTCGAATCCAGCAC
GACCCACCATCATTTCAGAATCCCAGGTCGTGTAGCTCAGCTGGGAGAGCACCACCCTTACAAGGAGGGG
GTCACTGGTTCGATCCCAGAAACGACCACCAGTTTGTAGAAAAAATGATTTAGTTGGGTCGTTAGCTCAG
TCGGTAGAGCAGTTGACTTTTAATCAATTGGTCACTGGTTCGAATCCAGCACGACCCACCACCATTTCAG
AATCCCAGGTCGTTTAGCTCAGCTGGGAGTGCACCTCCCTTACAAGGAGGGGGTCACTGGTTCGATCCCA
GAAACGACCACCAGTTTGTAGAAAAAATGATTTAGTTGGGTCGTTAGCTCAGTCGGTAGAGCAGTTGACT
TTTAATCAATTGGTCACTGGTTCGAATCCAGCACGACCCACCACCATTTCAGAATCCCAGGTCGTTTAGC
TCAGCTGGGAGTGCACCTCCCTTACAAGGAGGGGGTCACTGGTTCGATCCCAGAAACGACCACCAGTTTG
TAGAAAAAATGATTTAGATGGGTCGTTAGCTCAGTCGGTAGAGCAGTTGACTTTTAATCAATTGGTCGCT
GGTTCGAATCCAGCACGACCCACCATCATTTCAAGAATCCTAGGTCGTTTAGCTCAGCTGGGAGAGCACC
TCCCTTACAAGGAGGGGGTCACTGGTTCGATCCCAGTAACGACCACCATATTCGCAAGCAAGGCTGAGAT
ATCAGCTGCGGTTGCAGAGAAAGTCCCCACTCAGATGGGGATTTTTTTTGCCCGCAATTCAGTACTCCAG
CTTCTTCTGTTGCCGTTGTGGCGAGCCGAACGCCACCTCTTATAGTGCCACTCTGCCACTCTGCCACTCT
GCCACTCTGCCACTCTGCCACTCTGTCATTTCGCACATCCTCTACTCTGCTCTGCCGAGATTGCCCGACT
GCACTCCTGTCCATCGTGATGCTGCTTATATGAACGCCTGTAATAAAGTAAACGCCAGCAATAAAAAGGG
CAGGCCAAAGCCTGCCCGCAAAGATGCGCAAGGTGTTACATGATCCCGCGGATCAGGATCCCGCTGATCA
GCAGCGGCGCCACCACGCGCCAGCACCAGAGCAGCGCCAGACGCAGGGCGGGTGACAGGCCCGCAGGCAA
TATGGCATCACCCAATTGCCAGCCAAGCAGCAGGGTCAGTATCAGACCAAACAGCGGCATCATCAGGTTG
GAGGTCAGATAGTCGAGCAACTCGAAGATGGTCTTGCCAAACAGGGTCAGATCGCTCCATGGGCCGAACG
ACAGGCTCACCGGAATGCCGGAGACCATGATAAGCAGGGTCAACACCCGGCAGGCACCACGGCGCGACCA
GCCCCACTCCTCGGTGGTGAAGCGCACCAGATGCTCCAGCATGGAGATGGAGGAGGAGAGCGCGGCGACC
AGCAGCAACAGGAAGAAGACCACCCCCAGCCCCTGACCGAGCGGCAGATGACTGAACACGGCTGGCATGG
TCATAAAGGTGAGTCCTGGGCCCGCGGTGGGATCGATGCCGGTGGAGGCCAGCGCCGGGAAGATCATCAA
GCCCGCCAGCACCGAGATAAGGCTTGCCAGTGTGACCACCCAGACCCCGGAGCGCAGCACCCCTTCGCTA
TTGGGCAGGTAGGCGCCATAGGTAGTGTGTACCCCGAGCCCGATGGAGAGGGAGAAGAAGGCCAGCCCCA
GTGCGTCCAGCACGCTCTGGCCGGTCAGCATGGAGAAGTCCGGCAGCAGCAGCTGGCGTACACCCGCCAT
GGAGCCCGGCATGGAGAGACCGACCCCCACCAGCAGCAGCATCATGATGAAGAGCGCGGGCATCATCCAG

CGCAGCGCCCGCTCCACCCCTTTCTGAATACCCCCCTGCACGAACCACCAGGTCAGGCCCGCGAAGATCA
GATGGGTCAGCACCGGCCACCAGGGGTTGCTGACATAGCCGTTGAACAGGGCGGTGAGGGCGCTCCCCTC
GCTCAGGTTGAGCTTGCCAGCCATCGCCAGCGCGGCGTAACCCACGGTCCAGCCACCGACCACGCTGTAG
AAGCTGTAGATAAAGAAGCCGCAGGCGATCCCCATATACCCCATCCAGCGCCAGTTCGGCCCCACCAGTT
TGCGAAAAGCGCCGAGCACCCCGCGCTGGCTATGGCTGCCGAGCAGGGTTTCGCCGACCTGTACCGCTAC
CCCCAGGGTGAAGCTGAACAACAGAAACACCAGCAGAAAGGCGCCACCGCCATTGGTGGCGGTCACATAG
GGAAACTTCCAGATGGCACCAAGGCCAATCGCTGTGCCTGCTGCGGCCAGCACATGACCGAGACGGGAAG
ACCACTGAACCTGGCTCATACAACCTCCGGGTAACACGACATCGAAGCAATACTCCTGAGTAGCAATAAA
CGACAGACCATAAAAAAGGCCGCCTGAGGCGACCTTTGCAATGAACATTCAGAAAAACTGTGCGTACAAG
GTCACCTCTCTTGCGAGCTAAAAAATCGACCAAACAGCACGAGGATGGCAGACATGAGGTGACAACCTGA
CTAATGAATGAGGAAACAGCCTAAACCATGGAAAGAATAATTGACACATAAAATCAAAAATTTTTTATGT
GTGCCGCTGTCACCACTCTGGCTATACGTACAACTCAGTGGGGGAAAACCTCGCCGGGCCGTATCTTTTC
TACCTTGTGGCTATTTACTGGGCGATAATCGACGTAGCTTCTTCTGGAACTAGTGACCGCTACATGGCGC
TGACGGGAACAACGATGAGACAGGATGTCGTATGAGATGGATGGTGCTGCCCTGGTTACTGATCAGCCTG
AGTGTGTCGGCGCAGTCCGGATTAACCCTGCTGAGTCACGACTTACCCCCCTTTACCGAATACCGCGATG
GCAAGCTCGATGGTTTCGCCATTCGTTTGATTGATGAGGTGCAGGAGGAGCTTGGCACCCGCTATCCGGT
GCGGATTTACCCGCTCAAGCGGGCGCTGGCGTTGGCACGGGTCGAGCCCGGCTATGGACTGTTCGTGGTA
CAGCGGCTCCCTGAGCGGGAGTCCCATTTCAAATGGGTTGGCCCGCTCTTTATCAATCGGGTCTTTATCT
ATCAGGCGCCGGATAGTCGCCACCCGCTGACCTCGCTGGCGCAGCTGCGAGATCTGCCCAGAGTGGGGGT
AGTGCTGGGTAATGCCGATGACGTGCGGCTGACCCATGAGGGCTACACCAATCTGGTGCGCTACAAGACA
GTCGGGGAGGCGATAGAGCGGCTGATGCTGGGCAAGATCGATGCCTTGCCGATGGCGGAGCTGGTGATGG
AGGCCACCCTCGACAAGATGGGGTTATCCCGGCGCAGCATCGTCAGCAGTGGCGTGCTGCTCCATCAGGC
CGGTCTCTATATCGTCTTCTCCAAGGGAACGGATGATGCCGAGATCACCCGCTGGCAGCATGCCCTGGAG
GCCGTCAGAGCCGCCCAGGGGCGGGCCGATGAGCCCACCCGCGAAGAGTAACCGTCACACCAGACCGCGA
ATCAAGATCCCGCCGATCAGCAAGGGGGCAATCCAGCGCCAGCAGTTCATCAGCAGGTGATGCCAGTGGC
TGGCAATCCCCTGCGGCAGCGCCTTGCGTCCCAGCTTCCAACCCAGCAGCAGCGTCAGCATCAGGCCGAA
CAGCGGCATCATCAGGTTGGAGGTGACAAAGTCCAGCAGCTCGAAGATGGTTTTGCCAAACAGGGTTGCG
TGTTGCAGCGGGCCAAACGACAGGCTGACCGGAACACCGCTAACCATGACGAATCCAGTGATGGCGCTGC
AGGCCGTGCGGCGTGACCAGCCCCAGGTCTCGCACAGGAAGCACTGCAAATGCTCCAGCAGGGAGATGGC
CGAGGAGAGCGCCGCAAAGACCAGCAGGGCGAAGAAGATCACCTCAAGCACCTGACCGAATGGCAGCAGC
TGAAACACCGCCGGCATGGTCATGAAGGTGAGACCGGGGCCGGAGGCCGGGTCGATACCGGTCGAGGCCA
GCACCGGGAAAATCATCAGCCCCGCCAGTACTGCCACCATGCTGGCCAGCAGCACCACCCAGAGGCTGGA
GCGACCAACCCCTTCGCTGGTCGGCAGATAGGCGCCGTAGGTGGTGTGAATGCCCAGACCGATGGAGAGG
GAGAAGAAGGCCAGCCCCATGGCATCGAGCACGCCGGACATCCCCAGCTTGCTGAAGTCGGGCATCAGGA
AGTGACGTACTCCCGCCATCGCATTGGGCAGGGTGAGGCCAAAGGCGACCAGCATCAGGATCATCAGGAA
CAGGGCGGGCATCATCCAGCGCAGGGTACGTTCCACTCCTTTCTGGATCCCGCCTTGTACCACCATCCAG
GTGAGGGCTGCAAACAGCAGGTGAGTGAGGATTGGCCAGACGTCGCCGCTGACATAGTCATTGAAGCGGG
CGGTGAGGGCTGCTGCATCACCATGGTTGAGATCGCCGGAGATGGCCATGCCGGTATAACCCAGGGTCCA
GCCGCCCACCACGCTGTAGAAGCAGTAGATGAGCAGGGTAGAGATCACCCCCATTACCCCAACCCATCGC
CACTGTTTGCCGAGCAGCTCGGAGAAGGCTCCGACCACCCCGCGGCGACTCATGCTGCCGAGCAGGGTTT
CTCCCATCAGTACCGCCACCCCCAAGGTGAAGCTGAAGAGCAGAAAGACCAGCAGGAAGGCGCCGCCGCC
GTTGACGGCGGTGACATAGGGGAATTTCCAGATTGCGCCGATACCGACCGCGGAACCCGCTGCGGCGAGG
ATGTAGCCGAGTCGGGATGACCATTGGGACTGCTTCATAAGAACTCCAATCGAGCGCCCCTTGGCAAGGG
CAGAATGGCGCTCATGTTACGGAGGTCACGGCGCAATCACTACGACGGTTATCAGGTTTAAATGCGATTT
TTTGGCACGATTTGCTAATTTAATTCTATTTTTTGCCCAATCCTGCCAACAAATAAAAACCGGAGCGAGA
TTGCTCCGGTTTGGTCTCTGACAACTGCTCGTCGGTGGACTAGATCACCCCGAGCTCCCGCAGCCGCTCC
ATCAGGTAGCTGTGGGCGGTATGGCGCTCGGAGAGCACCACCTCGGGCCGCGGATGGAGGAACAGCGGCA
GCGAGATGCGTGACTTGCTGGCACTGCCACCGGTCGGGTTGATGACGCGGTGGGTGGTGGAGGGATAGTA
GCCGCGAGAGGCCTCCTGCAGCATGTCGCCGATATTGATGATGAGGGTGCCAAAGTCACACGGCACATCC
ATCCATTCGCCGTCGGAACCCTTCACCTGCAGCCCTGGCTCGTTGGCGGCGGGCAGAATGGTGAGCAGGT
TGATATCCTCGTGGGCGGCGGCGCGGATGGCGCCCGGTTCCTCGGTGCCATCGAACGGCGGGTAGTGCAG
CACCCGCAGCAGGGTCTTCTGGCTCTCTTCGATCATCGAGGAGAGGGGGCAGCTGTAGTGAGCGGCGATA
TCGGCCGGGGTGTACTGCTCGACCCAGCCCAGCAGCTCGGCGGCCAGACCGTTGGCGAGGCGGTAGTACT
CCATCGCCTGCTCCTTCAGTTCTTCCGGCATCTGGCCCCAGGGGTAGATGTGGAAGTACTCCTTGATGTC
TTTCTGGCTATGGCCCTTGGCCACCTCGGAGACCGACGGCGGGAAGTAGCCATCCTGGGTCTCGCGGTTG
AACAGGAAATCCTGCTTCTGCTCGCTCATGAAGAAGCGATACCAGTTGTCGTAGATGGACTGCACCAGCT
CTTTCGGGATGGGGTGGTTGGTCAGCACGCCAAAGCCGGTGGTTCGCAGGGATTCGGTGAAGCGTTGGGC
CGCATCGGGTGAGCGGTAATCGACGGTCAGGACTTTCATTGTTTTTGTTGCGCGCCAGTTAAAAGATGGC
GCAGTTTACCTTGCACCTGATAACAAGGAAAGCGGGTTGGCTGCCCCGTCTGGCACGGCGTTGAATAGTG
ACAAAAACATCAAATAAACCGCGCCAGACCGCACGGGAGCACATCCCTTGTTATGGCCTTTTATCCTCTC
TAGAATGCCGGTCACTATCTTTTCCGGAGTCTGTTCATGTCCCCCAAAATCCTGTTGCTGACCCTGCCGG

TATTGCTGGCGGGATGCAGTGCCCTGTCAGAAGATGAGTGCCGCACCATGAGCTGGTACAACCTGGGTTA
TCAGGATGGATCGGAAGGCAAAACCCGTCAGGCGACCCGCGATTACGTTTCGTCTTGCAGCGAATATGGC
CTCAAAGTGGATGAAGCCGAGTGGAAACGGGGCTACGACAAGGGGCTGGAGCTCTACTGCATTCCCGAGC
TGGCTTACAGCAAGGGCAAGGAGGGGCAGGCCTATCTGGGGGTATGCCCCAATGATGCCAGCTTCCTCAA
GCAGTATCAGCGTGGCTACGAAGAGTACAAACTGGCTGCCCGCCTGCGGGAGATCAGCGACGAGCTGGAG
CGTACCGAGAGGGATATCGATACGCTGGAGCGCAGCATCCGCAACGAAACCAACACGGAACAGCGTGACT
ATTACCGCGCCAAGCGCGATCGGGCCATTCGCCACTACGAATCCCTGCGCCGGGAGTACAATCGTCTGCG
CTATCCGGATCGGGTGATCCAGTTCTCCTTCGGCGGTTAATCCCTCTCTTCATGGCCAGCCCGCGTGCTG
GCCATGTTGCATCCTGTCAGCTCAGGGCAGAACCCAGCGCTGCAGCGCCAGTGCGATACCATCTTCGTTG
TGGTCGGCCGTGACCCGATGGGCATGGCTCTGGATCTGCGGGGCCGCATTGCCCATGGCCACCGCCAGCC
CCACCTGCTCGAACATGCTGATGTCGTTGTTGTTGTCCCCAAACGCCACCACCTGCTCCATCGCAATCCC
TTCGCTGGCTGCCCACTGGGCCAGCCGGTTGCCCTTGCTGCAACCGGGCTGTACCAGCTCCACCGCATAG
GGGGCCGCCCAGTCGCGGGTAAAGGGCATCTGCTCGACTACCTCGCCCATAAAGGTGTGCAGGCGTGCCG
GATCCTGATTGAACAGCTCCAGCTTCCAGACCGGGTTATCGAGCCAGCCTGCCAGCTCCTCGACCGGGTG
GAGGGCGATCTTGAGGTGGTCCGGCTGATTGGCCGACCAGTGGCGGATGCGGGTGACATGCTCTTCACAG
CCGATGTAGCCGATCCCCTCGCTCAGGTGGAACAGGGCGCCCATCTGCTGCGCCTCGACCGCGGCCAGCA
GCTCGGTAAGCGGGGCAGCCGCCAGCGGGTCGCCGCTGATGATCCGGTTCTGCACCGGATCATAGAGATA
GGCGCCATTGCTGCAGATGAGGGGGGTATCGAGGGCCAGCTCGTGGTGGAAGGGGCGTGCCGTCATGTAG
TGGCGCCCCGTGACCAGCAGCACCTTGATGCCGTGGGCGCGGGCCTGCGCCAGGGCTGCCCGGGTGGCCG
ACGAGATCTTGTGATCCCGGGTCAGCAGGGTGCCATCCATATCGAGCGCGATGGCCTTGTACTCACTCAT
AGCTCCTCCAGCATCGAACAAATCTGCAAAGAGCCTTACCGTCCCAGACCCGGCGCCCCGATGCAAGTCT
TGCCCGCGGCCGAAGCGCCGAACTGTGATCCCTTCGTGGCTCAGGTGCAGCTGGCCAGCTGTTGACGACG
GTCGGCACGGCGGCGCAGCAGATAGAAACCGGCGGCACTGGCAAGGGAGCAGAGGGTGGCGAGATAGAAG
GGCCACTCGGTACCGGCCAGCAGCACCAGACTGCCGAGCAGCACCGCCAGGATCTCGCTGCCCACGGCGA
GGGCGCGGTAGAGCCCCATCGCCTGCCCCTGCTGCTCCGGGTGACTGCGGTTGGAGATCGCCACCGAGGT
GGCCAGCTCCCCCAGGCTCATGCCGATGCCAGCCGGAACGAAGGTAAGCGCCAGTCCCCACCAGTGCACG
GGCAGGATAAAGAGCAGCATGCCGCTCGCCATCACCAGATGGCCGACAATGCCGAGGGAGCTGCCGCGCC
AGTGACGGGCGATGCGGGAGCCAAGCCAGGAGCCCGCCATCATGGGCAGGCTCACCAGCACCGCCGCCTG
CGCCAGCTGGGCCGGATCCCAGGACAGCCGCTCCACCAGCCAGATATTGAAGTAGGTGAAATAGAGCTGC
ACGGCGCCATAGCTGATCAGGGTGAGTGCGAAGTAGGGCAGCAGGGCCGGTTGCAGCAACAGCTGGCGGC
TGGAGAGTGTCGCGGCCTGAAGGGTGGCCGCCACCGGCGGACGATAGGGGATCAGCCAGAGCACCAGCAG
CAGGTTGAGCAGGGTCATTCCTACCGCCAGCCAGGCGGCCAGACTGTAATCCCCCGAGATCACCGCGGCG
TAGCCCCCGATCATCGGCCCCACGACCCAGCCCAGATTCATGCCGATGTTGATCCAGGCGAAGCTGCGCG
CCTTGTGCTCCTTGCCGGCGAGATCCGCCGCCATCGCCTGCACGATGGCGATGTTGCCGTTGCACAACCC
CTCCACAATCCGGCTGAGCAGCAACAGGGGGAGGGAGCGCCAGGCGATACCGAGTGCCATCAGGGCATAG
CCTGCCAGTACCCCCGCCAGCGAGAGCAACAGCAGTGGCTTGCGGCCATAGCGGTCGGAGAGGCGACCGA
GCCAGGGACTGCCCACCAGCTGGAACAGCGGGTAGAGCCCCATGATGACTCCCAGCCACACCTTGCGGCT
CCAGTCGGAGGCTTCCGGCGTCAGCATCCCCTGCGCCGGATCCAGCAGCAAAGGTGCCAACACCGGGGAG
GGAAGACTGAAGCCTGCAACTGCCAGCGTGACCGTCATGACCAGCACCCAGAGGGGATTTTTGTGCTCGT
GTTTCTCCATGCTGCACCCATTAAGTAAAGTTATTAGTTTATTTATGCTGGTTCATAGACGGGATTAAGT
AAAGTGAATTGTTGATTTAATAAAGGGGGCTCCCCATAATGGGGTCATGAACGCAAGCACCGACAAGATC
CTGTTTCTGCTCAAGAGCCATGGCCCGCAGAGCGCCGCCGCGCTGGGGGAGCAGTTGCAGATGACCTCCA
TGGGCGCCCGCCAGCACCTGCTCACGCTCGAGCAGGAGGGGTGGGTCACATTCAGCGACGAGGCGCGCGG
GCGCGGTCGTCCCGTGCGGCTGTGGCAGCTCACCGAGCAGGCCTGGCAGCGCTTCCCCGATAGCCATAGC
GACCTGACCGTGCAACTGATCGACAATGTGCGCGAGCTGTTTGGCGAGGAGGGGATGGAACGGCTGATCC
AGCAGCGAGAGCAGCAGATGTTGACGCGCTATCAGGAGGCGCTGGGGTCCCATGGCCTTTCTGGCCGGCT
GGCGGCGCTCAGGGAGCTGCGTACTCGCGAGGGCTACATGGCGGAGATCCTCCGCGAAGAGGATGGTAGC
TGGCTGCTGTGGGAGAGCCACTGCCCCATCTGTGCGGCGGCGCGCACCTGCCGCGGTTTCTGTCGCAGCG
AACTGACGCTGTTTCGCCAACTGCTGGCGCCGGCGACGGTGGAGCGGGAGCAATATCTGCTGGAGGGGGC
ACACCGCTGCCTCTATCGCATCACGCCGCCCGCGTTCTGATTTCGTCAAACGCCCAAAAACAACAAAGCA
GGCGCCGGGATGCCTGCTTTGTTGCCTTCGGGGAGGGGGTTCTCTCGTTCCAGCCCGTCGTTTCCCGATA
AGGTAAGACGGCTGATGGGGCTCTTGACCCGGTAAGGTCCTTCACCGCAGAAACCCGGTCAGGTATCTGC
TTTGCCACCGGAAGATGTAACCATAGTATCCTTCCCGTGCCGGTGAGTGGTTGCAAAGTAACCACTTATT
TCGATTGTTTTTGCAATACTTCACCCTTAATCTAGGAAAAGTGGATTAGTCATTGCAACGAGGAAGGGTA
AATGCTGAAACTTGATCGCATAGACAGACATATTCTCGAGCTGATGCAGGAGAATGGCCGCATCAGCAAT
CTGGAACTGGCCGAGCTGGTCGGGCTCTCGCCGTCCCCCTGCTCCCGCCGGGTCAAGGCGCTGGAGGATG
CCGGTCTCATCGACACCCACGTTACTCTGCTCAACGCCCGCCAGCTTGGCCTCACCCTCACCGCCTACAT
TCACATTTCCATGGACAGACATACGCCGGAGCGGTTCGAGAATTTCGATCGCGCCATCAGCGAACTGCCC
GAAGTGCTGGAGTGTGATCTCATCACCGGTAATGATGCCGACTACCAGCTCAAGGTGGTGGTGCGCGACA
TGGAGCACTACCAGCACTTCCTGCTGGACAAGCTGACCCATATTCCGGGGGTGACCGGTGTGCGCTCCAG
CTTCGTGCTGCGCCGCATCAAGCACAAGACGGCGCTGCCGCTCAACCATCTGGGTTGAGCCCGCGCAAAC

CGCTTATAGAAACAGAAAGAGGGGGCCAGTGGCCTCCTCTTTTGCTATTCGCTCTTGGTTATTCGGTCGT
GGTTATCGGGTCTGGATTAGCTGTCGCGGTGACGGGGCGACTGCCGTGGTCAGACGCTGACCTGGCTGAT
GTCTTCCACCTGCAGCAGCCAGCCGCTCTGCTCCTGCTGCGGTTCGCCGCTAACTCGCAGCCGCTCGCCG
GTCGGCACCCACTGCCCCTCGGGCAGGGAGACCAGCAGGCTCTCGCTGCCGTTGTCGAATAGCCAGCGAT
CTTCGCCAAGCGATTCAATCAGATAACCTTCCATCTCCACTCTGGACTCGGCTTGCCAGGATGGCGCAAC
GGAATCACGGCTCTCGATGAAATCCCCCTGAACACCAAAACTGGCTAGCCCCCACAGAATGGAGAGCCCG
ACGATGGATGTTTGCATCTTGTCTCTCCTCTTTTGGAACTGTAGGGCCAGTATGGGAACAGAAAACTGAG
CGCACCATGAACAAGGTGGCAAAGTGCTGGCGAGATCGCGAAAAAATCCGGTCAATTGGCCGGCAGAGTC
ATTTTATGGTGTCTGGATCACACTATTTGGCGGCTTCTTCTGCCATCCGTTCAGCTCTTGGCTTATTCGT
CCACATGGCTACCCCGCGATGATCCCCACAGATCCCCTCTGCCACCACGGCGCGAACCCGGGTGCCATCT
TTGCAAATGGCCTGCCAGGCGTCGGCATCATCGATGGCGAGCGAGTTGGGGGGCGGCGTGGTTTGGCAAG
CTGTGAGCAGCAGGGCGCACAGGGGGAGCAATTTTTTCATGGCGGACTCCTCGGGGTAATGGGGGACTGA
GAGGATGGGGATGGCCTCTCCATCCGGGCTTCACTCCCCAGCATCAGTGATAACCGGTTCAGGGGCAAGG
GGATGGGTCACCGACAGGCGCAGGTGCCGTGCAGCCGAGGCTTGCCCTGTGCCCTCCTTGGGCCTAAGGT
GGGGCAGTTTTACTGCCTGATTGAAGGATCGTTTTCATGTCCACTCTCTATCCCCGCGTCGGGGTCGGCG
TCATCCTGACCAACGCCCAAGGCCAGGTGCTGCTTGGCAAACGCAAAGGGAGCCATGCCCCCTACTGGTC
GATCCCGGGTGGTCATCTGGAGTTGGGGGAGAGCTTCGAGTCGGCGGCGATCCGCGAGGTGGCCGAGGAG
ACGGGACTTGAGATCCATGGGCCCGAGGTGGTGGCGGTGACCAACAATCTGGAGACCTGGCGCGAGAGCG
GGTTGCACTATATCTCGGTGACGCTGCACGCTAAGGCCGATGGTGAGCCGCAACTGCTGGAGCCGGAGAA
GTGCGAAGGCTGGGTCTGGTGCGATCCGTGCGCGTTGCCCGAGCCCCATTTCGACGCCAGCCGTCAGTCC
ATCGCTTGCTGGCTGGCGGGACGCTGCTATCTGCCGGGTGATATGCCCATGGTGTCGGCCGAATCAGCCT
GATTGTCATAAAACGATCATCGCGCTGTCATAATATCCCCTCTCCATCGGGAGAAGAAGGATGAGGGAGA
AGAGCGTGGGGCAGGTATTTATTCAGTTTTTATGGCTGGGATGCATAAGTTTTGGCGGCCCAGCCGCTCA
TATCGGCTATTTCCAGCGCACCTTCGTCCAGCGGCTCGGCTGGCTGACACAGGTCGAATTTGCCCGCTTG
CTGGCGCTGTGCCAACTGCTGCCGGGGCCCGCCTCCAGCCAGCTGGGTTTCGCCATCGGTCGTCATCGCG
CCGGTCTGGGCGGGGCGCTCTCTGCTTTTGTTGGCTTCACGCTTCCCTCATTTCTTCTGCTACTGGCGTC
TGCGATCGGTATCGGTCAGCTGGGCAGCAATTTGTGGCTTGATGCGGCGCTGCACGGCCTCAAGCTGCTG
GCGCTCATCGTGGTGGCCGATGCGGTGCTCACCATGAGTCGTCAGTTCTGTGCCACGGGAATGACCCAAG
GGATTATGGTGGCGACCGCTGCCGCACTCTGGTGGCAGCCCGGTCTGCTGACTCAACTGTTGATGCTGGC
GGGAGCGGCGCTGATCTGCGCCCGCAGCCAGTGCGGGGCGGGTAGTCGCCCAGCCTCCACCGAGTTGCCT
TCTGCCGCCAGCAGTCAGCCTCATTGGCCGACCCTACTGCTGTTTGGCATCCTTTTTATCGGCCTGCCGC
TGCTCGGTAGTCCGCTTGGCCAGCTGGCTGCCGACTTCTATCGCGCCGGTAGTCTGGTGTTTGGCGGTGG
CCATGTGGTGTTGCCGCTACTGCAAGAGAGCGTGGGCCACACTCTCAACGAGCAGCAGTTTCTGACCGGT
TACAGCCTGGCTCAGTTGGTGCCGGGCCCCATGTTTACCCTCGCCACCTATCTTGGTGCCCAGTTGCAGC
CCGAGATGCCGATGATCGGGGCCCTGTTGGCGACCCTGGCGCTCTTTGCGCCCGGCTTTCTGCTGCTCTG
GGCGGTGGGCCCCTGCTGGCAACAGTGGCTGGCCCGGCCCCGTCTGGCGGGGGCCGTCACCGGAATCAAC
GCCGCCGTGGTGGGCTTGTTGCTGGCGGCCCTCTATCAGCCGGTGTGGCAGAACGCGGTGCTGGCGCCGA
CCGATCTGGCGCTGGCTGCCATCGGCTTTTATCTGCTGCGGGTGGTGAAGCTGCCGATACCGGCCATCGC
TGCCCTGTTGGTGATGGCTGCTATCCTGCTGGCGTGAAGCCGGTGAAATCCCGATAAAAACGGCGCCTCC
GTGATGGAGGCGCCGTTTTTATTGCAATGGGTCAGCCGTTCAGACCTCGGGATCGAAATCCAGACTCAGG
CTGTTGACGCAGTAGCGCTGGCCTGTCTCGGTCGGTCCGTCGGGGAAGACGTGACCGAGGTGAGAGCCGC
ACTGGCGGCAGGTGATTTCCACCCTCTTCATGCCGTGGCTGTTGTCCTCGGTGTACTCCACCGTCCCCTC
GATGGCCTTGTCGAACGAGGGCCAGCCGCAGCCGGAATCAAACTTGGCTCCTGAGTGAAACAGCACGCTC
TTGCAGCAGACACAGAGGTAGTCGCCGCTCTTGCGATTGTGGAGCAGGGCGCCGCTGTAGGGGCGCTCTG
TGCCCTTCTCCCAGCAGACGTGAAACTGCTCGGCGGTCAGTTTGCTGCGCCACTGTTCCGGATTGTTACT
GCTCATACCGACTCCTTGCCATCCATACCGGAGCCCAGCATAGCGGCTCCGTAAAGCTGACTCCAGCATG
CCTCTTATGCTTTGCCTGTATCTGTGATCGAGGTGGCAACCATCTCCTCACCGGATGCAGGGAGAATTTC
CTGCTGCGGTGGCTGGGCATCCTCCGCCGTGCTGGCCAGCGCCACCCGCTCCAGCAGTTTGAGGGAGTGG
GCCCGGCCGACCGCCACCTGCCGTTTGATGTCATCGGGCAGGAAGGGCAGCCCATCGTTAGTGGCGGTGT
CGATGGTCTGGATCCACTCCAGCCCTTCCCCCGGCGGGGGCAGGCGGAACTGGATATCGTAGTCGCTGGC
GTTGAACAGCACGTACCAGCGCTCGCCGACACAGTTGATCATGCCGATATCCATGGCGAACGCCTGCCCC
ATGGGGGCGTGCCAATCCTGATCGGTGAGCTGGTGGCCGTCCGGGTGATACCAGTTGACCTGATGGCAGC
TGGACGGTGCGCCATTCCAGCTGTCGTCGCTCAGCTGCAGGTTGGAGAAGACGCAGGACTCTGCCCGCAG
CGCCATCATCTGCTGGGTGAAGGCGAACAGCCGCTCATCCTCCGGCCGCCACTGCCAGTTGACCCAGCTG
ATCTGGTTGTCCTGGCAGTAGGCGTTGTTGTTGCCAAGCTGGCTGCGGCCCATCTCGTCGCCGGCGAGGA
AGTGGGGAGTGCCTTGGGAGAGCAGCAGGGTGGCAATCAGATTGCGCTTCTGCTGCTGGCGCAAGCTGTT
GACCCGCGGATCCAGGGTCGGCCCTTCTACCCCGTGGTTATAGGAGAGGTTGTTGCCGTGGCCATCGCGA
TTGTCCTCACCGTTGGCCTGATTGTGGCGCTGGTTGTAGGAGACCAGATCCTCCAGGGTGAAACCGTCGT
GATAGCAGAGGTAGTTGACGCTGGTGTGGGGGGAGCGCCAGTTCTTCGGGAAGATATCCCGCGAGCCTAC
CAGGCGGGTGGCGAAGTCCCCCATCTTGCCCGCATCACCGCGCCAGAAGGCGCGCACCGTATCGCGGTAG
CGGTCATTGATCTCCTGCCACTGGCTCGGGAACTGACCCAGCCGGTAGCCGAAGGGGCCTATGTCCCAGG

GTTCGGCGATGAGCTTGACCTGTGACAGCACAGGGTCGGCCATCACTGCCTTGAAGAAGGCGCCCATGGG
CTCGAACTCCCCCGCTTCCCGCGCCAGTGTCACCGCCAGATCGAAACGGAAGCCATCCACCTGCATCTCG
GTGACCCAGTAGCGCAGAGCATCGAGCACCAGCCGCAGGGTGTTGGGGTGATCCAGATTGACGCTGTTGC
CACAGCCGGTCACGTTGGCGTGACGGGTGTAGTCCGGGCCGTGGGGGCCCGCTTCAAAGGCGTAGTAACC
GGCGTTGTCAAAACCCTTGAACGAGAGGGTGGGGCCGTCGAAGCCCGAGGCGGCAGTGTGGTTGTAGACC
ACATCCAGGATCACCTCGAGCCCGGCCCGGTGCAGCTCGCGCACCATGGTCTTGAACTCGGTCACCGCCT
CGTCTGCCCTGGTGCTGTAACGCGGCTCCGGGGCGAAGAAGGCGATGGGGTTGTAGCCCCAGTAGTTGGT
GAGTCCCAGCTGCTCCAGTCGCGGTTCCGACATGAAGGCGGCCACCGGCATCAGCTGCACCGAGGTGATG
CCGAGGCTCTTCATATGTTCGATGACCAGCGGGTGACTGATGCCAAGGTAGGTGCCCTGCAGCGCTTTGG
GGATCCCCGGGTGCTGCTTGGTAAAGCCCTTGACGTGGGTCTCGTAGATAATGGTGCGGGCCGGACTGAT
CCCGGGTTTGCCAACCCCTTGCCAGTCAAAACGGTCATCCACTACCACCGACTTGGCAATCATCCCGGCA
CTGTCACCCTGATAGAGGGCTTCATCCCAGTAGGTGGGACGACTGAGCGCCTTGGCGTAGGGGTCAATCA
GCAGTTTTTGCGGATCGAACAGCATCCCCTCCTGCGGGGTGCCATAGACCCGATAGCCGTAGCGCTGACC
GGCCTTTACACCGCGCACGTAGCCAAAGCGATACTGGCCACGGCGGCCGGGCAACTCCAGCCGCATCAGC
TCCTGCTCCTGCTCGTCGTACAGGCAGATCTCCACCTTGTCCGACTGGGGGGCCCAGACGCAGAAGTGGC
AACCGTTCTCGTCAAGGCGGGCCCCCAGCGGGTGGCCCACCCCTTTTTCCATTACAAGCATCAAGACTCC
TGTTTGATAAACAAGGTCGCCAGCGGCGGCAAGTCCAGCACCATGCTGTGTGCCATCCCCTGCCAGGGGG
TCGGCTCGGCAGCGAACACCAGACCTACATCATAGTTACTACCCCAGTAATACTCGCTGTCGGTGTTGAG
CACCACCCGATAATTGCCCGCAGCTGGGACGCCGATCCGATATTCGAGACGGGGCACCGGGGTGAAGTTG
CAAGCGACCACAATAGCCTGCTTGCCCTCTTTGTCCGCCCGCAGCCAGGCAAAGATGCTGTTGTCGGCAT
CGTTGTGATCCAGCCAGCGGAAGCCGGTCTGCTCATAATCGGCCTGATACAAGGCAGACTCATGACGGTA
GAGGTGGTTGAGATCGTGGATCAGCCGCTGCTGACCGGCATGCTTGGGATATTGCAGCAGATGCCACGGC
AGCTGGGCATCGTGGTTCCACTCGTTGCTCTGGGCGATCTCGGTGCCCATGAAATTGAGCTTCTTGCCCG
GGTGGGCGTACATGAAGCCCATATAGGCCCGCAGGTTGGCGGCTTGCTGCCACTCGTCGCCCGGCATCTT
GTAGAGCATGGAGTGCTTGCCGTGCACCACCTCGTCGTGGGAGAGCGACAGGATGAAGTTCTCGTCATAG
TGGTAAACCATGGAGAAGGTCATGTCGTTGTGGTGGTAGCGGCGGTGGATCGGCTCTTTCTGCATATAGG
TGAGGGTGTCGTGCATCCAGCCCATATTCCACTTGAAGCCAAAGCCGAGGCCGCCGAGGAAGGTGGGGCG
GGAGACACCGGCAAAGGCGGTGGACTCTTCGGCTATGGTCATGGCGTGGGGATACTTGCCGTAGACCTCC
TCGTTGGTCCACTTCAAGAGGCTGATCGCCTCGTAGTTGTGGTTGCCGCCATCGACGTTCGGTACCCACT
CACCGGCGTTGCGGGAGTAGTCCAGATAGAGCATGGAGGCGACCGCATCCACCCGCAGGCCGTCGATGTG
GAACTTGTCGAGCCAGAACAGGGCGCTCGCCACCAGGAACTGGCGCACTGTGTCGCGGCCGAAGTCGTAG
ATGTAGGAGTTCCAGTCCGGGTGCCAGCCACGGCGCGGATCTTCATATTCGTAGAGCGGGGTGCCATCGA
AGCGGGCCAGCCCGTGGGCATCGGACGGGAAGTGGGCGGGCACCCAGTCCAGAATGATGCCGATGCCGGC
CTGGTGGCACTGGTCGACGAAGTATTTGAAATCATCTGCGGTGCCGTAGCGGCTGGTGGGGGAGAACATG
CCCACAGGCTGGTAGCCCCAGGAGCCGCTGAAGGGGTGCTCGGAGACCGGCAGCAGCTCGATGTGGGTGT
ACTCCATCTCCTTGACGTAGGCGACCAGCTCCACTGCCAGCTCGCGCCAGCTCAGGCTGTCGCCGTTGGG
GTGGCGTTTCCAGGAGCCGACGTGCAGCTCATAGATGGAGAGCGGTTGCTCGCGTTTGTCGTTGTGCTGG
CTGGCAATCCACTCGGCATCCTGCCACTGGTACTGGGCGTGGTCATAGACCACGGAGGCGAACGACGGAT
ACTGCTCACTGTAGAAACCTACAGGGTCGCTCTTGTGGGGCAATCGGTTGCCCATGGGATCCTTGAGCTC
GAACTTGTAGCGATCCCCGGCCTTGAGGCCCGGCACAAACAGCACCCAGTGACCGCAGAGGCTGCGCTGC
ATCGGGTGGCGGCGGCCATCCCAGAAGTTGAAATCGCCGATCAGGCTCACTGCGGTGGCGCTCGGCGCAT
AGACCGCAAAACGCACCCCTTCAACCTGCTGGCCAAGATGCTCCACGGTGCGCAGCTGGGCGCCAAGGGT
GTGGTAGAGGTTTTCCGGCTGGGCGGTCAGTTCGGCCAGGCCTGCCCAGGCGGCATCCTGAAACTGATAG
GGGTCGATGACCTCGGTGGTGGCATCTTGGTAGCTGGCGATCAGCTGATAGGGGAAGGAGTTTTTGCGGC
GGGTGAAAACAGTTTCAAAAAGACCCGATTCATCGGTGGGCGCCAGGGTGGCAACGACTTTGCCCGACTT
GAGATCCTTGACGCGGACTTCGAGGGCATCAGGCAACCAGGCGGTCAGTTTGAGGCCGGGCCCATCCGGA
TTGGCCTGCAGGCCTAAGTGAGAGAAGGGATCGGAACAGCGGGCAGCAACCAAACGTTCAACGAGCATCG
AGATTTCCTTTCATGACAGAGCATCCACTCCGGCCGAAGCCGGAGTGGATTTAGGTGAGAGCGCTGTGGG
AGACAGCGTCTCAACGACTGGCCTCATCACGGGCTTGGCTCATCTTGCCAGCCAGTGCCTTGAGTGCCTC
ATTGGCAAAAATATCTTCCAGATCAGCGGAAAGCTTGCGGCGCCAGTTGGGGTATTCGTAACTGGTGCCC
GGTACGTTGACCGGCTTGTCCATCTCCAGCCAGTCTTCCAGCTGGGTACTGAACAGGGCGCAGCTGCCGC
GGGACATGTGGATCTGCAGACCGTGGTTGAGTTCGGTGTTCATGCCGACCCAGTTCACGTCATGACTGAT
GTTGTCAGAGATGACCTTGTGGGCATGCAGGCTGTCGAGGATCCGCTGTTTGGCCTGGTGACGATCAGCA
TACAGAGTCTTCAATACATCTTCATCCGGATACAGACCCAGTTGACGGCCGAGCTCCAGGTCATCGCAGT
GCCAGAAGCCCTTGAGGGTCGGCATGTCGTGGGTGGTCAGGGCCGACATCGCCTGCTCGGTGTAGTGCGC
CGGAGAGATAAAGCCGCCATCTTCCTTGGAGCGCTCGAAGAAGAATACCTTGTAGGAGTGGACGCCGTTC
TCTTTCAGAGTCACGTCGATCCCCTCCGGCACGGTGCCCAGATCCTCGCCGATCAGCAGGCACTGATTGC
GGTGGGATTCGAGGGCCAGAATGCCCAGCAGATCGTTGACCGGATAGTAGATGTAGGCGCCCTTGGCGGC
GGACGCTCCGGGCGGTACCCACCACAGGCGCAGCAAGGCCATCACGTGGTCGATGCGCAGTGCGCCGCAG
GAGCGCATGTTGGCACGGAACAAGTCCACCATCGGCTGGTAGGCCGCTTCAAACAGCTTGTTCGGGTTCA
TCGGGGGCAGGCCCCAGTTCTGACCGAGCGGGCCGAGGATATCGGGCGGCGCACCGACCGAGGCTTTCGG

GCAGTAGAGATCTTGGTTGGCCCAGATCTCGGTGGAGCCTTCGGAGACGCCCACTGCCAGATCCCGGTAG
ATACCCATCACCATGCCGGCAGCCTTGGCACGGGCATCGGCTTGCGCCAGCTGCTCGTCAGCCAGGAACT
GCAGATAGAGGTAGAAGTTGACATCTTGCGGGTGCGCGGCCATCCAGGCGGCAACGGCAGGATTGTGGTA
ATCGCGCAGCTCCTGCGGCCATACCGGCCAGCCCCAGGCATTTTCGCCTTTGGCATAGAAGTGGGCCTGC
AGGGCATCAAATGCTGCCTGCTGTTGCAGGCTGTCGCCACCGGCGGCCACGAAGGCATCAAAGGCGGCCT
GACGCTCGGCGCTCAGGCTGGCTTCATCGAACACCTGACGCAGCATGCCGAGTTTGGTCTGGATCACGCC
GGTGTAATCGACGTTCTCTTTGGCGCGCAGATCGGCCAGATGTTGCTGGAAGGCAGGACTTGCCACTTCC
GCTTTCAGGCGAGCGCTTTGCTGGAATTCGGGCACCGCTTCCACATTGATATAGGCCACGTTGAGCCAGC
GACGGGAAGAGGGGCTGTAGGGACTGGCGCTCTCCGGATTGGCCGGATAGAGGGCGTGGATGGGGTTGAG
GCCGACGAAGTGGGCACCCCAGGCGGCCGCTTTCTCGACCAGCTGGCCCAGATCGCTGAAATCACCGATA
CCCCAGTTGTGGCGGCTGCGCAGGCAGTAGAGCTGCACGCTCGGACCCCATACCTTGCGGCCATCGGCAA
TCGGCGCCTGTTTGTAGCAGGCCTTGGGGGCGACGATGAGGCGCATGGTGGCCAGTGGTTCGTCATTGCC
CTCTTCCAGCAACACCAGCTGGTGATAACCCAGCTCCGGGGCCATATCCAGAGTGACACGATAGGCCTGG
CACTCCATCTCTTCAAATTCGGCCACGCCGACCAGCTCACCCTCGATGGGGGTGAATTGACCGGACAGCA
CCGCACCCTGCTCCTGCTGGAGCTGCCAGGTGAGGGCGTCGTTGACGAATTCGATGGGGAGGCGAACCTC
AAGGGTAACGGCGTCGCCGGTGCGAACCACCATCACAGGATCCAGCGCCCGCAGCCAGTGCTGGCGATGC
TCCTCTTCCAGCTGCTGCACCAGTGCGGCCTCGTCATCGACGCGGTAGCCCATGGCGCCCAGCATGGCGG
CCTTGCTCTCTTCGGATACCTGGGCCGGGTGGCCCCAGGCATCGACGTACTCGCTGGCGATACCTTTTGC
GGCAACCAGCTGTTCAATCAGGGTGGTCATAGTTTGAGAATTCCCGCTGTGGATTCTATCTATACTATTT
TGTTTGATTTTTATGGGGTGGTACTTGAACAGTGGCTCTTTGGCCATGCTAAACCGCGCACATTTTTACA
TATCTCTGTGAATGGTTGTGTGACCGAGTAGGCACTATTGTTGATATCTGCGCGATGCCACGATCTGGCA
CTTTTTTCGTCAAAACGTGGTAATCAGTTACATAAACAGAAAATTAATTTCACAGTTTTTCATGATATAG
CGCAATTTCAGACAGAATGTTGGTTGACTCACCGGAGAGTTCTAGTAAATTTACAAGCGGAATTTTCTTT
GACTACTGAACAGTTAGGTAACTAAATATGACTATCAAAGTAGGTATTAACGGTTTTGGCCGTATCGGTC
GTTTCGTATTCCGTCTGGCTTGCGAGCGTGCAGACATCGAAGTTGTTGGTATCAACGACCTGATCGATGT
TGACTACATGGCTTACATGCTGAAGTACGATTCAACTCACGGTCGTTTCAACGGTACCGTTGAAGTTAAA
GACGGCAACCTGGTTGTAAACGGCAAAACCGTACGTGTTACCGCTGAGCGTGACCCGGCCAACCTGAAGT
GGGGCGAAATCGGTGTTGACGTTGTTGCTGAAGCTACCGGTCTGTTCCTGACCGACGAGACTGCTCGTAA
GCACATCGCTGCCGGTGCCAAGAAAGTTGTTCTGACTGGCCCGTCCAAAGACGCCACCCCGATGTTCGTA
ATGGGTGTTAACGACTCTACCTACGCTGGCCAGGACATCGTTTCCAACGCTTCCTGCACCACTAACTGCC
TGGCCCCGATCGCCAAGGTTCTGAACGATACCTTCGGTATCGAGTCTGGCCTGATGACCACCGTTCACGC
TACCACTGCTACCCAGAAGACCGTTGATGGTCCGTCTGCCAAAGACTGGCGCGGTGGCCGCGGTGCGGCT
CAGAACATCATCCCGTCCTCTACCGGTGCTGCCAAAGCTGTTGGCGTAGTTATTCCGGAACTGAAAGGCC
TGCTGACCGGTATGGCTTTCCGTGTTCCGACTCCGGACGTGTCTGTTGTTGACCTGACCGTTAACCTGAA
GAAAGCTGCCACCTACGCAGAAATCTGCGAAGCGATGAAAGCTGCTTCCGAAGGCGCTATGAAAGGCGTT
CTGGGCTACACCGAAGACGAAGTTGTATCTACCGACTTCCTGGGTGAGCGTCAGACCTCCGTATTCGACG
CCAAGGCTGGTATCCAGCTGAACGACAAATTCGTTAAAGTTGTATCCTGGTACGACAACGAAATGGGCTA
CTCCAGCAAAGTTCTGGACCTGATCGCTCACATCTCCAAGTAATTTGGGATGCGCAATCGCGTAGCTTGA
AAAAGGCGGCCATTGGCCGCCTTTTTGTTTTTCGGTTTCCCCTGATGACAGGAGTCTACATGCAATCCAT
TCGTCCCCTGACAGCCAATTGCAAGCTTGCCAACAATGCGGCCGGTCTGCCGGTTCTCACCATCAACAAC
GACTATGCCAAGGCAGAAATCAGCCTGTTTGGCGCCCATGTGCTGAGCTATCAGCGTCATGGCGAGCCAG
CCTCCATCTGGTTGAGCGACAAGGCGGTGCTGGATGGCAGCAAGCCTGTTCGCGGTGGTATCCCGATCTG
CTGGCCCTGGTTTGGCCCGGCGCCCGCCCGCGTCGGTAGCGGCAAGCCCTCCCACGGTTTTGCCCGCACC
ACCCTCTGGATGCTGGACGGAGTCTCCGATCACGCTGATGGCACGCTTGTTCATCTCTCCCTGCGCGATA
GCGATGCGACCCGTGCCCTGTGGGATCACGCCTTCGAGCTGGAGCTGGATGTACTGGTTGGCAAAGAGCT
CTCGCTGGTGCTGACCACTCGCAACACAGGTGCTCAGTCGCTGACTTATAGCGGTGCGCTGCACACCTAT
TTGCAGATCAGCGCACCGGAGGCGGTAAGCGTCACCGGCCTGGGTGAGCCCTATGCCGACAAGCTGACCG
GGCAGGATGCCAAGCAGCAGGGGGCATTGACCCTGAGTGGGCCGCTGGATCGGGTCTACTGGCAGCCAGA
GGCGCTGGTGAGCGTGCAGGATGGTGAGCGCGAGACCCGGGTTGTCAGTGGCAACCACGACAGCATGGTG
GTCTGGACCCCCTGGCTGGAAGGGGCTACCGCCATGGCCGACATGAGCGATGACGGCTATCTCACCATGC
TCTGTGTCGAAGCGGCCATCGCCGGCGAGGCGGGTGTGACCGTCGCGCCGGATGAAGAGCACAGCCTCTC
CACCGTAATCATCTGAGTTGGATGAGGTTCAGATAAATAAAGAGGGCTCCCGCGGGAGCCCTCTTTGCAT
CTCGTGACGGCGGCAATCAGGCGTTGCCCTGGGTCTTGAGCTTGAGTTCGGCGGCGAAGTTGAGCATCCG
ATCGAGGGGGATCAAGGCTTTGACCCGCAGATCTTCGTCGACATGGATCTCGTGGCGTTCGGCACCATCG
GTGAGGGCCTCCTTGATCGCCACCAGACCGTTCATTGCCATCCAGGGGCAATGGGCGCAACTGCGGCAGG
TAGCGCCATCGCCACCGGTCGGTGCTTCCACCATCTCTTTCTCCGGACAGGCCTGCTGCATCTTGTAGAA
GATGCCCCGATCGGTCGCCACGATCAGCTTCTGCTGGGGCAGCTCCTTGGCCGCCTTGATCAGCTGACTG
GTGGAGCCCACCGCATCGGCCAGCTCGATCACCGAGGCGGGGGATTCCGGATGAACCAGCACGGCGGCGT
CAGGATTCTGCTCTTTCTGCTCGCGCAGGGCGCGGGCCTTGAACTCGTCGTGGACGATGCAGTGGCCTTG
CCAGCGCAGCATCTGCGCCCCGGTTTTCTTCTCGATATAGGCTCCCAGATGGCGATCCGGGCCCCAGATG
ATCTTGTGACCCAGACTGTCGAGGTGTTCGACGATCTCGAGTGCGATGCTGGAGGTCACCACCCAGTCAG

CCCGCGCTTTGACCGCAGCTGAGGTATTGGCATAGACCACCACGGTATGGTCGGGATTGGCATCGCAGAA
GGCGGAGAACTGCTCGATGGGACAACCCAGATCGAGGGAGCATTCGGCCTCCAGAGTCGGCATCAGCACC
CGCTTGTGAGGGCTCAGGATCTTGGAGGTTTCCCCCATGAAGCGCACCCCGGCGACAATCAGGGTTTGCG
CCTCGTGATCGCGGCCAAAACGGGCCATCTCCAGCGAGTCACCGACGAAACCACCGGTGGCTTCGGCCAG
CGCCTGGATCTCGGGATCCGTGTAGTAGTGGGCAACCAGTACCGCTTTGCGCTCCTTGAGCAACGCCTTG
ATTTCTGTGGTCAAGGTCGTTCTCTGCTCGGCACTCAGGGGAAGAGGTTTGGCCGGAAAGGGGTAGTCCA
TCGCGACTACCGGCGGGACACTGCTCAGTGCATTGCTCATTGCGACTGTTCCATCAACGCTCGGGAAGGC
AAAAATTATAACCAGAAGCCTGCCACAAACCCATCAACAAATCGGGTGAAGCGCAAGTGGGCGAATATAC
TCAAGTAATTCGTTGTTATTCTGCCATAAGGAGTTGTCGCATGAAGCAACGTTCCCTCAAGTTCAAGTTG
TTGTGCCTTACCATGGGGCTGTTTCTGCTGACCGGGCTGGCCATGACCCTGTTGCAATCGGCCTCTTTCA
GCTCCCTTCGCAACATGGTGATGACCCAGACCAGCGATGCACTGGAAGAGGAGGTCTCCCGCAGCCTGCA
GTATCAGGCAGAGCGCTATGCCATCCAGATTGCCTCGCTGATGCAGAACTCCTATCAGATCCCCCTGACC
GTGACCGCCCAGATTGAAGCCGGGATGGCCAAGCCGGAACTGCGGTTGTCGCGGCCCCAAGTGGAGTCTC
TGCTCGGCAGCAGCCTGCAGCAGGCGAGCGGGATCAGCTCCATTTATGCCCAGTTTGAACCGGATGGCTA
TGATGGTGAGGATGGCAACTGGCTGGGCGGGGCCAGCCACTCGGTGGCAGGCAAGGGGAGTCTGGAGGTC
TATTTCACCAAGGATCAGGGAGGGGCCGTCGCTCAGCAAGCGGTGGACGCCGCCGCCAGCGAGGCCAAGT
TTGATACCTCCCTCAACGAGTTCGGCATTCGCAACAGCGAGTGGTATCTCTGTGGTCGCGATACCCGCCA
GCCCTGCCTGATGGAGCCCTACCTCTACGAAATCAGCCCGGGCAACAAGATGCTGATGACTAGTCTGACG
GTGCCCGTGCTGCGTGACGGCAAGTTTGTCGGGCTGGCCGGGGTGGATATGAACCTGCCCATCTTCCAGC
AACTGGCTGAAAACCTTGGCAAGAGCCTCTATGACAACAAGGCGGATGTGACTCTGGTCAGCAAGATGGG
GCTGATTGTGGGTAGCAACCGCTATGCCGACAAGCTGGGTCGTCCGCTCAAGGAGGTGGGGCTGACCCTG
CCTGACGGTCAGCCGGTCAGCAGCGACAGTGACTTCATTCTTCATCAGCCCATCCGGGTTGAGGCAGCCA
ATACCCAGTGGTGGTTGATGATCAAGGTGCCCAAGGCGCTGGCGCTGAGCAAGGCGCACGCCATCAGCAA
TGAACTGGGTGCCCTGCTGGTGGATGCCCAGCGGCAGCAGATCATCGCCATGGGCGGGATTACCCTGTTG
GTGCTGGGGCTGCTGGTCTGGTTTATCCAGACCATCACAGCGCCACTTTCTCTTATCAGCCGCCATGTGG
GCCATCTCTCCAGCAACGAGGGGGACCTGACCCAGCAGATGGAGATCGACACCCATCAGGAGCTGATCGA
TCTGGGGTCCCAGCTCAATGCCTTCCTCGGCAAGCTGCGGGGCATGGTGCAAATGAGCAAGGGGATCGGT
CAGCAGGTCTACCAGCAGGCGCGGGAGATGAAACATACCGCCGACACCATGCGCAACAGTCTGGACGAGC
AGAATCTGGAGCTGGAGAGCGTGGTGAGCGCAATGCACCAGATGAGTACCACGGCAGTCAGTGTGGCAGG
TTATGCCGAGCAGGCAGCGCAGGAGTCGGAAGCGGCGACCCACCACATCAATACCGCCCAGCAGACCCTC
AGCAATGCCCGCAACGAGATCCATACTCTGGTCGGCGATATGCATGAGGCGGATAAAGCGGTGGCGCTGG
TGGCCCAGCGCAGCACCAATATCAGCCGGATCCTCGATGTGATCCGCGCCATTGCCGAGCAGACCAACTT
GCTGGCCCTCAACGCTGCCATCGAAGCGGCGCGGGCGGGTGACATGGGCCGCGGCTTTGCGGTGGTGGCG
GACGAGGTGCGGGCGCTGGCGACCAAGACCCGTGAATCGACCGACGAGATTGGCCAGCTGATTGGCAGCC
TGCAGACCGAGGTGAGCAGCAGCCAGCGTCTGATGAATACCGGCATCGAGCGCTCCACCACCACGGTGCA
GGGTACCGAGCAGGCGTTCGAGGCGCTCAATCGGGTGGTGGCCCAGATCCAGCAGATCCACGACCACATC
AGTCAGGTAGCCACCGCCGCCGAGCAGCAGAGTGCGGTGAGCGATACCATCAACCAGAACCTGATGCGGA
TCGGCGATACCGCCAACGTGATTGGGCAGGAGGCCAGCTCCAGCCATCAACTGAGCGAGGCGCTGGAGCA
AGCGGCCACGGCGCTCGCCAGTCAGCTCGATCGGCTGCGTACCTGAGTGGCAGCCAGAGAGACAAAAGCC
CGCGAGCGATCGCGGGCTTTTTTATGACTTGATGGCAGAGTTGCGTCAGTTGCGGGGTTCAACCAGCAGG
ATGTGGGAGTCGGCCCCCACTACCCTGACCTTGCTGCCTGCTGGCAGCGACTCGTCGCAGCGCACCGTCC
AGACCGTATCGTCCAGATGCACCCGACTGGTGCCTTGGGCGACATCATCCAGCAGGGTCAGCTCGCGGCC
CATATAGCTCTGCATCCGCTCGTTGATGGTGCTGGCGGCATCCTGACCCGAGTTGTCACGCTGGTGCTGC
CAGCGCCACCAGACCCAGGTGGTGAGCAGGGATTGCACCGCAAACAGCAGCAACTGCGCCTGCCAGCCAA
AGTGCCAGATCAGCAGCAGCAGGCCCACTTCCAGAGCGGCGATGCCGGTCCAGAGCAAAAAGCCCACCGA
GCCGAGCACCTCGATGGCCAGCAGAATGAAGCCGAGCCCCAGCCAGTGCCAGTGGGTCAGCCCCTCTAAC
AGGGCTGTCATGACTTGCTGCCCTTGCCATCTTTGTTGAACAGCTCGGCCATGCCAGCCACCGAGCCAAT
CAGGCTGCCCGCCTCCAGCGGCATCATGATGATCTTGCTGTTGGGGCCTTCACCGATGCGGGCCAGTGCC
TCGGTGTACTTCTGAGCCACGAAGTAGTTGATGGCCTGCAGGTCTCCTTCCGCAATCGCCTGGGAGACCA
TATGAGTCGCCTTGGCTTCCGCCTCGGCTGCCCGCTCCCGCGCTTCGGCGGCGAGGAAGGCGGCCTGACG
CTCGCCTTCGGCTTTGAGGATCTGGGACTGCTTCTCCCCTTCCGCTTTCAGGATCTTGGACTGGCGCACC
CCTTCGGCTTCCAGTACCTCGGCCCGTTTCTGGCGCTCGGCCTTCATCTGGGCGTTCATCGCTTCGACCA
GCGCCAGCGGCGGGCGCACATCTTTAATCTCGATGCGGGTCACCTTGATGCCCCAGGGGGCGGTGGCGGC
ATCCATGGTGCGCAGCAGCTTCTCGTTGATGGTGTCGCGCTGGGAGAGCATCTCGTCCAGCTCCATGGCG
CCGAGCACGGTCCGCATATTGGTCATGGTGAGATTGCGGATGGCGCTGGTGAGATCGTTGACCTCGTAAC
CCGCTTTGCGGGCATCCACCACCTGCACAAAGCTGATGGCGTCGATGGTGACGTTGGCGTTATCGCGGGA
GATCACCTCTTGTGCCGGGATGTCCAGCACCTGCTCCATCATGATGATCTTGTGGCCGACCCGATCCACG
TAGGGGATCAGCAGGTTGAGACCCGGGGTCAGGGTGCGGGTGTAGCGGCCGAAGCGCTCCACCGTCCAGT
TGTAGCCCTGAGGCACGATCTTGATGCCCGCGCTCAGGGTGGCGAGCACCAGGAACACGAAAATCCCTAG
CACAATCAATGATTCATTCATCATTTAGCTCCTTATGAAACACAGTCCCTAGCCTATGGTCTGGCGTCAG
GTACCACAAGTGACTTCGTACGCCTTTGCCATGAATCGGCTTTGCTAATTACCAAAACGCATTAAAAAGC

ATTTTTTATTATTTTGAGGGATAAGCTGCCCTGCCTATAGTCGCCCCATATTAGTCGTGTTCCGAGGGAT
GGGCCGAAATGGATTATTTACCTATGTTTGCCAAGTTGGAAGGCCGCCCGGTACTGCTGGTGGGTGGGGG
CGAGGTTGCCCTGCGCAAGGCGCGCCTGCTGCTGGCAGCCGGTGCCCGGCTGACCTTGGTCTCCCCGGAG
CTTGAACCGGAATTTGCGGAATTCACCGGTCGTTTCACCCATCTGGCCGAGCGCTTCACTCCGGCCCATC
TGGCAGGCCAGATTTTGGTGGTGGCGGCGACCGACAACCTCGAAGTCAACGCCCTCGTTTATCAAAGTGC
CAACCAGCTGGGTCTGTTCGTCAACGTGGTGGATGACCCTAAACGCTCCAGCTTTATCTTCCCCTCGATC
ATTGATCGCTCGCCGCTGATGGTGGCGGTCTCCAGCGGCGGCAACGCGCCGGTGCTGGTGCGCCTGCTGC
GCGAACGGCTGGAGAGCCTGCTGCCGCGCCATCTTGGCGGGCTGACCGAGCTCTCCGGTCGGGTCCGTGA
CAAGGCCAAGCGGGTGCTCTCCTCGATTTCCGATCGTCGTCGCTTCTGGGAGCGCGCCTTTGCCTCCAAC
ACCCTCGCCAGCCTGATTGAAAAAGAGGATTGGCAAGGGGCCGAGCAGTGGCTGAGTGATGGCCTCGACC
AGGCGAAAAGCGAAGTGGGCGAGGTGGTCTTGGTGGGGGCGGGCCCCGGTGATCCCGGCCTCTTGACCCT
CAAGGCGCTGCAACAGATTCAACAGGCTGAAGTAGTGCTATACGACCAGCTGGTCTCGCCTGAGATCCTC
GATCTGGTGCGCCGCGATGCCACTTTGGTGTCGGTCGGCAAGAAGGCGGGCGCCCACAGCGTGCCGCAGG
AGGAGACCAATCGCCTGCTGGTGGAATACGCCAAGGCGGGCAACCGGGTGGTGCGGCTGAAAGGGGGCGA
TCCCTTTATGTTTGGCCGGGGTGGCGAGGAGCTGGAAGTGCTGGCCGATGAAGGCATCCCCTTCTCGGTG
GTGCCCGGCATCACGGCCGCTGCCGGTGCCACCGCCTATGCCGGCATTCCGCTCACTCACCGTGATTACG
CCCAGAGCGCCGTCTTTATCACTGGCCACTGCCAAAAAGAGGGCAGGGAGCCCGACTGGCAGCAGCTCGC
CGCCACCAGTCAGACCCTGGTCATCTACATGGGGCTGATGCGCTCCGAGCATATCCAGCAGCAGCTGGTG
GTTCATGGCCGAAGCCAAGCCACCCCCATCGCCATCATCGAGCGCGGCACCACATCCCGCCAGCGGGTGT
TGACCGGCACGCTGGCCGACTTGGCCGAGCTGGCCAAACAGGCGGTGAGCCCGTCGCTTATCGTCATCGG
CGAAGTGGTTGCCCTGCGCGATCGGCTGGCCTGGTTTGGAGAAAAAAGCGGGGAAAAGAGCGGCCAGCAG
CAGCGCGCTAACCCGGATCTCGATGGCTGCGACCTCAAGCTGGTGCAGCTGGCCTGACCCGGAAAAAGAT
ATTTGATGCGGCTGCCAGCGCGGCCGCGCACTGAGCCACCCAGCGCCCCGAGCAGGCGCATAAGCAATGA
CCCGAGGACCAACATGGACCGTGAAAGATTGACGCACTTGCAGCAGCTGGAAGCCGAGAGCATCCATATC
ATCCGCGAGGTGGCTGCCGAATTTGAAAACCCGGTGATGATGTACTCCATCGGCAAGGACTCCTCCGTCA
TGCTGCACCTGGCCCGCAAGGCCTTCTATCCGGGCAAGATCCCCTTCCCCTTGCTCCACGTCGACACCGA
CTGGAAGTTCAAGGAGATGATCAAGTTCCGCGACGAGACCGCCAAAAAGTACGGGCTGGATCTCATCGTC
CACAAGAACCCGGAAGGGCTCGCCATGGGCATCAACCCCTTCGTCCACGGCAGCGGCAAGCACACCGACA
TCATGAAGACCGAGGCGCTCAAGCAGGCGCTCAACAAATATGGCTTCGATGCCGCCTTCGGCGGTGCCCG
TCGCGACGAGGAGAAGTCCCGCGCCAAGGAGCGGGTCTACTCCTTCCGTGACAAGTCCCACCGCTGGGAT
CCGAAGAACCAGCGCCCGGAGCTGTGGCGGGTCTACAACAGCCAGGTCAACAAGGGGGAGAGCATCCGGG
TGTTCCCGCTCTCCAACTGGACCGAGCTGGATATCTGGCAATACATCTATCTCGAGAACATCGACATAGT
GCCGCTCTACTTCGCCGCTTACCGTCCGGTGGTGGAGCGCAACGGCATCAAGATCATGGTGGATGACGAG
CGCATGCCGCTGACCGCCGAGGATGAGGTAAAGCAGGAGCTGGTGCGTTTCCGCACCCTCGGCTGCTATC
CGCTCACCGGGGCCATCGAATCCGATGCCACCACCTTGCCGGAAATTATTGAAGAGATGCTGCTCACCAC
CTCGAGCGAGCGGCAAGGGCGGCTGATTGACCACGATCAGGCCGGCTCCATGGAGCAGAAGAAGCGTCAG
GGATATTTCTAAGGAGTCGTCAGTATGAACAGCCATATTCAGACCGCCATCACCGAACAGGGCATCGAAG
CCTACCTGCACGCCCAGCAGCACAAGAGTCTGCTGCGCTTTCTCACCTGCGGCAGCGTGGATGACGGCAA
GAGCACCCTTATCGGTCGCCTGCTGCACGACTCCCAGCAGATTTATGAAGATCAGCTCAAAGCGCTGGAG
TCCGACAGCCAGAAGCTTGGCACCACCGGCGAGAAGCTGGATTTGGCACTGCTGGTAGACGGCCTGCAAG
CGGAGCGCGAGCAGGGCATCACCATCGACGTGGCCTACCGCTATTTCTCCACCGCCAAGCGCAAATTCAT
CATTTCGGATACCCCGGGCCATGAGCAGTACACCCGCAACATGGCCACCGGCGCCTCCACCTGCGATCTG
GCCATCATTCTGATCGATGCCCGCAAAGGGGTGCTGGATCAGACCCGCCGCCACAGCTTTATTGCGAGCC
TGCTCGGCATCAAGCAGTTCGTGGTGGCGGTCAACAAGATGGATCTGGTGGAGTTCAGTCAGGAGGTGTT
CGAGCGCATCAGTGCCGATTACCGCGAGTTCGCCAAGAAGCTCAGCGTCGACACCATCCATATCGTGCCG
GTCTCGGCGCTGGACGGTGACAACGTGGTCAATCCAAGCGACAAGCTGGCCTGGTACGAAGGTGAAACCC
TGCTGGCGCTGCTGGAATCCGCCGAGGTAGAGCGCGAGCTGGAGCGTCATCCGGTGCGTCTGCCGGTGCA
GTATGTGAACCGCCCCAACCTCGATTTTCGCGGCTTCGCGGGGACGCTCGCTTCCGGCATCCTGCGGGTG
GGCGACCGGCTTGCGGTGCTGCCGTCGGGCAAAGAGAGCACAGTCACCCGCATCGTCACCTTCGACGGTG
ATCTGGAGTACGCCCTGCCGGGTCAGGCCATCACCGTCACCTTCGCCGACGAGATCGACATCAGCCGCGG
CGACCTGCTGGTGGATGCTGCCAAGCGGCCGCAAGTGACCCAGAATGTGCTGGCCCATATCGTCTGGATG
GGGGAGGAGTCCCTGCAACCGGGCCGGGTCTACGACGTCAAACTGGCGACCAAGAAGAGTCGCGGTCAGG
TGGAGGCTATCCGCCATCGCATCGAGATCAACAAGCTGGACGAGTTGGACGCGAGTGAACTCAAGCTAAA
CGAGATCGGCCTGTGCGAGCTGAGCCTCACCGACCCGGTGGCGTTCGACCCGTATCAGGAGATCCGCGAT
ACCGGCAGCTTTATCCTGATTGACCGGCTGACCAATGTCACCGTCGGCGCGGGCATGATAGTGGAGGGCT
TGGCCGCCAAAGCGGTAAAAGGGCACTACAGCCAGTTCGAAATCGAGCTCAACGCCCTGGTGCGCAAGCA
CTTCCCGCACTGGCAAGCCCTTGCCATCGGTAACGACACAAGCAAGGAGTAAGCCCATGACCTGGCAACA
ATTCCTGGTGCTGGGTCTGCTGGCTGCTCTCGTGGCCCTGCTGATCCAGGGCAAACTCCGCCCGGCGCTG
CTCTTCTCGGGCGCCGTGCTCATCACCTACCTGAGCGGGCTGGCGGATATCAATGCCGTGGTGGCGGGCT
ACACCAACAGTGCTCTGTTGACGCTGGTGCTGTTGCTGCTGGTGTCGCTGGCGGTGGAGAAGACCCGCAT
CATGAGCTGGTTCGCCAATCTGGTGGGCACGGGTTCATTCAACAAGGTGCTGGTGAAGGTGTGGTTCTCC

ACCGCCTTCCTGTCGGCCTTCGTCAACAACACTGCCGTGGTGGCCTCCATGCTGGGAGCGGTCAAACGCA
ACCCCAACCATGCGCCGTCGCGGCTGCTGCTGCCCATGTGCTTCGCGGCCACCTTTGGCGGCGTGCTGAC
CCTGATCGGCACCTCCACCAACCTGATCGTCAACAGCTTCCTCATCAAGATGGGGGCGCCGCCGCTCGGC
ATGTTCGACTTCTTTATGGTGGGAGCGGGCACCCTGCTGACCGGTCTGGTGGCCGTGCTGCTGTTTGCCC
GTCACCTGCCGGAGCAGGATACCGAGATGAGCCGCGAATCCGGCTACTTTCTCGAAGCCAAGGTGGGCGC
CGGTTCGCCGCTGGCGGGGCGCAGCGTCAAGGAGAACGGCCTGCGCAGCCTGAAAAGCCTCTATCTGGCG
GAAATCATTCGCGGCGATCAGGTGATCTGCCCGGTGCAGCCGGAACATGAACTGCACGAAGGGGACGTGC
TGCTCTTTTGCGGCGATGTGGAGAGCGTCGGCGTATTGCAGGAGATGAAGGGGCTGGATCTCTACGGCCA
GCACCGCCTCAACGGCCAGCATCTGGTGGAGGTGATCGTCAGCCACAGCTCGCGGCTGGTGGGTCAGACC
ATCAAGGATGCCGAATTTCGTACCCACTTCGATGCGGTGGTGCTGGCGGTGCGCCGCGGCGAAACCCAGC
TCACCGGCGGCCTCGGCCAGATTGAACTGCACGCCGGTGATACCCTGCTGCTGGCCCCCGGCCCCCAGTT
TGCCCGCAACAAATCGCTGGGGCGGGAGTTTGTGGTGGTGAGCGGGCTGGAAGCCTCCACCCGTCTCGAT
GGCAAGCGCAGCGCCGCCGTGGTGCTGGGTTTTCTCGGGGTGCTGGCACTGTCGGTGTTTGATCTGGTGC
CGCTGTTCAAGGGGCTGCTGTTGATGCTGGTGGCGCTGCTGCTCACCCGCATTCTGACCCTGGACGAGAT
CCGCCGCCGCTTTCCGCTGGATATCTGGCTGGTGGTGGGTGGGGCGCTGGCCATCGCCGATCAGCTGGAA
AAGAGCGGGCTGGCCAAGCTTATCGCCGACTGGCTGATGGGGGAGTTCAACGGTGCCAGCCCCATCATCG
CCTTTATCGGTATCTATGTGTTTACGCTGGTGCTGACCGAGCTGATGAGCAACAACGCAGCGGCCGCGCT
GGCCTTCCCCATGGGTTACCAGCTGGCGCTCAGCATGGATGTCTCGACCATGCCGTTTATTCTGGCGGTG
CTGTTCGGGGCGAGCTCCAGCTTTATCCTGCCCTACGGCTATCAGACCAACCTGATGGTCTATGCCGCGG
GCAACTACAAGATGCCGGACTACCTCAAGCTGGCCCTGCCGGTCTCGCTGGCCTATAGCCTCGGGGTGAT
TGTGATGGTGCCCCTGCTGTTTCCGTTTTAAGGCGTGAGCGCCAATCAAGGCGTGTTGGATGAGTAATGC
CATGGCATCAGCACGTTGTCGGCGGATAGTCCGAGGCGGCTCCCCGTGGTGCCGGACAGTTTAAGTTTTG
AGGCGTGAGCGCCAAGCAAGGAGTGTTGGATGAGCAATATCGTGTGGCACAAACATGCCGTGGACAAACA
GAGCCGGGCCGAGCTTAAAGGGCAAAAACCGCTGGTGATCTGGTTTACCGGGCTGTCGGGGGCGGGCAAA
TCCACCCTGGCGGGGGCGCTGGAGCAGGCGCTGGCGGCACAAGGCAAGCACACCTATCTGCTGGATGGGG
ACAATGTGCGCCACGGCCTCTGCGGCGATCTTGGTTTCGATGATGCGGCCCGTCAGGAGAATATCCGCCG
GGTCGGCGAGGTGGCGAAACTGATGGTGGATGCGGGCCTTATCGTGCTTACCGCTTTTATCTCGCCGTTC
CGTGCCGAGCGGGCGTTGGTGCGCAGCCTCTTGGGTGAAGGTGAGTTTGTCGAGGTGTTTGTCGATGCGC
CGCTCGCCGTCTGTGAAGAGCGCGACCCCAAGGGGCTCTACAAGAAGGCTAGGGCAGGGGAAATTACGAA
TTTTACCGGTATCGACTCCGCCTACGAGGCACCGGAGCAGCCGGAAATTCATCTGCTGAATGCCGGTAAA
TCGGTGGAAGTGCTGGTGGATGAGCTGCTGACCGCCCTGCGGCAGGGGAACTACCTGTAGCTGGATCGGA
AGAACCTATAACAAGCGCCCGGCGCCCCTTAAGCTGGCCACCACAGGCGCTTATGGGTAGGCGGCCACCA
CTATAAGCAAAAGCAGGGAAATTTTCGGGAAACCCATAAATTTGGCTTATAGCGGTGCCGATGAACCTTG
AGCAGCTCCAAGGCTGCCGATATCAATCCCGCAAGGGGAGCTCGCGATTTTTTGCTTATGTCGTTGCGGG
ATTATTCGCGATTGTCCGAATACGCTTCGCTATCCGAACCTACGGGCTGTACACTTAGTTAGTTCTATTT
TTTTGCCTCAATTTTGGTAACAATACTATAGAACTATATAATTCCCTTCATGCTTACATCCCCATACTCT
AATAAATGTTCAATGTACTTTTCATTTGGTTTTGCACCGGAAATAGATGCAAGCTCGTTGCCAATATCTG
TAAATGGAAATGCACTTAGTAGTATTCCATTACCTTCGTCAGGCTCCGAACTAGTGAGGTATTTTTTATT
AAAATAGTCTAGCGTAATAATGGTTTCATCATTATCATCGGCTCCGTACCAGATACTGCTATTGTTAAGA
AGACCAATGCTTTTGAGATGCAGCTCTACATTAGTCGTTAATCCGTGTGCTCTAATAAATTCATAATAAT
TGTGAGAGAAATTAGGTAATAGGTTGGCACCATCTTCAGACTTGAATGAAAACTCACATAATAGTGTAAA
CATATGAGCTTCTTCTGAAGTAAGTGATTTGACTGTTTGAAGTGTTCTTGGCTTAAATGTACCAGGATTA
GCCACTTCTCCGGCAAGCAATTTTGCCCATATTTGTTGCATTTGAATGTTGCCAATATCTTGACAGAGTT
CAAAAAAATTTACTATCCAGTCTTCATCAACTGGATTGGTATCAACAGTGTTTGGTAGCTCATTTGATGC
TCCTCTAACTATAGAATCAATATTTATTTGACGTCTATTTTCTCGATGCCTTAAGCGCTCCATAGCCCGT
AAGGATATTTCATCAGTCTCGCATTTTGCTTCGGTTAGAATTTTCATCACCTTCGCGTCTGCTCTTGCGT
TACGAATTTTTTTAGTGGGCTCATATATTAATCCAATGCCTTTTGAAACTGAGTCAATCAATTTTGTTAA
AGGTTCTGAAATTCCTTTCATATCAACCAACGAAAACCCAGTGTTACTCATAATTATTTCCTGTATAGAT
GATGAGGAATTTGTGTGAGACGTAGTGGTGATTAAATGGATGGGGGGGTATTATTTAGTCTTCAATATAA
TGTATCTCGCATATTGGGCTAAAAGTTATAGGTTAGTGCTGAACGAAGTGAAGTCCAACGTTTACCGCGA
AACGTATCTGCCAATTTGTTTTACCGAACGACCCAATCCACATTTATTTCGCTCTGGTTGAGGTGATCAT
GCTCATCTGTTGGGCTTCTCTACGCTCTGCGCCAACCTGCAAAAACCACGCTACCGCCCCTGAATTTCAG
CCTCCAGCTTGTTACCTGCGATTTGGCACTGGCTTGACTCATGGTTGACTCCTGCGAGAGGTTGCCAAGC
GGCTATGTTACCGAACGGGGCTGGGGTGAAAAGTGCAAGCGGGCGGTTTTCTGAGCAAGATCAATAAATT
AATAAATAACGGACGGTGATATAACCGCAGCACCGCAGCACCGCAGCAGCCTATCCGCGCAGAGTGAGAT
TGGAAGGGGAAAGCGGGAGTGTCGCCAGCTTGTCGACAGACATTAAAAAACCACATGGACCCAAGGGCCC
ATGTGGTTTTTCTGCGGTGATTGCTTGTCTTTATCTGGCTTGGCGTACCGGCACGGCGCGGGTGGCGGCC
ACTTCGTAGCGGCTTTGGCTTGCCGGAGCGCCCTTCTGGAACTGGCGTACCAGCCGGTCGAGATCGCTTA
ACTGGTTTACCAGCTGGGTGATGCCGTGCACGGCGTGCTGGCTGCCGACGCTGGAGTCATCGGCCAGGGT
CTTGATGCTGAGCACGTTGCGGTTGATCTCGGCGGTGACGTGGAACTGCTCCTGCACCGCCTGGGCAATC
TGGCCGCTGCCGGAGGCGACCTGCTGCAGCATATTGTTGATCTGCTGGAGAATGGTGCCGGTCGCCGAGG

CTTTCTGCTGGCAGCTTTCGGAGAGTTGGCAGCCCTTCTCCATCGCCTGTTCTGCATCCCCGGCGCTGGT
TTGCAGTTCGGTGATCATCTTCTGGATCTCGCTGGTCGAGGATTGGGTCTTGATGGCGAGGGTGCGGATC
TCGTCAGCCACTACCGAGAAACCCCGACCCGCTTCGCCAGCCCGGGCCGCTTCGATGGCGGCATTGAGGG
CCAGCAGGTTGGTCTGGTTGGCGATGTTGTTGATCACATCGAGGATGCCGTCGATATCGCGGCAGTGGCC
GACCAGCGCACTGATCACGCTCTTGGAGGTGGTCAGCTCGCTGTTCATGCCCGCCACGGCCGCCACGGTT
TCGGCCACGCTACTGTTGCCGCTCTTGAACAGCACGGCCGTTTCATCCAGCAGGCTGGAGGAGTCAGTCG
AGCTTTGTGACACCTCGCGAATGGATTCCGACATCTCGGTCACGGCAGTGGCTACCTGATTGGTTTCGGC
CTGCTGCTGTTGCAGGTTGTCGGTGATGGATTCGGCGTTGCGCAGATCCCCCTCGGCGGCCTGCAGGATC
TTGCTGCAAGTCTGCTGGGTGCGGGCCAGCACGGCGTTGAACTCGGCCTGATTCATCAGCATCGAGAGTT
CGATGGCGGCGATGTTGTCCACCTTGTTGGTGTAGAGCAGCTGCATCAGAGGGTTGTCAAAGCGGCTGCG
GGCCATCTTGTTGAGCTTGCCGAGGCGGCGGGAGAGGGCATTCAGGGTGATGGCCGCTATCGCCAGCGGC
AGGGCGACCAGCGCCAGTTGCCAGTTCATGTCGTGGTTGTAGCCCAGCGCAAACATGGCGAGCAGGCTCA
GCAGCAGGCAGAGGTTGATGGTCAGGGTATGGGAGAAGGTGGGCAACTTGAGCGCCAGCGGCAGCTTGTT
GTTGCGAAGGTCGGCATAGACCTTCTCGGCTCGCTGTTTGATCTCCTCGCTCGGGGCGGTGCGCACCGAC
TGATACTCCACCACCCGGCCGCTGGCATCCTTGATGGGGGTGACAAAGGCGCTGACCCAGTAGTGATCGC
CACTCTTGCAGCGGTTCTTCACCGGCCCCATCCAGCTCTTGCCTTCGCGGATATGGTTCCACAGATCGGC
AAAGGCCTGTTTCGGCATGTCAGGATGACGTACCAGATTGTGGTGTTCCCCCACTAGCTCGTCGAGCTGG
AAGCCTGCTATCTCGCAGAACTCATCGTTGGCGTAGGTGATGCGGCTCTCCAGATCCGTGGTGGAGATCA
GGCTCTGGTGGTCTGGATAGAGACGTTCTCTTTGGGTTACCGGTTGATTGATTCTCATGGCCTTACTCCC
GAGCTGTGATGGTTAGATTTTGATCTACATCAAGACATCTGCAAGGGGTGGGCCAGCTGGCAAAAGCTAA
TGACTGATGTGTTATTAGTGCCGTTTTGGCAGGAAAAGAACGTTGCTGTTTGCAATATGCAATGGAGCAG
ACGGGGTGTGCCTTTGCGGCATCTGCAATTTGCAACCTATTCTGGTCAAGCCCATGAGCTGTGAGCCAGA
AGCTTGATGCGAAGGAGCCACGAACCGACGGGGCATCCGCTGCCCCGCCAGAGATCAGGGGAGAGGGTCG
GGAGCGGGTGAGGAGGGGGCGCTGACGAGCTGCATCACTTCAATCTGGATACCCTGCACCAGCACGGTTT
TCAGATCCTGCAGGTACTCCTCCAGCGGCGACTCTTGCAGCAGCTGGGCGAGCTCGTTGCCCACCGGGGT
GAGCCGGTAGTAGAGCAGGGTGATGTTGTTCTGCTTGCACTGCAGCCGCCAGCGCTGGTTGCCGAACTCC
AGCTCGACCCCATCGGCGGGCAGCGGGCCGGACTCCAGCTCAGCGCGGTGCAGCAGCCCCAGCTCAAACA
GTTGCAGCAGGGCGTTGTAGGGCAGTCGGTATTTGCCAAGGCCAAGGCGCGTTGCCTTGGCGCGGCTCAG
CAAACTGGCCCCCTTGTGCAGCCCCAGCAGCAACCGCTGCTCGTCGCTGCCGGTGTAGTGGCAGGTCAGT
GAACAGATGCGCTGGAACAGCTGGGCCTCGCGCTGGGTCATCTCTTTGAGGGTGGCTAGCGCCCGAATGG
AGAAGCGGCCCGGCGTGGCGACTTCCAGCGCGAAGATCCGCCCCCACAGCTGCTGCATCGGCACCATGCT
GATATCTTCCGCCAGTTGCAGGAAGCGGGTCAGCCAGTCGCTGTCCATCTCGCCGCCAGCTGCCGTCTCT
TCGCAGTGGCGGGAGGCCATCACCATGATGGCTTCCAGATTGCGCTGACGGGCGGCCTGATCCGCCAGAT
GGCGAAAGGTGGCGCGCTGTTCGAAGGTGCTGTCGGTGTTGCGGGTGAGCATGCCGTCGATGCCGCGACT
GCGGGCCAGACGCAGGGTCTGCTCCTGGCTGCTCAGGATCGAACTCTCTTTTTTCGGGGGGGGGAGGTCT
GCCATGCTGACTCCATTCGCACAGGTTCTGGAGCTGAATGTTACTCCAACCGGGATGCCAAGATAACAAA
GCGGCCTCCGTGGAGGCCGCTTTTTTTGCAACGGGAGAATTACTCCTCGTAGACACTCACCTCGACCGCT
TCCATCGAATAGGAGGCGGTGGCCATCTCGTGGCTGGAGCTGACAGTGCGCATCTTGCCCTTCACCCAGA
TGGCATCCCACAGATCATCCACCGGCGCACCCTTGGGGTAGCTCACATAGATCACCTGATTGGTGGGGGG
CGGCGGCACATGGATGCAGGCGCCGAAGTAGGGCACCAGCAGGAAGGCGGTGATCTTCTTGGCATCCCCT
TCCAGTGGCACCACGAAGCCTGGAATGCGCACATCCTGGTTGTCCAGCTTCTTGTTTACCCCGCCCACCG
GTTGCGGCACCGACATGGGATCTTCGTGATTGACCTGGGGCGGTGGCAGCAGTTTTTCCCCTTCCGGGAT
GAGCACATCCCAGTCGATGGTCTTGTAGTCGGCGGCCAAGGCGGGCAGCACCAGCAGGCTGGCGAGCAGC
GCCACAATTTTCCACTTCATCATCTGTCCTTCTGTTTCTATTTTGGGCGTCTCGCTAGACGCGAATGCTC
ATGCCATCGCTCAGGCTGTAGCGGTAAGCGCGGGCGGCAGGGAGCAGGCCGATCACCATGCCTGCCAGCC
ACACTACCCCCAGCAACTGCCACTCGTAGGTGCTCGGTAGCCCCAGACTCAGTTGCAGGCCATAGTGGCT
GAGCAGCCAGGGCGAGGCAAGCCCCTGCCCCAGATAGAGCACGGCGACCCCGAGAGCGATGCCGACCGTG
GTCAGCGCCATGGCCTCCAGCGCCAGCAGCAGGAACAGGTGGGCAGGGCCCGCACCGAGGGATCGCAAGA
TGGCGAGCTCCCGGCGCCGCTCGTTAAGCCCCGCCAGCAGGGTAGTCAGCATGCCGATAAGGCCCGCGAC
TACCACGAAACCGGCGATGACCGAGAGGGCGGTTTCCGCCACGCTCATCAGGCTCCACAGCTCCTGCAGG
GCGGCCCCCGGCAGGATCGCCATCAGCGGCTCTTTGGGATAGGTGTTGACGCTGCGTTGCAGCTGAAACG
CCAGGATCCGGTTGCTCAAGCCCACCATAAAGGCGGTGATGGTTTTCGGGGTGAGATCCTGAGCCAGCGC
CTGCTCCGGCGTCACGTTCTTGCTGTGGCGGCCGGTGTCCCAACCGAGATGGATCGCCTCTATCCCCGCC
AGCGGCACATGGATGGTGCGATCGATGGGCGTACCGGTCGGTGCCAGAATACCCACCACCTTGAACGGCA
GATTGTCGTGCTGGCTGAACGAGGTGTTGCCAGCGCCGTGGGCGATGACGATGGATTGGTCGAGGTGATA
GCCGAGCTTCTCGGCCACCTGGGCGCCCAGCACAGCTTTAAACGGCGTGTCGAATGGCCGCCCTTCCCGC
AGTTGCAGCGACTGTTGCTGACCGTACTTCAGGTGGGTGAAGTAGTCGCCATTGGTGCCGAGCACTCGAA
ATCCCTTGTGGGAGTCACCAAGGGAAAGCGGGATGGTCCAGGCGACGCCCGGTTTCTGCTTGATGGCCTG
ATAGGAGTCCCACCCCACGTTGTTGGTGGGGTTGCCGATGTGAAACACCGAGTAGAGCAGCAGGTTAACC
TGGCCGGAGCGGGCGCCGACGATGAGATCGGTCCCCGAGACGGTGTTGGAGAAGCTTTCACGGCCTGGG
TGCGCACCCGCTCCACCCCGAGCAACAGGGTGACGCTGATGGCAACGGCCAGCAGGGTGAGCCCGGCGGT

GAGGCGGCGGGCCCAGAGACTTTGCAGGGCGAGCTTTAGCATGATGCCCTCCGGTTGAGCTGTTGCAGGT
TTTCGACCCGCGGGAACAGGGGTTCGAGATAAGGGTCGTGGCTGACGAAGATCAGGGTCGAGCCCTGCTT
GTCACACTCCTCGAACAACAGCTTGATAAAGGCGGCCCGGTTGTCGGTGTCGAGTGCAGAGGTGGGCTCG
TCGGCGATCACCAGCGGTGGAGAGCCGATCAGCGCCCGTGCGGCGGCAACCCGTTGCTGCTGGCCAATGC
TGAGAGCATGGACGGGGCGATGCAGCGCCTCGTCCGGCAGTTGCAGCTCCGCCAGCAGACGGCGCGCCTC
CTGCTCCGGACTCCCTTGCAGGCGGGCCCGTTTGGCGGGCGAAAAGGTGAGGGCAGCGGTCACGTTGTCC
TGCACCGAGAGAAAGGGCAGCAGATTGAACTGCTGGAAGATATAACCCAGATTGGCCGCCCGGAAGTGGT
CACGGGCACGGCCAGAGAGCCGGGCCAACGGTTGGCCCAGCACCTCCAGGGTGCCGTGGTTGGCGGTCTG
GATCCCGGCCAGCAACCCGAGCAGGGTCGACTTGCCAGAACCCGACGGGCCCTTGATAAAGACCCGCTCA
CCCTCGCGGATGGTGAGGGCGGGCAGATCGAGTACAGCGTCGCCATCTGGCCAGGCAAAGGCCAGATCGC
GGATCTCAACCACGGCTTTTTTCATGGCGTTACCAGCTCAGCTTGTTGGCGGACGGGGACAGCTCGCTAG
CGGTCTGGCCAGTGGGCAGGATCCCCTGCACGCTCAGCTTCTCAAGGCTCGGATAGACGCTGAACAGGGT
CGCCTCAAGACCGGTCAGTTTGGCTGGTGTAGCGCAGGTGTAGGTGTACATGGCGCCCATATCGGCGTGA
CCCGCTTCGTCATGATCATGGTGGTCTTCATGTTTGTCGCTGGCAGCCTTGTCATGGTCATGGTCATGGT
CATGGTCGTGATCATGCTCTTCTTTGGCTGCTTGCAGCTCCTGCTGGGTCAGTTTGCAGCCAGCGGCCGG
GTCGAAGCGGAACAGGGTGTCAGGCTGCTGGAGGCGGGCGACCGCTTTGGCATATTGCGCCTTCTCTTCA
TCGCTCTTGGCGGCGTGCTCGAAGCCCACCAGATCGGCTGCGGGTGCCTGCAGTTCGATCATCAACTGGT
CGCCATCGACCACCAGATTGAGGTGGCCGTGGCCATGCTCGTGGGCGCCGTGGCTGTGGCTCTCGTGTTC
GTCGTGATTTGCGTGTACGCCAAAGGCCGCAGCGGCCAGCAACAGAGTAACTGCTTTCATCTTTAATCCT
TCTTTCAAAACAAATAGTGAAATTAAACAAAGGGATAGATAAAAACAGAGGCGGGCGGCGCACGGATGGC
ATAGCTGTGAAGCGTGGCGGTATCCGCCGAGCGTTGTATGGTTGCAACTACGGGCAGAACAGAGGTCGAA
CCGGGGAAACAATAGGGGGTAGCCGTGATGACCTGTTTGCCATCCAGATGGAGGGCGCAAACCAGACAGT
GATGGTCGTCGTGGCCGGGCAGGGCGGATTCAAACTGGTGCCGGGCAAGGGCAGGCTGCAGCCATAACCA
GCCCAGCAGCAAGATCCAGATTTTCAGCTTGATACGACCCATAGACCCTTATGGAAAGGGCGCCATCAGC
GCCCTTTCTCTCTGACTTAGAGATGGGACTTGATGAGCCCCATCACGGTAGCAATGTTGTTGGCATCGAG
CTGGCCATCTTTGGCAAACAGCACCTTGCCCTCTTTATCCAGCAGAATGATCGCCGAGCTTTCCGGCGTC
AGATCCCAGGCGTTGCGCGCCATGCCCTTGGCATCCAGCACCATGGCGGACCAGGGGAACTCTTTCTTGC
TGTCCTCGGCACTGCTCTTCACGAAGCCGGAGGTGCCCCAGATGGCGTCATTGCTGTTGATGACGGTAAC
GGTCTGGTACTTGTCTTGCGGCAGCTTGGCGGCCTTTATCGCCTCGATCATCGGGGCGTTCAGCTCCTTG
GCACTGGAGCGACCGGCAATATGCTGGATCAGGAACACCTTGCCGGTGAGCGAGGTGCTCTGCCACGGCT
GGTAGCCGATATCCTTGCCGTTCAGTACCAGCTCACCCTTGTCACTGATATTGACCAGCGGCACCGGGTT
GCCATCCTTGAAGTTGTGAGCAAAGACCAGAGATGGCAGCAGCGCCAAAGTAAGTACGAGATGTTTCATC
CTGATGACACTCCATTTGTTATGTTATAACGAGTTTCGGATTGAGGGAATGGGCGAGCTTCATCCTAAGC
TGATCGGATCTGTTTAAAAAGTGTTAAATAAGATCGCAATTCAGAAGCGTCGGCGCCCCGCCAGTCAGGT
GGTTGGCTGGCAAGGGCGGCTAGGATGGCATGACATCGATAAAAATCAATGAATTGGCTGCTGGTCTGAG
CTGTCTGCGTCAGTAGAGCGACTCGCTGATGAGGGGGATCTTGGCCATGGTCATCTGCTGGGCCAGCAGG
GACGAGCCGGTCAACAGCACATGGCGCTGATTGTTGATCACCACCACATCACCGGGTACGCCACTGTCTG
CCGTAATGGCCAGCCAGGCAGGGGAGACGCTGCCGATGCTGATATCCGGATAGATGATGCTGAGCACATA
TTGCGGCGCCAGCTCTGCCAGCAGGCTGGCCATCAACAGCGCCTTCTCGTCGCTGTCGGCCTTGTTATCC
TGCAGTGCCTGCAGCGGCGGTGCGAAGTGATCCAGCTGCTCCGGCTGGGTGGGGATGGCGTTCAGCCACT
CCTGCAGCAGGCTGACGCTGGTGGCGAGATCCTTGGCCGCAAATTGCTGCGCCAGTGGCTTGAGGTGCCC
TTTGAAGTGGTAGAAGAGCTGGGAGTAATCGGGTCGCACGCAAGGCAGTTTGCGATTGGCCGGATGGACG
CAGGGTATCACCCTCATCTGGTAGAAGGCATCCTCCTGATACTGCTGGTAGGCTTGCCAGAACAGCAGCG
ACTCCATGCTGTCGTCCCGCAGGGTGAAGGCCTGCTCCAGATTGCGAAATTTGGTAGCGGTCTCGGGGCT
GCTCTGATTGATCAGGTTGATGCTGTTGTAGAGCCGCGGGCTGATGTAGGCGTAAAGGGTCTCCAGCGGA
TTGGTGAGAGAGGCCTTCAGCAGGCTCATCTCGTGGCGACTGGCGGCCATCACCTGATCGTCGGTGGTCA
GCTGGAAGTGCTTGCGCTGGCCATCGGTGGATTGCCACTGCCAGCTCATGTTGATTTCGCTGGCGTTGCT
TGTGGCGCCAAGGGTGGTGCTGAGAGTGAGGCTGAGCAACAGCGCGGGGGTCAGCTTCATGGTGGTGCTT
CCTGCCGTGGACGATAATTAACAACTAGGTGAGCCGGCACTACTTTGGTGATGTTTCGTTTTGTGATCTG
GATAACAGATTGATGTGACTTTTAATTTAATTGTGAAATTAAGTCACTCTGGAGGGGTTATGAAGCCTTG
GATCATAGTGCTGTTTGGGCTGCTCAGCCTGCAAGCCATGGCGGGTCAGGTGATCGTCAACAAGGGGATG
TCCGTTGCGGATACCTTTGCCCTGCGCGATCAGCTGGCGCGCGAACACGAATGGCAGGAGTGGCTGCGCT
ACCAACAGGCCATCAAATGGCTGGAGGTGTTGCCGGTCAACTGCGAGCTGATGAGCCAGCCGGGCGGCGG
CTATCGCTGTGGCGGTGATTACTATCGGCCTTACCAGAAAGATGGTCGTGAAATATACATTCAGGGCAAT
CCTGCCGAAGATGCGGCCGATCAGCTGCCGCAAATACCAATAAAAAAGGCGCCATAAGGCGCCTTTTTTA
CAAGCGAAGTGACGCTTACTTGGCAGCGTAACGCTCGGCAGAGGTCTTGATCTCGGCTTCGGCAGCGGCC
TTGTCGCCCCAACCTTCCACCTTGACCCATTTGCCCGCTTCCAGCTCTTTGTAGCGCTCGAAGAAGTGCT
GGATCTGGGCTTTCAGCAGTTCCGGCAGATCGTTCACATCCTGGATGTGGTCGTACTGCTTGGTCAGCTT
GGAGTGTGGTACTGCAACGATCTTGGCATCTTCGCCAGATTCGTCGGTCATCTTCAGCACGCCAACCGGG
CGGCAGCGAATGACGGAACCGGCCAGCAGCGGGTAGGGGGTCGGAACCAGTACGTCGACGGGGTCACCAT
CCAGAGAGAGGGTCGCGTTCACGTAGCCGTAGTTGCACGGATAGAACATCGGGGTAGACATGAAGCGATC

GACGAAAATAGCACCGCTGTCCTTGTCGACTTCGTATTTGATCGGGTCGGCATTCTGCGGGATTTCGATG
ATGACATAGATATCGTCGGGCAGGCTCTTGCCAGCCGGTACCAGGTTCAGGCTCATGATATTTCCTTCGT
TGTTAGTATCAGGTTTGCAGGCCGATATTATAGCGATGTGATGTTAAATGACCATATTTCAGCGGATATC
GCTAACGAAGAAGGGGGATGTTTCCGCTTTGCGGCGCTTTGATGTGTGCGGAATGCAACAAAACGGCTGT
TTTGATGCCCTGCCGCTGACCTTGGTCACCGGCAGGGCATGTGGAGTTGCGAGCAAAGGGGTCAGAGCGA
GTAGGGGAGTCTCGACTCATTGACCAGTCGTGACAGGGCTGTTACGACATGAGGGTCAAACTGGGTGCCG
GAGCGGGACTGGATATACTCCATGGTCTGCGTCAGCGTCCAGGGCTCCTTGTAGGGGCGCTTGCTGAGCA
GGGCATCGAATACATCTACTACCGCCACAATCCGCGCCGACAGCGGGATCTGCTGGCCAGCCAGCTGCTG
CGGGTAGCCCTTGCCATCGAAGTGCTCATGGTGGCCCCGGGCGATCTCTGCCCCCAGCGAGAGGTAGCTG
GTTCCCTCCACCATTTCACCCGCCTTGTGCAGGATATTGGCGCCGATGCCGGCATGCTGCTCCATGATGG
CCCGCTCTTCCGGGTCGAAACGGCCGGGCTTGTGCAGGATATGATCCGGGGTGCCCACCTTGCCCACATC
GTGCAGGATGGAGGCCATGCCGATCATCTCGACAAACTCCGGGGTTAGCTGCTCGGGATAGGCTGCCATC
TGCTTCAATTCGGCCACGATGGCATCCGAGAGTTTCTGCACCCGCAAGACATGCTCGCCGGTATCGGAGT
CGCGAAACTCTGCCAGCGCCGCCAGCGCCACCACGGTGGCCTCCTGCGCGCGCAGCAACTGGTTGTAGAG
ATAAAGGTTGTCGTAGGCCGCCGAGATGCGCTGACAGAAGACATCCAGCAGATCGCACTCGAGGGGATCG
AGAGGCTGGGTCGGGGTAAAACTCAGCACGAACTCGCGACCATTCTGGGCGGCGATATAGAGGGAGTCCT
GTGGATGTTCAAAGCGGTTGCAGCGCGCCGCCAGTGCCTTCATGACCGAGTCATTGAGGGTGGGAAAGTC
GGTGATCTTGCCATCATCCAGCAGAGTCTCGTAGTCGCCGGTGGCGGCGAGGATCTCCAGCCCCTGTTCG
CCGGGACGGCGCTCGGCGCAGATAGCCCCTTCGGTGCCCACATCGAGAATGGCGCTGATCTGTTTCAGCA
CGCCGGAGGCAAACTCCTTGAGTGAATGAAGATGGTAGAGATCGCCCGCTCCTTCCAGAATTTTGCCGAG
CCCCTGACGGCTCTTGTCGATGGCACTGAGGGTTTCGTATGAACGCAGGGATGCGATGACAGTGGTGTAG
AGCTTTTGTACCGTCAGCTCGGTCTTGGTCTTGTAGTCATTGATGTCGTAATCGAGGATCACCCGCTGCT
CGGGTGCTTGGCCGGGCTGGCCGGTACGCAGCACGATCCGCACCAGCGAGTTGTGCATTTCGTTGCGGAT
CCGGTGCACCAGCCGCAGACCTGCGTCTTCTGTCTCCATCACCACATCGAGCAGGATCAGCGCAATCCTG
GGATTGGCCTCCAGCATACGGCCAGCTTCGGCGCCGCTGTAGGCATTGAGCAGCTCCAGCGGACGGGATT
TGTAGTGAATGTTGGAGAGGGCCAGGCGGGTGGCATGATGGATATCCGGCTCATCATCCACGATCAGCAC
TTGCCATGGGTTGAGTGCCTTGGGGACCACGACCTCCTCATCATCGAGTTCGATGATGTCGTCGTCCAGC
AAGATATCGTCCTTGTCTTGGGCACTGCTCATATCGGTTACGCCTCCGGCGCTCAATGAGTATGTGAACA
GTATGCACGATCACATCCGCTCGGATCATCAGGATAAGTAGATATCGTGAGCTGGCTCCGCCTCTTTATC
GCTACTCGAAAGGTTCCAGATACCCCTGGATCGACTTCTTCAACCAGGCCAACACCTTGCCGTGATAGGC
GTTTTCCGCCAGATAGACCCCATAATCTCGCCACTGCAGGCTGAACTTGTGTTTCAGTGTCAGCAGGGAG
CCTTGTCGCAGCTGGCTCTGGATCAGCGGCGAGGGGAGCACCGCCCAGCCCAGTCCGTCCAGCACCGCAT
TGCGCATCAACTCGAAGCTGGAGAGACCGATATGCTGGCCTCCCACAGGTAGCAGGCTTTTTACCGAAAA
TTCATCTACATACGAGAGGGTAACCTGGGTGTGATCGGTCAGATCCTGTTGTTTGACCCGCTTCAGTCCG
GCCAGCGGATGGCTGGGCGCCACCACCATCAGGGTGCGGATCGGGTTGAGCACGTCGATATTGAGGCCGC
TGCGCTCCTGATCTTCGGTAATAAGGCCAAAAGCGGCATCGACGAATCCCTCTTCCACCAGCGCCGGCAG
TTCGGGTGGGGAGGCGAGCACCAGCGAGATACTGGTGCCGGGAAACTGGCTGTGCAAGGCGGTCAACATC
TCGCGCCATACCGGTTCGGGAATCGCGTCATCGCGGGCGACCCGTAGCGCTACTTCGGCCCCCGAGGCGT
AATGTTTGCACTTCTGCTGCAGGCTGTTGGCTGCCTGCAGCAGGCGAGTGCAATCATCAAGCACCAGCTC
GCCTATTTCGGTCAGGCGACTCTGATTGGCACCTCGTTCAAAAAGTTCGACGCCAAGCTCAACTTCCAGC
GCCGCGATGGCGGTGCTGACGGCGCTGCGGCTTTTGCCGAGGCGACGGGCGGTGTTGGCAATAGAGCCGC
TATTGGCTGACTCTACAAAGAGTTCTATCTGATATAGCTTCATGTTTTACAGGCCATGGCAGAGTTTTTC
ACCATCACCTTAGCGTGCCAAACCAAGGCAAGGCAAACCTGTGGCGTCGGAATAATCGACGCTGAGCGAT
TGTCGCGAGTTGCTTCTCCCCGTTTACCATCTCTCCCGTCGAAGCAACAAAGGCATCTTATATGTTATTA
AGTCGTATGTTTCTGGCCCGGCTGTTTTTGACCGGCGCCATCGTGACCGAGGTCGCCGGTACCTCCAGCA
TGGCCATGATCCCCGAGCAGGGGGCTGGCTGGATGAATTACATCCCGATGTGGCTGTTGATCGGTTTCTC
GTACTGGTTGCTGGCGAAAGCCGCCAAAACAATCTCCATTGGTATTGCGTTTGCGCTTTGGGAGGGGCTC
GGGATCACCCTGATCACCATAGTGTCGGTCATGTTCCTTGGTTACGATGTGACCGTTCAGGAGCTGATTG
GCCTGGCTCTCGCGATAGTGGGGATTGTTATGGTGACCATGGGGGAGACCCATGATGAGCCCGCAGACAA
GTCAGTAGAAGCGGAGGCCACAGCATGAGCCCGTTTGTGATTGTATTGGGTGCCGCACTGATCGATATCG
GTGCCAACATGGCCATCAACCGCTCCAAGGGTTTTCGTTACAAGGGGTGGGGATTCCTGGGGATCATGCT
GGTACTCGGCGCCTTTACCCTGCTCTCGGAAGCGGTAAGTGGCGGCCAGATTGACCTGGCTGTTGCCTAC
GCCACCTGGGGGGCTATCGGTATCGTCGGTACGGCGCTGGGCGGTTTGCTCTTCTTCGGTGAGCGGCTCA
AGCCCATCGGCTGGGCTGGCATGATGGTGATGGCGCTGGCTGTCGTTATGCTGACTACCGCCTGATAATC
TGAAGCAATAACAAAAGCCGCCCGGTAGCAACCGGGCGGCTTTTGTTTGGCTGATTGCTTGGTCGATGCA
GGCTCAGCGCGGCGCTTCGTAGAGCTCCAGCGGCAGGCCGTCGGGGTCGGCGAAGAAGGTAAAGCGCTTG
CCGGTCAGCTCGTCCACCCGCACCGGTTCTACCTCGACCCGATGGGCCAGCAGGTGGGCGATGACCCGGT
CAAGGTCGCTGACCCGAAAGGCGAGATGGCGCAGGCCGCACGCCTCGGGGCGGGAGGGGCGCTTGGGAGG
CGAGGGGAAGCTGAACAGCTCCAGCTGGCTGCCGTCCGGCAGGGCCAGATCCAGTTTCCACGACTGGCGC
GCTTCGCGCAGGGTCTCGGCGATGATGGGCAGCCCCAGCACCTCGTGGTAGAAGTGGCGCGAGCGTGGGT
AATCGCTGGCGATGATAGCCACATGGTGGATGGCGTCAAACAGCGGAAAGGTCATGGTGTTTCCTTGTCA

GCAGCATAGTGAACGGGCAGCCTGCGCTGCCCGTGTCGTTGATCTAGTTGAAGAAACCGCCGAACCAGTC
ATCCAGCGCACTGCGCGGCGATGCACTGCTGGCGCAACCGGCTGGGGAGCCCATGGTGGAGGCTTTGGCC
GGCACCTGCAGGCTGCCCGGGCAGTCGCTGGCGACGCGGGTGCCGCTGGCGCGGGAGAAGTTGGCCCAGG
TGACCCCTTCCGGCATCTTCAGCCCCAGACTGTTGACGCCGCGCTGGCTCATGTAGCCGCTAAAGAGCTG
CAGTGCCCCGCTGGCACCAGTCAAGCCCGCCGGGGTGTTGTCGTCGCGACCGACCCAGATACTGACCAGC
TCGTTGTTGTCCATCCCGGCAAACCAGGAGTCGCGCAGCTCGTTAGTAGTACCTGTCTTGGCCGCCATGG
TGGCGCCCGGGAACTTGGCACTGAGCGCGCGGGCTGTGCCCTGACTCACCGTCTTGGTCATGGCGAACAG
GGTCAGCCAGCTTGCTTGCTGGTCGGTGACCCGCTTGGCGGTCTGATCATGCTGATAGAGCAGGCTGCCA
TCCCCGTTGACTACGGCGCGAATGGCAGACAGCGGCTGGGTTAGCCCCTGATTGGCGATGGGCAGATACA
TCTGGTTCACCTCCAGCGGGGAGAGGGCCACCGCCCCCAGCACCAGCGACGGATAGGGCTGGATCTCCTG
CTGGACTCCCAGCTTGTGCAGGGTGTCGACCACTTTTTCCAGCCCCACCTGCAGACCCAGATTGACTGTC
GGCACGTTGAGTGAGCTGGCCAGCGCATCCATCAGCGGCACGCTCTGGCGGAACTTGCGATCGTAGTTTT
GCGGCGTCCAGACCTGACCATCCTGACCGCGAATGCGGATCGGCTGATCTTTGAGTGGGGTCGCGAGCGT
CAGGCCGTTGGAGAGACCGGTGAGGTAGACCGCCGGTTTCACCAGCGAGCCGATCTGGCGGCGGGCATCC
AGCGCCCGGTTGAACCCGGCGTAGCGGGGATCGCGGCCACCCACCAGCGCGACCACTTCGCCCTTATGCC
AGTCGGAGACCACCATGGCCGCTTCCAGCTTTTTCTTGCCGCGCAGTTTCTCGATATCGGCGAGGCCCGA
TTCGACCGCCCGCTCGGCCGCGTGCTGGGCGATGGGGTCGAGACTGGTGAAGATCTTCAGGCCAGACTGC
TTGAGCAGTTCGGCACCGAAGCGGCTCTGGATCTCTCGCTTGAGCAGTCCCATAAAGGCCGGGGTGCGAC
CATAGCTCATCTGGCCACGGGCGATGATGCCGAGGGGTTGGCGGCTGGCCAGCTCATATTCGGCCTGGGT
CAGGCTGCCGTGATCCATCAGCAGCTTCAGCACCAGATCGCGGCGGGTCTTGGCCCGCTCCGGGAAGCGC
CAGGGATCGTAATAGGAGGGGCCTTTCACCAGACCGACCAGCAGGGCCACCTGATCGATATCCAGCTCGT
TGACCGGAATGCCGAAGTAGAAGTAGGAGGCGAGGCCGAAACCATAGACCCCTTGCGAGAAGTTCTGCCC
CAGATAGACCTCGTTGAGGTAGGACTCCAGGATCTCGTCCTTGCTGTAGCGATAGTCGATCAGCACCGCC
ATATAGGCTTCCTGCAGTTTTCGCCACAGGCTGCGCTCACGGGTCAGGAAGAAGTTTTTGGCCAGCTGCT
GGGTCAGGGTACTGCCCCCCTGCACGGTGCGACCCGCGATCAGGTTGGCGAACATGGCGCGCATGATGGC
CACTGGCGAGACGCCGCCATGCTGGTAGAAGTCGCGATCCTCGGTGGTGAGCAGGGTCACCAGCAGGCTG
TCGGGTACTTCGGCGATACGCACCAGCAGGCGATCTTCCCGATCTTCGGTATTGAGGCGATCGAGCAGCA
CAGGGTCCATCTGGGCGTAACCGAGCTGGCGCTGGTTCTCCAGCGACTGGATCCCCTCAAGGCGCGGGCC
GTTGAAGGTGAGGCGCAGGCCGCGGGCTCCATCGGCGCCATCATAGAACTCGAACGGACGGCGAATAAGC
TCGATGCGGTTGCCGTTGATGGTGTATTCACCCGGCGTGTGAGGCTGGCGCACCTGCTTGTAGCTCAGCA
TGTTGAGCTCGCGCAGCATCTGGTCCCGCGACAGGCGCTGGCTGGGGTAGAGCTCCAGCGGGCGGCTGTA
GACCATCACCGGCAGCTGCCACTTCTCGCCATCGAAGCGCTCGCGCACCTTGGTATCGAGGTAGATGCCA
AACACCACCATAAAGGCCGCTACCACCAGGGAGAGCTTGAAGCCAAGCCAGAACAGCTTGCGCCAGATGG
TGCGTTTGGGCGCTGCCTTCTTGGGGGCACGCTTGCGTCGTTGGGTTGCCATAAATCCTCTTTGCTTTCC
TTTCTACTTGTTATGTCGCATTCGCGAACGTCCGAGCGGGATTAACCCGTCGAACCTAGATGCCCATCTT
CTTCTTGGTGACCCTGGTCGGCATCGCTTCCAGCGGGTTATCCGGCCAGTAGTGGCGGGGATAGCGCCCC
TTCATCTCTTTCTTCACCTGCTCGTAAGAGCCAGCCCAGAAGGCGGCCAGATCGGCGGTGATCTGCAGCG
GTCGCTGGGCGGGGGAGAGCAGCTCCACCACCAGCGGTACCCGGCCATCGGCCACGCAGGGGGTCGAGCC
TTGCCCGAACATCTCCTGAATGCGCACCGGGATTACCGGTGCCTGCCCCGGCTGATAGCGGATCCGCACC
CGGCTGCCGGTGGGCGCCGCAAAGTGGCTCGGCAGCGCCTCGTCCAGCCGTTTGGGCAACGGCCAGGGCA
GGGATTGGCGCAGGATGTCGCCGAGGTTGAGCTTTTTCAACTGCTCCAGCCGGGTCATGCCGCTCACCGC
TGGCAGCAGCCACTGTTCTAGGTCGGCCAGCAGGCTGTCGTCATCCATGGCTGGCCACTCCTCATCAGGC
AGCCACTCACGGGCGCAGCGCAGACGGGCCAGCAGCCCTTCGCTGCTCTCGTCCCAGGGCAGCACATGCA
GCCCCTTGCGGCGAATGCCTTGCAGCAGGCAGCGCCCTTTCTGCTCGTCAGAGAGTTCGGTCAGCGGTCG
CTGGCTCAGCACCAGCTCGCCGATCACCTGCTGGCGTTCGGCACGCACCCGCTCTTCCCGTTCATCCCAG
TCGAACCACTGCCGTTCGCTCACCAGTTCCGGCAGCAGGCGCACCAGCTCGTCCAGATCGACCTGTTCGG
CGATAAAAATGCGGCTGCCGCGATCGTTCTGCCCCATCTCGATGGCGATCAGCGCCCCTTGTCCCTGACA
GGGATGACCTTCTACCAGATCGCAGCTCACCCCACCACTGAGTTGATAGCGACTGCCACTACGCAGTTTG
CCGATGCGATCCGGCCAGGCGAGGGCACAGAGTAGCCCCAGCCACTGACCCTGCGCATTGGCAGGGCGCA
CGCCAAGCCGCTCAAACCAGCGGGCCGCCCCCTGACGCAGGGCACTTTGACGGCGGTGGAACAGCACAGA
GAGGCGCTCCGATCCTCGCAGATCTTCTTCCTGCAACGCCACCAGATAAGCGGCATCGGCGACAATCCCT
GTCAGACCCTCGGACTCCAGCTCGCGGGCTCGCAGCAGCATGCGGGCGAGGCGAGGGTGGGTACCGAAGG
CGGCCATGGCGCGGCCAGCGGCAGTGAGTTGTTGCTCACCCTCCGGCTTCATGGCGCCAAGAGCCACCAG
CAGCCGCTGGGCGCTGTCCACCGCAGCGGCGGGGGGCATGTCCAGCAGCGGCAGATCTTCCACCTTTGCC
CCCCACTGGGCGGCGTCCAGCAGCAGGCCGGTCAGCTCCTGAGTGAGAATATCTGGCGGGCTCTGCTCGG
CGAGCCGCTCCTGCACCTCGCTGCTCCAGAGCCGGTAGCAGACGCCGGGGCCGAGACGGCCAGCGCGGCC
TGCCCGCTGGGTGGCGGAGGATTTGGCTATCTGGCGAGTCTCCAGTCGGGTGACGCCGCTTTTCAGATCA
AAGGAGGCCCGTCGTTCCAGCCCGCTATCGATCACCACCGAAATCCCCTCGATGGTGAGGGAGGTCTCCG
CCACATTGGTGGTGAGCACCAGCTTACGGCGGCCCGCAGGCGCCGGTTCGATGGCCGCCTGCTGGGCCGC
CATATCGAGGCGGCCGTAGAGGGGAGAGAGGGTCACGTCGTCCGACAGTTTGTCTGCCAACCATTGCGCC
AGTCGCTCGATCTCACCCTGTCCCGGCAGAAAGACCAGCAGGCTGCCATCTTGCTCGGCCAGCGCTTCGA

GTACCACGGCGCCGACCTGCGGCTCCAGCCACTGCTGGCGATTGGCGGGGCGGTAGTGGTAATCGATCGG
GAAACCGCGCCCCTCGGAGCGAACCACAGGGGCATCCGGCAACAAGGTCTCGAGTGCCATGCCATCCAGG
GTTGCGGACATAATCAAGAGCTTGAGGTCATCGCGCAGCCCTTGCTGGCTCTCGATGGCGAAGGCCAGCG
CGGTATCTGCGTGCAGACTGCGTTCGTGGAACTCATCGAAGATGACCAGCGAGACGTCAGTCAGCTCAGG
GTCTTGTTGCAACATCCGGGTCAGCACCCCTTCGGTGACGATCTCGAGCCGGGTTGCTGAGCTGGTGCGG
CTCTCACCGCGTACCCGCAGGCCGATGCGCTCGCCCACCTTCTCCCCCAGCTGGCGCGCCAGAAAAGTAG
CGATATTGCGCGCCGCGAGGCGCCGTGGCTCCAGCATGATGATGCGGCCGGGCAGCCGGTTCTGACGCAC
CAGCTCCAGCGGCAGCAGGGTCGATTTGCCCGCGCCGGGTGGCGCCTGCAGGATCACGCTGGTGTGGCTG
TCGAGGGCGGCAAAGAGTTCGGGCAATACCGAAACGATGGGGAGCTGGGACAAAGCGATGAGCACCTTCT
GTAGCAACCGGTAAAACGGCGCCTATTCTGCCACAGCCACCCGTGCCACCGTGAGTGGATGTGCGCAGAT
GGTCACTCGGGCGGGAGGCTGGCGAGACATGGCCGGATGGCTGCCATAGAATGGGCCCATCCAATGAGCG
TGAAAAAGGCATTATCACCACATGGCCAAACTCTTCTTTGCCCTGCCCGTCAAGGAGCTCGCCCCCGAGC
TCATCGCCTGGCGCGATCAGCTGCCCTGGCCCGGTCAGCCGGTGCCGCAAGCCAACCTCCACCTGACCCT
GGCATTTCTGGGTGAGGCGGACGAGTTGACCACCCGCCGCCTGATCGCCGCCGTAGAGCGCCAGCACTGT
CCGCCGCTCTCCATCAGGCTGGATGAGACCGGCTGGTTCAGCCGGGCCAAGGCTGCCTGGATTGGCCCGA
AGGAGTGGCCCAACGAGCTGACCGTGCTGGCGAAGGCGCTGCGCCGCCACGGCGAGAAGTTGCGCCTTGG
CAATGGCGAGCAGGGTTATCGCCCCCATGTCACCCTGTCGCGCAAGGCGGGTGAGGCTCCGGCCATCCTG
CCTGCCCCAAATTTTCTGCTGCAGGCCGATGAGTTCTGCCTCTATCAATCCATCTCGACTCCGGACGGGG
TGCGCTACGAGCCGCTTGCCTGCTGGCCGCTGCGCGCCCGGGTAAAAGAAGACGCACGGAGGGCTAACCC
GTGATCTTTGATCCCCCCCTGCAATCGGGCCAGCTGGTGGCCCGCTACAAGCGCTTTCTGACCGATGTGC
AACTCGATAACGGCGAGGTCATCACTATCCACTGCGCCAATACCGGTGCCATGACCGGTTGTGCCGATCC
GGGCACCCGGGTCTGGTACTCCACCTCCGATAACCCCAAGCGCAAGCTACCCAACAGCTGGGAGATCGCC
GAGAGCCCGGCGGGCCACTTTATCTGCGTCAACACGGCGCGCGCCAACCAGATTGCCCGGGAGCTGATCG
AGCAGGAGGCCATTGCTCCGCTGGCGGGATACAGCCGGTTGCGCACCGAGGTGAAGTATGGCGAAGAGAA
CAGTCGCATCGATCTGTTGCTGGAAGATGACGAGAAGGGCAATTGTTACATCGAGGTAAAATCGGTCACC
CTGCTCGACGAACGTGAACATCCGGGTATGGGCTACTTCCCCGATGCGGTCACCGCCCGTGGTGCCAAGC
ACTTGCGCGAGTTGATGGCGATGAAAGCGGCGGGCCACAGGGCCGTGCTGCTCTTTATGGTGTTGCACTC
CGGAATAAGCCGGATGCGCCCTGCAACCCATATAGATCCGCACTATAGTCTGCTAATTGAGCAGGCAATC
GCGGCGGGGGTTGAAATTTTATGCTATCGGCCCCATGTTGGGGTGCAAGGCATGGTGGCTCAAGGGTTTG
TCCCGTTTGAATCAGGTCACCTGTTACCCGAGGGGAGTGCATAAATATTGGAGTGGGTAACATTATTGGT
ACGGCTCGTTTGCTTACCCGTTACCCTTCTGCTATAGATGTCGCCCTCAATTTATGGATGGCACGGGTAG
AGTGCTGAATTAGGAGACAGGGCATGCCAACAGGCGAAAACAGGAAATCTCTGGGCCCCCTGGCCATTGC
GGGTGTCGCGCCTTACCAGGAAAAGCCGGGTGAGGAGTACATGAATGACCAACAAAAGGCGCATTTTCGC
AAGATCCTGGGTGCCTGGCGCAACCAACTGATGCAGGAAGTTGATCGTACCGTCGACCATATGAAAGACG
AGGCTGCCAACTTCCCGGATCCCGTGGATCGTGCCGCGCAAGAAGAAGAGTTCGCTCTTGAACTGCGTAC
CCGCGACCGTGAACGCAAGCTGATCAAGAAGATCGAGAAGACCTTGCAGCTGCTTGAAGACGATGATTTC
GGTTACTGCGAACACTGCGGTATCGAGATCGGTATCCGTCGTCTGGAAGCCCGTCCGACTGCCGACCTCT
GTGTCGATTGCAAGACGCTGGCCGAGATCAAGGAAAAACAGATGGCCGGTTGATCCCGACCCTCTGGTAT
ATGAAAAAGGGAGCTCGCGGGCTCCCTTTTTTATTGAGCTTTTGGCGGTCGCACCCCAGAATGCAGCCTC
AATGAATCGCTTACCCGAATAGTCATGCCAGTGAATTCCCCATCCTCACCCCTTGCGCGCCCCTATGTCG
GCCGCTTTGCTCCCTCACCCTCAGGCCCGCTTCACTTCGGTTCGCTGATCGCCGCCCTCGGCAGCTTTTT
GCAGGCCAGAGCGCAGCAGGGACGTTGGTTGGTACGCATCGAGGATATCGATCCGCCGCGAGAGATGGCG
GGGGCTGCGGCCCTGATCCGCAAGACCCTTGAGGCATATGGCCTCGAGTGGGATGGGGAGGTGATGTTCC
AGAGCGCGCGCCACCGCCGTTATGACGAGATCATCGACCAGCTCTATCGGGCGGGGGATCTCTACTGGTG
CCGCTGCACCCGGCGAGAAATCATGGCCGCGGGCGGTCTCTACAGCGGCCACTGCCGCACCCTCGGGCTT
GCGGCTGAAGGTTGCGCCGCCCGGCTGCGCCAGCACCATCCGGTCTACCACTTCGACGATCGTTTGCAGG
GCCATATCGCGGTCCCTGCGGCGCTCGCCGAGGAGGATTTCATTATCCGCCGCCGCGACGGGCTCTACGC
CTACAACCTGGCGGTGGTGGTGGATGATATGGACAGCGGCATCACCGAGATAGTGCGCGGCGCCGATCTG
CTGGAGCCCACGGTGCGCCAGATTGCCCTCTACCAGACCCTAGGGGCACCGGTGCCCGACTGGGTGCACC
TGCCGCTGGCGGTGCTGGCAGATGGCAACAAGCTCTCCAAGCAGAACCATGCGCCTGCGCTGGCGCTTGA
TGAGGTGCGCCCGGCACTGTGGCAGGCGCTGCACTTCCTCGGCCAGTGCCCGCCGCCCGAGCTGATGGCG
AGCCGGATCGACGAGATCATCCGGTGGGGTATCGCCAACTGGCAGCTAGAACGGGTGCCAGCAAGCGAGA
AGATCCCGCTCGCGGATTGCTGAGTCGACCAGGTGATTTTTGTTTGATTGAGCGTAAATTGCCGCTGTTG
ATTGTGATTTTACCGGGATGATCCGTTTCAAACGGGATCAATTGTCAGCACGGTTTTTCATTTGGTCAGG
TGTGAGCTATGTGCAGATTGAGCGCTATTTTCGCTGGCAGACGGTTTCATGCGGGATCAATTGTCAGCAC
GGTTTTTCATTTGGTTAGGCGTGAGCTATGCGCAGGTAGACCGCTATTTTTTGGCCCGGTTTGATTCAAA
TGAGCTGAAGTAGCCACATGGTTTTTCATTTGTGCAGGCGTGAGCTATGATAGGCCGCTATTTTTTGGCC
TCCCGTCAATTTAGAAGTCATGAAGAGGTGTACCATTTTTTCCCAGATCGCAAACTTTTGCCGCAAGGTG
CTCGGCAAGGAAGACGGCGAGCAGTCGGTGGAATCCCCCATTCCAGCCCGTGTCGCCGGTCAACGTCGTA
CCTTAAGCCGTGATCAGCACCCTATTTCGCGCAAAGAGATCAGCGAGAATGCACTCAAGGTGCTCTATCG
CCTGAACAAGGGCGGCTACGAAGCCTACCTGGTCGGCGGCGGAGTCCGGGATCTGCTGCTCGGGAAGACG

CCCAAGGATTTCGACATCGCAACCGATGCGACGCCGGAGCAGATCAAGGAGCTGTTCAGCAACTGCCGCC
TGATTGGCCGTCGTTTCCGCCTAGCCCACATTGTCTTTGGTCGTGACGTGATCGAAGTGGCCACCTTCCG
GGGTCACCACCATCAGGTCAATACCAGCAAACATGTGTCGGCCCAGTCTGATGAAGGCATGCTGCTGCGC
GACAACGTCTACGGCACCATCGAAGAGGATGCCGAGCGCCGCGACTTTACAGTCAACGCCCTCTACTACA
GCGCCAAAGATTTCACCCTGCACGATTTCGAAGGGGGTCTGGAAGATCTGGCCGAGCGCAAGCTTGAGCT
GATCGGCGATCCCGAGACCCGTTATCGTGAAGATCCGGTGCGGATGCTGCGCGCCGTGCGCTTTGCCGCC
AAGCTGGATATGACCATCAGCCCGCGTACCGCCGCCCCCATCAAGGAGCTGGCGCCACTGCTGCAAGACA
TCCCGGCTGCCCGCCTGTTTGAGGAGACCCTCAAGCTGTTCCTGGCTGGCGACGGTCTGGCGACCTACAA
ACTGCTGCGCGAGTATGGTCTGTTCCAGCAGCTCTTCCCGCAAGTGGCTGCGCTGTTTACCCCGCACGGC
AACTCGCCCTACGAGCAGTTTATCGAGCAGGCACTGATCGATACCGATACCCGGGTTCGTGAAGATAAGC
GCGTCACTCCCGCCTTCCTCTACGCCACCTTGCTGTGGGGCTGCGTCGAGGCCCGTGGTCGGGTGCTGGA
GAACGAGAGCGGCCTGCCCTGGTATGACGCCTTCATGCTGGCGATCAACGAGGTGCTGGACAATCAGGTG
CGGATCATCGCCGTTCCCCGCCGTTTCACCACAGATGTCCGCGACATCTGGGCACTGCAACAGCGTCTGA
CCCGTCGTCAGGGCAAGCACCCCGAGCGCGCCATGGAGCATCCCAAGTTCCGCGCGGCGTTCGACTTCCT
GTTGCTGCGCAACCGGGTCGAGCGTGGTCTCGGCGAGCTGGCCAGCTGGTGGGAGCGCTATGTTGCCTCC
AATCCGGATGTGCGCCCGCAACTGCAGCGTGAAGCGACCCGTCGCCCGGCCAGCGGTGAGCGCGGCGAGG
CTCGCAGCCACGATGGCGAGAAGCGCAGCAGCAACAGCCGTAACCGCCGTCGCCCCCGTCGCCGCAAACC
GAAAGTGGCCGAGTGACAACAGACGTGATGACCGAGGTCTTCATCGCCATTGGTTCCAACCTGTCTGATC
CGCTGGGTCAGACTCGTCGCGCCGTTGCCGCGTTGGCAAGGTTGCCGGAATCTGTGCTGGTGGAGGCCTC
GTCGTTTTACAGCAGCCGCCCCATGGGGCCTGCTGACCAACCGGATTACGTCAATGCCGTGGCCCGGCTC
AATACCCGGCTCGCGCCCCTTGCGCTGCTGGATCAATTACAAAAAATCGAGCTGGAACAGGGACGTGTGC
GCAAAGATGAGCGGTGGGGGCCGAGAACTCTGGATCTCGATCTGCTGCTCTATGGGGATCAGGTGATCAA
CCATGAGCGTCTGATCGTACCCCACTACGGCATGAAGGAGCGGGAGTTCGTGCTGCTGCCCCTTGCCGAA
ATTGCGCCTGCTCTGGTGCTGCCCTGCGGCACCCCGCTCGCCCGACTGGTTGCCCGCTGCCCGCACAATG
ATTTGGCCATCATTGCCCCGCGTGCAGACGTACAGGAGCCTCTATGAGCAAGATCACCACCGCCAGCCTG
CTGAAGATGAAGCAGGAAGGTCAAAAGTTCACCGCCATCACCGCCTATGATGCCACCTTTGCCAAGCTGT
TCGACGACGAGGGGGCCCACGTGCTGCTTATCGGCGATTCGCTGGGCATGGTGCTGCAAGGGGGTCAGGA
TACCCTGGCGGTCAACATGGATGAGATGGTCTATCACACCCGCTGTGTGGCGCGCGGTGCCAGCAAGGCA
CTGGTGGTCGCCGATATGCCGTTCATGAGCTACGCCACCCCCGAGCAGACTTACCAGAACGCCGCCCGCC
TGATGGCGGCCGGTGCCCGCATGGTGAAGATGGAAGGGGGCGACTGGCTGTGTGACTCCATCCGCCACCT
TACCCGCAACGGGGTGCCGGTGTGTGGTCACCTCGGCCTCACTCCGCAATCCGTTCATGTCTTTGGTGGC
TTCAAGGTGCAGGGGCGCGATGAGTTTCAGGCACAGGAGATCTACCGTCAGGCCCTCGAACTGCAAGCGG
CCGGTATCCAGCTGCTGGTGCTCGAGTGTGTGCCCACTGCGCTGGCCGAGCGCATCACCAAGGCGCTTCG
CATCCCGGTGATCGGCATTGGTGCCGGCCCCGCGACCGACGGTCAGATTTTGGTGATGCACGATGCCTTT
GGCGTTACCTCCGGCTATGTGCCCAAGTTCACCAAGAACTTCCTGGCCGAGACCGGCGATGTGCGTGCCG
CCATCCGCCTCTATGTGCAGCAGGTGAGCGAGGGGAGCTTCCCCGGCCCGGAGCACTGCTTCAACTGATC
CTAGCTGTTGCATGATGGGGGCTGCGGCCCCCGTCTCTGCTTGTCAGCCTTGCACAGTTAATCCGTATAG
CTAATCCGTATCTTATTGATTCATCTAAAGGTGAACTGCTGATAGCCATGTTCGTGCCCCATCCCGCCCG
AGCGCTATCTGTGGATGCAACCCGTCTCTCTTTTTGATTTATCGGAAGGTGAACTGCTGATGTTGGTCGT
AAATAATCCCGTCGTTCTGCGTGAGCAGATAAGCCAATGGCGCCGTGAAGGGCGTGCCATCGCATTCGTG
CCCACCATGGGCAATCTGCATCAGGGCCACCTGACGCTGGTGAAGGAGGCGCACAGCCACGCCGAGAAGG
TGGTGGTCAGCATCTTCGTCAACCCGATGCAGTTCGATAAGGCCGAGGATCTTGCCAACTATCCGCGCAC
TCTGGAGCAGGATTGCGCTGCGCTGGCAGCGGCCGGTGTCGATATGGTGTTCACCCCGACCCCGGAGATC
ATGTATCCGCAGGGGCTGGCCAGCCAGACCTTCGTCGAGGTGCCGGGTCTCTCCAATCTGCTGGAGGGGG
CGCTGCGCCCGGGTCACTTCCGTGGTGTCAGTACCGTCGTCACCAAGCTGTTCAATCTGGTACAGCCGGA
TGTGGCCTGCTTTGGCCAGAAGGATTACCAGCAGTTGGCGCTTATTCGCAAGATGGTGGCCGACATGGCG
ATGCCCATCGAGATCGTCGGCGTGCCGACCGTGCGTGCCGAAGACGGGCTGGCACTCTCTTCCCGCAACG
GCTACCTGACTGCGGCCGAGCGTGCCATTGCCCCCGAGCTGGCGCGCACCATGAACTGGATCGCCGAGCA
GATCGAAGCGGGTGACCACCACCTGCCCTCCTTGGTGGCGCAGGCCAGCCAGCGTCTCGACAACGCCGGT
TTTCGTACCGATGCCATCGACATCGTCGATGCCGCCACCCTCGAATCCGCCACTGACGAGAGCGCACAGC
TGGTGATCCTGATGGCCGCTTATCTTGGCAAGGCCCGCCTTATCGACAATCAAGTGGTCACGCTGCAGCA
AGCCTGAGCCCGCCACAACCAGGAGGACCCCATGTCCAAGAAGGTTTACTGCATTGCCCAATTCCTGCCC
AAACCCGGTCAGGAGCAGGCCCTGTTTCAGGTGCTGCAGGCGCTCGAACCCAACACCCTGCGTGAGGATG
GTTGCCTGCAATATCGGGTAACCCGCCACATCGCGAGCCCGTTTGCCGAGGGGGAAAGTTTTCCCATCGT
GTTCAACGAGATTTGGCAAGATATGGCCTCTTTCGAGGCGCACTGCCAGCGCGCGGAGATCAAGGCTTTC
TTCGAGCGTTACTGTCTGGCGGAAGATGGTTTGGCCGCGAAGTGGAATGTCTGCGTCTATAGCGACGAAC
CCGAACAGTACGACGCCCCCGTAAAGAAGTAATTCCCCCGATTGCCAGCGGACGCCTTCCCGGGCGTCCG
CTGCTTTTTCACGCCTGCACTCTCCTGCATCCCGGCGTTTTGTTCTGCGGCCCTGCCCGCATCCCCCTTC
GTTTCAATCCACTTTCGGCCTGATGGCCGCTACAGGTATTCATGATGTTTGTCCTGATTGCATCGCTTGT
TTTCTGTCTGCTCTGTTTTGCTCTCACCCGGTTGCCCACAGGATTGCCGCTCTCGCGCAAGGTGCTTGCG
GGTCTGCTGTTTGGCGCCCTGTTCGGGCTGGCTCTGCAACTGGGCTGGGGCGCCGATGATGCCGGCGTCC

AGCAGACTCTGGAGTGGATCAATATCGTCGGCGGTGGCTATGTCAGCCTGCTCAAGGTGGTGATGATGCC
GCTGGTGTTCACCTCCATCCTCTCCGCCATCAACAAGCTGGAGCAGGCCCAGTCCCTCGGCAAGATCAGC
GCCGTGCTGATCGGCTCCATGCTGACCCTAGTGATGATCGCCGGTGTGGTGGGGATGACGGTGACCCATC
TGAGTGGTCTCACTGCCGAGGGGCTGCTGAGCCACGGTAGCGACACGGCAGCCCTGCAACAGAGCATGAG
CTCCATCGACAGTGCCCAGACCTTGCCTGCCTTGCTGCTCTCGCTGGTGCCCACCAACTTTGCCGCCGAT
ATGGCGGGTAGCCGCGGCCTTTCGGTGATCGGCGAGGTGATCTTTACCGTGCTGGCCGGGGTTGCCCTGC
TGCAGTTGAAGGCGGAGCATCCGCAGGCAGGGGCTCGCGCCGCCGTGGCCATCGACACCCTGCAACTGTG
GGTGATGAAGATGGTGCGGGTGGTGATTGCGCTCACCCCCTATGGCGTGATGGCACTGATCGCCCGGGTG
GTGGCTCACTATCCCCTGAGCGAGATCCTCAGCCTGCTCGGCTTTATTCTGGTCTGCTACGTCGCCATTC
TGGCGATGTTTGCGGTACACATGGTGATCCTGCTGCTGATCGGTCAGAACCCGCTGCACTATTTGCGGGA
AGTGTGGGGCGTGCTCACCTTCGCCTTTATCTCCCGCAGCAGCGCCGCCTCCATTCCGCTCAGCATCGAG
ACCCAGCAGCGTCTTGGTGTGCCCGCCAGCATCGCCAACTTCGCCGCCTCGTTCGGTGCCAACCTCGGCC
AGAACGGCTGTGCCGGCATCTATCCGGCCATGATGGTGGCGATGATTGCCCCCATGCTCGGTATCAACCC
GTTTGATCCCCTGTTCCTGCTGACTCTGCTGCCGGTGATTGCGCTGGGCTCCATCGGGGTGGCTGGCGTC
GGCGGTGGCGGCACCTTCGCCGCGCTGATCGTGCTCTCCACTCTCAACTTCCCCATCGCACTGGTGGGGG
TAATGATCGCCATTGAGCCGCTGGTGGACATGGGCCGTACCGCTCTCAACGTCAACGGCTCCATCATGGC
GGCCATGATCACCAAGCGGTTGCTGGGCAAACTGCAGCTGGCCGAGACGCAGGTGCTGGAGCAGCAGGCC
GCCAGCTAACCCCAGCGTTCCCCATGTCTAGTCCTGAAATAGGTTTACACATTTTCAACTAGCATTTCTC
TACTCAGAACCCCCTATGTACCGCATCGGGGGTTCTGTATTTCAACGCATGATGCGGCCGCTCCTCGTTG
TAAATATCCACCGCTTCACGCACCATTTCCCGCGCTTGCATCAAATCCTGCGGGCTTTGCAACAATAACT
CTCCTTTCAAAATCCCGTTCACCCGCTCCGCCAACGCATTCTGGTAACAGTCATACCCATCCGTCATCGA
GCATTTCACCCCGTACCGTTCATGTAGTGACTGATACAGCCCCGAGCAATACTGCACGCCGCGGTCTGAG
TGATGGATGAGTTCACCGCCGCCACGCCGTTGCTTCAGTGCTTTCTTGAACGCCATCGCCACCGACTTGG
CGTGCATCCCTTCGTGCACGTGATGGCCCACTATCTTGCGTGAGTACGCATCCGTCACCAGGCTCAGGTA
CAGCGGCCCGCTCCTGGCTGGCAGATAGGTGATATCAGCGACCCAGACCTGCTCCGGTGCTACCGGCGTA
ACCTGCTCTGGCCCCGCCTTGAGCAAGTTGGGATGACGGTAGAAACGGTGAAAACTGTGGGTGGTCTTGT
GATACGCCCGCTTAGGCTGCACCAGCAAGCGGTGCTCGGCCAATATCTGAAACAGCCTGTCCCGCCCTAC
CTTGAGCCCGCCATCCTCCTGACCCTGCAGCAGATAATGCAGCTTGCGAGTGCCCACCCGAGGTTGTCGC
AGTCGAACCTGACGCACGAAGCGCACCACCTGTAGCCCTTGTGCATGGCGTTCGTCGGCGACCCGATTGC
GCTTGTAGTAAGCCTGCCGGCTTATCCCCAGAAACAGGCAAGGTTAGTCTCTCGACTTGCCGCTGCGAGA
GGACTTGCCGGGTCGCTTTTTTACGATGGAGACGCCGTAGTCATTCTTCAGTACATCGACGACGGCCTCA
AAGAACTGGGCCTTCTGACTCATCAGCTCCAGCTGTTGCTCGAGTTCCTTGATGCGCTGTTCTGGCGTCT
GGTTGTCGGGGTCAGGCATGGTGATGCTCCTGCCGGCACGTACAGAAGCCCCCTGGCTCCAATCTTGCCG
ACCATGTTTACGCAACCATACCAGAACGGTGGAGCGGCCCTGAATGCCATAACGCACCTGAGCCTGTTTG
TAGGTGAGCTCGCCTTTTTCAATCTTCTCGACCAGTGACAGTTTAAAAGTCAGCGAGTAACCGTGCTGTG
TCCGTTTCACACCTTGTCCCATCGGGTTCTCCATTTATTGGAGGGAAAGGTGTAAACCATATTCAGGACG
GGACACCAATATGAAAGAAGCCCGCAGTTGCGGGCTTCTTTATGTCGACAGTGTTGTCTTGTGAGCAGAC
GCAGTTATTCGGCAGAGACGGGGGAACCGGCGCCTTTCCAGGCGAAGATGCCACCTGGCACCCGATAGAC
CTGTTGATAGCCCTGAGTCACCGCCCAGGCGGCAGCGTTGTGGCTGCGGCCGCACTTGACGAAGCCGCAG
TAGATCACCACAGGGCGGGACTTGTCTTCACCCAACAGCGCCTGGAACTGCTCCGCCGTGCCGCTCCCCT
CGACAATCTCGCTCCAGTTGTCACCGGCCTTGGCCTCCTTCACGAAGACAAAGTTCTTGGCGCCCGGGAT
GTGCTCTTTCTTGTAGGAGTCCTCATAGGGCATGGTGTCGATGATCACCAGCTCCTCGCCCTTGTCCATC
CGGGTTTTCAGCTCGGCCACGGTCAGCAGCTGATAGCCCCCCTGCTGCGCCTCCTGTGCCAGCTGCACAG
CCGATTGCTCAAGCTTCACCTCCTGCTCGAATTGGTTGTCGCCACAGCCCGCGAGGGCAAAGCTGGCGGC
GATCAGCGAGGCCACCAGAGTGCGGGATCGCAATGTCATCATGTCACTACTCCTTTGGAACGGAAAAAC
GGGGCGGGAAGTTCGCAGCAGGGCGCCCGCAAGCGTCAGTAGCAGCAGATCCCGCACAATGGCCTCCGGC
AGCGAGCTGAGATCAGCCCTGCCCAGACAGCCGCAAGAGATGTCGAGCCCGATGGCAACGCCATAGCCCA
ACACCGGAATAAACAGGGCGAGCAGCCCGATCACCAGCCAGAGTCCCCAGCGCCGGTTGGCGATGACCGC
AAGCCCTGCGCAGAGCTCAAGGGCAATCAGCCCCCAGGCGGCGAACGGGATTAGCGGCGGCCAGATCAGG
CCAAAGGCGCCGATCTGCTCGATGAACAGGGCGGGGGAGACCAGCTTGAACCCGGCGGAGGCGAGGAAGA
ATCCTCCCAGAGCCAGATTGGCCAGCATGCGCAACAATGCAGGCATGTTCTACCTCTTTATAGAGAGGGA
TGAATTCTAACAATTCCCGATTGAGCCGCAATGACTAAACCCATGCCAAACAAGATAAATCTCGATTTTG
CGAGACAGTGACCAGAATGGGGGCGCTAGACCCTGATGGTGGCGGTTGTGGAGGCGGGATGCTGTGGCTG
TGTCGATTGCTCCCTGTCGTGCTGGTGGGTCAGCAGTTCGGCAGCCAGCTCGATGGCCCGCTCGAGCAGT
AGCAGCAGGCTGTCGACCGTCAGGTGATCGAGCCACTCGCGGGCCCAGATCAGGGTCTCTCCTTGTTCGG
TCTGATCCAGCAGAAACACTGGCTCGTCGCCAAGGTGGTTGAGCAAGGGCGTGTTGGGCATGGCGTGGCC
CGGTAGCAGCAGATAGATCACCAGATGGCCCTCATCCTCTGCCAGCGTCAGCACCAGTTCGTCGTTGATG
GCGAGGGTGGTGGCTGTCTGTCCGGCCACCGGGAGCGGAAGTCCAAGTTGTGTGTACAGGGCGGTGATGA
GAGCGTGATACATGGCCAAACCTCGGGCAGGGAGAGAGTGACACCCTATGCGGCTGATCGGTGGCTGCTT
ATCACTTGTCGTCATTTTTTTAGCGTTTTTGGTCGGCGGGTCGGGTGATTGTGCCGAAATTGTGAGCTGA
CAGACATCTTTTACGGGTCAGTCCGAATCCTAAAAAAGCGGGACCATCGCTGACAGGGTGACAAGGGGGG

GCGGCGTACGCTGAGGATCCCCTCTCTCATCAACGCAGGTTTCTTGTCATGAAAATTGCCTCCCCGGATA
TCGATGTAAGCCGTCATCAGCAGACAACCCCTGATGCGACACCTCCTGCTACTGCTCGCCTTGGCAACGC
CAGACTACGGCTGCTGGATGCCGGTAACCGGGAGTTCGAGAGGGGGATCCGCCGCGGTATTGGCCATCGG
CAGAGCCCGGAACTCAAGGCGGTCAAGCAGGCAGTGCGGCACTATCAGGCCCAGCCAACGCCTTCACTGG
CAGGGCTGGCGAAGATTGAACAGGCGCTGCAGTTCTGGATCGACTCGGGCAAGAACCGCTCTCGTCAGCA
GGATCCTGTGTTGCAAAAACTGGTCGATGACATTGCTGTCCGCCGTCAGGCGTTGGCGCAGGGTGGCAAG
CCTCCCTCATTTCAGCTGGCGTGGGTCTGCAACGAGCAGGGGAGGGTCAATCAGCCTATCGAGAGTAACC
AGCAGATCCGCCAGTGGTGCAGCGAGTTGGCTCGCCTGCAACAGGAGGGGGCCAACGACTTGTTCAAGCG
GGAGCTGCAAGGAGGGCTCGATCTGATGTTCGGGGTGAGTGAGGGGATGATGGCGCGGCGGCTGGAGCGG
GCGCCGACCCGTGAAGAGGTCGCTACAAGTCACGAGTGGCAGCTGTTTTGCGGGACCTTGCGTCAGGCTC
TGCCCGCACTGGGTGAGCTCAAGCCCCAAGCCACTGCCGAGGAGAATGGTCGCGATATCCTGCTTACCCA
CAGTCAGTTGCTGGAGCTCGGCCACCTCAAGGAGGTTCTGGATCTCAGCATTCAGCAGCAGGTGAGCAAG
CAGGGGGAGATTGGCCTGCTCAGGACCGAGCTGCTGACCGCGCTGGTGGATGGACTGGATGAGCCCTGGC
TTGAGCGCGAGGCGCAGGAGAAGGCGCTCTGGCAGGGAGAGGCCGCATCAGGAAAGTACTATCAGAGTGG
TGTCCGGGCCGAGCTCAAGAGCCCTCTCTCTGCTGCCAGCAAGGAGCTGAAACCGGGTGAGCCCGTGAGC
CACAAGGCCTTTGGCGGGCTCGGGCTGGTCAGTGCCAGTGCGCTGGTGGAGGCTTTCGCCAAGATCGAGA
TTGGCAAGTGGGAGGGGCGTCGCGATACGCCGGTAGGAGAGTTTGCCGGCAAGGTGGCTCTGACCCAGGA
GAACAAGGCGAGTGCCAAGGCCGAGCTGGTGATTGGCAAGGCGGAGGGCTCATTGGTGTTCAATCCGGTG
AACAAGATCGTCAATGATACCGACGGATCGCGCTACACCCCGGGGCCCCATGACCTTAGTCTGCTGCGCA
TCGGGGCCAGTGCCGAGCTGGTGAGCAGGCTGGGGCTGTCGGTTGAGGGGAGCTGGACTATCGGCAACCT
GCTTAACGCCAAGCTGGCAGGCTCTGCCGAGGTGGTCGCCAATGCCAAGCTGGAGGCCGGTGCTGCGGCT
ATCATGACCCCAAATGGGCCGGTGGTCAGCGTGACTGCCAACGCCTCGGCTTTTGCTGGTATGAAAGCTG
AGGGGAGTGCCCAGCTCAATCTGCTCAAGAGCCAGTATGCGGATGCCTATAGCCTTTCGGCCAAGGTACG
CGCCTCCGCCAATCTGGGTGTCGGTGCATCGGCCGGTGCCACCGCGGCTTTCGGGCCACAGGGAGCCAAG
TTCCATACCACCCTTGGCGTGACACTGGGGGTGGGGTCCGATGTCGGTTGGGAGGGCGGAGTCAACGCCA
TGGCCACCAAGGCTTATGCACTGGAGCTGGCCGCTCGTGCGGTAAGCAACTTTGATCAGCGCAAAGGGGA
GCAGCTAGATGGACTGGCGATGGCTGCAGGGAATGAAAACCGTCTCTTCGAACTGGCCCAGCGGGTCGAT
AACCGGATTGTCACTCTCGCCACCCAGTTGGAGGAACTGCAGCTGGCGGCCAGCGAAAACCGGATCCGGA
TGCAGGAGAGCCTGATCGATCCCGAGCTCGCTGCTGGTCGCACCGCCATGGCGCAGGCTATCGACGATGC
CTTCGGTCAGGAGCGGGTCGATGAGCAGGGGGGCGTCACGGTTCCCGAGCGGGCTGCCGAGCGCAACGAG
AAGCTTGAGCTCACCGCCCGGTTGCTCAAGGATCACGCCATCCGGGTCTAAGTCATCCCGAGGCTGGCAG
GACAATGAAAACGGGCACCGCGAGGTGCCCGTTTTGGCAATCAGTGGCGCAGTCCGGTACCTCGGGAGAT
AAGCCACCAGGCGATACCGTAGAGGGCCACGATAAAGCCGATGATGATGAGGTAGGCGGTGCCGAGCGGC
ACATCGGAGATGCCGAGGAAGCCGTAACGGAACGCGTTCACCATGTAGATGATGGGGTTCACCTGGGAGA
CACTCTGCCAGAAGGGGGGGCAGCAGGCTGATGGAGTAGAACACCCCGCCCAGATAGGTGAGCGGTGTCAG
CACGAAGGTGGGGATGATGCTGATGTCGTCGAAGGTCTTGGCAAACACCGCGTTGATAAGGCCACCCAGC
GAGAAGAGAATCGCGGTGAGCAGCACGGTGAAGCAGACGCTGAACAGGTGGTGGATCTGCAGCGGCACGA
AGAACAGCGACACCAGGGTGACGATAAGGCCGACACAGAGGCCGCGCGCTACGCCACCGCCCACGTAACC
GGCGATGATGACGTAGTTGGGCACCGGCGCGACCAGCATCTCCTCGATATTGCGCTGGAACTTGGCGCTG
AAGAAGGAGGAGGCGACGTTGGAGTAGGAGTTGGTGATCACCGACATCATGATGAGGCCGGGCACGATGA
ACTCCATGTAGGTGAAACCGCCCATGTCACCGATGCGCGAACCGATCAGGTTGCCGAAGATGACGAAGTA
GAGGCTCATGGTGATGGCCGGTGGCACCAGGGTCTGGATCCAGATCCGGGTAAAGCGGCTCACCTCCTTG
GCCAGAATGCTCTTGAAGGCGATGTAGTAGATGGCCAGATTCATGCTTTTCTCCCCTCTTCGACCAGATT
GACGAACAGCTCTTCCAGCCGGTTGGCTTTGTTTCGCATGCTCACGACCTGCACCTGCTGGTTCGACAAC
TGCTCGAACAGGCTGTTGAGTGACTGGCTCTTGTCGAGATCAACTTCCAGGGTGCGTTCATCCGGCATGC
GGCCGTTGAACTCTGGTACCTCAGGTACGGCAGAACCTGGCGCAAGGTCGAGCAGGAAGGTCTCGCGACC
CAGCTTGCCCAGCAGATTCTTCATGCTGGTGTTCTCGATCAGACGACCCTTGTCGATGATGCCGATGTTG
CGGCAGAGCATCTCGGCCTCTTCCAGATAGTGGGTGGTGAGAATAATAGTGACGCCCTGCTCGTTGAGAT
CCTTGAGGAAGGTCCACATGGAGCGGCGGATCTCGATATCGACCCCGGCGGTCGGTTCATCGAGGATCAG
CAGTTTGGGTTCGTGCATCAGGGCGCGGGCGATCATCAGACGGCGCTTCATGCCGCCTGAGAGGGTGCGG
GCAGGCATGTCGCGCTTCTCCCACAGATCGAGCTGGCGCAGGTACTTCTCGGCGCGGATCTTGGCTTCCG
CCTTGGGCACGCCGTAGAAACCGGCCTGATGGGTGACAATCTGGATCACCTTCTCGAACTGGCTGAAGTT
GAACTCCTGCGGCACCAGTCCGAGCTGGGCCTTGGCCAGTTCGAGATCGCTGTCGATGTCGTAGCCAAAC
ACCTTCACCTTGCCGGCGCTTTTGTTCACCAGCGAGCTGATGACGCCGATAGTGGTGGATTTGCCCGCGC
CGTTCGGGCCTAGCAGGGCGAAGAAATCCCCCTGCCTGACGGTAAGATCGATGCCTTTAAGGGCTTCGAC
GCCCCCCTGATAGGTCTTTTTCAGGCCGCTGATTTCCAGTGCGTTCATTGCAACCTCTGTGATGGTTGAA
TCCGGCTCGATACAGAAAAGGCGGCCAAGGCCGCCTGCACAGAGTAAAGGGCGCAAGGCCGCTTCACCCG
TGATATTCCTGAACCTTGCTTACTCCTGGGGTACCACTTTGCCGATAAACGGCAGGTTGCGGTACTTCTG
GGCGTAGTCGATGCCGCAGCCGACCACGAATTCGTCCGGAATGGCGAAGCCAATCCAGTCGACCGGCACC
TGCACTTCGCGGCGGGAGGGTTTGTCCAGCAGGGTGCAGATGGCTAGGGATTTCGGCTCGCGCAGGGAGA
GGATCTCGCGCACCTTGTTGAGGGTGTAACCGGTGTCGATGATGTCTTCCACGATCACCACATCCTTGCC

CTTGATGTCATCGTCCAGGTCTTTGAGGATGCGGACGTCACGGGTGCTGTGCATGCCGGAGCCGTAGCTG
GAGGCGGTCATGAAGTCCAGGGTGATGGGCAGCTGGCACTGGCGGCAGAGGTCGGCCAGGAAGATGCAGG
AACCGCGCAGCAGCGCAATCATCACGACTTCGTCAGAACCCTGATAGCGGGCGGTGATCTCTTCACCCAA
TTTGGCGATCCGGGCGGCTACTTCAGCCTCGGAGATCATCACCTCGACGGTGTGTTTCATCACTTACTCC
CATAAGGCCAAAGGGGATTTGGCCGTTCATGAATATCAAAACCGATAATTCTACCACTCATGTTGAAGGG
TGCTAGAGATACGTGGTGCTTTTATCGGCTTATTTTAATTGATAGAGTTAGCAGGCACTCAGGCATGATT
AGTTAAAATACTATAAACACCATAATCAATAACTATTAGATGATAAAAACTATGGAACCACGCGCAGAAA
TAAGACGTCGTACCCGGCTGTCGCCGGAAGCTCGTCGCTCCCAATTGATGGAATGTGCCATCGAGGTGTT
CTCCCGTCGCGGCATTGGCCGTGCAGGTCACGCCGAGATAGCCGAACAGGCCAAGGTCTCTGTGGCTACC
GTCTTCAACTATTTCAACACCCGCGAGGAGTTGGTAGATGAGGTGCTGGCCGAGATCGAACGCTTTGTGA
ACCAGATGCTGTCACAGGCATACGGTGACCAGGGCTCCATTTTCGACAAGATCCAGCGTCATGTACGCCT
CTCTGTCGATGCCGCCTACGATCAACCGGATTATGCCGGTATCTGGCTGGAGTGGAGCTCATCGGTTCGG
GAAGAGGTATGGCCCCGCTATACCCGGCTGCTCGATCAGTGCCTTGAGCTGATCGCCAGCGAGCTGCAAA
CCGCCATGGATGCAGGGGAGATCACCAGCGTGCTGTCAGCACAAGATCTGGCCCGCTCTCTGACCGGTTT
TGGCTATGTGATGATGCAGATGATCAATCAACCTCAGCGCCCGGTGCGCCAAGAGGTGACTGATTTCCTG
TTCAAGTACGTGACGGCGCCAATTCAGGCTCGCTAACACTGCTTAACGAAAAAACGGCGTCACTCATTGA
GTGGCGCCGTTTTTTTATGGCCGGATATGGCGGTCACCTCAATGGTGGCCATAGCCCCAGCGGTGGGTCA
ACTCATGCTCGACACCCAGATGATCGAGGATGCGGGCGACCATAAAGTCCACCAGATCCTCGATGCTTTT
CGGCTCATGATAGAAACCGGGGGCTGCGGGCATGATGGTCACCCCGAGCCGTGCCAGTTTCAGCATGTTC
TCCAGATGGATCGCCGAGAAGGGGGTTTCGCGGGGCACCAGGATCAGTTGGCCGCGCTCCTTGATCACCA
CATCCGCTGCCCGCTCCAGCAGGTTGTCCGAGGTGCCGTTGGCAATGGCCGAGACAGTGCCCATTGAGCA
GGGGCAGACCACCATCTGTTTGGGCGCGGCCGAACCGGAGGCCACCGGCGAGAACCACTCCTCCTTGCCG
CAGGCGACAATCTGCCCCTCCTTGGCGCCATATTGGCGACAAAGATAGGCAGAGAGCTCTTTCGGCGAGC
TGGGCCAATCCTGTTGCTGCTCGGTCTTGAGCACTACTCTCGCCGCCGATGAGGCGAGCAGATAGACCCG
ATAGTCCGCCTGCACCAGACACTGGAGCAGGCGCAGGGCGTAGGGGGCGCCGGAGGCGCCGGTCAGCGCC
AGGGTGATCGCTTTGTTCATAGTTCGCCGTGGTAATCCTTGAGGCGGGCGATCAGCTTGTCATGAATGCC
ACCGAAGCCGCCGTTGCTCATGATCAGAATATGGTCGCCATCGCGGCTCTCGGCCACCAGCCGGTTGATC
AGGGTCTCCAGGTCGTTGAATACCTTGGCCGGACGGGTCATATGGGCCGTCACCTCGCCGAGATCCCAGC
CGATGTTGGGCGGCTGGAAGAAGAACACCTCGTCGGCGCCATCGAAGCAGCCAGCCAGCTCCTCCTTGTG
AACCCCCAGCTTCATGGTGTTGGAGCGTGGCTCCAGCACCGCCAGAATACGGGCTTCTCCTACCTTGGCG
CGCAGGCCGCCGAGGGTGGTCTCGATGGCGGTGGGGTGGTGGGCAAAGTCGTCATAGACGCTGATGCCGG
CCACCTCGCCCTTGAGCTCCATCCGCCGCTTGGGGGACTTGAACTGGCAGAGGGCTGCGATGCCATGCTC
CACCATCACGCCAGCATGACGTGCGGCAGCGATGGCCATCAGACCGTTGTTGACGTTGTGCTCGCCGATG
CACTCCCAGTGCACCTCGCCCTGTTTTTCACCATCCAGCCACACCTCGAAGGCGGAGCCGTCATCGGCCA
GCTTGACCGCTTTCCACTTGGCGTCATCCTGACCCACCAGCTCCATTTCGCTCCAGCAGCCCATCTTGCG
CACGGCGGTGAGGTTGTCATCGGCTTGCGGGAACAGCACCCGGCCGTTGCTCGGCACGGTGCGCATCAGG
TGGTGGAACTGGCGCTGGATCGCGGCGAGATCCGGGAAGATGTCCGCGTGGTCGAACTCCAGGTTGTTCA
TCACCAGGGTGCGCGGGTGGTAGTGGACGAACTTGGAGCGTTTGTCGAAGAAGGCGCAGTCATACTCGTC
CGCTTCGATGACAAAGAAGGGGGCAGAGCCGAGACGGGCGGAAACCGGGAAGTTGCCCGGCACGCCGCCG
ATCAGGAATCCGGGCTCCAGCTTGGCGTACTCCAGCACCCAGGCCAGCATGCTGGCGGTGGTGGTCTTGC
CATGGGTGCCGGCGACGGCCAGTACCCAGCGATCCTGCAGCACGTGTTCCAGCAGCCACTGGGGGCCGGA
GATGTAGGGTAGATTGCGGTCCAGCACATATTCAACACAGGGGTTGCCGCGGCTCATGGCGTTGCCAATC
ACCACCAGATCCGGCGCCGGATCGAGTTGGGAGGGGTCATAGCCTTCGGTCAGTTCGATGCCCTGTTGCT
CGAGCTGGGTGCTCATGGGGGGATAGACATTGGCATCCGAACCGGTCACCCGGTAACCAAGCTGTTTTGC
CAACACCGCCAGACCACCCATAAAGGTGCCGCAGATCCCGAGAATGTGAATATGCATACCGCTTCCTTTC
GCAGTAAAGGGAAGTGCGCATTCTAGGGGGTGTTGCGGTGGTGGCACAAGCCGCACTCATGGTGAGAGAC
CCACAGATGCAGCACTGGAGGCAGAAATCTTGGCGCTGTTGGGATATGCGAGATTGGAATGGGTTGGAAG
GCTTTATTTGAGACTTGATATTGTGTCGCACTTTCTTACAATTCTTCGGTTTTGTGACGCACGCTACAGT
TTTACGATATTGCCCTTTTCCAAAGGAGAGTCCCCATGGGCAAGTTCCTGCTTTGCTATCTGGTCGGTGG
TCTGGTGGCCATCAGTTGGGCTGCGCTGCCCGCTCACGGTCCCTGGGATCAGTGGGACCTCGCGATTTTT
CATACCGTCAACGGCTGGCTCGGCCAGTCGGCTCTGTGGGCCGATCTGGTGGCCATCACCAATAACCGCC
TGTTCGATCTGGCGGTGCTGGCCTGTATGGGGCTGATCCTGGCCCGCTGCTTCTTCGCCCGGGATTTGGC
GGGTCGTCGCCAGCTGGTGGCAATGGGCATAGTGATGTTGCTGAGCGCACTGGTGATCAACCAGCTTGGT
CACCGATTGCCGGTGGAGCGACCCAGCCCGACCCTGATGGTGGCCGATGCGCTGCGGGTGACCCAGATCA
GCGCTATTCCGACCAAGGACTCTTCCGGTGACAGCTTCCCCGGCGATCACGCCCTGTTCCTGATGATCTT
CGCCGGCTACGCCTTGCGATATCTGCCGCGCTGGGCGGGAGTCACCGCATTGCTGATGGTGCCGATCTTT
TCCGCGCCGCGCATTCTGGCTGGCGCCCACTGGTTTACCGACGTCTACGTCGGCGCGCTGGGGCTGGCGA
CCCTCTGTCTGCCCTGGCTGCTGTTGACCCCGCTGGCGGATCGGCTGATAGACCGCATTGCCCCCCGATT
GGCCCGTGTGCCGCTGCTGCGCAGTCAGACCCACTGATTGAACGTCGCAAAATAAAACGCCCCGACATGA
AAATGTCGGGGCGTTTTGTTATCCACCCGCGAGAGATCAGCCTTGATAGCCGTTCGGGTTGTTGCTTTGC
CAGCGCCAGGTATCGACCATCATCTGCTCCAGACCGCGCTCTGCTTGCCAGCCCAGCTCGGCACGGGCCA

GCGTCGGCTCTGCCCAGCACTCGGCGATGTCGCCCGGGCGACGGGGTTTGATCTGGTAGGGAATGGGTCG
GCCGCTGGCCGCTTCAAACGCCTTGACCATCTCCAGTACCGAATAGCCCTGACCTGTGCCCAGGTTGTAG
GTGAAGACGCCGGTGTCACTCTTGATGCGTGCCAGTGCTTTCAGGTGACCGATGGCGAGATCCACCACAT
GGATGTAGTCGCGCACGCCGGTGCCATCCGGGGTCGGGTAGTCGTTGCCAAATACCCCCAGCTCCTTGAG
CTTGCCTACACCCACCTGGCTGATATAGGGCAGCAGGTTGTTGGGGATGCCGTTGGGATCTTCACCAATC
AGGCCGCTCTCGTGGGCGCCGACCGGGTTGAAGTAACGCAGCAGTACGATAGCCCAGCGGGGATCGGATT
TGGCGAGATCACGGAGGATCTCCTCCACCATCAGCTTGGAGCGGCCATAGGGGTTGGTGGCGCTGGTGGG
GAAATCTTCCCGCAGCGGCACCGAGGCGGGATCGCCATAGACGGTGGCCGAGGAGCTGAATACCAGCCGG
AAAACGCCGGCCTTGGCCATCTCTTCGCACAGCACCAGAGTGCCGCTGATGTTGTTCTGGTAGTAGGTGA
GCGGGATCTGGCTCGACTCCCCGACCGCCTTGAGACCGGCGAAGTGGATGACCGATTCGATCTGGTGGTT
GGCGAACAGCTGCTGCAGGCAGGCTCTGTCCAGCACATCCCCTTCGACGAAGGTGACCGGCTTGCCGGTG
ATCTGCTCGACCCGCTTGAGTGACTCCGGTGAGGAGTTGGAGAGGTTGTCGAGTACCACGACCTGCTGCC
CCGCGCCGAGCAGCTCAACCAGGGTGTGGGAGCCGATATAGCCGGCGCCCCCTGTGACCAGTATTGTCAT
TTTTTATCCCGTGTTCTGGTGAAACTGGGGCAGAGTCTAGCGTCCCTGTCTACTTGGGAAAAGGTTGGGA
TACCGATGACGCCTTCACCACGCCTCAATAATCGAGACTGATATCCCCTTTCGCGGTGCAGGAGCAGGCC
AGAGCAACGCCGCTCTCCAGATCCTGAGCACTCAAGGTCATCACGCTCTGGCGCTCGATGTCACCTGTGG
TGGTGCAACGACAGCTGCCACATACCCCGGCGCGGCAAGCGGCTATCATGGTTTCGCCCGCTGCTTCCAG
CGCCGCCAGCAGAGTCTGACCCGGCAGGATTTTCACCTTTTTGCCGCTCTTTTTCAGGGTCAGCCAGAAG
TGGCCGCTGGTGGTCGTGGTTGGCTCTACAGGAGGAAGACCAAAGGACTCCTGATGCAGCTGCTCCGGCG
GCAATCCCAGCTCTGCCAGCATGGTGCTGACCGCCGCCATATAGGGGGCCGGGCCGCAGAGATAGACATG
GCGTTCGCGCAGATCTGGGGCCAGTTCGGCCAGCATGGCGGGGGTGAGTCGGCCCACCCAGGGATGAGCC
TTCGGCGCCTCTTCCAGTATCAGGGCATGGAGCACGCCTGAATGAGCCGCAGCCAGAGTAGCCAACTCGT
CTGCGAAGATCACATCTGCTGGCGAGCGGGCGCTATGGATAAAGCGGATATCGGCATCCGGCCGTTTGGC
CAGCTCGTCCCGCAACATGGCCCACATGGGGGTGATGCCGGAACCGGCGCTCAAGAGCAGTGGTCGTTCG
CAGGGCAAGGCGGTCAGGTTGAAGTCGCCAAAGGGACCATCCACCCGGATCTCGTCGCCCACTTGCAGGG
TCTGGTGCTGATGGCGAGAGACCCGTCCCACATCCTTGATGGTGACCTGCCAGCATGCATCCTGCGGGCT
GGAGGAGAGGGTATAGGCGCGGCACTCCGGCAGGCCATCGATCTCGGTGTGCAGGGTGATGCACTGGCCA
GCCAGTACGGGAGGGAGGCTGCCGGAAAGCGGCGCCAGCTGCCAGCTCACTACGTCGTGGGTGTTCTGTT
GGCGGCCAATGCAGGTGAGCGTGAGAGAGGTCATCACAAGGCTCCTTAGGTTTGAAACGGTTCGAAAAAC
GTCTGGTGAAAAAGCCGGATCCAGAAAAAAGGCGAGCCGGGCGGCTCGCCAAACGCTTACAACATGAAGT
GCCGATCAGGCCGCCAGGATCTCGGCCAGGTCAGCCTCGGCCGTGGTGGTCCCTTTCAAGCCAAACTGCT
CCTCCAGCACCTTGAGCAGAGCCGGGGTGAGGAAGGCGGGGGCGGTCGGGCCGGTACGAATGTCCTTCAC
CCCGAGGGAGAGCAGGGTGAGCAGAATGACGATGGCTTTTTGCTCGAACCAGGAGAGCACCAGCGTCAGC
GGCAGATCGTTGACCCCGCACTCGAACGCCTCGGAGAGTGCCAGCGCCAACTGGATGGCGGAGTAGGCAT
CGTTGCACTGACCCACGTCCAGCAGACGCGGAATGCCGCCGATGTCGCCAAAGTCGAGCTTGTTGAACTT
GTATTTGCCACAGCCCAGCGTGAGCAGCAGGCTGTCGCGCGGCGCCGCCTTGGCAAACTCGGTGAAGTAG
GCGCGCTCGGCCTTGTCGCCGTCACAGCCACCCACTAGGAAGAAGTGGCTGATCTCCCCGGCCTTGACCT
TCTCGATCACCGTGGGTGCTGCCTGCATCAGTGCGTTGCGGGCAAAGCCGATGGTGATGAAGTGCTCGAG
CTCGGTGTGCTTGAAGCCTTCCAGCGCTTGGGCCTTGGCGATCACGGCGGAGAAATCATCCCCCTCCAGA
TGGGTGACGCCCGGCCAGCCGACGATGGAGCGGGTGAAGATGCGATCCGAGTAGTTGCCCACATTCGGGT
CGATGATGCAGTTGGAGGTCATCACTACGGCGCCCGGGAAGTTGGCAAACTCTTTCTGCTGGTTCTGCCA
GGCGGAGCCGTAGTTACCCACCAGATGGGGGTACTGCTTGAAGAAGGGATAGGCTAGCGCAGGCAGCATC
TCACCATGGGTGTAGACGTTGATGCCGGTTCCCTTGGTCTGCTCCAGAATGAGCTTGAGATCCATCATGT
CGTGGCCGGAGACCAGAATGCACTTGCCGGAGATGGGGGTTACGCGTACCTGGGTGGGTTCCGGATGGCC
GAAGGAGCTGGTTTCACCGAGATCCAGCATCTCCATGATTTTGAAGTTGAGCTGACCGATCTCCATGGCA
CAGCTGAACAGCCGACCCATTTCGCTCGGGTCTGTGCTGAGCCAGCTCATGATGCGGTGGAATTCGGCGG
CCACCTCGTCACTCTGCTGATCCAGCACCCGGGCGTGCTCCATATAGGCGGCGGCGCCCTTGAGGCCGTA
AAGACAGAGCAGACGCAGCCCCATGATGTCTTCGTTCACTTCGCTCTTGCCCCGATTGGGGGCGGCTGGT
GCGGCATGGGCAAGTTGTTCCAGCAGTTCGGCACCGGGTTCGAAGTAAGCGGACTGTGGCAAATCGTTGA
CCTGCACCGCCATGGCGGTCAGACGATCGGCCAACTGCTGGCGATAGGCGAGGGCCTGATTGACGTAGGC
CACGATGCGATTCGAGTCGAAGTTGACGTTGGTGAGGGTGGCGAAGAAGGCCTTGGGCACGAAAGCATCG
ATCTCGCGATCCATGATGCCCTGCTCGCGGGCGGCCAGCGCCCAGGCGCTGAGCCCCTGCAGGGTGTAGA
TCAGCACATCCTGCAGATCCGAGGTTTCCGCCGTCTTGCCGCACATACCTTGTGCGTAGGCACAGCCGTT
GCCTGCCGGGGTACGAATTGTCTGTTCACATTGCACACAAAACATCATGCCTCTCCTAATTGGACATTTC
AGTTGTCTGTTTTGTGGTGATTCTATTCCTCTCGCGAAAAACTTAATAGGCATTCAATTTGCCAATTAAG
ATTCCCTTGATGCAGATCAAAAACAAAAAAAGCCGCCCAAACAGGGCGGCAAGCGGGTGGATAAAAGGAG
AGACAACTCAGGTTTGCAACAGAACATAACAATCAGACGGATTGGGCCGTGGTCTGGTCAGGAGCTTCCG
GCCCGGTACTTACTCAAGCAATCATACGTACGCTGTAGTCAGCTTGGTTCACTATCAGGCCAGATAAGCG
CGCAGCATCCAGACCAGTTTTTCCTGCTGCTGCACATAGTCGGAGAGGATGGAGGCAGTGCCTTCATCAC
CGGCTTCGGCGGCCTGACCCAGAATACGGCGCTGGGCGACCAGCAGTTCGCGGAAGCTATCCAGCAAGGA
TTCGACACAGGCGCGGCCATCGTGCAGCCCTTTTTGCTCCGGGATGGCAGAGGCTTTCAGGTATTCACTG

AAGCTGTGCATTGGCACGCCATCCAAGGTCAGGATCCGCTCGGCCAGCGCATCGACTTTCAGCAGCAGGT
CGTTGTAAATCTCTTCAAATTTCAGGTGCAGTTCGAAGAACTGGTTGCCACGGATATTCCAGTGGAAGCC
GCGCACGTTCATGTAGAGGATCTGGTAGCTGGCCAGCAATTTGTTCAACTCGGCTGCCAGGGCTTGTGAT
TGAGCAGTATCAAGACCAATCGGGCTTTTCATCATTCACCTCCAGATTTGGAAATCGGTTTTAAATTCTC
TTTCGGAACGCAGTCAGAATAACGCCTTGCAAAATGCAGAGACAATTGCTTGTGCCTAACTGTTTGATAG
CTAAAGGCTATCAAACAGTACGCAACAACAGGGGCAATGGATGAGGATTAGGGGAAGTATTTTTCCAGCA
GCGCCATCAGGATGGCCTGGCACTCGCTGTCCGGTTCGCTATCGACCTTGCTGGCGCGAAAGTGGAGCTG
AAAGGCGCCCATGACGGCGCGGCTCTGCTCATCCCACTGGCCGCTCCGGGGCAGGCCATAGCCATAGAGG
GCCAGCTGCTGCTGCCACATGGCCGCATCCCAGTCGGGCAAAGGGCTGGCCAGTAACTCGGCCACCCGAT
CTTCATCGGGCCAGGCGCCGACTCCGTACGTCAGATATAGCTGGCGCCAGGGGAAGTGAGGGCCGGGATC
CTGCTTGCGCTCGGGGGCGATATCGCTGTGGCCCAGCACCTGGGTCGGTGGAATCTGGTAGCTGGCGACC
AGCTCCCGGCTGAGGGCGCCGACGGCAGCTATCTGCTGCGGGGTATAGGGTTGCCAGCGGCGGGCATCGG
GGGCCAGCGGCTCATCTTCCGCTGGATAGCCGAGGTTGACGATCTCGATGCCAAGGGAGCTGGCATTGAG
CCCGGCATATTGGTGCCAACGGCTGCGTCCGGCATGCCAGGCGCGCGCGCTGTCCGGTACCAGCTGATAG
ACGGTGAGCGGTGTCTCGCGGTTGTCGCGGGGGATCAGGTAGTGGGCGCTCACCTTGTGCTCCGGCTCGG
TCAGCAGGCGCAGGGAGTTGGCATCATCTTCGTCGGTGTAGTGGAGGATCAGAAAGGCTATCCGCTCGTT
CTGGTTGGCGCTCTGATAGCGGGTAGATAGCTGGTACTCGGACGGTGTGCAGGCGCCCAGCAGCAGAACC
AGTGGCAGAAAGAAGAGGGGAAGAAGACAGCGCCAGCGCCATCCCCCTGCTGCAGCCAGGTTGCTGAGCG
TGGGAGGAGTGAGTCGCTGGCACATGTCGAAGGCCCGGTCTGGTCAGTCGCGCGGCTGGTTAGCCGCGGT
ATTTGGCCGTGAGACCGATCAGGAAGTTGCGCAGGATCTGATCCCCGCAATCCTGATAGTGCTTGTGATC
CTTGGAGCGGAACAGGGCACTGAGCTCGGACTTGGAGAGGTTGAAGTCGGCGAGCTTGAGGGTGCCGAGC
ATGTCCTCTTCCTTGAAGTTGAGACCGATGCGCAGCTTGCGCAGGATCATGTTGTTGTTGATCCGGTTCG
GGATCACCTGGGCCGGTGCATCTTCGCGCGCGCCGCGGCGGGCGATGATGAAACCGTCGAGGAACTGGCT
CAGGGCCGAGTCGGGACAGGCAACATAGCCTGCCTCTTGCTCTTCACCTTCCGCCGCCTCTTTCATCAAC
CAGCTGTGCAGTTGGGCGTGAGTGACCGTCAGGTTACCCTTGGCAAACATCTCGACCATCTGGTCGTTGC
TGATGGTGAGGGCGTAGCGCAGACGGCGCAGAATATCGTTGTTGGTCATGCTGACTCCTGTGTAAACGGC
GCAACTTTACCATCTGGCGGCCCTCGCGTCAGAAAAAGCGGGGGCCGGTACAGTTCAGGTCAAGCTCATC
AAGCCTTGCCGAGATAGCGGGGCGCCTCCTGGCGCACCAGTTCCAGCGCATCTTGCAGCTCCAGCAGCTC
GGGTTCCAGCTCGCTGACCGGCACGCCGTCACGCAGCTGTTGTTCAAGCTGGGAGAGCAGCCGTTGCAGC
CCCGGCACCCCGGAATAGGCACAGCCACCGTTGAGGCGATGGAGCTGGGCCAGCACCTCGCTATCTTCAA
TTTCGCCCGCCAGTGCGCTATCCAGCACCGGCTCGACCTCGTCAAAACTGGCCAGCAGCATGGTCAGCAT
CTCTCTGGCGAGATGCTCTTTCCCCGCCGCCTGTCGCACCGCCAGTGCCCAGTCGATCTGTTGGGCACCA
TTATTCTGGTGACGGCGATGGGCAAAGTCGGTGATGAGCTGGGCCAGCATGCTCTCGTCGATGGGCTTGG
CCAGATAGTCGTCCATCCCCTGGCGGATCAGCCGCTCTCGCTCTCCGGGAATGGCGTGGGCGGTCACCGC
CACGATCGGGGTGGTGGTGTTGAGGGACTGGTTGCGGATCGCCTGGGTGGCGCTGATGCCATCCATGATC
GGCATCTGGATATCCATGAAGATGATGTCGAACGGTTGGCTCTGGGCCTGTTTGACCGCCTCCTTGCCGT
TTTTGCACACCACCACCTGACTCACCATCTCCTTGAGCATGGCGGCGATCAGCTTGAGGTTGGCGGCGTT
GTCATCCACCGCCAGCACCTTGATTGGCTGCAACTGGCGCGGCGCAGGCTGGGCGGGCAGCTTCTGGCGG
CTCGATTCCGGCGAGAGCAGGGCGTGCAGCAGCTTGCGGTGGTTGACCGGTTTGGAGAGGCAGTGCTGGG
CGCCATGGGCCAGCATCGCTTCATAGAGCGCCGGCTCGTGGCTGCTGAGCAGCACTATGGTGTTCTGCTG
CTGGCCGTTCAGTTTGTCGAGACGGCCGAGCACTTGCTGCGGCGTATGCAGGGTGCTGCTGCCGATGATC
ACCATCTCCTGCTCGCTCATCTCGGGCCAGGGATCATCGCTGGTCAGTGGGTGCACATCGAGGTGCCACT
CCGCCAGCAGGGCGAGCAGGGAGTCTTGCGCGAAGGGATCCGGTTCCAGCAGCCAGAGCCGTTTGCCACG
GATCCGATCCAGCGGCAGTTGATCGGTCTGGGGCAGGGGTGAGACCTCCACATCCAGACTGAACGAGAAG
ATGGAGCCCTTGCCGAGCTCCGATTCAAAGCGGATCTGGCCGCCCATCTGCTGCACCAGCTTCTGGGTGA
TGACGAGGCCCAAACCCGTGCCGCCATAACGGCGGGAGATGGAGGAGTCGGCCTGATTGAAGGCCTGGAA
CAGCTGATGTTGCTGCTCTTGGGAGATGCCGATGCCGGTATCGCGGATCTGGACGTTGAGCCTCACCTTG
TGGTTGCCACTGCCGGTCTGCTCGATATGGACATCCACGTTGCCCTGCTCGGTGAACTTGATAGCGTTGC
CGGTGAGGTTGGTCAGCACCTGTTGCAGACGCATGGGGTCGCCGGTGAGGTAGTCCGGCACATCGGCATC
CACCCGCAGGGAGAGCTCCAGTTGTTTGTCGTGGGCGCTGGGGCCGAGCAGGTGCATGGTCTCGTTGAGG
GTATCGCGCAGGCTGAACGGGATATGTTCCAACTGCAGCTTGCCCGCTTCCAGCTTAGAGAAGTCGAGGA
TGTCGTTGATGATGCCAAGCAGGTTGCGCGCCGATTTCTCGATGGTCTGCATGTAGTCGGTCTGGCTCGG
GGTCAGGCTGGTTTTCAGCAGCTGGCGGGTAAAACCGATGACCCCGTTGAGCGGGGTACGCAATTCGTGG
GACATGTTGGCCAGAAACTCCGACTTGACCCGCGCCGCCTCCTGCGCCCGCTTCTTGGCCATGTCGAGTT
CGACGTTCTGGATCTCGATCTGCTCCAGGGTTTCGCGCAGGTCAGAGGTGGCCTGATCGATATTCTGCTG
CATCTCTTCGTGATATTCGGAGAGCGCCTTGGCCATGGCGTTGATGCCGTTTTTCAGCATATCCAGCTCG
CCGGTGAGCTGGCCGCTGACCCGGGTATCGAGCCGCCCCTCCCTGATCTTGTGCACCGCCTGCACCATGT
TGGTGATGGGTTGGGTCACGCTCTTGACCAGACGGAAGCCAAACAGGGTACTGAAGGCGACTCCGATGAG
CACCATGATCACCGCAAAGAAGGTGTCGCGGTATTGCAGCAACATGGCCCTGTCGGTCGCCATCTGCATC
GAGATGTAGCCGATCATCGGCGGCTCTACATCGCTTGGCAGCGGGAAGCCATCCAGACTGGTTTCAGCCT
GGATCGGGGTGCGCAAAATGATGTCGTCTCCCTGAAATGTCACGCTGGTCAAATCGGGAATAGGCTCACC

ATCGGGCAGCCTGAGCCGGGTGAAATCCCGGTGGTAGTTCGAGGTGACGAACAGCTGGTTGTCCTGGGTG
AAAACCGCGATGGACTTGATGAAGGGGGAGTTCTTGCGATGGGTCAGGCCAATCAGCCGCTTGAGGGATT
CGCGACTGTGCTGGGTCATCCCGTATTCACTGGCGATTGCCAGCGGTTCGATGATGTTGATCCCCTGATC
AATGAGGTTGTTTTCCAGTTGCTGGTAGCGGTGGAAAGTGAAGTAACCGGCCATCAGGCCACCGATGATC
AGGGTCGGCAGGATGGTGAAGGCGAGAACGCGTGCTCTCAGGCCATATTTGGTCATACGAACAGGCCACC
AGTGAACAGGGTTGGCGATAGGGTTGATGGCAATACACGGGCTCTGTGGCCGTATTTGGTCATACTATTA
TTCTTGTGGGACAATACCCGGCAGCAGATTCCACAATCATGACATAGCCACCTATAGGCACCAAGTTTTA
TGGCCCAGTTTTTCAAGCCCCAAAAGAAGTCCACCCAGCTCCAACGCATTGAATTTACGATAGACACCCT
CGATCACCACTGCGTCGGTATCGGTCGCCATCAGGGCAAGGCGATCTTCGTCGAAGGGGCACTGCCCGGC
GAGACCGTCAAGGCACGCATCACCGAAGAGAAAAAACAGTATGGTCACGCCGCCTTGCAACAGGTGGTGA
CCCCGGCGGCCAATCGTATCGCTCCCTTTTGCAGCCATTATCGCGAGTGCGGCGGTTGCAGTGCCCAGCA
CATGTCGGTTGCCGATCAGCAAGCCGCCAAGGCGGCCGGACTGGTCAGCCTGTTCGAGCGGTTGGGCAAC
ATCAAGGCGCCGGCGCTGGAGCCGGTACTGACCGGTGAAGATCGTGCCTACCGCCGGGTCTGTCGTCTGG
CCATCAAATTTGACAAGAACGGTCGCTGCACCCGGGTCGGTTTCCGCCGCCGCAACAGCAACGATCTGGT
GGAGATCGACGGTTGCCCTGTGCTGGCTGAGCCGCTCTCTGCGTTGATTGCCCCGCTGCGTGAATGCCTC
AATCGCCTCAAGAGCCAGCGCGAGCTGGGCCATGCGGAGCTAATCCAGGCCGAGCAGGGGATCATGATGC
TGCTGCGTCACACCGGCCGTCCCAATGAGGCGGATCGCGCCCTGCTGGTGGAGTTTGCAAAAGCGCAGGG
GATCGACCTCTATCTGCAGGCGGCGGATGATCGCATCGAAGCGCTGCAGCAACAATTTTCCCCATCCTAC
TCGCTGGATGGCCTGTCTCTTGCATTTGCTCCCGGTGATTTCATTCAGGTCAACGGCCCCATCAACCAGA
GCATGGTTGAGCAGGCGCTGACATGGCTCGGGGCGAGCAAGGATGACAAGGTGCTCGATCTCTTCTGCGG
CATCGGCAACTTCACCCTGCCGCTGGCCCGTCAGGCTCGCGAAGTGGTGGGTGTCGAAGGTGAACTGGCC
ATGGTGCAGCGGGCCGAAGAGAACGCCCGTCGCAACGGCATCAGCAATGCCCGTTTCTACAAAGCCGACC
TGAGCGATGACATCGTCGGCATGTCCTGGGCGCGTGAAGGGTTTGATCTGGTGCTGCTGGATCCGGCTCG
CCCCGGCGCGCTGGAAGTGATGGGACACGTCGTGAAACTTTCACCCAAGCGGGTGGTCTACGTCTCCTGT
AACCCGGTCACCCTGGCGCGGGACAGTCAGGTGCTGGTGAAAGGGGGTTACCGTCTGGTGCGCCTCGGCA
TGCTCGACATGTTCCCTCACACCGGTCATCTGGAGTCCATGGCGCTGTTTGAACGGGAGTAAATGACTCA
TCAGTACGGCGATGCGGATATGTAATCTGGCATGTCTGCGTCTGTTTGAGCTGGTGTGGTATATGAAGTA
ATCCTGCTCATCAGTACGGCGATGCGGATATGTAATCCGGCATATCTGCGTCTGGTCTGTTTGAGCTGGT
GTGTATATGAAGTAATCCTGCTCATCAGTACGGCGATGCAGATATGTAATCTGGCGTGTCTGCGGCTGTT
TGAGCTGATGTGATTGGGGTAATCTAGACCCTCGTCGTTCGGCAGGGCCTGTTGGCGCGCTCTATTGTCT
TTCTGGACAATATGCCTTGAAAAGTGCGCTGGATATCCCCATTTGAGGCAATCTCATGAGGGGAGAAAGT
GACAGCTTTCGGCCCGATAATTTAAAGGATTGATACCATGGTTGCGGTTCGCGATATTCATTTGAAAGAC
AACTTCACCCTGGAAGAGTGGGTCAGCTCGCTCTCCTTGAGTGACAGTGAAAAGGATCAGCTGCGAACCG
TCTACCAGTATTGTCTGACGCTCGGTGACGAGGCGCTGACCAGCAAGCTGCTGGTGCGCGGCATCGAAAT
GGTGGGCATCCTGCAGATGCTCAGCATGGATATCGGTACCCTCAAAGCGGCCATCATCTACCCCTTCGTC
GAAGCCGGTCTCATCAGCCAGGAGCGGATGGACGAGGACTTCGGCTCCAAGATCGCCAAGCTGGTGGAAG
GGGTGCTGGAGATGGAAGCCATCCGCTCCCTGCAGACCCTCCATCGCAGCGAAACCTCGCCGGAGCAGGT
CGACAACGTGCGTCGCATGTTGCTCGCCATGGTCGAGGATGTGCGCGCCGTGGTGATCAAGCTGGCGGAG
CGGATCGCCTGCCTGCGGGAAGCGAAGACGGCAGATGAAGAGACCCGGGTGCTGATGGCCCAGGAGATCA
CCAACATCTATGCACCGCTCGCCAACCGTCTGGGCATCGGCCAGCTCAAGTGGGAGCTGGAAGATCTCGC
CTTCCGTTACCTGCATCCGGATACCTACAAACAGATCGCCAAGCAGCTCGACGAGAAGCGGCTCGACCGC
GAGCGCTACATCCGCGAGTTTGTGCAATCCCTGCGTAATGGCCTGAAAGAGGCGGGAGTTGACGCCGAGG
TGTACGGTCGGCCGAAACACATCTACAGCATCTGGCGCAAGATGCAGAAGAAGCACCTCGAATTCAACGA
GTTGTTCGACGTGCGCGCGGTGCGGGTAGTGACCAAGCGGTTGCAGGATTGCTATGCGGCGCTCGGTATC
GTGCACACCCAGTTCCACCACATTCCTCGCGAGTTTGACGACTATGTCGCCAACCCCAAACCCAACGGCT
ACCAATCGATCCACACCGTAGTGGTGGGGCCGGAGGGCAAGACGGTCGAGATCCAGATCCGTACCGACCA
GATGCATCAGGATGCCGAGCTAGGCGTCGCGGCGCACTGGCGCTACAAGGAAGGGGCACAGGCTGGCGGC
AAGGCCAACACCTTCGAAGACAAGATCGAGTGGCTGCGCAAGCTGCTCGCCTGGCAGGAAGATCTCTCCG
AGAGCGGCTCCCTGCTCGAAGATCTGCGCAGTCAGGTGTTCGAGGACAGAGTCTACGTCTTCACCCCGAA
AGGGGATGTCATCGACCTGCCGGCAGGCGCCACCCCGCTCGATTTTGCCTATCACGTCCACAGCATGATC
GGCCACCGCTGCATCGGCGCCAAGATTGATGGCCGCATCGTGCCGTTCACCTACGCGCTGCAGACCGGCG
ATCAGGTGGAGGTGATCACCCAGAAAGAGCCTAACCCGAGCCGCGACTGGATGAACCCCAACGCCGGCTT
CCTGCGCTCCAGCCGGGCGCGGGCCAAGGTGGCGACCTGGTTCAGAAAACTGGATCGGGACAAGAATATC
GTGGCCGGTCGCGAGCTGTTCGAGAAGGAGCTGGATCGCCACAACCTCAGCATGTCCAAGATCGACAAGG
GCAACATGCTGCGCCGCTTCAATCTGGAGAGCACCGACGATCTGCTGGCCGGTATCGGCAGTGGCGATAT
CCGCATCAACCAGGTGCTCAACTATCTCGACACCTGTTACAACAAGCCGACGGCGGAAGAGGAAGATCGC
CGCCTGCTGGAGAAGCTGGAGCAGAAGGCCAATGCGCCGATGCGCCACAAGCCCAAAGACCACATAGTGG
TGGAAGGGGTCGGCAACTTGATGACCCATATTGCCCGCTGCTGCCAGCCTATTCCGGGGGACTCCATTCA
GGGCTTTATTACCATGGGGCGTGGCGTTTCGATCCACCGGGAAGATTGCGAGCAGTTGAAAGAGCTTTCC
CGTCGCAACCCGGAGCGGCTGATCGATGCGGTGTGGGGGGAGAACTACTCCGGCGGCTACGGCCTCACCA
TCCGGATCATCTCCAACGATCGCTCCGGCCTGCTGCGCGATATCACGACCGTGCTGGCCAACGAGAAGAT

CAATGTGATGGGGGTGCGCTCGCGCTCCAACGTGCGCGAGCAGACCGCGGAGATCGACATGGAGCTGGAG
ATTTACAACATCAATGCCTTCAACCGGGCGCTGGCCAAGCTCAGTCAGCTTAACGACGTGATCAGCGCCA
AGCGGCTTTGATAGCGAACCGACCGGCCCCAGTGGCCGGTCTGCATTTACGACCCTTTTTGAACGAATCG
AAAGTGAATATCCCATGTCCCAACGTTACGATCTGCCTCAACTGCTCGACATAATGGCTCGCCTGCGCGA
TCCGCAAAACGGCTGTCCCTGGGATCTGAAACAGGACTTCCAGACCATAGTGCCGCACACCCTGGAAGAG
GCCTACGAGGTGGCCGATGCCATCGAGACCGAGGATTGGCAGGGGCTGAAAGGGGAGCTGGGTGACCTGC
TGTTTCAGGTGGTGTTTTACGCCCAGCTGGGTAAAGAGCGGGGCTTGTTCGACTTCGCCGATATCGTCGA
CACGGTGAGTGAAAAGCTTATTCGTCGTCACCCCCACGTTTTTTCCGATGCAGACCTTGCTAACGAGCAG
GCAGTAAAAGCGAACTGGGAGGCGGAAAAAGCCAAGGAGCGCGCTGCCCTCGACAAAGAGAGCGTGCTGG
ACGATATCCCGCAGGCGCTGCCTGCCCTGACTCGCGCCGCCAAAATTCAGAAACGCTGCGCCACCGTCGG
TTTTGACTGGAAGACGTTGGGGCCTGTGGTGGACAAGATCCACGAGGAGATCGACGAGGTGATGGAAGAG
GCATTGATGGCCGATGTGGACGAGGCGCGGGTGAGCGACGAGCTCGGCGACCTGTTGTTTGCCACCGTCA
ATCTGGTGCGTCATCTGGGCAAGGATCCGGAGCAGGTGCTGCGAGGCGCCAACGCCAAGTTCGAACGTCG
TTTTCGTCAGGTTGAGCAGATAATCGCCGCAGAAGGCTTGAAAATGACCGATTGCACCCTCGAAAAGCTC
GATGCCGCTTGGGATAAGGCGAAGGAAACCGAGCAAAGTTGATTTGGCGCAAGATGGGGGGGCGGTGAGC
CTGATAGATTTTTGCTCATTGTTTGGGAGAGGGGTTTTTGGTATACTGTTTCCCCGTCCTGCTTCATCCC
CGAAGCACCCCTTAAAATTCCCTAAACTTCTTTCAGGTTCAGCATGACGACCAAATATATTTTTGTTACT
GGTGGCGTTGTTTCATCCCTCGGCAAAGGCATTGCCGCAGCTTCTCTGGCAGCCATTCTGGAAGCCCGTG
GTCTGGATGTGACCATCATGAAACTGGACCCGTACATCAACGTGGATCCGGGCACCATGAGCCCGACCCA
GCATGGTGAAGTGTTCGTAACCGAGGACGGGGCCGAAACCGATCTGGATTTGGGCCACTACGAGCGTTTC
ATCCGCACCAAGATGACCCGTCGCAACAACTTTACCACCGGCCGTATCTACGCCGACGTACTGCGTAAAG
AGCGTCGTGGTGACTATCTGGGCGCCACCATTCAGGTTATCCCGCACATCACCAACGCCATCAAAGAGCG
GGTGATTGCCGGTGCCGAAGGCCATCAGGTTGCCATCGTCGAAGTGGGCGGTACCGTAGGTGACATCGAG
TCCCTGCCGTTCCTCGAAGCCATTCGTCAGCTGGCTGCCGAAGTGGGCCGCAACAACGCCATGTTCATGC
ACCTGACGCTGGTTCCTTACCTGGCTGCTGCCGGTGAAGTGAAGACCAAGCCGACCCAGCACTCCGTTAA
AGAGCTGCTCTCCATCGGTATCCAGCCGGATGTGCTGATCTGCCGCTCCGACCGTGCCATTCCGGCCAAC
GAGCGCGCCAAGATCGCCCTGTTCTGCAACGTGCCCGAGCGCGCCGTCATCTCCATGAAAGACGTCGACT
CCATCTACAAGATCCCGGCACTGCTCAAGTCCCAGAATCTGGACTCCTACTTCACCGAGCGTTTCGGTCT
GGAGTGCAAAGAAGCCGATTTGTCCGAGTGGGAACAGGTTATTTACGAAGAGGCCAACCCGACTGGTGAA
GTGACCATCGGTATGGTCGGCAAGTACGTCTCCCTGCCGGACGCCTACAAGTCCGTCAACGAGGCGCTCA
AGCACGGTGGTCTCAAGACCCGTCACTCCGTCAACATCAAGTACATCGACTCTCAGGATATCGAGACTCG
TGGCGCCGAGCTGCTGGAAGGTCTGGACGCGATCCTGGTGCCGGGTGGCTTCGGTGAGCGCGGTGTGGAA
GGCAAGATTCTGGCTGCCCAGTATGCCCGCGAGAACAAGATCCCCTACCTGGGTATCTGCCTCGGCATGC
AGGTTGCTATGATCGAATTTGCTCGCAACGTAGCGGGTATGGAAGGGGCTCACTCCTCCGAATTCAAGAA
AGATTGCGCGTATCCGGTCGTCGGCCTCATCACCGAGTGGGTGGATGAAGAAGGTAATGTCGAGACCCGT
ACCGAGAAGTCTGATTTGGGCGGTACCATGCGTCTGGGTTCCCAGCTCTGCCACCTGGTCGAAGATTCCA
AAGTGCGTCAGATGTACGGCAGCCCGACCATTTACGAGCGTCACCGTCACCGTTACGAAGTAAACAACAA
GCTGCTGCCGCAGATTGAAGCGGCAGGTCTGAAAGTGACCGGTCTCTCCGCCGACAAGAAGTTGGTGGAA
ATCATCGAGATCCCGGATCACCCCTGGTTCGTGGCCGCGCAGTTCCACCCGGAGTTCACCTCTACTCCTC
GTGATGGCCACGCTCTGTTCGCCGGTTTTGTGAAGGCGGCAGGCGAGTATCAGAAGCGTAATTTGAAGTA
ATTGAAAATAAATGCGGTTGGGGCACTGTGCCACAGCCGCTTTTTTTGGCATATTTTTGTTGTTTTTTTA
CGAAACCTGAGGAAATACACATGTCCAAGATCGTTAAAGTGATCGGTCGTGAAATCATCGACTCCCGTGG
TAACCCGACCGTTGAGGCCGAAGTTCACCTGGAAGGTGGTTTTGTTGGTATGGCAGCCGCTCCGTCTGGC
GCGTCCACCGGTTCCCGCGAAGCGCTGGAACTGCGTGACGGCGACAAGAGCCGTTTCCTGGGTAAAGGCG
TGCTGAAAGCCATCGAAGCCGTAAACGGCCCGATCGCACAAGCTCTGCTGGGTAAAGATGCCAAGGACCA
GGCCACCGTTGACCAGATCATGATCGACCTCGACGGTACCGAGAACAAATCCAAGTTCGGCGCCAACGCC
ATCCTGGCTGTTTCCCTGGCTAACGCCAAAGCTGCCGCTGCTGCCAAAGGCATGCCGCTGTACGCCCACA
TCGCCGAGCTGAACGGCACCCCGGGTGTTTACTCCATGCCGCTGCCGATGATGAACATCATCAACGGTGG
TGAGCACGCCGACAACAACGTCGACATCCAGGAGTTCATGATCCAGCCGGTTGGCGCCAAGACCCTGAAA
GAAGCCGTTCGCATGGGCGCAGAAGTGTTCCACAACCTGGCCAAAGTGCTGAAGTCCAAGGGCTACAACA
CTGCAGTTGGTGACGAAGGTGGTTTCGCTCCGAACCTGAAATCCAACGCCGAAGCGCTGGAAGTTATCGC
TGAAGCCGTTGCCGCTGCCGGTTACAAACTGGGCACCGACATCACCCTGGCTATGGACTGCGCTGCTTCC
GAGTTCTACGACGCAGAGAAGAAAGAGTACAACCTGAAAGGCGAAGGTCGTGTCTTCACCTCCAACGGTT
TCTCCGACTTCCTGGCTGACCTGACCACCAAGTTCCCGATCGTCTCCATCGAAGATGGCCTGGACGAATC
TGACTGGGAAGGTTTCGCCTACCAGACTGCTGAACTGGGCAAGAAAATCCAGATCGTTGGTGACGACCTG
TTCGTAACCAACACCAAGATCCTGAAGCGCGGTATCGACAACGGCATCGCCAACTCCATCCTGATCAAGT
TCAACCAGATCGGTTCCCTGACCGAAACTCTGGCTGCCATCAAGATGGCCAAAGACGCTGGCTACACTGC
CGTCATCTCCCACCGCTCCGGTGAGACCGAAGACGCGACCATCGCCGACCTGGCTGTTGGTACCGCTGCT
GGCCAGATCAAGACCGGTTCCATGAGCCGTTCTGACCGTGTTGCCAAGTACAACCAGCTGATCCGTATCG
AAGAAGCTCTGGGTGCCAAAGCTCCTTTCCGCGGTCTGAAAGAAGTTAAAAACCAGGCTTAATTTGCTGG
TTTGCCAGCCTCGGCTGGAATGAAAAAAAAACCGGAACCCAAGGGTTCCGGTTTTTTTATGCCTGTTGTT

CAAGCCAGCGTTGCAGATGCTGCACAAACAGCGCGGGCTGCTCGATGGGGTAGAGGTGCCCTACGTGCGG
CACCTGCAAGCGATCGCAGGCGGGAATGGCGTTGCCAATAGCCTCACAGACCAGCGCAAACAGCGCTCTG
GTATCTGCGCCCTGCACGATGCAGGTGGGCATGTTGAGCATGGCAAGGCGCCCGGGTGACCAATCTGGTC
GCTCGCTCTGGCTCAACTGCTTGGGCAGGCTATCGGCTTCTGCCAACTGTGCCTGCTGCCACTTGAGCGG
TTGTGCAGCAAAATAGCCTGCATGACCACCCGAACCATCGATCAGGGTCGCCACGGCCTCTTGCAGCAAG
CCTTCCGCAACCTGGCCAAACACGGGGCCAAACATGGCTCCGGCATCGGCCATAAAGGCAGCGATCCCCT
GTTCATCGAGGGCTCCAGGGCAGCCTGGCTCCACCAAATAGAGGCTTATGAATTGCTCCGGAGCCATCAG
GGCAGCGGTCAGGGCAACATCCGCCCCGTATGACCAGCCCGCCAGATGCACTGGCCCGCACGGCAATTGT
TCCAGCAGGGCAAGCAGATCACTGGCGTGGGTCTCCAGACCAAAAGGCCCGTTTTTGCCCGCCTCGCCAA
AGTGGCGCAGGGTGGGAGCGTAGACGGTGTAGTGACGTGAGAGCTGCTCGATATGTTCGAGCCAGAGGGT
ATGGTTGGCCAGCGCGCCATGGATCAGCAACAGTGGCTCGCCCGAACCGGCCTGCAGGCAGGGCTGCGGG
CTGTCATTGACGATAAAGGTGTGAGTCTGTGATTGCATATCAACGCCTCCCCGGCGTGTCATAAGAGCAG
AGGAGTATATGACAAGCGCCCCGTAGTCGGTAAGTGGGCGCATTCAGGCTGGCGCTAGCCACTATAAGAA
AAAGTTGGGTCATTATCAGAAAACCAATAAATCCAGTTTATAGTGGCACCGACGAGCCTTGGGGTGAACC
CAAATGGTCGATATCAACCCCGCAAGGGGATCTTCCGATTCTTGGTGATGTATTGGCGGGGATATTCGAG
ATTGTCCGAATAGGCTGCGCTATTCGAACCTACGAGCTTGAGTAAATTGCTAGATTTCGTTCTGAGAAAA
CCACATGTATATGTTGTTGTCTTTAAATTCTTTCAACACTTTAAGCGCTTCAAAAGCATCCAGTCTAGCC
ATTTCAACTGGATCATAGACTTGTTTATCCATCATCTTCTTCTGAAGAGAACCATCTTTCAGCCCCACTT
TTGCATGAACATATGGATTTCTTATTCTCCTAAGTGAATCAAGTTTTTTATGTAGGTCAGGTGTGACAAT
TGATTGATCTTTAGCTGCGTCGATAATTTTCTTAAATGATGATTGAGCAAGTTCATCTTGGCCCATCATA
ACAAAGCGTCCAGACAAACTGTGTTCTATAAAAGCTTGAGATAGTAGAACTACTGCCATAAAATTACCAG
AGACATAACTATTTGCTACCTCGTGTAGTGCTAGTAATGATTGATACCCCCCCTCAAGAAGCTGATCGCC
TTGAGGACCAAATAATGTGTTAAGCTCTCTCAATCGCCCCAGCCTATCTTCTCTCGAGTCTTTTTCATCT
TGTAAAAACTGCTGTGTAACAGAGTTTAAAAACTTCTCTAAATTCATCACGCATACCTTGAGTTAATATC
AAATTTAGTGGTGGTTAATACCAATAATTAATTCTGGAATCAAACCGAAATAGACAAGATTCCTTAGGCT
ATTCCAATTTTTTGGATGATTATAATTTTTACGATAGATATTACCAACTAAGGTGTGACTAGAGGGAAAT
TTGGATGTCTAGGTTTTCCTCTGATGTTCAATAGGACTGCAAACGCTACGTGTTGGCAAATCTATAATTA
TTTTCTCAATTGCCAGTGCTTATTGCTTAAATTAACTACACCTTGAGAGCTACCAAGGTGGCCCGATTTA
AGGAAACGTGTGCCGTAAATATGTATGGAGATGGTATTCACCTGACAGTTGCCCACAAAAGGGAGTGGTC
ATACGGCTTCAAACCGGATAACAAAACGGTACCCCTACCGTGTTATCCGGAGTCACCGTATGACCACCAA
CGAGAAAGTAGCACGTCGCAAACTCAGCCTGCTAGAGCTTGCCAAAGAGCTCAATAATGTCAGCAAAGCC
TGCAAGCTCATCGGCTACAGTCGTCAGCAGTTCTACGAAATTCGCCGTAATTACCAGACCTATGGCGCCG
AGGGATTGCTCGACAAATTGCCCGGCTGTAAAGGGGCTCATCCCAATCGGGTCGCTCCCGAGATTGAGCA
GGCCATACTCGATTACTCCCTCACCAGACCAACTCATGGCCCGCTGCGGGTCGCCCAAGAACTGGCTCTG
CAAGGCATCAATGTCAGTGCCGGCGGCGTGCGTGGCGTGTGGCAACGTCATGACTTGCTCTCCAAACACG
ACCGCTTGTTGCGCCTCGAGAAAACCCACCGGGAGCAGACCATTGAGCTCAATGACGAGCAAATTCGCCT
GTTAGAGCGCTTCAGCCCCGAGTTTCGCGAACGCCAGATTGAGGTTCACTACACCGGGGAACTGGTGGCG
GTCGACACCTTCTTCGTCGGCGCGCTCAAGGGGGTCGGTAAGGTGTATCTGCAAACCGTGCTGGACTGCT
ATAGCCGCCACGCCTGGGGGCGGCTCTACACCAGCAAACTGCCCATCACCTCGGTCCATGTGCTTAACGA
AACGGTGCTGCCATTCTTTGAAACCCACGAGGCACGGGTCTATACCATCCTGTCGGATAACGGGCGCGAG
TTCTGTGGGCGCCCTGACCACCATCCCTACGAGCTGTTCCTGCAACTGGAGGGGATTGAGCACCGAACCA
CCAAGGTGCGCAGGCCGCAGAGCAACGGCTTTATCGAACGGCTGCATCGAACCTTGCTGGATGAGCACTT
CCGTATCAAGGGGGGGACGACCTGGTATGAGTCGGTAGAGCAGATGCAGACAGACCTGGATAGCTACCTG
GAGCACTACAACACCCAGCGGCCTCATCAAGGCAGGATGATGGAGGGTCAAACCCCCTACACCATGTTCA
AGATGGGTCTGAAATTGATACCGAAGGAAGTGCGCACTAAAGTAGCGTAAACAAGACACCGGTTTGAGGC
CGGTGTCAGGTGATAACTAGATCTGTACATTTTGCCAATGCATATTCAAGAACTAGATCATCTCTCCTTA
ATTTTAGTTCTTCGTAAGCTTTATCTAATACAGTTGCTACATCGACCAAACCGAATAATATTTCCTTACT
CTCGGGGCTGATATTTAGTTTCAAAAGCGGAGCTATATCTTTAGAACTACCTAAGATTCCAATCCATTCA
CTGATTTTTTCGTCAAATGAATTGAACGCATTGTTAGCTTCAACAGCATTTCGTTCTAACTCTGATAACA
TTTTCACCAAACTCCAAGTTATTACTTATATCTATAATTCGCATTAAGGCGTGAGTGACACAATGCTAAA
CATTCTGCAATCCACTTTAAAATAAACGGTCATTAAAATTCCACTCACTAATGATTACTATTAAGTTCGT
TACTATTTGTAATTAATGATGGCTGAACCGCAGAATATTTTCTAGAAGTTTTTCCTGCTTAATGTCGTAT
GTCCATTTTTAGTTCAAGACTAAAGTATTAGAGACAAGCTACGAGCAAAATGTAGTTTTAATCTTATATA
TCATCATCATTAAAAGAGCTCTCAATTAGAGTAGATTTTTTATTATTTCGTATGTTGGACTAAAAATAGT
AGGTTGGTGCTGAACGAAGCGAAGCCCAACTTTACCGCGAGAAGTATCTGAAATTTTTTTCAATCGACCC
GATCAATGTTTATCCCGCTCTGGTTGACGTGATAATGCTCATCTGTTGGGCTTCATTAAGCTTTACGCCA
ACCGACACAAACCACGCTACCGCCCCTGAATTTCAGCTTCCAGTCTGCTGCGGTTCACCAACGGGATGGG
GTTGGCATGGCTCATGGGAGACTCCGACGAGGCAATTGTTGCCAAATGGCAATATTAACCATGGCGCTGT
GGAGAGAAAGCTCAATATTGAGGATTTCTGTGCAAGATCAATAAATTAATCAAATGTCACCTACGATTGA
TGACGCGCCGAGATAGCGTGAACAACCATTCTTGAGCTATCACCATAGAATATTCATGGACATATCTGGC
TTGAACCAGCATAAAACTCTTTGTTATTGTCCGCATCCCCCGCTGAAATAGCTTATTTATCTATAAATCA

ACAAGATAGCATTATGCGGTGGCGTCGCGGGGGCATCTGCTATCGGGAAGGAGAGTTATGAAGTCGCAGT
TCAAAGTGGCGCTGGCCATGTTGGCCCTGTTCGTCGTCCAAGGCTGCACAGCCCCGGATTCATCCACCAA
GCGCAATGTACCCGGCCCGGTGCTGACGGTGCATTACGAAAGTGCCCCCCTGATCCCCGTCACTATCGCT
GGTGAGCAGTACCAGTTTTTGCTCGATACCGGGGCCAGTATCACCTTTATCGATCGCCGTCTGGCCAGCC
GCTTCAAGACGCTGACCTTAAGCGAATTGCCCAGGGCCTATCAGGCTGATTTTGCCACTGTGAACAGTGT
TTCGAGCCAACTGGGCAGCGAGCAGCTTGGCTTTGTTGAGCCGCAGCCCTTCTCGGTGGGGTCGGTCACC
ATTGATGACAGCGATCTGTGGGCAACCGTTGATTTCGATCTTTTCTCCCAGTCTCTCGGCAGCCGTATCG
ATGGCATCCTCGGGATCGACACCATCCGCCGTTTCAACTGGATGTTTGATCGCGACAAGCATCAACTCAC
GGCTTCACTGACGGCACCGTCGGCAGCCACGTTCGAGCGTTGTATCGGCTATGAAGACAGCTACAACCGC
TCCCCGGCAGTGTATCTGACCCACGGTGAGAGCAGCGTGCGCATGTATCTGGATACCGGGGCCGATCAGA
GCTATGCCGACCATGCCTTTATCGACTTTATGGCCAGCCAGAAGGCGATCTCTGGCGAAGATGCCACGCC
ACGCAAAGGGGTGGATGCCAGCGGTATGGTGGAGGGGCGTGACCATATCCTCACCGGCCTGCACTTTGAT
GAGCGCCAGCTCGGTGAGCTACGCGTGTCGGAAAACAGCAATGAGCAGTATGGTCTTGGGCTGGATTTTG
TTAACCGCTTCAATCGCTTTGCGATCATGCCCGGGCGGATGATGTTCTGCTACGACGCCGACAGCCTGGC
GCGCAAAGTGCCGCCAGTCCTGCGCAATATCGGGGTGCGTTTTGATGGCGAGCATCTGCAGGTCTACTAC
AGCGACGAACGGGATCTTGCCCCCTATGGTCTGGCCAACGGCGACATTCTGGAGCAGATCAACGGGGTGG
CTTATCGCCCCCTCGAGATCGACAAGGTGCGTGAACTGCTCAGCACCAGCAAGCCGGGGTCGCTCAAGTT
GCGGATTGTGCGGGAAGGAAAACCGCTGGATATCGCCATCTAGTCAAGGTGGGGCGGCAAGATGAGCCGC
CCCTATGAGCCTCAGCCCTTGGTCGGATTTTCCATGCAGGTGAGGGTTGGCTGGCCGTAACGCTCCAGCG
TGGCGTCGTCCCCCTTGGTGAAGAAGGTAAGGTTTTGCCCTTCATAACGGGCGCCGCTGGCGCTGGGGGC
TTCATAGGCGAGCACTTGTTGCTGCTTGTCGCCCTCGCTCAGATTGATCACCGCGGCGGGCAGCTTGGCA
TCGTTATAGAAGTAGGTAGAGAGCATGACGCTCTTGCCGCACTTGTAGTTGACCGGTGCCGGCACCATCA
GCTGGCCGCCGCTGATCTGCAGTTCGGTGATCCGCAGCAGGTAGTTATCCTCCACACACTGGCGCAGATC
CTGCCCCTTCCAGCAGTCGTTGCGACCCTTGATCCAGCCGCGCTGCATGGCACGCTCGGTCTTGAGCTGT
TCGGGAGAGAGCGCTGCAATCGCTTTGGGGTAGAGGGCCGCCAGTTCGTTATCAAGGGCGGCCAGTCCGG
CATCCTTGCAGATCAGGGTTTCGACCGACCCGGAGGCTTTGGTGCAGTCAAAGGAGGGAGTTGCGGCGCT
GGCCAGCAGAGGGGCAAACAGCAGGGTGGCAAGCAAGGTCTTGTTCATCGATAGATTCCAGCAATGAGGA
GGGCATATCTGCCTACTACAGCACACTCTACAGGGCAATGCAGCAGGGGAGCTGAGCGCACTTGCACAGC
TGAATGGCTGCAAAGCGGAAAAACCCGACACTCCTGGCCGGAATGTCGGGCTTTTTGTGGCCGCGATAAA
GGGTAGGCGGGTTGCCCGGTTGATCAGGCCAGCGCCAGTTGCTGACTGCGGGCGCGCCACAGCCGCTCGT
GGAAGAAGAACACCACGGTATTGATGGCCGGTTCGATCATCGCCATCACACCACCGGTGAGCAGCTCGCC
GCTCAGCAGGAAGGCCACCCCGAAGGCCACCGAGAAGTGGACCACCGCGAAGCTGCTGGTCTTGGCCTGC
AGGCTGTGATTTGGTTTGAGCTTGTGCCAGCCAAGGTCATGAAAGTAGAAAGCGACCGTGTTGACGGCGG
GCTCGATCAGGGCGATCAGGCTGGCGGTCAGCAGCTCACCGGTCAGCAGGTAGGCCACGGTAAAGGCAAC
ACCGAAGTGCAGAATGGCAAAGCTGATGGTCTTTTTCATGATGGCGGCTCCCTCTGTTGATGATGAGAGT
ATCCGCCGAAATGAGATCAAATGAAAACTATTCTCATCTATTGCCAGAATAGAGAGAACCTATCAAAGGG
CGAGTGGCCGGTGCGGTTCAGCGGTTGCCAATCGCAACCAAGGCGATAAAGTACAGGTTCTTCACACTTG
CCCATGAAAAAGGATCTTTCATGAATATCAATGCCACTCTGCTTGGCCAGTTCATTGCGTTGCTGATGGT
CATCGTCGGCCTCATCGCCTGGCGACTGGCGCGCCGCAAGAGCGAGCATCCGGCGCTGATCACCCTTATC
TGCTCCCTGCTCTGTCTGTTTCCGCCCTTTGGCCTGTTGGCCCTGTTGCTGGTTGCGCTGAAACAGGATC
TGCCCACCCGCTCATAGGGCGCGCAGGTTCAGGCTGCCGCACACACCGGCCGCACCAATAGCAAAAGCCC
CGTGATGGGGCTTTTGCGTTTGAACTCGGTGAGCAGAAGACCTTGGACACCCATCTTTTGGGTTCGGGCT
ATTCTACGCCTTGCTCAAAAAGGTGGGTGTCCCAGATTTGCCACTAGGCAAGAACTATATTTATCAAGTA
AACAATTAAAGCAGTTGTCGCAAATATAAATGACCATTGAGACAGGCTGCTAAAGCGTCTAATTATCCAG
TACTTAGCAACTTTGTCATCGAAATTATCCTTGTCTTCAGATATGTAATAGCCAAATGAAAACAAAGCAT
TAAGAACTAGAGCTGTAATATAAAATATTGTAGAAAATTGTAGTAATTGACTAGCCGAGATCAACTCAGC
TTTATCAACAACAGCTAGAACGAACGCAAAGCTCGCTCCCGTGAAAGTCGGATAAATGATTTTGCCTAAT
GCCATTTTAGCCTCTGTTTTATAACGCCTTGCTCAGCGGCAATTTAAAGTGGCACGTTTTTGCGAATGCA
AAACAAGTGACGCTTTAAATTGTCCGATGGAGCTATTTGTTAGGCTCATGTTTTTGTTAAATCACTTAAT
ATCCAGTCGAATTCTTCAAAATCCTGGAAAAATAGTTCCTCTGGGTTTGTCGTTTCATTTTCATATTCGT
CAAAATAATATCTTGCCGCCCAATCATCCATAACAAAGGCGTGTGGCCGCTGTTGAAGATGCATATATAA
ATATGGGAGTTTTTTACTAAAATAGATTTTTTGTGATTCGGTTACTAATTGATAACTAGCTATTATGCAA
TCACGGTAATCCCAAATAAAATACTCAACCATATCCTTTACTACTTTGTACGAAAAAGTAAGAGATCTTT
CTAATTTATTATTCTTAAGATATTCAACATGCCGATCATACCAATATGGTAGATATTCCGGTTTCTTTAG
TAGCAAATAGCGCGACAAGAGCGTAAAAAAATTCGTAGTGCTGAATAAGCATGTACTGAATACTTGTTGAG
CAGAATTCGTTATCGCCTTTTCGCTTACACATTAAAACCAATGGACCGAGAGAGTTATACGACATAATTG
ACTTTTCTATTGCACTTTCAATTTCAGGCCACCTTTTTTCGCGTATATAGGTCATTCTGAATAGAGCAAG
ACCGCTTTCTGAAAAATCTGAATCTTTTGTCAGGATCAGTGAGGACGTTTTATTATTCTTACTAAACCTT
CCTTCTCTTTGATTTATTTCGACACTAGACCAATAAACATGCTTATTACTTAAATCTACGACTATAAGAA
AAACAGGTACTGGAAGCCCTATCCAATAGTTAATAGTTGATTTTTTATTCCGCTATAAGTGAAAAATCCC
TTGTCATTAAATTGTACTGAGTTAGCGCTCTTCACTTGTAACGCGACTAAGTCACCAGTAACTTTTCCAT

TCTTTCCTACTAATTCCACATATAGGTCAACTCCGTAATCCCTTTCAGTTAATTCTCTTACTATCCATTC
CTTTGGCAATACAGATGATAAGGCACTAAAAGAAATGCTTTCGGATATATGTGTGTCGATTCTTTGTGGG
TATTCCATGACTTATAAGAGCCTAACAGTGATTAGATGGAAGGCGGTATTAATCCGTTAGCAGAGCATTC
CATATAACTCCGTAACATTTTCACTGCCGCCAAGTAAAAAAATCATTAAATCATAATGTTACAAACTTGT
TCTCGTGCTGAGCGTATTCGCTTGACTTCCTCTTTTTGCGCCATCGTTACTACTGTGTGAAAAATCAGTG
CTCAGCGCAATCTTGGAAAGTGCCGCCAAAGCACGCCTGCGTGAGCGGGTCAGAAATAAAAATGGGGCCG
TCACTATGTTGACTGGGTAGTCGTGGGCAAGGTTCGCTTATCCATTCCATCTGGCATGGCTACCGCCCCT
GAATTTCAGCCTCCAGTCTGCTGCGTTTAACCAATGGGGTGTGGCTGGCATGGCTCATGGGGTGACTCCG
ACGAGGCTCGTGTTGCCAATTGGCTATAGTACCGGCCCCTTGGCCAAGGGAAAGGGCGATGCGGGGATTT
TCTGAGCCAGATCAAGCAATTAATAAATCGCGACCGGTTGGCGCTGCGAGTGGAAAGTGCGACCACTGTG
TTGCTGGATAAAAAGTGTTGGCGGGTAAAGTGTCGGTGGGTAAAAGCGCTGGCAGGTAAAAAACAAGAAG
CCCCGGAACGGGCCTTCTCTTTGGCGGCATAGCCACTATGAACGGCTGGGCTGATCGTCTCGCTGGCGCG
TTAACCGGGCGCCGGAGCGAGCTGGTGCGGGCCTACTCTTCGACCTTGTTGAGCCACTCGACCTTGTATT
GGGCGCCGGCGCCCTGTGCCAGCTTGGCGCACAGCGGTTTGAGGAAGGCTTCAATCTGCTCGTCCAGCAT
CCAGGGCGGACTGATGATCAGCATCCCTGAGCCGTTCATGCCCCAGTCGGCAGTCTGGCCGGCCACTTCC
AGCTCGGCCACCAGGACGTTACCGAAGTTCTCGCGCTTGATGGCGCGCAGCATGTCGCGGGATTTGTCCG
CCTCTTGCCCGAGGATCGGATACCAGAGGGCGTAGATGCCGGTCGCCCAGCGTTTCTGCGCCTTTTTCAG
GGTGTCGACCACGGCGAAGTAATCTTCCTTGAGCTCATAAGGCGGGTCGATCAGCACCAGCCCGCGGCGC
GGGGTCGGTGGCAGCAGGGCGATCAGCCCCTCGAAGCCGTCACGGTGGTGCACGGCGACGCGAGGGTCGT
AACCCATGTTGGCACGCAGGTCTTCGACCTCGTTGTTGTGCAGCTCCATCAGCGCCAGCTTGTCCTGCTC
GCGGGTGAGGCGACGGGAGAGCTCCGGCGAGCCGGGGTAGTAGTGCAGCTCACTGTTCTGGTTCATCTCC
TCGATGACGCCGAGATAGGGGGCCATGGCAGACCACTGGGCCCGCTCGTCCCACAGGCGGCCGATGCCAT
CGGCATACTCGGCCTTCTTCTGGGACCAGTCGCCGCGCAGGTCGTAGAGACCGCCACCGGCATGGGTGTC
CAGCACTATGAAGGGTTTCTCCTTCTTCTTGAGGGACTCGATGATCAGGGCCTGAACGGCGTGTTTCAGC
ACGTCGGCATGGTTGCCGGCGTGAAAGGCGTGGCGGTAGCTCAGCATATTAGATCTCTGAGGGGGCCCTG
CTGGGCACCCAGTAACGATAGATAGTTCAATAAATTGCGGCTGTTGCCGCGAAACAGAGAGAGGCTGCGC
AGGGTCTGCAACAGGACGCCCTGTTGCAGCCGCTCCAGCTCCAGCTCGTGGGCCAGCATCGACTGATACT
GCAGCTCGTCGAGTCGCGTCTTGGCGCTCTGGCGCAGGGCGCGCCACGGGGTCAGCACCGCTTCGGTCTG
CACCAGCGCCGGTTGCAGGTCGGTGAGATCGACCACGATGCCGAGTTGATCGAGCCGGTGCAGCAGCAGC
GCCAGATTGACGTTGCCCCCCAGCTCCTGGACGGTGAGCCAATCCTGGCTGCGCTCACCATAGACTTTGC
CGCTCCAGTGCCAGAAGCCGATCGGCGTCGGCAATTCGTGGACTGCGACCCGCTCGCTGGCCATCAGCTG
TTCGTCACGCAAAGAAGGCTTGCTCCATGGTTTCCAGCTCTTCGGAGAGGCTCATCCACTCCAGCTCAAC
TTCTTCCAGCTGCTCCTTGAGGGGGCCCTGGGATTTGAGCAGCTCGGAGAGCTTGGTTTTAGCGCCCTCT
TCGTAGATGGCGGGGTCAGCCAGTTGGCTCTCGATGTCGCCAAGCTTGCCTTGCAGCTTCTCCATGCTCG
CTTCGAGCTTTTCCAGCTTCTGGCGCAGGGGACGGGTTTGCTGGCGGAACTCCGCCTCGCGCCGCTTCTG
ATCCTTGCGCGCCACTGCCGAGTTGGCAGAGACGGGGCCATCCGCCGGTTTGGCGTTGGCCTCTTTCTCC
TGCTCGCCGAGCCACTTGTGGTAGTCGTCCAGATCGCCGTCGAACGGCTCGACCCGGCCACCGTGCACCA
GATAGAAGTCATCTGTGGTGGTACGCAGCAGGTGACGGTCGTGGGAGACCACTACCATGGCCCCTTCGAA
GCCTTGCAGCGCCATGGTGAGGGCGTGGCGCATCTCCAGGTCCAGGTGGTTGGTCGGTTCATCCAGCAGC
AGGAGGTTCGGCTTTTGCCAAGTGATCAGCGCCAGCACCAGCCGCGCTTTTTCGCCACCGGAGAAGGGGG
CCACCTTGTCGAGCGCCTTGTCACCGTGAAAACCGAAGCTGCCCAGATAGTTGCGCAGCTCCTGCTCGGT
CTTGTCCGGCGCGAGGCGGGCCAGATGATCGAGCGGGGAATCATCGAGACGCAGGGTCTCCAGCTGATGC
TGGGCGAAGTAGCCGATGCTCACCCCGGCGCTCACTTCGAGCTTGCCGGAGAGCGGCGGGTTGGAGCCCG
ACAGCATCTTGATAAAGGTGGACTTACCGGCGCCGTTGCGACCGAGCAGACCGATGCGCGAACCCGGTAC
CAGATTGAGTTTGATCTTCTCGAGGATCACCTTGTCGCCGTAACCGGCAGAGAGGTTCTCCATGGCGATC
AGCGGGGTCGGCAGGGCCGAGGGCTCGCGAAACTCGAAGCTGAATTCCGAGTCGGCGTGGGCGGGCAGGA
TCTTCTCCATCCGCTCCATCGCTTTCAGGCGGCTCTGGGCCTGACGGGCCTTGGAGGCCTTGTAGCGGAA
GCGGTCCACATACGCCTGCATGTGGGACATTTCCCGCTGCTGCTTCTCGTACATCGACTGCTGCTGGGCC
AAATGTTCGGCGCGCTGCAGTTCGAAGTCGGAGTAGCCGCCGGTGTATTCGTTGAGCTTGCCGTTCTCGA
TATGGATGATGCGGCTGATGACCGAATCGAGGAAATCGCGGTCGTGAGAGATGAGCACCAGCGTGCCCTG
ATAGCCCTTGAGCCACTTCTCTAGCCAGATCACCGCATCAAGATCCAGGTGGTTGGTCGGTTCATCGAGC
AGCAGCAGATCGGAGGGGCAGAGCAGCGCCTGGGCGAGGTTCAGGCGCATGCGCCAGCCACCGGAGAAGC
TGCGCACCGGCGAGCCGAGTTGCTCGTCGCTGAAACCGAGGCCGTGCAACAGTTCGGCGGCGCGGGCGCG
AATGCTGTAGGCGCCGATGGTATCGAGGCGACCGTGCAGCTCGGCCACCAGCAGCCCATTGTCATCCTGC
TCGGCTTGCGCCAGCTGGGCTTCCAGCGAGCGGAACTCCTTGTCGCCGTCGATCACATACTCGAGCGCGG
AGCAGTCGAGCGCCGGAGTCTCCTGCGCGACGCCTGCGATCTGCCAGCCAGCGGGCAGGCTGAAGGTGCC
GCTGTCGATGGCCAGCTCGCCCCGTACCAGTGCAAAAAAGGTGGACTTGCCACAGCCGTTTTTGCCCACC
AGACCCACTTTGTGGCCGGGGTGAATGGTGGCGGAGGCGTTGTCCAGCAGCTTGCGGCCGCCGCGGATCA
GTTCGATTTGACTGGCAACTATCATGGGTGGGATCTGCTTGCTCAAAATGGAGGAGAAAAACTGGCGCGG
ATCTTACGTGAAATGACGCTGATTTGCACTGTAGCGAAGGGCTGGCCACTGGCTGGCACTGCAAAAACAT
GCCATAACAGAGAAACCAGTTTGGTTACGGGAGGTTAGCATGCATATCAGGGATATTATGACGACCCGGG

TCGCCACCGTTTCAATGGATGATCGACTCCGAGTCATCAGGGATATCTTCGAGCAGGCACACTTTCGTCA
CCTGCTGGTGCTGGAAGAGGAGGAGCTGGTGGGGGTCATCTCGGATCGGGATCTGTTCCGCGCCATCAGC
CCCTATCTCGACTCCGAAGCCGAGATGAGCCGCGATACCGAAACCCTCAACAAGCGGGCCCACCAGATCA
TGAGTCGTCAGCTCGTCACCATCGCTCCCCATCTCACCGTGCGCGACGGCGTCAAGCTGATGCTGGAGAA
GGGGGTCTCCTGCCTGCCGGTGCTGGAGAATGGCGCCCTGGTCGGCATCATCAGCTGGAAAGATTTTCTC
AAGGCGGCGCTGGAGAGTGACGCGCTGGTTTGAGTCTGGCACTAAGCACCATCAGCTGACAGAGAACAAA
TAGAGAGAGGGGAGCCAAGGCTCCCCTCTCTCTATATTGGCTTTGCCGACAATGCCGGTTTGCACGCCGC
CAGCATCATCACGGCTCGGGCTGTCCTGCGACAAGACAGGAGGGGCCGCTGCCGGAGAGGTGCGGCTGAA
GATGAGTGCGGATCTCCTGCAGGGTGCGCTGGTACTGCTGGGCATGTTGATCGAGAAACGCGGGGCTGTG
CTTGAGACGCCGCTCCAGGGTATCAACCAGCAGCGCCACCCTGGGGGCGGCCATACCCGCTGGCCAACAG
TGTTGCAGCAGCTCCAGCAGTTGCCAGGCATCGCCCGGCAGAGCGGGGAACTCCTCCAGCAGCTGTAAGC
CCGCTTGCAGGGCAGTCTGATACTGGCTGGCCTGCATCGCCTTGTGGCGCAGCTGGCGCAGTTCGCTCAG
CCGCTGCTCTCTCGCCGTCAGGCGCTGGCCCAACTCGGCCACCAGATGGTGGAACAGCTGGCGGGTGAGC
GGGTGGGGCGCCACCCTGACGCGGTATTGGGTCAGCTGGGTCAGGTAGCGCTGGGCGTGGTGGGGCAAAC
CGCAGAACTGGCTGACCGCAAAGCCGAGCAGCAGGGTGAGGTGGTTGTCGCTCCCCTCATCCGGCTGGTG
GTGGGCAATCAGGATCAGCGCCTCGTCGAAGCGGTAATCCAGCCCCAGCATCAACGCCTGCAGCAACTCA
TATTCGTGGGGTTGCAGATGGACGGTGCGTTTGAGGTGCTCCCACTCCTGTTGCAGGGAGCGGATCCGGT
TGCTGGAGGGGGCGTGCCACAACACCGCCAGACTGGCATTGAGCATGAAACTGTCGATGGCGAGCCGGAC
GCGCTGTTCGCTGAAGTGGCGCAGGGTCAGGCTGTTGGCCTGCTCCAGCACCTTCTCCTCATCTTGCAGC
ACCAGCCCGAGCAACATCGCCAGCTGATGCCAGCGCGGCTGATGGGGGCGGATCTGCAGCAGCTCTCCCA
TGGTCTCCCAGGCCGCCAGCCAGTAATGCGGATGCAGCTGGCTGGCGAGTTGCAGCTCAAGCCGGGTGGC
CATCGAGATGCTGGCTTGATGGCGCTGCAAGTCGGTCTGGGCGCTTTGCAATTTGCCGAGCCCCAGCATG
GCGTAGAAGAGCCCGGCCCTTGGCCAGCTCGCCTGATATTCGTTGGCACAGAGGGCATAGTGCTGGGCGG
CAGTGATCCGCTCGGCTGATTGCAGCAGCAGATAGCCCTTGAGCCGATCCAGCCAGGGGGTCAGCGAGCG
ATTCTCCCGTTGAAACAGCAGGTCTTCACAGAGGGCGGTAGCCAGTTGCCACTCCCGCAGCAGAATGGGG
GCGGCCAGCGTGCGGGTCAGGTTGCTTAACTTCACCAGCTGGCCGAGCACCGCGGTGAGCTGCTGGGTCA
TGAAGGGGAGGGGCAGATAGAGGGAAAAGTAGAGATCCGAGGGGAGAAACTCCTGCTCGCTTTGATCGGC
ATCGAGCAGCAGCAATACGCAACCGGGTTGCAGCAGGCCGAGATAGTGCGCTTCGTGCAGCAGCCGGCCT
CCGTCAAGATCCTGCTGCTTGTCCTGCTGACAGATGATGGCGTCGAAGGCGTGCTGTTGCAGCAGGGCCA
GCAGGCCACTGCGCTCGTGGTTTGGATGGCTGTGGTAGTGGATCCGGTCGGCGGGCACCCCCTGTTCGGT
CAGTTCCCGGGTGATGGCGCTACGGAGCAGGGGATCCTCTTCGAGCAGCAGTATCGTCATGCAGGTACTT
CCTGTGAGCAAACCATTAGGTTGATTATGGTAGACCCAAGAGGAACATAGGCACAGCACTCGCTGAGTGA
AAAGCTGACAATAGCCAGCAGGGTGCTGGAGGGCGCACTATGGGGTGTTATTTCAATCGGTTATGCCACC
TCTCAGGGTGGCAACCTGTTATTGCCGAGGGCGGGCCTGCGTCCCGAAGGGGCAGTCGCTCTGGCGGATC
AGCCGCGACCCCTTGCCCTGTTCCCCCAGCCAGCTTAATTCCAGCTTGCCGAGGCGGCCCAGATGGCGCA
GCTCGAACTCGTAGCGCTGGCAGAGGTTGTCCTGAGGCAGGCTCGCCATCCCCGCTGGCAGTCGGTATTG
ATAGCGAAGGAGTGGCCCATCTTCCAGCTTGCTGGCCACCGGCACCAGCCCCTCTGGGGTGGGCTGTGGC
GCCCCGAGTGCGCGGCGCAAACTGAGCGGCGCATCGATCGCCTTTTGATAGTGCAGGCCATTCATCGCCG
CCAGCTCCTCTTCCAGCCGTTGCAACTCTCCTTCCAGTCCATTGAGCGCGGCCCCCAGCTGCTGGTGGGT
CGTTGACGAGGCGCAGCCACCCAGCAACAGGGTGGCAAGCAGCAGGGCGGCTCTGATGGGGGGCACGGCA
TCTCCTTGGCAATGACGATGAAGCGTGAAGGTCAGGTCGGCTGGCCCTGACTATAGCAGGGGATTGCTGG
GGGGCGTCCCCTGCAGCAGTTGCGCCAGACGGGCCAGCCCCTCGCCGACCCGGGCGTCGGTGGCGGGTTG
GCTCAGGCTCAGGCGCACCCCCTGCGGAGCGGCAAACTGACCGGCGGCGAAGTGTTCCCCGCTCGCCACG
CCAACGTCAAGGGCATCGGCGGCAGCGGCAAACTGCTGGCTGCGCCAGGGTTCAGGCAGCGGTAGCCAGG
CGTGCATGCTGCCAGGCTGCCAGCGGGCGGCAGGCAGCAGGTGGCGCAGCAGTTCGCCACGGCGTTTGAG
GATCTCTCGCTGCTGCTCGACCAGCGGCATGATCTGGCGCTGACTGACCATTTTGCAGGCGAGCTCGATC
AGCAGCGGGCTCACCATCCAGCTCGACAGCCGCATGGTCTGGGCGAAGGCGCTGCGCAGGGCAGGGGGCG
TCAGCACGAAGCCCACCCGCAGACCGTCGCAGGCGATCTTGGCCAGACTGCCCAGATAGATGACCCGCTC
CGGCGCCAGATTGACCAGAGGGATCACAGGTTGCTGGGGCAGCAGGCCGTTGACGTCATCCTCGATGATC
AGCAGATCGTGGCGACGGGCGATGGCGACGATGGCCTCCCGCCGCGCCAGACTCATGATGGCGGTGGTGG
GGTTTTGCAGGGTCGGCGTGAGGTAGAGCAGCTTGGCCCGGCGGGTGCGGCAGGCGGCGTCCAGCGCCTC
GGGCAGCAGCCCCTCCTCGTCCATCGCGAGCCCCACCACCTGATTCTTCAGCTGGCGAGCGTTACCCAGC
ATGCCGGGGTAGCTCAGCCCCTCGCACAGGATGGTCTCCCCCACGCAGCCGGTCGCCAGCAGCGCCAGCA
TGATGCCGTGCTGGGCGCCGTGGCTGAACAGCAGGCGATCCTCACTGCAGGCCACCCCTTGCTCCGCCAG
CCAGTTGACGAACTGCTGGCGCTGATCCGGTCGTCCCGCTTCTTTGTCATAGCCGAGCAGGGCGTTGAGA
TCCCCGTCCGCCACTTCCTCCAGCATGGGGCGAATGATGGCCGCCCGATCCAGCTGCACCGGCAGGTTCT
GCCACAGCTCGATGCGGTGCCCCTCCTCCTGGCGGATCACCCACTCATTGGCGGGATCCGTGTTCGCTCC
CTTGACCCAGGTGCCGTGGCCCACCCGGGCGGCCAGCAGGCCGCGCCGCTCCGCCTCGCTGTAGGCGCGG
GTGACGGTGCCGACGGTGACACCCAGACGATCCGCCAGGGAGCGGTGGGTAGGCAATCGGGTCTGCGGTG
GCAGCTCGCCATTCGCGATCCCCTGTTCGATCGCTTTGACCAGCTTGAGGTAGAGGGGGCCATCGAAGGC
TGCAAGGTCGGGTGTCCAGATTGTCATGGTGACAATAAATCCATTGATCAAAATTTGTTGTCAATATAGC

GTGAATTTCAACCTGATAACAACCAAATAGCACACATTGTATCGATACAATGTGGCAAGCAAGGAGTCAC
AATATGACGATGGAGTGGTATGTCACCCTGTTCGGTTTTGCCGTACTCACCTGCGGTACGCCGGGGCCCA
ACAACCTGATGCTGACGGCCTCCGGCGCCAACTTCGGGGTGCGTCGCACCCTGCCACATCTGCTCGGGGT
CGGATTGGGGCAGCCGGTGCTGCAACTGGTGCTGGCGCTGGGGCTCTATCCGCTGTTCGAGCGCTGGCCC
TTGCTGCGGCTCGGTTTGCAGATCTTCGGTAGCCTCTATCTGCTCTGGCTGGCGTGGCGTATCGCGGTGG
CCGGGGCGCCGGGGGATCCGGCCCGGAGGGCCCGTCCGCTGACCATGCTGGAGGGGCTGGGCTTTCAGTT
CCTCAATCCCAAGGCGTGGATGATGGGGATCTCCGCCATCAGTCTGTTCAGCCTGCCCGGCGCCAGTTAC
TGGTCGAGTCAGCTGGCAGTGATGGTCATGTTTGCGCTGGTGGCCCTGCCGGTCTGCTTTGTCTGGGTAC
TGTTCGGCCATCAGCTGAGCAACTGGATCAGCTCGGATCGGGGCTGGCGGCGGCTCAACGGCTCCCTCGG
TCTGCTGACGGCGCTCTGCGTGGTGATGCTCTGGTATTGAGCCGTGCTGCGCTGATGGGGGAGGTGGCCG
AGAAAGAGGCATTTCTGACTTGTCGCCACGGGGTTGGCTCTGCATATTAGAAGGCTTTCCCCATAACGGG
CGCGTTGCATCGACAAGAGGTTGAGATGGATCTGAACTGGTTACTGCCCCTGGCGGGGTTTGCCTTGGTT
ACCTGCGGCACGCCGGGGCCCAACAATATGTTGCTCACCAGCGCCGGGGCGGCGCAGGGTTTTCGCCGCA
CGATTCCCCTGCTGGTGGGGGTAGTGGGGGGGATCAATCTGATGATCCTCGCTACCGCGCTGGGGCTTGG
TGTGGTGTTCGAGAAGGTGCCGCTGCTGCACGATGGCCTCAAGCTTATCGGTTCGGCCTATCTGCTCTGG
CTGGCGTGGAAGGTGGCGACCGCCAGCGGCCCGGCCGAGGGTAACCGGCCGCTTATTCCCGCTCATCAGG
GGGCCATGCTGCAACTGCTCAATCCCAAGGCATGGATGATGGCGCTCTCCGCCATCAGCGGTTTCACCCT
GGCGGGGGAGGCTTACTGGCCCTCCGCGCTCTGGGTGTTGGTGACCTTCTTTATTACCGGCCTCTATACC
GGGGCGTTCTGGGTGCTTTTTGGTGCCCAGGTGCGGCAAATCATTCGCACCGCCCGGGGCTGGCGGCGTT
TCAATCTTGGCATGGGGCTGGCGACGGCCGCCTGTGTCGGGATGATCTGGATTTAGTCCGGGGTTGACCC
GGGCCTTTACCGGCTGTCTTGTTGGCCCTGCGGGGACTTTTTATCTCGCCCCCGTGACCTTGCCTGCAAG
GTGGCGTGGTTCTGCCCTCCCTTGGGATGAAAGGAGTTCATATGAAATGTATCGGTCTGCTGGGTGGCAT
GAGCTGGGAGTCCACCGTCAGTTACTATCAGGCGCTCAATCGCGGGGTGCGGGCGCAGCTTGGCGGTTTG
CACTCGGCGCGGGTGCTGCTCAATAGCGTCGACTTTGCCGGTATCGAGCGGTTGCAGCATGCCGGTGACT
GGCCCGCTACCGCCCGCCTGCTGGCGGCGGAGGCGCGCAAGTTGCAGGATGGCGGCGCCGATTTCTTGCT
GATCGGCACCAACACCATGCACAAGGTGGCCCCCGAGATTGAGGCGGCCATCGATATCCCGCTGCTCCAT
ATCGCCGATGCCACCGCGGCCAAATTGCAGGCGGACGGGATAACCCGGGTCGGCCTGCTCGGCACCCGCT
TCACCATGGAGCAGGATTTTTACAAGGGGCGCTTGCAGGAGCGCTTCGGGTTGGCGGTGCTGGTACCCGA
TGAGGCCGGGCGGGAGCGGGTACACAGGATCATCTATGACGAGCTCTGTCTGGGGGAGATCCGCGAATCG
TCGCGGGCCGAGTATCTGGCCATTATCGAGGAACTGGCGGCCGCGGGTGCCGAGGCGGTGATCCTCGGCT
GTACCGAGATTGCCCTGCTGGTGGGGGATGCCCGGGCGGTGGTGCCGCTCTACGACACCACTGCCATCCA
TGCCGAAGCGGCGGTCGCGCTGGCACTGGCAGTTGATTAATAAACTGGTCAGATCTCACTATTCATGCTT
GTCTCATACCCCTTGCCATAAAAGCGTGTAAAATTGCGCCCTGAATTTTCCCCGGCCAACGCGGGTTGGC
TGCGGTTTCCTAGCATGCAAGGCTGACATTGTGACTGAAGTAGATAGATTCGCGGATATTCGCCCCTACC
GCGATGAAGAAGTGCCGGCAGCCATCGAACGGCTGATCCAGGATGATGAATTTATCGGCGCCATCGCCAA
GTATCGTTTCGGTTCCCTGATGAGCTGGCTGGGTTGGGCCATCCGCCCGATGATCCGCCTGTTCCTGCGC
CGCAAATGGTCAAAAGTGACCACTGTGCATCAGGTCCAGATGGGGGTTACCTGGTACATGTCCCGCATGA
TCGCCACCTCCACCAAGGGGGTGACCTTCTCCGGGATCGAGCATCTGAAGAAAGAGCAGGGCTATCTGTT
TGTCTCCAACCACAGAGACATCGCCATGGATCCGGCCTTCGTCAACTGGGGGCTGCACACCCACGGTTTC
GATACCGTGCGCATTGCCATCGGTGACAACCTGCTGCGAAAACCCTGCGCTACCGAGCTGATGAAGCTCA
ACAAGAGCTTTATCGTCAAACGCTCGGCCAAGGGGCCGCGGGAGATGATGAAGGCGCTCGGCGAGCTGTC
TGCCTATATTGGCAACTCGGTTCACGAAGGTCACTCCATCTGGATCGCCCAGAAGGAAGGGCGCGCCAAG
AATGGCGATGACAAGACAGATCCCGCCATCCTCAAGATGTTCTACGTCGACGGCAAGAAGCAGAATATCG
ACTTCGTCGACTACATGCGCAGCCTCAATATCGTGCCGGTCAGCATCAGCTACGAGCTGGACCCGACCGA
CAAGGCGAAGGCGCGCGAGCTCTACGAGAAGTCGGTCAACGGCAGCTATGAGAAGGGCGAGTTCGAGGAT
ATCGAGAGTATCGTCGCCGGTATCGTCGGCATGAAGGGGCGGGTACATGTCTCCTTCGGCGCGCCGATCA
CCGAGGGCTTCAGCGATCCGGACGAACTGGCCGCGCTGATCGACAAGGCGATCTGGAGCAGCTACCACCT
CTTCCCCAGCAACTATCTGGCGGCCGATGTTTCCGGCAAGGCCAATACCATCGAACAGGCGGCCTTCCTG
ACCCGTCAGTCAGAGGTGCCGGAAGAGCACAAGGCGATCTGGCGAGCCATGTACGCCAAACCGGTGGAAA
ACGCCAAAAAGGCTTGAATGAAACCGGTTGTTAAACGGTTTTGCGAAAAAAACGGGCGCTGATGCGCCCG
TTTTTGCGTCTGGTCAAAGTCGAGGGGGAAAGGGTGGTGAATAATATGGTTTCAGCTGGTACGATCACTA
TGAAAATGGGATTATGTCTCTTGGCAAGGTGCTGACGAGTGTGTAACAGTGCCCATATAGCTGAACATGA
GCAAAAGCTCTAAACCGGTTTGCACGGATTGCAGACGGCATTATTGAAGGACCTGTCACAGGTCCTTTTT
CATTGGCTGGCAAAGATGCCGCCCAGATATACCTAACAGGAAACAGACGCCATGATCACCGGTAGCCTGA
ACACCCTGCAGCGGACGCCGCTGCCCGCCCCGCTGGCCCGCATTATGGCTGAGGTGGTGAAGAGCGTGGC
GCACTGGGGTGACGCTCCGCTCGGCAAGACCGAGATCGATGGCCTCAATCTGTTCTGTCTGGTGAGCGAA
GATCTGACCGAGCCAGCCGCGGATCGCCGTGGCGAATTCCATCAGCAGTATCTCGATATCCAGTTGCTGC
TGCGCGGTGAAGAGTGGATAGGTGTGGCCCCCACGACTACGTGAGCGACGGCGCCGACCATCCCCATCC
CGACCTCTGGTTTGTCGATGACGAGCAGACCAGCTACCTGGCGCTGCAACCGGGCGATTTCGCCATCTTC
TGGCCGGGCGAGCTGCACCGCCCGCTCTGTACCTTCAACCAGCCCGCCAAGGTGAAGAAGCTGGTGGTCA
AGGTACACAAGGATCTGCTGGCTATCGCCTGAGCATTCCCATTGAGGCGAGCCCTGCGCAGCTGATGCGT

TGAGACTCGCAAGCATTGAAACCAGTGATACGCACCACAAGTTGCGATGACTCTTTCAAGAGCAAGAATT
GAAAAATTGGACGCACCCTCCTGCGACCATAAGCCCCCGGCAGGGGAATCCCATCCTCTTTTGGATGACA
CGACACTGTCGGCATAAAGCCGAAGCACAGGAAGATGTAGAAAATGAGCAAGTTGGTATTGATCCTTAAC
TGCGGTAGCTCTTCCCTGAAATTTGCCGTAATGGACGCCGAAACAGGTGAGGACATGCTGTCCGGCCTGG
CCGAATGCTTCAATCTGGATGATGCACGTATCAAATGGAAACTCCACGGGGTCAAGGGCGAAGCCATGCT
GGGAGCGGGTGCCGCTCACCAGGAGGCCCTCGACCATATCGTGGGTCACATCCTGCCGCAGGATCCGACT
CTTGCCGAGCAACTGGTGGCCATCGGTCACCGAATCGTACACGGTGGCGAGCAGTTCTCCCGCTCCGTGC
GCATTGATGACGGCGTGATCGCCGGTATCGAAGCCGCCATTCCGTTCGCCCCGCTGCACAACCCGGCGCA
CCTCATCGGTATTCGCGCCGCGCTGAAAGTGTTCCCGCAGCTGTCCGAGAAGAACGTGGCCGTCTTCGAC
ACCGCCTTCCACCAGACCCTGCCGCAAGAGGCCTACCTCTATGCGCTGCCGCACAAGCTCTACCGCGAAC
ACGGCATCCGTCGTTACGGTGCCCACGGTACTTCTCACCGCTACATCGCGGAAGAAGCCGCGAAAGAGCT
GGGCAAGCCGTTGAATGAGCTGAACATCATCAACTGCCACCTGGGTAACGGTGCCTCTGTCTGCGCCATC
AAGAACGGCGAATCCGTAGACACCTCCATGGGTCTGACTCCGCTGGAAGGTCTGGCGATGGGAACCCGCT
CCGGTGATATCGATCCGGCCGTGGTCTTCTTCATGCATGACCAGCTCGGTTACAGCGTTGAGCAGATCAA
CCACACCCTGACCCGTGAGTCCGGTCTGCTGGGGCTGACCGAAGCGAGCAGCGACTGCCGCTACGTCACC
ACCAACTACGCCACCGACGCCGGTGCCAAGCGTGCGCTGGATGTCTTCACCTACCGTGTGGCCAAGTATG
TCGGCGCCTATGCCGCTGCGATGGACGGTCGTCTGGACGCCGTGGTCTTCACCGGCGGTATCGGTGAGAA
CGCCGCCATGGTGCGCGAGATGGTGCTGAACCGTCTGGCCCTGTTCGGCTTCAAGGTGGACAAAGAGGCC
AACAACGCCGCAGTACTGGGTGGCGAAGGTCGCATCACCACCGCCGACAGCCGTTGCGCCATGGTGATCC
CGACCAACGAAGAGCTGGTCATCGCCCGCGATGCCCTGAGCCTGGTCTGCTAAGTCAGCCTCGCTTTGAA
ACAACGCCCGGCCTGTGCCGGGCGTTGTCATTTCTGGCGTTCACCGATTTGACATGGGGGAGGGGCGCGG
TACCCTGACCACCACTGTTTTTGCAGCCAGCGGGCTGCCCGGTTCGGGAGAGAGCAATGAAAGAGGCGTT
TTTTAACTGGTTGTCGGGCCTTGGGGTGGATGTGACCAGCCTGATGGCGCTGGTGATCGCGCTGGGCTTT
ATCGTCATCACCTCGTTGCTGCTGCACATGGCGCTGCACCGAGTGTTGCTGGTCCGGCTGGTGGGGATCC
TCGGCAAGGCCAAGCAGGTATGGCTGCCGCCCCAGTTGCAACAGCGCCTCTTCAACCGGCTCGCCTTTGT
GCTGCAGGGGGCCGTGCTGCTCGGTCAGGCGGAGATCTGGCTGCCCCGCCAATCGGATAGCCTCAAGCTT
ATCGAGATGGTGGCCCAGCTCTGGATTTTGCTGTTCCTGCTGCTGGCCTTCTTTGCTCTGCTCGACTGCG
TTTTATCGGTGAGCCGTTACACCCGTCTTGGTCGGGATTTGCCCCTGCGCGGCATCTTCCAGGGGGCCAA
GCTGGTAGGCAGCATTCTGGTGGGCATCCTGATGATCGCCCTGCTGCTCGGCAAGTCGCCGCTGTTCCTG
TTCAGCGGTCTGGGTGCCATGACCGCGGTGCTGATGCTGGTATTCAAGGACCCCATCCTCGGGCTGGTGG
CGGGGATCCAGCTCTCCGCCAACCGGATGCTGAGCGTCGGTGACTGGCTGCAGATGGACAAGTACGGCGC
CGATGGCGAAGTGACCGATATCGGCCTCACCACGGTCAAGGTGTCGAACTGGGACAAGACCATCACCACC
ATTCCCACCTATGCCCTGATCTCCGACTCTTTCAAGAACTGGCAGGGGATGACCGAGTCGGGCGGCCGCC
GCATCAAGCGCAGCGTCAATATCGACATGACCAGTGTGCGCTTTCTGACGGCCGATGAGCAGTTGCAGCT
CAAGCAGGCGCAGCTGCTGGCACCCTATCTCAGTCGCAAGGAGCAGGAGCTGAGCGCTTACAACCAGCAG
CTGAGCGATGCCCTGAGCTGCCCCATCAACGGTCGTCACCTCACCAACCTCGGCACCCTGCGCGCCTATC
TGGATGCCTACCTGCGCGCCCACCCCGGCATTCGCAAGGACATGACCCTGATGGTGCGCCAGCTGGCACC
CACGTCAGACGGTCTGCCGCTGGAAATTTACTGCTTCACCGCGACGACCGCATGGGCCGATTACGAAGGG
ATCCAGGCCGATATTTGCGACCATATCTTCGCCATCATTGAGCAATTTCATCTGCGGTTGCATCAATCTC
CTACAGGTTACGACATGCGTACCTGGCAACGGGGATAAACAGGGAAACCGGACGGGTCGGGGACGCGGAA
AAAACCGCATATTAATCATCGACCCGTCGCCACTTGTCAGTTGAAATTTGCCGTGCAGAGGAGAAAAATG
ACGGCCTTCTCTTCGCGCCCCTGATCCCCTCTTGATCAGCTGCCAGCCCAGAGACGTCACTTCAAGTCAT
CATCATGGGAAAGTTATGAAGCTCCTTCGCAATAGTGTGGCAACACTCTGCTACAGCCTGCTGCTGACCG
CTGCGCCGGTTTTCGCTGTTGAATATGCTCTGCCTGCGGCCAACAGCCGTCTGGTAGGGGAAAATCTGGA
ATACGTGGTTCCGGCCGAAGAGAACGGCCAGCCGCTGGAGGCCATTGCGGCCAAATTCCAGCTGGGGCTC
ACCAACATGACCGAAGCCAACCCCGATGCGGATCCCCTGCTGGTACAGACCGGCGAGAAGCTGGTGATCC
CCCAGCAACTGATCCTGCCGGATGCACCCCGTGAAGGGATCGTGCTGAACGTGGCCGAGATGCGGCTCTA
CTACTACCCCAAGGGTAAAAAAGTGGTGGAAGTGCTGCCCATCGGCATCGGCCAGCTCGGCACCGATACC
CCGGAAAAACTGGGTGACCAGCGTGCAGCGCAAGAAAGCGGGCCCGACCTGGACCCCGACCGCCAAGATGC
ATGCCGAATATGCCGCCCGTGGCGAGAGCCTGCCTGCGGTATGGCCGGCCGGCCCGGACAATCCCATGGG
TCTGTTTGCCCTCTACATCGGCAAGATGTACGCCATCCACGGTACCAATGCCAACTTCGGTATCGGCCTG
CGGGTGAGCCACGGCTGTGTGCGCCTGCGCAACGATGACATCGCCTATCTGTTCAAGCAGGTACCGGTCG
GTACTCGCGTCCAGTTCGTCAACCAGCCGATCAAGGCCTCTCTCGAGCCCAACGGTGCCCGCTATCTGGA
AGTGCATCAGCCGCTGTCGCGCAGCGAAGAGGAGTTCAACTCCAAGCTGCCGGCACCGCTGCCGATGACT
CCGGCGGTGACCCGCTTCATTGCCCACGCCGACAGCGACTCCCAACTGGTGAAGCAGGCGCTGGAGCAGC
GCAACGGCATGCCGATGGCGATCAACCGCTAAGGGCAAATCCCCGTCACGACAAAGGGCGAACAGCATGC
TGTTCGCCCTTTGTGTTTCTGCTGTGCTGACTCAGCTTCGCAGCAGATTTGCCTGCAGCTCGCTGCCGAG
CTGATCGAGCAGCTGATAGCGCGCCTTGTAGCTGGCCCGCTTGCGGGAGGGGATCTCCTCCAGCGTCTTG
CGGGGCACTTCCAGCGGCAGGCTCATCCAGAGTTCATCTTGCACGCAGCCCTGATGCTCCTGCCACAAGC
CGTCGAGGTCGAACTTGACCCGATCGGCAGTGCGTTTGCTGCTCTGATAGACGTGGCTTTGGGTGCGGAT
CCCGAGCAGGCTTTTGCACGCCAACCCCCGGGCCAGCTCGGCGGCCATATAGACCAGCAGGGACATGGGG

CGCAGCCCGCCGCACGCCTTGGTGACCAGCTTGATCTCCTCGCTCTGCCCCTTGCCGCCCTGCAGCGAAC
AGATCTCGAGGGTCGGGTTCGGCGCGGGGCGCAGATTGAGGGCCATGTAATATAGCGCCGCGTCACCCAG
CTTGAGGGTCAGGCTCATCTCCCCCTCTTTGCCCATGTGGGTTTGGTAATCCAGTTGCAGGGTGAGGGGG
GCCGTCAGCTTGTCGCTCTGCCACTGGGCCAGCAGCAGGCCGCGCCCGCTGTAGATTGCCAGTTGTTGCG
CCTTGGGCAGCTGACGTGCCAATTGCTGGTAGTGGCCGCTGATAAGCCCGGCTCGCAGGCGCTTGCGGAG
CCCCTTGTGGCGATAGGGGTGGATCGGCTTTTCCAGAATATCGGGGAAGCGGCTGATCGCCTGCGCCAGC
AGCGGTTGCTCCTGCTGCTGGCGATAGAGGGCGCGGGCGCCGGGATTGAGCCAGAGACGCCCCACATACT
TGGCCCGTCGGATGAGATGACCCGACTCCTCCACCGGATGAAGGCGGCGGGCGAGGGTGCGCAGGTGTGA
CAGATGAGATGACATGGTGCCGACCTCTGACTGGTGTAATGAGACAGGGCGGGGAGTGTAGGAGCGGGGC
GCTGGCTTGTAAAAAATGGATAGTAACAAAATGTGAAAACATAACGCTCAGTAGCGGCTGGCGCTGCGAG
ATGACCGTCACGGCCACGGCAGGAAATCTGGCGCCATCCTCTTGATGCGGCAGATGATTTGATGCGCAAA
GGTTTTTATTGTGTTTTATGTTTGTTAAAAGTGGTTGAGCTGGCGCGAACGGCTGATTAAGCTAGGCCGG
TTTTTTAGGGATGAGCCCGTCACCAGAGCGGGCAGAAAATGACACAAAACGGAGTTTTGCATGACAGTTA
GCACACCCATGTTAATCACCTTCCTGGTGTATATCCTCGGGATGGTGTTGATCGGCTTCGTCGCCTATCG
CGCCACCCGCAACTTTGATGACTACATTCTGGGCGGCCGCCGGATGGGGAGCTGGGTCACAGCTCTCTCT
GCCGGCGCCTCCGACATGAGCGGCTGGCTGCTGATGGGGCTGCCCGGCGCCGTCTTCGTCAGTGGCATCT
CCGAGAGCTGGATCGCCATCGGTCTGGTCATCGGTGCCTACCTCAACTGGCGCTGGGTAGCGGGTCGCCT
GCGGGTACATACCGAGAAGAACCGCAACGCCCTGACCCTGCCGGACTATTTCACCTATCGCTTTGAAGAT
CACAGCAAGGTGCTGCGCATTCTCTCTGCGCTGGTGATCCTGCTGTTCTTCACCATCTATTGCGCCTCCG
GCGTGGTGGCCGGTGCCCGTCTGTTCGAGAGCACCTTCGGCATGGATTACAACACCGCCCTCTGGGTCGG
TGCCGCCGCCACTATCACCTACACCTTCTTCGGCGGCTTCCTCGCGGTGAGCTGGACCGACACTGTGCAG
GCGACCCTGATGATCTTCGCCCTGATCCTGACCCCGGTCTTCGTCATCATCGCCGTCGGCGGGGTGGACA
GCGCCATGATCGAGGTGGCCGCCAAGAGCGCTACCAACCTCGACATGTTCAAGAATCTGGATGTGATCGC
CATTGTCTCGCTGATGGCCTGGGGCCTTGGCTACTTCGGCCAACCACACATTCTGGCCCGTTTCATGGCG
GCGGACTCCCACCACTCCATGGCCAATGCCCGCCGAATCAGCATGACCTGGATGGTGTTCTGTCTGGCTG
GCGCGGTGGCCATCGGCTTCTTCGGGCTGGCCTACTTCGCCCGCTTCCCGGAAGTGGCGGCTGGAGTGAC
CGGCAACCCGGAGCGGGTCTTTATCGAGCTCTCCAAGATCCTGTTCAACCCCTGGATCGCCGGGGTGCTG
CTCTCCGCCATTCTGGCGGCGGTGATGAGCACTCTAAGCTGCCAGCTGTTGGTCTGCTCCAGCGCCATCA
CAGAGGATCTCTACAAGCCCTTCCTGCGCAAGAACGCCTCCCAGCGTGAGCTGGTGTGGGTGGGGCGCCT
GATGGTGCTGCTGATCGCGGTGATTGCCATCGCCGTCGCCATGAATCCGGAAAACCGGGTGCTGGGGCTG
GTGAGCTACGCCTGGGCCGGTTTCGGTGCCGCCTTTGGTCCCGTGGTGCTGCTCTCTGTGCTCTGGCCGC
GAATGACCCGCAACGGCGCGCTGGCCGGCATGCTGGTCGGTGCCATCACCGTCATCGTCTGGAAGCAGTT
CGGCTGGCTGGGGCTGTATGAGATCATTCCGGGCTTCCTGTTCGCACTGCTGGCCATCGTGGTGGGCAGT
CTGCTGGGCGAAGCGCCCTCAAAAGCGATGCTGGAGCGTTTCCGCGATGCCGAGGAGGAGTACAACACTC
CGGCCGAGAGCGCGGGAGAGCCAGTCGTGGTGAACTGATCCCCCTTACCGCTTTCTGATTGAGAGCCCCG
CCCCCGGACAAGGAGCGCCCTGGTTGGCCATCCTTGCACCGGTTTGGCGGGGCTTTGTGCTATCTGATTC
ACTCTTGGGCACTTTTTTATGTTCGGCCGGTGGCTCCCGGGTGCTCGCTTTCTTTTGTCTTGGCACCGAA
TATGGGACACTAGGCCGCCTGAAGGAGACCTAGTGCATGCGGCTATTCACCCTGATCCTGATCCTCCTGC
TGGGGGGACTGCAATATGACCTCTGGCTGGGGAAGAACGGGCTATCCGATTATCAGAACCTGTCGGAGGC
CATCTCGCAGCAACAGCGGGACAACCAGACTCTCAAGGATCGTAACGACCTCATTTACCGCGAGATAGAT
GATCTCACCTCTGGCCTCGAGGCCATCGAGGAGTTGTCGCGCAACGATCTGGGTTACATCAAGCAGGGGG
AGACCTTCTACCGGATCATCCCCAAAGAGAAAGACAAAGAGACAGCGCCGGACGATGAGCCCTACGATGA
CTGATGCCAGCCGAGTATCCGGCCTGACGGCGATCGTCCCTGCCGCCGGTATCGGCAGCCGGATGGGGGC
CGATTGCCCCAAGCAGTACCTGCAACTGGCGGGTAAAACCATTCTCGAACATACCCTGGAGCGGCTGCTC
TCCCACCCGGCCATCGCCCAGGTGATCGTGGCGCTGGCGCCCCATGATCGCTGGTTCGAGGCCCTGCCCG
TGGCGGCCGACCCGCGCATTGTGCGGGTCGATGGAGGCGCCGAGCGCGCCTTTTCCGTCCTCAATGGCCT
CCATGTGGCGCAAGGGGAGTGGGTGCTGGTGCACGATGCGGCCCGTCCCTGTCTGACTCACGGCGATCTC
GATGCGCTGATTGCCGCGGCCATGAACTGCGGCGGTGCCATTCTCGGCAGTCGGGTGCGCGATACCATGA
AACGCTCCGATGGGGAGGGCAATATCATCGCCACCGTCGAGCGCGAGCAGCTCTGGCATGCCCTGACGCC
GCAGATGTTCCCGACCCGTGCCCTGAAACGGGCGCTCGAAGAGGGGTTGCTGCTGGGCGCCACCATCACC
GACGAGGCCTCCGCCATGGAGCGGGCCGGATTCGCGGTGCGGATGGTGGAGGGGCGCGCCGACAATATCA
AAGTGACCCGGCCGGAAGACTTGTCGCTGGCGGGGCTGTTTTTAAGCCAGCAGGCATAAGCCCCTCAGTC
AGTACAGACCGTTCAGAGCAGAGTGTCTCGAACAAACAGGCCCGAACAAGGAGTTAGCATGCTCCGGATT
GGAGATGGTGTGGACGTACACAAAATTGATGGGGTTGGTTTCAGCATGTTGGGAGGTTGCCGCTGATGAT
TCGCATTGGTCATGGTTTTGATGTCCATAAATTTGGTGGTGTTGGCCCCTGCATGCTGGGTGGCGTGCCG
GTTCCCTACGAGCAGGGGCTGGTTGCTCACTCCGACGGCGATGTGGTGCTGCACGCGGTAAGCGATGCCC
TGCTGGGGGCTATCGGTGCCGGTGACATCGGTCGCCACTTCCCCGATACCGCTGCCGAGTTCAAGGGGAT
CGACAGTCGCATTCTGCTGCGTGATGTCTTTGCCCGGGTACAGAAGGCGGGTTATGCCATCGGCAACCTG
GATGTGACCATCATCGCTCAGGCCCCCAAAATGGCCCCCCATATCGACGCCATGTGCGCCGTGCTGGCCG
CCGATCTGCAATGTGATTTGAACCGGGTGAACGTCAAGGCGACCACCACCGAACAGCTTGGGTTTACGGG
GCGCAAGGAAGGGATCGCCACCGAAGCCGTCGTCCTACTGGTGAAACAATAATGCTGGAACAACTTGCCT

ATCTGCACGGGGCGCCCACCGCCCGCGGTATCCTCAAGGCCGAGGCCGCCGACTTCGTGGTCAATGAAGA
TCTCGGCTTCGAGCCCTGCGGGGAAGGGGAGCATATCTTCGTCCGTGTGCGCAAAACCGGCGAGAACACC
GCCTGGGTGGCGGGTCTGCTGGCCGATGCGGCCGGGGTCAATCGCAATGCGGTGACCTGGGCGGGCCTGA
AAGATCGCCACGCGGTGACCGAGCAGTGGTTCGGCATTCACCTGCCGGGCAAGGCCGAGCCGGACTTGTC
GGTCATCGAGAGCGACAGCATCCAGATCCTGCAAGCCAGGCGTCACAACCGCAAATTGCGGGTCGGCTAC
CTCAAGGGCAACCACTTCACCCTGCGGCTCACCGGTCTGGAACAGGCCGACGGGCTGGAAGCGCGCCTGC
AGGCCATCGCCACTCAGGGCGTGCCCAATTACTATGGTGAGCAGCGTTTTGGCCGCGATGGCAACAACCT
GGAAGCGGCCAAGGCGATGTTTGCCGGCAAGCGGATCAAGGATCGCAACAAGCGTTCCCTCTACCTCTCT
GCCGCCCGCAGCATGCTGTTCAACGCCATTGTCAGCGCCCGTATCGAGCAGGGGCTGGCTCATCAGCTGC
TGGCCGGTGATTGCGTGATGCTCAAGGGCAGCCACTCCATCTTCAGCGAAGAGAGCGTGACCCCCGAGCT
GGAAGCGCGTCTTGCCTCCTGCGACGTGCAGCTCACCGCCCCCCAGTGGGGCCGTGGCCGTCTGGCCAGT
CAGGGCGCCGCTGCCGAGTTCGAACAGGGCGTGCTGGCCCCTTACAGCGATTGGTGTGACGGGCTGGAGA
AAGCCGGGCTGGATCAGGACAGGCGCCCCCTGCTGCTCAAGCCGGAGCAGATGAGCTGGCAGCTGGACGG
CGAGGCGCTGACCCTCTCCTTCTTCCTGCCGGCGGGCGCGTTTGCCACCAGCGTGGTGCGCGAGTTAATG
CAGGCCGAAGAGGCCGATCACGGTTTCAGGAATCAGACCGATGCGAATTCTGGTCAGTAATGATGATGGT
GTGCATGCCGAGGGGATTCGTGCCCTCAGCGAGGCGCTGCGCGCCTGCGGCGAGGTCATTGTGGTGGCGC
CGGATCGCAACCGCAGCGGCGCGAGTCACTCCCTGACGCTGGAAGTACCGCTGCGGGTTACCCGCATCGC
AGAAAACGGCTTTAATGGGTGCGAGAGCTATGCGGTGAAGGGGACGCCGACCGACTGCGTGCATCTGGCG
GTCAACGAGCTGGTGCGTCCTGAACCCGACATGGTGGTGGCCGGCATCAACCACGGTGCCAACCTGGGGG
ATGATGTCATCTACTCGGGCACGGTCGCCGCGGCCACCGAGGGGCGCCATCTTGGCTACCCCTCCATCGC
CATCTCGCTGGTAGGCAAGACCCACTTCGCCACGGCGGCCCACTATGCGGCCCTGCTGGTGAAAGGGTTG
ATGAAGCACCCCCTGCCTGCCGATCAGATCCTCAACGTCAACGTGCCGGACTTGCCCCTCGAGCAGATCA
AGGGGATCAAGACCACCCGCCTTGGCAATCGCCACCGGGCGGAGTCGGTGATCTGCACCGAGGATCCCCG
TGGCCAGCCCATCTACTGGATCGGCCCCCCCGGTAGCCAGCAGGATGCCGGTGACGGTACCGATTTTGCT
GCCATCGAGCAGGGCTATGTCTCGATTACCCCCCTGACCATCGATATGACTGCCTATGACAGTCTCGCCG
GTCTGGGGGCCTGGTTAGATCTGCAGGGATGAGGGTGGTGTGATAGTACAGCGCCTGTTAAATCAGCTCA
TTGCGCAGGATATTCGCGATTTTCGCGTGCTGGCGACCATCGCGAAAGTGCCGCGCCAGCTGTTTGTGGA
CGAGGCCATGGCGCACAAGGCGTGGGACAACACCGCCCTGCCCATTGGCCACGGCCAGACCATTTCGCAG
CCCTATATGGTGGCGCGAATGACCGAGTTGCTGATGCAAAACAATCCGGCCCATGTGCTGGAGATTGGCA
CTGGCTCGGGCTACCAGACGGCGATCCTCGCCCATCTGGTGGAGCATGTGTATACCGTTGAGCGGATCAA
GTCGCTGCAGTTCCAGGCCCGGCGCCGACTGCGCCAGCTCGATCTGCACAATGTCTCGGCCAAGCACGGC
AACGGCTGGCTGGGGTGGCCCAACAAGGGGCCTTATGATGCCATTCTGGTGACGGCCGCGGCGAGCGAGA
TCCCGACGGCGCTCACCGATCAGCTGGCCGAAGGGGGTCGACTGGTGTTGCCCGTGGGTGACAGCCAGCA
GACCCTGCAACTGATCGAACGATCCGGATCCCAGCTGACCAGCCGTATTCTGGAGCCGGTACGTTTTGTT
CCCCTGATTGACGGAGATGTTGAGTGAAGTTGTTTTCCCGTTTGTACGTACTGGTGATGCAGTGGGCTCG
CCACAAGCATGCCCCCTGGTATCTCTCCATCACCGCTTTCATCGAGTCGGTATTCTGGCCGATCCCGGTG
GACGTGATGCTGGCACCGATGGCGCTCGCTAAGCCCCACAAGGCGTGGCACTACGCCACGCTGGCTACCG
TCTTCTCTGTGCTGGGCGCCCTGTTTGGCTATGCCCTTGGCTATGCCCTGTGGGATCCGGTCGTGCTGCC
ACTGGTGGAGTCTGTGGGCTATCAGGACAAGATCGAGATTGCTCGCAACTGGTTTGAGCAGTGGGGGATC
TGGGTCATTTTCATTGCCAGCTTCACCCCTATCCCCTACAAGGTGTTTACCGTGACGGCAGGGTTGTTGC
AAATGGCTCTGCTGCCCTTCATCATCACCTCCCTCATCGGTCGTGGCATGCGCTTTTTCCTGGTTGCGGG
CCTGATGCGCTGGGGTGGCGAGAAGATGGAACGCAAACTGATGCACTACGTAGATGTGCTCGGTTGGCTC
TGTATTGGTCTGGCCGTGGTGGCCTATTTCTGGTTTAGTCGTTAAGGATCCATTATGCGTTCGCGTATTT
GGCAAGGAAGCAGTGTCGTGGTGTTGTCATGGCTGCTGCTTGCCTGCTCCTCCAGTAAAAATGCAGCTCC
GGTAGATGGTGTTGATGAGGGGCAGTTTGTCTCGACCATTCCGGCTCGCCAAAGCGGTGCCCAGGTCAGC
GCCCCCATTCAGGGCGGTAGCTATGTGGTGAAGCGGGGCGATACCCTCTACTCCATCGCCTTCAACTCCG
GCAACGACGTACCCTCGTTGGCCAGCCTCAACAACATTAGCCCGCCCTACAACATCTATCCGGGGCAGCG
ACTGCGACTCGATGGTTCCAGCGCCAGCATGAGCAGCAGCCGCAGTGCCGGTACCTATGAGGTGCGCCGC
GGCGATACCCTGAGCAGCATCGGCCGCCAGTTCGGCATGACGCCGCAGGCGCTGGCCCAGAGCAACAATC
TGAGCGCCCCCTATGCGCTGCAACCGGGTCAGGTGCTCAATGTCCAGACTGCTGGCGGCGGTTCGACCCA
GATGGTGGCTGCGACTCGTCCGGCAACCGTGACACCGGTGGCCGTGAGTCGTCCGAGCAGCGGTCCGCGT
CCGTTGGCCGAGCTGAGTGGATCTGGTCAGACCACGCCGTTCATCTCTCAGAAATCCGTTGCTCCGGCAG
CACCGAAGGAATACGCTCAGGTCAAAGAACAAAAAACAAACAACTCCCAGTCGAGACTTGCTTGGCACTG
GCCCGCAAAAGGCCGGATTGTCGAGGGGTTCTCTGTTGCAGAACAGGGTAACAAGGGGATCGACATAGCC
GGCCAGAAGGGGCAACCCGTCTACGCCGCCAGCAGCGGTAAAGTGGTATACGCTGGCAGTGCTTTAAGGG
GGTACGGCAAGCTGATTATCCTCAAGCATGATGATGACTATCTCTCCGCTTACGCACACAACGACGAGTT
GCGGGTGAAGGAAGGGGACAGCGTCAAGGGGGGCTCGGTCATTGCCAACATGGGTAGCACGGACGCGCCG
GATGTGCGATTGCATTTCGAGATCCGGTACAGAGGCAAGTCGATCAATCCGATGAGCTATTTGCCAAAGC
GTTAACCCCAGAGAGACCGTCTTACTCTGGTAACGTCAGGGAGACACACCATGAGCCAAGAACAACTGGT
AATGCAGGATGCAGAGTTAGAGTTTGACGCTGAACCCGAATCTCATGAAGTAGAAGAGTTGCAGGAGGTA
GCCGCCGAGGCGGACGAGACTATCTCCGATGAGTTGCTGGCACCGGAACTGAACAAGGTGATGGATGCCA

CGCAACTCTATCTGGGGGAGATAGGTTTCTCCCCGCTGCTCACTGCCGAAGAGGAAGTCTACTACGCCCG
TCGTGCCTTGCGCGGTGACAAAGCCGCCCGCAAGCGCATGATCGAGTCCAACCTGCGACTGGTGGTGAAA
ATCGCCCGTCGTTACAACAACCGCGGCCTGGTGCTGCTGGATCTCATCGAAGAGGGCAACCTCGGTCTTA
TCCGCGCGGTCGAGAAGTTTGACCCCGAGCGCGGCTTCCGTTTTTCTACCTATGCCACCTGGTGGATCCG
CCAGACCATCGAGCGGGCCATCATGAACCAGACCCGCACTATCCGTCTGCCCATTCATGTGGTCAAAGAG
TTAAACGTTTACCTGCGCACCGCGCGCGAACTCTCTCACCGTCTCGATCATGAACCGACCGCCGAGGATA
TCGCCTTCGCGCTGGATCGTTCGGTGGACGACGTGAACCGCATGCTCAAACTCAACGAGAAGATCACCTC
GGTGGATACCCCCATCGGTGGCGATCGCGACAAGGCATTGCTGGATGTGCTCTCCGACGAGCGCGGCATG
GGGCCTGAGCTGGAGTCACAGGAAGATGATATGCGCAGCAGCATTGTCCACTGGCTGGAGGAGCTCAATC
CCAAGCAGCGGGAAGTGCTGGCCCGTCGTTTCGGTCTGCTGGGGTACGAGCCCGCCACCCTTGAGGATGT
GGGAGCCGAGATTGGCCTGACCCGCGAGCGGGTACGTCAGATCCAGGTCGAGGGGCTGCGCCGTCTGCGG
GAGATCCTGCTGAGCCAGGGGCTCTCCATCGAGGCGCTATTTCGTACCAATTAAGTCCGCACCAACTGAT
CAGCGCATCAGCCAAATAGTACGAGGGGAGCAACAGCTCCCCTCGTTTTTTATTGCCTGTCGTCTGGCGA
TGGCTTCGTGGTTATACCAGCTCAGCCAGCATGGTGTCGGCGTAGGGCACCAGTGCTTCTCCCTTCTTCA
CCTTGGTGAGGTAGTCCGGGTTGGCGATAAGCGGACGACCGATGGCCACCAGATCGAAGCGGTTGGCCTC
GATGGCCGCAGCGGCGCGCTCGGCGTCGTAGTTGCCCACCCCGACCAGATTGCCCTTGTAGTGGGCACGC
AGATAGTCGGAGGCGCGGCCATCCAGATAATCGAAGGTGAGGTTGTCATCGAAGATGCCGAGGTGGATAT
AGGCCAGCGGGCGATCGTTCAGCTTGCCCAGCAGGTAGTCGAACACGGCGCGATCTTCCGGGCTGCCCGC
CAGATGGACATAGGCACCCGGGGAGAGGCGAATACCAACCCGATCACCGCCGATGCGGGCGCTGATGGCA
TCGACCACTTCCAGCGCGAAGCGGCTCATCTTTTCCGGGCTGCCACCGTAGTTGTCGGTGCGCAGGTTGG
TGGAGTGGTGCAGGAACTGGTCGATCAGGTAGCCGTTGGCACCGTGGATCTCTACCCCGTCAAAGCCCGC
TTCGATGGCGTTGGCACCGGCAGTCGCGTAATCCTGTACCAGCTGGGCGATTTCCGCCTCGGTCAGGGCG
CGCGGCACCTGATAGGTCAGCTCGCGCATGCGGGGCACAGTGCCCTCGTGCGCCACGGCAGAGGGGGCCA
ACACTTCAGCCTGATGGAAGAAGGGGTGGGAGAGGCGACCGACGTGCCACAGTTGGGCGAAGATCTTGCC
GCCTTTGGCGTGTACGGCGCTGGTCACTCTCTTCCAGCCATCGATCTGCGCCTGGGTGAAGAGGCCCGGG
GTATTGGGGTAGCCCTGCGCGTCGGGGCGGATGATGACGGCCTCGGAAACAATCAGGCCGATGTCGGCGC
GGCGGCCGTAATAGGCAGCCATCTGCTCGGTCGGCACCAGACCTGGGCCTGCCATGCAGCGGGTGAGCGG
GGCCATCATGAAACGGTTGGCCAGGGTCAGAGTGTCATTGAGTCGATACGGCTGAAACAGGGTATTCATG
ATAGGTTGTCCTGAACGGGTTATTCTTGAACGTGCATTCAAGACGTTTTTTGCGTCGGGCGCAAGCTTTT
TTTGAATGCGCGTTCAAGAAGGTTGGCCCGAGCGGGCGGCTGGGGTAAACTTGCCGCCCTTTCCAGCCAC
TCTTTATCGGCGGGCCCTGTTGCCCCTTGCCGGTGAACCATTTCCCACGGGAGGAACCATGCGGATAGCC
GAGTTTGATCGGGACGAGGTACTGCGAAATGCCATGGAAGCGTTTCGCGCCAAGGGATATGCCAAGACCA
CCATGCAGGAGCTGGTGGCTGCCACCGGCCTGCACCCGGGCAGCATCTACGGCGCCTTTGGTAACAAGCG
AGGCCTGCTGCTGGCGGCGGTGGATCATTATGTCGAGGGCAAACGCACCCTGCGCCGCTCCCTGCTCGAC
AACCCGAGCCCGCTGGCGGGCCTTGCGGCCTATCTCGACCGGATTGTGGGGGAGGCGCTCTGCGGCTCCT
GTCTGGTGACCCGCACCATGATGGAGCTGAGCGAGCAGGATGAGGAGCTGACCCAGCGGGTCAAGGGGAT
CTATGGCGAGCTGGAGCAGGATCTGGCCGAGTTGCTGACACAGGCGCAGGCCAAGGGTGAGCTGGCCCCG
GATAGCAATATCCACACCCTCACCGCCCTGCTGCTGATCAACTTGCAAGGGCTGGTCACCTTTGCCCAGT
GCCGCCCCGATCGCCCCCTGCTCGACGACGTAGTCGCCCAGCTGATGCGGGCCCTGCGGGCGTAACGCGC
GCCCGGAAACAACCATTGCTCCTGCGGGAGCCATGGTTGTTTCCGGGGATGCCACACGCGCCGGGTTGTT
TGCTCCCTTCTCCCCTTGCGGGAGAAGGGTTGGGGATGAGGGGGAGGGCTCTGCTCAGCGCAATGAGAGC
CAAATATGGGCCAGTACGACCTCCATCCGACTCAGCACATCGTTATTCCACACTCGTATCACCCGAAATC
CCTGCTGATTGAGATAGGCTGTCCGAGCCTCGTCATGCATCTGGTTCGTTTGTGCTCCATGTTGCGAACC
ATCCACTTCGATGACCAATCCCTGTTCAAGGCAGATGAAATCCACCACATAGGGGCCGATGGGCATTTGG
CGGCGAAATTTCAGCCCCGCCAGACGGCGACTGCGCAGCTGTTGCCACAGTTTCTGCTCTGCCAGCGTGG
CATCCCGTCTCAGTCGCTTGGCAAAGAGGTTACTCTCCATAAATTGTCCCCACTCTGTATCGCCTTATAG
ATATAGCCAGCTTCCCATCTGTTTACCGGTTGGATATTGCATTCCCTCACTCTAGCCCTCTCCCGGAGGG
AGAGGGAACAGATCGAGTTGTTGTCGGGATTTGGTTGATCAGCACTGTGTCTATTGCCTTATTCGAGCTG
GGGAAGCTGGATTGGGTGATAGCAGATGGCGATAACTTTTCGAATGTATTGGGAATCGCCTGGCTGGCAG
ACTGGCTGAATGGTTGTCAGCGATGCTGCAAGTGGTAGCGTAACTGCTCCCTTCTCCCCTTGCGGGAGAA
GGGCTGGGGATGAGGGGGGAGGGAGCCGCCCTCACCCTAACCCTCTCCCAGAGGGAGAGGGAATTGATCG
AGGTTCGGTTTGGGCATCATGGTCGGCCAACAAGCTCCTTCATCAAATCCTTGAGCCACACCAGTGCCGG
TTGTTGCGTATGCACCGATGGGGTATGCCAGACGCTGCGGCCTTACCTCGGCCCCACCAGCTCTTTCATC
AAATCTTTCAGCCACACCAGTGCGGGTTGCTGGGCATGCACGGGGTGCCAGACGCAGTTGAGGTGGTAGT
CGGGCACGTTTTCGGGCAGCGGCAGGCTTTTCAGCGGCCAGTGGGTGGCGAGCAGCTCGGCCACCATGCT
GGGCAGCACCAAGAGGTGATCGCTCTGGCTGACGATGGCGGCGGCGGACATATAACTCTGGCTGTTGACC
GAGATGGTGCGGCTAAAGCCCTGACTCTTCAGATAGCGGTCGAGGCTCGCCTCCGAGGTGCCAAGGGGCG
AGTGGAACACATGGGGCATGGCGATAAGCTGCTGCAGGGTGATGCGCTCCTGCCACTGGGAGGGGTCATC
CTCGGCGCCGGCGGGGGCCGATTTGGCAATCGGCAGATCGGCGCGCACCAGCCCGGCCAGCGGTTCGGTG
AGCCAGGTCTCCTTGCCGAGATACCCCGGCACCTGCATGTACTCGTCAAACCCCAGCACCAGATCGATCT
CGCCGCTCGCCAGCTCGGCTTCGGGCAGGGTGTCGCGCAGAATGCGGATCTCGATGGTGACCCCTGGCGC

CAGCCGTCTGAGCCGCTGAATAAGCGGCGGGATCAGCACGCACTCCACGTAATCTGTGGTGCAGAGCACG
AAGCGCCGCTTCGACTGGCTCGGCTCGAACTCTTCTTCCTCGGTGAGCTGCTGCTCCAGCAGCCGCAGCG
CCTGCTGCACCCCGAGGTGCAACCGCTGGGCGCGCGGGGTCGGCAGCATCCCCTGCGGGGTGCGCACCAG
CAGCGGGTCGCCGAGAAAGGTGCGCAGCCGGTTAAGGGAGTGGCTCATGGCGGGCTGGCTGATAAAGAGC
GCTTCGGCGGCGCGAGTGACGCTGCCCTGCGTCATCAGGGCGTCAAAAGCGAGCAGCAGATTGAGGTCGA
GCTGGTGCAGTTTCATGGGGGGGATTATTCGCCGTGTTTATGGGACAATCCATCCTATTCATTTGATGGA
TGGGGGCGCAACGTCTTAATCTGCGACCCACTCTTATTTTCCCGGGTTCCCGCGCCGCATCTGGCGTAAA
CGGGGGCCTCAATCCATGCAAGGAGCCTTGCGATGAGCATCAAGACCATTAACTCCACGCCGGCCGCCGA
CGGCTTCACCATGCCCGCCGAGTGGGCGCACCAGCAGGCCGTCTGGATGATCTGGCCGTTTCGCCCGGAC
AACTGGCGCGTAGCCGGTCGCTTCGCGCAAGAGACCTTCGCCAAGGTGGCCGATGCCATCGGTGGCGCGA
CTCCGGTCTTTATGGGGGTACCGGCCCAGTTTATGGAGCAGGCTCGCGCCATCATGCCCGCCCATGTGAC
CCTGGTCGAAATGAACAGCGATGACTGCTGGGCTCGCGATACCGGCCCCACCATCGTCACCAACGCTGCC
GGCGAGTGCCGTGGGGTGGATTGGGGCTTCAACGCCTGGGGCGGCCACAAGGGCGGTCTCTACTACCCGT
GGAATCAGGATGAAGAGGTCGCAGCCCAGATGCTGGCCCAGCACGGCATGGCTCGCTACGATGCGCCGCT
GATTCTGGAAGGGGGCTCCATCCACGTCGATGGCGAAGGCACCTGCCTGACCACCGCCGAGTGCCTGCTC
AACGAAAACCGCAACCCGCACCTGACCAAAGAGCAGATCGAAGCGCACTTGCGCGACTATCTGGGCGTCA
CCAGCTTTATCTGGCTGGACGAAGGCGTCTACATGGACGAAACCGACGGCCACATCGACAACATGTGCTG
CTTCGTGCGTCCGGGTGAAGTGGCCCTGCACTGGACTGACGACCATAACGATCCCCAGTACGCCCGCTCC
GTGGCCGCCCTCAAGGTGCTGGAAGCGGCAGTCGATGCCAAGGGTCGCAAGCTGAAAGTGTGGAAGCTGC
CCCAGCCGGGCCCGCTGTTATGCACCGAAGAGGAGTCTGCCGGCGTCGAGAGCGGTACCGGTGTACCGCG
CGAGGCAGAAGGCCGTCTGGCCGGTTCCTACGTGAACTTCCTCATCACCAACGATCGCATCGTCTATCCG
CTGCTGGATGCCGCCACCGATGGCGAAGCCCAGCGCATTCTGGAAGAGATCTTCCCGGATCACACCGTGG
TCGGCGTGCCGGCCCGCGAGATCCTGCTGGGCGGTGGCAATATTCACTGCATTACTCAGCAGATCCCTTC
AGGTCAGTAAGCTGGTCGAAAAGTGTAGAAATTCAAAACCCCACGCCGTGGGGTTTTCTCTCTTGATGGT
GTGATGGTCGCCGGCGCTGATGCGGCGATCATCATGACAAACAGTCACCCTTTTACAGGAGATCAAAATG
ATCGAAGTGACCGAGGTTTCCATTGCCGAGCTGCGTGCCGCGCTCGAATCAGGCCGCACCACAGCGGTCG
AACTGGTCAAGGCCTATCTGGCCCGGATCGAGGCCTACAACGGCCCCGAGACTGCCACCAAACTCAACGC
CGTGGTGGTCGCCAATCCCGATGCGCTGAAAGAGGCCGAGGCTTCCGATGCTCGCCGCGCCCGTGGCGAG
ACCTTGAGCCCGCTCGACGGCATCCCCTACACCGCCAAAGACAGCTATCTGGTCAAGGGGCTGACTGCCG
CCTCCGGCAGCCCGGCTTTCAAGGATCTGGTGGCCCAGCGTGATGCCTTTACCGTAGAACGCCTGCGCGC
CGCCGGTGCCATCTGCCTTGGCAAAACCAATATGCCGCCGATGGCCAATGGCGGCATGCAGCGCGGCGTC
TATGGCCGTGCCGAGAGCCCCTACAACGCCGACTACCTGACCGCGCCGTTTGCCTCCGGCTCCTCCAACG
GCGCCGGTACCGCCACCGCCGCCAGCTTCTCCGCCTTTGGTCTGGCCGAAGAGACCTGGTCGAGCGGCCG
TGGCCCTGCCTCCAACAACGGTCTGTGTGCCTACACCCCGTCCCGTGGCGTCATCTCGGTGCGTGGCAAC
TGGCCGCTGACTCCGACCATGGATGTGGTGGTGCCCTATGCCCGCACCATGAACGACCTCTTGGAAGTGC
TCGATGTAGTGGTCGCCGACGATGCCGATACCCGTGGCGATCTGTGGCGTATGCAGCCTTGGGTGCAGCT
GCCCAAGGCTTCCGAGGTGCGTCCGGCTTCCTATCTGGAACTGGCAGCCAAGGCCGATGCCCTCAAGGGC
AAGCGCCTCGGCGTGCCGCGCATGTTTATCAACAAGGATGAACTAGTCGGCACCAGCGAAAAACCCGGCA
TCGGCGGCCCGACCGGTCAGCGCATCCATACCCGCGAGACCGTGATCGCCCTCTGGGAAGAGGCCCGTCA
GGCGCTGGAAGCCGCTGGCGCCGAAGTCATCGAAGTGGACTTCCCGCTGGTCTCCAACTGCGAAGGTGAC
CGACCCGGCGCGCCGACCGTGTTCAATCGCGGCATCGTGAGCCCGGAGTTTCTCAATGACGAGCTGTGGG
AGCTCTCCGGCTGGGCGTTCGACGACTTCCTGCGCGCCAACGGCGATCCCAAGCTTAAGCAATTGGCCGA
TGTCGATGGGCCGAAGATCTTCCCCCACGATCCGGGCACCCTGCCGAACCGCGAGGGCGATCTGGCGGCG
GGCATGGACGAGTACGTCAATATGGCCAAGCGCGGCCTGAAATCCTGGGACGAGATCCCCACAGTGCCGG
ACGGCCTGCGTGGTCTGGAGAAGACCCGCAAGCTGGATCTGGAGGACTGGATGGACGGGCTCAAGCTCGA
CGCCGTGCTGTTCCCGACCGTCGCCGACGTCGCCCCGGCTGATGCGGATGTGAATCCGGCCTCCGCCGAT
ATCGCCTGGAGCAACGGCATCTGGGTTGCCAACGGCAACCTCGCCATTCGCCACCTCGGCGTACCGACCG
TCACCGTCCCCATGGGGGTGATGGCCGATATCGGTATGCCGGTGGGCCTGACCTTCGCCGGTCGCGCCTA
TGACGACAACAACCTGCTGCGCTTCGCCTCGGCCTTTGAATCCACCGGCAGCAAGCGTATGGTGCCGCCA
CGCACGCCGCCGTTGGTTTGATCGCTACAGCTGGCCTTGTTGTTAGATCAGGCACAAAGAGAGAGGGAGG
CATCATGCCTCCCTCTTTTTTTCGTCCATGTTCTGGCTTTGGCCATCACACCCGGATGTTCTGGCCCGCA
CCCATTTCGCTTCTTGTCACAGGCGGGGTCTGCTCATCTTGGGACAAGCTGGCCTCCCACTCGTGGGCCT
TCTCCAGCAGATCTGCGAGGCTCTCCCTGATCTGCTGCGGACGATAGTTCCCCAACGCGAGTTGGGAGAA
AAACGATACCCGCTCGGTGCCCGGATAGATGCCGACCCCGTAGCCATGGCGCAAGGGGGCGAGGGCATGC
TCGCCAAAGCGCAGGAACAATTCGCGAGACAGGCGCTCGGGAAGATCGCTCGCCGTGGTCACCGAGAGCA
CCAGCGCCATTTCGTCGGGCTGAGCCACGATATTGACCACAAAGGGGGCGCTCGCTTCCTCTGCTGCTCG
CTGGATCTCGAACGTGCAGACGCCGTTTTCATCGAAGGCCAGCGGCTCGGCCGACAGCTCGGCCACGATT
TTCTGATAGATATCCAACACAACACTCCAGTCCGGTTGCCCGGTCGTCTTCGAAGGGGCAGGGCAAGATC
ATAAAGAGACGGCCCGCCACCGAGCGGGCTGGGCCGTCATCCATATTCATGGCAAGGGTTACACCTTGAT
ATTGAGTGCTCGGCCCTCTTCCAGCTCGAACATCCGGACATCGCTCACCGGATTGTCGTTAAAGCGCACG
CTTTTCTTCATCTTGGCGGCGACGTTCTCCCCGTCCGATGAGCCAGTGCGCTCTTCGACCAGCGGTGAGG

TCTTGTTGGCGCGGGTGTTTGCCTCGGTCAGGGCGTCCAGTTCGGCGGTGGCTCTTTGCTCAAACAGCTG
GACGAAGCTGCGATTGAACTCGGCATTGACCTTGGCGTTGAACGCCTCCGATTCATGGCCCAGGGTATTC
AACGCCGAACTGAGTGCCTCACCCGAGAGTGACTCGTGCAGGGCGAACGTCCGCTCGATCACCCGACTGC
TCAGCATGCTGAAGATGGCATCTTTACCGTTGATAAAGCTGGAGAAGTGCTGCTGCACAAAGCTCAGGGA
GTCCTTATCCAGCGTCTCCAGACCGAGGCGCTTCTTGTTGGCATCTGTGATATCGGCCATGGTCGCCGTT
TTGGGGTCGCTACCCGGCTTCAGATATTGGGCCTCGTTCTTGAACTCGCTGACGGGCTGGTTAAAGGCTT
GCTGCACAATCTTCTTGGCTTGGCCAAGTTCGGTCAGCTGCCGCTGCACCTCGGACTGTATTTGAGTGGC
CCGGGTGGTGAGCGCACTCTCCCCCTTGGTGGCAGCGCGGCTAAAGAAATCGACCACCGCATTTTTGATG
CGGCCCAGCGTGCTTAAATGGGCCAGGTCCCGTTGTACGGAGACATCCCCACTCTTGCCGATATGAATGG
TGTACTCCTTGCCTTCACCCAGATGAAGGGCAAGCTTCTGATTGCTGCCGGGATTGACGTTGGCGAGCTG
CAGGCTGTCGATAGCCTTCAACATCACCGGGGTAGCTCCATGAATAGCCATGACCTTGGTTCCTTATCTC
ACTAACAAGCGATACATCTGGCTTGCATCATACCCGTCAGTGAAAACGCCGTTTATTAGCCATGGTTGCA
CTATTTTAGTTTGGGGGTGGTCTGCGCCGGAGGCTCCTGTGATGGATACAAGCTGCTGCGCTTCGCCTTG
GCCTTCGAGTCCACCGGCAGCAAGCGTATGGTGCCGCCACGCACGCCGCCGTTGGGGTGAATATTCCGGA
TTGGTTCGGTGATAAATAAAACAAAACCGCTCATGTAATATGGGCGGTTTATATTTTAGTGGAGGACTTA
TTGTTTCTTTGTAAAATATATCGCTGAAATTTTAAAAAATCAGAGATAATGAGCCACAATCTCACGGCTA
ACATTTTAATCGGTATGGGATTTTTTCCTTAAAACCATGTGGATGGTTGATAGATAAAAGTAAAACATCC
AGCTTAACCATATTGCAGCATCGTAATCTGTCTTTTGTCTGTCATTATGATGCCTTATTCCAAAGTTGTT
GGCTATATTAAATATGTCTTTTTCATCTTTCGTTGTAAGATGCTCTTGTAACTGAGGTCTTAAATATTCC
AAAACATCGGCTAGGTCTCGTACTGCCTGACGTCTGTTATCAATGCTTGCGCCATGTCTACGAAAACTAG
TTGTAGCCGCATTTATTCTATTTATTATGTTTTTGTCCTGGGATGGAATTTCTGCAGAGAATATCGCCTC
GAAGCCCTTCTCAGGTATATGAAGCACCTCTCCACTATCTGCTAGTTCAAACTTCACTCTATAGTGGCCA
AGCACAGTATTTATTTTTTCTTTAAATTCAATCTGACCATCTTCTTTACTAAATTTTTCCCAATGCATCC
CACATTCACCCCATGAATGCATTACTCCATCAATTGGTTTTGAAACCTTTTGGTAGAGGTATTCTATGAT
ATCTAGAAGATCGTCCTCACTATATGATTTGCAATATAACTCAATTGGCCATAAATACTTTTTTCTGACT
GACAATAGAATGTCTAGTTCGACATCCTTTATATTCCCTGTTATCGGACCAGCATCGACACAGTTAAACC
CAAAGGCCTCATCGAAGTAACCTTCCGACATAAATTGATTGAAAACTCTAACAAATAGCTCAACGACATC
CTGTAGTTCAAGACCATTGAGATTTGGATTGACTCCAATTCTTTGAGAGTAGAAAACATGAGACATATCA
CGCACCTAGATAATTAATATTTGAGTCATTATAAAGCTTGTCATGATTTATCTGAAGCTCATTAGGGCCG
TAGGTTCGAATAGCGAAGCGTATTCGGACGATCGCGAAAGGGTGTAGCCGAGAGGCCTCTGGAGTCCAAG
ATTTTCTGTCAGTCTTGTCATGACTTGATGTGCAGCGGTAAACGGACGATTACGCTGCGCTAATCGACTC
TACATTATGGGGCTTATTGTTACCTCCCCCTCATCCCCAGCCCTTCTCCCGCAAGGGGAGAAGGGAGCAA
ATATGCTCCGCTGATTGTGCTTTACGTACGAGGTGTGGGTGGGGGTATGAGGGGTTTGGAATTGGCACGA
ACGATCCCCTCTCCCTCGGGAGAGGGTTAGGGTGAGGGGCAGCTATCAGTGTTGTCGGTTCGTTTTGAGC
CTTTACCTCTCTCCACTCTGCGTATCGCCAATCGCCATCTTTGTCGTGGTTCGCAGTCGATCTCCCCCTG
ATTTTTTTCTGAATTCCCCCAGACTTCACCTTCTCTTCACACAACCTCATTTTCCCCTCACAAATCCCCC
GTATCTTCTGCCCACTGCTTAAAAGGCCCGGCTTGCCACAGGGGTGGCGAGGGGGCGGTTGGTTCGAGGG
AGCAAGATGATGAAAGGTTGGATGAAGTGCACATTGGCCGGGGCCGTGGTGCTGATGGCGAGTTTCTGGG
GTGGCAGCGTGCGGGCGGCTGGGATCGAACTCAAGCAGGTGAGTGGCCCTGTCTATGTGGTGGAGGACAA
CTACTACGTCAAAGAGAACTCGATGGTCTATTTCGGGGCCAAGGGGGTGACGGTGGTGGGGGCGACCTGG
ACGCCGGATACCGCCCGCGAGCTGCACAAGCTGATCAAACGGGTCAGCAGCAAGCCGGTGCTGGAGGTGA
TCAACACCAACTACCACACCGACCGGGCGGGCGGTAACGCCTACTGGAAGTCCATCGGGGCCAAGGTGGT
GGCGACGCGCCAGACCCGGGATCTGATGAAGAGCGACTGGGCCGAGATTGTCGCCTTTACCCGCAAGGGG
CTGCCGGAGTATCCGGATCTGCCGCTGGTGCTGCCCAACGTGGTGCACGATGGTGACTTCACGCTGCAAG
AGGGCAAGGTGCGCGCTTTCTACGCGGGCCCGGCCCATACGCCGGACGGCATTTTTGTCTACTTCCCCGA
CGAGCAGGTGCTCTATGGCAACTGCATCCTCAAGGAGAAGCTGGGCAACCTGAGCTTTGCCAATGTGAAG
GCCTATCCGCAGACCATCGAGCGACTGAAAGCGATGAAGTTGCCGATCAAGACGGTGATCGGCGGTCACG
ACTCACCGCTGCACGGGCCCGAGCTGATTGATCACTATGAAGAGCTGATCAAGACGGCGCCGCAGTCATA
ACGGCTGCGCGACAGACTGGCAGTAAGCCGTGCCATCGCTGCAATCAGCAGCCATAACGGCACACCACAA
AACGCCCGCTTTCAGGAAATAAGCGGGGGTTTTGCTGTTGCGGCTTTTCTGCGGCCACTTATTGCGGGCG
TTGGCACTGCGAGATTAAGGCTGTTCAAGGGAGGGAGGCTTTTATAAGATGGCCCTCTCTCTTTTCCCTG
AAAGTTATCCATTTTCGTTATGCAATACGCCTCTCTCGCGCGCCTCAAGCAGGCCTTTATCACCGGGATG
TGGGCCTCGCTGGTCAGCATCGTTATCGGCTTCGCCTTCAAGGTGTGGCTGGCGCAGTGGGTCGCCAAGG
GGGAGCTGGCGCTGTTCAACAGCGTGGTGGATTTGATCTCCCTCTCTTTTATCCTGATGACAGGTTTTCG
CTCCTCCATGGTGGTGAGTTACTCCCAGACCAAAAACGATCGGGACATCATCAATATCTTTCGCTACTTC
CTCATCGGCATCGTGCTGCTCACCTGGGGGGCGGTGATCCCCTATATCAAGCACGGGCTCCATATCCGGG
TGGAGTATCTGCAACTGGTGGGGGTCATCTTCGGGCTGGGGCTCAAGGTCTACTTCACCAATCTGGTGGC
CATGTATCGGATGTATCAGCTCTCCAACCGGGTCACCTGGCTGGAGCCGCTGGCCAACGGTCTGCTGTTT
ATGGCCTGCTATTTCGGGCTGGGGATGGATGCGCTTGCCTCGATCTTCTACGGGCTGACCTTCTCGTCGC
TCGCCATCGCCGGTTATATGTATGTTTCGCGCCGCCGGGCCATCGCCACCAAGCCGCTGGCCAAGGTGGC
GCTCGGCCCCGAGATGGTGAGCTATGTGAAGAAGAGCTTTACCGCATCCCTGGAGGCGGGGGCCAGCATT

CTGATGATTTACATCACAGTGCTGCTCACCATCCGTCACTTCAGCATCGACGAGCTGGGGGATTTTCAGG
TGGTGGTTCGCCCCATCTTCACCTATATGACCACCTTGTTTGTCTTCCCCATCTACCGCTTTGTGCTGCC
GGAGCTGGCGGTCAATCTGCGCAATGGCGATCACCGCCAGATAAGGCTGATCCGGCGCTGGATCTTCCGT
CTCTCCGCCATCGTCAGTCTGGCGTTCTTTGGCTTGATGCTGCTGGCGGGCAAGCCGCTGGTTTTGCTGT
TCTTCCCCGCCGACTACGTGGGTGCTGTGCCGGTGCTGTTCCACTTCTCCATCTTCTTCATTTTTATGAT
GCTTAACGGCTATCAGCTCGCCTATATCAAGGCTCACGGCCACTTTACCCAGAGCCTGCTGATCCGGCTG
GCTGGCATAGTGGCGCTGGTCGCCGCCTACTATCTGGTGGCCGAGTTCACCGCCAACGTGGTGGCGATTA
TCGTGGCGCTGGGGCTTGGCTATATGGTGATGTTTATCCTCTCGACCCTGGCAGAGCGGCAGATCTTGCG
ATCCCTGCCACTGGTGCTGGCGACCCGCTAGGGTGTTACTCGCCAAGAGCTGGCCCCTGTCAGATAACAA
AAAACGCCCGCAGGGACTTATACCCTAAACAAGCATAGTTCAGCGGCATATTGACCGGAGGCCTTTCAGA
TACTGCATTTCCTGCATTTTTGTGATGTATGTAAGAATACATCCTAATACATCATAACTCTCAAGCGCCG
ACGGTCCTGTTGGCGCTTTTTTATGCCGAATCTCCTAACACCCTACGAATTGCCCAAGCAAAACCAACAT
CGAATATTGTGCGACCGCGCTCGTTAACGATTTCACCATGGCTGCCTTCTTTCAAACCTCGATCTTCGAG
ATAGTCTGCCGACACGGCCTTCTGTAATGCCTCACGCTCAGAGGGGGTCAGTTGCGCAGATAATTCAACC
ACAGCCACTGGTGCACCAGTCGTAGACGTGATTGTTGCCCCCAGCGGACGCCGGTCCACAACCACTTGTG
TATGAGCTTTGAGGGTATTGAGCTGCGCCTTGAGCTTGTCACGCTCGGTGACGATGGCCTGCATGATAGA
ACGAATGGCCGGATCCTCGATGCGCATCAGGTAGTCGTGGCTGGCTAGCGTCTTGGTAGGCTTGGGGGTT
GCCGGACCTGCGTAAGCCGCCCAAGCCTCGATGAGCGCTTTGTAGTCGGCGGATTGGGCATTGTAGAGTG
CTCGGCCCTTCATGATGCCATCGGCTTCAGCCAGACGGCCAATAGTTGGTACAGAGAAGTCTCGTGACTC
TGCCTCATACTGCTTTCGACAAAGTTCGTGCATTCTAGTGAGGTTGTTTCGACGGTTAGAGCGCTCAGCC
TTAGCAAGCAGTGACGCCAACACGGTGTCAGGGTGAATATCCGTTGTACTACTCATGATCTATCCCATTT
CAAGGTCGAGTGTCCGGACGGGAACGAGGCGGTGTGACAGAGTGCCGGCAACTGCGCGGCAAGGTCGAGA
TCTAGGTAGGCACTGAGGCGTTCACCTGCATCCATTAGGCGGATGACTTCACGTTGGCCGAGCGCCGGGT
TCAGTGGGTTCATCTGCTGTGCTAGGTAGCGCATAAAGGCGTTACCGGCAAGCAGTTGTTCCTGTTCACT
CAGCAGCATGAACACTGGCGGCAACTTGTCGCGGTAGAGGACGGCATCCAACAACTGGCTGCGTCGAAAC
ACCGCCTTGCCCGGCTCCAAGTCTGGATAGATCTCGACGCTCTCGCAGACACCGGCCAGTTGTAGAAGCT
CCGACTCGGTTTCTTCAAAGACCGCCTGCACATCGCATAACGTGCCAGCCGCTACCAACTGTGTGCTGTC
GCCAGTGTGTGTTTCGAATGCAGCTTTGCAGCGTTCGATGAGTCGCCAACAGGCTACCAAATCTTCTGCT
GCGTCACTAAAGCGTTTCATAGCGCTCTCCCAAAGCCGCTCAGCTTGCCTGTAAGCATCTAGCCGGGTGA
ATGGCTTGCCAGTTGCTTCTGCATCGGCTTTGTCCTTTTTCAGGGCTTGGAGATCGTGTTCTCGGGTAAG
ACAGGTATTACGCGCCTCGTCAAAGTGGTAGGCCAGCGTATTGAAGTGGCCTGCCAGAGCTGGCAGGTGC
TGCGGCGCAGTCACAAACCAGCGACAGCGTACACAATTGCGGCTGCCACCTGGAACAGGAGCAAACTTGG
GGCGTGATGTCACTCCAATATTTGGCCCTCCGTTATAGCAGCCGCCCACTGCGCCGTTGTCTTCCTCTTC
GCTGGTGTTGCCACCCACTAGGCATAGGCCGTGGTGCATCAGCATCCAGCCCACGGGGTTACGTGCTGCC
GGATGCTGCGGAATAGCTGCACTCAGAGACGGCACACTGTTGCAGATCGCTTGTTCAAGGAGCACCTCAT
GTTCAGTGTCGAGCAGAAAGGTCTGGATGCTCTCTTCCTTTGCTGCCGCCAACCGCTCCACCGCACCGAG
CAATACATCGCGGATGTGCGTCGCCCCCGGCTTGGTGTAGTACAGTGTCATTAATAGGCGACTGTGCCCG
ACGAGTTTTTGCAGGATAGGGAACGGCACCTGCCCTTCCAGCGCCAACGCGGTGATGAGGGAAACGCGCA
GACTATGCAGGGGAAAGAGGGTTGTTATTCTGTTGTTCTGCTCCTCTGGCAGAGGAAGAAAGCGAATTGG
CGTGCCGTTGTGATGAGTCTCCCCCCGACCTGCGAGCCGCCATTCCAGCGCTTCCAGCAGGGCAAACCAG
CTGGAAAACATAGTTCTATCCGAAAGTGGGAAATAACGTTCATCCTCGCTGACCTCTGGCAACCGGAGCA
GGAAACACGCGTCTGGATAGCTTGCGAGTTGCACTTCGCTCTTCGCTATAATGTGACGTCCATCCAGTGC
AGACCAAGGTGTGCGCCGCGAGATAGGGTTGTATTTTTCTTGCCAATTGCGCAGTTTTTCCAGCCAGTAA
AACACATCCTGATGTACCGGGCCGCCAGAGGACCACGGCAGGGCGTACCCTTTCTCGGGGCCTGATTTGG
CGATATCGGCGGTTTTATTGGTGTTGATGTAGAACACCGTCGAGACCGCCTCGCCATCAGCCAGCGCGGT
GCTACGGCGAAACACACCTTGTTGCAGTGGCCGCCGCTCGCTGCCTTGTGCGAGTCGGCTCGGGCTCAAC
TCCCAAGCGCCGTCTGCGTAGCGCCAAGTATCCGCTTCTCCCGAGTCGAGCATGCGGACCTGAAAAGTGC
GCAGTGGCAAGATGAGCTTTACCAACAACGCCACCCAGCGCACCGGGCTCCACATTTCCAAGCGTGTTCC
ATTGGCTATTGGGCGTTCACGCCACACACAATCCGGATCGTTCCGGTCAATCTGCTCTTCGGTTACCTCG
AACCAGTCGGGGGCGCCGCGGCCTCGTTGCCCAATCTCTGCCCCCATCGCACTTTGCGCCCACAACCAGT
TTTGGAAGTGAGGTCCAGCTGCCAACATCTGGCGCAGTTCATCAATGTAGCCATAGGGTAGTGGTGAATG
TACGCTCTCGTCACGCTTAGGCAAGCCGCTGTGGGACATCTGAGGCACGGGGTTACAAAAGGCTGGAGAA
ATGACGGGTTGACCATCATCGCCGGGCGCGCTGAAATCACGCAGCAGAACAAAGTGCAGGAAGGCGTGAA
TGCAGTTGTTGTATCTGATGCCTTTCTCCGAATTCGGGCAGACTGTCTGATAAAAGTCTGGCAGAACTGT
GGTACGCGTCAAAAGCACTGTCGGGTCAAGCGGTAGGCCCTGCCGGATGAGGTAACGCTCGAAGAATGAC
GATAGAGATTCCAGTCTCGCATCAACCCCGCGTGTTTCCCCTTTGAGCCACTCCACTGCTAGGACTCTCC
AGGGTTCGAGTTGAGGATACTCCCGAGCGACCCAGCCGAGGGATGTGTCCGTGGCGCGTTTTACACCTAC
CTTTCTTGGCACAGGGTGCGTAGATGCAATGGCTACGGTTGTTTTCTTCTTATCGGGCATGGAAAATTCT
CAGTCGTTAAGTTCAGCGTCTGGTAGCAACAGGTCCTGCATGGCGGCAGTTTCCACCTTCGCAACCCGTA
ACCGCTGCGCAGCGGCTTCCAGTACAGCCAAGGCCTCGCGGGTAGAAGCCTGCGTGTAGACATCTTGGCT
CTCAAGCGATGCGTGGTGCATGAATCGCCGGATAAGGGACTTGTCGATACCCGCGTTTTTGAGTCGACGA

CCGTATGCGTGTCGGTGACCATGTGGCGTCGTGCCCAATTTTTTGCTCACTGTGAGACCGATGCGCTCGC
AGGCGGCTGCGTGGGCTTTGTTGTACTGAGTTAGCGTATACATGGCACCGATAGGCTCACGTGCGAGGTT
GATGAAAGCAAAGGGGTGATTACGTTCATTGTGCGCCACTTGCTTTAAGTAGCGATACCAGCAGTGCAAG
AACCACTCGCCGTACTCCGGTTGAAACCAGTAAGCCTGCTTGTAATAAGCCCCATCGTGCGTACCGCCTT
TCCAGCCAGCATGTCGGCGGTCCAAGAGGGCAGTGCGGGGGACAAGGCCAAACTGCTGGGAGAGGTATTC
AGCTCTATTACCCTTGCGGGGTCGTCCTCGTGCGTCACGCCAATCCCTAGGCGCAGCCCCATGGCTTGGA
TGGTGAATGCGCACTAGGGCCTGATTTGGGTTGGCAGGGTCAGGTAACACATCCTGAATATAGAGGTGGA
AAGGCTCCGACTCTCGGAAGCCTGCACCGTGCAGCAGCAAGGTGATAAGCATGCCCCGATAATCCTGACG
TCCGGCAGGGCAGAACCCCTTAACCAGAAGCTCTTCGAAGCGCTCTTCTGGAAAAGCTGGCGGCTCGCCA
CGCTCAACCTTGGGTAGGCGCTGTCCTCTGATACGATGTCCGGTCTGGGTTGGTGAGGCATTGGCTGCCC
AAGTATGTCCCAAGAATGCTTTACTGCGCCGATACTGGTAAGCGATTTCATCGGTCTGCCGGTCGAAGGC
TCCACTGGCGTAGCGCGGATTGACCTGTGCCAATGCAGGGCGCATTTCGCCTAACCAATCGAAGAAGTCG
GAGAGGTGGGTAATGATGTGAGAGGCATCTTGTGGCGAACGCGGTGCCCAACAAAGCCAACTAGGGTCAA
GTCCAGTCTCACGGTCAAAGGTGCCTGTGTAGAGCCGCTGGGCAAAGTTCTGGAACAAATGATAGGTATT
GCGTTCAGCAGGGTTGGTCTGGAAATACTCAAGAAACATCCGGACAGAGCGGGTCACCTTCAACATCCAA
GTGAGGCTGCGGTCATGGCTGCGATGGAGGCAGTAGTCAAGCAATGGTTCCAGCACCCCTGCTGGGGTCA
GCAAAGCAGGGAGCTCCGTGTAGACACCCGTGGCATCGGCATAAACACGGGCTTTGACTGTTACGAACAT
TCCATCACCTATCTACCCAAGTAACGTAATCAAGTGGTCACAGCTGTAAAAACAGATGACGAGCCACATT
AGACGCTTATTGGACAACAACGTACATACTCTTCCTCAAACCTCAGTACTTTGGCATCTTCAGTTCAAGC
GACTCGGTTTTCACAGATAACCTGCTGTTACGGTTCCACTCGTTACAGAGGGTTGAGGTCCAATGGAGTG
AAGACGTACGCTAAGGCCGAGCCCCGTGCAGCACGGTAGCAAAGATAGCCATCCATACGGCTCCCTTTAG
AATAAGCACGGCGGTAAACAGTGCCGCTATATGTCACGACTTTATTTATTTCCTTCCATGTTGTTGGGTT
GAGTTTTATTTCTACTTTTAATCCATCTAACAATCAATAAATATGAAAAGTAAAGCGCAGCAAATGAAAC
CACCGAGCCGAAATAAAGAAAGTAAACCATGTCGTAAACGCCACTGACCAAGCGGCTATACTCAGTGCCA
ATTAAATCAAATTCCAAACCATACTTTATATTGACCAGTACGAGAATCAATATAATGCTCATGATTATAA
AATGCATTATAAAGGGGGCTGCAACCCCTACTGTTTTAAGGATTTTTTTAATCATTGCTGCTACCTAATG
AACCACCAAATGTTCCTGGGGAAACGCCGCTTGAACTGCTACCTGCAACACCAAAACTATTGCCAGAGGA
ACCAAGGGTTTCTCCAATAGGCCCTGCACTTTTCACTGGGGATGAGCCCTCACCGTTTGGTGATGGCTGC
CTTCATGGCCGAAGTCACTTCAACCACAAGCATGTCAGAGCTTGAGGCCCGTTGTGAAAGGTGGCGTTCG
AGCGATGATGGTATGACGAAATCCTCTGTATACGTGTTTCCTAGAGGCCCATTGGCAGTGAAGGCACTTG
TCCCTGCCAGCTTCAATGCATCAACAGAAGTCGTTGTGCAGTTACTACCAGTTAGATCGTATTTATTTAT
CACCCTGCCAGTTTCTTTTATTTTATTTTTTTCTGGCTGGGTGGAACTGGAAAGCCATTTTTTTTGGAAG
ATTGCCAACGTCAAACCTTCATCTACATCCATGATTTTAAATGCTCTCGCACTCATGCTGTATAGCTCGT
GCTGATAATACTCACGAGCTTTGGCAAAGCTATATTGAATAAGCACACCATCACCTACAGGGGATATAGG
ATTACCATCAAATCGACCGTAGGTGTATACGTGCATACTCCATTCCTTGTGGACTGAAACAAAGGCATGT
CCGGTTCCCTTTGTTTCAGTCCACAGAAATACACCGTTTGCTAAAATATCGCTGGGCTTTTCGCAAACTT
TATTCTCTATCCAATCCCCCTTGTTCTTTTCAATGGGGGTGATGCTGCCGCGATCATCTGATCGAAAGTT
TGAAGTCTTCTCGCTTTCCCATTCGCTTTCTCCCCAGCGCTGCCATCCACCATTCCACCACTGTCCACTT
CTACTCGGCTCCACATTTCCTCCACAGCCTTGCTTCTCAAAAAAGCATTGTGCCATAGGCTCTCAGCGAA
TCCGTACCTTTTCGTTCTATTGGACTTCAAACCTCTTGCATTGAATCCACATAACATGACTATTTATGGG
ATAGCTTCATTAGCTAACAGAAAAAGAAAAGCCCCTACAAGAGGGGCATTCAATAGAAATTCCAACCGAA
AAGCCTTTACGATGTATCTCTAGTTGGGGGGAATAACGTGCGGGCGTTTTTTTATTGGCGGTGCTGGGCG
GCTTAGCGTTTGCCCTTGCCACCCTTGCTGCCAGCGAGGGCCTGCTTTCTGGCGCGCTGCTTCTGGGCCG
CCTTGCTGTTGCGGCGCGGCTCGGGCTCAAAGCGGGTGAGATCGGGCTCAAAGCCCGGAAACCACTGCTG
GGGCAAGCGGGTGTCGAGCACCGCTTCCACCTTCTCCAGCAGCGGCGCATCTTCCGGGCTGAACAGGGTA
ATGGCGAGCCCCTTGTTGCCGGCACGGCCGGTGCGCCCGATACGGTGGACGTAATCCTCGGCCACAAACG
GGAATTCGAGGTTAATCACATAGTTGAGATCGCTGATATCGAGGCCGCGGGCTGCCACGTCGGTGGCGAC
CAGTGCCTGCAGGGTGCCGGCGCGAAACTCCAGCAGCACTTTTTCTCGCGCGCTCTGCGACAGATCGCCG
TGGAAGGCGAGGGCATTGATGCCCGCTTTGCCAAGCTGCTGGGCCAGCTTGTCAGCCCCCTGTCGGGTGC
GGCTGAAGATAAGTGCCGGTGCCCACCCTTTCACTTTCAGCATGTGTTCGACCAGCGCCAGCTTGCGATC
GCTGTCCACCGTGTAGACCCGTTGTTCCACCTCGGCCGCCGTGGTGTTGCGCGGTGCCACTTCAATCAGC
TCGGGGTCGCGCAGCAGCACCTTGCAGAGGGCAAACAGGTTGTCGTCGCAGGTGGCGGAGAAGAGCAGGG
TCTGGCGATCCACCGGGATCTGTTTGAGCAGCGCCTTGATCTCGTCCATAAAGCCCATGTCGAGCATGCG
ATCGGCCTCGTCAAACACCAGATGGCGGAGGCTATCCAGGCTCAATGCCCCCTGACGCAAATGGTCGAGC
AGACGGCCCGGGGTGGCTATCAGGATATCCACCCCGGCTTGAAGCGCCTCTACCTGAGCCGCGATACTCA
CCCCGCCGTACACCAGCGTGCTGGTGAGACCGGAGCCCTGGCCGTAGCGCGCCACACTTTCGGCGACCTG
CACCGCCAGCTCGCGGGTCGGCACCAGCACCAGCGCGCGGATCGGGCGGGGCTCTGCTTGTGGCTGTTGT
AGCAACTGCTCTAGCAGCGGCAGCACAAAGGCGGCGGTCTTGCCGGTGCCGGTCTGGGCACCCGCCATCA
GGTCCCGCCCTGCCAGAATGACGGGGATGGCATGCGCCTGAATGGGGGTCGGTGCGGCATAGCCCAGTTC
GCTCAGGGTCTGCTGCAAGCGGGGGGAGAGGGCCAGTTCGGCAAAAGAGGCAATGGACATAGGGGCACCA
GTCGGAGTTATCGCGGCCTGCGGCCACGATGCAGTGAGTGATCGTGGCCGCGATGGTAACAGAAGCTGGG

GGAGCGGCAGAAGGCGGCTATGCCTCTGCGTCGTCCGGCAACGGCCGGATCAGCCGGATCCTTTTGCCCT
CGTAGCAGGCGATGTTGTGCTTGCCGTGCTGTTTGACGTGGTAGAGCAGCTCGTCGGCGAGATGCACCGC
CTCGGTGACGGTGAGGTAGTCACGAATGCGGATCACCCCGGCGGAGAAGGTGATGGGGGTGGGCAGCTCG
GGGATCCCCAATGGTCGATCCAGCAGGCGGCCAGCGGAGTGGATCATCATCTCGTCATCCTCCATCCCCT
TGAACAGCACGATAAACTCCTCGCCGCCCCAACGGATGGCGCTGTCCACCTTGCGGATATTGCTTCTGAG
GTGGCCGGCAAAGGCGACCAGTGCCAGATCCCCGACGCCATGGCCATAGGTGTCGTTGATCGACTTGAAG
TTGTCGATATCCAGGATCATCAGGTAGTCGTGCTCATGGAGCAGACTCTGGCAGAGCACATCCCTGCGTG
GCAGCCGGGTCAAGTGGTCGGTGTGCGCCAGGGTGCGCAGCCGCAGGTAGATCTGCTTCCAGGCCACCAT
GCCCAGCAACAGCACGATGAGATAGACCTTGAAGGCCGCACTTTGCAGGGCCAGCTTGACGAAATCGAAC
CAGCTGTAGGACCACTTCAGATAGAGATCATCCCCCGCCTTGGTGCGCATAAAGGGTTTGCTCAGGCAGA
GATGCCTGGGGCGGCAGGGCTCGGCGGCTTCAAACGATATGTTGGTGCGGGTCGGGTTGATGGTGTCGAA
GGGTCGCAGCACATCGTTAAATGCTTCATCGACGGCGATATCGATACCGAGCAGCCCGTATTTGTAACCG
CCGCGCCCCGATTGATGAATGAAGGGGAAGTACATCACTATGGTGTTGCGCATGCTGAAGCTGTCGATCT
CGATATCGGAGACAAAGGCTTCATCAGTGCTGCACAAAGGGCGGCCGGGCGCAAAGCTGGCGCAACCGAA
GTAGTGATACCAGGCGGGCAGTGTCATCAGATCCACCTTGATCGGCGCCAGTCTGGCGGGCAGCACATTG
TTGTCGGCCACCATGCCGATCAGCCGGTAGTCATTCCAGATGGTGTAGATATTGATCACCTCCGAATAAG
AGAGGGCATAGCGGAAGGTGTTTAGGATATTGGCTGCCGGGTATTGCAGCGGAATATCGGCAAAGCGGTA
TTTGCTTTTGAGGTCATACCCGTAGAGATAGTCGCTGCCGACGCTATTCGGGGGCGGTGGCTGATCGAGC
AGATGGTGCTGTGCCAGATAGCTGGTGATGTTCTCCACCTTGAGGATCTTGGCTCGCTGGTAGAAGACCT
CGAACACGTTCTTCTGTCTGGCGGTGATGAGATCCATGTCGTGCTGGATAGTGAGATAGGTGTAGTAGAA
GACAATGCCCAACCCGGTCAGGGCGAGCAGGGCCAATCCGGTCACTTTGACGATGAACTGGCGAGCAAAC
GCCGGTGGCCAGCGCAAGGCTGGGGGATTACCTGAAAATAAGGTCATAGGTGTCTGGGTGCAGTACCGAT
AGGGGCGCGGGCGCCATACGGGCAAAGTATACGCGAAGGAAATCCCGACAGGATCCACCGACAAACCTTG
CCAGAAGCTGAAGCCCTCACCACTTATTTATCCCTACTCCACAATCAGCACGCTCTCGCTGGCCAGATGG
TGCAGCCACTCCCTGACGATCAGCAGCACGGCGGCAAAGTGCTCGTCGCGGGGCAGCAAGCTGGCGGCGA
GGGGATCCCCTTCCGCCATGGCGGCCAGCTGGCCAAAGGCTTGCTCCACCAGATCCCGGTCGAGCTGGTT
GTCCCCCAGCACTTCGGCCAGTGATGGTTGACTGCGCAGGGTCAGCTGCAAGCCGGGCGGGATCTGCGGG
GCGATGCGCATCAGCTCATCCTCCACCACCATGATGGCCCGCTCCAGTTCGAGCGGGGTGGGCGGCGCGT
GGCGAAACGGTCCGGGCTGGATGGCGTGGGTGCCAAAGGCCAGCGGGTAGTGTGACGGGGCCGCATCTGG
CTGGCTCAGCCAGAGCTGACTGCTCTGTTCACCAATCTCCAGCAGCAGGGTCATCATCTCCGGTACGGGT
TGTGTCATGGTGCTGAGCTCCTGTATTGTAAACGTTTTCCCGTTCTGCCTTTGATACCCCCGCTGTCGCC
ATTTGGCGAGCACAATTTCTGCGAGTCGTCCCTCACTTGGTTGCAAACCCCTTGCAAAAAAATCCATTGG
TGGAGGTCTGGATGTCTAAATGGCTATCGCCGTTTCGTTATCCTCAGATGATTCAGGAGTTCAGATATGG
ATATTCGCACCGCTCCCCGCGCCATCGCGCTCGCCCTCATGCTGGCAAGCGGCATCGCCGTCGCCAATGA
AGCGCTGGTGATCCAGACCGATTTTGGCCTCAAAGATGGCGCCGTGTCGGCCATGAAGGGGGTTGCCTTC
GGGGTGGATCGCACCCTGCCGCTCTATGACCTGACCCACGAGATCCCGGCTTACAACATCTGGGAAGCCT
CCTACCGCCTCTACCAGACCCTGCAGTATTGGCCCAAGGGCACCGTCTTTGTCAGCGTGGTGGATCCCGG
TGTCGGCACCGAGCGCAAATCAGTAGTGCTCAAGACCAGGAGCGGCCACTACATCGTGACCCCGGACAAC
GGCACCCTGACTCTGGTGGCCGAGCACTTCGGCATCGAGGCGGTGCGCCAGATCGACGAGAAACTCAACC
GCCTCAAGGGTTCCGAGAAGTCCTACACCTTCCATGGCCGCGATGTGTACGCCTATACCGGCGCACGGCT
CGCCTCCGGCGCCATCAGCTATGAGCAGGTCGGCCCGCTGCTGCCCGCCGAAGTGGTGAAGATCCCCTAT
CAGCCTGCGGTCGCCGAGAAGGATGGCACCCTCAAGGGCACCATCCCGATCCTCGATGTGCAGTACGGCA
ACGTCTGGACCAATATCGACGATGCCCTGCTGACCAAGGCGGGGATCAACAAGGGGGATAACGCCTGCAT
CAAGATCCGCGAAGGGGATGTGGTCAAATATGACGGCAAGGCGCCGTACGTCAGCAGCTTCGGTGACGTG
CCGGAAGGTCAGCCGCTGGTCTACCTCAACAGCCTGCTGCAGGTGTCGGTGGCGCTGAACATGGACAGCT
TCGCCGCCAAGCATCAGGTGCAATCCGGTGCCAACTGGCATATCAGCCTGAAGAAGTGCTCCTGAAGATT
GTCGGGCTTTCGACAAGCGGGTTTGAAGCAAGAGAGGGCAGCCAAAGAGGCTGCCCTCTCTCATTGGGTG
GTAAGGCGTTGCTGATAAGGAGTTGTTGTCAAAAAGCATATTAATTGAATGCAATTGATAATGATTGGTA
TTTGATTGGATAATCGCGCACTTGTCTGACCACCCAACCATACCGAATGAAAACCAGCCTTTTACTGCTC
AGCCTGCTGGCTCTGACCGCATGCAATGACAATCACAGCAATACGTCTGTGACCCCCTCACCCGATGTGA
CCCCACCGCTCGTCAAGCCGACCGAACCGCCCATCACGCCGCCAATAGTACCGCCGGAAACTCATACCCG
GGTGCCTCTGACCATTGAGGTCAATGTGGCCGATCGGCTGGGGGCGGGGGCACAGCTCGAGGTGAGACAG
GTTGCCATCGGGATCCGCGTGCAGCAGGCCATCGAACCGGACCAACCTGTCAGCATCACCTTGCCAGCAG
GTCTGCAACTGGATCAGCCGCTGGAGCTGCTCGGTAGCGACGAAGGCTATCTGCTGCGCGCGGTATCGGT
GCGGGTGGCGGATCTCTCAGCCGGGATCACGCTGGCGCGCCGCAACAGCGTGCCGCGGCTGGGGTTGAAC
GAGGAGGAGACGGCGATCTTCCTGCTGGCGGATCAGAGCGGAGATGGCTCTCTCAGTCAGGCCGAGTGGG
GCAGGGCAGAGGAGATCCGCCTGCAACAACAGGCGGCTCTTCAGGATTATGCAGCCTTCCTGCTGGCCCA
GCGCCAGCCGGGTGCCGTGCTCGACTATGCCGACAGCTGGCAGATGCTGCTGGCCCTGCGCGATGACAAG
CAGGCGCGCAGCTGGTATCAGCGCAGCAACAGCGCCCAGATCCAGTCCGCCCGCACCCAGCTCTATCCCG
CCAATCCGAGCCAGCCGGACGAGCCGGAGACAGCGGATCTGCTGCGTCTCGATGAGCAGGGGCTGCTGGT
GGTGCGTGGCCCCTGGCAGTGCCTGCACGATGTGCGCACGGTCAGTGGGGTCAAGGGCAGCCAGTACTGG

CTTTATGGCAGTAACACCAGTGCGTTGAAGATTGCCAGCCAGCCACAACTGGCTGCGGGGCTGACCGCTT
GCGGCCGCAGCGACTGGCGCCTGCCGACCCTCACCGAGTTCGAACGGCTGCTGAGCAGTGACAAGTCGGC
ATGGCGCTATCCCAATACCTTTGCTGTTGGCCTGCCCGCCCAGATCTGGCTGCAGGATGGTGCGGGTCAA
CCCCGTCTCTATCAACTGGCAACCCGAACCTGGCTGGATGAGGGAGAGGGGAGTCTGCTCTGGTATGCGC
TGGCACCCTATCCGGTGCGTCCCTCCGTGGTGCGCGACAGCAGCAAGCCGGACATGGCGGCCCTGCGCGC
CCAGTACAGCGGTTCATCGGCCCAATGGCCTGCCGCCCGGGTGGATGAAGGGGTGGAATGGCAGGAGCTG
GGGCCGCTGCCGGCGGTTCCCTTCCCGGCCGACAACCCCTATAGCCGGGCCAAGGTGGTGCTGGGTCAGC
AGCTCTTTTTCGACAAGCAGCTCTCCAAGGGGCGGGACATCAGCTGTGCCAGTTGCCACGATCCGCAAAA
GGGGTGGAGCGACGGCCTGAGCGTCTCGGTCGGTCATCTGGGTCAGAAGGGCAATCGCAACGCCCCCTCC
ATCCTCAACAGCGCCTTCAGCTCGACCCAGTTCTGGGACGGGCGGGTCGCCACGCTGGAGGAGCAGTCGC
TTCACCCGATCCAGAATCCCATCGAAATGGCGCTGAGTCATGACGAACTGCTGGCTCGCTTGCAGGCCAG
CCCCGACTATCAGGCTGCTTTTGCCACTGCCTTTGGCGATCAGGGGATCACGCTGGAGCGGATCGCCAAG
GCGATCGCCACCTTCGAGCGCACCATCATCAGCCGCGACAGCGATTTTGAGCGCTTCGCCAAAGGGGATA
GTCAGGCGCTCAGTGACAAGGCGCTGTGGGGGATGCACCTCTACCGCACCGATGGCCGCTGCATGAACTG
CCACTCCGGCCCGCTGCTGAGTCAGCACGGGTTCGAGAGTGTGGGGCTCACCTATTACGGTCGTGGCAAG
CTGGAAGATCGCGGGCGCTTCCTGCAGACCCGCAACAAGGCAGATATCGGTCGCTTCAAGACCCCCAGCT
TGCGGGACGTCGCCTTTACCGGCCCCTATATGCACAACGGCATCTTCCCGATCCTGGCCCAGCGTATCGG
CGGCTCGGTGCAGGGGGTGGTAGCCATGTACAACTTTGGCGTGACCAAGTGGAGCGGCGGGGTCGGTTTC
CCGACCGGTGCGGATCAATATGATCCCTTCTTCCCCAAAGCCTCCGATCACCTTCATGCTCTTGGCCTCA
GCAACGACGAGCTCGATGCTATTGGCGAGTTTTTGCGCAGTGCCTCGGCCGAGCCACGCCGCACTCCGGC
CACGGTTGCCGAGCTGGGGGTGAGTCAGACGGATGACCCGGCTCCGGTGGGAGCGGTGATCACCGGCTTG
GCGGTGCTGCCTGTCCAGCCTCAGCTGGAAATGGGGCGCTCACTCAATCTGCAACTGCAGTGGCAACTGA
GCGATGGATCTCTCGGCCCGCTCGATGGGCCTGTTGAGTGGCGCAGCGAACAGCCGGAGTTCGCCACCGT
GAGTCCACAGGGCGAGGTGCGCGGTGTGGCGGTCGGTACGGCACGACTGGTGGCGACCACGGCTCGTCAC
ACGCTGACCGTGACGGTGCAGGTGGTGGATGCCCAGCCCGATTTTGCCCGGGGCTGTCGTCAACCCGAGC
TGGTGGCGGGCAAGCTTGCCTTTGTCTGCCAGCTGACCAGGGACGAGGCGGATCGTCTGTCCATCTCCTA
TGACGGTGTGAGCAGTGACAGCGATGGTGACTACAATCCCTTCCCGCTGGAGTATGTGACCATGGGTCAG
GCGCGGGCGCGGGATTACTGCCAGCAGCTGGTGGCACGCGGTCATGATGACTGGCGTCTGCCGACCCGGG
CCGAGCTGGAGGCGCTGTTTGCCGCCTACAACAAGGATGGCGCGACGGATCAGTTGCTTCAGCAGCAGGG
GTGGCCGCTCTACAACCGCTACTGGAGTAGCGATGCGACTGCCAATGACGATGGTGAGTTGCCGCTGCTC
GACCTGAGCACTGGTGATCCGCTGTGGGATCTGGCGGATGAGGCCCATGCCGCCACCTGCGTCAGGGCCA
TCTAGCCTTCATAGATAGTCAGATGATAAACGGGCGCCTCGGCGCCCGTTTTGTTCTCTGGTAATAAAGA
AAGGGATCAGAACTCGTTGGAGAGCGCCTTGTACCCTTTGACCAGATCGATATTGGTGTGAGCCACGTCC
TCGGAGAACTCGGCGGCATCCACCGAAACCCGCGGGATATGGGTCAGATCGCTCTGCTGGTTGATGCGGG
TGGTGGAGGGGACGTAGAACTCCGGCGGCAGATCGCAGCCATCGATCACCGAGTTGTGACGCACCACGGC
GCCATCGCCGATATGGCAGTTGAACAGCACGCTGTTGAAGCCGATAAAGACCCGGTTCCCCACTTCGCAT
GGCCCGTGGACGATGGAGCGGTGGGCGATGGAGGTGTACTCGCCGATGGTGACGGCGGCGCCGGATTTGG
AGTGGATCACCACGCCATCCTGAATATTGGAATTGGCGCCGATGACGATGGGTTCCATCTCGCCGTTGGC
ATCCACTTCGTCGGCGCGGATCACCGCATAGGGGCCGACAAAGACATTCTCTTTGATGATCACCTTGCCG
CAGATGATGGCGGTCTTGTCGATATAGGCGGATTCGTCGATGACAGGCAGATGGCCGGAGGGATTCTTGC
GGATCATGTTACATCCTGTGGTTGCCAAGAGGGATTATCTGGTGGTGGACGAGGAGCCTACCGGTCGGTC
AGCTTACCCCTGCTCGCCCTCTGGTGAAACCCCTGACTGGTCTGACCCTTGTCGTATGCCGTTTCGGCAT
GGCTGTTTCCCGCCGCCAGAATATGGGAGCATGGCTGTCATCCCGAGTCGCGTTAGGTGGACATATAAAG
ATGAAGAAGATGATTGCCCTGTTGGCGGGTGGTTTGTTGCTCTCCTCGGCCGTGCAGGCCAAAGGGGAGG
CGGGGGAGTTTGACTATTACGCCATGGCGCTTTCCTGGTCGCCCGAGCATTGCGCCGTCAAACCGGCCGA
TCGGGACCAGTGTTCCCGCAAGCTGGGCTTTGTGCTGCATGGTCTTTGGCCCCAGTACGATCGCGGTTAC
CCCAGTAACTGCACCCGCGAACGGCTCAATCCCGATATGGAGAGCGAGTTTGCCGATCTTTACCCCAGCC
GTTTCCTCTATCGCCATGAGTGGGAGAAGCACGGTACCTGCTCCGGCCTGAGCCAGCAGGCCTTCCATCA
GCTGGCGAGCGATTTGCGCCAGAAGGTGAAGATCCCGGCGGCCTATCAATCCCCGGAAGAGCCGCTGCGC
AAAACCAGCTTCCAGCTCAAGGCCGATCTGGCGAGCGCCAACGCGTGGCTGGCTCCCGACAACATCACGG
TGGCTTGTGCCGATGGTGGCCGCTTCCTGCGGGAGATCTATATCTGCGTCAATAAAGAGGGGACGGATGC
GGTCACCTGCTCCGCCGAGATGCAAAAGCGCGAGCGTCGCTCCTGCGGTCAGCCGGATTTCCTGCTGCGC
AGCGTGCGTTAAGCCTCCACTCTCTTCATCGGGCCCTTGCGGGCTTGATGTGTAACCACAGCTATCGGAA
AGGAGTCTGACAGATGGATGGCAACAACCGGTTTGGCCCGACAGAGCGGGCCCTGCTGCTTGGGGTAAAA
GGGGTGGGGCCGACCGTTATCGGGAGGCTCGAACAGCTTGGATACCATACCCTGGCGGGATTGGCCGAGG
CCGACACCACCCATATCGTCCAGCTGGTGGCCAGCATGCTGGGCTCGACCTGTTGGCAGAACAGCCCGCA
GGCGCGCAGTGCCATCAATGGCGCTATTGCGCTGGCCCGCGCCCATCTGGCGACGGATGCTACAAAGGGG
GTGAATGGATGAGCCTCTTTACCGAGTTGGGAATGCGTTATCCCATAATCCAGGCGCCGATGGCCGGTGT
GCAGGGCAGCGAGCTGGCGCTGGCCGTGACCGGGGCGGGTGGCTTGGGTTCTTTGCCTGCCGCCATGCTC
ACACCAGCAGAGCTGCGCCGCGAACTGGAGCATCTTTCCCAAAGCGGCCTTGGGCCATTTAACATCAACT
TCTTTTGCCATCAGCAACGGGAGCCGGATCCCGTTGCGCTGGCCCGCTGGCAGCAGCGCCTTGCTCCCTA

CTATGCCGAGTTCGGTGTAAAGCCCTCAGCCGTTACGACGGTGCCAGGTCGAGCCCCCTTCAATGAGGAG
CATGCTGCCATGGTGGCCGAGTTCAGACCGGCGGTGGTCAGCTTTCACTTCGGCCTGCCCGCTCCCGAGC
TGCTTGCCCGGGTGAAAGCGGCGGGGGTTAGGGTCTTTGCCAGCGCCACCACGGTGGCAGAGGCCGTATG
GCTCGCTCACCACGGCGCCGACGCCATCATCGCCCAGGGATTGGAGGCGGGTGGTCATCGCGGCCATTTC
CTCTCTGATGATCTCGCCCTGCAGCAGGGCACCTTGACCTTGCTGGCACAGATCCTCGCGGCGACTGATC
TGCCGGTGATTGCCGCGGGCGGTATCGTCGATGGCAAGGGGATCGCCAACGCGCTGGCAATGGGGGCGAG
CGCCGTGCAGATGGGCACTGCTTTTCTCTGCTGCCATGAAGCGACCACCTCGGCGCTGCATCGCGCCGCC
ATCCATTCACCAGAGGGCCAGCAGACGGCACTGACCAATTTGTTCAGTGGCCGCCCGGCGCGGGGTATCG
TCAACCGTCTGATGCGTGAGCTGGGCCCGCTCTCGGATCTTGCCCCTCACTTCCCCTATGCCAGTGGCGC
ACTGGCACCGCTGCGTGCTGCCGCCGAGAAGGCGGGGGGCAGTGGCTTCTCTCCGCTCTGGGCTGGGCAG
GATGTATCGGGCTGCCGGGATCTTCCCGCTGGCGAGCTGATGGCGTGCTGGGTCAAAGAGGCTGGATTGG
GATAAGCACTGCTATATACGCCTCTTTATATAGAAGCGGCCGTTGGTGCAGTGCGTCGGACGCGACGGTG
CTGCGATGATGGATGAGTGCTTGTCCCCTTGTGAGGCGCTTATCCGGAGACAAAACCCCGCACTTCACCC
ACTCCTGTGGATAAGTCTGTATAAAGGCTGTGTGTTGATATGAATAACTAATAAATTGCACTTTTTTGCA
ATTTTATTGCGATAAAGCCTTGCCAATGTGAGCTGGATCTCTATAATGCGCCTCCATCGACAGGGCAAGC
GGCAACGCAAACAACCTCGTCGGTAACCTCGAAAAGCCCTTGAAAATAAGGCTTGACTCGAGAAGGCGGT
TGAGTAGAATTCCACTCCCGCAGCAACACACTGTTGCGTCGCTCTTTAACAATTTGAATCAAGCAATCTG
TGTGGGCACTCACAGCATCGAGCATCAAAAACAATTTTTGATTTTCAATGTCTGGTGAAGTGACCAAGAC
GATTTCTTAGCAATAAGAAGTCGAACAGTTTATTTCAGCAATTCATTGAGCCGCTTTTCGAAGCAACCAA
ACTTAAATTGAAGAGTTTGATCATGGCTCAGATTGAACGCTGGCGGCAGGCCTAACACATGCAAGTCGAG
CGGCAGCGGGAAAGTAGCTTGCTACTTTTGCCGGCGAGCGGCGGACGGGTGAGTAATGCCTGGGGATCTG
CCCAGTCGAGGGGGATAACTACTGGAAACGGTAGCTAATACCGCATACGCCCTACGGGGGAAAGCAGGGG
ACCTTCGGGCCTTGCGCGATTGGATGAACCCAGGTGGGATTAGCTAGTTGGTGAGGTAATGGCTCACCAA
GGCGACGATCCCTAGCTGGTCTGAGAGGATGATCAGCCACACTGGAACTGAGACACGGTCCAGACTCCTA
CGGGAGGCAGCAGTGGGGAATATTGCACAATGGGGGAAACCCTGATGCAGCCATGCCGCGTGTGTGAAGA
AGGCCTTCGGGTTGTAAAGCACTTTCAGCGAGGAGGAAAGGTTGGTAGCTAATAACTGCCAGCTGTGACG
TTACTCGCAGAAGAAGCACCGGCTAACTCCGTGCCAGCAGCCGCGGTAATACGGAGGGTGCAAGCGTTAA
TCGGAATTACTGGGCGTAAAGCGCACGCAGGCGGTTGGATAAGTTAGATGTGAAAGCCCCGGGCTCAACC
TGGGAATTGCATTTAAAACTGTCCGGCTAGAGTCTTGTAGAGGGGGGTAGAATTCCAGGTGTAGCGGTGA
AATGCGTAGAGATCTGGAGGAATACCGGTGGCGAAGGCGGCCCCCTGGACAAAGACTGACGCTCAGGTGC
GAAAGCGTGGGGAGCAAACAGGATTAGATACCCTGGTAGTCCACGCCGTAAACGATGTCGATTTGGAGGC
TGTGTCCTTGAGACGTGGCTTCCGGAGCTAACGCGTTAAATCGACCGCCTGGGGAGTACGGCCGCAAGGT
TAAAACTCAAATGAATTGACGGGGGCCCGCACAAGCGGTGGAGCATGTGGTTTAATTCGATGCAACGCGA
AGAACCTTACCTGGCCTTGACATGTCTGGAATCCTGTAGAGATACGGGAGTGCCTTCGGGAATCAGAACA
CAGGTGCTGCATGGCTGTCGTCAGCTCGTGTCGTGAGATGTTGGGTTAAGTCCCGCAACGAGCGCAACCC
CTGTCCTTTGTTGCCAGCACGTAATGGTGGGAACTCAAGGGAGACTGCCGGTGATAAACCGGAGGAAGGT
GGGGATGACGTCAAGTCATCATGGCCCTTACGGCCAGGGCTACACACGTGCTACAATGGCGCGTACAGAG
GGCTGCAAGCTAGCGATAGTGAGCGAATCCCAAAAAGCGCGTCGTAGTCCGGATCGGAGTCTGCAACTCG
ACTCCGTGAAGTCGGAATCGCTAGTAATCGCAAATCAGAATGTTGCGGTGAATACGTTCCCGGGCCTTGT
ACACACCGCCCGTCACACCATGGGAGTGGGTTGCACCAGAAGTAGATAGCTTAACCTTCGGGAGGGCGTT
TACCACGGTGTGATTCATGACTGGGGTGAAGTCGTAACAAGGTAACCCTAGGGGAACCTGGGGTTGGATC
ACCTCCTTACCTTAAGATGTCGAAGTTGTTGAGTGTTCACACAGATTGCCTTGATTCAAAGTAGTTAGAG
CAAAGACCTGATGCGAAAGTGTCAGTGCTTATCTGGCTTCGGTCAGATGAGAGAAGCCCTATCATTGGGT
TTTGGGATGTGAATAATGGCGCTCGGCCTCGCAGGCTCGGCACTCGCCATTACCCAAAATCTGCACTGCT
GACGCAGCGCAAAGATGATTTTGGGTCCCCTTCGTCTAGAGGCCTAGGACACCGCCCTTTCACGGCGGTA
ACAGGGGTTCGAATCCCCTAGGGGACGCCACTTCTCTTCTTGCTAACAAGAATGCAGAGTTAAGAACTGA
TTCTTAACTCTGTTTTCTTCGGCCTTGCGCCGTTGCAAACATGCTCTTTAACAATCTGGAAAGCTGATTT
AAAAAGTAGTTCTCAAACATTTGTTACAAGTGCTTTGGAAACTTCTTGGCGAAAACCAAATTTTATTTGG
TCCTTGTTGTACGACAACAAGCAAGCGTTGTTTCGACGACACTTCTTGGGGTTGTATGGTTAAGTGACTA
AGCGTACATGGTGGATGCCTTGGCAGTCAGAGGCGATGAAGGACGTACTAACCTGCGATAAGCTGTGAGA
AGTCGGTAAGAGACGCTATTACTCACAGATTTCCGAATGGGGAAACCCACCCGAGATAACTCGGGTATCT
GTTACTGAATACATAGGTAACAGAGGCGAACCGGGAGAACTGAAACATCTAAGTACCCCGAGGAAAAGAA
ATCAACCGAGATTCCCTCAGTAGCGGCGAGCGAACGGGGATTAGCCCTTAAGCATCTTGGAAGTTAGTGG
AACGGTCCTGGAAAGGCCGGCGATACAGGGTGATAGCCCCGTACACGAAAACGACCTTGATGTGAAATCG
AGTAGGGCGGGACACGTGACATCCTGTCTGAATATGGGGGGACCATCCTCCAAGGCTAAATACTCCTGAC
TGACCGATAGTGAACCAGTACCGTGAGGGAAAGGCGAAAAGAACCCCTGTGAGGGGAGTGAAATAGAACC
TGAAACCGTGTACGTACAAGCAGTGGGAGCCCTTCGGGGTGACTGCGTACCTTTTGTATAATGGGTCAGC
GACTTACATTTTGTAGCGAGGTTAACCGTATAGGGGAGCCGTAGGGAAACCGAGTCTTAACTGGGCGTCT
AGTTGCAAGGTGTAGACCCGAAACCGGGTGATCTAGCCATGGGCAGGTTGAAGGTTGAGTAACATCAACT
GGAGGACCGAACCCACTAACGTTGCAAAGTTAGGGGATGACCTGTGGCTGGGGGTGAAAGGCCAATCAAA
CTCGGAGATAGCTGGTTCTCCCCGAAAGCTATTTAGGTAGCGCCTCGGACGAATACTACTGGGGGTAGAG

CACTGTTTGGGCTAGGGGGTCATCCCGACTTACCAACCCCATGCAAACTCCGAATACCAGTAAGTAATAT
CCGGGAGACACACGGCGGGTGCTAACGTCCGTCGTGAAGAGGGAAACAACCCAGACCGCCGGCTAAGGTC
CCAAAGTTCTGGTTAAGTGGGAAACGATGTGGGAAGGCTCAGACAGCTAGGATGTTGGCTTAGAAGCAGC
CATCATTTAAAGAAAGCGTAATAGCTCACTAGTCGAGTCGGCCTGCGCGGAAGATGTAACGGGGCTCAAA
CCAGGCACCGAAGCCGCGGATTCACACTTATGTGTGAGTGGTAGGGGAGCGTTCTGTAAGTCTGCGAAGG
TGTATCGAGAGGTATGCTGGAGATATCAGAAGTGCGAATGCTGACGTAAGTAACGATAAAGGGGGTGAAA
AGCCTCCTCGCCGGAAGACCAAGGGTTCCTGTCCAACGTTAATCGGGGCAGGGTGAGTCGACCCCTAAGG
CGAGGCCGAAAGGCGTAGTCGATGGGAAGCAGGTTAATATTCCTGCACGACTTGTAATTGCGATGGGGGG
ACGGAGAAGGCTAGGTGGGCCAGGCGACGGTTGTCCTGGTGAAAGTGCGTAGGTGGTGTTTCTAGGCAAA
TCCGGAGACACAACACTGAGACACGAGACGAACACACTACGGTGTGGAAGCCATTGATGCCCTGCTTCCA
GGAAAAGCCTCTAAGCTTCAGATTACAAGTCATCGTACCCCAAACCGACACAGGTGGTCGGGTAGAGAAT
ACCAAGGCGCTTGAGAGAACTCGGGTGAAGGAACTAGGCAAAATAGAACCGTAACTTCGGGAGAAGGTTC
GCTCTTGACAGTGAAGTCCCTTGCGGATGGAGCAGTTGGGAGTCGCAGTGACCAGATGGCTGGGACTGTT
TATCAAAAACACAGCACTCTGCAAACACGAAAGTGGACGTATAGGGTGTGACACCTGCCCGGTGCCGGAA
GGTTAATTGATGGGGTTAGCGCAAGCGAAGCTCTTGATCGAAGCCCCGGTAAACGGCGGCCGTAACTATA
ACGGTCCTAAGGTAGCGAAATTCCTTGTCGGGTAAGTTCCGACCTGCACGAATGGTGTAACCATGGCCAT
GCTGTCTCCACCCGAGACTCAGTGAAATCGAATTCGCCGTGAAGATGCGGTGTACCCGCGGCTAGACGGA
AAGACCCCGTGAACCTTTACTACAGCTTGGCACTGAACATTGAACCTACATGTGTAGGATAGGTGGGAGG
CTTTGAAGGCGTGACGCCAGTTGCGCTGGAGCCGTCCTTGAAATACCACCCTTGTATGTTTGATGTTCTA
ACGCAGGGCCCTGAATCGGGCTCGCGGACAGTGCCTGGTGGGTAGTTTGACTGGGGCGGTCTCCTCCCAA
AGAGTAACGGAGGAGCACGAAGGTTGGCTAATCCTGGTCGGACATCAGGAGGTTAGTGCAATGGCATAAG
CCAGCTTAACTGCGAGACGGACAGGTCGAGCAGGTACGAAAGTAGGTCATAGTGATCCGGTGGTTCTGAA
TGGAAGGGCCATCGCTCAACGGATAAAAGGTACTCCGGGGATAACAGGCTGATACCGCCCAAGAGTTCAT
ATCGACGGCGGTGTTTGGCACCTCGATGTCGGCTCATCACATCCTGGGGCTGAAGTCGGTCCCAAGGGTA
TGGCTGTTCGCCATTTAAAGTGGTACGCGAGCTGGGTTCAGAACGTCGTGAGACAGTTCGGTCCCTATCT
GCCGTGGGCGTTGGATGATTGAAGGGAGTTGCTCCTAGTACGAGAGGACCGGAGTGAACGAACCTCTGGT
GTTCGGGTTGTCACGCCAGTGGCACTGCCCGGTAGCTAAGTTCGGAATCGATAACCGCTGAAAGCATCTA
AGCGGGAAGCGAGCCCTGAGATGAGTCATCCCTGACCCCTTGAGGGTCCTAAAGGGCCGTTGGAGACCAC
AACGTTGATAGGTGGGGTGTGTAAGCGCGGCGACGTGTTGAGCTAACCCATACTAATTACCCGTGAGGCT
TAACCATACAACACCCAAGAAGTGTTCTAAGGCTTGTAGCAAATACGAACGAATTACTTACGTCAGCTTT
CTCAGATTGAAGAATTTGCCTGGCGGCCATAGCGCCGTGGAACCACCTGATCCCATGCCGAACTCAGAAG
TGAAACGCGGTAGCGCCGATGGTAGTGTGGCATTCGCCATGCGAGAGTAGGACACTGCCAGGCACTCAAT
ACAAGAAACCCCGCTCATCGAGCGGGGTTTTTTATTGCCTGCAATTCAGTAAGTCGCCTGATAATTGCCC
TGTTTTATTCCTCTCCCTTCCAATTCCTAGTCTTCCCCCGTCGGTTTTACTCATAAAAAAACAGCCATGGC
AGCGTAAAATAGCGCTTTATTTCGTCGGAGATAGCCCATGTTTTGTCCAAAATGTGGTGGTCAAACCCTG
CAGAGCGTCAGCCCCAAAGAGTTTCGCTGTGGCTGCGGTTTTCATTTTTTCCAGAATGTGGCGACGGCTG
TGATGGTGGCGCTTTGCTGGCAGGACGAGGTGCTGGTTGCGGTGCGTGCCCGCAATCCGGGCAAGGGGTT
ACTGGATCTGCCAGGCGGATTTGTCGATCCCGGCGAGTCGCTGGAGGCGGCTCTGGTGCGCGAGTTGCAG
GAGGAGTTGGGGCTCGATGTTAGCGTTCAACCTTTTATCTATCTTGGTTCATACCCCAATATCTATCCCT
ACGACAGTATCACCTACCACACCTGTGACACCTGTGACACCTTCTTCGCCATCAGGCTGAATGAAAAACC
GGTCATCCAGCCAGCTGATGATGTGGCTGCCTGTCAGTGGCTGAAGATCCGCGATGTCCCCTTGCCCCGC
TTTGCTTTCGAGTCGACGCGCACCGCGATGGCGCGGTTGCAGCAATCCCCTTTGCAAGCCTGATCCCTGC
TGATGACGATGGGGATTGGCAGCGACCATGCCTGTCTCATCCTTGTCCGCTTTTGTCCCGCAGACGCCAT
TCGTCAGACGGTGAGTGGTATAGTCTGCAGTGGATCCGGATTCAAGGAGCATATCGTGAAGAAGATAGTT
CTCATCTTGCTCGGCATCGCCGTGGCGGCCTTGCTGGCCATAGTGACGCTGATAAGCCTTATCGACCCCA
ATCAATTCAAGCCCCAACTGGCTGAACAGGTGCGCAAGAGCACGGGCCGGGAACTGGTGATGGCGGGGGA
GATTGACTGGCGTTTCTGGCCGAGTCTCGGCCTCTCGCTGGAGAAGGTGGCTCTGCGCAATCCGGCCGGT
TTTGCCGAGCCGGATCTGGTTCGTTTCGAACAGGGCGAGGCCTCGGTGGCCCTGTTGCCGCTGCTCTCAC
ATCGTCTGGAGATTGGCAAGGTGACCCTGAGTGGCGCCCATCTCTTTATCCAGACCAAGGCGGATGGCAG
CTCCAATCTGAGCGGACTGATCAAAGATGCCACTGCTGATGCAAGCGAACCGGTCGTCCCCGCAACCCCT
GCGCCAACCGCTGATAGCAAGCCCTGGCAGATCAGCTTGCAGGGTGTCGCGCTCTCCCAGGCCAGTGCGC
TGGTACAGGATGATCGCCGCGGCACCTCGCTGCGACTGGATCGCCTCGATCTCGATATGGGGCTGCTGGC
CACTGGTCAGTGGGTACCCGTGACGCTGGCCGCCAAGGGGAGTGCCGACAAGCTCGCTTTCGATGTTAAA
GGGCAGACCCAGATCAAGCTGGCGCAGGAGGTGATGGCCAGTGAACTGAAAGACTTGAGCCTCAGTGGCA
GCCTGAGTGACCCGTCCCTGCGACTCGACAGTTTCTCCATCAAGGGAGACCGACTGGCCTTGGGTGAGTG
GAGCAACCTCACGTTGGCGCTGAAGGGGACCAAGGGTGAGGGGCAGCAAGCTTCATTGGCTGGCTCGCTG
GAAGGAACACTCAAGGGACGGCTCGATAAAGAGATGAAGCTGGCCGAGCTCTCCGATGTGCTGCTGACGG
CTGCGCTGGAAGGTGATGCGCTGCCGCGCCCGCAGATGAAGCTGAAACTGGCCGGGTTTGCCCGTGCCGA
GCTCGACAAGGAGTTGATTACCCTGAGCAAGCTGGTGATGAGTGCTGATGAGGCGCTGCTGAGTGGTGAT
GGAACCGTGCAGCTTGGCGCTGTTCCCGCAATCAGCTTCGATCTCAAGGGGGAGAAGCTGGATCTCGACA
AATGGCTCGCCAAATCGGTACCGACCACACCTGCGCAGGCGAAAGAGGACAAACCGGCGACCGGTGTTGC

CAACACCGCTGCAAGTGACAATAAAGCGGTCACCGGCAAGAGCGAAGCCCTCTCCGCGGTTGAACCGGAT
CTGGGCGCGCTCAAAGAGGTGGATCTGGATGGTCGGTTGCAACTTGGCAGCCTGCGCCTGAAAGGGCTGG
ATCTCGGTGCTGTCGATCTGCAGGTGGCTCTGGCAAAAGGGCTGATGACGCTCAAACAGTTCAGCGCGAC
CGTGGCTGACGGTCAGGTAAGCGCGAAAGGGGTACTCGACGCACGCGAGCAGCCCGCCACCTACAAGGTG
CACAAGCAGGTGGCCGGAGTGAATATTCGCCCCTTGTTGCAGACTCTGGCTCAGAGTGATCTGCTGGAGG
GCAAGGGCGATCTCGATGTGCAGGTGCAGGGACGCGGTCTCTCTGCGTTGGCACTGCGCAACGGAATGCA
GGGCAAGGTGACCTTGAAGCTGAGCGACGGCGCCCTGCATGGCATCAACCTGCCGGAGATGATCCGCGAG
GCGCGGGCCACCCTCACCGGCAAGGGGGGTGAGCAGGTGAAGGAGGCGCGCAAGACCGACTTCAGCGCCC
TCACCGCCAGCTTCCAGATTGCCGACGGCATCGCCCGTAGCAACGACATCCAGCTCTTTGCCCCCGCCTT
GCGGGTCAAGGGCGAGGGGCAGACGGCGCTGGTGCCGGAGAGCCTCGACTTCCTGTTCCTCACCTCCATT
GTCGAGAGCAGCAAGGGGCAGGGGGGCAAGGATGTGGATGAGCTCAAAGAGATCACCATTCCGGTGCGGA
TCGGCGGTCACTGGCAAGCCCCCAGCTACCAGCTCGATGTGAAGGCGCTGCTTAGCAACAACAAGCTGCT
GGAGGATAAGGCGCGCAAGGAGGCCGAACGCGGCCTCAAGAAGCTGCTGGGTGACAAGGCCGAGAACGAG
GCCGTCAAAGGGGTGGCCGACCAGCTGCTCAAGGGGCTGTTCAAATAGATGACGGTTCATATGGGCTGGC
GGGAGCCGCGCCAATGCCGTTAGCGGTTGACCGGATGTCAGCGCCATTGAAAAATGGCGCTGACATTCTT
TATGGCGCAGTGACCTGCTGTGCCTTGATCCGGAGTCCTCTCGAGTTGAACCCATGCGCCTGATCCTGCT
GCTCTGCTCCCTGATCTGCTACGGCATGGCGTATCCCGTGCTGGCCGGGCCGCTGGCTGATCGCCTGGCG
GGCGAGCTGGCCAGGCAGGCTCCCGAACAGGCGCTGCCTCTTGACGAGGGGCAGCGGCTCGATCGTCGCC
TGATCGCTCCCGACTCCCTTCATCCTGCCTGGCAGCGCTATCCGCTGCGTCAACTGCAGGCCACCTATCG
CTATCAGCAGGAGTGCGGGACAGAGAATGAGTTGCCCGCCGAGTGGCTGCCGCTACTGCAGGCGCTTTGT
GGCCAAGGGGCTGTACCCTCGACCCGCTGGTTTGCCGCTCACCCCGTTTATCCGCTGGGTGGCTCCAGCG
CTGCGCGCTGGCTGGCTGGTCATCCGGTGGCCGGGCTCGATGCTCTGCTCCATGTGCGGGAGCGGCGTGA
TCAGCTGGGGCTGCTTGGCGAGCTCGGCGATGACAACCTCGATGCCCTGCTGCGGGGTGAGCGCTGGCTG
CTGCAATCGGGTCAGCTCTGGCTACTTGCCGATGGGCAGTGGCAGCCGCTGGCCGAGCGGCTTGGTGTGA
CGCTGGATCGCCGTACCGATGGTCCCTGTCAGGAGCGAGTCGGAGCGATTTGCTTCAATGCTCTCCCCAC
GCTTGGCTGGCAGTGGCTGGCGCTGGCCAGCTCGTTTGGTTTTATCCTGTTGCTGGGGTGGGCCAGCTGG
CAGCGCTGGCGCCTGCTGCGGGAGCGGCGTGTCGCATTGCAGATGCTGACCCACGAGCTGCGCACTCCCA
TCATGGCACTCTCCGGCATCAGCGAGGAGCTGCGCCACGATTTCGACCGGCTGCCCCCTTCCGCCCAGCA
GAGCGTGGGGCAACTGCTGGGCAGTGTCGCCCGTCTGCATCAGCTGGCGCAGGCCAGTCGCCACTATCTG
GCGGCAGAGACTCTCGAGGATGAGCGGGTGGCGGTCAGTCTCACTGAGTGGCTCACCCTGGCTTGCGAGC
GGCATGACGCCATCTTCTGCCTTGAGCGGGAGATGACTCTGGCGCTCCCCTTCTACTGGCTCGATCTCGC
CCTCGACAACCTGCTGCGCAACGCCAGCCAGCATGGCCGCGCGCCGGTTCGAGTCAGTGCTCGCTGGCAG
GCGGGGCGTTTGCTCCTCGCGGTCAGCGATGGCGGCGAGCTGCCGGGTTACCGGCTCGCCACCCTGCTGC
GCCGCGGTGCTCGTGCCGAGGGTTTGGGGCTGGGGCTTGCCATCGTGCGCCATCTGCTGCGCCGTCTTGG
CGGGTGCCTCGCCCTATCCGGCCCGCCCACTACCTTCACCCTGACACTGCCCTGCCAACTCTGGAGCGAC
ACCGAATCATGAAGACCATCCTGCTTGTCGAAGACGAGGCGCTGCTGGCGGAGAACCTGCAACGCTATCT
GACCCGCGAGGGGTATCGTTGCCTCTGGTGCGACACCCTGGCAGCCGCAGAGCAGCAGTGGCTGGCGGCG
GATCTGGTGCTGCTCGATCGCACCATGCCGGAGGGGGATGCCCTCGACTGGCTGCCCCAGTGGCTGGCCC
GCAAGGCGCTGCCGGTGATCGTGCTCACTGCAAGGGTGGAGGTGAGCGATCGGGTGGCCGGGCTCGATGC
GGGGGCGCGCGACTACCTGACCAAGCCCTTCTCCCACGACGAGCTGCTGGCCCGGATCCGGGCCCAGTTG
CGTCAGCTGGGGGATGGCACTCAGGTGGTGGGCGGCTTGCAGCTTCATCCCGCCCGGATGCAGGCGAGTT
GGCAGGATGCGGAGGTGAGCTTCACCCCTACCGAGTTTGCCCTGCTCACCCTGCTGGTGCGAATGGCGGG
TCGGGTGCTGAGCCGGGAGGAGATCCTCAATCGGGTGTGGGGCTATCAGGCGTTTCCTTCCACCCGCACG
GTCGATACCCATATTCTGCAACTGCGTCAAAAGCTGCCCGGGCTGGCTATCGAGACGGTGCGCGGTATCG
GCTACCGGCTGGAGGTGACAACGTGAAGCGCGCAACGCTGGTTGGGCTGCTGTTGGTTCTAAGCGGCGGC
CTTCTGAATGCAAGCAGCGTGATGGCAACAGAGGGGCTGGCCACCGATCGCGCCGACCCGCAGCAACCGG
CTACCCTGGCGCTGTTGCAGGGGGATAACCGGCACGCCTGGCAATCTTTGCAGCAGGCATGGCCCGCCCT
CAACAGCGAAGCCGAGCGCCGTAGCTGGCAGGGGATGCTGGCCGCGCTGAGTGCCCAGCACTGCGGCAAG
GATTTCCCGCTGACCTTGCCTGATGGGGTGAGCGAGCTGCGCCTTGAGCTGATCCAGCGCGATGCGCCGC
TGCTGCGGGACTATCGGGTGCAGTTGACCGGGGAGGGGCCCATCACCACCGCGGAACTTATCGATTCCGC
TGGCCGTGACCGACTGGCAGGTGCCCAGTGGGAGGCGGAAGAGAACTCCGGAGTCAGGGTTGTCGGGGCC
GATCTGCCGACGCCTTTACCTGCCGGACTCTATCAGCTGCGGCTGACGGTGGCGGGCAAGGAGTGGCAGG
TGGCGTTGCCGCTGCCTGCGGTGCAGGATCTGGACTGGCTCTCCCGCTCGCCACAGGCGGTAGCCAATCC
CCCCGCCAATCCCGCTTTGTGCACTCCGCTCTGGCTTGAGCAGACTGTGCTGGCCCGCCCGCAATACTCC
CTGCTCTGGTGGCACCGGATTGCGACTGATGAGCCGGTGAACTGGCCAACCGCTACGCCCGACAGTTGGC
GTACCCTCTCGCTGGTGCAAACCAGCCAGCGCGGCCAGCTGGTGTTGCAGCTGAGTCACAGTCAGGCTGG
GCCGGTGGACTAATGGTCGTCGTGATGGTCAGGTCACCGCAATCCTGACATTGGCCTGACAATCGCAGGC
GGCCAATCTGCTGAAATGGCGATTCCCGTGTGTTATCAGGAATCACCGACCATGACTCTTCTTACCGAAT
TAGCGAGCCGGATCCGCTCGCTGCTGCTGTTGATGCCGCTGCTCTCCCTGCCTGCCGAGGCGCTGACCCT
GGCCAACGATCAGTGGCGTATCGAGCTGGATCCCGCCACCCTGGCGGCCGAGGCTGTGCTGCCATCGGGC
CAGCATCTTGCCATCTCTTCCCCCGGCCCGCGCCAGCCGGTCACCGCTCTGCAGCAGGAGGGGGAGCGTG

CCAGCTGGCTGACCCGTGCCGATGGCGAGGTGCGTGCCTCTGCCCGTCTCGAGGGCAACCGTCTGATCCT
CAGCTTGAGTCGTGCGAGTGCTGGCGAGCTGGTGTGGCCGCGGGTGCCGGCAGGGGCGCAGGCGCTGATA
CTGCCGATCCACGAGGGTTTTCGCATCCCGGTTGATCATCCCAAGTGGCGCAAGGCGCTGGCGCAGGAGT
ATCAGGGGATCAACACAACCGAAAATCTCACCCTGCCGCTGGTCGGGCTGGATCACGGCGATCAGCAGCT
GGCGATCCTCTTTGCCAACCCCTTCAACAACCGGCTCACCTTTGCCCCCAAGCAAGATGGCATTGCCCTG
ACGGCGACCCATAGCGTCAATCGCCTCAATCTGACCGACCCCTATGAGGTGGCGGTGTCGCTGCAACCGG
GCAACGACTGGCTGGCGGCGGCCAAGGCCTATCGCAGCTGGCTGCAGGAGCGGGGCGAGCTGGTCTCGCT
CGAGAGCAAGCTGGCGGCAGTACCGGATGGCAAGCGGCTGATTGGTGCCTCTCACCTCTATCTCTGGGGC
GAGCGACTGCTGGTGCCTCAGGATGTGAAGAGTTGGCCCGCGCTGCGCCGCCTGATCCCCGCCGACTGGC
TCAAGGGGGAGAGTAAGAGTGCGCTGCAAGCGCCCGATCTTGAGCGCAACCGCTATCTGCAAAATCTGGT
GCTGACCGGAGTTGAGCAGGCGCTGGCGCTGCGCCATCCGGGTCAGCAACCGGCCGATTTCGCTGCCCGG
CGCACGCTGGCGGCCACGCAACTGGGCTCTGCGCTCAACGAGCCGCAGAGTTGGGGCGATGGCAGTTCGG
CCAAGATGATCGCCGCTCTCAAGCAGGCTGGACTACCCAGACTCTGGCTGGGTCTGCCCCAGTGGACCGC
CGGTTTTGCTGCGCCAGAGGGGATTGAACTTGCCAAACAGGCGGGCTATCTCATCGCCCCTTACGACTCT
TACGATACCGCGCTGCCGGAGGGGAATCGCCAACAGAGCTGGCTCACCGCCCAGATGGGGCAGGATATCT
ACCTGCGCTGCGGCATCATGCAGGAGAACGGCCGGCGCAAGAGCGGCTTTCAAAATAGCGGCGTCTACAC
CAATCAGGCCTGTGTGCGCCCGGTGATGGAGCAGCGAGTGCCCAAATTGCAGCAGGCGAGCCACTACAAC
AGCTGGTTCCTCGATGTGGCGGCGACCGGCATGGTGTTTGACGACTTCGACCCGGCCAAGCCGACCACTC
AGGCGCAGGATGCGCAAAACCGGATGGCCGGGATGACGTGGATAGCCAAGAGTCAGGGGGGGCTGGTGGA
CTCCGAGGAGAGCGGGGCGGTTGCCAACCGCACCGCGGCCTTTGCCCACTGCCCCCAGACCAGCGGCTTT
GGCTGGCAGGATCCCGATATGCGCCGCAACAAGCGCTCTCCCTACTATCTGGGGGCCTGGTATCCCGACT
ATCAGCCTGCATTTTTCTTTCGCCAGAGCCACCTAAAACCTGAGTATCAGGGGCTCTACTTCGATCCGGC
GCTGCGGTTGCCGCTGTTCCAGAGTGTCTTTCACGACAGCATAGTGACCACCCACCACTGGACGATGGAC
AGCCTCAAATTCAGGGAGAGTCGTCAGGTCACCGAACTGTTGCAGCAGCTCTACAACGTGCCGCCCCTAC
TCAACCTCAGTCTGGATAGCGCCAGTGCGCGCATTCCCTACCTCAAGGGGCTCGATGCCTTCTTCCGCCC
GCTGCATGAGCGGCTCTACAACCAGCCCCTCACCGGTTTTCGCTGGCTCGACAAACGAGGAGTGGTGCAG
CAGAGCGAGTTTGCCGATGGCACCAGATTGGTGGCCAACTTCGGCCCAGCCACTGAGCAGGCAGGGATCA
AACTGGCCGCTCAAAGCGTGCTGGCGCTGTTGCCCGATGGCAGCCAGCAGCAGTTTGTCTCGCAACCGGA
TGGCGTCCGCTAATCTGGCGTTCTGGCTTTTATTTCATCAGATGATGCTGTGGCAGAACTTTTCATGTAT
AGATGAACGTTCTGCAAAATAAACGAGAGGCAGTTCTGATGATTGAGAGTGAAGTGATTAGAGAGGTAAA
CCATCCGGCGTGGCCGCTGGTCTGGCAGATCTATGGCGAGAGTTTTCCCCGCTACGAGCAGCGCCAGTCA
CAGCATCAGCCCCAGAAGATGCGGGATCCTCGCTATCACTGCCAAATCTTTTATCAGGACGGCCTGCTGC
TCGGTTTCATCTTCTGGTGGGCGTGCGGCGAGCAGATCTATATCGAACATCTGGCCATCAATCCGGCGCT
GCGCGGCCGCAACTACGGCAGCCGCCTGCTGGCCGAATTTTGCGAGCGGGCAGGCAAAACCGTCATTCTC
GAGATCGATCCGCCAGAGGATGAGATTGCCATTCGCCGTCTGCGCTTCTATCAGGGGCTGGGCTTTTGTC
TCAACGACTATGCCCATCTCCACCCACCCTATCATCCTGATTATCAAGGACATACGCTGCGGGTGTTAAG
CTATCCGGGGCTCCTTGAGGAAGAGGCCTATCAGCGTTTCAACAGCATGCTGGTGGAGACGGTGATGGAG
CACGGCTGAGCAACCGTAAGGATGCTGATTTGCCGATAAGAGAGGGGCCCGACTGAAGCAATTCAGGCGG
GCCCTTTTTCATTGCCGCTGGCGGGAATTACTCCCCCAGCAGCTTGTGCCAGACGGCGAGCACATGGCTG
CGTTCGTTTTGCAGCTGATCGTCGCTCACCTTGCGGGAGATCTCCGAGAGGTTGAGGCGATGGCCGAGGT
TGCGGATCTCCAGATAGGCCTGCTTCAGCCCCTCCGCCTGCTCCAGGGTCAGCACCCCCGCCATGACGCA
CTCATCGAAGATGCGCACGTTATCAGACCAGCGGGTGAGGGCGTCGGGTTCACTGCAGGCGTGGGCCAGC
ACCAGATACTGGGCGATAAACTCGATATCTACCATGCCGCCGGGTGACTGCTTGAGGTCGAACTCGCCGG
CACCCGCCTTGAGCAGGTGATCCCGCATCTTGTGGCGCATCTCGCGCACCTCGCGGGCCAGAGTCGGCAG
CTCCCGCTCCCTGGCCAGCACATCGCGGCGGATGCGGGCAAACTCGGCAACAATGGCATCATCGCCATAG
ATAGGGCGGGCCCGCACCAGCGCCTGATGCTCCCAGGTCCACGCCTCGTTTTGCTGATACTGCTCATAGG
CCGAGAGGGACGACACCAGCAGGCCGGAGTCGCCGGAGGGGCGTAGCCGCATGTCGATCTCGTAGAGGAT
GCCGGACGGGGTGCGGGTGCTGAACAGGTGCAGGATCCGCTGGGCGAGACGCAGGTAGAACTGGCGGCTG
TCGATGGGCTTGCGGCCATCTGTGTAGCTGTTGGGGTCGCCGCCGTGCAGGAACACCAGATCGAGATCCG
AGCCGTAGCCGAGTTCGATGCCACCCAGCTTGCCATAGGCCACCACCGCAAAGCCGCGCTGGCCACTGGT
TGCCACCTCGGGCGGCACCCCGTAACGCTCGCTCATCTGGGCCCACGCCTGATTGACCACCTCCTCGGTG
ATCGCCTCCGCTAGCCAGGTGAGGTGGTCGCTCACCTTCATCAGCGGCAGGGCGCCGGCGATGTCGGCCG
CCACGATGCGCAGCAGCTGTACCTGCTTGAACTGGCGCAGAGCCTCCATCTGCTGCTCCACGTCCTCTTC
GGGGATGCGCAGCAGGAACTGGCGCAGTTGGGGCTTGTACTGATCGAGCGGGGTGGGGTGATAGAGGTGC
TGGGGATCGAGCAGCTCATCCAGCAGGATGGGGTAGCGGGCCAGCTGTTCGCTCACCAGATGGCTGGCGT
CACACAGCCGCACCAGCTGGCGCAGGGCGCCCGGGTTTTCCGCCAGCAGTTGCAGATAGGCGCTGCGGGT
GAAGATGCGAGTCAGCAGCTCGCACACCCGCTCGAACAGGCGGGCAGGTTGCTCGCTGGCCACCACCAGC
CGCAGCAGCTCCGGCATCAGCCAGTCTAGCGCGATGCGCCCCTGCGGCCCTACCTGGCGGCGCTTGTACT
CCTCCTTGAAGCGCAGCAGCGCGGCGCAGAGCGGAGCGGGCTCGACTACCCCTTGAGCAGTCAGCAATTT
TTCCAGCTCGGCGGCGTCGAGCTCGGTGCGCCACAGATCGAGCCAGAGCTGCTCCAGATGGGCGGGGGCC
TCTTTCTCTTCCCCCACCACGGCGGCGAACTCCTGATGGACGGCGGCCATCTCCTCATCGAGATGAGTCA

TGAAGGCAGCCCAGTCGGTAAAGCCGAGGGCGGCGACGAGCCGCTGGCGATCCCGCTCCTCGGTGGGCAG
GGTCTGGGTCTGCTGATCGCCGATCTCCTGCAATATGTTCTCGACCCGGCGCAGGAAGCGATAGGCCTCC
AGCAGGCGATCGCAATGGGCAGATTCGAGGGCGCCGCATTGGGCGATGGCGGCCAGCGCTTCGGGCAGGT
GGCGCACCCGCAGCGCAGGTTCGCGCCCGCCGCGAATAAGCTGCAGCGCCTGGGCGATAAACTCCACCTC
GCGAATGCCGCCAGCCCCCAGCTTGATGTTGCCCTCCAGTCCCTTGCGGCGCACCTCGGCGGCGATCATC
GCCTTCATCTGGCGCAGGCCGTCGATGACGCCGAAGTCGATATAGCGGCGAAACACGAAGGGGCGCAGCA
TCTCGGCCAGTTCAACCGCGTGCTCGCATTGGGGGCCCAGTACCCGCGCCTTGACCATGGCGTAGCGCTC
CCAGTTGCGGCCGTGGTGCTGGTAGTAATCCTCCATGGCAGCGAAGGAGATGGCCAGTGGCCCCGCATCG
CCAAAGGGGCGTAAGCGCATATCGACCCGGAACACCTGACCATCCTGGGTGGGCTGGTGGAGGGCGTTGA
TGATGCGCTGACCGAGCTTGATAAAGAACTGCTGGTTGGCGAGCTCGCGCCGCCCGCCGACTGTGTAACC
GTTCTCGGGGAAGGTGAAGATAAGGTCGATATCCGAGGAGAAGTTGAGCTCGCCACCGCCGAGCTTGCCC
ATGCCGAGAATGAGCAGCGGCTGCGGGTTGCCGTTGCCATCCATCGGGGTGCCCAGCTCGCCGCACTGTT
TGGCGTAGAGCCAGTCGCGGGCCGCGCAGATGAGCGCATCCGCCAGCTTGGTGAGGTGGATAAAGCTCTC
TTCTACTGCTGCGTGGCCCAGCAGCTCGCGCCAGGCGATCACCAGCATGCGGCTGCGGCGGAACTGGCGC
AGGATGCGCTTGAGCGCCTCTTCATCACTCACCTCGGCGAGCAGGGAGGTGAGGTCACTCTGGTAGGCGG
GCCAGCGTTCGACCCGCTCCAGATCCCCGGAGGTGAGCAGCTCGGTCAGCAACTCCGGCTGTTTGAAGAG
AGAGTCGGTGACAAAATCGGAGAGACCGAGCAGGGTCAGCAGGGTTTGGCGCACCGGCTCGGGCTGATTG
CCATAGTCGGGGGCCAGTTCAGTCAGCCGTTGCCACTGGCGCTGGGCCAGCTCCTGCAGCAGGGGGGAGA
AGGCGGAGAAAGAGGGAGAGTGCTGGGCCACGGTTGCCGTCCTTGTGGTTAACGAAACTGCCGGATAAAA
AGCTGGCTGCCAATCTAGCACTGTCAGGCCCCCGCGTCACAGCGCAGGGACGAGAAGCATGATCCGGCGG
GAATTATCAGCAGCCAGTCATTCAGGTGCGCTGGTAAATGGCGGGATCGTAGATCTCCATCAGGCTGCCC
GGGTTGGCCGGTGGGGCAAAGCCGAAACTGGCGTAGAGGCCGTGGGCATCCGAGGTGGCGAGGGAGAGGC
GGCGCAACCCCTGCAACTCGGGATGGTCGACAATGGCGGCCACCAGCGCCTTGCTGTAACCGCGGCCGCG
ATACTCAGGCAGCACGAAGACATCGGCCAGATAGCCAAAGGTAGCCTTGTCGGTGATGACCCGGGCAAAG
GCAACCTGCTGACCATCGACATAGCCGCCAACGCAGAGGGAGTGGTTGATGGCCCGCACCACAGTGCTGT
GGGGAATACCGCGACACCAATAGGCCTCTTCGGAGAGAAAACGGTGGATCAGCGGCAGATCGAGCTGCCC
CTTGTCGTCACTGATCTCGATGGGTGAGTGCGTCGTGCTCTGCTGCATGTTGGCTCCTTGCCGTGGGTCA
GAAATGAGAAAGGCCCCTCGCGGGGCCTTCCAGTCTAACCGGATTGGAGCGGGAAAGTCAGGCGAGCTTG
AAGGACTGGATCTGGCCATCCAGTTCCTGGCTGTGCTGCTCCACCAGTACCGAGGCCTCCTGCAGTTCGC
GCAGCACCACCACGGAGCCTTCGGCGATCTGCTTGACCTGCTCCAGATTGCCGCGCATCTCTTCGGCGAC
CGAGCTCTGTTGCTCGGAGGCGGCGGCGATGTGGCGGTTCATGTCGGCGACCCCCTGTACCCGCTGCACT
ATGGTGGCGAGCTGGGTACCGGCTTGGGTCACCTGTTCCACCCCCTTGTCGGCCTGCTGTACGCTTTGGG
CCATCAGCTCACCAGCCTGATGGGCATTGCCCTGCAGCTGTTCGATCATCTTCTGGATCTCGACGGTGGC
GGCCTGAGCCCGGTTGGCCAGTTCACGCACTTCGGAGGCCACCACGGCAAAGCCGCGACCAGCTTCACCG
GCGCGAGCCGCCTCGATTGCAGCGTTCAGGGCCAGCAGGTTGGTCTGCTCCGAGATGCCGCGAATGGTGT
CGACCACAGAGCCAATGCGCTCGACCCCCTGCTCCACCTCGTTGACCACGGTGGCTGAGCGTTGGATGTT
GCCGGAGAGGGTGTCTATGGTGCTGACGGTGTCGGCGACGCAGCGGCTCCCCTCTTCCGCCAGCTGGCTG
GTTTCGGCGGTGGCGTGAGAGGCCTGATCCGCATGGGCGGCCACTTCGCGAATGGCGCCGACCATCTGGC
TCATGGCGCTGGCCAGCTGTTCGATCTGGGTGAACTCTTCGTTGATCGCTTCGTTGGCCTCTTCCATGCA
AAGGGTCACCTGGGAGGCGATGTCGTTGAGCTCCTTGCAGGCGGCGCGCTGGTTGCCAAACACCTCTTTG
AGGTTCTCCTGCGAGTGGGAGACGGCCTTGGCGATGTCACCGAATTCGTCACGAGATTTGAGCAGTACCT
TGTGGCTGAGGTCCCGCTTGGCGAAGCGCTCCAGCAGGCCCACCAGATAATGCACCTGCTTGACCATCAG
GCGGGAGCCGGTGAGCAGCAGGATCACGAACAGCAACCCCATGATGGCGGTCTCCATCAGGCCAGTCCAG
AGCAGGCGATTGGAGATCGCATCCGCCTTGGCGGGATCGAGCCCTTCGCTGAGCAGGGTCGCTTCCAGAT
TGTTGGCGTCAAACCAGAGTTTTGTCAGTAGCAGGAGGGTACTGAACACCATCAGCAGCACCACTTTGGG
TACCAACCGGATGTTGCTCATTCTTTTCTCCAGCAGGTTCAGCTGCATTGTCATTCTCCTTTTAAAAGCC
GTTTTTTCCTTTGCCCTCAGGACTATCGGCATTATCTTCTCTTTTATTAATTTGCACTGTGCTTAAAGTT
GGAAAATGTATGCGACATCACTTAACTCATCTGCGGGCGATGGATCTGCTGATGGCATTTCTGTCGATTA
TCTCGGTTGTGCTGGTGACGGCCCGCTGGTGGGGCCATGAGAGCCTGACACCACAGACCCTGCAGCTCTT
CTATGACCTCGACAGTCTGATCTGCTACTTCTTCCTCGCGCACTTTGCTTATGGCTGGATCCGGGCCTCC
GATCGCAAGGCCTATTTCAAGGCCAACTGGATCTATCTGCCGGGTAGCCTGCCGATGGCGGAGCAGTTGC
GCTGGGCGCGGTTGATCCAGCTGTGGCGGGTCTATCAGCTGTTCAAGGAGCACCCGGATCTCTGGGCCAT
GTTCCGTCGCGAGCGCAACGAGACCACCCTCTCCGGTATCCTGTTTCTGTTCTTTTTGCTGCTGGGGGTG
GGCTCCGGCGTCATGTACTGGATTGAGTCAGGCCAGCCCGGCAGCCAGATTGAGACACCCTATGACGCGT
TCTGGTGGACGCTGGTGACCCTCTCGACAGTGGGTTACGGGGATCTGGTGCCCAAGACAGAGGAGGGGCG
GTTTGTCGCCAGCCTGCTGATCCTGTTCGGGGTCGGTCTGTTCGGTGCCATCAGCGGTTTTATGGCCAGC
CTGTTTTTGCAGCCCAACAAGGGGGATGGCGAGACTGACAAGTGGCGTCATCACTACAACAACCAACAAG
AGGAGCTGCTGCGGGAGATCCGCGCCCTGCGCAGCGAGGTGCAGGAGCTGAAAAACAAGTGAGACCGCAG
ACGGGCAAGTTGCGAGGTGACTCGCAACTCTGCGCGTATTATCCCGGGGGAGGTGCTTTTTCTTTGCTTG
TCCTTTAACCTGCTGGCGGCTTAACATTCAACCTCTTGTCCAAACCTGAAAAAGAGAATCGATCGACCAT
GCGCGCCCTTCTCGGGATTTTGATCTGCTTGTGTGCCCAACAGGCACTGGCCGATACCCGTTATGTTTCA

GACAACATCTTTACCTATATCCACAATGGCCCGGGTACCCAGTACCGGATCCTTGGCAGCGTCAAGGCTG
GTGAACCGCTGGAGGTAAAGGCGGTGAACAGCGAGGCCGGTTTCACCCAGGTGGTGGACGGTCGTGGCCG
CGAGGGGTGGATCAAGAACAGCGAACTGCAGAGCCAGATCAGCCTGCGCGAGCGTCTGCCCCAGGTGGAA
AAAGAGCGCGATGAGCTTAAAGATCGCCTGCAGAACCTCAACGGTGACAACGAGAAACGCTTCACCGAGA
AGGATGGTCAGATCGCTGCCCAGAGCAAGGAGATCGCCGCCCTCAAGGCTCAGCTGGTCAGTCAGGGTGA
GGAGATGAGCAATCTCAAAGAGCAGAATGATGCCCTGACCCAGAGCTACGACAACCAGGAGCACGACATG
CAGATGGACTGGTTTATCCGTGGTGGTGCCATGGTGGGGGCCGGTATCCTGCTCGGCATCCTGCTGCCGA
TGCTGCCCCGTCGTCGCAGCCGCGGCGATCGCTGGATGAACTGACAGCATCTGGCTGAATGAATTGCTGA
GAAGGGTCGCCGTGGCGGCCCTTCTTTTTTGGGGCGCATTGGGCGGAATGCGTGACTGCTTTGCGATAGA
GAGGCGGTGGCAGATCGTATACAAAATGAAGTCAGATGACCCTCACGCTTGCTGGCGTATTAGCTTCAAA
AGGGAATTCATGGGGGGAATGAGCGATCTTTGTTAAACATTGGCAACAAATTCATTTCATATAGGGTGCA
ACATTGTATACATTATCGGCGTTTCGGCGGTGCGTCCGCCTTTGTCGATATCAGCCCTAGGGCGTACAGG
AAGACCCCATATGTTCAAAGCTACTCAAGTGCTTGCCGCTGACTATATCCCCTCCGATCTGGCCGCCCTG
CGCGAAGCCATCACCCAGAACTACGTGGTGGATGAAAATGCCTATCTGGCCGAACTGTCGGCCATGTTGC
CGGGGGATGGCGCCTCGCTGGAGCAGGTGACCCAGCGTGCCCGTACCCTGGTGACCAAGGTGCGCAAGGA
GAGCGGCTCCGGTGACGGTATCGACGCCTTCCTGCAGGAGTACAGCCTCGATACCCAGGAGGGGATCATC
CTGATGTGTCTGGCCGAGGCGCTGCTGCGCATTCCCGATGCCGAGACCGCCGATGCCCTGATCAAGGACA
AGCTCTCCGGCGCCAACTGGTCCTCCCATTTCCGCAAGAGCGACTCCACTCTGGTCAACGCCTCCACCTG
GGGGCTGATGCTGACCGGCAAGATCATCAAGCTCGACAAGAAGCTCGACGGCAACCCGGCCAACCTGCTG
GGGCGCATGGTCAACAAGCTGGGGGAGCCGGTGGTGCGCTCTGCCATGTATGCCGCCATGAAGATCATGG
GCAAGCAGTTCGTGCTGGGGCGTGACATGAAAGAGGCGCTCAAGGCGAGCCGCAAATCCCGCGAGCTGGG
CTACACCCACACCTACGACATGCTGGGCGAATCGGCGCTGACCATGCAGGATGCCGACAAGTATCTGACC
GATTATCGCAACGCCATCGAGGCTGTCGGCAACGAGCGGATCAGCGACGGCGGCCCGGCCCCCTCCATCT
CCATCAAGCTGTCGGCGCTCCATCCGCGCTATGACGTGGCCAACCGCGAGCGGGTGCTCGGCGAGATGTG
CGAGCGGCTGGTGGGGCTGGTGCGGCTGGCCCGCGACAAGGATGTGGCCATCAGCATCGATGCCGAGGAG
ATGGACCGCCTCGAGCTGTCGCTGGATCTGTTCGAGCAGCTTTATCGCTCCGACGCCAACCGTGGTTGGG
GCAAACTCGGCATGGTGGTGCAGGCCTACTCCAAGCGCGCCCTGCCGGTACTCTGCTGGCTCACCGCACT
GGCCCGCGAGCAGGGTGACCTCATTCCGGTTCGCTTGGTAAAAGGCGCTTACTGGGACAGCGAACTGAAA
TATGCCCAGCAGGCGGGCCTGCCCGGCTACCCGCTCTTTACCCGCAAGGCGGGTACCGATATCTCCTACC
TCGCCTGCGCCCGCTACCTGCTGAGCGAGCCGACTCGCGGCTTTATCGCTCCCCAGTTCGCCACCCACAA
CGCCAATACCGTGGTCAGCATTCTGGAGATGGCTGGCGATCGCCAGTTTGAATTCCAGCGCCTGCACGGT
ATGGGGGAGGAGCTGCACAACGCCGTGCTGAACGACAATCCCGGCCTGCACTGCCGCATCTATGCGCCGG
TCGGCGCCCACAAGGATCTGCTGCCCTATCTGGTGCGCCGCCTGCTGGAGAACGGGGCCAACACTTCCTT
CGTTCACAAGCTGGTGGATCCCAATACCCCGGTGGACTCGCTGGCCGAGCACCCGCTGCGCACCCTCAAG
CGCTACTCCGGCTTTGCCAACGACCGCATTCCCCAACCGGTGAACCTGTTCAGCGACCGCAAGAACTCCA
GCGGCGTTAATATGAACATCCTCTCCATCCAGCGCCCCTTCTTCGCCCGCTTGAAAGAGCTGGAGAGCAA
GCAGTGGCTGGCGGGCCCCATCGTCGATGGCGAGACCCTCAAGCGCAGCGAAGCCCGCACCGTGCTGAGC
CCGCAGGATGTGAGCCTCACCGTCGGCAAGATCCACTGGGCCGACGAGCAGGCGGTGGAGCAGGCCTTGG
CATCCGCCCACGCTGCCTTCCCGCGTTGGCGTGACACCCCGGTAAGCGAGCGTGCTGCCGCGCTGGAGAA
GCTGGCGGATCTGCTGGAAGCGAACCGCGAGCAGTTTGTCTCCCTCTGTACCCGCGAGGCGGGCAAGCTG
CTGCAAGATGGCATCGACGAGGTGCGTGAAGCGGTGGATTTCTGCCGCTACTACGCGGTGCAGGCGCGCA
GCCTGATGGGGCAGGCGACCCTGCTGCCCGGTTACACCGGCGAGCAGAACGAGCTGTTCCTGCAGGGGCG
CGGGGTGTTTGTCTGCATCAGCCCGTGGAACTTCCCGTTGGCGATTTTCCTCGGCCAAATCGCTGCGGCG
CTGGCCACCGGCAACACTGTTATCGCTAAACCGGCGGAGCAGACCGGCCTTATCGCCCATCTGGCGGTGA
CGCTGGCCCATCAGGCTGGTATTCCGGGCGACGTGCTGCAACTGCTGCCCGGTAACGGTGCTACCGTCGG
CGCCAAGCTGACGGCGGATCCGCGCATCGGCGGCGTCTGCTTCACCGGCTCCACCGAGACCGCCAAGCTC
ATCAACATGACCCTGGCCCAGCGCGATGGCGCCATCCTGCCACTCATCGCCGAGACCGGCGGCCAGAACG
CCATGATTGTTGATTCCACCGCCCTGCCGGAGCAGGTGGTGCGCGACGTGGTGAGCGCCGCCTTCCAGAG
CGCCGGTCAGCGCTGCTCGGCCCTGCGGGTGCTCTACCTGCAGGAGGAGATTGCCGAGCGGGTGCTGGAG
ATCCTCACCGGCGCCATGCAGGAGCTCAAAGTGGGCAACCCGGCCGAGCACAACACCGACGTCGGCCCGG
TGATCGATGCCGATGCCAAGACCGGTCTCGATGCTCACCTCGCCTACATGATCCCGCGTGCTCGCCTGAT
TGCCCAGGCGCCGATGCCCGCCGCGACCATGGAGGGACACTTCGTCCAGCCGACCGCGCTGGAGATTGGC
TCCATCCGCGAGCTGGTGAAGGAGCAGTTCGGTCCCATCCTGCACGTGGTGCGCTACGCCACCCGGGATC
TGGACAAGGTGATCAGCGACATCAACGGCACCGGCTATGGCCTGACGCTGGGGATCCACAGCCGCAACGA
ATCCCACGCTGAAGAGCTGGCGAGCCGTATCAACGTCGGCAACGTCTACATCAACCGCAACCAGATCGGC
GCCATGGTCGGGGTGCAGCCGTTCGGCGGTCAGGGGCTCTCCGGCACCGGCCCCAAGGCGGGTGGCCCCC
ACTATCTCGGGCGGTTTGTTACCGAGAAGACCCGCACCATCAACACCACTGCAGTGGGCGGCAACGCCTC
CCTGTTGGCGATGGGCGATGCCTGATCCGCGTCGATAGAGGCCGGATACGGGATACCGGATAACAGCGGG
CCGCCTTCGGGCGGCCTCTTTGTTGGTATGCTCCTCTTGCTCGCCAGTTTTGGCCCCGGCGGGGAGAAGA
TCGGAGACGATTTTGTTGTGGCGGCGGGGTCGTCAGTGGCGCTTGCCAGTCGTAAGATGGGGCCCATTCG
CGCTATTTTGCCCCTGCTGCAGCCTGCTGTGACCGTGGCGTTGTTTAATCTGACTTAACGAGAGTGTTTA

TCCGATGTCATTAACTGAACAAGCCCGCTGGTTTGTCGTGGCGGGTGACCATCAAATCCTGCTCGATGAG
GCGGGTCAGGTGCCGAGCGGCGTGAAGGCGTGCCTGCCCGCAGCGCTGGCCGGTGCCCGTTTCGAGCGTT
TTGATGAGTGGCAGGGGCTGCCCTGCTATCTGCTCGATCTGGGGGCTGAGGCCGACACGAGCGCCATGAG
CCCGTTGCGCCAGCTGATGGTGGCGGGAGACGAAGAGGGCTTTCGTCTCGCAGGTCGTGCCTGGCAGCTC
GTTACCTTCCGCCGCACCCACCGTTTCTGCGGCGAGTGCGGCGCCCCCATGACCCCGAAGGCGGGGGAGT
GGGCGCAGGTGTGCCACCTCGGCCACACGGCCTATCCGCGTATCTCCCCCTGCATTATCGTGGCGGTGCG
CAAGGGGCCGGAGATCCTCTTGGCGGCCCATCGCCGTCACTATCAGGCGGACGATCCAATGTATACCGTG
CTGGCCGGCTTTGTGGAGGCGGGGGAGAATCTCGAGCAGTGCGTGGCGCGGGAGGTGTTCGAGGAGAGCG
GCATCCGGGTGCGCAATGTGCGCTATGTGGCGAGCCAGCCCAGGCCCTTCCCCCACAGCCTGATGATGGG
CTTTACCGCCGACTACGAGAGCGGCGAGATCCTGGTGCAGGATGACGAACTGGTCGCTGCCGATTTCTTT
GCCGCCGACCGGCTGCCGCGTTTGCCGCCTCACGGCACCATAGCCCGGCGGCTGATCGAGTTGTCACTGG
CTGGCGAGGGGTAACGGCGCTTATCTTTTCGCCAACCTTTTTGCACTTCATTTTAAAAACGGGGCGCTCT
GGTTTGCGCCCCTTCACATCTTTGCAACCGATCACCCATTTGCCACGTTTTGGGCAGTCCGCTTGACTGG
GCAAAGTGGTATTATGACCGCCATTTTATGCGTGATACGAACTCAGGAACTGGGATGAAAGAGCTGAAGA
ACGATCGATACCTGCGCGCCCTGCTGCGTCAGGACGTGGATATGACCCCGGTATGGATGATGCGCCAGGC
TGGCCGTTATCTGCCGGAATACAAGGCGACTCGCGCCCAGGCGGGGGATTTCATGTCTCTGTGCCGCAAT
GCCGAGCTAGCCTGTGAAGTCACCCTGCAGCCGCTGCGTCGTTACCCGCTCGATGCCGCCATCCTCTTCT
CCGACATCCTCACCGTGCCCGATGCCATGGGGCTGGGTCTCTACTTCGAGCAGGGTGAAGGCCCGCGCTT
CGAGCGCCCGATCACCTCCATGGCCGACGTGCAGGCGCTACCCATTCCGGATCCGGAAGACGAGCTGGGC
TATGTGATGAACGCGGTGCGCACCATCCGCCGTGAGCTGAAGGGCGAAGTGCCGCTGATCGGCTTCTCCG
GCAGCCCCTGGACCCTGGCCACCTACATGGTGGAGGGGGGCAGCTCCAAGGCGTTCACCAAGATCAAGCA
GATGATGTACGCGGAGCCGCAGACTCTGCACCTGCTGCTCGACAAGCTGGCCGACAGCGTGATTGCTTAC
CTCAACGCCCAGATCAAGGCGGGTGCGCAGGCTGTCATGGTGTTCGACACCTGGGGCGGCGTGCTCACCC
CGCGCGATTACCGCGATTTCTCCCTGCAGTACATGCACAAGATTGTCGATGGCCTGATCCGCGAGCACGA
CGGTCGCCGCGTGCCGGTCACCCTGTTCACCAAGAACGGCGGTCAGTGGCTGGAGCAGATCGCCGCCACC
GGTTGCGATGCGCTGGGTCTGGACTGGACCACCGACATCGCCGATGCCAAGCGCCGGGTCGGTGACAAGG
TTGCGCTGCAGGGCAACATGGATCCCTCCATGCTCTACGCCACTCCGGCTTGCATCCGTGAAGAGGTCGC
CTCCATTCTGGCCGGTTTCGGTCACGGCAATGGTCACGTCTTCAACCTCGGCCACGGTATCCATCAGGAT
GTGAACCCGGAACATGCCGGTGTGTTCGTCAACGCGGTGCACGAGCTCTCCGCCCAGTATCACGGCCGCT
AAGGCCGGGGTGAGAGAAGAAGAGTTTGTTTGATATCCGGGGCCGCAAGGCCCCGTTTTACATTAAAAAA
TGGTGCTGCGACACGAGAGCGTGGCGCCAGTGTAGGAGGCAAGATGAGCAGCAAGTCACTGGATTGGGGT
ATCGATCTTGGCGGTACCAAGTGTGAATGCGTGGTGCTGGATGGCGACGAGGTGCTGCTGCGCCACCGCA
TCCCCACCGAGCGGGCGGGCGGTTATGAGCACATGATCGGCCAGATCGCCAAGCTGGTAGCTGAGTGCGC
CGAGAAGATTGGTCAACGCCCGACCGTCATCGGCATGGGCACGCCGGGGGCGCGGGATCCGCAGACCGGC
CTGATGAAGAACTGCAACACCACCGAGCTCAACGGCAAGCCGTTCAAGGAGGATCTGGAGCGCAAGCTGG
GCGTGCCGGTGCTGATCGCCAACGACGCCAACTGCTTCGCGCTGGCAGAAACCCATCTCGGTGCAGTGCG
CCAGCACCATCCCGATGCGAAAGTGGTGTTCGGCATCATCATGGGCACCGGGGTCGGCTCCGGCATCGTC
ATCAACGGCCAGATCCTCAACGGCCACCACGGCATCGCCGGTGAGTGGGGCCACAATGTGCTCTCCCCCG
ATGGCCCCGAGTGCTACTGCGGCAAGCGTGGCTGCGTCGAGACCCTGATCAGTGGTCCGGCGCTGGAGGC
CTGGTACGAGGCCAAGGCCAAGCGCCGCCTCTCGCTCGCCCAAATCGCGGCTGCCACCGCCCACGATCAC
ATCGCCAAGCTCACCATCGACCGGCTGCATCTGCTGTTTGGTCAGGCGTTGGCCAACGTGGTCAACATTC
TCGACCCGGATGTGATCGTCATCGGCGGCGGGGTGGGCAATGTGCAGAGCCTTTACAGCGCCGGACGGCA
GACAATTTTGCCCTTCCTCTTTAATCCCCGCTTCGCAACCCCCATTATTGCTCCTGCCCTTGGTGACAGT
GCCGGGGTATTCGGGGCCGCACTGCTGGCCCGCGGTGAATTCAAGGAGGCCCTGCTGTCGTAGCCGGGGC
TCCCGGGCGGCCGGTGCGCCGCCCTGTTGCTGGTTCAAGGAAACGGATTCTCGATGATCAAGATTGCCAT
CAATGGTTACGGGCGGATCGGTCGCAACGTGTTGCGAGCCCTCTATGAAAGCGGGCGCGACAAGACCATC
AAGATCGTCGCCATCAACGAGCTGGCGGCCCCCGAGGCCATGGTGCACCTGACCCGTTACGATACCAGTC
ACGGTCGCTTCCACCATCCGGTGCAACTGGCGGGTAACAGCATGCTGGTGGGGGAGGATCTCATCTCTCT
GTTTGCCGAGCGGGATCCGTCAAAGCTGCCGTGGCGGGCGTTGGGGGTGGATGTGGTGCTCGATTGCACC
GGGGTATTCGGCTCCCGGGCCGATGCCAAGCTGCATCTGGCTGCGGGGGCGGGCAAGGTGCTCTTCTCCC
ACCCGGCGGATGCCGATGTGGATGCCACCGTGGTCTACGGGGTCAACCATCATACCCTCACCGGAGCGGA
GCGGATCGTCTCCAACGCCTCCTGTACCACCAACTGCGTGGTGCCGGTGATTGAAACATTGCATCGAGAA
TTCGAGATAAATTGTGGTACTATTACGACAATTCATTCGGCCATGCATGATCAGCAAGTCATCGATGCCT
ACCACAGTGACTTGCGCCGAACCCGCGCTGCCAGTCAGTCCATCATTCCGGTGGACACCAAGCTGGCAAA
GGGGCTGGAGCGTATCCTGCCCCACTTCGCCGGCAAGTTTGAGGCGATCGCGGTGCGGGTGCCGACCATC
AATGTAACAGCGATGGATCTCAGTATTACTGTTCGTAAAAAAGTGACAGTTACTGACGTAAATCAAGCCC
TGCAACGGGCATCCAGAGGTACATTACACGGTATTCTGGATTACACGGAAGAACCTCTGGTCTCCGTAGA
CTTCAATCATGATGCACACTCCTGCATCATTGATGGTACCCAGACCCGGGTGAGCGATGCCAACCTCGTC
AAGATGTTGATGTGGTGCGATAACGAATGGGGCTTCGCAAACCGCATGCTGGATACCACCCGGGCCATGA
TGGCCGCAGGCTGAGCCTGCCCCGGGCGAAAGGTTACTGTTTTTAACATTACCTGGAAAATATAGAGGAC
TGAATATGTCTGTTATCAAGATGACTGACCTGGATCTGGCGGGTAAACGCGTTCTGATCCGTGCTGACCT

GAACGTACCGGTAAAAGACGGCAAGGTCACCTCCGATGCACGTATCGTTGCGACTCTGCCGACCATCAAG
CTGGCTCTGGAAAAGGGCGCCAAGCTGATGATCACCTCCCACCTGGGTCGTCCGACCGAGGGTGAATACA
ACGAAGAATTCTCCCTGGCTCCGGTTGTCAACTATCTGAAAGACGCTCTGTCCTGCCCGGTTCGCCTGGC
CAAGGATTACCTGGATGGCGTAGAAGTCGCAGCCGGTGAGCTGGTTGTACTGGAAAACTGCCGCTTCAAC
AAAGGCGAGAAGAAGAACACCGAAGAGCTGGCCAAAAAATACGCCGCCCTGTGTGACGTCTTTGTAATGG
ATGCGTTCGGTACTGCTCACCGCGCCGAAGGTTCCACCTACGGTGTGGCTCAGTTCGCTCCGGTCGCTTG
TGCCGGTCCGCTGCTGGCGGGTGAACTGGAAGCGCTGGGCAAAGCCATGCTGAAGCCCGAGCGCCCGATG
GTTGCCATCGTTGGCGGTTCCAAGGTCTCCACCAAGCTGACCGTTCTGGAATCCCTCTCCAAAATCGCTG
ACCAGCTGGTTGTCGGTGGTGGCATCGCCAACACCTTCATCGCTGCTGCCGGTCACAACGTCGGCAAGTC
CCTGTGCGAGCACGACCTGATCGACACCGCCAAGAAACTGGCTGCCGAGACCAACATTCCTGTGACCACC
GACGTGGTTGTAGGTGCCGAGTTCTCCGAGTCTACTCCGGCGACCATCAAGTCCGTCTCTGACGTGACCG
ACGGCGACATGATCTTCGACATCGGCCCGGATTCTGCCAAGGCCCTGGCTGACATCATCATGAACGCCAA
GACCATCCTGTGGAACGGTCCGGTTGGCGTGTTCGAGTTCGACCAGTTCGCCGAAGGCACCAAGGTTATC
GCTGAAGCCATCGCAGCTTCTCCGGCCTTCTCCATCGCTGGCGGTGGTGACACCCTGGCTGCTATCGACA
AGTTCGGCATCGCTGACAAAGTCTCCTACATCTCCACTGGCGGCGGCGCCTTCCTAGAGTTCGTAGAAGG
CAAGGTACTGCCGGCTGTTGCGATTCTGGAACAGCGCGCTAAAGCCTAATTGACCCGGAACGGGGGGCAA
AAGCCTCCCGTTTCGTTTATCATGTGCCCGCTGTGCCGGTTCATGATGAACCCCCCATTTTTTATTAACG
GCATACGAGACAGGACGATTAAACATGTCTAAGAAAATTTTCGACTTCGTAAAACCCGGCGTGATCACTG
GTGATGACGTTCAGAAAGTGTTCGCCATCGCTAAAGAGAACGGCTTCGCTCTGCCGGCCGTAAACTGCGT
TGGTACCGACTCAGTGAACGCAGTACTGGAAGCTGCCGCCAAGGTTAAAGCGCCGGTTATCGTTCAGTTC
TCCAACGGTGGTGCCGTGTTCACCGCCGGTAAGGGTCTGAAGCTGGAAGGTCAGAAAGCTGCCATCCTGG
GTGCCATCTCTGGTGCCAAGCACGTTCACGCTGTTGCCGAAGCTTACGGCGTGCCGGTAATCCTGCACAC
CGACCACGCTGCCAAGAAGCTGCTGCCGTGGATCGACGGTCTGCTGGAAGCGGGCGAGAAGCACTTTGCT
GAAACCGGCAAGCCGCTGTTCTCCTCCCACATGCTGGATCTGTCCGAAGAGTCTCTGGAAGAGAACATCG
ACATCTGCTGCGAGTACCTGACCCGCATGGCCAAGATGAACATGACTCTGGAGCTGGAACTGGGTTGCAC
CGGTGGCGAAGAAGACGGCGTCGACAACAGCCACATGGATCAGTCCGCTCTGTACACCCAGCCGGAAGAT
GTTGCTTACGCTTACGAGCGTCTGTCTAAGATCAGCCCGCGTTTCACCATCGCTGCCTCCTTCGGTAACG
TACACGGTGTATACAAGCCGGGTAACGTCAAGCTGACCCCGTCCATCCTGGACGCTTCCCAGAAGTACGT
TTCCGAGAAGTTCGGTATTCCGGCCAAGTCTCTGGACTTCGTATTCCACGGTGGTTCAGGTTCCACTCTG
GAAGAGATCCGCGAGTCCATCTCCTACGGCGTAGTGAAGATGAACATCGACACCGACACCCAGTGGGCAA
CCTGGGAAGGTCTGCTGAACTTCTACAAGAAGAACGAAGCTTACCTGCAAGGCCAGCTGGGCAACCCGGA
AGGCGCCGACAAGCCGAACAAGAAGTACTACGATCCGCGCGTATGGCTGCGTGAAGGTCAGACTTCCATG
ATCGCTCGTCTGGAGAAAGCATTCTCCGACCTGAACGCCATCGACGTACTGTAATTCGTTACAGCACCTT
GATTGTATGAAAAGGCGCCCTTCGGGGCGCCTTTTGCATTTCTGTGATCCGCACAGGTTGGTGAGTGGAG
GAGGAACTGGGTATTATCGAAACAACTTTGCTTATTTTATTAAACAATTTGCAACCAAGGCAGGATGCTG
AGTATGCAGGCAGGAAAGCACAACGGATATCAGGAGCACGTCGCCTCCTACTACGCGGCCACCCGCAACG
ACTCGCAACAGTGGCCCGAGCTGGAGGGGGAGCACAAGGCTGACGTCTGCATCATCGGCGGCGGCTTTAC
CGGTCTCAATACCGCCATCAATCTGGCTGATGCCGGTTACAAGGTCGCCTTGCTGGAGGCCAACCGTATC
GGCTGGGGGGCTTCCGGGCGCAACGGCGGCCAGCTTATTCGCGGTATCGGCCATGACACCAGCCAGTTCG
CCCGCTGGATTGGTGAGGATGGGGTGCGCGAGCTGGATCTGATGGGGCTGGAGGCGGTGCAGCTGGTGCG
CGAGCAGGTCGAGCGCTTCAATATCGAGTGCGATCTCACCTGGGGCTACTGCGACCTCGCGACCCGTCAG
CGTCATCTGGCTGGCTTCGAGGAGGATCTCGACCATCTGGCGAGCCTCGGCTATGAGCACGAGCTAAAGT
TGGTCCCGCGTGAAGATATCCATAGCGTGGTGGGATCGGATCGCTATATCGGCGGCCTCATCGACATGGG
CTCCGGCCATCTCCATCCCCTCAATCTGGCGCTCGGCGAGGCACGCGCCGCCGCCAGTCTCGGGGTTTCC
CTGTTTGAACACTCCAGAGTGACCCATATCGATTACGGCAAGCAGGTGGAGGTGACCACGGCTCATGGGC
GGGTCACCGCCGATTATCTGGTGATCGGCTGCAACGCCTATCTCAACGACCTCAATCCAGAGCTGGGAGG
GCGGGTGCTACCTGCCGGCTCCTATATCCTCGCCACCGAGGTGCTCGATCGCCGCTTGCGCCAGCGATTG
CTGCCGCAAAACATGGCGGTGTGCGATCAGAATGTGGCGCTCGACTACTACCGCCTCTCTGCCGATGGCC
GCCTGCTGTTTGGCGGCGCCTGCAACTATTCAGGGCGAGACCCGAAGGATATCAAGGCCTTTATGTTGCC
CAAGATGCTGCGAGTCTTCCCCGAGCTGGCTGGCACTGCCATCGAGTTTCAGTGGGGCGGCATGATCGGG
ATCGGCGCCAACCGGCTGCCGCAGATTGGCCGCTTGAAGGAATACCCAAACGTGCTCTACGCCCAGGCCT
ATTCGGGTCACGGTCTCAACGCCACCCATATGGCGGGCAAGCTGGTGGCCGAGGCGATCCGCGGCGAGAG
CCGGGGTTTCGATCTCTTTGCCAGCGTGCCCCATATCACCTTCCCCGGCGGCACGGCGCTGCGCTCCCCG
CTGTTGGCACTGGGTATGCTGTGGCACCGTATGAAGGATATCTTCTGACCCCCATTGTCAGCCTGTGCAG
TGATGGCTGAACTAAGCCCCCTTGCCAGCCCTTGTTTATAGCGGGTTGGCACTATCGTTCCGCCTGATAG
ACCAAAAGATGCATTCGCGCTAATTTCGTCCGATTGGCGCTATCTTTTAGGTGTGAGAGCGGTTATCTGC
CAAGGAACAAGGATTTGTCAGATTGCCGGAATAGTTGCCGAGAAAGGGCCGAGGATGGGGGTGAGGAGGG
CCAAGATGGAGATAAATGTGGTCGCGTCAGGCGGGCTCAGCCCGCTGGCGCAACGGCAGGGTTCTCCCGC
TGTGCAGGCACAGGAGCGGGCTGCAACTTCACCATCAACGGGCAGTGACAGCCTGGTGCGCGACCTTGGC
GCTGTGCTGGATGAGCTGGGGCTGTCACCGCAAGGGGATAGCAGCGGCGGTGGCACTGTTTCAATCAGGG
ATTCGCTGGAAGCGTTCATCAACACCATGATGGGCTCCCTGCAACAGCAGCAGGAGCAACAGTTGGCATT

GCAGCAACAACAGCGGCAAGAGCAGGAACAGCGCAAACTCGCCATCGCCGACAGCAGCGAAAACCCGCTC
AAGCGGGATCTGGAGCGCCTGCTGGAGACGCTGGAACAATAGGGCGGACAGGGAAAAGAGAGCGAGCAAG
GAGTCGAGCTTGAAGCGGTGAACCCCTTGCAGAGCCAGCTGACCAAGCTGCTGGAGAGCAGTCGTGCGGC
AGAGAGCGGGGTCACCCTCAAGAAGGTGTTGTCAGGGTTGGCTAGCAAGCTGCCGAACGAACCTGCCGAG
CGAAAAGGGTATCGGGTCGATACCCAGGTTTGAGCTGGTTGGCAACGGCAGCCCCGCCGTTGCAGAGCAT
AAGGGGCGGAAAGCGCTTTATTGGGCCGGTTCAGCCAGCCGCCACCCCTGTTCGTCCTTCATCATCCACA
CCTCGGCCTCTTGTGGTAGCACCTGGCCCGCCTCGAACTCGCCATATTTTCGCTCGAGCTCCATCAGCCC
CATCTCCTGCTGGAAGGGGCCAACCCGGTCGTAGACCAGGTCCTCGTCGTGCAACACCTGCAACTGATCG
AGCCCCTGCTTGAAGTGGAGCTGGTAACTCACCTTGACCAGATAGACCCCCTCCATCCGCTCCTCCTTGC
CGAGGATGGTGAAGTCGCTGATATCGAACAGGTTGGCGTCTGTCTCGGCCAGTAGCTTGGCGGTGACCTG
CTGGCGGATATCCTCTTCACCGGGCCCTGCACTGCAGCCATAAAGCCAGAGGGCTAGCAGGGGGATGAAC
CACTTTTTCATCAAAACTCCGGACAAGTGCAAAACGAGAGGGGCGATTGTGCCACGCGAATGCGCGGCGG
CAAACCTCGGCTCACGGTTCCAGTGAACGTTCCAGCACGATGGCCTCGGCCCGATAACCAGCCAGCTCGC
GGCGATAGCACTCGGTGAAGCCTCTGCGCAGCCAGAAGGATAGAGCACTCTCATTACTGGCAATCACTGA
CAGGCGAAGCCGGGTAGCCCCCCATGAGCTTGCCAGCGTGCAGGCGTGAAGATAGAGCGACTGACCACGG
CCACTCCCTTGCCAGGGTTCCCCCACCAGTAACAGACCCAGATAGGCGGTGTCTGGATTGGGGTAGCCGC
GCAGCAGATCAATGACAGCGGTGAGTTCTCCCTGCTCCCAGCGGCCGATGAGATGTTTGTCGGAGAGAGG
CAGGCCGGATGGGCAGGCGACAAGTTCCTCTTCTCCTGCCTGTGCAGAGAGAGGGTCATTGCTCACCGCC
GCCTGATAGGAGGGGGCCTGCTCGAACAGCCGCTGCAAGGCAGGTGCATCGCTTGTCTGCAGCCGACGCA
ACTCAGTGTGCATCACTTACCATCTCGAGATGTTTGGCAAAGGGGATAGGCCCCATAAAACCGGTGACCC
GCTGGCCTGTCAGCTCCTTGCCCTCGCGGTCGAAGAAGATGAGGGTCGGCAGGCCCAGCACATTGAGTTT
GTTGAGCAGCTCCACATCGGCATCGTCGTTGGCGGTGACATCCGCCTGCAGCAGCACCATCTGGCCAAAG
CGCTCGCGCACGGTTGGGTCACTGAAGGTCTTGTGCTCGAACTCCTTGCAGGCGACGCACCAGTCGGCGT
AGAGGTCGAGCAGCACCGGCTTGCCGCGGGCGGCGGCCAGCTGCACCTTGAGATCATCCAGCGTCTTGAT
GCGGATAAACTGCGGCGCCGTTTGACTGGCATCCGCCGTGGCGGCAGCAGGGGGGGGCGGTTGCAGCAGA
TCCTTGCCCACTACCACGGCGCTGATCATCGCCAGCAACAGCACGAAGCCGCGCACGCTTTTGGCCACCG
AGTGGGGCTGGTGCTGGTTGTGGTGATAGAGATAGCCGCACAGCAGCAGCCCCCAGCCGAGCCAGGCGAG
AGTGGCAATCTGATCGCTCCAGAGCCGTGACAGCAGCAGGATCGGCACCGCCAGCAGAGCAAAACCAAAC
AGCTGCTTGATCACATCCATCCAGGCGCCCGCTTTGGGCAGCAGCTTGCCACCTGAGGTGCCGAGCAGCA
GCAACGGCAGCCCCATCCCCAGTGAGAGCGCATAGAGCGCCGCCCCGCCGAGCCAGAGATCGCCGCTCTG
GGCTACGTAGATCAGCGCCCCCGAGAGCGGTGCCGTGGTGCAGGGGGAGCAGACCAGCCCGGAGATCATC
CCCATGATGGCGACCCCGACTACCGAGCCGCCCTGCTGGCGGTTGGAGAGGCCGGAGAGCCGGGTTTGCA
AACTGCTCGGCAGTTGCAGGGTGTAGAGCCCGAACATGGAGAGGGCCAGCAGCACGAACATCACCGATAG
ACCGATCAGCACATAGGGGTGTTGCAGCGCCGCCTGGAATTTCAGCCCTGCCGAGGCCACTACCAGCCCC
AGCAGGGTGTAGGTCACCGCCATCCCCTGCACATAGACCATGGAGAGCAGGAAGGCGCGACCGGTGGAGA
GGCGATGACCGGCGCCGGCGATGATGCCGGTCAGGATTGGGTACATGGGGAAGACGCAGGGGGTGAAGGC
GAGCCCCAGCCCCAGTGCGAAGAAGGCGACGATGCTGAGCCAGAACCCCTGATTGCCGAGGGCAGCGGCC
AGCTGATCCTGCTGGCTAACCGGTGCGACGCTTTGTGCCGTTTCAGCAGTGGACGCTGTGGTTGCCGTGA
CCAGCGGGGCCACATCGATCAGCTTGTCGGTCGGCGGGTAGCAGAGGCCGTCGGTACAGCCCTGATAGCG
CACCCGCAGGGTGGCATTTTCCCCCACCTCCTTTAGCGGCACCGCGATGCTCACCTGCTGGTAGTAGACC
CGGGTCTTGCCGAAGAAGTCATCCTCGTGCTCGGTGCCTTCCGGCAGGGTCGGCTCGCTGAAGGTGAGGT
TGTTCCCCTTGAAGCGCAGGCTGTGGCGATAGAGGTAGTAGTCGTCGGCGATGTGGAGGGTCAGCAGCAG
CTGATCGCCACGCTGTTCGCTCTCGATCTGAAAGGCCTCATCGACCTGCAAAAATTTGGGTTTGGCATTT
GCTTCAATGCCCAGCGAGCTGAGCAGATCGGCGCCGCTGGCGTGGGCAGGGGAGCTGCCCACCGCTGTCA
TCAACAGGGCACAGAGCAGCAACAGGGGAGAGAGCAAGCGGGTGAACGTCATGAGGTCTCCCCATTGAGC
CAGTCGAGATAGGCAGGTAGTCCGGCACTTACCGGCAGGGCCAGCAGCTCGGGCACCTCATAGGGGTGAT
GGGTTTGAATGCATGCCTGCAGCTCGGCAAAGCGCGACTGGCGGGATTTGATGATGAGCTGGATCTCGGT
GGCGCGCTCCACCTTGCCTTGCCAACGATAGACGGAGGTGACGCCCGGCAGTTGATTGATGCAGGCGGCC
AACCGCTGGTTCAGCAGCTGTTCGCAGAGAAGGTCGGCACTGGTTTGATCTGGGCAGGTGCAGAGCACCA
CAATGGCGTCGGTCATAAAGCGGATCTTCACCTCGTTGGGGTAAACCGGGCAGGCGCCCTTGCCGTCTCA
TCCCTGTCTGCGGATGAAGGTGAAAAAAGCGCCTGAAAATCAGCGGCTACTATACCCCAGAGCCGCTTCA
AATAGCATCTGTAGCCATTTGTGAGCCAGCGCCAAAAATTTGTGGGGGGCATGAGATAGACGCTGTCATG
GGGACAGGGCAGAATGAGCGGGCTTGTTCCCAATTGACGTAAGGAAATGTATGACCCAACAGAATGAGCA
GCAGAGAAACCGCATGTTGAGCCTGCTGCGCGAAGGTGAGCGCCGCATGTTGGTTCAATTGGCCGGGCTG
CTGCGCACGACCGCCGATGAAATCAATGCCGAGCTGGAGAAAGAGGAGCTGATGGCCACCCTGGAGCAGC
CCATTACAGTGGAGTATCTCAACGGCGTGGTGCAGCACCATCTGTTCGAGCGCAAACACAAGGGAGATAT
GGCGGTTGCCCAGCAGATCCTGAGCGATTATCAGAGCGAGCTGGAAGCGCTGCAGGCCGCTCAGGCCCAG
CCTGAAGATGCTGCCGCTGCCGACGAGAGCGGCGAAGAGCTCAAAGCCCAGGCTTGAGCGACGGGCAGGC
CTGAATCCTTACCTCTGAATCAGCCGGCCTGCCAGCGCTGACCCCTCTTGCGGCTCCATCATGTCTGCCA
TGATGGGGCCGTGTCGTTTCTTTTGTTGTCCTTCACTTCTCGGTTTCAATCCAACGGATTCGTGCCTTAG
CTCGCCTCTTGCGGCATGCCGTGCGGCACTTCGCGGGGGATGGGGAACACCTTGTCATAGCCCCAGTTGT

AGACGAAGGCATAGATCAGATAGAAGACGACGAAGCCCAGATCCAGCACCAGCGCCTCCAGCAGGCTGAC
GCTCAGCCACCAGGACATGACCGGCAGCGCGACGATCAGCAGACCGCCCTCAAAGCCGAAGGTATGCAGC
ACCCGTTGCCACAAGGTCTTGTGGGTATGGCCCTGATATTTGAGCAGCAGCTTGTCGACCCCGATGTTGT
AGATGTAGTTCCAGACGGTGGCCAGCACCGAGAAGAACAGTGCCAGCGGGCCCATATGGTTGAGCCCTAC
CCCGGTAACCCAGCTGGCCAGCGGGGCGGCAATCAGCAGGCCAATGAATTCAAAGCCAATGGCATGGCGC
AGGCGATCGTTACGGGTACGCATAAGTCACTCTCTCAATCAGGTTCATTCACGGATTGCTGGTTAATCTA
TAGATCTTTAGCGATAGATTCATGTTGGTATCCATCGCTAAAATAGATGGATGAGAGAGCTTGCCGGAGG
GCCATAAGATGACGCCATCACTGGAACAACTGAGGATCTTGCTGGCGGCCGCCGAAACCGGCTCGTTTTC
CGCTGCCGCCCGCAAGCTGGGCAAGGCGCAGTCGGTGGTGAGCAGCGCCATCGCCAATCTGGAGATCGAT
CTGGCGCTCACCCTGTTTGACCGCAGCGGCCGCTATCCGCAGCTGACGGAGGCGGGGGCCCGCATGGTGC
AGGAGGCGGGGATCCTGCTGGCGCAGAGTGAACGGTTGCAGTCCATTGCCGGGGAGCTGGCGGCCGGGGT
GGAGACTCGGCTCACTCTGGCCATCGATGATGACTCTCACCTCCCCTGGCTCGGCACCCTGTTGGAGGAG
TTCGCCACTCGCTACCCGACCGTGGAGCTGGAGTTGCTCTTCCCGCTGATGGAGCTGGAGTTGCTCTTCC
CGCTGATGGAGGATGTGACCGAGATGCTGGTGACCGGCCGGGCCCAGCTCGGCATCAGCTATCAGAAGGT
TCATCCCCAGCGGGAGATTGTCGCCCATTCGCTGGGGGAGGTGACCATGCCGCTGGTGGTGTCACCGGAT
CATCCATTGGCCCGCAAGCAGCCGCTGCGGGAGGTCGATCTGCAGGGGGCACGCCAGCTGATGGTGACCG
GCCGACGGGAAGGGAGCGAGCGCCAGCGGTTTCGCATCTCGGCTCAGGTGTGGTGGGTCGAGGGGGATCT
CGGGGTGCTGGAGCTGGTCAAGCGGGGCCTTGGCTGGTCCGCCATTCCGGAATTTCTGCTGCATCAACCG
CTGAAGCGGGGCGAAGTGGTGGTGCTGAAGCCAGACTTTATCGCCAGCCACGCGCTGGCGCTGGAGTTGC
AGTGGCATCGCGCCCGCCCGCTGGGGCAGGCGGGGCGCTGGCTGAAGGAGGCACTGCTGGCGCGGGTGCC
TCGCTAGCCGACCTTACTCGGTCAGGGCGTAAGGCAGGGGTTGCAGGGTGAGGCGGGAGCCTTCGTCCTG
CTTGAGGCGGAATTGGGCGTCAGCCTCGGTATCTTTCGGCAGTACGGCGGTGAGCCACGCCTGCCCGGCC
TGTTGCCAGGCGTTGAGCACCATGCCGCTGCGGCGCCAGTTGTCACCCAGTTGCAGTTCCAGGGTATCGC
CGCTGGCGACCGGCTCACCGGTAGTCCCCGCCAGCAGGAACAGGGCGCGGTTGTTGGCGCCGCGATACTT
GGCGCGGGCCACCGTCTCCTGCCCCATGTAGCAGCCCTTGTTGAAGCTGATACCGTCCAGCGCCTGCAGG
TTGAGCATCTGCGGGATGAACTCCCCCTGATGCACCGCTTCCATGTGTGGCAGGCCCGCCTTGATCTCGA
GCCCCCACCAGAGTGACTCGTCACCTTGCGGCAGGGCGTCGGCTTGCTGATTGCTCACCAGCAGCCAGCG
ATCCTGTTCGATCTTCACCGCCATGCCGCCGTCGATGAGACCGCTCTGCTCAAGGCCAAACTTGGTGGCG
ATCCAGCCATCGCTGCCCGTACCGGCGATCCCGGTGGCGTGGCGTTCGTCGGCGGCAATCTCCACCTTGG
AGAAGACGGCAAATTTCTTCAGCTCCGGCAGTTGGCGCTCCAGTACAGAGGGGCTGGTCAGTAGCAGCAG
ACTGTCGTCGAGGCAGAGCAGGCGGAAATTGCTCCACAGCTTGCCCTTGGGATCACAGTGGCCACCCGGG
GTCTGTTGCCCCGGTTGCAGGGCGTTGACATCACAGGTGACCTGACCTTGCAGATATTTGACGCGATCCT
GGCCGCTGATACGGGTGATGGCCAGCTTGGTCAGGGAGAAGCGGGTCGGCTCGGCAGGAAAAACAGGTGG
TTGCAGGCTCACGATGGTATTCCAGAGAGAAAGTATGTGAGCTGCATGGTAAAGACGTAACTAATGTTTG
TCAGCCGGAAAATCCAGCTGGTACCATCCCCCGGTCTTTGTAAGGAGAGTGCCATGTTGAGCCCAGTTGA
AAAATCCCGCCTGAAGTGGGCCTGCCGTCGCGGTATGCTCGAGCTGGATGTGATCTTTATGCCCTTTTTC
GAACACGAGTTCGACTCCCTGAGCGAAGCCGATCAGCAGACCTTTATCCGCCTGCTGGCGAGCGACGATC
CGGATCTGTTCAAATGGTGCATGGGTCATGGTGTCCCTGCCGATCCCGCATTTGCCGCCATGATCCCGAT
GATCCTGGAGCGCAATCAGGCGCGCCACGACCGCCGGGGGTAATACCGTGGAGCGGGTGATCATGACGGT
TCACCCCTCCCGCCATCAGCAATGTTTGCTGGTGGCCCTCTTTTTGTTGCTGCTCTGGTCGGTGGCTGGC
CTGATTGCCGGTTGGCAGTGGGCACTGTTGCTGCCCGTCTGGTTGGTGTCGCTCTGGCTGAGCTGGCGTG
CGCTCGCCGCGCCCCCGTTCGAGCTCGTCTGGGATGGCCAGTGGCTACAGTGGCAAGGTCGTCGCTATCA
GCTAGACAAGAAAAGCCGCATTCTGCCCGGTGTGCTCAGGCTGGCGCTTTGTCCCGATCCGCAAGAGGGT
GGAGCCGTGTCGCCACGGCAACTGTGGCTCTTCTCCGATGCCCTTCCCCCCGAACATTACCGCCTGCTGG
CGCGAGCCATCCACTTTCTGCCGTCCCGGCGCTGACGCCTGCTTTCCGTGATGACCATGCGCTGATTTAT
CGCGATAAGCTGTTGCCATGTGGCTGGCGACACTCTTTTTGTGGCTGAAAAGCGGTTGAAAACAAGTGAC
CCCGCCGGGGCGGGGTCACTGATTGCTTTAACGTTTTGCGGGAAGGGGGATTAATCCCGCTCCGGGGTCA
GCACGGTCGGTTTGGAGTCCGGCAGCATATCCGGATGATCCAGGTTGTAGTGCAGGCCGCGGGACTCCTT
GCGCTGCAGGGCGCAGTTGACGATAAGCTCGGCCACCTGCAGCAGGTTGCGCAGCTCCAGCAGGTTGTTG
GAGACGCGGAAGTTGGCGTAGTACTCCTGCACCTCCTGCTTGAGCAGGTCGATGCGGCGCTTGGCGCGGG
CCAGGCGTTTGTTGGTACGCACTATCCCCACGTAGTCCCACATCATCAACCGCAGCTCGTGCCAGTTGTG
CTGGATCACCACCAGTTCGTCAGAATCTTCCACCTTGCTCTCGTCCCACACCGGCAGGCGCGGCGGCTCC
ACGCTCATCGGCAGCTTGGCCAGAATGTCGGCCCCCGCCTGATGGGCATAGACCACGCACTCCAGCAGGG
AGTTGGATGCCATGCGGTTGGCGCCGTGCAGACCGGTGTAGGTCACTTCACCTATGGCGTAGAGGCCGGG
GATATCGGTCTGGCCGTTGTTGTCGATCATCACGCCGCCACAGGTGTAGTGGGCGGCCGGCACGATGGGC
ATCGGCTCCTTGGTGATGTCGATGCCGACCGCGAGGCAGCGCTCATAAATGGTCGGGAAGTGCTTGATGA
TGAAATCGGCCGGTTTGTGGCTGATATCCAGATACATGCAATCCGCCCCGAGCCGCTTCATCTCGTGGTC
GATGGCGCGGGCGACAATATCCCGTGGCGCCAGCTCGGCCCGCTCGTCAAACTCCGGCATAAAGCGGCTG
CCGTCCGGGCGGCGCAGATAGGCCCCCTCGCCACGCAGCGCTTCGGTCAGCAGGAAGTTGGGGTCGTCCG
GGTGAAACAGGCTGGTGGGGTGGAACTGGTTGAACTCCAGGTTCGCTACCCGGCAGCCGGCCCGCCATGC
CATGGCGATGCCATCGCCAGAGCTCACATCCGGGTTGGAGGTGTACTGGTAGACCTTGGAGGCACCACCG

GTGGCCAGTGCCACGAAACGGGCGCGGATCACCTCGACCTGCTCGGCGTTGCGGTTCCAGACGTAGGCCC
CCAGCACCTTGTTGCCCGGCAGGTTGAGCTTGCGGGTGGTGATGAGGTCGACCGCGTTGAAGCGCTCCAT
CAGCTGGATATTGGGGTGGGCACGAACCTGATCGATCAGGGTCAGTTGCACCGCCTTGCCGGTGGCATCG
GCCGCGTGGAAGATGCGGCGATGGCTGTGGCCCCCTTCCCGGGTGAGGTGGTACTGGGATTCACCATCGG
CGTTCTCCTCCTGATCGAAGGGGACCCCTTGCTGAATGAGCCACTGGATGGACTCGCGGGCGTGGCGAGC
GGTGAATTCGACCACTGCCGGGTCACACAACCCGGCGCCCGCGTTGAGCGTGTCGCTCACGTGGGATTCG
ATGCTGTCGGTTTCGTCGAAAACGGCAGCGATGCCTCCTTGCGCATAGAAAGTGGCCCCTTCACTGATGG
GCCCCTTGCTCAGAACCAGGACTTTGGCGTGGTCTGCCAATCGCAACGCGAGGGACAAACCAGCGGCACC
GCTGCCAATGATCAGTACATCGCAAAGGTATTCAACACTTGCTTTCATAAGTATCATTGGCCTGGATTAA
GAACTGGCACCATGGTAGCCCAAGCGAGGGCGAGGATGCAGCCTGCATCACATTTTTTTCAATTGAAGCG
AACTTTCTCTGATTCTCCCTGTCTGAACTTGTGCGCTTAGGAAGGGCCGGAAAAGAAAAACGATAACCTG
ATGGGGGTTTTACGGCTCTGATGAGCGACCAGAATCTACTGGACGAACAGCTGGTTGAACGGGTCCAGCG
CGGCGATAAAGCAGCTTTCAACCTGTTGGTAAAAAAATACCAGCATAAGGTGGTCAACCTGGTTGCCAGG
TATGTCAACAATCCCGGTGATGTGCCCGATGTGGCGCAAGAGGCCTTTATAAAAGCCTACCGTGCATTGC
CGACATTCCGTGGCGAAAGTGCTTTTTACACCTGGTTGTACCGAATTGCCGTGAACACGGCGAAGAATTA
TCTCACCTCCCTGGGGCGCAGGCCGCCCAGCAGTGATGTGGAGGCTGACGAAGCGGAGTCTTATGGTGGT
GGTGAAGCCTTGCAAGAGGTCTCAACCCCCGAAAACCTGGCGCTGACTGACGAGATCAAACGCACCGTGT
TTTCAGCCATAGAGGCATTGCCGGAAGACCTGAGGACAGCTATCACCCTTCGCGAACTGGAAGGGCTGAG
CTACGAAGAGATTGCCGAAATCATGGATTGCCCAGTGGGTACGGTCAGATCCCGTATCTTCCGGGCGAGG
GAAGCAATCGATAAAAAGCTGCAACCTCTGATCGAGAATTAACTGGGGAAAGACGGGTTAACTTATGGCA
AATAAAGAGCAAATCTCTGCCCTGATGGATGGTGACCTGAGTGACGCAGAGGTACTGAACGAGCTGGAGA
TGGACTCCGACTTGCAAGATACCTGGGGTCGTTACCACCTGATCGGCGATGCCATGCGGGGTGACCTGCC
GGTCAATCTGCAACTGGATCTCAGTGACAGCATCATGGCTGCGCTGGAGGATGAGCCCACCATACTGGCA
CCCAAGCCGGTCGAGACGGCTCCCGTGCTGCAACCTGCCGTTGCACCGGTCAAGACAGATTCTAATGTGG
TTCCGTTGTTCCGCCGTGTCGGTCAGCAGCTGGGTCAATATGCCATTGCGGCATCCGTGGCCGCTGCCGT
TATCTTCGGGGTGCAGCAGTATCAGGGACAGGATGGCGTTCCGGCCAATCCGGTGCTCAATACCATTCCA
ATCGGTGGCAGTGCGGCACCTGTGAGTGTTCACTATCCGCAACAGGATGGCGCCCGTGCCCGTCAGCAAG
GGCTGACCGAACAGCAGATGCAGGAGCAGCGGGAGCGGATCAACGCCTTCCTGCGTGACCACCAGTTGCA
GCAGCGCCTGTTGCAAGACAGACAGCTCCAACAATAACGAATTGTCAGGGATACAAACGTGCGCCAGTCC
AGCCGTATTTTGCTGGCCTCTCTCCTGCTAATTAGTGCCAAGGCCTCGGCCGACGATAAGTCGGCCGAGG
CCTTGTTGCAGCAGATGCAGAGTGCCGTGCAGCAACAGAATTTCGAGTTGTCCATGGTCAAGGCCCGTCA
GGGGCGTCTTGAACCGATCCGGATCAGCCATGCCGTCATCAACGGCAAGGAAGTGGCCCACCTCAGTTAC
CTTGACGGCAAGGCGATCGAGTATCTCCAGCGCCAGAACGAATACACCTTCTTTGAAAACAGCCACGATC
CCTACACCCTGAAGGATGCCCGCTTCCCCGGCATCTGGTCGACCCTGGTGAAGATGCCGCTGGCGCAGGT
GCTGGAGAGCTATGATCCAGTGCTGGCCGGTCGCAGCCGGGTCGCCGGTGTGGTGGCGCAGGTAGTGAGG
CTGGTGCCTAAAGAGGCCGACAAATATGGTTTCGTGCTCTGGATTGATGAGCAGAGCCACTTACTGTTGC
GGATCGACATGGTCGAGCGGGAAGGCAATCTGGTGGAGCAGGTACTCGGTGTCGAGCTGGATCTGCAGAG
CAAACCGGCCCCCTGGCTGGTGAAGCTGGCCAACAGCAAGCAGCCTCCGGCGCTGGCGCTGGAGGATGCC
TATCCGGCGCCGCAACAGCAGTTGAACTGGCAGATCACCTGGCTGCCCGATGGCTTCAAGGTGCTGTCGC
AAGATCGGCACCAGCTGGTGACCACCAGTATGCCGGTGGATTACATGATGCTCTCCGATGGGCTGGTGGA
TCTCTCGGTCTATGTCTCCCGGGTCGATCCCAAGCAGGCGGTGCGCCAGCAGCTGATCCGCCAGGGAGCG
ACCTCGCTGGTGAGCTACGTCAACGAGGTGGGGGTCGAGATCACCGTGGTCGGCGAGGTGCCTGCCGAAA
CCGCCAAGCGCATCGCCGAGTCGGTCCAGCCGCTGGCCCGCAACGAAGCAGTGCAGTAACGGGGGATGCG
GGGTTTTGCCCCGCTTGCCGAGCGTGGCGAGTCGTGGTCAGATCCTCTGATTGTGTGAACCCGAGGTGTT
GCCGTGAATGAGATGAGTGAAGAGCTGGCGACCGTGGTGGCCATTGAGGGTGACCATGCCTGGGTGGAGT
GCGAGCGTCGCTCCGCCTGTAGTGGTTGTCAGCAGCAGTCCAACTGCGGTACCGGCACCGTGGCCAAGGC
GTTTCCCCTGAAAGCTCCCCGCTTGCGGGTCAGGCTGACCGCCGAGGTGCGGGTTGGCCAGCAAGTAAGG
CTCGGTATCCCGCAGGAGAGTCTGCTGCGCGGGGCTGCGCTGGTCTATGTGCTGCCGCTTTTCTGTCTGT
TGGCCGGCGCCTTGCTGGGGCAGCTTTGGCTGGCGCCGCTGCTCTCGGGCGGGGAGGGGATTACCATTCT
CTGCTGCCTGCTGGGGGGCGTGCTGGGCTTTTTACTGGTTCGTTACTTTTCCAACCGCCTGGATCAGGGG
CGTTACGGCCCGCAGATGCTCGGCGTGGTCGTCCCTGTCCGCATCCTCCCCTGACCGAACAGGCGCTATT
TCACTGGCGCTGGCGACTAAAGAATGGCCGTCAGCCGGGTTATCCAGTAAAATCGCGCCTCCATAACTGA
CCTTAACTAATTTTGAGTGACTCTTGCTCGATGAAACACATTCGTAACTTTTCGATTATTGCGCACATCG
ACCACGGTAAATCGACCCTGTCGGACCGTCTGATCCAGACTTGCGGTGGCTTGTCCGACCGCGAGATGCA
GGCTCAGGTGCTTGACTCCATGGATCTGGAGCGCGAGCGCGGCATTACCATCAAAGCACAGAGTGTGACC
CTGAACTACCAGGCACAAGACGGTAACACCTACCAGCTGAACTTTATCGACACCCCGGGCCACGTGGATT
TCTCCTACGAGGTCTCCCGCTCTTTGGCGGCCTGTGAAGGGGCGCTGCTGGTGGTGGATGCCGGCCAGGG
GGTTGAGGCACAGACTCTGGCCAACTGCTATACCGCCATGGAAATGGATTTGGAAGTGGTGCCGGTGCTG
AACAAGATTGACCTGCCAGCCGCAGAGCCCGAGCGGGTTGCCGAGGAGATCGAAGACATCGTCGGTATCG
ACGCGGTCGATGCGGTGCGCTGCTCCGCCAAGACCGGCCTGGGTGTTGATCTGGTGCTGGAAGAGATCGT
CGCCAAGATCCCGGCACCGGAAGGTGACCCGGATGCCCCACTGCAGGCGCTGATCATCGACTCCTGGTTC

GACTCCTATCTGGGCGTTGTCTCTCTGGTGCGGATCAAGAACGGTTCCCTGAAGAAGAACGACAAGATCA
AAGTGATGAGCACCGGTCAGGTGTGGGGTGTCGATCGTATCGGTATCTTCACGCCCAAGCAGGTGGATAC
CCAGGGGCTGGGGGGCGGCGAGGTAGGTTGGGTTGTCTGCGGCATCAAGGACATCCACGGCGCGCCGGTG
GGCGATACCCTGACTCAGGCCAAGAACGGTGCCGATAAGGCGCTGGCTGGCTTCAAGAAGGTGAAGCCGC
AGGTTTATGCCGGTCTGTTCCCCATCTCCAGCGATGACTACGAGTCGTTCCGTGACGCCCTCGACAAGCT
CTCCCTGAACGATGCCTCCCTGTTCTTCGAACCAGAGAGCTCCACGGCGCTGGGCTTCGGCTTCCGTTGT
GGCTTCCTCGGCATGCTGCACATGGAGATCATTCAGGAGCGTCTGGAGCGTGAATACGATCTGGATCTGA
TCACCACGGCGCCGACCGTAGTGTACGAGATCGAGCTGACCGACGGCTCCACCATCCATATCGACAGCCC
GGCCCAGATGCCCGCCATCACCAGCATCAAGGAGATGCGCGAGCCGATCGCCGAGTGTCATATCCTGCTG
CCGCAGGAGTACATGGGCAACGTGATCACCCTCTGTATCGAGAAGCGTGGCGTGCAGACCAACATGGTCT
ACCACGGCAAGCAGGTGGCGCTGACCTATGAAATCCCGATGGCGGAAGTGGTGCTCGACTTCTTCGACCG
TCTGAAATCCACCAGTCGCGGTTACGCCTCGCTGGATTACGGCTTCAAGCGTTTCGAAACCTCCGACATG
GTCCGTCTGGACATCATGATCAACGGTGAGCGGGTCGATGCTTTGGCCATCATCACCCACAAGGACAACG
CCCAGTATCGCGGTCGTCAGGTGGTGGAGAAGATGCGCGAGCTGATCCCGCGCCAGATGTTCGATATCGC
GATTCAGGCGGCCATCGGCAACCAGATCATCGCTCGCAGCACCGTCAAGGCTCTGCGTAAAGACGTAACC
GCCAAGTGTTACGGTGGTGACGTGAGTCGTAAGAAGAAACTGCTGAACAAGCAGAAAGAAGGTAAGAAGC
GGATGAAGTCCCTCGGCCGTGTCGACGTGCCGCAGGAAGCCTTCCTCGCGATTCTCCACGTCGGAAAGGA
TTAATAGATGGCAAGCACGTTTTCCCTGATCCTGGTGGTGGTCTGCGCGATCACCGGTGTTATCTGGTGT
CTGGACAAGATGATTTGGTCCAAGCAGCGCGCCGCCAAGATTGCCGTGGCTCGTGCCCATGGCGGTGCCC
ACCTGGATGAGAAGACCCTGGCCAAGGTCGCCCCCGTGCCGGTCTGGATTGAACAGACCGCCGGGGTCTT
CCCGGTCATCACCTTTGTGCTGATCCTGCGTTCGTTCATTTTTGAACCGTTCCAGATCCCGTCAGGTTCC
ATGATGCCCACCCTGCTGGTGGGCGACTTCATCCTGGTGGAAAAATTTGCCTATGGTCTGCGGGACCCGG
TGACCAACACCAAGTTCCTCGAGACTGGCGAGCCCAAGCGTGGCGACGTGGTGGTGTTCAAGTATCCCCT
TGAACCGCGCGTTGACTACATCAAGCGGGTGGTCGGCATGCCGGGTGATCGGGTCATCTATCGCAACAAG
GAGCTGATGATCCGGCCCAAGTGCGAAGAGCAGGAAGGGAAAACGTGCCCGGGCTTCAAGAAGCTCGACG
TCAAGTTTGAACAAAGAGGCGAATTTACCCAGATGGGGATCCCCCTTGACCGCTATACCGAGCAGTTGGG
TGATGTCTCCCACGAGACCCTGCGCAATCCGCTGATGCCCGACATGGTTGGCCGTTACTATCGCCAGCCC
GGCACCTACCCCGACGAGTGGGTGGTACCGGAAGGCCAGTATTTCGTGATGGGCGACAACCGCGACAACA
GCACCGACAGCCGATTCTGGGGCTTTGTGCCCGAGCAGAATCTGGTCGGCAAGGCGGTCGCTATCTGGAT
AAGTTTTGAATTCGAGCGCGAAGAAGGCTCCCTGCTGCCAAGTTGGGTACCGACAGGTGTGCGCTTCAAC
CGCATTGGCGGAATCAAGTAAATGAATATCAAGATGAACAGACTGCAGTCCCGTCTCGGGCATGTCTTTG
CGGATCTGGAACTGCTGACCCGCGCGCTGACTCACCGCAGCGCCGGTTCCCGTCACAATGAGCGACTGGA
ATTTCTGGGTGACTCCATTTTGAGTCTGGTGATTGCCGACGATCTCTTCCACCGCTTCCCGCAAGCGGCG
GAAGGGGATCTCAGCCGGATGCGCGCCACCCTGGTGCGCGAGAAGACCCTGGCCGAACTGGGCCGGGAGT
TTGACCTTGGTGATCACCTGATCCTCGGTCCCGGCGAACTCAAGAGCGGCGGTTTTCGCCGCGAATCCAT
TCTGGCCGACACGGTCGAGGCGGTGATCGGTGCCGTCTATCTGGATACCAATCTTGAGACGGTGCGGACA
CTGCTGCTGAGCTGGTATGCCAGCCGCCTGGAAGAGATCAAGCCGGGCATCGAGCAGAAAGATCCCAAGA
CCCGTCTGCAAGAAATTTTGCAGGGAAGCCGCAAATCCCTGCCGACCTACACCGTGACCAACGTCAAGGG
CGAGGCACACAACCAGGAGTTCACTGTCCAGTGTGACGTGGAAGGTCTGGATGGTCCCCTCGTCGGGGTC
GGCACCAGCCGTCGCAAGGCCGAACAGGCCGCGGCGCAGCAAGCATTGGAAAAACTGTCATGACAGATAC
CACCTACTGCGGCTTTGTCGCCATCGTGGGCCGCCCGAATGTGGGCAAGTCCACCCTGCTCAACAAGCTG
CTTGGCCAGAAGGTCAGCATTACCTCGAAGAAGCCCCAGACCACCCGTCACCGGATTTTGGGGATCGACA
CCGAGGATAACTACCAGACCATCTATGTAGATACCCCGGGTCTGCACATTGAAGAGAAACGGGCCATCAA
CCGCCTGATGAACCGTGCCGCCACCAGTTCGCTGGGTGATGTGGCCATGGTGGTGTTCATGGTCGACGGT
ACCCACTGGACCAAAGACGACGAGATGGTGCTGGGCAAGCTGCGCAATCTCAAGTGCCCTGTGGTGCTGG
CGGTCAACAAGATTGACAACGTCAAAGAGAAAGAAGATCTGCTGCCCCACCTCGAGTGGCTGGGACAACA
GATGAACTTCGCCCATATTCTGCCCATCAGTGCCGAAAAGGGCACCAACGTGGAGAAGATCCGCGAGTGG
GCCAAGGATCTGCTGCCGGAAAACGTCCACTTCTTCCCGGAAGATTACGTGACCGACCGCTCCTCCCGCT
TTATGGCCTCCGAAATCATCCGCGAGAAGCTGATGCGCTTCACCGGTGAGGAGCTGCCCTACTCGGTGAC
GGTGGAGATCGAGCGCTTCAAGGTGGAAGAGAACGGCCTCTATCACATCCACGGCCTGATCCTGGTCGAG
CGCGATGGCCAGAAGAAGATGGTCATCGGTAACAAGGGCGAGAAGATCAAGACCATCGGCACCGAAGCGC
GCCTCGATATGGAGCGTCTGTTCCAGAACAAGGTGCACCTCGAGCTGTGGGTCAAGGTCAAGTCGGGCTG
GGCTGATGATGAGCGCGCCCTGCGCAGCCTCGGGTATGGCGACGACTGATTGCTGACTCCGGCTTTCGTC
ATCCACAGTCGGCCCTATCGGGAGACCAGCCAGCTGGTCGAGGTCTTCACCCAGGATAGTGGCCGATTTA
CTCTGGTGGCTCGCGGTTCTCGCGGGCCACGCTCCCCTCTTAAGGGGCTGCTGCAACCCTTTACCCTGCT
CACCATCGCCTGGCGCGGCAAGGGCGATCTCAAGAACCTCACCCAGGTCGAATGCCCGGATCACTCCCTG
CGTCTCGGCGGCGATCGTCTCTTCTACGGCCTCTATCTCAACGAGCTGGTCTACTACCTGCTGGAAGCGC
ACACCCCGTTCCCAGAAGTATTCGATGCCTATGCCCGTGCCATCATGGCGCTGGCCGATGGCGAGACGCC
GGAGCTGCCGCTGCGCCGCTTCGAGTTCCTGCTGTTGCAGGCGCTCGGCTATGCGGTGGATTTCGAATAC
ACGGCGGACGACGAGTCGCCCATCGAGCCGACCCTGCTCTATGGCTTCGAGCGTGAATTGGGCTTTGTTG
CTTGCGAGCGGGCGCCGGATCCCCGTACCCTGTTTCTCGGTGCCCACCTGCTCGCCTTTGCCGAGGGACG

TTTCGATACCCCTCCCTTGCTGCAGGCGGCCAAACGTTTCAGCCGATTGGCGTTGCAACCCTATCTGGGC
AAGCGCCAGCTAAAGAGCCGGGAGCTGTTTTTAAAACGCAAAGCCAGCATCCAAGGCTGACACAGATTAC
AGACATACGGATATTGGCGCGGTGTGATTCGCTGACCGCGCAGTCAACAGATGAAGCCTTTCAAGGAGTT
CTCAATGAGTGAAATCTATCTGGGTGTGAACATCGACCATATCGCCACCCTGCGCAACGCCCGTGGCACC
CAGTATCCGGATCCGGTACAGGCCGCGTTCGTGACCGAGCAGGCCGGGGCCGATGGCATTACCGTGCATC
TGCGTGAAGATCGCCGCCACATCACCGACCGTGATGTGGAGATCCTGCGCCAGACTATCCAGACCAGAAT
GAACCTGGAGATGGCGGTGACCGAAGAGATGATCGGCATCGCCTGCCGCATCAAGCCCCACTTCGTCTGT
CTGGTGCCGGAGAAGCGCACCGAGGTGACCACCGAGGGCGGTCTGGACGTGGCCGGTCAGCTGGACAAGG
TGACTGACGCCGTTGCCCGCCTCTCTGCTGTGGGTGCCCAGGTATCCCTCTTTATCGACGCCGATCCCAT
CCAGATCGATGCGGCTGCCGAGAGTGGCGCCCCCTTTATAGAGATCCATACCGGCCGTTACGCCGATGCC
ACCACCGATGCCGAGCGCAACGCCGAGTTCAAGCGTATCGCCGCCGGCGCCTCCTATGCTGCTGGCAAGG
GGCTCAAGGTCAATGCCGGCCACGGCCTGCACTACCACAACGTCAAGGCCATCGCCGCCATCCCCGAGCT
CTATGAGCTCAACATCGGCCACGCCATCATCGGCCGCGCCGCCTTCGATGGTCTGGCCAAGGCCGTGAGC
GATATGCGCCTGCTGATGCAGGAAGCCCGTCAGGGGATTTGATCCGCTGGATAGGTCAGCGGCCTGAGAC
CGGCCGCTAGAAATAACAAGGGACGCCACGGCGTCCTTTGTTGTTTTTAGGTCTATTAATGCGGGGCTAA
TGAGTTGGCGGCACCCGCGCAGTAATAACCCAGCACAATCGGCTATAAGCAGTTGATGTCAAAAGGGTTA
CACTGGTTGACCCTGTGAGCTGGACGAGAATGTTATGGATCATCCCTTGTACCACCCCGATATCACCGAA
CTTGAACCTGTCGCCCGCGCGGCGGGAGCGGCCATCATGGCCATCTACAGCCAGCCCTTCGCGGTGGAGT
ACAAGAGCGACGAGAGCCCGCTGACCGCTGCCGACAAGGGGGCTCATGAGGTGATCGTTCAGGGGTTGGC
ACAGCTGACTCCGGATATTCCCGTACTGTCTGAGGAGTCGGGGACCGAGGTGATGGCGGCGCGCCACGGC
TGGTCCCGCTACTGGCTGGTGGATCCCCTCGATGGCACCAAGGAGTTTGTCTCCCGCAACGGCGAGTTCA
CCGTCAATATCGCGCTGATTGAAGAGGGGGCACCGCGCTGGGGGCTGGTCTATGCCCCTGTGCTGGACAA
GCTCTGGTATGGCGGTAAGGGCGCGGGTTCCTGGCGCATCGCCGATGGCAAGCGCGAGCCTATCCAGACC
CGGCCTCATCGTGAAGGCGAGGCGTGGCGAGTGGTGGGCAGTCGCAACCACCTGTCGCAGGAGACCCTCG
ATTTCTTGGCTCCTTTTGGCGAGATCGAGCTGGTATCGATGGGCAGTTCGCTCAAGTTCTGCATCATTGC
CGAGGGGGGCGCCGAGCTCTATCCGCGCCTCGCACCTACCTGCGAGTGGGATACCGGCGCTGCACAGGCG
GTGCTGGAGGGGGCGGGTGGCAGCGTCACCCTGCTCGATGGCTCACCCATTGCCTACAACAAGCCCGATA
TCCTCAATCCCTGGTTTGTCGCCAAGGCATAAACGAGATATCGGGATGCAAAAAGGCAGGACTGGAATGA
ACTTCCAGTCCTGCCTTTTTTATTTCGCTATTCGCTTTGCGGTCAGAAGACCTTTTTGAACGGCTTGATG
GTCACTTTGGCATAGACGCCAGCTGCCACATAGGGATCGGCATCGGCCCAGGCTTTGGCGTCAGCTTGCG
AGGCAAACTCGGCGATGACGGTGCTGCCGGTAAAGCCTGCATCGGCTGGATCCTCGGCATCGATTGCCGG
GTTGGGGCCGGCGGTCAGCAGCCGACCTTCATCCTGCAGGGCTTGCAGGCGAGCCAGATGGGCAGGGCGA
ACTTGCTTGCGCAGTGGCAGGCTATCGGGGACATCTTCGGAGTAGATGAGATACCACATGAGACAATCCT
TTTCTGATGAGGGTTACTTTTTGGGTTCGTCTTTTTGCTGGGACAACTGCTCGGCAGGGATGTGGCGATA
GAGATAGACGCCGCTCAGCAGGGTAAAGACCAGCGTCATCCCCAGCAGACCGAACACCTTGAAGTTGACC
CACATCTCTTGCGGCAGGTTGAACGCCACGTAGAGGTTGAGCAGGCCACAGACGGTAAAGAAGCCGACCC
AGCCGAGATTGACTGTGTCCCAGACCTTATCGGGTGCTTGCAGCTCCTTGGCCAACATCTGTTTGATCAG
ATTCTTGCCAAAGCCGTAGCGGCTGATAAGCAGACCCAGTGCGAACAGCACGTTGATGACAGTCACTTTC
CACTTGATGAAGGTGTCATCGTGGAAGAAGACGGTGAGGCCGCCGAAGAAGCCAACCAGCAGGAAGGTAA
AGAGCTGCATCTTCTCGACCTTGCGATAGCGGATCCAGCTATAGGCGAGCTGCAGACCGGTGACCACTAC
CAGTGCACCAGTGGCCATGTAGATGTCGTAAAACTTGTAGACCGCGAAGAAAACGATAAGTGGAATAAAT
TCAATAAATTGCTTCATGTCTGGCGCCTAGTCCTCAAAGATGGCCTGCAAATTTCGCCCAGTCTACCTGC
ATGGCGGACAACGGCAAATGAATAAGACCGCTCATTTTTACGCCGGTTCCGCGCTGTCGCCAAACAGTTG
ACGTGCAATGGAGATCCCCAGTCGCTGCTGATCGGCCAGCAGGTCGCGGATATGGCGCTCTTCAATCAGG
GGATTGAGCAGCACGGCACGCAGCAGGGTAAGCGGCTGGGCACCGTAGCGCACCACGGCCCGCTGGGTGC
GGGAGACAAAGCTGTGCCCCTCGGCCCGCTGGGCTTTTTGCAGTGCCACGTTGAAGTCGTTGATCTGTGC
ATTGGCCGCTTCATCCAGCACCTTGCCCTGCAGGTGGGGTGGAATGCAGCGGTAGGAGAGCAGGTTCATT
ACCGGAGCAAACATCAGCTCGAAGGCCGGATTTCTGGTAATAAGCTCGGCCATCAATCTGGCCCGGTCGT
AGTTGGCTTCGATAAGCTCGGCATAGCCTTGCTGGCCGAACAGCTGGAAGGCGGCGTCCAGATAGAGGGC
GTTGGCCGGGCGGGTGCCCTCCAGCGTAAAGCGCCCCTGATCAAACGAGGTGGCTCGGATGGCGTAGGGG
GCCTCCCGCTTGACCGCCATCATCAGGTCCGGCTGGCGGCAGAGCAGCATGCCGGTACCGAGCGGCACCA
GCAGCTGTTTGTGGCCATCCAGAGTGACGGTATCGGCCCGCTCGATCCCGGCCAGCAGGCCTTGATAGCG
GGGAGAGAAGAGGGTCGGGCCACCCCAGGCGGCATCCACGTGGAAGTGGATCTGCTCGCGCTCGGCGATG
GCGGCCAGTTCCGGCAGCGGATCGATGGAGCCAAAGTCGGTGCTGCCCGCCACGCCGACCAGCGCCAGTA
CTTTCAATTTCTGCTCTTTGCACTGGATCAGCGCCTGCTCCAGCGCAGCCAGACTGAGCCGGTTCTGTTC
GTCAGTCTCAAGGCGCCAGACGCTGCGAGCGCCCAGTCCCAGCAGATCCATCCCCTTGTCGAAGGAGTAG
TGCATCAGGCGGGAGCCCAGAATGACGGCGCCCCGATAGCCCTGTTCATAGAGGGCGAATAGGTTGTCGC
CACAGGCGCGGTTGCGGGCCAACCACAGTGCCGTGACGTTGGCGATGGTGCCGCCGCTGGTCATGACGCC
GAGGGCTGCATCCTTGGCATGCATCTGCTCGCCGTAGAAGGCATCATCCCTGCCATAGATGAGGCGGTGC
AGCTTGGCCAGCACCTCCCGCTCGAGAAAGCTGAGCAGACGGGAGGACTCCATCTTCATCGGATTCTGGT
TCAGCATCACCAGCAGGCGCGACAGCTCGGCAGTATAGGCCGGCAGTGGTGCCGTCATGTGGCCCATGTA

GCGTGGCGAGGTGAGATGCGAGGCACCGGGCACCAGAGTAGCCAGCCGCGCCAGATACTCCTCGATAGGT
TGGCCGACTGCAGGCATGCGGGATTGATCGAGCTGGTGGGTGAACAGGTCGCGCTGATAGGGAGCCTGAC
TGTCGTCGGAGTGGTGGAAGCTCTCCAGCAAGGTGTTCATCGCCTCGCCAAAGGCGGGATCCGCTTGTGC
AAACAGCTCAGTCAACTTCATCAGGTTCTCCAGACCAGGTCACCGGGGTAGGCGTCTATGCCGCTTGTCT
CGGGCATCGGCGGCAGAGCATACACCAAAGGGGGGCGGGTGACAGCCACCCCATTTTTTAAGCGAACACT
TGTACTCCGAAATGGCGCTGGGTAGAGTGAGCCACAGTTATTGTGGAGTCGATGTTATGAGTGGTGTGAT
GGATTACTCCCCCCGGGAGGAGCTGGCAAACCGGCTGAGTCACGGTGCGGGATTGTTGCTGGGGATTGTC
GGGCTGGTGCTGTTGCTCATCAAGGGATGGGGGCAGGGGACGCAGGCGCTGGTGAGTTACAGCCTTTATG
GCGGCAGTCTGGTGCTGCTCTATCTGGCCTCGACCCTCTATCACAGCATGGGGTCAGGGCGGGCCAGACG
CTGGTGCAAGGTGTTTGATCACTGCGCCATCTATCTGCTGATTGCCGGCACCTATACCCCCTTCCTGCTG
GTGGCGCTCGATACGCCGCTGGCGAAGGGGTTGATGGTGGTGATCTGGGCCCTGGCGCTGGCTGGCGTAG
TGTTCAAGCTGGTGTTTATCAATCGCTTCAAGAAGTTGTCGCTCTTTACCTACCTGATGCTGGGCTGGCT
GTCGCTGGTGGTCATCTATCAGCTCTACATCCATCTGGCCGGTGAGGGGCTGCTGCTGCTCGGGCTTGGC
GGCCTTATCTACAGCCTCGGGGTCATCTTCTATGTGGCCAAGCAGATCCCCTATAACCATGCCATCTGGC
ATCTCTTTGTGCTGGGTGGCAGCGTTTGTCACTTTATGGCCATTTACGGCTACGTCGCCTAACGTCTTGC
AACTAGCCCTTAATTGGTATTTTTAGCGCGTAAAAGGATAAATTCGTGCGGTATCACACAGTTGGCATCA
GCGTTTGCTGACAAGCTAGGGCCAGTTCAATTCGCAAAGGATGCACGAAATGAGTGAAATCGCGCTTTCC
ATCAGCATGTTGTCGCTGGTGGCAGTGCTAGGACTCTGGCTGGGAAACTGGCGAGTCTATGGCGTTGGCT
TGGGAATAGGGGGGGTGCTGTTCGGCGGCATCACAGTAGGTCACTTTGCAGGCGCCTCCGATCTCGCGCT
GGATGCACAAACGCTTCACTTTATTCAAGAATTTGGCCTGATCCTGTTTGTCTATACCATCGGCATCCAG
GTCGGGCCGGGTTTCTTCTCGTCATTGCGCAGTTCGGGTCTCAAACTCAACGCCTTTGCCGCCTTGCTGG
TCATCCTTGGCTGCGTGGTGGCCGCTGGCTTGCATCAATTTTTCGATGTGCCGTTGCCGGTCATTCTGGG
GGTCTTCTCCGGTGCCGTCACCAATACGCCATCTCTCGGTGCGGGCCAGCAGATCCTGGCCGAGCTGGGC
GCCGCGCCCGGTTCTACCGGTTTGATGGGGATGGGCTATGCGGTTGCCTATCCGTTTGGTATCTGTGGCA
TTTTGCTCACCATGTGGTTGGTCAGGGTCTTCTTTCGCATCAAGATCGACGATGAAGCCGCCCAGTTCGA
GCTACAAACCGGTAAAACCAAGGAGAGCTTGCAGACCATCAACATCGCGGTGCGCAACCCCAATATGCAC
GGCCTGATGCTGAGCGAGATCCCGAGTCTCGACGAGTCTGATGTCATCTGTTCACGGCTCAAACGGGGCG
ACGAGCTGATGGTGCCACGGCCGGAATCCCGTATCGAACTGGGTGACTTGCTGCATCTGGTGGGTGAGCG
CCACGCGCTGCACAAGGTGTTGCTGGTGTTGGGGGAGGAGGTGGAGACCTCGCTCTCGACCCGCGGCACC
GATCTGCGCGTCGAGCGGGTAGTGGTGACCAACGAGCAAGTTCTTGGCAAGAAGATCCGCGACCTGGATA
TCAAACAGCGCTATGACGTGGTGATCTCCCGTCTCAACCGGGCTGGTATCGAGCTGGTGCCGACCAGCCA
GACCAGCCTGCAGTTTGGTGACATCCTCAACCTGGTTGGCCGTCTCGACGCCATCGAAGCTGTCACCAAT
GTGGTCGGCAACGTGCAGCAAAAGCTGCAACAGGTGCAGATGTTGCCGGTATTTATCGGGATTGGCCTTG
GCGTATTGCTCGGCTCCATCCCCTTCTATCTGCCCGGTTTCCCGGCCGCCCTCAAACTGGGTCTGGCGGG
GGGGCCGCTGGTGGTGGCGCTGATCCTGTCGCGGATTGGCAGCATCGGCAAGCTCTACTGGTTCATGCCC
CCCAGTGCCAACCTAGCGCTGCGGGAGATCGGGATCGTGCTGTTTCTGGCGGTGGTCGGCTTCAAGTCCG
GTGCCGGCTTTGTCGATACCCTGATCAACGGTGATGGCCCGGCCTGGATGCTCTACGGCATGGCGATCAC
CCTGATCCCGCTGCTGGTGGTCGGTGTGTTGGCGCGCCTCTACGGCAAGATGAACTACCTCACCCTATGC
GGTTTGCTGGCCGGCTCTATGACTGATCCTCCCGCGCTGGCCTTTGCCAACGCCATGCACCCGACCAGTG
GCGCCAGTGCGCTCTCATACGCCACTGTCTACCCGCTGGTGATGTTCCTGCGGATCATCTCGCCACAGCT
GCTGGCTATCATCCTCTGGGCGGGTGTATAGGGTTAATCTTGACGGGAGCATGCTTTGTCATGCTCTCGT
CATCATTCTGTAAATTATTGATAATTTTGGTTTTTACTATTATCACAACCTAGTCTTACACACTTCTGTC
TGGCTGAACAGTTCAGCCTCAAATTTCCCTATCCTTTCGAATTTTCGAGCTGGTTATTTTTGCCATGTGT
ATGCACAGTAAGATGTGGCTTGTTGCATTCAAATCAGGATTCAGGAGAGAACATGATTACCAATGCCGTT
ACCTTGGAACAGGGCGTAACCGTAACCCAACTTAAAGGACAAATTTATCTGGTTGCCGAAGATGGCAGCC
GGAAGCTGTTGGCTGAAGGTGATGTGCTGCCGAAAGGGGCGGTGATCATGTCGCCGGATGGCGCCAGTTT
CATGGGGGGCGGACAGTCGTTCACTGTGCAACCGGCCAACGAACAGAGTGAGCCTGCCGAAGAGGGGGAT
GCCCCCCAGTTGGCGCAAAACGGTGCTGCAGGTACTCCTGATGATATCAGTGCTCTGCAACAGGCCATTT
TGGGGGGCGCGGACCCGACCCAGGCCTTTGAAGCCTCTGCCGCAGGCGGTGCGCCTGCTGCCGGTGGTGG
CGGTATTGGTGGTGTCGCCGGTGCCAGTGGCAACGGCGGTTTTGTCACCATCGACCGTACCGGTTCCGAA
ACCATCGCTGAGGCCACCTTCGATACCACCTACAACGCCAATGGTGAGCCGCCGCTTGGTGCTGCTGGCG
AAGATGAGGTATTTGACCTTACACCCCCAACCATCTCCGTTGTTGCGCCAGATAACACCAATGACACCAC
TCCGACCCTGACCGGAACAACGGATGCTCCCGTTGGCAGCACCGTAACCTTGCTGGTCACCGATGCAAAC
GGTAATCAACAAACCCTGACCGCGATCGTCACACCCACTGGCACCTTCACCGTGGATGTGGTTACCCCCT
TGGCCGAAGGCTCTTATACCGTTACCGCCACAGTGACAGATCCTGCGGGTAATTCCGGAAGTGCGACTGA
TTCAGGGAGTGTGGATGTAACAGCTCCAGTGATCACCGTCGAAGCACCGGACAACAGCACCGATGACACA
CCAACTATCACAGGTACTACAGACGCCTCCCCAGGCAGCACAGTAACCATCTTCATCACCGACAGCAATG
GGGATAATCAAACCCTGACCACCATAGTTAATAACGATGGTTCTTACACGGTTGATGTCACCAATCCATT
ACCTGAAGGTGGTTATACTGCAGATGCCAGCGTCACAGATCCTGCCGGAAACATAGGTACCGACAGAGAC
AATGGTGATGTGGTTTACCCTGAAGTCAGCCTTAGCGCCAATCAAATTGATGTCAGCGAAGGAGGCATAG
CAAGCTTTAATGTAAGCTTGGATCAGATTGCGACAGAAGATGTTACCGTTAGTTTCAGCTATTCAGGCAC

AGCCGCTGATGGCAGTGACTTTGTCGGTGTGGTCAGTGTGGTTATCCCCGCCGGTCAAACTAGTGTGACC
TTCAATATAAGTACAATTGATGATGCTTTTTACGAAGGTTCCGAAGACTTCAATATCAGCATCAGTGGCG
TGAGTGGTGGCAATGCCACCATTGGCCCGAATAGCAGTGCCAATACCAATATTATTGACGACGGCACCGG
TCCGGGTCCGAACCCGGATGATGATCGCCCATCGGTCTCTATCACCAATGCAGGGACTCTCAACGAAGGC
AGCACTGCGCTGTTCACCGTGAACTTGTCTGCGGCGGCCGCCACAGCCACCACGGTCAAACTGACCCTCA
ATACCGGGGATACCGAAGCCGGTGACATCGGCACATTGGAATACTTTGATGGCACCAACTGGGTCGGTGT
CCCGGCCGATGGTATTGTCAGCCTCGCGGCGGGCTCGACCGCGTTGCAACTGCGTGTCCAGACGAATGAT
GACAGCGTCTATGAAGGGGCAGAAAGCTTCAGCGTCACCGTGACTGGGCAAACCGGCACCACAGGCACCG
CTACCGGCAGTGCCAGCATTGTCGACGATGGCACCGGTCCGGGTCCGAACCCGGATGATGATCGCCCAAC
GGTCTCTATCACCAATGCAGGGACTCTCAACGAAGGCAGCAGTGCGCTGTTCACCGTGAACTTGTCTGCG
GCGGCCGCCACAGCCACCACGGTCAAACTGACCCTCAATACCGGGGATACCGAAGCCGGTGACATCGGCA
CATTGGAATACTTTGATGGCACCAACTGGGTCGGTGTCCCGGCCGATGGTATTGTCAGCCTCGCGGCGGG
CTCGACCGCGTTGCAACTGCGTGTCCAGACGAATGATGACAGCGTCTATGAAGGGGCAGAAAGCTTCAGC
GTCACCGTGACTGGGCAAACCGGCACCACAGGCACCGCTACCGGCAGTGCCAGCATTGTCGACGATGGCA
CCGGTCCGGGTCCGAACCCGGATGATGATCGCCCAACGGTCTCTATCACCAATGCAGGGACTCTCAACGA
AGGCAGCAGTGCGCTGTTCACCGTGAACTTGTCTGCGGCGGCCGCCACAGCCACCACGGTCAAACTGACC
CTCAATACCGGGGATACCGAAGCCGGTGACATCGGCACATTGGAATACTTTGATGGCACCAACTGGGTCG
GTGTCCCGGCCGATGGTATTGTCAGCCTCGCGGCGGGCTCGACCGCGTTGCAACTGCGTGTCCAGACGAA
TGATGACAGCGTCTATGAAGGGGCAGAAAGCTTCAGCGTCACCGTGACTGGGCAAACCGGCACCACAGGC
ACCGCTACCGGCAGTGCCAGCATTGTCGACGATGGCACCGGTCCGGGTCCGAACCCGGATGATGATCGCC
CAACGGTCTCTATCACCAATGCAGGGACTCTCAACGAAGGCAGCAGTGCGCTGTTCACCGTGAACTTGTC
TGCGGCGGCCGCCACAGCCACCACGGTCAAACTGACCCTCAATACCGGGGATACCGAAGCCGGTGACATC
GGCACATTGGAATACTTTGATGGCACCAACTGGGTCGGTGTCCCGGCCGATGGTATTGTCAGCCTCGCGG
CGGGCTCGACCGCGTTGCAACTGCGTGTCCAGACGAATGATGACAGCGTCTATGAAGGGGCAGAAAGCTT
CAGCGTCACCGTGACTGGGCAAACCGGCACCACAGGCACCGCTACCGGCAGTGCCAGCATTGTCGACGAT
GGCACCGGTCCGGGTCCGAACCCGGATGATGATCGCCCAACGGTCTCTATCACCAATGCAGGGACTCTCA
ACGAAGGCAGCAGTGCGCTGTTCACCGTGAACTTGTCTGCGGCGGCCGCCACAGCCACCACGGTCAAACT
GACCCTCAATACCGGGGATACCGAAGCCGGTGACATCGGCACATTGGAATACTTTGATGGCACCAACTGG
GTCGGTGTCCCGGCCGATGGTATTGTCAGCCTCGCGGCGGGCTCGACCGCGTTGCAACTGCGTGTCCAGA
CGAATGATGACAGCGTCTATGAAGGGGCAGAAAGCTTCAGCGTCACCGTGACTGGGCAAACCGGCACCAC
AGGCACCGCTACCGGCAGTGCCAGCATTGTCGACGATGGCACCGGTCCGGGTCCGAACCCGGATGATGAT
CGCCCAACGGTCTCTATCACCAATGCAGGGACTCTCAACGAAGGCAGCAGTGCGCTGTTCACCGTGAACT
TGTCTGCGGCGGCCGCCACAGCCACCACGGTCAAACTGACCCTCAATACCGGGGATACCGAAGCCGGTGA
CATCGGCACATTGGAATACTTTGATGGCACCAACTGGGTCGGTGTCCCGGCCGATGGTATTGTCAGCCTC
GCGGCGGGCTCGACCGCGTTGCAACTGCGTGTCCAGACGAATGATGACAGCGTCTATGAAGGGGCAGAAA
GCTTCAGCGTCACCGTGACTGGGCAAACCGGCACCACAGGCACCGCTACCGGCAGTGCCAGCATTGTCGA
CGATGGCACCGGTCCGGGTCCGAACCCGGATGATGATCGCCCAACGGTCTCTATCACCAATGCAGGGACT
CTCAACGAAGGCAGCAGTGCGCTGTTCACCGTGAACTTGTCTGCGGCGGCCGCCACAGCCACCACGGTCA
AACTGACCCTCAATACCGGGGATACCGAAGCCGGTGACATCGGCACATTGGAATACTTTGATGGCACCAA
CTGGGTCGGTGTCCCGGCCGATGGTATTGTCAGCCTCGCGGCGGGCTCGACCGCGTTGCAACTGCGTGTC
CAGACGAATGATGACAGCGTCTATGAAGGGGCAGAAAGCTTCAGCGTCACCGTGACTGGGCAAACCGGCA
CCACAGGCACCGCTACCGGCAGTGCCAGCATTGTCGACGATGGCACCGGTCCGGGTCCGAACCCGGATGA
TGATCGCCCAACGGTCTCTATCACCAATGCAGGGACTCTCAACGAAGGCAGCAGTGCGCTGTTCACCGTG
AACTTGTCTGCGGCGGCCGCCACAGCCACCACGGTCAAACTGACCCTCAATACCGGGGATACCGAAGCCG
GTGACATCGGCACATTGGAATACTTTGATGGCACCAACTGGGTCGGTGTCCCGGCCGATGGTATTGTCAG
CCTCGCGGCGGGCTCGACCGCGTTGCAACTGCGTGTCCAGACGAATGATGACAGCGTCTATGAAGGGGCA
GAAAGCTTCAGCGTCACCGTGACTGGGCAAACCGGCACCACAGGCACCGCTACCGGCAGTGCCAGCATTG
TCGACGATGGCACCGGTCCGGGTCCGAACCCGGATGATGATCGCCCAACGGTCTCTATCACCAATGCAGG
GACTCTCAACGAAGGCAGCAGTGCGCTGTTCACCGTGAACTTGTCTGCGGCGGCCGCCACAGCCACCACG
GTCAAACTGACCCTCAATACCGGGGATACCGAAGCCGGTGACATCGGCACATTGGAATACTTTGATGGCA
CCAACTGGGTCGGTGTCCCGGCCGATGGTATTGTCAGCCTCGCGGCGGGCTCGACCGCGTTGCAACTGCG
TGTCCAGACGAATGATGACAGCGTCTATGAAGGGGCAGAAAGCTTCAGCGTCACCGTGACTGGGCAAACC
GGCACCACAGGCACCGCTACCGGCAGTGCCAGCATTGTCGACGATGGCACCGGTCCGGGTCCGAACCCGG
ATGATGATCGCCCAACGGTCTCTATCACCAATGCAGGGACTCTCAACGAAGGCAGCAGTGCGCTGTTCAC
CGTGAACTTGTCTGCGGCGGCCGCCACAGCCACCACGGTCAAACTGACCCTCAATACCGGGGATACCGAA
GCCGGTGACATCGGCACATTGGAATACTTTGATGGCACCAACTGGGTCGGTGTCCCGGCCGATGGTATTG
TCAGCCTCGCGGCGGGCTCGACCGCGTTGCAACTGCGTGTCCAGACGAATGATGACAGCGTCTATGAAGG
GGCAGAAAGCTTCAGCGTCACCGTGACTGGGCAAACCGGCACCACAGGCACCGCTACCGGCAATGCCACC
ATTGTCGACGACGGCACCGGTCCGGGTCCGAACCCGGATGATGATCGCCCATCGGTCTCTATCACCAATG
CGGGGGCTCTCAACGAAGGCAGCACTGCGCTGTTCACCGTGAACTTGTCTGCGGCGGCCGACACAGCCAC
CACGGTCAAACTGACCCTCAATGCCGGGGATACCGAAGCCGGTGACATCGGTACATTGGAATACTTTGAT

GGCACCAACTGGGTCGGCGTCCCTGCCGATGGTATTGTCAGCCTCGCGGCGGGCACGACCGCGTTGCAAC
TGCGTGTCCAGACCAATGATGACAGCGTCTATGAAGGGGCAGAAAGCTTCAGCGTCACCGTGACTGGTCA
AACCGGCACCACAGGCACCGCTACCGGCAATGCCACCATTGTCGACGACGGCACCGGTCCGGGTCCGAAC
CCGGATGATGATCGCCCATCGGTCTCTATCACCAATGCAGGGACTCTCAACGAAGGCAGCACTGCGCTGT
TCACCGTGAACTTGTCTGCGGCGGCCGACACAGCCACCACGGTCAAACTGACCCTCAATGCCGGGGATAC
CGAAGCCGGTGACATCGGTACATTGGAATACTTTGATGGCACCAACTGGGTCGGCGTCCCTGCCGATGGT
ATTGTCAGCCTCGCGGCGGGCACGACCGCGTTGCAACTGCGTGTCCAGACCAATGATGACAGCGTCTATG
AAGGGGCAGAAAGCTTCAGCGTCACCGTGACTGGTCAAACCGGCACCACAGGCACCGCTACCGGCAATGC
CACCATTGTCGACGACGGCACCGGTCCGGGTCCGAACCCGGATGATGATCGCCCATCGGTCTCTATCACC
AATGCAGGGACTCTCAACGAAGGCAGCACTGCGCTGTTCACCGTGAACTTGTCTGCGGCGGCCGACACAG
CCACCACGGTCAAACTGACCCTCAATGCCGGGGATACCGAAGCCGGTGACATCGGTACGCTGGAATACTT
TGATGGCACCAACTGGGTCGGCGTCCCTGCCGATGGTATTGTCAGCCTCGCGGCGGGCACGACCGCGTTG
CAACTGCGTGTCCAGACCAATGATGACAGCGTCTATGAAGGGGCAGAAAGCTTCAGCGTCACCGTGACTG
GTCAAACCGGCACCACAGGCACCGCTACCGGCAATGCCACCATTGTCGACGACGGCACCGGTCCGGGTCC
GAACCCGGATGATGATCGCCCATCGGTCTCTATCACCAATGCAGGGACTCTCAACGAAGGCAGCACTGCG
CTGTTCACCGTGAACTTGTCTGCGGCGGCCGACACAGCCACCACGGTCAAACTGACCCTCAATGCCGGGG
ATACCGAAGCCGGTGACATCGGTACGCTGGAATACTTTGATGGCACCAACTGGGTCGGCGTCCCTGCCGA
TGGTATTGTCAGCCTCGCGGCGGGCACGACCGCGTTGCAACTGCGTGTCCAGACCAATGATGACAGCGTC
TATGAAGGGGCAGAAAGCTTCAGCGTCACCGTGACTGGTCAAACCGGCACCACAGGCACCGCTACCGGCA
ATGCCACCATTGTCGACGACGGCACCGGTCCGGGTCCGAACCCGGATGATGATCGCCCATCGGTCTCTAT
CACCAATGCAGGGACTCTCAACGAAGGCAGCACTGCGCTGTTCACCGTGAACTTGTCTGCGGCGGCCGAC
ACAGCCACCACGGTCAAACTGACCCTCAATGCCGGGGATACCGAAGCCGGTGACATCGGTACGCTGGAAT
ACTTTGATGGCACCAACTGGGTCGGCGTCCCTGCCGATGGTATTGTCAGCCTCGCGGCGGGCACGACCGC
GTTGCAACTGCGTGTCCAGACCAATGATGACAGCGTCTATGAAGGGGCAGAAAGCTTCAGCGTCACCGTG
ACTGGTCAAACCGGCACCACAGGCACCGCTACCGGCAATGCCACCATTGTCGACGACGGCACCGGTCCGG
GTCCGAACCCGGATGATGATCGCCCATCGGTCTCTATCACCAATGCAGGGACTCTCAACGAAGGCAGCAC
TGCGCTGTTCACCGTGAACTTGTCTGCGGCGGCCGACACAGCCACCACGGTCAAACTGACCCTCAATGCC
GGGGATACCGAAGCCGGTGACATCGGTACGCTGGAATACTTTGATGGCACCAACTGGGTCGGCGTCCCTG
CCGATGGTATTGTCAGCCTCGCGGCGGGCACGACCGCGTTGCAACTGCGTGTCCAGACCAATGATGACAG
CGTCTATGAAGGGGCAGAAAGCTTCAGCGTCACCGTGACTGGTCAAACCGGCACCACAGGCACCGCTACC
GGCAATGCCACCATTGTCGATATTAATAATCTCCCGACTACTAATGATTTAGAGGTAATTGGTACTGAAG
ATGATGCAAGCATAAGTATCCCCCCCCTCTCGGGCTCAGATACCGACGGCACGTTAGCCAGCTTTGTAAT
TGATACTTTGCCACTTAATGGCACCTTGTACCTCAATGGCGTTGCCGTCACGGTCGGTCAGAGCATCTTG
GTTGCCGATGCTGCTAGCCTGACCTTTACGCCAAATGCCAACTGGAATGGTGACACCAGCTTTACCTACC
ATGCAGTAGATAACAATGATTTCGCTGACTCAACTCCAGCTACAGTCAACATCACAGTGACCCCATCTAA
TGACGGAGTTTCTTTAGGCGGACTCAATGCCCAAGGTGGTGAGTTAACTGTTCACGAGGCTAACCTTTCA
ACGGGCTCTGCCCCTAATGGCAATGCGCTGACTCAGACTGGTAACTTTAGCTTTACCTCTGTAGATGGTT
TATCTTCTGTCACAATTGCCGGACAAACCTTTAATTTGACAGCGTTACTTGCGCTAAGCACCAATATGGT
GAGCATTACAACCCCTTATGGTGTTTTGACCCTTACGGGGTTCACTGGTGATGCAAACGGTGGCTCTATT
AGCTATAGCTATAGGCTAAAGGACAATGTCGATAATGACTCTGCGGATGGGGCTACTAATACTAGCTATT
TTGATAGCTTTGCTGTAACCGTTGTGGATACGGATGGCAGCCAAGCCAATGGTAGCCTTGATGTGAAAAT
TGTTGATGATGTCCCTAATGCCAACAATGACAGTGGCAGTGTCGATGTGATTGTAGATAGCTTTATAGTC
TCTGGCGTGGAGGCAAATTGGACGTCATGGACAAATGGCACAAGCGTAACCAGATTCGATGGTAACAGTA
ATCCTAATGGTGGTGGCACTGACAATGATGATGGTTTAGATCAGATCAGATGGGGGAATCCAACGAATAC
CTATAAATCTGGTTATGGTTTTATCGATAACGACAGTGCATTAAATGGTCAGTTTGCCTTAAACCAAGAG
ATTATTTTGGGCACGTTTACCCACTATAACTTCCCAATATCTTCTGGTGGCGCTATCACTAAAGCAACTA
TGGATGTTACTTTCTCGGTGACTGATGCCTATGGGGTAGTTACACCAGTAACTCTCAACGTCAACTTTGA
CCATAATGAAACACCGAACGACAACAACGATCCTGAAGCATCAAAAGATATCATTAAAGTCGGCAATACC
AATGTAACCTTTGAACATCAGGGGCAAGTTTATACGCTGCAAGTAATAGGCTTTAGAGTTCCTGGTACTA
ACCAAGTTGTTACTGAAATTAGAACGGCTGAAAATGCGGCAAGCAGTTATGAACTAGTTGTACGTATAGT
TGCTGGTAATGGCTATGAGTTACCCTCTACTGAGGGTAACGTGTTAGACAATGACATCATCGGTGCTGAT
AACGATCTCACGGTAATTGGTGCTGCTGTGGGTAACCAAACAGGGTCCGGTACCTCTAGCAACGTAGGAT
CTATTATCAATGGCCAATACGGTACGTTAATAATATACGCTGGTGGTAGTTATACATACACAGTAACAGC
CAATGCCTCTGCGATTCCGGCCAATGCAACGGAAACCTTTACCTATACTATGCAAGATGCTGATGGTGAT
AAATCCAGCGCATTGCTCACCATTAATGTAAATGCCGTTAATGTTAATGCAAGTAAAGCGGTTCAAGATC
AAGGTATTGGTTCCGAAGATACTGCTGTAATTGGCAATGTGCTCAATAACGATGGCAATAAAAATACATC
AGTCACACATTTTACAGTAGCGAACGATACCACTCAGCATACTGCAGGCAGTAAAATAATATTGGCAGAG
GGTGAACTAACCCTCAATGCTGATGGTAGCTACACCTTTAAGCCCGCAGATGATTGGAATGGCCAGGTTC
CAGTCGTCACCTACACCACGAATACTGGGGCAACAAGCACACTAACCATAGTGGTAACGCCTGTTGATGA
TCCAACGGTGACTGAATCAGATAGCAACACCGTTGCCGAGGATACTCAAGCGGAAGGTAACGTACTGAAA
AATGATACGGATGCGGATAGCACATTATCCGTGACCAGCTTCCAAGTGAATAATGAGATCTATACCGCTG

GCAGTACTTGGTATCAATTGCCGGAAGGTAAGCTGCAATTAAAGGCCAATGGTGAATACAGCTTCGAGCC
AAAAGAGCATTGGAGTGGTTCATTACCTGAAATCACCTACACCACTGATACTGGGGCTACAAGTACCTTG
AATATCACTGTTCAAGCAGTAGCAGATGCCCCTAATCTGACAATCAATGGTTACACCTCCGTTGCGGCTA
TTAACTTTGAAGATGCCAAGATTACAGGTGATTGGAGTGGTGTTAAGGCAGATGACATTAAGGGCCTAAA
TACCATAGGTACTTGGCATACCAGTAATAGAGGCGGGAAAGTTGAAATTGGTTATGAAAGTGTCTACGTC
TCTGGTGGTTCCAATAGTAATAAAGTCATGGAAATTGAGTTTAACAATGGTGATAAAACCTTGTATACCG
ATATCCAAGCGGATGCTGGTCGATTTTACGAACTCGGATTTGATATTGCGGCGCGCTCAGGTAGCGTAAG
CACCTCTGGACTGACTATTAAACTGATCCCGCTAGATACTCAAGGCAACCCACTAAATGCTCAAGCTATT
ACGCTATATGACTTCGATCCCACTAATGCGAATTGGTTAAGAGATCAAAAGATTACCTTACCCATTGGCC
AATCAGGTAAATATCGTTTGCAGTTTGAAGGTGATAATGGGGATTCTTATGGGGCCTTGCTGGATAATTT
AGCATTTAAAGTGGTCGACAATATGGGGTATCGCGGTGAGTTTATTAAACTCAGCAATATATCGTCATCA
CTTAAAGATACTGATACATCAGAAACGTTGAACCTTAAATTGCAGGGCTTGCCAGAAGGCGCTGTGCTGA
AAGATGGTTTGGGCAACCTGGCGACGGTTGGCGCAGATGGCACTGTGGATATTACCAACTGGGATAAATC
CAGCCTGCAGATTAAAGTCGCCAACCATGGCAACTTCACCATCACAGTTGTAGCTACGGCGACAGAAGCC
AGTAATCAAGATACTGCGCAAAGCACTGCTGATTTCCAAGTTACCGTTCTGCATAAGAATAATGTTGTCG
GTGGTCAGAGTGTAGATTCATTCATGATGACCGATTGGAGTGGTGATACAGCCCAGTTTGCCGTTAATTT
AGGAGGATACGGCAGTGTCGCTTCATCATCCAAGACTCAATATTTTGCTAAAGACACCGAGCTACTAATT
AATGCAGGCGATAGTAATGATTACATCGACCTTGGTGTTTCAACTGCTAACAATATCGTGAACACTGGTA
GTTCGTTGCCCAATGTTAATAACCCTGTGGTTACTCAAGATGAGTTACTTAGCAGCCAATTTATGACTCA
GGATGTTATTACCAACACCGTAGGTACTCTTAAGGAAGATGCGCGGCAGCCACTGCAACCCAAAACAGAC
ACAGTCAATTTGGGGTCTGGCGACGACATAGTCAACGGTGGCGAAGGTAGCCAGGTCGTTCATGGCGGCA
GTGGCAATGACCAACTGATTGGTGGTGAAGGTATTGATGGTTTACGTGGTGGTTTGGGTGACGACATTCT
GATTGGTGGTCTTGGCAATGATGTCCTGCGTGGTGATGAAGGTGCAGATATCTTTGTCTGGAACAAAGGG
GATACGGTATCCGGCTCTCAGACCACGGATTACATCATGGACTTCAACAAGGGGTCAGACACCAACAACC
TTTCGGAAGGTGACAAGCTGGACTTGAGTGATTTGCTTGATTCTTACAATCAGAATGAGCTGACTTCTTT
ACTCTCTGTTAGTCAGGGCGACGATGGTGTGCATCTATTTATCAGAGAAGACAGTAGTTCTACCTCACCA
ACTCAAGAGATTGTGTTGATGAATCATACTTTCGATAGCTTGACTGGGGTTTCTGATACTACGACCACTC
AGGTTCTCGAGTACATGTTGAACAACATACTGGTGATCGACAAACCTCATGGCTAACACGAAAATACAGT
AGAAAAACAAAAGCGGCAGAGTAATCTGCCGCTTTTTTTATCTTTGCAGGGAAGAGAAATCAGTTGCCGA
GGGTGGCGAACAGGCTCTGCTCCAGGGTGCCGCGGGAGACACAGACCGGCTTGATGGACGGGGCCGGTAC
GCGCTCGCCTGAGGATTCAACGAAGAACAGCTCGGTGTTGCCCTTGACGTTGACGGTGGCCACGCTGCTG
TTGCCGCTCTGGGTGATGATCAAGTTGATCTGCAGGTTCGGGTTGCCCAGTGCAACTTTGGAACCCATGG
TGCCGCAGTCCAGCCACTCCAGTCCGTCGGCTGACTGATTGACGTTGGCGGTCATCAGGCCGGAGCCACG
CTGGCTGTTTTCGATGGTGAGGCCGTGATCGGTGAACCACTTCTCGGCACGAGCCCACACCAGATCCACC
GGGATCGCCATCTCCTGAGTGTTGTTGGTTGCCTTGGCAATTGGCGGGTTGTAGCGATACTCCTCACCGC
CGTTATTGTCGCCAGCACAACCAACCAGCAGCAGCGCCAATGCGCTCCCCATAATCTTTTTCATCTTGTT
TCTCTCGTCCATATCACCAGGCCATTGGCCAACCGGCGCCTATTATCGCCGGAGCGACAATCTCTGTCAG
CAACCATCCAGCGTTCGATGTTATGGGGAGGTGCTCGGGGTGCTTTACAAAATTGGTTTGCTATCATAGA
TTTGCTCGAAATTCCTCCATAAGCACCCCTGTGACCAACATGCTCACCCCGGATCTGCTGCAGCAGATCA
TCGACCAATTGCCAATGAGTATCTTCTGTCGAGATAGTCATGGGCGTTACCTGTTCGCCAATCGTGAGTT
CGCCCGTGAAGCCAAGCTGGAGTCGCCTGCCGAGGTAGTCGGCAAGACAGATGCAGATATGCCCTGGGGC
GATCTCTTTCGCAGTGAGGATGAGCAGCTGCTTGCTGGAGGCGTGCCGATGCTGTATCAGCAGCTCCATT
GCAACCGGGGCGGGAATACCTGCTGGATGGAGGTGCACAAGGTGCCTCTTTACACCCCGCAGGGAGAGGC
GTGTGCAGTGTTTGGCGTGGTTAACGAGATTGCCCAGCGCAAGAGCCTGGAACAGACGGTCAAAAAGCAG
CAGTTGTTGCAGCGTTTTCTGCTCGATGCCATTCCTGACCTGATCCGTTTCGATGATCACAACGGGGTGT
TGCTGGAGTGCAATCAGGCGTTTCTCGACTTTATCGGCCAAGGGGCGGATGAGGTGCTGGGTCAGCAGCT
ACCGTTCCGGCTGGCGGCGCCGCAGGGGATGGGCCACGGCGAGTGCTGTCTGCTGGATGCCAGCGGGCAG
GGGCGACACATGGAGATCACCCGGGTCGATGTCCCCGATGGTGACGGTCACTCCCTCGGGATCCTGACGC
TGGGGCGTGACATCACCGGCCTGCGCACCACTCAGGCGCAGTTGCAGCAGGAGCAGCACTACGACAGTCT
GACCGGACTGCTCAAACTCTCCCGGTTCTTGCAGACCAGCCGCCATCTTGGGGGACGCAGTGCCACATTG
GTGCTGGTCGATCTGCACCATTTTCGTGAAATCAACGATCGCTTCGGGATCCGGATTGCCGACCGCCTGC
TCAGCCAGGTGGCCAAGCGGTTGCAGCAGTTGGTACCGCCGCGTTCCCTGCTCTGTCGGGTCGCTGCCGA
TGACTTCAGCCTGCTGCTGCCAGATTTCAACGAGCCATTGCAGGGGTGGAGCCAGCATCTGCAGCAGCAG
TTGATGGAGCACTATCAGGTGGAAGATCACCGGATCCAGATCCCGGTGTTTCTGGGGCTGGCCCAGGGGC
GGGCAAGCGATGCCGAGCGGTTGCTCAGTCATGCCGAGGCTGCGCTGGCGCAGGGCAAACGGCAGCAGCA
GTACTGCACCCTGTTTGATCCCGAGCTGCATGCCCGGCTCAAGCGGCGTCAGCTGATCGCTGCCCAGTTG
CCGCTCGCTATCAAGTCCGCCCAGCTGGTTGCCGTCTATCAGCCCATTATCTGTACCCGCACTCACCGGC
TGCACGGCGCCGAGCTGCTCTGTCGCTGGCCCCATCCTGAGCTTGGGATGATCTCCCCCGACGAATTCAT
TCCGTTGGCGGAAGAGATGGGGATCATCGGCCAACTTGGCCAGCTGATGCTGGAGCAGGGGTGTGCCCAG
CTGGCGCAGTGGCAAACTGCGCAGCCGGATATGGTGCTCTCCATCAACCTCTCGCCGCTGCAGTTCCGTG
ATCCGGCGCTCTGTGAACGTGTCTCTGCCTGCATCGCTCGCCATGGTCTCAAGGCCAGTCAACTGGAGCT

GGAGATCACCGAAGGGGTGTTGATGGAGAACGCCGACGAGATTGAGACCAACCTCGACAACCTGATTGCC
GCCGGTTTTCAGCTGGCCATCGACGACTTTGGTACCGGTTACTGTTCACTGGCCTATCTGCCGCGTTTGC
AGGTGGCGACCCTCAAGCTGGATCGCAGCTTCACTCAGGGGCTGGCCAGCAATAGCGCGACCACCGCTAT
CGTGCGCTCGGTCATCGGGCTGGGTCATGAGCTGGGGATCCGGATCACCGCCGAAGGGGTGGAAACACCC
GAGCAGCAGTTGTGGCTGACTGAGGCGGGGTGTGATCGGTTACAGGGCTATCTGTTCAGTCGCCCCCTGC
AGCCGGCGGAGTTTGCTGCCAGCTATCTGGTGTGAGAGATCGTCAGAAACAACAAGGCCACCGCAAGGTG
GCCTTGTTGTTTTACGATGGAGGCAGGATCAATCCTGGAAGTAGGTGCCCCAGCCATCGTAATCCACCCC
ATATTTTTCCAGGATGGGCAGCATCTTGGCGATATCGGCGACGATCGCTTCGACATTCAGCTTGCACTCG
ATGGTGGCATCGAAGCAGAAGATGGTGGCGCCGTCATCCAGCTCCATCTCTTCGGCATCGGTCACTTCAA
AACCGGCCTTGAACAGGTCAACCGCTGCTTTTTCCAGACGGTCGAAATCCTGGCAGGCGAAGTGGTGCTC
GATGTCGTAATCCGCATCGGGATTGCTGCCATCGGCCAGCAGTTCGTTGACGATCTCGGTGGTCTCATCA
CGCCACTCTTGCATATTCAGACTCATGTTGCCTCCAGGCGGTATCATTGCGGCAAAGCATACTCGCAGAG
TGGATTCGGCTCAATGCGGCGGACCCACTGAACCTCGAATTTCCATCATATTGGCGCAGCCTGCAGGGTG
TTCAGAGGCCTGCAAGTACGGCAAACAGGACTGAAATGGATAATAAATGGCCGCTTCGTTCGGTTGTTGA
GCGAACGCGCTTTTTATCGTGCCATCATGGTTGACACTCTGCGCTGGAAAGGGTTTAATGCGCGCCGTTG
CCCAGATAGCTCAGTCGGTAGAGCAGGGGATTGAAAATCCCCATGACAGTTTCAATACCACAGCTCTGGA
CAGCACACCCTACAAGGCTTCGCTTTCAATGCGAGGCCTTTTTGTTATGCGTATCTGGTCAATCTCTGGC
AAGGTTTGCCACTATACCCTGATTGAAGATCTACATATTGCTGGTTTGACTTCACAAGCATCATTATCCA
CACCGCCCCCTATCGCGGCACTGACCCCATGGTTCAGTGCCTTCGTCTTTCCTTTCAGGTCTTTTATCAT
TTATTTGAGTCGATAGATAAGATAACATGAAGACATGTGGTTTCACCTGGAGCATATCATGTCATACAAT
GTGAGATTCTCATCTCTTGATGAATCAGAACTGAGTCAATTTATTACGCCGATATCATCAGATAACGCAT
CTTTTTTTGACAACATAGATAGTCGCCATGACAAATACCTTCCAGCAATTAATTACTTTGCTGACTTAGT
AAATCAATCAAGAACATCTGCAGAGCTCCTTCATCTAATAAGAAACCCTCAGCTTCCAGCCAATCTTAGG
ATGAGTCTACTAAAATTATTTAGAAGGCTCATATGTGAGCACTGCGACACAGAAGCTACAAAGAAAATTA
AAACTCCAACAGAATTTTTTGTCACAGCTTATGGAAATCATTTCCAACCTATCGAATACGTTAAGAGTAG
ATTAATTCCATTAACACCCAGCATGCAGTCAATATTATCTGTATTACTTGGTGAGTATGATAATAGAGGT
AAGTCAGGCTATGGACTGACAGAGCGCTTCTTTGATTGGTTCCAGTTAAAGTTTGGTCATGAATTTACAA
TCGATGGCCCTACTGGCGCAGGAAGAGATATACAACTAAATACTATCCTCCCTGATTTTCATGAAGAATA
TCCATGTGATTTTATAATCAGAAAAGGTCAAAGAATTTGCGCGGTTGGTTTTGCAAGGTATGACAGTACA
CGTGGGGGAGCGCAGTCTGACGATCGTACGGGTGGTAATAGTGATAAAGTTACCAAAGCTATCGAGTACA
GCGATAGAACTGGGAAAAAAATTCAAGCTGATATTCCTATCCGATGGACCAGGTCTTCTACATAATGATAC
CTTGTTTGAGTCAAGAAAATTGGATGTACTGTGGAATGACAATGTCAGAGTAACAACATTGAAGTTGTCT
GAATATATTATAACGAAAGATTGGTTACTTTCATAAAACCATGGCCATCGAATTCAATTATCATGAATTC
GATGGCCAAACGTTACAACTATAGTAGCTCTTTAATCAAACTAGGTGATATTTCATCATCCACAATCAAT
TCAATCATGTCACCACTATTTGTCATTTTTATATAGCTGTCATTTTTGTTTTCAGGGGATACATAATCAC
CCATCTTTTTCAATCCATGTTTAAATCGAGAGATGGTGGCCTTTACACCCTCCATGGACTGATCAATTCC
TATGAATTTCCTATCTAATTCACAAGCTGCCTGCATGGTAGTTCCAGAACCACAGAATGGATCTAAAACA
ATATCTCCCTTTTCACTGGATGCGCTCACTATGAGTTTCAACATGTCAATGTTTTTTTCTGTTGGATAAC
CGGTTATAAGCTTACTCTGATGATGTGCATCTCTGTACTCATCCCAATAATCTGTGACAGGAATTGTTTT
ATTTTCAGATAGATATACCTTCCGACGCGGGTTGCCATTTTTTGACCAATGGATATGACCCTTTCGATCC
AGTTCATCCAGTTTTGATGGCTCTAGCTGCCAGTGTTTTCCTGGTGGGGGAAGCATTCCACGCCATTCTT
TTCCAGTTTCACCATTCCTGATTCCAGGAGCATGAACAGGCACAAGCTTATACCGACCATCAGCATCAGA
CTTAGTATATTCTTTATTCACCCATTCCTCAGATGCTACTTTGCCTGGAGCACTGAAGTATGTGACTGAA
GTTTTTCCATAAAACAATATATAGTCGTTAAGGTTTTGATATTGTTTTCTTGTTGAGTTTTTTGAACTAC
ATTTTCTTCTTACAATTAAGTTTTTGAAATTTTCTTCTCCAAAAACCTCATCCATAATAATTTTCAAATG
AAAGAGTCTTTGATGGCCGATGTGGATATACACACTCCCGTCTTTAGATAACAACTCCCTCATCAAAATA
AGACGACGCCTCATCATTTCTATCCATGGGGCAAGGCCTAAATTGTCTTTATATGAATGCTGAAGACTTC
TAGAGTGAAAATCTAGTCCAGTATCATATGGTGGGTCTGTATATATGAGCTTAATTTTACCAGTGTAATT
CCGCAAAAGAAATTGCATGGCGTAATAGTTATCAGCCCAGAAAAGAGAGTTAGTTGGTGCCCCACAATCG
CTTAGGTCTTTTCTGCTATTTGCCAAAACAAAGCGGTGCATAGTACTTGCAATTATTTCTTCGGTAGATG
CCTTCCCTTCATATGAAAGCTCCACTTTTGAAGAGCGCTCAGGCAAAGGGCACTCATTCCTAACCACTGA
AACTTCCGATGCAAGAATAGCCTCATCAGACACCGTCTCTAACTTGTTAATACCAGTTGCCATGATTACC
CCTCAGATTATCTTTCCAATTATAAATCAAAACCAACTTTAATCACAACAGAAAATAAATTTAAGATGGA
AGATAAAGAGAATTCTCAGCTTTTTTAAATAAGAACGTGGAAAAAGCACTAGCAGCTTATGGCTACTAAG
CCACACATTACAAATCAAAATATCAAAGTATTACTGAAGAATATCCAACAAGCTTCAAGAAAGATGCAAT
AATAGAATACTTTAACATCAAATAAATGTGTGCTGTGGTAAAATGAGAGCCCTGCAAGAGGTAATATTAA
AAAATCACATACTCTCCTTAAGGGAGTAACTCAACTCCCACCAGGAGTGCCATAACGAAGTTTTTATAAA
TCCAAACTAAACTCCCTTTAACAAAAAACACTTTCAATACAACTCACATTTGGTATGTAAATTACAGTCC
TGTATAGACGATACATCTAATGGTTAAAAGTGTATTTAATCATAAATACATGCGCCGTGTTAGATATATT
AACATAGCAGACCAAGAGTAAAATTTTCACGGCCAACACTTCCTTGATTTAAAAATGCTAAAATATAACG
TAAGAAAGCTATTGCCGTAGTGAGTGGGATTATGGCCGTGCCGCAGCAACGGCGGAAAGCTAATAACACA

TTTCCATTTATCAATTATAGATGGTAATATACCATCTATTCTTGGGCATTTTATAGAGAGCTGATTATGT
ATGTTAAAAGGGTTCATTTTTTACTTCGATATAGCGACAAGCTTGCAAGTGGTTCCGACACTCTTGCAGA
GCACAGAGCGATAATAGAAAGTAATGGTTGCGTTTGGTTAGGGAAGTTTGGTATTGGAATGAGCTCTCGT
TTCGCTGAACTAGCGATTAATCAGATTAGAATGAAGCAGCCTTGTTTCTTGTATCTTATGCATGGTTCAA
AATTAACGGCCAAATCAAACATAATTGATATAGCGACAGCAGGCACAAATCGAGGGGAAATACCAAGTCG
AGAGCCCAACCTGACTCCATTTTATTATAGAAGTAGAAAGTGCTCAGTCTGGTTCAAACTTGATACGATG
GATGACATTAATAATGAAGAGATTAAGAAGTTATGGTTATACAACAATCCAGCATCACATCCTAGCCTTG
TTGGCATGAGAGGCTTAATTTATCTGACACCGGATAACAGCAAAACAGAATTAAATGTAAAGGGGGATCT
TAAGCCGAGTATTTATACAGAGGGTTTTTTTGATTGAAAAAGAAATAAAGTAAAGCAGAGCCTAAATCAC
CATTAAGTTATAGCAAATACATGCTCGTGATTAATCATGGCAGCTCTGCCTATTTAATAGCTGATATGGA
GTCGGCTGGACCTCTACTACCTCACAGTTACATTAAAACAACATACTCTTGAGACCAAAAACATTATTAA
ATCAGAGATGATAATCTACCATATTCTCCGATAGATACTTGACTCCAAGCGCATGGTCATTACACTGATG
TGAGTTCATTACACTTATATAAGTGTATCACACAGTTATTCTAACACATATAAATTAAATCACTAAAAAG
CCATGCTTATTAACTGCATGTCAAACACCGAATACTCTATCGATTTAGATATATATAAATCCTTTGATAT
CCAATAGCCATTTCGGTTATTTAAATTCAAAGGAGAACATCCATGTCTGAACTTTCATTATTCGATGCCA
TTGGCGAAGGCAAAAACGCGACTCAGGATTATCTGGATACCCTGTCGCGGGCACTGCTGCGCCATCATCA
ACTGGTCAAAGCCATCCGCATCCACGGTCGTTATGTACCGGAATCCTTGCCAATGCCCGGTCTGCCGGTA
TTGGTTGCCATCTTCAGTCACACCAATACCTACACCTTGCACCAACTGCCCTCTACTCAGATAGGGCAGT
TGGTGTTGTCAATTGCTCGCTTCTCCGGTCAGGCCACCCATGACCTGGACAACGGCATCTTTGAAGAGGT
GTGGGACATAGGTGACAGCAGCTTCACCACGGACAGCGCCGAGCAGGCTGTCTACTGGTTGCCGTTACCG
GAGCGCTGGATCGCTCTGGAGGGCACACAAGTGGCGCTGCACGATAAAGACCATCCGGTGTTCAAGTTGC
TCAACGTCACCGCCCGGAACGCGGGTCCGGTCCTGGATATCTGTGATGGCGTGGTCAGTTTGGCGCTAGA
GTCAGAGTGGGATGGGGAGTGGGCCGCATGACCGAGATCAACCGCTCCTCGCTACTCGCCAAGCTCAAAC
ACATCAAGGGCATGAAGCTGCTGGTTGAGCAGGCCGTCTTGCTCTACCTGTTGATGGCCGACGAGCAGAC
GCCTGTGTGGGTCAAGACCATCGTGGTCAGCGCACTCGCCTATTTGCTCTGTCCGGTCGATGCCGTACCG
GACTTTATCCCCGGCGTCGGCCTCACCGATGACCTGGGGGTCATCAGCGGTGCCATTGCCTCGTTAGGCA
GTCATATCCATCAAGGTCATCGCAGCAAAGCGGCTGACTTCTTCAAGTAATCCCTTCAGAAGGAATTTCA
TCATGGCTAAAGCCACACCACCGGCAGCCTCGGCTGCTCCGGTCTGGACTGTTATTAAATCTGCTAAGGC
CCCGAAGATCAGCGCACGGGCCAGTGGTTTGCTGCACTACGATGTGGGCAAGAACGATGAAGGTCGTTAC
GCCCTTCGCATTACCGCCAACGACACGGGCGGCCTGTTCAGCAAGCACTGGTTATCGCTCGATGACATCC
TGGCACTGCTGGACATCCTCAAGGATGCGCCATTCAAATCGGTGGCGCTCAGGGCGCTGTTTGTTCGAGG
CAGTGCCAATAACCACGGTTTCCTCGCCGCCATCCTGCGGGCTGAAAAGTTACTGGTCGCCGCCGAGCCC
AACTCACCGTTCCACCGGGTCGGTCTCTCTCTCAGCGACTGGTCAGCACAGTTGGATGAACCGGCCAATA
TGGATAAACAGGCTCGCAAGGGAAAAGCCAAGGGGCAGAAGGGCCAAGATGACACGACCGATGGGATAAC
AATCGAGGAAGGCAATGAGCCCCCGTTTCTTGAAACAGCCAACGACCAGGCCGGTTTGTCAGACAAGATG
GATGAGGCCGGGGCCGAAACGGGATCCGAGACCGGCGACGAGGTAGCCGGGCAGGAGACCCAGCCATGAG
CAATCGTGACATAGCACTGAACTGGCAGGGGCTCCCCCTGCCGGTTTCACCCCGGCTCGCCATATGCCTG
ACCGACCTGCTGAACAGGCACCATCCCACATGGTCACAAGCCCGAGCTGTCACGATGAACTTTCGTGATC
CGGGTTATGGCCCGGAAAGTGGTGGCTTTCATCCGGTGGAAATCCGTCTGCAACGCCGAGGAAATCTATG
GTCCTTGGTCTATATGACGGACTTTTCCTATGTCGGCATGGGTGACTATGCCGAATTGGCCAAGGAGGTG
GATTTTGACTTCTCAAGCCAGGAAGGGCTGGTTGCCTATGCGCATGTGATGCCGTTGTCTGAGCTGGGTG
AGTTCTATGAATTATGGGAGGCCAATTTCCTCACCTACCTGTCGCTGGGTGTCTACGCGATCAGGGTAGC
CACTGAATGATCCCTCCCTGTTAATGCGCAAATTTCACGGGTTCACGGATTGGCGTCGAATTACGCAGT
ATCTCCAAGGGAAAAACGCGCCCCGTTTCCCCCCGTTCGTGCGGGGCGGGCACGGGGCTCGCCGTGAATG
TGCGGGGCAACCCGACCTCTTCATTAACTCACCCATTATTTCCGCAAATTTCGCGGGGTTACGGGGTTGA
GATGGAAAAGCCCAATCACTCAAAGAGAAAAACCAGCCCCGTTTTCCCCCGTTCGTGCGGGGCGGACACG
GGGCACGGGGGGATTTTTACGGGATCGGACCGGGTCGCGACCGGGGCGGCCATTTGCTCACTCACCTTTC
CCACGTTGTCATCGTGCCGGGCCAGTCAGGCCAAGGCGCGATGAGCAACGACCATTTTAATCGCCAAGCC
AGAGACGGGGATTAGACCTCCTTTCCACTGTGAAGGGGCCTATGCCGTGTAGAACACAGCTCACATGTTG
AGGAGCGAACAATGAACGAAACACCCACCCGCTTTAAATTTACCAACCGGAGAATTGCCGAACTGCCCCC
TAACCCAGATGACGCCCGCAGTACCGAAGCGGAGTATTCCGATACCGAAGTGACCGGTCTGCGCTGTCTG
GTGGGCAAAGGGGAAGGCCGTCGCAAGTTCCTACTGCGTTATCAGATACACGGGCGCAAACGGGCCATTG
CCCTCGGGCACTATCCCGCGTTGGATGTCAGCGCGGCTCGAAAGCTGGCGCTCGACTACAAACGCAAGAT
CGCCGAAGGCGTTGATCCGCAACAGGCCAAGGAGGACAAGCGCAACCAGAAGACCCTGAGCAATTTGTTT
CAGGAGGACTATCTCCCCTATCTGCAATCGAGAAATCGCAGTTGGAAGACGGACGAACATCGCTTCCGTA
ATCACCTACGGCCAATATTTGGCAATCTGATACCGGAAGAGATCACCGTTACCCGCATCCAGAACATGCA
ACAGATGCTGCTCAAGCGCCTCACCCCGGCCACCTGCAATCGAGTCTTGGCATTGCTCAAGGCCATCATG
ACCTGGGCAGCCCGTGCCGGACTGATCAAAGAGAATCCCGCCGCCCTTGTGTCGTTACTGCGAGAGAACA
ATCGACGCGAACGCTACTTCACCCCCGATGAAATACGCCGCCTGTTCCGGGCTGCTGACCAGCATGAGAG
TTTCTATACCGGTCAGTACGTCAAGATGTTGCTGCTCACCGGCTTGCGCCGGGCCGAGCTGGCAAGGGCC
AAATGGCAGCACTACAACAAGGAGGACAAGACCCTATACATTCCCCACACCAAGAACGGCCATAGCCGTG

TGTTGCACCTGAACCATATGGCGGTGGAGCTGTTGAACTACTTACCGGTGACAGAGGGAAATCCCTATCT
GTTTCCAGGCAAGCGGCAAGGGCAGCCATTGCAGAATCCCACCAAGGGTTTTCAGCGGATGCTGGCGCGA
GCCGGTATAGATAAGGAGGGGGTTTGCTTGCACACCTGTCGTCATTCGGTCGCGGCGCTGATTGTCTCCA
ACGGCGGCACCCTGTTTGATGTGCAATCCCAGTTAGGGCACCGGAGCAGCCAGACATCGCTGCGTTATGC
TCACCTGCATAACACTCGGATGCAGCAGACCAGCCAGAAAATAGCCAACTGCATCCATGATGCCCTGACC
AGTTCTGTACCATCCAAGCCGCTGCCACTTCCCGAAGAGGTCGAGTGGGTTCAGGCCAGCTAGGAGCATC
GGTATCTCACGGCAAAGTGAGATACCGATCCTTGCAATTAACCCGCACTTTGAGTGCTTTTGCCGCTATC
CCTGATAAACGGGATCAACCGGCTTGTCACCTCATATTATCAGTCTGAGTGACTCCTTATCACTCACATG
ATTCATACAGGTATAATCTGGCAGTTAGCGACTGTACCAAATATTAGGCTCTTGGCCCTGCAATTTCCCA
TAGCAAATCATTCAAATATTCTGAGTCCGGCTTTGTTGCAGGATCGAATGAACTTGTTCGTTGCAAAAAA
TATTCTATCTTTTCTTTAATAAAATCGTTTATCACGGGAACTCTTGGCTCAAGACCTAATTCCTGCGATG
CCTTTTTCCTAGAAAGCAACGTTAGAACCTCTTCTTTAAACTCAGCAGACACAGTGGCCGCATTGAGAAG
CTCCTCAAATTTCATGGGAGGTGGGCTATTTTTAGCCTCGACCCACATACATGCAAGTAATGGCCGCAAG
ACATAAAAATACTTCTTGATTTTCACCTGCTCACTCTGCAAATACTCACGGTTATTACCTGTCGCCATGT
GAAGATAATGATAAATGGATGAGACAGGGGAAAAATACTTGTCTGCTGCTTGGCGCAACAAGTTAGTTGC
ATTTGTTTCTTCCCTGTAAACAATTGGTGAGCGTAGCCATTCGAAAAACACGGGATTGGACTTATTTAGC
AAAAACAACGCCTTTCTAATATCCCAGCCTGAATAATCATCTACTCCAACAATCGGTAGTTCAATGACAT
CGCGCTTTGGCAAAATATTGAAATACCAGCTTGGTTTATGTCGATACACGAATCGAACATCGTAATCACT
ATCTATAGACTCAAAACCCCAAGCCCTAGAACCAGATTCAATAGCGAATAATATTTCTACATCATATTCA
CTTTCAATGTGAGACAGTTTTTAATAATATCCTTTCTGATTTCCGGAACAATAGCGCTCATCTACAAAC
CTCGATTCTTTTAATTCAATCATCTTAGATAAATTTGTTATTAAACTACATTTTTGTGAGTTTGACTG
TGAATAGTCACCAAAAGCCAAGTTCACTGACATACTTAGATCCCCGATACTGATATCACGGTTTAGCCTC
CCAAGGAGCAGTTTAGCCCGTCCTCCTCAAAAACAGCCACAGCCTGCCCTGACCGGCCTTCCTGGCCTG
CTTAACCGCTTCTCGCCGGTTGATAACAAAACCCCTCACCTGATGACAAAAGCCCATCGTCGATGATGGG
TTTTGCGAAAAACTGCCTGCCAGTCAATTCGTCACGCCGTGTCGCGGCCTGTTTGGATAATCCGCGACAC
GGTTATTCCCCTTTTGTGATGGTATGCACGAACACGACAAGATTGCTCAGCGCCTCGGTATGCTCCTCAC
CCGCCTGTACAGCGGTGAGAAGCTGCACCTGAGTGATCTGGCTGCCGAATACAACGTCACCGTGCGCACC
CTGCAACGGGATTTCAATCAACGCCTCAGTTATTTACCGATAGAACACCGCAGCCAGCAATACTGGCTGG
ATCCTGAGTTTCTCAATCACAAGAACGACAGAAAACTGCGAAAGCTGGCCGAACAGCTTGGCATCACCGA
CCTGTTCCCCCACGCCAGTGAACATCGTCTGGGTCAACTACTGGACAGAGGCAACCCCAGTATCTTCGCC
ATCAAGGGGTTCGATGGTCAATCAGCCGACTTACTAATCCACCAGATACCGGTATTAGAGCAGGCACTGA
CCCAGCATCTGCAATGCTGGCTCATCACTAAAGACCAGCGCAAGCAGCTGTTTAGCCCCTATCAGCTCGT
CAACTACAAGGGCACCTGGCACTTGGTCGGTGTCAGCGAGGGGAGGTTGCAAGCCTATCCGCTGAGCTCC
ATCGCGCTGGTTGAGCTATCGCAACAGACCTTCACCCGTGACGCGTACATTCAACGACAGATCGATACCG
ACCCGACGCTCTGGCGCTCCGGCATGATCGAGGTGCTCCTCAAGGCCAGTGCCGCCATTGCCGTCAACTT
TCAGCATCGCCCGTTGCTGCCCGAGCAAGAGATTGTCAAAACCCTGGATGACGGTGCGCTGTTATTGAGC
AGTCGCGTACTCGATGCCAATCAGATTCTGCCCGTCGTCAAATCTTGGATGCCGGAACTCACCGTGATCT
CGCCCGGCTTTATCCATGAGCAGATCGTCCGTGATTTACGCCATTTATTGACCCTGATGAGTCAGGCTTC
ACCCTGATTCATTTGTGACAAAGATCCCAGTTTATACATCTGGCGACAGCGCCTGACATGCCACGACACC
ACCTGTCGCCGCTGTTCTGCATTGTCGTCAACCATCAAGCAGTAGCAATCCAAGGAGTGATGCATGAGTA
AAATGATCCAGATGAAAGAGTCCACCGCAAAGCGCGAGGTGAAGCAGGAATACGGCCTGACCGAAACGGA
TGGCCAGGCTTTCTGGGATCGGGTGAACCGCGTGATCGGTGAATCCCGAGAGGAAGTCGATAATGAAGAC
CGCGTTCCTGCCGGATATGAAGTATATCGATAACGTCAACGCGTACATGTTAGTTGGTCATTAGTTGACT
CCTGTGAGCGATATCTGCCACGAAACGCTCGTAACGACAAAGCGGCCAGTTCTACTGGCCTCTTTTTTTT
GGACCGTTCGACAGCACCGGCTGAGATCACACCAGTGAGCAATACCGGGGTAATGCCCGCATGGCGAGGC
ATCGGTATCGCGGTTTAGCGACCCAATGAGCCGTTTTGGCTGCGCCGCCCTCCAAACCTCCGCAACAAGA
TCATCCTGGCCTTCTCGGCCTGCTAAAACGTTTCTCCCCTTGCTGCCTGCTTTTGCCACCGGATCCCATA
AAAGCAAAGGGGCCAGTTCGCACTGGCCCCCCTTTCATTTTTTAGAATTTTTCGACTTCCACCGATATCT
CTGACATGTCTGGTGAGGATTCAACCATCACGCACCCCGTCTCGCTCGTCGCGCGGCGGGTGAAACTCGG
AGCGGGGGAGCCAGGGGCCGTTGATGAACCCGTACTGCAAGGTTGGACGAGGCTCTCAGTCCGTTCATCA
AATAGCGGGCTTCTAAATTGAAATACCTTGTTCACCGAGAATACGTAATAATTACCAGGAAGGCTGTCTA
TTACGATCTCTTTGACATATCGTCCGTCAGCATAGTGCACTATGTACCCGTTTTCACGAAGCCACTTCAT
GACCAGCTTCGGCTTGCTTGTCAACAGTTTCTGAAGCTGGGTCATTTCGACATAAAGCTTTCCGTCTTCC
GTGAATCCTAATAGCTCCCGCACTTCCATGGATGGCTTGCCTTTGTGGATCTTTCTTCGTTGCTCGGAGA
GCGTATCCAATAACCGATAAGCCTCTCTGGTGACATCATCCATGATGCCTTCCATCGACATGAGCCACCG
TCGGAAGCATACCAAAACTGCATTATCTGCATACGCCACATCAAACGGCAGAATACCTAGCTCAACGGCC
AATCGCCCCGCTGCCATGACGTTTGCGAAGCAACCAGCACCACGAGCAGCAAGAGGCGGAGGGTTTTCTA
ATCCCGAGCGACTCATCAGCCACTCTTGGCACTCTTCACGCTTAGCAGCAAAGATAACCGCTAAATCAGC
AGGGGTATCGATGTCTGGCATATTCAACAGCGCCTGAATATATGCAGGCATCGCCGTACCGTAGTACTGA
GAGGTTGCAGCTTTAAGGCCATTCACCCGGCAACGAGTCAGCTCAAGGCTCTCACCCGCCAGCGCGACAT
CCTCCGCCATCATCTCAATATCCAGCAGTCTGACGAATATCCCCCCTTTAGTGCCCGCCTTGCTCCGCTC

TAGCAGTTCCTTGAAGGACTCCTCCCCCGAAGAGGGAGCACAAACATTCGATGCCAATGGGCTCCTATTA
CCCGTGCCCTTCAAATTGCCAAGCACTCTCGACTGTCCACCCGTCACCGAATAGGCAAACTGAAGCGGAT
CGTTACTAGACAATGAGCCCAGTTCATCCAAACAAATGCCAATGCCAGTTCGTTGTTGAAACATGCCTTT
GAACCCAGCAGCTGTTCCGTCCCACTTCACAATCGCCGTTGCGGGGCCCGCACTTGGGTCAGATGCCACG
CCTGACACACTCTGCCCCACTTGTAAAAGCGTCGTTTTGCCACAAGAGCTTGCACCATTGAAGTGAAAAA
TCATGGTCCCTGTATTTGATAACTTCATGAATACATTGGATAGATTTGCCATGATTGCAACGAGCATGGT
TGGTGACTCTTTGACAGACAACGCAATGTTCTTTTGCCAATCCTGCAGCGTTCCTTTCGAGTGGATAGCG
TCATGCTCACCAGGCGCGGACAGCAGCTCGTGCTGAGGACCACTATAGCCCGTTTTGTAAATTGACCGAC
CTACCCAGACATAGCAGGGATGTTCGCTAACAAACCCCGCTTGCAGATGCTGATGGACCACGCTGATATC
TTGGATACTGATGCTGGCGGACATGTATGACAGCAGCCCCTCCTCTCGTGCACCTGGGCATTCAATCCCC
GCACGGTATAGCGCAGCCCTCACTGTTTTGTAGTCCTGAATCACCCTATCGAATAATAGGTCGATGCGCT
GGCATTGCTTCCGCTCATCCAGATACATGAAACGAACACCACTGATCTCTGTACTGTCAGCTTGGAGTAT
CGATACCGGCAGCAATGGCTTACCGAGTATCGTTTTTGGCTTGCCATGATCGCTAGCTAATTTCCCCCAC
GGGGACATCGCAAACCCACCAGGAATAAGCCTCGAATAAACCGGTTTTTTCATCTCCGGCTCAGATCTGG
AAAGCACCAAGCCATCTGGGTCACACACAGTCCCATCAGAGGCAATCGAGCAACGCCCCTCGTTGTACAA
CCTGTATTCTGCTAAATATCGATCCAGCATAAGAAGTTCATGACGAACGAAAAGCTCATATTGAGTCTCA
TTGTCGGGGTGTGGCTGACAACATTCGTGCTGTTCGGACAATGGGCTCACAGCATCGGGGACAATATCCG
CTCGATGAGCATCACCATGGTGTGCATCAAGGTTCGTTTCCTCCACCAGTAACGGCAGCGAGTCAGGAGC
GACTGCAGGGTGCGATTCTGGCGCTGATGCCTCCTGATCCGAGTCCGGTACCGAGTCTGTCGGCGGTACA
CCCTGACTTGCCATCCTATCAAGAGATGCTTGAGTCCCTGGACGACGGGCAAAAAGCCTCGGGGGGTTAT
CACTACTAGATCTGGGTCTACGCATAATTCTTTATGGGGGAGTCTCCTCCCCCATTCTCCAAAAATTGTT
AACAGGTGATGCGTGGGAGGTCCCACAAGGTGTGTAATTCGTTGACGGCCTTGACCAGCGTGGTGTCGTG
GAGGCGTGCGTATCGCTCAGTCATTTTCGGCGAGCTATGGCCTAGGACTTTTTGCAGTACATCCAGCGAG
CAACCTTGGTTTAACAGCGATGTAGCGACGGTATGGCGGGCGTCATGCATGCGCACTCCTGATAGTCCGC
AATCATTGCAGACCCGGTACCAATACCGCCGAGGTTCGGAAATCTGAGAGACACTATCCTGGCCTCTAAA
GAGCAGACCGGTTGTGCCATGGCGCTGGAATTGGAGTTGAATCAACTCCAGCGCGAAATGATTAAGTGGT
ATGAGCCTTTCTCGACCAGACTTACTGGTGGGCAAATACGCAATCTTTCGATTGAGATTACAATCTTGAA
GCTGTAGCGCTCGTGCCTCACCCAGCCTGCACCCTGTTGCAAACAGGAACAGCAGAAGCCTAGCTTTTTC
ATCGTCGATGTCTTTCATCAAACGATGGACCACCTTATTCACCACCTCATCAGGCAACCCCTTGGGTGGC
AAAACAGCTTCATGCTTTGCCGGAATACCTGCTGCTGGCGAGGACTTGAGAATGCCACGTTCTACAGCGA
AGGACATGATGGCCTTTACCTGTGCCAAGTAGCGGTTCCGCGTGGCGACAGAGCACGTTTTATTTGATTC
TGTAAGAAAATCCTCGATGTCGAAACGGGTTACTCTGTTTAATGGCTTAAGCCCTAGAGCTGCCACGATC
GGCTTTACCCGAGACTCGATGGTTTTATATGATCTATGAGTTGCTCGTACTGAGGGCAGGTATTTATCAT
GTATAAACTGTTCTACTGACAAGCTCGAAAGGTAGGCTTGATTGAATCCCGCTGGATCTAGTACAAACTC
CGCTTTTAGCTTTCTTGCTTGTTCACGTATCTCTTTAACGCTGACTTCGTCCGTGCTCGCAATTTTTAAC
CGATAACGGACTTTATTTTTGGTGAAACTAAGACAACCAACCTTGCGACCAGTGGCATAAGTAATCCACG
TAAGGGAACGCTCGTATTTATCTTTGCTTTCACGCTGCTTTGAACTGCACTCCTCCAACTTGGTACTATC
TACAGTTGGAGCTACAGCCTGTTCGGGCATCGCACCTCCTATCATCTAGAGCCCCCACCGTGGTGACTCA
TGACAAGAATGTTGGGCGAAGAAAAACCGAAATGGAAGACCAGCTCCAGAGCGTAACCATCCGATAATGC
CTTATAAAAACAGACTACCCTGCACATGGTATGTTTTATGCGTGGTGTTTTTCTCTTGTAGCAGTTGAAT
CGAAATCAGTTTTTGCCCCCCCTCTAAAGAACCAGAAAAAAGAAACTCAATAGAGTCTGATTTTGTGGCAG
AAAAATATTTTTATTTTTTTTGTTCCCCCCGCCGCACTGTACGATTTTCGAGCTAAAACACTGCATTGAG
CACAAGTATTTACTTCCCCCTACAGCTCTTGCTGTAGGGGGTTATCACTCTTCTTCAAACGCAGATACGG
AAGTTGAGCGACCGGAGGTCCTTCGGTATCGACGCACCAAACCCTGTCTGGTCAATTTCTGGTAACTTTG
CAGCGCACAAAAAAACATGCAGGTTAGTTCATCCGTTTTTTGACAAAAAAATGGGCTCACTTGTTGGAGGCT
CGGGATGGGTGAGAGAGCGAAAAGGTAATTCTATAAAGGGAAAAAAACCTCGATAGCAGAACCTGCCGACA
ACCGGATCCTGGTGTGGAGTCAGGGGCTCCTTGCACACTATCTCGCCACTTAGTTCACCCTATGCAAAAA
CATGCCAGAAAGTGGGGCTACAAGGTTGACACCCCAGTCGAGAAAGGGTTTAATGCGCGCCGTTGCCCAG
ATAGCTCAGTCGGTAGAGCAGGGGATTGAAAATCCCCGTGTCGGCGGTTCGATTCCGTCTCTGGGCACCA
CAATTCCTCCTTAGTTCAGTCGGTAGAACGGCGGACTGTTAATCCGTATGTCACTGGTTCAAGTCCAGTA
GGAGGAGCCAAATTCGAAAAGCCCGCTCAATCGAGCGGGCTTTTTCATTTTTAGTTGCTGTTGCATGCTG
CCAAAAAGAGCGTTGCGCCACAGGTGGCACTGAACAGCAGCATTCCGGTCTGCAGCATGCTGCCAGGGCA
TGGCTTCTTCTCTCTTTTTCTGGCCCTGATCCACTTCGGTTTCATCACATTCCCTTATCTATTTTGTCGG
CCTGAATGGCTGCACTCCTGTGCAGGCGAGCCATCTTTGTTGAATGGCGCCATGGCGTCAATCTCCCCCG
CAAAACAATTAATTCAGCACCGTTGAAGAGCGGCTTGTCTGGTGATGTTTCGACCTTTAAGCCATTGTTA
ACATTTTGGAAAACCGGAGTGACCCTGTGGCTGGGCGAGGATATGATGGGGTCAATAATGTTCCCATCCT
GACGGAGAGATCCCAGATGAACAGCCCCTTGAGCTATGTACTGGACGGCATTCATTGCGAGCCGCACTTT
TTTACCGTTCCCCTCGATCATGATGAGCCGGATGGTGACAGCATTACCCTGTTTGCCCGCACCCTCTGTC
GTAAAGACAAGCTCGATGAGAAGTTGCCCTGGCTGCTGTTTCTGCAAGGTGGGCCGGGATTCGGGGCGCC
GCGCCCGACGGCTAACAGTGGTTGGCTCAAGCGGGCGTTGCAGGAGTTCAGAGTGCTGCTGCTGGATCAG
CGTGGCACCGGGATGTCGAGCCCCATCCACGGGCATAGTCTGGCCAAAATGACCCCGGCTGAGCAGGCGG

AGTATCTCGGCCATTTTCGCGCCGACAGCATAGTGCAAGATGCCGAGTTTATCCGGCTGGAGCTCAGTCC
CAAGCATCGCTGGAGCCTGCTGGGGCAAAGCTTTGGTGGTTTTTGCTGCTTGACCTATCTCTCGCTCTTC
CCCGATAGCCTGCGGGAGGTTTATATCACCGGCGGCATCGCGCCGATTGGCCGCAGCGCCGAGGAGGTTT
ACCGCGCCACCTATCAGCGGGTGGCTGACAAGAACCGAGCCTTCTTCGCCCGCTTCCTCCACGCTCAGGG
GATTGCCAACCGGTTGGCCAACCATCTGCACCAGCACGATGTGCGGCTGCCCAACGGCCAGCGCCTGACG
GTGGAGCAGTTCCAGCAGCAAGGGCTGGATCTCGGCGCTACGGGTGCTTTCGAGGAGCTTTATTACCTGC
TGGAAGAGGCCTTTATCGGCGACAAGCTGAGCCCGGCCTTTCTCTATCAGGTGCAGAGCATGCAGCCATT
CAATACCAACCCGCTGTTTGCCATTCTGCACGAGGCTATCTACGCCGAAGGGGAGGCGACTCGCTGGGCG
GCAGATCGGATCAGAGGGGAGTTTCCTGCGGTGAACTGGGCGCCCGGCAAGGATTTCGCCTTCACCGGCG
AGATGGTTTACCCCTGGTTGTTCGAGCAGTTCCGCGAGTTGCTGCCCCTGAAAGAGGCGGCTCATCTGCT
GGCCAACAAGGCGGACTGGGGGGCGCTCTATGACCCGGCCCAGCTGGCCCGCAACCAGGTGCCGGTAGCC
TGCGCCGTCTATGCCGAGGATATGTATGTGGAGTTTGACTACAGTCGCGAAACTCTGCGTGGCCTTGGCA
ACAGTCGCGCCTGGATCACCAATGAATATGAGCACAACGGTTTGCGTGCCGATGGTGAAGCGATTCTTGA
TCGTCTGATCGCGTTGAATCGCGACCGTTAATGGCCGTTATCGGCAACCATCGCGGGAGCCCGGACTCCC
GCGATGGTAGTGATGGATGGTTGTTATGAAAGCGTGACTCCCTGAACCTTGACCCGTATTCGCCAGACGA
TGAGTGGATAGCCGGCTGATTAATTTCCCCCTCTTTCACTGTTTCTGTCGATCCCTCACCATATACTTGC
CAAACGACTCTGTATTGAGCCGCGGTCAAATCGATTGACCAACCCGTGCTCGTTACCATTAGAAAGTCGC
TCTGATGTTCTGTATCCCGCGTATCAGATATGCCATCTCTGCATGCCCCGCTATCCGGGGGAGGGGATGG
TCGTGCTTTTCAATCAAGATAAGGATGTATGATGAGAAAGTCTCTGTTGGCCTGCTCCCTGCTGCTGTGC
CCAGCCGTGTTTGCTGCCACCCCCCATTGGGAATATAGCGGCGAGGCGGGGCCCGCCCACTGGGCCAAAC
TGACCCCGGAGTTTGGTCAGTGTGCGGGCAGCAATCAGTCGCCGGTTGACCTGAACGGGCTGGTTGATGC
CAAGCTGGCCCCGCTGGCACTCCACTATCAGGCGGGTGGCAACACAGTGGTCAACAACGGTCACACCGTG
CAGGTCGGTTATGCCCCGGGCAGCACCTTGCAACTCGATGGCACCACGTTTGAACTCAAGCAGTTTCACT
TCCACGCCCCGAGCGAAAACCTCATCGAGGGTAAATCCTATCCGCTGGAAGGGCATCTGGTACACGTCAG
TGGCAAGGGAGAGATCGCGGTAGTGGCGGTGATGTTCGAGGCGGGCAAGGCGAATTCGGCACTGGCGGCA
GCCTGGAGCGCGTTACCTGCCAAGGTGGGCGAGATCCAGGCCCTCAAGACGCCGCTCGTTGCGGAGCAAC
TGCTGCCCGAGAACCGTGACTACTATCGCTTTAGCGGTTCCCTTACTACGCCTCCCTGTTCGGAAGGAGT
GCGCTGGCTGGTGATGAAGCAGCCGGTTGAGGTCAGTCAGGCCCAGATCGACGCCTTCAAGGCGGTGATG
CACCACCCCAACAACCGGCCGGTGCAGCCCCTCAATGGGCGCGTGGTACTGCAATAACACAGCGTTGCAA
TAGCGCTGCAGATAGCAAAAACAACAAGAGGGCCGCGAGGCCCTCTTTTACATAGCGAGGTATCGGCGAG
CGATCAGAGGCTGGCGGCGATGCGGGCGCCGAGGGCCGCGTTGTTCAGTACCAGCTGGATGTTGGCGGCG
AGCGAGTTGCCGCCAGTCAGCTCGCAGACGCGGGCCAGCAGGAAGGGGGTTGACGCTTTGCCCTTGATCC
CCTGCTCGTCCGCTTCATGGAGCGCCCGGGCAATAGCTGCGTCGATATCGGCTTTCGGCATGGCGTGTGC
GGTCGGGATGGGATTGGCCACCACGGCGCCGCCCGCCAGTCCCAGCTCCCACTTGAGGCGCAGCGCCTCG
GCGATGGCTGCCGGACTCTCCAGTCGATAATCAACCTTGAAGCCGCTCTCGCGGGTGTAGAAGGCGGGCA
ACTCTTCGGTCTGATAGCCGATCACCGGCACCCCTTGTGTCTCCAGATACTCCAGTGTCAGCCCGATATC
GAGGATCGATTTGGCGCCGGCACAGACTACTGCTACTGGCGTTTGTGCCAGCTCCTGCAGATCGGCCGAG
ATATCGAAGGTCTCCTGCGCGCCGCGATGTACCCCGCCGATACCACCGGTGGCAAAGACCCGGATCCCCG
CCATGGCGGCGATGATCATGGTGGAGGCGACCGTGGTGGCCCCCATCCCCTTGCGCGCCAGCACGAACGG
AATGTCGCGGCGGCTGCACTTGGTCACGTCGTGGCCAGCCCGGCCCAGCGCTTCCAGCGCACCGGCATCG
AGACCCACTTTGAGGCGGCCGTCGAGGATGGCGATGGTCGCCGGAATGGCGCCGTTATCGCGCACTACCT
GCTCCACCTGACGGGCCGTCTCCACGTTCTGCGGATAGGGCATGCCATGGGAGATGATGGTGGATTCGAG
GGCAACGACCGGTTTGCCAGCAGCCAGCGCGGCAGCCACTTCCGGTTGGATATCGAGGTAAGCGTTCAGC
ATGAGTACTCCTCCAGTATGCGGTCAACGGCGGTCTTGCTCAGACCGGAAAAAACGGTATCGGAACAATT
GACGGTGAGTGCGGCGCAGCCCAAGGCAAAGCGGGTGGTTTGGGCAATGTCGAGCTGTTGCAGCCAGGCG
TGGGCGAGGCCAGCCATAAAGGCATCCCCGCCGCCGGTGACGTTGAGTACCGGACTGCCCAGCACCGGCA
GGTGGCCCTGCTGCTCGCCATCGCTGTAGAAGATCCCCTGTTCACCCAGACTGAGGAACAGTCGCTTGAC
CCCGGCGCGGTGGAACCAGGCGGCCACCATGGGCCAGGTTTCGGGCCCGGCGATGGTCATGCCGCACAGC
TGCTCGGCTTCGCTGCGATTGGGTTTGAGGGTGTGGATCTTGCCAAGCCAGGGGCGGATCTTCTCCACCT
TGGCCACCGAAACGGTATCGACAAAGATGGCGTGCTCACCTTGCCGCTCGAACAGCCAGTCGAGGGTATG
GATGGCCAGATTGGTATCGAGCACCCAGAGCCGGGCACCGCCGAGAAAGCCTGCGAGGGGGGGCAGGCGG
GCAGGGGTGAGCTGCTCTATGATGGCCATGTCATTGATGGCGCAGCTCATTTCGCCGCTGCCATCATGCA
GGCTGAGATAGCAGGAGGTGGTGTGACCATCGAGCACCAGAGTCTGGCGCACATCGACGCCGGCCCGCTG
GCTCACTTCCAATAGATGGTGACCATACTGGTCGTTACCGACAGCAGTCAGCAGTCGGGTATCGCTGCCG
AGCCGCGCCAGATTCTCGGCGATATTGCGGCCAACACCACCGGCCGAGGTGCGCACCTTGCCCGGGTTGG
AGTCGCCGAGCAGCAGGTTGTCGTGGCTGGTGCCGCAAATATCCATATTGGCACCGCCGATCACCACCAC
GTAGGGACCCTCTCGCAGTACATAACCCCTGCCTTGCACATACCCCTGCCGGATCAGGCTTGAGATGTGG
CTGGCCAGCGCAGAGCGGCTGATGCCGAGGCGATCGGCCAGCTCCTGCTGGGCAATCATGGGATCCTGAC
GCAGCAGGGCGAGGATCTCTTGTTCACGCTCAGTCATAGACAAACATCCATTTACAAACGTTTGTCTATT
ATTGCTGGGAAACGTTTGCTGGCTATCAGCTTGTACAAGATTTGTCGGCTTGCGCACAAAAAATCGACAT
TCAAAGCTGGGTTAGCAAGCGGTTTAGCCGGGGAGGGGACTTTTAACGGGTCGGTGGCCCTGCAACGGCT

TATAATGGCCGCCGCAAGATGAGGAGAGATGATGGAAAAGAGTGCATTCTGCTGCGTGGGGCTGGTCAAT
CCCAAGTCTCCGGAAAATGTCGGCTCAGTGATGCGGGCTGCGGGTTGTTACGGGGTGGATGAGGTCTACT
ACACCGGCAACCGCTTCGAGTTGGCGCGCCGCTTTGCCACCGATACCAAGCAGATGGTGGAGAAGATCCC
GCTGCTCGGGGTGGATGATCTGATGGCGTTTGTCCCGCAGGGATGCGTGCCCGTGGTGGTCGATCTGATC
GATGGCGCTACGCCGCTCCCCGACTATGTCCATCCGGAACGCGCTTTTTATATCTTCGGGCCGGAGGATG
GCACGCTCGATCCCGCCCTCTATGGCGCGGTGAAAGATGTGGTTTATGTGCCGACCCGGGGTTGCATGAA
TCTGGCCGCCTCGGTCAATGTAATCCTCTATGACCGCTTGGCGAAGGGATTCCGGCAACCCTGAGTTGTG
GCGTGGCTACGGTGACTCAAACCACAATGGAAAGGTGTTGCTCATGGACAGGTTGCTGGCGGTGGCCGCT
GTTGACGGGGCTGCAGCATCCTTCTCTTTCCCTATGGAGGGATCAGTTTTCGCCGACCTTGACCGCCTGC
TCCTCGAGAAAGTCGCGGTAGTAGTCATAGAGGTGATAACGGCGATATATCTCGGCCAGCGTGCCGCGAC
GGATCAGATTCACCATCTCCTGATCCCACAGCTGCAGTAGCCGGTCGCCCTGCTCGGTATCGGCAAAAAT
GGGGTAAGTCGGTATCCAGCGTAAAATGGTGCGGTACATATTGGCCGTGGTCTCATCGGCGGAATGGAGC
ACGCCAGCGGTCAGGTAATAGCGATAGCGCCCCTTGCCAATCAGCTGCAGGGCCATCTCATGACTGTCGA
TCTCGTGCCACTGCATGGTGATCGGAAGATATTTATCCAGCACCCATCCTCGCCGCCAGAGTACGGTCTC
TCCCTCCAGGCTGCGCTCTCCCCGCCATTGTGGCTGCCACTGCTTGAGCGTGAAGACGGTCACATCATCG
GCGGAGGCTGGCTGACGGGGCTGTTTGACCCCCTTGATCTCGTCCAGATAGACACTCATCGCCATATCCC
CCTCTTGCCTGGCTACCATGGCGACGGCGCGTTTGTAGGGCACGATATGGGGCACGATCTGGTAGCCGTG
GGGTTGGTAGATGGTTCGCACCAGGTCGAGATAGATACCGTGGCCATCCGGTTGGCAGAAACCGGGCCAG
CCATCGCAATAGACGTGAATATCGGCTGTGCGAGCGTTGGCGATCGCACACAGAGAGCAAAGCAGCAAAC
AACCGATGCGCAGCATGGTCAGCATTATCCTCGCAGGGGTGAGGAGAGCACGTATCACCTGATTTATACC
ATGCATTGATCGCGGCGGGCGGAGAAAACATCTCGCATCTCCATTTTGCGACAGGGTCGAGGGTTATTTG
CGGTCGGATCGTTATGATCGAGTAAAGATTCAATAGCACCCCAATCAGTAGAGGAGACTGCATTATGCCA
TCGATCAAGACTCTGCTCTGTCCAGTCGATTTTTCCCAAATGTCTCGCGCGGTACTCGACTATGCCGTTT
TCATGGCACAGAGCCATCAGGCCCAGCTCAAGCTGATCCACGTGGTTGATCAACTGCACGGTTTTGACAG
TTACAAGCTCCTTCATATGACGGCCATCGAGATCACTCACGAGATGGAGCGTCAGGCCAGAACCCAGCTC
AAAGAGCTGGTTGCCACCCTGCCGATCCCGGCCACCTTTGATATTCGCTTTGGCCGCGCCGCCGATGAAA
TTGTCATTCAGGCCAAGGAGGATGAGGTTGAGCTGATCGTGATGGGCAGCCACGGTCGCTCCGGTATCAG
CCATCTGCTGGTAGGCAGTGTCGCCGAGTCTGTGGTGCGTCATGCACCCTGTCCGGTACTGGTGGTGCGC
CAATAACGCGCCACGTTTAGAGTGAAAAGCGTAACAAACAAGGCCAGCGATCGCTGGCCTTGCGTTTTCT
GGTGTTGTGCCCGCTAGCCGTGAGCGTAGATGACACTCCCTCCCTTGATGGTATCTATGATGGCCCTGGC
CTGCTCTTTGGGCAGGGCGGGGCAACCCCAGCTGCGGCCCAGCTTGTCATATTTGCGCAGATGGGTCGGG
CTGGCATAGGCGGCGCCATGTACTACGATGGCCCGCTTGCGGGCATTGCTGTTCTTGCCGGGGCTGAGCC
CGTCCAGCCGCAGGGAGTAGCCATGTTTGCCCAGATAGGTTTCTGCGGTGCGATAGACGCCAAGAGAACT
TTGGCGGGAGTTGAGCTGGTTGGAAAAGTGTTTTGCGGCCAGTTCACCTGAGTTGCGGCCATGGCTGACC
CAGGTGTGATAAAGCAGCTTGTGGCGCTTCATATCGATGACAAACAGTCTGCGCTGGGTGGATGGCTTGC
TGTAATCGATGATGGTGAGAATGGGTTTGCGTTTGTGGCGCACAGTTTGATAACTGTTGAGTGCCTGACG
AAAGGTCTGTTTATCCAGCTTGCCGGCCAGACCCAGTTGACGATAGAGGCTCTGATCCCAGCTGGCATCT
GCCGTGCTGCTCCAGATGAGCGATATCAACAGCAGTGACAACAACAGCCACTCGCGTTGCCAGTCTATGT
TCCCGCGCGCTTGTCTCATCCGTGAATATCTCCTTGTCACTCCCTGTCAACGACGCGGATATTGCCATCA
ATCCGCTCGATAGTTATATGCAAGGTGCTGAAAAGGCGCAATAAAAAAACCGGCCGCAGAGCGGCCGGAA
GTAATTCAAACAGTAGACATTGGTATCACAGGGTATAACTGACTTCCAATTACTTAGAAGTTGTACTGCA
GGGCAACCGCGACTACGTCATCGGTGTTCTTGCCCAGAGAGTTGGTGGACTTGCCATCTACGGAGTCCAG
CAGGTTCAGCTTGTAATCAACGTAGGCGTTGAAGTTCTCGGTGAAGTTGTACCAGGCACCGATAGATACG
TACTCTGTGTAGGTATCATCGATACCGCGAGCGGAGTCTTTCACGTCAGTGCGCAGGTAGGCCAGAGAAG
GCTTGAACTTGCCGATGGTGTACTGGGCAACGGCTTCGTAGCCATCCATCTTCTCGGCGTAGCCGTTGTT
GACGAACAGGTGGTCTTCACCGTGGGAGTAGGTTGCAGCCAGGTAGAGGTCGCCAGTTGCATATTTGGCG
CCGAAGCCCATCAGCTTAGCATCCTTGTCGCCACCGAAGGCGGCGTAGCGAGCTTGCTCGTCAGTCTTGC
CAGCCTGGTTATAGGCAGCACCCAGGCTCAGGCCCATATCGAAATCGTAGTTGGCAGATACTGCGAAGCC
TTCAGCAGAACCTTTGCTATTCCAGCTTTCGCCGTTGTAGGTGTTGCCGTTGAAAGATTCAGTGATTGCA
CTGCGACCGGCGTTTTGCGGATCGTTGTTACCGGTGAACTGGGCACCCAGAGTCAGACCCTGATAGGTGT
AGATGTACTGGGCCAGACCGTTGGTACGGCCGGTACCGAATACTTCAGTATCTTTGCCCAGGCCGTCACC
GCCCCACTCAGGCAGGATGTCGGTATAGTTGTTGACGGCCTTGGTGAACAGACCATCCTGACGACCATAG
CTGAAGGCGCCGAAACGGTCGTGCTTGATGCCGGCATAGGCATAACGCAGCTCGTCGTTGTCAGAACCGG
AAGTCGGCAGGTTGTACTCGGCAAAGCCGAACGCCTTCATCTCGGGAGCAATCTTGGTCTCGCCAGCAAC
GTGCAGACGGAAGTAGCTCTGGTCACCTTCGGAACCGTTGTCGTCAGTACCGTAGTAAGCACCTTGTGCA
CGGCCGCCGATTTCCAGCTTGGTATCGTTCTGGTTGTAAACGGTAGCGGCATGGGCGCCGCTCGCCAGCA
GCAGGCTGGAGATGGCAATAGTCAGTGCAGACTTCTTCATATGCGTTATTCCTCTGATGTAAACACTTCC
GAACTTGTTGTCGGTTCTGTGAAATCGGAAATGGTCCGATAGCCACACGGTAGGCGTCCCAACCTTGACT
GGGCCTGAATCCATCGTTGTTTAATTGAGGTGACGAGGTCGTTATAAAGACTGGGTGTGACGGTTTTTGA
CAAGATAAAAAGAGATTAAATTCAATGAATTATTTTCTAGTGGCTTTTTTGACATCAAGGCTGTTCTGAG
CAACAAGGTAGATGGCTCAAGTTGTGAGCTATATCACACAGGGGAGAATGGGGCGGGAAAAACGGGGGGG

GGGGAGCCTTGCAAGGCTCCCCCCAGTGATCAGAAGGGCTTTTGCCACAGGGTCAGCAGCAGATCCTGTT
CCAGTTCATTCGGACCCTGTTGAGCGAAAGCGTGACGCTGCTCACCGGTTGCCCGCCAGCCAAGACGGCT
GGTCTCCAGAATAAAGCTGCGCATGGCCTGATCCATGGTGAGGCTGAACTGGCAGCGCTGTTGAGCCATC
GGATTGAGGCCGCCGAGGGTTGGCCAGCTGAGAGGGTGTTCCATGCTCACCGGATTGAGGCTGACCCGCA
ATTGCCACTGATGACGGGGACCCGGCTGCAGATAGAGCAGATAGCCACCGGGGGCCAGCCAGCTGACCAG
CTGTTCAGTCTTGACCTTGAGATCGTTGACCAGCAGCACCTGAGCAGAGCCCGGTTGCAACGGCGCGCGA
GCGGGATCGGCAATGACAAACTGGCCGTCCGGCTGCGCTTTTTTGGCGGCAAAGATGGTGTTCTTGGCGA
TATCAACCCCAGCAAACTGCCAGCCGGGCAGCCGCTCTGCCAGCGCGCGGCAGTAGTAGCCTTCACCGCA
CCCAAGATGGATCAGCTCTTGTGGGGCAAGGGGAGCGAGCAGGCCGGCCATGGCCTCAACCAGCGGCAGT
TGGTGGCCAGCTTCCAGGAAATGGTGCTTGGCGCGCATCAGGGCGCGGCTGTCGCTGTCTTTGGGGTTTT
TCTTGCCGGGGATGAGATCCAGATAACCTTCCGGTGCCGGATCGAAGTGATGCTTGTTGTCGCAATAGCG
CCCCTGGGAGGCTTCATGAAGATGGAGCGGACTGCGGCAGCGGGGACATTGAAGGTGCATAGTAACTCTC
TGGGCGAATGCCGGAACAGTGAAGGGCGTTCGGGATTATAGGGGAGAGGATCAAAAAAGGCTGCCGTAGC
AGCCTTTTTTATCCCAAGGCCTCAGTCAGGCCTGCGGACGGGTCATCAGCGAGTGCCAAATACCACAATG
GTCTTGCCGTGAGCAGAGATGAGCTCCTGCTCTTCCAGCATCTTCAGGATGCGGCCCACGGTTTCACGAG
AGCAGCCGACGATCTGGCCGATCTCCTGACGGGTGATCTTGATCTGCATGCCATCCGGGTGGGTCATGGC
ATCCGGCTGTTTGGCCAGGTTGAGCAGGGTCTGGGCGATCCGGCCGGTCACATCCAGGAAGGCGAGGTTA
CCCACTTTCTGGCTGGTGTGTTGCAGGCGCTTGGCCATCTGGCCAGAGAGACGCATCAGAATGTCCGGGT
TGACCTGGATCAGCTGGCGGAACTTCTTGTAGGAGATCTCTGCCACTTCGCAAGAGCCTTTCGCGCGAAC
CCAGGCGGAGCGCTCGGGGTTCTCGTTCTCCTCGAACATCCCCATCTCGCCCAGGAAATCGCCCTGGTTC
AGATAGGAAAGGATCATCTCTTTGCCCTCATCGTCCTTGATCAAGACGGCCACAGTGCCCTTGACGATGT
AGTAGAGGGTCTCCGCTTTTTCACCCGCATGGATCAGGGTGCTCTTGGCGGGATACTTATGAATGTGGCA
GTGCGACAAGAACCATTCCAGGGTGGGATCGCTTTGCGGTTTGCCAATGACCATGAGAATTCCTCTGTAG
TGTCTTTTTATGGACTTGTGCGTCCGGGCAAGCTGCCAATCTTGCCATTCCTTGTATTGGTCGCAGTAGG
ATAAGAGCCTTTGCCTGTGCTGACAAGGCTTTGCGGGCTTTTGACAGAGCCCTTTGAGTAATGCCTGCTG
CGCCGCTTCGGCCATTCTAGGGAAGATAGAGGCCGGAGATGTTGATTTTGATCTATTTCCATCCCAACTC
CCCCTGTTCGGCAGAGATAAGCGACTGGGTTATGCTAGGGATCCATCATCATGATTAACAGGAGAGAGTG
CGAGATGAAAGCCAAGGTCAGCTGGGTGGAAGGGATGAAGTTTGTGGGTGAGAGTGAATCCGGTCACAAG
GTGATCCTGGATGGTGCCAACCCCGGTGAGGGGGCCAGCCCGATGGAGATGATCCTGCTGGCGGTCGGTG
GCTGCAGCTCCATTGATGTGGTCTCCATCCTCGAAAAAGCGCGCCAGTCGGTGACCGCATGCCATGTCGA
TGTCAGTGGCGAACGGGTCGATAGCGTGCCCCGGGTTTTCGAGAAGATTAATCTGCACTTCGTGGTGACG
GGCAAGGGATTGGCAGAGAAACAGGTGGCCAGAGCGGTCGATCTGTCGATGGAAAAATACTGTTCTGTTT
CCCTGATGTTGGAAAAAGCGGTACAAATTACCCATAGTTATCAAATTGTTGAAGCTTGATAACGATAGTT
TCGTTGGTACCCTGGCATAAGGCAGTTACGTAAGGCCACTGCACAAGGCCATGACATAAAGCCATTACAT
AAGGAAAGTGCAATGAAAAAATTGATCGCTCTGCTTCCCCTTTTGGGCCTGGCGGCGTGCAGCAGCCCGC
AACCTACCCAGTATCAATACAGCCAATACCAGTGTGGCGAAGAGATGCTGGCCGTTACCTATGATGCTCA
GGCCGACATGGTGCAGTTCCCCTATGCCGGGGTTTATCACAAGCTGGCGCGTGTGGAGTCAGAGTCTGGC
GCCAAATATTCTGACGGCCTGACCACCTTCTGGAATCAGGAGAAACAAGCTCTTCTCACCCAGAAAGGGG
TCACACTGCTGACCTGTACGCTTAAATAAGTGACCACAGATAGCTGAAGGTGTGCAGCTTTGTGCCCGGG
TCTGGCCTAGGCCGGATTGGGTCAATAGGTTGAGACCACAAAAAACAGCTTGCCGGGAAGCAAGCCGTTT
TTCTATCCATCCGTTATCACTAAACAAGCGCTAGTTGGTGGGGACGCGATTGAGCAGCTCGGCCGCGAGC
TGCTGGCTGAGTGCCTGACGCTGGCTGTCTGCAATGCTCACATCGCTGGTCAGCTGGATGATGCTGCTCC
AGACCGCTTTCTGGGTTTTGCGATCGAGCAGCACGATGGCCAGCGACCCTTTGCGCTGGTTGGCCGAGGC
ATATACCCCGGGTGTCATGCCAAGGCGCGCGAAAATCTCGCCGTCACTCATATCCTTGCCGCCCGCGACG
CCGATGGCGACCCACAGATCCGCCTCGTCGAGCGGTGCGCTTTGCCATCCCTTGCCATTGAGCTCCCGGC
TTACCGCCTGCTGCATTGGCTCAAGCCATCCTTCCGCCAACTCACCGGCAAATTGATACTGCTCGGCCAG
GGCGTAGCGCGGGGTTGCACCATAGGTCCAGTTGCCCATCGGGCGAACCAGCATGGTGCCGGACTCCAGC
GTGGGGGCCGCCGGCCCGCTGCTGCAACCGCCAAGCAGGGCGAGCAGTGCCACAGAGAGATAAGCAAGCC
GCATCGTGAACTCCTTTGAATCAGGTTATTTTGCCGGTTTCGATCAATTGCTGGATGAGCGGGCGCAGGA
TCAGCTCCATCGCGAAGCCCATCTTGCCACCCGGCACCACTATGGTGTTCATCCGCGACATGAATGAGCC
ATCGATCATCGACAGCAGGTAGGGGAAATCCACCTGCTTGATACCGCGAAACCGGATCACCAGCATACTC
TCGTCCAGACTCGGGATCACCTTGGCGCTGAAGGGGTTGGAGGTATCCACCGTCGGTACTCGCTGGAAGT
TGATATGGGTGCGCGAGAACTGCGGGGTGATGAAGTTGATGTAGTCATCCATGGAGCGCACGATGGAGTC
GGTCACCGCCTCGCGGGAATGCCCTCGCTCCGAGGTATCGCGGATCAGCTTCTGGATCCACTCCAGGTTG
ACGATGGGCACTACGCCTATCAGAAAATCCACGTGGCGCGCCACATTGTGATCCTCGGTGACCACACCGC
CGTGCAGCCCTTCATAAAACAGCAGATCGGTATTGCTGGGCAGATGCTGCCAGGGGGTGAAGGTACCGGG
CATCTGGTTGAAGGGCACTGCCTCGTCAAAGCTGTGCAGGTAGCGGCGATACTGGCCATTACCATCCTGC
CCATAGCCCTGAAAAAAGCTCTCCAGCAGCCCGAAGTCATTGGCCTCAGGGCCAAAATAGCTGATATGAC
GCCCCTGTTCGCGCGCCTTGCGGATCGCTACATCCATTTCCGGTCGGGTGTAGCGGTGAAAACTGTCACC
TTCCACCACCGCCGCGGTATATCCCAGTTGATGGAACATGGAGCGAAAGGCGTTGGTGGAGGTGGAGGTG
CCGGCACCGGACGAACCGGTAACAGCAATAATGGGGTGCTTGGCCGACATGGGATTCTCTCTTCCTTGGG

AATTCCGTTATAAGGTCCCGATTGCGGTTGGGTCAAGACTGCTCGATGAAGCGGTCTTTCATCACGATAT
TCACCGATTCATGCAGCTCGCTATAGACGATGACAGCTTTGCCTTGCTGTAATTGTTCTCTCACTGCATC
GACTTTGACGCCAAGCGGTACATCCTGCTCTCCATATTCTGTTCCTTCCTGCAACACAAAGTGTTCGATC
AGGTTGGTCAGGGTGTCGCCGTCCAGTTCTTGCCAGGGAATGATCATTTAAAGGTCTCACAGCAAAAGAG
GTGATCGTCAGGCTATTGTTGTGTTGAATCCTGCGAATGCAATCACTTTTTACCTTTTCACCGGAGTCTG
AGAGATGTTGTCTGAAGCGCAGCGCAGCGCGCCGTTAACCCTGACTGAGCATCTGGACAGCGCACGTCGA
GAGGCGATTGCCCGAGGCATGGTGTGGTTCTGGTTGTTGAACTGGCTGGTGGTGTTGTTTCTTTCGATAC
GCTATGAGGTTTACAGCCAGGTCGGGCTCGCCAGTGATGACAGCTTGCCCTGGCGCTTTCAGAGTGAGCT
GGCGCTGGTCATGGTGGTGCTATTGCTGCTACCCGTTGTCAGCCTGTTTTGTGACCATCTGCAGGATCGC
CTCTGGTTACGGATGGCTGCACTGGTGACGGTGCTATGGGGCGGACTCTGGACGCTGCATATCTATACGA
TTGGTCAGGTGGAGCTGCGGGGAGGGGTCAGCTATGCCAACGGGATCATGCGGCTCAACCTGCTGGCTGC
GCTCATTGCGTTTTATCCCGATCGCAAGGTTTTTATGGCTATCTCTTGGGCCCGATGACGGTGGGTGTG
GGGTTGGAGCTCTTTTTACGCGTCGATTTTCCCCAGATCTACCTGCTTGGCGTGTTGTTTGGCGTGATTG
CGCTGGAGGTCGGCCGCCGCATGCTGCACCGCTGGTTTGAGTTGGCGATCATGCGGGAGCATGAAAATCT
GACGCTGCTGCGCAAACTTGATCAGCTCGCCAAGCAAGATCCTCTGACCGGTGTCGCCAACCGGCGGCAT
TTCAATATCGAGCTGGATCGCGCGTTAGCCCATAGCCAGGAGAGCGGCGCTCCGCTATCGATCATCCTGA
TCGATGTGGACTATTTCAAGCGCTTCAACGACCACTATGGCCATCAGGTGGGGGATATGTGTCTCAAGGA
GGTGGCACAAGCGCTCAACCATGCGGTACGCACGCCATCTGATCTGGTTGCCCGCTATGGCGGGGAAGAG
TTTGTGCTGCTGCTGCCCAATACCGACCGTCAGGCGGCGGCCAGCGTGGCGCAGCGGCTGCAAGATGGCT
TGGCCAGCCTGCAGCTGGAGCATCTGGCGTCCGATGTTGCCCCTTGGGTGACGGTGAGTCAGGGGATTGC
CAGCAATGTGTCCGGCGAAGGGGAGAGCCAGTTGCTGGAGCGGGCCGATCAGGCGCTCTATCGGGCCAAG
GAGTCGGGACGCAATCAGTTCTGTGTTGCAGAGTGACTCTGTTCGGCAATCCAGCGACTGATCCGCTCAT
CCAGCCAAAACCGCGGCCGCCAGGGCGTGCCGTGCAGAAAGCCGACATGGCCGCCACGCTGGTTCAACTC
ATAGCGCACCGTGGGCGAGAGTTGCTCCGGCCGGGGAATGACCGCGTGTGACATAAAGGGATCGTCGGCG
GCGTGCAGGATCAGAGTAGGGATGGGGATCTGAGCCAGCAGCGGTAGCCCCGAGCAGCTCTGGTAGTAGT
GGCTGGCGCTGTGAAAGCCGTGCAGCGGCGCGGTAACCAGCTCGTCAAAATCGCGCAGGGTGGCAATCTG
CTCCACTTGTCGAGGTTGCCAGCGTTCACTCGCTTTCTGCTGCTGGCTGATCTTGTTGAGCAGGTTGCAG
CGCATGGTGCGCAGCAGATAGTTCTGATAGACCTTCGAGAAACCCTGATTGACCCGCTCGGCGCAGCTTG
CCAGTTGCAGCGGGGCCGAGACCACCACCGCCGCTTTCAATGCCGCAGGACAGCCCCGCGCCAGCAGATT
GACCAGCATATTGCCACCCAGTGAAAAGCCGATGGCGATCAGCGGTTTGCCCGGGAAGCGCTGGCTCAGC
TCGGAAATCAGCTCCTGCGCATCACCGATGGCGCCGGAGTGATAGGCCTGCAGGTGGCGATTGGGCTCGC
CGCTGCAACCGCGAAAGTGCATCAGCACGGCCTCTCTGCCCTCTTGCTGCAGGTGGGCGAACAATCCCTT
GGCATAGTGGGAGTGAATGCTTCCCTCCAGCCCGTGAAACACCACCACCAGTGGCCGTTCATCAAGGCAG
ACCTCCCCGCTCCAGGCGAGGTCGACAAAGTCCCCATCCTGCAGTTCAAACCGCTCCGCCACAAAACGGG
CAGGGGTGCGGCGCAGCCATTTGGGCAATATGGTCTGCAGATGAGGATTGCGTGCCCACCAGGGGGCGTG
GAAGTGGCTCTCGGTCAGCATGGGGTCAGTCGGTCTGGGTCGAAGATAAGGAGGACGGGAGGTGTGTTCC
TCCCGCCAGTCATTGGCTTATTTCAGCAGGCGATCGTGATGTTCGGCGCCGCGCAGCATGGCCTGGATCA
GCTCATTGGCTTCGAACTTGGTCAGCGCCTCATGGGCTCCCACCTGATGGGCCCGCTCCACACTGATCTC
GCTGGAGAGGGAGGTGTGCAGGATGATGTAGGCCCCCGCCAGTTTGCTGTCGCTCTGCACTTCGAAGGCC
AGCTCGTAGCCATCGAGCCCGGGCATCTCGATATCGCTCACCAGAATGTCGATCGGGGTCTTCTGGCTCG
CCCGCGTCTGCATCAGGGTCAGTGCATCCTTGCCATTGGTGCAGACCTCATAGGGGATATTGATGTAGTC
GAGGGCGGCCATCAGCTGACGGCGAGCGACCGAGGAGTCATCGACCAGCAGGATCCGCATCGGCTTGATC
CGCTCCCGCTGCACATCGGTCAGCACCGGATAGAGGTGGCTCGGATCATCCGGATAGACCCGGGAGAGGA
TCAGCTCCACATCCAGCAGCTGGATCAGCTGGTCATTGACCCGGGTCACGCCGGTTACGAACACATTGTG
ACCGAGAGAGGCGGGGGGCGATTCGATATCGCGCCAGTTGCACTCGGTGATCTTGTCGATGCCCCGTACC
AGAAAGCCCACCAGGGTGCGGCGGCAGTCGGTAATGATGATGAAGCAGTGGGGCATCTCCTCCTTGCTGA
TGGGGCGATAGCCCACCGCCATCGCCATGTCGATCACCGGAATGGTGGAGCCGCGCATATTGGCAGCCCC
CAACACGGTCGGGTGCGAGTGGGGCATTGCCGTCAGCTGGGTGTAGGGCACTATCTCCTTCACCTTGAGG
GTACCGATGGCAAAGGATTGTTTGGCTGAAAGGTGAAACAGCAACATGCCCTGGGATTGATTGGCTTTGC
TCGTCATGGCAGATGTCCTGTTGGTCGAAACGTTGGCAGTCACTTCAGGCCTCTAGCATAAAGGATTACG
GGGGGAATGGCCCTTCCTTGTTAACGACCATCTGGCAAAAAACTGGACTCATTCAGCCAATTTAGTTCGC
GCATTCACACCTGCGTCCGGTTATTGATCACCATCCTCGCCAACGCCCCACTGCTGGCAGGCCGCTTGCA
ATGCGGCCTCATCGGCATAACCCTGCTTGCTGGCCACCTGCGCGGGTTTGCTGCCGCCCTTGAGCAATCT
GAGCGCCTGCAGCAGTTGCCACTGTTGTTGCAATTCACTCCCTTCCACCGGCGCATCGGCTTGTTGTGCC
CACTGTTGCAGAGACTCCGGTTGGCCATTGGCCAGCGCTTGCCAGCAGGCTTGCAGCTCACCGTGACCCT
GCTGGCTGATGGCACACTGTTGCAGCTCATCCAGGATCACCTGCAGTCGCTGACCGTAGGCCCCTTGCCA
CTCTTTTGGACGTTGCCACAGAGCCAGCGAGTCGAGCAGGGCATCGAGCAGGGGCGTGGCCTGCAGCCAG
CCAGCCTGCTGCGGCTGAGCCACGCGCAAAGAGACATTCAGCTGATCGTACTCACAACCGCTGAGGGGGC
TGAAGGCCGCGAGGGCATCGGCGCAGAGCCAGAAGCTGCTGCCTGCCGGCAGCAACAGATCATGTTGGCC
CAGTCTCAGCAGCGCGGCGCCCCGGGTGATCCGCAACAGCTGCCCGACCGGAGTTCGTTTGCGGTTGCCG
GCGTGCAGGCCCTGGCAGGAAAAAATGGTGATACTCGATGGCGTGATGCATGTAAAAACCCTGATATGAT

GCTGCCTGGAGGTAACAAATATGGAAACCAGACGTAAAAAACGCTTCATTGCCGGTGCTGTCTGCCCGGC
GTGCGGCAAGTTGGACAACATGATGCTGTATCTGGAGCATGGAGTCGAGAAGGTGACCTGCGTCGCCTGT
GGCGATACCCAGGTACAAACGCCTGCTGAGGTCGACAAGCAGACTCGCGAGGCGGAAGAGGTGATCGGGG
TGTTTCGCCCCTGATAAGGGAACGGCGGGTGGAATGCGGGTCTCCTCGCTGACGCTGTTGCCGTTTTTTT
CGTCACTCCGTGGCGCCGCACGGCATATGCGCGGCCCGCTTTTTAGGGTATTCTGAGCCCGTTTTTTTCT
ATCCGCAGGATCCTGACATGAAAGTAGCTCCTCTGAGCGTGGTTACGCTGGAATACACAGTCACCGACGA
GCACGGTGAAGTCATCGACAGCACTGTCGGCAAAGAGCCGCTGGTCTATCTGCACGGCACCCGTTACCTG
GTCTCAGGCCTGGAAGCCGAGCTGGAAGGTCGCGCGGTAGGTGAAGCCTTCGAAGTGACTCTGGCACCGA
GCCAGGCCTACGGCGAATATGACGAGACACTGGTACAAGAAGTACCGGGCGAGCTGTTCGACGGCATGGA
AGTCTCCGAAGGGGATACTTTTGTGGCTGAAACCGACGATGGCCACCGTCCGGTGACCGTGGTTGAAGTG
TCCGAAGAGTTCGTCAAGGTGGATGGCAACCATCCGCTGGCAGGCGTAACTCTGGGCTTCAAGGTCGAGA
TCAAGGACGTGCGTGCTGCCACTGCCGAAGAGCTGGCTCACGGCCACGTTCACGGTGCCGGTGGTTGCGG
CCACGATCACGGTCATGACCACGATCACGGCGGCTGCTGTGGCGGTCATGGTCACGACCACGATGAGGAG
CACGGCCAGGGCGGTTGCTGCGGTGGCGCTGGCCACGGTCACGATCATGGCCACGACCACGGTGACGACC
ATGGTCACGGCGGCTGCTGCGGTGGCGGCGGTTGTGGTGGCAAGGGCCATCACCACTGATCACGGCCACG
CCGATGATAAAAAACCCGCGATAATCGCGGGTTTTTTATTGGCTGACAGTGATGCCGAACCATGTTGCCT
GACCAGAGATAGTGCCGGATCAGTAGTGCGGCGGCGGGGTCTCTTCGGCCATGCTGGCAATCTGGCTGGG
TTGTACGCTTTGCAGTTTGTCGATCAGCAGCTGGATCTGGTATTTGAGCCGATCGAGTTCACGGCCCTGG
GTGGTTACCTCGTCATTGAGCTGCTCTATGGTGTACTCCGCATAGGCGAGACGGGTTTCCAGGGTTTCGA
TGCGATCGTTAAGTTGCTGACTCATGGGCCTTCGGGTCCTTACTTGATTGTCCAGATTTCGGCCAGACCA
CTGGAGCTGGCACTGACAAGGTGGCTATTGTCGCGCAAAGCGACGCCGTAGACCACTGCGCTGGGCGGTT
TCACCTTGGGTTGCGGCGAGACGCGCCAGCTCTGCAGCAGGGCACCATCGCTCACTTGCCACAGGGAGAG
CTGACGGGAAGGGGCGCCAGTGATGAGCCACTTGCCATCGTTGGCAAAGCGGGCGCTGGTGTAGACCTCG
AAGCGGTGATCATATTGCAGCTGACTCACCTCCTTGCCGGTTTTCAGATCCCAGATGCTGGCCCGGCTGC
TGTCGGCGGTGAAGGCGTAGCGGCCGCTCGGCTCCAGTCTGGCCATGATCACCCGGCCACCATGGTTGAA
CCGCCAGATCACCTGCCCGCTACGGGTATCCCACAGATAGGCGCTGTAGTCGTTGCCGCCGGTCAGGGCG
TAGCGACCGTTGGCGGAGAGATCCACCGTATTGACCTGTTCGGTGTGCCCGAGAAACTGCAGGCGACGGC
CGCTTCGGGTGTCGAGCCACTCAGCTTTGCCATCTTCCCGCCCGATCAGCACATAACGACCGTCGGCAGA
GAGCGCGATGTCCCTCAGCCTGGATTCAGGAAGTGAGTAGTAGCCTTGTGATTGCCCGTCGTTCAGCCGC
CAGGTAGCAAAGGTGTCTGGGGTAGCGGTGACGGCATGGCTGTTGCCTTCGGAAAAGCGGGTAATGAATA
CCAGATCTTCCGCAGAGTCGGATTGCTTCCAGCGCCAGCGTTGACGGTTCTGTTCCAGATCCCAGACGAT
GATGCCCTGGCTGATGCTGGAGACGATGGCCTGCTTGCCATCACTGGATAGATCTGCGGTCAGCAGACCC
GTGCTGGCGAGGGTTATTTGCTGGTTGGGTTGGGAGCTCTGCTCGCAACCGGTGAGCATTAAACAGAGGC
TCACCAGATAAATGGCACTTTTTATCATTTTTGATGTCTCACGCTTTTGTTACCTGCGATCACGGCGTTA
GTATAAGGCGCTTATCACGATAAACCTCTCATTTCACGATGGAACCGAGAGCGGTATTTGGAGTTTGGAG
AAACTGATGAACAAATTTCTGAAGGTGTCCCTGTTGGCGGCGGCCGTTGCCGTCGGTCTGACTGCTTGCC
AAAAAGACGAGAAGCCTGCTGCGGCCAATACCGCCGAAGTGAAGGCAGAAGCCAGCAAGCCGGCAGAGGC
GACCAAGGCCGAAGCCAAGAGCTTTGAAGAGCAATCTGGCTACGCAATTGGCCTCTCCATGGGTCGTTAC
ATCGCCAATACCCTGGAGCGTCAGCAGGAACTGGGCATCAAACTGGACAACGCCGTGATCCTGAAAGGGG
TGACTGACGGTCTGGGCAAAGAAGCCAAGATGACTGATGAAGAGATCCAGAAGGTACTGCAGGATTACGA
CGCCAAGATCAACGAGCTGACCAAGGCCAAGGCCGAGAAAGACGCCGTTGAGAATCTGAAGAAGGGCGAA
GAGTTCCTGGCTGCCAACGCCAAGAAAGAGGGTGTGAAGAGCACTGAATCCGGCCTGCAGTACGTGGTCG
AGAAGATGGGCACTGGCGCCAAGCCGAAATCTACCGATATCGTCAAGGTGCACTACACCGGTACCCTGAC
CGACGGCACCAAGTTCGACAGCTCCGTCGATCGCGGCGAGCCGGCTACCTTCCCGCTCAACCAGGTTATC
CCGGGTTGGACCGAAGGTGTTCAGCTGATGCCGGTTGGCTCCAAGTTCAAGTTCTTCCTGCCGTCCAAGC
TGGCCTATGGCGAGCACGGTGCTGGCTCCATCCCGGCGAACGCCGTGCTGGTGTTTGACGTTGAGCTGCT
GGCTATCGAGAAGCCGACTGCTGACGACGCCAAGAAGTAATCCCTTCTTGTAGTTAAAAACGGCTCCTTA
AGGAGCCGTTTTTACTTGTGTATTCCCTGCAATCGCGGGATAACAGAGCTGTCTTGGCGTTGGGTTTGCG
CGCGAAAATAAGGCAAAATAGATCGCAGAATCAAATACAAGGATGAGTCCAGTGGCTGAAAATAAATCGC
GTGTCCTGCTGGTGGAAGATACCCGTAGTCTGGCGGTGGTCTATGAACAGTACCTGCGCCAGGAGGGGTA
TGAGGTGGTGCTGGCCGACTGTGGTCAGCAAGCGTTGGCGCAGTTGCTGGCAAGTCCTCCCCCCGTGGTG
CTGCTGGATCTGGAGCTGCCGGATATGTCCGGTATGGATATTCTGCAAATGATCACCGAACAGCAGTTGC
CCTGCTCTGTGGTGGTGATCACCGCCCACGGCTCGGTGGATGTGGCGGTGGAGGCGATGCGCCTCGGTGC
CTTCGATTTTCTGACCAAGCCGTTTGACAGCAAGCGGCTCTGCGCCACCGTTCGCAATGCCTTGAAACAT
CAGCAGTTGAGCTCGCTGGTTGCCCACTATCGGGAGAACTTCGAGCGCAGCTCCTTCTTCGGTTTTATCG
GCGCCTCCATGTCGATGCAGGCGGTCTATCGCATCATCGAGAGCGCGGCGCCCAGCAAGGCTACCGTCTT
TATCACTGGCGAGAGCGGCACCGGCAAAGAGGTGTGTGCCGAAGCGATTCACCAGTGCAGTCCCCGCAGC
GAACAGCCCTTTATTGCCCTCAACTGCGCCGCCATCCCCCATGATTTGATGGAGAGCGAGATCTTCGGTC
ATGTGAAGGGCTCTTTTACTGGCGCCCAGGGGGATCGCAAAGGGGCGGCCAGTCTGGCCGATGGCGGTAC
CCTGTTTCTCGACGAGATCTGCGAGATGGACCTGGATCTGCAGAGCAAGTTGCTGCGCTTTATCCAGACC
GGCACAGTGCAGCGGGTCGGCAGCGGCAAGCTCGAGACGGTGGATGTCCGCTTTATCTGCGCCACCAACC

GGGATCCGCTGGTGGAGGTGAAAGCGGGCCGTTTTCGCGAGGATCTCTACTATCGGCTCCACGTCATTCC
CCTCACCCTGCCACCGCTGCGGGAGCGGGGCGAGGATATCCTGCTGCTGGCGCGTACCCTGTTGCAGAGC
TACGCCAAGGAGGAGAGCAAACGCTTCAAGGATTTTGATGTCGAGGCGGCGCGAGTGCTGCTCGACTACC
CCTGGCCTGGCAACGTACGCGAGCTGCAAAACGTGGTGCGCAATATTGTGGTGCTCAACGACAAGGAGCT
GGTGAGCCCGGATATTCTGCCGCCACCGCTCAATGGGGTTCGTCCCCAGCTGCCGGCTGCGATTGCGGCA
GCGGTGGCATCGGCTCCGACCGCCACCGGGGTGGCCAACGGGCCAATTCGTCCGCTCTGGCTGGTGGAAA
AAGAGGTGATCGAACAGGCCATCGCCAGCTGTGACGGCAATATTCCCAAGGCGGCGGCACTGCTCGAGAT
CAGCCCCTCCACCATTTACCGTAAAAAGCAGGGGTGGGAGGAGTCCAATCGGGCCTGAACCATCTTGCCA
GCAGCAAGGGCGGCTAGCGTGCCGCCCTTTCTATTTGGGGATCAGAGTTTGGCGAGGCGCTTGAGGCGG
GCCAGTCGCTCCCCCTCGTTGGCGCTGAACCAGCGGTCCTGCGACAACAGAAACCAGGCGCGGGAGAAGT
ATTCCGCGGCCAGCGGATCGTCCAGTACCAAGGCATTCTCCCCCAGCTCCTCGAACAGATAACCATCCTC
AGGTTCGCCAGCCTCGGCCATATCGGCCTTGAGCTGGTGCAGCTCTGCCAGCGCCTGCTGGTGCTGACCC
TGGGCCCGGGTCAGGCGAGCCAGACTCCAGCGGGCGATAAACGCCTTGGCCGGCTGGTTATGTTGCAGAT
GCCAGGCCAGGCACTTCTGTTGCTGGATTTTCGCCTCCTCGAAGCGGCTCAGTTCGAACAGGGTCCAGCC
CTGATTGTTGTAAAGGGGGGCCAGCCAGCCGCGCACCTTCTCGTCACTGCTGCGTTCCGCCAGCTCCATC
GCCAGTTGATGCCAGCGGCTCTGCTCTTCCAGCGGTGCGGCGATGGCGATCATGTGGGCGGCGTCGATGG
CAAGATAGGTCTCCTTCTCCTTGAGCCCCCATTGCCAGGCTTGTTCAAACAGTGGTTTGGCACTGGCTTT
GTCGCCCGCCGAGTTAAAGGTGCGGCCTCGCTCCAGCAGGGCCCGGATCCGTGCTTTGGGGGTTTTATCG
GTGAGGCGAGGTTCTATCTCGTCGAGCAGCTGATGGGCTTCGGCAAACTGGCGCTGCAGCGAATGGGTGC
GGGCAATTTGGGTCAGCAACTCCAGTTCGAGTTGCAGGTCGGCGGCGGCGCGCGCTTCGGGAAGAAGGGT
ATGAAAACGCAGGGCGCTGGCGGCGGGATCCTGATAATCCCACAGCGCCATGAAATCGGGCGGATTGTCC
ATCTGGCCTCCTTGCTGGTTATCCTGTCACCAAGATGCCTGCAGGCCATTGTTTGTACAAGGGGCGCCCG
TCACAGAGCGCCCCTTGAGTCATGTTAACGGCTGTGGCCAGTTGCTCAGTAGACCATAGCCAAGCTCATC
AGCATCAGACCGAGGGCGGCAAAGGCCCCCTCCTTGATGGCGAGCCGTGGCACCAGCGCCTTGGGCAGGG
CCGGAAACAGGCTCAGGGCAAAGCCAAGGCCCGGCATCAACCAGTGGAATGCCGGATCGCGCAGGGCGGG
ATCGGCGGCTCCATTGATGATGATCAGCAAAATAAATAGGGTGAAGGCCGGCAGGATAAACCAGCGTACC
CCACCGGCGGCGACCGCCCGCAGCGGCGAAATGCGATAGAGCAGCATGGTCACAAGGAACAGAACAAAAG
GGGCCAGCAGATAGAGCGGAGCGGGGTTCATAACATCTTTCCTGAAAACTGATCGTCAAAAAACAGGGAG
TCCGGTGCAAGAGGCGCGGGATTTAATCACTCTTCGCAGGTAATCACCAGCAGCTCGGGTCCGGGCTGTG
AAGAGGATCCCGGATAAAGGTTTGCTAGCGCGTCTTAATGCAACCAGAACCAGGCTGCTGCAAATCCCAG
CCAGCTCAGCACCAGCAAGATCCAGGGCAGGGGATGCTGATGGCTTACTTCAACCAGTTCCAGCTGATCG
TCTTGTTCTTCGATTTGGGGCGCGCCCTGTGGCTGCCTGGCTTTGGCCTGCAATTTTTTCTGGCGGCTCG
CTTCGCGGGCCCGCGCCTTCATCTGTTTCTTGTATTCGTCGATGCCTTTCTGGATCCCTTGTGCGATAAG
TTGGGTCTGTTCCTTGGTCTGCCCCGGCTTCTGGTTGGCACGGGCAATTTTCATCGCCTCCTGCTGGGTC
TCGGGGGAAATCTTGTCGTACTTGGCCATCAAATCATCTCCTCGGGCATGGCTGGCTGGGGAGTATGCCA
TAAAGGCGCGTCCGGGTGGCGCCTCCTTGGTGCGGGAGGGGAGCAAGTGAGTGACTGCTCAACAAAAAAC
TGATAAATCAGCAGGAAGCCGGATGTTGGGCCGAAGATCACAATTAGCAGAATTTGTGAGTCATCGATGA
AAAACGGTCATCTGTTGTTTGTTATATTGGCGCCCGACCCGCTGGACAGGGTTGATAAATAACAAAACAA
TCAAGGTGATATGGAAATGAATAATAACTTTATCAAAGGCGGCGTGGCACTGGCCGTGCTTGCTGCACTG
ACTGGCTGTAATACCTCGACCAATGACACCGTTGCACCCTGCACCGAAAATGTCTGCAAACTGACCATAC
TGCACACCAACGATACCCATGGCCGTTTCTGGCACAACGCTGAAAATGAGTATGGTATGGCTGCCCAGAA
GACTCTGGTCGAGCAGCTGCGTGCCGATGCCAAGGCTGCTGGCAGTGACGTACTGGTTCTTTCCGGTGGT
GATGTGAATACCGGAGTGCCGGAGTCCGATCTGCAGGATGCCGAGCCTGACTTCATGGCGATGAACAACA
TTCGCTACGATGCCATGGCAGTGGGCAACCATGAATTCGACAACCCCCTCAGCGTGCTGGAGAAGCAGCG
CAAGTGGGCCCAGTTCCCCATGCTCTCGGCCAACATCTACGACAAGGCCAGTGGCAAACACTACTTTGAC
CCCTACAAGGTGTTCAAGTTGGAGAGCGGTCTCAAGGTCGCCGTACTGGGTCTGACCACCGAGGATACCG
CCCAGCTGGTGGATCCCAACAACGTGCAAACCCTGGAGTTCCGCGATCCGACCAGCGAGGCGGCCAAACT
GGGCCAGCAGATCCGCAGCAACAAGGAGGCCAATCTGGTCTTCGCCATCACTCACATGGGCCATTACGAG
AATGGTCAGCACGGCAGCAATGCGCCGGGCGATGTAGAGATGGCTCGTGCACTGCCGGCAGGGACTCTGG
ATGCCATCATTGGTGGCCACTCCCAGAATCCGGTCTGTATGGAGCCGGGTACCACCAACAAGTACGCCAA
TTTTAAACCGGGTGATAACTGCCAACCGGACCAGCAAAACGGCACCTGGATCATGCAGGCCCACGAGTGG
GGCAAGTATGTGGGCCGTGCCCAGTTTGAGTACCAGAATGGCAAGTTGACCCTGACCAGCTATGACCTGA
TCCCGGTCAACCTGATCAAGCGCAACGCCAAGGGCGAGGCGATCGATGCCAACGGCACCGTGACCAAGGA
TCCAGCCAAGATGCAGTTCGTACAGGACGAGATCACCAAGGATCCCGAGCTGTATAACACCCTGCTGGGT
TATCAGGAGAAAGGTCAGCAAGAGTTGAATGTCGTCATTGGCTCGGCAGATGGCTTGCTGGTTGGTGATC
GCAACTCGGTTCGCAACAAGCAGACCAACCTGGGTCGCCTCATCACCACTGCGACCAGCAGCAAGCTGGG
TGTCGACTTTGGTATCGTCAACTCAGGCGGTGTGCGCGACAGCATCCCGGCCGGTGATATCACCTACCGC
AGCGTGCTGCTGGTGCACCCGTTTGGCAACACGGTCAACAAGGCTGTCATGAAGGGAAGCGATCTGGCGA
CCTATCTGGGCCAGGTGGCAAGCAAGACTCGCAATACCGGTGGTTATGCCCAGTTTGGCGGTATCAACAT
GGCGGTAGATTGCCAGGCCAAGAGCGTCAATATCACCAGTATCGGTGGCAAGGCGTTTGATCCGGCAGCA
ACTTACAGCTTCGCGATCCCGAGCTTCAGCGCCTCCGGTGGTGACGGCTATCCGAAGATCGATACCATCA

ATGCCGGTTTGGTCGATGCTGCCGTGCTGAAGGAGTACATCGAGATCAAGAAGACCATCAACGTGGCCGA
TTATCAGCCAGGCAATGAGGTGAGCTACCTGAACTCAGCCTCTACCGAAGGGTGTAATTGATTCGTCAGC
GGGGCGCATCGCCCCCAGATATGATCCCAAAAGGCAGCCGGTGGCTGCCTTTTGTCGTTGTTTGGATGGC
GGTTACAACTCTTCAATCATCAATGACGATAGCCATCGCCTGTATGAGGTCAGGTGAGGGGATGAAATGA
AAAGAGGCGGCCCGTGGGCCGCCTCTTGCGTAGTGACAATCTGAACCTTACAGCTGCTCGATACCGACGA
TATGCCAGGGGGCATTGTTCTTGGCCAGATCGCGCTCGAGGTGCCACACCTCTTTGATATCTTCTTCGAC
CTGCTCCTGACGGTCCATGTAGCGGCCGCTGAAGCGCACGCTCACCTGAGCCCAGTCGCTGCCGTAGTCG
GCGCGTACCAGCTGGGTATCGACGTACATCACTTCCGTATGCTGGTCGCCAGTCAGTTCGGCGCGTTCGG
CTTTGAGCTGCTCGAACAGCTCGGGGCTGACGTACTCGCGCACTTTTTCCAGATCGTTCTTGTTCCACAC
CTCTTGCAGGGTGCGATAGTGATCGCGGGCACCGGCCAGGAAGCCGTTCATGTCAAAGCCCGGCGGCAGG
CGGAACGGCACATCGCTGTCGGCAAAGCCGGTGGCTTGTGGTGCGCTGTTGCTCTGCTCAAACTGCTGCG
GGGCAGCGGATTGCGGTGGCTGATAGCCCGCATAGGCTGCCTGTTGTGGCTGTGGCGTCGCGACCTTGCC
CTTGCGCAGGGTACGGATCAGGAAGACGGCACCCAGCGCCAGCAGGGCGATCAGCAGGAAGTCCATTCCT
TGCAGACCTTCAAAGGCACCGCTGCCCAGCAGGTAGGCAAACAGGCCGCCAGCCAGCAGGCCGCCCAGCA
GGCCACCCATCATGCCGCTCTTTTTCGGCGGAGTCTGGGCATTCAGTGTCGGATTCTTGTTGTCGACCGG
TTTGGCCGCAGGCTGGGCCGGCGCAGTTTTGTAACTTTTACCAAAGGATTTGCCGCCGCCAAATTTCTTG
GCTTCGGCGTGAGGTGCCCCCAGGCCAATGGCCAGGATCACGGCAAACAGGGTCAGCATCTTTTTCATTT
TGACTCTTTTCACTCTCGTCTGTGGGTGGGGCATGCTCCGCTTTGGCAAGCTCAGCAAGGCGCATGGCAG
GCTGCCTGTCCCTAATTGTGTCGCAGCTTGTATTCATCAATATCACCTCTGGCATGAATTATTCATGAAC
GAGGTAGCCGCGATGCGGCGTCAAACAAGAGGAGTAAACCGGATGAAAAGTAGAAGATTGAGTCGCAAGG
AGCGCAAAATGCGAAGCAAAATCAAAATGGTCAAGGTAGAGCAGACTCTGGTTTGGCCCTTTATGATGGT
ATGAGCCGCCAGATAGGGCTCGCCGTAGTTGGCTTTCTTTTGTAAAATTCCCGTCCCCTTCCCGCTGCGG
TTTTTATGTGTATGGCAGAGCAAGTGACGGCGAAAAAAGACGCCAAGACACAACAGCTGAAAGTTCCTCC
CCACTCTTTTGAGGCCGAACAGTCCGTACTGGGCGGCCTGATGCTGGATAACGATGCCTGGGATCGGGTT
GCCGAACGGGTGATCGATCAGGATTTCTACAGCCGTCCGCACCGGTTGATCTTCCAGGCCATGACCCGCC
TCTCCAACGCGGGCAACCCCATCGACCTCATCACCCTGCAGGAGGAGCTGGAGCGCTCCGAACAGCTGGA
AGAGGCGGGCGGCTTCGCCTATCTGGTGGAGATCGCCAAGAACACCCCGAGTGCTGCCAACATCAACGCC
TACGCCGAAATCGTGCGTGAGCGGGCGGTGGTGCGGGAGATGATCTCGGTCGCCAACGAGATCGTCGAAG
CGGGCTATGAGCCACAGGGCCGAACCTCCGGCGATCTGCTGGATCTTGCCGAGAGCAAGGTATTCAAGAT
TGCCGAGCAGCGCACCAGCGCCAACGAGGGGCCCCAGCCCCTGAAGCTCATCCTCGAGCAGACCGTCGAC
AAGATTGAAGAGCTGTTCAAGACCCCCCACAACGGGGTGACCGGGGTATCGAGCGGTTACAACGATCTGG
ACAAGATGACCGCCGGTTTGCAGCGCTCGGACTTGATCATCGTGGCGGCCCGTCCTTCCATGGGCAAGAC
CACCTTTGCCATGAACCTGTGCGAGCATGCGGCCCTCACTGCCGACAAGCCGGTGCTAATCTTCTCGCTG
GAGATGCCCTCGGAGCAGATCATCATGCGTATGCTCGCCTCGGTGGGGCGCATCGATCAGACCAAGGTGC
GTACCGGTCAGCTCGACGACGAGGATTGGGCGCGCCTCTCCTCCACCATGGGATTGCTGCTGGAGAAGGG
CAAAATGTATATCGATGACGCCTCCGGCCTCACTCCGACCGATGTGCGCTCCCGTGCCCGTCGGGTGGCA
CGCGAGCATGGTGGCCTATCCATGATCATGGTGGACTACCTGCAGTTGATGCGGGTGCCGGCGCTCTCTG
ACAACCGTACCCTGGAGATCGGCGAGATATCCCGCTCCCTCAAGGCCTTGGCGAAAGAGCTGCAGTGCCC
GGTGGTGGCGCTATCCCAGTTGAACCGAAGCCTGGAACAGCGGGCCGACAAGCGCCCGGTCAACTCGGAT
CTTCGTGAATCGGGCTCCATCGAGCAGGATGCGGATTTGATCATGTTTATCTATCGTGATGAGGTTTATC
ACGATGATAGCCCGGACAAGGGGATTGCCGAGATCATTATCGGCAAGCAGCGTAACGGCCCTATCGGCCG
GGTGCGCCTCACCTTCCAGGGTCAGTTCTCCCGGTTCGACAACTACGCCGGTCCGGCGTTTGACGACGAT
TATTGATAAGTAACAATCATGGCAATCCTGTAGTCAGGATTGCCACCACCGCTCTGACCCGCTTGCCATT
TATTGAGAGGCAGCCAGCCAGAGCCTGTTTTCCCGGTTCGACAATTATGCCGGTCCGGCCTTCGACGACG
ATAAGTGAGTGGCGGTCTTCGGCCCGATGAGGGGGAGACCATTTGTGCCCTCGTAAGGGAAAACCATGAA
AGCGGCTACTGCCCAAATCAATACTGCGGCCTTGCGCCACAACCTCGCCGTGGTCAAGCGCCACGCCCCC
GCTTGCAAGATCATCGCTGTGGTCAAGGCCAACGCCTATGGTCACGGACTGCTGCCGGTCGCCCGCACCC
TGGTGGATGCGGATGCCTATGCGGTGGCCCGAATCGAAGAGGCGCTGATGCTGCGCTCCTGCGCCGTGGT
CAAACCCATCGTGCTGCTGGAGGGCTTCTTCTCTGCCGACGATCTGCCGGTGCTGGCGGCCAACAATCTG
CAGACGGCGGTTCACACCTGGGAGCAGCTCGAAGCGCTGGAGCAGGTCGAGCTGCCAGCACCCGTGGTGG
CCTGGCTCAAGCTCGACACCGGCATGCACCGCCTCGGAGTGCGTGCCGACGAGATGCCTGCCTTTATCGA
GCGACTCGGCAAGTGCAAGAACGTGGTGCAGCCGTTCAACATCATGACCCACTTCAGCCGCTCCGATGAG
CTGGTGCAGCCCACTACCCGCGAGCAGATCGATTTGTTCAGCAAGTTGACCGCACCGCTGGCGGGCGAAC
GGGCCATGGCCAACTCGGCGGGCATTTTGGCCTGGCCAGACTCCCACTGCGACTGGGTTCGCCCCGGTGT
AATCCTCTATGGCGTGTCGCCCTTCCCCAACACGGTAGCGGCCGATTATGACCTGCAGCCGGTGATGACC
TTGAAAACCCAGCTGATTGCGGTGCGGGATCACAAGGCGGGCGAGCCGGTGGGCTACGGCGCCAACTGGG
TGTCAGAGCGCGATACCCGGCTCGGGGTGATCGCCATCGGCTACGGTGACGGCTATCCACGCATGGCCCC
CAATGGCACTTCCGTGCTGATCAACGGCCGCATCGTGCCGCTGGTGGGGCGTGTCTCCATGGATATGAGC
ACGGTAGATCTTGGGCCCGGCGCAACCGACAAGGCCGGTGACGAGGCGGTACTGTGGGGCGAGGGGCTGC
CGGTGGAGCGGGTGGCCGACGAGATTGGCACCATCCCCTACGAGCTCATCACCAAGCTCACCTCCCGCGT
CTTTATGGAGTACGTCTGATGTGGCAGCAGTGCCTGCTGACGTTGAAGCCCCGCTCGCGGGGCTTTCATC

TGGTGACTGACGAGCTGGTGGCCAAGCTGCCGATGCTGGCGGATTACCGGGTCGGCATGGCCCACCTGCT
GCTGCAACATACCTCCGCCTCGCTCACCCTCAACGAGAATTACGATCCGAGCGTGCGCAGCGATATGGAG
GCCCATCTGCACCGGCTGGCGCCGAAGGATGCCCCCTACTATCGCCACACCTACGAGGGGCCGGACGATA
TGCCCGCCCACATCAAATCCTCCCTGCTCGGGGTGAGCCTGCAGCTGCCAGTTCGTGATGGTCAGCTGGC
GCTCGGTACCTGGCAGGGGATCTGGCTTGGCGAGCATCGCGTCGATGGCTGCGCCCGCAAGGTGCTGGTG
ACCCTGCAGGGTGAGTAATCAGGCTGCAACCAGCTGAAAAAATGGATAAAAAGAGGGATGGCGCCGAGCC
ATCCCTATTTTATTTCTGCGGTTTTATGAGCTATCCGCTTGCCGGTGGAGCTCAGCGCACCTTGAACGGC
GTCAGCTCGGCCAGCGGGCTCGCGTCCATAAAGGCTTGCGGCTCCTGACCGTGTTTGAAGATCTTGAAGT
CCTGCTCGCGGGCGCTCCAGTACTCCAGACGCTCGCTGCCGTTTTCTCCGGTGATTTGCAGCAGACTCGC
TTCGCGCAGGGCCACCACATATTCGCTCGGGTTGATGGCGCAGAACTCGGCCAGACGCTCGTCGCGGGTC
TCGCCCATGTGGCCGCTGATACTGGCGTCCAGATAGTGCGGGTTGATCTGCAGCGGGAACAGGCCCAGCG
CGGGCAGCACGGCGGCGTTGCAGACCGGCATGTCGTTGGTGGTGCGGATGCTCGGGGTCGCCACGTTGCA
ACCGGCGCTCCAGCCCACATAGGGGACGCCCCGCTCACGCACGGCGCGCTGGATCGGCACGATCAGGCCA
TTTTCATGCAGCAGCTGATTCAGGCGCCAGGTGTTGCCGCCACTGATAAAGATGGCGTCGGCCTGATTGA
CGGCCGCGACCGGATCATCGAAGTGATGAATGCTGATGGTCTCGATCCCGAGGCTCTCGGTAAGTTCGTT
GGCACGGGCATCCCAGTCGCTGCGGATCACCGCATAGGGGATGAGCAGAATGCGCTTGACCTGTTTGCGA
GCCAGCAGGCTTTGAACCCGATCTCGGGCCCAGCCGAGCAGACCCGGAAATTCGTTGGCTTTACCGTTGC
TAAGCAGAAGCAGTTCCATCAAATCGTTCCTCAAAAAGTGAAAGCGGAGGAGGGAGGGCCACCGGATCCG
CCGTGCGGCCGGATGGCAAGAGGAGGCATGCTACCACCATTGGCGCGGCGGTTCTGTGATCGCGAGGCCC
ATTCAACGGCCATGGAGGAGTTATGGATGTATAACTCAATGCCAAGTGGTGGATTAATAAGCAGCGCTCC
GCTCGCTGCCATCGCCAATAAAAGACCCCGCCGCAGCGGGGTCATGGCAGAGTTAGTCGAGTTTGAGATC
GACCCACACCATGCGGTGATCCGAGCTGACCCCTTTGGCGATGCCGAGTTTCGCATCATAGACCAGGTGA
TAACCGGCCTCGAAGCTGGCTGGCCAGAAGACGCCGCTCTTGACCGCGTTGAGGTTGGCGGAGGGGATGA
CGTGATCCAGCTGCAGCACGCTGGAGTTGGTGATCCGTTCCGGGTTGGGGCGAGTGAGGTTGCGTTTGCA
GTCAGCCCCTTTGCTGACACACTCCGGACCCCCTTCGCTGACCGGGATCAGGGTGCCATTGGTGATGGCC
TGATTCATCAGGGCGTTGTCCAGTAGTGCCTGAATGGCGCTCAGGTCGCCGTCGCCGGTTTGCGGATCGG
CGTTGAGATCGCCTGCGATAACGAACTTGGCGTTTTTGGCCAGCCCACCCTTGGTGCCCTTGTCATCGGT
CATGTAGCTGAGACCCTTGACGTAGTCGTTCCAGAAGGCGATCTCGGCACGGTTGTGCTTGACGGTGTGA
CGCGCCGCGTTGGCAAAGATTGGCGGGGTCGGGTGGGAGATCAGGAAGTGAATCACCTCTTCCCCCTTCT
CGGTCTTGATCCGTACCGGCACATCGGCGTGGTTCTTGGAGGAGAGCGGGTACTGCTGCCAGGCTGCGTC
TGAATACCAGGCATCGCCACACTGGCGGCTGGCCGGGATCTTGACCTTGGGATTGTTGCAGTCATCGATG
ATGGGGTTCTGCTCGCCCGGCATATCCTTCCACTTGAAGTTCTGGAAGGTGCGGATCTGCTTGGTGTCGA
TGGGGTATTTGGACATCACCGCAAAGGCGTACTGGCCGTGGTAGTTGCCAAAGCCCCAGGCATCGTCGGG
GCCGCTGTTGACCTTGCCATCCAGATTGAGGTCATAACCGCTCATCAGGCCGGTGTTGGTGGCGAAGTTA
TGCATCACCGGATAGCTGATGGCGGTCACTTCGGCATGCTGGGCGTGGGCCAGATAGTTGTCGTTGAAGG
CTTTCAGATCGGCGGTGCTCTCGCCTTTGCCGTCGTTGTCGAACTCGTTGAGCAGAAACACATCCGGACG
GGTGCGCTGGATGATCTCCGCCACGTTGCGGATCTGCTGCACGTTCTTGGCTTTGGTTTCCGTGGCGCCG
CTCAGGCTCCCTTCGGTGAGGCGGGCCAGCAGGGCATCCTGCTCGGTGCGGGTCAAGGCCAGCTCACCGG
TCAGCATACCGGCGGCAGTGCGGTCAAAAGAGAGGTTGAAGGTGGCAAAACGAACCATGGGATCAGCTGC
CTTTGAGTCGTTGTCGCTGTTGCAGCCAGCCAGCATCCCGAGGGAGATGGCGGCAGCCAGTAGGGTCTTG
TTGATATTCTTCATTGTAATTATTCCGTAGCATCAATGAGTTATGCCTTCTGTGAGGCGGCGCCATTATA
AATAGACGGCGATGCTGCGGGGATTTTGCCTGAGCAAAGTGTGAGGATATTCACACCACATAGCTGGCGA
TGATGAATGTTAGCAGGGCGATATGGGGTGTGAAAAACCGGTTTATACGCTGCAGTTTCCGGGATGAATG
GCGCATTTCCTGCCGTTACCGCACTCTGAATTTGTCACACCACCATCGGCAGGCCGCTTTCTGGATGGTG
GATCACCTGTGCCGGATAGTCATAGAGACGGGCGATGAGCTCAGGGGTGAGCACCTCGGCAGGCGTGCCA
TCAGCCATCAGCTTGCCCCGCTCCAGCATCACCAGCCGATCGGCATAGCGCGCCGCCAGATTGAGGTCGT
GCAGCACCACCAGCACTGCAGTATTGCGGCTTGCCAGGGCGCGCGCCATGGTGAGCAGCTGGTGCTGGTA
TTTCAGATCCAGCGCCGAGGTGGGTTCGTCGAGCAGCAGCAAGCGGGGTTGCTCGGCTTGCTCGGTCGGG
GAAGGGGCCTGCCAGATCTGCGCCAGTACCCGGGCAAACTGTACCCGCTGCCGCTCGCCGCCGGAGAGCC
CCGGATAGAGCCGCTTGGCCAGATGCTCGACCCCGGCATGGGTCATGGCCGCCGCCACTATCTCGTCACG
CCGCGCCGCCGGTTCACTGTGGGGCAAGCGCCCCATGGCGACCACTTCCTCGCAGAGAAAGGGGAAGTTG
AGGGAGGAGCTTTGCGGCAATACCCCGAGCCGGTGGGCAAGCTGGCTGCTGTCCCACTGCTGGCGATCCG
CGCCAAACAGGGTGATGGAGCCCTCATATTCCAGCTCGCCGGTCAGGCACTTGAGCAGCGAGCTCTTGCC
CGCCCCGTTGGGGCCGAGCAGCGCGGTGAGGGTGCCGCTCTCGAGACTAAGATCGAGCCCATCCAGGATC
TGGCGCTGGCCACGGCGCAGGCTGAGTCGTGTGCAGCTCAACAGGGGGGAGGAGAGTGAGGATGAGAGTG
GTTGCATAACAGTCCGTATCACAGGGCGCGGCGGCCTTTTACCAGCAGCCAGATGAAAAAGGGAGCGCCG
AGCAGGGCGGTGATGATGCCCACCGGCAGCTCGGCCGGGGCCAGCAGGGTGCGTGCCAGCATGTCGGCCG
CCAGCAGCAGGGCGGCGCCGAGCAGAGCCGAGAGCGGCAGCAACCTGACGTGGTTGGGGCCTGCCAGCAG
GCGCACCAGATGGGGCACTACCAGCCCGACGAAGCCGATCATCCCCGATACCGCCACCGCCACCCCGACG
CCCGCTGCGGTGAGCAGAATCAGGCGACGTTTGAGGGATTGCACATTGACCCCCAGATGACGGGCCTCTC
CTTCACCCAGCAGCAGGGCGTTGAGGGGATTGGCATCGCGCATAAAGAGCCACAGCAGGGCCGCCAGGGT

GAGCAGGGCCAGCGCCACATCGGCCGGTTTGCCTGCTGCCAGCGTTCCCATCTGCCACAGGCTCAGGTTG
CGCAGCATCTGATCGTCCGCCAGATAGGTGAGCAGGCCGATCACCGCGCCGGAGAGCGCCGTGATGGCGA
CCCCGGCCAGCAGCATGACGGTGACCGAGGTGCCGCCCTCACGGGTGCCGAGCAGATAGACCAGGGTGGT
GGTGAGCGCGCCGCCGAGAAAGGCGAGCAGCGGCAGCAGACCAACCCCGATGGTGGCTTGCAGCTGCTGG
CCGAGGGGGGTCAGCAGCACGATGGCGAGCGCGGCACCGAGCGCCGCACCGCCGGAGACACCTATGATGC
CGGGGTCAGCCAGGGGGTTGCGAAACAGTCCCTGCATCACGGCGCCACAGAGCCCGAGGATCCCCCCCAC
TGCGATGCAGAGCAGGGTACGCGGTAGGCGAATTTCCTGCACGATCAGCAGTTTGTGCGACTCAATCCCG
CTCTCCTGCCCGGCAAACAGCGCCCGCAGACTCTCGCCCAGGGTGAGGGTCATGGGGCCGGTTGCCAGAG
AACCGAGCAGCAACAGTGCCAGTGCGGCCGTGGTAAAGGCCAGCAGCCAGGAGAGGGGTAACCGCATCAT
GACCCCTGCTTGATCCACTGGGCGATCTGGTTCGCCTGTTGCAGGGAGTTGAGGCTCAGGCCCCCTTGCA
GGGCGTGGCCGTCGATGGCGTGAATGGCGTTACGCTTGCCAGCCGGGGTGGCGGCCAGGGCGGGCACTTC
GGCCAGCACCTTGGCCGGATCGGCATATTGCTGCCAGTCACGACCGCTGACCAGTACCAGATCGGGCTGC
ATCTCGATGAGGGATTCTGCGGATACCGGCTTGTAGTTGCGCAACTCGGCGACCGGATTGATGCCGCCCG
CCAGCGAGATCAGCGCGTTGGCAGTGGTGTCCCCCCCGGCGATGCTGGTGGGTTGCCCCTTGTGCAGCAG
CAGGAAGGCGACCTTGAGCGGTTTGTTCTGTTTGGCTTGCGCCGCCAGCTGAGCGGTCTGGGCCTGGATC
TCTGCCTTCAGGCGATCGCCAGCGGCTTTGTCGCCCAGCAGGCTGGAGAGGGTGTCGATACGCTGGTTGA
GGTTGGCGAGAGTCGGCGCGGTATCCAGCACCTCCACCTTGACCCCGGCACGGCGCAGTTGATCCAGGGT
CGGGGCCGGGCCCATCTCGTCGCTGCCCACCAGTCGGGTGGGGGAGAGGCTCAGGATCCCTTCGGCGGCC
AGCGCCCGGTGATAACCCACCTTGGGCAACCCTTTTGGCAAGGGACTGCTGACATCGGTGGCGATCAGGC
TCGATTCACCGCCAAGGGTCAGAATGAGTTCTGTGGTGGCGGGGCCGATACTGACGATACGCTCTTTCTG
TCCGTCGGCGGCAACGGCAGAGAAGGGGGGCAGTGAACTGCAGAGCAGGGTGATGGCGAGGGCAAGACGA
CGGCCCATGCTAACCTCCGAAGGGGTGAATGAAACGGGTGTGCCGCCGAGAATATCAAGGCTTTGGAAAA
AATGCACTGATAATGATAGCAATTATCAATTGCATTGGTTTCGGTGCTTTATTAGAGTAGCCGCCATGAT
AAACAGCGGGACAACATGGTCCCGCAAAGGGAGAAAACATGAGCATTGCACAACGCATTCACGCGCTGCT
GGAGCAGGATCCCGGCGCTCATCCGTCCACCCTGGCGGCCCAGCTCGCCATCAGCGAATGGGAGGTGGTT
CGCCATCTGCCCGCCGAGCTGGTGACCCTGCTGCCCGCCGATCGCGCACAAGGTCTGCTGGAAGATCTGG
CCGACTGGGGCCCGGTGACCACCATCGTCGAATCAGAAGGTTCCATCTTCGAGGTAAAAGCCCCCTTCCC
GAAAGGCAAGGACGCACGCGGTTACTACAACCTGATGGGCCGCGACGGTGAGCTGCATGGTCACCTCAAG
CTCGACAACGTGGCGGGGATTGCGCTGGTCAGCAAGCTGTTCATGGGCAAGGAGGGGCACTCCTTCCAGT
TCTTCGGTCACTCCGGCCGCTGCATTTTCAAGGTCTATCTGGGTCGCGACGAGAAACGCCAGCTGCTTGC
CGATCAGGTCACCCGTTTTATGGCGCTGCGCCACAACTCTCAAGAGGAAGTCAAAGCATGAGTGAACGTC
AGGAGCGTCTGCAGAACCGCCTGCAACCGGAGATCCGCGAGTTTCGCGACAGCTGCCGCACCCTGCAGCT
CGCCACCGTAGATGGCGAAGGCAATCCCAACGCCAGCTATGCCCCGTTTGTGCTGCAGGAAGATGGCTAC
TATGTGCTGATCTCCGAGATCGCTCGCCACGCCCGCAACCTGCAACAGGTGCCGCGAGTATCCCTGATGC
TGATCGAGGATGAGAGCGGTGCCCGCGAGCTGTTTGCCCGCAAGCGCCTGACCTTCGATGCGGTGGCTGA
AGTGGTGGCCCGTGACGACGCCCGCTGGAGCAAGGCGGTGGCGGCGCTGGAGGGGCGTTTTGGCGAGATC
ATCAAGGGTCTCTCCAACCTCAAGGATTTCAAACTGTTCCGCCTCAAGCCGGAGCAGGGGCTGTTCGTCA
AGGGCTTCGGCCAGGCGTTCGCGGTCTCCGGTGACGAGCTGGTGGATTTCGTCCATCTGGTGGAAGGGCA
CAAGCGCATCGACAACGGTGCCGAGCTCACCAGCCCGGCCGATGCGCCGGTCTGACGATCCCTGTCGACC
ACGACAGCCGTCATAACAAAACGGGCAGCCACTGGGCTGCCCGTTTTTATTGCGTTATCTCATCCGGCCT
GTTCAATCCTGCCAGCTGGCCTTGTTGGGAAAAGCGCGACGATCGGCGTCAGTGGCTGTTTCGTTGCACT
CTCCCCGCACCTTCCAGGAGGTCTGGCTCAGTACGATGTATCCCTGGCGGCAATACTGCTTGGTCGCCAG
GGTCTCGGTCAGCAGGTTCTCCTGTAACGCCTCTCGCTGTTCCAGCAGCCGCTGGCTCTGGGCCGCCGAG
AGACCGCGCGGCAAGGTTTCGTTGGGCGAGGCTTCAAAAGTGAAACGCTTGCTTCCCTCCTCGTTGATGT
GGGTCAGAAACAGGGGGTCCGGCCCGTCATACTTCTGTCCGGGCAAGCTGGAGCAGGCCGACAGCAGGGG
AACAATCAACAACAGGGTGGCAGACAACAGGCGCACGACAGACTCCAGTGAGAAACGAGGCGAGGCTCGC
AGATTAGCACGGCTGCAGGGCGGGTGTTACTGCCAGCAGCTCGTCGGTGGTGAGCAGGGTCACATTGCGC
CCCTCCGGCAGGCTGAGGTGGCGCCACATCCAGTGATGGTGCTCGGTGATCTGGGCCGCGCTCAGGTGGG
GGCGATCGGCGGTGGTGTGACCATCGCTCAATACCAGCACCTCGTACCCATGGGAGGCGGCACTACGCAC
CGTGGTGTCGACGCAAAAATCGGTGGCGCAGCCACAGAGAATGAGTCGATCGACCGGCTGCTCGGCCAGC
AGGAAGGCAAGCTCGGTATCGAGAAAGCTGTCACAGGCCGTTTTGCGCACCCGGCTATCTCCTTTGGCCG
GTTGCAGCGCACTGTGCAACTGCCAACCCGGTGTGCCCGGTTCCAGTTCGGTACCGGGGGGCGAGTCATG
CTGGATATAGATGACCCGTCCCTGTTGCTGCCTGACATGGGCCGCAGCCCGATTGATATTGGCCAGCACC
TGCTCCTGGCGGTAACGGGGCTCGTTGAACAAGCCTTGCTGCACATCGATGATCAGTAACACATCCATAT
TTTACCCGCCGTTTCCTTGAAGATTAGGGTTGCAGCCACTGGCTGCAGTCACGACTGCGGGATTGATGCA
GCGCGCCCATATCTTGCTTCATCTCGTCACTGTTTTGCCAGCGCTTAGGCAGCGGCATGATGGTTACCTG
CACATCGGCTGCGGGATCTGCAACAGAGGTCTCATCTGTACGGGGGACGACCAGCGCTACCTTGAGGGCC
GGATTGCGGCTGGCGATGCGACTGGCGAGCCCAAGCCCCCCCTCGACGTGGAAATTGCCGGCGATATGCA
TGATCTGCTGGCCCGGGTGCTTGGCGAGATAATCCACCATGCTCTCGGCCATGGTGTCATCCCAACTGGT
CTGGGCGGCGAAGCGGCGGGCGTTGCTGTCGGCATCGCCATGGTGCATGGAAGCCATAAATTTCTGGCGA
TAGGGGTCATCCCCCAGCGTCAGTTGGCGGGCCAGCTGGCTGCGACGCGACGCGGGCAGTTTGTCGAGCC

ACTCAGCCCCCTCCTGACCGACACAGCTCACCAGCGGTTTGGGGGCGTTGGCGGCGATCACCGGCATCTG
GTGAGCCTTGGCAAACTCCACCAGCGGGCGGTAGTCGCTCGGATAGTTGGGCCAGGCGTTGCCTTCGCGG
ATCAAGGCCGCTTCACCAATCTGGTTCGCCAGATAGGCATCCAGCGTCTTCTGATCGGCGCGGCTGAACT
GCTCCATGGAGAGGGCCAATGACCGCCCGTGCCGCTGGCCAGCAGTGTGGAGCCCGCTCAGCAGGCGCGA
TTGCAACAGATGCACGGCGGGATGGCTGTGCAGCTCGCCCACCAGAATGATGTCGGCGTCGGCAATGCGC
GCGATCGCCTGCTCCAGCGTCAGGTCTGCGTCTCCCTCTCCCTCGCTGCTTAGCTGGTAGTCATAGAGCG
TCTGCAACTGTGATGACTGGGTCGGTTGGGTGGGGGTGGCACAGGCTGCCAGTAGCAGCGGCAGGGCGAG
CAGGGGAAGTCGTCTAATCATGGGATCTCACGGCTGATATGCCCGGATGCCGGCGCATCTCGGGGGGCTA
ATAGAGAATAAGGGCAACACAGGGCGACAGTGTAAAACCAATTTGGTTCAAAACAAATGAGAATGATTTC
GTTTCCTGTTGATCTTTGTCGGGAGCCGGGTAATATGCGGTCTGTTTGATAATAGTTCTCAATAAGGATT
CGCTGTGACCCATCATAACGCACTCTCTCTGGTTGCCCTCGCCGTGGCAGCCGGTCTCTCTTCCCAGGTC
TTTGCTGCCACCCAGAAGGCGGATGAAGTCGTGGTCGTCAGCGGCTCACGGATGGAGCAAAAACTGGAAG
ATGTCTCCGGCCCCATCTCGGTGATCACCGCCAAGCAGATTGAAGAGCAGGTCGTCACCAACGTTGCCGA
CCTGTTCCGCTACGAACCCGGTGTGACCGTGCTCGGCGGTCAGGGTGATGCGCAGACCTTTATCATCCGC
GGCATGGGGGAAAACCGGGTCAAGGTGGTGCGCGACGGTGTGCGCCAGAACGATGCCTACAGCAAGGGCG
GTGTTGGCCAGAGCTACTTTGATACCGATATGATCAAGCAGGTAGAAGTGGCCAAGGGGCCCGCTTCTGC
CGCCTACGGCTCCGATGCGCTGGGTGGCGTGATTGCCATCACCACCAAGGATGCCAGCGACTTCCTCAAG
GGCAAGGATAGTTATCTGGATGTGACCAGCGGTTATGCCTCAAGCAGCCACCAGAAGATGGCGGGCTTTA
CCGGCGCCTTGCGCACTGGTGAAGTGGAGAACCTGCTGCGCTACACCTGGCGCGATGGCGGTGTGACCCA
GAATTTCGACTCCACCAAAAACGACTTCGATATCGGTACCCACGCCCTGCTGCTCAAGAGCAAGTGGAAC
CTCTCCGATGAGCAGTTCCTCAAGCTGACCGTTGACTACCTAACCGATGGCGAGGAGCCGGATGCGGTCA
AGCTCAACAAGGTCGGTACCGGCACTGCCCGTGTCTTTGATCAGCCCATCACCGACAAGCAGACCGACAA
CCTCTCAGTGGTGCTGGATCACGGCATCGCGCTGAATCAAGGCTGGGCCGATCGGATGGATAGCAAGGTC
TATTTCGCTCGCACCAAGCAGACGCTGGATCAGTACGCCTCATCCAAGTATCCGGTCAGCCCCGGCAACC
CCTATGTGACCAAAAACTCGCTGGATCACAACGTGTTCGATCAGAAGAACATCGGCGCCCAGCTGCAGTT
CCACAAGGCGGTCGCCAACCACCGCCTGGCTTGGGGGGCTGAATACGAGCACACCGACAACGAACGTACC
CGTTACAAGGGGCCGACGACTCCCGGTGATTTTGTCGGCTACAACGAGCTGAGTTTCCCGGCTACCACCA
GCGAGCGCAGCGCCCTGTGGGCCTTTGACGAGATCAAGTTTGGCGAGCGCTGGGTACTGACCCCGGGTGC
CCGTTATGACTACTACCGGATGACCCCGGACAACGATCCGGCCTACACAGGCGACAAGCTGCACAAGTTC
TCCGAAGGGGAGTTGTCACCCAAGCTGGGTCTGGTGTTCAAGGCCCACGAGGCGGCCAACCTGTTCGCCC
AGTACAGCCACGGTTTCAAGGCCCCCATGTATGACAGCGCCTTCTCCACCCTGAACCATCAGGCGTACGG
CTATCGCATCGAGCCCAACCCGAACCTCAATCCGGAGAGCAGCGACGGCATCGATCTTGGCGTGCGTGGC
AGCAGCAACGGCTTCAGCTATGAAGTAGCCAGCTTCTACAACAAGTTTGAAGACTTCATCGAGATGGTGG
AAGTGGGTAAAGAGGGGCGCACCGCCGTCTACCAGTACCAGAACCTGAGCAAGGCGACCACCAAAGGGGT
CGAAGCCAAGGCGGATTACTGGCTCAACGACATCGTCAATGTCTGGGGCAACCTCGCCTACATCGAAGGC
AAGGATGGGGATGGCAACTACATCAACAGCCTGAGCCCGCTGAATGGCTCCGTCGGTGTGCGTCTGGAAC
AGTCCCAATGGAACTTCAACACCGCGCTGCGCTTTGCTGACGACATGGACAAAGTCCGTAAAGACAGCAA
AGGCAACGATTACGTGAAGAGCGCCGGTTGGGGTGTGGTAGATATGTACGCCCAGTTCAACCCGATGACC
GACCTGCAGCTCAACGTCGGGGTCTTCAACCTGTTTGACAAGGAGTACACCAGCTACGAGCGCATCGCCG
GTCGTGATGCAGGTTCCGACAACAGCCTGATGACCGAACCGGGTCGCAACCTGAGTGCACGGGTCAAATA
CGTATTCTAAAAAGACAACACACGCAGTGATCTCGGGCTGTTGCCCGCACCGGATCCCCGCCCTGACAGG
CGGGGATTTTTTATACATGTAGCCAGGTGTGAGGTGACCCGCTCACACCATGTTATTCATTGGCTTCGCC
TTTTTCTTTGTGTTGGCGAGATATTATGCTATTTGATTCATTGTTAATGACATTTAGTAATTCTTTACAA
TGGGTGTGCCTAGCTTTTGACTTCGACATCGAACTTTTTCCTCGACACCGCCGACGCTCTTGAGCTGATT
ACCCGCTTCAATGAGGCCTCTGACGAGCAGACCATAGTGGCCCTGCTGCGTACCCTTAGCCAGCAGATGG
GTTTTGACCACTTCCGGCTGGGATTCATCTTTCCCAGTTCGATTCAGCGACCTGACGTGAGGATTTTCAA
CGGCTGTCCTGCCGAATGGGTACAGACCTATGACAAAAAGCGTTTCTTTACGGTAGATCCCGTTGTACGC
AAAGGGATGAGCCAGAGTACCCCTATCCTCTGGGCCAACCTGATCACCGAGTGTTGCGCTCAACAAGATA
TGGCGGGGCTTGAGGTGATGTTGCAGGCCCATGAGATGGGATTGTGCGACGGGATCACGATTCCCTGGCA
CGGGCCGAATGGCCATGTTGGCTTGTTGTCATTGATCACACGCACTACACGAACTGAGCATCAGTGGCTG
ACGGCTACTCCCTTTTTAAGCTGGCTGGCGGTGCATGTCTTCGAGGCTGTGGCCCGTGTTTGTCTTGCCG
TGAAGCTTCCTCAGGAAGAACTGACCCTACGGGAGCTGGAGGTGTGCCAGTGGGCTGCCGAAGGCAAGCA
AGTTAGTGATATTGCCCAGATCCTGGGGATCAAGCCAAGAACAGTTACCTTTCATCTCGAGCGAATCGCA
GAGAAGCTGGGGGCGAGCTGCAAAAATCAGGCAATATCCTGGGCGCTGATGCAGGGGGTTGTCAGACTAA
ACATTGATACGGCACAGATCGAAAATGTGGATGATAACAGTGCATAATGCTTCCTAACGCAAAATGTTGC
TCATTACGTAGATGAAATTACTAAAAATGTCAGTTTTGCCAGTGGGTGTGATGATATTGCGCAAGTTATT
GCATCTATGCGCGTAGAGAGAGAACAGATGTAAGGTAATTGACTGATAAATAAAGAATAAAAAAGTGGCA
TGGTCGCTGCTAAAGGTTGGTCGAGTGCTATCTCGTTTAACCAATCTATTTAGGAGCATACTCATGCCAA
CTCCATGTTATATCAGCATCGAAGGTAAAACCCAGGGCAATATCACCGCAGGTGCCTTCACCTCCGATTC
CGTCGGCAACATCTTCGTACAAGGCCACGAAGACGAGATGCTGGTACAAGAGTTCCAGCACGTCGTCACC
GTCCCGACCGACCCGCAATCCGGTCAGCCTGCCGGTCAGCGTGTCCACAAGCCGTTCAAGTTCACCGTTG

CGCTGAACAAGGCTGTGCCGCTGATGTACAACGCACTGGCTTCCGGTGAGATGCTGCCGAAAGTGACCCT
GAAGTGGTACCGCACCTCCGTTGAGGGCAAGCAGGAGCACTTCTTCTCTACCGTGCTGACCGATGCCACC
ATCGTTGACATCGACTGCCAGATGCCCCACTGCCAGGACCCGGCAAAGTCTGACTTCACCCAGCTTATCC
AGGTCTCCATGGCTTACCGCAAGATTGACTGGGAGCACACCGTTGCCGGTACTTCTGGCGCGGACGACTG
GCGCGCGCCCATCGAGGCGTAAGCCTTGCTTCGGTACTTTGGTATTCCTTGTAAGTAGTTATGTGTCTTT
GAGCCGGTTGTTCAACCGGCTTCTTGTACTGTGCATTTTCTCCAGCCTGGCCAATGGGTTGGCTGGAGCT
TTTCTGTTTTGCCATCCCCCTGCCGGGGTTTGATGCCCTGCCGGTGAGTGCGGAGGGATGGCAAAGCACT
AAAGGGTGTCCTGTGATGCAGACCCGAACCGCTCGTAACCTGTTAACCCCCTTATCTCTGGTGGCCCTTG
CGGCACCGGAGAGGCGGTAGCGTGGCTGCGCGGTGAGGGCGTGTATGGGCGCGAGAGTTGGCAAGGGCTC
AGGCACTGCTTGGCCGTGACAAGAGGGGAAAGCCATGGCTAATCAAACCGGATTGCAATTTACCGTCAAG
GTGGGGGCGTTGCCCGAGAGCACCTTTGTGGTGGCCAGTTTTCAGCTGGATGAGGGGCTGAACCGGCCGT
TCAACCTGCAGCTGGAGTTGGCCAGCAGCCAGCCCGATGTCGACTTTGGCGCCGTGCTGGACCAGCCATG
CGAGCTGATGGTGTGGTACAACGGCGAGCTGCAGCGCCGGGTCTGCGGGGTTGTGAGCGAGTTTGCCCAA
GGGGACAGCGGCTTTCGCCGTACCCGTTACAGCCTGCAAGTCAAACCGGCCCTGTGGCGACTGGGCCTGC
GCCAGAACTCCCGCATTTTTCAGGCGCAAAAGCCCGATGAAATCCTCAGTATCCTGCTGCAAGAGCACGG
CATCACCGACTACGCCTTTGCCCTGAAAAACGAACACGCCCAGCGGGAATACTGCGTGCAGTACCGCGAG
ACCGACCTCGATTTCATGAACCGCCTTGCTGCCGAAGAGGGGCTGTTCTACTTCCACGAGTTTGAAGCAG
GCAAACACCGCATTGTGTTTGCCGATGACGCGGCAGCACTGACTGCTGGCCCCGAGCTCTTCTTCAACCT
CGGCAACCGCTCGCTGGAACAAGGCCCTTACGTGCGCCAGTTCCACTACCGCGAAGCGGTGCGACCAAGC
GATGTAGAGCTGAAAGATTACAGCTTCAAGACCCCGGCCTATGGCCTCTCCCACAAAAAGCAGGGGGCTG
AGCTCGACCATCAACGGGACACCTACCAGCATTTTGACTATCCGGGTCGCTACAAGCAGGACCCGAGCGG
CAAGGCGTTTGCCCAGCATCGGCTCGATGCGTTGCGTAACGATGCGGTGGCGGGCAGTGGCAAATCCAAC
AGCGCAGCGCTCTTGCCGGGTCAGCATTTTTCATTGACCGAACATCCGAACGCCAGCCTCAACACCGAGT
GGCAAATCGTTCATATCCGCCACACCGGTGAGCAACCCCAAGCCTTGGAAGAAGAAGGCGGCAGCGGCCC
GACCGTCTACCACAACGAATTTGGCGTGGTGAAGGCGAGTACCACCTGGCGCGCTCGCATCGGCAGCCCC
GAGGCCCCCCACAAGCCGATGGTGGATGGCCCGCAAATCGCCATCGTGGTGGGCCCCGAGGGGGAGGAGA
TTTACTGCGACGAGCATGGCAGGGTGAAGCTGCAGTTCCCGTGGGACCGCTACGGCAGCTCCAACGACCA
GAGCTCCTGCTGGGTGCGGGTGAGTCAGGGCTGGGCCGGTGGCCAGTACGGCATGATGGCCATCCCGCGC
ATCGGCCATGAAGTTATCGTCTCGTTTCTGGAAGGTAACCCCGACCAGCCCATCGTGACGGGTCGCACCT
ACCACGCCACCAACCGGCCACCTTACGACTTACCGGCCAACAAGACCCGCACCGTGCTACGCACCGAAAC
TCACAAGGGCGAGGGCTTTAACGAGCTGCGTTTTGAAGACCAGGCGGGGCAGGAAGAGATTTATATCCAC
GGTCAGAAAGACCTGAACGTGCTGATAGAGAACGATGCGGCGTGGCACATCAAGCATGACGAGCATCGTG
ACATCGACAACGAGCGGGTGACCCGTATCAGAAAGGTGCCGGGCAAGGATGGCGCTCCGCCCAGCCTTGG
TAATGACCACCTGACAGTTGAAGGTGAAAAACGCGACCACATCAAGGCGGACTATTCACTGACGGTGGAT
ACCTCCCTGCACCAGAAACTGGGTCAATCCCTGTTGGTGGAAGCGGGCGAAGAGGTGCATATCAAGGTGG
GTGACAAGCTGGTGCTGGAGGCCGGCTCAGAGATCACCCTCAAGGGGGGAGGCAGCTTTGTGAAAGTTGA
CCCGAGCGGCATCAAGCTGGTGGGCCCCGCCATCAAACTCAATGCCGGCGGCAGCCCAGGAGCTGGCTCT
GGCTGGGCTGGTCAGGCTCCCGTTACTCCCAAAGGGGTTGAGGTGGTCAAAGCCCCGGAATTGGTTGAAC
TGGTCAAGGCGATTGCCACCGAGAAGGCTATGGAGGCGTTGCTCAAGGCGCTGGAGCCCGATAGCGATTA
TCAGCCCTCTGCTTGATGTTCGACGAATAACTTAGGATTGAATGACAAAGATGAAAATCGTCTATCCCAT
GCAACTGGCGGGTGAAAATGGCTCCGGCGAGATTGCATCCCTCGATGAATTTATCAAGAAGATCAATGGT
CTCAAGAGCGGTACCTTCCCGATAGGCCGCAACCGTATCTGGCACGGTGGTATCCACTTCAGTAAAAGCG
GAGGCTGGCACCCGAGCGGCGCAGTGCGCGCGATTGCTGACGGGGAGATTGTGGCCTACCGACTGGCGAC
TAAACCAGCCAAGGCGACCCGTAGCTCCGAAAAGGGCAAACCAGAGGGAAAGATTGACTTGTATACCTCC
CCTTCGTTTTGTCTGGTACGTCATCGCTACGAAGCCGGTGAGCAGAGCAAGAATCAGCTGACCTTCTACA
GCCTCTACATGCACATCGCCTGTGAGAACAGCTACAACAGTCCCGAGGCTGCGCGAGTAACGGTCAAGGG
GACAGGTGTGTCCACCTATGAACCGGTGGCGCAGGGAGACCCCCTAAAGCTGACACGCCGACTGTCGGGT
AGTAGTCCTGTGTATGCCAAAACAGGTGCTGAGGTGAGGTTGCTCCAGCAAACCCCGAGCAACCTGCTCA
ACCACAGGGATGAAGCCCACAATTATTTTCTGGTGCACTACGTCGATGAACCGAACAGCCCGTTCTATAT
CGCACAGAGCCAGTTACAGCATGAATATCCGCAAAAGCCGACGTGGATGACCCCACCAGAAGGCAAACCG
GCCCGCCACAAGGTCCCGAGTAATACATGGTTACGTAAAACTGCTGACACCACAGTGGGTAGCATGGGGC
TGCCTGCTGGCAGTGAGGTGGTGGTATCCACTGAGCCACCACAGATGGTGAACCTCAATGGCGGCACGAC
TGAGTTTCGTAAAGTACAGGTTTTCAAGGTGGGCAGCGGCACTGTCAAAGACAGTGCCAATCAGGTGATG
ACCAATGCCAGCAAAGGTGCCGTTGGCTGGCTGGCAAAAAGCAAGATGGGGGCCGGACTGACCGCAGAAC
CGAGTATCCCTGTCGAATTCAAAGACGATGCGGTCGTTGACCGCAGCGCAAATCCGATACCGGTGCAGGC
CGGAGAGATCATCGGTCATTGGGGTGAGCATGAGCTAGTAACTGCCGGTGACAGCGGTTTCGAGAAAGAC
GCAGATAGCAAAGCCGTGCACTTTGAGGTGTTTGTGGCAGAGAGCGACAAACAGGCGCTTGAAGATTGCA
TCAATAACAAGGCGCGAGTAACCGGTGGCCAAGGCTACCTGCTGGTAAAGAAAGAGGTCACTACCTATCG
TCTCACCAGCGATAGCAAGCATGGTTTTCACGAGGTTGCCAATTTTGGGCCGCTGGTCTTACCGTTGGCG
GTGAAAGAGTCTGATATTGTCACTCACGGTGCCAACAACTTTGTGAAGGTACGTGAGCGGACTGCTGCGG
ACGGGGAGTTGGCTGGCGAGTTCGTATTGCAGGGCGGTGATGTCGAAGTCATCAGTCAGCACGACTGGCA

CAAGCTGGGCGTCAAGCTGGTCGATGGCAGCAGCGATGACGATGGATTTTTGGACAAGGCTGATACCGAG
AGTGAAGAGCCGCAGCAGAAAGAGGCGAGCAAATTTTTTAGTACCCTCTATGACAAGCTGGTGACAGACG
GAGACAACGATGGCACCCTGAGCGGCAATGACATCAAGGCCGCGCTGGCCGATGAGGAGTTGGCGGGCAA
GTTGCGCATGTTGTTTATCAAGCACAAAAGCGAATGGGTAAAGCCAGGCCAAGAGTGGCCGCGCTTAAAA
CAGGAGTTGGCTAAGCAGCCAAAGCTCTATGAATATGCCATGCAGGTACACAACAATATGGCGTGGATGG
AAGATGCCAGTAAGATATTGGGTGATACCAATCCTTGGTTTATTCATCCGGCAGGGATGATAGAATTGGT
TAGAAATCAGCATGGCTTTAATATTGATCGCCAGTACTTTTTTGAAAAATATAAAGATTTTTACCCTCTA
AGTGAGAGCCAAAGAAGAAATATATCATTGATGTTTGATGCAATCGAACAGTATTATTCTGATAATGTCG
ACTCTTGCAATGAACGAAAAGTTGCATATATGTTAGCCACTGCAAAGTTAGAAACTGGTGATACTTTTGC
TCCAATATCAGAGGTTGGGGGAAGGAATTATTTTGAAGGTATGTATGATTATTTCCTTGCTAAAAATGAA
GATAGAAAAAAAGTGGCAGTCGCGCTTGGCAATATTCAGCCTGGCGATGGCGTCAAATTTCATGGCCGTG
GTTTTGTTCAGCTAACAGGTCGCTCCAATTATGATAAAGCCGGTAACTATTTAGGTGTTGATTTGCTGAA
TTCTCCAGAATTGGCCAAAGACCAAGTTATTGCAGGTAAAATTATGTGTTGGGGTATGGAAAACGGAATT
TTTACCGGTAGAAAGTTAGATCAATATTTTTCAGCTAGTAAAGCAGACTATTATAATGCTCGTAAAATAA
TAAATGGGTTAGATAGGGCGCAAGATATAGCGGATTATGCTAAGAATATATCCTCATGTATTTTAATTTC
AAGGTAAACACATGTTTTATTCACGAATTATGTTATGTTTATGTGCATTTTGGGCCTCACTCTCAGTTTT
TGCTTCACCTAGCTTTGATTGTGGAAAGGCAGAGTCATCAATAGAGAAGATGATATGCTCTGATAAGGTT
TTGTCAGAATTAGACAGCTCTCTATCCACTCAATATAAATATGTGCTGAAGACCAACCCTAGCTCGGCGT
CTTCAATAAAGGATGAACAAAGGACGTGGTTGCGTGAAAGGAAAAAATGCACCGATAACAGCTGCTTGAT
TTCAATGTATGAAAAACGAATTAAATTTTTTCATGACAAGGGAAATGCTAAGTTAAATGTTGTGTTGTCA
CCACCAAACGTTATTGTAACATTGAGTGGCCAACGTCTTGTTATTAAAGATGTAGCACCACAGGGAGGAA
CAACATTCTCAACTTTAATTGATGGTGAGAATAAATTTTCTGTATGCACAGAGTATCTTGCATCTGCAAT
AAAGAGAGTTGAGTGCAATGTTTTTAAAAGGATTGGTGAAAAAATATTTTGGGCTTTACATAGTGAAGCT
AAAAGAGACTATGATTCATTTTCAGCTATTTTCATGAAATTGGACGACATGTCATACAATGAAGAAGAAC
AATATAACGTCGCAAAAAAAAATAGATGGCTATCAATGTCTTTATAAAATTAGTAGTTCAACAACCAATGT
GATTGAAATTGATCGAAATGGCGTGCTAGTAAAAAAAATCCAAGTTGAGTCTTTCTAACGAAGATTTTTAT
AGCAATCTTGGACAGATTTCACTTGATTATTTATGATGCATCTTGTTAATTAAAATGCATTTCATTATTT
GAACGTTACGGTGAGTTGTTAACTTTTCAGTGTGATTTGGATGATGTCGAAAAATAAAAAAAAGTACATC
ACCTAATACCTTGATGATGTGGGTAATTGTTAATTTCACGGTAAGTTAAAGAAGAGTCTCTGACAGAGAC
TCCTCTTGCTTCATTATGGAAACTTAGTTCAAAAAAAGCAGTTTCTCTTATGTTTTATAGCAGTTCAGAT
TAAATTTCACAATCAAGCTCTGGAGCCTTGTTAGGTCGGGGAAATATCTGATTTTTTTTGTGGGTGGAGT
TAATGTCATCTTTGCTTTTTATATTTTTGTTGTCGTTTTCTTGTGCTGCACAATCAGAGGCTTCCATTCA
GGGTCATGAGGGGAAACTATTTTTCGTAGACAAGGTTGATGATACCATCGGCGACGATGTTATCGCGGTA
TTTTTTGATAACAATAAGAGTGGGAGGTATTTTGTTGATTACCTTCCTTATTTGTCTTCTAAGGGGGAAA
TACAGGATTCCTTCTACTTAAATACTGATAACTTAACGAGTAAATATTTTGTTGTTCAGGAGTCACAAAT
AGACTCAGACACAGGAATGAATTGGTCTAATTATTTTAATGTCATTGTTTTTTCTTATCTCAATGGTAAG
TTGATTAAAGATGAAAAGTTAACGTCATATTTTGGCTCTGGTGGAAACATTGTAAACCCTAACGGCGCTA
ATAGTATAACCTATTCCTTTCCATATTATAAAAAGGAAAGGATTTTACAAGCAGTGTCTAAGCCTGAATT
TGATGCATGGATGAAAGGGCAAGATGTAAAATTAAAAATAAAAGGGAAGGGTTATTTTTTCCTGAACCAC
TGCCAATTAACAGAGCTAAGTCTTATCTTGTCGAAGGTGATGTTGTCCTGAGGAAAAAACATGAGTCAGG
TTGGGATTATGTCGGCTATAAATCAACTAAAGGACGAGAATATTCTGGCTGGATGCCTTGTTCTTTAACT
GGAAGCTGTTAGATGATATGTGAAGTAGGTTTGATTAATTGCAACTAATTAGGCTTGTGTATAACTTTCC
AAGTGGAAGTTATTTTTGACAAGGGAAGATAGGGACATTTATTTTTTGGAGTGTACAGGCATAGTTATCA
CCTGACACCGGCTTAACGCCGGTGTTTTTTTATCGCTACAATACAAATAGTTTTGGTGCAGGATAGCTCG
AAGATTTGTTGTATCCAGATGATTTTTCGTTGTGAGGATGGATTTAAATTTGAATCCGAGATGCAGATCC
TGGTGATGAGAAAAGTGATTAGGGGACAAAAAAGAAACCCGCCATGGGCGGGTGGAAGGATTGCAACACT
AACCAGGAGGAGGTGTTACCAAGTAGACCCCACCTCTTCGGTAATAGTTCAATTTTATTGAAGTTTTTTC
GTTTTTTGCGCGGGGTTTCCATCGGGATCTGCGGCGGAGGATGGGTTATCATTCTGCTCCCGTCATCCGG
TTGCAGAGGATCTATGCCCGCGTTTTCCTGGTTAGCGCTCTTTTTCGGCGCCTTCTATTTTGTCTACGGT
GCTTACTTGCCGTTCTGGTCGCTCTGGCTGGAGGGGGTGGGCGTCAGCGCCGAGATGATTGGTCTCCTGC
TGGGAGCGGGGATGGCGATCCGCTTTGCCGGCAACCTGATGGTGATGGGGCAGATCAAGGGGGCGGGCCA
TCTGCTGCCGGTTACCCGGTTGCTCTCCCTGCTCAGTCTGCTGGCGTTTCTCGGCTTCTATCTCAGTCAT
AACCTCTGGTGGCTGGTGGGCTTGACCCTGCTGGCGAACTTTATCTATCCGACCCTGATGCCGGTGGGGG
AGGCGCTGGCGACCCGCATGGTGGTGCAGGCGCACCTCGATTACGGCAAGGTGCGGCTGTGCGGCTCCTT
TGCCTTTATCGTCGCCTCTACCCTGGTGGGGGCGCTGGTGGGCAACTTCGGTAGTGACTGGATTTTGCAC
ACCATGGTGGCGGGGCTGGTGCTGATGCTGCTGCTCAGCTGGCTGCCGCTCTATCCGGCGCCACAGGATA
GTCAGGGCGAGCGCGCCAAGGCGTCCTTGCTGGCAACCCTGCGCAGCGCGCCGGTGCGTCGCTTCCTGCT
GCTGACCGCTCTGCTGCAGGGGAGCCACGCCGCCTATTACGGTTTCAGCGCCATCTACTGGAAGGCACAA
GGGTATAGCGGCACCATCATCGGCTATCTCTGGGCGCTCGGGGTAGTGGCGGAGATCTGCATGTTCGCCG
CCGACAAGCGTTTCTTGCAGCGCTTTGGCGCCCAGAGTCTCTTTATCGCCGGTGCCATCGGTTGTGTGGT
GCGTTGGGTATTGCTGGGGGCCTCGACCGAGTTGTGGGTACTGGTGCTGGGGCAGTTGCTCCACTCGGTC

ACCTTTGGCATGAGTCATCTCGGGGCTGTGCGTTTCATGACTCGCCAGCTGCCCGCCGAGCAGTTGATCC
CGACTCAGGCGCTCTATGCGGCCCTTGGCCTCGGCATGACGGTGGCGGCCCTGATGGCACTTTGCGGCGT
GCTGTTCGAGCCTCTCGGCGGCGGCATCTTCTTCCTGATGGTGCTGGTGGTCTTGCCGGTCTTCTTCCTG
CGGCTGCGCCCCTTTGCGCCGTCTGCGCAGGAGAGGGCTCCGGCCTGATATTTGTCTATTTGAATCATCA
CCTAACAAAAGGGAGAGCCACGGCTCTCCCTTTTTCGTTACTGCGGTTTGTGGCGCTACTGCTGCTCGCC
AAGATAGAGGCGGCTGAGACGGCGCCAGGTATCCGAGCCGATGGTGCCGGCAAGCAGCTCATTGGATGCA
GCATCAAAGCGGCCATCCGGCAGCCAGCCCAGTTGGGGCAGCAGTTGCGCCATCGTCACGTCATTGTGCT
GCAGCTCCCGCTCCAGCGCTTCCCAGAAGGCGCTCCCCTTGACGTAAAAGAGCGGGTAGTAGGAGTGATC
GCCCGCCAGCACCTCGCGAGCTGCCCGATAGAGGCCGCTCTCGCTATGAGGTAGCAGTTTGCCCCGCTCG
GCCAGCTCGTTGACCGCCGTGACCTGATGCAGCCGAAAGCGGCGCAGCGCCTTGATGGCGAGGTACTCGG
CGAGGCTCTCGTTGACCCAGGTGGGCTGTGGAGCAGTACGGCGAGCGCTCAGGATATGTACCGCCTCGTG
GGCCGAGGTGCGCAGCAGGCGCAAGGGGGCCTGTGGCGTCGGTTTGCCAGCCTCGGTGGGGTAGTTGGCA
ACAAACACCTGATCTCCGGCGGCGCCGCCGAGAGAGCGGGTTGCCACATCCCGCCCCAGCCACACCAGTT
GCCATGGGTGAGTGGGCGGATCCACATCAAGCTGGAAGGCCAGATAGTCGATGATCTCATTCAGTTGCTG
CTGCAAGGCCGGCAGATCGAGCTGATTGGCGGTCTCGTCATGGCTCAGCACCAGCCGCGGATCCGGCTTG
GTGACGGGCACTGTGTCCAGACCAAACGGCAGGATCAGCGGCGGTTGGTTGACATCCGGCAGCGGTTGGC
AGCTGCCATCCGGCAGACAAACTTTTCCTTGGCGACTCCCCATCTGGCGCACAAAGTTACCCCACTCGGT
CAATAGCCACTGGTCAGGGGCAAACAGAAGCGACTGTTGGGCCGAGGCATCTGCACCCTGTGCCGGAGCG
GGTGTGAGCGTGATCTGCCAGCCGATCTCTTTGCAAGAGAAGGGCTGGTTGAGGGCAACCGGCTGGTTGT
CTGCAAGGCAGATCAGCGCGGGGGAAACCGGCGCGGCACTGCCCGGCAACTGGCGGGCGGGGAGCAGGGT
AAAAGGGGGGCTTGCCCTGCATGGTGAGGCGCACTTGCAGCACATTGGCGCTGGCGCACTGCTCGATAAGG
TAGCGGGGGCCGTTCGCGTCATTGCCAGCGCAGGCCCACAGCGGCAGCAGCCACAGGGCAAACAGGGTGA
TATGACGACTCATAAGAGGGTTAGCGCTCCTGCTCGTAGCCCAGCAGATCGCCGGGCTGGCATTTGAGTT
CACGGCAGATGGCCTCCAGGGTGGAGAAGCGCACCGCCTTGGCGTGGCCATTCTTGAGGATCGAGAGGTT
GGCCTCGGTGATGTCGATGCGCTGGGCCAGCTCGCGGGCGGTCATTTTACGCTCGGCCAGCAGGGCATCG
AGTCGGATGATAATGGGCATACGTTCCTCACACCGTCAGTTGCTGTTCATCGGCCAGCAGTTTGGCCTCG
CGCATGACCAGTGCCAGCGCCAGTACGATCAGTCCCGCCAGCAGTACCCGGAAATCTGAGGTACTCAGCG
CTATGGTACCGAGTACTTCCGGGCCTCTCATGGTGAGCACCGTATCCAGCAAGGGGGTCAGGATCAATTC
GACCAGGAAGGTTGTACAGAGCAGCAGTCCGATCTGCCGATAGCGGCGGATCTGCTCGAAGGTGAAGAGC
AGACCGGCGCGGTAGCCGCGAAACAGCTGACGCAGTTGCCAGAGCATCATCATAAAGACCAGATCCGGCA
ACAGCTGCACCAGGGTGCCGAGCAGCCGGGTGGAGAAGTGAAGGGGATAGTCGGCAGGATGGATGCCGGG
GCGGCTCAAGATGGTGTTGAGCTTCTCCTGCAGAAAATCGGCGCCACCGAAGGGGGTCCACACCTCCAGC
AAGGTGATCAAAGGGGTAGCCAGCAGTGCCAGCAGCAGCAGCCATTCGATCAGCATGCTCGAGCGTTCGA
GTTTGTTGGAGATCATCTCATTCCTCACTGTGATGGTTTTATCTGAAAGGTAAAGTTTATCGTTTATCAA
TATCCACATCGGCAATGTTTATTGTTTTGCGATATACAGTCAAGATAAAACTTATTGAAAAACGATGATT
TTAATCTGTCCGAAGGGGATGACTTTTGGCTGCAGCCTCTCTAGACTGGCGAGGTTGTATACAAAAACTC
AACATGGATAAGGAGTTATCGATGCACCAGGGATGGCTGCTGATCGGATTGTCGCTCACCTATCTGGGGC
TGCTGTTTCTGATCGCCTTCGTGGCAGACAAGCACAAACGGCACCGTCTCAAGGGGCAACCGCTGTTCTA
CAGCCTGTCGCTGGCAGTCTACTGCACCTCCTGGACCTTCTTCGGCACCGTGGGGCAGGCGAGTGAATCC
CCCTGGTCGCCGGTGCCCATCTACCTTGGCCCCATGCTGGTCTTCCTTTTTGGCTGGCGGCTGCTGGCCC
GCCTTATTCTGGTGGCCAAGCGCGAGCACATCACCTCCATCGCCGACTTTATCGCCGCTCGTTACGGCAA
ATCCCAGCGGCTCGCCATGGTGATCGCCCTCATCGCCATCATGGGGATCCTCCCTTATCTGGTGTTGCAG
CTCAAAGCCATCGTCACCGGGCTGGATCTGCTGATGGTCAACAGCGGCGGCATCAGCCCGACCAGCAATA
CCAGCGAGTTGGCGCTCGGGGTTACTCTGCTGCTGGCGCTGTTCAGCATCCTGTTCGGTACCCGCCATCT
CGATGCCACCGAGCACCACAGGGGGATGGTGGTGGCCATCGCTTTCGAGTCGGTGGTCAAATTGCTGGCC
TTTATCGCGGTGGGGGGCTTCGCCCTCTGGCTGATCTTGAGCAAACCCGGCGAAGCTCACGAGCAGGTGA
CCCGCGACTTCTTTGCGCAGGTGGTGGCGGTGAGCCCCGGCAACCTGCTGGAGCTTGCCATCTACACGGT
GCTGGCTATGTGCGCCGTCATCTGCCTGCCGCGCCAGTTTCATGTGACTGTGGTGGAGAACAATCAGGGG
CAGGATCTGCACTGGGCGCGCTGGATCTTCCCGCTCTACCTGTTTGTGATGGGGCTCTTCATCTGGCCGC
TGGCACTGGCGGGCAAACAGTGGGTGGGTGCCGATATGGCCTCTGATACCTATGTGATTAGTCTGCCGAT
GGCCCTCGGGTTTGATGGCATGGCGATGCTGGCGTTTCTCGGCGGCACCTCGGCGGCCACCGGCATGGTG
ATCGTCTGCACCATCGCGCTGGCGATCATGGTGAGCAACGATCTGGTGCTGCCGGTGCTGCTGCGCCGCT
TCTGGCGGCAGGGGCGCGACGAGCGGCTGGTGCGGCTGCTGTTGCAGGTGCGCCGTGGCGCCATCCTGCT
GATCTTGCTGGCGGCCTGGGGGCTCTACCTATGGCTCGGCGATCTCACCAGCTTGTCGCGCATCGGTTAC
CTCTCCTTTGGCGCGGTGGCCCAGTTTGCGCCGGCCCTGCTGCTCGGCCTCTACTGGCGTCACGGCAATC
GCAAGGGGGTATATCTTGGGTTGAGCCTCGGGGTGAGCCTCTGGTTCGTCACCCTGCTGGTGGAGAGCGG
GCTGCTGGCGGGCTCGCCCTTGGCCGAGCTGCTGGCGCCGCCGGACTGGCCTGCGTTTCGCGAGCTGAGT
CTCGGGGCTTGGTGCATCTTCCTGAGCCTGCTCTTCAACCTGCTCGGCTATGTGGCCGGTTCCTTGCTAT
CGCGCCCTGCGGTGAGCGAGCGGCTGCAGGCGGCCAACTTTGTCGGCAAGCCGAGCCGCGATACCGCCGC
CCTCTATCAGGCGCGGGTCTCGGTCAAGGAGCTGGAGATGCTGGCGGCCCGCTTTGTCGGCTCAAGCCGG
GTCAAACGCGCCTTTGGTCGTTTCGCCGGTGAGCGGGGCGGCACCCTTGCTCCGCAGATGCAGGCCTCGG

CCGAGCTGATTGCCCACACCGAGCGGCTGCTGGCGGGGGTGTTTGGGACCTCCTCGGCGCGGTTGGTGCT
CGCCTCGGCCCTGCAGGGGCGCAACATGCAGCTCGAGGAGATCGCCACCATAGTCGATGAGGCCTCCGAC
GTGTTTCGCTTCAACCGCGGCCTGCTGCAGGGGGCGATCGAGCATATCGGGCAGGGGATCTCGGTGGTGG
ACAGGGAGCTTCGGCTGGTGGCCTGGAACCGCCGCTACATCGAGCTGTTCCACTATCCGCCCGGGCTTAT
TCAGGCTGGGCGTCCTATTGAGGAGATCATCCGCTACAACGCCCAGCAGGGACTCTGTGGCCCCGGTGAT
ATCGAGGATCACGTGGCGCGGCGGGTTGCCTTTATGAAGCGCGGCAGCGCCCATATCTCGGCGCGGGAGC
GCCCGGACGGGCGGGTGATCGAGATGCAGGGTAACCCCATGCCCGCCGGTGGTTTCGTCATGACCTTTAC
CGACATCACGCCGTTTCGCGATGCGGAGCGGGTGCTGCGCGAAGCGAACGAACATCTGGAGGCACGGGTG
GCCGAGCGCACCCGTGAGCTCTCCGAACTCAACCGCCAACTATTGCTGGTGAACCAGCAGGTGGAGCGGG
CCAATCAATCCAAAAGCCGTTTTCTGGCGGCGGTCAGCCATGATCTGACCCAGCCTCTCAATGCCGCCAA
GCTGTTTACCTCCTCCCTGCTGGAGATGTTGCCCGATGCTGGCGAGCAGTCCCGCATTGCCCGCCATATC
GATGATGCGCTGGGGGCGACCGAGGATCTCATCACCGATCTGCTCGATATCTCGCGGCTCGAAGCGGGCA
AGTTCAAGGCCAAGAAGCTCGATTTTGCCCTGCGGGATCTGCTCGACAACCTCAAGGCGGAGTTTGGCGT
GCTGGCCCAGGCGGGGGGGATCCACTTCTCGGTGGTGGAGAGCAAGCACGCGGTGCACAGCGATGTGCGG
CTGCTGCGCCGGGTGCTGCAGAACTTCCTCACCAATGCGTTTCGCTACAACCCGGGCGGCCGGGTGCTGC
TTGGTTGCCGCCGGATGGGGGATAAGATCCGCATCGAGGTATGGGACAACGGCCCCGGCATCCCGCAGGA
CAAGCAGGAGGCGATCTTCGACGAGTTCTCCCGTCTCGATCACTCCCGTACCGCCAAGGAGCAGGGGCTG
GGTCTGGGGCTTGCCATCGCCCGTGGCATCTCGCAGGTGCTCGGTCATCAGCTCACATTGCAGAGCTGGC
CGGGCAAGGGATCGGTCTTTGCGGTGACCCTCAATCTGGCGACCCGGCCGGTCACGGCCCATCAGCTGGC
GGCGCCGGTGGTGCGTGACAGCCAGCTGGAAGGTGTAGCCGTGCTCTGCATCGACAACGAGCGGGACATT
CTGACCGCCATGAGCACCCTGCTTGGCCGTTGGGGCTGCGAGGTGCGCTGCGCCGTCGATCTGGCCGAAG
CCGAGGTGCTGGTGACAGACGGGTTTGTGCCGCGTCTGGTGCTCTCTGACTACCACCTCGACGACGGCAA
GACCGGGTTGGAGGCGCTCGCTGCCCTGCAACGGGTGTGTGGCGACGAGCTGGGGGGCATCATCATCAGT
GCCGATCGCAAGAGCGAGTTGCAGGCGCAGATCCGCGAGCGGGGGTATGGCTACATCAGCAAACCGGTCA
AACCGCTCAAATTGCGGGCCCTGATGAACAGCCTGCTGCTGCGCTAGCCGCATCCGGAGCGGCAAGCCGT
GAACCGGCTCACAAAACCGCGCAATGATCCTCTGGCCCGTGGTCATGGATGAATGCCGAAAACAGAGATT
TATTGGCGAAGATCTCTTCATCTTCCAGCGCAATCGCCGCCAGTTACAGTATTGGCTTGCCACTGTCGTT
CAACGCCGACAGTGGCAGACCGGATTGGCTCACTCTATAGTCGGGATGCGATTTTTTGCATCGCAAATGA
AGTAACCGAAGGGCAAACCGATCGCAAGGTCGGGACGCAAAGCTTCCGGTCCAGGGATGAGCCGTACTTG
AGATAGCGGGGCCACCACAGGTTGTTGTGCACCTTTGCCAAGAGGCCGCACTGCCGTCAGCGTTACCTGT
CAGGGCGTAGCCCTTTTCGTTGCCAACATAACGAAATAGGGAAATATAAAAAATGCTAAGTCCAAAGCCT
ACCATGCTGGCCCTTGCGGTCGGTCTGTGCTGCGCCTCGTCCGCTTATGCTGCGGCGCCCGGCAAGCCGA
CCATCGGATGGGGCCCCACCAAGTTTTCCATCGTTGAAGTCGATCAGGCGGCTACTGCCTACAACAATCT
GGTCAAGGTAAAGGATGCCGCCGATGTGGAGGTGAACTGGAACCTCTGGAGCGGCGATCTGGGCCAGAGT
GCCAAGGTGCTGCTCAATGGTCAGGAGGTGTGGTCCGGCCCCTCGGCCGCGGCGGGCAGCGCCAAATTCA
AGGTGAAGAAAGGGGGGCGCTACCAGATGCAGGTGGCGCTGTGCAACGCCGATGGCTGTACCCTCTCTGA
TGCCAAGGAGATCGTGGTTGCCGATACCGACGGCAGCCATCTGGCGCCACTTAAGGCGCCGCTGCAGGAG
AACAACAAGCCCTACGCCAACAAGTCCGGCAAGGTGGTGGGGACTTACTATGTGGAGTGGGGGGTCTATG
GCCGCAAGTTTACGGTGGACAAGATCCCGGCCCAGAACCTGACCCATATCCTCTATGGTTTCACCCCCAT
CTGCGGCGGGGATGGCCTCAACGACAGCCTCAAGGAGATCGAAGGGAGCTTCCAGGCGCTGCAACGGGCG
TGTGCCGGCCGGGCGGACTTCAAGGTGGCGATGCACGATCCATGGGCCGCCATCCAGATGCCGCAAGGCA
ACCTCACCGCCCATGACGAGCCCTACAAGGGCAACTTTGGTGCCCTGATGGCGCTCAAGCAGGCCTATCC
GAACCTCAAGATCCTCCCCTCGGTCGGCGGTTGGACCCTCTCCGACCCCTTCTATTTCCTCGGTGACAAA
ACCAAGCGCGATGTGTTTGTCGGCTCGGTGAAAGAGTTCCTGCAGACCTGGAAGTTCTTCGACGGTGTGG
ATATCGACTGGGAGTACCCGGGCGGACAAGGCTCCAACCCGAAACTGGGCAGCCCGAGTGATGGTGCCAC
TTATGTGGCCCTGATGAAGGAGCTGCGTGCCATGCTCGACGAGCTGGAGGCCGAGACCGGTCGCCAGTAT
GAGCTCACCTCGGCCATTGGTGCGGGGGGCGACAAGATCCAGGATGTGGACTACAAGGCGGCCCAGCCCT
ACATGGACCACATCTTCATGATGACCTACGACTACAACGGCGCCTGGAGCAACACCGAGCTGGGTCACCA
GACCAACCTCTATCCGGCAAGCTGGGATCCCGCTACCAAGTACACCACCGACAAGGCGGTGAATCTGCTG
CTGGCGCAGGGGGTTGAACCGGGCAAGCTGGTGGTCGGGGCCGCCATGTATGGCCGTGGCTGGACCGGGG
TGAGCGGTTATCAGGGCAACAACCCCTTCACCGGCAAGGCGACCGGCAAGGTGAAAGGCTCCAGGGAGGA
CGGGGTGGTGGATTACCGCGACATCGTCAACAACCGGATGGGGGCTGGCTGGGAGAAGAGCTATGACGAG
GTGGCCGAGGCGCCCTATCTGTTCAAGGCGGCGACCGGCGACCTCATCACCTATGACAACGAGCGCTCGG
TCAAGGCCAAGGGGCAGTATGTGCTGGCCAACAAGCTCGGTGGCCTCTTCTCTTGGGAGATCGACGCCGA
CAACGGCGACATCCTCAATGCCATGCACGAGGGGTTGGGCCACGGCGAGGGGACGACTCCACCAGCCAAC
AAGCCGCCGCTGGCCAACGCGGGCAGTGACCTCAAGGTGACCGGCCCTACCGAGGTGACGCTGGATGGTT
CGGCCTCCCACGATCCGGAAAACAGCGCGCTCAGCTACAGCTGGAAGCAGATCTCCGGCCCGCAGGCTGC
CCTGCTTGATGCCACTCAGGCCAAGGCCCGTGTTGTACTCGATGCCGTGTCCGCCGACATCAATCTGGTG
TTCGAGCTGACGGTGACCGATGACCAGCAGTTGAGTGCCAAGGATCAGGTTGTTGTCACCAACAGGGCGC
CGCAGGCCAATCTGGCGCCTGTGGTGAGTGTACCTGCCACGGCCTCTGTGGAGGCGGGCAAGCAGGTGAC
CATCAAGGCGACCGCCTCCGATCCGAACGGTGATCCGTTGACTTATCTGTGGAGCGTGCCGGCAGGCATC

ACCGCAACGGGTCAGAACAGCGCCACTCTGGTGGGCACCGGCCCGAGCGTGATCAGCGACACCGGCTATG
ACCTGAGCGTCACTGTCTCCGACGGTGGGCTGGATGCCAGTGCGGTAACCCGTCTGACGGTCAAACCGGC
CAGCAGCGGCGGTGGTTGCACGGCGACCGATCCGGATGCGGCCAACTGGCCAGTCTGGAAAGCGGCCAGC
ACCTATAACAGCGGCGACAAGGTGAGCCACAACCAGCTGGTATGGCAGGCCAAGTACTGGACCCAGGGCA
ACGAGCCTAGCCGCACTGCGGATCAGTGGTTGCTGGTGAGCAAGGTGCAACTGGGTTGGGATGCCAATGT
GGCCTACAACGGTGGCGAGCTCACCACCCATAACGGCCGTCAGTGGAAGGCGCAGTACTGGACCAAGGGC
AATGAGCCCGGCAAACACGACGTCTGGCAGGATGTGGGAGCCGCCAGCTGTAAATAAGCATTGAATGGTG
GCGGGGCATGCCGTCATGCCCCGTTTTTTCTCCCCGCTTCGCCACATTCTGGCGGGTCTGCCAATCGGTT
TCCCCACCCAGCTTCTCTTTTGTGCCTGCAAAGCAGCCCTTCCGGGCTAGTGATTAATGCTCTCCCCGAT
AGCTGGCATCTGTGCCGCACGTTGCTCTTTGTGGTGCCGCTTGTGTTGGTACATGTCGGCATCGGCTCTG
GCAAACGCAGCGGCAAAGTCGCCGCGATAGGGGGCCATCCCCATGCTGCACGAGATAGTGGGGGAGGCGC
TGGCCAGCATGGCCGTCAGCCTGGCCATCGTCTGTTGCAGCTCGGTCGGTGTCAGAGTGGTGAGCAGCAC
AAACTCATCCCCGCCGAAGCGGGCGGCAATCATTCCCTGACCGAAGTGCTGGCGTATGCCATCTGCGATA
AGGCGCAGGGCCTGATCACCTGCCAAATGGCCACTGCTGTCATTGAGCTGCTTGAGATTATCCATGTCGA
TCAGCAGCAGGGTGCCTTCCGGCTGCCCCTGCAGAGCGAGTTCGAATTGCTGGCGATTGGCGAGCCCGGT
CAGCTGGTCGGTATTGGCCAGCTGATAGATGCGGCGGGACTGCTGATAGATGGTGATGGCGTGGCGAATA
TCCCGCGCCAGGATCGCCATCAGGTGGCTGTCGTAGCGGGTAAAAGCATTACGTTTGCTGCTGTCCATAT
TGATCATGCCGTAGAGCTGACCATCGATATGGATCGGGGTCGCCAGCGTGGCCTGGATGGGCAGGTCGGT
GCAGTTGACCAGCAGATCTCTGTCCTGTTCGGACAGGGGCATGGTGGCATTTATCTTCGACAGGTCGTTG
ATCAGCAAGGTTTTGTTGCATCGTCCCTGAGTCAGCTTGTACTGAAAGGTGTGCTCCAGCGGCAGATTGA
TCCGCCGCAGTTTCACCAAATCCATGCCGATGGCGGCTTCATATCTCAACATGCCTGTGCCCGGCAGCAG
CTTGATCAGGCAGCCCATCTCGGCTCCCGGGATCAACATCACGGCTCGTCTGAGCAGATCCTCCAGAAAG
GCCTCTTCGGTCTGGTATTGCGCCGAGTTGCGGGCCAGCTCGGCGGTGGTTTCGTTGATCTTGAGCGCCT
CACTCAGATGCTGCCACTGGCGGTGCAGATGGTAGCGGTAGTGGCGATAGAGCCAGAAGGTGCAGATAAG
GTAGCTCTGGACTAGCAAGGTGCTGGCAAGCGGCGCCCTTTGCCAGCCATTGAGGCTGTTGATAAGCCAG
ATCATCCCGGCCAGTGGCAAGGCATAAATCGTCAAACGAACAAATTTGTCGTATCTGGATAACCGGGTGC
GTGAGGGCAGTTCGCTGGGCATGAAGACACCTGCTGGCCATGCAAAGCGGCGATTATAAATTCATTAGTT
GCGATACAGATGGGTCTTTTGTCGCATATTGTTCTTACTCATTGATTTTTCTCAATTTGGTGTGATGCAG
ATCAAAAAAATAGTGTGAGGTATCTGGTTGGAGGTGATGAGATTAGGTCTGCATATCCGGCTGTTTTTTA
AACATTAATTTCTTGAAAGTAGGTAATTGTCGGGGTTGGTAACCGGTGGGCTGGCAATGGTCCGATTTGC
TTACTTATCAGAATTACCCTACAGTCAATCGGCGATTATTTATCGCCAGAAATAACGTTGTGACAAGGGC
AAATCGATTGAAAAGTCGGGACGCAAAGCTTCCGGTCTAAGGGTCAAGACGACCTAAGATAGCGGGGCCA
CCACAGTAGAATGCAGGCTGCCTTTGCCAAGGGGTGGCCGTTTTTGTGTCACCTGTCGTGTTGCGTTGCC
CTTTCGTTTTTCCCATAACGAAACGAAAAGGAAAGCAATATGCTAAGTCCAAAACCTACACTGCTGGCCA
TGATGGTCGGCACCCTGCTCGCCTCCTCCGCCGCTTATGCGGCAGCTCCCGGCAAACCCGCCATCGGTTG
GGGCCCCACCAAGTTTGCCATCGTCGAAGTCAGCCAGTCCGCGACCGCTTATAACGAGCTGGTCAAGGTG
AAGGATGGCGCCGATGTGACCGTCACCTGGAATCTCTGGAACGGCGATATCGGCCAGAGTGCCAAGGTGC
TGCTGGATGGCAAGGAGGTGTGGTCCGGTCCCTCTTCCGCCGCTGGCACAGCCACCTTCAAGGTGCACAA
GGGGGGCCGCTATCAGATGCAGGTCGCGCTGTGCAACGGCGATGGCTGTACCCTGTCTGATGCCAAGGAG
ATCGTGGTCGCCGATACCGATGGCAGCCACCTTGCGCCGCTCAAGCCTGCCTTGCAGGAGAATAACAAAC
CCTATGTCAACAAGTCCGGCAAGGTGGTCGGTACCTACTATGTGGAGTGGGGGGTCTATGATCGCAACTT
CACCGTGGACAAGCTGCCGGCCCAAAACCTGACCCATATCCTCTATGGCTTCACCCCGATCTGTGGCGGT
GATGGCCTCAACGACAGCCTCAAAGAGATTGCCGGCAGCTTCCAGGCGCTGCAACGTGCCTGTGCCGGGC
GCAAGGATTTCCAGGTCGCCATTCACGATCCCTGGGCCGCGCTGCAAAAAGGGCAGGGGTCACTGGTCGC
CTGGGATGAGCCCTACAAGGGCAACTTCGGCAATCTGATGGCCCTCAAGCAGGCCTATCCGGACCTGAAG
ATCCTCCCCTCAGTCGGTGGCTGGACCCTCTCCGACCCCTTCTTCTTCATGCATGACAAGGCCAAGCGTG
ACGTGTTCGTCGCCTCGGTCAAAGAGTTCCTGCAGACCTGGAAGTTCTTCGACGGTGTCGATATCGACTA
TGAGTTCCCGGGCGGCGACGGTGCCAACCCCAATCTGGGGGACAAGACCAAAGATGGTGAAACCTATGTG
CTGATCATGAAGGATCTGCGCGCCATGCTCGACGAGCTGGAGGCCGAGACCGGTCGCACCTATCAGCTCA
CCTCCGCCATCGGTGCCGGTTACGACAAGATTGAGGATGTGGATTACAAGGCTGCCCAGCAGTACATGGA
CCACCTCTTCGTGATGAGCTACGACTACAACGGTGCCTGGAGCAATACCTCCCTGGGTCATCAGGCCAAC
CTCTATCAGGCCACCAACGGCTCGGCCAACAAGCGCTACAACACTGATGCAGCGGTACAGCAACTGCTGG
CGCAAGGGGTCGACTCCGAGAAGCTGGTGATCGGTGCCGCCGCCTACGGTCGCGGCTGGAAAGGGGTGAG
CGGATATCAGGGCAACGATCCCTTCACTGGCACCGCCACCGGCAAGATCAAGGGCACCTGGGAAGATGGC
GTGCTGGATTATCGCCACATCGTCAACAGCCATATGGGCGCAGGCTGGGAGTACAACTACGACGAGACTG
CCGAAGCGGCCACCCTGTTCAAGCCCTCCACCGGCGAGCTGATCACCTATGACGACGAGCGCTCGGTCAA
GGCGAAAGGCCAATACGTGCTGGCCAACAAGCTGGGCGGGCTCTTCTCCTGGGAGATCGATGCCGACAAC
GGTGACATCCTCAACGCCATGCATGAAGGTCTCGGCCACGGCGAAGGGGTCACTCCGCCAGCCAACAAGC
CGCCGGTCGCCAACGCGGGCAGCGATGTGGCGGTGACCGGCCCGGCTACCCTGACGCTGGATGGCTCAGC
CTCCCACGATCCGGAAAAAGGCTCTCTGACCTACAGCTGGAAGCAGGTTTCCGGTCCGCAAGCCGTGCTG
GGTGATACCACGCAGGCCAAGGTGCGGGTCGATGTGAATGCCGTGAGCGCAGATACCGCGCTGGTGTTCG

AGCTGACGGTGACCGATGACCATCAACTGACCGCCAAGGATCAGGTAGTGGTGACCAACAAGGCGCCGCA
ACCCAACCTACCACCAGTGGTCACTCTGCCCGCCAGCGTCAAGGTGGAAGAGGGCAAGCAAGTTACCATC
ACCGCCAAGGCATCCGACCCCAACGACGACGAGCTGATCTATCAATGGCAAGTGCCTGCCGGTATCACGG
CGACCGGCCAGAGCAGCGCCACCCTGGTGGTCACCGGCCCGGCGGTGGATACCGAAACCGCTTATGACCT
CTCCGTGACCGTCTCCGATGGCGCACTCGATGCCAACGCGGCCACCCGTCTCACCGTCACGCCAATCAAC
GAAGGTGGTGAGGGTGGCTCTTGCAACACCACAGATCCGGAAGCAGCCAACGTTCCCGCATGGCAGGCCA
GCAAGGTCTACAACGGCGGTGACAAGGTGAGCCATAACCAGCTGGTATGGCGTGCCAAGTACTGGACTCA
GGGTAATGAGCCGAGCCGCGCCGCCGATCAGTGGGCGCTGGTGAGTCAGGTTGATCTGGGCTGGAACGCA
GCCGTTGCCTACAACGGCGGGGATCTCACCAACCACAACGGCCGTCAGTGGAAGGCCAAGTGGTGGACTC
AAGGCAATGAGCCGGGCAAGCACGATGTCTGGCTGGATATCGGGGCAGCAAGCTGTAACTGAGGCCTCCT
CACCGATGAAGGGGCATGGCGACATGCCCCTCATTCCCGATAGCAATATGAACGAGAACCCGACAATGAC
CACAAATGTGACCCGAATCGCTGCCCTCATCATGGCAACCCTGGCGGCTGGCCCCCTGTCTGCCGCCACC
TCGCCCCTGATATTCAATGACACGACACCGCAAAGCGATCTGACCGGCAGTCTGGCTGCCAGCGTGCAAG
TTTGCCCAGAGCCAGATCCTGCCGTCTCATCCCAAGGAGGGGGATCGCCAACCCATCCTGACCAGCCTGC
GCAAGAGCCTGCTGCTGGTGAAACCACTCCAGGCCGACGGGGTGACCCCCATGACGGTGGAGGCCCGTGA
CGGCAGCGGCAAGCTGCTGGGCACCCTGACCTTGTCGCCGCCATCGGCGCTGCCAGAGACTGTCTACCAC
CTCGCGGGGGCTCCAGAAGGGGGAGTGAGTTTCATCCCTGAGAGCGGCACCACTGCCGTCATCAGCAGCA
GTGCTGATTTGGCCAAACTGAGCGACAAGAGCGGCGCCTTCCTCAAGGATCGGCTGACCGGGCGTGCGCT
GGTGGAGATCCAGACCGCCGACGGCCGCTGGGTTCGGGATATTTACCTGCCCGTGAGCCCGGAGCTGGAG
GGCAAGATGGTGCGGGTCCGCTCCAGCGCCGGTTATAACTCGACGGCCTTCTATGGCGAGCGACAGGTGA
CCGTCTCCCGTGGCCAGACCCTGCAGTTCAAGTTTGCCAATGGGCAGTGGTTCCGCGAGGGGGAGCTGGA
GAACAACCGAATCACCTATGCAGCGGATACCTGGAGCGGCGAGCTGCCTGCCGAGTGGATCCAGCCCGGA
CTGAACCTCTCCGTTCGTCAGGGCAATCTCAGTGGTGGACTGCGTGACATCAAGGTCGGCGCCCCCGGCG
AGCTGCTGCTGCACACCATCGACATCGGCATGCTTACCCCGCCACGGGATCGCTTTGCTTTTGCCAATGA
CAAGGAGGCACATCGCGAATACTTCCAGACCATTCCGGCCAGCCGGTTGATTGTCAGTCAGTATGCACCG
CTCTCCCTGCCTGAGGTGATGCTGCCCAATGGCACCTTGCTGACCGATTTTGACCCGAGCGAAGGGGGCT
GGCATGGCGGCACCATGCGCCAGCGCATCGGCAAAGAGCTTATCTCCCACGGCATCGACAATGCCAACTA
CGGCATCAACAGCAGTGCCGGTGAAGGGGAGGGAAGCCATCCCTTTGTGGCGGCCCAGCTGACGGCGCAC
AACAGTCGCGGCAAGTATGCCAATGGAGTTCAGGTGCACGGAGGTTCCGGTGGCGGTGGCATCGTCACGC
TGGATGATTCGCTGGGCAACGAGTTCAGCCACGAGGTGGGCCATAACTTCGGCCTTGGCCACTATGTGGA
TGGTTTCCGCGGCTCGGTTCATCGCAGTGCCGATCAGCTCAACTCCAGCTGGGGCTGGGACAGTGACAAA
CACCGTTTCATCCCCAACTTCTCGCCGACCCACACCAATGAGGATGCCTGTCTGGATGGCCAGTGCCAGC
CACCGTTCGATGGCCGCAAGTTTGGCCACGATGCGATGGCGGGCGGCCGCCCTCTCTCCGGCGCCAACCG
TTTCACCCTCTATACCCCCAATTCGGCCGCCATCATCCAGCGCTTTCTGGAGAGCAAGGCGGTGTTCGAT
GCCAACTCGGCCACCGGCTTTAGCAAGTGGAACAGTGCGACGGCCAGGATGGAGCCCTATCAGCACACAA
TCGAGGGGATCGAGAAGGTCGATGCCCCCATGGATGCCCTGAGCGAGGCAGGACTGAGTGCCCTGCTGGC
GGATTACGGCTTGGTCAGGGTCGCCATGTGGGATGGTCGCTGGACCCGTGATATCCGGGTGCCGATTGCC
TCTGCGGACAACCGTGGGCGCAGTCTGACCATCGATCACGGGGCCGGTTACAACAGTCGCCTCTTTATCA
ACGGCAAGGAAATTGTGGTGAACCGGGGCTTCAAGAAGAGCTTCACCTCGGATGGCCAGAGCTGGGTGGA
GGTCTCCCCCATCGATACCAAGGTTGCGCGCAAGCCCGAGCAGTTCGGCGTGCCGGTGACCACCCTGGTG
GGCTATTACGATCCGCAAGGAGCCTTGCGCAGCTACATCTATCCGGCTCTGCACGGCGCCTACGGCTTCA
CCTATCCGGATGACAGCAACACCCTGTCCGGCAATGACTGCCAGTTGCAGGTGGAGACCCGCGATGGTCT
GCAGCGCTTCAGGCTGGCCAATCACAGAGCGGCCAGCAGCGTCATGAACAAGTTCCACATCAACCTGCCC
ACCGCCAGCGAGCCCGGCCGCGCCACCATCCAGTGCCGTGGCCAGACACTGGTACAGCAGAGCTTGCTGC
CACCGCAGCAGATCCTCTCCTTTACCGTCAACGGGCGGCCGCTGGAGCAGGGCGTGGTGGAAAACCAGCC
TCCGGTGGTGACCGTGCCGGCGCAGCTTGGCGGCATCGGCGGTGAGTGGCTGACCATCACTGCCGAGGCG
AGGGATCCCGAGGGCGATGCGCTGAGCTACCGCTGGCACTTCCCGGCGGGCTGGCAGGCGGAAGGCGAGA
CCAGTGCAAACCTGCGTTTGCTGCCGCCGGCAGTCACCACCAGCGAGCAACATGAGCTGAGTGTGAGCGT
CTCGGACGGGGTACATCAGAGCGAAGGGCGCGTCGTGCTGACGCTGGCGCCCGTGGTCGACGGCTCTTGC
AACACCACCGATCCGGAAGCGGCGAATGTTCCCGCATGGCAGGCCAGCAAGGTCTACAACGGTGGCGACA
AGGTGAGCCACAACCAGCTGGTATGGCGTGCCAAGTACTGGACTCAGGGCAATGAACCGAGCCGCGCCGC
CGATCAGTGGGCGCTGGCGAGTCAGGTTGAGCTGAGCTGGATCGCGACCGTTGCCTACAACGGCGGGGAG
CTGACCAACCACAACGGCCGTCAGTGGCAGGCCAAGTGGTGGACTCAAGGCAACGAGCCGGGCAAGCACG
ATGTCTGGCTGGATGTCGGGGCGGCAAGTTGCCCCTAGGTTGAGTCCATGATGAAGATGGCGGAGCCTGT
TCAGGCGGGCTCCGCCTTTTGCCACAAAACCATAGAGAGCAACAATAATGAAAAAAAACCGCATCACCCT
TCTGCTTGCCGGCATGATGCTGAGCGGCCACCTGTGGGCGGCCGAGGCATACGACATCAACAAATCCTAC
CCGGGCGGCAGCCAGGTCAATTACAACGGCCAGGTTTTCGAGGCCAAGTGGTGGGTCAACCCGGGTCAGA
CTCCCGGCATGGCGGCCGCCAATGAGTGGGATACCCCGTGGAAGCTTGTCGGCGAAGGGGAAGTGACCCC
GCCGGACACCGGTGGCGATGACAAGCCCAATCCGCCAACGCCTACCCCGCCGGATGGCAGCACCACCCCC
GTCTATATGAGCCAGGCCGAGCTGGATGCCAAAGAGAAGGCGCTGACCGACTTCCCGGCGATGGAAGCGG
TCAAGGCCTCCATCGCTACCCGGGACAATCTGGTAGTCGAGGCGGTGCAGCCGGGTCGTGCCGACAACCC

GGGCAACGTCAAGCGGGTCGAAAGCCTGCTGAGCGAGAGCCAGTTCAACTACCTCTTCCCGCTACGCGCC
CCCGAGTACACCTATCGTGGCCTGTTGCAGGTGGCGGCCAAGTTCCCGGCCCTGTGCAGCGATTACAGCG
ACGGCCGCGATGCTGACGCCATCTGCCGCAAGACTCTGGCGACCATGTTCGCTCACTTCGCTCAAGAGAC
CGGCGGCCATGAAAACTGGCGCCCGGAAGCCGAGTGGCGTCAGGCGCTGGTCTACGTGCGCGAGATGGGA
TGGAATGAGACCATGCGCGGCGGTTACAACGCCGAGTGCAGCCCGGATGTGTGGCAGGGTCAGCAGTGGC
CGTGCGGCACCTTCGATAATGGCGAGTTCAAGAGCTACTTTGGCCGCGGCGCCAAGCAACTCTCTTACAA
CTACAACTACGGCCCCTTTTCCGAGGCGATGTTCGGCACTGTGCGCACCCTGCTCGACCGCCCCGATCTG
GTGGCCGACACCTGGCTGAACCTCGCCAGTGCGGTGTTCTTCTATACCTATCCGCAGCCGCCCAAGCCCT
CCATGCTCCATGTCATCGATGGCACCTGGCAGCCAAACGAGCACGACAAACAGAACGGTCTGGTGCCGGG
CTTCGGGGTCACCACCCAGATCATCAACGGCGGGGTGGAGTGCGGTGGTCCGGTCGAGCATGCCCAGTCC
CAGAACCGCATCAACTACTACCGCGAGTTTGCCAACTACCTCGGAGTGGCGGTGCCATCCGACGAAGTGC
TGGGTTGCAAGAACATGAAGGTGTTTGACGACAATGGCGCCGGTGCCTTGCCCATCTATTGGGAGCAGGA
CTGGAGCTATGTGGCAGAGAACCCCAACGGCGGCAAGAGTTACTCGTGCAAGCTGGTGGGCTACCAGACC
CGTTTCAGCGCCTTCAAGGCAGGGGATTACAGCCGCTGCGTTCAGCACTTCTATCCGGAGGTGGTGATCG
AGGATAACGGCGGTGGCACCAACCTGCCACCGGTGGCCAATATCACCGGCCCGGCGCAGGCCGAAGGTGG
CAGCAAGGTGACCCTTTCCGGCCAGCACTCCAGCGATCCGGAAGGCAAGCCACTCTCTTATGCCTGGACT
ATCCCGGCTGGAGCCTCTGCCCCGGTACTGGATCAGGTAACGCTGGAGTTGACCCTGCCCAAGCCGGCCA
GCGATACCAAACTGGAGATCAAACTGACGGTGACCGATGAAGGGAGCTTGAGCCGTAGCGCCACCCATAG
CCTGCTGGTGAAAGGGGAGGAGGGGGGCGTAACGCCGCCTGACTATCCGGCCTATAAAGAGGGGACTCCC
TACAAGGCGGGCGAGCGGGTCAGCAATAGCGGTGCCAGCTACGAGTGCAAACCGCATCCCTACACCGGCT
GGTGTGCCGGTGCCGCCTGGGCCTATGAGCCGGGCAAGGGCACGGCCTGGGCCGATGCCTGGATCAAGCT
CTGAGCCGTTTCAGGGTTACAAGGCTTGCCGATTAGCCAGCGCACTATCCAGCGCAATGACAATCCGGAG
CAATAAAAGATGGGGCCTGCGGGCCCCATTCTTGTTTGGTGGAGTTCAGGTTTGGCCGTGGAACATCTGG
CCCGGACAGGACAGCGCCTCGAGAGGGCTGTGTGATGATGTGTGTTGTTTTTTTTATTACATTTTCCGTG
AAAGGCTTTCAGCAATTATTTGCCGTAGCGGAAGTAGTATTCACGGGTGGAGGGGGGCATAGGCATCTTTC
TCTACAACGCCGTTCTTGGTGGTTGCACCGCGCAGGTGGAACAGGACTGCCAGATTCTCAAACATGGTTT
ACCTCATCGCTCTGTCATTTATGGTTATGGTGTGTGACCTGGGTCACGGTTTGATCCTATGGTCATTTGT
GAGCCAGATCAACTTTCTGTGTTGTAAATTTACAAAAATCATGGAAAGGGTTTTCAAATGCAATGCCCTT
TATGGAAAGGCTTTCCCGAGGTGACGGGGGAGAAGCTGATTACCAGCCAGCATAGAGAGGTCTGAGAGCG
GGAGCGTTGCACGGGGAAAGAGGCTTGCTGCACGGCCTCTCCTGTATAGGTGCTTGAGCCGATTTGTGAC
ATAAAACAGAGAGATAGCACGCGTCTTGCTGGTAAAAACAGCGAACTGCTGCAATTTTGACTAAACGAAT
CAGTCGTGGTTATTTTAGGGTTGACAGCAAAGCGGGTGATGCCTATCATGCGCCTCCGTTGCCGGAGATA
ACTTCGTCAGCGCCGAGGTGAGTTGGGGTTATAGCTCAGCTGGGAGAGCGCTTGCATGGCATGCAAGAGG
TCCGCGGTTCGATCCCGCGTAGCTCCACCAAATTCTCCTCGAACAATCTGGAATCAAAGGGGTTATAGCT
CAGCTGGGAGAGCGCTTGCATGGCATGCAAGAGGTCCGCGGTTCGATCCCGCGTAGCTCCACCAGGTTCC
TGATTGGCATGAAGTACCCAGGGCAGTGCGGTGTGTATCACCCAAGCCGACCTTTGTGGGGTTATAGCTC
AGCTGGGAGAGCGCTTGCATGGCATGCAAGAGGTCCGCGGTTCGATCCCGCGTAGCTCCACCATTATTTA
GGATGAATGGCCGCCAGAACCATTCATTCAGTACCGAATTTGGGGTTATAGCTCAGCTGGGAGAGCGCTT
GCATGGCATGCAAGAGGTCCGCGGTTCGATCCCGCGTAGCTCCACCAAATTCAGAGTGACTGCCGGTCAT
TCACACAAATCGGGGTTATAGCTCAGCTGGGAGAGCGCTTGCATGGCATGCAAGAGGTCCGCGGTTCGAT
CCCGCGTAGCTCCACCAAATTCTTGTTACAAGAAGCAAAAAGGCCACCGCATCGCGGTGGCCTTTTTGTT
TTCTCCATCATTTATTTTCTCCATACGTCCGCGCCTTTCATTTGTAAATCCAATGACCTGAATGAAAAAG
CCCGCGAGAGTCGCGGGCTTTGCTTGCACTATTCAAATGCGCCTGTGGTTAACGGCGACGTCGCCAGGCC
AGTGGCAACAGGGCCAGCAGGCCGAGTCCCAGTGCACCACCGCCTGAGGAGGTGTCCGGTTTGGTGGCAT
CGCTCTCCAGTCTCAGATCGACGATGATGGGGTCATGGTCGGAGGCACTGAAGGGGTTGTCAGACTTGAG
CAGGGAGCCGGTGTACTGGCTGCCGTATTCGAACAGGGAGCTTTCGAAGGAGTTGATGTGCCAGTCGGCG
ACACCAGCCACCTTGCTGGCCAGCGCCGGGTTGGCGAGGGCATAGTCCAGGGTGCCCAGCTCCCCGTCGT
AGCTGTAGGAGATGGCTTTCTCCTTGTTGAATTTGACGTTGAGATCGATCAGGCCGTAGCTGCGAGTCAC
TTCGCGACCGAGCTGTTCATAGGACTGCTCACCGATAAAGGTGTGAGAGGCCGATACGATCTTCCGCTCG
CTGGTGGCCGGGTTGTAGTCGGTCAGCACCCGGATCGGATCTTCCTTGGCGTAGGAGTTGAAGTCGCCCA
CCAGCAGCACCTGGCCCGGCAGTTTGCTGACCGCCTCACCCAGCACCTTGGCGGCAGAGACCCGGAACTC
GGTGCACTTGCCCTGCAGGTCGGCCGGTTCTGTCTTGCCATCGCCATTTTCGTAGCAGCCTGACCCCTTG
GATTTGAGATGGTTGACCACCAGCGTCAGCGGCTCCTTGCTCTTGGCGAGGGTGAAGGTCTGAACCAGCG
AGTCGCGCTGACCGGCTGTCTTGGCCACACCACCAGTGGTGTAGCGCTGCTCCGGCAGCTTGATGACGCT
GGCATCTCCGCTGGGGGTGAGCAGGGCAGGACGGTAGAGCATCGCCACCATGATAGCGTCACCGCCGAAG
AACTTGCCATCGGTAAGGGCAGTGACCGGCAGACGCACATAGGCGTAGTGTTTGCTCGCATCCTTCTGCT
GTTCGTTGAGGCGGGGCACCAGATTGTGGAGGGCCGAGTTATCCTCGAAGCCGTTGTTCTCCATCTCCAT
CAGGCCAAGCAGATCGGCATCCATTGCGGTGATGGCATTGACGATCTTGGTGCGCTGCTTCTCGAACTCG
GGTTGGCTCTTGGCACCCCGGTTGCAACCTTTGGCGTTGTCGGCATCCGCCTGATCCTTGCACAGGATAT
TGAGTGCGCCGCCAATGCTGGAGTGGCTGGTGAAGTAGTTGAGCACGTTGAAGCTGCCGATCCGCAGATC
GCTGCCAGCGAGGCGAGCCGGCGCGTCTTGACGATCCTCTTCAGGGCTGCGCAGCAGGGCGCCGGCAGTC

AGGGTGACATCGCTCGGTACCACCAGACGGAAGGCATCCTTGTTGTAGACGATCACTCCCTCCAATCCAT
TTAGCTGATCACCAATGCGCAGATATCCCTGATCGGCATCCCAGCTTGGGAACCAGGGCAGTACGCCATC
CGGTGCTTTGTAATCAGACTCAACTACCAGACGGTTGCTGCCATTTTTGGTGGCCAGCGCGACAGCGTCC
GCACTGTTGGCAACATGAAGTTGGGTCGGTTTGATCAGCGGGGTGTTGTGGGCCAACTCTACGTTGTTGC
GATAGACCTTGTAGTCGAAGCCGTAGTTGCGGGTCAGGTGCAGGTCACTGCTGCTGTCGAGACGTACCCG
CATCCCTTCGTGGCGATTGAGTGCCTGCGCCAGCGTCTCCCCTTCATTGACCTGCAGTACGCTGGTCGGC
ACATTCAGCCCTTGTGCGGTGACTTGCAGGCGGGCTTTGCTGGATTCGAAGGTGGGCTTGAGCTGGGTGC
CGCCGTAATACTCCTCGACCTTGGCTTCGAGGCAGAGTTCAGACCCCGGTTTGATGTCTTTATATTTGGT
CGCGAAGTCCTTGTCGTTCAGATAGACAAAGATGCCATCAGAGGTGGCCGGATTGCCATCGCCCACCGGG
TCGGTCAGATAGAACCCCTTGGTCAGGCTTTGGCCCAGCGTCGTCACGATACCGCGAACGTTGAAGGTGC
CCGGGCTGATTTTGCCGGTCACGTTACCGTTCTTGTCGGTCACGGTCTGGGTAGCCAGCGCGCTGGCGGA
GCCTGTACCCTGAATGGTCGGGATGGGGGTGAGTTGAGCCGGATCGGACGGGCAGACAAATGCCGCATGG
GCGCCTGAGGATGCAAGTGCCAGCCCCACGGCCAGCGACAGCAGGGTACGAGTACCTTTCATAGGTGTTC
CTTTTTTATTTTTGCCAGTGATTCCATAGCCTTGGCTCGTTGACCAAGGGCGCGCATTGTCACATTTCTG
GCATGTCATACCAGTGAGCCAACGCACATTAAATGAATCTGGGAAGGTGCGGGAAAGCGTTTTTATGATC
CCAATAACGATATGATTTTTTTTGATAATTCAATCAATTTTAACCCTGTGATCATGACGCTTTCGTGGCA
GGGGATAATTCTCTTTGAGAATAAAGATTGAGGGGATCTTGTCGCCTTCCTATACTGGGTTCGTTCCTCA
CGGAACCCAGGTAAAGGGCAAACCGGCAGTGATGCCGGGGCGCAATGCTTCCGGTCTCGATCGTGGATCC
AGATAGCGGGGTTTCCCTGGGTGAGGGGTGGTTTTTGTGGCAGGTCCATTGCCAGTTGTCTGCGCCCATC
ATGCAGGTTGCGATTGTTCTTTGCCTTGGTGACCAAGCCAAGACAAAACAAGGAAGATAACAATATGCAA
CAGCTCTTCAGACCCAGCAGGCTTGCCATGCTGGTCGCCCTTGCGGCTGCGCCCGCCTGGGCTCAGGCCG
CTTATCCCGCCTATAAATCCGGTACTGCCTACAAGGCTGGCGATATCGTCAGCAACGTCGGTGCCCTCTA
TGAATGCAAAGAATTTCCCTATTCAGGCTGGTGCGGGGCATCCCCCTATCACTATGAGCCGGGGGTAGGT
GTAGTCTGGGCAGATGCCTGGAAGCCCTATGGTGGCGAGGTGCCCCCTCCTGCACTCGGCGTAGCCGTCA
GCTCGCCAGCTGCCGGTTCCAGCTTCAACGAAGGAGCCAGCGTGGCGCTGGCAGTGACCCTGAGTGGCCC
GGTAACGGCGTTGGCCAAGGTGGAATATCTTGTCGACGGCAAGCTGGTCGCCACGGCGAGCGCCTCGCCC
TGGAGTGCCAGCTGGAGCGCCGCCGGAGTGGGGCCCCATACCCTCAAGGCTCGCGCGCTGGATAAAGATG
GCAAGGTGCTGAGTGAGGCGGATTCGAGCTTCAACGTCGCCGCCGTGGTGGCGCCAGAGGCGCCCAAGGT
GAGCATTAGCAGCCCGGCCAGCGGCGCCAAAGTGACGCTGGGTCGCAGTGTGGCGGTGAGTGGCAACGTC
ACCGATGCCAACAACGATGTGGCCAAAGTCGAGCTCTATGTCGACGGCAAGAAGGTGGGCGAAGATACCA
CTGCCCCCTGGCAGGTGAACTGGACGCCGGATGCCAAAGGGAGTGCCGCCCTCAAGCTGGTGGCCACTGA
CAAGACCAATCTGAGCGGCGAGTCGGCGGCGGTGACCGTCAATGTCGAGGAGGCCGCGCTGCCGCCCACC
GGTGGCAACCTCTCTTGTGACATTCGCCAGATCTATCGTCCGGATGGTTACGAGTGCATGGGGGATGACC
ATGCCCGTCGGGTTATCGGCTACTTCACCTCCTGGCGCACCGGCAAGAACGGCCTGCCCGCCTATCTGGT
GAGCGATATTCCATGGAACAAGATCACCCACATCAACTATGCCTTCGCGGCGGTGGATGAGCAGAGCCAC
ACCATCAAGGTGGACGATGCAGCCACCAAGATGACCTGGGATGGCGTGCCGGGTGCCGAGATGGATCCTG
AATTCGCCTACAAGGGCCACTTCAACCTGCTGTCGAAGTACAAGAAGCAGTATCCGGACGTGAAGACCCT
CATCTCGGTCGGGGGCTGGGCCGATACCCGCGGTTTCTACACTGCCACTACCAAGGGTGACTGCTCGGTC
AATACTGCAGGTATCAACACCCTGGCTGACTCGGCGGTGAGCTTTATCCGCCAGTACGGTTTTGACGGGG
TCGATATCGACTACGAATACCCCACCTCGATGAAGGATGCGGGCAATCCCAACGACTTCCCGCTCTCCAA
CCAGTGCCGGGCCAAGCTGTTTGCCAACTACGAGGTGCTGATGAAGACCTTGCGGGCGCGGCTCGATAAC
GCCGGTAACGAAGATGGCCGCAAGTACATGCTGACCATCGCCTCACCGGCATCGGCCTATCTGCTGCGCG
GGATGGAGAACTTCCAGGTCACCCAGTACCTCGACTACGTCAACCTGATGACCTATGACTTCCACGGTGC
CTGGAACCACTTTGTCGGTCACAACGCGGCCCTGTTCGACAACAATGCCGACCCCGAGCTCTCCCACTGG
GGCGTCTATACCCAGACCCAGTTTGGCGGTATCGGTTACCTCAACGCGGCCTGGTCTGCCCACTACTTCC
GCGGTGCGCTGGCGGCTGGCAAGATCAACGTTGGCGTGCCCTATTACACCCGCGGTTGGCAGGGGGTGAG
CGGTGGCAGTCATGGGCTGGGTGGCAAAGCAGCCCTGCCGAGCCAGAACGAGTGCCAGCCGGGCACAGGT
GGCAGCACCATTCCGTGCGGCAACGGGGCGGTCGGCATCGACAACATCTGGCATGATCTCGACAAGAACG
GCAAAGAGATCGGCGGTGGCGCCGTGCCCATGTGGCATGCCAAAAACCTGGAGCATGCAGCCAGCCTCGG
CATCACCACATTGCCAAGTTATGGCACTGCCTGGGGTCTGGATCCCAACAATCCGGCTCACGTGATGCAG
GGCAAGTATGTGCGTTACTACGATGACAAGGCCTACGCGCCCTGGCTGTGGAATGAAGAGAAGAAGGTGT
TCCTCTCAACCGAAGATGAAGAGTCCATGGGCCACAAGCTGGACTACATCATCAAGCGCGGTCTGGGTGG
CGTGATGTTCTGGGAGATGGCGGGTGACTACGCCTTCGATCCGGCCAAGAACGAGTACGGCATGGGCAGC
ACCCTGACTACTCTGGCTTACGAGAAGTTTGCCAAGGCAACTCCGTACAACACCAAGCAAAACGATCTGG
CCGCGCCGAGTGCCCAGCTCGATATCCAGCTCAGCCTCTCCGGCTTCAAGGAAGGGGACTCCAACTATCC
GATCAACCCGAAACTCAAGCTGGTCAACAAGTCGACCCAGACCATTCCGGGCGGCGCGCGCATCGAGTTT
CTGGTGCCCACCTCCACCTCTGATACCATCACGGATCAGAGTGGCATGGGGTTGAAAGTGGTGGAGAGCG
GTGGTAACGACAACTCGGAAGGCATTGCCAACGAGAAGGACTTCCACAAGGTCGCCATGAACCTGCCGGG
CTGGCAGACTCTGGCTCCAGGGGCCGAGATTGAAGTCGCCATGACCTACTACCTGCCCGCCGCTGGCGTG
CCGAGCGGTATGCGCATCATCAGCGGCAGCCAGACCATTGGTCTCAAGGCCGAGTTCCCGACCCTGCCGG
AGGCCGTGCTGGGTTCGGGTGGCGGCGAGAATCCGGGCACCAGCTGCAGCAGCCAGAACGTCAATCCGGC

GGCCTACAAGGCCTATCCGAGCTGGCCGCAGGGGGATCATGCCAATGGCGGCGATCGCATTACCCATAAC
AAGGTAGTGTGGCAAGCAAACTGGTGGACCAGCAGCGAGCCCAAGGCAACCGATGGTAGCTGGAAGTCTG
TCTGTAGCTATTGATTTTAAAATCAATGAGATAAGTGAGGCTTCGGCCTCACTTTTTTTGTCTGGTTGGT
CGCTCTGGTTGAAAATGCGCCTGCCTTTCCTGGCGGGCAGCCTCTATAATAGAGTCGATTAATAATCAGA
TGGATATCAGCGTATGGCGAGTATATTGGATTCCGTTGATCAACGAACTCAACTGGTGGGTGAAAACCGC
CTCGAGTTGTTGATGTTTCGTCTCGGCACCCGTCAGATGTTCGCCATCAACGTCTTCAAGGTACGGGAAG
TGGTCAAGTTGCCCCATCTGAGCAAGATGCCGGGCTCCCATCACAATATCAGCGGGGTGGCCAATATCCG
CGGCACCTCGATTCCGGTGATCAACCTGCGCAGCGCCATCGGTATGCGCCCCATGCCCATCGACAGTGAA
TCCAACCTCATCATCACCGAGTACAACCGTACCATTCAGGGCTTTCTGGTGGGGCAGGTGGCCAACATCG
TCAACATGACCTGGAAGGATATCCTGCCGCCACCGCGCTCTGCCGGGCGTGCCAACTACCTCACCGCCAT
CACCCGCATTCCTGAAGATGGAGTGGATCAGATCGTCGAGATCATCGATGTGGAGAAGGTGCTGGCGGAG
ATCATCACCTATGACATCCGCATCTCCGAGGAGGTGCTGGATCGGGAGATCCTGCCCGAGATGCATGGCC
GCAAAGTGCTGATCGTCGATGACTCCAGCACCGCCCGCAATCTGGTGCGCGAGACCCTGGGCCAGCTTGG
GCTTGAGGTGATCGAGTGTCAGGATGGTTTGCAGGCGCTGACCCTGCTCAAGGGGTGGTGCGATGCTGGC
AAGAAGGTGACCGACGAGATCCTGCTGATGATCACCGATGCCGAAATGCCGGAGATGGACGGCTACCGCC
TGACCCACGAGGTGCGCAGCGATCCGCGTATGTCCGATCTCTTTATCACCCTCAACACCTCCCTGAGCGG
CAACTTCAACGAAGCCATGGTCAAGAAGGTTGGGTGCGACCGTTTTATCTCCAAATTCCAGCCCGATTTG
CTGGTCGGCGTGGCCCAGGAGCGGCTGCGTCAGGTGCTGGAAAGCTGAGAAGCTGCCTGCCGGTTATGCT
AAATCAATAACAACACATCCCGCTTCGGCGGGATGTGTTGTTATTGGGGATTGGCGATGGATTGGTTGAA
TCAAGGTGCCACGCAGAGATCGTCGACCATTCAAAACATCCAAGTTTGGCGGGATATTTTGTTATGGGAT
ATTGGCGATAGACGGCGGGCTTATTGGCTGAGCAGCGGCTCCATAAAGCGGGCAACCTTATCGCGGATTT
GGGGCTGGGCCGCCGGGGTGGGATGAATACCATCGTTCATCATAAGTTCAGGGCGCAGCGCAATATCATC
CATAAAGAAGGGCAGCAGGGGCAGATCGTTAGCCTGGGCCAACTCGGGAAAGAACTGCTCGAACTGGCGC
AGATAGCGGGCACCGTAGTTGCGGGGCAGCTGGATTTGGGTCAACACCACACGGGTCTGCTGCTCCTTGG
CGAGCGCGATCATGCTGGCCAGATTCTGGCGGGTAATGGTCGGTGCGAAGCCGCGCAGGCCGTCGTTGCC
TCCCAGTTCGATCAGCAGCCAGTCCGGCTTGTGCTCCTTCAGCAGAGCGGGCAGACGGGCCAGCGCTCCT
TGGGTCGTTTCACCGCTGATGCTGGCATTGAGCAGGGTGTGTTTGCCCCCCTCCTCCTGCCATTTCTGGT
TGAGCAGGGCGGGCCAGCTCTGCTCAACCTGCATCTGATAGCCGGCGCTCAGGCTATCTCCCAGTACCAG
CAGGGTCTGGGCCTGAGCCGACGAGAGCAGGCTGCCGAGGCCAACGAACAAGGCAAACAGAATACGCACC
ATCAATTCAACTCCCGCTATGGATTGTCATCAAGGGATTGGAACAAAGGATACAGAGCATGAGTTCAACC
CCCATTATTGTCGTCAAGGGCCTCGGCAAATCGGTTCGCCTCGGCCAGGAAAGCCTCACCATCCTTGAAG
GGATCGATCTGCAAGTCAATAGCGGCGAGACGGTGGCTCTGGTCGGTGCCTCCGGCTCCGGCAAGTCCAC
CCTGCTGGGATTGCTGGCCGGGCTCGATCTGCCGAGTCAGGGGGAGATCGAGATCCTCGGCAAATCCTTG
GGCGAGCTGGATGAGGAGGGGCGGGCCCGGTTGCGCGCCGAACAGATCGGCTTTGTGTTCCAGTCGTTCC
TGCTGCTCCCGACCATGACGGCGCTGGAGAATGTGATGCTGCCCGCCGAGTTGCGCGGCGAGCGGGATTG
CGAGCCAAGAGCCCGTGAACTGCTGGCGGCGGTCGGGCTTGGCGAGCGGTTGCACCATCTGCCGCCACGG
CTTTCCGGCGGTGAGCAGCAGCGGGTGGCCATTGCTCGCGCCTTTATGACCCGTCCCAGCCTGCTGCTGG
CAGACGAGCCGACCGGCAACCTCGACAGCAAGACCGGCGAGAAGGTGATCGAGCTGCTGTTTGAACTCAA
TCGCAAGCACGGCACCACGCTGGTGGTGGTAACCCATGACCATCAGCTGGCCGAACGCTGCCAGCGGCAA
GTGGTCATGACGGCGGGTCGGCTGGAGCGCGAGGTGCCCCATGATGTGGTGTAACGGGGTGAGCTGCGGG
TCAGGTGGGAGATGCAAGGGATGATGCTGTGGCGATTGGGATGGCGACTGCTGCGCCGGGAAGGTTTCTG
CGGCGAGCTGCGCTGGTTTGTGCTGGCGCTGGCCCTGAGCGTGGCGTGCGTCTTGAGTGTTGCATTGGTA
GCGGATCGGCTCGATGCCGGACTCAAGGCGTCGGGCCGCGACTTTATCGGGGCGGATCGGGTGCTGCGCA
CTGCCGCTGCTCTGCCGGATGGTTGGCTGGAACGAGCTAAACAGGATGGCTTGGCGGTGCAGACCACCGT
CAGCTTCAACAGCATGCTGTTTCACGGTGATGCGTTGCAGCTTGCCTCCATTCGGGCGGTGCCCGATGGT
TTTCCCTTCTATGGCAAACTCACTCTGGAACCTGCCCGTGCCCCTGCTCCCGGCGAGATCTGGCTCTCCG
CCCGGGTGATGCAGCTGCTCAAGGCCAAAACGGGGGATGAACTGGAGGTAGGCAATACGGTGCTCAAGGT
GGCAGGCCTGCTTGGCGAGGAGCCGGATCAGGGCTTCAGCCCCTTCCTGCTGGCGCCGCGGGCGCTGATC
CACAGTGCCGATATCGAGGCGACCGGCGCCCTGCTTCCCGGCAGCCGCCAGCAGTGGCGCTATCTGTTCA
AGGGGCCGCGGGAATCGCTGGCCCGTTACGAGCAATGGTTGTTGCCGCAATTGCAAGCCGGACAGAAGTG
GATCAAACCGGATGACAGCGAATCCCAAGTGGGTCGCTCGCTGGCCAATGCCGAGCGTTTCTTCCGGCTT
GCCTCGCTAAGCGGGGTGTTGCTGGGGGCGTTGGCGATGGGGATTGCGGTGCGCCACTTTGCCGAGCGCC
AGACCAATATGGTGGCGCTGCTCAAAACCCTGGGGGCATCCCGTCGCTCGCTCTGGCAACTGATGGGAAC
CCTGCTGCTGTCGCTGACGCTGATCGGTGCGCTGCTGGGTCTGGTAGCGGGCACCGTCATGCAGTGGATC
ACCTTGCAACTGCTGGGGGACCTGCTGCCGCCGGATCTGCCGCCCCCCTCCTGGCGTCCCTTTGCCCTAG
GCGTGGCGGTGGCGCTCTTTATCACTCTGCTTCTCGCTTTTGTGCCTTTCTTGCGGCTGCTGAAAGTGCC
GCCGCTGCGGGTATTGCGCCGGGAGCTGGAGGCGGGTATTCCCCCCTGGCTGGCCCTGCCGGTCGGCCTG
TTCGGGCTGTTTGCGCTGGTCTGGGGCTTTACCGCCGATGTGCGGCTGGCGCTGGGTCTGGTGGGTGGCA
TGGGCTTGCTGATGGGGCTGTTGGCCCTGCTGGCAGCCCTGATGTTGCGGCTGGGGGCAAGAGTGAGCGG
GCCTCATGCCTTGCGGCTGGCTCTGGCCCACCTGTCGCGCGAGCACAGCAGCGGCCTGTTTCAGCTGGCG
GGCTTTGCGCTGGCATTGATGCTCTTTGGCTTGCTCTGGGCGGCCCGGGTCGATCTGCTGGATGAGTTTT

CGGCCCGTCTTCCCGTCGATGCGCCCAACCGTTTTCTGGTCAATGTGGCCGAGAACGAGCGGGAGGGGAT
CCTGAGCGAACTGGCGGCAGCCGGGGCCACCACTTCCGATTTCTATCCCATGGTGCGTGGCAGGCTGACC
CATATCGCCGGTGAGGCGGTGGGCGAGGGGGCCAGCAGTGGCCGCGAGGGGGTCGATCGGGAGCTCAACT
TCACTACCACGGTAACCCTGCCAATCGACAACAAGCTGGTGGCCGGGAAGTGGCTTGCCGGACCCGGCGA
GGTGTCGGTGGATAGCGAGCTGGCCAAGCGGCTCGATATCAAGCTGGGGGACATGCTCGCCTTTACCATC
GAGGGGCGCAGCTTTGGCGCCAGGGTCACCAGTCTGCGCAGCATCAAGTGGGACAATATGCGGCCCAACT
TCTACATGATCTTCTCCGAGGATCTGTTGGCACCCTTCTCCCGAACCTGGCTTGGCAGCTACCGGCTGCC
CGAGGCGGGGCGCTCTGCCGAGGTGGAGTTGATCCGCCGTCACCCCACGGTGAGCCTTATCGATGTGGAT
GACCTGATTGCGCGGCTTACTCAGGTATCGGAGCAGGTGAGCCGGGCGCTGGCGCTGATGCTGGGGCTGG
TGACAGTGGCCGCACTGCTGGTGCTGCTGACCCAGACCCAGGCCAGCATGGCCCAGCGTCGGCGAGAACT
GCTGCTTATGCGCACCCTGGGTGCCAGCAGAGAACTGCTGAAAAAGATGCTGCGTTGGGAGCTGGTGGCC
AGTGGTGCGCTGGCGGGGCTCTGTGCCGCCATGGTGGTGGAGCTCTGCTCTTTTGGCCTGCAGTGGTGGT
GGTTCGAGGGGCTGTGGCAGTTCCACTGGGCCATCTGGCTCGGCCTGCCGACGCTGGGGGCGCTGCTGGT
GACGCTGGCGGGGCAGGGAATGCAACGTCAGCTGCTGGCTGGCACCCTGAGCGATCGGTTGCGGGGTCTG
GGTCAGGGACTGTTGTAATCCCGACAGCGCGAGCCGATGCACACAAGGTGCAATAGCGCAGCCACAATAA
ACAAGCAGCCCGGCCATTGGCCGGGCTTCTTGTTATGTGAGGTGCTGACGAGCGGACGCCGCGAGCGGGT
GTGAGTTAGCGGGGCTCGCGCAGCAGATTGGTGAGGGCACTCGGCAGCTCGGGATAGCTGAAGTGAAAAC
CGGCCTGTTGCAGCCGTGCTGGTATCACCTTTTGTCCGGTCAGCAGCAGATCGGCCGCTTCACCCATCAC
CAGTTGCAGCAGGGGGGGCGGGCACGAAAAAGAGGTGCGGGCGGTGCAACGTGGTGGCAAGGGTCTGGCTG
AACTCCCGGTTGCTGACCGGATGGGGTGCAGTGCCGTTGAAGATGCCGTTGCATTCTTCGTGCTCCAGCA
GGAAGAGCATGGCGCGCACCAGATCCTGCACATGGATCCAGCTCATTATCTGGCTGCCTGACCCCATAGG
GCCGCCGAGCCCGAGTCGGTAGGGAGGGAGCATTTTGGGCAGGGCGCCGCCATCCATGCCAAGCACCAGC
CCGATCCGCACGATACAGACTCGGGTTTGGCGGCTCTGGGCTTCCTGCGCCAGGGTCTCCCACTGCTGGC
AGAGCTGGTGGGTAAACTCGTCGTTGGGAGAGCGGCACTGCTCGTCGAGGGTTTCGGTGCCCTGTCTGCC
ATACCAGCCGATGGCGGAGGCGTTGATCAGCACCTTGGGCGGCGTGCTGGATAGCTTGATGAGATCTACC
AGTTGCTCGGTCAGTAGCCAGCGGCTATCGCAGAGCAGCTGTTTGCGCGACTCGCTCCAGCGTCCGGCGG
CGATAGGTTCACCGGCCAGATTGATTACCGCATCCACGTCATTGAGGTCATCAAGCCGGTCGAGGTTATC
CAATACCCTGATATCGTGGCCGAGCAGGGAGTAAGCGCGGCTCCCCTGCCTGCTCAATACCACGACCTCG
TGATTGACCTTGAGATGGGCAACCAGCTTGCGGCCGATAAAGCCGGTTCCTCCGGTAATAAGAATCTTCA
TCGCTGCACGCTCCTGCTGCCGTTTGCTCTACTCTAATGGCGTCGTCCTCCTGCAACAAGTGGACGAGCT
TGGCATGTGTTGTTCACTAGACTGGTTTTAACTCTATCTTTTTCTTACCAGATATTGTTCAACAATAGTT
TGAATTGATTGATTTTTGTTGGGTGGAGATTTGTTGATCAATCGCTATTATGGCGACCCTTTTCGCCTGT
CCACCGAGGCTGTCACCCATCATCCGAGCATCAAAGAGGCCGCCATGCCATTTCGTCGTTTGAAACTTCG
CCAGCTTTACTCGAGGATTCTGTTGGCGCTGCTGATGGGGGGCGGGATCCTGCTGCTGGGGGTTGTTGCC
GTTATCTGGCAGACGGTGACGGAGATAGAGCGTGATGCCGATTCACGGTTGCAGCATGCCCGGATGATGT
TTGATCGCTCCATCGGCCATGCCCGGCAGGCGGCCATCAATGTGGAGGGGATGCTAGGTCACTCGTGCAA
GGAGGCGGCACAACTGCTGCGCGAACAGGTGGCCACTGTGCCCGATGTCCGTTCCGCCAATCTGGCTGTC
GGCAGCCACATCTACTGCACCTCGCTCTATGGTGACTACGATGGCCCCTTCAATCCTGACAGTTATGTGG
ATGGCAGGCTGCAACTGCTGCCAGGCAATGAGGTCACACCGGATCGCCCCTTGATCGTGCTGCGTCATGA
AACGGACAAGGGGAGTGTGCTGATCGGTGTAGATGGCTACTACCTGCGCAATACCCTGGAGATCTCCAGC
AAGAACTCCCCGATGGCTCTCGTGGTGGGGCAGAGGGCTCTGTTTGGATCAGGTCAGGTCGGGGCGGCGC
CCTCTCCGACAGAAGAAGGTTATATTGCCCTCGCTTCGGCCGATTTCCCCTATCAGGTCGTCACCCGGGT
CACGGTGCATGACTACTGGAGCCACGCATGGCGCTACTCCAGATATACCTTGTTCCTCTCTCCGTTGCTG
GCGCTGCTGATTGGATTCGGCACCTTCAGGCTGATGGGGCGTAGCAGCTCGCCAGCGGAAGAGCTCAAGC
GGGCGTTGATCGAGGAGGAGTTTATCCCCTATCTGCAGCCGGTGGTCTCCGGTGAGGATGAGCAGTGGCG
TGGCTGTGAGGTGCTGATGCGCTGGCAGCATCCCAAGCAGGGGATGATCTCGCCGGATCGCTTTATTCCG
CTGGCGGAAGATAGCGGGTTGATCGTGCCCATGACCAGCCTGTTGATGGCGCAGGTGCGCGATCACTTCT
CCCCTTATGCCCGCAAGTTGCCGGCAAAGTTCCATTTTTGCTTCAATATCAGCGCCAAGCACTTTCAGGA
CCTTGACCTGTTGGCTGACTGCAAGGCGTTTCTTGACGCATTTAAAGACAATCCCGTTGATTTGGGGCTG
GAGCTGACCGAGCGTGAACTGCTGGTGGCTGATGAGACAACCGCTCAGATGTTTGCCGAACTGCGCAAAC
TGGGCGTCTTGATCTCTATCGATGACTTTGGCACCGGTCACTCCAGTCTCACCTATCTGCAACAGTTTCA
GGTCGATGCACTCAAGATTGACCGGAGCTTTGTGGAGATGATCGGCACGGATGCACTCTCCAGCCATATC
GTTGAAAACGTGATTGATCTGGCGAAGCGACTTGAATTGATCCTGATCGCGGAGGGGGTCGAGAAGCAGT
CGCAGGCGGACTACCTGCGGGCTAGGCACGTTGACTATCTGCAAGGGTATCGTTACAGCCGCCCCATTCC
GATGAAGCAGTTTTGCAAGACCCTGTTTGGCTGAACGGTTGAATGGGGAGCATAAAAAACCAGCGATCGC
TCGCTGGTTTTTTGTTAATACGGTTTGGCTTGGGCGTTCAGAGCATGCGCTGGAGGGCGCGGGCCAAGCG
TTTGACCCCCTCCTCGATGGTCGCTTCATCGACGCAGGAGAAGCTCAGGCGCAGGGTGTTCTTGATCTCG
GGGCGCACGTAGAAGGGGGCGCCGGGTACGAAAGCCACCTTCTCCTTGATCGCCTCGTCAAACAGCGCCA
TGGCACTGATGTGGGCTGGCAGGGTCAGCCAGAGGAACATGCCCCCTTCGGGTCTGGTGTAGCTGACGCC
GGGCGGGCAGTGACGGGCCAGCGCGGCCTCCATGGCGGCGCGCTGGCGGCCATAGACCTCCTTGATCTTC
TCCAAGTGAGCGTCGAGGGAGTTGTCGGTCAGGAAGCGGTGCAGCACCTGCTGGCTGAAAGCATTGCTGT

GCAGATCGGTCGCCTGCTTGGCGATGGTCACCTTCTTGCGCAGCCAGTCCGGCATCACCATCCAGCCCAG
ACGGAAGGCGGGCACCACGGTTTTGGAAAACGAGCCGAGCAGCACGGTATTGTTTGGCGCCAGCTTGGCA
ATCGGGGGGAGATGCTCCCCTTCGAAGCGCAGCTCGCCGTAGGGATCATCTTCCACCATCAACAGCTCGT
GGCGCACCAGTCGCTCGGCCAGTGCCTCGCGGTTAGCACGGCTGTAGGAGACACCGCTCGGGTTCTGGAA
GTTGGGCACGCCGTAGAGCAGCTTGGCATTGGACCCGCTTGCCAGCAGCGCATCCAGCGCCGCCAGATCC
AGACCCTCTTCTTCAAGGGTGATCGGCTTGAAGGTGGGCTGGTAGACAGAAAAAGCCTGAATGGCGCCAA
GGTAACCCGGCTCTTCGATGATGAGATCCTGACCCTCGTCGATCAGCACCTTGCCCAGCAGATCCAGCGC
CTGCTGGGAGCCGCTGGTGATGAGGATGTTGTCCGGATTGACCTGCATGCCGTGGCGGGCCAGATAGCGG
TCGGCAATATACTGGCGCAGCGGGGCGAAGCCTTCGGTCGCCGCATATTGCAGGGCGGCAGCCCCCTGTT
CGCGCAGCACATCCTGACAGGCCTGATCGATCGCCTTGACCGGAAACAGGTGGGGATTGGGCAGGCCACC
GGCGAAGGAGATGATCTCCGGATTGGCGGCAACTTTGAGGATTTCACGGATAAAGGAAGGTTCAACCTTC
TCGAAGCGCTGGGCAAAAAACGGCTGCATAGACAATTCTCTCGGCATGTGGGCGGTCGCTCCATTCGACC
GCCAGGTTATCCGTTTGTATCAGATTTCAGAAATGAGGCAATGATTGTCACAAAAATCGCCAAAAAAGGG
CTGGAACGACTTTACTGCGGATTATATTGGACGGTTAGTCGGTGTGGTGATGCCAGTGAGGGAGCTGGGG
AGCAGCTGGCTGGAATATTTGTTGCGCGAAACACTTGCGCCTTGGGCCTATCTTGGGATAATTCGCTCCC
CTGTTTTCGCGGGTCGATGAAATGACGAACCGCGAGCGGTCCCGCTCGGGATCGAACACAAGATGTATTA
CCCAGATTGATGAAATAACGAGACCATTATGCCTGTGACCCAAGATGTATTTAACGAAGACGGCAAGTTC
ATGCGCAAGATCCGCAGCTTTGTCCGTCGCGAAGGCCGCCTGACCAAAGGGCAGGAGAAGGCCCTGGAAG
AGCTCTGGCCGGTCATGGGCATCGATTTCGAGCCAAAGCAGCTCGATCTGGTCGCACTGTTTGGCCGCGA
GGCACCTGTGGTGCTGGAGATCGGTTTTGGCATGGGCGCCTCTCTGGTGGAGATGGCCAAGAATGCCCCC
GAGAAGAACTTCATCGGCATTGAAGTGCACTCCCCGGGCGTGGGCGCCTGTCTGGGTACCGCTCAGGAAG
CGGGCGTCACCAATCTGAAAGTGATTTGCCACGATGCGGTGGAAGTGCTGGAGCACATGATCCCGAACGG
TTCACTCTCCTGCCTGCAGCTCTTCTTCCCGGATCCCTGGCACAAGAGCCGTCACCACAAGCGCCGCATC
GTACAGCCTGCGTTTGCCCAGGATATTCGTCAGAAGCTGGCCATCGGTGGCGTGTTCCACATGGCGACCG
ACTGGGAAAACTATGCCGAGCACATGCTGGAAGTGATGAGCGCCGCCGAGGGTTACGAGAACACCTCTGC
CACCGGCAACTGGGTGCCTCGTCCGGACTGGCGTCCGCTGACCAAATTTGAACAACGTGGCCACCGTCTC
GGCCACGGGGTTTGGGATCTGATTTTCAAGCGTGTCAGCTAAGCTGGCACGTCAACACACCTAGCGTATC
AACTAACTTGAGGAGCTATCATGGCCAATCGTAGCCGCCGCCAGCGCAAGAAACTGCGCGTCGATGAATT
CCAGGAAATGGGTTTCGACATCAGTTTCAACTTCCCGGTCGGCACTGCCGAAGAGACCATCGATGCGGTA
GTCGATGCGATGATCGATGAGGTTGTTGAACCCCGTAAACTGGCTTTTGCCGGCTCCGGTCACCTGGCCT
GGGAAGGGATGATCTGCACCCAGCAACTGGGCAAGTGTACCGAAGAAGACCGCGCTGCCGTCAAATCCTG
GCTGGAAGGCAAGGGGATGGAAGCCGTGGTCGTTACCGAGCTGTTCGACCTCTGGTACGGTGAACCGGCC
TGATACAAGGCTGCCGCATCTGGCGCCTTGTTGTGGGTAACTGAAAAATCCTGCCGCTGGCGGGATTTTT
GTCAGTGGGGATGGGATCTCCACCGCTGTTTTAGTCATGGAATCGCTTATTGCTTATGGTGTTGTCATCC
GAATTACTGGCCAGCATCCTTGAAGAGGTGCGCCCGTTGCTGGGACAAGGCAAGGTGGCCGACTACATTC
CGGCACTGGCTCAGGTACCGGCCGATCGGCTGGGTATCGCAGTCTGTACCGTCGAGGGGGAGTTGTTCAC
CGCCGGTGACGCTTTTGAACCCTTCTCCATCCAGAGTATCTCCAAGGCCCTGAGCCTGACCCTGGCACTC
ACTCTCTATCAGGAGGAGGAGATCTGGGCCCGTGTTGGCAAGGAGCCCTCCGGCCAGCCCTTCAACTCGC
TGGTGCAGCTCGAGTTCGAGCAGGGGATCCCCCGCAACCCCTTTATCAATGCCGGGGCGCTGGTGGTGAG
CGATCTGCTGGAGACCCGCCTCACTGCGCCCCGTCAGCGTACCCTTGAGCTGGTGCGCCCGCCTTTCCGGC
AATCCGGCCATCATGGCGGATCAGGTCGTCGCCCGCTCCGAATATCAGCACTCTGCCCGCAACGCCGCCA
TCGCCTATCTGATGAAGGCCTATGGCAACTTCGAAAACGAAGTGGACAAGGTGCTGCAAAGCTACTTCAA
TGCCTGTGCCATCCGCATGAGCTGCGTCGATCTGGCGCGGGCATTCATTTATCTGGCCAACCGTGGTGTG
CCACTGGGCGAGAGCGTACCGCTGCTGCCCGCCCGCACCACCAAGCAGGTCAACGCCCTGCTCGCCACCT
GCGGTCTCTATGATGAGGCGGGCGATTTTGCCTATCGGGTCGGCATGCCGGGCAAATCGGGGGTCGGCGG
CGGTATCATCGCCCTGATCCCGGGCGAGCTCTGCGTCTGTGTCTGGTCGCCCGAGCTCAACAAAGCCGGT
AACTCGCTGGCGGGCACGGCGGCGCTGGAGCTGCTGGCCGAGCGGCTGGGTCGCTCCATTTTTTAACCTT
GTTCACTATGAATCGAGAAATCACCATGAAACGACATCTTCGCTTCCTGCAGGCCGCGCTGGCGACCTTA
CTGCTCTGGTGTGCCCTGCCCGCCGTGGCAGGGGAGTTGCCACCGCTCTCGCTGGCCGATGCCAAGGCCA
AACAGGCTGTCATCCTGGATACCCGCGCCAGCTACTTCTATCAGGGGTGGCCGATGGAAGGGGAGAAGCA
GGGCGGTCATGTGGCGGGTGCCGAGAACCTCTCTGCCGAGTGGAAATACAGCGATGAAGAGTGGCCAAAG
GCGCTCGAGATCAAGGGGCTGAAAGCAGATCGTCCGGTTGCCCTCTACGGTGCGTCCCGCGAAGTGGCCG
AGGTGGCCCGTCTGCTGCGCAAGCAGGGGATCAAGCAGCTGTTCGAGTTGCAAGGGTGGCAAGCCGCACC
GCGCGAGTCGTTGGCACGCTGGCAGCAGCTGGTCTATCCGCGCTGGTTGGCGGATCTGCAAGAGGGCAAG
CCGGTCGCCGCAGCGCCCAAGGGTGACTGGAAGTTGTTTGAAGTGGATTGGGGTTCACCCAAGGCCTACC
TCATCAGCCACATCCCGGGGGCCGGTTATATCGATACCAACAGCCTCGAAGAGGAGCCGCTGTGGAACAA
GGTCTCTGACGAAGCGCTCAAGAAGCTGCTGCTGGATAACGGCATCCGTCACGACACCACCGTTATCCTC
TATGGTCGCAACACCATGGCGGCTGCCCGTGCCGCGCACCTGATGATGTATGCGGGGGTGGAAGATGTGC
GCCTGCTGGACGGTGGTCTGGATGCCTGGTTTATCCAGCACCTGCGCACCGAAACCGGTCTGCCCAACAA
ATATGAGCCGGTGAAGGAGTTTGGCGTGGCCATTCCCGCTCACCCCGAGTACTACACCACGCTAAGTCAG
GCGAAAGAGCTGCTCAAGAAGCCCGATAGCGCGCTGGTCAGTGTGCGTACCTGGGACGAGTTTGTCGGCA

ACACCTCAGGTTACAGCTACATCAAGCCGAAGGGCGATATCCCGGGGGCCAAATGGGGTCATGGTGGCGT
CGATGCCAACAGCATGAGCGACTTCCACAACCCGGACGGCACCATGAAGCCGGCCCGCGAGATCCTCGCC
ATGTGGGACCAGTGGAACATCGAGCCAACCCAGCAGGCGGCGTTCTACTGCGGTACCGGCTGGCGCGCCT
CCGAGGCCTTCTTCTACGCCTGGCTGATGGACTGGAAACAGATCAGCGTCTACGACGGTGGTTGGTACGA
GTGGAGTTCCGATCCCAAGAACCCGACCGTCACCGGTGAGCGTCAGCCTGCCAAGTCCTGATTACGTGCT
TGCGCTGAAAAGAAGAGGCCGGACAGAGATGTCCGGCCTTTTTCATTGCGCTCGCCACCCGGCCAGATAA
CTAGGCTAAGTCGCTGATTATTGACGGCGATCCCGCTTTGCTCATCCCGCCATCCCGTGCCGTGCTATTG
AACTAGGCTTCAAGAAACATCCTCGTGGTGAGGATGATCGCAGAAGAGGCCGCAATGAAAAAACTGATGA
TGATCCTAGTGTGCGCCCTGGGGCTGGTGGCATGCAGCTCCCAATACATCATGAGCACCAAGGATGGCAA
GATGATCACCACAGAGAGCAAACCCAAGCTGGACGAGAGCACCGGCATGTATCGCTATTACGATCATGAA
GGGCGCGAGGTAATGATCAAGAAAGATGACGTGACCCAAATCATGGAGCGCTAAGCGCTCTCTTGCCGCA
AGGGCCGCTATCTGCGGCTCTTCTCTTTTTGCCGAAAAGTCGCGCTGTTTTCTCTGTTTATCCCCGCACA
TTCGCAGCAAATTCAGCCGTGATACAGCTTCCGTTATGGTTTAACCCTTATCATCACACTCCATTTTTA
TTGGAGGTGTTATGTCCTTGATGTCTATCTGGAATTCCCGTTTTTCTGCACGCAGCTTGCTGGGTGGTGC
CTTGCTGGTGGTGGCGTCAGCCAGCCATGCCGGTGACGATCAGTTCACGCTGAGGCGGACTATTTTGCTG
GGAGCCGGTGATCATGGTGCGACAATTCCTTTGGTGGTTTGCCGTCAGACCAAATATATCAAGGTGAAGG
CGGAGCGTGATTTGACCCTGGATCGGGTGGTGGTGACCTATGGTGGTGATCGTACCAAGACCATTCGTTT
CGATCGGGAAATGAGCAGTGATAAAGAGACCGAGTGGAAATCACTGGGCTCGCGCCGTTGTGTGAAAAAA
ATCGAAGTGTACGGTAATTCAGAGCGCAGCAAAGCGGGCGTAAAAGTATTCGGCCGCAAATAATTGATGC
GGTTTTTCTGCGCAAACAGATGAGTTGAAAATAATGGCGAGCCGTGGCTCGCCATTATTTTTTATGGTCG
GGTTCGCTATTTTTTCGGCCGAAATGGTTTGATGACGGTTTCATCGCACTCGAGATACGGCCCTTCCATC
AAATCGATGCAGTAGGGGATGGCGGGGAAGACTGCATCCAGACATTCGCGGATTGATTTGGGCTTGCCCG
GCAGATTGACGATCAGGGTATCGTCGCGCAGTCCGGCAGTCTGGCGCGACAGAATGGCAGTCGGCACAAA
CTTGAGGGATTCGGCCCGCATCAGCTCGCCAAAGCCCGGCATCATGCGATCACACACCGCCTCGGTCGCT
TCCGGCGTGACATCCCGCTTGGCCGGACCGGTGCCGCCTGTGGTGACGATGAGACAACACTGCTCCTCAT
CCGCCAGCCGGATCAGGGTCGCTTCAATCTGCTCCTGCTCATCGGGGATCACCTGATAGACAGGTTCCCA
CTCCGAGGTCAGATAGGCGTTCAGGGTGTCGATGATGGCCTTGCCGGAGAGGTCTTCATAGATGCCCGCG
CTGGCGCGATCGCTGACGGTGACGATACCGATTTTTGCCTTGCTCATGGTCTGCTCTTGAATGTCCGATA
AAAGTGGCAATTAAAATAACATAATCAGTGAGATATGTTAAACCGTCAGGAGATGACGCGGGTCGGTGAG
GGAAGAATAAAGTGTCGAATGAAGCAAAATAAGGCCACCCGGATTTTTATTTGCAATAAAAAACAGAAAA
TCACAACCTAAAATGTATAAGTGTTTTATCTCGCCTGAATTAAAAACAATCGCTTGTTTCCAAACACAAT
TAAAATAGATAGACACCCTCGTAGTGACCATTGTCCGGTTTAGTCGCCAGTGGATGGATTGAACCCTTTT
TAGCCGCGTCGTATATACAGATTTATATGGCTGGGACGTGATGGTGTGGTACCGCAGTGTGTTGCCAGCA
ATAGTCGCCAAGGAGGGCATATGGAGTTGCCTGATTTTGACACCCTGAAGGAGTTGGCTGCGAGTGAGCC
CTCCCGGCTGGATGCGATTCTGGAGCAGCAGATCGACCAGTTGATGGCCCGAGCGAGCCCCGATCAGCAG
CGCCGTCTGCGCGGGTTGCAGTTCAAGATTGATGGTCAGCGCCGTCTGGCGAAAAACAATCTTGATAGCT
GTATCCGGATTACCAACATGATGCGGGAATCTTTTTATTTGATGCGCAGCGAAATGAGCCAGTTCAGAGA
AGAGGGGGACCCTCCGCCTGAAAGCGATGGCAGTGGTAAAATAATCCGTCTGGCGGACTATCGACGTCGC
AAATAATCTGCAATAACCCCCTTGAATGATATTTCAGCCAAATCGGTGAGATCATGATAAAGGGAATTGC
TGGCGACCTGATTGTTATGGTTTTTAATGTTAGTCGTAATGTATTTGTGATGTAATTTCTTTTCTCCTTC
TTGTAATAACTAATTAGTCAGGTTGTGATAGGGGTGGATGAATAACAGTAAAAGTTAGGCTAATATGGCC
GAAATTTTTATTGCTACGACTCGAAGGGATTATGAGAAGTATCACACCAAGACGGGTGTTGTCGCGGGAG
CAACAGACAGAGATCACCCGCATTATCGTCAAGGTGGGTCAGATGTTGGCCCAGTTCGGGGCGGAGAGCC
GGATCATCGAGCAGACCACGGTGCGGCTGGGGATGGCGCTGGGGCTGGAGAGTGTCGAGATGGCCATCTC
TTCCAGTGCCATCGTGCTCACCAGTCTCTATCAGGGGGGTTGCGTCACCACTACCCGGCGGGTGCGCGAA
CACGGCATCAATATGCAGGTGGTGTGCGAGATCCAGCGGATCTGCATCATGGCGGAAAAAGAGCTGATCG
GTGCCCGTGAAGTGCGCCAGCGCCTCGAAGCCATCGTCCCCTTCCACTATCACCCCTGGCTGGTGGTCCC
CATGGTGGGGCTCTCCTGTGGTGCCTTTAGCCTGCTGTTTGGCGGTGACTGGCCCATCTTCCTGGTGACC
TGCTTCTCGTCTGCCGTGGCGATGCGGGTGCGCCAACTGATGGCCCGCCATCACCATAGCCCGCTCATCA
ACTTTGCCGCCACCGGCTTTATTGCCACCTTGCTCTCCTCCAGCGCCACCTTTTTTAACTGGGGTGACCA
GCCTTATCTCGCCATGGCGGCCAGCGTGCTGCTGCTGGTGCCGGGCTTTCCGCTGATCAATGCGGTCTCC
GACATGGTGAAGGGCTACTTCAACATGGGCTTGGCCCGCTGGGGGACTGCGACCCTGCTGACCATCAGCG
CCGCCATAGGTATCATTTTGGCCATGTCGGTCACGGGGATCTGGGGGTGGAAAGTATGATGGATTTGCTC
TGGCTACTGGCCAAGGATGCCTTCTGGTCCTCCATCCCAGCGGTGGGGTTTGCCATGCTGTTCAACGTGC
CACCACGGATGCTGAAGTATTGCGCCATGGGCGGCGCGCTGGCCCACAGCCTGCGTACCTTGCTGATCCA
CTACGGTATGCCCATCGAGTGGGCGACGCTGGCGGCGGCGACCACGGTCGGCTTTGTCTGCGTCTACTGG
TCGCGCCGTCTGCTGGCACCGCGTCCGGTGTTCAGCGTTGCCTCCATCATTCCGATGATCCCGGGCAGTT
ATGCCTTCAAGACCATGATTGCGGTGGTAGAGCTCAACATCAACGGCGTGACCATGGAGTTGATCCAGAG
TGCGGTGGAGAACGGCCTCAAGGCGCTCTTTATCGTCGGTGCCCTCAGTTTCGGTTTGGCGATCCCCTCT
CTGGTCGTGTACCGTAATCGCCCCATAATCTGACAGACGGTGAGAGGCCCCATGAAGATTTCCATGATTG
CCGCCATGGCCCACGACAGGGTGATCGGCAAGGACAACCAGATGCCCTGGCATCTGCCTGCCGATCTGGC

TCACTTCAAGCGTGTCACCCTCGGTAAACCGGTATTGATGGGGCGCAAAACCTTCGAATCCATCGGTCGT
CCGCTACCCGGACGGCGCAATCTGGTGATCAGCCGCAATCCCGGCTATCAGGCCGAGGGCATTGAGGTAG
TGGGCTCCGTCGAGGCGGCGCTGGCTCTGTTGGCAGGCAGCTCGGTGGAGGAGTTGATGGTGATCGGTGG
TGGTCATCTCTATGCCGAGATGTTGCCGAGCGCGGATTGCCTCTATCTGACGCAAATCGATCTGGCGGTG
GAGGGGGATACCCGTTTCCCCGCTTTTGACGATGGCCACTGGCAACGCGTTGATTGCGAGAGCCACCCTG
CCGATGAAAAGAACCCGCACCCCTATAGCTTCGAGACCTGGCAGCGCCGCTGACGGACGAATGCTAATGG
GCAAAGAACAACAAGGGCATCTTCGGATGCCCTTGTTGTTCGGTTGCTTTTTCAAGACGGGATTAGATGC
CTGAGCCGTCGTTCTCGCCATTTTCCTCGTGCAAACCGCACTCGCGCTTGAGGCCGAAGAAGCGGGTCTG
CTCCTCGCTCATGCCCGGCTCCCACTTGGCGGTGGTGTGGACATCGCCCACCGAGAGATAACCCTGCTCC
CACAGCGGGTGGTAGGGGAGGTCGAACTCCTTGAAGTAGTAGAAGACATCCTTGTTGCTCCAGTCGATCA
CCGGCAGGAACTTGAAACGGCCGCGCTGGATGCCCAGCACCGGCAGCTTGCCACGGCTGCCGGACTGCTC
CCGGCGCAGGCCGGAGAACCAGGTCTGGGCCCCCAGCTCACGCAGGGCGCGGTCCATCGGCTCCACCTTG
TTGAGCTGGTTGTAGCGGGTGATCCCCTCTACCCCCTGCTCCCACAAGAGACCAAATCGAGCCTCCTGCC
AGGCAGGGCTGAGTTCGGCCCGATAGATCTTCAGGTTGAGCGCGAGGCGCTCGGTCAGCTCGTCGATGAA
ACGGTAGGTCTCGGGGAAGAGATAACCGGTATCGGTCAGCACTACCGGCACGTCGGCCCGCTCGCGGCTC
ACCAGATGGAGCATCAGTGCCGCCTGAATGCCGAAGCTGGATGAGAGCACATGCTCCCCCGGCAGATTGG
CCAGGGCCCAGCGCACCCGCTCGGGGGCGCTCATGGTATCCAGCTCCCGGTTGAGGGGCGCGAGTGCCTC
GACTTGCTCTTCACGGCCGAGGGCCAGCAGTGCCTGCAGGTCAAGGCGGCCATCTTCAGTGACATTAAGC
GGCATATTAAGCGGCATAGAAGTCCCTCGCAGAGTCGATAACGGGGGCAATTACCCCAACCCGGATCACG
AAGTCGCCAAAGCATTCGCCCTGATTGCGCTCGGTGGCCCAGCGGCCGATCAACGCGTCGAGTTCGGCCA
AAATCTGCGGTTCGGTGATGTTCTCCTGATAGAGGCGCGGAATGCGGGTACCCTCCAGGTTGCCCCCCAG
ATGCAGGTTGTAGCGGCCCGGCGCCTTGCCCACCAGACCCACTTCCGCCAGCATGGCGCGGCCGCAGCCG
TTGGGGCAGCCGGTGACCCGGAAGATGATGGCGTCATCCGCCAGCCCATGTTTGGCCAGCAGCCCTTCGA
TGTCGGTGACAAAGGCGGGCAGCATGCGCTCGGCCTCGGCCATCGCCAGCGGGCAGGTGGGCAGGGCCAC
GCAGGCCATGGATTGCTTGCGCTGCTCGCTCACGCCTTCATCCAGCAGGCCGTACTGGCGCGCCAGCCCT
TCGATGCGGGCCTTCTCGTTGGCAGGTACGCCCGCGATGATCAGGTTCTGGTTGGCGGTCAGGCGAAAAT
CCCCCTGATGCACCTTGGCGATCTCCAGCATGCCGGTCTTGAGGGGCTTGCCCGGGAAGTCCAGCAGGCG
GCCGTTCTCGATGAACAGGGTCAAGTGATGCTTGCCGTCGATCCCCTCGACCCAACCGAAGCGATCGCCA
CGGCTGGTGAACTCATAGGGGCGAACCGGCCCGAACTGGATGCCGGCGCGGCTCTCCACCTCGGCCTTGA
ACGCTTCGACGCCGACTCGCTCGAGGGTGTACTTGGTCTTGGCGTTCTTGCGGTTGACCCGGTTGCCCCA
GTCGCGCTGGGTGCTGACCACGGCGGCCGCCACCTCCAGCACGTGGGAGAGCGGAATAAAGCCAAAATCG
CTCACCTTGCGCGGATAGGTGCTGGTGTCGCCGTGGGTCATGGCCAGACCCCCGCCCACCAGCACGTTGA
AGCCCACCAGTTTGCCACCTTCGGCAATGGCCACGAAGTTGAGATCGTTGGCGTGGATGTCCACGTCGTT
GTGGGGCGGGATCACGACCGTAGTCTTGAATTTGCGCGGCAGGTAGTTGGAGCCCAGGATCGGCTCCTCG
TCCTGCCCCAGTTTTTCACCATCCAGCCAGATCTCCACGTAGGCGCGGGTCTTGGGCAGCAGGTGCTCGG
AGATCTTCTTGGCCCACTCGTAGGCCTCCTGATGCAGTTCGGACTCCACCGGGTTGCTGGTGCAGAGCAC
GTTGCGGTTCACGTCGCCGGCGGTGGCGATGGAGTCGATACCGGTGCTGTTGAGGGTCTGGTGCATCAGC
TTGATGTCGCGCTTCAATACCCCGTGGAACTGGAAGGTCTGACGGGTGGTCAGGCGGATGCTGCCGTAGA
GGCTGTGCTCTTCGGCGAACTTGTCGATGATCTGCCACTGGGCCGGGGTGATGATCCCACCAGGCAGGCG
GGCACGCAGCATGACGTTGTGCAGTGGCTCCAGCTTCTGAGCGGCGCGCTCCGGGCGGATGTCGCGGTCA
TCCTGCTGGTACATGCCGTGAAAGCGGATCAGCTGGAAGTTGTCGCCGTTAAAGCCGCCGGTGAGCGGGT
CCTGCAGATCTTGCTCTATGGTGCCGCGCAGGAAGTTGCTCTCGCGCTTGAGGCGCTCGTTGTCGGCCAG
CGGCCCTTCGACCGGTCCCGCTTTTGGGTGAGAAAGAGGTTGTTTGCTCATCAATAGACATCCCTTTGAT
AACGTTTGGCACGACGCAATTCGCTCAAATACTCTTCTGCTTCCTCACGGCTCTTGTGGCCGTGTTCGGC
AATCACATCCAGCAGGGCCTCCTGCACATCCTTGGCCATCTTGTTGGCGTCGCCACACACATAGAAGTGA
GCGCCTGCTTCCAGCCACTGGTAGAGCTCAAGCCCTGCCTCGCGCAGGCGGTCCTGCACATAGATCTTGT
TGGCCTGATCCCGGCTGAAGGCCAGGGAGATCTTCGACAGCAGCCCCGATTTCACATAACGCTGCCACTC
CACCTGATAGAGGAAATCCTGGGTAAAGTGGGGGTTGCCGAAGAAGAGCCAGTTCTTGCCTTCCGCGCCC
TGAGCTTCCCGCTCCTGCATAAAGGCGCGGAACGGGGCGATGCCGGTGCCCGGGCCCACCATGATGACGG
GGGTGTTAGGGTTTTGCGGCAGGCGGAAGTTGTCGTTGTGCTCCACAAACACCCGCACCTCGCCATCCTC
AATCAGCCGATCCGCCAGATAGGAGGAGGCGGCACCAGAGCGCACTGTGCCATCTTCCTGTGGGTAGCGC
ACCACGCCGACGGTCAGGTGTACCTCCTCTTCAACCTCGCTCTGGGCAGAGGCGATGGAGTAGAGGCGCG
GGGTGAGGGGGCGCAGCAGGCTGACCAGCTGCTCGGTCGTGAGGGCGGTCGGGAAGCGTTTCAGCACATC
CACCACCTGAGCGGAGGCGACCAGTGCATTGATCTGGGCCTTGTCGCCAGCCAGATCTTTGAGCGCTGCG
TTCTCCGATATGTCAGCGAGCCCTGTGATAAAGCCGCCGTGCAGCCGGGTCAGTTCGAAGTGACTGGTCA
GTGCGGTGTGCAAGCTGCCGTGGGCTGTGGCTTCATCACCAGAGAGGCCGGTCAGGGCGAGCACTTCCCC
CACCAGGTCGGCGTCATTGTCGAACCAGACGCCGAGGGCATCACCCGGTTGATAGGTGAGGCCGGAGTCG
GCCAGATTGATCTCGATATGGCGGATATCCTTGGTGGAATCGCGGCCGGTGATCTTCTGATTGACCGACA
GGCGGGCAGGGAAGGGGTTCTCCTTGCTGTATTGGCTGTGGCCAACCGCCTGCTGCACCGCGCTGGCTAC
GCTGCTGCTGGCCGCTCCGGTAGAGAGGGTCGCGGTGATGGCATTGAGCGCCTGTTCGCCCCACGCCTTG
GCGGCATCCTGATAATCCACATCGAGGCTGGCGAGCTCATAAACCCGCTCGGCCCCTGCGCTGGCGAGGA

AGTTGTCAAAATCTTTGCCGGTCTGGCAGAAAAATTCGTAGGAGCTGTCACCCAGCCCCAGCACCGCGAA
CCTGAGGCCGTCCAGCTTGCCAATCTTGCCTTTTTAAGCTGCTCGAACAGATCGACGGCGCTCTCCGGC
GGTTCACCCTCCCCGTAAGTGCTCACTACCACCAGCAGATGGCTCTCTTTCTTGAGCTGCTTGGGTTTGT
AGTCGGCCATCGACGCAAGGGTGACGGGGAGGCCACGTGCCTCGGCCTGCGCCTTGATGGCAGTGGCGAC
ACCTTTGGCGTTGCCGGTCTGGGAGCCGTAGAGAATGGTCAGGCTGCCGCTGGGAGCCGCGACTTCTGCG
CCTGCGGTCGCAACGGGCTGGACGCCCGCCTGGGAGAGGCCGTAGAGATAACCGCTCACCCAGGCCAGTT
GCTGGGTATTGAGGGTCGAGAGTACCTGACCCAGCTGACGTTGTTGCTCGTCAGAGAGGGGGGAGAGGTT
GAATGGGGAATCCTTCAATTGCATCACACGCGTCTTCCATGGTCTGATTTTTGGCCAGATTAAAGGGCGT
GTTGGATAACAACAAAGAATAAAAATTTCTTCTTTATTCTATTAATGCATTAAAGGGGCAGAGGTACGCC
TGTTCCCGCAGCAAAAGAGGGGCGTGGTATAAAGAGCGTTAAATGAGAGCCACTTGATAACACAGGAAGT
GTCATGTCGAATATCCGATTGTTTCATGTCAATGCGTTCAGTCAGCAGCCCCTTGGTGGCAATCCCGCGG
TGGTCGTATTGGGAGCAGCCCTGCCTGATGCCAGCCAGTTGGCGATGGCGGCCGAGTTCAATCTCTCCGA
GACGGCCTTTCTGGTGCAACTTGGCAGGGCCCATTACCAGTTACGCTGGTTCACGCCGAAAGTCGAGGTT
GATCTCTGCGGTCATGGCACCTTGGCGGCTGCTCATGTGTTGTGGCAGACAGGAGCGGTAGCTGCACACG
AGACTATCCGCTTCGAGACCCGTAGTGGTGAGCTGCTGGCCCGGCGCGGAGGGGAGTGGATTTGGCTCGA
TTTTCCGCGTATCGCGTTGACGCCACTGACGCTGGATCCCGCCTATGCTGCCGCGCTTGGTTGCCAGCCA
CGACAGACCTTGGCGGTCGGGGCCAAGTTCTTGCTGGAGCTGGCAAGTGAAGAGGAGGTCCGCGCTATTT
CGCCTGATTTCCATGCCCTGCGCGCCCTGCCGGGACGGGGGCTGATAGTCACTGCTCCGTCGAGCCGGGC
AGATGATGATTTCGTCTCCCGCTATTTTGCCCCTTGGGTTGGCGTGGATGAGGATCCGGTCACCGGCTCC
AACCACAGTGCTCTCATGCCCTTCTGGGCTGAACGGCTCGGCAGGCAGCAGTTGCGGGCCCGTCAGGTTT
CATCCAGAGGGGGTGAGCTCAGGCTGGAGCTGCGGGGTGAGCGAGTATTGATGGCGGGTCAGGGGTTGAC
CCTGTGGCAGGGGGAGTGGTTGCTGCCCCTCGCCAAATAACACGTTACGTGACACATGCCAATAAAAACG
CCCCGCAGTTGCGGGGCGTTTTTATTGGCTATCACGCTTGGGGTTGACCCAAGGGATTTAGCTGACTTCT
GTGGGCCTTACTTCTGCGGACGCAGGGCCGGGAACAGAATGACGTCACGGATGGTGTGGCTGTTGGTGAA
CAGCATGACCAGACGGTCGATACCGATACCCTGACCGGCAGTCGGCGGCAGGCCGTGTTCCAGTGCGGTG
ACGAAGTCGGCGTCGTAGAACATGGCTTCGTCGTCGCCAGCTTCTTTCTGATCAACCTGATCCTGGAAGC
GCTTGGCCTGGTCTTCTGCATCGTTCAGCTCGGAGAAGCCGTTGGCCAGCTCGCGGCCACCGATGAAGAA
TTCGAAGCGGTCGGTTACGTCCGGGTTCACGTCGTTGCGACGAGCCAGCGGAGAAACGGCAGCCGGGTAC
TCAGTGATAAAGGTCGGTTGCAGCAGCATGTGCTCCACGGTCTCTTCGAAGATCGCGGTGATCACGTGGC
CCAGCTCCCAACCCTTCATCAGCTCGATGTGCAGACGCTTGGCAACGGCCTTGGCGCTCTCGAGGGTAGC
CAGATCATCCAGGGTCACATCCGGATTGAACTTCAGGATGGACTCGACCATGGTGAGGCGCTGGAACGGC
TGACCGAAATCGATGGTCAGGCCCTCTTCGCCCTCTTTGGCGTAACGGATCTTGGTGTCGCCCAGAATGT
CCTGCGCCAGGGTACGCAGCAGCTCTTCGGTCAGGTCCATCAGATCGATGTAGTCGGCGTAGGCCATGTA
GAACTCGATCATGGTGAACTCGGGGTTGTGACGAACCGAGATACCTTCGTTACGGAAGTTGCGGTTGATC
TCGTAGACACGCTCGAAGCCACCGACCACCAGACGCTTCAGATAGAGCTCCGGAGCGATACGCAGGTACA
TGTCGATGTCCAGCGCATTGTGGTGAGTGACGAACGGACGCGCGGAGGCACCACCCGGGATGACTTGCAT
CATCGGGGTTTCCACTTCCATGAAGCGCTTTTCGTTGAAGAACTTGCGGATACCGGCAACCACCTGGGTG
CGGATCATGAAGGTCTTGCGAGACTCTTCGTTGGCGATCAGATCCAGGTAGCGCTGGCGGCAGCGAGCTT
CCTGGTCGGTCAGGCCTTTGTGCTTCTCGGGCAGCGGACGCAGTGCCTTGGTCAGCAGGCGGATATCGGA
AACGTGAACGGAGAGCTCACCGGTCTGGGTCTTGAACAGGGTACCTTCGACACCCACGATGTCGCCCAGA
TCCCACTTCTTGAACTGCTCGTTGTAGAAACCTTCCGGCAGGTCGTCACGGGTGACGTAGACCTGGATCT
TGCCGGCCATGTCCTGCAGGGTGGCGAAAGAAGCCTTGCCCATGATCCGGCGGGTCATGATGCGACCGGC
AATCTTCACCCGTTTACCCAGGCTCGTCAGCTCCTCGGCGCTTTTGTCGTCGAACTCTGCGTGCAGATCG
CCAGAGAGGCTGTCGCGACGGAAGTCGTTGGGGAAAGGATTACCCTGGGCGCGCAGGGCGGCCAGCTTGG
AACGACGCTCGGTCATCTCGTTGTTGAGTTCGAGAGTTTGGTCGACTTCCGGAGTGATGGTTTGTTCAGA
CATGGTGATTCCTGCTTGTTTACAGACCTGATTTCAGGCTGGCTTCGATAAACTTGTCCAAGTCACCGTC
GAGCACCGATTGGGTATTACGGGTTTCGACGCCGGTGCGCAGATCCTTGATACGGGAGTCATCCAGCACA
TAGGAACGGATCTGGCTGCCCCAACCGATATCAGACTTGGTCTCTTCCAGGGCTTGTTTCTCAGCGTTCT
GCTTCTGAATTTCCAGCTCGTAGAGCTTGGCTTTCAGCTGCTTCATACACTGGTCTTTGTTTTTGTGCTG
GGAACGGTCGTTCTGGCACTGTACCACGACACCGGTCGGAACGTGGGTAATACGCACCGCGGATTCGGTT
CGGTTAACGTGCTGACCACCGGCACCGGAGGCGCGGTAGACGTCGATCCGCAGATCCGCCGGGTTGATCT
CGATATCGATATCGTCGTCGATCTCGGGGTAGACGAACACAGAACAGAAGGAGGTGTGACGGCGGCCACC
GGAATCGAACGGAGACTTGCGAACCAGGCGGTGTACGCCAGTTTCGGTACGCAGCCAGCCGAACGCATAT
TCGCCGGTGAACTTGATGGTGGCGGATTTAATGCCGGCCACGTCGCCTTCGGAGCATTCAATCAGCTCGG
GCTTGTAGCCGTGGGCATCGCCCCAGCGCAGGTACATGCGCAGTACCATGTTGGCCCAATCCTGTGCTTC
GGTACCACCGGAGCCGGACTGGATGTCGATATAGCAGTCGGAGCCGTCGTGCTGGCCGGAGAACATGCGG
CGGAACTCGAGATCCACCAGCTTGGCTTCCAGCTGTTCGGATTCGGCGATGGCTTCGTTGAAGGTCTCTT
CGTCTTCCCCTTCCACGGCCAGGCTCACCAGCATCTCGACGTCATCCACGCCCTGGGTCAGGGTGTCGAT
GGTGCCGACCACGTTTTCCAGCGCTACGCGCTCTTTGCCCAGCGCCTGTGCGCGCTCGGGTTCATTCCAT
ACGTCCGGCTGTTCCAGCTCGGCGTTGACCTCTTCTAGACGCTCTTTCTTGGCGTCATAGTCAAAGGTAC
CCCCTAAGAAGCTCGGTCCGCTCAGACAGCTCCTTAAGTTTATTTAATACGGGATTAACTTCGAACATCG

TGAAAACTCTCAGGGTCGATGTGATTTAAAAACGGGATATTCTAGCCAATTGTGGAAGGAATAAACAGGG
TTGCCTTGGCTTGTGCGGCTCATGCCGTCAATTTCTGGTGCACTTTTCTCCACCTCGGTAGAAAATATCG
CCCGCAGACCCATTTTCAGGTTCAGTTTTGGCCATTCGGGTCGCTAATGACTACGTGTAGTGTTGTGCAA
TTTAGGGAAAAACCCGCTCCTCACCTCAATAAGAAGAACACATGATGAAAAACCTGTCACTCAAGCAGAA
GATACTGTTGTCAGTGGTGATCGCCCTTTCGTTGGTGATTTTGTTGCTCTCCTGGCAGAGCTATAGCAGC
CAGAAGTCCCTGTTGCTGCAAGGCAGCCAGGAGCAGTTGCAACGGCTGGGTACCCAGCAGGCCGAGCGTG
TCAGCGACTGGCTCGCCGCCCGTCGCGACGTGATCCAGGCGCTCGGCAACAAGGCCGGTGCCGACCCCCT
CAATGCCTTGCAACAGGCACAAGTTTCAGGCCGTTTCCAGCTCACCTATTTCGGTGAGAGCAGCGGCAAG
ATGAATGACTCCGATCCCTCCATCAACCGCGATGGCTACGACCCGCGCTCCCGTGGCTGGTATCAGGAGG
CGATGAGCAAAGGTGGTCTGATTGTCACCAAACCCTATCTCGATGTGGCTTACAACATCATGGTGGTCAC
TCTGGCCCAGCCGGTTTCTGGCGGGGTTGTCGGTGGTGACCTCTCCATCGCCTCGCTGGTGGAAGACGTG
ACCAAGATGCAGCTGCCTGCCGACGGTTTCGCCATCATGATGCACAAGGATGGCACCATCATCGCCTACA
AGGATGCCGCCAAGGCGATGAAACCGGCCAGCGAGATCGACAACGATCTGACCAACGCGCTGATCGAGCA
GAGCAAGTCGAGCAAGGAGCTGGTGCCAGCTTATTTTGACAACGAAGGTCGAGACAAGCTGCTGTGGGCG
GCCGATATCCCCGACACGGATTGGGAGCTGGTGCTGGTGCTGGACAAGGCCGCGCTGGAAGCGCCACTCT
CCTCCCTGCTGATGACCCAGCTGGGGATGGCGCTGCTGGTGCTGGTGGGTAGCATCCTCGCTATCTCCTG
GCTGGTCAGCATGCTGCTTGGCCCGCTCACCAAGGTCTCCCAGGCACTGGCCCGCATTGCCGATGGTAAC
GGTGATCTGACCCAGCGCATCAAGATCGACGCCAACGACGAAGTGGGCCAACTGGCCAACAGCTTCAACC
GTTTCGTCGGCAGCCAGCATCAGCTGATCGGTAATATCCGTCAGCTGGCCAACGAGTTGAACGCCGATGC
CGAGCGCAGTCTGGTGACCAACCAGGCCGCGGTGGACGAGCTGCAGCGTCAGCAGCAGGAGGTGACCATG
GTCGCCACTGCGGTCACCGAGATGGCGAGCGCCACCATGGAGATCGCCGGCAACGCCGAGAACACCGCCG
CCGCTGCCCAGCAATCTGCCCAGAGCAGTGAGCAGGGCAAGATGCTGGTCAACAAGACCCGCCAGTCCAT
CAATGGTCTGGCCGAAGAGGTAGGGCAGGCGACCGAGGTGATCGGCGAGCTGAGCCGCCACGCGCAGGCG
ATCACCAGCATCCTCTCCACCATTCAGGGGATTGCCGAGCAGACCAACCTGCTGGCACTGAACGCAGCCA
TCGAAGCGGCCCGTGCCGGTGAGCAGGGTCGTGGTTTTGCGGTGGTCGCTGACGAGGTGCGTGTGCTGTC
ACGTCGTACCCAGGACTCCACCCAGCAGATCCAGTCCACCATCGAGACCCTGCAACAGACCACCGCCCGC
GCGGTGAGCCTGATGCAGACCAGTCAGGGGCTGGCGGACAACAGCGTGCAGGATGCCGATCAGGCAGTGC
GCGCACTGGAAGAGATCACCAGCGCCATCTCCCTCATCTCCGACATGGCGGGTCAGATCGCCACTGCCGC
CGAGGAGCAGACCCAGGTGACCGGCGAGATCACCCAGAACACCACCGCCATCAAGGATGTGAGTGACGAG
ATCACCGCCGCCGCCATGCGCGATCTGGATCAGGCCCACAGCCTCAAAGGGCGTGCTGCTGACCTGAACC
AGCAGGTGTCGACCTTTATCCTGTAAGGTGCAACTCACGGCCTGTTCCGGCTGAGATAAACAAGCCCCGC
CCTCTGTCGAGTGCGGGGCTTTTTGTTGTTCGGAGGAGTGCGGGATGTGGGGACTTCGGGTTTTCTGAGT
TGCTCGCGGGTCATCCTGGTGCCGGATACGTGCCCCTGCGTCAGCTGGCGAGTAGAGAACAAACAAAGAG
CCCACCGAAAAGTGGGCTCTGATCATATACGTCTGACGGAAAGGGAATTACAGGAATGGCATCGCCATAA
ACAGCTTGATCACCATGGCGTTGATGATGTCGATAAAGAAGGCCCCCACCATGGGTACTACCAGAAACGC
TAGGTGGGAGGGACCGAAGCGCTGGGTGATGGCCTGCATGTTGGCAATCGCGGTCGGGGTGGCCCCCAAG
CCAAAGCCGCAGTGACCTGCCGCCAGTACGGCCGCATCGTAATTTTTGCCCATCACCCGGAAGGTGACGA
AGATGGCGTAGAGCGCCATCGCCACTGTCTGGGCGGCCAGCAGGATGAAGATGGGCAGTGCTAGGCTGGC
CAGATCCCACAGTTTCAGGCTCATCAGTGCCATGGCGAGGAACAGCGACAGGCTGACGTTGCCCAGCAGG
GAGACTTCTCGATCGCTCACCTCATGCCAGTTCAGCAGGGTCAGGACGTTGCGCAGCAGCACGCCGACAA
ACAGCACGCAGACAAAGATCGGCAGCTCGAAGGCGCCCCCTTTGAGGGAAGCAGAGAGCAGTTCGCCGCC
CTTAAGGCTGATGGCGATCAGCGCCAGCGTCTCGATAACGCTGAAAGTGGTGAGGGGGCGCTCCATGTCG
GGCATTTCGAAGCCCTGAGGGGCCTCATCCTGATTGTGCTCCTCACCGGGCACCTGCACTTTCTTCACCA
GATAGCGAGCCACCGGGCCGCCAATCAGGCCGCCCAGCACCAGACCGAAGGTGGCACAGGCGATGGCGAG
TTCGGCGGCGGATTGCACCCCGTATTTCTCGGAGAAGGTGGCACTCCAGGCCGCACCGGTGCCGTGGCCG
CCGGAGAGAGTGATGGAGCCGGCCAGCAGCCCCATGTGGGGATCCAGACCGAGCAGGGTCGCCAGACCGA
CACCCAGACTGTTCTGCACCAGCAGCAGCCCGGTGACCACCATCAGGAAGGTAACCACCGCCTTGCCGCC
GCGTTTCAGGCTGGCGAGATCGGCGGAAAGACCGATGGAGGCGAAGAAGGCCAGCATCAGCGGGGTTTGC
AGACTGGTATCAAATTGCAGCTCCATCTGCAGGGTGGAGCGCAGCAGTAGCAGTACCAGCGCGACCACCA
GGCCGCCGGCTACCGGTTTGGGGATGTTGAAGGTACGCAGCAGCCCGACTCGGGTAACCAACACCCGGCC
AAGCAGCAATACCAGAGTGGCGGCGACCAGCGTTGCGTAGGAATCAAGTTGTAACATTCAGTACTCCCAT
GTTGATTGGCTGTGTCTTTCATAGCAAAAGACTTACAGATTCAGCCGGGATGAAAACAGTGTGGCTTATG
TGCCAGCAAAGAAGGGGAAACATCTCCCCGCCCCAGGAAAGATGGGGCTTGTGCGGGCCTGAAATGCTCA
GGCATCCAGATGAGATTTTAGTAAAATCCGGCGGATTGTAGCAAAAAGCATCGTTTAATTCCATCTTTGT
CGTATTAATGCTCGTTTTAGTGGTTTATTGTGACATTTAACACTATGTGGGTAGAGGGCAGAAAAGCCGA
AAAGGGAGCATGGAACGGCTGTCATTTGGGGCGCCAGTCTCTATAATTCGCCGGTTTCCGCTGCACGCAG
AGGCGCTGGCGGGCGTTGACATTGTGGTTCACCCCGACATGGTTCATGTTTTGGCGGTGTTTACTTCATC
AAGAGGAGAGGGTGTGAAGAAGTCTATCTACATTGCCTACACAGGCGGCACCATTGGGATGCAGCGTTCC
GATCACGGCTACGTGCCGATGGCGGGCTTTATGGAAGACTGTCTGGCGCGGATGCCCGAGTTCCACCGCC
CCGAGATGCCGGATTACCATATCCACGAATATGCGCCCCTCATCGACTCCTCCGACATGACCCCGGCGGA
TTGGCAGCGTATCGCCGAGGATATTCGCGACAACTACGAGAAATACGATGGCTTCGTGATCCTGCATGGC

ACCGACACCATGTCGTTCACCGCTTCGGCGCTCTCTTTCATGCTGGAAGATCTGCACAAGCCGGTCATTA
TCACCGGCTCCCAGATCCCATTGGCCGAGCTGCGCTCCGACGGTCAGCAGAACCTGCTCAACGCCCTCTA
TATCGCGGCCAACTACCCGATCCACGAAGTGACGCTGTTCTTCAACAACCAGCTCTATCGCGGCAACCGC
AGCAAGAAGGTGCACGCCGATGGTTTCCACGCCTTCGATTCCCCCAACTATCCACCGCTGCTCAATGCCG
GGATCGCCATCTCGCTGGAAGCGGGTGAGTTGGGCGTGCCCTCCGCGCGGCCGCTGGTGCTGCACGACAT
CACCCCCCAGCCCATCGGCGTGGTGACCCTCTATCCGGGTATCTCCAGTGAGGTGATCGCCAATATCCTG
CAACAGCCGGTGAAGGCGCTGATCCTGCTGTCGTTCGGGGTCGGCAATGCGCCGCAGAACCCGGCCATGC
TGGCGCTGCTCTCCGAGGCATCGGCCCGCGGCGTCATCATCGTCAACCTGAGCCAGTGCCTGCACGGCAA
GGTCAACATGGGTGGCTATGCCACCGGCAATGCCCTGTCGCGGGCCGGGGTGATCTCGGGCTTTGACATG
ACCGCCGAAGCGGCGCTCACCAAGCTCCACTTCCTGCTGAGCCAGGACTTGCCCTCCCACCGCATTCGCG
AGCTGATGCAGCAGTCCCTGCGCGGGGAGCTGACTCCCTAAGCGATGCGGCCCTCACCCTAACCCTCTCC
CGGCGGGAGAGGGGATAGATCGAGGTGCTGTTGGCCACGAGGCTTGCCATGAAGAGCGTTGGCAGGTGGC
CCGTTCACTGTTCGCAGGTGCGAGCCTGCTCCCTTCTCCCCCTAGCGGGAGAAGGGTTGGGGATGAGGGG
GATGAGTCATCCTGCATAGATAGGAAAAGAGGGGAGCCATAATGGCTCCCCTCTTTGCTGTGGCCGCCGC
GAGCGGCCTGCCGAGTCAGGTTCAATGGTTAAGGTCGATCCGCACTATCTCTTCCTCGTCGCGATACTGA
CGCTTGAGCTCGCTCTTGCTCTTCATCACGATCTCGCCGTCACGACCTATGTTCATGTGCTCGGGGTTGT
CGAGATGCATCGCCTGCCAGGCCATCACGGCTTGCAGGGAGGTGGCCTTCTGCTCGTCGGTGAGGGGCTT
GCCATCGGGCCACTTGCCCAGCTCGACTGCGGTTTTGAGCCGCTCATACACCTCTTCGGGCATCTGCCTG
ATAGCTTGTAAAAACTCACCCTGTTGAAACGACATTTGGCCTCTCCTTGAACTATTGAGCCATAAACAGC
GGGGTCTGATGGGCCATGCAGGTAGACCCCATCGGTCTATGCCGGTAGATCTTGGCGGACTACGCCAGCC
ATCTGCTCCATCACCCTCACTACCTGACAACTGTACCCGAACTCGTTGTCATACCATACGTAGAGCACAC
AGTGATCCCCTTCGGCGATGGTCGCCTGGGAGTCGACGATGCCCGCATAACGGGAACCTACCATGTCGGA
GGAGACCAGCTCGGTACTAGCGCTGAAGTCGATCTGGCGATGGAGGGCCGAGCTTAGCGCCATCTGCTGC
AGATAGGCGTTGAGGCTCTCCCGATCGGTGGCCTGCTCCAGCGACAGATTGATAATCGCCAGCGAGACGT
TGGGGGTCGGCACCCGGATGGCGTTGCCGGTCAGCTTGCCCTTGAGCTCGGGCAGCGCCTTGGCCACCGC
CTTGGCGGCGCCGGTGCTGGTCATCACCATATTGAGGGCGGCGCTGCGACCGCGGCGCTCGCCTTTGTGG
TAGTTGTCGATGAGGTTCTGATCGTTGGTGTAGGAGTGGACCGTCTCCACGTGGCCGTGGCGGATGCCGA
ACCGCTCCTGCATCACCTTGAGCACCGGGGTGATGGCGTTGGTGGTACAGGAGGCGGCGGAGATGATCCT
GTCACCCGGGCCTATCACCTCATGGTTGACCCCGAACACCACGTTTTTCATCTCCCCTTTGCCGGGGGCG
GTCAGTAGCACCTTGCCGGCACCGCGCGCTTTCAGGTGCTCGGAGAGGCCTGCTTCGTCACGCCAGACGC
CGGTGTTGTCGACGATCAGCGCATCGTGAATGCCGTAGGCGGTGTAGTCGATGTCGGCCGGGCTATTGGC
GTAGATCACCTGAATGAAGGTGCCGTTGGCGATGATGGCGCTGCGCTCCACATCCACCTCGATGGAGCCG
TTGAAGGGGCCGTGCACTGAGTCGCGGCGCAGCAGGCTGGCGCGTTTCTCCAGATCTCCGTCGCGACCAC
CGCGTACCACGATGGCGCGCAGGCGCAGCGGATTGGCAGCGCCGGTACGCTCGATCAGCAGGCGGGCCAG
CAGGCGGCCGATACGGCCAAAGCCGTAGAGCACCACGTCGGTCGGCTGCGGCATCTGGCTGCGGCCAAGG
GCCGGTGCCAGTCGCGCTTGCAGATAATCCTCGATCCCCGCCTCATCCTGATGATCCTGCCAGTAGCCGA
CTGCCAGCTTGCCAAGGTCTACCTCGGCGGCGGTGGGTTTGAGGGCAACGATGGCCTGAAGCAGGGGGAA
GCTTTGCTGGATGGGGAGGGGAGCGCCCTGGTGGCGGCGGACGACCCGGTGGGCCTTGAGGATATCGATG
GTGGAGGCGTTTTGCAGCGGGCGGCCGTAGAGGGTGATCTCGACCCCTTGTTGTCGGTAGAGGCGACCAA
TCAAGGGCTGCATGGCTTCGGCGAGCTCCTGGCTCTCCTGCCACGCTTGCAAGTAGTGCTCGTGGGTCAT
TGACCAGATCCTTTATTTCACGTTGTTGGTGTAAATAGAAAATTCGTCGAATGCAGATCAGTGTAGGGGT
GTATATTTCGTTATATTATTCTGCAATCGCGGCCTTATATGGCGCGCGCATTGTAATGGATATGTAACCG
GTTTGCTAGCCGGAGGTGGCCCGTAAAGGCGCCAGTTGTTACCAACCCGCGTTACCAACAAGATGTGAAC
CATGGCCAATTTCCCCCTGCGACAGACCTTCATCATGCTTTCAGTCGGCCTTGCCGTGGCTGGCTGCAAT
GACGATCGGATCCACAATATCTGCAGCAACCATGCCGAGCTCTGTCAGGATCTGGTGGACGATGGCTGGT
GTCGCTACGAGCGTACCGATGTGATCCGCAGCCGCTTCTATCTCAAGGAGGAGGGAACGGATCGGCGCAA
GTACGAGCTGATGCGCAACCTTGAGCGCTATCTTAAGTGTGCCGAGCGCTCTACCAACATCGAGTATAAG
AATGCCCGCGAGCAGAAGAGTCCACGGGTGGAGGGAATGCTGGCGGCCGGGGATCAGTTGGCCCAGCTCG
ATGCGGCGACCCGCAATTCACAAGATCCCTACCTGCTGCTCTGGCACTGGACCAACAACACCAACGAGTT
GGCTCGTCAGCAGTTTATGGCGCTGGAAGGCACCAAGGCGCTGGATGAACCCGAGCTGCAACTGGCGCTC
GGCGGTATCTACGCCAAGAGTGACCCCGAAAAGGCCATTGCCACCCTGCAGCACGGGCTCTCCCTCTATC
AGGAGGGGGACAAGGTGAACAGCCGCATGTTCACCTCCCTCAGTACCCTCTATATGGGCCAGAAACAGTA
TGGGTTGGCCTATCTGTGGGGCAAGGTGAGCGAGTCGTTTCAGGATAGCCCCGAGGTCTCAGCCAAGCGT
TTTGGCATCTACCACACCCTGAGCAAGACAGAGCAGGATGGTTACGATGTGCGGGCTGCCGAGATTGTCG
AGCAGCTCAAGCATGGCAACTACCGGCCCTGATGCAGTGATGACGACGTTTTCTCCGGCCAGCTGCTGGC
CCAATCCGTTCGATCTCTCTTTCGTTCATGGCGAGGTACGTACCGATGGCCGTACTCTGGAGCGCCTCAC
GGTGCGCGCCGTGGTGCCCCGTGGCGACGAGTTGCTGCTGGTTCACTCCCGGGTCAACGGCGATCTGATG
TTCCCCGGGGGCGGCATTGAAGCGGGGGAGAGCCACCATGTGGCGCTCGCTCGCGAGCTGCTGGAGGAGT
GTGGTGCCGAACTGCGGGAAGTGGGTGGCCTGCTTGGCGAGACCCGTGAATATCGCGCCGCCCGCGAGCC
GGGGTTTGATGCCTACTGCATCCGCTCGCTCTACTACTTGTGCCGGATTGGCGATCAGCTGGTGGCCCCG
CAGCCACAGCCCTACGAGATCCGCCTTGGCTTCGTGCCCGGCTGGTTTGCCCTCGACGAGGCGCTGCAGA

CCAACAAGACCCAGCTGGCGGGGCCCTGTCCCCAGTGGACGGCGCGGGAGACCCGGGTGCTGGCCCAGCT
GCAACGCTGGCATCAGCAGGGGATCTTCGACAACGATTGTTGAACACATCAAACGGAAACGGTCAGTGAT
ACCAACAAAAAGAGCGCCAATAGGCGCTCTTTTTTATTTCGAGGGAGGAAGTTATTTGACCTCTGTCATT
TACATCGCTTCGTTGTACTCTCGTAGCTAATGTCAGCCGTTAGAGCAACGCTCTTCTGTACCAGCCCATC
AATCTGGCAGCTGATGCCCATTACCTATAATGTTATTTTGTATTGGATTGACATAGATGCCGGTGCGCAA
GATCCCGAAAAATTACCGCAATGTCACAGGGGTGGCCTCTCATCGCAAGTCCACTGGACGGGCTATGTTC
GAGTCTACCCTCGAGCGTGATTTCTTCACACTGCTGGAGTTCGATCCAGCAGTCGAGAGCTTTGAAGTGC
AGCCTGTTACCGTTCATTGGCTTGATGAATCGGATAAGGAACGCATCTATACCCCAGATGTATTGGCTTA
CTTCCACAGCCCTCATCATCCAACTACCCTCTACGAAGTCAAATATCGTAGTGAACTCAAAGAACTTTGG
CCGGAGTTGAAGCCCAAGTTCAGAGCTGCCTTGCGGTTTGCCCGTAGCAAAGGATGGCGGTTTAAGCTCA
TCACTGAAACAGAAGTGCGTGAAGTCAGACTGAAGAATGCGAAATTCCTGCTTCCTTTTGTTAAGAAAGG
GCCTGTAGCAGAAGTTGATATGGATATTCTGGACGATGCGATCCAGACGCTGAACCAATCAACACCTAAA
GACCTTCTGGCCCACGTATATCAGGATGAATGGAACAGAGCGCGTTTACTGCCTACGCTCTGGTACCTGA
TAGGAACCCGTCAGATCGCTACCGATCTCGATCAACCTCTCACCATGTCGAGTACCATTTGGAGGTTGAA
CGCATGAGTCATCAACAATTGGTTCTTCAGCCCGGTAAGCGGGTCAGCTACCAGGGACGCTTATTCCGGA
TCGTTCAACTGCTGTCTCTGCAATTGGCTGAGTTGATTGCCGAAGATAACCACGAAATCATTAATGCCAG
TATTACGGAACTACAGGCTCCAGAGGCTGAAGTCATTAAACGACCTGATTTGACACTGATTGATGACAAA
GCTTGGGAAACTGCTCGCAGCCGTATGAGTATCATCAAGCCGCTGCTCAATCTGTCATCCAGAACCAGAG
AACAGGTGGCAGCCCACGGCCAGAAACATTCGGTTTCAACCAACACTCTGTACGGTTGGATCAAGACCTA
CGAGAGCTCCGGCGTACTATCATCGCTGCTACCCAAAACCCGCCGGGACAAAGGAACGACCAAACTGTCG
TCTGAGGTTGAAGAGATCATCAGAGAATCAATGGCGACAGACTTCCTGACGTTGCAGAAAAAGTCTGTTC
AGAAGCTTTGCGATGAAATCATCAAGCGTTGTATGGGGGCAGGACTAGAGCCACCGCACGACAACACCAT
ACGCAACCGTGTTAAGGCTCTCTCGCCTGAGATAGTCGTCAGTCAACGGGAAGGCCGCAAAAAAGCAGAC
CTGTCATTCAAGCCACACCAAGGTTCTTTTCCAGGTGCGGATTGGCCGCTATCTGTTGTACAGATTGATC
ACACCAAGCTCGACATCATTCTGGTAGATGATCACTACAGGCAACCGATAGGTCGCCCCTGGATCACACT
GGCGTTCGATGTGTACAGCCGTATGGTGTCAGGTTTCTATGTGTCGTTTGATCCGCCCAGTGCTTTATCT
ACAGGCCTGTGCCTGGCTCATTCAATTCTGCCAAAAGACAAATGGTTGGCTCAGAACGGCATAAAAAGTG
AGTGGCCAGTGTGGGGACTTCCGACCACTATTCACGTTGATAATGCGAAGGAGTTTCGAGGTGAAATGCT
CCAGAAAGCTTGCAATGAATACAACATCAACATTGAGTGGCGCCCGGTAGGCCGACCGAATTTTGGTGCT
CACGTTGAACGTGCTCTCGGGACGTTTTCAAAAGAAATCCATACCCTGCCTGGCACTACCTTCTCCAACA
CATATTCCAGAGGCGACTACAAATCGGAGAAGCATGCAGCGCTGACCTTATCGGAGTTTGAAACTTGGCT
GTCTATATACATTGCTGATGTTTACCACCAGAAAATTCATTCAGCTTTGGGGGCTGCACCTATTGATGTT
TATAAAAAAGGAATTCTGGGTGATGAGCAGCAGCCGGGATGTGGCCTGCCGCCTAAGATAACGGATGAAG
ACAGACTGCGATTGGACTTCATGCCATTTGAGCTCAGAACCATTCAGGACTATGGAGTGGTTGTAGAGGA
AATTCATTACTACCACGATGTACTGCGGCACTGGATAAACGCCCCAGACCCAAAAGATAGCAAGCGGAAA
CGCAAATTCACCTTCAGGTGCGATCCTAGAGACATCAGTGTGATCTGGTTTTTTGATCCTGAAATCCAGA
CCTACTACCCGATCCCTTATCGGGATACTTCTCACCCCGCGATCAGTATCTGGGAACTACGGGAAGCCAA
GAACAAGGTTACCTCAGACAAACGCCGTCATCTTAGTGAGCGCAGTATTTTCGATGCCTATGACCGTATG
CGTGAGATAGAGGCCGACGCAATGGCCAAAAGCAAGGCTGCGCGTCGAGCTAACCAGCGGCGAGCTCATC
ATCAACAGATTGAAAAGCCGTTGATTACTACACTATCAGATAAGAGCCCTGTAATAGAACGCTCAGTGAT
AAGTGAACCAACTACGACTATACCGGATATTATGCCATTTGATGAAATGGATGACTTATCATGATTGCGT
ATAACCATTTAAACGATTCAGCGGTTGAGACATTGTGTTTACCGGACGCAGATAGAATTCTCAAAATACG
ATCAGAGCGATGGATCGGTTATCCAAAAGCAAAACAGATCCTCGCCAAACTGGAAGACCTGCTGATTTAC
CCCAGAACTAACCGGATGCCAAACCTGCTGATTGTAGGTGATACCAATAATGGCAAAACAATGTTGGTAC
AGCGCTTCAAAAACTTACATCCTGCAATCGACAACCCATCCGGAGAAGGTATTCAGGCTCCAGTCCTTTT
GGTTCAGGCTCCACCAGTACCGGATGAAGGACGATTTTATAACGCTATCCTTGAAATGCTTTTTGCCCCA
TATCGCCCCAGTGAACGTGTAGACAAGAAGCAGTTTCAGGCGCTCAAATTACTCCGTTATGTTGGTCTAA
AAGTACTGATTATCGATGAAGTTCACCATATTTTGGCGGGTAATATGAATCGCCAGAAGGCATTCTTGAA
CGTCATAAAATATCTGGGTAACGAACTTCAAATCAGTATCATTGGTGTCGGTACCAAAGATGCTTTTCGG
GCCTTGCAAACTGATCCTCAGCTTTCAAACCGGTTTGAACCGGTCACTTTACCTCGATGGGAAATGGATC
ATGACTTTCAGAGGTTAGTGGCAAGTTTTGAACGGATGCTTCCTCTAAAACAAGCTTCAAATCTCGCTCA
GCAGGACATGGCATCCAGCTTGTATGCCATGTCGGAAGGCTATATCGGTGAACTGTCACGCTTGCTATCG
GAAGCTAGCGTACTGGCAATTAATTCAGGAACTGAGACAATTTCAGCAAAAATTCTCCAGCAAGTAGATT
GGACTCCTCCATCTAAGCGTAAACGTCAGTTGGAAGGTATGCGCTGATGTTGTCGGGGACTCTTTGGCCC
GCACATCCCCATCCATTGCCAGGAGAATCATTGTCCTGCCTTATCGTTCGCACAGCCCACGCAAATGGAT
TGAAGGTGCAGACATTCTGTGATCAGGCCTTTGGCAAAGAATTTCAGGTCTGGAACCGGGACATAGATAG
GAATGCGCCTGACTGGCTACTGAATATCATGAGCGGGAAAGCGGGAACCCCAATCAAGCAGATCAGAAAT
ACCACTTTCAGGCTTTATGAGAAGCGGTTATTCCCGATTTTACACATGGCTAGTCACCTTCGCTGGGTCA
TGCCAATCAAGCATCGTCATCGTCTTAATACTGGTTATGCTATCCAATATTGTCCACAGTGCCTAGCAGA
GGATGAAACTCCCTATTTCAGGCTTGCCTGGCGTTTGGCACTCTATACTTTCTGCCCAAAGCATAGGGTT
ATGATGGCTGATCGATGCCTCCATTGCGGCGCTCCAGTAGCTTTCCACCGGATTGAGTTAGGTAAGCCGC

AGCGGTCAGATGTTGATACGCTGGATTGCTGCTGGCAGTGTGACGAAAAACTGAGCAGCATAACCACACA
GCCTGTCATTATCACACCAACGCTTGTGGACAGGCGGTGGACCCGTATTCTGCAATCCATTGACCGACAA
TTTTATCCAGCAGGAGCATTGAACTACAGGAATCTGGCCCTGCTCCATCAGATTTGTCGCCTGATGGGCT
CAAAAAAATATGGCTACCAGCTTATACAATACGTTAGCAAGAAAGGGCTATACCCTTTAATAAAAGTATC
GGTACTGTCACTTGCTTTTGAGCAGAGGTCAGTGCAAGAACGGCATTATTTTCTACAGTTCGCATTTTGG
CTTACCACACATAAAAAGAAAATAAAAGTGGCGATCAAAGAGAGAATTATTCCGATTAACGTCTTGTATC
GAGATTCTGATAATCATCTTAAGAAATACGTCACGATAGAGTTACTGACTTTTCGTATTGGGTAGGAACT
CTCTCTGACCCAGTCAGAGTGAAATGTTTGCGACCATATGAATATCACCCCCTTTTCCTCGGAGTAAAAC
CAATGGCAGCACCGTGTTGCTAGGCTGATGAAGGAAGGGGAAACCCGTCACATACAAGCATGTCACACCT
ATAAACCTTATCTTGTTGCCGCCTTTTACTGATGAGAACGAGTGGTACTATATGCTTCCAATGACGGGCT
ATCATCGGTAGAGTTCGAGAGGCAATACCTATCTCAACAGCAAGTCTAGGAATCTCAAGGCTATCCAGAA
AGTTTTGGATGTCAACGGACGAAGATAGCGAAGATGTTGGTATTGCTACTCATTGGAGGAGAGTATGCCA
GACTTAGATGTTTTGATTATTCAAGATCTAGAAGTGCGCACAAGACGCTTCCTTGATACGATAGGCGGTT
ACGAAGGTTTTGAGTTTCTGTCAGCAGGTGGGTCAGCTGCTGTATTCAAGGTTACGAGGAAGGGTAGATT
GAGTGCGTTCAAGGCATTTAACCCCGAGTTTTTCTCTGGTTCTTACGGCGATGCAGAAAGGCACCGCTTG
GAGGTGCAAAGGCGTTTAATTGGTCATAGATGTGAATCGTTAATCCAGACATATCATATTGCAGAAGCGG
AAGGAACCGCGTTCATTGAAATGGAGTTTATCGAATGGCCGCAATTGACAAAATGTCTAGATAAAGTTCC
CGATGAAGCGATAGTAACACTTATCTCCCAACTTGTTGATGCTGTGCGGTTCCTCGATTCTTTGAAAATT
GTTCATCGAGATATCAAGCCTGACAACATTCACGTCTCACCCGACTTTCAACATTTAAAATTACTTGATC
TTGGGGTTGTCCGTGAATTTGATTCTGTGGATTCAAGTGATGCGTGCTCGACCGATAATGGCAATCAACG
ACCGTTTTTGGCAACAGCTCAATACAGCTCTCCGGATTATCTTTTTCGTCTGGATGAGCCGACCAGCAAA
CTATGGCGCGGGTTGAACATATACCAAGTCGGTGCTGTATTGCATGACTTAATTAAAAAAGAGCCTCTTT
TTAAGCACGAAATGTCGCTTGGTAATAGGTGGCTTGTCGCAAGGGCCGTTCTTACAAAGCAACCCTCATT
TGAGGATTCAGTCTCAAATCGCCTTCTTGCCCTAAAAGCACTTGCATCACGTTGCTTAGTTAAGGATCTA
GACACACGAATAGCTCTAGTTGGTTGGGAGGATTTTCAACTTGAAGGTGCAGTAGACTCAGTCAGAGCTT
TGCGTGGCAGGCTTGCAAAAGGAAATATTGCTGCTAGGAGAAGATCTAACGAGTCCGTAGATGCACGGAA
CAATTTTGAACGTACTGAACTTATGAAGCGATTCTCTGACCAAGTCCGTGCAGAGCTCATTCATATCTGC
GGAAGGCAGCTCCCGTTAACAATGATCTACCTGTCACCTGGTGAGTCAACGGAGGTAAAGTATGAGTTAA
CTGTTGATCAACAGCTATCGATTAGTTGTCACATGGAGTTCAACTGGCAAGAGGGCCTATATCAGAGTAG
TGCTAACTTATTGATCGGTGTACAAATAATCTCTTCTGTCAGTAGTGAGGCTATGCCTGCAGTTTTATAC
CAAGTTGCAAGTGTTGTAGGAATTCAAGAAACGGAAGGTGAGGCTATAACTCAAACAGTTGATAAGTTAG
CTAGCCTGATATCTATTGCACTAGATAAAATCGAAGTTACAGATAATCCTGCTAGCTTGCATGGCTTGAA
TATTCAGGTTCCCCAAAAAGTTGAAATGTGATAATAGAGGATAGAAGAATGATGAATAAAAGTTGGTGGC
GTTCAAAAAACGAACTAGATGATGATCAGAAAGCATTTATTCAGTTGCCTGCCCAAGGTCGTTATTCTTT
AATTGGGCCGCCAGGATCAGGGAAAACAAATTTACTGCTTTTACGAGCGCAGTTCATTGCTGGGACAGGT
GAAAAAAACGTACTGATAATTACATATACCAAAGCATTGGCTGACTTTATACGTACTGGAATTGGGGCAA
CTGGCTTAATATCTCCTGATCAAGTCAGAACGTATCACTCATGGGCATTTTCTCATATTCTTGAAAATCT
TGGCCAGCGTGTACTCCCTCGTGAAACAGATTTCGATGATTCTGCTAGAGATGAGGTTGTTCGTCTTGTT
CGTGAGGCTAATTCAAAGTTACCTAGCAAAAAATTATTTAGTGCAATTTTTGTGGACGAGGCCCAAGATC
TAAGTGCAGATGAGCTTGATGCTCTCTTGTGTTTGAGTGATAAAGTAGCTATTTGCGGTGATGAACATCA
AGGTATCTATCGGAGAAATGGATTATCTATTATTGACAAGCTTAAATTGCAACCTCACTCGCTGAAACGA
CACTTCCGTATTGGCCAAAGAATAGCAAGAGTTGCGGATAGGTTGATCCCACCAGAACGTCAAGAAGATA
GTCTTGAGGCAATGTCAAACTACGATTTAAAATCTCAGGGCGAATCGAGCGCAAATTTATACTCATGTGA
GACAAGGGATGAGCAATTTATCCTAATGCTCGAAAAAATAAAAATTCAACTTGATGCATTCAAGGATGAG
GGGATTGGGATTTTCTGTGGAAAAAGAGAGTCGCTAGAGGATCTTCGGTCTCGCTTCAATGATACTGATC
TTGCAGAGCAAGTTTGTGTACATGGTATCGATGAAGATGCTAGCTTCTCTGGTAAATACATGATTCATGT
GCTGACAATGCATGCAGCCAAAGGAACCGAATTCCGTGCCGTTCATCTTTTTGGGATTGAAGAATTGGCA
AGATATCCTCTCAATCGTTCTCGTCTTGCCTATACCGCAATCACGCGAGCCAAGACGGCTCTCAATGCAT
TCCGAACAGGGAGCACAAGTAACGGTATAGAGAATGCATTTGCTGAACCCCGTCATATGGAATTAGACGA
CCTTTTCCCTGGTGACAAGACATGAAAAAATATGAAATTATTTGGACTTTAGTCGATAGAGAGGATCTGC
TCGATGTTTTTCTTGGTGAGCATGAGCCACTCTTTCACAGCGAAATATTTCCAGAATCAGGTTTATATGG
ATCTAAACTTGCCATTGGATATAAAAATACGCAACACCAACCAGCATTTGTATGTCCTTCACTAATAGTG
ATTCCAGACAGCTCGGCTACAGAGACACTATCTTGGCTTCGCACATTCTCTAACGATGTTTCACCCCTTA
GCCAATATGGAAGGGTTCTACTTCTTAGTGAATGGCATCAATATGATGCACTGCGTGATGTCCTAGATTT
TTCTGAGCCAAGATCCTATCGTTGGGCATCTATGATTGTTGGTGAAGCATTATGCCAGATAGAAGGTGAA
GCTAATTTACACAATCTTCCTTTATCGCGCTTTGCTGGTTGTTTCACTACTCCAATTGCGAGAGCCGCCA
TTATCTGGGGGCACAAAGATGTGACTACTGCTTGTGCGGATAGGTTACGACTACTGGAAACTGATCACCG
GTTTGCACGTCGATCAGTCAGTGTGGAACAATTACATCCAATTTGGTCAATCGCGAACTCTAACATCTAT
AGCAACCTATCCCCATACGAGGCTGTTGATATAGTGATGGAAATTGCACGAACTTACTTCAGATCTAGAA
ACCAAGATAATAGTAATATTTCGTCATCAATTCTGACGAAAGAATTCCACGGACTAAATAGTGACTCAGT
GGAAGAGCGTGTAAGCACGTTCAATAAACTCAGTTCGCACATTTTATATAGTGCTCAATCAAGTATTGGA

ACGACACAATCAGCCATGATTGACGTTGTCCTTGCTGCAGCTGCTTTTTTGGTTGGGAGAAGTACGTCAC
ATGTTTTCTTGTTACAGCGTATACAGAAAGTTGCACCAACAGCCGCAGCATGGTTTGGTGCAATAGCTGC
GCTGGCTGGTCCGCGTTTATGGGATAAAGCTTGGCTCAGGTTAGCAAAAGGGGCAGAACGATTGCTACGC
CCAGAGTTCAGTTGGCTAGATGTTCCTAATGCTGATATCTGTTGGGGAGAATTTATATGGCTTGCTCGAA
CTTTTGATAAATCTGATATATTTCTGGATTTGCCAAAAATGCTTTCTCGAACATTAACTATAGAAGTTGT
TCCTGGATCTACATGCCAGTTTCGTATTGGTAATGGAACTGCAGAAATAGAATCAAAGCCTACACAAATT
CCATCAGACCGTGAACGTGAACTCTCTGATATTGTTGGTCAATTTCTTGCTCTCGCTAAGTACGTTGAAA
ATAAAGTTGAGCGTTCTTCTTTGAGTTCACACGTGCAGTCACAACTGTCTTTAGAATCTAAAGGGAATTC
TGATGTGAATAAGAATCAACGAACGAAGAAACCAAGAAAAAACTCTATAGAAAAATAATTTAGTAGCGTG
TAGATAGAACACTGACGATGTCGTTTAAATGAAGGAGAGCAAAGCTCTCCTTCATTTATTTTAATCATTT
TTGGTAAGAGTTAGTTTTATTAGGCGGCGTATAGACGTTTTTGTACGGTCTCTGAGAATGAGCGATGATC
AGTCCAAGTATGTCTAAGGCTGATGATCTCAATGCCAGAACTTTTAGTATGAATATTTTGCCATACACCT
TTAGCCATCGAACACTGAAAAATTATATTGTAAAGCTCTTCATCACTTAGCTTTTTCAGCCCTGATGAAA
TCAAAGAAACTGTGTAATTTTCACCCTCTCTCATTTCAAATTCTATTAGATTAAAAAAACATCCATCAAA
ATATAAATCTGCAATTATGACACCACGCTTTGTTTTGGTTGATGAGTCCAAATATGCCCATTGCCGCCGT
TTATGAGGTTTGGTTAGTGGCATATTCTCCAAAGCAGGATTAACTTGGTGAATGCTGGCAGATATATGAT
TATACCTATTGAGTTTATGTATGGCAGCAATTAAGGTGTCAAAACTAGCTTTAGCACCTTTCTTCCGTGA
AAAACTAGGTGAAATATTAGCTTTTTGAATATTCTTTTCTTCAAGACCATAGCCACCAAGTCCAGTCCCG
AGTGCTTCTACAGCAAAGTCTTGTAAAGATGATCCTGCGCTACGGTACTCACATTCCAATTTTTTTGGTT
TATCTGGTTTTCGGCCATTTAATATCCCAAATCGATCGCTTGGAAGTGCAAGTATCACATTTGACAGGCT
TTTATTTGTGTCTGTTCCACTTTGCAAAGGTGGATCTTCAGCAGTTTTGGTATTAGTTCCTTTTTGGCGT
GAGTATGGTTTTTTTTCTTCTTCGGGGCGATCTGTATCACTTTCTGCTCCGGTGCCATCATTATCTCTGA
TGACGGTTAATGTACTGTACGGAAATGGAGCAGAGCAACGTTCTAAGGACAATATTAGGCAACGCCATGG
GGAGTCATCTGATTGTTGAATCCACTTAACCCTTGCTTCTAAATTTGTTTTACCGGTAAATGGAAAATTA
GATTCAATATGCAAGAATTTATGGTTAATCGTATTTTTAAGTACAGTATCATGAATCTTGCAATATGATA
AGAATGCCTCTTTAGAGTGGAGAATTCTGGCAATTATCCACCCTTCTTCATCAGAGATATGATCTCTCAA
ACCAAGAAAAAACCTATCATCTTCGGCTCGATAATAAGTTCGACTGACGTTTATTATCGAATCTGGATTA
TGTTGTAATGATCCAGAGAAAATAGCGTGAGCCAAATTCGTAGAAACTGCATAGTAAAATCTGACTAATT
CCATCACTGGGATGAGGAGAGCAAATGGATCGCCATTCCGTTCAATGGCTATCAATTTACTTCCGGCACC
AGCTTTACCAATACGATATGACTTATATGGAATTATATTCTTGCCGTTTTCTTTGTGTGTTGCCGTAATA
ATTCTAGTTGTCTGTGGCGAAATTATCAGATTTTTGATGATTTCATATTTGCCTGCTAGGATTTCCTGAC
AAAGACCGTCTCGCCATATGGATCCTGTGGAAACCAATGGCAATTGTCCAATACCAATATTGATAACTTT
TTGCTGTTCTACATCAGTTTGGTTAACTGATGCGCGCCTGTCTCCGGGGATCCCAGATGGGTTACCGAAG
AAAGGGCTAATTACTACTTGGAAGAGCGGCTCAGTAGTTATGTCCGGATTAGGGTGCACAGCTCCTTTCC
AATCAACTCGCCAGAATTTCCCATCCATGGGAAATTCTTTGATCTGGGGTATCCTTGTTATTGCCATTCC
TGAGCCTCGTAGTACAAAACATCAGTGTTAGTCCTATTTACTGATGTAGAGTACTATTTAGCCTTGTAAC
CCACAACCTCTTCGCCCTTGGCTTGCAGATCAGCATGGTAGGAGGAGCGAACGAAAGGACCACAGGCGGC
GTGAGTAAAGCCCAGCTCCATGGCGACATCTTTCAGCTCGTCAAACTCTGCGGGCGGCACGTAGCGCTTG
ACCGGCAGGTGGTGGCGGCTCGGCTGCAGATACTGACCCAGGGTCAGCATATTGACGCCGTGAGCGCGCA
GATCCTTGAGTACCTCGACGATCTCTTCGTTGGTCTCGCCAAGGCCCATCATCAGACCGGATTTGGTCGG
CACGTGGGGGTGCATCTCCTTGATGCGGCGCAGCAGCTCCAGCGACCACTTGTAGTCGGCACCCGGGCGG
GCGACGCGATACATGCGCGGCGCAGTTTCCAGGTTGTGGTTGAAGACGTCCGGCGGGGTTTCGCGGAACA
CCTCCAGCGCCTGCTCCATCCGGCCACGGAAATCCGGGGTCAGGATCTCGATGCGGGTCTGCGGGCTGTG
CTCGCGGATCTCGCGGATGCAGTCGGCGAAGTGCTGGGCACCGCCGTCGCGCAGATCGTCGCGGTCCACC
GAGGTGATCACCACATACTTGAGGTTCATCTCCTTGATGGTCAGCGCCAGCTTCTTCGGCTCTTCCGGAT
CGAGTGCCAGCGGGCGGCCGTGGGCCACGTCGCAGAAGGGGCAGCGGCGGGTGCAGATGGCGCCCATGAT
CATGAAGGTGGCGGTGCCGTGGTTGAAGCACTCCGCCAGGTTGGGGCAGGAGGCCTCTTCACAGACCGAG
TGCAGCTTGTTCTTGCGCAAGGTGCTCTTGATGTGTTCGATCTTCTGGCTGGAGGGAGGCAGCTTGATCC
GCATCCAGTCCGGCTTGCGAAGGAGTTCTTCGTTGGGATCCGGCATGAATTTCACCGGGATCAGGGCCAT
CTTGTCGCCATCGCGCAGCTTGACGCCCGGCTCCATACGAACGGGTTTGCTCATGATTCACTCTCGGTGT
TTGTTATTGTTTGATAGCCAATCAGGCGAGCCAGCTCGGCCACCAGAATGGATTGCGCCTCGGCCACGCT
CTGCGGGCCGCCCAGGGCGCTGGTCTGGGTCATGGCCATGCCCGCATAGCCGCACGGATTGATGTGCAGG
AAGGGGGTCATGTCCATGTTGATGTTGAGGGCCAGCCCGTGGAACGAGCACCCCTTGCGGATGCGCAGAC
CCAGCGAGGCCAGTTTTGCCTCCATGCCAAGGTCATTTTTCACATAGACCCCGGGGGCATCGGCCTTGGC
ATAAGCCTGGATATCGCTTTTGGCGAGGGTATTGATGATGGCTGTCTCCAGCGCGGTCACCAGCTCGCGA
ACCGAGAATTTGCAGCGGCGGACATCCACCAGTACGTAGAGCACCAGCTGGCCCGGGCCGTGATAGGTCA
CCTGACCGCCGCGATCGCTCTGCACCACCGGGATATTGCCCGGATTGAGCAGGTGTTCGGCCTTGCCGGC
CTGGCCCTGGGTGTAGACGGGATCGTGCTCGACCACCCAGAACTCGTCCGGGGTGTCGCTGGTGCGGTTG
TCGGTGAAGGCTTTCATGGCGTCCCATACCGGCTGGTAGGGGCGGCGACCCAGCTGCCTGACAATCAGGC
TGGACTGGGCGGGGATCTGGGATGACATGGCGTCCTCGCGGCGTGCTGCGGCCAAAAACAGTCGCCCCGC
AGAACGGGGCGAGAGAGAAATTACAGTACGTATTTCACCAGCTCGATCTTGCCAAGGGCGTGGTACATCG

CTTCGATGTGCTCCTTGCTCTGGGCGGTCACGGTCACGCGGACCGAGTGGTAGTTGCCCTTGGAGCTGGG
CTGGACTGTCGGGCTGTAGGTGCCGGGGGCGTGCTGCTGCAGCACTTCAACCACCATTTCCGGCAGTGCC
GGGTCGGCAACGCCGAGTACCTTGAACGGGAATTTGCAGGGAAACTCCAGCAGTTCATCAAACTTGGTAT
TCATTTACTCACCTTTTGCGTCATCACCGGGATGACTTCATCTTGGATGGCTAGTGGCCGATGGCCCGTG
GAGGCCGCCATTTCAAGTCTATATGGGCACGGCTCATAACTATATGGGCGCTGCCCGGACCGGGATCAAG
GCCAGGATCGGTCAGACAAGGATGGAAGTGCTGCTTTTTACCACAAATCAGGGTAAGGCGGAACCTGCAG
TTTACAGGGTTATTCACTGCCTTTGCCGGTCACAGACAGCAGCGGCGCCTGTTGGCGCCGCTGTTTGGTG
AGGATTGTGCGTTGGATTACTTGAACCAGCTGGTGACCAGCAGTACCAGATAGTCCCACAGACGGCTCAT
CAAGCCACCTTCCTGCACCTCTTCCAGGGCAACCAGCGGCACCTGCTTGATCTCTTTGTCGCCCTGTTTC
AGGAACACGGTACCGACGCGCTGCCCCTTGGCCAGCGGCGCTTTCAGCTCGCTGTCGAGCTGGAAATCAG
CCTTCAGGTTGTTACCCTGACCGCGGGTGACCAGCACGGAGACATCTTTGTCGACGCCCAGTTTCACTTC
CGGTTTGTCGCCCATCCAGATCTTCTGGGTGACCAGCTCGGTGCCTGCCTTGTACGGGGTCAGGCTCTGG
AAGAAGCGGAAGCCGTAGGTGAGCAGTTTTTTGCTCTCGGCGGCGCGGGAGGCTTCGCTGCTGGCGCCCA
GTACCACGGCGATCAGGCGCATGCCTTCGTTGTTGGTGGCAGAGGAGACCAGGTTATAGCCGACCTGGCT
GACGTGGCCGGTCTTGATGCCATCCACTTGCAGGGATTGATCCCACAGCAGGCGGTTGCGGTTGTGCTGG
GTGATGCCGTTGAACACGAAGGATTTCTGGGAGTAGATCTTGTACTCCTCCGGCAGGTCGCGGATCAGCG
CCTGACCCAGACGGGCCATGTCGTGAGCGGTACTGTAGTGACCTTCCGCATCCAGACCGTGCGGGTTCAT
GAAGTGGCTGTCATTCATGCCGAGCTTGGCGGCCCAGCTGTTCATCAGGCTGGCGAAGGAGTCGGTGCTG
CCCGCCAGGAATTCGGCCATGGCGACGCAGGCGTCATTGCCGGACTGGATGATGATGCCCTTGTTGAGAT
CATCGACGGAGACCTGCTTGCCCACCTCGATAAACATCTTGGAGGAGTCGGAGTAGTTCTTGGACCAGGC
GTTCTGGCTGATGGTCACCATGTCGGTGCGCTTGATATTCCCCTTCAGCAGCTCCTGGCCGATGATGTAG
GAGGTCATCATCTTGGTCAGACTGGCGGGCGGCAGACGCTCTTCGGCGTTCTGCTCGGCCAGCACCTGAC
CGGAGTAGTAGTCGATCAGAATATGGGCCTTGGCGGAGATCTCCGGCGCGGCGGGAACCACCATGGGCAT
GGCATTCGGGGTCGGCACCGGCGTATTGGCCTGGGCGGTTACGCTGATCAGTGAGGCCAGAAGAACTGAA
CGGGCAAATTTGGACATCTTGAAATTCCTGCGAGCCAGATGGGGATAAAATGCGGGCCTAACTATACCAG
ATGCGCCCGCAGGTTATAGCCCGAGATGGCGATCAGAACTGGTTTTTTATCCATCACTCACTGTCATCAC
GCCATAAAACGACGTGATGACAGTGAGTAAGCGGTTAGTCACATCCGCGGTAAAAGGGGGCGATCAGGGC
ACGATAAAGGCCTGTTCCAGCCCGCCCTGACGTACTTTTTCCAGCACACTTGCCTGCTGGTCGGCGGCGA
CCGGGCCAATCAGCACCCGGAAAATGGCGCCATGGGCATCGATCCGGTAGGGCACGCCGTAACGCTGGGT
CATCACCTTGCCCATCGCCTCGGCCCGATCCCGGGAGCCGCTGGCCAGTACCTGGATCATCTTGCCATCC
CCACTGCTGGCAGGCGCTGCGGCCTTGGCGGGAGCTGGCTTGACAGGTGCCGCTTTGGCGGGCTTGACGG
GAGCGGGTTCGGCAGCGGGAGTGGCCATCGCCAGCGCCGTCGGTTCACCCAGAAAACCATCTTGCGACAG
CGTGTTGGAGGCCGCAGCCGGAGTGGTTGGAGTCATTTCGATGGGCTTCATCGGCTCCATGATCACCATC
TGATCCGGGGTGTCGCTGATCAGCTCCAACCGTACTCGGGCCTCACCGCTGTCGACCATCTCCAGCTGCT
TGGCCGCTGCGTAGGAGAGGTCGATCAGGCGCGGGCTGTTGAACGGGCCGCGATCGTTGACCCGCACAAT
GGTCTCCAGCCCGTTGTCCAGATTGGTAACCCGCACATAGCTAGGGATGGGCAGGTTGCGGTGGGCGGCG
CTGAACAGCTTGCTGTCCAGCACATCGCCGTTGGCGGTCTTCTGGCCGTCCAGATCCGGCGACAACCAGC
TGGCGGTGCCCTCTTCGCTGTAGTGTTTGATGTCGCGCCACACCTCGTGCTTCTGCTTGCCGATCCAGTA
GTCCCGGTTGCCGCTGGCACTCAGGGACTCGGGGCGCGGAATGGCGCCAGTGGCTTGCGGGATCTCGATA
ACGGGTGGCTTGCTGACCGGTGCTTGCGGCTCGACGGGGGCGCTCTGTTCCGGCTGGCTGCTACAGGCGG
CCAGCAACAAGCTCAGGCTGAGCGGGAGGAGTTTGTGACGAAGATGCATACGCTTCCTGAATGGCTTGGC
TAAGTTGATACACCGCCATCGCATAGAGTGGGCTGCGATTGTAACGGGTGATCACATAAAAGTTATGCCG
TGCGACCCAGTATTCCGGTCCATCGGCTTGGGCAAACGCCAGCAACTTGACCGGCGTCTGTGGGGCCAGC
GGCGTGGCGAGCTCAATCCCTGCCGCCGCCAGTTCTGACCACTGTTGTGTTAACTCGGGCTCAGGCCTGA
GTAGAGGTTCGACTGCCATCAACCCGATCTGGGCCGGTTCCACCACGGATTCGCCCCAGCGCCACTGGTG
GCTGGCAAAATAGTTGGCCACGCTGCCGATGGCATCGACCGGGTTGGCAAACAGGTCCCGCTTGCCATCC
CCGTCAAAGTCGACGGCATAGTGGCGATAGCTGGAGGGTATGAACTGCCCCATCCCCATGGCGCCGGCAT
AGGAGCCCTTGAGGGCATCGAGTTGCCACCCTTCCTCTTTGGCCAGCACCACCAGCTGGGCAAACTCCTT
GGCAAAGAAGTCGCTGCGCTCCGGGTAGTGGAACCCCAGGGTGTAGAGGCTATCGAGCACCGGATGACGG
CCCATCTGGCGACCGTAGAAGGTTTCTACCCCTATGATGGCCACGATGAGCGAGGCGGGCACCCGATAGG
TCTGCTCCGCGCGGGCCAGTGTCTCGGCATGTTGCTGCCAGAACTGCTGGCCGTCACGGATGCGATCCGG
GGTAACAAACAGCGGCCGGTAGCGATGCCAGGGCTTGGCCTCCCACGGCTTGCTGATGGTGTCGAGCACC
TCCTGCCGTAACTGGGCGCGGGCGACCGCGGCCTGCAGCTCCGCCAGCGGCACCTGCTGCTGGTCGGCGA
GCAGGGCCAACCGATCCGGCGACACGGCGGCGGTCGCCATCGTGGTGGCGAGGGAGAGCAGCAATCCAAC
CCGTCTCATCAGGCGAGCAGTCTTCTATGAGTCTGGATGGACATCAGTATGCCAAAGCCGGCCATCAGGG
TCACCATGGAGGTGCCGCCATAACTCACCAGCGGCAGGGGCACCCCTACCACCGGCAAGATGCCGCTCAC
CATGCCCATGTTGACGAACACATAGACGAAGAAGGTGAGAGTGATGGAGCCGCCCAGCAGTCGCTCGAAG
CTGTTCTGGGCCTGCATCGAGATAAACAGGCAACGGCTGATGACGTAGAGGTAGAGCACCAGCAGCAGGG
CAACCCCTACCAGACCGAACTCTTCGCTGAATACCGCGAAGATAAAGTCGGTATGCCGTTCTGGTAAAAA
CTCCAGCTGGGACTGGGTCCCTTGCAGCCACCCCTTGCCAAAGACGCCGCCGGAGCCGATAGCAATCTTG
GACTGGATGATGTGGTAGCCTCGGCCGAGCGGGTCTTTCTCCGGGTCGAGCAGCATCAGCACCCGCTGGC

GCTGGTAGTCGTGCATCAGGAAGAACCAGAGCACCGGCATAAAGGCCAGCATCAGCAGGACCGCCAAGCC
GATCAGCCACCAGCTGAGCCCCGCCAGGAAGATGACGAAGAAGCCGGAGGCGGCCACCAGAATGGAGGTG
CCAAGGTCAGGCTGGGCGGCGATCAGCAGGGTGGGCAGCAGCACCATGATGAGGGCGATCACCACATGGC
TGAACTTGGGGGGCAGCGAGTGGCGACTGAGCCAGGCGGCCACCATGATGGGCATGGAGAGCTTCATCAC
CTCGGAGGGCTGGAACTTCATAAAGCCCAGATCGAGCCAGCGCTGGGCCCCCTTGCCGATGTGGCCGAAC
ACGTCCACCGCAATCAGCATCAGCACCACCACGGCGAACAGCGGCACCGACCAGCGGGCGTAGAGGGAGG
GGGGCAGCTGGGCCAGGCCGATCATCAGGGCAAAGGCGAGACCGCCGCGCACCAGCTGGCGCACGATAGA
GTCCATATCCTGACCTGAGGCGGAGTAGAGCACCACCATGGAGAGGGTCATCACGGCCAGCAGACCGAGC
AGCAGGGGCAAGTCGATATGCAGCTTGTACCAGATGCTCTGCTGGCGGGCAGAATTACTCATTGGGGGCG
ACCTCACTCGGTTGGGACGGCTTTTCCGGTGCCGGTGGCAGTTGCGGATCAGGGGGCAGCAGATAGGCAT
CCAGCATTTGGCGTGCAATGGGCGATGCAGCTCGACCACCGCCACCTCCGATGGAGTTTTCCAGTACCAG
AGCTACCACCGCTTTGGGTGCCTCGAAGGGGGCATAAGCAACGAACAAAGCGTTATCGCGATGCTCCGCC
TTGACCGTCTTGATGTTGTAAACCTGATTTTCCTTCAAACCGACTACCTGGGCTGTGCCCGATTTGCCAC
CTGAGGAGTAAGGCGCCTTGGCAAACGAGTGACGGCCACTGCCTTCACTGCCATGGTTGACTCGCCACAT
CCCATCCTGCGCCACTTTCCAGAAGCTGTCGTTCTTGAGCTTGAGGGCGATATTGGGGTTGATGGGGGCA
TCGACCACCCCCGCCTTGGAGGCGGTGCTCTTGAGCAGGTGCGGGGTCACATCGTGACCGTTCTGGGTCA
GGATGCTGATCGCTTTGGCCAGTTGGAGCGGGGTGGCGCTCCAGTAGCCCTGACCGATACCGACAGAGAT
GGTGTCACCCTGATACCAGGGCTGGCGCCAACGGGCCATCTTCCAGTCACGGGACGGCATGACACCCTTG
GTCTCTTCATAAAGATCGACCCCGGTGTACTGACCGAAGCCAAACTTGGTCATGTAGCTGTGGATCTTGT
CGATGCCGACTCGATAGGCGAGATCGTAGAAGTAGGTATCTGCCGAGACCTCGATGGCGCGATAGACGTC
GAGCCAGCCATGACCCCAGCGGCGCCAGTCGCGAAACTTCTTGGTGGTACCGGGAATGGAGAAGCTCGGG
CCGCCGAAATAGCGGTAGCCCGGGGTGATGGCCCCCTCGTTGAGACCCATGATGGACATCATGGGCTTGA
CGGTGGAGGCAGGGGCATAAATGCCCTGAGTGGTGCGGTTCACCAGCGGCCGCGCCGGATCGTTGAGGAG
CGCCGAGTAGTTGGCGCTGGAGATGCCGGTCACGAACAGGTTGGGGTCGTAGCTGGGGCTCGACTCCAGG
GCCAGGATGGCGCCGGTTTTCGGGTCCATCATCACGATGGCTCCGCGCTGGCCAGCCAGCAGTTGCTGGG
CCTTGAGCTGCATCCGGACGTCGATATTGAGGTAGATATCCTTGCCCGGAATAGGTGGCTGGAACTTGAG
GGTGCGGACAATCCGGCCGCGGTTGTTCACCTCGACCTCCTGATAGCCCGCCACACCGTGCAGCTCATCC
TCGTAATACTTCTCCACCCCCTGCTTGCCGATATCCTTGGTGGCCGCGTAGTTGGCGAGTTTTTCTTCCT
TCTCGAGGCGCTCCACGTCACGGGTGTTGATCTTGGCCACATAGCCCAAAGCATGGGTGAGGGTGTCACC
AAACGGGTAGTAGCGTTTGAGGTAGGCCTCGATGCTGGCCCCCGGGTAGTTGTGCTGGTTGACCGAGAAG
AGGGCCACCTGATATTCGGTCAGCTTCTCGTAGAGGGCGACCGGCTTGAAGCGGCGCTGGCGCTTCACTT
CGGCGAGGAACTTCTCCTTGGTGCCCTCTGGCAGACCCAGCAGGGTGATCAGCTGGTCGGCGGTTTTTTC
GAGATCTTTGGTCTCTTCCGGCACAATTTCCAGACTGTAGACCGGGCGGTTTTCCGCCAGCAGCACGCCG
TTGGCGTCATAGATGAGGCCCCGTGGTGGCGCCACCGGTACCACGCGGATACGGTTGGAGTTGGAGCGAG
TCTGGTAGGTGTCGTAGGACTCGACCTGCAGATAATAGAGATTGCCCAGCAGCACCAGCATCAGCACGAC
GATACCGATGTAGGCTACCAGGGTGCGCCGGAAGAACAGATTGCCCTCGGCACTGTGATCGCGCATTTCG
ATGCGTTGTTTCAGCATGCCTTTACCTCAAGACCCTTGGGGGCAATAAGAGCAGGAACTGCATTGTTCAC
TCTCTGTGATAGGGGTGAATATGAGAGGTGAGACTCCAGAGACGCATGGCTCACTCTCTATGATACGGAT
GATTGGTGGTGATGCTCCAGGCCCGATAGAGGCTCTCTGCCACCAGCACCCGCACCAGCGGATGGGGCAG
GGTGAGCGGTGACAGTGACCAGCTCTGTTCAGCGGCAGCCTTGCACTCGGGGGACAAACCTTCCGGCCCG
CCTACCAGCAGTGCGACATCGCGGCCATCGAGTTTCCACGCCTCCAGCTGGGTAGCCAGCTGCTCCGTGG
TCCAGGGCTTGCCCGGAATGTCGAGGGTGACGATGCGCGAGCGCCCCGCAGCCGCCAGCATAGCTTCCCC
TTCCTTCTGCAGGATGCGGGGAATGTCGGCATTTTTGCCCCGTTTGCCTGCGCCAATCTCGACGAGTTCA
AACGGCATGTCTTTGGGGAAGCGGCGACGATACTCTTCGAAGCCATGCTCTACCCAGGCGGGCATCTTGG
TGCCCACCGCAATCAACTGGATCTTCATGGTCGTTACACTTGTGCTCTTATTGCGTATTCATTTGAGCGG
CACCGCTCCACAGCTTTTCCAGTTGGTAGAAGTCACGATGTTCATCCTGCATCACATGCACGATCACCGA
GCCCATATCTACCAGCACCCATTCACCGCTCAGTTGCCCTTCCACGCTCAGCAGATCCAGACCCGCGTGG
CGGGCCTCGCTGGCCAGATGGTGGGCAATGGCGCTGACGTGACGACTGGAGTTGCCGGAACAGATGATCA
TGGTGTCAGTAATGCTGGATTTGCCGCGTACATCGAGGGTGATGATGTCGCGGCCTTTCATGTCATCGAT
CTTGTCGATGATAAAGGCATGCAATTCTTGGCCTTGCAATGAGTCTCTCCTGTATTCCGGCGCGCAGCGT
TTGCGTCAGGGCGGCGGATTATATCATGGTCACTCCCGTTTCCCATCGGGTGGGGCGGGATTACGACAGG
GGCTAGCGGCGGTTGGCGCGGTAGAGCCCCTGTTGATCAATATAGTGCGCAACCGACTCGGGCAAGAGGT
AGCGGGTGTCTTGTCCCGCATCCAGCAAGGCTCGCAGTCGGGTGGCTGACAGTTCGATGGGCTGGTTGTC
CGCCAGCCAGATATGACCGGCCAGACGCTGATGCAGCGCGTTGGCATCTGTGGTGTGATGGTGCGCCAGC
AGCTGCGCCACCTCGGCCGGATAATCCGGTTGCCAGCCCGGCCGTACGCTCACCACCAGATGGGCATGGT
CGAGCAGCTCCTGCCAGCGGTGCCAGCTTGGCAGGCTCAGCAGTGAATCCATCCCCATCAGAAAACAGAG
CGGCGTATCGGGCAGTTCATGACGCAGCTCGATGAGGGTATCGATGGTGTAAGAGGGCTTGTCCCGTCTC
AGCTCCCGCTCATCCACCACAAAGTGGTGGTTCTCTGCCGCCGCCAGTTTGACCATGGCAAGGCGCTGTT
CGCTCGAGCAGAACGGATTGGCGCGGTGGGGCGGGATATGGTTCGGGATCAGCCGCATCTCGGCTAACCC
GAGGGCATCGCGCGCCTCGATGGCCGGGCGCAGATGGCCGATATGGATAGGATCGAACGTGCCCCCCAGC
AGGCCAATGGGCGCCTTCAGCACGGGCTGTTCTCCATCACCAGCGGCAGCGGGGTGCCATCACGAAAGCC

GAGGGCAATGGTCTGCAGCATCAGCCAGGCCTGCTCGCTATCGAAGCGGCGCTGGGCATCGTCCAGCTGG
GCCATCAGTTGCCACAGTTGCTGCAACTTGGCAAACGGCAGTCGGGTCAGCGCTTGCTGAATGAGCTGCT
GGCGGCTCTGCCAGATGCGCAGGCGGCTGAAGTGCTCACCCAGCGGCTGGCCGTTGCGCATCGCCAACGA
CAGCTCGGTCAGCTGCTCCAGCTCGCGGCAGAGGGTCCAGGCCAGCATCCCCGGCTCGGTGCCTTCGGCC
CGCAGGGCGGCCAGAATGCGCTGGGCCCGGTTCACCTTGCCCTCCAGCAGGGTATCGACCAGCTGGAACG
GGGTGAAGTGGGCGTGGCGGATAATGGTCTCTTGCAGATAGGTGACGCTGATGGGCTGGGGTGGCGAGAG
CAGGGTCAGCTTCTCGATCTCCTGGCTGGCGGCCAGCAGGTTGCCCTCGTAGGAGTGGCACATGAAGTTG
ATGGCATCCGGCAGCGCCTTGAGGCCGAAACGCGCAAAACGGGCGCTCATCCAGCGCGGGAAGAACTTGG
GCTCCGGATGGTTGACCGGCACATAGGTGCCAATCTGGTAGAGCTTGTCGAACCAGGCTGATTTCATCTG
GGTCTGGTTGAGACGACCGCCACTCAGCACCAGCAGCAGGTCGGGATGAAGCTGCTTGCCAATTTCGCTG
ATGCGGGCAGCGAGATCCTTGTCCACCTTGGCGGGCAGCTCCAGCTCGATGATCTGGCGGGCGGAGAAGA
GGCTCATGGCCTGACAGGCATCGTAGACCTGATTCCAGTCCAGCCCCTGTTCCACCACGAAGGTGTGGCG
CTCATCGAAACCCTGCTTGCGGGCGACCTGACGGATGGCATCGATGGCCTCCAGTCGCAGCAGGGGCTCG
TCGCCAAAAACGAGATAGCAGGGCCGAAGCCCTGCTTTGAGGTGGTCACCAAATTGTTCCGGGTAGATCC
GCATCAGAAGCTGACCTGACTCAACTGCCGGATCACCTGATTGGCCGCCTGCTCCTGCATTTCGCGGTTG
AGCTGCTCCTGCTCACGGGAGGAGGCGAGCGCTGCATCCGGCTTGTTGAGGAAGCTGCGGTTGAAGTTGA
TGGGGAAGACCTGAGTCGGTTTGCCCGGTACCGAGATGGTGAACTGGGTACCAAAGCTCAGCACATATTC
GGTGTCCGTCCCCAGCGCGTTGACCGAGGCAACCTGACTGGTGTTGCCTACGCCGCCCAGAGTGAGCACC
GGAATGCCCTCCTTGTCGGCCAGGGTCACGCCAGAGGCCTTCAGACGGGTAGTAACCATCCGGTAGAAGT
CGCTCTTGTTGTCGCCCACGACCTTCATGGTCATCAGCTCGCTCGGAATGCCGTTGCCGCGCATGTGAAA
GCCGCAGCCTTGCAGCAGCAGACCAGCCAGCACGGTGGCCATCCAGCGGGTGAAGATGGTGCCCATAGAA
AATCCTCTTTGCATTAAGGGAAAGCGGCGGGCGCAGGGCCCGCCGTTATCATCCGTTTGCTGTTACAGAT
TGGCAATGATCAGTTGGTAACCAATCAGTTGGCAGCCAATTAGTTAGCGACAATATTGAGCAGCTTGCCA
GGTACATAGATCACCTTGCGCACGGTCAGACCGTCGGTGAACTTCTGCACGGAAGCATCGGCCATCGCCA
GCTTCTCGACGTCGGCTTGGCTGATCTCGGCCGGCACGGTCAGTTTGCCGCGCATCTTGCCGTTGATCTG
CACCACCACCAGCTTCTCGGTCTCGACCATGGCCGCTTCATCGGCCACCGGCCAGGCTGCGTGATCTATG
TCGTCGCCCTTGCCCAGCATCTTCCACAGCTCGAAGCCGATGTGCGGGGTGATGGGGTAGAGCATAACAA
CGACAGCTTCCAGCGCTTCCTGCATCAGGGCGCGGTCCTGCTCGTCGTTGCCGAGCTTGGCCAGATTGTT
CATCAGCTCCATGATGGCGGCGATGGCGGTGTTGAAGGTCTGACGGCGACCCACGTCATCGCTCACCTTG
GCGATGGTCTTGTGCAGTTCACGGCGTACCGCTTTCTGCTCGTTGGTGAGAGCAGCCACATCCAGCGCGG
CAACGGCGCCACCGGAAACGTGTTCGAAGGTGACGCGCCACAGGCGACGCAGGAAGCGCTGGGCACCCTC
TACGCCGGAGTCGGACCACTCCAGGGTCATCTCGGCCGGAGAGGCGAACATCATGAACAGACGGACGGTA
TCGGCGCCGTAACGCTCGACCATCAGCTGCGGGTCGATGCCGTTGTTCTTGGACTTGGACATCTTGGTCA
TGCCGGAGTGGAGCACTTCGCGGCCTTCGTTATCGATGGCCTTGGTGATGCGACCCTTCTCGTCACGCTC
TACCTTGACGTCGGTCGGGCTGACCCAGACGTTGCCGCCCTTCTCGTCTTTGTAGTAGAAGGCGTCGGCC
AGCACCATGCCCTGGCAGAGCAGGCGCTTGAACGGCTCGTCGCTGTTGACCAGACCCGCGTCACGCAGCA
GCTTGTGGAAGAAGCGGGCGTAGAGCAGGTGCATACAGGCGTGCTCGATACCGCCGATGTACTGATCCAC
CGGCAGCCAGTGGTTGGCAGCAGCCGGATCCAGCATGCCCTTGTCGTAGTCCGGGGAGCAGTAGCGCGTG
TAGTACCAGGAGGACTCCATAAAGGTGTCGAAGGTATCGGTCTCGCGGAACGCTTCCTGACCGTTGTAAG
TGGTCTTGGCCCACTCGGCATCAGCTTTGATCGGGCTCTGGATACCATCCATCACCACATCTTCCGGCAG
CAGCACCGGCAGTTGGTCTTCCGGCGTCGGCACCACTGTGCCGTCAGCCAAGGTCAGCATCGGGATGGGG
GCACCCCAGTAACGCTGACGAGAGACACCCCAGTCACGCAGACGGTAGTTGACGGTGCGCTTGCCTTTGC
CCAGCCCTTCCAGCTTGCTGGCGATGGCATCGAAGGCGCCCTGGAAGTCGAGACCGTCAAACTCGCCAGA
GTTGAACAGCACGCCCTTCTCGGTGTAAGCGGCGTCGCTCACGTCGACGTCGCCATCAACCTGCTTGATC
ACCGCCTTGATGTCGAGGCCATATTTGGTGGCGAACTCGAAGTCACGCTGATCGTGGGCTGGAACCGCCA
TCACGGCGCCGGTGCCGTAGTTCATCAGCACGAAGTTGGCGACCCAGACTGGTACCTTGCGGCCATCCAG
CGGGTGAATGGCATAGAGGCCGGTGGCCATGCCCTTCTTCTCCATGGTGGCCAGTTCAGCTTCGGCTACC
TTGGTGTTCTTGCACTCTTCGATGAAGGCGGCCAGCTCAGGATTGTTTTGGGCTGCCTGCAGCGCCAGCG
GGTGACCGGCAGCGATACCGACATAGGTGACGCCCATGAAGGTGTCCGGACGGGTGGTGTAGACCTCCAG
CTGCTCAGCCTGACCCTCGATAGCGAAGCTGATGTTGACCCCTTCGGAGCGGCCAATCCAGTTGCGCTGC
ATGGTCTTGACCATCTCCGGCCAACCTTCCAGATTGTCGATGTCGCTGAGCAGCTCTTCGGCGTAGTCGG
TGATCTTGATGAACCACTGGGGGATCTCTTTCTGCTCCACCGGGGTGTCACAGCGCCAGCAGCAGTTGTC
GACCACCTGCTCGTTGGCCAGTACGGTCATGTCGTTCGGGCACCAGTTTACGGAGGAGGTCTTTTTGTAG
ACCAGACCCTTCTCGTAGAGCTTGGTGAAGAACCACTGCTCCCAGCGGTAGTAATCCGGTTTGCAGGTGG
CGACTTCACGATCCCAGTCATAACCCAGACCCAGCATCTTCAGCTGGTTCTTCATGTATTCGATGTTTTC
GTAGGTCCAGGGCGCCGGTGCGGTATTGTTTTTGATCGCGGCGTTCTCGGCCGGCAGACCGAATGCATCC
CAACCTATGGGTTGCAGCACGTTCTTGCCATTGAGGCGTTGATAACGGGAGATCACATCACCTATGGTGT
AGTTACGAACGTGCCCCATGTGTAGTCGACCAGACGGATAGGGGAACATGGACAGGCAGTAGAACTTCTC
CTTGTCTACCTTCTCCACGGCCTTGAAGGTTTTCTTGGCATCCCAGTGCTTTTGCACTGCTGGTTCGATT
GATTGGGGAACGTATTGCTCTTGCATTGGTGACATCCGGATCTGTGAAATGAATAGATAAATCAGCTGGG
AAATCGCAATAGAATACCCATATCCTGCGGCGGCAACAACCGCCAGCGTCCCTTGGGCCTTAAGCCGGGC

CAGATCAGGGTTTAGTAGAGGAGTTAGAGCCATGGACAAACGTCAAAAAGGGTACGAAGCCTTCATTGCC
GAGCTGCAAAAACAGTGGCAAGAGACTGAAGAGTTCCAGGAGGAGCGCCTCAACCGGATGATTGCCAAGG
TTCAGGCCTATCTGGAGGCGGCCAGCGACCTGACCCAGGACGAACTGGCGCTCATCGCCGAGTACGTGAA
GCGGGATCTGGGCAACTATGACGAGGGGCGCCATGACGATAGTGCGCAGGTGGAAGAGTCCGCCTTTATG
CTGGCCCTGAAAAACACCGCCTGGTCCTGGCTGGCGGATGTGACCGACCGCGCCCAGGTTGAGTGGCGCG
AGCTGGCCGACGAGCTGGAGCACAAGGGCGTCTATGAGGCGGGTGACTGGGTAGGGCTCGGGGTCATGGT
GTGCGACCAGTGTGGCTGGCGCCATACCGTGCTGCATCCCCAGCAGCTCGATACTTGCCCCGAATGCGGC
TGCGCCGAGTATCATCGCGAGCCGCTCTCGCCCTGAGTCATGCCATCAAGGCGGACGAGATAACGAGCAA
AAAGGCGCCATCAGGCGCCTTTTTCTTGCTTGCAGGTTTCGTCGGGGCGTTTACTTGCCTGCGACGGAGA
GGCCACCAAAACGGATAAGCGGGGAGTAGAAGCCCTTGCCGTCGTTGTGGTGCAGGGTATCGCCGACCGC
TTCCAGCTCGCCCAGCAGGCGGTAGAAGTTGCCTGCTACCGTGATGCCGCGCACCGGCTGCAGGCGAACT
CCATCGCGGCAGAGGAAGCCCGACGCGCCAAAGGAGAAGTCACCGCTCACCGCATCGGCACCGGAGTGCA
GGCCATCCAGCTTGACCAGCTCCAGATAGTCGCCGCTTTGCACCTCCGCTTCCCTATGGGGGCCCGCGCC
AAACACCAGATGGCGAGCGGTCACATCGAGGGCGCTGCGGGCACCGCGAGCGGCCGAACCGTTGCTGGCG
ACTCCAAAGTGGCGGGCGGTGGCGCTGTTGTGGAGCAGGGTTTTCAGCTCCCCTTCGCCAATCAGCAGCA
GATCCGAGCTGGCAGCTCCCTCGCCATCAAACGCCTTGATGTTCATGCCGCCCGGCATATAGACCCTGCT
CTCCAGAGTAAGCCAGCGCGACGCTACGCTCTGGCCGACCTTGCTGCGCCAGGGGTTGATCCCCTGCTGG
GCCCACTTGCCCGACAGCACACCCTGAAAGCAGCCAAACAGGCTGGCGAAGCAGTTGGTGCTAAAGATGA
CGCTGTAGTGACCGCTCGGCACAGCTTCACCGGGCAGCAGACCATCGGCGATGGCGTAGATCTGCTCGAT
CAGGGCCTGACTGTCCAGCTCGTCGAAGCGGCGGCCATACTGCCCCTGCGAGTGCATCGACTGGCGGCCA
TCCCGTTCCACCAGCGCCGAGGTGTAGCAGCCGACGCTGAACTCGCCGTGCTGGCAGAGGGTGCCCTGAC
TGTTGGCGAGCCAGAGCTGGCTCTCCCCTTCGAAGAAGCTGTTGTAGGGGGCGCCGCTCACATCGGGCAT
GGCCAGCATGTCAGATTCCAGACGCAGGGCCAGAGCGATCTTCTCCTCAACCGGCGTGGTATCGGGCTGG
TTGATCTCGGCGCAATCTGTGGTGATCCGGCTATTGGCCACCGAGATGCGCTGATCTGCATCCACCTTGG
CAAAGTGGCTGGCATCGAGCGCATCCTGCAGCATGGCATCCAGCGCCAGCTCATCGAGGGATTCCGAGTA
GGCGATGCCGACTCGGCCCTCTTTTATCAGGCGAATACCGAGTTGCTGGCTGCTGGTGACCTTGTATTCA
CCTAGCTCGCCCTTGTCTGCCTTGAGGGAGAAGGCCTTCTCCTGATTGGCGATGATGTCTGCCTCGGCGC
CGAGGTCGGCCACCTTGCCTAGCAGGCGGGTGAGCAGTTGGTTCATGTCAAGTTGGCTCATCAGGCGTTC
CCTCCCACCAGGATATTGTCCACTTTCAGCGCCGGCTGGCCCACCGAGGTGGGGACCGAGCCCGAGACAG
AGCCGCACATACCGGCGGCCAGCGCCAGATTGCGGCCCACCATGGAGATCTCTTTCAGCACCTGCGGGCC
GGTGCCGATCAGGGTGGCCGACTTGAGCGGTTTGGTGATCTTGCCGTGCTTGATGAGGTATGCCTCCTGC
ACATTGAAGTTGAACTCGCCGGTGCCGGGCTGTACTGCGCCGCCCCCCATCTTCTTGGCGTAGATGCCAT
GTTCGACGGTGGCCAGCATATCGTCGAGGCTGTGGTTGCCCGGCTCGATATAGGTGTTGCGCATGCGCGA
GGTGGGGGCGTACTTGTAGGATTCGCGCCGCCCGGAGCCGGTGCGTGCGTAGCCTGTTTTGAGGGCACCC
ATTCTGTCCACCAGAAAACCGGTCAGTACGCCGTCCTTGATGAGCTGGGTATGCTGGGTCGCCATCCCCT
CGTCATCGACGTTGATGGAGCCCCAGGCGTTGCTCTGCAAGCCTTCATCCACCGCATTGACCACCGGATT
GGCGATAAGCTGGCCCATCTTGTCGTGGAACACCGAGGCCTTCTTGGCTACCGAGGTGGTCTCCAGCAGG
TGGCCGCACGCCTCGTGGAAGATGACGCCGCCAAAGCCATTCTCCATGATGACCGGCAGGGTGCCGCTCG
GGCAGGGAGCCGCGCCCAGTTTCACCAGTGCCTGACGGGTGGCGGTGAGGGCCAGCTCGCGCGGATCGAT
GCGATCGGCATGCTCCCACCCCATCAGGGCGCCGGGGGCCTCGTAACCAACGCTCTGCTCGCTACCTTCC
ATGGCGATGGTCTGGGCCATGATGCGGTTGTAGTGGCGGCGATCGGCCACTTGCACTCCTTCGCTGTTGA
AGATCTCCACCTCCTGCTCCCAGCCCAGCACGTTGCCGGAGAACTGGCTCACCTTACTGCTCTCGGCACG
GGCCACCGCATCCATGGCGTGCAGATAGGCGATCTTCTGCTCCAGCAGCTTGTCTACCGACAAGGGCAGG
GCCACCGGGTTGCGATCGCTCAGGCGGGTGAAAGCGAAGGCGGTGGCGGTCACCACGGGGTCGCGGCGGT
CTTTGGCCGCCAGCAGGGTGACGATGCGCAGCAGCTCGTCGCGGCTGGCTTGGTTGGTGTAGCCGTAGAG
CACCTTGTGGCCGTAACAGAGGCGCACGCCGATGCCGAAATCGAGGCCACCGCTGATGTTCTCTATCTGG
CTGGAGAGCAGGCTGAGCTGGCTGCGGCGCTTGCGCTCGACAAACAGCTCGGCGAAATCGGCACCCAGCG
CCAGCGCGTGATCGAGCACGTCACGGGCAATGGCGGGGGCCAACAGGGAGAATGGTGTCATAGAATATCC
TTTTCTGGTCTGTCCGTTTGCTTCCGACATCCGGCTCTGATTGTTATGGGATGTCGAGTCCATTGTTGCA
GACCAGCACAAAAAAGTCGCTATAGCGTCATGCGATGATACCGATTGAGAAAATCGGTGCGATAACTGCG
CCCGGCGGCCAGGGCCAGGCGCTCAGGGTCGTTTTGCCAAAACGTGGGAAAGATCAGAGCAGGGCGTGGA
TTTCGCTCAGGATCTGCTCGACCCACTGCTCGATGCGCGGATCGGTCTGGTCATATTGGTTGGCATCATC
GAGGGCCAGCCCGACGAAGTGACGGCCATCGTCGATCAGTGCCTTGGAGGCATCGAATTCGTACCCTTCG
TTGGGCCAGTAGCCCACCAGCTTGACCCCTTTTGGCACCAGCTGATCGTGCAGCATGCCGAGGGCGTCCT
GGAACCATTCGCCATAACCGAGCTGATCGCCGAGGCCAAACAGGGCCACGATGCGTCCCTCCAGATGGAG
ATCGGCGATATCTTCCCAGCGGGACTCCCAGTCCTCCTGCAGTTCGCCAAAATCCCAGGTGGAGATGCCG
AGGATCAGGATCTCGTAATCCTCCATCCGGCGCAGGGAGACATCCTTGATATTGTGAATATCCACCAGCT
CGGGGCCGATGAGGGCGCGGATCTTCTCGGCTGCCATCTCGGTGTAGCAGGTGGTGGAACCGTAAAACAG
ACCAATCTTCATCGCTCTTCTCTCTGTTGGCGCTTTCTCGCTTGCTTTTGGCGCGTTCTCGCATGCTCGT
GTGGGCTGCGCCATGTTGGCAATACGATGGGTAACGTAAGGGGAGGGCGCCAGTCTAGCAGATTGCCATT
CGACCTTCACCGCCCGGCAAGGGGTGTGGCAAAAGTGCCTTATCTTTCCAATGAAAGTGCCTTGTCTTTC

CAAGGAGTTGGCGCGTTACAATGGGCCATCGATCCCCTGATTGCAGCGCACCATGAGCAAGCCTGACCCA
CATCCCCTGATTGAACCCTTCCTCGACGCCCTCTGGCTGGAGCGGGGATTGTCTGACAACACGGTCAGCT
CCTATCGCAGCGATCTGGAGAAGTTCTCCCTCTGGCTCGACGAGCAGGGGAGCTCGCTGCTGCTGGCGGG
GATGGACGAGATCCAGCACTACCTCGCCTGGCGGGTCGATCATCAGTTTGCGGCTAGCAGTACGGCCCGC
TTCCTGTCGGCCCTGCGCCGCTTTTACCAGTATCTCAACCGCGAGAAGCTGCGCCGCGACGATCCGACCG
TGCTGCTGGAGGGGCCCAAGCTGCCGAAAAAACTCCCCTCCGATTTAAGCGAAACGGAGGTGGAGGCACT
CTTGAGTGCGCCGCTGGTGGATGACCCGCTGGAGTTGCGGGACAAGGCAATGCTGGAGCTGCTCTACGCC
ACCGGCTTGCGGGTTTCCGAGCTGGTGGGGCTCACTGCCGAGCATGTTAGTTTGCGTCAGGGCTTGGTGC
GCGTAGTGGGCAAGGGCAACAAGGAGCGGTTGGTGCCGATGGGCGAGGAGGCGATGCACTGGCTGGAGCG
TTACTACCGGGAGGCCCGCACCCTGCTGCTGGGGGGCACCAGCTCGGATGTGGTGTTTCCCTCCAAGCAG
GCCCGTATGATGACCCGCCAGACCTTCTGGCACCGCATCAAGCTTTACGCGCAGCGGGCCGGGATTAACG
GCGAGCTCTCCCCTCACACCTTGCGCCACGCCTTTGCCACCCATCTGCTCAACCACGGCGCCGACCTGCG
AGTGGTACAGATGCTGCTGGGCCATGCGGATCTCTCCACCACCCAGATCTACACCCATGTGGCCAACGAG
CGACTCAAGGCGCTGCACGGTGAGCACCACCCCCGCGCTTAAGCCTGTTGCAGGCGATTTTTGCCCGCTT
GTTTAGCCTGATACAGGGCTGCATCGGCGCGGGCCATCACACTGTCTGGGGCATCATTCAGGGATTCGGC
AATGCCACCGCTAAAGGAGACGCTTAAGCCCTTTTCACGCCAATCCTGTTGCCCCATGCTGATACGCCAC
TGCTCCATCAACTTCTGCGCGTTGGCAATATCAGTCTGTTCAAAAATAGCGACCAGCTCTTCCCCCCCAA
AGCGGCACAGGGTTGCCTGTTCCTCCAGCAATTGGCTACCAAGCTGGGCAGTGCGTTTCAAAACGGCATC
ACCGATGGGATGACCATAGGTATCATTTATTTTTTTGAAGTTATCAATGTCGATGATCGCCAATATATA
TTGGTTATTTTCTTTATGTAGAGTGGCGAGCACCTCTTCCAAATGACGGCGGTTGTACACGCCGGTTAAT
GCATCACGCAGCGATTTTTCTGTTTCGGCCTGCATTTTGTCTGAAATCATCACTATTTCATCCTCCAGCT
CCTCTATACCAGCCAGCTTGGGGATAATAATTTGTTGCCCTGGAGGGGTGATACGCAATTGTCTAACAAC
TTGTACTAATGTGCTGATGGTATGCTCCAGACGTTGCCTGAAAATAATGGCAACCAGTGTGAATATCGAT
AGTGACAATATCAGAGTGATAAATATGGTAGGCAATATTTCACCAATCAGAATATCGCTCTTGAATGGAA
ATACAGTAATAACCAGCCATGATGGGTTGTTGTGAAGGGCGTGATATGAAATGAGATCACCATTTTCACT
CATTAAGGTACCATTCCTGCCTTTCAATTCGGAAATCCATGGTTGGTCAACTCGACTGCCGTTTTCCTGA
GGGTCGCTGTGAGCCACAATCATCTTGCTGCCTCGGTCAAAGAGCATATATACAACATTGTTACTACTCT
CCAGAATCCGCCCTATACGTGCTGAAAATACCTTCAAGTTCAGGTCGATGCCAATCACGCCAAGAAATTC
CCCCTGGTTATTGAAAACAGCATGGGAAAGGGTGACGACCGGCGTTTTTTCAATCGCGTCAATGTAAGGG
GAACTCCAGATATTGATGCCGGGATGGTCGATGGCATTGCGATACCATGGGCGTTCCGATTCTTTGAACT
CAATCGGGGTAGGATGAAGAGAGAAATTGATATGCTTGTTATCCTGAAGCATGTAGTACACGGTTTTTAT
TGGGCCATCAGGGTAAAGAAGGTATTGCCAAATGTCGCGATAACGTTGTTTTAATTCCGGGGGAAGATCT
CCATTATTCTCAGTAATGTCGGCAATGATGATATTTATTTGCCTCTCAACCGAAGAGAGTGAGTTATCCC
ATTGCTGCGCAGTACTATCGTTAAAAAATCTGGCGGTGCTTAGCAAGGTGTTTTTCTGCATAACAGAAAA
AAGGTACGCACTACCCAATGTGGTCAAAACAAGTGGCAGCGCAAGCAGCAAAAGAAAACGGAGAATAAAG
CTGTGATTGGAGTTTGCAGCATTGGAGGTTTGCATAGTCACGACGCCTATATAACTTTACGCCATCTAAC
CATAGCCCATGGGCATCGTGACACACATACACACTAGTCTGGGGAGTCGGCTTAACGCTCCTGTTGAGAT
TTCAGCAGATCACGGATCTCGGTCAGCAGCTCCTGATCCTTGGTCGGGCCTGCCGGGGCGGCGGCTTCCT
CTTCCTGCTTTTTCTTGAGGGTGTTGATGGCTTTCAGTCCCATGAAGATGGCAAAAGAGATGATCAGAAA
GTCGATGATGGTCTGGATGAACTTGCCATAGGCGATAACTACGGCGGGTGTTGCACCTTCTGCGGCCTTC
AGGGTGACCGCAAGGTCGCTGAAGTCCACGCCGCCCAGGATCAGGCCGATGGGCGGCATGATCACGTCAC
CGACAAAAGAGGAGACAATTTTGCCGAAGGCGGCACCAATGATGATACCGACCGCCATATCGATGACGTT
GCCGCGGGCGGCAAAGGCTTTGAACTCTTGAATCAGACTCATTATTTCCTCCGAAATTAAACAAGATAGC
GCGTTGATTACCCTTGCCAAAGGGCGAACTCTTGTCAGGAAAAACAGAGGCTAAGCCGCGTTGTTCCTGT
CGTCCTGACTAGCTTGCGGGCGTTATGAGTCCTTGGCAATCGGGGGGAGCCATTTTTTTGGTACAAAATG
ATATTAATTTTTTGGTAAGTAGCTGGTTGAATGATGAGCTGTGCTGCTGTAGTTTGCTGGGTCGATTCAT
TCATACCACGTGAGTAAACAAGTGAAGTATTTACTACTGGGAATGATGCTTTTGTGCACATTTGCTGCCA
GAGCGGGGATGGATACCCAAGCCTTGAAACAGACCATCACGGCCCGGCTGGGGGTCCCCGTCTATCTGGT
TGAACCGACCCCAATCGCGGGGTTGTATATGCTGGGCACCTCACAGGGGGTGCTCTACAGCGATGCCAAA
GGGGATTATGTGGTGCAGGGGGTCATGCTGGATATGACCCGCGACATGAAGAACCTGACCATCTCGGGTA
TGCGCGAGCAGCGCCGACTTGGTTTGGCGCAGGTGGCTCATGCCCCCATCGTGCTCAAGGCTCGGGATGA
GCGTCACCGGGTTGCGCTGTTTCTCGGCGAGCAGGACGCAAAGCGCCGTCAGCTCTCCGGCACCTTGCAA
CACCTGCAGGCATCCGGGGTGAGCGTGGCACTCTATCCGGTGATTGATCACGCCGAACGGGCCGCCGACT
GGTGCAGCGATCCCCTGCTGCAAAATGATCCGTTAAAAGCCTATTTGCCTCAAACGGCATGTAGTGAGAC
CCTTATCCAAAATATTGGCCTGAGCCAATGGTTGGGGGTCAAGGTACTGCCGGCCTGGGTCTCCTCCGAC
GGGGATCTGGTGCGCGGGTATCAGAGCCCCGAACAGCTGCTCAAGATACTGGATCATATTGATGTCCCTG
CAAATCCGTCGTCGTCCGCGAGTTGATGATTCCCATCTGCCTGCCACCCTGCATCCCCTGCTTCGCCAAA
TCTTCGCCAGCCGTGGCGTAGAGGATCCCGCCCTGCTGGAGCGCAGCGCCAGCCAGCTGTTGCCGCCCCA
GCGCCTCTATGGGATGGAGCAGGCTGTGCCGCTGCTGGCGGATGCCCTGACTGCCCAGAAACGGCTACTG
ATTGTCGGCGATTTCGACTGCGATGGCGCCACCAGCTCTGCCCTGTGCGTGCTGGCCCTGCGCGCCATGG
GGGGGCGCCATATCGATTTTCTGGTGCCCAATCGCTTCGAGTTTGGCTACGGTCTCACCCCCGAAATTGT

CGAGCTGGCGGTGGCCCGTGGCGCCGAGTTCCTGATCACGGTGGATAACGGCATCTCCAGCATCGCCGGG
GTGGCGGCTGCCAAGGCGGCCGGCATGCAGGTACTGGTTACCGATCACCATTTGCCGGGTCAGGAGCTGC
CTGACGCCGATGCCATCGTCAACCCGAACCAGCGCGGTTGCGATTTCCCCTCCAAGTCCCTGGCCGGGGT
GGGGGTAGCCTTCTACCTGATGGCGGCCCTCAACACCCATCTGCGTCAGCTCGGCTGGTTCGAACGGCAA
GGGTTGCGCGCGCCCAATGTGGCGGATTACCTCGATCTGGTGGCCCTCGGCACGGTCGCCGACGTGGTGG
CGCTCGATGGCAACAACCGCATTCTGGTCCATCAGGGCTTGCAGCGGATCCGGGCCGGGCGCTGTCGCCC
CGGCATTCAGGCGCTGGTGGATGTGTCGGGCCGCGATGGTCGTCGCCTCACCGCCGCCGACCTCGGTTTT
GCACTGGGGCCACGCCTCAATGCGGTGGGCCGTCTCGATGACATGTCCCTCGGGGTCGCCTGTCTGCTCT
GCGATGATCTCAATCTGGCGCGCCAGCTTGCGGCCGAGATGGACAGCCTCAATCAGGAGCGCAAGGAGAT
CGAGCAGGGGATGCAGCAGGAGGCCCTCGCCACCCTGGAGCAGATCCGGTTTCGCGATGGCGAGGTGCCG
TCGGGCATAGTGCTGCACCGCAACGAGTGGCATCAGGGGGTGGTGGGGCTGGTGGCCTCCAAGGTGAAGG
AGAAGTACTACCGCCCTGTCATCGCGTTTGCCGAGAGCAGCGAGACCGAGCTCAAGGGCTCCGGCCGCTC
CATCCCTGGCGTGCACTTGCGCGATGCGCTGGAACTGCTCGATACCCGCCACCCGGGCCTGATGGGCAAG
TTTGGTGGCCATGCTATGGCGGCGGGGTTGACCCTCCCCAAGGCCAACATTGAGGCGTTCGGTCGGGCCT
TTGAGGCGGTCATCAGCGAGCTGGTGACGCCGGAGCTCTTGACCGGGGTGCTGCTCACCGATGGCGAGTT
GTTGCCGGATGAGCTCAATATCGAGCTGGCGGAGCTGATTCGCGCCTCCGGCCCCTGGGGTCAGGCCTTC
CCCGAGCCGCTGTTCGACGGCGAGTTCGTGCTGGTGCAGCAGCGGCTGGTGGGGGAGAAGCACCTCAAAA
TGATGCTGACAACCGATTCTGGTCACGCGGTGGATGCCATCGCCTTTGGCGTCGATCTCAAGCGCTGGCC
CGATGCCTCGGTCAAGCGGGTGCGGCTTGTCTACCGGCTTGATGTCAACGAGTGGCGCGGCAACCGCAGT
GTGCAACTGCTGGTGGAGCATCTGGAGGTGGCCGGTCTCTGAGCCGGTAGCATCGCTGCTCTATCGCTGC
TTTATCCCAGATAGCGCTGACAACGGCCACCCTCGGGTGGTCGTTTTTGTTGGCGGATCTCTGGAAAAGG
GCGTTGACCTGTCTATAGCTTTACAGTTTAAGCTCGTCTCTATCGGGTCACCTGAGGGCCCGCACCGTGA
GTGATTGCCATGAAAATATCCGAACTGGCCCGCCGCTGCGGCCTCGCCCGCTCCACCCTGCTCTATTACG
AGAAGCTGGGGGTGATTGCCGGCACCCGGGCCGCCAACGGCTATCGCCACTATGACGAGGGGGATCTGCA
GCGACTGCAGATGGTGCAGGCCCTGAAGGCAGGTGGTCTCAGCCTAAAGCAGTGCCTCGCCTGTCTGGCT
GGCGAGATAGATCAGGCGGCATTGCAGGCCCGGGTCAGGGAGCTGGATGAGGAGCTGGCGCGGATGCAGC
GCGCCCGGGATCTGTTGGCGGATCTGGCAGGGCTGCGCCCGCGCTCCGGTGAGGAATTCAAGGCCTGGCA
GCTGCAACTGCAGCGCGAGGCACCGGAGGCCTACTTCGCTTGGGTGATGAAACAGGGTTTAGCGAGAAA
GATCGCTACCACTTGCAATGGTTGAGCAAAGACATGAATGAACATGAGCGTTACATCAAAGACTTTATCC
AGCTGCTGGATGGCATGAGTTACTGGGGGCCGGGGGATCGCGCCTTCACCCAGCAGCAGTTTGCCGCCTT
GCCCATCCGGCCGCGCCGGATCCTCGACATGGGCTGCGGGCGCGGTGCCTCGACCATGGCGCTGGCTCAG
GTGACCGATGCCACTATCGTAGCCATCGATCTGGAAGAAGAGGCGCTGGCCGCCGTGGCGCACTCCGCCC
GTGCAGCGGGCTTTGAGCATGTCTCCACCCTCTGCGCCAACATGGCGGCACTGCCGGACGATCTGGCCCC
GGCCGACCTCATTTGGGCAGAGGGGGAGCGCTTACGTGATGGGGGTTGCCAATGCGCTGAAGGCGTGGCGT
CCGCTGCTCGCGAACCCGCAGGCTTGCATCGTGCTCAGTGATGCGGTCTGGCTGACCGATAACCCGCCCG
AGGAGGCGTTGGCATTCTGGCAGCAGGATTACCCCGCCATGCAGACGCTGGCGGGCCTGCTTGAGACGGT
ACAAGCGGCAGGCTACCGCTGCCTGTCACACACCCCATTGCCGATGAGTGCCTGGAACAATTATCTGGAC
CCCATCGAGGTCAATCTGTTGCGCTATCGAGACGAGCTGGGGGAGAGTGCCGCCTGGCAGGACTTGAGCC
GGGAAGTAGCCATCCACCGCCAATATCTGGGCAGTTACGGCTATGTGATCTGCTGCCTGCAGGCGATGTG
AAGAGGATGGCTATTGCCATTGAGGGTGAGCCAGTGATCGGACGGGGGAGCAGATGCTCCCCCATTTGTT
TTAGTTTTTAAATGCCAAGTTCAGTGATAATTGAGTGTTTAGTGCTGATTTGCATGATTAGGTGAATATC
AAAATCCAATAAAGCGATGGCTTGTGATATATTTATCCTCGCATTCCTGTCATCCCTGAAGGAGCTCGAT
CCTTGCCTCTTGCCAGTCGCTTACCCCCAAGCCGCATACAGCACGGTGCAATGGCCTTTGCCATCGCCTG
CATTCTGTGGCTCGCTGCTTTTGCCGCAGCTGGGCTGAGCGCCGTTTCGCAGGGGCCATCTGCCGGGGCG
ACGGCGGGGATGGCACTGCAAGACCCGATCAACCAGCGTCTGGTCGACACGGTCGGCTTGCTGCCGGGCA
ACCAGTGGCGCCACGGGGTCGAGCAGGGGCGGCGGTTGGCGAAGGAGCGGGAAGGCCATCTCTATCAGAT
GCTGCTGGCCTGGTTCCAGTACGACATCCTCGGCCTGCTCTGTGGCCTGCTGTTCTTTGTGCCGCTGATC
TATGTCAGACAAGCTTGTGGCCACGTTGGCTATCTGCATAAACGGCAACTCTGCGGCCACCATTTGCAAT
TTCGCTTCTGTCACGGCGACTAGCTCACCCTGCTGGCGGACGCTCATCCACATTTTCACACGGCTTTTAA
CGCTCCCGATTGACTGACGGGGCAGGCTGACACCTGCTGCCTGCGCTCTATCGTCCGGTGCTGCGTCCGC
GCTTTCTGTTGTTTCGTCCATGCTGCTGGCCATCGGCCAGAGGCAGACTTTCTCTTTGCGCAAACACCTG
GTGAGCACACCTATGGACTACTACCTCAACCTGTTTATTCACTCAATATTTGTCGAAAACCTCGCCCTGG
CCTTCTTTCTGGGGATGTGTACTTTTCTGGCCGTCTCGAAGAACATCAACACCGCCTTTGGCCTTGGCAT
GACAATCCTGGTGGTGATGGTGATCGCCATTCCCCTCAACAACTTGATTTACCGCCATATCTTGCGGGCA
GGCAGTACCTTTTTCGGTGAATTCGATCTGGGATTTCTCGATTACATCATCTACATCGGGGTGTTGGCGG
CGCTGGTGCAGGTGATGGAGATGGTGCTCGACAAGTTCGTTCCGGCGCTCTATCAGGCACTCGGCATCTA
TCTGCCGCTGTTGACGGTGCACTGCGCCATCTTCGGCGGGGTGATGTTTATGGCGCAGCGGGATTACGAT
TTTGTCGAGTCACTGGTCTACAGCGCAGGCTCCGGGCTTGGCTGGCTGCTGGCCATTGTCGCCATGGCCG
GACTGCGCCAGAAGCTGAAGTACGCCGATGTCCCCGAGGGGCTGCGGGGCATCGGTATCACCTTTATCGT
GGCAGGGCTGATGTCCCTCGGCTTTATGTCCTTCTCCGGCATATCGCTATGAGGGGATGACAAAAAACGA
GGCGCCTGCGGGCGCCTCGTTGTTTATCAGGGATTGCGGATGGCCATTACAGCTCGCGGGCCCAGGGGTG

AGCCTTGGGGCGCAGCGCCAGCGCCAGACCCAGCAGCAGCACGGTCAGACCGTAGAGGGGCCAGCTGCCA
AAGCGCATGTAGGGGGTCTCGCCGTGGGCGGGACGCACTTCGCTGCGCAGCACGCCCGCTTCAAACTGCG
GGATCTGGCTGATGATGCTGCCATCGATCTCGGTCACGATAGTGATACCGGTGTTGGTGTCCCGCAGCAG
CGGACGAGCCAGCTCCAGCGCGCGCATCCGGGCGATCTCCATATGTTGCCAAGGGCCGATGCTGGTGCCG
AACCAGGCGTCGTTGGAGACGGTCAGCAGGAAGTCGGTATCGGTATTGACGTTGCGGCGCACCTCTTCCG
GGAAGATGATCTCGTAGCAGATGGCGGCGGCAAACTTGAGCCCCTTGGCGATCAGGTTTGGCTGCACTTC
GTCACCCCGGCTAAAGGAGGACATCGGCAGGTTGAAGAAGGGGGCGATGGGGCGCAACAGATCTTCAAAT
GGCACGAACTCGCCGATGGGCAGCAGGTGGTACTTGTTGTAGCGGTTGGAGTGGTTGTAGTAGTAGGACT
CCTTGCCATCCGCATCCTGCACCCCCATGCCGAGCACGGTATTGTAGAAGCGCTGTTGCTGCTCGTCGTA
GTGAATAATCCCCGCCAGCAGGCCGGTATCATTCACTTTCATCGCCTTGTCCAGATTCTCCAGATAGGGG
CCCATCGCCTTCTCGATAGCCGGAATCGCCGACTCTGGCCAGATGATGATGTCGGCATCCTGATTCTCGC
GGCTCAAGTCCTGATAGGTGCGTACCGTGGGGGCCAGCGCCTCCGGATCCCACTTGAGGGACTGGGCGAT
ATTGCCCTGCACCAGTGCCACCTTGACCGGCTCGCCACGGGTGACCCAGTTGAGCTGCATCAGACCGTGA
GCACCTGCCACCAGCAGGGCAGGCACCACCAGCCAGAGCGGGGAGCGGGACTTCCACACCAGCCAGATAG
CGGAGGCGGACAGCAACAGGGCGAGGGTGATGCCCTGCACCCCGAGAATGGGAGCAAATCCCTTGAGGGG
GCCGTCGATCTGGCTGTAACCAAACCAGAGCCAGGGGAAACCGGTCATCACCCAGCCGCGCAGCCAGTCA
GCCACTAACCACAGGGCCGGGAAGGCGAGCAGCCAGCGGCTCCAGTGACGGCCGCCAAAGAAGCGGGCGA
AGGCCCAGCAGGCGAGCGTCGGGTATAGGGCCAGATAGGCGGAGAGCCCGGCCAGCAGCACGAAGGCGAC
CGGTAGCGGGATGCCACCAAACTCGGTCATGCTGACGTGGATCCACCACAGCCCCGGCAAAAAGAGTCCC
ATGGCGTAGCCAAAACCGCGCCAGGCGGCCTGTTTCGGCGTGGCTTTGTCCAGCAGGGCGTAGAGGCCGA
GCAGGGAGGGGATCACCAGCGGCCAGTAGCCGAAGGGGGAGAAGGCCAGCACGGCGATGGCACCAGAGGC
CAATGCACTGAGGCTTAACAGAATTTTGGAAGTAACAGGGGATAATCTCACGGGCGATGGTGCTCGTCTT
GGTGTGCAACTGGGGTCGAATGGCCGGTATCTTAACCTGTAAGCGGGGCAACGGCGAGCCTCGATGCCGG
TGGCAGAGAGCACAACGCCCCGAGGTCGGGGCGTTGTTTCACACATCGAGCGGATCTGCCTATTGCGGCT
CGGATTGCGCCGCCGCGTGGTGTTCGGGGATCTTCACCTGCAACTGCTGCAGACGGCGCCGGTCGGCATG
CATCACCTTGAACAGGTAGCCGTCCAGCTCGATCTCCTCGCCCTTCTTGGGCAGGTGGCTGAAGGCGTGC
ATTACCAGACCACCTACCGTATCCACCTCTTCATCGCTGAATTTGGTGCCGAAGAAGTCGTTGAAATCCT
CGATCTCGGTCAGGGCACTGATGGAGAAGACCCGCTTGCTGAGGCGGCGGATTTCGTCCGGTTCATCCTC
GATGTCGTCAAACTCGTCGTCGATCTCGCCAACGATCAGTTCCAGTATGTCTTCGATGGTCACCAGACCT
GAGACGCCACCAAATTCGTCGACCACGATGGCCATGTGGTAGCGCTCTTCACGGAACTCCTTGAGCAGAC
GGTCAACCCGTTTGCTTTCCGGCACGATCACCGTGGGGCGCAAGATTTTCTCGAGCTGCAGCGGCTCGCT
GGCGTGATGGCCGCCAAACCCGAAGGGGAGCAGATCCTTGGCGAGCAGTATGCCCTCCACATGATCCTTG
TCCTCGTTGATCACCGGGAAGCGGGAGTGACCGGACTCGATGATCACCGGCAGGATCGCATCCACCGGTT
GGGACTTCTCGATGGTCACCATCTGGGAGCGGGGGATCATGATATCGCGCACGCGAAGATCGGCGATCTC
CAGCACCCCCTCGATCATGTCCTTGGTGTCCTGATCGATGAGGTCGCGCTCTTCGGCATCGGCTATCACG
TCTACCAAATCATTGCGGTCTTTTGGCTCGCCCTGGAAGAGTTGGCTGAGTTTGTCGAGCCAGGTTTTCT
TCGGCGAGCCGGTACTAGGGTGATCGTCGGTCATTTTCTCTCTTGGTTACTCGTATGAACACACTTGAAA
TAGTTCGGGTTATATAAGGATGGTCATCTGCTGTTTCAAGACGATGACCATCCGGTTCTTTACTCTTCGT
CGTTCAGATAAGGATCGGCAAAACCGAGCTCCTGCATGATGTCGCGCTCAAGCGCTTCCATCTCTTCGGC
TTCCTCATCCTCGATATGGTCATAACCTAGCAGATGCAGACTGCCGTGCACAACCATGTGTGCCCAGTGG
GCCATGAGGGGCTTGCCCTGCTCCTCGGCCTCGCGCTCTACCACCTGACGGCAGATCACCAGATCGCCGA
GCAGCGGCAGCTCCAGACCGGGTGGCGCCTCGAACGGGAAGGAGAGCACGTTGGTGGGTTTGTCCTTGCC
GCGATAGGTGAGGTTGAGTTCGTTGCTCTCCGCCTCGTCGACGATGCGCACCGTCACTTCTGCCTCCTGC
TGGAAGCCGAGAATGGTGCCGTCGAGCCAGCCTTGCAACTGGGCTTCGGTGGGCAGGCCGTCAGTGCTGG
CACTGGCCAACTGCAGATCTAGGGTTACGCTCATTGCTCACTCTCTTTGCTGGCGTTTGTTTGGCCGGCC
ACCTTCTCGGCGGCCTGTTTGGCGTCGAACGCCTCATAGGCCTGCACGATGCGGGCCACCACCGGGTGGC
GCACCACGTCTTTGGACTCGAAGAAGTTGAACGACAGACCATCGACCCCTGGCAGCACTTCCAGCGCGTG
GCGCAGGCCGGATTTGGCGTTGCGCGGCAGGTCTACCTGGGTGATGTCACCGGTGACCACTGCCTTGGAG
TTGAAGCCGATGCGGGTCAGGAACATCTTCATCTGCTCGGTGGTGGTGTTCTGGGCCTCATCGAGAATGA
TAAAGGCGTCATTGAGGGTTCTTCCGCGCATATAGGCGAGCGGCGCCACCTCGATGATGTTGCGCTCCAT
CAGCTTCTCGACCCGCTCAAAGCCCAGCATCTCGAACAGGGCGTCGTAGAGTGGGCGCAGGTAAGGATCC
ACCTTCTGGGAGAGATCCCCTGGCAGGAAGCCCAGCTTCTCACCCGCTTCCACCGCCGGACGAGTCAGCA
GAATGCGGCGAATTTCCTGACGCTCCAGCGCATCCACCGCCGCAGCGACCGCCAGATAGGTCTTGCCGGT
ACCGGCCGGGCCTATGCCGAAGCAGATATCGTGGCGCACGATATTGGCAATGTACTGGGCCTGATTTGGG
CTGCGTGGCTTGATGAGACCGCGCTTGGTCTTGACCGTCACCTCCTTGCCATAGGGGATGCTCGGCGCCG
GTTCGTCATCCTGCTCCAGCACCCGCGACTCCTGAATGGCCAGATGGACCATGTCGGGAGTGATGTCGGT
CACCTTGCCACCCTTGAGGGGCTGGGTTTCCACATAGAGGTGCTTGAGGATGACGGCGGCGGCATCCAGC
TGGGCCGGTTTGCCCACCAACTGGAAGTGGCTGTCACGGTAGCTGATCTCGACGCCGAGGCGGCGCTCCA
GCTGCTTGATGTTGTCATCGAAGGGGCCGCACAGGCTGGCAAGGCGCTGGTTGTCGGCGGGCTCCAGATG
CAGATTCAGGGTCGAGATGTGTCGGCTCAAAATATCCTCGTCTCAAGGCGCTCCCCATCCGCCGGGCGAA
TGGGGAGGAGGGGATTAATGGGGAGTGAAGGCGGCAACGCCCAGCTCGTCCGGTACGTTATCCGGGCGAC

GGGCCAGGATTTCGCTCGGGGCGGTGGCGATCCGCAGGTTCATCTCGCTCTCGCCGCGGATGAACTTGCC
GCGCAGGGAGTTGGGACGCACCTCGGTGATTTCCACGTCGGCAAAGCCGCCGATCAGGGTGTGCAGGCCT
TCGAAGTTGACCACCCGGTTGTTCTCGGTGCGGCCACACAGCTGCATCGGGTCGAGCTTGGAGGGCCCTT
CCACCAGGATGCGCTGGGTGGTGCCCAGCATGGCACGGCCAATCTGCATCGCCTGGTTGTTGATGACGTG
CTGCAGGCGGGCCAGACGGGCTTTCTTCACTTCCATGTCGACATCGTCTGGCATGTCGGCGGCCGGGGTG
CCCGGGCGCGGACTGAAGATGAAGCTGAAGCTCATGTCGAAACCCACATCCTCGATCAACTTCATGGTTG
CTTCAAAGTCTTCGTCCGTCTCGTTCGGGAAGCCAACGATGAAGTCGGAGCTGATGGTGATGTCGGGACG
GGCGGCACGCAGGCGACGGATCTTGGATTTGTACTCCAGGATGGTGTGCGGGCGCTTCATCATGGTCAGA
ATACGGTCGGAGCCGCTCTGCACCGGCAGGTGCAGGAAGCTGACCACTTCCGGAGTGTCTTTGTAGACTT
CGATGATGTCGTCGGTGAACTCGATCGGGTGGCTGGTGGTGTAGCGAATGCGATCGATACCGTCGATGGC
GGCCACCAGACGCAGCAACTCGGCGAAGGTGCAGATGCCGCCATCAAAGGTCGGACCGCGATAGGCGTTG
ACGTTCTGGCCGAGCAGGTTCACTTCGCGCACGCCCTGCTGGGCGAGCTGGGCGATTTCGTAGAGCACGT
CGTCCAGCGGTCGGCTCACCTCTTCACCGCGGGTGTAGGGCACCACGCAGTAAGTACAGTATTTGGAGCA
GCCTTCCATGATGGAGACGAACGCGGTAGCCCCTTCGGCGCGCGGTTCGGGCAGGCTGTCGAATTTTTCG
ATCTCGGGGAAGGCGATATCAACCTGGGCACCATGGCCCTCTTGCACTTCCTTGATCATGGCGGGCAGGC
GATGCAGGGTCTGCGGGCCGAACACGATATCCACATAGGGAGCGCGCTGGCGGATGGCATCACCCTCCTG
AGAGGCAACGCAGCCACCCACGCCGATCACCAGACCGGGCTTGTTGGCCTTGAGCTTTTTCCAGCGTCCC
AGCTGGTGGAACACCTTCTCCTGCGCCTTTTCGCGGATGGAGCAGGTATTCAGCAGCAAGACATCTGCCT
CTTCGGGCTCTTCGGTAAGGGTGTAGCCATTACTGGCATCCAACAGGTCGGCCATTTTGGACGAGTCATA
CTCGTTCATCTGGCAACCCCAGGTTTTAATGTGAAGTTTCTTGCTCATTGCTTCTCTTGACTTGTTTTGA
AATCGGCATGGTCAAAGGACCGCGCATTTTACTCCGCAATCTGGATACTGCCTAGCTAATATCCAGCTGG
CAATGGTGCTCACCCGATACCTGTTGCCATCAAGCTCAATAAGCTTTCTCCTTGATTTGACGCAGCAATT
CCCCGTTTGTCTCACTGTTTATCAGTCGCCCTTGCGCTATCATGCAGCCATCCGGTTCACGTCCGGGCGC
TGTCGCCGGACGCACCCTTTCAGGAATAAATGTATGGAACAGTGTGATATCGCCATCGTCGGCGCCGGCA
TGGTGGGCGCCGCCACCGCCTGTCTGCTGGCGGCTCAGGGCCTCTCGGTGCGGGTCATCGAGACCCAACT
GCCGGAGCATTACGCGCCGGAGCAGCCGCTGGATCTGCGGGTCTCCGCCATCAGTCAGGCTTCGGTGGCG
CTGCTGGAGCAAGCTGGTGCCTGGCACCATCTGCAGCAGATGCGGCTCTGCCCCTATCGCCGGCTGGAGA
CCTGGGAGCTGGACGGTTTTGCCACCCGCTTCAATGCCGCCGATCTCGGTCTGCCCCAGCTCGGCTACAT
CATCGAAAACCGGCTGGTGCAGCTCGCCCTGCTCAAACGGATGGAGGATTTCCCCACCATCCAGACCCAC
ACCCCGGCGGCGGTGACCAGCCTGCGCCAGAGTGCAGATGACGCTGTGCTGACTCTGGACGACGGCACTG
AGCTGGCGGCCCGCTGGGTGCTGGCTTGCGATGGTGCTGAATCCCACACCCGCAAGCTGGCGGGTATCGG
CGTATCGCGCTTCGAGTACCGCCAGCACTGCATGCTGATCAATATCGACACCGGCTTTGCGCAGGAAGAT
ATCACCTGGCAGCAGTTCACCCCGAGCGGCCCGCGCGCCTTCCTGCCACTACCAGGCCAGCACGGCTCTC
TGGCGTGGTATGACAGCCCGGCCCGTATCCGGGCGCTGGCGGCCATGAGCAACGAAGGGCTTGCCGCCGA
AGTGCGCCGCCACTTCCCAAGCCGGCTCGGCGGATTTACCGTCACCGGCAAGGGGAGTTTCCCGCTGGTG
CGTCGCCACGCCAACGACTATCACGTCGGTCGGGTGGTGCTGCTTGGCGATGCTGCCCACACCATCAACC
CGCTGGCTGGGCAGGGGGTCAACCTCGGCTTCAAGGATGTGGCCTGCTGGGCTGATCTGCTGCAAGGAGC
TGGCGCCGACTGGCATCAGCTGGCGTTGGCCGAGCGTTACGAGCGACGCCGTCGCCCCGACAACCTGCTG
ATGCAGTCGGGAATGGATCTCTTCTACGGGGTATTCAGCAACGAGATTGGCCCGCTCAAGCTGGCCCGCA
ATCTTGCTCTCAATCTGGCGGACAAGGCGGGCCCGCTCAAGGAGATGGCGCTGCGCTATGCACTGGGGCT
GGTTTGACAGGTAGAAACCAGGTAACACGGGCTGAATTCGCGAAGGAGCAAAGGTGATGAACAAGGCACT
GTTGGTGATCGATTTTATCAACGATATTGCCCATCCCGACGGGCGCATTGCCGCCTCCGCAGCCCACGTG
GTGGAGCAGGATGCCATTGCGCATGCCAATCAGGCGCTGGCCCATGCCCGCGCCCACGGCTGGCTGGTGG
TGCTGATCAAGGTGGGCTTTGACGGCGGCTATCAGCTGCAACCCAAAGGTTCCCCCATGTTTGGTCGCGC
TCACCAGCAGGGTGCGCTCTCTTTGGCTGACAGCGGCACCGACTTTCATCCTGATCTGGATGTGCAGCCC
GGCGATCTGGTGCTGACCAAGCCCAGGGTCAGTCCCTTCTATGGCTCGGCGCTGGAGCCTGCGCTGCGGG
CCAATCACATCGACCACCTTTATCTGTGCGGGGTGAGCACCAGCTGGGCCATTCAGGCGGCAACGCGGGA
TGGTCACGATCGCGACTACACCATCACCATTCTGGAGGATGCCTGCGCCGCCGCAGATGCCACCGAGCAT
CACACCTCGCTGCGGATGCTGGGTCGCATCGCCGAGATCATCAAGGTGTCCCAGCTGGCTTGATCCTTAA
CGCAGGCTGCGCCCGATCACCGTTTTGAGATTGACTCAGCGGCTCCCTGTCGGGCCGCTGCGCTTTTGGG
CAACATGATTGTCGGGCAAGATAGTGCTCACATGGATAACAACAAGGATGGACAGATGCGTTATCAGGGA
CGGATCGCGAGCTGGAACGAGGCGCGCGGCTTTGGATTTATTACGCCGGAGCAAAATGGCGCCGAGCAGG
CAATCAATGGATCACGCAGTGCAGGGCTGTTTGTGCATGTTACGGCGCTGCAATCAGATGGCCGGGTGCC
CGAGCCGGGGGAGCGTGTCACCTATCTGATCGGCAGTGGCCAGGATGGCAAACCAAGGGCGTTACAGGTC
TTTTTCCCCGATCGCCCGCTCACGTTGGCGGATGCCGTGCCGTCGGCCGCATCATCAGCGGCGCCATCGT
TTCGGGCGGCAAGTCAGCCGCCACGACCCACTTCTACCAGAGAGCGGAGTAACCCCTATCGACGTCGCAA
GAGCAGCTGGAGTGGCAAATTGATCCCTCTGCTGGTGCTCGTCGGCCTCTTTTCCATCTATTCCCGTTTC
TCGGCCGAGTCGATGGCGCCATTGCCGGTCTCATCTTTCAATCAGGAAGAGATCAGCACCTCGGTCAGCA
CGCTACAGACAACGACCTTTATTCGTCAGTGTGATGGTCGGCAGCATTGCTCCCAGATGACCTCCTGCGA
GGAGGCAACCTGGTTTTTACAAAACTGCCCTGGTACCAAGATGGATGGGGAAGGAGATGGCATCCCCTGT
GAAAATCAGTGGTGTGGTCATTAAATGTGACTTATCAGAGACATATGCTCATGATGTGCTCTTGTTATCC

GGAACAGGGAAACTGGCCTGAAACCTTGCCGGATAGTGTTCGATGGTGAAATTTGGTCTCAGTTGGCTAA
GTGGGAATGGGGTGCTTTACTCTATCTGCTGACTTAATTGTTAATAGATGGCGGTATGCCACTTTTGCCA
CAGGATGGCCATCATGGTGGATGATATACAGAGGCCGCGAAGTCACTTCTCCGAGCGGGCTCATTATCTG
GCTGCGCTGCTGGATTCGCTGGAAGACCAGATATCGGTGATAGACCGGGATGGAGTGATCCATTACACCA
ATCTCGCCTGGGAGCTGTTTGGTGAACAAAATGGCATGCCCAAGGGATATAACTGGCTCGGAGTCAATTA
CATGGGGGCTTGTGACGATGAAGCCGAGCCCTCGGGATTGGCTGCCGTGGAGGGGATCCTCCGGGTTATC
CGTCATCAGCAGGAGAAGTTCTATCTGGAATACCCCTGCCACAGCCCCGACACCCAGCGCTGGTTTATGA
TGCGAGTAGCGCCGGTACCCGGGCTTGATGCCGAGTATTTTTCCATCGCCCATATCAATATCACCGAGCG
CAAGCTGCTGGAGCTGGAAGTTGCCAGCCTCTCCCAACATGATCCCCTGACCGGACTGGCCAATCGACGG
CTGTTCAACAGCTTTTTGCATAACGAGTGGCGGCGCAGCATGCGGGAACATACCCCGCTCTCCCTCATCA
TGCTGGATCTGGATAACTTCAAGGCGTGCAATGACCATCTTGGTCATCAGATGGGGGATGGCTCCCTCCA
GCTGGTGGGCCATCTTCTGCAATCCTTCTGCCAGCGTCCGGCGGATCTGCCCTGCCGTTTTGGTGGTGAC
GAATTTGCCCTGATCCTCGGCAATACCGACCAAAATACGGCCCAGCGGATTGGAGAGGAGATCCGCCAGG
CCATCAACGATCTCGACATCGTGGTGGCAAATAATATTCAGCTTAGCGCCAGCATTGGCATTGCCACCTA
CACGGCAGAGCAACCGCTGGAGGGAGAAGGGGCGTTGCTGGCCGCGGCCGATGCGGCGCTTTATTGCGCC
AAGGGGGCGGGTAAAAACCGTAGTCACTGCGCCAGCTGTTGAGCCGCATTGCCGCTTTTGTCCTGCATGC
GCCCCCTTGATGGGGGCGCACTGTTTTTTGGCTGGTCACGATGTTAGCTACCAGCAAGGGTTGGTTTCAG
CGCTCGGGTGTGGCCGACACAGGCAGGGGATGGCTGGCGATCACCTGCCTGACGAAGTGATCCCGGATGG
GGTCGGCAAAGTCCGGATTCCATACCATGCTGACCTCCCACTCGGAGCTGGGACCAGAGAGAGGATAGAG
GGTGACATCGGGCCCTGCGATAAAGCTGACGCTGCGCGGCAGGATGGCATTGCCGATGCCCGCCGCGACC
AGCGCCACCAGCGTCTGGATATCCTCGGCCTCCTGGACCATGCCGGTCAGCCGATTGGCGCCGAGGAAAC
CGGCGATCTGCTGGCTGAGCCCGGGGCAGCGGTGGCCCACCATCTGCAACAGGGGTTGACTGGTCAGTGC
CTGCTGGACGTCGAATGCGGCCGGGTCTGGTTCGGTCATTCGTGTCGGCACCGCCAGCACCAGCCGGTCG
ATCAGCAGTCTGTGCTCCTGTAACTGGGGCATCTGAGGTCTGCGCATAAAGCCAAGCTGCAACTGGCCGG
AGAGCAGCTGTTCCTGCTGGATGGCGGAGGGCATATCCTGCAGATGGACCATCACGTCCGTGACCTGCTG
TTTGAAGCGGGCCACCAGCGCAGGGGCCAGTGCGAAACTCGACAGACCGAAGCCAATCTTGAGCCGACCG
GCTACCCCGCTCGCCACAGCCAGCGCATGGCTTTCGAACTCCCGACCGTGCTCGACCAGCGCACTCGCCT
GAGTGAGCAGCTGGGCGCCGAGCGCCGTCAGCTCGGCACCGTGGCGACCCCGCTTGAACAGGGTGCAGCC
CAGCGCCTGCTCCAGCCCCTGGATCTGCTTGGTGAGGGCCGGTTGGGTGATAAAGAGGCGGCTGGCCGCT
TCCCGGTAGTTGGCGGTGTCGGCCAGCGTCACAAACGCCTGCAGTTGCCTCAAGTCGAATTTCATTCCAA
TTGGTTATCAAAAGTGGCGATTTATTGATTATAAATTATCTAATCGCCTTGTTCTAATGGCTCGGCAAGG
GGGGAATCGCCCTCCTCTGAACCGGAACAAGGAGCCATATCATGTCTACCCAACGCTATCTTTTTTTAAT
CAAGGGCACGCTGGTGCTGGCTCAGCCATTGGTGGCCCCGGAGACGGGGGAGCTGCTCGCCAGCCTGTTG
CAACAGGGGTTTGTGGTCGGCCCCTTGCAAGTGTGGGCTGGCAGTGCCGAGCAGGCGCTCGCTTGTTATG
AGCGTGCCACCCAGCAGCAGGCCTTTGCCGATGCGCTCTCTGGCTCTCTGCCCAAGCTGGCCGGGGTGTC
TGTATGAGTGCCTGTATGAATGCCTGTATGAGTGCTGGCATGAACACGATCACCCTGTCGGTATCCGCCC
TGCTGCTCGATATGGATGGCACCCTGGTCTGCTCGACCGGTGATGTGGAGAAGGTGTGGCGGCTCTGGTG
CCAGTATCATCAGCTTGCCCCAGAGCCGGTACTGGCTATGTGCCACGGTATGCGCTCACGTGAGGTGATC
CGGGCGCTGGCCCCGCAGCTGGATGTGGAGCGGGAGGCAGCGCGCCTCGACGATCTGGAGATGGAACATA
CCGGTGGCGACGCCATCTTTGGGGCTGGCGAGTTGCTGCGCCGCTTGCCGCCAGAGCGTTGGGCCATCGT
GACCTCCGCCAGTGAGCGGGTTGCCCGCCATCGCCTGAGTTGTGCCGGTTTGCCGCTGCCCCACGTACTG
GTGGGGGCCGAAGCAGTGATCAATGGCAAACCCGATCCCGAACCCTATCTGCTGGGGGCTGAACGGCTTG
GCGTGTCGCCCGATTGTTGTCTGGTGTTCGAGGATGCCCCGGCCGGTATCGAGAGCGCCCTGCGTGCAGG
TTGCACCGTGGTGCAGGTAGGGGGCGATGCGCCGCTCAATCCGGCGGTGGTGGCGGTGATCCGCGACTGG
CGCGAGGTGAAGGTGGAGATTGGAGAGACATCCCTGCTGGTGACGCTGCCCGCCTGAGCGGTAGAGCGTA
TTACGACCGCAGATTGCGGTATGAACCATCGCCATCAACACTGTATGCCGCAATAAAAACGCCCCCGAGA
TGCGGGGGCGTTTTGCTTTCTCAACGGTTATTTGAAAACGCGGAAACGGGTGGCATCGTCGATAAAGGTT
TTGTCACCGGCCGGTTGCTTGATACTGCCAAAGGGCATCTGGGCACGCAGCAGCCAGCTGGCGGGCAGGT
TCCACTCCTTGCGTACCGCCTCGTCGATCAGCGGGTTGTAGTGCTGCAGGGAGGCACCGACTTTCTCCTG
CGCCAGCGTTGTCCAGACCGCAAACTGGGCGATGCCGGTGGCATGTTCCGACCAGATGGGGAAGTTGTCT
GCGTAGAGGGCGAATTTTTGCTGCAGATCCTTGATGACGTCGGTGTCTTCGAAGAACAGCACGGTACCAA
AGCCTGCGCGAAAGCTGCCATCTACCTTGGCCTCACTCTGGGCGTAGGCCTCCGGCGGCACAATCTTTTT
CAGCTCGGCTTTGACGATATCCCACAGCTTGTGGTGCTGGGCGCCAAACAGGATGACGGCACGGGAGCTC
TGGGAGTTGAAGGAGGAGGGGCTGTGCCTGATGGCATCCTGGATTAGGGTCGTCAGCTGCTCCGGCGTAT
GGGACACCTGGTTGCCCAGGGCATAGATGGTACGACGTGCTTTGATCTGTTCGATAAACGGACAGCTCAT
AACATCTCCTTGTTCTGCTTGGCGTTGCAGCCGGTGATGGACGGTTTGTCCAGCGCCTGCCGAATCAGGT
ATAGACTATAACACCCGCGATGCAGAGCGGGGTTAACCCGGGGTAACTAGCGTTTTTCCTGTCAGTTGAT
ATTCGTTTGCGAACCTTGACGTTTCGTTAGTGTTGGAAGTGTCATTTAAGGGGCGCGAAAGTGATATTAA
TCAATGATTCACTCTCCTTTCTCTCCCACAATCCCCGTATGTTTTCTTGGTTCGGATGTGATGTGAGCCG
TTCGTATCTCAATAAACGCTACTATGTGTTGGTCCCCCTCTCGGCATTGATTGGTTTGATGATCCTGATA
GTGACGATCATCATGGCTATTGCCAGCTATCTGAGGGATATCGACATGACGGTATCCGGCTTCGGCCACA

TGGCATCCTCCGTTGAAGCGTCCCTGATCCACGAGATGGTGAAGGTCAATGAGCGGCAACTGGCAGTGCT
GGAGGCCTCGCTTGACAAGGAGAAGATCGCTCGCGGCGGGCTGGCCGATTCCCCCATCTGGGCCATTGCG
CACAAGATCAAACCAGGCAACCACTACATCTACTTTTACAACCTGAACGCGGATCGTATCGACAGCTACC
CGGACTGGCTACCGCCCCCCAGTTATCAGGCGGCGCTGCGACCCTGGTATCAGGTGACCACCATGCCGGG
CGACGAGCTCGTCTGGTTTGGCCCCTATCCTGAATACAATACCAATGAACTGATACTCAGCCTGATTAAA
CGGGTGCGGGACGATCAGGGGCAACTGCTGGGGCTGCTGATGGTCGACATGTCATTCGACTCGCTGCAGC
AGGCGCTACAGCGGACGGTTGGCAGCAATCAGGCCGCACTCTATCTTACCTTGCGCGACAGCGGCCAGCT
GGTGGTGGGCAGCAATATGGATCTGCTGGCAGATTACCCCATCGAGCAGCATCAGGGCCAGCAGTGGCTC
GGTCTGGATGTGTTGTGTCATGGTACTTTCCTGAAGCGCGAGCTGGCGGACATTGGCTGGGATCTCAATA
TCTATCTGCCGCCCGTGCTGTTTCGCAACAGCCTCTATGAGGCGCTGCAGATGGTGGTTTTGCCATTGAT
CTCCCTATTTGCCATCTGGTGTTGCACGCTGAGTCTGCTGGTGCGGATCTTCCGCCAGGAGCAGGCGCTG
GTGTCGGGTTCACTGACCGGTATCGTGCGCAATCTGGAGAGTTCTCCCCCCATCACCAAGGCCAATACCT
GGTTTGTGCGACGCAGCCTGAACGAGATCGATCAGGTCAGGGCCAGCTTCCTCAAGGGGCAGGATGCGCT
GTTGCGGGACCCTCTGACCGGTATCATGAACCGGCGAGCCTTCGAGCAGCACAAGAGCGCGCTGGAGCTG
AGGGGGGAGCGCTTCTGGCTGTTGCTGTTTGATATTGATCACTTCAAAGGACTCAATGACCGTCGGGGTC
ATGCCTTCGGCGATCAGGTGCTGTGTCGGGTGGCCAATTGCCTTGCTGATGTGCTGGGGGTGAGCACGGT
TTACCGGATTGGTGGCGATGAGTTTGCCGCTCTGCTGCAACAGCCGCAGGATGAGTTGGCGGAGAAGCTC
AATTGTCTGCTTGACGTGGTGCGACATCAACAGTGGCGTGAGCACAAGGGGCAGGTCACCTTGAGTATCG
GCGGCGCCTGCCATTCACCCGTCGCCGACCCCATCTTCGAGCGTGCTGATGCAGCCCTTTATCGTAGCAA
GGAGGCTGGCCGGGACTGCTGGCATCTGGCGGATGAGGCTGCTGACTGACAGCTTTTGTCCCAGCGCGGC
GGGTCTGTGCCGGGACACGACAACCAGATTGTGGGGCGCCCAGCCGTAATAGTGTGGCTTTTTGGTGATT
TTAATCTTTAAATTCAATCGCTTGATTTTACTCTTCCATTTTTGGCAGTAGCCGTGCGGCCATTGACTGT
CGCATCCCTCATTTCTGCTGGCTGGCGAACCCCGTGGCGCTTTGTATAGTGCCGCTGAATATTAATCAGG
TTTGACAGGAGTACCCATGGAGATGAATTCGCGTGCTCTTCGCGCACTGGCACTGCTGGGCGCCTTGCTG
GCTGGCAGCACGACTGTGCACGCCTCCGAGACCCTCAATACCATTTTGGGTGGCGGCGCAGGTGGTGTGG
CGGGCACCATGATCGGCAAGGAGCTGGGGGGAGATACCGGTGCGCTGGTAGGTGCGGCCATTGGCGGCGC
AGCCGGTGGTGCGGCGACGGCCAACAAGGGCAACAAGAATGAAGCGGCCCTTGGCGGCGCGGTGGGGGCG
CTGGGCGGTGCCGCCATCGGCAAGAGTGTCGGTGGTGATACCGGTCAGCTGATCGGCGCCGGGGTCGGCG
GGGCGAGCGGTTCGGCCATCGGTGCCAAGACCGGCGATGGCCACAAGAGTCAGGATCGCTATTACGATGA
TGACCACCATCACAAGCACCACAAAAAGCACAAGAAGCACAAAAAACACAGATGGCATGACGACGATTGA
TCGCCATCTGTCGTGAGTGGTGGGGATGAAACAACGGGGCCAGTGGGCCCCGTTTTGCATGTTTGCTCAG
ACCAGCCGGTGCTGACGGCGACCGGCGGCAAAGGCGTTGATGTTGTCGATCAGCTGATCGGCGAGACGCT
GCATCGACTCTTCGCTGGCCCAGGCCACATGAGGGGTCAGAATGAAGTGGGGGTATTGCAGCGCCTTCAT
CAAGGGATGGTCGGGGGGCGGGGGTTCGACGCTTGCCACATCGAAACCGGCGCCACCGAGGCGGCCGTTG
GCCAGCGCTTGCAGCAGCGCCTCCTCATCCACCAGTCCGCCACGGCCGACGTTGATCAGCAGGGCGCCCG
GTTTCATCAGATCCAGCTCCCGCTCGCCAATGAGGTTGCGGGTGTAGGGGGTGAGCGGGCAGTGCAGGCT
GATCACATCCGAGCTTTGCAGCAACTCGTCGAGGGGCAAGCGATCCTCGTCGTGACTGGCGCCGACCTGG
CTCTGGGCGTAACGCACTGTCATCCCCATCCCTTTGGCCCGCTCGCCGAGCGCCTGTCCGAGCGTCCCTT
TGCCGATGATGCCAAGCTGCTTGCCGTGCAGATCCATGATGTTGTGGTCGAAGAAGCAGAACTGGCCGCT
CTGTTGCCAGCGCCCCTCCAGCAGCGACTGGCGCCAGCAGAAGAGGTTGCGCGACAGGGCCAGCATCAGC
GCCATGGCGTGCTCCGGCACCGAGGGGCCCGAGTAGTTGCGGATATTGCACACCCCGACGTTGGCGGCGC
GGCACGCCTCCAGATCCACGTTGTCGCTGCCGGTGGCGGCGAGCGCGATGAGTTTGAGCTCTGGCAGCTG
GGCCAGTTCGGCGGCGCCGATCCGTACCTTGTTGATGATAGCGATGCTGGCATCTTTGAGCCGTTCCACC
ACCTGTTCCGGGGTAGTACTTGGATAGCTCTGCCAGTGGTGGGGGAAGTCGGGGTGATGCAGGGTGATGC
CCGCATCCAGGGTATCGCTGTCGAGAAACACAATCTGCTGCATGGACCTTCCTGCCATTGTTCAGGTTCG
AGAGGCCATGATGCCAAAGAGGATGCGGTCAGGGCCAGCCTGCCGCTGTGTCATGGCGGGGCTTTATCCG
GCAGATGTGAGCTGGCGCGCGCTTTATTGCGCCACGGTGGCGGCAGGTTTAGAGCCAGCCCAGATGGTTG
AGCCAGCCCTGAATGCGGGTGGGGCTCTCCTCCAGCTCCATCAGGGCGTAGGGGCGATAGTTGAGCTTGT
TGCTGCGCCGGCTCTCGCTTCTGCGGGCCTCGGCGGCCAGCAGTGGCCAGCGACCGCCATCCTCCTGATA
GAGATCCAGGGTGGGGATGGCCGCCTGAGCCACCTGTTTGGCCAGGATCTGATTGCTCTGCTGATTGGGG
AAGAAGCCATCGAGCAGCACCAGCGCGTCGGGGGCGGGCAACTGTTCGCTGGCGATGAGGTTGGCAGTCC
AGGCGGCGCCGGTACCTTGCGCCAGGATCAGCTTGAAGCCCCCCTCCTGCAGCTTGGCATCCGCCAGCTT
GCTGATCCGCTCGGCAAATTGCTTGCGAAACTCATCGCTGTTTTTCGCTTTTTTTTCGGCGGCTGGGTCC
AGTTCGGTCTGTTGGGGGGAGGGCAGCAGCAGCAGGGTATCAAAGCCCAGTCGGCCGAGGGCGCGAGTCA
GTTGCCAGAGCTGGCCGCTGTGCTGCCAGTCGGGCAGTAAAATGGCGAGCCCCAGATAGTTTTCACTGTC
GTGTTTGGCAAACAGCACCGTCTGTTTGTCGATCTGGGTAGCATCGGGCAACCCTTTCCAGTCGCTCGCT
TGCCAGTGGTCGAGGGGCATAGGTTCGGCAACGGGGGGCGCAACCTCGCTCGCCTCTGCCCCATGGCCAG
CCAGCAAGGTCAATAACAGCAGTGATAAGGATAATCTGGCCATAGTGTGCTCCCCCGGTTATCTGGGTTA
GCGGCGGCTGCGGGATTTTCTGAAGGCGGGCGGTGTGATGGCAACCCGGAGGCAATAAAAAAGCGAGCCG
TTGGCTCGCTTTTTTGTAATCAATTCAAAGCGGTCAATTAGTTGACGCGACGCTTGAATTCACCGGTGCG
GGTATCGATTTCGATCTTGTCACCGATCTCTACGAAAGCGGCAACAGCCAGCTCGTAGGTAGTGCCTTTC

AGACGGGCAGGCTTGGTCACTTTACCGGAGGTGTCGCCACGGGCTGCCGGTTCGGTGTACTCAACTTCGC
GCACGATGGTGGTCGGCAGTTCTACGGAGATGGCTTTCTCGTTGTAGAAAGTCACTTCACACAGATCTTC
CATGCCGTCTACCAGGTAGTTCAGCACGTCACCCAGGTTGTCTTTCTCTACGTCGTACTGGTTGTACTCG
GTGTCCATAAAGACATACAGGGGATCGGAGAAGTAGGAGTAGGTGCACTCTTTACGTTCCAGCTGAACAA
CGTCCAGCTTGTCGTCGGCCTTGAAGACGGTCTCGGTAGCGCTGCCGTTCAGCAGGCCCTTCAGCTTCAT
TTTGACCACGGCGGAGTTACGGCCGGATTTGTTGAATTCAGCCTTCTGAACCACCATCGGCTCGGTGCCG
ATCATGACTACGTTACCAGCGCGGATTTCCTGTGCGGTTTTCATGTAAAAAATTCCTGTTCGTGAAACGG
ACGGATAAAAACTGCCAGATTATATACGGCTTTCTAAAAATTTCACCAACCGTGTGACGAGATCCCCATC
CCGGAGCAACTTCTGTGACCAGCTTCGCCCATGTTGCTGCCAAATGGCGCTATATCGGGGCCATTGGGCC
CAAAGTTCGCCGCAATCTTCGCCACGGTTGAGAGCCAGATTGAACCGGCGCAGCCATTGGCCGCACTCGG
CGGGTAGATCTGATAAGTAGTGGTCAAGGAACTGCTCCAGCTTGACCAGATGGGCTTCCTCCTCCTGTTG
GTAGATGTGCCAGAGGAAAGGACGAGCCGCCCACTGGGCCCGTACGAAGGAGTCCTCCCCACGCACCAGA
TTGAGATCGCAGTTCCACAGCAGACGATCGTAACCGGCCTGATCGGTCATCGGCAGCAGCTTGATGGTGA
GGTTGCCCGCGTGGAGCAGATCCCCCGCTTGTGCGGTGGTGATGGCATCTGCCAGACCGGCACCGGTGAG
CACATCGTTGAGAGAGCGCCCCAGCGGCAGCAGCAGGGTGACGGGCGTGGCGCTCTGGCACCAGCTCTCG
ACCAGACTCTTCAGCGCATCGCTCTCGTAGGTAAAGAGGCTGACCCGCAGCTCGTTCTCTTGTTTTGGCG
GCAAACCGAGGCTGGCCCAGTAGGCGGTAAGTCCCGCATCATCTTGCTGCCACTGCTCCCGTTCGGTGAT
GAGGTCGTGCTCGCACAAAAGGCCGCCGGTTTTAGCGGTAAAGCCCGGGAAGAAGAAGTATTTGTTGAGG
CTCTGGTTGCCAACTCGCTGTGGCGAGGCGAGGCAGTGACAATCCTCGACCCAGCTCTCCGCCGAGAGGT
ACTCCAGATTGATCCAGCAGGGGGGCTTGGGTTGGCTCGCCATCAGTTCTATGAAGGGGGTGGGCAGTTC
GCAGGCAAAGGCTTCGATCACCACCCTGGCCGGTGTCATGTCGCTCGGCAGGGACTCATGCCAGTGCTGG
ATATAGATGCCATCGACCCACTGGCCATCCTGCGCCGGGTCGAGCCCCGGACAGATGCGGGCGAAACTTG
CCAGATCGTCCACCCAGAGCCGCACCTGAACCTCATTGCCATGTGCCGCAATATTCCCCTCCCGTTTTAG
CTGGCGGGCAAGTCGCCAGGTGACGCCGATGTCACCGAAGTTGTCGACGACGCAACAGAAGATGTCCCAG
TCAAACGGGGGCAGGGGAGTTGTCATGGCTCAGGATCCCGGATGCAAAAAAACCGGTGCGCAGGGTAGCA
CACCGGTTGACTGGCTTCTAACCGTCAGCGTGTGACGGCGGTCTGTGGCAGGTTACTCCTTGCCGTAGAC
GTTGTTCTCTTGCTCGCGTACCCGGATAAAGGTGGTGCGCTTGGTGAGCTCTTTCAGCTGGCTGGCACCC
ACGTAGGTACAGGTGGAGCGCACGCCGCCCAGAATGTCGCGCACTGCGTTCTCGACCGGGCCGCGGTAGG
GCAGCAGTACGGTTTTACCCTCGGAGGCACGGTACTCGGCGACGCCGCCGGCGTGCTTGTCCATGGCGCT
GGAGGAGCTCATGCCGTAGAACTTCATGAAGGGTTCGCCGTCTACATCGACGATCTCGCCACCGCACTCC
TCGTGGGCTGCCAACATGCCGCCCAGCATGACGAAGTCGGCGCCGCCGCCGAATGCTTTGGCGACGTCGC
CCGGGCAGGTGCAGCCGCCGTCACCGACGATCTGGCCGCCGAGGCCGTGGGCCGCATCGGCACACTCGAT
GATGGCGGAGAGCTGCGGGTAACCGACACCGGTCTTGACGCGAGTGGTGCAGACGGAGCCCGGGCCGATG
CCGACCTTGACGATGTCGGCGCCGGAGAGGATCAGCTCTTCGACCATCTCGCCGGTCACCACGTTGCCCG
CCAGAATGACGTGGTTGGGGCAGGCTTCACGAATTTTGCGCACGAACTCGACGAAGTGTTCGGAGTAGCC
GTTGGCCACGTCGACACAGATGAAACGCAGCGCTTCGGACATGGCGAGGATCTGGCGGGTCTTGGCGAAA
TCTTCAGCCGAGGTGCCGGTGGAGACCATCACATGGCCCAGCAGCGCCGGGTCGGTCTCGTTGACGAAAG
CTTGCCACTGCTCAAGGCTGTAGTGCTTGTGTACCGCGGTCATCATCTCGAAACCGGCCAGCGTGCGTGC
CATGGCGAAGCTGCCGACGGTATCCATGTTGGCGGCGATAACGGGTACGCCTTGCCATTGGGTCTGAGTG
TGTTTGAAGGTGAACTGACGGGTCAGGCTTACTTGGGAACGGCTCTTGAGAGTAGAGCGCTTGGGGCGGA
ACAGGACATCCTTGAAACCGAGCTTCAAGTCTTCTTCGATACGCATTACAAATTCCTTATTGAGCCTTGT
TGAAAGTCACTTTTGCAGTGGCAAATTTTCGGCAGGCGCAAAAAAACCGGTGGTTTTCAGGCCCTCCGGT
TTACAACAGTGTAGGCAAAAATCCCTTCAGAACAAGACTGATAATTTGTAAATCCTTGTGGTAGCCTTTG
GCGTCACAACCTGAGGTTAACCACCAGCTTCATCCAGCACCGTTCGGTGCCTTGCTCTCCTTCGATATTC
CCGCCCGCTTGGGCCGCCGTGCGGCCACTCTTTGTCGTCTCTCTCCTGACCCGCCAGATATTATTCAAGT
AGCTGAAATAAATCGGATTGACCCACAATGGGACTGTGGTAGATCTGGGCGCAGGATTGACAAGGCTGGG
TCGTAAAATGGGCAAGATGATGGTGGGGATCACCCTGATGCTGGCACTGTGTGGCAGTGCGCTGGCTGAG
ACCCCGACCAGCTATGGCTGGATCGAGAAGGGGCTTATTTTGCCTACCGGCGTGGCGGTGAAGATGAAGC
TCGACACTGGGGCGCTCACCTCTTCCCTCGATGCACGCTCCATCACCAGATTCAAACGGGATGGCAAGCC
CTGGGTGCGCTTCATGCTGGTGGTGACCGATGCCGACAGCGGCAAGCTAGTCAAACAGCAGTTCGAGCGG
CGGGTCGAGCGCAGCGTTACGGTGCGCGGGGCGGGCGGCATGGAGCAGCGGCCAACGGTGACCATGTCCA
TCTGTCTGGGGGACAAGGTGTATGAGGAGTGGTTCACCCTGCGGGATCGCAGCAAGATGATCTATCCGGT
GCTGCTGGGCCGACGTCTGCTGGCGGAGCTGGGCCCGGTGGACTCCAGTCGAACCTTTACCGTACAGCCC
GCCTGCCGTTAGGGGCAATTTCGGCGTGAATCTAAAAAACGCCGTTTCACAGCTAAATCCCCGCCCCTCT
ACCAGAGGTAGCATCTCCACCATACCCTGAAGCGTGCGAGCTCGTTCGCCAGCAAGGGCTTGAGCGTGGA
GTGCATCATAGTGAAACCCTATCAACAAATAGTATCTGAATGGCTATTGCTCGCGGGCGAACAGCAGGGC
GTAGCCTTGAGTCTGGGCAGCGATGGCCACTGCCGTGTGCCATTCGGTCTGGATAGCGAGTGCCTGGTCG
AAGTGCCCGATAACGATGAGCTGCAGGCATGCTACCTCTATCTCCCCTTGCTACGTCTGCCTGACGAGGC
GGTCAGTCAGTTGCAGCTGACCCGCTCGGCCCTTGAGCTGAACATGTTTGGTTTGACCACGGGGGGAAGC
CAGATCAGTCTGGACAGTCGCAGCAACCATCTGGTGCTGACCTTTTCCTCTCTTATCGAAATGCTCGATA
AAACTCTGTTTATGCAGACCCTGGGCGAATTTCTTGATCTCGGCGTCACCTTGCAGACGCAATTGCAGGC

GTTGAACGCCCGGCAGGAGACAGCTGCCATTCCCTCCCCGTTATCTGGCGTCATGATCCCGTGTTGATGG
ATCGTCATTGGTATTAATCAGGAGTAGAAGATGTCTATTCAAAGCATTGGACAACCCCAGCGTGCTAGCG
CTGTAGCCTCTCATGAGCATGGCGATGTGCAATCCTCATCGCGCCAGCACTCGGTTCATCACAATCTGGC
CTCTCACTATACCAGTCTCGGCTTCGGCTCCCGGGTTCAGCCCGGCAAGGTCTTGAATACCTCGCTCAAG
TGGTTGACCCTCAGTGCCCCGGCTCGCAAGATTGGCATCGCCATCCAGGGCGGCCATGATTCCCATGCCC
AGAGTGCCAAGGGCAAAGCCCGTTACTGGACGGGCGCCATCATCAAAGGGCTGGCCAATATCACCGGTGG
TGTCAGCAGCGTGGCCAGCGGTGCGGTGACGGCTGGGGTGGCCGGGGTGAAAAACCTTGGCGAGTCAGCG
GCGCGTCTGGTGGCTTCCTCCTCTCTGGGGTTGGCGGGTAGCGTGATGAAGGGGGCCTCCCATCTGCCGC
TCGGTATGTCAGAGCGGTTGCAACAAGGCTCACAGCAGGCCTTGAGTGCCGCCAAGGGGATCATCAAACC
GGCCTTCGTGGCGAAGGCGCAGCAGGAACCGCTGACTGCCGAGGGGGCGAGAGCGGTGATGGATGCGGCC
CAGCTGGCTCGGGTCAGCTGCTCCAGCAGTTATCGGGAGATGCCGGCAGGCTACAGTCACGCCACGCTGG
CAGATATTCCCCCTTCGATATTGGCCCGCCGTGAAGATGGTTCCGGTGGCACCGGGAGTGCCCTGACACT
TCATGTCACCCCGGGCAAGAACGAGAATGACCCCTTTATCCTGACAGGTGATGCTTGGTCTGCGCTCAAG
GTCGCCGTTTACAAGCGTGGCGATACCATGGTGCTCTCGTTTGTGGGCACTCAGCCTGCCAAACGCCCCG
GCACCCTGAAGAGCGATGCTGGCGCGGCCCTTGGCATCAAGGACAGCGCGTTTCAAGATGCCGACCGGCT
GGTCAAGGCCTTTGCCCAGCAGTATGGCGGCAAGGTCGAGGTGCTGGGCCACTCTCTGGGGGGCGGTCTG
GCGCAGTATGCCGGCATCAAGCATGGACTCAAGGTTACGGCGTTCAACAGCATGGGACTGCATGTCGGGT
TGCGGGATCGCCTCGCTCACAAGCTGGACGATGCCAATGTCACCCACGTCAACACCAGCGGGGATCCGCT
GAGCCAGAAGGTGGAGCACGGTGTGTTCGGGCTGGATGCCTCTTCCCAGGTGGGCAAGCGTTATGTGATT
GAACACTCTGGCGGGCATCGCATGGAGGCGGTGACGGCAGGTCTGGATGCGCTGCTTGCCTGATCTACGG
GTGATGGCGCCGCAACAAGTCGTGTAGCGTCAGCGTGCGAACTATCCATGAACAACGCCTCCCTCGGGAG
GCGTTGTCATTTTCGGATCAGGGCTGGGAGTGGGTGTGGGTCGGTAGCCAGACGCTCACCTCCAGCCCCC
CTTCCGGTCGGTTGTGGACATCGAGATCGGCGCCGTGCAGTTGGGCGATGCGCGAGACGATGGCCATGCC
AAGGCCGACGCCATCCCCCTGCTGGGGATTGACCCGAAAGAAGCGTTCATACAGCCGGGGCAAGGCACTG
GCGGGAATGCCGGGGCCGTTGTCACTCACATCCACCCTCACCCGGTTGCCCTCTCGCTGAACCCGCACCC
TGATCTCGCCCTCTGGCTGGGTGTAGCGCAACGCATTGTCGATCAGGTTGCTGAACAGCAGCTCGAGCAG
GGTGGCCTGCCCCATCACCAACTGCGGCAATTCACACTCCAGCGACAGCTGCTGATCTTTCTTGAGCGCG
ATGGGAGCCAGCGTCGCCAGGGTGCCTTGCAGCAGGTGGCCTATCTCCAGCTCGGTCAATACCAGCCCCT
GCTGGTTGTCGATACGGGCCTGGGTCAGCAGTTGGCGGATCAGCCGATCGCTGCGGTCGAGGGCCTGCAG
CATCTTGTGCAGGGATTGCTGGCGGCTCACTTCATCTTCGGCCGCAAGGGCATTTTCACTGTGGATCCGC
AGCACCGCCAGCGGGGTGCGCAGCTCGTGGGCCGCCTCGTTGGTGAACTGCTTCTCCCGCTCTATGGTCT
CCGCCAGCGCCTGCATTAGCCGGTTGATCTCGTTGGTGAGGGGGGTCAGCTCCTGAGCACTTATCTTGAG
GCTGAGCGGGCTCAGGTTGGCGGGGTGACGTTCGCTGATGGCCTGGGCCAGCTGACGCAAGGGGGCCAGC
CCGCGTGTGATGAGCCACCAGAGCAGGGCCAGCAGGAAGGGCAGGGTGATCAGCAGCGGCAGCATGGTGA
GGGTGGCCATCTTGCTGGCCAGTTCGCTGCGCTCGTCGTCCCGCTCGGCCACCACCAGCCAGGTCTGCTC
CTGCTGGTTGTAGAGAGTGAAGGTGCGCCACTGGTGATTATCTTTTTCGACCGTGTCAAAGCCGATAGCC
AGGGTGACCAGCGGCTGATCCGGGGCGCTGGGGGCGCGCAGCAGCAAGCCGCCCTTTTCATCGAGCAGCT
GGAAGTAGAGATTGCGTTCGAACTCGTGGCCGTAGGGGGTGATCTCCCGATCCTCGCCGGTCAGGTTGAG
GCTGGGGTCTTTTTGCCACTCATCGGGATTTTGCGCCTCCCACTCCTGATAGACGGTGCCGTTGGCGGGC
AGTTGCCCCTGCTGGGCCATGTAGCGAGCCAGCAGCTGGTTGGTGATGCGGGCCGACTGTGCGAGGCGGG
CATCAAACAGCTCCTCCATTTCGTGGCTCGCCTCCATGTAACCGATAAAGGCGCTGATGGTGAGACTCAG
GCTCACCAGCGTCAGGATACCGGTGAGCAGAAAGCGGCGAATGGAGCGCGGGCGTTTCATCGTGCGGGTG
TCCGGCTTGGCTGTTCGAGCTGGGGAATGATGTAGCCGACCCCCCGAATATTGCGGATGAGGTCGCTGTA
GAACTTCTTGCGCAGATGGTGGATATGCACCTCTACCGCGTTGCTCTCGGCGCCTTCATCCCAACCGTAG
AGGCTCTGTTGCAGCTGATCCTTGCTCAACACCCGGCCGCTCTGGCTGATCAGCTCCTGCAGCAACTTGT
ATTCATGGGGAGTGAGTTTGACCGCCTCCCCCTGATAGGTGACCTGCTGGGATTCGGGATAGATGAGGAT
GTCGCCATACTCCAGTACCGCCTGAGCCTGCCCCTTGCTGCGGCGAACGAGGGCGCGCAGGCGGGCGTTG
AGCTCCTGCAGGGCGAAGGGCTTGACCATGTAGTCGTCGGAGCCGGCATCGAGGCCGACCACCCGATCGT
CCAGCGCATCGCGGGCGGTGAGGATCAGCACAGGCAGTTGGTGTCCTTCACGCCGCAACTTGCGCAGCAG
GCTGAGGCCATCGATGTCGGGCAGGTTGAGATCGAGGATCAGGGCGGCGAATTCGTCGCTCTTGAGGGCT
GCCAGAGCGGGCAACCCCTGTTGCAGCCAGTCCACCGTGTAGCCGCTATGGCGCAGTGCATCCTGCATGC
CATCCCCCAGCATCACATCATCTTCCACCAGCAGGATCCGCATTCTTTTCTCCCTGTTTGTTGGGTGTGG
CGATCTGGCTCAGCGCCAGGGCGTGCCGTGGCATAAACAACCCTGCCCACCAGTATGCAAGCTGGTGGGC
AGAGTTTAAATAGTTCTTTGATCCATCAGGGTTTTATAGCTTGGCCAGCCGATCGCGGATCTCCATTCTG
CGCCCTTCGTCAGCCACTTCGCGGCCCGGGCGGGCCGGGGCGGCCAGCGCCTTGTCGAGATAGGTTTTGG
CCTCGGCTTTGCGACCCTGATCCAGCAGGAAGTCACCGTAGAAGAAGTTGGGATCGATGCCATCCGGATT
GATGGCCAGCGCCTGCTTGAGCAGCTGCTCTGCCTTTTCGTCATCGCCAAAGCCGACCGGCCAGCCGGGC
ACCTGATAGTAGAGGGAGCCTAGGCTGGTATAGGCGGAGCCATCCAGCACCTTTGGATCCTGTTTGAGTG
CCAGCTCCAGCTTGGCTTTGGCCTCTTTCACCAGCCCCAGCGCTCCCAGCCCGCCCTTGGCGCCAGCCCA
GGTGCTCTTGACGATGGCAGACCAGATCAGCAGATCGGCGCGAAAAGGCTCCTTGGCGCTGGCCGCTTCG
GCCTGGGTGCTCAGGGTTTCGAGGCAGCTCTCTTTCGCCTTGCCGGTCTGTTGATACTGGCAGACGGCCC

ACTGTTGCTGGATGGGGACGAGGGCATCATTCGCGGCCATGACTGAGGTGGATCCCAGCAGGATGGCGAT
GGCACAAGAGGTCACGGATTGACGGAAAAAATTCATATTAATGCTCCTTCTTGGTCGGATCGGGCGGGA
CAAGTCTGGTTGTGGCGCACTTTTGGCGCCAGCATCAGCCGTCCCTGGCTGAAGATGACGGGCATAACGT
TTGATGATTGGCAGCTGTCTGGCCAGCGCCTTGTCGACGATGCCAGGCAGCAGGGCATTGAGCCGCACGA
ACAGTTTTTCCGGCCAGCCGAGCCAGCTGCGGCGGGTTTCGTTGCACAGGGCGATGACCGCCTCTTCGGC
CACCTCCTGCGGGCTGTCGGTCTGGGTGCCGAGCGCCGCATTCATCTCGTAAGCGGCCTCAGAATTGAGC
TTGGTGCGGGTGGCGCGGGGTGCAAAGTGCAGCACCTTGATGCCGCTGCCCTCCAGCTCCCGTCCCAGCG
CTTCGCTAAAGCCGCGCAGGGCAAATTTGCTGGCGCAGTAGACGCTGTAACCGGGGTAGCCGATGCTGCC
CAGGCTCGAGCCCATGTTCATGATGATCCCCGGCTTGCGCATGGTTGGCAGCAGGGCACGGGTGAGCTGG
ATGGGCGCCTTGATATTGAGCAGCAGCTGGCGCTCCACCTGCTCGAAGCTCTGATCTTCCAGCCAGGCAA
ACTGGTTGCAGCCAGCGTTGTTGATCAGGATGTCGATCCCGTCCTCCAGCGCAGGGTGTTGCAACAGCTT
GGCCCGCTCCTGCGCAGAGCCGAGGTCGGCGAGCACCACCTGATGGCGCTCGGGATGAGGCAGTGACTTG
CACAGCCGCTCCAGTGCCGGGCCGCGACGTCCGTGCAGCAGCAGGTGTGCCCCCTGGGCGGCCAGCTCCT
GCGCCAGCTCCTCACCTATGCCACCGCTGGCGCCGGTGAGCAGCACGAGTTTCCCTTCAAGGTTCATGTT
GTGGGTTCCCTTTTTGCTGTCTGCTGCCGACTTACGGCGTGGCGGTGAGGTTGTAGAGCATCTCGCCGTA
GAGGCGATAGACCACCTTGGTGGAGTGGATGATGGCGGCCTGATCCGCCGGGCTCTCGACCTTATCCATC
AAGGATTCAAAGAATTTGAGGTGCTCCTGATCCAGAGCGCCGTGGGAGCTCAGGTAACTCATCGCCTGCG
GTGGCAGGCCGAGTCCCTTTTTCAGCTGGTCGGCGACGGTGAGGGCGATGGCGACGCTGGTGCCTTCCAG
TACCTGCACCATGCCGAAGAAGCCCATGGGGTTGCCGCGTTGGATCTGGTCATAGAGGAAGGCGACCATC
AGCTCGATGGGCAGCCCGGGCTGGCCGTGGCGCACCGTCTCGGCATCACCGCCACAGGCGCGGATATCGC
TTAAGATCCACTCCTGATGGCCATACTCCTCGTCGATGTATTCGGCAATGGCGCCGCGCACCCACTCGTA
CTCCTGACCCAGCTTGCCGCCGGTTGCCATCAGCAGCGGAACTGTGTGGCGCACGTGGTAGTAAGCCTGC
GCCAGAAAGGCGAGATAGGTGTCACGGCTGATATCGCCACGGCGGCAGGCTTCGATGATGGGGGCGTTGA
ACAGATGCTCACGCTCCTGGGCCGTGGCTTGTTGCAGAGTCTGATAGAAGCTCATTACGATTCTCCTGTA
AGGCATTGGCTGATTTGCCGGGAAAAGTGTTGGTAAATCTCGTTGCGACGGGGGCGCCCGTTGGCGGTCA
GCAGGCCGGGATGCTGGTCGAGTGCCACCGGCAACCAGTGGTGCAGCCGGGCATAATCGGGCAGCCGCTG
GTTCAGACCGGCGATCTGGCGTGGCAGATCCGCCTCTTTGCCCGGCAGGGGCTGGATCAGCGCCACGTTG
GCAGGCTGACCATCGCCAAAAACCACGATGCGGGCGATGGCGGGGCAGAGCTGGGCCTCGGCTTCCACCC
ACTCTGGGGAGAAGTTGCGGCCAAAGGCGGTGATCTGGACATTCTTCTTGCGCCCGGTGATATGGAGGTA
GCCCTCATCATCCAGATGACCCAGATCGCCTGTGGCAATCTGTTCGGGAGTCGGTTCGTTGCTGCCGAGA
TAGCCCAGCATGGCACTGCCGCTCACCATAATTTCGCCGTCGCTGGCGATGGTGATCTGACAGTGGGGCA
GGGGGCGGCCGACGCTGCCGGGCTTGTCAGCATCCTGGCTGTTGAGCGCCACCACGGAGCCGCACTCCGA
GAGGCCATAACCTTCGTATACCGGCAGGCCGAGCTGGCGGGCATGTTTGATCAGGTCGGGTGCTACTTTG
CCGCCACCGACGGCAATAAAGCGCAGGCTCTTGAGCTGCTGGGCCAGCGTCGGGGTGCTGGTGCAGAGCG
CCAGCAGCAGGCGCAGCAGTTCGGGGACCAGCACCAGACTGTGGGTCGACCAGCGCAACAGGGCCTCCCC
CAGCATGGCTGGATTGAGCGAACTGGATCCGGTGAAACCGAGCTCTCCGAGGGACGGAATGCGGCTGCAG
GCGCCGGTCAGCAGCGGCACATAAAGGCCGGTGATGTTCTCAAGCAGGGTGGCCAGCGGCAGCAGGGTCA
GGTGTTTTTCCACCTTGGCTGGCGCGACCCGCTCGGCCAGCGCATGGCTCACCGCCATCATCTGGCTCAG
GGACAGGCAGACGCCCTTGGGCTGGCCCGTGGTGCCGGAGGTGTAGGTGATCTTGGCTGTGCCCTTGGGC
AGCGCCGGTCGATTGGTCGGTGTATGGCGCCAGAGTGGCAGCTCGCCTGTGATCAGCGAGAGCGGCTCGG
CGGCATGCCACCCCTGATGATGTGGGCCCACCAGCGCATCGGCACCGCTGCTATCCAGCAACCAGGCCTG
CTGGGCTTGACTGAAGAAGTGGGGCACCGGTATGACCACGATCCCGGCTTTGGTGCAGGCGAGATCCAGC
AGCGCCCAGGGCAGGCCGTTGTCCAGTTGCAGCGCCAGACGGCTGATGTCGAGCTGTTGCAGCTGGCTGG
CCAGACGCTGGATCTGTTGCCACAGGGTCTGGTAATCGAGACGCTGCAGGCTGCTCAGCAGCGCAGGGGA
TTGCGGATTCTCGTGGGCATGTTTGGCGATGGCGTCGAACAGGCTCATTGGGCGCACCTTTTTTGATGGG
TCTGGTTATCACTGCTCTTGCTGGTCTCGTCAAACAGGGTCGGCATGGGGGCCAGCAGATTGAGCAGCAG
GCTGCGCTGGGCCAGCACCTCGCGCCCCTGATTGATGTCGCCCACCATCACCTTGGGTTGGCAGCGGTAG
TACTCACCCCAGGCATCGGCGTCGGCCCCCACCTGATGGGCCTGGGCGGGGGCCAGTTCCACCGGATTGA
GTTGCAGGCGGGCAAAGCTGTTGCGCAGCTTGGCCGTGCCGGTAAAGACCACGTAGTCGAGGGCGTTTTC
ACACAGCAGACGACACAGGGCCGCAAACATCAGGCGGGCATTGCCCGGTTCGCTGCAGGCCAGATTGCCC
ACTTCGATAATGCGGTTGCGTGCCGGAGAGACCCCGAGGCGGGTGTTGATGGCGGCCTCGATGGGTTGCT
CCAGATACTGTTCCAGATAGAGAGGCCCCTCACTGGCATGATTGAGACCACAGGCGCCGACCAGCGTCCC
ATCCGCCCGATAGAGCCCCAGCAGGCAGGGGAGAAAATGGGGGATGCGGGCGTTGAACTCGAGGGTGTAG
GCTGCCTGGATATAGCTTTGCAGCGCCATGACCTGCTGCGGGGACTTGGCTAGCAGCAATCGGTAGGGGG
CCTGGATCTCGATGGTCATGGGTGACTCCTTCTCTTGCGATGGTTTGAGAGTAAAAGGGCAAACTTAAGA
AACCCTTAAGGAGCAAAAAAAGTTGTCAGCGAGGCAGCGGGCAGGCAATCTGCGCCGCTGTTATCCAACA
GGAAGCCCCCAAGAGGAACCGCCATGTCATCCACCGTCAGCTGCCCCCTGTGTGCCAGCCATCGCAGTTA
TCCCCTGCTGGTGGCGGGCAAGCGCTACCATCGCTGCCTCGCGTGCGAGCTGGTCTGGCTGGATGAGGCG
GATCATCTGGATGAGGCGGCGGAGAAGGCGGTCTATGACGGTCACGACAACCAGGTTGATGATCCCCGTT
ATCGCACCTTTCTGATGCGGGCATTTGGCGAGGTACTGAGTCGGGTGCCAACCCCTGCCTGCGGCCTCGA
TTTTGGCTGCGGTCCTGGCCCTGCGCTGGTGGCGATGGGGCGTGAGGCGGGTTACGACATGGTTGGATAT

GACAAGTTCTATGCCGACTTTCCCGAGTTGCTGACGCGTCAGTATGACTTCATCACCAGTACCGAGGTGA
TCGAGCACATTGCCGAACCGCGTGCCGTGCTTGACCGGTTGTGGGCCTGTCTCAGGCCCGGCGGCATGCT
GGTGCTGCAGACCCAGCGGGTGCTCGGTGACGAGCGGTTCAAGAACTGGCGTTACCGCCACGATCCTACC
CATATCGTCTTCTTCTCCGAGGCGTCGTTTCAGGTGTTGGCCGCCAGCTGGCAGGCCGAACTCAGCTTCC
CCCACGCCGATGTGGCGGTATTGCAGAAAGGCACGCCCTGAGGCGTGCCTTCGTCTGTTACTCGGCTGTT
TCTTGCTCGGCTCTCGGTTACTCCTTGTAGCCCTTGTCCTGCCACTGGTAGTACATCAGGGCAGATTTGA
CGCCAATTGACTTGAACGGCTCTGGCGGCAGCCACATCGGCAGACTCTGCTCCGCGTGGTTGAACCAGGG
GTGCTCCTTGCCTTGATAGAGGCTGGCCACGATGTCGCCAAACAGGAAGGAGAGGTTGATGCCGTGGCCG
TTGTAGCCGAGGGCGTAGTAGATGTTCTTGTGATCCCCCATTACCCCCACCGCTTCGGTGCTGTCGCCGG
TCATGCCGAGCACCCCGTCCCACACCTGCTCAAATTTGATCCCCTTGAGGGCCGGATAGAGGGTCACAAG
CTCGGTCATCATCATCTCGCTGACCCGGTTAAGATCGCCCTTGTAGTTGAGGCCGTCATTGAAGGCGTAC
TCGGCATTGCCGCCACCGATGACGATACGGTTATCCGGGGTCAATACCAGATGGAACAGGATGTTGCGGG
TGTCGTAGTAGGGCACCCGACTCTGCCACTGGATGGCGGCCAGCTGCTTTTTGCTGAGCTGCTCGGTGAC
GGCGCTCTGGGCGTGTACCGGAAAGACCCGCCCGCTGAAGTAGCCAAGTTTTGAGGTATAGCCGTTGGTC
GCCAGCACTATGCTGTCGGCGGTCACCTTGTACTTGTCGGCTACATTCAGCTCGATCTGCTTGCCCTCCT
TGATACCCAGCACCGGGCTCTGCTCGTAGATGGTAACGCCCGACTGCTCCACCGCCTTGCGCAGGGCATT
GACCAGTTTCATGGCGTGTACCTGACCGCCATTGGGGTCGTAGACCGCCCCCACGTAGCGGTCGGTGCCC
AGTTTCTCTTCGGTGGTATCGGCGTTCCAGTACTGCAGCGGGATGCCGAGGCGATTGGCGGTTTTCACAT
AGTCGCGGTACTCGGCGATATCCTCCTCATCGAGGATCGCCTCCACATAGCCATTGAGCTTGAGGTCGGC
CTCGACGCCGGTTGAGGCCACCAGCGCCTGCAGCTTGCGCATGCTGGCGACGGTGAGATCGTAGTGGCGC
TTGCTCGCTTCCCCCTCTTCGTAGAGATCCATCACCTCGGAGCCCGGCAGCACCATGCCGCCATTGCGGC
CCGAGGCGCCGCTGCCCACCTGCTTGGCTTCGTAGATGGCGATCTTGAGCGACGGGTTAGCCTTGGCCAG
ATGCCAGGCACTGGAGAGGCCGCTGTAGCCGCCACCTATGATGGCGATATCCACCTTCATGTCTTGCGCC
AGCGGGGCACTCTCCTCTGCCGGTTTCTCCTTGAACCAGTAGCTGGTGTTGGGCTCAAAGTCGCTCATCT
TGGGGCCGCAGGCGGCGAGGAAAAAACAGCTGATGCACATGGTCAACGCTCGTTGGTAAAGGGGGGTCGG
ATTCATCTTCTACTCCGGTTACAGATACAAAATGTCAAATCAGTTCATCGGGTTGGGCGGTGTTGACCCG
GCGGCCTATGTCTCGCAGCCGGGCCAGCTGATCGGCAAGACCAGGCCCCTCATCCAGGCTGGTGACAAAG
TTCCAGAGGTAGGTCATCACAGCATAGACATGGCCTGCCGAGACCTGGGGCTGGGCCATCAGCATGGTGA
TGGCCAGCAGGAACAGCAGGGAGGCGCTGATGCCGATCGCCAGAAAAGCGAGTGCCTCCCGATCCGAAAT
TCGGATTCGGAGCCAGGCCAGCGCGCGGTAGTGGCGGGCGAGGCTGGTTGACTGGGCATGGGTGACCAGT
TTGACCTCGTGTTCCAGCCGATCGTTGAGCCGCTCGTGCAGCAGCTGGTTCTTGCGCGAAAAGCCGGGCA
GCAGCAGGCCGAACAGCAGCAATATCCCCATGCAGGCGATGCCGACCCAGAACTCGATCACCATCAGCAT
CACGGCTGCCCCGACGATGGAGATCAGGCTGGTGGCCAGGATCGGGATATGTTTCTCGAAGAAGTCGACA
AACTCCCGCGCCAGGGTGACCCGCGCCACTATGGTAGATTGCCCCTCCTGCTGCTGGCGTTGTGTCAGGA
TCACCGGCACCGCCAGTGCCGCATAGATATGGGTAAAGGCGCGGGTGTCGACCGCCCGGCGCAGGGCACC
GATGAGCCAGAACAGCAGGACGATGAGGGCGTAGCTTGAAGCCTGCCACCACTGGCCCTTGACGATGGCG
TCGATGGCAAAGCCGGCGAACAGGGGATAGACCACCAGCAGGATGTTTTCGAGGGCCACCAGCGAGAAGG
TGCCCGCCAGCTTGAGGGGATTGGCCCGTGCAATCGCCCCCAGCGAACGACCGGCCGAGTGTGGCAACGG
CGTGGCCTGCTCGTCGCAACTGGCTGCCGAAGTGGTTGGTGTTGTCATGGGTTATCTCCTAAGATGTCGG
AAACGCTTAGTTTGAGCGACTGCTCAAAGTTTGTTTGAGCGACTGCTCAAAGTCAAGCAGATCGATTGAT
ATCGCAGGAGGTCAGATGGCCCGCCCATCAGCAGAACAGCGCAAAGAGATGATCATCGAGGCCGTATTGG
CCCTGTTTCGAGAAAAGGGGATGGCGGCATCGTCAACCCGTGATATTGCCGAGCGCACCGGTCTGGCGCG
CAGTCACGTCTACCACTACTTCAAGGATTGGAAGGAGCTCTGCCTCTGCGCCGTGGAGCGTTTCACCTAT
CAGGAGATCGCCGAGCAGCGGAAGTGGCTGTTGCCGCTGCCGGCCCGCGAAGCGTTGCCGCTCTTTGTGC
GGGACAACCTGCCGACCAGTCAGGATGCCTCCTGGGCCATCTACCTCGATGCCTGGAACGAGTCACTGCG
CGACCCTGTGTTTGCCGAGAGCTACCGCAAAATCATTCTGGCGTGGCGCGAGGTGCTAGAGCTGATCCTG
CAAAAGGGGATCGACAGCGGCGAGTTTGCAGCGGGTGATGCCCGGCGACTGGCGCGTCAGATCACCTCCC
TCATCAACGGCTATGCCGATGACCTGATTCTGGAGCCGACCCCCGAGACGGTAGAGGCCACTTATCAAGA
GATCATGCTGGTGGTCAATCAGCTGGTCTTGCCCCCGGCCCATTCATAACGGCGCAGGTACACAAGGGGG
GAGGCGTGCCAGCGTGGTGCCTCCCCCGGTGTTGTTGGCCATCCGCCGGGCGTGCGATGGCCAGATAACA
GGGAGCGGTTGTTGATGTCGATTACCCATTTGTCACTGCAAGATGCCCGTCATCTGCAACTGGCGGCGCA
GGGGCTGCTTGCCTCCCGTCGCTGCAAGGCGACCCCGGCCGATCTGTTGGCCACCATTCGCCGCATGGCG
CTGCTGCAGATCGATACCATCTCGGTGGTGGCACGCAGCCCCTATCTGGTGCTCTTTAGCCGCCTTGGCC
AGTACGAACCGCGCTGGCTGGAGCAGTTGCTGGCCGACGGCGAAAGCTTCGAATACTGGGCCCATGAGGC
CTGCTTTGTGCCCCGCGAGGATTACCGGCTGCTGCGCCACCGGATGCTCGACCCCGCCGCCATGGGCTGG
AAGTTTTCGGCCAACTGGCTCGCCACCCATCAGCAGGAGATTGGCCAGCTTATCGAGCGCATTCGCCAGC
ACGGCCCGGTGCGGGCTGCTGACTTTGAACGCACAACCGGCAAGGGGAATGGCTGGTGGGACTGGAAACC
GGAGAAGCGCCATCTGGAGGTATTGTTTACCAGCGGTCAGCTGATGGTACGGGAGCGGCGCAACTTTCAA
CGAGTCTATGACCTGGCCGAGCGGGTTATCCCCGAGTGGAGCGACCAGCGGGATCTGCCGAGCGTCGAGC
AGGCGCAGCGGGATATGGTGCGCGCCAGCTGCCGGGCGCTGGGGCTGGTCAAAACCGGCTGGGTGGCGGA
CTACTACCGGCTGCGGCGCGGCAAATATGACGCCCTGCTCCATCAACTGGCGGACGAGGGGGAGCTGTTG

CCGGTACGAGTGGAGGGGTGGCAGCACGGCGCTTTTGTGCACGCCTCGCTGGCCGATGAGCTGGTGCAAG
CCAAGGAGGGCCGTCTCAAGGCAACCCATACCACCGTCTTGTCGCCGTTCGATCCGCTGGTGTGGGATCG
CAAACGGGCCAGTGAACTGTTCAACTTCGACTATCGCATCGAGTGCTATACACCGGCCCCCAAGCGCCAG
TATGGCTACTTCGTGTTGCCCTTGCTGCATCGTGGCAAGCTGGTGGGCAGGATGGATGCCAAGGCCCATC
GCCAGCAGGGGATCTTCGAGATCAAGAGCCTCTATCTGGAAGCCGGGTTGAGAGTGACCCGCACGCTTGC
ACAGGATCTGGCGAAAGCCCTGCAAAAGCTGGCTGACTGGCACCAGACGCCGCAGCTTCGTTATGGTGCC
ATTCCGGCCAATTTGCTGGCGTTGTGGCATGAAAGCGCCCCTGCAAAGTGAGCTCTATCCCTTCTGGGCT
AAGCTGCAAGAGGGATAAACAGTCCGGGAGCGGGAGATGACACGAGTGGCAACATGGTGGGCGGGATTGG
TGATGGCCATCTTGTCCCCCGCGCTATGGGGGCAAGAGCCGCCAGTGAAGATCTGTCTGGGGGAGGGCAA
CGAGTGGGCCCCCTTTACCTACTGGGAGCGCAAGGACGGGGTGCCCGATACCAGCCGCCTGACCGGTTCG
GCCACGACGCTGGTGCTCGATGCATTGCAACAGAGCGGGCTCCCGCACCAGATCCGCTATCTGCCCTGGG
CGCGGGTGCAGCAGGAGCTGGCGGATTATCACCAGAACGGTCTGTGCGAGCTGACCTGGGATGCCAGCTA
CAAGCCGGAGCGGGCCGAGTTTGCCTTCTACAGCGTTCCCCTCTATTACACCCATCTCGGTTTCTTCTAT
CTCAAGCGCCGCTACCCCGAACCGCCGGATCTGCAGACGGTCAATCGCTCCCGGGTGTGCGGGGTGATTG
GCTACAACTACACACCCTACGGGCTTGCGCAGGAGCCACGCCGGGTCAAGCAGTTGCAGCAGGCGCTCGA
TATGCTGGAGCGGGATCGCTGCGACTTTCTGCCGAGCGAAATCGAACCTCTGGTGGCTGGCATCAGCCTT
GGCATCTACCGCAGCGAGAGCGGGTTGCTCAATCTCGCCCTGCCCAGCCGCAAGGGATTCTATCTGCTGG
TGAGCAAGGGCTCCCCCCGCGGTCACCAGCTGGTGAACATCCTCAATCAGTCGCTGGTGGCATTTCAGGA
AGGGGGGCATACGGATGAGGTGATGCGCCGCTTCTTGCCACCGATGGAGTAATAGCGAAGGGTTGCTGTC
ATTCAGGGGGAGCAGATAAACAGAGGGCCAGCCGGTGCAAACCGGCTGGCCCTTTTTTACGTGAGAGCTT
GTCAGGCTGCCAGACGGGTCAGTACCTTCACCAGCAGCTCGATGCGCGGGGCAATGCTGACCAGATCCAG
ATACTCCTCGTCACTGTGGAAACCTGCACCGATAGGGCCCAGACCATCCAGAGTGGGGATACCGAGGGCG
GCGGTCAGGTTGGCATCAGAGCCGCCCCCCACCTCTTGCCAGCTGATCGGCAGTCCCAGTTCGGCGCCAC
ACTCTTCCACCAGCTGCATCAGCGCCTGAGTACCCTCTGACGGCACCATGGCCGGTTTGTGGGCCTCGCG
GGTCAGCTGGATGGTGACGCCGTCCAGATGGGGCCGCTCGGCCATGGTCAGCAGATGATCGTTGATCTGG
TGGTACTCCTGATTGTCCCAGAAGCGGACGTCTACCACGGCGCAGGCATGGGCCGGCACTATGTTGGCGC
CATTGCCCCCCTCGACAATGCCGATGTTGAGGGTGGTGCCGCTATCGAAGTTGGTCAGGGCGTTGATGGC
CAGGATCCAGTGGCCCAGCTCGGTGATGGCGCTGCGCCCCTTCTGCGGTTCGTTGCCCGCGTGGGCTGCC
TTGCCGGTGAAGTCGATGCGATAACGGGCCATCCCCTTGCGCGCCTTGACCAGCGAGCCATCGGCTCGGG
CCGCTTCGGCCACCAGCACCTGTTTCGCCTGTTTGGCCACCGCTGCCAGCCAGTCGCTGGAGTGCAGGGA
GCCGATCTCCTCGTCCGGGTTCATGCAGACGCAGATGCTCAGGCGATCGCGCACCTCGCGGGGCAGTTCA
CGCAGGGCATAGACGATGTTGAGCAGGCCGGATTTCATGTCGGAGACACCGGGGCCGTAAGCGCGCTCAC
CATCGATGCTCATCGGGCGGGCAGCGGCGGTACCGAGGGGGAAGACGGTATCCATATGGCCGATCAGCAT
TACATCGATCTGCGCCGCATCAGGCTGGTTGCGGATCTCCAGCCCCACCCCGGCGGCGCCGCAGTCGACC
CGCTTGATCTGCCAGCCGCCCATCGTCTCATATTTGGCGGCCATCAGGTCGGCCACTGCCGCAATGCCCG
GCTGGTGCAGGGATCCGCAATCCAGATTGATCAGCGGGGCCAGTTCGGTCAGGTATTGATCCAGAGAAAA
AGTCATTGTTTGCTCCACTATCAGGTTCGGGTCAGAGAAGGATGTTCATCACCAGCATGGCGCCGAAGGC
GTTGAGTACCGAGATGGCGACCATGATGGGGATGTAACGCCCTTCGGTGCCGATGCAGCCGAGGATGCGG
CCCATGTACTGTACCTGGGAGCCCATCAGGTAGATGGCTGGCGCCAGAATGGCGATGTGGGTGCCGTTGA
GGATGCCCTGATCGAACAGGGTGATGACCACACCGACTGCGCCCCCCATCGACATCCAGGCGCCGATCAG
CACGGCTGCCGCCTCACCGGGCAACCCGAAGATGGCCATGACAGGGGAGAAGATGGCGCCCATGGCATCC
AGCGCGCCGGTGATCTGCAGCGCCTTGATGATGACGAAGGCCATCAGTACGTTGGGTACGGTGGAGGTGG
TGGCGATGACCCAGCCCTTCTTGGCCCCTTCGACGAAGATATCGGTGACGATGGGTTTCTTGATGTTGGC
CTGGCTCATTTGGCTTGCTCCTGAGTGTTGAGGGTCTGCTGGGTGTTGTTATCGGTCTGCTTGCCTTCGC
TCATATTGAGGTAGACCCGGAACAGGTTGGCCCCGACAAATTTGAAGGCGAACATCACGATGACTGCCAG
ACCGATGGAGGAGCTGACCGCCGGGCTGCCATCCGCCAGCGTCAGGGTGAACAGCACGGCGCCGGAGGAG
AAGAAGTTGACGATGGTGGCACCAGCGGAGAACTGGAACATGGTGAACACGTCGGTTTCACGTTTGGTGA
TGAGCCCTTCATCCTTGAGCTGGCGGGTCATGGCGGCACCGGCATCGGTACTCTGCAGGGAGGCAATAAG
GGCGAGGCCGGTGTTGCCCGGGATCCCCATCAGCGGACGCAGCAGCGGGGTCAGCAACTTGCGGGCCGCT
TCCAGCGCGCCGTAGTGCTCCAGCACATTGATCATACCGAGGGCAAACATCACGGTGGGGATCAGGGTCA
GGGCAAACAGAAAGCCGTCTCGCGCCCCGTTGCCTCCCTTCCCACGCATGTCGGTCATGGCGGTCTTGAT
GCCGTCACCATTCTCGCTGACGCTCTGGACCATGGTACCGAACGAACCGTTCAGCGTGGTGAAATCGAAT
ACCCCGTACCACTCGCCGGATTGCAGCAGTCCGGAGAAGAAGACCACGGCGAAGGCCAGTGCCAGGTAAC
TGCCTATGCTCACCGAGCGTGTTTTGTCTGTCATTAGCTAGCTCCATATGAATAATTATTTAATCTGAAT
AATTATAATTGATGTGGATGTCACTAAATTGATGGTGGTCAAAATGGTTTGCTACAAATGACGAATGTAT
TGGGGTTATGTGACTGTGATCGCCATATGATTGGACGATAATCTGGTTTTTATCGAATTGTTTGAGTGAA
TAAAAAGCAGGGGCGGGATAGCGGGGCAATAAAAAACCGGGCACGAGGCCCGGTTTGGTCAATCAGTCAG
ATGTGATTACTGGAGTACCAGCACCATCAGCAGGCCGACGGTAATGGCAGATACCAGGAAGGCAAAGCCA
TAGAACACATAGTGGTGCAGTTTGGTGGTGTGGATCCCCAGATCGTGCAGACCGTGGTAGATACGGTGCA
TGCCGTGCCAGACTGGCAGGGCGATGATCACCAGCAGGATGCAGGCACCCCACCAAGAGCTGGCGAACGC
GTGCACGCGGTCATAGCTCATGGTTTCCACGTCCATGATGCCCATGGGCACCAGCAGACCGGTGATGAGG

ACAATGACCGGCAGCAGCATGGCAACGACCATGCCACCCGCACCAAACAGACCCCAGTAAATTGGTTCGT
CAGAACGTCTCATTGTTTTCTCCTCAGGCCACGATGATCAGGACAAGCAGGGACACCACCGCCAGCGCCA
CATACTGGGCGAGAACGATCGGCTTCTCCGGTACCAGGTCTTCACCACGGAAGATACGCATGGCTTTCGG
CGCCAGGGCGAACCAGGTTTTGGCATGGAACAGGCACGCCGCCAGAGCGACGACATGGAACAGGATAGCG
ATCGGGCTTTGCAGGGATTCCAGCCAGCCGTTGAACGCTTCCTGACCCTGAACCAGGCGCATCAGGCCCC
ACATCAGCACAACGGCGTAGATGGAGACGAAGATACTGGTGCCTTCACGGATCATGTAACCGGTGTAGAA
GGCGTTCTTCAGCCACCAGTCTTTCTTCATTTCGCGGACGTAAGGTTTACGTTTGCTATCAGTGTTGTTC
ATGGTCTCAATCCTGCTCTCAATCTGGCTTGAACATGGCGATCATGTAGTCCTTGGCGCTTTCGACCTTG
CCAAGCTGGATGGCCGCCGCCGGATCGACGCCCTTCGGGCACACTTCGGAGCAGTAGCCCACGAAGGTAC
AGCCCCAGACGCCTTCGTCCTGGCTGATGATGCGCATACGCTCTTTGGCACCAGCATCACGGTTGTCGAC
GTTGTAGCGATAGGCCAGAGCCAGTGCCGCCGGACCGGTGAACTTCTTGTTGATGCCGTACTGCGGGCAG
GCTGCGTAGCAGAGACCACAGTTGATGCACTGGGAGAACTGCTTGTATTGCGCCATCTGGGCCGGAGTCT
GGATGTACTCGCCATCACACAGGCTGCGCGGCTCGCTCGGGATGATGTACGGCTTGATGCTCTCCAGCTT
CTCGATGAAGTCGGACATGTCGACCACCAGATCGCGCTCGATCGGGAAGTTGTTGAGCGGCTCGACGGTC
ATCTTGCCCGGCAGGTAGTCGCGCAGGAACGCCTTGCAACCCAGCTTGGGCACACCGTTGACCATCATGC
CGCAAGAGCCGCAGATCGCCATCCGGCAGGACCAGCGGAAGCTCAGGGTAGAGTCCAGGTGATCCTTGAT
GTACTGCAGGGCGTCCAGCAGAGACATCTCTCTCTGGTATGGCACCTGGAAGGTTTGCATCCAGGGCTCG
TTATCCTGTTCCGGACGGTAGCGCAGGATCTCAATTTCAATCATCTCAGCCATTCTTCTTAGCCTCGTCT
TGTTTCTCACCCTCGGAGCCGTACACGCGTTTTGCCGGCTGGGACTTAGTGATCTTCACATCGGAGTAAT
CGATGGAGGGCGTTTCGCCATTTTTGTAGAACGAGAGCGTGTGCTTGAGGAAGTTCACGTCATCGCGCTC
TTCGTAACCATCCAGACGCTGGTGGGAACCGCGGGACTCTTTACGATTGATAGCGGAGTGCGCCATGGCT
TCAGCCACGTCCAGCATGTAACCCAGTTCGATGGCGTACAGCAGATCGGTGTTGAAGACAGAGGACTTGT
CGTTGATCTTGACCTTTTTGTAACGGGCTTTCAGCTCGTTGATCTTGTCGATGGTGCCCTGCATCAGCTC
TTCGGTACGGTAGATACCGACACCGGCTTCCATGGACATGCCCAGCTCGTTGCGGATATCGGCCGGGTTT
TCGGTACCGTCTACATCCATCAGGGAGAGGTGCTGCTTGATGAGCGCCTCGGCCTTGGCGGCCAGCGCTT
CGGTGTTGCCATGCTTCTGAGTCTTGGCAAATTCACCTGCCTGCTCACCGGCCAGCTTGCCGAATACCAC
GATCTCGGACAGGGAGTTGGAACCCAGACGGTTGGCACCGTGCAGACCGACGGAGGCACATTCGCCCGCG
GCGTAGAGGCCGGGCAGACGGGTGGCACACTTGTTGTCAGTCTCGATACCACCCATGGTGTAGTGGGCAG
TCGGACGTACCGGGATAGGCTCTTTGGCCGGATCCACGTTGACGTAGGCTTTGGCCAGTTCGCAGATGAA
CGGCAGACGCTCTTTCAGGTACTTCTCGCCAAGGTGACGCAGGTCGAGGTGAACCACATCGCCCATCGGG
CCCTGAATGACGCGGCCTTTCTGCTGCTCTTGCCAGAATGCTTGGGAAACGCGGTCGCGCGGACCCAGCT
CCATGTATTTGCTCTTCGGCTGGCCAATCGGGGTTTCCGGGCCCAAGCCGTAGTCTTGCAGGTAGCGGTA
GCCATCCTTGTTCAACAGGACGCCACCCTCACCACGGCAACCTTCGGTCATCAGGATGCCGGTGCCCGGC
AGACCGGTCGGGTGATACTGGACGAACTCCATATCACGCAGCGGTACGCCGTGACGGTAGGCCAGTGCCA
TGCCGTCGCCGGTGACGATGCCACCGTTGGTGTTGTAGCGATAGACACGGCCAGCACCGCCGGTTGCCAC
GATCACGGATTTGGCCTGGATGAAGCGGGTGATACCGTTCTGGATGTCGAAGGTCAGTACGCCCTGGACA
CGGCCGTCGTCCACGATCAGATCAAGGCAGAAATGCTCGTCGAAGCGCTTGATGCTCGGGTATTTGACGG
AGGTCTGGAACAGGGTGTGCAGAATATGGAAACCGGACTTGTCTGCGGCGAACCAGGTACGGGGAACCTT
CATGCCACCAAACGGACGCACGTTGGCTTTACCATCCGGCTTGCGGCTCCAGGGGCAACCCCAGTGCTCC
AGCTGGATCATCTCTTTGGTTGCGTTGTTGACGAAGTATTCCACTACGTCCTGCTCGCACAGCCAGTCAC
CACCGGAGACGGTGTCGTTGAAGTGGTTTTCGAGGGAGTCGTCATCACGGACGACACCGGCGGCGCCGCC
TTCGGCAGCAACCGTGTGGCTGCGCATCGGGTAAACCTTGGAAATCAGGGCGATGTCCAGGTCTGGCTGT
GATTCGGCTATAGCGATAGCGGCGCGTAAGCCGGTACCGCCAGCACCGATGATGGCCACATCGGTCTTGA
TAATATCCACATCTGCCTCCTTAGGGATAGATTTGCTGGGCACGATTTTGCTCTATGAAAAAAATTCAGG
CTTGATGCAAGTCAAACTGCTAGGTTTTATTCCCTTTTTATTGGTTATTTTTCTATAAAACACCCAAATC
CCTGGATGAAAATGTCGTTATTATCGTCATTTTTTGGTGTTACTGAGCAGTTGAACACCTAGCTACGAGT
ACTTTAACTTCTGGATTGTACGTAGTTTTCTTTCAATAAAATGCGAATCACCTCTCTTTTTAGGTACTCG
CGAAAAAAATTTTAAGTTTTTTATTGCAATTTTTTAATGAGAATTATTGGCATTCATTTGTTCGGATAGA
AATAGGTAATCCGAAATGCTACGGTGACCACGGCGGTGATCTGATAATCCCAGACGAATTACAAGGGTTT
CAAGGCGGGGTATTTGGCGGGTAATGTGAGATTACTCACTGAAAATTAAGCGCTATTTAGAAAAATAAAA
GGGGCCTGTAAAGCCCCTTTTGCTCATGCTCAAATGCGGAACATGCCGACCTGATTGCTCAGTTCACCCG
AAATATTTTTAAGTTGTTCAGAGAGATGGCGTGAGCTGTCTGCATCCACTGCCAGTTCGTCCGAGACATC
TTTCACTGCCTGAATGTTGCGGCTTACCTCGTCGGTGACGGCCCGCTGCTCTTCAGCCGCGCTGGAGATC
TGGGTCGCCATGTCGGAGATCTGGTTGATCGAGGTGGTGATCTGCTCCAGCGCGTGGGTGGCGTTGTTGG
CGTCATCCACGCTGTTCTGGGCCAGCAGCTGGCCTTCGTGCATGGTGCTGACCGCCCCCTGGGTATTGCG
TTGCAGGGTTTCGATCATATTGCGGATCTCGACGGTGGAGCTGTGGGTACGCTGTGACAGGACGCGCACC
TCGTCGGCTACTACCGCAAAGCCGCGACCCTGCTCACCGGCACGTGCCGCCTCGATGGCGGCGTTGAGTG
CCAGCAGGTTGGTCTGCTCGGCGATGCCCTGAATGGTGGAGAGGATGGTGTTGATCTCCTGAGCGTTTTT
CTCCAGTTCCTGAATGATGTTGGAGGCCTGTTCCACCTGGGTGGCCAGATCGGTGATGGAGCGCTGGTTG
CGGGCGATCACCTGCTTGCCATGCTCGCAGCTGCTGGAGGAGCTGCGGGCCGCTTCGGCGGTCTGCTCGG
CGTTGCTGGCGACCTCGACTGCGGTGGCTGACATCTCGTGTACCGCAGTGGCGATCTGGGAGATCTCGTT

TTGCTGGCGTGCCAGCCCCTGATTGGCCTTCTCCGCCATGCTGTGAGAGGACTTGGCCTGCTGGTTGAGC
TGGCCGGAGGAGTTGGAGATATCCTGCACCATCACATGGATCCGCTCGATGAAGGTGTTGACGTGTTTGG
CAACCGCGCCCACCTCATCCTCACGTTCAATCTTGATCCGGCGGGTCAGATCGCCGTTGCCACGGGAGAG
ATCGGCAATCGCTTCGCCCAGGGTTTTCAGAGGGGCCAGCGAGCTGTTGATGGCGACATAGGAGAAGCCT
GCCACGATCAGTATGGTGATAAGCCCCTGAATGAGGGAGGAGGTAACCATGCGGCGTACCGACTGGTAGG
CGATCTCTTCATCCACCATCATGCCGTAATACCAGTTGGTATTGGGAATGGCATTAAAGTCGTAGACCTC
TTTCTTGCCGCTGATCACCAGCTCGTTGATCATGCCGTCATGGGCGACCTGATTGATGTAGGCGGCAGTC
AGCTCCGGTGCCAGAGCGGTAGCGGATTTCAGGCTCAGGGAGGCATCGGGGTGCGCGATGATGGTGCCAT
TGCTGTCCACCAGCATGGCGTAGGTGCCCTCGGTGGTGACCGCCAGAATGTCCGAGATCAGTGAGCTGAT
GGAGACGTCACCGGCGATGACCCCCTGGGTATTGCCACGCTGGAACGGCTCGGCAATGGTGACAATCAGC
TGGCCGGTGGAGATGTCGGCATAAGGGGAGGTGATGGTCATCTTGCCAGCAGCCATTGCGTCTTTGTACC
AGGGGCGTTGACGGGGGTCATAACCGGCGGGCAAGTCGGTTGGTTTGCTCTGGATCATTTTGCCGTCTGC
GGTACCGGCATAGACCAGGTCGAAGCCACCGGCATTCATGGACTGGGCCAGATGACTGATGGGCTCATCT
TCTTTGGCGAATGCTTCCTTGGTGGTGCTGACCATGCTGATCCGGGCGTCGACCCAGCGGGAGATCCCCT
TGACGTTGGCCATGGAGCTTTCGTCGATGATGTTCCAGACCAGTCGCTGGGTCTCGCTGCGCAGTTGCGA
GGTGTTGTACCAGACCTGCAGGCCGGTGATCACCAGAATGATGACCCCGATAGCCAGAACAAGCTTGTTT
TTAATGGATAAGTTCATAGTTACATCCTTGATGAGCGTCAGTCTGAAGTGTGTGGCATCTGATTATTGTT
ATAGCCTTGTTACTATCGGCCGTAGTCGAACTTTTTTTACCCCGGTCAACAAATTAAAGTTAGTGCCTAA
ATGGGGGCTTATCCATACGCGTATCGTCCCATCATGGTGAGATAAAGCGATCCGGTCGATTGGTGAAGAT
TCCATCGACCCCCATGCTAGAGAGCGTTTCATAATCTTCTGGATAATCAACGGTATAAACCAGAACTTTG
AGCCCCCGGCGATGTGCATCATCGACCAGCGCGGCGTCGATGAAATCAACATCGCAATTGATGGACCAGG
CCCCCAGCTCGTCGGCAACCTGCGCCAGGTTGAGCGGCAGGTTGGCGGTCAGGGCACCGAGCCGGATATC
GGGCCGCAAGGCGGCAAATCGGGCCAGCTCCGGGTGATGGAAGGAGGAGACCACCCACTGCCCGGGAGTA
AAGCCAAGCTCGGCCTCTGCCCGTCGGGTCAGGGCAGCCACCGCATCGGCGGTATGAGTCCCCTTGAGCT
CGATATGCAGCTCGCAGCGTCCGGCGATGATCTCCATCACCTGCCACAGGGTCGGGATCACCTCTCCCTC
ACCGGCATCGAGCTGCTCCAGATAGGAGCGGGATTGTTCGGTCAGCACCCCCTTGCCGTTGGTGCAGCGC
TCCAGCCGCCGGTCGTGGAAGACCCACAACTCGCCATCGGCCGCCTGCACGTCGATCTCGATGGCTGATG
CTCCCAGCTCCAGCGCTTTGGCCATCGCCTTTAGGGTGTTCTCTGGCGCCAGACCGCTGGCGCCACGGTG
GGCAATAATCTGCATGTTTTGCTCCTTTTACCTCGCGGATCAGCCGCGGCTCAGGTGAATGTCGATCTGT
TGATGGGGCAGGGTAAGTCCCTGCTGCTGCATCTTCTCCAGCACCAGCGAGTGAACCTCGTGAGTGATGG
GCATCCGGTGATCCATGTTGTTGACGTAGAGGCGGATCTCGTAGATCAGTGCCGAGTCGGTGAATTCGAT
CAGGAAGACCTCGGGCGTCGGCGTCTCCAACACCAGGGTCGATGCCTGGATGGACTCTTCCAGAATGCGC
TGCACCAGTTTCGGATCCCGGGTCAGGCCGATCCGGATCCGCAGTTTGACCCGGGTGATGGCATCGGAGA
GCGACCAGTTGATGAACTGCTCGGTGATGAAAGCCTTGTTGGGCACGATGATCTCCTTGCGATCCCAATC
CACGATAGTCGTCGCCCGGGTCTTGATCTTGGTGACGGTGCCGGTGAGATCGCGGATGGTGACGGTGTCA
CCGAGCCGGATCGGCTTCTCAAACAGGATGATGAGGCCGGAGACAAAGTTGGCGAAGATCTCCTGCAGAC
CGAAACCCAGCCCCACCGAGAGGGCGGCGACCAGCCACTGGGTCTTCGACCAGTCGATGCCGAGCATGGA
GAAGCCGGTGAGGGCACCGATCAGGATCACCAGATACTTGCTGGTGGTCGTGAGGGCAAAGCCGGTACCC
GGGGAGAGGCTGAGGTGTTGCAGCAGGGTCAGCTCCATCAGCCCCGGCAGGTTGCGGGCAGTCACCACGG
TCAGAATGAAGACGAAGGCGGTCAGCATCAGATCCTGCAAGGTGATGGCCGAGAGCTGCTCGATGCCGCC
AATCTTGCTGCTCACCTGCCACACCTCGATATTGCTGAGGAAACTGAAGGCGGAGTTGAGATCCGACCAT
TGCACCACCACCAGCATGGTGAAGCCCAGCATCAGCAGGGTGCGCACCAGTCCCAGCGACTGGGCGCTGA
TGGTATCGAGATCCAGCTCAGTCTCTTCCACCACGTCCGGGGTCTCGGAGCTGAGCTCGTCATGCCCCTC
TTCCCGGCCGCGCTGGGCCAGGATCTCGGCCCGCTTGCTCTTGGCGCGCTCGAAGGCGAGGCGGCGGCGC
TGGATCAGCATCCAGCGTCTGGCCAGATAGTAGACCAGCAGGCAACCCAGCCCCATCAGCAGGCTCAGCT
CCAGCTGGCGCAGCAGGGTGCGCGAGGTATAGAAGTAGCCAAACAGGGCGCCAATCACCGCCAGCAGCGG
CGCCAGCATCAGGCTGTTCCAGATGAAGTGGTGTAGCAGATGGGGCTTGGTCATGTCGGTCTGGCCCCAG
GTGAGCGGCAATCGCTCCCGGTTGAGGCGGGCAAAAAAGAGCAGCAGCCAGAGTGCACCGATCATGAAGG
CCATCCGGCCGAGGGAGTCGTAGAAGGCGTGCTCCTGATAGCTGTTGGCCATCCCCTGCACAAAGAAACT
CGGGATCAGCACCAGCAGCAGGGTGCGAAACTGCCCCCATACCTTTTGCACCTGTTCCCGGGGCCAGCGG
AAGTGGTGGATCAAAATCCCCCGCTCCAGCGTCAGGTGGCGGGCCATCACCAGTGCCAGCAGCAGAAACC
AGATCTCGCCAAGGCCGCGTGCTACCGCCACCGCAAACGGGTATTGCCAGGCCCCTTCGAGCAGGCCGCG
CACCATCAGTACCATGGCGGGCAAGGGGAGGGCGGCAAGCAGGGAGCGCAGCAGCAAGCGGCTGGTGAGG
CTGAATTTGTCCTGGGTCACCTTGCCGATCCGCGGCGCAATATGGTCGCAATAGAGGTTCAGATGACGGG
CCAGCTTGAACCAGCTCCAGGCCAGCACCAGCAGACCAATACCGGCCAGCGTGAGACTCAGCGGGCTGTT
CTCGGCGATGGCGTAGGGCAGTTGCAGCCAGTTATCCGGGTTGAAGGCCAGGGTCAGCGCGGCGGGAATA
CCGAGCATCACCTGTGGGGTAAAGGGGCGCACATCCGGCATCCAGAACAGGTGGCTGGCGCTTATTTCGC
GGATCTCGTCGATCTTGCTGTTTTGACGGCTGTAGAGCAGTTTGAGGCGGGTCTGCTCCTGAATGAGGGT
GTCTGAGGCGTCGTTGAGCCGGGTCAGCAGTTGGCGCCGGGCCTTGAGCAGGTTGTCGAGCACCGCTTGC
TGCGGCCGATCCAGCGTCACCTCCTCTTCGGCCATCAGCTTGTCGCGCAGCTGGCGGGTATCCTTGAGCC
CATCCTGCTCGCTCTCGTACTGGTACTTGGCCATGCGGGCGCTGACGATGGCGGACTCCAGCTTGTCGAG

GGGAAACTGGGGAGGCAGGTCGGAGAGTTTGCTGCGCAGGTTCTCGCCATAGGCATTACTGATCTGCAGC
CACTCCAGCTGTTCGCGTACCGAGTTGACCAGCTCATTCAGTTCAAACAGATTCTTGTCGACGCTGCGCT
GCTCGTCCTGCACCCGCTCAATCTGATCTTCCAGTGTGCCGAGCTGGATGGAGAGCGCCTGATTCTGCTG
TTGCTGCTCCAGCAGCAGCGGCATGTCGGTGGTGAGCTGTTTGCGCAGCCGCTCGCTCTCCTCCAGCGCC
TGCTCGGTCTTCTCCCGCCGCAGCTGGTTCTGTTGGGTCAGCAGGCTGGCTTGCCACTCATCCTCGTCGG
CGATGGCCAGATTGAGCTCCTGCAGCTTCAGCTTGCCGAGATCGTTGCGCTGCTGGGCCGAGAGCTGCTC
CAGCTCCAGCATCTGGATCCGGTCGGAGAGGCTCTGCTCCTTCATTCTGGTCAGCAGCAGTTGGGCCTCC
TGCTGGCGATCCGATTCGCTGCTGAAGCTCAGTCGCTCCAGCTGGCTGCGGGTCAGTTGCAGCTGTTTGC
GCAGATCGTCCACCCGCAGGTGATACTCCTGCCCGGTGCTCTCCAGCTGGTTGAGGCTGTCCATCACCCC
CTGCCGCTCCTTGCGCAGGTTGAGCTGGCGGTTGCTGCTCAAGCCTATGGCCTGGCGCAGCGCCTCCTCC
TTGAGGCTCGCCAGCGGCGGGCGAGCAGCGGGCTGATAGTTGTGCAGGCTCTCCTTGAGGCGCGCCGACT
CCTTGGGGTAATCGATGAGGATCTGCTGATAGGCTTTGCTCTGCTCCCGGTAGCGCTCGGCCTCTCTGGC
AAATTGCAGCGCCTGCTGGTAGCTCTCCCGCAGCTTCTGGTTTTCCGGGGTATCGCTCTTGGGCACGCTC
TGCAACAGGGATTCGGCCTGGGCAATGGCATCGTCGGCGCGCACGGCTGGCGGCAACAAGATAAGGGAGA
GCAGCAAGAGGGTGAGCAGGCGTTGCAACATGAGGCTCGTTTCCGTTCAGAAGAGGTGGCCCTAGCCTAA
AACAGGGCAGGGAGGCGGGTAAAGCAGTCTATATTAATGATTCATAACACAAATAACAGGAAGTCGTGCT
GCCGTTTCGCTATGATCCCGGCCGCTTTCTCTCCACCGAGTCGAATATGTCAGCTGTTGATCTGGGCACA
GCCCCGCTATCTCGCCTTTTCTGGCGTTACGTAACCCCGACCGTGGCGGCCATGCTGATCACCGGCATCT
ATGTCACCATCGATGGCATCTTCGTTGGCCATGTGCTGGGTCAGGATGGCATGGCCGGTCTGATGCTGGC
TTATCCCATCTGCGCCGTGCTCTATGCGGTGGGGGCGCTGATCGGCATGGGGGCCTCGTCGCTGGTCTCC
TTCCATCTGGGGCAGGGCAACCCGACCAAAGCCCGCCATATCGTCGGCAATGCGCTGGTGATGGTGTTGC
TGGCCTCGGCACTGCTCAGTTTTATCGGGATCCGCTATGGCGAGCCCATGCTGGCCTGGATGGGGGCGGA
GGGGGAGATCTTCACCGCCGGGCTCTCCTACCTGCAGGCCTACGCCAGGCTGGGCGTCTTTGCGGTGCTG
TCGATGGCGTTCAGCTCGCTGCTGCACAACGATGGCCGCCCCGGATTTGTCACCCTCATCATGGTAGTCG
GGGGGCTGCTCAACGTGCTGCTCGACTATCTGCTGATGGTGGTGTTTCCGTTCGGACTGGCCGGGGTGGC
GACGGCCACTATGCTTTCCCAAGCGGTGACGGGCCTCGCCTGCCTGTGGCACTTCTTCACACCGCGCAGC
CAGCTGCGGATAAGTTGGGACACCTTGCGCCCCGATCTGCGGCTGATGGGCGAAACGGTGCGCCTCGGGG
TCTCCAGTTTTCTGATGTACCTCTATCTCTCGGTAGTGCTGGCGCTGCACAACAAGGCGTTGCTGGCGGT
TGGCACTTCGCTGCATGTGGCGGCCTACGGGGTGATCAGTTACACCGAGGCCTTCTTCTATCTGGTGTTT
GAGGGGATCGCCATGGGTATCCAGCCCATCGCCAGCTTTAACGCGGGCGCCGGTCACTGGCGGCGGGTGC
TGCGGATCCGCAATCTGGCACTCGGAGTGACGTTGGGGATCGCCCTGTGCGCCATGTTCCCGCTTTATCT
GTGGCCGGAGGCGGCGGTCTATCTGTTTGCCAGCGACAACGCCACCTTGCTGCCGGTGGCGAGCCTCGGG
ATCTGGCTCTACTTCTGGGGGCTGCCGATGGAGGGCTTGCTGCTGGTGGGGGCCACCTACTTCCAGGCCA
TCAACCGGGCCCGCATCGCTTCCCTGCTCACCGGTGGCAAGCTGGTGCTGATCGGCGGTTTCTTGTGGGG
ATTCTCGACCCTGTGGGGCGTGCCCGGGGTCTGGCTTGCATTGCCAACGTGCAGCTCATTGCTGGTGTTG
GTGATGTGGCGGGCGATGCATCGGGAGTCCGGCGCCCACGCGCTGGCCGGATAGGTAATCAGGTTCGACG
TGCAGATAGCAGACAGGCAGCCATGGGCTGCCTGTTTTGTTAGAGGATGTTTTGTTAACGGGCTCTGCCG
ATTCAGGGCTTGAAGGCGCCGATAAAGATGGCGGGATCGACCCGGGTGTCGTTGAGGCTGACGTTCCAGT
GCATATGGGGGCCGGTAGCGCGGCCGGTCGAACCGACTCGCCCGACGATGCTGCCACGCGGCAGGCTCTG
ACCGAGCTTCACATCGATCTTTGACATGTGGCAGAACATGCTGATGAGCCCCTGACCGTGGTCGACAAAG
ACGGTATTGCCATTGAAGAAGTAGTTGCCGATCAGGATCACCTTGCCGGCGGCGGGGGACTTGATCGGAG
TGCCGGCGCCCACTGCGAAGTCGAGGCCGGAGTGGGGATTGCGCTCCTCGCCATTGAAGAAACGACGCAG
GCCAAACGGGCTGGATAGGGGGCCGTTGACCGGCTTGTCGAACATCAGGTTGCTCGGCTGGTTGGGGCTG
AAGGTCTGGTAAGCCCGAGTCTGCTCCGCCAGCTCGCGGTTGATGCGGGTCATATCCTCGGCGAGTGGAT
TGACCTGACGGCTGTTCTTGAGCTTGATGTGCTGCTCGCGGTAGTGCTTAGGGGTCACGGTGAAGCCAAG
CGTGCGGCCATCACCGGTGGTCAGCAGCTGGCCCCCAAGCGGGTTTTTGAGAGAGATGCCGACGATGGCG
ATCCACTGGCCATCTTCCTGCACGACCAGTACGGGCTTGCCCTGATAATGGGCTTGTGGCGCCGTGGATG
CCTTGCCAAGGGGCACGACGGCCACGCCGCCTGGTACCGGATGGTTGAGCAGGCGACTGATAAAACCCTC
GGCCAGGGCTGACTGGCTGGCCAGTAACAACAACAGACAACACACAACTCTCATGACGATACCACTCGAT
GTTAAAACGCCCGGGATAGCGGGATGACAACCCCCTGTCCGGCATTCGCCGATCACTAAAGGGTGATCAC
GACACGTTGGCCACGACAGGTGCGGTTGGAGGAGGGGACAGATTGTGCCTCGTTATAGTCGGTGCAGCCA
GAGGCGGGGCGTGGCTGACGCCCCTTTTGTGCATAGCTGCGTCAGGTTTTATGTCGTGGTCGGGTTGCTT
CGCCATTGAGTCGCCCCCGCCCTAGCTCTAGAATCTGCTTCCGGCACTGATGCCACCGACATTCCGAGGA
GACGATATGAACGGATTACCCCCTTTGCGCACCACTCCGGCCCGGTACACGACAGGTCGTGGCTGGCTCT
GGGTTCGCAGCGGGTTCGATATCTTCAACAAGGGGATGGGGATCAGCGTAGCCATGGTGCTGGTCTGGTT
TATGGTGGGCATGCTGCTGGAGCAACTGCCGGCGGGCGGCCTTATCTCCCAGCTGCTCTACATGGTGTGG
GGCGCGGGTTGGGTGGCGGTCGCCGCTCGGGGTTATCGGGGGGAGCCGCTGCAGTTTGCCGATCTCTTCG
CCGGTTTTCGCCACAAGTTGACCCCGCTGATGCTAGGCGGTCTGCTGGTGTTGCTGCTGTTTAGCCTGGT
GGGCCTCGTCTGTCTCGGTCTGCTCTCCATGTGGGGGCTGCTCCCGCTGCTGACGCAGGGTCCCGAGAGC
ATGGTGGTCTCAGCCAGCCAGTTGCAACAGCTGCTGCTGGTGTTGCTGGTGGGGATGCTGCTCATCATTC
CCGTGCTGATGGGGGTCACCTTTGCTCCGGCGCTGATCTACCACCACAATATCGGCATCATCCAGGCGGT

ACGGCTGAGCTTCTTCGGCTGCTGGCGCAATATTTTTCCCTTCTTCTGGTGGGGGCTGCTCTCAGCCATT
TTGTTGCTGTTTGGCGCTGTGCTGTTGCTGGTAGGTCTGCTGGTGGTGATCCCGGCGCTCAACTACTCCA
TCTATGTGGCTTATCGGGATATTTTCCTGGACGAGAGTGCGCAGCCGGAAGCGCCGCATTCAGAACCATT
CGGATTTGAAGCCTGATCAAGGGATCAGAGCGAAGAAGGGGCCCGCAGTGGCCCCTTCGTTATGTTTGGG
TGTGCGGAATGCTTACTCCTGCCAGATGGGATCCGCCGCCATGCGGGCCAGCAGAAAATCGAGGAAGCTG
CGCAGCAGTGGCGACATCTGCTTGCGGGTGGCATACACCCCGTAGACGCCGAGCCGCTTGGGTTGCCAGT
CGGCCAGCAGGGGCACCAGTTCCCCGCGCTCCAGATAGGGTTGTGCCGAGTAGCGGGGCTGCAGGCTGAT
CCCGGCACCTTCCAGCGTTGCTTCCAGCAGCAGGTTGGAAATATTGGCGCTCAGGTTACCGCTGACCGGC
ACTGACTCGGGACCGTCCGGTCCATTGAACTCCCACAGGCTCTTGCCAAAGTAGGTGTAGGTGAGGCAGT
TGTGCAGCGCTAAATCCTGCACCTTGTGTGGCATCGGATGACGGGCCAGATAGGCCGGGCTGGCACAGAC
TACCGAGTGGCAGGTGCCGAGACGGCGAGCCACCAGATTGGGGTCGAGGTCGTTGGTGATGCGCACCGCC
AGATCGATCCGCTCCTCTACCAGATTGACGGTTCTGTCGAGCAGCAGAATGTCGATGGCCGTCCCCGGGT
ATTGCGCCAGATAGTCGTTGGCAGCCCCGACCAGCAAGGCTTCTGACAGGGAGTAGCTGCTGGTGATCCG
CAGTTGTCCCTTGGGGGCGTCATCTCCCTTCACGGCGATCTGCTCCATCTCTTCGCCGAGCGCCAGCATG
GCGCGGGCCCGGTTCAGCGCCTCCTCTCCTGGCCCGGTCAGACTCAGACGACGGGTGGTGCGGTGCAGCA
GGCGGGCGCCCATCCAGCTCTCCAGCTCGGCCAGATAGCGCGACACCATGGCGCGCGACATATCCAGTTT
CTCTCCGGCGCCGCTCAGGCTCCCTTGTTCGACTACGCTGACAAAGACCCGCAAGGCGGTCAGTCGATCC
ATGTACGGCTCTCTATATGTTCGATATGTGCAACAGTGATGCGCTTTTTATGCTGTATTTGCACCTTAAG
TGATCCAATACACTGTGTCCATCAAATGAACCCCCGAATACTTAATGAGGAATAGATGATGAACAACACC
CTGATCCGCAACCTGACTCTGGCGACCACCCTGCTGAGTGGCGCCGTACTGGCCGCCCCCTTGAGCGTGA
CCGTCTACAACCCTGGCGAGAAGGCGATCTTCCCGGTCTCCTCCTCACTGGTGACCGGTGACAAAGAGGC
CATCCTGATCGATGCCCAGTTCGACGTGCAAAATGGTCAGGCGCTGGTCGACATGATCAAGCAGAGCGGC
AAGAAGCTGACCACTGTCTATATCAGTGCCGGTGACCCCGACTTCTACTTCGGTCTGGAGCCTGTCATGG
CTGCGTTCCCGGATGTCAAAGTGCTGGCCGACCAGCACGTGGTTGACCATATCAACCAGACCAAGGATGC
CAAGCTGAGCTACTGGAGCCCGATCCTCGGCAAGGGGGCACCGAAGAGCTTGACCGTGCCGCAAGTGATG
ACCGCAAGCCACCTGACGCTGGATGGCGAGAAGATTGAAATCAAAGAGATGAATACCCCGAATGCCTACC
TCTGGGCCCCTTCCATCAAGACTGCTTTTGGTGGTGTGCCGGTTTACCACGGGGTGCATGTCTGGATGGC
CGACAGCCAGACCAAGGCGGCCCGCAGTAACTGGGTCAAGGCGCTGGATAACCTGCTGGCCCTGAAACCC
GAGCGTGTGGTACCGGGCCACTTCTTGGGCACTGCTCCCAAAGGCACTGCCGCTGTGAGCTTTACCCGTG
ACTATGTAGAGCGTTTCGAACAGGAGCTGGCCAAGGCGAAAAACTCCGGTGAGCTGATTGAGGCTCTGAA
GAAGGCCTATCCGGCACTGCCGGTTGACGATGGTCTGGCCATCGGTGCCAAGGTCAATACCGGCGAGATG
AAGTGGTAAGCCACTGAGCCTGCTTTCCCTGTGTTTTTTTACCGGGCCTAAGGGCCCGGTTTTTTATCCG
AGATCGAAATGAGAATGAAGATGAGCGAACAACAAACCACCCTCCATTATGTCTATGACCCGCTCTGTGG
CTGGTGCTACGGTGCCGCCCCCCTGTTGCAGGCGGCAGCCACCATGGCCGGACTCAAGATCGAGCTGCAT
GCGGGCGGTCTCTGGCTGGGTAGTCGCCGTCAGCCGATGGGCGAGGCATTGCGTGATTATGTGCGCCCCC
ATGATCAGCGGATCGAAGCGCTGACTGGCCAGCATTTTGGCGAACGCTACTTTAACGAACTGCTGCTGCG
CGAAGGGTGTCTGCTGGACTCCGAGCCGCCGATCCGTGCGGTTTTGGCCGTCACGACATTGGGGGGCGAT
GGGCTGACTATGTTGCACCGGATCCAGCAATCCCACTATCGGGACGGTATCTGGATTGGTGAACCTGCGT
TTCTCGCCACGCTGGCGGCAGAGCAGGGGATTGCAGCCGAGGAATTCCAACAAGCTTATCTGCAGGCCCC
CTTGCTGCAACATCTGGCAGATAGCCAGGGCTGGATGAAACGGCTGGGCGGTCAGGGTTATCCTACTTTG
GGTATAGAGCGAGGGGGAAAACTCGAAAGGATAGAGGTCAACCAGTATCTGGGGGAGCCGGAGTTGCTGA
TCCCACGACTGTTGCGGGCAATAGAGTAAACAGTTAATAGTCTGCGGTAGTTAAAGTGGCCCTCTCTGCG
TGTCGATAGCGATATATGGTGCTTATGCAAACTACAAAAGCACAGCTCTACGGCTATGCTCAATTACAAA
AACATGAACACATAGCTCCTATCAAAACCGACATTAAACGATGACACGCAGTTAAGGATGAATCTATGCT
CCAGAATATGTCCATAGGCCAGAAGTTGAGTACTGGCTTTGCGGTGCTGGCACTTTGCGTATGCCTGCTT
GCCGGGTATGGCGTCTCACGACTGTCGTATCTCAATCAACAAAACACTGTATTGGCAGAGAACCTTATCC
CTTCTGTTCGCTATGCAAGTCAGATGCATATTGCACTGCTTGATGCTCGGCGTTTCCAATTATCTATGGT
GATCGCGATGGGCTACAACGACAGTGCGGATATTGAACGAACCCAACAAGGTGTGATGGATTCAAAGGCA
CGTTTTGAGCAGGCTGCGGCTAATTACGGCGGATTGGCTTTTAATGCTGCTGATGAAGAGCAAACCTTTA
ACGCGTTGAAGACTGCCGGAAATGCCTTTTTTACAGCGCATGACCCGCTATATCAGGCGGTGAAACAGAG
TAGTGACAAGACAAGAGAGATTTTGCAAGGGAATCTCAGAACCACGCTGGAGCAGGCAACAAAATTAACG
ATGGATTTGCGTGAGATAAATGAGCGTATTGGCAAGGAGATGGATGCCCAAGGTGAAGAGGCATACACCA
GCTCCAAATTCCTCAGTATCTTGACTGGCGCAGCGACTCTCATTTTCGTGGTGTTGATTGCATGGTATTT
GAGTCAGCAAATCAAACGACCCGTTAATCGCTTGCTGGAACTTGTTGGTAGCGTCGCGGCTGGTGATTTG
ACTCATCGGGTGGAGTTGAATGCGTTTAATCAGGATGAATTAGGACAACTGGCGAAAGGCTTTGCGCAGA
TGCAAGAAAACTTGCGCAAACTGGTTGGGGAGGTATCCGGTTCTGTTGTACAGATGAGCTCTGCGGTAGA
AGAGATCAGTGCCGTAGCAAGGCAGTCAGCCAACAATATGAATGCTCAGCAACATGAGCTGAACCAGCTG
GCGACGGCGATGAACGAGATGCAGGCTACGGTGCAGGAGGTTTCTCGCAACACCAGTGATGCGGCCAGTG
CTGCCACCTCGGCCAGTGAGACGGCCGAACTTGGTGCGCAAACGGTGAATGACTCCATTCAACGTATTGA
ACGGGTCGCCGGTGCCATCGAGAGTACCGCGGTGGTGATCCGCCAACTGGGCGATGACAGTCGCAATATC
GGCATGGTGCTGGAGGTGATCCGCGGCATTGCCGAACAGACCAACCTGCTGGCATTGAACGCCGCCATTG

AAGCGGCCCGTGCCGGTGAGCAGGGACGGGGGTTTGCGGTGGTGGCCGATGAGGTGCGTACTCTGGCCAA
GCGCACTCAGGACTCCACCTCCCAGATCAATACCATCATTGCCGAGTTGCAGCAGCGTGCCGAACAGGCG
GGCAGTACCATGCAACAGAGTCAGGAGCTGATGAACACCACGGTTGCTACCGCGCGGGAAGCGGGCGAGT
CTATCTCCCAGATCAGTGGCTCGGTCAGCAGCATCTCCCACATGAATATCCAGATTGCAACGGCCACCGA
AGAGCAGGGGGCGGTGAGCGAGGAGCTCAACCGCAACGTGGTCAATATCAGCCATGCTTCCGAAGAGGTG
GCCGCCGGTGCCACCCAGATGGCTCAGGCGTGCAACGAGCTGAACCATCTGGCTACCCAACTGCAAGATA
TGGTGCGCCGTTTCAGGGTGTAATCCCTCGGTGAGTCAAACAGCCCGATTACCACAAGGTGATCGGGCTT
TTTCTGTTTTGGAAGGGCAGCCGGAGAGGTTCACCGCGAGCTGATGACTTGTCCAGCCGGACGATATTGT
GCATGCTGATGGCCCATTTTCCAGGCAGGACTCTTGCTGTATGAATCTTCGCGATCTCGAATATCTGGTG
GCGCTGGATGAGGAAAAACATTTTCGCAAAGCCGCTGAACGCTGCTTTGTCAGTCAACCCACCCTGAGCG
GCCAGCTGCGCAAGCTGGAGGATGAACTCGGGGTCATTCTGATCGAACGTACCTCGCGCAAGGTGCTGTT
TACACCGGCTGGCGATGCCATGGCCCATCAGGCGCGTCGGGTGCTCAAGGAGGTGCGCGAGCTCAAGAGC
ATCGGTCAGCACTTCTCCGAGCCGATGTCCGGGGAGATCCATATCGGTTTTATTCCGACCGTGGGCCCCT
ACCTGTTGCCCCGTATTATCCAGACCCTGCGTGAGCGTTTTCCCCAGCTGGAGTTCTACCTCTATGAGGA
GCAGACCCAAACCCTGCTCAAGCGGTTGGAAGAGGGGGAGCTGGATTGCGTCATTCTGGCCGAGATGCCG
GGGATGGAGGGGTTTGGTTCAATCCAGCTCTATCATGAGCCGATGTGGTTGGCGGTGCCTTGCCATCACC
CCGAAGCACAGGCCAGGCAGGTGCCACTCGGCAATCTCAAGGGCAAGAAATTGCTGATGCTGGCCGATGG
CCACTGTTTGCGGGATCAGGCGATGGGATTCTGTTTCGCTGGCGGGATTGGTGAGGATCAACGCTTCAAG
GGAACTAGTCTCGAGACCCTGCGCAACATGGTGGCCGCCGGTAGCGGCATGACCCTGGTGCCAAAGCTGG
CCGTACCTGCCGATCATGATGAAGGCGGGGTCAGTTATCGTCCCGTGGTTGACCCTGTGCCGGGGCGTAC
CATCTCGCTGCTCTATCGCCATTACTCGGTGCGTCGCCCCTGCTTCAATGAGCTGGCGGCCTGGATCGCC
ACGCTGATGAGCAAGCAGCTGGCGTAATAAAAAACCGGCAGCCAAGGCTGCCGGTTTTGCTTCTGCCGCG
ATAACGACTTAGAAGATATCGTCGGTAGAGACAGGCCCTTCTACATCGGGGTTGTCACCGCCGTAGACCT
GATTGCCATCCGAGCTCGGTGCTGCGTAGCGGGTCGGCTCGGTCCCTTCCTTGAAGTATTCGTCGGTTCC
GCCACTGGTTGCCAGCAGGCCGGTTTCGCTATCAATCTTCACCGTCATGATCCCCTCGGGAACCGGCATC
TTGTGCTCCGGCAGCTTTTTGAGGGCGACTTTCATGAAGTCGATCCAGATAGGCTGCGCGGCGCCGCCAC
CAAACTCGGCACGGCCAAGGCCACGCTGGTGGTTGTCAAAACCGATCCAGGAGGTGGCAACGATGTCCGG
GGTATAGCCGGAGAACCAGGCATCCCGCGATTCGTTGGTGGTACCGGTTTTACCGGAGATGTCGTGACGT
TTCAGGTCGCGGGCTGCACGCCAGCCGGTACCACGCCAGCCGTTGCCACCCCAGATGGCTGTGCTCAGGG
TGTCGGTGATCAGGAACGCACTCTGGGCACTGATGGTATGCGGGGCGATGGTTGGCGCGAGAGGGGTCTG
CGTGCCATCTTGTGCGGCTCCATCAACAGTTGGCTGCGGCAGTGTGCTCTGCTCGGGTGCAGCCTGCTGT
TCGCAGGTGGTGCAAGCAATAGCAGGGTTGGCCTGATAGAGGGTGTTCTCAAGACTGTCGGTCACTTTGT
CGATGAAGTATGGGGTCACCAGGAAGCCACCGTTGGCCAGAACCGAGTAACCACGTACCACTTCGAGGGG
GGTGAATTCGGCAGCGCCCAGCGCCAGCGACTCATGGGGAGAGATGAAGTCGCGGGGGAAGCCAAAGCGG
GTTAAACCGTCGATATAGGTATCCAGACCGATGGCGCGCATCAGTCGAACTGACATCACGTTCTTGGACT
TGGCCAGTCCCAGACGCAGCGGAGTGGGACCATCATAAACGGCCGGGGAGTTCTTGGGCTGCCACATGGG
CCCTTTTGACGGATCCCACTGGTTGATCGGCGCATCGTTGATGAGCGACGCCAGGGTATAGCCGTGCTCC
AGCGCGGTCGCATAGAGGAAAGGCTTGATATTTGAACCGATCTGGCGACGCGCCTGATCGACCCGGTTGA
ATTTGGATAGTTCGAAGCTGAAGCCGCCAACCACTGCTTCCAGCGCGCCATTTTTCGGGTTCATGGCAAC
CAGCGCGGCGTTGACGTCCGGGATTTGGGCCAGCAGCAACTCTTCGCCCTCTTCCCGCACCCAGATCTGG
GCGCCGACTTTCAGCACATCGCGCGCCGATTTCGGGGCATAGCTCTGACGGTCGTCGGTAATAAAGGAGC
GGGCCCATTTAATACCGTTCCAGCCGAGCTCTGCTTCCTTGCCATTTTTCAGTACCAGCGTGGCGCTGCG
ATCACCAAGCTTGGTCACGACCGCCGCCATCAGTGGATCATAAGTGGGCTGCTTGCCAAGGTGAGCGACG
ATCTGCTCGTAATCCCAGGCTGTCTCGCCAGCCTTCCACAACTGAGCCGTCGGGCCACGATAGCCGTGAC
GCATGTCATAGGCAAAGACGCCATCCAGCAGCGCCTGTCTTGCCGCCCGCTGACGTTCGGCAGAGACGGT
AGTGTAGACGTGCAGGCCCATGGTATAAGCATCTTCACCGAACTGCTCGACCATCTTCTGGCGCACCATT
TCCCCCAAATAGGGGGCGTGCAGGGTCACTTCGGCGCCGTGAAAACGGGCCTTGATCGGCATCTTGGACG
CTTCGTCAAACTGGGCCTGGGTGATCTTGCCGGTTTCCAGCATCCGGCCCAGCACCACGTTGCGGCGAGC
AAAGGCGCGCTCGGGGGAGCGGATGGGGTTGAGCATGGAGGGGGCTTTCGGCAGACCTGCAATGATGGCG
ATCTCGTCCAGTCCCAGATCCTTGACCTCTTTACCAAAGTAAACCTGTGCCGCAGCACCGATCCCGAAGG
AGCGATAACCGAGGGGGATCTTGTTGAGGTAGAGTTCGAGGATCTCGTCTTTTGAGAGATTCTGCTCGAT
CCGCCAGGCCAGGAAGACCTCCTTGATCTTGCGGATGATGGTCTTCTCATTGCTCAGGAAGAAGTTGCGG
GCAACCTGCTGGGTGATGGTACTGGCACCCTGACGGGCATGGCCGGAGACAGCCCAGACGGATGCAGCTC
GAATGATACCAATCGGGTCAATGCCCGGGTGCTCATAGAAACGTGCATCTTCGGTCGCCAGTACGGCATC
GATCATGGGTTGCGGAATTTCGTCCAGACGCAGCGGAATGCGACGAATTTCGCCATATTGGGCGATCAAC
TCCCCATCCCGGCTGTAAACCCGCATCGGGGTTGCCAGCTTGACGTCACGCAGTGCGGTGACATCGGGCA
GTTGGGGTTTGATATAGAAGTAGATACCGATGAGGCTGGCAACACCCAGCACAGCACCCAGCATCATGAC
AATAAACAGGCGAACGAGCCATTTCAGCATGGAAGAACTACCAATCGTCTTAAAATTAGCCAACGGGCGC
AGAGTATAGCCCTTTTGCCATCGGGAATTCGAGGTTTTCCTGTGGCGAGCCACCCACCTCAAAAAGGATG
CGAGAGGTTAGCACTACAGGGAGCAATTACCAAGGAGGGCCACTTGCCATGAAGAAAGCGAGATGGGTCA
CTTTTCGACATAAACTTTATGACATTGATAGTTAGAGCATTTAGGATGAGTGAAGCACTCCTCTGAGCGG

GTGTCCTACACTCTGTTCAGGAATAAAACCAAATAACGCATGGATGTGTATATGTTTGGGCTTTTTAACA
AGGGATCCCTCCTCCCTCTGGTGGGCATTGATTTCGGCAGCCAGACCATCAAGGCCGTTACCCTCAGTGG
CCGACCCGGCAAGCTTCATCTCGAATCGGTCGCCGATATTGCCACCCCGAAAGGGACGCTGGTTGACTAT
CAATTGCAGGATATCGAGCGCGTCAGTCAATCTCTCAAGACCCTCAAGCGCCTGATCCCAGCTCACAGTG
ACTATGCCGCCACTGCGGTGACAGGTTCCAACGTGATAACCAAGGTGATCCAGATTGATGCCCGCTTCAG
TGACAAGGAGCTGGAGAATCAGGTGCAGCTGGAGGCCGAGCAGATCATTCCCTTCCCCCTCGATGAAGTG
AGTCTCGATTTTGAGGTACTGAGCACCAACAACAGTGAAGATCGTCAGGATGTGCTGCTGAGCGCAGCCC
GCACCGAGAGTGTCAACGGCCGGGTCAGCGCCCTTGCCGAAGCCGGTATTACCGTCAAGGTGGTGGATGT
GGGCGCCCACGCGCTGGGCCGTGCTGTGGTGGCCTGTCTGCCCGAATTGCAGGAGAGTGAGCGACCGATC
GGGGTGATAGATATCGGTGCCAGTGCCATGACCTTTGCGGCGCTGGTCAAGGGAGAGGTGATCTACTCCC
GCTTGCAAAACTTCGGCGGCGATCAATACAGCCAGGCGCTCTCCTCCTTTTATGGCATGACGCTCGATGA
GGCAGAGCAGGCTAAAACCAAAGGTACCTTGCCAGTTGACCATGAGCTGGATGTGCTGATGCCCCACATC
AACTCGCTGCTGCAGCAAGTGCGGCGCAATATCCAGCTGTTTTGCAGTTCATCCGGTTATCGTGACATCG
CAAAACTGGTGCTGACGGGGGGAGGCAGTCTGCTACCCGGTTTGTTGGTACAGGTAAAAGAAGAGGTCAA
TTGCGAGGTGTTGCACCCGGATCTGTTCGCCCTGTTTGGCAAACCGAAAGGGGAGGCCGAGCTGGTACAT
GGTGCTAAATATATGACGGCATTCGGGTTGGCTCTGAGGAGTTTCACACCATGTCAAATATAAACCTTCT
ACCCTGGCGTGAAGCCCGGGCCCAGCGCCAAAAGAAACAGTTCGGGGCCTTGCTGGGGATTTTTGTGCTG
GCCACGGTCGCACTGGGATTTTTTGCCAACTGGCTGGTTGATCGCCAGATCGTCGCCCAGCAGCAGCGCA
ACCAGCGGCTGGTTCAGGAGATGACTATTCTGGATGCCCAACTGGGGGAGATCCGTCTGCTCAAAGAGCG
TCGCAAGGAGCTGATCGATCGGATGCAGCTCATCGAGCAGTTGCAGATGCGCCGCAATGTGCCCGTTCGC
CTCTTTAATCAACTGCCTTCACTGGTGCCCAACGGGGTTTACCTCAACACCCTTTCGATGCAGAACAATA
TTATTGATGTTAATGGCAAGACCGAAGCCTATGGGCGGGTGGCCAGCATGATGCGGCGGATTGATGGTTC
CGGTTGGTTGGGCCAGGCAAAGATAAGCACTATTTTTGCGGCTGATGTTGCGCCAGTGTCATTGAGCCAG
TTTTCCATGATGTTCCTGATCGCCAACAATGCTGCAGCTGCAGGGAATGTGGCCAAGGAGCAACAATGAA
CCTTCAGCAACTCAACGAGTTGGATATCAATGACATAGCAAGCTGGCCAAAATGGGCAAAAGGGGTCTTC
ATCTTTTTCTGCTGCGTGTTGACCGGCGGCGCCATCTACTACTACGTGATTGCCAACTCACTGACCAGTT
TGACTCAGGAAGCGGGGAAAGAGACCGAACTGAAAGCCCAGTTTGAAAGCAAGGCGATGCTGGCTGCCAA
CCTGGGGGCATACAAGGCGCAAATGGTTCAGCTTGAGCAACTGGTTGATACCCAGCTCAAGCAGTTGCCC
AATACCCATGAGGTCGCCGGTCTGCTGGACGATATCAGCTTTATTGCTACCGACAACGGCTTGAAATTGA
ACCGGATCAACTGGGAACCCGAGGTAAAGCACGAGTTTTCAACCGAGCTACCAATGCGTATCGAGGTAGT
GGGCACTTACCATGAAATTGGCAAGTTCACCGCTGACATGGCCGCCTTGCCCCGTATCGTCATCCTTGAC
TCCTTCACTCTGGGTCAGAGCAAGGATGAAGGGGAGTTGATTTCGATGTCGATGCTGGCCAAGACCTACA
AGTACAACGGTAAAACAGTGGAGCCGAAACAATGAGACGCATCTGCTGGTTATTGCCTGCCCTCTTGCTG
GCGGCCTGTGGTGGTCAGGAGGACATGGATAGCTATGTCGCCCAGACCAAAGCAAGGCCACCCATACCTA
TCGAGCCCTTGCCCGAGCTGAAGCCTTTTGCCCCTATGCACTACCAGCAACGCGAGTTGCGCAGCCCCTT
CATGGTTCCCCAGCCTGAGGCGACGGTCGGAAGTACAAGCACTATCAAGTCCCAGCCGGATTGTGCTCAG
ACCCTGATGGATCGTGAGAAAGAGGTGCTGGAAAATTATTCGCTGGCTAGTCTGTCGATGCATGGTTCTT
TGGGTAAGGCGGGTCAGTTGTGGGCCTTGATCAGCACCCCGGATGGCAAATCGATGCGGGTCGGGCTCAA
CCAGTATATGGGGCTTGATCAGGGGCGGGTCATCAAGATCACAGACACTACAGTGGATTTGCTGGAAACC
ATTCCGGACGGGAAGGGGTGCTGGGTAACAAGAGAAACCCAGTTGGCGATGGCCAATGTGCAAGAAAAAC
AATAATCAGGGACGAACAGCAATGAGAACAACAATAGAGAGTGTATCCAAAGTGGCTTTGCTGTTTTGCG
CCAGCGCCCTCAGTCAGGCGTGGGCAGTGGCAACCCTGCAGGAGATCAAGGTCAATCCGCTGCTTGCCGA
TCAATTGCTGGTAGAGCTCAGTTTCAGTGAGCCTGTCACAGGCTTTACCGATCGTCTCTCTTACCAACCC
AATCAGCTGTTGTTGCATGTTCCCGGTGCGGTAGGGGCGCTGAAAGTGAACCCGCTGCCGATCCAGCGCC
TGGGGGTAGATAATATCAAGGTTGAGGGTAAGGGCGCAGGTCTGGATATCAAGATCGCACTCGATCAGCT
GGCCCCCTATCAGGTTCAGCAACAGGGGAACAAATTACTGGTGTCATTGGGCGAGGGGGCCTCTTCCGGG
CTCATCAATGGTCAGAGCGCGCTGATTGCACCCGCAACCTCGGCTACTCCTGTGCCAGCGCCCTCTGCGA
TGGTGGCCACACCCTCTCCTTCGGCACTGCTCAATCCTCAGGTCGTCGCCCGGTCGATGCCTGTTGCGCC
CGTGACTACGCCGGTAGCAACACAGCCTGCCAGCAAGCCGGTGCTCAACAACTATCAAAGCAGTGGGGGT
GGCGCCTATTTCAACTCGGTTACCGGTGTGGATTTTCGTCGTGGCAAGGATGGTCAGGGGGAGTTTCTGG
TCACGCTGGATAACAGCTCGGCCGCCGTCGATGTGAGCAGCCGAGGCCAGACCGTACTGGCCAAGTTTCA
TGGCACTCGTGTACCGGATGGCTTGCTTAATCTGATCAACGTGCAGGATTTTGCAACGCCGGTCTCTCAG
GTTGAAGTCTCCCGGCAGGGCAGTGATACCCTGTTTGAACTGTCGATCAATGGTCAGTTTGATTACCGTT
ACGATCAGGCGGACAAGCTCTTCATTATCGAGGTAAAAAAGCGGACGACGGCCACGGCATCCAAGCAGTA
TCAGGGCAAACCCATCTCATTGAACTTTCAGGATATTCCAGTCCGTACCGTATTGCAGCTTATTGCCGAT
TTCAACAACCTTAACTTGGTAACCACAGATTCGGTATCAGGCAATATTACCCTGCGTCTGGATGGGGTGC
CGTGGGAGCAGGCGCTCGACATTATTTTGAAGGTGCGGGGTCTGGATAAGCGGCTGGATAACAACATCTT
GCTGGTAGCGCCTGCAGAAGAGATCGCAACACGCGAGAAGCAACAGCTGGAGAGCCGAAATCAGGTTGCC
GATTTGGCTCCGCTCTATACCGAGTATCTGCAGATAAACTATGCCAAGGCCTCGGAAGTGGCTGCCCTGC
TCTCTTCCGAAAGTACCAAGTTGCTGTCACCCCGTGGTGCGGTGAGCGTGGACGAGCGAACCAACGTGCT
GGTGGTCAAGGATACCGCCGATGTCATCAGCAGCATCAAACGGATGCTCGATATCCTCGATATTCCGGTC

AAGCAGGTGGTGATCGAGGCGCGGATGGTGACCATTGACGACGGCTTTGATGAATCGCTCGGGGTGCGGT
GGGGAGTGACCAAAAACGATGGTCATGGCAACAGTACGTCCGGTTCCATCGAGGGGAATGATGGCTCTGG
TAACAGCAATGGTGGCTCGGCGATCAACCCTCGGCCATCAGCAGACGATCGGCTCAACGTCAACTTGCCG
GTGACCAATGCGGCCGGTACTTTGGCCTTCCAGGTTGCCCGTCTGGCTGATGGCACCTTGCTGGATCTCG
AGTTGTCGGCGTTGGAAAAAGAGAGCAAGGCCGAGATTATCGCCAGTCCGCGGGTGACCACTGCGAACCA
GAAACCGGCATTGATCGAGCAGGGTACCGAGATCCCCTATGTGGAGTCCTCTTCCAGTGGCGCTACCTCG
GTTACCTTCAAGAAGGCGGTATTGAGCCTGAAGGTGACCCCGCAGATCACCCCTGACAATCGGGTGATTC
TCGACCTGACCGTGACCCAGGATACCAAGGGTGAAACGGTACCGACCGGTACCGGTGATGCGGTTTCCAT
CAACGCCCAGTCCATCACCACTCAGGTGCTGGTGAACAATGGCGAGACCCTGGTGTTGGGCGGCATTTAT
CAGCAGACCATCAAGAGCGATGTAACCAAGGTGCCGCTGCTGGGTGACATTCCCGGATTGGGGGCCTTGT
TCCGCAAGACCACCAGCGAGAACAAAAAGCGGGAATTGCTCATTTTCGTGACGCCCAAGATAGTCACCGA
CGCCTTCTGAATGCTTTACATGTGAGGGTAAAACACATATAACCTTACGCCCCAATGAGGCTGGGGCGTA
AGGGCTGCGGTTGCCAGTCCCGATATCTATTGAGATAATCCCCCTCCTGATCCATCGCAAGATAAGGTTC
ATAGGTACCCCGATCCAGTATTCATCGGGGTCGTACAGTTTGTAATTACTAACGAAAAGACATGGCTGAG
AAACGCAATATTTTCCTGATAGGTCCCATGGGTGCGGGCAAGAGCACCATAGGTAGACATCTGGCAGAAC
AACTGCATATGGAGTTTTTCGACTCAGATCATGAGATTGAGCGCCGCAGTGGTGCTGATATCGGCTGGGT
GTTCGATGTTGAAGGCGAAGAAGGTTTCCGGATTCGCGAAGAGAAGGTGATCGGCGAATTGTCAGAGCAG
CAGGGTATCGTGCTGGCGACGGGTGGCGGTGCCATCAAGAGTCGCGAAACCCGCAACAAGTTGTCTGCTC
GCGGCATCGTGGTCTACCTCGAAACCACTATCGAAAAGCAGCTGGCGCGCACACAGCGAGACAAACGCCG
CCCTCTGCTACAGACCGAAGAACCGGCACGCGAAGTGCTGGAACGTCTGGCACAAGAGCGCAATGCCCTG
TATGAAGAGGTTGCCGACTTTGTTGTGCATACCGATGACCAGAGCGCAAAAATCGTTGCCAATCAAATTG
TAAAGCTGATCGGTATGTAATGTAGAGGACGTCCATGGAACGGTTGAAGGTCGAACTGGGTGAACGCAGC
TATCCCATCGAGATAGCTGCCGGGTTGTTGCAGCGCCCTGAGGCGCTGGTATCGACCATCAAGGGCAAGC
GGGTGATGATAGTGACCAACACGGTCGTTGCTCCGCTTTATCTTGAACGGGTAACCGCTCTGCTGTCGGG
TTTTCAGGTTGAACACCTGATCCTGCCTGACGGTGAGGCATACAAGAACCTGGCGACCTTTGAGCAGATC
ATGTCTGCTCTGCTTGAAACCAACCATGGGCGCGATACGACACTGATCGCTCTGGGGGGCGGCGTGATCG
GGGATCTGGTGGGCTTTGCGGCAGCAAGCTATCAACGTGGCATTCCTTTTATCCAGATCCCGACCACTCT
GCTCTCTCAGGTTGACTCCTCTGTCGGCGGTAAAACCGCGGTCAACCACCCCCTCGGCAAGAACATGATT
GGGGCTTTTTATCAGCCCAAACAAGTTGTTATCGATACCGAATGTCTGAGTACCTTGCCAGCCCGCGAAT
TTGCCGCAGGGATGGCCGAGGTGATCAAGTACGGCATCATTTGGGATGCTGATTTCTTCAGCTGGCTGGA
GCAGAACATGGTGCGTCTGCAACAGCTGGAACCTGCCGCGCTGGCCTACGCCATCAAACGCTGCTGCGAG
ATCAAAGCCGATGTGGTCGGTCAGGATGAGACCGAGCACGGCGTCCGTGCGCTGCTCAATCTGGGCCATA
CCTTTGGTCATGCCATCGAGGCCGAGCAGGGGTATGGCAACTGGCTGCACGGTGAAGCGGTATCCGCTGG
CACCATGTTGGCAGCCTGGACTGCGCAGGCCCGTGGCGATATGAGCGAAGCGGACGTTGCGCGAGTGCGC
GCTCTGCTGCTGGCCGCCAATTTGCCCATTCATGCGCCTGCCGAGATGGATTTCGCCGCCTTTATTCGCC
ATATGCGCCGTGACAAGAAGGTGCTGGAAGGCAAGCTGCGTCTGGTGTTGCCGGTTGGTATCGGCCATGC
GCAGGTGGTGGGGGATGTGTCCGATGCCGAGTTGCTGGCGGTGATCGAGTCTGGTCGTGACGAATAAACT
GCTAAAACTTCCTTCCCAGCGGCAGCTGGTTGACCGGCTGCTGCATCTGATTGAATTCAATCACCCTTTC
ATTTTTCTCTCCGGTAAACCCGGTTGTGGTCGCGGTACCCTGTGCGAATCCCTGCTCGGCGTGTTGCCGG
AAAAAGTGCGGGTGGTAAGCCTGATTGGTACACCTGCCATGAAGATGGTGGATGTGCGCCAGCTGTTGCT
GCCACAGGTGGTAGCGCGCCCGCTCTTCAATCCACAGGATGCGCTGGCGGAGAGCTTCTTTCGCATGCTG
GAGGGGAGCAAGCCAGCCACCCTGTTGCTACTGATAGAGCGGGCCGACCAGTTGCCAGCCGAACTGCTGG
GAGAGTTGTGGGCGATCATCCGTCATAACGATACCCTGGCGGCGCCCCATCAGCTGGCCATTATTGTCTC
GGGCAGTGACGTCTGGTGTCGTACCCAACGGCCGCAGCTCAAAGGGCGTGCCATGCCAGCCCTTGAGCTG
GAAGTGCCGCCCCTGAGCCCTCCCGAGCAGCGCATCTTCTTGTATGAAAAAGCCAAAGAGCTCAAGATCC
CGGCGACCCTCTTGCCCAAGCCGCAAGTGGAGGAGATCCTGAAAACGGCGAACGGACATCCGTCCACCAT
CATGCAGTTACTGGAGGATATCATGACCGACAGAAGGCCCAAGAAGCGTCAGGCGGAGTTGCCTGTCAAG
AAGATTGCCACCGCTATCGCGCTGGTCGCTGGCGGCCTGCTGGCGCTGAGCTACCTGCTGCCTGCGCTGT
TTGACAATGAAAGCAAACCGGCACTGCAGGATCCGGCGCAAAACACGGCATTGCCGATCCCGGTAGCGGG
AGGCGATCCCCTCACCGCTGGCGGTTCGAATACAGATTCCGCTGCACAAGGCGCGCAAAACAGCGCTGCA
GCCAGTAATCCCAATGGGGTTGTGAAAGATTGGCAGCCGGAGGCGAAAGTGCTGCCCAAATCGGTGGAGA
GCGAAACTGTGACCACCGAATCAACCAATTACGAAGGGCGTCGGGTGGTGATCTCTGACGAGGTCGTGCA
AAAGCTGATGACCGAACCCAAGGTGAGCGGCGCACTGCCTACTGACGTGGTGGACGAGCTGACCGGCAAG
AACCCGAGTGGCACAGCCCCCAGTGGTGCAGTCGCAACCACAGCACCAGCCAGCACTAGCAAGGTACAGA
ACCCGGTGACAACCGCAACGCCTACGGCCACCACCAAAACCGCCAGCAACGAGCCGGTTGCCGCGCCAGC
ACCCAAGGTGGCCCTGACACCGGTCGCCACCTTGAACAAGAAGCCGCGTAACCACTACAGCGTTCAGCTG
ATGGGGGCGAGCAACACCAAGGCGGTGGATCAGTTTGTCGCCGAGCATGGTCTGGCGGGCAAGGTATGGG
TCTATCAGACCAAATTCCGTGGTAGCCCCTGGTATGTGGTGCTGCAAGGGGATTATGCCGGTCTGACCCA
GGCCAAGAGTGCCATTCGCAAGCTTCCGCCAGCTTTGCTGAAAGGTCAGCCCTGGCCCAAGTCGTTTGCA
CAGGTACAAAAAGAGCTGAAGCAATAGCCTTGATACCGGATAAGGGGGTAGAATCGTCCCCCTTTTTGGG
TCTTGCCTTCTTACACACCGGATTTCCATGAAAAAAACACGCGCTTTTTTAAAATGGGCCGGGGGAAAAT

ACTCCCTGGTTGAAGAGATTGCCGAGCGCCTGCCGGCCGGGCGTGTGTTGCTGGAGCCCTTCGTCGGGGC
CGGGTCTGTGTTTCTCAACACCGACTACGACGCGTATGTGCTCAACGACATCAATCCGGATCTGATCTGC
CTGTACAACCACCTCAAGCTGACGCCGGACAGTTTCATTGCCGAGGCGCGCAAGCTGTTTGTGGCAGAGC
ACAACCACAAGGCGGCCTATTACCGGCTGCGTACCCAGTTCAATCAGACCGAGACCACCTTCGAGCGGGC
ACTCTTGTTCCTGTTTCTCAACCGTCACGGTTTCAACGGACTGTGCCGCTACAACAAGAAGGGCGGCTTC
AATGTGCCGTTCGGTTCCTACAAGAAACCCTATTTCCCGGAAAAAGAGCTGTGGGCCTTTGCCGAAAAGG
CGCAGAAGGCGACCTTTATCTGCGAGAGCTACGCCGATGCCATCAAGCGTGCCGAAGAGGATTGGGTTAT
CTACTGTGATCCACCCTATGCGCCGCTCTCCACTACGGCGAGCTTCACCACCTATGCCTCCGGTGGGTTT
ACCCTGGATGATCAGGCGATCCTGGCGCGATTGGCCCGCCACACAGCGGCTCACAAAGGGGTGCCGGTGC
TGATCAGCAACCACGATATCGAGCTGACTCGTGAGCTTTATCGCGGTGCCCGCCTCGACGAAATCCTGGT
CAAGCGCACCATCAGTCGCAACGGCGGCACCCGCAACAAGGTGGCCGAGTTGCTGGCGCTCTATCCCCCC
GGAATCGAACCTGAGCAGGGCTACTATCCCAGCGACAGTGAACTACTGCCACTGGCGTAACAGACCATGC
GGCGCCCAAGGGCGCCGTTTTTTATGGGCGAGTCCGCGCCCGTGCCGATGCCCCAAAACCCGGGTGGCTG
GGGGAGGGGAGGCGATTGCCCCATTTACCCCGCATTCGAGTCGTTTTCGCCCGCCTTTTACGTTAAAATT
GCGCCGTTTTTTCTCCCTGGCTGCCAATGGATGATGCTGATGAAAGACTTTCTGATTGCCCCCTCGATCC
TCTCTGCCGATTTTGCCCGTCTGGGTGACGATGTAGCCAAGGTACTGGCTGCCGGTGCCGACGTGGTTCA
CTTCGACGTGATGGACAACCACTATGTCCCCAACCTGACCATTGGCCCCATGGTTTGTCAGGCGCTGCGT
GACTACGGTATCGAGGCCCCCATCGACGTGCACCTGATGGTCAAGCCGGTGGATCGCATCATTCCCGATT
TTGCCAAGGCGGGTGCTTCCCTTATCACCTTCCATCCGGAGGCGTCTGACCACGTCGACCGTTCTCTGGG
CCTGATCAAGGAGATGGGCTGCCAGGCGGGTCTGGTGCTCAATCCGGCCACCTCGCTCTCCTGCCTCGAG
TATGTGATGGACAAGCTGGACGTGATCCTGCTGATGTCGGTCAACCCGGGCTTCGGTGGTCAGAGCTTCA
TCCCCGGTACCCTCGACAAGCTGCGTCAGGTACGTCGCCTGATCGACGAGAGTGGCCGCGATATCCGTCT
CGAGATCGATGGTGGGGTGAAGGTCGACAACATTCGCGAGATCGCCGAAGCGGGTGCCGACATGTTTGTC
GCCGGCTCTGCCATTTTCAGCCAGCCTGACTACAAGGCGGTGATCGACAAGATGCGTGCGGAGCTGGCTC
ATGTCACACGCTGAGCGCGCATTTGATTTGGTGCTGTTCGATCTGGACGGCACCCTCATTGACAGTGCCG
CACAGCTGGCGCTGGCGGTCAATCTTACTCTGGCTGATCTGGGTCTGGAACAAGCTGATGAAGTGGTTAT
CCGTACCTGGGTCGGCAATGGTGCCGACAAGCTGATCCAGCGGGCGCTCGCCTATCGCGAGGCCGATCCC
GAGCTGTTTGCCAAGGCCAGACCGATCTTTTTCCAGCACTACAACGCTTGCCTGCTGCAAGGGCTGGCGA
TGTATGAAGGGGTGGCGCAGAGTCTGCGCCGTCTGCAAAGCCTGGGCTACAAGCAGGCGATCGTCACCAA
CAAGCCGAGCGACTTTGTCGCCCCTATTCTCGATGCCCTTGGCATCAGCGATTGTTTCGAACTCTGGCTT
GGCGGCAACTGCGTGCCGGTCAAGAAGCCGAGCCCGGAGCCCTTGCTGCACGCCTGCCAGGAGTTGGGGG
TGAGCCCGGCTCGCACCCTGATGGTGGGCGACTCCGAAAATGACGTGCTGGCGGCGAAGGCGGCTGGTAT
GAAGGTGGTGGGTCTCACCTACGGCTACAACTATGGCCGCCCCATCGCCGACTCCCATCCAGACTGGGTA
TTCGAGCAGTTTGCCCAGCTCGATGCACTTTTGGTCTAATAGCGCAGCTGGGCTGGCTGAATAACAAGGA
TTAATACTCTCATGACTAAACCAATCGTATTGAGCGGAGCGCAACCCTCCGGTCAACTGACCATCGGCAA
CTACATGGGGGCCCTGCGTCAGTGGGTCAACATGCAGGATGAGTTCGACTGCCTCTACTGCATCGTCGAT
CTGCACGCCATCACCACTCGCCAGGACCCGGCTGCCCTGCGCAAGGCTTGTCTGGATGCGGCCGCCCTCT
ATCTGGCGGTCGGTATCGATCCCAAGAAGAGCACCGTCTTCATCCAGTCTCACGTGCCGCAGCACGCCGA
GCTGGGCTGGATCCTCAACTGCTACACCCAGATGGGTGAGCTGTCGCGCATGACCCAGTTCAAGGACAAG
TCCGCCCGTTTCGAGAACAACGTCAACGTCGGCCTGTTCGGTTATCCGGTGCTGATGGCGGCCGACATCC
TGCTCTATCAGGCCAATCAGGTGCCGGTCGGCAACGACCAGAAGCAGCATCTGGAGCTGACCCGCGACAT
CTGCACCCGTTTCAACAACCTCTATGGTGACGTGTTCACCATGCCGGAGCCCTTTATTCCGACCGAAGGG
GCACGGGTGATGAGCCTGCAGGATCCGACCAAGAAGATGTCCAAGTCGGATGACAACGAAGCCAACTTCA
TCGGCCTGCTGGAAGATCCCAAGCAGATCGTCAAGAAGATCAAGCGTGCGGTGACCGATTCCGACGAGCC
GGCGGTGGTGCGTTTCGACCCGGAAAACAAACCGGGAGTTTCCAACTTGCTGACCCTGATGTCCGGGGTG
ACGGGTCGCTCCATCCCCGAGCTGGAGCAGCACTTCGAAGGCAAGATGTACGGTCACCTCAAGACCGAAA
CCGCCGAAGCCGTGGTTGCGCTGCTGGAACCGATCCAGGCCCGTTTCCACGAGTTGCGTCAGGATGAGGC
CCATCTGATCGCGATTCTGGCAGAAGGCGCACAAAAGGCGCGCGAAAGAGCTGAAAAAACCCTGACCAGC
GTATATGATGTAGTCGGCTTCGTTCCTCGAGCCTGACATACTGGGAAGGTGTAGTAGAAGAGAGCTGGGT
ATTTGCCTGGCTTTCTTTATTTTCTGCCCGGCTATTTTGTTTAATTTCTTTATTTCAACCTGGGTAATAT
CCCCAAGAGAAAGCATGCCTGTTCAACCAACTATTTCTGCTGAAGAGCTGAGTGACAAAGGTCACTGGCT
ACGTAAATTTCGCAAGGCGAAAAACCTCAATACCCTGCAGTTGATGGTCTCCAATGCCATCGACAAGCAC
CATAAAACCCCTGCCGTTGCTGCCGCCATCTATCTGGCCGAGTGTCAGCGTGAACGGGAAATGGAACAGG
GTCGTTATCTCGATCGTTAATTCTCCGCTGTTGGTTATCTCATGCTGTTGTTGATCGATAACTACGACTC
TTTTACCTGGAACTTGGTGCAATATTTCGGCGCCCTGGGACAAGAGGTTGTGGTCAAGCGCAATGATGAA
CTGACGCTTGATGCCATAGCGCAGCTCTCTCCCCGTTTTCTGGTGATCTCTCCCGGTCCCTGTACGCCCA
ATGAGGCGGGGATCTCTCTCGATGCCATCAGCCACTTTGCTGGCAAGCTGCCCATTCTCGGCGTCTGTCT
CGGTCATCAGTCGTTGGCGCAGGCCTTCGGCGCCCGCGTAATACGTGCCCGTCAGGTAATGCACGGCAAA
ACCTCGCTTATCCGCCATCGCAATGAGGGGGTGTTCAAGGGGCTCAACAACCCGCTGCGGGTGACCCGCT
ACCACTCCTTGGTGGTGGAGCGCGACACGCTGCCCGACTGCTTCGAGGTCACCGCCTGGAGCGAGCATGC
CGATGGCTCGTTTGACGAGATCATGGGGCTGCGCCACAAGACGCTCCCTGTCGAGGGTGTTCAGTTCCAT

CCGGAGAGCATTTTGAGCGAGCAGGGCCATCAGTTGCTGGCCAACTTTCTGGCGCGTTCATAAAAAGTTG
TGATCGCCTTCTCAAATTCGAGAGAAGGCAAAAAAAATTTCCCTGACACTTTATTCACCCCCTCTCCTGC
CCAAATTATCTCTTGCCAAGCCCCCCTGAGTCGTTATGCAATGCGGTTGCCTAAAATCTCAAGAGCAATC
GAACATTGTTCCTTATAGGCCGTCTTGTTACAGACTTTTTACTTTATTTCTGTGATAATCCCGTCACGTT
TACACGGATTTAACTTTTGGGCGGTGGATTATTCTGTCGCCGAATCGAAGGAGAGCTATATGTCAGAGTT
GTTGGCAGTGACACGTGAAGTGTTTGATGAAGTGATGGTTCCCAACTATGCACCCGCCAAGATCATCCCG
GTGCGTGGTGAAGGGTCACGTGTCTGGGATCAGGAAGGGCGCGAGTATGTCGACTTCGCCGGTGGCATCG
CCGTCAACGGTTTGGGTCACTGTCATCCCAAGCTGGTCGAGGCCCTGAAAACCCAGGGCGAGAAGCTGTG
GCACCTCTCCAACGTCATGACCAACGAGCCGGCCCTGCGTCTGGCCAAGAAGCTGGTCGCCGCCACCTTC
GCCGACAAGGTCTACTTCTGCAACTCCGGCGCCGAAGCAAACGAGGCTGCCCTCAAGCTGGCACGCCGCT
ACGCCATCGAGAAGTTTGGCGAGCAGAAGACCCAGATCATCGCCTTCAATCAGGGCTTCCACGGTCGTAC
CTTCTTTACCGTCAGCGTCGGTGGCCAGGCTGCCTACTCAGATGGCTTCGGTCCCAAGCCCGGTGACATT
ACCCATGTTCCCTACAACGATCTGGCGGCGCTGGCCGAGGTGATCTCCGACAACACCTGCGCCGTGGTGA
TGGAGCCGTTGCAAGGGGAGGGGGGCATCATTCGTCCTACCGACGAGTTTGCCAAAGGGGTGCGCGAGCT
GTGCGACAAACACAATGCCTTGCTGGTGTATGACGAAGTGCAGTCAGGGGTCGGCCGGACCGGTGATCTC
TATGCCTATATGGGTCTGGGCGTCACGCCGGACATCCTCACCAGCGCCAAGGCGCTGGGGGGCGGTTTCC
CCATCGGCGCCATGCTGACAACCAACGAAATCGCCTCTTACCTGAAAGTGGGCACTCACGGCTCTACCTA
TGGCGGCAACCCGCTGGCCTGTGCGGTCGCCGAGGCGGTGATCGATGTGATCAATACTCCGGAGGTGCTG
GCTGGCATCAAACAGCGCGAACAGCTGTTCCGCGATGGCCTTGAAGCCATCAATGCCAAATACCACTGCT
TTAGCGAGATCCGCGGCAAGGGGCTGTTGCTGGGCGCTGCTCTGAGCGATGCCTACAAGGGACGCTCCCG
CGACTTCCTGCTGGCCTCCATCGATCAGGGGCTGATGGCACTGGTGGCTGGTACCAACGTGGTTCGTTTT
GCCCCTTCACTGGTGATCCCGGAAGCGGACATCAAGGAAGGGTTGGCCCGCTTCGAAAAGGCGGTTGCCC
AGGTGGTTAACGCCTGATTCCAAATTGAGGTGGGGAGCTCTCCCGGCAGGAGCCGGGACCCCACCTGTCG
TCAATGAAAGGGAGTTGTCATTTATGTTGGTTATCCGTCCCATCGAGCAAAAAGATTTTGCCGGGCTCAA
GACCTGCGCCATCGAGTCCGGCACCGGCATGACCTCGTTGCCCGTCAACGATGAGCTGCTGCAGCGCAAG
ATCGATGCCTCTACCCAGACTTTTTCCGACAAGCCCGCCGGCAAGGGGGACTGCCTTTACTTCTTCGTGG
CCGAGGATCTGGAGACCGGCGAGATGGTGGGCACCTGTGCCATCGATGCTGCGGTTGGCCTCTCCCAGCC
GTTTTACAACTACCATATCGGCAAGGTGGTGCACTCTTCCCCCAAGCTCGGCATCTACAACGTCGTTGAA
ACCCTGACCCTCTCCAACGACTACACCGGCAACTCCGAGCTCTGCACCCTGTTCTTGCGCGAATGTGCCC
GCGAGGGGCTCAACGGCCGCTTGCTCTCCAAGCACCGCTTTCTGTTTATGGCCGAGCACCCCGAGCTGTT
TGACGAGACCGTCTTCGCCGAGATGCGCGGCGTCTCCGATGCCAACGGTCACAGCCCCTTCTACGAGTGG
CTGCAAACCCATTTCTTCTCGATGGATTTTCCCACCGTCGACTATCTGACCGGCATCGGCAAGAAGCGCT
TTATCGCCGAACTGATGCCCAAGTACCCCATCTACGTCAATTTGCTGAGCAAAGAGGCGCGGGCCGTTAT
CGGCCAGACCCACGAGAAGACCCGCCCGGCCATCAAGATGCTGCAGGACGAGGGCTTCGTGAACCGTGGC
TACGTCGATATTTTCGATGCGGGCCCCACCGTCGAGTGCGACGTGAAGAATATCCGCAGCGTGCGCCGCA
GCAAGAAACTGCGGGTGCTGGTGGGCGAGCCCATCGGTGGCAAGACCCACCTTATCTGCAACAGCCATTT
CGAGCATTACCGCGCCTGCTACGGCAATATCCGGGTCGATCTCGACAAGCATGAAGCCATCATTCCGCCC
AAGATGGCGGCGGCACTCAAGGTGCAGGATGGCGACATGGTGCGGGTGGTTGACCTCGACTGAGGCAAAC
AGGACAAGCAAACCGATTATCAGACAGGCAGCCGAGGCTGCCTGCCAGCGACAGGGAGTGAATGGATGAG
CCAGTTAGTGCAATTTATCGACGGTCAGTGGCTTGCCGGTGAAGGCAAGCCGTTCGAGTCAAAGGATCCG
GCCAAGAACGAGGTGATCTGGCAGGGGGCTGCCGCCAGCACGGCGCAGGTGGATACAGCCGTCAAGGCCG
CCCGTGCCGCTTTCTACCACTGGGCGGACATGAGCCTTGAGGATCGTCTGGCCATAGTGCGTCGTTATGC
CGACCTGCTCGGTGAGCAGAAAGAGGCGCTGGCCCTGACTATCGCCCGCGAAACAGGCAAACCGCTGTGG
GAGACCCGTACCGAAGTGGCCGCCATGCAGGGCAAGATTGCCATCTCCATCCGTGCGCACGACGAGCGTA
CCGGCACCGTTGAAAATGCCATGCCGGGCGCCAAGGCGTTTGTCCGTCACAAGCCCCACGGTGTGGTGGC
GGTGTTCGGCCCCTACAACTTCCCGGGCCATCTGCCCAACGGCCATATAGTCCCGGCGCTGATTGCGGGT
AACGCCGTGGTGTTCAAACCCTCCGAGCTGACCCCCATGGTGGCCGAAGCCATGGTCAAGGTGTGGCAGG
AGGCCGGATTGCCCAAGGGGGTGCTCAATCTGGTGCAAGGAGAGGTCGAGACTGGCAAGGCGCTGGCGGG
CAATCCCGACATCGACGGCCTCTTCTTTACTGGTTCTTCCCGCACCGGTCATTTCCTCCATCAGCAGTTT
GCCGGTCAGCCGGGCAAAATTCTGGCCCTCGAGATGGGCGGCAACAACCCGCTGATCGTGAAAGATGTGA
GCGACATCGACGGCGCCGTCCATGCCATCGTCCAGTCGGCATTTATCACCTCGGGTCAACGTTGCACCTG
TTCCCGCCGTCTGTTTGTCGAGCGCGGTGAGCGGGGCGATGCCCTGGTCAATCGACTGATCGAGGTGGTC
AGCCAGATCAAGGTCGGCCTCTATGACGCCGCCGAGCAGCCGTTCATGGGCTCGATGATAAGCGAGAAGG
CAGCACAGGGCATGGTCGAGGCGCAAGCCAACCTGCAGCGCCTCGGTGGCGAGAGCCTGCTTACCCTCAA
GCACCTGGAAGCGGGCACAGGCTTTGTCTCCCCGGGTATTATCGACGTGACCTCAGTCAGCGAGCTGCCT
GACGAAGAGTACTTCGGCCCGCTGCTGCAACTCATTCGCTATGACGATTTCGATGCTGCCATCGATCAGG
GCAACGCCACCCGCTACGGTCTCTCGGCCGGTCTGCTGGGGGACAAGGAGGCGGACTGGCACCATTTCTT
CAAGCGGATCCGCGCCGGTATCGTCAACTGGAACAAGCCCATCACCGGTGCCTCCAGTGCCGCGCCGTTT
GGTGGCATCGGCGCTTCCGGCAACCACAGGGCAAGCGCCTACTACGCCGCAGACTATTGTGCGTATCCGG
TTGCCTCTGTCGAAGACAGCAAAGCCGCTATGCCGGATCAGCTGTCTCCTGGTTTGACGTTTTAAGAGCA
AACGGGGATGCCAATGCAGCAGAGATGGCTCAACGCCGCCGACTACTGTGTCATATCCCCGGTTGCCTCC

GTTGAAGACAGCAAAGCCGCCATGCCGGATCAACTGTCGCCCGGCCTGCGCTTCTGATAACGACATGGGG
CATCCAGCGGTGCCCCCATCTGTACCCGCAATCAAAGGAAAGAGAGATGCAAAAGGATATCGACACCCTG
TTCAGCCATCTGTGGGACAACTACATCAAGGTGACCCCGAGCGCCCTCAAGGTGCACACCCTGCTGGGTG
GCGGCGAGCCCATCGTCAACGATCACGTGGCGTTTCGTACCTACAACCTGCCCAAGCTGGGGCTGGAGAA
GCTGGCCGCCCACTTCCTGGCGCTCGGTTACGAGCAGAAGGGCGAGTACGTTTTCAAGGCCAAGAAGCTC
TATGCCAAGCACTTCGAGCATCAGGATCCGGACGCTCCCAAGGTGTTCATCAGCGAGCTGAAGGTGGAAG
AACTCTCCCCAGCGGCGCAGGCCATCATCCACAAGCTGGCGGATCAGGTGCCCGATCATCTGACCGATAC
CCCGGCATTTCTCTACACCGGTGCCCCCTGGCAGGTCTCCTATGCCGATTATCAGACTCTGCTGGCGGAG
AGCGAGTACGCGGCCTGGATGGCAGCCTGGGGCTATCGTGCCAACCACTTCACGGTCAATATCAACAAGT
TGAAGGACTTCGATACCATCCATCAGGTCAATGCGGCGCTCAAGGCGGCGGGGTTCGTCCTCAACAGTGT
GGGTGGCGAGGTGAAAGGGGACCCGCAGGTGATGCTGGAGCAATCCTCCACCATGGCCGACAAAGCAGAG
GTGCCGTTCAGCGATGGGGTGCGTACCATCCCGAGCTGCTTCTACGAGTTTGCCCTGCGTTATCCGCAGG
CCGATGGTGTGCTCTATCCGGGCTTCGTCGAGGCCTCGGCGGACAAGATCTTCGAATCGACGAATGCCAT
GTAACGGGAGCCACCCTGTTTCAGTCGCAACGCCTCCTTTGTGGAGGCGTTTTGTTTGGCGCAGAGTGAC
GGTATCGGGTGTTGTCTGAGGCTGCGTGTGGAGGGGATTTGGACGGCGCCTAATCGGTAAATGTTTGTTG
ATAAAAATGTGAACGAGATCACTTGAGACGACACAGATTGACGCTGGCGGTGGAGCAAAAAGGAGTAGAA
TGGCGCTTTATTTTTGCGGGGCCAATCGATGCCTAACTATCACTTTTTCAAGCAGGGTCAGGCGCTTACC
TATCTAGATGCCAATGCGCCCAGCTATAGCGATGAGCGTCGTCAACTGGTCGAACAGGGATTTGCCGCCA
TCGCCGCCCCCACCTTTGCAGATACCCCGGCCGAAGCTCTTGAGCTGCTTCGCACTCATCAGGCACTACA
GGATGAGGCGCAGGCCAACGCCCTTTGTCTGACGGTTGCCGCTATTCCGGCTGCGATCGCAACTCTGTCC
GGGCACTGACGCTGCCGCTATGCTCACGTGAATAAAAAAATGGTGCCACTGTCATGACACCATTTTATCG
GTCTGTCTGCTTGCCCTGAGTACTTTACCGCTGGTGCAGGTCACCTTCGATAACCAGCACGGTATCCATC
CCCGCCGCCTTGCCAGCCTCAAGCCCGATCCGGGTATCTTCAAACACCAGACAGTTGGCCGGATTAGCGC
CGAGCAGCTCGGCCACCCGTAAAAAGGTGTCGGGGTGCGGTTTGTGGTTGGTGACATCATCGGCGCTCAC
CACCACAGAGATGTAGGCATCGAGCCCGGTGCTTTTCAGGATGGAGTCCGCCTGATTGCGCGGTGAGCCG
GTGCCAATCCCCATCTTCACCTTGCCGTGATAGTGCCTGACCAGCTCCCACATCTTGGGAAAGACGCTGA
CCTTGTCGATATGTTCCATATAGAGGGCAATCTTGCGACGGGTAAAAGCCTCGACATCCACCGTCAGCCC
CTGCTGCTCGGCCAGCATGGCGATGATCTTGCGGGTGGGAATGCCGCCATACTGATAGAGCTGATCCCGC
TCGAAGGGGATGCCGAATTCGGCGCAGGTAAGCTCCCAAGCATCCAGATGAAGGGGCATGGAATCTACCA
AAGTGCCATCCATATCGAAAATCAGGGCATCGTAGTGATCAAACCCGTACTGCTCACGCTCGCTCATGGG
GGCTCCTCGAATCAAATAGAGCAGGCTAGCAGAAACGGTCGACGTTTTGTGGGATCGGCCTCATGGTTGG
GCAGATTGTGGCCGCCGTTTTTTCTATCTCTTGCTGATTTGTGAGACAATAATCCGTTCATTGCCGGATA
CTTTGGCATATCTGATCAGCGAGTCCGCTTATGACCTCGAAAATATCCCCCAAAACCATCTTCTCCAGTG
TGGCGCTGGTGATCGCGCTCGGTTCACTGGAAAAGAGCATAGTCACCACCCCGCTGCCCATGATTGGCGC
CGAGCTGCATGCCGGTGCCGCCCTCACCTGGGTGGTCACCGCCTACCTGCTGGCGGCTACCGCCGTGCTG
CCCCTCTACGGCAAGCTGAGCGATCTCTTTGGCCGGGTGCGGATGCTCAATATCGGCATCGGCCTGTTTC
TGGCGGGCTCGGTGGCCTGCGCGCTCTCCCAGGATCTGCCGACCTTGCTGGCAGCGCGGGTGCTGCAAGG
GCTGGGGGGCGCCGTGGACGGCAGCGATGGCGCCCCCTTCCGACTGCATCTCGACAACCCGGGGGGCATC
CCCCCACAGGTTCTTGCCCCGTTCGCTCGACCAGGCCGACGACTGCTCAGCCATGGCCGAGCTGGGGGTG
ATGGGGTAGATGGCGATGACTTCGCTGGTGCGGTACGCCACCGAGGCGACTGCGTTATTCCCGTCTGTGG
TGATCATGTGTTTTCCACTTTGCTATGCCAGTTTGAAGGCCGATAGTTGCATCGGCAATGGATAGGTCGC
TGGCATAGTAAGAGGGGCAATGAGAGCGTATCAAACGGGATAAATAATCAATTAATAAGTCAGTAATAGT
GGTTAATTTAATCCCGCCGCGTGGGGGCATGATTTTCTGCTGACAGCCGATGGATGGCGATTGACGGGCC
ATTGGCATGGCCCGTCAGCACGGGGTTAACCCTGATCCACTTGCAGCCAGATGGCGCACTGATAAGGGGC
GAAGAGCTGCAGCGGTTCGCCTGGGTTATTGGCCAGCAGCAACCGCATCTCATCTGCCACCTTGTCGAAG
TGGGGCATCGGCAAGGGGTTCGCCGTGAGATTGGCGAGCAGCAGCAGGGTGTGCCGCTCTCCGCTCGCCG
GATCCTGCCAGTGGCGGCGATAGCTGATACGCTCGGGATCATCCCCATCCAACAGCTCGAAATTGCCGTG
GCGAACGACAGGGTGAGCCTTGCGCAGGGCGATGAGCTGGCGATAGCAGTGAAACGGCGAGTCGGGGCGG
GCCAGCTGCTCGGCGGCATTTATCTCGAGATGATTGGCGTTGAGGGCGATCCAGGGCGTGGCCGTGCTGA
ATCCGGCGTGGGGGCCGGCGTCCCACTGCATCGGGGTGCGGGCGTTGTCGCGGCCGATGGCATCGAGCCG
CCGACTCAGCTCGGAAGGAGCCAGCCCTGAGTGGCTGGCGCAGTAGTTGATGGCCTCCACATCGCGCAGC
TCGTCCGGCTGCCAGTGGCGGTTGGTCATGGCGAGCTCCTCCCCCTGATAGATGAAGGGAGTGCCCTGCA
TCAGGTGCAGCACCATGGCCCACAGCCTGGCGCTGGTTTCCCGCCACTCAGGCCTGTCATCGCCAAAGCG
GGAGACGGCGCGAGGCAGGTCGTGGTTGCCGAGAAACAGGCTGTTCCAGCCACGTCCGTGCAGCCCTTGC
TGGTGGCGGGCCATGGCTCGCTTGAAGGCGAGGGGATCAAAGCGGGATGACCACTTGTCACTGCCCAGCC
CCAGATGCTCGAAGTTGAACACCATGCTGAACTCGCTGCCATCCGTACTGGAGTAGCGCTGCATATGGGC
TAGATCGGCTCCCCAGGTCTCCCCCACCGTAATGATCCCCTTGCCGCCGAAGCTGGCGGCATTGAGCTCG
CGGATATAGTCGTGCAGGTGCGGGCCGTTGCTCATGGCCAGTCGGTCCCACTCTTTGCTCACCATATCGA
TCACATCGAAGCGAAACCCCTTGATCCCTTTGCCCAGCCAGAAGTTGATCACCTCTGCCATTTCGGCACG
CACCGCCGGGTTGTCCCAGTCCAGGTCGGCCTGACTGGGATCGAAGTTGTGCAGGTAGTAGCGATCAAGC
GCTGGCACATACTGCCAGCCGCTGCCGCCAAAAATCGAGGTGATTGGGTGAGCGTCAACGAAGGCTTGGT

CGCGAAACACGTAATAGCCCTGATAGTGGGGATCCCCGGCCAGTGCCTGCATAAACCACTCATGCTCGGT
GGAGGTGTGGTTGGCGACGATATCCATCATGATGCCGATGCCGTGAGCGTCTGCTTCGGCTATCAGCTGC
TCCATCTCGGTCAGGGTGCCGAAGGCAGGGTCGATGGCACGGTAGTTCGCCACGTCATAGCCGTTGTCCC
GCTTGGGGGAGCGATAGATGGGGGTGAGCCAGAGCATATCGACCCCGAGGGTGGCCAGATAGTCGAGGCG
CTGACGGATGCCGTTGATATCGCCCATGCCGTCGCCATCGCTGTCCTGAAAACTCATGGGGTAGATCTGG
TAGATGACACAGCTATCGAGGTCGATATCGGGCGTGTGGAGGGGATGCGGGTTCATCAGCGTGGCCGGTT
GGGGAGGAGTTATTTCTATCTTACGCAGATGCGCGGGCCCTCGACAGGGCAGGGGAAGCCTTGAAAAGGG
TTCCATTATTGGCTGATTGCCAAAAGTGCGGGAAGGGTCACAAAGGTGCCATCCCGGGCCACGGCTGTGG
TCGGGATGACACGGCACTTAGCGCTGATGCACCGCAGCGATACAGGCTATTGAGCAGCCTTGGCCTGCTC
GGCGTTTTGTTCCTGCTGGCGTTCCGCCTGCGCCTCCAGCTCCTTGCCGAGGAAGTAGTTCAGGAAGGTG
CGGATCACCGCGATGATGGCGAGGCGGATCAGCTCCTCCTTGCTCGGTGCGACCGTGGTGGCCAGGATAT
CGGCGGCCAGCTGGAACTCCAGCGCCAGAATAAGCCAGCTGCCAAAGCAGAGGCGAATGCGCGGAAAGCC
GGGCTGGTGATCCCGCACCCGTGCCGCCAGCCAGAGAGTTTTAAAGAGGCCAATGACCACACAGGCGATG
GAGATGATCTCCAGCAGCAGCTGGAACAGCTCGACCCCGTGCATCATCCCCTCTTTCAGGCTTGCTATCT
CAAACACTCGGGTCACCTCCCTGATTCAAAAGAGGACACTATTAACACATCGCTCGGCAAAGGCCAAATT
GAAGCCACTTAAGGCTTGTGGGGGAAATGAGCTGATGATCTAACGTTGGGCTCCACTTGACCACTTTTGG
TATGCACAGCACAATCCTTGCTCTTTCATGTAGTTAAAAGGTGAAGAGGCATTTTCATGACGCAGATGCA
GAACCCAGCCATTGGCATTGATCTGGGGACAACCAACAGCGCGATCGCAGTATGGCGTGAGGGCCAGGCT
CAACTCATACCCAATGCCCATGGCGAGGATCTGACGCCGTCGATTGTCAGCATTGGTGAGGACAACACCC
TGCTGGTGGGTCAGGCTGCTGCTACCCGCCTGCTAACGGACCCTGCCAATACAGCGTCTCACTTCAAGCG
TTTTCTGGGCAGTGAGCGGCGTTATCGACTGGGCAAGGAGCAGTACACACCGACCGAGCTCTGTGCATTG
GTTCTGGGGAGCCTGAAGTCGGATGCCGAGGCGTGGCTCGGCCACCCTGTGCGCGAGGTGGTTATCTCGG
TGCCAGCCTACTTTGGCGATCAGCAGCGCAAGCAGGTGCGACTGGCGGCAGAGTTGGCCGGGCTTGATGC
GGTGCGCCTTATCAACGAGCCGACGGCCGCCGCCATGGCTTACAGCCTGCATGAGCAGCACAGCCGGCGT
TTTCTGGTGTTTGATTTGGGGGGCGGTACCTTCGATGTGACCGTGGTGGAGTATCAGGATGGCATCATGG
AGGTGCGTGCCTCGGCGGGAGACAATCGGCTAGGTGGCGAGGACTTTACCCAAGATTTAGTCGATGCGGT
GCTTAGCAGGCTCGATACCGATCGCGGTGAACTGACGCTCTCCCAACTCGGGCAAGTGTATCTGGCCTGC
GAGCGGGCCAAACGTCATCGCAGTGGTAGCGGCCTTGATGTCGAGCTTGGTGCGGAGTGGAGCCGGACGT
TCGGTTTCTCCGAAAGCGAGCTGGCCGAGACCTGGCGCGATACCCTCACCCGGTTGCGTAAACCTCTGAG
TCAGGCGCTCTCCGATGCGCGCCTTCGGCCCGAGGAGGTGGATGAGCTGATCTTTGTCGGTGGAGCGAGC
CGCCAGAGCGAGGTGCAACAAATGGCGGTGCGTCTGCTTGGTCGCTTTGGCCGCCACGAGCTGGATCCGG
ATCGGGTGGTCGCCATGGGAGCTGCTATCCAGGCTGCCTGCCGTTTGCGTGACAGTGCGGTTGAAGAACT
CATCATGACCGACGTTTGTCCTTTCACGCTGGGCATCGCCGTCAGCCAGGGTGACCAGCACGGCATCTTT
AGTCCCATCCTCGAGCGCAATACAGTTGTACCAGCTTCACGAGTGGAGCGTTATCGCAGTGGCCACGAAC
AGCAGAGTGCTGTGTGCATCAGCATCTATCAGGGGGAGCGCTTCTGGGTGCGCGACAACCTGCATGTGGA
CGATCTCGAGATCCCGTTGCCGACCGGCAAGGGCATTCAGGAAGTGGATGTGCGTTTCAGTTATGACATC
AACGGCATGCTGGAGGTTGACGTTACCCTGGTGAATTCGGGTGAGCAGTTCCAGAAGGTAATAGACAGAA
GTCCGGCTGGCGTGACCGAAGAGGGCAAGCACGAGAGTCGAGAGCGTCTGGCCCGTCTCAAGCAACATCC
GCGCGATGCGTTGCCCAATCTGACCCTGAGCGAGCGATTACAGCAACTCTATGAAGAGAGCATCGGTGAC
GAGCGGCGTCAGATAGAGCAGTGGCTGCTGCAATTTGAACGGGTCCTGGCTGGCCAGGACCCGCAGCTGA
TCCGCTCATTTCGAGAAGAGATGAACGCTCTGCTCAAGCAGTTGCACTAGTCGAGAATGTCGCTCAGCGA
CATCCGGCCCTGTTTGTAGAACTCCCTGGGCCAGCCACGGCGCAGTGCGCCCGGCCTTAGCTGACTGCTC
ATGACTAGGTGGCCGAGCCACAGGGGGACCGCCAGCCAGGCGCTATTGATGTAGAAGGCGATGGCTAGCC
AGATCAGACTCCAGACCAGATAGTTTCGACTGGTGCGCGGACGTGTCTGGGCCCGGCAGATATGCCACAT
CAGATAGAGGTTCATGAACAGCATCGCTGCCATATCCAGACCGAGGGAATAATATTCCACCCAGTTGGAT
GAGCCGGAGGCGAGGACTTCGCGTAGGGATGGTTCCAGTGGAAACGCGAAGTAGGGCAGGATGGCGGACA
GTATGCTGACGAACATCAGCTGTGAACCGCTTTGTAGCCTGAAACGCTTTTTGGCGTGTGCCCTGTCATG
TTCAAGTTGGGCGTTGAGCTTGTCCTCCAGATAGCTCTGTACCAGAGATCGCACCTCCTGCTGGGCTGGC
GTGAGGCCATCCGGTTGCTTGTAAAGCGGGTCTAGTTGACGGGCCTGAAAGCCATTTCTGCCAAAATCAT
GAGCCAGCATGGCCAGGGCATAACAAACGGGATAGACAAACTTGGCTGCCGCTTCGTTCGGGCGGTGTTT
CTCAAGTTCTTCCAGCGTGGTGATATTGTCAGGGTCCTGTTGCAGGTAAGCGTAAATTAGTGACTCTTCG
GCATAGGGATAGACTTCCAATGCTAATCCCTGTGCCTGGCGCAGTTTTTCGAGTCGTTCGCGCAGTGGCG
TCAAAATATAGCCATAGGACTCATTGCTTGCCTTGTGGCTGAGCAACAGGTTCCACAGCACTATGGTGCA
GGCCAGATCCGGTTCGTTATCCAGTCGTCGATCAGGGCTGCCAGTGCGGGTAGTTCGTTCAAGCGGGCTC
GTTCGAGAAGCGCCTCGTCGCTGACATCAGAGCGTTTAAGAAGTTCTGCCTGGTTGCGCAGTTCCAGCGA
CAGCACGTACTGCTGCGGCCAGCGGGCGCAGGCGTCCATGATGGCAGCTTGGTATAGGGTGGGCTGGGCA
CAAAGACGCAGGAGGGTGGAGAGCGGTGTGTCCTCCTGACTGGTTGGCAGTGAGCCCACCGACTGGCGAA
TGGCGTCGAGATGCTGTTTCGGCGTCCAGGGTCTGGCATAGTAGAACTGCGCCCAGCGAAAACAGCCGGG
CCAGCTTTCGCCATGCCAGGCATCAGCGAGCGCCGTATCGCTGTAGAAGCGTTGATGAGAGAGACGGGCG
CCCAAATGCATCCCCAGCATCATCCGCTCATCCCATCCTTCATTGCTGGCGCGCTGGGCGATTGCTTTGA
ACCAGGTGTCAGGCAGCAGCGGATAGCGGCGCAGGCAATCGAGCCACTGCTCCGGCGTAGCGCAGGCAAT

CTGCTCGGCACTGGGGGGCGCGATACTCAACCAGTCATCGTTGCCATAGCGGGCAAGCAGTGTATCAAGC
AGGGGCAGTGCTGGCCGCTCGGCCAGTTTGCGGCGAGCGTGGCGTTGCAGCAGGTGTGCGAGGTACCGCC
AGCCGCCCTCGGCCTCGGCCAGCTCGGGCTGTGTCAGTGCCGCTTCCAGTGCCTGCCAGGTGCCGTATTC
GCTGTGCCACCATAGGCGGGCCAGGGCACCACGTGTGCCGGGTAGCGCGTCCAGCGCCAGGGCGGTGAGC
GGCATATTGTCAAACCCCAGCATCTGGTCGTGCAGCCACTCTTCCACCGGGTCCGGTTGCTGCCAGGGCA
GCATCAGCCACTGGGGTTCCCAATAAGCGCTGCGCGAGGTGGGCCACTTCTGGGCGGTGGCGAATGCCTG
CAGCCAGCGGGACAGATCCTGATCGCTGTCTAGATGCCACTGGATAAGGTGATTGGCGAGCAGCGCCTCG
TATTCCCGCTCTTCCAGTGCCTCGGCGACCTGCATCACCAGCTGTTCCTTGCCGCAGCGCAGGCTGGCGT
CGGAGAGCAGGTGCCAGTCATCTTCACTGAGGTGATCCTGCTCCAGCAGAGCAAGTGTGAGCGCAAGACG
GTGGGTGGGATCACCGTCAGTACCGGCGGCATGGAGCGCGCGCAACTGCAGCCGGAGCAGATCCGGGTGG
TCACCGACGATGGGGAGGGCTGAATGATAGATGAGCCCTAACACGCTGTTGCGATCACCTGCCTCGAGCG
ATAGCAGCAGTGGCTGATAGAAGGCCAACACGGCGCGTTGACGGGCATGGTCCAGGGTGGCAAGCCACTC
CAGAGGGGCTGCCAGCGGCGCCTGACGCCACCAGTCGAGAAATTCCCCCTTCTTTTGCATGTCGCCGGTA
AGACGCAGTAGTGACGCCCAGTCGAGCGCTTGCCAGAGACGCTCGATCACCGGTGCTGGCAACCATTGCC
ATTGGAAGATGTGTTGCAGCACTTCGTCCGACAGCCGGCTCTGCTGAACCAGCGAAAGTGACTGCAGTGA
TGTCTGCCACTTATCCCATTCGGTCATTACAAATCGCCGGCTGGCATCGCCAAGCAGTTCAAGCAGAGCG
GTCAGTTCAGCGGCGTCGGCCGGACGAACCAGCTCGGTCTGTGGCTCAGAGTCATCACTCATGGCTGGAC
GCGGCTGGCTGGCCTCTTCATAAGCCTCGCGCAGCAGGCGGAAGCCTTCGGGATCGTCTTCCGGGTGGCA
GTCACGCAATTTGATATGATAGGCGCGCTTGATGGCGGCCAGATCGTTGGTCTCTTGAAGACCGAGACGT
TGCCAGATATCCATGTTTTATTCCTGCGGATCGCTGTTGAAGAGGTCAATGAGGCTTTGGGGTTGTTCTA
TGTCGGGTAGGGGGACCTCGTACCAGCTTTGTCCGTGGATTGGGTTCTCCTTATCCCTGAGCGCATCGGC
CAGCATGGTCCTCATATCCCTTGCAATCCAGCCGTGTAGCAGAGCCTGGGCGGCTTGATCCGGCTCCCCC
TCTTCATGACTGAATTCCCATAGGCAGCGGCCGGCATACCAGGCATGAGTGTACTGTTCCCAAGAGCCAT
AACGTCGGCGGGCTTGATGGGCTAGGTGGTTGTGGATAAAGCCGAACTCCTCCTCGCTGACCCAGCCAAA
GCGAAGTCCCTCCCGGGTGAGGTACGAGGCGCGCCCATAGTCCCATGCCTGCACACCATGGGGGCCGATA
TAGGGGGCGGTACGAGCCACAAAATCGAGAGAGGCGCGGTGACGCGGCGAGGTTACGCGCTCTCGCATGG
TTTGCCATTGGCTCGGTTGCAGGCAGTGAAAGGCGGTGTACTCCTCAAGCATGAGATGCCCGTGACAGTC
GCCAAAGGCCAGTTGACGAACCTGCCGGTGCAAGTCGAGGCGATTCTCGATGCCCCAATCCTCTGCGAGT
GTATCCGGCTCTTCGAGCCCCTCTCGCAGGGAGGTAGGCTTGAGGTATTCCCGCCACTCGGTCTGGAAGG
CGATCTGTGGGGAGGTTAAAGTCAGCCACCATCGTTCGTGTGGGGCAAGATTGGTACTATCTAACATGGG
GCTCACCAAGGGTGCATTTGAGTCAGCTGGCAACTATAAAGGCGTGCGAAAATGGATGCAATTGATCCTC
TGTTACTGGTTGCGCAACCAGTCGGAGAGTTGTTCAATGAGCTGACGCGCCGCGGGGCTGACTCCGGCCA
GTTGGGAGAAGCCGTGTATAACACCCAGTTGCCGCTGACAGGTAGCTGTGACTCCGGCTTGCAGCAGTTT
GCGATAGAGGGCTTCCCCTTCATCGCGCAGCGGATCATATTCCGCCGTTACGATATGGGTCGGTGGTAAT
CCTGACAGGTCGGAATAGTGGAGCGGGCTGGCTTCGGGATGAGTGGCGGGTAGCTCGCCCAGATAGGCGT
GAAAACCGCTTAACAGCATGTCGCGGGTGATGAGGTAGTCATCCCCTAGCTGCCGATAGCTGTCACTGTC
GCCCGCAGCATCGAGCATCGGATAGATGAGCAGTTGGGCGGCAGGCAAGGGGGCGCCTCGCTCCTTGAGT
CGCAAGGTGGTGATCAGGGCTAGGTGGCCGCCGGCACTGTCGCCCGCAAGGGCAATCCTTGCCGGATCCC
CATGCCATTTCTCCACATTGGCCATGATGGCGAGCGTGGCTGCCAGTGCATCGTCATGTGCTGCAGGATA
GGTGTGTTCAGGGGCAAGGCGGGTATGTACCGCGAACACCAGTGCTTCGGCGCGATTGCACAGCATCCGC
ATCTGGCGGTCGTGGGTGTCGAACTCGCCGCTGACAAAACAGCCGCCATGAAAGTAGATCAGGGCCGGAC
GGCTTGCGCTTGAGGTTGGTGCTGGTTGATAGAGGCGAACGGAATAATGGCCATTTTGCCACTCTTCGAC
AGCGCCAACCTCTTCCCGTTCACCACCCAATATGGCTGAAGCAAGATAGCCTTCACGCCGCTCCTGCCAG
CTCAGGCTGCTTGCCTTGGGGCGGCCTGCTGCAATGAACTCTGCCACCATGGCTGCAATGGCCGGTTCAA
GTTGTGTATCCATAACGCACCAATAACTGTATATAAAAACAGTTATTTTGCGCTTTTAGCCAAAGAGCGC
AACCGGCGTTAGCTGAGGCAGCTCACAAAATCAGGCTTCAAGCAGCTGGAATTCAATCACATAGAGCGGT
GGCAGATCGGGGTAGATGTCGCGGATCACATCTTTAAGCTCGGGGAGGGTCATGTTCTCCTGACGGGCAT
GCTCGTCATTCAGTTCATCGAAGGCCACTGCGTTGACGGAGATCACCTCGATGGTGCCGAAGGGCTGGTG
CTCCGGGTTGGTGAACAGGGCCAGCCGCTGGCCGGGCTGCCAGTTGGCCTCGCTCTCGTCGCGGATGGTG
ATGGTTTTGCGGCCCGCCTGAATATCGGCGACGAAGCGGCTGAAGAAGGTGAATTCGGGATAGGGATGGG
CAGACATGGTGACTCCTGACAGGGTGAATCGAAATGAGGTGAATGGAAATCAAAGATCGAGATAGGCGCG
CAGCCTGGCCAGATCGGCGATAACAGGGCCGTCGCTGCTGTCGGCGACTATGCCCTGCTCGACCAGCTCC
TTGAGGGCGCGGCGGTAGACCCGCTCGGTGGTGCCGAAGCGTGCTGCCTCGTGGGCCCGCTTGCGCGATC
CCAGCATCGGAGTGCGCTGGCGCTCACGCCATAGCTCATAGGCGATGTTGTAGGCGATGGGGTGGAGCAG
CCGGCTGGTGGTGATCTCCAGGGTATCGAGATAGTCGCTGGCAAGCGCGGCGGCAAAGAACAGGGTCAGC
TCGGGTTCGGCCAGCAGCCGCTGGGCCAGCTTGTCGGCGCAGATGACCCGGGCGTCCAGCTCGGTCTCGG
CCACCACGTTCCACTGCACCGGGGTGGCGCTGAAGAACTCCATTTCGCCGAAGATCTGGCTGTGACACTC
AATTTCGCCAAGCTGGAAGCGGCGGCCAGAGTGTACCGTGCTGTTGAGGGAGACCCGGCCATGTTCGACC
AGATAGAGCTGCTCGCTCTGCTCCCCCTGCCGCATGATCAATTCGCCCGGCAGGTAACGGCGCAGAAACA
GGGTGCAGGCGTCGAGCGCCGCGGCAAAGCGGGGTTCAAGCTGCTGGAGTTCGGCGTGAAAACGGCCGAG
GGGGTAGGGCTGTTGCTGCATGACTTGCCTGTCGCACCGGATGACGAGGGCCATGAGGCTAGCAAGAGTG

CGGGCCGGATACCAGCCCGCACGGTAGCGGATGTGCCGCAGTTAACGGTTTGTGCTTTATTGCACCAGAG
CGTGGAGGGCGGCGGCGATGGCTTGCTGCTCACCGGCCATCATCGCCTGTTCGGCGCTGGCGTAATCCGC
CACCCCTTGTTGCACATCCTGCTCGAACAGCACCACGTTGCAGAGCACGGCGGCGACCCGCACCTTGCGC
AGGCGACCCACCGTCAGCAGGGAGGCGGTCTCCATATCGGCGCCGAGCACGCCACGGGCATGCCAGTAGC
GGCACACCTCCTGCTCGTCATCCCGATAGAAGCTGTCGTGGGAGCGCACCAGCCCGTGCCAATGAGGTTG
GCCGCTGGCCGCCACCTGATTGAGCAGGGCCGCTTGCAGCCAGATATCGGCGCAGGCGGGGTACTCGGCG
GGCAGATAGGCCTGAGAGGCCCCCTCATTGCGTACCGCTCCTTCCACCACGATAAGGTCGCCGAGCCCGA
TGTCGTGCTGCAGGGCACCGGCGGAGCCGACCCGAATAAGCGAGCGGGCACCGGCTTGCACCAGCTCCTC
CAGCGCTATGATGGCCGACGCGCCGCCGATGCCGGTGCTGCAAACCGTGATGGGCAGCCCCTGATAGCGG
CCGTTGATGAGGCGAAACTCCCGGTTTTGCCCCAGCACTTCACACTGTTCGAGCTGGCTGGCGATGCGAT
CGACCCGGGCCGGGTCGCCGCACAGTACCACCCGCTCGGCAATCTGGTCGGGGCGGCACTGGAGATGGGG
TAATAGTGTCATGGCTTACAGACCTTGTGGCTGGCGCTTCGCGGCAATCACCCAGAGGCGAGTGCCGTTG
GCGGCGATAAACAGCAGGATCAGGTATTCCAGCGACATGGCGTAAACGCCCTGCGCGGCATAGACACCGA
TGCTGATGATGTTGATGACGACCCAGAGGATCCAGTTCTCCACGTATTTGCGGGTCATCAGGATCTGGGC
GACGACCGAGAGCACCGTCATGCTGGCATCCCAGAACGGGAAGGCATCAGGGGAGGGCTCGGGGGTGGCA
AGGTTGGCACCAAACAGGTTGAGCAGGGAGACCGAGGTGCGTGCCAGGGTGGCGAAGACCGGGTCGATAT
AGCGGGTCAGCAGAGCGATGGCCAGCACGCTCACCACAGCAGTGACGATAAGCTTGGATCTGCTGAGCCA
GCGGATCTGCAGGGTATCCCCCTGATCGTTGGCCGGGTGGGTCCAGGCGTACCAGCCGTAGACGTTGGCG
CAGAAGAAGAACAGCTGCAGCAGCAACAGCCCGTAGAGCTGGATCTGGAAGAAGATGATGGCAAACAGGC
TGACGTTGATCAGACCAAACAGGTAGTTGATGGTCTTCTCCTGACTGGCGAACCAGATACAGGCCAGACC
AAACAGGGTGCCGATGGCTTCAATCCATGACATGGCATAGCCGCCACCCAGCGGTATGGTCACTAGGGTG
TTATTGATGCTGAACAGCTCGAATATGCTCATTTTCTGATCCTTGGGCATGAGGGTTAGTATGCGAGGGC
GCCATTTTAAGGGGTGAGGGGGAGGAGGGGCAGGACTTTTGTCCGCCCCGGGCTTGGCCCATATCAAAGA
AATCGTCGCTGCTGCCCGGTTATTCCAGCAGTGATGGGTTATGGGGCCCCGGCGGGCCGGACTTTATTGA
TATGTAGCAAGATGATAGCGCCGTGGCTGTGGTCTGATAGAGTCCACTCTCCGGGAGGAGAATCACGTGA
TAAAAGGAGGTTGTATTGAATTCCGAGCTTGTCTGGGTGCTCTGTCTGCTGGCTGGCGCCATCTATCTCT
TTATGACCAACAAGGTGCGGATGGACGTGGTTGCCCTGTTGATCATTATCCTCTTTGTGCTGAGCGGCAC
CCTCACCCTGCCGGAGGCGCTGGCGGGCTTTAGCGATCCCAACGTCATTCTGATAGCAGCCCTGTTTGTG
GTCGGGGAGGGGTTGGTGCGCACCGGCATCGCTTATCAGGTGGGGGATGCGTTGGTGAAGGTGGCGGGCA
GCAGCGAGACCAAGCTGCTGGTGCTGCTGATGGTGGCGGTGGCGAGTCTGGGAGCTGTGATGAGCAGCAC
CGGGGTGGTGGCCATCTTTATCCCGGTGGTGCTGAGTGTGGCGAACCGGATGGGGATCCCGGCCGGTCGC
CTGATGATGCCCCTCGGTTTTGCCGGGCTGATCAGCGGCATGATGACGCTGGTGGCCACGCCCCCCAACA
TGGTGGTCAACAGCGAGCTGATGCGCCACGGCATACATGGTTTCGGGTTCTTCGATTTCACCCCCATGGG
CGTGCTGATCCTGGGGCTGGGCATCCTCTACATGCTGCTGGTGCGTGGCTCCTTGGTGGGTGAAGGGGAA
GAGGGCAAGGGCAAGCAGACCGGGCGCCGTACCCTGCGCGATCTGATCCGAGACTACCGATTGGCCGGTC
GGGCCCGTCGTCTGGCGCTGCGGCCGGACTCCCCGCTGATTGGCCATACCCTGGATGAGCTGCAACTACG
CGCCCGTTATGGTGCCCACGTCATCGGGGTGGAGCGCTGGCGCAAGTTCCGCCGGGTGATGGTGCCGGTG
TCGGGGATCAGTGAGTTCCAGGCGAAGGATGTGCTGCTGATCGACATGTCAGACCCCGAGGTGGATGTGC
GGGCGTTTTGCACCGAGGCGCGGCTTGAACCCATGATCCTGCGCGGGGAGTATTTCTCCGATCAGGCGCG
GGAGGTGGGGATGGCCGAGGTGTCGCTGATCCCTGAGTCCAAGCTGCTGGGTAAAAGCGTGCGTGAGGTG
ACCTTCCGCTCCAGCTATGGCCTGAGCGTGGTGGGGCTGCGCCGCGATGGTGAGGCACTGGCAGGCAAGC
TGGTGGATGAGCCGCTGCAGATGGGCGACACCCTGCTGGTGGTAGGGAACTGGAGCCACATCAGACAGTT
GCAGACCCATAGCCGCGACTTTCTGCTGATGGGGCTGCCCGCCGAAGTGGACGAGATGGCCCCCGCCCGC
AGTCAGGCGATCCATGCCCTCTTCTGTCTTGGAGTGATGATCCTGCTGATGGTGACCGATCCGGTGCCCA
ACGTGATTGCCGCGCTGATCGCCTGTCTGCTGATGGGCAAGTTTCGCTGCATCGATATGGAGAGTGCCTA
CAAGTCGATCCACTGGCCGACCCTGATCCTCATCGTCGGCATGCTCCCCTTCGCGCTGGCGCTGCAAAAA
ACCGGCGGGGTGGATCTGATCGTCGGTGGCCTGATCAATGCGGTGGGGGAGATGGGGCCGCGGGTCATGC
TGGCCAGCCTGTTTGCCCTCTGCGCCGTGATCGGGCTCTTTATCTCCAACACCGCGACAGCTGTGCTGAT
GGCGCCCATTGCACTGGGCACAGCCCAGCAGATGGGTGTCTCGCCCTATCCGTTTGCCATGACCATTGCC
ATCGCCGCCTCGGCCGCTTTTATGACGCCGGTCTCGTCACCGGTGAATACCCTGGTACTGGGGCCGGGCA
ACTACAAGTTTGCCGATTTTATCCGTATCGGGGTGCCATTCACCCTGTTGGTGATGGCTGTGAGCGTGGT
AGCGATCCCCTGGTTCTTCCCGTTCTGATAACCGGGATGATGAATGTGTGCACCAGCGGCCTGCGGGCCG
CTTTTTTATGTCTGGGAGACAAGAGTGGCAAGGCGTGCTCGGAATCAGTGAAGAGTGATGTGGACGGGAA
TTAACGTGGAGATGGGTTGATAGAGGAGCGAGAGAAAAACTGGGGAGAGGCAAATTTCAGATACAAAAAA
ACCAGCGTGAGCTGGTCTGTTTGGTACTGCTATTAATGGTGCGGAAGGAGAGACTCGAACTCTCACGCCT
AGGGCGCCAGAACCTAAATCTGGTGCGTCTACCAATTTCGCCACTTCCGCAATATTAATGGTGGCTATGA
CGGGATTCGAACCTGTGACCCCCGCATTATGAGTGCGATGCTCTAACCAACTGAGCTACATAGCCGTTTG
TTATCGACCACTGATGTGGAAGAGAACAGAAAAATGGCTGGGGTACTAGGATTCGACGCTAGGGATGGCG
AGATCAAAACCCGCTGCTTTACCGCTTGCCGATACCCCAACAGTATTAATGGTGGCTATGACGGGATTCG
AACCTGTGACCCCCGCATTATGAGTGCGATGCTCTAACCAACTGAGCTACATAGCCGTTTGTTATCGACC
ACTGATGTGGAAGAGAACAGAAAAATGGCTGGGGTACTAGGATTCGAACCTAGGGATGGCGAGATCAAAA

CCCGCTGCCTTACCGCTTGGCGATACCCCAACAGAAATAATGGTGGCTATGACGGGATTCGAACCTGTGA
CCCCCGCATTATGAGTGCGATGCTCTAACCAACTGAGCTACATAGCCGTTTGTTATCAACCACTGATGTG
GAAGAGAACAGAAAATGGCTGGGGTACTAGGATTCGAACCTAGGGATGGCGAGATCAAAACCCGCTGCCT
TACCGCTTGGCGATACCCCAACAGTGCTCCTCAACAGTCATGAGACTGGCAAAGAACTTTTAAAAATGGC
TGGGGTACTAGGATTCGAACCTAGGGATGGCGAGATCAAAACCCGCTGCCTTACCGCTTGGCGATACCCC
AACAGCGCTCCTCAACAGTCGCGAGACTGGCAAAGAACTTTTAAAAATGGCTGGGGTACTAGGATTCGAA
CCTAGGGATGGCGAGATCAAAACCCGCTGCCTTACCGCTTGGCGATACCCCAACTAGAGATGGTGGCTAT
GACGGGATTCGAACCTGTGACCCCCGCATTATGAGTGCGATGCTCTAACCAACTGAGCTACATAGCCAAC
CTTGCTTCCGGTGAGTCGTTACCTCGTCACCGTGCGGGCCGAATTATGCGTATCTGGCTCTGGGGCGTCA
ACCCTTTTTTTGCCAGAAAAACGCAATTTGAGTGCGTTTGACCAGTTATTGGTCTCTGTTGCGCAAAAGC
CCAACAATCTGGTTTTTTTTAAGACATTGCCATGATTGCCCGGGCAATGACAGATAAAAAACAGGCCAGC
AAATGCTGGCCTGTCATGGGATTAGACGTTGAACAGGAAATTCATGATATCGCCATCTTTGACGATGTAA
TCTTTCCCTTCAGCGCGCATCTTGCCCGCTTCCTTGGCGCCTTGCTCACCCTTGTAGTTGATAAAGTCTT
CAAAGGCGATGGTTTGGGCGCGAATAAAGCCTTTTTCAAAGTCAGTGTGGATCTTGCCTGCCGCTTGCGG
GGCGGTGGCACCGACCGGAATAGTCCAGGCGCGCACCTCTTTCACACCGGCGGTGAAGTAGGTCTGCAGG
TTGAGCAGCTGGTAGCCGGAGCGGATAACACGGTTCAGGCCCGGCTCTTCGATACCAAGGTCAGCCATGA
ACTCGGCGCGATCTTCGTCGTCCAGTTCGGCGATGTCCGCTTCGATGGCGCAGCAGACCACGACCACGAC
CGCGTTCTCGGCGGCAGCGATTTCACGCACCTTGTCCAGATAGGGGTTGTTCTCGAAGCCATCTTCCGCC
ACGTTGGCGATATACATGGTGGGTTTCAGGGTCAGGAAGTTGAGGTGGCTCACGGCGGCCAGCTCTTCTT
TATCCAGCTTCATGCCGCGGATCATCTGACCGTTTTCCAGCGCGACCTTGATCTTCTCCAGCACTTCCAC
TTCCAGCATGGCATCCTTGTCGCCGCCTTTGGCACGCTTGGACTGGCGGTGAATGGCGCGCTCGCAGGCG
TCCAGATCCGACAGGGCCAGCTCGGTGTTGATCACTTCGATATCGTCAGCGGGAGAGACCTTGCCGGCAA
CGTGAATGATGTTCTCGTCGTCGAAGCAGCGCACTACGTGGCCGATCGCTTCGGTTTCACGGATGTTGGC
CAGGAACTGGTTACCCAGACCTTCACCCTTGGAGGCACCGGCAACCAGACCGGCGATGTCGACGAATTCC
ATGGTGGTCGGCACAACGCGCTGCGGATTGATGATGGCAGCCAGCTGATCGAGACGGGGATCCGGCATCG
GCACCACACCGGTGTTCGGCTCGATGGTGCAGAACGGGAAGTTGGCGGCTTCGATACCGGCCTTGGTCAG
CGCGTTGAACAGGGTGGATTTGCCTACATTGGGCAGGCCCACGATACCGCATTTAAAACCCATGGATTGT
TACCTTACTTGATGGATGCTGTTGGGGGTCGCCCGCAGGCGGCCCATGTCATGCTTGTTCTGGTCGTTGC
TCATGTACCGGCACAATGCCCGTACAGGGTCAAACCGACCTAACCCTTTTCAGCCTTGAAGCTGTGCAGG
CGATTCATGGCCTTGGTCATATCTTGTTTGAACAGGATGTCGGTGGCACGCAGGGATTCGTCCAGCGCGG
CGTCGATCAGGTTCTGTTCACTGCCGGGGGCCTTGGTCAGGACGAAGCCAGCCACCTGCTCTTTGCTGCC
GGGATGGCCGATCCCGAGACGCAGCCGGAAGAAGTTATTGTTGTTGCCCATCTTGGCGATGATGTCCTTG
AGGCCGTTATGGCCACCGTGACCACCACCCTGCTTGAACTTGGCAATGCCTGGCGGCAGGTCCAGCTCAT
CATGGGCAACCAGTATCGCTTCCGGTTCAATCTGATAAAAACGCGCCAGTGCGGCCACCGCCTTGCCAGA
GAGGTTCATATAGGTGTTCGGGATGAGCAACCGCACATCCTGCCCCTCGACCAGGATGCGGGCAGTATGG
CCGAAGAACTTGGGTTCTTCACGCAGGCTGACATTGTGCCAGCGGGCCAGTTGCTCCACATACCAGGCGC
CCGCGTTGTGGCGGGTCTTGGCATATTCCGGGCCGGGGTTGCCCAGTCCGACGATCAGTTTGATTTCGCT
TGTCACGCTTGATGTCTTCACTCGCAGCTGCCGTGGGCACACTACCCAGGGCAATAGGACGCAATATTCT
AGCCGCCGAGTGGCGATCACTCAACCAATGCGCATGTTATGGCGGGATTTGCAGCATTATTGCGGCCAAT
GGGTGGCTAACTGTGGGCAAACGGGCCTCTTGCAAGAGGCCCGTTTCAGTTTACCGATGGATTGTGCCCG
GTTGACTACCGTCGTTATTTGGTACGCAGCAGGGTGACGGTCTCTTCATCCAGTGCGGTCAGGGTGGGAC
GGGCATCGCGCACATACTCCACGAAAGAGGCTGCATCGGTGCCGCAATCCACGCCGCTGGCTTTGCAATC
GAGGGCCTGGCCCGGGGCATAGACGGCGCTCAACAGTTCACCGTTTTGTGCCACCTTCAGCACCGGGAAG
GCGGTCAGCTTGCCATTGACGCTGGCGATATCGACCCGCTCGCTCTGGTGGCGCTGGGCATTGGCCAGTG
CCTGCCAGCCATCGTTACCGTTGGCGCTGTAGGCGTTCATCACCACCCGATAGCGCTGGCTATCCACCAG
ATCCTGCCACTCGCCGCTTGCGGTCTTGCGCTGCAATTGGGTCAGGGTGCCCGCTTTGCCTGCGTCGGTC
TCCTGGAAGGTGTAACGCAGGTGGCCGCCATAGGGGAACTTGCCGGCATGGGCGCTGGCGGGCAGGGTGG
CTGTGATGGTCTCGGTCAGCAGGGCGCGCACCTCGGCGCCAGTCAGGGTCAGCACCGAGAGCTGGTTCTT
GAACGGCAGCAGGGTGAGGGCACTATCCCCCTCGCGCCACTCGCCGGCAGGCAGGTCGGCGCGCACGCCG
CCCACCGCAACCAGAGAAAAATCGACCGGGCCGCCGGTCAGGGCTTGCACTGCTGGGGTATTTGCCCAGT
AGAACTCCCCTTCGGCAACCAGCGGCGCTACATCTGAGCCGTGGCTGTCGCTGCCGTTGTCGCCGGGGCG
ACGGGCATTTTGCAGCTGCTCGGGGATGGTGCCGATCACCGGGCCGTAGGCTTTCTCCAGCGCCGGTTTG
TAGTGGCTGTCGATGATGCCGCGCAGGCGCGGATCTTCATCCACGGTGACCAGATTGGGCTGGTTGTCGA
CAAACTGGCTTGCCTCTTTGCTACGGGCGTCGTCGAGCGCTTGCTTGCGCGCCGCATCGGCGAAGAAGTC
GCGGCTGGCCAGCAGGCTGTTGTGGCCCTGGCAGTCGGTCACCTGACCTAGGCTGTCAAAGGTCACCTGT
GCCAGACCGATGGCCTGGGCGTAGGAACCTGCCTGCACCACGCAGGTTTTGCCGATGCCATCCGGATTGG
TCACCAGCTTGGCGTAATCGCCGGTCTTGCCCCAGCCGATATTGCGAAAATCGCCGAGCAGGCTGTGGGA
GTGGCCGCCGACGATGGCATCGAGACCGTTTACCTTGGCGGCCAGCGCCAGATCCCGCTCGTTGCCGATG
TGGGTCAGCGCCACGATCTGGTGGATCCCCTTGGCTTGCAGCATGTCGACCGTGGCCTGAGCCGAGGCGA
CCTCCTTGCCAAAGCGCAGCTTGCCGACGTTGGGGGCGATATTGCCCATGTCTTCCAGTACCAGACCGAT
GACGGCGACCAGCTGTTTGCCTTGCGGCAGGTTGTCGAGATCGTTGACCGGGCTCTTCTGGTTGCCGTCA

AAGGCGTAGACCACGAAGGGCTTGAGGTTGTCAGCCTTGCTGAGGGCCGGATCGGCCTTGGTGTCGAGGT
TGGCGGCCAGCACCGGGAAGTTGACCCCCTCGATGAAGCGGGCCAGCTTCTGGTTATCCAGATCGAACTC
GTGGTTGCCCAGCGCCATGGCATCGAGGCCGAACTGGCTCAGCAACTCGGCGTTGGCCATCCCCTCGTTG
AGCTTGAAGTAGCCGCTGCCCTGCCAGGCATCGCCGCCGTGCAGCAGCAGCAAGGGCTGGTTCTTGGCCA
GCGCATCGGCCTGATAGGCTTTCGCCATGGTCAGCAGCCGCGGGTAGCCGCCAAAGCGGGTATAGAGGGT
GTCCGTCTCCCCCTGCGGGGCAAAGATCTGCCCTTTGATCTCGGCATTGACCGGATCAAACTGGGAGTGG
GTGTCGTTCATGTGAGCCAGGGTCAGCACGAAGGGCGGGTTCTCTTTGACGGGCTGACAGGCGAACAGGG
CGGAGGTGAGGGTCGCGAGCAGGAGGGGACGCAAGCAATTCATGGGTAAATCCTTAAGCCTGCCGGGGGC
AGGGTCGTACCAGATAGCAGAAAGTTGGAAAGAGAAGGTCCAGAAAGACGAAAACCGCCTGATGGCGGTT
TTCGTTATTGCTGAATCTATCAATGCTCAAACATGGCAGAGATGGACTCTTCGTTGCTGATGCGACGAAT
GGCCTCGGCCAGTACAGTTGACAGCGTCAGCTGCTTGACCTTGCCGACCGCTTTCATCTCCGCAGTGAGC
GGGATGGAGTCGGTGATGATCAGTTCATCGATCACGGAGTTCTTGATGTTCTCGACGGCGGAACCGGAGA
AGACCGGGTGGGTAGCGTAGGCGAATACACGCTTGGCACCGCGCTCTTTCAGGGCCTCTGCGGCTTTGCA
CAGGGTGCCGCCGGTGTCGATCATGTCATCAACGATGATGCAGTCGCGGCCGGCAACATCACCGATCAGG
TGCATGACCTGGGAGACGTTCGGGCGGGGACGGCGCTTGTCGATGATGGCGATGTCGGTGTCATCCAGCA
GCTTGGCGATGGCGCGGGCACGCACGACGCCACCGATGTCCGGGGAGACAACGACAGGAGACTCGAGGTT
CTTGGACTTCATGTCTTCCAGCAGCACCGGGCTACCGAACACGTTGTCTACCGGAACGTCGAAGAAACCT
TGAATTTGCTCGGCATGCAGGTCAACGGTCAGCACGCGATCCACACCGACGCTGGAGAGGAAGTCAGCGA
CAACCTTGGCGGTGATGGGTACACGGGCGGAGCGGACGCGGCGGTCCTGACGGGCATAACCAAAGTAGGG
GATAACGGCAGTGATACGGCCAGCTGAAGCACGACGCAGAGCATCCACCATGACGATCAATTCCATCAAA
TTGTCGTTGGTCGGGGCACAGGTGGATTGAATGATGAAGATATCGCCACCACGTACATTCTCATTGATCT
GTACGCTGATCTCGCCGTCGCTAAAGCGACCTACGGCAGCATTGCCGAGTTTGATAAACAGGCGATCTGC
GATCTTCTGGGCTAGTTCCGGCGTTGCGTTACCAGCAAACAGCTTCATGTCAGGCACGGTTTGAAACCTC
GGGGTTGCATCCAGTATGTGGGGGAGTCTGAAATCAGACCCTATCGTTTTGGGGCAAACGATTTATCAAG
ATAACGTACAAGGTGTCTGCTGCCCAATGAATGTGATGATACCGGTAACCCGAATCCGTGGCCGAGTGCA
AAACCGGCCACGTTAATCATCATCAATACCGTTCGGTAAAATTGGCTGGGGTACTAGGATTCGAACCTAG
GAATGGCGAGATCAAAACCCGCTGCCTTACCGCTTGGCGATACCCCAATCATTTGAACTGTTGCAATGTG
GAGAAGAGAGGTGATATGTTCTCGCCCTTGGCGACAAACCCATCAAGTCCCTTCGGCACGCAGGCCAACA
CTTCCCGAGCAGTTACTTCGTCTTCAAATTGAGCAAAGACGCAGGCTCCTGTGCCCGTCATTCTTGACGG
CGCGTATTCTAGCAACCAGCCGAGCGCATTGGCAACCTCGGGGTGGCGCTTTTTCACCAGTGATTCGCAA
TCGTTTTTCCACTCGCCTTCCAGCAAGTTGTCCAGCGCCATTTTCGGCGTGGCTCTCGGCAGGTCCGGAT
CCTGGAATACGGCCGCGGTCGCCACATGGCAATCGGGCTTGAGCATCAGATACCAGGCATCGGGCAGTTC
GACCGGTTGCAGCCGCTCGCCCACTCCTTCGGCAAAGGCCGCCTTGCCACGAACAAACACCGGCACATCG
GCCCCCAGCTGGACGCCGAGCGCAGCCAGTTCATCTTCACTCAAACCTGCTTGCCAGAGGTGGTTAAGCG
CCACCAGCGTGGTCGCTGCATCGGATGAACCGCCGCCAATACCGCCCCCCATGGGCAGCACTTTCTCCAG
CCGGATATGGGCCCCTTGCGGGCTCGGCAGGGTTGTTTGCAGCAGGCGCGCCGCCCGGATAATCAGATTC
TGCTCATCCGGCACCCCGGGAATGGCCGGGCTCAGGGTGAGAGTGTCGCTATTCGGTATCACTTCAAACT
CGAGCCAGTCGCTGTGGTTGAGGAAGATAAATAGGGTCTGCAGCTCGTGATAGCCATCGGGACGACGGCC
ATTGACGTGTAGAAACAGGTTGAGTTTGGCCGGTGCCGGCCAGCGTGTGGAAGCCATTAACGTGCAAGAG
TCCATTGGTTGATCTGAAGCTTGAGGCGGCTGTTCTGGCCGGTCATGGTCAGCTGGTGCGGCAGGCGGTA
GCCATCCTGATCCCGATAATCGCCATAGACGATCTGCCACTGGCCGTCGCGCACGCTGGCCAGACTGCTG
TCGGGGTTGAGTTCAAACTCTGCCTGACCTGGCAGCCCCTTGATCCACTCTGGCAATCCCGCAACCGGAA
TATTCCAGCCGGTCAGTTGATAGATGAGCGCTTCGGCATCCTGGCTCTGGTGGCGATTGCCGTCGCTGTC
GAGCAGGGTGATCTCTCCCTTGCTGCGGGAGAGCTCCAGAATGCTGGTGCCAATCAGGCTGGTGAGGTTG
AGGCGATAATCCTCCCCATCCTGCTGCCAGTTGAGGCGGGCACTGCCCTTTTGCTGGGGGGTGATGATGG
CCATCTTGCCAGAGAGCTGCCAATGGGTCAGCTGCTCGAGACGGGCCCGCTCCTGGTTCCAGTTCACCTG
GTCACGCTGGGGCTGGATGGTGGCACAACCGGCCAGCAACAGCAGGCAGGTGAGGGTAAAAATACGTAGC
AGCAAGAGACGGCTCCAGAGAGAGGAATTTGCCACAGTATACGGGGCGGCCGTATTGCTTTTAAAGACTT
ACCCTTTCTCGTACAATTGGCAGCCTTTGCACCCAGCGTATGAAATGATCGCCGCCCACTCATGAGTTTG
CTTGCCCTCGGAATCAACCACAACACCGCCAGTGTCGCCTTGCGGGAACGGGTCGCCTTTGGCCCGGACT
GCATCGATCGCGCCTTGCGTGAGCTGGTGAGCCAACCCGGTGTCAGCGAGGCGGTGATCGTCTCCACCTG
CAACCGGACCGAACTCTATTGCAATCTGGAGGAGCAGCGGCAGGAACAAGTGCTGCGCTGGCTGCAACAG
TTCCACGGGCTGGACGCGGCCGAACTGGTCACTGCCATCTATCAGCATCACGATGAAGAGGCGGTGCGCC
ACCTGATGCGGGTGGCGTGCGGGCTCGACTCGCTGGTGCTGGGGGAGCCGCAGATCCTGGGGCAGATCAA
GCAATCCTACGCCCATGCCCAGCAGGCGGAGTCGGTGAAGGGGGCGCTGGAGCGGTTGTTCCAGAAATCC
TTCTCGGTGGCGAAAAGAGTGCGTACCGATACCGAGATTGGCGCCAGTGCCGTGTCGGTGGCCTTTGCCG
CCGTCAGCCTGGGTCGGCGGATCTTCTCCGATCTCTCCCAGACCAAGGTATTGCTGGTCGGGGCGGGGGA
GACCATCGAACTGGTGGCCCGTCATCTGCGCGAGCAGAACGTCACCAAGATGATGGTGGCGAACCGCACC
CTGCAGCGTGCCCAGACCCTGGCAGCCGAATTCGGTGCCCAGGTGATGACGCTCGAAGAGATCCCCGACT
ATCTGCACGAAGCGGATATTGTCATCAGCTCGACCGCGAGCCCGCTGCCCATCATCGGCAAGGGGATGGT
GGAGCGCGCGCTGAAGGCCCGCCGCTTCAAACCCATGTTTCTGGTGGATATTGCCGTGCCGCGCGACATC

GAAGCCGAAGTCGATGAGCTCTCCGATGCCTATCTCTACACGGTGGATGATTTGCAGGGGATCATCGAGC
AGAACCTCGAAACCCGTAAACGGGCTGCCGCACAGGCGGAAGTGATCGTGCAGGAAGAGCGCGACGAGTT
TATGGGCTGGTATCGCGGCCAGAGCTCGGTCGACCTCATTCGCGATTATCGCCATCAGTCCCAGCAAGTG
GCTGATGAAGAGTTGCAACGGGCCCTGCTGGCATTGGCGCAGGGCGAGAGTGCCGAACAGGCGCTCAAGG
CGATGGCCCATCGCCTCACCAACAAATTGATCCACGCGCCGACCCAGGCGTTGCGCCAGGCTGCCTCCCA
GGGGGATCAGAGTGAGCTCTCCCGCTTGCGGCAGGCGCTGGGGTTGTCACAAGAGCCATAATTTTCGGGC
AACCTGCCGGTTGTCCCGTCCTCCATTGAGAATGCAAGAGTTATGAATCCGTCACTGATCAGAAAGCTGG
AAGGCCTCATCGAGCGCCACGAAGAAGTACAGGCCATGCTCGGCGAGCCGGATGTGGTCTCGGATCAGGA
TCGCTACCGCGCCCTGACCCGCGAATATGCCCAGCTGGAAGATATCGTTCACGCCTTCCAGCGTTACCGT
CAGGCGCAGGATGATCTTGAGACCACCCAGATGATGCTGGAGGAGGATGATGCCGACCTGCGCGAGATGG
CGCAGGAGGAGCTGCCGCTGGCCAAGGCCAATCTGGAGGCACTGGAGCAGGAGCTGCAACTGATGTTGCT
GCCCAAGGATCCCAAGGATGACAATAACTGCTATCTGGAGATCCGCGCCGGTGCCGGTGGCGACGAAGCG
GCCATCTTTGCCGGTGACTTGTTCCGCATGTATTCACGGTTTGCCGAGCGTCAGGGCTGGCGCGTCTCCA
TCGTCAGCTGCAATGACGGTGAGTACGGTGGCTACAAGGAGGTGATCGCCAAGATCGACGGCGAGCGCGT
CTATGGCCGCCTCAAGTTTGAATCCGGTGGTCACCGGGTACAGCGGGTGCCGGAGACCGAATCTCAGGGC
CGGGTGCACACCTCCGCCTGTACCGTCGCCGTGTTGCCGGAAGTGCCGGAAGCCGAGCAGATCGAGATCA
ACCCTTCCGATCTGAAGATCGACACCTTCCGCGCCTCCGGTGCCGGCGGTCAGCACGTCAACAAGACCGA
CTCGGCCATTCGCATCACCCACCTGCCGACCGGTCTGGTGGTGGAGTGCCAGGACGAGCGCTCCCAGCAC
AAGAACCGCGCCAAGGCGATGTCGGTGCTCTCCGCCCGCCTGCAAGCGGCCGAGGATGAGCGCCACCGCG
CCGCCGAGCAGAGCACCCGCCGCAATCTGGTGGGCTCCGGCGATCGCTCCGAGCGCATCCGTACCTACAA
CTACCCGCAGGGCCGCCTCTCCGAGCACCGCATCAACCTGACCCTCTATCGCCTCTCCGAAATTTTGGAA
GGGGATCTGGACTGCGTTATCACGCCGCTGGTGCAGGAGTATCAGGCCGACCAGCTCGCCTCGCTGGCCG
AGAGCAATTGATGCAAATGCAGAACCGGCAAGCTCGCATTCAGACGTGCCTGATTAAGGTGGGACGGTAC
TGATGCAGATCCAGCAGGCTCGTGCCCATATCATGACCGTACTGGCTGAGGGGGAGTCCCCTCGCGCCGA
TGCCGATGTGCTGCTCTGTCATTTGCTTGACTGCCGGCGCAGCTATCTGATGACCTGGCCCGAGCGCGAA
CTGGATGCCGCCCAGCAGGCCACCCTGCAGACGTGGCTGGCCCGTCGTCTCAACGGCGAGCCCATCGCCC
ATCTCGTTGGCGAGCGGGAGTTCTGGTCGCTGCCGCTGAAAGTGAGCCCGGCGACCCTGATCCCGCGCCC
TGATACCGAGGTGCTGGTGGAGCAGGCGCTGGCGAAGATCCCGCAAGGGCCCTGCGCCGTGCTGGATCTC
GGCACCGGCACCGGCGCCATCGCGCTGGCGCTCAAGTCCGAGCGGCCCGAGGTCGATGTGTGGGCGGTGG
ATCGGATGGCCGATGCCGCGGCTCTGGCCCGCGAGAACAGCGCCGCACTGGGGTTACCCATCGAGGTGCG
CGACGGCAGCTGGTTCGAGCCCCTTGGCGAGCCGGATCACGACGAAACGCCACGATTTGCGGTGATTGTC
TCCAATCCCCCCTATATTGATGGCGCCGATCCCCATCTCGAGCAGGGTGACGTGCGCTTTGAACCCCGCT
CGGCGCTGGTTGCCGATGACGCCGGGCTGGCCGATATCCGCCACATCGTGGCCCATGCTCCCGCTTATCT
GTTGCTTGACGGCTGGTTGCTGCTGGAGCATGGCTGGGATCAGGGGAAGGCGGTGCGTCAGTTGCTGCGC
GATGGCGGTTACCGCGAGGTAGCCACTGTGCGCGATTACGGGGATAACGACAGGGTGACGCTGGGCCTCT
GCCCGCTGCGCTGACGGTTCCTCACTGTTACACGGCGGGTTGCCTGTGCGCCCCCATGCAAGGATTGAAT
ATGGTTTTTGTGCTCAAACACCTGCACCTCGCCCTGATTGCCGGGGCCGTGCTGATGTTTATGCTCCGCT
TCTACTGGGTACAGACCGCCACTGGCCGAGCCTATAGCGCGCCGGTGATGAAGGTCAGCGGCTGGCTCAA
CGGTCTGGTGGTGCTCTCCGGCGTATTGCTCTGCATGGTGCTGGATCTCAACCCGCTCAATAACGGAACG
CCCTGGCTCAGCGAGAAGCTGATGGCCATTCTGGTGCTGGTATTTCTGGCGGCGATGGCGCTGCGGCTGG
CCAAAAGCAATCTGGTGCGCTGGTGCTCCTTTCTGGGGGCGCTCGGCTGGATCTACTTTATTGCCAAGTT
GGCAGTGCTCAAACAGACGAGTGTGTTCGGCTAAGGCATGAAGAGAATGTTACACATAGACGAATGGACC
GATTTCGAAGCCCACGACATCACCGAGCTGGCGTTGTCAGTCAGTGACGAAATTTATGGCTCGGTCGAGG
AGGGGATCCGCGAGTTGAACCTGCTGGACGAGTGGCTGGACGAGCAGATCGAAGAGGGCACCTGCGACGA
GGTGCGCCAGCAGTTGCTGCAAGGCTTCTACCACAGCCTCGGTTTTCATGGCGATTGGCAGGATTATTTC
TCTGCCAACAATTGCGATCTCAATCAGGTGCTGGTTCAGCGCAAGGGGATCCCGGTCAGCCTCGGCATTC
TCATCATTCAGCTGGGGCGCAAGCTCGGGCTCGATGTAGAGGGGATCTGCTTCCCCGGCCACTTCCTGGT
GAGCTTTGCCGGTGGCGAGGGGGAGGTCTATGTCGATCCCTTTACCGGCGATGAGCTGAGCCTGGCCCGC
ATCGAGCTGCTGCTGCGCGGCGCGTTGGGGGACTTGGCGACCCTGACCCCCGACTACCTGCAACCCTCCG
GCCAGTGGGAGATCATCGAACGGCTGCTCAACGTCAGCAAGGGTGCCCTGCTGCGGGAAGACCGCATGCC
CGAGGCGCTGCGTTGCAGCGAGCTGTTGCTGCGGATGAAGCCCGGCGATCCGCTGGAGACGCGGGATCGC
GGCTTTATCTACGAGCAGCTCGACTGCCCGCGCCAGGCCGCCGACGATTTTGAATATTTTATTGAACAAT
GCCCGGACGATCCGGTCGCCGATGTGCTGAAAGTGCAGCTGGCCGCGCTGGATCTGAGCCCCAAGCCCCT
CCATTAAGGGCGGCTTGAAACTAAACAGAAGAATATCAAGGCCGCTGCGTCCGGTCGCAACGGAGTAACT
TGGCTTCAGGCCCTTTTATTAAGGATGTCTTGGAGTTGAAACAGAAGAATATCAAGGCCGCTGCGTCCGG
TCGCAACAGAGTAACTAGGCTTCAGGCCCTTTTATTAAGGATGTCTTAGATGGAACAGAAGAATATCAAG
ATCAACCAGATCGACGTTGCCAACGACAAACCGTTCGTGCTGTTTGGTGGCATGAACGTGTTGGAATCCC
GCGACATGGCGATGGCGGTGTGCGAGAAGTACGTGGAAGTGACCAGCAAGCTGGGCATCCCGTTTGTCTT
CAAGGCGAGCTGGGACAAGGCCAACCGCTCCTCCATCCACTCCTACCGCGGTCCGGGTCTTGAAGAGGGG
ATGAAGATTTTCCAGGAGCTGAAAGCGACCTTCGGCATGCCGGTCATCTCCGATCTGCACGAACCCCATC
AGGCCAAGCCTGTCGCCGAGGTGGTGGACGTTATCCAGCTGCCCGCTTTCCTGGCGCGCCAGACTGATCT

GGTGGAAGCCATGGCCCGCACCGGCAACGTTATCAACATCAAGAAGCCACAGTTCCTGAGCCCAAGCCAG
ATGAAGAACATCACCGACAAGTTCGAGGAGTGCGGCAACGACCAGCTGATCCTGTGCGAACGCGGTGCCA
ACTTCGGCTACGACAACCTGGTGGTGGACATGCTGGGCCTGGGCGTGATGAAGAAGAGCACTGGTGGCTA
TCCGGTGATCTTTGACGTGACCCACTCCCTGCAGTGCCGTGATCCGCTGGGTGCCGCCTCCGGTGGTCGT
CGCGAACAGGTGACCGAGCTGGCGCGCGCCGGCATGGCGGTCGGTCTGGCCGGTCTCTTTATCGAATCCC
ACCCGGATCCGAAAATTGCTCGCTGCGATGGCCCGAGTGCCCTGCCGCTGGAGAAGCTGGAAGGCTTCCT
CAAACAGATGAAGGCCATCGATGACCTGATCAAGGGCTTCGAACCCCTCGATACTTCGGCCTGATCCCGA
CGCTGTCATGCCTGATAAAAATGCCCCGCAATGCGGGGCATTTTTATATCTGCCATATTTAGTTGAAGAC
TCAAATTAATCTGCGAGCAAACAATTATTGGTTGTCGTGGTGTTAATGGTGTTAATTTTGTTGTGTGATT
AATGTCTCTTAATTTTTTTTATTACATGTAAAGTGGTCACCGTAGTAATGCAGAAACAGCATGGATCGG
GTGAGGTACGCAGATATCTCGTCCGGCTATCTGCATGTTTATTGCTGCCATAACAATGTGCAATTAAATT
AACAGGACGTTAATAATGCGAAAGAAACTATTTAGCAGCCTCTATTTTCAGGTATTGCTCGCGATTACCC
TCGGGGTATTTCTGGGGCATGTCTATCCGGAACTGGGCGCCGATATGAAGCCCCTCGGTGATGGCTTCGT
CAAATTGATCAAGATGATCATCGCCCCCGTTATTTTCTGTACCGTGGTTACCGGCATTGCCGGGATGGAG
AGCATGAAGGCGGTCGGCAAGACCGGCGCCATTGCTCTGCTCTACTTCGAGGTAGTCAGCACCATCGCCT
TGATCATCGGGCTGTGCGTGGTCAACCTGTTACAACCGGGTGCCGGCATGAACGTCGATCCGGCAGCGCT
GGATGCCAGCGCCATCTCCGCCTATGCGGAGCAGGCCAAGTCCCAGGGCATTATCGCCTTTTTGCTGGAT
ATTATTCCGGGCAGTGTGATCGGTGCCTTTGCCAGCGGTAATATCTTGCAGGTGTTACTGTTTGCGGTAC
TGTTCGGCTTCTCCCTGCATCATATCGGTGAAAAGGGACAGGTTATTTTCGGCGTCATCGACAGTTTCTC
CAAAGTCATATTTGGCATTATCAATATGATAATGAGACTGGCGCCGGTCGGTGCATTCGGGGCAATGGCA
TTTACCATCGGCAAATATGGAGTAGGTTCACTGGTACAGCTGGGCCAGCTTATTGCCTGCTTCTATCTCA
CCTGCCTCTTCTTTATCTTCATTGTGCTGGGCAGCATCGCTAAAGCCTCAGGTTTTAGCATCCTGCGTTT
CATCTCCTACATTCGTGAGGAGTTGCTGATCGTGCTGGGGACCTCCTCTTCCGAGTCGGTACTGCCGCGG
ATGCTGGACAAGATGGAGAGGCTGGGTTGCCAGAAATCGGTGGTGGGGCTGGTCATTCCGACCGGGTACT
CCTTTAACCTGGATGGCACCTCCATCTATCTGACCATGGCGGCGATTTTTATCGCCCAGGCTACCAATAC
GCCGCTGGATCTGTTCCAACAGATCACCCTGCTGGTGGTACTGCTGATCTCCTCGAAAGGGGCGGCGGGG
GTAACTGGTAGTGGCTTTATCGTGCTGGCGGCGACTATTTCGGCGGTTGGCCATCTGCCACTGGCTGGTT
TGGCGCTGATCCTGGGGATCGACCGTTTTATGTCCGAAGCGCGGGCGTTGACCAACCTGATCGGTAACGG
GGTTGCCACCGTGGTGGTCGCCAAATACTGCAAGCAGCTGGACGAGAAGAGAATGGATGCAGTGCTCGGC
GGCAAGGTCGGGCAACTGGAAGCGGACAGGGCGTAAGCGCTGTTTCCCCCAGCACGGCTGCGTTGGCTGG
TGCGGGGGCAATAAAAAAACGGCCAATCGGCCGTTTTTTTGTGCTGATACCGCAAGTATCAGCTCTTCAC
TGCAGGGCGCTGATTACAGGCTCTGGGTGAAAGTACGGGTAACCACGTCACGCTGCTGTTCCGGGGTCAG
GGCGTTGAAACGCACTGCGTAGCCAGATACACGGATGGTCAGCTGCGGGTACTTCTCCGGGTTTTCGATA
GCATCTTCCAGAGTTTCGCGACGCAGCACGTTGACGTTCAGGTGCTGGCCACCTTCAACAGCAGGCTCAG
CCTGAACCGGCAGTTCGCGGTATTCGAACTGACCCAGGTCAGCCAGTTGCACTTCTTGATCGGCAGCGAA
AGCATCGCCTTTGACGCAGATGCAGCGAGCTTGTTGCTTCTCTTCGTCCAGCAGCCAGAAAGAGTTCAGC
AGAGCGGTATTGGCGGCCTGGGTGATTTGAATGCCTTTGATCATGGTATGACTCCTCAATGGGAACGCGA
ATCCAACTGTAAGGAAATTACCCTGATGTATTTAAGCTACCGTCGATGGCGATGACTTAAAGGGCCCTTG
CCTTACACCTTTTCTTTGATGGGGTCATATATACCACGGTGGTAATATTACCAAAATTGATCTAGATCAA
GAAAATCCGACAAGGCACCATTTCAATGGGATTAAATTCTTGAGTTGAATCAAAAATAGGTCATTTTTCT
GATTTTGTAAGATATCCAAATTATGCATCTTTTATCTCGTTAATCTAGTAATTTAACTACATCTTTTGTG
CTTTTTTCTGAGGTTAATTGAGAGGTTGGCTACACTCAGCAAAAAAAGCGAGGAGAGTGCCGGGTGAATA
ATGGGTGGCGAAAGTGGGCATGGCTGGCCCTGTTTTGTTGCTGGGCAAGCAGTGTTGCGGTGCGGCCGA
GACGGTGCGTATTGCTGTGGGCGAGTGGCCACCTTATATCAGTGAGGCGCTGCCCAATCAGGGCTTTCTC
TGTGAGGTGGTGCGCCAGGCCTTTGCCGAAGAGGGGCTCGATGTCAGCTTTCACTTCTATTCCTGGGCCG
AGGCTTACCGACTGACTCTGGAGGGGCGCTATGTGGTCACCATGCCCTGGTTTCGCTCCCGTGAGCGGGA
GCAGCTTTTCTATCTCTCCGCCTCGCTCGGCCATTTCACCACTCGCCTGATGACGCTGACCGGTACGCCC
GCGGTCAATCTGGGTGAGCTGAAACACGAGCCGGTCGGGGTGACCCGTGGCTACTTCTATGGCGATGGTT
TTGCCGCCCGTCAAGCCGGGCTCACCCTCGAGTGGTCAGATTCTGATGAGGAGAGCATGGGCAAGCTGCT
TGACGGGCGGATCCACTATCTGGTGATGGATGAGCAGGTGGCACAGACTCTGTTCCAGCAGGCGCTCGTT
CGTCATCCCCAATCGCTGTCACTGACCGGTGCCGCCGACCCCATCTGGTCGCAACCGGGATACCTGCTGA
TGCGCAAGGCTGACCCTGAGGCGCGCCGTCTGATGCTGCTGTTCAATACCGGGCTGCGCAAGCTGGAGCG
CTCGGGGCGCCTCGCCAGGCTCTGGAAAGCGTGAGCCACGGTTGCGTCTTGCGGGCTGGCCCCCTATCCT
GAGCCCTTCGCCGTATCAAGTGAGGAGGCCGGGGTGCTGACCTGGAGTGATGTGATTGGCTCTGAGAAAG
AGCAGGACTATTTCAAGAGTACGCTGGCAACCGTCAGAGCCGAGCGGGAAGCGGGCAAGGTGATCTACCC
GCCAGCTACCGATGTGTTCAATGCCTTCAAGCTGACCGAGCTGGATCAGGTCAAGGTGGTGATCCTGGGG
CAGGACCCCTACCACGGCCCCAATCAGGCGCACGGGCTCTGCTTCTCCGTTCTGCCCGGCGTGCGTACGC
CCCCCTCACTGGTCAACATCTATAAAGAGATGCAGCGTGACCTGCCAGATTTCGTCACACCCAATCACGG
CTTTCTCGAATCCTGGGCCAAACAAGGTGTCTTGCTGCTCAACACTGTGCTGACGGTGCAGGCCGGTCAG
GCCCATTCCCATGCCCATCTGGGATGGGAGACCTTTACCGATCGGGTCATCGAGCAGATCAATGCCAGTT
GTCAGGGTGTGGTGTTCCTGCTGTGGGGTGCCCACGCCCAGAAGAAGGGACGTTTCATCGACCGCCAGCG

CCACTATGTGCTGACCGCGCCGCACCCGAGCCCACTATCTGCCCACCGCGGTTTCATCGGTTGCGGCCAC
TTTAGCGAGACCAATCGCTTGCTGGAACAACAGGACAAGAGTCCGATAGAGTGGAAATCTGTCTGCATCC
CACTTGATTGAGTGCAAAGTTCGGGCGACAAATGACCGTATACTCAGGGACATAAGATTTATTCAGAGCC
AAGGAGTGCCCATGTTATCCAGTCTGCACAAGGACCATCTCAACATCGCCCGACTTTTGGAGCTGCTCAA
GCACAAGCTGGCAGCCATCCGTGCCGAAGAGCCCATCAGCTATTTTCTGGTGCGCGATGTCATCGACTAC
CTGCGGGAGGTCTCTGACAAGTACCACCATCCCAAGGAAGACCTCATCTATGACTACTACCTGAAATATC
GGGTGGTGGAGGGTGAGGTCGCCAACCGCCTGCAGGAGGAGCACGTCAAGCTGCTGGAGGCCGGGGCCGA
TCTCAAGGAGATGGTGGAGATGATCCTGATGGATGCGGTGATCCCCCTCGATCAGTTCACTACCAAGCTG
GAGCGCTTCGTGGAGCTGCAAGAGGCACACATGAACTATGAAGAGGGGGTGATCTTCCCCCATCTGCGTG
ACTCCCTGACCGAGGATGACTGGCGTCATCTGGAGCAAAACTGGCAGAACCGCGCCATTGGCGACCCCCT
GTTCGGGGCTGAGGTGAGCCAGCACTTTCAGGCACTGGCCAAACGCCTCTCTTTGCCGCTCACGACCGGC
TAAGACGAGCCCGAACCCTCAGCGCGGCGGGTATTGTGTGAGTGCAACTTGAGTGAAAACAGAAAGGCCT
CCATTCGGAGGCCTTTTGCATGGTGGAGCAAGCTTACGCCTGTGGTTTCAGGAAGTTGCGATAGAGCATA
TAGAGGTGGGCCGAGCTCCAGCTGAAGTTGCTGGCCCCCTGCATGGCGCCGGTCTCCGGATTGTAGTTTT
CGCGGATGGGGCCGCTGCCCGCCAAGCCCTCTGCGTTGTTGAACAGCTTGTTGACCAGAGCCTCGGCATC
GCTGCGGTAGCCGTAGTTTTCGAGCCCCTTCACCCCGAAGTAGAACTGATCGAGCCATACCCGGCCGCGC
CAGTAGATATTGGGGTCGTAGGCCGGGTTGGTCAGCGCGGCTGTGCCCAGCGGGATCTTTGTGTTGAACT
CCTGGGCGTTGAGCATCACCTCGCGCACCCGTGCCGCTTTCTCCTTGTCGGCGACTTCTGCCCAGAGCGG
GCTCCACCCTTCCGGGCCACGGCCGCGGGCTGTCAGCAATTTGCCGACGCAGCCGTTGCCGTCAGCAACA
TCCCCCTGAGCAATCTGGCGATCGTAATAGAAGCCTGATGCCTCATCGAACATGCAGCTGTTGATGTAAC
CAGCCAGCTGGGTCGCGCCATCGCGGTAGCGGCTCGCGTCGTCCGGCTTGTCAAGCAGGTCGGCCATGTC
TGCCAGAATGCGTTTCTCCTTGGCCAGGAAGGCGTTGAGCTCCACTGACTCCTGATCGATGGAGAAGCCG
ATCAAATCGCCATTGTCCTGATGGTTTTCAAAGAAGGCGACGTTCCAGTCCTGACGTGCCTTGGCCAAAT
CTCCGCCATAGGTCTTGTCGGCATAGCGTTGCAGCTGGCCCGGTTCGATAAAGCCGAAGCGGGCGGCGTT
GTCCATGCCTGACTCCCAACCGGCACCGTGCTGGGCACCGATATCCATGTCGCTGTACCCGCCCACGTTC
AACACCTGTTGATAGAGCGGCATACCGGCGCAGGCATACCAGCCATCCCCCTCACTGGTACAGCTGGAGA
GATCCAGCCCGGCGGGAATACCGTTGGAGTACTGCACTTTGAAGGTGATATTGCCGTACTCGTCGTTGTG
CTCCACATGCTTGGTGGCGCCATATTCGATGATGCCGTTGCGGTTGTTGTCCCGCGCGCGGTACCACCAG
TCGTGGTAGGCCTGCAGCTTGGGATACATCTCGGCGATAAAGGCCTTGTCCTTGGTGGCGCTGTAGATTT
CCCAGATGGCCCAGGCGGAGAGGGGCGGCTTGGTGTCGCGCTCGTTCCAGTTGCCGCCATCGCCACCGCG
ATCCGCCAGCTTGTTGTAGAAGACGGCGTCGATCACCATGCCCGCATCCTGTGGTCGCACCGGATCATCC
GGCTGTACCTGATAGTCATACATGGCGCGCACGTTGTTCTTGGCTACCTCCGGGTTGAAGTGTGCCATGG
CATAGGCGTGCTTCCAGCTATCCCAGGCCCAGACGCCGTCGAACCAGCGTGCGGTATTGGAGGGGGTTAC
CCCGTCGTGGAGGATGGCGCCAGCCGGGGAGCGCCAGTTGCCGTTGAGGGTCTCCATCGCCTTGACGGCG
ATGCGACGCTCCGACTCGGGGATCCCCTGATTGGAGAGACCGTTGGCAAGGTAGCCCTCCCAGCGGCGGA
TGGAGTCGGCAAAATAGCTGGCCGGGTCGGCCAGCAGGGCCTGACGGCTCTGCTGATGGCTGCTGGCGTC
ATCTGCGGAGTGGAGATAGCTCTGCAGGGTGTAGATATCCTCGCTCCCCTTGGCGGCCAGAGTAATGCTG
GCGTCACTTTGATAGCCCAGTGTCTTCTGGTCGAGGGTGGTGCGGCTTGGCAGGGTGCGGTCGATGCGAT
AGCGGGCGCTGCCGCTCTGCATGATGTTCCAGGTGCTGCGCAGTTTGCCGAACTGGAATTCGATTCCTTT
GTCGCTCTCGCTGATGGTGCGAGTCCAGCCTGGATATTTGCTGGCAATCGTATCCTTGGCATCCCATTGA
TTGAGCAACTCGCCCTGCCAGTTGAGGTTGAGGGTGAGCGGTGCATCGGTCTGATTGATTAGTCGGGTGC
GGATAAGGGCGGTGCGGGCATCGCCATAACGCAGCTCCAGCTCCAGCGTAAAGGGCTCGAACTCGAAGCG
TTGCACCAGCGCACCGGGAATGGCGTACACCTCCTGCTTGCGGGCGCTGGTGAGCGGGAACGGGCGGCCG
CTTGCATCGCTCAGGGTGAGCTTGTCCAGCTCGCTGGCAAAGAAGAGGCTGTACTCCTCGGAGATGACCA
TGGGGCCGGTAAAGCCCCCCCACTCCTTGTCGCTGGCAGGCAGCAGGAAACCGTGCCATGCCCCCAGATC
GAGCAGCGGGTTGTATTTGAGGTTGGAGTAGCTGTCAAAGTCGCGGAATTGGGTCGGTGTTCCCCGACGG
TCGATAACGTCCTTGTACTGGATGGCCTGCTCCGGTTTGGCGACTGTGCTGCTCTCTGTCTGGTTATCGT
TGCAGGCGGTCAGCCCGAACAGGGCGGTGCCGAGGGCTACCGAGAGTAAAGTTTTACGAAACATTTCTCG
CTCCAATTCCGTTTATGTAGGTTAAAAGGAAGATTTTTTACGCTTTTTTTTAGTGAAAAGGTGTTCGGTA
GCCATTGTATGGCAAGGGGAAAGGGAGCGTTGGCGCGAGATCACACTCTGAAGAGGCGGCAATGTGAGCG
GGATCACCGAGTTACATCAGGTTACATAGAATGGTATTTAAGTAATATTTTACTTTTCGTTGCTCGCCAA
TCTGGCAGGGCAGGTGTGCAGGTATTGGTGGGGCGTCAGGCCGAACTCCTGTTTGAAGCGGGCGGCAAAG
CGACTGGGGGAGTCATAACCGCAGGCGAGGGCGACCTCCAGCGGGGTCAGGCCCTGTTGCAACAGGTTGA
GGGCGTGGCTCATGCGTACCTGAGCCAGCAACTGGCGAAAGCTGCGACCTTCGGCGGCCAGCTTGCGCAC
CATGGAGGCGCGGCTCATGGAGAAGTGGGGGGCCACCGCCTCCAGGGTGTGGTCGATTCCGGGTTCCACC
GCCAGGTAGCGTGCCAGCCGCTCTGCGAGGGTCATGGTGCTGGCGGGAAAAAGTAACCCGAGGGCGTTGG
CGGCCCGCAGCTCGGCATAGAAGCCGCGCAGCAGTTCGGCCTGGGTCGCTTCGCTCAGTCCCCGTTCGGC
CATGGTGCTGATGAGTTCAAAGCAGAAGGCGAGCCCGGGGGTGACCTTGACGGCAGGTTCCTCCAGATCG
GCGTGGGAAGGGGCTGACTCGGCCAGCCATTCTGGTGGCGGCGGAGTGAGCAGCGTGATGGCGTGGGAGC
GAAACTTGCCCTGCTCCGGCTGATTGACGAAGGTGAGCTGGTGGCCTGCCGGGATCAGCAGCCACCGCTC
CCTGTCAAACGACAGACGTCGCTCGCGCCACCACAGTTGCTTGCTCCCCTGGGTGACCCAGATGAGGGTC

GGGGCGTAGATGAGCACCCGGTGTAGGGGCTGCTGGCGGATCCCGAGATAGTGGCTCTGTTGCAGCAGGG
AAGTGCTCATCCGGTTGGCTCCTAGGGGGTGACGAAGGTGGCGGTCAGGGTGGCTTTGCCGAGCGCCATG
CTGTTGAGCATGTAGCCTACCATAGCCGGTGGGGTATCAGCCTTCACCTCGAGATCATGGGGCAGGGCCC
AGGCGGTGAACTGATAGCGGTGCATGCCGTGACCAGCGGGCGGGCAGGGGCCGCCGTAACCCTGGTTGCC
AAAGTCGGTGCGGATCTCCAATCCCTGCTTGAGCTTGCCGGAGGCATTGCTGGCCAGTTCGCTCTGGGTG
GCAGGCAGGTTAACCACCAGCCAGTGCCACCAACCGCTGCCGGTAGGGGCATCGGGGTCATAGGCGGTGA
TGGCAACACTCTTGGTGCCTGCCGGCAGATCCTGCCAGCTAAGTTGGGGCGAGTGGTTGTCGCCGCTGCA
GCCAAAGCCGTTGAAGCTGAACTGTTTGTCCATCAGCTGGCCTTCGTGAATATCTGTGCTCTTCAGGGTC
ATGGCAAAGGCTGGCATGGCCGTCAGGCAGAGCAGTGGCAACAGGGCACTCTGTTTGAGCAATTTCATCG
GGTGACTCCTCGTGTGGTAACGGGTTCACTTTAGCGAGAGCAGGGCAGAATAATATTGCAGAACGGCTCA
ATGTGAGCTCGAATGGTGAATTAATTGAGCCTTTTGGTGGTTTTATGGCAAGAAGGTAAACGGCAGCCAT
AAAAAAACCGGCATCAGGGATGCCGGTTTTTTGTTTGACGATGAGAAGTGCGGCTTAGCGCAGGATCATC
TCGTCACGGCCCGGGCCGACGGAAACCATCGGAACCGGTACGCCGAGCAGCTCTTCGATACGCAGCACGT
ACTGCTGGGCAGCGCGCGGCAGCTCTTCGAAAGTGCGGCAACCGGTGATGTCTTCGCTCCAGCTTTCCAT
CTGCTCGTAAACCGGCTTCAGGGCAGCGGTTTGCGGCCAAATTGGGTTCTCGGTGTGGGCACCTTCGTAG
GCCACGCAGATCTTCAGATCCGGCAGACCGGTCAGGCAGTCCAGCTTGGTCAGAGCCACTTCGGTGGCGG
CTTGCAGCTCGACACCGTTCTTGGTGGCGACGGCGTCGAAGTAACCGACATCACGGGGACGGCCAGTGGT
AGCGCCGAACTCATTGGTGATCTTGCGGAACTCGTCCTGGTTCTCCATGGCGGTCAGCAGGGTGCCGGTA
CCGACAGAGGAGCTGAATGCCTTGGCCACAGCGATGACGCGCTCCGGGCGCAGACCCGGCAGACCGCCAC
CGATACCGGCGTAGGCGCCGGATACGTGGGAGGAAGTGACCCAGGGGTATTCGCCGTAGATCAGGTCACG
GCCAGCGCCCAGCTGGGCTTCGAACAGCAGGGTCTTGCCTTCTGCCTGCAGCGCTTTGAGCGGCATGCTG
ACGTTGCAGACTGCGTCGATCCAGGGTGCAGAGACTTCCAGCAGCCAGTCGGCCATCTCTTGGGCAGTCT
GGGTGAATTCGAAAGAGGGATAGATAGCCTTCATCTGCGGCAGTTTCCAGTCCAGCATGAACTGGATGCG
CTCGACCAGCACGTCCGGCTGTTTCAGCCAGCCAACCAGGATCGCTTTTTTCATGACGCGATCGCCGTAG
GCAGGGGCGATGCCCTGACGGGTGGAGCCATAGGCGCCGTCACCCAGACGTTGCTCTTCCAGGGTATCTT
CCAGTGCGTGCAGCGGCAGGCACAGGGTTGCGCGGTCAGAGATGGCCAGCTTCGGAGTCACGCTGGAGGC
TTTGACTTCCTCGAGCTCGACGGTCAGTTTCTCGGGGCTGATCACCATGCCCGGGCCGAGGACAACCAGG
CAATCCGGGTTGAATACGCCACTCGGAACCTGGTGCAGCTTGAAGGTGCCAAGGTCGTTGACCACGGTGT
GGCCAGCGTTGTTGCCGCCCTGGAAGCGAATGCTGGCGCCAGCTTGACCTGCCAGATAATCCACGATGCG
GCCTTTGCCTTCATCACCCCAGTTAGCACCTACGACTACGATAGACGACATGACTCTTTCTCCTAAGACG
TTAGGCGTCCATCCTAGGAGCCTGATGCGGATTAGAAAAATTAATTATCTTTATAATTCTGATAAGAGAA
CCATATAATTATGGCCAATTAAGCGGCAAACCGTCCGATTTTGCCTGGCAAGCCCCGCACATTGCGGGTT
GAGGGACTCTTATGCTGGATATTCATTGGTTGCAAACCTTTGTCACTCTGGCCCGATTGCAACATTTTGG
CCAGACCGCCACCGAACTGCACATGACCCAACCCAATGTCAGCCTGCATATCAAGCGGCTGGAGCAGGCG
ACTCGGGTGCGGCTGATCGAGCGCAGCCCGTTTCGCCTGACTCAGGCGGGGGAGCGGCTGCTGCAAAGCG
CCGAGCGCACCATTGCCGAGTTGCAGCTCTGCCAGTCCGATCTCAATGCCCTCAACCAGCTGAGTCAGGG
GACGCTGGCCATCGCCGCCAGCGACATCATCTCCCGCCTGCTGCTGATCCAGCCGTTCCAGCGCTTCAAG
CGGGAGTTCCCCGGCATCGACCTTACTTTGTTCAACACCACTTCGGATCAGGCTGCCGAGCTGGTCAAGG
CGGGCAAGGCGGATCTGGGTTTTGTCATGGCGCAAAAGCGCAGCGAGCCGCTCTTCTATACCGAGCTGCA
ACAGGTGACCTGGTGCGCGCTGGGGCACGGCCTTGCCCGCTGGCAGGCGCGGGCGGTGGCGGGAGAGAGC
GATCCCCTCGACGAGCCGACCCTGATCCTGCTCGGTCACGACACCCGCACCCGGGCGCTGATCGACCCGT
CCCTGCCGTTGCTGGCCTTGCCCCCCTGCCGCATCATGGAGGTGGGCAGCGTGGACGCCCAGCTCGATTG
GGCCGAGGCGGGCTTTGGTGTCGCCATCGTGCCGGATTTCTCCCTGCATCCGCGCCTCAACCTCACCGCG
CCGGTCACCAGCCTCGGCGACTTTCCCGGTACCAGTCTGGGCTACGTGGTGCGTCAGAATCAGGTGTTAT
CGCGGGCCATCAAGCAGTTGCTGCACTGGGTTGAGGAGGAGATCCAGAAGCCCAACCCCTATCTGACCCG
GGTGATGGAGCCGTAGCGATACGGCAAGGTGAGCAGGTCAATCGACTTCGCCACTATCGAGCGCATATCA
ACAATCAGGGCAGCCGAGGCTGCCCTGATGCATTTGTGGGCGCTTACCGCTCAGGGTTGCGGCGGTGTGG
CTGCGCTGGTGGATGGGGAGGGCAGTGTATCGGTTTTATCCTGCTGCGAGGTTTCGCCAGTCACCGCGCT
GCTGGCAGGCTGGATTGCCTGCTCTTGCGGCGCAGTGGCCGCAGAGTCGCTTTGCGGCCACTGAGTCGAA
ACGGCAGCCGATGGTGCTGGCGCAGTTGACGCTGCGGCAGAAGGAGCCATGCTCTCACCCTCGGTGGTTG
GCGTAGCAGTGAGCTCGGAGGTGACCTCAAGTGCCTCTTCGGGCAAGGGATTAACCTCTGTGGCTTCTGT
TGCCACTGCGGCACTGCTGGTGGCGGTCGCGTCAGAGGGCGCAGTCGTCCCGTTGGCGGCACTCACCTGC
TCTGTCGTGGGCACCGCCTCCGAGGCCGATGGCGCTGGTGTTAGCGGCTCCGGCGTCAGCTGTTCCGCGG
TTAACTGCTCGGGGGTCAGTGGCAAGGGGGCTGGCGGTTGATCCGACATCCCAGCATGGAGGGGCAGCTC
GGTTTGCGCCTGCAACTGCTCGGGCGTCACCTTGCTCAGGGCAGGATGCAGCCGACTGAACTGTTTGACC
CGGGCCAGCAGGGTATCGCGCACGGTACGGACGATGGGGTCCGGCAGGCTGGCCAGCGCCACCAGTTCGG
CACTCATCCCGATATTGTTGATGGCGGTGGTCTGTCCCGGATAGAGGTTGGCGGCGATGCGGCTGCGCTG
GTAGTAGGGGTGTTGTTTGAGCAGCGTGTCGATTTGCTCCCCTTCGATGGGGATGAGGCGCACCTTGCAG
CGGCTGATCAGATCGCCGACTGCCAGACTGGGGTGACCGGCAACCACAAACGCGGCATCCAGCGAGCCAT
CGCACAGCCCTTCCAGCGGTTCGCTGTCCGATTTGAGCTGATAGGCAGCAAACTGCCCGGGTTGCCATCC
CATGGCCGCCATCAGGGCCTGGCTGGTGATCTGCTCACCGGCGCCCTTGTTGCCGGGATAGACCCGCTTC

CCTTCGATATCTGCCAGGGTGGTGATGCCGCTATTGGGGGCAGCCAGCAGGGTCAGCGACTCCTGATAGA
GGCGAAACAGGCTGCGCAGCTCATTGTTGGCCCCCTGCCCGCTGAAGGGGCCGGTGCCGTGCAGGGCGTG
GTGCAACACATCGGACTGCACCAGCGCCAGCTGTACGTCCCCCTTGTCGAGTGCCTTGAGGTTGGCCATC
GAGCCACGGGTGCTCTGCACGGTGCAGCTGTCCCCATGCAGGGCATGGCCGGCATTGAGCACCCGGCAGA
TGGCGCCACCGGTCGGGTAGTAGACCCCGTTGAGCGGGCCCGTTCCGATAGTCAGCGCCTGGCTGCCAAA
GGGGAGCAGCGCAGAGAGTGCACAGAGGGGGAGCAGAAGGTGATGCAAGATCCGTAGAGACATAATTGAC
CGTTGACTGAGTTGACCGCTGGCTGATGACACAGGGAAAAAGGGATGGGGCTTGCCCATCCCGTTGCTGG
CTGATCAGGGCTTGTTGTGGTTGAGGATTGCCCGCTCCATCCGCGCCAGCGCGTCGGTCAGCCGTGCCCT
GGTGCAGCCGAAGTTGAGCCGCACGAACTGACCGTCGCCAAACTGGGCGCCGGGGGAGAGACCAACGCCG
GCCTGCTCGAAGAAGGCGATGGGATCGGCAAGGCCAAGGGCGCTCACATCGATCCAGGCCAGATACGTCG
CCTGATGACTGGCCAGCTTGACGCCGGGCCAGCGGGTAACCGCTTGCTGGATCAGGGCCAGATTGGCTGT
CAGATAGTCGAGTTGCTCGGTCAGCCACTGTTCGCCCCCCTCATAGGCCGCTTCGGCGGCGACAAAGCCC
AGCAGGTTGACGTCGGCGCTGATGCCGCGCATCGCCTGCTGTAGCCTGAAGCGCAGACGGGGGTTGGGGA
TAATGGCGAAGGAGCAGCAGAGACCGGCGATATTGAAAGTCTTGCTCGGGGCCATCATCACCGCGCAGCG
CTCGGCTGCTTGTGCACTGATGGCGCCGTAGGGAATGTGGCGGGCGTTTTTATCCAGCAGCAGATCGCAG
TGGATTTCATCGGAGCAGATCACCAGATTGTGGCGCGCGGCGATGGCATCGATGGCCAGCAGCTCGTCAC
GGGTGAAGATGGTGCCGCCCGGGTTGTGGGGGTTGCAGAGCAGCAGCAGATCTGCGGCGGGGGCCTGACG
CTCCAGCTCTGCCAGATCCAGCACCCAGCGACCCGCCTCTTCAACCATCGGCACGGTGAGCAGGGGACGA
TCATTGAAGCCCGGCGCCTGCAGGAAGGGGTAGTAGACCGGCTTGGGGGCGATGATACCGCGGCCATGCT
GGCTCCACGCCTTGCAGGCAAGGTTGAGCCCCGGCACCACGCCGGGCAGGAACACCAGCCATTCGGGCTC
GATGGCCCAGCCATAGCGGGTCGCCATCCGTTCGATAATAAGTTCGATCAGGCGCGGCGAGGGGCGTGAA
TAGCCGAAAATGCCGTGGGCGACCCGTTTGGTCAACGCCTCTATCACGGACGGGGGGGAGACAAAGTCGG
TATCGGCAACCCACATGGGCAGCACATCCTGACCCTTGTACTTGTCCCATTTCAGGCTCATGGTGCCACT
GCGGTCTACTTCCTGATCAAAATCAAACATGCGAACTCCTTGTGCTGGCAGGATGGGCTTTCACTCTACG
GGGTCGCGGCCTCCCTCGCAAGGCGCACACCCGCTTATGCCATCCTTGAGCCCGCTCTTGATAGTGACAT
AAGATAGGCGCCGAATTTTCATACGGCTGCCGCCCGTTAGCCCGTATCGTCCCTCCAATCAGTGGTAACC
CTATGAGCTTGTCGATAGCGGCGGTCAAACGCCCTGACCCCGTCTCTCTTTCATTTCTTGCCGTGACCTT
TATGACCGGCGTCGCAGTGGCACTGCAGGTGCCTACCTTAAGTCTGTTTCTGGCCGAGGAGGTGCAGGTG
CGCCCCTTTCTGGTCGGGCTCTTCTATACCATCAATGCGGTGGTGGGCATTCTGGTCAGCCAATGGCTGG
GCCACCGCTCCGACAACAAGGGGGATCGCAAGCGACTGATCCTGCGCTGCTGCTTTGCCGGGGTAGCGCT
CTCCCTGCTGTTTGCCTGGAACCGCAACTACTGGCTGCTGGCGAGTCTGGGGGTGATGCTGGCGGCGCTG
GCGGCCACTGCCAGCCCCCAGTTGTTTGCTCTGGCGCGGGAGTATTCTGACAGCCGCAACCGGCGGGCGG
ACATGTTCAGCTCGGTGATGCGGGCTCAGTTTTCGCTGGCCTGGGTCATAGGTCCGCCCATCGCCTTCGC
GCTGGCCATCGGCTACGGTTTCGAGCTGATGTACCTGGCCTCGGCACTGGCCTATCTGCTCTGTGCGCTG
GTGGTGTGGCGCTGGCTCCCCTCTCTGCCGCTGCCCAAGCTGGCTGAAGAGAGCGAGCGGGTCAGCAGCT
GGCGGGATCCGGCGGTGCGGGCGCTCTTTATCGCCTCCACCCTGATGTGGACCTGCAACAGCATGTACCT
CATCAATATGCCGCTCTACATCACCCGGGAGCTCGGGATGGATGAGAAGCTTGCCGGCATCCTGATGGGC
ACGGCGGCGGCGCTTGAGATCCCCTTCATGCTGCTGGCGGGTTACTACACGGCGCGCTTTGGCAAGCGCC
CCATGATGTTGCTGGCGGTGCTGGCTGGCGCGGCCTTTTACACAGGTCTGGTGACCCTCACCGACCAATA
CGCCCTGATCGCCTTGCAGCTGTTCAACGCCATCTTCATCGGTATCGTGGCGGGGATCGGCATGTCCTAC
TTTCAGGATCTGATGCCGGGGCGAGCCGGGGTGGCGACTACCCTGTTTGCCAACAGCATCCGCACCGGCT
CCATCATGGCTGGGGCCATCGCTGGTGTGGTGGCGGAGGTGTGGAGTTTCCACGGGGTCTTTATGGTGGC
GTTGGGATTGGCACTGGTAGCACTGGCCGCCAGCTGGCGGGTGCCCAACGTCTGACTGGGGACTGGCTGT
TATTTGTCGTGCTGGGGCTGGGCGTGCGGGAGCGGCGCTGATTCGCCCTGATATTCGTTGCGCAGGCAGC
GCAGCAGCAGCTCGGTCGCGGGCCCGAGAGATTCGGGGGAGAGGGTCAGCAGATAGGTGTAGAGGTGGCG
CTCGTTGCCGCTTTCTAGATCCAGCTGTATGAGTGACCCTTCCGCCAGCTCCCGCTCTACCAGATGGCGC
GGCAGCCAGGCATAGCCGAGGCCGAGGCGCAGCATGGCGAGCACTTCGTGGTAGTGGGTCGCCTTCCAGC
CGGTGTCATCCGCATCGTTCCAGTCCACCGAGCGGTGAATGGGGGGATGCAGGCTGATCTGGTTGTGACT
GGTCAGCACATCGCTGCGGATGGCCCCCTCCTCTTGCGCCAGAGGATGGCTGGCGGCGCAGAGGGGCAGC
AGGCACACCTCGTCCAGCAGCAGACCGGTGCGATCGCCGGGCAGGTGCTCGGAGAGCATCAGATCCCCCG
GTTGCAAGGCGTTGAAGTGGCAGTGAATGCGTTCGTGCAGGTTGACCCGGCAACCCCGGCTGCGGGGATA
GAAGCTGGCCAGCGCCCGATAGAGGCGATCCCTTGGTTGCAGCGCCGAGACATAGAGGTTGATCTGCGGC
TCCCATTGCCGCTCCAGGTTGTTGGCCAGATTTTCCAGCTCCTCCACCTGTTGGCTCAACTGACGGGCCC
GCTTGAGGAAGATCTCGCCCGCCGGCGTCAGCACCGCCTTGCGGCCGCGCACCTCCAACAAGGGCACCCC
CAGACTCTGCTGCAACTTTGCAACCGCATGATTGAGGGAGGACTGGCTCTTGTTCAGCGCCTCGGCCGCC
TGGGCGTAGCCGCCGCAGTCGACCACCGCCTGAAAGATCCGCCATTGCTCAAACGTGCTCTTGGGACGAA
ACATAGTTCAACCTGATTGGAGATAGGATGGTCAGTGGTTCGAACAGGGAGCCTGTGCCCCCTATTCAAT
TTAACCCCTGCCGCACAACACTCTGGGTATTTTATTGATTTATTTGGGCTGGGCCACGCAACTTGTTTAT
TTTGGCTGAGCCACAAAGGTCTGGCCGGTTTTGCCACGGCTATCCGACCCCATCAGGTAGAGGTAGAGCG
GCATCAGATCGGCTGGAGTCTTGAGGTTCATGGGATCTTCTGCCGGGAAGGCGCTGGCGCGCATCTTGGT
ACGGGTACCGCCCGGGTTTAGGGTATTGACCCGCACACCGGTGTTCTCAAGCTCGTCGGCCAGCACTTCC

ATCATCCCCTCGATGGCAAATTTGGAGATGGCATAGGTGCCCCAGTAGGCGCGCCCCTTGCGTCCCACGC
TGGAGGAGGTGTAGACGATGGAGGCATCGCCCTGCGCCTTGGCTGTCTGGCGCAGCAGCGGCAGCAGCGC
CTGGGTCAGCAGGAATTCTGATTTGACGTTGACCTGCATCACCTCGTCCCACTCCTTCTCCTGAATATGT
TCAAACGGAGAGAGGGTTCCAAGCAGACCGGCGTTGAACAGCACGCCGTCGAGGCGGCCAAACTGCTGGG
CGAAGGTTTCGGCCATGCCGCGGTAGTGGGCGGCGGTGGCCCCTTTCAGATCCACCGGCACGATGGCGGG
CAGCGGATAACCGGCCGCTTCAATCTGGTCATACACGGCTTCGAGTTTGGCGCTGGTGCGGCCGAGCAAG
ATCACGGTGGCACCGTGAGCGGCGTAGGTGAGGGCGGCTTCGCGGCCGATCCCGTCGCCGGCACCTGTGA
CCAGAATGACTTTATCCTTGAGCAGATCGCGGGGTGCCTGATAATCGAGCATGACAACTCCTGTTAACAA
ATTCCGGCCATAAGTGCATGGCAATCGGCGCCAAGTGTACTGATCCGCGCGCCTGTTTGACCAGCCAGCG
GCAAGAAAAATGTGAAGAAAATAGAGAGGTTGTGATGCTCGGGTTGGTTTTTCCGGGCTCTGGCTGCGAT
TTTTTGCGCGCATTCACCAAACTCCCTAGAATGTGACGAAAACCGATCCATTCTGGAGTCCATCGTGACA
TTTCTGACCGAATATGGCCTGTTTCTGGCCAAGACAGTGACCTTCCTGCTGGCCATCGGCGCCATCATCC
TGATGATTGTCAGCACTCGCCAGCCGAAAGCGCGCAAGGGGGAGCTAGTGGTGACCGATCTCTCGAGCGA
GCTGGAGCAGGGGCAATTTGCGCTCAAGAGCGCGCTCGCCAGCAAGACCGAGCGCAAGGTGCTGGAAAAG
CAGCAGAAAGAGGAGCAGAAAAAGCGCGCCAAGGCGGGGGATGAGCGCAGTCGCCTGTTTGTGCTCGATT
TCAACGGCAGCATGGATGCCAAAGAGGTGGCCTCTCTGCGCGAAGAGGTGAGCGCGGTGATTGGCGTCGC
CCAGCCCGGCGACGAGGTACTGCTGCGGCTCGAATCCGGTGGCGGCGTGGTGCATGGCTACGGCTTGGCC
GCTTCTCAGTTGCAGCGGTTGCGCGATCGTGACATCAAGCTCACCGTGGCGATTGACAAGGTGGCGGCGA
GTGGTGGCTACATGATGGCCTGTGTGGCAGACAAGATCCTGGCTGCCCCCTTTGCCATCGTCGGCTCCAT
CGGGGTGATCGCCCAGATGCCGAACTTCAACAAGTTGTTGAAAAAGCATGACATCGAGTTCGAGATGCAC
ACCGCCGGGCAGTACAAGCGCACTATCACCATGTTCGGCGAGAACGACGATCTGGGGCGCGAGAAGTTTC
GCGAGGAGCTGGCGGCGATCCACGAGCGCTTCAAGGCCTTTGTGGCCGAACACCGCCCGCAACTTGATAT
CGATCAGGTCACGACCGGTGAACACTGGCTGGCGAGTCAGGCCAAGGGGCTGGGGCTGGTGGATACCCTC
TGCACCAGTGATGACTATCTGCTGGCGCAGGCCAGCCACCACAAGGTGATCGGCATCAGCTATCGCAAGC
CAAAAAGCCTGACCCAGAAGCTGGGGCAGCAGGGCGCGCAGGCGCTCGAGGCAGGACTTGGGCGGCTGTG
GCAGCAGAGCCCCTGGCGCTAAGCCTGGCCCTCTGGCGTTAGCCAGAGGGAAATCCCGCGATAAAAAGGC
CAGTCGATGCCCATCGGCTGGCCTTTTTGCATCCGGTTGCACCTTGTCGGTGAGCGAGGGTTGTTTCTAT
TGAGACCCTCTGTTACAACTGAATGCTTTCCCCGTTTATGACAGGATCTTGTGGTAAGAGCTGCGGGGCA
TGGAGCAAAGCGATGTCATGGAGCACCTTCAGGCAATTTACCCCGCTGGTCTGGATCGTCATTCTGGGCA
CCTTTATGGTGCGTACCAGCTACTACATGGTGTGGCCCTTCCTCTCCATTCTGCTCTATCGGGAGTATGA
CCTTTCGGCCACCTTCATCGGCCTGTTGCTGGGGGGCTCAATGGCCCTCTCTACCCTGGTCAGCTTTTAC
GGCGGCTGGCTCTCTGATCGCTTCGGGCGGCGCAGCATACTGCTGGCCGGTTGTCTGGTGGCGATGATCT
GTTTCGGCCTGCTCGGTTTTTCGCACGAGGTGTTCTGGCTCGGAGTCGGGGTGATGGGCAGCGGCCTCTC
GTCGGGGCTCATCGATGCCCCTGGCAAGGCGCTGATGGCCGACAGTCTGGCCTCGGCCAAAGCGCGGGAG
CTGGCACTGCACCTGCGCTATTTTCTGCTCAACCTGGGGGCCGCGCTGGGGCCGCTGCTCGGGGTGACCC
TCGGGATCAACGCCCAGCAGGAGACCTTCCTGTTTCTGGTGGTGAGCTATCTGCTGCTGGCGCTGGCCTT
TGGCTGGGGATTTCGCCGGGCTGGCAGCACAGGCAAGCCGGGGACTGCCGGGCCCGGACTGCGGGATGCC
ATCAAGGTGTTGCTGGCGGATCGCGCCTTCCTGCTGCTGGTGATGGCCAATCTCCTGATGGTGCTGGTCT
ACTCCCAGTTTCACTCGCCGCTGGTGCAGTACCTGACCCGGGCTGGCATGCCGCAGGTGGCCGAGCTGAT
TGCCCTGCTGGTGGCGACCAATGCCCTCACCGTGGTGCTGCTGCAATTTCCGCTGCTGCGATTGTTGTCG
CGCTGGCCGGTGCGGGTACGGCTTCATATCGGTATGGCGCTCTTTCTGGCGGCCCAGCTGCTCTTCGCGT
TGGGGGACCCGATGGTGCGCAGCCACTGGTTTGGCGCCGTGCTGCTGCTCAGCATCGGCGAGACCATTCT
GTTCCCCCTGCTCAACGTGCTGATCGACCAGATGGCCCCCGCTCATCTCAAGGGGAGCTATTTTGGTGCC
AGCGCGCTGGCCGGGTTGGGAGGGGCCGCAGGTGCCCTGCTGGGAGGCTGGATCCTCGAGCAGTGGAGCG
GTCAGTTGCTCTATGTGCTGATGGCGCTGCTTTGTGTGGCGATTTTTGCGCTTTACGAGTGGGGCGCCAG
AGTACAGCGTCCCGCTGTGGCCACGTCTGTGGTTGAGCGGAGCTGACCCGGTTAATGCGGGGATCTGTGA
TGCTGAATGTTCAGTGCGTGCTACCCGGCGTCTTCTCTTTGAGCGCCTTCTTGGCTGCGTACATCCTCTC
GTCGGCGAGCCGGATCAGGTTTAGCCCGTCGTCGTAGTGCTCCGGCCCGGTCTGCACTATGCCGATACTG
AGGGTGAGATCGCCCAGCTGTTCCTTGACGGTGTTGACCAATCGTTTGAGATAGATATCCCGCGCCTGAC
TCTCGTTGCAGTTGGGCAGGATCAGGCAGAACTCATCTCCGCCATAACGAAAGCAGCTGTCTTCGGCGCG
AAAGACCTGACGGATGCAGTTGCCGACGATGCGCAGGATCTCGTCTCCGCGCTGGTGACCTTCGGTATCG
TTGATTTGCTTGAAGTTGTCGATATCGAGATAGGCGATGGCCATGGGTTCGGCACGGCGCTGACAGGTGC
GCAGGCTCAGCAGCAACAGCTCCTTGAGGTGGCGAACGTTGAGCAATCCGGTTAGAGCATCGGTGCGGTT
CAGCTCCTCCAGTTGCTTGGTACGCTCGCGTACTTTCTGCTCCAGACTCTTGGCATAGTTCTCCGACTTC
TCTCTGGAGGACTCTATTTCCGACACCAGACTGCGAATGTAGGTTTCGAAGACCAGAGTGATGTCGAACA
GAAAGAGTTTGTCCAGGGCGGCCAGCATGACGAGGCGCTCCGGTTCATCGCGGATCTGCTCGAACATATC
CTCGATCAGCAGAGCTTTGAGGGTGTTGATGGCGGCCAGATAGAGCTTGGGTTCGACCCCGATCCGCTTG
TGAACCAGCCCGATCCGAAGCCGGTTGTTGACGTACTCCAGATCGTAATGGCCGCTGAAGAGATCGAGCA
CATAGCGCCGTTGTGCGGTGCGCAACCGTATGAGGGTATCGGCATCCCCGATCAACAGGGCGATTTCGGT
GATGGCCGTCTGCTGCTGGTAGAAGCGATCCACCAGGGCGTCGATCCGTGGTTCTATCTTGCTGCAATAA
CTTGCCAGCCGCGCCACATCCTGTTCATTGAGCAGCAATAACTCCTTGCGATGGGCAATTTCAAAGTCGG

TGATCCTCATCTGCTCAAGCAGGGTCTGTTCGGTCGGATTCATCGTCGTCCTCGGTTTATTACTGGGCGT
TACGCTGTCCGTGAGGTGGGACAAACAAGCAGGTGTGGCAATTGATAGTGTAGTCAGTGAGGCTGGGGCG
CGCGCAATGCCCCCGCGTGGCGGGGGCTATGGAGGGTCAGGTGAAGGCTAAAGGGGCAAGAGATCCGGAG
GGGAGATCAGAAGGTCAGCACCTTGTCGGCAGCCAGGGTCCACTGGGCCAGCATCGGCAGGGTGCCGACC
TCAATGCCCTCGATCAGCGGCAGGGCGGTAATGCCACGGGCATCGGTGCAGGTCTTGCACAGGCGAATGG
TGACGCCTTGCGCCAGCAGGATCTCCAGCATCTGGCGCAGGTTGTATCCCTCGGCCGGTGTCTGACCGGC
CAGTGCTGCACTTACCGCATCGGACATCAGGAACAGCTTGAGGCTGGCACCACCCTGTTCGTTGAGGGCG
ATAGCGAGGCGTAGGGCGTTGAACAGGGATTCACTGCCGTAGGGGGCACCGTTGGCAATGACCACAATTT
GTTGATTCATAACAGACTCCCGATTGGAAAGAGCGCCATTGTAGAACCAACAGGGTGCCAATTGCGTGAT
GTTGCACAACTTCTGTCGCACCTTTTTCCCGCTTGCTTGACCCTGCTGCCGATCGAGGTTTTTATGGTCA
GCTCGCAAACAAGACCGGATGAATCGAGATGTGGAAAGTATTGGGAGCCGGCTTGCTGCTATTGGCGGGT
CAGGCCTTTGGTAGTGAGGCGGTCGGTTGCAAGGCCCGTCTGCAGGCCGTCAACGACCAGCTGGTGTTTG
CCAAAGCACACAACAATGCCGGGCAGATTGCCGGGCTGGAACAGGCAGCGCGCAATATCAAGGCCAACTG
TACTGATGAAGGTCTGCTCAAGGAGCAGAAAGAGCGGGTTAACAAACTGCGCGGCGAGGTGGAGGAGCGC
CTGCTCGAGCTGCAGGAGGCGAGAGTCAGTGGCAAACCGGAGAAGATTGCCAAGCGGCAGGCCAGGCTGG
AGGAGTCCCAGACCCGGATGCTGGAGGCCCAGCGTGAGCTGGACAAACTGATCCGGCTGGTCGGCAAGTG
ATGGCCAGCCGCTGCACGCAGGATCAAAAAAGGCCGATTGCTCGGCCTTTTTTTCGTTTTACGGTCTGTG
CATCAGCTACTGCCTGTGCATCCGCACAAATTTTTCCATCAACTGATCTTCGGTCTCCACATGAGCGGGA
TCCCAGATGATGCAGTCGATAGGGCAGACACTGATGCAGGTCGGCTTCTCATAGTGACCGACGCACTCGG
TGCAGCGACTGGGGTCGATCTCGTAGATCTCCTCACCCAGGCTGATGGCCTGGTTGGGGCACTCGGGATC
GCACATGTCACAGTTGATGCACTTGTCGGTGATGAGCAGCGCCATCTCAGGCCGCCGTGTGCGGGTTGCG
GGTATCCTGGCTGTCGCCCAGATTGCGCAGCAAAATGCCGTAGTTCACATCCAGATCTTTCGGGACTGGC
ACATAGACCACGTGACCGTTGCCCGGCGCGGTATCGATAAGCTCGCCCTTGCGGTTTTCCATCTGCTCCA
GATTGAAACGGACGTTGCCCTGCGGGGTCATCAGCTCCAGATTGTTGCCCACCAGGAACTTGTTCTTCAC
CTCGACCTCGACCATGTCACCTTTGCGACCGGTGATCTCGCCGACAAACTGTTGGGTGTCGGAGACGGAG
TAGCCGTAGTCGTAGTTCTGGTATTCGCTGTGGTTGTGACGGCGCAGGAAGCCCTCGGTGTAGCCGCGGT
GGGCCAGATTTTCGAGGGTGCCCATCAGACTGCGGTCGAACGGACGACCCGCCACCGCATCGTCGATGGC
CTTGCGGTAAACCTGCGCGGTACGGGCGCAGTAATAGAAAGACTTGGTGCGTCCTTCGATTTTCAGGGAG
TGAACGCCCATCTTGGTCAGTCGCTCGACGTGCTCGACGGCGCGCAGATCCTTGGAGTTCATGATGTAGG
TGCCGTGCTCGTCCTCGAACGCGCTCATGTACTCGCCCGGGCGGTTGGACTCTTCCAGCAGAACCAGCGC
ATCGGTCGGTTTGCCAAGGCCCAGGGTGTTGTCCGGACGCACTGCGATGGGCTCCTGACGGTGGACGATC
TGGCCCACGTCATCTTCTTTACCCTCGTGCACCTTGTACTCCCAGCGGCAGGAGTTGGTGCAGGTGCCCT
GGTTGGGGTCACGCTTGTTGATGTAGCCGGAGAGCAGACAGCGGCCGGAGTAGGCCATGCACAGCGCCCC
GTGAACGAACACTTCCAGCTCCATCTCCGGTACCTGCATGCGGATCTCTTCGATCTCGTCCAGTGACAGC
TCACGGGAGAGGATGGCGCGAGTCAGGCCCATCTGATGCCAGAACTTCACCGTGGCCCAGTTGACTGCGT
TGGCCTGCACCGACAGATGAATGGGCATATCAGGGAAGGCTTCACGCATCATCATGATAAGACCGGGGTC
GGACATGATCAGCGCATCGGGCTTCATCTCCACCACCGGACGCATGTCGCGGATAAAGGTCTTGAGCTTG
GAGTTGTGGGGCTGGATGTTGGCCACCACATAGAACTGCTTGCCCAGTGCATGGGCTTCGTTGATGCCCA
GTTGCAGGTTTTCGTGATCGAATTCGTTGTTGCGTACCCGCAGGCTGTAGCGGGGCTGACCGGCATAGAC
GGCATCGGCGCCATAGGCGTAGGCGTAACGCATGTTCTTGAGGGAACCTGCGGGGGAGAGCAATTCTGGT
TTAAACATGGCGGACTCTTTATATCTGATTGCAAATCAGGACGATGCCACCTGATGGCATCGTAGTGGTT
GAGTTGCGGTGCAAGAAGCTCTCAGAGAAGAGGCTCAATGCAACGCGACCCGATGACGGCATATCCGGGC
CTATCTACAGAAGATGCGCCGGATATTCGGGGTGCGAGATTGTACTCGCCCCCACGTCATCATGCAATTT
GGTAGGAATATCATGCGAATGGAAAATCAGTTTTTGGGTAATCTGTGGTGGTGGGTTAGCGATAGTTGTT
GATGTTCAATTATCCATTGCGAGTTGAAAATCGGGAAACTGTTCGCTCATAAAATTACGGGGGCTGTGGC
CATTGCCTGCGCTGGCCAGCCATTCCCAATCCAGTCTTGGCGAGCCCGCCTTGAAGCGTTGATAGGCAAC
GCCCTGATGAATGGTCAGAAAGAGGGTCGTGGGGAGTCCCAGCTTGTTGCTGTAGCGCACCATCAGATCA
CCCTGATAGTCGTTGCTGATTCTGACTTTGAGCGCCTGCAGTGTCAGACCGGTGGCGTGACCATGCTCGT
CCCAGACTTCAAACATGATGTGAACACTCTGATGTCGATGATGCCACGATGAGCAAGATGGGATGCAATT
GCAACTGTCAGATCTGACAAGGTCGGAAAAATGTGATGAGAATGGCAGAAAGCGAAAACCCGGCGCGAGG
CCGGGTTTTCTTGAATGATGGTGGAGGGAGAAGGATTCGAACCTTCGAAGGCAGAGCCGTCAGATTTACA
GTCTGATCCCTTTGGCCGCTCGGGAACCCCTCCACGGGGGCAAAGCAAATTGTATTCAGCCACTGCAATC
AGGGAAATGGTGGAGGGAGAAGGATTCGAACCTTCGAAGGCAGAGCCGTCAGATTTACAGTCTGATCCCT
TTGGCCGCTCGGGAACCCCTCCACGGGGTATTTCCTGATTGTCACTACTTTTGCACTTGAATGGTGGAGG
GAGAAGGATTCGAACCTTCGAAGGCAGAGCCGTCAGATTTACAGTCTGATCCCTTTGGCCGCTCGGGAAC
CCCTCCCCAAGTGCGAGGCGCATACTATCAGATCCAGAGGCTTTGTGAACCCCTTGATGAAGCATCTGGC
AGTAAAAAATGACTGTTCGTCGATTTTTTGATACATCCAGCTCAAAATTCAAGCTTATCAGCAAGTTGTG
CGCTGCTTTTCTGCACAAAACAGCATCCGTGCAAGTTGGCGATCGCGCTGAAGAGAAGGGAAATAGCAGA
AGACCGCCAGCGTCGGCGGTCTTCTCGGGCATCACATCTAGCGCTGGAACGGGTAGACCGAACCCAGTTT
CAGGATCTGGGTCAGCTCGTCCAGTGCGCTGCGGGACTCCTGCACCAGTGCCGGATCCCGCAGGTCATCG
AGGGTCAGGCGGTCGTGGTAGTGACGATCGACCCAGCCATCGAGGCGGGCAAACTGGCTGTCGTTGATAA

GGCAGGCCGGATTGACCGCAGCGAGTTCCTGATCGTTCATGGCAACCCGCAGTCGCAGGCAGGCCGGGCC
GCCACCATTACGCATGCTCTGTTTCACATCCATATAGTGGACCGCCTTGATCGGAGTCTCCTGCGTCAGC
AGCTTGCCAAGATAGGCCGACACCGCCGGATTATCGCGGCACTCGGTGGGGGCGATAATGGCCATCTCGC
CACTCGGCAGGGTGAGGATCTGGGTGTTGAACAGGTAGGATTTCACCGCATCCAGCACGGATACCTCGGC
GGTTGGCACTTCGATAAAGTGCAAATTCCCCTCGCCAAACTTGCCGCTGATCTCGGTCAGTACCGCTTCG
GTGTTGAGGAACGCTTGCTGGTGGAAGAAGAGCACGTTCTGGTTGCCCACCGCGATCACGTCGTTATGGA
ATACCCCCTGATCGATCACCTCCGGGTTTTGCTGAATAAACACCACGCTCTCCTCGCCAAGGCCATGCAG
GCGGGCGATGGCCTGGCTTGCTTCCAGAGTCTGGCGGGCCGGGTAGCGCTTCGGCGCCGGGCGGGAGTAG
TCAAAGGCGCTGCGGCCATAGACAAACAGCTCGACCCCCGCCTCACCATAGGCGCGACAGAGGCGGGTAT
GGTTGGCCGCCCCCTCGTCGCCGAAGTGCTCATTCTCCGGCAGATGGAGGTGGTGGCTGAAGTGGCGATC
ATTGTTGAACACGGCACGCAGGATACGGCCCGTGACGTCGGGCTCCAGGGAGCGGTGAAACTTGTTGGTC
AGGTTGGCGGGGGTGAAGTGAATGCGGCCATCCTGGGTATCGGCACTGGGAGAGACGGTGGCGGCGTTGG
CGGTCCACATGCTGGAGGCCGAATAGCAGGCAGCCAGTACGGCAGGAGCCTGCTTGGCGGCCTGCTCCAG
CACCCGGGCATCGTTGCCGGTGAAGCCGAGGCGGCGCAGGGTGGCGAGATCGGGACGCTCTTGTGGCGCC
AGTACCCCCTGGGTCATGCCAAGCTCGGTCAGCGCCTTCATCTTGCGCAGCCCCTGTTTGGCGGCCTCTT
TCGGATTGGAAGCCTCCTGGGCATTGCTCTGGGAGGCAACGTTGCCGTAGGAGAGGCCGGCATAGTTATG
AGTCGGGCCGACCAGACCGTCGAAATTGACCTCGAAGTGCTTCATTGAGTACATCCTTTCGATTTTTATG
ATGGGTAGCGGTTCCTTCCGCGCGCAATGCGTCGGGGTTGGGCCATTATAAAGAGATTTTATGTTTTTGT
AACAAGCCGGTAACCAGGAGACGTTTTTATGTCGTTTCAGATATAAAAAGAGGGGCGGTATGGCAGAAAA
TTGAAAAAGCGGTTGCGTCCGGCCTGTTGCCTTCGCATTAATAAATAAAGAAGCAATAAAAGAAGCCGTG
TGGTTCGGGCTGGTCAGGCAAATTTGCTCACAAGATTGCTTTTTCGCGCCAAATCTCTTATACAGCCCCA
CAAAACCGCACACCAACCCCGTCGGGTGCAATGAGGATAATAGTTACAGCATGGGCAAATCACTGGTCAT
AGTGGAGTCTCCGGCCAAGGCCAAGACGATCAACAAATATCTGGGCAAGGACTACGTGGTGAAATCCAGC
GTTGGTCATGTTCGCGATCTGCCCACCAGCGGCAGCGCCAGCACCGAGCCCAAGAAACCTGTCATTCGCG
GCATCAAGTTGAGTGAATCCGAGAAGGCTGCCAAGGATCGCAAGGCCCTGTTTGCCCGGATGGGTATCAA
TCCGACCGCTGGCTGGCAGGCTAACTACCAGATTTTGCCCGGCAAGGAGAAGGTGGTCAGCGAGTTGCAA
AGTCTGGCCGAGAAAGCCGACACCATCTATCTCGCAACCGACTTGGATAGAGAAGGAGAGGCGATCGCCT
GGCACCTGCGCGAGATCATCGGTGGCGACGACAGCCGCTACAAGCGCGTGGTGTTCAACGAGATCACCAA
GAGCGCCATCAAGGAGGCGTTTGCCCAGCCGTCCGAACTCAATATTGACCGCGTCAATGCCCAGCAGGCC
CGTCGTTTCCTCGACCGCGTGGTGGGTTACATGGTCTCCCCGCTGCTGTGGAAAAAGCTGGCTCGCGGTC
TCTCCGCCGGTCGGGTACAGTCCGTTGCGGTGCGTCTCATCGTCGATAAAGAGCGCGAAATCAAGGCGTT
CGTTCCGGTCGAGTTCTGGGATCTCAATGCCGACCTGCTGACCGCCGACAAGAGCGATCTGCGGATGGAA
GTGGTCAGCCGCAATGGGGCTGACTTCAAGCCGGTGACCCAGGCCGAGACCTTTGCCGCTGTCGCAGCCC
TGGAAGGGGTCGACTACAAGGTGGTGAACCGGGAAGACAAGCCGACCGGCTCCAAACCGTCCGCGCCGTT
TATCACATCTACCCTGCAGCAGGCGGCCTCTACCCGTCTGAGCTTCGGGGTCAAGAAGACCATGATGATG
GCCCAGCGTCTCTATGAAGCGGGTTACATCACCTATATGCGTACCGACTCCACCAACCTCAGCAAAGAGG
CGGTTGAGGCGGTGCGTGAATATATCGGCGAGCAGTATGGCGCGGCCTATCTGCCAGCCGAGCCGAATCT
GTATGGCGCCAAGGCCAATGCCCAGGAAGCGCACGAAGCGATCCGTCCCTCCAACGTACAGGTGACAGCC
GAGATGCTGGAAGGGATGGAGCCGGATGCCATGCGTCTGTACGATCTCATCTGGCGTCAGTTCGTTGCCT
GCCAGATGACCCCGGCTCAGTACGACTCCTCCACCCTGACTGTCAAGGCGGGTGAGTTCGAGCTCAAGGC
CCGTGGCCGTACCCTGCGCTTCGCCGGTTGGACCAAGGCGCTGCCGCCGATGGGTCGCAAGGGTGAAGAT
AGTCAGCTGCCGGCGGTGACCGTGGGCGAGGTGCTGAAGCTGATCAAGCTGGATCCGCGCCAGCACTTCA
CCAAGCCGCCTGCCCGTTTCACCGAAGCTGCGCTGGTGCGCGAGCTGGAAAAACGCGGCATCGGCCGTCC
GTCTACCTACGCCTCCATCATATCCACCATCGTCGATCGCGGTTATGTGCGGGTCGAGAGCCGCCGTTTC
TTCGCCGAGAAGATGGGTGAGATCGTCACCGATCGTCTGGTGGAGAACTTCGTTGAGCTGATGAACTACG
AGTTCACCGCCAAGATGGAAGACAAGCTCGATGGCATCGCCGAAGGGAGCCTTGAGTGGAAGAAGGTGCT
GGATGCCTTCTACGCCGAGTTTACCCAGGAGCTGGAGAAGGCCGACAAGGCGCCCGAAGAGGGCGGCATG
CGTGCCAACCAGATGGTGATGACCGACATCGACTGCCCGGATTGCGGCCGCAAGATGGGCATTCGTACTG
CCACCACCGGTGTTTTCCTTGGCTGCTCCGGCTATGCCCTGCCGCCGAAAGAGCGCTGCAAGAAGACCAT
CAACCTGGTCTCTGCCGACGAGTTTGTCTCCGCCACTGACGGCGAAGAGGCGGAAACCGAAGCGCTGCGC
GCCAAGCACCGCTGCGGTGTCTGTGGTACAGCGATGGATGCCTACATCATCGACGACAGCCGCAAGCTGC
ACGTCTGTGGTCGCAACCCGGATTGCGATGGCTACGAGATCGAGAAGGGCCAGTTCAAGCTCAAGGGCTA
CGAAGGGCCGAGCATCGAGTGCGACCGTTGCGGCAGCGAGATGCAGCTCAAGAACGGCCGTTTCGGCAAG
TACATGGGCTGCACCAACGAGACCTGCAAGAACACCCGCAAGATCCTGAAAAACGGCGACATCGCGCCGC
CGAAGGAGGATCCGGTGCCGCTGCCGGAGCTCAAGTGCACCCAGTCCGATGCACACTTCGTGCTGCGTGA
CGGTGCTGCCGGTCTGTTCCTGGCGGCCAGCAACTTCCCCAAATCCCGCGAGACCCGCGCGCCGCTGGTG
GAAGAGCTCAAGCGCTTCAAGGATCGCATCTCTGCCAAACACAAGTATCTGGCCGGTGCCCCGCTCACCG
ATCCGGACGGCAACCCCGCCATCGTACGCTTCAGTCGCAAGACCAAGGAGCAGTACGTGATGAGCGAGGT
CAACGGCAAGGCGACCGGTTGGACCGCTCATTACGACAACGGTACCTGGCAAGAGAGTTTGCCAAAGGCT
AAAAAAGCCTGATGAAAAGAGGCCCCGACAAGGGGCCTCTTTGCTTATGGATAGCGGTATACTGAGACAG
CTGCAAGGAGTGCAGCTGTCGTCAATCGCGAAGTAGACAAGGAGGTTGAGATGGAGATTAACGAGATCCG

GCCAGGCATGAGCTGCATGACCCGTGAAGGGGCGGCCTATGTGCTGGAGGTTGACCGGCAGAGCCTGCTG
GTGCTGCTGCAGCGCGCGGATGAGACCATACCGGTGCAGGTGCGCAGCGACGAGATCCTGGCGCCGCTGG
AGTGACCCGGCTGTGCTCCCCTCAAAACGGCAGTAGAAAAAGCAGCTCGGCCAGATCGGGCTGCCATTGC
CACTGCTTCATTCTGACCCGCCCACCGATCACCACGACCCCTTCTTGCTGCAGGCGTCCCGTCTGTTCGG
CAAAGCTTGGCGATCCTTTCGGTAACGAGATGGTGCCCGCCGCGCTCAGCACCCGATGCCACGGCAGTAC
CTTGTCGGTTTCCCGCAGCACCTTGCCCACCAGTCTTGCCCGTCCGGGCAACCCGGCCAGATCGGCCACC
TGACCATAGCTGACCACCCGGCCTGCCGGGATCAGGGCGATGATCGCTTCGATACGGGCAGCTTTGTTCA
ATGGGACAGGACTTGATTCAATGCTCATAAACCCGATTCGTCAAAAACAACAGAAGCGCCATTGTGGCGC
TTCTGTCATGGGGCTGGCAATGATTCAGCCGAAGGTCTGGCTAAGCCCCTTCAGTTGCTTGGCCCGTTCC
AGCAGAGCCTGACTGGAGTGATCGACATCCAGCATCTTCTGACTGTTGGCGTGGGAGTACTCGGAGATCT
GGTGGATGCTGCGGCTGATCTCCTCCACGACCGCTTGTTGTTGCTCTGCAGCGGTGGCGATCTGGGTGGA
GTGTTCGGTGACCAGCTCTATCTCTGCCAGCACGGTATGGAGGCTGCTGGCGCCCTGGCGGGTCATCTCG
GCACATGCGCGGGTCTGTTGCAGGTTCTCCTGCATCATCCCCTTCCAGCGTCCCAGCGTGTGCTGGATCT
GCTTGATGCTGCCCTGGATCTGCTCGGTCGCCTTGTGGGTGCGGGTCGATAGAGTACGCACCTCGTCAGC
CACCACCGCAAAACCACGGCCCTGCTCCCCGGCGCGAGCGGCTTCGATGGCAGCGTTGAGGGCCAGCAGG
TTAGTCTGCTCGGCGATGCCCTGGATCTCGCCCATCACACTGCCAATCCGTTCGGATTCGCTGGCGAGCT
CTACCGCCGAGTTGAACGCCAGCTCTGCCTGTTTGGCGAGCTGTTCGATCTGTTGCTCGGTCTGGTTTAA
CTGCTGGTTGGTTTGTTCACAACGATTGCGAGCTTCGTTGACCTGATCGTTGGAGTCTTGAATGTTGCGG
GCGATCTCGTGAGCTGTGTTGGCCATTTGAGTCATAGCCGTAGCCATCTGCTGGGTCTGGGCATCTTGCT
CGTTGATATCGAGACAGGCCTGATGAGAGGCCTGCTTCAGATTGATGGCCAGGATTTGCAGAGGTGCCGT
GGCATCGTTGATCCGCCCCAACACGGTTCGGATGCGGGCTTGCAGCATCTTGATATGAAAATCGGCAATG
GCGCCGGGGTTATCACCGGAGTAGACCAGCCGGGTCAGACTGTCATATTCACGGCCCAGTTGCTGCAGAT
AGTGGGGCAGGGCAAAGAGCTCGCGCCAGTAGGCTCCGCCAATAAGCAACAGGGGCAGCAGCATCAGCAG
GACGGTCATAGCCCCTTGGCTGGCGAAGGCCCAGCCCATCAGGCCGAGCAGCAGCAGGGTAATGGCCGGA
GTGCGCACTCTGGCAAGAGAGGGAAGGTTGCTGCTGTTGCCGCGCTCGGCCTTGAGCAGGGCTTGGTAGG
TCTGAGCGGCGATGCGCTTGAATTGGGGCTCGGGCTTGCAGCGTACCGACTGGTAGCCGCTGATCCTGTT
GCCTTCATAGATGGGGGTCACATAGGCATCTACCCAGTAGTAACGGCCATCCTTGCAGCGGTTTTTTACC
AGACCGCGCCAGGGTTTGCCCTCTTTGAGGCGGGCCCAGAGATCGGCGAAGGCGGCCTTGGGCATGTCGG
GGTGGCGTACCAGATTGTGGTTGTGACCGACCAGCTCGTCAGCGCTATAGCCGGCGATGGTGCAAAAAGC
CGGATTGGCATAAGTGATCACCCCGCGCAGGTCAGTGGTGGAGACCAGTTGTACGTCGGCAGGAAACTCC
ACTTCGCTATCCAGGGTATGGACATCTCTGCGGGCCATAGGAATACGCTCCGATGTTCTTGTTGATAATG
TTGTCGTTGTTATTGTGGCAGATCTGGAGGTGGCCGCAGGCTCGCGCCAGTAGGAGGCAGACCAGCTTCA
TTGGGGGCTGAATTTTGCCAATAATGGCGGATGAATGGTAGGGGGAAGTTGATGTTTTGTTGGCTAATCG
TAAAAAAGGCGGCCGATTGGCCGCCAAAGTAGAGACAACATTACCTCTGTGGCATCCGGTAGCGCACCTG
CAACTGATCGAGGATCTGCAGCTGACCCGGCAGGCGGGCGGCGAAGTGCTCCCAGCCCTTGTGGGCGAGA
TCGCTCCAGGCCACCTCGGCCGTGGCCAGCATACGCGGGAACAACATGTAGTCGAGACGATCACGGCTGG
TGATCAATTCTGACCACAAGGGGCTCAGCACCCCGAGGATGTTATCGTTGGCATGGAAATCGGCTGGGGC
AGGGTCACACGAGTAGATCTGCTCCAGATTGAGGGTACCCGCCCAGTAGAGGCCGGGTTCATGAATATCG
GCGCTCCAGGCCAGATCCAGATAGAGGAACTGGGCGGGCGCCATCACCACCGGATAACCGGCTGTGGCAG
CATCCAGCCCCGCCTGAAAGCTGGTCCAGGCACAGATGGCCGCTTTGCGGCTCACCTTGTCGCCATGCAG
GATCTCCTCCCAGCCGATCATCTGTTTGCCTTTGGCGGCCAGATAGCGCTGGCAGTGGCGCAGCAGATGG
CCCTGCAGCTCGCGGCAGTCGGCGTAGCCCTGTTCGGTCATCAGCTGTTGGCTGGCGGGGCTGTCGGTCC
AGACCCCGGTCGGCACCTCGTCACCCCCCATGTGGATCTGGGGGCCGGGGAAGAGTTCGCACACCTCGTC
CAGCACTGCCTCCAGGAAGCGGTAGGTGCCGGGCAGGGCGGGGTTGAGCACGTTGTCATCGAAATGCTGG
ACCGAGCGGTAGCGGGAGTGATCCGCCGTTTCGATCAGCAGCTCGGGCAGCGCCTTGATGGCGGCGTGGC
AGTGGCCCGGAATGTCGATCTCGGGAATGACGGTGATGCCGAGCTCACCGGCATAGCTCACCAGCTCCTT
CACCTCGCTCTGGGTGTAGTAGCCGCCATAGGGTTCGGCGCCGCCGCTGAGCTGGGGGCCGATCGCCAGA
TTATGGCCGCGCCAGGCACCCACGTCGGTCAGCTCCGGGAAGGTCTTTATCTCGAGTCGCCACCCCTCGT
CATCGGTCAGATGCCAGTGGAAGCGGTTGAACTTGTAGAGGCTCATCAGTTTGAGCAGGCGCTTGATGGT
GGCCGGAGTGTGGAAGTGGCGGGCGCAGTCGAGAAAAATGCCGCGAAAGCCAAAGCGCGGGGCATCCTCC
AGCGTGACGCAGGGCAGGCTCTCCCCATGTTGCAGATACCACTGGGCCAGAGTGACCAGCCCATGACGCC
AGCCCGTTTCGTCGGCGGCGGCAATTACCACCTCCTGCGGCGTGATGGTGAGTTGGTAGGCTTCGGGGGC
CAGTTTGGCCTCGGTCAGCCGGATTTCAGCGTCGTGATCCTGCTCCAGCGCCAGCGACGTCAGCTCGTTA
AAGTGGGCCAGCAATTTCGGCACATCGCGGGGGGCGCTGACATGAGCCCGCTCGGCCAGCCGGAAGTTCC
CCTCCAGCAGCACCTGATGGGCCGGTTGTGGCACTATACCGGGCTGGGGCTCGACATCCGGCAGGTCGGG
GCGGGTTACCGGCGAGTTCTCCAGTCCGAGATCCTGCGCCGCCAGCGTCACCGGTAGCAGCTGGGCACCC
GTGGTCGCCTCCGGCAGGCTGAGAAAGAAGCCCTGCGGCATGTCGGAGAGCTTCTTGATGGGGGTCTGGC
GGCAGCAAAAGCGCAGCTCGCAGCTGCCAGTTTTTGGGACAATCACCTTGTAGCTGCCGCTCTGGCGCTC
CAGCTCCCCCCCTTGCAAGGAGTCGGGGTCGATATGGCGAGCCAGACAGAAGTGCAGTTTCCACCGGGTA
TCCGGCATGCGGGAGAGGGTGAAGGTGAACCAGACATCCTCGCTGTCACGTCGGGCGACCTGGGTGTGGA
TCAGGGGTGTAGACATGGAAAATCCTCTGATTGGGCAAACAGGATGGCGCCATCGAGCGCATCTGCCTGG

GGGGGGATCAGGTTGAGCTGGCCGAGACGGGGAGCGAGGCGGGTGCCAATCCCCCCCAGCAGGGCGATCT
GTCCGGCTCCGAGCTGGGTCATTCCCTCCAGCAGGATCCGCAGCTGTTCGCAGGTTTCGTCCAGCAGCTG
ATTGGCGAGCTCGTCGCCATCGCTGGCTGCATCGAATACCCAGGGGGCAAAGTGGCCATAGTCCGCCGGA
ATGGCGCGGGCGGCCCAGCAAACCACCTCGGCTTGATCCCGCTTGAAGCGATCCATCAGGCGGATGGCGA
GGGTGCTGGGGGGCAGAATGCCGTCGTGGCAGAGCAGGGATTGCTGGATGGCGCGCAGCCCCAACCAGGC
GCCGCTGCCTATGTCTGAAATGGGGAAGCCGCGGCCGGAACAGGTGCGGATCTGCCCCTCCTGATAGACC
AGCCCCACCGAGCCGGTACCGGCGATCAGAATGGCCCCCTCACGGCCGCCAAAGGCGCCGAGGCAGGCAC
CGAAGGCATCGGAGGTGAGCCTGACGCTGGCAAAGGGGAAGGGCATCTCGAGCAGGGCGTGATAGCAGTC
GGCCAGCTCGGCGGAGGCGAGCGCCAGCCCTACCCCCATCTGCTGTTCGCTCTCGATAGCGATCCCGGCG
GCAAGGCGGGCGCTGGCGATGGCGGCCAGCACATTGGCGTGGGCATGGGGGATGCCCAGCTCCAGATTGG
AGCCGGCCGCGCGCCCCTCTCCGAGCAGCTTGCCCGCCCGATCCCGAATGCGGGCGCGGGTGTGGGTGCC
CCCGCCATCGACGCCGACCCAGAACTCAATCATGGTGACCACCCTGCTCATTCCGATCACTCAGGGCGCT
CTGCCAGCTGATAGCCAGGGTAAACCAGGCGGCGTGGGGGATCCACTGCTCGCCCCAGCGCCAGTTCTGA
TCCAGCCGATCCTTGTACTCCTCCGGAATAAAAGCCACGTCGTTTTCGTCATCGAATCCGGCGGTGATGC
CGTTGCAGATCCCCCCCTGCGCATTGGGATACCCGGCGGTATAGGCGGGGTTGTTGATGCCGTGACCGGC
CAGCATGCAGGCGTTGAAGGGGTTCTGCCCGAGGATCCAGTCGAGCTGGCGCTGGCCGTAAGTGAGCAGC
GGCTGGCTCTGGCGGGGGAAGTGGCGGGCGCCAGCAAAGGCCATGGTCGCCAGAGATGCGAGGCGGGCGT
TCTCCCCCTGCCACCAGTAACCGGATTCGTTGTTGTGGGGCATAAAGAAGCTGACGCGCGGCGCTTCATC
CACCCCTTTGACATATTGGCGGGCGAGGGAGAAGGGGTTGCCCGCGCGGGCGCAGAGCGCCAGCTCGGCG
GCCAGCGCCTGCTCCACCACGGTTTGTACCCTAGCCTTGTATTCCTGATCCGGCTCTACGCCCAGATATT
CCAGCAGCGCCAGCACCGGCAGGCCTGCCTCAGCGGCGTGGTAGTAGGGGCGCGAGCCATCCTCGTTGGC
CGACCAGTAGTGACCCAGCTCAGGATGGGAGCGCTGGCGGGCACAGAGCTTGCTGGCGCGAGCTCTCGCC
TCATTCAGCCACTCGGCGCCGAAGGTGCGGGTCAGCTCCACCGCTGCCAGCAGGGCGCAGTAGTCGTCGA
TGATGTTCTCGACCCCGTCATCCAGATAGGAGAGGTTGTGTTCACGCAGATGCAGGTAGCCGCGTTTGGC
GGCCTCGGCGTAGCGGCTGGCATCGAAGTCGCCGCTGACGCTCTCGCGGGCAGCGCGGGCCAGCGCGGCG
ATGGCCATGCCGCCCCCCTGGCGGAAACCCGCTTGCCAGTTGGCGGACTTGAGCCCCTGCTGAGTGGCAT
AGGCGCACAGCTCGCGCTGGGCAGGGTCCTTGCTCCACTTGTCGAACAGGGTCATGTAGAAGAAGCCCGA
CTCATCCTGCATCCGGCAGAGGAAATCGGCGCCGTGCAGCGCCTCGTCATTGAGCCGCTTGCAGAGGTTC
ACCCCGTCGATATCGCGGCGCGGGGCCAGCAGTTCCCGGCACTTGAGCAGAGCCCACACCACCATGGGGG
TCTGCTGGGGGTTGAGGTAGTTGGCGTAGGATAGGTGGCTCAGGTACTTGCTCATGTCGCCGCTGGCATC
GAACCAGCCGCCGTGCACATCTTTGTGCTGGTCGGTGCCAAACAGGCGGGCGCTGCGGTCGGCCCGATCG
AAGGTGCCGCTGGCGCGCTGCCCCTTGAAGTAGTGGATGACATCGGAGAGACAGCGGCTGGCCAGCAGAT
CCTGGCCGATGGCAAAGCGGGTGGAGCGGACTATGCCTTGGGCAGTGATCGCCTCCAGCCGATAGTGGCC
GGGCTCCTTGAAGGCGGAGAAGTCGGCGCGCCAGTAAGCGCGGCCCTGCCAGCCTGGGACGCTGCCAGCC
GGTTGCAGCGGAGTTTCAAACAGGATCTGGCCATCATCAAGGCGTTGCAGCCTGACATAGCCGCCGAGAT
CAGGGGCGGTCGAGGTTTGCAACAGGGCGACCTTGTGTCCGTCGCACTCGTAGCCCACCTGATTGATCAA
GAGTTCCATCAAGGTATTACTCCTGTGGTTCACCGCCGGGCGGTGAGGCACATCACGGGGCTGTGGCCGC
CCCGTATCGAGGCATGGCCGGACTGTGCTGTCCGGCCGGTCTAAGGCTCACCATCACTCGTAGAGGTAGC
AGCGCACGAAGTGGTTGTCGGCAAGCTTGGTGATGGGGGGCAGCGCCTCCTTGCAGCGGGGCATCGCCTG
ATTGCAGCGCCCGGCAAAGGGGCAACCGCAGGACTCCGGTGTCCAGAGCGGGATCTCCCCCTTGCGGCCT
CCCTCCAGCTCGGCGTGGATGCTCTTCTCCGGGTCGGGCACCGCCGAGATGAGCAGCTGGGTGTAGGGGT
GCTGCGGGTGATGCAGGATCTCGTCCACCTCGCCCCACTCCACCATGTGACCCACATACATGACCGCCAG
ATCTTCCGCCACATAGCGGGCGGTGGCGATGTCGTGGGTGATGTAGAGCATGGAGACACCGAGCTCGTTC
TTCATCTGCTCCATCAGGTTGAGGATGCCGATGCGAATGGAGACGTCGAGCATGGAGGTGGGCTCGTCCG
CCAGCACCACTTCCGCCTCCACCGCCAGATTGCGGGCGATGTTGACCCGCTGGCGCTGGCCGCCGGAGAG
CTGGTGCGGATACTTGGCCGCCGTTGCCTTGGCGGGGGTGAGCCCCACCTTCTCCAGCAGGGCGTAGACC
ATGTCGGGCAGCTCTTTTTTGTCGATCACCTTGCTGTGCAGCAGCAGGGGGCGGGCGATGTGGTGATAGA
TGGTGTGGGTCGGGTTGAGCGAACCGAACGGATCTTGCCACACCATCTGCACGCTCTGGCGATAGTCCAG
CAGGGCGGTGCCCTTGTTGAACTCCTCCAGATCGTGGCCGCGATAGAGAATGCGCCCGGCACTGAGCTTG
TACATCTTGGCGATGATCTTGGCGATGGTGCTCTTGCCGGAGCCCGACTCCCCGACCACCGCCAGGGCGC
GGCCCCGGTGCAGGTTGAAGGTGATGTCGGAGAGTGCCCGCATCTTGCCACTCTTGATGGCATTGGAGTT
GATGGGAAAGTCCTTGTAGAGGTGCTGGACTTCGATGATGGGTGCATCGGTTTTGGCGATTTGCATGACA
GACATCCTTAACAGCCAGCGGCCTTGTCCAGATAGATCACGGAATCTTGTTGCAGTCCCTGCTCCTCATA
GAGGTGGCAGGCGGTCTGTTGTCCGGGGCGGATCTCGGCCAATCTGGTGTACTCCTGACGGCAGCGATCG
TGCACCTTGCCGCAGCGGGCCTGAAAGCGGCAGCCGACCGGCACCTGCAGCAGGTTGAGCGGGGTGCCCG
GGATCCCTTCCAGCACCTTCTTCGGCCCGTGCAGGGGCGGGAAGGAGTTGCCGAGCCCTCTGGTATAGGG
GTGCAGCGGTGCGGTCAGGATCGCCTTGGAAGGGGCGACCTCCACCAGTTCACCGGCATACATGATGCCG
ATGCGATCGCAGAACTCCACCATCAAGGAGAGGTCGTGGGTGATGAACAGAATGGAGAAGTTGAACTTCT
CTTTCAGGGCATAGACCTTCTGCAGGATCTCCCGCTGCACCACCACATCGAGTGCCGTGGTTGGCTCATC
CATGATGAGCAGCTCGGGGTTGAGCGCCATGGCGATGGCGATCACCAAGCGCTGACGCATGCCGCCGGAG
AACTGGTGCGGGTAGTCCTTGAGCCGGCTCGGGTGGATATCGACGATGGTGAGCAGCTCTTGGGCGCACT

TGATCGCCTCGTAACGGGTCATGCCGGGGTTGTGGGCCAGCAGCACGTCGCAGAACTGCTCCTCCATCCG
CAGCACAGGGTTCAGGGCGTTCATGGCGGACTGGAACACCACCGACACCTGTTGCCAGCGATAGGAGCGC
AGCCGCTCGTCGTTGTAATGGAGGATGTTCTCCCCCTTGAACAGGATCTCGCCACCGCTGATGTAGGCGG
GCGGCTTGTGCAGCCGGGCCACCGAGAAGGCGACGGTGGATTTGCCGCAGCCCGACTCACCGGCGAGACC
GAAGATCTCGCCCTGTCTGATATCGAAGCTCACCGAGTTGACGGCGCGCACGTCGCCATTTTCGGTGATG
TAGTCGACGCAAAGATTGCGTACTGACAGCAGGGGATGCTTGTTCATGCTGTCTTCTCCTGTGCGGCAGA
GAGTGGTGTTGCGACCGGGGTGGCAAGTTTCTTCCAGCGGTCGAGCCCTTTGTGGGAGCGCAGCTGCGGG
TTGGCAATCTCGTCGATGGCGAAGTTGAGCAGGGCCAGACCGGCACCCAGGGTGGCGATGGCGAAGCAGG
GGGCCAGGATCTCCCACCAGGCGCCGACCAGAATGGCGCTGGAGGTCTGGGCGTTGTAGAGCATGATGCC
CCAGCTCACCACAGTCGGGTCGCCAAGCCCCAGGAACTCCAGAGTCGCTTCGGTGACGATGGCGTAGATG
ATGGAGCCGATAAAGCTCACCCCGATGATGGAGATGAGGTTGGGCAGGATCTCCACCAGAATGATGCGCC
AGGCCGGCTCGCCCAGCACTTCGGCGGCAATGACGAACTCCTTCTCGCGCAGCGCCAGGGTCTGGGCGCG
GATGACCCGCGCGCCATAGGCCCACGAGGTCAACCCTATTATCACGGCAATTACCGCCGGTGACGCCTCC
CCGATAAAGCTCGCCAGCACCAGTAGCAAGGGGAGGTTGGGGATCACCAGCACCACGTTCATCAGGAAGG
TGAGGCATTCATCCACCAGACCGCCGAAGTAACCGGCAGTGATGCCGATGGCGACGGCCAGGGTGCAGAC
CAGCAGGCCAGCACTGAAGCCCACCATCAGTGAGATACGGGCGCCGTTGGTGAACTGGGCCCAGATATCG
TGGCCCATGCGGGTTGAACCCATCACGAACTCGGCACTGGGCGGCTGGTGCGGGCGAGCCACCCGTTTGG
TCGGCTCGTTGCTGGCCAGAATGGGGGCAAAGAGGGCCATCAGTACGAAGGCAACCACGATGGCGAGGCC
ACAGGCAGCCTTCTTGTTGCCCAGCAGGATTTTGAGAATGGTCGGCATACGCATCTTATTTGCCTCCTGT
ACGCAGACGGGGATCCAGGAAGACGTAGAGCACGTCGGCCAGGAAGTTGAAGCTGAGCATGGTGATGGTC
ATGATGAGCAGCTGCCCCTGAATGAGCGGGTAGTCGCGGGCCAGAATGCCCTGATAGAGGGTCAGGCCCA
GACCGGGATAGTTGAAGATCACCTCGGTGATGAGGGAGCCACCGATGACGAAGCCGAGCGCCATGGAGAG
GGCGGTGACGCTGGGCAGAATGGCGTTGCGGGCGCCGTAGTTGAAGATCACCCGGTTGCTGGAGAGCCCC
TTGGCCTTGGCCATGGTGATGTAATCCTCGCCCAGCAGGTTGATCATGTTGTTGCGCATGGTGATAAGAA
AGCCCCCCACCTGTACCACGGCCAGGGTCAGCACCGGCATGATGGCGTGGGTCATGATGCTCTTGATGTG
CTCCCAGCCCCAGTCAGCCTTGATCTCCGGGCTGTAGGCGTAGCCGATGGGCAGCCACTCGAGGCTGACC
CCGAACAGGAACAGGGCGAGCAGGGAGACCACCACCGGCGGAATGGCCTGCATCACCAGGGTGAGCGGGG
AGAGAATGCTGTCCATCCGGCCGCCGCGATACCAGGCGGCAAAGATGCCGACGATGGAACCGAGGGAGAA
GCTCAGGATGGTGGAGGTGCCGACCAGAAACAGGGTCCAGCCGATGGCACGGCCCAGCACGTCAGAGACC
TGCTGCGGGTAGAAACGCACCGAGGTGCCGAGATCCCAGGTGAAGACACTCTTCACATAGGTGAGAAACT
GGGTGCCGATGGGGCCGTCGACGAAGCCGAAGGTGGCTTTGAGGGCTTCCAGGGCGGCGGGTTCCATCAT
GGCGCCAGCGCGGGCAAACATCACGGAGACAGGATCCCCTGGCATGGCACGGGGTAACAGGAAGTTGATG
GTGGCGGCTACCAGAAAGGCAACCAGATAGAAGGAAAAGCGTCTCAGTATGAATCCCATGATAGTACCTT
TTACTGCAAGTCTGCCCCGCGTCGGGTATAGCTACCCCAACCCCGGCAAAGTCGGGCAAGAAGGGGATTA
ACGGGGAAGATGGCCCCCCGCAGGGGGCCGGGATTGTTTAGCTGTTTGGCTTGAGGGACAACACGTGCAG
CAGACGCTCAGGGGTGTCCGGGTGGAGCTGAGGTTTGGCTACCGGGTTATCAGCACTGAACCAGCCGTTG
AAGCGCTTGGTGCTGTACTCGTACCAGGTCGGGTTGTTGAAGACCGGAATGATGGTCTGGTTGGCACCAA
CACGATCCTGGATGGCGAACATGATCTCCTTCTGCTTGGCCGCATCGGCGGTCTGGGTGAAGTCATCGAT
CAGCTTGTCGAGCTCGGGATCCTTGTAACGAGGCGCGGCGAAGCGGTTGCCACGGTCGCCCATGTTGCGG
GAGTGGAACGCAGTCTCGAAGTACTTGTGCGGGTTGGCACCGGCAAAGTAACCCTGCAGGGCGACATCGA
AGTCACCGGTGATCAGGTTCTCGGTCCAGACGGTCGCTTCCGGGGTGGCGGTCTTGGCGTTCAGGCCCAG
TGCCTGCAGCCCTTCTACACCAATCTGAACTGTGTTGACCCAGTCGGTCCAGCCGTTCGGTACCAGTACC
TTGAACTCGATCTTTTTGCAGCTCGGGGTGTCGAGGAACTTGTCGCCGTCACAATCCTTGTAGCCTGCCT
CTTTCAGCAGGGCCTTGGCTCCTTCCAGATCGAACTTGTTGTACTTGCCGTACTTCTTGTCCACTTCCGG
GTTGTTCCAGGAGTCAAAGGCTTTGCCAAGACCTGACGGGTACTCGTTGACGGTGGGGTAGCCGTAGCCC
GCGATATCCACCATCGACTGGCGGTCCATCGCCATGGAGAAGGCGCGGCGGAAGTTGATGTCGTTGAAGG
CTTCGTTATTGCCCGGGTTCTTGGTCTGGAAGTTGACGTTGAACGCCACGGTACCGGAGGCCGGGAACCA
GTATTTGTTGTTCTTCGGATCGTTGCCGACGTAGAGGCGCTCGATATCCGGCACGAAGGAGCCGAACCAG
TCGATGTCACCTTTCATGACAGCAGCCAGCACCTGGTCGTTGGTGGCCATCTGCGGCATACGCAGACAGT
CGATGGCGAGGTTGGCGTTATCCCAGTAGTGCGGGTTGCGGCACTGGGTATAGAGCTGGGCGGAGAAGTT
GTCCACTTCGGTGAAGGGGCCAGTACCAACCGGCTTGTCGTTGGTGAAGGCAACCGGATCTTTCACCGAT
TTCCACTGATGCTCCGGCACTATGGGTACCAGCACCAGATCGTTCACCACGTTGGTGTTGACCTCGGTCA
GGTCGAATTTGACCTTGTTGGGGGCCAGCTTCTCGACGCTCTTGATCTGGGTCCAGATTGAGCGCTCATC
CAGCGCCTTGTTGGCCTTGACCAGATTGAAGGAGAAGATGATGTCATCGGCATCAAAGGCTTCGCCGTCA
GACCACTTCACGCCGTCACGCAGATCGAAGGTGACGGATTTCAGGTCATCGCTGAACGCATAGTTGGTGG
CCAGACGATAGACGGGTTTGCCCCCTTGCATATCGTTGAAGATCACCAGCGGTTCATAGATGAAGTGACG
GGTGGTGTGCAGCAAGCCCGCTTGCAGGAAGGGGTTGAAGTTCTTGACCCAGGCGGTTTCCTGCTGTGCG
ACCATGGTCAGCACGGTTTTGTTTTCTGCGGCCATGGCGTTACCGGCGGCACCGGCAAGCATCATGGTTC
CCAGCATTACAGCGGTAGAGATTTTGGATACTTTGGCAAGCATATGTTTTCCTTTCTTATTTTTTGGCAT
TCCCGAGGGTTCGCCTTGTTTTGTGTGAGCACCGGTTTCGCATGCTGATGAAATCGGCCCTCTGTGTTGG
TCAGGTGTCTAACCTTTGTCGTTCTGTTTTCCAACGCCCTAAGCAAACCAGAATTAACAGGAAAACAACA

ACTATTGATTTCGTTGTTTTCGTAAAAATTGACGGTGTTACGTCAATCTCGTCAAAATCGATTTTTATAT
GTTTAAAATCATTGCTTTGCGTTATTTGCTCTCGCGTAGAAAGTGGCTGTGAAAATGGCGTATATGACAA
ATTTGTGAATTGGATCTGCTCTCGGTTTCGGGGTTGTGCGGGGCTTTGTTTTGCATAAAAAGAAACCAGA
TTGCCAATTGTGATCCGGCGCCCGTTTAGGGCGTCGTGTCACGGCTTGCGGTTGGCGAGCTGACGCAGGG
TCTGGGCGCTGTGCTGCAACTGCTGGCGCTGGTGGTCATTGAGCGGACAGTGGCCCAGCACATACTCGGT
GGTGGCCACGACCGTCTTCCAGCGTGGCGACTTGGGGAGGGTGTCCAGACTCAGGTATTTGTCGAGGGTG
CGGGTCTGCGGGCTGGATCTGTCCATATAGACCCGCCACAGCTTGCTCTTCTCTGCCAGCACAATTTTGC
CCTTGCCGGTCCCCTGCTGCCAGGCATCCAGGGTGAGCTGCATGGTCTCTACCAGCGCACGGCGCAGCGG
CTCGTCATCCTGCTGGCGAATGAGGTCGTCGGCCAACGAACGGAACTCGCCACCCAGGCGGCTCAGCTCG
CCCAGCAGCTGTTCATCTTCGATGATGGCTTGCCGCGAGAGGTTCTTGAGGGCGTTCTCCAGCGCCATTA
CCCGGTTCTCATCCTGACGCAGCTCGGTACTCAGAGTGAGGGCGAAAGCGGCCTGTCCTCCGAGCGCGAT
GGGCAGGGCACGGGCGTTGAGCACATCTTTTTGCCCGGCCACCAGAAACTCCAGATTGTGTTCCCGCTCG
CTGGCAAGGTCGGGCCACTGCTGTTGCAGCGGGGTGGCGAGCCACTCATTGAGGGGCTGCCCCTCCATGG
CTTCCGGCTCCTGCCCCAGCAGGCGTGCCATCACCCGGGAGGCAAACAGCACTGTGCCCTGCTCGTCGAT
AAAGATCATGGGGTCGGCCCCTTCGCCGAGCAGGGCCAGCAGCCGCTGCTGATCCTGATGCAGCCGCTCC
TGCAGGGTGAGGCGATGTTCGATCTCCTGCTTCAATAGCCGGTTCTCTACAAGCGAGGCGCGTCCGGCCT
TGGCTTCCAGCAGTGCCTTGACCCGGGCAAACAGCTCTTCTTTGCAGAAGGGTTTGACCAGATAGTCGTT
GGCACCGGCCCCGAAGCCCTCCTCGATATCCTTGGGCTGGTTGAGGGCGGTGAGCAGCAGCACCGGCAGC
ATATCTTTCGGGTGCTGCTCGCGCAGGTGACGGCAGACGTCAAAACCGGAGAGGGTCGGCATCATTACAT
CGAGCAGGATGAGGTCCACCGGCTCCTCGGCCAGCTTGGTCAGCGCATCGTGGCCGTCGCTGCTGGGCAG
AATGCGGTAGCCGCAGGGCTGCAGCAGATGGCGCAGGATGTGCAGGTTGATGGGCTCGTCATCCACCACC
AGAATGAGCGGCGCATCCTCGGGGGGAGGGGGCAGATCGTCGCTCTCCCAGTCCGGCAGGATCAGCGGTT
GCTGGCGCACCAGCTGATGCTCGATCTGGCGGCTGTCGAGAGTCAGATTGGCCGCTTTGCCGGTGGCAAG
GGGCAGGGTAAAGGCGAAGGTAGAGCCAACGCTCGGTTCGCTCTCGACAAACAGCTCGCCCCCCATCAGG
TGCACCAGCTGGCGAGTGATGGAGAGGCCAAGGCCGGCTCCCTGCTTGCTTACCTCCGGGCTCATCTGCA
CCAGCGGCTCGAAGATATGTTCCAGATGGTCGGGGGCAATGCCGATGCCGGTGTCGGTGACCTTGATGCG
CAGGAAGTTGTTCTCGACGATGGCACTGAGCACCACCTTGCCCTGCGGCGTGTACTTGATGGCGTTGCCC
ACCAGGTTGTAGAGCACCTGTTCGAGCCGCTGCTCGTCGGCCAGCACCGGCGGCAGTTCGGCCGGGCTGA
TATCTACCAGGGTGAGGGGTTTGGCCTGCACCAGATGCTGGCAAAGATCCAGCACCAGCCGGATAACCCC
TTTTGATGAGACGGGCTGGGAGTGGATCTCCAGCTGGCCGTAGCGCATCTGATGGTAGTCCAGCAGATCA
TCCACCAGACGGGCCAAGCCGTCGGCGCTCTTGATGATGAGGCCGAGCTGCTTTTGCTGCCCTTCGGTCA
AATCCTGCTGACTGGCGAGCATCGACTCGGCCATCCCCTTGATACCGTGAATGGGGGTGCGCAGCTCGTG
GGAGGTGTTGGCCAGCAGTTCATCCTTGAGCTTGTCCGCCTGCTGCAGGCTGAGGTTCTGCTTGTGGGTC
AGCTTGACCTGCTGTTGCAGCTCCTGGGTCTGCTGTTCGATCAGTTCGATCTTGTCCTTCACCGAGCGCT
GCATATGGCGAAACGCCTCGGCCAGCCGGTCGATATCATCGCCGGTGCGCTCGATGGCGATGGGGTGATC
CAGCGCGCCGTCGGCCACCTGCTCGGCTGCTTCGGTCAGGGATCGCAGCGGCGCCGTGATGGAGTGGGAT
AGCCAGCGGGAGAAGAGGATCAGCAGCAGCACCGAACCCGCGCCCAGCACGATGACCAGCGTCTCCAGCT
GGCGGATCGGGGCATAGGCTTCCCGGGTGGAGATCTCGGCGATAAAGGCCCACTCGGTGTCGAATACCTT
CACCTGGGTGTAGGCGGCCAGCACCTCGTCATCCTGATAGGAGTGGAACAGCCCGACCCCCTGCTCACCG
GAGAGAGTCTTGGTGATGAGCGGCGTTTGCAGCGGCCGGAAGTTGGGGCTGAAGCTGTTCTCGACGCTGA
ACTCCGGCGCCAGCCAGGCATCGGCGCGCATCCGCTTGTCAGGGCCGATCAGGATGGTCTCGCCGGTCTC
GCCCAGCCCCTCCCGCTCCGCCATGATCTGGTTGAGCTTGTCCGGCTGCAGCTCGAAAATCACCACACCG
CGCACATAGTCGTATTGCATCACGGGGGCCGCCATGTAGGCGACCACCTGCCCGGTCAGCTCGTTGCGGC
CAAAATCGGTGAATTCGAAGATCTGCTGCGGCTTCTGGCCGCCATCAAGGCGTCGTTTGATCTTGGCGAA
GGTCTGGCCCAAGCCACTGCCCTGATAAGGTCCGGAGAGCAGATTGGTGGCAAAGTTAGGGTGCTTGGTG
ACCGAATAGACCACATCACCGTTGAGGTCCACCAGAAAGATGTCGCTGTAGTTGGACTTGCGCACCATGT
CGGCATAGCCGCGATGAAAGCGCTGGTGCACCCGGCCATACATCTCGTCGGCGAGGATAAGTGCCGTTAT
GGAGGGTTCGATCTCCACCTGACTGTCCGGTTTGCGCAGTTGATCCCAGGAGCCGGGCAGATAGCGGGCC
TGGGCACGGCTGCGGGCCTCCTGCGGTGTGGTGCCCAACTTCTCGAAGGCGCCGGTAAAGCCATAGAAAC
GGCCGATACTGTTGCCGGCAAAATCCTGCTGGGAGAAGTTGATGATCTGCTCTTTGAGGTTGTTGAAGTA
ATCCTCGAGCTGACTCTTCTTGATATTACGGACGGAGACCAGATGGTTGGAGGTCTGGGTGGTGAGATCC
TTGCCGTGGCTGTATAGGAAGAAGAGGGTGATGACCAAAAAGGGCACCATGCCCAACATGAAAAAAGCCA
GCATTAACTGCGGCCTGAGCCCACGAAGCGTGAAGATCCATTTCATCTTGTGATTTTCAATTGAGAATCC
GGTTATTAAGCCGCTCAAGTCTCGAACGAAAGCGTAATCATTTCAACGACTTTATCCCAAATTCGCTTGG
TGGGTCACAGCTGGCTGGAGAAAAATGCAGCAAGGCGTTTAAACCCTGAGCAAACAGTCTGCATCAAGGC
TTAAATTGCGAAAAAGGGGTTGTGCTGGCGCTCGTGGTACGTATAATGCGCCTCGTTGTCAGGCAACTGG
CACGAAAGCAGTAACGCGGGAATAGCTCAGTTGGTAGAGCACGACCTTGCCAAGGTCGGGGTCGCGAGTT
CGAGTCTCGTTTCCCGCTCCAATTTTCCATCACACCAACGATGGAATTCACTTTCAAGACGACCAGTCTT
GTCAGCATGGCGCGTTAGCAAAGCGGTTATGCAGCGGATTGCAAATCCGTTTAGTCCGGTTCGACTCCGG
AACGTGCCTCCATCTTCTCTGTCTTTTCCCTGATTCATCACTATTCGAGCTGTGAAGCTTGGTCAGTATC
CTTGCTGTACCAGTAACTGTTCGCAGCCAGCCTGTTTCCACTCCCGCTCGAGATAGTGGCTCACATCCGC

CATGTTTTGCCCCCGATATTGAAACTGCACCTCGGCACTGTGCTGGTTGAGATCCGCAATCCGTTGCTTC
AGTGTGCCGCATTGCCACAGGCTGACGGTGGGCACGCCCCGTTCGGCCAGCACGATGGCATCTTCCCGAT
TCAGTTGCCACTCCTCCAGTTGTGCCCGTGTCAGGGCAACCATCAGCACCAGCAGTGCCACTTCAGGCAG
TTTCATGCGTGTCTCCCTACTGCATGTTTTTCCCCTGATAACTGTGGCAAAGCGCACGATTTTCGCAAGT
TTGTTAACTGTTGGTTAATGACGTAACCCGTTTTCAGTATTTCCCGTTTTTCACCATTTTTTTATAAAAA
AATGCGTTGTGATGTAGTAATCCTACATGGGTTGGGGTAATCTTTGCTCAGTGGTCTTACCAGTAAGGAG
TTACATCATGTTTCATCCCTTTTCCCTGACTCAGATTATTCTGCGTCGCATTTATGTATTGCTGGTGGGC
ATTCTGGCTTTCCCCGTGATGCTGTTTCGCAGCGATCGTGCCCGCTTCTACAGCTATCTGCACCGGATCT
GGAATAAAACCAGCACCAAACCGGTGTGGCTCAAAATGAGCGAGCGTGGCGAGCGGATCTTCTACTAAAC
CCCATTCGCACCATGTGCAAAAAGAGACAGCCAAAGCTGTCTCTTTTTTTATCTGCTTGCCGCAACTCCT
GGCTCCCAACACCACTCAGATTGTTTATTTCTTGTTTCTTTAAAGTTGCATCCAGCCCTGCCTGTTGGCG
GGGCTTTTTTATGTCTGACGCATCTCTTTTGATGGAAGCTGTGAAGAAGATTAACGGGATGAAAGCACCA
TATGGCACTTTCCTCGTTCATTAATAGGTGGTCGTCGAGAAATTTTCAATGATGAAATAGCTGTTTAAAA
TCAATTGATTGAGTGTCTGTCTTTGGATTTTTTAACTCTTTTTGTGTTTTTTTGAAAGGGAATATCCCGT
TTGCCGGTTCTATTGCAGGTACCCGGTTGCGACAAATCGGGGATATCAGCAACATCCAACGACATCAAAA
GGAACATGAACATGAATGTTAAAAAAAGCACTGCTATCGCCTCCGCCATGACCGGTCTGCTGGCGCTGGG
CGCCCAATTTATCGCGGCACCAGCAATGGCTGCCGATGAAAAGGAGAAGTGCTACGGCGTGGTCAAGGCC
AGCAAAAATGACTGTGCGACCAAGAGCAGCTCCTGCGCCGGTACTGCCAAGGAAGATGCTCAGGGTGATG
CCTTTGTGGTGATGCCCAAAGGGCTGTGCGAGAAGCTGGTCGGCGGTTCGCTGCAACCCAAGTAAGAGAC
ATCGGGCAGGGGATGGCCAACGGGCCGCCCCTGCATCTTCTCTGGTTGGCGAAAGCGAGTCTTGCGATGA
ACCCTTTGATTGGCATCGGATTACGCACTCCCCATTACGATGAGATCCGGCAAACCCTGCCGGCAGTCGG
TTGGCTGGAGGTGCACAGTGAAAATTACTTCGAACGGCACAGCCAGGGTTTTCAGGTGCTTGCCGAACTG
GCGCAGCACTACCCCGTCAGTCTTCACGGCGTTGGCATGTCCCTCGGTTCTGCCGACCCGCTCGATCCGC
ACCATCTGCAGCAGTTGTCGACACTGGTAGCGGCGATCAAACCGGTACGGATCTCCGAACATCTCAGCTG
GGGCAGCATTGGCGGGCGCTATTTCAACGATCTGCTGCCGGTGCCCTATACCCGCGCCTCCCTGCGCAAC
ATGAGCGACAAGATCCAGCAGGTACAACAGACCCTGGGTTGCCGCATGCTGATTGAAAATCCCTCCTCCT
ATCTGCAACTGGCCGGGGAGATGAGCGAGTGGGAATTTCTAGCCGAGCTGCAGCAGCGCAGCGAGTGCGG
CATCCTGCTCGACCTCAACAATCTCTATGTCAGTGCCTTCAATCACGGTTTCTCTTGCGATGAGTATCTG
GCGGCGATCGATCTCGCCACCGTCGGGGAGATCCATCTGGCCGGTTATACCGAGAAGCAGCTGCCGGAAG
GCTCTCTCTATATCGACACCCACAGCGCCCCTGTGGTGGAGCCGGTCTGGGAACTCTATCGCCAGATCTG
CCGCAAGCAGGCCATTCCCACCCTGATTGAGTGGGATCTCGAGATCCCGCCGCTGCCGGTCTTGCTGGCA
GAGGCGGCGCAGGCTGGTGCCATTCTGGATGAGGCGTGCGCCATGAGTGCACCACGGGAGGTAGCCCATG
GCTGAACTGGCACAGCTGCAACAGCGCTTTGCCGATGGTCTGCTGGGGGCGCCTGACGCCATTTCCCCTC
ACATCAGCTCCCGGCTCTTTTCTGCCGAATCTGTGCTGCAGGTCTATCGCAACCACTTCATCCTGAGCCT
TGGCGAGGTGCTGGCGAGCAGTTATCCCGCCGTCAAGGCCATGGTCGGAGATGACTTTTTTGCAGCGGCG
GCCCGTGGCTTCGTATTGGCCGAGCCGCTCAGGGAAGGGAGCGTCATGCACTATGGCGCCGGATTTGGCC
AATGGTTGGCCCGCTTGCCGACCACTACCGGACTGCCCTGGCTGGAGGCGCTGGCGCAGTTCGAATGGCA
GCTTGAGCGTGCCAGTCTGCTGCCGCTGGAATCCCGCTGCTGGCCAGCTGAGCGTCTGGCCGCATTATCG
CCGCAACAATGGGAGCGGCTGCGGCTATTACCAGCCACGGATCTGCTGCTGGTTGCCAGTGATTATCCGG
TGCTGGCCCTGTGGCAGATAGCGCTGCACGGTGGTGAAGCGGTGGAGGTGCTTGATGCCCCCGTCTGGTT
GGCATTGAAGAAACAGCCGGATTGCCGGGTTGCCCCCTTGCCCCTGACGGCGGGTGAGTGGGCGTTGTTG
CAGGGTTGCCTCGCCGGTACGGCGCTGGTCGAGCTGCTGGCGGCCGACCCTGCGGCCAGTGAACATCTCA
CTCGCCTGATAACCCTTGGTCTGCTGGTGGATATGGAGGTAATGCCATGATGGCAAACAAGTGGCTGGCA
CTGGCCAGCTCGAGTGGTGATTTGCCGCGCTGGAGCAGCGCTGGTGTGCTGTTGCTGGCGCGGCTGTGGG
TCGCCTCGGTTTTTTTCACTTCAGGCTGGCTCAAGCTGACTGCGTGGGATTCCACCCTCTATCTGTTTGA
ATCCGAGTATCAGGTGCCCCTGCTGCCCTGGTTGTGGGCTGCCTATCTGGGTACGGCGACCGAGCTGCTG
TTGCCGCTCTTCCTGCTGGCGGGGCTTTTTACTCGCCCGGCCGCCTTGCTGCTGTTTGGCTTCAATATCA
TGGCGGTGATCTCCTACCCGACCCTCTGGGCGGGTGGCTTTTACGATCACCAGTTGTGGGGCTGGATGCT
GCTGATCCTTATCATCTGGGGCGGCGGTGTGCTGAGCCTGGATCACTGGCTGACACGTCGCAGCCATTGA
TTTATCAGTGATTTCCCCAATGACAAAGAGCGCCACAAGGCGCTCTTTGTCATAATGACGGCCGGCGAAA
TTTCCCATGAGAGAAAAGGAGCAGCCAATGATCATTCGTCAGGCAAGGGCCGCAGATATTCCCACACTGG
TGGCGCTGCGCATGGAGCTGTTTTGTGAGGTCGGTGAGCTGGTGACGCCGCAGGCAGACCCGGCCTTGTG
GCAGGCCACCCTTGACTATTTCGCCCGGGCGATGAGTGAAGAGAGTGCTTTATCCTGGCTGGCTGAGGCC
GATACGCAGGAGGGCGGCAAACAGATCGTGGCGGCAGGGACGCTGGCATTATTTGAACGGCCGCCCTATC
CCGGCAATCTGGCGGGAGTGGAGGGGTATCTGCTCAACATGTATACGCGGCCACAATGGCGCCAGCAGGG
GATCGCCACGGCGCTGCTCGATGCCATAACGGCCTTTGCCAGAGCGCGTCAGCTGGGCAAGGTGTGGCTC
CATGCCAGCGAGCAGGGGCACCCTCTCTATGAGCGGATCGGCTTTGTCGCCAATCCGGCCTATCTGGAGT
GGCAACCGGCGGTGGAATAAGGGTTGCAGCTCTGCATGAGTGGCGTTTGGTCGGTGAATTTGATTTACAG
CAATAAATATGGCGTCAGATGGGATCGCGGGAAAGCCGCCAACTATGCTGATGGAGCACGCCACCATTAT
CTTATTGATCCACTGCGTGGTTTGGCCATGGTGGGTGCCAGTCTGGAGGATACGTTTCGATGTTCAATAG
CAAACTGAAAGCCGAGTTGCAGCAGTGCCAGGATCAACTTCTGGAACAGCAGGGATTTTTTGACGCAGTG

CACGGCAGCGTGGCGACCATCACCTTTACGCCGGATGGCACTGTGCTGGCCGCCAATGATCTCTTTCTCA
ACGTGGTAGGCTTCAGTGCGCCCGAGGTTATCGGTCAGCATCACCGTATCTTCTGCGACAAGCTCTATGC
CCAGAGCAGCGACTATCAGCAGTTTTGGGCGGATCTGAAGCAGGGGCGCTCCCGTACAGGGGTGTTCCAG
CGCTTCAACAAACGGGGCGAGGCGATCTGGCTGGAAGCGACCTATTTTCCGGTCAAGTTGCGGGGCGTGG
TGACCAAGGTGATCAAGATCGCGGCCGATATTACCGAGCATCACCTGCAGCTGCTGAGCCAGCAGGCGGT
GGTGGCGGCGCTGGATCGGTCGCTGGCGGTCATCGAGTTCACCCCCGGCGGGGAGGTGATTGCTGCGAAT
GGCAATTTCCTCCATACCATGGGCTACACCCTGGCGCAGGTGCAGGGCAAGCACCACCGCATCTTCTGTG
ATGACCACTTCTATCAGGAAAATCCCCACTTCTGGGAAGAGCTTGGGCGCGGCCAGTTCAAATCTGGCCT
CTTCTGCCGCCAGAATAGCCATGGCAGCAAGGTGTGGCTGGAGGCGACCTACAACCCGATCCTGGATGAC
AACCGCCGGGTGGTGAAGGTGATCAAGTTCGCTAGCGACATCACCGAGCGGATCAATAAGAGCGATGCGG
TGCGCGAAGCGGCCATGCTGGCCCACGATGCGGCCCGGGAGACCCTGAACTGCGCCGAGCGCGGCGCGGG
TTTGCTCTCCTCGGTAGTCGATACCTCCAGCCTGATTGCCAGCCAGCTGACTCACAGCATCGGCCTCATC
AATCAGCTCAACGAGCAGTCGCGCAGTATCGAGGCGATCGTCTCCACCATCAGCAGCATCGCCGATCAAA
CCAATCTGCTGGCCCTTAACGCAGCCATTGAGGCGGCGCGGGCGGGGGATCAGGGGCGTGGTTTTGCGGT
GGTGGCCGACGAGGTTCGCCAGCTGGCGGCCCGTACGTCTCTCTCTACCGACGAGATCGCCAAGGTGGTA
CAGAACAACCGGGAGCTGACCGCCAGGGTCACCTCCGAGATGTCTGAAGTCGCCAGCAGCGCCGAGCTTG
GCAAGCAGCAGGTGGGCGAGGTGAATGAGGTGATGAGCGACATCCGCCGGGAGGCGAACAACGTCTCCAG
CACCGTCTCTGATCTGGCCATCTGAACCCCCTGACTTCCTGTCATGCCGGGGCATTGACTTATATTTGAG
CAGTCAGTGCCCCAATCTGAACCCCTCCTGTATCGTCCCGCCATTGTGAATTGTCCTCTTCTGGCCCCTT
GATTAGGCTTGCCGCCTCATTTATTTCAGAGCAGGAACAGAACAAGATGGCATTACAACAGAAGGGGCGG
GCGGCCGCGTTGCTGATGATGGTCGCGAGCATGGGTCTGGGCGGCTGTATGGGGCAGATGGGGCTTTCCG
GCATGGTCACCAAGGGCAACCTGAGCGTGGTGGATAACCGCTACGGCCGGGCCGGTGTCTTTATGCTGCT
TTCCCCTGTCTACGGTCTGGCCGCGACCGCTGACCTGTTCATTTTCAATACCATCGAGTTCTGGTCTGGC
AAGAACCCCATCACCGGCAAATCCCCGGCAGTGGTGGACATGAAGGCCAATCCGGTGATCAAGGTGAACC
AGCATCTGGATCCGGCCCTCAAGACGGTGCCGTTGGCCATGCTGCCACAAGGGGTGCGCGAGGTGGAGCT
CAGCTACCCGGACGAGCATACCGCCCAGATGGAGGTGCACTATCTGGATGGCCGCCGCAGCATGATGCGT
GGCGAGAAGCGTGGCGAGTTGATGGATATCTACTTCGATGGCCGCTTTGTATCTACCCTGAGCCGCGCCG
AGCTGGAAGAGCGGGCGAATCAGGGCAAGGCTGCTGCCTAAATTCAACTGAATCCAGATCGATCCAGCTT
CAGATGGGGTATACCTAATCTGCTGGCAAAATCGCCATGATGTCTAAGGGAGTAGACTATGACGGTGAAT
GGATTGCGATTGGCGGAGTTGATTGGTTCCCTCAGTCATGCGCTCGATATGACTGAGGGGCAACCAAGAG
GACACTGCATTCGCTGCTGCTGGATAGGTTCCCGTCTGGGGGAGCGGCTGGGACTGGACGCCCGGCTGCG
CCATGATCTCTACTACACGCTCCTGCTTAAGGATCTTGGGTGCAGCAGCAATGCCGCCCGCATCTGCGAG
CTATATCTCACCGACGATCTTCACTTCAAACGCGATTTCAAACTGGTGGATGGCTCCCTCTCCGAGGTAG
TCAATTTTGTGCTGGGCCACACCGGCTTGCAGGCGGATCTGCTGGGACGTTTTCGCAGCCTGTTTCATAT
CTTTCGCAACGGTGACCAGATTGCCAACGAACTGATCCAGACCCGCTGCCATCGGGGGGCGGATATTGCT
CGCCAGCTGCGTTTCAGTGACGAGGTGGCCGATGGCATTCTCTCCCTTGATGAACACTGGAATGGCAAAG
GTCGGCCCCTGCAACTGGCTGGCGAGGCGATCCCGCTCTTCTCCCGCATTGCCCTGCTCTGCCAAGTGGT
GGATGTGTTCCACGCGGCCGCCGGCCCTGATGCAGCGCTCAAGGAGCTGCGCGAGCGCAGCGGCAGTTGG
TTTGACCCGGCGCTGGTTCGCGAATTTGAAGCTCTGGCGGACGATGATTTCTGGCAAGGACTGGCCGATC
CCCGACTGACCGAGTGGGTGCTGGTCCACCCGGCCGGACAGGGCGAAGTAGCGCTGGATGAGGATTATCT
GGACGAAATCGCGGCGGCCTTCGGGCAGGTGGTGGACTCCAAAAGTCCCTACACCGCGGGTCACAGCGGC
AGGGTTGCGCTCTATACCGACATTCTTGCCACCCAGATGGGGTTGTCCGAGTCGCGTCGACGCTGGCTCA
AGCGGGGCGCCTTGCTCCATGATGTGGGCAAGCTCGGGGTGAGCAACGCCATTCTCGACAAGCCGGGCAA
ACTCGATGCGCAGGAGTGGGCGGCGGTGCAGATGCATGCCGCCTATACCGAGGAGATCCTGAGCCGTATA
GGCGCCTTCGAGGAGCTGGCGCAAGTGGCGGGCGCCCACCATGAGCGGCTCGATGGCAAGGGCTACCCCA
AGGGGCTGAAAGGGGACGAGATTACCCTTGAGACGCGCATCATCACCACAGCCGACATCTTCGATGCCAT
CACCGCCAAGCGCCCCTATCGCGGCGCCATCCCAGTGCCGCAGGCGCTGGAGATGATGGCGGAGAATCTG
GGCACTGCGATAGACCCCGACTGTTTTGCCGCCCTGCAGGCGGGTATTCAGGATTTCTCCCTCTACCTCA
CCACCGAGCAGCCGCTGGCCAATGCCAGTTAGAGTTGCTGATCAACGGCCCAAGCGGCCAGTCAGACAGC
AAAACGCCCCGCAACTGCGGGGCGTTTTGCATTGGGTCTAGTCCGGCTGCTTGCGATGGGGCGGGGTGAG
GATCAGTCCCAGCAGGCGGCGCAGGTGGGTGAGCTGTTCGGTCAGCTGGACGTTGAGCCAGAGGTAGCCA
TTGATGGCGGCCTCGGTATTGGGGGGGAGCGATACCTGGGTTGCTACCTCGTGCAGGCGGGCGATGGCGG
CATCTGCGCCGCGACCATCGCCGGTTTGCATCATCAGGGCAAGCTGGTTGATCGCCTCCTCGGTGGCCAG
ATGGCAGGTTTGCAGCGCAGCCGTGCTCTGCATCAGGAAGTGGCTCTCCCGATCGTTCCAGTAGGTGTTG
GCCAGCATCTCAAGAGTGCAGAGGGTGTTGCGCATGGTGACCTGCACCGCATCAAACAGGGCGACGCTGT
GGCGTGTCTCCTTGGCCGCAGGGGCCAGCAGGGCCCGCAGTTTGCCGGTTTGGGCCAGCAGTTGGGAGAG
ATTGTGCTTGAGGCGCGGGCGCTCCAGCACATTGGGGGAGAGGTGGGTGTGATAGAGCCGCCCCGACTGC
TGCAAGATCTGGTGCAACTGCAGGCGCCAGTGGCTGTAGGCCCGCTGGGGATAGATGCTGCAAAAGAGCA
GTGCCAGCAGGGAGCCCAGCACCACGTCACCGCTGCGCCAGAGGGCGGTATGGATATCCCCCGGCTGGGC
TCCCATGGTGACGCCGAGGGTGATGCCGATCAGCAGTCCCACATAGGGGCGCTTGCCGAGCGCCAGATAG
CCGCACAGGAACATGATGCAGCCGCACCAGATCAGGGTGAGCGGCAGGGAGTAGGTCTCAAGATAGAGTG

CCACCACCCCGCAGCTTGCGCCGAAGATGGTGCCCACCACCCGCTGCAGGGCACGGGAGAGCACATTGCC
CCAAAACGAGATGGGGCCCATCACCACCACCAGCGTGATCAGCGGCCAGGTACTCTCCGGCAGTTGCAGC
TGGCGGGTGAGCAGAAAGGTGAGCATAAAGGCGAGCGCAATGCGCAGGCCATGGACGATGCGATAGTGGC
CGTAGACCAGCAGATCGAGTCGGGATAGGGGGGCATCAAGACGCACGGTCTGCCTCCTGTCAGGTGGGGG
ACGGGGCGCCGTGCATCCGGCCAACGCCCCGTCGGGATTCAGGCCAGTCGTCCCTGCAGGGGGTGGATCC
AGACCCGCTCGCCAACGGCGATCCCGGGTTGATCCGGTACGATTTCAATCAGGCAGTTGGCATCGGCCAT
GGAGCTGAGAATGCCGGAGCCCTGTGGCCCTGTGGTGCGCACCCGCAGCTGGCCGTTACGGTCCTGATGA
AAGATGCCGCGCAGAAACTCGGTGCGCCCCGGGCGGCTCTTCATCTTCTCGTCCGCCAGTGCCGAGAGGC
GCAGCGGCTGCCACCGCTTGCCTTGCAAGCGGGCGATGGCGGGCTCGACAAACTGCAGCACGCAGATCAT
CGCCGCTACCGGATTACCCGGCAGGCCGAAGAAGGGGGTCTGCCCCAGCTTGCCAAAGGCGAGCGGGCGG
CCCGGGCGCATCTGGATGCGCCAGAAATCGATCTGGCCGAGCTCCGCCAGCACCAGCTTGATAAAGTCGG
CATTGCCCACCGAGACTCCGCCCGAGCTCAGCACCAGATCTGCGCTGGCGGCGGCCTGCTCCAGCTGCTG
GCGCAGAATGGTCTTGTCATCGGGAATGATGCCAAGGTCCATCACCTCGCAACCGGCGGCGCGGATAAGG
GCCATCAGGGTGAAGCGATTGCTGTCGAAGATATGGCCGGGGGCGAGCTGCTCGCCCGGCGCCTGCACCT
CGTCGCCGGTACTGAACAGCGCCACTTTCAGCGGGGTGCGCACGCAGAGCGATGCCTGACCGAGTGATGC
CGCCAGCCCCAGCTGGGGGGCACCAAGCCAGGTACCGGCCGGGATGGCGACCGCACCTTTGGCGAGATCT
TCGCCGGCGAGGCGCACATTCTGCCCTGCGCGGATCGGTGCGGTGACCGTGACCCACTTACCTTCAATCT
GCGTCTCTTCTCGCATCACCACGGTATCCACCCCAGGGGGCAGCGGGGCACCGGTCATGATCTGCACCGC
CTGACCGGCCTGCACCGGTTCATGGCGCTGCTGGCCTGCCAGCACTTCCCCCACCAGTTGCCAGCGGCCG
GCGGCGAGATCGTCGCTACGAAGGCCAATGCCATCCATCGCCGAGTTGGCGTGGGGCGGCACATTGATGG
GGCTTATCAGATCGGCGGCCAGCACGGCGCCGTGGCACTGGGCCAGCGGTTTTTCGGAGCAGGCCGTGCT
CGGGGTGAGGGCCGCCAGAATGGCATTGCGGGCCGCTTCGACCGAGCGAAAGCCCTTGCCCGCCTCCGGC
TCCTTGCTCTGGCAGCCGTCAAGCCAGCGGCAGACGAAGTCGGCAATGGCCGCCAGATCGTTGATATCGA
GGCAGGGCAGGCTTGTCTGCTGTTGTTCGCCACTCTGTTGGGGCTGATCGCTGGCCAGCGCGATGATGTC
CCGGTCTTCGGGGAAGAGCAGCGGCTTGCCAATCTCGGCGCGGTGCAGCTCGATTTTCGGGAAGTGTTCG
TGCTTAAACCCCTCGACCAAGAGCAGATCGAGCTCTGTCTGATCGAAGCTGGCCAGCAGCTGGCGAAAGT
CCGCTTCGGCCAACTCGGTCTCGCCGTGCGTTGTTTCAGTAATGCGAGCATGACGACGGCGGGAGGCAAC
CATCATCTGCTGGGCGCCTGCCTTGCGCAGGCGATAGCTGTCTTTGCCCGGCTGGTCGATATCAAAGTCG
TGGTGGGCGTGCTTGAGCACCCCGATGCGCAGGCCGCGATCCACCAGCAGGGGGATCAACTGCTCCAGCA
AGGTGGTCTTGCCGGTGCCGCTCCAGGCGGCGAAGCCGAGCAGGGGAAGAGTGGGGGCCGGGGTCATGAG
GTGACTCCTTGTTGGGGGTCGATAGCCGGAGCAAGCAGAAGGGCCTCGTAGGCGGCCAGCTCGGCAGGGC
TATTGAGATTGATAAAGGCATCCGGCTGATCGGCAAAGCTCACCACCGCCATATGGTGGCGGGCAAACCA
GATATCGATTTTGCGCTCTCCCTCTGCCAGCGCAGCGGTGAGGGAGGGGAGCAGGGATTTGTGCAGCAGG
GCGACCACGGGTTGCAGCCAGTCGCCGTCGTGGGCCACTACCAGCTCGGTATCCGGGGTCAGGGCGGCGC
GAAAGCGGCTCATCAGATCCAGCGGCAGGGCGGGGCCATCGCAGGGGACAAACAGCACCCAGTCGTGACG
GGCGGCAGCAAGACCTGCCAGCATGCCCGCCAGCGGGCCGAGATAGTCGTTGTTGTCATCGCTGATGACC
GGGGCCAGCGCTTGATAGCGGGCCTGACTGCGGTTGGCGTTGATCAGCACCTCATCCACCTGGGGGCGCA
GGCGCGCCAAAACATGGTCGATAAGAGGGCGTCCCGCCAGTGCTACCCATCCCTTGTCTTCGCCCCCCAT
CCGGGTGGCGCGGCCTCCTGCCAGGATCACTGCTGATACGGCAACGGGATCAGAGTGGCTGTCGCTTTTT
GAAGTCATGTCGCTCGATGATTTTTAAAGGGGTCGTCTCTTGCAGCTGGCCACGGGCGAGGGTCCATTGC
CGCTCGCACAGGGAGGTCAATGCGTTGCTCTGGTGGCTGATGACGACCAGGCCGCTGCCATGTTCCCGCA
GATCTTGCGCCAATGCGGCCATCAGGGCAACCGAATGTGTGTCCAGATTGGCGGTGGGCTCATCCATCAG
CAGAAAGGCCGGTTGCAGCGCCCAGGCTCGAGCCATCGCCAGCCGTTGCCGTTCGCCACCTGACAAAATG
CTGATGTGCTCCCGCGCCAGCCCCTCCAGCTCGACCCGGCGCAGCGCCTCGAACACCCGGATCTCGCTGC
CTAGCTGTTTATCGAGCGCCCAGGCCACGTTGTCGTGCACTGTGCGATCGAACAGATAGGGGGTCTGGTG
CAGATAGGTGACCCGGCCTGGCCCCAGATCCCGCTTGAGCAGCCGGTTGAGCCGCTGTTGCCAGCGCCCC
GGCAGATTGGTGTGACCGCGGGTGGGTGCCAGCAGCCCCGCCAGGATTTTGAGCAGAGTGGTCTTGCCCA
CCCCGTTGGCTCCATGCAGCCAGATGGCATCCCCCGGTGCGATGGCGAGCCGGTCAATCTCGAACAGCTG
GCGCTCGCCAAAGCGCATCGTCAGCTGGTGGGCGGTCAGGGCCATGGGGTACTCCTGTTGATACACAGTG
GGGGCATGGTCAGTTCTTCAGATAGCTGTGGCCGCGACAGGCCGTCAGCAGCAGGTTGAGCAAGAGGGCG
AGGGTGAGCAGCACTATGCCGAGCGCCACCCCCTGGGTGAAGGCCCCTTTCTGGGTTTCCAGCGCGATGG
CGGTGGGGATATTGCGGGTAAAGTGCAGGATATTGCCCCCCACCATCATGGAGCAGCCCACCTCGGTGAC
GATACGGCTGAAGGCGCCGATGATGGCGGCCAGCAGGCCAAAGCGGCACTCGTGCATCAGGCCGATCAGC
GCCTTGGGCCAGGAGACGCCCAGCGTCAGGGCGGTCTCCCAGGCGCGCCGGTCACTCTGGACAAACGCCG
CGTGAGCCATGGTGGCGATCACCGGAAAGGCGATCAGCATCTGCCCTACCACCATAGCCTGCTGGGTAAA
GAGCAGCTTCCAGTCGCCAAAGGGGCCGGCCCGCGACAGCAGCATATAGAGCAAGAGGCCAATCACCACG
GTCGGCACCGATTGCAGGGTATTGATGCAGGAGAGCCAGAGCCAGCGGCCGGGCAGCGGCAGATAGGCCA
GCGCAAAGCCCAGCAGCAAGGCGGGCAGCAGCACCAACAGCAGGGCGATGCAGGAGACGGAGAAGGAGAC
CCCCACCACCTGCCACAGTTCGGTGTCACCACTGAACAGCAGGGCGAGGGCCGCCAATGTCGTGTCGATC
AATGTCGGCATCAGACCTCTCTCTCATCGTGCAGGGCGAGGGCCGTCAATACGGTCGTTTCGATCAAGGT
CGGCATCAGGCGCCTCCCTCAGCGAACCGGGGGAGGGCCATCAAGGTCATCGCAGGGGTCAGGGTTGCCA

TTATTTCCCGTTGGCATCCGCCACAAACAGCGCTTGACCGGCTACCTTGAAGTCGCCGATCAACTTCTGG
CCATGCTCGGAGACCAGCCAGTTCTTCAGGGTGCGCGCCCCTTCGGTGTTGAGGTCCGGGTTGCGCTTGG
GGTTGACCAGGATCACCTGATAGGGATTGAACAGGCGCTTGTCACCCTCCAGCAGCACGGCGAGATCCAG
CTTGCTCTGGTAGGCGAGCCAGGTGCCGCGGTCGGTCAGGGTGTAGGCACCAAGCTCCGAGGCCATGGTC
AGGGTCGGGCCCATCCCCTGGCCGATGGATTTGTAACCGGCAAACTCGGGTTTGACGTCAGCCGCCTGCC
ACAGGGTCTTCTCTTTGATGTGGGTGCCGGACTCGTCACCGCGGGAGATAAAGGTCTGGCCCTGCTCCTT
GATGGCCTGCAGCCCCTTGGCCGCGTCGTGCAATTTGGCCAGACCCGCCGGGTCGTTGGCCGGGCCGACG
ATAACGAAGTCGTTGTACATCAGGTGCACCGGCTCGATGCCAAAGCCTGCGTCGACAAACTTCTTCTCGG
CAGAGGGGGCGTGGGTCATCACCAGATCCGCATCGCCATTCTCGCCCATCTTCAGGCTCTGGCCGGTACC
GGCCGCCATTACCTGCACCGGATAACCGGTCTCCTTGGTGAACTGGGGCAGCAGCCAGCCCAGCAGACCG
GACTGCTCGGTACTGGTGGTGGTGGCGAGGCGGATCGGCGCTTTTGTGGCATTAGTATCCGCTTCGGCGT
GGGCCGCACCGGCGATCAGCAGGCTGGTGGCGAGGGAGAGGGCAAAACGTTTGAGGCTGGTTTTTATCGT
CATCGTGATGCAACTTTTTCAGGGTGGCTGAGGAGAGGGCAAGAGTAAGCAAGCAGGGTGCCAAACTGTT
CCTTTGCCGGTGCATTCACGGTTTGCCGTTTAAAAACAGAAGCTTATTAGAATCAATTAGGTGAGCTTGT
TATCGCTTTTAATAATTGGTGTTGGTGCTTTTGATACGGTAAGGACAAAATGTCCCACTAAGGTTCCGGT
TATCGGGACATTCACCGACCGTACGTGCATCCGAGGTACTTTTTTCCATGACACTCCCTCCCAGCCGCTG
GTCTGCCCTCTCCCTGCTTATCGTTGACGATGAGCCCGGCATGCGCAGCTTTCTGGCCAAGGCGCTGGCC
AAGCGTTTTGCCCAGATTGAGACTGCCGCCAGCGTGGAGGAGGCGGAAGCCCTGCGCTGCCGCCTTCACT
TCGACCTTATCATCGCCGACATCCGGCTGCCGGGGCGCTCCGGCATCGAGTGGCACGAGGCCATAGACCC
GGCCACTCGCCGCAGCGACATCATCTTCATGACCGGTTTTGGCGATATGGAGACTGCCCTCAAGGCGCTG
CGCCTCGGCGCCAGCGACTTTATCCTCAAGCCCTTCAACCTCGATCAGATGGTGCAGGCGGTGGACAGAG
TCATCGAGCGCCGGCAGATGGAGCGGGAGAACCTGCTGCTGCGCCGGGAGGTGAGCAAGCTGTTCCCACC
CACCCTGATTGGCGACAGTGCCAAGACCCGCGAGCTCAAGACGCTCATCGCCAGAGTGGCGCCCTCCAAT
GCAGCCGTATTGGTGGAGGGGGAGTCCGGCACCGGCAAGGAGCTGGTGGCCCGCGCCCTGCATCAACTCT
CTGGCCGGATGGGAGCCTTTGTGCCCCTCAACTGCGCCTCCATCGCGCCGGAGCTGCTGGAAAGCGAGCT
GTTCGGCCACGTGCAGGGTGCCTTTACCGGCGCCCGCAAGGGGCGTGACGGCCTGTTCCGGGTCGCCCAT
CAGGGCACCCTGTTTCTCGACGAGATCGGCGAGATGCCGCTCGCCATGCAGGCCTCATTGCTACGGGCGC
TGGAGCAGAAGGCCATCCGCCCGGTCGGTGCCGAGCGCGAATTGGCGGTGGATGTGCGCATCGTCGCCGC
CACCAACCGCAACCTCAAGGCCGAGGTGGAGGCGGGCCGCTTTCGCGAAGATCTCTTCTATCGCCTCAAC
GTGGTGGCGCTGCCGGTCGCCCCGCTGCGGGAACGGATCGACGATATTCCCGCGCTGGTTCACCACTTTA
CCCGCCAGCTCACCGCCGAGATGGGGATGGGCTCCTTGAGCTGGACCCACGAGGATGTGGTGGCGATGCA
GGCCTACCCCTGGCCCGGCAACGTGCGGGAGCTGCGCAACCTCATCGAGCGCTGCCTGCTGCTGGGCAAG
CCCCCGGCGGATTACTGGCAGAGCAGCAAGCCCGCCGAACAGGGGGCGAGCGAGGAGGGTTATCCGTTGG
CATGGCCGCTGGCGGAGGTGGAGAAGGCCCACATCCTGCAAGTGGTGGCGAGCCATGATGGCAACAAGTC
GGCCGCCTCCCGGGTGCTCGGGGTGGCGCGCAAGACGCTGGATCGCAAATTCAAGGAGTGGGCCATCGAA
TGAGGAAGGCTGATCGGATGGGGTGGCGATGAAACTGACCCTGCTCTCCCGCTGCATGCGCGCCCTGTTG
CCCCGTTGCGTTCGCACCAAAGTGCGCTGGCGCCTGCTGCTGCTTACTTCGGTGCCCATCATGCTCACCC
TGGTGGGCATGATTGCGCTCACCCTCTACTGGACCCTCACCTACAGCTGGCAGAACCTGCTCACCACGGT
GCGCAGCGATCTCGGGGTGGCCCATCACGCCATCACGGTGCAGCAGCAGCGCCAGACCGAGCATCTCGAG
CATCTGCGCGATGCCTGGGCTTTTCAGCTGCGGCTGCGGGAATCGCCGGATCAGCTGCATGACTGGGTGA
TGCCGCTGGCGGCCAACTATGGTCTCGATTTTCTACGCTTGCGCCGTGGCCCCGAGCTGGATCAGCTGAC
GGCGCAGGAGCGCGCCGCCCTCACGGCCGGACGCAGCGTCAGCCACTTTGTGGTCTGGTCGCAGCAGGAG
ATGCTCGCCCTCTCCCCGCAACTGGCCAAGCGGGCCGTGCTGTGGCAGCGGGATGGCACTAGGGTGGTGG
AGAACAGGGGGCTGATCAGCCTCTCACTGACGCCGGTGATGGGCCGCGACGGCTCCCTTTGGGCCGTGCT
GGAGGGGGGCGAGCTTGTCAACGGCAGCACCCGGCTGGTGGATGAGCTCAAGGTGCAGGTGTTTGCCAAA
AACACCCTGCCGGATGGCAGTGTCGGCACCGTCACCCTGTTTCTCGACGATCTGCGGGTGAGCACCAACG
TACCGGTCAGCAGCAGCCGTCAGGTGGGCCGGGCGGTGGGCACCCGGGTCTCGCTGCCAGTGCGCGAACA
GGTGCTGGGGCGGGGGGAGATGTGGGTTGACCGCGCCTTCGTCCACGATGCCTGGTATGTATCTGGCTAC
GAGCCGCTGCTGGGAGCCAGGGGCGAGCGGATCGGCATGCTCTATGTGGGCTTTCTCGAGTGGCCCATTA
TCCGCACCTACCTCACCAATCTGGTGGAGCTGGGCTCCGGCGTAGTGCTGCTGTTGCTGCTCTCCGGCCT
GCTGGTCTATCGCGGCGCGCGGGATCTCTTCTCCCCCATCGAGCGGATGCACAAGGTGGTGCGGCAGGTG
CAGGCCGGGGAGTCCGCTCGCATCGGCCCGCTGGCCCTCGACCCTGACCACGAGCTGGCCCAGCTCGGTC
GCCAGTTCGACGCCATGCTGGACAGGCAGGAGGCGCATCAGCAGCAGATGGCCCGCAGCGCCGACGAGCT
GGAGCAGAAGGTGGCCGAGCGTACCCAAAGCCTCGAGCTCAAGACTGCCGAGCTGGAAGATCATATCCAT
ATGCTGACCGAGGCGCGCCAGCAGTTGCTCATCAGCGAGAAGCTGGCGGCCCTTGGCGAGCTCTCTGCCG
GTGTGGCCCATGAGATCAACAACCCGCTAGCGGTGATCCTCGGCAATGTGGAGTTGATGAAGCTGGAGCT
GGGGGAGGCGGCCGAGCCGGTCAGCGAGGAGCTGGCGCTGGTGCTGGCCCAGATCGAGCGCATTCGTGCC
ATCACCCGCAGCCTGCTGCAATACTCTCGCCCTGGCGATTACGAGACGGCGCCTGTGTGGCAGCACCTCA
ACCCCATCATAGAGGAGAGCCTGACCCTGGTGCGCTCCGCCCTGCGTCAGCAGCAGGTGGAGCTGGTGGC
CGATCTCAAGGCCCAGCAGCCCATCGAGGCGGATCGCCAGCAGCTGTTGCAGGTGCTGATAAACCTTCTG
GTCAACGCCAGCCACGCGCTGGGGGAGCAGGGGGAGATCCGGGTCGAGAGCCGCGACTGGGTCGATGAAC

TGGGTCAGCTGCGCGGGGCAGAAGTGCGGGTGATCGATAACGGCAGCGGCATCGAGGCGGCCATTATCGA
CAAGATCTTCGATCCCTTTTTCACCACCCGCGCCACCGGCACCGGGCTGGGGCTGGCCCTGAGCCACCGC
ATCATCAGCCGCTGGGGCGGCGAGCTGTTGGTGGACTCCACCCCGGGGCAGGGGAGCTGCTTTACCGTAC
GGTTGCGCAGCCATGCCCTGCTGCCCAGCGGCAGCGAGCCCGCGAAACTGGAAGGGTGGCGCATCGCCCT
CAATGCCGAGGGGCGTGAGACAAGCGATTGATGCTGTTATAAAAAGGCCCGTGATAAAAACAGCCCGTCA
ACATGGTTGGCGGGCTGTTTTGTCTTTGGGTGAGTGGTTCTTTACTGCGGGCTCCACACCAGCTTGCCGC
TTTGGGTCAGGTCATAGCCGCCGAGGCGGGCCGCCAGCTCGCGTCCCTCGGGGGATTGCAGCCAGCCGAT
CAGCTGTTGCAGCAGGGTGCGAAAGAAGACCCCCTGCGGCATCACCAGATCGAAGCACTCCTGCGACAAG
GGCATAAAGCCGAGGCCAAACTCGGTGGCGGTACTCTGGGCGCCGGGCCCGACATCCGCATCGCCGCGGC
TGATGGCGGCAGCCAGTTCCCGCTCGCTATTGACCTCGAGGGTGCGCTCCAGCCGGGTCAGCGACTCCCC
CTGGGTTTGCAGCCACTCCTGCAGGGCGCGCTGGCTGCCAGCCCCCTCCTGACGCATGGCCCAGCGCCAG
TCGAGCGCCTCTTTCGCCTCCTTGGGTTGCAGCCCTTGCCGCAGCACCAGCCCCTGCACCCGCCTGAATC
CATGAACGATCACCCATTGCCGGTGGCCCTGATACTGCTGCACCAGCGCCGGGTGGCGCAGGTGCGCCTC
CTCGGCCTGCCCCCAGTGGATGGCGCAGAGATCCACATGGCCGCGGGCCAGCATCGCCAGCCCCTGACGG
GTACCGCACGGGGTGTAGCCCACCAGTGCCTGGGTGCCGAGCTGCTGTGCCAGCCGGCTGCTGGCGTAGT
GGAGTAGTACATCGTCGGAGCCCGCCAGCAGCAGCCGGTCGTTCATCACCCCCTCGTGGCAACTGCCGAG
CAGCCAGCGATCGAGCAGGGAGCGGGGAAACAGCCACTTGCCGGTCGCCTTGGTGGCGGGGATGCGCGCC
TCGTTGGCGAGCTGGTAGACCTTCTTCTCGTTGAGATCGAGGTATTCCGCCACCTGCTTCACGTTCATAA
ACTCATTCATGGCTTAGTCCTGCATCCCGCGACCGGCCGGACTGGGCGCCAGCACCGGCGGCTTCTCGTC
CAGCACCATATGGTCGTGACCGCTGAATACCTCGAAATGACGCCCCTTGGCGCGGGCAATCATGGTGATA
CCGAGCTGGCGGGCCAGATCCAGTCCCATCTGGGTGACGCCGGAGCGCGACAGCAGCACCGGAATGCCCA
TCTGTGCCACCTTGATCACCATCTCCGAGGTGAGCCGCCCCGTGGTGTAGAAGATCTTGTCGTGGCCAGC
CTCGCCGCGCATCCAGAGCTCGCCCGCCAGGGTATCCACCGCATTGTGGCGCCCCACATCCTCGACAAAG
GAGAGGATTTCGCTACCGCGACAGATGGCGCAGCCATGCACGGCACCCGCCATCTTGTAGGTTTCGTTAT
GGGCCGACAGCGCTTTGAGCAGGGCGTAGAGGGTGCTCTGTTTAAGCTCGGGCGCCGCCAAAACCACCCC
TTCGAGCTTCTTCATCACGCTGCCATACATGGTGCCCTGACCGCAGCCGGAGGTGACCGTCTTCTTCTCC
AGCGACTTGTCGAGATCGGCTGCCTGCTGGCTGCTCACCACGGCGGCGGCCTCGCTCTCCCAGTCGACAA
TCACCGACTCGATGGCTGCAATATCCTCGATAAAACCCTGATTTTTCAGATAACCCAGCACCAGCGCCTC
GGGGCGGGCCCCCAGCGTCATCAGGGTCACTATCTCCTTCCAGTTGAGGTAGACAGTCAGTGGCCGCTCA
CAGGCGATTTGGCGGGTGTTCATTGTGCCCTGCTCATCCATCACCTCGACGGCCATGGTGAGCTGGGCGG
CGGCATTGCTTTTGATAAAGGTCGGTGTCATGGGCCCCTCGCGACCGGTTGCCGGGATCTAAGGGGAGGG
ATACAGCAAGTTTTGTTCCAGATTAATGAGACGGACCAAATGTCAGAGTGATGGTACGCAAAGCATAATT
ATGTTGTCTTAATAAAAGCGAGCAGGCTCCCATTCTGACAAAGAAATAAGTAGCCATACTTTTCTTTGAA
AACACAGATGGTTAGACTTATGAAAAACTAATTTTTAGGTGTGAAAATTAGCATTTAAATGGATATGCTG
TTGTAGTTTTTTTGCCTCTCGACGCTAATATTCTGGATTAAGTATGAACGAAGAAATTGAGAACTTATGT
AATGCGTTGGATAGCCTCGGTGAGGCTGTGCAGAATGGATGGAGCGATAATCGAACACTCAATGAGGCTT
TTGGGTGGCATCATCCGGTCTTAGATAGAAAGGAATTAGCTTATTTACCCATATCTCTTGCCGATAAAAT
TAGACGTGTAAAGTTAGAAACTGATGATGAAAAATTCCTCCAAATAATCGATGGCATTCCTGAAAAACTT
GAGAGATTAAAATCTTATACATTGCCATATTTTTATAATGGCAATGGACATCAAGCAATTCCTGCCTATA
TCGACACTTTAAATTGGATATCCAGTGTTGTTGAGCCTTATATATCATGGGTTGTATCCAATGATCCAAA
TTCAATGCCACCAGCCATATCGCGTCGTTTAAAAGGGTTGCAAGCCAAAATTCAGCAAATTGATGTCGAC
CAAGATAGGCTATATAAACAAATTAAATTAATTAACGAAGCAACGGAAGCAGCAGAGTCATTACCATCAG
ACCTGGAAGACTTGAAAGCTGCGAGAAAAAAGGTTGAATCCATAGGACAAGATGTTTTGAAGACATCTTC
AAAAGTTTCTATGGCCAAAGAAGAGGTTGAAGGACTTTTAAATTTAATCACAGAAAATAAATTAACATCT
GACCAGCTAGTAAGTAATTGTGAGGATGCATATCGCATTACAACCAGCAAAGGACTGGCTGGGGCGTTTG
ACCAGCGAGCTAAACAACTAGCCAAGTCAATGTGGGTATGGGTTTTTGGTCTGCTATGTGCATTAGGCGC
AGCATGGTATGTAGGTGCTCAGCGTATTGAAATTCTCACAAGTTCATTATCTACTCCAGACCCCAAATGG
GGTATGGTATCGATGCATTTTATGCTGGCTGTGGTTAGCCTTGGGGCACCACTCTGGTTTGCTTGGTTGT
CAACAAAACAAATAGGTCAGAGATTTAAGCTTGCAGAGGATTATGGCTTTAAAGCTTCTGTCGCAAAAGC
TTATGAGGGTTATCGAAAAGAAGCTTCAAGAATTGATCAAAAACTAGAGGCTCGCTTATTTGAGTCTGCC
TTAACAAGGGTTGAAGAGGCTCCATTACGACTTGTTGAAGGTACATCACATGGTACTCCATGGCATGAGC
TTGCTGAATCAAGTGGCTTTAGAGAAGCATTAAAAAATATACCTGAGTTTCGAGATAAGGTTTTTTCGTT
AGCGCAATCAATGGTTGATAAACCCGGACGAAGTCAGCCCAAGGATGAGAGTAACGCTGCATAAATATTG
GCCATCCGATATAACGTATGTTGGATAAAATTGTAGGTTGGTGCTGAACGAAGTGAAGCCCAACGTTTAC
CGCGACACCTTATCAAGGCAATAATCGGATAATTCAGCGTTATTCGATTATTGCCATTCCTCGATCAGAT
ACACAGGCTGATTGGTTTTACTCCGCATGTTGGGCTTCGCTGCGCTCTGCGCCAACCTACGCCCGCGCCA
ACCCATACTCCTCATGACGGGCCAACCCGCATTAAAGCCTCCATCCACCACCAAACCTGCCTTGGCCCCA
CTCTTGCTATGTCTTATCCCATTGATGGCCGTGGTCGCCCTGAATCTCCTGCTCTGATCCGCCCCGAACG
GGGTGAATCACGGGGGAGGGTGGCCCGGCAGAGACCTTCTGATGGGAGCCGTGACAGTGCAAGAGACAAC
CAAACCCCTCTCCCCGCAGCGCTGGCTGCAACTGGAGTTAAAGGCCATCGACAGTCAGGTTGGCCGCTGG
CAGAGCCGCCGTTTCGAGATTGCGGCGCTCCACCCCGCCGAGCCCGAGGCGGCCAGCGCCTTGCCCCTTA

CCCTGTTTCGCGATGAGCGGGGGGATTATCGCTTCAATTTGAGCTCCGGCTCGCCCCGCCTCTTTGTGAC
CGGAAGCCGCGTGGACAATGGCTTTGTGGCCGATGGCCTGACCTTGAGCCAGACTGTGGCCGCCGCCTAT
ATGGATGGCGAGCGGCAGGTGCTCTCCTGCCCGCTGCCCGAGGCGCTGCAAGCCTGGGTGGAGGCCTTTA
TCGGTCGCCACGGCGAGCTGCTGGAGGCCCGTCGCAAGGGCAAGAAGGGCAAGGGACGGGCGCAGGATCA
GCTGGGAGTCGATACGGTAGATCAAGCCAGCTGTGGCGCCGTTAAAGCCACCGGCCCCGATGGCCGCGAG
CGTGGCAGTGACAGCGGAAATCGCGCCCATGAGTGAGTTCCTCAAGCGCTGGTCGTCTCGCAAGAGTGAG
GCGCGCAAGCATCAGGCATTGCAGCCACGGGAAGAGGTTATCCCCGAGGCATTTGCCGCCCCTATCGAAG
GCAGTGGCGAAGGTGAAACACCAGATAAATCGCTGGATAGCCCCGTAGTCAGCACTGACAAGGGGAGCTG
TGAATCTCTTGCCGTTGAGGGGACCGAGAGTGGCGAGGCGCTGCTGAAAACAGCCCATCCGGGCAAGGCC
AAGCTCCCCGACAAGGAGGCGCTGCGGGCCATGTTTCGCACCCATAAGCCAGACGGGCTCGACGACTACA
CCCAGGATTTCAGCCAGCCCGAATTGCTGCCGGCAGCCCTGACCGCCGGGCTGCGCAACTGGCTGGTGGA
TCAGGTGATCGCCCCGGAGAGCGAATCGGCCAATCCGGCCAGCGGATCCGCTGAACCGGTGGTGCTTGCC
AGCGCACCTGCCGCCGACGAGCTGGCTGATAACCATTCTCAAACCCACACCGATGATCGGGACATTTTGG
TTTCCTCTGGTGGACAAAATGTCCCAACCAAAGGTGAGGGAATGGAACCCTCAGGATCCTGACTCCCTGT
CAGTCACTTTTTTCCCGGGGGAGTTGGCTCGCCTCTTGCTCTCTCGGCTTACCAACAAGCGCCCATCCCA
TGAGGTGGCGCGCGCAGGAGTGTATGTGAGCACTGAACAAACATTGCCAAATCTGGCCCCAAATCTGGCC
CCAAATCTAGCCCGCGAGGCGGGGGCGGCCGGGCATAACGCGCGCGCTCGCGCCTATGCCCTGCAGCATA
CGGTCAAGACCGACAACCTGATCCCGGCCACTATCTCTTACCAGAGTCAGGGGCGCCTGCTGCTGGTCGG
CCCGGAAGATCGCATTCGCCGCGCCGCCAGCCTGCTGCCTGCCGGGGTGAAGCCCACCCTGCTGGTGATC
GAAGCGGTCCGTGATGCCGAGGCGGCCGATCTCGAAGCCATTTTCGATGCCACCGCATCCCTCGCGGCCC
TGACCCTGCGCGAACCGAGCCTGACAGGGTATCTGGGGCAGTTCCAGCTCACCGGCCTCAATGGGCAGGG
TGAGCGGGTTAATCTGGCGGCGCTCTGCTTCCCACAACAAGGCTTTCCACAACAAGCGGCTGCTGTCAGT
GAACAGCCACGCGAACCGCGCTTTGACCTGGTGGCCGATCTGGGCCGCGCCCCGCTCTTTGCGCTGGAGC
GCCCGCCGGTCGGTTATCTGCATCTCTTTGATGACCAAGGGCTGGCCGAACAGCTTGCCGAGCTCTGTGC
GCTGACCGGTATCTTCGACAAGCCGCGCTATTTTCGCCTGGACCCCGAGGCCTGCGCCTTTACCGCCCGC
GGCGTGCCCGGTTGCAGCCGCTGTCTCGATGTCTGCCCGACCGATGCCCTCAAGCCCATCAATGGTCGCA
TCCAGATCGATCCCCATCTCTGTCAGGGCTTTGGTAGCTGCGCCTCTGCCTGCCCGACCGGGGCGATTGC
CTATCATCAGCCCGATGCCAACACCAGCGGCGACTACCTGCTGCGATTGCTCAAGCATTATCGGGAGGCG
GGCGGCGAAGCGCCCCAGCTGTTGATCGCCGGTGAAAGCGAGCGCGAATGGGTGGAAACGGAACTCACCC
GCCTGCCCGCCAACTGGCTGCCGGTCTGGGTGGAGGAGAGCGCGAGTTTGGGTATCGAGAGCTGGTTCGC
CGCCCTTGCCTATGGCGCCAGCGCGGTGCGCATCGCGCTGGGCGACGATGCCCCCGCAAGCGTCCGTGTG
CTGGTTGAGCGGGAACTGGCGAGCGCCGCCGTGCTGCTGGCGGGCGCCGGTCTTGCTGCCAATCGTATCG
CCCTGTTTAACGGCGATGCCGAAATGGATGAGTCTCTTTACGCGCAGCCAGCCGTTGCCCTGTTGGATAA
GCCGCTGAAAGGAGACAAACGGGAAAACCTGTTTGCCGCCTTCGATGCCCTCTGGCAGGCCAACGAAGGG
AGCCGCGAGCCGCTGGCGGTGCCTCACGGCGCCCCATACGGCAGCGTGGAACTCAAAGAAGCAGACTGCA
CCCTCTGCATGGGCTGCGTGGCGGTTTGCCCGAGCCGCGCCCTGCATGCGGTGGGGCACACGCCCGGCCT
CAACTTTATCGAGCAGGATTGCATCCAGTGCGGCATGTGCGAAAAGGCGTGCCCCGAGCAGGCCATTGTG
CTGGTGCCCCGCCTGCAACCCGTGCCCGAGGCGCGCCGCTCCGTGCAGAGCCTCAAGGCCGAAGAGGCGG
CCTGCTGCATTCGCTGCTCCAAGCCGTTCGCGCCCGCCTCGATGATCCGCCGCATCCAGCAAAAACTGGC
CGGTCACTCCCACTTCCAGAACGAGGCCGCCCAGCGGTTGCTGATGTGCGAGGACTGTCGGGTCAAGGAT
GTGTTTGCGGCGCTGGCGGCGGATCCCGTCTCCCAGCTCAAGGTATGAGGGGCCAGTGATGAGTGAACAA
CATAACGAAAAGGCTGGCGAACTGACCATGACCGAACTGGAACAGAGTAGCGCCGATCTCTACGCCCTGC
TGGCAAGCCTCTGGCGCGCCGCCCCCCGTGGCGAACAGCTCGCCTGGCTGCAAGGGTTGCAGGTTATGCC
GGGCCCCTTTGCCAGAGGGCTGGGGCTGGTGCAGCTGGCAGCCAGTAGCCACCACGAAAACGAGCTTGCC
GAGGAGTATCAGGATCTCTTTATCGGGCTGGGGCGCGGGGAGATTGTGCCGTTTGCCTCCTGGTATCTCA
CCGGCTCTTTGATGGAGCTGCCGCTGGCGGTGCTGCGCGCAGACCTCCAGTCCCTCGGCTTCGAGCGCCA
GAGCGGAGTGTGCGAGCCGGAAGATCATCTGGCCGCCCTCTGTGAGGTGATGGCGGTACTGATTGGCGAG
CAGCACCCGGAGCGCAACCGCTTCTATGAGCGCCACCTTGCCCCCTGGGTACTCGCCTGCTGCCGCGATC
AGCAACAGGCGAAAGGGGGCAGCTTCTACCGGGCGGTCGGCGCCCTTGCCGAAGATTTCTTCAAGCTGGA
AAACGAATTGCGCCTGCCGTTTGGCGCCTGCGTGGTGCCGGGCTGAACGGCCCTATCTGCGCATAACAAG
AGCTGCGCATAACAAAACGAGCAGACGAGATATCCCATTGGACGCGAAGCTGGCGCACAGGCGCCAGCAA
GAACAACAACAGAGGTCACAAGAATGAGCGATGAACAGCTGAAAGACCCGTCCCGCCGCAATCTGATCAA
GGGAGTTGGCGTGGTCGCCACCATAGGCGTGCTGGCCGGTGCCACCGGCCGGGTGGTGGGCAAAGAGGTG
CTGACCCACGACACCGAGCCTGACAAGGGCTATCGGGAGACCCCCCATGTTCACGACTTTTATCGCACCC
TGCGCGGCACCGACTGAGCCGGATCAGTGAAGGAGAACTCATGAAACTGACAAAACGTGCCGAGCTGCCC
CAGGCGGGCGAGCAGGGCAATGAGGCCATTCGCGGTCAGGGGCTGACCCGTCGCGCCTTTATGGTCAACT
CCGGTCTGGTGGCCGGTGGCGCCGTGGCCGCCGGTTGGCTGGCCCCGGCCATGATGCAGCGGGCCGAGGC
CAAAACCGTGGCGGCCGGGGTGCCGGTCGAGGTGAAGCGCACCATCTGTTCCCACTGCTCGGTGGGCTGC
GGCGTCTATGCCGAGGTGCAGAACGGCGTCTGGACCGGTCAGGAGCCGGCCTTTGATCACCCCTTCAACC
TTGGCGGCCATTGCGCCAAGGGGGCTGCCCTGCGCGAGCACGGCCACGGTGAGCGTCGCCTCAAGTACCC
GATGAAGCTGGTGGGTGGCAAGTGGCAGCGCATCAGCTGGGATCAGGCCATCGAAGAGGTGGGCAACGAG

GTGCTGCGCCTGCGCAAGGAGTCCGGCCCCGACAGCGTCTACTGGCTCGGCTCCGCCAAGCACTCCAACG
AGCAGGCTTATCTGTTTCGCAAGATGGCCTCCCTGTGGGGCACCAACAACGTGGATCACCAGGCGCGGAT
CTGCCACTCCACCACGGTGGCTGGCGTCGCCAACACCTGGGGCTACGGCGCCATGACCAACAGCCTGAAC
GACATGCACAACAGCAAGTCGATCATGTTTATCGGCTCCAACCCGGCGGAGGCCCACCCGGTCGCCATGC
AGCACATCCTGCTGGCGAAAGAGCGTAACGGCTGCAAGATCCTGGTGGTCGACCCGCGTCGCACCCGTAC
CGCCGCCAAGGCGGACATGTACCTCTCCCTGCGTCCGGGCACCGACGTCGCCTTTATCTGGGGCATGCTC
TGGCACATCTTCAAGAACGGCTGGGAAGACAAAGAGTTCATCCGCACCCGCGTCTACGGCATGGACGAGG
TGCAAAAAGAGGTGGCGCGCTGGACCCCGGCTGAAGTGGAGCGGGTAACCGGCCTCAACGAACAGCAGGT
CTATGGCGCTGCCAAGCTGCTGGCCGACAACCGCCCCGGCTGCGTGGTCTGGTGCATGGGCGGTACCCAG
CACACCACCGGCAACAACAACACCCGCGCTTACTGCATCCTCGAACTGGCCCTCGGCAACGTGGGCAAGA
GCGGGGCGGGCACCAACATCTTCCGTGGTCACGACAACGTGCAGGGGGCCACCGACTTTGGCGTCACCTC
CGACTCCCTGCCCGGCTACTACGGCCTGGAAGAGGGCTCCTGGAAGCACTGGGCCCGGGTGTGGGATGTC
GATTACGAGTGGATCAAGGCACGCTTCGACAACAAGGCCTACAACGGCAAGCTGCCGATGAACAACAACG
GCATTCCGGTCTCCCGCTGGGTGGACGGGGTGCTGGAGAACGCCGATCTCATCGAGCAGGGCTCCAATAT
CCGCGCCATGTTCTACTGGGGCCATGCGGTCAACTCCCAGACTCGCGGCCCCGAGATGAAGAAGGCGATG
CAAAAGCTCGACATGATGGTGATTGTCGACCCGTACCCCACCGTCGCCTCGGTGATGAACGATCGCACCG
ACGGCGTCTACCTGCTGCCGGCCACCACCCAGTTCGAGACCTATGGTTCCGTCACTGCCACCAACCGCTC
CATGCAGTGGCGCGACAAGGTGATTGAGCCCCTGTTCGAATCCAAGGTCGACCACGAGATCATGTATCTG
CTAAGCAAAAAGCTTGGCTTTGCCGACCAGCTGTTCAAGCACATCAAGGTAGAGAACAACCAGCCGCTCT
TGGAAGACATCACCCGCGAGTACAACCGCGGCATGTGGAGCGTGGGCTACACCGGCCAGAGCCCGGAGCG
GCTCAAGGCGCACCAGCAGAACTGGCACACCTTCCACAAGACCAACCTCAAGGCCGAGGGTGGCCCGGTC
AGTGGCGAAGTGTACGGTCTGCCCTGGCCCTGCTGGGGTACCCCTGAGATGAAGCACCCCGGCAGCCACA
TCCTCTACGACACCAGCATTCCGGTGTCGGAAGGGGGCGGCGGTTTCCGCGCCCGCTTCGGTATCGAGTA
TCAGGGCGTCTCGCTGCTCGCCGAAGACTCCTGGCCGAAGGGGAGCGAGGTGGAAGACGGTTATCCCGAG
ATCACTGCCGACCTGATGAAGCAGCTCGGCTGGTGGGATGAGCTGACCGAGGCCGAGAAGAAAGAGGCCG
AGGGCAAGAACTGGAAGACCGATCTCTCCGGCGGTATCCAGCGGGTCGCCATCAAGCATGGCTTCGTGCC
CTACGGCAACGCCAAGGCGCGCTGCGTAGTGTGGACCTTCCCGGATCAGGTGCCCAAGCACCGCGAGCCG
CTCTACACCCCGCGCCGGGATCTGCTGGCCGACTACGCCACCTGGCCTGATTCGAAGAGCCTGTGGCGCC
TGCCGACCCAGTACGCCTCGGTGCAGGAGAAGGACGTCAGCAAGGAGTTCCCGATCATCCTCACCTCCGG
CCGTCTGGTGGAGTACGAGGGGGGCGGCGACGAGACCCGCTCCAACCCTTGGCTGGCTGAATTGCAGCAG
GAGATGTTCGTCGAGATGAACCCGGCGGATGCCAACGATGTCGGCGTGCGCGACGGTCAGGATGTGTGGG
TCGAGGGGCCGGAAGGGGGCCGGGTCAAGGTGAAGGCGCTGGTGACCCCGCGGGTCAAGCCGGGGCTCGC
CTTTATGCCGTTCCACTTCGGCGGCCACTTCGAGGGCAAGGATCTGCGATCCAAATATCCGGAAGGGTCT
GACCCCTATGTGCTGGGGGAGTCGGTCAACATAGTCACCACCTATGGTTATGACCCGGTCACCCAGATGC
AGGAGACCAAGGTCACCCTGTGCCGTTTTCGCAAAGTAGGAGCCTGATCCCATGGCCAGAATGAAATTCA
TGTGTGACACCAAGCGCTGCATCGAGTGCAACGGCTGTGTGACGGCGTGCAAGAACGAGAACGACGATGC
CCTGGAGTGGGGCATCCAGCGCCGTCGGGTGGTGACCCTCAACGACGGCCTGCCGGGGGAGACCTCCATC
TCGGTCGCCTGCATGCACTGCACCGACGCCCCCTGCATGGCGGTCTGCCCGGCCGACTGCTTCTATCACA
CCGACGACGGCATAGTGCTGCACAACAAGGATCTCTGTATCGGTTGCGGCTACTGCCTGTTTGCCTGCCC
GTTCGGGGCGCCCCAGTTCCCGAAGACCGCTGCCTTTGGCGATCGGGGCAAGATGGACAAGTGCACCTTC
TGCGCCGGTGGCCCGGAAGAGGATCACAGCGAAGCGGAGCGCCAGAAGTACGGCGCCAACCGCATTGCCG
AGGGCAAGCTGCCCATGTGTGCCGAGCTCTGCTCCACCAAGGCGCTGCTGGCAGGGGATGCGCAGGTGAT
CTCCGATATCTTCCGCGAGCGGGTTGCCTATCGTGGTGCCAAGGATGCGGCTTGGGGTAATGGCGATGAG
TTGTTCCACGATGCCAGCAAGCTGAACAAGCAGGGAGGGGCGGCATGAAAACCTGGCTAATGGCCCTGTT
TGCTGCCCTGCTGCTGGTGAGTACCGCAAGTTTCGCCGATGCCGAGAAAGAGGCGGTCGGCTTTGCCGGC
GCCGATTATTGGCGGGCGGTGAAGGATGGGCAGGAGGGGGTGACCACTTCCCGCTCGCCGGAGCACGGAG
TGCTTATCTCGGTGCCGGGCCAGCTCTGGTTCGAGGTGAAGACCAAGTGGGTGTCGCCGCTCGGTGCTAT
CGCCATCTTTGGATCCTTGGCCATGTGTGGCCTGATGTACCTGGTGGTGGGGCAGACCAAGCTCTCCAAA
CCACGCACCGGGCGCAAGCTCAAGCGCTGGAGCAGGCTGGATCGGGCGCTGCACTGGACCACGGCGGCCA
TGTTTCTGACCCTCTCCGGGTCGGGGCTGGCCATCATCTACGGCAAGTACTTCATCAAGCCGGTGGTGAG
CCTCGGGGTGTGGGAGAACTGGATCTGGTTTGCCAAGGTGTTCCACAACTACGTGGGGCCGCTGTTCTTC
CTCTGTCTGATGGGGGTGCTCATCAAGTGGTTCCGCCACAACATCGTCAACATGGTTGATGTGCAGTGGT
TCATGAAGTTTGGTGGCATGCTCGGCAAGCACAAGGGCAGTCACCCCTCGGCGGGCTTCTCCAACGGCGG
CGAGAAGGCGATCTTCTGGCTGCTGATCTGGTTTGGCGGCATTGTGGTGGCGACCGGGCTGTTCCTTGAT
TTCCCCATCTTCGGCCAGCTGCGCCGCCAGATGGAGTGGGCCAGCTTCATCCATATCGTAGGCGCCTTGA
TCCTTATCTGCGGTTTTATCTTCCATATCTACATGGGTCTGTTCCAGCTGGAAGGGGCGCTGGAGGGGAT
GGTGACCGGCGAGGTGGACGAGACCTGGGCCAAGGAGCACCACGACCTCTGGTATGAAGAGGTGAAGCAC
ACCCTCGATGAGCCCAAGGCGGGCAAATCAAGCTGATGGTCGACAAAGAGTAAAGAGAAGGGGCGCTGTG
GCGCCCCTTTTGATGGCGGGTCGATGGTGATGGCAAATTGATTGAGCGATGCCGCAAACGGCGATGGTTT
TTTGGGCCACCTTGAGCAACAGTAACAGGGGTTCCATTCACACAAGGGATGACAAATGATGAGAAAAGCA
TGGCTGGCAGCGGTATGTGCATCTGCGCTCTTCGGTGGTATTGCGGGCGTGGCGCAGGCGGCTGACGGGA

TCAAGACCCAGCAGGTGCAGTTCAAGAAGGGGGAGTCAGGGGCAACCATCAACGGCACCATTCAAGGTAG
CCAGACCCTCGATTACAAGCTGACGGCCAACAAGGGCCAGAGCATGGTGGTGATCCTGACCGGCAAGCCC
TACTTCAATATTCTGCCGCCAGGCAGCAAGGGGGAGGCGATGTTTATCGGCTCTACGGAAGGGAACAACT
TTGAGGCGACCCTGCCCGCCAAGGGCACTTATACCATTCGGGTCTACCAGATGGGGGCGGCCGAGAGCAG
CAAGGCCAAGACGCCGTTCAAGCTTGAGGTGGGCATCAGCGGCTAATGCCAGCAGCTGACAAGCATCATG
GCGGTGGCGGCCACAAAGGGCCGCCACCGTTGTTTTATCCTCTCTGTTTTTGCCTCGCCAAGGTCAATAT
TCCCCCCGGGAAAGCTGTTCGAAGTAGCGCTTATAACGCCCGCTGGCGATGGCCTGCTGCAACTGCTGAT
TGAACTGATCCCGCAGTTTGGCCGAATCCGGCATTTTGCGCGGGAACATCACGAAGCTGTCGTTGCGCAG
AAAAGGGGTCTGACTGTGGGTGATGAGTGCCGCGCTGGCGGGGTCGAGCTGGCTCTGCAGCTCGGCCTGC
CCGACTCGCAGCTCTTGCGGATAGAGGTCGATCCGTCCGCGCAGCAGCTTGCCGAAATTCTGTTTCACGT
CGGTGACTTTCTGTTGGGTTACCTTGCCATCGGCCACCATCTTGTCCAGCTCGGGCCCGTAGCTGTAGGC
GATATCGCCCCCCAACCGCATGCCGATGATCTGCTCAAGGCGCTCGGCCTGCAGCTGCCGCGATTTGAGG
GAGAAGAAGACAAACTGCTCCTGCAGCACCGCTTCGCTAAACAGAAAATCCTGCTCCCGCTCCGGTTTGT
GCATCCAGACTGCGGTGGCGTCGTAGAGCTGGCTGTTGGCCGCCAGCGAATAGACCCGGCCCCAGGGGGC
GAAGTGGAACTTCACCTCATAGCCCGCCTCCTTGAACAGATCGCTGATAAGGTGGGCCACGGGACCGTAC
TGTTTTGACTGCTCGGAGACGAAAGGGGGCCATTCACCCACGATGATGTTGAGCACCCGACCCGGTGCGG
CGAGAGCCGACTGGCTGGTCAGAAACAACCACAGAGCAAGCAGCGGGATCAGGCGCATGGCAGGAAGAAG
GGGGCGCAGGCTGGCAGAGGCGAGGGCATCAGGTCACTCCCGGCTGTGATGCGGTGAAAGGGGCTGGGTT
GCCCGCAATGCTAACATGAGTCTCATTCCTTGCCGTGGATATTTGTCTCGCCGGGGCCGCCAGACGCCTA
TTTTGGCCGCAGGGTATGCCCAAGCTCACAAAACCGATCGAGTAAACGTCATATGATTGATTTCATTATG
AGTCTTGCTCTCCTGCCGAACGGCAATTTCACGTAAATCAAAGAGAATGACTATGGCTACCGGACAATAT
CTGCTGTGGAAAGATGCACAGGGACAGGATTTTCGCCCCGTTGCACTGACCGGCACTGATCTGCTTAACA
ATCGATACCTCAACAAGGGCACGGCCTTCACCGAACAGGAGCGGGCCGATTTTGATCTCGATGGTTTGCT
GCCGCCTCAGGTGCAAACTTTTGAAGCCCAGCTCGAGCGAGTGTATGAGGGGTTCAAAAGCGCTTGCAGC
GATATCGAGAAGTACCAGAACCTGCGTGCCCTGCAGGATCGCAACGAGACTCTCTTCTATGCCCTGTTGT
CACGCCATGTGGAGGAGATGACTCCCTACATCTATACCCCGACCGTGGGCAAGGCGTGCCAGGAGTTCAG
CCACCGCTTCCAGAAGCCGCGCGGCCTCTACATCACCCCGGACAACGTGGATGACATGGGCAGTCAGGCC
CGTCACTTCGATGCCAAATCGATCCGCATCATAGTCGTCACCGACAGCCAGGGCATTCTGGGGATCGGCG
ACCAGGGTGTTGGCGGCATGGGCATCCCCATCGGCAAGCTGGCGCTCTATACCCTGGGGGCCGGTATTCA
CCCCGCCTGCTGCATGCCCATCGCGCTGGATGTGGGCACCGACAACCAGAAACTGCTCGATGACCCCATG
TATCTGGGCCGCCCGCACAAGCGTATTCGCGGCAAGGAGTATGAAGAGTTCATCGACAAGTTCGTGCAGG
GGGTCAAGCGCCACTTCCCCAACGCTGTGCTGCAGTGGGAAGACTTCAGCAAATCCAATGCCTTCAACAA
TCTGGCCCGCTACCAGCAGTCATTGCCGTCATTCAACGATGATATTCAGGGCACCGGTGCCGTGGTGCTG
GCCGGTATTATCGGCGCGGTCAAGGTCAAGGGGGAGACCCTGGGCCAGCAGAATTATCTGGTCTATGGCG
CTGGCTCCGGCGGGGTTGGTGTCGCGGATCAGATCTGCGCGGGGATGATCCGCGAGGGGATGGATCCGCA
GGCGGCCCGCGATCGCATCTACATTCTGGATACCCAAGGGCTGGTGTGCGACAACCGGGACGGGCTCGAC
GAGTACAAGCGTCGCTACGCCAAACCGGGGTTGCTGCTGGCGGGCTGGGATACCGAGGTGCCCGGCAAGG
CCAATCTGGCCGATGTGCTGCGTCATGTGCCCATTACCGTGCTGCTGGGTACCAGTGGCTCGGGCGGTGC
CTTCCAGGAAGAACACATCCAGCTGATGCTGCAGCATTGCGAGCGCCCCATGGTGTTCCCGCTCTCCAAC
CCGACCGCCAACTGTGAAGCGCTGCCGGAAGATATCTACCGTTGGAGCAATGGCAAAGCCATCGTTGCAA
CCGGTAGTCCGTTCAAGGATGTGAGCTACGAGGGGCAGGAGTTCCGGGTTGGGCAGGGCAACAACGTCTT
TATCTTCCCCGGTGTAGGTCTGGCGGCCATCGTCAGCCAGATCCGCGAGATCACACCCGATGTATTCACT
ACTGCCGCCTTTGCGTTGGCCGAGTGCGTGAGTGAGGTGGATCTGGCGAAAGGGGCCGTGTATCCCCGCA
TTCGCGAGCTGCGCAGCATCTCGGTGCATGTGGCCACCGCCGTGCTCAAGGATATCGTGCGCCGAGATCC
CTCCCACCCGCTGATCGGTCAGGATCTGCAGCAGCATATCCTCAACCACATGTGGGAGCCGGTTTACCTG
CCCTATCGTCGGGTATAACCGCGTATTCACCGCCCGGATAACGGGTCGGTAAAGATGACAAGGGGGTAGC
GCTGCTACCCCCTTTCGTTTACCCCGTGGCATTCACTAAGTTCGCAATGGCCGCCAGCGCCCTTGCTGAT
TGCGGCAGGCGGTATCCTGCCACTGCTGGCTCGCCTTGCCGATATAGAGTTGCTCGCGATAGTCGCGACA
CCACTCCCCGCTGGTATGGCGGAAGGTGCGTAGCGGGGTGACGCTACCCGAGTGACGGTTAGCCGGATTG
AACCAGGTGAGGCTTTGCCCTTTGGTGCGGGTTTCCAGCGCCTTGGCAAGGGCCTGATTGGCCAGTTGAA
CATCTGCCTTATCCATTTGATGGTAAAGCGGCGGAGTGTCGACCGTGGCGCAGCCAGCGAGGCCGAGCAG
GAGGAGTGGTAACAGATATCGCATTATGGTTGCCCCTGTTGGCGAAAACCGTTGTTGAGCAGCGCCCGCT
CCTGCTCATCGGTAAGCACTTGCTGGCGATCGTCGACCGTGCCATCACCCCTGAGCTTGTTGCCGTTGGC
ATCGACAGCATCCACGCCGTTGTCATCGACCGTGCCGTCACCTCTGAGCTTGTTGCCGTTGGCATCGACG
GCATCCACGCCGTTGTCATCGATGGTTCCATCGCCTCTGAGCTTGTTGCCATTGGCATCGATGGCATCTA
CACCGTTGTCATCAACGCCATTGCCAAATCCGCGGCCACCCCCATGGCGTTCTCCCCCTGCATGTTTTTC
TCCACCAGCATGCCGTTCACCTCCACCATGCTGCTCTCCGCCGGCATGCTCGCCTTGGCCGCCACTGTCA
TCGCTGCCCCCATCGCTGTCGCTGCCACCGCCGCTGTCGCTGCCTCCGCCGCTGTTGCTGCCACCACCGC
TGTTGCTGCCACCACCGCTGTTGCTGCCACCACCGCTGTTGCTGCCTCCGCCGCTGTTGCTGCCTCCGCC
GCTGTTGCTGCCCCCATCGCTGCCACCGCCGCTGTCGCTGCCACCGCCGCTGTCGCTGCCACCGCCGCTG
TCGCTGCCACCGCCGCTGTCGCTGCCACCGCCGCTGTCGCTGCCACCGCCGCTGTCGCTGCCACCGCCGC

TGTCGCTGCCACCGCCGCTGTCGCTGCCACCGCCGCTGTCGCTGCCACCGCCGCTGTCGCTGCCACCGCC
GCTGTCGCTGCCACCGCCGCTGTCGCTGCCACCGCCGCTGTCGCTGCCACCGCCGCTGTCGCTGCCACCG
CCGCTGTCGCTGCCACCGCCGCTGTCGCTGCCACCGCCGCTGTCGCTGCCACCGCCGCTGTCGCTGCCAC
CGCCGCTGTCGCTGCCACCGCCGCTGTCGCTGCCACCGCCGCTGTCGCTGCCTCCACCGCTGTCGCTGCC
TCCACCGCTGTCGCCACCACCACTGCTGCCCCCACTGCCACCATCGCCATCTTTGTTGTGAACGATCAGC
TCATTGGCGATATAGCTGTGGCTGCCATCGAGCAGCAGATTGAAGAGTGCGGTAGTGGGAGACGCCTCGA
TCCAGCAAAGTGACTCAACCAGCAGTAATTCCGGCTGGGATTTCAGGGCCAGATTGCCCGTCACCATAAC
AGGTGCGGGGCGACACATTATCTGCATGCCGGTAAATAGCGGCAGCACCGCCAGTTGCGGGTTTTCGGTT
CGTGTCATGGCCGGTGAGATGGCTGCCCAGCCCCGAGTGGTCAGCAGAGGGTGCTCTGCGGTAAAGAACG
GGGGCAGATCATTGATCCCATAGAGTCTGCGCTTGCCTAGTATGACCCGTTCGACCGCCAGGATCCGGTT
ACTGCCGCCGTGCTGATCCAGCACCTGTTCACCGACCCGGAGCGTCTCTATGGGGCGGGTGGAACCATCG
GCCATCGTGATGCGAGTGCCGGCGATAAAGCAGGACTTGGCATAGGCCTGATTCATTTGCCACTCATTGT
GGGCGAAATCATAACTGAACGGGTTCGCAAACAGGGCGAGAGCGATGGCCGAGCCGATCAGCAATCTGCG
CTGGCTGGCCTTGAATGGGGGCTGTGACATGGGGACTCCTTGTCCATCTTGATTCACAGGATCCATTATA
GAGCGCCCCTTGGTGGCGGCATTTTTGACCATGAACACAATAAACATGATGGAAAAACGATTGGCGTTCG
TCACAATAATGCGTAAAATCGCATACTCGATGCTTGTAGCAACGAATCCCGCCTAAAAAGCCAGTTTCAT
GCCCTTCCCAAGCAGTAAAAGGTAACAAGTGACGCATTTGTGTTCATTTCGCGCCTTGAAAAAGTCTTGT
GATAACTGAAAAGTGATAAATATCTCCATCAGTTATCAAAAAATTAAGGACATCCATGTTTTCCTTAGTC
AAGCAAAGTGTCGCCGGGCTGCAAATCCTGTTTGTCGCCTTCGGTGCCATGGTTCTGGTGCCCCTGCTCA
CCGGACTGAACCCCTCCATGGCCCTGCTTGGCGCCGGTGTGGGCACCCTGTTGTTCCAGCTCTGCACCCG
CCGCCAGGTCCCGATTTTCCTCGGTTCCAGCTTTGCCTTCATCGCCCCCATCATCTACGCCACCCAGACC
TGGGGATTGCCATCCACCATGTTTGGCCTGTTTGCCGCCGGTTTCATGTATTTCATTCTGGCTGCGCTGA
TCCGGGCAAAAGGGATGGGGTTTGTGCACAAATTGCTGCCCCCCGTGGTGATTGGCCCGGTGATCATGGT
GATCGGTCTTTCTGTGGCGCAGGTTGCCTGCAACATGGCGATGGGCCGCGCCGGTAGTGAGCAGGTGGTT
CCTGCGGGAACTGCGCTGACACTGGCCGGTATCTCGCTGGCGGTCACCCTGATTGCAGCTGTGTTCTGCA
AGGGGTGGATCAAGCTGATCCCCATCCTGTGCGGCGTGATTGCCGGTTATGTCTCGGCGGTGTTCATGGG
CCAGGTGAGTTTCGATGGCATGGTGAACGCCCCCTGGCTGGCGCTGCCCCACTTCGAAACCCCGGAGATC
AACTGGGCTGCAGCGTTGTTCATGTTGCCGGTAGCCATCGCCCCCTGTATCGAGCATATCGGTGGCGTGT
TGGCGATTGGTGGCGTGACTGGCAAGGATTACACCAAGGATCCGGGTTTGCATCGTACGCTGGCCGGTGA
CGGTATCGGCGTCTGCTTCGCCGGTTTCATCGGTGGCCCACCCGTGACCACCTACGCCGAAGTGACCGGT
GCCGTGATGATCACTCGCAACTTCAATCCGGTCACCATGACCTGGGCTGCGGTGTTTGCCATCATTCTGG
CCTTTTTTGGCAAGTTCAATGCGCTGCTTACCTCCATCCCGATGCCGGTGATGGGCGGTATCATGCTGCT
GCTGTTCGGATCCATCGCTGCCATCGGTCTCAAGACGCTGGTGGAATCCAAGGTGGATCTGGGACTGTCA
CGCAATATCGCCATCGTCGCCGTGACTCTGACCGTCGGTGTCGGTGCGTTGGAGATGAAGATTGGCGACT
TCGCGCTGGCCGGCGTTGGCCTGGCCTCTGTTGCCGCCATCCTGCTCAACCTGATCCTGCCTGCGCACTC
CGAAGAGGGTGTGATGGACGCGGCCAAGGACTGAGTTCCACCAGACGTTTATTGTTGATTTGGAGAAGGC
CACCTCGCGGTGGCCTTCTGTTTTTCGGGTATCTCGCGCCACTTTTTGGCTCGACGAAGCTCATCTGGAA
TCTAATTTGGAAGCAGATGAGGGGGTTAGCATTCTCATTTGACGTATCTCTTTTCGTGACCTTAGCTAAA
GGACAGGTTTGGCTCTGAGGTCTCCTCGAGCAGTATGGGAAAATGGATGTTGGCGTTTTATATCATTTCT
CTCCCAGACCCCTGTCATAAAACGATGCGCTTCTTCTTTGCCACGGCGCTGTTGTATGGCAGAGGCTTAT
CCGCCCCACTCCCACTCCCCTTATGCTGCCCTGGCATGTTTCCTCCTCGATTTGTTGTGAGCCTCCCTCG
CCGGTTGCGTTTCGTTGCGCACAGGCAAAATCCGGATCTGTTTGCTGCTGCACATGGGTGGGCGGCGAGC
CGTGAAAGCCGGCATGACCCCAGTGGGTCAGACGTGCACCGCCATCGGTAACGGCGGATGTTCAGGGATG
AATGGCAAGCAAAGCGAGGTTGCAATGAACTATCAACTGCTGTGGAAAAGTGCAATTTTTTCAGTCTCTT
CCATGATGGCGATAAACGCTGTTCAGGCTGCGCCACTGAATGTGATGACCGAGGATTACCCGCCATTCAA
CATGCAGGGAGAGGGGGGCAAGATTGTCGGGCTGTCGACCGAGGTGGTCGAAGAGCTGTTCAAGCGTGCC
GGGGTCGATTACAAGCTGACCCTGATGCCCTGGAAGCGGGCGTACGAAGATACCCTCGGTACTCCCAATA
CCGCCCTCTATTCCACGACCCGCACTCCTGAGCGGGAAGCACTGTTCAAGTGGGTCGGGCCGCTGGTCAA
TAACAACTGGGTGTTTTTCTCCAGCTCGACCGCCAATATCACGGTCAAGTCGCTGGAAGATGCCAAGCAG
TATTCGGTGGGTGGCTATAACGGCGATGCGGCCGCCGAGTATCTCAAGGCCCAGGGATTTGCCAAGTTGC
AGCTGGCGGCCAACGACACCGCCAACGTGAAGAAACTGGAGGCGGGCCGCATCGATCTTTGGGCAACCGG
CCAGTATCTGGCGCCCTATCTGGCCAGCAAGGAGAAGGTGGCCTCACCCAAGCCGGTCTTCGTGTTCAAG
GAAACCCAGATGAGCTTGGCGTTTAACAAGGACACGCCCGATGAGCTCATCACCAAGCTTAACGAGATCC
TCGCTGCCATGAAGGCGGATGGCACCATCGACAAGATCAATGCCAAGTACCAGTAACAGTACCAATAGCA
AGTGCCGCTCAATGGCACTGCGCCGGCTGCCGTAAAACCGGGAGGAAACCATGGATTGGAAAAGTCTGAC
GTTCAGGATTGCCGTGTTGACCTCCCTGTCGGTGAGTCTGCTACTGGTGTTGTTTGGTGCTTACAACTAC
AACGCGGTCAGGGCGGAAAGCTACAGCAAACAGAGTGCGCTGGTCGAACAGGCCATCGGCCGGCTGCAGC
TCTCCCTGCCCGGCCCGCTGTGGAATTATGAATCGGCCTTCATCGAGGCATCGCTCAAGTCCGAGCTGGC
GGCCGATATCCTGAGTGCTTTGCTGGTGGTCAACAAGGACAAGCTGGTAGCCGGGTTTGCCCGTGACTCG
GCGGGGGACATTGCCCCCGTGACAGCCATGCCTGCCGATGTGTCTGCCTACGGAGGGTATGATCTGGTCT
ATGACGACAGCGGTCAGAAAAACACAGTGGCCCGATTGCTGGTGCAAACCAGTGATGCCGCCATTCAGGC

CCAACTGCGCCAGCAGTTGATCACTACCGTCTGGCAGATCCTGGTGCTCGATCTCTGCATCATCGCCCTG
TTGATGGTGCTGATTCATCGCAATGTGCTCAGGCCGCTTCATCAGATGAATCAGGCGGTGTTCGATCTGG
CCAAAGGAGAGGGGGATCTGACCCGGCGTCTGAAGATCAGCCGTCAGGATGAGATGGGCGCGCTGGCCAC
CAACATCAACCACTTTATTGCTAAGTTGCAGGAGATGGTGGCGGAGATCAACGAGTTCTCTGCCGGTGTG
TTGCAAACCGCTGAACTCTGTGACTCCCTCTCGGCAGATACTCGGGTAGGCAGTGACGCCCAGCAATCCG
AGCTGGATCAGGTCGCTACCGCCATTACCGAAATGAGTCAGGCAGTGCAGGAAGTTGCTGGTAACGCGGT
GAAAACCTCTGGCGCTACCGATGAGGCCAACCTCTATGTGGAGCAGGGTTACAATCTGGCGGGCCATGCC
AATGAGGCCATCCATCAACTGGTGGGGGAGGTGGAGCGAGTCGCCACCCTGATGCGCACCCTTTCGACCG
AGAGCAGCAGCATTGGCACCATCATCGAAGTGATCAACGGCATTGCCGAGCAGACCAATTTGCTTGCCCT
CAACGCGGCCATCGAGGCGGCGCGGGCGGGTGATCAGGGGCGCGGTTTTGCGGTGGTGGCGGATGAGGTA
CGCACCTTGTCGGGCCGTACTCGCCAGTCGACGGAGGAGATCAGTGCCAGTATCAAACGGTTGCAGCAAG
CGACCCAGAATGTCGGGGCCGGAATGCAACAGAGTCAGGAGAAGGCATCCCGCGCCGGAATCGAGGCGGC
AGATGTGCAGATATCGGTGGAGAAAATTAAAAATCAGGTCAGCCTGATCCGCGAGATGAACATGTATATC
GCCCAGGCATCGGAAGAACAACACTCGGTGGCAGAGGCGGTAAGCCATAGCGTCAACCGGATTGCGACGC
TCAACAGCGAGAGTGCAGCCAGAGTGGCAAAGGTTGCCGAGGCCAGCAGCGATCTGCGGCAGTTGTCGGC
CCAGCTTGACACCCTGCTCAAGCAGTTTCGGGTCTAGTCACTTAACTTCGCCCCGTTGGATCATCCTGCG
GGGCAGGCCGGTTCCCATTAATTTGAGGCGTGGCGAGATGTCGTGAGGGGCAAGGGGCCTTGTCCAGCAG
AGCTTTTCGTCATGCCTATGTCACTGTTCAGCTGAGATTGGCATGACCGTGATAGGCGAAACTCGCAACA
TCATGTTCTGCCCGATATGCTGTCCGACAGCGCTGGACGCATATTTTCATTTTCCCCGCTCATGCCTGCC
TGCATCTATTGTGCCGCTGATCCCGACCCTTTAGAATTTCGTTTAATTACAGCGAGTCCTGAGCCGCCAT
CTGGCTTTATCAGGTGTGGAATCCCATCTTCTTCATGATGAATGCAAATAACAGGTGGTTGGTATGACGG
GGGTTGCGCCAGGCAATAACATATCAGCCTATCACTTCGGTGATTATGTCTTTGATGTCAAAAGGCGAGA
GTTGATCTTCTGTGCCGACAAGGACAACTTGCAAAGCGCCCGGGTGGTGCGCTTGTCATCGGCAGAGAGC
CTCATTTTCAGTTATCTGCTCGATCACCATGACGAGGTGTGCGACAAGGATCTGTTGCTAGAAATCGGCT
GGTTGGGTCGCCCCATCAGCCCCAACTCACTGAACGTGGCCATCGCCAATATTCGTCGCCACTTCACCTC
ATCCCCCCAGACGTTGGATATCAGAAGTACCCCGAAAAAAGGGTACTCCCTGCATCTTAACTGCAAGATG
AAGAGCTATTACGGCAAGGCACTGCCAACAGAGAGTGAGCCCTTGCCCGAGCCGATGAGCGAGCCCACGC
CTGCAGCGGCTGCGCAGCCACCGTCGACATCACCAAGCTCCGACCACGAGGTCATCAAAGAGCCTGCTGC
TCCCCGTTCTTTTGGTGAGCGCGCCAAACTTGCCTTTCTCAGCGGTATCGGGCGGCCAATGCTGGCAAGG
GGCCTGATATACGTCAATCTGCTACTGGTTGTGCTGGTGGCGTTTGTCGCCAGCTATCTCCATTTCGAGT
GGCTCCATGTGCACTGCACCGACTCGCCGACCGGCGTTGTTTGCCAGATGGAGGCACGGAGCGACGTGCC
ACCAGTCAAACCGGCATCGCTGGTGCTTGTCTCTGGCCACCAGGTTCGCGTGCTTTCCTATAAAGAGCTC
GAAAAAACGTTGTAAGGGATCTCTGATGAAACGAACGCTGAAAAATACCTTGCTGCTGTTGCCACTGCTT
GTCGTGCTGCTGTTTGTGGTCGGGATGTTTATCAACAGTCTGGGCTGCCATTACGACCTGAAAATGTCGG
CCAATGGAGTCAACTATCACGGGGTGTGCCGCTGGGGCAAAATCACCGTGATCGAAGAGGAGGAGGCGAC
CAGGGAGAAGTGGCGGGTGAGCGGTTTTCAACTGACCTTCTTCAACCAGCTTGTTTACCTCATCGACAAT
CGTACCCAACTGCACGAATCGGGTAACGATGACTCCTATCTCAATGAGTACAACCTGATCCAGTCGCAAT
ATGCGCTGGTCAACTATGCCTGGGTGCCGCTGACTGAAAACAAGGTGGCGATCTTCCAGCGCGATCCGGT
GCACGAGGTTGTGATTGCCCAGCGAGATGGCTGGTTCGATCTCTACCGCTGGTTCTTCCCCCCGGCACCG
CTGGTGCCTGTTCAGCCGCAAAGCGCAGGTGCCGGGCGTCCCGCTAGTTTCTAAGCAACTTGTACCAAGG
GCCGGTGATATCGCAGTGGCCTGTCTCATCACTGTTTTGCCAGCCCAGCCGATGGTAGAAGGCTACTGCC
GCCGTGTTGCGGGTCGCAACCTTGAGCGAGCCGTGCGGCGAATCAGCAAGGCCCCGCTCCAGCAGGGCGC
GCCCTATCCCCTGACCGTGCCAGTCGCTTGACACATAGAGGTGATGGACGAAGTGATCGGGCTGCCAGAT
GGCGATAAAGCCGCAGATATTGCCCCCCTGTTCGGCTACCCACACCTGTTCACCTTTGGTCTGTTCCTCG
AAATCCTCGAGTTGGAAACGGGCAGGATCGACCCAGTGAAAGGTGTGGCGGCGGCTCTTTAAAAACAGCC
GGGCCAGTTGGGGAATGTCGTGTTCTTCCATGAGCCGGATCATGATGTCCTCCCGGATCAAGCCAATGTT
CAATCAGGCCGCAGCATAGAGGCAGGTTAATTGATGCAAATGACAAGGGTGTGATTAAGCACTGTCCATT
GTATTTGCCATGGGGCTATACTGCCGGAGTCCCTTGTATGGATGGCTTGAGGATCGCTGATTTATGGACA
ATAAACTGATTGAAGGCAACATTTTTGTTCCGGCGCACATAGACGATGTCTGGCACGCCTGGACTACCGA
AAGCGGTCTGCGCAGTTTTCTGGCTCCCGAATGTTTGATGGTGGCCGAACCGAATGGTCCTTTCGAGGTC
TACTTCAGGCCTGATGCCCCGCTGGGTGAACGTGGCAGCGAAGGGTGCCGGGTCATGGCCGTCATGCCCT
ATGACCTGCTCAGTTTCAGCTGGAATTTCCCGCCCAGCCTACCCCATATCCGCCAGCAAAAGACCCAGGT
TTGCCTGCGCTTTGTGCCGGAAGGGCAGGGCACTCGCCTCTACTTCCTGCAGAGCGGTTGGGCCAATGAT
CCGGATTGGGATCAGGGTTATGCCTATTTTCGGGAAGAGTGGCTGGAGACCAGCCTGCCACGGTTGCAGT
ATCGTTTTACCCACGGCCCCATCGACTGGAACAATCCCCCCGACAAGGCGCAGCTGAAGGCGTTGGCCCA
GTAATCCCGGATATTCAGACATAAAAAAAACCGGCCAAAAGGCCGGTTGAAACAGTTCAGGCTTGTCAGG
TGACAAACGTATCCAGCGGATGAGAGTCGCTTTCCACTCCTTGTGAAGCACAACACTCTTGGTTACGTGC
GGCTACTTTATGCCATTCACACTGAATCGAGGCTGAACCATGGATGATCCCGCCTCGTTCCCCTCTTCAA
TCAGATAGTGATCCCCATCGACTGGCGCAAGTAAGCGCCCGCACCGAGCAGCCCCGGCCCTTCATGGGTG
ATCAGAAACACCGGGATCTCCTTGAGGTAGGACTTGAAGCGCCCCTTGTCTTCAAAGGCGACCCGGAAAC
CGGAACCGGTAAAGAACTCCTGGAAGCGCGGTACTATGCCACCTGCGATATAGACGCCGCCAAAGGTACC

CATATTGAGGGCCAGGTTGCCGGCAAAGCGGCCCATCAGCACGCAGAACAGGCTCAAGGTGCGACGGCAA
TCGAGGCACTCACCCGCCAGCGCACGTTCGGTGATGTCTTTGGGGGCGAATGGCTCAGGCTCACGGCCAT
CGGATTTCACCAGCCCGCGATAGAGATTGACCAGACCCGGGCCGGAGAGGAAACGCTCTGCCGAGACATG
GCCCAATTCGCCTCGCATTACTTGCAGCACCGCATCTTCCTCTTCGCTGTTGGCAGCAAAATCCACATGA
CCACCTTCACCCGGCAGGCTCAGCCACTGCTCACCCGCCTTGACCAGATGGGCGACCCCAAGGCCGGTGC
CCGCACCATAGATGGCGATGGGCTTGCCCGCTACCGGCGCGGCGCCGCCCAGCTGGATGCGATCCGATTC
AGCCAATACCGGCACAGCCATCGAGACCGCAGTAAAGTCGTTGATCACTTGCAGGCGTGCCAGCCCGAGA
TTGCTCTGCATCTCGCTGATGGAGAACTCCCAGCTGTGGTTGGTCATGGCGACCCAGTCACCGTTGATGG
GGCAGGCGATGGCGATGCAGGCTTCAGCCACCTGAACCTTGTGTTCCTCCAGATAGAGGCGTACCACGGC
TTCAAGGCTGGGATGGGCCAGACCTGAGTAGGTCTTTGCCTGAGAGATAGTACCGGTGGCCAGCTCACAC
AGGGCCAGACGCGCATTGGTGCCGCCGACATCGCCGACCAGCGCATGGGTATGCTGCATAACAACTCCTT
CGTTACAGGATGGGGACGACTCTGATGTCCCGAGATTATTGTTGGATTATGACGGCCCATTCTATCCGAG
CTCAGGCTTGCTCACCCGCCGGATCGATACAAACGTGCACCTGCTCACAGTGTTGTTGCTTTGCAACATT
TGTAAGCGCTTTCATGGCTAGGTGACGTTTTGTAACGAAAAGTACGAAATAATTAGAGATGAGTCATACT
TTGTATGGCAGGCGTTAGTTGCGATAACCGCTTGTCTTGTATAGTCCTTAGCTTGAATGGAAACTTGCTT
AGCCGTAGATTGAGTGCAAAGACACTGAAGCTAAATTACAAAAACGCCTGGTGGTGATGATGAATACCCT
TGATAAAATTACGAACAACTTGGAAAGTTTCAGTAAATCAGAACGCAAGGTTGCCGAGGTGATCCTGGCG
TCTCCCCAGACCGCCATCCACTCCAGCATCGCCGCTCTGGCCAAGATGGCCGATGTCAGTGAACCGACCG
TCAACCGCTTTTGCCGCCGTCTCGATACCAAGGGCTTTCCTGATTTCAAACTCCATCTGGCCCAGAGCCT
GGCCAATGGCACCCCTTACGTAAATCGTCATGTGGAAGCGGAAGATGGGCCCGATACCTACACCGCCAAG
ATTTTCGAATCCACCATGGCCTGTCTGGATGCCGCCAAAAACAGCTTGGATATCTCCGCTATCAACCGCT
GTGTCGATATCCTGACCCAGTCCAAGAAGATCAGCTTCTTCGGCCTTGGCGCCTCCTCCGCCGTTGCCCG
CGATGCCCAGAACAAATTCTTTCGCTTCAACATTCCGGTAGTGAGCTTTGACGATATCGTCATGATGCGC
ATGAGCTGCATCAACAGCAGCGAAGGGGATGTGGTGGTGCTGATTTCCCACACCGGCCGCACCAAGGCGT
TGGTCGAGATCGCCGCGCTGGCTCGCGAGAACGACGCCACCGTGATCGGCATGACCGCCAAGGACTCCCC
GCTGGCCCGCGAGTGCAATCTGGTGCTCTCCATGGATGTGCCGGAAGATACCGATGTCTATATGCCGATG
GCATCCCGCATCGCCCAGCTGGCGCTGGTGGATGTGCTGGCGACCGGTTTTACCCTGCGCCGGGGGATGA
AATTTCGCGAAAATCTGAAGAAAGTAAAAGAGGGATTGCGCGAATCCCGCTTCGAAACCCCGCACTCTTC
CTGAACCGTCGGGGGCTCAGGCCCCCGACTGTCTATGCCACCCGCCTTGGTGTAGTATTGTAATTTAATA
ACAAAAACTACCCAGATGGTTTGCAAACGGGTGCATCGAGCCATGGGCTTGATGGGGCAGAATCTCAAGT
CATATCAACTTCATATTGGAGTCTTTCATGCCTAGACGTACAAAGATTGTAACCACACTCGGTCCGGCTA
CCGACCGCGAAGGGATCCTCGAAGGGATCATCCGCGCCGGGGCCGACATGGTGCGGATGAACTTCTCTCA
CGGCACTGCTGAAGACCATATCCTGCGCGCCAATCAGGTGCGTGACATTGCTGCCAAGCTCGGTCGTCAC
GTCGCCATCATGGGTGATCTGCAAGGCCCGAAAATCCGTGTTTCCACTTTCAAGGATGGCAAAATCTATC
TGGCCATCGGTGACAAGTTCATTCTCGACGCGGCGCTCGGCAAGGGTGAAGGTTGCCAGGAGCAGGTCGG
TATCGACTACAAGAGCCTGCCAGACGATGTGGTACAGGGCGACATCCTGCTGCTGGACGATGGCCGTGTG
CAGCTGAAGGTCGAGCAGGTTGAAGGCATGCGCATTCACACCACGGTGACCGTGGCCGGGCCGCTCTCCA
ACAACAAGGGCATCAACAAGAAAGGGGGCGGTCTCTCCGCTCCGGCCCTGACCGAGAAAGACAAAGAAGA
CATCAAGACCGCTGCCCGCATGAATGTGGACTATCTGGCTGTCTCCTTCCCGCGCTGTGGCGATGACCTG
CGTTACGCCCGCGAGCTGGCCCGCGAAGCCGGTTCCAATGCCAAGATCGTGGCCAAGGTTGAGCGCGCCG
AAACCGTCGCCACCGATGAGGCGATGGAAGACATCATCCTCGGTTCCGATGCCGTGATGGTTGCCCGTGG
CGATCTGGGTGTCGAGATCGGTGACCCCGAGCTGGTTGGCGTACAGAAGAAGCTGATCCGCCACTCCCGT
CGTCTGAACCGTGTGGTGATCACCGCGACTCAGATGATGGAGTCGATGATCAATGCGCCTCTGCCGACTC
GTGCAGAGGTCATGGACGTGGCCAACGCCGTGCTGGATGGTACCGATGCGGTGATGCTCTCTGCCGAGAC
TGCTGCCGGTCAGTTCCCCATCGAGACCGTGACCTCCATGGCGAGCGTCTGTGTCGGTGCCGAGAAGCAC
CCGAGCCTGAACGTCTCCAACCACCGCATGAGCTACACCTTCACTTCCGTGGAAGAAGCTGTAGCCATGT
CCACCATGTATGCGGCCAACCACCTGGAAGGGGTGAAGGCGATCGTGGCACTGACCGAGTCCGGCACCAC
GCCGCTGCTGATGTCCCGTATCAGTTCTGGTCTGCCGATCTTCGCTATGTCTCCCCACAACAAGACCCTG
TCCTGGACCAATCTCTACCGTGGCGTCACCCCGGTGATGTTTGACGGTGACGAGACCAAACCGGTCTACG
AAGTGTGCCGCGACGCGCTGGCCGTGCTCAAGGAGAAGGGGCTGCTGCAGAGCGGCGACATGGTGCTGCT
GACTCACGGTGACAAGATGGAAACCGTGGGTAGTACCAACACCTGCAAGGTGATGATGGTCGAATAAGCC
CGTCCGGTTTTACTGTGCTTAACAAGGCTGCTCCTCTTTGATGAGCAGCCTTTTTGTTAACCGCAGTCGG
GCTGTGGCGGTATAAACAGGACTCAATGATTGCTTAACCGGATTCCCCTTCCGATAATGCGGGAGTGATT
TTCAGCGGGTAGCCTGTTGTAGGTGATCGTCTCAGGTAACCCAGACACTCGACAATACGGGAGTGAAAAA
ATGAACAAGCGTAACTGGTTGCTGGCCTTGTCACTGAGTCTGGCCTTCTCACCCTGTTATGCCGACTGGG
CCAAGCTCAAGGCCGCAGCGTCCGATCTGGGAGCGGCAGTGAGCGAGACCAGCAAGGAGGTGTGGCAGGA
TGTCAGCGATTTCTCCAAGAAGAGCTGGGCTTCCATCTCTGCATGGGGTGAAGAGGCGTTCAATACCGCC
GGGGTCTGGACCGACAAGAGCATCGCGACCGGCAAGGAGTGGCTGAAGGCAGCCGACAAGGAGCTCAACG
AGATGCTCAACCCCAAGACGGCGAAAGAGGCGAGGATCGCGATCAATACCATGGCCGACACTGCGCTGAT
CCGGTTGTTCAACGAGCAGCCATCAGCCAAGTTGTTGTTTGACAAGGCTTATGGTTATGCGGTGTTCGAT
TCGCGCAAGTTCTCCCTGATGCTGCATACCAATCAGGGGGCCGGGGTGGCAGTCAATCGCAAAACCGGCA

AGCACACCTATATGAAGATGTTTGGTGCGGGTCTGGCGGCCGGGATTGGCGGCAAGTTCTATCAGCAGGT
GATCCTGTTTGAAGACAAGGCCAGATTCGATGCCTTCGTGACCCAGGGGTGGGAAGCTACCTCCGAAGTG
GGTGTGGTGGCCGGCAAGGAGAGCGCCGAGCTGACCGCCAAATACAACGGCGGCATGGCCATCTACCAGA
TTGGCGAGAAGGGCTTGCTGCTCGATGCCAATATCTCCGGCTCCAAGTACTGGATTGACAAGGACTTGAC
TGAAACGAGCCGCTAATCCCGCCGTTATCGGTTGCTCGCTGCACGCCATGCGCTTGTCGTGATCTCGCCA
ACAGGCCCGCAGTGCGGGCCTGTTTGTTCTCTCGGTGTCAGTGGGCTGAATAGACCTGCTTGCCCTTGAA
CATGGTCCAGAGCACCTTGGTCTCCTTGAGCTCTTCGCTTGCTACCTGTTCCAGATTGCGATCCAGCAAG
ATCAGGTCAGCACTCTTGCCCGCGCTGATGGAGCCAATCTCTTTCTCCCGTCCAATTACCTTGGCGGCGT
TGAGGGTATAGGCCTGCAAGATGGCCTGACGGCTGATCTGCTCGCTGGCGGGGGGCAGCAAGCCAAGCTC
ACCTTGGCGGGTGATCGCGGTATACATGGCGAGGAAAGGGTTAGGGCTGGAGACCGGCCAGTCACTGGCA
CCGGCGATAATGGCGCCGCTTTGCAGCAAGCTGCCTGCCGGATAGAGGCGGGCTAGCAGTGCCGGGGGCA
CTTTTCCTTCAAGCAGCAGGGTGGTCGCCACATTTTTACCGGCCCACAAGAGTTGCATCGATGCGGCCAC
ATTGAGCGGTTTGAAACGGGCAATGCTGGCCGGGCTCACCACCTCCAGATGGGTGATGCTGTGCAGCAGG
CGGTGATCCTGATTGTGGGCCCGGGCATAGGCGATGGCATCGAGGGATTCACGGACGGCCCGATCGCCGA
TGGCATGAAAGTGAGTGATAAGTTGATGGGCATCCGCGGTGCGGATCAGCTCGTTGAAACGCTGTTTGTC
CAGATCGTCCTTGCCGCTGTAACCGGGACGGTTGAGGTAGGGCTCGCTCAACTTGGCGGTCTGGGATGGA
TACTCGATGACACCATCTTGCAGGATCTTTATCCCCACCAGTTTAACGTCCGGGGCATCTTGAAACTGCT
GTTGCAGCGCCAATACGTCATCAATGATGGCGGGTGAGGCATGTATCCCCACCAGTGCCAGTGCGGATAC
ATGTCCGGTCAGCTTTCCTTCCCGGGAGAGCATGCTGTACGCCGGCAGCAGGCCGGTATCTCGGCGATCC
GGCTCGGCGTTGAAGATGGGGGCGAGGGGGCGAATGTTGCTGATAGGGTCCATCCAGGCGGTAATGCCCA
CCTGATTGAGTTGTTTGGCGGCAGTCATGATGGCACTGGCAATCGTCTCGTCGCTGACGGGGGGCAGCGC
CTTGAGGATGGCATCCCAGCCGCCTTCTGAGGCAAAGCCATTGAGCTTGCCGGATGGGGTGAGGCCAAAA
TTGCCTTTCAGCGGAGCATAACGACCGTCAAGGGTGTCGGTATCAATGCCAGCCTTGCGCAGCATGGCCT
GATTGGCCCAACCGGTGTGGGCATCCGAACCGGCCAGCACTACGGGTATTTGCCGGTATTGCGGGCCATT
GAACACCTGATCGAGCAGGGGAAGCTGATGCCAGTAGTCGAGGGAGACATTGCTGTATAGCTCTACATCG
CCCAGTGTCAGGCGGGGATCATCCTTGCTGCGGGTCAGAAATTGCTGCACCTGCTCAACCTCACTCATGG
CATCGGTAAAGTTAACCGTCATCCCCTGAAAACCGGCAAATGCCGCATGAACATGGCTGTCGATGAGCCC
CGGCATCAGGTACTTTCCTTTCAGATCAAGGCGCTTGGCATGGTTGCCTAGGCGGGCGCTGACCTCCTGA
TAGCTGCCGACTGCCACAATCTTTTTACCTTCAATGCCGACCGCTTCCGGGTGAGGGTTCGCCGGATCAC
CACTGTAGATGGTGCCGCCAAAGATCAGCGTCTGGCTCTGTGACTGGGCATTGGCGGCAGCGGGAAGCGT
CACCATCAGGCTGGCGAGAAACAGCGTTTTGATATTCATGGGCATGACTCCGTCGCTTCGGTGCCCGAAG
GCACCGAAGTGGGTAAATGGAAATGAGGTATCGGCTTATCGGTTGATCAGCGGGATACAGATATCAAACA
CGTAATGCTCCCGGTCGGGGTCCGTGCCTGCGTGGTATTGCTCGTAACACGGGCGATCGTCGCAGTTCAG
CCCTTGGCCCACTACCCAGCGAAACGCCGTTTCCCAGCAAGCGACCAGTTGATCGCCGCGAGCGATAAAC
TGACAAACCGCATAGGGGCCCGCGGCGATGGTCTGGGTGGTTAACTGGCGCCCAGCGGGGATCTCTGGCC
CCAGCTCGATGCAGACGTCAGTACGGCAACGGGTCTCATCGGTGATATCGGGGATATCCCAATAGAGACT
CAGGGTACCCGCCGGTGTTATCGCCTCGTCTGAGGCGAACAGCGCGAGCAAATCCCGATGAGCTTGCTCG
CAGGTCTCTTTGCCATAGGGGCCCAGGCGGCGCATGTACGCCACGCGCCTAGCGGGCATCTGCCTGACGT
TTGCCCCCTGCAACAGACCCGGGTGTTCGGTGACCGGGTGCATGACCAGGCCATCATAGGTGTCAAAGGG
AAGGGGATCGTTTCCAATCTTGCTTTTCCAGTTTTCCCCCTGCTGCTGGCGGTATTCCGAAGGACTCATG
GCGAAGTGCAGGCGAAAGGCCTTGGCGAAATTCTGCGAGCTGGAGAACCCGGCGGCCAGTGCGAGGGTGG
TGATGTCGCTCTGGGGGGAGGCGAGCAAGCGACGGGCAGCGCGCTCCATACGCAGGCGCCGGGTATATCC
GGCAATGGTCTCGCCGACCATCGTCTTGAAAATACGGTGGAAGTGGAAGGTGGAGATGGCTGCCGCTGCT
GCGACCTCATCCAGGCCCGGATTGTCATCCAGATGGTGGCTGATGTAGTCCATCGCCTTGAACACGGGTT
TGCGATAATCCAGAGCGGACTCCTTGCCAGCCAGCAAAAGTGGGCCTCAGAGTCGGATTTAATCAGAGCC
ACCTGCCGTTGTCGAACCCGTCCGTCCTGGTCTGGCCACTCATCCTCGTCGCGTTTTTAACGCATTGGCA
CTCTAATTGCTTGTTGCATGCCAGCGTCAATGATTTGTCTGGGGATGGAAAATGGAAATTAGTGCAAGCA
GCCTGATGCAAGAGATGCAGGCGCTCAAGATCGAAGCAGGCGCAACGCCGCTGGCACAACCCGGTCGTCA
GGTAACCTCCGACTTTGGTCAGCTGCTGCAACAGGCGCTTGGCAACGTCAACGAACTGCAAGGCGCCGCC
AATGACCTGCGTACCCGTTTCGATATGGGTGACAAGTCGGTGGATCTGGCCGAGGTGATGATCGCGGGCC
AAAAATCGTCCATCGCCTTTGAAGCCACGGTACAGGTGCGCAACAAGATGGTCGATGCCTACAAGACCAT
CATGAACATGCCGGTATAAGTGATTACTGAACATCATGAACATGTCAGCAATAAGGACATAAATCGTGGC
TACTGACCAAAACGGCGATCTGCTGATAAACGATGAGGGCACCGCCGCCCTCGATGAAAATGAGCAGAAG
AGTTCGGCGCTGGGCTTCCTGTCCAACACCGATGTGCTGCGGCAAATCACCCTGATCCTGGGGCTTGCCA
TCTGTCTGGCCATTGCGGTGTTTATCCTGCTGTGGGGCAAGGAGCCGGACATGCGTCCCCTCGGCACCTA
CACCACCCAGGAGCTGGTGAAGACTCTCGATTTCCTCGATCAGCAGAAGATCCCCTACGAGGTGGATGGC
AAGGGCGTGCTGGTCAACGCCGAAAGCTACCCCACTATCCAGCTGGCCCTGACCCGTGCCGGTCTTGCCA
ATGCCACCGATAGCAGCATCGGTGGTGAAGATATCCTGCTGAAAGATTCCAGTTTCGGCGTCAGTCAGCG
AATGGAGAGCGAACGTCTGAAGCTGAGCCGCGAGCGCCAGCTGGCGAGCGCCATCGAGCAGTTCCAGAAT
GTGGCCAAGGCCCAGGTGCTGCTGGCTATCCCGAAAGACAACGTGTTTGCCCGCAACGAACGCAAACCTA
GTGCCACCGTAGTGCTCAACCTGAAAGGGGCGGCGCTGGGGCAGGGGGAGGTGGACTCCATCGTCGATAT

GGTGGCCTCCGCCGTACATGGCCTTGAGCCGACCCGCGTCACCGTCACCGACCAGAATGGCCGCCTGCTC
AACTCCGGCTCGCAAGACCCGATCTCGGCCCAGACTCGCAAAGAGTTTGCCATGCAGCAGAAGCAGGAGC
TGGAGTACAAACAAAAAATTGACGCCATCCTGATCCCGGTGCTGGGGGCCGACAACTATACGGCGGAAGT
GGATCTCTCCCTCGACTTCTCCCAGCAGGAGCAGACCCGCAAGACTTACAACCCGGACCTGCCTGCAGTG
CGCTCCGAGATGACCATGGAAGAGAACAGCGCCAGCGGCAGCAACGGTGGTGTGCCCGGCGCCCTCTCCA
ACCAGCCCCCTGCCGCCTCCAATATTCCCGAGGAGGCGACCGGTGGTGATGGTGCCAGCGCTGCTGCTTC
GGGCCGTTCCCGCAAAGAGGCGACCCGCAATTTCGAGCTGGATACCACCGTCAGCCATACCCGTCGCCAA
ATGGGTGGCATTCGCCGCATGACTGTTTCGGTTGCCGTGGATTACAAAGCGGTGCCCGGTGCCGACGGGG
CCGTGACTCGTGAGGCGCGCACCCAGGCAGAACTCGATACATTGCGCCGTTTGCTGAGCGGTGGCCTTGG
CTTTGATGTGACCCGTGGTGATACCCTTGAGGTGGTCGCGATTCCGTTCAATCGCCCAGAGCTGGATACC
GTGGCCGAGGTGCCGCTCTACGATCAGCCCTGGTTCTGGCGTGCGGTGCGGATTGCCGCCTCGGTACTGG
TGATCATCGTCTTGATCGTCACCGTGGTTCGCCCCATGCTCAAGCGACTGCTCTACCCGGATGCCAAACC
GGAAGGGGAAGTGGATTTCGACAACCACACCGTACTGGGTGGCGACGACGAGTTGAGTCTGCTGTCAGCA
CAAGTCGAGAACGATCAGGTATTTGGTGTCCGCGATGGCCAGCTGAAACTGCCGGATCTGCACCGTGATG
AAGACTTGCTCAAGGCGGTACGTGCGCTGGTGGCGAACGAGCCGGATCTGGCCGCTCAGGTCATTAAAGA
ATGGGTAACGAACAAAGATGCCTGAAGAAAAGAAGAGTCAAGTCACCGGCAATGACATAACCGACAGTCA
GCGCCAGATCCTCGACCAGATGTCGGGAATGGAGATGGCTGCGGTGTTGATGCTGAGCCTCAATGAAGAG
GATGCCGCCCAGATCTTCCGTCATCTCGAGCCCAAGCAGGTGCAGCGGCTCGGTATGTCGATGGCCTCCA
TGTCCGACTTCAGTCACGATCGGGTGGCGGCGGTGCACCGTCAATTCATCGACGATATCCAGAAGTACAC
CAACATCGGTATCGGCAGCGAAGACTTCGTGCGCAAGGCGTTGGTGGCGGCCCTTGGCGAGGACAAGGCG
AGCAATCTGGTGGATCAGATCATTCTCGGTTCCGGCGCCCGTGGCCTCGATTCGCTCAAGTGGATGGATG
CCCGTCAGGTGGCCAGCATCATCCAGAACGAACACCCGCAGATCCAGACTATCGTGCTCTCCTATCTGGA
GCCGGAGCAATCCGCCGAGATCCTGAGCCAGTTCCCGGAGCAGGTGCGCCTCGATCTGGTGATGCGTATC
GCCAACCTTGAAGAGGTACAACCGGCGGCACTGCAAGAGTTGAACGACATCATGGAGAAACAGTTTGCCG
GTGCGGCCGGTGCCCAAGCAGCCAAGATGGGCGGCCTGAAGGCAGCCGCCAACATCATGAACTATCTGGA
TACCAATATCGAAGGCCAGCTGATGGACTCCATCCGCGAGTCGGACGAAGAGATGAGCCAGCAGATCCAG
GATTTGATGTTCGTGTTCGAAAACCTCATCGATGTGGACGATCGCGGCATCCAGACCATGCTGCGGGAAG
TGCCGGGGGATCTGCTGCAACGCGCCCTCAAGGGCTCCGACGATCAGATGCGCGAGAAGATCTTCAAGAA
CATGTCCAAACGGGCGGCCGAAATGCTGGCGGACGATCTGGAGGCCATGGGGCCGATCCGGGTCTCCGAA
GTGGAAGCGGCCCAGAAAGAGATCCTCTCCATCGCCCGTCGTCTGGCGGATGCCGGCGAGATCATGCTGG
GCGCCGGTGGCGGCGAGGAGTTCCTCTAAGCCATGGCCCATAAACAGAACCGCGATGCCAAGCCCGCATC
AGGCAGTGGTGAGGAGCCGCGCAAAGCATGAACAACAAACTGCGTGGGTATGTGCGCCCCGAGGGTGATG
ATGCCCGGGTTGAGACCTGGGGCTGGCCCGAGATGGAGGTCGAGGTAGAGGCGGGAGCGCCTACCAATGC
TCTTGGCCTGGCCCCTGACTGGTATCAGCAGGAAGAGGAGGTGGTGGAGCCGGAGCCCGAACAGGAGCCG
ACCCCCAGCATCACCGCCGAAGAGCTTGAAGCGATCCGTCAGGCCGCCTGGGAAGAGGGGATGGCTGAGG
GGCGCGAGTCCGGTTATGCCAAGGGAATGGAAGAGGGCAAGCTGGAAGGATTGCAACAGGGTCATTCTGC
CGGTCTCGAACAGGGGCGCGAGGAGGGGCTGGCTATGGGCCGCGAGCTGGTGGAGCAGGAGATCGCCCAC
TGGCAGGGGCTCGCCGCGCGCCTTGCCAATCCGCTTAACGAGCTGGATCAGGCGGTGGAGCAGCAGTTGG
TCTCGCTGGTGATGCAACTGGCCCGCGCGCTGATTCGTCACGAAGCGGAGACCTCCCCCCGCATTCTGCT
GGAGGCGCTTAAAGAGGGGCTGGCCTTGCTGCCTGCCGCCGAACAGGGGGTGACTCTGATGTTGCATCCC
GACGATATCGCGCGAATCGAGCAGGCATTTGGCGCCGAAGAGTGCGCCAAGCGCCACTGGCGATTGCAGA
GCGATCCCACCCTTTCCCCCGGCGATCTGCAACTGGCGACCGAGCTCTCCAGCATCGATCTGACCCTCTC
CGGGCGCATCGATCAGTTGCTGCGTAACTTCTTGCAGGACAATGCGTCGCAGAAACCGGCTTCGCAGGAC
TCACGCCCATAACCGATGCCGGACGTTGCCGTCCGGCTGCACTTTTTAGCTCTCAGGAGGCCAGATGAGC
GGCTCGCTACTGAACCGGTTGCAAGGGTACAAGGTACGCGACAGCGCCCCCAAACCGCCGGTCGCCGGTA
AATTGACCCGGGTGGTGGGGCTGACCCTCGAAGCCATCGGCTGCCGCGCCGCCATCGGTGCCCTCTGCCG
CATCGATACCCTTGATGGGTCGCTGGAGGCTGAAGTGGTCGGTTTTTCTGGCGATCGCCTCTATCTGATG
CCGAGCGAGCAGCTCAAGGGGGTGATCCCCGGTGCCCGGGTGGTGCCCATCACCGAAGAGCACGGCATTC
CGGTTGGTATGAACCTGCTTGGTCGGGTCATCGACGGCATCGGCCAGCCACTCGATGGCCTCGGCCCCAT
TCTCTCCTCCCAGACCGTGCAGTTTGCCCAGCACCGCATCAACCCGCTTTCTCGCCGCCCGATCCATCAG
CCGATGGACGTGGGGGTACGCGCCATCAACGCCGTGCTGACGGTAGGGCAGGGCCAGCGGATGGGGCTGT
TTGCCGGTTCCGGTGTCGGTAAATCGGTGCTGCTTGGCATGATGACTCGCGGCTCGGTGGCAGATGTGGT
GGTGGTCGGCCTCATCGGTGAACGGGGCCGAGAAGTGAAAGAGTTTATTGAAGACATTCTGGGAGAGGAG
GGCCGTGCCCGCTCCGTGGTGGTAGCGGCTCCCGCCGACGCCTCGCCGCTGATGCGGCTAAAGGGGTGTG
AAACGGCGCTGGCCATCGCCGAGTATTTCCGCGATCAGGGGCTCAACGTGCTGCTTTTGATGGATTCGCT
GACCCGTTATGCCCAGGCCCAGCGTGAGATCGCGCTGGCGGTGGGCGAGCCGCCAGCGACCAAGGGTTAT
CCGCCCTCGGTATTTGCCAAATTACCGGCGCTGGTGGAGCGTGCGGGTAACGGCAGTGACGGCCAGGGCT
CCATCACGGCATTCTTCACCGTACTGACCGAGGGGGATGACCTGCAAGATCCCATTGCCGATGCGGCGCG
GGCGATCCTTGATGGCCACATAGTGTTGTCGCGCGAATTGGCTGATGCGGGGCACTATCCAGCCATCGAT
ATCGAAAAATCCATTAGTCGGGTCATGCCCATGGTCACCTCGCCGGAACATATGGATTTGGCGCGTACCC
TCAAGCAGTTCTACTCCCTTTATCAGCAGAACCGTGACCTCATCACCATCGGTGCTTACAGCCAGGGTTC

CGATCCGCGCATCGACCGTGCCATCATTCAAAAACCCTATCTGGATCAGTTCTTGCAGCAGGGGATGCGC
GAGGTCATTCACTACGATGATGGCCTGCAAGCCTTGCAGATGGTGGCGATGCCATTGATGCGCTGATGAT
GGTCGGTAACAGAGAGGTCGACTATGAGTAAAGGGTTGGTATTGTTGGCCGATCGGCTGGTGGAGGCGGA
GCAGCAGTGTGCGCTGGCGCTGGTGAAGGCGCAGCAGGATCTCAAGCTGTTCATGGATCAGCAAAATGCC
CTCAACCAGTACCGCCAGATCTATCACCAGCAGTGGACCGATCGCGGCCTGCAGGGGATTTCACCCCAGC
AAAACACCCAGTATCACGCCTTTATCAACAAGCTCGAACAGGCAGCGACCCAGCAGTACGAAGGGGTGTT
GCAGGTGCGTCAGGCGCTGGAACAGCGCCGTGAAGAGTGGCTGGCGGCCCAGCAGCGGCGCAAGGCGGTA
GAGCTGTTGCTGGAGAAGCAGGCGAGTCGCGAGCAGCAGAAAGCCCTGCGTCAGGAGCAGAAGATGCTGG
ATGAGTACGCCATGTTGCGACGCTTTCATCAATCTGGCCTGTAACTTGCTTTGATCTGGACATTGTCGGC
GGCCATCTCTTTGGGGGCCACCAGAATTCAGGGATCCCATCATGATGCAGACACTGTTGACACAAACGCC
TTCGGCTCCGGCCAACGGGTTGGACAAAGCGCCTCTGAGCGGTTCCGTCGAGAGCAAAAGTATCGATGGG
GGAACTGGCAGCGACAGCAAGGGGGATTTCGCCACCCTGATTGCCAAAGCCCGGAACAGCGAACAGGGCG
CCGCCGAGGCCACTCAGGGCGGTAACCATGTCCGATCAGCCAAATCTTTGGCGACCGAGAGCGCCTCTCC
CAAAGAGGAGACCGGCAAGGAGCAGGATCCTGACGCCGACAACAAGGTGGAGGAGGTCCCTGCCACCGAC
TTTCTGCTGCGCCTGCAAGACTCCCTCAAGCTCGATACCTCGCTAGTGACTCCCCAGCTGACGGTAGCAG
CCCAACAGAGCACCACTGTGGTAGACGGCAATCCGTTGCCGCAAGAGGGTGAGCTGGAGGGGATTGCGCT
TGATGAAAGCGAGCAGACTCCTGCCGCTGCCAGGATTGCCCAACAGCTTGCAGGTCAGACCGGGACGCAG
TCGCCGCTGGCGACGACCGCCAACTCGACTCTTGATACCACCGTATCCTCCTCAGCGGCGCGAGTGGTGG
CGAAGGGCGATGCCTCTGCTGCCGTTCTCTCCTCTGAAGGTGAAGATGCCGCCACTACTGCGGATGGGCA
GGCCGGGGAGAAAGCCTCTGCCAAGGGCGAAAACCTCGGGGTAGATGGCAAGCAGACCAAGCCCCTCACG
GGGGCAGAGCGGATGGCCCAGCTGGCCAAGGTGCTGCCGGTCGATGAGTCGGCAAGCACCGCCAAATCCG
CGGAGTCACTCGCTGCAAAAGAGGCTGGCGACAGCAAATCCGATCTCGCCGCTAGCAATGCTTTGACTAA
CAAGGGGCAGGCAACGCAAGCCGCGACTCAGGGGGCCGCCCTCAAGGCCTTCAATGAGGGGCTCAAGCAG
CCTGAGGCGGGTGCTGCAACCACTTCAGCAGCTGCGGATGGAAGTGGGGTAGCTGGCAAGGGGGCAGAAA
AGGGGGCCGCCGAGGGTAGCCAGCAGCCAGCCACCGCCGCTAATCAAGGTTCGGCGCCCGTGAGGACGGC
GCAGAGTGCCGGAGAAAGTGCCGCAGCTCAGAGCAGCGTAACGACAAATGCGACGACTCCGACCGATAAC
GCTGCCGCCCCCGTGATGGTGGCGCCGCAGCAGGTGTTGGCACAGGCCGAATCTCAGGGGCCGCAAGCGC
CGCTGTCGACTATTTCAGCCGGCATCAAACAGATGGAAGTGACGGGCACCGACGAGGTACGAAAAGCGGT
TTCGCTTGAGGTAAAACCGAAGGCCGGTGAGGTGGAGAAGAGCCTGGTTGCGACGACATCTAGCAGTGAG
AGCAGCCCGAGCCAGACCGTTCAGCATGGCCAGAACAGCCAGACTCAGCCACAGGTAGCCGATAGCCGGG
CGCCCGCCGCTGAGACGACGTTGCGCCGTGAGTCGCAGAATTTACCCCATTTGAAACTGGCCAGTCAGGA
AGCACCTGCCGAATTGCACCAGAAGGTCAATGTGATGCTGGCCGAAAAACTGCAGCAGGCGGAAATTCAG
CTCGACCCCCTCGGCCTTGGCAAGATGAAAATCCAGATCCAGATGGGAGCCGACAGCCAGGCCAACGTCC
ATTTTGTGGTGCAACATGGACAGACCCGCGAGATGCTGGAGCAGGCGATGCCCCGGCTGCGTGACATGCT
GGCAGGGCAGGGGATCCAGCTTGGTCAGACGCAGGTGCAGCAGCAGTCGCAACAACAGCAGCAACAGGGC
CAGCCCACCTTTAACGGGCAGGGGCAGCAAGGTCAGAGTAGTGGTCAGCCGCGGTCCGGTAACGGGCTGG
TTGAAGGGGAACTCACCACTTCCACCCCCAGTTTGCTGGTGGAATCGACAAACGGTTCCGGAATTGATTT
CTATGCTTGATGCAATCCCGAGTAAGTTTATGATATCGAACAAGTTTGAGACTAGGGGGGACAAGGGATG
GCTGACGATAACGAGTTGACGCTAAAAGACCCGGCTGCGGGCAAGAAAAAGAAGCTGATCATTATCATTG
CCCTGGCGGTAATTTTGCTGGGCGGTGGTGGTGCTGGCGCCTTCCTGATGCTCTCCGGCTCCGATAAACC
GCCCGCTGAAGCGGCGGAAGGTGCCGAGGCCACACCCAAGGAGGAAGCGCCTAAACCTTCCCTCTATGTC
GGTATGCCGCGCCCCTTTGTCTTCAACGTCGCAGGTGCCCAGCGGGATCGACTGGTGCAGATCAAGATCC
AGCTGATGGTGCGCGGTACTGCCGACGAGACGCTGGCCAAACAGAATATCCCCCTGATTGAAGGCACTTT
GCTGCGGGTATTCAGTGCAGCCAGTGCCGAACAGCTGATGACCTTTGAAGGCAAGGAGAAGCTGCGCAAG
GATTCACTGGAAGAGTGCCGTCGGGTGCTCAAGGACCTGGTCTCTTCCCCCGTTATCGAACAAGTGCTGT
TTACCGGCTTTGTAATGCAGTAGGTGAAGCGTGAGCGATCTGTTAAGTCAGGACGAAATTGATGCCCTCT
TGCACGGTGTCGATGATGTCGAAGAGGAAGAGATAGGCGGCGCTGGAGACCCCGGCGTAGCCCAGTTTGA
CTTCTCCTCCCAGGACCGCATCGTCCGTGGTCGCATGCCGACGCTGGAGCTGGTGAATGAACGTTTCGCC
CGCCATATGCGGATCAGCCTGTTCAACATGATGCGTCGTACCGCCGAGGTCTCCATCAACGGCGTGCAGA
TGCTCAAGTTTGGCGAGTATGTTCATACCCTGTTTGTGCCGACCAGTCTCAACATGGTGCGCTTCCGCCC
GCTCAAGGGGACGGCACTCATCACCATGGAGGCTCGACTGGTCTTCATCCTGGTAGAGAACTTCTTCGGT
GGTGATGGCCGTTATCACGCCAAGATTGAAGGGCGCGAGTTTACGCCGACCGAGCGGCGCATTATCCAGA
TGCTGCTCAAACTGGTGTTTGAGGATTACAAGGATGCCTGGGCGCCGGTGATGGACGTGGGGTTCGAGTA
TCTCGACTCCGAAGTGAACCCGGCAATGGCCAACATCGTCAGCCCGACCGAGGTGATCGTGGTCAGCTCG
TTCCACATCGAGCTCGATGGTGGCGGCGGCGACTTCCACGTGGCGATGCCTTATTCCATGCTGGAACCGA
TTCGCGAGCTGCTGGATGCCGGTGTGCAGAGTGACAAGGGGGATACCGACGTACGTTGGAGCAAGGCGCT
GCGTGACGAGATCATGGGCGTCAAGGTGGAGCTGCGGGCCAAGCTGCTGGAGACCGAACTCACTTTGCGT
GAACTGATGGAGCTGCGACCCGGCGATATCATTCCGGTCGATATGCCGGAAAATCTGCTGGTTTTTGTCG
AGGACTTGCCCACTTTCCACGCCAAGATGGGCCGTACCAAGGATAATGTGGCGCTCAAGATCACCGACAA
ACTCAAACGTCCGGAAGGGGTCAAGACGGAGATGCATCTGGTGACCCGGGGCGGGTACGAATTGATGGC
GAGGCCGAGCTGGAAGAGCTCGAGCGCAGTATCGAAGATCAACAGCGATAATTGACGGTTTATTACGGTA

AACGGTCACGAAGTAGCATAAGGAAACAAGATGAGCGGAACTGAGGAAGAGGATCTGATGGCTGATGCCT
GGGCTGCGGCTCTGGAGGAGCAGGTGACGGCGGAAGCCAAGCCAGCCCCGCTGGAAGAGTTGAAAGATGA
TGCCCCCATCAGTGCCGAGGAGCGTCGCAAGCTCGACACCATTCTCGATATTCCGGTCACTATCGCCATG
GAGGTGGGCCGCAGCCAGATCAGCATCCGCAACCTGTTGCAGCTGAACCAGGGCTCGGTGGTGGAGCTGG
ATCGGGTTGCAGGCGAGCCGCTGGACGTATTGGTCAACGGTACCCTGATCGCTCACGGCGAGGTGGTGGT
GGTGAACGACAAGTTCGGTATCCGCCTGACTGACGTCATCAGCCAGATTGAACGGATCAAGAAGCTCAAA
TGATGCGCCTCCTGTTGTTATTGCTGCCTGTTCCGGCCTTTGCCGAGACAGCGGCATCTGCCAACGTGCC
TAGTCTGAGCTCCTGGTTGCTCTCCAGTCTGATGGTGATTGGTCTCATTCTGGTGCTGGGCTTTCTGCTC
AAGAAGAGCAAGCTGGCGACCGCCATGGGGGGCGGCCAGATGCGGGTCATCGCCTCGTTGCCGGTGGGTT
ACAAAGAGAAGCTGCTGGTGGTCAAGGTGGGCGAACAGCAACTACTGGTCGGCGTAACACCGCAGCAGGT
CAACTTTCTCTACCGGCTGGAGGAGCCGCTGGATGAAAACCAGCCGCAGGCGTTTTCCCAGCAGCTTGGC
AAATTGATGGGCAAGCATGAAAAAAGCTAACGGCTTATGGCTGAAAACGGGGCTGCTGGTCAGCCTGCTG
ATACCACTGTTTGCTCCTGGCCTGCGCTGGCACAGGACGCCATGTCGGCGGTGACCGTCAAGACCATGG
GGGATGGCTCTCAGGAGTACAGCCTGACCCTGCAAGTGCTGGCGCTGATGACCGCGCTCTCCTTCTTGCC
GGCCATGGTGATCATGATGACCTCGTTTACCCGCATCATTGTGGTGTTGGGTATCTTGCGTCAGGCGATA
GGTCTGCAACAGAGTCCCTCCAACCAGGTGTTGATCGGCATCGCCCTGTTCATGTCCTTCTTCATCATGT
CGCCGGTGCTGGATCGCATCAATGATGAGGCGTTGCAGCCCTATCTGGCCGAGACCATTCAGGCAAAGGA
GGCGCTGGAGCGGGCCGAGTTGCCCATCCACCAGTTCATGCTGGCCCAGACCCGGATAAAGGATCTCAAC
ACCTTTATGGAGATCGCCAACGTGCAGGTGGAGAAACCTGCCGATGTGCCGTTGCGGGTGCTGATCCCGG
CCTTTGTCACCAGCGAGCTGAAAACCGCTTTCCAGATTGGTTTTATGCTGTTCTTACCGTTCCTGGTCAT
CGATCTGGTGGTCGCCAGTATTCTGATGGCGATGGGGATGATGATGCTCTCGCCGATGATCGTCTCGCTG
CCCTTCAAGCTGATGCTGTTTGTGATGGTGGATGGCTGGAACCTCATTCTGGGGTCACTGGCCAACAGTT
TCGGACTGGGTGCCGGTTAGCCGGTCGAGCGCAAGGAGCCCGAACATGAGTCCTGAAACCTTTGTCGACG
TCTTTCGCGAGGCGCTCTGGCTGGTCAATATCATGGTCAGCGCCGTGATCCTGCCCAGCCTCGCTATCGG
GCTGGTGGTAGCCATTTTTCAGGCGGCCACCTCCATCAACGAACAGACCCTGAGCTTTCTGCCCCGTCTC
ATCGTGACACTGTTTGCGCTGGGTGCTGGTGCTCACTGGGGTCTGCAGAGTCTGATGGACTTCTTTGTAC
TGATGGTCAATCAAATCCCCGAGGTGGTGGGATGACCTACACAACCGCTCTGATCATGGAGTGGTTGTCT
TCCATCCTCTGGCCGCTGGCGCGGGTGAGCAGCCTGCTGATGGTGATGGCGGTGTTCGGCAGCCGCCTTG
CTCCTGCGCGGGTACGGATGGGGCTGGCTCTTGCTATCACCTTTCTGATTGCCCCGTTGCTTCCGAAAAT
GCCCCCTATCGAACTCTTCTCTGTCGGCAGCTTTCTGGTATTGGGCCAGCAACTGCTGATCGGTATTGCC
CTCGGCCTGATGACTCAGTTTCTAATGGAGAGCTTCGTGATGGCCGGTCAGGTCATCGCCATGCAGACCA
GCCTGGGTTTTGCCACCCTGGTGGACCCGATGAATGGCCAGTCGGCACCCGTGGTGGGTCAGTTTTATCT
GATGCTGGCGACCCTGGTGTTTCTGGCGCTGGATGGCCATCTGGTGATGTTGCGGCTGGTGATCCTCAGT
TTTGAAACCTTGCCGGTGTCGGACAGCGGACTTACCTTGCCCGCCATCCGTACCCTGATCGGTTTTCTCG
GGGTGATGTATCAGGCCTCACTGGTGATGGCCCTTTCTGCCATCGTCGCCTTGCTCTTAATCAACTTCGC
CTTTGGCGTCATGACCCGGGCGGCGCCGCAGCTCAACATTTTCAGTATCGGTTTTGCGGTCAGCATGATG
TCGGGTCTGCTGATCCTCTGGCTCACTATCGGCGGTTTCCTCGGTCACTTCGACATCATGTGGGAGCGGG
TGCAGGAGGTCAGCTGCGAGCTGATCAACACCCAGTGCTTCGGAGGCGCTCATGGCTGATACCGACCAGG
AACGTACCGAACAACCCACGGGCAAACGACTGCAACAGGCCCGTGAAAAAGGGCAGATTGCACGTTCAAA
AGAGTTGGGGACCGCCAGCGTTCTGCTCTCGGCGGTCTTTGGCCTGTTGATGCTCAAGGGCTCGCTGGCG
ACCGCCATGGTCAAGGTTGTCACCATGGGCTTTACTCTGGACAGGGAGCAGGCTTTCGATCCCAATACCA
TGATCGCCATGCTGCCGGCGCTGCTGGGGGAACTGGTGCTTCCGCTGGGACTCTTGTTTGCACTGGTGGC
GATTGCCGCTTTTATCGGCAACACCCTGATGGGGGGGATGAACTTCTCCACTGAAGCCATGATGCCCAAG
ATGAGCAAGCTGAGTCCACTCAGCGGCATCAAGCGAATGTTCGGGGTGCAGTCACTGATTGAGCTGGTCA
AATCCATCGCCAAGGTGGTGTTCATCACCCTGTTTGCCTGGTGGATGTTATCCAGCCAGTTCAATCACCT
GCTCAACCTCTCCATGGAGGGTTTCCCCGGCGGTATTATCGATGCGCTGGAGTTGTTTCTCTGGATGTTG
ATCATCCTCTGCTGCGCCCTGCTTCCCATCGTTGCCATCGACGTGCCGTTCCAGCAGTGGAACCACATGC
GTCAGCTCAAGATGACCAAGCAGGAGGTCAAGGACGAGTTCAAGGACAGCGAAGGCAAGCCCGAGGTAAA
GGGCAAGATCCGCCAGTTGCAGATGCAGATGGCGATGCGTCGAATGATGGGGGATGTACCCAAGGCTGAT
GTGGTGATCACCAACCCGACCCACTACTCGGTAGCGCTCAAGTATGATGCGGGCAAACCCGGTGCCGCGC
CCAAGGTCGTGGCCAAGGGCACAGATGAAATTGCAATGAAGATCAGGGAGATCGCCAGAGAGTACGAGAT
CCCCATCATGCCGTCACCAGCCTTGACCCGGGCTATTTACCACTCAACCGAGCTGGGCCACGAGATCCCG
GAAGGGTTGTTTGCGGCGGTTGCCCAGGTGCTCGCCTATGTCTTCCAGCTCAAGAAATTCCGCCGTGGTC
AGGGGCGCCGTCCCCAGCCGCTGGCTAAAGATCTGCCTATTCCGACCGAATATCGGAAATGACAAAGTTC
TGACGTAGTCAAAATAAATCCATTGCATTTCAATTTCTTGTATTGTTGGCTTGATGTTTGCTTTAATCTG
GCACCAGCAGGTTGAAATTGAGAAGCGCGATGGAATTCAAGGCAGTATTGGGCCAGTTCAAAGAGTACCG
GGGAGTATTGGGCAAAGGCCTTGGTACCCCTCTACTGGTATTGGCCGCGCTCGGCATGGTGGTGTTACCC
ATCCCGCCGCTGATGCTGGACGTGCTCTTCTCCTTTAACATTGCGCTCTCCATCGTGGTGCTGCTGGTAT
CCGTCTATACCCGCCGCCCCCTCGAATTTGCTGCCTTCCCGACCGTACTGCTGCTCGCTACCTTGCTGCG
GTTGGCGTTGAACGTTGCCTCGACCCGGGTGGTGTTGCTGGAAGGTCATAATGGCAGTGCGGCGGCCGGC
CATGTGATCGAGGCGTTCGGTAACGTGGTGATCGGCGGCAACTATGCGGTCGGCATCATCGTTTTCACCA

TCCTGATGATCATCAACTTTGTGGTGATCACCAAGGGTGCCGGGCGGATCGCGGAAGTGAGTGCCCGTTT
TACCCTTGATGCGATGCCGGGCAAACAGATGGCCATCGATGCCGATTTGAACGCCGGTCTCATCAATCAG
GAAGAGGCGAAGAAGCGTCGTCAGGATGTAACCCAGGAAGCTGACTTCTACGGCTCCATGGATGGTGCTT
CCAAGTTTGTCAAAGGGGATGCCATTGCGGGCATCATGATCCTCTTTATCAACATCATCGGCGGCTTCAT
CATCGGGATGATGCAGCACCAGCTGACCTTCAGCGAATCTGCCGAGATCTATACCCTGTTGGCCATCGGT
GACGGTCTGGTGGCGCAGATCCCCTCCCTGCTGCTCTCCATCGCCGCCGCCATCATAGTGACCCGTCAGA
ATACCGATCAGGACATGGGAACTGCGGTGCTGGGTCAGCTGTTTGACAATCCCAAAGCCCTGATCATCTC
CGCCGGTATTCTGCTGATGATGGGACTGGTGCCGGGGATGCCGCATTTCGCCTTCCTGAGTCTGGGGGCT
CTTGCCGCCGCTGGCGCCTGGTGGTTGCTGCGCCGAGAGAAACTGGCGGCCGCCAAAGCGGCCAAGGGTG
AGCTGCTGCCTGCCACTGCCGAACAACCCCATGAGCAGAAAGAGCTGAGCTGGGACGATGTGATGCCGGT
GGACATCATCGGGCTGGAAGTGGGTTATCAGCTGATCCCGCTGGTGGATCGCAATCAGGGTGGCGAACTG
CTCAACCGGATCAAAGGGGTGCGCAAGAAACTCTCGCAAGAGCTGGGCTTTCTGGTCCCGGCCGTCCACA
TTCGCGACAACTTGGATCTGGCGCCAAACCAATACCGCATCACCTTGATGGGGGTCAGTACCGGCGAGGC
CAATGTCTACCATGATAAGGAGATGGCCATCAATCCGGGGCAGGTGTTCGGCCAGATCCAGGGGATAGCC
ACCCGGGATCCCGCCTTCGGACTGGAAGCGGTCTGGGTTGCCAAGGAGATGGTCTCTCAGGCACAGACGC
TGGGGTATACGGTGGTAGATACCGCGACCGTGGTGGCTACCCATCTGAGCCAGATCCTGACCAACCATGC
AGCTCTGTTGCTGGGTCATGACGAGGTACAACAGCTGCTCGACATGATTGGCAAACATCAGAGCAAGCTG
GTCGAAGGTCTAGTGCCGGAAGTGATCAGCATGGGTAACCTTGTGAAGGTATTGCAGAATTTGCTCAATG
AAGGCGTGCCAATCCGCGATATGCGCACTATCCTGCAGACCCTGGTTGAATACGCGCCGCGCAGTCCGGA
TCCGGAAGTGCTCACGGCCGCTTGCCGGATTGCCCTGCGTCGCCTGATTGTGCAGGAGATTGCAGGCCCC
GATCCCGAGCTGCCGGTGATTACCTTGGCGCCGGAATTGGAACGTATATTGCATCAATCCCTGCAGGCAG
GTGGCGGTGATGGTGCCGGTATTGAACCGGGTCTGGCAGAGCGGATGCAGCGTTCGCTGGTGGAAGCCAC
TCAACGACAAGAATTGGAAGGCCAACCGGCCGTGCTTCTGACATCAGGGATCTTGCGCAATACCCTGGCG
AAATTTGTCAAGAATGCCATTCCTGGATTGCGCGTTCTGTCTTATCAGGAAGTGCCCGACGACAAGCAGA
TCCGCATCGTCAGTGCGGTAGGGCAGAACTAGTTAGCGGAGACGACGGGTGAAGATTAAGCGGTTTTTTG
CCAAGGATATGCGAACGGCTCTGGCCGAGGTCAAGGAGACTCTCGGGCCTGACGCCGTCATCATGTCCAA
CAAGAAGGTCACCGGAGGGGTAGAGATAGTCGCCGCAGTCGATTATCAATCCCAGGTCCCGGGGCCCAAA
GATGCGCCGGTTCGCCGTCAGCTGAACGATGAGAGCGTCAATATTTCGTCTGCTGCACGGCAGATGATCC
GCCCCGAGCCGTCGCCGGCGAAGGAGCAGAACGAACATTATGCCGATACCCTGGCCGCCTTGCTGGCGCG
CCAGCAGAAGCTCAAGCCGGGCAATGGGGCGCCAATGGGTAATACCCAATCGGCCGGTCTGGCCGCTGCC
AATGCAGCGCCCAGAACATTGGCAGAGCAGGGGCAGTGGGGCGCAGCCCCCGAACCGGCCAATCCCAAGG
CCCGGGTTGCCTCCGGCCAGCGCGCCCCTCAACCTGCCCAGCGGGATCAGGAGATGGAAGGGATGCGCAA
GGAGATGGCCAGCATCCGCAAACTGCTGGAGCATCAGATATCCGGGCTGATGTGGCAAGAGGTGGAGCGC
CGCGAGCCGATGCGGGCCCTGCTGATCAAGGAGCTCAAGAAGCTGGGCTTTGACGATGCGTTTGCCGATC
AACTGGCGGGACTGATCCCCGAAGATATGTCCATCCATCAGGCGATGGCCCAGTTGGCCGAAGTGCTGAC
GGCCCAGCTCAAGATCAGTGAGGACGAGATCATGCGTCAGGGCGGCGCGGTTGCACTGCTTGGCCCGACC
GGGGTGGGCAAAACCACTACCATCGCCAAGCTGGCTGCTCGTTTCGCCATGAAGTATGGCGCCGAGCAGG
TGGCGCTGATCACCACTGACAACTACCGGATTGGTGCCCATGAACAGCTGCAGACCTATGGCCGCATCAT
GGGTTGCCCGGTACGTCAGGTGCGTGATGCTGAAGAGCTGGCCAATGCCCTTTATCAGTTCCGCCACCGT
CGACTGGTACTTATCGATACGGCCGGGGTGGGTCAGCGGGATATCCGCCTGACCGAGCAGCTCGATACCC
TGGTCAAGAATGCCAAGGTGCGGATCCGCAGTTATCTGGTGATGTCGGCAACGTCCCAGCGTCGGGTGAT
GCAGGAGGCGGTTGACCATTTCCGGCGTATTCCATTAAGCGGCTGCATACTTACCAAATTGGATGAAAGT
CTGAATCTGGGCGAAGTGATTAACGTGTGTATCCAGAATTCATTGCCCATCAGCTATATCACTGACGGAC
AAAGGGTTCCGGAAGATATTCAGGTTGCCAATGCGGCGCAGCTCGTCGGGGCTGCCATGGGCTCGCTGGA
GCGAGAAACAGAGGAGCCCTATTTCTGGGGCGCAGGCTTTGGCGAGGCCGAAGATTCGGAGTTTTATGAG
TAACATGTATATGGATCAGGCGAGTGGTCTGCGCAAAATGCGTCAAAACAATCGAGTAAAAGTAATCGCC
GTTTCCGGTGGTAAAGGTGGCGTGGGCAAGACCAACGTGACCCTGAACGTGGCCGGAGCCATGGCCGCCC
AAGGCAAGCGGGTGATGGTGCTGGATGCCGACCTGGGTCTGGCCAACGTCGATGTCATGCTGGGGCTGCG
GGTTCATCGCAATCTCTCTCATGTCCTGGCGGGTGAATGCACCATCGACGATATCATCGTTGAAGGCCCT
TACGGCATGATGATCGTGCCTGCGACCTCGGGCACCCAGTCCATGGTCGAGCTTTCTCCGGTGCAGCATG
CCGAGCTTATCCGCGCCTTCAGCGAGATGAAGACCCAGGTCGACATCCTGCTGGTGGATACGGCTGCGGG
GATCTCCGACATGGTGCTGAGCTTCACCCGCGCAGCTCAAGACATCATGGTGGTGGTCTGCGATGAGCCG
ACCTCCATCACCGATGCCTACGCCCTCATCAAGATCCTCTCCAAGGATCACGGCGTGTTTCGTTTCAAGG
TGGTGGCCAATATGGTGCGCAGTCTGCGCGAAGGGCAGGAGCTCTTTGCCAAACTGACCCGGGTCACGGA
TCGTTTCCTCGACACCTCCCTGGAGCTGGTCGCCTGTGTACCCTATGACACCAATCTGCGTGCCGCCGTG
CGCAAGCAGAAGCTGATTGTCGAGGCGTTTCCCAAGTCACCGGCCGCACTGGCGTTCCGGGCCCTTGCCA
ACAAGGCGGCGAGCTGGCCGGTTCCCAACCAGCCGGGCGGCCATCTGGAGTTTTTCCTGGAAAATTTGTT
GCAGAAACCGGCCATAACACAGGAAGGTTCCCGTGAATAAAGCCCAAGCGTACTTGCGTCATCAGGATTC
CTACGTGCTGGTGGAGCGTCATGCTCCCCTGGTCAAGCGAATTGCCCAGCATTTGTTGGCTCGCCTGCCC
AGCAGTGTCTTGCTGGATGACCTGATCCAGGCAGGTATGATTGGGCTGCTTGAAGCCTCCCGCAACTTTG
ACGGTAGCAAGGGGGCCAGCTTCGAGACGTATGCCGGGATCCGCATCCGAGGCGCCATGCTGGATGAGAT

TCGGCGTGGTGACTGGGTGCCCCGTTCAGTGCATCGCAACAGCAGGGCTATTGCAGAAGCGATCGAGAAT
GTCGAGCAGACACACGGGCGTGACGCGCGAGATACCGAGGTTGCTGCCGAGATGGGGGTCTCTATCGCTG
ATTACCATGCGATGCTGCAAGACGTCTCCTGCGGCAAAATTATCGGCATCGAAGATTTGGGGGTCAGCGA
GGATGTGATCGGCAACCCCGATGACGCCCCTTCAGGCTCGGGATTCGATGAATTGGCTGCCGAGCGATTT
CAGATTCAATTATCCGAGCAAATTGGTCGTCTACCAGAGAGAGAAGCTTTGGTTCTCTCTCTTTACTACG
ATGAAGAGCTGAATTTGCGTGAAATTGGCGAGGTTTTGAATGTCAGCGAGTCGCGTGTGAGCCAGATCCA
CAGTCAGGCAATGCACAGATTGCGTGCTCGTTTGAGAGATTGGCATCTATAAGTGTTTGTACTGGCTACA
CTGAGATGAAACTCTAAGGCATAATCCGCACGACACAACCCGTTTTTAGCATCATGTAAGTGAGCCACTC
TGGTGGCTATTTAGGAGGGAATTTTGGACAAGAACATGAAAATCCTCATTGTTGACGATTTTTCAACGAT
GCGCCGGATTATCAAGAACTTGCTGCGCGACTTGGGATTCAACAATACCCATGAGGCGGATGACGGCAAT
ACAGCGCTGCCGATGCTGAAAAATGGTGATTTTCAGTTTGTAGTTACCGACTGGAACATGCCTGGCATGC
AAGGTATTGACCTGCTTAAAGCCATCCGTGTCGATGAGAAGCTCAAGCATCTGCCGGTACTGATGGTAAC
CGCTGAAGCAAAGCGTGAGCAGATCATTGAAGCCGCCCAAGCTGGTGTTAACGGTTACATCGTCAAGCCC
TTCACCGCAGCAACGCTCAAAGAAAAACTCGATAAAATCTTCGAACGAATTGGCTAAGGAGCTGCTGGGA
TGATGGCCAAAACGAGAGCAATGATTTCGCTCGACCAGGCAAGGATGCTTGTTGCTCATCTAGAGCAAGG
CGAATTTGAGCTGGCCGATGAACTGCTTGCCGAAGTATGTGCCCCCAACGCAGCCGATCTGTTTGAAAAA
GTAGGTCAGCTGACCCGACAACTGCATGATTCACTTCAAGAATTTCGCCTCGATCCCCGTATTCCCGATT
TGGCTACTCATGATATTCCCGATGCTCGGGAACGCTTGAGCTACGTTATCGATATGACCGACAAAGCGGC
CAATCGCACAATGGATGCGGTGGAAGCCAGTTTGCCGATAGCGGATCGCCTGAATGACAATATCCAGCTG
GTGATGCCGAACTGGAATGCCCTGATGTCTCGTGACATGAGTGTGGGGCAGTTCAAATCCTTATGTCACC
AACTTGATGATTTTATTAAGGCTTCAGAGTCTGATGCCGATAAATTACGGCAACTGCTGACAGAAATCCT
GATGGCACAAGATTTTCAGGATTTGACAGGTCAGATGATCCGCAAGGTCATCAAGCTTGTTCAGGAAGTT
GAAACAAAATTAATAGAAATGCTGACCATGTTTGGTGAGGCTTCTGCTGAGCATTCAACTCGTAGTATTC
CTGTCAACGGGATTGAAGCAGAGGGCCCTATCATGAACCCCGAGGCTCGTGCCGATGTGGTCAATGGTCA
GGATGATGTGGACGATCTGCTGTCTAGCTTGGGATTCTAATGGAGTGACGCATGGCCTTCGAAGTTGATG
AAGACATACTGCAAGACTTTCTGGTCGAAGCATCCGAAATATTGGAACAGTTGTCTGAACAACTGGTCGA
CTTGGAAAAGCGGCCCGATGACAAGAACCTGTTGAATGCCATCTTTCGCGGTTTCCATACCGTGAAAGGG
GGGGCAGGTTTTCTGTCGCTCGGTGAACTGGTTGATGTATGCCACGGTGCGGAAAAACGTGTTCGATATCT
TGCGTAATGGCAAGCGAACCGTTACTGCGGAGCTGATGGATGTAATCCTGCAAGCGCTGGATGCCATCAA
TGTCATGTTCGCCCAGGTTCAAAATCGCGAAGCACCCAGCCCTGCGTCAGATGATTTGCTGCATGACCTC
CATGAATTGTGCAAGCCAGAAGGGGAAGAGCAACTGCGCACTGCTACCAGTGCGGCTGCGGCTGTAGAAT
CTGAACCTGCAGCACCGGTAGTTGTTGAAGAAGTTGCTCCTGAAGTTGTTGAACCCGCAGCCATCAGCAG
CGAGCCTGCTCCTGTCAATGGCAGCGGCTCGATCGATGAAATTTCTGCAGACGAATTCGAGCGTCTGCTG
GACGAGCTCCACGGCGTGGGTGGGGCTCCGACCAAGACGGCTGCAGCAGCCCCAATAGCGGGTGGCTCTG
GCGATATCACTGATGATGAGTTTGAGGCCTTGCTGGATCAGCTCCATGGTCAGGGACAGCACATGGGGGC
GCCAGAGGTAAATCCTCTGAAGTCGGTGCAAAAAGAGGTGGACGAGCTTATCGACGATGATGAGTTTGAA
CGTCTGCTTGATGAATTGCATGGTCGTGGTCAAGGGCCTCAGAGTACTATGGCACCTGCCGCACCTGCCG
CACCTGCCGCACCTGCCGCACCTGCCGCACCTGCCGCACCTGCCGCACCTGCTGCGGTGACTCCGCCCAA
ACCGGCCACTCCTGTTGCCAAACCAGCACCGGCAGCCCCCGCAAAACCCGCTGAAGCAGCCAAGGCACCA
GCGCCTGCCGCCAAGGCTGCGCCTGCGCCAGCAGAAAATAGTGCCGTGCAAAGCGATACGACGGTGCGGG
TGGATACCAAGACCCTCGATGTCATCATGAACATGGTAGGTGAGTTGGTACTGGTTCGTAACCGGCTGGT
GAGCTTGGGTATTGCCAGTAATGATGAAGAGATGTCAAAGGCGGTTGCCAATCTGGATGTGGTAACGGCT
GACCTGCAAGGTGCGGTGATGAAAACCCGCATGCAGCCAATCAAGAAGGTATTTGGTCGTTTCCCGCGCG
TGGTACGTGATCTGGCCAGAACCCTCAAGAAAGAGATTGAGCTGGTGATGGTGGGCGAAGAGACAGATCT
GGATAAAAACCTGGTAGAGGCGCTCGCCGATCCTCTGGTTCACCTCGTTCGCAACTCCTGCGATCACGGG
GTCGAAATGCCGGATGTGCGGGAGAAAGCGGGCAAGCCGCGGCAAGGGACTATTACCCTTAGCGCCTCCC
AACAAGGTGACCATATTCTGCTTTGCATCGAGGATGATGGGGCAGGGATGGATCCTGAGAAACTCAAGTC
CATCGCAATCAGTCGCGGTGTTCTGGATGCAGATACTGCCGCTCGCATGAGCGACAACGATGCCTATAAC
CTGATTTTTGCTCCGGGCTTTTCAACCAAGGCCGAGATCTCCGATATCTCTGGCCGTGGTGTCGGAATGG
ATGTGGTTAAAACCAGCATTGTCAGCCTCAATGGGTCGGTGCACATCGATTCCACTTGGGGGAAAGGTAC
CCGTCTCGAAATCAAGGTTCCGTTGACGCTAGCCATTTTGCCGACGCTGATGGTGGAAGTGGGTGAGCAG
ACCTTCGCACTTCCCCTTGGTTGTGTGAACGAGATTTTCCATCTGGATCTGAAAAAGGCCAACATGGTTG
ATGGCCAGCTCACCATCATTGTGCGCGAGAAGGCGATACCGCTCTTCTATCTGCATAAATGGTTGATTCG
TGGCGCCAACAAGCGTTCACGTCAGGATACCGGACATGTGGTGATCGTGCAGATTGGCACTCAGCAAATC
GGGTTTGTGGTAGACAGTCTGATTGGTCAGGAAGAGGTGGTGATCAAGCCACTCGATAACCTGCTGCAAG
GCACCCCTGGCATGGCCGGGGCGACCATTACCAGTGATGGTGGAATTGCTCTTATTTTGGATGTGCCGAG
TCTGCTCAAAGCATATGCGCGTAGACATTAATAGCTCAGGCCCAAGGAGTTAGAAAAACAACAATGGCAG
TCAAGGTATTAGTTGTCGACGATTCGAGCTTCTTTCGCCGTCGGGTGAGCGAGATCATCAATCAGGATCC
CATGCTGACGGTGATTGATACGGCCCAGAATGGACGTGAAGCGGTGGACAAGGCGATGTCGCTGCGCCCT
GATGTGATCACCATGGACATCGAAATGCCCGTGATGGATGGCATAAGTGCGGTGCGGGAGATCATGGCCA
AGTGCCCAACCCCCATTCTGATGTTCTCCTCCCTGACCCATGAAGGGGCCAAGGCTACGCTGGATGCGCT

GGACGCCGGTGCCCTGGACTTCTTGCCCAAGAAGTTTGAGGACATTGCCAGAGACAAAGATGAAGCGGTC
AGACTGTTGCAACAGCGGGTGAAGGAGATCTCCCGCAAGCGCTTCCTGATGACATCCGCTGCTCGTCCCA
AAGCACCTGAACCGGTTGCTCGTGTTCCTCTGGGCAGCACAGCGCCCAGATCTTCAGATCGTCAGCCTGA
CCTGCAACGCACTGTGCCAAGTGCCTCATTCAAGCGCAGTGGCAAGAGTTATCAGTTGGTGGCGATCGGT
ACTTCTACCGGTGGACCTGTTGCTTTGCAAAATGTGCTGACCAAGTTGCCGGGGGATTTTCCCCATCCCA
TCCTGCTTATCCAGCATATGCCAGCCACTTTTACCGCCGCGTTTGCTGCCCGTCTCAACGGGTTATGTCA
GATTGGCGTCAAGGAAGCGGAAGATGGCGATGTGCTTAAACCTGGGCATGCCTACCTTGCTCCCGGTGGT
AAGCAGATGCTGTTGGAAGGTCGTGGTGCAGGGGCCCGCATTCGCATCGTGGATGGCAACGACAAGGTAA
ATTACAAGCCTTGTGTGGACATCACCTTTGCCTCTGCGGCCAAAACCTATGCCGACAAGGTATTGGCGAT
TGTGCTGACCGGTATGGGGGCTGATGGCCGTGATGGTGCCAGACTGCTGAAAGAGCAGGGATCAACCATC
TGGGCTCAGGATGAGGCTTCCTGCGTGGTATATGGTATGCCCCAAGCAGTAGCCAAAGCGGGGATTGCCA
GCGAATCACTCCCTCTGGACAAGGTGGCCCAGCGTATTCTGGTTGAGTTGGGGCGCTAATCATGGGCTTG
CTAGGTCTGCTCCTGGCATTGGGGGCTATTGCCATTGCCCAACTGTGGCATGGCGGCTCCCTGTTGACGC
TGGTAGACGGCCCCGCTTTTTTGATCGTGCTGGGTGGTACCGTTGGTGCAGTTACCTTGCAGACTCCAGC
CAAGTACATCCTGCTGGCGTTGCGCCAGCTGTTATGGATTTTTACCCCTCCCCACCAAGATCTTGCGACG
CAGGCCGAACGATTGCAGTCATGGGCCGTCTTTGCGCGCCAGCAAGGGCTGCTTGCCCTTGAAAATCAGG
CCGAGCAGGAGGAAGACAACTTTACCCGTCAAGGGCTCGGCATGATTGTCGATGGGGTCTCCACAGAGGA
TATCCGTATGCTGCTGGAAGCCGATATCGATATCGAGCAGGAGCGCAATGAACATGCCGCCAGGGTATTT
GAAGCCATGGGGGGGTACAGTCCGACCATCGGGATCATCGGTGCCGTGCTGGGCCTGATCCAGGCCATGT
TTCATCTCGAAGACCCGTCAGCACTGGGGGCGGGGATCGCAGTGGCATTTGTGGCAACCATCTATGGAGT
GGGGTTTGCCAACCTCATCTACCTGCCTATCGCCAACCGGCTACGTGCATTTCACTATCACTACGCACTC
TTTCAGGAGATGACCCTGGAAGGGCTGCTGGCTATCGCCCATGGTGAAAACGGCTTGCAGGTTGAACGCC
GGCTCAGGGCCTATTTGAAAGGAGAGTAGCCATGCGCAAGCGCTATCGGTTGCACTTGCAGGGTCGCGAG
CATCTTGACCGCTGGCTGGTCTCCTATGCCGATTATATGACGCTGATCTTTGCATTGTTCGTGGTGCTCT
ACTCTGTTGCGCTGGTGAACAAGGACAAGTATCGCGCGGTGATTGATGGTATGAGCCAGGCGTTCAGTGT
CATGCAGTCTGGCGGCAATGGCTTGCTTGAAGGGCAGGGTAATTCCCTGCTGGATAACCCGGTATCTGCC
TCTCCATCGTTGCTGGAAAGCCCAGCATCCCTCTCTCCTTCACTTGCTCCTATTTCTGGTGTGCCAGTGC
TGCAAGATGGCGCGACACTTGCAAAACTCGATGTGCAGTTGCAGCAGAGCATGGGCTCGCTGGTGGATGC
CGGTATCGTCAAACTGACGCAAGATGAGAATTGGCTGACGGTTGAACTCAGTTCCGGCTTGTTGTTTGGC
AGCGGCAGTGCCTTTTTGGGACCAAACGCTGCACCGGTCATTAATACTCTTTCAAGTATTCTAAAACCGG
TCGATAACTATGTAAGGGTTCGGGGTTATACCGATAACCAACAGATAAATAATGAAATTTATCGTTCTAA
CTGGGCGCTTTCTGCTGCTCGGGCCGAAGCGGTACTCAATGGTTTGATTGCAGATGGGATCGCCCCTCAG
CGTCTGGCGTTCGAGGCATATGGCGAGTTTTCCCCCTTTGCTGACAACGGCAGCGAGCAGGGCAGACTGC
AGAATCGCAAGGTTGTGGTCGCTATCTCCAAATTTGCCTGGGTGCCGCCGGCACCTGTGGTTGCAACACC
GACGGTAGTTGCTGAACCGGTTCCTGCTGTACAGCAGGCGGTGGATGCAGGAGAGATAAAGGTGATCACC
TTGCCCGGTGGTGGCATACGCATAACAACAAGGCAGGATTGATACAGTGATTGTTTGGACGGTTGCCAAC
CAGAAAGGTGGTGTGGGTAAAACCACCACAGTGGTCTCGCTTGCGGGGATCCTGGCCCAGCGTGGCCAGC
GGGTGCTGTTGATTGATACCGACCCCCATGCTTCGCTGACTGCATATCTTGATTTTGATTCAGATCGCCT
GGATGGAACCCTCTATGAGTTGTTTCAAGCTGTCAAACCGACAGCCGAGCTTGTCAATAAACTGACGCTC
AAGACCAAGTTCGAAAATATCAACTTACTGCCAGCATCTATTACTTTGGCGACCCTTGATCGGGTGATGG
GCAATCGTGAGGGGATGGGACTGGTACTGAAGCGGGCACTGCTGCGCATTCAGGATCAATATGATTACGT
GCTGATCGATTGCCCGCCAGTGCTGGGAGTGATGATGGTCAACGCGCTGGCTGCCTGCGATCGCATTCTG
GTGCCTGTCCAGACCGAATTTCTGGCCCTCAAGGGGTTGGAGCGGATGATGAAAACATTCGAGATCATGC
AGCGTTCCAAGCGTGACAAGTTTCGCTATACCGTGATCCCGACCATGTTCGACAAGCGCACCCGTGCTTC
TTTGATGACCTTGAAATCGATCAAGGATCAATATGGGGATTCGGTGTGGAACGCGGTGATCCCTATTGAT
ACCAAGTTTCGGGATGCAAGCTTGCTGCATATCCCCCCGTCTATCTATTCTCCCGGGAGTCGTGGAACCT
ATGCATATGAAACGCTGCTCAACCATCTTGATGCCCTTGAGCGGCAACGGGTTCATGAGGTGACGGCATG
AGAGATACCATTCAAGTACGGGCGATGGATGACTATTTCCACTCCCTGCTGCTCGATGAGCCACTGGTGC
TGGATGAAGTGGAAGAGGTTCAATCTGCCCCTGTTTTACAGTTGCAACGTCAGCCTCAAAGCGTCCCCGA
GGTAGTCGCCGCACCATACCTCGAAAGCGATCCACGGCTTGCCCAGCTCGGTGAGCTATTGGCTCAGGTG
GGACAAGTCCAGTTCGAAAATGATGCCTTCACCCAGGAGATAACACTCACCGCACCTCTGGAGATTGTGG
CGGAGGAGTCACTGTTGCCGGTGGAGCCTGAGTGGCCTGCGGAGGTGCTGCTCCCCGAGGTTGCGGAGCC
CCATACTACTATCCTGACCGAGACAGCGCTGGAGGTTGCTCCGGTAGAGGCCAGTGAACCGGTTGCTTGG
GAAAATATCGATACCGGTAAAGAGTTTCAGGCCCTTTTCTTTGAAGTCGCGGGCGTGACGTTTGCGGTGC
CGTTGACCGAGCTGGGTGGTATTCATCAGCTGGGCGAGATCACCTCCCTGTTTGGCCAGCCTGGCTGGTA
CAAGGGGATCATGACCTCCCGCGAGCAGAAGATGAATGTGGTCGATACTGCCCAGTGGGTCATGCCGGGT
CAGCATCTCGAGATGGATGATTACAAGTATTTGATTATGCTTGGTGAATCGCCGTGGGGATTAGCCTGCC
ATCATCTCAAAGGGACTGAATTGTTACACCGTGACCAAGTGAAATGGCGCCATCAGGAGGGCAAGCGCCC
CTGGTTGGCGGGCATGGTCAAAGAAAAAATGTGTGCCCTGTTACACGTCCGTGAACTGCTCCTGTTGCTG
GAGCGTGGGGTTAACATAGACGGTCGCTGATAACTTCTGCAATCGAGCTGTTTTATTGCTAGTTTTAGCA
GTAGACGTTTTCATAAAAGGAATAAAAACATGACAGAAAAGCGCAATATTGCACAGAATCTGGCCGAAGA

TGAAGTGCTGCAATGGGTAACCTTCCAACTGGAAAACGAAACCTACGGCATCAATGTGATGCAGGTTCAG
GAGGTGTTGCGCTACACCGAAATTGCACCGGTGCCGGGTGCGCCTGAGTACGTACTGGGGATCATCAACC
TGCGTGGCAATGTGGTGACTGTCATCGATACCCGTTCCCGTTTCGGTCTGCCTTCCGGTGATGTGAGTGA
AAACTCCCGGATCGTGATCATCGAGGCCGAGAAGCAGGTGATCGGCATCATGGTCGACAGTGTGGCGGAA
GTGGTTTATCTGCGCTCTTCCGAGATCGATGCGGCACCTGCGGTCGGTACTGAAGAGAGCGCCAAGTTTA
TTCAGGGGGTGAGCAACCGCGATGGGCAACTGCTGATCCTGGTTGACTTGAACAAGCTGCTCTCCGATGA
AGAGTGGGAAGATATCGGTTCCCTCTGATAAGTAAGGTGATGTTTTGATTATCGAGATGGTTGCCTTGGG
GGTGTCCCTGATTGCCTTGGCGTTTGCCTCTTATATCTGGTTCAAGAGCAAGCGCAGCAAGCAGGAGTCC
GAGGACAGTGTGGCAACCCTGCAAGGTCAGTTGGAAAGCTTGCGCAAGCAGATGATGGAACTCCATACCG
GCGCCATTGGCATGGGGCAGCGTCTGCAGTCGGTGGAAGGGGCGATGCAGCAGGTCAATGAGCGCCAGCA
AGAGCTGACCATGCAGGATCCCGAACGCAGGCTCTACAGCCGAGCGGCCAAGATGGTTGAACTGGGCGCC
GATCTTGATGAAGTCATGAGTGAGTGCGAGTTGCCAAAGGCAGAGGCCGAGCTCTTGATATCGTTGCGAA
AAGGTCGCTCTTGATGATTCAGGAAGGCAATTGCCTTCCTGAATTTTATGGCGTGCAGCCGCTTGTCCCC
CAACTGCGTTGAGCAGGCGGTTTCTACCGTATTTTCTTTGTGATCCCGGTCGCCAGTCAGTTCAATTGTT
GCATCTTTGAAAAGACGTATTCCAATACTGTGTTAAGATGTAGACCGCTTCATTTTCTCTGAAAAAATCC
CCATGCTGCTTCAAGTTAACAATCTGAGCTGCGTGCGCGAAGATCGCACTCTGTTTGAACACCTCTCTTT
CTCTGTTGCGCCCGGCGATCTGGTGCAAATTGAAGGACCCAACGGGGTCGGCAAGACCAGTTTGTTGCGG
TTGTTGACCGGGCTGTCGCAGCCATTTGCCGGTGAGGTGTGCTGGAATGGGGAGAATATTCGTCACTGTC
GCGATGAGTATCATGCCAATCTGCTCTATCTCGGCCATCAGCCCGGTGTCAAAGCGGCGCTGACCCCTTT
TGAAAACCTGAAGTTCTACCAGCAGTTACATCACCCGCAGCAAACAGAGACTGACTTGTGGCAGATATTG
GCCCGGGTTGGGCTGGCCGGATTTGAAGAGAACCCGACCGGGCAACTCTCTGCCGGCCAGCAACGCCGGG
TAGCTCTGGCCAGACTCTGGCTGTCACAGAAACCGGCACTCTGGATCCTCGATGAGCCCTTTACCGCCAT
CGACAAGCAGGGGGTCAAGGTGCTCGAGCAGCTTTTTCTGGCCCACGCTGAGCGGGGCGGCATGGTGATC
CTCACCACCCATCAGGATCTGACCCTGATGCAGGGACGGCTGAAGACTATCTCGTTGATCCCCTTCGCCT
CGACATTGCAGGAGTAACGCCATGTTGCGCGCTGTTATGCACCTTATTCACCGTGAACTGCTGATGGCTC
TGCGCCAGCGTTCCGATATTCTCAACCCGCTCTGGTTTTTCCTCATCGTCATCACCCTGTTTCCGTTAGG
GATTGGCCCGGAGCCCAAACTGCTGGCCCGTATTGCCCCGGGGATCATCTGGGTAGCGGCGCTATTGGCG
GCCATGCTGTCGCTGGAGCGACTGTTTCGCGACGACTTTGCCGATGGCGCGCTGGAGCAGATGATGCTGA
TGCCGCACCCACTGAGCTTGCTGGCGCTGGCGAAAGTGGTCGCTCACTGGCTGCTGACCGGATTGCCGCT
GCTGCTGATCTCTCCCTTGCTGGCGATTTTGCTGTCGCTGGATATGAACACCTATCTGGCTGTCGTCGCG
ACGCTGGCACTTGGCACTCCTGTATTGAGTCTGCTCGGCGCGGTCGGGGTCGCGTTGACGGTGGGATTGC
GCAAAGGTGGGGTATTGCTCAGTCTGCTGATCCTGCCGCTCTATATTCCGGTACTGATTTTTGCCACCTC
AGCCATCGATGCCGCGGGGATGAGCTTGCCCTATGGCGGGCAGCTTGCCATTCTGGCCGCCATGTTGGTG
GGGGCTTTGACACTGACGCCGCTGGCTGTGTCGGCGGCGTTGCGGGTCAGCCTCAACTGATGGCCGTGAG
CTGAACCGGATTTGGCTCCGAACGGAGTCTTTTTGCAAAAAAATAATGTTGCGATGGATCAAGGAGAGTGC
CGCTTATTGCTTTCGGGGTATTTGATGCTGCCCTGCGGTCGGTATGGTAGGCCTCGCCAAACGATTAATA
ACAAAAAGGTTTTGTGAGCTATGTGGAAATGGTTGCACCCTTATGCCAAACCGGAGCGTGCCTATCAGCT
GGCAGGCACGCTGTTGCCCTGGTTTGCGGTGATTAGTCTGCTCTCGTTTGTGATCGGTACTGTCTGGGGC
CTCGCGTTTGCTCCGGCAGATTACCAGCAGGGAGATTCGTTCCGGATCATCTATATCCACGTGCCTTCCG
CCATTCTCTCCATGGGGGCCTACTCCACCATGGCGGTGGCCGCCTTTATCGGCCTGGTGTGGCAACTGCG
GATGGCGGATATGACGGTTGCAGCCATTGCGCCGGTCGGCGCCGTATTTACCTTTATTGCCCTCTTTACC
GGTGCCGCCTGGGGCAAGCCGATGTGGGGAACTTGGTGGGTGTGGGATGCGCGGCTCACCTCTGAGCTGA
TCCTGCTGTTTCTCTACCTCGGTGTGATAGCCCTCTACAACGCCTTCAGCGACAAGGTGGTCGCTGGCCG
TGCGGCCGGCATTCTGGCACTGGTTGGGGTGATCAACCTGCCCATTATTCACTACTCGGTGGAGTGGTGG
AACACCCTGCATCAGGGGGCCACCATCACCAAATTTGACAAACCCTCCATTTCACCCGAGATGCTCTGGC
CGCTGCTGATCATGATCCTGGGGTTTGCCACGTTTCTCGGGGCCCTGACCCTGATGCGTTTTCGCAATGA
ACTCCTGATGCGGGAGTGGCATCGCCCCTGGGTCAAAGAGATTGCAGAGCAGGAGAAGTAAGCATGCACT
TTGCCTCTTTCAGTGATTTTATGGCGATGGGCGGTTACGCCTTCTATGTCTGGCTCTCTTTTGGCCTGAC
GCTGGTGTGTCTGGTCGGCATCATCATTTCTACCCGCATGAAAACCCGCAGCCTGCTCGCCGAGTTGTGC
AGCAAGCAGGCGCGTGAGGCCCGTCGCAAGGCGGCCCAGCAGATGGAGAACACCCTATGAACCCGAGACG
CAAAAAACGCCTCACCATCATTCTTGCCATCACCCTGGGTCTGGCGACCGTCAGCGGGCTGGTGCTCTAC
GCCCTCAGTCAGAATATCGACCTCTTCTACACCCCGAGCGAGCTGGTGGAGGGGAAGGGGCCGGACAAGA
TCAAGCCGGAAGAGGGGCAGCGCCTGCGCATCGGTGGATTGGTCGTGCCGGGGTCGGTGCAGCGGGATCC
CCAGAGCCTCAAGGTTGCCTTCAAGCTGGTGGATAACGGCGGTCATCTGGTGAAGGTCGAGTTTGACGGC
ATCCTGCCGGATCTGTTCCGTGAAGGGCAGGGGATTGTTGCCCAGGGCACCCTCAAGGATGCCACGACCG
TAGAGGCGTTCGAGGTGCTGGCCAAGCACGATGAGAACTACATGCCGCCGGAAGTGGCCGATGCCACTAA
CGGCATGCACTTCAAGCCGGAGTATACCGAGGCGCAGTTGAAGGGAGCCAAGCAGTGATCCCTGTGGTGT
TTGCCTTTTCCGCTTTGCCTGCCGTGCGTGGTGCGGCTGTTGTCACCGTTCAATTGAAAGGAATGCCTTC
ATGATCCCGGAATTAGGTCAGTTTGCGCTGATCCTGGCCTTTGCCACCGCCCTGCTGCTGGGGAGCTACC
CACTACTGGGCGCTAGTTGGGGGCGTCTTGGCATGATGTCGGCGGCCCGTCCGCTCGCCTATGCCCAGTT
CTTGCTGCTGCTGCTGTCTTTTCTCTGCCTGACCTGGGCCTTTATCGATAACGACTTCACCGTGCAGTAC

GTGGCCACTAACTCCAATAGCCTGCTGCCGCTCGCCTATCGCATCTCGGCGGTGTGGGGGGCCCACGAAG
GTTCCCTGCTGCTGTGGGTACTGACCCTGGGTGGCTGGACTGCGGCGGTGGCCATGTTCAGTCGCCGTCT
GCCGCTCGATGCTGTCGCGCGGGTACTCGGCGTGCTGGGTCTGATCTCGGTCGGCTTCACCGCCTTCGTG
CTGTTCACCTCCGATCCCTTCACCCGCACCTTGCCCTATTTTCCGGTCGATGGCCGTGACCTCAATCCGC
TGCTGCAGGATCCGGGTCTTATCTTCCATCCGCCGATGCTCTACATGGGTTATGTAGGCTTCTCGGTGGC
ATTTGCCTTCGCCATAGCCTCATTGATGACCGGTCGTCTCGATGCAACCTGGGCCCGCTGGTCGCGCCCC
TGGACCATGGCTGCCTGGGTATTCCTGACCCTAGGCATAGTGCTCGGCTCCTGGTGGGCCTACTACGAGC
TCGGCTGGGGCGGTTGGTGGTTCTGGGATCCGGTAGAAAACGCCTCGTTTATGCCTTGGCTGGCGGGTAC
CGCTCTGCTGCACTCGCTGGCGGTGAGCGAGAAGCGCGCCACTTTCAAGGCGTGGACCGTGCTGCTGGCC
CTCTCCGCCTTCTCGTTGAGCCTGCTGGGTACCTTCCTGGTGCGCTCCGGCGTGCTGGTGTCGGTCCATG
CGTTTGCCTCCGATCCGACCCGCGGCCTGTTTATTCTCGGCTTCCTGCTGGCGGTGATCGGTTCATCCTT
GCTGCTGTTTGCCTTCAAAGGCTCGCAGGTGAAGAGCCACGGCAAACACGAGCTGTGGAGCCGCGAGACC
CTGCTGCTTTGCAACAACATCATGCTGGTGGCGGGTCTGCTGACCGTGCTGCTCGGTACCTTGCTGCCGC
TGGTGCACAAGGAGCTGGGGCTGGGGAGCATCTCTATCGGTACCCCCTTCTTCAACCACATCTACAGCTG
GCTGATCATCCCATTTGCCCTGCTGCTGGGGGTTGGCCCGCTGTTTCGCTGGCGTCGTCAGGAGCTGGGG
GAACTCAAAAGCAAGGTGGTCGTGGCACTGGTGCTGAGTCTGGCCGCAGCCTTGCTGTTGCCGCTGCTCT
TTGCCGAAACCTTCAAGCCGTGGGCGGCGGTAGGGATTGGTCTGGGCGCCTGGATCATCGTCACCAGCGT
GCAGGAGACCTGGGTGCGTGCTACCCACAAGCACAGCTTTGCAGTCGGTGTGCGCAAGCTTGGCAACAGC
CACTGGGCCATGATTCTTGGCCATGTGGGGCTGGCAGTCAGCATCATAGGTATCGCCTGCACCCAGAACT
ACAGCATCGAAAAGGATCTGCGAATGCAGGCGGGTGATCGAGTGCACTTTGCCGATTACGAGTTCGTCTT
TGCCGGGATCAAAGAGAAGAACGGCCCCAACTACGATGGTTTCAAGGGCGTGCTGGAAGTGCACAAGAAT
GGCAAGCAGGTCGCCCTGCTCAAGCCCGAGAAGCGGATGTACAAGGTGAGCCGGATGCCGATGACCGAAG
CGGCCATCGATGCCGGTATCACCCGCGATCTCTATGCGGCGCTGGGGGAACAGCTCGACAACGGCGCCTG
GGCGGTGCGGATCTACTACAAGCCGTTCGTGCGCTGGATCTGGTTCGGCGGCGTCTTTATGGCAATTGGT
GGTGTGCTGGCGATGCTGGACAAGCGTTACCGTTTCGGCCGTCGTGAGGAGGAGGCCAAGGCATGAACAA
AAAGACTTGGTTGTTTTTAATCCCCTTTATCGTCTTTTTGCTGGGGGCGGTGTTCCTGTTCAAAGGGCTC
TACTCCGATCCCACCAAGCTTGAGTCCGTGATGGTGGGTAAAGAGGTGCCGGTGTTCACTCTGCAGGACA
TTTACGATCTGAAAAAGCAGCACGATCGCAGTATCCTGACTGGTCGCCCCATGCTGCTCAACGTCTGGGC
TACCTGGTGCCCCACCTGCTATGGCGAGCACACCTACCTCAAACAGCTGGCAGGGCAGGGCATTCCCATC
ATCGGCATGAACTACAAGGATGAGCGTGACAAGGCGATCAAATGGCTGGCGGAGCTGGGCAACCCCTATC
AGGTCAACCTCTATGATCCGGACGGCATGCTGGGGCTGGATCTCGGGGTATATGGCGCACCGGAGACCTT
CTTTATCGACAGCAAGGGGATCATCCGGTATCGCCATGTGGGGGATATCAACCCCGAGAACTGGGCGTCG
ACCCTCAAACCCATCTTTGATGCCATGAAGTGAGGGGAGTCCTGATGAGAGTATTTATTGCTTCCCTGCT
GCTGTTGCTGGGATGGGTGGTGCAAGCGCCCGCCATGGCCGCTATCGATGTCTACACCTTCGACAACGAC
AATCAGGAGCAGGTCTTCCGTGAACTGACCAAGGAGCTGCGTTGCCCCAAGTGTCAGAACCAGGATATCG
CCGACTCCAATGCTGGGCTGGCCAAGGACTTGCGCGACAAGACCTACCAGATGGTGCGCGAGGGTAAGGA
TAAAGAGGAGATCGTGGATTACATGGTGGCCCGCTACGGCAACTTTATCCTCTATAACCCGCCCCTGATG
GCCTCCACCCTGATCCTCTGGCTCGGCCCGCTGCTGGTGATCCTGATCGGTGGCGCCGTGGTCGTGATGC
GCAGCCGCCGGCCACCTGCGGTGAAAAAACAACTGGATAACGCCTTGAGCGAGGAGGAGCAGCGCCGACT
GGCCGAGCTGCTCAAAGAGGAAGAGAAACAATGACCGCTTTTTGGATAGTTATTGCGGGCTTGCTGGTGC
TGGTGAGTCTGGCGCTCGTCGTGCCGCTGTGGCGTGGTGAAGGCAAACAGAGCATCAGTCGCTCGGCGCT
CAACAAACAGCTCTATCGCCAGCGGTTGCTGGAGATTGGCGAAGAGCGCGAGCAGGGTGTCCTGGCTGAG
GAGTCCGACTCGCTGGTCGAGCTGCAACGAAGCCTCTTGGATGATATCCCCGATGTGGAGCAGACTGCCC
GCAGTGGCAAGAGTCTGATCTGGATCCCGGGTGTACTGGTGCTGTTGGTGGTGAGTCTGGGGCTCTACTA
CAAGTTGGGCTCCTGGCAAGAGGTTAGTCGCTGGCAAGATGCCAGCAGCCGTCTTGGCGAGCTGAGCAAC
CGTATTCTGGTGGAACGCGATGCCCAGGTGACCGAGCAGGATCTGCTGGATTTCACGCTGGCACTGCGTA
CCCGCCTCAAGGATGAACCGAACGACTACCGCGGCTGGCTGCTGCTGGGACGTTTGGCGCTGGATGGCAA
TGATCCTGAAATGGCCAGCGAGGCGCTGGAGCGGGCCTACACGCTGGCTCCCCAGAAATCGCTGGTGGCG
GTACCCTACGCGCAGGCGCTGATGATGACGGGGGATGAGGCGCAAGCGGATGCCCTGTTGCAGGCTGCTA
TCGCGCAGGATCCGGACAATATCGAGGCGCGTTCCGTCTATGCCTTTATGGCGCTGCAAAAAGAGGACTT
CCAGACCGCGCTGTCTCGCTGGCAGGCGATGTTGCCACTGATGGAGAAAGGTACCCCCCGTTACGTGATG
GTCGAGCGCTCCATGGAGTATGCCCGCGCGCAGCTCAAGCAGCGCGGCATCGAGGTGACGAATTCAGGCG
CAGCCAAAGCGGGCGCAGTTGAGGGACAACCGCTGGCGGTGAAGGAGGGGGAATTCCCCATCCACGTCAC
GTTGGCGGCGGGCATTCAGATGCCGGAAGATGCCCATCTCTTCGTCTTTGCTGTGGTGCCCAACGGGCCG
CCCATGCCCATCGCTGTGAAGCGTATCGCAGGGCCAACCCTGCCAGTTACCCTGTCACTCGGGGATGGCG
ATGCCATGATGGAAGGCAGCAAGCTGGCGGCCTATCCGGAGCTGCAGTTCAAGGCGCGTCTCTCGCGCGG
TGGCAATGTGATGAACAAGGAAGGAGCCTTCGAAGGGGTATCCGTGCCGGTCAAGACGGCCGAGATCCCT
GCTGCTCCGATTGACATCCGCATCGATCACGCCCTCTAATATGCCTTTGGATAACCCGGCTCCTGGATAG
GAGCCGGGTTATCTATACTTCTGATAAATCAAAATAAAACGTCAGGACAATCATATGTATCTTCGTTCAG
GGGCCATACTGGCAGCCCTCTTGCTGGCTGGCTGTGCCGGTGGCCCACCCAAGCCTGGCCCAGAGAGTCT
GGATCTCAATCCCGGCATGAATAATCCGGGTGCCACCAGTACGGCGGCCGATCCCCGCGATCCCTTCCAG

GGGGCCAACCGGGCCATGTGGGCGGTCAACTATGATGTGCTGGAACCCTATGTGGCACGTCCTGTGGTAC
ATGCCTATGCCCGCTACATTCCGGTCGGGGTGAAGGATGGCATTGCCAACTTTGTTGCCAACTTTGATGA
GCCAAGCAGCATGGTGAACCATCTGATCACCGGTGATTTGAAGGGGGCGGGCACCAACCTGGGGCGCTTT
ACCCTCAACACCACGGTCGGTCTGCTGGGTATCTTCGATGTGGCCAAACATGCCGGCCTCGAGCGTAACA
AGCTGGAAATGGACACAGTGCTGGGCAGGGCCGATATCGGTAATGGTGCCTACATCATGGTGCCTGTTTA
CGGCCCGACTACCACCCGGGAGCTGGTTGGCGATGCGATTGACAGCTTCTACTTCCCCTATGCCCTGTTA
ACCTTCCCGATGAAGGTTGCACATTGGGCACTGGATGGCTTGGGCACACGTGCCAAGCTGATCGATCAGG
AGCGGATCATCGACAACTCCCTCGATCCTTATGCGCTGACCAAGGACTTCTACCTGCAATATAACCAGGG
CAAGGTGACGGGCAAGCAGACCGAGCTGAAAGCGTCTCAGCAAGAGCAGGCTGACGATGCCAATCTGGAT
GAATACATGGACGAGCTGGACCAGTAAGCGGGTCATCCCGATATGTAAAAGGCGCCTGCGGGCGCCTTAT
TCATTTTCGCAGCCATATCGAGATAAAAAAACGCCCCGCAGTGCGGGGCGTTTTGGCAAGCGAGGCTGAT
TAGGCAGCCAGATTCTTGGCCACGAATTCCCAGTTAACCAGAGCCCAGAAGGTTTCCATGTACTTCGGAC
GCAGGTTGCGGAAGTCGATGTAGTAGGCGTGCTCCCACAGGTCAACGGTCAGCAGCGGGGTGGTACCGGC
TTCAGTCAGCGGGCAGCCAGCGTTGGAGGTGTTGACGATAGCCAGAGAGCCGTCAGCTTTCTTCACCAGC
CAAGTCCAGCTGGAACCGAAGTTGCCGATGGCAGATTTGGTGAAGGCATCTTTGAACTCGGCGAAGGAGC
CGAATGCCTTGTTGATGGCCTCAGCCAGGGCGCCAGTCGGCTCGCCACCGCCATTCGGGGAGAGGCAGTG
CCAGTAGAAGGTGTGGTTCCAGATCTGGGCGGCGTTGTTGAAGATGCCGCCGTTGGAGGTCTTGATGATC
TCTTCCAGAGACTTGCCTTCAAACTCGGTACCCGGCACCAGATTGTTCAGGTTGACCACGTAGGTGTTGT
GGTGCTTGCCGTGGTGATATTCCAGAGTTTCCTGGGAGATGTGCGGTTCCAGAGCGTTGATTGCATAGGG
CAGAGCGGGAAGTTCGAATGCCATTTTCTACTCTCCATTGGCTGGACCGTGCGAAGGCGACGGTTGCAAG
ACATTGCGTACTCTTTTCTATATGTGAAAAGAGTTGTTTTTAAAATGATGGGGCTGGGTAGCCCAAAGCC
AAGGGATTGTAGCAGTATCAAAGATGGCCTAACAACTCTTGATTGGCGCCATGATCGCGATCAATCCTGT
GTGGGTTCTTTGCACAATTCGAGCCGGGTCACCCCTTTGTCAGTTTGTGCTCGGGCAAGATTGCAGTAGA
ATGGCCGCCGAAATCGCTCTAGTGAGGCACCTATGGAAACTATTGAAAAGATTAAGCAGCAGCTGGCTGA
CAACCCCATCATCATCTACATGAAGGGCTCCCCCAAGCTACCCAGCTGTGGCTTCTCTGCCCAGGCCTCC
CAGGCTCTGATGTCCTGCGGCGAGCCGTTTGCCTACGTTGATATCCTGCAAAATCCGGATATCCGCGCCG
AATTGCCAAAATTCGCCAACTGGCCGACCTTCCCCCAACTGTGGGTTGAGGGTGAGCTGATCGGTGGCTG
TGACATCATGATCGAGATGTTCCAGGCTGGCGAGTTGCAGGCTCTGATCAAAGAGACTGCTGCCAAGTAC
AAGAAAGATGATGCTGCAGCCGAGTAATCGGTTCTGTGTTGTCGAGAGGGAGCCTTATAGGCTCCCTTTT
TTATTGTCCCGGTTCTGGCAGGAAAGCAACTGATTGCCGGTTGCAAAAAGCAACGCAATTGCCCTGAAAA
ATCTAATCTTTACGGCTTTGTTTTTGAGTTGTTAATTATATTGTTTGCCAATATCTGATTACTTTTTTTA
ATCCATTTGGATTCAAGTGTTGCTGCCATCACATTTTTTGATAATTTCACCGTAACGCAGTCTCTTTGTA
GATTTTTTTGTGTTTATGCTCACATTAATTGCAAGTGATAGAACCTAGTATCGCTTCGCCCTGTGAAATA
AAACCCTACAAATTAATAAATAAAAAGAGATTTGCGATGAAGATGTATAGCAATCTGCCCATTGGTCTTC
ATGATATTACTGAAGCCAAAAAAAGACTGACCAATCATATATACAAGACCGCGCAACTACCCTCTAACTA
CATCAGCGAACGTTGCCATGGCGAGGTTTATCTCAAGCTTGAAAACATGCAACGTACAGGCTCTTTTAAA
ATTCGTGGTGCCTTCAACAAACTCAGTTCATTAAGTCTGGAAGACCGACAAAAAGGAGTTGTCGCCTGCT
CGGCGGGCAATCATGCCCAAGGTGTATCACTCTCCAGTGCTATGCTGGGAATTGATGCCAAGGTGGTCAT
GCCTTGCTGTGCCCCCAAATCAAAAATTGCTGCAACCAGTGACTATTCAGCCAAAGTGATCCTCCATGGA
AATAGCTTCAATGACACCATTGCCAAAGTCAGCGAAATCGTGGAGATGGAAGGCCGCGTATTTATCCCCC
CCTTCGACGATGCAAAAGTGATTGCCGGCCAGGGCACCATTGGCCTGGAAATTATCGAAGATTTGTATGA
TGTCGATAACATCATAGTGCCGATCGGTGGCGGTGGTTTGATTGCCGGTGTTGCCATGGCTATCAAATCT
ATTAATCCGACCATCAAGGTGATTGGTGTGCAATCCGAGAATGTGCACGGTATGGCGGCCTCTTTCTATG
CCGGTACCATGACCAATCACAGAACCAGCAGCACGCTGGCCGATGGCTGTGATGTGGCAAGACCCGGTTC
ACTGACCTTCGAAATCGTCAAACAATTGGTGGATGATATTGTACTGGTCACCGAGGATGATATTCGTCAT
AGCATGGTTGACCTTATTCAGCGCAACAAGGTGATTACCGAAGGGGCGGGAGCTCTGGCCTCGGCAGCGC
TGCTCAGTGGCAAGCTGAAACATTATGTCGAAGGTAAAAAGACCGTCAGCATTATTTCGGGGGGCAATAT
TGATCTGACCCGGATCGCCGAAATTATCGAACAGAACAATATTCGTCAATTTAGCCTGTAAGGAATGCAG
TCATGATATTAGACAGTGTTGTCACCGGCAGTAAAGAGAATACGCGTGCATGGCGTAAATCGGATACCAC
ATGGGGATTAGGTCTGTTCGGTACTGCTATCGGGGCGGGGGTCTTGTTCTTTCCAATCCGGGCCGGATAT
GGTGGCCTGATCCCAATATTAATCATGCTGGTGCTGGCTTACCCCATTGCATTTTATTGCCACAGGGCGT
TGGCCAGATTATTTCTTTCTGGCAGTAATCCATCGGCAAATATTACCGAGACCGTTGAAGAACATTTTGG
CAAGATGGGTGGAGTGGTAATTACCTTCCTCTATTTCTTTGCCATCTGCCCACTGCTGTGGATATATGGT
GTGACGATCACCAATACCTTTATGGCATTTTGGGAGCATCAACTACAGTTGGTTCCGCTTAATCGAGGTG
TGGTTGCACTGGCATTGTTGATGTTGATGGCATTCGTGATCTACTTCGGTAAAGATCTGATGGTCAAGGT
GATGAGCTATCTGGTCTTTCCCTTTATTGCCAGTCTGGTCGTTATCTCTCTCTCCCTCGTCCCGTACTGG
AACAGTGCCGTCTTTGATCAGGTCAATCTGAGCGAGATATCGATGTTTGGTCACGATGGTATTCTGGTTA
CCGTCTGGTTGGGGATCTCCATCATGGTCTTCTCCTTCAACTTCTCGCCGATTGTCTCCTCTTTCATTGT
CTCCAAACGAGAAGAGTATGAGCAGGAGTTCGGGCGTGATTTTACCGAGAAGCGCTGCTCGAAGATTATC
TCCGGTGCCAGCATGCTGATGGTGGCCGTGGTGATGTTCTTTGCCTTCAGCTGCCTCTTTACCCTGTCAT
CGCAAAATATGGCGGAAGCCAAAGCCCAGAACATTCCGGTGCTCTCCTATCTGGCCAATCACTTCTCCTC

AATGACGGGCAGCAAGTCTGCGTTTGCCACCACTTTGGAGTATGCGGCGTCCATCATTGCGCTGGTTGCC
ATCTTCAAATCCTTCTTTGGCCACTATCTGGGGACGCTGGAAGGGTTGAACGGCCTTATCCTCCGCTTTG
GCTACCAGGGCGACAAGAGCAAGGTTTCCAGTAAAAAACTCAATATGGTCAGTATGTTGTTCATCATGGG
TTCGACCTGGCTGGTCGCTTATGTGAACCCCAACATCCTCGACCTGATTGAGGCAATGGGGGCGCCAATC
ATCGCTTCCCTGCTCTGTCTGCTGCCGATGTATGCGGTGAGCAAGACACCGGCACTTGCAAAATATAAAG
GTCAGGCCAGCAACCTGTTTGTCACCATCATCGGTGTGCTGACCATCCTGAATATAGTTTCCAAGCTGTT
CTGAAAACCTTTTCTGCAAGTCATCAACCATTCAGGCCCCCCTGGCGGGGTCTGGATGGTGCAGTCAGAC
ATGAGCAATAACCGGTTCTTCGTTGGCCACAATTATGTGGGCCCTGGGTAAGGCTTGCCGTTTTTTGACT
TTCTGTTTAGGGCGAAATGCCCTGACTGCAGTGACTCGACACTATTAATCTCGAGGCGATGTAATGAAGT
TTGATTCGTTGGTTCTGGTTATCAATTGTGGCTCTTCGTCGGTCAAATTCTCGGTACTGGATAGTCAATC
CTGTGAGGTGGTGATATCCGGTATGGCTGATGGTATTGATATAGCCCATCCCCAATTGCAAGTGGGTAAA
GAGCCCGCAATGTCATTACCTGAAAATAACCACACCTGTGCATTGGCTGCCATCGCGGCGGAATTGAAAA
AACGGGAGCTGCTGGATGCCATTGCACTGGTTGGTCATCGAATTGCTCATGGTGGTAGTGAATTTAGTGA
AGCCGTGATCATTGATGAACATGTATTGGCCAAAATCAAGCAGGTGTCACCCTTGGCACCCCTGCACAAT
CACGCCAATTTAAGCGGCGTGATGGCCGCCCGCCAGCTATTTCCCAACCGGCCACAAATAGCGGTATTTG
ATACCGGTTTTCACCAGACCCTGAAGCCGGAAGCCTATATCTATGGGCTGCCTTACCAATATCTGCAGGA
TTACGGCGTGCGCCGCTATGGCTTCCATGGCACCTCCCATCGCTATGTCGCCGAGCAGGCTTGCGTATTT
ATGGGGCTGGATGAGCTGCATGCTGGTCTGGTCATCGCCCATCTGGGCAATGGCGCCTCTATTTGCGCGG
TGCAGGATGGCAAGAGCGTCGATACCTCGATGGGGATGACGCCGCTGGAGGGGCTGATCATGGGCAGCCG
CAGCGGCGATATCGATTTTGGCGCGCTGGCCTATATCGCCAGCAAGACCGGGCAATCCATCGAGGATATT
GATCGGGTATTGAACAAGCAGTCCGGCCTGCTGGGTATTTCGGGGCTCTCCTCGGATATGCGGGTATTGG
AAGCTGCCTATCACGAAGGACATCCCGGGGCGAAATTGGCCATTCACGCCTTTGCCCATCGTATTGCCCG
CCATATCGGTGGTCATGCCACCTCATTGCTCCGACTGGATGCCGTTATTTTCACCGGCGGTATTGGCGAA
AACTCGGCCTTTATCAGAAAGCTGGTATGCGAACGACTTCAGGTATTTGGCGTAGCGATCAGCGAGCCGT
TTAACCAGCGTCCCAATAGTGAGGGCTGCCGGGTAATTTCGGCGGCAGAATCCATGGTACTGGCGGCGGT
TATTCCCACCAATGAAGAGAAAATGATCGCGCTGGATGCCATTCGTCTGGGGCTTAAGGTTCGTCAGGCC
GAGTGCGTCTGACATCTTCCACTGTAGTGTCATGAGTAGTGAATGAGGTTTATTTGATGAAAGTTGATAT
GCAATTGGATCAGGATAAATTCCGTCAGGCATGGCAAGGGTTTGCCGGTGAAAACTGGAAGCACGAGATC
AACGTCCGTGATTTTATCCAGCAGAACTACACCCCCTATGAAGGTGACGAACAGTTTTTAGCAGATGCCA
CCGAAGCGACCACCACTTTATGGAACAAGGTGATGGAGGGGATCCGCATCGAGAATGCCACCCACGCGCC
CGTTGATTTTGATACCAATATTGCCACCACAATCACCGCCCACGGGCCGGGTTATATCGAGCAGGGGCTG
GAAAAAATCGTTGGCCTGCAAACCGACAAGCCCCTTAAAAGAGCACTGCATCCATTTGGCGGCGTAAATA
TGATCCGCAGCTCTTTTGAAGCATATGGCCGGGAGATGGATCCCGAATTTGAATATACCTTCAGCGAATT
GCGCAAAACCCATAACCAGGGAGTGTTCGACGTCTACTCTCCCGAGATGTTGCGCTGCCGAAAATCAGGA
ATTTTGACCGGTTTGCCGGATGGTTACGGCCGTGGTCGCATTATCGGAGATTATCGTCGGGTCGCGCTCT
ATGGCATAGATTATCTAATCCGTGAGCGGGAGCTGCAGTTTGCCGATCTGCAACCGGCGCTTGAGCGGGG
AGAGCAGCTGGAGCAGGTGATCCGCCAGCGCGAGGAGCTGGCCGAGCACAAGCGGGCACTGGTGCAGATC
AAGCAGATGGCGGCCAACTACGGCTTTGACATCTCCGGGCCAGCACGCACGGCGCAGCAGGCGGTGCAGT
GGCTCTACTTTGCCTATCTGGCTGCGGTGAAGTCGCAAAATGGCGGGGCCATGTCCCTCGGTCGTACTGC
CACCTTCCTCGACATCTATATCGAGCGGGATCTTAAGGCGGGGCTTATCAGCGAGCAGGATGCCCAGGAG
CTGGTTGATCACTTCATCATGAAGATCCGCATGGTACGTTTCCTGCGTACCCCCGAGTTTGACACCCTCT
TCTCCGGCGACCCCATTTGGGCCACCGAGGTGATTGGTGGCATGGGGCTGGATGGTCGCACCCTGGTGAC
CAAGAGCTCCTTCCGCTACCTCCACACCCTCAAGACCATGGGGCCGGCGCCCGAGCCCAACCTCACCATC
CTCTGGGCGGAAGGACTGCCGCGGGCCTTCAAGACCTATGCGGCCCGCGTCTCCATCGATACCTCATCCC
TGCAGTATGAGAACGATGACCTGATGCGCACCGACTTCGAGAGCGATGACTACGCCATCGCCTGCTGCGT
GAGCCCCATGGTGATCGGCAAGCAGATGCAGTTCTTCGGTGCCCGCGCCAACCTGGCCAAGACCCTGCTC
TATTGCATCAACGGTGGCGTGGACGAGAAGCTGAAGATCCAGGTTGGCCCGAAAGTGGCGCCCATCATGG
ACGAGGTGCTGGATTACGAGACCGTCATGGCCAGCCTCGACAGCTTTATGGATTGGCTGGCGGTGCAGTA
CATCAGTGCGCTCAACCTTATTCACTACATGCACGACAAGTACAGCTATGAGGCTTCCCTGATGGCGCTG
CACGATCGCGACGTCTATCGCACCATGGCCTGCGGCATTGCCGGTCTGTCGGTGGCGGTCGATTCGCTGG
CGGCCATCAAGTACGCTCGGGTCAAGCCGGTGCGCGATGCTGACGGGCTGGCGGTCGATTTTGTGATTGA
GGGGGAGTATCCCCAGTACGGCAACAACGACGAGCGGGCCGACTCCATCGCCTGCGATCTGGTCGAGCGC
TTCATGAAGAAGATCCAGGCGCTGCCGACCTACCGCAATGCGGTGCCGACCCAGTCGATCCTGACCATCA
CCTCCAACGTGGTCTATGGCCAGAAGACCGGCAACACCCCGGATGGCCGCCGCGCCGGTGCCCCGTTTGC
GCCGGGGGCCAACCCGATGCACGGTCGGGATCGCAAGGGGGCGGTCGCTTCGCTCGCCTCGGTGGCCAAG
CTGCCGTTCAAATATGCCAAGGACGGGATCTCCTACACCTTCTCCATCGTGCCGGGGGCGCTCGGCAAGG
AGCCGGCCAATCGCGAGAACAATCTGGTGGGGCTGATGGATGGCTACTTCCATCATGAGCGGGATGTAGA
AGGGGGCCAGCACCTCAACGTCAACGTGATGAACCGCGAGATGCTGCTGGATGCCATCGAGAATCCGGAC
AAGTATCCCAGCCTGACCATCCGGGTCTCCGGCTATGCGGTGCGCTTCAACGCCCTGACCCGCGAGCAGC
AGGACGTCATCTCCAGAACCTTCACCAACATGATGTAACTCTGATGCCCGGGGTGAGCAGGTTTGCTCCG
GGCCTACTTTTTGTTTGTCAGCACGAGGAAGTAACAGATGCGACAGGTTATCACTACCAAAGCGGCGCCA

GGCGCCATCGGCCCCTATGTGCAGGCCACCCGGATTGGCAACATCCTCTATACCTCCGGCCAGATCCCGC
TGGACCCGGTGAGCGGCGACATTGCGGAGGGTATCGAAGCACAAACCCTGCGCTCCCTGCAAAACGTCAA
GGCGATTGTCGAGGCGGCAGGCGGTGAGGTGCGCAATATCTTCAAGACCACGGTGTTTGTCTCGGATTTG
AACGACTTTGCAGCGGTAAACCGGGTCTACAACGACTTCTTCACCGAGCACGATGCCATGTATCCGGCGC
GCTCCTGCGTAGAAGTGGCCCGCCTGCCGAAGGATGTGAAGATCGAGATAGAGGCGATTGCTGTGTTGGA
GTCCTGACGCGGCGATCCCGGCCAATAAAAAAGAGCGGCAACCCGAGGGCTGCCGCTCTTTTTCATCGAA
CCCGCCGTGCGAGATAAGGGCGCAAGGAGTGGGGCATTCTGGATCAGGCGATCCGCTCGATGCGCGCCAG
ATAGAAACCGTCGAAACCGGTAGCCGCCGGACTCACGGTGTGATCACCAAGCAGGCGGAAGTTTTCGTTG
GCGGCGAGGAACTTGTCCACCTGTTGGCGGTTCTCCTCGGGCAGGATGGAGCAGGTAGCGTAAACCAGCA
TGCCGCCCACCTTCACCATCTGGCTGTAGCGCTGCAGGATCTCTTCCTGCAGGGCGACCAGCACCGGCAG
ACGCTCGGCGGTATCGCGCCACTTGGCATCCGGATTGCGCTTGAGCACGCCAAGGCCGGAGCAGGGGACG
TCCAGCAGCAGGCGATCCGCGCTCTCTTTGAGGCGCTTGACGGTCTTGCTGTTGGTGATGACCCGGGTCT
CCACGTTGTGGGCGCCGGCGCGGCGGGCACGCTGCTTGAGGTTCTCCAGCTTCCACTCTTCCACATCCAT
CGCCAGCAGGCGGCCTTTGCCTTTCATCATGGCGGCCAGATGCAGGGTCTTGCCACCGGCACCGGCGCAG
GCGTCGATCACCCGCATGCCGGGCGCGACCTCGAGTGCGGCAGCAATGTGCTGGGAACCGGCATCCTGTT
GCTCGAACCAGCCGTCGGCAAAGGCGTTGGTGCGAAACAGGGCGGCATCGGAAGTGACTTCCAGAGCGCT
CTCAACCCCTGCTACCGGCACAGTGTTGACCTGCTCTTTGGCCAGAGCGGCTTGCAGCTCCTCGCGGGTG
CACTTGAGGGTGTTGACCCGCAGGTAGCGCTTGGGCATGGCGGTCAGGGCGGCACGCTCGGCAGGCCAGG
CTTCACCCAGCTGCTCGCTACCCAGTTTCTCCAGCCAGCTCGGGCAACCATCCATCAATACCGGGTTCTT
CTTCGCCTCGGTCAGACGGCGGCGGAATGCCTCTTCATTGAAGCGATCCAGGGTCGGGTGGTTGGGCTGG
GCAATTTTGTGGAAGGCATGCCAGCTGTGCAGCAGCGGCCAGACATTCTTTTCGGCCTGACGCACCTGAA
TTCCGGTGAGGAAACAATAGAGGCTGAGACGGCGCAGCAGGTCGCCGGTGACCAGGGCAATCCTTGCCTG
TTCTTGCGGTTTGAGTTCGATGCCTGAGAAGTGACGGGCATAAGCGCGGTCGAGGGTAAGACCCTGACCG
AGCACATCGTCAACAATGGCAACGACCAGATCCCCGAAACGGGGCAACAGTGGGGATTTGAGCATGAGAT
TAACCTTGGCCAGATAAAGATGAACCGCACCTTTGTGAAGAGGAGCAGTTCTAAAATTGACAAATCCGCG
CAATGATAGGGGTTAGACGGGGTTGGCGCAACACTCCCCATGGGTGAGTGGCTGAAACAGTGGCGCCGGG
CTTTTTGATTCGGCGGGATAATGATGCCGGTAATGGTTCAAAAAAGTGCATGACTACAAGGATGACAAAG
ATGCATGTGAGCAGTAAGTGTTCGTTGGCCTTGTTGGTGGCCAGTTTGTTGGGGTCAACCAGCCTGATGG
CCAGCGAGTGGAGTACGGTAGCCGCCAGAGCGACTGCGGAGCAAGGTTATGAGGTGCGGGCCAATCAGTT
TCGGGTGGCCAAGGTGCCGCTCGACTATTTTGCCGGGCTGCAAGCCGGGGCGTGTGAGCTGACCCTGCCG
TTGCCCGATGGGGGGGAGGTCACCTTTGCTCTGGAAGCCTACGACTTGCTGCCTGCAGATCTCGTCGCCA
AGTATCCGCAGATCCGCACCTTCAAGGGTCACAACCCGGCGCATCCGATAGAGACGGGGCGCTTCGATCT
GGGGCCGCAAGGTTTCCACGCCATGTTCAGCCATCAGGGCAGGATGGTGTTTGTCGATCCGCTGCGCAAT
GCCGAAGGCTACGCCATCTATTACCAGCAGGATGCCCATAGTCGATTGGAAGAGGCGGCAGATGAGGTGA
TAGGGCCCAAGCTCAGCAAACTGGTGCGCAAGGTGCAAGTGGATGGCAATGTTCGCAAGCGCTACGTCAT
TGCCATTTCGGCGACAGGAGAATTTACACAATACCACGGTGGCTCCAAAGCGCTTACCATGGCGGCGATT
GCGACCTTGCTCAACCGCGTCAATCAGGTCTATCAACGGGATGTGGGCGCCGAATTCGTGCTGGCCAGTG
GCAATGACAACCTGATCTTTACCGATCCGGCCAGCGACTTGTTTGCCAATGATAATTCGGATGCGCAGAC
CAATATCGGGGTACAGGCTTATTACTTTACCAATATGCAGCTCGGTAATTTTGATATCGGCCATGTCCTG
AATACCAATGGCGGCGGTCTGGCCGGATTGGCAGTGCTCTGCACCAATCAGAAGTCTGGTGGGATGTCGG
GTTCACCCACACCGGTTGGAGATGCCTTCTTCGTTGACTATGTGGCTCATGAAATTGGCCACCAGTTTGG
TGCTGATCATACCTTCAACGGCACTACCGGGAGTTGCGGTGCTGGTACCCGGGTTGCGAGTCAAGCCTAT
GAGCCTGGTAGTGGCAGCTCCATCATGGCCTATGCCGGTATTTGTGGTGATGAGAATATCCAGACTCACA
GCGACCCCTATTTTCATAGCAAGTCCATCGAGCAGATGCGTGCACACATGGCGACCGTATCGACTTGTGG
AACCACACTCGCGTTGAGCAATAATGCCCCGCAGGCAGCTGCTGGGGGGGACTATGTGATCCCTGCCAAT
ACGGCCTTTATGTTAAAAGGGGCTGGTGGAGATCTGGATAATGATTCTCTGCTCTACAACTGGGAGCAGA
TCGATCTGGGTACTGAATCGAGCAGTGCCGCCACCATGATTGACGATGGCTCCCGGCCGCTGTTTCGTTT
TGTCTCTCCCACGGCATCGCCGGAGCGGATCCTGCCGAGTTTGCCATCCCTGCTCTCCGGCACGCTTGCC
AAAGGAGAGGCCTGGCCGACTACCAACCGGGATCTCAATTTCAGACTGACGGTGCGTGATGGTCAGGGTG
GCGTTGCCAGTGATGACATGAAAGTGCAAGTGATCAATACCGGTGCGGCATTTCGCATCACCAACCCGTT
TTACGGGGTGACTCTCACTCCGGGGGCCAGTCAGGCAGTGGCATGGGATGTGGCGGGCACAACTCTGGCG
CCGATCAGTTGCAGCAAGGTGGATATCTCGGTGACCCAGGATGAGGGCGTAAGCTGGACAATGCTCGCCG
CCGGACAACCCAATAACGGCGCAGCAACGGTAACGTTGCCTGCCGATCTCACCAGCAGCGTCAGGCTGAA
AGTGGCATGCAGCGACAATATCTTCTTTGCCATCAGTCCCCTCAAATTGCCTCTGGTGCAAGGTGGCTCG
ACTTCAGGTGGTGGTGGCGGGGCGCTCGGATTGTGGACGCTGGCTCTGGCGCTGCTCGGCTGGCAACGGA
GGCGTACATGAAGGTAGTCCCTCTGTTGCTGCTTGTCATGCTCGGCGGTTGTCAGGCCGATGCCAGCACG
CTGGAACAGGAGCTGAGTGCAAACCTCGCCAGACAGGATTACCGCCTGATAGTGATAGCCGGGCGGGGAG
AGTTTGCCCCCGGTATTGCAGCCGAGCAGCAGGCAGAGGCCAAGGCGCGCTGTGGAAAGCGTTATCTGGA
TGGATTGGGCGATGTGATAAGGCCCGGTCAGCAAGAGATACATACCAAGTTGAGTGCTTACGCCAGCGAG
TATAACCAGCGTATGGTGATTCATTGCCCAATAGCATCGGGCGCTGGCAAGCAGTAATACACCAACGAAC
CAATCCAAATGGAGGGGATATTCATCCCCCTATCTATCCGTGAAGGCGCCAATGGCGCCTTCACTTTTTC

CGTTGTTGAATGGGGCCAGCCTGTATTGCTTGAGATGGGGAGCGCGGGTATAGCACGATTTTTCAGCGTG
ACAGAAGTTTTGTGCGAATTTTCTTTTCTGTCTGTCACGCAGATTGTCAATTGAGGCTGCGCCATACTTG
TTGTCACCAAGGTAGGGCATGGAGGCTCTATGAAGCACGGCATTGCGGTGATTATCACCAGTTTGTCTGT
TCAGGTTGCAGCAGAACCCCAGTGGACCTTCATGGATGAGAACTTGGCAAGGGGAGGGGTCTATGATGTG
CGAGCTGAAAAGGTCAGAGTGGTGGTTCCTGACAAGCCTTTTGAACCCGGGCATCTCGCCAGTGTATTGA
CCTTGCCATTGCCGGATGGCACTACGGTGCAGTTTGATGTTCATCCCAATGCCTTGCTCCCGGAGCCTTT
GGCCCAGCGCTACCCTTCTATCAATACTTACAGTGGGAAGGCTATCGATCGGGCGGCGGATACAGGCCGA
TTTGATGTCACCATACATGGTTTTCACGGTATGTTTACCCATCAGGGGCGTACCTTTTATGTCGATCCGC
TTCAGGACGGTTTCTCTTATGCCGTTTATGAACAGCAAGATGCCTGGCGGCGACAGGATGTGGATCAGGT
GATTGATCCTCATGGTATCGAGCGTGAGCGAGCTGTGCTGGTCGATGGCAATTTGCGCAAGCGCTACACC
ATCGCCATCTCGGCGACTGGGGAATATACCCAGTATCACGGTGGTACCAAGGTTGCAGCACTCTCGGCCA
TTGCAACACTGCTTAATCGGGTCAATGAAGTCTATCGCCGTGATGTGGGGGCCGAGTTTCAGCTTGCAGC
TGATACGGATAATGTGATTTTCACCGATCCTGCGACGGATCCTTTCACCAACTCGACCCAGGATATCGCA
ATCAACCAGACCCAACAGGGGGTGTTGCTAAATCCTTCTTTTGATCTGGGCCATCTGCTGGCGACATCAG
GCGGCGGGGTGGCGACCCTGAGATCTTTATGCAACCCTTTCAGCCGTTCACGGGGCATGACCGGAGGCTC
ACAGCCTATCGGCGATGCATTCTTTATTGATTATGTGGCTCACGAATTGGGGCACCAGCTGGGGGCAGAG
CACTCCTTCAATGGCATGGCCGACAGTTGTGCCACGGGTCGCAAGGCTAGCTCAGCCTGGGAACCGGGCA
GTGGCTCGACCATCATGGCCTATGCCGGGATCTGTGGTGAGGAGAATCTGCAGGTTAACAGCACACCTTT
CTTTCACAGCAAGTCGATCGAGCAGATGCGGGCTCTGATGGCCTCTCTGCCCTCCTGCGGCACCGACCTG
GCGCTTGCCAATAACGCGCCGGTTGTGGATGCGGGCGGCGATAGGGTGATCCCTGCCAATACGCCTTTCA
CTCTTGTCGGTAGTGCGCTGGATCTGGATGGCCATCCCCTCAGTTACACCTGGGAGCAGGTTGATCTTGG
CAATGCATCCTACAGCGTGGCAACCATGGTGGATGATGGTACCCGCCCGCTGTTTCGCTTTGTTTCACCA
ACATCGGATCCCCATAGAACATTGCCCGCCATGAGCTCTCTGATATCGGGTGTCTTGGCCAAAGGAGAGG
CGTGGCCTGCGACCAATCGCAATCTCCAGTTCCGCCTTACCGTACGTGATGGTCAGGGGGCGGTGAGCAG
TGATGAAATGGTGGTTCAGGTGATCAATACCGGCGCGGCATTTGCCATCACAGCTCCGCAGATCGGCGCC
ATGATGGCGGGTAGCACTCAGCTAGTGACATGGAATGTTGCCGGGACCGATCAGCCTCCTATCAATTGTC
CCAGCGTGGATTTGGCACTGACACAGAATGGTGGTTCTACTTGGACTACGCTGGCAAGCGGGCTTGCCAA
TAACGGTTCGGCCAGTGTGGTGTTGCCTGCACTGTTGACCAGTTCGGCCAGGCTGAAGCTGGCATGCAGC
AATAATATTTTTTTCTCTATCAATGGGATAAGAGGTAATGCGTCTGCCACTACGGAAAGTGCAACTGTCA
TTACCGACAACAATGCGGGTGGTGGCGGTGGTGCAGTGAATCTTATCGTGATCCTCCTGATGGGAGGGGC
AGCCGGATGGCGCAGGTATTACTGTTAGCCGTCAATTTGCTGTTTCTATATATCTGCGCAGCGCCTGTTG
TGGCCGCATCACTGACCGAGGCAATAGATGAAAGTCTTGCTGCTCAGGATTATCGGCTCATCGTGCGTGG
CGGGCGCGGCGGTATGGCTCCGGGGGTTGTGGAGTCATTGCAAGCAGAAGCGAGAACCCGGTGCGGAGTC
CGTTATCTGAAAGGGTTCGGTGATGTGATTGAGATGGGTAAGGCGTCGGAGTTTGAGCAGCGGATTGACT
ATGCCACCCGATATAACCGACAGATGCTGGAAAAATGCATGCCGGAGGCGATTTCAAGGCCGTAGTGGCT
GTTCAACAACGGCGTGTAAATCGGCATGATTGGAGTTATAACTCTAATATTCCAAACATAGTGATAGCCT
CAGGGGAATGATTCGATGACTCACATATGAGCCTCCATATAATTTTTTTGGAGTTTTAACTCTATTAAAA
GAACTGCTGAAAATGCTAACAAAATGAGCTGTTTATAAACTTTAACTCACTAACCTTATGATTTTAAATG
AAATAAAATTCATTTAATTTTAATTGAAAAAGTTGAATTGTGACTTGTGTCACGTTATTCTCGCCGGCTG
TTCAGTCAGGTGATTAATAATGATGAAGTTCACAATAAATGCAATCTCAGTTGTCATCGCAATGTCAGCC
AATATCGCGCTGGCATCCCCATGGCATATGATCGAAAAACGCAGCAAGCAGGCCGATGAACCTGTAAGGG
CTCGCGCATACAATATCTTTGAGGTAGAGCCGGATTTTTTAGAAAATCTGCGCTTGGGCCAAACCGTGAT
TACGCTACCATTGCCTGATGGCAAGATGGTGCAATATGAGTTGGAAGCCTATTCCGTATTGCCGCCTGCG
CTGGCAGCGAAATACCCAGATATCATGACCTATAAAGGACATGAAGTGGGCACCCCCAGTAATACCGGTC
GTTTTGATATTTCTCCGCGTGGTTTTAACGGCATGTTTATGCATGCCGGAGAGCAGATATTTGTCGATAT
TCAGCGTAATGGTACCTACGCTGTCTATTACAAAAAAGATGCACATTCAATGAATGGCAAGAATATCAAG
GAGATCATTGAAAAATCCGAGCTGAAAAAAAAGTTAGCATACAAAACTGAAGCGGCTCGTAACGAGGCTA
GCTATAGCAAGCCAGAGCTTACCCAGTACAGCATTGCATTCTCTACTACTGGTTCATATACCAGGTTCTT
TGGTGGCCAAGCCCAAGCATTGGCGTCACTGACAACCTTGACTAACCGTTTAAACGAGGTATATGAAAGG
GACTTGGGCGTTCGTTTTGTATTGGCAGATGGGAATGACAATATTATATTCGAAAATCCTGTTGACGACC
CTTTTGTGAACGGTAAATATCCAAAATTTAAAAACATCAGCACTATAAATGCTGTGGTTCAGAGAGAGAA
GCTGAATAGTAATGACCTTGGCTATTTCGATATTGGTCATGTAGTAACGCAATCGGAAAAAGATGAGGGG
GATGCACAGCTAGGGTCATTGTGTGATAAGGTAGACTCATATGCATCATATAGGTCTAACGCGGCTACAG
GAAACAAAAATCCTGTTGGAGATGCATTTTTTATTGACTTTGTGGCGCATGAGATTGGCCATCAATTTGG
TGCCGACCATACCTATAACGCTAATAAGGGGCAGAGAGAGTCCTCCATGGCATGGGAGCCAGGTAGTGGT
AGCTCCATCATGGCTTATGCGGGACTGATGGGGGAGCAAAACGTACAGCTCAACAGCGATGCCTATTTTC
ATAGCAAGTCGATTGATATGATGCGCAAGCATATCAACAACCGTGAAGCTCAAGCAAAGAAGAGTGAGAT
CCTATTTGTTGGTGCGCCATTAGTGCTTGCTCCTATTTTTTCCTCGCGTTGTGGCACTGCTATCCCAAAC
CAAAATGGTGATAATCAGGCGCCAGAAATTACCGATGTGCCAGCAGAACATGTGATCCCCAAAGGGGTGG
CATTTAGTCTGAGTGGCAAGGGTAATGATATTGACGGCGATACCCTTACCTATACCTGGGAGCAGGTCGA
TCTGGGGGATGCAACGACGAGTGGCGATCAATTGAGTTCAGGTCGCCTTGCAACCGGCCCCCTGTATCGT

TACACCGCGCCGACCACTTCCCCGGTTCGTCACTTTCCTTCGCTGGCTACCCAGTTGAAAGGGGAAGCTG
AGAAAGGTGATGTCTGGGTCAATGTGCCGCGCACCCTTAACTTCCGTCTGGTCGCCCGTGATGGCAAAGG
CGGTGGTGCCTATGGTGAGACCTCGGTCGAGGTGGTCGATACAGGCGCACAAACCTTCAAGATTGAACCG
GCATCTGACCAGCGTATGCTGGTTGGTAGCCCGACCACAGTGAATTGGCAAGTAGCTGGCACGGACCGTC
ATCCTGTCAACTGTCAGCGGGTCGATATCAGTTATAGCAACGATGAAGGCAAGAGCTGGCAGCCGCTGGT
CAATGGCGTGAACAACAATGGCGCCCATGAGGTGACCATTCCGGCCAATGCTGGCAGCGCTGTTCGCCTC
AAGCTGGCCTGTAGCGACAATATCTTCTATGCCATTACGCCAAAACTGGCGACCAAGAGCAGTGATACAC
CGGCTCCCCAGCCTGAAAGCAATGGCGGCGGCGGTGGCGGCGGTTCGACCGGTCTGTTCGTGCTGCTGGG
CCTTGGTATCGCTGCGTTGCTGCGTCGTCGTGAGATCATGAGCTACTAAACCAGATAACCCAAGGGTGAG
ATTACCCATCTTTGGTAAATTGATGAACCAAGCGAAGAGGCGTTAGCCTCTTCGCTTTTTTTGTATGGTT
TACAAGGTCGCTTTATCGAGCGCACAATGCCTGCGTCACTCTCTTGATGGTCGTGGAACCATTATTTATT
TGAAGGAAAATCAAGATGATGGATGTTGCTGATCTGCGCCGTGAGTATACCCGTGGCGGGCTGCACCGGG
CCGATCTGCCGGCCGAGCCGCTGGCGCTGTTCGAGAAGTGGTTGGCACAGGCTTGTGAGGCCAAACTGAC
CGATCCCACCGCCATGGTGGTGGCGACGGTGGATGCCGAAGGTCAGCCCTGGCAGCGTACCGTGCTGCTC
AAGCACTACGACGCCCAGGGGATGGTCTTTTACACCAACATGGGCAGCCGTAAGGCGCAGCAGCTGGAAG
GCAATCCCCGTATCAGTCTGCTCTTCCCCTGGCACACGCTGGACAGACAGGTTCATGTGACCGGTCGGGT
GGAGAAGATGAGCACCTTCGAGGTGATGAAGTATTTCCACAGCCGTCCCAAGGATAGCCAGATCGCTGCC
TGGGTCTCCAAACAGTCGACCCGCATCTCGGCCCGCGGCGTGCTGGAGGCCAAGTTCCTCGAGCTCAAGC
AGAAGTTCGCCAATGGCGAAGTGCCGCTGCCGAGCTTCTGGGGCGGATTCCGGGTGGTGATCGAGACGGT
GGAGTTCTGGCAGGGGGGCGAGCACCGGCTGCACGACCGCTTCTACTACACCCGCGACGGGGATGGCTGG
CATATCGAGCGTCTGGCTCCCTGAGCGCGCTATTCAAATCTGTTTTCACAAGGAGTGTGTATGCGTCTGT
ATGGTGATCTGCGTTCCGGCAACTGTTACAAGGTGTGGCTGCTGGCCCGCTGGCTGGAGCTGCCCCTTGA
GTGGGTAGAGGTGGATATTCAGGCGGGGGATACCCGTACCCCCGCATTTCTGGCCATGAACCCCAACGGC
AAGATCCCGGTGCTGGAGATTGGCCAGGGCGATACGCTGTCGGAATCCAACGCCATCCTCTTCTATCTGG
CGCAGGGCTCGTCTTTGTGGCCAACCGATCCGCGCCAGCAGGCCGAGGTGCTCAAATGGATGTTCTTTGA
GCAGTACAGCCATGAGCCCTACATCGCGGTGGCCCGCTTTATCGTTCACTATCTCGGCCGACCCGAGTCG
CAGGAGGCGCGGCTCGACGGTCTGAAAGCCGGTGGCGAGAAGGCGCTGGCGGTAATGGAGCAGCAGCTGA
AACAGAGCCCCTACCTGTGCGGTGATGCGCCTACCATCGCCGATATCGCCCTTTATGCCTACACCCACGT
GGCAGAGGAGGGGATCTTCTCCCTGCAGGGGTGGCCCGCTATCCGCAGCTGGATCGCCCGTATCGAAGCA
CACCCCCGTCACCTGACGCTGCCAGCGTCCTGTCAGGCCCGCTGATGGAACTGCTGATCGAGGCCAGTCT
GTGGCAACCGCAGTGGGCTGCGCTGTTGCTGCACTGGCAGCAGCAAGGTCATCGCTGGCAGCTGCTGCTC
GGCAAAGAGGCGGCGGCCGCCCTCGATCAAACCGATGCTCCCTGGGCGAGCAGCCCACCTGACGGGGTGC
TCTGCCCGGGGACCCTGCTGGCTGCCTGGCTCGATGGGGATCTGCTGACGGATTTTCATGTTGATCCCAG
CCGCCAGATCCTGATCAGCGCCTCTACTTCCCTATTGACCCTCGCCAGGGAGCAGGGACTGCTCACCCTT
GGCCCTGTGGGGGCTGATCTGCCGCTCACCCCCGAGGCCGATTTGGGCGCCGTGCTCAATCGCCTGCTGG
CGCGCCGCGTGACGGTGCCGACCCTGGCTGAGGGGCAGTCGCTATCAGGTGTCGTATTGCGACCACTGCA
GGCCGCCGATGAGTGCGAGATAGTGCGCTATTGCAGCGACGAGGCGCTGGCCCGCTATACCCTCAATATT
CCTCACCCCTATCCGCCGGAGGGGGCCCGCGACTGGCTGGCCTTCAGCTGGCGCAAGGCGGCGCTTGGGC
TTGGCTGGAGCTGGGCCATCACCTTGCCAGAGGAGCAAGGTGCGGCGCTGCTTGGGGTGATCTCCCTGCA
CTGGAATGGCGAGATCGCCTGGTGGGTGGGCGTGCCCTGGCAAAATCGCGGTTTGGCGACCCGGGCCGCC
AGCTTGGTCAAGGGGTTCGCATTTGACCAACTTAAACTGCCTGCGCTGACGGCTCGCCATATGCCAGACA
ATTTAGCCTCCGGCCGGGTGATGGCAAAAGTGGGCATGCACTATCGCGGGCGGCGCCATGTCAGCGGTCG
CCAGCCATGTGAGGTGAGCTACTGGCGGCTGGATCGCGAACCCTCCTTACCCAATCCCTTGCCCGAGGAA
ATTTCCCGCTCGCTGGCAGATGAGCGGATCGCCGTGGTGATCCTGCGCGATCCTGTAACGTTGGATCGGC
AGAGTACTAACGGCAAGCTGGCCATCTCGCTCTTTGTGGATGAGAGGGGCGCGGGGGAGGAGTCCTGTTT
TCTGCGTTGCCCGCCGTATCTTGAGCGGTTGCTGGCGCAGCACTGTTATCCCGTCGATCTGCTGGAGCAG
GCCGAACCGGAGCAGTTGTCCCATCTTGGCGGCGTCTTGCTGAAAGAGAGGGATGAACAGGGGTTGGCGT
GGTTGCTGCAACTGTCCGCGCTGCAGCGTCAGGGGCCCGATCTGCTCAGCCGCGAGGAGCGCGTATCACG
GCTCCACTGGTTCAACCAATTGATGGCGCAGGCGCTGGGCGGCAACGGTAACTCTTTGCAGATACGCTAT
CAGCAGTTACGGCTGCTGGTGGAGCTGCCGGAGCTGGTCGATGAGCTGGATGGCTGTTGGCATCAAACGC
CCGAACTGACCTTTGATCGGTTGGCGCGGGAGGCTCCCGCCCTCTGGCTGGCCTACCGCGAGGCGATGAA
CAGGGTCACCCCGGCGACGCTAACCGCCTTGCAGCAGCAGTTTGCCGCTCGTTTTCCTGAGTGCACCCTC
CCATTTTTGGACAAAGGTACTCAGAATGACCAGCCGTTGTGTGGAATAATGCCCGCTCTGCTGTACGAGG
AGTAATACCTGATGTTGAGAACTGAGCGCCCCGGTGACATGCTGCCGGTCTATGAGCTGCTGAGTGCCGC
CTTCGGTCGTACCGATGAGGCCGAACTGGTCAACCGCCTGCGTGAATGTGGCGCAGCCGTGGTGACCATG
GTCGAAGAGGAAGAGTACGAGTTCATGGGGCACCTGATGCTAAGCCCGATCACCATTAACGGCATGGAAG
GGCCCTGGCTCGGTCTGGCGCCGGTCGCGGTTCATCCCGACTGGCAAGGCCAGGGGATTGGCTCGGATCT
TATCCGTGAAGGGCTTGATACGGCGCTCGAGATGGACTGGAAAGGGGTGGTTGTACTCGGGGATCCGGCC
TATTACAGCCGTTTCGGTTTCCGCCCGGCCAGCGAGTTTGGCCTGCACTGCATCTATGAGGTACCTGCCG
ACTGCTTTATGGCGATGGAGCTGCAGGAGCGCGGTTTTTCGGGTGTGCAGGGTGAGGTGCTCTATCACCC
CCTGTTTGATGAACTCCCCGACGAGCTGCCGGACGCGCAGGCAGAAGTGTCCCATTTATAGCGAGACGTC

GCAGCGACAAGCCATAGCGAAAAAGCCCTCATTGAGGGCTTTTTCATCTTGTATTGATCCCTGCCGGGCT
TATTCGAGCAAATTGGCTCAGGCAATGATGTCGAGCTGGGTGCCCTTGCGATTATCTTCGCCCACCATGT
CACCCATCTGCTGGGCGAGCGGGCTCTGCTGGAATTGCATATAGTCGCTGAGATTCTGCTGGCGCTGGTA
GCGAGTCGCAGCCTGATACATATCAGCCGTGCTCGGGCCCGACTTGCTGTCGCTCTCCTGACCGCTGGCC
TCCAGGTAGCGCTCCACCACGGCTTTATCTTGCTGGTTAGCCGTATAAGAGACCGCCATGGCTCTGCGAG
CCGTTGCTCTCTCTTCGCTCTGTGCCTGCACTTCATCCTTCTTGGTCAGTGCTTCGTTACGGGTCTGACT
GACCTGCTCGGGTGTGACAGCGGGAGAGGAGGGATATTTGCCGTAATCGGGCAGCGGCATGGGCCGGGAG
GTGATTTGCATGGTGGCGCTTCTCTTGTGAACAGGGACGGACGCATGATACCCATCCATTTTAGTTCAAT
AAATCACCACCTGTCGGTTCATCAAGAGTGGGTTCAGCTTCGCGCATCGATTGTTAATTGAACCGCCTCT
GAGTGACAAAAAAGGCGCCATAAGGCGCCTTTTTATCCTTCGGTTACTGCCGGATCAGTTGAAGAACCAG
TACCCGCGGTTGACCAGTCGGGTCAGCAGTTGCAGGAAACCGGCCTGATCGGCCAGCTCCACCATGTCGG
CCTGAGTGATGATGTCCTTGTCGCACAGCAGGGAAATGGCTGCCGCATCTTCGCTCTGCAGGGTCCAGGC
TTCGCCGTCGATATAGCACTGCTGGCTTTCGCCGCTGAACCAGACGGTGCGCAGACCCGGCACCTTGATG
GCAGGTTCGCCCTGGGTCAGCAGGTCGGCTACCTCCTCGGCGCTGTAGTCCGGCTCGACCGGATTGACGT
CCAGATCGTGCTTGGCCTCGGAGATCATGGTGCCGAACCACTCCTTGAACAGGGTCTCGTCATCGAGGGC
CTGTTGCATCAGCTCGCGCAGACGGTGCAGCTCGTGGGGCTGGATCTCGCCGTGACGGGCACGGGGCTTG
AGATCGGCATCGCCGTAACGCTCGGTACGCACCTCGTTGTCGATCAGATGATCGGCAAAGGAGGAGATAA
GCGCCTTGGCATCCGGCGCGCGGAAGCCCACCGAGAAGTTCATGGAGGGCTCGATGGCGTAGCCTTCGTG
GGGGAATCCGGGTGGAATGTAGAGGATGTCACCCGGCTCCATGATGACGTCGATGATCGCCTCGAACGGC
TCGCAGTGGAGCAGTGCGGCGTGGGCGGCGAACTCGTTGAGCGGCTTGGCATCGCCGACTCGCCAGTGGC
GTTTGCCCAGACCCTGGGTGATAAACACATCGTAATTGTCGATGTGGGGGCCGACACCCGCATGGGGGGT
GGAGAAGCTCACCATCACATCGTCAAAGCGCCAGCCCGGGATGAACTGGAACGGCACGGCCAGCTCGTTC
ACCTCGGGAGCCCAGTGGTTGCAAGCCTGTACCAGAATGGTCCAGTTCTCTTCGCCCAGATGGTCGTAGG
ATTCGAAGGGGCCGTGAGCGGCATCCCACTTGCCGTCGAAACGGGTGACGAGGCGGGACTCGACCACCTC
TTCCATGGCCAGACCCGCCAGCTCATCCGGGCTGATGGGATCCTGAAAGTCCTTGAAGCCACCCTTGATA
AGGATCGGGCGCTTCTGCCAGTAGTGCTCGAGGAAGTGAGCGATATCCAGATTGAGTTCGTACATGCTGT
TTCTCGTGTCTCGCAAAGAGGTTTTGCCCAAGGCAGGCTTGGGAAAAGCCACTCGGGATCCTGCCGTGGC
AGGGAAAATAACAGGGGCCCTGTGGCATATCGTGCACAGGGCCCCTTCGTCACTGCCGGTTGGCTTACTT
CAGCAGATCGACCAGACGCTGGGCGTCGCCGATGTAGTTGGCCGGGGTCAGCTTCTTCAGCTCAACCTTG
ACCGCTTCCGGCAGCGCCAGGGTGTCGATAAAGGTACGCATCCCTTCAGCATCGACGCGGCGACCACGGG
TCAGCTCCTTGAGCTTCTCGTAGGGCTTCTCGATACCGTAGCGGCGCATCACGGTCTGGATCGGCTCGGC
CAGCACTTCCCAGTTGGCATCCAGATCGGCGGCCATGGCGGCGTCGTTGGCTTCCAGCTTGGAGATGCCC
TTGAGGGTCGCCTGATAGGCAATCAGGGAGTAACCCACCGCCACACCCAGGTTGCGCAGTACGGTCGAGT
CGGTGAGATCACGCTGCCAGCGGGAGATGGGCAGCTTGCTCGCCAGATGCTGGAACACGGCGTTGGCCAG
ACCCAGATTCCCTTCGGAGTTCTCGAAGTCGATGGGGTTGACTTTGTGCGGCATGGTGGAGGAGCCAATT
TCGCCGGCGATGGTGCGCTGCTTGAAGTGACCCAGGGAGATGTAACCCCACACATCGCGGTCGAAGTCGA
TCAGGATGGTGTTGAAGCGGGCCATGGCGTCGAACAGCTCGGCGATATAGTCGTGCGGCTCGATCTGGGT
GGTGTAGGGGTTCCAGGTCAGGCCGAGGGAGGTCACAAACTCCTCGGAGAACTGGTGCCAGTCCACATCC
GGGTAGGCGCTGAGGTGGGCGTTGTAGTTGCCGACTGCGCCGTTGATCTTGCCGAGGATCTCGATGGCCA
TGATCTGCTTGAACTGGCGCTCCAGACGGTAGGCGACGTTGGCCATCTCCTTGCCCATGGTGCTCGGGGT
CGCCGGCTGGCCGTGGGTGCGGGAGAGCAGCGGCATGTCACGATACTCGTGAGCCAGGCGCTTGAGCTCG
CTGATCAGCTTTTCGCAATAAGGCTTGATGACCTCTTCGCGAGCGGTTTTCAGCATCAGACCGTGGGAGT
TGTTGTTGATGTCTTCCGAGGTACAGGCGAAGTGGATGAACTCGCTGACAGCCGCCAGCTCCGGGTTCAC
TTCGACTTTCTCTTTGAGGAAGTACTCCACCGCTTTCACATCATGGTTGGTGGTGCGCTCGATCTGTTTG
ATACGGGCGGCGTCACTCTCGTTGAAGTTGCTGACGATGCCGTCGAGCAGGGCGTTGGCGGCCGCACTCA
GGGCTGGGACTTCCGGGATCCCGGCGTGGCTGGCCAGTTTCTGCAACCAGCGAACCTCGACTTCAACGCG
AAAACGCAGCAGGCCGAATTCGGAGAAGATAGGGCGCAGGGCATCGGCCTTGTCGCCATAACGACCGTCA
ATCGGGGAAACAGCAGTCAGCGCGGACAGCTCCATGGGGTGAACTCCTGATGATTTGTGTGGGGGATCCG
TATCCACTCGCACGTGGGGCTGAGTCGTCATCAAGGCGGATGTGCGGCTATTTACCCTGATGTCGACCTC
GCTTGCCCGCACGCGAGCCGATGATTAGTAACGTTTGGCCAGCTCCACCATCTTCTTGCGAGCGAAGATG
ATCTGGGTGCGGCTGCCACCGAGCTGACGCCACAGCACGCAGGCACGAATGCCTGCCAGCAGCAGGGCGC
GCACCTTGTGCTGCACCAGCGGCTGCTGCAGGAACAGCGGGGTACCCGCCACCTGGATACGCGGCCCGAT
GGGGCTGATAAGATCGCTGTAGATGCTCGCCATGTTGGCCAAAATCTGCTCGTCGAGCAGATCGAAGTGC
TGTTGCTGGCGGCCAATCTGGCTGATCCGCTCACCCAGCATATTGAGAATGTCCTTGCGCTTGGCCAGCT
TGCGCTCTAGCGCAATCAGGCTCACCACGTAGCGGGTCAGCTCGGCATTCTTCTGGCTGCCGTCGGCACC
CAGCTGCTCGACCAGCGCACGGTAGCCGTCGCGAATGGCCAGATGGGTGTTGTTGAACACCTCGAGCGGC
TGGCTCGGGTTGGTCACCAGAATGCTGGAGAGGCTCTCGCGCAGGGCGGCCTCGTCACAGCTGCCGTCTC
GGGCCACCTTCTGCACCAGATACGCGGCCTGGCAGATGCCGGCAAAGGCCATTGTTCTGTCTTGGAATTT
ATCGCTCACGTCTGCTGCCTTCTGTTAAGGATTCAAACCGGATGGCTGAAACGTTGTTCGATGATGCCGC
CGCCCAGACACACTTCCCCGTCGTAGAAGACGGCGGACTGGCCCGGGGTCACCGCAGCCTGTTTCTCGTC
GAAGATGACCCGGATGGTCTCGTCGTCGATGGGCTGGATCAGGCAGGGAATATCCTGCTGACGGTAGCGG

GTCTTGACGGTGCAGCGACGCGGCTCGCGAATGGGAGTGCGATCCACCCAGTGCAGCTGGCTGGCGATGA
GGCCGTCGGAGTAGAGGCGGGGATGTTCGCCCTGCGCCACCACCAGCACGTTGCGCTCGACCTCTTTGTC
GACCACATACCAGGCCTCTTCGGTGGCATCTTTGCGACCACCGATACCGAGCCCCTTGCGCTGGCCCAGG
GTGTGATACATCAGACCCTGATGCTCGCCGATCACCTTGCCATCCACGGTCTCGATTTTACCGGGCTGGG
CGGGCAGGAACTTGGCCAGGAAGTCCTTGAACTTGCGCTCGCCGATAAAGCAGATACCGGTGGAGTCTTT
CTTCTTGGCGGTGATGAGATCCAGCTGATCGGCGATGCGGCGCACCTCCGGCTTCTCCAGATCGCCAACC
GGGAACAGGCTCTGACCCACTTGCTTTTCGCTCAGGGTATAGAGGAAGTAGCTCTGATCCTTGTTGGTAT
CGAGACCGCGCAGCAGGCGCGGGCGGCCTGTGCTGTCGTCACGGCGCACATAGTGGCCGGTGGCGATGTA
GGTGGCGCCCAGCTCTTCGGCGGCAAATTCGAGGAACGCCTTGAACTTGATCTCCTTGTTGCAAAGGATA
TCGGGGTTTGGCGTGCGGCCAGCCTTGTACTCTTCGAGGAAGTGCTCGAACACATTGTCCCAGTACTCGG
CCGCGAAGTTGATAGTGTGGAGTTTCATCCCCAGCTTGTCGCAGACGGCCTTGGCATCAGCCAAATCCTG
GGCGGCAGAGCAGTACTCGTCCGTGTCGTCTTCTTCCCAGTTCTTCATGAACAAGCCTTCGACCTGATAG
CCCTGCTGCTGGAGCAGGTAGGCCGAGACGGAAGAATCCACGCCGCCGGACATGCCGACGATCACCTTGA
TTTGGCTGTTGTCTGTCATTAGTCGAAGTTACCAGTTCGTTTAAACGGGGCGTATTCTACCAGAGTTTTG
CGCCCCCGGTCACATATCCATGCCGCAGTTCCCATCAGGAGCCTGATAGCAGGAAATCGTTTTTCTGTTG
TTGGCCTTTGTCACCGGAAAAGAGTGTCAAATGGCCTCAGATAAAGGCCTTGAGGGCCGAGAGCGGCAGC
CGTGTCCCCGCCAGATAATCCTGAATGCACTGCCATACCAGGGGGCTGCGCAGGGCCGGTTTGCATTCAG
CTATTTCTTCCAGAGTGAGCCAGTGGCAAGCCAGCACATCGCCGTCAGGATCCTCTGGATGGTGCTGACC
GGGCGCTTTTTCAAGGTCGAAGATGATGGCTGTGCGCACATAGGTGGCCTCGCTGTCGGCCGGTTTGTAG
AGATAGGTACCAAGCCAGGCGGTCGGCGTGGCGGTCAGCCCCGTCTCCTCTTTGAGCTCGCGGCAGGCGG
CGGTGAGCAGATCTTCACCGGCCTCCACATGGCCGGCGGGTTGGTTTAAGCGGCGCTGGCCTTTTATCTC
CTCTTCCACCACCAGAAAGCGGCCCTGCCACTGCACCAGGGCGGCGACCGTCAGGCGCGCCTCCATGCGG
GCGTGGCTGGTGGTCGAGTGCTCTGCCTGATTGCTCATCATGACTCCTTGTCGTTGTTATGGGGATTGCC
CGCTCGGGCGGGTCTGCGTGCCGTTCCTCGCCCGCTGCGGGGTTTGCCGCCGGTCGGGTTCGTGGCTGCT
CGCTCGGTTGGGTGAGTGCTGCGGGGGCGTTCGTCCCGTCTCGGCTGGCTGGGCCGCCGTGGTGCCTCCC
CTGCCGGTTTGGCCGGTTTGGCGGTATGAGCGCCTGAACGGGATGAGGAGGGACGCGGGCGGCTGCTGGA
GCTCGGCACCAGCTCGGGGGCGGGCAGGCTGCGGCTCTCGCCGGGGGCCAGACCATCCAGGGTCCAGTCG
CCGATGGCGTAGCGAATAAGGCGCAGGGTGGGGTGGCCGATATGGGCGGTCATCCGCCGCACCTGCCGGT
TGCGTCCCTCGATGATCTTGATCTCCAGCCAGCTGGTGGGGATCTCCTTGCGTTCGCGGATCGGCGGATT
GCGCGGCCACACCGCAGGTTCATCCATGATGCGCGCCCCGGCGGGCAGGGTCATGCCGTCATTGAGCTCC
ACACCGGCGCGCAGGGCGGCCAGTTGCTCCTCGCTCGGAATACCTTCCACCTGCACCCAGTAGGTCTTGG
GGGTCTTCTCCCCCGGCTGGGTGAGGCGCGCCTGCAGCTTGCCATCGTTGGTGAGCAGCAGCAGCCCCTC
GCTATCGCGGTCAAGCCGACCTGCGGCGTAGATGCCGGGCTCGCTGATGTAATCCTTGAGGGTCTCGCGG
CCCGCCTCGTCGGTGAACTGGCAGAGCACCATGTAGGGCTTGTTCAGCAGGAGCAATTTGCGATCCGCTG
GGGCCAGCTCCGGTTTGCGGGGGGATGCATGGCGAGGTGCGGCTGGTTTGGCCGCCGTGCGCGGGGCGTT
ATCGCGCGGCGAACGGCTGACAGAGAGACGGGGGCGGGGAGATTTCATCGGATACACACTTCAGACAGAC
GATGGCAGCGAGTGTACCACAGGGTGCTGAGGGCAGGATGAATGGCTGGCGTTACAGCAAGCAGTGCCCA
GAGCCAATTCGCCTAGCTCAGCAGGCTCTCGATGACGATAGCCTGACGGGCGGGGGCCGAGCCCGCCAAC
AGCAGGGCGTGGCTGACCACATCGTCGATCATCGCCTGCGCCAGTCGGGTGAGGGGGCGCAGGGTCTCCC
GTCCGCTACCAACCGGCACCGCCAGCAGAATAATGGTCTGCACCGGCCCGATTTGAGAGCCCCACTCGAT
GGGCTCCTTGCAGCTAAGCAGGGTCAGCGCTGGCTGGCTGATCCCTTCGCTCAATACATGAGGCATGGCA
AGGCCGGGGCGGATGATGGTGGCCGAGACCTGCTCGCGCCGCTCTACCGCAGCGGTCAGTGTGGCGCGGT
CGGTGATCAGCTCGGCGGGCAGCAGATCGATCAGATGCTCGAGCGCTTCGGCTTTGGCCAGCGGTTCGGG
GCTGTGGGCCAGCTGGCGCAAGGCAAAATGGTAGTCGGGCAGCGCGTTGGCCAGTCGCAACTCCGCCAGC
GCGGCGGTGCGCGGGCGCACCAGCGGTTGACCCAGCTCCCCGCACCAGCAGGTGAAAGCCATGTGGGCCA
GTTCGGCATCGCACCCTTCAATTTGCAACTGGCAGAGATCGCCGGGCTGGGTGGCGAGGGTCAACAGGGA
GAGCTGGTTGCTGGCGGCAACGGTCTGACGCCGGGTAAGGTTGGCGCAGCGGATGGTGGATTTGAACATC
CCCGCCAGCCGGTTCAACTGCTTGGCCTGGGTGATGCTCAACCCCTGCTGGCAGTAGAAGGTGATCTCGC
GGGTCAGCATCAGGCCGGGATCGGCAGGGCCTTGAGTACGGCGTTGGCATCCAGCAACACAGTTTCCAGT
CTCACTTGATGGATGGTGCAGCCGAGGAAGCGCTCTTTCTGCTCAATCTCGATATCGGAGGCAATCAACA
CCAGGTCGGCGCTGGCAATATCGCGGGCGGTCAGCATGTTCTCCATGCCCATGGCGCCCTGAGTCTCGAC
CTTGATCTTGAGTCCCAGCCCAGTGGCGGCTTTTTGCAGGGCATCGGCAGCCATATAGGTATGGGCAATA
CCGGTCGGGCAAGCGGTGACGGCGACGATCTTCATCTGCTCTCTGTGATGCTGACAAGGGGGAGCAAAAA
GATAGCTCCAGCGGGGGGCTTTTACCAAAAGTCCATATGAAAAGTGGGATCTTGATGGGGATATGCCCGT
CCCTTGAACGGCAGTGCAGGCTGATATGGGCCAGCTGGGAGTGGGGTTGCCCCAAACAACAAGGGCCACC
CCTGTGGGTGGCCCTGAACAATCTGCGTCTGTTGTTATGGCTATCAGGCGGTCTGGGCAGCCAGCTCGGC
GGCTTTCAGTTCGCGCTTGATGCGGCGGATCTTGCGCTCTTCGGCAATCGCCACGCAGGCCATCAGCACC
AGACAGGCGGCTGCTGCCATGTCCAACGCGGCGAAGGTGCCGGTCCAGCCGGTCAGGCCGAAGATGGGGG
TACCATCGGCGATCATGCCCAGACCCAGCTTGGCGAAGCTGTCACCAATCAGATAAGCGAAGGTGCCCCTT
GACGCCATCGGCCACGCTGATGCCCTGCTTGGGTACGAAGCCCACGGCGGCCACGCCGATCAGCAGTTGC
GGGCCGAACACCAGGAAGCCCAGGGTGAACAGGGAGCCCAGGAACATGAACTGGGTGGTGGCGTGCTGGT

AGAGACCCAGAGTCGCGATGATGAGCGCCAGTGCCACACAGGCAACCAGACCACGACGGCCGTTGGCCAG
ATCGGAGAGGTAGCCCCACATCAGGGTGCCGACCAGAGCGCCCACTTCGAAGAAGGTAAAGCCCTGAATA
GCGACGTCTTTGGAGAAGCCGAGCTCCTGGTGGGCATAGACGGTGGACCACTGGTCGATACCGATACGCA
CCACGTAGAGGAAGATGTTGGCAAAGCAGAGCAGCCAGATCACCTTGTTCAGCAGCACGTACTTCACAAA
GATCTGCCACTTGGTCAGCTTCTGCTCCTCGGTGGCGATATCCTCTTCGCTCGCCACTTCGTCAAACAGC
TCTTCCACCTTGCCAAGGCCGTAGGCTTCCGGCGAATCGCTACCAAAGCGCAGGCCGATAAAGCCGACGA
TCAGCGCAATGATGGAGGGGAAGATGAACATGCCGAGCACGTGGCCATCAAACAGGTAGTTGGCACCGAA
CAGTGCCACACCGGCCGCACCGGCACCGCCCACATTGTGGGAGAGGTTCCACATGCCCAGATAGGTGCCG
CGCTTGTTGCGGGGAGTCCATTTGGTGATGGTGGAGTAGCTGCAGGAGCCGCCACAGCTCTGAAAGAAAC
CGCTCAGGGCGTAGAAGAAGATCATGAAGTAGAGGCTGAAACCGGTGCCGCCCATGCTGGCACTGAAGCC
CAGCATGCACAGAGCGGAGAGGATCAGCATCAGTGGCAGGAACTGCTTGGTGTTCTTGCCATCGGCATAG
TAGGAGACGATGGTCTTGCCTACCCCGTAGGTGATGGAGAAGCCAAGGCCGATCATGCCAAGCTCGGTCA
TGGAGAGACCGTACTCCTGGATCATGTCATTTTGCGCAATGTTGAAGTTCTTGCGGATCAGATACATGGT
CAAATAGCCAATAAATACAACCAGATAAGATTGCATAAACGGCTTGAACCAGAGCTTGCGCCGGGCTTCG
ACCGGCAGGTCGAGGGTGGGGACCCGGACCTGTTGGAGCAATTTCAACATCTTCGTTCATCCTCTGCGAG
ATAGACGGTATTTCCGCCGCGCCCCTCATTTGTAGATAGGTTGTTCGAGGTCACGCCACGGAAGGGGGAA
CGGAACGTCGGGGCTGTCAGCCGTTGCTGCGCTCGACGCTCCATCCATATAAACCCGGGCCCGGCACGGG
ATGACGGGGCGCATTGCCCATCGCGGTGCGGGTCACGACATCAGGTGGGGTGACTCTAATAAGAGGGGCA
AAAAATGGCTTGAGAGCCATCCCTAGTGGAGCTAGGAAATTTTCCTAGTTTTTGCGCTTTCGCGGACAAA
GCGTGAGAAACATCACACCGCTGACGCCGGATTGCTGCCATTTCAGCCAGTTTGGCGCTCTGTGTGAGTG
CTGGGCTGGGCTGGGCTGGGCTGGTGGGATGGATAGGCCAGAAGGGAGCCAGGGAAAGGCAAGGTGAGAG
GTGAAGGGAGAAACGAGGAAGAGTGCTTATCGACCACTATTTCTCGGGGGGCGATTTTGGGAAATACGGC
TTGCCAGCCATCCCAAAAAACAAGAGGCCATCCCGTGGGGTGGCCTCTTTTATGGTCTAGGAGCCTCGAT
GGTGAGGGGGGGGACTCAGGCCGCCTTGGCCAGAGTTACCTCATCACTCTTTTTGACCCGCAGTTTTTTC
AGGGCAACCGCCACCACGCCGGTGACGATGGCACCGCAGATCATGCAGAGCAGGGCCAGCAGCGGCTTGT
TGACCGCACCCAGCAGGGCCACGATGGGGCCACCGTGGGCGACGCTGTTGGTGATGCCCAGCAGGAAGGC
CATCACGGCGGCGGTCATGCTGCCCAGCACGTTGGCCGGAATGACGGCCAGCGGGTCACGGGCGGCGAAC
GGAATCGCACCTTCGGAGATACCCACCAGACCCATGGCACCGGCGGCTTTACCCGCTTCGATCTCGGACT
CGTCGAAGATGCCGAGCTTGCGACCGAGCACGGTAGCCAGCGACATGCCAAGGGGGGCAGCCGGGATGGC
GCAGGCCATGGCACCCATAAACTGGGTCTGACCGGAGGCGATCATGCCGACGGAGAAGAGGAAGGCCACC
TTGTTGACCGGGCCACCCATATCAAAACCAGCCATACCACCCAGCACGATACCCAGCAGCACCAGACTGC
CACCGCTCATGGAGAGCAGCATGGCGTTGAGGCCAGTCATCATGTCGGCAATCGGGGCGCCGATGATGAA
GATGAATACCGCAGCGATAAAGAGCGACCCGACGATGGGGGCGATCAGGATCGGCACCAGCGGTTGCACC
ATCTTGTGGTACTGACGAGTGGCAATCCAGCGCACCAGATAACCGACCAACAGGCCCGCGATGATGGCAC
CGATAAAGCCGGTACCGGCGTCAGCGCCGTAGAAGCTGCCGTTGTTGGCGATCCAGCCGCCGATAAAGCC
CGGTGCCAGTGCCGGACGATCACCGATGGCATAGGCGATATAGCCCGCCAGAATGGGGATCATCAGGGTA
AAGGCGGCCACGCCGACGTTGAGGATCTGGTTCCACATGCTCCCTTCCGGGATCTGCATGCCGCTCGGGG
TCGGGTTGCCGCCGATGGCCAGTGACAGGGCGATAAGCAAACCACCGGTCACCACGAACGGGATCATGTG
CGATACGCCGTTCATCAGGTAGCGATAGAGGTCCGAGCTGCCGCCGCTGGCGCGCTTCTCTTCACCTTTT
TTATCTTCTTTGCCAGCCACGTAGGCGGGGGCTTTCAGGGCCTGCTCGATCAGACCTTTGCCATCCTTGA
TGGGGGCCTTCACGCCGGTCTTGATGAGCGGCTTGCCGTTGAAGCGGACCATGTCCACCTGCTTGTCGCA
GGCCACCACGATGGCGGCGGCGCGGGCGATCTCGTCCGCGGTCGGGCTGTTCTTGACGCCGATGGAGCCG
TTGGTCTCCACCTTCACCTCATAACCCAGCTCGCGAGCGGCGCGCTCCAGCGACTCGGCCGCCAGATAGG
TGTGGGCGATACCGGCCGGACAGCCGGTGACACCGATGATCAGCCCCTTGTTGGCCGCAGGCGCGGCAGC
CGCTTCCTCTTTCTTGGCCAACAGCAGTGCCAGCGCATCGGCCGGGGTTTTGGCTGCCAGCAGGGCATCG
ATAAAGCCCTCTTCAATCAGCTTGGCGGAGAGCTCGGCCAGCACCTCGATATGATGGTTGGCGCCACCGG
CCGGGGAGGCAATCATGAAGAACAGCTTGGACGGCTTGCCATCTTCGGCGCCGTAGTCGATGCCGTTGCG
GCTGATGCCGATGGCCACCGCCGGGGTGCTGACCGCCGCACTCTTGGCGTGGGGCAGGGCAACACCCTCT
TCAAAGCCGGTGTTGTCCAGCTCTTCACGGGCCCACAGATCCTTGACGAACTGCTGCTCGTCGCTGACCT
TGCCTTGCGCGACCAGCAGGCGGGCCATCTCGGTGAAGACCTCTTCCTTACGTGTCGCTTTCAAATCGAG
ATTGATCAGATGTTCATTGATCAAGGTGGTTATCATGGCGCTCTCCCCGCTCCGTTGCCGGAGCCTGTTG
GCATGCCGCCCTTGCAGGCTGGCATGCAGACAAAATTGACCCGACTATTATCACGCCGGGGCAGAGGTCA
CCCTATCGGACAAATGTGCGAATAACTGGATTTTTGTGCGCCATCACCTTGAGTGTGACCCTGATCGCAA
AGCTGAACAGCTGCTGAAATGGGGGCGGATTGAATCGCTCATATTTTTATTGATGATTAAAATCAATTGG
TTGTGTTTGTTCGTGGTTCAGCTCCTTGGCCGTAAGGTGAGTTTTTACTCAGTGGATGGGAGCGCTCTTT
CTATAAATTTGTGAGGTGAAAGACGGCGGCGGGATGTGAGCCAACGCACAGAAATGGCCCCGCTGGCGGG
ACGCGAGGGGCGGACGCAGGGGAGGCGTGAGCAGAACAAGCTGGAGAAATGGGTTGCACCATCGATGCCA
GACAGCCCACCGCAACGGGCAAGAAACCCCACAGCCATAGGCGCACCGTAGGGTGCAATGAACAAAGTGA
ATTGCACCGCCTGTTTGGGCAGGGAGTGTGGTAGCAAGAGAGGGGATGGCTGAAAAGAGAGAAGATGGCC
AGTTCACGTTAGCCAACTGGCCACTCGCTTAGCGGTGAGTGGCCAGTACTTCAGCATGAACACCTGAACA
TATTTATCTGTTCTAAATGTAGCGGAGTTTCAATATACGGTTATTTAAGCTGATATCAAATGAACTTCGG

TTACAGATATGGCTTTACCTGGCTTCGAGTTTCTACCATCTGGTTTTACAGCAATTTGATCCCCCTCTTT
TAATCGACACCATTCATTCCAGCTACCATTTACTAACTTATCATAATGAATGAAATAATCTTGGCTTAGT
TCATCAGTCGTAAACATAAAGGTATTAGGGCCAGAGTTGCGGCCTTTTGGTATGTTTTTATTTTTGTTT
TTATTAATGTTAGCTCTGTGATGTCCTGTTCACTTATTCCATTTGAAAAATATGCCGGTTGGGCTGTTTT
GGTATCTCTATTAAAAAATGGATTGCCAGAAATATCTAGTTTTGATAGTTTTTTCAATAGTTTTGTCGCG
GAGTCTTGCTCGGAAAAGCTTGCGTGCTCAGTAGTGTTATTGAGATGTTTTGCTATAAATGCTAGCGCCT
CATTAGCAAAGGATTTATTCTCTTGGTATTCTTTTCTCACTAAGCAGTAAGCGACTTCATTGAATATGTT
CTTGGCTTGTACGCAAGCGCTTTTTATATAGCCATCATTGGTAAATATGTCATCCAATATCCTTATTGAG
CTGGAAAGACATTTTATCGTTCCTTGGATGTCTGTGTTGACTATATTTTCTGACTTTCTTTTCCATGCGC
TAGCTCTATATGCGCCGAGCATTCCTCTGAAATGTTTTGACTTATTCCAGTTTTTAGTTTTATCTAGAAT
TACATCATGCTTCAATCCATACAATAGTGATAGCATATAACCACTATTAACATGATAGGCAAATTGAGAG
TCATCATCTTTTACATCACCCCATCCATCATCAATTAAACTTTCATATATTTTTTCTGCTGCATCATAGT
CACTCTGAGTATGATGTAACATCGCTAAGAAAAGCTTGCTATTAGGGTTTTTATTATCTATTTCAATAGA
CTTTTTATAATGCAATATTGCATCATCTTGAGCTTTAAGGGCAGATAGAATTCTTGCATATGCTCTTTGA
TATGATGCATCGCTTTCATATATATGCTCGACTTCTTTAAATTTTTTATATAGAGGGATTAGTTTTTCAT
TCTTTATTTTGAACCCTCTTATTGTTGAGTTATTGAACTCAGTAAGAAGAATTTTTAAGCTTTCATTAGT
TTCAGTTGGGATGTAGTCCCAGTGATACCAAGCTAGCTTTTGACTTTCTTGGATATTGTCAATTTGTCTA
GCCAGCACTTTACGTTTGCTTATTTTAGCTTGTATATCGCTGCGAATTGAAATGTTTCTAGCATTGGTTA
GTAAAAGCTCTCGAATAGAACCACTTAGACTAAAAGTTTCTTTATCCTCATCAATGATTCTATTTATTAA
GCTTGTATTTGATAGCTCAGCAATTCCAATAGCTAATTCTTCTTTAGAAAGGCTTAAAAGATCACATAAA
TCAAGCCTGGTGCTTTTATCGTCAACAAATAGAGCCTCTAGAATTTTTATTGATGTTTCAGAAATGTTCT
CAATTAAATTGTTATAGGAGAAGCTTGCAATTTCTTTATTTGCTACTTCAATGGATTTTGGAATTTCTTT
GCCAGACAAGAATAAATCCACAGTCAATCTGATAGCTAGTGGATTACAATGGCAGCTATTTGCTATTTTT
TCATAGATAGCATTATCTTGGGCTTTACCACCTCGTCTTGTTGAGTAAAGTCTGGCCATCAATGCCGCAG
ACTTATCTTGGAGTGGGTTTAAAGAGGTAATGCTAGCATTTGATATTGTAATTCGACTTGTAACAAGCAC
TCGCCAGCTTGCAGGAAGAGAGTAGTTAAACTCCTCAAAATCTTCTGGAGAATCTACAAGTAGGGTTTCT
AAATTATCAATGAAAAGTAATATTTTTTCATTTTTATATTTATTCATGAAAGAGTCAAATGATGGAATAT
CTTCATCAAATATGCAGCTTGCTTCTAATGCTAGTTGATCTTTAATTTCTGCGATTGTTTCTACAGCATC
AAGTTTTTTTACTCCTTCTGCTGTTAGTCGCTCAGTCTTCAATGAAATAAAAACACAAGCATCACACCAA
GTTTTTGTCTCAGGGATAGACATTTGTTCATTTATCAAGTCAAGAGCTAACGCTGTTTTACCAAGTCCGC
CAGGTGCGACTAAAGCCAATGTATTTACTCTAGGATTTTTGAGTGACTTTAACAGTAATTCAGCTTCCTT
CTGGCGCCCAACAAGCCCTGTAATTGCGTGTTCAAATTTGGCTGGGATATTATTTGGAATATTCCAAATG
ACTCTTTCTAACCATTCCTCGGGGGGGTCTGTAATGTCTCCATTTTCTGCAGATATGAGAGCTCTTTTGA
CTTCATCCATACCTAAGACATCAATCAATGGATCAGATGATATTGATGCGACCTTATACCAATATGTATC
AATAAACTTCCTGTTTGGATGTGATATTATATTTCTTATTTCATAAACATCATATATTACGAATAGTTCT
TTGAGTTTGCTTATATATTTATGGCTAGTTGTATCTTTTGTTATGTCGAGAATTATTTGAAATAACTCAT
CGAGGTATGTCGCTTCAATTACATCTGTTATCGAGTCAATATTAATACACCTATTTCTAGAAAGCTCTCT
ACTTGCAATATCCTGAATTAATTTATCGGATATATTGTCTACCGTGAGCTCTCTAGACACGACATAATTT
CCAAGTGCCATTTCTAGCTCATGGAGAACCATTGCAGTTTTTCTTCTATTTACCTCAGGGTTCATAATAT
CCTCAAAGATTTCTTTAAAAGGTTTTCAAGTGGAGCATATTAATCTTGATTGATAAATCAAGAAACTGCA
GATGAGTAAAAACCTGCCAGAGATCAATAAAGTTGTCATTAGGTGGTCTTTGTTTGAACAGTTGGTGGGG
TTTACGCCTCAATACTCCAGAAACTTCTCCCCGCACCGCTCGCAAATTAGATGTTTTACATGGGTGGCCT
GATAGGCGAGGGTCTGACCGAAGACCGCATTGGTCAGGCTGCTGGAGCGAAAGGCTTGCCAGAAGGTTTT
GCGCGGATCCTTGGGCATGATGTCCTGGCTGTGATCGCGGGCGACCAGCACCACGATATGGCGGGTACGC
TCGCCGCATTTGTTACAGCGGTGGATTTCAGTCGGCGGATTCTGGTCGTGCGGGTGGCTCATAAATGGGC
TCGTGAAGTGAGGCAGGCGCCCATCATATTCCTCTCCACTTGGCTCTCAATCTCTCCTTGCCAGCGCAGT
GAGCAGGCTCACATTTTGCGCTGCTGCGGGCTTGCCCAGCCAGCTCAGGCCGATGCCCGTTTCTGCGATT
TGCTGGTGAGCGGTGATGATATGGCGGGTGGGGCGAGCTCGGGCGCCCAGCCAGTTCAGGCCGACTCCCG
CTTCTGCGGTTTGCTGGTGAGCGGTGATGATATGGCGGGTGGGGTGAGCTCGGGCTTGTCCAGCCAGCTC
AGGCCGATGCCCGCTTTTGCGCTTTGCTGGTGAGCGGTGATGATATGGCGGGTTGGGACGAGCTCGGGCG
CCCAGCCAGTTCAGGCTGATTCTCGCTTCTGCAGATTGGGGGTGATGATATGGCGGTTGGGGGCGAGCTC
GGGTTGTTGCATCCGGGTCAGCAGGCGCCTTGCCGCCTGACGGCCGATTTCGCGGGCCGGTGAGCTGACA
AAGGTGAGGGCGGGGGAGGTGAGTTCGGCCTCCGCCACATCGTCAAAACCGAGCAGGGCCACCTGCTGGC
CGATGTAGTTGTCCTTGCCCACGGTGCGGCCGCTCGCCTCGACGCCATAGATGGCGCCGAGCGCCACCTC
GGGGTAGTGGCAGAGCACGGCGGTCACCTTGGGGTGCTGGTGCAGCAGCTGGGCGATGGTGTCGGCAGCG
CTGCGCTGGGTGCGCTCGCACTCCAGTACCCACTCCGGTTTGAAGGGCAGGCCATATTGCAGCAGGCTGG
CACAGTAGCCGCCGATCCGCTCGGCGCGGGTGAGGGAGTCGGCCCGCCCGCCGACATAGGCGATATGGCG
GTGCCCCTGCTCGATAAGGTAGGTGGTGGCCTGCTTGGCGGCGTGGGTGTTGTCGGGGCCGATAAAGTCC
ACGTCATCGCGATAGTAGGAGCGGGCGGCACACACCACCGGCAGCTCGGAATCGAGCAGGGTCTCCAGGG
TTTTGGCCGCCGCGCCGCGCACCGGATTGAAGATGATGCCCGCCACCCCTTGCCGTGACAGGGAGTGGAT
GCTCTGCTGCTGCCGCTCCGGGCTGTGGCCGCACTGGGTCAGAAACAGCAGATAGCCCTGCTGCTCCAGC

ACCTCGCTCACCCCGGCGGTCACCTCGGTATAGAAGGGGTCGGTGATGTCGCGCAGGATCAGCCCCACCA
GATTGGACTGCTGGCTGCGCAGGTTGGCGGCGGCGCTGTTGGGCACATAGTCGAGCTCTTTTATCGCCTG
CTGCACCCGCTCGGCGGTGGCCGGCGAGATGCGCCCCTTGCCGTTGAGCACCATGGAGACGGTGGTGACC
GACACTCCCGCCCGCTGGGCGACATCGGTGATCTTGGTGGCTTTGGCAGAGAGGGATTTGGGCGACATAG
GGCGAACGGACGATGGCATGTTGAGAGCGAGGCCCCAGCATACCTGCTTGGGTTGCCAAGTGAAACGTTA
CATCACCATCTGACCGATCCGGCTCACAAATTTGGATCCTCCTGCCAGCCAGCCCCCTTATTCTGTGATC
CTGTCGGCACTTGTAAATTGATAAAACGTTTTATCATCCAGCAAAACCTGATGCTGACCCGCGCCTTTTG
CTTCGAGATTGCTTCGAGATTGCCTCGAGATTGCCTCGAGCGGGGGAGGGCAGCCGCAGATATTGTTGTT
TATTCACAAGGAGCCGTTTCATGGCCAAGCCTCAAGCCAAATCCACCATCTGGGAGTTCCTGCAGAGCCT
CGGCAAGACCTTTATGTTGCCGGTCGCCCTGCTGGCCTTCTCGGGCATCCTGCTCGGTGTCGGCAGCTCG
CTCACCAGCGATGCGGTCAAGCAGAGCATGCCCTTCCTCGACAATACCGTGCTGCAACTGCTGTTCCTCT
GGATGACCAAGGTGGGGCTGGTGGCCTTTATCTACCTGCCGGTGATGTTTGCGGTGGCCATTCCGCTCGG
GCTGGCGCGAGAAGAGAAGGGGGTCGCGGCCTTTGCCGGTTTCGTCGGCTATGCGGCGCTCAACCTCTCC
ATCAACTTCTACCTGACCGTGGCCAACGTCATTGGTGATGCGGCTGCGGAGAAGGCCTACGGGGTGAAAT
CGATCCTCGGCATCAGCTCCATCGACACCGGGATTCTGGGGGCCATCATCGTCGGCGTGATCGTCGCGAA
GCTCCACGCCCGTTTCTACACCTACAAGATACCCGATGCGCTCGCCTTCTTCGGCGGTGCCCGCTTCGTG
CCCATCATCACCGCGCTGGTGCTGGGTGTGGTCGGTCTGGTCGTGCCGTTCGTCTGGCCCTGGTTTGCTG
CCGGTATCAACGGCATCGGGGCGCTTATCTCCCACGCCGGTCCCTTCGGCCCCTTCCTGTTCGGTACCGG
TGAGCGTCTGCTGCTGCCGCTCGGCCTGCACCACATTCTGGTGGCGCTGATCCGCTTTACCGAAGCGGGC
GGCACCCAGCTGGTATGCGGTCACGAGGTGAGCGGCGCCCTCAATATCTTCTACGCCGAGCTCTCCTGCG
CTGACACTTCCGGCTTCTCGGTCTCTGCCACCTCCTTCCTCTCTCAGGGCAAGATGCCTGCCTTCCTCGG
CGGCTTGCCGGGCGCGGCGCTCGCCATGTATCACTGCGCTCGCCCGGAAAATCGCGGCAAGATCAAGGCG
CTGCTGGTTTCCGGCGTGGTGGCCTGCGTCATCGGCGGTATCGCAGAGCCGCTCGAGTTCCTGTTCCTGT
TCGTGGCGCCGGTGCTCTACGTTATTCACGCCATCCTGACGGGTCTGGGCTTTATGACCATGGGGCTGCT
CGGCGTCACCATCGGCAACACTGACGGTAACCTCATCGATTTTCTGGTGTTCGGCGTGCTGCAAGGCACC
GCCACCAAGTGGTATCTGGTGCCGGTCGCGGCGGCCATCTGGTTTGCCATCTACTACAGCGTGTTCCGCT
TCGCCATCGTGCGCTTTAACCTGAAGACCCCGGGCCGCGAAATCGAGACCAGCGAGACTGCCCAGCCCGC
AGGCAAGAAGGCCGGCAACAGCGGTTACAACGGTGAAATCATCCTCAACGCCCTGGGCGGCGCCGATAAC
ATTCTGGCGCTCGATAACTGCATCACCCGCCTGCGGATGTCGGTGCAGGACATGAGCCGGGTCGATGACA
AGGTATTGAAGGCAAACGGCGCCATCGGGGTGATCAAGCTCGACGAGCACAACCTGCAGGTAGTGATCGG
CCCGCAGGTACATCTGGTGAAAAACCAGCTGCAGAGCCTGATGGGGGCCTGAACCGGCCAATAAGAACCG
AACGGGGGGCGCAAGCCCCCCTTGTTTGCCTCTCCCAATATGTCTCCTGTTTATGGAGGGAGAAACTTCA
TGCACGATATTTATGACTTCGACCATGTGGTCGACCGCCACGGCACCTACTGTACCCAGTGGGACTATGT
GGCCGACCGTTTTGGCCATGCCGATCTGCTCCCCTTTACCATCTCCGACATGGATTTCGAGACCGCCCCC
TGCATTCGCCAGACCCTGGCAACGCGGCTCGCACACGGGGTATTTGGCTACAGCCGCTGGAATCACGACG
ACTTCAAGGGGGCGGTGAGCGACTGGTTTGCTCACCGCTACGGGGCGAGCCTCGACCCCGAGACGCTGGT
CTACGGCCCCTCGGTCATCTATGTGATTGCCCAGCTGGTGAGCCTCTGGTCGGCGCCGGGGGAGGGGGTG
CTGGTGCATACCCCCGCCTACGATGCCTTCGGCAATATGCTGGCAGCCAACGACAGGGTGCTGCTGCCCT
GCCCGCTTAAAAAAGAGGAGGGGAGTTACCAGATCGACTGGCAATGTTTCGAGCAGCAAGCGGCTCGCCC
CGATTGCCGGATTTTGCTGCTTTGCAGCCCCCACAACCCCACCGGGCGGGTCTGGACGCGGGATGAACTC
GGCCGGATGGCCGCTATCTGCCAGCGCCACGGGGTCAAGGTGATCTCGGACGACATCCATATGGATGTGA
GCTTTGCCCGCTACCTGCCGTGGAGCGAGGTGGCACCGGACGATCACTGGGCGCTGGTGAGCTCGGGCTC
CAAATCCTTCAATATTCCCGCCCTTGGCGGCGCCTATGCCTTTATTGCCAACGCCGAGGCGCGAGCCGCC
TATCTGCAGCGGCTCAAGGCGGCCCATGGCCTATCATCACCGCCCATTCTCGGGGTGCTGGCCCATATCA
GCGCCTATCGGGATGGCGGTGTCTGGCTCGATGCCCTGAAACGCTATCTGCACGGCAACCTCGCCCATGT
GGCGGCGCGGCTCAACGCCGCTTTCCCCGCCATCGACTATCGGGTGCCCGAGGGAACCTATCTCTCCTGG
ATTGACCTGAGCGGCCTTGGCATCGACTCCACTGAACGGATGGATGCGCTGCAAACCTTGCTGGTGGAGA
AATACCGGGTCGCCATCATGCGCGGCGACACCTACGGCCCGGAAGGCACAGGCTATATCCGCCTCAACGT
CGGCTGCCCGCGCTCAAAGGTAGACCAAGGGCTCGACGCCCTGATCGGCGCACTGGGGGAGGCGGTGTAA
GGGATATCCTGGACTAGCGATTGAGCAAGAAGACGACCTCCCCCCGGTAGTGGGGGGAGGTTGCAAGCTT
TGCCTGCCACGAGGGTTCCCACTCGCCCTGCTGCACCAGACCAACCTTGAATGGAATGGTGAGCTGCATC
ATGGCGGGTAGATAGGAGCTGTAACCCGGTACCGTATGTTTTCCCTGATAGGTCTGGATCACTATCTCAT
CGACCGGAAGGCTGTTGAGCTGCGCCACATCCCCCGTCTTGGCCCAGTCGAGCAGGCCCGTTACGCTGAG
TTTGCACTCTGCTGGCAACACTTGTCGCAACTGCCGCAAAGAGGTGGCGTAACGATCCAGTTGGCGGGTG
GCTGCATCAAAGTCGATCTGAATACCCGTTACTTTGGCTCCGGCCTTGCGCCACTCCTGATACAGCTTGA
CCAGCAACTTCTTGTGATCGGCAGAGAGCGGCAGCGTATTCACTCTCACGACAAGCCAGAGTTGTTCGGT
CTGAAGTGGCGTTGGAGCACGCCCCAGTTTGATAAAACGGGGGCCGGATGGCCTGTTCAGGATCTCCCCC
TGATGCAAATAGAGGGTTGTTGCCTGTGCCAGGACTGGCTGATGCCGGGTGGCAGACCAGAGCCAGAATG
CGTGATAGCTGCTGGCATCGACCCTTGCCTTAAGTGGCGCAGCAAGTGTCGATAACAGAATTAGCAGGCT
AGGCACGAGGGGTTGCTGCATGGTTTACCAGTAGTATTTGAGTGATTTAGCCCATACCGAGTTGCCATAA
CGTTGCTTGAGCGTGTTGAACCAGGCTTGCCGCATGCTCTTGGGGAGCTCCTGATCGTCACACCCGTTGT

AGCCTGATGGTGCGTAGCAGTTGATGGCTCTATAAAGGGCATAGCTCTTGTCTTCGGGCTCGGCTTTAGG
GTTGGCCATGACGGCCTGATATAGCTTTAGCCTGCTTGGTGCGGTGGCTGACTCAGGTTTTGACAACCCC
CAAACCATCTCTTTTCCTTGCCACAAATTGATCGCGGGATCCTTGTTGCGCAGATATTCACCCAGGCAGT
TGAGTGCGTGTCCGTCTCCGGGGTGTTGCAGCAGTGTGCCGAGAGTGGAGGCCAAATCACGGCAGTAATA
GCCCTGCGGTGTGCCTTCACCTCCCCACTTGAATGCTGACAAGAGCACATCTCTGTTGGGTACCGGTTGC
CATGGTTCATCCTTGGGTGGGGTAGCCTCCTTGTAGTCGGCCGGAATAAGTGTCACGTCTTTCAGGAACG
TTGCGTAATCCTCTGCAATCAGGTCTCTCACCAGCAAGGTATGCAGGGCGATTTGTCTTTCCAAGGCGCT
TGTGGCCTGCCGAGCCTGCTGACGAAGCAAGTCCGGATCTGCCGACTGTTTTAACACGACGGTGCGTATT
CGCAACTGAGTGACAGGGCTCTCTTTGTTGAAAATGGCCGCAGTATTGCCGGTACGTACCCACTGATCTG
CCAGCATGTTCTCCACGAATGCCTGTTGGGCTTGAGTGAGAGGTGCCGCTACGAGCGTCATCAGGGCTTG
CTCTGCCTGGTCAAACGCCTTCATTCCTTGCTGTGCCCAGATGCGCAGCAACTGGCGGCTAAACGCCAGG
GTTGATGCGTTTTGTCCTTCTGTTTTGGGCAACAGCGCCAGTACAGCATCAAACTGTTGGTTCTGCAGCA
ATCGATAAGCTTGCAGATAGGTAGCCTCTTCCGTTTTACCCGTCTCTTGCAGCTGTTGGATGGCCTCATC
GATCTGCTCGTCACTCCAGTTGCGTTTCTGGTCATAGCCAGTTCTGAGGCCACGCAGTGCATTGATAAAC
AGCAGCTCAGGCTGAGTTTTATCCTCGTAGGCGAGTTCATTTCGATAAGCATCTGCGCTCAGCAAAGAGA
GATCTATTTCGTTAACCAGCGCCTCAAGTTCCGGCAGGGAGTGGTCAGCGGCTATCGCTTCGCTATAGTT
ATCGCGCAATGAAGCCTGGTCACCCGCCATCCAGTTGATGCGTCTGAACATCCCCTTGGTCGAGTTGACG
TAACGGCCTTGCGGATAGGCCGCCAGATACGACAGCCCCTGTGTCATGGCCTGTTTCAGCGCCTCTTTAT
CACTCTTGGTGAAATCCGTATCACCATACTCGCTCTGCGCATCTTTCACCGCTGCATTAAGGTGTGTTCG
CATCACCATATAGTTCGCGGTTTCGGCAACCCACTGCGCTTTTGCCCCTTGCAGAGCCATAAAGCCGGTC
AATGCCTCGGAGAAATGACCTTGGTAGAAGTGGCGGGCCGCATCCAGATAAGCCTGCCACTCCCTGGCAG
ACTCGCTCATTGTGACGTCGGAAGACTGGGGGCTATCTTCCTGTTGACCAAGGAGCTGTAAGCGGGTTTT
GAGCAGAAGCGTGCGCTCGGTAGGTGACAGGTCACTCTGCTCAAGGGCATCGAGAAATTGACTGACAGAT
TGCAGCGAGTTGGAAATGGCTCGGCCGTAAAGGGCATCCCGACCAGCATAGTGTTCCAGCAAAGTCGGGA
GCGAGGCGTCATGGGCGGTGAGCTGGCTAACCAGCTGTTCACGTAGTTCCGGGACTTCCAACTCGTCGGG
AAGTGGCAGGCGTTGCAGAGAGAAGAGCTCCATATCGCGTTCGTTGGCCGGATCGGCCGGAAGCATAAAG
GGAAGGGAGAGTCGCTGCTTGTCAGCCAGCAGCATCAGCAAGTTGCTCTTGGTGTCATTTTCGGAAAGGA
AAAGCGGATAAGCAGGCAATGGACAGTCTGTCAGCCAAAAACTGCACCCGTCTTGGTCGAATGAGGCAAC
CGAAGGAAAAGCAGCAAGCGTGAGTGCCAGTGCCGAACAGTATTTCAAGATCTTCATAAGAGGCGGTATC
CACTATCTGCCGAGTGACAACTCCTTGAATCACGAGCCAAGAAGCCTGAAGCACAATGGCTGGCAATAGT
ACGCAAGTGGGTTGCAATCGACAACCTTTCAGTGGTTTATGATGGGCGTTCAAGCAGATAACAGAATGCT
GATGGCCTTACGTTGTAAGGCCTGCAATAAAAACGGGGCCATCAGTGATGGCCCCGTTCTGGTTGCGCTG
TTTTCCCTTATGAGCAGACGCCGATATTCAGGCGCGAATAGTCGTCGGGATACCGCAATTCTGGCGTGAC
ACGGCTGGTTCGATTGCGGGCTCGCTGAGCTCGGTAGCTGCTAGTGCAGTGCCTTGGGTCAGGGCTTCAG
CCTGATGCAGCATGCGGCTGACCATATCGGTCAGGAGATCCACATCCAGCTTGTCCAGCCACTCGCGACA
CCAGAGCAGGGTGCTTTGCTCGCTGTCTCTACTGAGTTGCAGCACAGGCCAGTCGGCCATGGCAGCGAGG
GAGGGGGTCTGGATATCCCCCAGCGGGATAAACAGGGTCACCAGTTGATCCCGCTCTCGAATATGAAGGC
TGAAGGGGCCAGCCTGCAGATCCAGCCCCTCTTCGTGCAGGCACTCCTGCGGCAGGGTTAGCTGGCAAAC
GAGGGTTGATAGCATGGCATGGAACATCATTAATCCTCCCGATGGGTGTTATCAAAGACCGACTCTGTGG
GCGGCCACAGGTTGGCGATAGGGGGGCGGGCAGGGGTACCGACCAGTGGCGCCCCTATGGTGGCGGCCCA
GGGGTCGACTACCTTGGCGTGTGGCCCCCAGCTTTCCGGCTTGGTGAGTTCACTCCCCTCGGCGCGGCCA
ATCACTACAAAGCAGTGGGTCTGGGCGTGACCTTGTGCACCACGATGGGCCAGCACTTCGATCCGCTGGT
CACTGTTGTGGCGCAGCAGTTCGGCCGCCGCCGAATAGGCCAGGGTCGAGCAGCAACCGGCCTTGGCGTC
ATCCACCGCCTTGGCGATGTTTTCCATGGTGCGCAAGGAGAGTGGCTGCTTGTTTCTGGTTATCTGGATG
GCGGCCATATTGGCGCGGTTGTTGGCGCGCTGGTTGGCTGCCAGGCGCTGGGGATCCTGCCCGAAGGCGT
TGAGTTTGCCGATGATCTTCTGATAGCTCGGGTTGTTGGGCTGGACAAAGGTCTGGGCGTCGGTGCAGGC
GTAGCTGCCCAGTTTTTCCAGTCCGGCCATGCTCTTTTGCAGCAGGGCCTGGGTGCCACCCGCCATCACT
TTCGGTTCATGTCGCGGGGCTGCTCTGGTGATGGCACCCTGTTCTGCCAGGGCCTGCTTCAGTTCAGTCA
GACGAGCGCCGCGTGCTGCCACCTCTTTGGGGTGGTGCTGCTCCCAGTGCTGGATTGCATCCTGCACCCG
CTGCGCTTTGCCGCTATCGGGGGCGCTTTGCAACTGGCGCAGGGCGCTGATGACTTGCTTGAGATCGCTG
CTCTGGAAGTGGCCGATGCCGCGCTTGACGGATTGTTCGAACAGCTTGTTACCCTTGGCCAGCAGCGGGC
TGGCCTCTGTCTGTAGGGGGGATGCTGGGCGGTTGAGGTGAATGTGTCCACCGATTGCTGGTAAGGCCAT
GATGTGATTCCCGACAGGGTTGACGATAAGGGGATGATACCGAGCCGTGACGGCGCGCTATATCAGGGCG
CGGTCGGGGATTTGTCAGTTTTCCGCGTGTTGTGCTGTTTGTTCGGCGATGTGGGCAGCGTGCTGCCCTA
CCACTGTGGCCCTTCGCTCTGGCGCCACTGCTCCAGCCAGCGCAGAACCTCGGCGGTCGCCTCAATCTGG
TCGGCGGGAATGTAGTTGTTGAGTTGGGCATCGCGATAGAGGGCACGGGCCAGTGGAATGCGTTGCAGCA
CAGGGATCCCTTCCTCTTCGGCAATCTTGCGCACCTGTAGCGCCTGGGCATCGGTGAATTTGAGGGTCAC
CACCGGCAGCGGGGTTTCTCCCCGCTCGTAGCGGATACCGATAGCGATATGGGTCGGGTTGGCGACGATG
ACCGAGGAGCGCTTGACGTCGGCGCGCATGTTGCTCGACTGCAATTCCTGATGGAACTGGCGGCGCTTGC
TCTTGATCTCCGGGCTCCCCTCCATCTCCTTGTACTCCCGCTTCACTTCGTCCTTGCTCATCTTGAGCTG
CTTGATGTGCTGGTAGTGTTCGAAGGCGTAGTCAGCGATGGCGATCACCACAAAGCCCATGGCGCAGACC

GCCATCAGCTGGCCCAGCATGCCCCCCAGCACCGGGGTGATGCAGCTCATGCCGCAGCCGGGCAACTGGA
GCATCGCCTGCAGATTGCCCTTTATGGTGACCCACACCAAGAGGCTCAGGAGACTCACCTTGAGCAGGGA
TTTGACGAACTCCATCAGGCTCTTGATGGAGAAGATCTTCTTGGCCCCTTCCACCGGATTGATCTTTTTG
ATATCGGGCTTGATGGACTCGCCACTCAGCAGTAAACCGTACTGGGCGAAGTGGGAGGCGAGCACCACCA
GCGCCGACACCGCTAGGATCGGGATGCAGAGATAGGCCATCTCCTGCATCAGGTTCTCCACCACATGGTT
GAGCGCCTGGGTAAAGGGGAGGTTGATGTGGGATTCCGGGATCATCATCAGCTTGCCCAGATGTTCGAGG
TAGTAGTCGGACATCGCCATCAGCATGCCCACCAGACTGAGGATGATGGCGGTGGAGACCACCTCCTTGC
TCTTGACCACCTGCCCCTTCTGGCGGGCATCCCGCAACTTTTTGGGGGTGGGTTGTTCGGTCTTCTCGCC
GCTCATCGCGCCTCCAGAATGGGGATCAGCAGTTGCAGCGGATCCATCACCGCCAGCACCTGTTGATAGG
CGTGGTCCATCATCAGCTTGATATAGATGACCAGCAGCAGGCTGGCGACGGCGCTTTTTATCGGCATCGC
CAGCACGAAGACGTTGAGGGAGGGGGCGAAGCGGCTGATCAGCGCCAGCCCGAATTCGGCTAGAAACATG
GCGATCAAGAGCGGCGCCGCCAGCAAAGCGCAGAGCAGCAACATATGGCTGAACTGGCTGTAGAAAAAGT
TGACCCACTGTTCGTCGATGGTGGGGAAAAAGTGCGCCACCGGCCAGCTGATGTAGCTCTGGAACAGGGC
GGCCAGCAGGGCGAGAAAGGCACCGCTGGCAAAGAAGAGGGTGATCAGGGTCTGGGTCAGCAGCAGGCCG
GTGGGACTGGTCTGGCTGCCGAGACTCGGGTTGAACAGGGACCCCATGGCGGCACCGCGCTGGTTGTCGA
TCAGAAACCCGCTCGACTCGATGGCCCAGAAGGGGATGGCGGCGACAAAGCCGATCAGCAGGCCGAGCAG
CACCTCCTTGGCGATCAGCATGGCCAGCGCCATGCCGTCGATCTGATCCGGCAAGGTGTCGGCGACTGTG
GGGTAGGCGTAGAGCGCCAGCGAGCAGACCACGCCGTTGCGGATCATCGCCCCTCCCAGCATCTGTTTGC
TCAGCACCGGCAGCATCACGAAGCAGCTGATAAAACGCGGCAGCAGCAGGGTATAGGCCAGCATCTGGGC
CTGCAGGCTCTCCAGCGTCATTGGATCTGCGGTACCTTGAACAACACCATATCGGCAAAGCTGTAGAGCT
CGTTACCCAGCCAGGCGGTGGTGGCAAACAGGGTGATCACCACGGCGATCAGCTTGGCGACAAAGCCCAG
GGTCTGCTCCTGGATCTGGGTCAACGCCTGCAGCAAAGAGACCAGGGTGCCCACCAGCGCCGCCACCAGC
ACAGGTGGCATCGAGAGGATCAGCACCAGCCAGAGCGCCTGGCTGGTGAAGTGAAGGATATCGGCGTGAC
TCATGCTAGCCTCCGTAACTGATGACCAGCCCGTGGGTCAGGCGGGCCCAGCCATCGAGCAGCACAAACA
GCAGCAGCTTGAAGGGCAGCGAGATGGTCATGGGGGAAACCATCATCATCCCCATCGCCAGCAAGATGTT
GGAGATGATGAGGTCGATGGCGATAAAGGGCAGGTAGATGAGAAACCCGATCTCGAAGGCGCGACTCAGC
TCGCTCACGGTAAAGGCGGGCAGCAGGATCAGCAGGCTGTCGGGCTCCAGCCGGTCGGCATATTTGCTGG
GCCAGAGCTGCTTGGTGCTGTCGATAAAGAAGGCGTGCTCCCGTGGTTTTATCTGCTTTTTCAAAAAGTC
GCGGTAGGGGGCAATCCCCTCATCGAGGAACTTCTCTACCGATTCGGATTTGGCCAGTGACACCTCGTTC
TGGCGCAGGTAATCCTGGGTGGCAAAGCCTACCGGCGCCATCACATAAATGCTGAGGATGATCGCCAGCC
CGTACATCGCCATGTTGGGGGGGATCTGCTGCACCCCGAGGGCGTTGCGCAGCAGCGAGAAGACCACCGC
CAGCTTGACGAAGGAGGTGGCCATCACGGCGATAAAGGGGAGCAGGGCCAGCAGCGCCAGCCCCACGATA
AGGTTTAGCTCGTCTGGCAACTGGATCATGACTGTGACTCGTTGTCGAGGCGTTCGACCCGTACACCCAG
GCGCCCCTGGATCTCCACCAGTCGGCCCACTCCCAGCGAATGGCCGTTGCTGATGATGCGCACTTCGCCA
TCGAGGGGCGTGCCAAGATCGATCAGGGCGCCCGGTTGCAGGCTGGTGAGGGTGTGCCAGTCCAGCGTCT
GGCGGCCCACTTCGAAGCTCACCTGGATCGGCAGTTGATCCAGTTTGGTCGGGCCGAAGGGTTCAGCGGA
GGCAGCATTATGCATGGCAGTCAGTTCCAGATGTTGTTGATGGGGTTGGCAGTAGGCAAAGGGGCGACCC
TCCACGCAGATCAGCAGTTGCTGACCGAGTTGTGGATTGGGTGGCGGCAGCAGCACATCGCCGATAGCGA
GGGTGGCGAGCTCATCCATGGTCAGCCCCATCTCGCCCCACTGCAGGGAGAGGGTGAGGGGCAGCGGCAA
CCGCTGCTGCCGTGGCCGTGGTGGCAACTTGCTCAGCAAGGGCTCGGGCTGCGACAGCCACAGAGCGAGA
CTGGCCCCTTCCCGTTGCAACAGCAGTTGCAGGGCTGGCTCATCGGTCTGAACGTTACTCTCCTGTCGGG
TCAGCGCCGAGCAGGTGATGCCACCGAGGGGTTGTCCCAGCCGTTCGATCAGTGCCAGTTGCAGCTGCTC
GGGCAGGCTGAACAGCGCCGCCTGTTGTAGATCCGGCGCGAGCCAGTACGCCAGCTCTGCGCCGTGGCAC
AACAGCGGCAAAGAGAGCCCCTGCCACTGCAGTTGCAGCCGGGTATCGAGTGTCGCAGGTGGCAAACCGA
TGGCGAGGGCCAGCTCGCCGCCACTCCAGCCAAAGTGCTGGCGGTATTGGCTCAGGGTCTGCTGTAAAGT
CAGCTCCGCCAGCGAGGCGGAGGTGAGGGCAATCGGTGTCATGCGTCGGCATCCCACTCTTCGTAGATGG
ATCGTTTATGGCGTGAGCCCTGCTCACTGTCGGGCTGGCCGAAGAGATCGAGGGCGACCCGCTCGCCGGG
ATAGAGGCGCTGCAGGCGGTCGACCAGATCGCTGCGCCCCTGATTGAGCAGCAGTTGCCCTTCGCTGGTC
GCTTGGATCTCGATCTGCAACTCGCCACCCTGATGGGCGATACGAATGGAGAGATCGCCCAGCTGTGGCA
ATGTCAGGCGCAGAAGCGGCGGACGCTCCGGGCGGCCCAGCTCCTGATAGATATCCACCGCCAGTCGCTC
CAGCAGCTGACCCAGTTCCCGATTACTTTCGGCAGGTGCCAGCCGTGCGAGCTGCAGATCCCCTGGGGTT
TTCACCTGTGGGGGGATCGGTTGACCACTGGCTTCGGGTATCTCGTCGTTCAGAGTGAGCGCATTGAAGT
CAGTGTCGCTGTTGGATACCTTTTGTTCGGCGAATTCGAAGACTTGGGCAAGAGAGGCTTCACCTTGTTG
AAGGGTGGCTTGATCTGAGCTCAGCGGCAGCGCCATACCGGCTGCGATCCCGATTGTTTGTAGATCATGT
TGTCGCGAGCCACTCTCTTTTTGCTCGGGGTGGTGCAATGGCTCTTTCATCAAAGAGGGTATCGCTGACA
GAGCGCTCTCTGGTGCCCAATCTCCAGCTCCCTCGAGGAGGGGGCGAGGGGCCGGTGTATTCAACATTGA
TGAAGGCACTGAACAGGCGTTTTCCGGGGCGGGTAGCGCCACATCTCTGGTTCCGCCCTCGTGCACGTTG
TCTTGTGCATCCCGCACTTGCTGCGATCTTTTGCTCTCGATTTCCTTCTGGTCACGTTCGGTCACCTCCG
GGAAGGTGGATAAACCGGATGCCTTTCTTGTGATGAGCTGTTGTGTCTGTAAATGCTTTGGCTGGCTGTG
AGAAGCAGCAATATCAGACTGCCGGACAGCATCGGCTGCCTGTTTTTTTTGCTGCTGCGTCTGTAATAGT
TGCTGAGGCCGAGGCTCCTGGGTCTTGCTCTGGCCATTCGTCCTGTGCTTGTCGGTTGCCTTTTGCTCCG

AATCAAGCCTCATCGCCTGCTCGAAACGCTGCTGTTGCGACCGATTGGCCTCAGCCATCTGGGTATCACC
CCGGGCTGTCACTGTGCCCTGTATCGGGAGGATTCGGTTCATGACTCGTTACATCCTGATCTGCTGGCGG
AACTCTTCCTGTTCCTGCTCTTCGTTGTAGTCACGCAGGGCTTGCTGTGAGGCCTGCTCCTGACGGTGTA
GTTCGGCAAATTTCTGTTGCTGCTGGCGATCGAGCTGCTCCGCCTGACAGATGGCAAATAGCCGCTCCTC
TTCGGCAAGGCGCCACTGGCGATAGTCGAGACTCTCCTGCTCCGCTTCGCGTTGGCGAACGCGGGCGAGT
TGCACCCGAGCCTGCTGGGCGGCGAGCTGCTGCTCGGCCCGATCCAGCCGCAGGTCTTTTATTCGTTGCA
GGCGGCCAATCATCGGGTCAGCGCCTCCAGAGTCGAGAGGGCTTCCGGCAGTGAGCTGGGTTCATGAGTG
CCTTGCCTGAGCCACTGGCGAATAGTCTCGATGCGGGCGATCGCCTCGTCGGCTACTTTGTCCTGCCCTT
TCTGGTATTCGCCGATCTGCAGCAACAGCTCCACCTCTTCATATTTCGCCAGCCAGCTGCGCAGTCGACC
TGCCTGAGCCTGATGCTCGGGCTCGATGATCTGGTTCATCACCCGGCTGGCGGAGCGCAGCACGTCGATG
GCCGGATAGTGGTTGGCCGCCGCCAGTTTGCGCGACAGTATGATGTGGCCATCGAGGATCGAGCGAGTCT
CGTCAGCAACCGGCTCTGTCATGTCATCCCCCTCTACCAGCACGGTATAGAGGGCAGTGATGGATCCCTT
GTCTGACTGACCGGCCCGCTCCATCAGGCGGGGCAGGGCGGCAAACACCGACGGCGGATAGCCCCGTCGG
GTTGGCGGTTCGCCAGCGGCCAGACCAATCTCCCGCTGGGCACGGGCAAAACGGGTGACGGAGTCCATCA
ACAGCAGCACCCGCTTGCCCTGATCGCGGAAATACTCGGCGATGGAGGTGGCGACAAAGCCCGCCTTGGC
CCGCTCCATGGCAGGGCGATCCGAAGTAGCCACCACCAGCACCGCCTTTTTGAGCCCCTCTTCCCCCAGA
TCCGCTTCGATAAATTCGCGCACCTCACGGCCCCGTTCACCGATCAGCGCCAGCACGGTGACATCTACCT
CGGCGCTGCGAATAAGCGACGCCAGCAGGGTGCTCTTGCCGCCACCTGCGGCCGCGAAGATCCCCATCCG
TTGCCCCTCACCGCAGGTGAGCAGGCCGTCGATGGCGCGAATACCGAGCGAAATGGGCTCGGCGATGCGC
TTGCGGCTCATGGGGGCCGGCGCATCCCGATAGACCGGATACCAGGCATCGGGCTCCGGCAGATGGCCAC
CGCCAAAGGGGTTACCGAGGCCATCGAGCACCTGACCGAGCAGATGATCGCCGACCCCGACCTGATGCAT
GGCACCGGTCGGGCTCACCTCGGTGTTGGAGGAGATGCCGAACATCTCGCCAAGGGGGGTGAGCAGCGCT
TGATGCTGGGCAAAGCCGATCACCTCCGCCTGCAGGGAGAGGGTGTTGTCCGGGTTGCGCAGATGGCAGA
GCTCGCCGATCCGCACCCCGGGGACGACTGCCTTGAGCAGGGTGCCGGTCACCTGGGTGACCCGGCCACG
GATCTGGATCAGGCGGCACTCGTCGATGGCGTGACGAAGCTGGTTGGGGATGTGGTCGAGGGAGAGGGTC
ATAACAATCTGCGCCATTGAAACTGGTGGCCACTATAGGGGATGGGGGGCTACGGGGATTTCAGCGCGAG
TCGGAACTTTTTAGAAAACCGTTGGGGGCGGGCTAAAAATTCACGGCCGATTTTGGCATAGGGAGGTGAG
GGCGATTTTTATAATGCCATCCTCGTCAATCCATTCGTCACGGGAGTTTCCATGGCCATTATCCAGAGCA
ACCCCTATGCCCCTGCCGCCCAGCCCGAGCTGCGCGGAGCAGAGGTCGCATCCCGTCAGGTACCCCTGCA
GGGTGGGCAATTTCAGGGGGAGCGGGTCGTGCTGGCCAGTGCCGCCCAGTCGCTGGCCGATGCCGCCGAA
GAGCTTACCTTTGTCTTTTCCGAGCGCGCTGAGCTTTCGCTCGCCAAGCGCAAGCTGAGTGACAGCCATG
CCCGCATCAGCGAGATCGAGGCGCTGGTGGGGGATTACCTCGACAAAGTGCCGGAGCTCGAACGCCAGCA
GAAGATCAAGGAGATGGTGAGCCACCTCGGCAGCGGTCGGCTGGCCAATCTCTCCCAGTTGCTGGCCTAC
CTCGAGAGCTATTCGGGGGAAGTGAGCGAGCAGTTCGAGGCGCTCTGCCAGGCGCGCAAGGCCCTCGCCG
GACGACCGGAGATGCAGGGCACCCTGGCGTTGGTGGAGCAGGCGCTGGCCAAGCTGGCGGACGAGCAGGG
CAGTGCCATCGTGCTGGGGGCGCGCATTACCGGCCCCGCGGCCGAAGCGGCCAAAGCGGGCGTGGGAGCC
GAGCAGGATCTACGGGATCTCTATCGCGATGCGGTGCAGGACTACCGTGCCCTTTCTGCCGCCTGGCGCG
ATATTCACAGCCGCTTTGGCAACGGTGCGCTGGAGAAGGTGACCGGCTTCATGATGAAGGCCCTGAGCGC
CGATCTCGACAGCCAGCAGCGTTGGCTCGATCCGGTCAAGCTGGAGCGGGTGATGAGCGACATGCACAAG
CTGCGGCTGCTCAATACTCTCTCGGCGCAAGTGGACGAGCTGTGGCAACGGGTCAAGGAGGGCTCGGCCC
ATGGCATACGGGCCTTCTGATCTGATGGGCGATCTGATCGCCCTGGTGGAGAAACGCTGGGCCAGCGTGC
GGGATGTGGAGCAGGTCGGTGTGGCTCTTGAGCTGTCAGATTGTCGCCAGCAGGTGCTGCTCTATCAAGA
GCTCAAGCGGCTGATCCGCCTGCTGCCGGTGGAGCTGTTCAGTGAGGAGGAGCAGCGCCAGAACCTGTTG
CAGTGCTGTCAGGGGGCGCTCGATAACGCCATCGAGCGGGAAGAGGATGAGTTGTCTGGAGAGTCGTCAT
GAACTGGATTGAACCCCTGCTGGTGCAGTTTTGCCAGGATCTGGGCATCACCATAGGGGATAACCCCCAT
TCGCTGATCCAGCTTGAACTGGAGCAGAGCGGCACTCTGCAACTGGAGCGCCATCAGGGGCAACTGACCC
TCTGGTTGGCCCGCGCCGTGCCCTGGCATCAGAGTGGCGAGGCGATCCGCCGCGCCATGACCTTGACTGC
CGCGGCGCAAGGGCCGGCACTGCCGGTGCGCAGCGGCTGGTTGGGGGAGGGTCAGCTTATCCTTTTCGTC
ACTCTCGATGAGCGGGCCGTGACTCTGCCCCAGCTCCATCAGGCAGTGACCACCCTGACACGGCTGCAGC
GAGAGGTGCTGGCGTCATGAGCCGGATCACTGCCGCGCATATCGGGATCGAGCAGCTCAGCGCCATCTCC
CTTGACGATCAGGAGCGCAGCCTGCCGGGGCGTTATGCCCTGTTGCCCGATGGCCAGTCCATCGAACCCC
ATATCAGCCGCCTCTACCCCGAGCGGCTGGCGGATCGGGCGCTGCTCGATTTCGCCACTCCGGATCGCGG
CTTTCACGATTTGTTGCGGCCGGTTGATTTCAATCAGGCGATGCAGGGGCTGCGCAGTGTGCTGGCAGAG
GGGCAGAGCCCAGAATTGCGAGCGGCCGCCGCGCTGCTCGAACAGATGCACACCGATGAACAACTGATGC
AGATGACCCTTCATCTGCTGCACAAGGTATGACCATGGTGCTTACGTCACAGCAGCAGGATGCGCTGCTG
CTCACCGGCTGGTTGCAACTGCAATATGGCCACCCCGACAAGGCGTGCGTGCTGCTGGCCGCCCTGCTGC
AGATCCACCCCGACCATCAGGGAGGGCGACGGACCCTGCTGGTGGCCCTGCTCAAACAGGGGGAGGGGGA
GGCTGCGCTGGCCCATGTCGATCAGCTGATGCAGCAAGGGGAGGCCGACGGCCCGCTCTGGCTCTGCCGC
AGCCGAGCCTGCCAGTTGGCAGGGCGGCTGGATGAAGCCCGTTTTGCCTATCAACAATACCTCGAACTGG
AAGAGCAGAATGAATCAACGCTCCCTTGAGCTGCTGCGCCGGATAGGCGAGCGCAAGGACATCATGCTGG
CGATCCTGCTGCTGGCCATCGTCTTTATGATGGTCTTGCCGCTGCCGCCGGTGGCCCTCGATATTCTGAT

TGCCATCAACATGACCATCTCGGTGGTGCTGTTGATGATGGCGGTTTATATCAATTCGCCGCTGCAGTTC
TCCGCCTTTCCGGCGGTGCTGCTCATCACCACCCTGTTCCGGCTTGCCTTGTCGGTGAGTACTACCCGGA
TGATCCTGCTGCAGGCCGATGCGGGGCAGATCGTCTACACCTTCGGCAACTTCGTGGTGGGGGGCAATCT
GGTGGTGGGGATCGTCATCTTCCTCATCATCACCATCGTCCAGTTTCTGGTGATCACCAAGGGCTCGGAG
CGGGTCGCCGAGGTGAGTGCCCGCTTTTCCCTCGATGCCATGCCGGGCAAGCAGATGAGTATCGATGGTG
ACATGCGCGCCGGGGTGATCGACGTGCACGAGGCACGGGATCGCCGCGGTGTCATCGAGAAGGAGAGCCA
GATGTTCGGCTCCATGGATGGCGCCATGAAGTTTGTGAAAGGGGACGCCATCGCGGGCCTCATCATCATC
TTCGTCAACATCCTCGGCGGTGTCACCATTGGGGTGACCCAGAAGGGGTTATCCGCCGCCGATGCGCTGC
AGCTCTACTCCATCCTGACGGTGGGTGATGGCATGGTCTCCCAGGTGCCGGCGCTGCTGATCGCCATCAC
CGCGGGCATTATCGTCACCCGGGTCTCCTCCGAAGAGTCTTCCGATCTGGGTACCGATATCGGCGCCCAG
GTGGTGGCCCAGCCCAAGGCGCTGCTGATCGGCGGTCTGCTGCTGGTGCTGTTTGGATTGATCCCGGGCT
TCCCGATGATCACCTTCTTTGCGCTGGCGGCCATCGTCACGGCGGGCGGTTACTTTATCGGCTTGCGTCA
GCGCAAGGCGCAGAGCAGCAACAGTCAGGATCTTCCTGCCGTGCTGGCGCAGGGGGCGGGAGCCCCCGCC
GCCCGCAGCAAGCCAAAACCGGGCAGCAAGCCTCGGGGCAAGCTGGGGGAGAAGGAGGAGTTCGCCATGA
CTGTGCCGCTTCTGATCGATGTGGATGCCGCTTTGCAGGCCGAGCTGGAGGCGATTGCCCTCAACGACGA
ACTGGTGCGGGTGCGCCGCGCCCTCTATCTCGATCTCGGGGTGCCCTTTCCGGGCATTCACCTGCGTTTC
AACGAGGGAATGGGGCCTGGCGAATACCTGATCCAGCTGCAGGAGGTGCCGGTCGCCCGCGGTCTGCTGC
GACCGGGCCACCAGCTGGTTCAGGAGAGTGCCTCCCAGCTCGATCTGCTGGGGATCCCCTACGAAGAGGG
GGCGCCGTTACTGCCGGGACAACCGACCTTGTGGGTCGCTAATGAACATCAGGATCGGCTGGAGAAGTCA
CGGCTGGCCAGCCTCACCACCGATCAGGTGGTGACCTGGCATCTATCCCATGTGCTGCGGGAATATGCCG
AGGACTTTATCGGCATTCAGGAGACCCGCTACCTGCTGGAGCAGATGGAGGGGGAGTTATGGCGAGCTGGT
GAAGGAGGCGCAACGCATCATTCCGCTGCAGCGAATGACCGAAATTTTGCAGCGGCTGGTGGGGGAGGAT
ATCTCCATCCGCAACATGCGCGCCATCCTAGAGGCGATGGTGGAGTGGGGCCAGAAAGAGAAGGATGTGG
TGCAGCTCACCGAGTACATCCGTAGCAGCCTCAAGCGCTACATCTGCTACAAGTACGCCAACGGCAACAA
CATCTTGCCTGCCTATCTGCTCGATCAGCAGGTGGAGGAGCAGATCCGTGGCGGCATTCGCCAGACCAGC
GCGGGCAGTTACTTGGCGCTCGATCCCACCATTACCCAGAGCTTCCTCGATCAGGTGCGCCATACCGTCG
GTGATCTGGCCCAGATGCAGAACAAACCGGTGCTCATCGTCTCCATGGATATCCGCCGCTATGTGCGCAA
GCTGATCGAGGGGGATTACCATGCCCTGCCGGTGATCTCCTATCAGGAGCTGACCCAGCAGATCAATATC
CAGCCCCTCGGGAGGGTCTGCCTGTGAGGGGGGACCCGTTAACCTCTGACCCCCTGATCCCCTGGCTGCA
AGCCAGGGGTGTGGCGGTGGTACCTCACCATCTGGGGGCAACCCCCATCCAGCTCGGCCACGCTTTCTGC
TATCGCCAAATTTATCTCGCCTGGCGCTTTGATCCTGCGACCCGACGGGTCTGGATCCTGCTGGTGCGCC
GAGAGGGGGAGCGGGCCGGACTGGCCAATCCCTTTGCAGCCCTTTATCTGCTGGCCGAAGCCACTCTGGC
TGTACTCGGGCCGGGCCATTACCTCTACGGCAACGTCGATGTCTTTCGTGGCAGTAGCCTGAGCAGTGAG
CGGCTCGGCCGCTTCTACCTGTGCTGGACGGGAGCTAGTGAACCCGAGCCCGGCTGGTTCATGCTGGCGG
CCGAGCAGGTCTGTTCCCTGCGAGATATGCGAAAACGACAAAAGCACGGCCTTGCGTGACAGGCATGTCC
AAAAGGGCCTCATAGAATAGGAGCCAAGATGAAACAACCGCGTTTTGCCGACCATAGCGAGACCATTTCG
CAGGCAGAGCAGGCGATTGCCGATAGCGATCATCGCAACGCTCTGTTGCAAGAGATGCTGGCTGGCCTCG
CTCTCTCGGATCAGACCTGTCAGCTGCTATTCGATGCGCCGACCGAGCAAGTGGCCGTGGCCGAGCAGGA
GTTGCTGGCTGAGATCCAGCGCAGACAGGCGTTACTGCCGGCACAGCCGGGAGAGGGCCGCAAGAGTCGC
CGTCCCACTATGATGCGCGGACTGATGATTTAAGGAGTCGTAATGAGCACTATCCCTGACTACAACACCA
ACCCGGGCGCGTTTGTCGGCTGGCTTGATGGGCAAGCGCTGGACGCATTGCCGGGCCATAAAAACCCCAA
GTTGACCGAACTGGTCGAGCTGCTCAAGGGCAAGATCACCATCAGTGCTGACCCGTCAACGGCGCTAAGC
AAGGAGCAGCTGGAGAAGTTGCTGGCTGCCTATCTGACGGATCCCGCCTCGATCAACGGGGGTTGGGCGA
TGGGCCAGTTCAAGGGAGGTCAAGATGCGGCCATTGCCGCCATCAAGGGGATGATCGAGCGGGGAGAAAA
ACAAACCCCGCCAGTCACCCACTGGACCATCCCTGAATTTATGCTGCTCTCCCTCAGTGCGCTGACCATG
GATCGCATCGATGACGATCTCATCACAACCTTTACCGGGGTGATGATGTTTCAGGACAATCAACGTAAAG
GGCTGCGGGATGAGCTGACAGAGATGAACGCTGAGCTGAAGATCTACGGGGTGATCCAGTCCGAGATCAA
CAAGGTGCTCTCTGCGGCGTCCAGCCAGACCTTCAACACCAATTTCAATCTGCTGGATTACAAGCTCTAT
GGCTATGAGTCTCTGGCCAAATTTATGGAAGGGGGCGAGTTCAAGCTGTTGTCAAAAATGTTTAGCGATA
AGCAGGTGAAAAAAGCACAGCAAGATTTCACCAATGCTAAAAATGAGCTGGAAAAAGTAATGTCGACCAG
CCTAAACCCCAAAATCCAGGCGGAAGCTAAGATCGATTATGAGCGTAAAAAAGCCAATTTTGAGAAGATC
GTAGCCACGCAGGTCATCACAGTTAAAACGTTCCTCGAAAGTGATCAAAAGAAGAGCGGCGCGATGAAAA
ATATCGAAGCCAGCTACAGCTATGACAAGGACAACAACAAGCTTGGCAACTTCTCCACCAGTGTGAGCGA
CCGTTCTCGCCCGCTCAACGATCAGGTCAGTGAAAAGACTACCCGCCTCAACGACGTCAGCTCGCGCTAC
AACGCCGCCATCGAGGCACTCAACCGCTTTATCCAGAAATACGACAGCATCATGCGCGACATTCTTGGCG
CAATTTGAGGAGAGATCATGCAGACCGACACCACCCTGACCCCGGAATATGAAGCAGAGCTGGAGGCCTT
TATAGCTGACGGTGGTACCCTGGCCATGCTGCAGGATATCTCTGGCGACACCTTGGAGCAGCTCTATGCC
CTGGCCTTTGGCCAGTATCAGGCCGGCAAGTGGGAAGATGCTCACAAAATATTCCAGGCTCTTTGCATGC
TGGATCACTACGAGCCACGCTATTTCCTCGGGCTGGGTGCCTGCCGCCAGGCGATGGGAGCGTTTGAAAC
GGCAGTTCAGAGTTACAGCTTTGGCGCCATGCTCGACTTAAAAGATCCTCGTTTCCCGTTTCATGCAGCC
GAGTGCCGGCTGCAACAAGGTGATTTGAACGGGGCTGAGAGTGGCTTCCACTCGGCCCGACTGCTGGCGG

ACACAGATCCCCGGCAGGCAGACTTGGCGGCAAGCGCCAAGGTCATGTTGGAAGCCATCGCAATCAGAAG
GGATCTGCAAGATGAACCCGATCAGTAATGAAAGACAGGTTTCCCCACTGGGAAACACAGGTGATATCGC
GGGCCTCATGCCCGTTACTCAACCTCACAGTAAAACACGGCTCGATGCCGCAACGGTGCCAGTTACCGGT
CAAGCTCGCAAGTCAGGGGTTTCGCTGAACGCACCTTTGGTGGTAAGCAATAGCCAGGTCAGTGCACAGC
AGCTTGGTGACATTGGCAAATTGGTGACCTCGGTGAACGAGAACTCCCGCTTGATTTTGCCGCACGTCAC
CGGGTTGGTGACCGCAGCCTTTACCTCGCCCGAAGCGTTCGAGATTGAGCTGGGCAAACTCACCAGCGAT
CTGGAGAAGACCCAGACCAAGCTGAAGCTTGATGATCTCCGGCAAGCTCGTACGTTGAATGAGAAGAAGA
TCGGCGAGAACGAGGCAAAGATTAAAGAGGCAGAAGAGAGTGCTCAGGAGGCCAAGAAGGCCGGTGTCTT
TAGCAAAGTTTTTGGTTGGCTCAGTGCCATTGCCTCGTTGGTGATCGGCGCCATCATGGTGGCAACCGGG
ATTGGTGCTGTCGCCGGCGCCCTGATGATCGCTTCCGGTGTGGCCGGAGTGGTCAATTCGGCCGTGCAGC
AGGCTGCAGCGGATGGCCTTATCTCCAAGGAAGTAATGGAGAAACTGGGGCCAGCCCTGATGGGGATAGA
GATTGCGTTTGCAGTGATGTCGGCCGTTGTCAGCTTAGGCCCGTTGGTTGGTCGCGCGATAATGAGTCTT
GCCAGCAAAATGGGTGGTAAGATCGCAGATGCCGCCGCTAAGATTGCTGCCAAAGCGGGTGAAATGACTG
CCAAAGCGGGTGAAATGGCTGCCAAGTTTGGTGCTAAAGCGACCGTCGAGGCGGTGAAGCTAACAGGCCA
GACAGCAAAAATCGCAACTGTGGCCACTCAGACCACTGATATAATTATTGATGTGAGCAGTGGCGCGGCT
AAATCGGCCCAGTCTGCGAAAGAGTCCGAGTCTCTGTATGCTCAGGCTGATCTTGCCATGAGCAAAAGTG
AGATGACCGAACTGCAAGGGGTCATCGACAAGCTCAAGGAGGCGATAGCCCAGATGATCGAGGCGTTCCA
GCAGACTATGGAGATGATCTTCCAGTTGATCAATGCCAAGGGCGACATGATGAACAACTTGGCCCGCAGA
CCGGCCGCTATCTAAGGAGTATCGACCATGATTAACAGTGATTATACAAATGACGTGACCCGTACCACCG
CGATGACCTCGGTAGATTTGGATGGGTCTCGCCAAGTTCGCAGTGCCGCCACTGCTCATGAGGCTGCCGC
TTCGACCCGCCCGCAAGGGTTAGAGAACAAGAGTGTTCCCGCCCTGCCACGCCCCTCAATCCCTTTCAAT
ACCAAAGGGGTGACCCAGATGGTTGCACAGTTGGACAGTGCGATGGAGATTATGTCCTTGCTGTTCAAGC
TGGCCCGTCAGGCCCGCGAAGAGGGGCTACAGGTTCGGGATATCGAAAACAAGCTGGTCATCAGCCACCA
GCAGGCGCAAGTGGAAGAAATGCGCTATGGTGCCAAGCTGATGCTCGTCATGGCGGTTGTCTCCGGGGTG
ATGGCTGGGTTATCTGCTGTTGCTGGCGGGTTTAGCTTAGCACAAAGTGCTAAGACCATTAAGCAGCAAA
AATCTCTTGACGTCAAGATTGCCGGTCGAGAGGCCTTGACTGATGCCAAGGTTGACAGCTTGAACTCGCG
AGGCCAGCAGGTTGACCGTGCTGAGGCGGGCAAGGTTTGGAAACAGGATCAAGTTGCAGACAGTAATGCG
AGCCGGTTGTTGGACAAGAAATTTGCTCGTAATACCGAGCGAACACAGGTATTTAATACCGTACTCCAAG
CTGTGGGGCAAACAGTTAACGCTGCGATACAGGCAGCTCAGGCCGAAAGTCAGGCTGAAGTCAAGGAATA
CGAGGTTGAGGCGACCATCAGCCAGACCGAGAAGCAGAAGGCCGAGGACAATATGTCCTTCAATGCTAAC
TTTATGAAAGAGTTGCTGCAATTGATGCAGCAGTATTCCCAGAGTCAGAATCAGGCTTTGAAAGCGGCAT
TCGGCGTTGCATAAACGCCATGGTAATCAAGGCGCTCATTCGAGCGCCTTTTTGTTTTTCCGGCCATTTT
TCTTAAAAGTACCGTCCTTGAGTGATATCCCGGATGGCGGTGGCTGGCTAACATGATTTCCTCTCTGCTG
GAGGAAATCATGGATGTAACTGTCATCATCAATCGACTGCTCGCTGAATTTGCCACCAAATATGGTTTGC
CATCCCTAGCTCTGAATCAGGAGGGGATTGCGGCGCTCTGTTTTGACGAGCAGTTGCAACTCAGCCTGAT
CTTAGTATCTGAGCGGGCTCAGCTGGTGTTGCAGGTTGACGTAGCCGAACTGCATCAGGTGGGCGAGGGG
ATTTTTCGTCAGTTAGCCAGCTTCAATCGTCACTGGTATCAGTTCAATCTTCACTTCGGTTTCGATGAAG
AGAGTCTGACGGTGCAACTCTATCGCCAGATGGCGGCAAGCCGGTTGACTCTGGCGTACCTTGAAGAGAG
TCTCGCCAGCATGCTGGAGCATGCCGAGTTCTGGCAGGAGTTGCTGCAACACGGTACTCGGGAGGCTGGC
AGCGGCGATCAGGTGATGGGAGTGCGGGTATAACCATGAAGATTCAGGAATCACAAGGCGCGCTGGCCCT
GCATGGGGTAGAGGCGGGCAAGGTAGGGGCATTTGCAGGCAGAACTGTGAGTGCCCGGTCAGCCAATGCG
AGTCAGGTTCCCCTGTCGGCACTGCTGCGACGCTCTATTACCCTCAATCAGGTCCAGGAGCTGGCGCTGC
AGCGACTGCAGCAGGGTGAGCACACACCGCTGAGTGAACGGCGGATCCGGGTGTTATGAGAGGTCTGCAG
TTGCTGATGTTGTCGGCCATGCTGACCGGTTGTGTCACCACCCCGGAGCTGAAGCCATCGCAACAGCAAC
TCAAGGGTGAGGTGCATTTCCCGCAAGCCTTGCCACGTCCGGCGACAGTGGAGGTGGTGGTGCTCAGCGT
GATCGGGGGGCGTCCGTTGCAAGTGGCTGCCACCCGTTATGAGGTGAATATGCTGCCATTGCTGTTTGAT
CTGCGTTTGACCCCTCTGCAACTGGCGGAAGGGGAGATCTATCTGCGTGCCCGTTTACGATTTATGGATG
GCACTGCGGTACAGGCTATGAGTCAACAAAACGTTTTTAAGATTTTTAATGACAAAAAAATGGTTATTCA
ATTGCAGCCAAAAGCTTGTTATCCACAATGTCAGTAGTGATTGTGTTTAAATAAAAAGCCTCAAATCAAT
CGTTTATTAATTTGTCAGTTAAGATTGAAGGTTTCTTTTTGTAACGAAGTATGTTTTTTTGTAAACACAC
CATTAAGTATGAGTCGTTGTACTGGATAAATAGTGTGTTAGCTCTAATTTAGGCTGGTTTTTCTGCTTG
TGACTGTTGTAGGATCCCCCCTCATTTCAATGGTGATTAATTTTTGAATTCACCAGCGAATGACTTGATT
ACAAAATAATAAATAAAAATAACAGCAATAATAAAGCACCCGAGAGCGACCCGCTTCCGGAGGTGTTACT
GGTACTACACGCAGATTCAGGAAAATAAAACATGAAAGGCATTACTACCACAGAGAAGGGCGATATGGCC
CTGCTCCAGTGGCGCATGTCTGCTTTTAACGTTATCGAACATCCGCAAGAGGGAATTTATATTCTCCTGG
AAGGATCGATGAAGTGGCAGGACTGTACCGATACCTATGAATTATCCCCCAACGAACTTCTGTTCGTGCG
CCGTGGCAACTACGCGGTCTGTACCGATGACGGCCCCTGCCGCCTGCTTTGGTTACCTCTGTCAAACTCC
TTTTTACAAGGCTTCTTGCAACGTTTCGGTTCACTATTGAGTGAAGTCCCCCGGCTGGAGGGGATGGCCC
CTACGCTGTTGCCGTTTCACTCATCCCCCTTGCTGACCCAATGTATTCAGGGGCTGCGCGGGTTGACCGA
ACATGAACATCCCCCCGCACTGGCTCAATTACGCACCGAAGAGTTGCTGTTTCTGCTCGCGTTTGGCGAA
CAGGGGCCTCAACTGATGTCTATTTTGCGCCAGCTGAGCAATCGCCAGGTCGAGCGGTTGCAACAGTTTA

TGGAAAAGCACTATCTGATGGAGTGGAAGCTCAGCGAATTCGCCAAAGAGTTCGGTATGGGATTGACCAC
TTTCAAGGAGCTGTTTGGCTCGATATATGGTGTTTCTCCACGCGCCTGGATCAGTGAAAGGCGGATCCTC
TTCGCTCATCAGTTATTACTCAATAGTGAAATGAGCATCGTCGATATTGCGATGGAGGCCGGTTTCTCCA
GCCAATCCTATTTTACCCAGAGTTATCGTCGTCGTTTTGGCTGTACGCCAAGCCGTGCCCGACACGGCAC
AGATTAACGCCGGACTAAAATAATCTGACGTTTTTTATAAGCCGCGGAGCGAGGCAATGGATAGAATAGC
CAGCATATCGAAACATCATTCCATTATTGCCCGCAGCAAGATGAGCAAGGCATAGCATGAGTCAGCAAGA
TCACCATTCACCTAACCAGGGCCTGTTTGCAGGTCGTCGGGTTACCGTTGTCCAGCCAGATACCCTGAGT
CGGGATCGTCTGGTCGGCCAGCTGTCCGTGCTCCGTTATCAGGATGCCGGCGTGATCACTTCGCAGCAGA
TGGCCCTGTTGCAACGACTGTTGCCAAGAACCCGACTGGAGAGCCTGTTGGGCTCCATCTGGTTTCAGCG
CCGTCTCGATGCCGCGCTGGCCGTCTCGCGTGAGGAGCTGCAACAGATCTTGCGTCTGGCAGGCAGTGAG
CGTTGTGACTGGATGCAACAGCTGGGGGATCGCATCAATCTGGCGGATCGTCCCTTGCTGTGGCACTGGG
TGCTCTATCCCTTGCACCGCTGGTGGGTTCAGCGTCTTGAACCACTTTACGGCGCCTGGCTTAACGAACT
GGAGCAGTTGCAGGTGATGCGCCGCCAGCTCAATGCCCAGGCCGTGTTCTGGCAGACCGTGGTCGATGTA
CCGGCGGATCTGGAGAGCCGGATCACCGATCAGCTTGAGCAGTTGAACCTGCGCGAGCAGGAGCTCACCC
GGCTGCAGACTGATTGTGAGACACGTCTCCAGCTGGCCTGGCCTGCATGGTATGCACAAACGAGCAAGGA
GGGAGACCCGGTTCACCTGATGCCGGTGCCGCTTGAGCTCGGCACATTCTGGCATGCCCTGCAGGCTCTG
CCCCATCAGGACGAGGCAGCCCTAACCCTGCATGAGTGGCTGGCCGGCCGGGGCATTGCCCTGGGTCAGG
ATCACTTCTACTGGCAGCCTCCTGCGCCCTAGATAGCCCGAGGAACTATGCAAACGCTACTGAATCGATT
GGCCGCAAACCTTGGCCAAGGGCCTTTTATTGCCGACGCACAGGGAAAGTACCATCTGCGGATCGATGGT
TATCCCCTCTTTCTGACCCCCAGAAACGGTGAGTTGCTGCTGAGTACCCCGCTTGTAAGCAGTCAGAGGA
ATGATGCTGAGGCCCATGATCCGGCGCTGCTAAAGCGTCTGTTGCAACAGGTGGTGGCCTGGGGGCGTCA
TGCCCCCCATGCCCTGGTGCTGGATGATCTTGGCAATTTGCAGCTGGAGGCGCGGCTCTCGCTGGAGTGG
CTCGACGAGGCGCTGCTGGCGGAGCGCCTGAGCCAGCACATCGCCCTGCTGGAGCGGTTGGAACCCCAAT
TGACCGAGGCCGCCTTGGCCCCCCAATGGAGACAGATGATATGGCACCCTTGAGTGTGCAACCCCTGCTG
CGTCCCCTGTTGTGGATCTGCCTCGCCGTGATGGCGTGGCAGCGTCCCGCCATGGCACAGGAGCTGGACT
GGGTGCCCATGCCTTACAGCTATGTGGCTGAAGGGGAGAGTCTGCGCGACGTGCTGGTCAACTTCGGTGC
CAACTACGAGGTCTCGGTGGTGGTTAGCGACAAGGTCAACGATCAGGTCAACGGTCAGTTTGAGCATGAC
GAGCCGCTCGCCTTTTTGCAGCAGCTCTCCTCCCTCTACAACCTGGTCTGGTACTACGACGGCAATGTGC
TCTACGTCTTCAAGAACAGCGAAGTGCAGTCCCGTCTCATCAATCTGGAGCAGACTGGCGCAGCAGAACT
CAAGCAGGCGCTGCAGCGAGCCGGGATCTGGGAGCCCCGCTTTGGTTGGCGCCCCGATGCGGATAACCGG
TTGGTCTATGTCTCCGGCCCGCCCCGTTACCTTGAGCTGATCGAGCAGACCGCGCTGGCACTGGAGCAAC
AATCCCAGCTGCGCAGCGAGAAGACCGGGCCGCTGGCCATCGAGATCTTCCCCCTCAAGTACGCCTCGGC
GACTGACCGCTCCATCCAGTACCGTGATACCGATGTGGCGGCGCCCGGTGTGGCGACCATTCTGGCCAAG
CTGCTGAGCGATGCTGGCGTGCAGATGGTCAACGGCGAGGGTAGCAAGGCAGCGACGGCCACCTCCGGTC
AGGCCATCGTGCAGGCTGATCCTTCCCTCAATGCGGTGATCGTGCGCGATGCTCCGGAGCGGATGGCGAT
GTACGGCAAGCTGATCGCCGCGCTCGACAAACCGGCGGCCCGCATTGAGGTGGCGCTCTCCATTATTGAC
ATCAATGCCGAGGATCTCTCCCAGCTGGGGGTGGATTGGCGAGTGGGGATCCGCACCGGTTCCAACCAGC
AGGTGATTATCAAGACCACCGGCGATCGCAAAGGAATTGAAGGGGGGGCCGCGCTCGGCAGTTTGGTGGA
TTCGAAAGGACTCGACTATCTGCTGGCCCGGGTCAACTTGCTGGAAAACGAAGGCAATGCCCAGGTGGTC
TCCCGTCCGACCTTGCTGACCCAGGAGAATACCCAGGCGGTGATCGACCACAGCGAGACTTATTACGTGA
AGATCAGCGGCGAGCGGGTGGCCGAGCTGCAGGGGATCACCTACGGCACCATGTTGCGGATGACCCCTCG
GGTGATCCAGATGGGTGACAGACCCGAGATCAGCCTGAGCCTCCACATCGAGGATGGCAACCAGAAACCC
AACAGTGCGGGGGCCGATGGCATTCCCACCATCAGTCGCACCGTGGTCGATACCATCGCCCGGGTCGGCC
ACGGCCAGAGCCTGCTGATTGGCGGTATCTACCGCGACGAGATGAGCGAGAATCTGAGCAAGGTGCCCTT
CCTTGGTGACATCCCTTATCTGGGGGCGCTGTTCCGTAGCAAGGACACCCAGACCCGTCGCTCGGTGCGG
CTCTTTATCATTGAGCCTCGTCTGATTGATGACGGTATTGCCCACTATCTGGCCCTCGGCAATGGTCAGG
ATCTGCGAGCCGGGGTGCTCGGGGCTGATGAGATCTCCAATCAGAGTCTCTCCCTTGGCAAGGTGCTGGG
AGGCGCGCAATGCCAGCCGCTGGCTGCAGCCCGTGACGTGCAGCAACTGCTGCGTCAGGGGGGCAAGAGC
TCGTCTCTCACTCAGTGCCGGATGGGCAATGAGCTGGGGTGGCGTCTGGTCGAAATGAACAAGGATCAAA
AATGTACACCGGCCGCCAGCTGGTGCGTGCAGGCGGGCAATGAGGGCGCGACCCGATGAGCTGGAAGTGC
CGCGTATATCGCGGGCTCAACCGCGGGGTAGAAGTTCCTCTGCCAGAGGGGCGTCTGGTCATCGGTTCCG
ATCCCCTCAAGGCTGATCTGGTGCTGGTGGATGAGGGGATGGCCCCCGAGCATCTGGTACTGCTGGTGAA
TGGGGAAGGGATCACCCTGCAAGCCTGGGCGGACGGTATTACCCCGACTCAGGACGGTGTCGCCCTGACC
GCGGGAGAGACGCTCAGGGCGGGGACGCGACTGGAGGCAGGCCCGCTGCTCTGGAGCTTCTGTGACAACA
GTCGTTCGCTACCCGAACAGCTTGAGGCTCTGGCGGCGGTGGTTGCTCCTTCTCGTGCGCCGCGGCGGCG
GACAGCGGGCGCCGATGTCTGGATGGGGGCCCTCTGCCTGAGTCTGGTGGTCGCCGTGGTCTTGCTGCTC
GGCACGGGCTGGTGGCATGGCGCCGACGAGAACGAATCCGCCCGCAGTGAGCAGGCATTGAAGCGCTTTC
TGGCCGCACCAAACTACCGTCAGGTGGTGCTGAACAGCAGTGATCCCGAGCTGTGGCAACTCTCGGGTTA
CGTCGATGAGAACAGCGCGCGTCTTGCCTTGCAGCAGTACCTCGACAGCAAGGGTTTCAGCTACCGGCTG
GAGCTGCGCACCATGGAAGATTTGCGGCAGGGTGCCGGCTTTATCTTGCAGAAGCTGGGTTATGAACAGC
TGCAGATCCGCAATGGCAAGGAGCCCGGCTGGCTCAGGCTCAGCGGTGAAATTCAGGGGCAGGATGGCAA

GTGGAGCACTATTGAATCTCTGCTCAAGCAGGAGGTGCCCGGTCTGCTGGGGGTTGAAAATCAGGTACAG
ATTGCCGGGGCTTATCGCAAGCGCCTCGATGCCTTGCTACAGGAGCAGGGGCTGGCTGGTGCATTGCGGG
TGCGTGAGGCGGGTGGGCGACTCGAACTCTCCGGCCAGCTGGATGAGCGCCAACTTGGCCAGTTTCAGCA
GGTCAACCAGCAGTTTCGCCGGGAGTTTGGTACACACCCGACGCTGGAGCTGATCAATCAGACCAGAACG
CCGCGTCAGGATGAGCTGGAGTTCGATGTGCGCAGCGTCTCGTTTGGCCGGGTGCCTTATGTGGTACTGG
CGGACAACCAGCGCTATCCGGTCGGGGGAGCAACCTCCAGCGGCGTGCGGGTGCTGGCCATTCGCCCCGA
TGCGGTGGTGGTGAGCAAGGGTAAGCAGCAATACATCGTTAAATTAAAAGGAGCCGAGCGGCATGATGAC
CAGTTTGGAAGCGCGACTGTCCGGCGCTGATCCCGCCTTTGCCCGTGAGCTGCATGAGCAGCTTGTTCAG
GCGCAGGGTGACGTGAAACGGCAACTGCTGCGCGGGGGAACCCCGCAACAGTATCGGGAGTGGCAACAAC
AGGCCGACGCCATCGAGGCGGGGCTCACGATCATTGGCAAGATAAAGGAGCACAACCATGGCTGATTACT
TTAAGTATGCGGACGGTACTACTAACACCCTCGATCAGGTGGCATCGAACCTCTCCGGGCAGGCCAATGA
CGCCAATACCGAGGTCAACAAGGCCATTGAGGCGATGAAAACCAATCCGGATAACCCGGCGCTGTTGGCC
GAGCTGCAGCACAAGATCAACAAATGGTCGGTGATCTACAACATCAACTCCACCGTGACTCGGGCCATGA
AAGACCTGATGCAGGGTATTTTGCAGAAGATTTGATGATGAACCCTCAACTCAAACGACAGCTGGCCGAG
CTTGCGCTGGCTGGTACCGGCCACCATTGCCATCAGGAGGCGGCTACTATTGCCGACTGGCTGGCTGGTG
CGCCGGAGATGACCGAGTGTGTAACGCTCATTCGCCTCTCCTCCCTGATGAACCGGGGGGATTACCAGGC
CGCTTTGCAGCTTGGTGGCGAGCACTGCACATCGGATATCGAGCCCTGGCTGGCCCTGTGCGAGTGGCGG
CTTGGTCAGCAGGAGGCGCTGGCTGCACGGTTGCTGCGACTGGAGCAGAGCGGTCAGCCAACCTTGCAAC
AGTTTGCCGCCGGCCTGCGTGAGCAGATGGCCTCATGAAAATTGAAGGCAGCGATCAGCTGGGGGGAGAG
CAGCCGCTGCGTCAGCCCCCCCCGCCCGAATCCATGGCTCAACGCCAGTTCGAGCGACTGCTGGCCAAGA
CTCCCGAGCCGGATCTGTTCGAACGCTGGCAACAGGGGGCCCCCCTTGAGGGTTTGCTGGCCAGTGTGGA
GCCCGCCGCCAAGCGTGAACTGCTCTGGCAGATCTACCAACAGGGCGACAAATCGGCGCCGGAGATTGGC
AAGCAACTGTTTGTCCCGGTCACTGACAAGTTGATCGCGCGTTTTGGCGAGCGTCAGTCGCCGGTGGTGG
ATGCCATTGATCTGCCGGAGTTGCGGGCCACCATGCGGGAGTTTGATCCCTTGGCATCGCGGCGGGAGAA
GGTGTTGCTGAACTTGTTGAGCGAGCTCAGGGACGGGCAGGGCGCTGTGCCTGCGGGACATCAATTTCTC
GATGCACTGGCCCGGCGCGAGCTGATGACCCTGATCCCGCTCAACGGCATGGTAGACAATCTGATGCGCA
ATTCGCACAAACTTGATCTGGAGGCGTGATGGAAATTACACAGTCTGCCCAGGCGGTGGTCACCAGCCTG
GATGAGTTGAAAGCGGTGGATCTCTCCTCGCCCCATATCGCCAGTTTTGAACAGGCGATGGGGAGTACAT
CCCAGGGGATGGGGAGCTCCCTGCTCGGGGAGCTGGGGGAGTTGAAGCAGCAGTTTGTCGAGGCCAAACA
GAACCTGCAAGCCGAGCTGGCCACCCCGGGGGATGATCCCAACAGCCTGATGCAGATGCAGTGGTCGCTG
ATGCGGATCACCATGCAGGAGGAGCTGATTGCCAAGACAGTGGGCAAGATGAGCCAGAACGTAGAAACCC
TGTTGAAGACCCAGTGAGGTAGTGTGAAGAGATCGATGCTGATGGGGGTGCTTGGCGCCTCGTTGCTGCT
CAGTGGCTGCAAAGTGGAGCTCTATACCGGGGTCACTCAGAAAGAGGGCAACGAGATGCTGGCGTTGCTG
CAGACCGAGGGGATCGCCTCGGACAAGCAGCCGGACAAGGATGGCCGCATCAAGCTGCTGGTGGAGGAGT
CTGACGTGGCGCAGGCCATCGAGACCCTCAAACGCAAGGGCTATCCAAAAGAGAGCTTCTCCACTCTGCA
GGATGTGTTTCCCAAGGATGGCCTCATCTCCTCGCCCACCGAGGAGCGGGCTCGCCTCACCTATGCCAAG
GCGCAGGAGATCTCCAATACTCTGTCGGAAATAGACGGCGTGCTGGTGGCTCGTGTCCATGTGGTGCTGC
CGGAGGGCAATGAGGGGATCAGCAAGAAACCCTCTCCCGCATCGGCCTCGGTCTTTATCAAACATGCGGC
GGATATCCAGCTCGATACTTACATTCCGCAGATCAAACAGCTGGTCAACAATAGTATCGAGGGGCTCGCC
TACGACCGGATCAGCGTGGTGCTGGTGCCCTCGGCGGATGTGCGCCAGAGCCCGCTGATGCCGCGCAATA
CCTCGCTCTTCTCCATTGAAGTGAGCGAAGGTTCAAAGTGGCGGCTTGGCGGCCTGATTGCCCTGTTGCT
GGCGCTGGTAGTGACCAGCAACCTGGCGCAATACATGTGGCACCGGAGCCGGAGTCGCGGATGACGGTCG
GTTTGACCCCCTATCAGCTGCGCTTTTGCCCGGCCAATTACCTCCATGAGGCGCACCTGCCCTCATCCTG
GGCGCAGGTGCCGGAACTCTTGCCAGAGTGGCGGACGCTGCCAGCCAGTAACGGCGCCTTGCTCGAGGCG
CTGGCATTGGGAATTGGCTACGAGATGCCCGCGCCTCTGGGGGGATTGGCGCTCTTTCCCCAAAGCGAAC
TGGTGTTGTTGTTGCGCCGTCTGGGGGCCGTGCTGCACGGAGAGGCGATCCGCCACTGCTTGCTGGCACC
AACGCTGCGCCAGATCATCGCCCTGCTGGGGGAGGAGGGGCACAGAGCCGTACTCTCCCAGCTCGACTTG
TTGATCGGCCCCTGGCCTGCGGGTTGGCAGCAGCCTCTGCCCGCTCAGCTCGATGAGGGGGTGCTGGAAG
AGGCGGGCTTGCAGTTCTGGCTGGCGGCTGCAGGGGAAGGCTACCCCGACTGGGCTCGCCGTCTCACCCT
GCGACTTCCCCTAGCGCCACCTGGGCCATCATGGCCTTTGGCGGCTGACCAGCTGCCACTGGCCCGGGCG
CTTTGTCTCAAGATTGCCAAACAGGTAACCCCCCAATGCTGCCATTTGTTGAAATAAAGAGTAACCATCT
GCAACTGGCGCCAAGCCAGCGTATTTTGCGTAGCCAGGATTACCTCTCCTACCTGAGCGCACAGGAGTTG
GTAGAGGCCGCTCGGGCCCAGGCGGCCGATATCGTGGCCGAGGCCCGCGCCGTCTATGAAGAGCAGAAAA
GCCTTGGCTGGCAAGCCGGGATTGACGAGGCTCGGCGCGAACAGGCAGTGCTGATCCACCAGACCCAGTT
GCAGTGCCAGGGGTACTATCGCACCGTGGAGCAGCAGATGAGCGAGGTGGTGCTGCAGGCGGTGCGCAAG
ATCCTGCACGATTACGATCAGGTGGCGCTCACCCTCAAGGTGGTGCGGGAGGCGCTCTCGCTGGTGAGCA
ACCAGAAGCAGGTGACGATACGGGTTCATCCCGAGCAGGTGCAGGCGGTGCGCGACCAGATTGCCCGGGT
GCACAAGGATTTCCCCGAAATCGGCTATCTCGAGATCTCCGCCGATGCCCGCCTCGATCAGGGGGGCTGC
ATTCTCGAGACCGAGGTGGGGATCATCGACGCCAGCCTCGATGGTCAGCTGGAGGCGCTCTCCCTCGCCA
TGAGCTCGATGCTGGAAAAGCGCGGTGAACAGCGAGCAGGTGCAGGCGGTGCGCGACCAGATTGCCCGGG
TGCACAAGGATTTCCCCGAAATCGGCTATCTCGAGATCTCCGCCGATGCCCGCCTCGATCAGGGGGGCTG

CATTCTCGAGACCGAGGTGGGGATCATCGACGCCAGCCTCGATGGTCAGCTGGAGGCGCTCTCCCTCGCC
ATGAGCTCGATGCTGGAAAAGCGCGGTGAACAGCAGGCTCGACTAGCCGGGGCGAGTGTCGCTGTTGAAT
AAGGCGGCTGAATATATGGCCGGCTACGTGAGAGAACATTTATCTGACAAGTCCCGCTGGTTTTTATTTA
GCGATACCTATCTGTGCACCCTACTCATCCATGGCGCCCGCGCCGTGCTGCGGGTGGTGAAGAGCAAGGC
ACAACACGCAGGTAGTTGGCTGGAGGGGGTCATGGCGCGGCGCAATCATAATGTGGCGTCGATAGCACAG
GCCAACAAGACGGCCCGTATCGTCTGGACTCTACTAGCCAATGATCGAGAATTTGATGCGCAATACGGCA
CAGGTACAATCCTGGCGGCGTAATGACAAGAGATATAGCAGAAGTAGAAAAAGAACCACCGATTGCACAG
GCAATCATGACGTGATGGCGAGACAGGTCAGACCGTGACTGGCAAAACCCGAGGTGTTCAAAGCACTTTC
GAGAGCGACAAACTGATAGAGGCGCCAGTCAGCGAATTCCATCAGGGACAGAGGCAGTAGCCTCGATCAC
AGATCCGGATCTATGACTGCAATCTCAACCTAACTCACCGCCATCGATTGAAGACTTGGCAAACTGGGGC
GACCATCTATGAATACCCTTGGCCTACCTGTCAGAAAGCCCCGAGTGTAGAGGTCACTTGGGGCTGGGGA
AGTCATGACTTGCTGGTTGATTTGGGCAACAACGCCCCAGACAACTCTTCGAAGACCAAGTAATTATAAA
AAATTGCGTCCATACTTAATATCTTGCGATTCTCTCACCTTTATAATTCCTTTCACATAAATGATGACCA
TCATAAAAACACTAAGGAAGAACACAATGGATATTTATCAAGAAAGTATCAGCTTTTTAGGTAACGGCGC
AGACTTTGATGAAAACGGATTGTTTCAATGTGAGTTGTCACCGGATAAGAATAACCATGATGACGCACTT
TTTTTAATAATATTTAAAAATGAAACAACCATTAACTTACACATGTCTCTCACCAGTCCAGTGGAGTTAC
CAACCCCTCTTCCAGAAACGATTGCCATTGCGATCGGTGAACATGCATTGGAACCTTTCCGCGGTGGTTT
TGGTATCGGTCTAATGCCTGATAGTCGAAGATTAACGATCTACAAGGTTATATCTCTCTCAAACAAACCT
CGTGATTATGTGCAGAACACCTTTCAGCAGCTGTTAGAAAGAATGGAGCAATGGCACAATTTTATTGAGA
AAGCGGATAGCGCGGAGACAATCCCAGAAAAATTATCTGCTGGGATCTTTATTTAACTTCTACTTCATCT
CTTATTTAAAACACAAGGTGAATATATGTCTATTATTCAAATCAATAGCCAACACCGAGGTAATTTAGAC
CTCGCAGATATTCAAAACATCAAAAGCAAAACAAAAGAAGGCGATATAGTTAAATTTGGTTCTGTATTCG
GTAAAGAGTACCGCGTAACTAAAAATAGTAATGGTGATGTTACTCTAAAACAAAAAGAAAATCGCAGCTT
TTTTAATCGCTTCTTTGGTAAAACCGATGCTTCAAAAAACAGTGATTTAAAGCTGAATCAGATGAATCAG
CAACTACATAAAAAAGAAGGCAGCGGCAACGTTAAGGTCTTAACTTTGACATACAACCAAGCAAACCAAA
AGATGCCGGAAGACACAAAGAACTACTTCCAGAGCCTGATTCAAAAAGGCGACTATGATGTGGTCTTGTT
TGCGGAACAAGAATCTAAATTGCTCGCGAATGATCTTGAGCTAGATGGCATGAACCTTCTGAGTCAAAAC
AAAATGAAAGTTATGACAAAAGGTCTTGGTGAAGGTATTAGTTACACTTCAATGTCTATTTTTGCAAAAG
ATGGCGTCGATATTAAAGTCATAAGAGAGTCTAAATACCGTCACGGTCTCGGTGGTCAAAACATGGAGTT
TTTCATGGGTATCACTGGTAACAAGGGGGGTGTAAAAACAGCTTTGGAGATCAATGATCAAGCCTTAACC
GTCATTTCTGCACACTTGGACTCTAACAAAGAAGTCAAACGCGAATTCGAAGGTAACAAGTTAATGGAAG
GGATCAATCCGAAAGAAGAGGTTCTGATCACTGGTGACCTAAATGAGCGTGAAAAACGTATCGCTGAAGG
TTCTGAAGTCATGTACGATCCTGTGGCACACGATGATACGCACCTAGCCAAGCATGGTTTTAAGTTCAAT
CAACTTAATAGCCACACCTACATGCAACTTGATAAGCACACCGGTAACATTAAACAGAAAGAAGGTCGCG
ACCGCCCTGACTTCGGTGAGCTCGATAATACAGGCTTAACCAACAAAACAGGCAACTTACAAAACCATGA
AACCAGTGTTATCACTGAAGGGTTTGAGAATATCAGCGACCATAAGCCAGTACAGTCTACTTTTGAAGTC
AGAAGCTTTTCTCAAAAGCTGATTGAAAACGCACAAACACATACAGCAGCAAACTTCAAAAATGAAGCTG
CATACTTAAATCCTGGTACTGATCCGGCAAAAGCGACGTTTGATGATGTGACCTTTGCTAACCAAGCTCG
TCTTGGTCTAGAAAAGTTGAGTAGCGATGAACAAGCATTTGTAAAAGAGAACTTCGCTAGATTTATCATC
GGTAAAGAAGCAATCTTTAGCCAGCTAACAAATGGCTTTATGGAAGAAATGGGGAAGCTTCACATTGCTA
ACCTGGCCACTGATCAACTACAAGCTCAACAAATTGCATTGAGCGAGAAATACGAACAGCTTAGCGATAA
AGTAAATGCTCAATTTAACCAGCAGTTCGTTGCCAATATTTAAGTGCAAGAGAGACTCAACCAGAGCACT
TATTTGTGAATGTAGTGATCAACTAAATCCAGACATTTGGTTAGGCTCAGTCTTGCCTTTGCTGGCGGTA
TTCCATCATTGTGTTGATGTGAACACTACCAGTTGTAGTTATCCATAAGGTAGTAACTGATATCCCGTTT
CGCTTTCAGCAGGCTCTCTTTGCATTAGTTTATCTAACTGCGAAAACTGATTGTGATTCAGGATTACCTG
CTCACTTATTTGTATGCATCTTATTGATTGTTATATATTTCGAAAATTAAAATCACTCATAAGATATTCT
AATGCCATTTGTCAATGGATAGGAATGGCATCACTCAATAGCGACATTAAACTCATGAAAAAAATTATTT
ACGCCACTACGCTAATGACTCTACTCTCCAGTGAAGTGGCTTATGCGAACAGTGAAAGCTACTTACAGAG
GGGGACACAGGAGGTCAACGTCCAAGGATTAGTCGATTTTGATGATGAAGATAGCTACTCTGTGGATCTT
AGTGCAAAATATGGCTATTTTATAAAGGATAACTGGGAACTTGGTACAAATATCACCACAGACTTAAGCA
AAAGCTACAAGTCTGGTGGCCTTGGTATATTTACTGAGTACTATTTCACCAATAGTACCAAATTTGTCCC
TTATGTTGGCCTCACCACAGAATTGATTCACGCCTACAATAATGATGACGAATTCAATAGTGATGATAAT
AGTTCATACACAAATTCATCTAGAATTACAACTGTAAGTATCAAAGCAGCGTTGGGCATGAAGTATTTCA
TTAGCTCTAACGTAGCCATTTCTGCTGAAGTGAACTACAACACAGCTACAGATAACAAGGCAAAGGACTC
TTTTACTAAATTTATAATTGGTACTAGCTTTTACTTCTAAGCATGTAGCTCATTATCCCATTGCGCCTTA
ATGACTGTATGGAGGCATGCGGCAGTGCCCCATTACTGGGCCAATAAATTGCAAGGGCTGGGGCATGCCG
TCAAGCTGATGGCGCCTCAGTTCGTTAAGCCCGATGAAAGACCAACAAGAACGATGCTGCGGATGCCGAA
GCTATTTGTGAAGCTGTGACTCGGCCCACCATGCGGTTCGTGCCGATAAAGAGCAGCGAACAATTGGCCA
TCCTAGCCTTGCATCGTGCTTGACAAGGCTTCATCAAGGCCAGGACAGCTCAAGCCAACCAGATCCGGGG
CCTGTTAGCAGAATACGGGTTCATCCTTCCTCAAGTCATCATCCACATAGGCAAACACGTTCCCGAACTG
CTGGAGGACAGTGAAAACGGCTTGCCAGAAACGTTTCGCCATGGCGTTGTTGCCCAAATCAGTTGTAACT

GCGCAGCTTCCCCAGCCCAAGCCAGCACTTTGCCATCGACTCGGAGATGAGCTCGGTCTTGCGTTCCTTG
AGACACGTCAGGAGGTACGTATTCATTCCGCCGAGTCAGGTTGCCACTTGTAGAAGGTGGCCGAGCTGAT
GCCATGCTCGTGGCAGTGCCAAGGGGGTGGCTTGATGATAGCGATGATCTGGGGATCGCTAAAAGGTGAT
GTTTTTATGAAGAACCTCCTGCATTTACAGTTCGGGAAAATTTTATTTATGAGCACCTCAATTTTTCGAG
GGGATTACCCCTCTCATCAAGGATCTCTGTGAAAATCCGGAAACTTCAATGTAATGACAAATTAATAATT
AACTAATGATTGGTTGATCTAGTATGGAGCTCAAAAGGTTAATGAGGTATAGTTCTCAACTGCACATCGT
CGCCTAAATCGATGTAATTGGATAGGGTTAGCTGAGGCCTCGGTGATCTGAACAACTGCACCCTCTTCAA
GTCTGCATCATGGGCAGTGAGGCAGCAGCCAGCTTTGCAGTGACAAAGCCATTGCAGCAGCCCTGATATT
CTAGAGGATTTTCAAACTCAGCCTGCGGGCTACTCAGAAATTACATAGCTGCGCAAGAAATGGAAAATAG
AAAACCAAGAAATGAGGTTGTGCTGATAATATCCAAGAAAGAGCTAGCTTACAGGAAGAATATATCGGGA
TACCAACCCGTTCGCTGGCAGAAACGGCGATGATTTGGTTTCAAGCTACGGCGGATGAGGAAAATCACTC
TGCGTATTACAATGGTAAGCGGGAGAAGCCATGCGTATATAAGCGCCATAAGTAAATTAAATACCCTTGG
TTGCCAGTCAGAAAGCCCCGAGTGTAGCGACTATCTGGGAATGGGAACACCATGACTCGTTAGCTGATTT
GAGTAAGAACGCTATTCAACACACTTCTTAGAGATAAATAAATGTCGAGTAAACTTTTATTTTTAGACTT
GGCGCGCGGTTTGGCTGCAATATTTATGGTGTGTACTCATGTAGTGAGTATTAACACAAAGACTCAAATT
GCAGAACATTCTATATTTGGAAAGGTTATAACATTACTAGGTGAAGCCCCTGCTGCCCCAGTATTCATGG
TTGTCATGGGGATTCTATACGCATATAAGAAAGAGCATCAATTTACGCACGATATAAAGCGCTCATTTTC
ATTGTTTGCTAAAGGGTACTACCTTAATTTATTACGATTAGCAGTCCCCTTTACCCTCTTCTTATTTTAT
ATGCCATTTGATATAAGTGAGCCGGATGATAAATTGACAAACTTTGCCGATGATATCATCTCGAATTTAC
TTGTGGTTGATATCCTACAAGCCGCAGGCCTATCATATTTGATAATGGCGATAGTCAATAAATTACAACT
TAATGATGTTTGCATAACGCTTCTGATACTATTGGTTTTGGTGTACTCCCCTTTTATATGGGGCCTTGGA
ACCTATGTTCCATTTTGGGGAAGGTTACTCGAACCATTATGGGGTATTAATGGGGAAATGGTTTCATTCC
CTCTATTCCCATGGGTCATCTATCCATTGATTGGCATGATCCTTGGTAGAAGATATAGTAATCCACTTAC
ACTAACAACACAGGTAATGGGGTATCAATTTGGTTTTGGTGTATGTTTATCCTTTATTGGTATGCTCATT
TCTCAAACTAATATTTCATTTCACTTTGGTGATTACTGGAGAACCGGCCCTGGTGGACTAGTATTATACA
TTGGATTTATTATGCAATGGCTTGCACTCATGTTCTTCATATCTCCATACATCCATAAAGGTCTTTTTAA
TGTCATTGGTTTTATAAGCACACACATCACCAATTTCTATATTGTACAGTGGGTCCTTATTTCAGCGAGC
ATTGTCGTACTTAATCAGTATAAACTAGATATAATCGAGTCACTTGTAGCTATTATCATAATAATCGTAA
TGTCATTTTTAATTTGCAAGAAATTACAGAAACACAATATAAAATTATAGTAGGAGAAAACTTATGAAAA
TATTTATGTATATACTAGGTTTGGTTTTATCTGGTGGTGTCTTTGCAAAGGATAATCGAGATGGGATTGC
CCATTCGATAATATCACTTAATCAAAAGATCATAAACTTCGATAACTCATTGAACTTAGATAACTCCATT
CGAGAGCAAGGCGTCGATTTTCTATACGATAGCTTATCTCTTGCCAATGAAGTTTTTATCGACAAGGCCT
CCATCGGATCTCCATCATTTGTTGATTGGATCACTACATCACGTAAAACAGCCGGTGATAACCCTTGGAC
ACGCTATCGCTCCACAATGATCAAAGGTGAGAGCAGTTATGTTTTAGAAGGTAAATTCGGTGATACGCTT
TACCTTGGCATTCAAGTTTATACATTACGTGATGGAAGAAACGTGGCTCTTCCAGAAAAAAATGTTTCAA
CAGATAACATCAAAGTTGGGGATAATGGAACTTTCCGTATCGAGATCGGTCCTACAGCTGCAGAAAATGG
GATCACAACTGATGCCAATGACTACATGCTAATTGTTCGTGAGTATTATGCTAATGGCGCCGTTAGAAAA
AATGCGCCAGCCGAGTTGATGATCAAACAAACTGATGGTGATTTTGACACTCCATTCATTGCTTCATCAT
CAGAACGAATTACACGAGCCAGTGCCTTTTTGACTAGTTTGGTTGAAAGCTCACTTGACCTCACATCCCA
AATGAGTAAAAGCAAAAATACAAGTGCAGATGTAAACCCTAGACCTGACCTCGTTCAAGCACTCTTTCCC
ACCACTGATAATCGCTATGATGGTTTCTACATTCAACTTGAAAATGACAAAGCAATTAAGCTGACTGGTA
AAATACCTGCCAATTTAAAGTATGCATCTATCACATATTACAACCCTTACTATGCTACTGTTGATTATGA
TAAGAATAAGACTTACATCACTAAAGATGAATTAGAATTGAATGCAGATGGCAGCTACGAAGTCTATATA
ACTAAGTTTGACATCAAAGACAAAAAGAATCTTATTACCACTCAAGGATATAATGAAGGCGTTATATCGG
TACGATACCTTGGAAAAGAAGAAATTGAATTTGAAACCAAGGTTATTGATGTAAATGATATACCAAACAA
AGATATTAAGAAGGATGAACGTAATGGTGGTGGCGGTTCAGTACCTGCTGAGCTGTTAATTTTAGGTGCT
GTATATATGGCAAGGAAGAGCTTGGTAAGTTGATGTCAAGTATAAAGCCACCGTGTATCGGTGGCTTTAT
GCCACCTCAATGCTGACAGGTATATGGGATTTCTGTTGTAAAGTTGATTACCTTTTCTACGGACACAACT
GCATCATAATCTTCCGCAAAACCATCGTCAGCTTTCCCTTCATTCTTTCCTTCGAATTCAATATAAACTT
CAGGATACAAGTACCCACGCTCAGTTCTAATTTTCTCTATTTCCTGCTCGACATCTTTCATAATTTGAGG
ATGCGGAGAATATAACCAAAAATCTGTTCTGTCTCCACAAGGGGTAAAGGCGCTCACTTCAAAGCCTTCT
GAATAGACACCTCGCCAAACATTTGGCTCTGTGACCAAACTTGAGCAGCCTGTAGCAAGTAAAGAAATAA
TAATTGACAAAAACAAAGTTCTCATTGATGACTCCCATTGAATTTAGGTTTGTAATTTAAGTCTATAACA
TGTTCCCTGTGCTATGGTAATCCTCCATTTTTAGGGGCATTCAAAAGTAGAGGTCATGCTGCCTGTGCCA
GGCGCAGTTTCGGCATGTAGCCATCCAGCGCCATATTGGGGCGCTCATGGTTATAAACCCACTGCCAGTT
CGTGGCAAACTCCTGTAGGTCATTCAGTGACTCAAACAAGTAATGGCCCAGCCAGTCGTAGTGCACCGTG
CGGTTAAACCGTTCGATGTAGGCATTCTGCTGCGGATTTCCTGGCTGGATATAGCCCAGGGTTATCCCCT
CCTGTTTCGCCCATCGCTTGAGCTCCTCGCAGATATATTCCGGGCCGTTATCACACCGGATCATCCCCGG
CTTGCCACGCCACTCTATCACTTGGCCCAGGGCTCGCTTGACCCACGAGGCTGGCAATGAAAAATCAACC
TCTATGCACAGCGCCTCGCGATTAAAGTCATCGCTCACATTAAACAGTCGCACCGACCGGCCATCACTGA
GCTGGTCATGCATAAAATCCATCGATCAGCACTGATTGGGCTTGTCGGGCACGGCCAGTGCGTCCGGCTT

TTCACGCACCAGTCGCTTCTTGGGTTTGATCCGCATGTTCAGTTCAAGCTCCCGATAAATCCGGTAGACC
CGCTTATGGTTCCAGCCAAACCCTTTGACATTGCGTAGGTAGAGGAAGCAGAGGCCAAAGCCCCAGTTCC
GTTGGGTGCTGGTGATGCGCCGCAACCAATCAGCAATCTCCTAGTTTTCATCGGCAAGCACGGGTTGATA
GCGATAACAGCCTTCACTGACCTTGAAGAGGTTGCAGGCCTGCCGGACAGAAATGGCGAAGTGCGCTACG
GCCTTGGTAGCCATCTCCTTGCGGGCAGATGGCGTTACCACTTTTTTGCTATGGCCTCGGAGATGATCTC
GGCCTTGAGGCGCTCCTCGGCATACATTTTCTTGAGGCGACGATTCTCCTCTCCCAGCTCTTTGAGACGA
GCCATGAGGGAGGCATCCATGCCACCGAATTTGGAGCGCCATTTGTAGAAGGTGGCGAAGCTGATGCCAT
GCTCACGGCACAGCTCAGGGACGGGCGAACCAGACTCAGCTTGCTTGAGAATGGCGATGATTTGGGCATC
GCTAAAGTGTGATGTTTTCATATAGAACCTCCTGCGTTCAAGATACGAGAAAATTCTACTTATGAGCACC
CCAGTTTTTCGGGGGGATTACCCGACATAGGCCCACTGCTCATCAAGTTCACAGATAAGCGCCACGTCAT
CGAACACCACCTTTTTGGAGCTTACTTGCCTTGGGGTGAGTTTTTTAGGGCGCGTATGACGGTGTTGATG
CCAATCTTGAGGACGCGAGCAGTATCACGAACACCTGAGCCGTTGAACGCCATATTTGCGGCCTTCATAG
GTGTAGGTGAGCTGAAACACGTGCGGACAGACGGGGCAACGATAGCGAACATGGCCAGCAGGGGTATTAC
CATGGCGATATACGTGGTCAGAGTTGCAACGAGGACAGTGAACGGGGATGGATGCCATGAAACTACGAGT
CTCAAAGACACCCATCATACCCGCTCAACCGATCGGAGGCATCACCCCTTTGTTATAGAAAACATATCAA
TGCTGGTGTGAGGTATCCTGATTTTCACAGACAGTCCCGATTGTTACGATGAGACAATCGGGGGTATTGA
ATGAAATCGAGAGAACACTATTCACCAGAGTTTCGGGCAGAGGCGGTTCGACTGGTATTGGAGCAAGGCC
TTAGCCAAGCTGAAGCCGCCAGAAGGTTGGGTATTGCCAAAGGCACACTGAGCAACTGGGTGGCCGCTGC
CAAAGGAGGAAAGGATACATCGGCCATTCCCGGTGCGCGTTCTGTCGCCGAGCTGGAGGTTGAAGTGGCC
CGGCTACGCAAAGCTCTCGCACAAGCCAACATGGAGCGCGACATCATAAAAAAATGTATGGTCTGCTCCTT
ATCTGCAAGCTTGAACTGATTTCCAGAAGTCGTTTGCGTCAATGTATCCGGCCTGTCTGTGAGCCGTTTT
CCGGCTCTGGCCTTGATGGGATCCGCACGTCATCGTCCTAATAAAGCTACCAGTCATGAGAGACTGCTTT
TTTAGCCAGGCTGCAACGTCGTGGGGTGAACGCTTCTGTCATCTGTTTTTGGCTCGCAAAACCGGGTATG
AGTTATATCTGTTAAACGGCTGCGCTTGCGTTGGGATAGGGTTGGTAATCCCTGCCGGATGTCAGTACTT
TCCAGCAAATTCGCATTATCTTGTTGGCCAAGGCGATAAGAACCACATGATGGTGATGGCCAGCCTCCAG
CCTAGCGACCCATTCGCCTAACGATGATTTGTCACGCTTCATGTGAATTTTTAGCGCTTTGGCGCCTTGA
ATAACCATTTGCCTTAAATAGCTATTGCCACGCTTACTGATCCCGCCGAGCGAAGATTTGCCGCCCGTTG
AGTATTGCCTGGGGACCAGCCCCAACCAAGCTGACAGGTCGCGGGCTTTCTTGAATTGTGAGCCATTGCC
AATCGCGGCAATCAACGCGGTTGAGACAATCGGTCCAATGCCGGGGACGGTACTGATTTTTTGGCACAAA
CCTGATGCGCTTGAGGCTTGTCCGAGCATGAGGCTCATGTCATCAATCTGAATATCCAGCTGTTGCCACC
GTTTTCTCAGGCGTAATACCAGGGCGCGGATAAAGTCAGGCAAGCCATTCTCGGCATCTTCAAGGATCGT
CGGCAGAGAGCGCTCGAAGACTTTTCTGCCGACTGGCACGGTGAGCCCATATTCCAGCAGCAACGCTCGC
ATCTGATTGACAGTGGCGGTACGTTCGACGATGAAACGCTGACGGACGCGATGAGTCGCTTGCAATGCCA
GTTGCTCATGAGATTTCAACGGGACACAGCGCATAGTACCTCTGCTACCAGCCTCGGCAATGGCGGTAGC
ATCGTTGAAGTCATTCTTGTTTGATTTGAGGTAGGGCTTCACAAATTGGGCAGGGATAATCTTGGGTTCG
AACCCCGCCTGTTGAAACAGGCGCCCCCAGTATTGCGAACCAGGACATGCCTCGAAGGCGACCCGACAGG
GTGGGGTCGTGCTAATGAACTGTTTCAGTTGTGAACGCGTGAGCTTTTGCTTGGTCAATACATGCCCCAT
GCGATCCATCGCCACCAGGTGAAACCAATTTTTGCCTAAATCGATCCCGTAAATGTAAATCTGCGCTTGA
GTCATGATGAGCCTCCTTGATGCTGGCTACAGCATAGCGGGGTGGGTGGAGCAGACCATCCCATTAGGCC
GCCGCGTACTTTGCTCAAGAGTCACTGCCAAGTACGCGTGGATAGCCAACCAGCAGGACCGCTATCCCAT
TTCCGTTATCTGTCGTGTGCTGGAGGTCTCTCGTGCGGGCTTCTACCACTGGCGCCAGCGTCCACCGTCA
CAGCGCCAACAAGAAGATGAACGACTCAAGGTCGCCATTCGGGCTGCTCACAACAAGACGCGCCAGACCT
ATGGCGCAGCGCGTTTACAAGCTGAGTTGGCCGCCGAAGGATTTTACGCCGGACGGACCGGATTGGGCG
ATTGCGCCGTGAAATGGGGTTGTCTTGCAAACAAAAGCGCAAATTCAAGGCGACAACCCATTCTGCTCAT
TCCCTGCCTGTGGCGGAGAATCTCCTTGGTCAGGTCTTTGCGCCGACGAAGCCCAACGAAGTCTGGACCG
GTGATATCACCTATATCGCCACCGACGAAGGCTGGCTTTATCTGGCTGGTCTCAAGGATGTGTTTACCTG
TGAGATAGTGGGTTATGCGATGGGGGAGCGAATGACCACCGGGTTGGTCAGTCAGGCGCTGTTTCGGGCT
GTGCAGCAGAAGAGGCCACCCGTTGGGCTTATTCATCACACAGACCGGGGCAGTCAGTACTGTGCCAAGG
CTTATCGGGCTCTCCAGGTACAATTTGGCATGCAGGCCTCCATGTCACGCAAGGGGAACTGCTTCGACAA
TGCCCCGATAGAAAGCTTCTGGGGGTCTCTTAAAAATGAACTGGTTCACCACCATAGATTCAAGACGAGA
GCGGAGGCCAAGGCAGCGATTCAGGAATACATAGAGATTTTTTACAACCGCCAACGTCGGCATTCACGAC
TTGGCAATGTCAGCCCGGCGGAGTTTAACAAGAGGTATTGGAGAACGGCTCAGGCTGCTTGATCCGGGAC
TGTCCGATCTTGTCAGGAGACCTCACGTTCTACAAATGGCGCTCCAAATTCGGTGGCATGGATGCCTCCC
TCATGGCTCGTCTCAAAGAGCTGGGAGAGGAGAATCGTCGCCTCAGACCTCACGTTTTAGCAGGATGCTA
TTAACTTTCTTGAAATACAAAAGAATAAGATGAAAACGGCTATTTTTTCCGTTTTCATCTTATTCAGTCGG
CAAATCACCAACCACAAATTCAGCCCATGCATCCATCATCGACCGTCGCTGGTCTAGCAAATCGGTGCGG
TGGTACGCAGCTTCTACCTTGTTTTGAACTGTGTGAGCCAATGCTCGTTCAGCAAGATCCCGTGCATAAC
CCTGCTCGCTACACCAGTCTCGAAAGCTGGAACGAAAACCATGTGCCGTTGCCAGACGGCCAGGAGTAGA
GCTTGGCGCCTTTACTCGTCGCAAAAATGTTGTTAGTACCATATCTGATAGAGGAACTTGATCGCGTGGA
GAAGGGAAGACCAATTCATCATGCAACCCCTGCAATCCCATCAGGATCTCGATGGCTCGAAGAGATAACG
GGACGCGATGCATTTGTTTTGCCTTCATTCGCTCCGCTGGCAAAGTCCAGATCTTTGCTTCCAGATCGAC

CTCTGACCATTTCATTGCCCTGACTTCACCTGAACGGCAAGCCGTCAGGATCAAGAACTCCAGCATTAAG
CGAGTAACATCGTAACGTTGTGTTGTCCTCAGATGCTGCTGAACAAAGGCTGGAATGTCACGCCAGGGCA
TGGCAGGTTGATGTTCGGTACGAACGGCTTTACTGGGTTGTAGTGGGAGTAAGTGATTTACCACATCGAC
CGGGTTTGCTTGGCAGTGACCGTGCGCCCAGCCCCAAGCCATTACCGCATGCAGCCGTTGTTTCACACGG
CTGGCAGTTTCAGGGATCTCCAACCAGATCGGCATTAAGACTTCAGCGATCTGTGCAGGAGTGATAGCGG
AGATTGAGTACTGGCCAATCATGGGGAATGCATACTGCTCGAGCGTCGAGATCCACTGTTTGCCGTGTTT
AGGATTTTTCCATCCAGGCAACAGTTTGGAGTGAACTTCTCGAGCCGCAGCCTCAAAAGTTGGGATAGAG
GGTTTGCTCTCTTCAGCACGTTTGAGTTCGAGTGGGTCAAGGCCATTGGCGAGTTGCTCTCGCATCTTCT
GTCCTTGCTTGGTGGCATCGGCTATGGATATCTCTGGGTAAGAACCAAGCCCCGCATTTCTGCGTTTGCC
GGTCACTGGGCTGACATAGCGAAGCACCCAGTTGCCACGACCTTTCGCTGTTGACGGATGAAGTGCAAGG
CCTGTTACGCCGCCATGTGCAATTGCTTTGTCACCCGGCTTGAGATTACGTGCTTTGGTATCGGTAAGAA
GTGCCATAGGGAAGATTCCTTGAGAATGTGGCATGCCATGGACTATGCCATCCATTGTGCCATTTAGTGC
GGGATAGAGTCATTTTTAGTCGGACGCTGTTGGACGATCTTTTAAGTAAGGCATTGATTGTAATTGGATA
TGTAGGATGTGACTGGTTTTGGCTGGACTTGATTCAGGCGGTCTCCCTCACCGCCAACTTGCAAAGCCCT
GATGGAAACATCGGGGCTTTTTGCTATCCGCGTTTTTGCGAGCGCTGTGGGCAAGCCGATGGCGATGGGC
GGCGGGCTCGGGTATAGTCCTGCGCCAATGGCGTTATCCTGTTTTGCTGACCCGTCGTGGCCCTAGTGGT
GGCGAGGGGCAGGCCCGTGTAAGGAGTGTTATGAAGTTCAGTCCCCTGCTCGATGAGATGATGAAAGCCC
TGCAGGTATTGCCTGGGGTGGGGCCCAAGTCTGCTCAGCGGATGGCCTTTACCCTGCTCGAGCGTGAGCG
CAGCGGCGGTTTGCGGTTGGCCCAGTTGCTCTCTCGCGCCCTGACCGAGATTGGCCACTGCAGCCACTGT
CGCACCTTTACCGAGAACGATCGCTGCGATATCTGCGCCAATCCCAAGCGGGAAGAGAATGGCCTGCTCT
GCGTGGTGGAGAGCCCGGCGGACGTGGCGGCCATCGAGCAGACTGGTCAGTTTTCCGGTCGTTACTTTGT
GCTGATGGGCCATCTGTCGCCGCTGGACGGCATAGGCCCGGAGGAGCTGGGGCTCGATATTCTCGAGCGC
CGTCTGAAGGAAGAGCACATCAGCGAGCTGATCCTCGCCACCAATCCCACCATTGAAGGGGATGCCACCG
CCTGGTATATCGCCGATATGGCCCGCACCGCCGGGGTAGAGGTGAGCCGCATCGCCCACGGGGTGCCGGT
AGGGGGCGAGCTGGAGCTGGTGGATGGCACCACGCTCTCCCACTCCCTGATGGGTCGCCAGCGCCTCAAG
TAAGGGGCGGGTGGCGGCAAGTCGCGAAAAACTGAACATAAAATCGGCGGTTACACGTCAACAGGGGGCG
AATGAGCAGGATTGCACATTCGCCCCCTTGATATTTGGATCGCTGTCCCCATCTAGGTTGGGCATATGTT
CAACCCGATGTTTCTGAGGATTGGTCCATGACCCAAAGTGTTCATGCCGAAACCCACGGCTTTCAGACTG
AAGTCAAACAACTGCTGAGCCTGATGGCTCACAGCCTCTACTCCAACAAAGAAGTATTCCTGCGCGAGCT
GATCTCCAACGCCTCCGATGCGGCGGACAAGCTGCGCTTCAAGGCGCTTTCCGATGCCTCTTTGTTCGAA
AACGACGGCCAGTTGCGTGTGCGTCTGGTGGTGGACAAAGAGAAGCGTACCCTGACCATCTCTGATAACG
GGATCGGTATGACCCGTGATCAGGTGATTGAGCACCTGGGTACCATCGCCAAGTCCGGCACCGCAGAATT
CTTCAAAAACCTCTCCGGCGATCAGGGTAAGGATTCCCAACTGATCGGTCAGTTCGGGGTCGGTTTCTAC
TCCGCCTTTATCGTGGCCGACAAGGTGACCGTGGTCTCCCGTGCCGCCGGTACTGCTCCGGAGCTGGGCG
TACAGTGGGAATCGGAAGGGGAAGGCTCCTTCACCGTCGCTGATGTGACCAAAGAGGGTCGCGGTACCGA
TGTCATCCTGCACCTGCGTGCCGAAGAGGACGAGTTCCTCGATGACTGGCGTCTGCGCTCCGTGGTGGGC
AAATATTCCGATCACATCAGCGTGCCTGTAGAGATGTACAAAGAGGGCACCCCGGATCGCGAAGAGGATG
GTGAGACCATCGTCGGCACCCCGGGCGAGTGGGAGCAGGTCAACCGCGCCACGGCACTGTGGACCCGCAA
CCCGAAAGATATCAAGGACGAGGAGTATCAGGAGTTCTACAAGCACGTCGCCCACGACTTTGAAGATCCG
CTGCTGTGGGGTCACAACCGGGTGGAAGGGGCGCAAGAGTACACCAGCCTGCTCTACGTACCGGCCCGCG
CACCGTTCGACCTCTACAACCGCGAGCAGAAGCACGGTCTGAAGCTCTATGTGCAGCGCGTCTTCATCAT
GGACGATGCCGAGCAGTTTATGCCGACCTACCTGCGCTTCGTCAAAGGGGTGCTGGACTCCAACGATCTG
CCGCTCAACGTCAGCCGTGAAATCCTGCAGGACAACAAGGTGACCGCCTCCCTGCGCAAGGCTTGCTCCA
AGCGCGTGTTGACCATGCTCTCCAAGCTGGCGAAAGACGATGCCGAGAAGTACGCCAAGTTCTGGAAAGA
GTTCGGCAACGTGCTCAAAGAGGGCCCGGCCGAGGATTACGCCAACCGCGAAGAGATCGCCAAACTGTTG
CGCTTTGCCAGCACCGCGGGCGAGGGCGAAGCCCAGACCGTCTCTCTCGAGGATTACGTGGGCCGCATGA
AGGAAGGTCAGCAGAAGATTTACTACATCACCGCTGACTCCTATGCCGCTGCCAAAAACAGCCCGCACCT
CGAGATCTTCCGCAAGAAGGGTGTCGAAGTGCTGCTGATGTGGGAGCGCGTGGACGAGTGGCTGATGAGC
CACCTGACCGAGTTCGACGGCAAGCAGCTTATCTCCGTCACCCGTGGCGAACTGGATCTCGGCGATCTGG
AAGACGAAGCCTCCAAGCAGGCGCAGGAAGAGGCCGAGAAGGCCAACGCCGGTCTGGTTGAGCGGGTCAA
GAAGAGCCTGGGCGATGCAGTGAAAGAGGTGCGTGTCACCCACCGTCTGACCGACTCCCCCTCTTGTATC
GTCACCGACGCCTACGGTATGAGCACCCAGATGATCAAGCTGATGCGCGCGGCCGGCCAGCCGGTACCGG
AGCAGAAGTACATTCTGGAGCTCAACCCTGACCATGCGCTGGTGAAGAAACTCGACACCATTGAAGACGA
GGCACTGTTCGGTGAGTGGGTCACCCTGCTGCATGAACAGGCCCAGCTGGCCGAGCAGGGCGGGCTGAAC
GATCCGGCGAGCTTCGTCTCCCGCATCAACCGCCTGTTGCTGCAAGCCTAAGCAGCAACGCCGGTAACAG
AAAGCCCACTGCATGCAGTGGGCTTTTTTGATCCCGCTGTGTGAGCTCGGCGAAACACAGCGGTGAGGGA
GGTGGCCAGCGCCTCTCTATTTGCGGTATTCGTTGTTGTAGTTCTGGATGAGACGGCGAGTCACCTCATG
CTGGGGATCGGCAAACACCTTGAGAGTCTTGCCGCGCTCCACCACCCGACCTTCGTGCATCACCAGCACC
TCGTCGCTGATATGGCTGACGATACCCAAATCGTTGGCTACCAGCACATAGCTCAGCCCCATGGTCTCCT
GCATCTCCAGCAGCAGATTGATGATCTGGGAGCGTACCGAGATATCCAGGGTGGAGAGCGCCTCATCGGC
CACCACTATCTTGGGGTTGAGGATCAGCGCCCGCGCCAGCGCCACCCGCTGCTGCTGACCGGAGGAGAGC

ATCTGCGGATAGAACAGGGCGTGATCCGGCAGCATCCCCACCATACGCAGGGTCTGCACCACCTTCTCGC
GGCGCTCCTCCTCGGTCATCTCGGTGTTGAGACGCAGCGGGGTCTCCAGGATCTGGCCGACCCGGATCTT
GCGGTTCAGCGAGGTGTTGGGATCCTGAAAAATCATCCGCAGCAGTTTGCAGCGAGTCTGGTAATCGCCA
TGCACCATCAGCTGGCCGTCGATGGCGATGTCGCCGCTGGTGGGTTCGATCATGCCAGCCAGAATGCGGG
CGAGGGTACTCTTGCCGGAGCCAGCTTCGCCGACGATGGCAAGGGTCTGGCCCACTTCAAGATCGAACGA
GAGGGGCTTGATCGCCTCCACCGCCTGACGACGGAATAGACCGGTGCGGTTCTGGTAGGTTTTGCTAAGG
CCTCTGACCTGCAACAGGGATGGCTCGATCACAAGCGGGGCTCCTTCATGGGGGCATTAACGGTCATCAT
TTTTTTGGCTCCTCCATGTTCAGCGGGAAGTGACAGCTGAACTGATGGCCCTTGATCTTGTTCATCAACG
GAGTTTGCACGCACTGCTTCTGGGCATAGGGGCAGCGCGGCCCCAGACGGCAGCCGATGGGCAGGTGTTG
CAGCGGCGGGATGACGCCGGGCAGGGTCTCGAGGCGCGACTTGTGGCGCACCAGCTTGTCAAAGTCGGGG
ATCGATTTGAGCAGGGCATCGGTATAGGGGTGATGCGGGGTCTCGAGGATCTGCTCCCGGGTGCCTACCT
CCACCATCTGGCCGCAGTACATCACGTTGATGGTATCGGTCAGGTTGGCGATGGCGGCGATATCGTTACT
GATGATCAGGATGGTGGTATTGCCCAGCTTATTCATCTTGTCCAGCAGTCGCAGGATCTGGGAGTGGGTG
GTGGACTCCATGGCGCTGGTGGGCTCGTCGGCTACCAGCAGACGCGGCTGGTTGGCAATGGCCATGGCGA
TCATCACCTTCTGGCACATGCCATCCGACAGCTCGTGGGGATAGGCGCGCATCACCTTGCGGTGATCCTT
GACGCCTACCCGGTGCAGCAGGGCGATGGCCTTCTTCTTGCGCCACTGGAAGCGCTGCCAGAATTGTCCC
TCGAACGAATCGGTCGGGATCGCCTCTTCCAGCTGGGTGCCGATCTCCTCGGAGGGGTCGAGACAGCTGA
TGGGGTCCTGGAAGATCATGGCGATCTCGCGACCCATGATGCGACGGCGCTCCTTGGGAGCCATGGTCAG
CAGATCCATGTCGTTGAAGCGCATCCGGTCGGCACGTACCGTCCAGTTGTCCTTCTCGATGCCGACGATC
ACCTTGGCGATCAGACTTTTACCGGAGCCGGATTCACCCACTAGACCGCGGATCTCCCCCTCGGTCAGGG
TCAGGCTCACCTTGTCCACCGCCTTGACCTTGCCTTGCGGTGTGTCGATCTCGATGGTGAGATTGCGAAT
GTCGAGCAGCGGCATCAGTCATTCCCCTCTTTGAGTGCTTCACGAATGCCTTCACCAACTAAATTGGTCA
CCAGCACGCTGAACAGGATGGCGATGCCGGGCAGGGTGACGGTCCAGGGGGCGAGATAGATGAGGTCGCT
CGAATCGGCCAGCATGGTGCCCCACTCAGGTTGGGGAGATTGGGCGCCGAGGCCGAGAAAACCGACCGCC
GAGATATCGAGAATGGCTGCCGACAAGGTGCGGGTGGTCTGGGCCACCAGGGTTTCGACAATGTTGGGCA
AAATAGCCAGCCGCATGATCCGGAAGGGGGGCGAGCCATCGAGGCGGCTGGCGGTGATGTACTCCTTCTG
CATCTCGGCATGTACTGCGTTGTAGGTGGCCCGGATAAAAGGCGGGATCAACGCCAGGGTAATGGCTATC
AGGGTATTGCCAAAACCCGGCCCCATGATGGCGACCATGATGATGGCCATCAGCAGGGAGGGGATGGAGA
GCAGGGTATCGAGCAGGTGGTGCAGTACGCTCGACTTGAGCCCCTTGCTCATGCCGCCGAGGATGCCGAT
GGCGGAACCCACCACCATGGCGATCACTACCACGACCAGCGCATAACCGAAAGTGAGGCGCGCCCCTACC
ACCAGCCGGGAGAGCATGTCGCGCCCCAGATCGTCGGTGCCGAGGAAGTAGTCGATGTTGCCGGTCGCCG
CCCATGAGGGGGCGAGCAGCAGCTTGTCGGTGTGCTGTTCATCGATGCCGTAGGGGACGACCAGCGGCCC
CACCAGCGTGATGAGCAGCAACAGCAAAAAGCACCATAGCCCCGCCATTGCCAACGGATTGCGGCTGTAG
GAGGCCCACGTCTGCTCAAGGGGGGAGAGGATCTTGAGCTCGGGATAGATGTTCGATTTATCTTGCATAG
GATACCTGGCTCCTGGTCACGCCATGAGCGCGTTCAATCTTGTTTGGCATACAACTCTTTGCGCCGTGCC
GGATAGATAAGGGTGGTCAGCAACTCGGTGCTGACGCTGGTCAGGATCACGAAACTGGCGACCACCAGGG
TTGCGCCGCGAATGGCGGCGTAATCCTGCAGGGCGATACTGCTGATGAGCCAGCGGCCGATCCCGTGCCA
CTCAAACACCACCTCGGTGATCATGGCGGAGGTGAGCACGCTACCCAGTTGCAGACCCAGCAGCGGCAGT
ACCGGCGGCAGGGCGTTCTTCAGGCCATGGCGCCAGACGATCTGCGCCTTGGAAAGGCCCCGGCTCGCCG
CCGCCTTGATGTAGTTCTGCTTCATCACGTCGATGAGGGAACTGCGCACATGGCGGATCACCTCGGTAGT
GGGCACCACCGCCAGTACCAGTGACGGGAGAATGAGGTGGCGCAGGGCATCGTGCAGCGCAGCTTGCCGC
CAGGGCTCGTTGCTGAGCAGCACATCGATCACCGCGATGCCCGTGATGGGGGGCACGTCGTAGAGCAGGC
TGATCTGTCCGGAGGCTGGCAACCAGCCGAGATCCAGCGAAAAGAGCATTACCACAAGCAGCGCCAGCCA
GAACACCGGCACCGCATAGCCGATAAGACCGGCAGTCATGATGGAGAGATCCAGCGTCTTGCCCTGTGAC
AGGGCCGCCAGGGTGCCGAGAGGGATCCCCACCAGCAGCGAGATGGCGAACGCTGCCAGACAGAGGATCA
GGGTCGCCGGGAAGTAGTGACGGATCTCGTCCAGCACCGGCAGCCCGGAGACGCTGGAGAGCCCCAGGTC
GCCTGCGAAAATGCGGCGCAGGAAGTCGGGATAGCCGCTCCAGAAGCTGAGCTGATTGCCGATCAGCCGA
TAGTCCAGCAGGTAGGCGACAAAGGTCAGCGCCAGCAGAGTGATAAGCAGCAGGTTGATGCGGCGCAAGG
TGTATAGAAGCATGCTTACTCCCTGTGAGCCTGGTTGAAGGAGGTGCCGCCAAAGGGCATCAGCGTCAGT
CCCTCGATGTCGCTGCGGCTGACCTGGGTACGCAAGGCGTGCGCCAGCGGGATCAGCGGCAGCTGTTCGT
TAAGCAGGGTCTGGGCATAGTAGTAGTTGCGCAGCCGGAACGAGAGCTGGGGCGTGGTGACCGCATCATC
CAGCAACTGATCGAAGGCCGGGCTGCACCAGCGGCTGTAGTTGTTGCCGCGCTCCACTGCCGAGCAGCTC
AGCAGCTGGCGGAAGAAGTTGTCCGGATCGGCGTTATCGGCAATCCAGCCGGAGAGCAGGCTGTCGTACT
GCCCCTTGGTGAGGCGGCCTTCGATCACCGGCCACTCCCGTTGCACGATCTTGAGCTTGATCCCGACCTT
GGCCAGATCGCTGCGGATCAGCTGGGCGGTCTTGACCGCATCCGGGTTGTAGGGGCGGGCGTCTGGCTGT
ACCAGCACCTGCATCTCGAACCCTTTCTCCAGTCCCGCCTCCTTGAGCAGCTGGCGCGCCCGTTTCAGGT
CGGGTTTGCGCTCGGGCAGGCTGGGGTTGTACCCCCACGAGAGGGTCGGCAACAGGCTGTTGGCGGGCAG
GCCGGTATCGAAATAGACCGCCTGCAACAGGTTGTCGATGTTGATGGCGCTGGCAATGGCCTGACGCACC
TTGACGTTGTTGAACGGCGGCTTGCGGGTATTGAGGGCCAAAAAGGCGACGTTCATCCCCGACTGGACCG
AGAGGCTCAGCGCCGGATTTTGCTTGATCACCGAGAGCTGGCTGGCGGAAGGGGTGCTCATCACGTCGCA
CTCGCCGGACAGCAGCTTGGCCAGCCGTTTGGCGGATTTGGGGGTAATGTCGTAGACCAGCTGTTCAATC

TTGGCATCGCCGCCCCAATAGCCGGGATGGCGCAGATAGCGGATGAACTCGTTGTGGCGATACTCCTTGA
AGCTGAACGGCCCGGTGCCGATGGGGCGGCTATCCAGCAGGGCTGGCGTGCCCGCCTGCAACATCTGCTC
GGCGTATTCGGCGGAGAGGATCACCGCATAGTCGCTGGCCAGGGTGGCGAGGAAGGAGGCGTTCGGCTGG
TTCAGCTCGAATACCACCGTCTGCGGCCCCTTCTTGCTCACCCGCTTGATCAGCAGATCTTGCCCCAGGC
TGTAGAAGTAGGGGTAATCGCCGCCGGAGACATTGTGGTAGGGGTGCTGCTCGTCAAGCTGGCGGGCATA
GGTGAAGACCACGTCATCGGCATTGAAAGTGCGGGTGGGCTTGAACCAGGGGGTATGGTGAAACGTCACC
CCCTTGCGCAGGGTCAGGGTATAGGTGAGGCCATCCTCGCTGACATGCCAGTTGGTGGCGAGCGCCGGCT
CCAGCGCCAGGGTGTTGGGGTTCACCTCCAGCAGGCGGTCGTAGAGCGGACGGGCACTGGCATCCAGTGT
CACCCCGGAGTTGGAAACATGGGGATTGAAGGAGAGGGGGGACCCCTCAGCACAATAGATGAGTCCGGTT
TTGGAGGCCTCGGGTTGCTTCTGGCAGCCGGTGACCAGCACCAGCAGACTCAGCAGCAAGGGTGTGAGCA
TTCTCATTGTTGAACCATGTTCTTATAGGGGTTTTGTACGCACGTTTTGATACAAATGTGCCAGCGCGAA
GGCTGGCGTGTTGCTGGCGTCGGCATCAACCGCACTCCTTCACTCTTGTTCTGCATCGTTATCGCCATCC
AGCAGCTGATATTTGCGTAGCATGCCACGGAACTGGTGATAGCTTAAGGCCAGCAGTTGAGCGGCTTTGC
GCTGATTATACTGGGATTGCTGCATCGCTTGCTTGAGTAAATTAATCTCGTACTCCGCCACCGCCTGCTT
GAGATCGAGCGGCAAGGCGAGGGGGGATGCAGATTGTACCTCGCTTCGGGCCTCTTGCGCAGGTTGCGTG
ACCTGTGGTCGCCAGGGGGAATCAAACGGGTTGAGCACCAGGGTCGAGAGCGGCAGCTGGGGATTGCCCT
GACGATAGATGCTGCGCTCCACCACGTTCTTCAGCTCCCGCACGTTGCCCGGCCAGTGGTAATCCAGCAG
CAGCTGGGTTGCCTTGCGCGAGAAACCGGCAAACAGCGGATAGCCCAGCTCCCGCGTCATGCTGTGGGCG
AAGTGTTCCGCCAGCATCAGGATATCTTCCCGCCGCTCCCGCAAGGGGGGGAGGGTGATCACATCAAACG
CGAGTCGGTCGAGCAGATCGTGGCGAAACTGCCCCTTGTCGGCCAGCGCAGGGAGATCCTCGTTGGTGGC
GCAGACCAGCCGCACATCCACCTTGAGGGGCTTGGCCCCACCCACTCGCTCGAACTCGCCATACTCGATG
ACCCGCAGCAGCTTCTCCTGCACCCGGGCGCTGGTGGTGGCCAGCTCGTCGAGGAACAGAGTGCCGCCAT
CGGCCCGCTCGAAGCGGCCCTGATGGCGCTTGGCCGCGCCGGTAAACGATCCCGCTTCGTGGCCGAACAG
CTCGGTTTCCAGCAGGCTCTCGCTCAGGGTGGCGCAGTTGATGGTGACAAAGCTTTGATCCCAGCGCGCC
GAGAGATAATGTAGGCGGTGGGCGATCAGCTCCTTGCCCGTCCCCCGCTCACCGATGATCAGCACGGGTT
TGCGCAGTGGGGCGAGCCGTGAGGCCTGCTCAATGATCTCGAGAAATGCGTTGGATTCCCCCAGCAGGCT
TTCCGTGGCCGTGGTCATAGTGATCCCTGTCAGTTGTAAAAGCTGGTCAATCTTACCAAAAGTTGGTGAT
TTTAATCATCACTTGCGCGAGGTATCTTGCAATCCGGCTATTCTGCCCCATATAAATCAACAAGTTAAAT
TTTGTTCCAGATTGGCACACAAGTTGCCATTGTCAATGGAGAAACAACGCCATGCCAATAACAGGAGTGC
AATCATGAGTATTTTTTCCCGTCTGGCCGACATCATCAATTCCAACCTGACGGCCCTGCTCGACAAGGCA
GAAGATCCCCAGAAGATGGTGCGCCTGATCATCCAGGAGATGGAGGACGAGCTGGTCAAGGAGCGCTCCA
ATCTGGCCCGCTTCCTCGCCAGCCAGAAAGAGATTGGTCGTCAGGTCGCCCGTCATCAGGAGCGGGTCGA
CGAGTGGCAAGCCAAGGCCGAGCTGGCCCTGACCAAAGGGCGTGAAGATCTGGCCCGCGCCGCCCTTATC
GAGAAGAAGAAACAGACCGAGTTGTCAGAGACCCTCTACCGCGAGCAGCAGGCGGTCGATAGCGGCATCG
AGAAGCTGGGTGAAGAGATCCGTCAGCTCGAAGCCAAGCTGGAAGATGCCCGCGCCCGTCAAAAGGCGAT
GGCCATTCGCACCGAAGCGGCCAGCAGCCGTCTCAATGTGCAGAGCCAGGTAGCCCGTGGCGAGAGCCAG
GCGGTGGTGAGCAAGTTTGAGCGGATGGAGCGTCGCATCGACGAAATGGAAGCGCGCGCCGATCTGGGTC
AGTCCGACAAGGCGCTGGCGCAACAGTTTGCCGAGCTGGAAGTGGACGATCAGATAAGTCGTGAGCTGGA
AGCCATGCGCCAGAAGCTGGGCCAGGGCGACAGCAAAAGCGAAGGCAAACAGGGGGAGTAACAGATGGAA
GATCTGCTGGGCGTATTGATGGTTCCCATGGTGGTCTTCATGGTGGTGGTAGCCCCCATCTGGCTGGTGC
TGCACTATCGTGCCAAGGGGCGGATTGGCGCCGGTCTTGCTGATAGCGAGCGGGAACAGCTGCAAGGGCT
GCTGGCTCGCACCGAGAAGATGCAGGAGCGAGTGGGCGCGCTGGAATCAATTCTGGATGCAGAAGTACCC
GGTTGGAGGAACAAGGTATGAGCAGTGTCAGTCGTGAAGGGCGTAACCTCTATCGCGATCCCCAGCGTGG
CAAGATAGCCGGGATCTGTGCCGGTTTGGCTGAGTATTTCGGGGTGGAGACCTGGATCGTGCGGCTGCTC
GCCATCAGTGGCCTTATCTTCGCCGGTTTCATTACCTTCACCGCCTATATCGCCGCCTGGTTCTTGCTCG
ACAAAAAGCCGGTCACCCTCTATTCGCAAGAGGATGCCGACTTTGCCGAGGTGCGGATGAAAGTGCGCAG
CTGGCAGGCGGGGATCACCCCTCATCAGGCGCTGGGCCGCATCGCGCAGGAACTTGACGCGCTGGAGCCC
AGAGTTCAGCGGATCGAGAAGCTGGTGACCTCCAGGGAGTTCACCCTGCAGCGGGAATTTCGCAAGCTGT
AGTGCTTGCTCAAGCACTCAATGTCGATGGCGGCGCCTCCCGTGATGCGCCACCATCGACACCAGCTATC
CAGATGCAACCACCAAGGGAGAACGGGTCAAGCCTTGATACTCAATAAACTGGAACAGAAGTTGACTCGC
CTGCAGAGCAAGGCGAACGACGTGGTGAACCGGGTGCGGGATCGCCATATCCGGCTGGCGGTGACCGGCC
TCTCTCGCAGCGGCAAAACCGCCTTTATCACGGCGCTGGTCAACCAGCTGGAACACGCGGCCATCGATGG
CCGTTTGCCGTTGTGGGATGCCCTGCGTCAGGGGCGCATCCTCGGCGCCCGCCGGGTGCCGCAGCTCAAT
GCCCACATCCCCACCTTTGCCTACGAGCGTGGCCTCGACTCCCTGTTTGGCGATCCACCGGCCTGGCCCG
AGCCCACCCGCGGTGTGGCCGAGGTGCGTCTCGAAATTCGCTATCGCACCCGTCATCCCCTGCGCAAGCA
TCTGGGGGAGATCTCCACCCTCTACGTGGATCTGGTGGACTACCCCGGGGAGTGGCTGCTCGACTTGCCG
CTCCTTGAGATGAGCTACGAGCAGTGGAGCGAACAGGTGCGCGAGCAGCTTCGTCGCCCAGAGCTGCAGG
CACTTGCCGCCAGCTGGCTGACACCCGGGTGGCAGGTCGATCAGCGCTTCGAAGAGCGGCCGGTGGCTGA
GCTTGCCGAGCGCTACACCGCCTATCTGCACGCTTGCAAACAGGAGCTGGGATTGCACCTTATTCAACCC
GGCCGTTTCGTGTTGCCGGGGGAGTATGCCGGTGCCCCCATGCTGCAATTTGTCCCCTGGGTGTGGAGA
TGCCTGTCGGCGAGCCCGCCGATGGCACTCTTTACGCCACTCTCAGGCAGCGCTTCGAGCAGTACAAACA

GCATCTGGTGCAGGGTTTTTATGAGCAGCACTTTGCCGGTTTTGATCGCCAGATCGTGCTGGTGGATTGC
CTGCAGCCCCTCAACGCCGGGGCGGCCAGCTTTGGCGACATGCAGCAGGCCATCGCCCGCATCATGGAGA
GCTTTGCTTACGGCAAGAGCAACTGGTGGCGGCGACTCTTCTCGCCCCGTATCGACAAGCTGCTGTTTGT
GGCGAGCAAGGCCGATCACGTCACACCTGAACAACATGCGCCCATGGTCTCACTGCTGCAGCATCTGGTA
CGCAGCGGTCGCGGTCAGGCCCGTTTTGAGGGGATCACCACCGAGTGTCTGGCGCTCGCCGCCATCAAGG
CGACGGAAGTTGGCAAGGGGGTGGCCAATGGCCGCGAGTTCCCCGCCATCCGTGGCACCAGCCTGAGCGG
TGAGCCGCTGCTGCTGTTCCCCGGAGAGGTGCCGCCCCATATTCCACCCGCGCAGTGGTGGAACAATCAG
GGCTTTGATTTTCAGGCGTTTCGCCCCATGCCCATGAGCCCCCATCAGGCGCTGCCCCATATCCGGCTCG
ATGCGGCGCTGGAGTTTCTGCTCGGGGATCACCTCGAATAACGGTGTGCGGTCTTGGCACCTGAGCCGAA
GGCTGCGCACAGGAGAACAATGCGATGAACGATCAGATAAAAGAGCGGCCACCGTTGCAGGGCAAGGTGA
TTCTTGAGCCTGCCTTGACGGTGGCCAGTGACGAGCGACCCCCCGCACCGGCCCAGCGGCTGGAGGAGGC
GAGCTTCGAGCGCCTCGACGAGCTGGACGAGTTCACCGAGGTGGAGAGCGCCCTCGTCATCAAACCGCGC
CGCCGCCATCGGCTACTGGCGTGGGGGCTGGGGGCGGTCGGTCTGCTCTCGTTGGGCCAGTATGGCGCCT
TTCTTTACGATCAATTCCTCACCACCCCGCTCTGGGGCAGCGCCTGGCTGGCGGCCTCGGCGCTGGTCGC
TGCGGGGACCGCCAGCGTGGTGGGGCGCGAGTGGCTGCGGCTGCGCACCCTCAAGCGGCGTCAGGATGTG
CGCTCGCGAGCAGAAGATCTGCTTGCTCATCAGGGAGTCGGGCAGGGGCAGGCCTTTTGCGAAGGGTTGG
CGGTCAAAAGTGGCGACAAAGGGCGCGAGGGGTATCAAACCTGGCTGGCCCAGCTCGATGAGAGCCACAG
CGATCGGGAGGTGCTGACCCTCTACAGCCAGCTGGTACTCGGCGAGCGTGACAAGCTGGCCCAGGCGCGG
GTGGCCAAATGGTCGGGGGAGGCCGCTGTTCTGGTGGCGCTCAGCCCGCTCGCCACCGTCGACATGATGC
TGATGTTGTGGCGCAACCTGCGGATGATCGAGGATATCGCCGATGTCTATGCCATCGAACTTGGCTACTG
GAGCCGCATACGCCTCATCCGTCAGGTGTTTCGCAACATGCTCTACGCCGGTGCCACCGAGCTGGTGACC
GAGGTGGGGATGGACTTGCTGGGCGCCGAGTTTGCCGCCAAGCTCTCTGCTCGCGCGGCGCAAGGGGTGG
GGGCCGGTCTGCTCACCGCCCGCCTTGGCCTGCGCACCATAGAGGCGTGCCGTCCGCTGCCCTGGTGTGC
GGACGAGAAACCGAAACTCGGCGAGCTGCGCAAGCGGCTGATCGGCCAGCTGGCGGGGTATCTCAATCAG
CCTTGATGGCTGCTGGAGATTAATAGCGGTTGTCACAATTTATTTACAACTGTTTCAGGCAGCCGACTCA
TGGGTCGGCTGCTTTTTTTGTTGCCATTTCGTTACAGTGAAACCGTGATGAAACGGCAAGGCGCCTGATG
TGGCGCCAGAGGATGGAACCGCTATGGCAACCTTCTACACCGCCCACCAGCCGGATGCTGACGGGGTGAT
CCACTACAGCAACGAAGAGCACGGCACCTGGCAGATCCTGATCGAACGCCAGTTCGCTGCCCTCGATGGC
AAGGGGTGTGACGAGTATCTGGCCGGTCTCGACAGACTGGGATTGCCCCGTGATCGCATCCCCCAGCTGG
CCGAGATCAACTCCGTCTTGCAGCCTGCGACCGGCTGGTCGGTGGCGGCGGTGCCTGCGCTTATCTCGTT
CGATCGCTTCTTCGCCCTGCTAGCCAACCGCCAGTTTCCGGTAGCGACCTTTATCCGGCGCCGGGACGAG
CTCGACTACCTGCAAGAGCCGGATGTCTTTCACGAGATCTTCGGCCACTGCGCCATGCTGACCAATCCGG
CCTTTGCCCACTTCACCCAACTCTACGGTCAGTTGGGGCAGCAGGCGAGCAAGGAGCAGCGAGTTTTTCT
GGCGCGGCTCTACTGGTTCACCGTGGAGTTTGGCCTCTTGGCTACACCTGACGGGCTGCGTATCTACGGT
GGCGGCATTATCTCCTCCATCGGCGAGACCGCCTATGCCCTCTCCGGCCAGCCGCTGCTGCAACCTTTCG
ATCTGTTGACCGTGCTGCGCACTCCCTATCGTATCGACATCATGCAGCCCACCTATTTTGTGCTGCCGAG
TCTCGATCATCTCTACCAGCTGACCGGTGAGGAGATCATGGCTGCTCTGGCGGAGGCCAGTCGGCTTGGC
CTCTATCCGCCACGTTTTGAGGTTGCCGCTGGCAAACAGGCGAGCTGACCAAAAGCAAACAACCCTATTT
CATTGTCATTAATTTTTCATTAATTCAAGCAGGAGTAACCCATGTCCGAACTGGCCAGCCAGCAGTGTGA
AGCGTGTCGCGCCGATGCCCCCAAGGTGAGCGATGAGGAGCTGCGCGAGCTGATGCAGCTTATTCCCGAC
TGGCAGCCGTTGGTGGAGGAGGGGGCGCTGCAACTGCGGCGGGAATTTACGTTTCGCAACTTCAAGGAGG
CGCTGGCGTTTACCAACCGCCTGGGGGAGCTGGCGGAAGCGGAGTTCCATCATCCGGCCATCCTGACCGA
GTGGGGGAAGGTGACCGTCAGCTGGTGGACCCACAAGATCGGCGGATTACACCGCAACGACTTCATCATG
GCTGCGCGTACAGACCAATTGCCCAGATAATCTGAGAACAGGTTGAGGCGGAGTATCCCCCTTCCTGCAT
TTCTGACCGAATGGGGGAAGGTGACCGTCAGCTGGTGGACGCACAAGATTGGCGGCTTGCACCGCAACGA
CTTCATCATGGCGGCGCGCACCGATCAGTTGCTCCGCTAATCGCACCCCACACCAGCGTCATCGGTTGAC
CCGGGCCTGCCTGCGAAAGCGGGGCGGGCCCGCATTTTTATGGGCTGGCGCTCGCACTGGGGGGATCGAG
ACTGTATTATCCGCTCATTCATAGAGTTAGCTAGGAAGTGTGTCCCGAATGCGTCTTGAAGTCAGCTGTG
AAGACCGCCTGGGCCTGACCCGGGAACTGCTCGACCGCCTGGTGGAGCACAATATCGATCTGCGCGGCAT
CGAAATCGATACCTCAGGCATCATCTATCTCAACTTTCCCGAGCTGGAGTTCAGTGATTTTCAGCATTTG
ATGCCGGAGATCCGCCGCATTCCCGGCGTCTACGACGTCAAGACCATCCCCTACATGCCTTCCGAGCGAG
AGCATCACGAGATCGAGGCGCTGCTCAAGGCGCTGCCGGATCTGGTCTTCTCCCTCGACAGCAAGGGTAA
GGTGACCCAGGCCAACCAGTCGGCCCTCACCACCCTCGGCTTGCCAGTGGAGGAGGTGCGCGGCGTGGCG
CTCGGTTCGCTGGTCAAGGGTTTCTCCTTCACCCGCTGGCTGGAGGCGAGCGAGATCAAGCCGCAGACCT
GCAAGCTCAGTTTCAATGGCGAAGAGTATCTGGCGGATCTGATGCCGCTGTTCGTGGCAGAAGCCCGCGG
CAAGCAGGCGCTGGCCGGTGCCGTGGTGGTGCTCAAATCTGCCCGTCGGGTCGGCCTGCATTTCAGCGCG
CTTCATGCGGTGGAAGTGGGGGGCTTCGAGCATCTGCAGGCCGAGAGTCCCAAGATGAAAGAGGTGCTGT
CGCAAGCGCGCAAACTGGCGATGCAGGACGCGCCGCTGCTGATCGTCGGCGAGACCGGCACCGGCAAGGA
GCTGCTGGCCCGCGCCTGTCACGGGGCCAGCCTGCGCTCCAGCCACCCCTTTATGGCCCTCAACTGCGCC
GCCATGCCCGATAACGTGGCCGAGAGCGAGCTGTTTGGTTATGCCCCCGGCGCCTTTGGCAACAACACCG
AGGGCAAGCGCGGCGTACTGGAGCTGGCAAGCGGCGGCACCCTGATGCTGGACGAGATTGGCGACATGTC

TCCCCATCTGCAGACCAAGTTTTTGCGGGTGTTGCAGGATGGGGTGTTCCGCCGGGTTGGTGACGAGCAG
GAGGTGAAGGTCAACGTGCGTTTCATCTGCACCACCCAAAAACAGTTGCTGGATCTGGTGCACGAAGGAA
CCTTCCGCGAGGATCTCTACTATCGCCTCAACGTGCTCAGTCTGGCGCTGCCCCCCCTGCGCGAGCGCAA
GGCGGACATCATGGTGCTGGCCCAGCAGTTTGTCTCCCGCTTTGCCAGCGAATTGCAGCGACCCCGTCCG
CGCTTTACCCGCAACATGGCCGACTACCTGACCGCCTACCGCTGGCCGGGCAACGTGCGCCAGCTGCGCA
ACTGTCTCTACCGCGCCATGACCCTGCTGGAAGGGGATGAGCTGGGGCCCGAGCAGATCGACCTGCCGGT
GGCGGCCGATACCATGCCGCTGATCGACGAGTGGTTTGAGGGGAATCTCGACGATGCGGTGAAGCGCTTC
GAGAGCCAGCTGCTGGCTCGGCTCTATCCTGCCTTCCCGTCGACCCGTCAGCTGGCCAAACGCCTGGGCG
TGTCGCATACCGCTATCGCCAACAAGCTGCGGGAATACGGCATCAGCAAGAAGTGATTTCTGCCTTGCTG
CCTGCCACCAGAGAGCCCGTTACCAGACGGGCTCTCCGTTTTCTTTACACGAACTCTGTAGTTGATGGCG
GGGTGAGACTTGTCAGGCCAGTGTCAGCCAACTTACTATCTGGGTTCATGTACCCGAACCGCAATGCCAT
AGCAGCGGTATTGTCAAGGCGACCACCTCCCGATCATGCTGTTACTCTTTTCCCCCCGTTTAACTGCCCG
TCTGGCGCTCCTGTTGGTTGCGTTTGGCCTGCTGTTGCCCCACGCGCTGGCGGAAACGGCCGCGGAGAGC
CGGGCGACGGCGGTCAGGGAGCTGGTGCTCGATATCATGAGTTACACCCGCTGGCCCACCGAGCCTGCGG
TGCTGCGCATGTGTGTGGCGGCGCCGACCGAATATGCCGGCGCCCTCTTTACCATCGATCGTCAGGCCAA
TGGCCGTACCGTGCAGGTCCATCGTTACGAGGCGAGCGATCCGGCCATCAGCGATCACTGCGATGTGCTC
TATCTGGGAGTGCTCGACGAGCCGACCCGGATTGCGCTGTTTGCCCGTCTGCAGGGACACCCAGTGCTCA
GCATCAGCGAATACAACCACGACTGTTCCACCAGCTCGGTGTTCTGCCTGCAGACCTCGCCGGACAGCGT
GCGGGTGCGGTTTAAAGTGAACCTCGATGCTCTGGCCCTGAGCGGAGTGCGGGTGCATCCCGCCGTGCTC
AAGCTGGCCCATGCCGACAAGGGGGTCCCATGAGACGACAGGTGAGCGCGGCTCCCCGCCGTCTGACCCT
GCGTCAGAGCCTGGGGCGAACCCATCTGGTGGCCGCCATCACAGCCGTCAGCGTGGCGGGACTCTTTTTT
ACACTGGTGGCCCTGCTGGTAGTGCGTTTTTACGCCGACCACAATCTGGAGCTGGTAGCCCGCGCCATCA
GCTACAGCGCCGAGGCGGCGGTGGTGTTCCACGATGAGGAGGCGGCGAGCGAGGCGCTCAGTGGCATCGT
TGCCAAGGAAGAGGTGGCCCAAGCCACTATCGTGCTGACCAATGGCCAGCTGCTCGCCAGTTGGGAGCGC
CCCAGAAGCAACCCGCTGGTGACGCTCGAAGACTATCTGGCCCAACTGCTGATGCCGGGGCCTGTCAAAC
TCCCCATGCTGCGGGGTGGTGAGCAGATCGCCACCATCGAGCTGCGCGGCCACGGTGAATATCTGTTGCT
GTTTTTGCTCTCGGCGCTGCTGGTGATGGTAGTTTGCCTGATGCTGAGCGCCATGGTGGCACTCTATGTG
GCCGACCGGATGCAGGTCTCCATCACCGCACCGCTGCGCAAACTGGCACTTGTCGCCCACTCGGTACGTC
GACAACGCACCCTGGGGATGCGGGCGCCACCGGCCGATATCGTCGAGATTAACGAGCTGGGGGATGACTT
CAACTCCCTGCTGGATGAGCTGCAAGCCTGGCAGGCCCAGCAGGCCCGCGAAAATGCCAGCCTGCTCCAT
CAGGCTACCCACGATGCCCTCACCGGACTGCCCAATCGCGCCCTGTTTGAAGCGCGGCTCGCGCAGGCCA
TCTCCCTCGGCGAGCAAGAGGGGTTTGCGCTGCTCTATCTCGACTGCGATCGCTTCAAGCAAATTAATGA
CACGCTGGGGCACAACGCCGGTGACGATGTGCTGATCGCGCTCTCAAGACGGGTGCAACATCAGTTGCGT
CCGGATGATCTGGTGTTTCGCCTCGGTGGTGACGAGTTTGCGGTGCTGGTTGGCCCACTCCAGCGACAGC
ATGAGGTGGAAGAGGTGATTGGCCGCATCCAGCAGGCGATGAGCGAACCGGTGGCGCTGCGTGACGGTCG
TCAGCTGGTGGCCGAGGTGAGTGTCGGCTTTGCCGTCTATCAGGCGGGCATGAGTGCCAGCGTGCTGTGT
GATCAGGCTGATGCAGCCATGTATCTGGCGAAACGGGCGCGCCGCAGCGAACAGCAAACCATCGCCTGAC
TGGCAAGAATAACAACATAAACAAGGAGCAAACCATGAAGATCACCCACCTGTTGAAAGTGACCCTGCTG
GGGTTGATGTTGTCGCTGCTGGCCGGTTGCCAGAGCGCGCCCAAGGGGTTGACCCCGGAGCAAATCGCCG
TGCTCAAGGAGCAGGGCTTTTATCTGACCGATGAGGGGTGGACGCTGGATCTAGCCAACCGGGTGCTGTT
TGCCAACAACGTGGGGGCGCTCAATCCCCAGACTCGCCAATCCGTTGAAAAACTGGGTAAAACACTGCTT
GGCGTAGGGTTGGACAAGGCGCGGGTGGATGGCCACACCGACAATACCGGCGAGAGCAGCTACAACCAGC
AGCTCTCCCTGAAGCGGGCCCAGTCGGTGGCCGATGTGCTGGTCTCGGTCGGCATGAAGCCAGCCAATCT
GGAGATCCATGGCCGTGGCGAGACCATGCCGCTGGCAGATAACAAGACCAGAGAGGGCCGGGCCGCCAAC
CGCCGGGTTGCCATCGTCATCAGCGATCAATAACCCTTTCATCGTTTCGTTACCCATAAAAAAAGAGCGC
CTGATCGGCGCTCTTTTTGATCGCGACAGCTGGGGTCGCGAGGGCATGTGCGTGCAGGGTTACTGCGCGC
TATTTTGCCCGTTATTGTCGTGATGGCTGCGCAGGCGGATGTTGATCATCTCCACCACCAGCGAGAAGGC
CATGGCGAAGTAGATGTAGCCCTTCGGAATGTGTAGGTCCATCCCCTCCGCCATCAGGGCGAAACCAACA
AGGGTGAGGAAGGAGAGGGCCAGCATCTTGATGGTGGGGTGGGCATCGACAAAATCACCGATGGATTTGG
CGGCAAACATCATGATGAGGACGGCGATCATGATGGCCAGCACCATCACCGGCACGTGATCGGCCATGCC
GACCGCGGTGATCACCGAGTCGAGGCTGAACACGATATCGAGGATGGCGATCTGGATCAGGGTGCTGATA
AAGCCCGAGGCCGCTTTGCTGACGCTCTCCGGCTCAGCCGCCCCTTCCAGGGTGACGTGGATCTCGTGGG
CACTCTTGCCGATGAGGAACAGGCCCCCCAGCAGCAGGATCAGGTCGCGGCCGGAGATGGCTTCACCCAG
CACGGTGAAGAGCGGATCGGTGAGGCGCATCACCCAGGCCAGCGAGAGCAGCAGCAGGATCCGGGTACCC
ATGGCGAGCCCCAGACCCAGAATACGCGCTTTTTGACGCTGCGCCTCCGGCAGGCGACCTACCAGGATGG
AGATAAAGATGATGTTGTCGATGCCCAGCACGATCTCGAGCAGGGTAAGGGTGGCGAGTGCGACCCAGGC
AGAGGGATCAAGAACCCATTCCAGCATGATTGAACCTTGGTTGAAAAATTGAAGCAGCGCATTCTAGCAG
GGGCGCCACTGCTTGTCCCAGAGGGGGGAAAAGATAAAAATGGTGGCCATCGCAAGGGAGGCTGGCATGT
TTCGATTAGGGTTCATTATTAATCCGGTCGCCGGGATTGGCGGTGCAGTGGGGCTCAAGGGCAGTGACGG
TGTGGTTGCCGAGGCGCTGGCCCGAGGCGCTGTGCCCAAAGCGCAGGAGCGGGCGCGGCAAGCTCTGTTG
CCACTGTGCGAGCTGAATGTCCCGTTCGAACTGCTGACGGTGGCGGGAGAGATGGGGGAGACGGTAGCCC

GATCTCTGGGATTGCCCTGTCGGGTCATCTATCAACCGGTCGGGGAGAGTACCTCGGCCGATGATACCCG
CCACGCCGCCGGGCTGATGCGCGAAGCCGGGGTCGATCTGCTGCTATTTGCCGGTGGTGATGGCACGGCG
CGGGATATCTGCGCCGCGGTGGGGGAGTCTTGCCATGTGCTCGGCATTCCCGCTGGGTGCAAGATCCACT
CCGGCGTCTACGGGGTGACGCCCGCCGCCAGCGGGCGGGTGGTGGCGCGGATGATCGCAGGGGAACTGCT
CAGCCTGATCGATGCCGAGGTGCGCGACATCGACGAGGAGGCGTTTCGCGCGGGCAAGGTGCGGGCCCGC
CACTACGGCCAGCTGAAGGTGCCCGGCGATCTGCGTTACGTACAGGCGGTCAAGCAGGGGGGAAAGGAGT
CCGAAGAGCTGGTGTTGGCGGATCTCGCCGCCGGGGTGGTGGAGCAGATGGAGCCCGATACCCTCTATCT
GATGGGCTCCGGCAGCACGGTGGCAGCCTGTATGGCAGAGCTCGGTCTGGAAAATACTTTGCTCGGTGTG
GATCTGGTGGAAAATGGCCGACTTATCGGGCAGGATCTCAGCGCCGCCGAGATCCTGACCCGGATCCGCG
GCCGTCGCTGCCGACTGATCATTACCCTGATAGGCGGACAGGGACATCTGTTCGGGCGCGGAAATCAGCA
ATTGAGTCCTGAGGTGATTCGGTGTGTCGGGCTGGAGAATATCCAGGTGCTGGCAACCAAAGCCAAGCTT
GCCGCCCTTGCGGGCAGGCCGCTGCTGGTGGACACCGATGATCCCGAATTAAACCGGGCGTTGCGTGGTT
ATCTGCGAATAACCACGGGATACAAGGATCAGGTCATCTACCCGGTGGCCAACCCGGAATAGGACGCCAG
GGACGCTGCTGGCCTGGCGCCAGAGAGCTGCAATCAACCGGATTGCGGTTCCATACCCCCGAATTTTTTG
CCGCAAATGAGGCTGCGTTACATGACGATTCACGATTTTGAGCACAAGTTGTTGGGACTGATCGACAGCA
TGGTTGCTACCGCGACCGACGATGAGCTGTTTGCGGGGGGTTACCTGCGAGGTCATATCTCATTGGCCGT
TGCCCGGGCCGAGCTGGAAGGCAAGACCCTGGTGCGGGACGTCAAGAGCTATGTGCTGCGCAGCCTCAAC
GAGGCGATCTTGCAAGGTGAGCTTTCTGAAGAGGATGAAAAACTGGTGCAGGCGATGTGGAGTCGTTTGC
AACAAGACGCAGAAGCTTGATCGGATACACAATTCAAACTGAAGTTTGAGTTTATTCTTCCCCTGAAGTT
AGTGAAAGGTTAATAATAAGTTTCGCCTTTTGTTTCAGGGATTAAACTATGGCCTCCTCCTCACGCATTG
CCAATAAACTGCCGCTCGAGATGCTCTATCACTGGGAGCGGCAGTGTCCTGATCGAACCTACCTGCGTCA
AACCATCAACCGCGAATATGTCGATTTCACCTGGGGTGAAGTGGCCGATGAAGCGCGGCGGATGGTGACG
GCCTTGCGTCACCTCGGTCTGGTGGCCGGTGACAAGGTGGCGCTGCTCTCCAAGAACTGCGCCCAGTGGT
TTATCGCCGATCTGGCGATGCAGATGGGGCAGTATGTGAGCGTGCCCATCTACCCGACTGCCAACGTCGA
TACCATCGAATATGTGCTGCGCCACAGCGAGGCCAAGGCGATCTTCGTCGGCAAGCTGGATGACTGGAAG
AGTCAGGAGGCTGGCGTCCCCGCCGATCTGCTGCGCATCGCCTTCCCCTATGACACCATGCCGGCCAGCC
ACCAGTGGGACGATCTGCTGGAGGCGCATGAGCCGATCCCTGACAGCCCGGTGCAGGCCCCTGACTCCCT
GCTGAGTCTGGTCTACACCTCCGGCAGTACCGGCAAGCCGAAAGGGGCCATGCTGAGCGTCGAGCGCTAC
GCCTGGTCGTGCGAGAAGCTGGTGGAGACGGTGAGCCTGAGCCAGGCCGATAGGGGCTTCTCCTACCTGC
CGCTGGCCCACATTACCGAGCGGGTCTACATCTACGGCGGAAGTCTCTATGGCGGCGCTACCATCGCGTT
CCCCGAATCCCTCGACACCTTTATCGAAGATGTCAAACGCTGCCGTCCCACCGTCTTTATCTCGGTGCCA
CGGCTGTGGGCCATGTTCCGCATCAAGATCCACGAGAAGCTGCCCCAGAACAAACTGGAGTTGCTGCTCA
AGATCCCGCTTATCTCCGGCCTTATCAAACGCAAGCTGCAAAAGGGGTTGGGACTGGATCAGGCCCGGGT
ACTCGGCTGCGGCTCCGCGCCTGTCTCCCCTGCGCTGCTTGAGTGGTATCTGAGCATCGGCCTCAAGGTG
ACTGAAGCTTGGGGCATGACCGAGAACCACGCCTACTCCACCATCAACTACCCGTTCCGTGCCGACAAGA
TCGGCACCGTCGGCAAGGCGGGGATCGGAGTGACCATCAAGATCTCCGACGAGGGGGAGATCCTCTGCCG
CTGCGAAGGGATGATGCTGGGTTACTACAAGGATCCCGAGCACAGCGCCGAGGCCATCGATGCCGAGGGG
TGGCTCCACACCGGCGATATGGGCAAGCTGGACAAGGAGGGTTATCTCACCATCACCGGCCGGATGAAGG
ATGTGTTCAAGACCGCCAAGGGCAAGTATGTGGCGCCGGTACCCATCGAGGGACTGCTGGGGCAGGAGCC
CATTATCGAACAGCTCTGCGTGATTGGTTACGGCATGCCGCAGCCCATTGCGCTGGTGCAACTGGCCGAG
AGCGCCATGAAGGGCAATCGGGAGGAGGTCAACGCCAGACTGGAAGCGGCGCGGGTACGGGTCAACGATC
AGCTGGAATCTCATGCCAAAATTCGCGGCATTCTGGTGGTGAAGACTCCCTGGAATATCGAAAACGGCGT
GCTGACCCCCACCATGAAGATCAAGCGTCACCTGCTGGAGCAAAAGTACGCCCAGGTCGGGGATCGCTGG
CCCTCATCCCAGGTGGTGGTGTGGGAGGAGTAAATCCCGTCTTTCCATAGCGCAACAAACAACAACGGCC
CTTGATGGGCCGTTGTTGTGTTTGAGGCATATCACTCGCCACTGTTTTATCGGCGAGTAAAAGCGCGTCC
GGTTAGATCTGCGCCAGCGCGGCGGCCAGATCGGCGATCAGATCGTCCGCATCTTCCACCCCGATGCTGA
AGCGGATGAGGTCGTCGTTGACCCCGGCGGCGAGGCGGCTCGCCTCGTCCATGGCGGCGTGGGTGGTGCT
GGCCGGATGGCAAGTGAGGCTCTCGAGATCCCCCAGACTCACCGCCTGCACGAACAGCTGCAGGTTATCG
AGCAGGGTGGCGCAGCGCTCAAACCCGCCTTCAAGTTCAATACTCACCATGCCGCCAAAGCCGCTGGCGA
AGGCGCCGAGGGCATCGTGGCCCGGATGGTTGGCAAGCCCCGGGTAGATCACCTTCTTCACTGCCGGGTG
GGCGGCCAGAAACTCGGCCACTTTCTGGGCATTGTGCAGGTGGGCATCCATCCGCAGCGGCAGGGTCTTG
ACCCCGCGCTGCAGCAGGTAGGCCTCCTGCGGGCCGATGGGGGAGCCGATATGTTTGAGGGCCACGGTGC
GGATCGACTTCATCAGGGCGTGGGAGCCCGCCACCACCCCGGCGATGATGTCGCCGTGGCCACCCAGATA
CTTGGTACCGCTGTGCATCACCACATCGACCCCGAGCTCCAGCGGACGCAGCAGGTAGGGGGTGAGGAAG
GTGTTGTCACACACAGTGATGGCACCCACCTTGCGGGCTGCTTCCACCACCAGCCGCGGATCCACCACCT
TGAGGGTGGGGTTGGTCAGGGTTTCGAACAGTACCACCTTGGTGGCGGGGCGGATGTTGGCCAGCAGATC
CCGATCGCTCTCGTAGGCGGTGACTTTTATGCCGGCGCGGCTGAAGGTCTGCTTGAGGAAGGCATCGGTG
CCGCCGTAGAGCGGGCCGATATAGGCCACCTCATCGCCGCTGTCGGCCAGGGCATACATGATGGCGCTCA
CCGCCGCCATGCCGCTCGCCACCACCAGCGCTTCATCGGCACCTTCAAGCGCCGCCAGTTTCTGCTCCAG
CTCGGCAGTGGTGGGGTTGCCAAAGCGGCTGTAGATAAAGCCGGGGGCTTGACCTGCAAAGCGCTCCTTG
CCCGCCTGGGCGTTGAAGTAGCTGAAGGTGCTGCTCTGGTAGAGCGGGGTGGTGAGGGCACCGGTCTGTG

GGTCGATCCGCTGGCCGCTGTGTACGCTCATGGTCTTGAATTTCATCGTGTTGCTCCGTGATGGCCAGGC
TCTGGCCATGACTTGTTTTGTTGTTCGTTGCTTGTTGTTTTTGTCAGCGCGGGTATGTGCATGCGGCTTG
TGGCTTATGCATATACCCGCCTGCGCATCTCTGCGCTGTTCAGGCCGGTATCGCTTCGCTCTTTGTGCGC
GGTAGGCGAGCGGGTTCCCCATAGTGGCGGGCCGCCACATCCGGGTGGGAGCGCAGCCGCCCCTTGAGTA
CGTTTTCCCCAACCTGATAGAAGAGCGGGTTGGTCGGGTCACTCTCCGGCCCTTGCGCCTGCGCTGCCAG
CTCTGGGGAGAGGCTGAGCAGCGGCACGGTGGCATCGAGCCGCGCCGCCATAAAGAAGGCGCAGGAGAGG
CGCGAGACGCCGGGCGGCGGACTCACCACCCGATGCAGGGTCGCTTTGAGATAGCCGTTGGAGGCCAGCT
CCAGCAGCTCGCCGATATTGACCACCAGCGCTCCCGGCAGGGGCGGTGCGGAGATCCAGCCGTCGGCCGT
CTCGACCTCAAGCCCAGAGTGATTGTCTTGCATCACCAGCGTCAGGTAGCCGGGATCCTTGTGGGCGCCG
ACCCCTTGACTGCTGCCACCCGCCTCTTGCCCCGGGTAGTGAATCAGCTTCATGTGTTGGTAGGGGGCGC
CGCGAATACTGTCGTCAAACACGTTGGCCGGTTGCTCCAGCACCTCGGCGAAGGCAGCGAGCAGGGTGAG
GGTGATATTGCTCAGCCGCTCCTGCCACGCCAGCAGATGGGCTTTCATCTCTGGCAAGGCGGCCGGCCAT
TGGTTGGGGCCGATCAGCCGCTGCCAGGGTTCACGGATCAGGGCCGCAGGCAGGGCTTGCGCCTCATTCA
TGATATCGAACTGCTCCCGCAGATCCGGCCGCGCTCGGGTCAGCTCGGCACCGACCCGGTTGTAGCCGCG
AAAGTGGGGGCTGTTGACCATCTGCACAGCCAACTTCACCTGCTCCGGCAGAGCAAAGAAGCGCTCTGCC
AGCGCCAGGGTCTCTAGCATCTCAACCTTGCTCAAGCCGTGGCCGACCAGATAGAAAAAGCCCACATCGC
GGGCGGCCGCGGCCAGCCTGGCCAACATAGGGCGACGCACCTCGGGTGCCCCGTTGAGGGCAGCCAGATC
GATGATCGGCAGTGGTGAATGTTCCATGTGCACCTCCTCACTCTTTCGTTATCTCTAGCGCCATCTCCTC
TGCTGCCTCTGGTGCGGGCAGGTCTGATGAGCGGCAAACGGGATGGCGCGACAGTGTGGCGGTATGCGCA
GCGGTGTGGCGCCATCTGGAATGAAGGGGATGCTACTGTTCGGTGTTATAAGACGGAAACAACAAATTAT
TCTTAGTTATATACAAAATTCGAAATGGAATTCGGTATGTAATGGGGTGGCTGCTTTTTGCGCAGCCATT
AAGGGCGGAATGGGAAATGATGGGAAGGAGAGTGATAACCAGAGGCAAGCTGCCCACGGTAAAAATGACC
AAAACAGAAAATGGCCAAAACAGAAGGGAGGCGGATGCCTCCCTTCTGGTTGAGCGGTTTTTCACCTTAC
CGAAAGCACAGGCAAGTGACCGCGCATTCTGGCCTTAACCGGTTGCAGGCTCTCAGTAAGCCAGGGTAAA
GCGCTGGCGCAGGTGAGCCGGTTTTTCCGCCTCGTTCAGCACGCCATCGGCCAGATCACCGGCGGTGATC
CCGGCAGGCTGTTCGCCACTAAAGAGCACCTGATCGCCACCAATCCGATAGCCGCCGCGCTTCTCGCCCG
GTTCGAGGAACACCGGGGGAGAGACGAAGCGCCAGTCGAGGGTGGTTTCACGACGCAGGGCGGTGAGACC
GTCGCGGGCCGCCAGTGCTCCCTGTTTCCACTCAGCCGGGAACTCAGGGGTATCCACCAGTTGCACGCCG
GGGGCAACCTCCAGACTGCCGGCGCCACCTACTACCAGCAGCCAGCTGTTGGCCGCTTTGGCTGCTGCGA
CGATCGCGTTGGCGCCCTTGAGGTACTGGTCGTAGATATCCGGTGTGGTCCAGCCCGGGTTGTAGGCGCT
GATCACCCGATCGTGCCCCTTGAGCACCTCGGCCAATGCCTGCGGGTTTTGGGCATCCACCGCCACGGCG
GTGACCTGCGGATGGGCGGCCACCTTGCTGACATCGCGGACGATGGCGGTGACGTGATGGCCGCGGCTGA
TGGCCTCGTTCAGGATGGCTGAACCGACAAAACCGCTGGCACCGATCAGGGCAATTTTCATGGGGAACTC
TCCTTCACTGTTGGAATGGGGCTATCCTACGCCGCCATTTATGATTTAATAATCACCTCAAAAATGCATT
CACTTATAAGCTGGATTTAATAATGGAACAGCTCAGACGTTACGCCATCTTCGCCGCCGTGGTGGAAGCG
GGCAGCATGACAGGGGCGGCCAAATCCCTTGGCATGACCCCCTCCGCCGTCAGTCAGCATATCAGTCAGC
TGGAGAGCCTGCTCGGACTGCCCCTGCTCCATCGCTCCACTCGCCGTCTTAGTCTGACCGAGGCGGGAGA
GGTGGTGTGGCAAGGGTGCCGGGCGTTGCAGCAGACTCTGAGCGAGACCGAGCTGCGCCTCTCGGAGGTG
CGCGACGCCCTGCTCGGGGAAGTGCGGATCACCGCCCCGGTGGGGATGGCGGGCCAACCGTTGGCACTGG
CCCTCTCGCCGCTGCTGCAGGCCCACCCCTGCCTTACCATCCAGATCATCGCCGACGACGAGAAGCGGGA
TCTGATTGCCGAGCGGATCGACGTGGCGCTGCGGGTCGGCACCTTGCCTGACTCGACCCTGGTGGCCCGT
AAACTGGGGCAAACCCGAATGCTGCTCTGCGCCGCTCCCGCCTATCTGGCGCGCAAGGGGACGCCGCGCA
GCCCGGAGGCGCTGCTGGATCATGACTGGCTGGCCGCCGATATGCTGAGCGGCGGGCTGACCTTCTTCGA
CGAGAAGGGGCAGGGGCACAGGGTCAAGCCCAAGCCGCGGGTGGTCTGCAACAACGTGCTGCCGCTGCGC
CAGTTCACCCTGGCGGGGCAGGGGGTGTCGCTGCAGCCGGAGGGGGAGATCGCCGAAGATCTCGCCTCGG
GCCGCCTGCTGGAACTGCTGCCGGGCTTTCGGGTGCCACCCATGGAGATCCACGCCATTACCCCGCAGCG
GGAGATCCCGGAGAAGGTGCGCCGGGTGATCGCCGCCCTGCGCAGCCACTTCGCCGAACCGGTGGGGGCC
GCGGCGACACGTCCGTAGCCGGGGCAAGGAAGAAGGCCGTTCCGGTTGGCCGGAGCAAACAAAGAGCAAA
CAAGGATCAAACAAAGAGGGCAGCCCGCGGGCTGCCCTCTGTCGATGCAAGGCCGGTGAAAACAGGCCCG
GCCTCACTTCATGTAGAGACCGCCGTTGATGGCCAGGGTCTCGCCGGTGATATAGGCACCCGCCTCGCTG
GCAAGAAACACCACAGCGGCGGCGATCTCTTCGGGCTTGGCCAGTCGGCGCATGGGGATGTCGGCCTTGA
TGCTGTCCAGGATGTCGGTGCGGATCGCCTCCACCATGGGGGTGGCGGTGTAGCCGGGGGCGATGGCGTT
GACCGTCACTCCGAAGCGGGCCCCTTCCGCCGCCAGCGCCTTGGTGAAGCCCAGCATGCCCGCCTTGGCG
GCCGAGTAGTTGACCTGGCCGAACTGCCCCTTCTGGCCATTGACCGAGGAGATGTTGATGATGCGGGCAT
CGCGCTGCTCGCACATGGCGTCAAACAGCGGCTGGGTCACGTTGTAGAGGCTGCACAGGTTGGTGTGGAT
CACCTCGAACCACTGCTCCGGGGTCATTCGCTTGAAGGCCCCGTCCCGGGTGATGCCGGCATTGTTGACC
AGCACATCCACCCGCCCTTCATCCTGCAGCAATCGGCCCAGAGTGTCGCGGCACATGGCGGTGTCGGCCA
CGTCCAGCGCCAGCAGCCGCACCTGATCCGGCCGAAAACCCTGCTCCCCGTACCAGTCGTGCGCCGCTGC
CCCGTTGGCGCTGGAGTGGGTGGCGATGATGCGATACCCCTGGGCCAGCAAGGCGGTGGTGATGGCGGAT
CCGATGCCGCCACGGGCTCCGGTGATCAAGGCAACTTTCTTCATCTTGGCTCTCCCTGCGTAAAAGATTC
GAACGAGGACTTTATGGGCTTTTTGAGGTAATTGATATATTTGTTTTCAAACAATCTGACAGGCAAACGC

GTCACTTTTGTGACAGTCTGCCAAGCTGGCTTGACGCGCTGGCCTAAGGGGCTGGCAAGGCCCTGGGCAA
GGGGACCGGGGTGAGCGCAGCGGCCAGCGCTTGCCGCCAAGGTCGCGGCCATGTTTAATGGGGCCCTTGC
GCGTAGCGCCTTGCCGACAGCCCAGATCCGATCTCGTCATGACCATCTTGTCCACCATTCCGGGCGCCGT
GCCCCAGCCGCATAGCCAGCTGACCCGCATCGAGCGGGTGCTCGACTACATCCACGCCAACCTGGATCAG
CCGCTGGCGCTGGCCGAGCTGGCGGAGCAGAGCTGCTGGTCGCGCTGGCAGCTGCAGCGGGTGTTCCTCC
ATGAGACGGGGCTGACGGTGGCCCACTATGTGCGCGAGCTCAAGCTGAGCCAGGCGGCAGAGGCGCTGCT
CTCTGGCCGCCAGCGGGTGCTGGATCTGGCGCTCTGTTACGGTTTTGGCTCAGAGGTGAGCTTCAGCCGC
GCCTTCAAGCAGCAGTTCGGCTGCTCGCCGCTGGCCTATCGCAAGCGGGGTTTGCGGCTGGGGCTGCGTA
CCCCCCTGGTGCGGGCTACCCTGCCGCAGCCCATGCCTGCCAGGCTGGTACAGGTGCGGATCGAGAGCCT
GCCGGGCTTTATCCTGCACGGAGTGAAGGGGCAGATCCGCGGGCTGTTTGCCGCCGCTCCCGACTTTCAG
CAGACGGTGCCCGCCATCTGGCGTGCCCTGCGCACGGCGGGCGGTTTGCCACCCGGGCAGGAGCGGGTGG
GGGTGGTGGATGTTTCTGGCGCCGGCGAGTCGCTCCCCTACTGGGCGGGCTGCGTGATGGAGGAGGGGGC
CAGCTTGCCTGCCGGTCTGGCGCATCTCCTGGTGCCGGCCCAGACCTACGCGGTGCTAAGCCACGTGGGC
CCGGTCAGCCAGCTGGGGCAGACCCTCAACTGGTTTATCCTGCACTGGCTGCCGAGCTCCGGCTATCGCG
GGCTGGACGGCTTCGAGCTGGAGCGTTACGCCCCCGATTTTGACGGCAACCAGCCGGATGCCCGCATGGA
GTATTGGTTGCCCGTCGCCCCTCTCTGAGAGGGCTATTCCCTTGCCCGGAGGATGCCGGGCACTGCTATC
TCTCTTTCCCTTATTTGCTGCGCCAGGCTTTGCTATCCATTCGACATCGCCTGCGTTGCTGCCGTCAGTT
GAGGTTGCCGGATGGGGCCAAACTGGCCGCATTTTCTCTCTTTTCAGCACCAACATGCCCCAAATTGTTA
ATTCAAGGGCAAAATGACCCAATAAAATGCAAATGAGATTTGTTACTATTCACTCATTGCATTCGAGGAT
CAGATGTACGCCACTCACCCCCCTTTGACCCTGGTCGCCGCGACTATCTCCGCCCTGTTGGCTGGCCCTG
TGCAGGCAGCTGCTGCCGAGCGCCCGACACAGACCCGGGTCGACGAGACCCTGACCGTGCTCGGCGAGAC
CTATCGCAACACCGCGACCAAGACCCGGCTCGACCCCCTGGAGACGCCCCAGGCCATTTCGGTGGTGGAG
GGTGACACCCTGGAACAGCGCGGCGTCAGCTCGGTGAGCGAGGCGCTGCGCTACGTGCCCGGCGTCAACA
CCGAGCTGCGGGGCGGGGCGGTCAACCGGCTGGATCTGTTCAACATCCGCGGCTTTGACAACTACCAGAA
CTTCTACGACGGCCTGCTGCTGCAGTACAACGACTGGAACCTGCAGCCCCAGATCGATCCGGTGGCTATC
GAGCAGCTGGAAGTGTTCAAGGGGCCCACCTCCGTGCTCTACGGCAGCATGCCCCCCGGCGGCATGGTCA
ACCTCATCGCCAAGCGCCCCCAGCGGGAGAGCAGGCACAGCGTCAGCGTGGCGAGCGGGACGGGCACCCT
CAAGGAGCTGACCCTCGACAGCACCGGTGCCCTCAACGATCAGTTTGCCTATCGGCTGGTGGGGCTGGCC
CGCCAGAAAGAGGGCCAGGCGGTGACCTCAGAGGAGGAGCGCTACGTGCTGGCGCCCTCTCTCGACTGGC
AGTTGAGTGACCGCACTCTGCTGAACCTCAACCTCTATTACCAGAAAGACCCGCAAGCCGGCATCTACAC
CACGGTACCGGCCAGCGGCTCGGTCAAATCCAACCCGCTGGGCCAGCTGGGCAGCGATACCTTCCTTGGC
GATGAAAACTGGAACCACTACAGCCGGGAGGTGACCCTGCTCGGCTACAAGCTGAGCCACGACTTCAACG
CCAACTGGCAGCTGCTGCAAAACGCCCGCTACATGGATGCCTCGGCCAACCAGCAGAACACCTACAATGC
GGCGCTGGCGGCCGATAACCGCACCCTGGCCCGCAACGCCTACCTGACCGATGAAGAGTCCCGCGGCTTT
GTCATCGACAACCAGCTGGCGGGCAAGGTGCAGACTGGCAAGGCCCAGCACAACCTGCTGCTGGGGGTGG
ATTACCAGTATCTGGATGCGCGCATTCTCTACCGGGATACTCTCGACTACTCTGCCCCGGCCATCGACAT
CTTCAACCCGAACCACCAGCAGATAGTGCCGGCAGACCTGGCCTTCAACTATCAGGACAACCGAACCATC
CGCCAGTATCAGAGCGGCCTCTACCTGCAGGATCAGGTGCGCCTCGATCGGTTGGTGGCCATCGGCGGCG
CCCGCTACGACCGCTACCGGATGGACACCGACAGCCGGACCCTCTATCAGGGGGCGGCGAGCAACACCCT
GGCCCAGATTGATCAGGACAACCTCTCGCTGCGCCTCGGTGCCCTCTACGAGCTGGATCACGGCCTCTCG
CCCTATGTGAGCTATGCCGAGAGTTTCGAGCCGGTGGCGGGGGCGGACAAGCAAGGCCAGGCGTTCAAAC
CGGCCACCGGCCGGCAGTGGGAGGGGGGGGCTCAAGTTCCTCTCCGAGGACATGAGCAAAACCGCCACACT
CTCGGCGTTCCACATCACCAAGCAAAATGCCCTGGTGACGGATCCGGACAATGTCTACGGACCCAAGTTG
CAGACCGGCGAGATGGTCTCCAAGGGGGTCGAGCTGGAGGGGCGACTCGATCTCACCAGCCGCTGGGATC
TGGCGCTGAGCTATACCCGTCAGGAGATGGAGATCACCCGCGACACTACCAGCCTGCAGGGCAAGACTCC
GGTCTGGGTGCCCAAGCAAATGGCTTCGCTGTGGAGCAACTACCTGCCGGCCGGCCCGCTGGAAGGAGTG
CGCCTGGGGGCCGGTCTGCGCTATGTGGGGGAGGCCCAGCTTGATGCCGCCAACACCGATACGGTGCCGG
ATTACCTGCTGATGGACATGGCTGCCTCCTACGATCTGGCCCAACTCAGCCAGCGCCTGCAAGGGGTGGA
GGTGTCACTCAGTGCCAGCAATCTGTTCAACAAGACCTACTACTCCTGCTACGACCAGAACAACTGCTGG
TTCGGCGCAGAGCGCAGCGTGGAGGCGCGACTCAAATACGTATTCTGACCCTGCGTCCCGGGCCGCGCCC
GGGACTCTCACACCCCATGAGCGGCGCCGGCCGTCGTGCCCGCAGCCCGGGGGCGCGGCGCGAGCCGGAT
CTGCAGGCGCTGGCGGATCACGGCGCCCTGTTCGCGGCCGCCGCCGGGCTTATCCCTTCCCTCAAGGGGC
GGCTGGTGGCTGCCGGCGAGGCTGCGGCGCTGTTGACGGCGACCAAGCAGGCCACGTCGGCCGAGGTGGC
GGCCGCGCTGCACGAGTATTGGCGGCTGGCCCATCCGGAGGCGGGTCCCGCCTACTGGCTCACCCGCAGT
TGGGGCATGCTCTGCTGGCAATCCATCTATCTGGCCATGGTGGCCGTCTATCGGCTGCGGGCCGTGCCGG
CGCTGGAGAGGCTGGGCCAGGGCTATCAGGATGGGCTGGTATCGGGCTTTACCCTGCCGGCCGAGCCCAT
GATCAAGGGGGAGCTGGAACTCTTGATCAAGACGGCGGGCGAGCGACTGCAGGCCCATTGGCAGGCGCTG
TTCGCAGCGCTCGGGCAGGTGCAGCGGCTGCGGCCGGGCTTCGTGCGTCCGCTCTTGGCGGACGATCTGC
TGGCGGCGCTGGTGCGGGTACCGGCGTTCTTTGACGAGATAAGCCACGCCGAGGTGGCGGCCCATGCACC
GCACTGGCTTGCCGCCTGCGGCCTGCCGGCCGAGCACCTGACGGGCTGGCAGCTGGCGCCCTGGCCGCCA
GCGGCCGGCTTGGTAGGTTATGTGCGCCAGCGCTGCTGCCTGCACTACAAGCGCCGCGATGGTGAGCTCT

GCGCCAACTGCCCGCGGCGGCGGGGGGCGGCGAGCTGTGAAGAGGGTGCTTGAATAGCGAAGCCCTGCGT
GATGACAAGCCGCCTTAAGGCGGCTTTTTACTGAGCTTTTAGGTCGCCCATCTTGCTGCGAATGCGCTCG
AACAGGCTCTGCAGGCAGGCTTGATCCCGATCGGCGGCCCGCACCGCGCCATACATATGGCGACGAATAC
CGCTGCCGAGGGGACGGGCGCAGAGCATCCCCTGTCTGACAAACTCGTCGCTGGCCCAGCGCGGCAAGGC
GGCGACGCCGAGTCCTGCGGCGGCCATTTGCAGCATCACCGAGGTGTTGTCCACCGGCTTGCAGCGAGCG
GGCTCCACCCCGGCGGGTTGCAAAAAGCGGCTGAAGATATCCATGCGGCTGCGCTCCACCGGATAGACCA
GCAGCACCTCCGGCCCCAGATCGCCCGGCTCGATGCGGCTCTGGTGGCAGAGGGGATGATCCGGCGCCAT
CACCAGAGTCAGATCGAAGGCAAACAGCGGCTCGAAGTGGAGATCGCCGCGGGCCTGCACGTCCGAGGTG
AGCAGCAGATCGAGCTGGCCGCCTAGCAGGGCATTGATGGCATCAAAGCCCGGCACTGACTCCACCTCCA
GATCCACCCCAGTCCACTGGCGGCGAAAATCTGGCAATAGCGGCAACAGCCACTGGATGCAGCTGTGGCA
ATCGAGCGCCAGATGCAGCCTGCCCGCATCGCCGCTCTGCAAGGCCTGCAACTGCTTCTCGGTCTGGGCC
AGGGCGGGCAGTACCTGACGGGCGAGGGCGAGCAGCTGGCTGCCGGCTGCGGTCAGCACCAGCGGGCGGC
TTTTGCGCAGGTAGAGCTCCAGATTGAGGCGGGTCTCCAGCTCTTTGAGCTGGTGGGAGAGGGCGGACTG
GGTGAGGTTGAGGGTCAGCGCCGCACCGGCCAGCGAGCCCTGCTCGGCCAGCGCGGTCAATATTTTCAAG
TGCTTGATCTCTATCCCTGCATTGCTTGGCACTGCCATAAGATGGCTCCTTGTGAGATTGCGTATGGCGC
TGAGGTTGCGGTCGGCGGTGGCCTGTTGCGCTTCTTGACTCCGGTACCTCTTGCACCTGTAAAAATCGGC
CTCTGTATCCTCGCGGTGAACCTGACTGGCGGTCAGTTGAGGATTATTTGAGAGCCACTTGAAGAGGATT
CAATAGCCAGCGTCACCTTATTTTGTCATTTTGGATGTGTAAACATCTGGATGGCTAAATCATCCAGTTT
CCCATCAGATGAATGAGCATAAGGAAATGAACCTATGACACGAGCACACACCCTGGGCTTCCCCCGTATC
GGCGCCAAGCGCGAGCTGAAATTTGCCCTCGAATCCTACTGGCGTGGCGAGACCACTCAGGCCGAACTCG
TTGTCGTGGGCAAGACGCTGCGTGAGCGCCACTGGCAGGCCCAGCACGGCGCCGGGGTCAATCTGCTGCC
GGTGGGCGACTTCGCCTGGTACGATCAGGTGCTCGGCACCAGCCTGCTGGTAGATGCAGTGCCTGCCCGC
CACCGCCATGGCGAGACCGATCTCGACACCCTGTTCCGGGTCGCCCGCGGCCGTGCGCCGACCGGCCCGG
CGGCGGCGGCCGCCGAGATGACCAAATGGTTCAACACCAACTACCACTATCTGGTGCCGGAATTCAGCCG
TGACCAGCGCTTCAAGCTGGGCTGGAGCCAGCTGTTTGACGAGGTTGAAGAGGCCAAAGCGCTGGGGCTG
CCGGTCAAGGCGGTGCTGCTGGGACCGGTCTCTTATCTGTGGCTTGGCAAGGAGAAAGAGAGCGGCTTCT
CGCGCCTTGAACTGCTCGATCGCCTGCTCATCGTCTATCAGGAGATCCTGGCCAAGTTGGCCGCCCAAGG
GGTGGAGTGGGTACAGATCGATGAACCTGCGCTGACGCTGGACCTGCCGGACGAGTGGCGGCTGGCCTAT
CTCAACGCCTACGAGCGCCTTGCGGGCCCTTGCAAGCTGCTGCTCACCACCTATTTCGGCTCGGTGGCCC
ATCAGCGCGACATCATCACCAGCCTCAAGGTAGATGGCCTGCATCTGGATCTGGTGGCCGCCCCCGAGCA
GCTGGAGACCCTGGTGCCGCATCTGCCCGCCCAGTGGGTCATCTCAGCCGGGGTGATCAACGGCCGCAAC
GTCTGGCGTGCCAATCTGGCCAAGCTGGCGCCGACTCTGAAGGCACTGAAAGCCAAACTCGGCGAGCGGC
TCTGGGTGGCCTCATCCTGCTCATTGCTGCACAGCCCGGTGGATTTGACCCTGGAGCAGGAGCTGGATCC
CCAGACCCGCAGCTGGTTCGCCTTCGCCTTGCAAAAATGTTTCGAGTTGGGGCTGCTCAGAGAGTATCTG
GATGGGGGCGAGCTCGACAAAATCACCGCCTACAGCCAGCCCATCGTGGATCGGGAGTCCGACAGCCGGG
TGCACAAAAAAGCGGTGCAGTCACGGCTCGCCAGCCTCACCAACAGCGATTTTGATCGCCACAGCCCCTA
TCCGGTCAGGGCAGAGCTGCAGCGCTCGGATCTCAAGCTGCCGTTGCTGCCCACCACCACCATCGGCTCC
TTCCCGCAGACCTCGCAGATCCGGGTATTGCGGCAGGATTGGCGCGCCGGTCGCATCGATGACAGTGCCT
ACGAGGCGGGCATTAAGGCGGAGATCCGCGATGCCATCGCGCGTCAGGAGGCCATCGGGCTGGACGTGCT
GGTACATGGTGAGGCCGAGCGCAACGACATGGTGGAGTATTTCGGTGAACTGCTGGATGGCTTTGCCATC
ACCCGCTTCGGCTGGGTACAGAGCTATGGCTCACGCTGCGTCAAGCCGCCGGTGATCACCCGCGATATCG
ACCGCCCTACCCCTATGACGCTCGAATGGACCCGATTTGCCCAGAGCCTGACCGACAAGCCGGTCAAGGG
GATGCTGACTGGCCCTGTGACCATCCTCTGCTGGTCCTTCCCGCGGGAAGATGTGAGCCGCGAGCAATCA
GCCCTGCAAATCGGGCTGGCCATTCGCGACGAGGTGGCTGACTTGGAAGCCGCCGGCATCAAGATCATCC
AGATCGACGAACCGGCGATCCGCGAAGGTTTGCCCCTGCGCAAGCAGGATCATCAGGCCTATCTCGACTG
GGCGGTGCGAGCCTTCCGTCTGAGCGCAAGCCCGGTGGCGGACAGCACCCAGATCCACACTCACATGTGT
TACAGCGACTTCAACCTCATCATCGAGGCGGTGGCGGCGCTGGATGCGGATGTGATCACCATCGAGACCA
GCCGCAGCCAGATGCAGCTGCTCGAAGCGTTCGAACGCTTCAACTACCCCAACGAGATTGGCCCGGGTGT
CTACGACATCCACTCGCCGAACGTGCCAAGCCAGAGCTGGATTGAGGGGTTGCTGCGCAAGGCGGCCAAA
CAGATCCCGGCCGAGCGGCTCTGGGTCAACCCGGATTGCGGCCTCAAAACCCGCGGCTGGGAAGAGACTG
AGGCCGCCCTCAAGGTGATGGTCGATGCCACCAGGGCGCTGCGGGCCGAGCTGGCGTAACCGGCAAGCCG
TGACCGCCATCGATAAGGTGGCGTGCTGACAAACGAACGAGAGAGGGGAAGCCATTGCTTCCTCTCTCTC
TTTTTTCGTTGGCCACTTTTCCACTGGATCCTGAACCTTGCCGCAGCCCGCTTTGCACTAGGCTCAGAGT
TGGCAGAAGTGGATGGAGTCGCGGCCATGAGAGTCAGTCTGGGGAGCGCAATCCTGTGGGGATTGCTGAT
GTGTCAGAGTGCATGGGCAGCGCCAGCGGCCGAGTTGAAGATGGTCTACTTTGACGACTTTGCCCCATAT
TCGTGGCGTGACGAACAGGGCAGGATGCGCGGGCTGATGATCGACGTGATGGATCGGGTGGCTGCGCAGA
TGGGGCTGCGGGTGCACCACGAAGGCTATCCGTGGCAACGGGCGCAAAAACTGGTGCGGATGGCGCAGGC
CGATGGCTTCGTGACCATCGCAACCCGGGAGCGGCGCAGCTATACCCTGATTGTGGAAGAGCCGGTTACC
ACCACAGTGCTAACCATTTTTACCCAGCGTGCCCATCCCCGAATGGCGGAGTTTCAGCGAGCCAGCCGCT
TGAGCGACTTCAAATCGCTTACCTTTCTCGACTATTTCGGTAATGGCTGGGGCAAGGAGAACCTGGCGGG
CTTCACCGTTCATCACACCATCAATGTTGATAATGTCTTCAAAATGCTGGCGGCGGGTCGGGGGGACCTG

ATGGTGACCGACCCACTGGTGGCACGTTTCAAACTCAACCAGCTGGGTCTCTCCGGGCAGGTGGTCGAGG
TGCCGCTGCAGCTTGCCTCTACCCCGTTCAATCTCTGCATCGGCAAGCACTCCCGCTTCAATACCCGGAT
AACCGAGTTCAACCGAGCCCTGCAACGGTTGCGACAAAGCGGCGAGTTGGCGCGTATCGTCGAACGCTAT
CGTTGATGCGAGGCCGCCATCTGCGGTTTGCCAACGGCGTGTCTATCTTGGCGTCATATCGGCGATGGTT
GTCCAGAAGGCATCGATCAAGGGCCCCTTGCTGTGGGGCGGACGGATAAGGCCAATGGTCCAGCAGGCTT
CCCCATCTCTAAGGGGCAGGGTGAGTACATCCGGGTGCTCTATGGTGGTGGAGGAGGGGACCAGAGTCCA
GGCGATCTCGGCTGCCGCGGTGGCAACACCGGCGGCGAAGTCGTTGACGTACTGGATATCTCGAAAACGC
AGCCCGCTCTGGGCCAGATAGTTCATCAGGTGATCGTAAAACGACGGCGCCTTGTCCCGACGCAGGATCG
CCAGCGGCTTGTCAACCAGATCCGCCAACCCCCTCACATCGCGGTAGCTGGCGGGCAGCACGGCGACAAA
GTGGGCTTCGACCACCGGCAGGGCGTGCCACCTCTTGGGGGCGGGCAGGCGGCAAAAACCGAGATCCAGC
TTGCCCTCACTCAGCGCCAGCAACTGGTGGTGGGTGGAGAGATCGTTGAGCTCTATGGTCACTTGCGGCC
GCTCGGCGCGAAAGCGGGCGATGGCGGCGGGCACCAGGATCTTGGTGGCTACGCCAAAGCCGATGCGCAG
GGTGCCGACATTGCCCGCCAGCACGTCGCGGGCGGTGCGCGAGAGTTGCTCGCTCTGGATGACCACAGCG
CGGGCATCCTCATACAGCGCCCTGCCCAGTCCGGTGAGGCGAGTGGTACTGGCGTTGCGCTCGAACAGCT
CGCCGCCCAGCTCCTCCTCCAGCCGCCTGATCTGTTTGCTCAGGGCCGACTGGGTAACATGCTGGCGCAC
CGCCGCCTGGCCGAAGTGCAGCACCTCCCCCAGAGTGACGAAGGCTTTCAGATCCCGTAGCTCCATCAGG
GCTCCTTTCATTCCTGTTGGGAATGTTGAGTGGTCAAATTTGTCATTGGACGGAATTTTAGAAGTGATGG
ATGCTTATGCCAATTCATTTCGGATGCCCCGACAGGGGGCGCCGAGTTATCAGAGATGGCTGACACCATC
AGCGCAGAAGGAGCAACACCAGATGCATCACCACAAGTTCAACGACTTCCCGAGCGATTTTCTGTGGGGC
GCAGCCTCCGCCGCCTATCAGGTGGAGGGAGGCTGGAATGCCGATGGCAAGGGGCCCTCGGTGTGGGATC
TCTTCACCAAGCTGCCGGGCAAGACCTTTGAGGGGAGCAACGGCGATGTGGCGGTGGATCACTACCACCG
CATGGAAGAGGACGTGGCGCTGATGGCCGAGATGGGGCTCAAGGCTTACCGCTTCTCGGTGAGCTGGCCG
CGCATCTATCCCACCGGTCGGGGGGAGGTGAACGAGGCGGGTCTGGCCTTCTACGAGAAGCTGATCGACA
CCCTCTTGGCCCACCAGATCGAGCCGGTACTGACCCTTTATCACTGGGATCTGCCGCAGGCGCTGCAGGA
TGAGTACGGCGGTTGGGAAGACCGACGCATTATCGAGGATTTCGAGCGCTACTGCGTGACCCTCTATCAG
CGCTTTGCCGGCAAGGTGAAGTACTGGGTGTCGCTTAACGAGCAGAACTACAACCTGACTAACGCCTATC
AGCTCGGCACCCATCCGCCCGCTGTGCAGGATCGCAAGCGCTTCTTTGCCGCCAACCATATCGCGTTTCT
GGCCAACGCCCGGGTGATCAAGGCGTTTCGCCAGCATGTGCCAAATGGCCTCATCGGCCCGAGCTTTGCC
TACTCTCCTGCCTATCCGGCGTCGTGCGATCCGAAAGATATGCTGGCCTATGAGAACGCCGAGGAGTTCA
ACAACCTCTGGTGGCTGGATGCCTACTGCTTTGGCCGCTATCCGCAGGCGGCGCTCGCTTACTTGCGAGA
AAACGGCGAGGCGCCGGAGATCCTGCCGGGCGATATGGAGTTGCTGCGAGAGGGGACGCCCGACTACATC
GGCGTCAACTACTACTACACCCTGACCTACACCGATAATCCCTTGGACGGTCAGACCCTGCAGCGGATCA
ACACCACCGGCAAGAAGGGTACTACGCCATCGAGCGGCATCCCCGGCCTCTACAAGACAGCGCCCAATCC
GCACCTTGAGACCAGCAACTGGGACTGGGCCATCGACCCGACCGGGATGCGCATCGCGCTGCGTCGCCTC
TCTTCCCGTTATGGTCTGCCGCTGATGATCACCGAGAACGGTCTTGGCGAGTTCGACAAGCTGGAGGCGG
GCGACAAGGTGAACGATGTGTACCGCATCGATTACCTGCGCAGCCACGTCAAGGCGTGCCAGGAGGCGAT
GCAGGATGGGGTGGAATTGCTCGGCTACTGCGCCTGGTCGTTCACCGACATCCTGAGCTGGCTCAATGGC
TACCAGAAGCGTTACGGCTTCGTCTATGTGGAGCGTGACGAGCAGGGGGGCTCCCTTCGCCGGATCAAGA
AGGCGAGCTTCCACTGGTACAGCCAGGTGATTGCGAGCCAGGGCAAGCAGCTCTGACGATGTATCGCGTC
GCGTAACCAGCGGGCCCGGGCAATCCCCGGGCCCGCGCTGTTTTTGGCGGGGGAGAGGGGGCAAGCCGGT
TTCTCACCGTGCGACTCATCCCTGTTTGCTGATTTTTGTCCTGCCAAAGCCGCCGACCCCTGCTATTATC
TGCGGCCGAACATGAGCCAATAAAAATCAGGATATCGTGATGACTGACCACACTTTTATTCCCGGCAAGG
ACGCGGCGCTGGAAGACTCCATCGAACGCTTCCAGACCAAGCTGCAAGCCTTGGGCTTTGATATTGAAGA
GGCCTCCTGGCTCAACCCCGTGCCGAACGTCTGGTCGGTGCATATTCGCGACAAAGAGTGCGCCCTCTGC
TTTACCAACGGCAAGGGTGCCAGCAAGAAGGCGGCCCTCGCGTCTGCCCTTGGCGAATATTTCGAGCGTC
TCTCCACCAACTACTTCTTCGCCGACTTCTATCTGGGCGAGCAGGTCGCCAACGGCGATTTCGTTCACTA
CCCGAACGAGAAGTGGTTCCCCATCGAGGGTGACCAGTTCCCGGCCGGTCTGCTCGACAGCTTCACTCGC
AAGTTCTACAACCCGGACGGTGAAGTGACCGCCGACATGCTGGTGGATCTGCAGTCAAACAACGAAGAGC
GCGGTATCGTCGCCCTGCCGTTCGAGCGTCAGTCCGATCACGAGACCGTCTACATCCCGATGAACATCAT
CGGCAACCTCTATGTCTCCAACGGCATGAGCGCTGGCAACACCAAGACCGAAGCGCGCACCCAGGCGCTC
TCCGAGGTGTTCGAGCGCCACGTGAAGAACAAGATCATCGCCGAGGCCATCAGCTTGCCGCTGATCCCGG
ACGAGGTGATTGCCCGTTATCCGGGGATTGCCGCCTCCATCGCGGCGCTCGAAGCGGAAGGCTTCCCCAT
TTACAGCTTCGATGCCTCTCTGGGTGGCCGTTATCCGGTCATCTGCGTGGTGCTGCTCAACCCGAGCAAC
GGCACCTGCTTTGCCTCCTTCGGCGCCCACCCACGCTTCGAAGTGGCCTTCGAGCGGACCGTCACCGAGC
TGCTGCAGGGCCGCGGTCTCAAGGATCTCGACGTATTCGTGCCGCCGTCATTCGATAACGAGCAGGTGGC
GGATCACCACAACCTCGAGACTCACTTCATCGACTCCTCGGGCCTTATCAGCTGGGACCTGTTCAAGCAG
GATGCGGACTTCGAGTTTGCCGATTGGGACTTCCGTGGCACCTCGCAGGAGGAGTTCGACCACCTGATCG
GCATCTTCCATGAGCTGGAGCAGCCGGTCTATATCGCCGACTACGAGCATCTGGGTGTTTACGCCTGCCG
CATTCTGGTGCCCGGCATGTCCGACATCTATCCGGCCGAAGATCTGCTGCTGGCCAACAACAACATGGGC
GCCAACCTGCGCGAAGTGATCCTGGCGCTGCCATCACTGGATTGGGATGCCGAGCAGTACATGGCGTTGT
TTGATCAACTGGATGAAGAGGGGCACGACGAGCGCACCCGTGTACGCGAGCTGCTGGGCATCGCCGCTGC

CAAGGGTACCGCGCTGCACACCCTGCGCGTTGGCGAGCTGAAAGCCATGCTGGCGCTGGCCGCCGGTGAG
CTGGAGACCGCACTGGATCTCATCGACTGGACCATGGAGTTCAACCAGTCGGTGTTTACTGCCGAGCGTG
CCAGCTACTACCGCGCCCTGCGTGCCTGCGTCGAGTTGTTCCTCGACGAGGAGCGCGACCCCGAGCAGTA
TCGCGCCGTCTTCACTCGCATGTATGGTGAAGAGACGCTGGCACAGGCATGGGCCAACGCCAGTGGCGAA
GCACGCTTCCACGGGCTGGAGGCGGCCGATCTGTCGCTCTCCCAGTTCCCGCTGCACCAGAAGCTGCTGG
CGGCCTACGAGAAGCTGCAGAAGGCCAAGCGAGCCCATCACTGGGCGTGATTGTTGCTGTAAAGGAAGGG
GCCGAGTGGCCCCTTCTGTTTATCGTGCGGCAAGGCACGGGATAGTTATTCAAAGATGAAATAAAAAACA
AAAAATACCCCTAAGGGGGTTGATGGCAGGGCAGGGGATGTGCTGTCGATCCGCAAAGATATCTTGCCAA
TGCCACATTTCCATGATCGAAAATAAATTCTTTAAAATCATTGCATTAAAAATGTGATATTTCTGTTTCA
GCTGATACCTGGTGTAAAGTGGCGTAGCCATCGCACTAAAATATTTGTAGTAAAATTACATTCGCACTCT
GATAAACTTGATCAATGTCAATTTTGGCTATTTGGCCCTTTGTTATGATTTCAACAGGCAAAACATTCAG
GTAGAAAATAATTGAAAGCTGAATCTCCATTCGATTGTCTCAAGCCCGAAGCCATCGCTGCACTGGCTGA
AGACATCACCTATGCCAAGGCTACCAAGCCGGCATACAAGGTCATTCCACTGGCCATCACCGCAGGCGCT
TTCATCGCCATCGCATTTATCTTCTACATCACAGTGACGACCGGAACCGGCTCCATGCCTTGGGGTATGG
CCAAGTTGGTTGGCGGTCTCTGCTTCTCTCTCGGACTTATTCTCTGTGTGCTGCTCGGTGCCGAGTTGTT
CACCTCCACCACCCTGACACTGGTGGCCAAGGCTGCCAATCGCATCAGCTGGGCTCAGCTGCTCAAGAAT
TGGGGTCTGGTCTACTTCGGTAACCTGATTGGCGGTCTGCTGATGGTGGCGCTGATCATCGTTTCTGCCG
AGTACACGGCAGCAGATGGTCAGTGGGGCCTTAACGCCCTCAAAGTGGCCCAACACAAAATTCACCACAC
CTTCTTTGAAGCGGTGGCGCTGGGTATCCTCTGTAACCTGATGGTATGTCTTGCTGCCTGGATGGCCTTT
GGTGCCCGCAGCGCGACTGACAAGGTAGTGGTTATGCTGCTGCCGGTTGCCATGTTCGTTGCATCAGGTT
TCGAGCACAGCATCGCAAACATGTTTATGATTCCGGTTGGTATCGCTATCCACAGCGTGGCTAGTCCGGA
ATTTTGGCAGGCCATCGGCCAGGATCCGGCAACCTTTGCTGACCTGACCGTATCCAACTTCGTCCTACAC
AACCTCATCCCCGTGACCATCGGCAATATCATCGGTGGTGGCGTGATGGTTGGTCTGACTTACTGGTTTA
TCTTCCGTCGTCATCATTAAGGATGATTGCGGGATACTACATCAATACCGTGTGAGGTAATGAAAAATGG
CAGAACTTAACGAACAGTTCCAAAAAGCATGGGAAGGCTTCGCGGCCGGTGAATGGCAGACCTCTGTCAA
CACCCGTGACTTCATCCAGAAGAACTACACCCCGTATGAAGGTGACGAGTCCTTCCTGGCTGGCGCGACC
GAAGCGACCACCAAGCTGTGGGACAAGGTCATGGAAGGGATCAAAATCGAGAACCGCACTCATGCGCCGG
TTGATTTTGATACTGACCTGCCCTCCACCATTACCGCACACGATGCCGGTTATATCGATCAGAGCCTGGA
GCAGATCGTTGGTCTGCAGACTGACAAGCCGCTGAAGCGCGCCATCATCGCCAACGGCGGTATCAAGATG
GTTAAAACTTCTTGCGAAGTGTACGGTCGTCAACTGGACCCGATGGTTGAGAAGATCTTCACCGAATACC
GCAAGACTCACAACCAGGGCGTGTTTGACGTTTACACCAAAGACATCCTGAACTGCCGCAAGTCTGGTGT
TATCACCGGTCTGCCGGATGCCTACGGCCGTGGCCGTATCATCGGTGACTACCGTCGTGTTGCTCTGTAC
GGTGTTGACTTCCTGATGGCCGACAAACTGGCCCAGTTCAAGTCCCTGCAAGCTGACCTGGAAAACGGCG
TCAACCTGGAAGCCACCATCCGTCTGCGCGAAGAGATCTCCGAGCAGCACCGTGCCCTGGCACAGATGAA
GGTGATGGCTGCCAAGTACGGTTGCGATATCTCCGTACCGGCCAAGAACGCCAAAGAAGCCGTACAGTGG
ACCTACTTCGCCTACCTGGCGGCAGTCAAGTCCCAGAACGGTGCAGCTATGTCCTTCGGTCGTACCTCCA
GCTTCCTGGATATCTACATCGAGCGTGACCTGCAGAAAGGCCTGATCACCGAGCAAGAAGCCCAGGAGCT
GATGGACCACATGGTCATGAAGCTGCGTATGGTTCGCTTCCTGCGTACTCCGGAATACGATTCACTGTTC
TCCGGCGACCCGATGTGGGCGACCGAGACCCTGGCTGGTATGGGCGTTGACGACCGTACTCTGGTCACCA
AGAACAGCTTCCGTATGCTGAACACCCTGTACACCATGGGCCCGTCCCCTGAGCCGAACCTGACCATCCT
GTGGTCCGAGAAGCTGCCGGTCGGTTTCAAGAAGTACTGTGCCAAGGTATCCATCGAGACCTCTTCCGTT
CAGTACGAGAACGACGATCTGATGCGTCCGGATTTCAACAACGATGACTACGCCATCGCTTGCTGTGTAT
CCCCGATGATCGTTGGTAAGCAGATGCAGTTCTTCGGTGCTCGTGCCAACCTGGCCAAAACCATGCTGTA
TGCAATCAACGGTGGTATGGACGAGAAGCTCAAGATCCAGGTCGGTCCGAAAACCGAGATGGTCAAGTCC
GAGTACCTCGACTACAACGATGTAATGGATCGTCTGGACCACTTCATGGATTGGCTGGCCAAGCAATACG
TTGCTGCGCTGAACATCATCCACTACATGCACGACAAGTACAGCTACGAAGCCTCTCTGATGGCGCTGCA
CGACCGTGACGTATATCGCACCATGGCGTGTGGTATCGCTGGTCTGTCCGTAGCGGCTGACTCCCTGTCT
GCCATCAAGTACGCCAAGGTCAAGCCGATTCGTGACGAAGATGGCGTTGCCATCGACTTCGAAATCGAAG
GCGAATACCCGCAGTTCGGTAACAACGACGCTCGCGTCGATGATATCGCCTGTGACCTGGTTGAGCGCTT
CATGAAGAAGATTCAGAAGCTGAATACCTACCGTGGTGCTGTTGCTACCCAGTCTGTTCTGACCATCACC
TCCAACGTGGTATATGGCAAGAAGACCGGTAACACTCCTGATGGTCGTCGCGCTGGTGCGCCGTTCGGCC
CGGGTGCCAACCCGATGCACGGTCGTGACCAGAAGGGTGCCGTTGCGTCCCTGACTTCCGTTGCCAAGCT
GCCGTTCGCTTATGCTAAAGATGGTATCTCCTATACCTTCTCTATCGTGCCGAATGCACTGGGTAAAGAT
ATGGAAGCTCAGAAAGCCAACCTGGCTGGCCTGATGGACGGTTACTTCCACCACGAAGCCAACGTAGAGG
GTGGTCAGCACCTGAACGTGAACGTGATGAACCGTGAAATGCTGCTGGACGCCATGGAAAACCCGGAGAA
ATATCCGCAGTTGACCATCCGCGTATCCGGTTATGCCGTTCGCTTCAACTCTTTGACCAAAGAGCAACAG
CAAGACGTCATCACCCGTACCTTTACTGCTTCTCTGTAATCGAGACGTAGTGAGCAAAAACCGGCGCCAA
GGCGCCGGTTTTTTTATGCGTGCGATTCAGGGGGAGTAATCATCCCCTGTGAATCGCCCCCAGTTCCATC
GCAACCACAACAGTTGTTACTGGGTATTTGAAGAGGATTAAGGGGAGGGGCTCACCAGGATTGACCTGGG
CTCCGTTTGTAATGCGATTATGCTTTATTCGCGCTCTAAGGGGATAGGCGGTTGCTTTGCCGGCCATAGC
ATCAGTGTTGCTATGGCTCGTGGAACAGCTGATCAGGTTTAAGGGGTGAGCAGAGACGCCATCGCGGGTA

TCAAGAAATACCCATAAGCCTGTGACTATGGAATCATTCTCTGCCTCTGCCTCTGCCTCTGCCTCTGCCT
CTGCCTCTGCCTCTGCCTCTGCCTCTGCCTCTGCCTCTGCCTCTGCCTCTGCCTCTGCCTCTGCCTCTTC
CTCTGCCTCTGCCTCTGCCTCTGCCTCTGCCTCTGCCTCTGCCTCTGCCTCTGCCTCTGCCTCTGCCTCT
GCCTCTGCCTCTGCCTCTGCCTCTGCCTCTGCCTCTGCTTCTGCTTCTGCTTCTGCTTCTGCCTCTATCT
CTCTCAATGCGCAGCTGGGAGGGGTTCTGGTTTAGAGATATGTGTGAAGCTACAAACCCTGCTTGCAGAA
ACAACAAGGGCTGCACATTGTGCAGCCCTTGTCTCTGGAGAATAGCGGGTTGAGCGTGTCAGCTGGCCCA
GGTGATGATGTGATCGATCCATTCACCCGCCTGATCCTCGTGAATGACTTTGCCACGGACGGATTGACCA
GCCTGATGCATCGCGGAACGACTGCCGGTCAGCAGAGGATGCCACTCCGGAAGAGCGTCACCTTCAGACA
GCAGGCGATATGCGCAGGTTGAGGGGAGCCAGTCATATTCGGTGATCTTGTCATGGGTGATCTGCAGACA
GTCAGGCTCTTTTTTGAAACGATTGGCGTAATCAGAGCACTGACAGGTCTTGGTGTTAAGCAAATTGCAG
GCGACATTGGTGAAAACCAGTTCGTCGGTATCTTCATCGATCAGCTTGTTGAGGCAGCATTTTCCGCAAC
CATCGCAGAGGGATTCCCATTCGGATTCAGTCATCTGCTGCAGACTCTTGGTCTGCCAAAAGGGGGCTTG
CATCATACCGCTCACTTATTACACCTGATAAAACGCGTGGTTATACCCCTTGTTACCCGGTATTTCAAGG
AGAGGGTGACTCCGGTGGGCAAATTGTCGTGATCAGCTCAAATGAATGCAATGACTTTCAGCCTCATCAT
ATTGCTCAAACGGGTTCGCTGGTGGGCAGGAAGTCCCGCAGGTTTGAATACTTGGGCAGCATGATTGTCC
AGTTCAGGTTTTATTTTCTGCTTCTTTATCCGTTACATCTTTTGATGCTTGTCTGCTTGCAGCAAGCAGG
GTCAAATGATCTCCGATGCCTACCGTCAGGCTGTTGCAAGCTTGTCTTTCAAATAAAGCCGCTGGTCACA
TATTGTTTGCGCCCTTCTGTCACTGCGACCCGCGGGAGAGCCTGCGTAGGCGTTCGTTTTTTGTCTTAAT
GATTGAGATAGATATCTTGCTCTGAATAGGCCAGCAGAGGTGTGCCGAGGTTAGTCCGTTTCGCGCCAAA
GAAGCGGGTGAGTACGCCAGATTGCTGTCCCAGTCGCTGCAGCTTGACGTGATCCAGCTCGATCTGCTCG
GGTAGCCAGGTAAATACGGCGGCGCAGTGACCATTGCCCAGACTGCGCTCCAGGGTTTGCTGCCAGTTCT
TGATTTTGGGGCTATGTACCACCAGCACTCTCTTGGGATCGATACCTCGCTCTTCCAGACGGCTGACTGT
GGGGCGTCCCGGCGGGGCGATCAACAAGATCCAGCCGGTATGAGCCTCCCCAAGCTTTGTCAGCTCGCTC
AGAAACTGATCATCAGACTGGATAGTCACACCATAATGAGCCAGCTCCGCTTTGCAGGCGGTTCTGGTTG
GCCAGGGCTTGCTCATGACACGATTCATCGCAAAGGGCAGTGAGTGCAGGGGCTGGTTCATATATGGGGT
TCCTGTATGAATATCCAAGCTGCATGTGCATACAGTAGTTCTATCCTGTGGCAGTGACAAGGTGTTTTTG
CTGAAAAATGTGATGGTGTTGGCATGTATCGTTAGTAAGGTTATGTTTTTAAACACTATTTAATATAAAG
CTGGTTGGGTGTTTTTCGTACTATGGACAAACCTTGCGTAACCGCCCATGGTTATACAGGTCATTTACAG
GAGGTGCCGCCATGGATATCAGTACCTTGCAACGTCTTGATGCCAACATCTTGCTGGGCATCATCAATGA
GAAGCTAAGGCTTGAGTGTGACGGGTTGGAGGAGTTGCTGGCCTATTACGATATGAGTGAAGAGCAGTTG
GCAGGACGTCTGGATCAGATTGGGTACCATTACGATCCATTGAGCAATCAGTTCAAAGCACATCTTTAAG
GGTGCAATGATTGCTTCATATTTATACGGTAATTTTTCAGGCACATTGAAACCGGTTTCAATGTGCCGTC
TTGCTGCCTATTTTCAGGGATTTTCAAGACTTGGCAATCTCTGAAAACCTATTCATGCCAGAATATTTTT
AATGTTGCATTAAGTTGCTGATAATAAATGAAATATTTGTTTTTGCTCGATCAATTAAAGGAAAGGGTTT
TCGAGCTTTTCAGGCTGTTTTTATCGGTTAAAGTGTGACTTGCGTTAACTTTTAATTCTGCCGCTTTAGT
TAGTATTCTCACGCCGGTACATATTTTGAGATAACAACAGCGCCGGTCCCGCAAGCGGGATAACAATTTT
ATTCGTAACGAAGGAATATGGTTTATGGCTACCAAGAAGAAGACAGACCAGGATCTCCAACTGGATAAAG
ATCAGCTCAAGGCGAGTATCGTGCGTCACCTGCGTTCTACCTTGGGAACAAGCGAACAGAAAGCCTCTCC
CGAAGCGTGGTGGAAAGCCACTGCTGCGGCTGTCAATGAACAGGTGTACGAGCGCCTGACCCGCACTCAG
CAGACTCATTTCAAGAAAGATACTCGTGCGATTCACTATCTCTCTGCTGAATTCCTGATGGGACGATTGA
CTTCCAACAACCTGCACAACCTCTCCCTCTACAAGACTTGCGAAGAGGCGCTGGGCGAACTCGGTCTGGA
ACTGACCGACCTGTGCGAACAAGAGCCGGACATGGCACTTGGCAACGGCGGCCTGGGTCGTCTGGCTGCC
TGCTTTATCGACTCGCTGGCCACTCTGAACTATCCGGCGGTTGGTTACGGTATCCACTACGAACACGGTC
TGTTCCGTCAGGAAATCCAGGATGGCCGCCAGATCGAGCGTCCGGACTCCTGGCGTGAATATGGCAACCC
CTGGGAGATCTGCCGCCCCGAGTCGGTGCAAGAAGTGCCGCTCTATGGCTACGTCGAGACCGTGTTTGGT
GACAATGGCGGCCTGAAGAAGGTATGGCACGCCGGTCGCAAGATTAAAGGGGTACCTTGGGATATTCCGG
TGGTCGGTTTTGGCGGTCACACCGTCAACATCCTGCGTTTGTGGGAATCCCGCGCCTCCGAGTTTTTCGA
CTGGGACGTGTTCAACGCCGGTGGTTACATCGACTCCCAGGCTGAGAAAGCCCAGGCCGAGACCATCTCC
AAGGTGCTGTATCCGAACGATGAGACCGATGCAGGTAAAGAGCTGCGTCTGATCCAGCAGTACTTCTTCT
GTGCTTGCTCCATCAAGGACATCATGCGCCGCTACAAGCGGGTCCATGGCAGTGACTTCAGCAACTTTGC
CGCGCAAATTGCCATTCAGCTCAACGATACTCACCCGACTGTCGCCATCCCGGAACTGATGCGGGTGCTG
GTTGACGAGGAAGATCTGAACTGGGATGCCGCTTGGGCTATCTGCTATCAGGTCTTCTCTTATACCAACC
ACACTCTGCTGCCGGAAGCGCTGGAGAAGTGGTCGGTCAGCCTGTTCGAGAAAGTACTGCCTCGCCATCT
GGAGGTGATTTACGAGATCAACGCCCGCTTCCTGAACGAGTTGGTGGAGCCCAAGTGGCCGGGCAACGAT
GCTATCAAAGCCAAGCTCTCCATCATCGAAGAGGGAGATGTGCGCAAGGTTCGCATGGGCAACCTGTGCG
TGATCGGTTCGTCCAAAGTGAACGGTGTGGCCGAGATCCACTCCAAGCTGGTGAAGGAAGATCTCTTCCC
CGAGTATGCCGAGCTGTGGCCGGAAAAAATGTGCAACGTGACCAACGGGGTGACCCCGCGTCGCTGGCTG
CTGGCGTGCAACCCGGAACTGGCTGGCCTCTACAACGAAGTAGTGGGCAAGGAGTGGCCGCTGCAACTGG
ACAAACTGCGTGGCGTGGTGAAGTTTGCCGACGACAAGGCGTTCCAGCAGCAGTTCATGACCATCAAGCG
TCACAACAAAGAGAAGCTGGTCAAGGTCATCAAAGACGAAACCGGTATCGATGTCAGTGCCGAGGCGATC
TTTGACGTTCAGATCAAGCGTCTGCACGAGTACAAGCGCCAGCAACTGAACCTCATTCACATCATGGCAC

TCTATCGTCGTCTGCTGGCCAACCCGGACTATGACATGCATCCGCGCGTGTTTATCTTCGGCTCCAAAGC
GGCGCCCGGTTACAAGCTGGCCAAAGACATCATCTATGCCATCAACAAGCTGGCGGATCGGGTCAATAAC
GATGCTCGTATCCAGGGCAAGCTGAAAGTGGTGTTCATGCCGAACTATCGCGTGAGTCTGGCCGAGAAGC
TGATCCCGGCAGCAGATGTCTCCGAGCAGATCTCCACTGCAGGTTATGAAGCATCCGGTACCGGCAACAT
GAAGATGGCACTCAACGGTGCAATCACCATCGGGACGCTGGATGGAGCCAACATCGAGATCGCTGAAGAG
GCGGGTGCCGAAAACTGTGCCATCTTCGGTCTGAACGTGGACGAAGTGAAGTCGCTGAAGGCGCGCGGTT
ACAACCCGTACGACTTCTACTATGCCAACTCCGAGCTCAAAGCCGTGCTGGACTGGTTTGATACCGACTA
CTTCACGCCGGGTCGTCCGGGTGAGCTGGCCTCCATCAAGCGCTCCTTGCTGGAAGGGGGAGACCCTTAC
CTGACCCTGGCGGACTTCCAGTCCTACTCGGATGCACACAAGCTGATCGATACTTGGTATCGGGATCCTG
CACTGTGGGCGAAGAAGGCCATCATCAACTCGGCCACCATGGGTAAGTTCAACTCAGACCGTTCCATCCA
GGACTATGTGGATCGGATCTGGGATCTGAAGGCCTGCAACATCGGCTAATCGGATTCATAAATGAGAAAA
CCCCCGCAATGCGGGGGTTTTTGTTTATTGGGGGGAGGTGGATCAGAGGATATCGCCAACGAAGTAGGCC
AGCAGGGCGAGCAACACGGTAAAGACCACGTTCAGAGCCATACCGGCTTTCATCATCTCCCTCTGGCGGA
TGTGGCCACTGGCAAAGACGATGGCATTGGGCGGCGTGGCGACTGGCAGCATAAAGGCACAGGAGGCACC
GATAGCGATCAACACCGACATCACCACAGGTGATACACCCAGCGCCTCGGCGACACCGGCAAACAGGGGA
ACCAACAGTGCGGCGGTGGCGGTGTTGGAGACCAGCTCGGTCAGGAACACCACGAAGGCGGCCAGCATAA
GCAACACAACAAAAATGTTGGCGTTGGAGAGTACGGCCGACAGATGGCTGGCCAGGAATGCGCTGGTACC
AGTGGCCTTGAGAATGGCGCTCAGGGTCAGACCGCCGCCAAACAGCATCAGCACGCCCCAGTCGGTGTTC
TGGTTGACGTCATCCCAGCTGGCGACACGGCTGACCACGATGGCCACGATGGCGCCCATGGCAATCAGGG
TGTCGAGTTGCGGAATACCGCCAAAGGCGTTGGAGATGGGTTGTGATCCGATCCAGAGCACAACGGTGGT
CAGGAAGATTGCCAGCGTCACTTTACGCTCGCGGGTCCAGTTGATGGTGACATCCTGGATCTCGACGCGG
TGGCTGAGACGCGGGCGGGTCACCAGATAGAGCAGGGCAATGCCGCAGGGCATCAGGATCAGCACAACTG
GAACACCGAATTTCATCCAGTCAGTGAAGCCAATCCCCATCTGGGCGGCTGCGATGGCGTTGGGTGGGCT
ACCCACCAGGGTGCCGATGCCGCCGATACTGGCGCTGTAGGCGATCCCCAGCAATACGAAGACGAAGGTG
GCGCGCTCTTTTTTGCTATCGAGGCGTGCCAGCATACCGATCGCCAGTGGCAGCATCATGGCGGTGGTAG
CGGTGTTGCTGATCCACATCGACATGGCGGCTGTGATGGTAAAGAGCAGGATGACGCCCCAACCCAGATG
ACCATTGGCCAGGTGCATCACCTTGCCTGCCATCAGGGTATCCAGTCCCTGTTTTGAGAGGGCTGCCGCG
AGGGCGAAACCGCCAAAAAAGAGGAACAGGATCGGGTTGGCAAAATCGGTGAGGGATTTGCTCATGTCAA
ACACCCCGAGCACGGTAGCCAGCAAGGGCACCAGCAGCGCGGTGATGGTGACGTGCAGTGCTTCGGTCAG
CCAGAGGATGGCGATAAAGACCAGAATGCCCAGCCCCTTGTTGACCATGGGGTCAAACGGGAGCCAGGCG
AGCATGGCGCAAAGTAGCAAGACATCCAGGATGACGATCCCTGATTTGATGTCCAGCCAGCGAGATGGAG
CAGAGGGTACAGCTGATGTGTGCATAATGTTCTCTCGGTTAATTTATGGTTGTTTTTGTGTTGCCAAGAT
TACATCCATTCACTTGTTTGAAACAGACTCACATGTTGAAAATCAGCCTCTACTCACATACTCATTGATA
AAAATGAGGAAATGAACACATAAATAAAACATGTGCGAAATATAAATCAAACATCCATGAACTGTTCCAG
CCATTGCTTGAACATGCTCTTGGGCAAGGAGCCATTTATCTGGTTCACCAGTTTGCCCTGTTGGAAGACC
ATCAGGGTGGGGATGCTGCGAATGGCGTAACGGGCGGCAATGGTGCTCTGTTGCTCGGTATCGACTTTGG
CAAAGCGCATCCGTGGTTCGAGTTCATGGGCAACCTCATGAAATACCGGAGCAAACTGCTGGCAGGGGCC
GCACCAGCTGGCCCAGAAATCGACCACGACTGGGATGTCTGAGTTGATCAGGGTATCGAAGTTATCCGCA
TTCCCGAGGCAAGGGGTCTGGCTGAACAGGAGGGCTTTGCAGCGCCCGCATTTGGCAGATGTATCGATAT
GGGTAACGGGCAGGCGGTTCCTGGTAAAGCAGTGGCTACAATAAGTAGTGATAAATGACATAATTGGTTC
CTTCTAAATCAGCTCTGGCCGGGGATATGATGAAAAGCCAGAATGAAGCAATCGCGAAACAATAGCGAAA
TATCAGAGCAATAACGGTAACACAGTGCCCAGCGGGAGTGGATAATTCCCCCATGGTTCATTGCCTGACC
GGATAATTCAGCGACCACCAGATGAGAGTCGAGACGGGATGAATAAAGGATCACGGAAAAACCGTGCTGT
GCCAGGGCTGATTGTTGCCCTGCTGCTTGCCTTTTCGGGCTACTGCTGGCAGTCGCTCAGTCAGCAGCAG
CAGGCCCACAACCTGAACGAAGCGGCCGAGGTGGTGCTGAACAGGATCAACGACGATACCCTCTATCTGC
TTGGGCGGGAGCAGGCCAATGACAGCAATGTCCAGCAGCGCCTGAACCAGTTATCCATCACCCTTGACGG
TTTTGTACGGGAAGTCAGAAGCGATCCCGAGCTGGTGCAAGACGACCTTTCCCAGGCTCTGCTTGATGAC
ATGGACAACTATCTGATGGCGCTGGCCAGGCTTCATGCCATTGAGATGGCTATCGAGTACCTCAATGCGC
CCCTGCAGGCCCAGATTAAAAAGGTGCGGGGGATAAGCCCGTCAGACAGTGCTCTCCATCGCGACCTGGA
GGAGTTGGCGCAACTGAGCAACTATCTGCAGCGCAGTTGGGACAAACGTGAACAGACTACGCTGGATCAG
CTGCAACAGCAGTTGCAATCCCGTTATGGCAACTTGCCGGAGATCGCGACCCAGATCCGCCTGAGCAAGG
AGATGAGCCAGCAGGTGATGGCCTATCACACCTTGCGTTACCAGCTCATCGACATGCATGTGATCGAGAG
TTTGGTGCGCTGGAAACTCGACAAGCTGGAGCGCGCCTCCTCTTTGATGAATCGCTTTTATCTGATGGTG
CTGTGCGGAGCTTTGATCTGCTTTGCGCTGGTGGCCTTCACCCTGTGGCGCCGTAACCAGAAGCTGAGCG
AACTGTCGCGCCAGACCGGCTTGCTGGCCCAGGCCAAAAGCGATTTTCTGGCCAACATGAGCCACGAGAT
CCGCACCCCCATGAATGCCATCATAGGTTTTAGCTCGCTGGCACTGCAGACTGAACTGGATCAACAACAG
CGGGACTATCTGGGCAAGATCAAATCCTCATCCGATACCCTGCTGCTGCTGATCAACGACATCCTTGATT
TGACCAAGGTGGAGTCCGGCAAGCTGACGTTGGAGGAGATCGACTTCGATCTCAGCGAGCAGCTCGATTC
GCTGGCGGGCATGTTTGCCGATCTGGCGGAGCGCAAGCACGTGGAGGTGGTTATTCGCAAGTCACCGGAG
GTACCGGTCTTCTTGCGTGGAGATCCCCTGCGATTGGGGCAAGTACTGGTCAATCTGGTGAACAACGCCA
TCAAGTTTACCGAACGCGGTGAAGTCGAAGTCTGCATCGAGCTGGCCAATCCTCACCCAATGCGGATCCG

CTTCAGTGTGCGCGATACCGGCATCGGCATTGCCGAAGAGAAGCAGACCCAACTGTTTCAGGCGTTTACC
CAGCTGGAGGCGGGCAATACCCGCAAATATGGCGGCTCGGGTCTTGGGCTCAATATCTCCCAGCGGCTGG
TGGAGCTGATGGGGGGGCGGATCACCGTAGAGAGCAAGCCTGGCGTCGGCTCTCTGTTCAAGTTCGATAT
CCCGATGGCGCAGGCGGTCTACGGCGTGGCTGGACGGAAGGTCTTCTTCGAAAACCAGCCGCTGGTGATG
GTGATGGATGACAACGAACTGGTGCTGGATCTGGCCCGGGACATTCTGACTCGGGCGGGAGTGCGAGTGC
TGCCGGTTAACTCCCTTTCCCGCGCCCGTCAGCTGCTCCGTGAAGAGGGACCGAATCTGCGGTTGGCGAT
CCTCGACTGGCGCATGGGTCAGGAGGATGGGCTCGATCTGGCGCTGGAGATGTATCAGCACAGCCAGTGG
CGGCGGATCCCCATCCTGATGATCAGCGCCTGGGCCAGAGAGGGGCTGCACGCCAGGATGGACAGCATGG
GGCTGACCCATTTTCTGCCCAAACCGATGACCGAAAATGCCCTGCTGGCCAAGGTGGACGAACTGCTCAA
CGGCAGCTCTTACGAGCTCAACCTGCGCAAGGCCGAGGCACAGGGGGATCAGCAACACTTCAAATCCCTG
TTGCAGGGTACCCGAGTGCTGTTGGCGGAAGACAACCGGGTCAATCAGCAGCTCATCATCGAGTATCTGC
GGCGCGTCGATGCGGTAGTGTGTGTGGTCAGCAATGGTCGGGAGGCGGTGGAGCGGGTCGCCGAGCAGCC
TTTTGACGTGATCCTGATGGATTTGCAGATGCCAGTGCTGGACGGACTCGATGCCACCCGCCAGATCCGC
AAGATGGTCGACAAACACGATGTGCCCATCATTGCCCTCACCGCCAGCGCCATGCCCGGTGACAAGGAGC
GTTGCCTGGGGGTGGGGATGAATGGCTATGTCACCAAGCCGGTGAGCAAGCTGGATCTCTACAACAACCT
GCTGCAGTGGGTAAAGCCGCAAGGGGTGGAGCAGGCGGGGATCATGGAGGTGAAACCGCCGGATATGATC
GGGATCCTCGATCTGCAGGATGCACTCAAGCGGCTGGAGCAGGACAAGGAGGCGTTGCAGATCCTGTTCA
AGCTGTTCATGTCGGAGCACAAGGACGATCTGTGGGAGATCCGCTCCGCTCTGCGCCGTCAGCAACCCGA
GGTGGCCAGCAAGCTGCTGCACACCCTCAAGGGGGTGTCGGCCAATATCTCGGCGGCCCGTCTGCAATTG
GTGGCTGGCGAGCTGGAGTGGCGCCTGCGGCAAAACGAGGTGCTAAGCGAGACGGATCTGGAGCAGTTGC
AACAGGTCTTTAGCCAGACCCGGGAAGAGGTGTCACACTTCCTGTTGACCGGTGAGCAGCATGAAAATTC
GTATAATCCCTATTCATCAGGGGATGATCGCATGTCACAGTGAATTAAATAGTAATTATTCTCAACCATC
CCGGTTCGGGATATGATGAGCTTTCGTTCGAATCGCCTCCAGCCGGAGGCGATTGTTTTCATTCTTCTAT
TTCTGCGTGGCTTCTCTGATGGCCGCTCCTTGGAGCGGTCACGGGCAGAGAGCGCTAAAGGTTGGTGGTG
TGAGCGTATTTCTACGATTGAGCTGGTTTTTCAAGGAGCAGTGGCCCCGTTATCTGGCTGCCATCTCCCT
GCTGCTGATGGTGGCCCTGTTGACCGTGATTCCGCCCAAAGTGGTGGGCTGGGTGGTGGATGGCATCACC
AGGGGTGGTCTCGACAATGGCACCCTGATGCGCTATCTGGCGGGGCTGTTTGGTCTGGGTGTGCTGATTT
ATCTGCTGCGCTACGTCTGGCGGGTGATGCTGTTCGGTGCCTCTTACCGTCTGGCCTATGTGTTGCGCAA
TCGCCTGTTCAGCCATTTCACCCGGATGAGCCCCGATTTTTATCAGCGCCACCGTACTGGCGATTTGATG
GCCCATGCCACCAATGACATTCAGGCTGTGGAGATGACCGCTGGCGAAGGGGTGCTGACTCTGGTGGACT
CCATCATGGTCGGGGTATTGGTGCTCTCCATCATGTGCAGCCAGTACTCCTGGCAGTTGACGCTGGTCTC
CTTGCTGCCGCTGCCGGTGATGGCCTATTTCATGAACCGCTTCGGCACCCAGATCTACACCCAGTTCAAG
GCGGCGCAAGGGGCCTTCTCCAGGCTCAACAACAAGACCCAGGAGGCCCTTTCCGGCGTGCGGGTGTTGA
AATCCTATGCGGTTGAATCGCTGGAAGATGAAGGCTTTGCCGAGCTGACCAAACAGGCGGGGGAGCGCAA
TATGGCTGTGGCCCGCATCGATGCCAAGTTCGATCCGGTCATCTATCTCTGTATCGGTTGCTCCTACTTC
CTCGCGGTGGCGGGGGGCAGTGTGCTGGTGCTGCGCGATGAGTTGACCCTGGGTGAGCTCACCAGCTTCA
CCATGTACCTCGGCCAGCTGATCTGGCCGATGTTTGCCATCGCCTGGCTGTTCAACATCATCGAGCGGGG
TAGCGCCGCCTACTCCCGTATCGAAAGCCTGCTGGGGGAGCGCAGCGATATCGAAGAGCCCGCCCAGCCG
GCCGCGTTTACCTCTGCCTTTCCTTTGCAGATCAAAGGGGTGAGTTATCAACTTGAGCAGCGCACCCTGC
TTGCGGATATCGACGTTACCCTCAAGCAAGGGGGGATGCTCGGGGTGGTGGGGCGCACCGGGGCAGGCAA
GAGCTCCCTGCTCAAGCTGTTGATGCGCCTTGCCAATCCGACCCACGGTTCGATTGTCATGGGTGGCGTT
CCGATTGACCAGCTGCCGCTCGCCACACTGCGCAGCCAGTTTGCCTATGTGCCGCAGGAGCCATTCCTGT
TTTCCACCACTATCGCCGCCAATATTGCCCTCGGCAAACCCGATGCGAGCCGTGAGGAGGTCGAACGGGT
GGCGCGCATTGCCTGTGTCCACGATGATATTGTGCGTTTCCCCAAGGGCTATGAGACCGAGGTGGGGGAG
AAAGGGGTGACCCTCTCCGGCGGCCAGAAACAGCGGCTGGCCATTGCCCGCGCCCTGCTGCTGGATGCGC
CCATTCTGGTGCTCGATGATGCGCTCTCGGCGGTGGATGCCCACACCGAGCAGCAAATTCTGCATGCCCT
CAAGGCCCATCGTCGCACCCTGATCCTGGTCAGCCATCGCATGACGGCGGTGGAGCAGGCCGACGAGATC
CTGGTGCTGGAGCAGGGAACCGTTAGCGAACGGGGTCATCACGGTGCCCTGATGGCGCACAATGGCTGGT
ATGCAGATATGGTGCGTTATCAGCGTCTCGAAGAAGCGGTGGAGGAGAGCCTGTGAATCCGACCATTAAA
CGACTGCTCGCCTACTGCGCCCAGTATCCTCGTTGGCTGATCCAGGCGGGGATTTGCCTGCTGCTTGCGA
CCGGCGCCGAAGTGGCCGGGCCGCTGCTGATCAAGCTCTTTATCGATGACTATCTGGCGCCTCGCAATAT
CGTGCTGCCCACCATAGCCCTGATTGCAGCGGGCTATCTGGGTTTGCAGGTGCTGTCGGCGGCGGGTTTC
TATCTGCAATCCCTGCGCTTTAACCGTATCGCTCAGGCGGTGGTGCAAACCCTGCGCGAGCAGGTATTTG
CTACCGCCATCCGTTTGCCGGCCCGCTACTTTGACAAGCATCGCTCCGGCTCGCTGATTTCCCGCATCAC
CAACGACACCGAGGCGATCATGAACCTCTATGTGCAGGTGATCGGCTTGCTGGTGCAAAAGGTGGTGCTC
TTGTGCGGCATCCTCATCTCCATGGCGCTGCTGGATCTGCGGCTGATGCTGGTCTGCTCCCTGTTGCTGC
CCGCCGTGGGCGGGGTGATGTGGCTCTACCAGAAACTGAGTGTGCCGGTGGTGCGAGCGACCCGCAGCCT
GCTCTCCGACATCAACAGCCGCCTCAACGAGTCGCTGCAGGGGATGCCGGTGATCCAGGCGATGGTGCAG
GAGCGCCACTTTGCCAACGCCTTTGCCGAGGTGAATCAGCAGCACTGGTCGGCACGGATCAAGAGCCTCA
AGATCAACGGCATTTTGCTGCGTCCGCTCATCGATCTCTTCTACATGATGGTGCTGATCGGTCTGTTGGC
GCTGTTTGCCCACGAGGGACTGAGCAGCACTGGTGCCATTCAGGTGGGTGTGCTCTACGCATTTATCAGC

TATCTGGGCCGGATGATCGAGCCGCTCATCGAGATGACCAACCAGCTTAACCAGCTGCAGCAGGCGATGG
TGGCGGGGGAGCGGGTCTTTGCCCTGCTCGATGAAACCCGCGAGAGCACCGAGGGCGAGCTGCGCCCGCT
GGAAGGGCGGGTCAGTTTCGAGCGGGTGAGCTTCTCCTACGATGGTGAGCAGCAGGTGCTGCGGGAGGTC
AGTTTTGCCGCAAAACCGGGCCAGATGCTGGCGCTGGTGGGCCATACCGGCAGCGGCAAGTCCACCATCA
TCAGCTTGCTGATGGGCTTCTATCCGCTGGTTCAGGGGCAGATCCGCTTCGATGATCACGCACTGGATAC
CCTGTCGCTGGCGGCGGTGCGCCGCAGCATCGGGTTGGTGCAGCAAGATCCCTTTATCTTCGTCGGCACT
CTGGCGGAAAACCTGCGTCTCGGTCGGGCCGGAATAGACGAAACGAGGCTCTGGCAGGCGTTGAGCGAGG
TGCAGTTGGCCGAGTTTGTGCGCAGCTTGCCACAGGGGATCAACACCTTGATGGAGGAGGGGGGCAAGAA
CCTCTCTGCCGGCCAGCGGCAACTGCTCTCCTTTGCCCGCGCGCTGGTGGGGGATCCGCGTATCCTCATT
CTGGATGAAGCGACGGCCAGCGTGGACTCCCAGACCGAGCTGGCGCTGTCGCAGGCGATCTCTGCCGCGC
GTCGTGGCCGCACCACCATCGCCATCGCCCACCGCCTCTCGACCATCGTCGATGCGGACGAGATCCTGCT
ACTCTCCCGCGGTCAGGTGAGCGAGCGGGGTACCCATGGTGAGTTGATGGCCCTCGGCGGTCACTATGCC
CAGCTGTTCGAGATGCAGAGTCAGGGGGCCTGGCTGGAAGAGAAGCGCGCCTGATCGGCACATTTGCTGT
AACAGCGACATGAGCGAAAAGAGGGAATCGGTGCGGTTCCCTCTGTTTTTTTGGGTTGGGCAAAAGGTCG
TAACCGTGATCCCTGTGCTTGTCGAATGGCGCAGATGCAGGCAACATGTTGTTTACATTAACTCATCGGA
TCAGTTCTCATATGGATTTGCAACGCTTCAAAGCCAATCTGGCGCTCCGGCTGTTTGCCTGGCGCTATAT
TCCCGTCATCGGGTTTTGTGCCCCGATCATCGAAGAGATGAACGCCAAGGCGCTGCGCATTCGCATTCCC
CTTGGCTGGCGTACCCGCAACCATCTGGGCAGCATGTATTTTGGCGCGCTGGCGACCGGCGCCGATCTGG
TAGGTGGTCTGCTGGTGATGGAGAAGGGGCGGCAGCGGCGCAAGAAGGTGCACTTCGCCTTCAAGGATGT
GCAGGGGGAGTTTTTGAAACGACCGGAGGGGGATGTGCAGTTCGACTGCGCAGCGGGGGAGGAGATCGAT
GCCATGATCGACGAATCGCTGGCAAGCGGTCAGCGCATCAATCGCCCCATCCGAGTGGTAGCCACCTGCC
CGTCATTGAACGGGGATGAGGCGATGGCGCGTTTTGTGCTGACCCTGTCGATCAAGGCCAGCTAGTGGGT
TGCCGGAGAGTGGCTCCCCAAGACGGGATTAGCCAATCTCCGGGCTGACGCCCTCTTTCACCACGACCTT
CATGCCGTTGAAGTTGACCACCTGTTCGGCCACGATCTTGCAGCGCTTGCGGGTCTCGACCTGACCGTCC
ACCTCGACCAGCCCTTCGGCAACGAGGGCTTTGGCGGCGCCGCCACAATCGGCCCACCCCTGATGTTTGA
TAAGGTCACACAAGGCAACGAAGGGGTGACCTTCGAGCAGAAACGTCTCCATGGGAAATCCTCTAGATGG
ATGAATGCCGGATACAGCAGGGCGACTATTTTAGGCAGTTCGCTCAGGCAAAGAAAGGAAGAGGGACTAC
TCGCTGCGAAAAAGTGGCCGATTGTTCTTGAGGTGGCGCTTTCGCAAGTCCTGATGGAGAGGTGAGAGGG
CCACCCGTGACGTATCGTGACGCACCTCGTCCCTGCTGGTGTGCCCACACTCTCCCCTGACTGTTAGGAT
CGGGCCACTCTCAATGGCCCAAGGAACTTTCCATGACACTCTCCCGCCTGCTGCTGGCATGCAGCGCCCT
TACTCTGTGCGGTGTCGCGCAGGCCAGTACTCCGGTGCAGGTCTCGCTGCCCGGGGTCAATTTGCCCGCG
TCCCATCAGGTGGAAGGGGCGCGCGCCTCGCTGCTCTACGGTCGGTCCGGTCAGGTGAAGGGCATTGATC
TGCCGATCTTCGCCCTCTCCGATGTGGATCAGTTCAGCGGCCTGCAGCTCGGGGTATTCCTGGGTGCTGG
TCGGGTGCGCCACCAGTTTGGCGGGGTCGCCATCAACGCCATCAACTGGCACGAAGGGCAGGATACCGGC
GTCAATCTGGGGCTGGTCAACTTGACCAATAACGTACAGGGGCTTAACTGGGGGGCGGTCAATATCGCCA
AGGGCAATACGCTGGCCAACGTCGGCTTCGTCAACTATGCCGAGCGTACCACCTTCCAGATTGGCTTTAT
CAATGCGACCAAGCACCTCGATGGCTTGCAGATCGGGCTGGCCAACTATGCCGCCAACGGCGTGTTCCCC
ATTCTGCCACTGGTGAACTTCCAGAAATCGTTCTGACCAAGGCGGTTCGCTTTGCGTTAAAGGCCTCTGC
TGATTACCCTGTGCCTCTTGCAACTTGTTACAGGCGAACAGAGGTAATTGATGACGGCAGGGCAGATGAG
TGCAATCGGGATCGGCTGGGATGTGCGTGGCTGGCAGGGCAGTGCCCAGGCGGTGGCCGTGGTGGGCTGG
CAGGCCGGTTCCAACCGCCTGCACTGGCTCGGGGTGTCGCCGCTGTTTCGTCTGAGCTCGCGGGTGGCGC
CAGATCTGGCCGCTCTGCTGCGTCCCGCCCTGCAAAACGAGCTGACCCTGATGCAGGTAGAAGCCTGCCC
GCAACTGGCGCTCGGTATCGATGCGCCGCTCGCCTTTCCCCGCGCCTTGCGGGATCTGCTCAACGGCCAG
CCACACAGCTGCGCCGTGCCCGAGCGGGAGATAGACAACCCCTACGCCTATCGCGACTGCGAGCGCTGGC
TGTATCAGCAGTACGGCAAAAAGCCGCTGTCGGCCACCTTCGACCGGCTCGGCAACAACGCCACCCTGGC
GCTCTCCATGCTGCCGCAGCTCTCTGATCTGCAACTGGTGCCGACAGTTGCCCCGAAGGCCAGTCGTGCC
GTGCTGGAGGTCTATCCGGCATTGGCCAAAGTGGGCGGCAAGGCCGCCCCGGCACGGCCCGAGCTGGCAG
CGCTGCTGCCTGCGGATGTGCTGGTCGACACCGATCGCTATGATGCGGCGCTCTGCGCCCTGATGGCGCT
GCAATATGCCGCTGGTGGCAAGGTGACGGTATTGCCTGAGCTGATTTCGCCACCCAGTGACATGCCGTGT
GATGAGGGATGGGTCTATCACTTTACCCGTCATGGCGTTGAGCACAACGGCATGTGATGGACGGTGACGC
GGCGCATCATGGCGCCGCTCATCCTGTCGATGCGGGCCAAATGGCGCAAAAAACCTGTCTTTAGTCAGGA
AATTGCCTGTTCATCCCGCGCCAAGGTGGCGCATCGTTATCTTCATCCGATATATTGCCCACCTTGCTGA
TCCTTTGCTGTGGTATTCCCGATGAAATCCGTTCTCTCTATTCAGTCCCACGTGGTCTTTGGTTGTGCCG
GCAACAGCGCGGCCGTCTTCCCCATGCGTCGCCTCGGCATGGATGTATGGCCCATCAACACGGTGCAGTT
TTCCAACCACACCCAATATGCCCAGGGGTGGCAGGGAATGGCGATGCCGGCGGGTCATATCAGTGTGCTG
GTGAAAGGATTGGGCAATATCGAGGTGTTGAGCCAGTGTGACGCCGTGCTGAGCGGCTACCTTGGTTCCG
CCGAGCAGGGGGACGAGATCCTGACCGTGGTCAACGCCGTCAAGGCGGCCAACCCCGATGCCATCTACTT
CTGCGACCCCGTGATGGGTCACCCGGAGAAGGGGTGTATCGTCGCTCCCGGCGTCACCCGTTTTCTGACC
GAGCAGGCGTTGCCGGTGGCCGACATGATGGCCCCCAACCTGCTGGAGCTGGAGACCCTCTGTGATACCC
ATCTGGCTGATCTGGATCAGACCCGCGCGGCTGCCCATCAGCTGCTGGCCCGCGGCGTCAAGCTGGTGCT
GGTCAAGCATCTCGGTCGTGCGGCGCAGGACTCCAGCCGCTTCGAGATGCTGCTGGCAACGCCGCAAGGG

GATTACCTGATTGCCCGTCCGCTCTATGACTTTGCCCGCCAGCCGGTGGGGGTGGGCGATCTTATCAGCG
CCCTGATGCTGGCGAATTTGCTGGCCGGTCACGATGCGGTCAGCGCGTTCGAGCGCACCAACGCCTCGGT
GGATGCGGTGCTGCAGGAGACCTGGCGCCAGCAGGCTTACGAGCTGCAACTCATCGCCGCGCAAGATGCC
TTCGCCCTGCCAGAGGTTGCAGTGCGAGCGGTGAAACTCCCCTGAGTCAACGCGGACTTTCGTCTATCCC
GACAAGGGGGTGGGATGCAGATTAAAAAGGCTTGCCCATGGGCAAGCCTTTTTACTGTTTGCACTGTTTA
GTGCGGCAAGCAATCAGATGCGGGGATGGCGGAGGCGATAACGCTCAGCCATGGCATTGGCCGCTACCAC
GGCGGCGTAGACATCCTGTTCACTCACTGTCATCGGCATGTTGCCCATGGTATCGAGGGGATCGCAGGCC
AGCTGCGCCACCTTGCGCCACTCGCTTTCATCAAAGTGGGTGAGCCCCAGTTCTTGCAGTGTCATGGGCA
GGCCTGCACAGTCGATGATGCGAAACACCTCATCAAGTTCATGTTGCGGGGCGCGCTCAAGCACAAGTTG
GGTCAGCAGGCCGAAGACCACCTTCTCGCCATGCTGGGCACGATGGAGCTCCGCGATCAGTGACATGCCG
TTGTTGACGGCATGAGCTGCGGCCAGTCCACCGGCTTCGGCGCCGACCCCGCTGAGGTAGATGGTGGCCT
CGATAACCTGCTCCAGCGCCGGAGTCGAGACATGGTGGCGTATCGCATCCATGGCCAGCTCGATGTTCTC
ACCAAGCAGCTCGTAACAGAGCTGTGCCAGACCAAGGCCAGTGCGTGAAGGGCGCTTGCCGACCAGATTG
AGTCCGTCTGACCGATAGCAGGCGCGCGCCTCGAAATAGGTGGCCAGGGCATCGCCAACGCCTGCGGAAA
AGAAGCGGGGAGGGGCGGCGGCAATGATGGCCGTGTCAGCCAGCACCACGTCCGGATTTTGCGGCAGGAA
CAGATAGCGCTCAAATTCCCCCGCTTGGTTGTAGATCACCGCCAGCGCCGTACAGGGGGCATCCGTGGAG
GCAATCGTCGGGAACAGTACAACCGGACGGCGCAGATAGAAGGCCACGGCCTTGGCGACGTCCAGCGTCT
TGCCGCCGCCGATCCCGACAATCACGTTCGCACCATATTGCTCTGCAAGGTGGGTCAGGCGGTGGATCTC
CTGTTCGGTGCATTCATAGCGAAACTTGTCGACCACTCCCCTGAGGCCTGCCTGCAGAAGCCCCGCCAAA
GCTTCTTCCCGTACCCGCTCCAGAATGAACTCATCGCTGATGAGTAACGCATAGTCACCAAAATCTTTAA
CGTGCTCATGGAGTTGCGGGAGAAGGTGTTGACCGATGATGAACTTTTTGGGGGAGGTAACTGTACGGGG
CAAAGTGGGCATCCATTATTCCTCAAGGCAGTGAACAACATAGATTTGATTATCAAATGGGTCTCTCACG
ATACGCCTGATGAGGCAATAGATGAAGAAGAAAAGGGGGGCGTTTAATCATTAATTAATCTTTCTGTTT
GTTTGGTTGTATTTTGGGGCGAGCAATTGGCGGTGTTGAGGCATACAGGCGCAATGGCTCTATCGTGGCA
ATCCGTCGCAAGCCATACTGCTGCAACAAACAGTCACGCTGGAAACAAGCAGGGCCGCTTGGTGCGGCCC
TGCTGTATTTGGATAAGGGATAGGGGATGGTCAGAGCGGTTTGTAGAAGATGACGGTCGCAGCAAGAGAG
CCATCTGAGTTGCGTGCACTTTGGTAGTTTGCTGCGCTATTAACTTCCACAATGTCCATCTGACATCCTT
GTCGTTGCGCTGTTATGTGGCTGGCTACCACCGCTGGTTCATATCAGGTGGGGACTTATGCTCGCCGCGA
GTACGGTTTGTCAGCGGATCAGCCCCTGCTCAACCAGCCAGGTAAACAGCAGGCGGGAGACGGGGGAGAG
CTCATCGAGGTGAGAGCTGTCGAGCCAGCGTAGCTCGGCAATTTCTGCCGAGGCGATCGGGGTGCCGCTT
GTTTCGCCGGTGTAACAGCGCAGTCTCACCTCGACTCCTGCCGGTTTGCCATCGGCCTGAGCGCTGAACT
CGCAGACAAAATGCAGGGTATCGGGTTGCAGGGTGACAGCCAGCTCCTCTTCAATCTCGTGGATCAGCGC
CGCCTCGTCGCTCTCGCCCGGTTCCCGCTTGCCGCCCGGGATGTAATAGATGTCCTTGCCGTGGGAGCGG
GCGCAGAGCAGTTGCTGATCTTTAAAGGTGAGCCAGGCGAGTTTGTCGATCATTGAGTTGCATCCTCTGC
TTGTGTGGTGTGTATTGTCTGCATGTACCGGGTATACAGGGCGCGGTAGGAGCGAGGCTCCTGCGCATTG
GGAGAAAGTCCCAACTGATCTGCCAGCGCGAGCAGTTGCTGGCGAAACGCCGGGAGGCGTTCCTCGTCAT
CGGTCATGATGGCGAGCTCGGCAAAATCCCCGATCTCGGCCAGATGATCCAGGGTGACGTGAAACGGGCC
AAGGAAGTAGATGCTGCGCCTTTTGGTGATCACGAGTATCTGCCGGTAGTTCATGGTCAAGAGCATGCTG
ACGGCCTTGTCCGCATCATTGATAGTGACCGCCTCGCAGCGATCCTTCTCGGGCCCCTTGACGATCCAGA
GCTGGATGCCGGAGGGCTGCATGGTGCGGATAGCCAGACTTTTGCCAGCCTGGGCAAGGGCATCATCCGG
GGTGTCGAAGTAGCGATCCTGCTCCAGATTATCCTCCAGCATGATCTCCGGATTGAGGGCGAGCAGTGCC
TTGAGGAAGGCGAGGCGATCCGTCAGCTGATATTTGAACTCGACTTCGAAGCGGCCCTGAAAGTGCGCCT
GTGACATCAAGAAGATCCCTCTGTGAGCCTGTGTGGCAGGCAGCCTAACAAAGATGAAGTTGAGTCCCAA
GGAGAAAGAGAGGATAGGCCGGAAACGCACTGTTTCCAGCGCGGTGACCGCGTAGCTGAATGAGTGCGCA
ATGGCTGGTTCGCCACAGACTTTTCGACCCTAAAAAAGCGGACTGGCGCACATCACGACAGGGCCGGACT
GGGCGAAGGCGGGGTCCTTGTGGTTTAATCTTGCCACTTTAGCGGTAGTAGGCGACGAGCCTGCTACCTT
GCGTTGGCAAGATGCAGGTGAGCAAGATGCAGATAGCAGCAGAGAGCAAGTTTATCGGACTCGGGGTCGC
GGGTAATTTCGCCGGTCATCTGGAGCAGGCGGGTGAAGCGTCGGACTTCGTAGCGGTAGTCGTGCGCGAC
ACCACGGCGCCCAAGGCGCTGTTTCCCTTTTTCGTGCCGGGCCATCCGGGTCAGCTCGGGGTATTCCCGT
TGAGCGACAAGGCGATCTTCCTGCCGGAAGCGGCGGTCAGCGGTGACGAGAAGGTGCAGATCGAGCCGGA
AGTAGCCCTCTGGTGCGAGCTGGAGTATGCCGGCAAGCAGGTGGTGGCGATCCATCCCCGCGCCTTTGGT
GCCTATAACGACTGCTCCATCCGTCGTCCCAACGCCAAGAAGATCAGCGAGAAAAAGAACTGGGGCGAAG
AGAGCAAGGGGTTGGCGGACAAGCTGCTGCCGCTTTCCGGTTTTTCTGCCGGCTGCGAGCTCGATGACTA
TCGCATCGCCTGCTATCTGGAGCGGGATGGTAAGCTGCACGCTTACGGGGTAGACAGCGCCGCCATCGAC
TACAGCTACTTCCACGGCAAGCTGCTCGACTGGGCCATCGACAAGTTCAATCATCAGCAGGATGAGGGGC
CGGCGGAGCATATTCAAGGACTGCTGGCGCAGGCGGGACATCCTCCCCATGCGCTCATCAGTATCGGTGC
CACCCGCTATACCCCGTTTGGCGAGACCCACTTCCTCAAACCGGGCGACACCTCCTGCGTGGTGGTCTAT
CCGGGCAGCCGTTATAGCGAAGCGGATATTCGCGAGGCGATCCGCAGCCGCAACTTTGGCGCTGATATGT
CGGTGCTGCTGCAAGCGGTGCAGGCGCGCTAATCACCGTTGCCAGCGGGAAGGTGTCTCTTCCCGCTGGT
TATCATATCGAGTCATTTCATGACGATTGTTCCGGTATCCCGGGCCGATTATCCGGCCCTGATCGACCTG
TGGGAGGCCTCTGTTCGGGCGACCCACCACTTCCTGCCCGAGGCCGAGATCCTCGCGCTTAAACCGCTTA

TTCTGGAGCACTACTTCGATGCGGTGACACTGCGCAGTGCCCGCACAGAAGAGGGGCACATTACGGGTTT
TAGCGGAGTGTACGAGGGCAAGCTGGAGATGCTGTTTGTGGCTCCCGAGGCGCGCGGTAGTGGCGTGGGG
CGTTTGCTGGTGGCCCATGCCATCACGCAGGGCGGCGCGACGCAGGTTGACGTCAACGAGCAGAACGAAC
AGGCGGTGGGCTTCTATCTGAAGATGGGATTCACCGTGACGGGTCGTTCGCCGCTGGATGGTCAGGGCAA
GCCCTATCCGCTGCTACATATGGCGCTGGCGCCGACCTGATCGTGCCTGATGAGGCACAGTACCCATAAA
CCAACGGGGGATGGCATCAGCCATCCCCCGTTTTTTTGATCGTGTTTGTTACTTGCTGGCGACCTTGTTC
ACTACCTTGATCAGCTGATAGCGCAGGGAGGGGGCATCGGCCGGCACATCGGTGAGCTGCTCTTTGCTGA
CTGTGAGCTGATACCGGTAGCCCGGCTCGAACTGGAAGCCTTCAATATTCTGGTAGAACAGCGTCCAGGG
CTGCTGCTCTTCACTGCGAACCTGCATGCACTGCATCGGGCCAACTCCGACGCAATCGACCAGCTGGCTG
TTGATATAGAGGGTCTCCCCCTGTGACGGGGCGGGGGCGCTCTGGCAGGCGGTCAGCAGCAAGGCTGCGC
TGATAAACAGTGGTTTCATAATGGACTCCTTAACGCGAAAGGGGGCATTAGAGCGGCAAACAAAAAGGCG
GTCAATGACCGCCTTTGTAACAAACCATATCAGCGCAGTGGTTAGCGCTGCTCTAGCGGAGTGAACTCGC
GCAGCGGGTGACCCACGTAGAGCTGGCGTGGGCGACCAATCTTGCTGTCCGGGTCTGAGTGGAACTCGTT
CCAGTGGGCAATCCAGCCAATGGTGCGGGAGATGGCGAAGATCACGGTGAACATGCTCATCGGGATGCCG
ATCGCCTTCATGATGATGCCGGAGTAGAAGTCCACGTTCGGGTAGAGTTTCTTCTCGACGAAGTAGGGGT
CGGAGAGCGCAATACGCTCCAGCTCCATGGCCACGTCCAGCAGTGGATCCTTGATCTGCAGCTCGGTCAG
TACTTCGTGGCAGGTTTCGCGCATCACAGTGGCACGAGGGTCGTGGTTTTTGTAGACACGGTGACCGAAG
CCCATCAGACGGAACGGATCATTCTTGTCCTTGGCCTTGGCGATGTACTCGGGGATGCGATCCACCGAGC
CGATCTCTTCCAGCATGCGCAGGCAGGCTTCGTTGGCACCACCGTGGGCCGGTCCCCACAGGGAGGCGAT
CCCCGCGGCGATACAGGCGAACGGGTTGGCACCGGAGGATCCAGCCAGACGAACGGTGGAGGTGGAGGCG
TTCTGCTCGTGATCCGCATGGAGGGTAAAGATACGATCCATGGCACGTTCAACGATGGGGTTGACCTTGT
ACTCTTCGGTCGGCACGCCAAACATCATGTGCAGGAAGTTACCGGCATAGGAGAGGGCGTTACGCGGCTG
CATGAACGGCTGGCCGATGGAGTACTTGTAACACATAGCGGCCAGAGTCGGCATCTTGGAGAGCAGACGG
AAGGCACAGATCTCGCGATGCTGCTCGTTATTGATGTCGAGGGCATCGTGATAGAAGGCGGAGAGGGCGC
TGACTACACCGCACATGATGGCCATGGGGTGGGAGTCGCGACGGAAGCCGCGGAAGAAGAAGGCGATCTG
CTCGTGCACCATGGTGTGGCGGGTGACCAGGCGTTCGAATTCGGCATATTGCGCCTTGGTCGGCGCTTCG
CCGTACAGCAGGATATAGCAGACTTCCAGATAGGTGGCTTGGGTGGCCAACTGGGCGATGGGGTAACCAC
GGTGCAGCAAGACACCCTGATCCCCGTCAATGTAGGTAATGGCAGATTTGCAAGAACCGGTCGCCATAAA
ACCGGGGTCAAAGGTGAAATACCCCTGTGATCCCAATTTTCTGACATCGATGACATCCGGGCCGATGGTG
CCGGAGAGGATCGGCAGTTCGACCGGATCTTTTCCGGGCAGGTGTAGGGTGGCTATCTTATCAGCCATAG
CACGTCTCCTTTGCTCTTTATAATTTGGTTCCAGTCCTACTCTATTACGACTTTTATGACGCTGGCAAAG
GTTGTTACCCATACCGAAGTGGCTGGAGCGCCTTATTTGAAACAGAGTTCCTTATCAATGTCAATTTTGC
TGGAACCCCATTAATGGCATTAATGAAATGTTAAATTAAATATGCGATCGAGTTTGCGGTTTTGTGTTTT
CTTTGTAGCTAATTGATTGTCTTCGGTTAAGGCCGCCGATATACTGCGCCACGTGGGTCATGGCTGCGGG
TGTTTGAAACGCTTTGGCAAAGCCTGATGTGAAACCGAAAACTCGCGTTTTGGCTTTTTCAGAGTGTTTT
GGTAACAAAAAACCAACAATCACCATTCCGTGCCCTCAAGTGTGAGTTAAAACAATAACTAACGTGCTCA
ATGGAGCTGAGTGGGCAAGCGTGAAAAACAAACAGAGACCGGTCAACCTCGACCTACAGACGATCAGCTT
TCCTGTCACTGCCATTGCATCCATCCTGCACCGTGTTTCAGGGGTCATTACCTTTGTTGCACTGGGCATC
TTGCTGTGGATGCTTGGTACTTCACTCGCCTCTCCCGAAGGGTTTGAAACCGTGGTTGCCATCATGGACA
ACTTCCTGGTCAAACTCGTGCTGTGGGGGATCCTGACTGCGCTGGCATACCACATTGTGGGCGGCTTGCG
TCATCTGGTCATGGATATGGGCTATTGGGAAGAGCTGGAATCCGGCAATCAGAGCGCCCGCGTGGCCTTT
GTGATCACCGCGATCCTGGCTGTATTGGCGGGAGTGCTTGTATGGTAACCAATTCTGCAACCTTCGGGCG
CAGCGGCGTTCACGATTACATCCTGATCCGCGCCACCGCCCTCATCATGACCTGTTATGTCCTCTATCTG
GTGGGCTTTGTCGCGTTCAACGACATCACCTACGAGGTGTGGGCAGGCTTCTTCGCCAAAACATTCACCA
AGGTATTCACCCTGTTGACCCTGCTCTGCGTACTGATCCACGCCTGGATTGGTCTGTGGCAGGTGCTGAC
TGACTATATCAAGCCGGTAGGGCTGCGCGGGGCGCTGCAATTTGCCCTGGTCGTGGTGCTGTTTGTCTAT
CTGATGACGGGTTTCGTCGTGCTGTGGGGTGTGTAAGGTGACTATTCCAACTCGTGAATTTGATGCGGTG
GTGATTGGTGCCGGTGGTGCCGGTATGCGCGCCGCGCTGCAGATTGCCCAGTCCGGCAAGAGCTGCGCTC
TGTTGTCCAAGGTTTTTCCAACCCGTTCTCACACTGTCTCCGCCCAGGGCGGGATCACGGTTGCGCTGGG
CAATGCCCACGATGACAACTGGCAGTGGCATATGTATGACACGGTAAAAGGTTCCGATTACATCGGTGAT
CAGGAAGCCATTGAGTACATGTGCAAAACCGGTCCCGAGGCTATCTATGAGCTGGAGAACATGGGGCTGC
CCTTCTCCCGTTTCGATAACGGGTCTGTCTACCAGCGTCCGTTTGGTGGTCAGTCCAAGAATTTCGGTGG
CGAGCAGGCTGCCCGTACTGCCGCTGCGGCCGACCGTACCGGTCACGCCCTGCTGCACACCCTTTACCAA
CAAAACGTCAAGAACAAGACCACCGTCTTCTCCGAGTGGTATGCGCTGGATCTGGTGAAAAACCAGGATG
GCCACGTGGTGGGTTGTACCGCCATCGATATGGAGAGCGGTGAAGTGGTCTACTTCAAGGCAAAGGCCAC
CGTGCTGGCCACCGGCGGTGCCGGTCGTATCTACCAGTCCACCACCAACGCCCACATCAACACAGGTGAC
GGTGTCGGCATGGCGCTGCGTGCCGGCGTCGGCGTGCAGGACATGGAGATGTGGCAGTTCCACCCGACCG
GCATCGCCGGGGCCGGGGTGCTGGTAACTGAAGGCTGCCGTGGTGAAGGCGGCTATCTGCTCAATAAAGA
TGGCGAGCGCTTCATGGAGCGTTACGCCCCGAACGCCAAGGACTTGGCCGGTCGCGACGTGGTGGCCCGC
TCCATCATGATCGAGATCCGTGAAGGCCGTGGCTGCGACGGTCCCTGGGGCCCGCACATCAAGCTGAAGC
TGGATCACCTCGGCAAAGAGGTGCTGGAATCCCGTCTGCCGGGGATCTGCGAGCTCTCTCGCACCTTCGC

CCACGTGGATCCGGTCAAAGAGCCGATCCCGATCATTCCGACCTGCCACTACATGATGGGCGGCGTGCCG
ACCAACGTGAATGGCCAGTGCCTGACTCAGGACAAGAACGGCAACGATGTACCGGTGGTCGGTCTGTTTG
CGGTGGGCGAGATTGCCTGCGTCTCCGTACACGGCGCCAACCGTCTGGGTGGCAACTCCCTGCTGGATCT
GGTGGTCTTCGGCCGCGCTGCCGGCATGCATCTGGTCAACACTCTGGGCGAGATGGAGCACGGCCGCGAT
GCTTCCGAGTCGGATCTGGATGCCGCGTTGGCCCGCTTCAACCGCTGGGAAAATTGCCGTGACGGGGAAG
ACCCGGTGCAGATCCGCAAAGACCTGCAGCGCTGCATGCAGAACAACTTCTCCGTATTTCGCGAAGGTGC
GGCGATGGCAGAAGGGTTGGCCGAGCTCAAGCTTATCCGCGAGCGCCTCAAGTCAGCCCGTCTGGATGAC
ACCTCCAAAGAGTTCAACACCCAGCGCATCGAGTGCCTGGAGCTGGACAACCTGATGGAGACGGCGTTTG
CCACGGCGGTTGCCGCCAACTTCCGTACCGAGAGTCGCGGTGCTCACTGCCGCTTTGACTTCCCGGATCG
CGACGATGAGAACTGGCTGTGCCACAGCCTCTATCATCCGGACACCGAGTCCATGACCCGTCGCGCTGTC
AACATGTCACCCAAGACCCGTGAGGCGTTCCCGCCCAAGGTCCGGACTTACTGATTGCGGAGCCAGCCCT
ATGAACGTCACATTCTCTGTCTACAGATACAATCCGGATGTTGATAACGTCCCCCATATGAAGGAATATC
GTCTTGATATTCCTGAAGGTTCCGACATGATGGTGCTCGACGCACTGATCCAGTTGAAAGAGCTGGATCC
CACGCTCGCCTTCCGCCGCTCTTGCCGCGAAGGGGTCTGTGGATCAGATGGTCTCAACATGAATGGCAAG
AACGGTCTTGCCTGTATCACCCCGCTCTCCGACCTGCTGAAGAAGGGCAATAACGTCGTTATCCGCCCCT
TGCCCGGCCTGCCGGTGATCCGTGATCTGGTGATCGACATGACCCAGTTCTACACCCAGTGGGAGAAGGT
CAAACCCTTCCTCATCAATGATGACAAGTTGCCGCCGGTGCGTGAGCACCTGCAATCCCCCGAAGAGCGC
GCCAAGCTTGACGGCCTGTACGAGTGCATTCTCTGTGCCTGCTGCTCCACTTCGTGCCCCTCCTTCTGGT
GGAATCCCGACAAGTTCATCGGTCCGGCCGGGCTGCTCGCCGCCTACCGCTGGCTGGCTGACAGCCGCGA
TACCGCGACCACCGAACGACTGGGGAATCTGGACGACGCATTCAGCGTGTTCCGCTGCCATGGCATCATG
AACTGCGTGAACGTCTGTCCGAAAGGCTTGAACCCGACCAAGGCGATTGGCCAGATCAAGTCGATGTTGC
TCAATCGGGCTGTTTAGTATCAGGGCCGGCCTGGTGCTGGCCTGACCTCTGGATAGCCATCCCACCCGTG
GCAGGATGGCTATTTGACCCCTACAAGCTGCGCAGCAGTAACAAGAAGTACAAGGGATAGAAATGCATAA
CGGCGTGATGAAGGCCTGGTTGGAGTCATCTCACCTGGCTGGTGCGAATGCAAGCTATATCGAGGATTTG
TACGAATCCTTCCTGGAAAATCCTGACTCCGTGTCCGACGAGTGGCGCAGCCTGTTTAGCGAGCTGCCCC
AGGTCAATGGCCATGCGGTTGAACAACCCCACTCCCAGGTCCGCGATTATTTCCGTCGTCTGGCCAAGGA
TCCCTCTCGCTACAGTGCCCCCGTCTCTGATCCGCAAGTGGATGCCAAGCAGGTGCGGGTATTGCAGCTG
ATCAACGCCTTCCGTTTCCGTGGTCACCAGAATGCCAATCTGGACCCGCTGGGCCTGTGGCAGCGTGAAA
CCGTTGCCGAACTGGATCCTGCGTTCCACAACCTGCAGGGCGCAGACATGGAGGCCACCTTCAATGTGGG
CTCCTACGCCATCGGTCGCGAGACCATGGTGCTCTCCGACCTCTACGCCTCGCTGAAGAAGACCTATTGC
GGCTCCATCGGCGCCGACTACATGCACCTCACCTCCACGGAAGAGAAGCGCTGGCTGCAACAGCGTCTCG
AATCCATTGAGGGGAAGGGGAGCTACACCCTCGAAGAGAAGACCCGTTTCCTCGAGAGCCTGACTGCCGC
CGAAGGGCTGGAGAAATATCTGGGCGCCAAGTTCCCGGGAGCCAAGCGCTTCTCGCTGGAAGGTGGCGAC
GCCATGATCCCCATGCTCAAGGAGTTGATCCGTCGCGCCGGTGAGCAGGGCTGCAAAGAGGCCGTTATCG
GTATGGCCCACCGCGGTCGTCTCAACGTGCTGATCAACGTACTGGGCAAGCGCGCTCAGGATCTGTTCGA
CGAGTTTGCCGGCAAGCATGGCGAATCCTGGGGTACCGGTGATGTGAAGTACCACATGGGCTTCTCCTCC
GACTTCGCCACCCCGGGTGGCAACGTCCATCTGGCGCTCGCCTTCAACCCCTCCCACCTCGAGATCGTCA
ACCCTGTGGTCATCGGCTCGGTCCGTGCCCGTATGGATCGTCGCGGTGACAAGGATGGCTCCAGCGTGCT
GCCCATCACCATTCACGGTGACTCGGCCATGGCCGGTCAGGGCGTGGTGGCCGAGACCTTCAACATGTCC
CAGACCCGCGCCTATGGTGTCGGCGGGACGGTGCGCATCGTCATCAACAACCAGGTTGGTTTCACCACCT
CCTATATTCGTGACCTGCGTTCTACCGAATATTGCACCGACATCGCCAAGGCGGTGCAGGCGCCGGTGCT
GCACGTCAACGGGGATGATCCGGAAGCCGTGGTGCTGGTGACCCAGATTGCACTGGATTACCGCAACACC
TTCAAACGCGACGTGGTGATCGAGCTGGTCTGCTACCGTCGTCACGGTCACAACGAAGCGGACGAGCCGA
GCGCAACCCAGCCGCTGATGTACCAGAAGATCAAGCAGCACCCGACCCCGCGCAAGATCTACGCCGATCA
GCTGGTGGCCGAGGGGAGCCTCGCTCAGGAGCGGGCGACCGAACTGGTCAACGAGTATCGCGAAGCGCTG
GATCGCGGCGAGCGGGTGAGCAAGGAGTGGCGGCCGATGGAGCTGCACTCCGTCGACTGGACACCCTATC
TTGGCCACGACTGGAACATGGCCTATGAGAGTGCCGTGCCAATGGATCACCTCAAGAGCCTCGGCGAGCG
GGTGAGCCAGTATCCGGCGACCCACGTGCTGCAGCGTCAGGTCGAGAAGATTTACGAAGACCGCCGTCTG
ATGGCCGCTGGCGAGAAGCTGGTGGATTGGGGCTTTGCCGAGACCCTGGCCTACGCCACTCTGGTGGACA
AGGGGTCCCGCATCCGCTTTACCGGTGAAGACTCCGGCCGTGGCACCTTCTTCCACCGCCACGCCGTGCT
GCACAGCCAGACCGATGCCAGCACCTATACCCCGCTGTGCAACCTGCACGACGAGCAAGGCCCGTTCGAG
GTGTATGACTCTGTGCTGACCGAGAACGCGGTACTGGCCTTTGAATATGGCTACGCTAGCGCCGAGCCGG
CTGGCCTCACCATCTGGGAAGCCCAGTTCGGCGATTTCGCCAACTGTGCCCAGGTAGTGGTGGATCAGTT
CCTCTCCTCCGGTGAGCAGAAGTGGGGCCGGATGTGCGGCCTGACCATGTTGCTGCCGCACGGTTACGAG
GGGCAGGGCCCGGAGCACTCCTCCGCTCGTCTGGAGCGTTATCTGCAGATGTGCGCTCAGCACAACATGC
AGGTGTGCGTGCCCTCTACCCCGGCGCAGGTGTTCCATATGCTGCGTCGTCAGGTGGTACGCCCGATGCG
CCGTCCGCTCATCGTCATGACGCCCAAATCCCTGCTGCGCCATCCGCTGGCGGTGAGCAAGCTTGAAGAG
CTGGCTGAAGGTACTTTCCAGAACGCCATCGGCGAGATCGATGCGCTGGATCCGAAAGCGGTCAAGCGGG
TGGTGTTCTGCTCCGGCAAGGTCTACTACGACCTGCTCGATGCCCGTCGCAAGGCCGAGCAGCAAGATGT
GGCACTGGTGCGAATTGAGCAGCTCTACCCCTTCCCGGAAGAGGAAGTGCGCGCCATTCTGGCCGATTAC
AGCCACGTCACCGACTTTGTTTGGTGTCAGGAAGAGCCGCAGAACCAGGGCGCCTGGTACTGCACCCGTC

ACCACTACGATGGCGTGCTGCCAGCCGGAGCCACCCTGCGTTATGCCGGTCGCCCCGCGTCAGCCTCACC
GGCCGTCGGTTACATGTCTGTCCACGCCAAGCAGCAAAAAGCCTTGGTGGAAGATGCCCTTACCCTGTAA
AGCCTCTGGCATAGAAGTAAAAAGGAACTGATTGGATGACTATCGAAATCAAGGTACCGGATCTGCCGGA
ATCAGTGGCCGATGCCACTATCGCCACCTGGCACAAAAAACCGGGCGATCTGGTCGCCCGTGACGAAGTG
CTGGTCGACATCGAGACCGACAAGGTGGTGCTGGAAGTGCCGGCACCGCAAGCCGGTGTGCTGGGGGATA
TTCTGCAAGGGGAAGGGGCGACTGTGTTGTCCCGTCAACTGATTGCCATCCTCACCGCCGCCCCGGTGGC
CGGTGAAGAGACCAAAGAGAAACCCGCTGAAGCAGTGGCCGATGATGGTGCCGATGGCTTGAGCCCCTCG
GTGCGCCGCCTGATTGGTGAGCACGATATCGACGTGACCAAGCTGACCGGTACCGGCAAGGGTGGTCGCA
TCACCAAGGATGATGTGGAAGCCTTCATCAAGGCCAAGAGCCAGCCTGCGGCAGCCGCACCGGTCGCAAC
TACAGCCCCGGCAGCCAAGGTTGCCCCGCTGGGTGGTCGCACCGACAAGCGGGTACCGATGACCCGTCTG
CGCAAGCGCATTGCCGAGCGTCTGCTGGAAGCGAAAAACACCACCGCCATGCTGACCACCTTCAACGAGG
TCAACATGGAGCCCATCATGGATCTGCGCAAGCAGTACGGCGAGATCTTCGAGAAGAAGCACGGCATCAA
GCTCGGCTTTATGTCCTTCTACGTGAAGGCGGTAGTGGAGTCCCTCAAGCGCTATCCGGAAGTGAACGCG
GCGCTCGATGGTGATGACGTGGTTTACCACAACTACTTCGACGTCAGCATCGCCGTCTCAACCCCCCGTG
GTCTGGTGACGCCGGTGCTGCGCGACTGCGACAACATGAGCCTGGCGGACATCGAGAAGGCCATCAAGGA
TCTGGCGGGCAAGGGCCGTGACGGCAAGCTGACCGTGGATGAACTGACTGGCGGCAACTTCACCATCACC
AACGGTGGCGTGTTCGGCTCCCTGATGTCCACCCCGATCATCAACCCGCCCCAGAGCGCCATTCTGGGCA
TGCACAAGATCCAGGATCGCCCGATGGCCGTTGACGGTAAAGTCGAGATCCTGCCGATGATGTATCTGGC
GCTCTCCTACGATCACCGTCTCATCGACGGACGCGAATCGGTAGGCTTCCTGGTCTCCGTCAAGGAGCTC
TTGGAAGACCCGACCCGTCTGTTGCTGGATGTCTAAGCCTGTGGCTCCCGGGCAAGGGGTTTGCGCCCCG
AGCCGGATGTGCTGCTGGACGTTTGAGTAATACCCGAGCGATTGAAAGATAACGAGGGGCCGCCCAGGCC
CCCATTCACAGGGGATTGAGAAACACCAGCCCCGGACGGAAATACCTACAACACGGATAGAGCATCTATG
AATCTGCATGAATATCAGGCAAAACAGCTGTTTGCCGAGTATGGCCTGCCGGTCTCCGAAGGTTATGCCT
GTGCTACCCCACAAGAAGCGGCCGAAGCGGCCGACAAGATTGGCGGCAATACCTGGGTCGTCAAATGCCA
GGTACACGCCGGTGGCCGCGGTAAAGCGGGCGGCGTGAAAGTGGCCAAGAGCAAGGATGAGATCCGTGCT
TTTGCCCAGAACTGGCTTGGCAAAAATCTGGTGACCTACCAGACAGACGCCAACGGCCAGCCGGTCACCA
AGATCCTGGTGGAATCTTGCACCGACATCGCCAAAGAGCTCTACCTGGGCGCCGTGGTTGACCGTGGTTC
GCGCCGCGTGGTCTTCATGGCCTCGACTGAAGGTGGCGTGGATATCGAGAAGATCGCCCACGAAACCCCC
CATCTGATCCACAAGGCGGCCATTGATCCGTTGGTGGGTCCGCAGGCCTATCAGGCCCGCGAACTGGCCT
TCAAACTGGGTCTGGTCGGCGATCAGATCAAACAGTTCACCAAGATCTTCATGGGTCTGGGCCAGATGTT
CCTGGATTGCGATTTCGCGCTGCTGGAGATCAACCCGCTGGTGATCACCGCGCAGGGCAACCTGCACTGC
CTGGATGGCAAGATCAATATCGATGCCAACGCACTGTATCGTCAACCCAAGCTGCGCGAGATGCACGACC
CGTCACAAGATGATCCCCGCGAAGCGCACGCTGCCCAGTGGGAGCTGAACTATGTGGCCCTCGATGGCAA
CATCGGCTGCATGGTCAACGGCGCGGGTCTGGCCATGGGCACCATGGACATCGTCAACCTGCACGGCGGC
AAACCGGCCAACTTCCTTGACGTCGGCGGCGGCGCCACCAAGGAGCGCGTAACCGAGGCGTTCAAGATCA
TCCTCTCCGACAGCAACGTCAAAGCGGTGCTGGTCAACATCTTCGGCGGCATCGTGCGCTGCGACATGAT
CGCGGAAGGCATCATCGGTGCGGTCAAGGAAGTGGGGGTCAAGGTGCCCGTCGTGGTCCGTCTGGAAGGC
AACAACGCCGAGCTGGGTGGTATCAAGCTGGCCGAGAGCGGCCTCAACATCATTGCGGCCAAGAGTCTGA
CCGATGCCGCGCAGCAAGTCGTCAAAGCAGCGGAGGCCAACTGATGAGCGTACTGATCAACAAGGACACC
CGAGTCATCTGCCAGGGCTTTACCGGCGGTCAGGGTACTTTCCACTCCGAGCAGGCGCTCTCCTACGGCA
CCCAGCTGGTAGGCGGCGTCTCCCCGGGCAAGGGCGGCACCACCCATCTGGGCCTGCCGGTGTTCAACAC
CGTCCGTGACGCGGTCGAAGCGACCGGCGCCACCGCCTCCGTCATCTATGTGCCAGCGCCGTTTTGCAAG
GATGCCATCCTGGAAGCGATCGACGCGGGTATCAAGCTGATTGTCACCATCACCGAAGGCATCCCGACGC
TGGATATGCTGGACGTCAAAGTGAAGCTGCAAGAGACCGGTGTGCGGATGATCGGCCCCAACTGCCCGGG
TGTGATCACCCCGGGTGAGTGCAAGATCGGCATCATGCCGGGCCACATTCACAAGCCGGGCAAGGTGGGC
ATTGTCTCCCGCTCCGGCACCCTGACCTACGAAGCGGTCAAGCAGACCACTGACGAGGGCTTTGGCCAGT
CCACCTGCGTCGGTATCGGTGGCGACCCCATCCCGGGCTCCAACTTTATCGACATTCTGGAGATGTTCCA
GAACGACCCGCAGACCGAAGCGATCGTGATGATCGGCGAGATCGGCGGCAACGCCGAGGAAGACGCGGCC
GCCTATATCAAGGCCCACGTCACCAAGCCGGTCGTATCTTATATCGCCGGAGTTACTGCCCCTCCCGGCA
AGCGGATGGGCCACGCTGGCGCCATCATCTCCGGTGGCAAGGGTACTGCCGCCGAGAAGTTTGCCGCGCT
GGAAGATGCTGGCGTGAAAACCGTCCGCTCCCTGGCCGATATCGGCAAGGCCCTGCGCGAAGTCACCGGC
TGGTAATTCCAGTTAGCCCGTTATCTCTAATAAAAATGCCGCCCGATGGGCGGCTTTTTATTGGCATGGT
TCGTAATTTATCAGGCATACCAGAATACGCTTCTCTATTTTAAACTACAGTTCTAAAAATTGATTTAAAT
CAAGCGCCAATGCAATGTGAATGTATTAAATATGAATTTTAACCATTGATGAAAAGGAAGGTGCACCGTG
CCATACGATTTAACAAAAAGATTGGTTGTTGGCTTGTCTTCTAGCGCGTTGTTTGACCTTGAGGAGTCAG
ATAATATATTCAGGACAGAAGGTGAAGAGTCTTATAGAGAATATCAGAGGGAAATGCAAGATGAACCATT
GGGCAGAGGTGTTGCATTTCCATTTATTCGTCGTTTACTTAAGTTAAATACATTGTGCCCACAAGATCCT
TTGGTAGAAGTAATATTGCTTTCACGAAATGATTCTGATACTGGGCTCCGAGTTATGAACTCAATTGAAC
ATTATGGCCTAGGTATTACGAGAGCTGTATTTCTACAAGGTAAGTCGCCTTATGTCTATATCCCTGCGTT
TGATATCGAATTGTTTTTATCAGCCAATTATCAAGATGTTCGACAAGCAGTTCTTGCTGGATACCCTGCA
GGTCAAATTCTGAGTGGTGATATCAAAGATGATACTGAAGACCTAGAGCTTCGAATAGCTTTCGACTTTG

ATGGCGTTATTGCAGATGATGAAGCAGAGGGCGTCTATAAAAGTTCAGGGCAGTTAGCTGATTTTCACTC
TCATGAGTCTGAGCTAGTTGATGTCCCACATAACCCAGGTCCACTGAAGAATTTTCTGCAGCGGATTTCG
GACATACAAAAACTTGAGGCTGACAAGAAAAAAGAGGATTCAAGTTATTTACCGATGCTTAAGGTATCAA
TTGTGACAGCAAGAAATGCACCTTCTCATAAACGAGTCATTAATACTATGCGCTCTTGGGGTATTTCTGT
AAATGAGGCATTTTTTATGGGAGGGGTGGAAAAGTCAAAAGTATTGAATATATTAAGGCCGCACATATTT
TTTGATGATCAAAAGTTACATTTACAATCGTCGTCTATTTTACCATCGGTACATATTCCGTTTGGCGTCG
CAAATAAAGATTTGGTACTTTAATAAAGTGATCTTACTCGAATAGAAACGAATATTCGGATGATCGGGGC
GTTAGTGTGTCGCTGTCGTTACCTGTGGCCAGAGTCGAATAGCCCAAGATTGTTATGCTGCTGCAACCAC
GTTGGCTAACCAGTGTCTCGCTGGTAAATCAGTTCTGCCGCTTTTTGGGCAACCAGAATGCGGGTTAACC
CAAGGCCATTGACACTTGCGGCAGCTTTCCTTCCTATCAGTTCTGGCTACCACGCCGGACCATAAAAATG
CCGACCAGGGGGTCGGCATTTTCAAGAAATAGGGTGTGGGGCAAAACAGTATGGGAAATGGTTGCCCTCT
ATGACCGGGCTATTTATCCGCCTTGCCCGCCGCATCGGCAAGTTTGGCCAGCTGTTTGTCATACCAGGCC
ACCGGGCGGTTCTCATCTTCCGCCCGCTCCTTGCGCCGGATGGCGTTGCGCACCGCCATGGCCCCCAGCC
AGCGAAACGGCTCGGGCGGAAAATAGCCACGCGGGCCGCTGACCAGCGCGCAGTCACGCCACGGCTTGTC
CTCCTCCCGCACTTCATCGCGCACCAAAGTTGCCAGGATCTGGCCGCCGATCCAGCTTTGCGCCACCCCG
TTGCCGGAGTAGCCGAGGCCATAGACGATATTGGGCTGCCCTCGCCAGTGGCCGAAGAAGGGGAGGCCAT
CTACCGAGCGGTCGGAGGCGCCGCACCAGCTGGCGGCGATGGGCACCTCTTTGAGCGCCGGAAAGAAGCG
TCGCAGGGTGCGGGTGAGAGGGGCAAGATAACGGCTAGGGGCATCGAACTCGGCCAGATGGCGGTTGGCA
AAGGCGAAGGTGTTGCCCCCCTTGCCCAGCATCAGCCGCCCTTGCGGGGTGGAGCGGTAGTAATGCACAA
AGGTGCGGCCATCCACCACGCTGCGACCGTGCGCAAGCTGCTGGGCAGCCAGTGCATCCGGCACGGGGTC
GGTTATCACCATATCGGAGGAGACCAGCACTATACTGCGGCGAAACTCGCGAAAGTGCTGCACCATGGCG
CTGTTGAGGGCCAGCACCACCTTGCGGGCACGCACCTCGCCCTGTGACGTTTGCACCCGTGCAGGCTGGC
CCGCCTCAAGGCGCTGCATGGGGGAGTGCTCATAGACCTCGACCCCCAGTTCCCGCGCCACCCGCAGCAG
ACCACGCACCAAGAGCGCCGGTTGCACCGAGCCCGCGTGAGGGCTGTAGAGCCCCTCCAGATGAACGTGG
GAACCGCTCTGGCTCGCCAGTTCACCCTCCGCAATCGCCTGCCACTGGTTAAGATCGGCTCGCTGTAGCT
CCCGCAATACCGGCTCCATCAGGCCGCGCTGGGCCTCGCTGGTGGCGGTGTAGCAGGTGCCCTCGACCCT
CAGCTCCGCTTCTATGCTGTGCGTTTCGCAGAAGCGGGCGATCTCGTGTACCGCCTGCTCGGATCTTTGT
ACCAGCCAGGCGGCCTGCTGCTCGCCAAAGAGGCGCTTTAACGTCAGGTATTTGGCCGACCAGGTGAGCA
GACAGCCGCCGTTTCGCCCGGAGGCACCGCTGCCGCAGCGATCTTTCTCCAGCACTATGACGTGCAGGTG
GGGCTCGGCCTGTTTAAGCGCGATGGCGCTCCACAGCCCGGTAAAGCCGCCACCGACGATGCAGACATCG
GCCTCGTGGTTGCCGACTAGGGGCGTGGGCGGTGCGGGTTGTTCGGCGGCGAGCGCTTCGGCCAGCCAGA
AGGGGAGAGTGGACATGATGACTCCTCGATAAGGTTATGGGTGGCGCCAGCGCTGGGTGCGATCCAGCAG
GGCGCGGCTCAAAAAGAGCTGGAGCAGCTTGACGGTGGCGGCGGCCAGCAGGATCAGGGTGGCCATGGCG
GCGGCCGCGGCGGTATCGCCGGAATCCTCCATGTTCAGCACGGCGATGGAGGCGAGCACGCTGTCGCTCG
AATAGAGGAAGATCACCGCCGAGGTGGTGGTCATGGCGTTGACGAACAGATAGATAAAGATCTCCAGCAG
TGCGGGCAGGGAGGCGGGCAGGGTGACTCGCCAGAACGCCTTCCAGAGCGGAATGCGCAATGAGATGGCC
ACCAGCTCCAGCTCCTTGGGCAGCTGCTTGAGGCTGGTCAGCGCCGTCATATGGCCCACCGTGTAGTAGT
GGATGACGCAGGAGAGCACCATCAGCGGCAGAGTGCCATAGAGAGTGCCAAAGAGCGGATTGCCGTTGAA
AAAGAAGATGTAACCGAGCCCCAGCACCATACCGGGTACCGCCAGCGACAGCATGGCGAGTAGATGCAGC
AGCTGGCGCAGCGGCGCGAAGTGGCGCCCCTTCTCCTGTGCCCAGGCCAGCACCATCACCAGCATGGTAC
CGATAAGCGCGGTATAGAGGCCAAGTTGCAAGGTGTTGGTAAAGGGCTGCCAACCGTAGACGCTGTTGCT
GTCAAAGGCGTAGTGATCCAGCGTCAGCTCGAGGTTGTAGGGCCAGAACTGCACCAGGGAGCCGTAAACG
GCCATCCCCACCAGCGCCAGAAACAGCAGGGCCCAGAGCGTGCACCAGCCGAGCGCCAGCAGATCCCGCA
GCCGATGGGGTTTGGGCTGATAGGGCACCGCCTTGGTGCTGCTCTGCTCCTGCATCTTGCTGCGCACTCT
GTGCTCCAGGGCAAAGCTCAAGAGCGCCGGCAGCAGCAGGGTGACGCTGGCAACGGCGCCCAGCGAGAAG
TTCTGCATCCCGATCACCTGCCGGTAGATATCGGTGGCCAGCACGCTGTACTGGCCGCCAATCACCTTGG
CCACCCCGAAGTCGCAGATGGTGAGGGTAAAGACGGCGATGGCGGCGCTCAAAAGACCGTAGCGAGCGGT
GGGCAGGGTGACGGTGACAAAGGTGCGCCAGCCGCTGCTGCCCAGCACCCGGGAGGCTTCATAGAGGCGG
GCATCGCTCTGACCGAGCGCAGTGATAAGGATCATCAGGGCGTGGGGGAAGCACCAGAAGCCCAGACCAA
TGACGATGCCGATAGGACCGTAGATGCTCTCGCCGCCGAGCAGCCCCTTGGCGATCCCCTGATTGCCAAA
CCAGTAGACCAGACTGATGGCGGGCAGCAGGGAGGGCATCAACAGCGGAATAAGGCCAATGAGGCGAAAC
AGGCTCTTGCCCGGCATGCAGGTGCGTGTGAGGGCATAGGCGTAGCCAAAGGCGATGGTCACCACCAGCG
CGGTGATCAGCACCCCGAGCCAGAGGGTGTTGCCTATGGTGCGCCCTAGATGAGGGGAGTCGAAGTAGGC
GCTGAAGTTGGCAAGGCCTATCCAGAGGCCGTTCTCATCCTGCAGGCTTTTTTGCAACATGGTGAGCAGC
GGCAGCAGCAGACCGCCGGTGAGCAGAGCGATGCCGAGCACCAGCAGGATCAGTTGCAGCAGCTGATCCC
GGCTCCAGCCGCGACAGAGCCGCTGCCAGCGAGAGTCTTGGCGGGCTGTCGCTGGCGGCAACAGGGAGGA
GGTCGCGAAGGGGGCTGTCATGGGCGATCTCCTTCAAACAGCTGCATCGCACCGGCGGGCCAGTGCAGCG
AGCAGCGCATGCCGCTGCGCCAGCCTCCTTGTTCGTGGCGGGGCAACTCGTGGGTCGGGCGCTCCACCAG
AATTTGCTGTGGGCCCAGATAGGTGTCGGCGGTGCAGATCAGTCGCTGGGCGGCGCCGAGAAACTCCACC
TGATCGATGGTTGCTTCAACGCCATGTTGCCCAAGGTGCGGCTCGTTCGGCGCGCTTAGCGTGATTGCCT
CGGGGCGGATCGCCACCTGCAATTTGCTGCCGGCAGCGGCCTGGTTGGCAAGTGTCAGCGGTTGTTCGTT

GAGGCGCACCTGATTGGGGCTCAGCACCAGGGTCTCGAGGAAGTTCATGGTACCGACAAAGCTTGCCACG
AAACGGCTGGCAGGCTGGTGGTAGATCTCCTGCGGGGTGCCCACCTGTACGATGCGGCCGCCCTCCATCA
CCACGATGCGGTCGGCCATGGTCAGCGCCTCTTCCTGATCGTGGGTCACCATGATGGTGGTGATGCCAAG
GCGCTGCTGCAGGGCGCGGATCTCGCTACGAAGATGGGTGCGTACCAGTGCATCCAGCGCGCTGAGCGGC
TCGTCCAGCAGCAGCAGGCCGGGGGAGAGGGCCAAGGCCCGGGCCAGCGCCACCCGCTGCTGCTGGCCGC
CGGAGATCTGGCTCGGGTATTTGTGGGACTGGGCAGTGAGATCCACCAGCCCCAGCCAGTGATCGACCCG
CTCCTTGATGAGATCCCGCGCCAGCCCCTGATTCTCAAGGCCGAAGGCGATGTTCTGGGCCACCGTCAGG
TTGGGGAAGAGAGCATAGGACTGAAACACGATGCCAAAATCGCGCTCCTGGGGCGGCAGGCGGGAGATAT
TGCGCCCTCCTTGCCAGATCTCGCCGCTGTCTGGCAGATCCAGCCCGGCGATGGCTCTGAGCAGGGTAGT
TTTGCCGCAGCCGGAGGGGCCGAGAAAGCAGATAAACTCCCCCGGCTCAATGGTCAGAGAGATCCCCTTG
AGGGCCTGAAAAGCACCGAACTGCTTGTTGAGATGTTGAATGTCCAGATAGGGCTGGGTCATGGTTCGAC
TCGCTACTGACTTGGTATATACCAGTTTGCGGGTAAAGTGTGACGGTCAGGTGATCGGGGGATGACAGCT
TGATGACAGTCAAATGACATCGGGTCGCCAGCAAGTGACTGGGCACTGTGCGAATGGACTAAGCATCGCT
AATTGAAGGCAATGCAAACTCGGGAAGTTAAAACCAAAAAGCGAAGGCCAACAGCTGTTGGCCTTCGACT
GGCAGTTTATTGGCAGCGGTGTGTCTGGCGTGAACAAACCGCCGCCCTTCACGCTTACTGTTTGGCTTCG
GTCTTGCCGTCGAAGCTGGCGCTCCACTGGGCCAGGATGGCGTTACGCTCGCGGGCTGCCCAGCTAAAGT
CGTTGTCGATCATCTGGCCTTTGATATCCGCCGGATAGCCCGCTCTCGGTTGGGCCACATCGGGGATGGC
AACCACGGCGAACGATTTGTTGTAGAGGGCGTTGGCCTGTTCGGTGGCGGCAAAGTCGAGCAGCTGTTTG
GCGGCTTCCAGCTGGGGGGTGCCCTTGATGATGGCGGCAGCTTCCATATCCCAGCCCGACCCCTCTTTCG
GGAATACCACCTCGATGGGGGCTCCCTTGGCCTTGAGGCTGGTGGCCGGGAAGTCGAAGGAGATACCGAT
CACCGTCTCGCCGCTGGCGGCCAGCTTGCAGGGGGCTGAACCCGAGTGGGTGTAACGGTCGATGTTCTGG
TGCAGGCCGTTCATAAATTGCCAGCCCTGCTTCTCACCCATGGTTTGTAGCCAGCCAGCGACGCTCAGGT
AACCGGTGCCGGAGGAGCTGGGGTTGGGCATGATCACCTTGCCCTTGTAGATGGGCTTGGTGAGGTCGGC
CTAGCTGGTGGGGGCCGGGATCCCCTGTTTCTCCGCCTCTACCTTGTTGAAGCAGATGGCGCTGAAGAAG
GCGTCCAGACCGATCCAGTGCGGCTCGGCCTTGCTGTCGCGAAAGCGGCTGTCGAGCTTCTCCAGTCCTT
TCGGTGCATAGGCATTGAGCATCTGCTGCTGATCGAGCTGCATCAGGCTGGTGGCGGCCAGTCCCCACAC
CACATCCGCCTTGGGCTGCGCTTTTTCTGCCAGCAAGCGGGCGGTGACCACGCCGGTGGAGTCACGCACC
CACTTGATGTTGATGTCCGGATGCTGGCGTTCGAATGCGGTTTTCAGCTCGTTGAGCTGCTCCGGTTCGA
AGGCGGTGTAGACGGTGAGATCGGTCGCGGCCAGTGCCGGGGCGGTCATGGCAGCCAGCACGGCGGCGGC
CAGCAGATGGGTTCCCTGTTTCATCCTCTTCTCCAGTTAAATTGGTTTGGTATAAACCAGATTGGTCGAG
AGAGAGGCTAGGGGATCTTTATGACGTGACCGTGACAGCCCCGTGACGTTTTGCCGTTCCTGATTTGTCC
GTGACTAATCCTGGGTATCCACCTCGATCTCCAGACTGTCGTGGCGCCAGTACTCCAGATCGAAATCGAG
CACCCGGCCGTGCTGATCGAAGTTGAGTCGCTCGAGGCGCAGGGCGGGCAGACCGGCAGTGGCCCCCAGC
GCCTTGGCCACTTCGTCCGGCAGAGCGGTGGGGAAGAAGCGCACCTGCATGCGGGCGTAGTGAAGATCGT
AATGGGCCTGATAGATCTCGGTCAGGCTGCCGTTGAGATCCAGCTCCAGCAGGCCGGGCACCCGCTCGGG
CAGGCAGTGGTTCTCGCAGTAGCAGATGGCGCGCCCGTTGGCGTAGCGCAACCGTTTTAGCAGAAACAGG
CTGTCGAACGGCTTGAGCTGCAGTTGCGCCATCAACGCGGGCGGTACCGCCTGACGGCTTTTCTCCAGTA
GTTTGGTGCGCGGCTCACCCCCTTGCTCCCGCACTATCTGGTGAAAGTTGGAGGTGCGTCTGGGGTTCAA
GCGAAAGCGTGGCGGGGTTACGAACCAGCCGCGGCGATCCTCCCGATAGATAAGGCCGTTCGCCTCCAGC
TGAACCAGTGCCTCGCGAATGGTAACTCGGGTGGTGCCATAGAGTTCCCCCAGCAGCCGCTCGGAGGGGA
GCTTGTCGTTGGGGGCCAGACCCCCCGCCAGGATCTGGTTGGCGAGGGCATCCTTGATCTGCAGATATTG
GGGTTTGCTCATGAAAATCCCTGGTTTTGCAATAAGTTGGGCAATGGGCTGGGCACAGGGCAGGTCATCT
GCCCGGTCGTTACACGGCAACATCAACATGCGTTGGTAGATACCAGCAGCGCCCCTGCTGTTCAGGCTTC
CTAGATTATCGCGATCCCGGCGCTGAAAATGAATTTTTTGTTGCACGTAGCGCCACCCCTTTGACCTTGG
CGGGGCAGTTACCCACAATGCGCTCATTCTGGTATATACCAAAAAGGAGCGAAGTCATGACCCATATTCC
TGCGGCCCCCGCCGCCGTCGATTACCTGCTGCTGACCCCGGGCCCCCTCTCGACCACTGCCACGGTGCGC
GCCGCCATGCTGCAAGACAGCTGTACCTGGGATACGGACTACAACCAGGGGGTGGTGGAGCCCATCCGCC
GCGAGCTGGTGCGCCTCGCCACAGGCCCTGAATATCAAAGTGACTACAGCGCCGTGCTGCTGCAGGGGAG
TGGCAGCTATGTGGTGGAGAGCGTGCTGGGCTCGGCGATAAGCGCCGACGAGTGTCTGCTGATCATCAAC
AACGGTGCCTACGGAGCCCGCATGGGGGAGATGGCCCGCTGTCTTGGCCTGCGCCACCACGAGCTGGATT
GCGGCGAGACCAACCGCCCCGAACCCGCCGCCATCGAGGCGATGCTGGCGCGCCACCCCGAGATCACCCA
TCTGGCCATGGTGCACTGCGAGACCACCACCGGCATGCTCAATCCCCTTGAGGAGGTGGCTGAGCTCTGC
CAGCGCCGCGGCATCCGTCTGATCGTCGATGCCATGAGCAGCTTTGGCGGTATCCCCATCGACATGGGGC
GCCTTGGCATCGAGTTTCTTATCAGCTCGGCCAACAAGTGCATTCAGGGGGTACCGGGCTTTGGCTTCGT
CATCGCCCGCCGCGCTGCGCTGGCGGCCTGCGCCGGTCGTGCCCGCTCTGTCTCCCTCGACCTGCATGCC
CAGTGGCAGACCATGGAGCAGCAGGGGGGCAAGTGGCGCTTTACCTCGCCCACCCATACCGTGCTCGCCT
TTGCCCAGGCCCTGCGCGAGCTGGACGAAGAGGGGGGCATTGAAGCCCGCCATCAGCGCTACGGCGAGAA
TCAGCGCACCCTGGTGGCGGGGATGGCTGCCCTCGGCTTTGCGCCACTGCTGCCAGAACAGTGGCAATCT
CCCATCATCACCGCCTTCTACTCTCCGGCCCACCCTGACTACCGCTTCGCCGATTTCTACCAGCGGCTCA
AGGCGCAGGGCTTTGTCATCTATCCGGGCAAGGTCTCCCAGGCCGATTGCTTCCGTATCGGCAATATCGG
CGATGTGACCCCTGAGCGGGTGCGGGATCTGCTCGCCGCCATGGCCAGCGCCTGCTACTGGCAAGGGGAC

GTGCGATGACCAGCATGCTGGCTCAGGGTCGTGATCTGCGTGGCCGCGATTTGCATGACCATAAGCTGCA
CGGCAGCGGCCGTGCCGACAGCGAAGGGGATATCAACCTCGGCGAGGGGCGGGCGCGCTGGTGGGCGGGC
CAGAGCGAAGATGTACAGGCCGGATTGGCGGAGGATAGCCGCTACTTTCTCCATCAGGCACTCTCCACCC
CCTGTCTGGATCTGATTGAGGGGGCGCAGGGGAGCTGGTTGACCAGTGTATCGGGCGCGCGTTACCTCGA
TTTTCACGGCAACAGCGTTCATCAGCTGGGTCACGGTCACTCCAGGGTGGTGGCCGCGGTTCAGCAGCAA
CTCGCCGATCTGCCGTTCGCGCCCCGCCGCTACACCCATCAGGTTGCCATCGACTGCGCCCGCACCCTGG
CCGAGCTGGCCCCCGGCGATCTCAACCGGGTGCTGTTTGCCCCCGGCGGCAGCGAGGTGGTGGGGATTGC
CCTGAAGCTTGCCCGTTTCATCACTGGCAACAGCAAGGTGGTGTCCCTCTGGGATGCCTTTCACGGCGCC
TCCCTCGATGCCATCTCGGTCGGCGGCGAGGCCTGTTTTCGCGCCGGTATGGGCCCGCTGATGGCCGGGG
TGGAGCGCATCCCGCCCCCCACCCGTTATCGCGGTGTCTGGTATCGCGGGGAGGGGGATGACCTGCACTA
CGCCGATTATCTGGAGTACGTCATCGAGAAGGAGGGAGGTGTCGGCGCCTTTATCGCCGAGCCGATCCGC
AACACCGACGTGCAGGTGCCCTCGAAGGCCTACTGGCAGCGGGTGCGGGAGATCTGCGATCGCCACAAGG
TGCTGCTGATCGTCGACGAGATCCCCAATGCCCTGGGCCGCACCGGCCACTGGTTCAGCTTTGAAGAGTT
CGGCATCGAGCCTGACATCATCTGTCTGGGCAAGGGGCTGGGGGGCGGGGTGGTGCCGTTTGCGGCGCTC
ATCACCCGCGACAGGTTCAATCAGGCGGCAGCTATTTCACTCGGTCACTACACCCACGAGAAGAGCCCTA
TCGGCTGCGCGGCGGCGCTCGCCACCATCAGTACCATCCGTGAAGAGGGGTTGCTGGCAAAAGCGCAGGA
GGATGGCCGTTTTATGGCGGCAGAGCTCGCGGCGCTTGCCGAGCGTCATCCGCTGATCGGGCAGGTGCGC
GGCATCGGTTTGCTCTGGGCGCTCGATCTGGTGGTCGATCATCAGAGTCGCATCCGCAATAGCCAGGCCG
CCGAGCGGCTGCTCTACCGCTGTCTGGAGCTGGGGCTCAGCTTCAAGGTCTCCCAAGGGAACGTCATCCA
GCTCTCGCCACCGCTCAATATCGCCCGCGCCGATCTGGTGCGTGCCATCACCATTCTCGATCGCGCCCTC
GGCGAGATCGCACTTTCACCGAAACAGGAACATCACCATGACTGAGTTGCAGACTGCCATCCACCCGACC
ACCGACGTGGAGGCGCTGATCCTCGACTGGGCCGGCACTGTCGTCGATTTCGGCTCCTTCGCCCCCACCT
CCATCTTCGTCGAGGCCTTCGCCCGCGCCTATGACTTCCCCGTTACTCTGGACGAAGCACGCCAGCCTAT
GGGCCTTGGCAAGTGGGATCATATTGCCGCGCTGGGCTGTCTGCCTTCGGTGGATGCGCGCTGGCAGGCC
CGCTTTAGCCACCCCATGAGCCACGCCGAGGTGGACCACATTTATCACACCTTTATGCCGCTGCAGATTG
CCGCCGTGACCCGCTTTGCCGCCCCCATCCCCGGTGTGCTGCCGGTGCTGGATGCTCTGCGGGGTCAGGG
GATGAAAATCGGCTCCTGCTCCGGTTATCCCCGTCCGGTGATGGAGGCGCTGGTACCTGCTGCGGCCGAT
CATGGCTATCGGCCGGATCACTGGGTGGCCACCGACGATCTCAAGGCAGGTGGTCGCCCCGGTCCCTGGA
TGGCGCTGGCCAACGTGATTGAGCTGGGAGTAAGCGCGGTGCATCGCTGCATCAAGGTGGATGACGCCGT
GCCGGGTATCAGTGAAGGGTTGAACGCGGGCATGTGGACGGTGGGATTGTCGGTTTCCGGCAACGAATTT
GGTGCCACTTGGGAAGAGTTTGCCGCCATGAGCGAGGCGGAGATCGCCGCCCGGCGCGCACCGGCTGAGG
CCAAACTGCGGGCGGCGGGGGCACACTATGTGATTGATACCCTTGCCGACATCGCCCCGGTTATCGCCGA
TATCAACCGCCGTCTGGCCGCTGGCGAGCGGCCGTAGGCCATCTGCTTGGGCTAACAAAAGCGCTTGGCT
GGCTTTAGATGCAACACGCCCCCATCCGGGGGCGTGTTGCATCTATAGGGGGAAGTTGTGCGCGGTCAGC
GGATATCGACCCGCATCAGGGGAGAGGGGGCGTTGTCGACAAAGTGGTGCCAGATATGGTCGTAGAGTGC
CTTCTGGCCGGGGAAACTCACCAGCGCCTTGGCCTCGGCCAGGGAACACCAGCGATAGTCTGTGTGCTCG
TCGTTGAGGATCACCGCCTGATTAGCCGGGCAATAGACCACAAAGACCGGCACTACCTCTATCGTATTCA
GGCTTGCCTCGTAAAACTGCTCCAGATACTGGCCGTTGTAGAGTTCGGTGACCCTGATCCCCGTCTCTTC
GCCAAACTCCCGAATAATGGTCTGCCAGCCGGTTTCACCCGCCTCCACCGTGCCCGCCACATGGCACCAG
AAGCCTCCCTTGACCCGCTTCATCACCAGCAATCTGGTTTCGCCATCGACAGTGGTAAGGGCAACGCCAG
AGACGGCCTTGCAGCGAATGGGGATCATGATCTTCTCTCCGGATTTGGGCTCTGTGATAGCCACGAACAG
TGTGAAGGCGTGTTAATTGAAATATGCCTCGGCGGGTAGGCCCATGACCTCTATTTCATTCAGGCCGCTT
TTGGCCAGATAGTTGGCCACCAGATTGAACCCCACCCAGTAACCGGCATATTTGGGAAATGGCCGGTTAT
CACGGCAGTAGAAAAAGTCTAAAAAGTTGAACTGCTTGCTGGATAGATGGGGTGTCATCTCTGCCAGGAG
CGATTGCCAGTCATGGTGATGAGACTCCTTGAACAGCGAGGGGAGAGTGCCACCATTGAACCTCTGCTCA
AAGTAGCAGGCCAGCCCTTCAAAAATCAGGTTTTCGGCCAGTGTTTCTGCTTCCACCTTGTTCTTCTTTT
GCACGATATGGTGGACTTCGTGGGCCAGCACGGCAGGCAATTCGGTGATGATCACCTTTTTGATATCAGG
CCGTTCCAGATCAAGCTGGAGTTCTATTCGATACATGTTGATGGCCCAGCCACCGATACCCGAGTCGGGA
GCATCCCCCTCGGCAAAAGGGCTGAAGGTGACATCCAGTTGGGAGAGATCGAACAAGGCATTCAATTTGT
TCGCCGTCTCGGCAAAGACGTGCCTAATGGTGTCATCGAGCCCATCGAATTTGCCCGAGTCGTTTAACAG
GGTGAGTTGCATCCAGACATCCTTGTCTATTGAAAGAGAGTGCTGAACCACTTGCGTGGTCGCGGCGCCA
TATCACCACGATTACCCTTCTTCAATCAAGGGGTTACCCGCCCATTATGCTTGCCGTCTGGATCGCCTCG
ATCAGCGCGGCCCGGTTGATGCGAGGGCGCTCGCCATCGAGCAGTTGCTGCCACAGGGCAATGGCCCGAC
TGTAGCGACCATGCAGGAAGTGATCGGAGGCGAGCAGCATCAGGGCGCTCACTTCCCCCGCGTCCTGCTT
GAGGGCGTTCTCCAGCAGGCGGGTTGCCTCGGGAGTCAGCTGCTGGCCCGCCTGATAGTAGAGCACGGTG
GCTTGGGCGGCTTGAGTCGCCGCGTTGGCGCCCCCCTCCAGCAGAGCGAGACGCTGGTAGGCGAGCAGGG
CGTTGTCATATTCGCCCCGATAGAGATAGAGCTGGCCGAGCGCCGCCCAGCTGTCGAGATCGGCGGGGTT
GCGCTGAAGTTTCTGATGCAGACTCCCCAGCTCCTTGTCTTGCAGATCCCGCGGGCTCAATCCCGCCAGC
GGATCGGGACGGGATGCATAATCCTGCCATGCCTGATAGCGGCCGCTGCTGAGATACACAGCGGCAACGG
CCGCGATGGCCAGGAGGCCAAGCAGCAACAAGGCCTGCCCGTGCCGGTTATTGCGGCCGTGATTGTCATG
CTGGCTGGCCAGTTGCGCGCGCAGGGCCAGCTCAGCCGCCGGGATGGTGTTGGCCAGGCGGGCGGCGCTC

AGCTCCCTGTAGAGTGGCTGGTCTGGGGAGAGGCCCGCTTGTTCTCCACGCATGATCAACAGATTCAGTT
CGCTGCGCGGGTCGGCCGCGGGTTGAGGGGGAGCCTCAACGATGGCCTTAACGACAGGGATGGGTTGCGC
CATTGTGCGCGTGAGGCGGCGAAACAGGGTCAACAGCGCCAGCCCCAACAGCAGCAGGGGACCGCCCCAC
AGCAGCGCCGTGCTGCTCTTGAAGGGGGGATCGTAGCGGACGAAATCGCCGAAGCGCTCTGTCATGCGGG
CAATCACCTGCTCATCAGACTGCCCCTCGTCCACCCAGCGGTAGACCTCGAGCCGCAGATCCCGAGCGAT
GGGGGAGTTGGACTCTACCAGATTCTGGTTCTGGCACTGAGGGCAGCGCAGGGCGCGGGCCAGCTCTTGC
GCCCGATTTTGCAGCTCGGGGTGGCGAAATTGGTAGGTATCGACCCCGCTGGAATATGCAGTGGCACTCA
GCGATATGCCGAGGGCCAGCAGGGTGCAGGCAAGAGAACGGCACATCATGGCTGTGTCTCCCGAGCTGAT
GTCGCAATAGATGGCGCAATAGATGGCGCAATCGCGGGTGCCGTTGTGGCGGCCTTTATCTGCGCATCAG
CTCGCGCCTTGTCGAGCAGGGGGGCAAACTGGCGTAGCCAGATCTCGTTGGTGAGCTCGCCGGTATGGCG
GGCAAGTAGGGCGCCATCGGCATCAAAGAGATAGGTCTCCGGCGTGCCGTAGACGCCGAGCTCCAGTGCG
AGTCGGCCGTCGGGGTCGGCCAGCACGGCGCGATAGGGGTTGCCCGCCTCGATGAGCCAGGCGCGGGCGG
CGTCCCCCTTGTCCCGGTAGTTGAGGCCATAGAGGGGCACGGCGCTGGCCAGCTCACCCAGCAGCGGATG
CTCGGCGCGGCAGTTGGCGCACCAGCTGGCCCAGACGTTAAGCACAAAGGGCTCCCCCTTGAGGCTGGCG
GTCTGGATCGGACTCCCGTCCAGCAGATTGTCGCTCTGCCACGCAGGCAGAGTACGGCCGACCGTCGCCT
CATCCTGGCTATAGGGATTGTTGCCAAGGCCGAGCCAGAGCAGGATGCCGAGGCTCAGGGTAAGGAGCAG
TGGAAGAAAGAGGCGCAGGCGGGATCTCATAACGCCTCCTGTTCGAGCCGGGGATGGGAGGCGCCAACCA
CAACGGGGCGGGAGGCAAGCAAGGGGAGACGGGCGAGCAGGCGCAGGGTGCCGCCCGCCATCATCAACAG
GCCGCCGAACCAGATCCAGCGTACCAGCGGCTTGTAGTGCAGCCGCATGGCATAGGCATCTGGCCCCATC
TTCTCACCCAGCACCACGTAGAGATCGCCGAGCATTCCCCACGCGATGCCCGGCTCGTTCATGTTCATGG
TGCGCACGCTGTAGTGGCGCCGCTCCGGCGTCAGCCGCGCCACATCTTTACCATTCTTGCGCACGCTAAG
CGTGATCCGCTCGGCGGTATAGTTGGGGCCGATCACCGGCTCGCGCCCCTCATAACGAAACTCGAACGCG
CCGAGCTGGTAGGGCTGGTTGGCACTTAGCACTGTGCCTCCCTCCTGACTGTGGTGGCTCACTTGGGCAA
TGCCGAGGGCGCACACCGCCACCCCAAGGTGGGCCAGCAAGCTGCCAAACCGATTGAGGGTGAACTCGCG
CAGCGACGTCGGTTGCAAGAGCGGGAGCAGCAGGCTGGCCACCAGCCAGAGGCTCACCATATTGGCGAGA
AGGCCAATCCAGCTAGCGGAAACCGATCCCTGTTCCAGATAGGCAAGGCTAATGAGCCCGCCGCCAAGCA
GGCCAAACAGCGGCGGCAACAGCGCGTTGAGTCGGGAATGTGCCGCAAGCTGCTGCCAGCGCAGGCGCGG
CAGCTGGGTCATCAGCCCCATCAGGCAGAGGGCCAGCGGCATGAAGATGGTATTGAAATAGGGGGCACCC
ACCGAGAGCGAACCCAGATGGAGCGACTGGCACACCATGGGGTAGAAGGTGCCGAGCAGCACGCTGGCGC
AGGCCACGCAGAGCAGCAGGATGGCGGCGCCGAGCAGCCCCTCTTTTGACAGCAAGCCAAAGCGGGCATA
GGGGGCGCGAATGTCCGCTCGCAGGGCAAACAGGGTGAGGGCGCAGGTCAATAGCAAACCGAGCAGCAGC
AAGAGGGTGAGGCCGCGGCTGGGGTCCAGCGCAAAGGCGTGTACCGAGGTGAGCACCCCGGAGCGCACCA
CAAAGGTGCCGAGCAGGCTCAAGGCGAAGGTGAAGATGGAGAGCAACAGCACAGCGTGACCATAGGCGCC
GCGCTTCTCGCAGACAATCAGAGCGTGCAGCAGAGCGGTGCCGAGCAGCCAGGGCAATAGGGAGGCGTTT
TCCACCGGATCCCAGAACCACCAGCCGCCCCAGCCGAGCTCGTAATAGGCCCACCAGGAACCCAGCACGA
TACCGGCGGTGAGAAAGACCCAGGAGCCGAGCGCCCAGGGGCGACTCCAGTGGGCCACGGCGCCATCCAG
TCTACCCGAATGGAGCGCAGCCATGGCGAAGGCGAAGTTGACGGCAAAGCCGACGTAGCCGAGATAGAGC
AGGGGCGGGTGGATGATGAGGCCGATGTCCTGCAGCATGGGGTTGAGATCTCGCCCCTCCAGTGGCCCCG
GGAATTGGCGGTCAAACGGGCTGGAGAAGATCAGGGTATAGAGGCCGAACAGCCCGACGATCAGCCCCAT
GATGGCCAAGACCCGGGTTGTGATCAGCGGATCGACCCGCTGGGAGCAAAGAGCCACCAGGGCTCCCCAC
AGCCCCAGCGCAAAGACGAAGAACAGCATGGAGCCCTCGTGGCCGCCCCACACGGCCGCCAGCTTGAAGC
CAAGCGGCAGGGCGCTGTTGGCGTGCTGGGCCACATAGCTGACGGTGAAATCATCCAGGGCAAAGCAGCT
GGCGAGCAGCAGCAGGGCGGCCAACAGCAGGATGGCATTGATCAGCGCCAGTGGCTTGGCGCAGCGCAGC
AGAGTTGGGGAGGCGAGCCGTGATCCCAGCCAAGGCAGTGTGCCCTGCAGCAAGGAGAGTGCCGCCGCCA
GCAACAGTGAGAGATAGCCGAGCTCCGCCAGCACATAACCTCCGGTTCAATCTTTGATCTCATTCATGTC
ATGCCACACACGCCGCGTCGTCTCCCTTTGGGGGAACACATTGCCAAGCAACGGGTGTGGCATCACCAGA
AATTTATTGGGCCAGCAACAAACCAGCGCCAGTCACCCGAAGGTGGCTGGCGCTGGTGATCATACCGTTA
TACGACTGTCATCTGGCCGGCATAAAGCACGAAGTAGCGCAGCAGCAGGATCCCCACCAGGGTCAGGCTG
CAGACCAGTACCAGATAGCCGTTTTTGGCCCGCAGCGGCGCGGGGGCCAGCAGGGCCAGCAACTGGGGCA
GCAGCATGCCGATGCCCACCACCCCAAGCCAGAACACCTGAGCCCAGAAGCCGCTGCCGATGGCGGCCCA
CATGGCTGCCTCCTTCTGGCCGCCACCGAAGTAGAGGCCGGTAAAGAAGCAGACCAGCAGCAACACCTCC
ACCGCCAGCACCGGCCGCTCGAAGCGGTGGACAAAGCTCACCCCCTGGCTATGATGGTTCTCCTTGAACA
GGGTGACCGCCAGCAAGATGGTGGAGGCGATGCCGGAGCTGACGCCGGAGATGAGGAACAGCACCGGCAG
CACCGGGTTGTTCAACAGCGGATAGGTCTTGAGCGCCGACAACAGAAAGCCGGTGTAGGCCCCCAAGAGC
ACGGCAAGCAGCAGCAACAGCGGCGCCAGCAGCCGCTCGATGCGGGCAAGCCAAATGATCAGATCATCCA
CAAAGGCGAACTTCTCTTGCAGCAGGTGACGACGCAATGTCTCAATCTCGGAGCGAAACACCACCGCCAG
CCAGGCGAACAGCACCGCCATGTAGACCTGAAACAGCATGACCCCCATCGACATCACAGAGTCGAACTGG
TAGTGGAACATCAGCTTCCAGAAGGTCCAGGGGCGGCCAGGTGGAAGATGAGGATCAGTAACCCCAGCA
CCACGCTCACCGGCGCCAGAATGGCGGTCGCCTTGATGATGCCGCTCTCGCTGCCGGCCCCCTTGCGGCG
CAACAGCACCGAGAGGGTGGTGGCCCCGGCGGAGATGCCGAGCAGAAAGAGATAGATGGCGATCGGCCAG
TCCCAGACGAGGGAAGGGAAGTGAAATGCCTCTTGCATGGTCATGACTTTATCTCCCCTTTGGGCACGCG

GTACATCTTGGGCGAGGTGCCGAGTGCCTGCTTGTAGCGATAGGTGGTCTCCTTCACCAGCCGTTGGGCG
ATGGGGCTGTCCGGGTCATCCAGATTGCCGAACACCAGCGCCTTGGTGGGGCAAGACTCGACACAGGCGG
GCTCCTTGCCCGCCGCTAGGTTGGTCTTGCGGCAAAAGTCGCACTTGTCCGCCACCCGGGTGACCGGGTT
GATAAAGCGCACCTGATAGGGGCAGGCGGCCAGACAATACATGCAGCCGACGCAGAGATCCGCGTCGACA
TCGACGATGCCATCCTCCTTGCGGATATGGGAGGCGCCGGTGGGGCAGACATGCACGCAGGGGGCGTTGT
CGCAGTGCTGGCAGGACTTGCGAAAGAAGCGGTACTCGGCGTCGGGAAATTCACCGATGGGACCTGAACG
GATGATCTCGAGGCGCGAGACCCCTTCCGGCACTTTGTTCACTTCCCGGCAGGAATCCATACAAGCGGTG
CAGCCGATGCAGGCCGTCTCGTCGTGGATCATGCCATAGCGTATCCCGTCGATGGTCTGGCTGTGGGCGA
GAGGGGTGGCAAGGGCGCGATTCGACCCCGTCATCATGATAAGGGCCCCCGCGCCCATGCCCGCCATAAA
GTCACGGCGAGAGCAGCTCATGATGCCTCCTTGCCGGTAGCCGGTTTGGACTGCTCCTGCCGGGCCTTGC
TGGCCTCATGCTGCTTGCTGTGGCAGTCGACGCAGAGCTTGATGCGGGATTTTTCCGGGATCCCCACCAT
GGGCTCCTTGGCCGGATGCAACTGGTGACAGCTGGTGCAGGTGAGCTTGGTGGCGTGCACGTCGTGGGCC
CAGAGCGCCTCGCGCAGTGGTTTGGGCTCGTGGCAGCTCATGCAGACTCCATTCTGCTCGCTGATGGGAT
AAAGCTTGATGCTCTCTTTATCCTTCAGGCTGTGGCTGAAGCGCATCACATCCTTGACCCCTTCCCTGTG
CTGGGGGGAGGGCTGGCCGTGGCAGTTGGTGCAGGTCACCGGTTTGAGGTTGTTGGGATTGGTCGCCTGA
CCATGCTTGCCGTGCATCCCCTCGCTCTGCTCCTTGTGACAGTCGGTACAGGCCTTGTCGGGATTGCGTA
AAAATTCCACCTCATGTGTAGCTGGAACAGGGGCGGAAACCGATTCGGCCGCCTGACCCTGCAGGCTGGT
GAGCAGCATGGCTGCCATCAGCATCAGTCGTAAAAAATCCATCGTTAAATCACCCATAGGAAGCCTCCAT
CCGCTGCGGGCCCGACTTGGGGCCGGACCCGCAGGCGCCGTTACTGTGCCAGCCGTCCGGCCGCTTTGGC
CTCGGCATCCCAGGCGGGGACCGTGGTCTTGAGGAACTGGGCCTTGTCGCTGTTGAACTGCTTCATATCC
ATGCCAAGGGCCGCTTGCGCTTTCTCCTTGGTGGAGATGTCGGGGATGGCGATTTCGTGGCTGATCCCCT
TGCTGGCCAACAGGCGCGCCAGTTTGGTGCGGGCGTCAGCGGCTTTATCCAGCGCGCCACCAAGTACCTT
GAGGGCCACTTCGGGGGCGTGCATCTGCACCCCGTGGGAGGCGATGGCGTAGTCCCAGCGCCACTGGGCG
TGACGGATGTCCTGCAGAATATCCTTCATCTCTGCCTCGGTGGCGCCGGCATCCCAGGCCGCCTTGGCTT
CAAAGTGGGCGTGTACCAGCTGCTTTTCGACCTTCAGCTTGGTTTCGATCACCGCCTCTTTGCGCTGCTT
GACGATCCCTTGCAGCATCTCCTTGCTCTGGGTGTGGCAGTTGCGGCAGGTCTGCTCGAAGCGATCGAAG
GGGTTACCCACCTTGTGATCGGTGTAGACCTTGCCCTGCTCGTTCTGCACCTTGGGCATGTGGCAGTCGA
CACAGGCGACGTTGTTCTGGCCGTGAATGCCGGCGCGCCAGGTCTCGTACTCGGGATGCTGGGCTTTCAG
CATAGGGGTCTTGGAGAGGGCATGGGTCCAGTCGGCAAAGCCGATTTCATCGTAATAGGCCTCCATCTGC
TCCACAGTGGTGCCCTTGTCCCAGGGGAATTTGACCGCCTTGGTGGGGCCGCTGAAGTAATACTCCACGT
GGCACTGGCCGCACACCTGGGACTGCTGATCAAAGCGCCCCTGCTCCTTGAACGGCTTGCCGATGGCGGT
CATGGCCCGATCGGCATAGGGACGGGAGAGGTGCAGGGCAGGTTTACCCTCCTTGAACTCCTTCGAGGCG
GTGTCGTGGCAGTCGGCGCAGCCGATGGTGTTGACTACTTCCGGCCCACCTTTGGCCCACATCCCCTTGA
AGTAGCCATCTTCGCCTTTTTCGTCGATGACGCGGGCCACATCCGGGCCTTTGCAGCTCCAGCAGGCCAT
CGGCAGGGGGCCATCTTCGGCGGTCTTGGGGGCGCCGGTGCGCAGGGTTTCGCGCACATCGGTCAGCGCA
TAGAAGTGGCCGCGCGGCTTGTTGTAATCCTTGGCGAAGGGGTAACCGCCCCACAGCACCACCATGTTGG
GATCTTCCGCCAGCGCGTCGGTGCGCTCCTTGCTTTCGCTGGTGGCTTGCCAGCTGGTGAACTGATCGGC
GTGATCCTTGGCGAACAGCTCGCTGCGCGCCTCTATCTTGCCATTGGTGGTTGCGTCATCAGCCAGTGCC
CAGCCGCTGGTGCCAAGGCTGGTAGTCAGCAGCAGGGTGGCGAGGCCCAGTTTGATATGCCTGTGTTGTG
AACCCACGTTTATCACTCCCAGATAAATGTTTTGGTCTCGTCTGACCCGTTCCATCTTGGTGTGGATCTT
CCCCCCGTCGGGCGGGGTGGGCAGACATTCGCTTGCGGGTGAATAAAACCGCGCAATATATGATTGCTCG
AGGGTTACCCTAACAAATAGGTAAATTTAATGATGATTTATCGGAGGGAGAGTTGTGCCAGATCAATTGG
TGCACAGGATTGATCGCTGAAATGAGATCTGGATTAAATTTTATGGAAACTCAACTTCTTGTTAACTGCA
GAGCATTAGATGGCAGGCGTTTGATCGATACAGTTACGTACTGGCAATTTTGCCAGCTGAGCTGGATTGC
TGCCCTATTGTTCACAGATTCTGGCAATGACTTATGCTGATAATTGACTGAATATGCAAATTCTCGGTGT
CGTGCTGTGAGCTTTCGACCTTCGGATTATGATCCATTTATTGAAGCTGCAGTGGATTTATCGTCAAACG
ACAGAAATGGTCACATGGATTCTGATAGACCTGTTAATAAAGGTGATGCGATTGGGGTCATCCAAATCTC
ATTTCATTTAAAAAGGCCTGTTAGATGCACAATCTTGTAAGTTATGGTCATTTTATGGCAATCATTGTCG
AAAATAGCGAAAAAACCCACTTAGTCTGGTGCGGTATGATTATTGAACAGACAAGTACCCTGTAGGTACT
GATGTTGGTGGTTAAGGTTTTGCTGGAGGTTGTATGGCAGAGTTGCAAATTGAAAAGTCCACGGTGATCA
CCCAACTCGAGGGCTCCGTGTTTGTCATCAGTGCAGATGGATCTGTCATGCGAGCAAAGGTGGGGATGGC
GCTGCAGCCGGGGGATCGCCTGCAATCCGTTCAAGGGAGCAATTTTCAGCTAGCCGAGCCGGGCAGTGAT
CCTGCTGGCGTGCAAGAAACGGCTGCCAAACCTGAGCCCGTCACGCCTGCTGCTGATGGGCAACCGGCCG
ATCCGGAACTGGCCTCATTGCAAGAGGCGATCCGTCAGGGTATTGACCCGACCGAATTGTTCCAGGAGAC
CGCCGCCGGTAACGCAGCCGCTGCGGGCAATATCGGTGGTGTAGCCGGTTCCAGCTCGGGTGGATTTGTG
GTGGTCGATCGTACCAATGACGCCACCCTGGCCGAGGCCGGTTTTGATACCGACCATACGGTGCAACCTG
TGGGCGATGAGCTGCTGTTTTCCGAAGAGCGCGATTTCTTTGCCGCCATCATCATCACCGAGCCCCAAAC
CAGCGACAACATTATCAACGCCGATGAAGCGGCTGGCGTCATCATTCGTGGTGTGATGGATGATGTGGAG
GTAGGCCAGCCAGTCACTGTGACCCTTATCGATCAGACGGGAAATTCACTGACGGTGACGACAGTGGTGC
TGCCCGGCCTGGTGTGGGAAGCCAATTTTGGCGATGTGACCGGCAAGCTGGTGGATGGCCCGTTGCAGAT
TGTGGCCAATACCCAGGATCAGGCGGGCAACAACGCCAGCGATACCGGACAGACTCTGCTCGATACCATC

ACCACCATTACCCTCGATCTCGCCGATGAGAGCGACACCGGCGCCAGCCAGATCGATGATCTGACCCGCG
ACAATACCCCGCTGCTGCAAGGGAAGGGGGAGCCCGGCGCCACCGTAACCCTGAGCCTTGGCGGCAGCGT
GCTCACCGTCCTGACGGTGGATGCCGCCGGTAACTGGCAATATCAGTTGCCGCAAACCCTGGCCGATGGC
CAGTACCAGTTTCAGGTCAATTCCGTGGATATTGCCGGCAACCGCGCCAGCGATACCCTCGTTATCAATG
TCGATACTCAGGCATCTATCGACATCGATGATCTCGATACCACTGCCCTGTTTGGCAACAGCAGCGCCAC
CCTGAGCGGCGCCACCAGCAATGTGGAGGCGGGTCAGAGTGTCATCATCACTCTGGTCGACAGCAATGGC
CAGCCGCTGTTGACCACCTCGGCACAGGTCGGGGCCGATGGCCGCTGGCAGGTATCAGGTCTTGATCTCT
CCGGTATCAATCAATCCTTTGAAGTGCGTGCCAGAGTCACCGATCTGGCGGGCAACAGCGCCAGCGATGG
GGCGCCGCTGATCGGTCAGAGCGAAACCCTGACGCTCTCCGAAGCCGGATTGGCCGATGGCCCGATCACC
GCGACCGGCAGTCTAAATACCGGTGCCGGCCAGGATGGCAATCTGCAAGTCCAGTTCGCCGCCGACCAGA
GTGCTTTGGAAGGGCTGGGGCTCGTCAGCCATGGCGCGCCTCTTGGCTATACCATCAACGGTCAGACCCT
GACCGCCAGTGCCGGTGGGGCGACTATCTTCACCCTGACCCTCAACAATGACGGCAGCTACCGCATCGTC
TGGAGTCAAAGCCTGGATCACGGTCAGGACGCGTTGCGCCTGCCGTTCAACCTCGAATATCGCGACAGCG
ACGGCGATCTGGTGAAGGCGCCGCTGGTGATCAATCTGGTGGACAGCAATCCGCCCATCTTCGAGATCCC
GGCCATTCCTCTGACCGAAGATGCCTTTACCAACGGTGCCGCCGTGGTGGGCGAGAGCAGCTTTGTGGTC
AAACACACCGCCGATCCTCTGCTGGTGGATTCGTTGGTGTTCAGCAATCAGAGCAGCACCCTGACTACCC
TCAACGGCTCGGGTCTTGCCAGCGATGGTCATCCGCTGACCTTTGAATTTGACGATGATCGCACCCTGGT
GGGTTACTACCTGGATGGCAGTGGCCAACGGGTGGAGGTGCTGCGTGCCGAATTGAATGCGCAGCAGCAG
GGCAGCGATCTGGCTGGGCGGGTGGTGGTCACCCTCACCGGCCCGCTCGATCATCAGGGCAGCGACACGC
TTTCCCTCGATTTGCAGGTGAGCGCCAACGAGATAGATGGGGACAGCACCAGCGCCAACCTGACGTTGAC
TGTCAATGATGGCAGTGATCCCGCATTGGACATCGACAAGGGGGTCGCCCTGCAAGAGGGGGGCAACCAG
AGCATCGATGGTCAGCTGCCGGTGACAGTGGGGAGCGATCGTCTGGTCTCCCTCAACTTTGAGGCGAGCC
AGCCGGCTTTGGCGGGGCTCACCAGCCAGGGCAAGGCGACGAGTTACGAGGTTAAAGACAACCAGCTGAT
CGTCAAGGATTGGCAGGGCAAAGCAATCCTCACCGTTACCATTGGTCTGGATGGCAAATACAACGTCTCC
CTGACCGGGGTGTTTGACGAACCGGAAGCGACCAACAGCCTCAACCTTGACCTGAAGGTACAGGGCTCAG
ATTTTGACGGCGACAAGAGCAATCTCGGCATCCTCAATATCACCATTACCGACGGTGCCTTGCCGCAGGT
TAATCCTGTCTCCCTCACGCTCACCGAAGATAGCAACTGGCAGCAGGGCCAGACCCTTGACGGTGATCTG
ACTCTCACAGCGGGCAGCGATCCGCTGGCCAGCATCACTTTCGATGCTAATCAGCCGGGGCTGCAAGGGT
TCACCTCGGGCAAGCAGGCCGTGACCATCACGGTAGCTGACCATCTTATCGAGGGGCGCACCCCAGATGG
CAAGCTGGTGTTTGAACTGACCCTCGGCAACGATGGCAAATACAGCTTTACCCTCCATCAACCGCTGGAT
CAGGGCAGTGCCGACAGCCTGCTCAAGGCGGGCTTTACCCTGCTCGACAGCGATGGTGACAAGGTCTCCT
CCACCATTACTGTGGCGGTGGGTGATGGTGCCAATCCGATCATCTCCGCCGTGACCGGCACCACCATCAC
CGAGATGGCGCAAGGGGAGACGGCTGCCGTCAGCACCATGAGTTTCTCGGTCGCGCACGGCAGCGATGCC
CTTGACCCTAACTCTCTTGGTTTTGATATCGCCGCTATCCAGAGCACCCTGACCGGACTGACCAGCCACG
GTAACCCGGTCACTTTCGCCGTTGATCCCGCAACCGGCCAGCTGGTCGGAACCGCCGGTGGCCAGGTGGT
GCTGCGCGCCGAGCTGAGCCTGGTCGATAACAACGGTAACTGGAGCGTCACCGCCAAGGTCACCCTGAGC
GGCGAGCTGGATCACAAGGGGAGTGAATCCCTCAACCTGCCCCTTGCCGTCACTCTGGCTGACAAGGATG
GCGATCGCATCGGTACCACGCTGCCGCTCACCATCGTGGATGGCAAGGCACCGAGCTTTATTCCGGGTGC
TGGATGAGGGGAACCTCACCGGCAGCAACAGCCTGAGCCAAACCGGCCACTTCGATGTGCAAGCCGGGTC
GGATCGGGTGGTCGAGGTGGCCTTTGCCGATGGCAGCCAGCAACCGGCGCTCACAGCCCTCGGCCAGCCC
GTCAAATACGAGCTGGTTGATGGGGACCCCGCCATCCCCGGTAGCCAGCTGATCAAGGGTTATGTGATGG
TGGATGGCCAGCGGGTCGAGGTGTTCGAGGTCAAGCTGATTGGCAGCCTCGATCAGGCTGGCAAGAACGG
GTTTGACTATCAGGTCACCCTCTATCAGGGGCTTCATCAGGGCGGCAACGCCGTGACCGAGCTGCCCATC
AAGGTGATCGTCAACGATTACGACAAGGCGGGCGGTAACAACGACAGCACCAGCGGCACCCTCAATATCC
AGATTGGGGAAGGGGAGAAACCAACCCTGACCCTGACCGATGTAACCCTCAATGAAGGGCGCTTCGATGG
CACCAACTCGGCCGTTGATGACCAGCAGACGAGCGGCAAGATCACCATCAAGGCTGACTCGGATCCGGTG
ACCGATGTGCGACTGACCCTCTCCGGTCAGGTCATGGATGCCAGCGGCAAGCCCATCACCCATAACGGCG
AAACCCTGACCTGGAAAGCGGTTGGCACCGATGGTCACACCTTCCAGGCGGTGACCAGCGGTGGTCTGGT
AGTGATGACGGTCTCCATCCCCTCCGTCCCCGCCAGTATCGCCGCCCATACCAGCGCCGAAATTGGTTAT
CAGGTAACGGTGCATACCAACCTGGATCACGGGGCAGACAACAAGCTGGAACTGAATCTGCCGGTCGAAG
TCACCGACAGCGATGGCAGCAAAAAATCAGGGAACCGCCACTGTCACGGTGACTGATGGCATCAACCCGAT
CATATCCGCCATTGAACCGGTTCGGGTCGAGGAGTCTGATCTCGATGCAGTGAACGGTCAGCATCCGGGG
ACCACACCTGCAGGGAGTGGAGAAACCGATAGTGGCAAGGTGACCATCACAGCCGGTTCGGATCGGGTTG
CCTCCCTTGAGCTGGATGTGGCTGCATTCAATCAGGCCAATCACCTCACATCGGGTGGCAAAGCGGTTAC
GCTGGCTGCACACAGCACCGGCTGGTATCTGGCTACGGCCGAGGGGGGCAAACCCGTTTTCTGGGTCCAC
CTCGATGTTAACGGCAGTTACAGCGTCCATCTGGTTGGCCAGCTTGATCACGGTGTACAGGGCAAGGATG
AGTTGACCATCGAAGTGCCGCTGTTTGTCACCGATCATGATGGCGATCGTAGTCCGAACGTAAGCGTGAG
CGTGACGGTACAAGATGACATGCCTATCGGCACTGTGCTGGCTCATACCCTGAGCGAGGGGAGCAGTACC
GTGACCGGCGATCTGCTGCCTACTGCCAATGAAGGTGCGGATGGTGCCCGTGTCGAGTCGATTACCATCA
ATGGCCAGACGTACGCCATCAATGGCTCTTTGATACAAGATGTGATGAGCCCCGATGGCAAGCAGGTGAT
GGGAACCCTGACCATCTCTGCCGACGGCTCCTACTCCTTCAATCCTGCGGCCGGTTTCAATCACGATGCA

AGTCAGCTCAGTCAGGTGGTGACAGCCCATCTGGTCGATGGCGATGGCGATCGTGCAGACAGCACACTGA
CTCTCAATCTGCAGGATGAAGCGGTGCGCTTTACCGTTAGCGCAGCCATTGGTAACGAAGATCAGGGGTC
GCAAGACCCGAGGCTGGGTATCCCTATCAACATGCAGCTCGATATTGGCGATTTCGATCGCGGTGAGCAT
GTGGATCAGTTGCTTATCCAGGCGCCTGCCAATGCCCATGGCACCTTCTATTACAACGGCGTTGCCCTCA
CCACCACGGCTGATGGCAAGTGGTTTATCGTGCCGCCAGCGGCCATGGTGACCAGTGATAATGTTCACTA
CACCACTCAGGGGGTGACCTTTGTGCCTTCAAGCAACTGGTCGAGCTGGAGCAATGGCGGTCAGCCGCTG
CACTTCCAGGTGCAACTGCAGGTCGGTGTCACCGAAGGGGCGGCACCTGCGGCTGTAACGGGCGATCTGG
CTGTGACAGTGCGCGGCGTGGCCGACAAGCCGATGTGGGACACAGCCAACACCACTCGCCACTATGACAC
CAATGAAGACAGCACGGGTATCAAGCTGGACGTCAAGGCGAGCCTGACTGACACCGACAGCTCCGAGACG
TTGACCTATCAGATCAAGCTGGAAGCGGGCAAGGGAACCCTGATGCTCAACGGAGTCGAGCTCAAGCCAA
ATAGCAGTGGCATCTACACCGTTTCGGCCGGCAACATCAACAAGGTGACTGTTGTTCCGGGCAAGGATTT
CAGTGGTGACATCAAGCTGTCGGTGACGCCGATCAGCACCGAGAAGAACCCGATCGCTCAGGGTCAGGGT
ACGGCGCAGGGAGACAAAATTGATCTGGTGATCAATGTCAATCCGGTGGCCGATGACGCCAAGCTCACCG
TGCGTGACATTCAGGGCAAGGAAGACACCCTGCTCGATCTGGGCAGCAAGATCGCCCTTGCTCATCTGGG
TGACAAACTCGATGGCTCCGAGCAGATGTTTGTCCGCATCAGTGGTCTGCCTGACGGTGCAACCTTGTTG
CTGGGTGGTTCCGCCGTTGCACAGAACATAGCCGGTTACTATGAGGTTCCTTTTGATCGGATTGGCGAAC
TCAAGCTGCAACCGCCGAAAAACAGCAACGTCGATTTCGATCTGACCATCAAGGGGGTGGTGAAAGACAC
GGCGATATTGACCGATGCGAACGGCCAGACCCATACCGTGGTGAACGAAAAAGAGACAGTCTCCCAGACC
CTTCATGTGGATCTGGTGGGGGTGGTGGATCAGCCCCATTTCGAGCTTGGCAATACCGTCTGGGAGGCCG
AAGGGACAGGCTATGCCACCACAGTGGGTGAGGATGGACGGGCACCGCTCAACTTTACCCTCACCTCGGG
CGAGTGGGTCGATACCCCGCATGACACTTCCGAGAGCCTCAACGTGGTGCTGCAAGGGCTGCCGGAAGGT
GCCCGGGTCTTTGATGCCAATGGCAAAGAGCTGACTCTTACCTTTGCCGGACTCGACGCCCATGGCAAGC
CGACCTATCAGGTCGATGTCTCCGATTTTGGCAACCTGCTTATTCAGCCGCCCCATAACAGCACGGCGGA
TTTGAACCTGATCGCCAAGGTGGTGGTGACCGAGAACGATGGCGACCACAAGAGCTTCGAGGTGCCGCTG
CATATCAAGGTGGAACCCGAGATTGACGCCACCGACTACAGCAAGACCAGCCATGGCCTGGAAGATCAGT
TCACCGTGCTGGACTGGCATCCGACCCTTACTGACGGCGCCGAGAAAGTCACGGCGCTGAGTCTCTCCGG
GGTGGAGGATGGCTACCAGATCTGGCTGCGAGTCGGCGGTGTCGAGAGCCAGCTGACCGTGGTCAGTGGC
AAGGTCACCCTGACTGCTGCCCAGCTCGACAGCCTGCTCAACGGTGGGCAACTGCTGGTGAAGGCGCCGG
AGGATAGCGATCGGGATACCACTATCCAGAGCCATGTTACCGTGACCCAGCATGATGTGGATTCGGGCGC
GGTGGCCGAGAAGGTGATCGATGGCAATCTGCATGTCGATATCCAGGCCGTGGTGGAGCCCGATGGCGTG
CTGACCCAGAGCGGTGGAGCTCTCGTGGTGCAGAGTGGGGCTGATATAGACCTGACTGGACGCTTCATCT
TTACCGACCTCGATCCCTCGAGCGTGGAGGAGATCGACTATCTGGTGATCAACCTGCCGGTGTTGAACGG
TGGTGCAGGCGATCAGTTCGTGGTGGTTGGCGGTATCAACAACGGTAACGGCAGCTGGATCGTGCCGGCG
GGCAATCTCGGTAGCTTCAAGCTGCATGCGCCGGACGGGTTTGTCGGCAAGGTTCAACCGATCCGCATCG
ATGCCATGGTGATCGACCGGGGTGATGATGGCGATGCCAGTGCCCACGTCAAAGTGAGCCTGGAAACCAG
TGCCGAATTTGAGAACAAGCCAGTGACGCCCAAGACACCGGCGGCGACTGTCGATATCGACGACAACGCC
GTGCTGGTGGGCAAGGAGGATATCCGGGTTGATTTTGGTCAGCAGCTCAAAGGCGTTATCCATATCGGCG
CCGCCGATCAGGCGAGCGACGAGGTGACCATTGTCCTCAAGGGGTTGCCAGCAGGTGTCACCATCAGCGG
CGCCGAGTACAACATCTCGACCGGTGAGTACGTCATCAAGGTGCCGCCGTCACTGGCGGGCCTGGAAGGG
TTGACCCTCGGCTTGCCACAGGACTTTGCCGGTTCGCTCAATATCGAGGTGAAGGTGGTGAATACCGATA
CCGCCAGTGGCGATATCAAGGTCGACAGCAGCACGGTTAGGGTCGATGTGGCCCCCGTGGTCGATGTGAA
TGGTGGTCGTGGCGAGCATCCGGAACTGACACTGACCGTGCAGGACACCAACCAGGATGGACAGCCGGAC
AATCTGGAAGATAGCAAGATCCACCTCGATCTCTCGGTGAAACTGGCCGATATCAGCCCCTCCATCGCGG
GCGGCGGTCTCGAAACCGTTGAGCGGGTCACCATCACGGTGGATGCCAAATATGGTTACTTCCTCGATGG
CAGCGGCAAGCCGGTCAACATTCTGGTTGTGAGTGATATGGCGGCACTTAAGGATTTAGTGTTTGTGCCC
AAAGAGCATTTCAGCGGTCAGGTACCGCTCGACATCAAGGTCGATATCGTCGATACCGCGCTGGTCGGCG
GTCAGCTGGTGACCGACAGCGGCAGTTGGAGCGGGCAGGTCGGCTTTGAGGTGAGACCGGTCAACGATCC
GGCCCAGCTCTCGGTGCATAGCGTCACCGGGAGCGAGGATGGCACGGTCAACCTTGCCGGATTTGGCGCA
ACCCTTATCGACAATGATGGCTCGGAGCAGATCGTCGCGCTGCAGATCAAGGGGGTACCGGATGGCTTCA
CCCTGTCGGCTCCGGCGGTCAACAATGGCGGCGGCGTCTGGCAGATCCCGGTGGGGACCGATTTCTCCAA
GCTGACCCTGATCCCGCCAGCTGATTTCAGCGGCACCGTGGAGCTGTCGCTGAGCGCCTTTACCCTAGAC
AAGGGGCTCACCATTCCGCTCGAAACCAGTGGCAGCTTTACCGTAACCGTCAATTCGGTGGGGGATGCCC
TTATCGCCGACATTCAGGGCCAGGCGAGCGGTACCGAAGGGGATGTGATCACGCTGGATCTGGGGCTGGA
GACCCGGGATAACCAACCAACCGGCGGCAATGCGGGTAATGTGCATGAGAATAAACCGGAGCAGGTGCGG
GTCACCATCGAAGGGGTGCCTGACGGTGCCGAGATCCGATTGCCAAATGGCGTGGCGGGGACGGTTGTTG
ATCTGGGTGGCGGTCGCTGGCAGGTCACCACCGAGGGCGGCAAGCTGGGTGCAGTCGAGCTGGTGACCAA
TGATGCCAACGGTGCACTGGATCTCAAGATCACCGCTCAGAGCCTCGACAACGGCGCCCTTGGCCCCGAG
GTCAATGGCACCATCCATGTGGATGTAAGCCCGGTCAACGATGCGCCAATCAACGTATTGCCAACGACCC
CTCAGGTCGCGCAGGAAGATCAGCCTTTTGTCATCCACGGTTTGCAGGTGAAAGATGTGGATGCGGGCAA
CAGCCCCATCAAGATCACCCTGTCGGTGGAACATGGCACCCTGACGCTGCCTGCTGGCAGCGGCGTGACG
GTAGTCGGCAACGGCACCGGTTCGCTGGTGCTGACCGGCACGCTGGCCGATCTCAATGCCTTGCTCTCTG

GTGGGGTCACCTATCAGGGCAACAGTGATTTCTACGGCAATGGCGCCCTGACCATGGTCACCAACGATCA
GGGGAACACGGGTAGTGGCGGGGCACTCAGCGATACCGATGTGCTGCCCATCGAGGTGCAGCCGGTCAAC
GATGCGCCGGTCAATCAGCTGCCTGGCAGCATGACGGTCAAAGAGGATGGCTCTCTGTCGCTCTCTGGCG
TCAGCGTGAAGGATGTGGATGCAGGCAGTGCGCCTGTCTCCATGGTGCTGAGGGTCGAGCATGGTGTGCT
GACCTTGCTGGGTGCAACCGGTGCCGTGATGGTGCAGGGGGCGGGTACCAGCGAGATCACCCTGGTGGGG
TCGCTGGCTGATCTCAACCAGTTGCTGGCAAGCAACCTGCACTATGAACCGGCCAAAGATTTCTGGGGAG
AGGATACCCTTACCGTCACTACCTCCGATCAGGGCAACACTGGTGCGGGTGGCCCGCTGAGCGACACCGA
TCAGGTGACCATCACTGTCACTCCCGAGCCGGATATGCCGGGTCTGACGGTGGATCAGCATGCGTTCCAT
GCCTTGCAGGGGGCCGTTATCCCCCTTGGCATCAATGCCAGCGTGGTCAATCCGGCCACCGGTGAACTCT
CCATCCGCATCAGCGGCCTGGATGGGGCACAAGTGCAGGATGAGCATGGTCAGAGTGTCGGTCATGCCGA
TAGCAATGGCGACTGGTTGATCCCGGCCGATCACTCGGTTCCGCTCTACTTCAGCGGTTTTGGTGACGGT
GACCATGTGCTGGGGATCAGTGCCGAGTCGAGTGTCGGCGGCTCAACCGTCAGCACGCCCCTTGAGACCA
TCACGGTGCACGGCCAGAGTGGTCATGAGCTGGTCGGTTCCGATCAGGGGGATTGGCTGTTTGGCTCCTC
AGGCGATGACCGTCTGCTGGGGGGCCATGGCAACGACATTCTCACCGGCGGGGCTGGCAGCGATCTCTTC
GTCTGGCAGCACGGGGATGAGGGGAGTCAGGGGCAGCCCGCCATCGATACCATCACCGACTTCCATCCGG
AACAGGGGGACAAGATCGATCTGGCTGATATGCTGCAAGGGGTCACTGGCAATGGTGTTGATGATCTGCT
CAAGCATCTCTCAGCCTCGGTGACCACGGGGAGCAATGGCCTGAGCGATGTGAATCTCTCGGTGGCGCCA
GCCGGGGATGGTCATGTGACCCAGCAGATCACCCTGAAGGATGTGGACCTGACCAGCTGGAATCTCACCA
GCACCTCGTCCCACGATATCCTGCAGAGCATGCTGGAAGATCAACATAGCCTGATCATCCAGCACCCCTG
ATAGCGTGCGTGAAGCGTAAAAAAGAAGAGCCGGCAATTGCCGGCTCTTTGTTTATCTCTGCGTGGATTG
TTTTTATGCGACCGCAAGGGCCGGGGGGATTCAGGCCAGCGCCTGGGCCAGCAGGTGGATCGGATGTTCA
CAGCGGAAGCTGGTGCTCATTTCGATCTGCCATTTGCAGGTCTCGCAATCGGTGATCACCAGATCGGGGG
AGAGGCGGTTGATTTGATCAAACAGCCCCTCACCGATGGTCTGGCTGGTCTGATAGTTCTCCGACTTGAA
GCCGTAGGTGCCGGCGATGCCGCAGCACTGGGACTCCAGCACCGTCACCTTGACTCCGGGAATGGCGCGC
AGCAATTCGAGGGTGTAGATGACGCCCCCCATCTTCTCCATGTGGCAGGGGGTGTGGTAGGCGATGTGCA
GATTGAGCGGTTTCATCGCCGGTTTGTTGCCCTTGTAGAACTCGTTCCACAGGAAGCGGGTCACCAGCGA
GATGCGATCCCGCACCTTGTGGTTGTCCTGCTCCAGCAGGTGGGGGTATTCATCGCGCAGGGTAAAGCCG
CAGGTGGAGGAGGTAGCCAGCAGCGGCATCTCGTTCCCCAGCAGGGAATTCTCCATCTGCTTGATGTTGA
AGGCCGCCTGCTGCTTGGCCTTGTCCATGAAGCCGTTGGCAATGAGCGGCACGCCGCAGCACTTTTCCCG
CTCCAGCAGCTGGACGCCGATGTTCATGGCGTTGAGCACCTGTACCAGCTCCTTGCCGAGCTGCGGGTGG
TTGTAGTTGACGTAGCAGCCGTGGAAGTAGGCGATTTGGCGCTCGAAGCGGGCCTGACTCGCTTTCTGCT
TCTTGTACCAACCGCGGAAGGTGCCCTGGGAGTATTTGGGCAGATCGCGGTGGGAGGAGACTCCCAGCGC
CTTGTCCAGCACCATTTTCATTGGCTTGAGGCCGGTGGTGAAGTTGACCACCGGCGCCATCAGGGTAGAC
ATGCTGCCCATGAGATCGGTGTGGGAGAGCACGAACTCGCGGATCCCCGGTTTGAAGCCGCTGTACTTGT
CTTTGGCCTTGGCGATGATATCGCCGATGCGCACCCCGCTTGGGCAGGCCACTTCGCAGCGCTTGCAGTT
GGTGCAGTGCTTGAGGGCGCTGTCAAAGAGCTCCGGGCTCTTGATCCGCAGCCGCTCGCCATCCGGGCCC
GCCTGTTTGGGGCCGGGGTAGGCGGGGTTGGCCTTGGCCACCGGACAGTAGGCGGTACAGACGGTGCATT
TGATGCACTGATCGAAAGTTTGGTTGGCGTGATCCAGTAGCATGTCAGTTCTCCTCCCGGCGATGATGAA
GACACGGGATATGGGACGTAGTAATCATTGGGCCGCGCCCAGAATATGGCCGGCAGCCTGCCAGCCGGTG
GCAACCGCAACACCGGAGCCGGAACCTTCACGAATTGGGTCGTAATGGGCCAAGACACTGCCTGCGCCAT
AGAGGTTGGTGAGCGGCTTGCCGCCACGCAGCACTCTGAGCTTGTCGTCGGTCTTGACCCCGAAGCCTAT
GAAGGGGTGGTGATCGAACAGGCGGCGACCGGCCCAGGCATCGCGCTCCTCCAGACTCAACACATCGGCA
TCGAAGATGGGTTCGCGAATGCCACGCAGACGGGACTCCAGCCCGCGGCTGAAGAAGCTGCCGGAGGCAA
GCACGAAGTTGTCTGCCTCGAACAGCTGATCATCGCCATTCTGGGTATGTACGCCGACCACCCGGTCACC
TTCGTAACGGGCGCCCAGCACCCGCTCGCTGGTGAGGAAGGTGCCGCCAAGGGCCATAAAGCGGCGCTTG
AGCGCCTCCTGCATCCGCATACCTATCAGAGACGGCGGCATGGTGGCCACCTCCTTGATGGTGCAGCCGG
TGCGCTGTTCCAGCTCCGCCAGACGCGGGCCAACCAGCCCCAGCGACAGGCAGGCGGGCAGCACGATATG
GCGGCAGCCGGGTACGCCGCTCTCCTGCACCATGCGGCTGATCTCGCGTGCCAGATCGGCCACCAGATGA
TGCTTGTCACACAGGCGCGCGATGTCGGCTGAGCGAAACTCGTGGGGATTGCGGCTGAATTCCGCCAGCT
GGGGCAGGCGGATTTCGCCGGTCAGAATGCGGCAGTGGGCAAAGCGCGGATGGGTCGCCAGATTGGCCGC
CGCCAGTGCCGGATGAAAGTCGCGAAAGCCTTCGAGGGTAGCCAGCAGTAGCACGTCCGGGATCGGTGCA
TCGGTGACGCAGGCGCAAGTATCGGGGGAGAGCCAGGTACGCTTGAGGGTGCCGATGGGGGTCAGGCGCT
GGTGATTGGTGTCATGGCGCACCAGCGGCAGCCCCTGCTCAGCACAGTGGCGCTGCAGATCGGCCAGACT
TGCCTCGATATTTTGCATCCCCACCTTGCTGTAGGGGTGATCCGGATGGGCTGCCATAAAAGCTGGCAGG
GCTGCGCGGGGATCCCCCTCGCTTTCGAGCAGATCGACCGAGCCGGAGGAGAAGTGCAGGGCACTCTGGC
CGGAGGCCATCAGCAGGGTCTTCTGTCCTGCCTCCACCAGCCGCAGAGCAGCCGAGAGGCCCGCCATGCC
ACCGCCGATAACGATATTGTCAAACTTCATGACGGCTCTCCTTGTGAGTGGTCTCGCTGGTTGGGTCGCG
TGGGGTATCGGCAACCAGCTGCGGCGTCGTCCATTCCCCCAGGCCAAACAGCCCTTCGTAGATCCAGTAG
GTGAACTCGGCCTCGCGCAGGGCATCGCCCCACAGAACGGGGCGGGTTCCCTTGTAACGCTCCTCAAGGA
ACTGACGCAGCATGACACAGGCCTGACGACCGTCCGCCTTGCCATACTCCTGCATCAGACCGGCGGCGCG
ATAGGCGCAGAGCTCCCCCTGACAGGGTCCCATGCCGAGGCGGGTGCGGCGGCGCAGGTCGATGAGGTTG

TCCACATCCAGCTCGCGCAGGGCGTATTCGATTTCGCCCGCGGTGACCATTTCGCACTCGCAGATCAGGG
CGTTGGCCTTGGGATTGTCACGGAAGAAGGCAATGACCCGCTCGCCGTGGCGGTACTGGGCCGACCCTTC
GACCGGCACCGAGAGGCCGGGAGCGGAGACCTTTTCCGGATCCCGTGAGCCGGGCAGGCTCAGCTCGGCG
GTCTGGCAGGGTTTGCTGTTACCGAGCTTCTTGGCCAGCAGATCGGTGGCCCATTCGGCCATCAGGCGGT
AGGTCATCAGCTTGCCGCCGGTGATGGTGTTGAAGCCCTTGAGGCCATCGCGCTCGGCGTGATCCAGCAG
CACGATACCGCGGCTGATGTTGCGGCCGGTGTCGTCACCGTCCACCGCCACCAGCGGGCGTACCCCGGCG
TAGGCGCGCAGCAGACGGGTGCGGGCCATGATGGGGGCGAGCTTGATCCCCTCGCGCAGCAACACTTCGA
CCTCTTCGGGTTCGACGGTGAGCTGGTCAATCTTGTTGTAATCGATGCGACTCGAGGTGGTACCGATGAG
GGAGATGGTATCGCCTGGCACCAGAATGTCCGCATCCGCCGGTTTGCGGGCGCGGTTGAGCACCAGCTGG
TTGATGCGGTAATCCATGATGAGCAGGGAGCCCTTGGCCGGGAACATGCGGATGCCGAGATCGGCGTATT
CGCAAATTTGCTGGCCCCAGATGCCGGCGGCGTTGATCACCTCCTGACACTCCACCTCGAATGCTTCGCC
GGTACGGGTGTTGATCGCCTTGACCCCGTGTACCCGATCCTGGCTGCGCAGCAGCCCCAGCACCTTGCTG
TGGGTGAAGATGTGCGCGCCGTGCTCTTTGGCGTCCAGCACGTTGGCAGCGGCGAGGCGGAACGGATCGA
CCGTGCCATCCGGCACATGGATGGCGCCGATCATCGCCGGGTTGGCGTTGGGCTCCATACGCAAGGCTTC
GCGCGGATCGAGCTGGCGGGTTTCGATACCGGCCAGGGCACAGGCATCCATAAAAGTGGTTTGGAAATTG
ATGTCATCTTCCGGCAGGGTGAGGAAGAGGCCGCCCGTGTCTTCGACACAGTGGCTGGCAATCCGCTTGA
GGATGCGGTTTTCCTGGATGCATTCGCGGGCGGACTCCTGATCCGTCACCGCGTAGCGGGCACCGGAGTG
GAGCAGGCCGTGGTTGCGGCCGGTGGTGCCAGCGGCGATATCATCGCGCTCGAGCAGGATGCAGTCGATG
CCGCGCAGTGCGCAATCGCGCATGATGCCGGCCCCTGTGGCACCGCCGCCTATGATGACGACTTGTGTAG
TTATTCTTTTCATTGTTGCCCGTCTCATCTCTTCCAGATGAGGCCAGTGTGGCCTACTTCTGAGTGGAAA
TGTTTGATATGGGCCAAATTTGTGCTCGCAAACGCTCATTTGCTCGCGCGTTAATGAGCATTATGTGACA
TTTGTCACGCTTGTTATCACATCGGTTTGTGGGCTGGTGGCGAATAACAGGCACAAAAAAGGGCATCGGA
TGCCCCTTGTCACTGTTTGACGAGTCTGGTGGGTGTGTGATGCGTCAGCCGCAGCACTTCTTGTGCTTTT
TGCCGCTGCCACAGGGGCAGGGATCGTTGCGTCCTGCCTTGAGGGGGGCTGGATCCTGCTCTCCCTCGGT
GTAGACCCAGCGGCCCTGATAACGCACGAAGCGAGAACGCTCATGGAGGCAGTGACGCTCATCCTCTTCC
AGAAACCAGGCTTTGAACTCCACCATCCCGGTTTCATCCTCCGGGCCGCCCTGGCTGGCGATGATCTCGA
GACCATCCCAGCGGGTGTCGATGCGTGAAAGCTCTTCGGCGGTGAGACCGCCAAGGTGGGCCGGGTGCCA
GCTATGGACCAGATACTCCCCCAGACCGAGCACAAAAGCACTGTAGCGCGAGCGCATCAGCTGCTCCGGG
GTGTCAGCCACTGCGCCACTGTGACGTGAGCCACAGCACAATTCGAGAGAAAGGGTTGAGCCGCAAGGAC
AAAGATTCATGATTTTCACGTTCTGGCCGATTTGTTTAAGGGAATTGTAACGAAAGCTGGGCTCGCCCTG
CCAGCACCTATACTGATGAGTAAGCAAGGAATTCCAATAAAAACAGCTGCAAGGATGTGAAAGATGACGC
TAAAGCAGAAAATCCTGTTACTGGGTGCAGTCCCCGTACTGTTGATGGCCCTGGTGGTTAATCTCAGTAA
CTACATGGTCTCCAAGAGCGATCTGGAGTCTGATCTGGTGGTGGCGAGGGAAAATGCCATCAAGGAGCGT
AAAGCGCTGCTCAGCAGTTACATCATGATGGCCAAAACCGCCGTCGATGCCGTGTATTCCCTGCCGGATT
CACCGGAAAATCGTCAAAAGGTCAAAGAGTTGCTCAGGCCGCTGCGTTACAGTAGTGACGGCTACTTCTT
TGTCTATGACTTCGAGGGCAACACCATACTGCTGCCGGTGCGTACCGAGCTGGAGGGGAAAAACCGCTGG
AACGACAAGGATGCCAAGGGCAAATTGCTGATCCAGGAGATCCTTAAATCGGCCCGTCAGGGGGATGGTT
TTACCGAATACTGGACGGCCAAGCCCTCCATCGGTCGCGATGCTCCCAAACTGGCCTTTACTCTGGTGCT
GGACAAATACCAGTGGGCCATTGGCACCGGTTTTTATATCGATGATATCGATAACGAGCTGGCAGCCCTC
CGGGCAGAGCGCGAGAGCAACATGCATGCTTCGTTGCAGAGCAGTGTGCTGGTGATCCTGTTGATCCTCG
GTATCACTCTGGCGGCGACCGTCATTGTGGGCAACCGGGTCACCAAGCCGCTGGCGGATGCGGTGCTGGC
ATTGAACGATATCGCCAACGGTGACGGGGATCTGACCCAGCGCCTCAAGGTGCAAAGCCAGGACGAGATT
GGCCAACTGGCCAGCGCTTTCAACCGCTTTGTCGAGCGGATCCAGTCGGTGGTAAGTCAGGTGGGCGAGA
CCAGCAACCACCTCTTTAGCGCGGTCGACAAGTTGCACCATTTGAGCGAGCACTATGACCATCAGATGCA
GGGTCACAGTCGCGAGACCGATCAGGTGGTCACAGCGGTGACCGAAATGAGCTCCACAGCGCAGGAGGTG
GCGGCCAGTGCCTCCAATGCCGCGACGGCAACCAGCGATGCGGCCCGTGAATCGGACGCCGCCCGCGGGG
TGGTGAGCGGGGCTATCAACAGTATCAACCGGCTAGTGGGGGAGGTGCATACCGCCTCCGGCGTTATCGA
GCAATTGGCACAAGAGACCGCCAAGATTGGCTCCGTGGTGGAGGTCATTCGCGGCATTGCCGAGCAGACC
AACCTGCTGGCGCTGAACGCTGCCATCGAGGCGGCCCGCGCCGGTGAGCAGGGACGCGGCTTTGCGGTGG
TAGCGGACGAAGTGCGTTCGCTGGCCGGCCGTACCCAGCAGAGCACCAAAGAGATCAACGAGATGCTGCA
ACGTCTGCAGGGCGGCGTGAAGCAGGCGGTGGAGGTGATGCAGGCGAGCGAGGATCGCAGCCAGGAGACA
GTGCAGGAGGCGAGCCATATCGCCAGCTCCCTCGACAGCATGGTGATGGCGGTCAGTACCATCAACGACA
TGAACATCCAGATCGCCACCGCCGCCGAGGAGCAGCACGCGGTCTCCGAGGAGATCAACAAGAACCTGGT
GGCCATTCAGCAGATTGTCAGCGAGCTGACCAGCGCGGCGGTGGAATCCAACAGCACCACCCGTGATCTG
GCGAGTACCGGTGACAAATTGCGCATACTGGTGTCGCAATTCCGTTACTGATCTTGCAATGGTCCGTGAC
GGCCACCACAAGATGCGGCCTGACCCCCGGTTCAGGCCGCACTTTATTTGGGGATTGACCAATAATGTGT
GAAAATTGCCAGCCTCTTTTTTTCAGCCTTTGCCCCATACCCTATGAATGAGATCGTGAAAGGTAGTATC
GACTTGATCCGCCAGCTCAACTACGGCCTGGTTTATGCCAGCCTGGAAGAGCACAAGGAGTTGTCGCGCA
CCGATCTCTCCCGCTTGACTGGTCTCTCTCCCGCCAGCATTACCAAGATCACCCGAGAGCTGCTCGACAG
CGGGCTGGTGGTCACCTGTGGTGAAAGCTCGGTGGGGCGTGGCCGTCGCCAGACCTTGCTGCGCATTAAC
GAGCGCCGCTTCCAGTTTCTCTCCATGCGGTTGGGGCGTGGTTATGTAGACATGGCGCTGTTCGACTTCA

CCGGTCGTTCGCTGGCGCGCCATCGCCACATTTTTACCGAGGCCGAGCGTGGCGAACTGCTGGATAGCCT
GGTTCACGCCATTCGCAGCTTCTTGCCGAAAAAGGCCCTCAACCTTGCCTGCATTGCCCTTTGTCTGCCG
GGACAGGTAGAGCGCCACTCCGGCATGGTCAAACACTTCCCCTTCTACGATCTGCGCAACTGGCCGCTGG
GGCCGACTTTGCAGGAGGCCTTTGCTGTGCCGGTGCTGGTGAGCGGCGACGTGCGCACCTGGATCCAGGC
AGAGCGCGAGTGGGGAGCCGCCAAGAATTGCGCTGATGCGGTGCTGGTGTTCGTTCACAACGACATCGGG
GTCGGCATGGTGGTCAATGGCCAGCTTATCGAGAGCGAGAATGCGCTGCTTGGCGATCTCAGCCATTTGC
AGCTCGAACCCTATGGCCAGCGCTGCTACTGCGGCGGCTTTGGCTGCGCCTGCACGCTAGTGACCAATCA
GGCGCTGGAGGCGCAATACCGGGATCTGCGCGAGCGGATGCAGGAGCACGACCTGCCCGAGAGCGTCACC
ATTCGCGAGCTGTGCGAGCTGGCGCTGGCGGGCAACAGCCTCTGTCAGGACATTCTCCATCAGGCGGCGG
GGCGCCTGTCGCGGGTGCTCTCCAACCTCATCTGTCTGCTCAATCCGGGCAAGCTGCTGCTGGGGGGGGA
AATCACCCGTGCCGATCGGCTGCTCTTCCCCATGTTGCATCAGTCGCTGGCGAGCCAGTTGCCGGCCGAA
TATCTGGTGCGGCTGCAGATTGAATCCACCCACTTCTACGAAGATCCCACCAAGCCCACCTCGGTGCAGG
TGCACAAGGCGCTGCGCGACGGCAGCCTGTTGCTGGAGCTGCTGCGCACCTGTCAGGAGTCCTGAGCACT
TTTTGCGGGGCGTGGGAATTGGCGAAAGGGGCAGTGAATGCTGTTCCATCGCCGCGCAACTGGGTACAAT
CCAGCCCCTTTGTGGCGGGCCGCTGGCGCCGCCGTTGATGATCTCCCTCTGTTTGTGACGCAAGATGAAG
CCTGTTCCTGATCACGCCGTCAACCGCCTGCGGGCCTGGCGCAAGAGTATTTCTACCCGACCGTTTCTCG
CCCGTGGTGGCACCCTGGCTCGTTGTCCCGCCTGCCAGCTGCGCGAGGATTGGTGTGCCTGCGAGTGGCG
CCCCGAGCTCAAAGCGGAGGCGGGCTTTTGCCTGCTGATGTACGACAGCGAGCCGATGAAGCCCTCCAAC
ACCGGCCGTCTCATCGCCGATGTGTTGCCGGATAGCACCTGGGCCTTTCTCTGGTCGCGCACCGAGCCCC
ATCCCGAGCTGCTGGCCCTGCTGGCCGATCCTGCCTGGCAACCCTATGTGGTTTTTCCCGCCTGCGAAAA
GGAGCCGGCCCGTTTGACCGGCGAGGTGACGCTGGCGCCGGGCAAGAAGCCGCTCTTTATCCTGCTCGAC
GGCACCTGGCCCGAGGCGCGCAAGATGTTCAACAAGAGCCCCTATCTCGACGGTTTTCCGGTGCTGGCCA
TCAAACCGGATGTGCTCTCCACCTACGGCATGCGGGTCGCCAACTGCGACGAGCACCTCTGCACCGCCGA
AGTGGCGGCCTGCGTGCTGGAAGTAGCTGGGGAGCTCAAGGCGGGGCAGGCACTGCAGCACTGGTTCAAC
TTGTTCAGCAGCCGCTACATGGCCGGGCGGGTCAGCCGTTTCCCCGGTGCCGATGAAGAGCATCTCTGTG
AGGCGCTGGCATCCATCAAGGAAGCGAACGTCGCTTCACCGGCTTGCTAACGGGCCCTCTGAGCTCAAAT
CAATGAAAATCAGGAATCAATTGGCTGCCACTGTTTCGTTAGATTCATGGTCAGTGGCTCGCTAGGATAG
CGAACAGCCTGTGCAGCCCCCCGCACAACGTCGTTTTGGTGTTCTCCGGTTGCTCGACCCTTCGGGCAAC
CCACTCTCGTGAGGTCTATGGATGGAATTGGAACTGGTTACCCTGTTTGCCCTGTTTGGTGTGGCGCTTG
TCGCCGGCTTTATCGATGCCATCGCTGGCGGCGGCGGTCTGCTGACGGTACCTGCTCTGCTGGCCACCGG
CATGCCCCCCGCGCTGGTGCTTGGCACCAACAAGCTGCAGAGCAGTTTTGGCTCCTTCTCCGCCACCTGG
TTCTATGCCCGCAAGGGGCTGCTGGAGTGGGCCATGATCTGGCCTGCGGTGATCTGCACTTTTATCGGTG
CGGCTATCGGCACGCTGGCGGTGCAGACCATAGACGCCGCCATTCTCGAGCGCCTGCTCCCTTTCCTGCT
GATGGCCTTTGCCTGCTACTTCTACTTCTCGCCGCGGGTAAGCGATGCCGAGAGCTCCCGCCGTCTGACT
CCCATGCTGTTTGCGCTGCTGGTGGGGGGCGGTGTCGGCTTTTATGACGGCTTCTTCGGGCCGGGGACAG
GTTCGTTTTTTGCCATCGGTTTCGTGGCGCTGGCGGGCTTTGGCATGGCGCGCGCCACCGCCCATACCAA
GCTGCTCAACTTCACCTCGAATATCGCCTCCCTGCTGTTCTTTGCGCTCGGTGGCAAGGTGGTGTGGAGT
GTTGGTTTTTGCATGGCGCTGGGGCAGTTTATCGGCGCCCGTTTTGGCTCCAAAATGGTGCTGAAAAAAG
GGGTGAAGCTGATCAAGCCGCTGCTGGTGACCGTCTCCTTGCTGATGTCTGCCAAGCTGGTGTGGAGCCA
GTATCCGGAGCTGTTTGCCTGGATCTGATGTGCGCCTTGCTTGCCGCATCGCAATTGTTAACGGGGCCCT
TGGGCCCCGTTGTCATATCTGGTGTTTTACACGGCTAGTGCGGCGTGTCGATGCAGTGGCGAAAGTGTTG
GTGCAGGGCGCTCACCAGCCTGGCGATCCGCTCTCGCTTTAGGCTGTTTGGCCGGTAGACCAGATGTAGG
GGGCGGCCCAGTCCGGGCAGTGGGCGCGCCATCTCCCCCTGTTCGCCCACCAGCCCCAGCAGGCCAGCGG
GGAGCAGCACAGGGCCAATACCTGCCTTGGCGAGTTCGATGAGCGCCAGATAGGAGTCGAGCTCCATGGT
GGGCTCCAGCCCCAAATCAGCCAGCGGTTCGCGCAGGTAGCGGTTGGAGGGGTTCTCCAGATCCATGGTG
AGGATCTTGCGCCTGCTTTCTGTGTTGGCTGGTGGCAGGCTCGGCTGCGCCTCGCCCACCAGATAGAAGG
GCTCCTCAAGCAACAGATAGGCGCCGAGGCCATGGCCGGGCGGGAGCCGACCGGCGCAGATGCCGAGCAG
GGCATCACCGCTGCGCACCCGCGCCAGAATGACCGGCGTGTGGTGGGTAGAGAGGGCAAGGTGGGGATCG
CGCTCCAGATAGTCGCGCATAAAGTGGGCGAGATAACCGGCCAGCAGGGTTTCGGAGCAGGCGATGGGGA
GGGGACTCTGATCCGCCAGATCCTGGCTATCGGCCAGCACTCCTTTCATCTCTGCCAGACTCGGGGCCAC
TCGCTCAAGCAGCTCCTTTGCCTGCGGGGTGAGCCGGATCTGGCGCCCCTCCGGCTCGATCAGCTTCTTG
CCAAGGCGCTGCTCCAGCTGGGCAATTCGCTTGCTCACCGCCGACTGGCTGATATAGAGGCGACTGGCCA
CCTTGGCCATGGTCCCTTCACTCGCCAGCAGGCTCAGCGTCTCCAGCCCTTCCAGTAACATCTCGCCCCC
TTGTTTATCGCCATCAGGATCGTCACAGATTGAGCGCTACCTTAGGGGCTGACTGGTTCAGGAGCAAGGG
TCGTTTGCTGCCTTGGCCGGTCGAATGCGCCATTCGCTCAAGGGCGGCGCGAAGATCACCAGATTGCCGA
TCAGGCTCACCACCACCCCGGCCACCGAGACCGCCTGCCAGACAAAGCCTTCGTAGAAGGTGGAGAGGGT
AAGCGCCACCAGAGGAAAGAGCACGGTGGCGTAGGAGGCCTTGCTGGCACCGATGCGCCCCACCAGGGTC
AGGTAGGCGGTAAAGGCGATCACCGAGCCAAACAGGGCGAGGTAGACCATGGCCGCCAGATAGTAGCCAT
CGGTCGGCACCACCAGTTGCTGCCCGAGCAATGTGACCCAGCCCAGCAACAGCACCACGCCGTAAACCAT
GCCCCAGGGCACCAGTTGCAGTACGTGATAGCCGTGGCGCTGGCCTCGGGCCGAGACCAGATTGCCCAGC
GAAAAGCAGAGGGTGCCGCCGCAGGCGAGCAGCAGCCCCTTCCAGCCGTTTGCACCCAATTGCGCATCTG

CCAGCACCGGCCAGAACAGCAGCAGGGTGCCAAAGAGCCCCAGCAAGGAGCCTTGCAGCCAGCGCAGAGT
GGGGCGCTTGTTCTCGAACAGCCAGAGGTTGAGGCCGTTGAAGATGCTGGCACTGGCAAAGATCACCGCC
GACATGCCGCTCGGGATATAGCGTGTGGCGTGGTAGAAGCAGAGGAAGTTGGTGGAAAAGAGCAGGGCAC
CGAGCAGTGCGGCGTAACGCTGGCCGTGCCAGGGGAGGCGCGGAAAGCGGCCGCTGGCGGTGAGCAGGGC
AAACAGTGCCACCGCCGCTAGCAGGAAGCGATAGAGCACCGACACCTCGATGGGAATGGGGCCAAGTTGC
CAGGCGATAGCAATCCAGGTGCTGCCCCAGATAAGCACGGTGGCAAGATAGAGCATCAGATTCATCACAG
TTCCGGTGAGTCAGGGAGAGATGACCCACTATGGGGCGAGCCCTGATGGCTGGATTGCACCATCTTGCGG
TTTTTCATCGTCACGCGCTGTCATCTGCGGGCGCGGTCGGTAAGATGGATGGCAGCGGCATAACATGTAC
ACCGGAACAGGCGGCAGGGGCGAATGAAAACAGCAGGGGTATTTGATGCACTGGTCAGCACCGGCGCCCG
GCTGGAGGACGCCTGCTGGCTCGAACCCGGGCTTGGCGTCGCCAGTTGGCGCAACTGCTATGACCAGACC
CGCTATCACAAGCCGGGCCACCACACCCTGAGCGTCTATCTGCAAGGAGGCGAGCAGACCGAGCGACTCG
ATGGGCCGGGTGGCCACGGTGGCACAGGCAAGGTGTGCATCATGCCGGATCACCACAGATCCGAGTGGTT
GGTGCGCGAAGAGTTTCGCTTCTTTCATCTCTACTTCACCCCGGCTCACCTCTCCCGTATCGCCGAGGAG
GTGTGTGACAAGGAGGGGCGCCACCTGCAACTGGTGGACAAGACCTTTATCGACGATCCGCAGGCGGCTT
CGCTGGTGCAGCGCCAGTTGATGCAACTGCAGTGGCAGCACGGGGTCGATCGGCTGGCGCTTTCCCACGG
CGCCAGGATGTTGATCCTCCACGCCTACTGCCACCACACCGAGGAGGCTCCCGGTCTGCCGCCTCTACGC
GGCGGGCTGGCGCCCGTGGTGATCAAGCGGGTGCAGGAATATCTGCAGGCCCATCTGGCCGATTCGGTCA
CGCTGGCAGAGCTGGCCCAGCAGGCGGGACTGAGCGAGTACCACTTCGCCCGCATGTTCGGCCAGAGTCT
CGGCTGCCCGCCCCACCGCTATCTGCTGGGGCTGCGGCTCAAACGGGCCAAGCAGCTGTTGGCTGGCCCC
GAGCCGCTGGCCAGTATCGCCAGTCAGTGTGGCTTCTCTTCCCAGGCCCATTTCGGCAATCGTTTTCGCG
AGGCGTTTGGTTTGACGCCGGGGCAGTGGCGGGCACAATTGTCATCTTCCCGTCACAGATAACCCTGAGA
CTTTGGGCAAAAGAGGTGTACTCTCTACCACTTATCCATTGAAAGGAATGCCCATTGAATTATCGCAAAC
GTCAAATCCGTACCCGTCATCTCTGGTTCAGCCACTGGAATGCCCCGCAAGATCGCACCGCTGTTGTTAC
CCCTCCCGAACCTGCAGTGCCTCACAGTTCACGTTGAAAAAGCCCCGCCTGCGATAGGATTTTTCCGGTA
ACTTTGCCACTATTGCCCCATCACAATGATGTGGACATGGCTGGATGTTTCTGGAACGTATTGAAGTCAA
AGGTTTTCGCGGTATAAATCGCCTGTCGCTCGGGCTGGATCAGACCACAGTGCTGATTGGCGAGAATACC
TGGGGCAAATCAAGCCTGCTGCGCGCCCTCTGGTGTTTGCTGGGGCAGGATGCCGAGCCCTATCAGTTCA
CCGCCGAGGATTTTCACCAACCGGAAGATCCCGAACTGGCCCCGGCGCGCCACTTGCAACTGGTGCTCAC
CTTCAGTGAGCACAGACCCCAGATGTGTCAGCACTCCCGTCGGCTGGCCCGTCTTTGCCCCTCCTGGGTG
CTGCACAAGGACAAATTCCACCGCATTCACTATCGCGCCAGCGCCGAGCTGCAGGCGGATGGCTCTGTGC
TCACCATTCACGATTTTCTCGACGGGGTAGGCAAGAGCCTGCCCATCGCCAATACCCACGAGCTTGTCTG
CATGCTCATCACCATGAACCCGGTGTTCCGGCTGCGGGACGCCCGTACCGCTCGCAACGGGGTGGAGACA
TTGCAGTGGGGAGATCTCTCCGAACATCGCCTGAGCGAGCTGGCGGACAAGCTGATCGACGAACCCCAGC
GGATCGGCGAGCCGGAGCTCAAAGAGGCGTTGCAGGCGGTGCGCCAGCTGATGGATCACTACTTCAACGC
CCTGGCCCCCATCAAGAACAAGCCGCGCAGTCAGCGTGACATCATCAATCGCCCCATGACATTGCGCAAT
CCGGGCAACCTGCATACCCTGCTGCGCCGCGCCGATAACCGTGCGCTGCAGCTGGCGATGGCGGGGATGG
CGGCTACCTTGTTGCAGGCGCGTGGCAACCGCGAGCTGGAGGAGGGGGCTCGCCCCATCATGATCCTGGA
GGATCCTGAGAGCCGGCTGCACCCGACCATGCTGGCGCTGGCGTGGGGGCTGCTGGAACAACTGCCGGGT
CAGAAGCTGCTCACCACCAACTCGGGGGACTTGCTCTCTTCCCTGCCGCTCAATCAGGTGCGGCGGCTGG
TGCGCCGTCAGCAGGATATCCTCTGTCACCAGCTGGGGGGCGAGCGTTACAGCAGCGACGATCTGCGCAA
GATCGCCTTCCACGTGCGGATCAACCGCCCCATGTCGCTGTTTGCCCGCTGCTGGCTGCTGGTGGAGGGG
GAGACCGAGATCTGGCTGCTCTCCGAGTTGGCGCAGATCTGCGGTTACAGTCTGCGGGCCGAAGGGGTGC
GGATCATCGAGTTTGCCCAGTGCGGCCAGTCGCCGCTCATCAAGGTGGCGCGAGATTTTGGCATCGAGTG
GCACCTGCTGACCGATGGCGACGAGGCGGGGCTCAAGTATGCCACCTCGGCCCGGGGCCAGCTCAAGGGA
GAGCGGGAACGGGACCGACTGACCCAGCTCCCTGCCGCAGACATTGAGCACTACCTCTATCACAACGGTT
TTGAAGCTGTGTTTCGCCGTGAGGCGGGAGTGGGTGGGCGCGCCATGTGGAGCGCCAGTCACATCATCAA
CAAGGCGATCCATCACCGCAGCAAGCCGGGAATGGCGCTGGCGGTGGTGGAAGAGGCGGAGCGGCTCGGC
GCCGAGCATATTCCGCCGGTGATCCGCCAGATGTTTGCCCGGGTGGTGGCGCTGGCCCGTGGTCAGGGCT
AACGCCACCACCGCATCCCGTCACTATGGATATGGCTGCCAGCAGGGCGCCCCTCAAGGTGGGCTCTGCT
GGCGGCAAATTGCCCCGGATTGTTTGCCCACGATTGTTTGTCGCGATTGGGGATAGCCGCCGGTTTGGGT
ATACTCGCCCCTCTCTGGCGCCCCGGTGGCGCACCTCGAATCTTTACTGCTGGAGGCGTCGTGTTCGTCC
AACTCCCTTTTTGGCTCTTTCCCCTCACCGGATTGATGGCGCTTGGCGCCCTGATCGCCCTCGGTATCGT
GCTGTGGCGCGGTGATATCTGTCCTGGCCAGCGCTCGCGCATCAGTCAGCAGCTCTTCTCCATCTGGGTC
ATTACCGCGCTCTCCCTGATGCTGGCGGTCGAGGCCAAGGCGGCCAGTTGGCTCATCTGGAGTGGTGGGG
CCGCACTGGTGCTCGGAGTCGGCTTGTCACTGGCCCAATCACGGCTGGAGGGAAAGCGTTCAGTCCCCTC
GACACTGCTCTGGCTACCGGCGTTGCCGCTCGCTGTCTATGGCATCGGGCTGCTGCAACTGCAGGGCTGG
GTGAGCGGTCTGCTGCAGATGGCGATGCTGGGGGCGGCCTTTGCCCACCTGATGCTGCTGCGGGCCCGTC
ATCGTCTCAAGGCATTCAACACCCTGTTGCCGCTGGCCGGTCTGGTGGGAGCGATGGCCAGCCTTGTTTG
GCTGGCAGTGCTGGTGGGCTGGCAAGGGGGTGAGGCCAATCTCGATGCCCTGATCCCGGCGGTGGTGACC
CAGGCAGCGCTGCTGGTGGCCTCTCTGCTGCTCTGGTTCAGCCCGCTCTATTTGCAGCGTGATACCGCGC
CAGTGGTGGTCTCTACCTCCCTGTGTGGCCTGCTGATCGCCCAGATTGCCGCTACCAGCGTGTTGCACCA

GCTGATCTGACACTGGCGCTGAAAAGAGAAAGCCCCGAGCCTTGGCTCGGGGCTTTTGCATATCTGGTTT
GCTGGAATCGCAGCAATCCATTAGCAGCAATCAAGCAGCATCTATCGATCAATAGAAATCGTCGGCCCCG
CAGATAAAGAGGCAACTGCTGCGCGGCTGGGCGAGATATTCGCGGGTCAGTCCCTCCAGCGGCATGCCGA
AGGTGCAGATATCTTGCGGCGAGGGGAGGGCGGTATCGAGGATCCGCCGCCAGTAGCCGCCGACCCCCTT
GGGGGGGCTCGGTAGGTTAAAGGTGAGGGGTTCCCAGTAGGCGTTGAACAGCACATAGAGCGCCAGCTTG
GTTTCGCTCGACAGCGCTGACATGGCGATGGCGTGGGAGTGGGGGCTGAAATCCGGTTGCCCGGCGTTGA
CCCCGTGCCAGCAGATCTCGCTGTGGCGCAGGATCTCGGTGAGCGACAGCGGCATGCTCTCCTGCTCCGG
GCGCTGGAACAGGTGTTTGCGGTACTGGATCAGCTCCTTCATAAAGCGGAACATCTCCTGACTGCGGGTA
TTGGGCAGCCAGTTCATCCAGCAGGTGGCATTGTCCTGACAGTAGCCGTTGTTGTTGCCCCGCTGGGTGC
GCAGCAGCTCATCCCCCATCAGCAGCATGGGGGAGCCCACCGAGAGCAGGGTCGCCACCATCATGTTTTT
GGCCTGGCGCATCCGCAGGGCGTTGATCTGGGGATCTTCCGTTACCCCTTCATGGCCGTGGTTCCAGCTG
AAGTTGTGATCGCAACCGTCACGGTTCTCCTCCCCGTTGGCCTCGTTGTGTTTGCTGTTGTAACTGGCCC
AGTCCCACAGGGTAAACCCGTCGTGACAGGTGACGAAGTTGATGCTTTTCTCGGGATCGGCGTGGTGATA
GTGGTAGATGTCGGGGCTGCCGCAGAGCCGCTCGACAAAGGCGGTGACGCTGTTGTCATCCCCGCGCAGA
AAGCGGCGCACATCATCCCGGAACTGGCCGTTCCACTCACGCCAGCGGGCGCCGGCCAGTGACCCCACCT
GATAGAGCCCCCCCGCGTCCCAGGCTTCGGCAATCAGCTTGATGTCGGCGATGTTGGGGTCGGTATCTAT
GGTGCGCAAGGTAGGCGCATTGGCCTGCGGCTGACCCGATTCATCCCGCGACAGAATGGCGGCCAGATCA
AACCGAAAGCCATCGACGTGCATCTCCTGACGCCAGAAGTGGAGGCTGTCCATGATCATCCGCAGCACCA
CCGGATGGGCGCCGTTGAAGGTGTTGCCGCAGCCCGAATAGTTGGTGTCCTTCTGGTTGTTGTCGAGGAT
GTAGTAGGCCTCGTTGTCGATGCCGCGAAACGAGAAGGTGGGGCCATCATCACCCCCTTCGGCGGTGTGG
TTGTAAACCACGTCCAGGATCACCTCGATATTGGCCTTGTGCAGCGCCTTGACCATGTCGCGAAATTCGG
TCAGCGGATCATCGCCGCAGGCGTATTGGGCGTGGGGGGCGAAAAAGGACATGGGGCTATAACCCCAGTA
GTTGGAGAGTCCTTTGGGCGCATCCTGAGGATCGAACTGGAACACCGGCAGCAGCTCGACGGCGGTGACG
CCGAGAGATTGCAGATAGGGGATCTTCTCAATCAGCCCGAGATAGGTGCCACGCAGCGCCGGATCGACCC
CGGAGCTCGGGGACTTGGTAAAGCCCCCCAGATGCAGCTCGTAGATCACCGAGCGGGAGAGGGAGTGAGC
GGGCAGTTTGTCTCCTTCCCAGTCGTAGTGGCGAGTATCGACCACCTTGTTCTTGGCGCAACAAGCCAGA
TTGCTGCCTGGACGGGCCGCCGCCCATCGATCGTAATTGGCGCCAAGCTCGATGCTGCGGCCGTAAGGGT
CGAGCAACACCTTCTCGGCATCGAAGCGGGTGCCGCCATATGGGCGCCACGGGCCCTGAATGCGATAGGC
GTAACGTTGCCCGACCGAGATCCCCTCCACATGGATATGCCAGTAGTAGGCGGTGCGGTTTAGGCGCGGG
GAGAGGGTGATCACCTCGGGGGTCGCATCGTCTGCGCTTGCGAAGAGCAGCAGTTCGACCCGGCTGGCGA
GGCGTGCCCAGATCGCAAAGTTGACGCCGTGACTGTCGGGCGTAGCCCCCAACTGACGGCAGTGACCGGC
GGAGATGCGCATATTCTACCTACCCAAAATGAGGAGTGTTCACCTTATTGAGTGTAGTGCCGGATGCCTG
GCAAGGACTCGATTGCCTGAAAAAACAGCATGATGCTGCTAATGACCGCACTATTTGTCCGATTAATCTA
CGAATAAACTTTTTTTGAGATATTTACTTGCAATCTGTGCGCTGGCGCCTATTATTGGCGACCTCAGACG
CGGGGTGGAGCAGCCCGGTAGCTCGTCGGGCTCATAACCCGAAGGTCGTCAGTTCAAATCTGGCCCCCGC
AACCATCTGAGATCCTGACCAGTCAAACCGGTGTAACCGGTTGGTGGGAAAGGAGAAATCGAACTGGCTA
CTGCTCGGCCACTTCGAGAAAAAATTCGGACGCGGGGTGGAGCAGCTCGGTAGCTCGTCGGGCTCATAAC
CCGAAGGTCGTCGGTTCAAATCCGGCCCCCGCAACCAACTCTTTCGTCAGCTTGCGATTGTGTAAACAGC
CGAAGGCAGACAAACGCCATCAAGCTACTGCTCGGCTTGAGGGTGACAATTCGGACGCGGGGTGGAGCAG
CTCGGTAGCTCGTCGGGCTCATAACCCGAAGGTCGTCGGTTCAAATCCGGCCCCCGCAACCAACTCTTTC
GTCAGCTTGCGATTGTGTAAACAGTCGAAGGCAGACAAACGCCATCAAGCTACTGCTCGGCTTGAGGGTG
ACAATTCGGACGCGGGGTGGAGCAGCTCGGTAGCTCGTCGGGCTCATAACCCGAAGGTCGTCGGTTCAAA
TCCGGCCCCCGCAACCAACTTTCTGTACGTTCCCTTCCTGCATTGCCAGTCATTGAACAATCCTTCTTGA
TGCTCGTCACCATCTGGTGATTGCTGCTATTTTTGACGCTTTTTCAAGACCAAAACTGGTCGTATCAACC
ATTTGTTGGGTATCATCAGGTATCTCTTCATGGATGAGGCACGCGCCTGTGATCACTCCCTTCACCTATC
TTCCTCCTGCTATTGAAAGCCGGGATGCCACGCTGGCCGAAGCCCTTGCCCAAGGGCTGCCGTTGCAACT
GCTGCTGATCGCCGAAGAGCAGAAGGAGTCCTTGCTTTCGCTCCCCGCCCTTGCGGGTCTCCAGACCCAG
CTTGCGCTGGGGAAAAAAGCGCCCTTTACCCTGATGCACGGCAATACCCTGTTGCAGGTGATGTTTGTGA
CCGGCAGTGTGACGCGGGATCACCGCCTCTATAAACAGATCAGAGGCTGGATTGCTCCTCTTTCTGGGCT
CAATGTGGAGCGCCTCGGGCTGCATCTGGAAGGTTTTTCCCCCTCCGAGCGAGTCATGCTGGCCGAAGTG
ATGTTGTCGGCCCTGCTTGCCGCCAATATTTCGTTGCCCACCAGCAAGCGCCGCAGCGAAATCAGCTGGA
GCTATCAGCAGATCAAGGTTTCACCCTCAGTGGTGCTGGACTTGGCGCGCCTCTACAGCGAAGCGGGGGG
CAACGGGCTGGCTCGCCATCTGGCGGTGTTGCCTGCCTCCGAGCTGACGGCCGCCAGCTATCGCGAGTTT
GTCAGCAAGCTGGCGCAAGATGAGGGGTGGCAGTGCCATCACTATGATCAGGCACAGCTGGCCGAGTTGG
GGGCGGGGGCGTTTCTGGCCGTTGCTCGTGGCTCGGAAGACAATCAGGGGGCCATGGTCAAGCTCAGCTA
CTATCCACCCAAGCCGGTGCGCCGCATTGCGTTGGTGGGCAAGGGGATCTGCCACGACTCTGGCGGTTAC
AACCTCAAGGTCTCCGGCAGCATGTATGGCATGCATCTGGACATGGGGGGCAGCGCGGTGGCGCTTGGGG
CCCTCTACGCCATCAGCCGAGCTCAGCTACCCTACGAGGTACACTGCTGGCTGGCCATTGCCGAGAATCA
TATCGGCCCTCACGCCTATCGTCCTGGCGAGGTGGTGACTGCCATCAATGGCACCTCCATCGAGGTGGTC
GATACCGATGCCGAAGGGCGAATGGTACTGGCCGATACCCTGGCGATGGCGGTGCAGGATACTCCTGATC
TGCTGCTCGATTACGCAACCCTGACCGGGGCCTGCAAACGGGCGCTGGGTAGCCGCTACAGCGGCGTCTT

TACCAATCGCAGCGAATGGATTACATCGCTGGTCAATCTGGGTCAGCGCAGCGGTGAACGAGTCTGGCCC
TTCCCGCTTGATGACGATTATGACGACAACATCGAAAGCGAGTGGGCCGATCTGTTGCAGTGTGCGCCGG
GAAGTTCGCCGGATCACATTGATGCGGCCCGTTTCCTCGCCCACTTCGTGCCCGAGCAGACCCCCTGGCT
GCACTTCGACCTGAGCGGTTTTCGCAACAAGGGGGGCAATGGGGTGGTCTCCAGCGAAGTGACCGGCTTC
GGCGTGCGTCTCACGCTGGATCTGCTGGCCAGCCCGCTTGGCAGAGTCGGTCGCCAAGCCTCTGAACAAT
CTGAATGATAACAAGGAGTTCTGTGAAACAACCAAGCTATGACTGGGTGCTGTTCGATTTGGATGAAACC
CTGCTCGATTTTCCGGTCGCCTCTGCGCTGACGCACACCCTGCAGATCTATGGCGTACAGGCTGATGACG
CCAGCATGGCGCAGTATCACGCCCTCAACTACGGGCTCTGGCAGCAGTACAACGACGGCGAGATCGATGC
AACGGCGTTGCAGCAGACCCGCTTCTCCCTCTTTGCCCAGCAGGTTGCCGCCGATCCCATGGCGATGAAC
AACACCTTTCTGGAGCAAATCATTGCCCTTAGCATGCCTCTCGAAGGGGTGGTCGAGACCCTGCAGCAGC
TGCGCAGCAAGGTGCGGATGGGGATTATCACCAATGGTTTCAGCGTGCCTCAGCGGGGGCGGCTCGGCAA
ACTGGGCTGGAGCGAGTGGTTCGAGCCGCTGGTGATCTCCGACGAGATCCGGGTGACCAAGCCGGCGCCA
GCTATTTTCCAGCACACCCTCTCCCTGATGGGGCAGCCGGATCCTGCGCGGGTGCTGATGGTGGGGGATA
ATCCGAAAACCGATATTGCCGGGGCGGCGGCGCAGGGGCTGGCGACCTGCTGGTACAACCCGGCGCGGCA
AGCGGGCGAGTGCGATGCCACCCACGAAATCCACCACTTCGCCCACCTTGCCGACATTGTGCTGGGACGC
TGATAGCCAGGGTTAACCCATCACGTTATCGGTGACCAAAACGTCCTGGCAAGTTGCAACCAGGGTTGTT
TGAGACAGGATTGGGTTTGCTTGAGACAGTAAGGGGAGCTGATGCTCCCCTTACTTAATGAGTGTTTTCC
TGTGAATGCAGGGATCTGAACTGATAGCGATCGAGGATCTGCTGATAGTCGGGACTCTGTTTCAATTGAT
CGATAGCCTGATTGATCCGCTGCAGTTGCGCCTCTGTGGTGCTCTCTTTGCTCAGCATCAGGTGAACCGG
CGTGACTTCGATACGCAGCAGGGTATGCAGCTGATCCTGCGCTTCATCCTTATCCTGCCACCCCTGCAGT
CCGTGGGGATCCAGAATAAACCCCTGGATCCGACCCCCTTGCAACATCTTCAGCAGCTGATCCAGCCGCA
GGCGCATCTGCAGACGGGGCTGCAGCTCGGGCCTTGCCATCAACTGGCGGAAGGTAGTGCCGTAATCAAA
GCCCTTGAGCACCCCGATATGGAAGTTGGGGGTGTTGATCAACTGCTCGAGGGTGGTGATCCCGCGCCAG
CGTTCGGCATCTTGCCGGCGGATGATGATGGCCGTCTCCTCTTCCCGATAGGGGTGGCTGAAGCGGGCAT
ATTGCTGCCGCTCGGCGGTGATATTGGCTGACATGGTCAGCTGAACCGTGCCGAATTTCAGCTCGTGCAG
CACGCGAGCCCAGGGCATTTCACTGAATTTGGCCTGATAACCGGCTCGTAGGAAGATGGCGTTAAGCAGC
TCCACATCAAGTCCACGGAGCTGTTGTTGTTCATTGCGATAACTGAAGGGCTGCCAGTCGACCCAGCCCA
CGACCAATGACTCTTGTGCCGTAGCCTGGCAACACAAACACCATAACAACAAGAGTCTGTACACAACGCA
TACCCTCGAACAGGCTGATACGGTTGAGTATTGGACAGGAACGACGGAGTAACCAGTGCTGAGGGGTGAG
TGGATGCACAGAGGTGCATCCCTCACAGTTCGTGGGCGTGGAATCCCTTGCGATAGCGGGCTTCGCAGCG
CGGGCAGCGTTGCAGAGTCGGATCCTGTTCCAGCTGTTCGATGGAAAGCGGCTCTTCACAATCGGAGCAG
AGGCCATAGAGGCCGAGATCCATCTGGCAGAGTGCCGCATCGATCCGCTTGAGGCGGGCCACGCTCTGCT
CAACCTTCGGCAGATCCAGCCGGCTGGTCAGTTCCACCAGTTGATCCTGCTCGCAGTGACCGACCTGTTC
GGCCCACTCGTCACGGCGGCAGGCTTTGAGCTGACCGATCAGCGTTTCTCGTACTTGGGCCCAGTCTGAC
AGCAGGCGATTACGCCAGACAGTCAACTGTGCTTGCGTCATTTCACACACATTATCCCCCACAGGACAGA
CGACAGATTTGGGGCAGTATAGGGATCCGATAACTCTATCAAGATGATATGCGTCAAGTTAAACGTGGTT
AGCCGTGGCTTTTGGCTGCTTGCAGCAGGGCATCGCGGATGGGAGCCGTCTCTTTGATCACCCGGATCTC
CTGAAACAGCAGGTCCGCTTCGGGATATTCCCGCTTGAGATAGCTGAACCACTGTTTGATGCGACTGGGG
TAGTAGTCGGCTTTTTCGCTGGCCAGATCCTGCTCGGTATAGCTCACCATCAACTGCAGCACCTCGGCCC
AGCTCATGTGCGGGCAGCCGGTTTTCATCACGTTGGCCAGATTGGGCAGGGAGATGGCGCCGCGCCCGAC
CATCAGGGCATCGCAACCCGAGACCCTGGCACAGGCGAGGGCATCTTTATGATCCCAAATCTCGCCGTTG
GCGGTGACGGGGATGGGCAGGGCGCGGCGAATGGCGTCCACATACTCCCAGTGAGCGGGGGCCTTGTACC
CTTCGCGCTTGGTGCGGGCATGAACGGCCAGCTCGTTGGCGCCCCCCTGGAGGACCGCCTGAGCGTTCTC
CAGATAGAGATCCTTGTCATCGTAACCGAGGCGAATTTTGGCGCTCACCGGCAGATGGGCGGGCACTGCC
TCGCGCACCGCCTTGACGATGCGGTAGATGGTCTCGGGCTCTTTGAGCAGCACCGCACCGCCCTTGCTCT
TGTTGACCGTAGGGGCGGGACAGCCAAAGTTGAGATCGACGCCGGGGGAGCCCAGCTCGATGGCGCGCAC
GGCGTTCTCGGCCAGCCACTCGGGGTGTTGCCCCAGCAGTTGTACCCGCACCGGGGTGCCTGCGCGGGTT
TTGCCATCTTGCAGCAGTTCAGGGCAGAGGCGGTAGAACACCTTGGCGGGTAGCAGCTGATCGACGACCC
GGATAAACTCGCTCACGCAGAGATCGTAATCATTGACGGAGGTGAGTACCTCCCGCATCAGGTGATCCAG
TACCCCCTGCATGGGGGCCAAAATAACGCGCATAACGGCTTTCCGACGAGAGTGGGGAGAAAAAGAGCGC
AGATTGTAGTGGGGTTGCGGCCCGCTGTCAGCACCCGCTCAAACTCTGTTAGGCTAGAGGGAGATAAATC
ACGCAAGGAGAAGGAATGAAGCGTTGGTGTCTGATGGCGCTCGGGTGGCTGGCATTTGCCACCGGTATTG
TGGGCATTGTGCTGCCGCTCTTGCCGACTACCCCCTTCATGTTGCTGGCTGCCGCCCTGTTTGCCCGCTC
GTCGCCCCGTTTTCATCGCTGGCTGTTGACCCATCCCTGGTTTGGCCCGCCCATCGTGGATTGGCAACAG
TATCGCGGCATTCGTCGTCAGGCCAGACGGCGCGCCATTATCTTCATTTTGCTCACCTTTTCGGTGTCGC
TGGCGGTGGTGCCACTGCTGTGGGTCAAGGGGTTGCTGGTTATCATAATGGTGATCCTGCTCACCTGGTT
AATGCGTCTGCCGGTGCTGGAGCCAGTTGCAATGGTGAAGTGAAGTCCCTAAGCTATTGCAGCAATAATG
CTATGGAGTCGGCCTGTCATTTTTGCGTGACAGGCTTTTTTATTTCCCGCAGAAGGGAATTGAGAAAAAT
GGCCACCCCTGGGTCAAAAAAATAAGCGGTCAACACATGAATCCGGAAACCCTGAAATTTATCGAAGCAA
GCATCAAGACCATTCCCGACTATCCGAAGCCGGGCATTCTGTTTCGTGACATCACCAGCCTGATCGAGAA
TGCCGAGGCCTTCAAGGCCACCATCGATCTGCTGGCCAATCATTATCGTGATCAAGGGATCACCAAGATT

GTTGGCACCGAAGCTCGCGGCTTCATCTTTGGCGCGCCGGTTGCCTATGCCATGGGGCTGGGCTTCGTAC
CGGTCCGCAAGCCGGGCAAGCTGCCGCGCGAAGTGATCGAGGAGTCCTATGCCCTCGAGTACGGCACTGA
CACCCTGCAACTGCACACCGATGCCATCGTGCCCGGTGACAAGGTGCTGGTCGTGGACGATCTGCTGGCG
ACCGGTGGTACCGTTGATGCGACCGTCAAGCTGATCCGCCGTGCCGGTGGTGAAGTTGCCGATGCGGCCT
TCATCATCTCCCTGCCGTCTCTGGGTGGCGATGCCCGCCTGACTGCTGCTGGCATCAAGGTAGTCTCACT
GGTCGAGCTGGCCGGCGAGTAATGCCTCTTCGGCCCGATCCTCTGCATCGGGCCTGCATTGAACCGGTGC
CCTTGCGGGCATCGGTTTGAAAATCAAGCGATCCCTCCCGCACTGACCGATAGCCGATACAGCCAGCCCG
CCATTTGTGTTAGCATCCGGCCTCAATGCCCTGTCTTCCCTGCATGATGGATTTATGAGCTATCAGGTTC
TGGCGCGTAAATGGCGCCCCCATACGTTTGAACAAGTGGTAGGCCAGCAACATGTGCTGACCGCCTTGAC
CAACGCCCTGGATCAGGGACGGCTGCATCACGCCTATCTGCTGAGCGGTACCCGCGGGGTCGGCAAGACC
ACCATCGCCCGTATTCTGGCCAAGAGCCTCAACTGCGAGCAGGGGATCAGCAGCCACCCATGCGGCGTCT
GTGATACCTGCCGCGAGATCGATCAGGGCAACTTTGTCGACCTGCTGGAGATTGACGCGGCGTCCCGCAC
CAAGGTGGAAGATACCCGCGAGCTGCTCGACAACGTGCAGTACCGTCCGGCCCGCGGCCGCTTCAAGGTC
TACCTCATCGATGAAGTGCACATGTTGTCGCGCCACAGCTTCAACGCGTTGCTGAAAACCCTGGAAGAGC
CGCCCCCCTATGTGAAGTTTCTGCTGGCCACCACGGATCCGCAGAAGTTGCCCATTACCATTCTCTCGCG
CTGCCTGCAGTTTCATCTGAAGAGTCTGGATCAGACCCAGATCGCCAAGCAGCTCGAATGGGTGCTGGAT
CAGGAGGGGCAGCCCTTTGAACCGCGTGCGCTGCTGGCCCTGGCCAAAGCCGCTGACGGCAGTATGCGCG
ATGCCCTGAGCCTGACCGATCAGGCGCTGGCCCACGGCAATGGCAGCGTGCGCCTCGACAGCGTGCTGGC
GATGCTGGGCACGCTCGAGCACCGCCATCTGCACCAGCTGCTGGAAGCCATTTTGCGGCAAGATGCCCCC
GCGACTATGGCCAAGATCACCGAGATCGCCACCCTGGGCCCCGATTTTGATCAGCTCCACGCCGAGCTGG
AAGGTTTGCTCCATCGCATCGCCATGGCGCAACTGCTGCCTGCCAGTGTGCAGGAGCAGGGGGCGGATGC
TGATTCCCTGCTGCAACTGGCCAAGGCGATGAGCCCAGAAGAGGTGCAGCTCTGCTACCAGATCGTGCTG
GGTGGCCGTAAAGATCTGCCCTGGGCCCCCGATGGCCGCACCGCGCTGGAGATGACCTGCCTGCGGATGC
TCGCCTTCTCCCCAAGGCGTGATGCCCTGCATCCGGCCAGCCTCACTGCGCTGCCCCCGTCAATGACGAG
CAATACCACTCCGGTAGCCTCCTTGCCGGGAAAGCCCCCGGCCGCTGAATCGGCCACACTGGCACCGATC
AGCGGCGCCGTGCCCGCGGTTAATGTCTCCCCGCCTGCGATGACGGATGACATTGGCAACCCCGCTGACA
GAGAGGAGGAGGCGCAAGCCGCCGAGCTGTTCAGCCAGCAGGATGAGCTGCTGCGCGAAGCCGCAGGCAT
GGGGTATCAACCCGAGCCCGCGTTACCCCATGTGCAGCCCGATGTGTCTGCGCACTCAGCCGAACAATCC
GCTGATGCTGATGATCGAGCACCTTCCGCAGCCGATATGGACTATTCCCGTAGCGAGCCGCTGACCGCCC
CCTTCGAGCCCGCGCTCGAAGCGACAGCTACGCCTGTTGCCGCTGCTGAACCGGTATCAGGGGTATCGAG
CATGCAAAGCCTGCTTGGCAAGCGAAACATGTTGCGCAGCCGCCAGCGTGGTCAGGATGCCCCGGCGGCG
CCCGCGCCGCGCGCGGTGCCGGTTGCGCCACCTCGTCCTGTCGCGCCCGCTACTGCACCGGCCGTCACCC
AACAGCCAGCTCCGGTCGGCCAACCGGCCGGGCAGGGGATGGCGACGATGGGACACTATGATGAGTTGCC
ACCGGTGGATCTCTATGGCGACGGTTACGAGTCGCAGGGTGACAACGGGGATTACGAAGAGTACCTCAAT
CAGGGCTTTGCCGAGCAGACTCCGGTGATGAGCCACGCCCCCGTCACGGCACCGGCCCAGCCAACCGATC
TGCCCCCCTGGGATCTCGACGGGCAGCCGAGCTATCGACCTGGCGAGCGCAGCACTGCGGCGCCTGCGGT
GACGCCCGCCCCAGCGACCCAGCCACCCGTTGCAAGCACGGCAGTGGCTGCTGCCCTTGTGACGCAGGAT
GAGCCCGCTCAGCCAGTGATGGAGACCATCGACTGGGATGAGCTCGAGAGCGACAGCGAGCCGCAAGAGG
GGGAGGTGACTCGCCTTATCCCCTCCTCGCTACTCAATAGTTGCGGCGACCCTTGGGCCGAGTTGATCGC
CCACACTCAGGTGGGTGGTCGGCTGCGCCAGTTGGCCATCAACTCGGTGATGACGCGGGAAGGGGACAAG
GTACTGCTAACCCTCAAGCCTGAACAGCGCCATCTGGTGAGCGACAAGGCGCGCGCCGATCTGGCCGAGA
TCATCGGGCCGGCGCTGGGTCAACCGGTGCAGGTCGAGGTGACGCTCGGTGTGGTACCTGATAAGGAGAC
CCCCCTCGAGATCGAGCACCGCCTCTATCTCGGGGTGCGCGAGCAGGTGACCCGGGATCTGGAGCAGGAT
CCCAACGTGCAGTTTTTAGTTCAGCGGTTTGGTGCCGTGCTCCATCACGAGAGCATCGAGCCCCTCAACC
GCTAAATTCCGTGGCCGGGAGCTCGCTTCCGGTTGAAAAGCAGTTTGGCTGTCCCCATCTAGGACAGACA
AGCCCACAGCCCGGCGCTGGATGCCGGATTTCAAGACGAGAGACGACATATGTTTGGTAAAGGTGGAATG
GGGAACCTGATGAAACAGGCCCAACAGATGCAAGAGCGTATGCAGAAAGCGCAGGAGCAGCTGGCTCAGA
TGGAAGTGACCGGTGAAGCCGGTGCAGGCATGGTCAAGGTCACCATGACCGGTAGCCACAGCGTCCGTCG
TATCGAGATCGACCCCTCCCTGATGGAAGATGACAAGGAGCTGCTGGAAGATCTGGTTGCTGCCGCTTGC
AACGATGCCGTGCGCCGTGTGGAAGAGCAGAACAAGGCCAAGATGGGCGAGCTGACCGGTGGCATGCAAC
TGCCGCCAGGTTTTAAGATGCCGTTCTAAGGCGACTCTCCCCGCGCATTCTTGCTCCCTTGTCGCTGGCC
ATTCGGGTCACGGCAAGGGGGACGGCCGAGAGACCCAAAGACGCAGCGTTATTGCTCAGGCGATACGCTG
CGTTTTTTATGGGCGCCATGCTGGCGCGGGCGGGCTCTAACTATCCCTTCTGGGGAGAGGTTGCCACAGC
GAAGGCCGATTACGCCATGAAACGCAGCAGCCTTGCTAAGGCGGCGCTATTTGTCGATACCCGCTATCGG
ATATCCCGGTTTGGCGTGGCCGGGCTGGATCGGCATAATGGCGGCTTGCCGTATCGAGGAGATTGTATGA
AATATATCGGAGCCCACGTCAGTGCGGCCGGCGGGGTGGAGAATACCATCGCCCCGCGCCCAGGCCATCGG
GGCCAACGCGTTTGCCCTCTTTACCAAGAATCAACGTCAGTGGCAGGCCGCCCCCTTGTCGGATGCCACC
ATCCGCGCCTTCAAGGCGGCCTGTGACAAGGCCGGTTTTCTGCCGGAGCAGATCCTGCCCCACGACAGCT
ATCTCATCAATCTGGGCCACCCGGATCCCGATGGTCTGGCCAAATCCCGCGATGCGTTTCTCGACGAGAT
GAAGCGCTGCGAGCAGCTGGGGCTTTGCTATCTGAACTTCCACCCCGGTAGCCATCTCAAGCAGATCCCA
GAGGCGGCCAGCCTCAAGCTGGTGAGCGAGTCCATCAACTGGGCGTTGGAGCGCAGTGAAGGGGTGACGG

CGGTGATCGAGAACACCGCCGGGCAGGGGACCAACCTCGGCTGGTCGTTCGAGCACCTCGCCACCATCAT
CGAGGGGGTGGAGGACAAGAGCCGGGTCGGGATCTGTTTCGATACCTGCCACGCCTTTGCCGCCGGCTAC
GATCTGCGCACTGCAGAGAGCTGCGCTGCTGTCTTTGCCGAGTTCGATCGCACGGTGGGTTTTCATTATC
TCAAGGGGATGCATATCAACGGCGCCAAGTGCACCTTCGGTAGCCGGGTCGATCGCCACCACAGCTTGCG
GGAGGGCAATCTGGGAGAGGCGGTATTCCACCACATCATGAATGATGATCGTTTCGATCGCATTCCGCTG
ATCCTCGAGACCATAGACGAGTCTATCTGGGGCGAGGAGATCAGCTGGTTGCGCAGTCTGGCGAAGTGAT
ACTCAGAAGCGAATCAAAGAGAGGGAGCCGTGGCTCCCTCTCTGCTTTTTCCGTTATTGGCGAGCGGGCC
TGCCAGCGGCGCTCCGAGCGGGCGTTACTCTGGGCGGCTCACCTCGGCATCCTTCACCTGAGCCCGGCAG
AAGTGGATAAAGTGGCGCAGGGAGGTGGAGTGGTACTTCTGCTTGTGCCAGATAAGGGAGAGCTGGCGCG
GGAAGCGACGGGAGAGGGGCAGGGCGATCAGCCGCCCATCTGCCAGGCGATCCGAGGCTGCCAGCCGGGA
GATAAAGGAGATGCCAAGCCCCTTCTCCACCGCCAGCATCACCGCCTCCAGGGTGTTGAGTTCAAGCCCT
GGTTGCCAGCGGGGCAGCTCGGGCTGGATCTGCTGGATAAACTGCTCGCGGCTGCCCGATTGCGCTTCAC
GCAATACCCAGGTCTCGCCCCCCAGTCGGGAGAGGGAGAGCTCCTTCTTGTTGGCAAGCGGGTGACCGGG
TGGGGCCACCACCAGCATCTCGTCCTGCAGCCAGGGTTCGGCGAGCAGGTCGGGGTGGTGGTTCTCCCCC
TCGATCAGTGCCATATCCAGCTCGAAGTTGGCCAGCGCATGGCAGAGCAGGTGAGTGTTGTTGATGGTCA
CCTTGGGGGGGAGCCCCAACTCCTGTTTGCTGCTGGCGAGCAGGGTGGGGAGCAGGTAGTTGCCGATGGT
CTGGCTGGCACCAAGGCGCAGCTGGCCGCTCGGCTCGGCATCGGGGCTGAACAGGTTCTCGATATCCTGC
ATCCGGGTCAGCAGCTCTTCGGCGGCCGGTTGCAGCAGGCGTCCCTCGTTGTTGAGCTGCAATCTGGGGT
GAATACGGTCAAACAGCGGGGTGGAGAGCTGGCGCTCCAGCTCCTGCAGCGACTGGGAGACAGCCCCCTT
GCTCAGGCAGAGCTCGTTGGCGGCAAGGCCAAGGTTTCCATAACGGGCAATGGTGGCGAACACTTTCAGC
TGCTGCAGGGTCAGTTTCATCGTGTTTGGTTTTCCTGAATGATTGATTTAATTCATTTAGATATCGTAAA
CGATAAGGGACCATTATCCTATCGCCATAGTCATCAATAGAAAGGTGTCATCATGATCGCAAGAACCCGT
AAATCACTGGCTGCCGCCCCAACCCCCATGGCGGGTCTGGCGCTGGGCATCGCCAGCCTTGGTTGGTGTT
GGGAAAATGCCGGTCACTTCGATGGCCGGATGCAACTGCTGGGCGCTGCCATTGCGGCCGTGTTGCTGGT
GATCCTGACCTTCAAGTTTCTGCTTCACCCCCGCCTGCTGTTGCAGGATCTCGCTCACCCCGTGGTGGGC
AGTGTGGTTCCGACCTATGCCATGGGCACCATGGTGGTCTCCAAAGCGCTCGGTATGATTGCGCCGCAAG
CGGGTGAGGCCCTCTGGCTGTTTGCCGTGGCGGTTCACCTGCTGTTCCTGGCCACCTTTATCTGGCATCG
CGCCAAAGAATTTGAAATCCACCACATGGTGCCGAGCTGGTTCGTGCCGCCAGTGGGGATCATCGTGGCT
GACGTCGCCTTCCCGGGTGGTCAGTTTGCCCCGCTGGCCAACGGTCTGCTCTGGTTCGGGATCATCTGCT
ACGGCCTGATGCTGCCGCTGATGCTCTATCGCCTGATCTTCAGCCATGAAGTGCCGGATGCCGCCAAACC
GACCATCGCCATTCTGGCGGCTCCTGCCAGCCTCTCTCTGGCCGGTTACCTGACCGTCAGTCAGGAGCCG
TCACTGCTGCTGGTGGCCGTGCTGTTGGGGATTGCCATCCTGATGACCGGCATCATCTATCTGGCCTTCA
TCAAACTGCTGCGTCTGCCCTTCTCGCCGGGTTATGCCGCCTTCACCTTCCCGCTGGTGATCGGTGCTAC
CGCCCTGTTCAAAGTGGCCCATCTGGTTGAAGGGTTTGCGCAAGGGAGTCACTACGTCGAGCAGATCAGA
CTGCTGGCCCACTTCGAGCTTGGGGTGGCTACCGTCATCGTCGGGTATGTGGCACTGCGTTACCTGATGT
TTTTTCTGCCGCAGCGTAATACCGAAATGAATAACGGCATGGCCCGCCAGGGTGCTCACCGTTAAATCTT
GAAGAAGATGTGACATCGGGAAATGGATAAGCAGTGCGCTCCGAGAGCGACGGGATAAACGACCAGTCAT
ACGATGGATGGACAAAGATCCTCCACCCTGGTTGTGTAACGAGCAAAATGCCCCGCAGTGCGGGGCATTT
TTTTATGGTTTTTTGATGTGGCGCCCGTGGCGGGCTATTTGCCATGGATTTAACTGGTATTACCTGTCAG
GACGACTACAATGCAGCACCACTTTTTCGCTCGACCCCAATACATGTCGTCCCAAGATCCCGTATTTGAC
TCCTCTTTTGAAGCCCGTTTTCTGCATCCCCGCTACTGGTTCAGCTGGCTGGCGCTCGGCGTGCTGATGT
TGCTCGCCTTTGTGCCGGTGCGATTGCGCGACCGTTTGGCCGAAGCCTTGGCTCCGCTGGTGTTTCGTAT
CTCGAAAAAACAGATTTATATCGCCAGAACCAATCTGGAGATCTGCTTCCCGGATAAGAGCCCGGAAGAG
CGGGATGCCATTGCACTGACCTCGATCCGGGTCGGCCTCAAGAGTCTGTTGGGATTTGGCGAGTTCACCA
TGCGCAGTCAGCGTTATCTGATGAGCCGGATGCAGGTGATCGGTTGGGAGCATATCGAGGCGGAGCGGAC
GGCCGGCCGTCCGGTGATCCTGGTGGTGCCCCATACCTGGCCGGTGGATGCCGCCGGTTACTGCTTCACC
CAGCGCGGGGTGCCCATGTGCACCATGATGAAACGGGCGCGCAATCCGGTGTTTGACTGGCACCTCAACC
GTGCACGGGCCAAGAACGGTTGCCGCGTCTATGAGCGCAGCGCCGGGATCAAGGCGGTGATCAGGACCAT
CAAGAGCGGTCGCAGCTTCTTCTATCTGCCGGATCAGGATCATGGCCGCGAGAAGAGCATCTTCGCCCCC
TTCTTTGGCTATCCCAAAGCGACCCTGCCTGCCTTGCCCCGTCTGGTGCAGGTCAGTGGCGCCAAGGCGG
TGCCGCTGCTCGCCTGCTATGACGAAGCGAACCACTGCTACCGGCTGGAGATTGAGGCACCGTTCGACAA
CTATCCCACCGAGGATGTGATGGCTGATACCTGTCGCATGAACGACATGGTGCAACAGCAGCTGATGCGC
CATCCGGGTCAATACATGTGGTTCCTCAAGATCTTCGATACCCGTGAAGATCAGGAGGGGCTGGATGGCC
TCTACGAGGCGGGGATCCAGCGGATCCGCAAGGGATTGTCGCCCCACGGCTGACCCAGAATTTGCGTTGA
TTGAAATGGCCCCAAGTGGGCCATTTTTTATGATAACTAGTCTGATCAGTGCCTTGCCGGTCGATGGCCA
GCCTCCCCAGGGTAGAAAAAAATGTCAGGATAACTACAATAACCGTTTGTTTTTGATGACTTGATGGTCG
CCATGTCTGCGTTAGATCCGGTATTTGATACCTCATTTCATAGCCGCCTGCTGCATCCCAAATGGTGGCG
CAGCTGGTGCGCGCTTGGCCTGCTCTCCCTGCTGGCATTTACTCCTGCGAGATTGAGGGATCGCTTTGCC
GATCTGGTCTCTCCAACCCTGCTGCGTTTTTCGAAAAAGCAGGCCTATATCGCAGATACCAACCTCAAGA
TCTGCTTTCCCGAGCTGGATGCCGCCGCACGGCAGGCGCTGCTGCTCAAATCGATCCGGGTCGGGCTCAA
GACCTTTATGGGCTTTGGCGAGCTCAGCTGGCGCAGCAAGGCGCGACTGACCCGCCGCATCAAGGTCACC

GGCTGGGAGCATGTGCAAGCGGAGCAGGCTGCAGGCCGCCCGGTGATCCTGGTGGTGCCTCACACCTGGG
CGGTCGACATGATTGGCCGCTACTTCTCCATGATGGGCATCGAGATGTGCACCATGATGAAGAGCCCGAA
AGATCAGGTGTTCGACTGGTACATCAACCGCGAGCGGGCCAAAGGGGGCAAGGTCTATGAGCGCAGCGCC
GGCATCAAACCGGCGATCAAGTCGATCCGCGCCGGTGCCAGTTTCTTCTATCTACCGGATCAGGATCACG
GCCGCGAGGCGAGCGTGTTTGTTCCCTTCTTTGGCCGCCCCAAGGCAACCCTGCCAGCCTTGCCCAAGCT
GGTGAAGCTGACCGGTGCCAGAGCGGTGCCCATCTTCGCCAGCTATGACGAGGATGAGGGGCGCTATCTG
CTGGAGATCGAGCCGCCGTTTGACCCTTATCCCACCGCCGATCTGCTGGCAGACGTCAATACCATGAACC
GGGTGGTAGAGATGCAGGTGGCGCGCCATCCCGAGCAGTACATGTGGTTTCTCAAGATCTTCGAAACCCT
GGAGCATCAGGAGGGGGATGACGGCCTCTATGAAGCGGGGATCCAGCGCATTCGTCAGGGCTTGCCGCCC
GAACCTTGAAAATTGGCCCGCTTGCGGGCCATTTTTGTTGGCACAGGCGCCTTTCGTATTTGGTCTGCTG
CTGGTGGTTTTTAAGGCGAACGGGTGGACAAAAACAGCGCAAACAGGGGATAACAGGGTGTCTTATGAAA
GGGAGAGGCAGGTGAGCGTGCGCGGAGCTATCTTCGAGAGCGGAAATAGGGTAAAAGGGCGGCCATGAAA
ATTCCAAATCGAATCCAACCTCTGGTTGATGACGGCCTGGTCGATGAGGTCGTCAGCCGGTTGATGAGCG
GCAAAGAGGCCGACGTCTATGTGGTGCGCTGCGGCGACGAGATCCGCTGCGCCAAAGTCTATAAAGAGGC
GTCCAAGCGCAGCTTCAAGCAGGCCGTGGTCTATCAGGAGGGGCGCAAGGTGCGTAACAGCCGCAGCGCT
CGCGCCATGGAGAAGGGCTCCAAGTACGGCCGCAAACAGCATGAAGAGGTGTGGCAGAACACCGAGGTCG
ACGCCCTGTTCAAGCTGGCGGCGGCCGGTGTGCGGGTGCCGCAGCCTTATGTCTGCCTCGATGGCGTCCT
CTTGATGGAACTTATCACCGATGAGGATGGCAATGTCGCGCCGCGCCTCAACGATGTGGCGCTTACCCCA
GAGCAGGCGGTCATCGACCATGCCAAGGTGCTCCGTTACGTGGTGCGCATGCTCTGCGCCGGCCTTATCC
ACGGGGATCTCTCCGAGTTCAATGTGCTGGTGGATGAACAGGGTCCCGTCATTATCGATCTGCCCCAGGT
GGTGGATGCCGCCGCCAACAACCAGGCCAAGGCGATGCTGGAGCGGGACGTCAACAACATGCGCAACTAC
TACGGCATGTATGCCCCCGAGCTGCTCAAGACCCGTTATGCCCAGGAGATGTGGGCGCTGTTTGAAGATG
GCAAACTCACCCCCGATAGCGAGCTGACCGGTCACTTTGAAGAGAGCAGCGCCGCCATCGATCTCGATTC
GGTGCTGCAGGAGATCGAGAGCGCCGAGGAGGAGGCCTATGCCCGTCAGGCCCGGATGAAGGCGGAGCAG
GAAGACAGCTACTAACGGTCTTGCCACGTTCGCAAAAAGAGCGCCTTCGGGGCGCTCTTTTTATTACTGC
TGCGGCTATGGTCGGCTGGGGCAGGGGGCTAGCTGATACATCTCCAGCCAGTTGCGATCCCCCGGATGGA
AGCGCCGTCCCAGCAACACGCCACCGGCTGCTGTAATGACCTTGCGGGAAGCCAGATTGTCTTGGTCACA
GCAGATCAGCACCGGCGCTGCCAGCACCTGTTGTTGCACCCAGCGCAGCAGCACGGTCGCGGCCCCTTCG
CCACGGCGAGAGGGACGGGTTTCGTAACCGATATGACCCAGCCACTGGCGGGTTGCCTCATTGTCACCGT
GACGAAGACGCAGGGTGGCGATGATCTCCTCGCCATCGAGGGCAAAATAGGTGGTGGTCGGCAGGTATCC
ACTGGGCAAGTTGCGCCCTTCGGCGTGGTCCAGCAGGCTCTGTAACCAGCCGTTCGGGTCAAATTGCGCC
ACCTGATAGAGCGGTTGTCCGTCTTCGGCGGATTCGGTGACATAGCGATGGTAAGCCGGAGCATCTGCCA
GTCTGGCCGGTCGGCATTGCAGAGCGAGCACTGCGCTCATGACAGGAGCGCGCAGGGCGGCAGCGGCTGC
GGCTCGTTGTGGTGGAGCTCCTGCACGCAGGCCCGTTGCCTCCCAGCCTGTTTGGCGGCGTAAAGGGCCT
GATCCGCGCGCAGGTAGGCCGCTTCAAACCCCTCGTCAGGGTGCAGCGAGGTGAGGCCGATGCTGAGGCT
GATGGGGCCACCGGCCAGCGCCCGGGTCGCCTCGGCGATCTGCTGTTGCAGGCTGTGACAGTAGTCGGGC
AGGGTCTGCGGGTCTGCCATCAGCAGCAGGGCCAGCTCATCGCCGCCAAGCCGACCGGCCATGCCCTCGG
CAGGTAGGGTATTGGCGATCACCCGACCCACGGCCCGCAGCATGGCATCACCGGCGGGGTGGCCGTGGCG
GTCGTTGATCTGCTTGAAGTTGTCCAGATCGATCAGCAGCAGCTGGGCGGGGCTTGTCACCTCGCGGGCG
CGCAGGGTCACCTGGCGTTCGAAATAGTGGCGGCTGAACAGACCAGTCAGCTCGTCCTGATTGGCGCGCC
GCTCCAGCTTGCGCTGGGTGGCCAGCAGGGCTTGCTGTTGGCGGGTGTGGACCAGGCTCAACAGCAGATA
ACTGCGGGCGATGGTAAAGAGCAGGCTGAGCAGGATCACAGGCCCGAGCAGCGGGTCGGTGAAGGAGTTG
GCCGCCAGCTCCGGCTGCCAGAGGTGTTTGGCGATCCGCAGCAGTTGCAACAGTAGTTGCAGGCCGAGCA
GCAGAATGGCAAAGCGCAGCACCCGATAGACGGGGGCCAGCCGCTGGTAGCGCCAGAGCTGCCAGATAAG
CAGTGCAATCTGCAGCGAGAGCACGCCGGAGAGTACGATGATCCGGCTATGCACATCAAAGAGGGGTTGC
TGCAGCGCGGTCAGGGCAAACAAAACGAAACCACCGAGGGTAACGGCGGGCAGCAGCCAGCCCAGTTGGT
CGGCTTCACGGCGAAAGAAGTGGCGCATCCCCCGTTCATAGAGCAGGGTGGTCGACATCAACAGCAGGTT
GGTGCAGAGAATATTGGCAAGGGATGCATTTCCCTCAAGGGGGCGCAGCAGCAGGCAGCAAAAAGCCCCC
GCGATAACCAGTTCCCCCGCTGACCAGTAGATCAATGCCCTGATCTGGCGATGGGTGTGCCACATGGCGG
CGAAAATGGTGGCGACCCCCACATGTGACAGCGTGACGAACAGCACCAGACTCAACAACTGGGCTGAGGA
AAACAGACTTGGCATCAAGGGGCTCTCTGGGAAGTCACCTGTGAACGAACAGGCGATGGGGTATCGCCCG
CTGGCAGTGGCCGATCACAGCATGAATCCAACCTATTATCAATGGGTTAAATGGCTGAATGTGCTCACCT
CCGACTTCTTGCACGATGGCAACTGCTGACTCGATTTATCAGCGCACAAGGAGCCATTTTATTGTCTTGT
CAGTCAATTGTGCTGCGTGAACGGTGGTTGTGTAGGTGGTAGGGTGAACCCAACTGCCTGACGTCGTGCT
AATCGCAGGTTGCAGCAGGCAAGCGCATTACGATGGCCTCCCTGTCCAGATGGGTCAGCAGGCGGCGCAC
ATCGTCGAGGCGGATGATCAGAGTAGCGGTGTTGACCAACGGATGGCACTGCACCTGTTCGGCCTGCCAG
ATGGCCTCGTCCACCACCAGCTCTACCGCATTTTCCCTGTCGCGCACCATGGCGAGCAGGGTTACCGAGC
CCGGGGTAAGGCCGAGCACCGCATCGAGCCGCTCGGGGGAACCAAAACTCAGTCGCTTGACGCCAAGGGC
GGTTGCCAGCGCCTTGAGATCGACCCGGCTCTCCTCCGGTGTTACCACCAGAAACAGCCGCTCGCTTTTC
GGGTCGCGCAAAAAGAGGTTCTTGGTCTTGGCGGCGGGCAGGTCGGCAGCAGCCTGCTCGCCTCCTCAC
AGGTAAACACCGGGGGATGGTCAAAGCGCTGGTAGGGGATAGCCAGCTGATCCAGCAGGGAATAGATTGC

CACATAGACTCCTTTTGTCGGCGAAGAGTGGTCAGAACGGGATGGGCGGGAATCACGACCGGATCGAGCT
TACCAGTTTGACTCCCGCCTCGCAGCCAGCGTGACGATTGCGAGCAGGCACTGTATGAACGTACAATAGT
CCCATTCTCGGACAGCCCCTTTACGCTTCATGACTGCAACCCAGCCCACCGTACCCGCCACTCCGCCCCT
CGACGGTTTTATCCTGACCCGCCACGGTCGGGACGTGCGCGGTCGCAACGGGCAGCCTGCTACTACCGAA
ATCGTGATGTGGCTCTGGAGCGCGCAGGGGCCGGTGCGGGTGGTGGTGCCGGGGCAGGAGCCGGTGTTCT
TTTTGCCACAGAGCCACATGGCAGAAGCCGCCGAGTTGTTCAAAGGGGCCGGGGTGAGCGGGCGGTTTCG
CCGCCTGCCGATGCACACCTTCGACGGTCGCCCCGTGGTGGGCTGCTATTTCGCTACCCTGAGCGCCTTT
CACCGCGCCATCGAGCTGCTGCTTATTCGCGGTCTGGAGCACTTCGAGGCGGATATCCGGCTGCCGGAGC
GGTTTCTGATGGAGCGTTTCATCACCGCTGGTGTGCGCTTCGATGGCCGGGCGGTGAAAAAGGGCGGCAA
GCAGGGCTACTGGGAGGTGAACGAGGCACGCTGCGCGCCGCAGGCGGTGACGCCAACCCTCTCCTGGGTA
TCGCTGGACGTGGAGTGTGCCATGGACGGGGCCCTCTTCTCGGTGGGGCTCTACAGCGAAGAGGATGCAC
GGGTCATCATGATCGGCACCCCGGAAGCGGATGCCGAGAGCTGGGGCACTCAGGTCCAGTGGGTAGAGGA
CGAGAGCGCCCTGCTGAGTGCGCTGGAGAGCTGGTTCAGCCTGCATGACCCCGATCTGGTCATCGGCTGG
AACGTGGTCAACTTTGACTTTCGCCTGCTGCTGCGCCGCGCCGAGCTTAACAAGCGGCGACTGCGGCTCG
GGCGGGGGCGCGAGCTCTGCTTCTGGCGCTCGTCACGGGGCGATCCCCAAACCGGCAACGTCATTATCCC
CGGGCGCATGGTGCTCGACGGCATCGATACCCTCAAAAGCGCCACCTGGCAGTTTGCCAGCTACAGCCTG
GATGCGGTGGCGAGCGAACTGCTCGGCCGTGGCAAGGATATCGAGGACGTGGCCAATCGGGGGGAGAAGA
TCACCGAGCTGTTCCACTCCGACAAGGAGGCGCTGGCCCGCTACAACCTGGAAGATTGCAAGCTGGTGTG
GGAGATCTTCGATTACTGCCACCTTATCCCGTTTGCCATCGAACGCGCCTCCCTCACCGGTCTTGAGCTG
GACAGGGTAGGGGGCTCGGTGGCGGCCTTTACCAACCTCTATCTGCCGCGGCTCCATCGTGGCGGCTACG
TGGCCCCCAATTTGCCGGCCGACGGGGGGCTGGCGAGCCCCGGCGGCTATGTGATGGACTCCAAACCCGG
TCTCTATCGCCATGTGCTGGTGCTCGATTTCAAGAGCCTCTATCCCAGCATCATCCGCACCTTCTGCATC
GATCCCATGGGACTTATCGAGGGGTTGCAACACCCCGACCACGCTATTCCGGGCTTTCGCGGCGCCATGT
TCTCCCGCGAGCGGCACTTCTTGCCGGAGCTCATCGCTACCTTGTGGGCGGCGCGGGATCGGGCCAAGGC
CGCCAGCAACAAGGCGCTCTCCCAGGCCATCAAGATCATCATGAACTCCTTCTACGGCGTGCTGGGGTCG
GGGGGCTGCCGTTTCTATGATCCGCGACTTGCCTCGTCCATTACCTTGCGTGGTCACAGCATCATGCAGC
AGACCCGCGAGTGGATTGAGGCGAAGGGGTTCGAGGTGATCTATGGTGACACCGACTCCACCTTTGTCCA
TCTGGGGCGCGAGACCAGCCCGGATGAGGCGGACGAGATTGGCCAGCGACTGGCGCAAATGATCAACGAT
AACTGGGCTGCACTGATCAGGCGCGAGTTCGACCTGCCGAGTTATCTCGAGATCCAGTACGAGACCCACT
ATCGCCGCTTTCTGATGCCCACCATCCGCGGGCTGGAGAAGGGCAGCAAGAAGCGCTATGCCGGGCTGGT
TGGCAAGGCCAGCGAGGGGAGCGAACAGCTGGTGTTCAAGGGGCTCGAATCGGTGCGTACCGACTGGACC
ATGCTGGCCAAGCAGTTTCAGACCCGCCTCTATGAGCTGGTGTTTCACGATGAAGATCCCAGCGGTTATG
TGCTCACCATGGTAGATGAGACTCGCGCCGGTCGCCACGACGATCTGCTCATCTACCGCAAACGGCTACG
CCAGAAGCTCGAGGAGTATCAGAAAAATGTGCCGCCCCATGTGCGGGCGGCGCGGCTGGCCGATGAGGAG
AACCTGCGCCGCGGTCTGCCCCAGCGCTATCAGCATCGGGGCGCCATCGCCTATCTGATGACCCTCAATG
GCCCAGAGCCGCTGGAGTACCGGCGCAGCGCCATCGATTACGAACACTATATAGACAAGCAGTTGCGGCC
CATTGCCGATGCCATCCTGCCCTTTATCGGCGAGTCGTTCGACCGGATCTGTGGTCAGCAGCTGGATCTT
TTCTGATTGGTGCAAGATGGTACTTTACTTCCCATAAATACTGGATAAAAATACAGTTATAAATCATGGA
GAAGTTTCCTATGCGTCTTGAAATCATCATCGACAGAAAACATCAGATCCCGACCTCGACCATGGAGGCG
CTGCGTCAGGAGCTGCTCAAGCAGCTTGGCGAAAAATTCCCCGACATCCATGTGCGGGTCGCGCCGGGCA
GTGCCATGGCCCTGACCGTTAGCCGTGCCAGCAAGGAAGACAAAGAGCTGGCCGAAGCCATGGTGCAGGA
GGTGTGGGAGAACGCCGAGAGCTGGATGCCGGAAGAGAACGCCGCCTCCTGATCCCGCACGGTCAGTGGA
TAACTCGGGCGTTGGATAAATGGGTTGCTGATAAACGGGCATGCCCGCTACTGATCCGTTGACCTTCGGA
CAACAAAAAACGCAGCCAGGTGGCTGCGTTTTTTGTGGGTGGTGAGAGGCTCGCGGTGGTTTTTCTAGCT
TTGGGTTGAGCCAGGCCCATTACAACCCCGCCCAGCTTTTCAGGCTCTGCTGCTGCACCCGGCGATTGGC
TTCGCGGATCTCCCCTTTGGCGCGCAGGTCGGCCAGCGCAATGGGCAGTGCGGTGCGGATCTCGTCACTG
CTCATTCCGGCTTTGAGGGCCGGGTGATAGGCCATCTTGAGCAGCACATAGTCCAGCCCGGTCAAAAAGC
TGTCGATGCTGTTGTCATTGAAGATGGAGGGAAACACCTTGTCCGAGTCGTTGGGCAGTCCCATCACCTG
GGTGAACTCCTCCACCACACAGTCGAGAAAGCGCCCCTTGGCTCTGCTGTAGTCTACCGGAATGATGATG
GTGGCACGGCTGATCTCGTGTTTCGCATTGGTTGCGAAGTTGGCAAGGCAAAGCCCCTCCTTGAGCGCCA
TCTTGACCGATGGATCCCCCCCCATGGTGCGGTTGGCCCAGCTCTTCATCTTGCTGTAGTCGGTCATGAT
CAGGGTGAGGTTGGGCTTTGCGGCCACCATGCTGATGGAGTGGCCGGTGATGCGGGCCAGATGGGCTGCC
TGTACCTTCACCACGTCGGCCTGCAGCGGCTTGTCACCCGATTCGTTGATAAGTTGCAGCCGGATCGGCT
GCTGCCAGCGGGCAAAACGGGTCTGCCTGGCTTCGCCATACTCCCGCTCCATGGCGATGGCCATAAAGCT
CTCGACCAGATAGCTGTCGCTCTGCCAGCGCTCATCGGCACGGGCGCTGTGGCCCGCGAGCAGAAGCAGC
AGGGCCAGCAGCCCGCGTGATGCGCGGCGGGGATAGTCAGCGCGGCCCAAGCTGGTCATTACCAAAGTGG
CAATAAAGCGGGTGGCACCAAAGGGGATGGCGGCAAATGACATGTTTCCTCCCTGAAACGGTTGTCAGCC
TAGCGGTTGGCCTTGTCGATATCGCGCCAGGTGAGCCAGAGCCTGAGATCAAACTCCAGTTGATGATAGT
CGGGCTCCATGTGCTGGCAGAGTTGGTAGAAGCTCTTGTTGTGATCTTTCTCTTTGATATGGGCCAGTTC
GTGCACCACGATCATTTTCAGGAACGGGGGCGGTGCCTCCTTGAACAGGGCGGCGATGCGGATCTCGTTC
TTGGCCTTGAGCTTGCTGCCCTGCACCCGGCTGACGTAGGTGTGCAGGCCGAGGGCATCCTTGATCACGT

TGATCTTGCCATCGAACACCACCTTTGATAGGGGCGCCGAGCTCTTGAGGTAGCGGTTCTTCAGCTCCAT
GGTGTAGGCGAGCAACGCCTTGTCGCTCTGGATCTGGTGGCTCTCCGGATAGCGTTTGGCCAGCATCTCC
CCCAGCTTGCCCGCCGCAATCAGCTGCTGTACCTGTGCCTTGAGGGGGTCTGGATAGCCGCCCAGATAAG
TCAGTTCCGCCATGGTGTCACTCGTTACATCAAAAAGAGCGTCATTGTAGTTATCTCGGGCGGGCTTGCC
TACTGCGGGCCGCTACTGCAAGGGGGCTCTGGCGCAAGAAAAAGGGCGGCCATTGCAACAATGGCCGCCC
TGCGGGCGTATTGACGATCAGCGTCGGCGATAGAGACCCGGATCCACCTCTTCATAGTGGCTGGTCAGGT
ATTGATAGAAGGCAAGATGGGAAGGGGAGCTCGGCTTTTTCAGCTCCTCGCTCACCCGATCCGGCTTGGC
GATGATGAACTTCGGGCTCTGGCGCTGGATATCGGCCAGCACCCGCTCGCGCACGTCATAGGGCGGATTG
ACGTTGGCAAAGAAGAGGTAGGCGGACGCTGGCTGGCGATCGGTCCAGAGATAATATTGCGGCTGGATGC
CATCGACCCAGATCTTGTCGCTTGGGACTGTCTCGGCCTTGATGCGATCGACCCGTACCTGCGCCGCCGG
ATTGAAAGGGCGACCCTTGAGGGTGCCTTCCCAGGCGTTGTTGATCATCAGCAGCAGCATGGCAAGCGTT
GCCAGCCAGGGGGTGGCACTGCGCACATCCCAGGCGCGCCAGAGGGGGATGAGGATCAGCACCGGCAGCG
CCCACAACAGGCTGGTGCCGAGCACCTCGTTGACCCGCTCGAGGGACCACTCCTTGGTGCTCCAGCCAAA
GGCGAGTTGCTGGGCCAGCAGCCAGCTGGCGGTGAGCACCACCAGCGAGATGCAGAGCAGGCCGATGGCA
TCAGCCTGCTGTTCGCTGCGATCCAGCAATACCGCCAGCGGCAGCACTGCCAGTTGCAGATAGAGCACGT
CGTAGTGATCGAACACATGGCCGGAGACCGAGTTGGCGATCAGGGTGAACAGCACCCCAAACAGGGTCGC
CAGCAGGGCGGTCAGATACCACTCCTGACGGCGGCTCAGCATCAGAATGATGAAGGCGGGCAGCAGCATG
ATAAGGCCGGTGCGCAGCAGGCCGACGGCGCCCACCATGGGGGAGAAACCATTGCCGTAGCTCCCCTCGA
AGAAGATGTCGAAGGCGTAGTATTTGAAGCCCGCCAGCAGATCATGCTGCTGGAAGTAGGCGTAAAGCGG
GCCGCCAATCAGCGCCAGTCCCAGCAGGCTGGAGATGAGCAGCAAGATGGCGTCACGCCACTTCTCATGC
AGACACCAGTGGCAGAACAGTACCAGATACCAGGCGCCCCAGAAGGCGCCGTTGTTGGTGCGCATCCAGG
CGACGATGGCGAAGGTAAGGGCTGCAACCAGCGCCGGCCACCAGCGAAATCCCTTGTCCAGGAGCAGGGT
AATGAAGGCCCACTGGGCGATGAGGGCGAGGTGGAAGGTCCAGTCCTCGGTCATGTTGCCGTAGTAGTAA
CGGGTGCCGTAGAGAGCCAGCACCGCCAGCATGGCGCCAAGGCGTGCCTTGAACGAGAGTCCGAGCTGGG
CAAAGGCCTGCCAGCTGGCCAGCAATCCGAGCCAGCAGAGCACCCACTCCAGCGCCCAGATCCCGACAAA
ACCCCCCATTCCATAGCCCAGCGCGTCGAGGGCGAAGATCATCGGCCCCTTGATGTCGATCATGTCGCGA
TAGAGCACGCCCCCTTCGCTCCACATCTTGCCCATCACCGCGAACAGCGAGGCATCAAAGCCCGGTGCCA
TGGCGTGGAAGAGCGGGTTGCGCAGACAGAAGAGCAGCGCCAGCAAGGCGGTCAGCAGCACAATGGCTGG
CTTGATACCCGGGTTGACGCGAGGGGGAGTGAAACGGATGGAGTGATTCATGAAGTCGCCTGTTCCTGCA
GCTGGTCTTGAAAGCGGGCCTATCTTGCCGAGCTAGTGAGTGCGATGCAATGGGGCGGGCCCGTGTGGGG
CAAATATTCTCGAAAAAGCCCGGTTCTTGCAGGGAACCGGGTGCTGGCTGCGGCAATTTTGGCCGTCAGG
GGATGTGGCAAAGCCATCCCCCCGATATCAACTGAAGTACTCAACCAGGGGCGGCTGCGCTTCCAGTGTC
GGCAGGTGACGGGCCCAGCGGATAAAGGCGTCGGCCAGGGTGATGCCGAGCTCTTGATTGTGCTGGGTCA
GGGTCCGCGCCAGTGCGGTGTAACCCTCCTTGCCGGAGGCGGTATAGGCGCTCGACACCCCGCGATAGAC
CTGTTCGGCATTGACCGCTTGCCACTGGCCGGGGGCGGCTTCCCACTCCAGTCGGGTGATGCGCTGGCCC
TTGGGCCGATCGGCCTGATAGGTGAATCGCACCCCCGCGGTGTAGGGGAAGCTGCCGTTGCCATTGCCGA
CCACGCCGTTGTTGGTGGCGTTGTCGATGGCATTCTCCAGCGCTTCCGCCAACTCGTGACCGTGCACCCG
ATAGAGGGTGAGGGGAATGGCAAACGGCAGCAGGCGACCGGCGATATCCGCTTCGCTCAGGGGACCCGGC
TCCAGCGAGCAGCGCACGCCGCCAGCATTGTGCAGGGCAAAGTCCACCTCGCCTACCTGTTCACGGGCGG
CGGTTAGCATGGCGCGGGCCACCAGCGGGGCGAGCTGGCTGCCGTGGGGCAGGGTGGCATCTGGCAGCCG
CTGATGGGGCAGTGACGCCGCCAGTCGGGTGACGATGCGCTGGCTCATGGTTTCAATCTCCGGCCGGTAG
CGTTTGGCCAGATGTTGTTCAAGCTGTTGATCCGGCTCGCAGAAGGTGAGCCCGGCGGGTGGGGTGGAGG
CAAATGGCCAGGGCTGCCAAGGGAGCCACTCCAGCTGTCCCTGACTTTGCACGACGCGGCCCGACTCATC
GAAGGTGAGCTCGCAGACACCGAGGCAGAGGGCGCTGTGGCCAGCGTGCAGGATAGCCACCCCGTTGTTC
ACCAGCGGATAGCTGCCTTCGCTTGGCAGACCCAGCGGCGCGAGATCGCCGAGCAGGCTGTGGGTATGGC
CACCGATGATGAGGCTGAGCTCGGGGAAGCGCTCGGCCAGCAGCTTGTCCTGATCCAGCCCGAGGTGGCT
CAGCAGCACGATATGCTGGATCCCTTCGCGCTTGATCTCCCGCAGCACCCGGGTGAGGGTGTCGGCGACC
GCGTGGAAACGGGTGTGGGGATCCGGGTTGGCGATGGCAGCCATCTGGGCGAGGGTGATGCCGAGCAGCG
CAAAGGGAACCCCGTCGATCACCTGACGATGCCAGGGCTGATGGGCGTCGTAGAGATGGGGAATATCCCA
CAACCCGAGGGTTGTGTCGGCAGGCTCCTGACTGTTATCCAGATTGGCGGCCAGCACAGGAAAATCGAGC
TGGCGCAAGAACTCCACCAACGGGCCATTGCCCAGATCGAACTCATGGTTGCCAAGTACCATGGCATCGG
GGGCCAGCATGGCGAGCAGGTCGGCGTTGGCGCGCCCTTTGAAGCGGTTGAAATAGAGAGAGCCCTGAAA
GGAGTCGCCACCGTGCAGAAACAGAAAACGCTGGCCGGCTGCTGCGGCTTTGGCACGCAGTTGGGTCAGT
CGGGTCAGGATCCGGGCATAACCGCCGCACTGGGTGCGCCATTGGCCTTCACCGACAGGGGAGAAGCTCA
TGGGCGCGCCATCGAAGTGGGAGTGGCAATCACTGAAATGGGCAAGTTGAACCGTAATACTCATCTCGGT
CCTCCTTAGGGGAAAATGAGCACCGAGTCTACTGCAAAGGGGGGGGAGGATACCAGCCCGGCAGGGTGCC
GGGCTGGTCAGCGATCAGGCGGGAACCATGATCTGGGTGGCGACGATCACCACCAGCAGGCCGACGGCTA
CCGGCATACTGGTGCGCTTGACCACTTCGAACGGCGAGAGCTTGCCCATCCCCGCACAGGCGACCACCAC
CCCAGAGACCGGAGAAATGGTGCGACCCAGGTTAGAGGCTTGCAGCATCGGGATTACCAGATAGGCCGGG
TTGATGCCAAGGCTGCTGGCCAGGTTGGGGATCAGCTCTACGAAGGCGTAGAAGGGGGCATTGCCTGAGC
CCGTGGTGAAGGCGGCCAGGGTGGTGATCACCACCAGCGACAGCATCATGATCAGGCCGCCGGAGCCGAG

ATCCTGCGCGTGAGCCAGCAGGGTGTCGATAAAGCCGATGGTCATCAGGCCCTGGGCAAAGACACCGGCA
CCGACCAACAGCATGACCACACCGGCAAAGGCGTCAGCCATGTTGCGCCAGCAGACCTGCAGACCGTCCA
GCAGCTCCTTGCCATTGCGGATCCGCACCAGCTCCAGCAGGCTGGCCAACACCATGCACATGACGATGAT
GGTGACGATATCCAGTTGTGGGCCCCATTTACCGTCAAACACCAGCACCCCAATGATGGGGGTAAAGGGC
AGGATGGCGTAGAAAGCGGGGGCGGTAGTCACCAGATCACCCGGTGCGACCGGCTCGTTGTGTTCACCGG
ATTTCTGGTCAAGGTAGCGCTGCCAGAAGAAGTGGGTCACCGCCATACAGGCGATCGCCACCAGCGAGAT
GGGCAGGGTCAGCTTGAAGGCAAAGTCCAGCAGCGGCATGTTGGCGGCCTGAGCGGCCATCACCACGTCA
CCCGAGGTAGGTGCCAGAATGATGGCGGCTGGCGAGGCGCACACGGCCGCAGCCGCGCCACGGGAGATCC
CCATGTTGACCATCAGCGGGAACAGGGTGGCCATCAGCAGCACGCCGAGGCCGGTAGCGGAGCTCACCGC
CAGCGACATGGCGCAGGCCACCAGATAGGCAGCCACCAGCAGGATATAGGGGGACTTGATGATGCCGAGC
GGTTTGCTGGCCAACTTGACCAGAATGGTGTTGGCGCCGATATGGCTCATGTAGCCGGCGAAGCCGCACA
GCACCATGATCAGCATCCCCAGTCCACCGCCACGGTCATTGAGCAGGAAGCGGACGTATTCGAAGATATC
GGTTGCAATGTAACCGGTGCTGACCACCTTGCTCGGCAGTACCGGCTTGCCCATCATGGCGGTGATGACC
AGCAGCAGCAAGCCGCCGCTCAGCAGCACCCCGGTCGCGGAGTATCCTTTGAAAATGGCACGGCCCACCA
CCAGGGTGACCAGGGTGCCAACGATGAGTTCCAGCATATGTTGCCTTCTGATTGGATGAGAGTGAAGGGA
GTCTATTCCGATAGGGGTAGAGTGGCGACTGGAAACAGGATGTTGTGTGAGCTAAATCAACGTTTAACCC
CGTGAACAGAATTAAAGAAAGGTAAAGGTACTTTACCCAGCGGGTTTGGCTACCTACCCTTGCCAGCCTT
GGTTGTCTTTTGAGGCTTTTATATGGCGATTGGTGATTCTGTAGGCATTAAACGCTCTCTTTTCCTGCTT
GCGATGCTATTTTCACCTACTTGCGTGTTTGCGGTAACCAATGACGGTTTTCATCAAACCGGTCCTCATC
AGGCGGTCGCCATGCGGCGTCTCATGGCTCCTCACTCTTATGGGGTACTGGTGGTTGCCGAGGGGAACAG
ACCCCGTTTTGCCGCGCTGGCATCCAAAACCACCGAAGCAAACTGCCTGGCCCGTCATGCCATCCGGGTA
GATGGGGTCGCGCTGCTGGTCACCCCGAGATTCTATGCCAAAGAGGGAAAACGGGGACTTTGCGAGCTGT
GGCTCAATGAGGGGGCAGATCAGGACTTTTTTGCCAACCGCCTTAAAAATGATCATTTTATTCAGATAGA
TGGTCATGACATCAATGTGAAAAACTATCACCTCGACTGGCAGCGCATCAGCCGTCATGCTCAATAAAAA
TACAGTTTTGGCCTTGTGCCATACTCTGCTCACGCAACCTGTGAGTGTGCTCTGATTTCTGGGCGCATCA
TTGTATTGCGCCGCCTCGTCGTCACACATAGGCTATACCCGTCATCTGTGTGGCGGTGGGTTTCACCAAG
GTTGGTACTCCGCCCGTGCAATCAAGGAAGGTAGTCTGGCATGGTCAGCAAGGTTTATGGAGTAGTGGGG
GTCGGGTTGTTGCTGTTGCCCCTGATGAGTCAGGCTCAGCCGAGCCGGCCGCAAGTGGTGCCAGAGTCCC
GCATCAATGTCCGTTTTTATGAGGTCAATGCAACCAGTCCGGAGCAGCTTGCCCGGGCGATTGCCCAGAG
TGCTCCCAAGGTGAATGGCAAGCCCGTACTCGGCAAGGCGAGTTATCAGGTGGACTGGCAGCTGGATACC
GAGCAGTTGCCGGGGCGCTGCCAGCTCAACCGGGTGACGGTGACCACCAACGCCACCGTGCTGCTGCCCC
GCTGGCGTCAGAGCGCGAACAGTAACGATCCTGCCCGCCAGCGCTGGGGGCGGCTGCTCTCCAGCATGTT
TGACTACGAATCCCGCTTCAAGGAGATGGTGCTGCAAGGGGCCAATCAGGTGGGTGGCGCTATTGCTGGG
CTACCCGGACATGCAGATTGCCAGGCGCTGCGCTATGCCGCATGGGCAGCAGGCAGTGGCGAGCTGAACA
AAATCAAGGGACGGCTCGACGGATTCCAGCGTCAGACAGGTTACGGCGAACAGCTCGGGGTGCACTGGCC
CAAGTGAGCGCTATCTCAAGACGCAATAGCCAGCATCGCACGATTTGATAAATAGAATGGCCGCCCGATG
GGCGGCCATTCTATTTGCTTGCAGAGTCCGGTTCAGACCAGCGCTTTGCCCTTGAGCAGCTTGCGTACCC
AATAACGGGCCGGGTGGAGCGCTTCCAGCAGATCGGCGGCCAGCGGCAGGGGCTCGCCGCAGATCTCGCT
GGCGAGCAGCTCACCACAGAGCGGCGCCGAGCAGAGGCCCCGTGAGCCGAGTGCCCCCAGCATATAGAGG
CCGGGGTGAAGGGGGTGCGGCTGGGCGTTGTGCTGATCATGCTGCAGCTGCGCATAGTGATCCGCCAGAC
CTTGCAATCTGGCCACTGGCCCCACCACCGGCAGGTGATCCCGACTGGCGCAACGCACCCCTACGCGCGC
CTTGTTGCCACTCACATCCACCTCGGCGGGCCAAGTTTGCTCCGGCAGGCAGGCTTGCAACCGCACCTGA
TTCTGGGTTTGCTCCTCGGCACGAAACTCAAGGCTGCTCTGGTTGCGGCCATAGCTGGCACCGATGCAGT
GGTGATCGTTGTGCATTGGCGTGAGGTAGCCATCGTAGCAGAGCACGGTATTGAGCCGGCTCAGGCTGGC
TGAGGTCGGCACATGGCTCACCTGACCGCGCACCGGATAGAGCGGCAGTTCGGCAAAGGGGAGTAGTGCT
GGCAACTGGTGACCGGCAGCGACCACCAGATTGGGGGCCTGCCAGTGGCGGCCATCCTGGCTTTCTACTT
GCCAGCCATCGGCCTGCTCGGCAATGGCGCTTACTTGGGTGTGGTACTCCACCTGCAGCAGACCACTGGC
TTGTGCTTCTTTGATTGCAGCGCGGGTGAGATCCGCAGGGCAGAGCCAGCCCCCCAGCGGGTAACTGACG
CCGCTGTGGCCGCAGGGCAGGCCAACCACCTGCTCTACCTCTGCTGTAGAGAGGGGATGCATCAGTTCGG
ACGGGAAGGGGCCCTGACTCATCTTGGCCAGCTTGGCTGCCGATTTGTCGTCATAACCGAGCTGGGTTAC
CCCGCACAGCGCAAAAGCGATGGGATGACGCTCGGCCAACGCCAGCAGCCGTTGGCGGGCATAGCCAAAG
GCGAGCGAATAGAAGCGTGACAGGGCATCGTGCTCGCCGTTAAGCAGTGGATAGAGGGCTCCTTGGCGGT
TGCCCGAGGCGCCGATGGCCGGTTCGCCATCTGCACACAGCAACGTCACCTTGCGACCCCGCTCCACCAG
AGAGAGTGCCGTCATAGCCGAGGCGATGCCGCCGCCGATGATCACTACCTCGCCAGCGCGTCCGGCAGGA
CGGGCATACCAGGGGGCGATGGTCTGCTGCGGCTGCTTGTCGGTGCGATCCCCCACCAGCATCTCCCGCT
TGCTACCGTGGCCCTTCACCTTCTTCATGGCAAAACCGGCGGCAATCAGGCCGCGACGGACAAAACCGGC
ACAGGTAAAGGTGGCGATGGAGCAATCGGGACGGGCGAGACGAGCGAGACCATCAAACAGCTCCTGTGTC
CACATCTCCGGGTTCTTGGCCGGGGAGAAACCATCCAGATACCAAGCATCCACCAGCCCCTTTGCCCGGT
GAGGCACCTGCGGCAGCATATCCTTGATGTCGCCAAACCAGAGATCGAGGCGAATGCGGCCACCCGCAAA
ATGCAGGCGGTGGCAACCGGAGACCGGCAGCGGCCATTGGGCGACAAGATCCTGACTCAAGTGGCCAAGT
TCAGGCCAGGCGGCCAGCGCCTTGCGCAGATCGTCCCGGGTCAGAGGGTATTTCTCGAAACTGATGAAGT

GCAGACGGGAGCCGTTGCCGGATTGAGGCGCCTGTTCCAGGAAGGCTTGCATTGTCGCCAGAAAGTTAAG
GCCTGTGCCGAAACCTGTTTCACCTATCACGAAACTGTCACTATCGTGGTGCGAAAATCGCGCAGGTAGC
CGGTTTTGCTGCAAAAAGACGTAGCGGGTTTCACTTAGACCGTTATCATTGGAAAAGTACACATCCCCGA
AGTCACTGGAGACAGGAGTTCCCGCTTCATTCCAGTCCAATCGGGCATGATGTAAGGATGTTTGACTCAC
GTTTTCCTCGGCTCGCGTCACAAAATCGCGGGCATTCTACGTGAAAGCAGACCAGTTTGGCAGCCGAAAG
CGGGTAGACTTGGCGCCAGTTTTTCCAGACGAGATGAATTAATGAGAAGAGCAGTGATCACCGGTATCGG
CGTCATCTCCAGTATCGGCAACAACAAGGAAGAAGTGCTGGCCTCCCTGAAAGCAGGTAAATCCGGCATC
ACCTATTCCGAGCAGTTCGAGCAGCACAACCTGCGCAGCCGTGTCTGGGGTAACATCAAACTCGATCCGT
CTGAACTGATCGACCGTAAAGTCATGCGTTTCATGGGCGACGCTGCGGCCTACGCCTACCTCTCCATGCA
GCAGGCCATTGAAGATGCCGGTCTGGCGGAAGATCAGGTCTCCAACGAGCGTACCGGCCTGGTGGTCGGT
TCAGGTGGTGCCTCCGGCAAGAACACCTCGGAATCTGTGGACATCGCCCGTGAGAAAGGGGTCAAGCGTG
TGGGCCCTTACATGGTGCCGCGCACCATGTCTTCCACCACCTCTGCTTGCCTCGCGACCCCGTTCCAGAT
CAAGGGGGTCAACTACTCCATCAGCTCCGCCTGTGCCACCTCTGCCCACTGCATCGGTCACGCGCTTGAA
CTGATCCAGCTTGGCAAACAGGATGTGGTCTTCGCCGGTGGCGGTGAAGAGCTGGACTGGTCCTCTACCA
TCCAGTTTGATGCCATGGGCGCCCTCTCCACCAAGTACAACGACACGCCGGAAAAAGCGTCCCGTACCTA
TGATGCGAACCGCGACGGTTTCGTTATCTCCGGTGGCGGCGGCATTCTGGTGATCGAAGAGCTGGAACAT
GCGCTGGCCCGTGGCGCTCACATCTATGCCGAGCTGACCGGTTACGGTGCTACCTCCGATGGCTACGACA
TGGTCGCGCCGAGCGGTGAAGGTGCGGTGCGTTGCATGAAGATGGCGATGCAGGGTGTTGGCAAGGTTGA
TTACATCAACACCCACGGCACCTCCACTCCGGTGGGTGATACCAAAGAGCTGGAAGCTATCCAGACTCTG
TTTGGCGCCAACGGTCCCAAGATCTCTGCCACCAAGGCGATGACCGGCCATGCGCTGGGCGCTGCGGGCG
TGCACGAGGCTGTCTACTCCCTGCTGATGATGGAACACGGCTTTATCGCCCCCAGCATCAACATCGAGAC
CCTGGACGAGAAAGCCGAAGGCCTGCCCATCGTGCGCGAGTACGAAGCGGCCGAACTCAACACCGTCATG
TCCAACAGCTTCGGCTTTGGCGGTACCAACGCCTCCCTGGTGTTCAGCAAGTTCAAAGCTTGATCATCCG
GTTGATATGAAAAAAGCGCCTCAGGGCGCTTTTTTTGTCTTCGGGATCTGCCATCACTCATTGCAGAGCA
GAGCAGAGCAGAGCAGAGCAGAGCAGAGCAGAGCAGAGCAGAGCAGAGCAGAGCAGAGCAGAGCAGAGCA
GAGCAGAGCAGAGCAGAGCAGAGCATCGACCATTGTGGAATAGAGGGAGTGGGTTGGTGGCAGATTTCAG
CGCAGATGGGGGGCACCACACCAATGAGCCTGCTCCGCACTTGGGAGAAAGGCAGATAGCAAGAAGGGAG
CCTGAGGCTCCCTTCTTTGTTAGCGGTAATGTGATAGCTGCTGGCTAGAACCAGAAGCCGATGATAAAGG
AGCCCGCAAGGGTCAGCATCACGTTGGCCACCGCATAGGTACCGGCATAGCCGAGCGCCGGAATGGAGCT
GTTGGCTGCCTCGTTGACCACATCCATCGCCGGGGCACAGGTACGGGCACCTGTGATGGCACCGAGCAGC
AGCGCCGGGTTCATCTTGAGCACCCAGACCCCGAACAGATAGCCAACCAGCACCGGCAAGGTGGTCACCA
GCATGCCGGAGAAGAGCACTACCGTACCCACCTCGCTGAGGTGGTCGAGGATGCCGCCACCGGCTTTCAG
ACCCGTACTCACCATAAAGACCGCAAGACCGAGATCCTTGGCCAATCTGAGCGCACCGGGCGGCACATAG
CCGACCGTCGGGTGGTTGGCCCGCAGATACCCCATCAGAATACCAGCCAACAGCAGGCCGACGGCGTTGC
CGAGACCGAATTCGATCTGACCGAACACCAGCGAAACGGAGCCGATCAGCAGACCGAGTACGAAGAAGGT
GGTGAAAGCCACCAGATCTGTGGTCTGGCTGTGGATGCTGATAAAGCCGATCTTGTTGGCCAGCAGTTTG
ACCCGCTGCTTCTCGCCGCTGATCTGCAGTACGTCGCCTTTTTGCAGCATGATGTTGCGATCGAACGGCA
TCTCGATCTGGGAGCGCACCACCCGGTTGAGGAAGCACCCTTTCTCTGTGAGGTTGAGCTCCACCAGATG
GCGGCCTACCACGGAGTCATTCTTTACCACTATCTCTTCGGTGACGATCTGCAGGTCAAGCAGGTTGCGG
TCGAACACCTCTTTACCGTTGCGGTAGTTTACATCCAGCTTTTCGTGGCTCTCGGGATAGCCCACCAGGG
CGATCTCATCCCCTTCCTGAATGACGGCATCGCCATCGGGACTGGCCAGAATGCCGTTGCGGCGGATCCG
CTCTACATAGCAGCCGGTATGGGGATAGATGCCGGTCTCCCGCAGGGTACGGCCACCGATCCAGGCTGCC
AGTTCGGGGCCCACCCGATAGGCGCGGATGATGGGCAGATAGGTCTTGCGACTCTCGCTGTCGGAGAGAC
CCCGCTCGCGGGCGATTTTTTGTGCCTCGGTATTGAGGTCGAGGCGAGCCAGCGAGGGGAGGTAGCGCAC
CACCAGCATCAGACCCACCAGACCCACCAGATAGGTCAGGGCGTAGCCGATGCCCATGTTGTCTAGCACC
GCCTGCATGTCGGTCTGATGGGGCAGGTTGAGCAGACCGCTGCGCAAGGCATCCTGGGCACCCACCAGCG
CCGGGGTCGAGGTGAGGGAACCCGCCAGGATCCCCGCCGCCATGCCCGGCCCCAGATTGAAGAACTTGGC
CAGCCCCACAGTCAGCAGCAGGGCGGTCAGCAAGATCACCAAGGTCAGGGTGATGTAGTGGATGCCGTCG
CGCAGGAAGACGCTGAAGAAGTGAGGGCCCGCCTCGATCCCCACACAGAAGATGAACAGCATAAAGCCGA
CGTTTTCAGTGGTGGCTGCAAATTCGAAACCCATCTGGCCGAACAGCAGGGCGGTAAACAGCACACCTAT
GGTGTTGCCAATCTGAAAGTTGCCAATTCGAACCTTGCCAAGCAGCAGGCCGAAGGCCAATACCACGAAC
AGCAGCAGGGAGTCGCTTTGATGTAGCAGTGTCACGAAATCTATGGTCATGCCCGGAAAAACCGAGGAAA
AAAGTGGGGTCGATATTGTGCCGGATCTATCCCCAATAAAGAAGCGCAAGGGATCGATTAATCGGACGTT
ATCGGACGCTATTTGTGGAACCGGCGGCGTGATAGGGAGACAAGCGGATGAAATCTGGCAATTTTTGCTG
TAACCGTCGTTGTCAATTAGTGAAATCTGCTGCCCGCGACCGAAGTGCTCTGGCGCAGGCCGATATTTTA
GATTCTGCTGTCCGGCCGCTTGGCGGGGATCAGTTTCAGCAGATTCAACCGATGGCCGCCATGGTTGGCT
GAGGGTCAAATCAGCTTGATATTTGCCCGTTCGCCCACAGGGAAAGGGTTGGCAAAAAGATACCTCTGAA
AAGTGCCAATAGAGCCTGTCACCCCGCTGTTCCTGAGGGGGCAAAAAAACAGCATAAAATCAGGTTGGCC
AGCAAAATGACTCTTCGCCGGGGGCCATCAAGGGCAATCCCGCGTCCTGACAGGGCTTGCTGCACTTACC
CACAGAATCTGTGGATAACCCTGTTTATGAAACTTGTTCATCGCCCTGAAAGCCGCGCCAGTACTGGGAT
GCAAGGGCTGTTCAAAAAATACCGGAAGAAAAATGTTTGTATAAAAATCAAATAGTTAGGTGCAACAGGA

GAAAACTCTGTCGAGCCGGAACGGGTAACAAAAGGGAGCGTCAGCAGCAAGAAGGATCTTGTGCATTAAA
ATTGGCAGGAGAGGGCACTAAAGTGGATAACTTGCACCGAGATGGTGAGGAATTGGCTCCCCCGACTGGA
CTTGAACCAGTGACATACGGATTAACAGTCCGCCGTTCTACCGACTGAACTACGGGGGAACATCAGGCAA
GGAGGAGCATCATACAGAAGGTGTTCGAGGTGGTCAAGCCATTCGCGGGATGATGGCACGGGTGTACCGC
TGCCAAACCCTCGGTTACACTGCTCCTCTGCTGTTTTGTGTCGGGAGTGTTCATGAGCAAATTGTTCAAG
TTAGCCAGCCAGTTTAAACCCGCCGGGGATCAGCCCACGGCGATTGCCCAACTGCTGGATGGCATCGAGT
CGGGGCTGGCCCACCAGACCCTGCTCGGGGTGACCGGTTCGGGCAAGACCTTCACCATGGCCAACGTCAT
CGCCACCCTGAACCGCCCGACCATGATCTTGGCTCCCAACAAGACCCTGGCCGCCCAGCTCTATGGCGAG
ATGAAGGAGTTCTTCCCCGACAATGCGGTGGAGTATTTCGTCTCCTACTACGACTACTACCAGCCCGAAG
CCTATGTGCCGACCACCGACACCTTTATAGAGAAGGATGCCTCGATCAACGATCACATCGAGCAGATGCG
ATTGTCGGCCACCAAGGCACTGCTGGAGCGGCGCGATGTGGTGATCGTCGCCTCGGTGTCGGCCATCTAT
GGTCTGGGGGATCCCCAAGCTTATCTGAGCATGATGCTGCATCTCAAAGTGGGGGATGTGATCAACCAGC
GGGATATCCTGCGCCGTCTCGCCGAGCTGCAATACACCCGTAACGACATGGCGTTTCAACGCGGCACCTT
CCGGGTGCGCGGTGAAGTGATCGACATCTACCCTGCCGAATCGGACAAGTTGGCCCTGCGGGTGGAGCTG
TTTGACGAGGAGGTGGAGCGCCTCTCCCAGTTCGATCCCCTGACCGGCGCCATCGAGCAGACGGTAGTGC
GCTATACAATTTACCCCAAGACCCACTACGCCACCCCGCGCGAGACCATTCTGGGGGCTATCGAGCATAT
CAAGGAGGAGCTGCGCAGTCGCCGCGAGCAACTGTTGTCTCTCAACAAGTTGGTGGAGGAGCAGCGCATC
AGCCAGCGCACCCAGTTCGACATGGAGATGATGCAGGAGCTGGGTTACTGCTCCGGCATAGAGAACTACA
GCCGCTACCTTTCCGGTCGTGCGCCGGGGGAGCCGCCGCCCACCCTGTTTGACTACCTGCCGGGGGATGG
CTTGCTCATCATCGATGAGTCCCACGTCACGGTGCCGCAGATCGGCGCCATGTTCAAAGGGGACCGCTCC
CGCAAGGAGACTCTGGTGGAGTACGGCTTCCGGCTCCCCTCGGCGCTGGACAACCGGCCACTCAAGTTCG
ACGAATTTGAAGCGCTGATGCCGCAGACGGTGTTTGTCTCGGCGACTCCCGGCCCCTACGAGCTGGAGAA
GTCCGGCGGCGATGTGGTGCAGCAGGTGGTGCGCCCAACCGGCCTGCTCGATCCGGAGATCGAGGTGCGT
CCTGTGACTACTCAGGTGGACGATCTGCTCTCCGAGATCCGCAAGCGGGTCGCCGTCGAGGAACGAGTGC
TGGTTACTACCCTGACCAAGCGGATGGCGGAAGACTTGACCGAGTATCTGGCGGAGCACGACGTCAAGGT
GCGCTATCTCCACTCCGATATTGATACGGTCGAACGGGTGGAGATCATCCGCGATCTGCGCCTTGGCAAG
TTCGATGTGCTGGTGGGCATCAACCTGCTGCGGGAAGGTCTCGACATGCCGGAAGTGTCGCTGGTGGCGA
TTCTCGATGCAGATAAAGAGGGCTTCCTGCGCTCCACTCGTTCGCTGATCCAGACCATCGGCCGTGCGGC
GCGTAACCTCAACGGCAAGGTCATTCTCTACGGCGATACCATCACCAACTCGATGAAGGTAGCCATTGAG
GAGACCGAGCGGCGCCGCGCCTTGCAGCATGCCCATAACCTCAAGCATGGGATCACCCCTAAAGGGCTCA
ACAAATCCATTGGCGATGTGATGGACATGGGGGGCAGCCGCACGGCAGGCAAACCAGGCAAGGGGATGCG
CAAGGCAGCCGAGCCGCAAGGGGAATACCATGTCCGCTCAGCCAGCGAGATTGCCAAAGAGATCAAGCGG
ATGGAGGAGCAGATGTTCCAGCATGCCCGGGATCTGGAGTTCGAGCAGGCGGCGGCACTGCGGGATCAGA
TCCAGCGGCTGCGCAGCGAGTTGATCGAATCATGAGCCGTAAACGCTGGCTGATTTGGCAACGACTTCGT
CGTCTCTATCAGGGCTTTCGCCATCCCTGTACCCCCTGGTGGGCGAGGGGCCTGGTGATCCTTATCCTGC
TCTATGGTATCAGTCCGGTGGATCTTATCCCTGACGTGGTGCCCTTGCTGGGGTGGTTCGATGATGTGAC
CCTGCTGCTGGTGTTGCTATGGGGCTGGGAGCGTTGTCTTCCCCCCGCAGTGCGCAGCGATCTGGAACAG
GCCTCCTAGTTCACCTTTTCAACACCACGCTCGACATCACTCTGTGCGCTCCCACCCTCGAAGGAATCGG
AAAAACGGTTTCATTTGAAGGTGGGCGCTGCGGGATGAAAAAGAGGCAGGCTCGGTGATTGTCGCCCGGA
TGGCATTGGCGTAGACTCCACGCCGTTCACTTTATGGGTATGGAAAAAATGGATCTGATCCTCTTTGCAA
TCGACTTTATTCTGCACGTTGACGTGCATTTGAAAGAGCTGTTCGAGAACTATGGCGTCTGGGTTTACGC
CATCCTGTTTCTGATCATCTTCTGTGAAACCGGGTTGGTAGTGACGCCGTTCCTGCCGGGTGACTCCCTG
CTGTTTGCCGCCGGTGCGCTTACCGTGGGCAGCGTGCTGGACGTCCATACCCTGGCGGCCGTGCTGATCA
TCGCTGCCGTGCTGGGCAACGTGGTCAACTACACCATTGGTCACTTCTTCGGCGAGCAGCTGTTCCGTAA
CCCGGACTCCAAAATCTTCCGCCGGGATTATCTGGAGAAGACCCATGCCTTCTATGCCAAGCATGGTGGC
AAGACCATCATCATCACCCGCTTCCTGCCAATTGTGCGTACCTTTGCGCCGTTCGTGGCAGGGATGGGGG
CCATGACCTATCCGCGCTTTTTGGCGTTCAACCTGGTTGGCGGCCTGCTGTGGGTACTCTCCTTCGTATA
TGCCGGCCACTTCTTTGGCAATCTGCCGGTGGTGCGTCACAACTTTACCCTGCTGATCTTCGGCATCATC
GGTATCTCCCTGTTGCCCATGGTGATTGGCGCGGTCAAGGCCAAGATGGGCCCTGCCAGGGCGTAATCAG
AAATACGGTCAGCCAGAACGAGAAAGGGGGGAGCCATCATGGCTCCCCCCTTTTTATCTCCACATGGCCA
TGACACTCAGTTACAGCAACGCCCTTTCACCAGATTGCCGATGGCCACTACGGTGAAGACCAACAGGTAA
GCGGCGAAGATGACAACCAGCCACTGGGGCATGGAGTAACCGAAGAACTGCCAGGTGATCTGGGCACAAT
CCCCTTCCGGCATAAAGAGCCAGGGGAGCCACTGATCGAGCGGAGCCCAGCTCGGGAAGTCGGCAAACGG
GCTGCAGACGTTGAACGGGGAGGGGTTAACCTGATAGTCCACATGCTTGAGGGCCAGCTGGAAGCCACGG
AAGGCACTGTAGCCCCACAGCAACATGGCACTCCAGCGAACATACCACTTATCCGGCGCCACCATGCCGA
GCAGACCAGCGGCCAACACACCAAGGGTCGCCAACCGTTCGTAAACACACATCACGCACGGGGAGAGGCC
CAGCACATGCTGGAAGAAGAGGGCGCACAACTCGAGGAACAGGGCTGATGCTGCCAACAGGCCCCAAGCC
AGCCGATGGGCGGCAATACGGCGGAGAAACTCGATCATCTCATTGATCTCTCAGAAAAAACAGTGGCTGA
ATTAGAGCAGAGCGGGGCAGAAAAGAAAAGGGCCCTGAAAGGGCCCTCGGAAACCGTCGCAACGGATTAA
TGCAGTGCCGGTTGTACCGCTGGCAGCGCACTCTCCATCACCAGCCATCCGTGCTGATAGAACCACTCGG
TGGCAGGACCAAGCAGCAATTCGACAGAGAAAAAGCCGATCAGCCCCAGCACCAGGGTGTAGGGCAGCGC

CATCCAGACCATGCGGCCATAGGAGAGACGCAGCAGCGGCGCCAATGCCGAGGTGAGCATAAACAGGAAG
GCGGCCTGACCATTGGGAGTGGCGACCGACGGCAGATTGGTACCTGTGTTGATGGCTACCGCCAGCAGAT
CGAACTGGGCGCGGTCGATGGCACCATTGAGCATGGCCGTCTTCACCTCATTGATGTAGACGGTGCCGAC
AAAGACGTTGTCACTGACCATGGAGAGCACCCCGTTGGCCAAATAGAAGAGGGCCAGCTGGTTCTCCGGT
TTGGCTGCCAGTACCAGCTGGATCACCGGCTTGAACAGCTGCTGATCGATGATGACGGCGACCACGCTGA
AGAAGACCGCCAGCAGGGCGGTGAAGGGGAGTGCCTCCTGGAAGGCTTTACCCAGCGAGTGCTCCTCGGT
GATCCCGGTCAGGGAGGTGGCCAGCACGATGACCGAGAGGCCAATCAGACCAACGGCAGCCAGGTGCATG
GCCAGACCGACGATCAGCCAGACCGCAATCAATGCCTGAACGATCAGCTTCGCCTTGTCTTGCTGGCTGC
GTTTTGCCTCTTCGTAGCGGTTGTACTCCTCCATGATGGCGCGCACCGTGTCCGGCAGGCGGGCACCATA
GCCAAACCAGCGGAACTGCTCCACCAGCACGCAGGTCAGCAGACCGCAGATAAAGACGGGGACGGTCACG
GGGGACATACGAATGGCAAATTCGGCGAAGTTCCAGCTCGCTTGCTCGCCGATGATGAGGTTTTGCGGCT
CGCCCACCAGGGTGCAGACCCCACCCAGCGCGGTGCCGATGGCGGCATGCATCATCAGGCTGCGCAGGAA
GGCGCGAAAATCATCGAGATCCTGGCGATTGAGCTCGGCGACTTCCTTATCATCGGTATGGTCATGGCTG
TGACCAAACTCCTTGCCGGACGAGACCTTGTGATAGATAGCGTAAAAGCCGGTGGCGACGCTGATCACCA
CGGCAATCACGGTCAGGGCATCGAGAAAAGCCGACAGGAAGGCGGACACCAGACAGAAGGAGAGGGAGAG
CAGGATCTTGGAGCGTACGCCGATCAGCAGCTTGGTGAAGACATAGAGCAGCAGCTGCTTCATGAAATAG
ATGCCTGCCACCATGAACACCAGCAACAACAGCACTTCGATATTGGCGACCAGCTCGTGTTTGACCTGAT
CGGCGGAGGTCATGCCGATCAACACGGCTTCGATCGCGAGCAGACCACCCGGTTGCAGGGGGTAGCACTT
GAGGGCCATGGCCAGGGTGAAGATAAACTCAACGACCAGTAACCAGCCCGCAATGAAAGGATCAAGGTGG
AAGGCAATCGGATTGATCACCAGAAATAGCAAGATGGCGCTTTTATACCACTGTGGTGCATTGCCCAAGA
AGTTCTTGGCGAATGCCTGCCCTAGGCTTATTGGCATGATAATGGGTCTCAACTTTGTTGTTAGGATCTG
CTTCTATGCAGTCAAGGATGATTCGACCTGTGCCTCCCGATGTTGGCCTTTATCTGTCTGATTCCTCGTG
ATTTTAATCAAAAAGCGTGACGATTATAAATGATAGCCGATGGATCTCTGCCCATGTGTCTGTTTTATAA
CCACCTTGTCGGGGTTGGCTGGCTCCCTCGTGGCGATTAGATACATATTCCCAACACTATATTTGCCATA
TGTGAGCAGAGGACTGACCTTATGCTTGCCAGCCATCATCGCTTTGATATCATTCGTCAGCCTTTTTACG
CATTACAACAGAGTCAATTCATGGTTATAAAAGCGCAGAGCCCAGCTGGGTTTGCCGAGGAATACATCAT
CGAGTCCATTTGGAACAATCGCTTTCCTCCCGGATCTATTCTGCCCGCCGAGCGTGAATTATCCGAGTTG
ATCGGGGTGACGCGGACGACCTTGCGCGAAGTGCTGCAGCGACTGGCCCGTGATGGCTGGTTGACCATAC
AGCATGGCAAGCCGACCAAGGTGAACAATTTCTGGGAAACCTCCGGCCTCAATATTCTGGAGACGTTGGC
CCGTCTGGATCAGGACAAGGTACCTGATCTGATAGCTCAGCTGCTCTCCGCCCGTACCCATATCTGCACC
ATCTTTATCCGCGGGGCGATCCGTCACAATCCGGAACAGGCCGCCGAGATCCTGCGTGGCGCCGATGGGG
TCGAGAACAACGCCCAGGCTTATGCCGAGTTCGATTATCGTCTGCACCACCAGCTGGCGTTTGCTTCCGG
CAATCCGATCTATGCTTTGATCCTGAACGGTTTCAAAGGGTTGTACAGCCGGGTTGGCAGCTACTACTTC
TCCGACAAGCAGGCCCGCGAGACCGCAGATGCCTACTACAAGACCCTGCTCAATCTGGCGGAGACCAAGA
ACCACGAAGCCGTGTTCATGACGGTGCGTCAGTACGGTATCGAGTCCGGCAAGCTGTGGACTCGCCTGCG
TCAGGACATGCCAAGCGATCTGTGCGACAACTGATCCCTTGCCTGTGCTGTCCAAGAAACGCGCCATTCG
GCGCGTTTTTTTATTCTGCCGTGTTTGTGTCTATCCTTGACTGGTGGTGGTAATTGTTTGAACTAAATGG
ATTTTTGGCCAAAAAGCTTGATCCAGCGCAAATGAACCCTGCATTTATCACTTTGGTGGCATAGGACCCA
ACCGCTGATTGAGGTCAAATGGAGGCATCAAGAGCGGAGCCAATATCCGCCAGAAGGAGCACACCTTTCA
CCGGATGAAGGACACAACAATGAGTCTTCTTGAACACACCATCTGGACAGTACTGGGATATGGGGCCATG
CCCTTTATCTTTCTGAGCGGATTCGTGGCGGTGGCAGTGACCTGTTGCATGCTGCTGAATGCGTTCGGGG
TACAGTCCGCCGAGAAGTGAGTCGCTTCTCCATGCCGCCAAATGCCAGTCTTCGGACTGGCATTTGTTTT
TTGTGTCTTTTAAAGCACTTATCCCGGCAGGGAAGGGGAAATCTTTGGTCAGGATGGTCGAACCTGCCGC
CGATTTTGATTACACTGGCGCCCGTATTTCCCACTCACCCTTCTCTTATCAGGTGTTCAAGGTTTTCTAT
GGCTCTCAAGGCAACCGTATTCAAGGCTCAGATCAGTTTGTCTGATATGGATCGCAATCTGTATCAGGAC
TTCTCCCTCACGCTGGCGCGCCACCCCTCCGAAACCGATGAGCGGATGATGATCCGGCTGGCGGCCTTTG
CCTGGCACGCTGCCGAGCGACTGGAATTCACCAAGGGATTGAGCGCCGATGATGAACCCGAGCTCTGGTG
CAAGAACTACTCGGACGAAATAGAGCTCTGGATCGAGCTGGGCCAACCCGATGAAAAACGGCTCAAAAAA
GCCTGCAACCGCGCCCGTCAGGTGGTGCTCTATCTCTACGGTGGTCGTGGTACAGCCGTCTGGTGGAAGC
AGAACCAGGGCAAGCTGAGCCAGCATGGCAACCTCAGCATCATCGAACTCTCCGACAGCCAGACTCTGCC
GCTCACCGCCATGGTCGAGCGCACCATGCAGCTCACCTGCACTATCTCCGATGGTCAGCTCTGGGTCAGT
AACGGCGCACTGGAAGTGACGCTGGATCCGGTGGTGCTGATGGGCAAGCCGGCCTGCAAACTGTAACGGA
CGCCGCCCATGTTCAGCGCCCTGCTCAATATCGTCATTCCGGTGTTCGGTGTGGTCGGGCTCGGCGCCCT
CTACGGGCGATTGCGACCGGGGGCTGCGCTCGGTTACGTCAATCGAGCCAATATCGAGCTGTTTACCCCG
GCGCTGGTCTTCTCCGCGCTGGTCAAGTATCCGCTGGCACTGGCGGAGCATATGCCGCTGATTGCGGCGG
GGGCGCTGGTTATTCTGTTACCCGGCCTGCTGCTCGCGCTGCTCAAACTCAAAGGGATTGAGCGGGCTGC
CCTGATCTTGCCCGCCATGTGTAACCTACCACTAAGAATGAAAAACCGGGAGCTGGTGAAAACAAGTGGG
AGATGGCGGGACAAACTCAGGAATGGTGGGGCTTGTAGCGTGATACGGTTAAACCAAGCCGTTTGTAGTG
GTCAACTAAATCCGGACACTTGGTTAGGCCAAACCTCGGCTTTTGCTGGCGGTATCCCGTCATTGTGGTG
ATGTGGACGCCGCCAGTTGTAGTAATTCATCAGGTAATAACTGATATCTCGTTTTGCCTCTCGCAAGCTC
ATGTATCCCATAGCCGGGAGCCACTCTGACTTCAGGCTTCTGAACAACCGTTCCATCGGGGCGTTATCCC

AGCAGTTGCCACGCCGACTCATGCTCTGGGTTATGCGATAACGCCACAGTCGTTGCCGAAATGCCCGACT
GCCATATTGGCTGCCCTGGTCGGAGTGAAATAGCGCACCGGATGGGCAACCCCGCTGCTGATAGGCCATC
TCCAGTGCTTTGATTGCCAATTCTGCATCAGGTTTATCAGACATCGCCCAGCCCACGACCCGCCGGGTGT
GCAGGTCAAGAACCGCTGCCAGGTAATGCCAGCGACCACCGGCCCAGACGTAGGTGATATCGCCACACCA
CACCTGATTGGGTTGCTGGACATCAAATTCGCGGGCCAGCAGATTGGGAATGTCCGGTCGTTCAGACCGA
GCGACCTGATAGCGATGAGCGCCCGGTTGTTTGGATACCAGATAGGCTTCCTTCATCAGGTTACGTACTT
TGAATCGACCAATCTGATACCCCAGCTCCCGCATCATCGACATCAAGGCACGGCTGCCCGCTGAGTTCCG
GCTTTCTTTGAACAAACGGCGCAACTCAGAGCGCTGCTGCATACGCTCACGGTTAATCGCCCGCCTGCGG
GCCAGATAATCGTAAAAACAGGAGGTGGGAACATCGAAGGCGCTGCAGACCAGAGTGATGGGGGCTTGCT
CCCTTAATCGGTCTATCAGCGCGTACGTGTGAACTCGTCCGCCATCAAGAGAGCAGTAGCCTAATGGGAT
GGTCTGCTCCACCCACCCCGCTATGCTGTAGCCAGCATCAAGGAGGCTCATCATGACTCAAGCGCAGATT
TACATTTACGGGATCGATTTAGGCAAAAATTGGTTTCACCTGGTGGCGATGGATCGCATGGGGCATGTAT
TGACCAAGCAAAAGCTCACGCGTTCACAACTGAAACAGTTCATTAGCACGACCCCACCCTGTCGGGTCGC
CTTCGAGGCATGTCCTGGTTCGCAATACTGGGGGCGCCTGTTTCAACAGGCGGGGTTCGAACCCAAGATT
ATCCCTGCCCAATTTGTGAAGCCCTACCTCAAATCAAACAAGAATGACTTCAACGATGCTACCGCCATTG
CCGAGGCTGGTAGCAGAGGTACTATGCGCTGTGTCCCGTTGAAATCTCATGAGCAACTGGCATTGCAAGC
GACTCATCGCGTCCGTCAGCGTTTCATCGTCGAACGTACCGCCACTGTCAATCAGATGCGAGCGTTGCTG
CTGGAATATGGGCTCACCGTGCCAGTCGGCAGAAAAGTCTTCGAGCGCTCTCTGCCGACGATCCTTGAAG
ATGCCGAGAATGGCTTGCCTGACTTTATCCGCGCCCTGGTATTACGCCTGAGAAAACGGTGGCAACAGCT
GGATATTCAGATTGATGACATGAGCCTCATGCTCGGACAAGCCTCAAGCGCATCAGGTTTGTGCCAAAAA
ATCAGTACCGTCCCCGGCATTGGACCGATTGTCTCAACCGCGTTGATTGCCGCGATTGGCAATGGCTCAC
AATTCAAGAAAGCCCGCGACCTGTCAGCTTGGTTGGGGCTGGTCCCCAGGCAATACTCAACGGGCGGCAA
ATCTTCGCTCGGCGGGATCAGTAAGCGTGGCAATAGCTATTTAAGGCAAATGGTTATTCAAGGCGCCAAA
GCGCTAAAAATTCACATGAAGCGTGACAAATCATCGTTAGGCGAATGGGTCGCTAGGCTGGAGGCTGGCC
ATCACCATCATGTGGTTCTTATCGCCTTGGCCAACAAGATAATGCGAATTTGCTGGAAAGTACTGACATC
CGGCAGGGATTACCAACCCTATCCCAACGCAAGCGCAGCCGTTTAACAGATATAACTCATACCCGGTTTT
GCGAGCCAAAAACAGATGACAGAAGCGTTCACCCCACGACGTTGCAGCCTGGCTAAAAAAGCAGTCTCTC
ATGACTGGTAGCTTTATTAGGACGATGACGTGCGGATCCCATCAAGGCCAGAGCCGGAAAACGGCTCACA
GACAGGCCGGATACATTGACGCAAACGACTTCTGGAAATCAGTTCAAGCTTGCAGATAAGGAGCAGACCA
TACATTTTTTAGAATGTCCTTCTCACGTTCGAGCCGGTTGATGCGGGCTTCCAGCTCCTGGATTTTCTGC
TGCTCAGGAGTTAGCGCTTTGCTGATTGGGGTTGTGCCACTGCGTTCGGATTGAAGCTGCTGAACCCAGC
GACGAAGTACGGTTTCACCAACGTCCAGTGAACGGCTGGCTTCGGGGACTGAATATCCCTGGTCGAGCAC
CAGGGATGCGGCTTCGAGTTTGAATTCGGCAGTAAATGAGCGACGTAATCTTGTCATTGAACACCTCAGG
TCATGGTGGCGACTGTACCACCTAAAATGGTGCCCGAGATGATTAGACCACTACACTACACCAGCAGCCA
ATTCCAGCCGCAAGATGTGGCCTGGCGCGGCACCGGGTACTTTGCTTCACCCAAGATGGCGAATTGGTTG
GCGAGACCTACAGCCGCGCATCGGATGGGGTCTATCGGTTCGATCTGCTGTGGCTGAATCGCCGTTATAT
GGTGGTTGCCCAGGATGACCCGGCATATGGTCCCGCCGACTACAACGCCGTAGCCGCCGACTATCAGGCG
CCAAAACCCTACCCGCCTGGTGGTGGCGTGGCGCCCGAGCCGTTCCCCCTGATTGCCCTGCTCAAGAGGA
AATAACCATGATCTCCTATGCCGAAGGGCTGCGCACCAGTCGCGCCCAGCTATTGGCCACGGCCATTGAT
ACAGGCAGCGGGGCCAGTGCCAAGCTGACCATCTATACCGGCACCAAACCGGCACCAGGGGCAGCTACGA
CTGACCAGCTGGCTCTGGTGGTGCTGACGTTCAGCCACCCTTGCGCCAAGACGATCGCTGGTGGGGTGTT
GACCCTGAAACCCCTTGCCGAACAGATGGCCACTGGCAGCGGCGCACCCACCTGGGGGCGCATTGTTGAC
CGGGATGGAGCCTTTGTGGCGGATCTCGATGTCGGGGTGCCCGGCAGCGGCGCCGATCTGGAGCTGCCCG
CAGCGGAGTTTTTTGCCGGTGCGCTGATCCGCATTAATACCGCCACTATCACTGAACCGTAACCGGGGGG
CCATATGGCCAGAAAGGACGCCAGCCTGGCGCTGCGTAAGGCTCGCAGCATCAATGGCCAGTTGGAGCTG
AACCAGTCCGACGTGGTGCGCCTGGTCGGCATCCTGAATAGTACCAACTTGCCGCCACGCCTGAGTGCGT
CAGCGGGTCTGGTGCTGTCATCGGTATTGGATGGCGCTCCGCATCGCTCCAGCGCCATGCTGGGCGGATC
AGTGGTGTTTGATGCGGTGCTTGCCAGTCATACCCATAGCCGTGGGGAGCTAGTCGGCGCGTTCGTCATT
GAATGCACCATGGCCAGCACATCCCGCGCCCCGCAGCCTGTGCTCGCCGGGCAGTATGACCAGAACGTAT
TCCGAGGCCCTGCCAGTGCAATGGGGGATGAGTGGGAGCGGGCAGATAGCCATTCCCAGGCCCTCAGTAG
CGAATGGCAGAAGGCCGGTACCGAACGGGCAACCAGCCGCTCCCTGTGGCAGCAGGCCGCCGCGCATCAG
CAGCAGGTGGCCGAGCTGGGCGAGCAGATGCACCAGACGTTCATGGCCAATCAACAGCGCTTTGCCGAGG
GGCTGCCGGTCAGCCAGCAGAACCGCCAGGGCTATGACAGCCTGGCCGCAGGCCATGTGGCGAATCAGTC
TCTGTGGGTTGAGGCGGCGCCGGTCAGCAGCTGGCGCCTGGTCGGGTTCACCAACCCGCCGCGCTTTGAC
AAGGCTTGGCAGGCTGACCAGTGGCAGGAGGGCATCCCCATCGGTAAAGGGGTGGCCGCTCAAGCCTGGC
ACCACGGCAAGCCGCTGATAGAGGGCTGGCGTGATGGCTGGGATGAGGCCATGTTGCCCACCTGGGGCAA
GACGCTGCCACCAGAACCACCCAAGCCTCCCATCCGTCCCGATAAGCGGGTGCTGCGGCTGGCGTTCGGG
CGCAAGCGCGACACGGCAGAGCTGGAGTTTGTCTGGCAGGGCAGTGATGCGGCAATCGTCATTCCAACCC
GGAGGGTTTATCTGGTGAGCAATACAGCAAAGATTGTGCGGGTACGCGATGGGCTCGATATCCCGGCCAC
AGCGGTGAGCATCGAGCTCGATACCTACTCCTGGGCGTGGCAGTTCAGCGCTCAAATTCCCCGTATCGCG
GCGGCTGCGCTGACCGATGAGGAAGACGTCAGCATTCATATCAACGGTCAGCAATGGGACTGCGCGTGCG

ATGGTTGGCAATCGAGCCAGAGTTTTGGCCGCGAGTCGGCAACGCTGACGGGTCGCTCTCGTACCGCTTA
CTTGTCACCGACCCATGTATTGGCACAGGCGGTGAGTGAGCCTGCTGCTGCGACCATGGCTCAGCTGGCG
GCCGCCGTATTGCCGGTGGGTTGGTCGCTGGATTGGCAAGCGGCTGACTGGTTGGTGCCTGCCGGGTTCT
TTAGCCTGGATAACCAGACCCCGATCGAGGTGGTCAGGTACCTGGCCGAGGCGGCCGGTGGTTTTGTGCT
GCCACACCAGCGCAACCGCCATCTGGTCATCAAACCGCGTTACCCCACAGTGCCATGGCAGCTCGATACT
GCGGAGGCCGATGTGGCGATCCCCCGCGCGATCATTACCACCCTGGGCAGTGACTTCCTGCCGGGTCATG
CCGCCAACGGGATCTGGGTCAGTGGTGGCCACCAGGGAATCAGTGCGCGGGTGGTACGCCAGGGGACGGC
TGGCGAGCAACAAGCGCCGACCATCACCCACCCGCTGGTATGTGATGTGACGGCCGCCCGTGCCCTGGGT
GTGGTGGGGCTGGCCAAGACCATGCCCAAGCGTACCCAGACCATTGAGCTGCCGTTGTCTACTGATACCG
GCTTGATCCTGCCGGGTGCGCTGCTCGCCGTGGACGGCTGGAAGGGTTACAACCGGGGCGTCAGGGTCTC
TGCTGCGCTGCAGAACAGGGCCATGACGGTGCGCCAGCAACTGAGTGTGGAGCGATTTGTATGAACCTGT
TTAAGCGATTCCTTGAGCTGGTACCGGGCGCTGATCCCTTGCTGGTTGGTACCGTGACCGCAGTAGGTAC
CACGACCACCACTCTCACCGCGTTGGCGGGTGGAACGGTCACAGTACGGGGCACTGGGGTAGCCATTGGC
AAGAAGGCGTTTTACAGGGGAGGGGAGCTGGCAGGAGAGGCGCCGGATTTACCGACCTATGAGATAGAGG
TTTAATGCCGGTTTAACCTGCTTATCAAACTGGGCGATTCCGGTGCCACCAAGTTTGATAAACTACTGGG
CCGGTAGCTTTTCATTTTCCCCGCAAGTCTTTTTCATTTTCGGCGGCGGGCTACATCAAGTGCAACCTGC
TCTATCTGCTGGCGGGGGGATTGTCGCTGCTGCTGGCGATCTGGCTGTTGCCGCTCAAGACGGAGTGGAT
CCCGCTGTTGATGTTGTCGGTTTCCTTGCCCCCTGCGGTCTTGAATTACATGCTCTGCGAGCAGTATCAC
TGCCAGCCGGACAAGGTGGCCAGTATCGTGCTCGGCGGCAATGCCCTCTCTGTACTGGTGATCCCGCTGG
CGGTCTGGTTGGCACTTCATCTGCCCGGCTAAGTCAGATTGCATCGCTGCGGCTCTTCTCGTGGAAACGG
TTCGTACCAAATCATTGCCTCCGTGATACATCTGTCGTCCCCGTATCCGGCCCTGAGCCCCAGTGCTAAC
GGCGACAGCTATAGTATGAAAAAGAGCTGACTGACACTGTTTAACCAATGCCAGTGAGGGAAACCGGATG
GGCACAAGTGAGCAATCTGATCCTGTGGGGCAGGCAAAATCGCGCTATGACCCGGCATTGCCTACCGGTT
ATGACTGGCGCAGACACCGCCGTTTTATCCTCAGTGGCGGTGGTTACAAGGCAGAATGCAGCCGTCGCAA
CTGGTTTTTGGAGTTGCTCAACCCGGTGCCCTATGTGGTAGTGCACGACATAGCCATCGGCGGCATCGGT
ATTCTGGGGGCCATACGGTTGAAAGAGGGGGATGAGCTAGTGATTGCCATTCCCTCTGGCGAGAAACTGC
GCGGTCTGGTCGTGAGATCCCAGCCAGACCCACTCTTTCCCAAACTCTATCGCACCGGTATTCGTCTGCA
TCGTTATCCAACGGCCGAGCTGTTTGCTCGCTGGCAAAGTTACATTCAGCCGCAAGATAAAGCGGATTTC
GACCATGAGTGCCAAGTCTTGAAACAGGTACGCGGCGCGTGAGCCAGATGCCATTTCAGTGGCTGTTTTC
CCCACCTTCAGCATGATTTGTCGATGGATTCCACAGATCAGCAGGCTTGGCTGTCCCGAGTGGTTTATCT
TGCTGCATGTGTTTTCATCTTCATGTTTTTATTGAATTTTATCTGTGCCTCTGAAGTCACGGAGGATACG
GATCATTTTCCCTTTTCGTGATCCCCCCCGAAGTAGTTCCCCCTGATATCCCTTAGTATCTGGCCAGCAT
GACTCCCTTTCGGGGGAGGATAACAAGAACAAAAAGGAATGATGGATGAGATCCGTGAGCAACCAGTGTC
TGTTGATGCAGATCCAGCCGACCATCTTGCGCTTTGCCAAGATGCTGGCGAGTGTGCTGCAGCTTGAGGT
CGAAATTGTGGATGCCGATATGGTTCGGGTGGCTGGCACCGGGCCCTATGGCAAGTTCTTCGGACGTCAG
CTGGAGGGCGACTCCCGCCTGCTGCGCTATGTGATTGATAACCAGCGGGAGAAGATCGTTACCCACACCA
GTGATGATCCCGTGTGTGACGGTTGTACCTGCAAGGAGAGCTGCCGTGAGCGCGCCTTCCTCGGGGTGCC
CATCATGGTGGACGAGCGCTGCATCGGGGTGATCAGTTTGGTGGCTTTCAGCGACGAGCAGCAGGCGCGC
CTCAACAACAATCTGCAGGATGTGTGCGACTATGTGCGCCACATCTCCACCATCTTTGTGGCCAAGTTGC
TCGATTCGCGCGGGCTGTCGGACGGGGTCAACAAGGTGTTCCTCAATCTGATGACCAACATGGATCAGGG
CTGCCTGCTGCTCGATGACAAGAGTCAACTGCTCTACGCCAACGAGGCGGCGCTCCATCATCTGGGTTGC
CAGCAGGAACAGCTGCAGGGGCGCGAAGTGGGGATCCGCCCGCTCACCTTCTCCCAGCAGGGGCTGAGTG
GTCATCTTCAGCACATCATCACCTTTGAAGGTCGTCAGGAGCTGATCATCGGCCAGCTACACCATGTGCA
GGGGCAGCAGCTCTTTCTGATGGCGTTTCACCAGTCCCACTCCGGTCAGGTTCAGGAGCCGGATCCGGAT
GTGCGGATCGATCAGCTGATCGGCGAGAGCAAGCCGATGCGCACCCTCAAACGACTGCTGCACCGCATCG
CCCAGAGTCCCTCCAGCGTGCTGATTTGTGGCGAGAGCGGCACCGGTAAAGAGGTGGTGGCCCGCGCCGT
CCATCGCCTGAGTCCGCGGCAGGACAAGCCGTTCATCGCCATCAACTGTGCCGCCATTCCCGAGCAACTG
CTGGAGAGCGAGCTGTTTGGCTATGTGAAGGGCTCCTTTACCGGTGCCTCCAGTGGTGGCAAGCAGGGCC
TTATTCAGGCGGCCCATCAGGGAACCCTGTTCCTTGACGAGATTGGCGACATGCCGCTCACCATGCAGGC
CAAGCTGCTGCGGGTGCTGGAGCTGCGGGAGGTGACCCCCATCGGTGCCAGCCGACCGGTACCGGTAGAT
ATTCGCATCATCGCCGCCACTCACCAGCATCTCGATCAATACATCGCCGAAGGGAAGTTTCGTGAAGATC
TCTACTATCGCATCAACGTCATCCCGCTCAACCTGCCGCCGCTGCGGGAGCGGGAAGGGGACGTGGAGCT
GCTGACCCACTACTTCCTCAACCTGCACACCAGCCGGATCGGTATCGTCTATCCCGGTTTGGCTCCCGAG
GTGATGGCGCAGCTCAAGGGGTATCGCTGGCCGGGCAACGTGCGCGAGCTCAGCAACCTCATCGAATATC
TGGTCAACGTTAGCCAGGCGGGCGAAGTGATTGAAGCCAGCCTGCTGCCCCCCAACATCATCCGCAATCA
GGAGCCCCATCGCGTGGCGGCCCAGTCGATCCCGGTGACCAATCTGGATGCGTCGTTCAATGTGCCTGCC
ACAGCTCCGCTCGCCGCCACGCCGGAGAAACTGCCTGAGCCCGTTCCGGGTGAGAGCGGTCTTGAGAGCA
TGGAAAAACAGATGATAGAAGAGACCCTGCAGCGGCTTGGCAACAAAAAACTGGCCGCACAGGCGCTGGG
GATTGGGATTGCCACCCTCTATCGCAAGATAAAGAAGTACGAGATTGCCGTGCAGAGCTGAAGATCTGGC
GGCATCTATCCGGCCCGTGTCGTTTATCGGATGACACGGGTTTTTTATGGGAACAAGGCTCTGCGCCGT
TGCCCGGTCGGTAGTGAACGGATTGGCGGGGCAGGGGAGTGGCTGGACAGAATGCAAAAAGCCCGCTCGA

TGAGCGGGCTTTTTTAATCTGGTGCGTCCTAGTGGATTCGAACCACCGACCCCCACCATGTCAAGGTGAT
GCTCTAACCAGCTGAGCTAAGGACGCAGGGTGCGCAATGCGACTTTTATTTCTTTATGCAGAAGTGGTGG
GTTTAATTGGATTCGAACCAACGACCCCTACCATGTCAAGGTAGTGCTCTAACCAACTGAGCTATAAACC
CACATCTGGAATAACTGCAAAGTATTGAAACTGAATGGTGCGTCCTAGTGGATTCGAACCACCGACCCCC
ACCATGTCAAGGTGATGCTCTAACCAGCTGAGCTAAGGACGCATAATCAGTGTTCTGTAATGGTGCGTCC
TAGTGGATTCGAACCACCGACCCCCACCATGTCAAGGTGGTGCTCTAACCAGCTGAGCTAAGGACGCATC
ACAGAAAAAGTGGTGGGTTTAATTGGATTCGAACCAACGACCCCTACCATGTCAAGGTAGTGCTCTAACC
AACTGAGCTATAAACCCACTTTCTCTTACTTCACTACGCCAGCGCTGCTGACGTGGTCGGCATACTACCC
ATGCCCCCCAAGGCTGTCAACAGGCCATACGCACAAAGGAGACTGTTTGCCCAGTTCTTGCGCAGCCTGT
TGATTTTGCAGGCGAGTTGCCGCTTGCCAAGCCCTGTTACTGAGATCGCGACAGGGTCGTGCAGGCTTCC
GGAGTGGCAAAACGGCCATAGATGTAGCGCAATCTCGCCCCCAGCATCAGGGCCGCACTGGTGAGGCCGA
CGATAAAGCCGATCCAGAAGCCCTGCGGCCCCATCCGTGGCACCAGCCAGTCGGTCAGACCCAGGATCAT
GCCTGTGGGCAGCCCCAGTCCCCAGTAGGCGACGATGGTGACGTAGAAGATCGCCTGAGTATCCTTGTAA
CCGCGTAGTGCGGCGGCGGCGACCACCTGCACCGAGTCGGAGATCTGATAGATGGCGGCGAAGAACAGCA
AAATGGCTGCCAGGACGATCACCTGCTGATCATCGGTATAGATGGAGGCGATCTGCTCGCGCAGCAGCAC
CGTCATGGCGGCAGTGGCAGCGGCAATGGCCATCCCCAGCATCAAGCCGGTGCGAGCCGCCACCCGGGCG
TGATGGGGCTGCCCTTCACCGATGCTGTGGCCAACCCGGATGGTGACACCCACCCCGATGGAGAGGGGCA
GCATAAAGACCAGGCTGGAGAAGTTGAGGGCAATCTGGTGACTCGCCACCGTCTCGGCACCGAAGGGGGC
CAGCATCAGGGCAACCACGGTAAACAGGGTCACCTCGCAGAAGATCGCCATGGCGATGGGGAAGCCCAAG
CGAAACAAGCGCCAGATCCGGCTACCGTTGGGGCGAGCCAGCTGGGAGAAGAGGCGGATCTCGGTGAAGT
GACGGGAGAGCTTCACATAGAGGATCATCGCCAGCAGCATGGCCCACAGCACGATGGCCGTGGCCACGCC
ACAACCCACCCCGCCCAGTTTCGGCATGCCGAAGTGGCCGTGAATGAAGATGTAGTTGGCGGGAATATTG
ACCGCCAGCCCGACAAAGCCGATCACCATGGTGGGCATGGTGTGGGAGAGACCCTCACTGAAGTTGCGCA
GCACCTGGAACATCACGAAGGCGGGCAGGCCCCACAGGATGGCGTGCAGATAGCCGGTGGTCTTGGCGGC
CATCACCGGCTCCACATCCATGAACTGCAACGCCAGCGGGCTGTAATAGAGGGCGATCATCGCCAGTGGG
GTGACGATCAGTGCCAACCAGAAGCCCTGATGAACGGCCGGACGAATTGCCTCCCGTTTACGGGCGCCAT
TGAGCTGGAAGACGATGGGGGTGAGCGCCATGATGATCCCCTGCACCAGCAGGATCACCGGTAGCCAGAA
GCTGGAGGCCACCGATACGGCGGCCAGATCGACCGCACTCACCTGACCCGCCATCACGGTATCGACGAAG
TTCATGGCGACTTGGGCCACCTGGGCGACCAGAATGGGGCTGGCTAGACGGACGAGCTGCTTCGCCTCGG
ACCAATAACGTTGCACTGACACCTCCGAGTGGATCAGATAAGAGTAAAAAAGAGCGGAAATCGGGAAGAG
CCTTTGCACTTTCGGACGAAAGTGCAAAGGGTGGGGGAGGCATTCTAGCCTGAAATTCAGCCAGGACGAA
ATAGTTGCCACCCCAAGGGGCAGGATCAGGCGTCTTCGTGATCGCTGATCAGCAGGTTGGCTGCGGCATA
GGCAGCCTGCTCGCGCAGCTCGGCGGGTACCCGCTCATCAGCGGCGACCGCGTTGAGGGCGCGGATAGCG
GCGGCGATGGCCTTCGGCACATAGCCCTGCTCGCCGGAGCCAATCTGGTTATAGGCGATACGGATCGCTT
CACAGCACTCATAAACCATCATGATTTGCTTCCTTGAAAGTAAAAAGGCGAGCAAATATAGCGGTTTTGC
TCCCATTTTGTCAGTCTCGCGGTTGTAATTTGCGGCAAGCCCGTTAGTTTGGTTACCCCCTTTTCCTGTT
TCGGCCTCTTTGATGCTGCTCTCTTCGCGCAATTCACTGGCGATCTTTCTGACGCTGTTTGCTCTTCTCG
CCCTGACGCTTAAGGATGGCGCGGACAAATATCTGGTACTCCATGGCTACTCCGTGATGGAAATGGTCTG
GTTTCGCTTCTTTGTTCCTTTCCTCGGTCTGCTGCTGTTGTTGCCAAGGCGCACCGTGTCGGCGCTGCTG
GCGGTGGATCGCTGGTTATTGCTGCGCTCGCTGCTGTTTCTGGTCTGTGCGCTGGTCTCGGTGATTGCGC
TGGCCCATGTACCGCTCAACGTCTACACCATCATTTGCCAGCTTGGTTCGCTGGCTTTCGTGGTGGCGGG
CGTGATCTTTTTCAGGGAGCGTCTGACCCGGGCCAAGGTAGCGGCCGTGCTGTTGGGGTTGTTTGGCGTG
CTGGTGGTGATCCATCCTGACGGGGGCGACGCGAACCTCTACTATCTGCTGCCACTGGTCATTGTACTGG
CCAACACCGGCTACAACCTCATCACCAAGCTGGTGGATCCACGTCTGACCACCCTCGATATCCTAATCGT
CACCTGCTTCATGCTGGGGATGGTGGCAATGGTCGCACTCTGCTGGCAGCCTGCACTGTGGCGCTTTCCG
ACGCTTGCCGACTCGCCCTATCTGCTGGCGCTACCGGTGGTGACCCTGCTCTCCCAATATTGCCTTATCA
AGGCGATGCAGTATGCGCAGGCCTCTACTCTGGCGCCTTTCTTCTATTTCCAGATCTTTTTTTCAACCCT
CTGCGGCTATCTGATGTTTGGTGAAATGCCGACCCTCAGTAGTATGGCGGGGTGTCTGCTCATCGTGCTG
GCTGGCATAGTGGTCATCCATTCCCAGCGCCGGACTCGTGTGTTGGCGCTGTCAGAGGCGGCGGAGCAGG
TGTGACAGTTTGTATAAAACGAATTGTGTAAAAAGCGGATTATGTAAAAGAAGCGGCCTCGGGCCGCGAT
AAAAACAGAGCGTGTTACCAGAAACAAAGAGGTGATGATGTTTACCGGAATTGTGCAAGGTGTGGCCGAG
CTGGTCGCCATCGAAGAGAGTGCTTCATTTCGTACCCACGTGATCCGCATGCCCAGCCGGGAGTGGGGGG
AAGGGTTGGCCCTCGGCGCCTCGGTCGCTCACAACGGCTGCTGCTTGACCGTCACCCAGGTCGAGGGGGA
TCTGGTCAGCTTCGATCTGATGCAGGAGACCCTGCGTCTCACCAACCTCGGCCTGTTGCTGGTGGGGGAC
AAGGTCAATGTGGAGCGGGCCGCCCGCTTTGGCGACGAGATCGGCGGTCACGCCATGTCCGGCCACATTA
TCGGGATGGCGGAAGTGACCTCGGTGATCGACACCCCCAACAACCGTCAGGTGTGGTATCGCCTCGCCCC
CGGGCTGATGAAATATGTGCTGACCAAGGGGTATATCGGCATTGACGGCATCAGCCTGACCATAGGTGAG
GTGCGCGAGCGGGAGTTTTGCGTTCACCTGATCCCCGAGACCCTGGCTCGCACCAACTTGGGTTGGGTCA
AGGCTGGCTGGCAGACCAATATCGAGATCGATCCCCAGACCCAGGCCATCGTCGATACGGTGGAGCGGGT
GCTGGCGGCACGCGGGGGTAATTGAAGGGAAAACGGCTTGGATTCAAGCACCTCATAAGCAAGGGCCTGC
AGTATGCAGGCCCTTGTTATTCATGTGGTGAAAATCTCTACTCGATTTTCTGTGACTCACAATCAAAACA

GCTGCTCGTCGTCTGCATCCACAAACAACTGCAAACAGTCGCGGGCTTCGTCGCGGTGAAGCCGCAGGTG
GATCGGATTGCAACAGGCCATGCAATCTTCGTAAAACTCCTGATCACCCTGGCTGGCATCCAGCTCGACC
CGGGTATGGTGGCCGCAGTGGGGGCAGACGATGCGCTTGCTGGTGTACTCGATCATGATAACCTCCTCGG
GCCCTGGGGCCGCCTATCTACCCTGATTCTAGTCGGGCAGCAGGGGGTTAACCGCGAGCAAACGCACGTT
TATGACAGAGATCAAGCCTGAGGGATGGCAAGGTGCAACCGGTTGGAGTGGTCGTGTGCGAGAGGAACAA
TCTGGTTGTGAATGACAGGTGAAAGGGGGGGATTGCCAACGGTTTTGTCTGGTAATAACAGGGGGCTGGA
GCCCCCTGCGGATATGAGAACGTGAGCGCAACAAGGCGCCGATAGGCGCTATTTGACCCAGCTGGCCAAC
ATGTCGGCATTCAGCTCGGCCAGTGAACCGAGCTGGTAGTTGGCGATGGCCAAACGTGGATCACCCACTA
AAGCAGGATCCGGCACGATCAGCGCAGTCATGGAGGCCGCCTTGGCCGCCAGCAGGCCGGTAAAACTGTC
TTCGATGGCCAGACAATGAACCGGCTCTACCCCCAGTTTCTCGGCAGCGTGGATATAGACATCCGGGTGC
GGTTTGCCGAAACGCTCGATTTCAGCGGAGTGCACCGCCATAAAGCGATCGCGAATGCCAAGTTTGCCGA
GCACCGCTTCTACCATCGCAAAGGGGGAAGAGGTGGCCAGACCCACTTTCAGGCCGTGGGCCTCGATAAG
GTCGAGCGCTTCCAGCACACCGGCTTTGGCCTGACCTTCGGAGAGGATCAGGGCGTTGACCCGATCCAGA
ATGCGCTGTACCACCTCTTCCTGAGTTGGGCCGCTCCACGGGCGCAGGCGATACCAGTGGGCAACCAGCT
GATCGATGCGCACGCCGATGGTCGATTCGCAATCCTCTACCGTGTGGGGGTGACCCAGTTCGGAGAAAAC
GGCGATCTGGGCCCGTTGCCAAAAGGGTTCCGAGTCGATCAGAACACCATCCATATCGAAGATCACGGCA
GCTAACATCTTGTACTCCTGACAAATTTGAAGAGGGGATTATAAGCCTGACGGGGAAATGCAAAAGGGGC
ATTGCCATGCCCCTTGCGCCCGATCACATCGGCAAACGTTTGTTTTGCTGTGGCAAAAAAGCCCTTAGAA
CAGTACTTCGACCTTGGCGGCCACGGCTTTGGCTTTCTCTACCGCATCCTGGCAATCCTCTGCGCGAGCC
AGCGCCACGCCAAGACGGCGGCGACCGGCGATCTCCGGTTTGCCAAACAGCCGCAGCTGGGCACCCGGCA
CCAGCGCCAATGCCTCACCAATCCCCTGATAGCGGATATTCTTGCTGTGCCCTTCGCGCAGTACCACGGC
GGAGGCACTCGGCCCGTACTGGGTGATGGTGCCGACCGGCAGCCCCAGAATGGCGCGTACGTGCAGGGCG
AACTCGGAGAGATCTTGCGAGATCAGGGTCACCATGCCGGTATCGTGGGGACGCGGCGAGACTTCGCTGA
ACCACACTTCATCGCCTTTTATAAAGAGCTCGACTCCGAACAGGCCGTAACCACCGAGGGCCTCGACCAC
CTTGGCGGCCACCTCTTTGGAGCGGGCCAGCGCCAGCTCGCTCATCACCTGTGGCTGCCAGGATTCGCGG
TAGTCGCCATCTTCCTGCCGGTGGCCGATAGGGTCGCAGAAGTGAATGCCATCGACGGCGCGTACCGTGA
GCAGGGTGATCTCGTACTCGAAGGGAACGAAGCCCTCGACGATCACCTTGCCACGGCCGGCGCGGCCGCC
TTCCTGGGCGTAGGTCCAGCTCTCATCGAGCTTGGCGAGATCGCGCAGTACGCTCTGCCCCTTGCCGGAC
GAGCTCATTACAGGTTTTACCACGCATGGCAGGCCGATGGCCTCGACGGCGGCGACAAACTCCTCCTTGC
TCTGGGCAAAGCGGTAGGGGGAGGTGGGCAGACCCAGGGTCTCGGCGGCGAGGCGACGGATCCCTTCCCG
GTTCATGGTGAGCTGGGTAGCGCGGGCGTTGGGCACTACCTTGACCCCCACTTGCTCAAGGGCAGCCAGG
GTATCGGTGGCAATCGCTTCAATCTCGGGGATGATGAGGTCGGGTTTAACCTCGGCAACCAGGGCACGCA
GGGCGTTGCCATCGAGCATGTCCAGCACGTGGGCTGCGTGGGCCACCTGCATGGCGGGGGCGTTGGCATA
GCGGTCGGCGGCGATCACTTCGATCCCGAACCGTTGCAACTCGATGGCGACCTCCTTGCCAAGTTCGCCG
GAGCCCAGCAGCAGGGCACGGGTGGCGCCGGGGCGGGTTGCGGTTCCAAACATACGATCTCTCCTGATGT
CGATGGTCATTGTTGATGATCGCTATCGCATGAATAGCGGACAAAAAAAGAGGCACGCATGGCGTACCTC
AGGAAACGGGTTTGTGAGCGCGCTGCTCCAGATGGGTCAGCAAGGTAATGGCGCCAATGATGATGGCGGC
GCCAAGCCACAGACGGCCGGGGGGCGCCCAGCCAAATACCAGCCAGCCTGCCAGCACGTTCATCGGCAGC
TTGGCAAAATCGAAAGGCTGCACGAAGGAGGCCTCCGCCACGCTGTAGGCGCGAGCGATGGACATGTGCG
CGAGTGCCGACAGAATGCCCGCTGCCGCCAGCAGCAGCCATTGTGGCTCGGTGGGCCACTGCCACTCCGG
CAGTGCCAGCATGACGTTGAACGGGGTACTGAAGATAAGCAGATACACCACTATGGTGTGGGATGATTCG
TTGGCTGCCTGATAGCGCACCATCAGGGAGTAGCCGGCCCAGAACAGCGCCGCCGCCACCGGCAGTAGGG
AGGCCCAGTTGAATTCGTCAGTCCAGGGGGCGAGGATCAGCATGGCGCCGGCAAAGCCCGCCAGGGTGGC
CAGAATGCGGGTGCGGCTCACCTGCTCCTTGAGCAGCAGCGCGGAGCCCAGGGTGGCAAACAGCGGCGAG
GTCATCAGCAGGGCGATCCCCTGCCAGATAGGGACGGGGTACGCCAGTGCCCAGAGCCAGAGCTGGATGC
CGATCACCGCCAACAATACCCGCAACAGGTGCAACCGGAACTGATTGGTCATCAGGGATTGGCGCAGGCC
ATGGCGAACCAGCCAGGGCAGCAGGCAGATCAGGGCGATCAGGTATTGATGAAAAGCGACTTGGGTCGAG
GGGAGGCCAAGCTTGAAGCTGACATACTGGCTCAGGCTGTTGACGGCGGCGAAGCAGAGTCCTGCCACCA
TCATCCAGACTGCACCGGCCAAAGGAGAGTGCCTGCGCGAGGGTATCATGGGATCCGGGTCGCTGGAAAA
AAGTGCGGCGGATCATAACGGCAATCGTTTGTTCTGCCAAGGGTTGAAGCGCGGCCGCAGGGCTGGCTGG
CGAGGAAGTGGCCTTGCATCAAAAATTTTGAACAAGTTGAGCATCTATTTTCGCTATCAGCAAATAAGCG
CAATGTTTAGAGTGACCTCCATCGACAGCAACGCTGTTCAACCGACAACCAAATGGAGTTTCACCATGGC
CAATATCCTCGTACTCAAATCCAGCATCCTGGGTCAATATTCCCAATCCAACGCCCTGATCGACGGCTTC
CTGGCTGACCATCAGGATGATGCCGTCACCGTGCGTGATCTGGCTGCCCTCAACCTGCCGGTGCTGGATG
GCGAGCTGGCCTCCGGCCTGCGCGGTGGTGATAACCTGAGTGAGCGTCAGTTGGCGGTGATCGCCCAGTC
TGACGAGCTGATCGCCGAGCTCAAAGCGAGCGACTTGGTGGTGATTGCGGCCCCCATGTACAACTTCAAC
ATCCCGACCCAGCTGAAAAACTGGATCGATCTGGTGGCTCGTGCTGGCGTCACCTTCCGCTATACCGAGA
CCGGTCCGGTCGGTCTGGTTGAAAATACCCGTGCGCTGGTGATCAGCCCCCGTGGCGGCATGCACGTTGG
CAGTGCCACCGATCTGGTCACTCCCTACATGCGTACCGTGCTCGGTTTCATCGGTATCAAAGAGGTGGAT
TTCATCTACGCCGAGGGGATGGGTATGGGTCCGGATGCCCAGAGCAAGGGGATGGAAGCGGCCAAAGCGC
AGCTTGAGGCTCTGGCTATCTAAGGGCTCTCTCTATAAGGGCTCTCTATCCTGTCAGGGGTTATCTCTTC

AAGGGGTTGCCCGCCAGATACCACAACGCAGCCATCAGGCTGCGTTTGTTTTTTGGCTAGATATGATTGC
GACAGACGTTGCTGCTGCAGAGAGCATCGCGGCAAGCGGTCGGTATAGACGTTCCGGCGGAGTCACTACT
CCATCATAAAGCGGCGACAACCGCGCACCAGCAGCAGCGCCACCGTGACCAGCAGGCCAAGCAAGAGGGT
GGTCAGCCACTGTTCGGTACTTTGCGGCAAGTGGGGCGTGAAGAGGCTGATCATCAACAGGGTACAGAGC
GTGATCAACAGGCTCTGTCGGGTCATCAATATCATGGGGTCATCCTTGGTTTGCTTACCTGAAACGATTT
TAATGCCCTCTTGTGACAGGGTGATGTATCCGTGCATCACCCTGTTGGGGCTCTTCAGACCATATCAGGT
TTTTACCGCAGAGCCAGCGCCATCACTGGCGCTGTGATCCCGATCCCGCTAGCTCTCTTTATCTGAACTG
TTTCTGCGCTGGCTGGTAGTGAAAATCGCCGCTTGCGAGCCGCGCTTCCAGTGCTGCCTGATCGATCTCC
TGTGCCTTGCAGAGATCATCCAGATCGTCATAGCGATCACGCAGTTGCATATTGACGACGCTCAGCAGCA
TGTTGATGTCCATGGTGGCGAACTTCTTCAAATCCATGATTATTTCTCCCGATCCAGTGATTGCAGCTGC
TCGATGGCGGCGCTGACACTGTGCAGATGCTGGCGGATCGCCTCGCGGGATTCGCTCGGCAGATAATCGG
TCAGGGTATGTTGCCAGCTGCCCGCTTCACTCTTGAGCTGCAGGAAGAGGGCGAGCCCGGCATTGGTGAG
GGAGACCAGGCTGATGCGTTTGTCCTGCTCGCTGCTCTCGGTAAAGATGATCCCTTTTTCGACCAGACGG
CGGATCTCGCGGGAGATGCGCACCTTGTCCATGCCGCTCGCCTCGACCAGCTCTTTCTGGCTGATGGGGT
AGTTGGGGCCGAGCACCATCATCAGGCGCCACTGCGGCACGGTCAGCTGGTAGCGCTGTTCATAAAACTG
TTCGAAGGTATCCCGTAGCATCTCGGCGGCATGTACCAACTGATAGGGCGGGTAGTGAGCGAGTAACGCT
ATGATTTTTGACATATTACACCCTGCTGTGACGGCTGCATCAGATGCAGTTTCTTTTGATACTTTGACAT
TGATGATACGAAACTCAAGCTGATTTGTCTGACCCGGTTGCGATGAAATGTGCGGTGGCGCACGCCCTCA
AGGAAAAACCGGGTCTGACCGACGGGATTTTAAGCCAACGCTGTTCAACCTTTACGCAACCAGATGAGGG
CGTTCTGCTTGAGCCAGGCCTCACGAGTGTGGATAGCGTAGAGCGCGGCCTCGTCGCGGCTGCCCGCTTG
CCAGCTTTGTGGCTCCTGTTTGGGCAGCTTGCGCTGACCCAGCAGGCAGGCCGGAATGTGCGGGTTGGCC
TCGATGGCGACCAGCAGATGGGGCTTGGCTGCCTCGGCACCCTCGGTAGCAAAGACCATCAGCGCCTTGT
TATAGAGCAACCAGCTGGAGGCTTCATCGAAGCGCACCAGCAGGGCGGCCAACGCCTGCCAATCCCCTTG
CAGTGCATAGAGGGTGGAGAGGTTGTAGCGGCACCCCAGTTCATCGGCCATGCAGATTGTCAGCAGCGAC
ATATACTCCGCTTTGGCCTCGCCCAGCTTGCCAAGGCGCAGCAGGCAGTCGGCGCGGCCGGCTCTGGCGG
CCAGCAGAGGACGCAGCTCGGGGGCACTCCAGCCGTGGCCGGCGATTTGCTGCCAGAAGGCTCCATCAAA
GAGCGGAGTGGCATCGGCGATGATCTTCTCGAACCAGCCGAGGCGGGCCTGATGATCGCGGCAGCTGTCG
GCCAGCTCGAACCAGGTCTGCCACTGTTGGGCCTGCTCGCCATTGAGGGCCACATCAGCGCTGGGGGCGG
CCTGATACTGGTCGAGGTTGAGCAACTGGTTGAGGGCGGTTTTCATGTTCTGGCGCTGGCGTTTGCAGAA
CTCGACAAAGGGGAGTTCGCTGGCGGCGGCGCTATAGAGCATCTGGGCCATCTGCTTGTCGCGAAACAGG
CTCAGCTCTGCGGCCAGACTGAAGCGGTACTGGGTGGTCGGGGCGTTCAAATCCTTCGGCTTGGGCCAGG
TGGCAAAACCCATGCTGAAGCCATAACACCATTGACCGAGGGGGTGGCTCTGTTCGAACACCTGCTCGGG
GTTATCGCTGGGCAGGCGGCACTGGCTGGGCAGGGCCAGCTGAGCGTCGTCGGCTTGGGATTCCAGCTCG
GCGATCAGGTTGTTGAGGGCTTCTGCGCTTTTTTCATCCAACTCGGCATCCTGTTCCAGCAAACCAGCCA
GCGGGCGTTGCCAGTCAGGGCTTTCCGGCCCCACCAGACGAGCACAGAGAAAACCGTGCAGGGCCGGGTA
GGTCATGTCCTGATCGCTGTGGCTCTGGATCCATTGATTAAGATGGCGGGTACGAGTGGATGGGTTCATC
GACAGTTCCTCTGGATTTGAACAGCCGCGAAGTCTACCTAAAAGTGGGGTTATTTGCAGCAAATGGCCGC
CTGTTTGGCGATTTAATCACCATTGGCGGATAAGATAGGGGCGAAAGGGCGGCTCGTGGTGGCTTGGGGA
CTAGGCCGGATCACCGCTCTTGAGGTAACATGCGCGCCCATAGGGGCCATGTTCAGTTGGCCCATCCGGG
TAATGAAAAAAGAGAGCACCATGATACAGATTGGCAAGATGAACACCCTCACCGTTGTTGAACTCGAAGA
CCGTGGCGCCTGGCTTGCGGCCGATGAGTACGGTGAACTGCTGCTGCCCAAACGTCAACTGCCGGAAGGG
GTGAAAGAGGGGGATAGCGTGCAGGTCTTCCTCTATCTGGATGCCGATTGCGAGCCGGTGATCACCACCG
ACAAGCCATTTGCCATGGTGGACGAATTTGCCGGTCTGAAAGTGGTCAATGCCAACAAGATTGGTGCCTT
CCTCGACTGGGGCATGAAAAAGGATCTGTTTGTGCCCAGCCGCGAGCAGAACAAGCCGATGCAGGTGGGC
CACGTTTATCTGGTGCGTCTGCAACTGGATAACGAAGGGCGCATGGTCGGCACCAGCCGCATCGAGCGTT
TCCTGACCCCGGCCGGCAGCAACTTCAAGGTCGGTGACGAAGTGACCGCGCTGCCGGGGGAGCACTCGCC
CCTTGGCATCAAGGTGATCGTCAACGGTCAGTATCCCGGCATGCTGTTCAAGAACGAGGTGTTTGGCCGG
GTGATGACCGGTCGGCCGCAAACCGCCTGGGTCAAGCAGGTGCGCGAAGATGGCAAGCTCGATCTGACCC
TGCAAAAGCCTGGTCTGGCGCGGGTTGATGATGCCGGTGAACGCATTCTGAGCCGGATGAAAAAGCAGGG
TGGCACTCTGGCGGTGGGTGACAAGAGCGAACCCGAGCTCATCTACAAGCTGTTCAGCATGAGCAAGGGC
ACCTTCAAGAAGGCGATCGGCGGACTCTACAAGCAGGGCAAGATTGTCATCGAAGATAACGCCATCACTC
TGGTGGCCACAGAGACCGACTCGGCAGTGGATAGCGAATAAGATGCCGAGCCCCTGTGTTGGCCTCTGCA
AGGCGAAAGATGGGCTTTGTCTTGGTTGTGGCCGCACCCTGACCGAGATCGGCCACTGGTCGGCTATGGA
TAGCAAGGCCCAGCTGGCGGTGATGGCCGAGCTGGATGGCACGAAAAGTACCCACCGCTGTCCGGGTTGT
GGCGAACCGGCCTATTGCGCCGTCTCTGCCGGTCAGACCATTGCGCAGTGCTGGTGCAGCCAGTTGCCGG
TATTACCGATGCAGGAGGCCAGCGCTTGCTGGTGCCGCCGCTGTCTGGCCAAGGCCATCGCGGCGCAGGA
TTAGCAGAGAACAGCAATAAGAGTGCACAGAAACAAGAAAGGCGCCGATGGCGCCTTTTTTATGCCTGCT
GCTGGCGATGCCAGCGCACATGCACTTGATCTTCGGTCAGCAGACGGGGCAGCAGGAAGGTGGCGAAGAT
CACCTCAAACTCATCCTGATGATCCAGCAAACCGACCCACACTTCGACCCAGCGCTCCATATCGACCGGC
TCACCCAGCTCGGTCGACCAGTCGGCATTGGGCAGGGTTGCCTCCACGGCGCCACGCTGCTCAAACTCCA
GGGTGATGTCGACTATTTGTTCGGGGGCCAGCATATCGGCGGCCTGCTCAAGAAACTGATCATAGGCCCA

TTCGACCAGCTGATCCGGGGTACGCTGTTCCATGAAATCACTCCAGATACATCGAGGTTCGCTACTGTAA
CAATTATTGGCTGTGGCGGCCAGACCAGTGCGCCGGGCAAGCCTGGCCTGTCCAAGTTGCCGTTGGGGGA
GTATGGTTAAGGCGCGGATCATCTGGCAGGGAGTAAGGGGATGAAGCAGCGGGGACGGCGGCAACAGATC
AAGCGACGGCGCGAGGCCTGGTGGTGGCACTATCTGCCGCTTCACCGCGAGACGGTGGGCGGTCATCTGG
GCCCACCGGAGAAACCCGATGAGCCCTGAGGATCAGTCGGCCTGATGCACCCGATTGAGGGTGATCTGGC
CCAGTAGCCCCATCTGGCGGCGGATCCAGACCGAGCGCTTCTGCACATAGGGTGACGGGGGCTTGACCCG
ATAGCGCCACGGATTGGGCAGGGCTGCGGCCAGCAGGGATGACTCGTAGCGGGTCAAGCGGCTGGCCGGT
TTGCCAAAGTAGGTGCGGGCGGCGGCTTCGGCGCCATAGATGCCGGGGCCGAACTCCACGATATTGAGAT
AGACCTCGAGAATGCGTGCCTTGTCCCAACCCAGCTCCATCAGCAGGGTAAACCAGGCCTCCAGCCCCTT
GCGCAAGAAGCTGCGATCACTCCACATAAAGAGGTTCTTGGCGGTCTGCTGGCTGAGGGTGCTGGCACCG
CGCACCCGTTCGCTGTGCTGGTTGTGCTTGAGTGCGGCGTTGATGGCATCCATGTCAAAGCCGTTGTGAT
CGGCAAAGCGCTGATCCTCGGCGGCGATTACCGCCAGTTGCAGCTCCTTGGAGATGGACTCCAGATCTAC
CCATTGATGGCGCACCTGGGTGATTTTGGCGGGCGGAAATAGAGCCCGCTCGATACGCCAGCTCCACATC
GGCGGATCAATAAAACGCAGCAAGGCGACCGAGCCGACCGAGCAGAGCAGCCCGGCCAGCGCGATCTTGC
CTGCCCACCTCAACACCCGGCGCCCAAACGATGCAGATGCGCGCACGGGGGCCACGGCGTCAGGGTCAGA
CTCCCACTGATAGTGGCTGGGATCATCGGGATCCGGAATTATTGGGGCAGCCATTCGATGTGGCAGAACT
TCTCGTCAACACGGCGGGAGAGCAGCATGCGGGCAAACAGGTGCTCATACTCTTGTTCTTCGTTGCCAAG
GCCGACCCAGATCTCGGCATAGTCGGTGTCGCTCACCGCAAAACCCACGTCCGCTTCCCAGTCATCGGCC
GGATCGGTTTCGGCCAGCAGGCCGCGATCGTTGAACTCCTGATTGAAGCGGGCGATCTCCTGCTCCGGCA
GGTTGTCAGAGGCCAGCTCCATAAAGAGTTCGAGGGCGGTATCGAGCAGTTCCTCGACGCTGTGCATTTC
GTCATTCATAGACATAAGCGGTTTGTTCCAGAAAAGAGAAAAAGGTGCCGGCGCAGATCACTGCCTGCGG
GGCGGCGCTATTCTAGCGGTCGGTAGACCCCTTGGCGAGATGATGCGAGCCGCTGTGACCTGTTTTTGTG
CCAATAAAAAACCGGATCCAGTGACAGGATCCGGTCTTCATTGGGGGAAGGGGTCACGCATAGGGCGTCA
GCTCATCCCTTCTTGTCATCGGCGAAGGTGATGTTGAGCTCCAGTACCGACAGCTCATCACTCTTCTGAT
CCAGTTGGACGGTTATCTTCTCCGGATCAATGTTGACGTATTTGCGGATCACATCGAGGATATCCTGCTT
GAGCTGGGGCAGGTAATCCGGCCCCCCTCTGGATGAACGCTCGTGAGCAACAATGATCTGCAACCGCTCT
TTGGCCAGTTGTGCGGTGTTCTGTTTCTTGTTGGAACGAAAATAATCCAATAATGACATGTTTAACTCCC
GAACAGTCGGCTAAAGAAGCCCTTCTTCTCTTCTTCCAGAAAACGAAATTCCTTGGCATCGCCCAGCAGG
CGGGACACCGCATCTTCGTAGGCCTGACCGGCATCGGACTCCTTGTCCAGAATGACCGGTTCGCCGGAGT
TGGAGGCGCGCAGCACCGCCTGGGACTCCGGGATCACCCCGAGCAACGGGATGGCCAAGATCTCCTGCAC
ATCCTGCACGCTCAGCATGTCGCCGCGGTTGACGCGGGTCGGGCAGTAGCGGGTCAGCAGCAGGTGCTCC
TTGATGGGGTCTTCGCCACGCTCGGCGCGACGGGACTTGGAGGCCAGGATGCCAAGGATGCGGTCGGAGT
CACGCACGGAAGAGACTTCCGGGTTGGTGGTGACGATCGCTTCATCGGCAAAATAGAGCGCCATCAGCGC
GCCTGCCTCGATCCCCGCCGGGGAGTCGCAGACGATGTAATCGAACTTCATCTCCGCCAGCTGATCCAAG
ATCTTCTCGACCCCCTCACGGGTGAGGGCATCTTTGTCCCGGGTCTGGGAGGCGGGCAGGATAAACAGAT
TTTCCGAGCGTTTGTCCTTGATCAGCGCCTGATTGAGGTTTGCTTCCCCATTGATGACGTTGACGAAGTC
ATACACCACGCGCCGCTCGCAGCCCATGATGAGATCCAGGTTTCTCAGACCGATGTCAAAGTCAATGACC
ACTGTCTTGTGACCACGCTGGGCTAAGCCGGTGCTCAGGGCCGCACTGGTTGTGGTCTTGCCAACGCCAC
CTTTACCCGATGTAACGACTATGATTCGCGCCATTCTCGCTTCCTTGTAACTCTATTTGATACGGTCTAT
TCGGAGTTGTTCATTGTCCAGCCGGACGTGTACCGGTTGCTGGATCAGGCCTGCCGGCAATGCATCGCTC
AGCTGGAATGTGCCCGCGATGGAGAGCAGCTCGGCCTGTAATTGCTGGCAATAGATCCGGGCCTGGGTGT
TGCCCCGGGCACCGGCAATGGCTCTGCCACGCAGGGTGCCATAAATATGAATGCTGTCATCGGCGAGCAC
TTCGGCTCCCGGACTGACGGACCCCAGCACCACCAGCGAGGTGCCGGCGGCGTAAATCTGCTGGCCGGAA
CGAACCGGTCCCACATGTACCTTGCTCGGGATCAAGGGGGCGGGTTCGGGCTCGGGAACCACCTCAGGGG
CGGCTGGCGCGGGGGCAGGCTCGGGTTCCGGTTCGCTCTTGCGACTCTTGCCCGAGGCCATTACAGCCAG
TCCCACCGCCTTGGCGGCTGTCTTCATATCTTCACTGCGCGCACCCGTGATGCCTACCAGCACAAACTCT
TCGGACTCCACCAGCTCCTTGAGCTGTTCGAAATCGGGAATATGTTCCAGTCGGGAAACATTGATCACCA
GCGGCGCACAATTAAAAAAGTAGGGTGCCTGGGCCACTTTTTCGGCCAACAACTGACGTATCCGCTCAGG
CTCGCCATCGCTGATATGCAACACAGAAATAGTGAAGGTGGTCCCTTTCAATTCGTTATCGCGCTCAACC
ATGAATCTTCCCCGAGGCAGAGATGCTAATCCGTTATTGTTATTCAATTCCTGATGTTATAGTTTGCCCC
CATCAGAGGCAATAAACTCGTCGGATTATATCAATTTCCATGTTGTGTGCAGTCTATAAAAGTCGCAAAA
AAGCCGAGACCTACCTTTTTGTTGAAAAAAGAGAGGATTTCTCCCGCGTTCCCGCGCCACTGCTGAGCGC
ATTCGGGCGTCCCGAGCTGGTGCTGATGACCAAGCTGGATCCGGCCAAGCCCTTGGGTCTGGTTGATGTC
GGCAAGGTGATGGCCGCGCTGACCAACCAGGGGTTCTACCTGCAGGTGCCACCGCCCCCTGAGAATTTGC
TGGAGCAGCATAAGGCCAGCCTGAAAAAACCATCCTGAATATCCAGCCCCGTGGTGTGTTCTGCCAAGCG
GGGCTAATTCTTTGTGACTCTGCATGACGCATTCCAGCCCCGGAAAGGTGGCCTGCGATTATCCCGTGAC
AGGATCTGGCCAAATTAAACCCTCCCTTTGGTGATATTCACCTTTCGCTGTTTAAATAGAAAGTCAATCT
ACTCCCAACACCATATCCTCAACCTTTGCTGGCGCTCACAAAAATAGCGAGTGGCGCGTACTCATATCCG
CCATTTCAACGGCTATTGGCTCATTCATCGATTATTTTCAGCCTCTTACTTGGGCGCTTTTTTGGCCTCC
ACTGCGCGATGAGTTATCCATCAAATGGGGTGCAAAGAGGGCGGTGATCACTGCGCTAAAACCAGTTTGA
GTGATGCGCTGTGGCCAAAATGATGAGCTAATCCCCTGTTTGTGTGCTTGATTGTTTGCCCTGATCACGA

AAGTGCCACATAGATGGCTATCAGCATGTAATTCGCAAGGGTGAGCGGTAACCGCATTTTGCCAGGCAAG
GGTATGCCCAAAGTGGAGGGGATTGAGCTGAGGGGCCATCTCGATATCGATTAGTTGTGAAACCGTGACC
GGTTCAAGATAGCTAGTAAAGGTTTCATGTAGGGGCGTAATGTCCCTTTCAATGCATTGAGAAGAGTTAA
TCAAGGTATGTAATTAGAGAGGCATTTAGACAAGGCAATCCGCATTGCGGTGGCGTAGCAGGGGTATATC
CGCGACGCCCTACAGTGAAATGGTCGGTATGCCGGTATAAGTGCCCGGCATCCCATTCGGGCAATAAAAA
AGGCGGGATATCCCGCCTTTGTTCAGATGAGGGTCGTAACCCTTATTTATTCGCAGGCAACCTGCATCAC
CTTGATGGCCAGACCGCCACGGGAGGTTTCACGGTACTTGGAGTCCATGTCCTTGCCGGTGATGTACATG
GTCTCGATCACCTTGTCCAGAGAGACGCGCGGCTTGGAGGTACGACGCAGGGCCATACGGGAGGCGTTGA
CCGCTTTCATGGCTGCGATGGCGTTACGCTCGATGCAAGGCACCTGTACCTGACCGGCAACCGGATCACA
GGTCAGACCCAGGTTGTGTTCCATGCCGATCTCGGCAGCTTCGCAGACGTGCTCCGGGCTACCGCCCATC
AGCTCGGTAAGACCGGCAGCGGCCATGGAGCAGGAGACACCCACTTCACCCTGACAGCCCACTTCGGCGC
CGGAGATGGATGCGTTCATCTTGTAGAGGATGCCGACGGCACCGGCAGCCAGATAGAAGCGGGTGTACTC
GTCGCGGCCAACCGGCTGGATGAACTTGTCGTAGTAAGCCAGTACGGCCGGAATGATACCGGCAGCGCCG
TTGGTCGGTGCAGTGACCACGCGGCCACCGGCAGCATTCTCTTCACTCACGGCCAGAGCGAACATATTGA
CCCAGTCAACGATGTTCATCGGGTCGCTCGAGAGGTTTTCGTTGGTGTTGAGCTGACGGTAGAGCGGTGC
GGCACGACGGGGTACACGCAGTGGGCCGGAGAGAACGCCTTCGGTACGGCAGCCGCGCTCGATACCTTCA
CGCATGGTGTTCCAGATCTTGCCGAACTTCTTGTAGATCTCTTCCGGAGTGTTGAAGCACTTCTCGTTTT
CCATCATCAGGGCAGAGATGGAGAGACCGGTTTGGCTGCAGTGGGCAACCAGCTCAGCGGCGCTCTTGAA
CGGATAGCGTACGGACATGTCGCCGCTGTCAGCCTTGCCGAAGTTCTCTTCGTCAACGATGAAACCGCCA
CCGATGGAGTAGTAGGTCTTGCTGTAAACCACTTCATCTTCAATCAGGGCAGTCAGCTTCATGCCGTTTT
CGTGCAGCGGCAGGGCTTCGTCGTGGAATACCATGGCGTCACGCGGGAAGCTGACGGTGTGGCAGTGCAG
GCCGATCGGCAGGCGCTCGGTCTGTTCAACACGGGAGATAAACTCGGGGATGGCATCGATATCGACATTT
TCCGGCAGGTTGCCAGCCAGGCCCATGATGATGGCAGTGTCGGTATGGTGGCCTTTGCCAGTCCAGGAGA
GGGAGCCGTAGACATCGACATTGATCCGGGTTACATCGCGGATCTTGCCGCCCGCGCGCAGCTCGTCAAC
AAACTGTTTGGCCGCTTTCATCGGGCCAACAGTGTGGGAAGAAGAGGGGCCTACACCGATTTTGAAGATA
TCAAAAACGCTGATCATGTCACTATTACCTCAGGGTAGAAGGGGGCGGCATGCCCACTCACAGAAATTTG
GGAGATCCATGGAACGCGCTTGTCAGCGCAGGTCACAGAACCAGATAACCGTAGTGTAACGGCTCCAGAC
CTAAAAAAAGTTGAAATAGATCTATTTTTGTTGAATCTGAGGCGCGAGTATAAAATTTATTTTTGTGCTT
ATCCATTTTGTTAAGCGTTTGCCCATAAAAATACCCGGCGATGCCGGGCATTTAGGAACCAAATGGAGTC
ATTTGCCGTTTTTTAACATTCGGTAATGTTAACGGCGAGGCCGCCACGGGCCGTCTCCTTGTATTTGGTC
TTCATGTCGTTGCCGGTCTCCCACATGGTCTTGATCACCTTGTCGAGCGACACCTTGTGCTCGCCTGTGC
CGCGCAGGGCGAGGCGGGCGGCATTGATCGCCTTGACCGCCCCCATGGCGTTGCGCTCGATGCAGGGTAC
CTGCACCAGTCCGCCTATAGGATCGCAGGTGAGGCCGAGATTGTGCTCCATGCCTATTTCGGCGGCGTTC
TCCACCATGGCCGGGCTGCCACCCAGCAGTTCGGTCAGTGCGCCAGCCGCCATGGAGCAGGCGACTCCCA
CTTCCCCCTGACAGCCCACTTCGGCGCCGGAGAGGGAGGCGTTCTTCTTATATAAGATGCCGATGGCAGC
CGCGGTGAGGAAGTAGCGGATCAGCGTCTCGTCATCCACCTTGTGAACGAAGCGATCGTAGTAGTGCAGC
ACGGCGGGAATGATGCCCGCAGCGCCATTGGTGGGGGCGGTCACCACCCGGCCACCGGCGGCGTTCTCTT
CATTGACGGCCAGCGCAAAGAGATCGACCCACTCCATCACCATCAGCGGATCCTTGTTGAAGTTGGTCTC
GCAGCTTAACTGGCGGAACAGCCCCGGGGCGCGGCGCCGAACCTTGAGGGCGCCTGGCAGCACCCCTTCG
CTTTTGCAGCCTCGCTCGACGCAGCCTTGCATCGCCTGCCAGATCTTGCGCAGCCCCGCTTTTATCTGCT
CATCGCTGCGAAATATCCGCTCATTGGCCAGGATCAGCCCCGAGATGGAGAGGCCGCTCTCCTGACAGTG
GGCCAGCAGTTGGGCGCCGCTGTCGAACGGGTAGGGGACAGGGTGTTCGGCATCGGACTTGGCGGCCTCT
TCTTTGGCGGTGGCAAAGTGGCTCTCTTCAATGATGAAGCCGCCACCGATGGAGTAGTAGGTCTGGCTGA
AGCGCAGGTTTTCCTCTTTATCAAAGGCCCGCAGAGTCATGCCGTTGGTGTGGGCCGGCAAGGTTTTGCG
ACGGTTGAAAATGATGGCACCGGTGCGGGGAAAATGGCTTGGTTGTTCGCCTGCCAGCTGCAGGTTCTGT
TCCTTTTCCACTCGAGCCAGAATGGCGGGAATCGCATCGATATCGACCGACTCCGGTTCAAAGCCCGACA
ACCCCAGAATGATCGCCTTGCCAGTGCCGTGTCCTTTGCCGGTCTGGCCCAGCGAGCCGTAACACTCCAC
TTCGACCCGTGTGATCTGCGGCAGATGGCCTTGCTGCTTGAGCTGCTGAACGAAGGCATAGGAGGCACGC
ATGGGGCCGACGGTATGGGAAGAGGAGGGGCCAATTCCGATGGAAAACAGGTCAAACACGCTGATCATGA
CATCAATCCGGTGATGGTTTATGTCTGTTTATTCTAGGATTTTTGCCATGGATAGGAAGCGGGGATGTGC
GGTGCTGTGACATGGATACAGATAATGGGGCTGGGGGGCCAATAAGGTTGAAGGGGCTGATCCCCTTCAA
CCGGCCTGATCAGTCGAGAAATTCGCACAGATAAGCTGTGGGGGTTTTCACTTCCAGTTTGAAGGAGGAG
TGACCGGCCACCAGGAAGGATTCACCCTGGCTGTAGGTGTGCCACTCATCTTCATCAGCCAGCTGGATGG
TGAGGGCGCCCTTGATCACTGTCATCCGTTCGGGGGCGGCGGTGTTGAACAGGTAGTCACCGGCGTTCAT
CACCCCGACGGTTGATTTCTCCCCTTTTTGTTCGAAACCGATGGATTTTACATTTCCGTCGAAATACTCG
TTAACTTTCAGCATCTTTACATCCCTGTATGTACGTTTGTGCAGCCAAACTAGCACAATCTTTTGCCAGT
TGTCATGGGGGATGAGGTAAAAATCGCCGAGACCGCTTTGAATCTTCACTTTTTCGCAAACTTATTAATA
GCCAAGGTTAATGATTTGCTGATTTAAAACAGCAATTAAATGTGAGATAACACGCGCAAACAGGGGAATG
GGCAGCCGCTCATTCCCCTGTTTGGTTAATTGCACTGGGAAAAGTGTGAATTTTGCTACATAAAGGAACA
TATTGTGTTAACTAAAGGACTCATTAAAAACATCGGCTTCCAGGTTGTGGTCGCCATGGTACTGGGCGCA
GTGGTCGGCGCGTTCATGGGTGAACAAGCCACCGTGTTTGCACCGCTCGGTACCCTGTTTATCCAGCTGA

TCAAGATGCTAGTGATCCCGCTGGTGGCGGTTGCCATCCTCTCCGGCGCCGCCAATCTGGGCGCCAGCCC
GGCTGCGGGCAAGATCGGGGCAGCGACGCTGGCTTTCTTCCTCGTGACCTCCGCGTTGGCCGTGTTGCTG
GCGCTGGTGATGGGGCAGATCTTCAAGCCGGGTGTCGGGGTCGATTTCGCTTCCGTTGCCTCCATGTTTG
GCGGTGACTATGCCGATAAAGGGGCGTTGCCGGATGCAGTCGCCACCTTGCTTGGCATGATCCCCACCAA
CGTCTTCAACTCCCTCAACGAAGCGAACATCCTGCAGATCCTGGTGTTCTGCATGTTCCTCGGCATTGCG
CTGGCCAAACAGCCGCGCGAGCGGGCCAAGCCACTGGTCGATGGGCTCAATACCCTGGTGGATGCCTTCG
TCTGGATGATCAACAAGGTGATGATCATCGCGCCGCTCGGGGTGTTCGGTCTGATGGCGGAAGCCATCGG
TACCTATGGCTTCGATGTGCTGACCCTGGTACTCAAGCTATTCGTGGTCTATGTGGCTGCCATCCTCATC
TTCGGCTTCGTGGTCTATCCGCTGCTGGTGATGATGTTCTCCAACACGCCGGTGATGAAGTACCTGTCTG
CCATGAAGAAGCCGCAGATCGTGGCCTTCTCCACCGCCTCTTCCATGGCGACCCTGCCGGTCAATATGGA
GACCTGCGAGCATGATCTGAAAGTGACCAACGCGACCGCCTCCTTCGTGTTGCCGTTGGGTGCCACTATC
AATATGAGCGGCAACGCCATCTATTACGGTCTGGTCGCCATCTTCTTCGCGCAGGTTTACAACATCGATC
TGAGCGTCGGTGCCTGGGCGGCCATCATTGTGACCTCCACTCTGGGGGCGATCGGTCAGGCCGGTGTGCC
GGGCCCAAGCTTCCTGGTGGTTGCCGTGCTGCTGGCGGCCGGGATCCCCATCGAAGGGTTGCCGCTGCTG
TTCGCGCTGGACCGTCTGTTCGACATGATCCGCACCTCCCTCAATATCACCGGCGATGCGGCCTGTGCTG
TCATTGTTGACCGCTTCAGCCCGGAATATGACCCAGAGCGCTGGAACAAGGCATAACTGCTTTGAGCTGG
GCTGAGATATAACAGCCTTAAGCTGACACTGCGTAACGCGAGCCCCTGCCATGATGGCGGGGGCTTTTTT
ATATCTGCCGGACAAGTATCAGAGCAAAGGTGTGCTCAAGAGATTGCTAACGGATGTAGCTGTGTTGAAT
GGAGAGTACAGGAGGGGAAAACATCAGTCGTGGGCATGGGCCGTATGGCGTCAACCGGCCTGTTGTAGAT
GCCGGGGTAGGGGATTGTCAAACGACGGGTAACAAAAAGGGGAGCCTTGCTCCCCTCGGATATGATCTGC
TGCTGGTTCTACTCCATCAGGCCATATGGTTAAACCATCCTGGTTACACCCTTGTGGCGAAAGAAACAGC
GTCAACGTGATGAAATCATATCTATGTTGACCATCTCGGCATTGTCACTGAAAAGCGCCAGTTGGTGGCG
ATTAAACAGACATGGCAAAGAGGGTCGGTCCGGATTGATGCCTCTCTATCCGGCTCAGAACTGTGACAGT
GAGGGCAGAGAGACAGCAACCAGGGTGAAAAACACCGTGAAAACGGCAGTAATGACAAACTCTTTCATTT
TTCTCTCCCTAAGTTGTTGGTTGATGTAGTCAAGATACCATAACAAGGGGGTAAAGCAAGGATGAAACAA
GGGTAAAGTTTGTAAAATGATGGATTTTATCAGTGTATGGGTTGAAGTTGTTGTTTTTTACATGTTGAGC
TTATTTTGTCACCCATTGCTACTCACCACCCATGAAAGGTCAATCAGTTATATTTATTGTGTTTAAATTC
AATGTCTTATGTGTTTTCTTGTTGGCTTTTGGGTGGGTTTTATATGGGTCTTTAAAAGGGTTTTATAAGT
TATCCCTCTCATAATGGCATCATTAAAGTCATTAAAAGCGTGCAAGAACATGGTTTGACGATTCCTGGCG
TAGATAGCCCATCCAAAAGAAGTCTATGGAGACAATCTGGTTGCCAGCCCTTGTCGCACCATCACTTTAT
CAGTCCAGAGTCTCCGCATTTTTACCGCAATACACTGGGTTCATAGGGAGGAGAGCGGTGCATCGGGGGG
ATACAGAGTGGCAGCTGGCTGTTTTATCACCACCAATTTCCCCGTTGGCCTCGGAGTAGACATGGCCGCA
GCAAGAGTGAGGTGCGCAATAGAGCAGAGGCGTATTCTCTGGCTGGGCTGGGCTGGGCTGGGCTGGGCTG
GGCGCGAGCAGCGATCTGCGTGGGCGGTGCACCTGCGTGAGTCATGTGATTGCAATACGAGATCAGAGCC
GGAGCATGGTTGGGTAAGATATCTGAATCCCTTCTACAAGATTGAGATATAAAAAGCGGCGTATGACGAG
GAGGCTGAACAAGCTCGTTTCAGGGTCGACAGAGCACAGGTCAACGCAATCCGGATCGCAGTAATAAAAC
ACATCCACGACGATACCGGAACTCTGTTCGTAGGCTGATGCCGGTTGCTGTGAGGAGGGGAAGGCTAGTG
ATCAACCAGATGGCGCAAGGGTTGGTAATCAGGCAACCCTGTAGTGAGGGGCACACAACGTGCCGATGCA
CCCGTTTTTGTAAGATATTAAAAAACCCTCCGACGGAGGGTTTTTGTCATCAACAGCCGGTTAAAAGCCG
CGTGGTTTAGAACAGGGTGGTGGAGAGGTTGTAAAGCTCCTGATCGAACGGACGCTTCATATTCTCGATG
CAGTCGATGATGTCGTGGTGTACCAGCTCGTTGCGCTGGATACCGACGCAGCGACCACCGTGACCAGCCA
GCAGCTGCTCAACGGCGTAAGCCCCCATACGGCTGGACATGATGCGGTCACGGGCAGTCGGGGCACCGCC
ACGCTGGATGTGGCCCAGGATGGTGGCGCGGGTTTCACGACCGGTCAGGGCTTCGATGTCTTTCGCCAGG
GCGTTCACGTCGGTAACGTGCTCGCAGATGGTGATGATGGCGTGCTTCTTGCCCTTGGCTTCGCCTTCCT
TTATCTGCTTGAGCAGCTTCTCTTTGTCGAAGGCCACTTCCGGCACGATCACATACTCGGCACCACCGGC
AACGGCGGCAGACATGGCCAGATCGCCACAGTGGCGGCCCATGATCTCGACCACGGAGATACGGTGGTGG
GAGCTGCAGGTGTCGCGCAGGCGGTCGATGGCGTCAACCACCACGTTCAGGGCGGTATCAAAGCCGATGG
TGAAGTCGGTACCGGCGATGTCGTTGTCGATGGTGCCCGGCAGGCCGATGCAGGGGAAACCCTCTTCGGT
CAGCTTCTTGGCACCCATGTAGGAGCCGTCACCACCGATGACGACCAGCGCATCGATACCGTGTTTTTTC
AGGTTTTCGATCGCTTCGGCGCGCACTGCGGGGTCTTTGAAAGCAGGGAAACGGGCGGAGCCGAGGAAAG
TACCACCACGGTTCACCACGTCGGAGACGCTGTGACGCTCCAGCTTGACGATACGATCCTGGTGCAGACC
GAGGTAGCCATCATAAATACCGTAAACTTCCAGGCCATGATGCAAACCGGCGCGAACCACGGCGCGAATG
GCTGCGTTCATACCCGGTGCGTCGCCACCACTGGTCAAAACACCAATACGTTTGATTTGTTTGGTCATGA
GTGCCTCTGGATCTGAGTTGTCAAATACGAATGTTTAAATAGACAAGCCACTTTTACTATATCGGCTTTC
ATATGGGATGCGGGGGCCACCATAGGAAAAGCGATACAAGAGAGGGGACGAGCCCCTCTCTTTGCCGTAA
TCAGCGATTACTTGGCAGCGCCAGCGATGATCTGGGAGAAGTCGTCCACTTTCAGGGACGCACCACCGAC
CAGGCCACCGTCGATGTCCGGCTGACCGAACAGGTCGGCTGCAGTGGCGCCAGTGACGGAACCGCCATAC
AGGATCTGTACTTTGGCAGCAACGGCGGCATCAAAACCGGCCAGGTACTGACGGATGTGAGCGTGAACGG
ACTGAGCGATCTCCGGAGTGGCAGTCTTGCCAGTACCGATGGCCCATACCGGCTCGTAAGCGATAACGGC
GTTGTTGAAGGAGGCGATACCGCAACGGTCGATAACGGCTTTCAGCTGGGTCTCAACCACGTTCTTGGTC
ACGCCGGCTTCGAACTGCTCCAGAGTTTCACCGATGCACAGAACCGGCACCAGGCCGCCTTTCTGGATCG

CTTCGTACTTGGCAGCAACCACGTCGTCAGTCTCTGCGTGCAGGGTACGACGCTCGCTGTGGCCGACCAG
AGAGTAGGTGCAACCAAAGGCTTTCAGCATGGAGGGGGCGTTTTCGCCGGTGAAGGCACCAGACTCGTGA
ACGTCAGCGTTCTGGGCACCGTAGGCAATCTTGCTGCCAGCGGTCAGTTGCTCAACCAGACCCAAAAAGA
TAACCGGCGGGCAGACGGCGACTTGCACAGAAGGATGAGCGGCAGCAGCCGGGGTCAGTCCTTTGATCAG
AGCTTCTACAGAAGCCTTGGTGCCATTCAGTTTCCAGTTACCCATTACCAGGTGCTGTCTCATCTTGTAT
ATCTCCTAGGCATTTGATTGCGCGACATTATATGAATTGAACCGAGCCCAAGATAGCTGACCCGGCGGCA
AATGACGTAATTTTTTTTCGCTTTCCTGATCCAAATCAGGAATGAAACGCTTGATCGCTCATTCGTTAAC
CTGCCCCCGCAAGCGAGCCCACCATACCCCCATCCATTCGTGGATGGCAGCTTCGGAATACATCAAATAA
CGCCCTTTGGGCAAATAACTGTAGAGCGGCTGGGGTACCTGGCTCTGCTTGGCGGTGTAGTCGGTGGGGG
CCGGCACGGCCTTGAGTCCCTGACCCTGATAGAGGGCCATGGCGCGCGGCAGATGGGTGGCAGAACTGAC
CAGCGCCACCTCTTTTCCTTTCAGATGTCGGCTGATACTGACCGCCTCATCATGAGTGTCTTTGTTGTTT
TCAAACAACGTCATTCGCGAACGGGGTATGCCGAGGCTCTCGGCCATCCGAGCGTTGACCTCGGCATTGG
AGAGGGTATCCCCCGACACATAGCCGGAGAACACCAGCTCGGCTTCGGGATAGGCCTGGGCGAGGCGCAC
CCCTTCGAGAGTCCGTGCCAGCGAGATATTGTTCTGCCAACTGCGCATAGGCACTGCTGGGTCGCTGACA
TGACCGTTGCCGAGCACCACGATGGCATCGATGTTGGGATGCTGGCTCACCTCGAACGGCGGGAAGGTCT
GCTCGAGCCCGCGGGCCAGTTCGTAGCTCACCGGCCGCATTCCCAGCAGGGCGACCAGCAGCAGCGAGAG
AGTCGCCAGCAGCTTGCCGGTTTTCTGAAAACGGGTGAACCAGAGCAGCAGCAGGGCGACCAGCAGCAGG
GAGAGGGAGAAGGGGAGCGGCATCAACAGTTGACCCAGCCACTTTTTGAGCACAAACACGGCAGACACTT
CCTTGAGTTTTCAGCAAATTCGGGATGTGACAGAATACCTCTCTTTTTTGGGATGGCCACAACCCTGTGG
CGCTAATGTTGAGTTTCTCCTTGAAGCACGATCAAAGTTTTGACGGGCGCGCCGACAAGTTCGCCCGCAA
CATTTACGACTCCACCAAGGGGCGCATCCGCACCACCATTGTCTGGAGCGACATCGAAGCCTGCCTGGCC
CGCCTTGGCAACCGTCCGCTGCGCATCCTCGATGCGGGGGGCGGTTTTGGCTATTTTGCCCAGAAGCTGG
CGGCCATGGGCCATCAGGTGGAGCTGTGCGATCTGTCAGCAGAAATGCTGGAACTGGCCCGCAGCCAGAT
CGCCGAGAAGGGGCTGGAAGACAAAATCCGTCTCATCCATTGCCCCATTCAGGATCTGAAGCAACACGTG
ACCGGGACCTTTGATCTGGTGCTCTGCCATGCGGTGCTGGAGTGGCTTGCCGAGCCGCGCCAGACCTTGC
TGGGTCTGTTCCCTTTCGTCAATGCCGGGGGCATACTGTCGCTGCTTTTTTACAACCGTCATGGCTTGCT
GTTCCAGAGCCTGGTGGTGGGCAATTTTGACTATGTGCGCCTTGGTCTTCGCAAGAAGCGTCAGACAGCG
CTCACGCCCACCAATCCCCAGCTGCCGGAGCAGGTCTATGACTGGATTGGTCGCGGGGGGCTGCGCATCA
TCGGCAAGACCGGGGTGCGGGTGATCCACGACTATATGCGTCACAAGCAGGATCAGGTGGAGAAGTTCGA
CGATCTGCTGGCCATGGAGCAGCAATATTGCCGTCAGGAGCCTTTTGTCTCCCTCGGTCGCTATATCCAT
GTGATGGCCCAGAAGCCGGCCAGCTGATTGCTCGCTGTTCACGTCAATGTGAACCATGTTTTCGATGCTA
CCGGATGTAGCCGCGCGCACGATTTTGCGCCCAATACAACAACGAGATGACAACATCATGATGAGTACAT
CCCGTACCCTGCCAGAGCGTTCTTTGCCGGAGCTAGTAGGGTGGGTCAGGCAGGAGGGGTTGGCGCTGAG
CCTGCCGACCGACCGGCTGGCCTTTCTGATTGCAATCAAGACCCTGTCCGAAGATGAGTCGGACCTGTCG
GAAGCGGCGCTGCACGACGCCTTTGGCTATGTCAGCAGCGCCTTCGGCCAGATGGACGAGACCCTCACCG
GTCGCGCCAACAACGCTATCAACGATCTCATTCGCCAGCAGCTGCTGTCGCGTTTCAACACCGACATGGT
GGCGGGGGAGAGCCTCTACCGCCTGTCGCGCCTGGGTGTGAGCATCGTCGACTTCTTTATGGATCAGCGT
CAGGTTGACTCCATCAAGCTCTCCATCCTGCTCGAGCACCTGGCTTCCGAGCTCGACACCGCCCGCAAGG
CGGCGCTTGAGACCAACACCCGCGAGCAGTGGGATGCCGAGGTGCTGCCGCGCCTCACTTTCTCGCTGGA
AGAGACGCTGGGGCGGATCGATCTCACCCAGCGCGCCATGGATGAGCAGCAGAATCAGGTCAAGAGCGAG
ATTGCGGCCCTGCTGACCCAGAACTGGGTCGATGCCATTCACAGCTGCGAAAAGCTGCTGCACGAGACCG
GCCAGACCCTGCGCGAGCTGCAAGATACTTTGGATGCTGCTGGCCACCGCCTGCAGGCGGGGCTGCTCAA
CATTCAGGAAGCGGCGCAGGGGCGCGACGATCTGTTCCACGTCGAGGAGCTGACTCACGCCTTGCAAGGG
CGCCTTGACGTCATCACCAGCTGGGGTCAGCAGTGCATCGAGCTCTGGTCCCGCTACGACAGACACGTCC
ACAAATTTATCCGCAACGCCATCGACATGGACAAGAACCGCGCCTTCAGCCAGCGGCTGCGCGAGTCCAT
CCGCAATTTCGAGCAACACAACTGGCAGCTGCGGGTGGCCGAAGAGCCCCGTCTGCTGGAGCTGCGCGAC
GAAACCATCGCTATCCACGACGAGGAGGTGACCGGTGAGGTGCCCCTCGAGATGGAGTACGTCGAGATGG
TGGACATCAACCAGGAGCTGGCCGAGCGTATCGAGCGCTATCTGGCCCGCTACCCCGAGCAGGGCCAGCA
GCTGGACTTGGCCGATGTGCTGCGCGAATACCTGCTGGACTACCCGGCCCATGCCCACTTCGATGTGGCG
CGTCTTCTGATCGACCAGGCCGTGCGCCTGGGCCATGCCAGCGGCGAGCGCGAGCTGCATCGCCAACCCG
CATGGAAACCCATTAACCAAGCTGGATCCAAGGTTCAGGCCTATGTCATTGATCAATACTGAGTTTCCCT
TGCCGCTGCGCCTTGCAGAGGCGATTGCCAACCCCTTGTTTCCTCGCATCGACACCGCCTTGCGCTCCGG
TCGCCACATCAGTGCCGACGAGTTCGAGCAGCATTCGGCGCTGGTCGAATATCACAGCGAGCTGGAGATC
TTCTACGGCCGCTATCAGGTGGAGCTGATCAAGGCGCCGGAAGGCTTCTTCTATCTGCGTCCGCGCCCGA
GCTCCGACATCGGCACCACTGTGCTCTCCGAGCTCGATATGCTGGTGGGCAAGGTGCTCTGCTACCTCTA
CCTGAGCCCGGATCGTCTCGCTTCGGAAGGGGTATTCGCCATGGCCGAGCTGCAAGAAGAGGTGCTGAGC
CTCGCCGACGAGAAGCAGCTGCTGCGGATGGTCAACAGCCGCTCCGGCGGTACCGATCTCGACAAGAAGA
AGTTGCTCGAAAAGATCCGTACTTCCATGCGCCGTCTGCGCCGCCTTGGCATGATCACCGCCCTTGGCAA
CGGGGATAAATTCCGGGTCAACGAATCGGTGTTCCGTTTCGCCGCCGACGTGCGTTCCGATGAAGATCCG
CGCGCCGTTCAGCTGCGGATGATCCAGGAGGGTGAAGCCATCTTCCACGACGACGAGATGCCGAGCAGCG
ACTCGCTCTTTGATGACATCGAGGCGGATCTCGATGAAGAACAACTGGATCTGGAGGTATAACCGGTGCG

AGGCAAGTTCAAATCCCTGACCATGGTCAACTGGAACGGCTTCTTTGCCCGGACCTTCGATCTCGACCAG
CTGGTCACCACCCTTTCCGGCGGCAACGGCGCGGGCAAGTCCACCACCATGGCGGCCTTTATCGCGGCGC
TCATCCCCGACCAGTCGCTGCTGCATTTCCGCAATACCACGGAAGCGGGATCGAGCAGCGCCTCCCGTGA
CAAGGGGCTCTACGGCAAGCTGCAGCGCGGCCACTGCTACTCACTTCTGGAAGTGATGAACAGCCGCGAA
CAGCGGATCTGGGTCGGCGTGCACCTCGAGCAGGTGGCCAACCGCGACAATAAGGTCAACATCACCCCGT
TTGCACTGGTGGATGTGCCGGAGAATCTGGCGCCGACCGATCTGCTGCTGGAGAAGCTCGATGGCGGCAA
GGGGCGGGTACGCGCCTTTACCGATCTGAAAGGGGCGGCCGCTGAGCTGGGGGCGCTGAAAGTCGCCAAA
TTCAATACGGTGACCGACTATCACAACTTCATGTTCGAGTTCGGCATCACCCCGAAGAAGCTGCGTGATC
AAAAAGATCGCGGCAAGTTCTATCGCCTGATCGAAGCCTCCCTCTACGGCGGCATCTCCTCGTCCATCAG
CCGTTCCCTGCGCGAATACCTGCTGCCGGAGAACTCCGGGGTGCGCAAGGCGTTTGCAGACATGGAGGCG
GCCATCTATGAAAACCGCCGCACGCTGGAGGCCATCAAGGAGACCCAGGGTCAGCGGGATCTGTTCAAGA
ACCTGATCACCGAGACCACCCATTATGTGGCCGCCGACTATGTGCGCAACGCCGCCGAGAAGAGCCGCCT
CTCCGAGCTGGCCCTCAAGGCGCGCCAGGAGCTGGCCGACAAGCGCCGCATTCTGGCCGAAGAGAAGCAG
CGCGCCATCTATCTGGCCGACGAGGTGGAGCAGCTCACCAACCGTGAGAAACTGCTGACCGACGAGCTGG
AGTCTGCCTCCGAGCATCTGGCCAAGGTGATGGCGGCTGTCAGCTTGCAGAAGAAGATCGAGATGTACCG
CGCCGATCTGGCGGATCTCTCTGACAAATTCGAACAGCAGCAGGCAGTAGTGGAGGAGATCCACGGCCAA
CTGCTGGAAGTGGAAGAGCGCAAGGAGCTGGCACAGGCAGAAGTCGACAGCCTCAAGACCCAGCTGGCCG
ACTACCAGCAGGCGCTCAATATCCAGCAGACCCGCGCCATCCAGTACCGTCAGGCGGTGCAGGCGCTGGA
GGCTGCCCGCGAGCAGTGCGAGCTGCCGGGGCTGACCGCCGAACAGGCGAGCGACACCCTGCACCAGTTC
CGCGCAGCCGAGCAGAGCCTCACCAGCCGGGTGCTGGGGGCCGAACAGCAATTGGCGCTGGCCAAGGCGG
CGGTGGCCGAGCATGAAGCGGCGCTGGCGCTGCTTTTGAGCATCGCCCCCGAAACAGTGCGCGAGGCCGC
CTGGCCCACCGCGCAGGCGCTGCTCAAGCGTCACATCGAAGACAAGGCTCGCATTGCCCAGGGGCAGGGG
CTGCGCGCCGCGCTGGCTCGCCTCGAGAAAGAGAGTGACAGCCAGAAGAGTCTCTTCAAGTTGCGTGACG
AGCTGCAGCAGGCCTCTGGCCAGCTGATTGCCAGCAGCGATGAGCTGGAGATCTTCCTCGAAGCCCAGCG
CACCCGTCAGGAGGAGCTCTCCGAGCAGCAAGCGGAGGTGAACCAGCGCCGCGCCAGCCTTGAGCATCAG
GGCTCTCAGCTGGGGCGCGATATCAGCGATCTCACCAAGCTGGCGCCGCGCTGGCTGAAATCGTTCGAGA
AGCTCGAGCAGCTGCGCGAGCAGAGCGGTTTGCCACTCGCCACTGCCGAAGAGCTTTCGGCCGGTATGCA
GACAGTGCTCGACAAGGAGCGCGAGTTCGAGCAGGAGAGCAATCGCATTGCGGCGCAGAAACAGGCGCTG
GAGCTGCAAATTCGCAACCTGCAGCTGGGGGCAGGCGAGGCGGATCCGCGTCTTGCCCGCATCGCCGAGC
AGGTGGGGGGCGTGCTGCTCTCCGATGTCTACGACGACATCTCCATCGACGATGCCCCTTACTACTCCGC
GCTCTACGGCCCGGCTCGCAGCGCTCTGGTGGTGCTCGACCTTGAGGGCGCCATCGAGCGGCTGAAAAAG
CTGGAAGATTGCCCGGAAGATATCTACCTGATTCAGGGCAACCCCGACTCCTTTGACGAGGATCTGGTGG
AAGCGGACGAGCTGGGAGACGCCGTGCTGGTGCGCACCAGCAAGCGTCAGGTGCGCTTCTCCCGCTACCC
CGAGCTGCCGCTGTTTGGTCGCGCCGCCCGGGAAAAACGGATCGAGCAGCTCGACCTTGAACGCGAAGGG
CTCATCGAGGGTTACGCCAAGGCCGCTTTCGAGCAGCAGAAGTACCACCGTCTCTACGGTCACTTCCGCG
ACTTTATCGGCCAGCATCTGGATATCGCCTTCCGCCCCGACCCGGAAGCCGAAGTGCAGGCCAAACAGGC
CGAACTGCGCGCGCTGCAAGGGGCCATCGGCGAGTGCGACAAGCAGCTGAGTGATGCCAAAGCGGCCGGC
GCCCAGCTGACTCGCCATATCCAGCTGGTGCAGGGGATGCTGCCCTTTGCTCACCTGTTTGCCGAGACAG
AGCTGGCGGCCCGCCTCGAGGCGGCTCACGCCGATGTGGAAGCCCTGAAACAGGCCGAAGCCTTTATCGC
CAAGCACGGCAAGTCGCTGGAAAAACTGGAAACCATGGTGCAGGTGCTGCGTCAGGATCCGCAGGATCTT
GCGGCGCTGCAGGCTGCCTATGATGAGGTGAGCGATCTGCTGGCCGAGCAGAAACGCCGGTGCTACGCCC
TCGATCAGCTGGTGGCCCGCCTGCCGCACTTTGCCTATCAGGATGCACAAGACCTGCTGGGCAAGGCCTC
CGAGATGAGCGAGCGACTCAAAGAGAAGCTCAAAGCCGCCGAGCTGGCGGCCCGCACCGCTGGCGAGCAG
CACAAGCAGATTGCCCAGCGCCACACCGAGGCGTTGCAGCTGCGTACCGCGCTGAACTCCAGCGTCGAGG
CCAAGCGCCAGACTCTGACCGAGTTCGAGCAGGAGCTGGCGGCCATGGGGCTGACCCTCTCTGATGACAT
GGAAGAGAAGGCGCGCGCCCACAAGAAAGAGATTGAAGAGCTGCTGATCCGCACCCGTTCCCGTCGCACC
AGCGCCGAGGCACAACTGCAGGTCACCAAGCGCGAGATCGAATCCCTCGGTGGTCGTCTCAAGCTCGAAG
GCAAGGATTACTACAGCGCCCGTAAATCCCTGGTGGGCCACAAGGCGAGCTGGTCGCGGGTGGTGCGGCT
GGCGCGGGAGACCGACGTCGAGAAGCGCCTCAACAAGCGCGAGCTGGCCTACCTCGACAGCGACGAGCTG
CGCTCCATGTCGGACAAGGCCCTCGGCGCCCTGCGGGTGGCGGTGGCCCACGACGAGGCTCTGCGCGATG
CGCTGCGACTGTCGGAGGGCAACCGCAGCACCGAGCAGAAGGTGGCCTTCTACGTGCAGGTCTACCGTTA
CCTCAAAGATCGCATTCGCAACGACATCATCCGCTCCGATGATCCGGTTGAGGCGATCGAGGAGATGGAG
ATCGAGCTGGCCCGTCTGGCTGACGAGCTCAAGCAGCGGGAAACCCATCTGTCGCTCTCTTCCCACGAGG
TGGCGGCCAAGATCACCAACATCATCCGCCGCGAGCAGAACCGCATCCGGGTGCTCAACCAGGGCTTGCA
GAACATCGCCTTCGGTCTTGTGCGCGGAGTGCGCCTCAACGTCAATATCCGCGAGGCCCACACCCGTCTG
CTGACCGCGCTGGCCGACCAAACCGCCATGCACAACGATCTGTTCGCCGACAAGGAGCTCAGCTTCTCCG
AGGCGATGGCCAAGCTGTTCCAGCGCCTGAACCCGCAGATCGAGCAGGGGGATCGCAGCTATCAGGTGAT
GGGCGACGTGCTGCTCGACTATCGCAACTATCTGGAGCTGGAGATCGAGGTGCAGCGCGGGGCCGATGGC
TGGCTGCGGGCCGAGAGCGGTGCGCTCTCCACCGGCGAGGCGATCGGTACCGGTCAGGCCATCCTCTTGA
TGGTACTGATCAGCTGGGAGGAGGAGTCCCGCCGTCTGCGCGGCAAGGATATCGCCCCCTGCCGTCTGTT
GTTCCTCGACGAAGCGGCCCGTCTGGATGGCAAATCCATCGCCACCCTGTTCGAGCTGTGCGAGCGGCAG

GATATGCAGCTGCTTATCGCGGCGCCCGAGAACATCAGTCCCGAGAAGGGCACCACCTACAAGCTCATCC
GCAAGGTGCAGGGCAAGCACGAGCATGTGCATGTGGTGGGTCTGCGCGGCTTCGGCTTTGCCGACGACAA
GCTGATTGCCTGAGAAGTCTCTGGCCCATCCCGACACGGGATGACCGGTAACTAGTTGAAAGGGAGGCAT
TTGCCTCCCTTTTTACATGGAAGATATGGCAAAGTAGCCGCACCATCGCTGCGGCCTGACAATAAGGCGA
AAAGTTGCTTGCGAACATGCCGCCAGTGTCACCGGCCGCATCGAAGATGCAAAAATAGCGCAGAGTGAAA
AAAGAAGAGAGGAGAGGGGAAGGTGCAATATATCCAGAAAGGGCTGCTGTTATCTCTTTGCCTGCTGTTG
GCGGCCTGTAGCAGTCGTTATGACGAGAGCCACGGCGGCAGCTGGCGCGGCTATAGCGAGACCGGTTATG
CCAGCTATTATGCCGACCGCTATCACGGTAAAAAGACCGCCAGCGGCGAGCGCTATCGCAACAACCTCAA
TAGCGCCGCCCATATGGAATTGCCCTTTGGCGCCATGGTGCGGGTCACCAACCTCGCCAACGGCAAGAGC
GTAGTGGTCAAGGTCAATGACCGCGGCGCCTTTAAAAACGGGCGTATTATCGATCTCTCCAAATCGGCTT
TTAGCACCATCGGCAATATTCGCGACGGGATTATCAAAGTAAAAATAGAGGTGCTCTAGGCTGGGTGAAC
CCGTTTATACACGAAAATGGATGTTGATGAATGATATTGGAAGGCTGACATCGACCAGTCAGCCTTTCGG
TAGATAAAGATATAACATTGATATGGATCAATATTTAATTCAGCTAAAAATGGATTTGTTATCAATGGAA
AAAATCGATGATCGTGATTGATATTTATTTTAGCTTATCTCGCCTGTTCGGCCTTACTATGGGGCAAACG
GAGGGATTAGTGATGATTATCTATGGTAAATATGGTGGTGCAGCCCTGAGAGCTGTTCATCTGATGGTGC
AGAATAATTATGGGCATGCCACCGCCTGGGATATGGCTATTGCTGAAGCATTTGGTGGTGATATCAGTAC
AGGAAAATTCAAAAATAATCCAAAGGTAACTTTTTTGACTATTTGCAGTCTTGGTTGTATCAGTTGCATC
ATGCCAGTTCATTATGTAGCTGCGACCAAAACCAGAGCATATGTGTTGGCTGCCATTGATTTATTGTCAC
ACAAGGATATTACCAAACCGATCAATCCCGAGCAGTTATGGCTGGAGGTGATCTCGGCCTGTAAGAGCGG
CAAGGTTGTGAAGCATAACAATCAGATGGATGTCATTCTGACACTCTGGTATGCCGGTGTGCTCAATCCC
GAGTGGGATAGGGATGCCATCAGGATAGATTATCGACGAATATAAAAAAGCCAAGGTAATGAATACCTTG
GCCATGAAACATCAATTGGTTCTGGTCAGGGATGCACTATCGGAGTGTGCGAATGGCTTGCCATACACCA
TCAGGTTGGTGGTTTGACTGTATTTGAGGAGATCTTTCTCTGAGAAGTCCAGAGTCATCGAGAAATCCGT
GGTGGAATCATTAATGGTCATATAATTGCTTTCCGCAATACCGCGCTTGCCCGTCTTGAACTCGATTTTG
TTACCCGCTTTGCCTTCAGTGACCACACAGACTGCACGGGGTATACAGTATGAGTTGTAAACAAGTTGAT
TAGTCTTGTCATAAATCAGATAGCCGCGTTGGTCATGAAATTTCCCATTATCGCTTTTTCTGAATACTTC
CTGTTTGTAATACATGGCTACCAGATATTGTTCGCTGGCATTTTTAGCATCGGCAGCCGGTTCAAAAGTA
ATGACTTCGTAAAAGGGTTCCACTGCCGGCCCCCCTTTGCCAACTTTGCTACCCTCCTGGCCGGGTGCTA
CATCTACACCACCAGCATCGGTGCTTTTCCATGTGCCAGCCAACTGAGCTAATGGACCAAAATCCAGACC
GTTGATAATAGTGCTGGACTCTGCGTGAGCGAGCGTGGCACAGGTAATTAATGCTATGGGTAAGATAACT
TTATTCATTGTGATAACTCCATCACTAATAATGACTTGCGAATAAATATGTTCAGCAAAGTAATTGTTAT
AACGCACCAAGGGTGTGGTCAATGATATTTAGTAATGGTGGTTCGTTTTTGTGATGTATGATTTGCTTTT
TCGTGGCTAAATAAATTGAATTTGATATAAGACAATGTTGTATTTTTTCATCGCGCTTCCTCAGATATAT
CGTGATAGAGATAAAGGAACATTGACATGATGCGATGATTATTATGTCAACGGCGTGAATATTTCACTCA
GCCCCATCAATTGAAGGCGATAGCCACTAACTCTGTGGCACCCTGACCCCTTTATCGAGATAGAGGCTGC
TGTAGTTGGCCCTGTGGGTGACAAACATCACCTGATGGCGGCTGCCGTCGGAGAGGATAAGGAAATCGCC
CGCCTGAGCGGGGCTCTGCAAGGTGATCTCCTGCTCGGGGGCGGTCAGGCGATCGACATCATCCTTGAAG
GTGAGGGTGTAAGAGAACATATGGCGCTCCACTGCTGGCTCATATCAAACACTCCGTTGAAGGTAGTTGC
TGGCATGGGCACGCGCGGCGCCCGATTGGCGCTGTTAGACCAAATAGCTGAGATGGGAAAGCGCGTGGTT
TCAGTCGGGCTGTTTTGCTGAGGTGTGCTCGAGGCGTTTACATTAAGGCCATCGGTATCAAAACGATGGT
AAAGGGGCGAAAAGGGGATGAAAAAACCGCCAGCTGGCGGTTTCTCTTATGCTGTGGGTCCTACTTCAGG
GCCTTCTTCAGCTTCTTGATCTTGCCTTCATGCTTGGCAATTTTCTCAAGACGAGCGGCGATCTTCTTTT
TGATCTCTTTCTTGCTTGCTTTGGCCATCGGTGGCGCTCCTTTGCAGTGGGGACACATGAAGTGTCAGTG
TGGGTCTTCCCTTTGACGTCCCAGAGTGCCACAGGGCTCCTTTGTGAACCAGTACTCTGTTGCTTTAATA
GCCAGCTCGCTATAACTGCAAGGGGCTGAGCCAGCGCAGCAGTTGCTGGCTTACCTGCGGGTTGCCGAGC
AGGGCCATATGGCTGGTGCGATAGACAATCAGGGTATGGGCAGGAAGAAAAGCCAGCTGATGGCGCGCCT
CGCTGTGCTGGCCGAGGGCGCTACGAAGGGGCACCAGCCCATCGCCAATCAGCCGGTCGGCCAGCAGGCT
GCGCTTGGTGGCTGTGGTCGCGGCGATGGCATAGCAGTTGACGCCGCCGGGCAAGGGGAGTGGCGCGCGT
GGGTCCTGTGAGGGGACTGCGGGGTCGGGTGGGGCAACGGCATCGATCACCTTGCCGTGGCGTAAATCCT
TGATGCCTGCACTGCGTAGCCGCGCCAGCCTGACGAAGGGTTCGCTGTAGGCGGTGGCGGGCAGCAACTG
CTCCAGCCAGTGACCGGCATGTTCGAGCGGTGCGCCGTGGTGGGGTGTGCCGAGAAAGATCAGGTTGGCA
AGGCGTGCAGGCCAGGTGAGCCCGGCCACGCCGGCATGGTGGCAGGCGCTGCGTGCAACCAGTCCCCCCA
TGCTGTAGCCCATTATCGTTATCTCTTTGAGCTCTCCCGGCCAGTAGGCGAACAGCTCCTCCAGCAACAG
GGCAAGTAGCTGCCCGTTATGTGCGATAGGCAGGCCCGAGTTGTAGCGCAAGTAAAGAGGCAGATAGCCA
AGCGCGGCGGCAAGTTGCTCCCCGTGATTGACCGGTTTGCCTTGATGCTCGGTCTTCCACTGCAGATTGT
TCATGCACAGGCCATGGATCATCAGCAGCACCCGTCCCTCGCGGGGTAGATCCTCCGGCATCGCTTGCCA
ATCGAGGGGCTTGCCCTGATAGCGCAGGGTCATGGGGGTGGCGAGGGCATTGTGGTGCTCGACCAGCTTG
TCCCCCAACACGCCGTTGAGCGTGGCGATCAGGGCTGCACGTTTCGGGGTTTCGGGGATCTGGTGGGGCG
GCGGTGTGGCTGGCAGGCTGCTGAGTAGTTGGTTGATACCGCTGCTTAGGGTGTTGGCGGCGCCATGTAC
CCCCTTGTAGACCAGCCCGGTGATCCCGGCTGTTTTTCCCTCATCCTGTCCCTTGAGCCCCATACCACTC
AGTACCGCTTGATGTACCGCTTCGGTGATCTCTACCGCGGCGGGCAGCGCCAGAGTCAGCAGGTGCGCCA

GCTCGCGGATATCCGAAAGATGCCAGGAAGAGGATGGCGAGGGGTCTGTGGCGTCGCTCATGGCAAGCCC
TTGTGAAAGCGGGTATCGCGTTGTCGTGCCGCGAAGAGCGCAGTGTGTCTCTATATCAAACCAGCTTGCG
CCCTTTGTGGCAGGTTTACCAGTAACGGGCCATGCATGGCGGGGCTGATAAGAACCGCTTGCGAAGCCGG
TCACGGTAATGACAGGGAAAATCTCCTCTACTTCGTCATATGAAGCCACATGGAGAGGTGAGGATGTCGC
TGTTTGATGAGGTGCGGGTACAGGATTTGTGTGCACGGTTGCTCGATAGCCAGAACGAGAAGGGGTGCAC
CCTTGAGAGCAGGCATGGCCGCAAGTTGAAAGAGTTGTTGCAGACGAATTGTGGGTTAAGACCTTTTGGG
CGAAGCGCTCGTCATGTATTAACCGAGGCGGGACGGGCTTATCTGATCAGACAGCTGGCGTTGGTGGCGC
CCCCCGAACCGAACAAGGGTGAGCTGCTGCAACGTTTGGGAGTGAGTTTGCCTGCACGGCTCAATCAGGC
CTGTTTTTATGCCCTCTGGCATGGGGACAGCAAGCATTCGCGGTCAGCATCCCTTGCAGCGCCACAGCTT
GCGGACTTGACGCTCACGCAAGATGAGGTGATCCGCATCCGCACCCTCGAGCCGCTCTCACTGGTCGATC
GGCAAGGCCAGCAGCACGATATGACGGTAATGATGGCACTACTGGGAGAGTTGGCGTTGCAGGAGCGGAC
TCTGGGCACGCTGGCGGCCATCGGCTGGCAGGGGGAGCGGGTCATTACGGTGGAGAACAAGGGGGCCTTT
GTCGACTACCCGCTGCAACCCGGGCAACTGCTGCTCTTTGCTCCCGGTCGCAATACCTCCCTGGCCAAAC
AGCTGATTCCGCTGCTGCCTGCGGGCGTGCAGTGGGCACATTTTGGCGATCTCGATCAACGGGGTATCGA
TATTGCCGTCGAGTTGGCGCGCGAGCTAAATCGACCGGCCATGCTCTGGCTTCCCTGTGAGTTGCAACCT
TATCTGGAGCACTATGCCCGCCCTGTTCGCACCTTTGCAACGGACAGTGGCAAGGTGCCCTGGCGTGTGA
CGACGCAGGCTGATCAGGTTGATGGTCATGGCACGCCGGCAGGTGTTCCCTGGCTCGATAAACTGATCAC
AGACGGGACTTGGCTGGAGCAGGAGGTTTTGATTCTGGCCCCTCACTGGCGGAGCTGGTCATTGGAAGAG
ATGGGCAATCAGGGGTGAAACGGGGAGCCTGTGCTCCCCGTTATTTCGTGGTCAGCTGTTTACTTGGCGT
TGATGGGCCCCTTGGTGCCGGCATCATTATCGACCCAGCCATCTTCACCCTTGATGCGGCGATCCCCCTG
AATTGTGTAGATGACCATGCCATCCTTGAACACCAGCGCGCCATCGGATTTCTTGGCATTGACCTTCTTG
TCGTTGACCCACACCTGATCCTTGCCATCGATGCGGATATTGGCCACGGTGGCGCCGGTTTGAGGGAAGG
TGGCGATCCAGTTGCCCGCATAGGGGCTGCCGCTGGTTGCCGCGGCCGGAGCATTGCCAACACCGGCAGC
CGCGTTCTCAGCCTTGGTTTTGTTGATATCGCAGCCATTGAGCTCGCTCACCTGAGTGCAGCCCGAGCGC
TCCAGCTTCTGCTCATAGCTCAGATTCAGACCGCCCTCCGCCGCAGGGGCGGCCGCGCCAAATCCCGCCT
TGGCATTTTCCGCTTTGGTTTTGTGAATGTCACAGCCCTGCGCCTCGGTGACCTGGGTGCAGCCGGATCG
CTCCAGCTGCTCGGCATATTTGGCACTGATGGCGTGGGCAGGGGTGATACCGAGCAGGGTCAGGGTGATG
AGCAGAGCAGCATTCCTGGACATAGATAATCCCTCAAAGATGTAAGTCATGCAGGTTCATCCGCGCAGTT
GCGGCAGTGAACGTCGGCCTATCCTGAGCATCCGATACAGATGCGCAGCTGGCGTATTCACGTTAGCACC
TTGGGCCAACCGCGCCCAGCGATGTTGCCGGAAGATAAGGCGAGAGGTGATACGGGAGTGCAGGGGAGGC
GAGATGGCCGAGACATTTCTGCAGAAGAGGTGGTAACAAAAGAGCAAGTCGCTGCACGGCATTATCATAA
TGATAACGACAGCCCCGCTGATATCATTTCAGCCCTTGAAACTGACCTTTGATGATAATTCTCACCGATG
CCGAGATATCGCCTCTGATTATTATCACTCGGCTATTTTATGGCTTGGCTGATCCGCTCACCTTTTTATT
GAATAAACCGCTTAAATAGCCGCTGATTTTGTGGGTGGCATCACACACTTTCGCCATTTATTAATATCTG
CCTGCATTTTATTGGTTTGGCACGGTTATTGATTGCCTCATATCGACGCTAACCGACGCGCTCACCCACA
ACCGGGATGCTGGTGACATCGTGAGTGAAAGGGGAGCTATCCATATCTCATCCAATGATTGGGGCGAACG
ATGAAAGCAGTTAACGAATTAATCAAAGAGATCAGAGCACTCAAATCCAACCTGCATGAAAAAGATTTTC
TGCTGACCTGGGAGCAAACCCCTCAGGAACTGGAGCTGGTATTGAAACTGGCCGAATCCCTCAAGACCCT
GCGCGCCGAAAATATCTGCACCAAACTGTTTAACAGCGGGCTCGGTATCTCGGTTTTCCGCGATAACTCC
ACCCGAACCCGCTTCTCTTACGCCTCTGCCCTGAACCTGCTCGGTCTGGCCCAGCAGGATCTGGATGAGG
GCAAATCCCAGATCGCCCATGGTGAGACCGTCCGTGAAACCGCCAACATGATCTCGTTTTGCGCCGATGC
CATCGGGATCCGTGACGACATGTTCCTCGGTGCCGGTAACGCCTATATGCGCGAAGTGGGTGCGGCACTG
GATGAAGGGTTCGAGAAGGGGGTGCTGCCCCAGCGTCCGGCGCTGGTCAACCTGCAGTGCGACATTGATC
ACCCGACCCAGTCCATGGCTGATCTGGCCTGGTTGCAGGAACACCTTGGCAGCCTGGAGAGCCTCAAGGG
CAAAAAGATTGCCATGACCTGGGCCTACTCCCCGAGCTACGGCAAGCCGCTCTCCGTGCCGCAGGGCATC
ATCGGCCTGATGACCCGTTTCGGTATGGACGTGACCCTGGCCCATCCGGAAGGGTATGACCTGATCCCGG
ACGTGATCGAAGTGGCCAAAGAGAATGCCAAGGCCTCCGGCGGCAGCTTCCGTCAGGTCACCTCCATGGA
AGAGGCGTTCAAGGATGCCGATATCGTCTATCCCAAGTCCTGGGCCCCCTACAAGGTGATGGAGCAGCGC
ACCGAGTTGCTGCGTGCCAATGACCACGCCGGCCTCAAGGAGCTGGAGAAACACTGCCTTGAGCAGAACG
CCAAGCACAAGGATTGGCACTGCACCGAGGAGATGATGAAGCTCACCAAGGGGGGCGAGGCGCTCTATAT
GCACTGCCTGCCGGCTGATATTTCCGGCGTCTCCTGCAAAGAGGGCGAAGTAACCGAAGGGGTATTCGAG
AAATACCGTATCGCCACTTACAAAGAGGCGAGCTGGAAGCCCTATATTATCGCCGCCATGATCATGGCCC
GTAAATTCTCCGCCCCGGCAGAAACCCTGGAAAATCTGATTAAAGAGGCGCAAAAGCGGGTGAAATAAAT
AGCAGAAGGGCAGGGTGAATATTCATCCTGCCCACGCTGTCGAATCCATTATTTCGAGTTTTCTCCCCGT
GGTATCTGAATAACTGATCTCCCGGCGAGGCAGCTCTTTATCAGGTAACGCCTAGTGAGGTAAGCATGTC
CCAATTTTCGCTGAAGATGGATATTGCCGATAACCGCTTTTTTACCGCCGAGCCATCTCCGCTGTTTTCC
CGTGTTGAAGCACAAAAGGCTCGCGCTTTTCATAAAAAACTGGCCGGCTATGAACCGACCCCATTGTGCG
ACCTGAAAGATCTGGCAGCCTATATCGGGGTGAAAAATATATTGGTCAAGGACGAGTCAAAGCGCTTTGG
TCTCAATGCCTTCAAGATGCTGGGTGGCGTTTATGCCATCGCCAACCTGCTCTGTGAAAAGTACGGGGTG
GCCATCGAGGATTTCTCCTTCGATCTCATCAAACGCACCATCAAGGAGCCGATGACCTTCGCCACAACCA
CGGATGGCAACCACGGTCGCGGCGTCGCCTGGGCCGCCAAGCAGGTAGGTCAGCATGCGGTGGTCTACAT

GCCCAAGGGCTCGGCCCAGGAGCGGGTTGACCACATCCTCAATCTCGGTGCCGAGTGCATCGTCACCGAC
ATGAACTACGACGACACCGTGCGTCTGACCATGAAGATGGCCGAAGAGCGCGGCTGGCACATCGTTCAGG
ACACCGCCTGGGAGGGTTACACCAAGATCCCGACCTGGATCATGCAGGGTTACTCCACCCTGGCCGATGA
AGCGGTCGAGCAGATGCAGGCCATGGGCATCAAGCAGCCGACCCACGTGCTGCTGCAAGCCGGGGTCGGG
GCGCTGGCGGGCGGGGTGCTCGGTTATCTGGTGGACTGCTTCGGGGCGAAAAATCTGCACAGTATCGTGG
TCGAGCCAGATCAGGCCGACTGCATCTACCGCTCCGGCGTGGCGGGGGAGATGGTCAATGTGGGTGGCGA
TATGCGCACCATCATGGCCGGTCTCGCCTGCGGCGAACCCAACCCCCTTGGCTGGCCACTGCTCAGAAGC
TGCACCACCCAGTTTATCTCCTGCCACGACAAGGTGTCGGCGCTGGGCATGCGGGTGCTGGGCAACCCCC
TTGGCAATGATCCGCGCATCATCTCCGGCGAATCCGGCTCGGTTGGCACCGGCGTGCTGGCGGCGGTGCG
TCACCACCCGGACCGCGCGGCGCTGATGGCTCGCCTCGGGCTGGATAGCAACTCGGTCGTGCTGATCATC
AACACCGAAGGGGACACCGATGTGACCCACTATCGGGAAGTGGTGTGGGAAGGCAAACATCCTGCCTGCG
ATTAACCCCGGCTGCGAGTAACTCCCTGCGAGAGCAGAGACTCGAATTGAAGAACTGATCGCGCCTCGCC
AACGGGGCGCGGTAACGGGCACAGGGCGCTGCCGACGAACAGGCCGCGCACTCTCAGTTGTTATGGAGAA
TCACAATGAGCAAGCAAATTCCGTTTGAAATGGTGTTGGAGAAGGCTTACCAGTACAAGGCCGAGATGAG
TCAGTTCCTGCGCGATATGATTGCCATCCCGAGCGAGAGCTGCGATGAAGAGCGCGTCGTGCTGCGCATC
AAGCAGGAGATGGAGAAGGTTGGCTTTGATAAGGTCGAGATCGACCCCATGGGCAACGTGCTGGGCTATA
TCGGCCACGGCCCGCACCTGATCGCCATGGATGCCCACATCGACACCGTGGGCGTCGGCAACATGGCCAA
CTGGAAGTTCGACCCCTATGAGGGGATGGAAGATCACGAGATCATCGGCGGCCGCGGCGCATCTGACCAA
GAAGGCGGCATGGCCTCCATGGTCTATGCGGGCAAGATCATCAAGGATTTGGGGCTGGAAGATCAGTACA
CCCTGCTGGTGACCGGTACCGTACAGGAGGAGGATTGCGATGGCCTCTGCTGGCAGTACATCATCGAGCA
GAGCAAGATCCGCCCCGAGTTTGTGGTCAGTACCGAACCCACCGACTGCCAGATCTACCGTGGCCAGCGT
GGCCGCATGGAGATCCGCATCGATGTGCAGGGGGTCAGCTGCCACGGCTCCGCACCGGAGCGCGGTGACA
ACGCCATCTTCAAGATGGGCCCGATCCTTGGCGAGCTGCAGGAGCTGAGCCACAACCTCGGCAATGACGA
CTTCCTCGGCAAGGGCACCCTGACCGTCTCCGAGATCTTCTTTACCTCCCCAAGCCGCTGCGCCGTGGCA
GACAGCTGCGCTGTCTCCATCGACCGCCGCCTCACCTGGGGCGAAACCTGGCAAGGGGCGCTCGACGAGA
TCCGCGCCCTGCCTGCGGTGAAAGCCGCCGGTGCAGAGGTCTCCATGTACCAGTACGACCGTCCGGCCTA
CACCGGTCTGGTCTACCCGACCGAGTGCTACTTCCCGACCTGGAAGGTGGAAGAGGATCACATCACCGTC
AAAACCCTCTCCAAGGCTTATCGCGAGCTGTTCAACAAGGAGCCGGTGGTGGACAAGTGGACCTTCTCGA
CCAACGGGGTCTCCATCATGGGTCGCCACGGTATTCCGGTCATCGGTTTTGGCCCGGGTAAAGAGCCGGA
GGCCCACGCCCCGAACGAGAAGACCTGGAAAGAGCATCTGGTCAAGTGCGCCGCCATGTACGCCGTGATC
CCCATGCTCTATCTGGCCGAGCTGGACAAGTAAGGGGGCCGCCGGGCGGTGACTCTCATCACCGCCCGTC
TGTTGCAAGTGCCCCGTCTCCATTGAGGCGGGGCTTTGCTGTTTGAAGTGATGTTCGAGGTGGTGGGGAT
GAAACGGCTGATCAAAAACGGTGTGCTGGTCGATGCACAGGGGGAATATCGTCAGGATCTGCTGATCGAG
AACGGGGTGATCCGCGCGCGAGCCGCCGCTATCCAGCCCGATGGCGAGACCGACGTGATCGATGCCAGCG
GCTGCTATGTGATGCCGGGCGGCATCGACGTCCATACCCACTTCAACATCGACGTGGGCATTGCCCGCAG
TTGCGATGACTTTTTCAGCGGTACCCGTGCCGCCGCCTGTGGTGGCACCACCACCATTGTCGATCACATG
GGATTTGGCCCGGCGGGGTGCAATCTGCATCACCAGCTGGCCCGCTATCACGACTATGCCGCCGATCAGG
CGGTGATCGATTACAGTTTTCACGGCGTTGTCCAGCATGTGGATGACGCCATCCTCCACGAGATGGCCGC
CATGGTGCAGGAGGAGGGGATCAGCAGCTTCAAGCTCTATCTCACCTACCAGTACAAGTTGGGGGATCAC
GACGTGCTGTGCGCCCTGGCCCGCCTCAAGGAGGTGGGGGCGCTTGCCACCGTCCACCCCGAGAACGATG
CCGCCATCGCGGCGCGCCGGGCGGCCCTGCTGGCCGCTGGCCATCATGAGCCTTGGTATCACCCGCAAAG
CCGCCCGCTGGAGTGCGAGGCGGAGGCGATCGCCCGGATGATCAATCTGGCGGGGCTCTCGGGTAATGCG
CCACTCTATATCGTCCACCTCTCCAACGGCTTGGGGCTGGAGTATCTGAAACTGGCGCGCCGTCAGGGCC
AACCGGTCTGGGTGGAGACCTGCCCGCAATATCTGCTGCTTGATGAGCAGTGTTATTACAGGGAGAACGG
GGTCGACTATCTGCTGAGCCCGCCGCTGCGCTCGCGCCGCGAGCAGGACAAGCTGTGGGTCGGTATCGCT
GCGGGCGACATCGATACGGTGGCGACCGATCACTGCAACTTCTCCCACGCCCAGCGCATGACCCTCTCGG
GGGGCGATTTCAGCCGCTGCCCCAATGGCTTGCCCGGGGTCGAAAACCGGATGCTGCTGCTCTTTGCCCA
CGGGGTATTGGGCGGGCGCATCTCGCCGTCCCGCTTTGTAGCTCTTACCAGCGCCAACCCGGCGCGCCTG
TTTGGCCTCTGGCCGCGCAAGGGCAACCTGCAACCGGGGTCTGACGCCGATCTGGTGATCATGGATCCCC
GCGGTGACACCCTGATTTCTCACCCACTGCTGCATGACAACGGCGACTACAGCCCCTACGAGGGGATGCG
CTGCCCCGGCCGGATCAGAATGACCCTGAGCCGTGGCGAGATAGTCGCTCGCGATGGCGAGTTTCTGGGG
CGGCGTGGTCATGGCCGTTTTCTGGCCAGAGCGCCCTTTGATCCGGCGTTGGCGCCGGAGCTGCACTCGC
CTTGCCCCACAGTGGCAGGCTGACAATTCGTTCGAAACACGCAGAGCAGCAAGGTTATGTGAACAACGAA
AGGTGGGCCGGTATTGCACCGGCTTGTCCCATCACTGGAGATAACAAGATGAAACAGAGAATCGTACTGG
CCCTTGGTGGCAATGCGCTGGGCAACAACCTGCCCGAGCAGATGAAGGCGGTGCAGAGCACGGCGGGCAC
CATTGCCGACCTGATCGCCCAGGGGCATGAGGTGGTGGTGACCCACGGCAACGGCCCGCAGGTAGGGATG
ATCCAGCAGGCGTTCGAGGTGGCGGCCCGCCACGACCCCAAGGCGCCCCATCTGCCCATGTCGGTCTGCG
TGGCCCTGAGTCAGGGTTATATCGGTTATGACCTGCAAAACGCCCTGCGCGAGGCCTTACTGGCGCGTGG
TATTCACAAGCCGGTTGCCACTTTGGTGACCCAGGTGGAGGTGGATAAAAACGATCCCGCCTTCCTCGAC
CCGAGCAAACCCATCGGCGGTTTCTTCAGCAAGGAGGAGGCCGAAGCATTGATGGCCAAGGGGGAGCGGC
TCAAAGAGGATGCCGGTCGCGGCTATCGCCGGGTAGTGGCATCCCCCAAACCCGTCGATATCATCGAGAA

GGAGACCGTCTCTGCCATGCTGGCTGCCGGTCAGGTGGTGATCACCGTCGGTGGCGGCGGCATTCCGGTG
CTGCGCGAGGGCAATCACCTGCGCGGCGCCAGTGCGGTGATCGACAAGGATTGGGCCAGTGCCAAGCTGG
CGGAGCTTATCGACGCCGACATGCTGATCATCCTTACCGCGGTAGAGAAAGTGGCCATCAACTTCGGCAA
GCCCGAGGTGCAGTGGCTCGATCAGCTGAGCGTGGGAGATGCCCAGTGTTTTATCGAGCAGGGGCAGTTT
GCCAAGGGCTCCATGCTGCCCAAGGTGGAGGCGGCGCTCTCGTTCGCGACATCCATGCCGGGGCGAAAAT
CCCTGATCACCTTGCTGGAGAAGGCCGCCGACGGCATTGCCGGCAAGACCGGAACCCTTATCAGCCGCTA
GGCGTTCCAGCTCGGGTTGAGTGTGATTGCGGGGGCCGTCGGCCCCCTTGTTCATTTTGGGGCAGTCATG
GTCAGCAGAAGATGAGCGGCGTAAGATGGCCGCCTTCAAGCGCGGGCAAGACGCCGAGAGCAGACCCGGC
GCAATGAGTGACAAGAGGTGAGTAACAAACAAATGAGCGGAAAGATCAATATGGCGGCGCTGGTGGGATT
GATCCTGCTCACCGCCATCTGGAGTTACAGCTGGATCGTGATGAAGCAGGTGATGCTCTACGCGGGGGCC
TTCGAGTTCACCGCCCTGCGCTGCCTGTGCGGATCTCTGGTCTTGTTGTTGGTATTGGCCCTGCGCGGTC
GCCGTTATCTCAAACCCACCCCGTTTCGCTATACCCTCGCCATCGCCCTGTTCCAGACCTGCGGCATGGT
GGGGTTGGCCCAGTGGGCGCTGATCAGCGGCGGGGCGGGCAAGGTGGCCATTTTGAGTTACACCATGCCG
TTCTGGGTGGTGATCTTCGCGGCGCTCTTTCTCGGCGAGCGGATGCGCAAGCTGCACTATCTGGCGGTCG
GGGTGGCGGCCTGCGGCCTGCTGTTGGTGATCCAACCCTGGTTGTTGCATCTTGAGTCCCTCAAGAGCGC
GCTGTTGGCGATCCTCTCCGGCATCTGCTGGGGGATCAGCGCCATCATCGCCAAGCGGATGTATGCCCGC
CATCCCGGGGTGGATCTGATGTCCCTCACCACCTGGCAGATGATCTATGCCACCCTGGTGATGACCGTGG
TGGCCGCGCTGGTGCCGGAACGCCCCATCGACTGGCAACCCTACGTGATGGGAGCGCTGGCCTATAGCGC
CATCTTCGCCACCGCCATCGCCTGGAGTCTCTGGTTGTTTGTGCTGAAAAAACTGCCAGCGGGTATCGCC
AGCCTCAGCACTCTGGCGGTGCCGGTGACCGGCGTGCTGCTCTCCTGGTGGCTGCTCGGGGAAAACCCGG
GGCCGGTGGAAGGGAGCGGCATCGCCCTTATCGTACTGGCGCTTCTCGTCATCAGCCGCAAGGGCAAGCG
CTCTGTGGTGTCGGTGGCGGGGGAGACCCGCATCAAGCACGGCTGAGTGGTGACTGGGCTGCCAGTGGCA
CGATAAACAGCACTCAAAAGAGCCCGCGACAGGGGACCAGCCCCTCGTTACAATGGCGTGGTCTGACTGT
GCTGGAGCACAGGTATCTGATTTATTGTGATTATTTCAACGAACCTAACGGCAAGGATGAGACCGTATGA
TGACGCTGGTGTGGTTGGCTGTAGGCCTGGTGTGTCTGGTGGTGGGGGCCGAATCTCTGGTGCGGGGCGC
ATCTCGCCTCGCCACCATGATGGGGATATCGCCACTGGTGGTGGGATTGACCATAGTGGCCTTTGGGACC
AGTGCTCCCGAGCTGGCTGTGAGCATCCAGTCTGGCCTCGAGGGGCAGGCAGGGATAGCGGTGGGGAACG
TGGTCGGCAGCAATATTTTCAACGTGCTGTTTATTTTAGGTATTTCGGCACTGATCGTACCGTTGACCGT
CTCCCAGCAACTGGTACGTCTCGACGTTCCCTTGATGATAGTGGTCTCTTTTCTGGTGTTGTTGCTGGGC
TACGATGGCCGTATTGCACCACTGGAAGGGGCGCTGCTGTTTGCCGGCATCATCGCCTACACCGTGTTTC
TCTTTGTCCAGAGTCGACGGGAAAACAAAGCGGTGCAGGCGGAGTATGAGCAGGAGTTTGGTACCCGTGA
AGCGAGTACGGCGGGCAACTGGTTGCGCAATCTGCTCTTTATTGCCGCAGGTCTGGGTCTGCTGATCCTC
GGCTCCCGCCTGCTGGTTGGCAGTGCAGTGGAGATTGCCCACTACTTCGGGGTGAGCGATCTGATCATCG
GCTTGACCATAGTGGCGGCCGGTACCTCCTTGCCCGAAGTGGTGACCTCCATCGTTGCAGCTCTGCGCGG
GGAGCGGGATATCGCTGTGGGGAACGTGGTGGGCAGTAATATCTTCAATATCCTCTGCGTGCTGGGGCTC
ACCTCCATCGTCACTCCCGGTGGGCTTGAAGTGTCGGCCACCGCCCTTCAGTTCGATATTCCGGTAATGA
TTGCGGTGGCGCTTGCCTGCCTGCCCATCTTCTTTACCGGTTACACCATCGCCCGCTGGGAAGGGGCCCT
GTTTCTGGCCTACTACATCGGCTACACCTGCTGGCTGATCCTCCACGCCACATCCAGTCCGCTGCAAGGT
CAGTTCAGTGCAGCGCTAACCGGTTTTGTGTTGCCGCTCACCGCCATCACTCTGGTTGTGCTGGCGGTGC
GAGCCTGGCGATTGTAACGAACCCGGTCGTGATGCAGGTTCTCCTGATGTAAAGGAGCTGCATCAACCGA
CAGGCCGAAAAAAGCCCCACCATCATGGCGGGGCTTTTTGCTTGCTGAGGGGACGCGAAGAGCCGTTATC
GGCGCTGGGCCAGATGCATCATGGCTTCCAGCACGGCGCCGCCGATAGCGCGCGCCTTGTCAGAGACGGT
GGAGGCATCGGCGCGCTCGCCACGCGGGTCGATATCACCAATCTTGAAGCCGACTGGCACTTCGATGCCA
TCGCTGAGCAACCCCCGCACCATGCCCGAGAGGGGCGCGATAATGGGGGCATCCCCTACATGGGCAATCA
CATCTCCCTCCTGCACCAGATCACCGAGCTTGATAAGGCTCTTCATCACCCCGGCGACGGGGGCGCGCAC
CACCCGGCGGGCGGAGTGCCCCTCGATCACCCCGGGAATGCCGGTGTTGGGCTGGGCGGGGCCCTGATAG
ATAACCCGCCCCAGATGATGACCGCGGTTGGTCTCGATCACCGCATCGCAGTCGCGCCCCGCCTCAAAGC
CCGGGCCGAGGGCGACCGTGATGGGGGCCATCTCCTTGTGGGTGCCGAGATTCTGCTTGGCGAGGATGGC
ATCCACCAGATAGCGGGGTTTGAGCTCGGCGAGGGTGGCACAGTCGGGGTCAACCAGTAGCGGAATGACG
CCCCGATCCAGCTGCTCGAACGCCTGTTCGACGCTCTCGACCCGCTTGGCACGCACTCCCTCAACGCTGG
TTTCACCGTCAAACATCCCCTGAGCGAAGGCAACGGTACGGCGGATCACGGTGGGCTTGTCGAGATCCAG
CATCACCACCTTGAAGCCGGCATTGTGCAGGCGCAGTGCCACCCCGGTGGCGATATCGCCGGAGCCACGC
ATCACCACCAGCTGATCCCGCTTGAGGCGCACATCGTCTTGCATCAGACCACCCGCTTTGCCGTTTTTCA
CTTGAAGCAGCTCGGCGAGGATGCTGATGGCGATCTCCTCGGGGGTTTCGGCTCCGATGTTGTAACCGAT
GGGGGCGTGCAGCTGCTGCAGTTGGGTCTGGCTCACCCCTTGCTGGCGCAGCGCCTGGGTGAAGGTCTGC
ACCTTGCGCTTGCTGGCGAGCAGCCCCAGATAGGTGAGGGGACGGCTGATGAGCCGATCCAGCGCCTCCT
TGTCCTGATGGTTGGTGGCGATGATGACGAAGTTGGCGGGCTCGATGGCCAGTTTCTCGATCACCGCGCT
GATGGTATCGGCCTGCATCAGATGGGTACCGGCTGGGAAGAGGTCAGGGTTGAGGCTCCCCTCGAAGCAA
TCCCCTACATGGACATCAAAGCCGAGCGGCGCCGCGGCATGGGCCACAGCCCGGTTGACGTGGCCGGCAC
CAATGAGCACCAGCCGTGGTCGCAGACCATAGACATCGATATAGACCGACATGGCACCACCGCAATCCGA
CCCGACCGCATGCTCACCGTTGCGTGCCATCCGGCCGTGGAAGATGCGCGGCTTGCGCTCGGCAATGGAG

GCCATCGCTTCCTCGATCACCAGCCGCTCGATCATGCCGCCGCCTATGGTACCGAGGATCTGGCCATCGG
CCATCACCAGCATCTGGGCCTGATGGCGCGGGGTGGAGCCCCGGCTCTCGATGATCTGGGCCATGGCAAA
CGGCGTGTTCTCCTGTTCCAGTCGGGCTGCGTGTGCAAACAGATTCATAAGGTACCTCTTTTCATCCTTA
AAACGTCAGGGGTGAGGCTAAAGCGCCTCCCCGTGCGGAGCTGTGCAGATCAGGTGACGGATGGGGGGG
ATTCGCGCACAGCCCCCTGCCAGATGGCGTGGATCTCGGGGTGGCGGCTCTGAAGCTTTATCAATTCCGC
CAGCCACGACGCCTCATTTTGATAATCGCCATTGATAAACCAGATCCGGCGCGCCCCGGGCGGGGTGCCC
TTGAACAGCCCCTCGGGGTGGGAGAGCAGCTTGTCAAACAGTGCCCAATCCAGTGGCTCACCCGCCGCAA
TCCCGTACAGGGCCTGCAATTCAGCCCAGCGGTGGATCTGCGCCGGTTCGGCATTGGCTCCCACCATTGC
GCCGCCCAGCAGGGCGATCACCCAGTCACTCTGGTGTGGAATGCAAGGTTCGTGCAAAGCGGGCACTTTC
AATAACTTGTGATGGGCGCCATCCGCTTCGACCAGGATGAGATCGAAGCGCCGCTGCCCCTTGATGAGAT
CAATCTGCTGGGGCGTGGGGCCCGCCACCTTTCCGGTGCGCTCGTCGAGATGGCTGAACAGGGCAACCAG
AGCCGGGCCATCCGGTAGCGCCTGACAGCGGGTAAGTTGACGAACGAGATCCGTCTCGATAATCAATTCC
CGATAGTGTGACGGGGCAGGCAGAAACATCCGGGTGGTGGTGGTAATTAGCACCCTTTTACCCTGTTGAT
AAAAATATTCCGCCAGCCAGAACAGGGTGCTGGTTTTTCCACCGGCCCCGCAGAGGCTAATTAAGGTGGT
ATGGCTATCAATATTTTTGGTGAGTGGGTTTAATAGCGGATCTATATCTGGAAATGCGACAGCAACAACG
TGTGGGTGCGACATAATGACGACCAAAATAAATATCGAAGGGAGTGGAAAACAACGTTGCCAGGAACAAG
AGCAGTGTGACTGTATAATAACGGCGGCGGGTTTATCTTCCCGAATGGGTACTTGGAAAATGATGCTACC
TTATTGCCAGGGGACAATGCTTGATGCAAGTCTCAAAAATGCCCTGGCTTTTTGTCAGCGGGTTATTCTG
GTGGTGGGCCATCGTGGCGAGGAGCTTCAAGCCCGCTACGGATCACGGCCGGATATTTTTGTCGTCCACA
ATCCCGACTATCGGCAGGGGCTGGGCAGCTCCATCCGCTGCGCCCTGGCGGCCAGTGACGCTGACTACCT
CTTTATCAGCCACGGGGATCTCCCCTGTATCCCGCGCGATGTCTATCAACAGCTCTGGCAGACACGGGGC
GGTGAGACACTCTTTCCCACCTGGCAGGGGGAGGCGGGTCATCCGGTGTTGCTGCCAAAATCCCTTGCCC
GCGAGTTGGCAGCGGCCCCGGTGCAGTCGTCGGTACGGCGCTGGTTGCAAGGGCATCCGCACCGTATGGT
GCCAGTTGACAGCCCCGCCATCCTGTTTGATGTGGATACCCCGGAGCGCTATCAGGCGCTGCTGGGTGGC
ATTCTTGAAAATGAGAGCGAGCCCACGAAATAACCCAATTTAAAAAGATAACGGGGAGATAAATGGCGTA
TCAAAGTGATATTTCTCGCACTTAATTTGATAGGGTGAGTTCTTTTTAATCAGCTCATCAATTGAATTTC
AGAGTTATTGATAACTACTCGCAATACCTATAATGGTGGCATCGTAAATTCTCATCATTAATTGGAGCTG
CCTCACGCTTTCACCCTAAAAATAGTTTTGTAGCTCGTTTTGTAACTGTAGTGGCCTGATAGTTGCTCCT
CCCCTTGCCAAGGGGTAAAGCCCGTGCGGATACCCCATTTATTCATACTGCCAATGGGCAGAACAGCTG
CAAGGAGATAGCCATGGGCGATATTATGCGACCCGTTCCGTTCAAGAACCTGCTGACCCGTATTTTTGCG
GAATATAAAGAGAGTCAGAGCATTTTCGGTATTCCCGCCGCGCAATTCTATCGCAAGCAGGACGAGCGCA
AGATCAAGGTGTTCGGTGAAACCTGCGAGACCCCCATTGGCCCGGCAGCCGGCCCCCATACCCAGTTGGC
CCAGAACATCGTCACCTCCTGGCTGACCGGCGGGCGCTTTATCGAGCTCAAGACGGTGCAGATCCTGGAC
CGTCTCGAACTGGAAAAGCCCTGCATCGATGCCGAGGACGAGTGCTTCAACACCGAGTGGTCAACCGAGT
TCACCCTGCCCAAGGCATTTGACGAGTATCTTAAAGCCTGGTTTGCCCTCTATCTGCTGGAGGAGGTGTT
CGACCCGCGCCTCGACGGAGAGGCCAAGTCTTTCATCTTCAACATGAGCGTGGGGTACAACCTCGATGGC
ATCAAACAGCCACCGATGCAGCAGTTCATCCACAGCCTGATGGATGCAAGCCAAAGTCCGAAATTTGCCG
AGTACCAGCAGGTGCTGCGCGAGCTGGTTGCCGACGAGCAGTTTATCGCCGAGCTGGGACTGGGGGCGCG
GCGCGATCGCCTGCAACAGCTGGTGGAGCGCATCCCGGCTGAGCTGGTGCACGGCGTTACCCTCTCCACC
ATGCACGGCTGCCCGCCCAACGAAATTGAGGCCATCTGCCGCTATATGCTGGAAGAGAAGGGGCTCAACA
CCTTCGTCAAACTCAACCCGACCCTGCTGGGTTACCCGCGGGTGCGCGAGATCCTCGATACCTGTGGCTT
TGATTACATTGGTCTGAGCGAAGAGTCGTTCGATCACGACCTCAAGCTGGATCAGGCCAAGGCGATGCTG
ACCCGTCTGATGGCGCTGGGAGCCGAGAAGGGGCTCACCTTCGGGGTCAAGCTCACCAACACTCTCGGCA
CCATCAACCGCAAAGGGGCGCTGCCGGGGGATGAGATGTATATGTCCGGCCGGGCGCTGTTCCCGCTCTC
CATCAACGTGGCGGCCGTGCTGTCCCGCGCCTTCGATGGCAAGCTGCCCATCTCCTACTCGGGCGGTGCC
AGCAAGTTCAACATCGCCGAGATCTTCGAGACCGGTATCCGTCCCATCACCATGGCCACCGATCTGCTCA
AGCCGGGTGGCTACCTGCGCCAGATTGAGTGCCTCAAAGAGATCGACGCCTCTGACAGCTGGGGCATGAG
TCAGGTAGATGTGGCCAAGCTGGAGGCGCTTGCCGCCAAGGCCCTCACCATGGATTACACCCAGAAGGAG
TGGAAGTCCGAGGAGCGGATCGAAGTGGGGGGCGGCCTGCCGCTGACCGACTGCTACGTGGCACCCTGCG
TCACCGCCTGCGCCGTGCATCAGGATATCCCAGAGTACATTCGTCTGATGGGTGAGGGGGAGTATGTGGC
AGCCCTTGAGCTGATTTATCAGCGCAACGCCTTGCCTGCCATCACCAGCCACATCTGCGATCACCAGTGC
CAGTACAACTGCACCCGTCTCGACTACGACAGCGCGCTCAATATCCGCGAGCTGAAAAAAGTGGCGCTGG
AGCGCGGCTGGGAAGGGTACAAGGCCCGCTGGCACAAACCGGCTGGCAGCGGTGACAAGCACCCGGTCGC
GGTGATTGGCGCAGGCCCGGCCGGTCTCTCCGCCGGTTACTTCCTGGCCCGTGCCGGCCATCCGGTCACC
GTGTTTGAGCGCGAAGCGGATGCCGGTGGCGTGGTTAAACACATCATCCCCGAGTTCCGCATTCCGGCCG
AACTTATCCAGCATGACATCGACTTTGTTGCAGCCCACGGGGTCAAGTTCGAGTTTGGCTGCGATCCGGC
GCTCACCGTCGACAAGCTGCACGCCCAGGGCTTTAGCTATGTCTTCGTCGGCATCGGCGCCGACAAGAAT
GGTGGCATGCGCCTCGAAGGGGGACACGAGCGGATTTACAAATCCTTCCACTTCCTGCGCAGCTTCAATC
AGGGCAAACCGCTGCCGCTCGGCCGCCATGTGGCGGTGATCGGCGCCGGCAACACCGCCATGGACTGTGC
CCGCGCCGCCCTCAAGGTGCCCGGTGTCAGCGATGTGACCGTTATCTACCGTCGCAGCGAAAAAGAGATG
CCTGCCTATCGCGAGGAGTATCTGGAAGCGGTGGAGGATGGGGTGCGTTTCTGCTTCCTCACCAATCCGG

AGCGTTTTGAGAAAGATGGCAAGCTCACCGTGCGGATGATGGCGCTGGGGGATCCTGACGAGCAGGGCCG
CCGCCGTCCGGTGCCCACCGAGCAGACCGAAGTGATGCAGATGGATGCCTTGATCACCGCCATTGGCGAG
CAACCTGACTGCGAGGTGCTCAAAGCGATGGGCATTCCTCTAGGTAGCGATGGCTGGCCCGAGGTGGATG
GCCAGACCGGCGAAACCAGCCGTCGCAACGTCTTCCTCATCGGTGACGTGCAGAGCGGCCCGTCCTCCAT
CGTCGGTGCCATCGGTGGTGCCCGCCGCGCCGCGGATCTGGTGCTCTCTCGCGAGCATATCGCCAGCCAG
CAGAGTGAAGTGACCATCCAGCCGTCGGGCAAGGATGAGATCTACCGCCGCAAGGGGAGCATCGCCATCA
CCATGGTTGACAAGAACGAGCGCGATGCCTTCGTGGCGCAAGAGGCGCACCGTTGCCTCGAGTGCAACTA
CCTCTGCAGCAAGTGCGTGGATGTCTGCCCCAACCGGGCCAACGTCGCCATCGCGGTGCCGGGCTTCAAA
GACCAGTTCCAGACCCTGCACCTGGATGCCTACTGCAACGAATGTGGCAACTGCGCCCAGTTCTGCCCGT
GGCAGGGCAAGCCCTATCAGGACAAGGTCACCATCTTCAGCCTGCCCGAGGACTTCGACAACAGCCGCAA
CCCCGGTTTCTATCTGGCTGACGGTGGTGAACTGCGGGTGCGTCAGGATGGCGAGACCTATCGCCTGACC
ATCGATGCCCAAAGCCGGATCAAGGATGCGCCAGCGGCCCTTGGCGACATGTGCCGGATCATCAGCCATG
TCCATGCTCATCACCACTACCTGCTGGGGGCCGTCGAGGCCTGAGCGAACCACGAATAAGCGAAGGAGCA
AACCATGTTTATTCTGAAGAACGTCACCGCTGTCCAGCTGGAGCCGACCCGGGTGCTGGAAGGGGTGGAT
ATCGCCATCGAAGGATCGCTTATCAAAGCGGTGGGGCCCAATCTGCGGGCCCTCTACCCGGAGGCCAACT
ATCGGGAGATGGGCGGCAAGCTGGTGATGCCGGGCATCGTCTGTGCCCACAACCACTTCTACTCCGGCCT
GTCGCGGGGGATCATGGCCAATATCGCCCCTTGCCCGGACTTTATCTCCACCCTGAAAAACCTCTGGTGG
CGGCTCGATCGGGCGCTCGATGAGGAGTCCCTCTACTACAGCGGCCTCATCTGCTCGCTGGAAGCGATCA
AGAGCGGCTGCAGTGCGGTGATCGACCACCACGCTTCGCCCAACTTCATCAAGGGCTCCCTGAATGTGTT
GCGCAAAGGGTTTATGGAGGCGGGCCTGCGCGGCATGACCTGCTTCGAGACCACGGACCGCAACGGCGGC
CTTGCCGAGCTGCAGGCCGGGGTGGAGGAGAACATCCTGTTCGCGCAGGCCATTGATGCCGCCAAGGCCA
AGGGGAACGAACCCTATCTGGTGGAGGCTCATATCGGCGCCCATGCCCCCTTCACCGTGCCCGACGAGGG
GCTGGCCATGCTGCGCGAAGCGGTGCAGGTCACCGGTCGCGGGCTGCATATCCATGTGGCGGAAGACCGC
TACGACATGGCCCACGGCCACCACCACTATGGCAAGGAGCCCATCGCCCGGCTGGACGAGTTTGGCCTTG
TCGACAGCAAGACCCTGATCGCCCACGGCATCTACCTGAGCCCGGCCGACATCGAGCTGCTCAACAGCCG
CGATGCCTTCCTGGTGCATAACGCCCGCTCCAACATGAACAACCACGTGGGCTACAACATGCACCTGCCG
GAGTATCGCAATCTGGCGCTGGGCACCGATGGTATCGGCTCCGACATGCTCGAAGAGCTCAAGTTCGCCT
ACTTCAAACACAAGGATGCCGGCGGAGCGCTCTGGCCCGACAGCTTTGCCCGGTTCCTCTGGAACGGCAA
CACCCTGCTGGAGCGCAACTTCGGCGCCAAATTTGGCCGCCTGGAGCCGGGTTACAAGGCCGACCTCACC
ATCTGCGACTACGCCGCACCGACCCCGCTGGCGCCGCGCAACCTCGGCGGCCATCTCGCCTTCGGTATGG
GCTCATCCTCGGTCAACAGCGTGATGGTCGAGGGACGCATGGTCTATCAGGATCGCGAGTTCGCCTTTGA
CGTCGAGCCCATTTATCAGCAGGCCAGCAAGGTGGCGAGCCGCCTGTGGCAACGGATGGATCAGCTGGCC
TAGGGCCACCCTCCACAGTTTTGGACCGGATTGCCCGCCCTGTGGATCTCCCCGGCGGCGGGCTGCAAGA
GTAAGGACGACACATGATTGAGCAATTCTTCCGACCCGAACAACTGGGTCAGGCTTTGGAACTCAAGGCC
CGCTTTGGCAACGACGCCGTCTACATGGGGGGCGGCAGCAAGCTCAACGCAGCCCCGACCCGCACCGACA
AGAAGGTCGCCATCTCGCTGGACAAGCTCGGACTTGGCCAGATTGAGCAACAGCACGGTCAACTCCACAT
CGGCGCCACCGTCACCCTGCAAGCCCTCAAAGATGACCCCCGTACCCCGGCCGCCCTGTGCGAGGCGCTG
GGGTTTGTCTACTCCCGCCATCTGCGCAATCAGGCCACCATTGGCGGCGAAATTGCCTGCCAGCAGCAGG
ACTCCCCGCTGTTGCCGGTACTGCTGGTACTCGAAGCCGTGGTGGTGCTGGAGAACAATCAGTTGCAACC
CATCGATGCCTATCTGGCGGGCCAGCGCAAGGAGCTGGTGCTGGGGGTGGTGCTGCCGGAGCCCGCGCTG
CGCTGCGCCACTCGTCGCATCAGCCGTAACGCCGATGGTCTGGCGGTAATGACTGCCGCAGTGGCCCTCG
ACGCCCTTGGCGAGATGCGGGTCGCCGTTGCTGGCGTTACGCCGCAGCCGATGCGCCTGCGTGATGTGGA
GCGCCGCGATCTGCACGATGCCGCGCTGGAGGCGGCCGTGAGCGAGCTGGTTGCACCGCGCGAGGATCTC
GGCGGCAGCGTGACCTACAAGCGCTATATCAGCGGGGTAGTGGTGGCGGATCTGCTGGTCGAGTGCCAGC
AGCAGGAGGTACAGGCATGATGGAACTCAACTTCACACTGAACGGTGCCCTGCGCACCGTCATTACCCCG
GCTGGCGAGAATGCCCAGCGGGTGCTGTTCAACCAGTGCCGGATGCACTCGGTGCGCAACAGCGACGACG
GCTTTGGCTTTGCCGGCTCAGATGCCATTCTCTTCAACGGCAAGGTGATCAACGCCTCCCTGCTGATCGC
CGCACAACTGGATGGATCAGAGGTGCGCACCGCCGAGTCGCTGGGCAGCTGGAACCAGCTCAGTCTGGTA
CAGCAGGCGATGGTAGATGTGGGCGTGGTGCAGTCCGGCTACAACGATCCGGCTGCGGCGCTCATCGTCA
CCGACCTGCTTGATCGCCATCCCAGCCCCAACCGTGATCAAATCGACGACGCGCTCTCGGGCCTGTTTCA
CCGGGACGGCGGCTACCAGCAGTTCTATCAGGCCATCGAGCTTGCCAGTTCACGGATGAAGGATCCCGAT
TATCTGTGCCAGATTGCCCCCGAGTTTCGTGACGACCTGCGCCATATCGGCAAGAGCTGCCCGAAAGTGG
ACGCCGCCAAGATGGTGCAGGCCAAACCCTGCTACGTGGAGGATCGGGTCACCGAGGATGCGCTGGTCAT
CAAGATGCTGCGCAGCCCCCATCCTCATGCGGTGATCACCAAACTCGACGTGAGCCGCGCCGAAGCCATG
CCGGGGGTAGTGCATGTCATCACCCACCTCAACTGCCCGGATATTTACTACACCCCGGGTGGCCAGAGTG
CGCCCGAGCCGTCACCGCTGGATCGCCGCATGTTTGGCCGCAAGCTGCGTCACGTAGGTGACCGGGTGGC
CGCCGTGGTGGCCGAGAGCGAAGCCATCGCATTGGCCGCGCTCAAGCTCATCGAGGTCGAGTATCAAGTG
CTGCCGGCGGTCATGAGCATTGACGAGGCGATGGCCCCCAATGCACCGCTGGTGCACGACGAGCCCATCG
TCTACATGGCCGGGGCACCGGCGGATCTGGAGCAGCAGAACGCCTGCTCGGTGCGCCGCGGCGACGAGCA
CATGATCATCAACTTCCCCATCGGTTCCCGCCCGCACGAGAACCTGGCCGCCAGCGTCCATGGCCAGATC
GGCGATGTGGCCAAAGGCTTTGCCGAGGCGGACGAGATTGTCGAGCGCACCTACGAATCGACCCAGGCCC

AGCAGTGCCCGACCGAGCCACACATCTGCTTCACCTACATGGATGGCGATCGTCTGGTGATCCACGCCTC
CACTCAGGTGCCGTGGCACGTGCGCCGTCAGGTGGCGCGCATCGTCGGCATGAAGCAGCACCAGGTGCAT
GTGATCAAGGAGCGGGTAGGGGGCGGCTTTGGCTCCAAGCAGGATATCCTGCTGGAAGAGGTGTGCGCCT
GGGCGACCAAGGTCACAGGCCGTCCGGTTACCTTCCGCTACACCCGTGAAGAGGAGTTCATCAGCAACAC
CTCCCGCCATGTGGCTAAGGTGAAGGTGAAAGTGGGGGCCAAGAAAGATGGCACCATCACCGCCATCGAG
ATGGATTTTCGTGCCAACACCGGTCCTTACGGCAACCACTCCCTGACGGTGCCGAGCAACGGCCCTGCGC
TCTCCCTGCCGCTCTATCCGTGCGACAACGTGCGCTTTACCGTCAACACCTACTACAGCAACATCTGTCC
GACCGGCGCCTATCAGGGCTACGGTGCGCCCAAGGGCAACTTTGCCCTGACCATGGCCATTGCCGAACTT
GCCGAGAAACTGGGCATCGATCAGCTCGACATGGTGGAGCACAACCGGGTGCATGAAGGGGACATCCTCA
AGGTGCTGGGTGCCATCGGCGAGGGCAAGATGCCTACCTCGGTGCCCCACGCGGCCAGCTGCGCCCTTGA
GCCCATCCTCAAGCAGGGCCGCGAACTGATCGCCTGGGATAGCCCCAAACCCGCCGACGGGGATTGGCGC
ATCGGTCGTGGCGTCGCCATCATCATGCAGAAATCGGGGATCCCGGATATCGATCAGGCCAACTGCATGG
TCAAGCTGGAATCGGACGGCACCTTTATCGTCCACTCCGGTGGCGCCGATATCGGCACCGGCCTCGATAC
CGTGGTGGCCAAGCTGACTGCCGAGGTGCTGCACTGCCCGCTCGGGGATGTGCACGTCATCTCCGGCGAC
ACCGACCACGCCCTGTTCGACAAGGGGGCCTACGCCTCGTCAGGTACCTGCTTCTCCGGCAACGCGGCCA
AGAAGGCGGCCGAGAACCTCAAGGAGAAGATCCTGTTCCATGGTGCCGCCATGCTGGGCGAGCCGGTAGC
CGATGTTGAGCTGGCGTTCCCGGGCGTGGTGCGCGGCAAGCTGGGGGAGGTGAGCCTCGCCAAGCTGGCC
CACAAGGCCGAGACAGGTACCGGCTTCGGTATCCTCGTGGGCACTGCCAGCTACATCACCTCTGAGCTTG
CCTTCCCGTACGGGGCCAACTTCGCCGAGGTGGCGGTCAACGTCAGAACCGGCGAGATCCGGCTCGACAA
GTTCTACGCCCTGCTCGACTGCGGCACCCCCATCAACCCGGAACTGGCGCTCGGCCAGATCTACGGGGCG
ACCATGCGGGCCATCGGCCACACCATGAGCGAGGAGATCTGCTACGACAGCAAGGGCATCCCGCTCACCC
GGGATCTGAAGAGCTATGGCGCGCCCAAGATTGGCGACATTCCGCGCGACTTCCGGGCCTTCCTGGTACC
GAGTGATGACAAGGTCGGTCCTTACGGCGCCAAGTCCATCTCGGAGATCGGGGTGAACGGCGCGGCGCCC
GCCATCGCAACCGCCATTCACGACGCCTGCGGTGTCTGGCTGCGCAAGTGGCACTTCACGCCGGAACAGA
TCCTGCGCGAACTGGGCAAGCTGGAGCAAAAAGTGTCGGCATAACAAAAGAAAAGGTGAGGGGCGGGCGC
CAGCTCGCCCCCAGCGCCTGACGGCGGAATGAGTACTCCGCCTACATTCTGTGTGATGGGACAAACACGA
TGTCTAAAACAACACGCAAGCACTCGGATCTCATTTACGAGCTGGAAGACAAACCCCCGTTTTATCAAAC
CCTGATGGGTGCAGTGACCCATCTGCTGGCCATTTTTGTTCCTATGGTAACCCCGGCGCTGATCGTCGGC
GGCGCCCTGGGCCTCTCCACCGAGATCACCGCCTATCTGGTCTCCATGGCGATGATCGCCTCCGGTATCG
GCACCTGGCTGCAGGTGAACCGTTATGGCCCCATCGGGTCTGGGTTGCTCTCCATCCAGTCGGTCAACTT
CTCCTTCGTGACCGTGATGATCGCCCTCGGCGGCGCCATGAAAAAAGATGGCATCCACGAGGAGCTGATC
GTCTCGACCCTGCTCGGGGTCTCCTTTGTCGGTGCCTTTCTGGTGATGGGCTCCTCCTTTGTGCTGCCCT
ACCTCAAGCGGGTGATCACCCCGACCGTGAGCGGGGTGGTGGTACTGATGATTGGCCTGAGCCTTATCAA
GGTGGGGATCATCGACTTTGGCGGCGGCTTCTCCGCCATGAGCAGCGGCACCTTTGGCAAATATGAGCAC
ATCGGGCTGGGGCTGCTGGTGCTCTGCGTTATCGTCGCCTTTAACTGCAGCCGAAGCCCGCTGCTGCGGA
TGAGCGGCATTGCCATCGGCCTGCTGGTGGGCTATGCGGTTGCCCTGATGCTTGGCATGGTGGATTTCTC
CGCCCTTGAAAACCTGCCGCTTATTACGGTGCCTATCCCATTCAAGTACGGTTTCTCCTTCGATTTTCAT
GCCTTTATGCTGGCGGGCACCATCTATCTGTTGAGCGTGCTGGAGGCGGTGGGGGACATCACCGCCACCG
CCATGGTTTCCCACCGGGATATTCAGGGCCCCGAGTTTCAAAAACGGCTCTCCGGCGGCGTGCTGGCGGA
TGGGTTGGTCTCGGTGATCGCCTCGGCCCTCGGCTCTTTGCCACTCACCACCTTTGCCCAGAACAACGGC
GTCATCCAGATGACCGGAGTGGCGTCACGCCATGTGGGCAAGTTTATCGCCGTCATCCTGGTGCTGCTGG
GGCTTTTCCCCGTAGTAGGGCGCTTCTTCACTACTATCCCGTCACCTGTGATGGGGGGCGCCATGGTCAT
CATGTTCTCCATGATTGCCATCGCCGGTGGTCGCATCATCATCAGCCACGGCTTCGATCGACGGGAGACC
CTGATCGTCGCCACCTCGCTGGGTCTTGGGCTTGGCGTTTCCTATGACCCGAACGTCTTCAAGGTGCTGC
CGGCGGGTATCTACATGCTGGTGGAGAACCCCATCTGCGCCGGCGGCATCACCGCCATCATCATGAATCT
GGTGTTGCCGCAAAGCCGAAAGCGCAAGGCGGCCGTCAGGGATGCCGAGGCGGTGGAGGTGATCCAGCTG
AGCGACAAGCCGGATGTGATCTTTACCGCCCGCAGTGCGGGCCAGCCATCGGCCAGCAAGGGTGAGGGTT
CACCAGAGCCAGTACCCGGGGAGGCGGTGGCGCCACAGGTGGAACGGATCTGACGGTTAAACCGGCAAAA
AACGTCAACAGAACAAGAAAAACAGGGAAACGGTTATGAAAAACGACAACTCTGTCAAAGCGGTACGCGG
CAGCTTCTTCGATATCACCCGGGTGGTGGGTCAGCCCGAGGAGATTGAGTCCAACGCCCGCCTTATCGAG
GATGGCCTGCTGATCATTCGCAACGGTTGCATCGACTGGTTCGGTGAGTGGGAGGAGGGGAAAGATTGCA
TTCCGGCCGAGGTGCGGGTGCGGGATTACCGCGGCAAGATGATAGTGCCGGGCTTTGTCGATACCCACAT
TCACTATCCCCAGAGCGAGATGGTAGGGGCCTATGGCGAGCAACTGCTGGAGTGGCTCAACCGCCACACC
TTCCCGGCCGAGCGGCGTTACAACGATCTCGAATATGCCCGCGAGATGTCCACCTTCTTTATCAAGCAGC
TGCTGCGAAACGGCACCACCACGGCGCTGGTGTTCGGCACCGTTCACCCCGAGTCGGTGGATGCCCTGTT
CGAGGCGGCCAGCCGCATCAATATGCGGATGATCGCTGGCAAAGTGATGATGGATCGCAATGCACCCGAT
TATCTACTCGACACCGCCGAGAGCAGCTATGTGCAGAGCAAGCAGCTTATCGAGCGCTGGCACGGCAACG
GTCGACTGCTCTATGCCATCACACCACGATTTGCACCCACCTCGACACCCGAGCAGCTGGCGATGGCGCG
GCGACTGCGCGAAGAGTTTCCCACCACCTATCTGCACACCCATCTGTGCGAGAACAAGGACGAGATCGCC
TGGGTCAAATCTCTTTTCCCGGCGCACAAGGGTTATCTCGATGTCTATCACCAGCATGGCCTGACCGGCC
ACAACTGCGTGTTTGCCCACTGCGTCCATCTGGAAGAGCAGGAGTGGGATTGCCTGAAGGAGACCGGCTC

CACCATCGCGTTTTGTCCCACCTCGAATCTTTATCTCGGCAGCGGGCTCTTCAAGCTGCACAAGGCGTGG
CGCAAGCAGGTGAAGGTGGGGATGGGCACCGACATTGGCGCGGGCACCACCTTCAACATGCTGCAGACCC
TCAACGAGGCCTACAAGGTGATGCAACTGCAGGGCGAGCGGCTCTCGGCCTATCAGGCCTTTTATCTGGC
GACCCTGGGCGGCGCCCATTCGCTGGGGCTTGACGAGAAGATCGGCAGCTTTGCCGTAGGCAAGGAGGCG
GATTTCGTGGTGCTCGACCCCGTCGCCACCCCGCTGCAGCAGCTGCGTTATGACAACTCCACCACCTTGC
GCGACAAGCTCTTTGTGCTGTTGACCCTGGGGGATGACAGATCCATCTACCGTACCTATGTGGATGGCCA
ACTGGTGCATGAACGGGGGTAGGGGCTGGAGTGGGCTGCCGCGGCGCAGCCCCAACGAATACGGCGGCTG
GCGTACCCAACAGGAGGCCAGTCTTGCCGCACCACAGGGGATCTCCCCGCATAGAACGTGAAATGGCCCG
GTTTTTTTGACCAGCAAGGCAAACGTTTGAGTCAAGGCTGCCTGCCCTGGTCTGTCTGTCGCATCCTCTG
CCACCATCGCCTGTTTCTCATCAAACTGGCGCGAGGCAGACCCGCAGATCCTGTCGCCATTTCGTCATGG
AACATTCAGGCGCGTGTGAAGGAGTTGGGTATGACCCAAGCAAACAGCAGGACAAACAGCGTGGAACAGC
TGGCCAATGATGACGCCAGCCAGAGCGCAGCAAAGCAACAGCCGCTCACGGCTCTTGATCGCTACTTTAT
GATCAGCGAGCGGGGCAGCAATGTCAGACAGGAGGTGCTGGCGGGCCTCACCACCTTACTTGCCATGGTC
TACTCGGTGATCGTGGTGCCGAGCATGCTCGGCAAGGCGGGCTTTCCTCCCGGCGCCGTGTTTGTCGCTA
CCTGTCTGGTGGCGGGGTTCGGTTCGCTCTTGATGGGGATCTGGGCCAAGTTGCCGATGGCCATCGGCTG
CGCTATTTCCCTCACGGCGTTTACCGCCTTCAGTCTGGTGCTGGGGCAGCAGATCCCCATTCCGGTGGCG
CTGGGGGCCGTATTCCTGATGGGGGTGCTGTTTACCGCTATCTCGGTGACCGGGGTGCGTAGCTGGATCC
TCGACAACCTGCCGATGGGAATTGCCCACGGCACCGGCATCGGGATTGGTCTCTTCTTGCTGCTTATCGC
CGCCAACGGGGTGGGGCTGGTGATCAAGAACCCGCTGGAGGGGCTGCCGGTGGCGCTGGGGGCTTTTACC
TCGATGCCGGTCATTATGTCCCTGATCGGCCTTGCGGTGATCTTCGGGCTGGAGAAGCTGAGAGTACCAG
GGGGGATCCTGCTGGTGATTATCGCCATCTCCATCTTCGGCCTGATTTTCGATCCGGCGGTGAAGTATCA
GGGATTGTTCGCCCTGCCGAGCCTCTCGGACGATCAGGGCAAGTCCCTCATCTTCAGTCTGGATATCATG
GGGGCCCTCTCGCCGCTGGTGTTGCCCAGCGTGCTGGCGCTGGTGATGACGGCGGTATTCGATGCCACCG
GCACCATCCGGGCGGTAGCAGGGCAGGCCAACCTGCTCGATAAGGATGGTCACATCATCAACAGCGGCAA
GGCGCTCAGTGCCGACTCGGTCAGCAGCATCTTCTCCAGTCTGGTGGGGGCGGCCCCCGCTGCCGTCTAT
ATCGAATCGGCGGCGGGCACGGCGGCGGGAGGCAAAACCGGCCTGACCGCGACCGTGGTCGGCGGCCTGT
TCCTGCTGATGCTGTTCCTCTCGCCGCTCTCCTATCTGGTGCCCGCCTATGCCACGGCGCCGGCACTGAT
GTATGTGGGGCTCTTGATGCTGAGCAACGTCACCAAACTCGATTTCGATGACTTTGTCGGGGCCATGTCC
GGGTTGGTGTGCGCCGTCTTTATCGTGCTCACCTGCAATATTGTCACCGGCATCATGCTTGGCTTCAGCT
CGCTGGTGATCGGCCGCATCTGCTCGAAGGAGTGGCACAAGCTCAACGTCGGCACCGTGGTCATCGCCCT
GGCGCTGGTGCTCTTTTATGCTGGGGGGTGGGCTATTTAGGGATAATCCGCCCGTTTGGGGTCGCTCTGG
CGGCCCCATTTTTATGCCTGCTATTTTATCCCTGCGTTGCTCAGCCTGCCTTGGCTGGCTATCACGCCTC
GGCCGTCTCTGCAGCTGGGGCCATCGCGATACCATTTTGATAAATCCTCCTACCAGATTGATAGGCTCCT
ACCCTTGCTCTCCTCTCTCTGCCGCTTTTTTCACCGAAGATGTGCCCCGTTTGCTCAGGGGCCACGCTTG
GCTTGTCGTTTCGGGCCAACAAAAGCGGGCTAGATGGGATCCCCTTCACACTTCTCCCCATTCTCCTGAC
GATGGCGGGAAACAGGCGCATATCTTGCTGTTTTTTATGCCGGTTCCAGATTTTTCCATTCGAAAAAGGA
CTTGCATATGTCCATAGGATTACCGCTCAAGCGGGTGGATGCCGAGGCCAAGGTGACCGGCAGCGCCCGC
TATACCGATGACAACATCATGCTCGGGATGCGCCACGCCAAGTACGTGCGAAGCAATATTGCCCACGGCA
AGGTGCTTGCCATCGATGCGACCGAGGCACTGGCCCTGCACGGGGTCGAAGCCGTGTTTACCCATGAAGA
TGTCCCCACCACCTGCTTTCGCACTGCCGGTCACGCCTGGTCCCTCGATGAGGCCAAACGGGACGTGAAA
GACCGGCGTCTGCTCACCGACCATGTACGCCACTTCGGCGATGGGGTCGCCATAGTCGTGGCGCGCGATC
CGCTGGTCGCCGAGAAGGCTGCCGCCCTGGTCAAGGTGACTTACGAGCCGCTGCCGGTGATGACGGATGC
CCAAAGCGCGCTGGCCGACGATGCCTATCCCATTCACCCGAGCGGCAATCTGCTGCGCCAAAGTCAGCTT
CGCGCCAATGACCCGGAGCAGGCCATTGCGGCGGCAGATCTGCAGTTTCAGGGGCACTACCGGACGCCGG
TGGTGCAGCACTGCCATCTGGAGACGGTGACCTGCTACGCCTACATGGATCAGCCGGACCACATCATCGT
AGTCAGCAGCACCCAGATCCCCCAGATAGTGCGCAGCCGGGTGGCTAAATCCCTCGGCCTGCCCTGGTCG
AGCGTGCGGGTGATCAAGCCCTATATGGGCGGTGGTTTTGGCAACAAGCAGGATGTGCTGGAAGAGCCGA
TGGCGGCCTTTTTGAGCTACAAGCTTGGCGGCGTGCCGGTCAAGGTTACCTTGAGCCGGGAGGAGTGTTT
TTTTGCCTCCCGCACCCGTCACGGTTTTGCCATCGATGGCAAGATGGGCCTCAACCGCGACGGCACCCTC
AAGGGCTATCAGCTCGACGTGCTCTCCAACACCGGCGGCTATGCCTCCCACGGCCACTCCATCGCCAGTG
CCGGGGGCAACAAGATCAGCTACCTCTATCCGCGCAGCGCCTTCGGTTATGACGCCAAGACCTTCTACAG
CAACCTGCCCACCGCTGGTGCCATGCGCGGTTACGGCGCGCCGCAGGTGATTTTTGCCCTCGAGTGCATG
CTGGATGATGCCTGCGCCGAACTGGGGCTGGATCCGCTCGATGTGCGGATCGCCAACGTGGCGCGCGAGG
GGGATGTCAATCAGGTCAACCAGAAGACCATCTACAGCGCCGGGTTGCCGGACTGTCTGCTGCGTGGTCG
CGAGATGTTCGAGTGGGATCGGCTCAAGGCCGAGTGTCTGGGGCAGGATCCTGCCTGCCGGGTGCGGCGC
GGCATCGGGGTTGCGTGCTTTAGCTACGGCTCCAACACCTATCCGGTGGGGGTCGAAATTGCCGGCGCCC
GCATGCTGCTCAATCAGGATGGCACCGTCAATCTGCAGATCGGCGCCACCGAGATAGGGCAGGGGTCGGA
CACTGTCTTTGCCCAGATGGCGGCCCAGACGTTAGGCATTCCGTTTGAGCAGATCCGGGTTATCTCGACT
CAGGATACCGACATCACCGCCTATGATCCTGGCTCGTTCGCCTCGCGTCAGAGCTATGTGGCGGCCCCCG
CCATCCATCAGGCGGCGCTGCAGTTGCGGGCCCGCATTTTGTCGCTGGCGGCCCAACTCTGCCATCAGGA
TGTGGGGTCATTGACTCTGATCGACGGTTACGTGGTGCTGGCCGGTGCGCCGGAGAAGGTGTTGATCAGC

GTCGCCGAGGTGTCGGTCAACGCCTACTACCACCTCACTATCGGCGGTCAGATCACCTCCGAGGTCTCCC
ACAAGACCACCACCAACCCGCCCAGTTTCGGATGCACGTTTGTCGATCTCAGCGTTGATATCGATCTCTG
CAAGGTGACCATCAATCGCATCATCAACCTGCACGATGCAGGCAAGATCCTCAACCCGCAGCTGGCGGAG
GGGCAGGTGCACGGCGGCATGGGGATGGGGATCGGCTGGTCCCTGTTCGAGGAGATGATCATCGACGCCA
AAACCGGCGTGGTACGCAACCCCAACCTGCTCGACTACAAGTTCCCTACCATGCTGGATTTGCCCGAACT
TGAATGCGACTTCGTGCAGACCTACGAGCCCCAGTCGGTCTATGGCCACAAATCCCTGGGGGAACCGCCC
ATTATCTCGCCGGGCCCGGCGATCCGTAACGCCATTCGCATGGCGACCGGGGTGGCCATCAACGAGCTGC
CTATCACCCCCAAAACCCTGTTCCGGGAGTTTGTCGCGGCGGGTTTGATTGGCGAGGGATCAGGGGAGGA
GCAACACCATGTTTGATATTGCCCGTTACTACAAGGCGCACAGCGTGGCCGAGGCGGTCGCCCTGCGTCA
GGCTGATCCCGAGGCGCGTTTGCTGGCCGGTGGCACCGATGTGATGATCCAGCTCCATCACCTCAACGCC
GCCTATCGCCATATCGTTGATATTCACGACTTGCCCGAACTCAAAGGGGTGTGCGAGTTGCCTGATGGGT
CGATCCGCATCGGCTCCGGCACCACCTTTACCGAGCTCATCGAAAACGAGCTGGTGCAGCGCCGCCTGCC
AATGCTGGCCGAAGCCTCTGCCACCATCGCCGGCCCGCAGATCCGCAACGTCGCCACCTATGGCGGCAAC
ATCTGCAACGGCGCCACCAGTGCTGACTCGGCGGCGCCTACCGTCGCGCTCGAGGCCGAGCTTGAGATTG
CTGGCCCTAACGGCACACGCCGCATCCCCATCGCCGGTTTTCACACCGGCCCCGGCAAGGTGGCGCTGCA
ACCGGCGGAAATTCTGGTGGCATTTCACTTTGCGCCGGATTCTCACCCCCATGTGGGCTCCCACTACTTC
AAGTACGCCATGCGCGATGCCATGGATATCGCCACCATCGGCTGCGCCGCCTACTGCCAGATTGAGAATG
GCCACTTCAAGCGCCTGCGGCTGGCCTATGGCGTGGCGGCCCCGACCCCGGTGCGCACTCCTCACGCGGA
AGCGGCGGCCGAAGGGCAACCCCTGACTCGTGCCACACTGGCCGCGATTGCCGAGGCGGTGGTGGCCGAT
GTGGCGCCGCGATCCTCCTGGCGGGCAGAGAAGGAGTTTCGCCTGCACCTTATCAAAACCCTGGCGCAGC
GGGTGGTCGCCTGCGCAGTCGAACGTGCCGGAGGCAAGATCCTATGATGCAACCGCAAAGCTGTCCCGCA
GTGGAGATCCAGTGCGAGATCAACGGCAAGCCTTACTCCTATGCAGTGCCGCCCACCATGTCGTTGCTCC
ATTTCCTGCGCGGGCAGGGGCTTATCAGTGTGAAAGAGGGGTGCAGTGTCGGTGAGTGCGGCGCCTGTAC
CGTGCGGGTGGATGGCACTGCCATCGACAGCTGCCTCTATCTGGCGGTGTGGGCCGATGGCAAATCCATT
CGCACCGTGGAGGGAGAGCGCAAAGGTAACGAACTCTCCGACGTGCAGCAGGCCTTTATCGACGAGGGGG
CGGTGCAGTGCGGTTTTTGCACCCCGGGCCTCGTGATGGCGAGCGCGGCTCTGCTCGACAAGACCGAACG
CCGTCCGCTGACCGATGCGGAGATCCGCCGCGGGCTGTCGGGCAACCTCTGCCGCTGCACCGGGTATCAG
AACGTGGTGCGGGCGGTGAAGAAGTGCTGCAAGGGTTGAAAGCAGTTTGCCCCTTCGGTGACGAAAGCCG
CGTAACAAGCAATAAAACCAAAGGCCCGTCTCGCAAGAGGCGGGCCTTCTCTCTTTGAGTCGTTAACCGG
GTTGATTACCCTTTTTGCGGCTCGATCACCAGTTTGCCATCCTTGACCGGAATGGTGGCGCCCGGGGCTT
GCTTCATGCGGATCACCCCCTCCTGACCGGCGAGGCGATAGGTCACGTCATAACCAATGGTGTTGACGCT
CGTATCCTGTACGGTTTTGCAGCGCTGCTCGGTGGTGGTGTAGGTATCGCCCGCCTGCATATTGCCCTGC
ACCTGATTGCCGGCATAACCTCCCGCGACCGCCCCGGCGGCAGTAGCCACCTTCTTGCCGCTGCCACCGC
CAAACTGGTGGCCGACCACGCCACCCAGCGCCGCGCCCACCACGGTGCCGAGGATCTTGTGCTCATCCTG
TACCGGCTTGCGGTGAGTCACGGTGACGTTGCGACACTCCTGGCGGGGAGTCTTGATGGTCTCGGTGATC
GGCTTGCTGGCTATCACCTCGGCAAAGGTGGGCTGGGGTGTAAAGAGGGAGCTGCTGGCCGCCGCCGAGA
GTGCCAGGGCCGAGGCGATGCCAATACCGATACCTGCCATCATGGATCTGTTCATCTTATGGTTCTCCTG
CATACCCATACGGGCTGTTCCAATCTGGCGCCATCTTGTCATAACCGGTTGGCCGGACAAGGCGAGGGGG
ATGGGTCTGCGACAGGCGCTGGCAAGGTCAAAAAACAGCCCCCCTGAAAAAGTGTCTGGCTTTGGCGACA
ACGGACTAAAAGTGACCGGTTTCCGGCGCATGGCGGGCTTTGCCAGTGTGATAAGGAGCAATTCGAGTCG
CAATTTGCAAGGCGGGCGGTGAGCAGCATGCCAGAGTGGCAAAGTGCTGACGGGGGCAGGTCAAGGGAGG
TCTTGAACAAGGGCTCTGTTCGTTGAGATGGCCATGCAAGTGGTTGATAAATGGCTCCAGCCCCATCGGA
TGTTGGCCGATAAGAGATCACGGCAAGCATTTTGCTTTGATTGGGTGAAGCAGAGGGTGAACGGGTCGGC
AGACAACTGCGTAAGTCCTGAAATGAAGAGGTTTACCCGAGGTCATCGGATCAACGAGGGGGAGATATGT
CAATGTTACCCAGCTGGATCACCCCGGCCATTCAGGCCAACATCATCACGGTGCAGCAGGCCGAACGTCT
GCTCTCATCCCTCTACCGGCCCCTGGCCGAGCAGGGAGAGGAGCTTCCCGCCGATATCGCCGACATCAGC
CTCAAGCTCTACTGCTTCCGCCAGCAGGGGCGGGTACGCCACTAAGGCTTACAAAAGAACACCCCGCGCC
AGGCACGGGGTGTTGTCACTCATGTATCACAGGCCCATCAGGCCAACATCACTTGCGCCACCAGCATCAG
GGCGCCAAATCCCAGCACAAAGCCCACCATGGGCAGCACGAACTTGACCCACTTGTTGAACTGGATGCCG
AGCATCTGCAGGGTGACCAGCACCAGTCCGGTGGGGGCCAGAAACAGCATGGCGTATTGGCCCCAGTTGT
AGGCCGACACCACGATATAGCGAGGGATCATCACGGTATCCGCCAGCGGCGCCATGATGGGCATGGAGAG
CACTGCGAGCCCCGAGGAGGAGGGCACCACCAGCCCCAACAGGGTGAACACCAGCATCTGCGCCACCGCG
AACACGGCGCCATGCATGCCGGAGACCAGATCGGAGGCGTAGGCCAGAATAGTGTCCGAGATCATCCCCT
GATCCAGTACCAGATTGACCCCCCGCGCCAGCCCGATGATGAGGGAGACGGCCACCAGCTCCGACGCCCC
CTGAGTGAAGGCCTCGACCGCCTCTTTCTCTTTGAGACCCGAGATAAAAATGATGATGATTGCGATGGTC
AGGAAGGAGGCCGCCATCTGCGGGAACCACCAACCGGCCATGGAGACGCCCCACACCATCAGCGGGAAGG
CGAGGATAAACAGGGTCAGAATGATCTTGCGGCGCAGGGTGAAGGGCACCCCTTCGTCCATAGAGCCCTC
TTTGAGAAAACGGGCGTGAAAACTCTCTCTGTCCTCATAGGTGTAGGAGAAGGCCGGGTTGGCTTTGATC
TTCTTGCAGTACCAGTAGAGATAACCGATCACCACCAGGGTGCCCACCAGACAGCCGACGGCGCGAAAGG
TCAGCCCTTCGGTAAAGGGAATGCCGGCGGCATTGGAGGCGATCACCACCGAGAAGGGATTGATGGTGGA
GAAGGTGGTGCCCATGGAGGCGGCGAGGAAGATGGCCCCTACGCAGACGATGGAGTCATAGCCGAGCGCC

AGAAAGATGGGTACCAGTATGGGGTAGAAGGCGACCGCTTCTTCCTCCAGTCCGCAGGTGGTGCCTCCCA
GCGCCATCACGAAGCAGACCAGCGCCACAAACAGGAACTCGTTGCCCCGGGTCTTGTGTGAGAGGGCGAC
AAGCCCCGCACTGAATGCGCCTGTCTTGTTGATGACGCCGATCAGCCCCCCGAGCACCAGAATAAAGACG
ATGATATCCGCCCCCTCTATGGTGCCCTTGACCATGCTGACTGCCACGTCACCGAGCCCCTTGGGCGCTT
GCTCCAGCCGTGCATAGGTGCCGGGAATGGCGATGGGCTTGCGGATGGTGCCGTTGGTAAATTGCTCTAC
CGCAATATTGATATTAAGTTTTGCCTGGGCATCCTGGGTTGCGGGAAGTCGGCTTATTTCCCCCTGTGGG
CTATTGACCATCAGGCTTTCACTGGCCGGATTATAAGAGAGTTTGGCATAGGCGCCCGCCGGAATTATCC
AGGTCAGCGCAATGGCCAGCAACATAATAAGAAACAGAATGGTAAATGCAGTAGGGAATTGTCTGCGTGG
TGTGGTATTCGGCACGGCACACATAAAATGCTCCTTGATAAAGCAGGTTTGGGCTCGGCGGAATATCGGG
TCGATAGGGTTTCTGCGTCATACATCCAAGGTACGCAAATTCACTGTGTATTGCGTAGTGATTGGCGAGA
AATCTGGCCTGATATTGGACATCCGGTAGATTGTTTCACGTTGTAACCGTAACCCGTGCGCGGCTGGATG
AACATGGTGCCATGCTCGCTTTATGGGATGGCTCACAAAATTTATTTTCCATGGATTTAACAGCTGATAT
GAAATAAAAGTTATTATGCAATAACTATTTCAGCTTTCGTTTTACGCGGCTTGCCTTAAATAAAGTTCAT
GAAAATAGTGCGTACCGAGATGAATGAAATAACACATAAAGCAGCTTTTACGGAATAATATTTCGGCATC
ATCTGTTTCCGTTATTCGCCAAAAACAGTCTGTGAAAAAAATGGCACGCTTTCCAGTAACGAAATATTTT
TTTGCAACGAAAACAGGGGGCCCCTGTTGGCAGCAGGCTGCCCCTGTCTAAGACGTTTTGCTAATACCTG
CCGCCGCAGGCTCACTGTTAGGCTGTCATCCCCATAAGAGACCAGTGACGGGCAGGATGCCTATGTCGCT
GTCTGATCTCTGAATCATCCCGAAACTGCCCGTTTTGTGCCACTTGTGAGGAAGGAATCCGATGACAGTG
CAGAACATCTATGAGCGCGACCTTGCCAAAACCGCCGCCAATTTTGAGGCCCTGACCCCCATCAGCTTTT
TGGGGAGGGCGGAGCGGGTCTATCCCGACTATCCGGCGCTGATCCACGGCTCGCTACGCCAGAGCTGGGG
CGAGACCGCCCGCCGTTGCCGCCAGCTTGCCTCGGCCCTGCGGCTGCGCGGTATCGGGGAGGGGGATACC
GTCTCCATCGTCGCGCCCAATATCCCGGCGATGTTTGAAGCACACTTCGGGGTGCCCATGAGCGGCGCCG
TGCTCAACACCATCAATACCCGGCTCGATGCCGAGTCGATGGCCTTTATCTTCCAGCATGCCCAGAGCAA
GGTGGTGCTGGTGGACAGGGAGTTTGGCGCCGTGGTGCGCAAGGCACTGGATCTGCTGGAGCGCCAACCG
CTGGTCATCGCCATCGACGATCCCCTCTACCGGGAAGGGGCGCTTGTCAGTGAACTGACCTATGAGGCCT
TTATTGCCACTGGTAACGGCGATGAACCCGGCTGGTTGCCAGAGGATGAGTGGCAGGCGATCTCCCTTAA
CTACACCTCCGGCACCACCGGCAACCCCAAGGGAGTGGTCTATCACCATCGCGGTGCCCACCTCAACGCC
ATCAACAATGTGCTCTCCTGGGAGCTGCCCAAACATCCCGTCTATCTCTGGACCTTGCCGATGTTTCACT
GCAATGGCTGGTGTTTCCCCTGGACGCTGGCGGCCACCGCCGGGGTGAGCGTCTGCCTGCGCCATGTGCA
GGGCGCTGCCATTTATGAGGCGCTGCACGAGCACAAGGTGAGCCATTTTTGCGCGGCGCCCATCGTGCTC
AATATGCTCAACAATGCCGATCCGGCGCTCAAGCAGGGGCTGGATCACCCCATCAAGGTGATGACCGCTG
GCGCTGCGCCCCCCGCCACCGTGATCGCCGGGATGGAGGCGATGGGGATTGCCGTGACCCACGTCTATGG
CCTCACCGAAACCTATGGTCCCTGCGTGTTGTGCGAGCCGCAAAGAAGCTGGCAAGGTCAGGATGCCACC
ACGCTCTCCCGCCTCAAGGCGCGCCAGGGGGTGGCATCCCCGCTGCAGGGAGAGATGCGGGTCATCAACC
CGGTCAGCGGCGAGCCGGTGGCCAAAGATGGCAAAACCATGGGGGAGATAGTGCTGCGGGGCAATGTGGT
GATGAAAGGCTATCTGAAAAATCCGGCGGCCAGCGCCGAGGCGATGGCGGAGGGGTGGTTTCGCAGTGGG
GATCTGGCGGTGTGGCACCCGGATGGTTATGTGGAGATCAAGGACAGATCCAAGGACATCATCATCTCCG
GCGGAGAGAATATCTCCAGTCTGGAGGTGGAAGATGTGCTCTATCGCCATCCCGATGTGGACGAAGCGGC
GGTGATCGCCATGCCCGATGAGAAGTGGGGGGAGGTGCCCTGCGCCTTCGTGAAATTGAAAGAGGGGCGA
GAGACTACCCAGGCGGAGCTGATCGCCTTTTGTCGTGAGCAGATGGCCCACTTCAAGGCCCCCAAACGCA
TCATCTTCACCCCGCTGCCGAAGACCTCGACCGGCAAGGTGCAAAAATTCGTGCTGCGCAAACAGCTGGG
GTAGCGGTCTGTTCGTAAGGCGCCAGCACAGACTTTTATAGAAGGGAGAAACGAGGGCGCTGGAGGGCTG
CTGGCCCTCCCGTTGTATGGCTAGGGAGCCTTGCAGGCGACGCAGTTGCGGCCGCTCTGTTTGGCCTGAT
AGAGCATCTGATCGGCGCGGGCGTAGAGGCTCTCCATCAGTTCCCCCTCGCCATTTTCCACCAGTCCGGC
GCTGATGGTGATAACCAGCTTATCCCCATCGGGCAGGGGCAGTGGGCTTTGCATCACCTGCAGGCGCAGC
CGCTCGGCCAGTGATGCGGCGGCCTGGAGGGTGGTGTTGGGCAGTAAAATGGCGAATTCGTCGCCGCCGA
TACGAGCCAGCACATCTTCGCTGCGCAGCACCGATTGCATCACCCTGCCCAGATGAACCAGCGCTCTGTC
CCCCAGGGGATGGCCGAAGCGATCGTTGATGCACTTGAACTGATCCACGTCCAGCACCATCAACACCGAG
GTGAGGTGGTGGCGCATGTCCAGACTGAGGTTCTTCTCGCACATCTCGAAAAAGAAACGGCGGTTGTAGA
GTCCGGTGAGGCCATCGCGCACCGACTGCTGGTGCAGCTGCTCCTTGAGCTGGTGCAGTTCGGTGATATC
GGAGGAGATACCGATCAGGGTGGGGGTGCCATCCCCCAACAGAAAGGGCACTTTGACCGACCAGTAGTAG
TGCTGATTGCCTTCGGGATCGGTCAGCATCTCTTCCCCGGCTTGCAGCTCGCCGGTTTCGAATACCCGGT
TGTCCAGTCGCCACAGCTGGCAGGCGCTGGCAGCTGGGATCACCTCATCATCGCGCCGATTGATGATCTG
CTCGGCAGGAAGGCCCAGCAGATCGGCGACCTTTTGGTTGACGTAGAGAAAGCGGTGCAGGCTGTCCTTC
ATATAGATATGGGCATCCACGTTGGCGAGGATGATTTCCAGCAGCTGATGCTGCTCCGCCATGCGGGCCT
CAATCAGCTTGTGTTCGGTGATATCGGTGGAGATGCCGCACAGACCGATCAGGTTGCCATCCTTGTCATG
GATGGGCTTTTTAACGGTCCAGTAGCAGCGCTTTTCCCCGGTGGATTTGAGGTAGTTGATCTCCTCCTGG
GCGATGGCGAGTTGCTGCTCCAGTACCAACCGGTCGTTGCGTTGAATGGTGTCGGAGAATTCGAGATCAA
AAAAGGCCCGATCATCCTGCCCCTGCAACTCGGCGAGAGTACAGCCAAAGAGACCCAGCACTGCCTGATT
GGCATAGGTGTAACGGCCCGCCAGATCCTTGGTAAACATGTAGGCGTCCGTCTCGTTGAAGATGGCTTTG
AGGTGATCCAGCTCATGCTGTAACTGGATAGAGAAGGGGTCATCCTTCATGGAGTTAACCATCCTTGGTT

AAGGGCGTCGTTGTATACAGCGTGAATATATGTACCAACTGTTTAAATTAAGCACTTCACAGGCCATATT
TCGCGTTTATTTCCGCAGGATCTGCGCCATGTCACACGATTGTCATCGTTGGTGTGGTCAAAGGAAATCA
ACTGAGTCCGTGGCTATGCAGATAACTGTCCCACTCATATCGGCCCTGCTTCGTGGGCCAGCAGCAAAGA
GATTGCCCGGCAGTCAAATACGCCAGCCCGAGCGGTATAAAGGGCAACAAACAGGTCTTTAATCAAACTA
TTTTCACTGTGTCCCTGATACCATTGCCGCACTATTTGGCTTGGGGAGTGCAGGATGGCAGAGCGAACAG
TATTTGACTGGCTGGAACCGATTACATGGGATGGGCCGGGAGCCATTGACGGAGTGGTGGAAAAGTTGGC
CGGTGATCGGCTGGAACGCGCTCGCTATGCCGAGTTTCTGACCAACTACCTCGCTGCCGAAGGCAAGCAG
CGCAATTATGTGCTCAACCTCAATGCCGAATGGGGGGCGGGAAAAACCTGGTTTATCAAGCGCTGGTATA
TGGAGCTGAAAGCGCACTATCCCACCGTCTATATCGATGCTTGGCAGCAGGACTTTTCCGATGATCCCTT
ATTGACGGTGATTTCGTCGATTATCGATCAATTGAGAGATGCAACGGGAAATAAGGTTGCCATACCTGAT
GGCATCAAGTCCAAACTATTGGGTTTGCTCAAAGTTGGCGGGAAATTGGTGCTCAAGTCAGCCATTAAAA
AAGCTGGATGGGAAGATAGTGACTTTTCTATGGAAGGAGAAGACGCAGACAGGCTGGTTGATACGCTTTG
TAGTAATCACAAAGAGCGTTATGAATCTATCCAGTATCTTAAACAAGAGATCCGGCAATGGGTTGCAGCG
GCGGTTTCAGCAAACTTAGATCAGGTGGATGGGAAAGGTCGTTTGGATTACCCCGCTTTTATTTTGATTG
ATGAGCTGGATCGCTGTCGTCCTTCTTATGCGGTAGAAATGCTGGAGACTATCAAGCATATATTCGATAT
TCCCAAAGTAGTGTTTGTATTAGCAACGGATACCGAGCAGTTACAGCATGCTATCAAGGTCATTTATGGC
GATGGTTTTGATGCAGAAAGCTATCTTGGACGATTTTTTCATAGACGATTTACGTTAGGCAGTATCTCTC
GGCGAGAGTTTATTCAGCAGGCAATTGTAGATAAAAAATTGCAGTTTGAACACGTCAATGTATGGCCAGA
GATCACTGATAGCCAAGTGTTGGTAAATGTATTGACAGCTGTTTGTGATCATTTTTCATTGAGCTTAAGA
AAAACAGAGCAAGTGTTGGATAGAACGATCGCTTCCATCGTTAATTACGAAGGTTGCACATTAAATTTGA
TTTTTCTTGTTTACCTGTTCTCTCTATGCGAGAAAAGCAATGGAGATTATCGTGCTGTAGCGGACGGCAA
AATGCCTACGACAGATAACCATCCTAAGCTAAAAGGTTTCAATACTCTTACAAGTGAGTATGAAGAACAA
CATTTTCAATGTTTTATAAAAGCAGGGAGTGCCATAGGAAGTTATATTGAGGATATCGATCATGAACGAT
ATCTAAATCCTTTTATAGATGGAATATATATGGTTTCATTAAAAAATATGTTAGCCATTTCTTATGAAAG
TCTATTGAAATCGACTTCAGATGATGAGTGTTCATATCGTTTTATCACGTTAGCAAAAGATTGTGTTAAG
CGAATGCGGAATTTGACTGTAGAAGAAAATACTATTTTGGCAGAAAAAGAACTATCACAGTTGGAACGAC
GTTTAACAACGTATAAAAATTTAGTTGAGCTAGCCGTGCATCTTGACTAAGTGTACAGATCTAGTTATCA
CCTGACACCGGCCTCAAACCGGTGTCTTGTTTACGCTACTTTAGTGCGCACTTCCTTCGGTATCAATTTC
AGACCCTTCTTGAACATGGTGTAGGGGGTTTGACCCTCCATCATCCTGCCTTGATGAGGCCGCTGGGTGT
TGTAGTGCTCCAGGTAGCTATCCAGGTCTGTCTGCATCTGCTCTACCGACTCATACCAGGTCGTCCTCCC
CTTGATACGGAAGTGCTCATCCAGCAAGGTTCGATGCAGCCGTTCGATAAAGCCGTTGCTCTGCGGCCTG
CGCACCTTGGTGGTTCGGTGCTCAATCCCCTCCAGTTGCAGGAACAGCTCGTAGGGATGGTGGTCAGGGC
GCCCACAGAACTCGCGCCCGTTATCCGACAGGATGGTATAGACCCGTGCCTCGTGGGTTTCAAAGAATGG
CAGCACCGTTTCGTTAAGCACATGGACCGAGGTGATGGGCAGTTTGCTGGTGTAGAGCCGCCCCCAGGCG
TGGCGGCTATAGCAGTCCAGCACGGTTTGCAGATACACCTTACCGACCCCCTTGAGCGCGCCGACGAAGA
AGGTGTCGACCGCCACCAGTTCCCCGGTGTAGTGAACCTCAATCTGGCGTTCGCGAAACTCGGGGCTGAA
GCGCTCTAACAGGCGAATTTGCTCGTCATTGAGCTCAATGGTCTGCTCCCGGTGGGTTTTCTCGAGGCGC
AACAAGCGGTCGTGTTTGGAGAGCAAGTCATGACGTTGCCACACGCCACGCACGCCGCCGGCACTGACAT
TGATGCCTTGCAGAGCCAGTTCTTGGGCGACCCGCAGCGGGCCATGAGTTGGTCTGGTGAGGGAGTAATC
GAGTATGGCCTGCTCAATCTCGGGAGCGACCCGATTGGGATGAGCCCCTTTACAGCCGGGCAATTTGTCG
AGCAATCCCTCGGCGCCATAGGTCTGGTAATTACGGCGAATTTCGTAGAACTGCTGACGACTGTAGCCGA
TGAGCTTGCAGGCTTTGCTGACATTATTGAGCTCTTTGGCAAGCTCTAGCAGGCTGAGTTTGCGACGTGC
TACTTTCTCGTTGGTGGTCATACGGTGACTCCGGATAACACGGTAGGGGTACCGTTTTGTTATCCGGTTT
GAAGCCGTATGACCACTCCCTTTTGTGGGCAACTGTCAGGTGAATACCATCTCCATACAGACTAAGTAAA
GCCGGGATAACCCGGCCTTACTATCCGCTTATTTATTATGCCGCTTATAAAGCCGCTGCACCACATCCTG
TAATCCCAGCCCTTCATGTTGTAGCAGCACGGTGAGGTGGTAGAGCAGATCGGCGGATTCGTTGATGAGC
TCTTCCCGATCCTTGGCCATGGCGGCCAGTGCCACTTCGACACCTTCCTCACCGACCTTCTGGGCGATGC
GCTTGGTGCCTTTGCCGTAGAGGCTGGCGGTGTAGCTGGTGGCCGGATCGGCCCCTTTGCGGGCTGCCAG
CACCTGCTCCAGCTCTGCCAGAAAACCGAGGGGCGGCAGCGGGTGGCCGTTAAAGCAGGAGGTAGTGCCC
TTGTGGCAGGTGGGGCCGACGGGGTTGGCGGCAATCAGCAGGGAATCCTGATCGCAGTCGGTGCTGATAG
CCACAAGGTTGAGCACATGGCCCGAGCTTTCACCCTTGGTCCAGAGCCGCTGTTTGCTGCGGCTAAAGAA
GGTGACCTGACCGGTGGTCTGGGTCTTTTCCAGCGCTTCGCGGGTCATAAAGCCCTGCATCAGCACCTCG
CCGCTGGCGGCGTGCTGGACGATGGCGGGGATCATGCCGTCACACTTTTCCCAGTCGAGTTGTTCGGCAA
GGGGGCGCGCGCTGGCGGTTGTGTGTGTGTTATCAGAGAAGTTATTCACGGATGGCGACTCCTTCGGTTT
TCAGCCAGCGTTTCAATTCGGGAATGGGGATAAGGCCCTTGTGGAACACAGAGGCGGCCAGCGCCCCGTC
CACATCGGCGAGGGTGAAGGCATCGCGAAAGTGTGCCATGGCACCGGCGCCGCCGGAGGCAATCAGCGGC
ACCTTGCAGACGCTACGCACCAGCTTGAGCTGGTCGAGATCATAACCCTGACGCATGCCGTCCTGATTCA
TCACGTTCAGCACGATTTCGCCCGCGCCGCGCTTTTGCACCTCCTGCACCCAATCGAGGGTGTGCCAGGC
GGTGGTGCGGGTGCGACTCTCGTCACCGGTAAACTGTTTGACCTGATAGTGGCCCGTGTCGGCGTCGAAG
TAGGAGTCAATGCCCACCACCACGCACTGCACGCCAAATCGCTCGGCCAGTCGGCTGATAAGGGTCGGGT
CGGCCAGCGCCGGAGAGTTGATGGAGACCTTGTCGGCGCCAAACTCGAGGATCTGGCGGGCATCTTCCAC

GCTTTTAATGCCACCGGCGACGCAGAAGGGGATATCGATCACCTCCGCCACCCGGCTCACCCAGCTCTTG
TCCACTACCCGCGCATCGCTGGAGGCGGTGATGTCATAGAACACCAGCTCGTCAGCGCCCTCTTCGGCGT
AGCGGCGCGCCAGCTCGACAATGCCGCCCATCACCTCGTGGTTGCGAAATTGCACCCCTTTGACCACCAC
GCCATCTTTCACGTCGAGGCAGGGGATAATGCGTCTTGCCAGCATCAGTTGCCTCCTGTGGTATCAGCAG
CGGGGGCTTGCCAGCAGGCGATGGCGTCACCCACCGAGAACTTACCCTCCAGCAGGGCGCGGCCAAGGAT
GATCCCCTTGACGCCGGAGCCCTTGAGCGCCTCGATATCCGCGATGCCGCCAATGCCGCCGGAGGCCTGA
AAGGCGACGCTCGGGTAGCGGGCGCAGAGATCCCGATAGAGCTCGACGTTGGTGCCAGCCAGGGTGCCGT
CACGGCTGATGTCGGTGCAGAGCACATGCTTGAGACCGGCAGGGGAGGAAACGCTCGATCAGCGCCTCGAT
GGTCACGCCGCTGTTCTCCTGCCAGCCTGCCACCGCGATATGGCGATTGCCCTGCTCATCGATATTGACG
TCCAGCGCCAGCACGATCTGCTCGGGGCCATATTTCTCCATCCAGCTGGCCACCAGATCCGGGGATTTGA
CCGCGGTGGAGCCGATCACCACCCGGCTGGCACCGGCGGCCAGCAGATCTGCCACATCCTGCTCGGTACG
CACGCCGCCGCCAACTTGCACCGGTGCTTCGGTGGCCGACAGCAGTTGGGTCAGCAGGGGCAGCTGGCGC
GCCTTGGCATCCTTGGCACCGTCAAGATCTACCAGATGGAGCTGCACCGCCCCCTGGGAGACATATTCAT
CGAAGCGGGCCTGCGGGCTGCTGCTGTATTCGGTCTTCTGCTCGTAGTCCCCCTGATAGAGGCGAACCAC
CTGACCGTTGATAAGGTCAATTGCGGGAATGATCATGCTAGTCACAGCTCCAGAAAATTTTGCAGCAAAC
GGGCACCGGCTGCGCCTGAGCGCTCCGGGTGGAATTGGGCGCCGAAGAAGTTGCTGTGCTCTGCAACCCC
ACCGACGATGGCGGTAAACGGCTGGCCATAGTCGCAGGTCGCCAGCGTCGCGTCGGTCACCTCCAGCGCA
TAGGAGTGCACGAAGTAGAAGTAGCTGCCGCTCGGGATCCCCTTGAGCAGGGGATGGGTCTCGTCATGCT
CAATCTGGTTCCAGCCCATATGGGGCAGGCGCATGCTTGACTGGTGTAAGGTGCCCACCTCCATCAGCCG
GATCTTGCCCGGCACTATACCAAGGCAGTCGATAAGCCGCTCTGACGAGTCATCCGTACCCATGCTCTCT
TCGGAGGCTTCCGCCAGCATCTGCATGCCTAGGCAAAAGCCCAGCAGCGGCTGCTTGGCGGCGCGAATAA
GGGGCACCAGCCCCCGTTCGTTGAGGTTCTTCATAGCGGCTACCGCGGTGCCGACGCCGGGCAGGATCAG
CTTGTCCGCCTGCTCGATATCAGCCGCCACGCTTGAGACCAGCGGCTGCACGCCGAGGCGTTCGAACGCC
ATTCTGACCGAGGCCAGATTTGCACAGCCGGTATCGATAATGACCAGCTTCTGCTTGCTCACATCCATCA
CAGCACTCCCTTGCTGGAGGGGAGCTCGCTCCCTTCCACCCGAATAGCCTGACGCAGGGCGCGGCCAAAG
CCTTTGAACAGCGCCTCCACCTGATGGTGGGTGTTGCCGCTGCCCACCTTCATCTGCAATGTGATGGCCA
TGGCGTCCGACAAAGAGCGGAAGAAGTGGGGCACCATCTCGGTGGCCATCTCGCCCACGTTGTCGCGGCC
AAAGCCGCTCGGGCAGTCGAACACGAAGTAGGGGCGACCGCTCAAGTCCAGCGCTGTGCTTACCTCCTGC
TCGGTCAGCGGGCGCGCATCGATCACCGCCTGCACCTCGTCCATCGCCAGCACGAAGCCGAAACGGCCGA
TACCGCGCTTGTTGCCAAGGGCCTGACGCAGCGCCTGACCCAGTGCCAGCCCAACATCTTCCACCGTGTG
GTGATCGTCGATATGCAGATCACCGCTGACGTTCAGCTTGAGCTGGAAACCTGCGTGGGTGGCAATTTGA
TCCAGCATGTGATCAAAGAAACCGATGCCGGTGGCGATCTGGTTGCCGCCGCTCTTGTTTGTGGCAGAAG
GGTCCAAATCCAGCACCACCTGGATGCGGGTCTCCTTGGTATAGCGTTCCACCGAGGCTACGCGCCCCTT
GGATAACAGCTGATCACGGATGGCGAGCCAGTCGTGATCCTGTGGGTGGTAGCGAATGCCGCCAATGCCC
ATATTCTCGGCGAGTTGCAGATCGGTCTGTCTGTCACCAATAACGAAGGAGTTGGCAAAGTCGATGCGAC
CCGAGGCGAGATACTCCTTTACCAGCCCGAGGTGGGGCTTGCGGCAGCTGCAACCGTCGGTGGGGAAGTG
CGGGCAGATCAGCACCTGTTCCCACTTCACTCCTTGCGATTCAAACAGGTGCATCATCAGGTCGTGGGGT
GGCTGGAAGTTCTCCAGCGGCAGGCTGGCGGTGCCCAGCCCATCCTGATTGGTCACCATCACCAGCACGT
AGCCAGCGGCCTGCAACTCGCGCAGCACCGGAATGACCATGGGCTCGAAGCGCAGCTTGGCGAGGCTATC
CACCTGCTTGTCGGTCACCGGCTCTTCAATCAGGGTGCCGTCGCGGTCGATAAACAAAAACGGGGTCTTC
ATCGCTACATCCTTGTCTTACAAAGTGGGAGAGGGCAGATCACGCAGTACGGCCAGCACGGCATCCATCT
CGCCCTGATAGCCGATGGTGATGCGAATGCTGTTGTCCAGACCCGGCTTGCTGGAGAAATCCCGCAGAAT
GATGCCGGCGGCCTTCATGGCGGCAAAGACGGCTGCGCCATCCACAAAGCGCACCAGCACGAAGTTGCCC
TTGTCCTCGAATACTTCCCTTACGCAGGCAAGCTTTGCCAGCTCTGCCTTGAAGGCGGCTTTTTGCTTGT
TGAGCTCTACCACTCGCTCGCGCATCAGCTCCAGCCCCATGGGGGAGAGGGCTTGCGCCGCGATCTGGGC
GATGGGCTCGGGGATGGGATAGGGGGCGATCACCTTGGCCAGCATGGCGATCACCTCGGGGCTTGCCAGC
GTAAAGCCGCAGCGGATCCCCGCCAGGGCAAAGGCCTTGGAGAGGGTGCGGGTCACCACCAGATTGGGGA
AGCGGGCCAGCAGGTCCACCACGGACGCCTCGGGGCAGAACTCGATATAGGCCTCGTCCACTACCACGAT
GGCACGATCCCTGGCTTTATCCAGCAGGGCGATAAGGCCGTCACGCCCCACCAGATCCCCGGTGGGGTTG
TTGGGGGAGCAGAGAAACACCAGCTTCACATCAGACAGACGGTCCGCGATGGCGGGCCAGTCGGGCTGGC
GGCTGGCGGTCAGCGGCTGCTCGACGATGCCGACGCCACAGGTCTCGGCGCTGATGGCGTACATGCCGTA
GGTGGGCGGGCAGATGAGGATCTGATCTTCACCCGCTTCGCAGAAGGTGCGGATCAAGAGCTCGATGGCC
TCGTCGGCGCCGCGGCTCACCAGCACCTGATCGGGATTAACCCCGGCATAGGCGGCATAGCCGTTCACCA
CCTCGGCCGGTTGGCACTCGGGGTAGCGGTTGAGGCGGCTCCCCTCGATGGTGAAGGGATAGGCCTGTGG
CGCCTCGTTGGCGTTAAGCCAGACGTGGCCCTTGCCGCCGATGCGGCGGGCGGATTGATAAGGGGTGAGG
GCCCGCACCACGCGGCGCGCCAGATTGGCAATGCTCATGACAAATCCTTCTGCTGTGCTGTTGGTTGGGG
CGCGGCGGCCAGGGCATTCAACCTAAGGGTGACGGCATTCTTGTGGCCATCGAGCCCCTCGGCCTGGGCG
ATGCGCTCGACAATGGGGCCAAGGCCGCGAATACCTTCGGCGTTCAGCTCTTGCACTGTGTAGCGGCGCT
GATAATCGGCAAGGCCCAGGCTGGAGCAGGTGCGGGCATAGCCGTAGGTGGGCAGGGTGTGGTTGGTGCC
GCTGGCGTAATCGCCGACCGATTCCGGTGTCCATGCTCCGAGGAAGATGGAACCGGCGTTCTCGAGTTTA
TCCAGCAGATCGCGGGGGGCTCGGGTCTGCACGATCAGGTGTTCCGGCGCGTAGGCGTTGGAGATGGCAC

ACGCCTCGGCCAGATCGTCACAGAGGATCACCAGACTGCTGCCGAGCGCCTGGGCGGCGATATCGCGGCG
GCTCAACTTGGCCAGCTGGCTCTTGATCTCCCCTGCGATGGCATCGGCCAGCAGGGGCGAGGTGGTCACC
AGCACCACCTGGCTGTCGGGGCCATGCTCGGCCTGAGAGAGCAGATCCGCCGCCACGAAGGCCGGATTGG
CGCTCTCGTCGGCAATCACCAGCACCTCGGAGGGGCCGGCTGGCATGTCGATGGCGGCGCCGCGCCAGTC
GCGGGATACCTGTCCCTTGGCTTCGGTCACAAAGGCGTTGCCTGGGCCGAAGATCTTGTCCACCTTGGGG
ATGCTCTCGGTGCCGTACGCCATGGCGGCGATGGCCTGGGCGCCGCCGATGGCGAACACCTTCTCGACCC
CGCAGGCGCGGGCGGCGAACAGGATCTCGTCGCTGGCGGGAGACGGGGTGCAGAGCACCACTTCACGGCA
ACCGGCGATAGCAGCCGGAATGGCCAGCATCATCACGGTGGAGGGGAGCGGCGCGCTACCCCCCGGCACA
TAGAGGCCGACCCGACTGATGGGCTGGGTGCGCAGCTCGCATACCACCCCGGGCTGGGTCTCTACCCGTA
CCAATGGCTGCATTTGGGCAGTGTGGAAGGTGCGAATATTGGCAATGGCTGCTTCGATGGCATTTTTGAT
GTCGTCATCCAGCCGCGCGCAGGCAGCTTCAATCTCATCTTCACTGACGCGCAGCTTCTCACGGGGGGCG
CGCTCGAAGCGCAGGCTCAGCTCGCGCAGGGCCTCATCGCCACGGCTACGCACCGCGGCGATGATATCCC
GCACCACGGCGCCGATATCCGCCTCGTCACTCAAGGCCGGTCGGGTCAGCACGTCGGCCTGCTGGGCGGG
GGAGAGGGTCTTCCAGATCAGGGTCTGCATGATGTCCCTTCCTTATTCCATCATTTTTTCGATGGGCAGC
ACCAGAATGGAGCTGGCACCCAGAGCCTTGAGCTGCTCCATGGTCTCCCAGAACAGGGTCTCGCTGGAGA
CGACGTGCAGGGCCACCTGATTGGTGTCACCGGCCAGTTGCATGATGGTGGGGCGCTCGGCACCCGGCAA
CAGATCGGTGATGGCATCGAGCTTGTCTTTGGGGGCGTGCAGCATGATGTATTTGCTCTCGCGGGCCTGC
TGCATCCCCTGAATGCGGGGCAGCAGTTTGTCGATGAGCTTCTGCTTGGCATCGGACAAAGGGTTGGGGG
CCTGTACCAGTACCGCCTTGGAGCGGTAGATGACCTCCACCTCTTTAAGGCCATTGGCTTCCAGCGTCGC
GCCGGTGGAGACCAGATCGCAGATGGCATCGGCCAGACCCGCACGGGGTGCCACTTCCACCGAGCCGCCC
AGCATCACGCTCTTGAAATTGAGCCCTTTTTCGTCAAAGAAGCGCTTGAGCAGCCCGGGGTAGGAGGTGG
CGATGCGTTTGCCGGCAAGGCTCGCTGGCCCTGTGTAGTTCACATCTTCCGGCACCGCCAGCGAGAGGCG
GCAGCCACCAAAGTCCAGCTGCTTGAGGGTTTTGACCTCGAAGGGCAGTCCCTGTGAGGCACGAATAAGC
TGCACCTCTTCCAGCACGTTCTCGCCGATGATGCCAAGATCGACCACCCCTTCCATTACCAATCCAGGGA
TGTCGTCATCACGCACCCGCAGGATATCGATGGGCATGTTCTCGACATGGGCGATAAGGCGCTGCTCGCG
CAGGTTGATTTTCAGACCACAGCTCTTGAACAGGGCTTGGGAGTCCTGGCTCAGACGACCGGACTTTTGC
ATGGCGATACGAAGACGTTGTGTTTCCATTGCTCGATTTCCTTCTAGTCAAAAACGAAAAAAACCCTCGGA
AGTGTGTGCTTCCGAGGGTTTATGAATCTTGCGGTCCTTTTGGAAGTCACCAGTTAAGTGTCTTCCAGCA
GTCAACCTCCTGAAAGACTAGTCGGGATGATGATGGTGGTGATGAGTCTTCAGGGTGGTGAACTTCATGT
AATCAATCCTTTACAAAATAAGATGTCGTAATTGACGCCTGATTTAAACTACCTGCCTTGACAGAAAATG
CAACCCGAAATTGTGATTTGATCGATTAAAGTGGCGAAAACGGTTTTCTGGTCGATAACTAGACAATAAC
CCCTGCAGGAGTCGCCATCATGCCCAGCCAGGACCTGTTATTCCCCATTTTGCCCCGGGCGCCCGCCGCG
CCCGGCAGCGAAGAGATCAAGCGGGAAGTGAATCAGATCAGCAAGAAGCATCAGCTGCGCAAGACCAGCA
CTGACAACAGTGACTCCCAGCAGCGCCAGCAGGCGTATAGCACGCCCGCCAAGCGTGATCAGCCCAAGGC
GGAGGAGGGTGATCACAAGCCGGACCCCGAGCATCAGATCGATCTGTTCGTCTAGGCAGATTATCTGTCC
CGCTTTTGCCGCGCTTTTGGCATAATGGCCGCTTTTGCTGCGGGACGCCCTTTTGACCATCCAGACCCTG
TTCAAGCCAGATGGCCCTCTGGCCAGCCATATCGACGGTTTCAAAGCCCGCGCTCCCCAACTGGAGATGG
CCGAAGCGGTCGCGCGCGCCATCAAGAGTGGTGGCCGTCTGCTGGTTGAGGCAGGAACCGGTACCGGCAA
GACCTACGCCTATCTGGTGCCTGCGCTGGAATCCGGCAAGCGGGTGGTGATCAGTACCGGCTCCAAGAAC
CTGCAGGAGCAGCTCTTCTATCGGGATCTCCCGACCATTACCGGCGCGCTCTCCTACACCCCGCCGGTGG
CCCTGCTCAAGGGGCGCAGCAACTATCTCTGCATCGAGCGGATGAACCGGCTCTTGAGCGAATCTCATCT
GCAAAAGCCTGAAATTCTCAACGATCTGGTGAAGGTCAAATCCTGGTCCACTGCTACCGATAACGGCGAT
GTGGGGGATATTCCGGGATTGCAGGAGAACGCCGAGATCCTCGCCCACGTCACCAGCACCAACGATAACT
GTCTTGGTCGCGACTGCCCCTATTACGACGACTGCCATCTGGTGGTGGCCCGACGCCGTGCCATGGATGC
CCAGGTGGTGGTGATCAACCATCACCTCTTCTTCGCTGATATGGCGGTGAAAGACACCGGATTTGGCGAG
TTGGTGCCGGAGGCGCAAGTCTATGTGTTTGATGAAGCCCATCAGTTGCCGGAGATCGCTACCAACTACT
TCGGTAAGTCGGTAGGCAGCCGAACAATTCAGGATTTGGCGCAGGATATCCAGCTGGCCTATCGGGCAGA
GGCCCACGACATGGCCCAGCTCGGCAAGGCGGCGGACCGGCTCGCTATCGCCAGTCAGGACTTGCGTCTC
GCCTTCGGGGTGGATGGCGGCCGCGGCAATACCCGCGACATGCTGCGCCAGCCGCTCTGGGGTCAGGCTT
TGGCTCGTCTCAACGATGCCATCGGGCTCTGCTACGAGGTGCTGAAACTGGCTCTCGGCCGTGGCGAGCA
GCTGGATCACGCCTTCGAGCGCATCAGTGAACTGCGCACCCGTCTTGGCGAGGTGCTGGCGGTCAACCAG
ACCGGTTTCTCATACTGGTACGACTGCTCACGGCTTCATTTCACCCTCAATCTGACGCCGCTGTCGGTGG
CCGAGCGATTCAGTGCCGAGGTGCAAAGACCGGAAGTGGCCTGGGTCTTCACCTCCGCCACCCTGACGGT
GGATATGCGTTTTGACCACTTCAAGGCCGAGCTGGGACTGGCGGGCAGTGCTGAGCTTATTCTCGACAGC
CCGTTCGACTATGGCGAGCAGGCGCGGCTCTGTGTGCCCCGCTATCTGCCGGAGCCCAACGCCTTTGGTC
GCGGCGAGCAGCTGGCCAATCTGATGATCCCGCTTATCAACAAGACACCGGGGGGCTGTTTCTTCCTCTG
CACCAGCCATCAGGTGATGCGTCAGGTGGCTGAGGTGCTGCGCCGGGAGATTGGCCGCACCGTCTTGTTG
CAGGGGGAGGACAACAAGCAGCGGCTGCTCAAGGAGTTTGTCGAGAATGGCCGTGCCGTGCTGGTGGCGA
CCAGCAGTTTCTGGGAGGGGATCGACGTGAGAGGGGCGGCGTTGTCGTGTGTTATCATCGACAAGCTGCC
GTTTGCCAGCCCCGATGATCCGCTGCTCAAGGCGCGGGTGGATGATTGCAAGCTCAAGGGGGGCGATCCC
TTCGCCGAGCTGCAGTTGCCCAAAGCGGTGATTGCCCTCAAGCAGGGGGTGGGCCGGCTTATTCGTGATC

GCAGTGATCACGGGGTGCTGGTGATCTGTGATCCACGAATGGTCAACAAACCTTACGGTGCCACCTTTAT
CAAGAGTCTGCCCGCCATCCCACGCACCCGGGAGTTGGGCACGTTGGGGAGTTTTTTCGATCGTACTGAA
CGCGGTGAATAAGTGTGCCGCCAGATAACAGATTTGAACAACGGGTGAGCATATCTCGCTTGCCGGTTAT
GTAATTGACTGATTTTATAGAGATTGGAAAGCATGAACGAGTTGAAGATCCTGGCCGTTGATACCGCCAC
CGAAGCCTGCTCCGCCGCCTTGCTGGTGGGTGACAAGCTGTTCTCCCGCTGGGAGGAGGCGCCACGGGAT
CACACCCGAAAAATCCTGCCCATGGTTCAGGCTGTGTTAGAGGATGCCGGGATAAGTTTGAGTGACCTCG
ATGCCATCGCGTTCGGCCGCGGCCCGGGCTCCTTTACCGGGGTGCGGATCGGCATCAGTGTGGCGCAAGG
TCTGGCGTTTGGTGCGGGTGTGCCGCTGATCGGCATCTCGACCCTGGCCGCCATGGCGCAATGCGCCTAT
CGAGTGGATGGCGCCCAGCAGGTGCTGACCGCCATCGATGCGCGGATGAACGAGGTATATTTCGGCCGCT
ATGAGATGATTGATGGCCGCATGCAGTTGGTGGGGGACGAGGTGGTGAGCGAGCCCGCGGCACTGGTTGA
CGTCCGTGGCAAGCTGGCGGGCCCTGTGACCTGCGTCGGCACCGGTTTTGAAACCTATGGCGAGACCCTG
AGTGGTCTGGCCGATGAGCTGGCGGTGAGCCAGGTGCGCTTCCCGGCGGCAGAAGACATGTTGCCGCTGG
CCCGCGCCGCCTGGCTGGCGGGGGAGGCTGTGCCTGTGGAGCAGGCGACGCCGGTCTATTTGCGTGACAA
GGTAACCTGGAAAAAACTGCCGGGTCGCGAATAAGGGCAGGAGCACTGACAGCATGTCGCTCTTGCCGCT
CATCAGCTGCAATATAAGCGGGTGGCCCGACGGGCCACGCCGGCAACAGGAGAGTGACATGATGCGTAAT
AGCAGAAGCGACATGATGAAACGCACTATGGTGGTCGGGGCGCTGGCTCTGCTGCTCGGGGCCTGCAGCT
CGATCCCCAAGGAGCTGGCCTATGAGCCAGAAAACCAGCTGGTGGCCTATCAGCCCGCCCTTGGCGGAAT
GGAGGGCAAACCGGCCCGCTGGAGCGGCGTTATCTCGGCGGTGCACAACAAGGCGGATCAGAGCGTTATT
GAAGTGGTCTATCTGCCGCTCAAGTCCAACGGTGTGCCGGAGCAGACCGAGCAGAGCCCGGGCCGTTTTC
TGGCCATCATGAAGGGTTTTGTCGATCCGACCCTCTACGCCAAGGGGCGCAGCCTGACAGTGCTTGGCAC
CATAGGCCAACCGGTTGATAGCCAGATTGGCGAGCACAAGTACCGCTTCTCGGTGCTCAATGTGACGGGC
AGCAAGCTGTGGCCGCCAGTCAAGGAGGTGGAGGTGCGCTATATGGCCCCCTACTTCTACGACCCCTTCT
ATGATCCCTTCTGGCCGAGACGGCCGCTGCGTCGTTAAGGCTTGCCATGATCAAGAGCATCAAAAGCACT
CTGCACGAACGTCTGCTCACCTTGGCAGATGGTCGGCAGGTGGCCCTGCTTGAGAATCAACTCGCGGGTC
AGGCTCAGCAGGGCAAACCCCTGCTGATTGCCCTGCACGGCTGGCTCGACAACAGTGCCAGCTTCTTGCC
GCTGGCGCCGCATCTGGGGGATTTTCACCTCATCTGTGTCGATCTGCCGGGGCACGGTCACTCCGATCAC
AAAGCCACCCCCTATGTCTTTGTCGACTGGCTGGACGATCTCTACCAGATCGTGCAGGCGGCGGGCTGGT
CGCGTTTCACTCTGCTCGGTCACTCCCTCGGGGCGCTGATCGCATCCGCCTATGCCGGGGTGTTTCCCGA
ACAGGTGGAGCGGTTGATCATGCTGGAGGGGCTGGGCCCGCTCAGTCAGCCCGATGAGGAGGTACCTGCC
CAGCTGCGACGTGCGATTTTAAACCGTAGTCGCACCCGGGAGCGCAGTGCCAACGGCTTTGCCTCCATCG
ATGAGGCTGTCGCCGCCCGTTGCAAGGTGGCGGATATCACGCCGCAGGCGGCGCGTCTTATCTGTGAACG
GCAGCTTGAAGAGCGGGCAGGGCGCTGGCACTGGCGCAGCGACCCCCGTTTGCGGGATCTTTCACCCCTG
AGAATGAGCGAAGGGCAAGCCCGAGCGCTGATGGCGGCTATCCGCTGTCCTGTGCTGTTGATTCAGGGGG
AGCAGGGCTTTGCTGCGCTTGAAGCCCAATGGCAGGCCCGTCAGGATGCCTTCCAGCAGATCGAGCGGGT
CATATTGGGTGGAAACCACCATTTTCATATGGAAAATTCGGTTGATACGGCCGTTTGTATCGTAAAGTTT
TGTCAAGTTCGGTAGAATCGCCAGCTTTCAATAATTCTAGGTTTGTTTTCTCCCTCACATTTTTCTGTTG
TTAGTTTGCTGTTAATTGATGTTGCGCTGTGTAAATATGTGCGGCATTAGAGCATCAGCTTAAAACAACC
GATTAAAATGGATGTGAACCGGGACGGAGGCAACACAGTGAGCGCACAGGTGCTAGTTATGCAGGAGAAG
TTTGTGGAAAAAGTCTGGCTGAAACGTTATCCGGAAGGGGTCCCCGCCGAGATCAATCCAGACCAATACA
GCTCCCTGGTCGAGATGTTCGAATCCTCGGTGACCCAGTTTGCCGATCAGCCCGCCTTCGTCAACATGGG
CCAAACCATCACCTACCGCCGGCTGGAAGAGCAGTCCCGCGCCTTTGCTGCCTACCTGCAAAACGAGCTG
AAGCTGGAGAAAGGGGATCGGGTTGCGGTGATGATGCCTAACCTGCTGCAATACCCCATCGCCGTGTTCG
GCATCCTGCGCGCCGGCATGATCGTGGTCAACGTCAACCCGCTCTATACCCCGCGCGAGCTGGAGCATCA
GCTCAAAGATTCCGGTGCCAAAGCCATCGTCATCGTCTCCAACTTCGCCCATACCCTGGAAAAAGTGGTC
TACGACACCCCGATCAAGCACGTTATCCTGACCCGGATGGGTGACAACCTGGGGCTGGCCAAGGGCACCC
TGGTCAACTTCGTGGTCAAGTACGTCAAGAAGCTGGTGCCGAAATACAATCTGCCCCACGCCACCACCAT
GCGTCAGGCCCTCTCCAAGGGGCGTTTCCTGCAGTACATCAAGCCGGAGATCACCAACGACGATCTCGCC
TTCCTGCAGTACACCGGTGGCACCACCGGCCTCTCCAAAGGGGCCATGCTGACCCATCGCAACATGATTG
CCAACGTCGAGCAATGCCTCGGCGTCTACGGCCCCATGCTGGAGCGCGGCAAGGAGTTCGTGGTCACCGC
GCTGCCGCTCTATCACGTCTTCGCCCTGACCGTGAACTGCCTGCTCTTTATGCGCATCGGTGGTTACAAC
CTGCTGATCAGCAACCCGCGCGACATCCCAGGCTTTGTCAAAGAGATCAAGAAGTACCCCTTCACCTGTA
TTACCGGGGTCAACACCCTGTTCAATGCGCTGGTCAACAACGAAGAGTTTCAGGGGATCGCCTTTGACAA
GCTCAAGCTGACCATCGGTGGCGGCATGGCGGTGCAGCGCGCGGTCGCGGAGAAGTGGAAGATGCTGACC
GATACCCCGCTGCTGGAAGGCTATGGCCTGACTGAGTGTTCGCCACTGGTGAGCGTCTGCCCCTACGATC
TGACCGATTACAACGGCTCCATCGGTCTGCCGGTCTGCTCTACCGAGATCCGGTTGGTGGATGACAACGG
TCAGGTGATCCACGCCCTCGGCACCCCGGGCGAGATGCAGGTTCGTGGCCCGCAGGTGATGGTTGGCTAC
TGGCAGCGCCCCGAGGCGACCGCCGAAGTGATGCAGGATGGCTGGCTCTGTACCGGTGATATCGCCGTGT
GCGACGAGCAGGGCTTCTTCAAGATCGTTGACCGCAAGAAGGACATGATCCTGGTCTCCGGTTTCAACGT
CTATCCGAACGAGATCGAAGATGTCGTTGCCCTGCACCCGAAAGTGTTGGAAGTGGCTGCCGTGGGTGTG
CCGCACAAGGTTTCCGGCGAGCTGGTGAAGATCTTTGTGGTCAAGAAAGATGCAAAATTGCAGGAGGACG
AGATTATTGCCCACTGCCGCAAACATCTGACTGCCTATAAGGTGCCGAAACTGGTAGAATTTCGTGACGA

ACTTCCCAAGACCAATGTGGGCAAGATCCTGCGCCGCGTACTGCGCGACGAGGAGATTGCCAAGCAATAA
CCGGACTGAAGCTGCAGAACCCTCTGCAAACCAGCACCGTCGTTATGCAAAACCGTGGGTCATCCCACGG
TTTTTTCTTTTTGGCCCTGATCAACCGCGCAGGCACAGATGCACTATCAACTTATCACTCAGCAAGCCGA
ACTTGAGCAGTATCTCGTTTCTCTGGCCGATGTTCCCCTGGCTATCGATACTGAATTCGTTCGCACCCGT
ACCTACTACCCCCAGCTTGGCCTGTTCCAGATCTATGACGGCGAGCATCTGGCGCTGCTGGATCCGCTGA
CCCTGGATCTCTCCGCCCTCTGGCAGCGACTGGGCCGGGAAGGGCAGATCGCCATCCTGCACGCCTCTGG
CGAAGATCTGGAGCTTATCCAGCATCAGGCGGGCCACCTGCCCAACCAGATGCACGATACCCAGCTGGCT
ACCGCCTTTCTCGGTTACGGCGTCTCGGTCGGCTTTGGTGCGCTGGTGAATGAATTCCTCGGGGTCGAGC
TGGAAAAAGATCAGGCGCGCACCGACTGGCTGGCCCGCCCGCTTACACCGCGTCAGCTCGAATACGCGGC
AGCGGATGTCTTCTACCTGATGCCGCTCTACGAGAAGGTGATGGCCAAACTGCGGGAAAGCGGCAAGTTT
GCCTGGTTCGAGCAGGAGTGTCAGAACCACTCTGCCCGCAAGACCCAGGGTAGCGATCCCCGCCAGGCTT
ATCTTGACATCGTCAACGCCTGGCAACTGGGTCGCCGCGAGCTCGCCATTCTGCGCGAGCTGGCCGCCTG
GCGCCAGCAAGAGGCGGTGCGCCGCGATCTGGCCCTCAACTTTGTGGTCAAAGAGCTGCACCTGTTCAAG
GTGGCAGAGCGCCGTCCTGCCTCTCTGCGGGATCTCAACGAGCTCGGCCTCTCGCCCATCGAGATCAAGA
TCCACGGCAAGCGGATGCTCGATATCGTCGCCAAGGCGCAGCAGAGCGATCCTGACAGCTGGCCCGAGCC
CATTCGCCGACTGGTGGACTTCCCCCAGTACAAGGCCGAGCTCAAGCGCATCAAGGCCATGGTGGAAGAG
AAGGCCAAGGCGAACAATATCCCGCCCGAGCTGATTGCCAGCAAGAAGATCATTCACCAGTACTTCACCT
GGAGCTGGCGGATGAACGACGAAGAGCGGGCCCGCAGCGACAAGCCGATGCTGCTGCAAGGGTGGCGTCA
TGAGCTGGTCGGTCACCTGCTGGCACAATAAGCCACCAGACGGTTGCTCCTATAAATCAATCCTCACCCT
CGGGTGGGGATTTTTATTGGGCGAAAGAGACAAACAGGCATGCTGAACTGGAGAACGCAGAGGGGACATG
GCTGGCCGGATGGCAGGAATCGAGAGTGAAAAAGAGAGCAGAGAAGAAAAGGCAGCGGCTAACACCGCTG
CCTTTTTTGATCACCAGATAGGCTGGGTCAGGTAGGGGTTGCTCGCCTTCTCGCGGCCGAAGGTGGAGCT
GGGGCCGTGGCCCGGGATAAAGACGATCTCGTCACCCAGCGGCAGCAGGGTCTCGGTGATCGACTTGATA
AGCGTGCCGTGATCGCCACGGGGGAAGTCGGTGCGGCCGATGCTGCCGGCAAAGAGCACGTCGCCAACCC
AGGCGAGCTGGTCGGCTTGGGAGAGCAGCACGATATGGCCCGGGGTGTGGCCGGGGCAGAAGTAGACATC
CAGCTCGCACTGGCCAAGCTGCACTTTGTCACCCTGCTCAAGCCAGCGATCCGGCGTGAAGGCGGGAGTA
TGGGCGAAGCCGAACATCTGGCTCTGCTGGGGCAGCATGTCGAGCCAGAAGGCGTCTTCCTTATGGGGGC
CGGTAATGGCGACGCCGGTCTTTTCCCGCAGCTCGGCGGCGGCACCCACATGATCCAGATGACCGTGGGT
CAGCAGGATATGGGTCAGGGTCAGGCCGCGTTTGGCGATGGCCGCCAGCAGTTTGTCCGCTTCGTCGCCC
GGATCGATCAGGGCCGCCTGATGGGTCTCGTCACACCAGATGAGGGAGCAGTTCTGGGCGAACTGGGTGA
CCGAGATCACCTCGAATTTCAACATAGAGTTCCTCGCTTGGGATGGGTCAGCCGCGCAGTGCGCTGGCAT
GCTGCTCGGCGAAATAGTCGAGGCAGGCCTGCTTGTCACCGCGAAACACGATGCGCTCCTGCTTGTTGCC
AAGCTGGTAGAGATACATGGGGTCGTAGTAGCGGGTTAGCAGCAAGGTGATCCAGGCCAGATGCAGTGCG
GTATCGCCGCTCTTTTCCTGCTCGGTGAGGGCGGTTTGCATCAGGGCATCCAACTCGCGGTGGGCCTGAT
CGCCGAGGCGGCGCTTGAGGCGGGCCAGCGCATCGGTGAGGTAGGCGGCAAACAGCGGCCAGCCAGCCTC
CTGGCCATACATGGCTTGATAGCGCAGCCAGAGATCGTGCACGTAATCTTCACGGATCTGCTCGGCCCGA
TCGATTTGCGGTACATCCACCACCACCAGCGGTGCCTGACTCATGGCCTCATGGAGAGAGAGGGGCAGGC
AGCAGCGGCCGATCAGGCGGCTCTCGTCCTCGACCACGAAGGTCTGCTCGCCGCGATGGCGGCGCTTGAG
CAGTTCGATGGCGAGGTTGTTTTCAAAGTTGATGTTGCTGGGCTGGCCGCCGGGCAGTTGACCGAAGGAG
GAGCCCCTGTGATGGGCGTGCCCCTCCAGATCCACCGCCTGGGTCACCTGATCGAGCATATGGGTCTTGC
CGGAGCCGGTCATGCCGGTCAGCACGCTCCAGTGACACTCGGCAGAGGCCTTGTCCTGGGTGTCGATCAG
GAAACGGCGCAGCTCCTTGTAGCCACCGGCGACGCGGGGGCGAGTCACTCCCGCTTCAGCCAGCCACTGT
TGCACGGTTTGCGAGCGCAGGCCGCCGCGAAAGCAGTAGAGCACGGCATCGGGCTGCTGGCGCAACTGCG
CCAGCCAGCCTTCGAGTCGCTCGGCACGTACTGCTCCCCCCACCAGCTTGTGTCCAAGCTCAATGGCGGC
GGCCTGACCCTGCTTCTTGAAGCAGGTCCCCACCTGGGCGCGCTCATCGTCTGTCATCAAGGGCAGATTG
GTCGAGCAGGGGAAGGCCCCCTCCTTGAACTCGATGGGGGCGCGCACATCAATCAACGGCACATCGTCCA
AAAAGAGTTGGGCGTAGTCCGTGGCCAGTGGCAATTCACTCATTGGACAATCTCGATAAAGCGGGAGCCC
TCAAGGGGCTTGATCTGGCCGATGGGTGAGAGCGCAAGGCCAGCTTCGCGCGCGATGGCGAGCAGCTCGG
CTTCGCTCTCTTTACCCACGGCGACCAGCAGGCCGCCGCTGGTTTGCGGATCGCACATGATGTTGCGGGT
GCGATCGTCCATGGCATCGAGCTTGTGGCCGTAGGAGTCGTGGTTGCGCAGGGTGCCACCCGGTACGCAG
CCCTCGGTGAGGTAGTAATCCACCTCGTCCAGCAGCGGCAGCGCCTTGAAGTCGAGGCGGGCGCTGACAT
TGGATCCTTCGCACATCTCCAGCAGATGGCCCGCCAGACCAAAACCGGTGACGTCGGTCATGGCGTGGAC
ACCCGGCAGCTCGGCAAAACGCTGGCCAATCTTGTTGAGGGTGCACATGGCATTGGGGGCCAACTGCTCG
TGCTCGGGCTTGAGCTTGCCCTTCTTCTGGGCGGTGGTGAGGATACCGATACCGAGGGGCTTGGTCAGAT
AGAGGGTGTCACCCACCTTGGCGGTGTCATTCTGCTTGATGGCGTCGAGAGGCACGATACCGGTTACCGC
GAGGCCGAAGATGGGCTCGGGGGCATCGATACTGTGACCACCGGCCAGCGAAATCCCCGCTTCGTGGCAC
ACCTGACGGCCGCCGTCGATCACCTGCTGGGCCACTTCCGGCGCCAGGGTATTGATGGGCCAGCCGAGGA
TGGCGATGGCAACGATAGGCTTGCCGCCCATGGCGTAGATATCGCTGATGGCGTTGGTGGCGGCGATGCG
GCCAAAGGTGAAGGGGTCATCGACGATGGGCATGAAGAAGTCGGTGGTGGAGACAATGCCCTGACCGTTG
CCGATATCGACCACGGCCGCGTCATCCTTGCTGCTGTTGCCAACGATCAGGGTCGGGTCGTTGAAGCCCG
GGATCTGGCTCTTGAGAATGGTGTCGAGCACCTTGGGAGAGATTTTGCAGCCACAGCCAGCGCCATGGCT

GTATTGGGTCAGACGAATGGAAGACATACTTACCCCTTGTTTGTGCACCGTGTGCAGAGGCTCTGCGGGA
GCCATCTTGGTCAATGCGCGGCCATCTACCGTCATGTAAAGAGCCGCAAAAAATAGGTGGCCTTGGCTGC
TTCTGGAAGCAGTGATGTCAGGCAGTCACGCAGGGCTGCCGGGTAGGACGGGGAAGAAGAGGCGACAGAT
GCGCCAACACAGCCCCGCCAAGGGCCACAGGACAGGGCATTATGCCACAAATGCCCCCTCTGGTTCGAAC
AGGAATTCACACAAAGCGAAGGGGATTGGCGGGTGGAGGGTTAGGGAGGAGGGCTCTGGCGTGAGCCGCT
CAGTTATCAGCATGGGTGAAGAGAACTCGCGGAGTTGCCCCGCAGAGGTTGGCTTAACCTCTGCGGGGCA
ATCAGCTGGCGAAAGCAGATTAGCCGGCTTGCTCCAGCACCGGCTGGCGAGCCTTGCCCCAGCGGCGCAG
GCGCCGTTGCAGCGGCCGCTCCAGCAGGTTGTAGCTCAGCGCCGAGAGCCCGATCAGCACCGCCAGCATC
AGCAAGGTTGCTACCACCCCCAGCAGCGTGTTGGTCTGGCCATACCACCATTCTGTGATCCCCGGTGCCA
GATAGTGGATGAGCTCGCAACCGGTGAGCAGCACCAGCGCATGGAGCAGGTAGATGGAGTAGGAGAGATC
CCCCAGCCAGTTGGTGAAGCGGTTATCCAAGAGGTGCAGCAGCGGGCTTTGGCGATCGCCCTGGCAGGCG
GCAATCCAGATCAGCAGCACGTAGGAGCTGATCAGCTGCAGTGTCAGTGGTGGCGTCATGGTGAACTGCA
TCAGCGCGAAGGGCAGCACAAAGGCGACCAGCAGCGGCAGATCATGCTTCATCATGGGCAAGCCTTTTGC
GGCCTTGACCCTCGGCAGCAGCCAGAGGCCAAGCACAAATTCACTCACCCCGCGCCCCACCGCCAAGGCG
CCGCTGGTGACATCCAGGGTGCCGCGACTGTGCACGATAAAGGCCAGCACCGTCAGCGCCAGCAGGGGGG
CGAGATGGCTCCAGCGACTGCGGGTGACCGCCAGCGGACTCAGCAGCGGGAACAGCATGTAGCTGATCCA
CTCCACGCTGAGCGACCAGGCAGCGAAGTTCCAGGTCAATCCGTGATCGGTTACCACCTGTAGCAGCAGG
AAGGCGCTCGGCAGCGCCTCGGCCGGGGTGAAGGGCGAACCGAAAGGCGGCATCCCTTCCCACTCCCACA
TTTTGAACAGGGGGCCGGCATAGAAGCCCACCCCGTTCTGCGCCTTGTAAAGCTCCCAGCCGACCAGCAG
CAGCAGGGTTACCAGGTAGAGGGGGTAGACACGGGTCAGCCGCAGCCAGAGAAAACGGCCATAGCTCACG
GTGCGGGGCGTGCGGGTGAAGGCCTCGCCATAGACATGGGCCAGCACCACGCCGCTCAAAATAAAGAACA
GGTCGACCCAGAGGTAGCCATTCTCGATGAGTTGGGTGTGGCCTGCGAGCGACTCCATCCACTGGGGAAA
GAGCAGCAAGCGGGCATGGAACAGCACCACCATCAGGGCAGCGATGCCCCGCAGCGGCGTTAACAGCGGC
AATGATTGATTCATAACGGGATCCCTTGTGATGTTCAGCTTGGCCTGACATCGATTTTTGTTGTTGTACC
GTCCATCACTCCTGATGGGACGGTTGCCCGGGCTTGGCTGCCGCAGGCGGGATCACACTACGGAAAAGAG
AGCCATACGGGTGTGATTTAAATCATAAATGCGCAGTTATTGTGCAAAAAAATTTAAACAGATAAAGACG
TGAGACGCAGGCAGGGAAAGAGGAGGAGAGAGAACGCCCATAAAGCAGAGAGGGTGGTCAACGGGGCGAG
GGTCAGGTCTATAGGCGTTAAGCAAAACAATCAGGCCCGCAAGGGGCCTGATTGATCACAAGGGGAGGGT
GGTTGCCGATCAGCGGGCGCGTACCACCAGACCCTTGAGGTAGAAGCCTTCCGGATAGGCGGTACCGATG
GGGTGATCAGAGGCCTGGGAGAGCAGCTCCAGGATCTGGGCATCGCGACCGGCATCGAGCGCTGCATCGG
CAACGATCTTCTGGAACAGGCTCTGCTCCATCAGGCCGGAGCAGGAGTAGCTCAGCAGCACGCCACCCGG
CGCCAGCAGCTGGAACGCCAGCATGTTGATGTCTTTGTAGCCACGGCAGGCACCCAGCAGCTGGGCCTTG
CTCTCGGCAAACTTGGGCGGATCCAGCACGATGACATCGAACTTCTCGCCCTTCTCGCGGTATTCACGCA
GCAGCTTGAACACGTCGTGACGGACAAACTGGGTATTGCTGGTATCCAGCCCGTTCAGCTCGGCATTCTG
GCGGGCGATATCCAGCGCATTCTGGGAGAGATCGACGTTGACCACTTCCTTGGCGCCGCCCTTGAGGGCA
TAAACGCCAAAACCGCCGGTGTAGCAGAAGCAGTTGAGGACGCGCTTGCCTTCGGTGTACTTGGCGGCAG
CCTGACGGTTGTCCCGCTGATCCAGATAGAAGCCGGTTTTATGGCCGTTGCGAATATCCACCAGGATCTT
GACGCCACCGTTCTCCTCGATGACCACCGGCTCGTTCGGGGTCTCGCCATAGATGACGCCGGTGCGCTCT
TTCAGCCCTTCCTTCTTGCGCACTGCCACATCGGAGCGCTCATAGATGCTGCACTCTGGATAGAGGGTAC
GCAGTGCTTGCGTTATCAGCTCACGCTGGAACTCGGCACCGGCAGAGAGGATCTGGCACACCAGAAAATC
GGCATAACGGTCGATGGTCAGACCCGGCAGGCCGTCGGATTCGGCGGCGCAGAGGCGATAACCAGTGAGG
CCCTGACGCTTGATCAGCGGATCGCGCGACTCCTGCGCATATTTGAGGCGACGGATAAAGAAGTCGAGAT
CGACGGTCTCATCCTTGTCGAAGGTCCAGACCCGGGCACGGATCTGGGAGCTGCCTGACCAGGCGCCACG
GGCGAGCCACTTGCCATCGTTGGCATAGATCTCGACGGTATCGCCGTCAATGGGGTTGCCCTGAACCCGC
TCAATCCCCTTGGAGAAGATCCAGGGGTGGCGACGCAGCAGGGATTTTTCGCGGCCTTTGACGAGATAGA
TGGAAGCGCTCATGGATGTTTGCCGATGGTAGAAAGAAAAGGGGCCACATTCTATGTGGCCCGCAGGTAA
AACTCAAACAACCCTATGTAATGGATGGGGTGGCCCTGATCTGACTCCTCATACGATCAGAGCTTAATCC
CCTTGAGCGCCCGGGTCAGCTCGCTGGCGGTGGCTTTCAGCCCTTGCGTGCCAGCCACCGAGGCGCTGGC
AGACTCCTCCACCTGCGCCGCCTCATGGCGCACTTGATCGAGATTGTGGGTGATCTCGGCGGCGACCGAG
CTCTGCTCTTCGCTGGAGGTGGCGATTTGGGTGCTCATCTCCAGTACCGCATCGCTGTGGTTGCTCAGCT
TCTCGATATCGAGCCCCACCTCGCTGATGAGTTGCTGGCTGCTGGCGGCGCGCCCTACAGTCTTTTCCAT
GATCTCGTTCAGCGCCCCGGTACCGGTCTGCAGCTCTTCGATCATCTGCTGGATCTCCACCGTCGCCTGT
TGGGTACGGCTGGCGAGGGTACGCACCTCATCAGCTACCACCGCAAAGCCGCGACCCTGCTCACCGGCAC
GGGCCGCTTCGATGGCGGCGTTGAGGGCCAGCAGGTTGGTCTGCTGGGAGATGGCGTTGATCACGTTGAC
CACCTCGTTGATCCGGTTGGCGTTGTGAGTCAAGCGCTCGACCGCGCCGGAGGCGTTGCCAATCTCGTCG
GCCAGGGTACGGATCGCCTCTATGGTACGGGCTACCCGGTTGGCGCCATTGGCCGCCTCTTCGCTCGACT
GGCGGGTTTGTTGCAGGGTGTCGTTGGCATTGCGCGCCACTTCGCGGATCGCTGCCGACATCTGTTCCAT
GGCGGTGGCCAGTGAATCCATGTGCTGGCGCTGGGAACGCGCCATGCTCTCGCCTTCGCGGGCATGGTCA
CGAAACTCGTCGGCGGCCATGGTCAGCGCTTCGGCAGACTCCTCCACCAGTTTGACGATTTGGTGCTGGC
GGTCGGTAAAGCGGTCGATGCTGCCCGCCAGGGTGGAGAACTCGTCCCGCACCGGGAAGAAGTTGAGTCG
GAAGGTGAGATCGCCGTCCGCCACGGTTTGCAGCGCCTTGTTGGTGGAGTAAAGCGCACCGGAGACAAAG

GTCATCATGTAGTGGATCACCAGCGCCATCAGGGCGGCCAGCGCGCCGATCAGGAGTAGGGTCGAGCCCT
GACCCTCCCACATTGAGAGGTTGCCGGTATCGAGATCACCGGTGACGTAGCCCGCCTGACCCGCTGGTGC
GGTGAAGCGGTAGCGATCGCCCTGCTGTTCGAGCCCGCTCACCCCTTGTGGCTGGATGCGTAGATCGGCT
GGCAGAGTGAGCGTTGGCTGCTGGCTGACCAGTTGCGCCGTGGTCTCGACCCGTTGTTGCAACTGCGCTA
CCCGCAGGGAGATCTCCTGCTGATGGCTGGCCGCCAGCATATACCAGCTGAGAAAGGCGATGATGAAGAG
AGGTAACCAGAACGCGATATGGAATTTTTCACTGATGGTGAGGTGAATGAGATAGCGGTCAACCCACCGG
AATTTAACTTCTTTCATAATGGAGCTGCATCCTGCAGAGATAGAGTGTCGGGCACATTATCGTCTCGGGA
ATTTTTTTCTTAAGCAGAAGGTGTGAGAAATGGGAGAGCAATCGTTTGTCATTCGGGTGTGGGGGCAGGT
GCAGGGGGTTGGATTTCGCTATTTCACCCGTGAGCGGGCGCTGCAACTGGGTTTGCGGGGCCATGCCTAC
AACCTGGAGGATGGATCGGTGGAGATCCTCATCAGTGGCCCCGCGCAGGGGGTGCAGATGATGCTGGGGT
GGCTGGAGCATGGCCCGCGCACCGCCAGAGTGACCCGGATGGAGTATGAGGAGGCGCTGCCGCCCACCGG
TCAGGGCTTTCACACCAACTGAATGGGCGGAGACGAGATGGACGCTGACAAGGAAAGTCTGTTGCTGACT
CAGGCAACAAGCTGGGTCGCCCGTTATCAGGAGGCGGCGCGGGCGGTGCGCGAAGAGGCCAGCGAACCGG
CCGTGCATGGCTATCGTATCGCAGCGCGCCGCTTGCTGGCGCTGCTCGCCCTGTGGCGACCACTCACCTA
TCATCCCGAGCTGGAGCGGCGTCTGCTGCGCTCGGTCGGGCGGCTCTCGGCGCTGCGGGATGCTCAGGTC
TACGCCGAGCGTTTTGGCAAGCCGCCTGCCAGCACGGGTAATACCCACAAATCGGCCTGTCGCGTGCCCC
TGTTGTCGCGGCGACTGGCCCGCTGGCTTGCGGCGGTGGCGCAGGTGCCGGATGCGCGGGCATTGGCGTT
CCTCTATCAGCAGCATCTGGCCCTGCGCCTTGATGAGGCGCAGAGCGCTCACGATGAACCTATTCCCTGC
GTTGGGAAATCTGCCAGCTCCCGTCAGCAACAGTTGCGCCGCTGGCATCGGCTGCGCCTCGAGATCAAGC
AGGCCCGTTATGGCGTGGAACTGTTGTTGGATCTGGGGAGTGGGGAGCCCGGCTGGTTGAGCACCCTGAC
TCACTGGCAGGAGCGACTCGGCCAGTTGCAGGATTGGCGGCAATGGCGGCGGAGGTTGCGAGCCGAGCAG
GGGAACAACCACAAGCAGGCGAAACTGCGGCAGCAGTTGAAGGGGAAGATCACCGCCCGTTTGCGTCAAC
TGGACTGCCAGCAGGCGGAGCTGGTGGCGCTCAAACTGACGATGCTGGCAGGCCACAACATGAATGACAT
GCCAAGTCGGCTGGTCAGGTATTCAGAACAAAATGTATCGCCCGCGAATCCCTGTTGTTGATAGCCCCGA
GACCGCAATCTGCGGAAGTCATCCGCGACCAGCATCTACAAAAACTATCCAGGCCAAACAGGTGAGACAA
AAAAAAGAGAGGCTGTTTGGCCTCTCTTCTCTATCTGGCTGTGGTCGCAGTCAGCGATATGCTTACTTGA
CGCGCATACCAGGCTGGGCGCCTTCCTGTGGCTCCAGGATCCAGAGATCCTTGCCGCCCGGGCCTGCGGC
CAGCACCATGCCTTCGGACATGCCGAAACGCATTTTGCGCGGCGCCAGGTTGGCCACCATCACGGTCAGC
TTGCCTTCCAGATCTTCCGGGTTGTAGGCGGACTTGATACCGGCAAACACCTGACGGGTTTCACCGCCGA
TATCCAGTTGCAGCTTGAGCAGCTTCTCGGCTTCCGGTACCGCTTCGGCCTTCTTGATCAGCGCGACGCG
CAGGTCAATCTTGGCAAAATCGTCATAGCTGATGGTTTCGCTGATGGGGTCATCAGCCAGCGGGCCGCTC
ACTTTCGGTGCCTGCTCTTTGGCCAAGTCTTCCTTGGAGGCCTCGATCATGGCTTCAATCTTCGCAGGCT
CGATGCGGGAGAAGAGGGCCTTGAACGGCGCAACGGTATGATCGACCAGCGGCATCTCGATGCCATCCCA
GCTGAGGGTTTCGGCCAGGAAGGCTTCGGCACGTTCGGCCAGCAGCGGCATGACGGGTTTGAGGTAGGCC
ATTAGCACGCGGAACAGGTTGATGCCGACCGAGCAGACCGCCTGCAGCTCGGCGTCAGCCCCTTCCTGCT
TGGCGATAACCCAGGGCGCCTTGTCGTCCACGTAGCGGTTGGCCTTGTCGGCCAGCGCCATGATTTCGCG
GATGGCGCGGCTGAATTCGCGGCTCTCATAAGCCTCGGCAATGGCGGTACGAGCATTGGCGAATTCGGCG
TAGAGCTCGGGTTCGGCGTAGGTTGCCGCCAGCTTGCCGCCAAAACGCTTGGCGATAAAACCGGCGTTGC
GGGAGGCGAGGTTGACCAGCTTGTTGACCACGTCGGCGTTGACGCGCTGCACGAAATCTTCGAGGTTCAG
GTCCAGATCGTCGATGCGGCTGTTGAGCTTGGCGGCGTAGTAGTAACGCAGGCACTCCGGATCAAGGTGG
TTCAGGTAGGTAGATGCCTTGATAAAGGTGCCCTTGGACTTGGACATCTTGGCGCCGTTGACGGTTACAT
AGCCGTGCACGTTGACCTTGGTCGGCTTGCGGAAGTTGGCACCTTCCAGCATGGACGGCCAGAACAGGCA
GTGGAAGTAGGCGATGTCCTTGCCGATGAAGTGGTAGAGCTCGGCGTCGGAGTCCGCTTTCCAGTAGCTG
TCAAAGTCGATGTCGCCACGCTTGTTGCACAGGTTCTTGAAAGAGGCCATGTAACCGATGGGGGCATCGA
GCCAGACGTAGAAGTACTTGCCCGGGGCGCTGGGGATTTCAAAACCGAAGTAGGGGGCGTCACGGGTGAT
GTCCCACTGCTGCAGGCCGCTCTCGAACCACTCCTGCATCTTGTTGGCCATCTCTTCCTGAATGGCGCCG
GAGCCACGCACCCACTCAGAGAGCCATTTTTCAAACTGCGGCAGGTCGAAGAAGAAGTGCTCAGAGTCCT
TCATTATCGGAGTCGCGCCGGAGACGGCAGATTTCGGATCGATCAGCTCAGTCGGGCTGTAGGTGGCGCC
GCAGCTGTCGCAGTTGTCGCCATACTGCTCCGGAGATTTGCACTTGGGGCAGGTTCCCTTGACGAAACGG
TCCGGCAGGAACATGGATTTTTCCGGATCGAACAGCTGGGAGATGGTGCGGCTCTTGATAAAGCCACCAT
TTTTCAGGCGGCCGTAGATCAACTCCGCCAGCTCGCGGTTTTCATCGCTGTGGGTGGAGTGATAGTTGTC
AAAGCTGATGTTGAACCCGGCGAAGTCGGCCTGGTGCTCCTTGGAGACAGCGGCAATCATCTCTTCCGGG
GTGATCCCCATCTGCTGTGCCTTGAGCATGATCGGGGTGCCGTGGGCATCATCGGCACAGATGAAGTGCA
CCTGATGACCGCGCATTCGCTGATAACGAACCCAGATATCGGCCTGGATGTGTTCCAGCATGTGGCCAAG
ATGGATGGAGCCATTGGCATAGGGGAGGGCGCAGGTTACCAGCATTTTACGAGGATCAGTTGCCATATTA
CGTTCTTGATGTGCTTGTTGGGATGATAGAGTGCCGTGCATGTTACCCGAATTGGGGGTAGCCTGCCTAG
GGTTGTGGCTGTGCCAACCTTGGCAGCACCTGCTGTTACGATGGCAGGCAAGCCACTGATTTGTGTGCGG
TAACCGTTCACCTCAAGATAATGACAAGATAATGAGGAGTTAATGGTGATTGATTCAGTAAAACAGATCC
TCGCCGAATTTAAGCCAGCAGGCTGGCACAAGGATCTGGTGGATGCCGGATTCGTTCGCGAGATCATCAA
TCAGGGTCAGGGGCTTACTATCCGGCTGGTATTGCCGTTTGCCGGTCTCTCGCTGCTCGATCAGCTCAAA
GAGAACTACGATGCCAGGCTGCGCAGCGCCACCGGCGCCGCCCGTATCGACTGGGCACTGGAGATTGACG

TCGCCAGCATGCCGCGGGCGCAGGGCCTCAATGCCGTGCAGGGCATTCGCAACATCATCGTGGTCGCCTC
CGGCAAGGGGGGGGTGGGCAAATCTACCACTGCCGTCAACCTGGCGCTGGCCCTGCAAAAAGAGGGGGCG
CGGGTCGCCATTCTCGATGCCGATATCTATGGCCCTTCCATTCCCACCATGATGGGCACCCTCAAGGAGC
GCCCAGTCAGCCATGACGGCAAGCTGATGGAGCCGGTGATAGCCTGCGGCCTCAAGAGCAACAGCATCGG
CTATCTGGTCTCCGAGCAGGATGCCACCATCTGGCGCGGCCCGATGGCGAGCAAGGCGCTGGCGCAGATC
CTCCATGAGACCCGCTGGGGCGAGGTGGATTATCTGGTGGTAGACATGCCGCCGGGCACTGGCGATATCC
AGCTCACCGTGGCCCAGCAGGTGCCGACTTCGGCGGCAGTGATCGTCACCACCCCACAAGATGTGGCGCT
GGCCGATGCCCGCAAGGGGATCGCCATGTTCAACAAGGTCAATGTGCCGGTGCTCGGCATCATCGAGAAC
ATGAGTTATCACGTCTGCAGCGCCTGCGGTCATCATGAGCCGCTGTTCGGTACCGGTGGCGGCCAGAAGA
TGGCAGAGCAGTATCAAGTGGCGCTGCTTGGCCAGTTGCCGCTGCACATCGACATCCGCCAACACATGGA
TGATGGCTGCCCGACGGTGTTCGGCGCACCGGAAGGGAGCCTGGCTCAGGCCTATTTGAAGCTGGCGCGC
AGGGTAGGGGCTGAACTCTTTTTCAGCGCAAAACCGATTGCGACCCCCCTCTATGCCATGGCGCTGGATG
AATAATCGGCGGTCGGTCACTTGAAAGGGAATTGAGGGTGGGCATGACCCACCCTTTTTTATATAATGGA
TCGGTTTAAACCATCCCCACTGCTCTTCTCTTTTAGGTATTTGTCATGTCTGATACTCAACATTCGTGTG
TGATCATCGGTATTGCGGGCGCATCTGCGTCTGGCAAAAGTCTTATAGCCCAGACCATTTATGAAGAGCT
GGTGGCCGAACTGGGCGCAGGTCAGATTGGTGTGATCACCGAAGATTGCTACTACCGTGATCAGACCCAC
CTGACCATGGAAGAGCGTGTCAAGACCAACTATGACCACCCCAACGCGCTCGATCACGACTTGCTGGTAC
AGCACCTGAGCCAGCTGGTCAAAGGTGACGCGGTTGCCATTCCCCAGTACTCCTACACCGAACACACCCG
GATGAGCGAAGTGACCCCGTTTGCGCCGCGCCGGGTGATCATTCTGGAAGGGATCCTGCTGCTGACCGAC
AGCCGTCTGCGGGATCTGATGGATGCCTCCATCTTTATGGATACCCCGCTCGACATCTGCCTGCTGCGCC
GTCTGGTGCGTGACGTGCAGGAGCGTGGCCGTACCATGGACTCCGTGCTCAAGCAGTACCAGAAGACAGT
GCGTCCGATGTTCCTGCAGTTTATCGAGCCGTCCAAACAGTATGCCGATGTGATCGTGCCCCGTGGTGGC
AAGAACCGCATCGCCATCGACATGCTCAAAGCGCGTATCCGCCACATGTTGATTGGTTAAGATGAAACAA
GCCGGGTGATGCCCGGCTTTTTTAAAAGCACAATTTGTCAGGAGAGTCGCCCAATGCGTCTGTGTGATAT
CGATATTGAACGCCATCTGGATGAAGGCAAAATCGTCATTGAGCCACGGCCCGGGGTAGAGCGGATCAGT
GGTGTCAGTGTCGATGTGCTGTTGGGTAACGAGTTTCGGGTGTTTCGCGATCATACCGCGCCCTATATCG
ACCTGAGCGGCCCGAGCAGCGAGATGGCCGATGCCATCGATCGGGTGATGAGTGACGAGATCCATATCCC
CGATGGCGAAGCCTTCTATCTTCACCCGGGTCAGCTGGCGCTGGCGGTGACCTACGAGTCCGTCACCTTG
CCTGCCGATATCGTTGGCTGGCTGGATGGCCGTTCTTCATTGGCGCGCCTGGGTTTGATGGTGCATGTGA
CCGCGCACCGCATCGATCCGGGTTGGTCCGGTCGCATCGTGCTGGAGTTCTACAACGGCGGCAAACTGCC
GCTGGCACTGCGCCCGAAGATGAAAATTGGCGCCTTGAACTTCGAGATGCTCTCCGGCGTCGCGGCCCGT
CCCTACAACAAGCGGGAAAATGCCAAGTACAAGAGCCAGCAGGGCGCCGTTGCCAGCCGTATCAATCAGG
ACTGATGACAGGCCGCACAGCAACGATCCAAGGGAGCACAAGGTGCTCCCTTTTTGCATCTGGAATCAAA
CGGATATGGGGTTGATGGTGCTGGTCGGATGAGATTGGTGGGGCTGGGACGTTTTATCACCACTTCTGCC
CGTTTTCCCGTCATAAAAACGTCATAAAAATGAAATTATTGGCTTAATTGTCAGCTATTTTTGCCCTTTC
CCGCAATCATTGATGGCTTTAATTAATCGATGGCAAGCCGCATGAAGACTGGCTCTAGGATATTTTTTCC
AAAAAGCTCTTTTTTTTTATGGCTGAAATATTTCAATGGTGGCTATGGTGTTGGCCGCCATCTGGCATAA
TCCGCCGCAACTCATAAACAGGCTTTTTTTTATGTTTGAAATGTTTAGTGGTTTGGGTTTGTGGTGGTCA
ATCGGTTTGGTATTGGCCGTTTTTTTTGTGCTTGCCTATGAATTTATCAACGGCTTTCACGATACCGCCA
ACGCGGTAGCAACGGTTATCTATACCAAGGCGATGCCAGCTCACCTGGCGGTCGTTGCATCGGGGATTTT
CAACTTTTTTGGCGTCATGTTCGGCGGTCTGGGTGTGGCCTATGCCATTGTCCATCTGCTGCCCATCGAC
CTGCTGCTTGGGATGGACTCCACCCAAGGGTTGATCATGGTCTTCTCCTTGCTGTTTTCCGCCATCGTGT
GGAACCTGGGCACCTGGTTCTTCGGGATCCCGGCCTCCAGTTCACATACCTTGATCGGTTCCATTCTGGG
GGTAGGCGGCGCCTATGCCTGGATCACCCATAAACCGGTGACAGAAGGGATCAACGTGGGCAAAGCGATC
GACATCATGCTTTCCCTCATCATCTCTCCCACCATCGGCTTCATCATCGCGGCACTGCTGCTGCTGGCGA
TGAAACGCATCTGGACCAGCAACAAGATCCACAAAACACCGGAAGAGCGTCAGCTGGTGGATGGCAAGAA
GCACCCGCCATTCTGGGCGCGTCTGACCCTGGTGGCCTCGGCCATGGGCGTCAGCTTCGTTCACGGCTCC
AACGATGGCCAGAAGGGGATCGGTCTGGTGATGCTGGTGCTGATCTGTATGGCGCCCGCCTATTTCGCGC
TGGACATGAACAGCCGCTCTTATGACCTGGATCGGACCCAGGATGCCAACCAGCGCATCATGGAGATATA
CCAGCGTAACCAGTCCGCCCTCTCTGGCGTCATCAACTTCTCTGCACCGGCAACGGCTCAGGAAGAGCTG
ATGACCCACTGCTCCGCAGATGGCGTGCTGCAGGCGATGGCCACCCTGGATGCCCGTCTGGGTGCCGTGC
GTACTTATGATCAGATGAGCCTGACCGACCGCCGCGAAGTGCGTCGTCTGCTGCTCTGTATCGATGACAC
TGCCCGCAAGGCTGCCAAGCTGCCGCTGCCCGCCAAGGAGCTGACCGATCTGGCCAAATGGCGCAAGGAT
CTGACCGCGACCGCTGAATACGCACCGACCTGGGTGATCGTCTCCATCGCGCTGGCGCTCGGTTGCGGCA
CCATGGTGGGCTGGCGTCGTATCGTTCATACCGTGGGCGAGAAGATCGGCACGACCGGCATGACCTATAG
CCAGGGTATCGCGGCCCAGATCACGGCTGCAACCTCCATCGGAGTCGCCAGTCTGACCGGTATGCCGGTG
TCCACCACCCACATCCTCTCTTCTGCGGTGGCGGGGACCATGATTGCCAACAAATCCGGCCTGCAGAGCC
AGACTATCAAGACCATCCTGATGGCCTGGGTGTTGACTTTGCCGCTGACCATGCTGCTCTCCGGTGGCCT
GTTTATCGTCAGCCACCATCTGTTTGGTTGATCTGCTGCGTTCATAACGCGCATCACAATGACAATGCCG
CCCGGTCGAAAGATCGGGCGGCATTTTTTGTGTCTATGAATTTTCAGTGGAGTTGTATCTCCACCTGATG
TTTTTTGTTCTTGAGGTAAACAGATAAGAAGGCTCTTCTTCATTATTTGATTGACGACTCGTTATGGTTT

TTCTGGCTGAACTTTCGTCATCGAATATCTCTTTCTAATAGTGTGCTTCTCTTGTGATTAATGGATTAAC
CAATTAGCGTGTTTTGGCGAGGTGCAGATGCACCATCTGATAAAGCGTCACTGAGGAGTAGATGATGAAG
CTGATGATCCCGCCCATGGTGGATTTTGCGACCGGCAGCTTGTCCGGTGCCGAACTGGTGGAGAAGTGTA
CAAGGTTGGCTGATCTGTATGGCTTGTTTGCCGATCCTGCTTCTGAAGCAGGGGTTGACCCGCAAACGGT
GCTCTATCGTGTGGCGATGTTGCCCAGCAGTGGCGTGGAGGGGGATCTCAACATGGGAGTGACCTATCTG
GAGGCAGGCCGGGTCGGGCAGGAATTTTTCATGACCCGCGGTCATATCCATGCTCGCGCCGAGCAGGCGG
AGTACTACTTCGGGTTGTGCGGAAAGGGACTATTGCTGTTGCAGGATATGGCCGGTGTTTGCCGGTTGGA
GCAGGTCTTTGCCGGCAGCGTTCATCATATCCCTGGCCACGTTGCCCATCGACTCGTCAACACTGGCGAG
ACGCAGCTCTCGGCGCTGGCGGTCTGGCCTGCCATTGCCGGTCATGATTACCGGCTGATGGCAGAGCGGG
GGTTTGGCCTGCGGGTGGTCGCAGGGAAGGAAGGGGAACCTTGTGTTGTGAGCAGCGAGGTGAGTGTCTC
AATCACCTAACCATCAGCCTGCATGGGTCGTGAGCGACCCCGTTAGCGGCTGCCGCAAGATTGGCGTACC
ACCAGGGTAGGGGGCAGAATGATGCTCTTGGCCGGCTCGTCGTTGCCGGCCATCCGCTCAAGCAGCAGCT
CGCCCGCCTTGCGGCCGATCTCCCGCGCAGCCACCGAGACGGTGGTGAGCGCCGGTTCGACCAGCGCGGT
TTCCGGCAGATCGTCAAAACCCACCAGTGCAAAGTTCTGGCCGATGAGCCGCCCCTGTTTGCGTAGCGCC
AGCATGGCGCCGAGCGCCACCACATCCTGATAGCAGACCGCCGCCGTCACCTCGGGGTGCTGCTCCAGCA
GCGCCGTCATGGCGCGGGTGCCCTCTACCCGGCTGGCGCCGCAACCGATGATCCACTCCGGTTTGGGCGA
GATGCTGTGTTCGAGCAGCTTGCTCATGTAGCCACCGAGGCGTTGGGAGCGGCTGATGGAGGTGGGGGAG
CCCCCGACAAAGGCGATATGACGGTGGCCGAGTTTCAGCAGGTGCTCGGTCGCCAGCTGGCAACCGAGAA
AGTTGTCGGTGCCAACAAAGTCGATCGCCACATCCGGCACCGGTCGCACCACCAGAATGGTAGGCATATG
GCGCCGCTGCAGGCTCTCGAAAAAGCTGTGGGGGGTGGGCTGGGCAGCACAGATCACCATACCGGCGGCG
CTGTTGCGTTGCAGCGAGTCCATAAACTGCTGCTGCCTTGCCGCCGACTCCTCGCTGTTGGCAAGAAACA
GCATCAGATCGTGATGCTCCATCTCCCGGCTCAGGCCCGCCGTCATCTCTGCATAGAAGGGGTTGGTAAT
GTCGTGCAGCAACAAACCGACCTGATTGCTGCTCTTGTTGCGCAGGTTGGCGGCAGTCTGGTTGTAGACA
TAGCCGAGCTCGTCGATGGCTTTGAGTACCCGCTGGCGGGTGTGGTCGGAGATGCGTCCCTTGTTGCGCA
AGGTCATGGAGACGGTGGCGGTGGAAACGCCCAGATGGCGGGCAATATCGGCAACAGTCACATTGCTCAT
GGTGTGGGTCCAGTCATGATCGTGTCATCAAGATATATCGATTAACCTTCATTTCGGATGAGAGAAGGTT
GCAAGTGAGCGATTGATCACGAATTTCACCGCCAATACCGATGATATATCCCAATGTTTGAGGTTAATCG
TGTTTATCAGGTTGTGGTTAACGGGTTAATCGATTTATCTCTGTCGTAGGTGTCAACGAGAGAGCAAGCC
AATGATTCATTCGAGTTACGTCTCCAACTACGACAAATATCCAGAAGTAACCGTTGTCGGATATGAACAG
GCCGCCTGCGCGGGATGGGACGCGATTGCCCATCAGCTGAAGGCCAAAGTGAACGGGCAGGAGAATGCGA
AAGTGGTACTGGTGATCGACTGCTATCACGGCGTGGATCAGACCGAGCTACGCACCCAGCTGCTGACCAG
ATTCGGGCCTCACACGCTCATCGATGTGGAACAGGCGCGCCTGCCCGAACCCAAAGTGCTGGCGATGCTG
GATCGCTTCATCACCGATGACAGGGTGTTTGGCGTGCTGGCACCCCACAAGATGGTGGAGTTTTTTGACC
CGGTCGCCTTGGCGCGCCTGCAACAGCAGATCGCCGAGATGGCCGAAGGTCTGGTGGTGGTGATGGGCAG
CGGCGCTTCGCTGGTGGCGCAAGGGGATGTGCGGGTCTATGCCGATCTTGCCCGCTGGGAGATCCAGCAG
CGTCTGCGCCGTAAAGAGATTGGCAACTGGGGTGCTGATAACACCGAAGAGGACATATTGCGCCGCTACA
AGCGCGCCTTCTTTATCGAGTGGCGGGTGTTTGACCGCCATAAACTGGCCCAGCTGCCCACCATCGATTT
TCTGCTCGACACCAACACCAGGGGCAAGCCGACCATGGTAACGGGGGCGGCACTGCAACAAGGGTTGCAT
CAGGCGGCGCGCCAGCCGTTCCGGGTGGTGCCCTTCTTCGACCCGGGGGTCTGGGGTGGGCAGTGGATGA
AGCAGGTGTGTGATCTGGACCCGGCCCAACCCAACTACGCCTGGTGCTTTGACTGCGTACCCGAGGAGAA
CAGCCTCTATCTGCGCTTTGGCGAGGTGAGGGTGGAGATCCCGTCGCAAAACCTGGTGCTGTGCCAGCCC
AGCGCCTTGCTTGGGGAGCGGGTACACGCCCGCTTTGGCACCGAGTTCCCGATCCGGTTCGATTTTCTCG
[truncated: 512,517 more chars]
